# Supplementary material for: Transcriptomic and proteomic landscape of mitochondrial dysfunction reveals secondary coenzyme Q deficiency in mammals
Source: eLife. 2017 Nov 14;6:e30952. doi: 10.7554/eLife.30952 (PMC5703644; doi:10.7554/eLife.30952)
Supplement: Supplementary file 9. — Time curves of differential expression analysis of each protein on the Lrpprc knockout analysis at different ages. [file elife-30952-supp9.pdf]

Supplementary File 9: Differential expression time-curve per protein Kühl *et al.*

Time curves of differential expression analysis of each protein on the *Lrpprc* time point analysis

Time curves represent the log2 fold change of L/L, cre / L/L

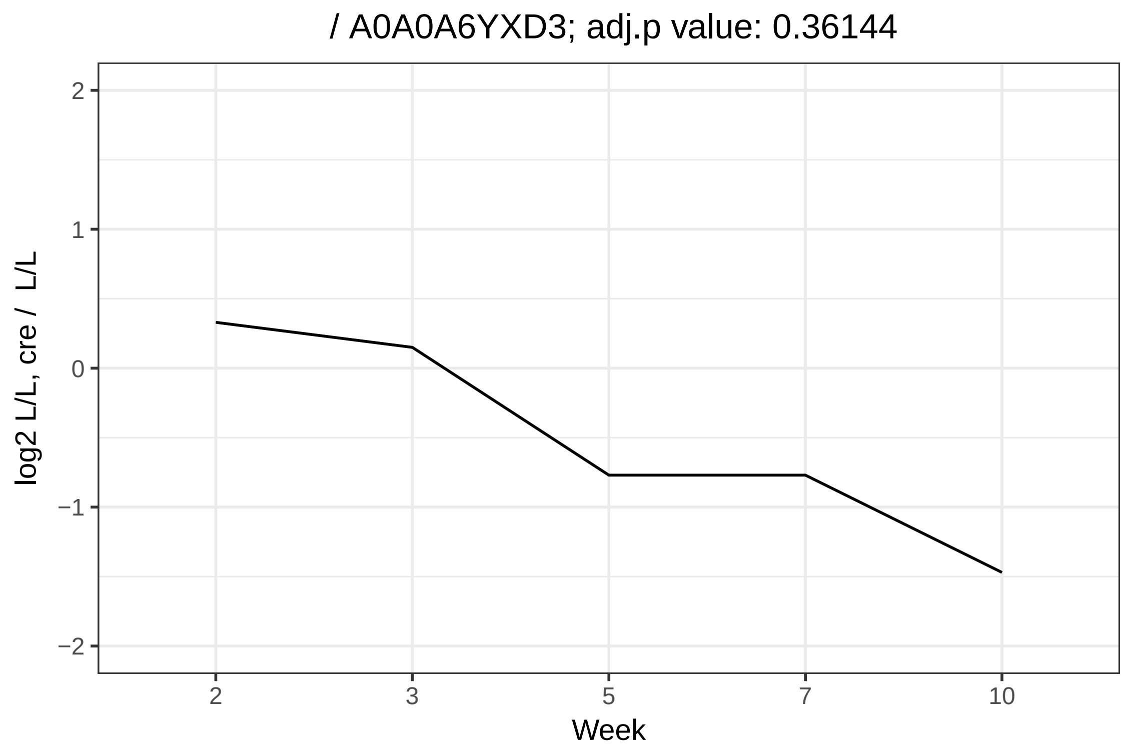
/ A0A0A6YXD3; adj.p value: 0.36144
log2 L/L, cre / L/L
2
1
0
−1
−2
2
3
5
7
10
Week

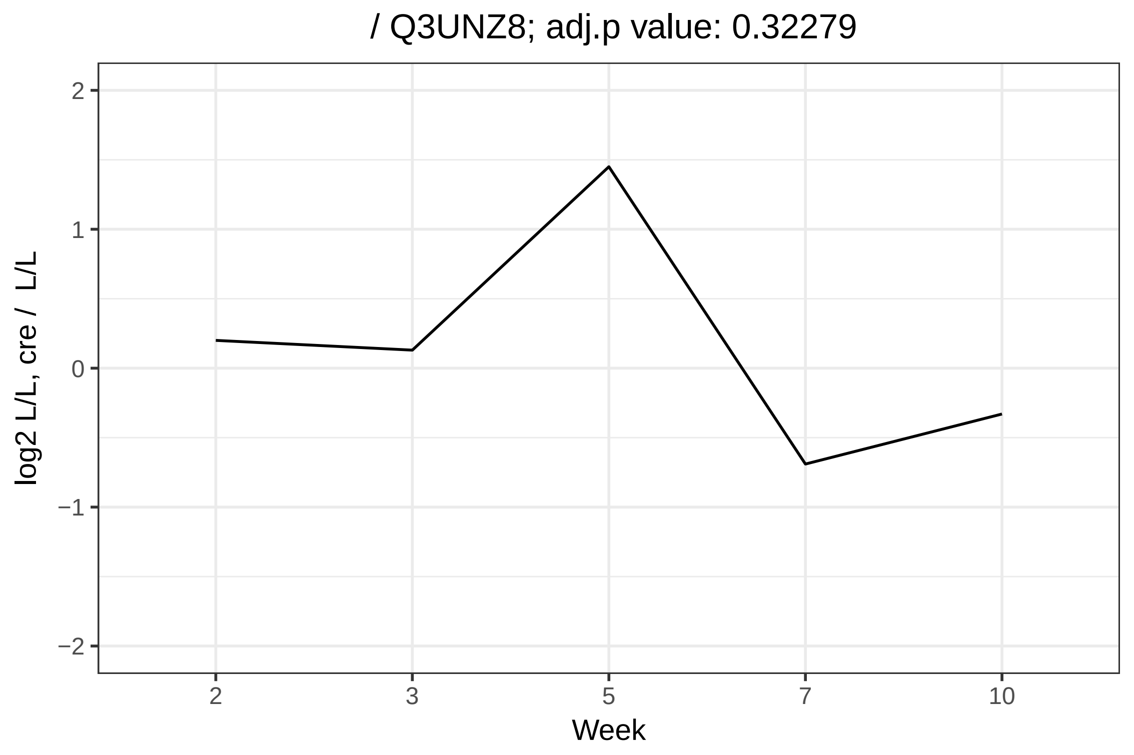
/ Q3UNZ8; adj.p value: 0.32279
log2 L/L, cre / L/L
Week
2
1
0
−1
−2
2
3
5
7
10

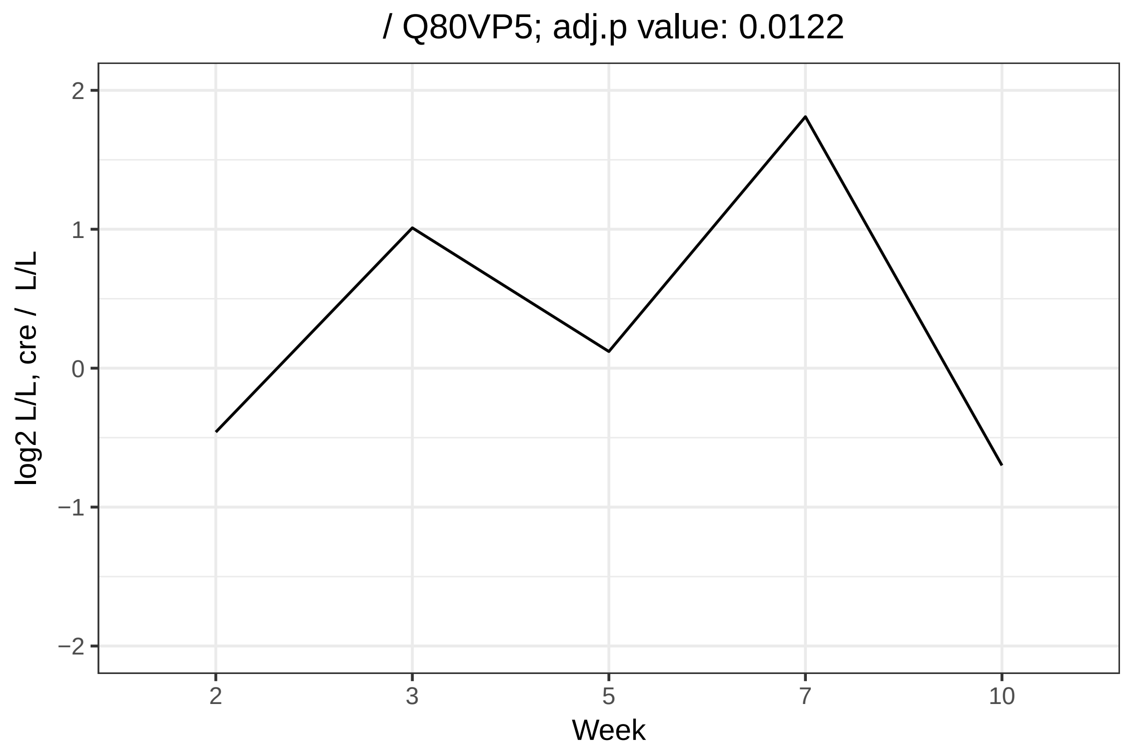

/ Q80VP5; adj.p value: 0.0122
log2 L/L, cre / L/L
Week
2
1
0
−1
−2
2
3
5
7
10

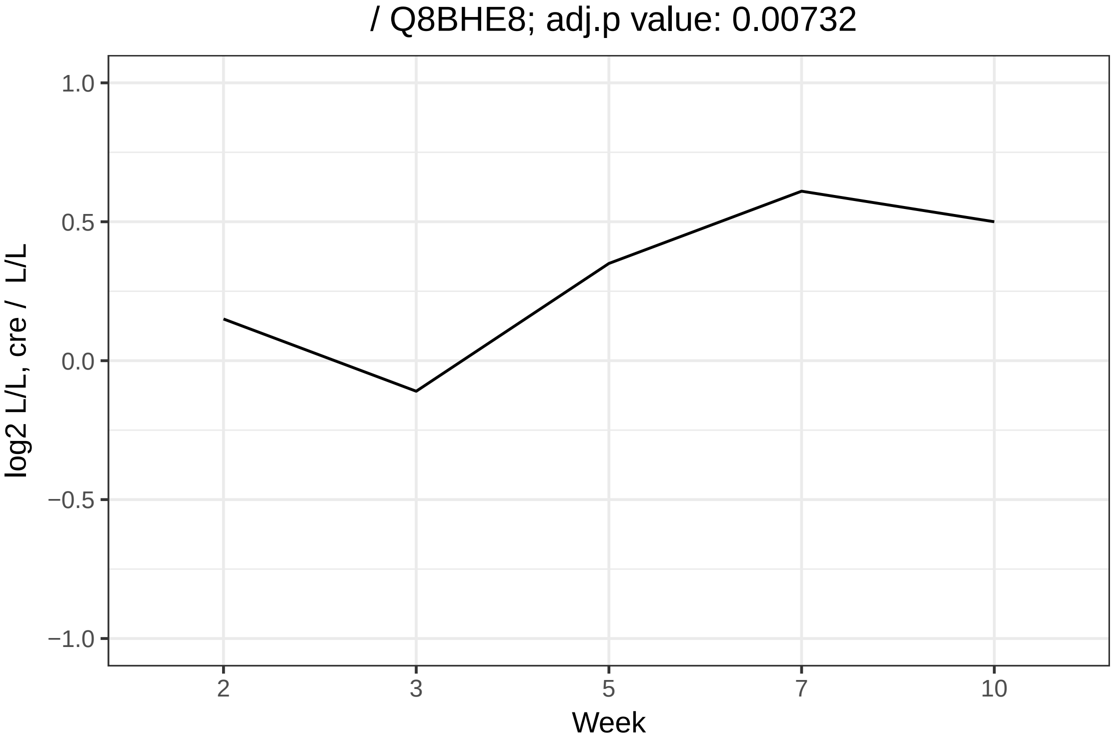
/ Q8BHE8; adj.p value: 0.00732
log2 L/L, cre / L/L
1.0
0.5
0.0
−0.5
−1.0
2
3
5
7
10
Week

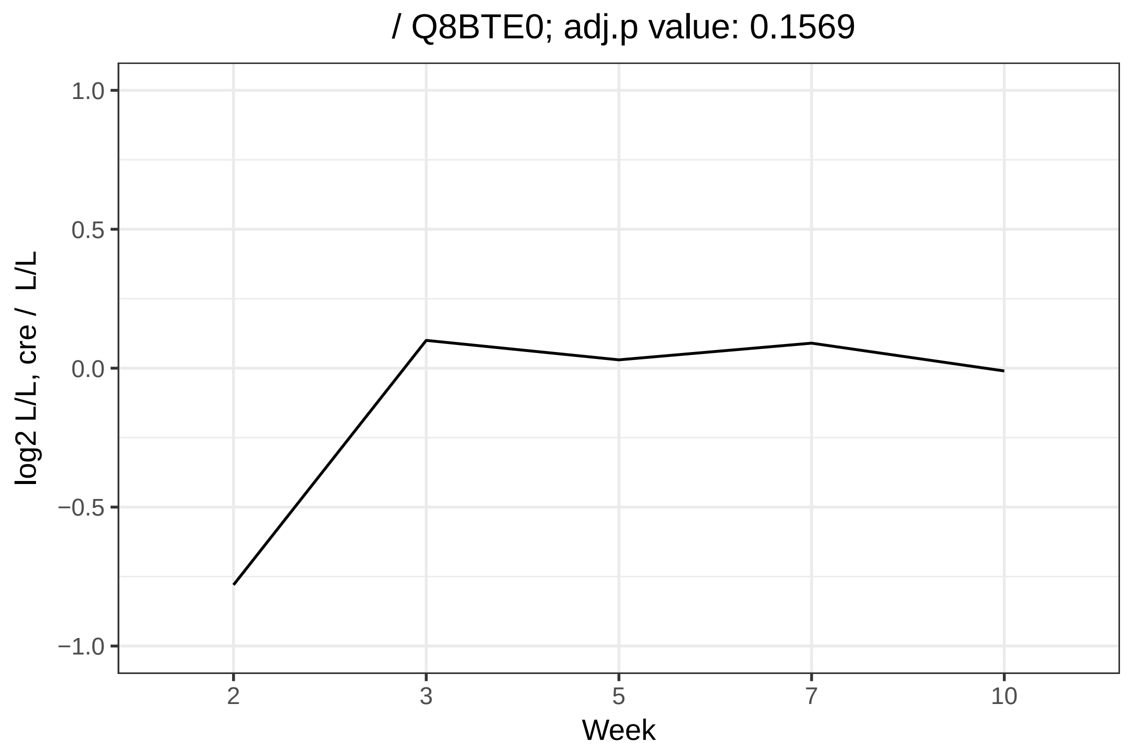
/ Q8BTE0; adj.p value: 0.1569
log2 L/L, cre / L/L
1.0
0.5
0.0
−0.5
−1.0
2
3
5
7
10
Week

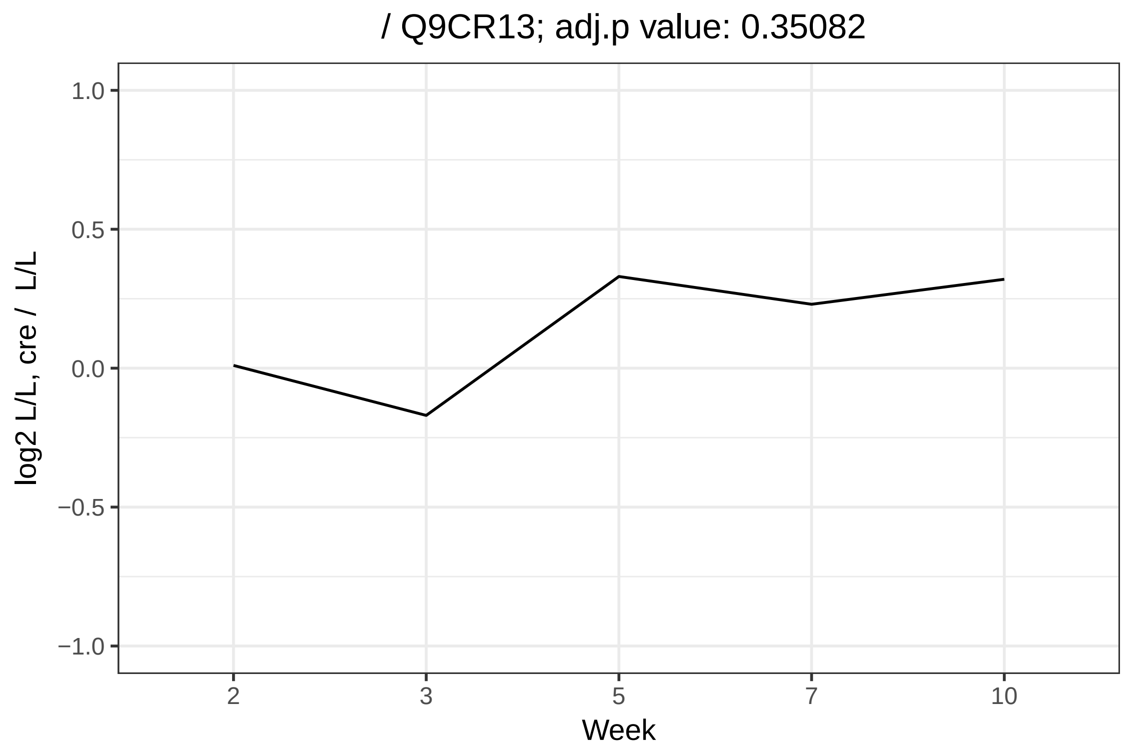
/ Q9CR13; adj.p value: 0.35082
log2 L/L, cre / L/L
1.0
0.5
0.0
−0.5
−1.0
2
3
5
7
10
Week

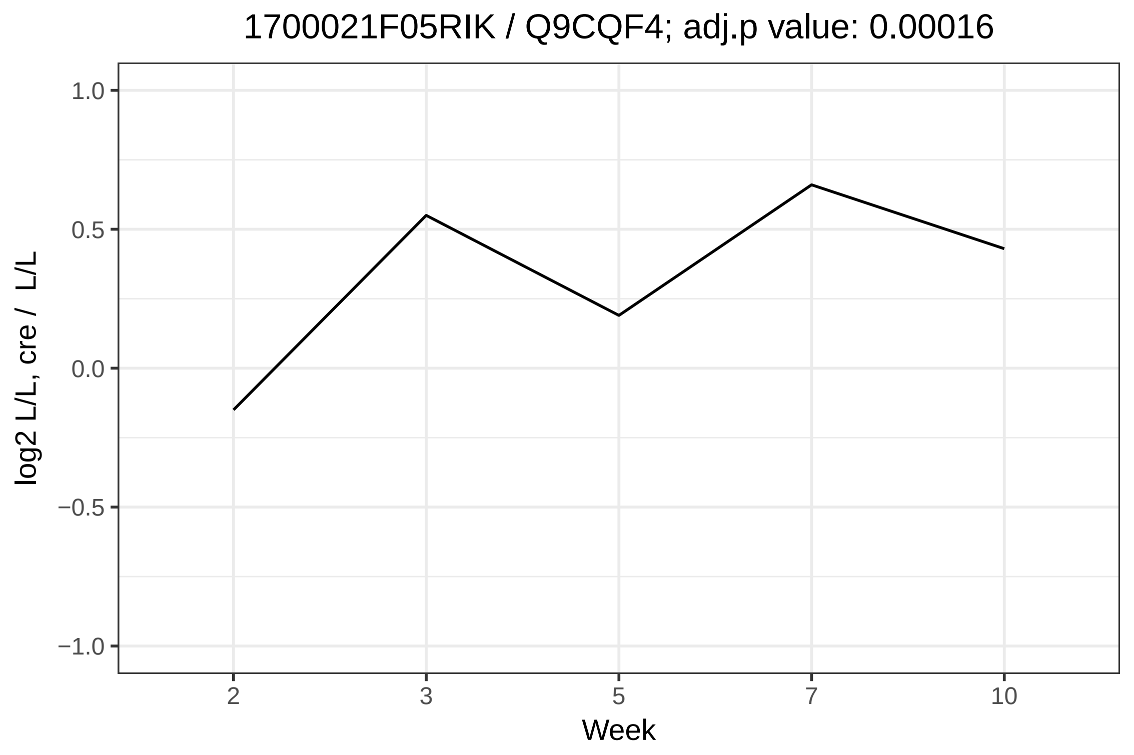
1700021F05RIK / Q9CQF4; adj.p value: 0.00016
log2 L/L, cre / L/L
1.0
0.5
0.0
−0.5
−1.0
2
3
5
7
10
Week

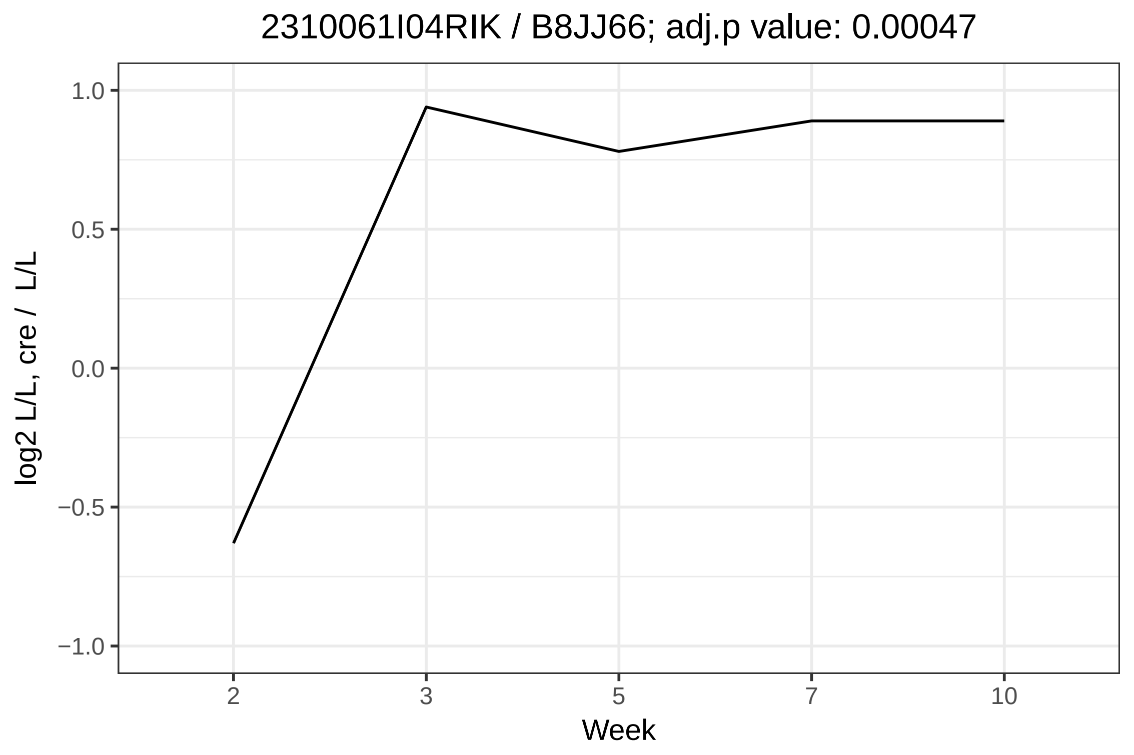
2310061I04RIK / B8JJ66; adj.p value: 0.00047
log2 L/L, cre / L/L
1.0
0.5
0.0
−0.5
−1.0
2
3
5
7
10
Week

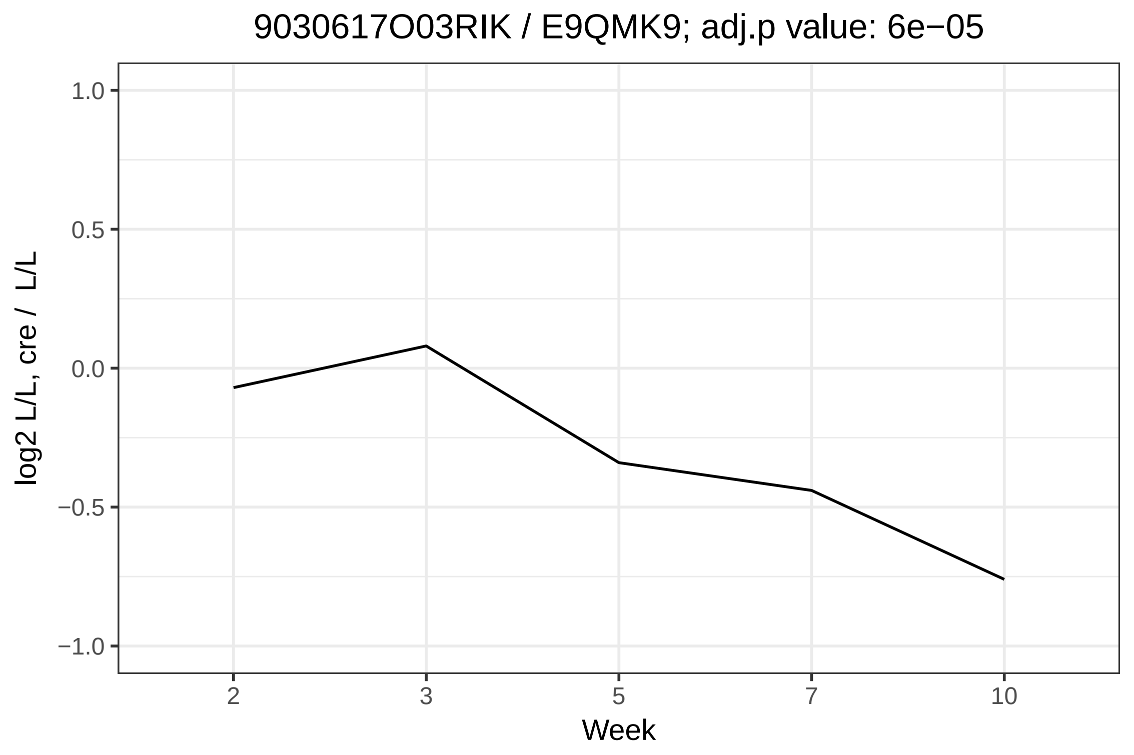
9030617O03RIK / E9QMK9; adj.p value: 6e−05
log2 L/L, cre / L/L
1.0
0.5
0.0
−0.5
−1.0
2
3
5
7
10
Week

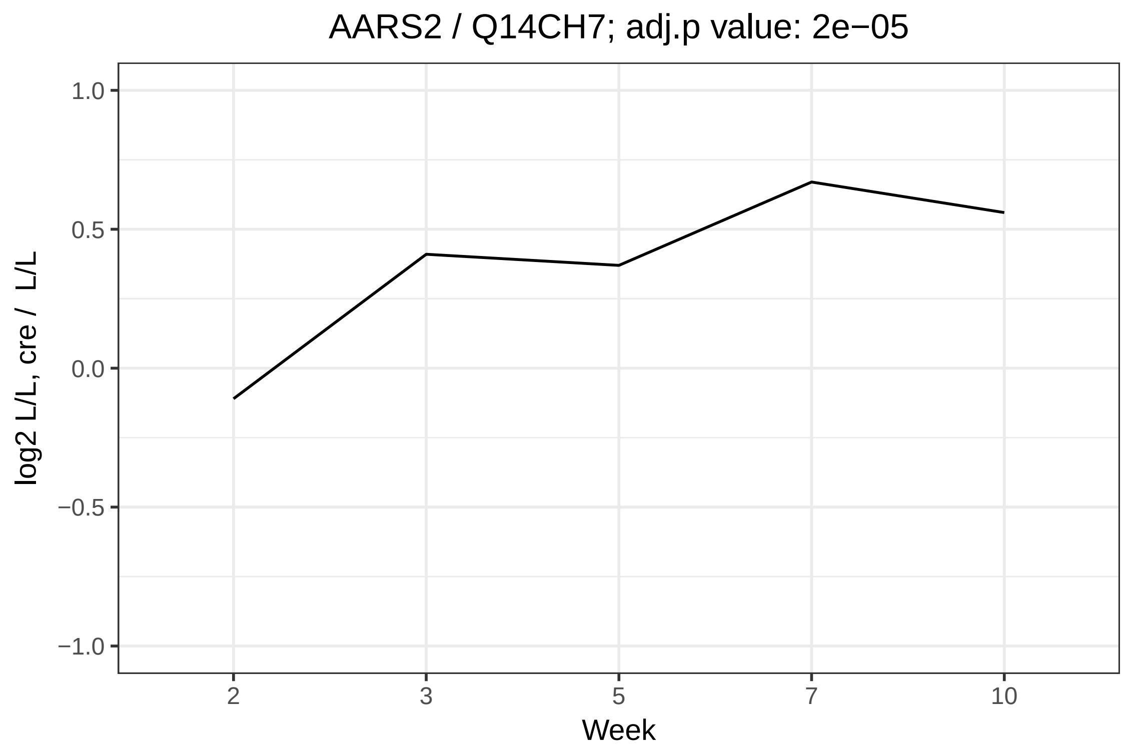

AARS2 / Q14CH7; adj.p value: 2e−05
log2 L/L, cre / L/L
1.0
0.5
0.0
−0.5
−1.0
2
3
5
7
10
Week

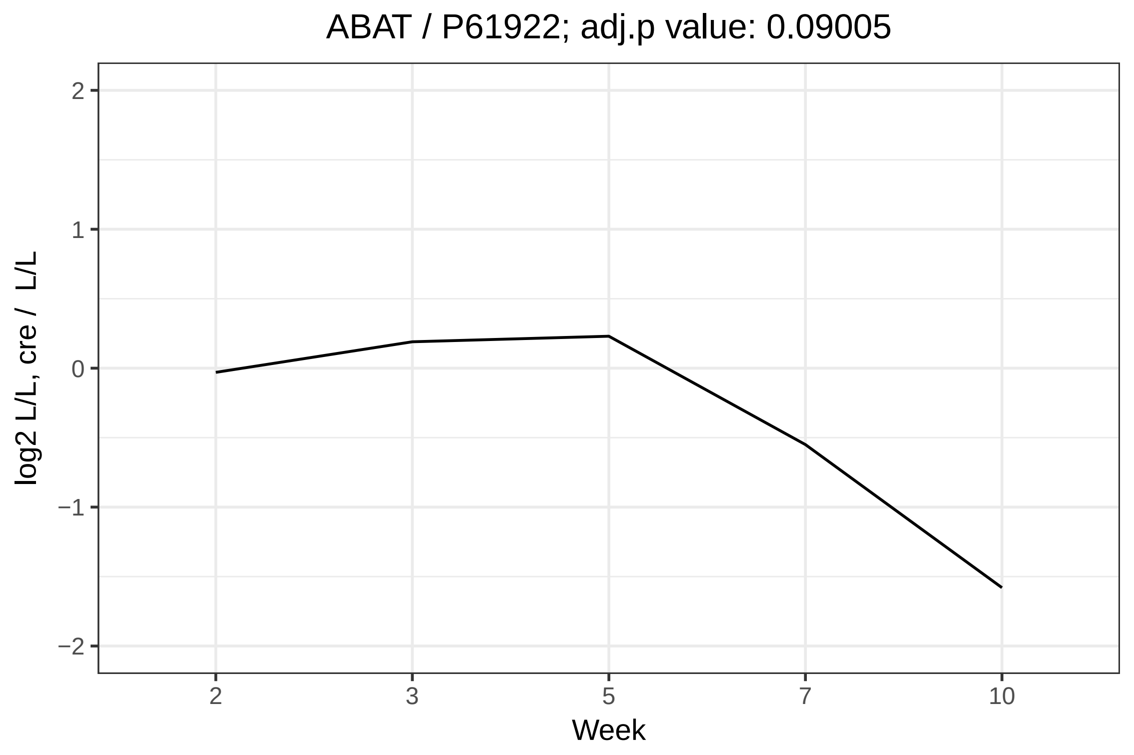
ABAT / P61922; adj.p value: 0.09005
log2 L/L, cre / L/L
2
1
0
−1
−2
2
3
5
7
10
Week

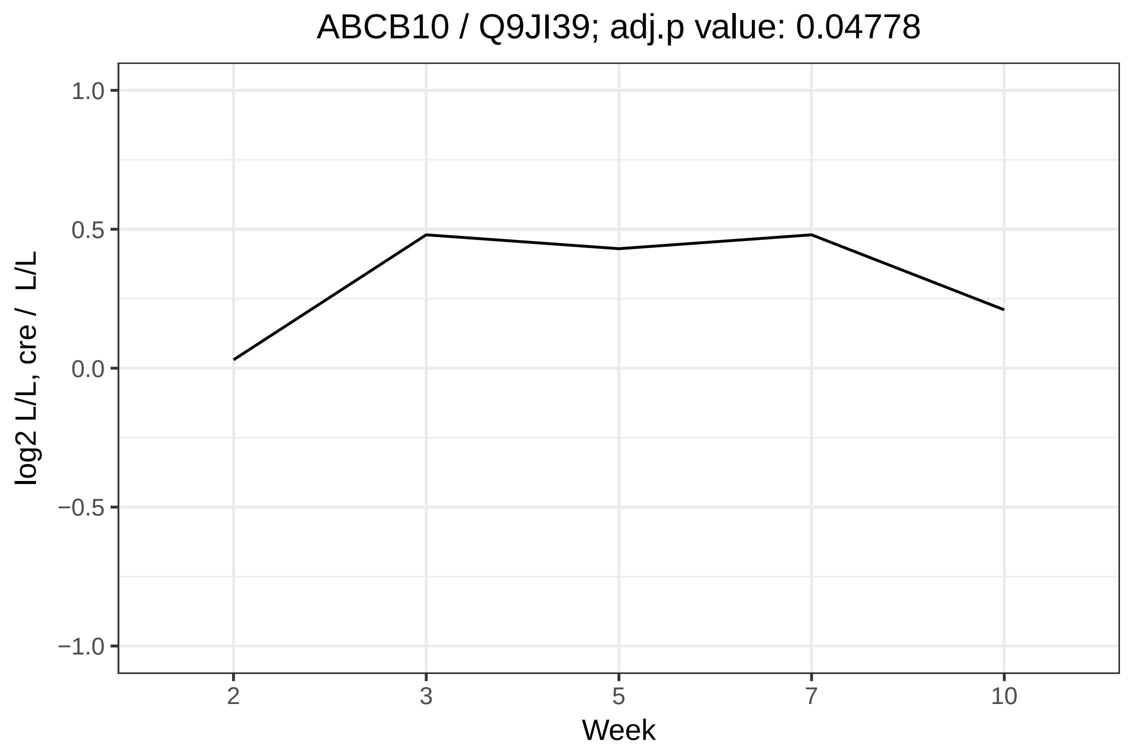
ABCB10 / Q9JI39; adj.p value: 0.04778
log2 L/L, cre / L/L
1.0
0.5
0.0
−0.5
−1.0
2
3
5
7
10
Week

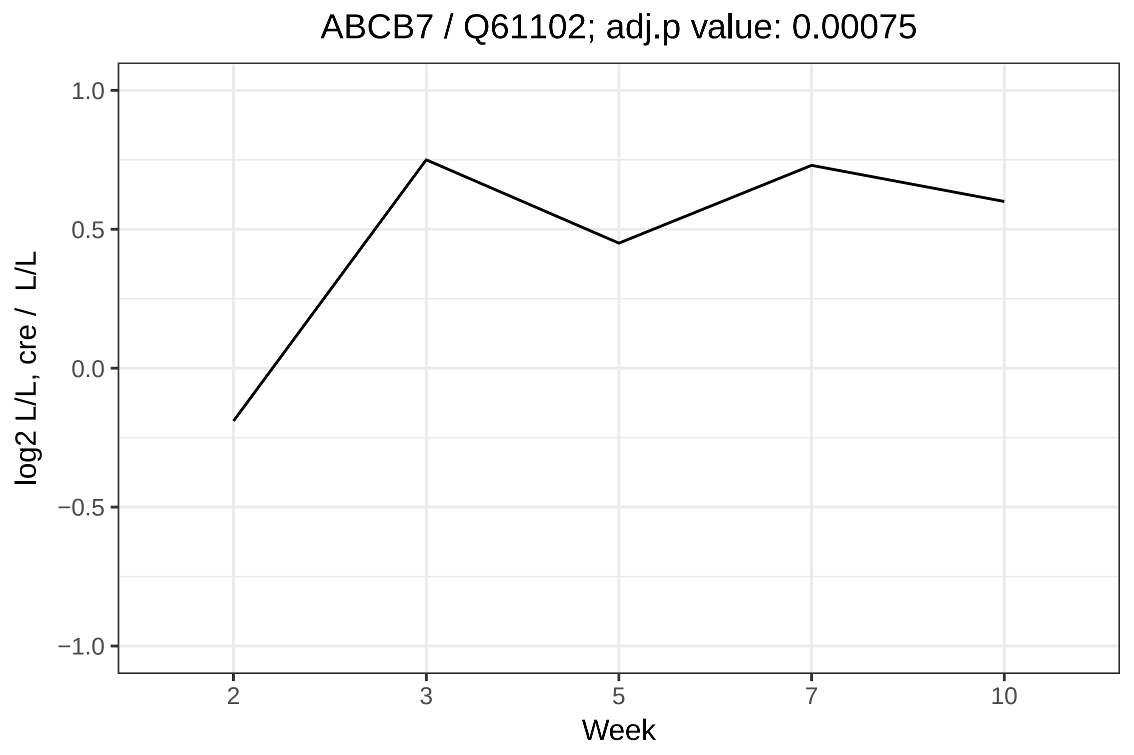

ABCB7 / Q61102; adj.p value: 0.00075
log2 L/L, cre / L/L
1.0
0.5
0.0
−0.5
−1.0
2
3
5
7
10
Week

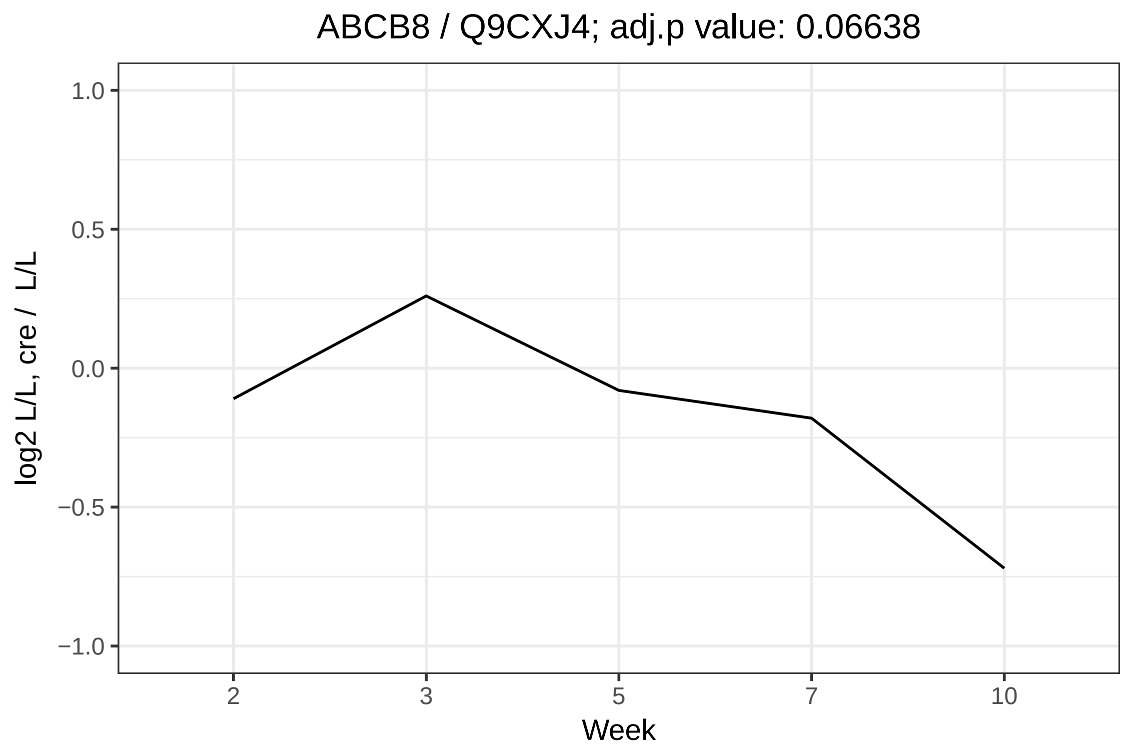

ABCB8 / Q9CXJ4; adj.p value: 0.06638
1.0
0.5
0.0
−0.5
−1.0
log2 L/L, cre / L/L
2
3
5
7
10
Week

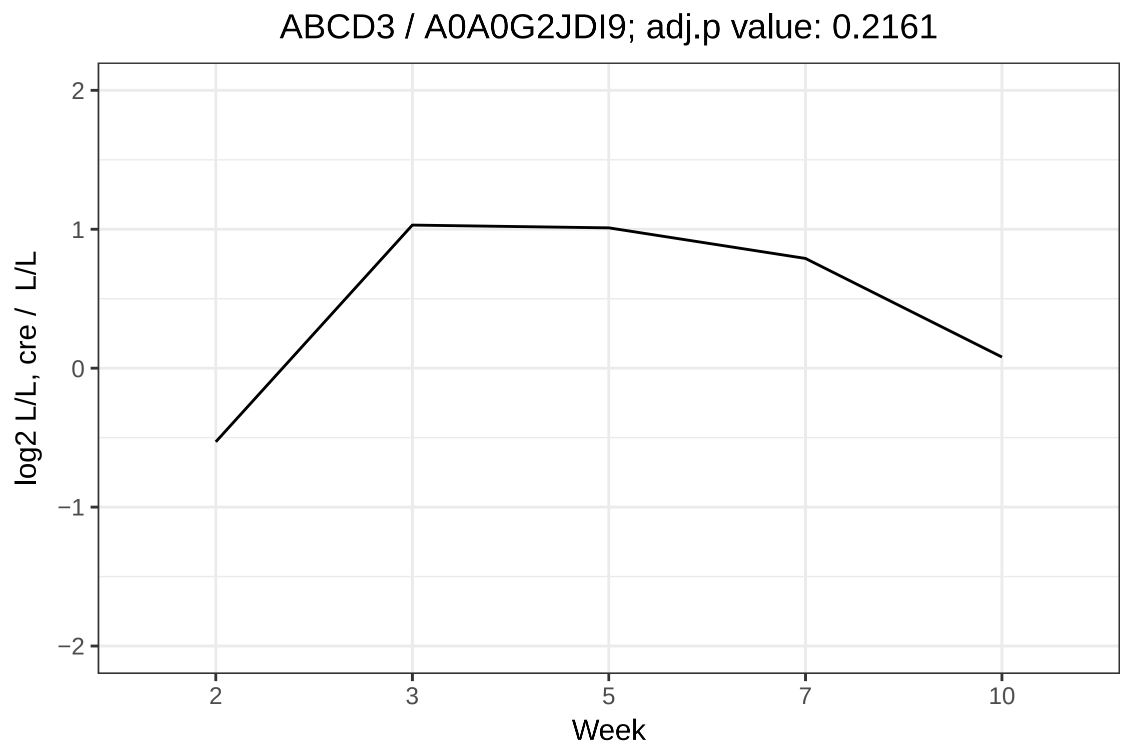
ABCD3 / A0A0G2JDI9; adj.p value: 0.2161
log2 L/L, cre / L/L
2
1
0
−1
−2
2
3
5
7
10
Week

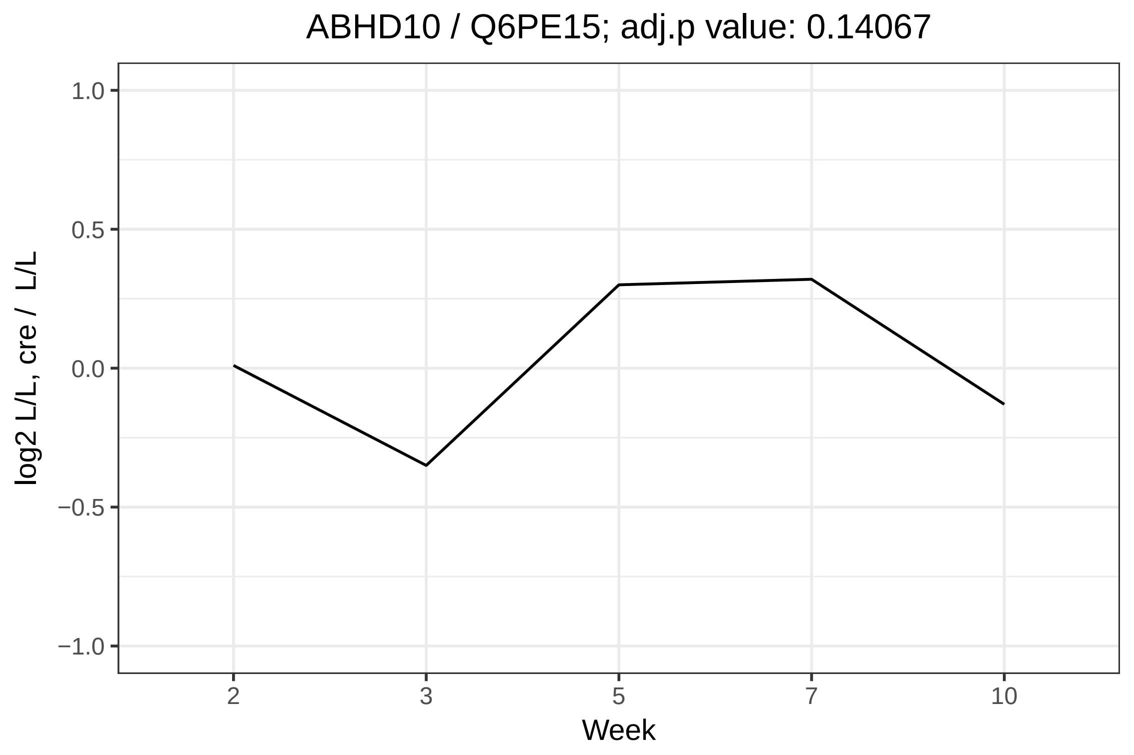
ABHD10 / Q6PE15; adj.p value: 0.14067
1.0
0.5
0.0
−0.5
−1.0
log2 L/L, cre / L/L
2
3
5
7
10
Week

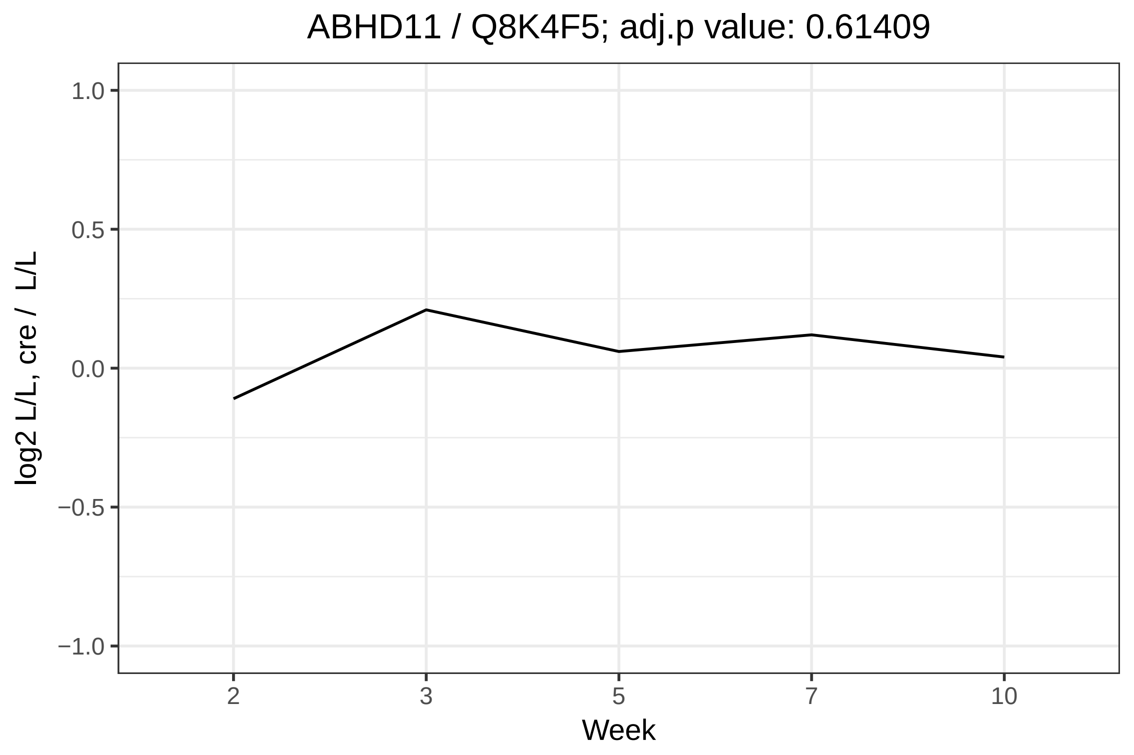
ABHD11 / Q8K4F5; adj.p value: 0.61409
log2 L/L, cre / L/L
1.0
0.5
0.0
−0.5
−1.0
2
3
5
7
10
Week

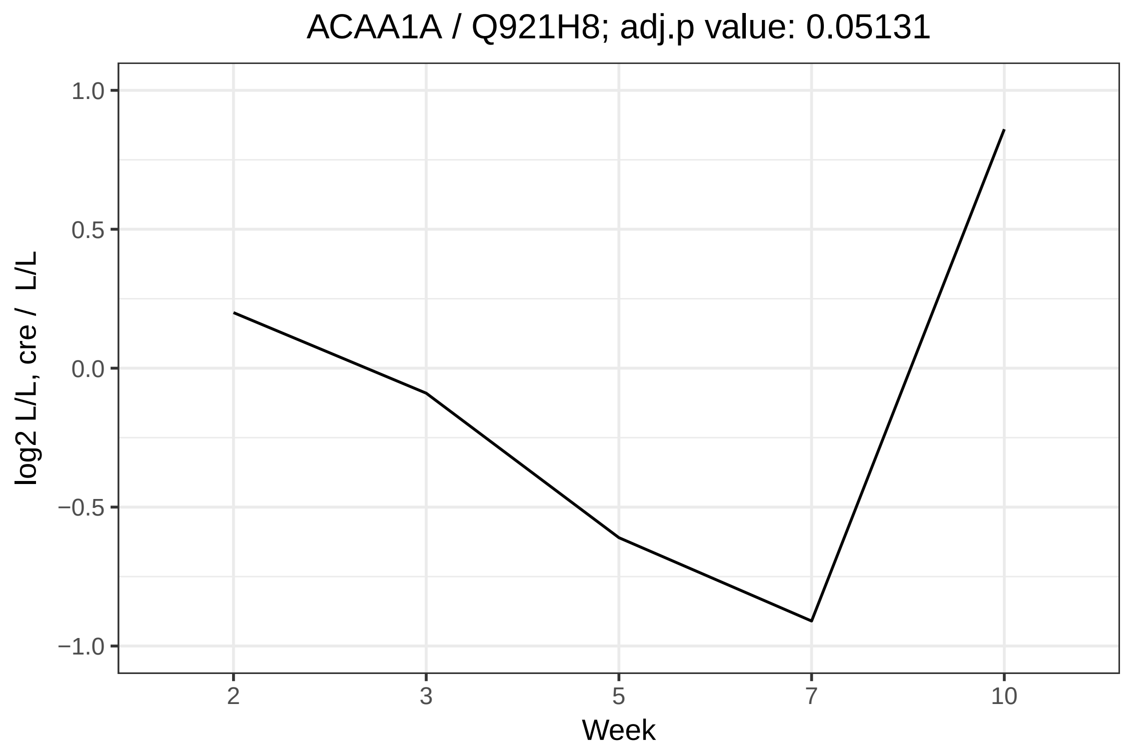
ACAA1A / Q921H8; adj.p value: 0.05131
log2 L/L, cre / L/L
1.0
0.5
0.0
−0.5
−1.0
2
3
5
7
10
Week

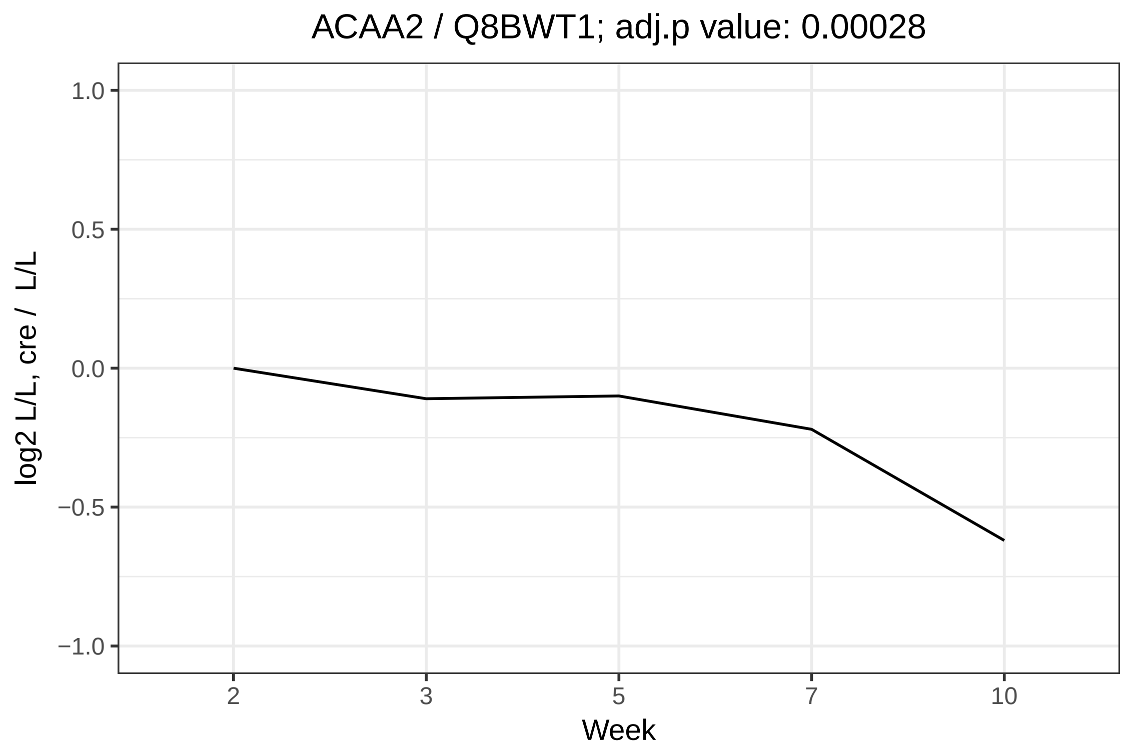
ACAA2 / Q8BWT1; adj.p value: 0.00028
log2 L/L, cre / L/L
1.0
0.5
0.0
−0.5
−1.0
2
3
5
7
10
Week

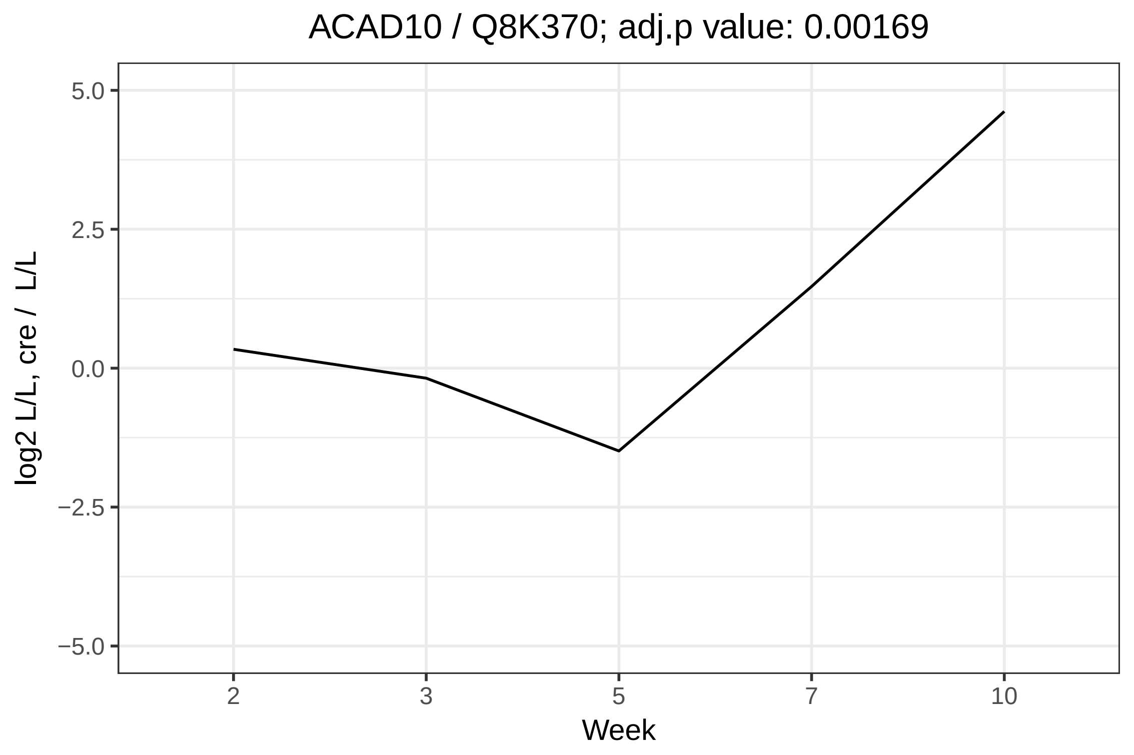
ACAD10 / Q8K370; adj.p value: 0.00169
5.0
2.5
0.0
−2.5
−5.0
log2 L/L, cre / L/L
2
3
5
7
10
Week

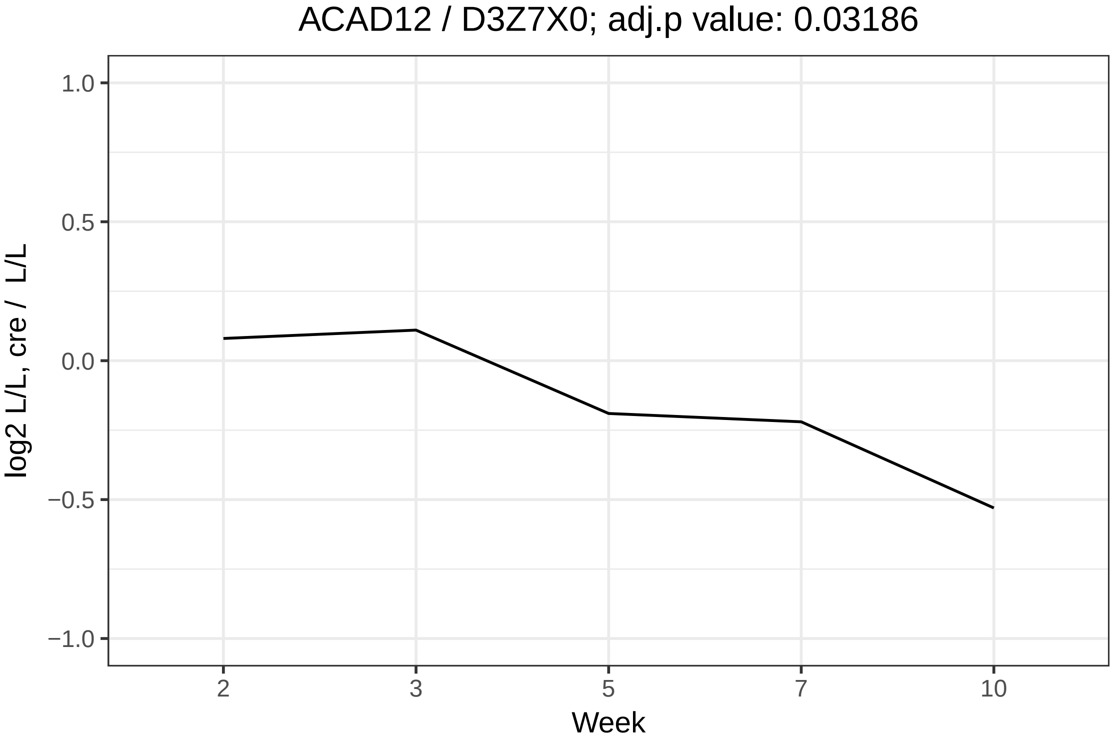
ACAD12 / D3Z7X0; adj.p value: 0.03186
log2 L/L, cre / L/L
1.0
0.5
0.0
−0.5
−1.0
2
3
5
7
10
Week

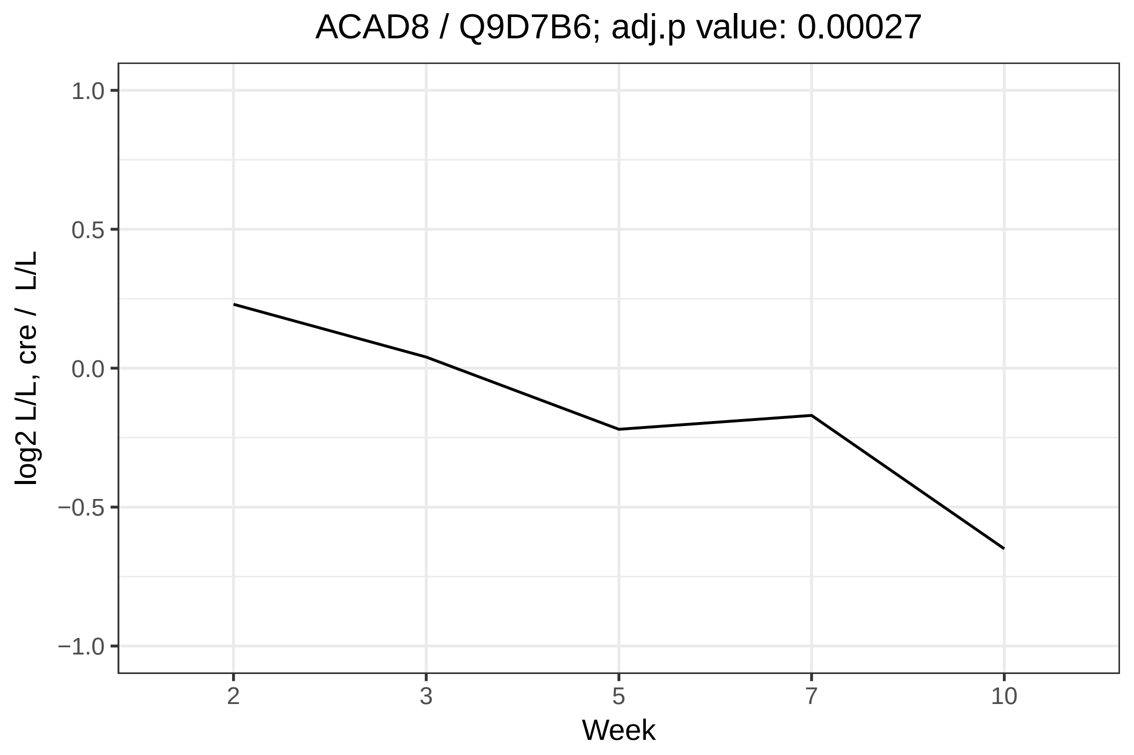

ACAD8 / Q9D7B6; adj.p value: 0.00027
1.0
0.5
0.0
−0.5
−1.0
log2 L/L, cre / L/L
2
3
5
7
10
Week

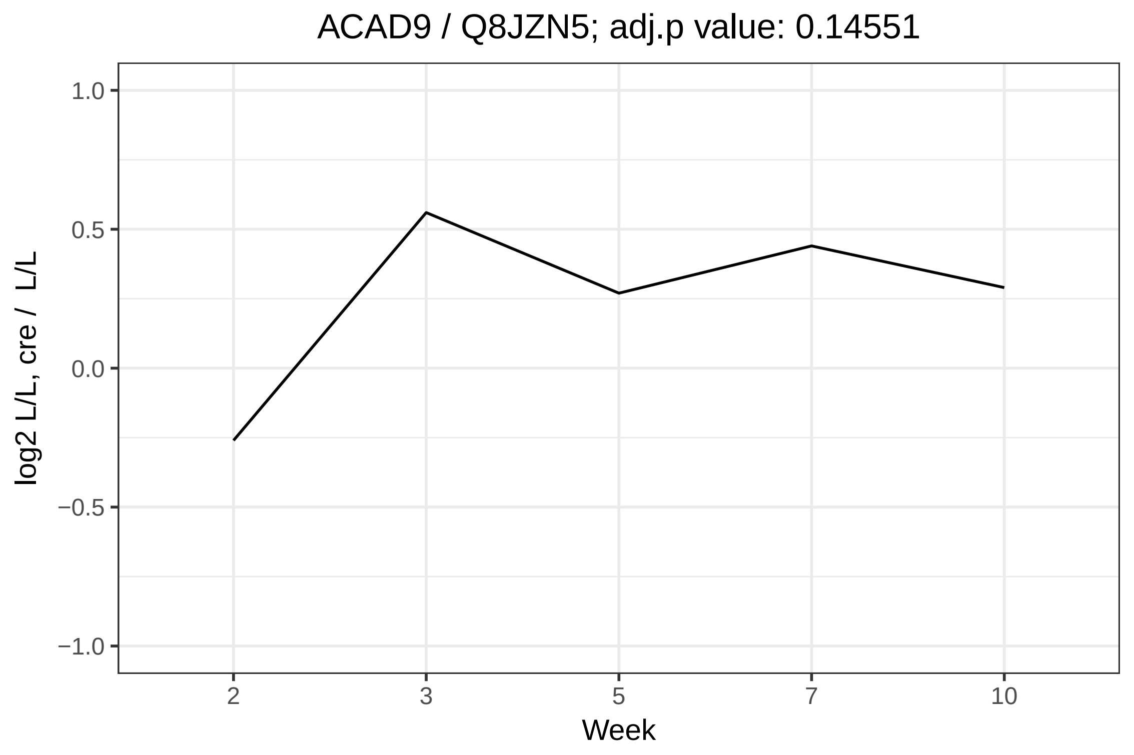
ACAD9 / Q8JZN5; adj.p value: 0.14551
log2 L/L, cre / L/L
1.0
0.5
0.0
−0.5
−1.0
2
3
5
7
10
Week

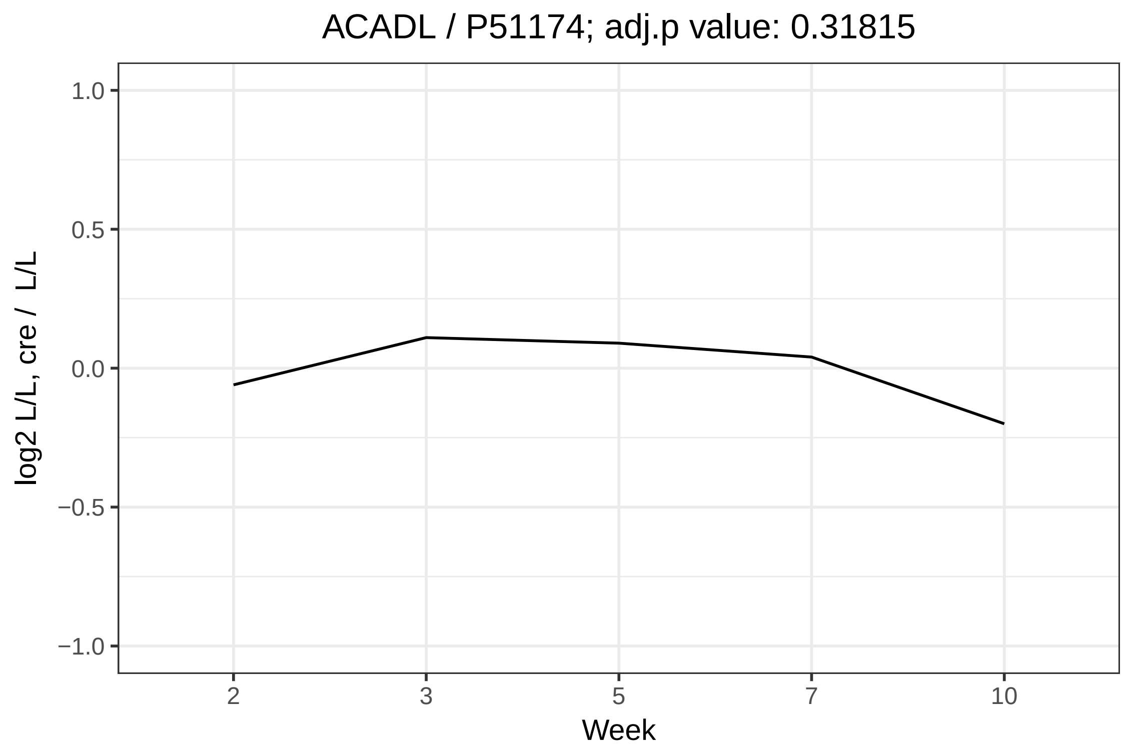
ACADL / P51174; adj.p value: 0.31815
1.0
0.5
0.0
−0.5
−1.0
log2 L/L, cre / L/L
2
3
5
7
10
Week

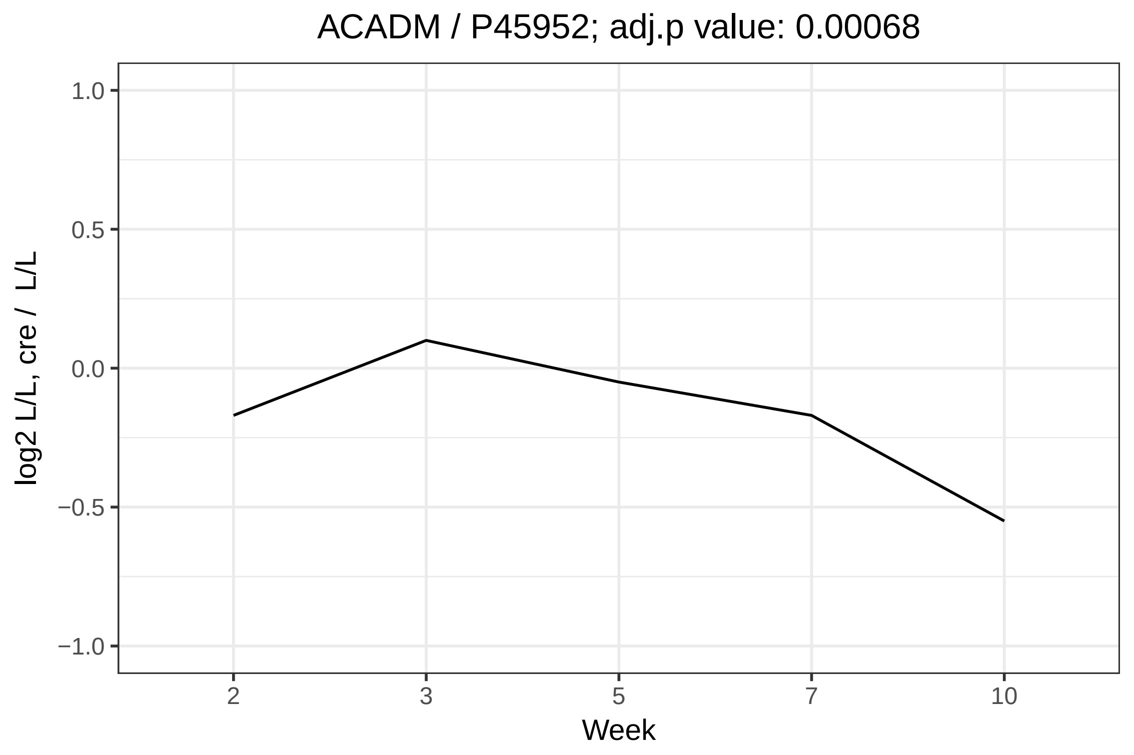
ACADM / P45952; adj.p value: 0.00068
1.0
0.5
0.0
−0.5
−1.0
log2 L/L, cre / L/L
2
3
5
7
10
Week

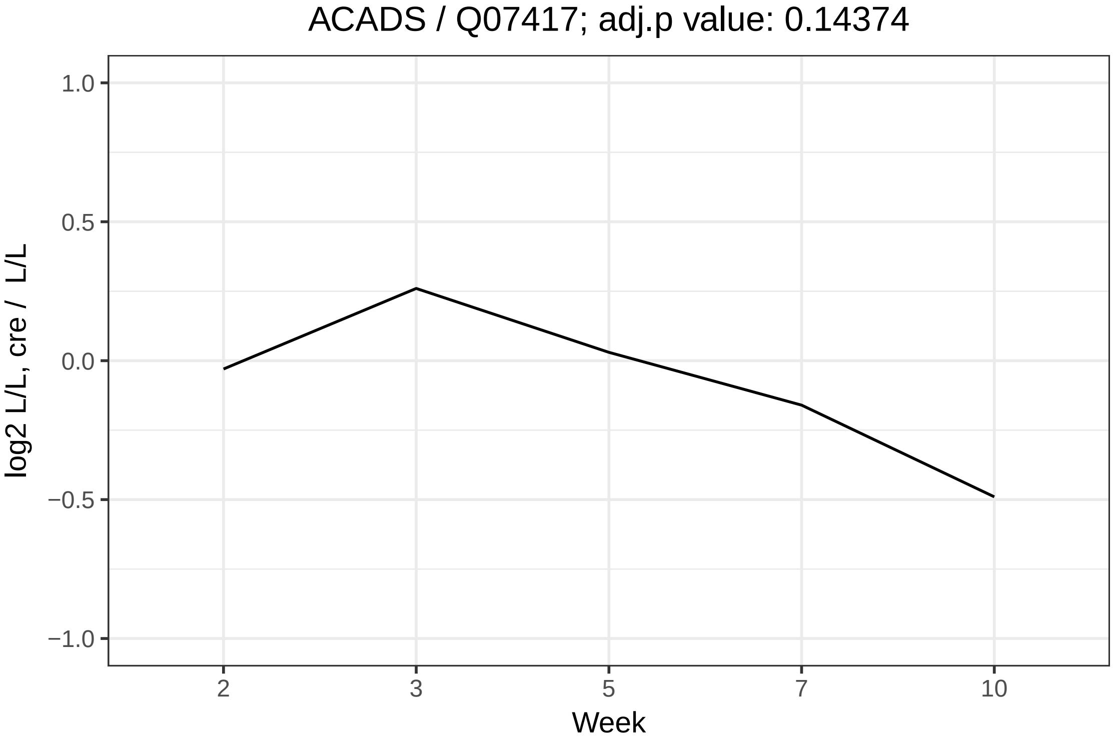
ACADS / Q07417; adj.p value: 0.14374
log2 L/L, cre / L/L
1.0
0.5
0.0
−0.5
−1.0
2
3
5
7
10
Week

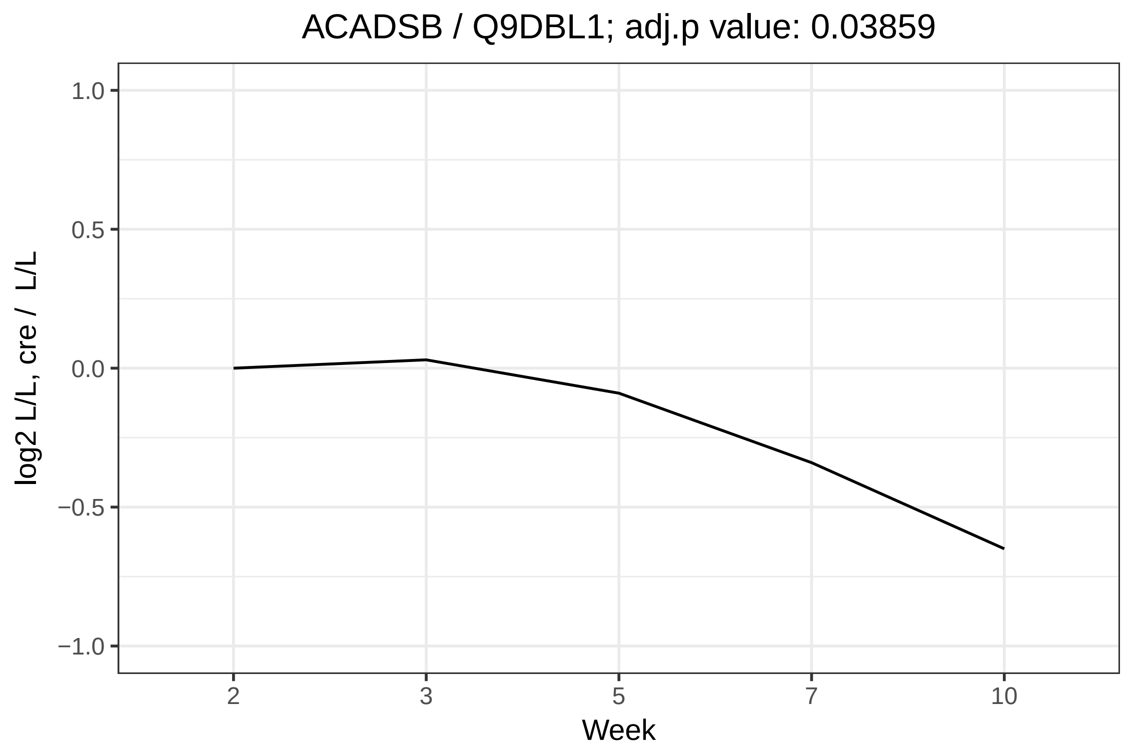

ACADSB / Q9DBL1; adj.p value: 0.03859
log2 L/L, cre / L/L
1.0
0.5
0.0
−0.5
−1.0
2
3
5
7
10
Week

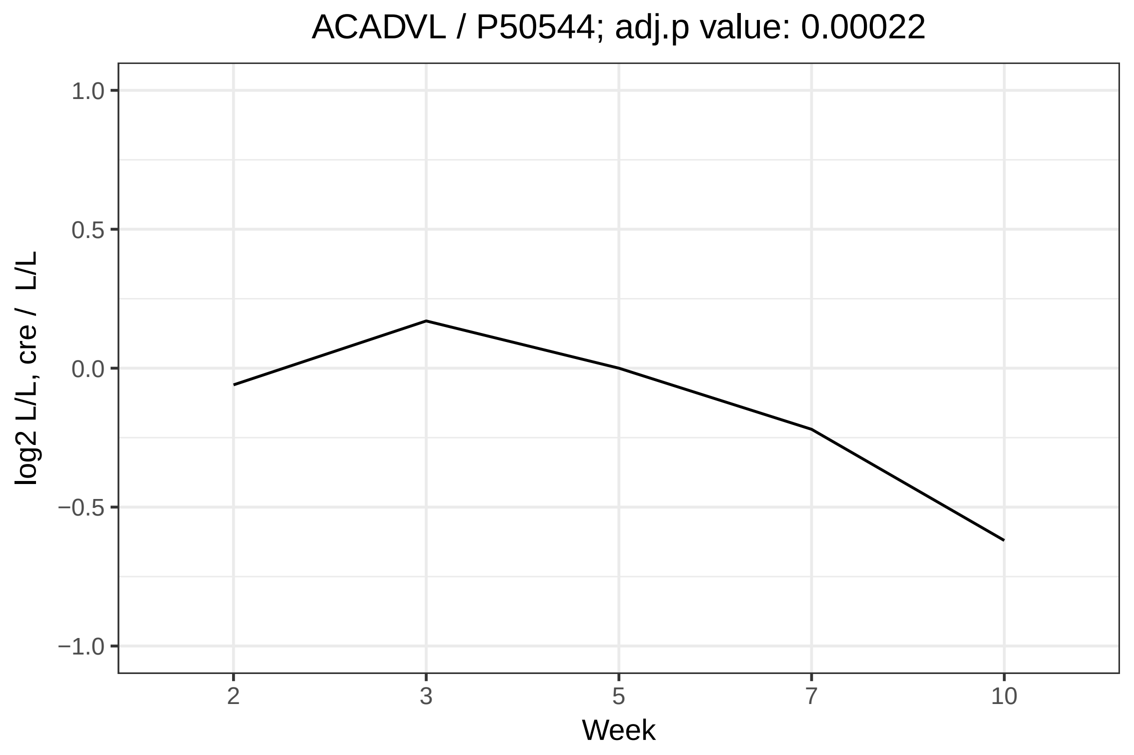
ACADVL / P50544; adj.p value: 0.00022
log2 L/L, cre / L/L
1.0
0.5
0.0
−0.5
−1.0
2
3
5
7
10
Week

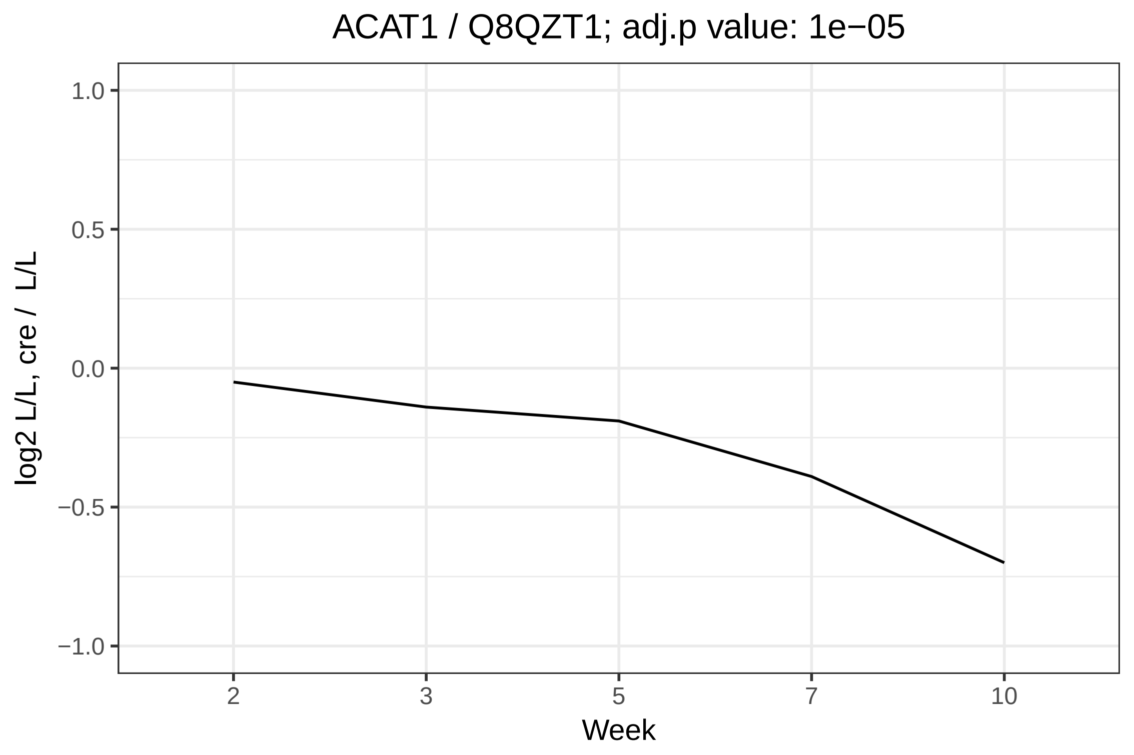

ACAT1 / Q8QZT1; adj.p value: 1e−05
log2 L/L, cre / L/L
1.0
0.5
0.0
−0.5
−1.0
2
3
5
7
10
Week

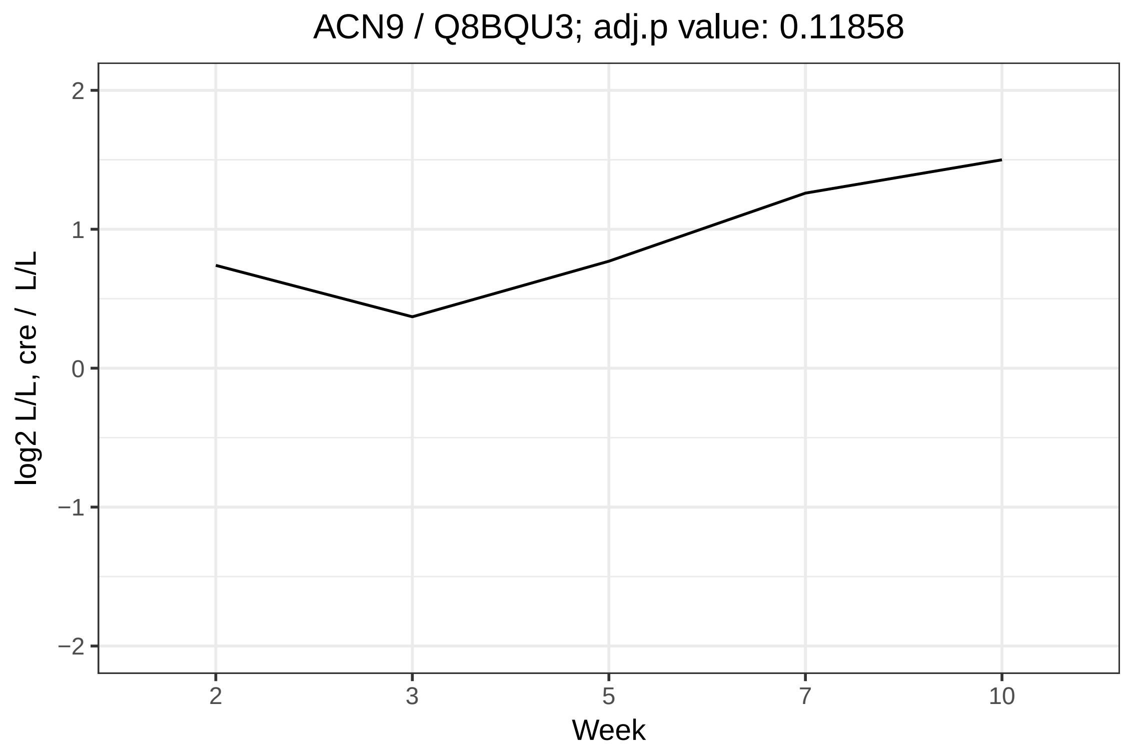

ACN9 / Q8BQU3; adj.p value: 0.11858
log2 L/L, cre / L/L
2
1
0
−1
−2
2
3
5
7
10
Week

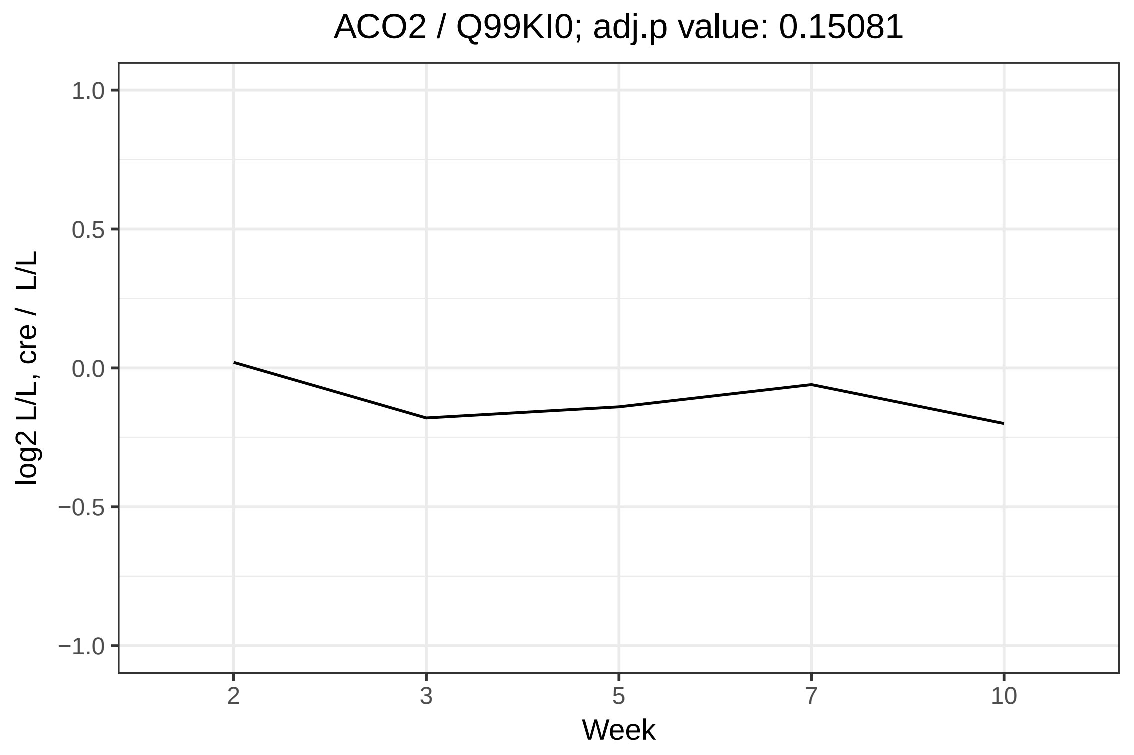

ACO2 / Q99KI0; adj.p value: 0.15081
1.0
0.5
0.0
−0.5
−1.0
log2 L/L, cre / L/L
2
3
5
7
10
Week

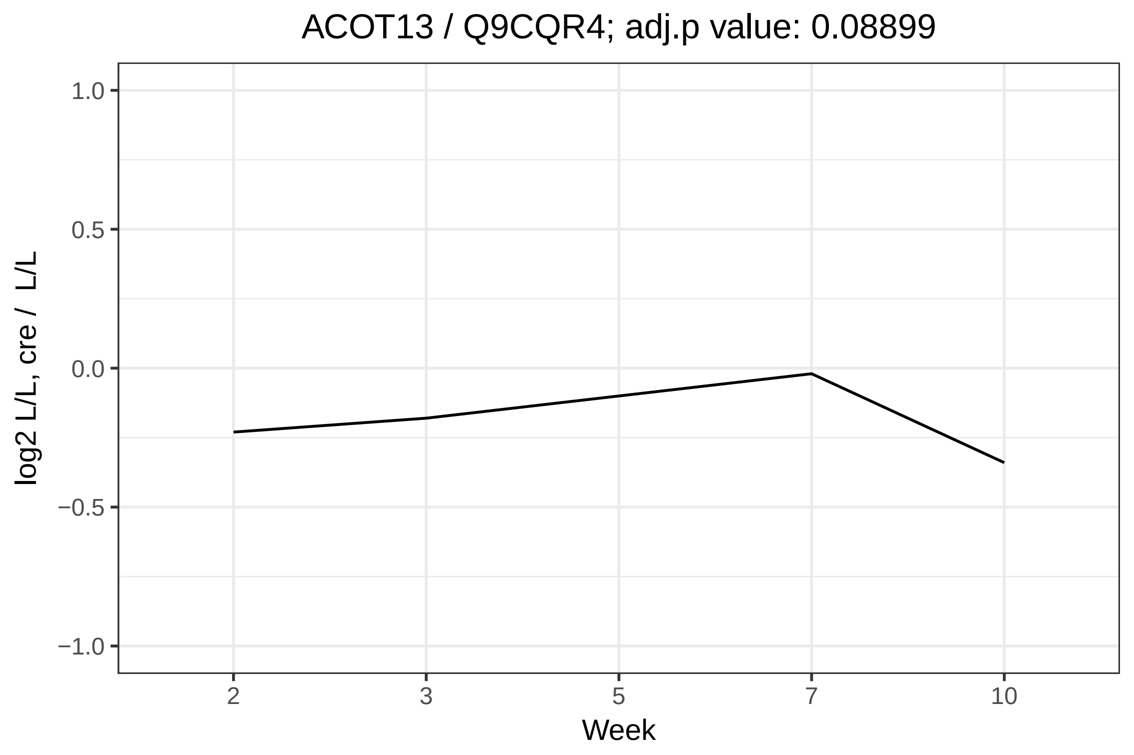
ACOT13 / Q9CQR4; adj.p value: 0.08899
log2 L/L, cre / L/L
1.0
0.5
0.0
−0.5
−1.0
2
3
5
7
10
Week

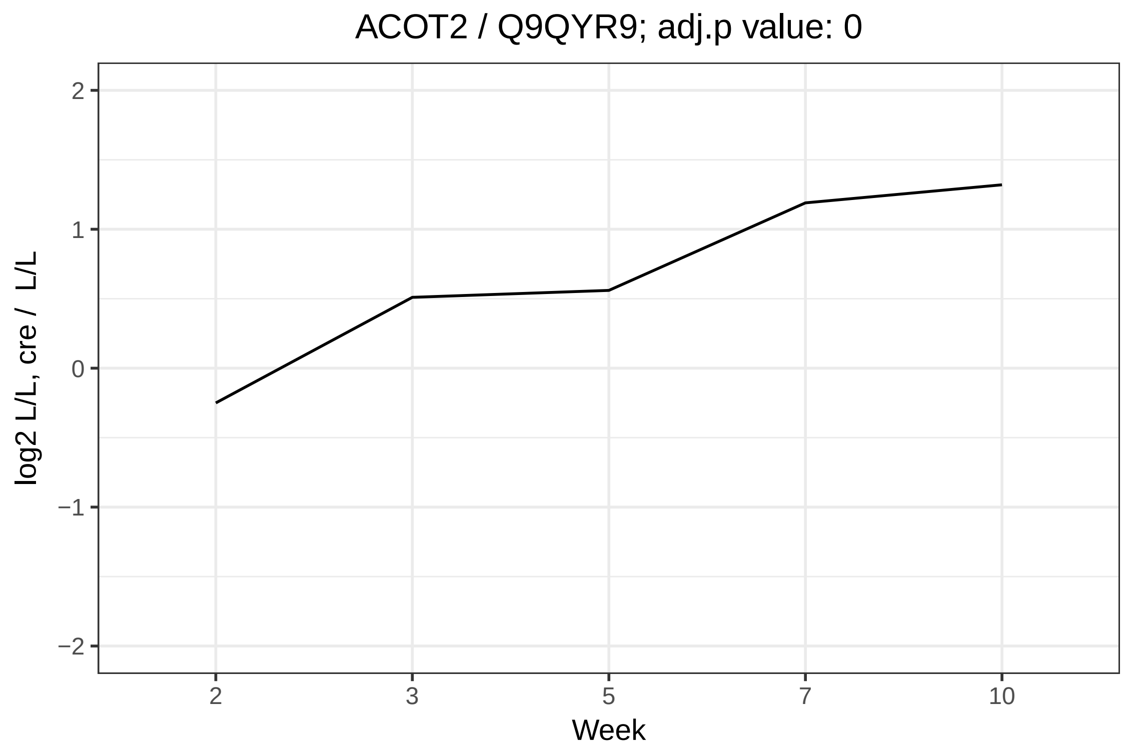
ACOT2 / Q9QYR9; adj.p value: 0
log2 L/L, cre / L/L
2
1
0
−1
−2
2
3
5
7
10
Week

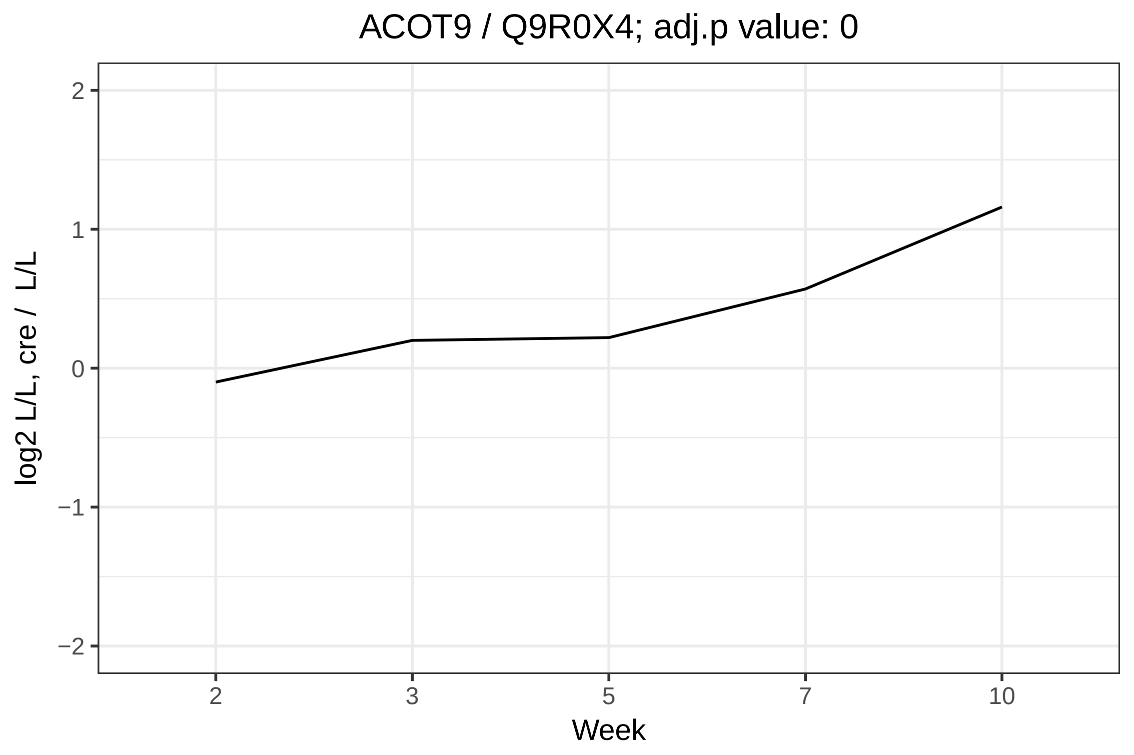
ACOT9 / Q9R0X4; adj.p value: 0
log2 L/L, cre / L/L
2
1
0
−1
−2
2
3
5
7
10
Week

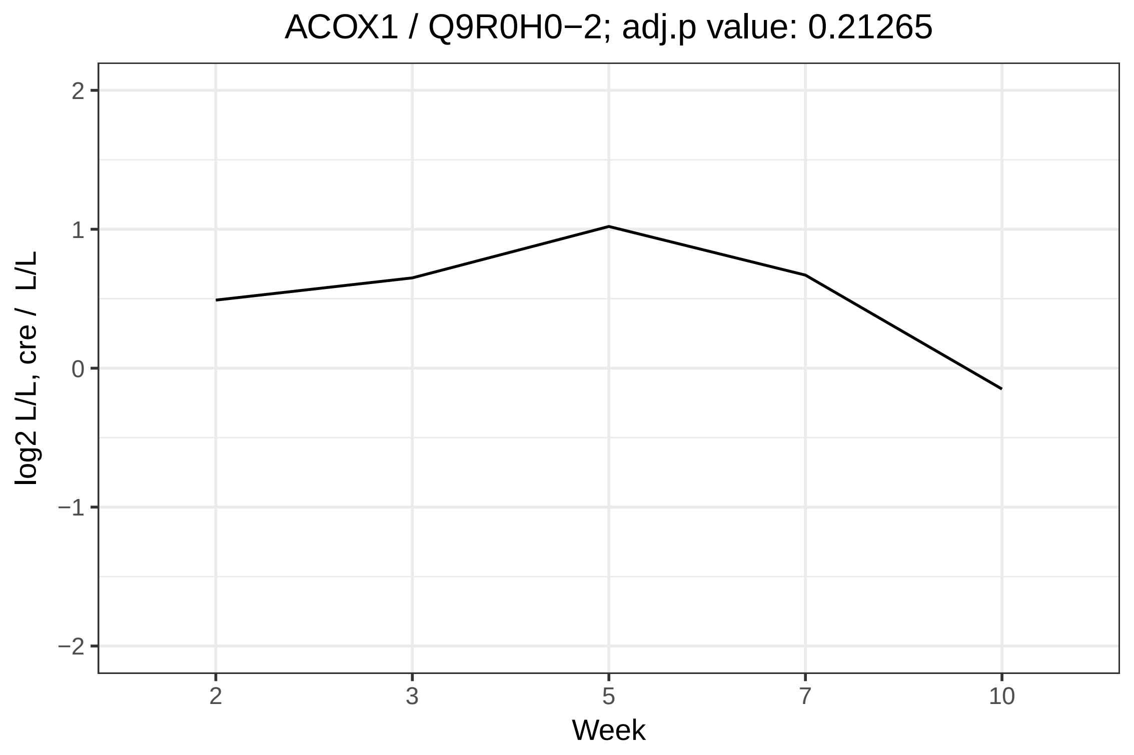
ACOX1 / Q9R0H0−2; adj.p value: 0.21265
log2 L/L, cre / L/L
2
1
0
−1
−2
2
3
5
7
10
Week

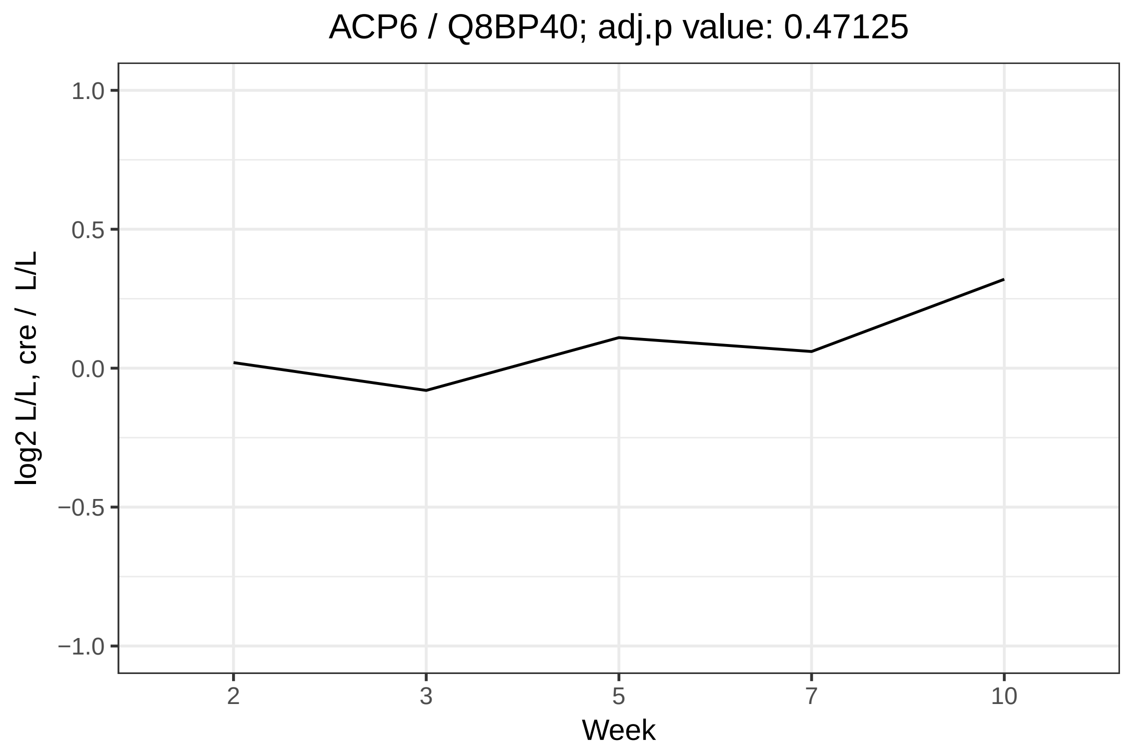

ACP6 / Q8BP40; adj.p value: 0.47125
log2 L/L, cre / L/L
1.0
0.5
0.0
−0.5
−1.0
2
3
5
7
10
Week

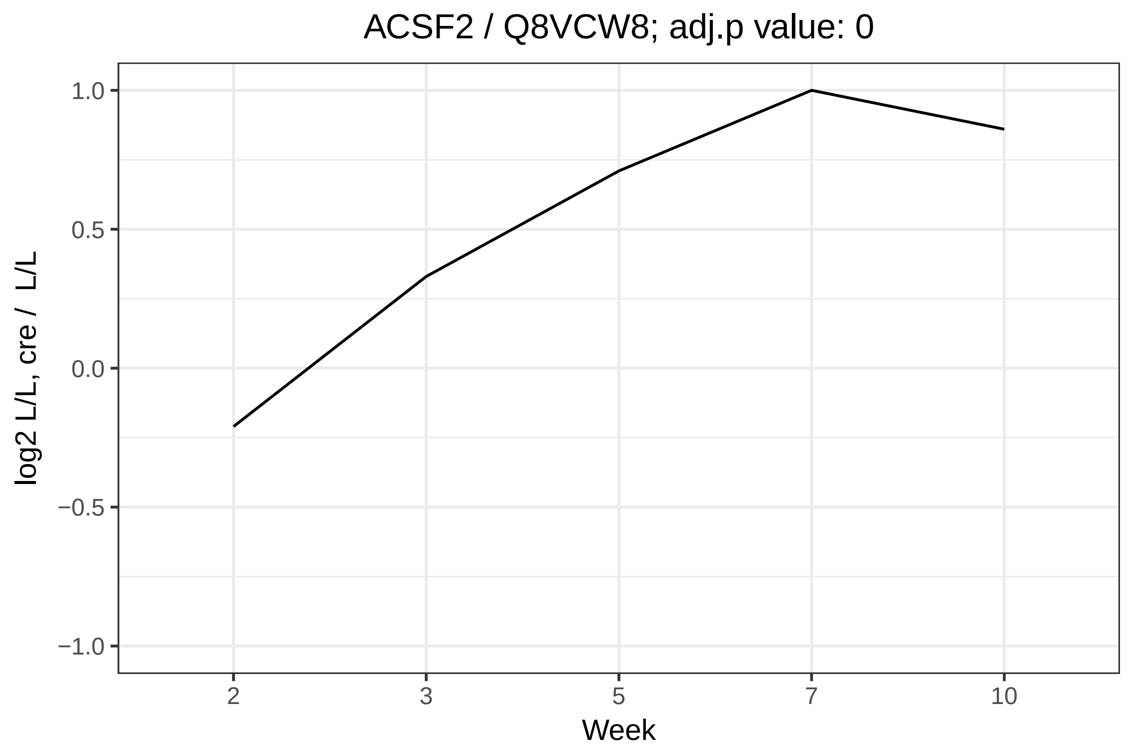

ACSF2 / Q8VCW8; adj.p value: 0
log2 L/L, cre / L/L
1.0
0.5
0.0
−0.5
−1.0
2
3
5
7
10
Week

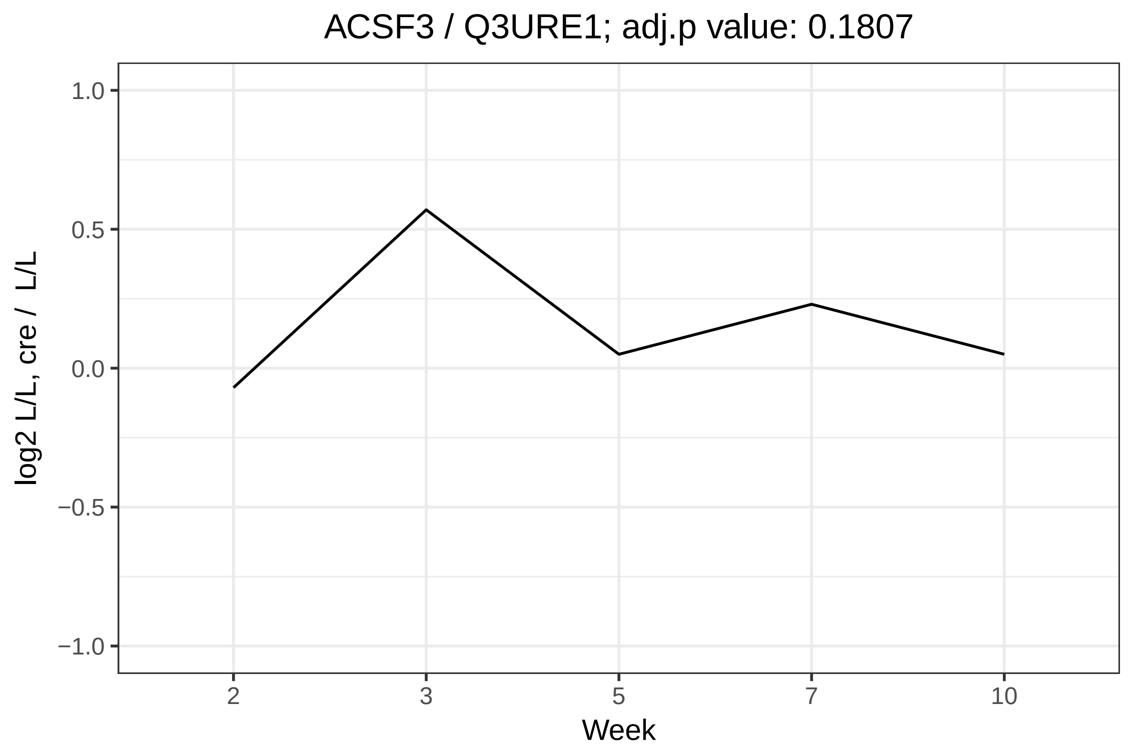
ACSF3 / Q3URE1; adj.p value: 0.1807
1.0
0.5
0.0
−0.5
−1.0
log2 L/L, cre / L/L
2
3
5
7
10
Week

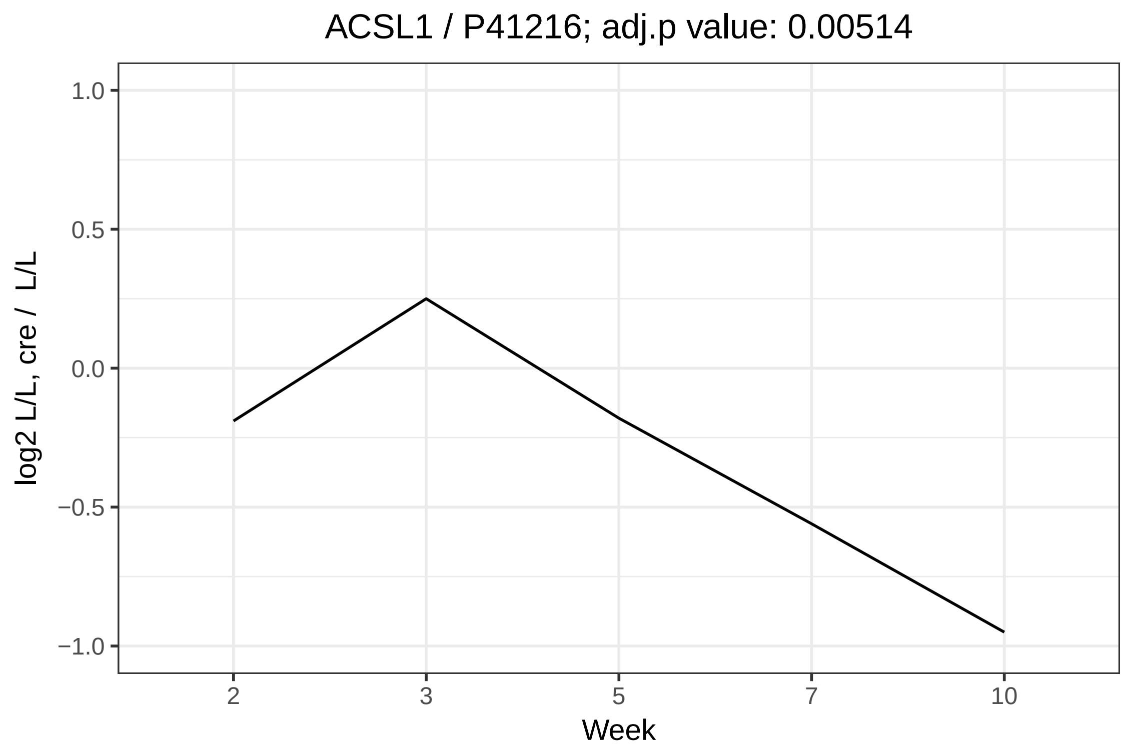
ACSL1 / P41216; adj.p value: 0.00514
1.0
0.5
0.0
−0.5
−1.0
log2 L/L, cre / L/L
2
3
5
7
10
Week

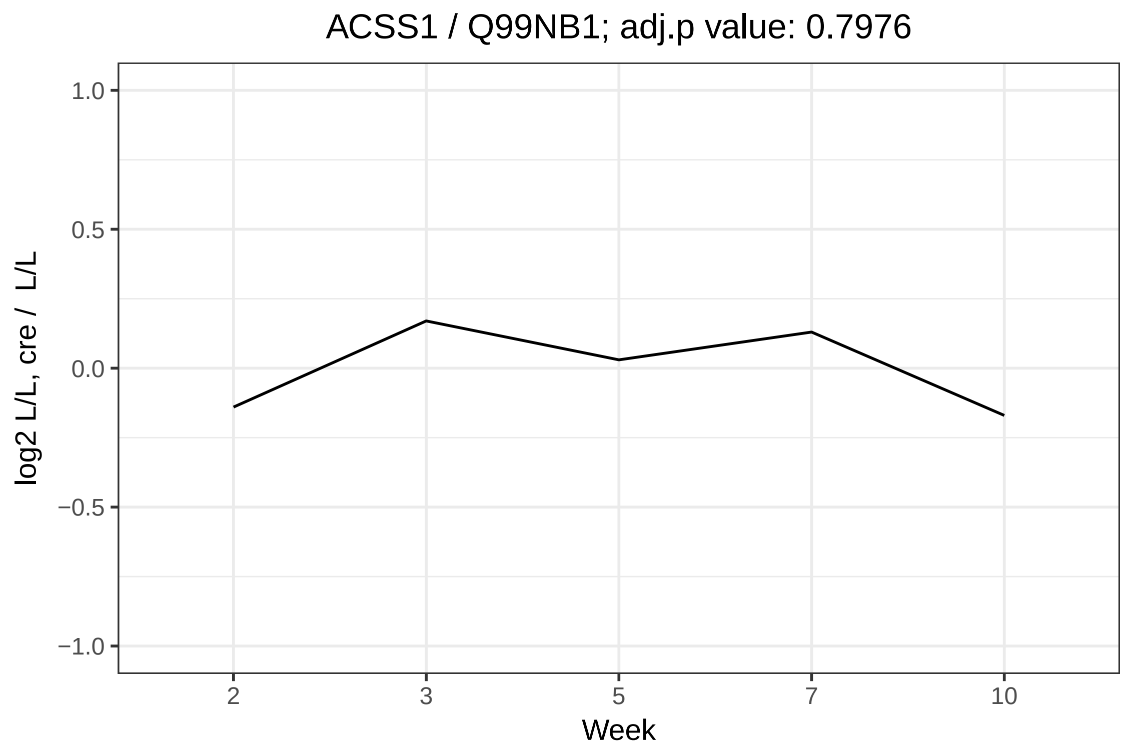
ACSS1 / Q99NB1; adj.p value: 0.7976
1.0
0.5
0.0
−0.5
−1.0
log2 L/L, cre / L/L
2
3
5
7
10
Week

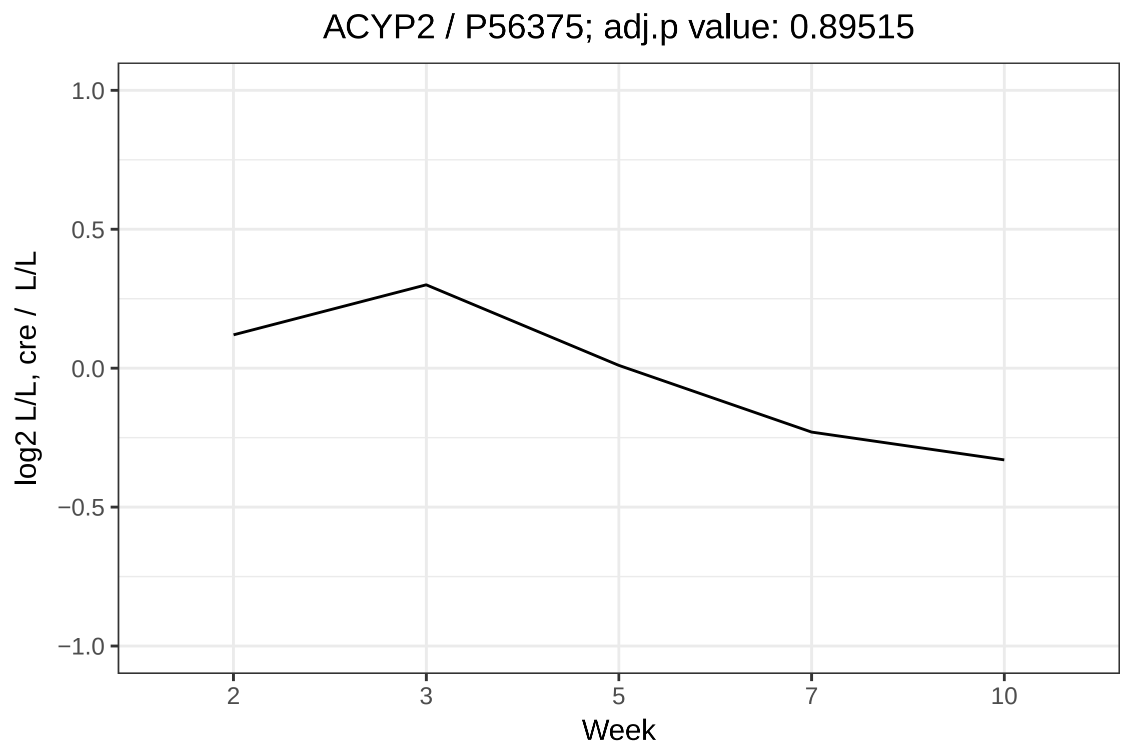
ACYP2 / P56375; adj.p value: 0.89515
1.0
0.5
0.0
−0.5
−1.0
log2 L/L, cre / L/L
2
3
5
7
10
Week

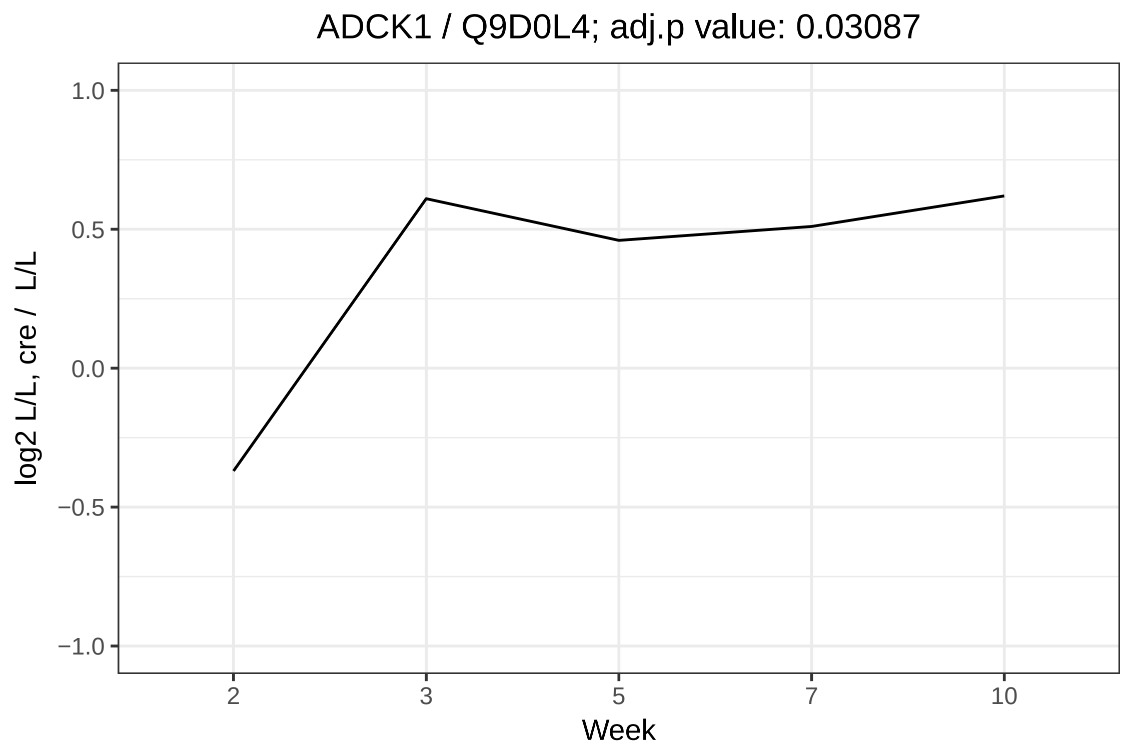
ADCK1 / Q9D0L4; adj.p value: 0.03087
1.0
0.5
0.0
−0.5
−1.0
log2 L/L, cre / L/L
2
3
5
7
10
Week

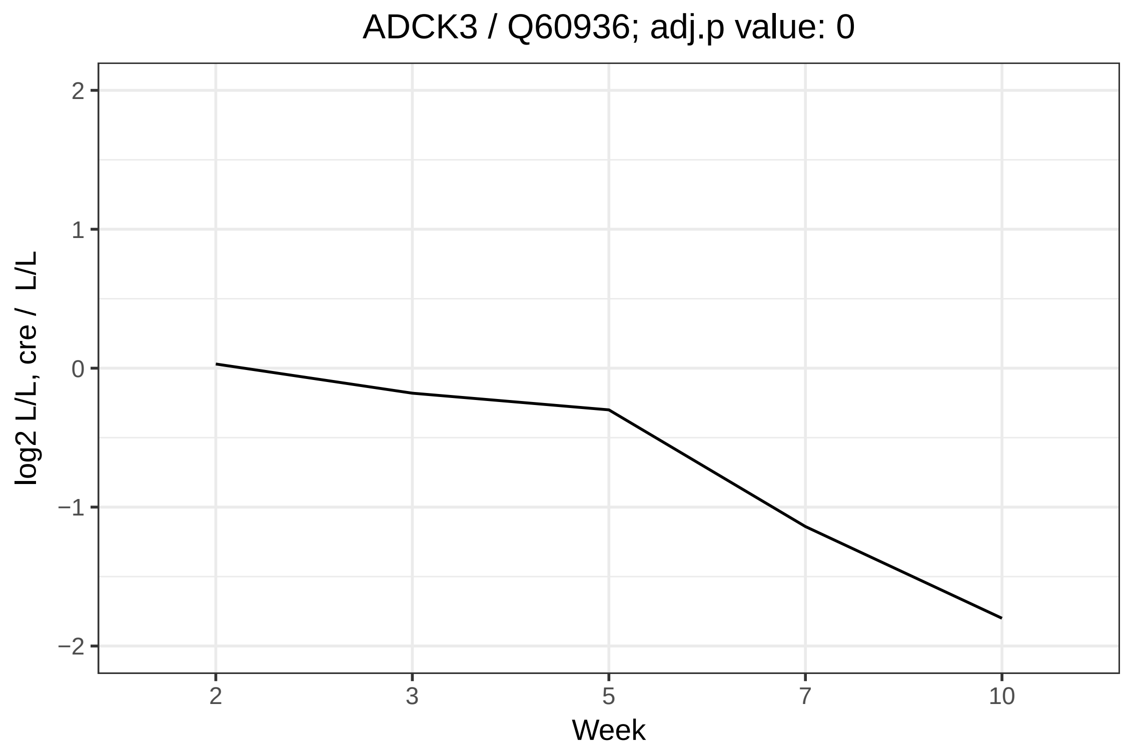
ADCK3 / Q60936; adj.p value: 0
log2 L/L, cre / L/L
2
1
0
−1
−2
2
3
5
7
10
Week

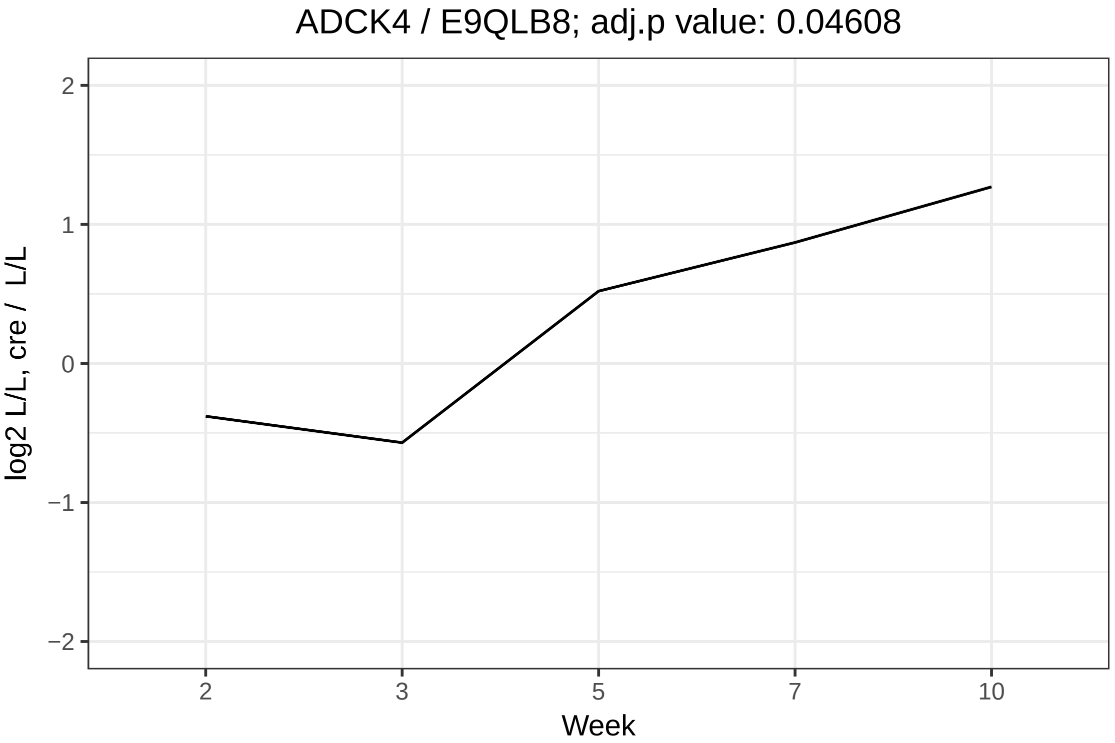

ADCK4 / E9QLB8; adj.p value: 0.04608
log2 L/L, cre / L/L
2
1
0
−1
−2
2
3
5
7
10
Week

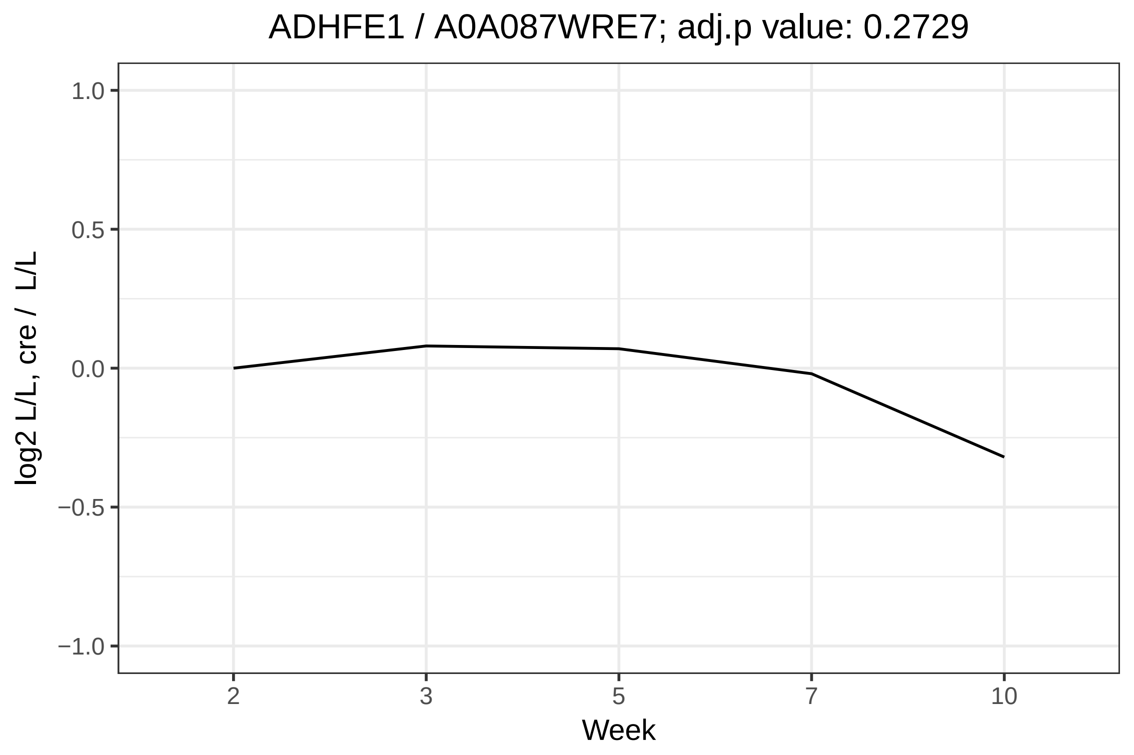
ADHFE1 / A0A087WRE7; adj.p value: 0.2729
1.0
0.5
0.0
−0.5
−1.0
log2 L/L, cre / L/L
2
3
5
7
10
Week

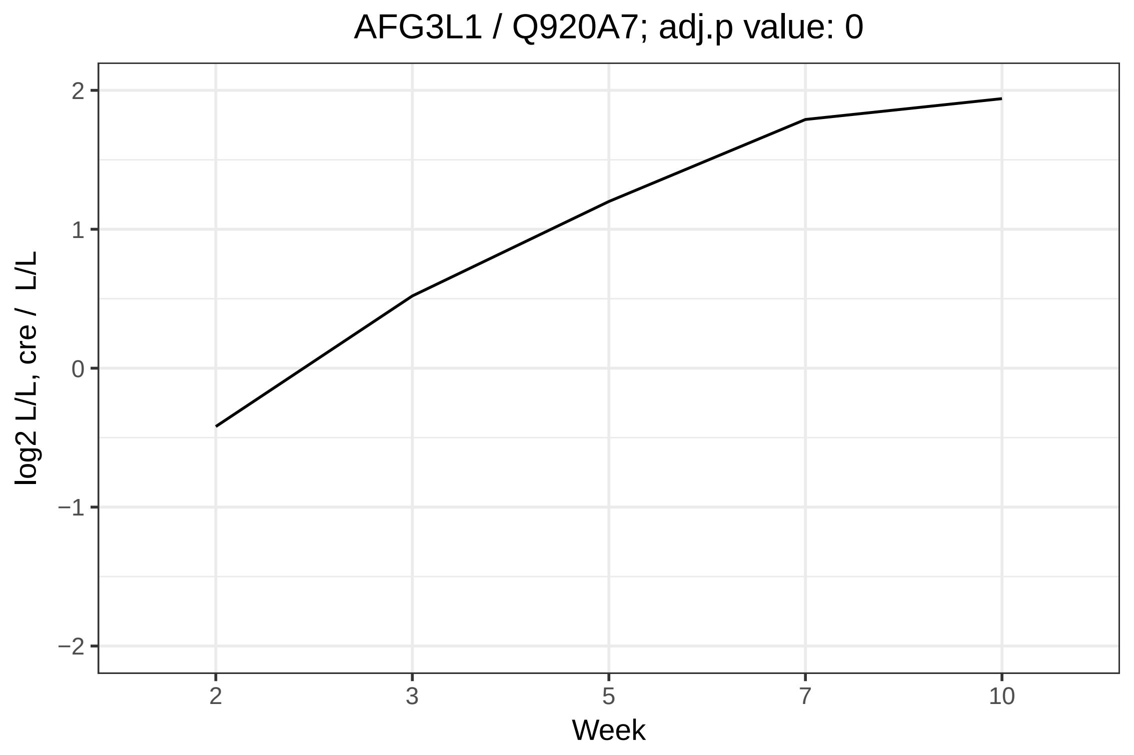
AFG3L1 / Q920A7; adj.p value: 0
log2 L/L, cre / L/L
2
1
0
−1
−2
2
3
5
7
10
Week

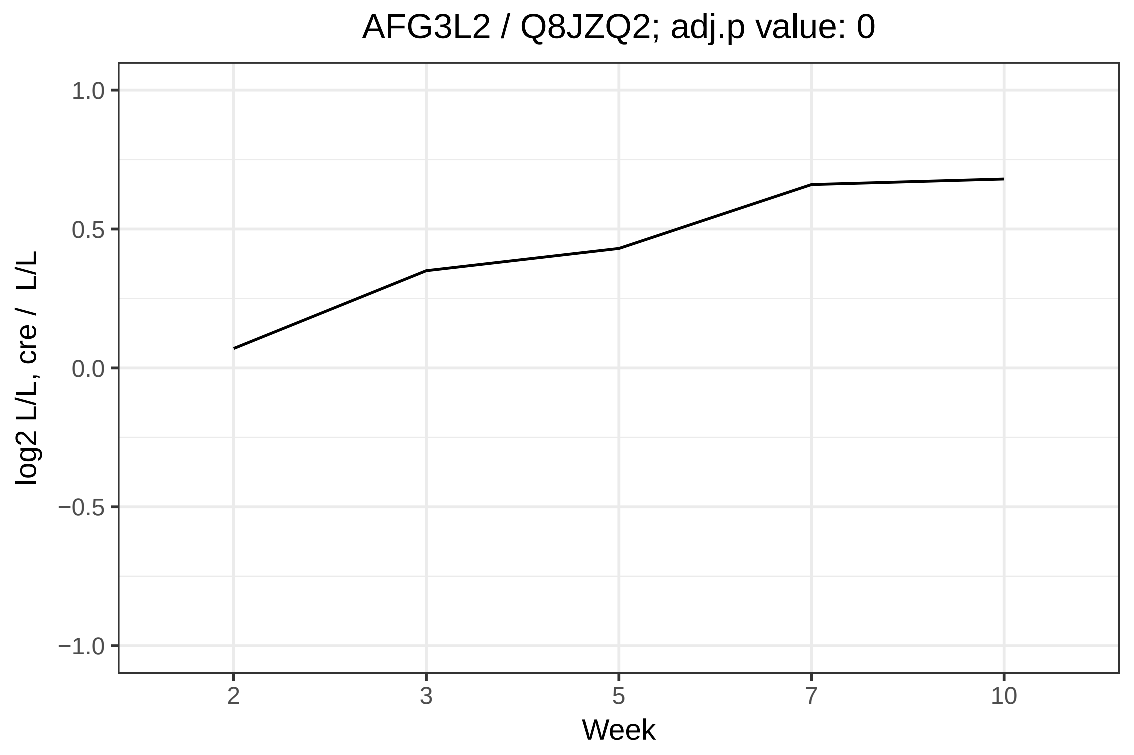
AFG3L2 / Q8JZQ2; adj.p value: 0
log2 L/L, cre / L/L
1.0
0.5
0.0
−0.5
−1.0
2
3
5
7
10
Week

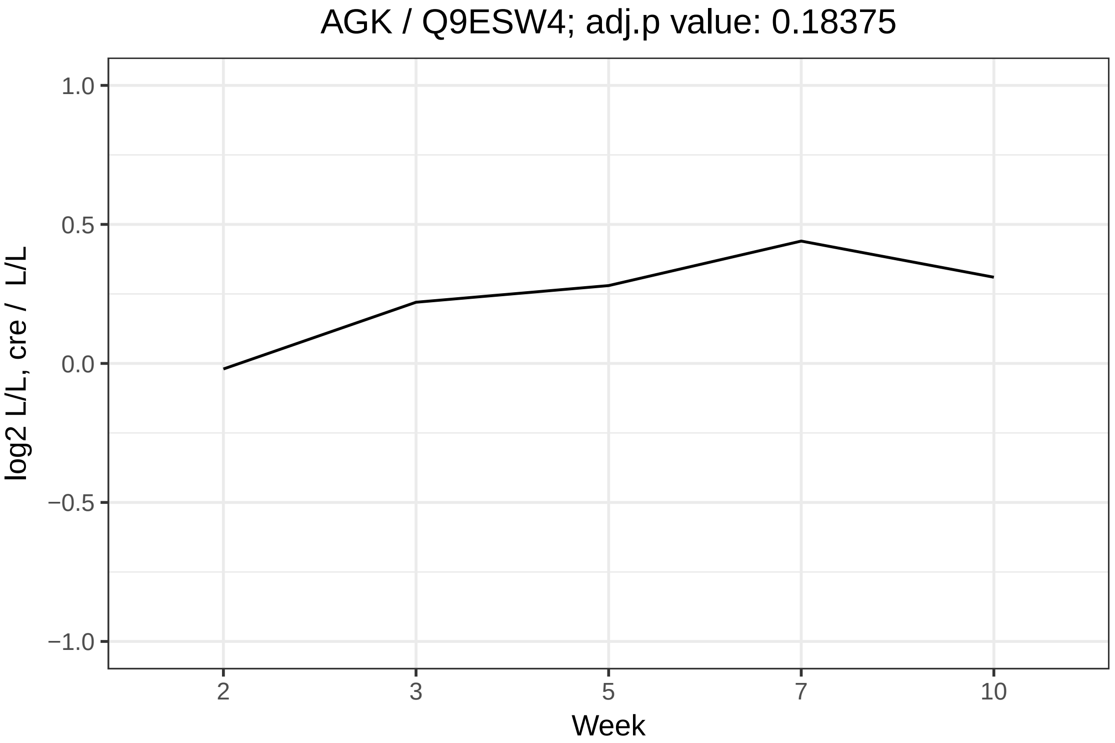
AGK / Q9ESW4; adj.p value: 0.18375
log2 L/L, cre / L/L
1.0
0.5
0.0
−0.5
−1.0
2
3
5
7
10
Week

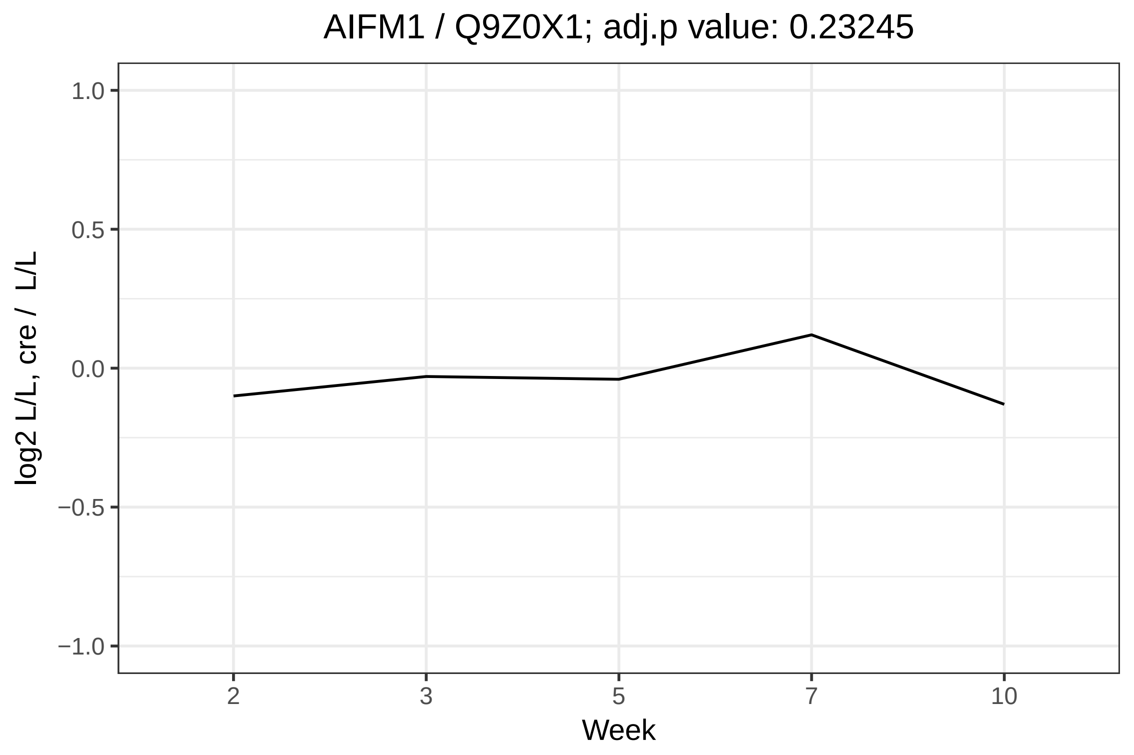
AIFM1 / Q9Z0X1; adj.p value: 0.23245
log2 L/L, cre / L/L
1.0
0.5
0.0
−0.5
−1.0
2
3
5
7
10
Week

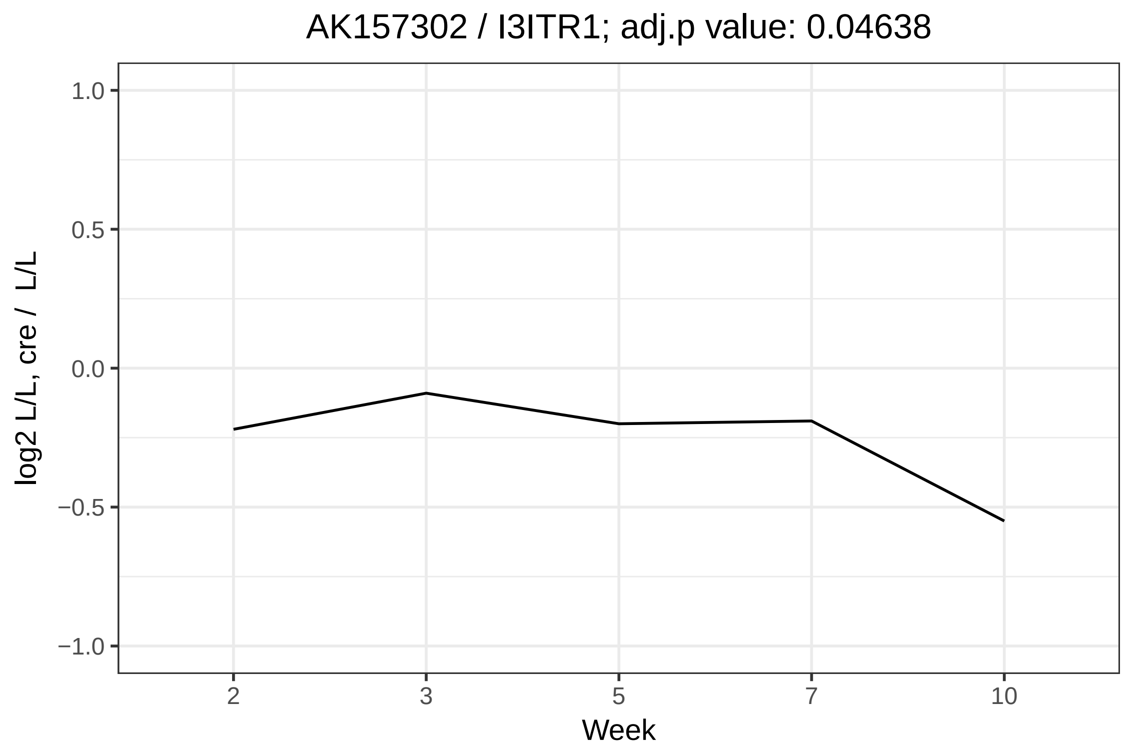
AK157302 / I3ITR1; adj.p value: 0.04638
1.0
0.5
0.0
−0.5
−1.0
log2 L/L, cre / L/L
2
3
5
7
10
Week

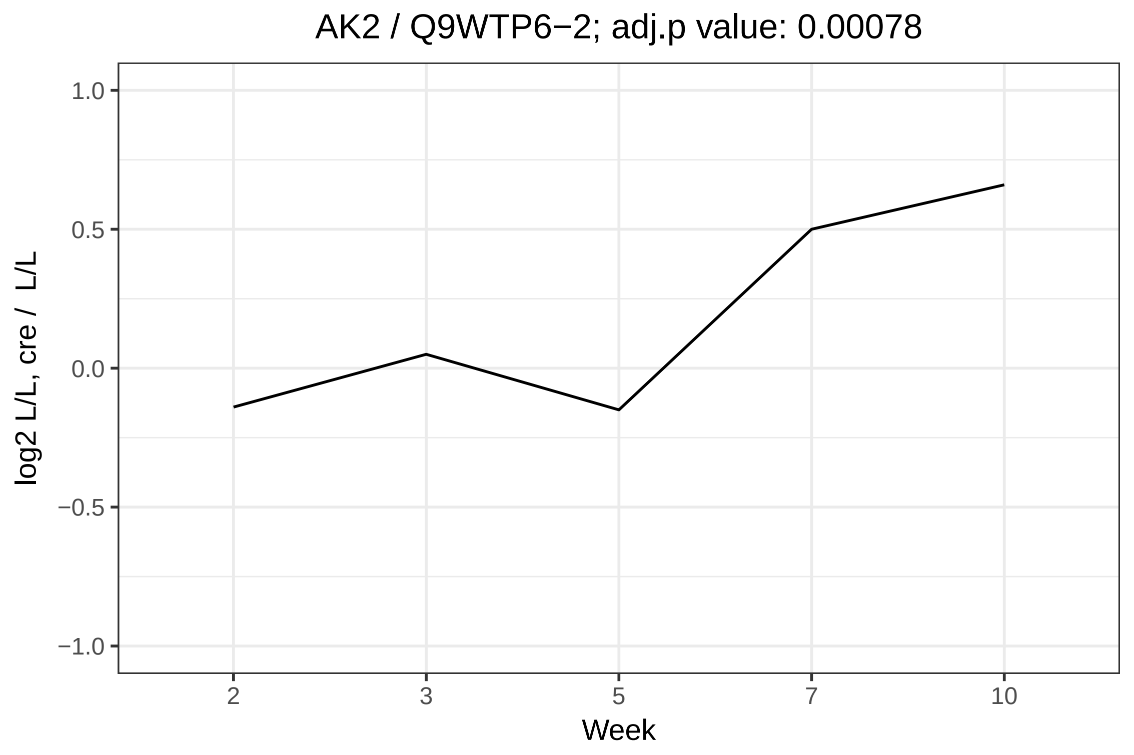

AK2 / Q9WTP6−2; adj.p value: 0.00078
1.0
0.5
0.0
−0.5
−1.0
log2 L/L, cre / L/L
2
3
5
7
10
Week

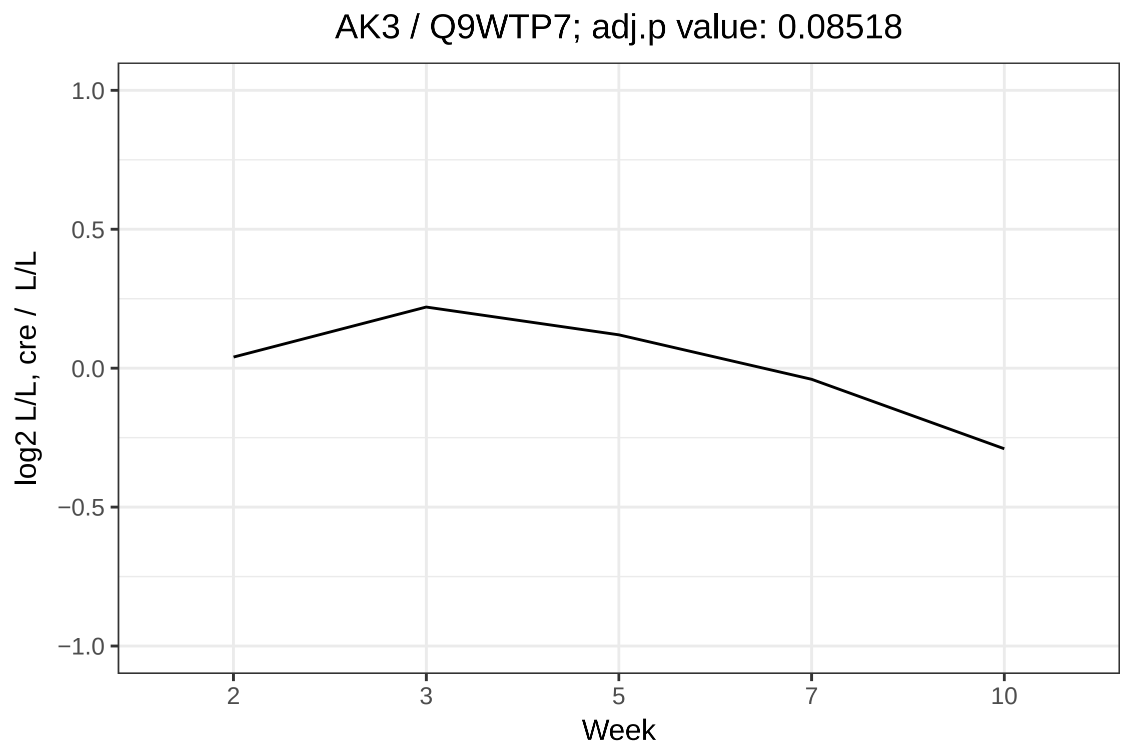

AK3 / Q9WTP7; adj.p value: 0.08518
log2 L/L, cre / L/L
1.0
0.5
0.0
−0.5
−1.0
2
3
5
7
10
Week

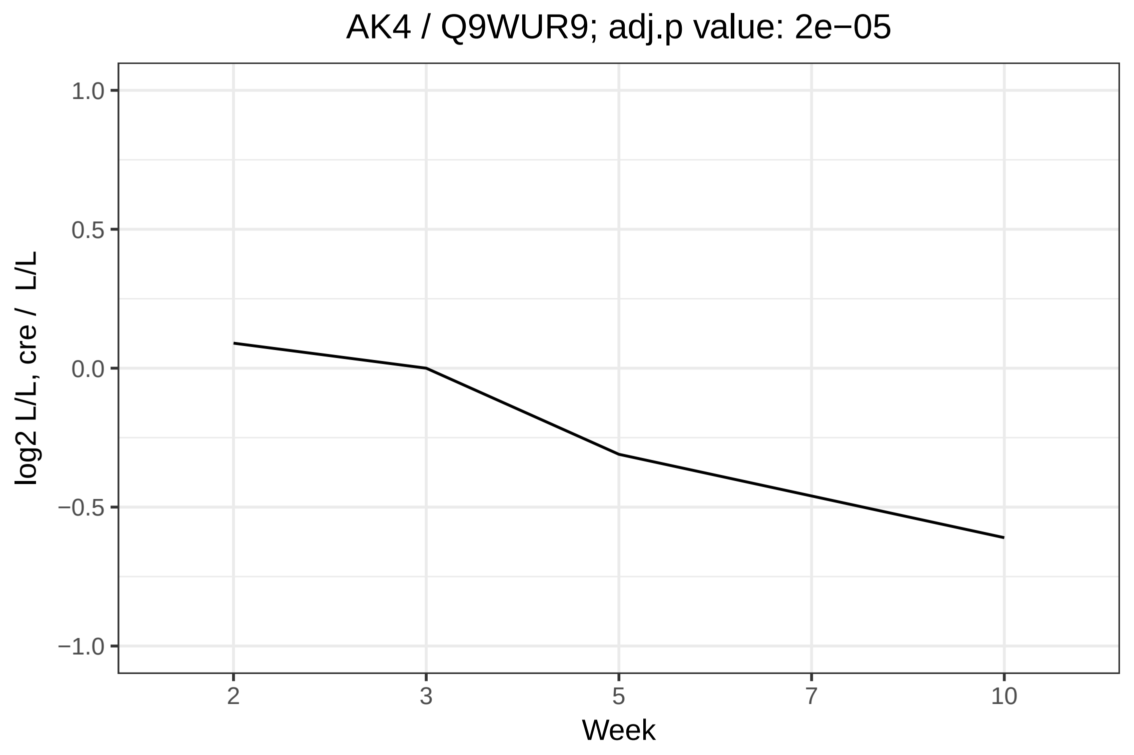

AK4 / Q9WUR9; adj.p value: 2e−05
1.0
0.5
0.0
−0.5
−1.0
log2 L/L, cre / L/L
2
3
5
7
10
Week

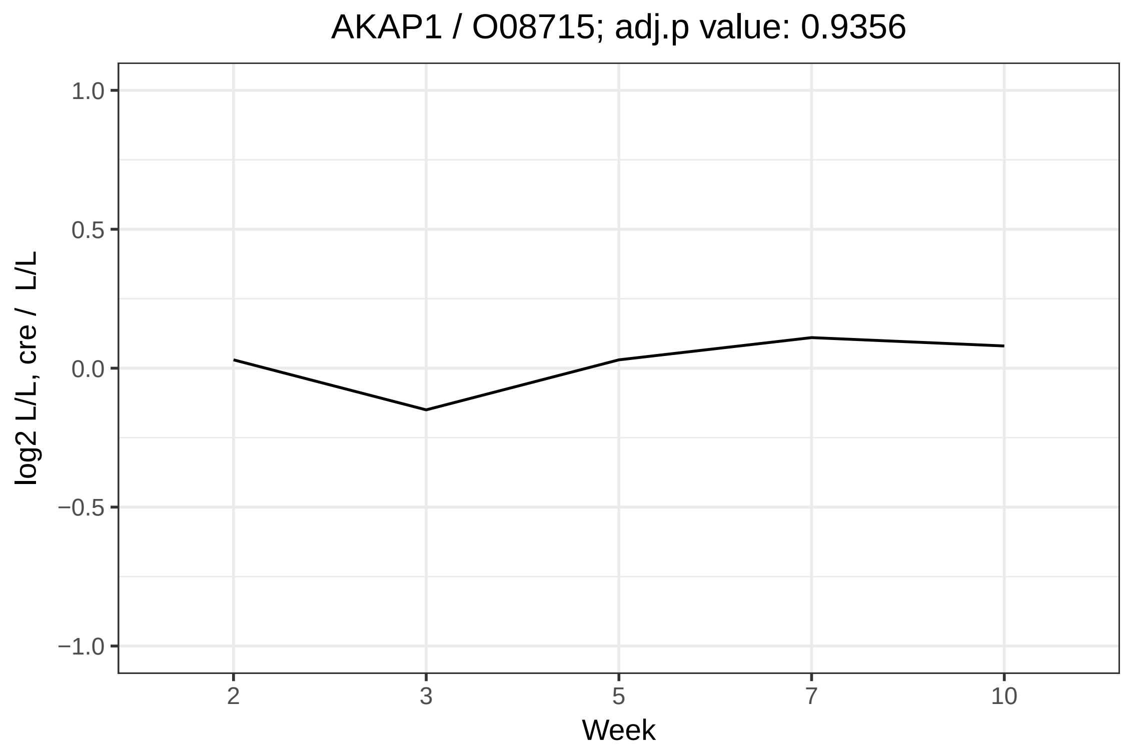
AKAP1 / O08715; adj.p value: 0.9356
log2 L/L, cre / L/L
1.0
0.5
0.0
−0.5
−1.0
2
3
5
7
10
Week

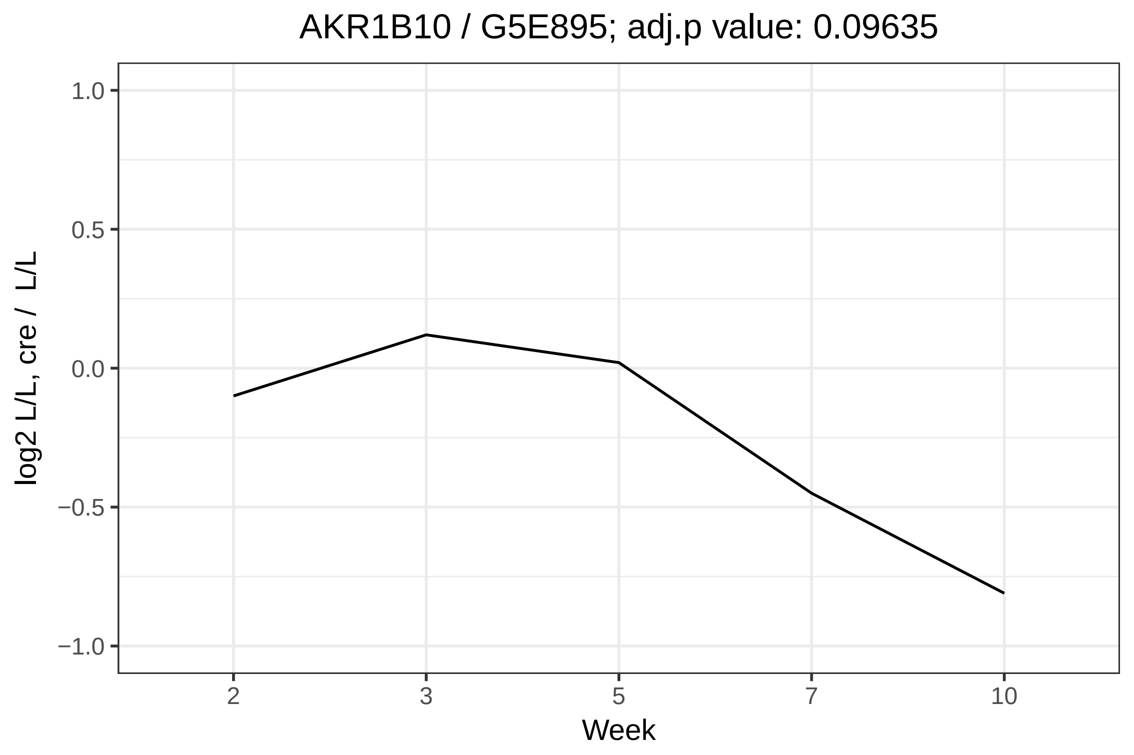
AKR1B10 / G5E895; adj.p value: 0.09635
log2 L/L, cre / L/L
1.0
0.5
0.0
−0.5
−1.0
2
3
5
7
10
Week

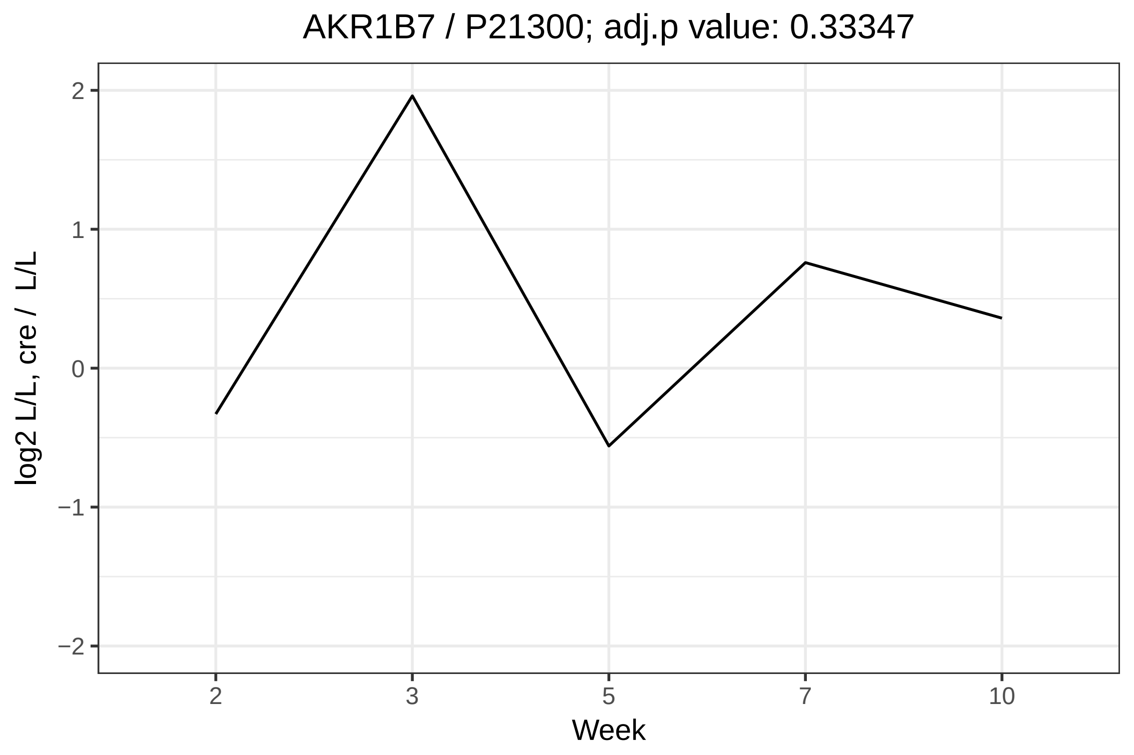
AKR1B7 / P21300; adj.p value: 0.33347
log2 L/L, cre / L/L
2
1
0
−1
−2
2
3
5
7
10
Week

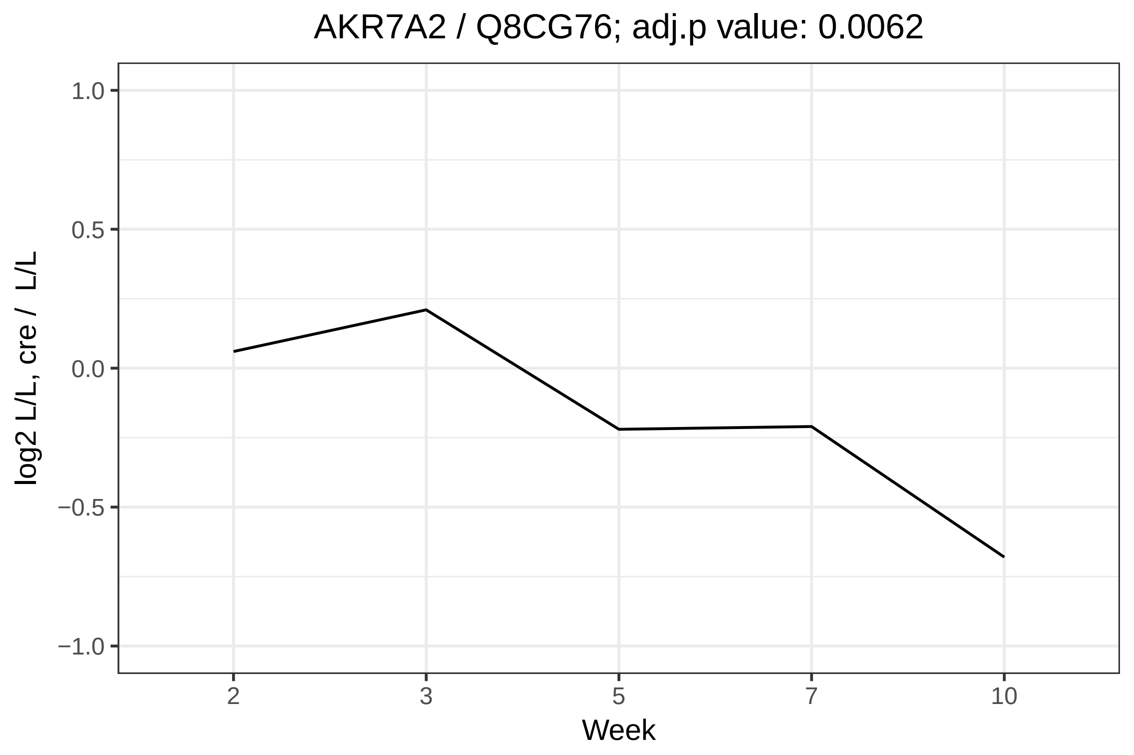
AKR7A2 / Q8CG76; adj.p value: 0.0062
log2 L/L, cre / L/L
1.0
0.5
0.0
−0.5
−1.0
2
3
5
7
10
Week

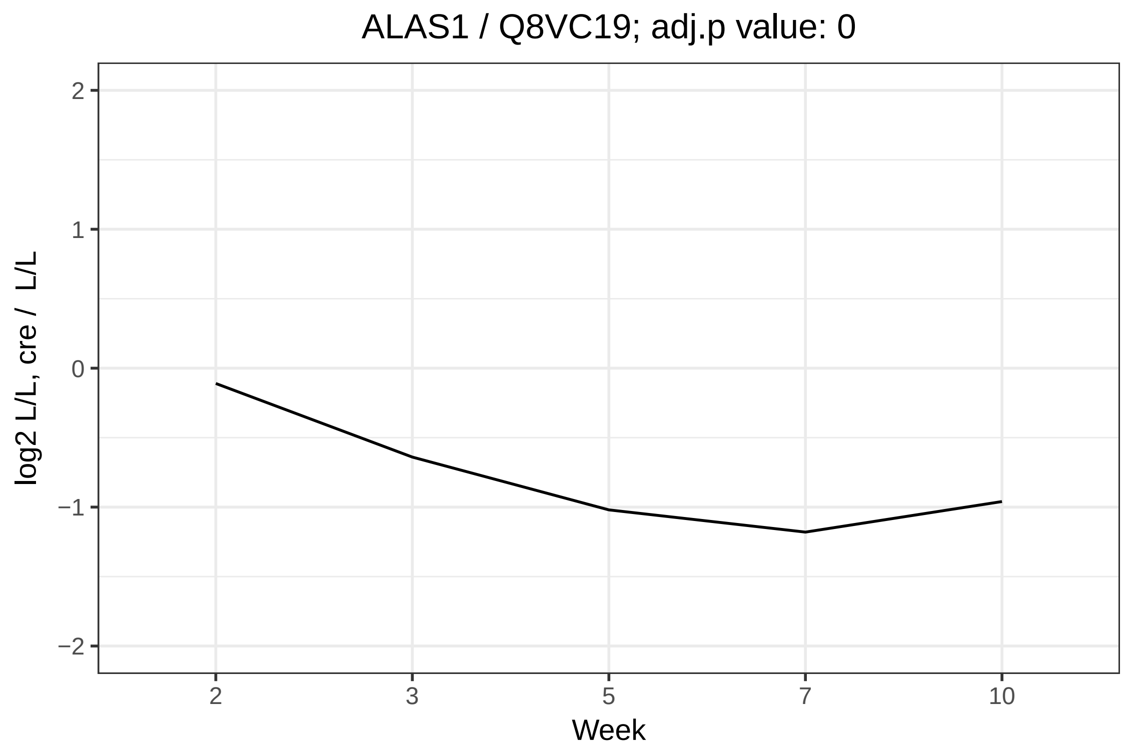

ALAS1 / Q8VC19; adj.p value: 0
log2 L/L, cre / L/L
2
1
0
−1
−2
2
3
5
7
10
Week

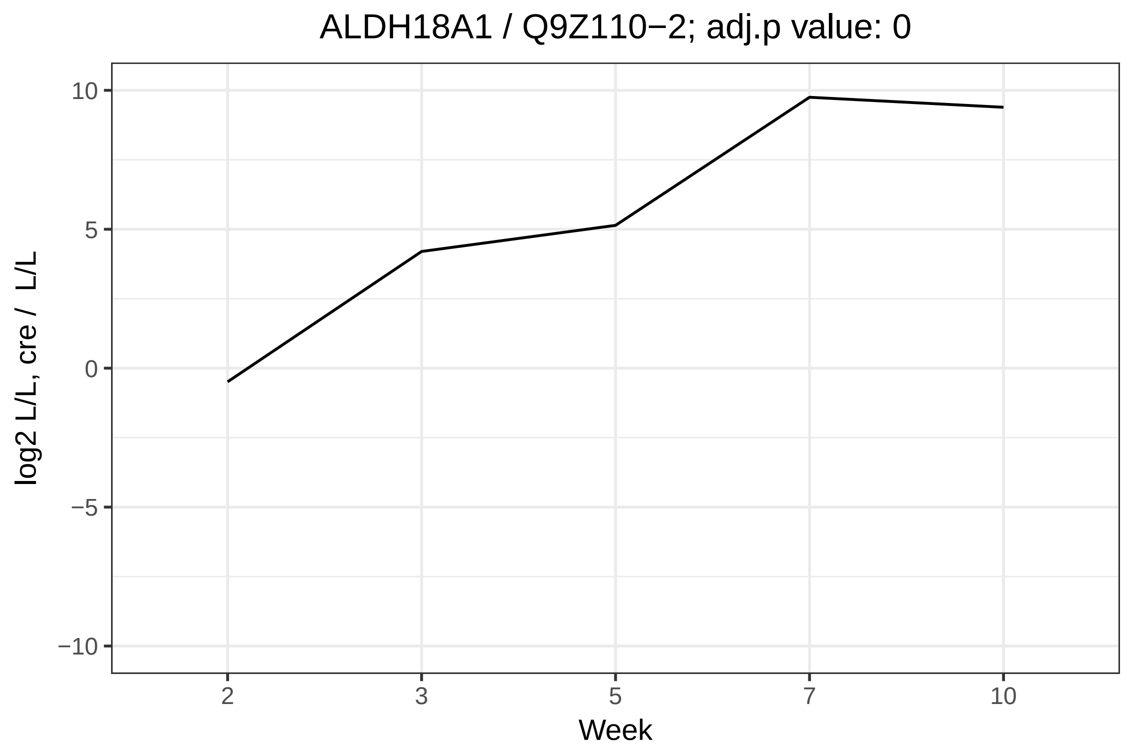
ALDH18A1 / Q9Z110−2; adj.p value: 0
log2 L/L, cre / L/L
10
5
0
−5
−10
2
3
5
7
10
Week

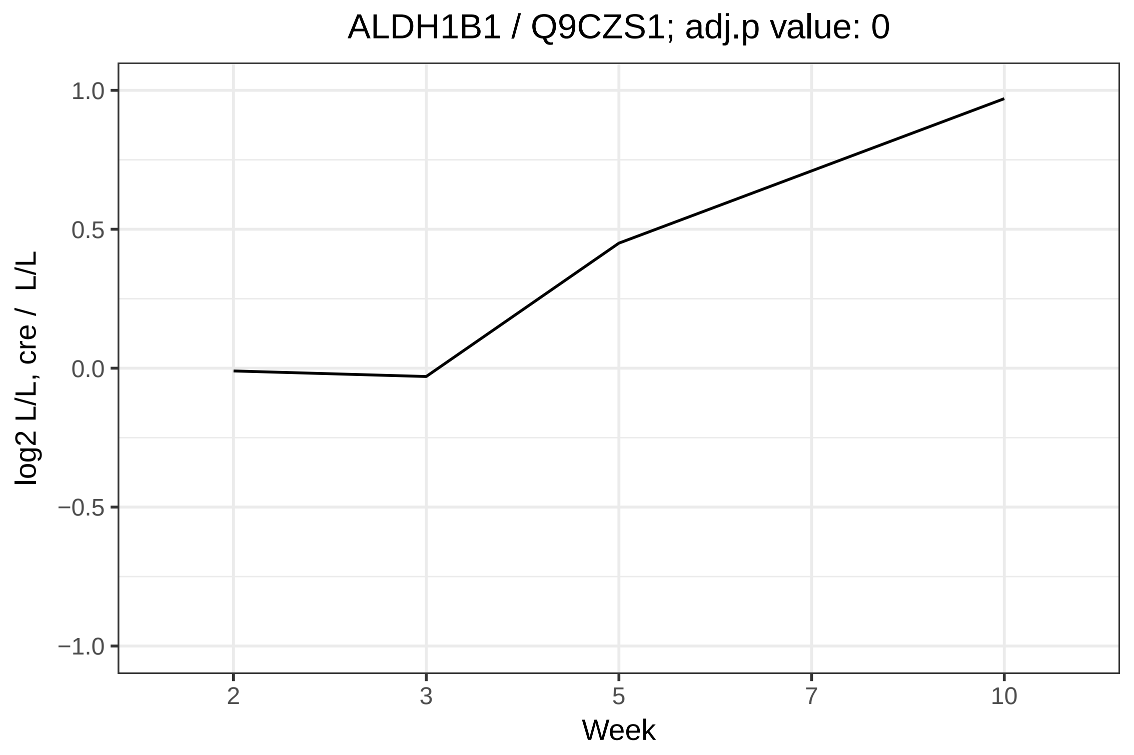
ALDH1B1 / Q9CZS1; adj.p value: 0
log2 L/L, cre / L/L
1.0
0.5
0.0
−0.5
−1.0
2
3
5
7
10
Week

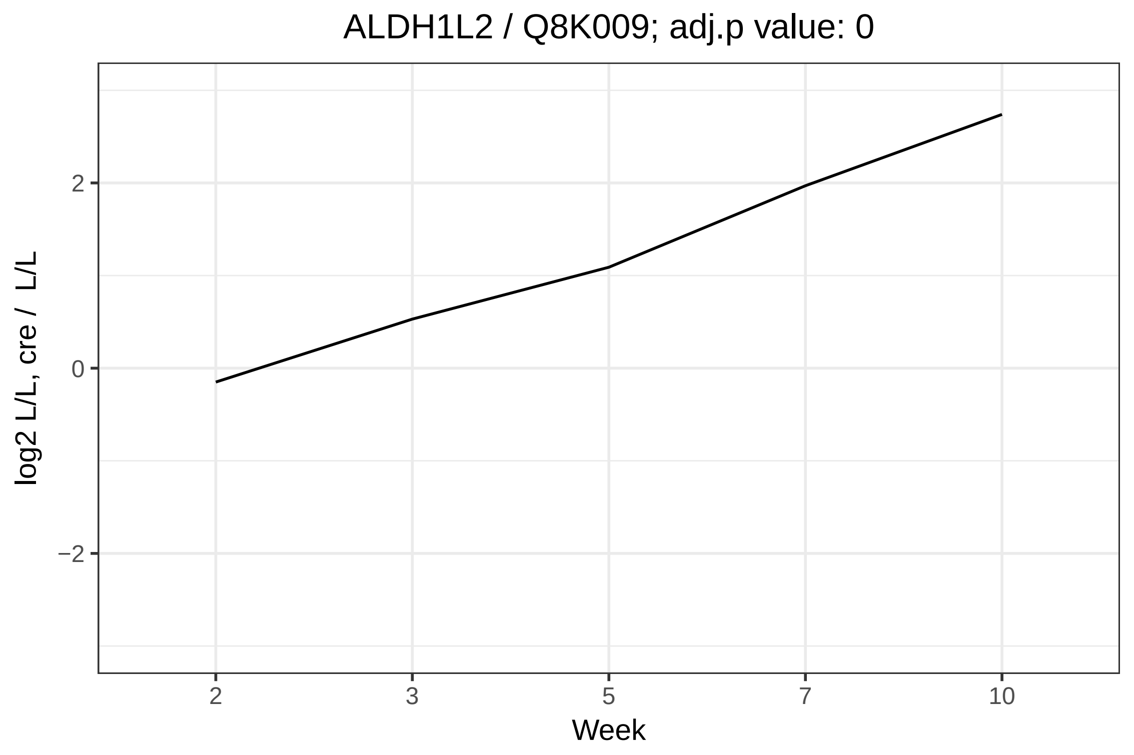

ALDH1L2 / Q8K009; adj.p value: 0
log2 L/L, cre / L/L
2
0
−2
2
3
5
7
10
Week

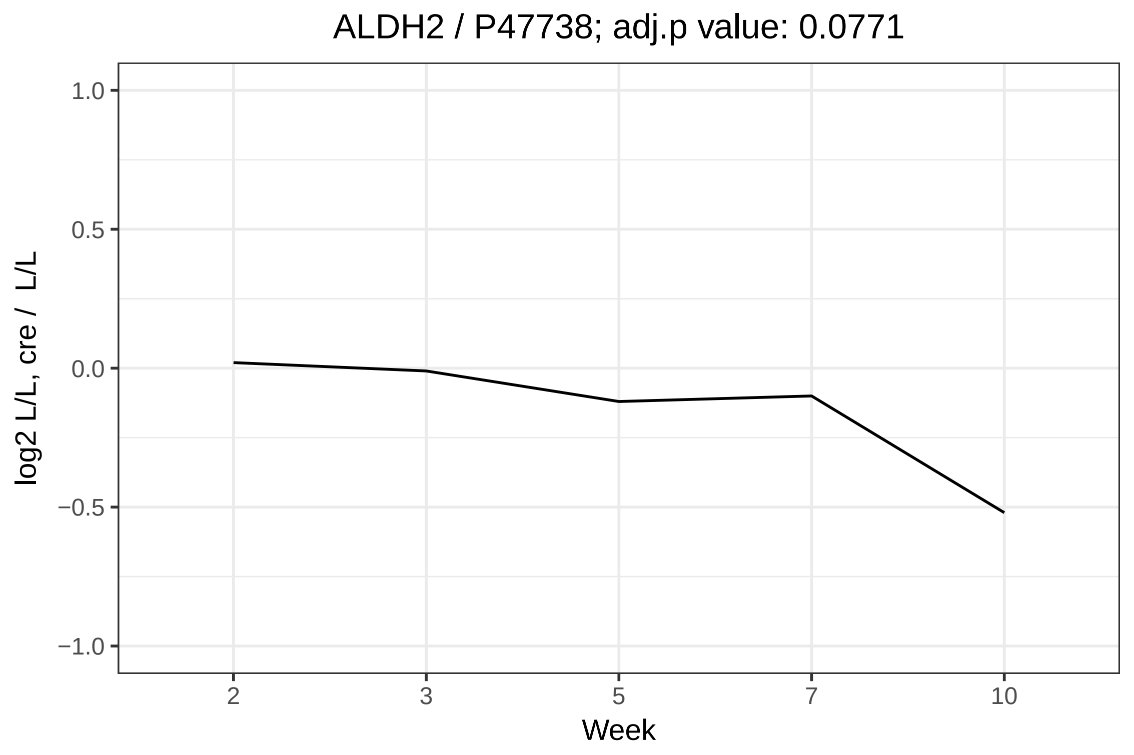
ALDH2 / P47738; adj.p value: 0.0771
log2 L/L, cre / L/L
1.0
0.5
0.0
−0.5
−1.0
2
3
5
7
10
Week

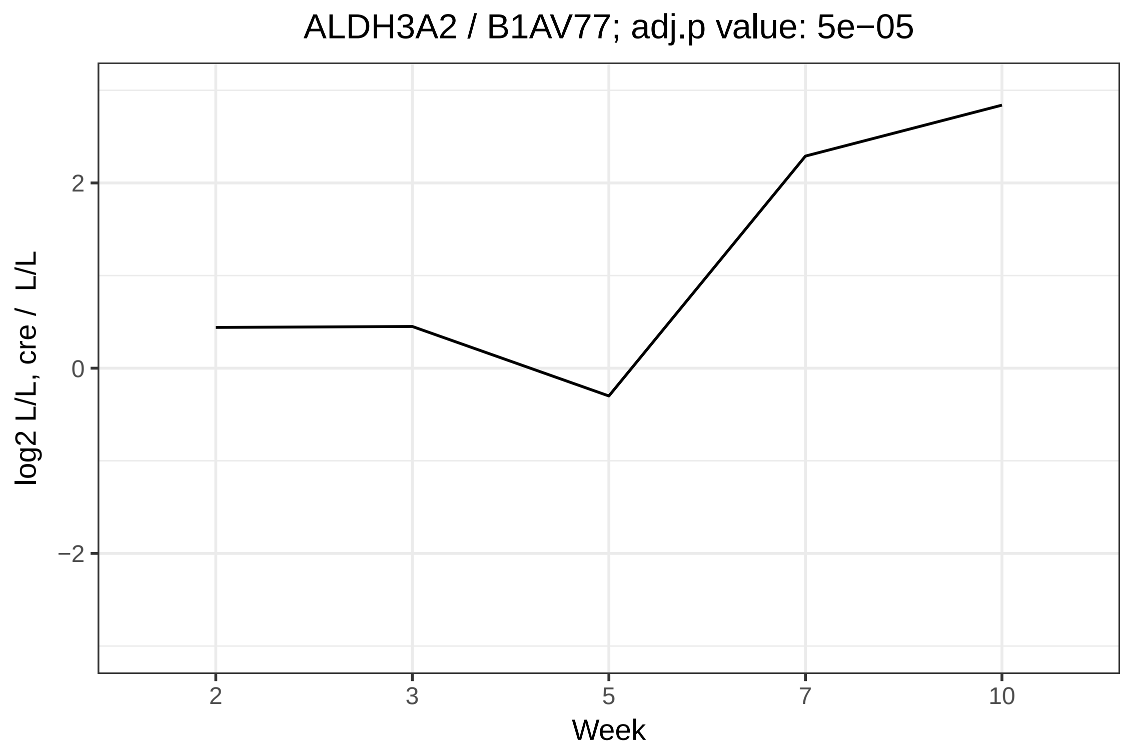
ALDH3A2 / B1AV77; adj.p value: 5e−05
log2 L/L, cre / L/L
2
0
−2
2
3
5
7
10
Week

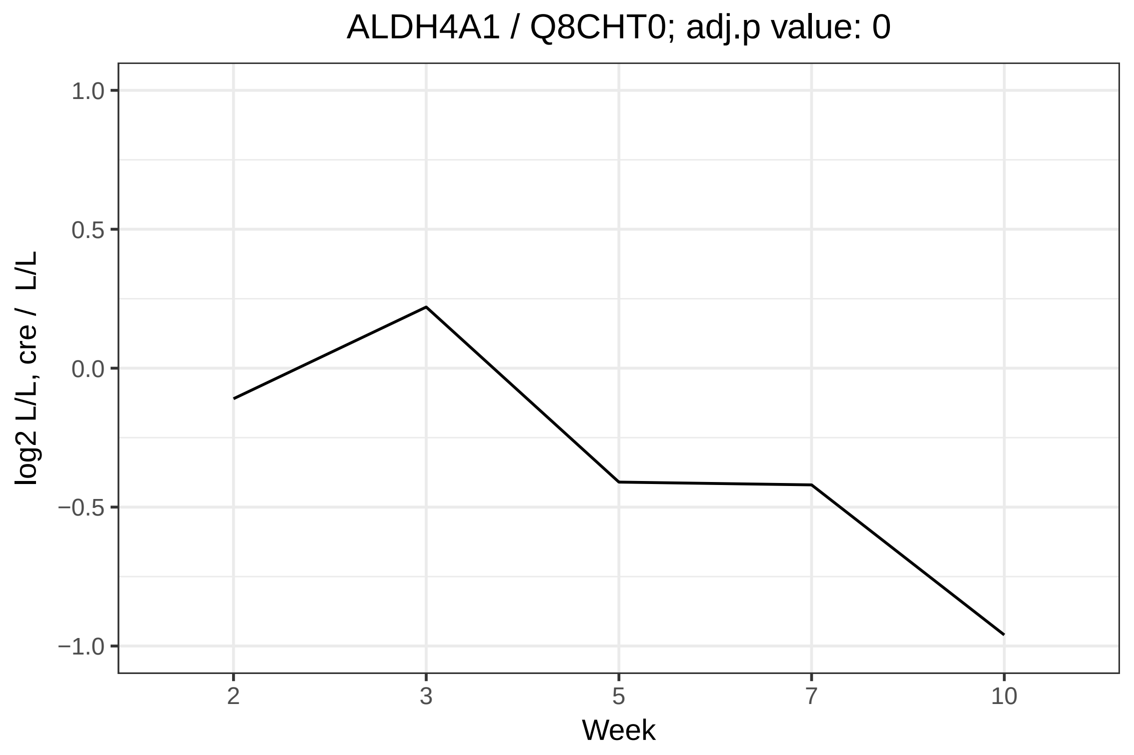

ALDH4A1 / Q8CHT0; adj.p value: 0
log2 L/L, cre / L/L
1.0
0.5
0.0
−0.5
−1.0
2
3
5
7
10
Week

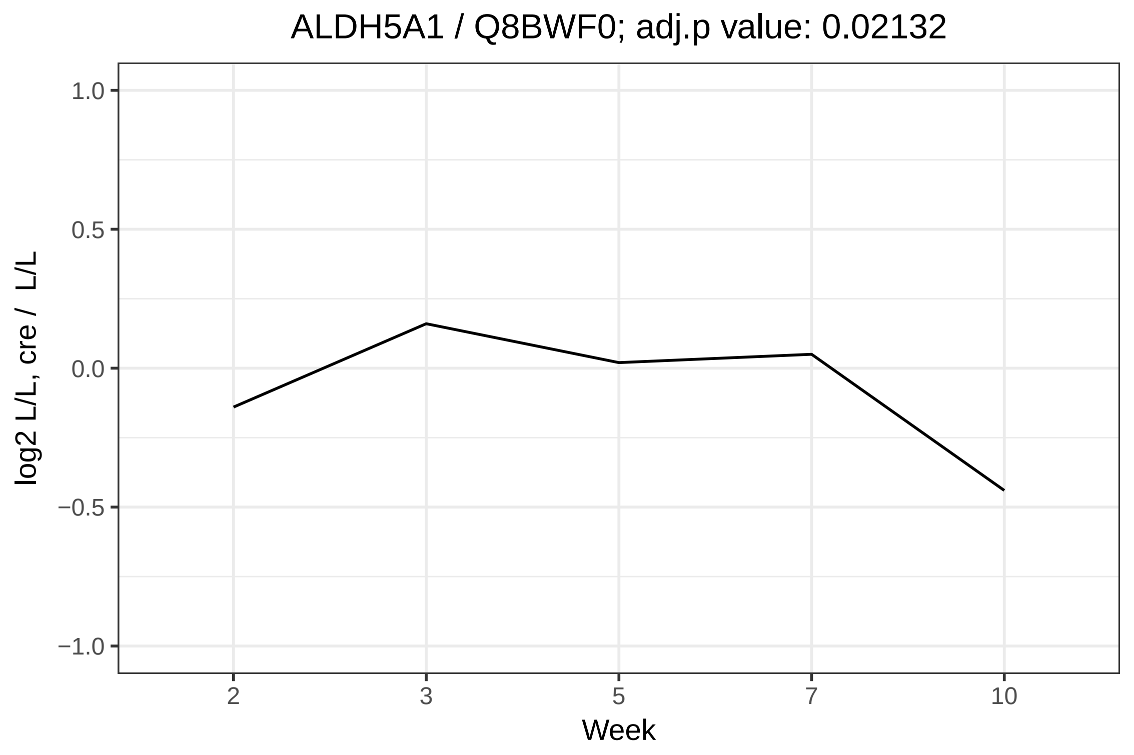

ALDH5A1 / Q8BWF0; adj.p value: 0.02132
log2 L/L, cre / L/L
1.0
0.5
0.0
−0.5
−1.0
2
3
5
7
10
Week

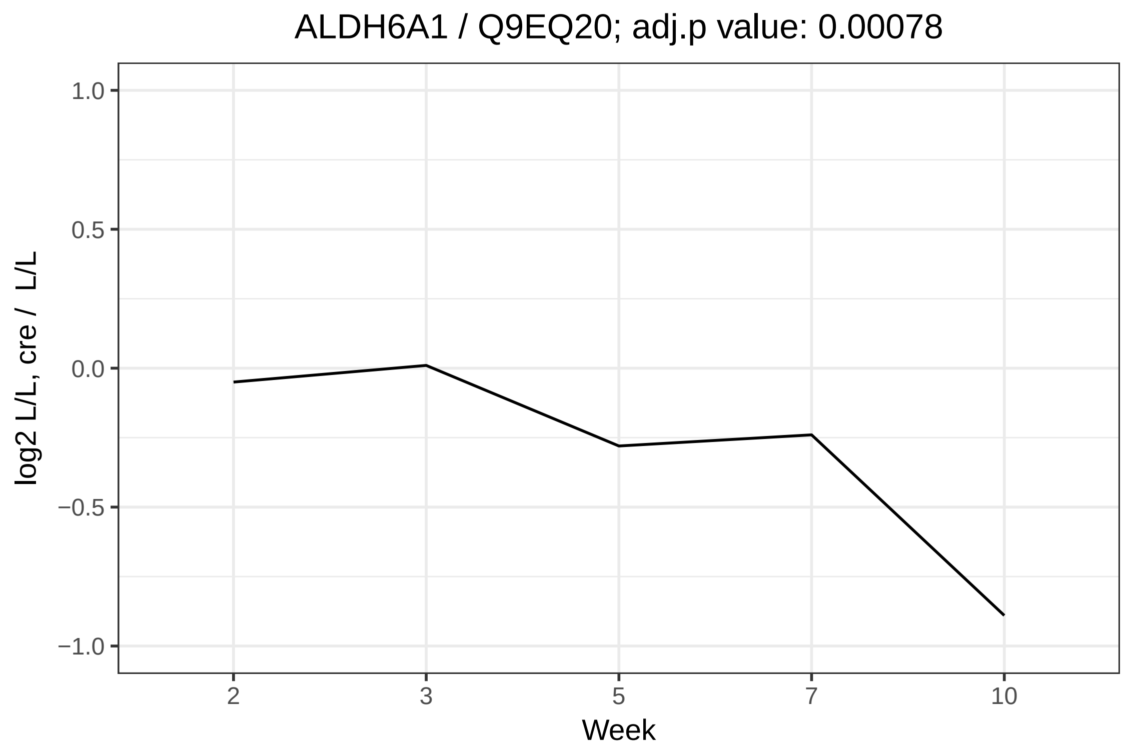

ALDH6A1 / Q9EQ20; adj.p value: 0.00078
log2 L/L, cre / L/L
1.0
0.5
0.0
−0.5
−1.0
2
3
5
7
10
Week

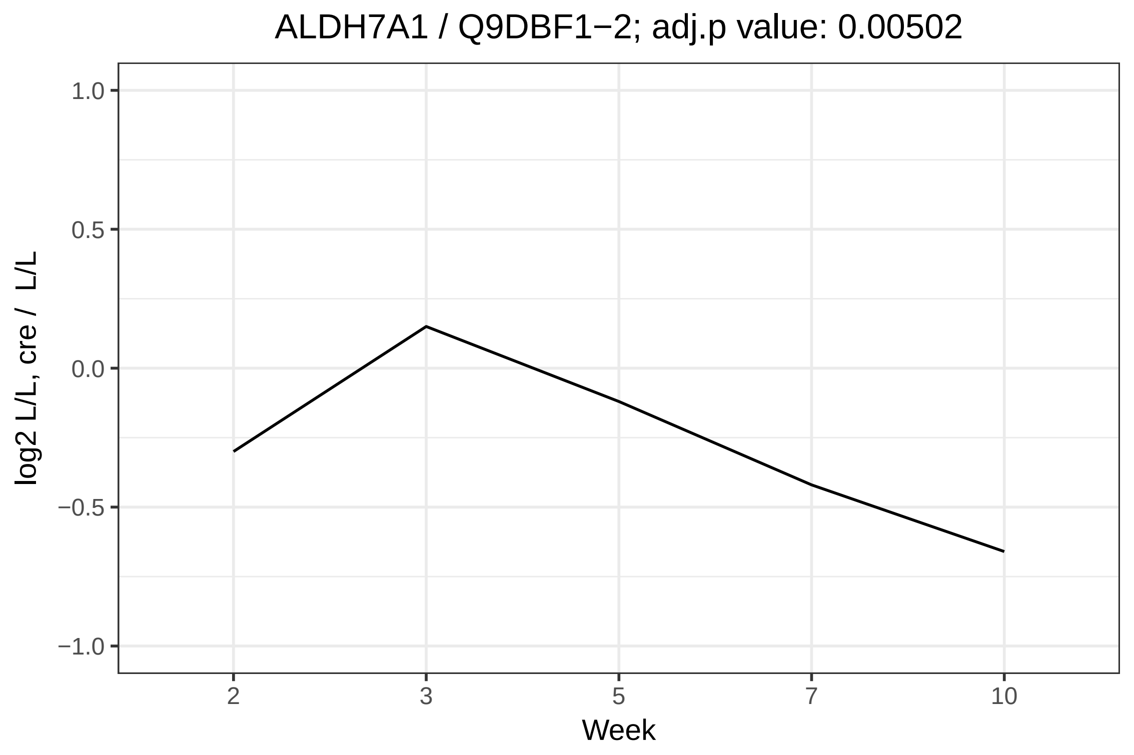
ALDH7A1 / Q9DBF1−2; adj.p value: 0.00502
log2 L/L, cre / L/L
1.0
0.5
0.0
−0.5
−1.0
2
3
5
7
10
Week

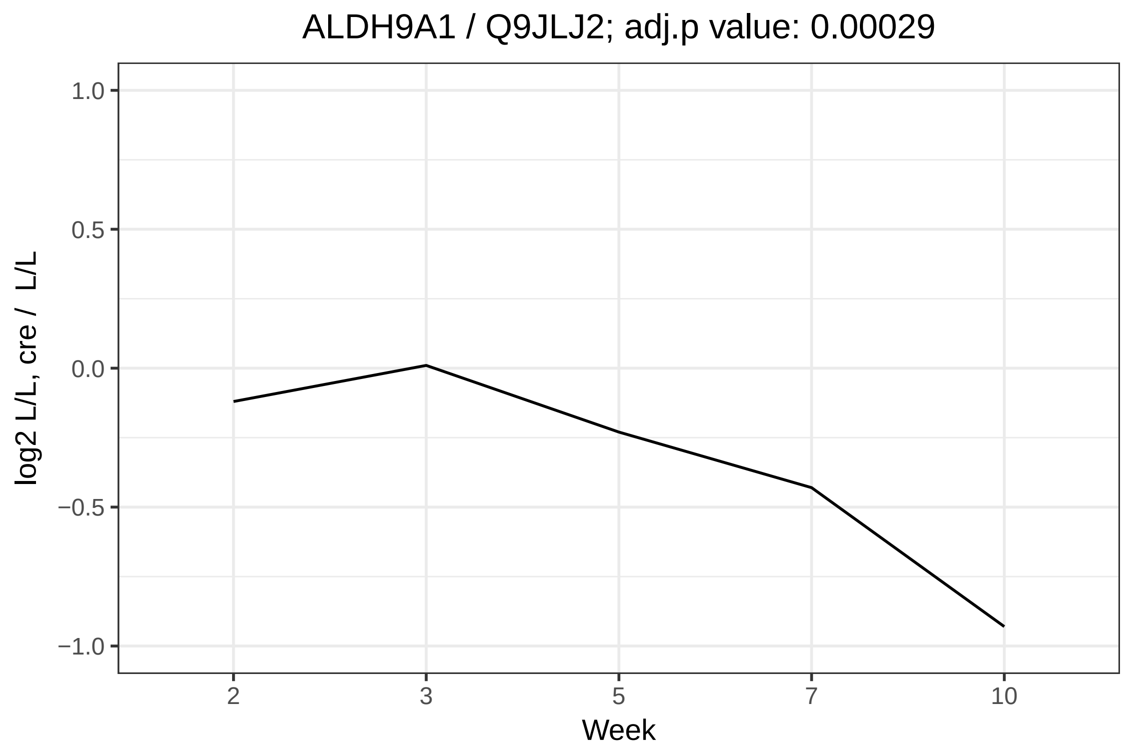
ALDH9A1 / Q9JLJ2; adj.p value: 0.00029
log2 L/L, cre / L/L
1.0
0.5
0.0
−0.5
−1.0
2
3
5
7
10
Week

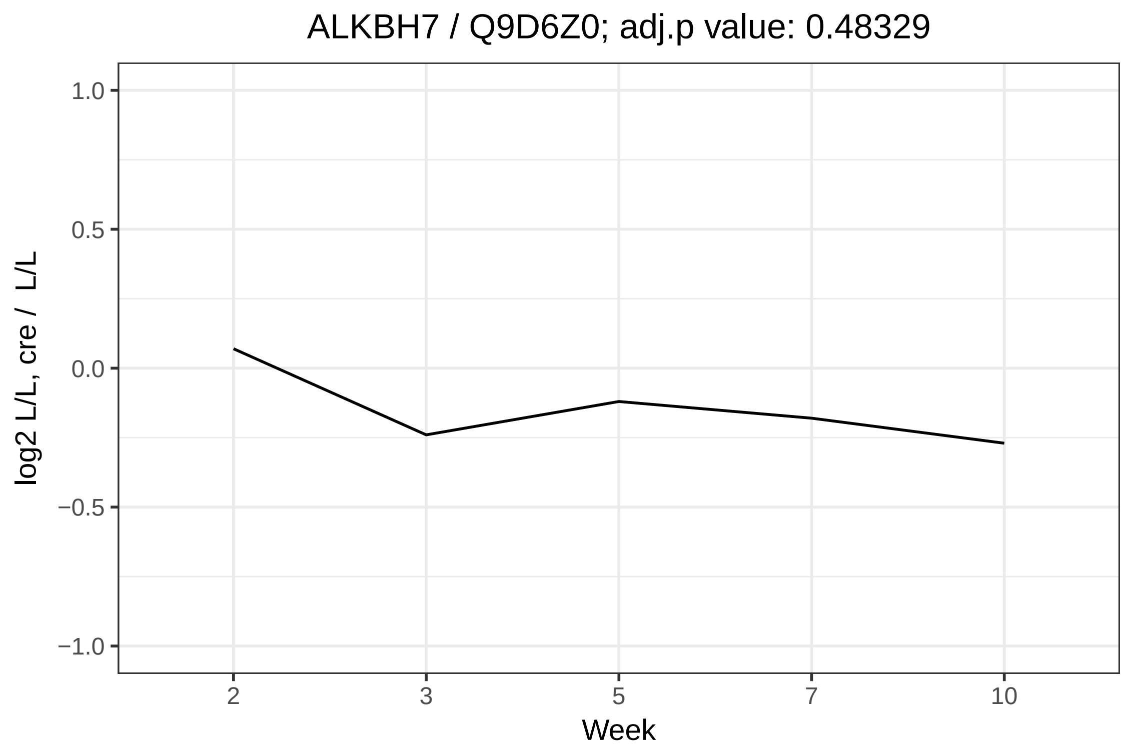
ALKBH7 / Q9D6Z0; adj.p value: 0.48329
log2 L/L, cre / L/L
1.0
0.5
0.0
−0.5
−1.0
2
3
5
7
10
Week

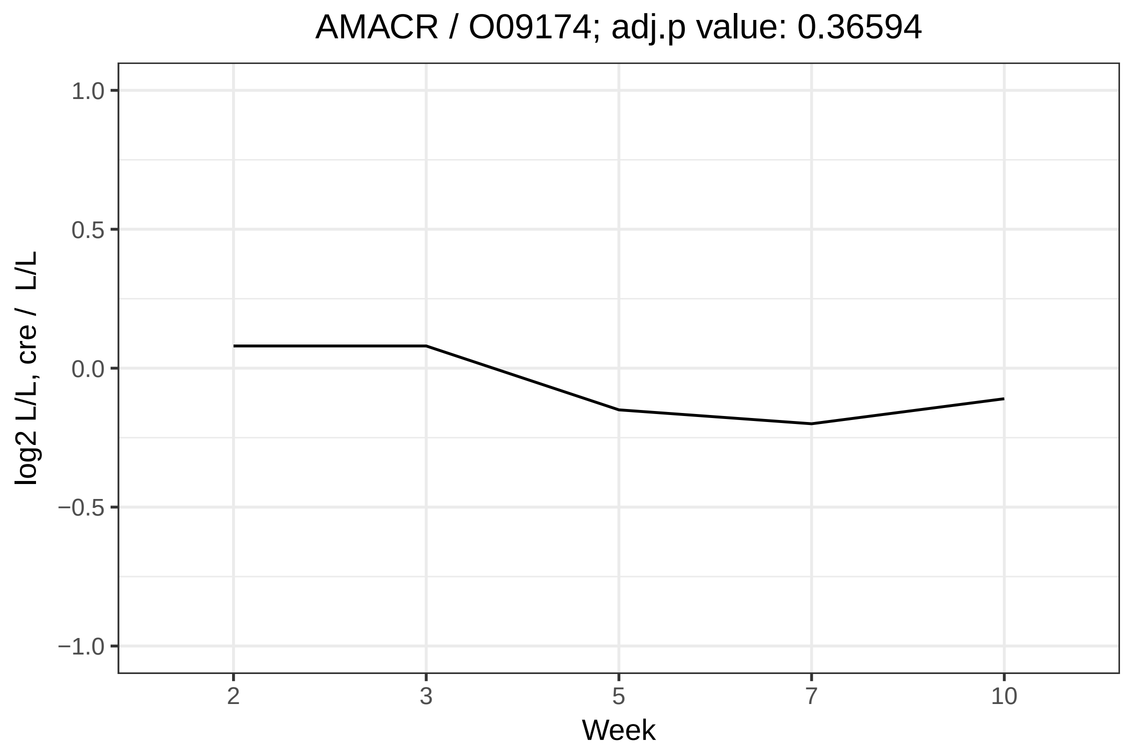
AMACR / O09174; adj.p value: 0.36594
log2 L/L, cre / L/L
1.0
0.5
0.0
−0.5
−1.0
2
3
5
7
10
Week

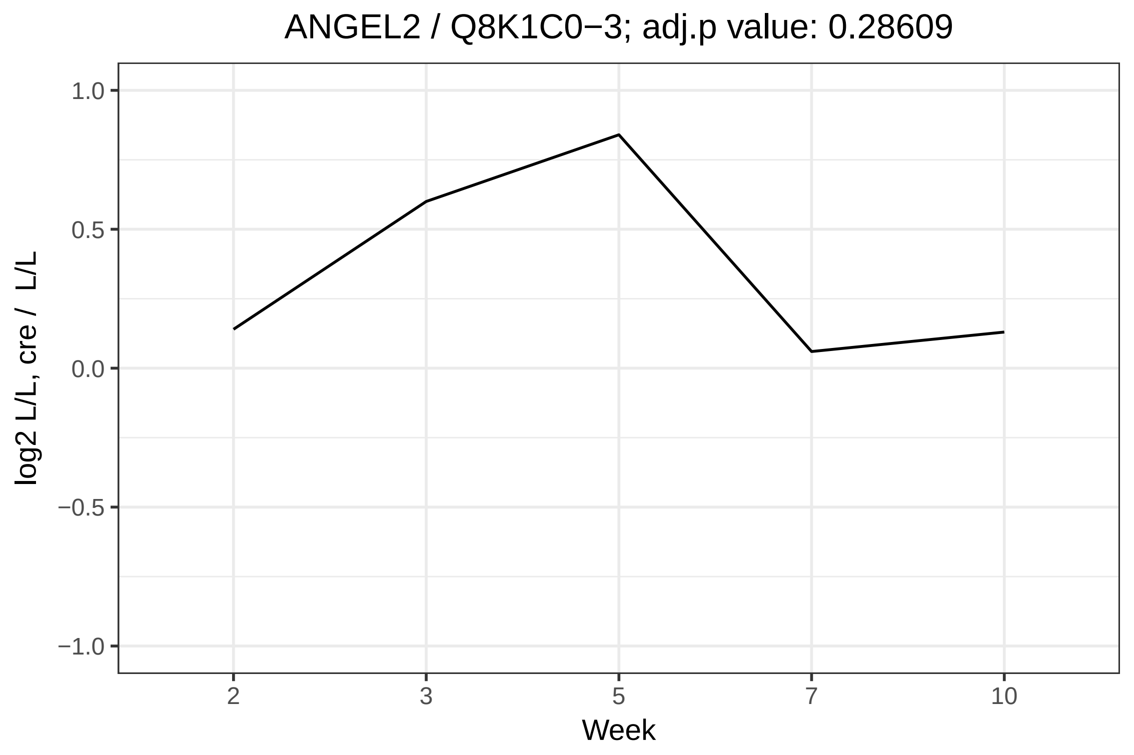
ANGEL2 / Q8K1C0−3; adj.p value: 0.28609
log2 L/L, cre / L/L
1.0
0.5
0.0
−0.5
−1.0
2
3
5
7
10
Week

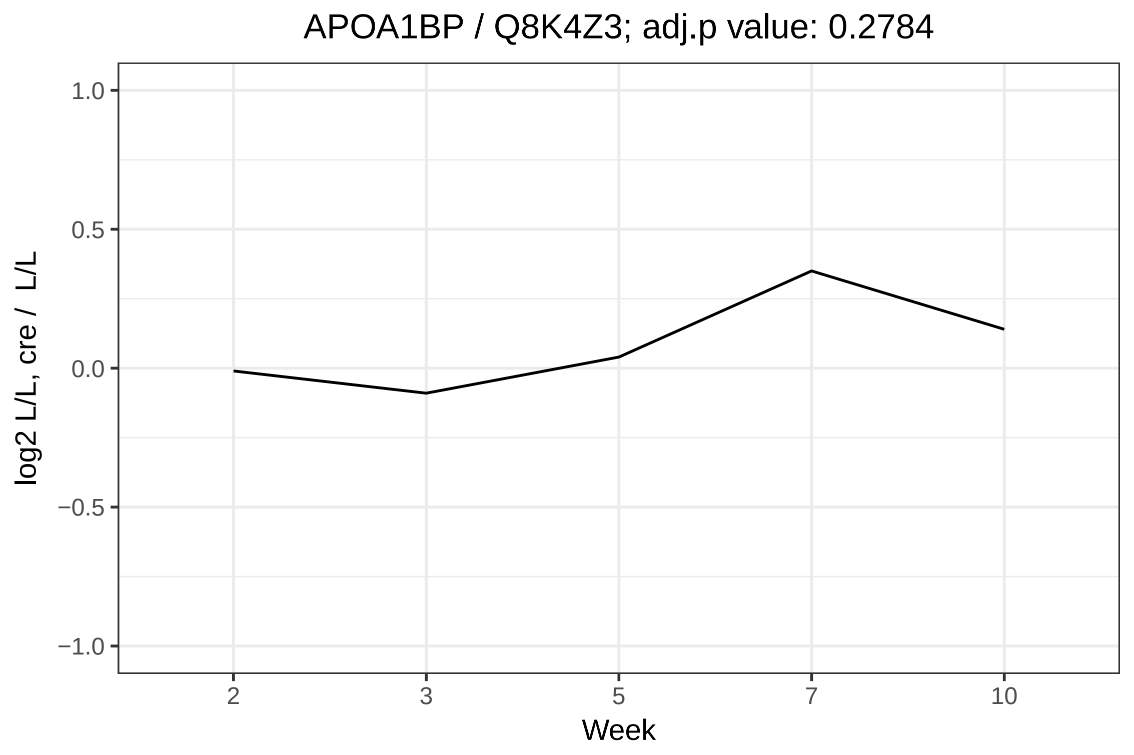

APOA1BP / Q8K4Z3; adj.p value: 0.2784
log2 L/L, cre / L/L
1.0
0.5
0.0
−0.5
−1.0
2
3
5
7
10
Week

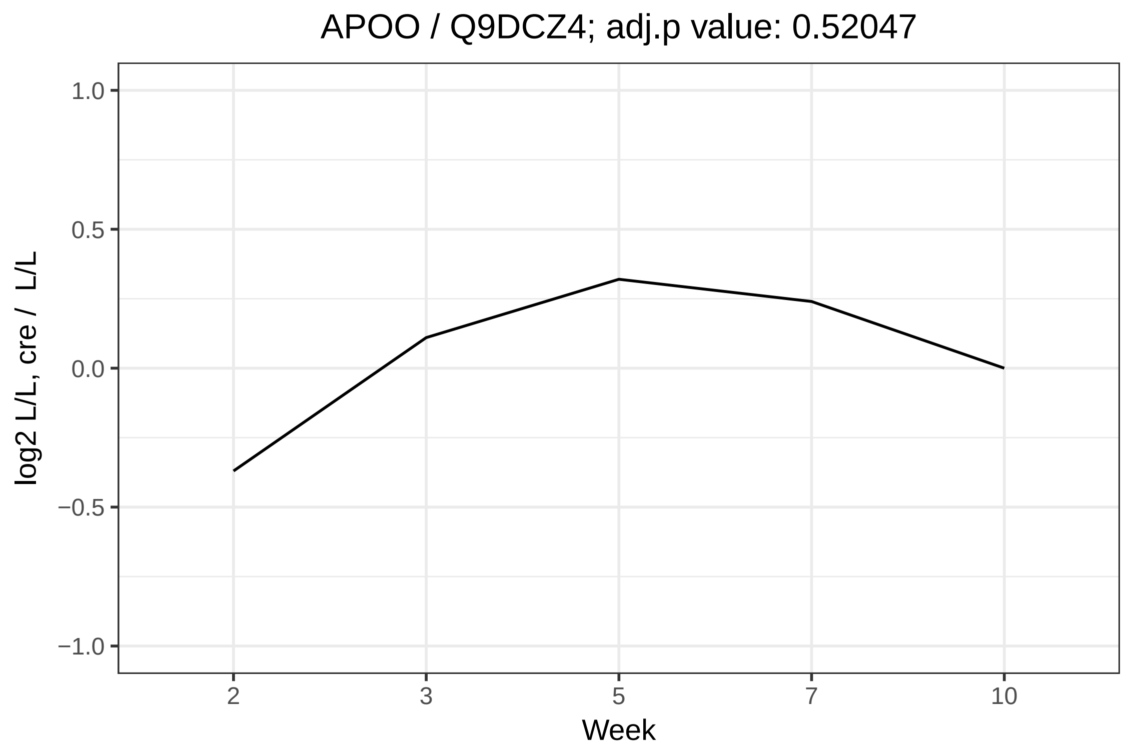
APOO / Q9DCZ4; adj.p value: 0.52047
log2 L/L, cre / L/L
1.0
0.5
0.0
−0.5
−1.0
2
3
5
7
10
Week

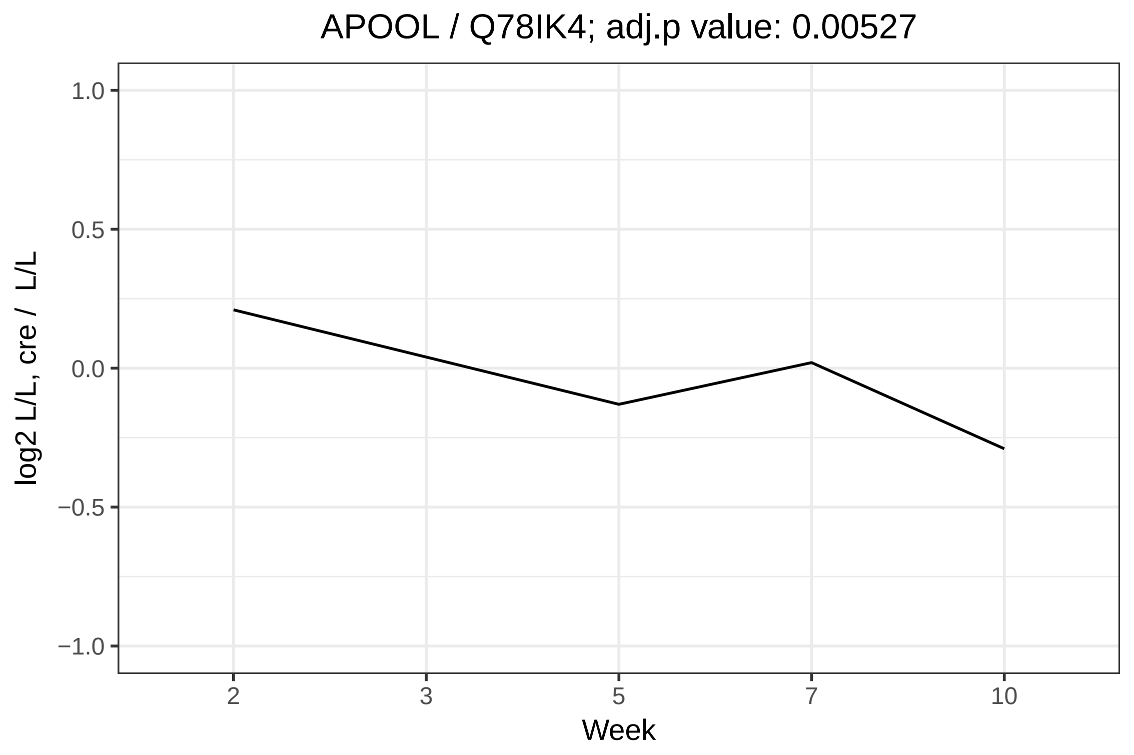

APOOL / Q78IK4; adj.p value: 0.00527
log2 L/L, cre / L/L
1.0
0.5
0.0
−0.5
−1.0
2
3
5
7
10
Week

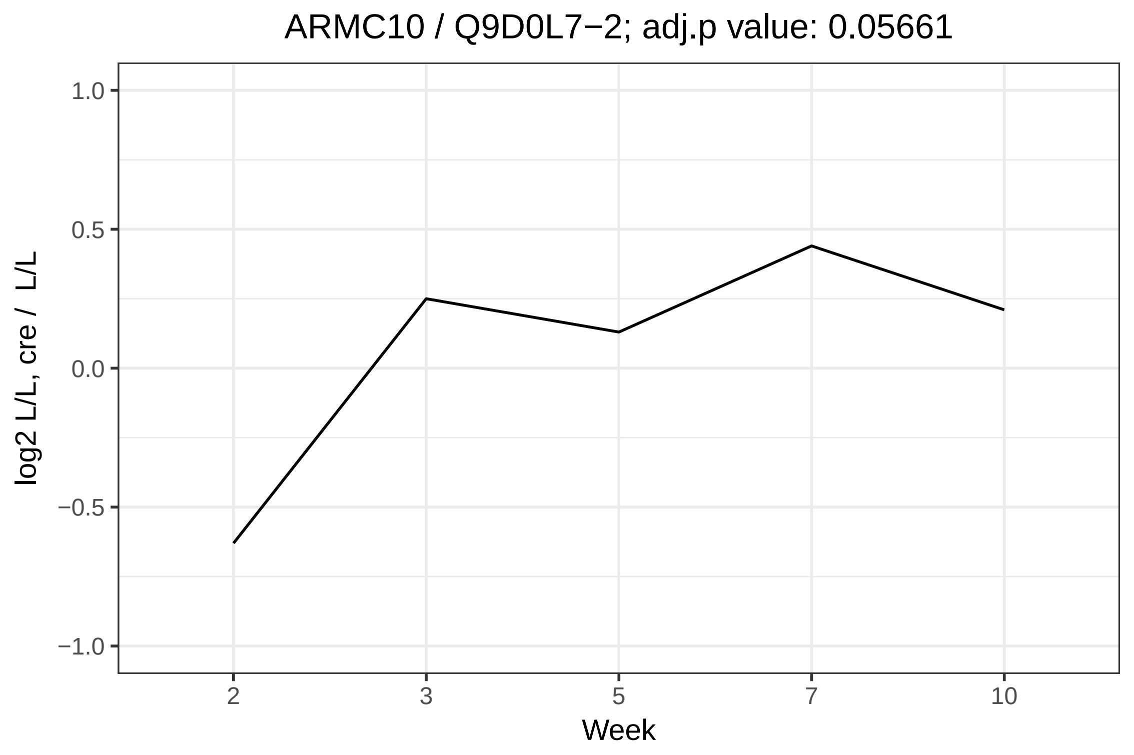

ARMC10 / Q9D0L7−2; adj.p value: 0.05661
log2 L/L, cre / L/L
1.0
0.5
0.0
−0.5
−1.0
2
3
5
7
10
Week

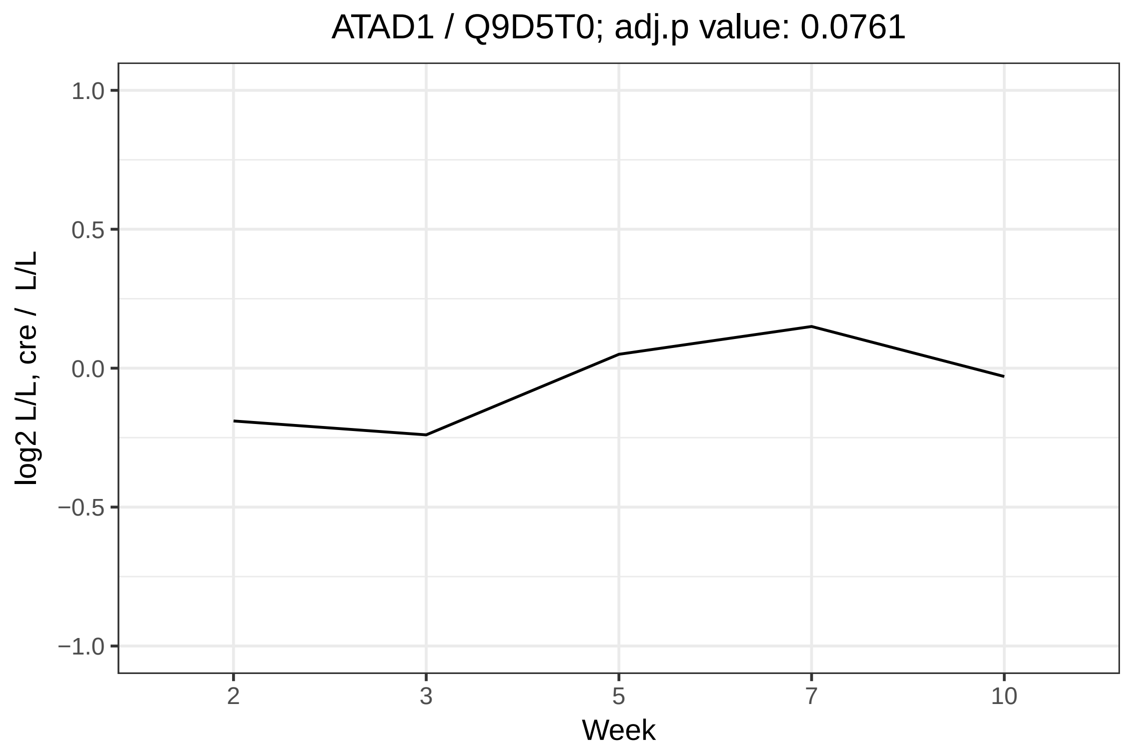

ATAD1 / Q9D5T0; adj.p value: 0.0761
log2 L/L, cre / L/L
1.0
0.5
0.0
−0.5
−1.0
2
3
5
7
10
Week

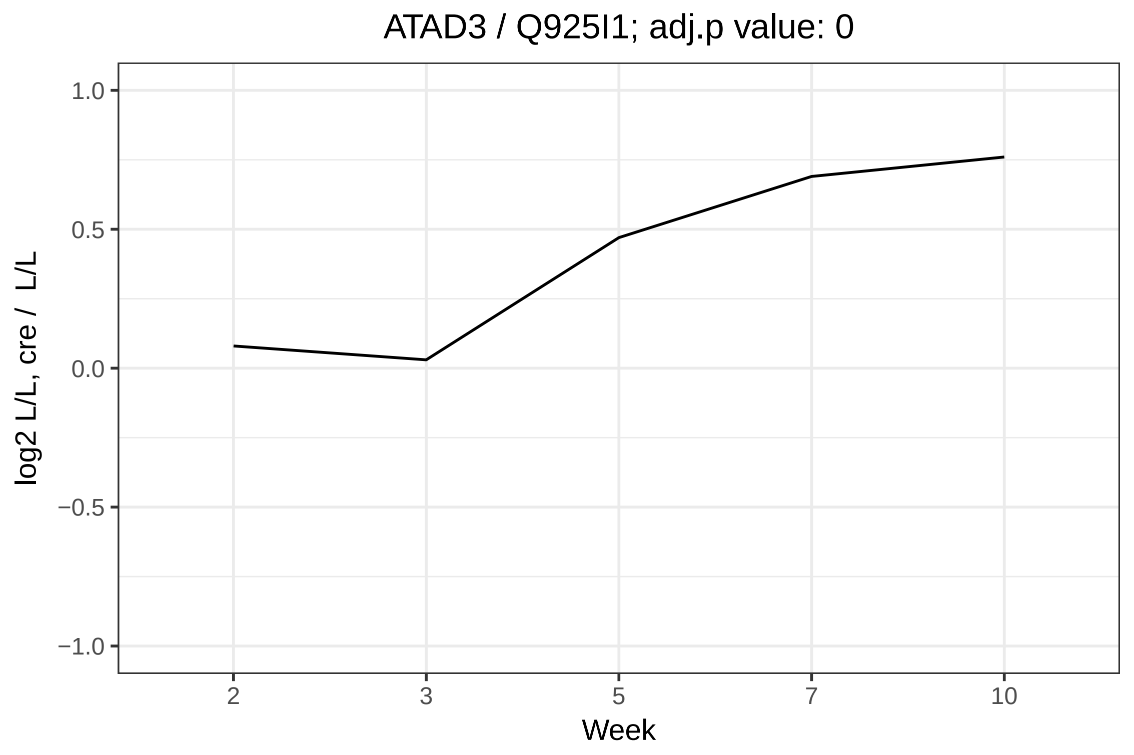
ATAD3 / Q925I1; adj.p value: 0
1.0
0.5
0.0
−0.5
−1.0
log2 L/L, cre / L/L
2
3
5
7
10
Week

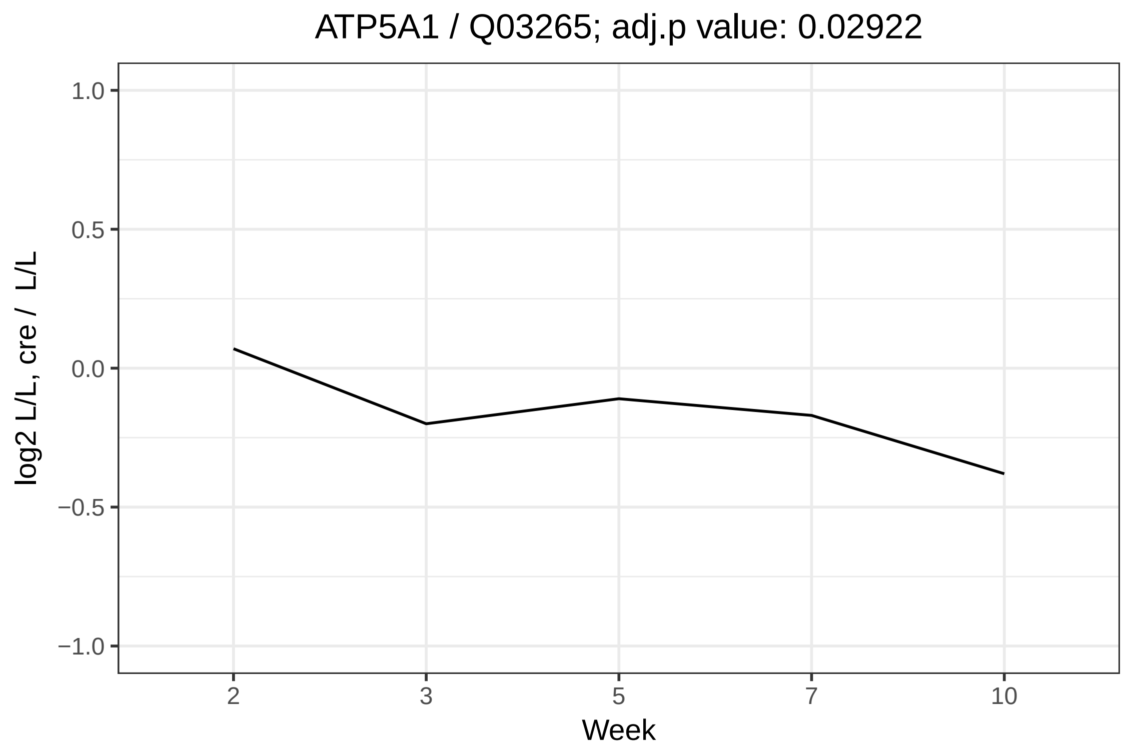
ATP5A1 / Q03265; adj.p value: 0.02922
log2 L/L, cre / L/L
1.0
0.5
0.0
−0.5
−1.0
2
3
5
7
10
Week

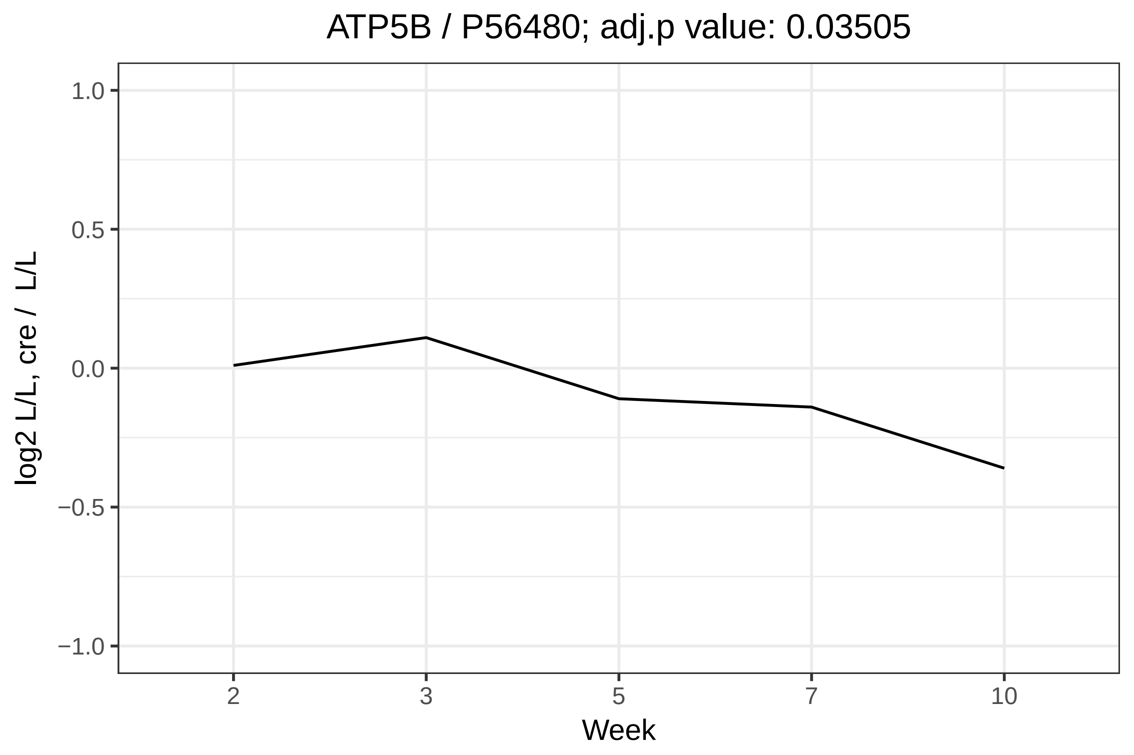
ATP5B / P56480; adj.p value: 0.03505
1.0
0.5
0.0
−0.5
−1.0
log2 L/L, cre / L/L
2
3
5
7
10
Week

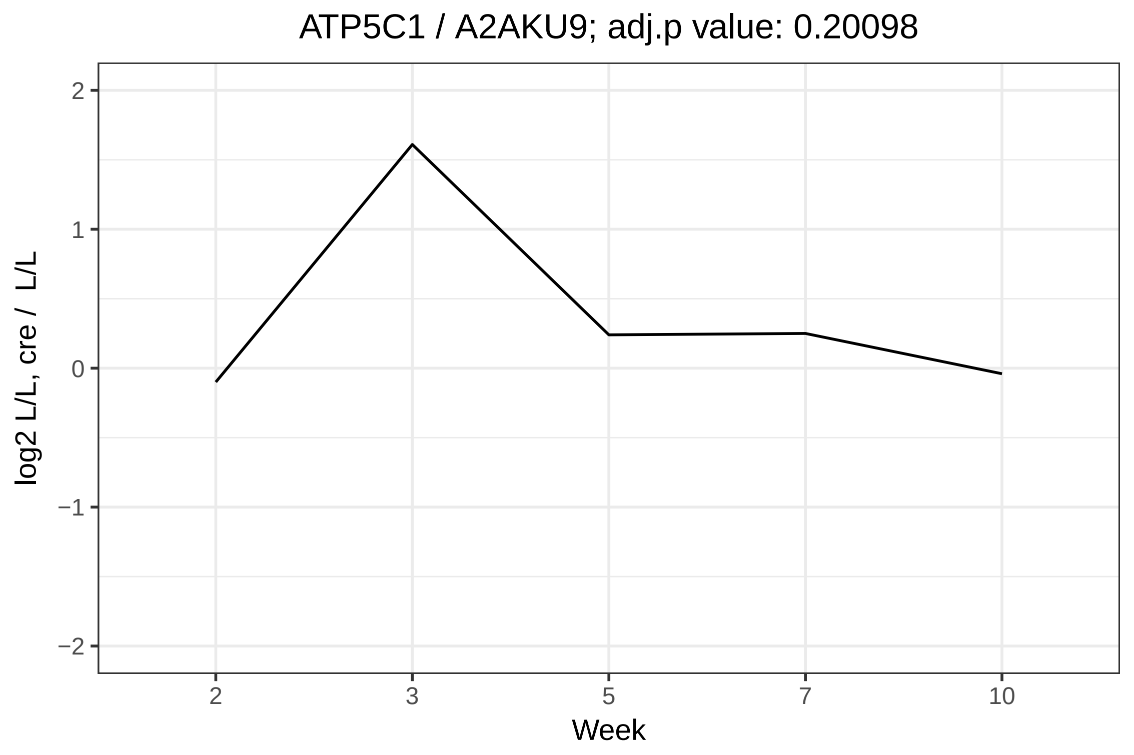
ATP5C1 / A2AKU9; adj.p value: 0.20098
log2 L/L, cre / L/L
2
1
0
−1
−2
2
3
5
7
10
Week

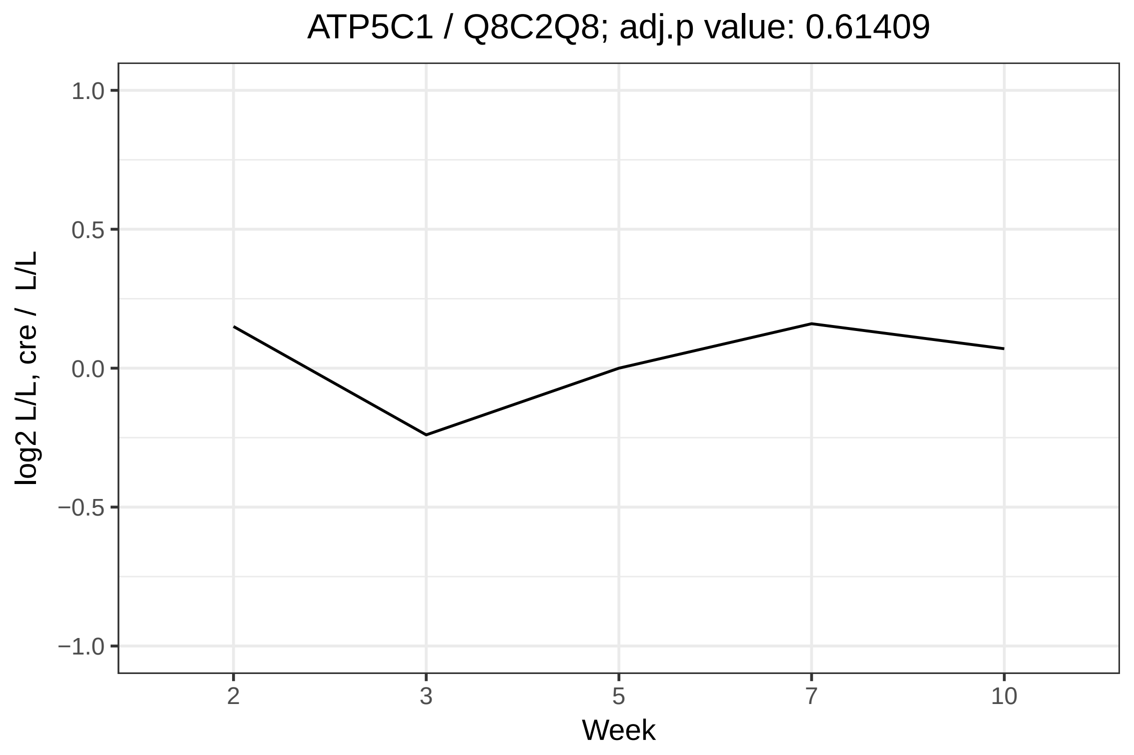
ATP5C1 / Q8C2Q8; adj.p value: 0.61409
1.0
0.5
0.0
−0.5
−1.0
log2 L/L, cre / L/L
2
3
5
7
10
Week

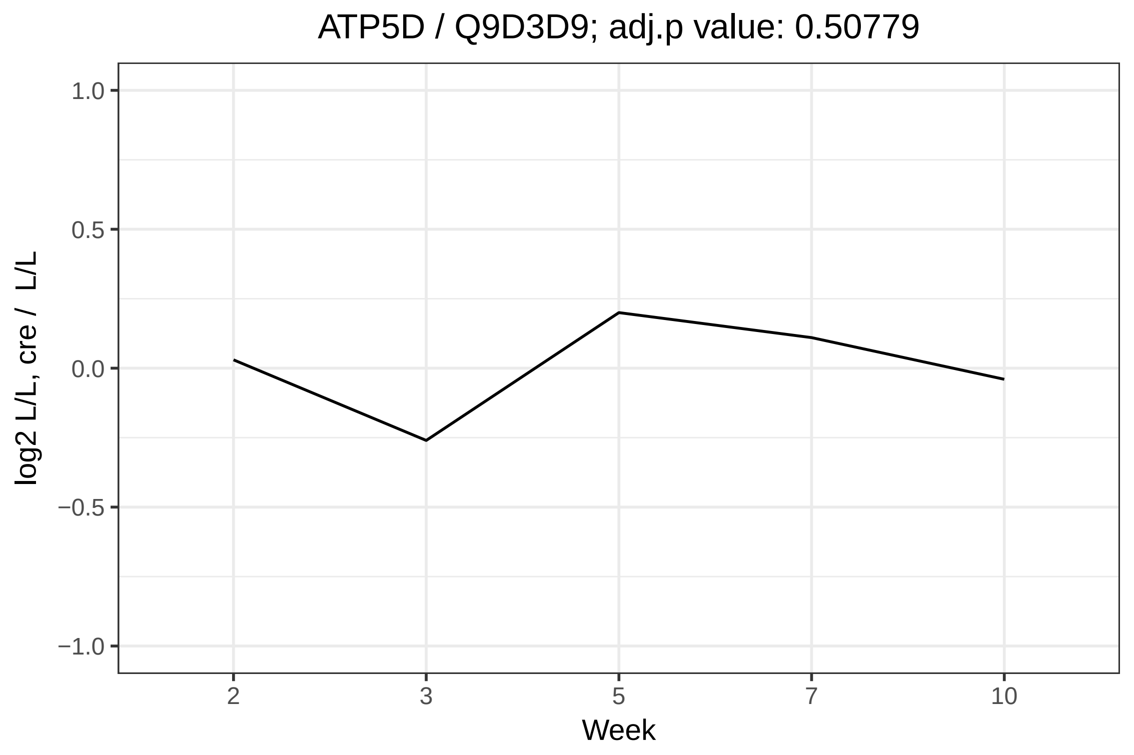

ATP5D / Q9D3D9; adj.p value: 0.50779
1.0
0.5
0.0
−0.5
−1.0
log2 L/L, cre / L/L
2
3
5
7
10
Week

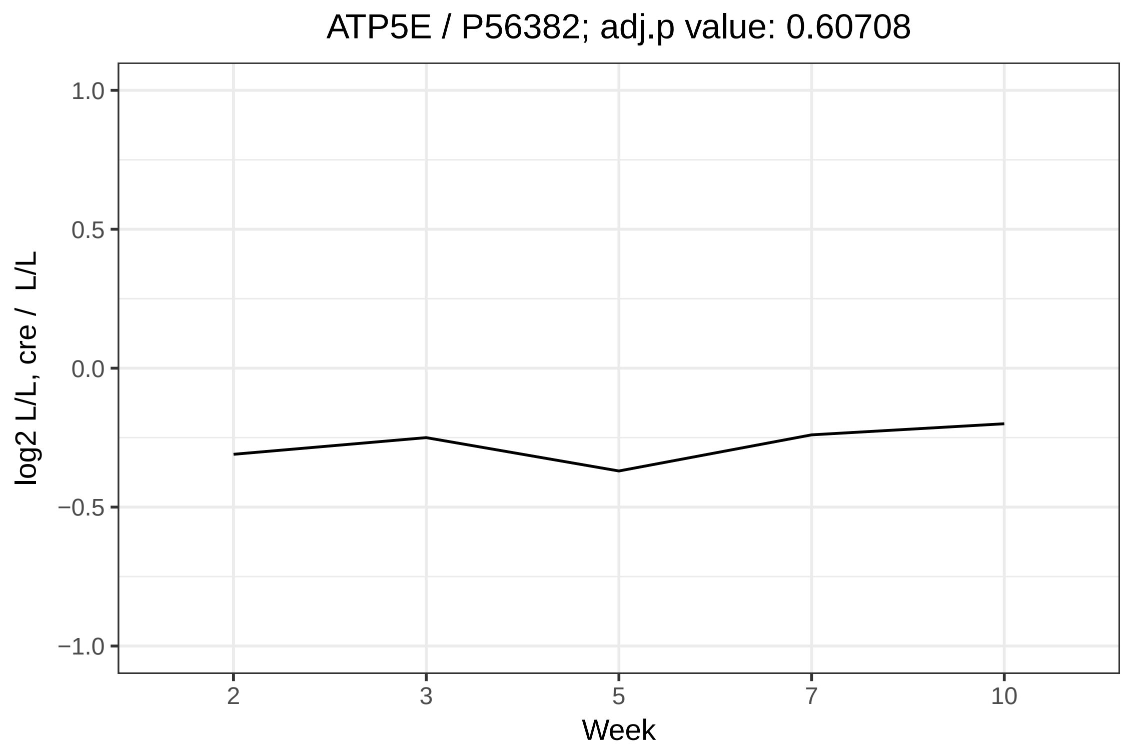
ATP5E / P56382; adj.p value: 0.60708
log2 L/L, cre / L/L
1.0
0.5
0.0
−0.5
−1.0
2
3
5
7
10
Week

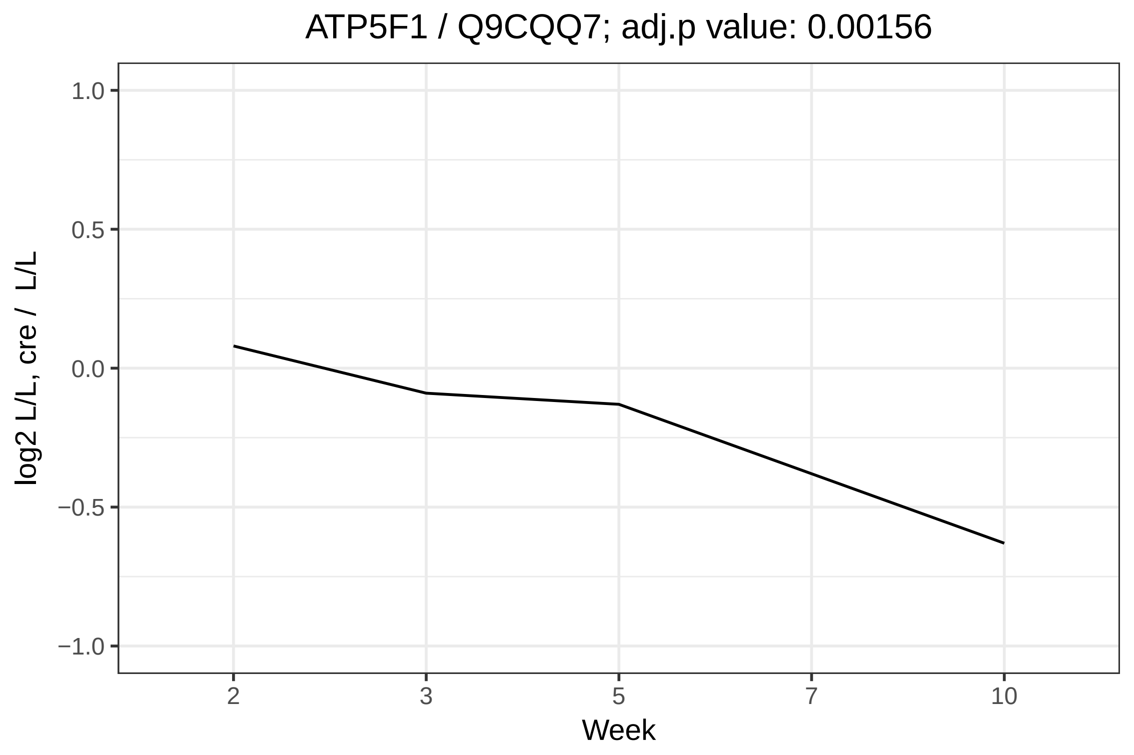

ATP5F1 / Q9CQQ7; adj.p value: 0.00156
log2 L/L, cre / L/L
1.0
0.5
0.0
−0.5
−1.0
2
3
5
7
10
Week

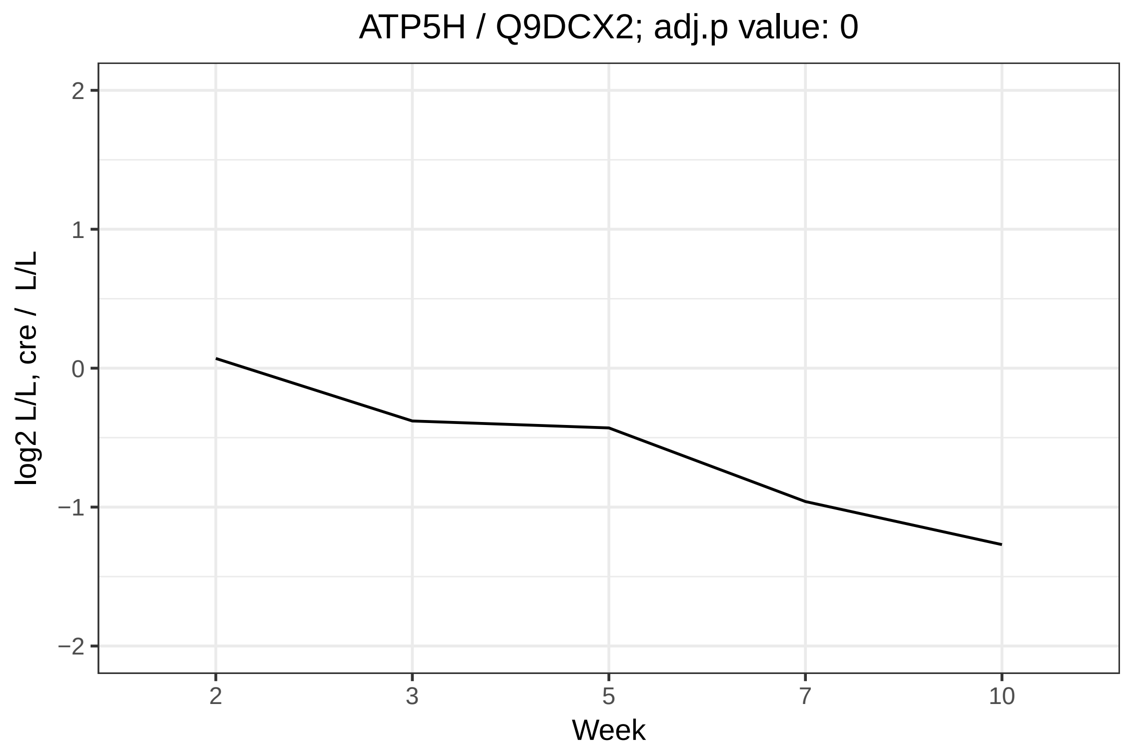

ATP5H / Q9DCX2; adj.p value: 0
log2 L/L, cre / L/L
2
1
0
−1
−2
2
3
5
7
10
Week

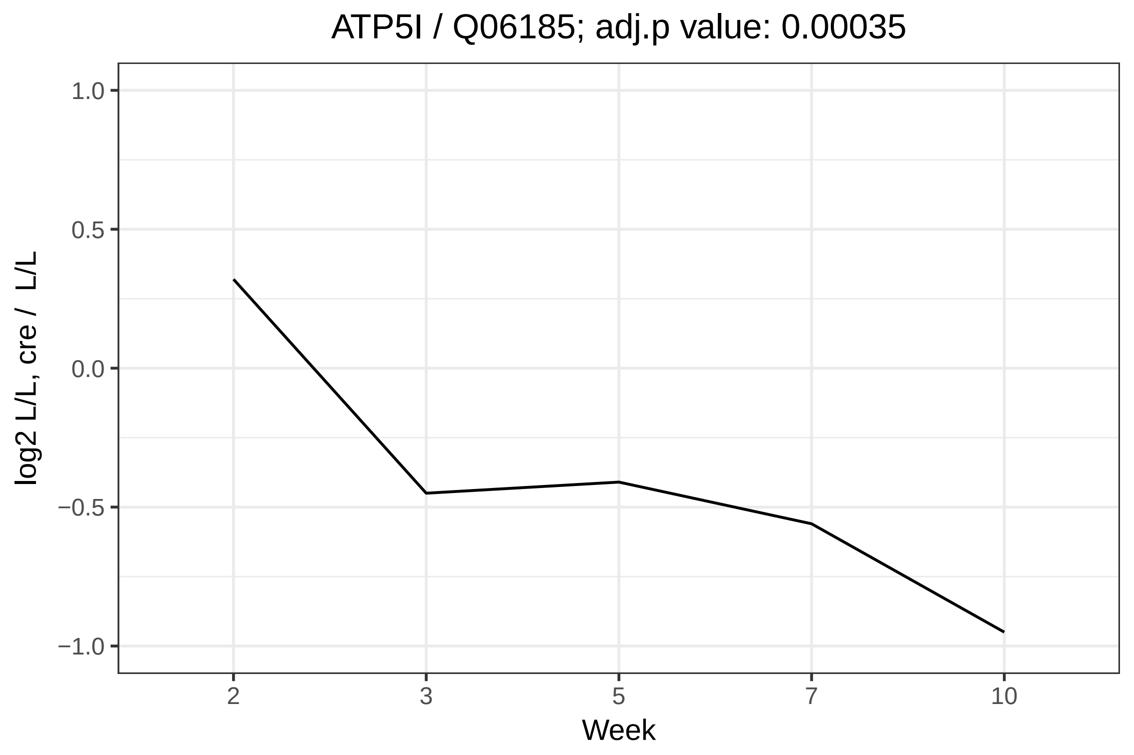
ATP5I / Q06185; adj.p value: 0.00035
1.0
0.5
0.0
−0.5
−1.0
log2 L/L, cre / L/L
2
3
5
7
10
Week

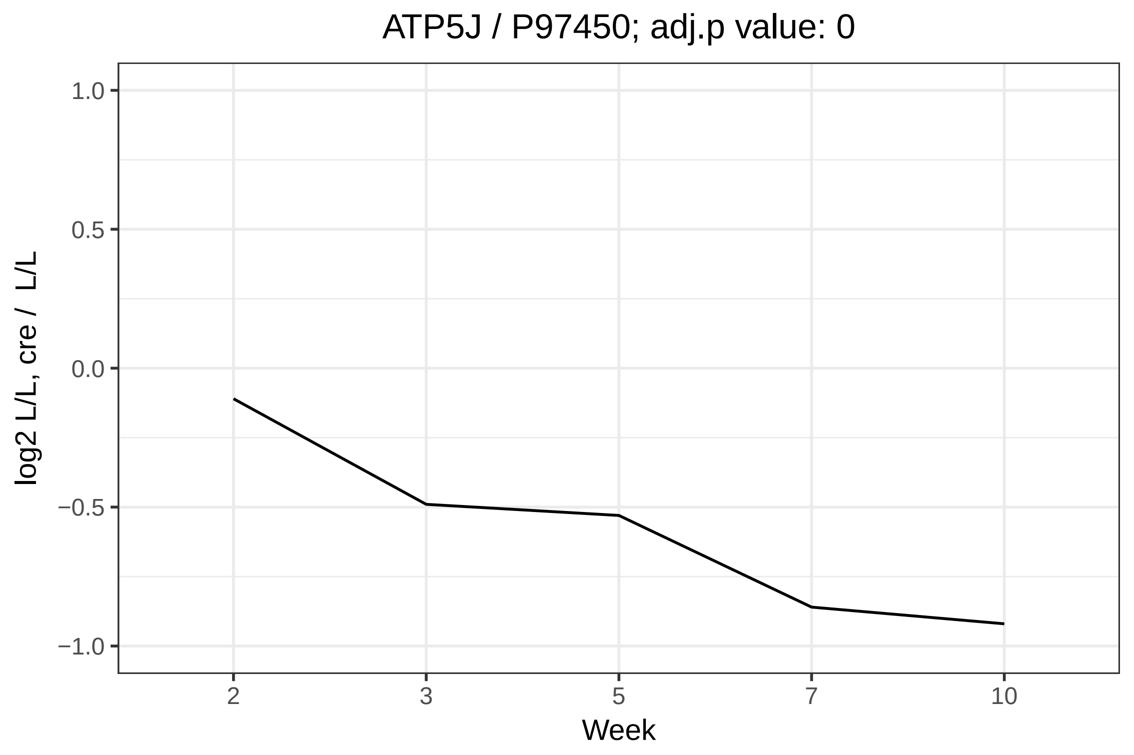
ATP5J / P97450; adj.p value: 0
log2 L/L, cre / L/L
1.0
0.5
0.0
−0.5
−1.0
2
3
5
7
10
Week

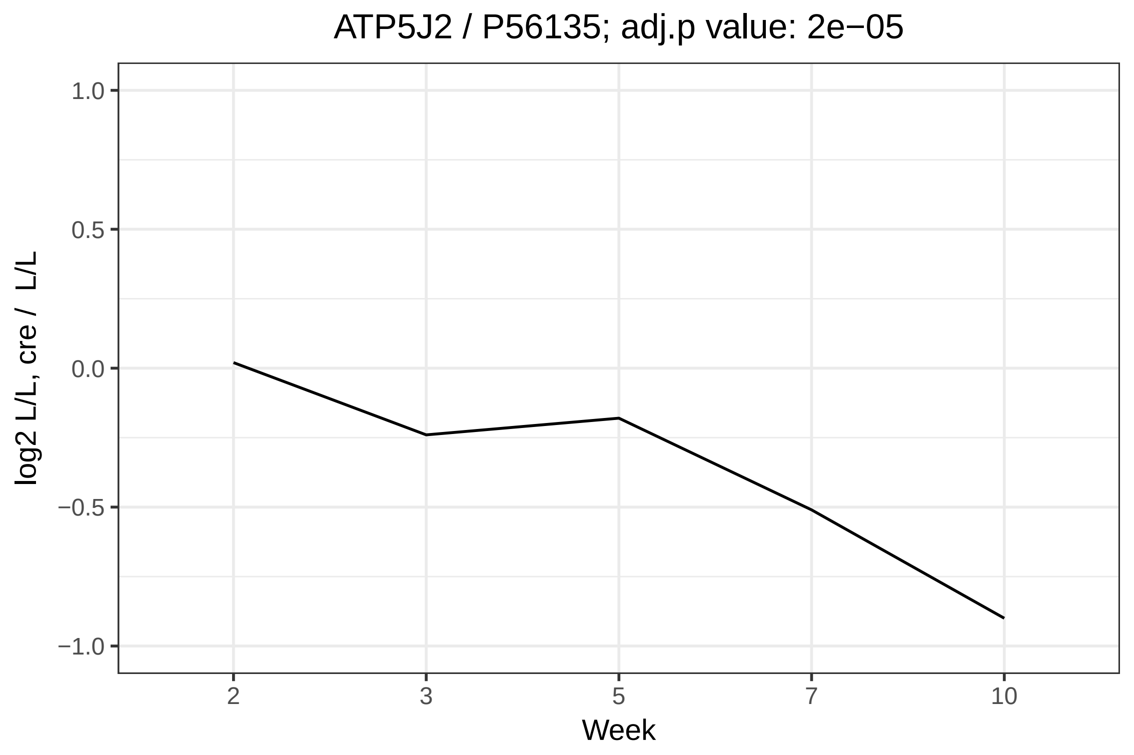
ATP5J2 / P56135; adj.p value: 2e−05
1.0
0.5
0.0
−0.5
−1.0
log2 L/L, cre / L/L
2
3
5
7
10
Week

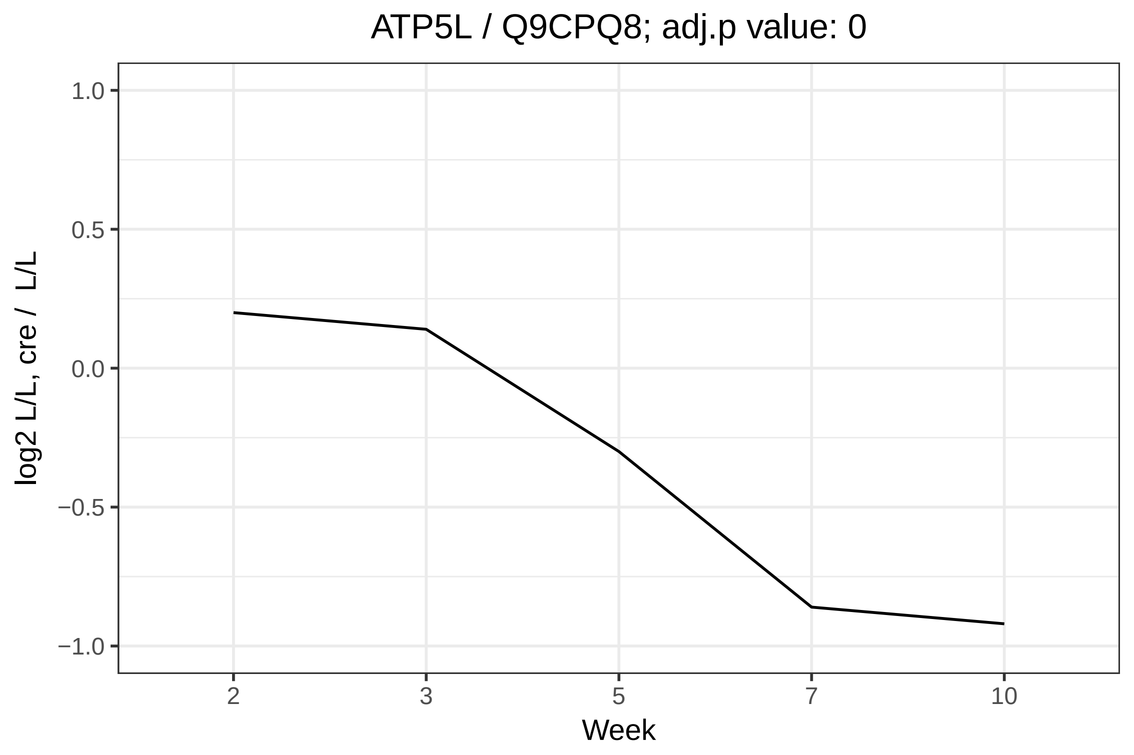

ATP5L / Q9CPQ8; adj.p value: 0
log2 L/L, cre / L/L
1.0
0.5
0.0
−0.5
−1.0
2
3
5
7
10
Week

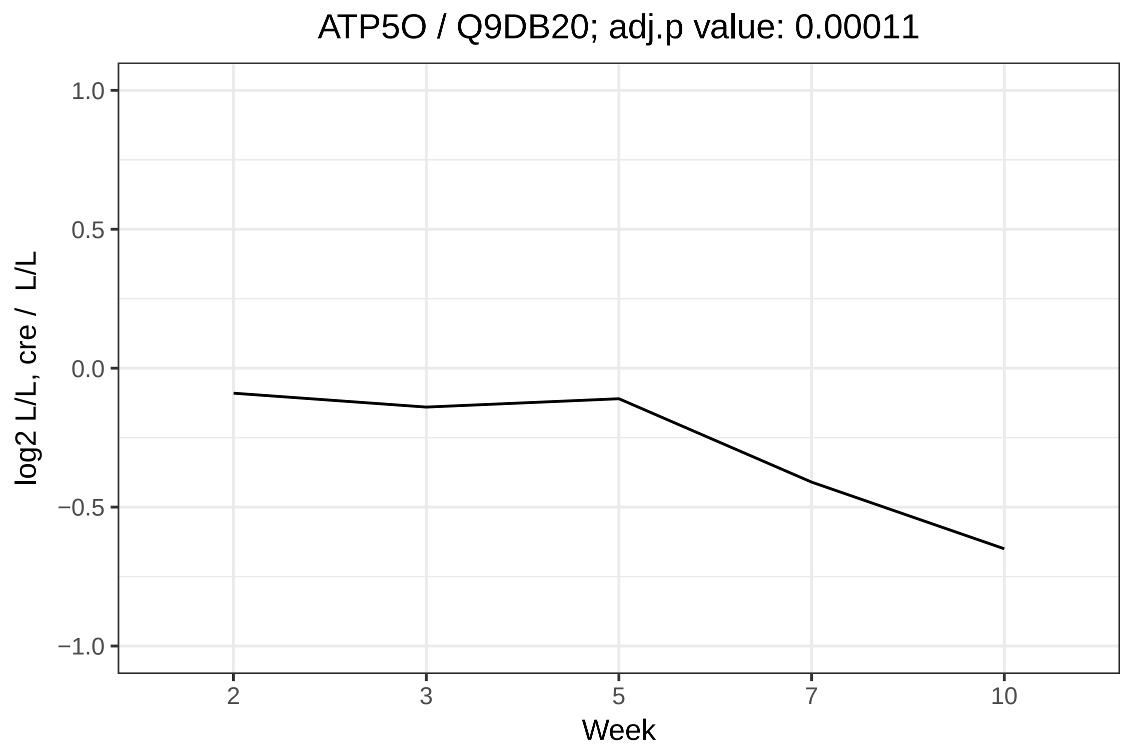

ATP5O / Q9DB20; adj.p value: 0.00011
log2 L/L, cre / L/L
1.0
0.5
0.0
−0.5
−1.0
2
3
5
7
10
Week

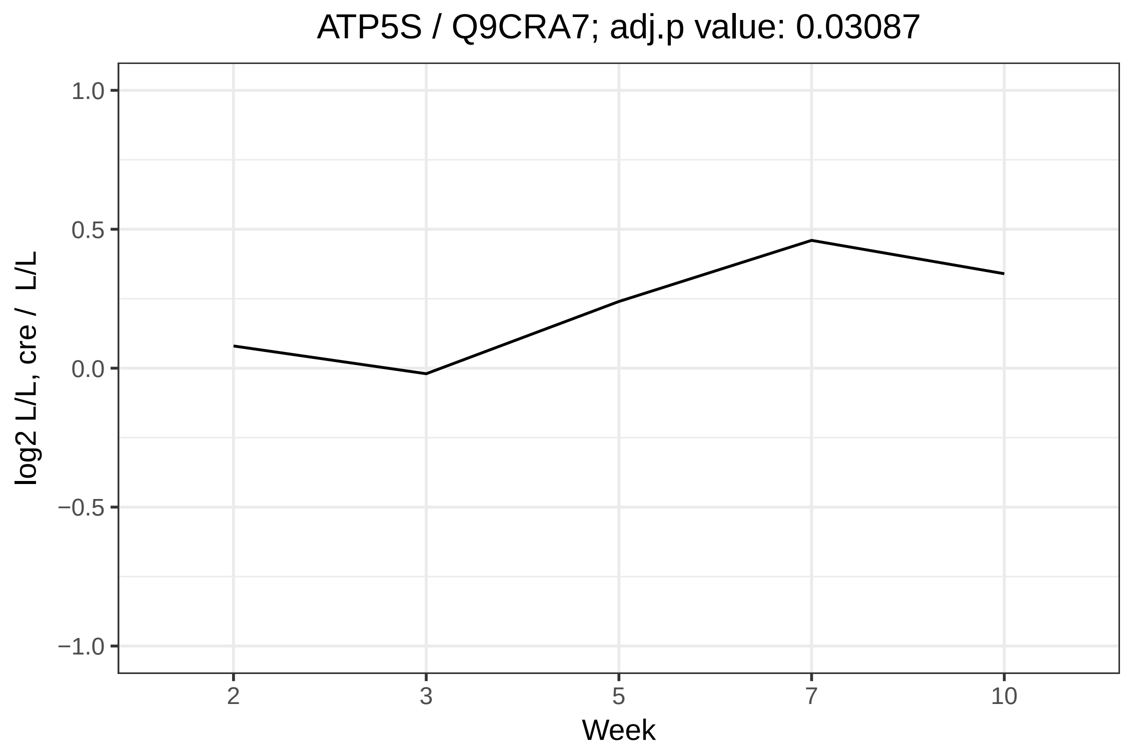
ATP5S / Q9CRA7; adj.p value: 0.03087
log2 L/L, cre / L/L
1.0
0.5
0.0
−0.5
−1.0
2
3
5
7
10
Week

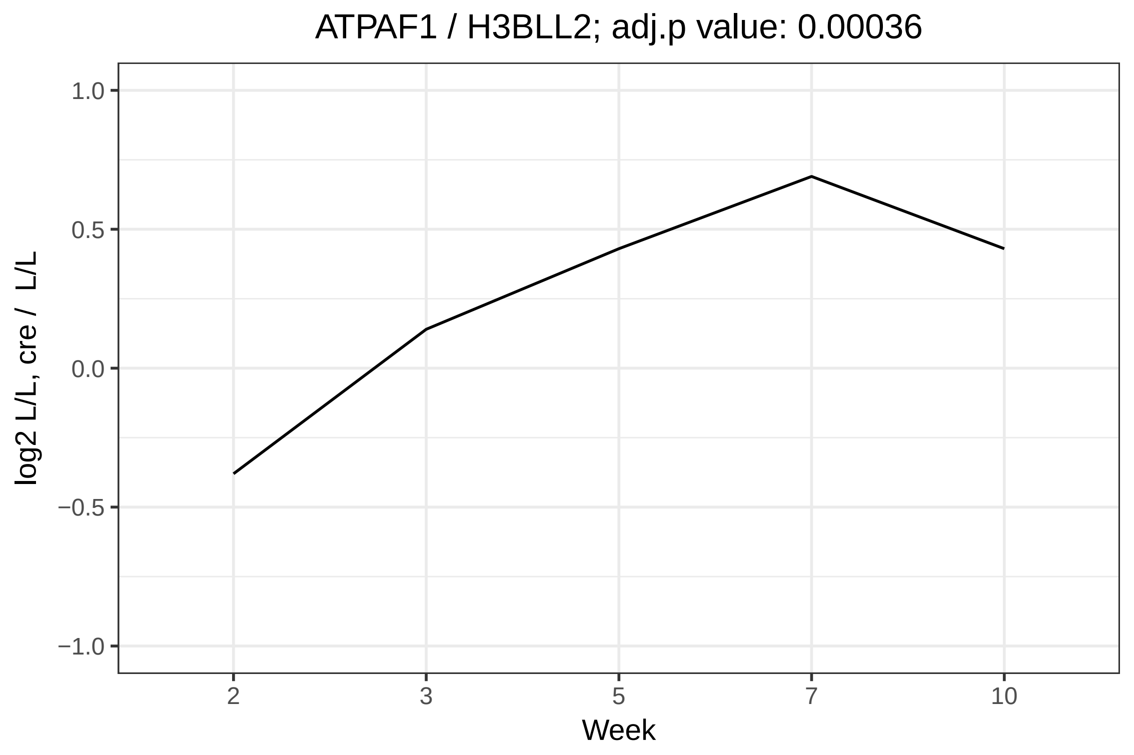

ATPAF1 / H3BLL2; adj.p value: 0.00036
1.0
0.5
0.0
−0.5
−1.0
log2 L/L, cre / L/L
2
3
5
7
10
Week

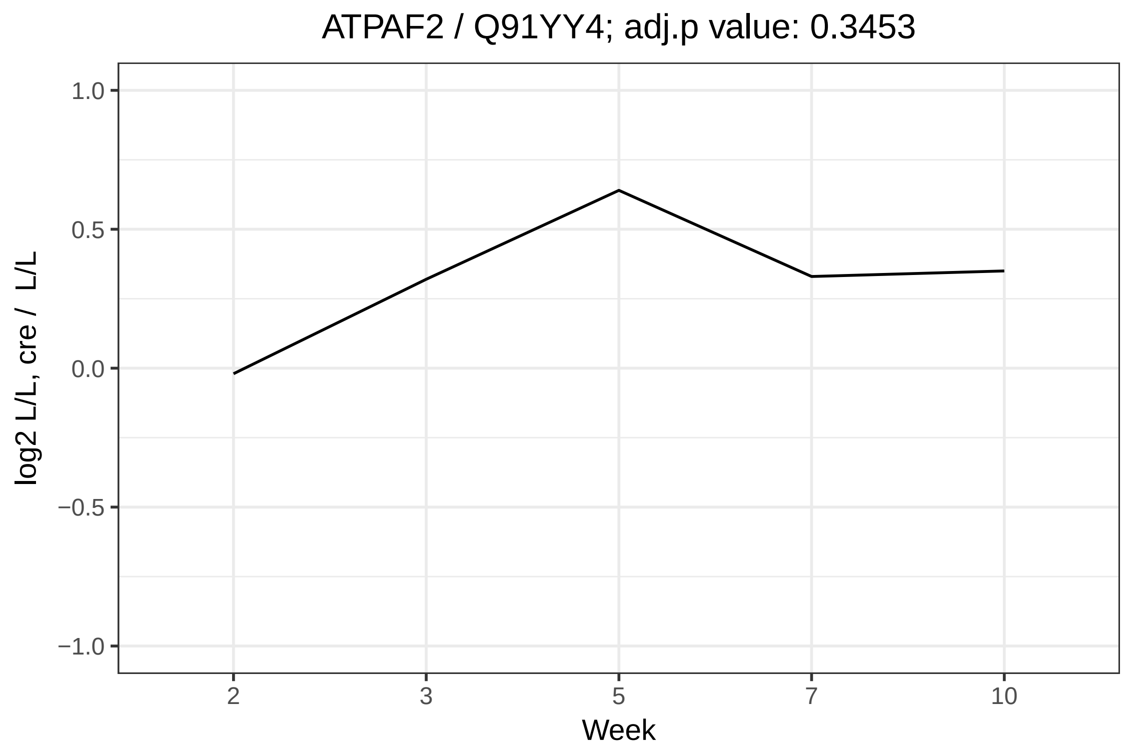
ATPAF2 / Q91YY4; adj.p value: 0.3453
log2 L/L, cre / L/L
1.0
0.5
0.0
−0.5
−1.0
2
3
5
7
10
Week

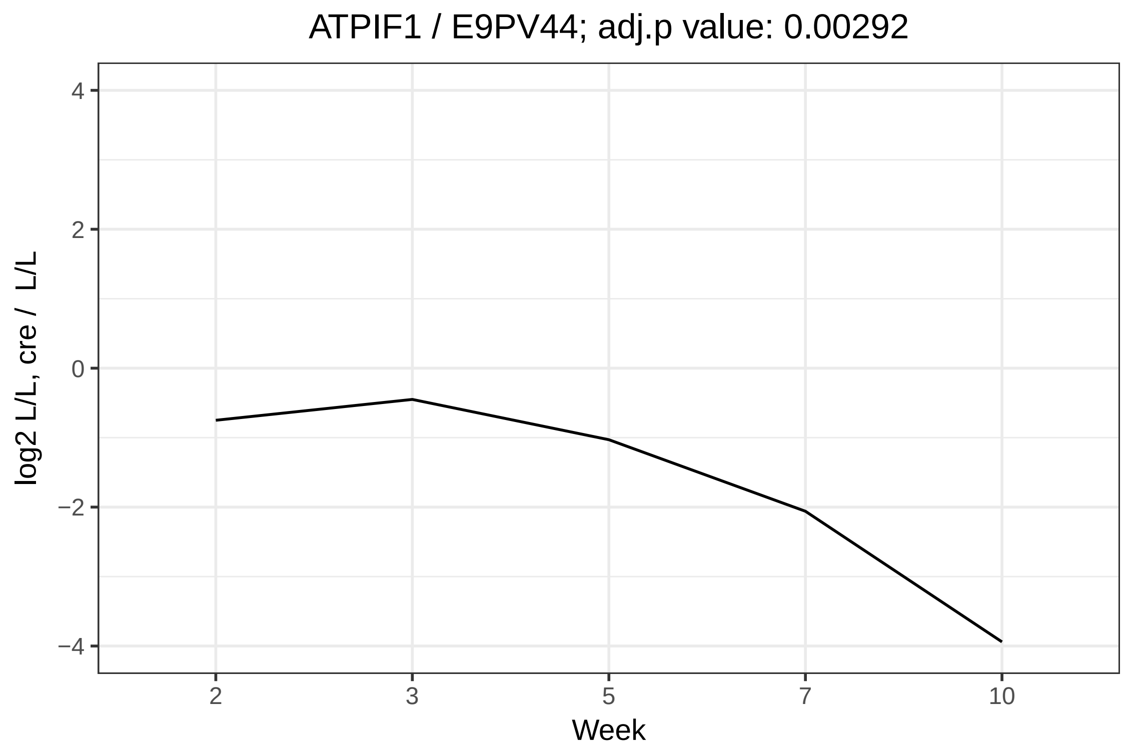

ATPIF1 / E9PV44; adj.p value: 0.00292
log2 L/L, cre / L/L
4
2
0
−2
−4
2
3
5
7
10
Week

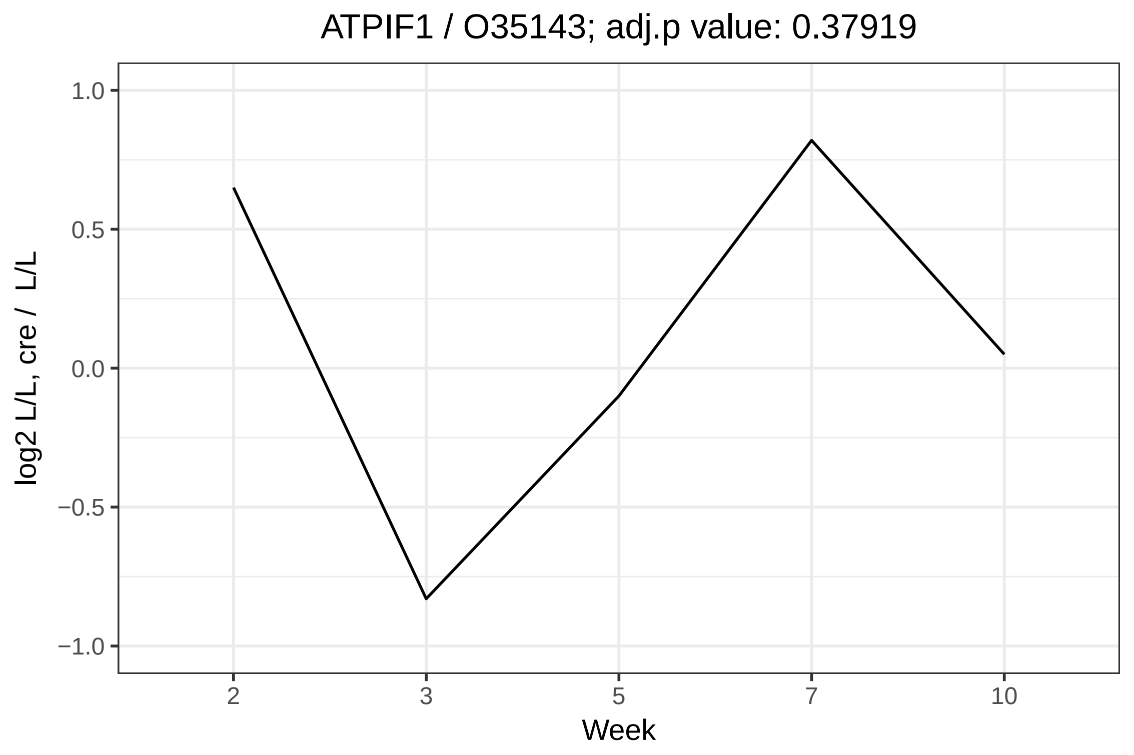
ATPIF1 / O35143; adj.p value: 0.37919
log2 L/L, cre / L/L
1.0
0.5
0.0
−0.5
−1.0
2
3
5
7
10
Week

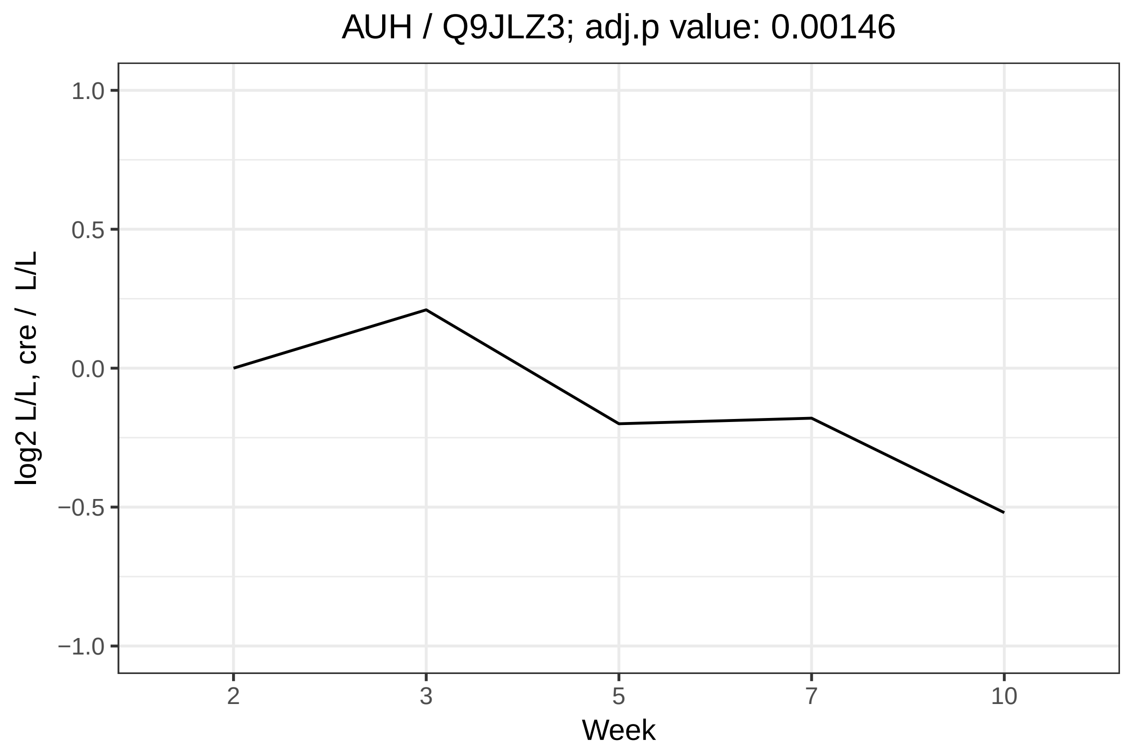
AUH / Q9JLZ3; adj.p value: 0.00146
1.0
0.5
0.0
−0.5
−1.0
log2 L/L, cre / L/L
2
3
5
7
10
Week

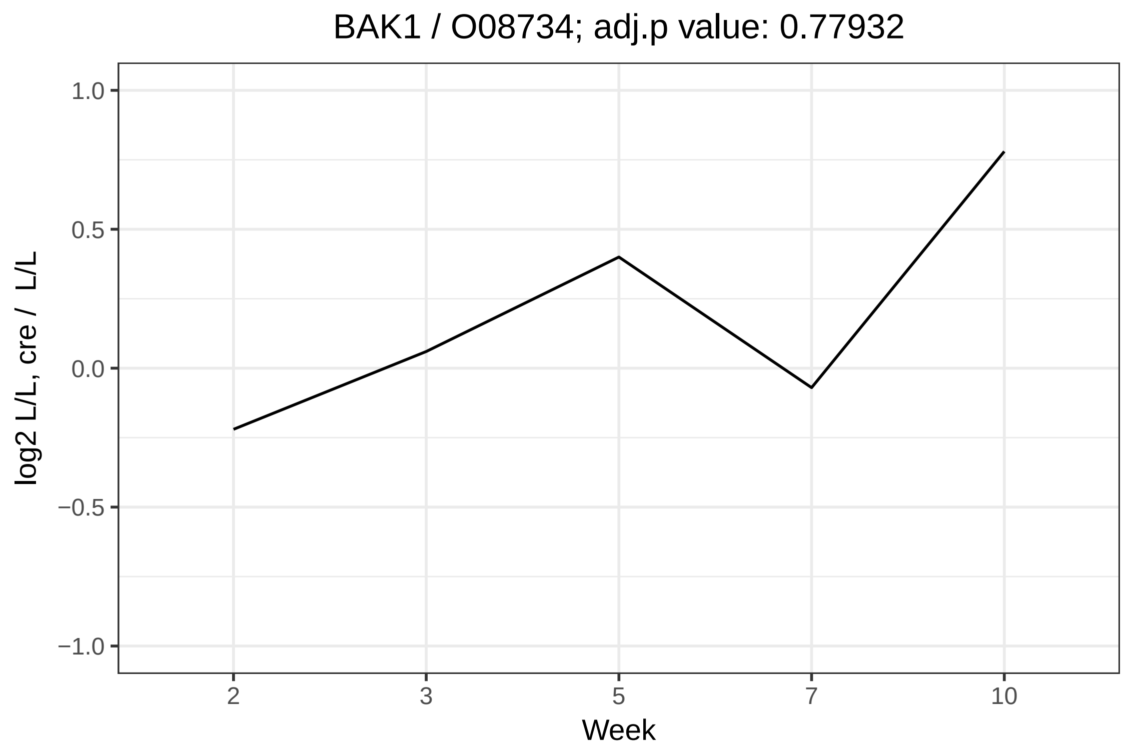

BAK1 / O08734; adj.p value: 0.77932
log2 L/L, cre / L/L
1.0
0.5
0.0
−0.5
−1.0
2
3
5
7
10
Week

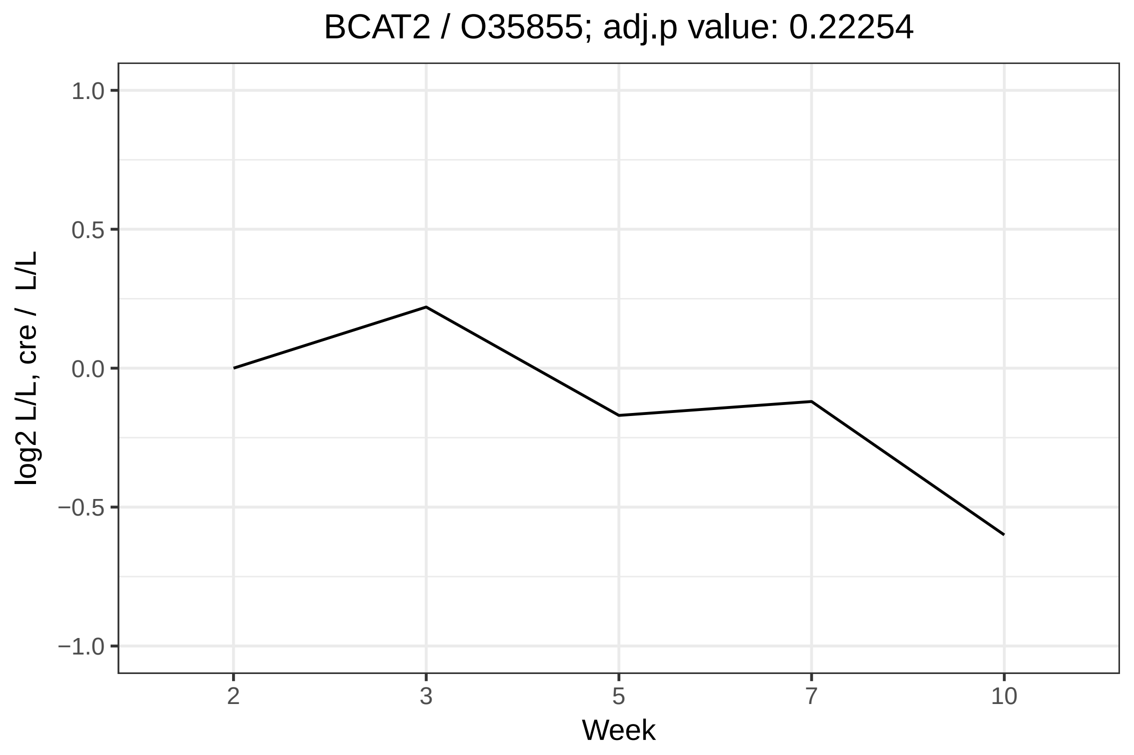
BCAT2 / O35855; adj.p value: 0.22254
1.0
0.5
0.0
−0.5
−1.0
log2 L/L, cre / L/L
2
3
5
7
10
Week

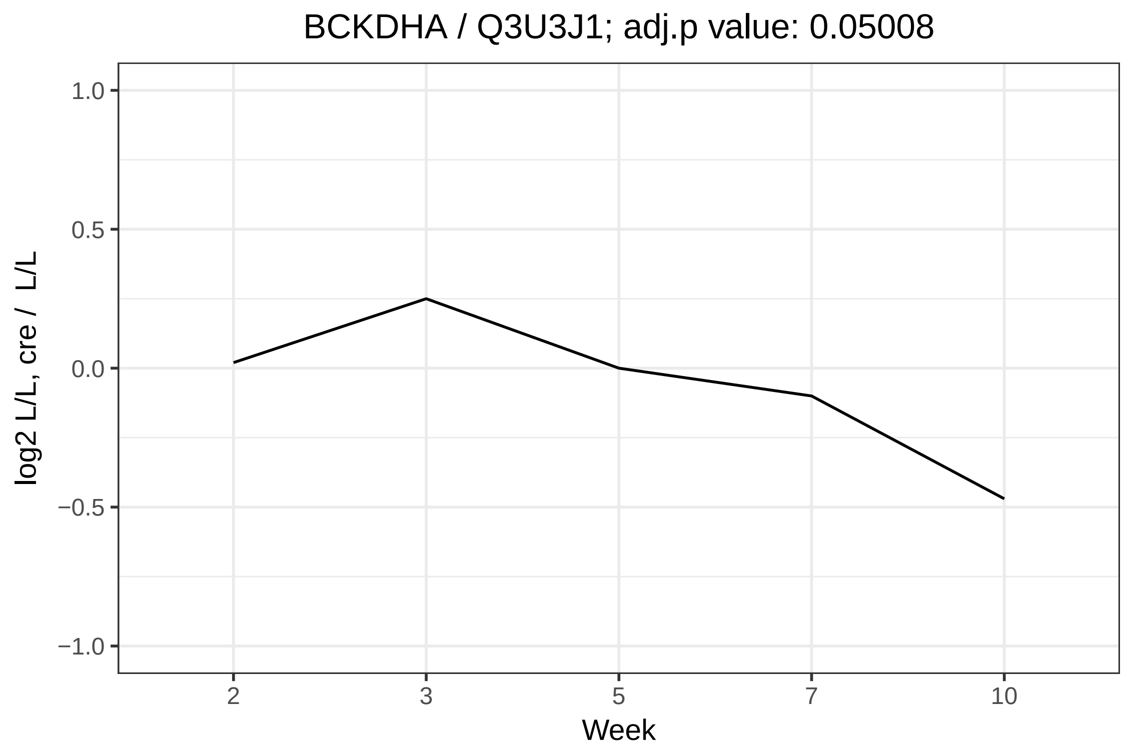
BCKDHA / Q3U3J1; adj.p value: 0.05008
1.0
0.5
0.0
−0.5
−1.0
log2 L/L, cre / L/L
2
3
5
7
10
Week

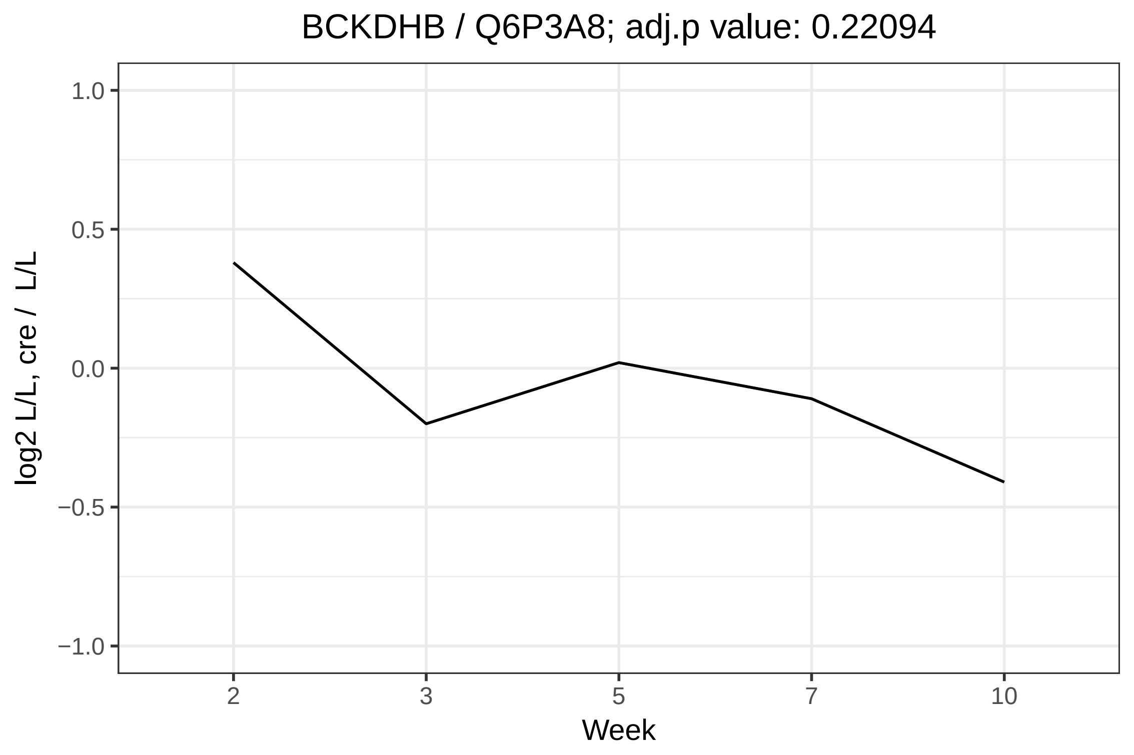
BCKDHB / Q6P3A8; adj.p value: 0.22094
log2 L/L, cre / L/L
1.0
0.5
0.0
−0.5
−1.0
2
3
5
7
10
Week

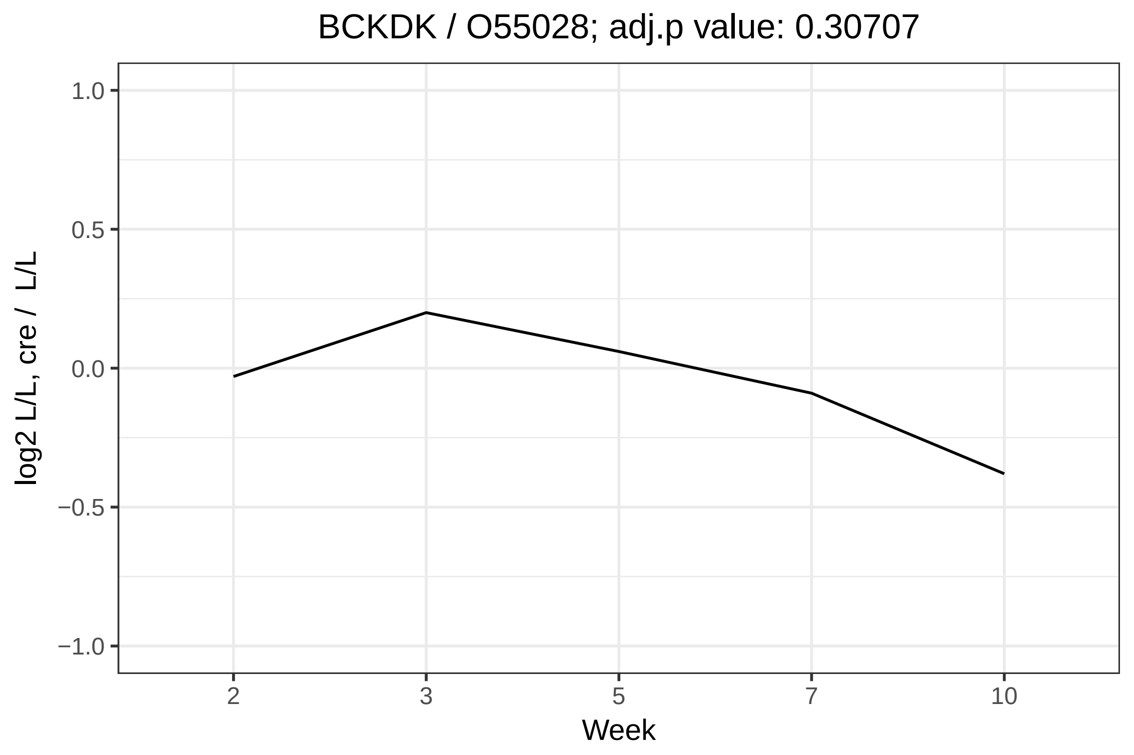
BCKDK / O55028; adj.p value: 0.30707
1.0
0.5
0.0
−0.5
−1.0
log2 L/L, cre / L/L
2
3
5
7
10
Week

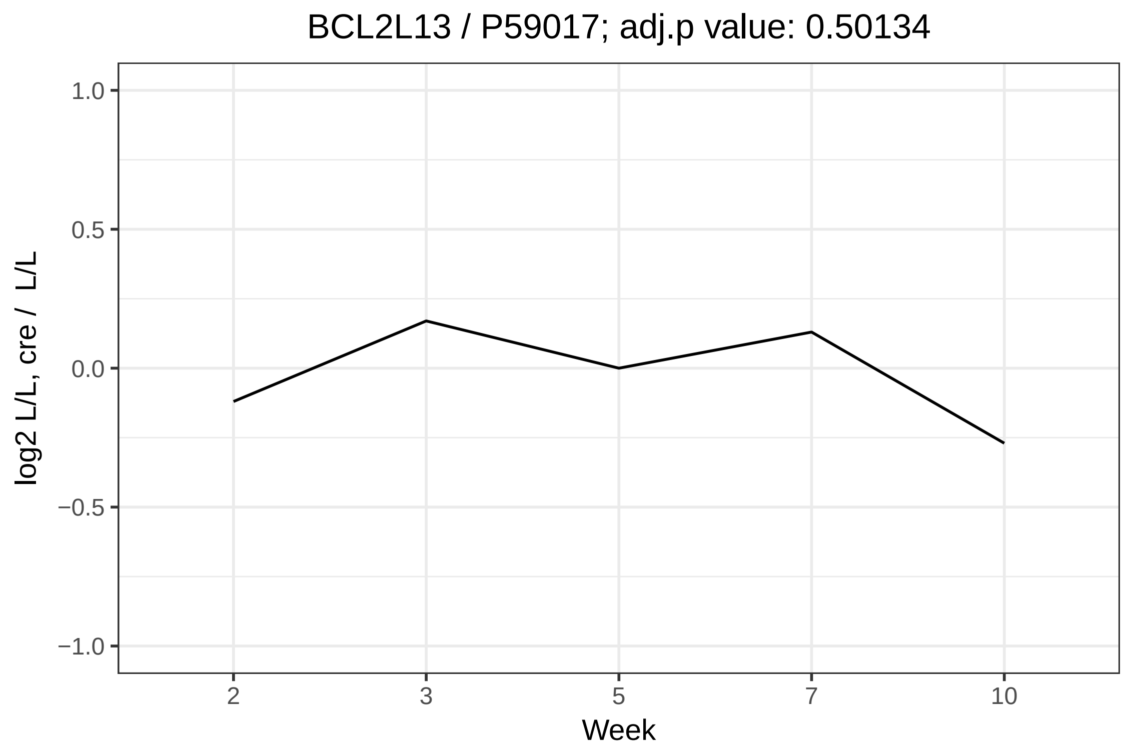
BCL2L13 / P59017; adj.p value: 0.50134
1.0
0.5
0.0
−0.5
−1.0
log2 L/L, cre / L/L
2
3
5
7
10
Week

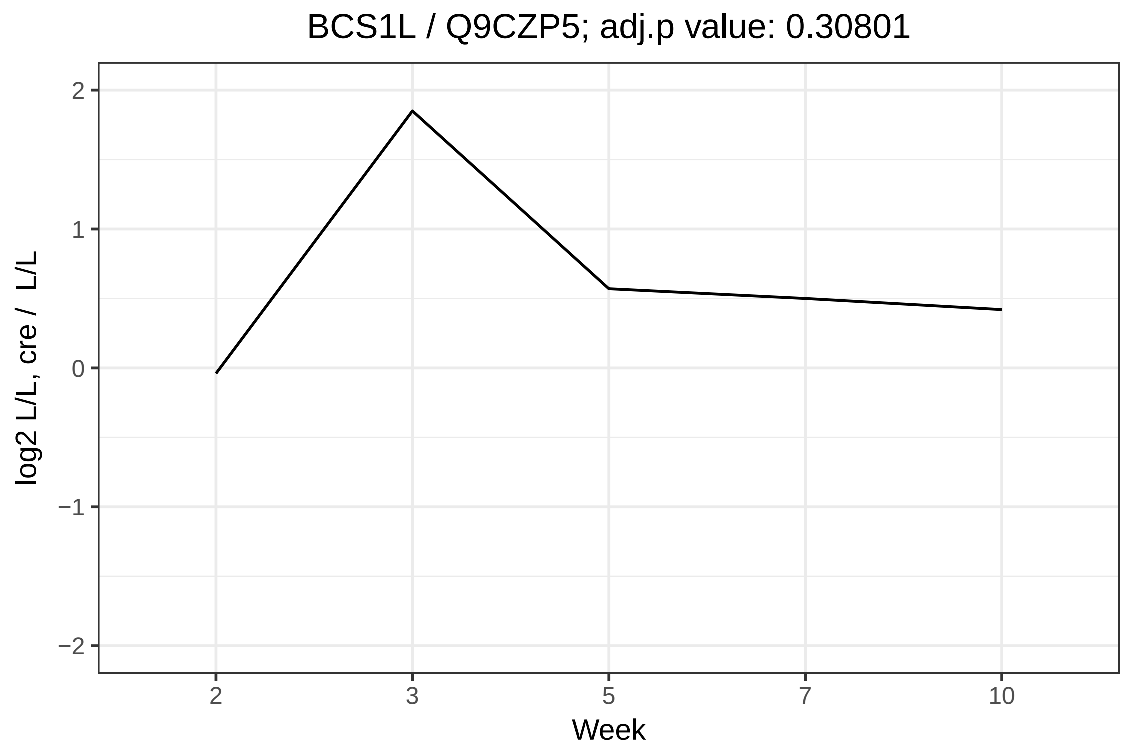
BCS1L / Q9CZP5; adj.p value: 0.30801
log2 L/L, cre / L/L
2
1
0
−1
−2
2
3
5
7
10
Week

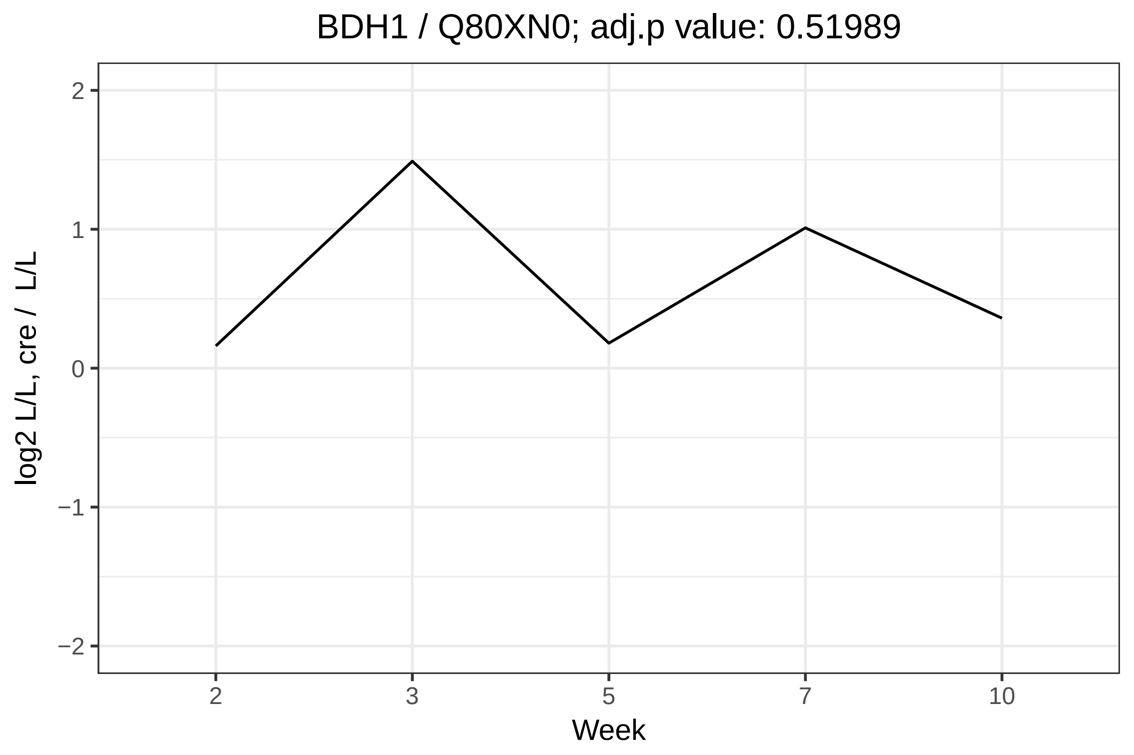
BDH1 / Q80XN0; adj.p value: 0.51989
log2 L/L, cre / L/L
2
1
0
−1
−2
2
3
5
7
10
Week

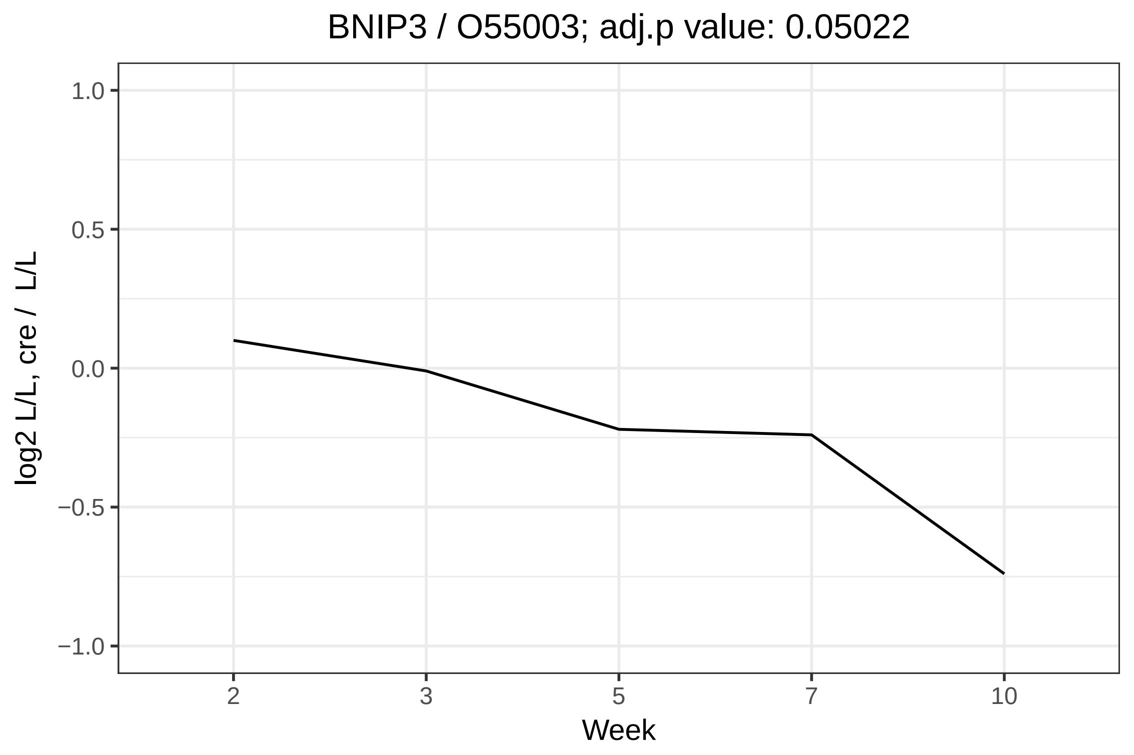

BNIP3 / O55003; adj.p value: 0.05022
log2 L/L, cre / L/L
1.0
0.5
0.0
−0.5
−1.0
2
3
5
7
10
Week

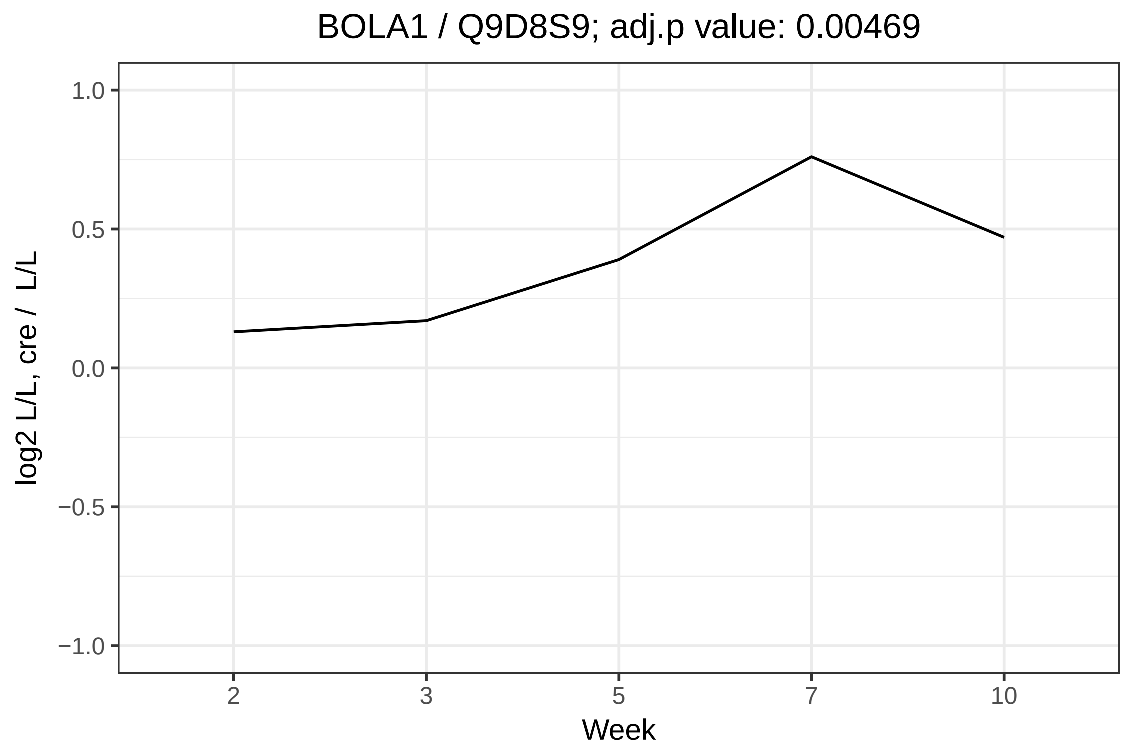
BOLA1 / Q9D8S9; adj.p value: 0.00469
1.0
0.5
0.0
−0.5
−1.0
log2 L/L, cre / L/L
2
3
5
7
10
Week

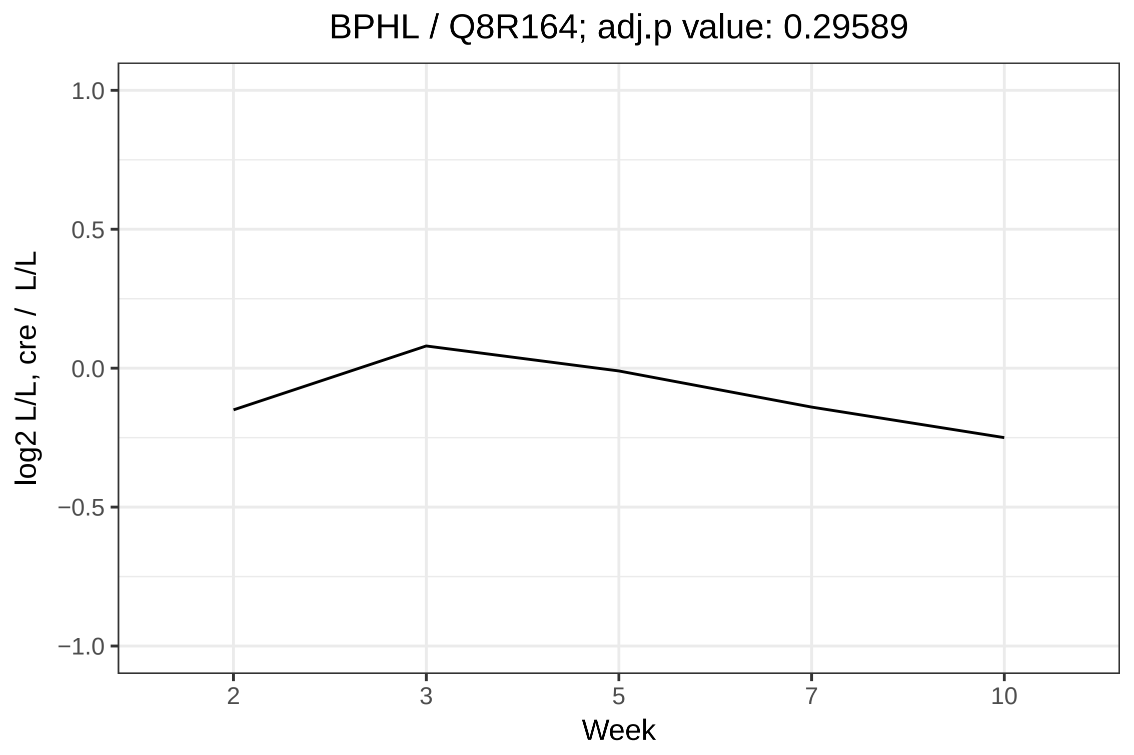
BPHL / Q8R164; adj.p value: 0.29589
log2 L/L, cre / L/L
1.0
0.5
0.0
−0.5
−1.0
2
3
5
7
10
Week

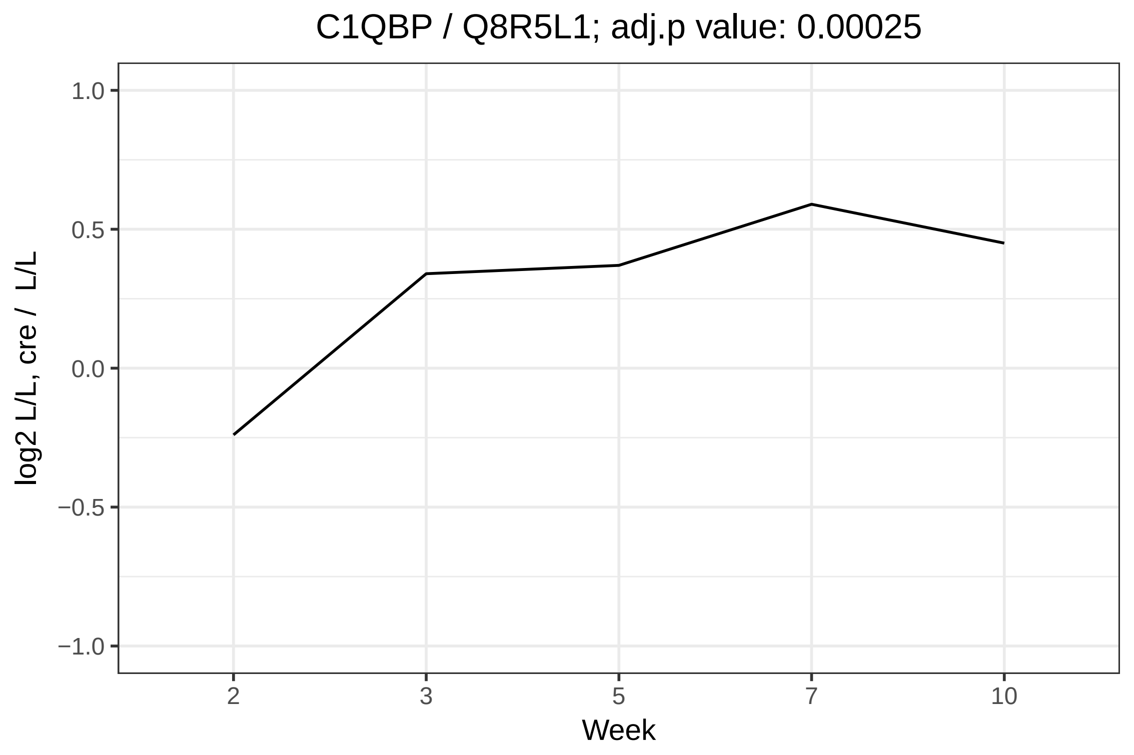
C1QBP / Q8R5L1; adj.p value: 0.00025
1.0
0.5
0.0
−0.5
−1.0
log2 L/L, cre / L/L
2
3
5
7
10
Week

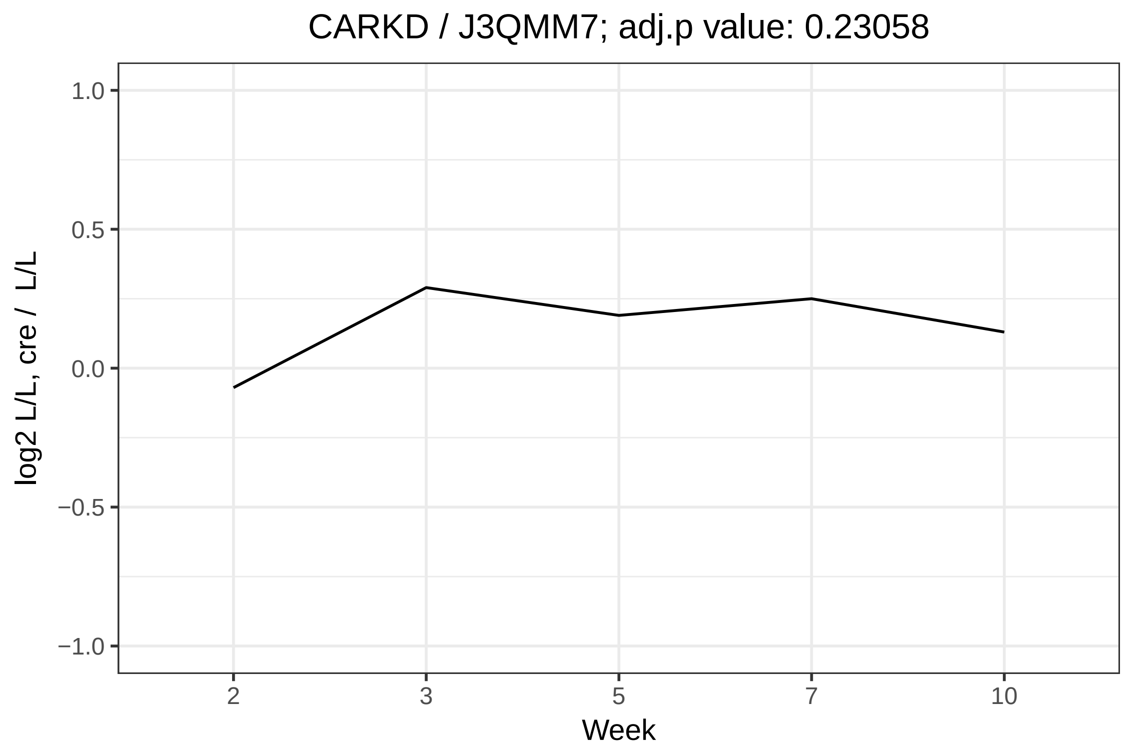

CARKD / J3QMM7; adj.p value: 0.23058
1.0
0.5
0.0
−0.5
−1.0
log2 L/L, cre / L/L
2
3
5
7
10
Week

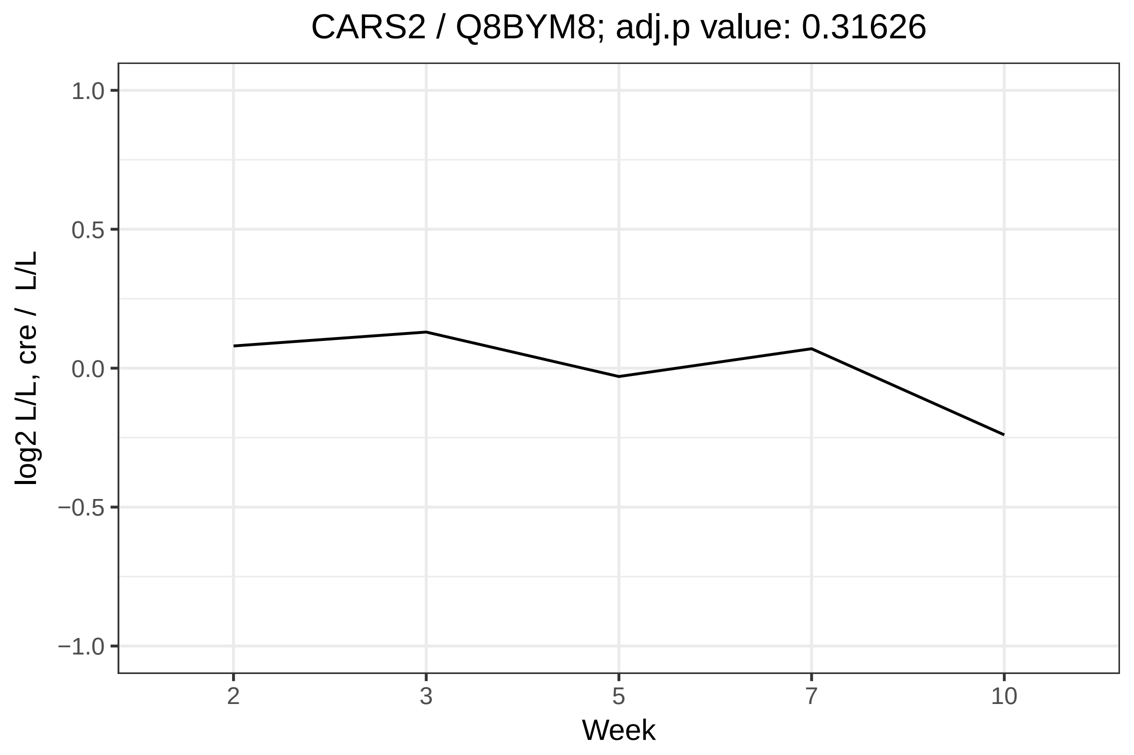

CARS2 / Q8BYM8; adj.p value: 0.31626
log2 L/L, cre / L/L
1.0
0.5
0.0
−0.5
−1.0
2
3
5
7
10
Week

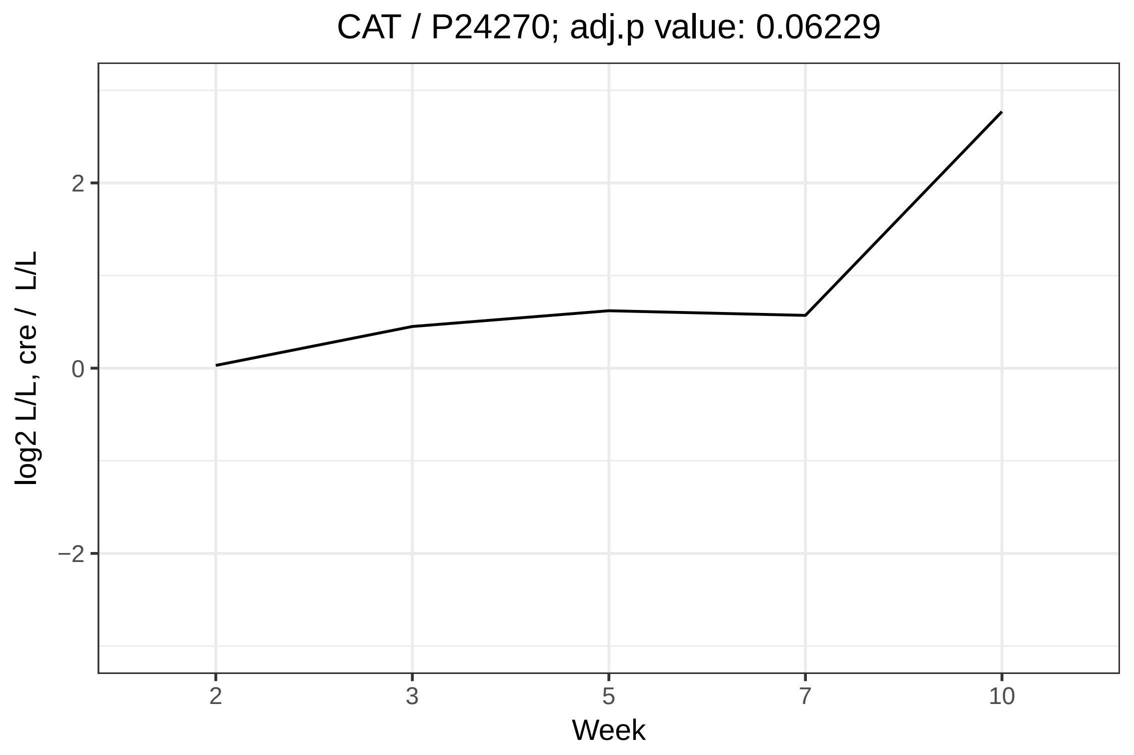

CAT / P24270; adj.p value: 0.06229
log2 L/L, cre / L/L
2
0
−2
2
3
5
7
10
Week

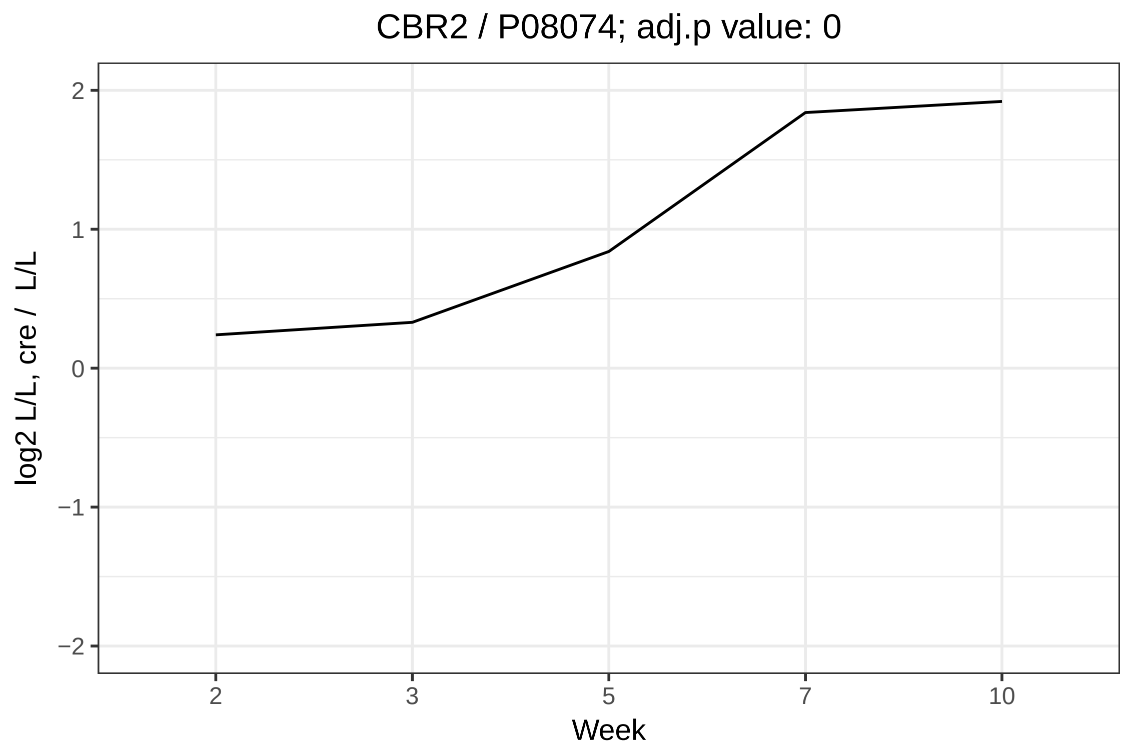
CBR2 / P08074; adj.p value: 0
log2 L/L, cre / L/L
2
1
0
−1
−2
2
3
5
7
10
Week

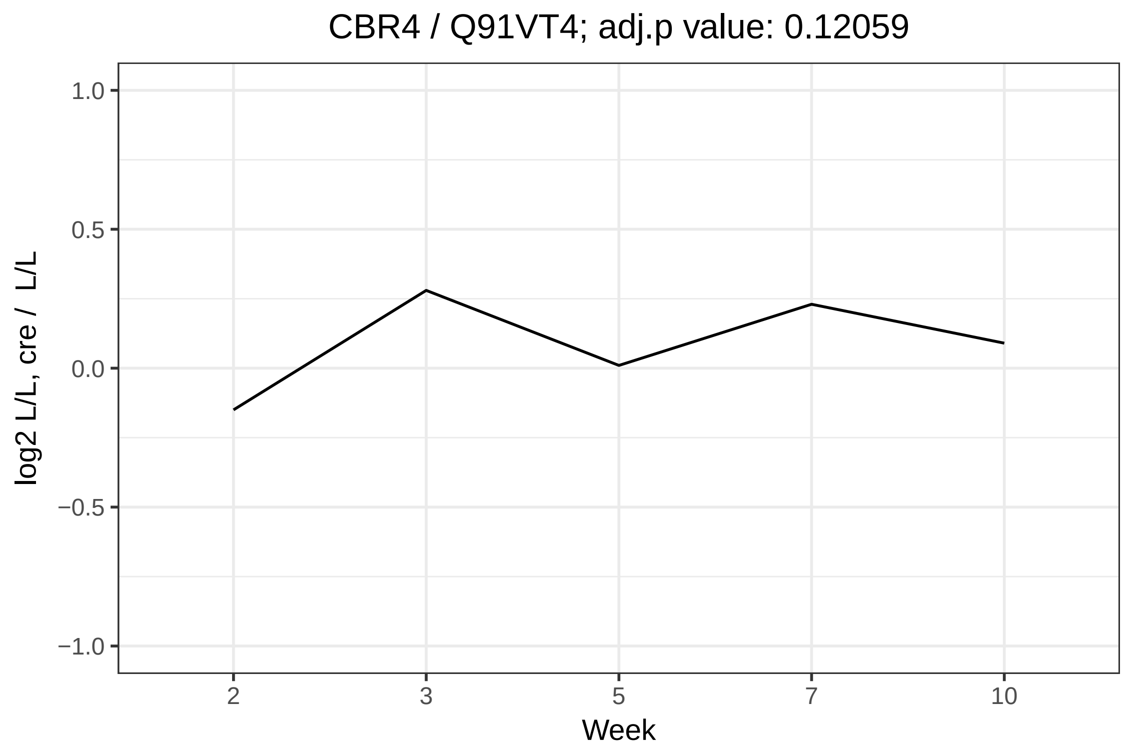

CBR4 / Q91VT4; adj.p value: 0.12059
1.0
0.5
0.0
−0.5
−1.0
log2 L/L, cre / L/L
2
3
5
7
10
Week

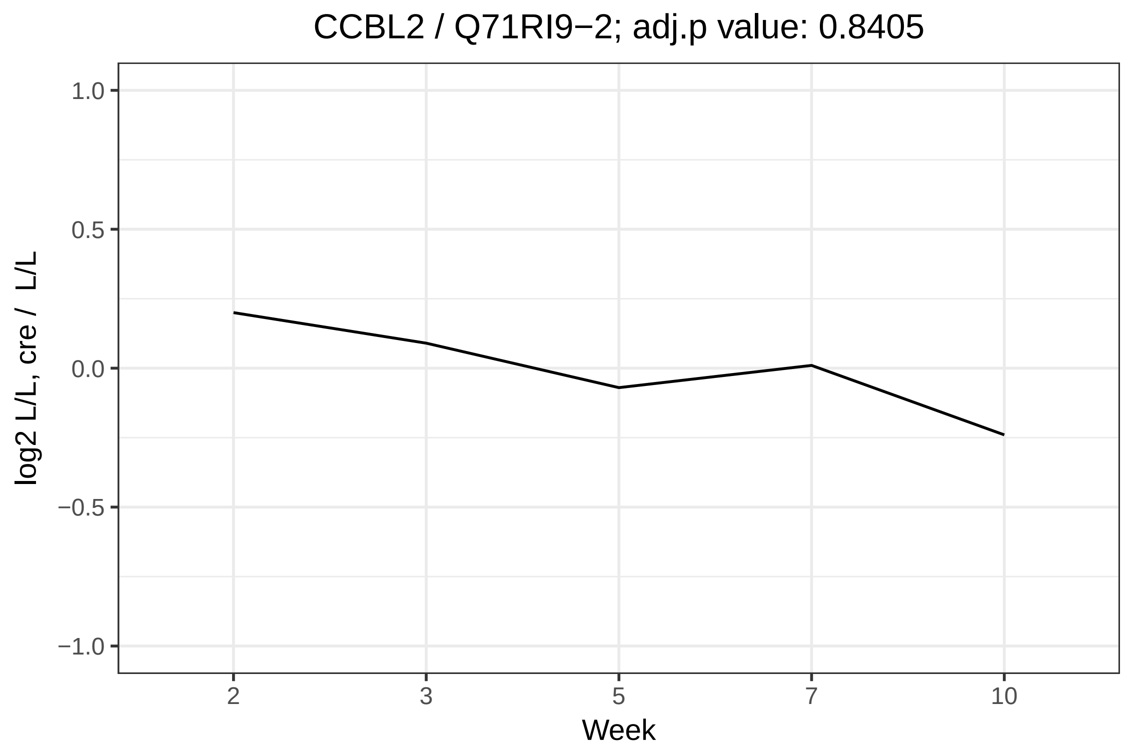
CCBL2 / Q71RI9−2; adj.p value: 0.8405
log2 L/L, cre / L/L
1.0
0.5
0.0
−0.5
−1.0
2
3
5
7
10
Week

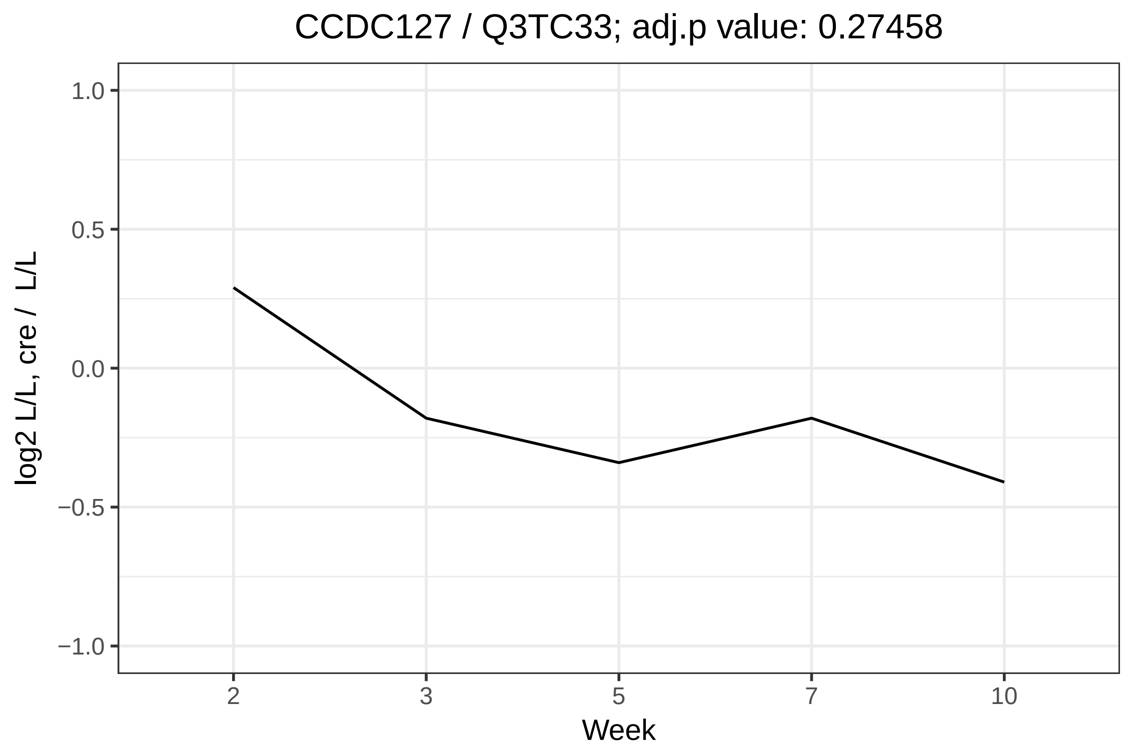
CCDC127 / Q3TC33; adj.p value: 0.27458
log2 L/L, cre / L/L
1.0
0.5
0.0
−0.5
−1.0
2
3
5
7
10
Week

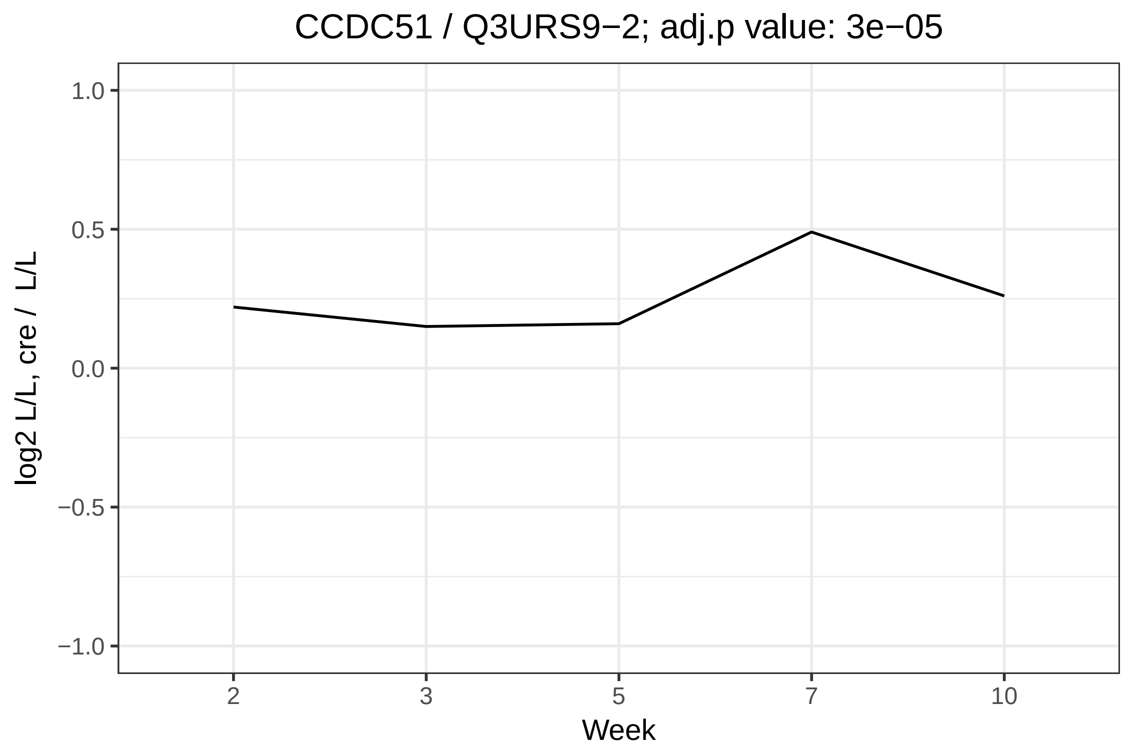

CCDC51 / Q3URS9−2; adj.p value: 3e−05
1.0
0.5
0.0
−0.5
−1.0
log2 L/L, cre / L/L
2
3
5
7
10
Week

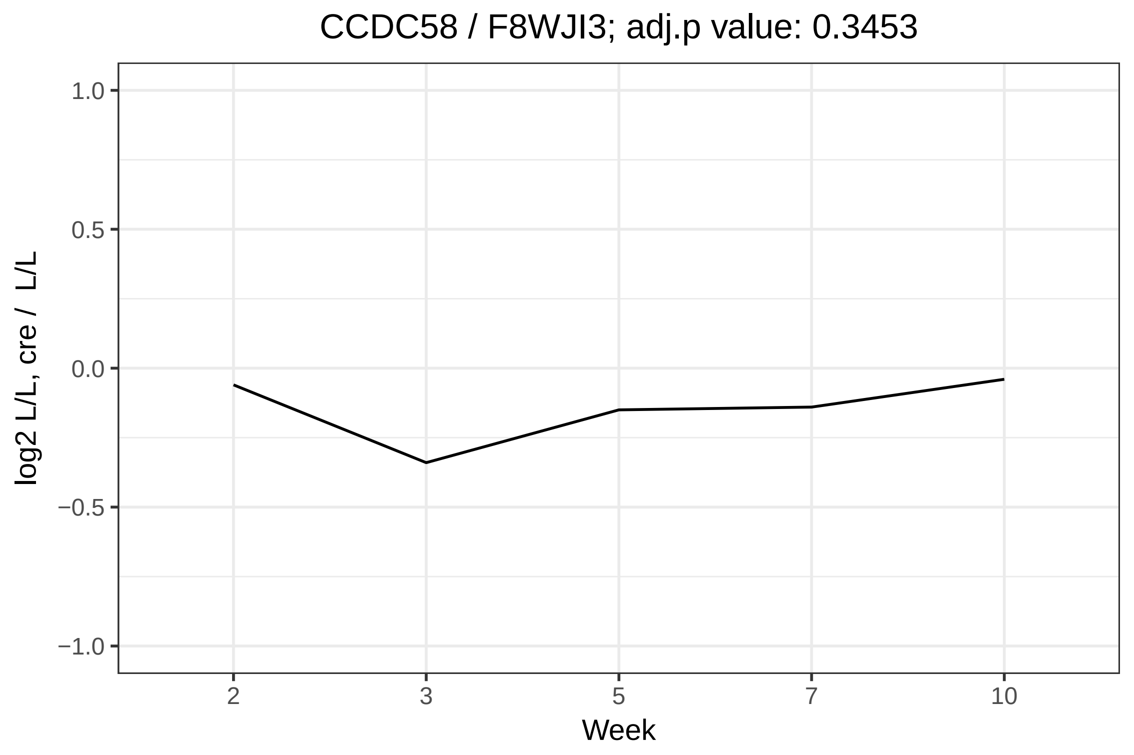

CCDC58 / F8WJI3; adj.p value: 0.3453
log2 L/L, cre / L/L
1.0
0.5
0.0
−0.5
−1.0
2
3
5
7
10
Week

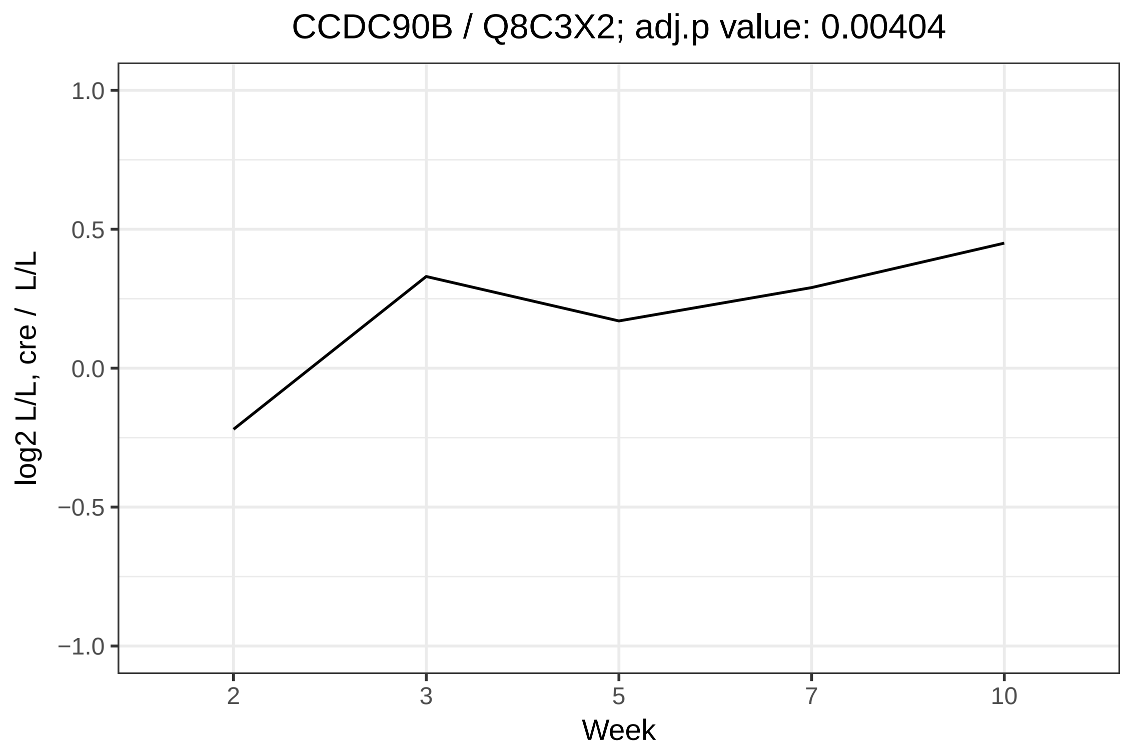

CCDC90B / Q8C3X2; adj.p value: 0.00404
1.0
0.5
0.0
−0.5
−1.0
log2 L/L, cre / L/L
2
3
5
7
10
Week

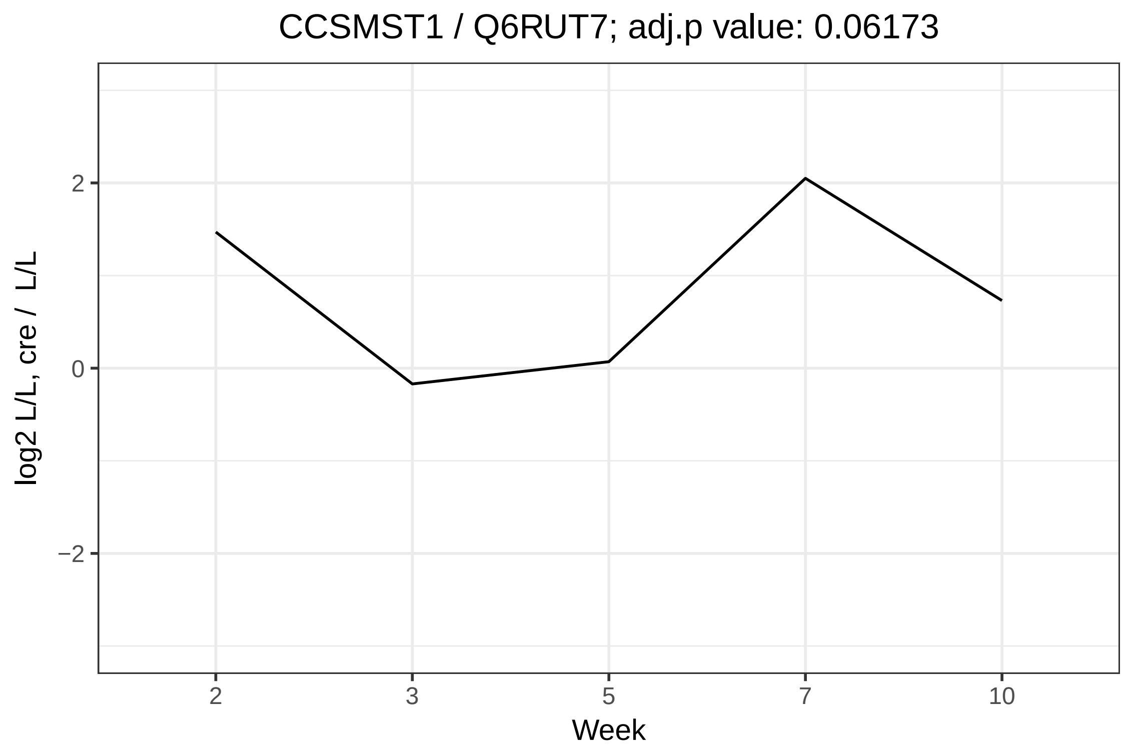

CCSMST1 / Q6RUT7; adj.p value: 0.06173
log2 L/L, cre / L/L
2
0
−2
2
3
5
7
10
Week

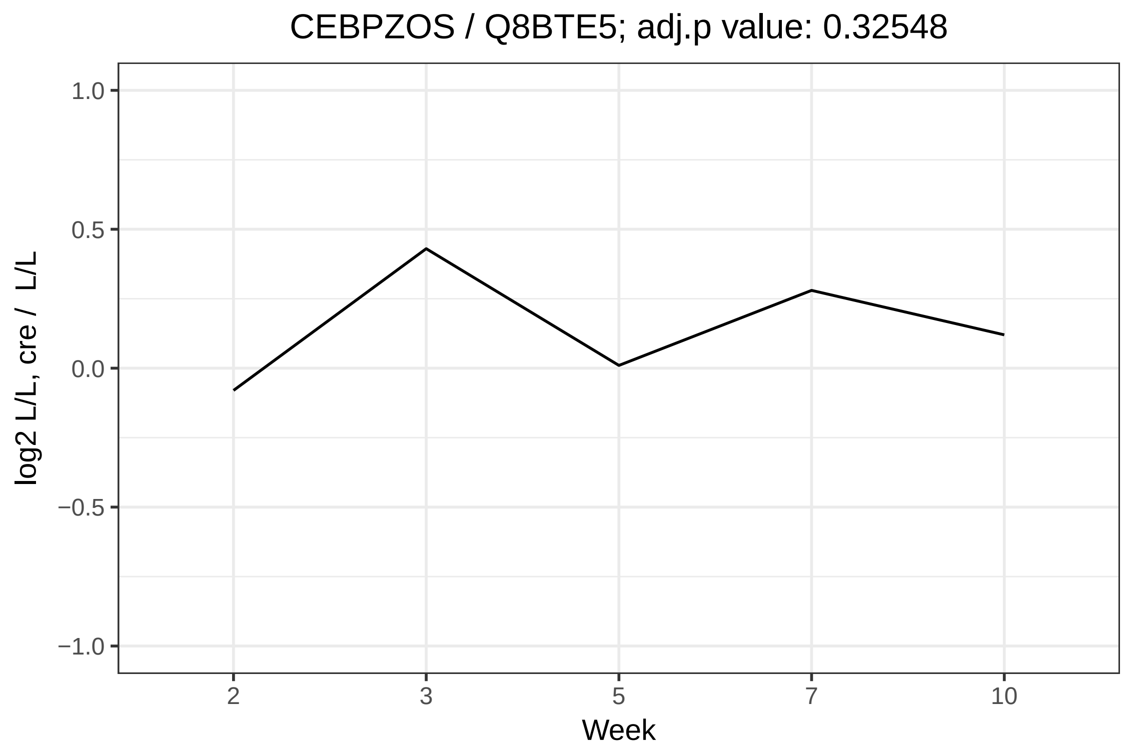

CEBPZOS / Q8BTE5; adj.p value: 0.32548
log2 L/L, cre / L/L
1.0
0.5
0.0
−0.5
−1.0
2
3
5
7
10
Week

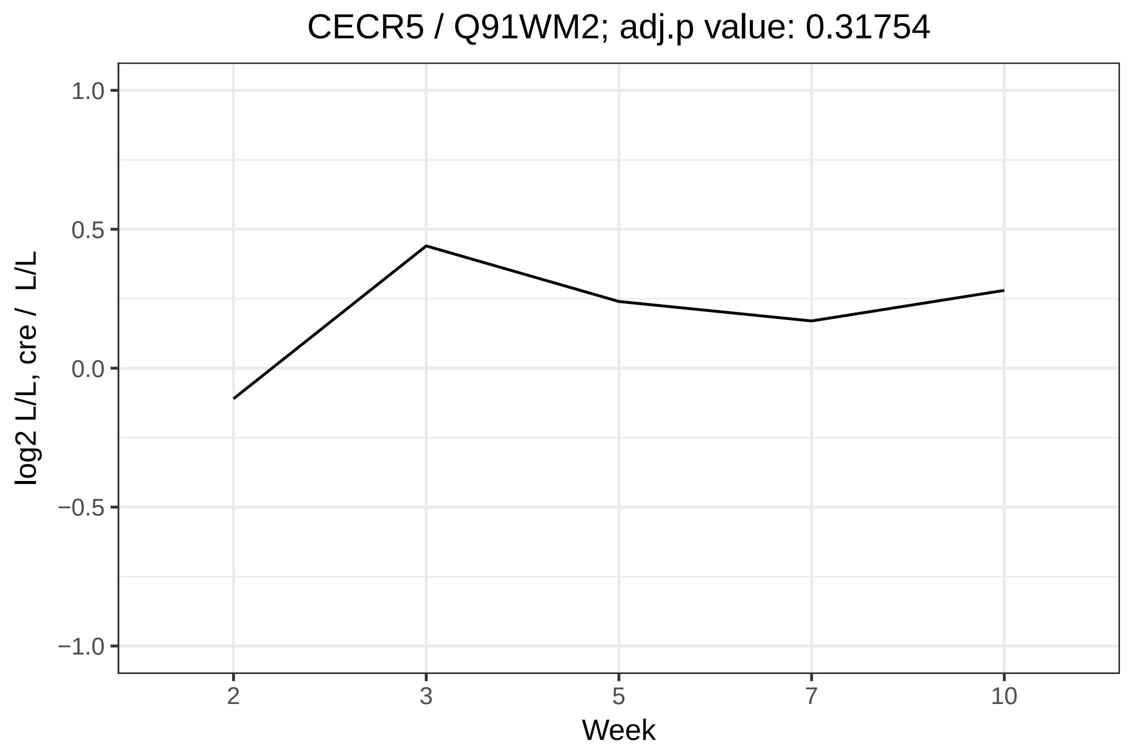

CECR5 / Q91WM2; adj.p value: 0.31754
1.0
0.5
0.0
−0.5
−1.0
log2 L/L, cre / L/L
2
3
5
7
10
Week

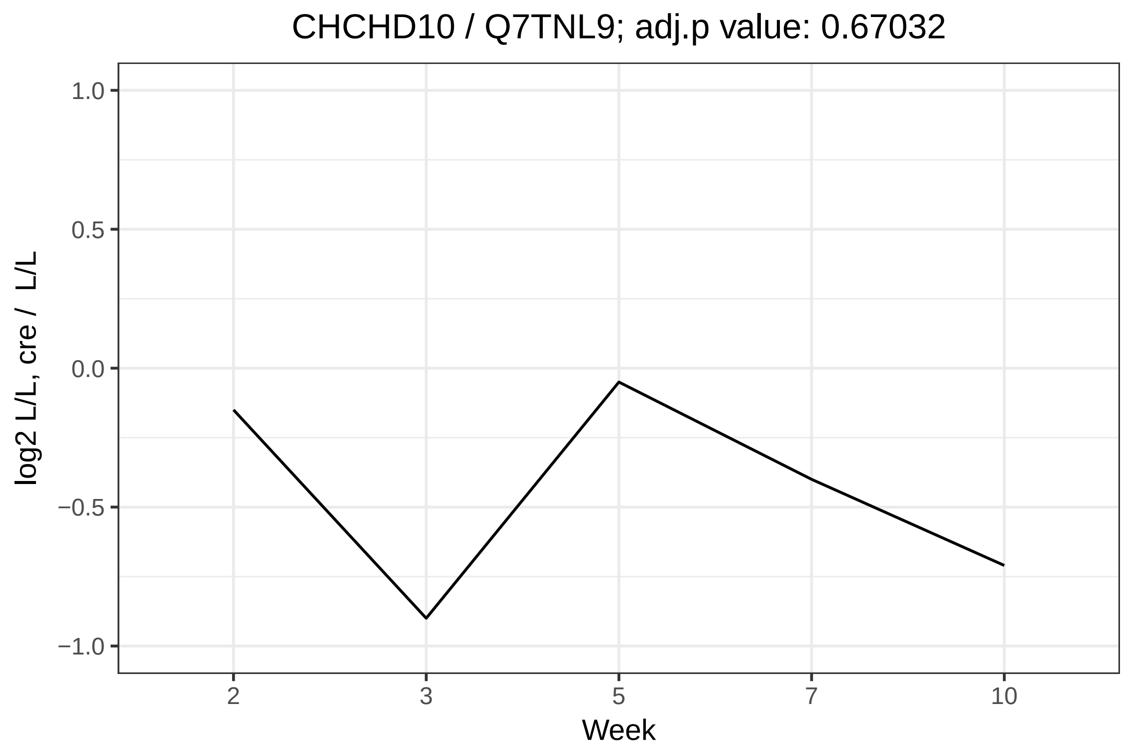
CHCHD10 / Q7TNL9; adj.p value: 0.67032
1.0
0.5
0.0
−0.5
−1.0
log2 L/L, cre / L/L
2
3
5
7
10
Week

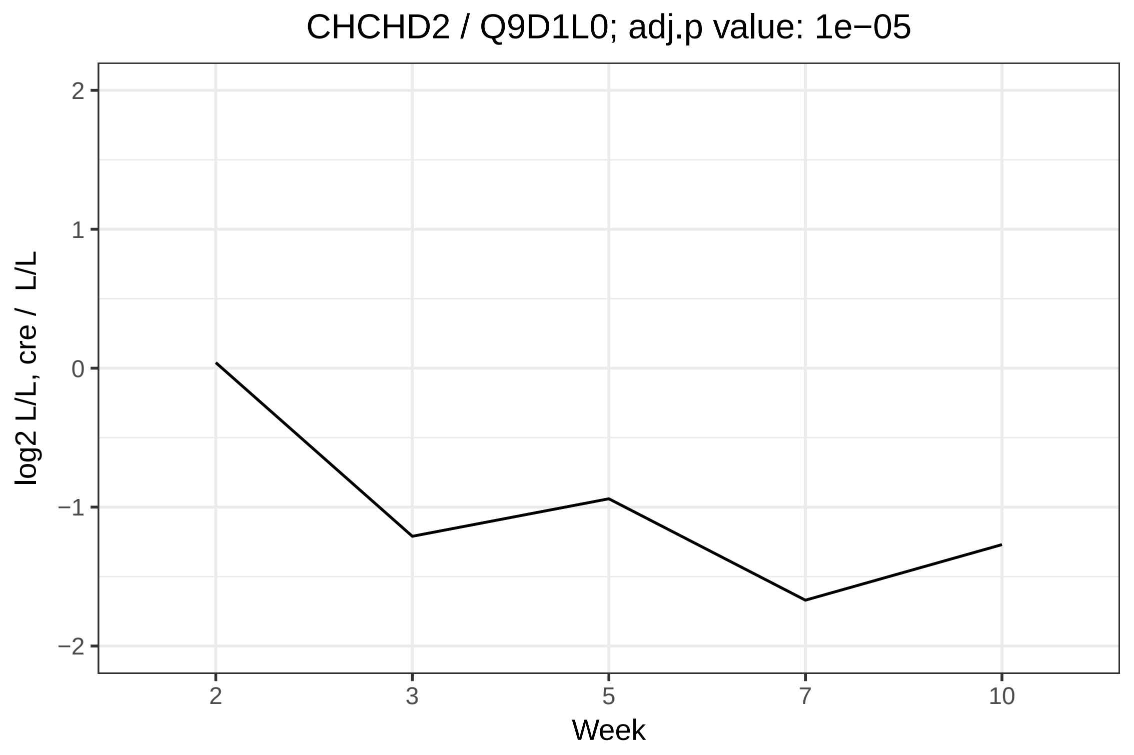
CHCHD2 / Q9D1L0; adj.p value: 1e−05
log2 L/L, cre / L/L
2
1
0
−1
−2
2
3
5
7
10
Week

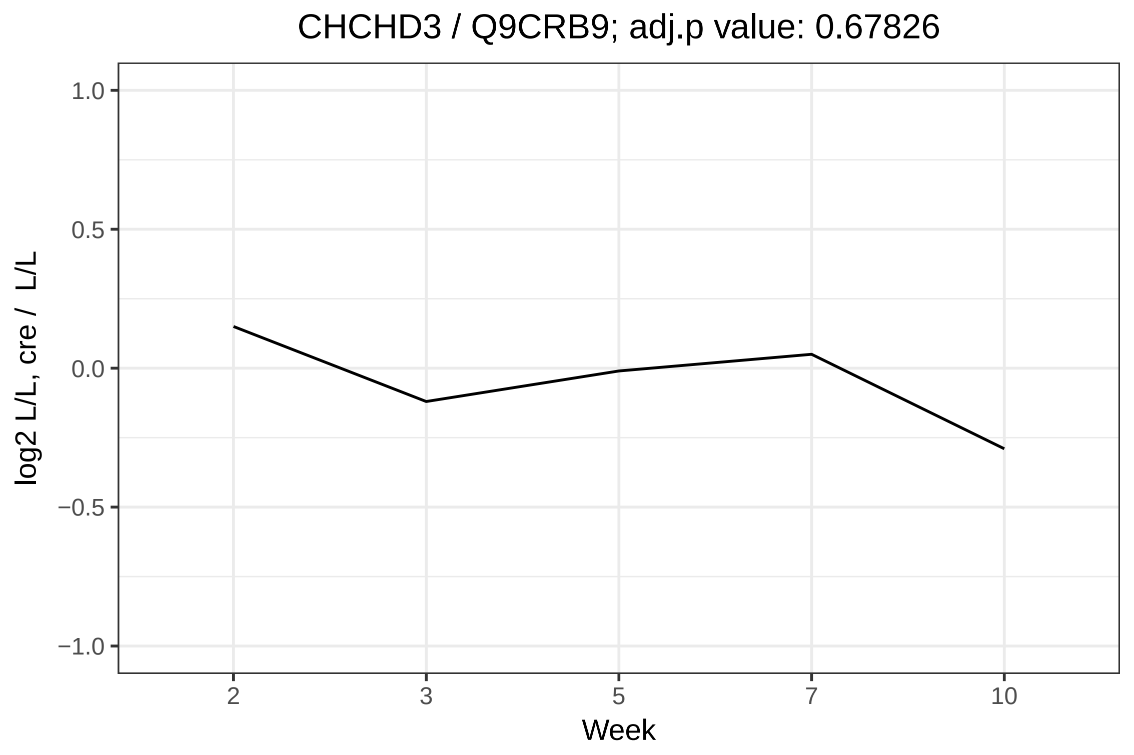
CHCHD3 / Q9CRB9; adj.p value: 0.67826
1.0
0.5
0.0
−0.5
−1.0
log2 L/L, cre / L/L
2
3
5
7
10
Week

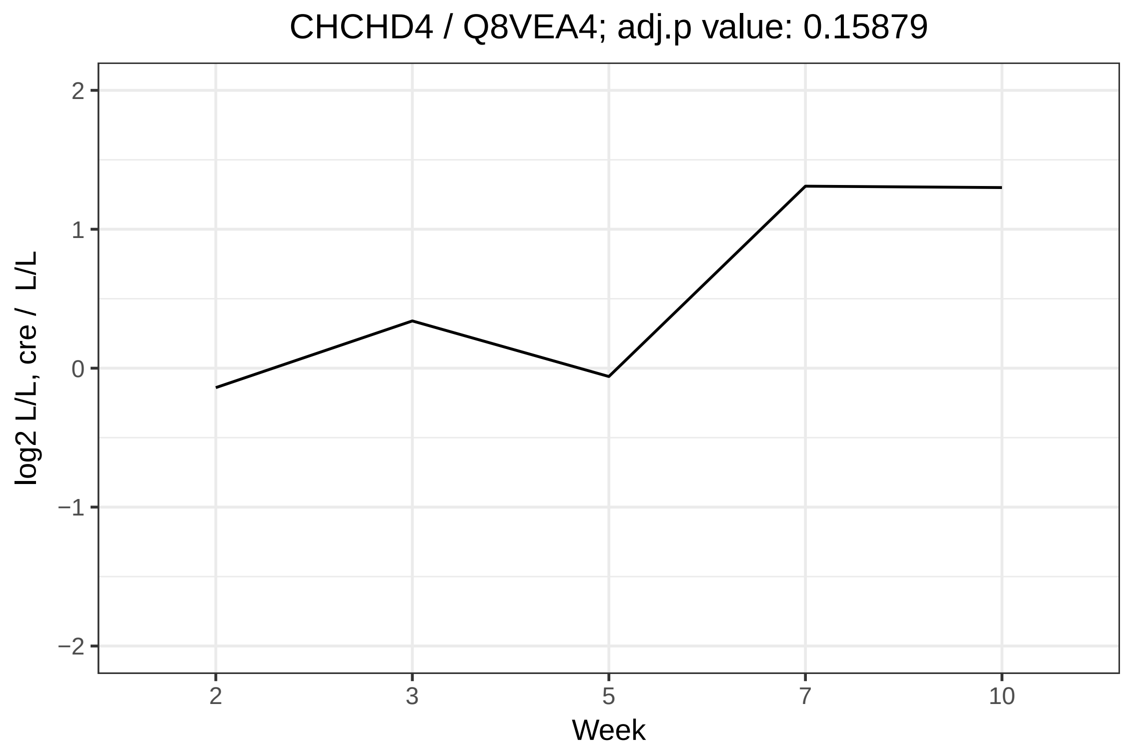

CHCHD4 / Q8VEA4; adj.p value: 0.15879
log2 L/L, cre / L/L
2
1
0
−1
−2
2
3
5
7
10
Week

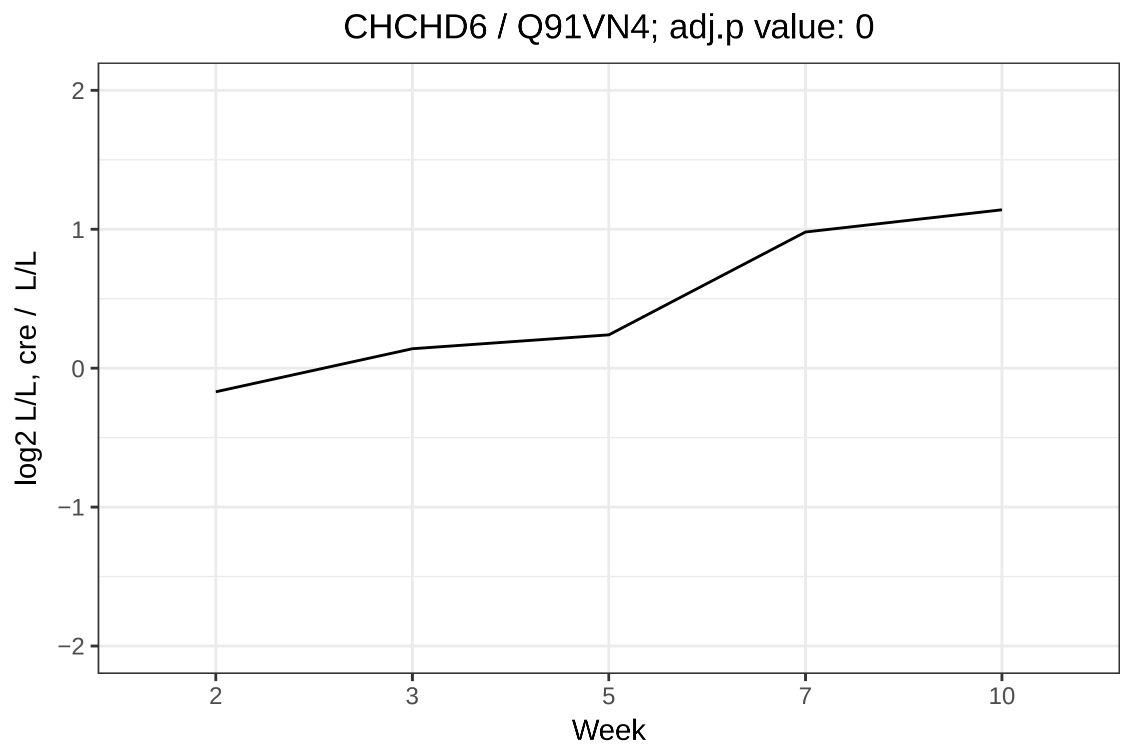

CHCHD6 / Q91VN4; adj.p value: 0
log2 L/L, cre / L/L
2
1
0
−1
−2
2
3
5
7
10
Week

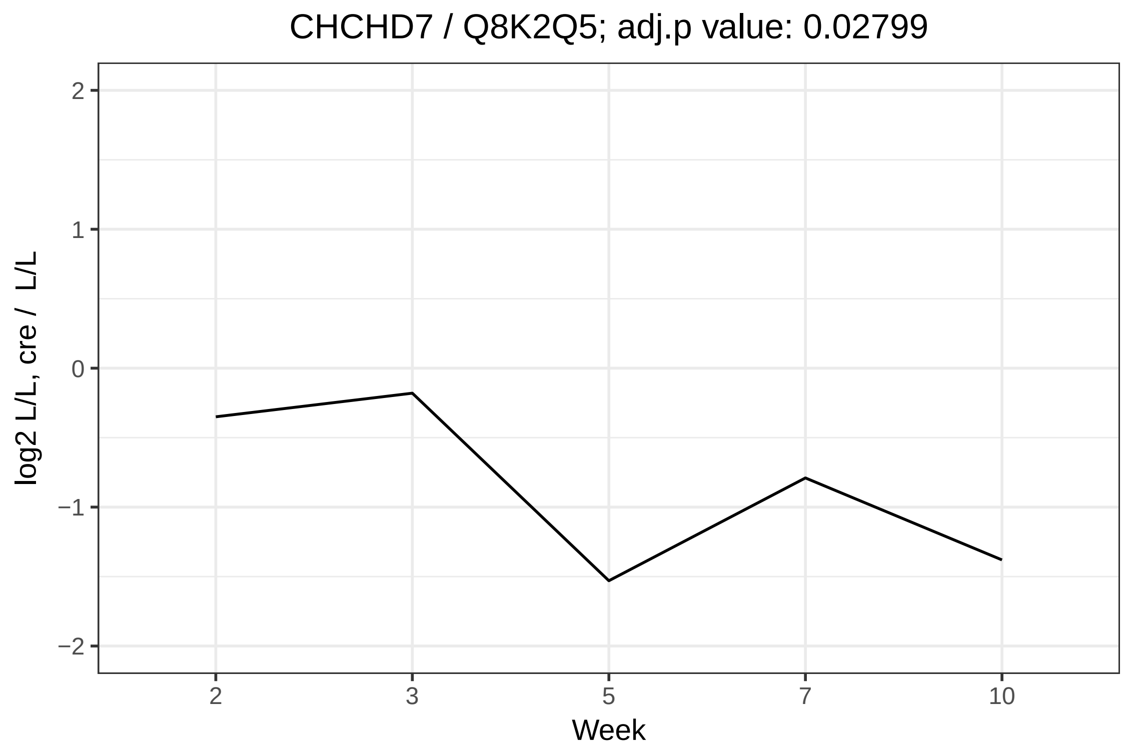
CHCHD7 / Q8K2Q5; adj.p value: 0.02799
log2 L/L, cre / L/L
2
1
0
−1
−2
2
3
5
7
10
Week

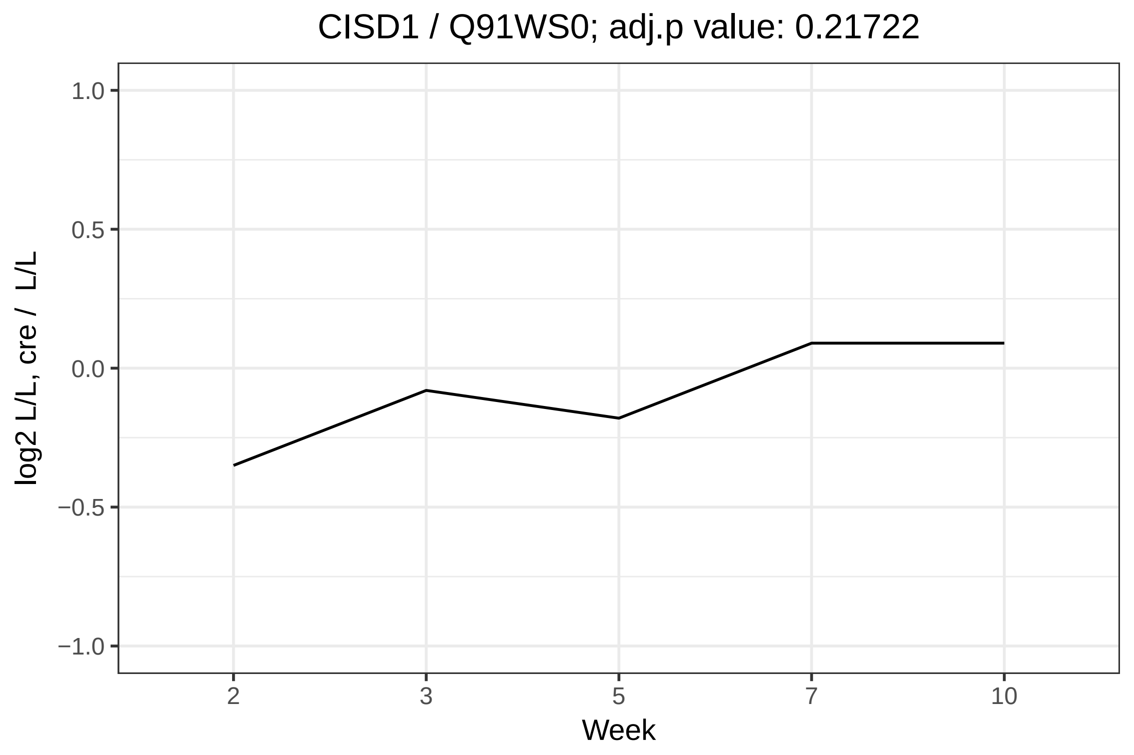

CISD1 / Q91WS0; adj.p value: 0.21722
log2 L/L, cre / L/L
1.0
0.5
0.0
−0.5
−1.0
2
3
5
7
10
Week

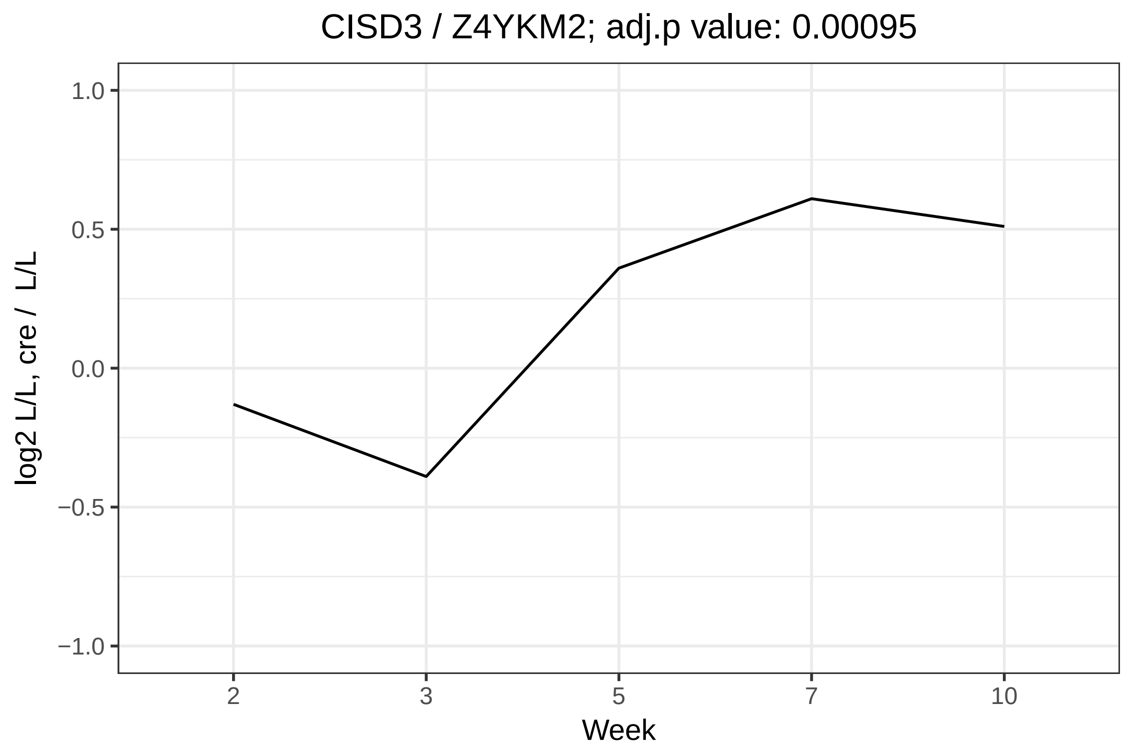

CISD3 / Z4YKM2; adj.p value: 0.00095
1.0
0.5
0.0
−0.5
−1.0
log2 L/L, cre / L/L
2
3
5
7
10
Week

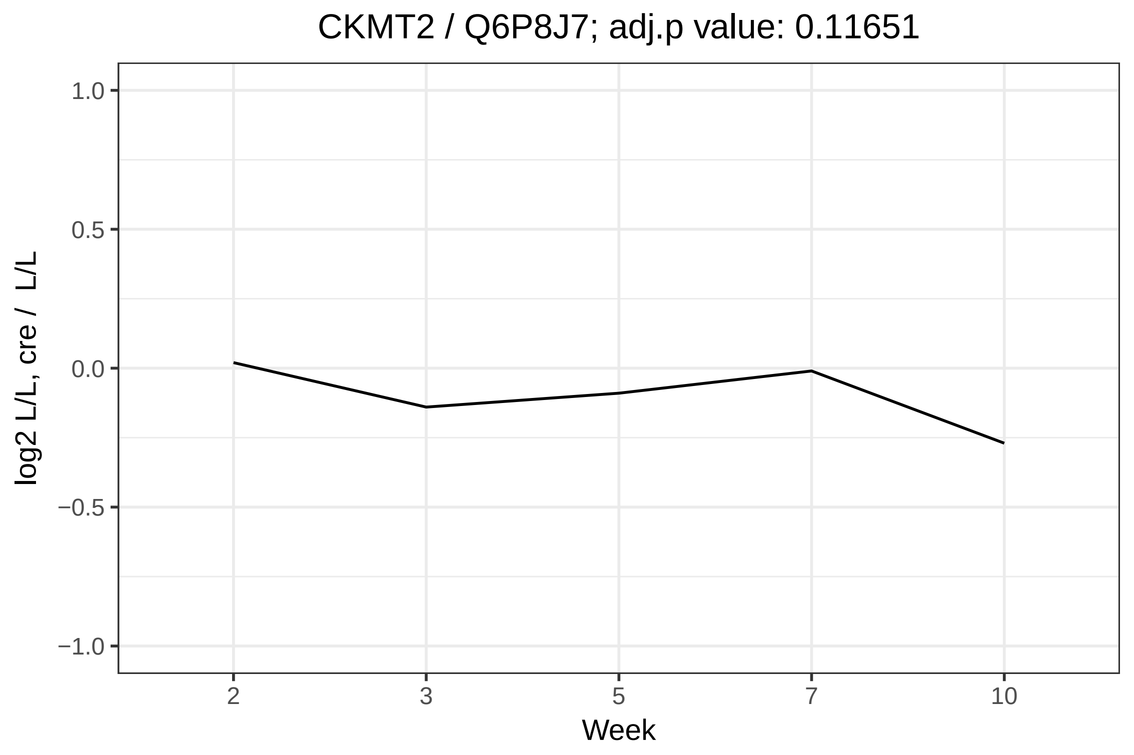

CKMT2 / Q6P8J7; adj.p value: 0.11651
1.0
0.5
0.0
−0.5
−1.0
log2 L/L, cre / L/L
2
3
5
7
10
Week

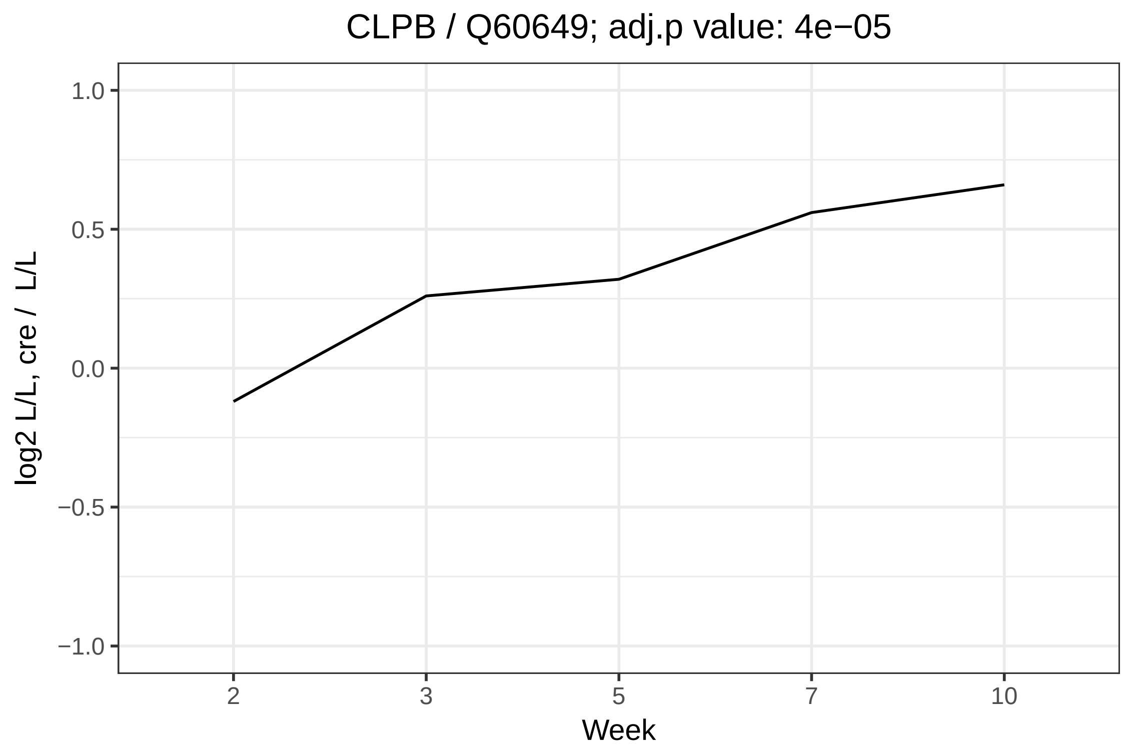
CLPB / Q60649; adj.p value: 4e−05
1.0
0.5
0.0
−0.5
−1.0
log2 L/L, cre / L/L
2
3
5
7
10
Week

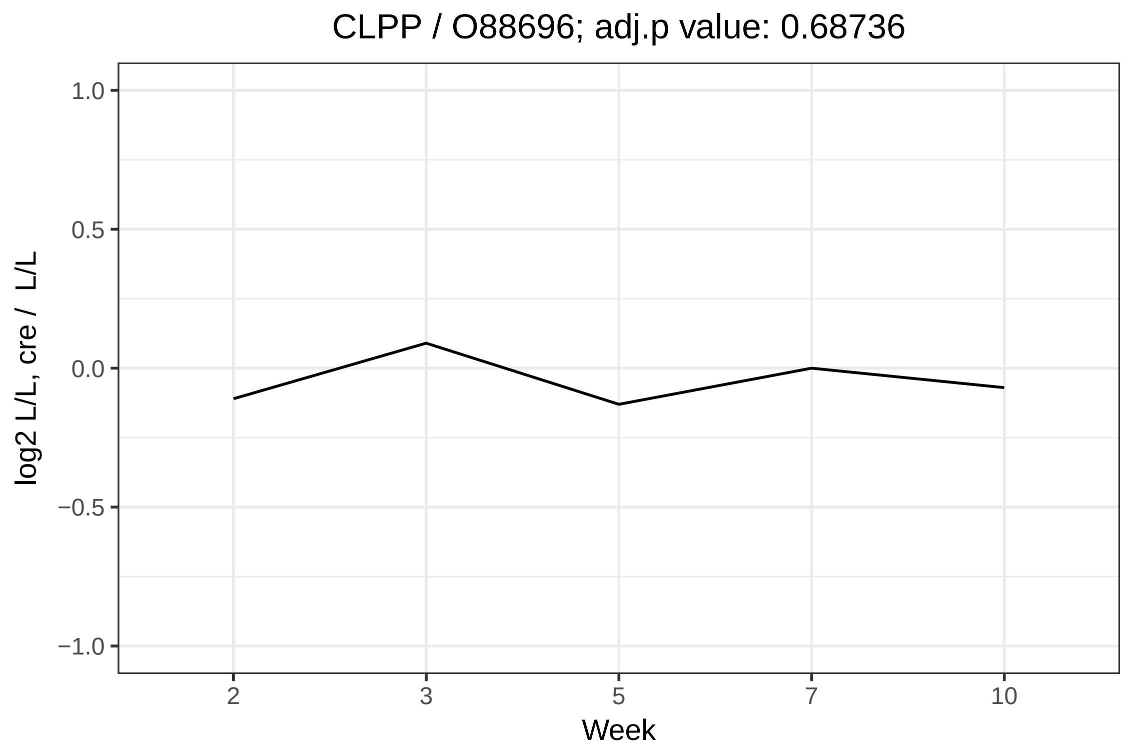
CLPP / O88696; adj.p value: 0.68736
log2 L/L, cre / L/L
1.0
0.5
0.0
−0.5
−1.0
2
3
5
7
10
Week

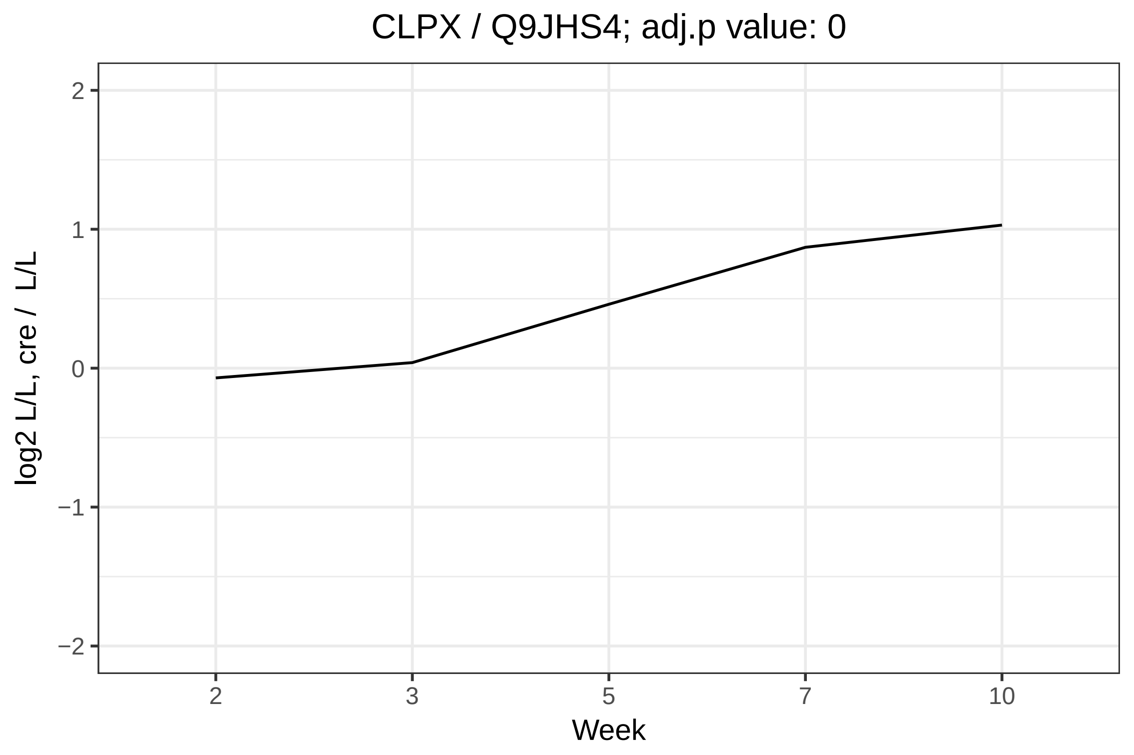

CLPX / Q9JHS4; adj.p value: 0
log2 L/L, cre / L/L
2
1
0
−1
−2
2
3
5
7
10
Week

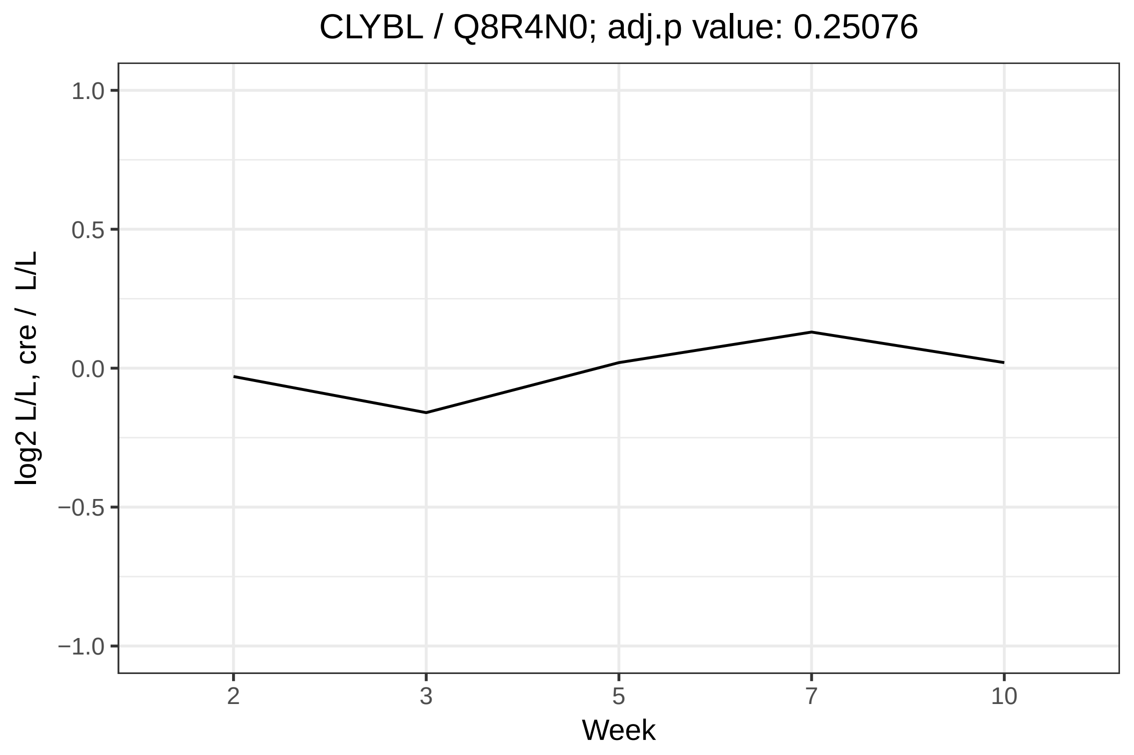
CLYBL / Q8R4N0; adj.p value: 0.25076
1.0
0.5
0.0
−0.5
−1.0
log2 L/L, cre / L/L
2
3
5
7
10
Week

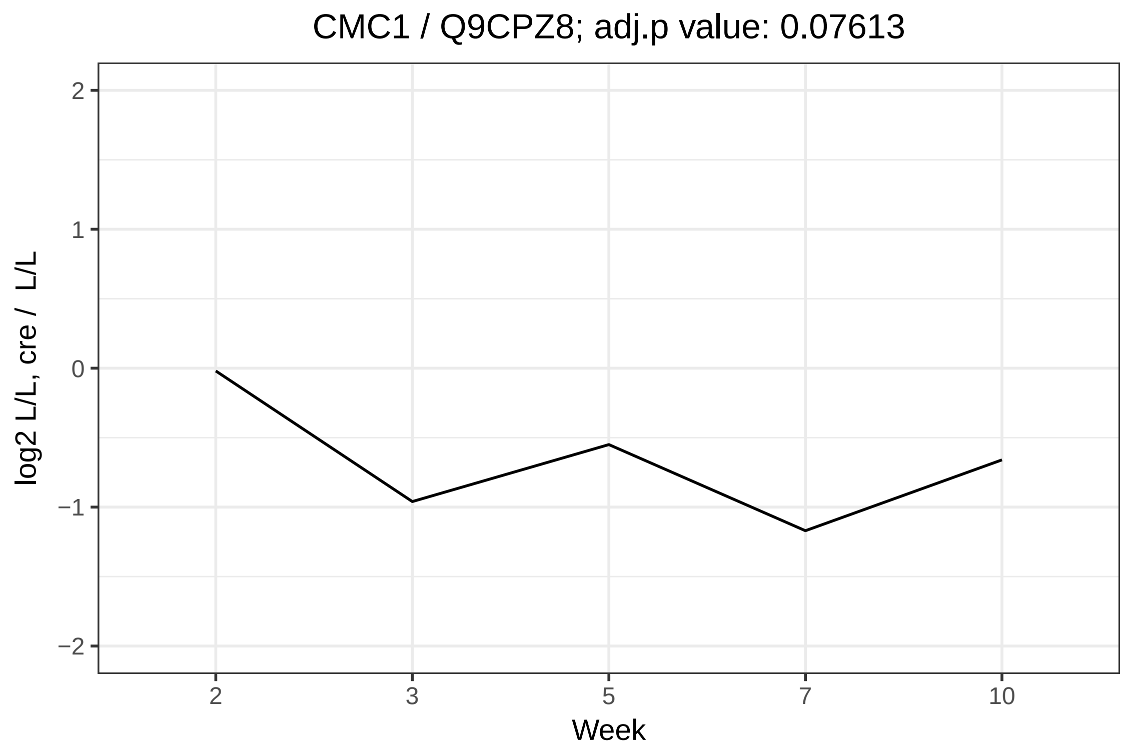
CMC1 / Q9CPZ8; adj.p value: 0.07613
log2 L/L, cre / L/L
2
1
0
−1
−2
2
3
5
7
10
Week

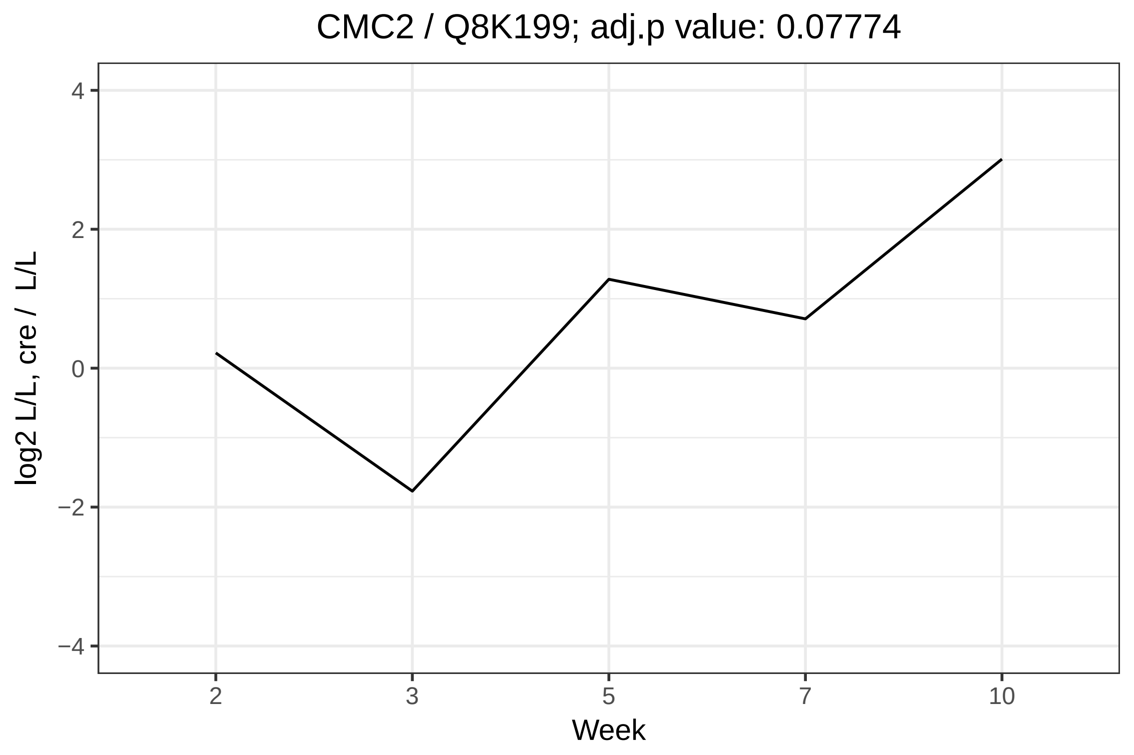

CMC2 / Q8K199; adj.p value: 0.07774
log2 L/L, cre / L/L
4
2
0
−2
−4
2
3
5
7
10
Week

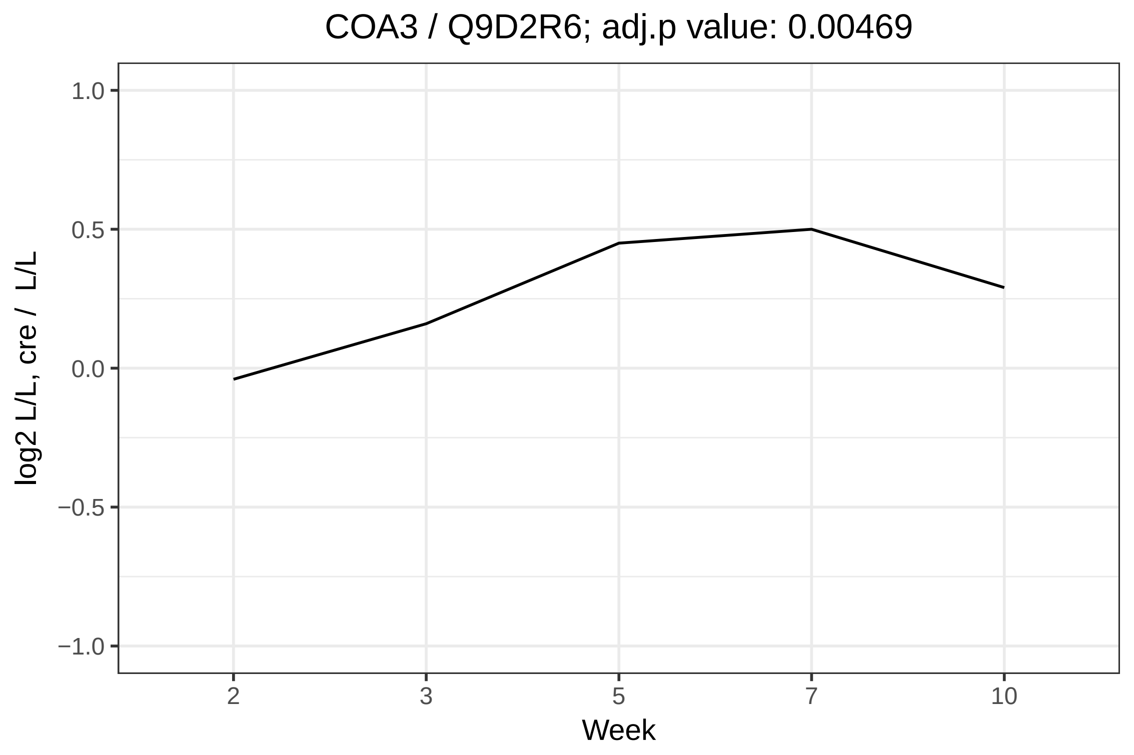

COA3 / Q9D2R6; adj.p value: 0.00469
log2 L/L, cre / L/L
1.0
0.5
0.0
−0.5
−1.0
2
3
5
7
10
Week

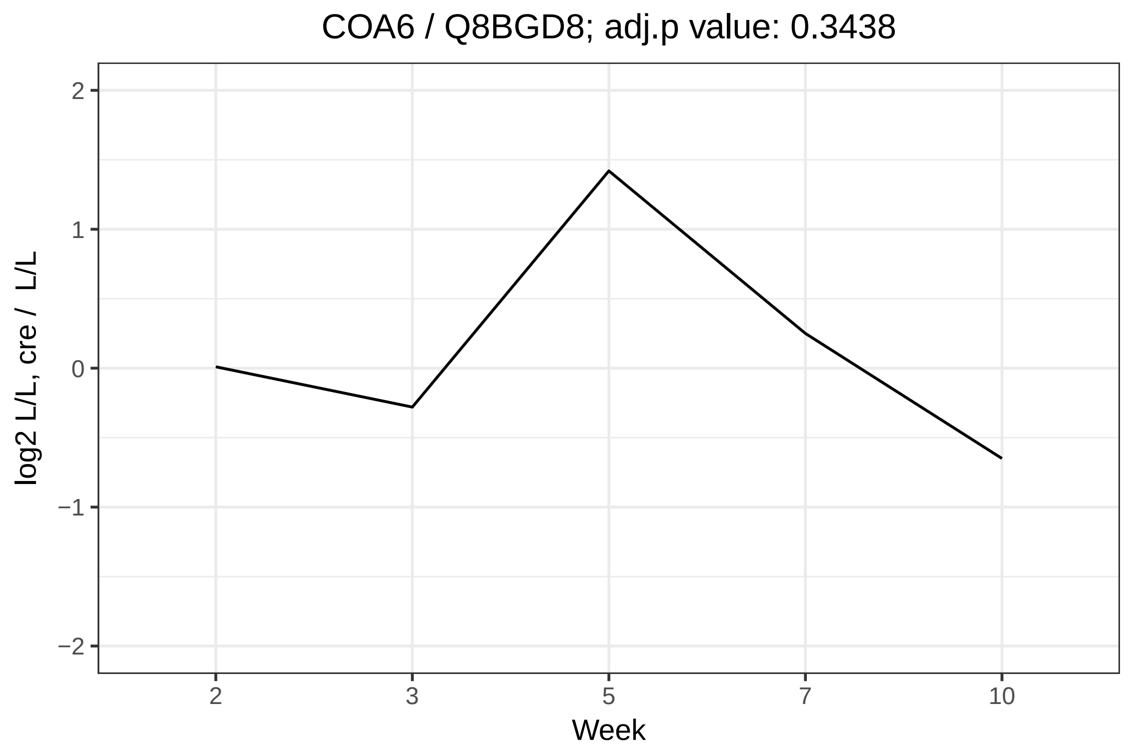
COA6 / Q8BGD8; adj.p value: 0.3438
log2 L/L, cre / L/L
2
1
0
−1
−2
2
3
5
7
10
Week

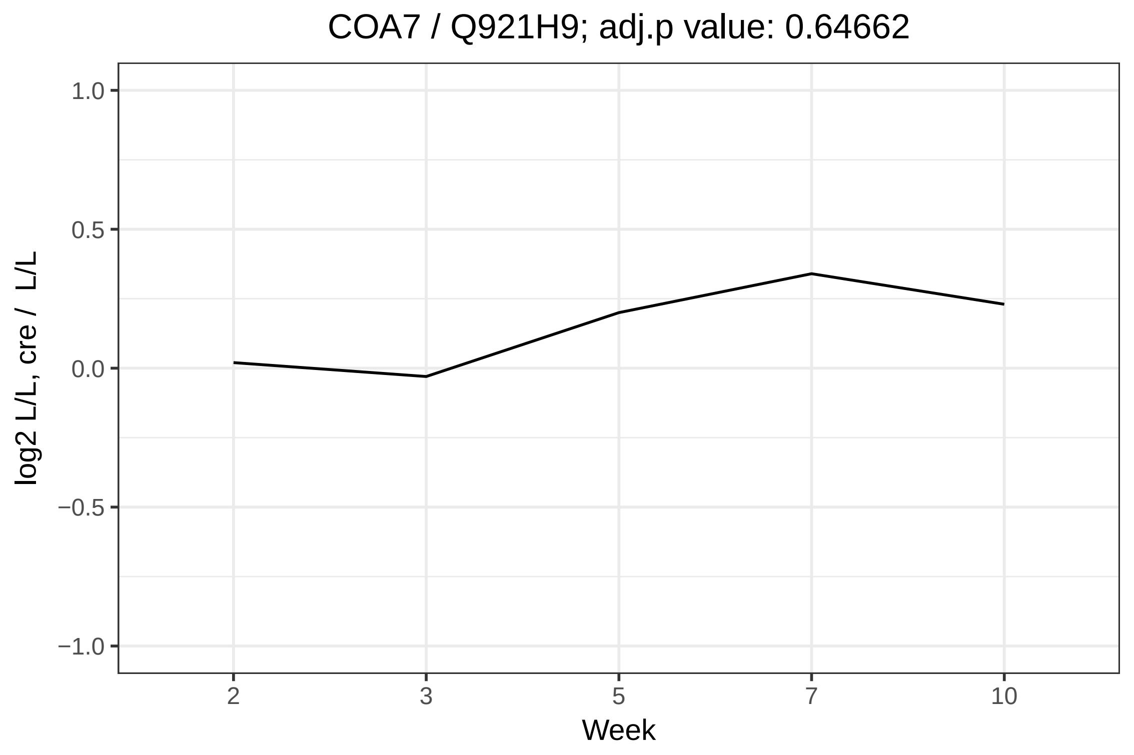

COA7 / Q921H9; adj.p value: 0.64662
log2 L/L, cre / L/L
1.0
0.5
0.0
−0.5
−1.0
2
3
5
7
10
Week

COASY / Q9DBL7; adj.p value: 9e−05

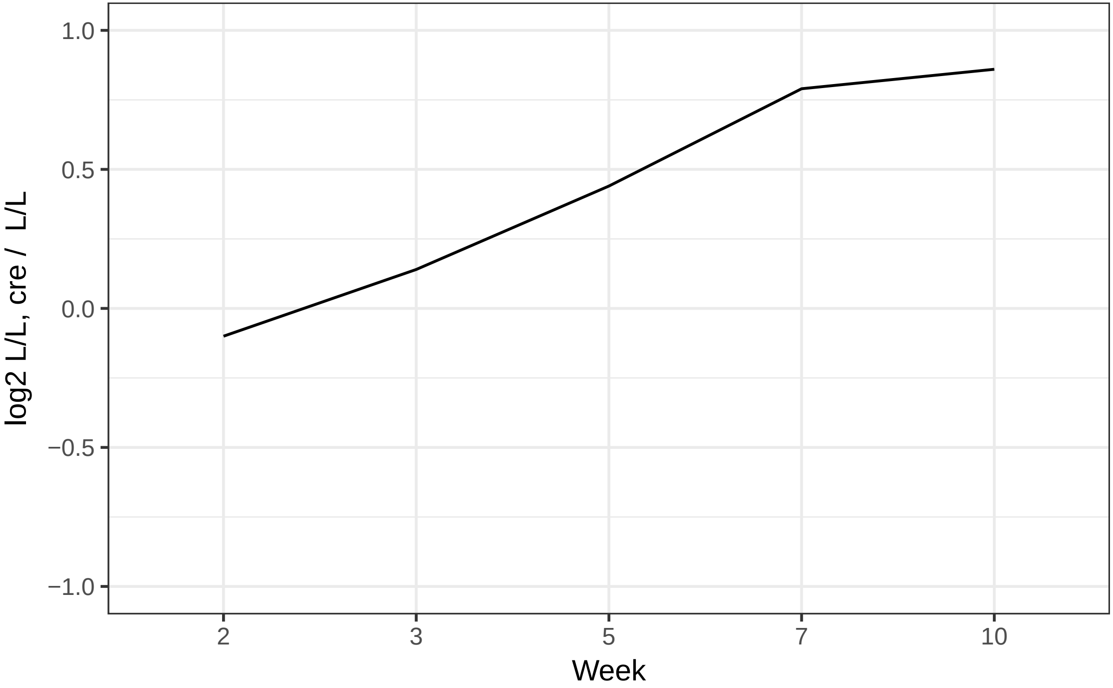

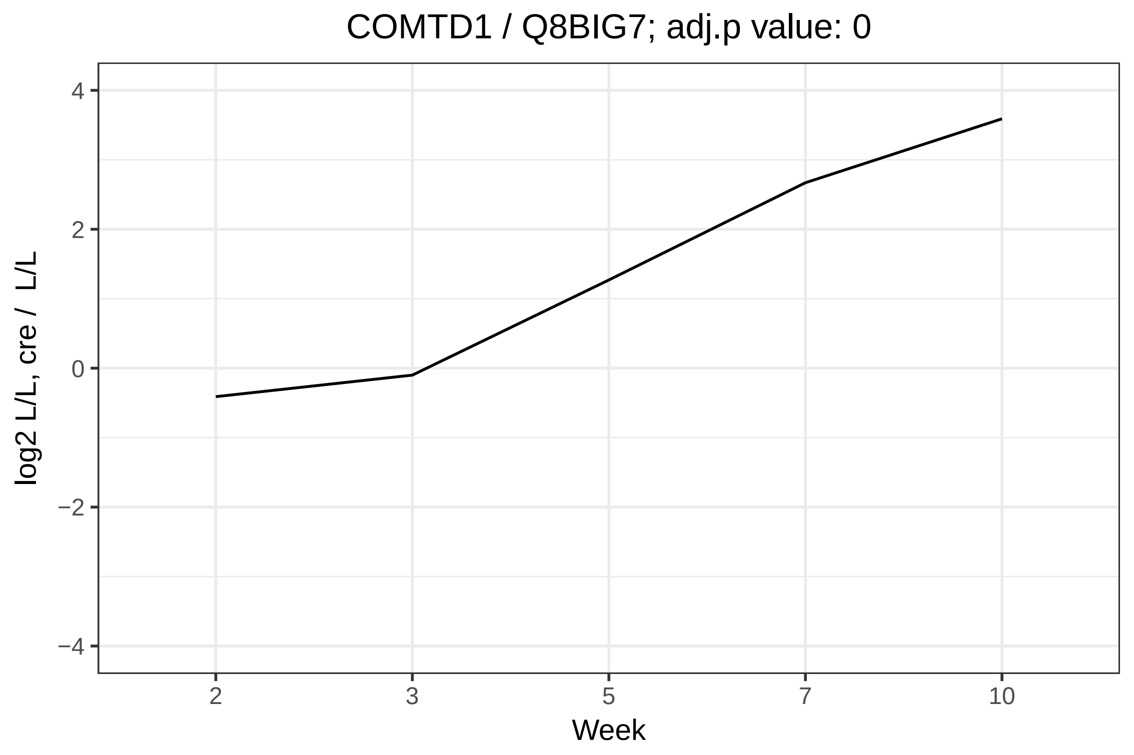
COMTD1 / Q8BIG7; adj.p value: 0
log2 L/L, cre / L/L
4
2
0
−2
−4
2
3
5
7
10
Week

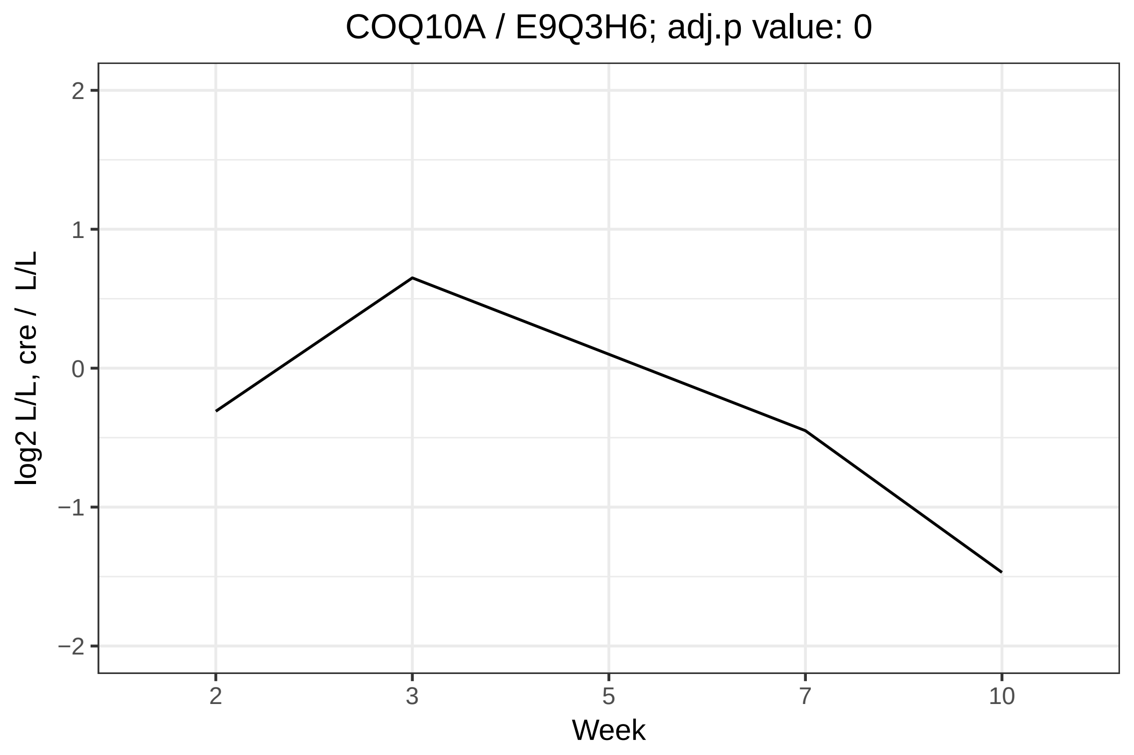
COQ10A / E9Q3H6; adj.p value: 0
log2 L/L, cre / L/L
2
1
0
−1
−2
2
3
5
7
10
Week

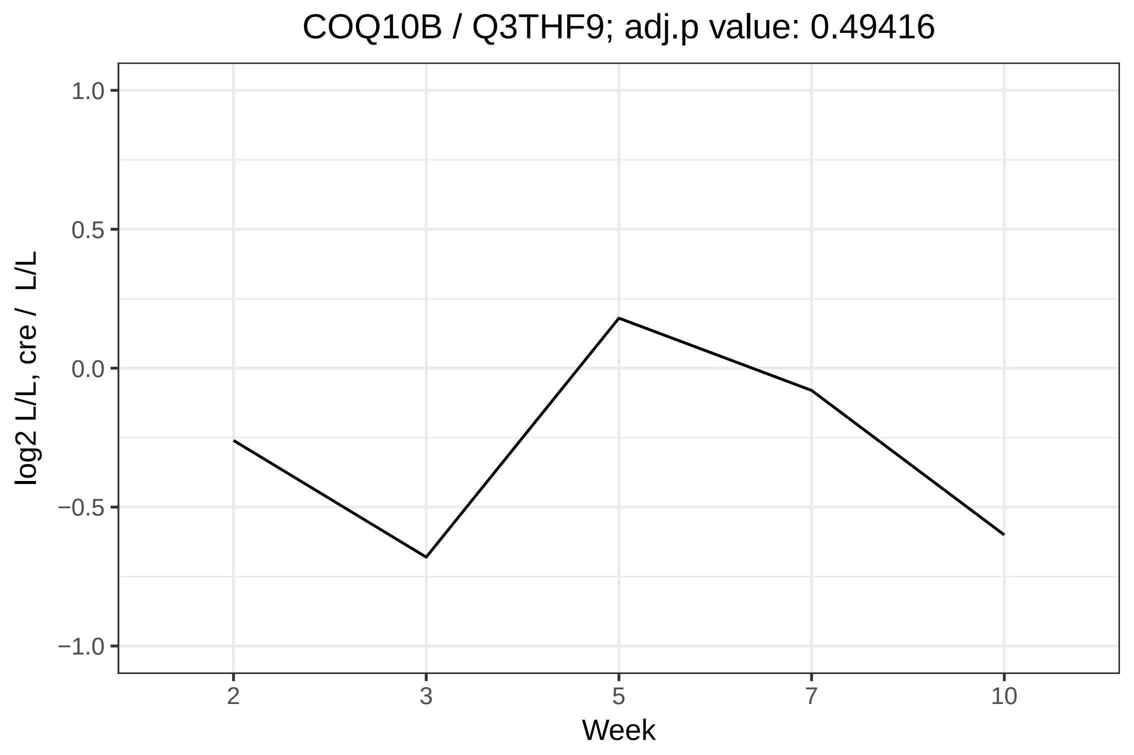

COQ10B / Q3THF9; adj.p value: 0.49416
log2 L/L, cre / L/L
1.0
0.5
0.0
−0.5
−1.0
2
3
5
7
10
Week

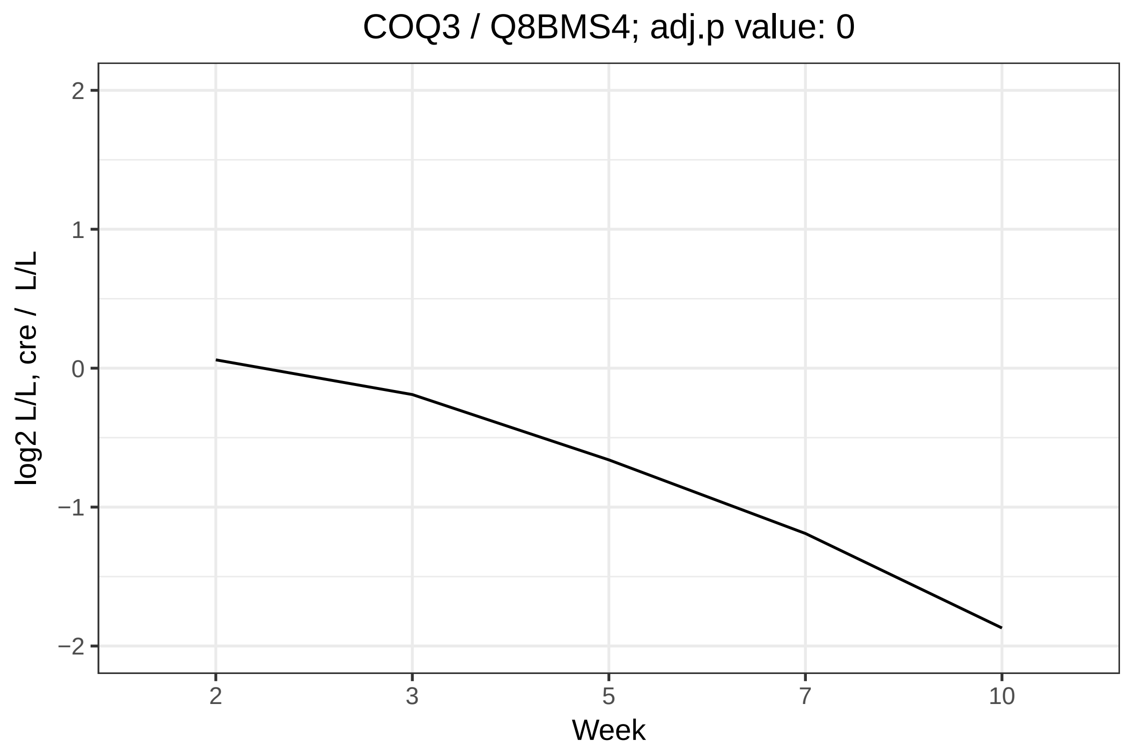
COQ3 / Q8BMS4; adj.p value: 0
log2 L/L, cre / L/L
2
1
0
−1
−2
2
3
5
7
10
Week

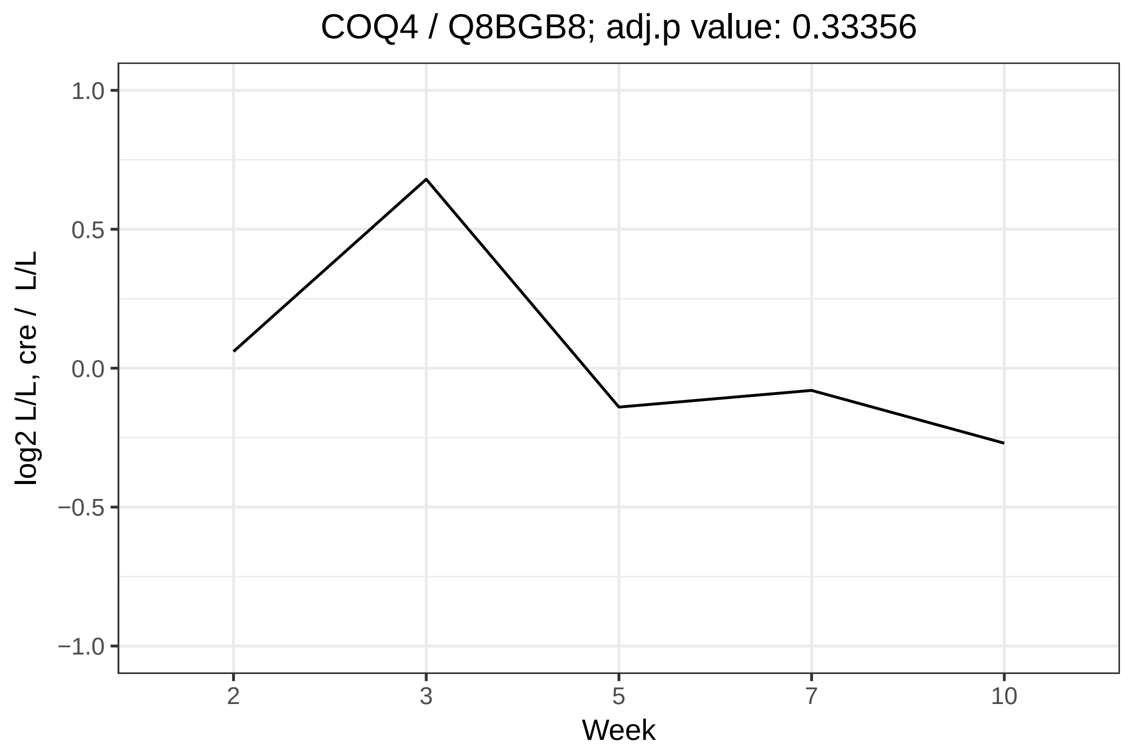

COQ4 / Q8BGB8; adj.p value: 0.33356
log2 L/L, cre / L/L
1.0
0.5
0.0
−0.5
−1.0
2
3
5
7
10
Week

COQ5 / Q9CXI0; adj.p value: 0

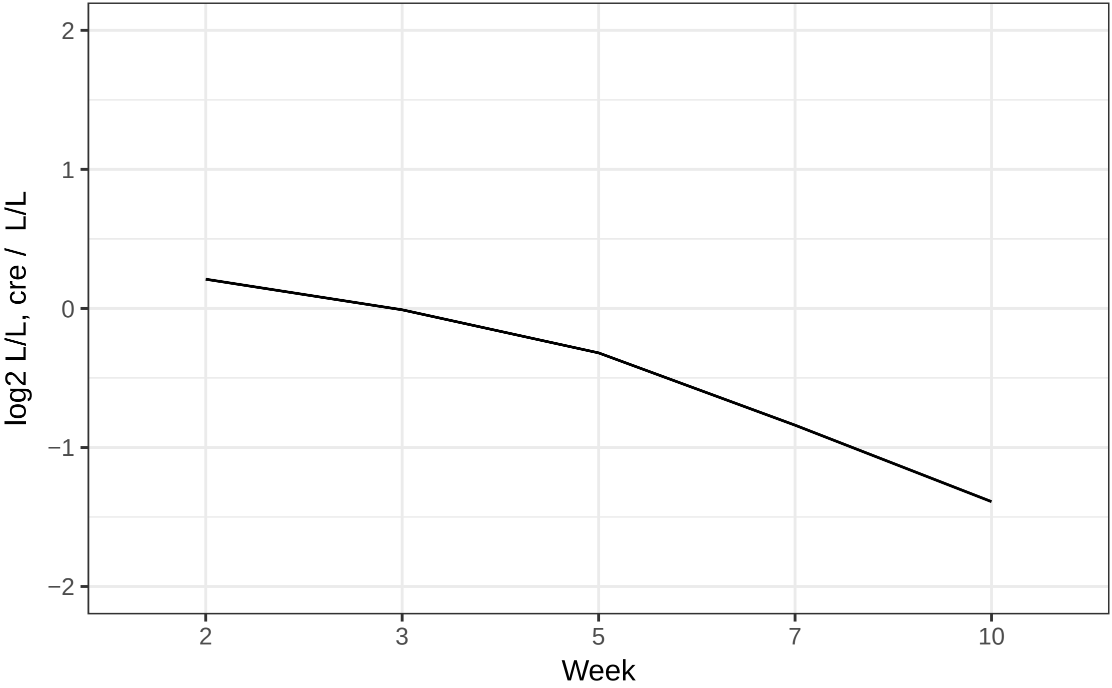

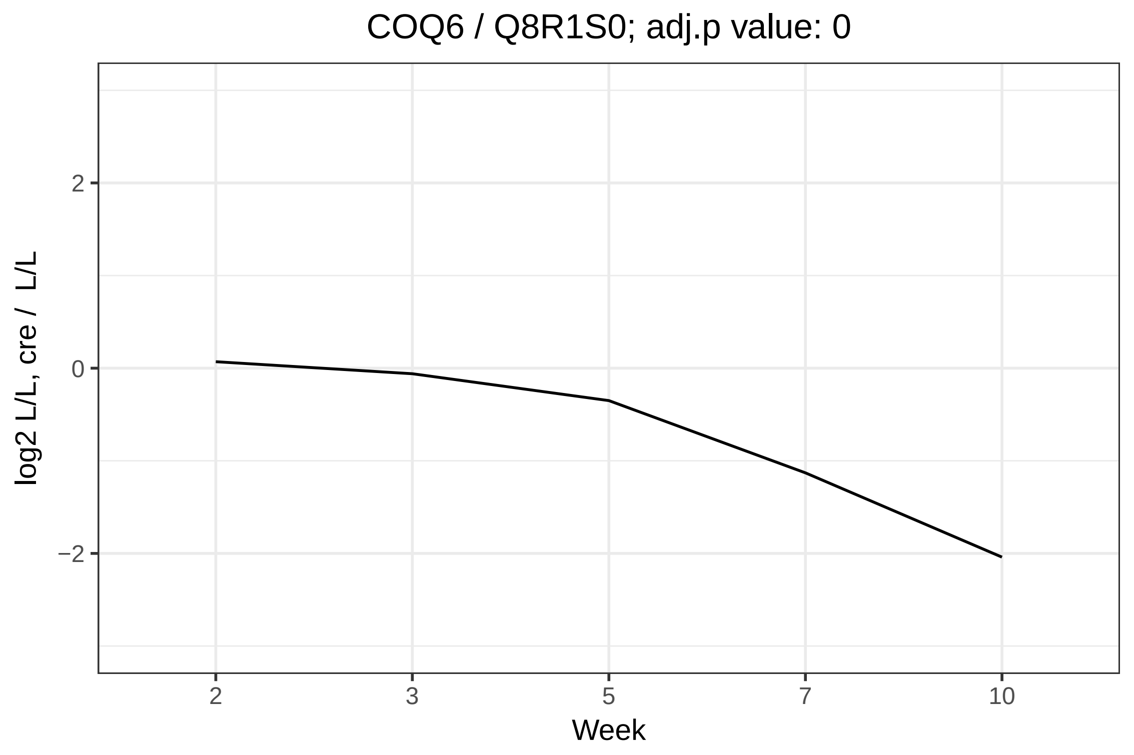
COQ6 / Q8R1S0; adj.p value: 0
log2 L/L, cre / L/L
2
0
−2
2
3
5
7
10
Week

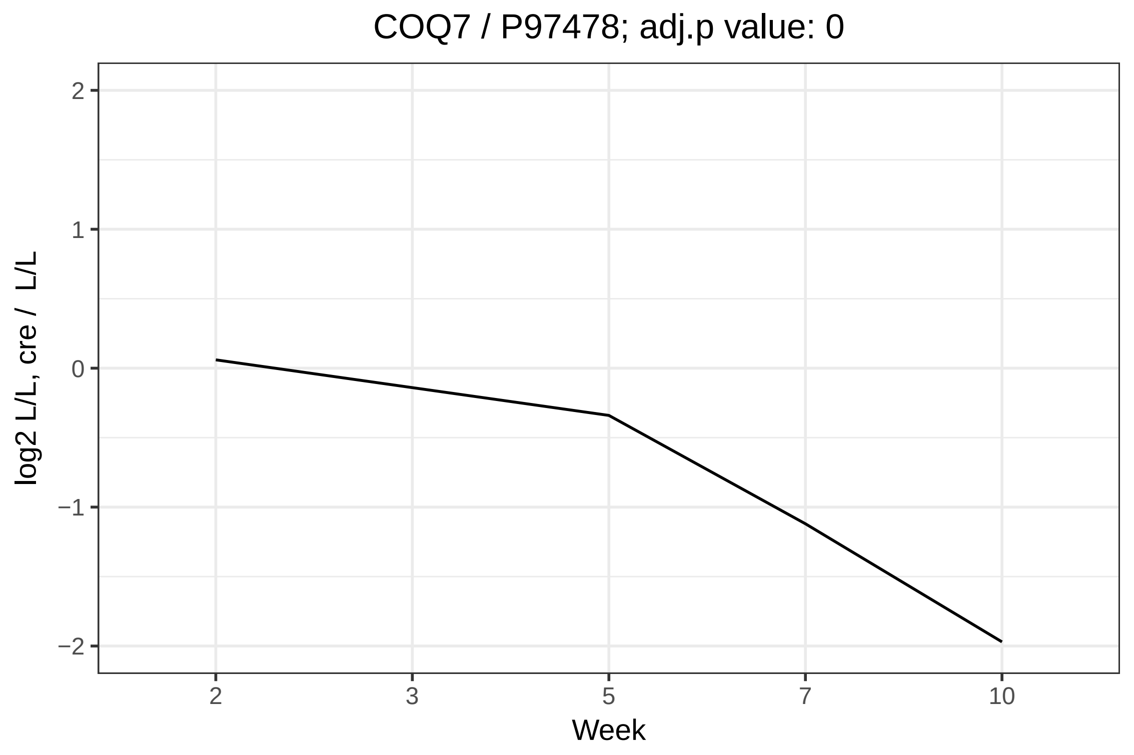

COQ7 / P97478; adj.p value: 0
log2 L/L, cre / L/L
2
1
0
−1
−2
2
3
5
7
10
Week

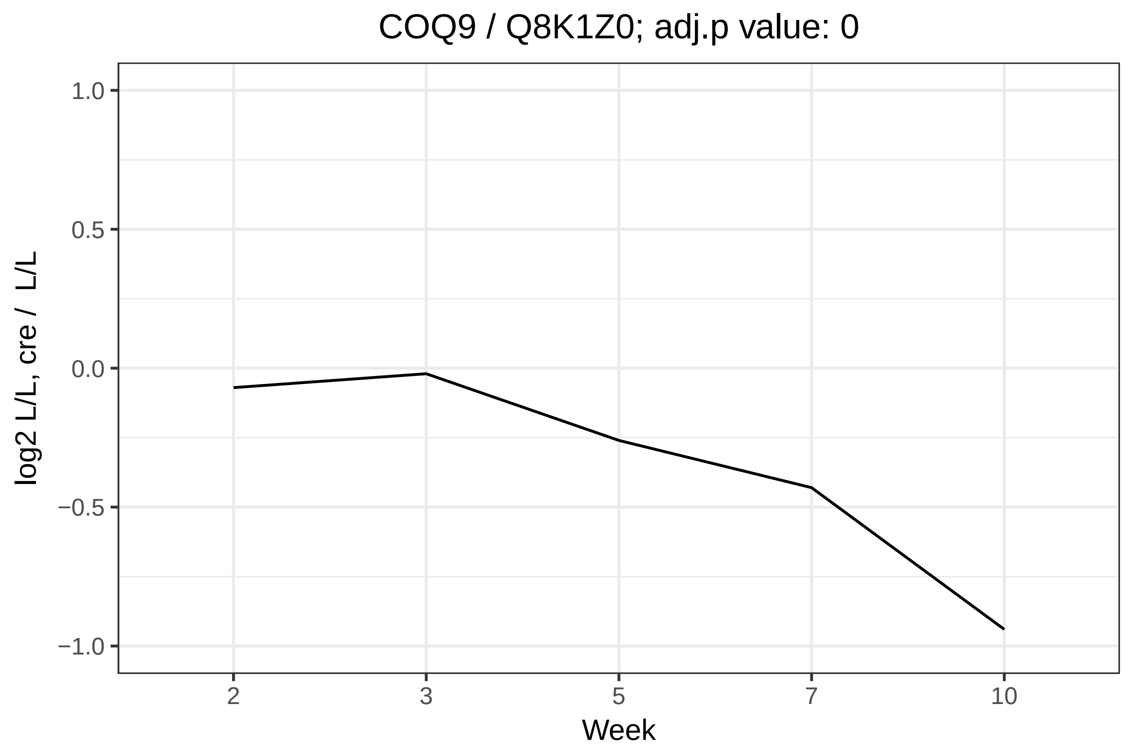
COQ9 / Q8K1Z0; adj.p value: 0
log2 L/L, cre / L/L
1.0
0.5
0.0
−0.5
−1.0
2
3
5
7
10
Week

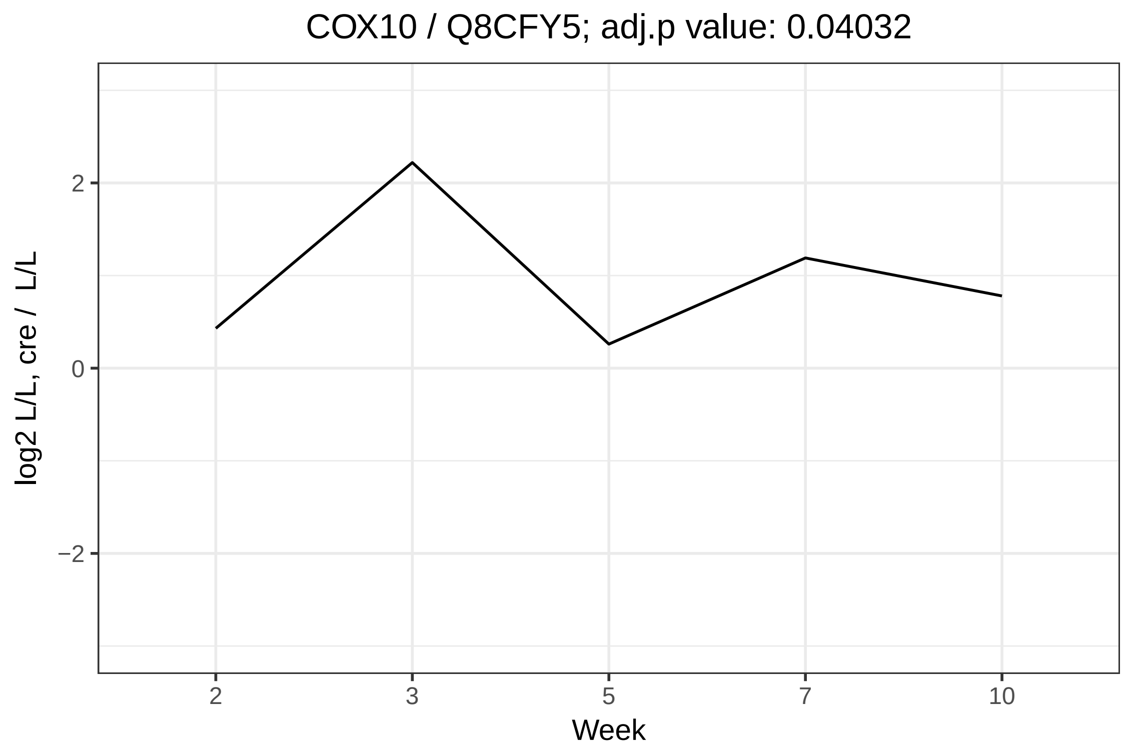
COX10 / Q8CFY5; adj.p value: 0.04032
log2 L/L, cre / L/L
2
0
−2
2
3
5
7
10
Week

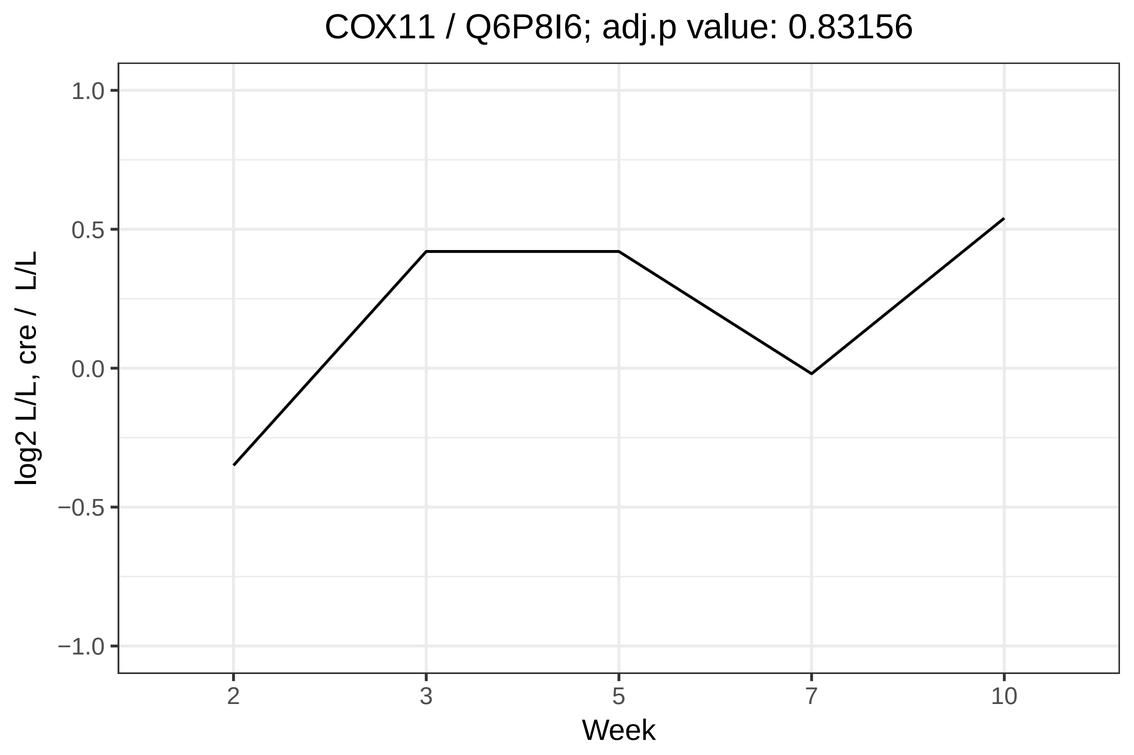
COX11 / Q6P8I6; adj.p value: 0.83156
log2 L/L, cre / L/L
1.0
0.5
0.0
−0.5
−1.0
2
3
5
7
10
Week

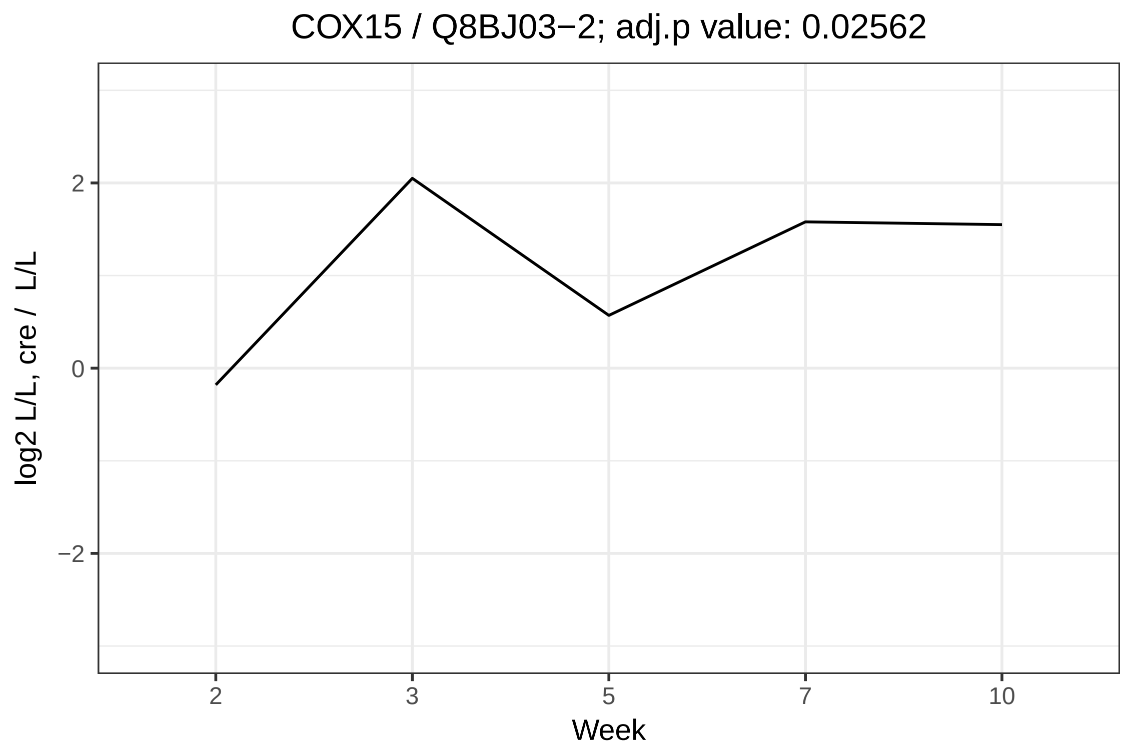
COX15 / Q8BJ03−2; adj.p value: 0.02562
log2 L/L, cre / L/L
2
0
−2
2
3
5
7
10
Week

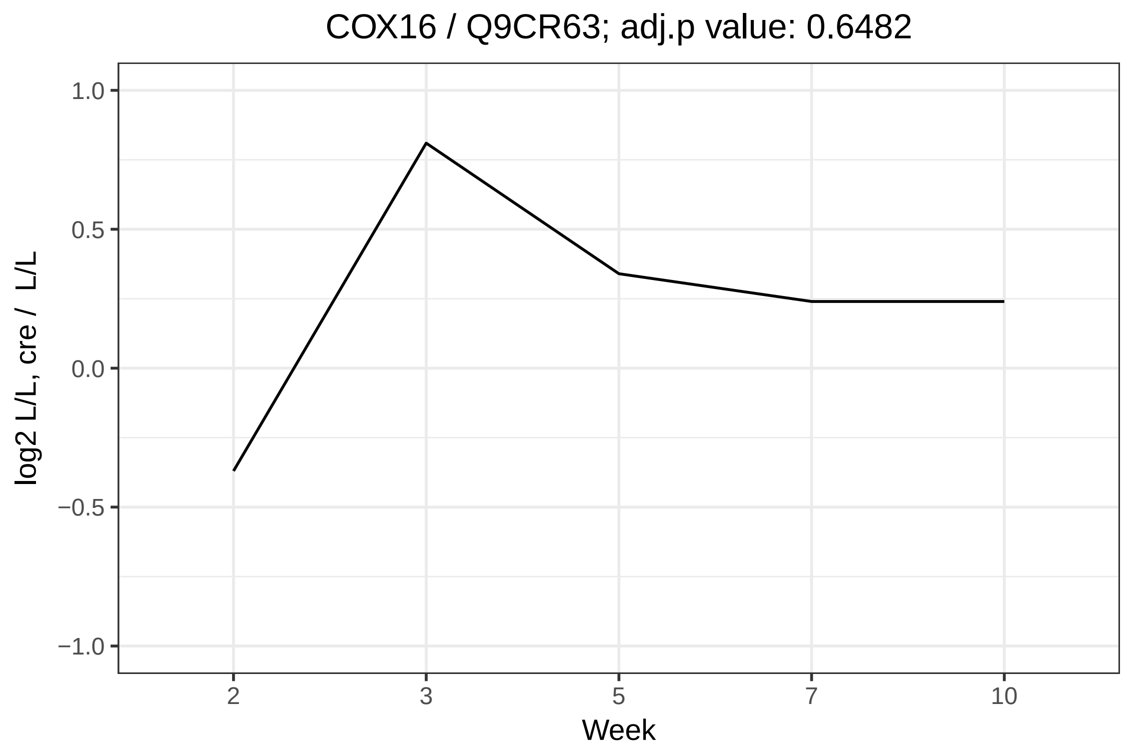

COX16 / Q9CR63; adj.p value: 0.6482
1.0
0.5
0.0
−0.5
−1.0
log2 L/L, cre / L/L
2
3
5
7
10
Week

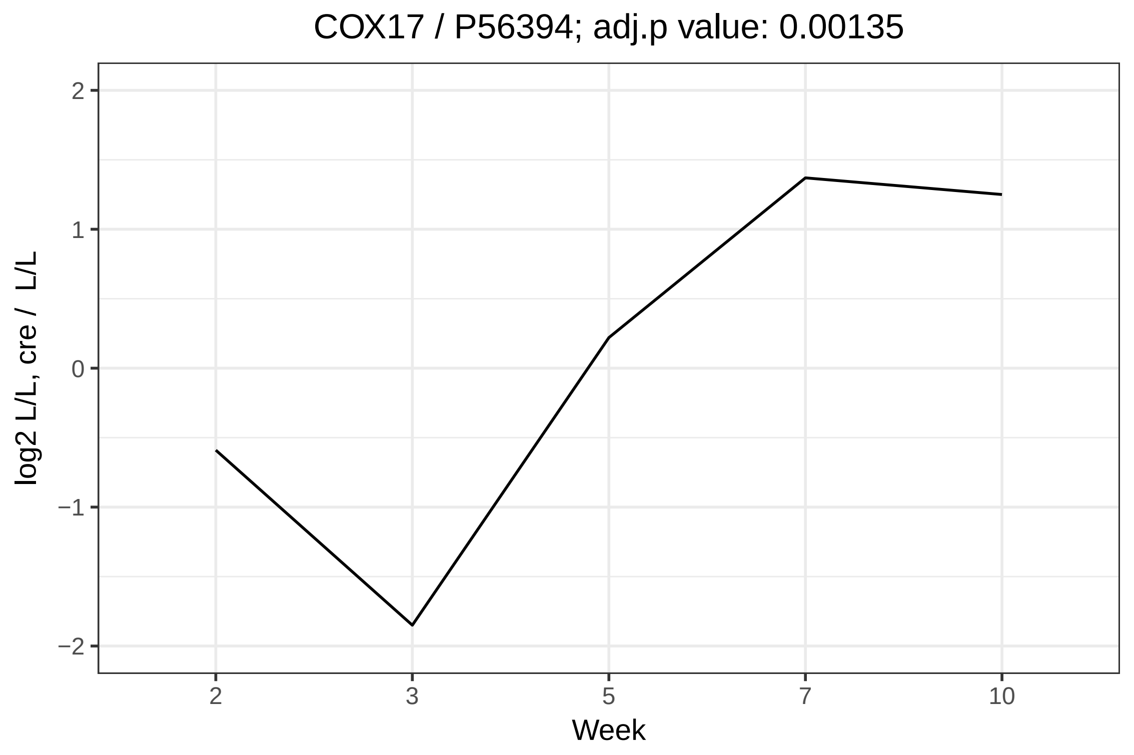
COX17 / P56394; adj.p value: 0.00135
log2 L/L, cre / L/L
2
1
0
−1
−2
2
3
5
7
10
Week

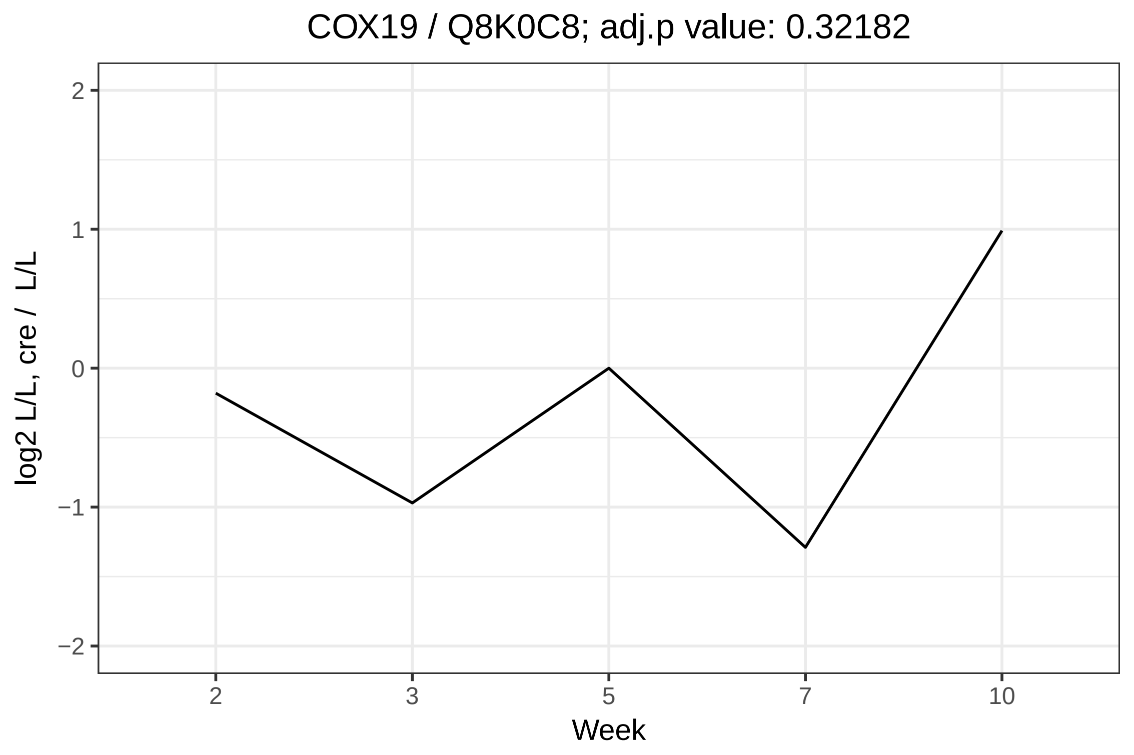

COX19 / Q8K0C8; adj.p value: 0.32182
log2 L/L, cre / L/L
Week
2
1
0
−1
−2
2
3
5
7
10

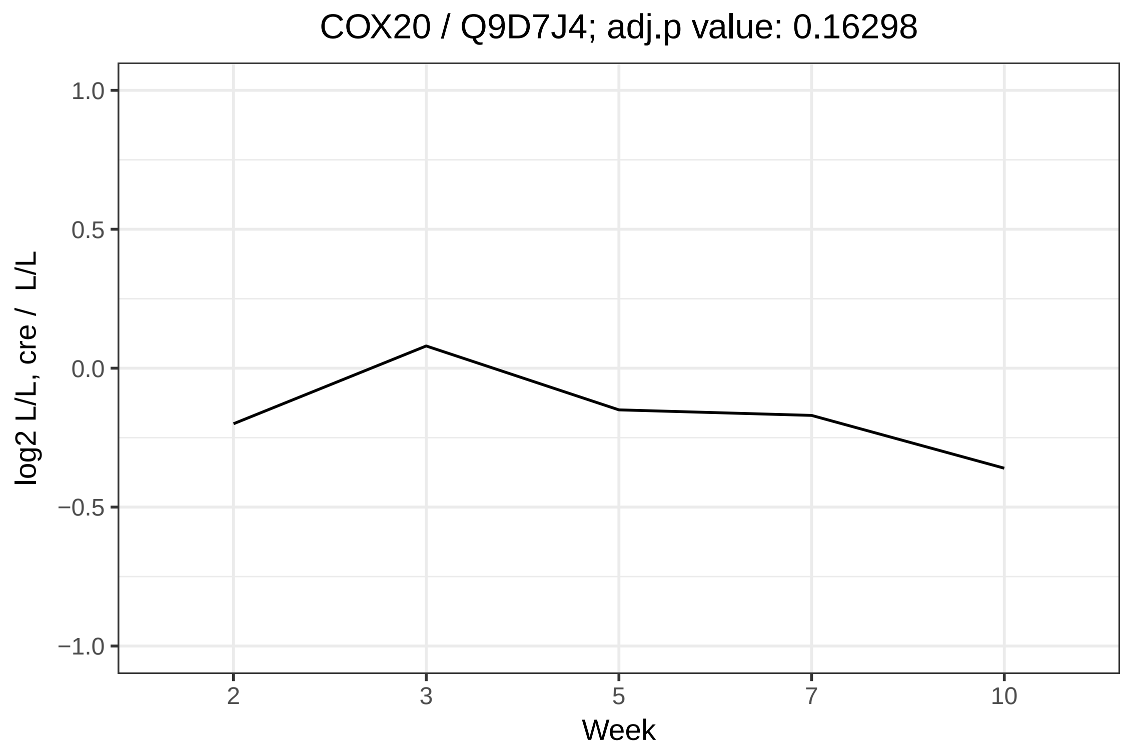

COX20 / Q9D7J4; adj.p value: 0.16298
log2 L/L, cre / L/L
1.0
0.5
0.0
−0.5
−1.0
2
3
5
7
10
Week

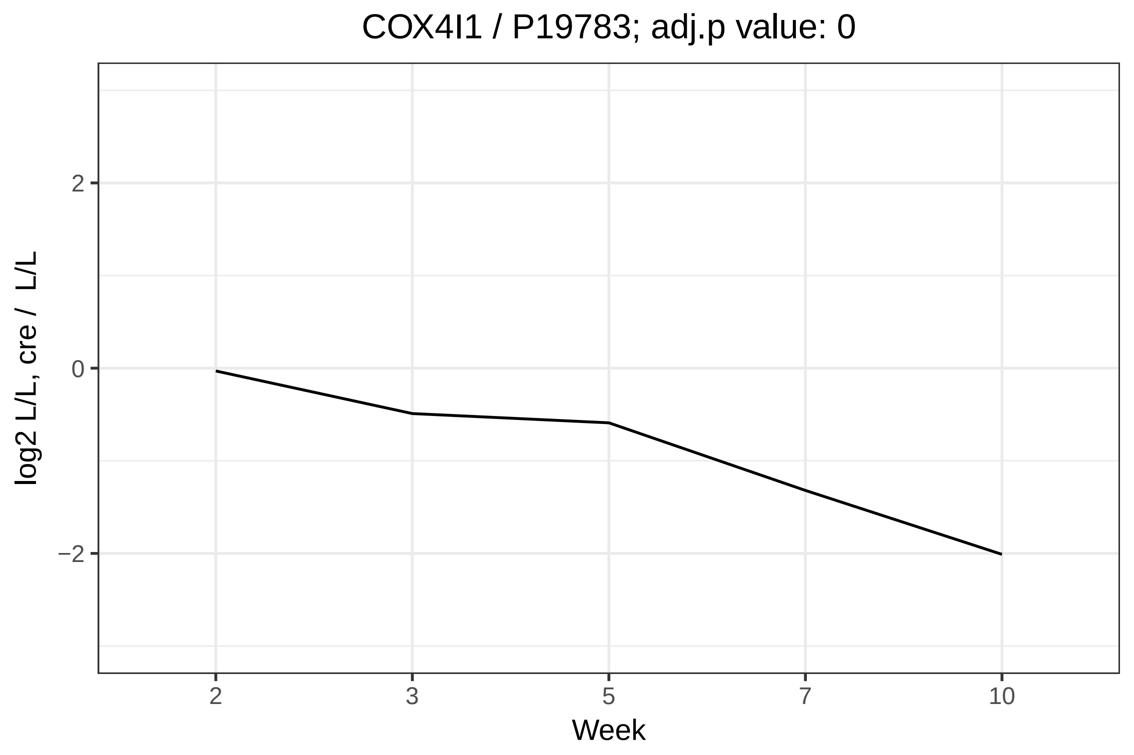
COX4I1 / P19783; adj.p value: 0
log2 L/L, cre / L/L
2
0
−2
2
3
5
7
10
Week

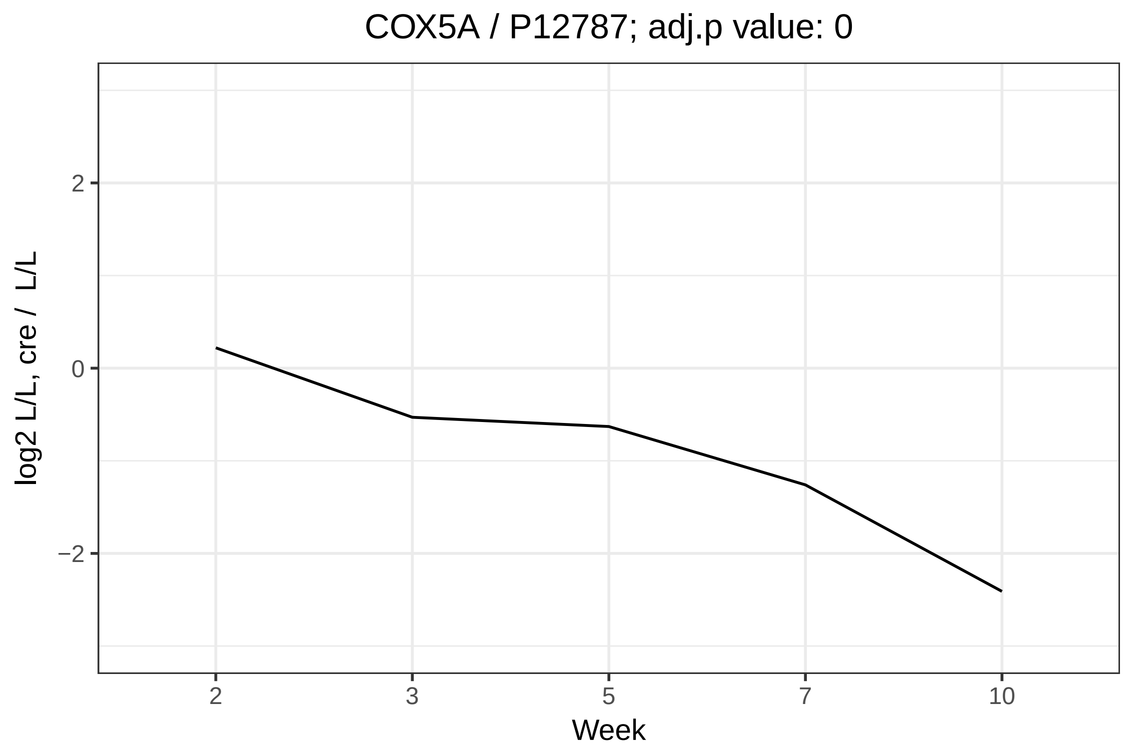
COX5A / P12787; adj.p value: 0
log2 L/L, cre / L/L
2
0
−2
2
3
5
7
10
Week

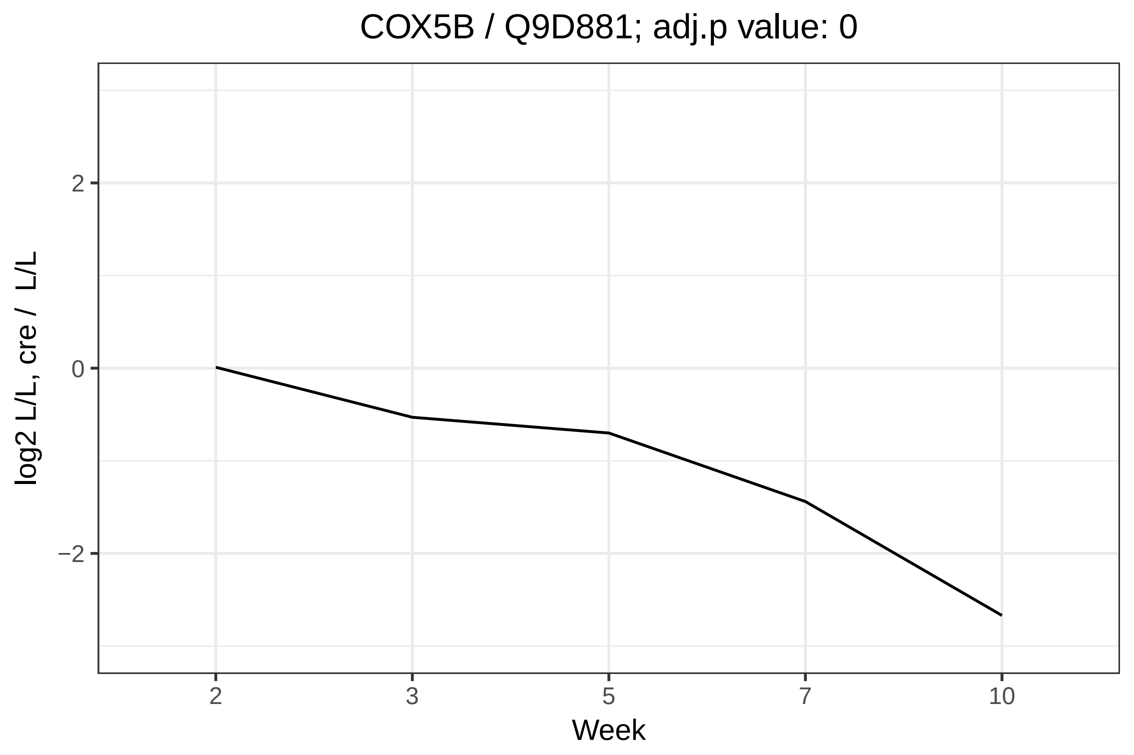
COX5B / Q9D881; adj.p value: 0
log2 L/L, cre / L/L
2
0
−2
2
3
5
7
10
Week

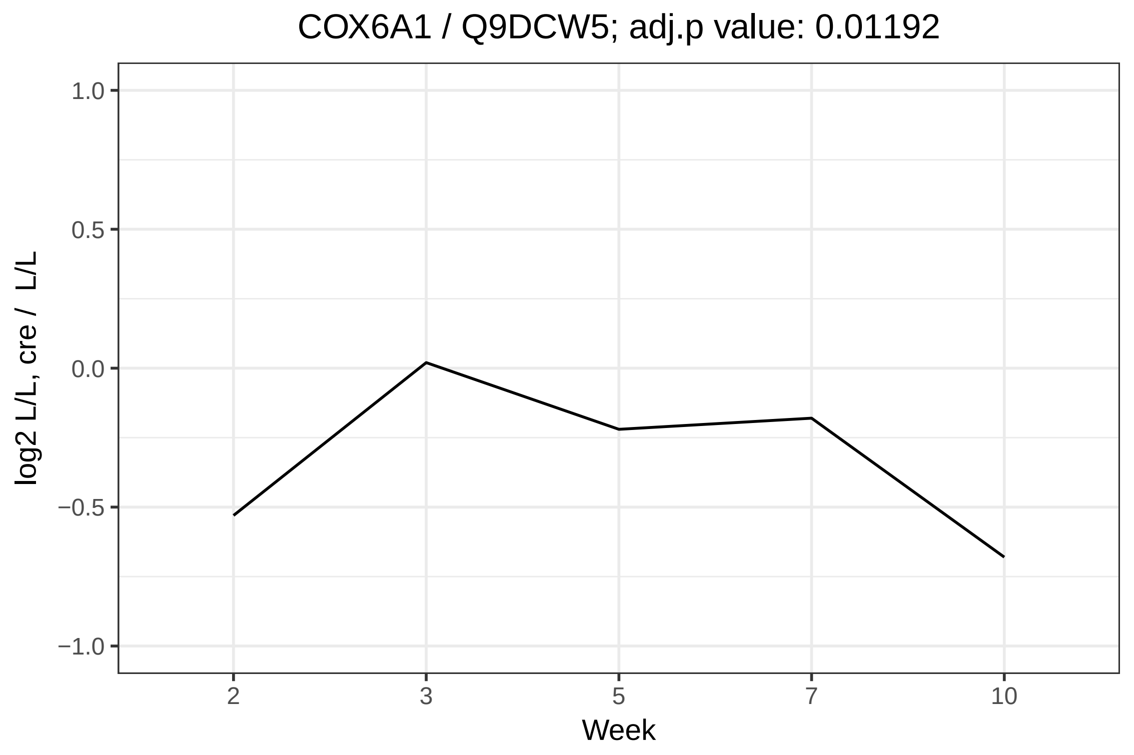

COX6A1 / Q9DCW5; adj.p value: 0.01192
log2 L/L, cre / L/L
1.0
0.5
0.0
−0.5
−1.0
2
3
5
7
10
Week

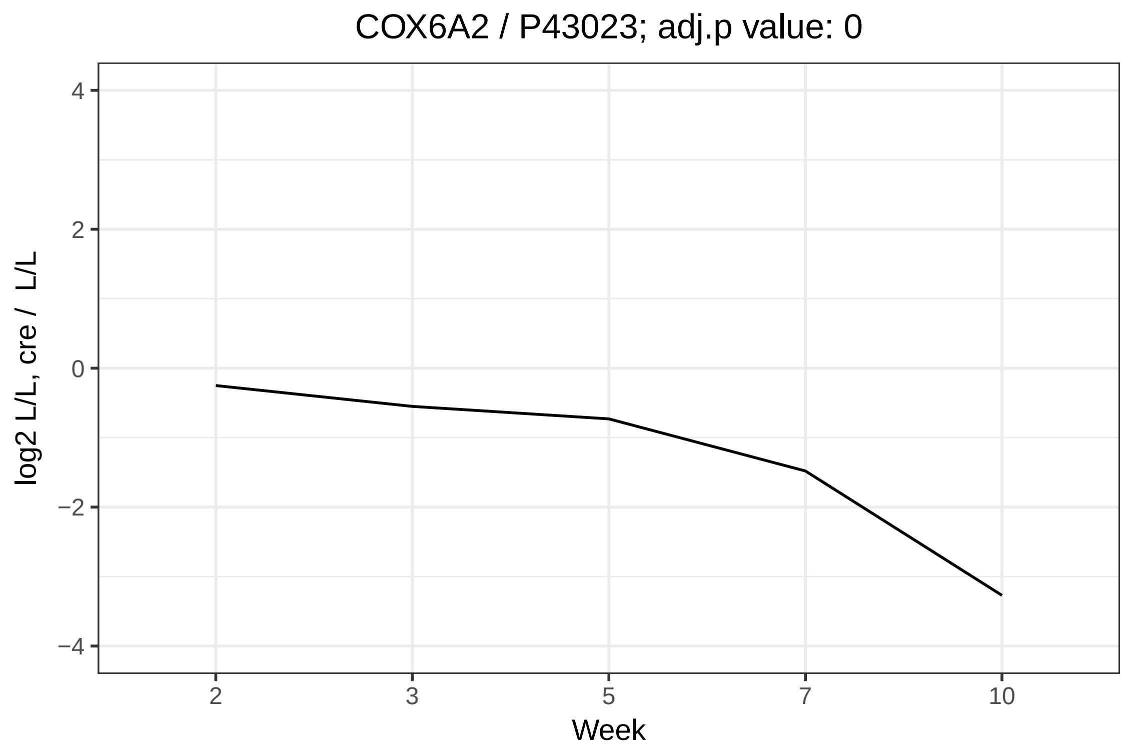
COX6A2 / P43023; adj.p value: 0
4
2
0
−2
−4
log2 L/L, cre / L/L
2
3
5
7
10
Week

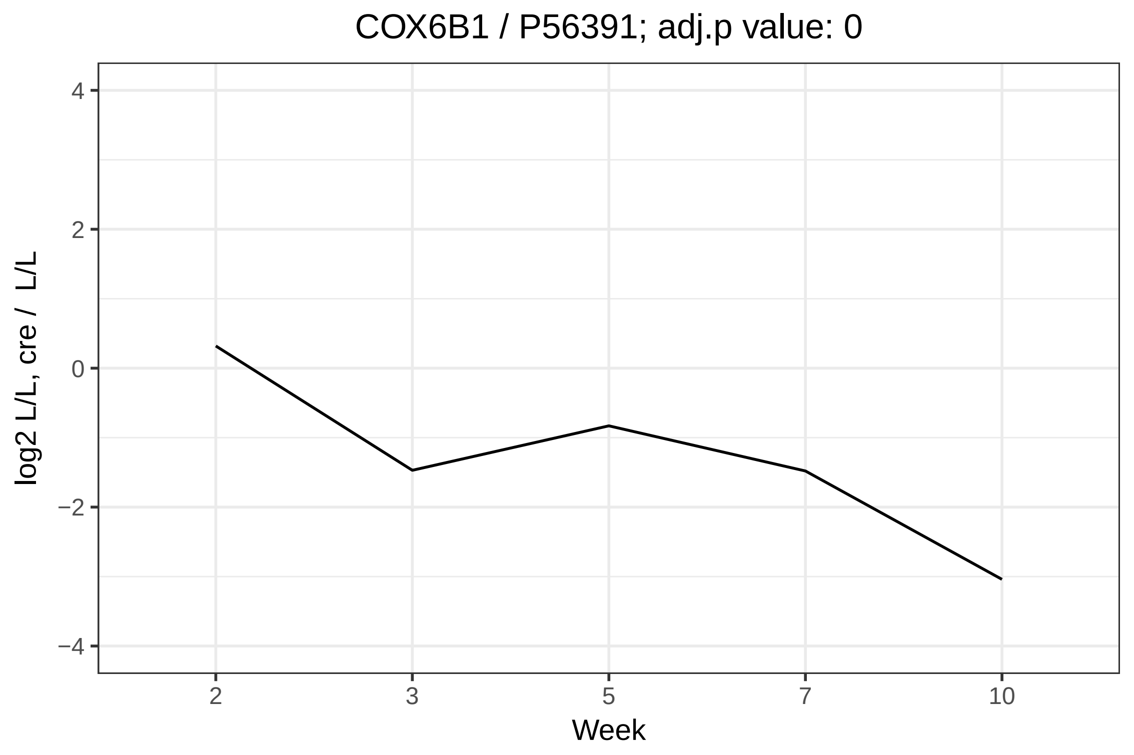
COX6B1 / P56391; adj.p value: 0
log2 L/L, cre / L/L
4
2
0
−2
−4
2
3
5
7
10
Week

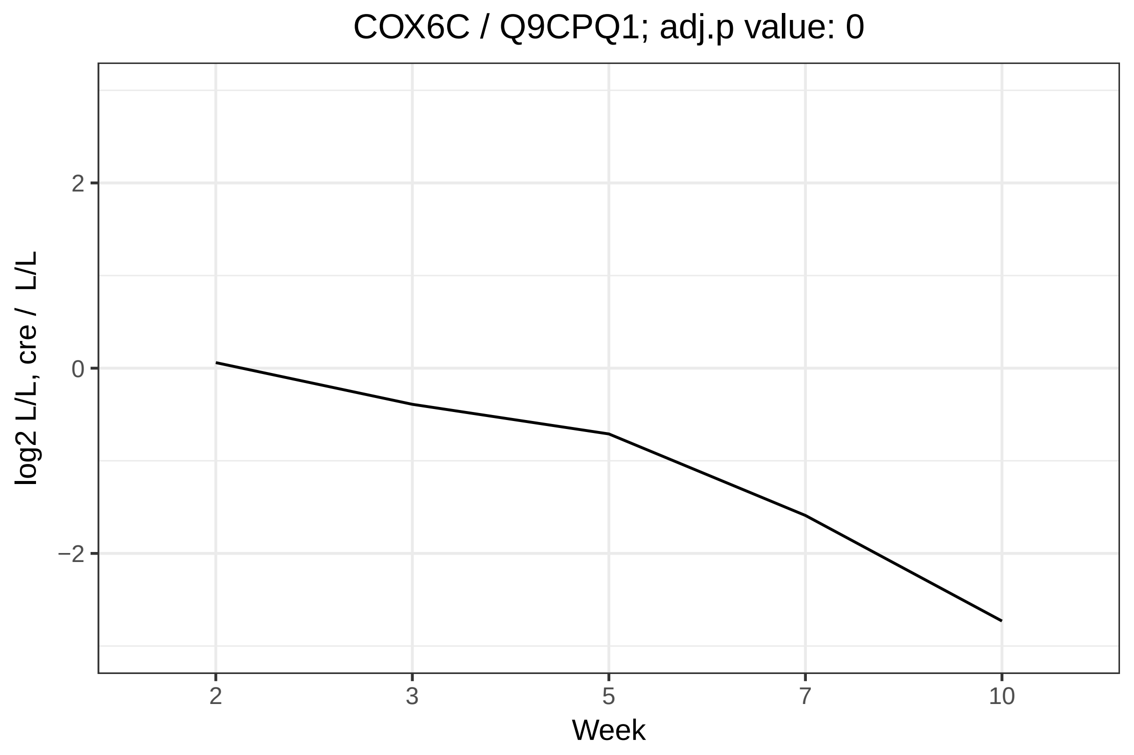

COX6C / Q9CPQ1; adj.p value: 0
log2 L/L, cre / L/L
2
0
−2
2
3
5
7
10
Week

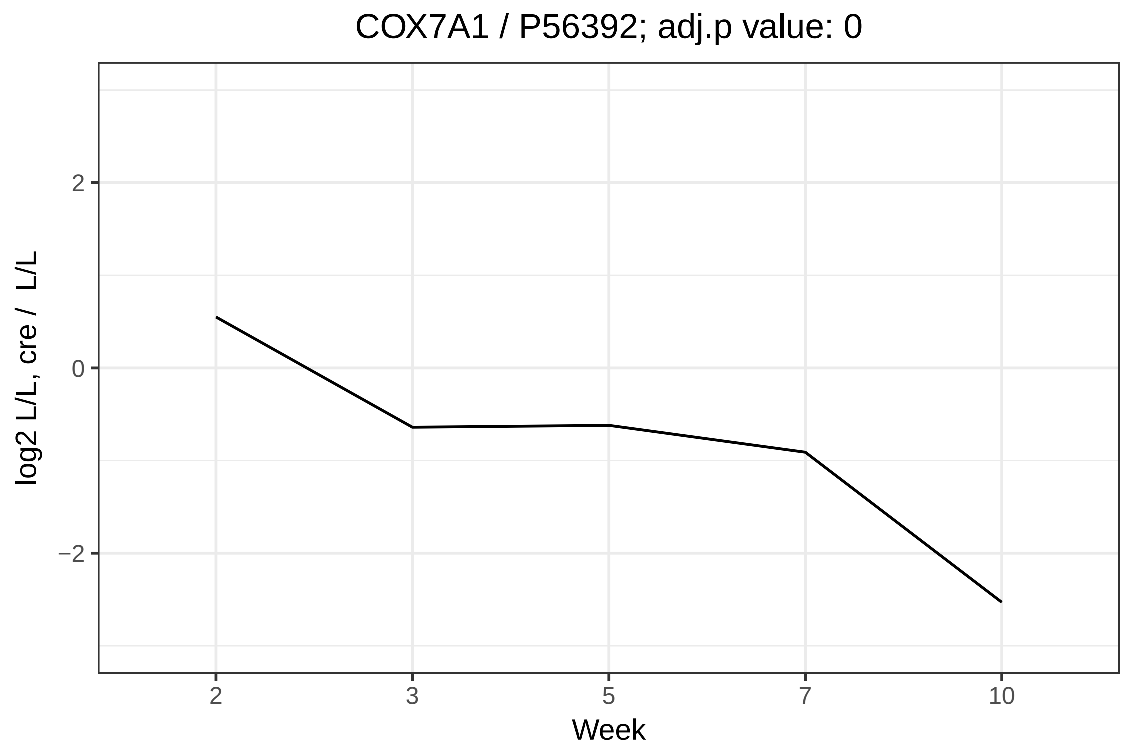
COX7A1 / P56392; adj.p value: 0
log2 L/L, cre / L/L
2
0
−2
2
3
5
7
10
Week

COX7A2 / P48771; adj.p value: 0

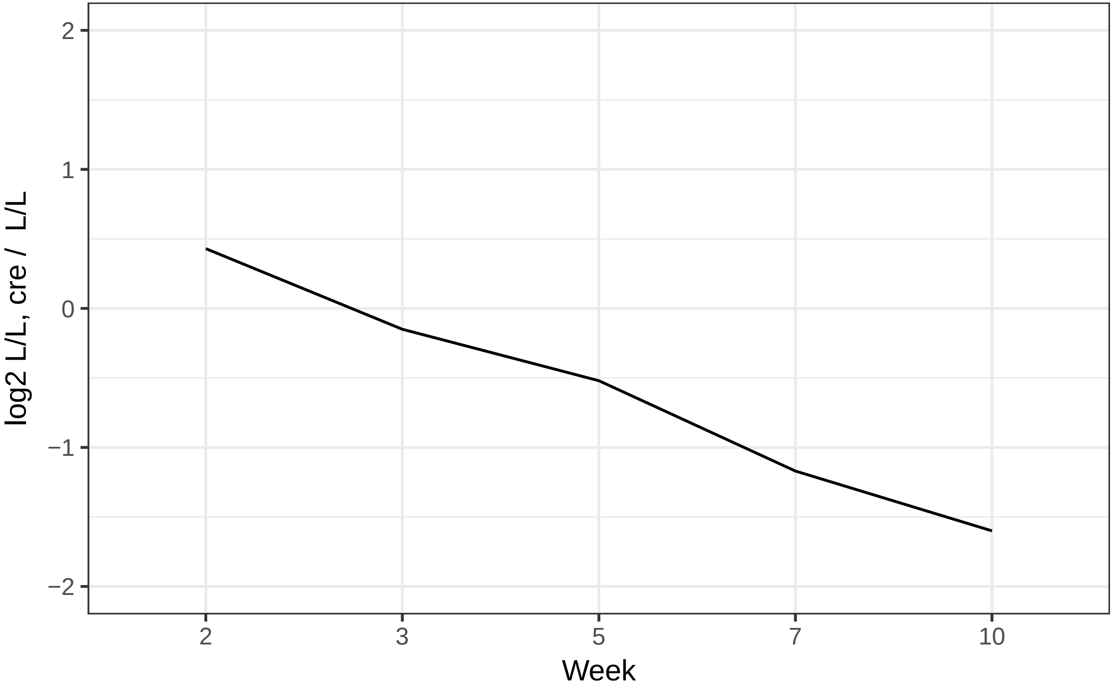

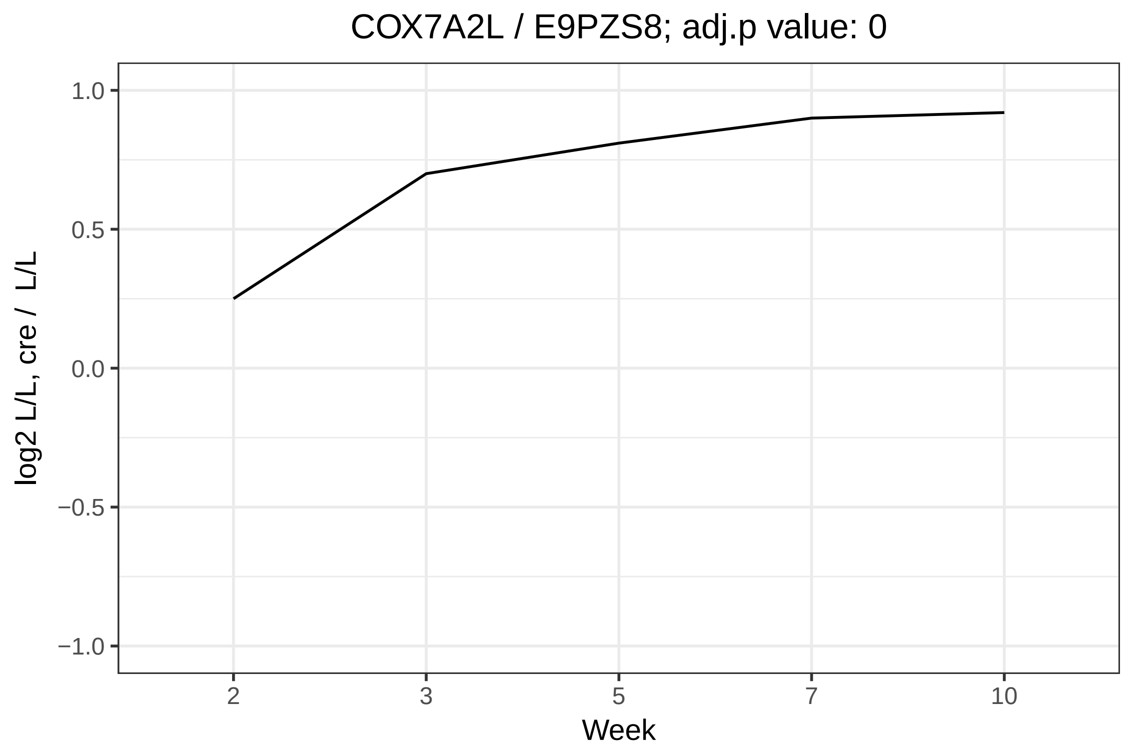

COX7A2L / E9PZS8; adj.p value: 0
log2 L/L, cre / L/L
1.0
0.5
0.0
−0.5
−1.0
2
3
5
7
10
Week

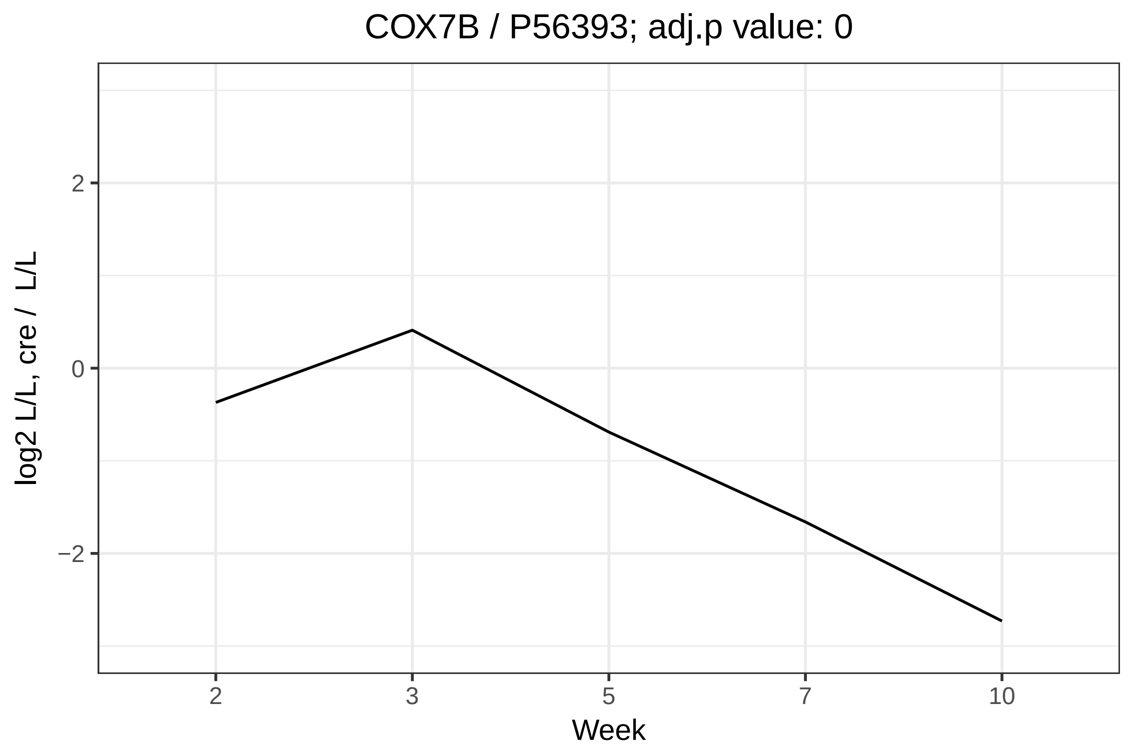

COX7B / P56393; adj.p value: 0
log2 L/L, cre / L/L
2
0
−2
2
3
5
7
10
Week

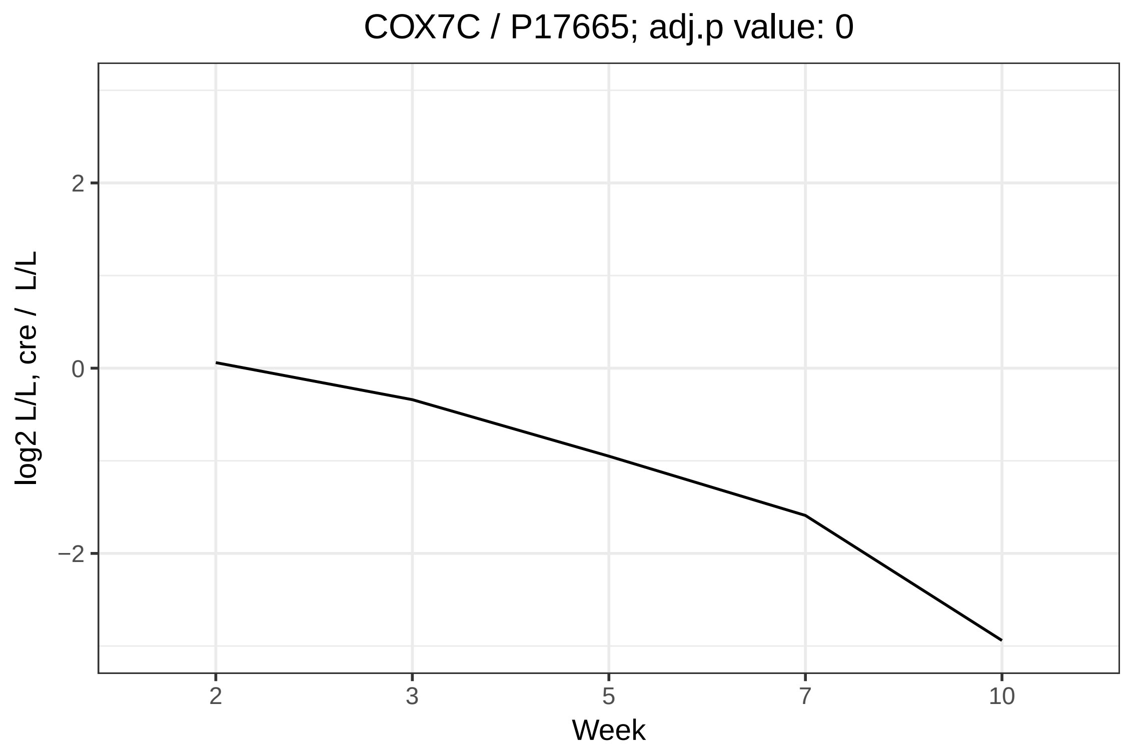
COX7C / P17665; adj.p value: 0
log2 L/L, cre / L/L
2
0
−2
2
3
5
7
10
Week

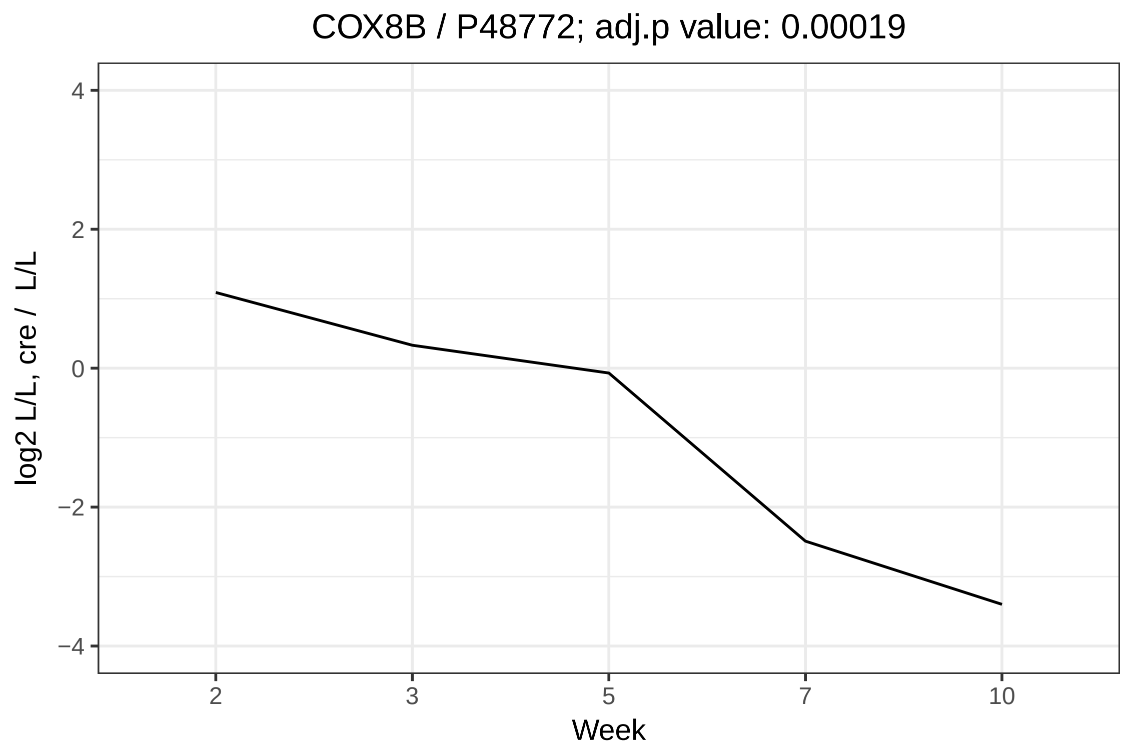
COX8B / P48772; adj.p value: 0.00019
log2 L/L, cre / L/L
4
2
0
−2
−4
2
3
5
7
10
Week

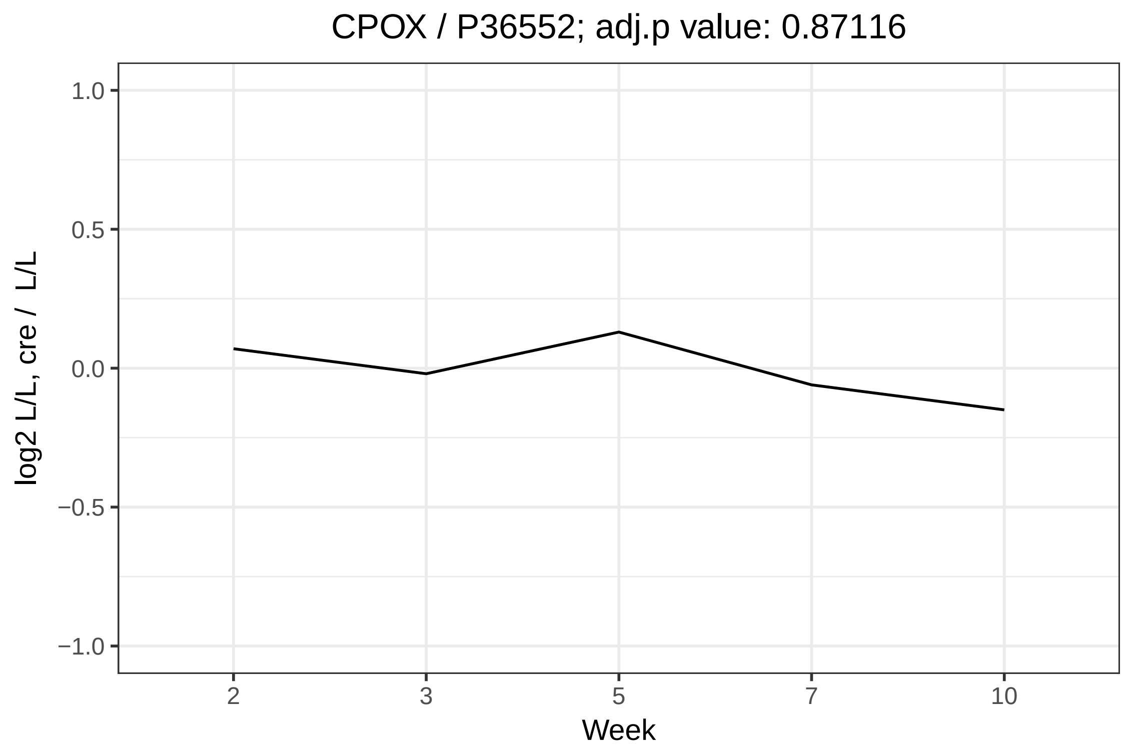

CPOX / P36552; adj.p value: 0.87116
log2 L/L, cre / L/L
1.0
0.5
0.0
−0.5
−1.0
2
3
5
7
10
Week

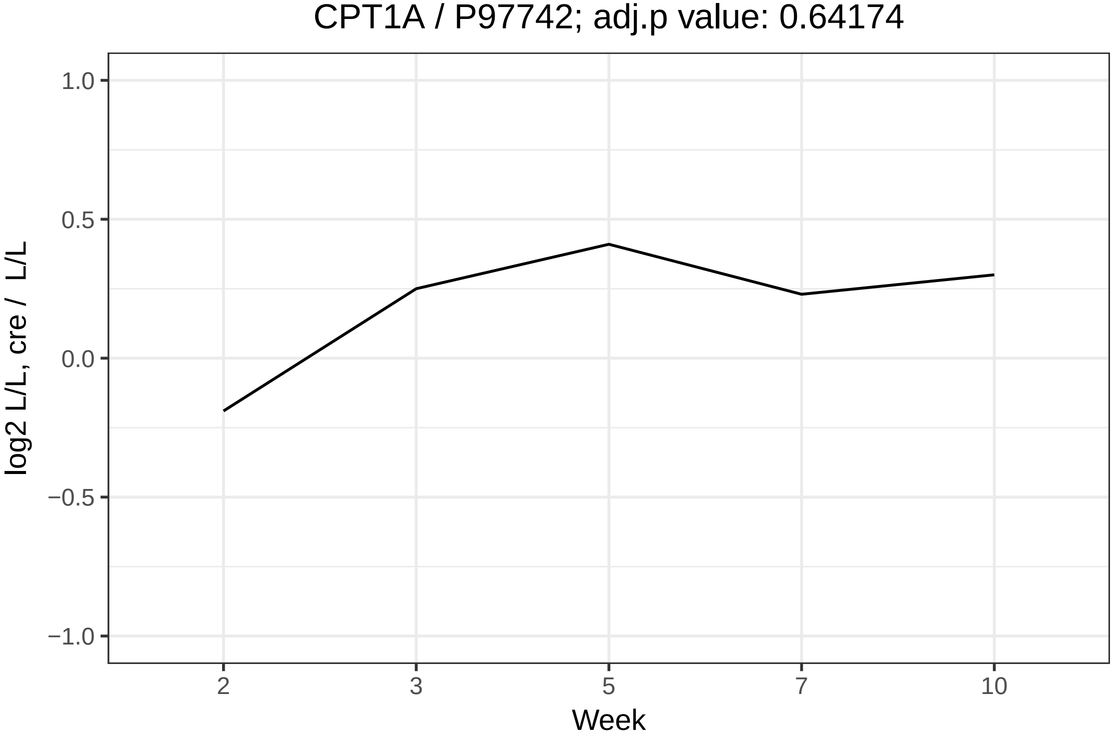
CPT1A / P97742; adj.p value: 0.64174
1.0
0.5
0.0
−0.5
−1.0
log2 L/L, cre / L/L
2
3
5
7
10
Week

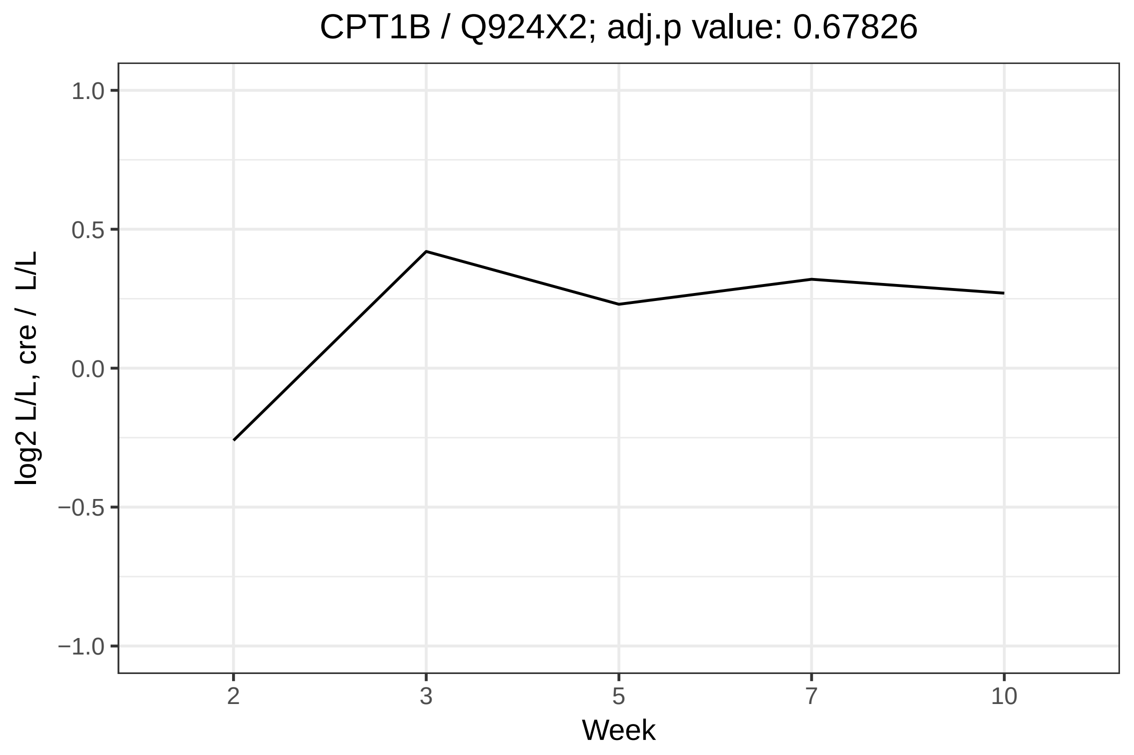

CPT1B / Q924X2; adj.p value: 0.67826
1.0
0.5
0.0
−0.5
−1.0
log2 L/L, cre / L/L
2
3
5
7
10
Week

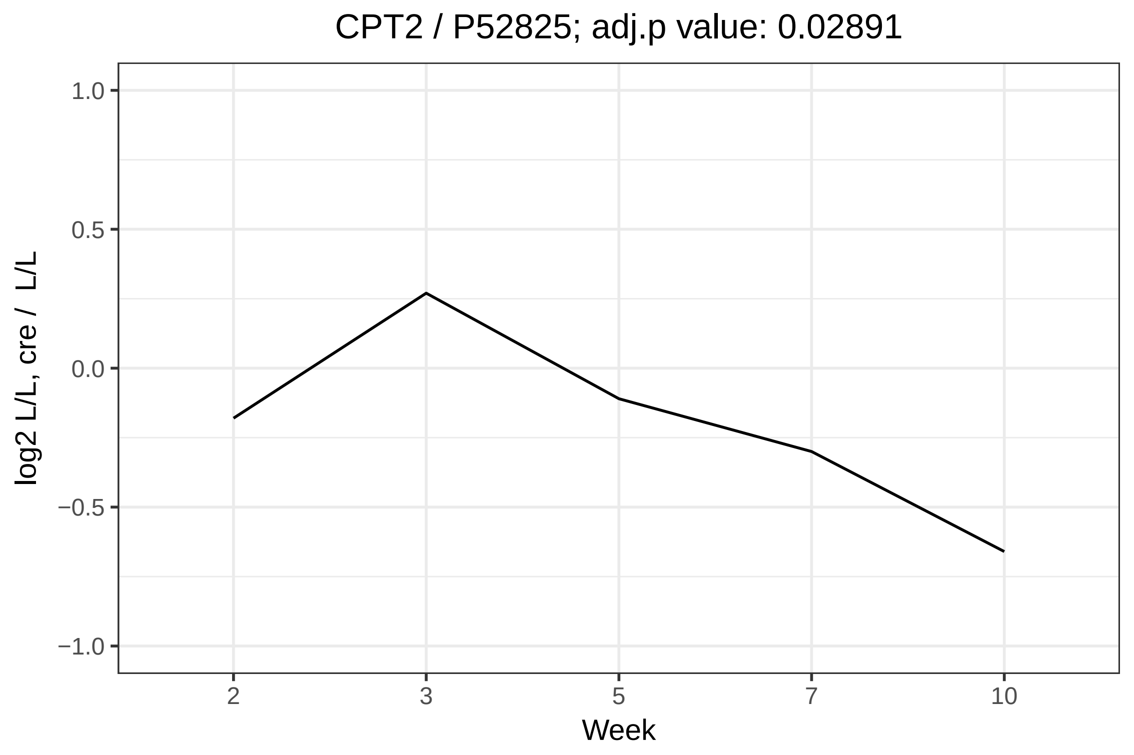

CPT2 / P52825; adj.p value: 0.02891
1.0
0.5
0.0
−0.5
−1.0
log2 L/L, cre / L/L
2
3
5
7
10
Week

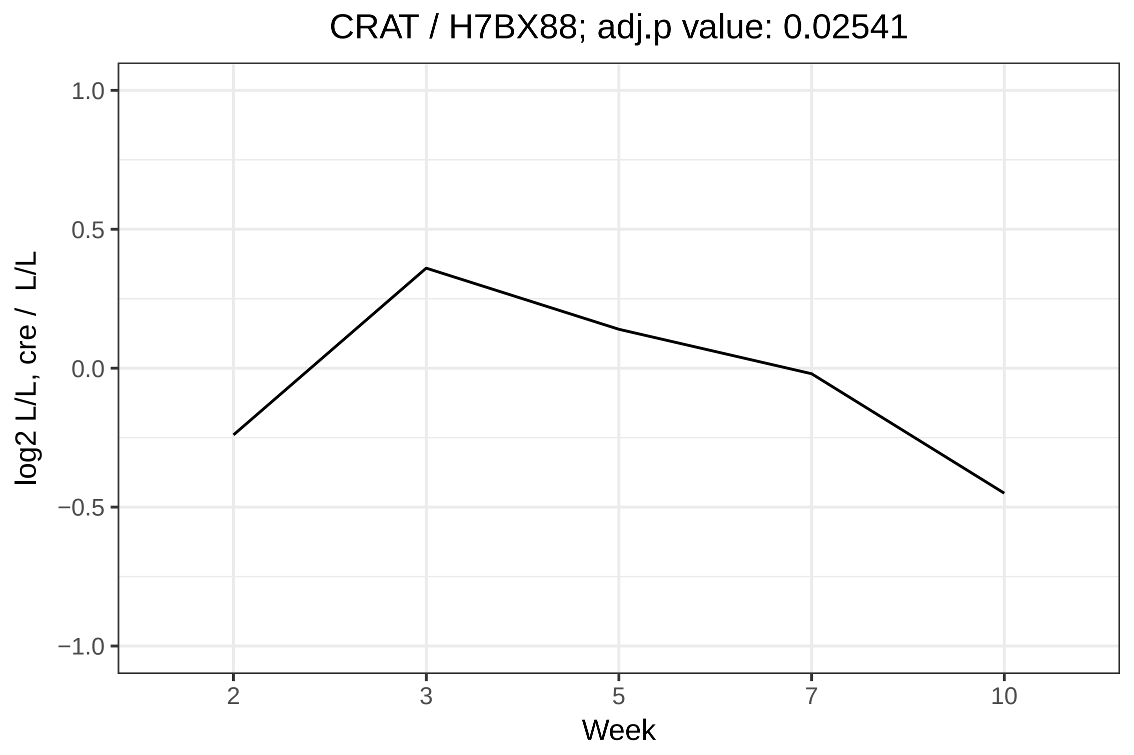
CRAT / H7BX88; adj.p value: 0.02541
1.0
0.5
0.0
−0.5
−1.0
log2 L/L, cre / L/L
2
3
5
7
10
Week

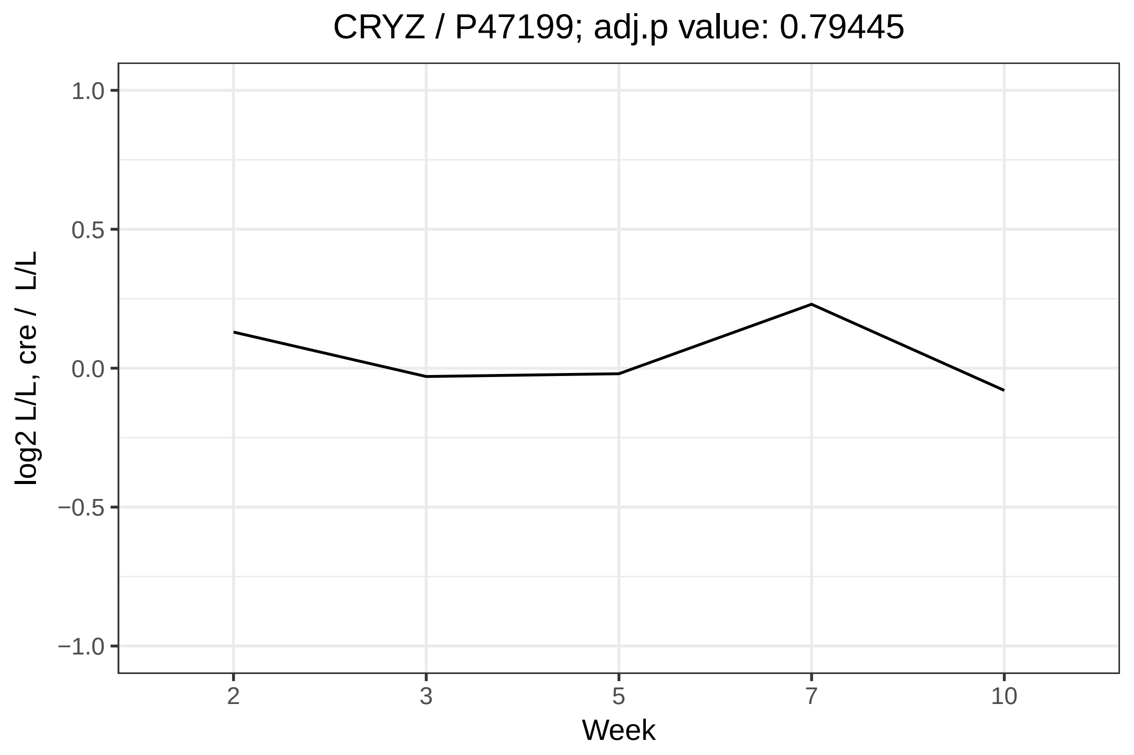

CRYZ / P47199; adj.p value: 0.79445
1.0
0.5
0.0
−0.5
−1.0
log2 L/L, cre / L/L
2
3
5
7
10
Week

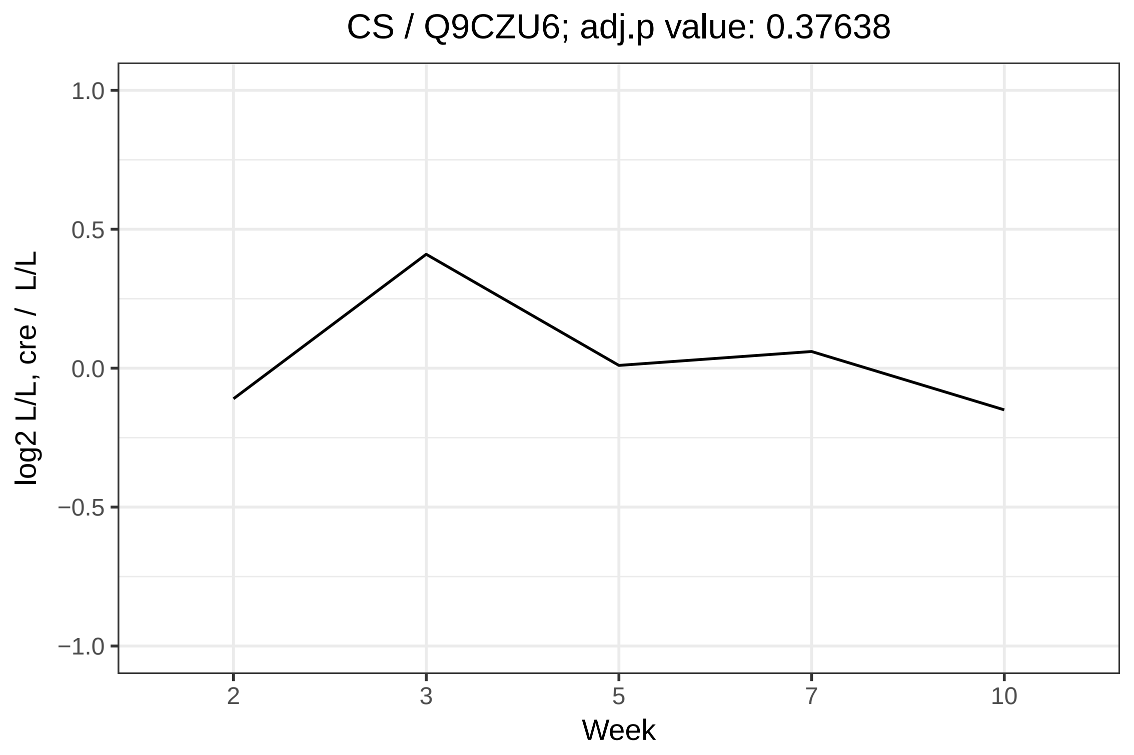
CS / Q9CZU6; adj.p value: 0.37638
1.0
0.5
0.0
−0.5
−1.0
log2 L/L, cre / L/L
2
3
5
7
10
Week

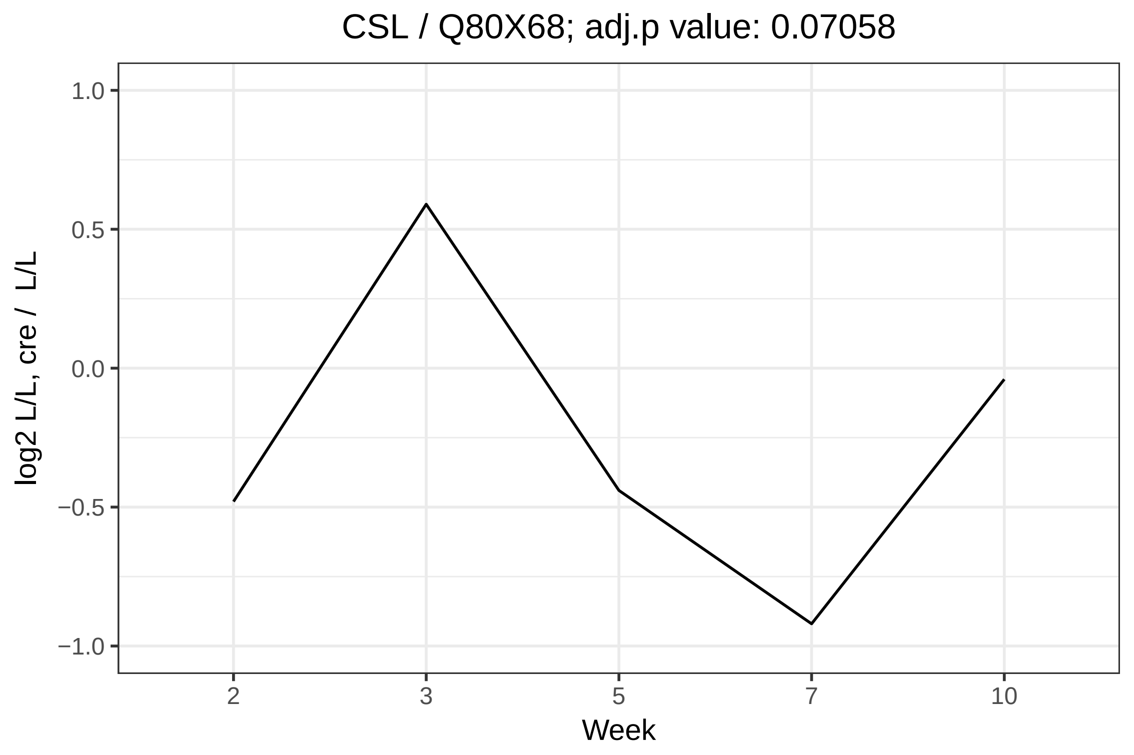

CSL / Q80X68; adj.p value: 0.07058
1.0
0.5
0.0
−0.5
−1.0
log2 L/L, cre / L/L
2
3
5
7
10
Week

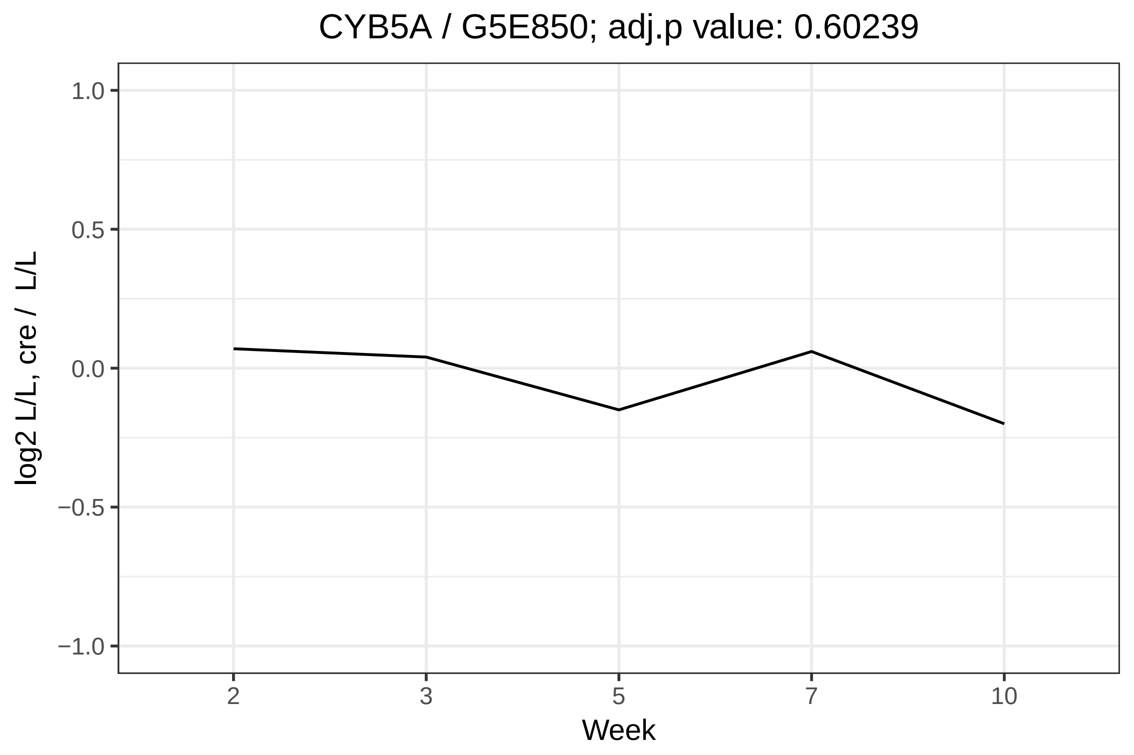
CYB5A / G5E850; adj.p value: 0.60239
log2 L/L, cre / L/L
1.0
0.5
0.0
−0.5
−1.0
2
3
5
7
10
Week

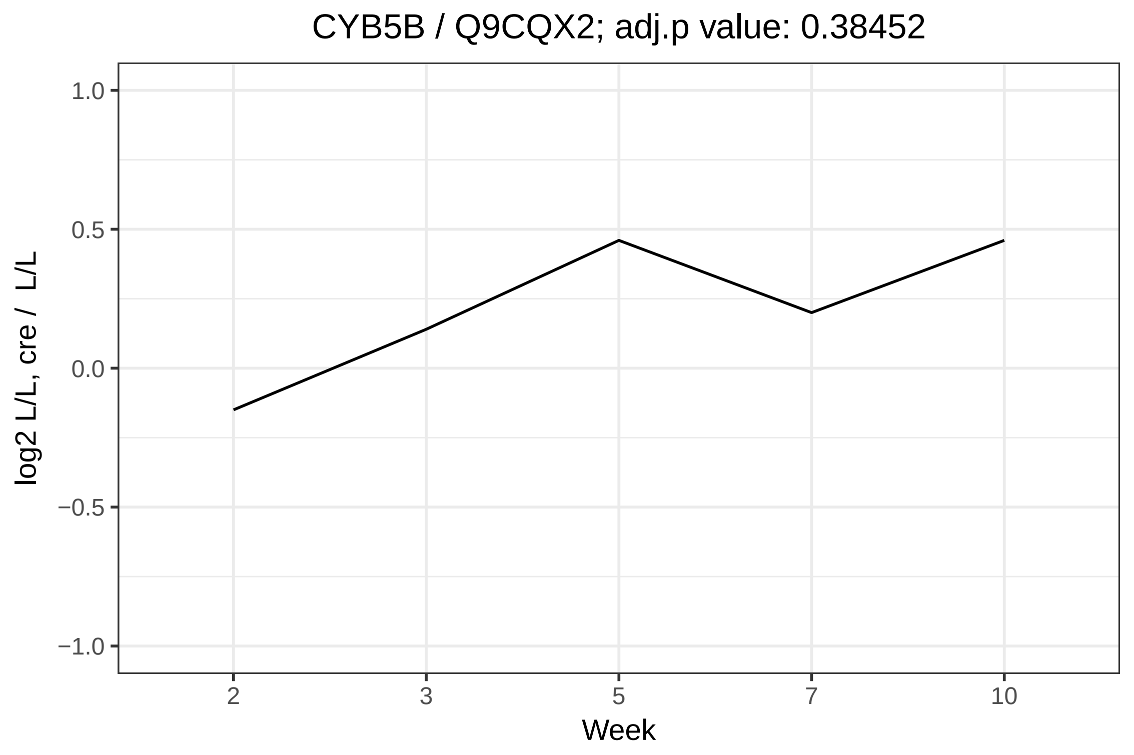
CYB5B / Q9CQX2; adj.p value: 0.38452
1.0
0.5
0.0
−0.5
−1.0
log2 L/L, cre / L/L
2
3
5
7
10
Week

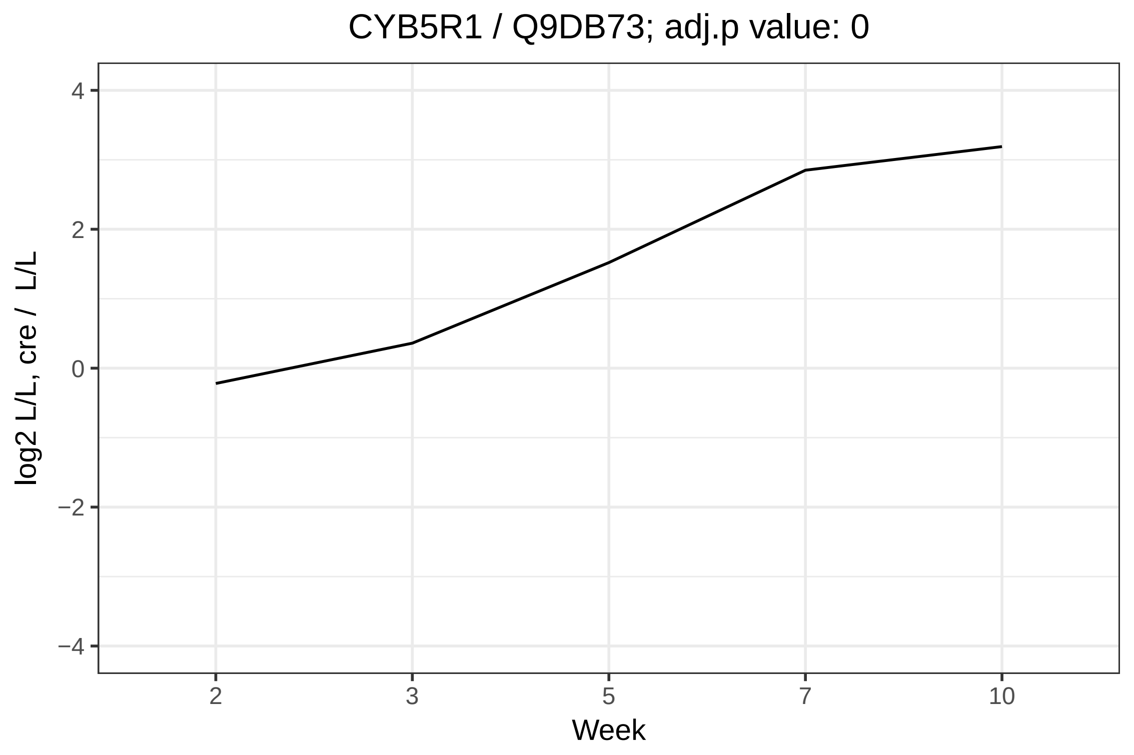
CYB5R1 / Q9DB73; adj.p value: 0
log2 L/L, cre / L/L
4
2
0
−2
−4
2
3
5
7
10
Week

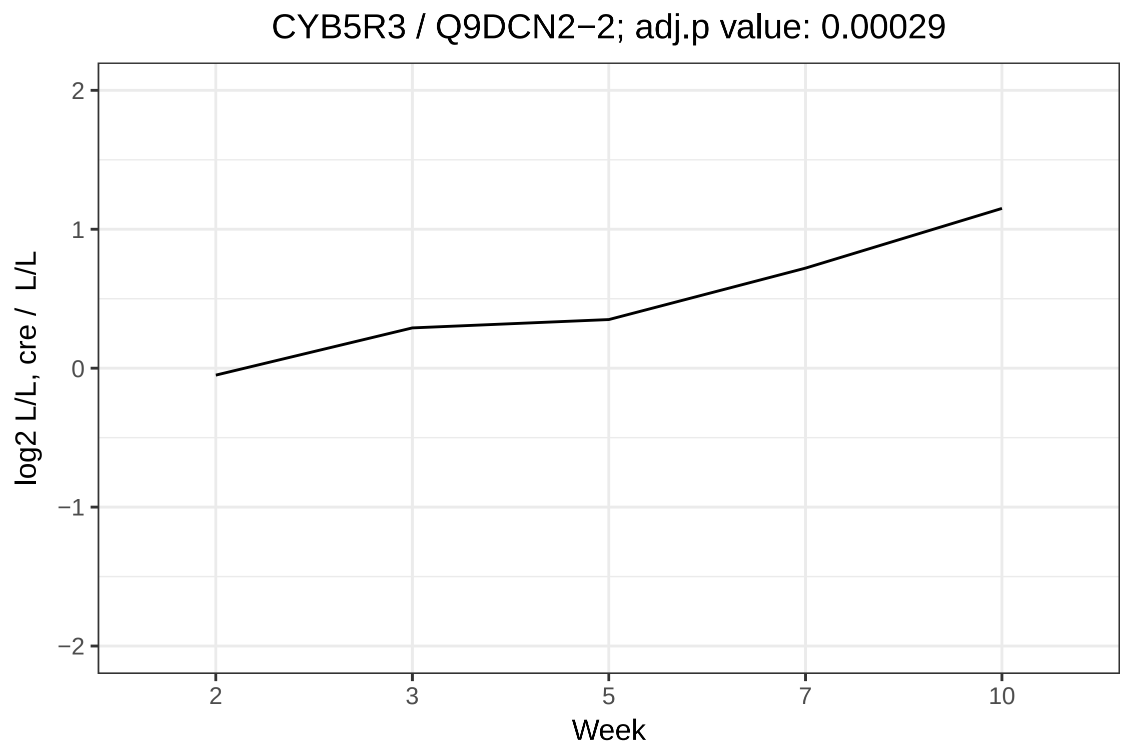
CYB5R3 / Q9DCN2−2; adj.p value: 0.00029
log2 L/L, cre / L/L
2
1
0
−1
−2
2
3
5
7
10
Week

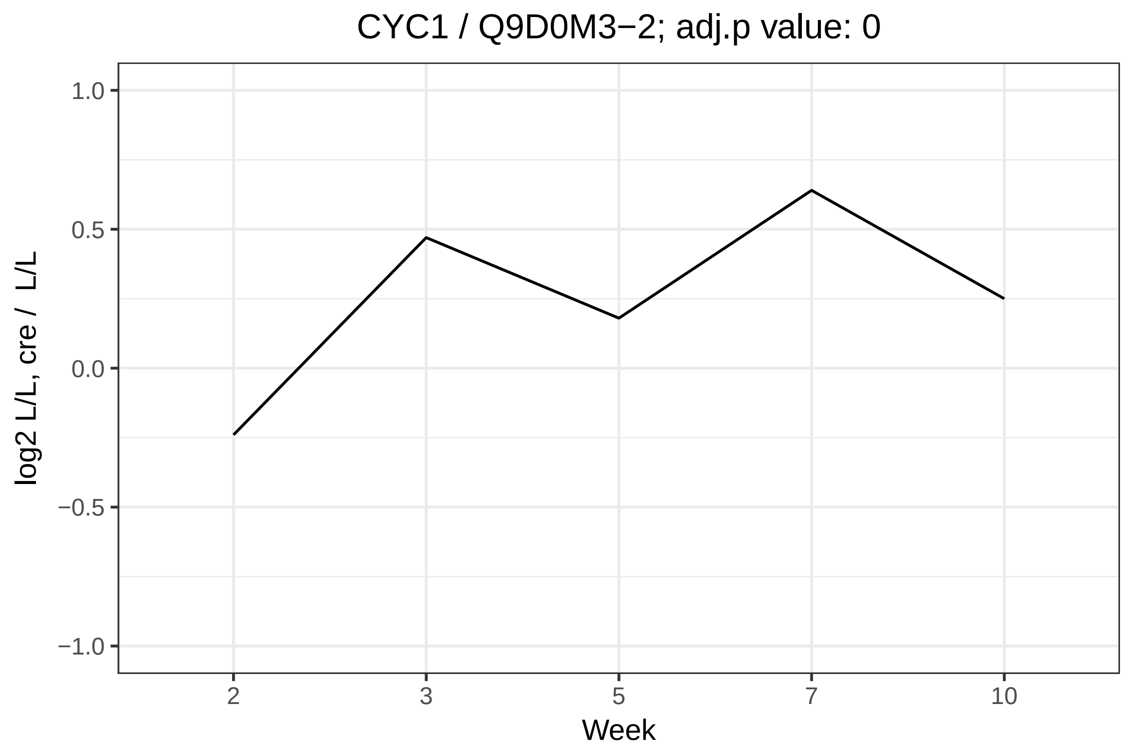

CYC1 / Q9D0M3−2; adj.p value: 0
1.0
0.5
0.0
−0.5
−1.0
log2 L/L, cre / L/L
2
3
5
7
10
Week

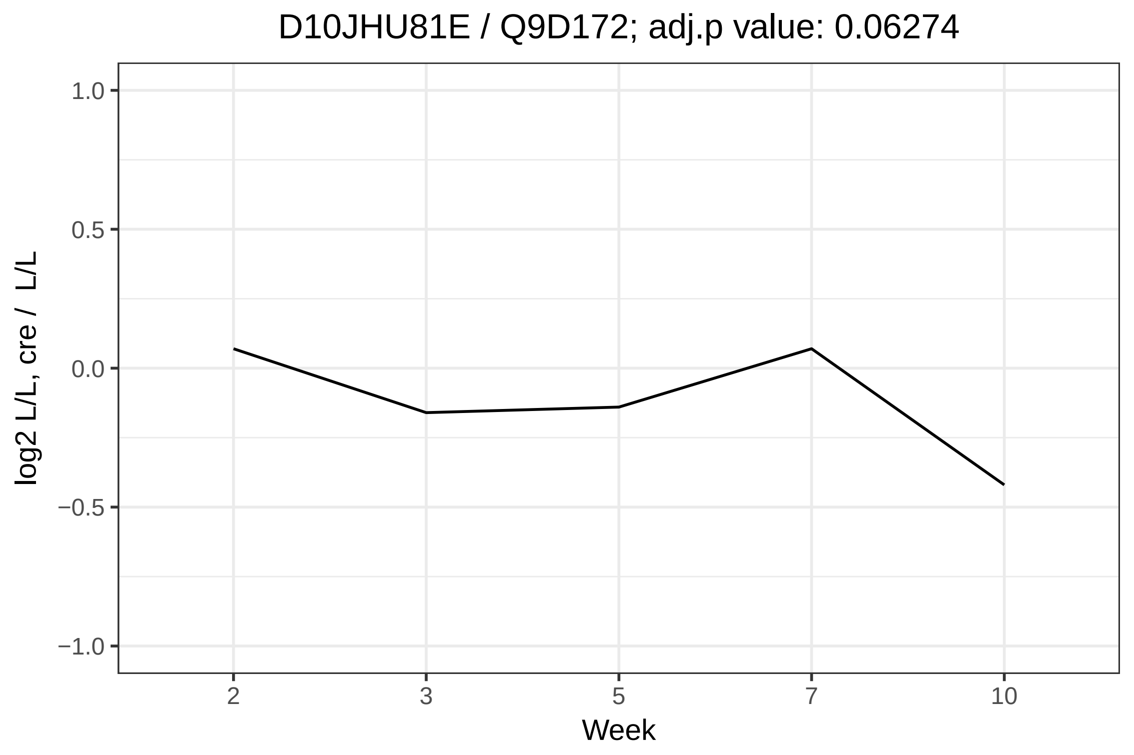
D10JHU81E / Q9D172; adj.p value: 0.06274
log2 L/L, cre / L/L
1.0
0.5
0.0
−0.5
−1.0
2
3
5
7
10
Week

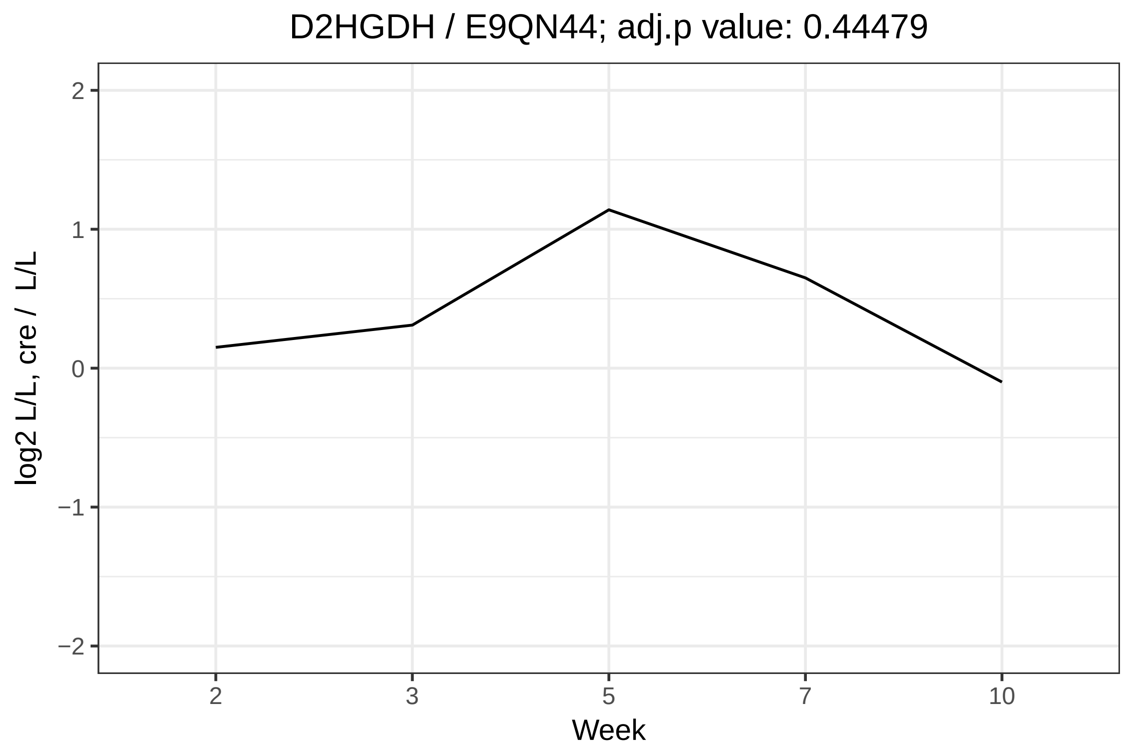
D2HGDH / E9QN44; adj.p value: 0.44479
2
1
0
−1
−2
log2 L/L, cre / L/L
2
3
5
7
10
Week

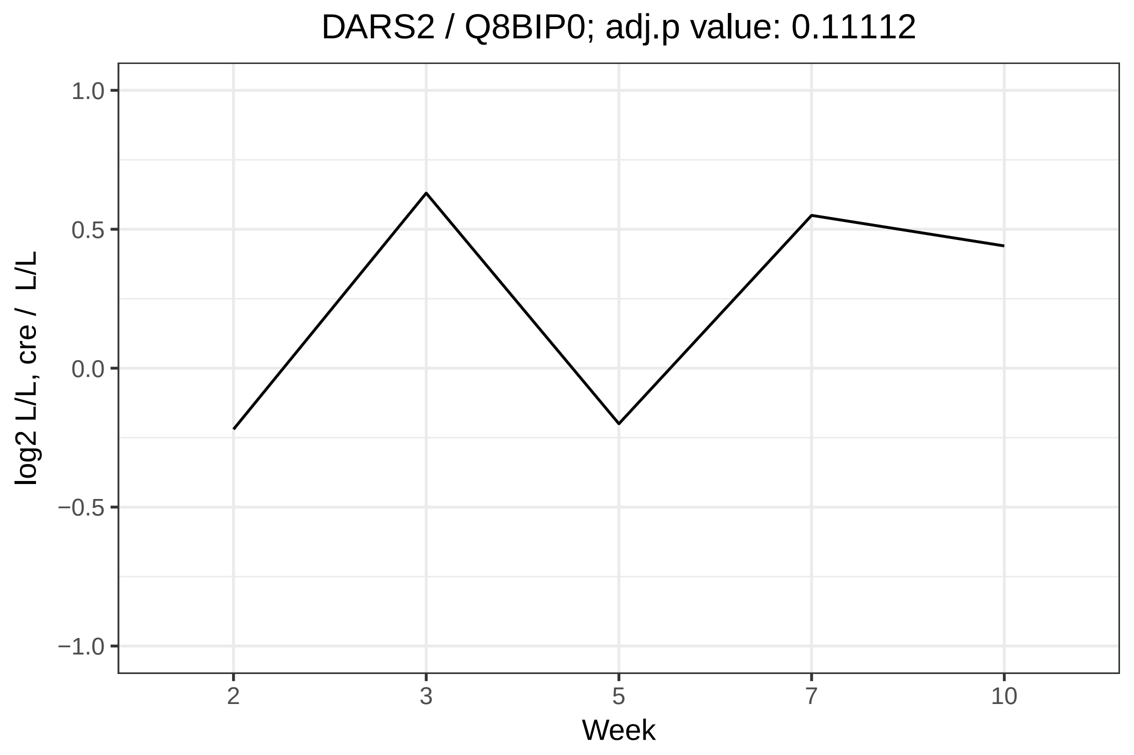
DARS2 / Q8BIP0; adj.p value: 0.11112
1.0
0.5
0.0
−0.5
−1.0
log2 L/L, cre / L/L
2
3
5
7
10
Week

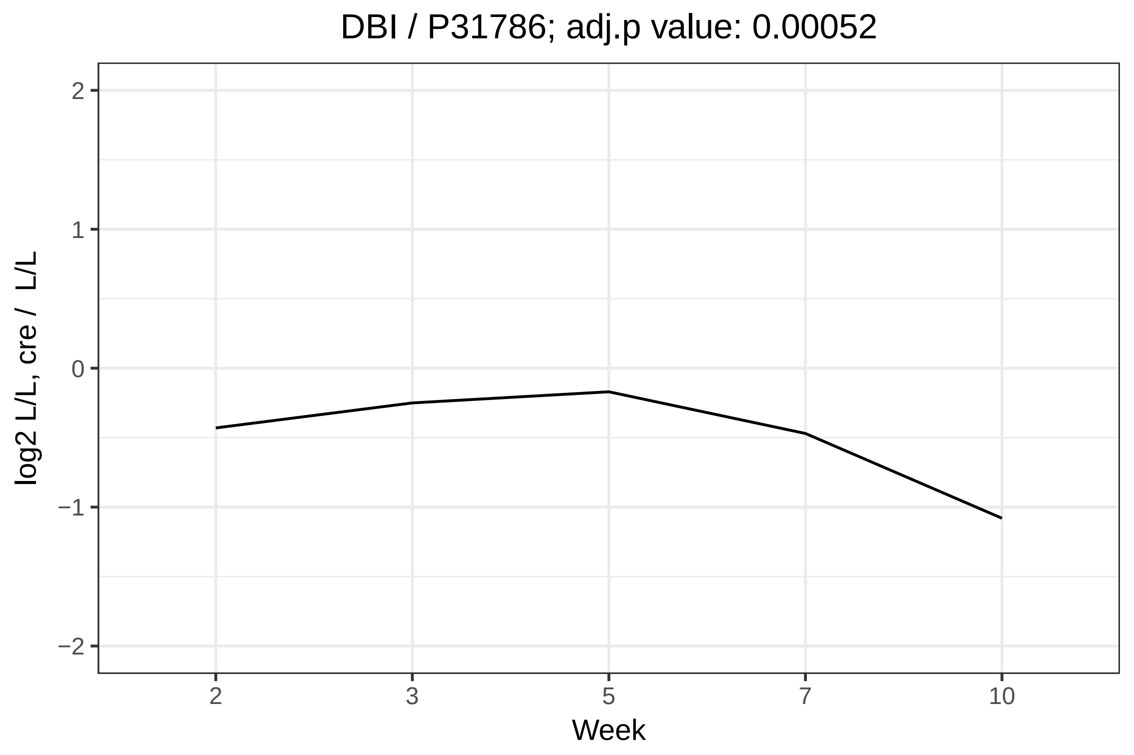
DBI / P31786; adj.p value: 0.00052
log2 L/L, cre / L/L
2
1
0
−1
−2
2
3
5
7
10
Week

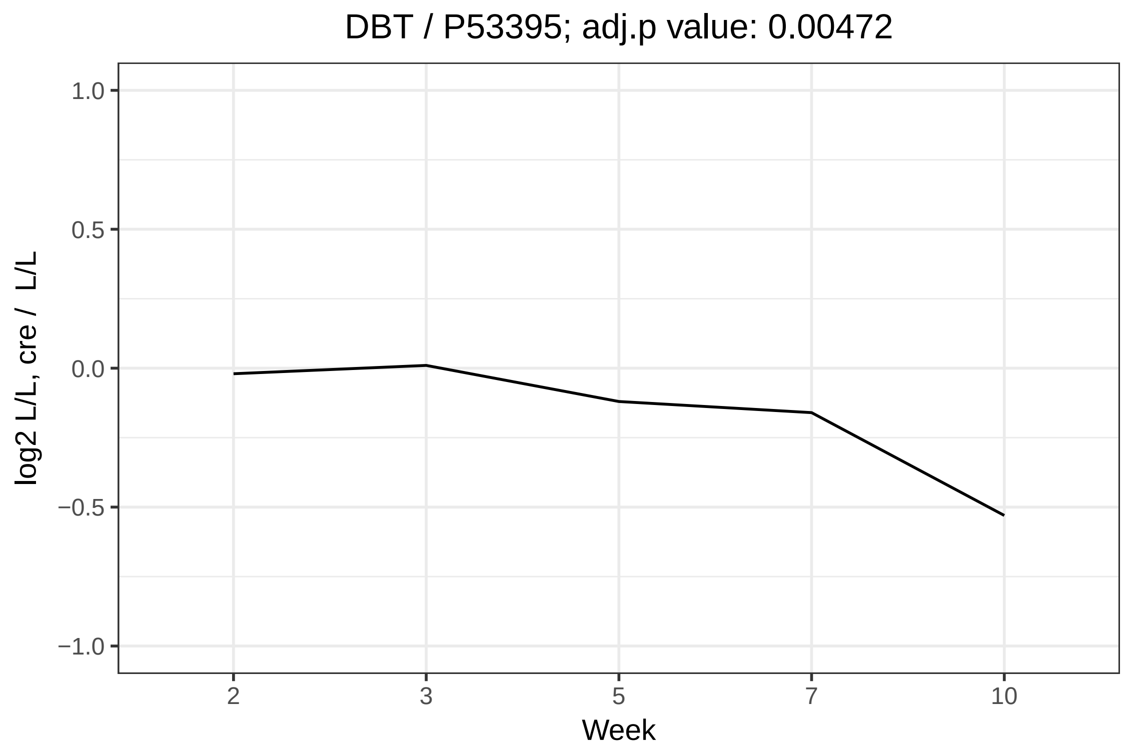

DBT / P53395; adj.p value: 0.00472
1.0
0.5
0.0
−0.5
−1.0
log2 L/L, cre / L/L
2
3
5
7
10
Week

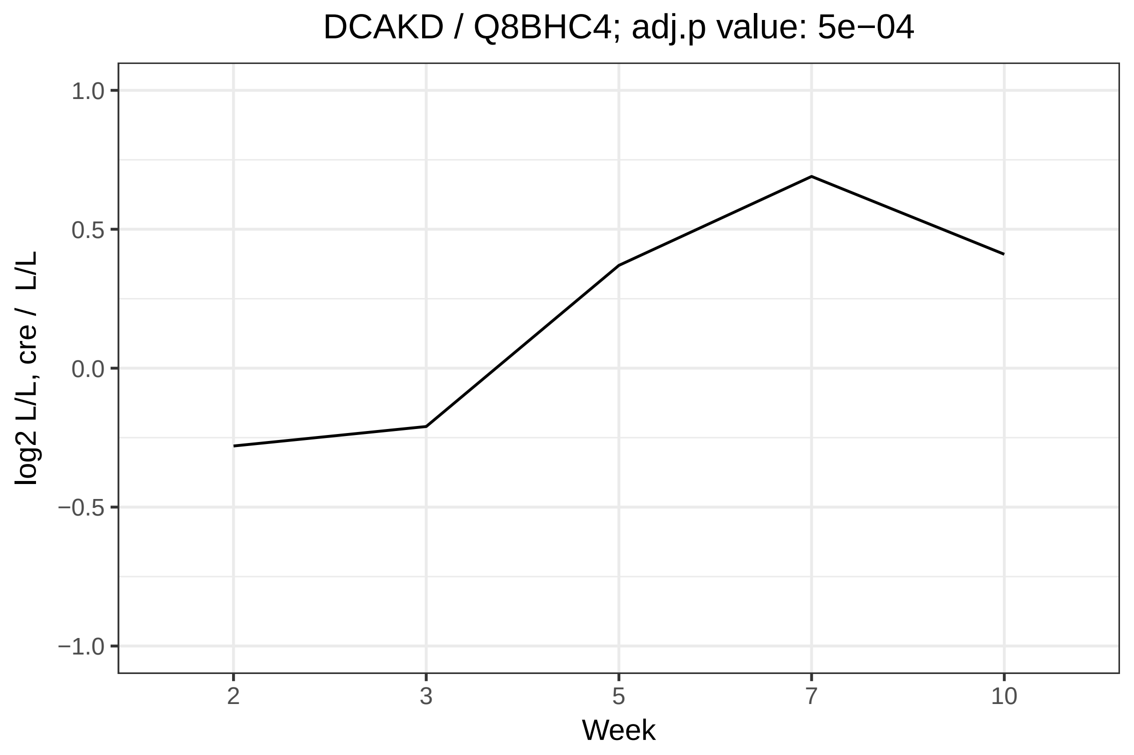

DCAKD / Q8BHC4; adj.p value: 5e−04
1.0
0.5
0.0
−0.5
−1.0
log2 L/L, cre / L/L
2
3
5
7
10
Week

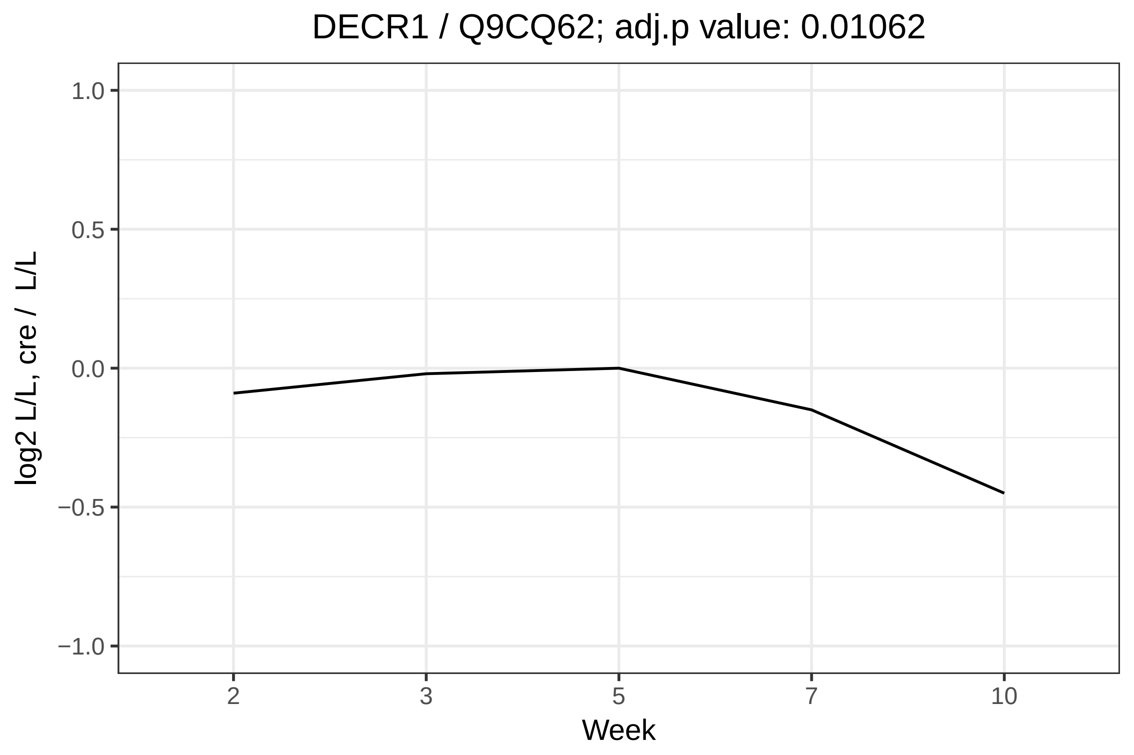
DECR1 / Q9CQ62; adj.p value: 0.01062
log2 L/L, cre / L/L
1.0
0.5
0.0
−0.5
−1.0
2
3
5
7
10
Week

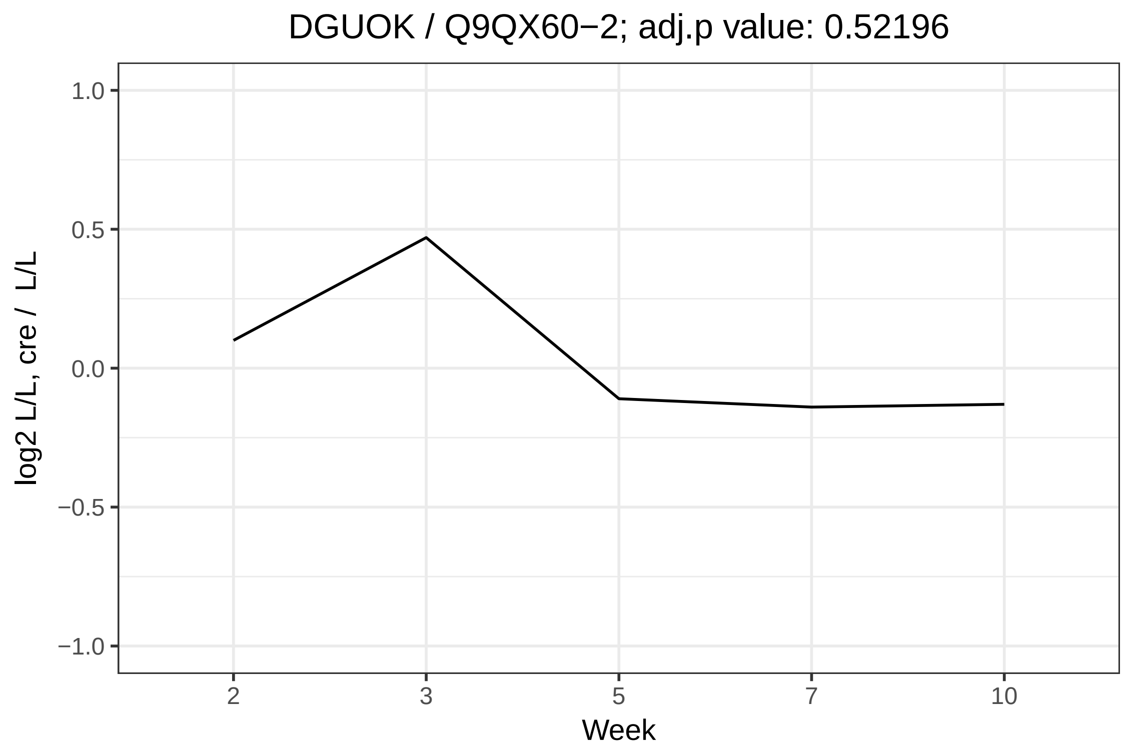
DGUOK / Q9QX60−2; adj.p value: 0.52196
log2 L/L, cre / L/L
1.0
0.5
0.0
−0.5
−1.0
2
3
5
7
10
Week

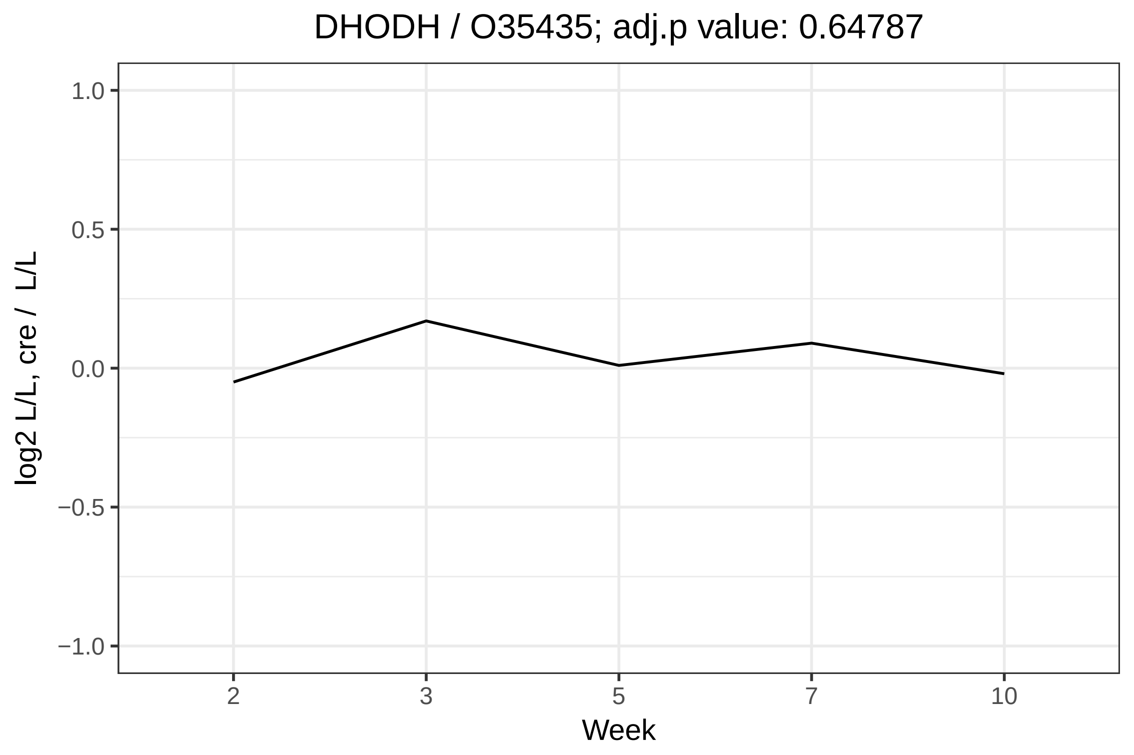

DHODH / O35435; adj.p value: 0.64787
1.0
0.5
0.0
−0.5
−1.0
log2 L/L, cre / L/L
2
3
5
7
10
Week

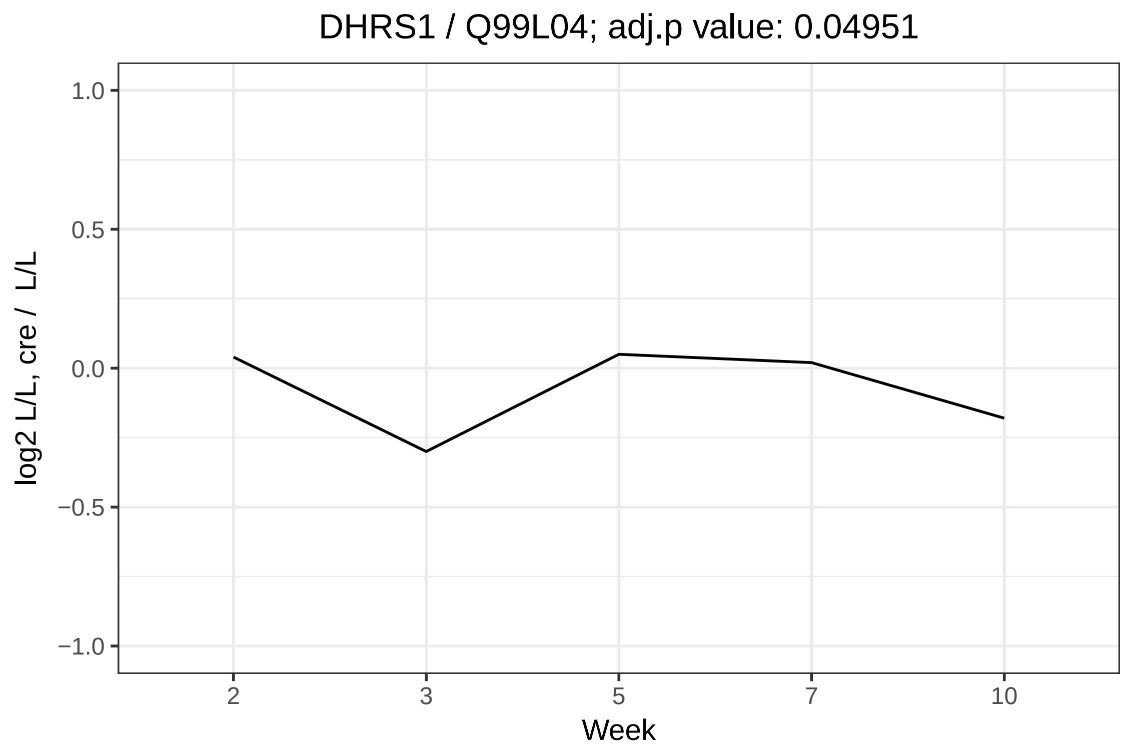

DHRS1 / Q99L04; adj.p value: 0.04951
log2 L/L, cre / L/L
1.0
0.5
0.0
−0.5
−1.0
2
3
5
7
10
Week

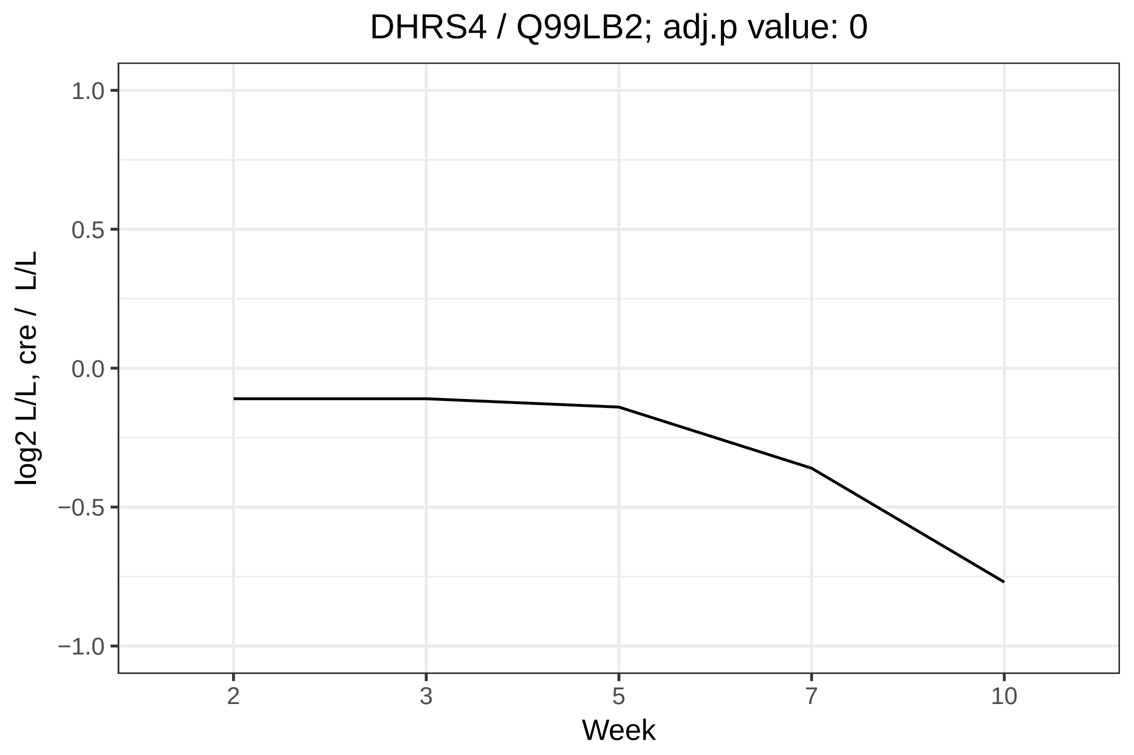

DHRS4 / Q99LB2; adj.p value: 0
1.0
0.5
0.0
−0.5
−1.0
log2 L/L, cre / L/L
2
3
5
7
10
Week

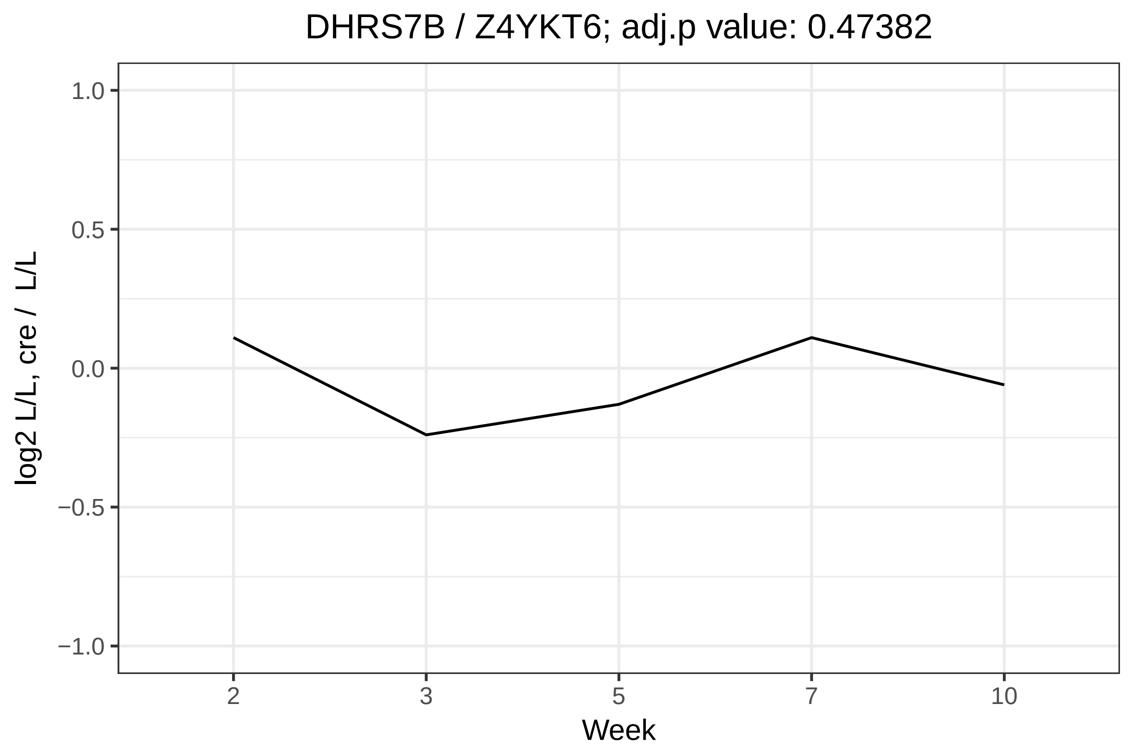
DHRS7B / Z4YKT6; adj.p value: 0.47382
log2 L/L, cre / L/L
1.0
0.5
0.0
−0.5
−1.0
2
3
5
7
10
Week

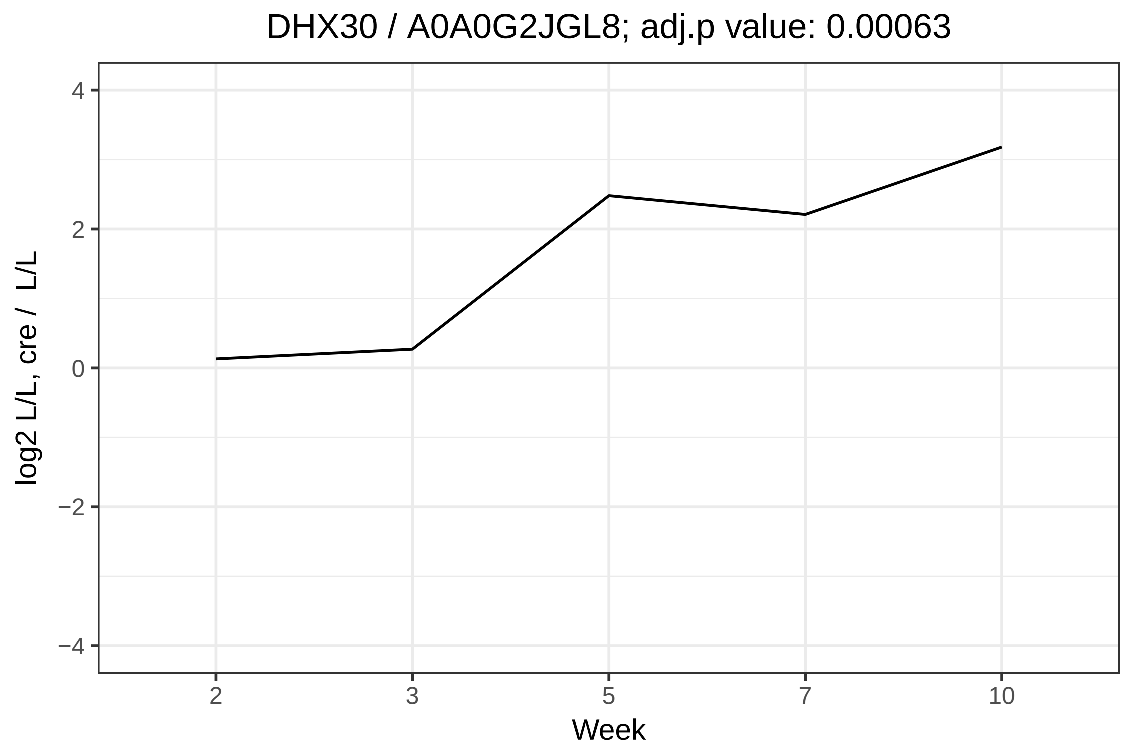

DHX30 / A0A0G2JGL8; adj.p value: 0.00063
log2 L/L, cre / L/L
4
2
0
−2
−4
2
3
5
7
10
Week

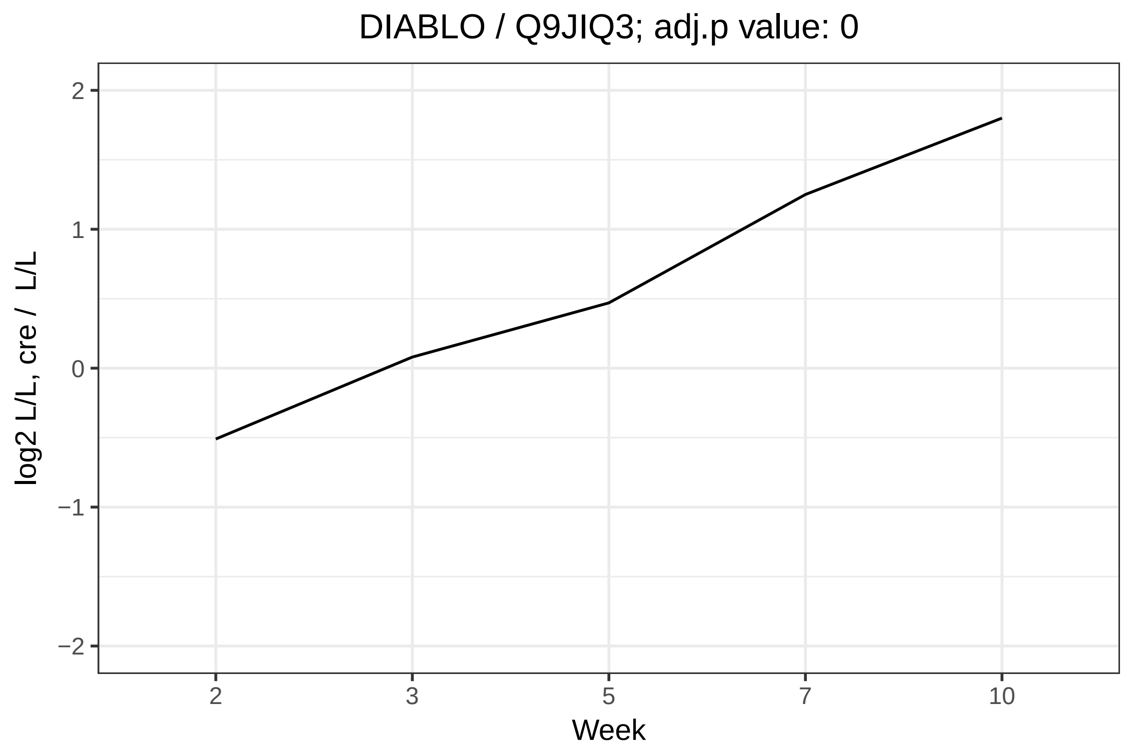

DIABLO / Q9JIQ3; adj.p value: 0
log2 L/L, cre / L/L
2
1
0
−1
−2
2
3
5
7
10
Week

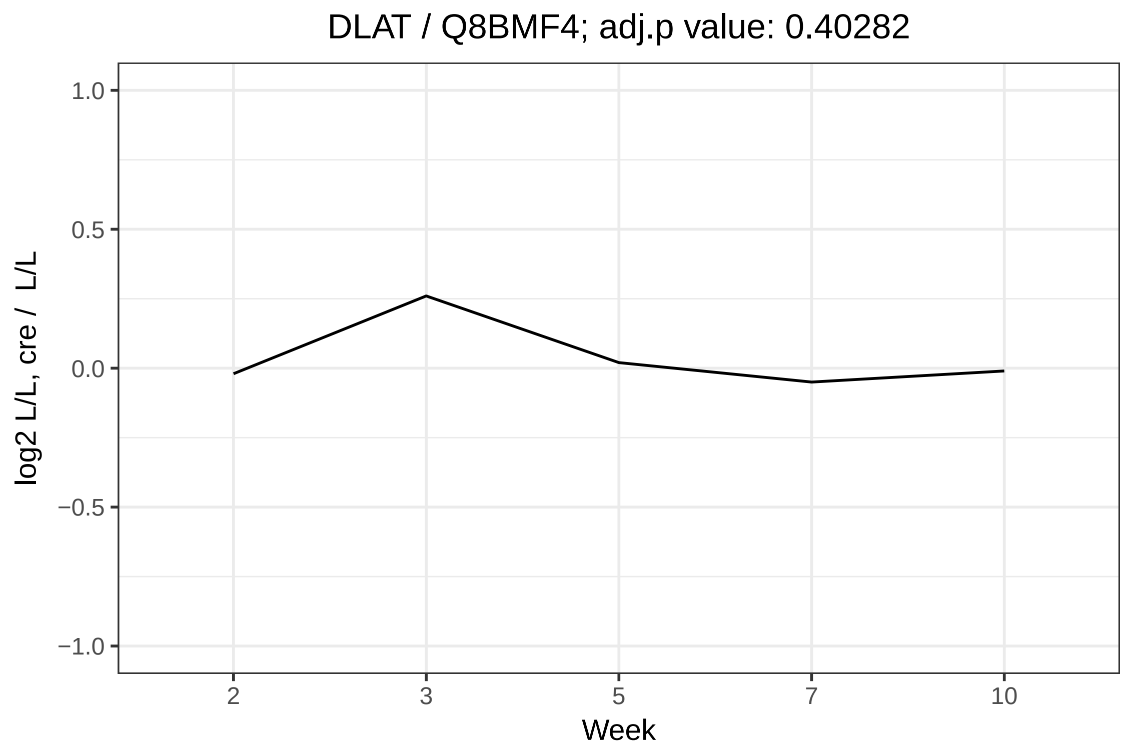

DLAT / Q8BMF4; adj.p value: 0.40282
1.0
0.5
0.0
−0.5
−1.0
log2 L/L, cre / L/L
2
3
5
7
10
Week

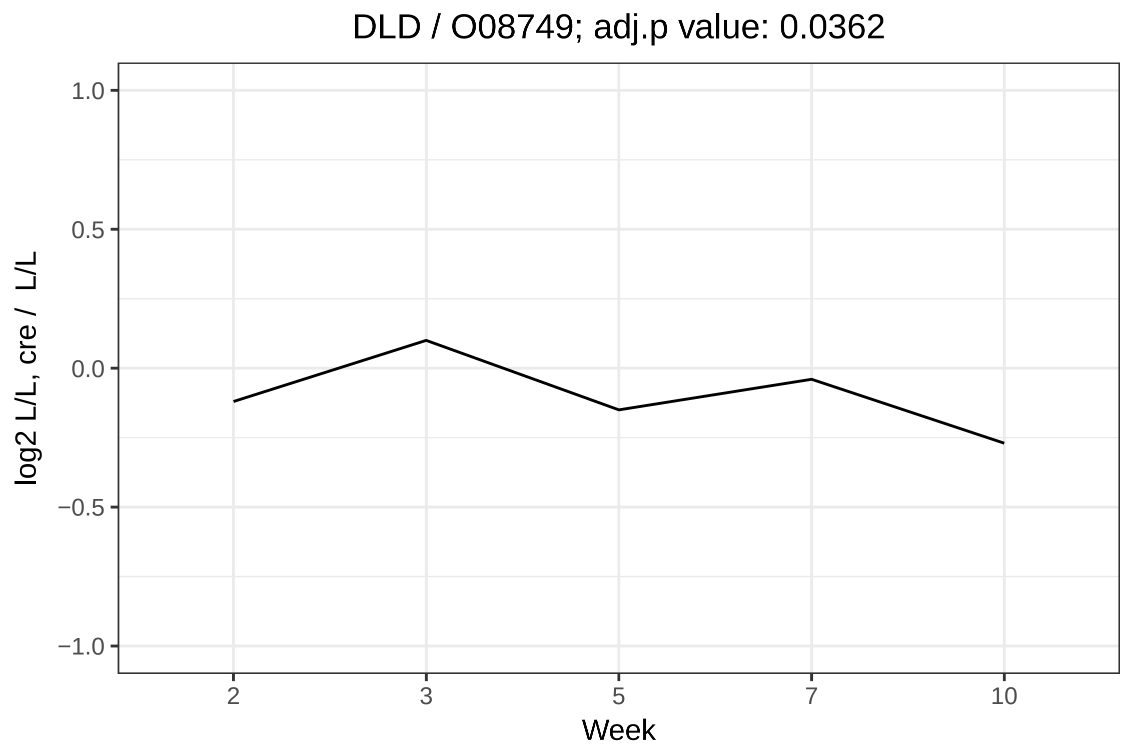
DLD / O08749; adj.p value: 0.0362
1.0
0.5
0.0
−0.5
−1.0
log2 L/L, cre / L/L
2
3
5
7
10
Week

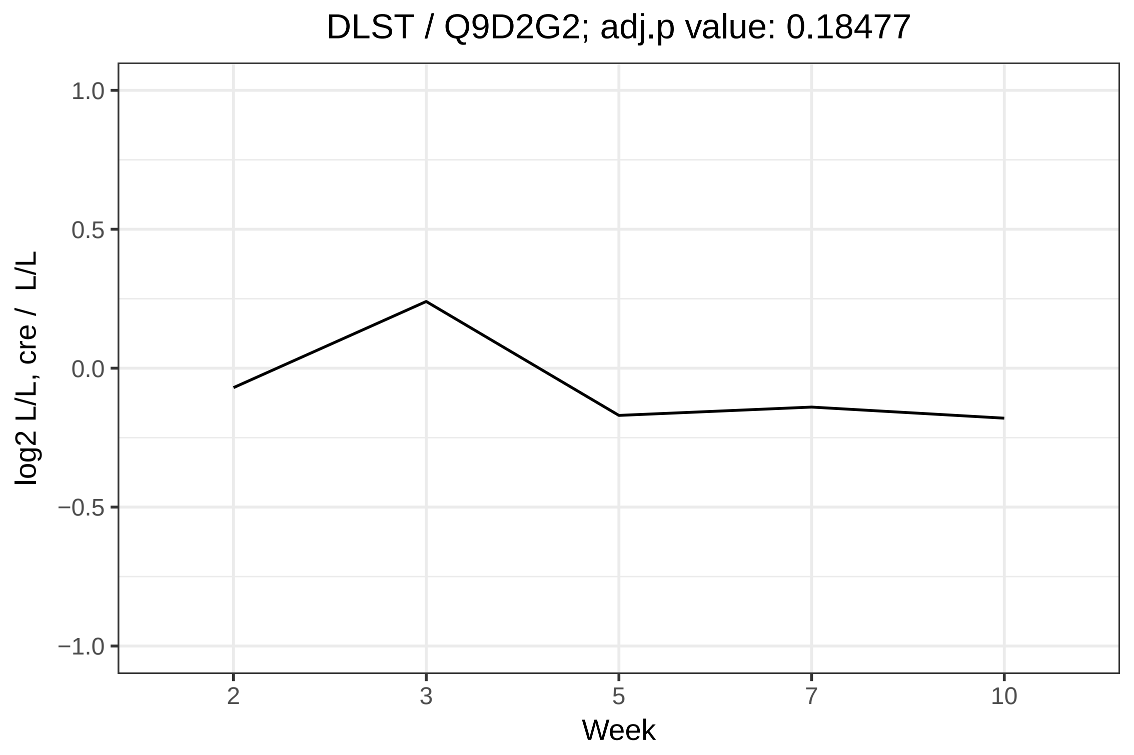

DLST / Q9D2G2; adj.p value: 0.18477
log2 L/L, cre / L/L
1.0
0.5
0.0
−0.5
−1.0
2
3
5
7
10
Week

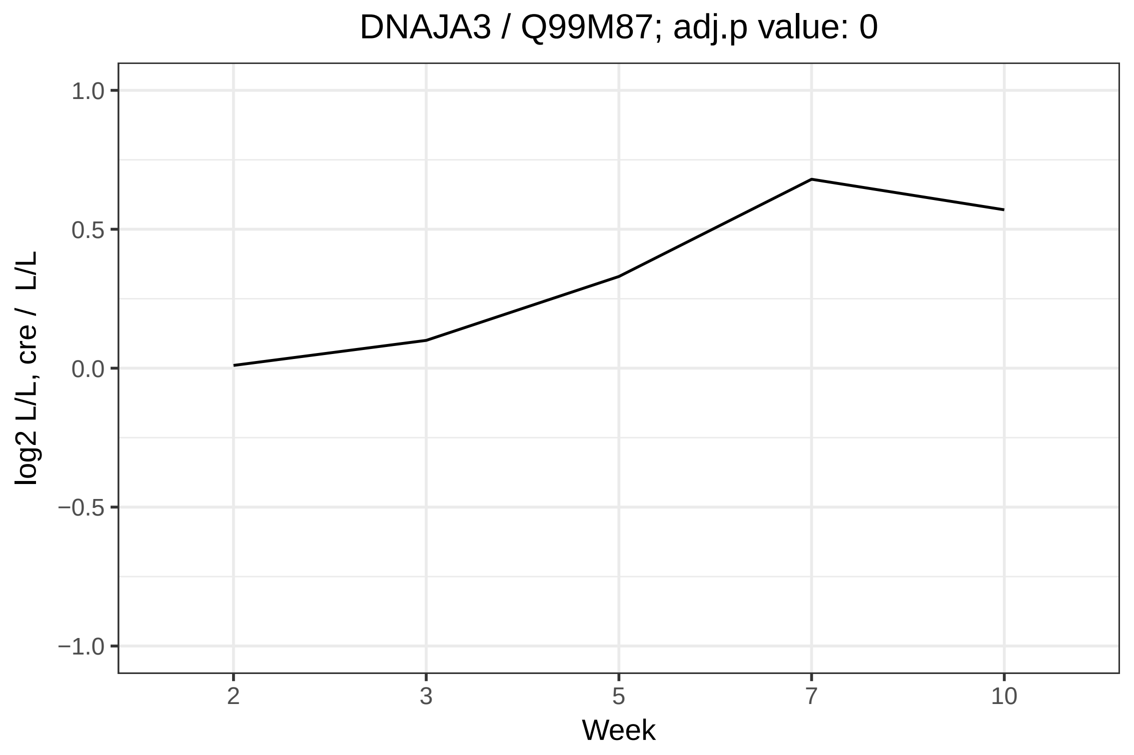

DNAJA3 / Q99M87; adj.p value: 0
1.0
0.5
0.0
−0.5
−1.0
log2 L/L, cre / L/L
2
3
5
7
10
Week

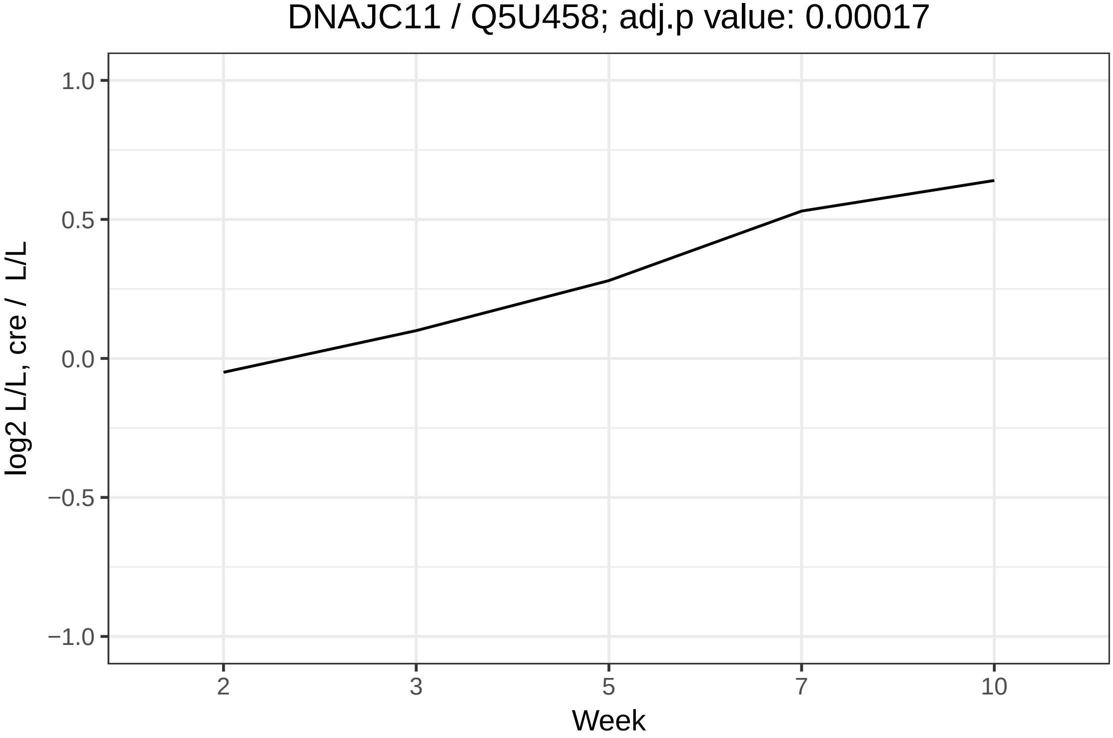

DNAJC11 / Q5U458; adj.p value: 0.00017
log2 L/L, cre / L/L
1.0
0.5
0.0
−0.5
−1.0
2
3
5
7
10
Week

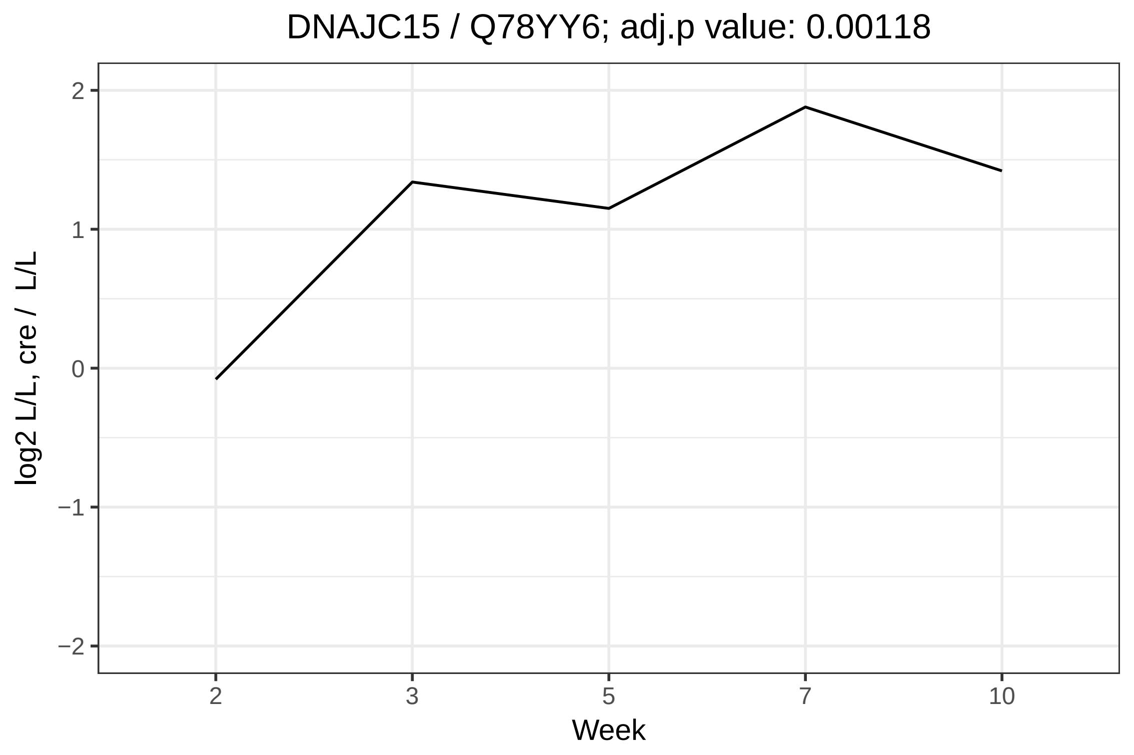
DNAJC15 / Q78YY6; adj.p value: 0.00118
log2 L/L, cre / L/L
2
1
0
−1
−2
2
3
5
7
10
Week

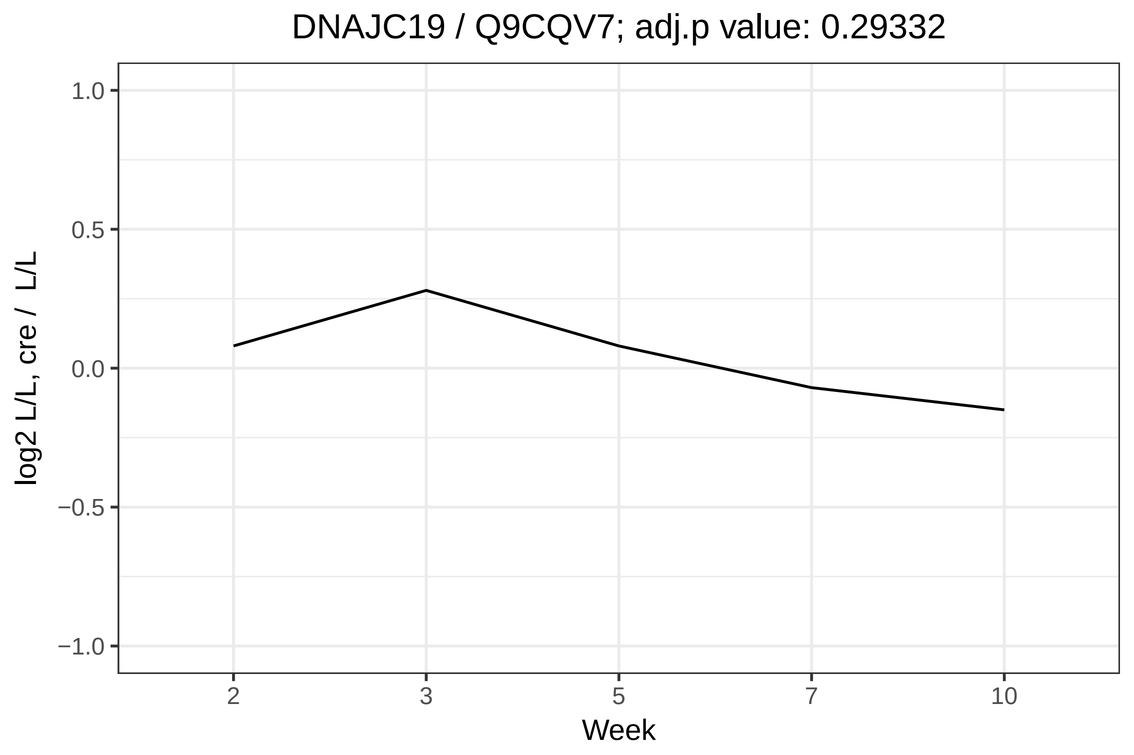

DNAJC19 / Q9CQV7; adj.p value: 0.29332
log2 L/L, cre / L/L
1.0
0.5
0.0
−0.5
−1.0
2
3
5
7
10
Week

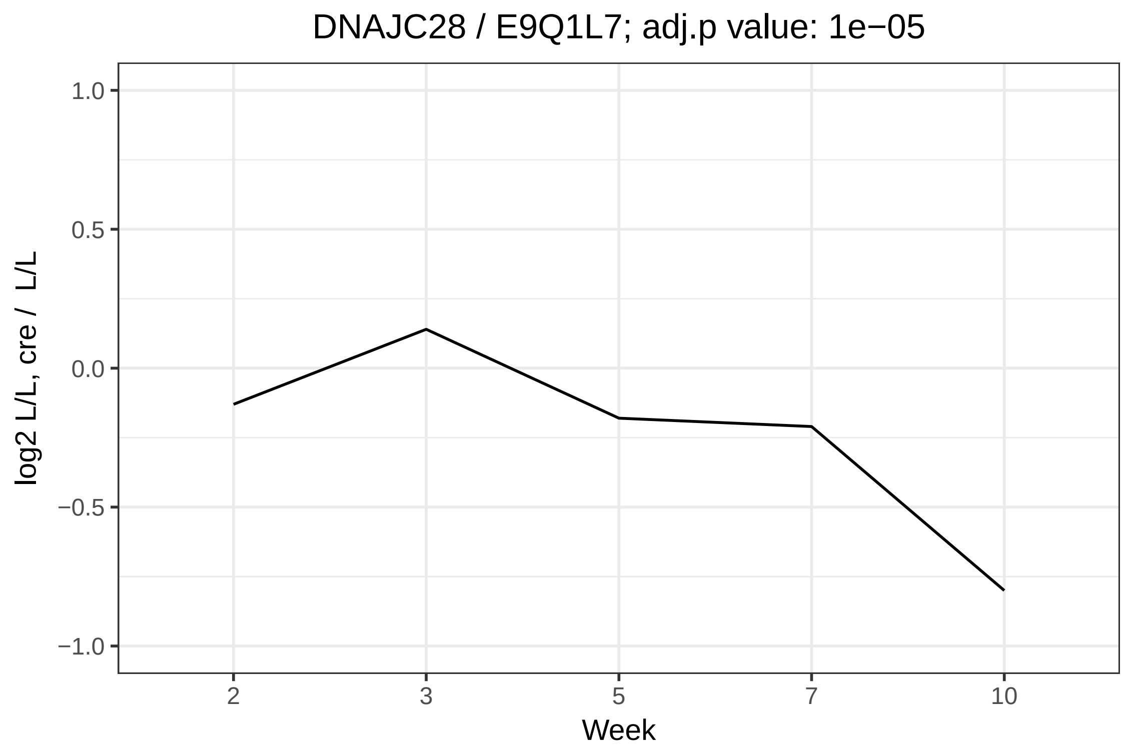

DNAJC28 / E9Q1L7; adj.p value: 1e−05
1.0
0.5
0.0
−0.5
−1.0
log2 L/L, cre / L/L
2
3
5
7
10
Week

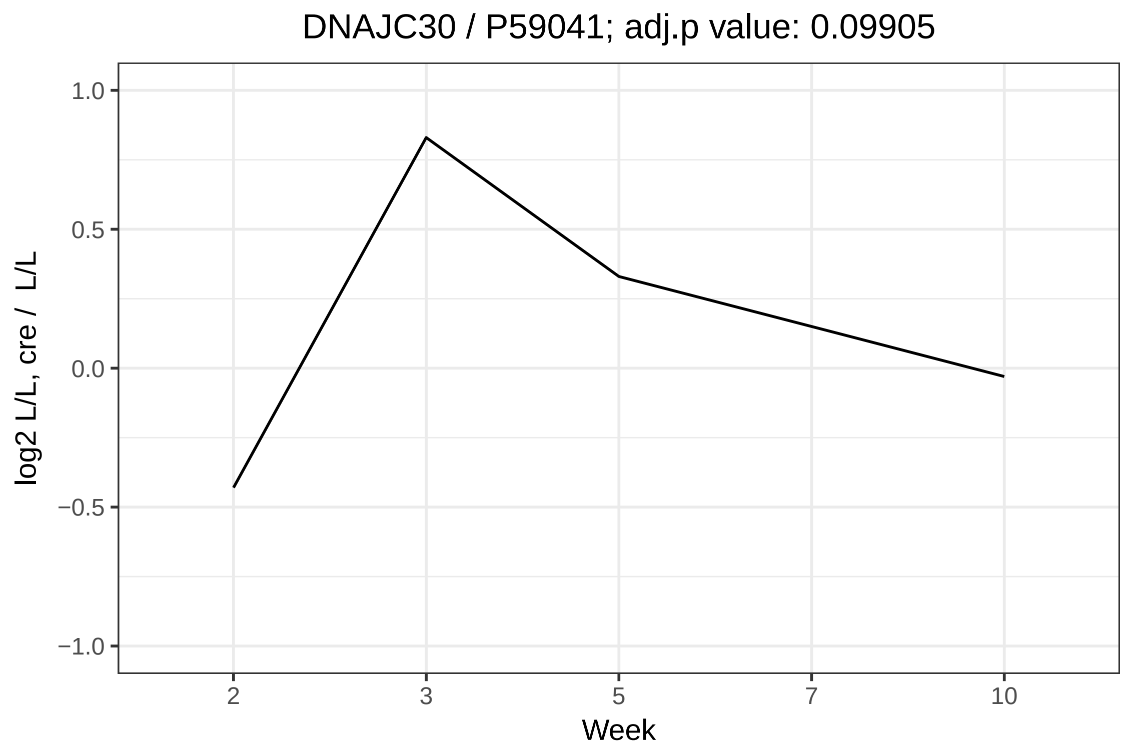
DNAJC30 / P59041; adj.p value: 0.09905
log2 L/L, cre / L/L
1.0
0.5
0.0
−0.5
−1.0
2
3
5
7
10
Week

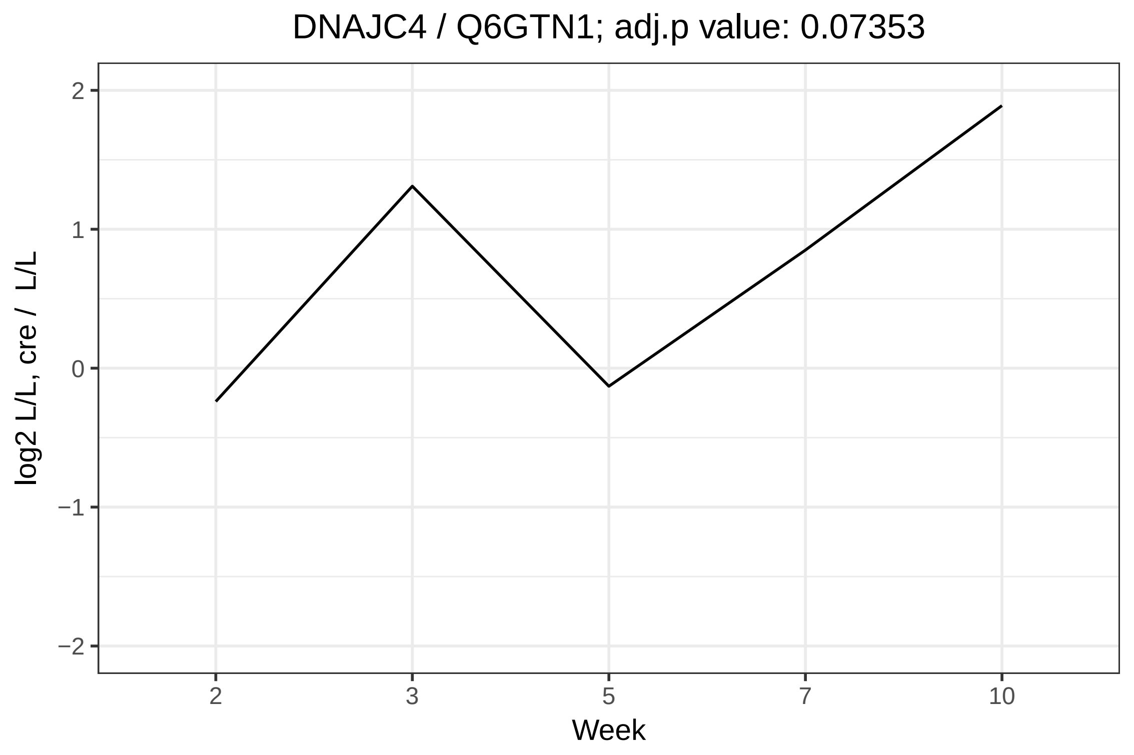
DNAJC4 / Q6GTN1; adj.p value: 0.07353
log2 L/L, cre / L/L
2
1
0
−1
−2
2
3
5
7
10
Week

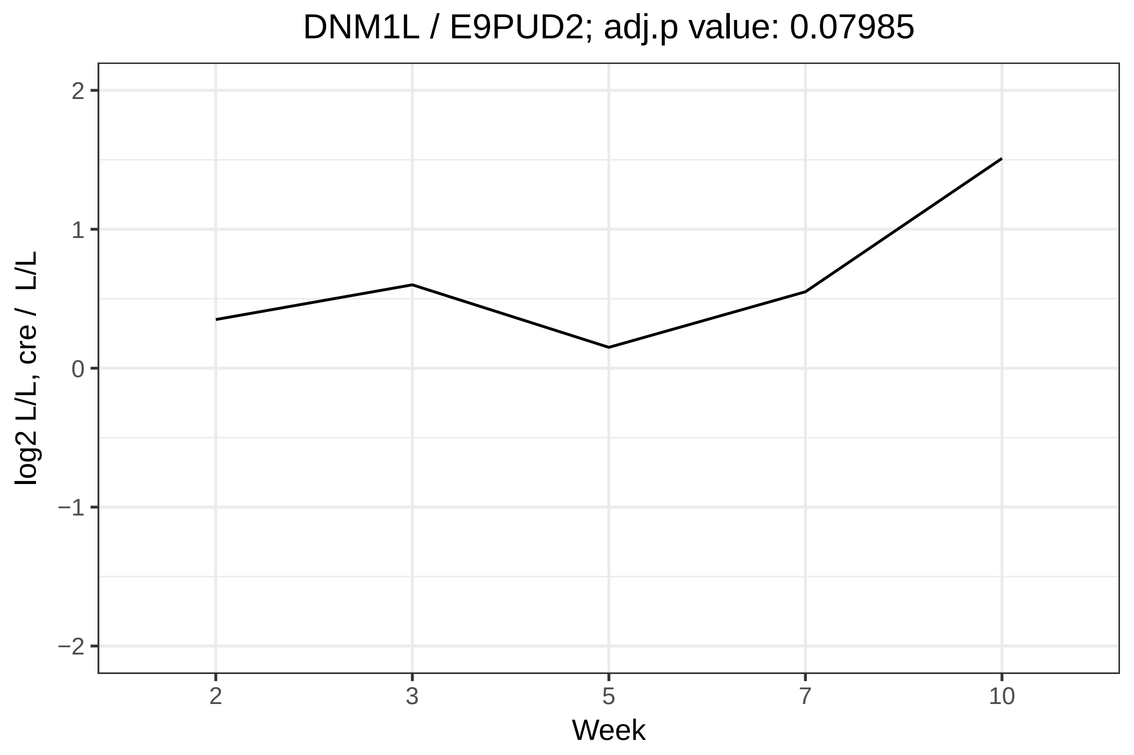
DNM1L / E9PUD2; adj.p value: 0.07985
log2 L/L, cre / L/L
2
1
0
−1
−2
2
3
5
7
10
Week

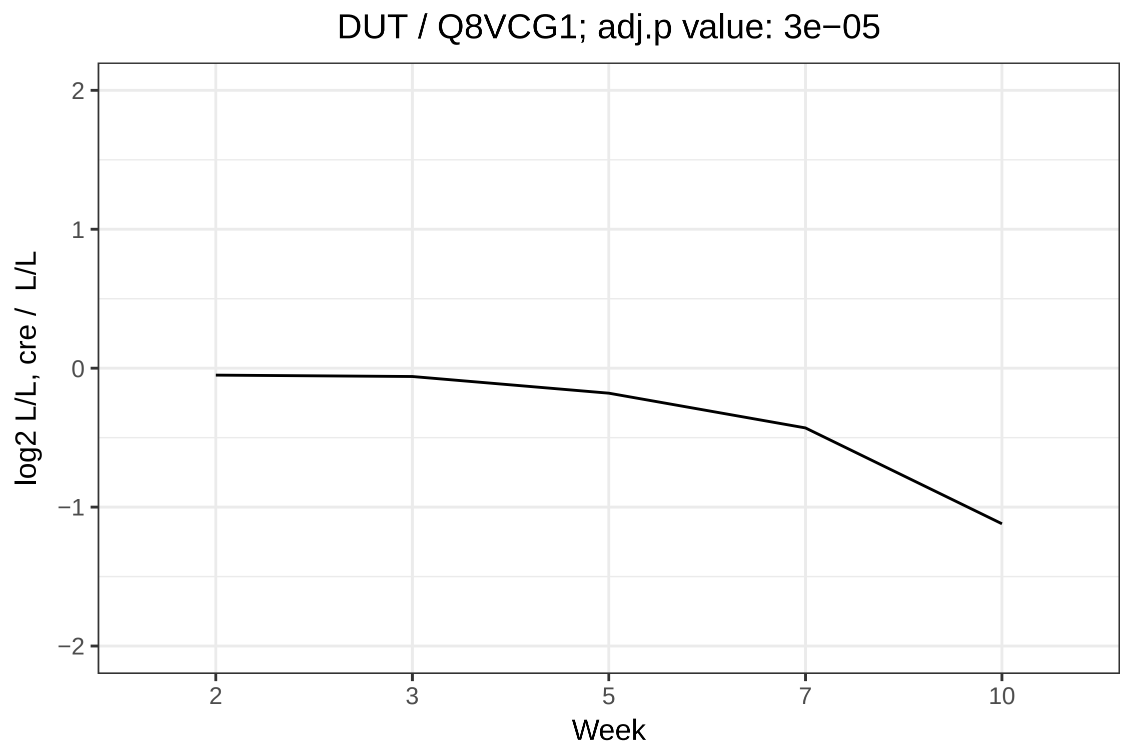
DUT / Q8VCG1; adj.p value: 3e−05
log2 L/L, cre / L/L
2
1
0
−1
−2
2
3
5
7
10
Week

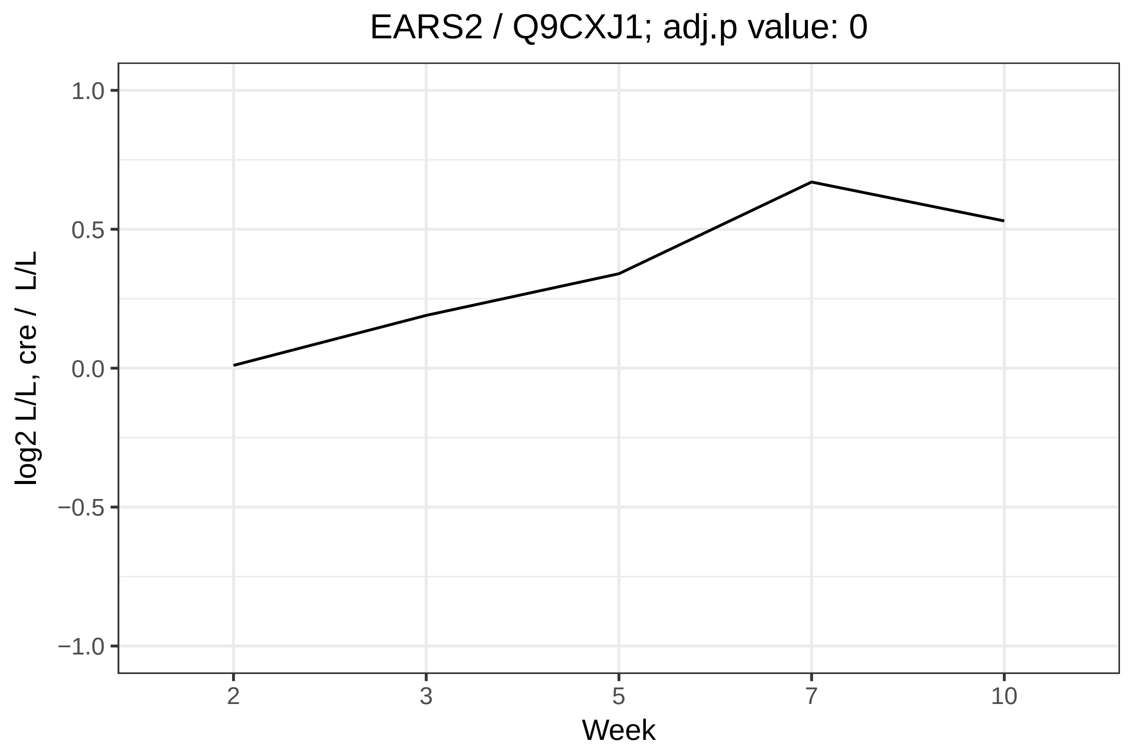
EARS2 / Q9CXJ1; adj.p value: 0
log2 L/L, cre / L/L
1.0
0.5
0.0
−0.5
−1.0
2
3
5
7
10
Week

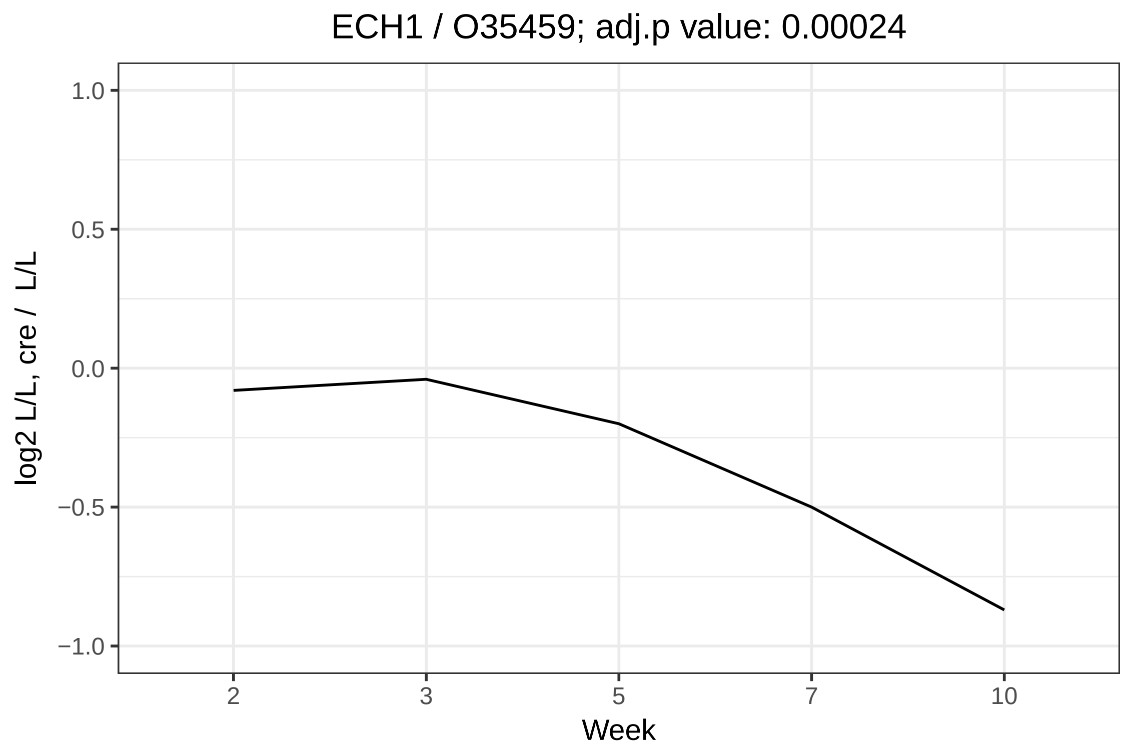
ECH1 / O35459; adj.p value: 0.00024
1.0
0.5
0.0
−0.5
−1.0
log2 L/L, cre / L/L
2
3
5
7
10
Week

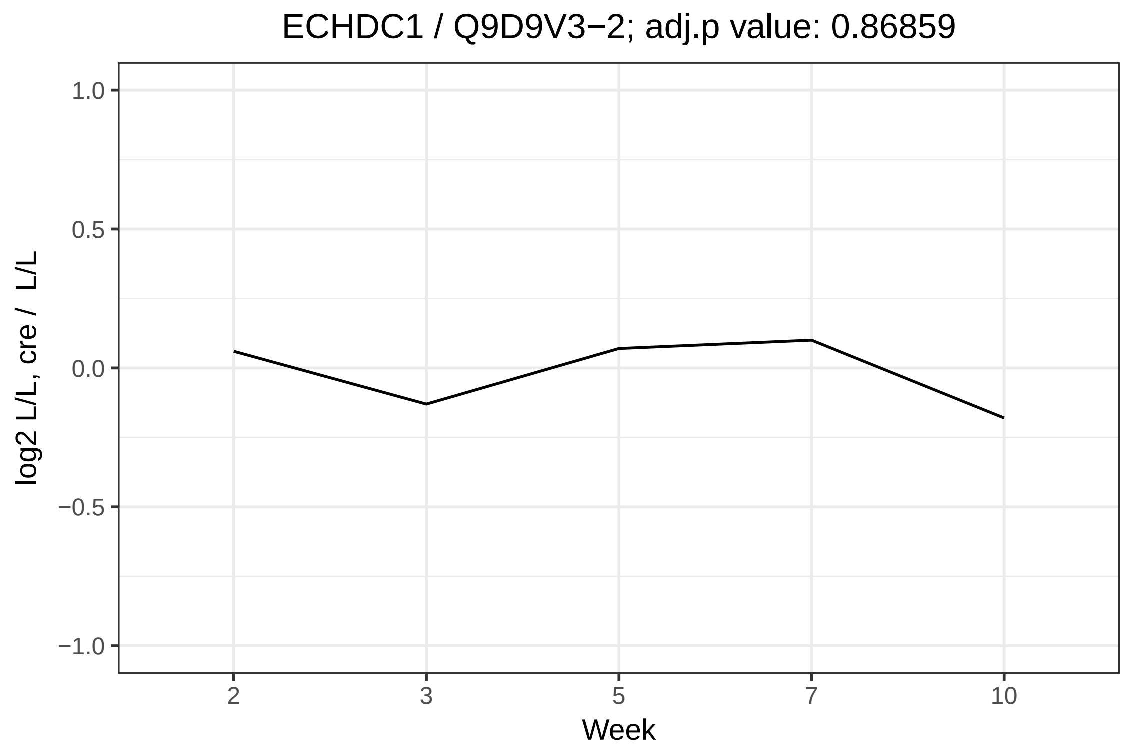

ECHDC1 / Q9D9V3−2; adj.p value: 0.86859
log2 L/L, cre / L/L
1.0
0.5
0.0
−0.5
−1.0
2
3
5
7
10
Week

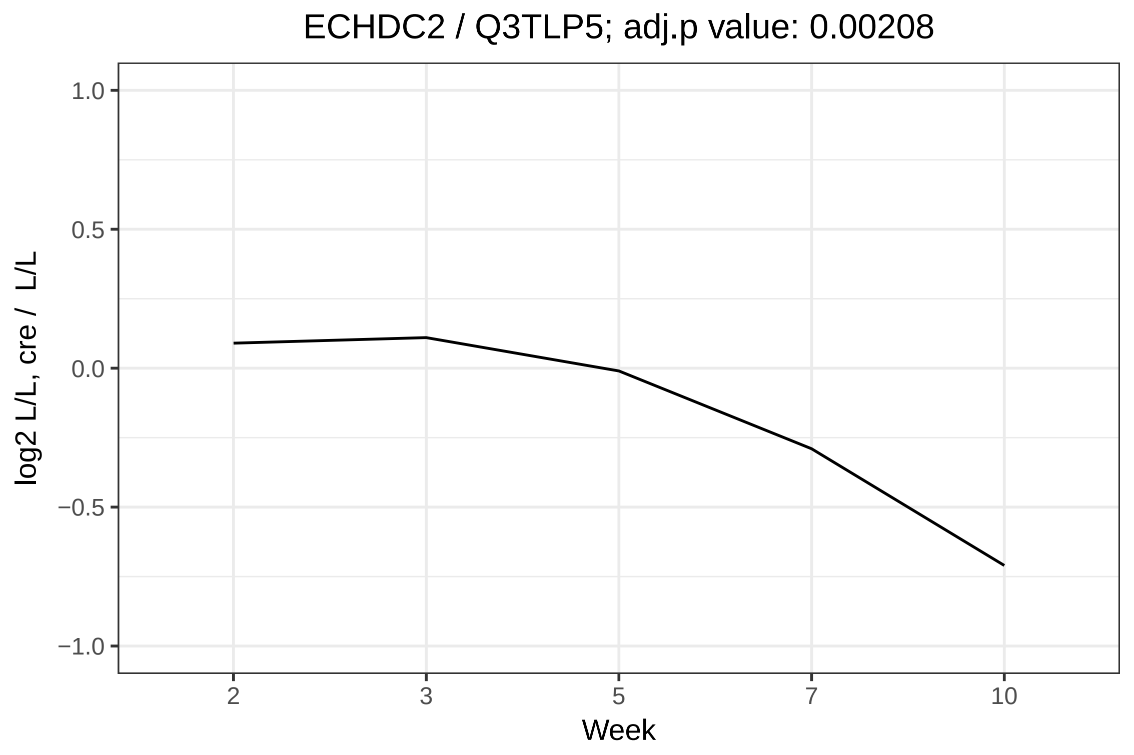
ECHDC2 / Q3TLP5; adj.p value: 0.00208
log2 L/L, cre / L/L
1.0
0.5
0.0
−0.5
−1.0
2
3
5
7
10
Week

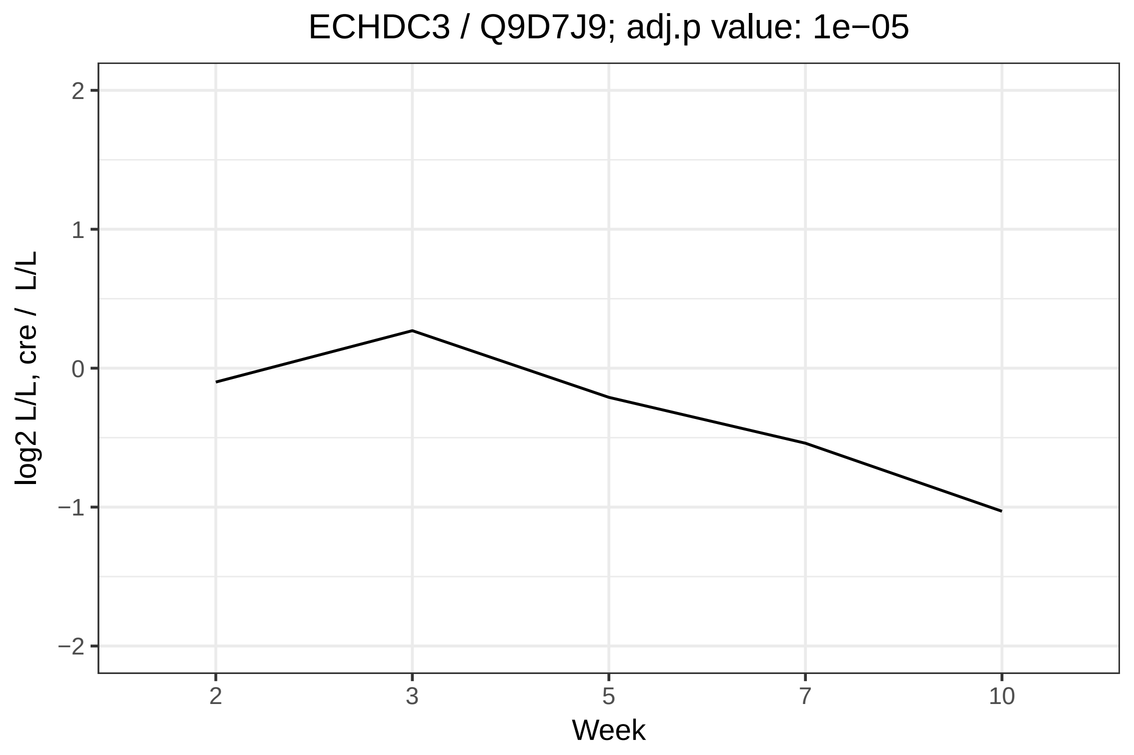
ECHDC3 / Q9D7J9; adj.p value: 1e−05
log2 L/L, cre / L/L
2
1
0
−1
−2
2
3
5
7
10
Week

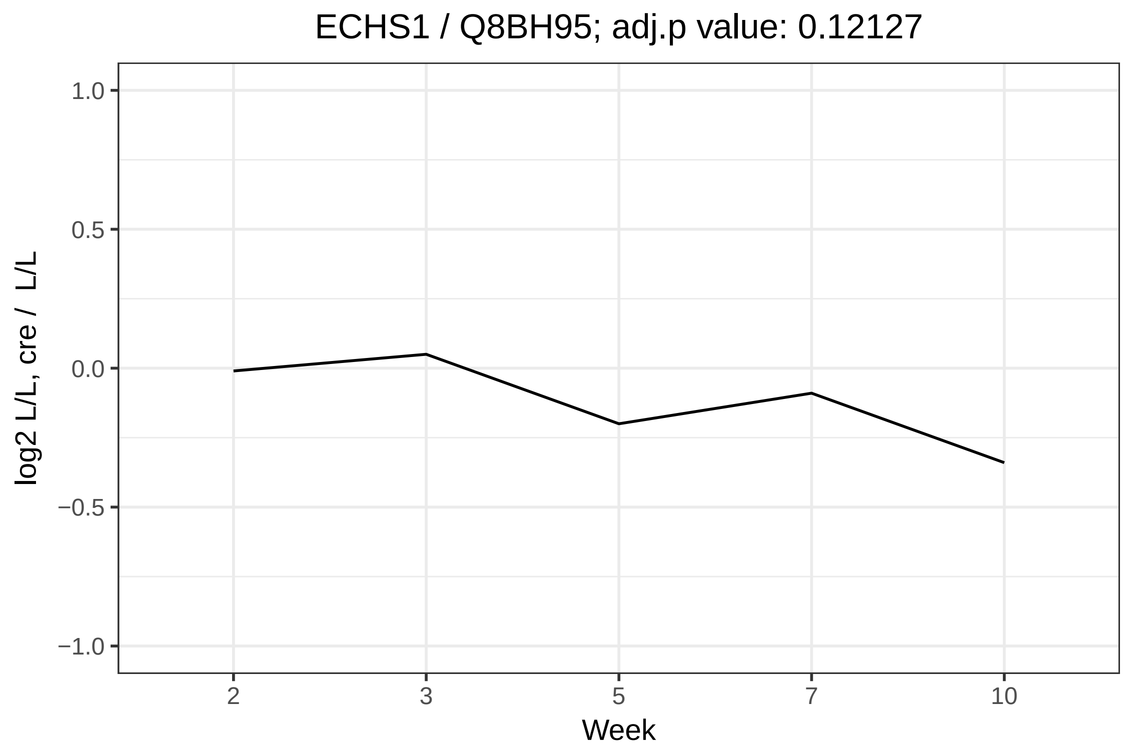
ECHS1 / Q8BH95; adj.p value: 0.12127
log2 L/L, cre / L/L
1.0
0.5
0.0
−0.5
−1.0
2
3
5
7
10
Week

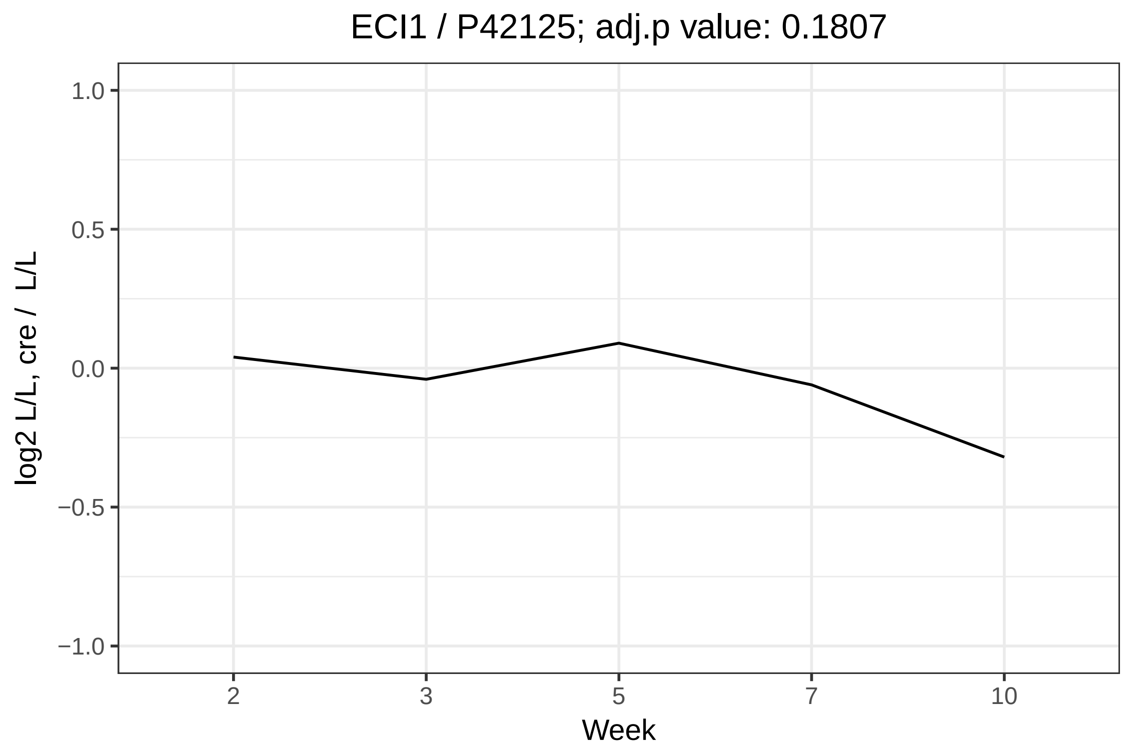

ECI1 / P42125; adj.p value: 0.1807
log2 L/L, cre / L/L
1.0
0.5
0.0
−0.5
−1.0
2
3
5
7
10
Week

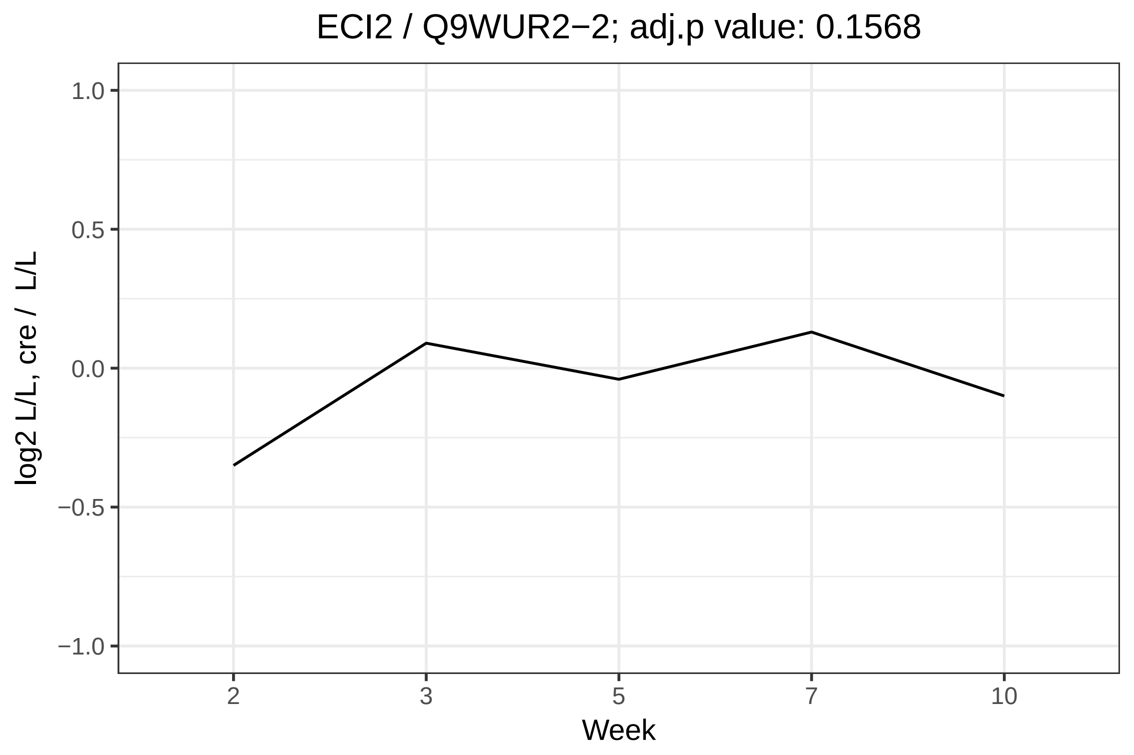
ECI2 / Q9WUR2−2; adj.p value: 0.1568
log2 L/L, cre / L/L
1.0
0.5
0.0
−0.5
−1.0
2
3
5
7
10
Week

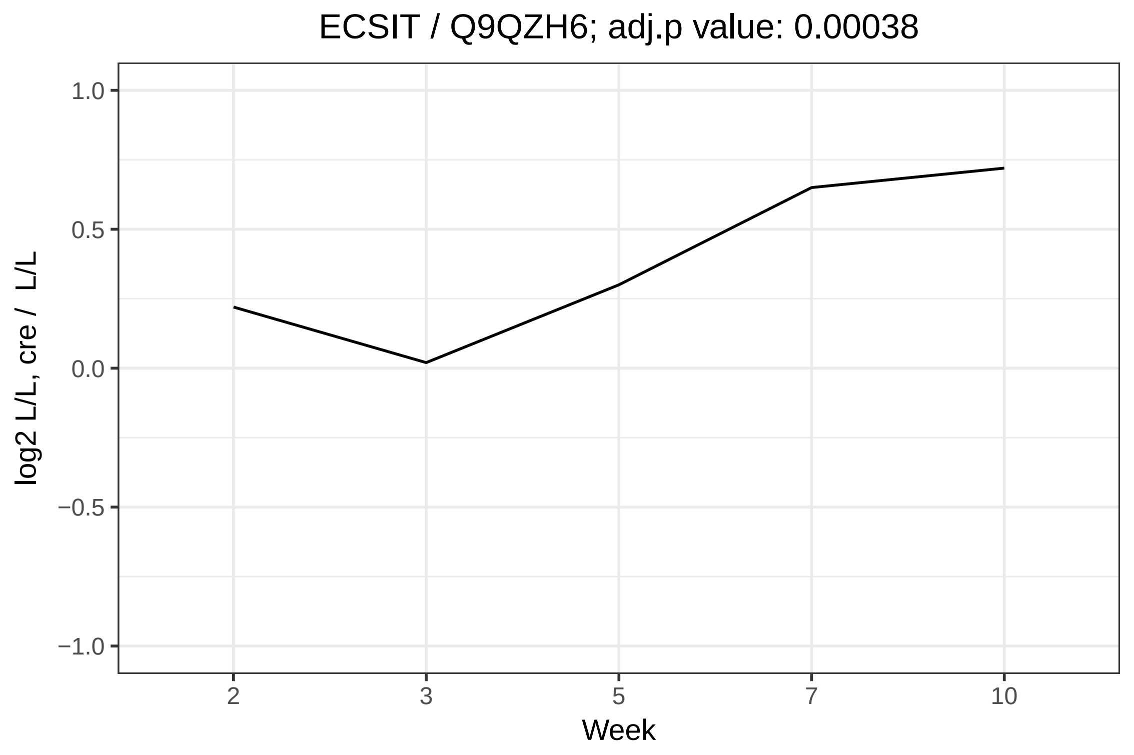

ECSIT / Q9QZH6; adj.p value: 0.00038
1.0
0.5
0.0
−0.5
−1.0
log2 L/L, cre / L/L
2
3
5
7
10
Week

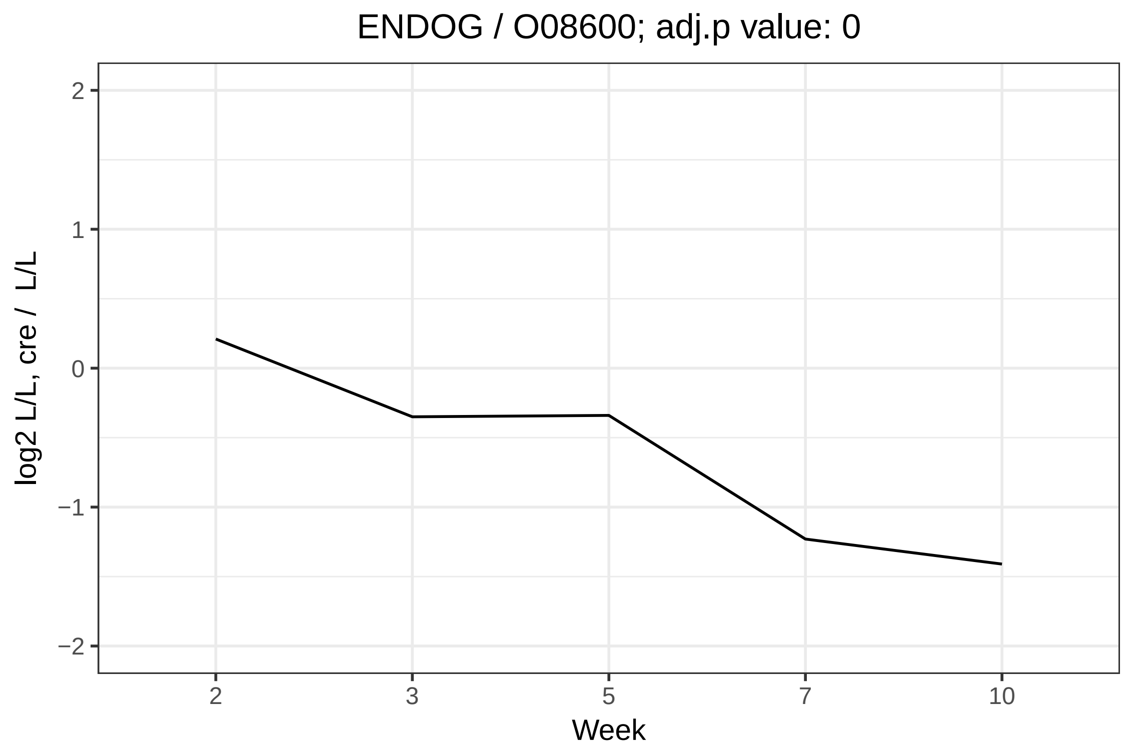
ENDOG / O08600; adj.p value: 0
log2 L/L, cre / L/L
2
1
0
−1
−2
2
3
5
7
10
Week

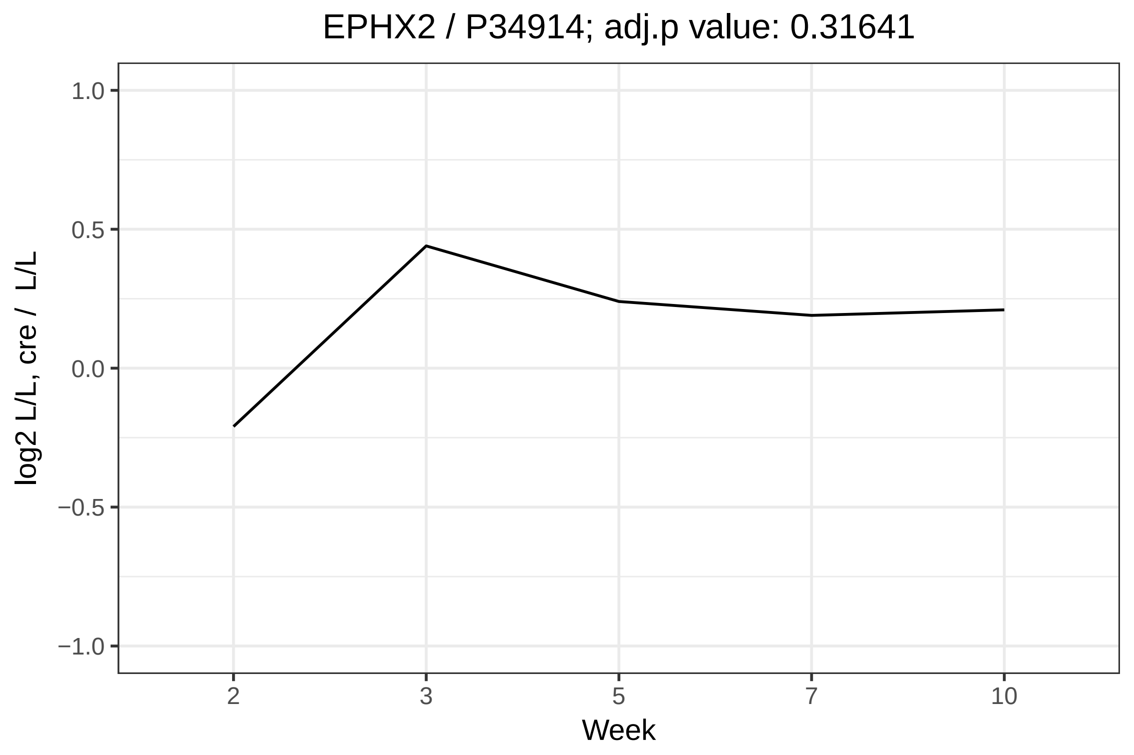

EPHX2 / P34914; adj.p value: 0.31641
1.0
0.5
0.0
−0.5
−1.0
log2 L/L, cre / L/L
2
3
5
7
10
Week

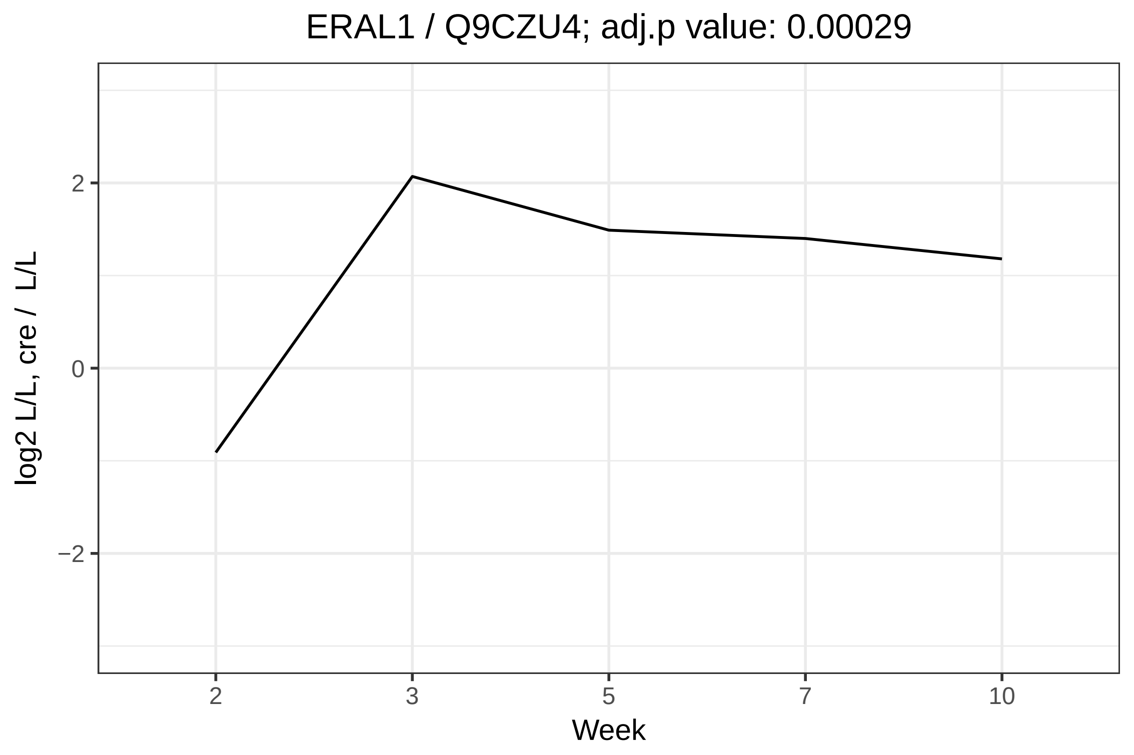
ERAL1 / Q9CZU4; adj.p value: 0.00029
log2 L/L, cre / L/L
2
0
−2
2
3
5
7
10
Week

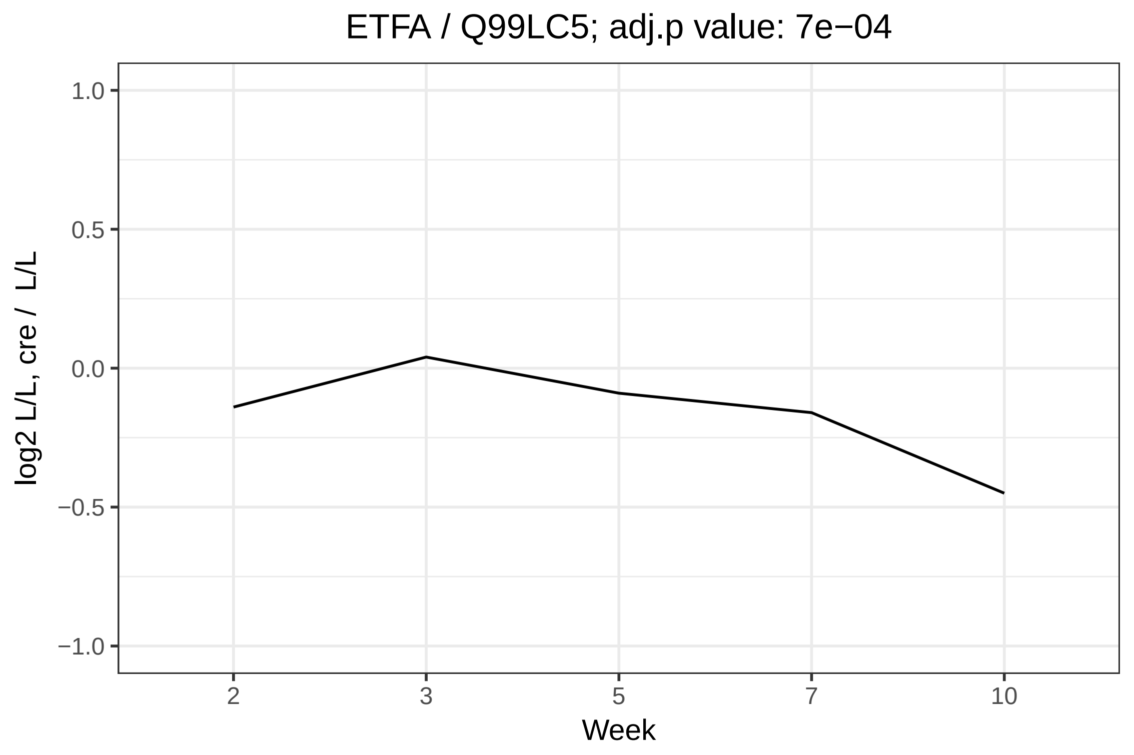

ETFA / Q99LC5; adj.p value: 7e−04
log2 L/L, cre / L/L
1.0
0.5
0.0
−0.5
−1.0
2
3
5
7
10
Week

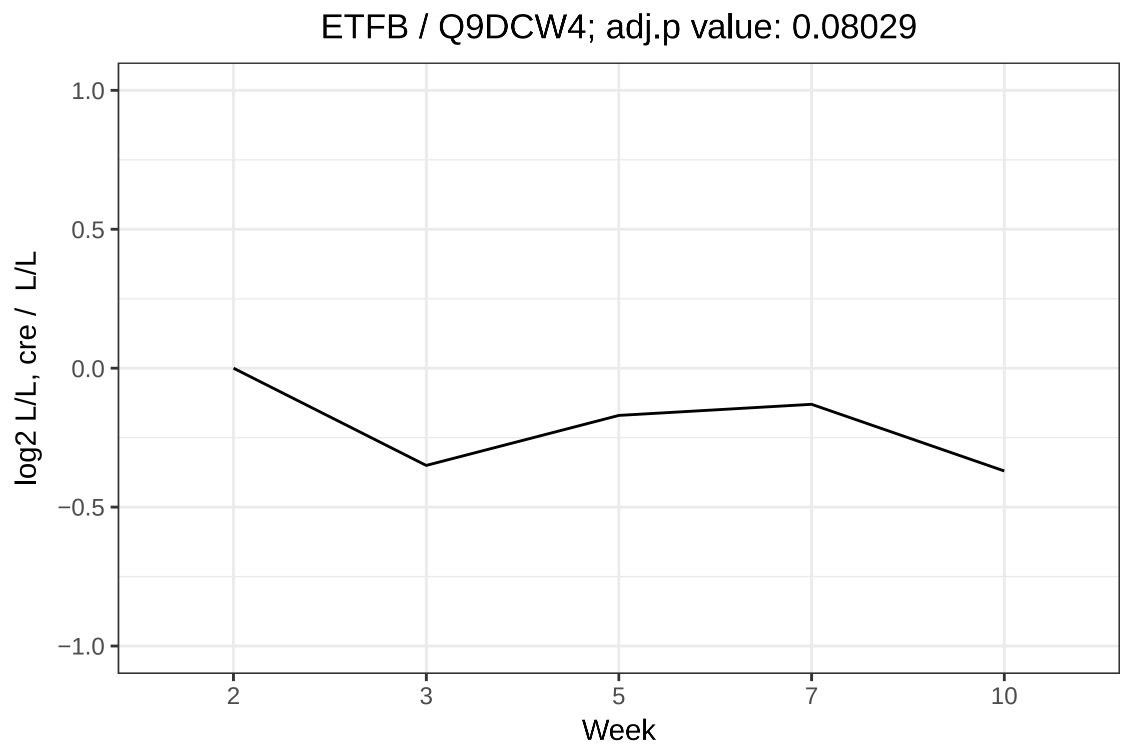
ETFB / Q9DCW4; adj.p value: 0.08029
log2 L/L, cre / L/L
1.0
0.5
0.0
−0.5
−1.0
2
3
5
7
10
Week

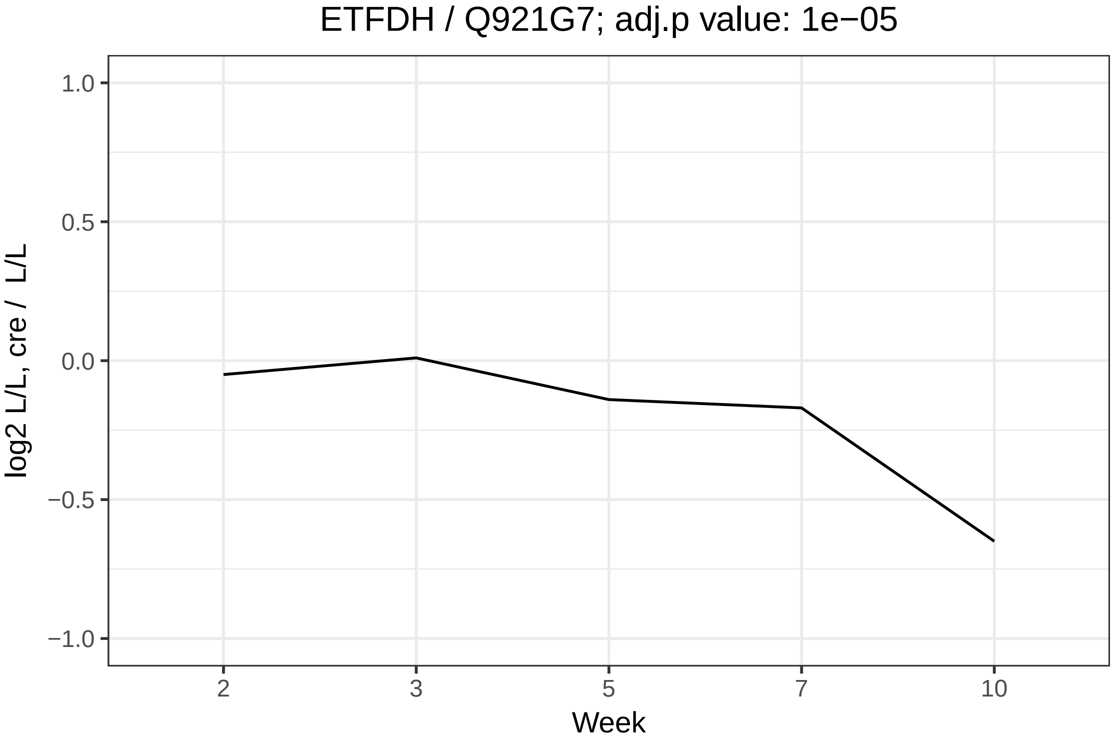
ETFDH / Q921G7; adj.p value: 1e−05
1.0
0.5
0.0
−0.5
−1.0
log2 L/L, cre / L/L
2
3
5
7
10
Week

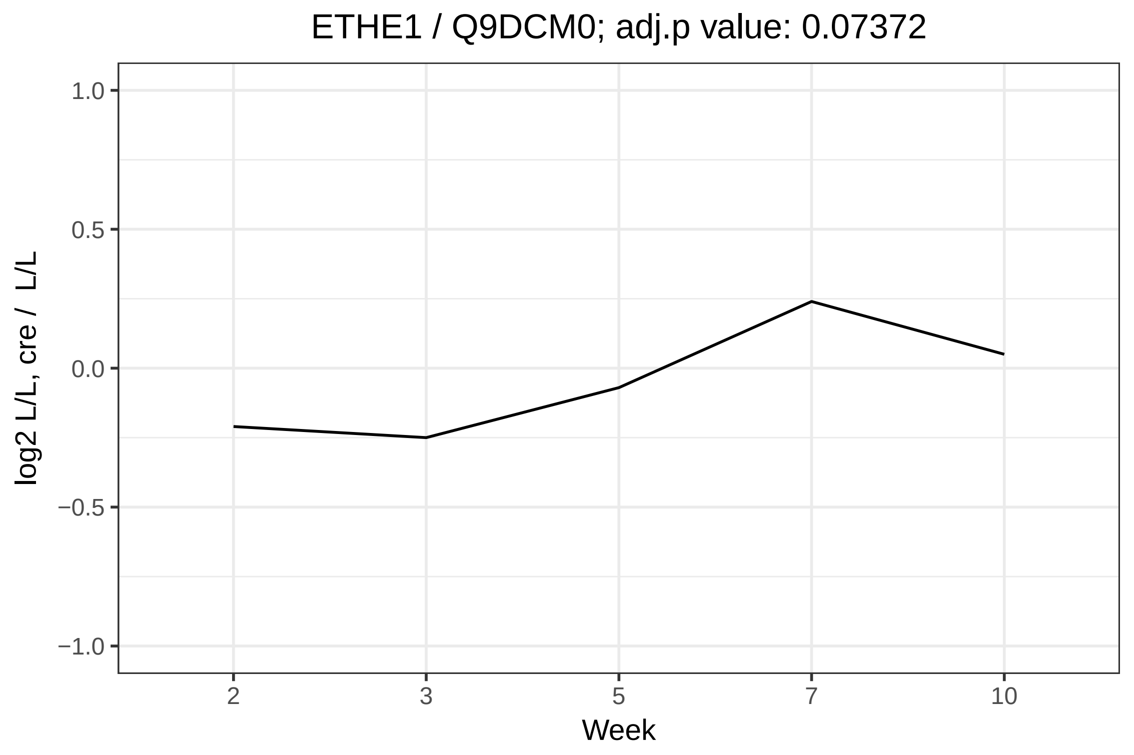
ETHE1 / Q9DCM0; adj.p value: 0.07372
1.0
0.5
0.0
−0.5
−1.0
log2 L/L, cre / L/L
2
3
5
7
10
Week

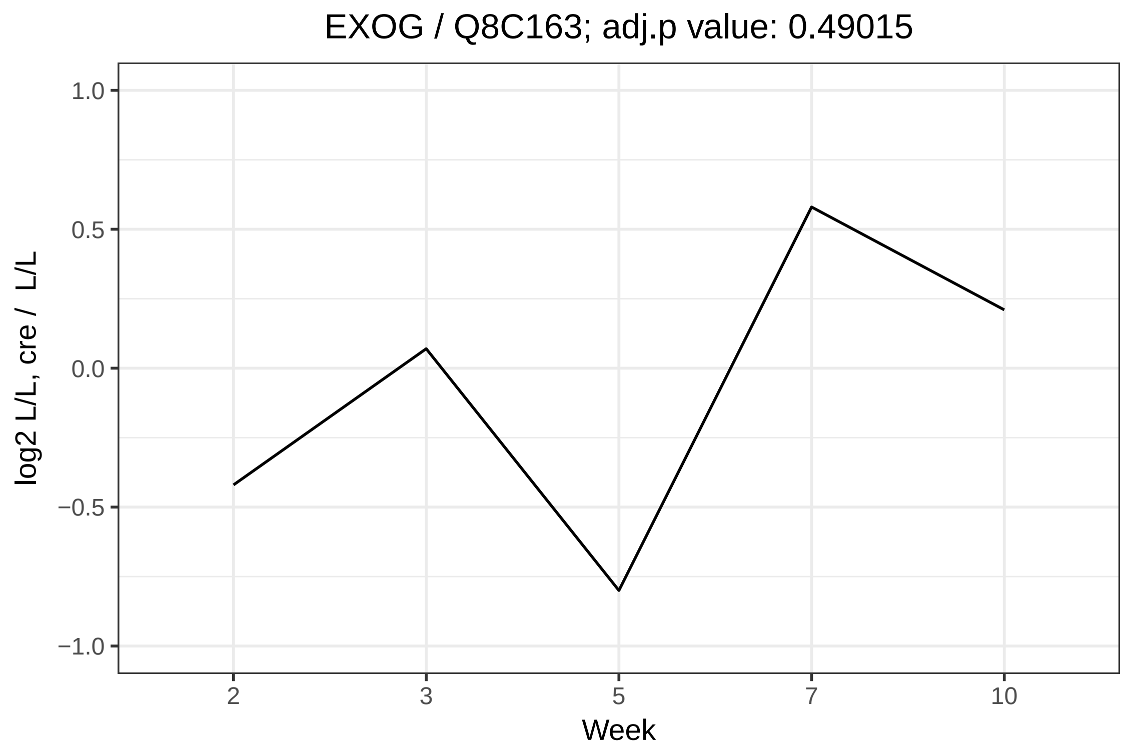

EXOG / Q8C163; adj.p value: 0.49015
1.0
0.5
0.0
−0.5
−1.0
log2 L/L, cre / L/L
2
3
5
7
10
Week

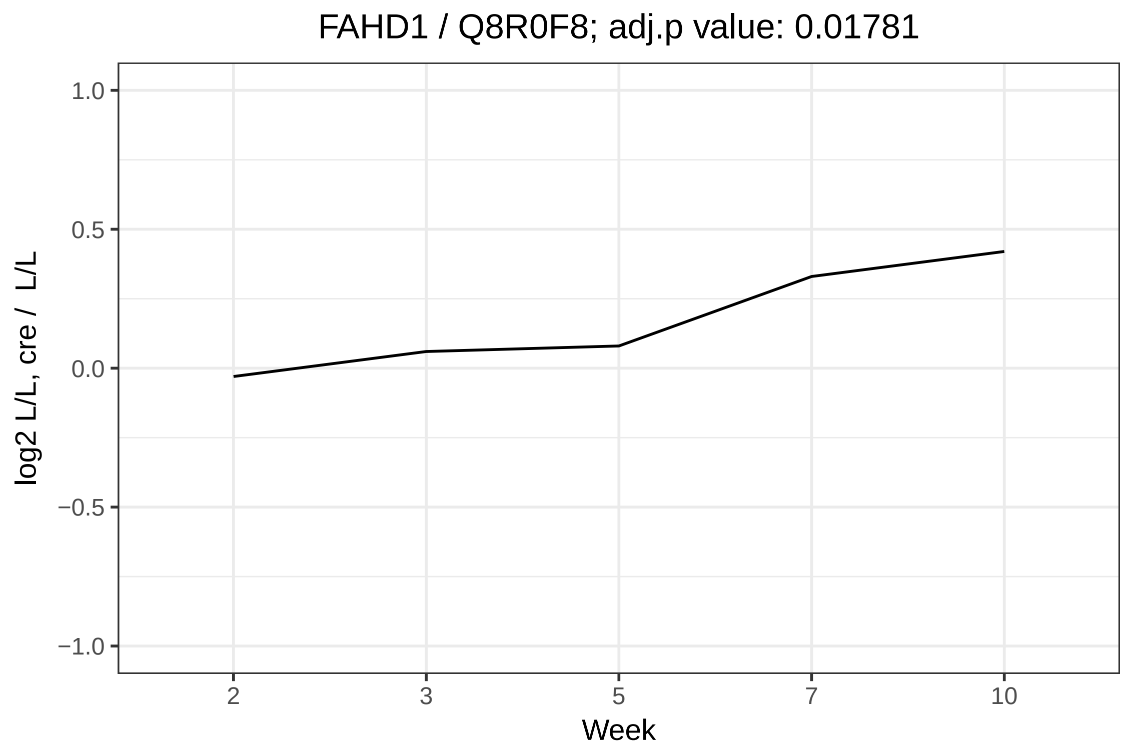

FAHD1 / Q8R0F8; adj.p value: 0.01781
log2 L/L, cre / L/L
1.0
0.5
0.0
−0.5
−1.0
2
3
5
7
10
Week

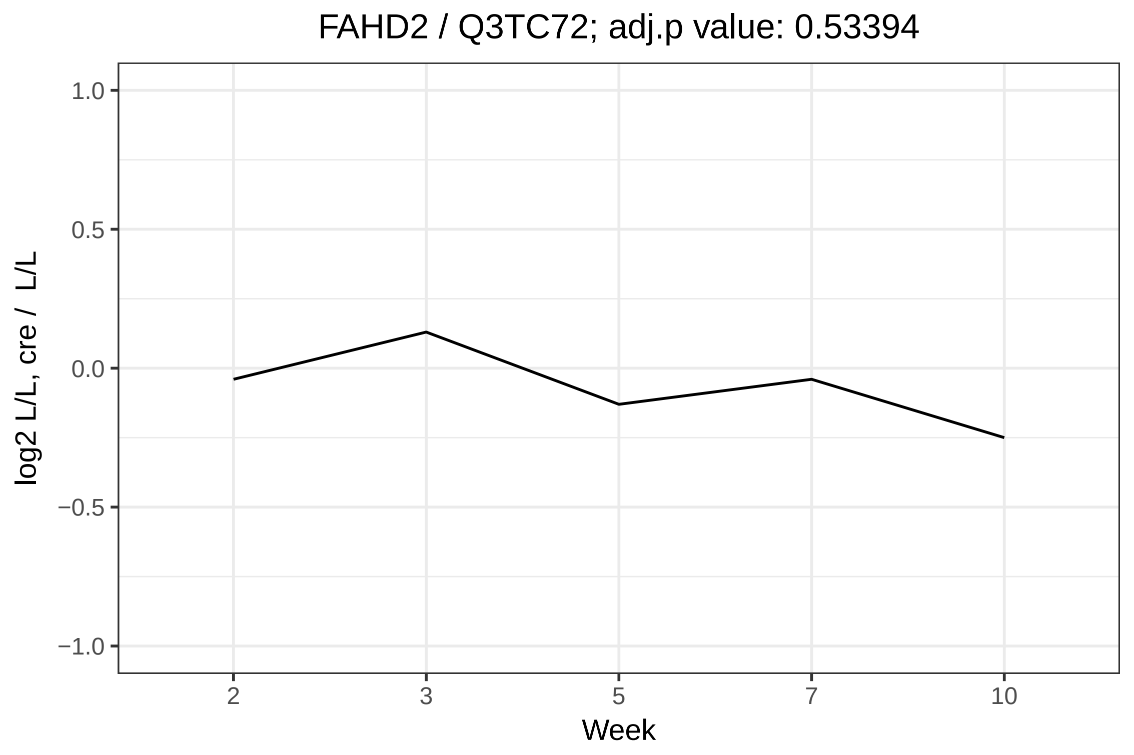

FAHD2 / Q3TC72; adj.p value: 0.53394
log2 L/L, cre / L/L
1.0
0.5
0.0
−0.5
−1.0
2
3
5
7
10
Week

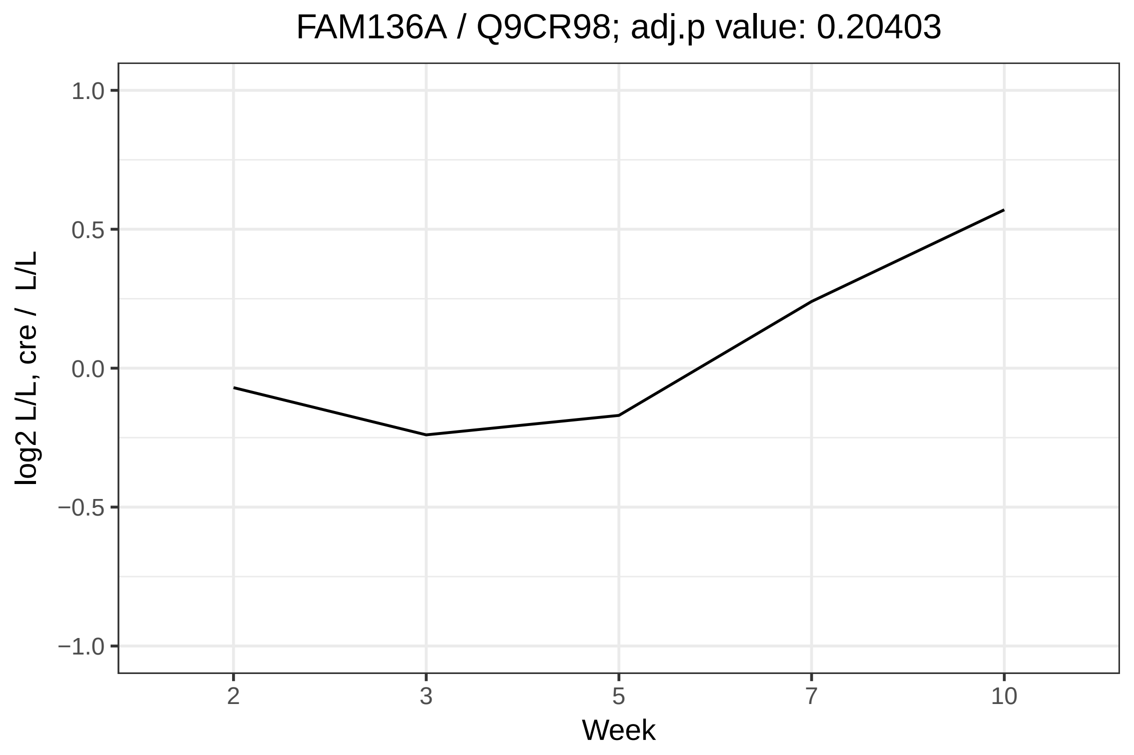
FAM136A / Q9CR98; adj.p value: 0.20403
log2 L/L, cre / L/L
1.0
0.5
0.0
−0.5
−1.0
2
3
5
7
10
Week

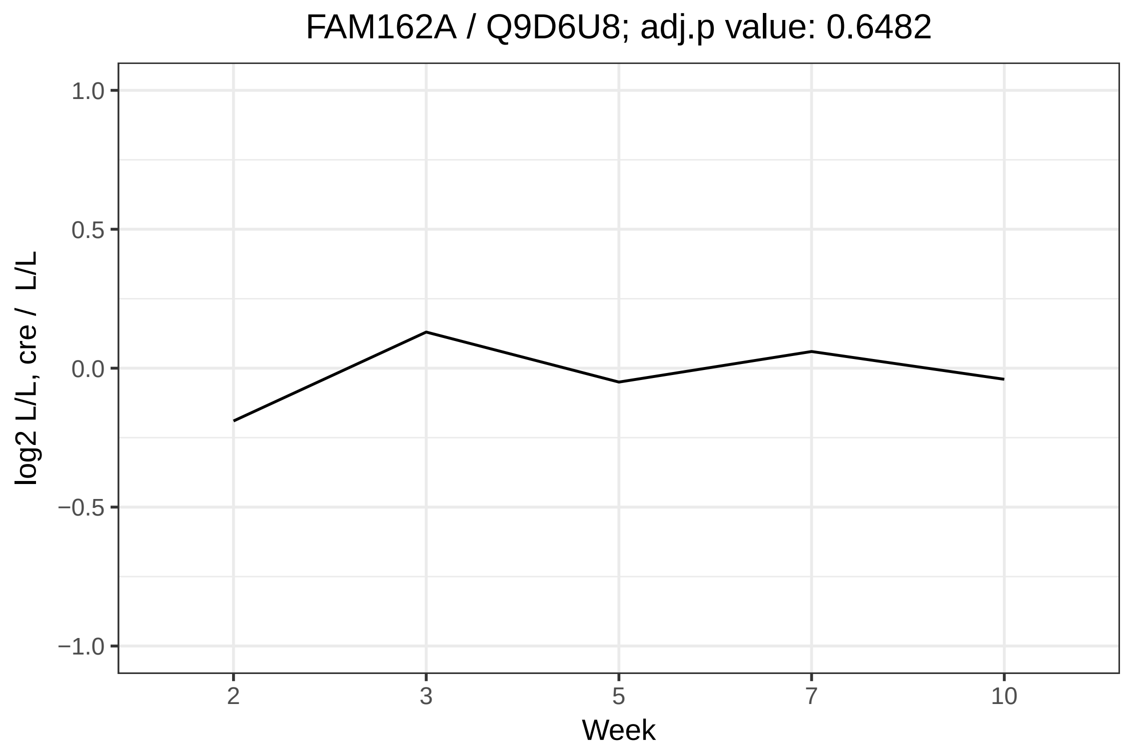
FAM162A / Q9D6U8; adj.p value: 0.6482
log2 L/L, cre / L/L
1.0
0.5
0.0
−0.5
−1.0
2
3
5
7
10
Week

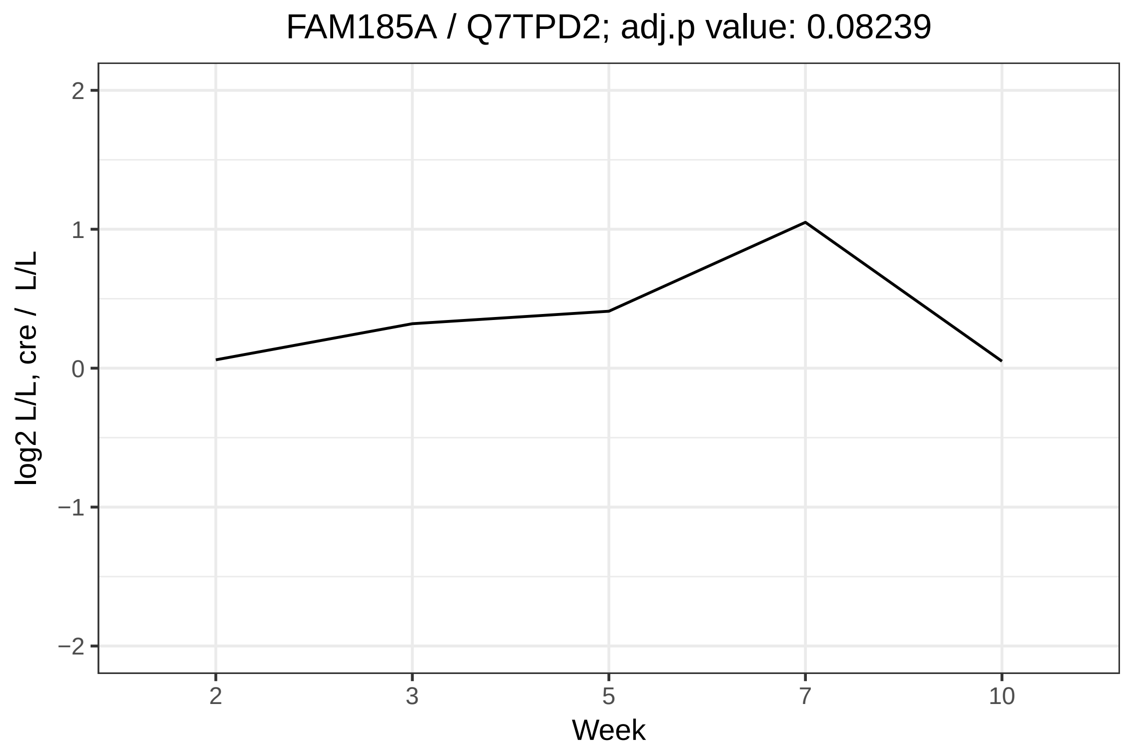
FAM185A / Q7TPD2; adj.p value: 0.08239
log2 L/L, cre / L/L
2
1
0
−1
−2
2
3
5
7
10
Week

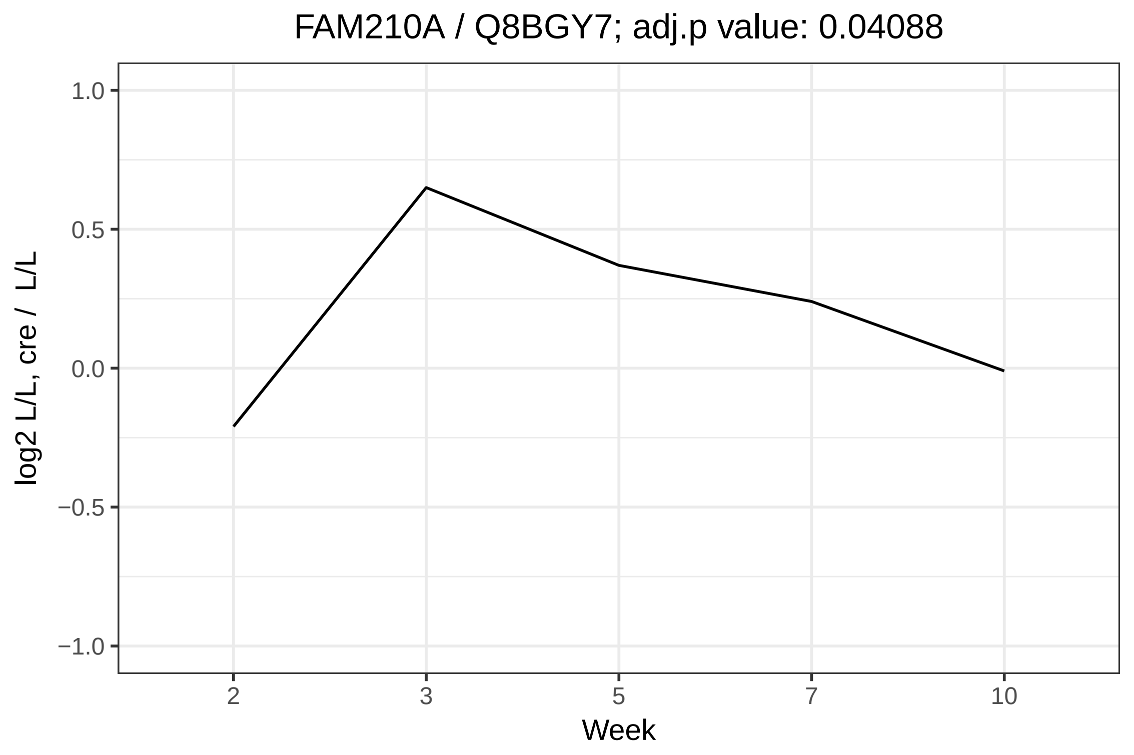
FAM210A / Q8BGY7; adj.p value: 0.04088
log2 L/L, cre / L/L
1.0
0.5
0.0
−0.5
−1.0
2
3
5
7
10
Week

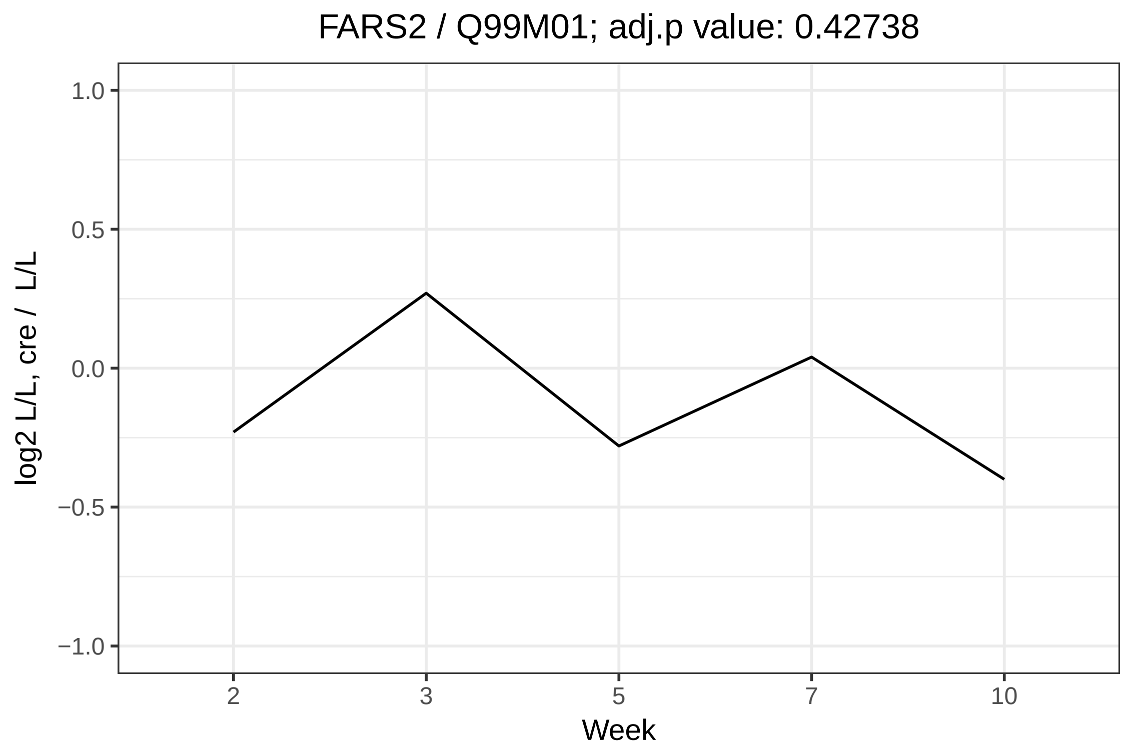
FARS2 / Q99M01; adj.p value: 0.42738
log2 L/L, cre / L/L
1.0
0.5
0.0
−0.5
−1.0
2
3
5
7
10
Week

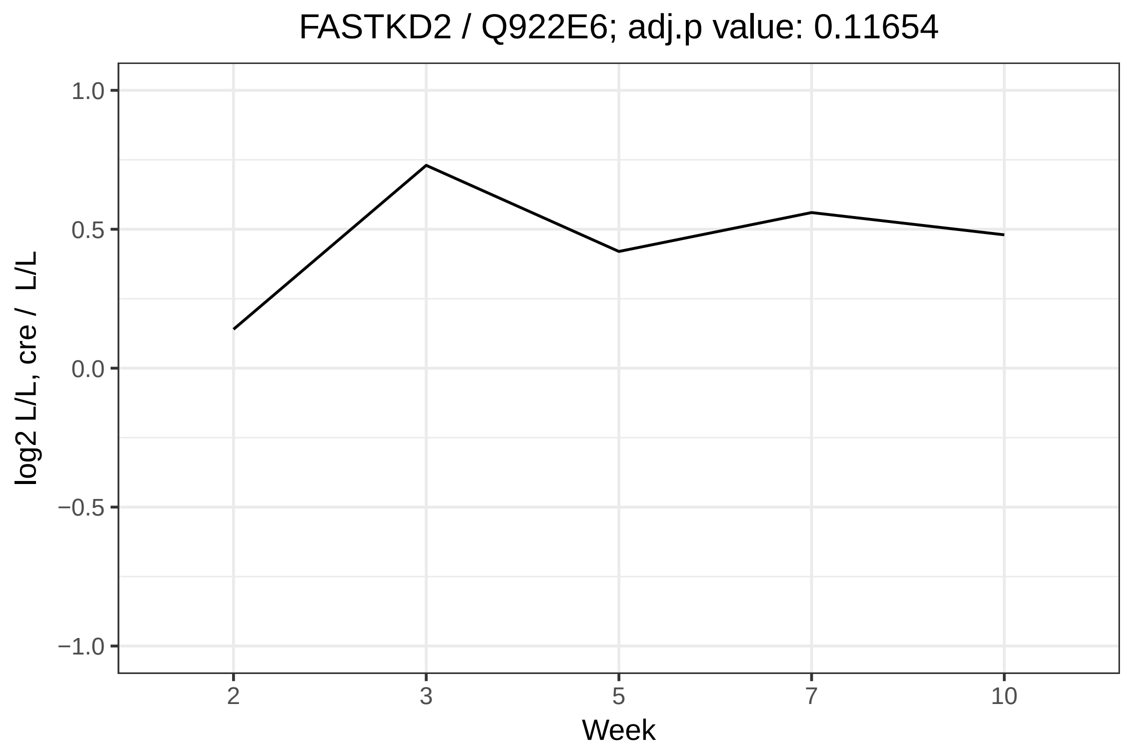

FASTKD2 / Q922E6; adj.p value: 0.11654
1.0
0.5
0.0
−0.5
−1.0
log2 L/L, cre / L/L
2
3
5
7
10
Week

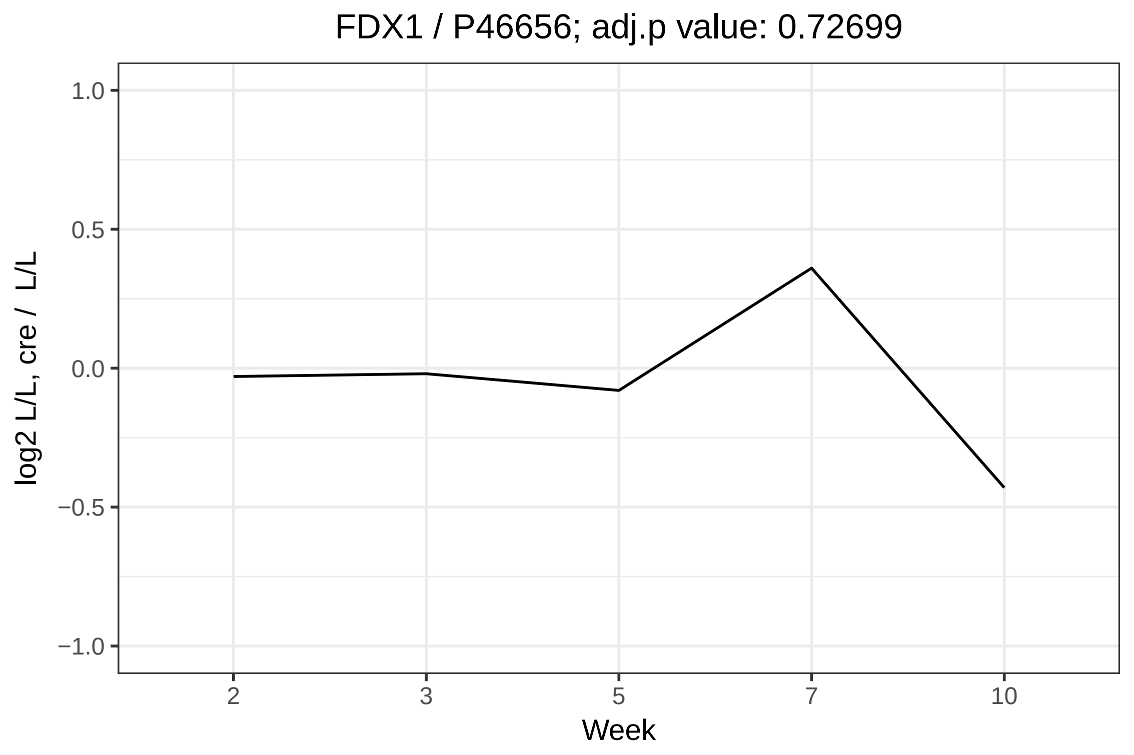

FDX1 / P46656; adj.p value: 0.72699
log2 L/L, cre / L/L
1.0
0.5
0.0
−0.5
−1.0
2
3
5
7
10
Week

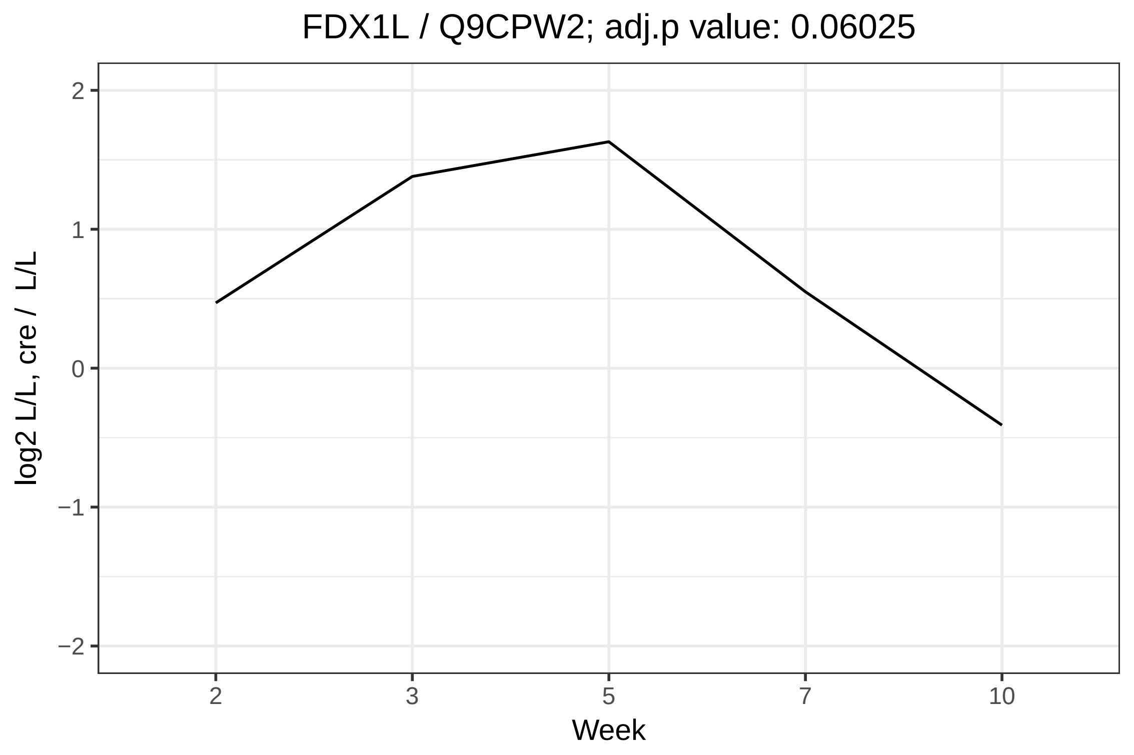

FDX1L / Q9CPW2; adj.p value: 0.06025
log2 L/L, cre / L/L
Week
2
1
0
−1
−2
2
3
5
7
10

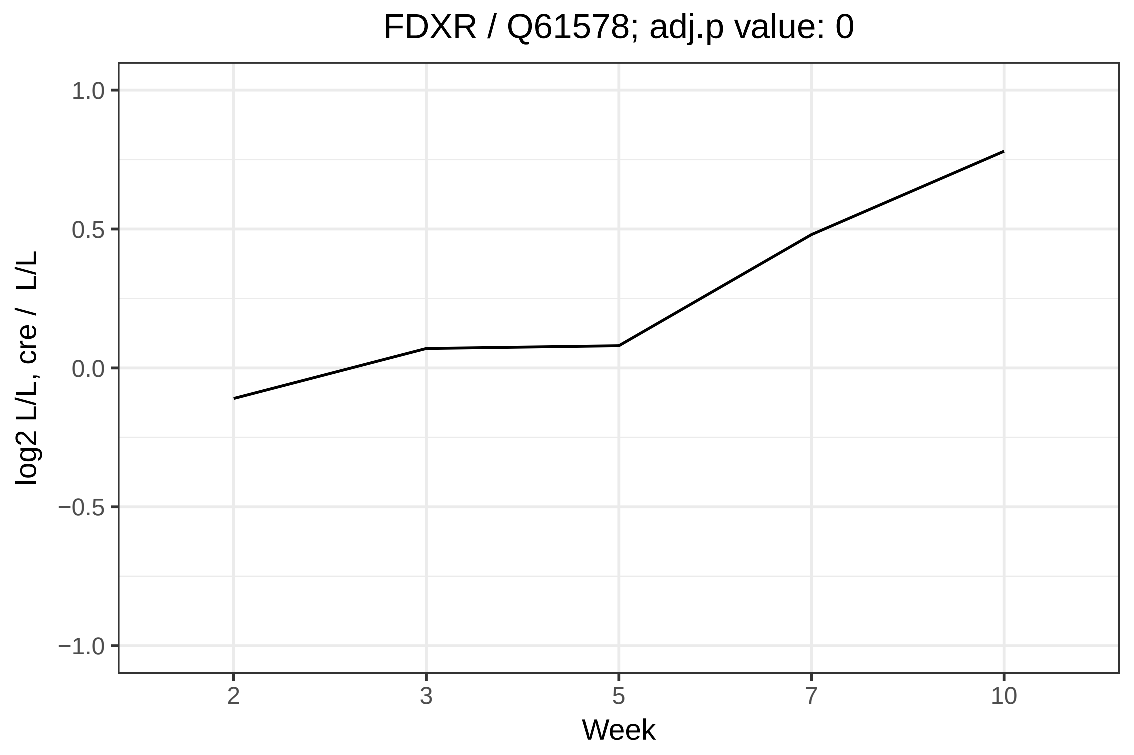
FDXR / Q61578; adj.p value: 0
log2 L/L, cre / L/L
1.0
0.5
0.0
−0.5
−1.0
2
3
5
7
10
Week

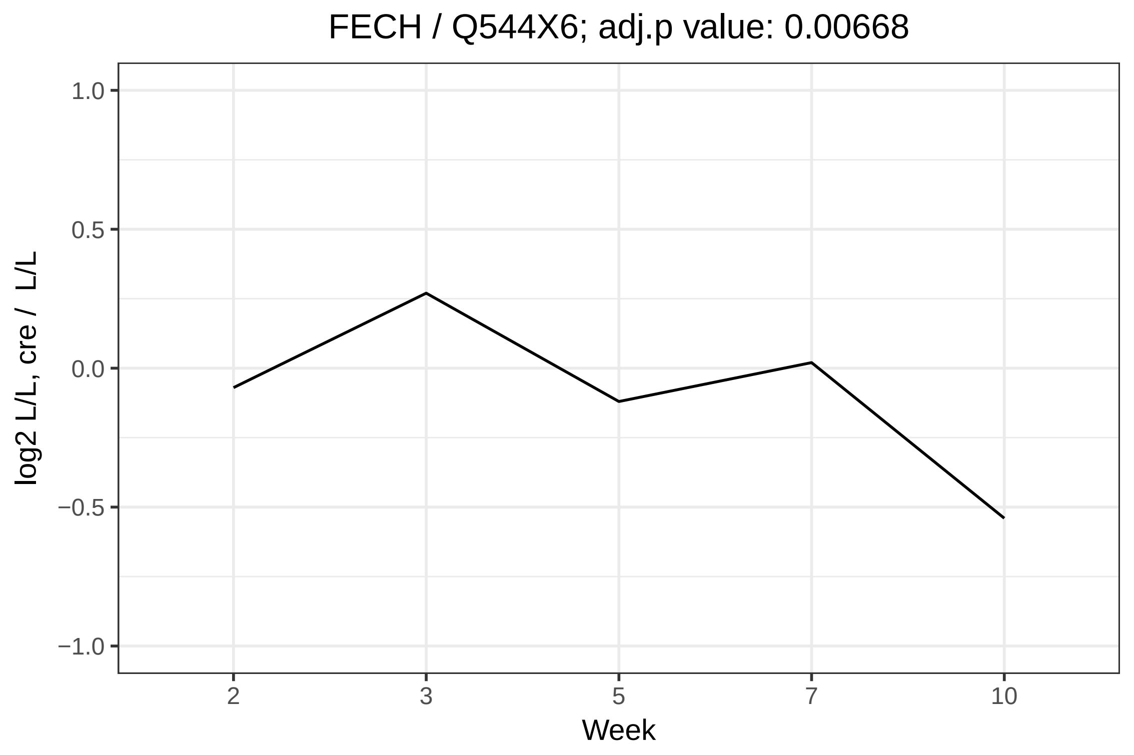

FECH / Q544X6; adj.p value: 0.00668
log2 L/L, cre / L/L
1.0
0.5
0.0
−0.5
−1.0
2
3
5
7
10
Week

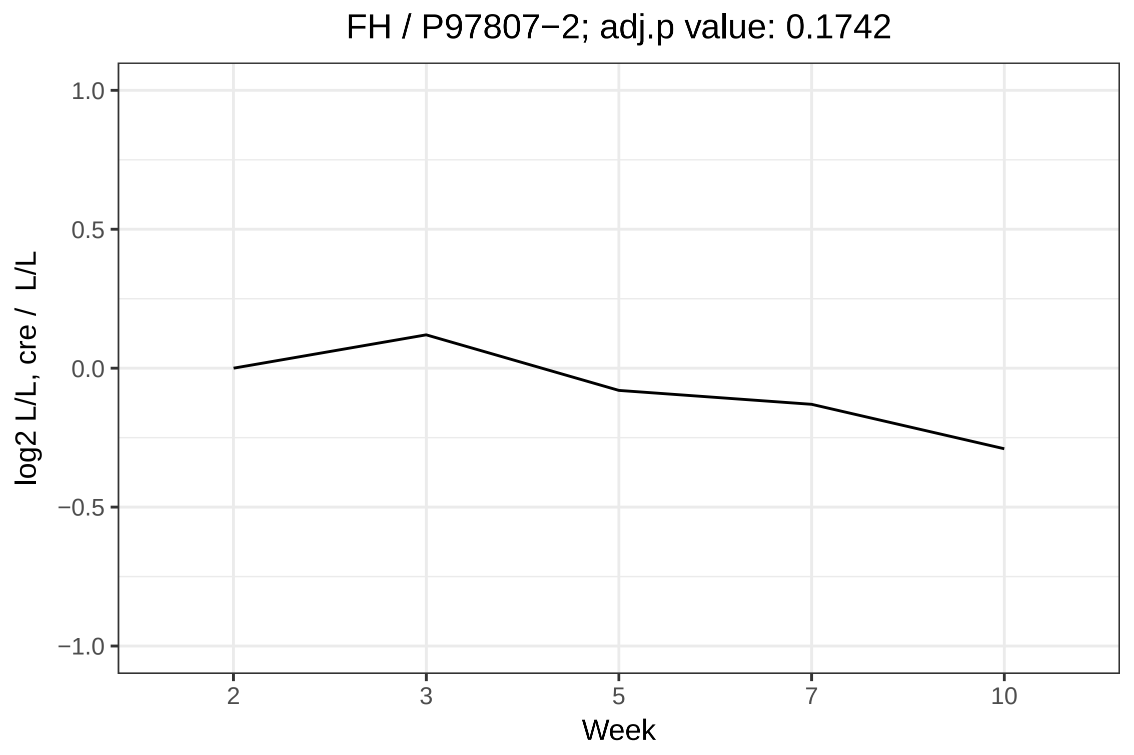

FH / P97807−2; adj.p value: 0.1742
1.0
0.5
0.0
−0.5
−1.0
log2 L/L, cre / L/L
2
3
5
7
10
Week

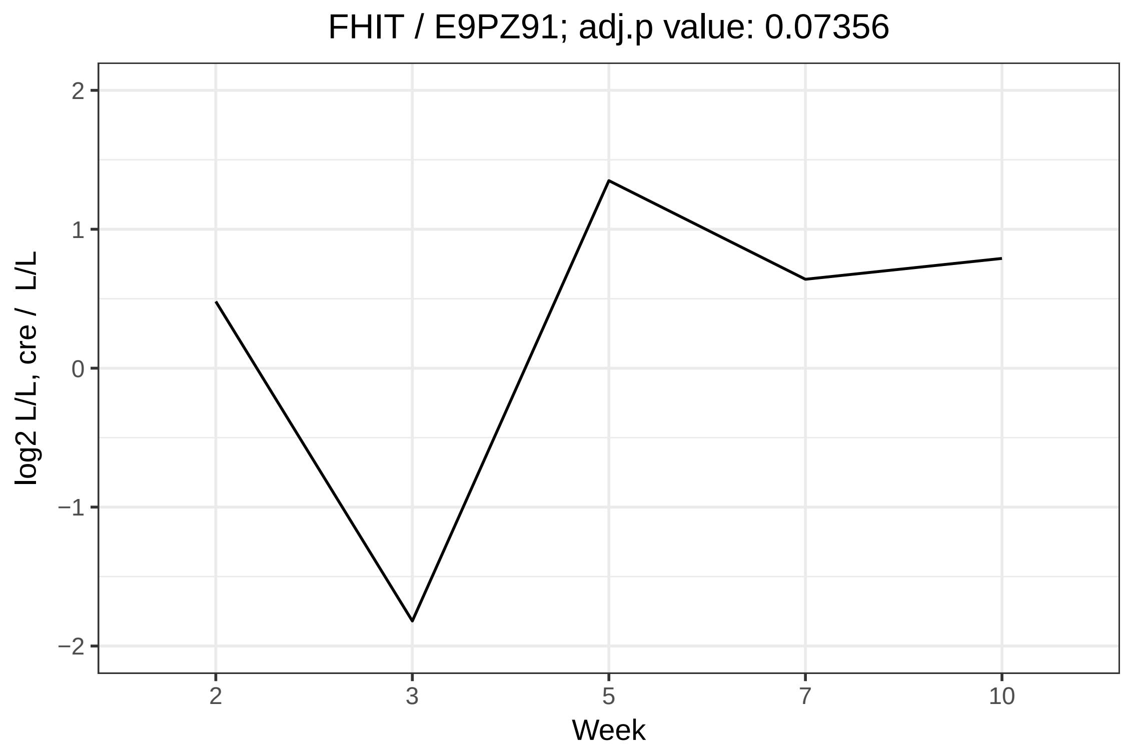

FHIT / E9PZ91; adj.p value: 0.07356
log2 L/L, cre / L/L
2
1
0
−1
−2
2
3
5
7
10
Week

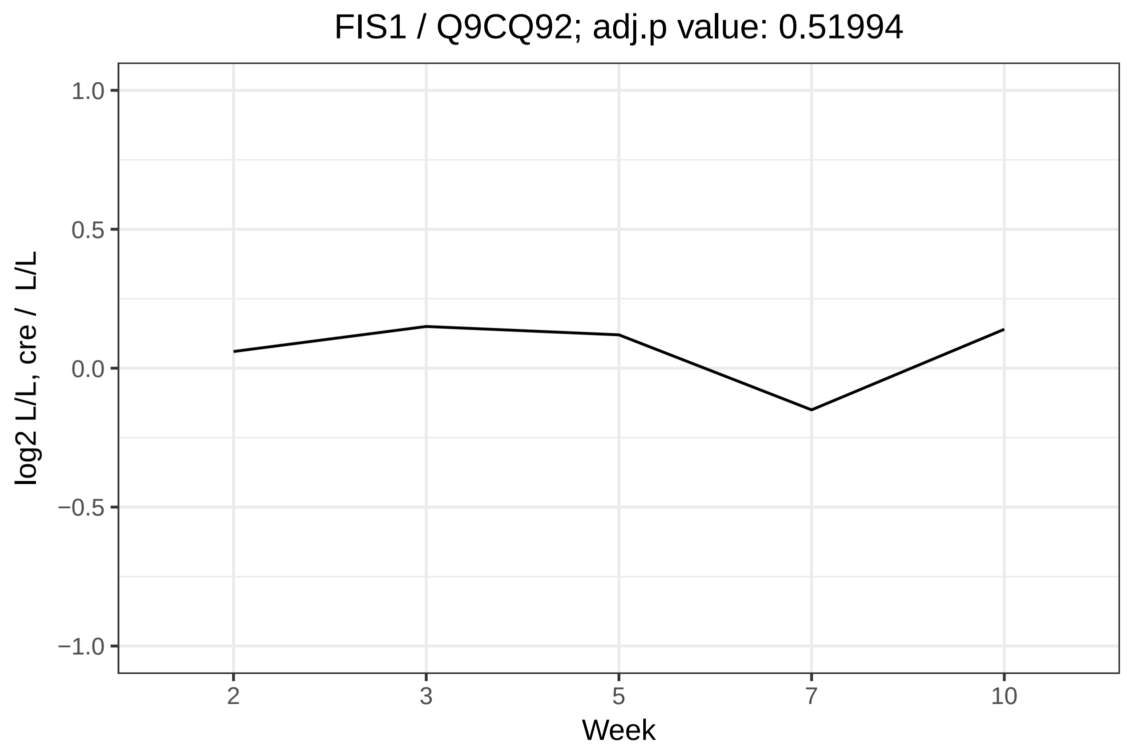
FIS1 / Q9CQ92; adj.p value: 0.51994
log2 L/L, cre / L/L
1.0
0.5
0.0
−0.5
−1.0
2
3
5
7
10
Week

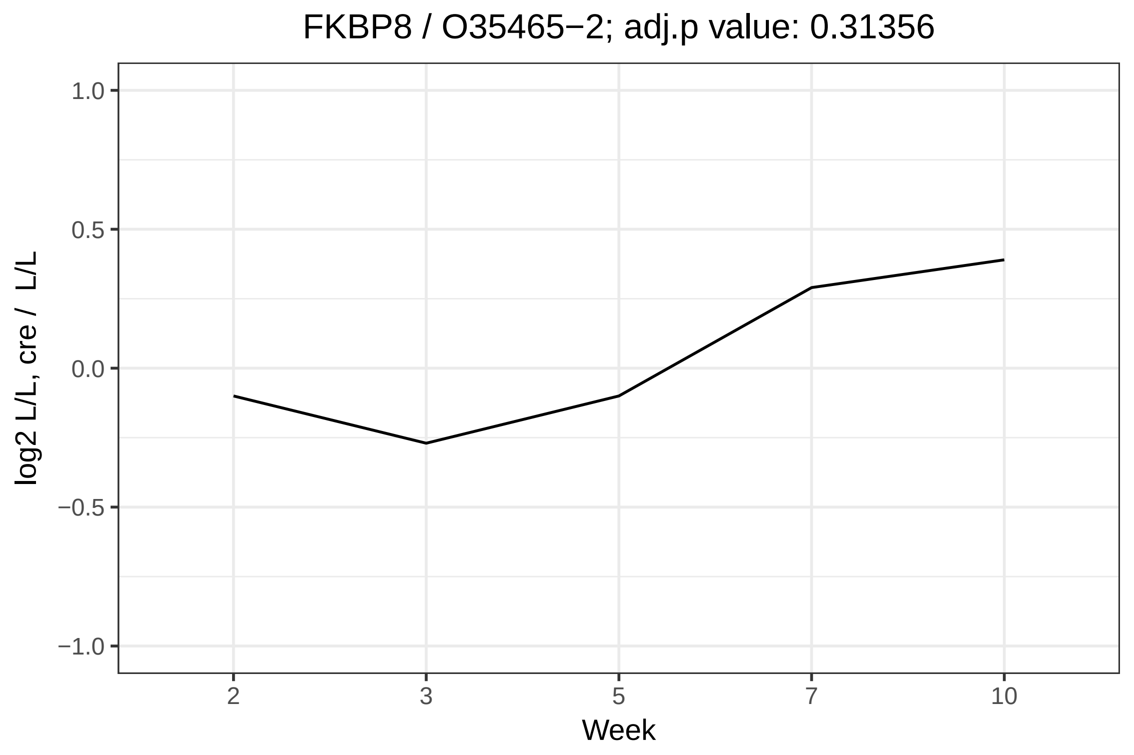
FKBP8 / O35465−2; adj.p value: 0.31356
1.0
0.5
0.0
−0.5
−1.0
log2 L/L, cre / L/L
2
3
5
7
10
Week

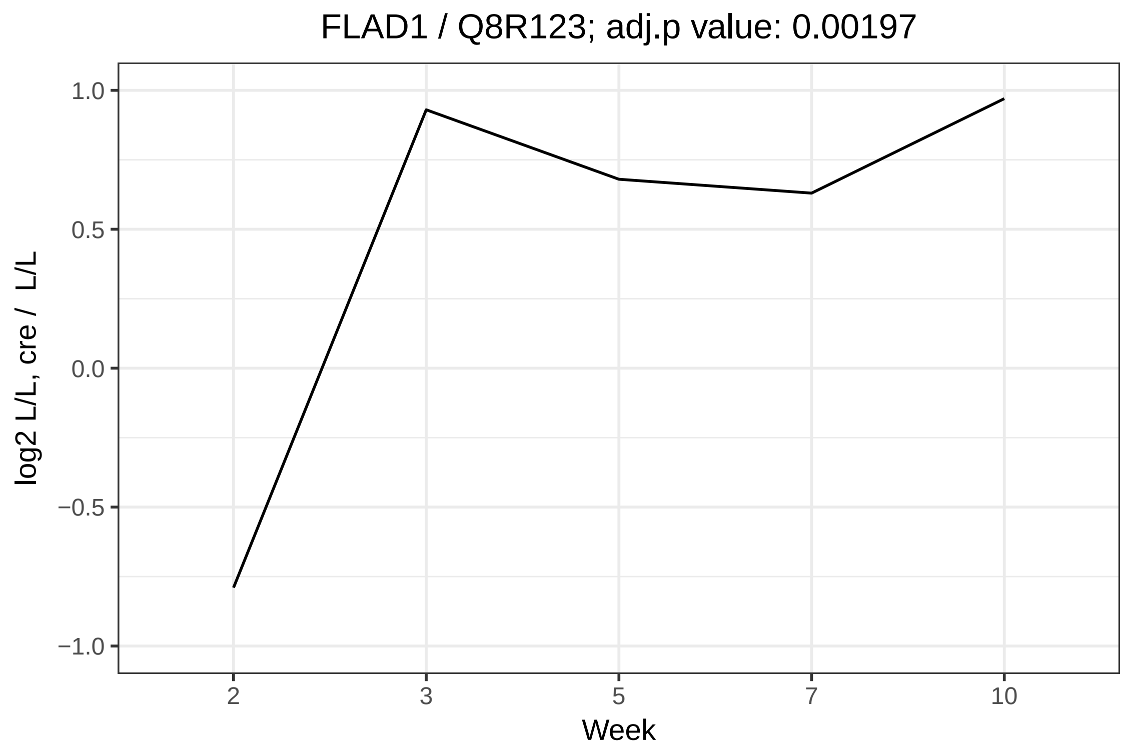
FLAD1 / Q8R123; adj.p value: 0.00197
log2 L/L, cre / L/L
1.0
0.5
0.0
−0.5
−1.0
2
3
5
7
10
Week

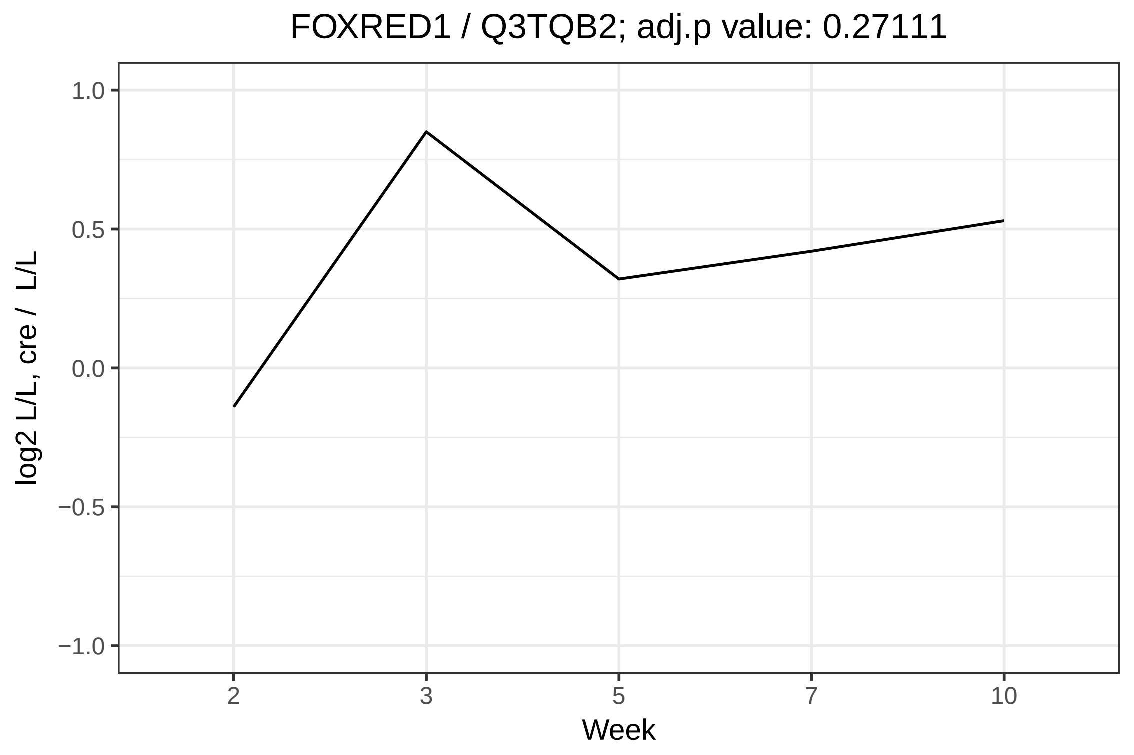
FOXRED1 / Q3TQB2; adj.p value: 0.27111
log2 L/L, cre / L/L
1.0
0.5
0.0
−0.5
−1.0
2
3
5
7
10
Week

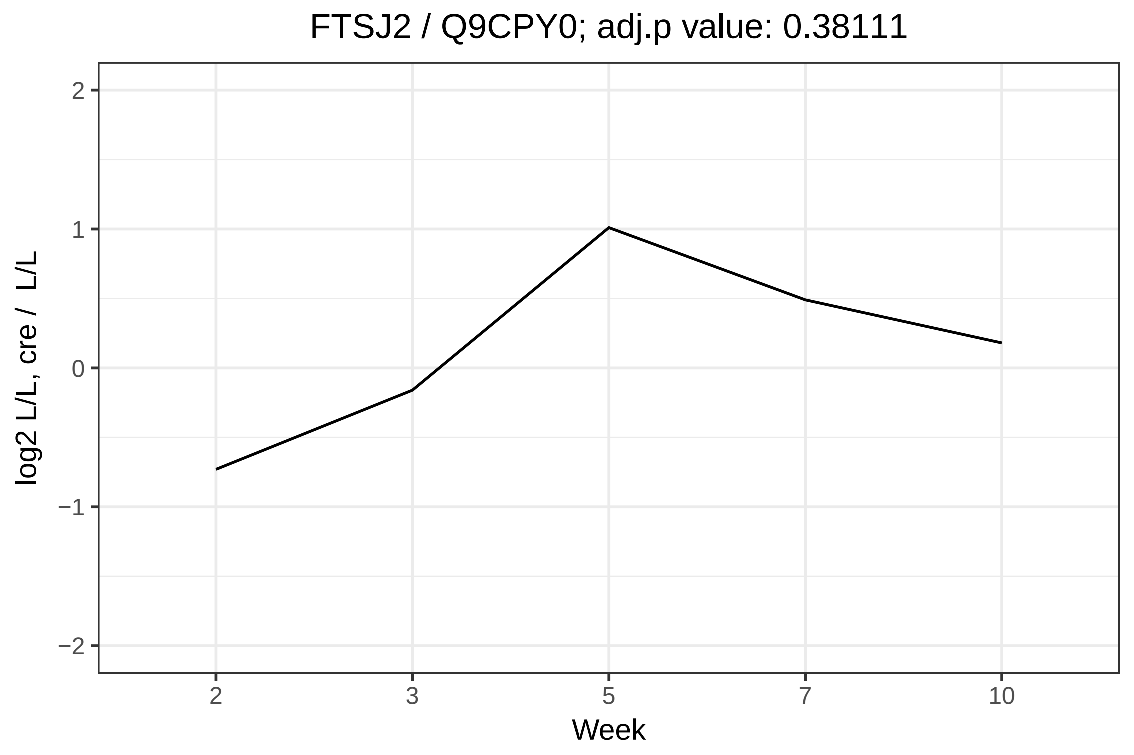
FTSJ2 / Q9CPY0; adj.p value: 0.38111
log2 L/L, cre / L/L
2
1
0
−1
−2
2
3
5
7
10
Week

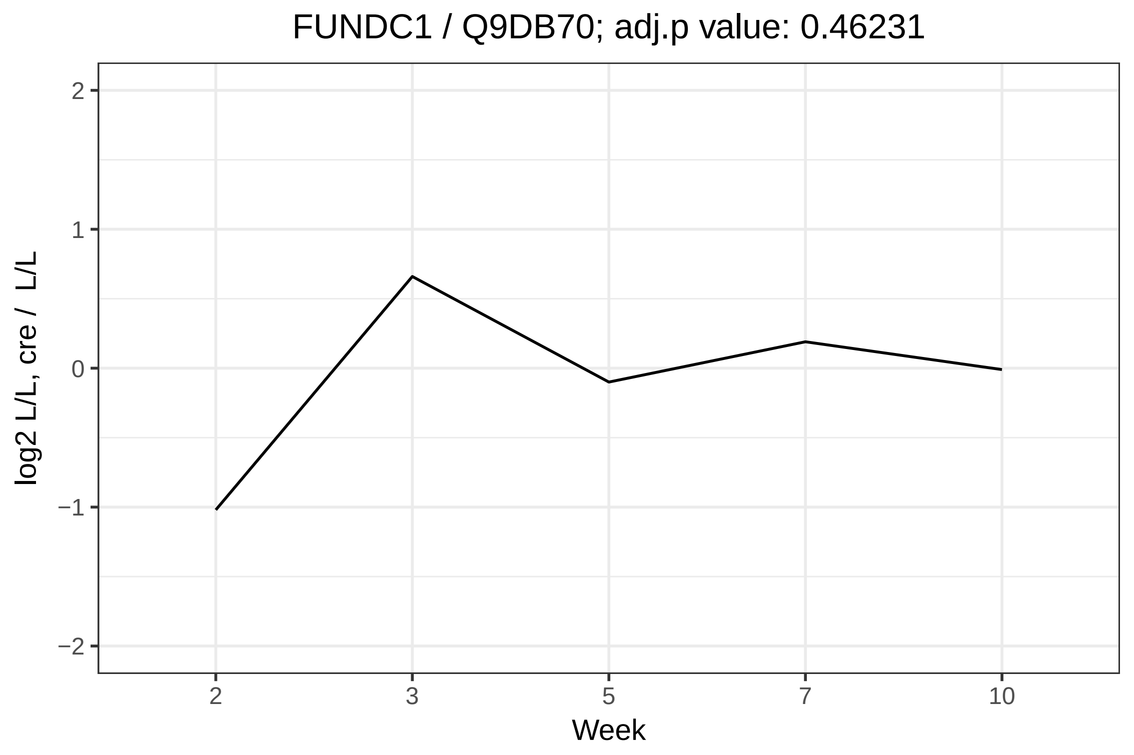
FUNDC1 / Q9DB70; adj.p value: 0.46231
log2 L/L, cre / L/L
2
1
0
−1
−2
2
3
5
7
10
Week

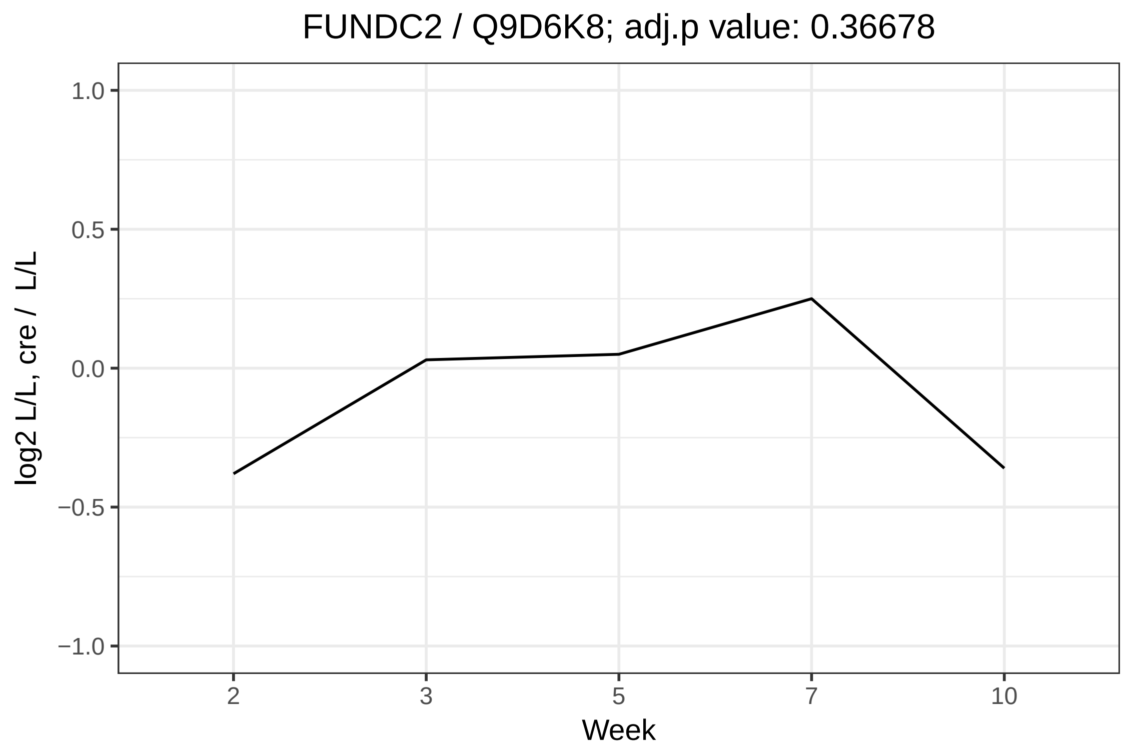
FUNDC2 / Q9D6K8; adj.p value: 0.36678
1.0
0.5
0.0
−0.5
−1.0
log2 L/L, cre / L/L
2
3
5
7
10
Week

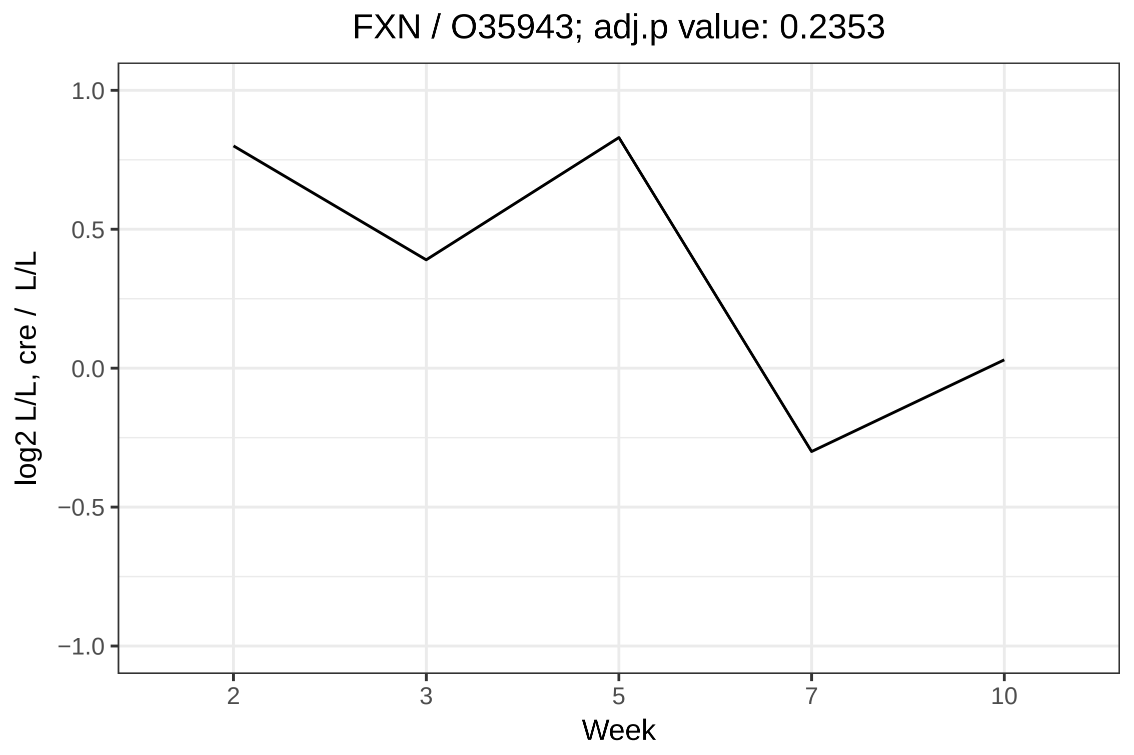

FXN / O35943; adj.p value: 0.2353
log2 L/L, cre / L/L
1.0
0.5
0.0
−0.5
−1.0
2
3
5
7
10
Week

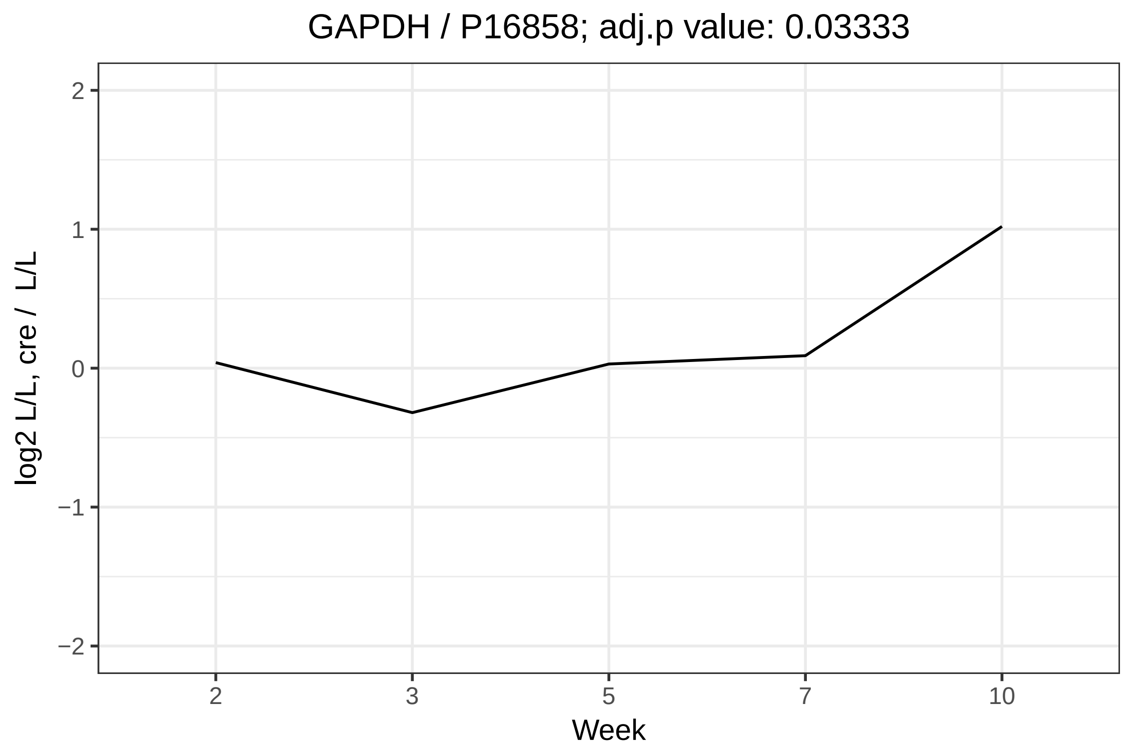

GAPDH / P16858; adj.p value: 0.03333
log2 L/L, cre / L/L
2
1
0
−1
−2
2
3
5
7
10
Week

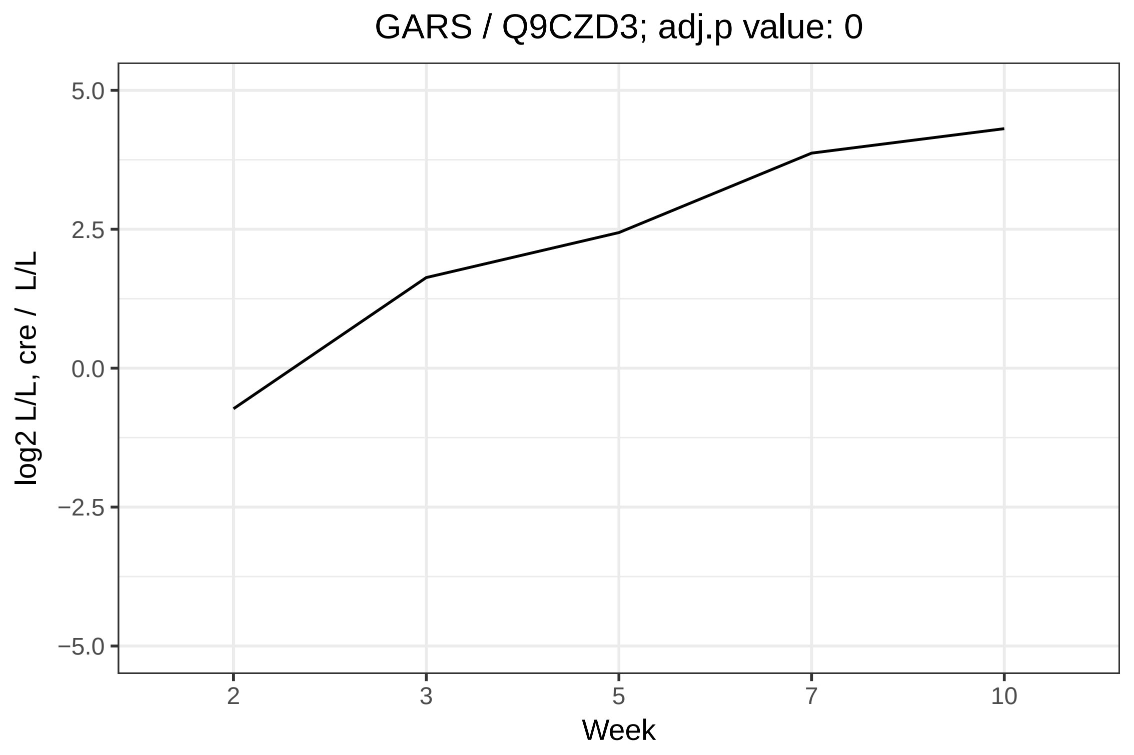

GARS / Q9CZD3; adj.p value: 0
5.0
2.5
0.0
−2.5
−5.0
log2 L/L, cre / L/L
2
3
5
7
10
Week

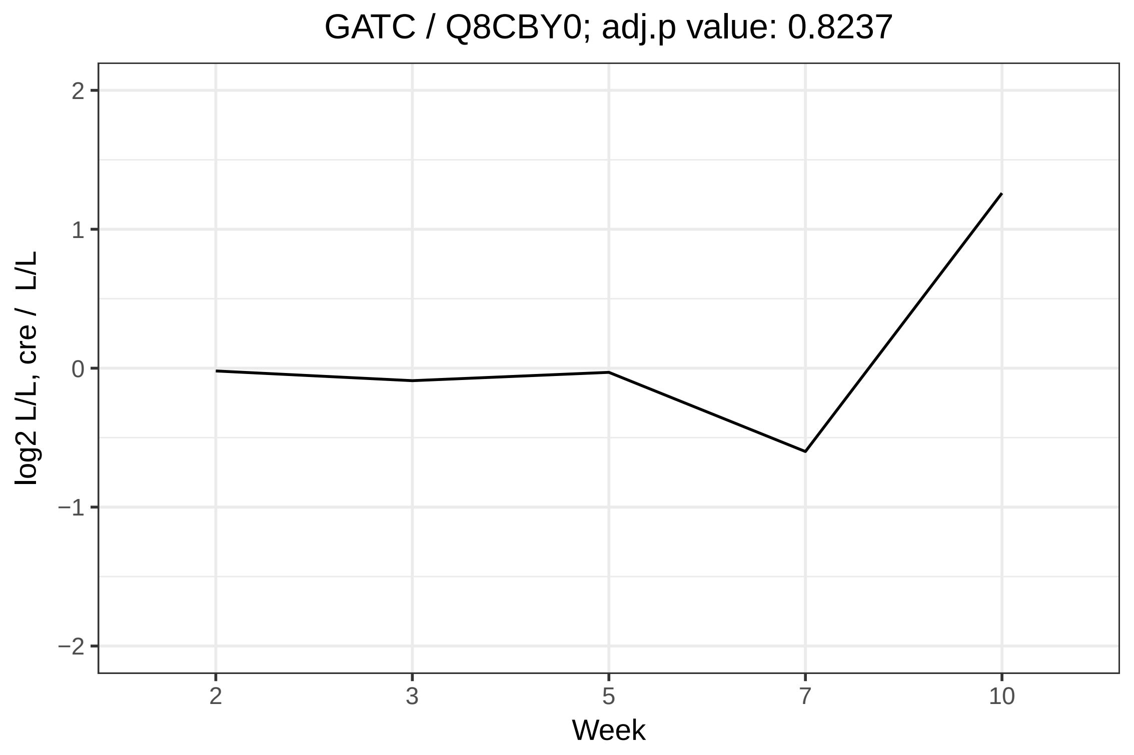
GATC / Q8CBY0; adj.p value: 0.8237
log2 L/L, cre / L/L
2
1
0
−1
−2
2
3
5
7
10
Week

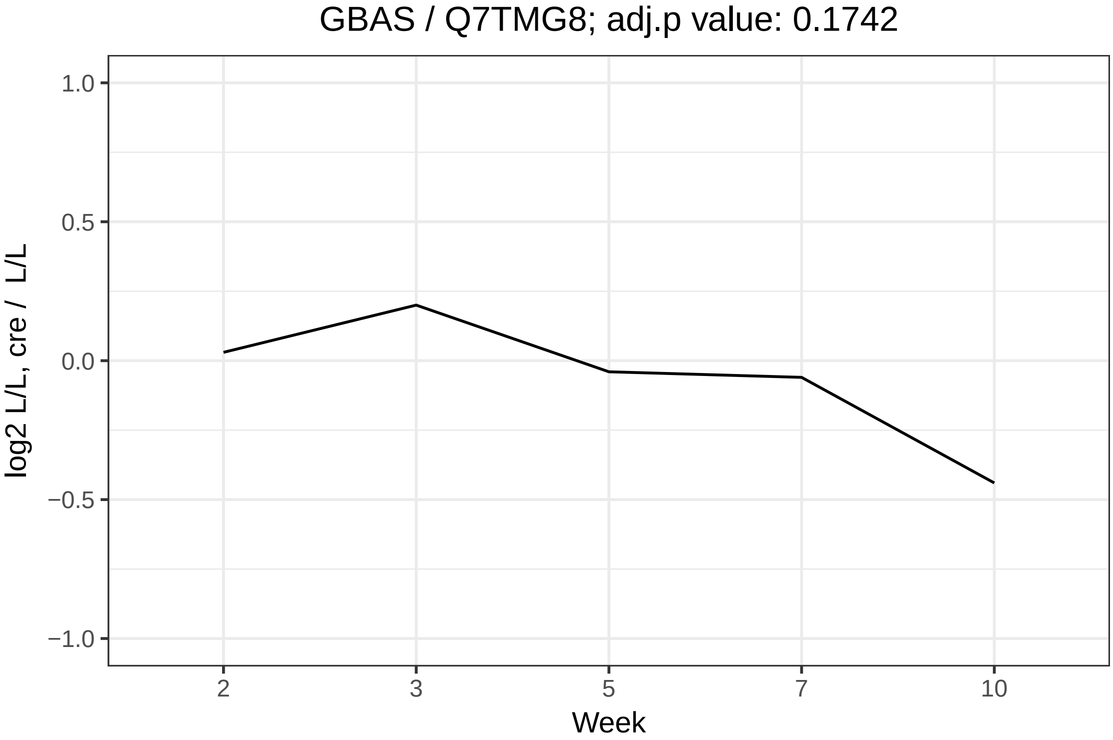
GBAS / Q7TMG8; adj.p value: 0.1742
log2 L/L, cre / L/L
1.0
0.5
0.0
−0.5
−1.0
2
3
5
7
10
Week

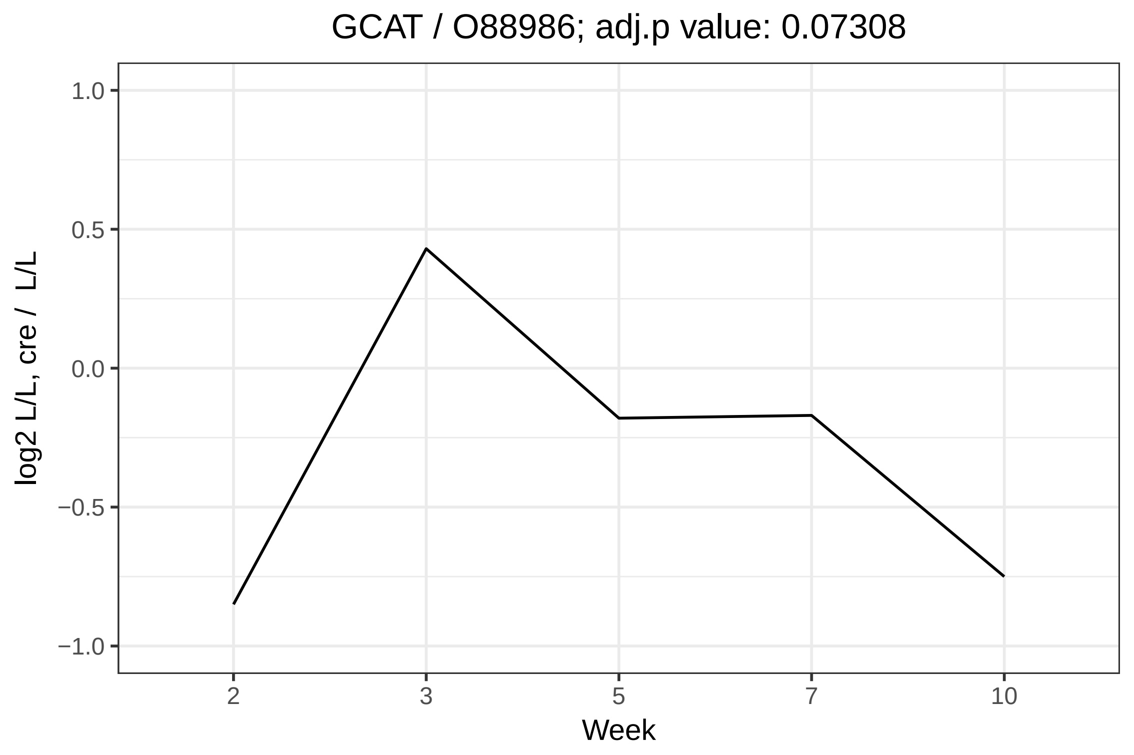
GCAT / O88986; adj.p value: 0.07308
log2 L/L, cre / L/L
1.0
0.5
0.0
−0.5
−1.0
2
3
5
7
10
Week

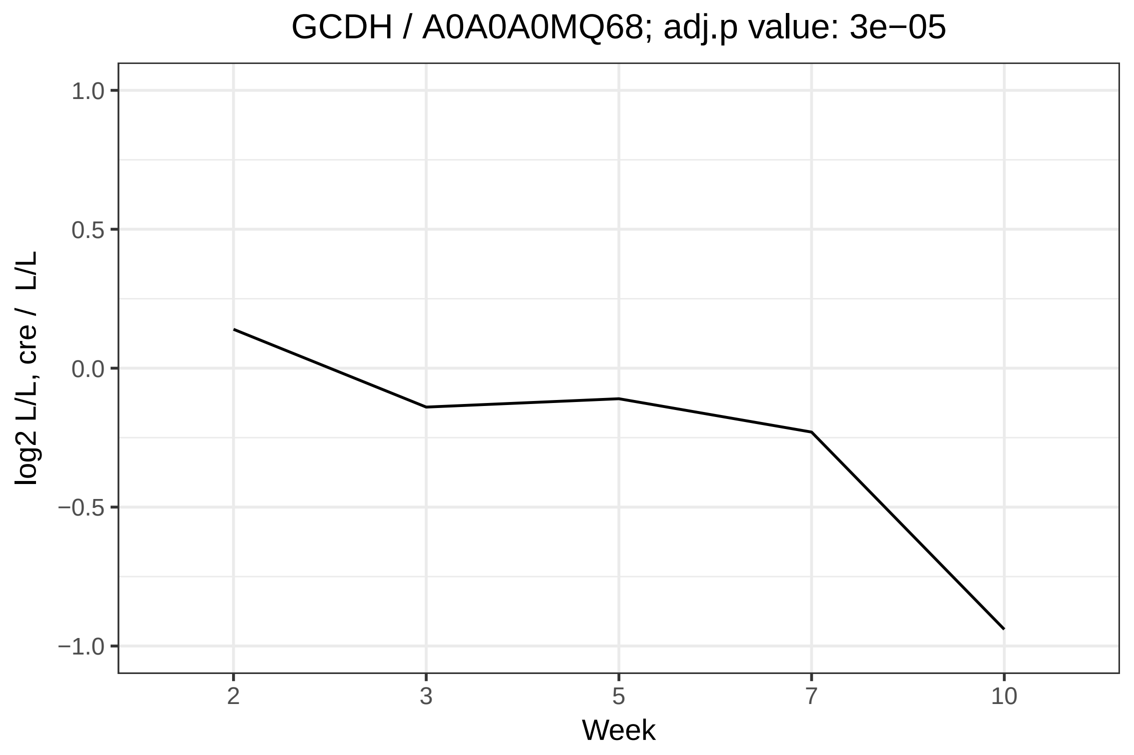

GCDH / A0A0A0MQ68; adj.p value: 3e−05
log2 L/L, cre / L/L
1.0
0.5
0.0
−0.5
−1.0
2
3
5
7
10
Week

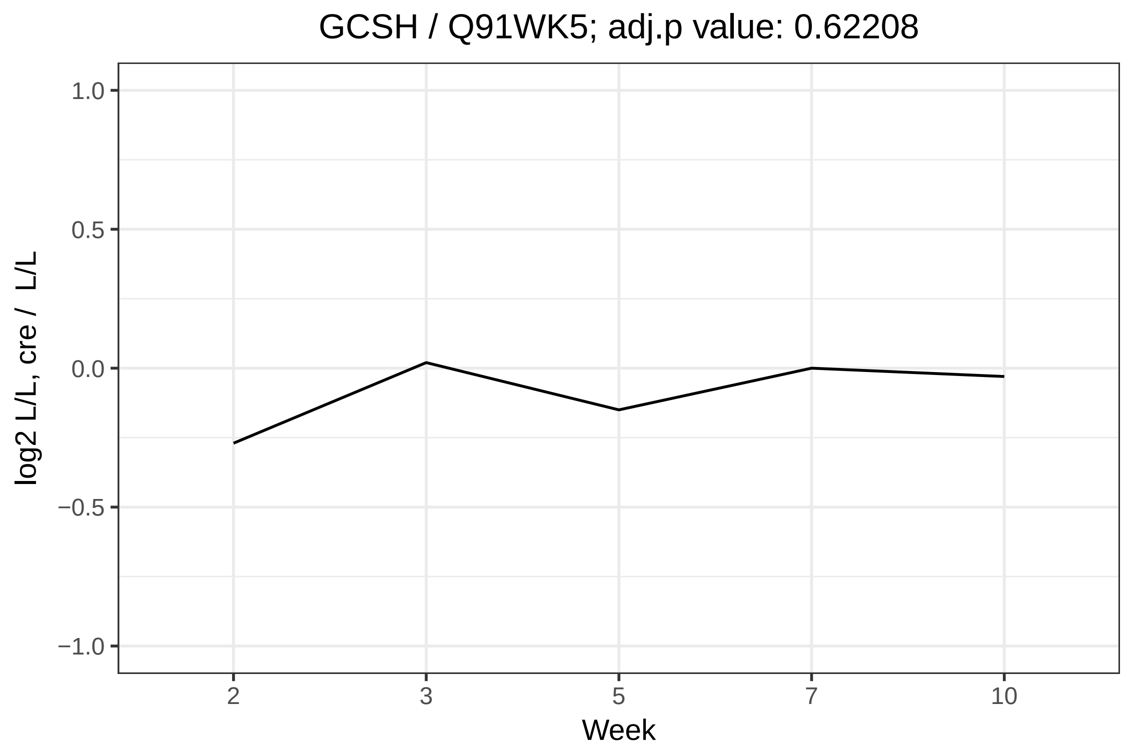

GCSH / Q91WK5; adj.p value: 0.62208
log2 L/L, cre / L/L
1.0
0.5
0.0
−0.5
−1.0
2
3
5
7
10
Week

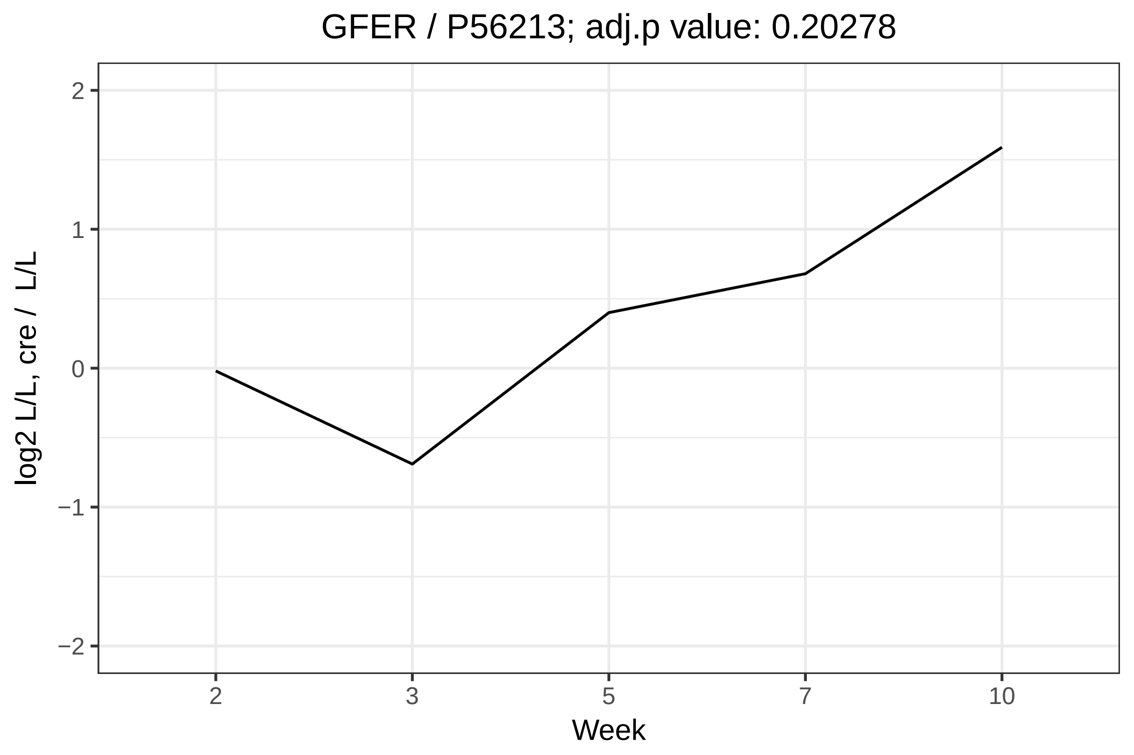
GFER / P56213; adj.p value: 0.20278
log2 L/L, cre / L/L
2
1
0
−1
−2
2
3
5
7
10
Week

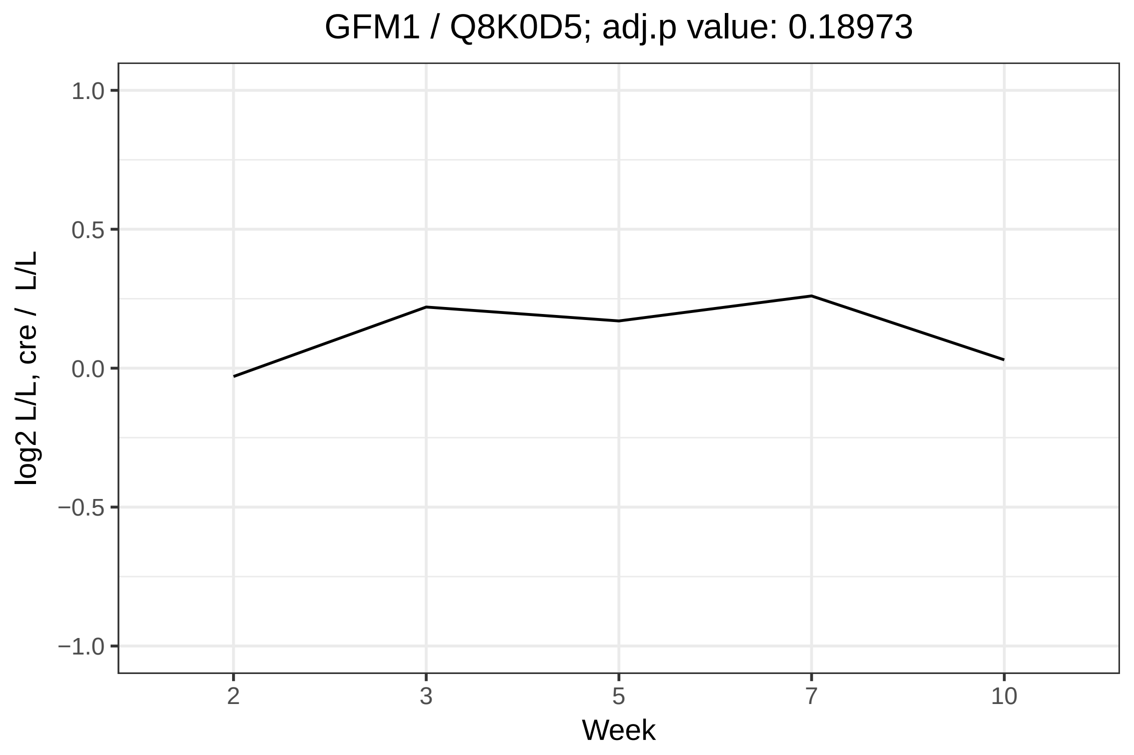
GFM1 / Q8K0D5; adj.p value: 0.18973
1.0
0.5
0.0
−0.5
−1.0
log2 L/L, cre / L/L
2
3
5
7
10
Week

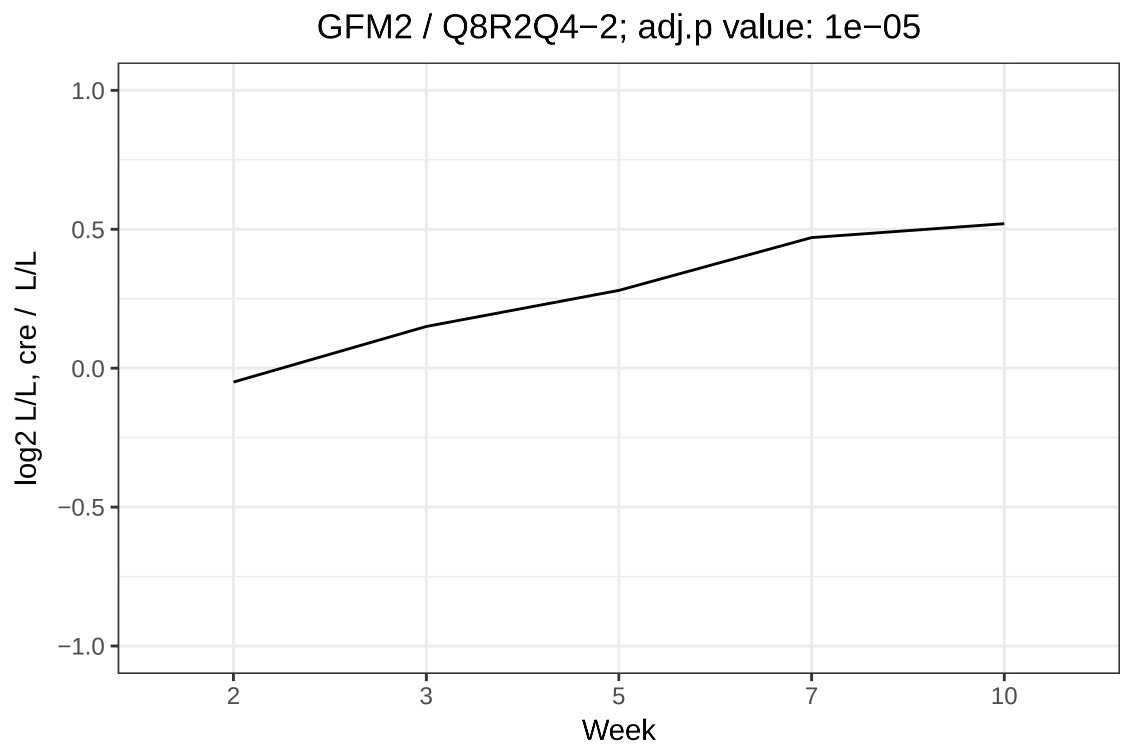

GFM2 / Q8R2Q4−2; adj.p value: 1e−05
log2 L/L, cre / L/L
1.0
0.5
0.0
−0.5
−1.0
2
3
5
7
10
Week

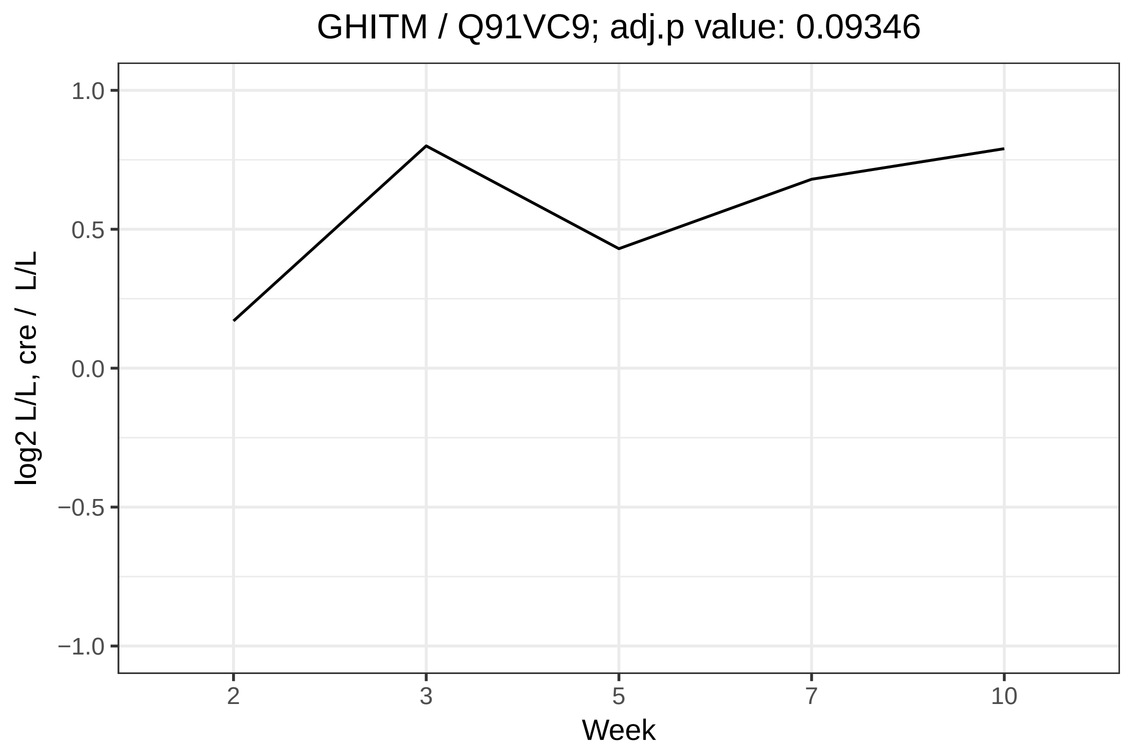
GHITM / Q91VC9; adj.p value: 0.09346
1.0
0.5
0.0
−0.5
−1.0
log2 L/L, cre / L/L
2
3
5
7
10
Week

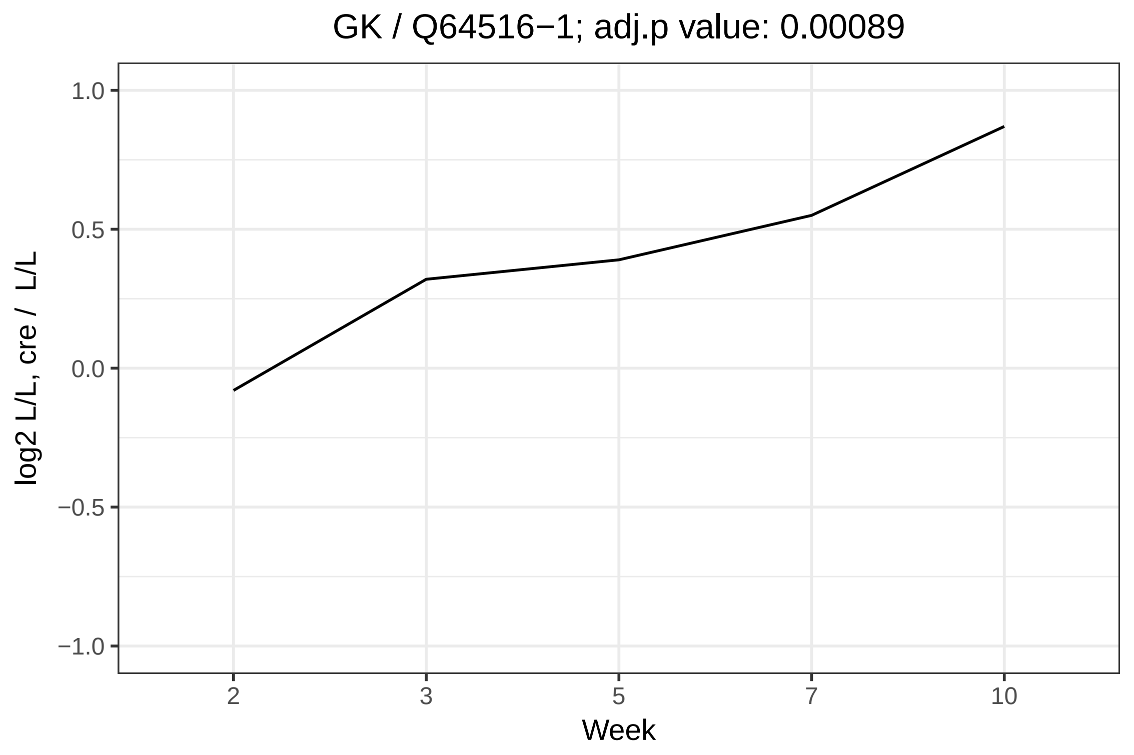
GK / Q64516−1; adj.p value: 0.00089
1.0
0.5
0.0
−0.5
−1.0
log2 L/L, cre / L/L
2
3
5
7
10
Week

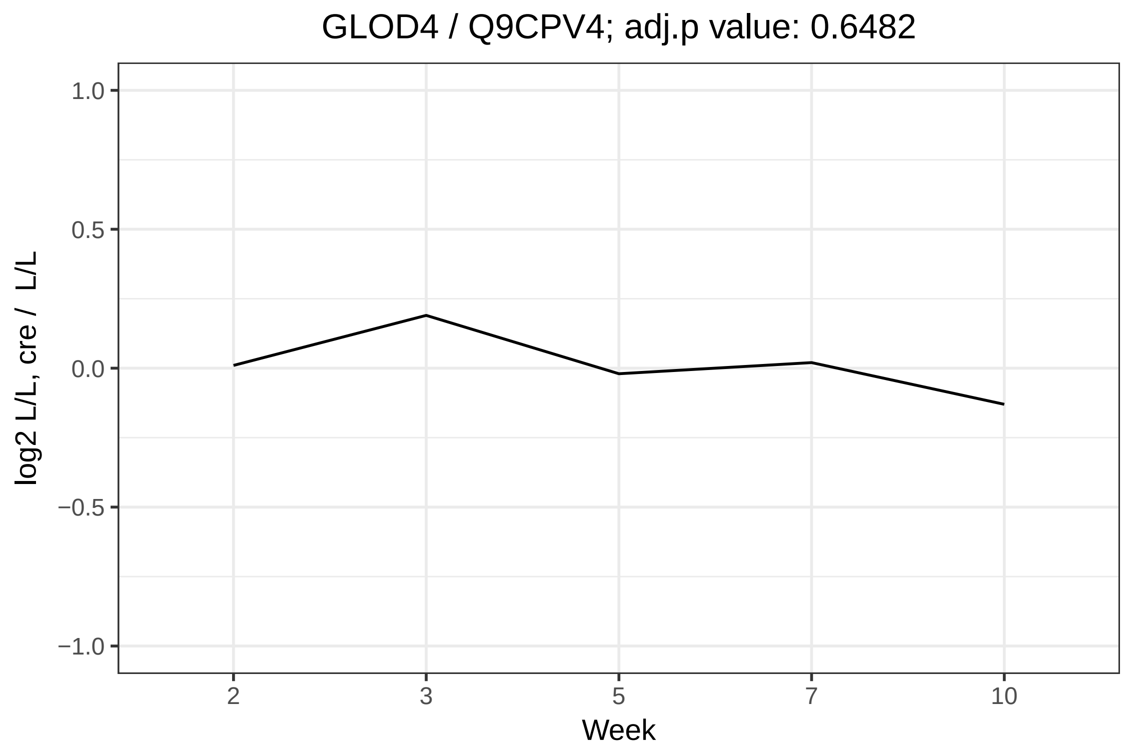
GLOD4 / Q9CPV4; adj.p value: 0.6482
log2 L/L, cre / L/L
1.0
0.5
0.0
−0.5
−1.0
2
3
5
7
10
Week

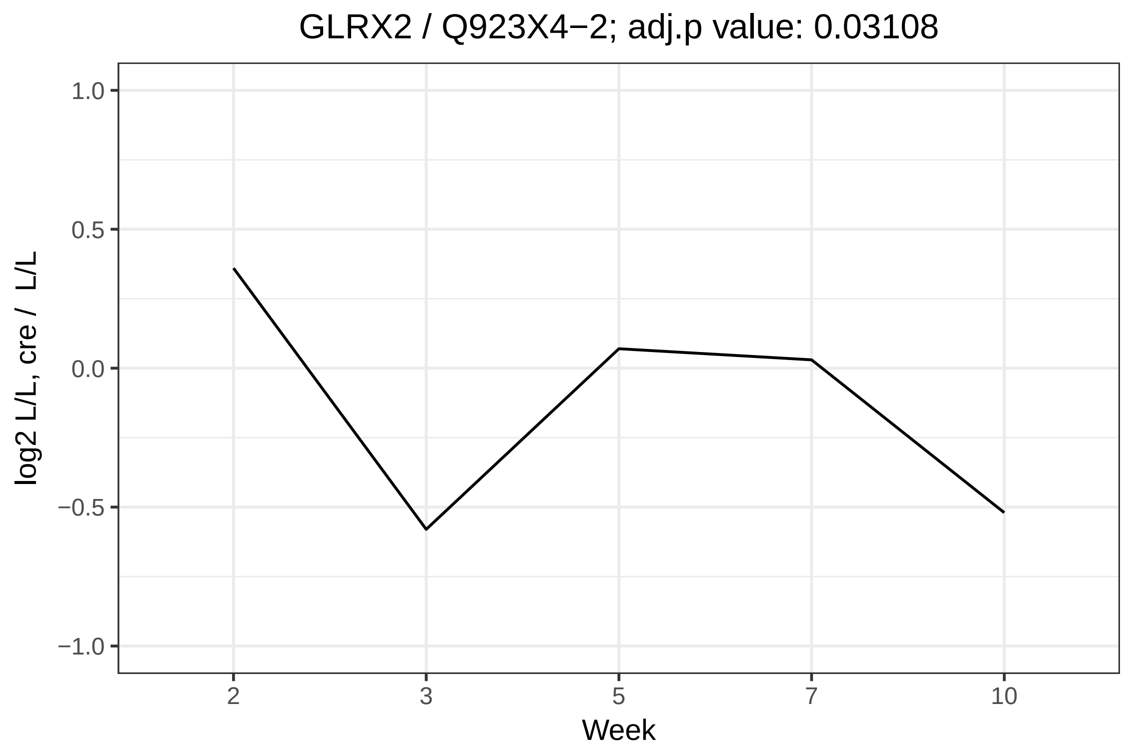

GLRX2 / Q923X4−2; adj.p value: 0.03108
1.0
0.5
0.0
−0.5
−1.0
log2 L/L, cre / L/L
2
3
5
7
10
Week

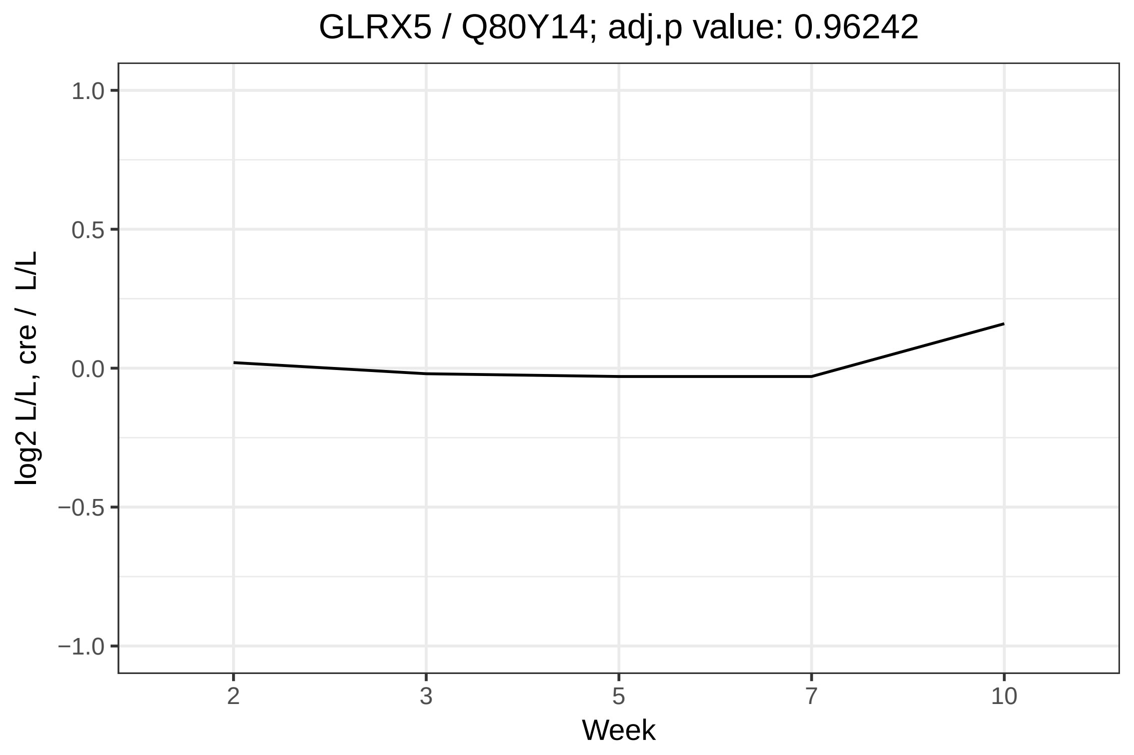
GLRX5 / Q80Y14; adj.p value: 0.96242
log2 L/L, cre / L/L
1.0
0.5
0.0
−0.5
−1.0
2
3
5
7
10
Week

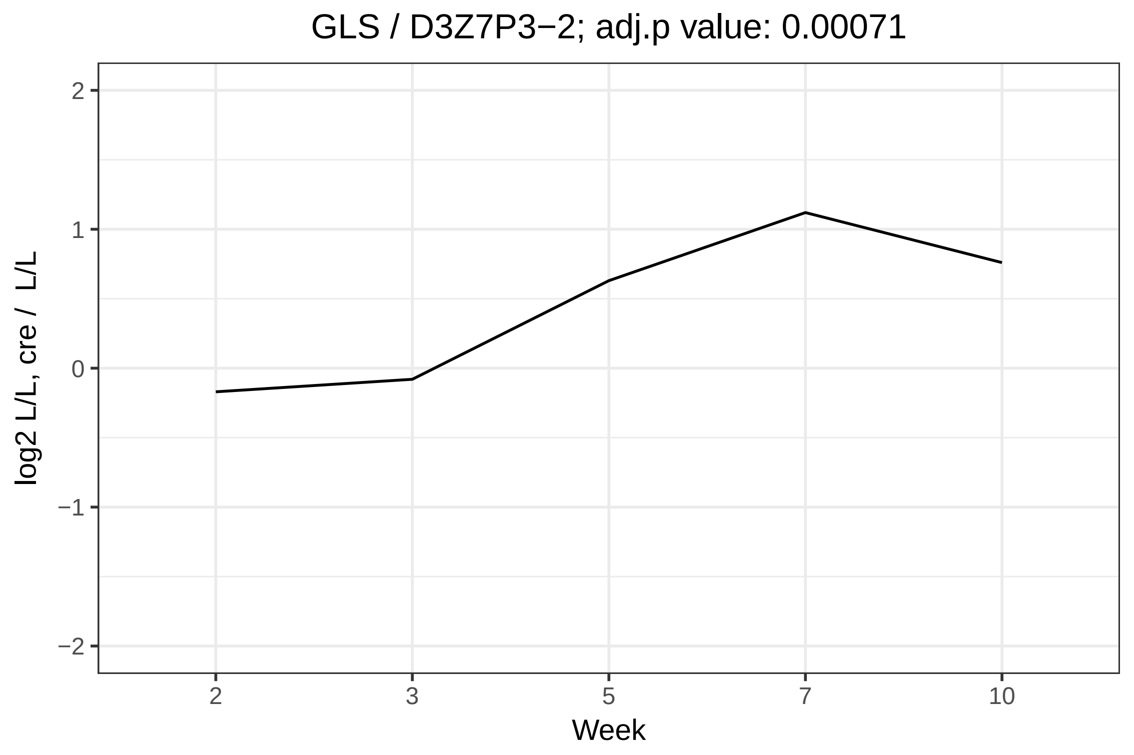

GLS / D3Z7P3−2; adj.p value: 0.00071
log2 L/L, cre / L/L
2
1
0
−1
−2
2
3
5
7
10
Week

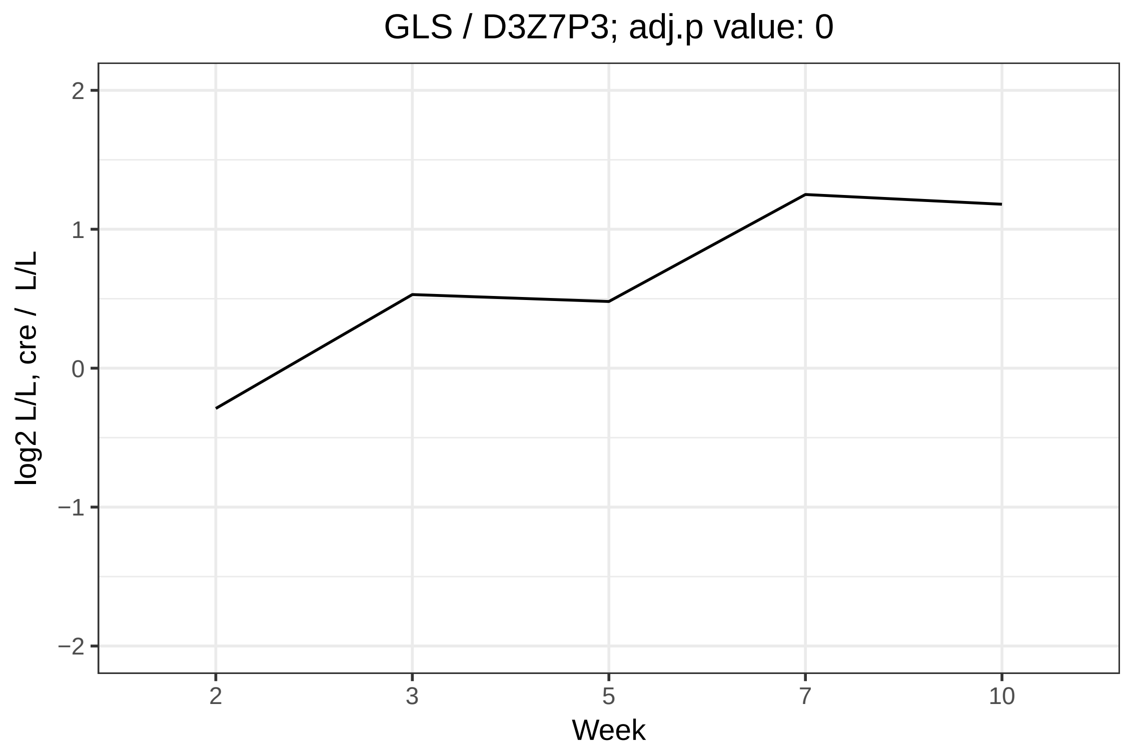

GLS / D3Z7P3; adj.p value: 0
log2 L/L, cre / L/L
Week
2
1
0
−1
−2
2
3
5
7
10

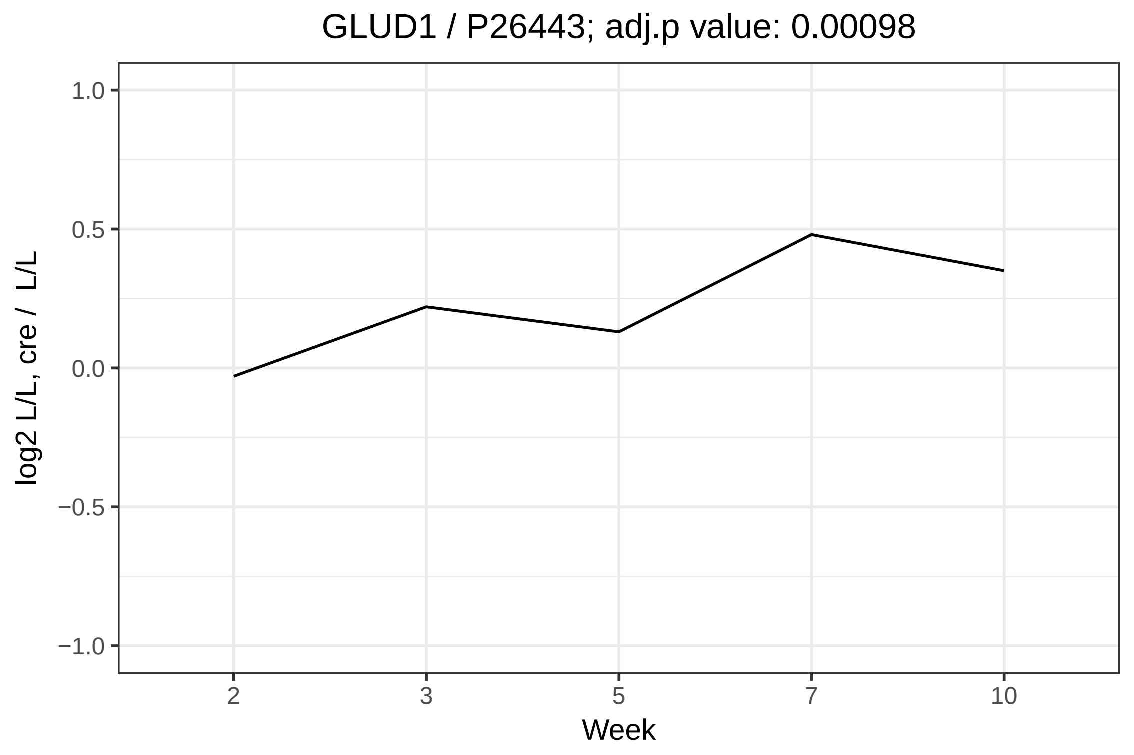

GLUD1 / P26443; adj.p value: 0.00098
log2 L/L, cre / L/L
1.0
0.5
0.0
−0.5
−1.0
2
3
5
7
10
Week

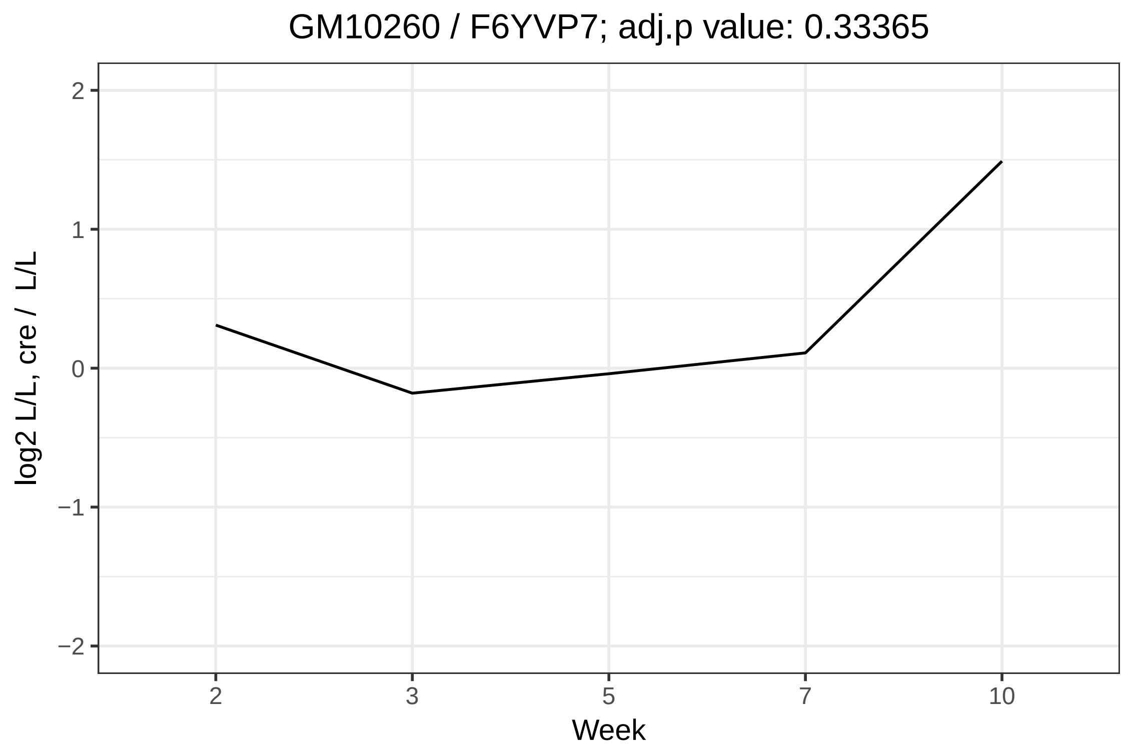
GM10260 / F6YVP7; adj.p value: 0.33365
log2 L/L, cre / L/L
2
1
0
−1
−2
2
3
5
7
10
Week

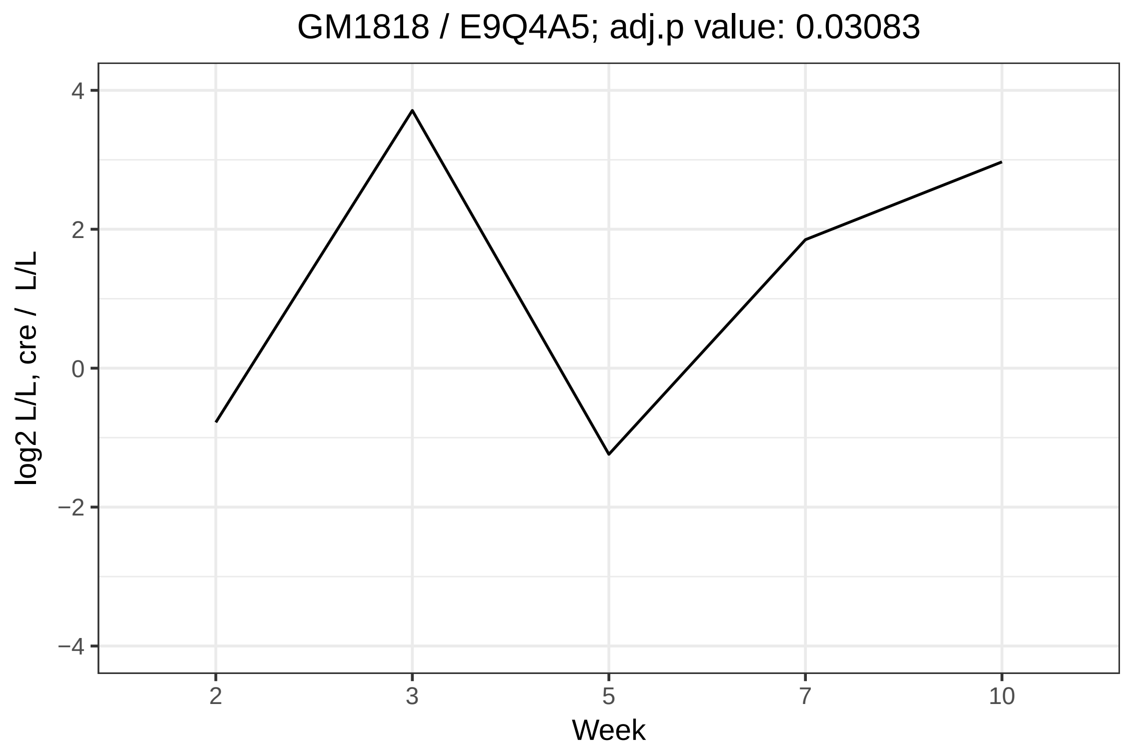
GM1818 / E9Q4A5; adj.p value: 0.03083
log2 L/L, cre / L/L
4
2
0
−2
−4
2
3
5
7
10
Week

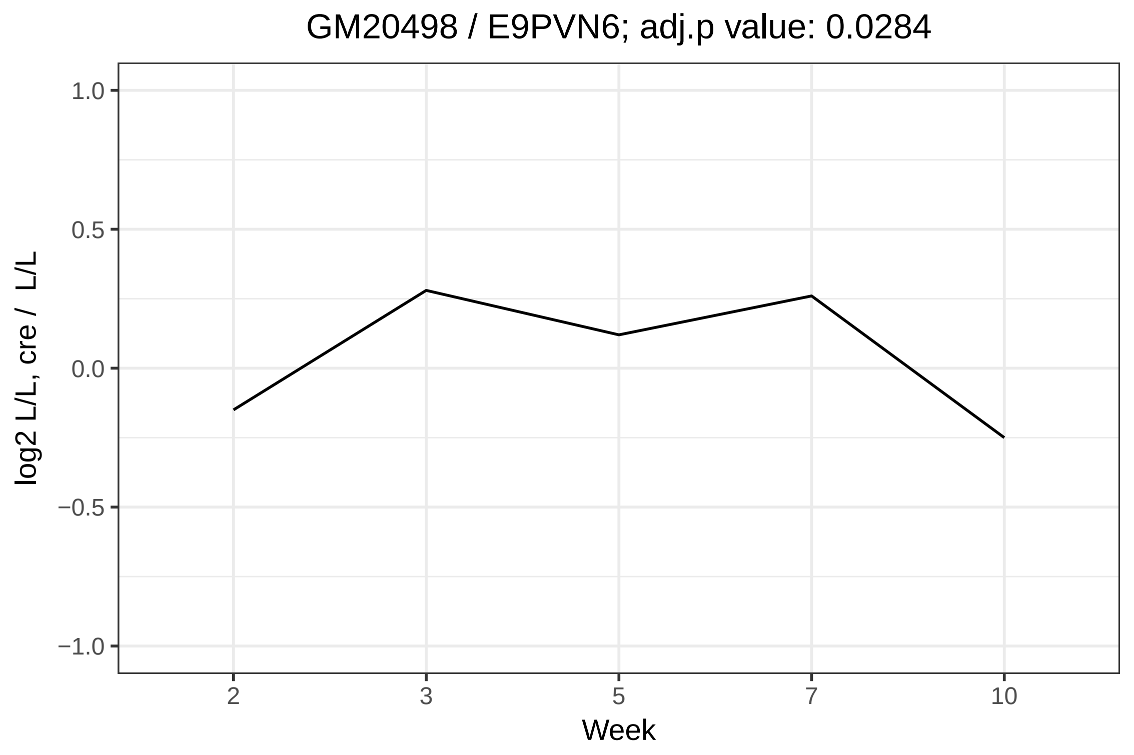
GM20498 / E9PVN6; adj.p value: 0.0284
log2 L/L, cre / L/L
1.0
0.5
0.0
−0.5
−1.0
2
3
5
7
10
Week

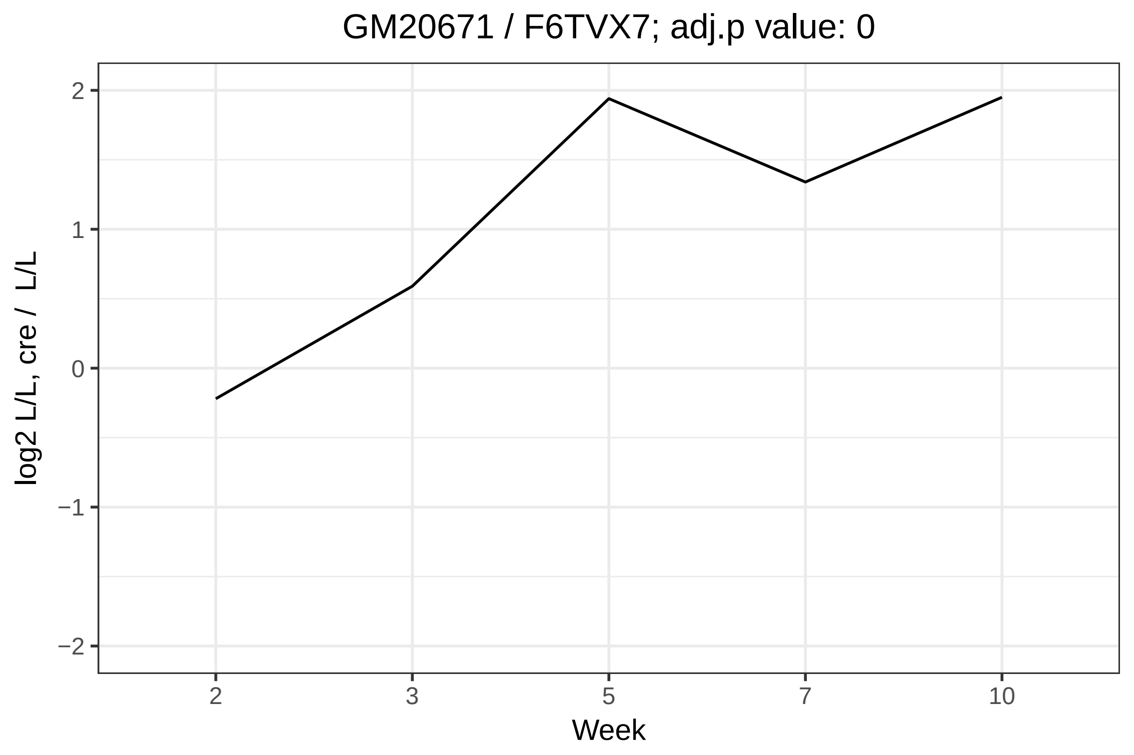
GM20671 / F6TVX7; adj.p value: 0
log2 L/L, cre / L/L
2
1
0
−1
−2
2
3
5
7
10
Week

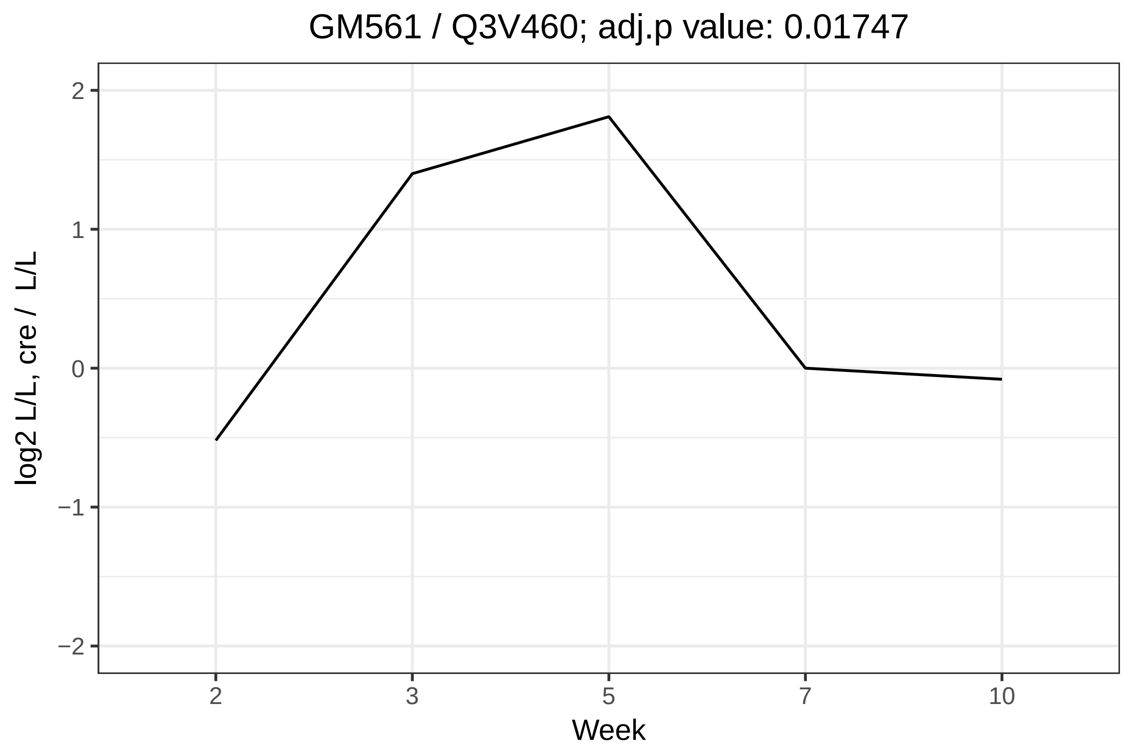
GM561 / Q3V460; adj.p value: 0.01747
log2 L/L, cre / L/L
2
1
0
−1
−2
2
3
5
7
10
Week

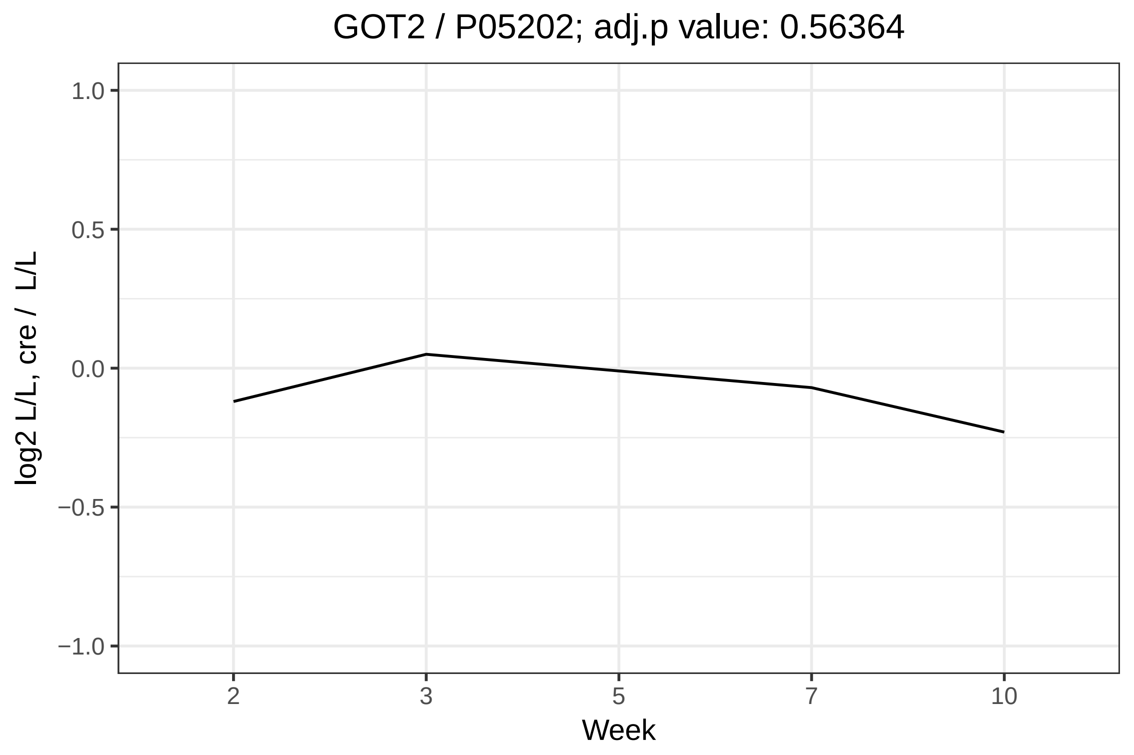
GOT2 / P05202; adj.p value: 0.56364
1.0
0.5
0.0
−0.5
−1.0
log2 L/L, cre / L/L
2
3
5
7
10
Week

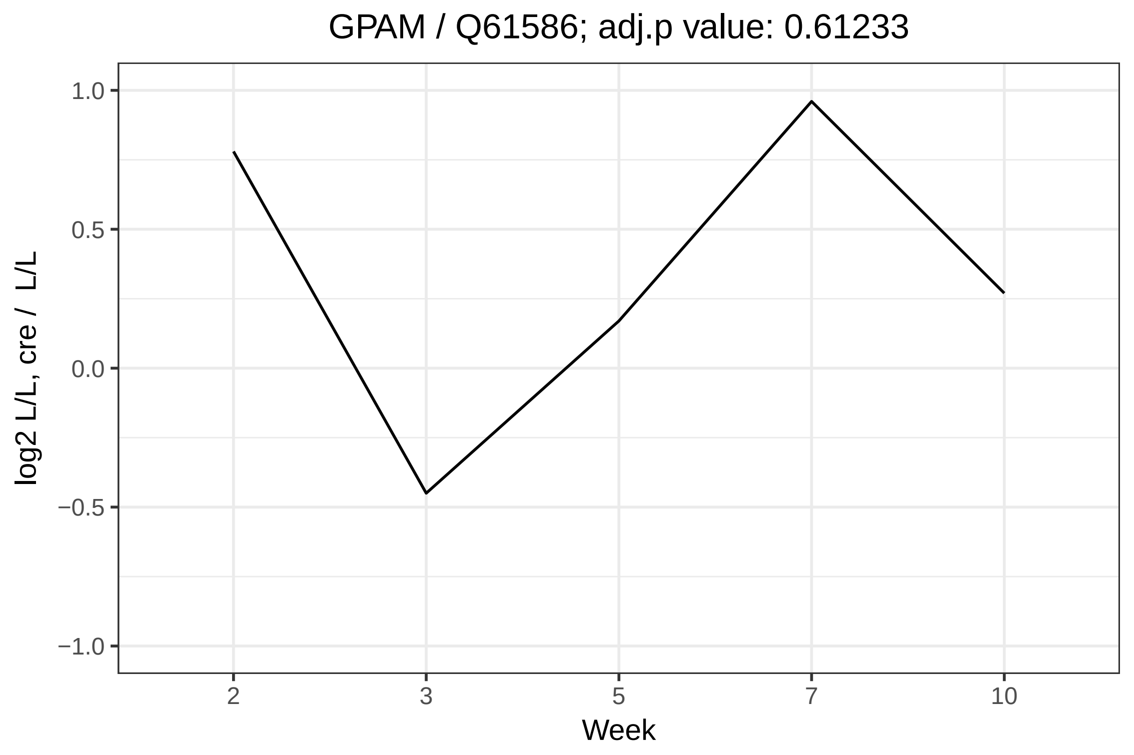
GPAM / Q61586; adj.p value: 0.61233
log2 L/L, cre / L/L
1.0
0.5
0.0
−0.5
−1.0
2
3
5
7
10
Week

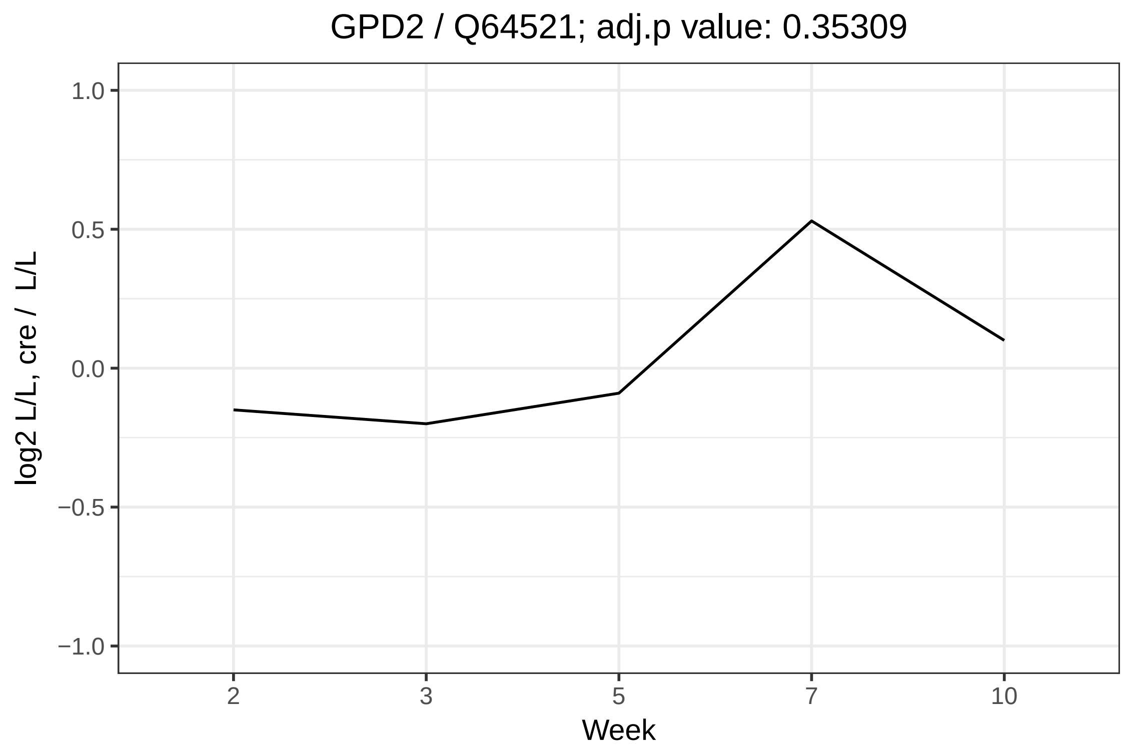
GPD2 / Q64521; adj.p value: 0.35309
1.0
0.5
0.0
−0.5
−1.0
log2 L/L, cre / L/L
2
3
5
7
10
Week

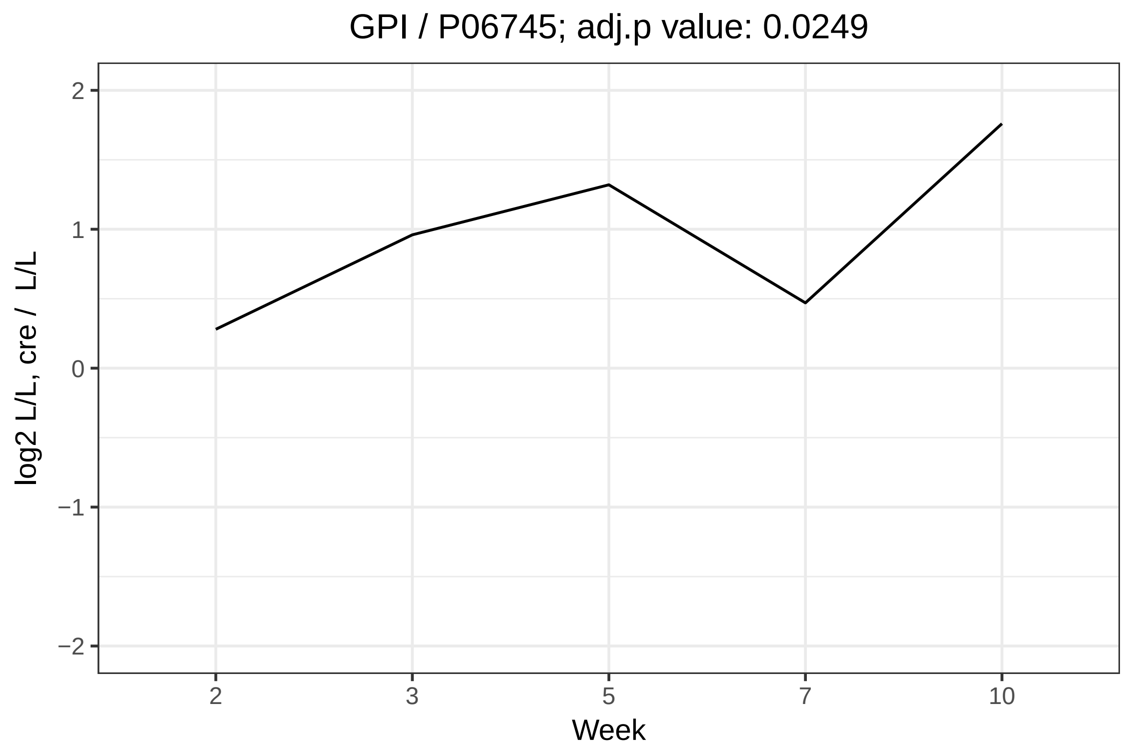
GPI / P06745; adj.p value: 0.0249
log2 L/L, cre / L/L
2
1
0
−1
−2
2
3
5
7
10
Week

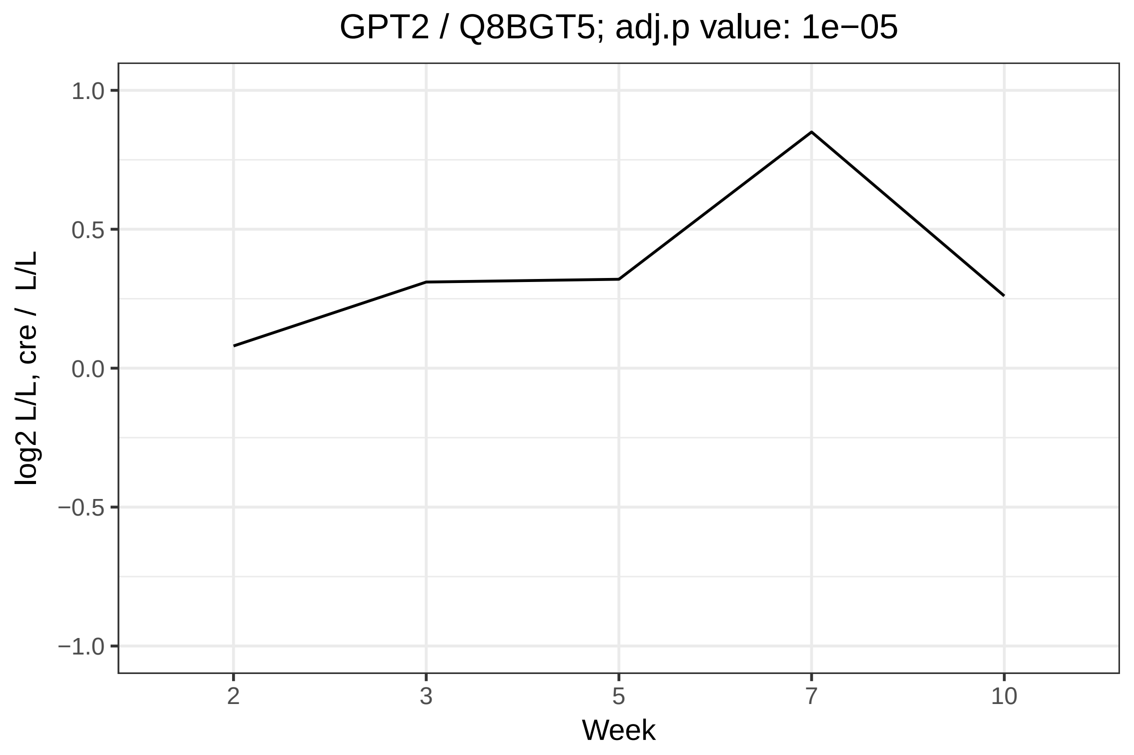
GPT2 / Q8BGT5; adj.p value: 1e−05
log2 L/L, cre / L/L
1.0
0.5
0.0
−0.5
−1.0
2
3
5
7
10
Week

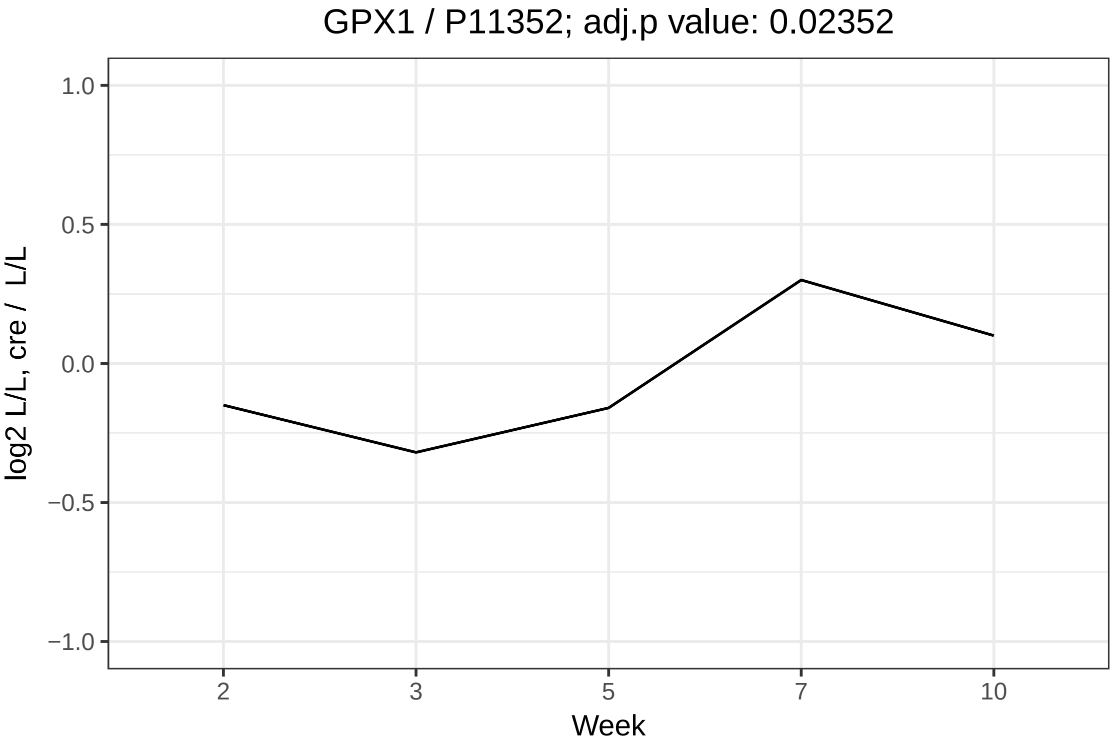

GPX1 / P11352; adj.p value: 0.02352
1.0
0.5
0.0
−0.5
−1.0
log2 L/L, cre / L/L
2
3
5
7
10
Week

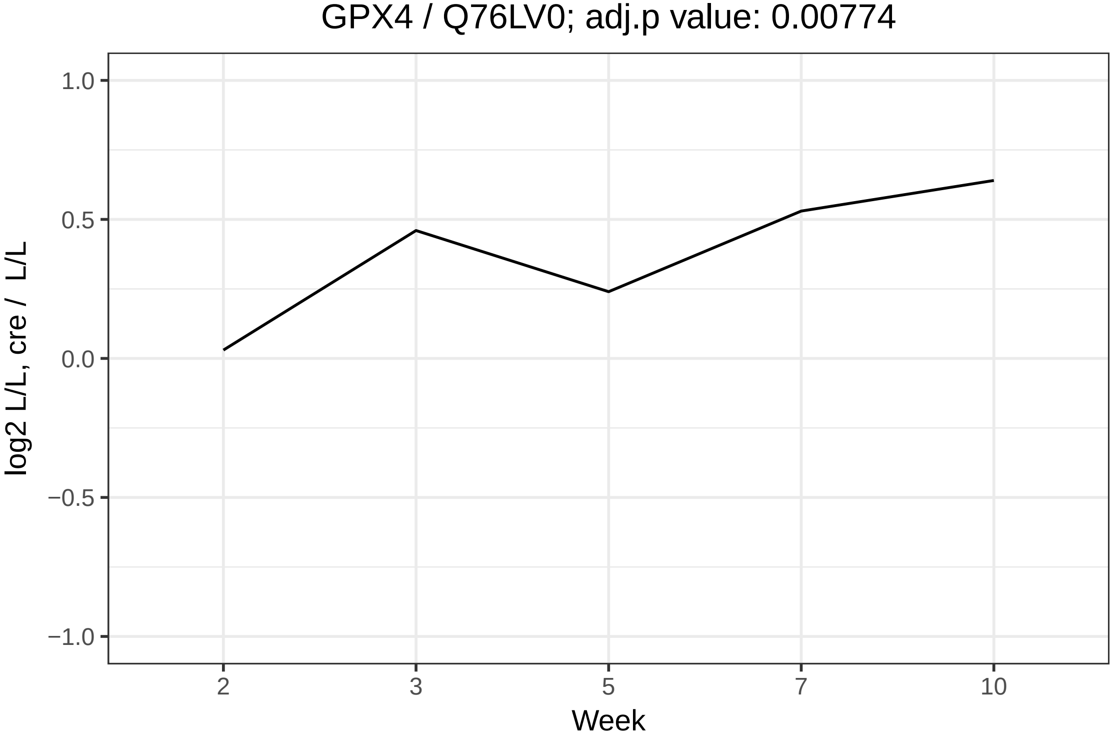
GPX4 / Q76LV0; adj.p value: 0.00774
log2 L/L, cre / L/L
1.0
0.5
0.0
−0.5
−1.0
2
3
5
7
10
Week

GRHPR / Q91Z53; adj.p value: 0.16301

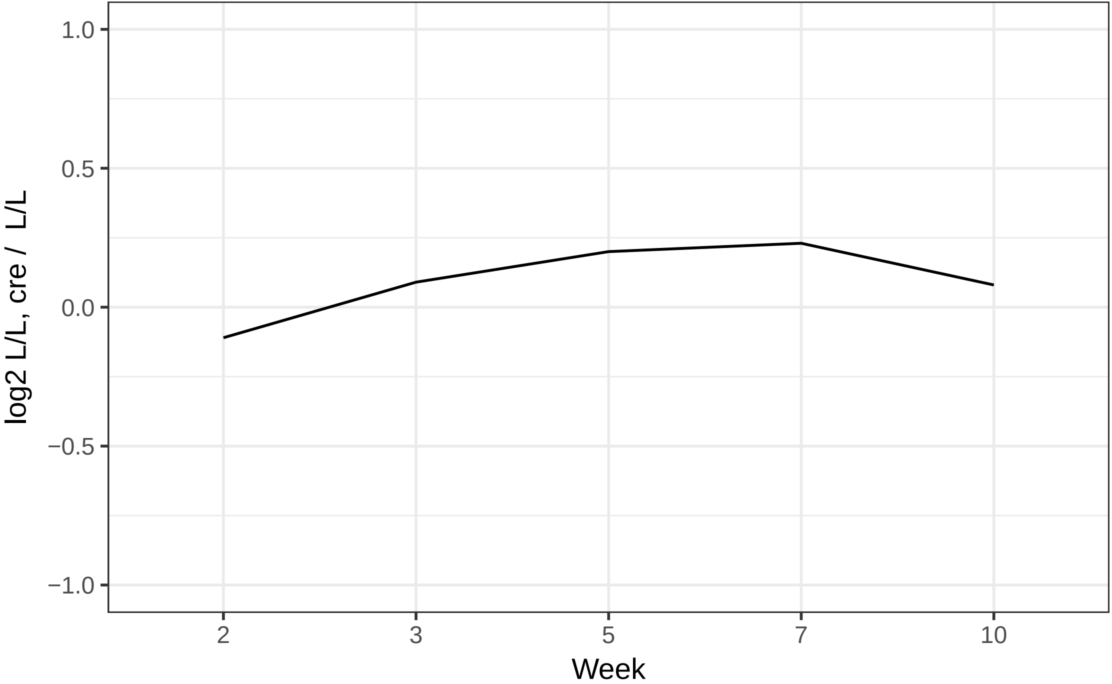

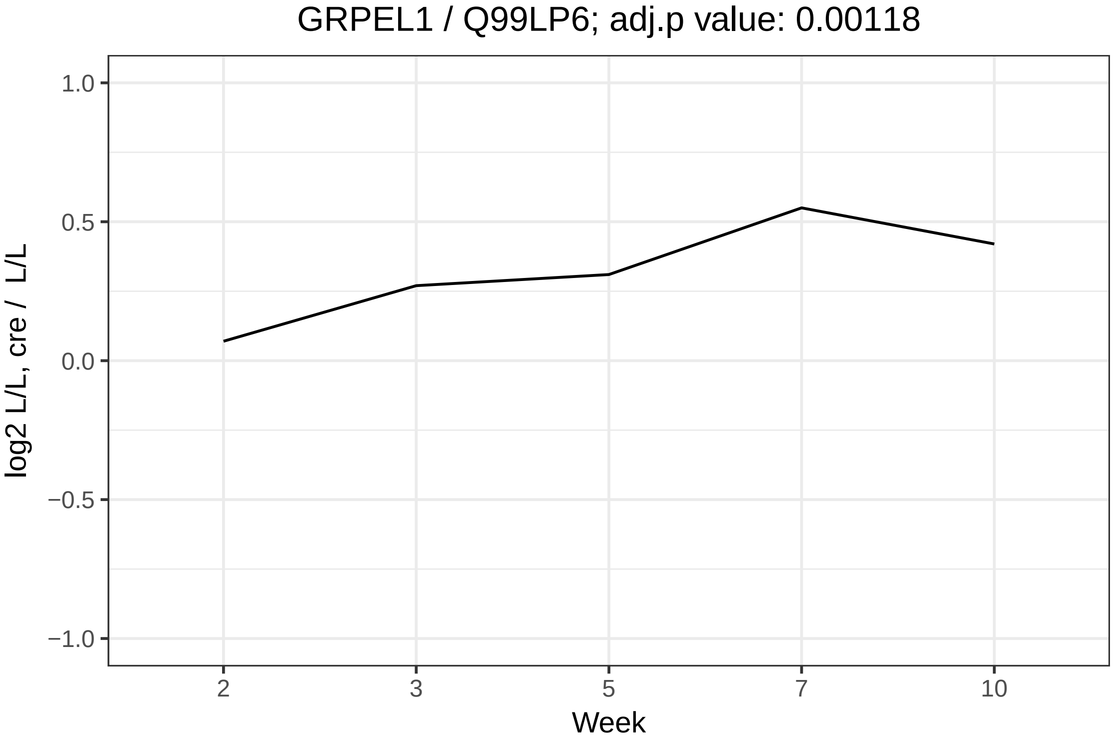

GRPEL1 / Q99LP6; adj.p value: 0.00118
1.0
0.5
0.0
−0.5
−1.0
log2 L/L, cre / L/L
2
3
5
7
10
Week

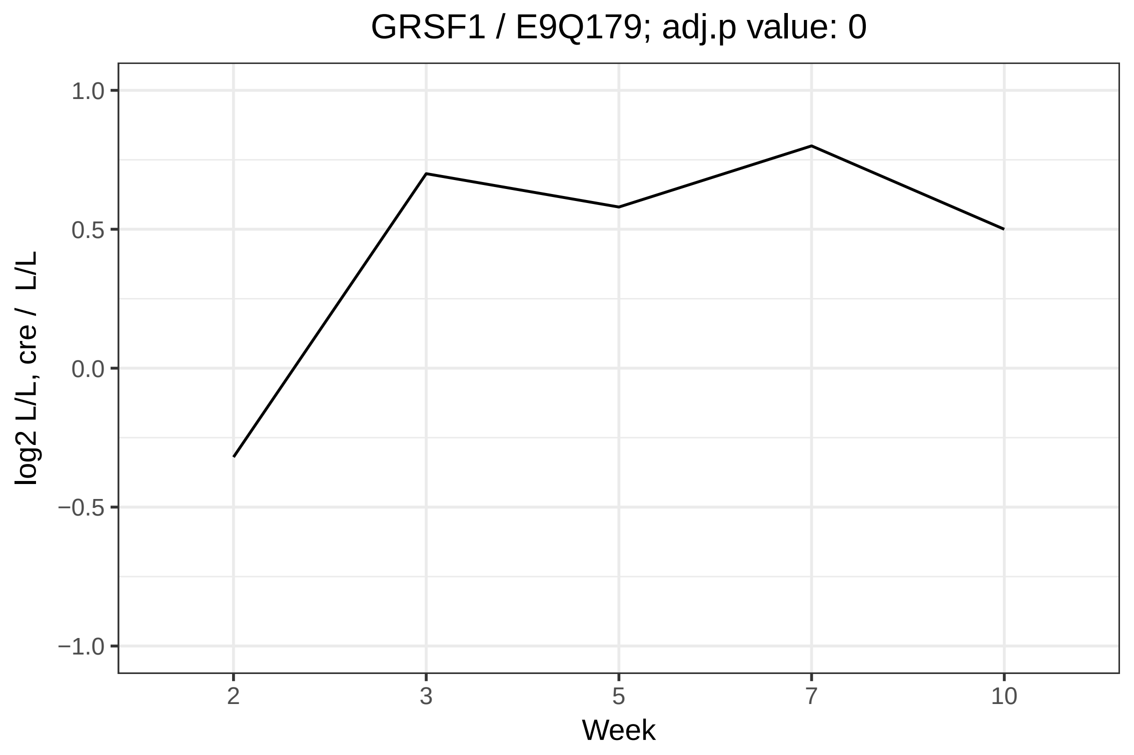
GRSF1 / E9Q179; adj.p value: 0
log2 L/L, cre / L/L
1.0
0.5
0.0
−0.5
−1.0
2
3
5
7
10
Week

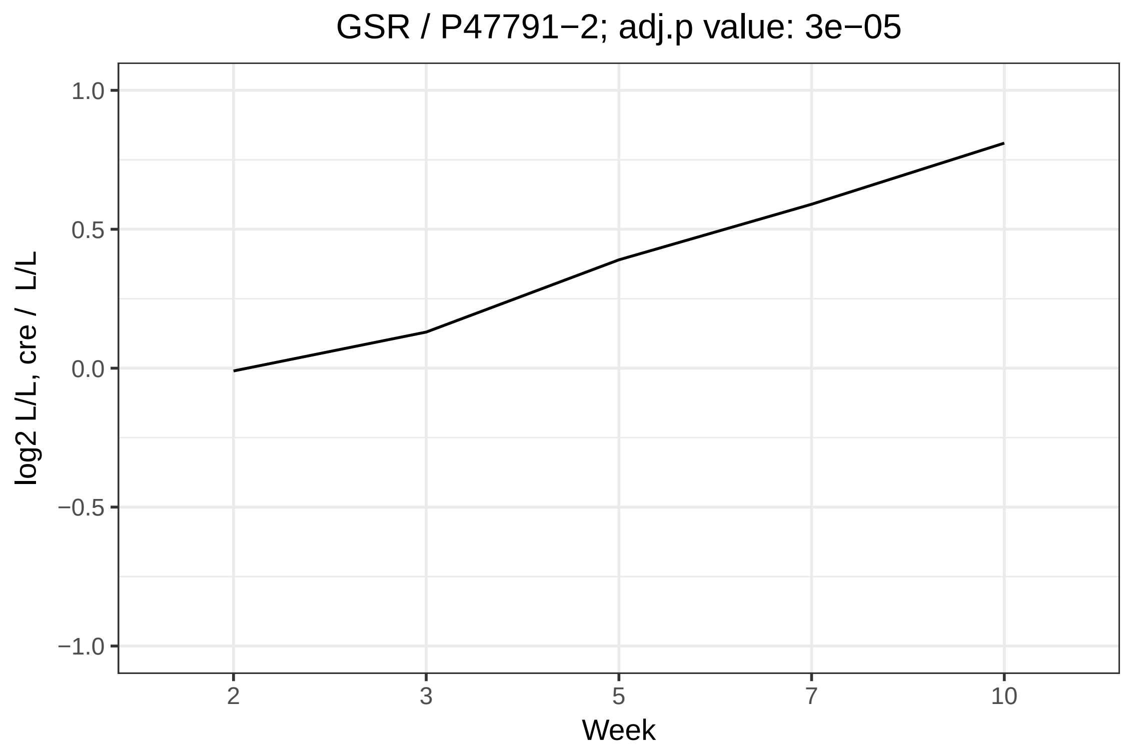

GSR / P47791−2; adj.p value: 3e−05
log2 L/L, cre / L/L
1.0
0.5
0.0
−0.5
−1.0
2
3
5
7
10
Week

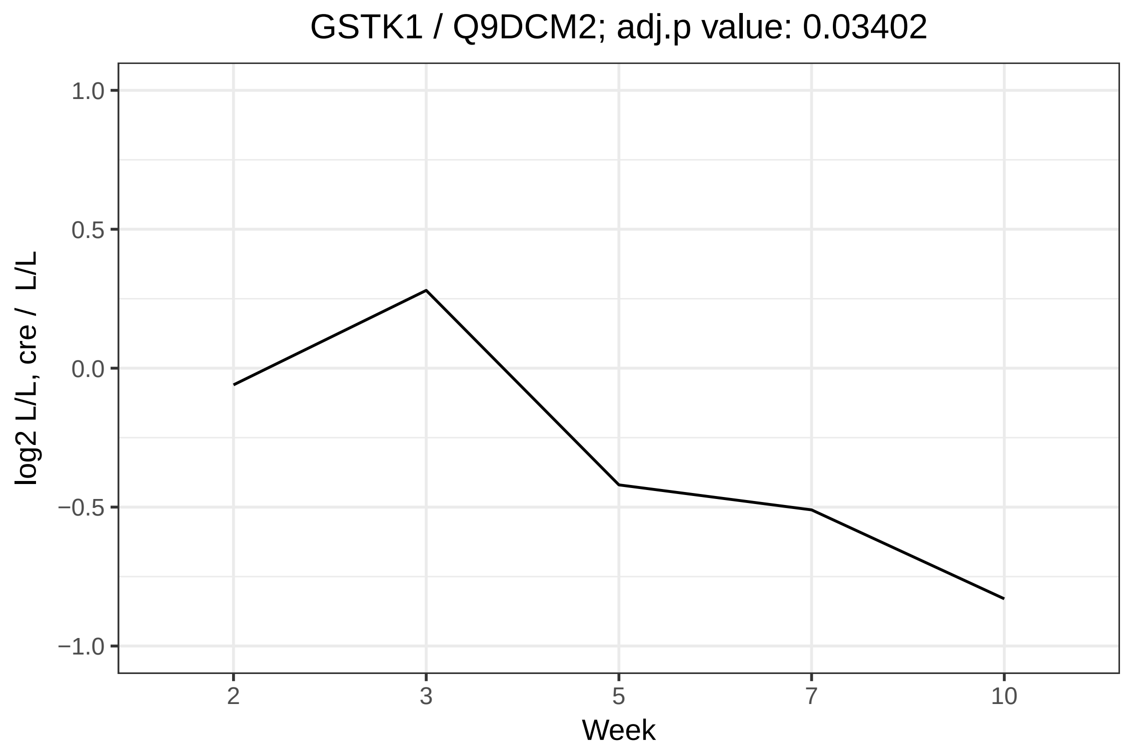
GSTK1 / Q9DCM2; adj.p value: 0.03402
1.0
0.5
0.0
−0.5
−1.0
log2 L/L, cre / L/L
2
3
5
7
10
Week

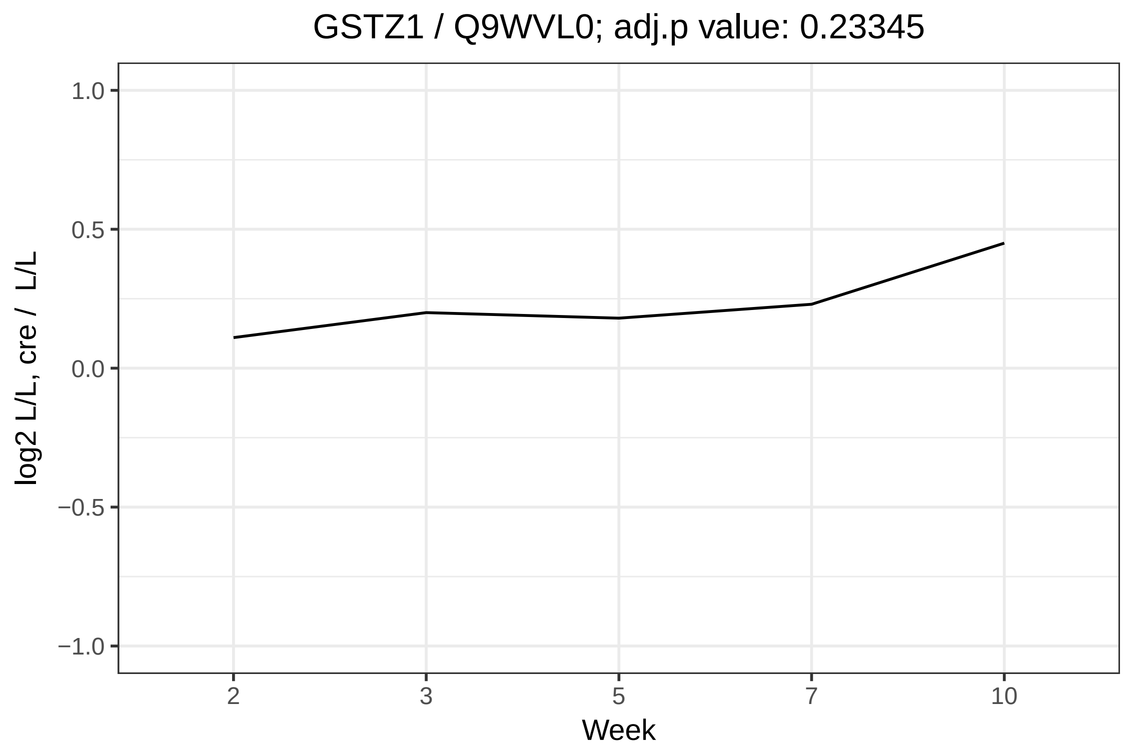

GSTZ1 / Q9WVL0; adj.p value: 0.23345
log2 L/L, cre / L/L
1.0
0.5
0.0
−0.5
−1.0
2
3
5
7
10
Week

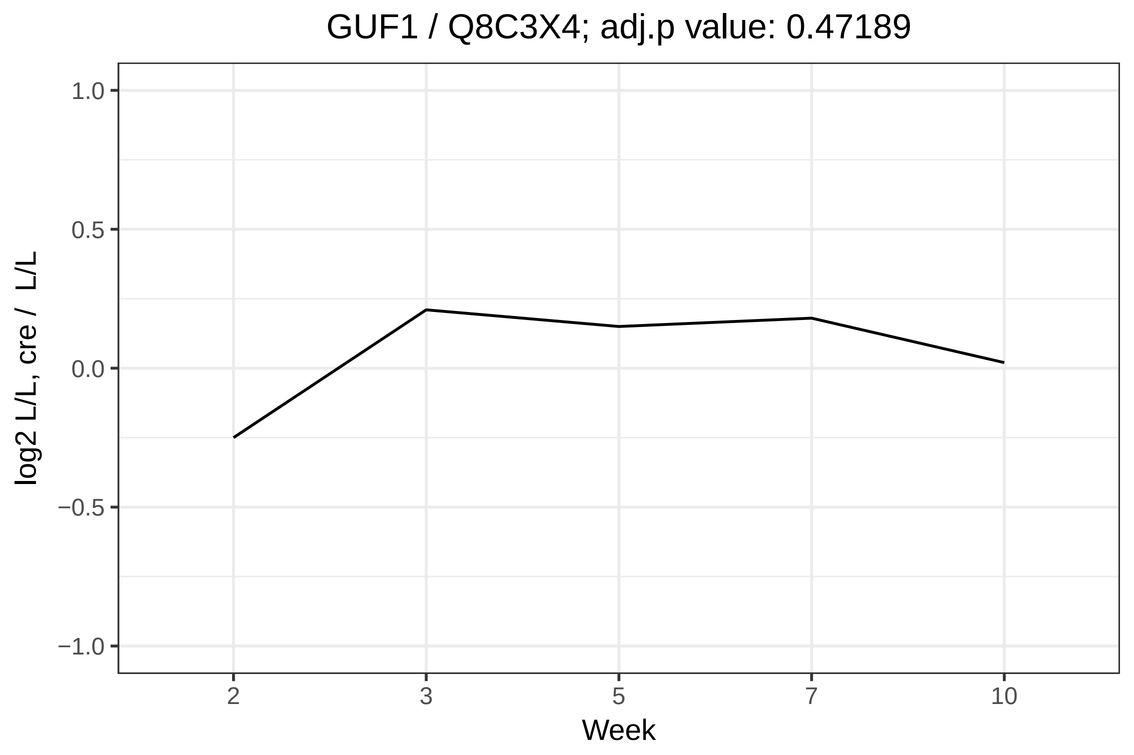
GUF1 / Q8C3X4; adj.p value: 0.47189
log2 L/L, cre / L/L
1.0
0.5
0.0
−0.5
−1.0
2
3
5
7
10
Week

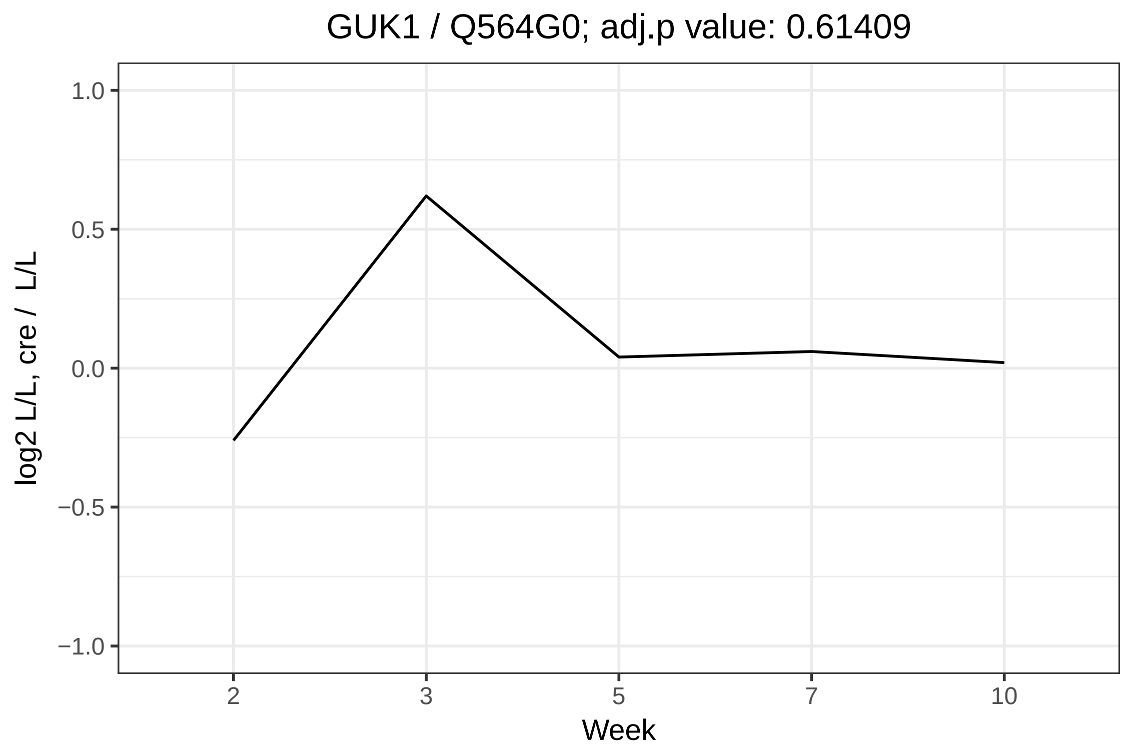
GUK1 / Q564G0; adj.p value: 0.61409
log2 L/L, cre / L/L
1.0
0.5
0.0
−0.5
−1.0
2
3
5
7
10
Week

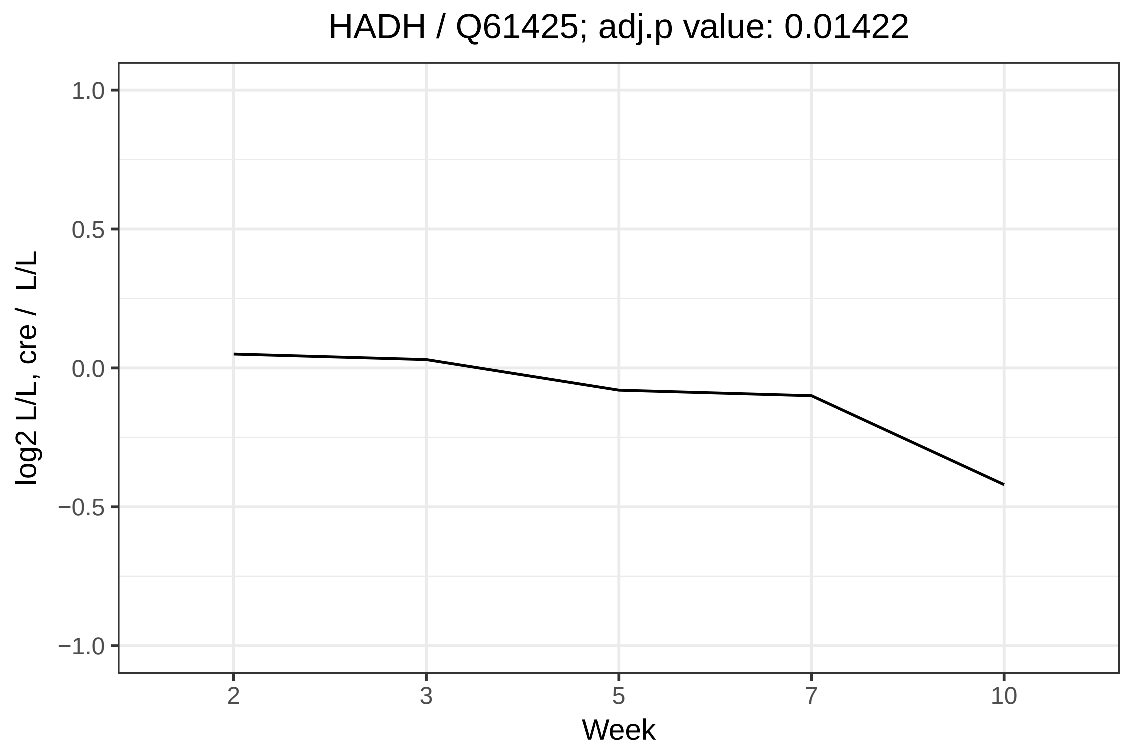
HADH / Q61425; adj.p value: 0.01422
log2 L/L, cre / L/L
1.0
0.5
0.0
−0.5
−1.0
2
3
5
7
10
Week

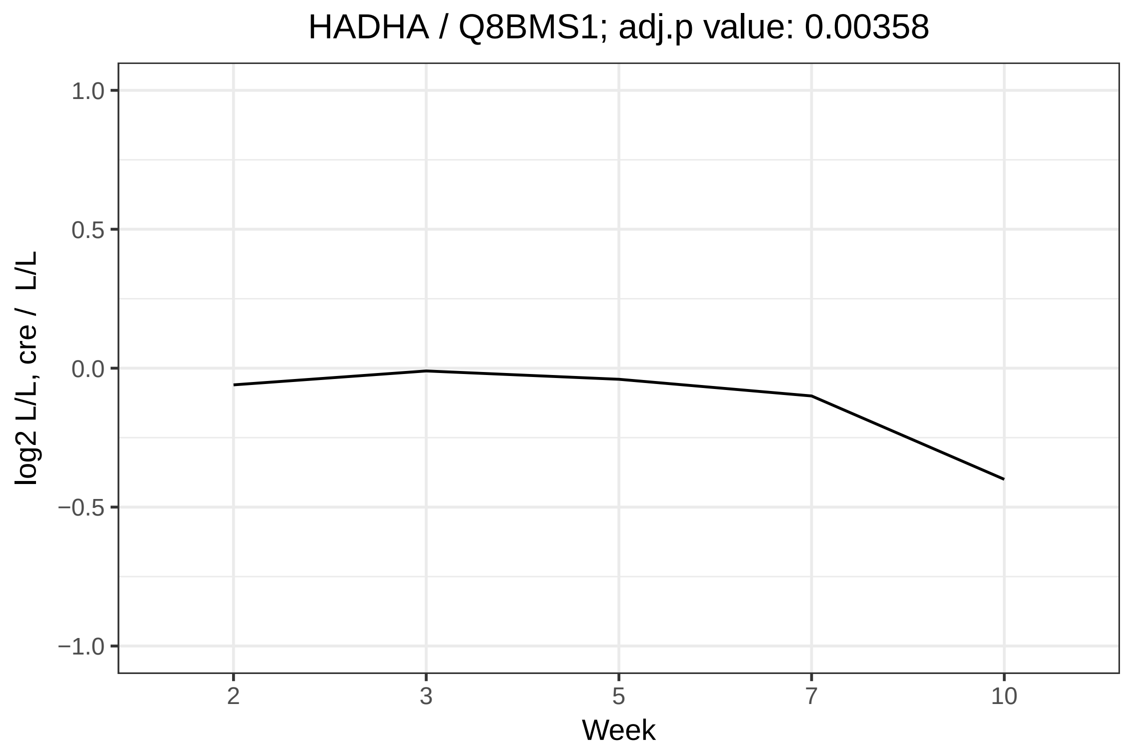

HADHA / Q8BMS1; adj.p value: 0.00358
1.0
0.5
0.0
−0.5
−1.0
log2 L/L, cre / L/L
2
3
5
7
10
Week

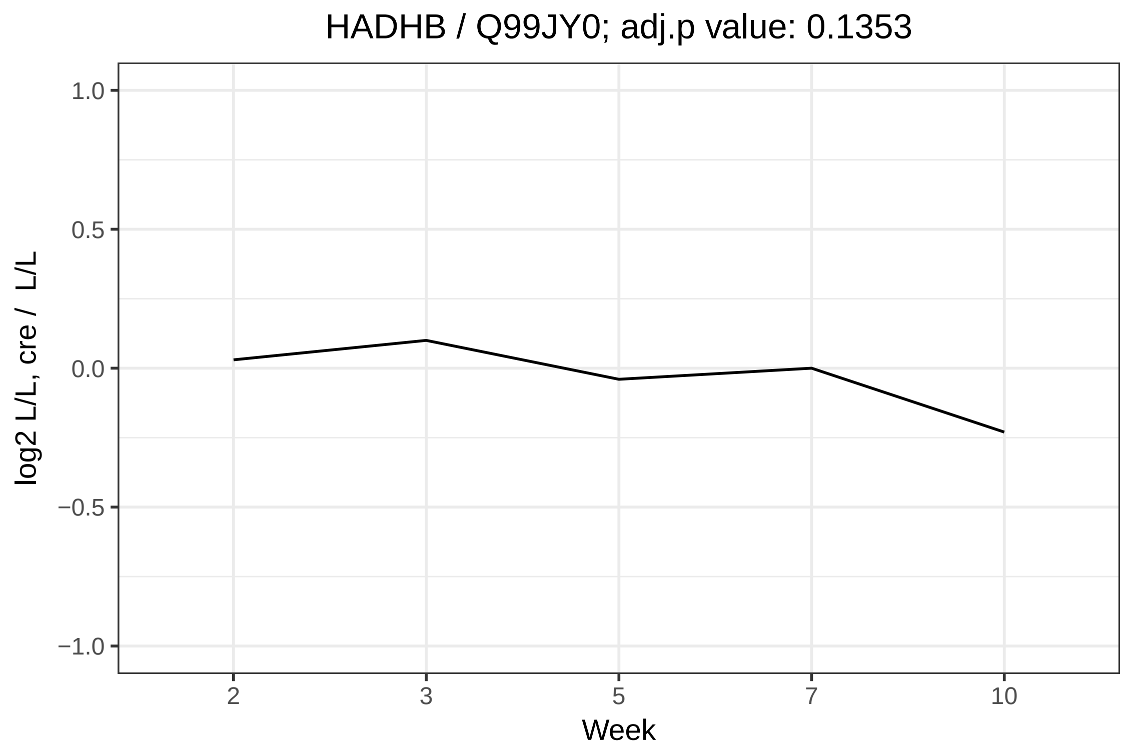
HADHB / Q99JY0; adj.p value: 0.1353
log2 L/L, cre / L/L
1.0
0.5
0.0
−0.5
−1.0
2
3
5
7
10
Week

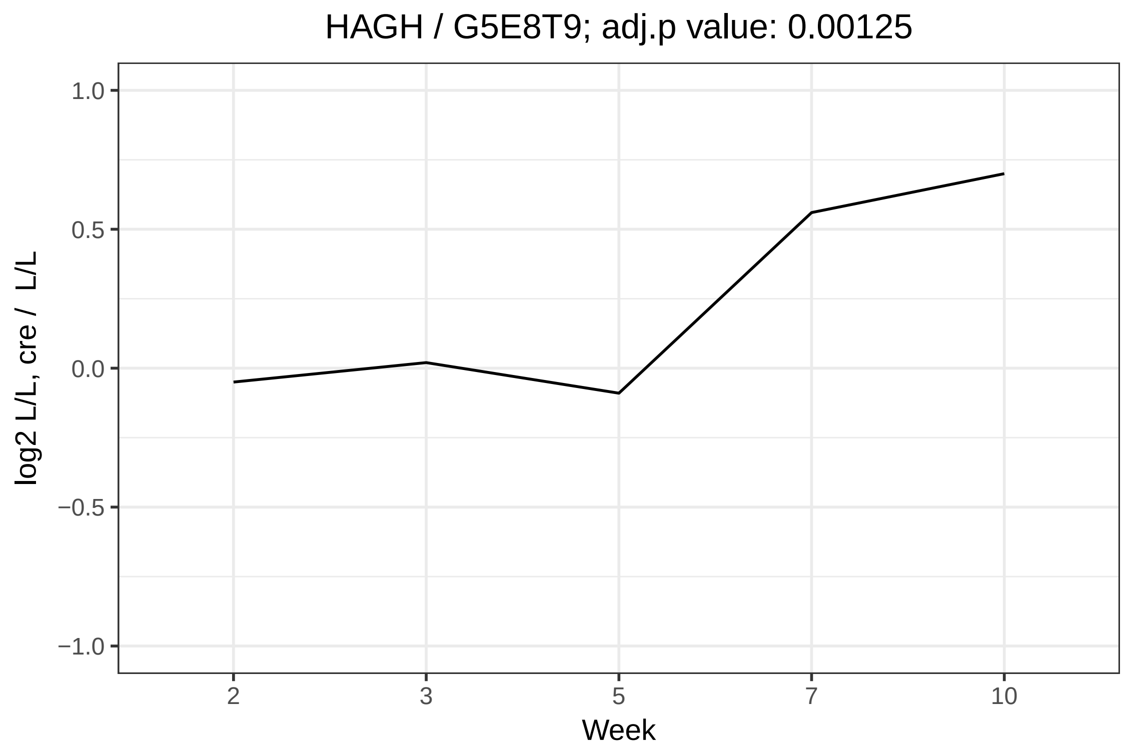
HAGH / G5E8T9; adj.p value: 0.00125
1.0
0.5
0.0
−0.5
−1.0
log2 L/L, cre / L/L
2
3
5
7
10
Week

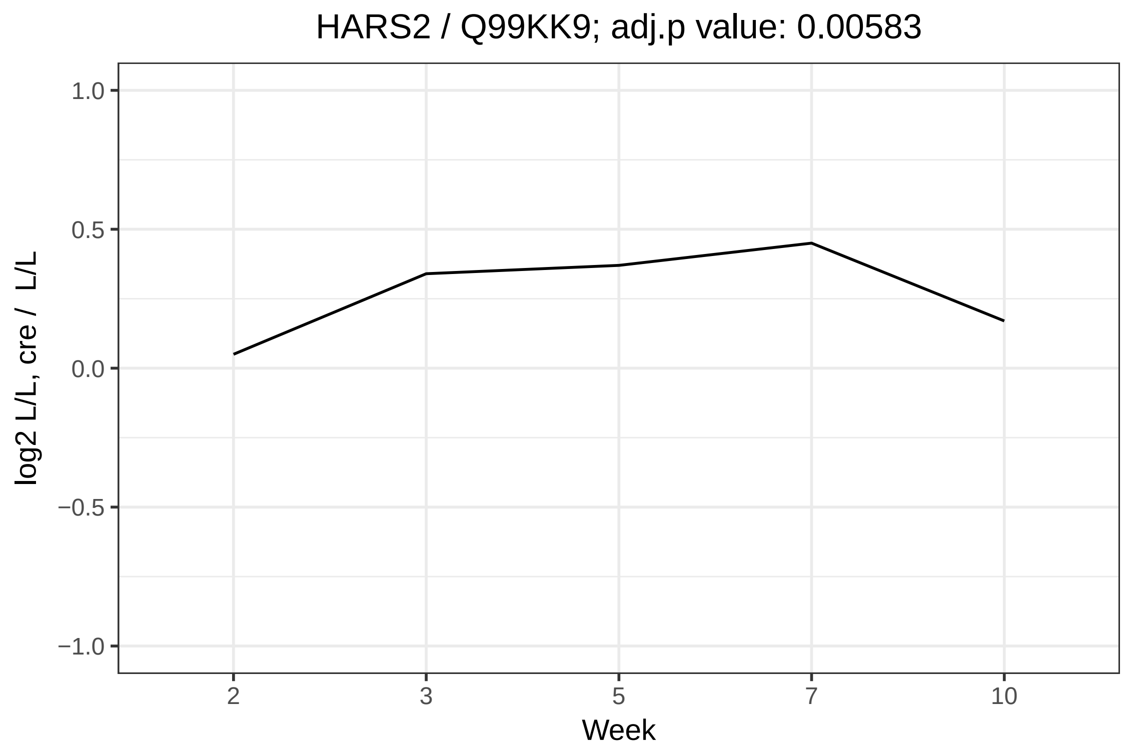

HARS2 / Q99KK9; adj.p value: 0.00583
1.0
0.5
0.0
−0.5
−1.0
log2 L/L, cre / L/L
2
3
5
7
10
Week

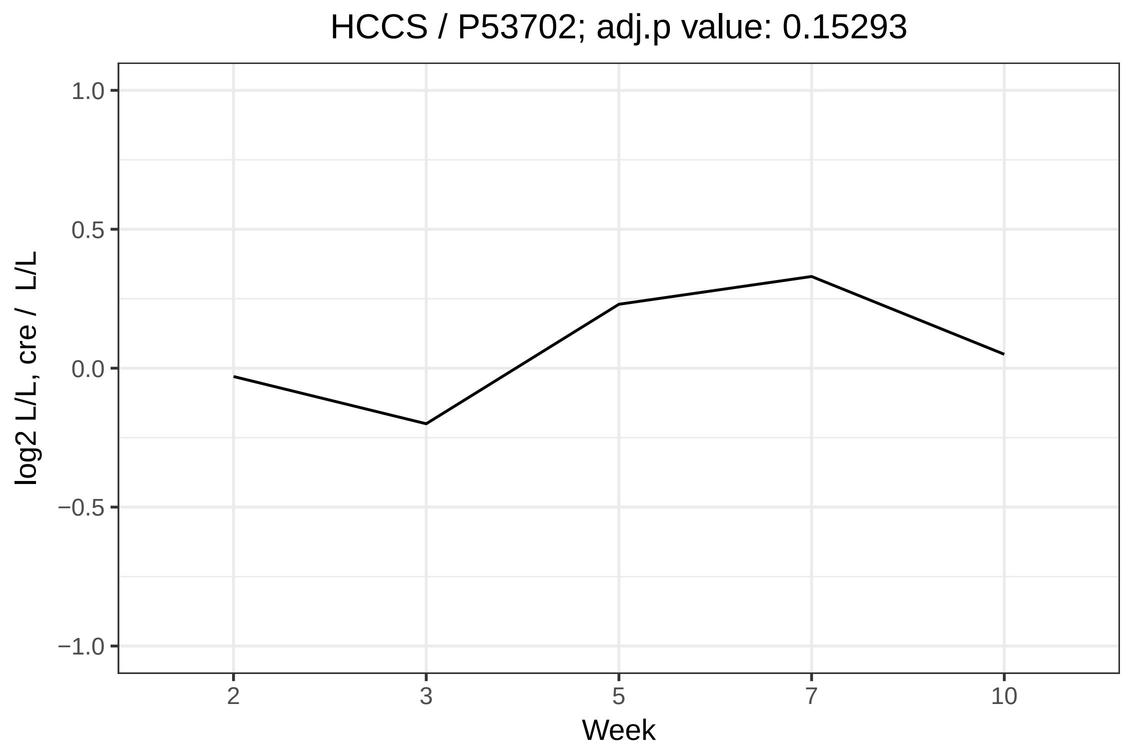
HCCS / P53702; adj.p value: 0.15293
log2 L/L, cre / L/L
1.0
0.5
0.0
−0.5
−1.0
2
3
5
7
10
Week

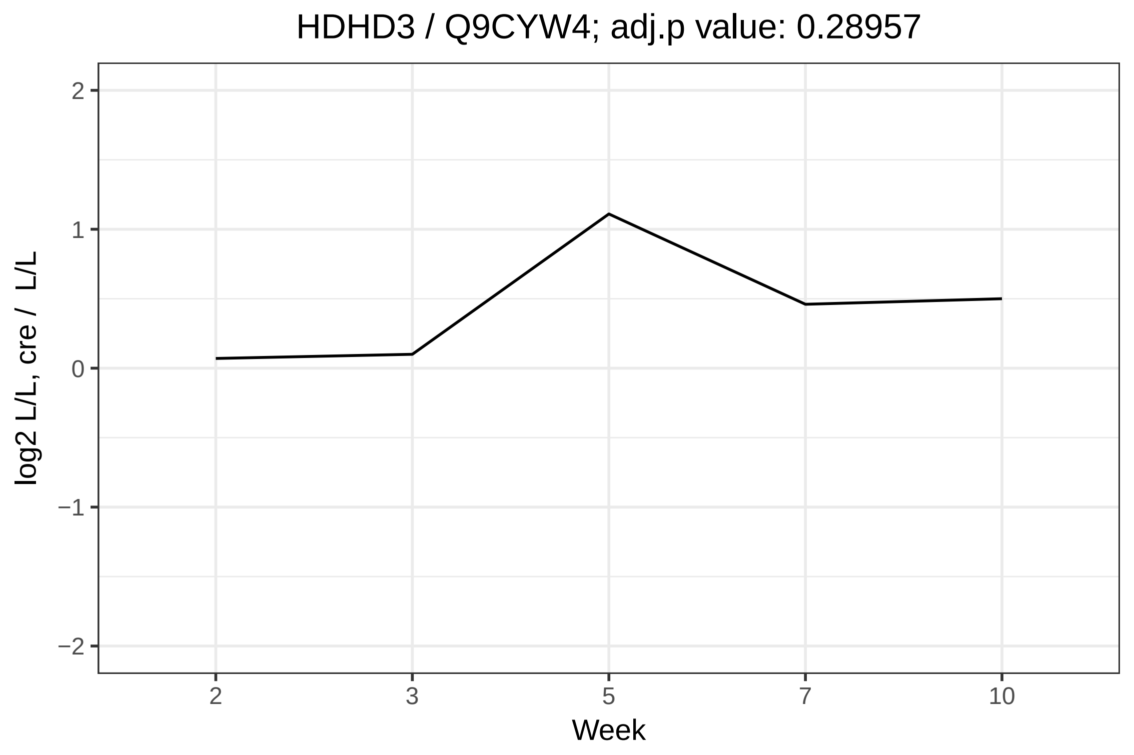

HDHD3 / Q9CYW4; adj.p value: 0.28957
log2 L/L, cre / L/L
2
1
0
−1
−2
2
3
5
7
10
Week

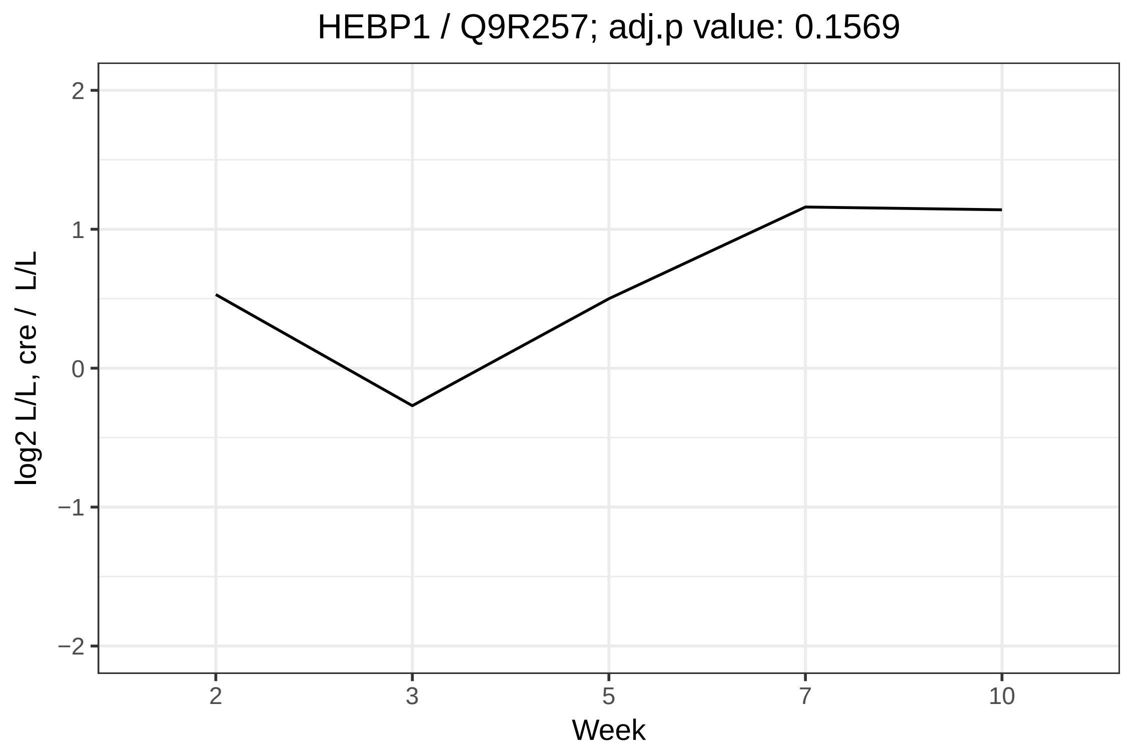
HEBP1 / Q9R257; adj.p value: 0.1569
log2 L/L, cre / L/L
2
1
0
−1
−2
2
3
5
7
10
Week

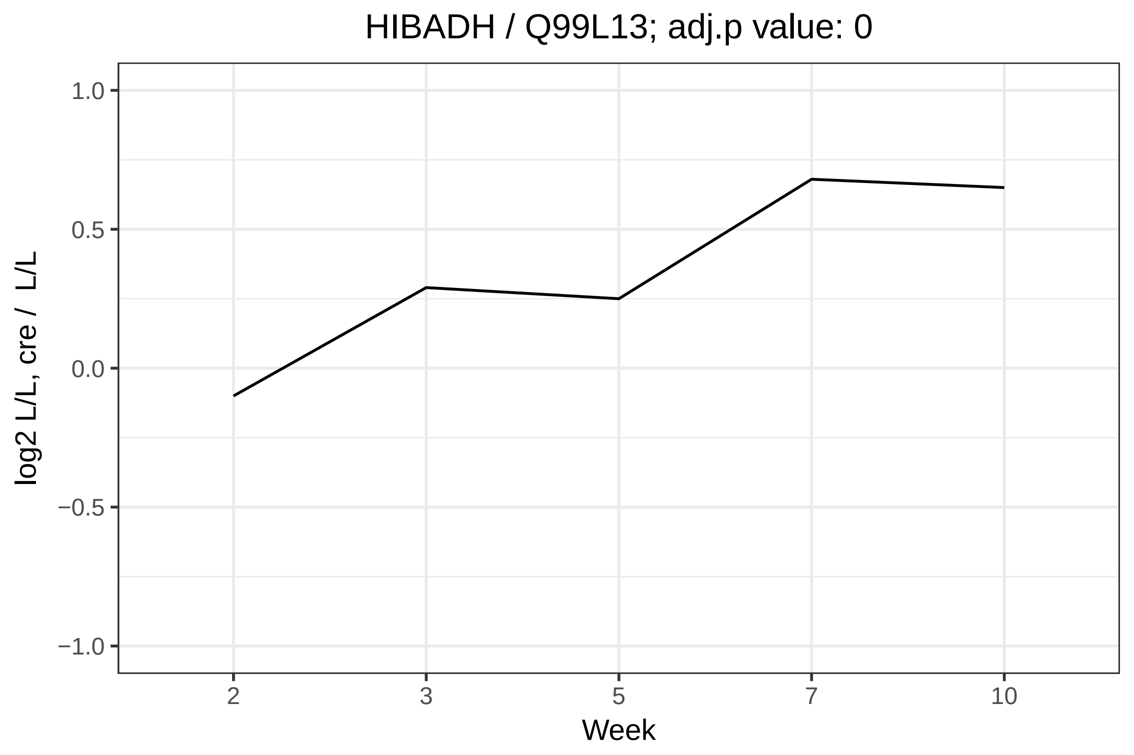

HIBADH / Q99L13; adj.p value: 0
log2 L/L, cre / L/L
1.0
0.5
0.0
−0.5
−1.0
2
3
5
7
10
Week

HIBCH / Q8QZS1; adj.p value: 0.05242

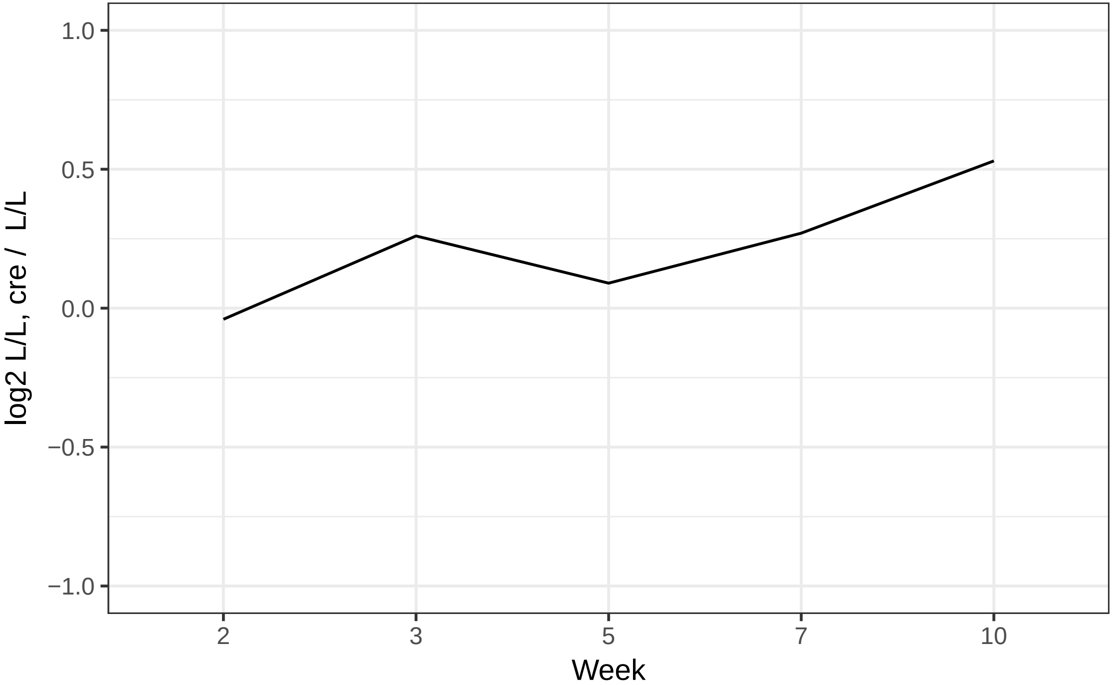

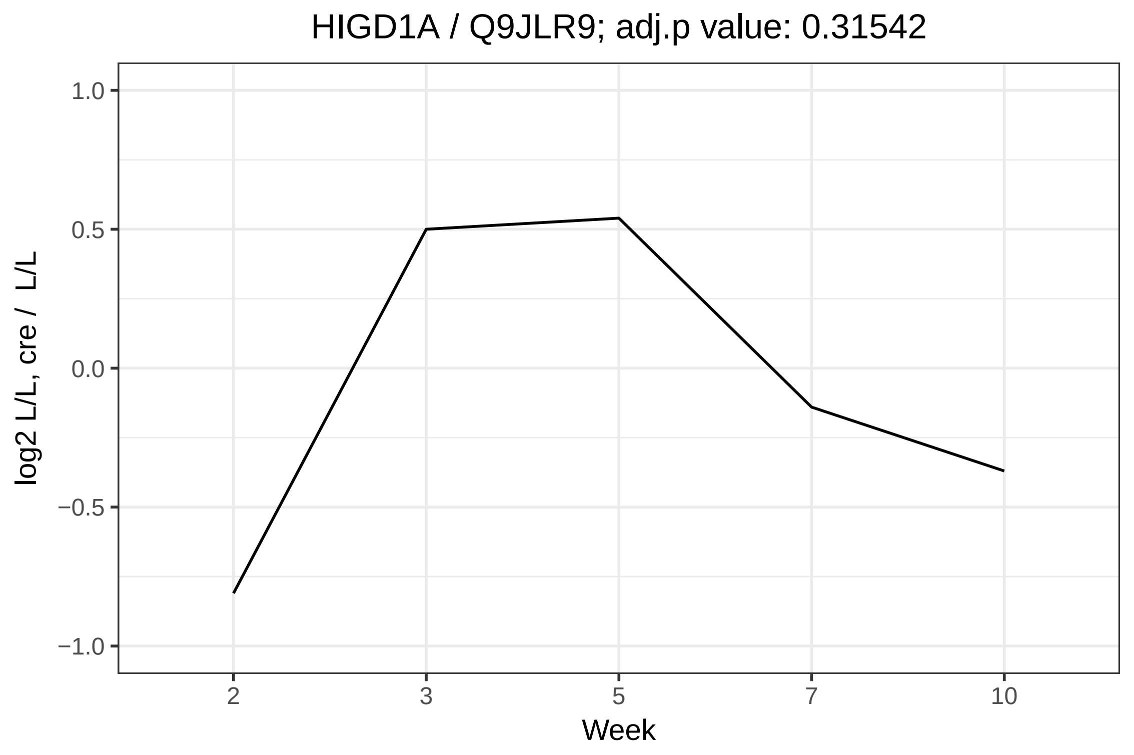
HIGD1A / Q9JLR9; adj.p value: 0.31542
log2 L/L, cre / L/L
1.0
0.5
0.0
−0.5
−1.0
2
3
5
7
10
Week

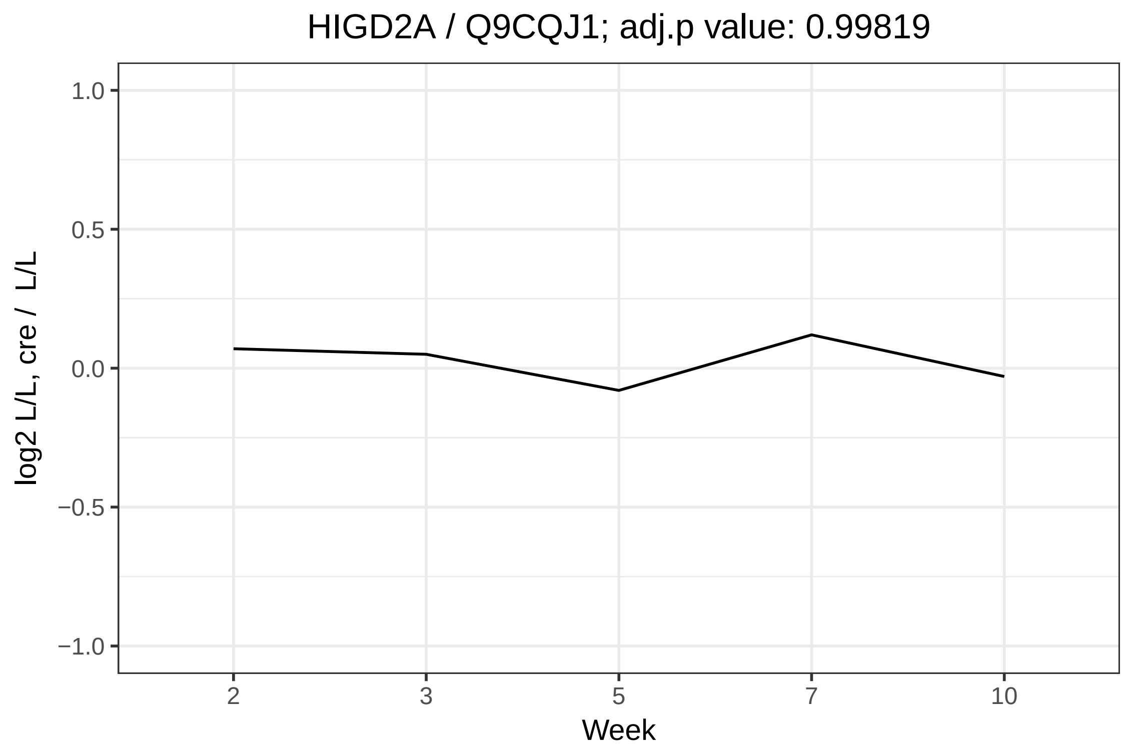
HIGD2A / Q9CQJ1; adj.p value: 0.99819
log2 L/L, cre / L/L
1.0
0.5
0.0
−0.5
−1.0
2
3
5
7
10
Week

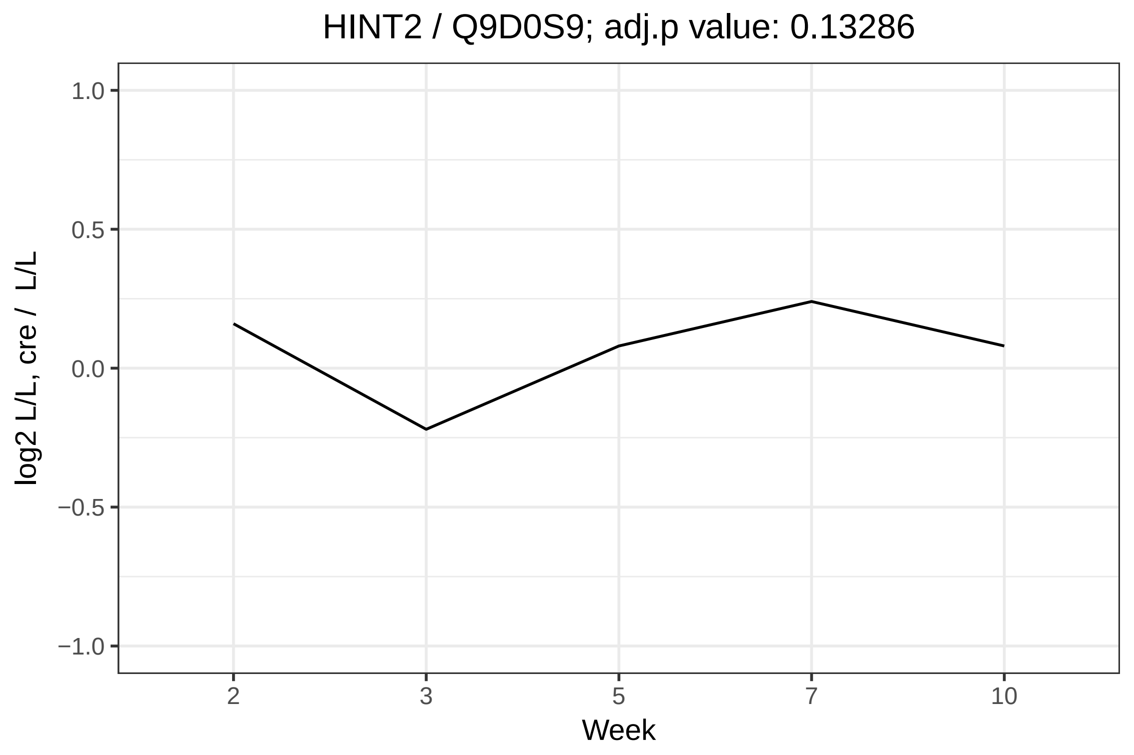
HINT2 / Q9D0S9; adj.p value: 0.13286
1.0
0.5
0.0
−0.5
−1.0
log2 L/L, cre / L/L
2
3
5
7
10
Week

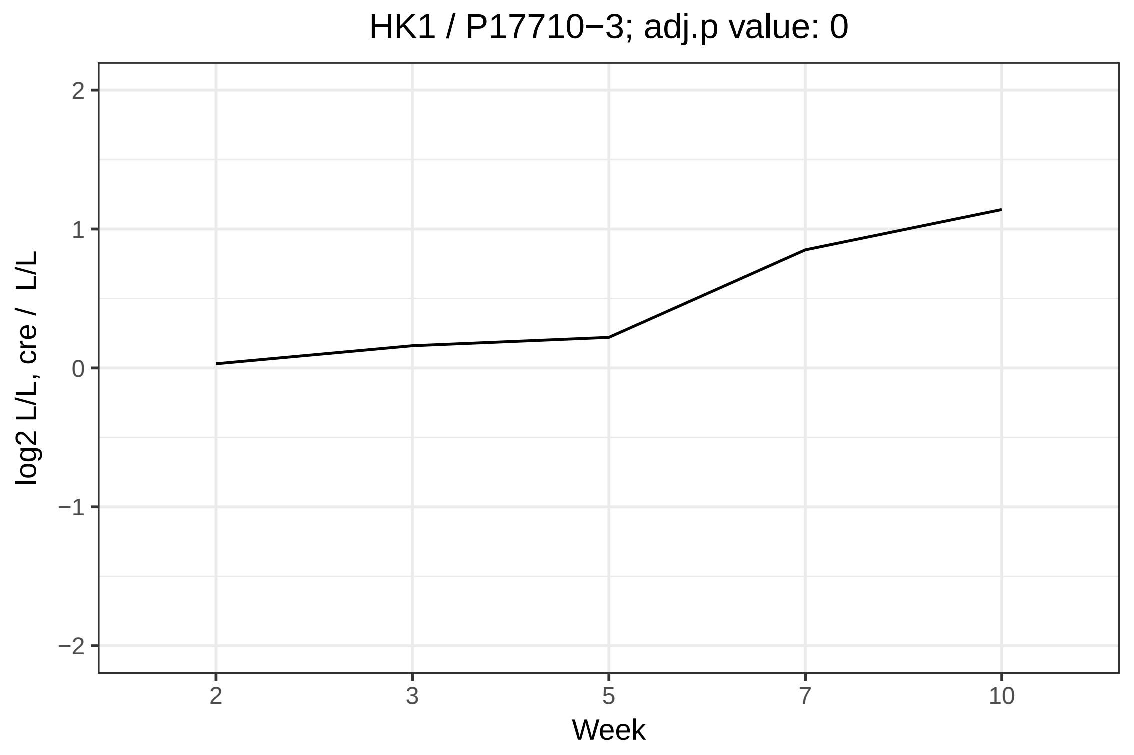
HK1 / P17710−3; adj.p value: 0
log2 L/L, cre / L/L
2
1
0
−1
−2
Week
2
3
5
7
10

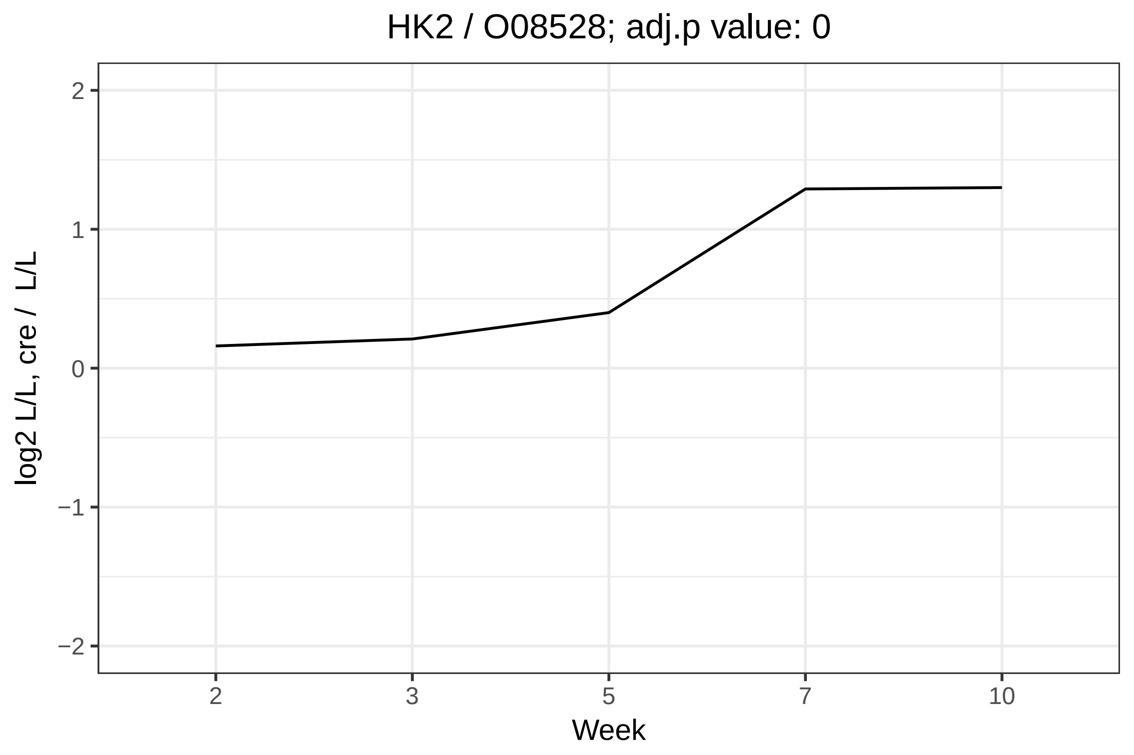
HK2 / O08528; adj.p value: 0
log2 L/L, cre / L/L
2
1
0
−1
−2
2
3
5
7
10
Week

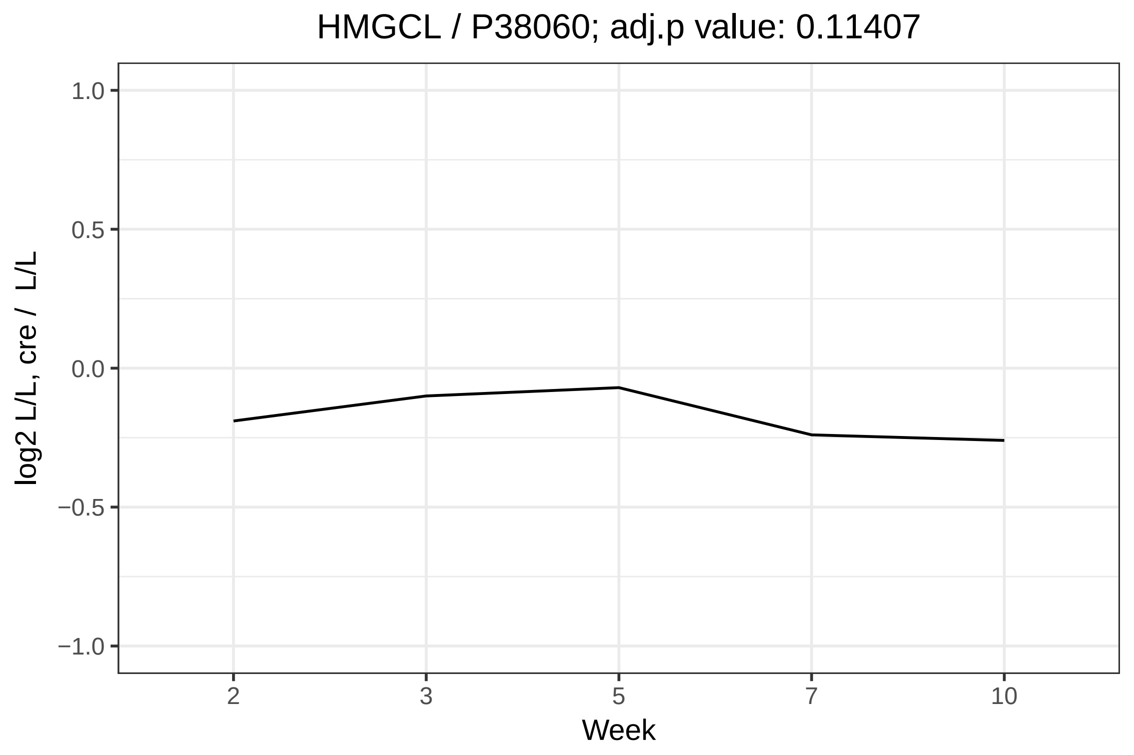

HMGCL / P38060; adj.p value: 0.11407
1.0
0.5
0.0
−0.5
−1.0
log2 L/L, cre / L/L
2
3
5
7
10
Week

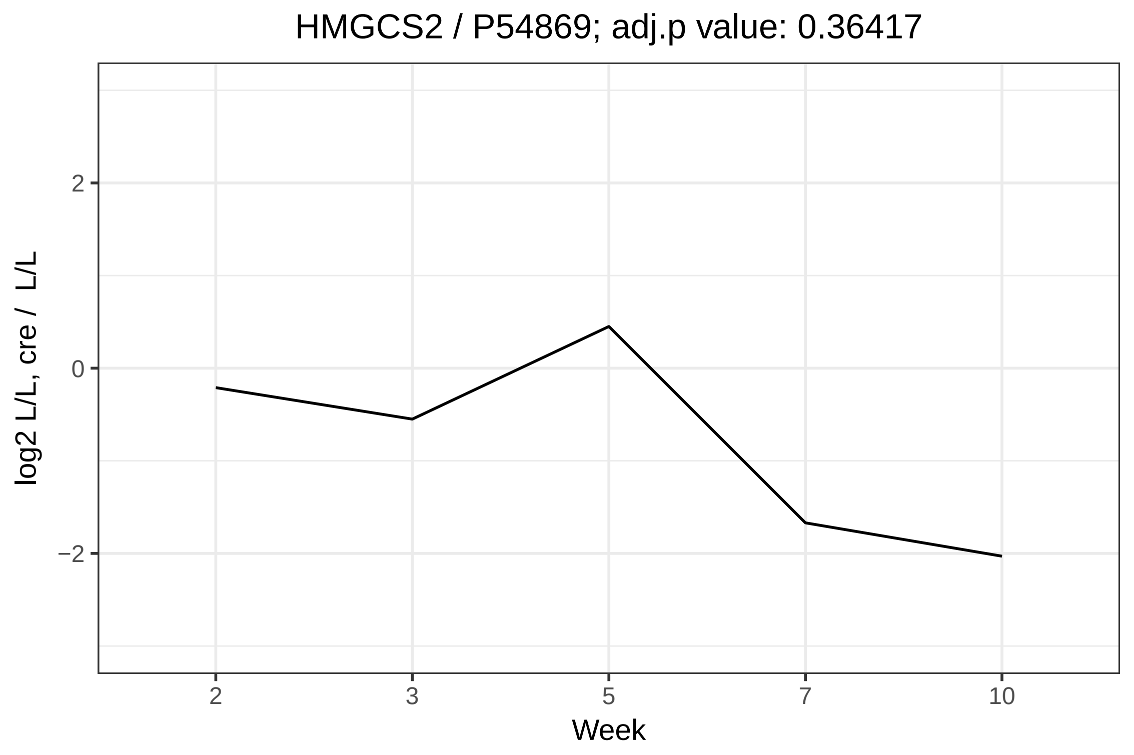
HMGCS2 / P54869; adj.p value: 0.36417
log2 L/L, cre / L/L
2
0
−2
2
3
5
7
10
Week

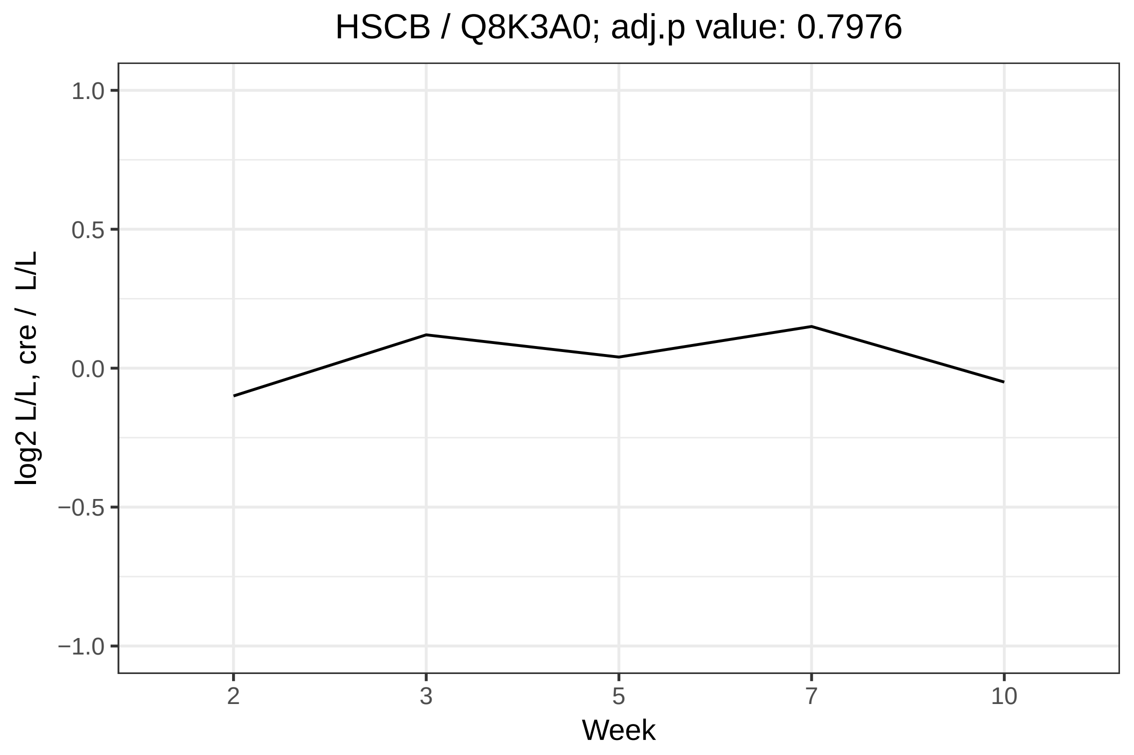
HSCB / Q8K3A0; adj.p value: 0.7976
log2 L/L, cre / L/L
1.0
0.5
0.0
−0.5
−1.0
2
3
5
7
10
Week

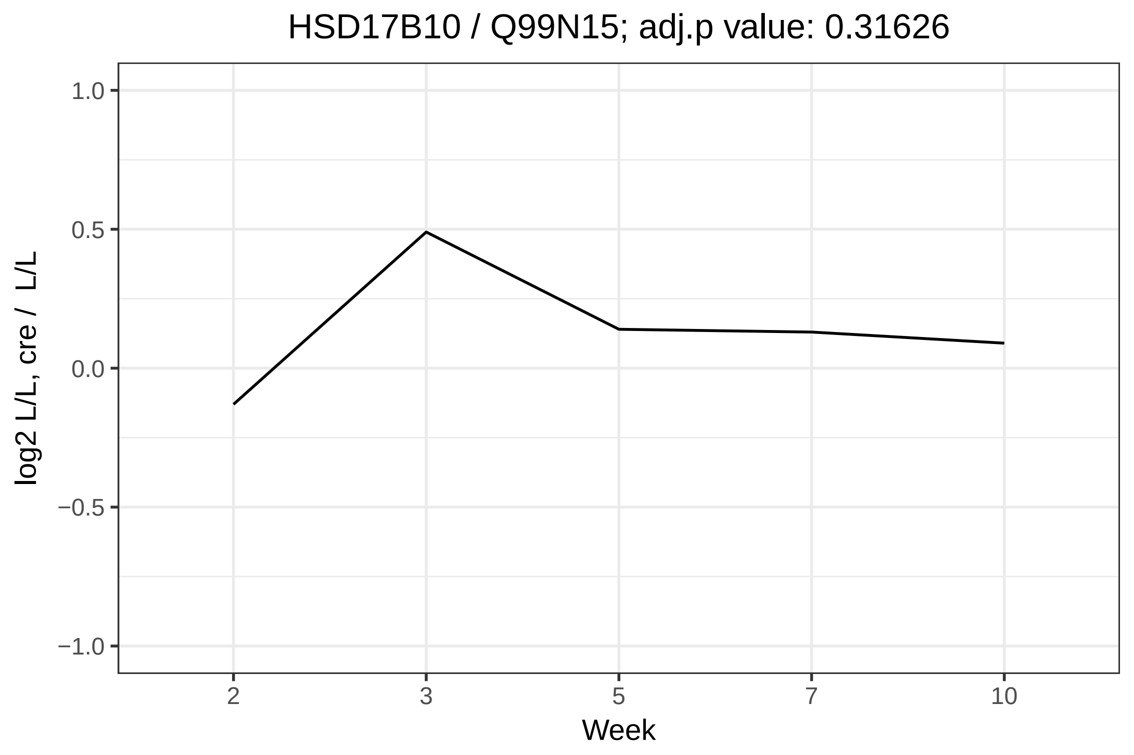
HSD17B10 / Q99N15; adj.p value: 0.31626
log2 L/L, cre / L/L
1.0
0.5
0.0
−0.5
−1.0
2
3
5
7
10
Week

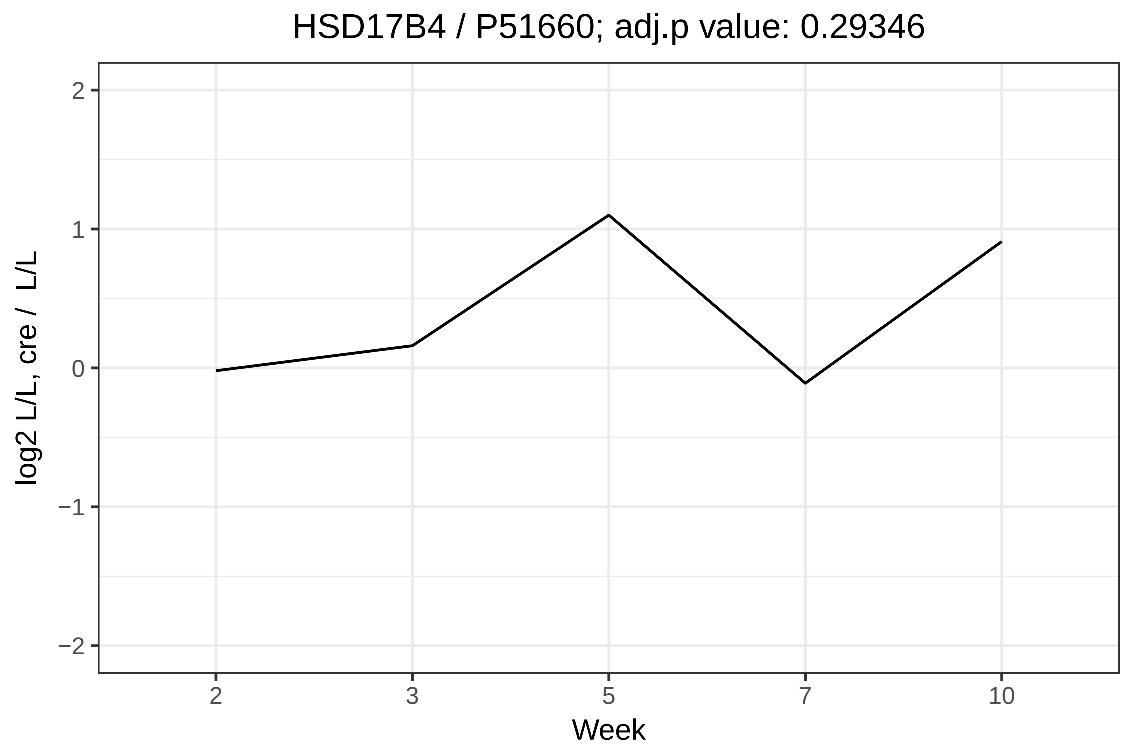
HSD17B4 / P51660; adj.p value: 0.29346
log2 L/L, cre / L/L
Week
2
1
0
−1
−2
2
3
5
7
10

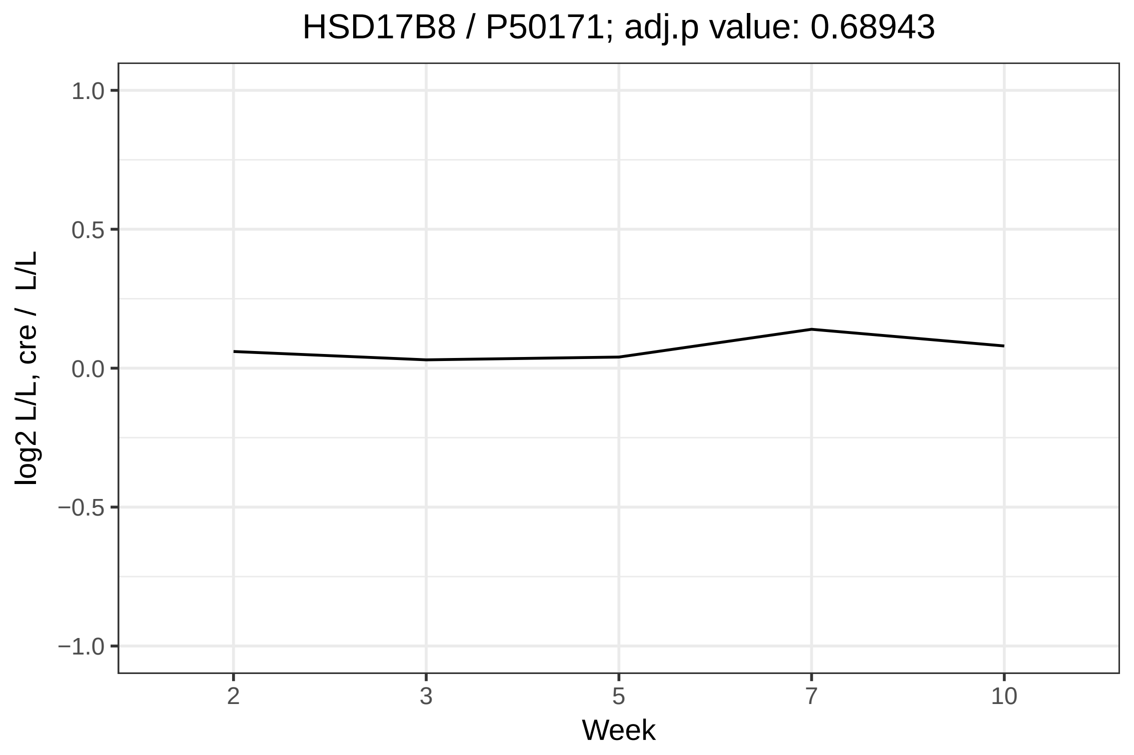

HSD17B8 / P50171; adj.p value: 0.68943
log2 L/L, cre / L/L
1.0
0.5
0.0
−0.5
−1.0
2
3
5
7
10
Week

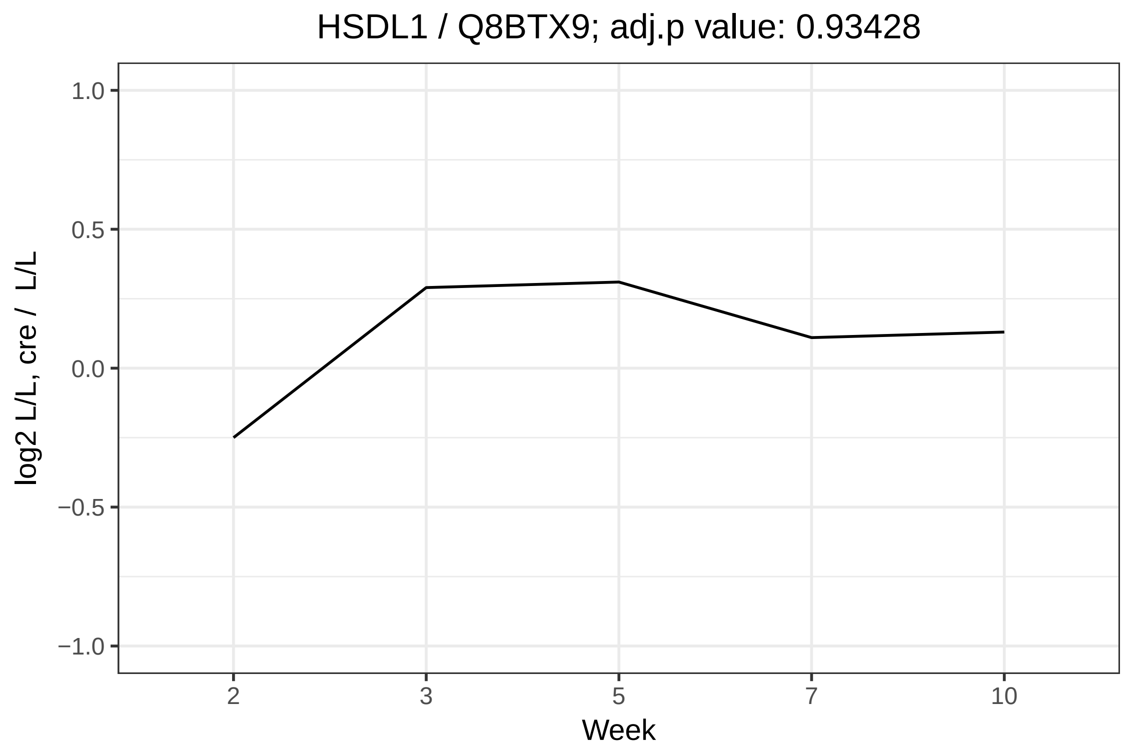

HSDL1 / Q8BTX9; adj.p value: 0.93428
log2 L/L, cre / L/L
1.0
0.5
0.0
−0.5
−1.0
2
3
5
7
10
Week

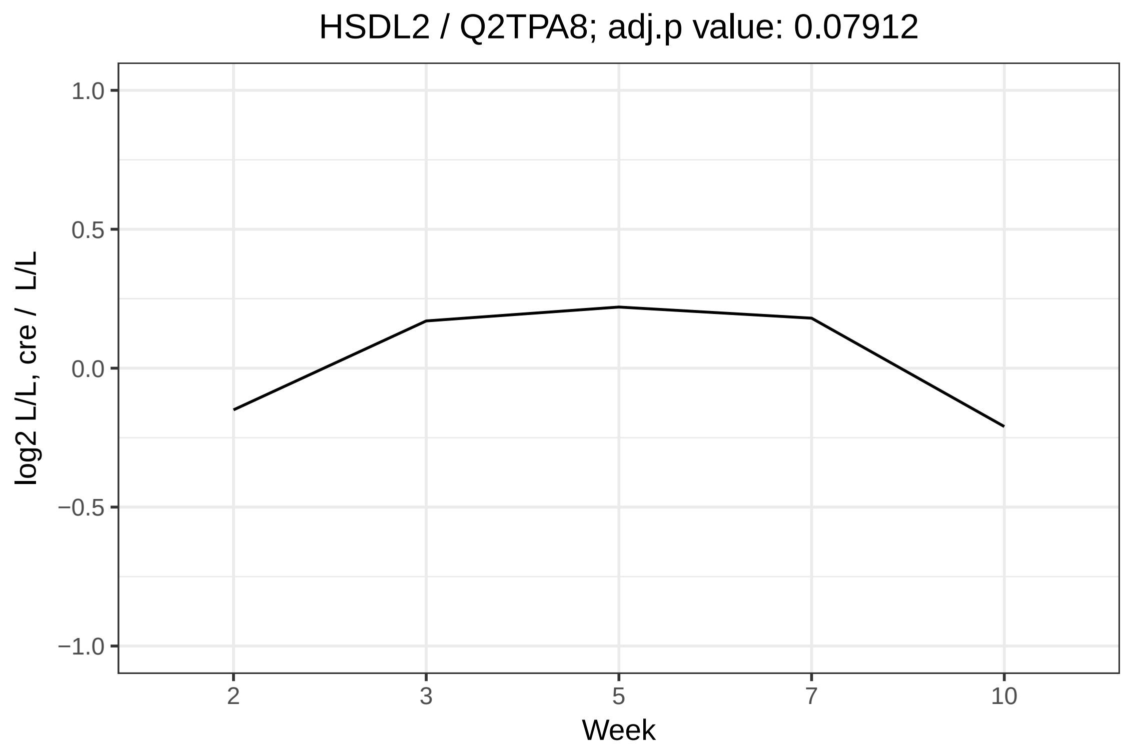

HSDL2 / Q2TPA8; adj.p value: 0.07912
1.0
0.5
0.0
−0.5
−1.0
log2 L/L, cre / L/L
2
3
5
7
10
Week

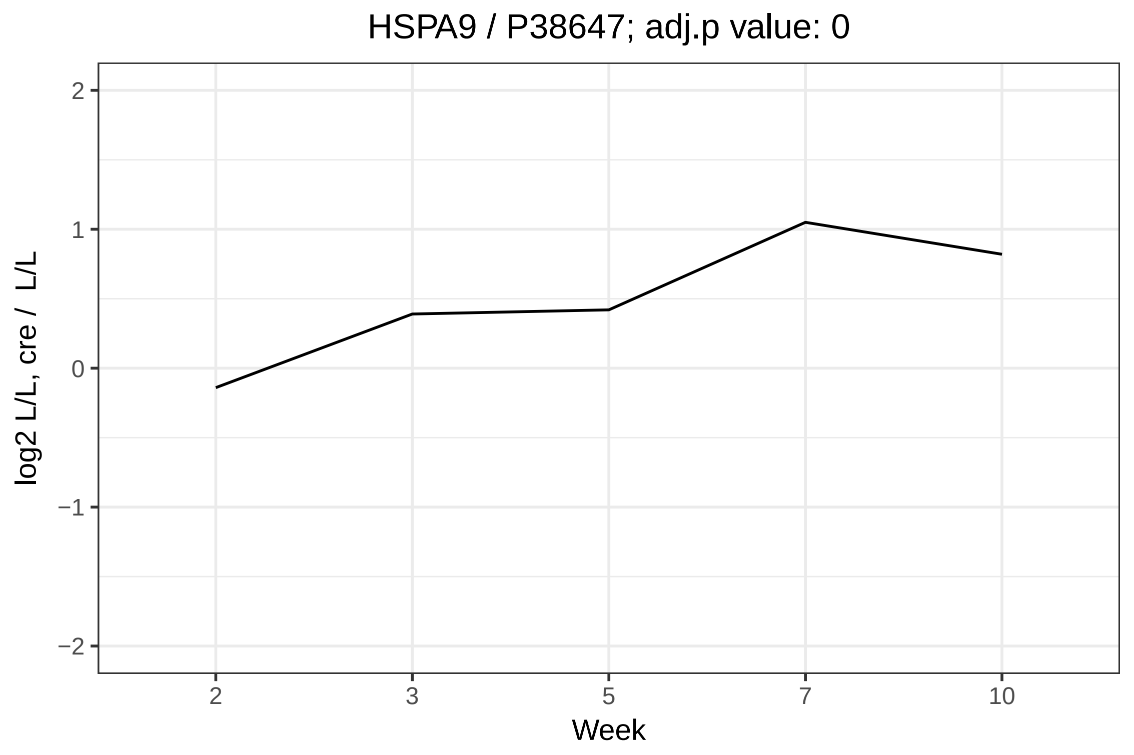
HSPA9 / P38647; adj.p value: 0
log2 L/L, cre / L/L
2
1
0
−1
−2
2
3
5
7
10
Week

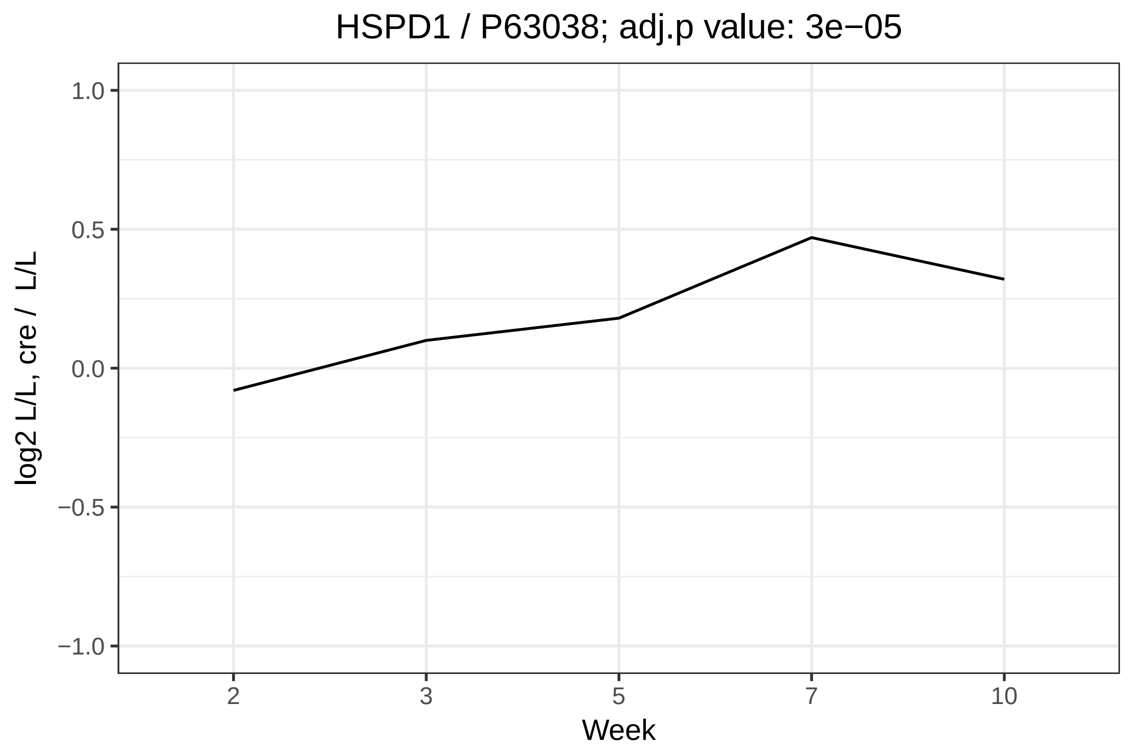

HSPD1 / P63038; adj.p value: 3e−05
1.0
0.5
0.0
−0.5
−1.0
log2 L/L, cre / L/L
2
3
5
7
10
Week

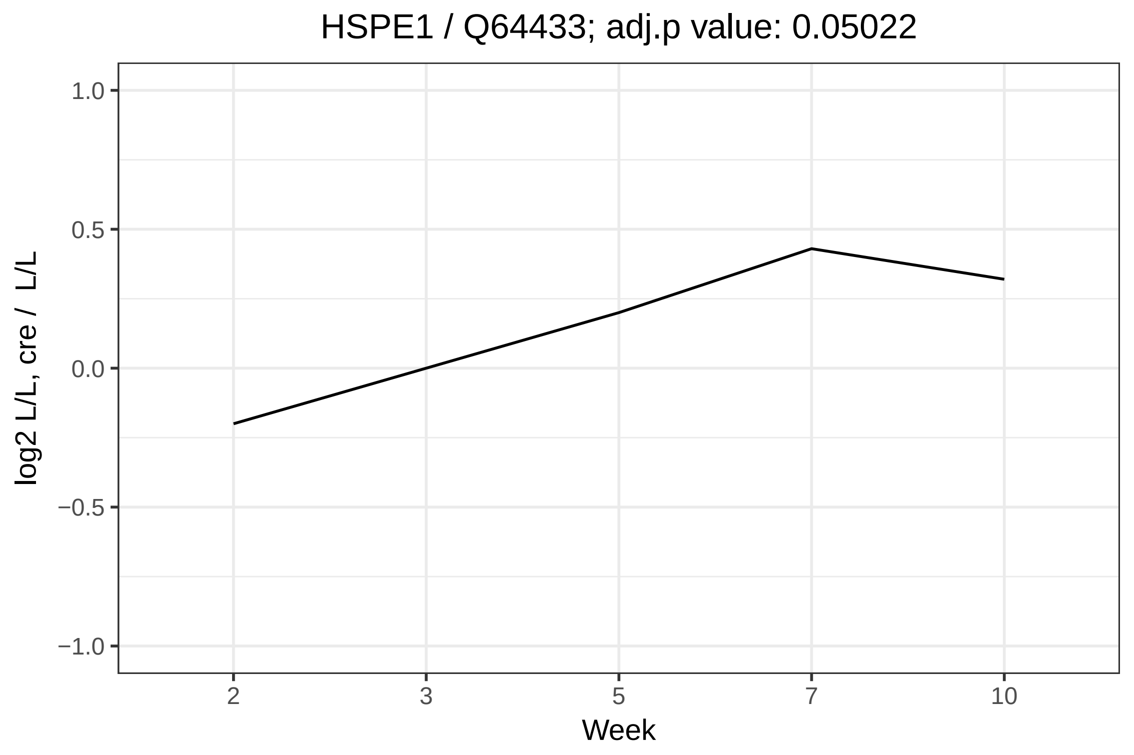

HSPE1 / Q64433; adj.p value: 0.05022
log2 L/L, cre / L/L
1.0
0.5
0.0
−0.5
−1.0
2
3
5
7
10
Week

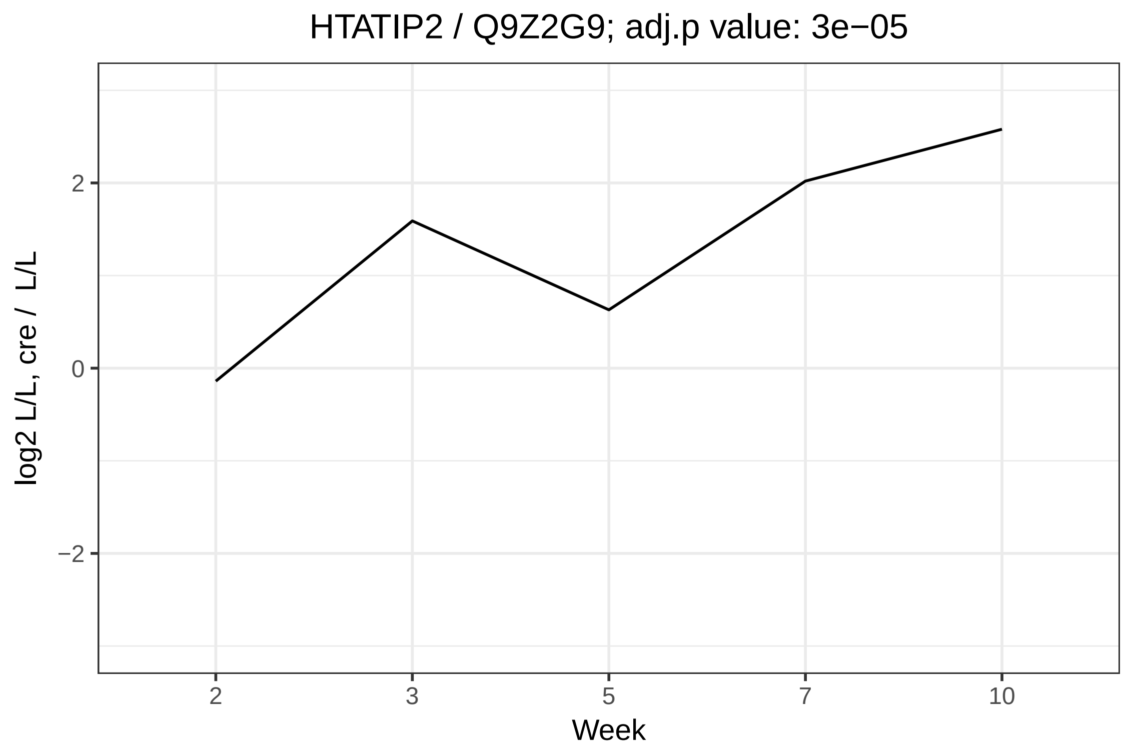

HTATIP2 / Q9Z2G9; adj.p value: 3e−05
log2 L/L, cre / L/L
2
0
−2
2
3
5
7
10
Week

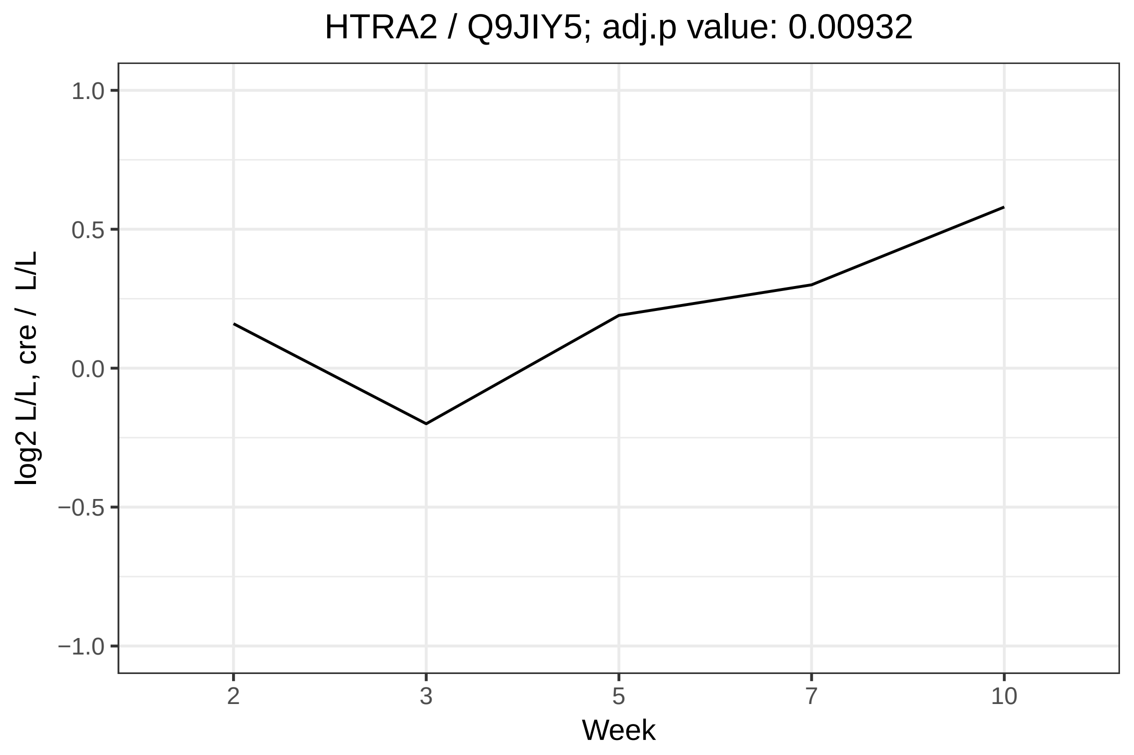
HTRA2 / Q9JIY5; adj.p value: 0.00932
log2 L/L, cre / L/L
1.0
0.5
0.0
−0.5
−1.0
2
3
5
7
10
Week

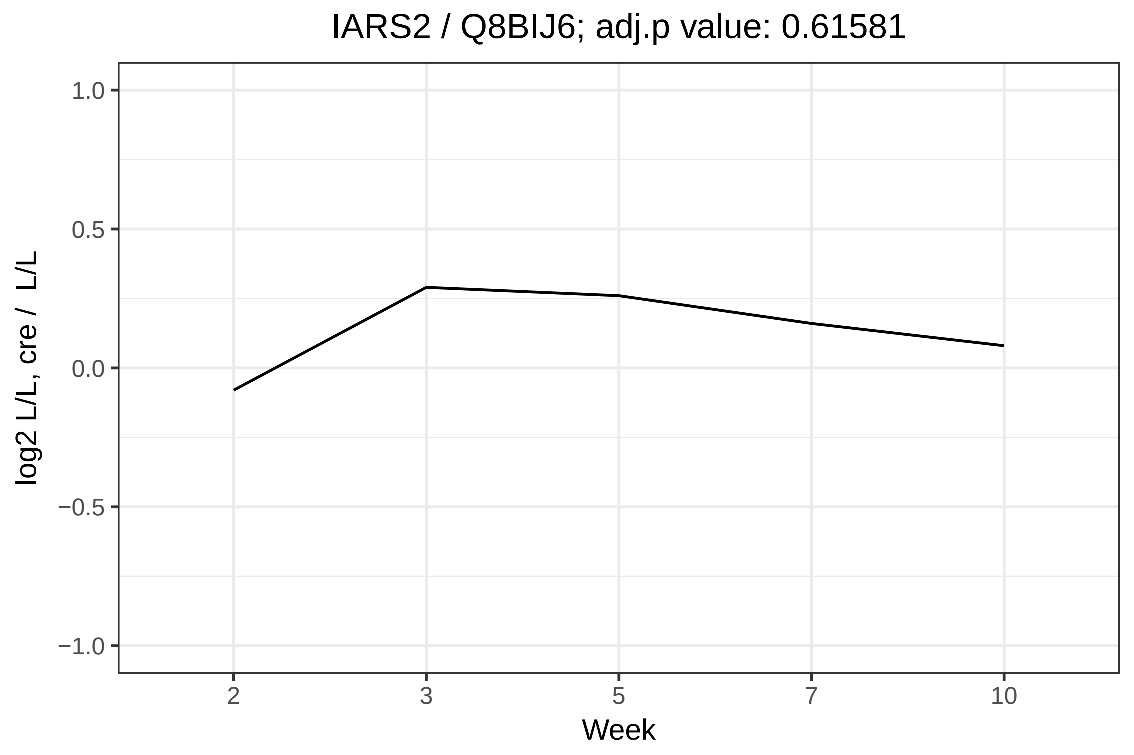
IARS2 / Q8BIJ6; adj.p value: 0.61581
log2 L/L, cre / L/L
1.0
0.5
0.0
−0.5
−1.0
2
3
5
7
10
Week

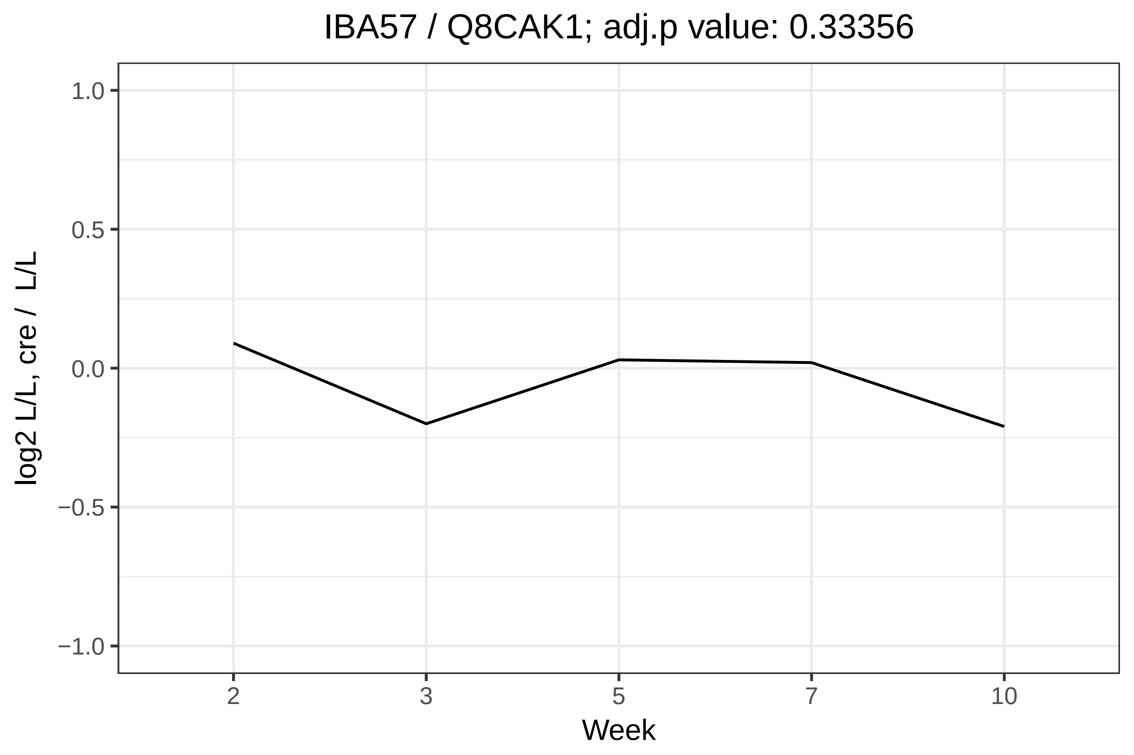

IBA57 / Q8CAK1; adj.p value: 0.33356
1.0
0.5
0.0
−0.5
−1.0
log2 L/L, cre / L/L
2
3
5
7
10
Week

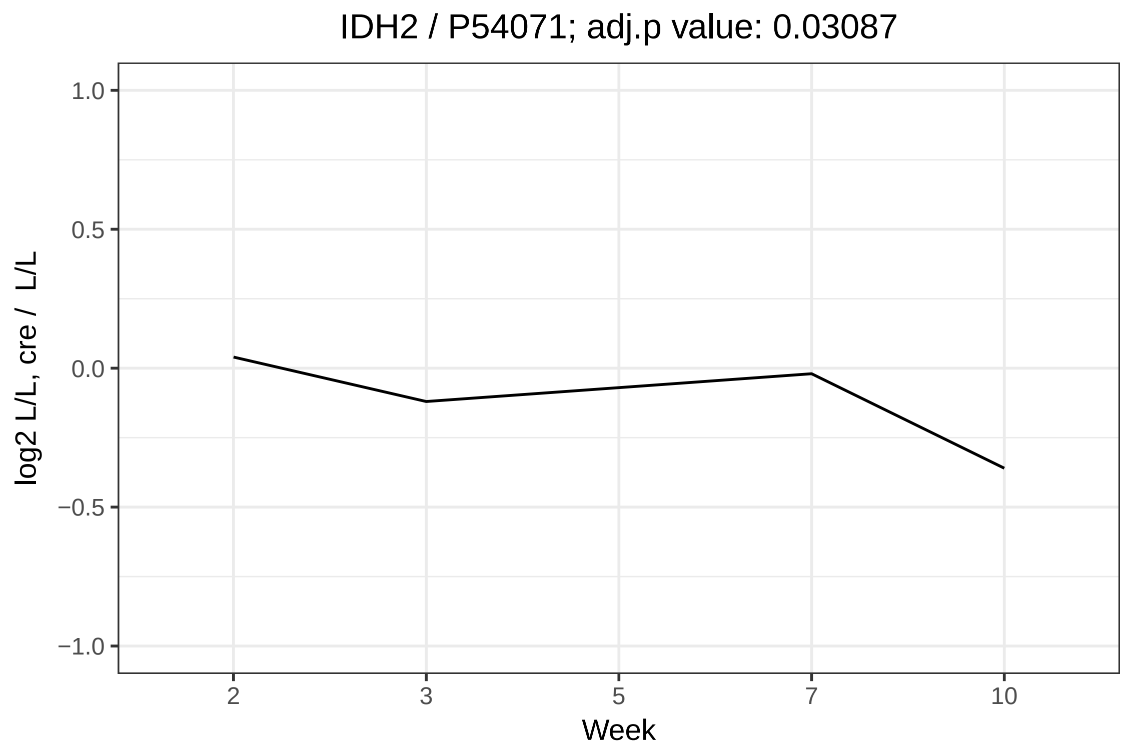
IDH2 / P54071; adj.p value: 0.03087
log2 L/L, cre / L/L
1.0
0.5
0.0
−0.5
−1.0
2
3
5
7
10
Week

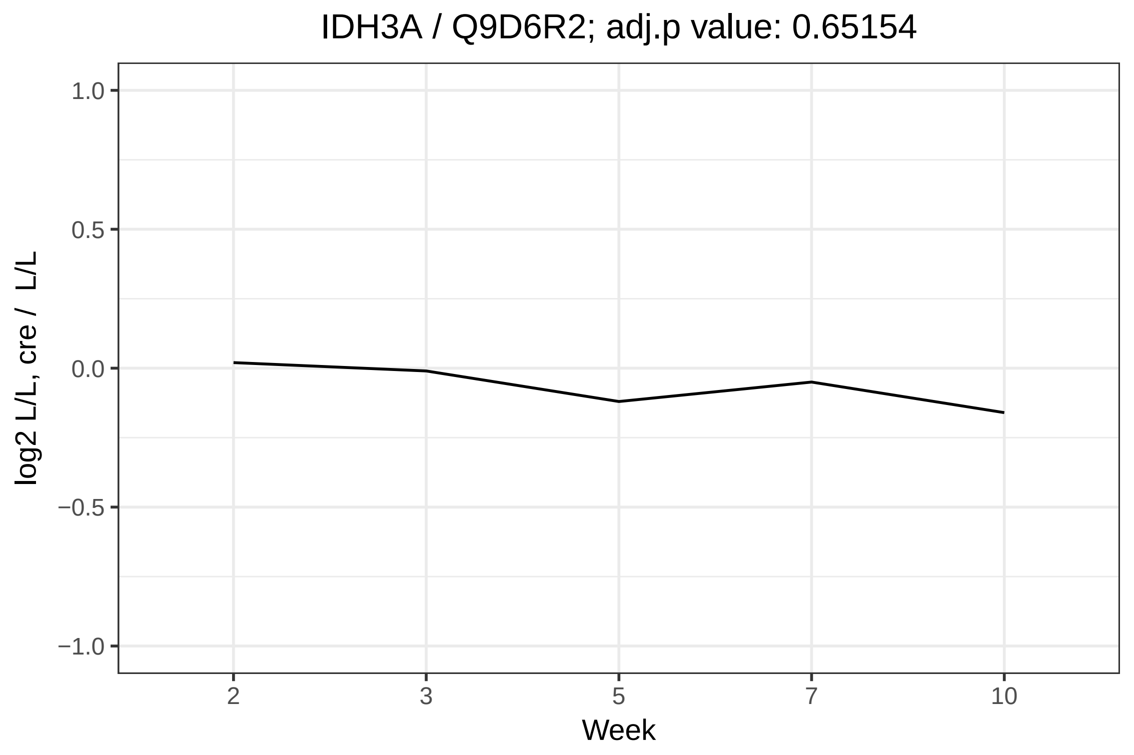

IDH3A / Q9D6R2; adj.p value: 0.65154
1.0
0.5
0.0
−0.5
−1.0
log2 L/L, cre / L/L
2
3
5
7
10
Week

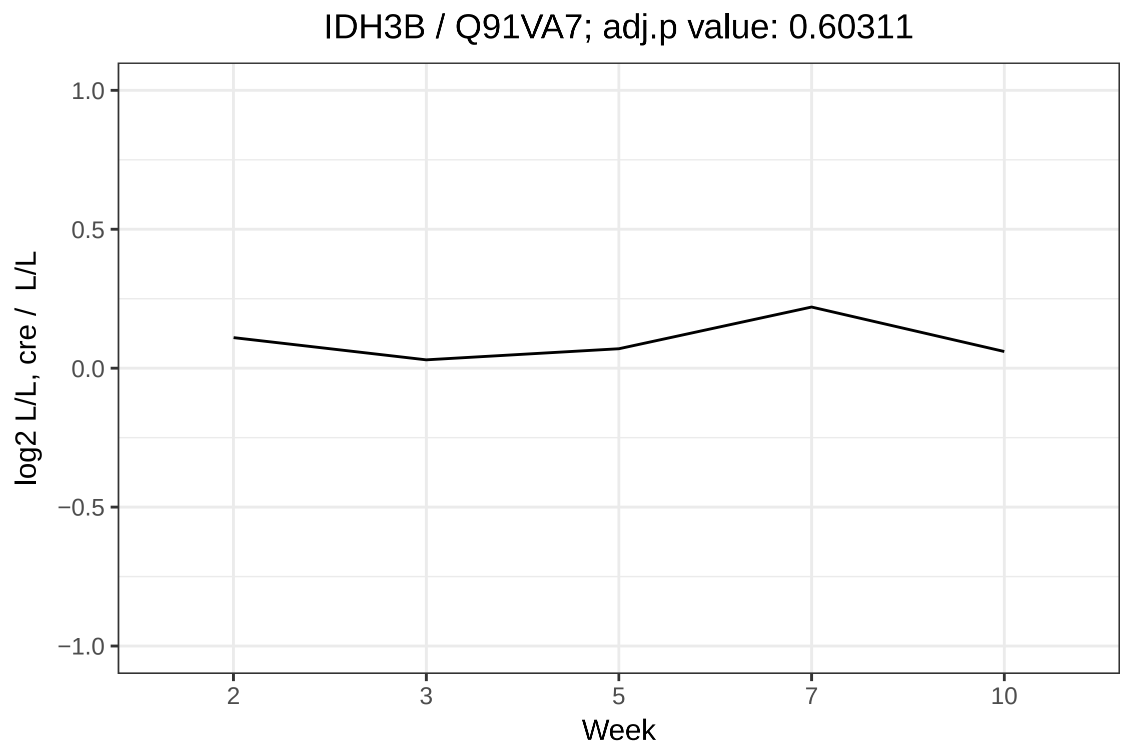

IDH3B / Q91VA7; adj.p value: 0.60311
log2 L/L, cre / L/L
1.0
0.5
0.0
−0.5
−1.0
2
3
5
7
10
Week

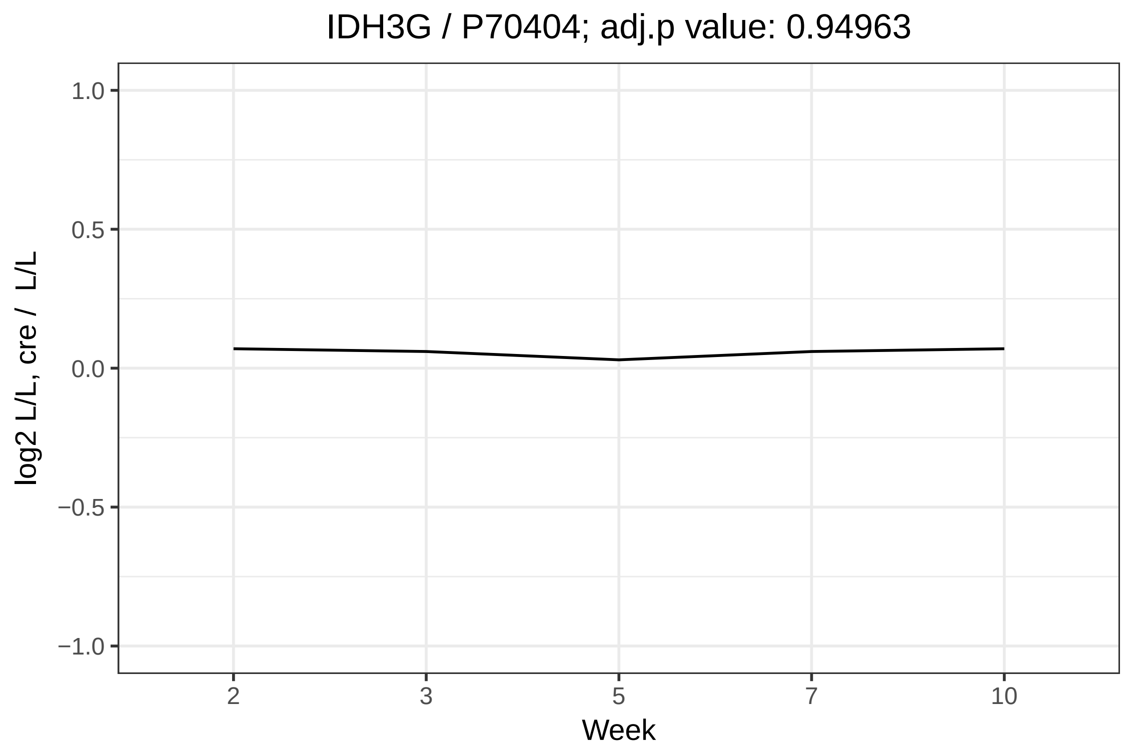

IDH3G / P70404; adj.p value: 0.94963
1.0
0.5
0.0
−0.5
−1.0
log2 L/L, cre / L/L
2
3
5
7
10
Week

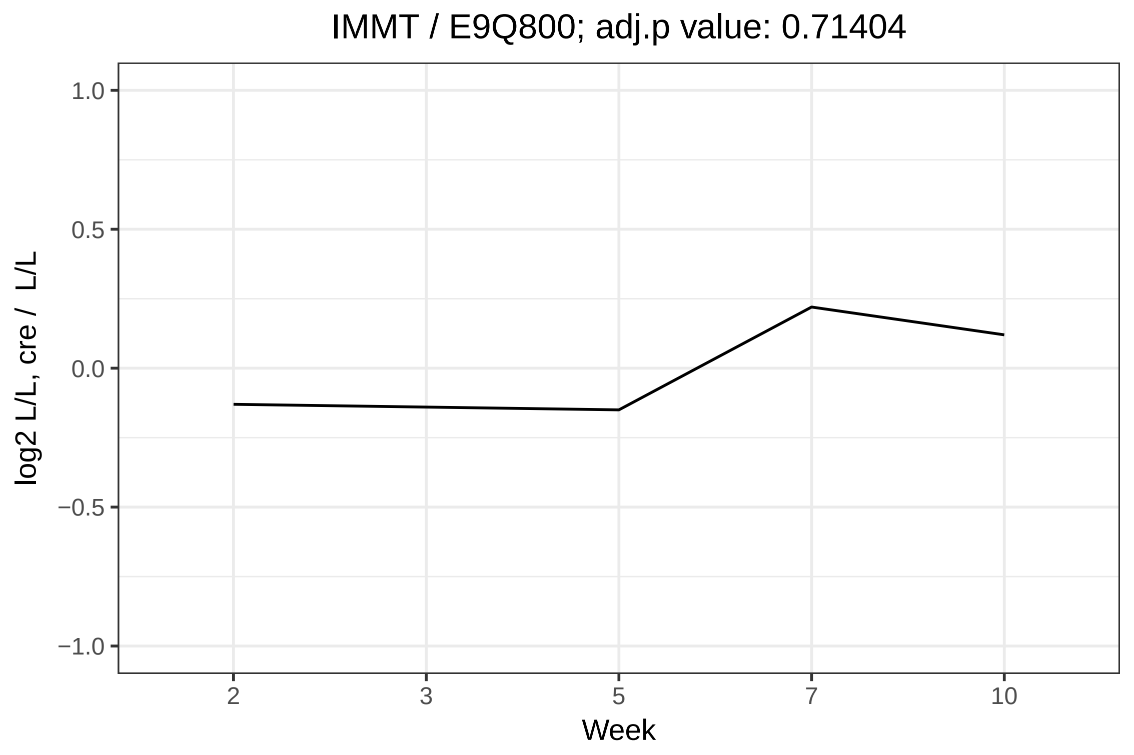

IMMT / E9Q800; adj.p value: 0.71404
1.0
0.5
0.0
−0.5
−1.0
log2 L/L, cre / L/L
2
3
5
7
10
Week

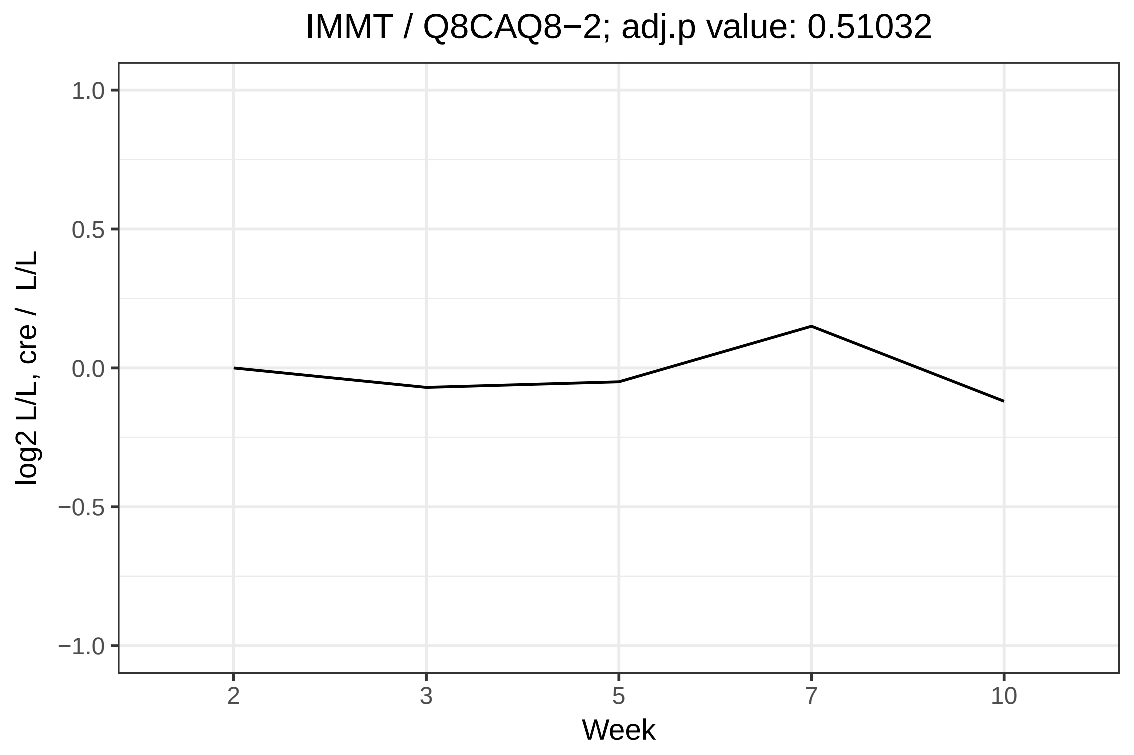

IMMT / Q8CAQ8−2; adj.p value: 0.51032
log2 L/L, cre / L/L
1.0
0.5
0.0
−0.5
−1.0
2
3
5
7
10
Week

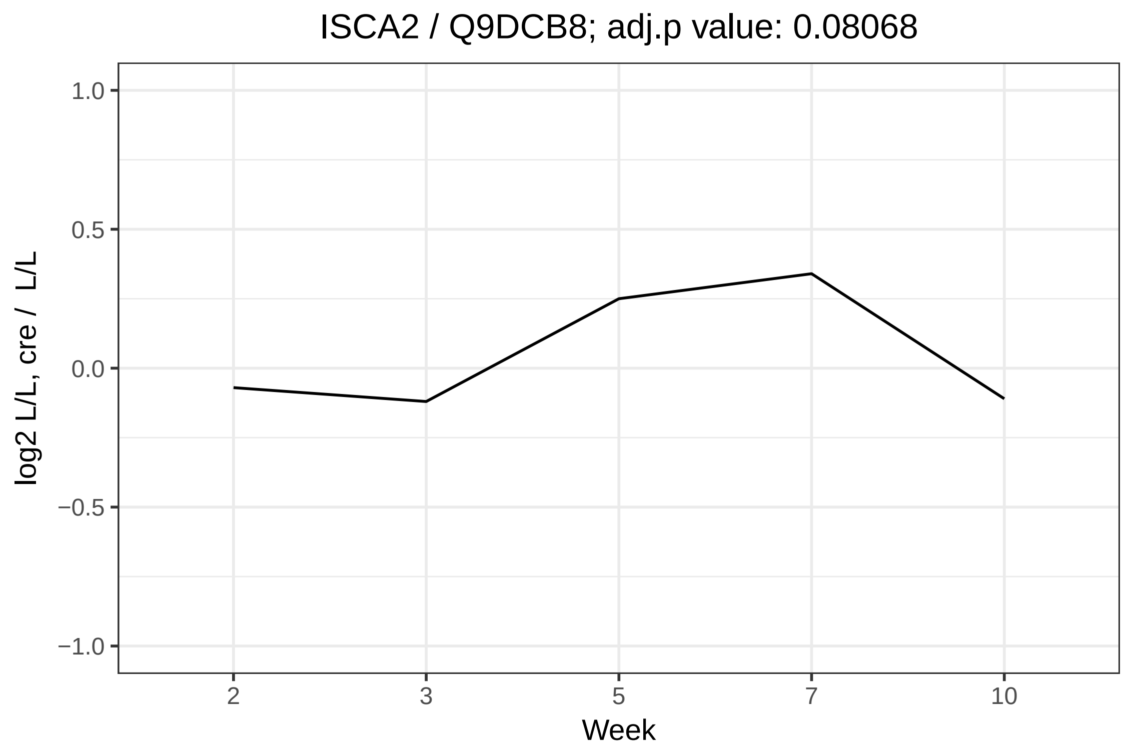
ISCA2 / Q9DCB8; adj.p value: 0.08068
log2 L/L, cre / L/L
1.0
0.5
0.0
−0.5
−1.0
2
3
5
7
10
Week

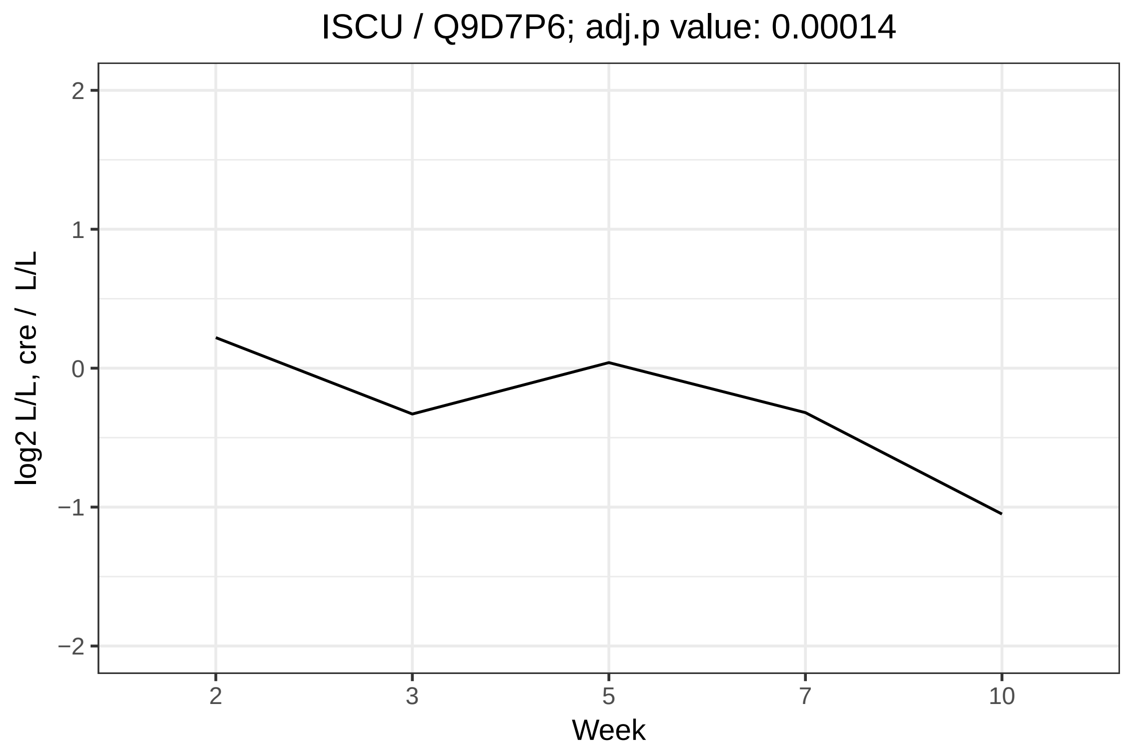

ISCU / Q9D7P6; adj.p value: 0.00014
log2 L/L, cre / L/L
2
1
0
−1
−2
2
3
5
7
10
Week

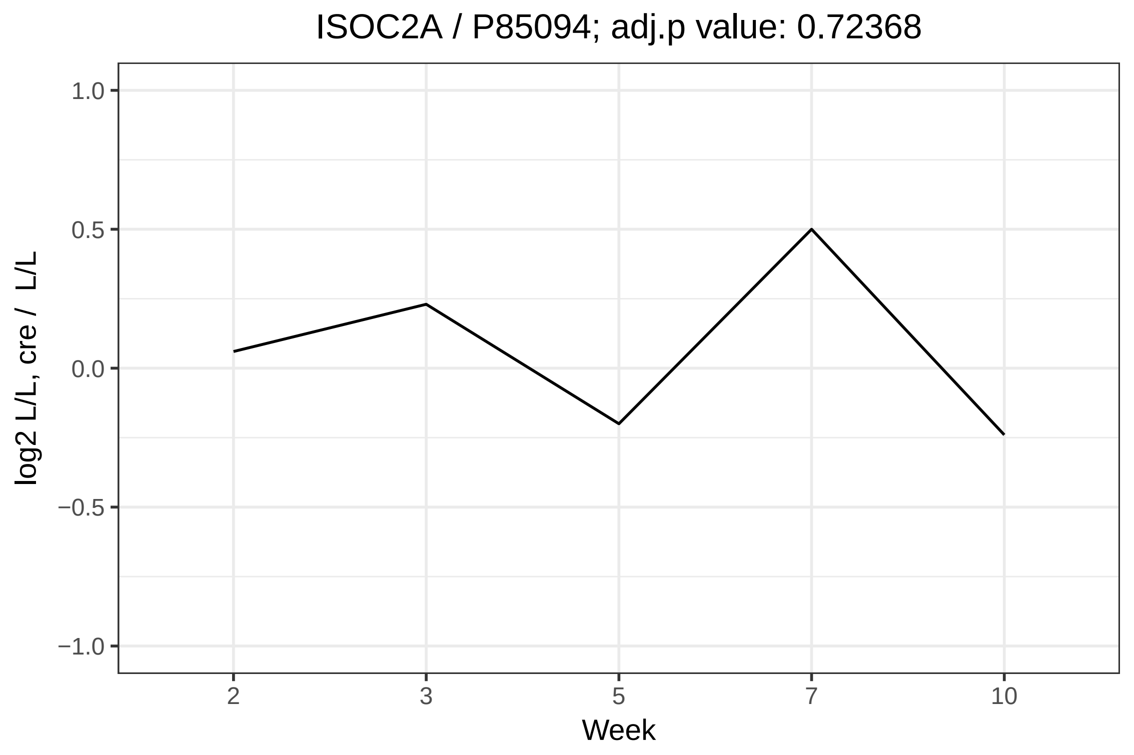

ISOC2A / P85094; adj.p value: 0.72368
1.0
0.5
0.0
−0.5
−1.0
log2 L/L, cre / L/L
2
3
5
7
10
Week

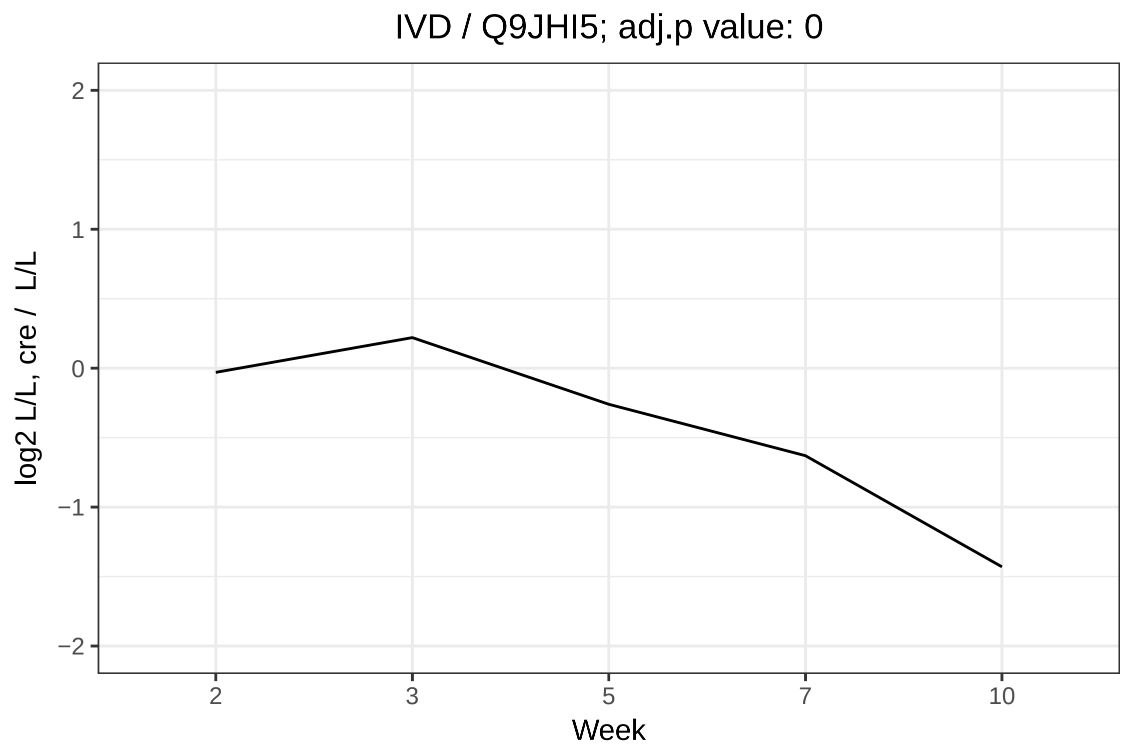

IVD / Q9JHI5; adj.p value: 0
log2 L/L, cre / L/L
2
1
0
−1
−2
2
3
5
7
10
Week

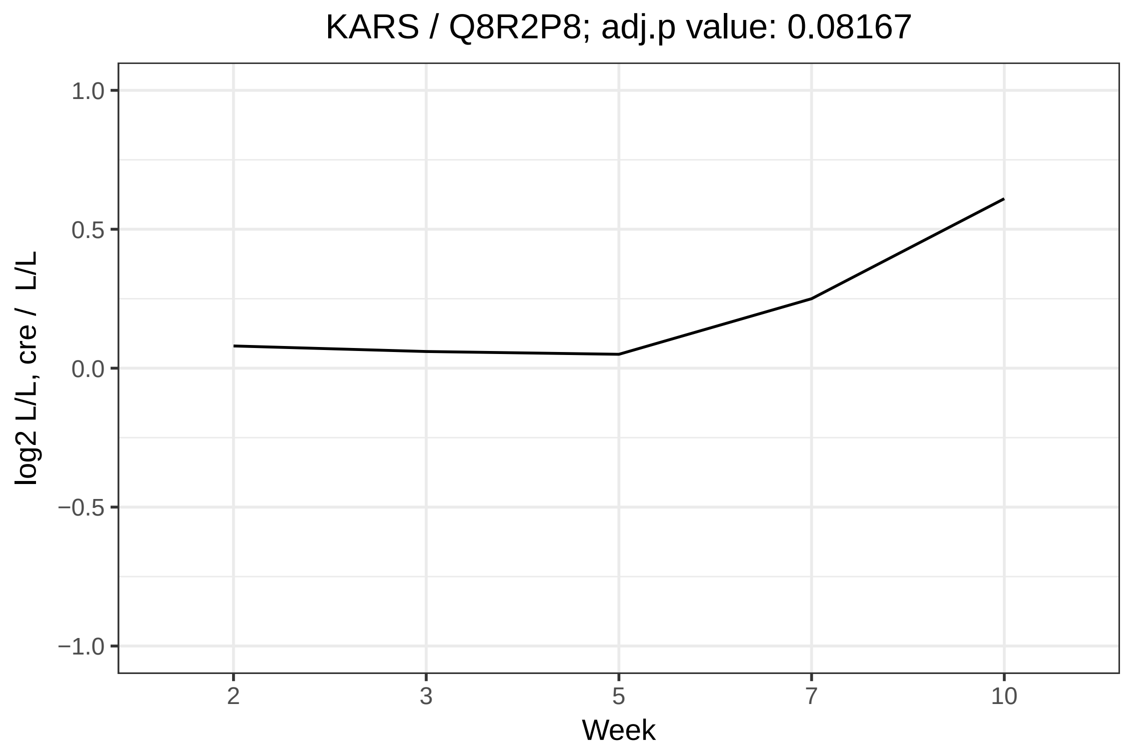
KARS / Q8R2P8; adj.p value: 0.08167
log2 L/L, cre / L/L
1.0
0.5
0.0
−0.5
−1.0
2
3
5
7
10
Week

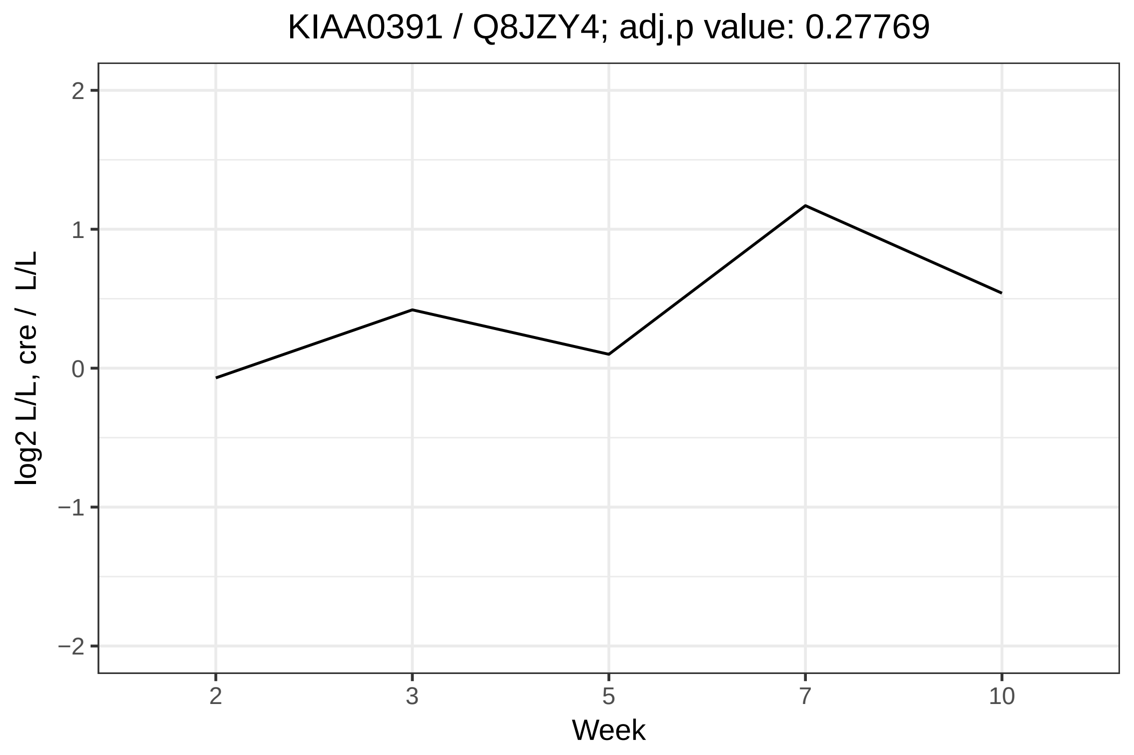
KIAA0391 / Q8JZY4; adj.p value: 0.27769
log2 L/L, cre / L/L
2
1
0
−1
−2
2
3
5
7
10
Week

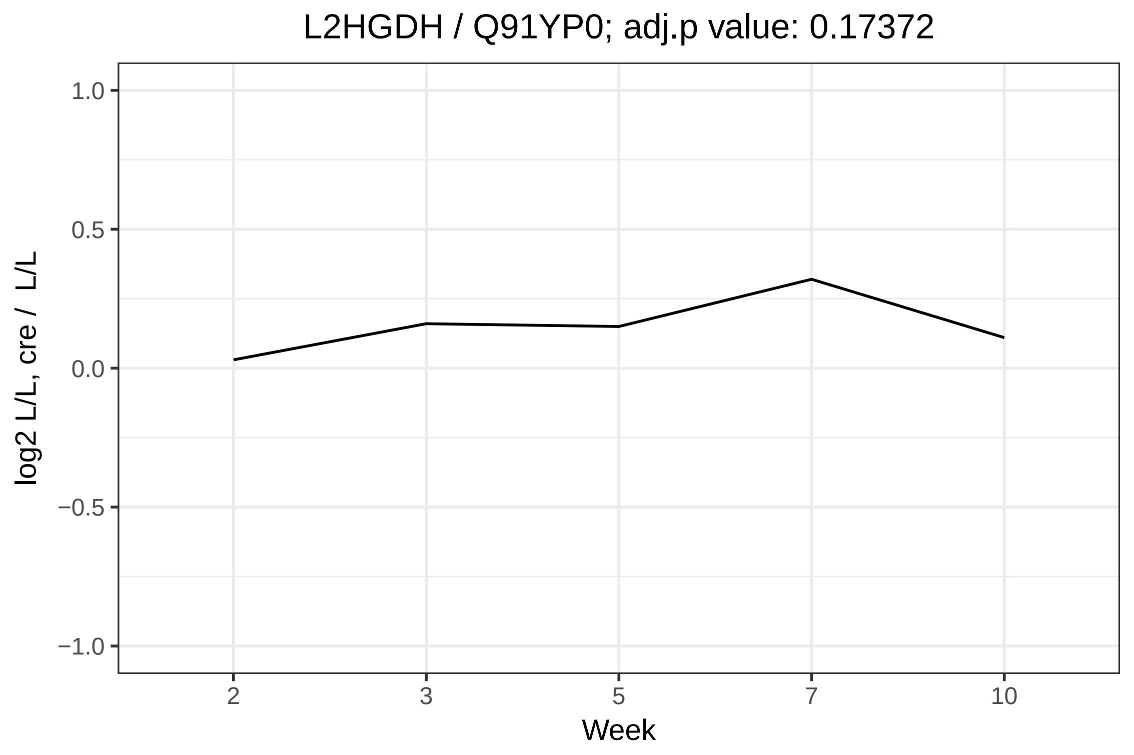

L2HGDH / Q91YP0; adj.p value: 0.17372
log2 L/L, cre / L/L
1.0
0.5
0.0
−0.5
−1.0
2
3
5
7
10
Week

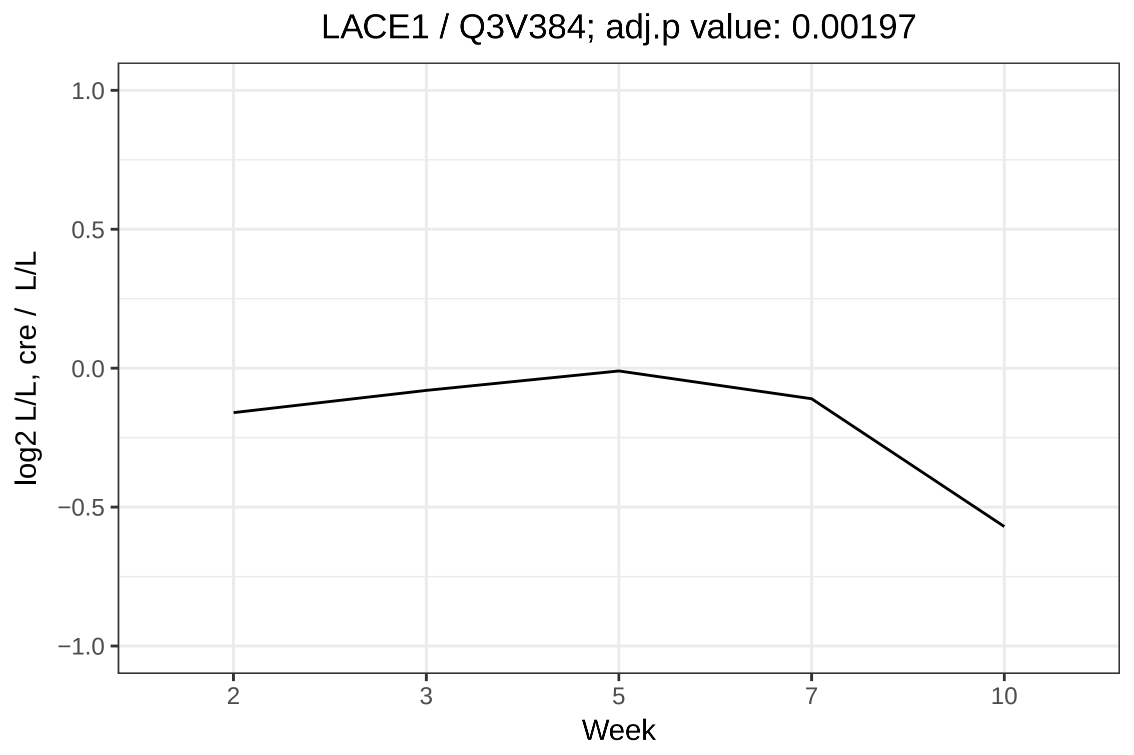
LACE1 / Q3V384; adj.p value: 0.00197
log2 L/L, cre / L/L
1.0
0.5
0.0
−0.5
−1.0
2
3
5
7
10
Week

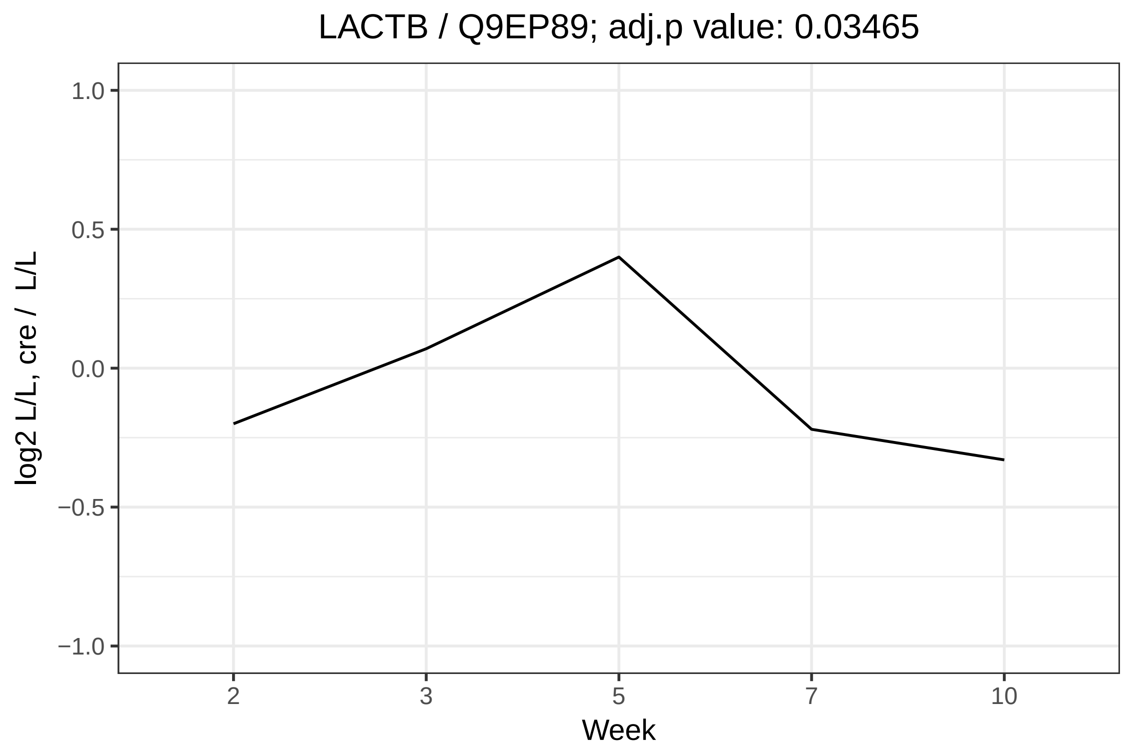

LACTB / Q9EP89; adj.p value: 0.03465
log2 L/L, cre / L/L
1.0
0.5
0.0
−0.5
−1.0
2
3
5
7
10
Week

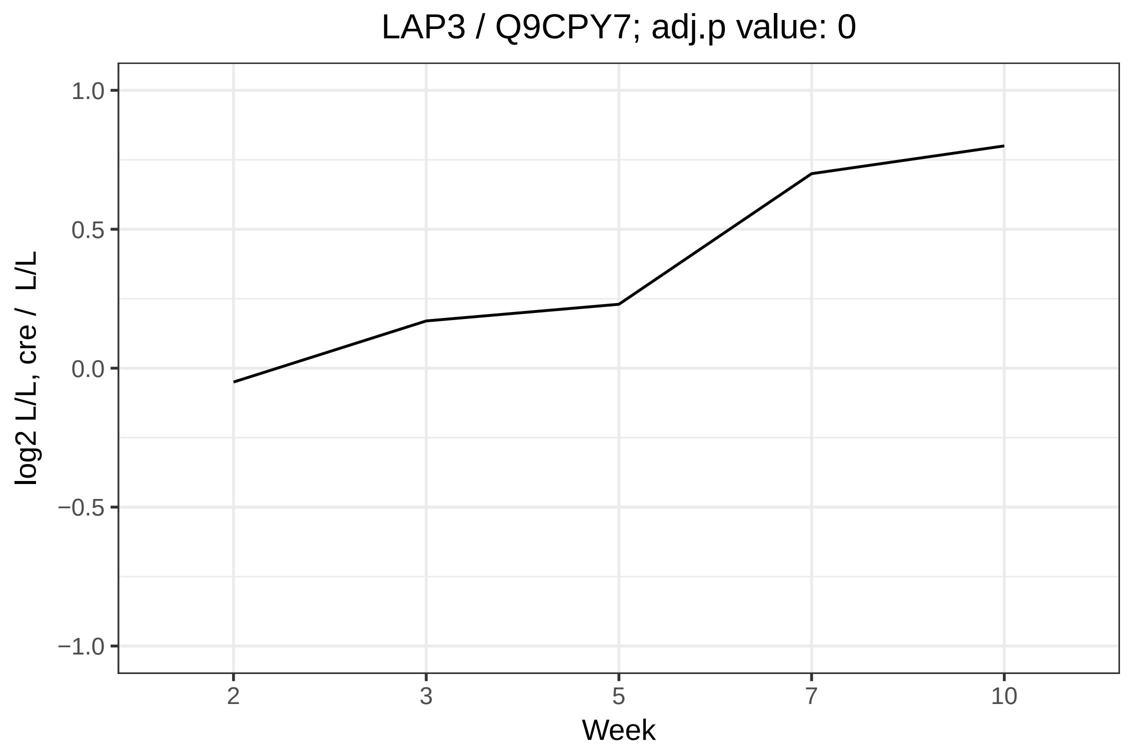
LAP3 / Q9CPY7; adj.p value: 0
1.0
0.5
0.0
−0.5
−1.0
log2 L/L, cre / L/L
2
3
5
7
10
Week

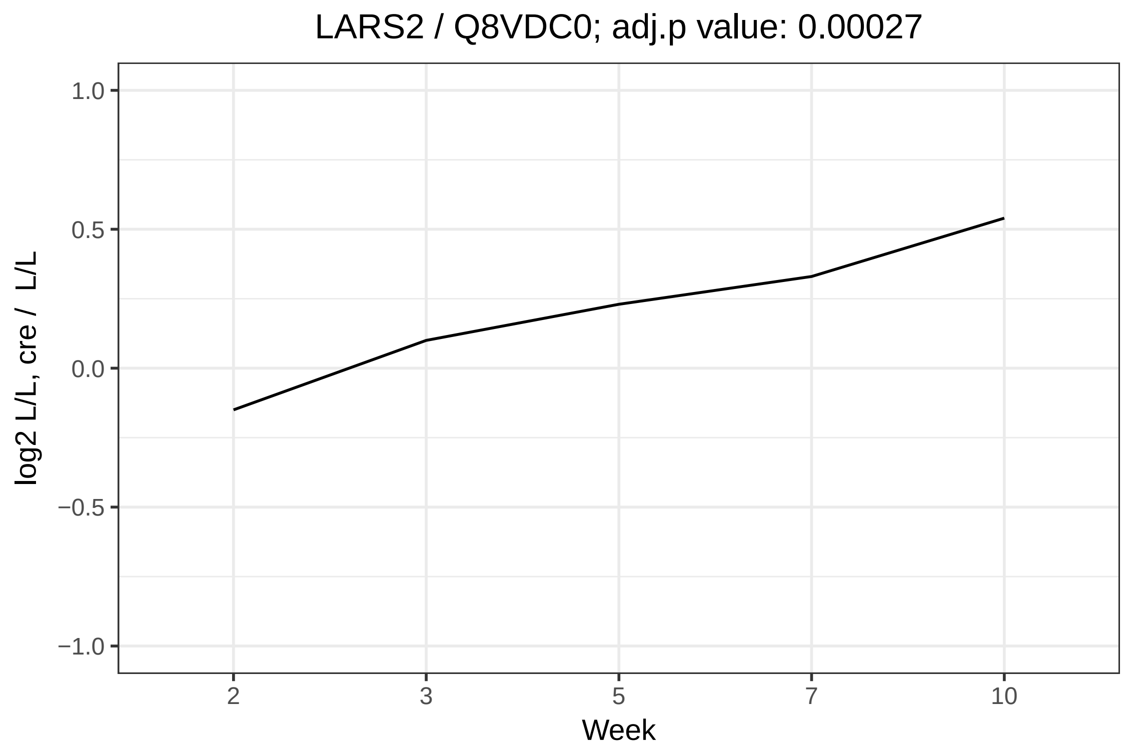
LARS2 / Q8VDC0; adj.p value: 0.00027
log2 L/L, cre / L/L
1.0
0.5
0.0
−0.5
−1.0
2
3
5
7
10
Week

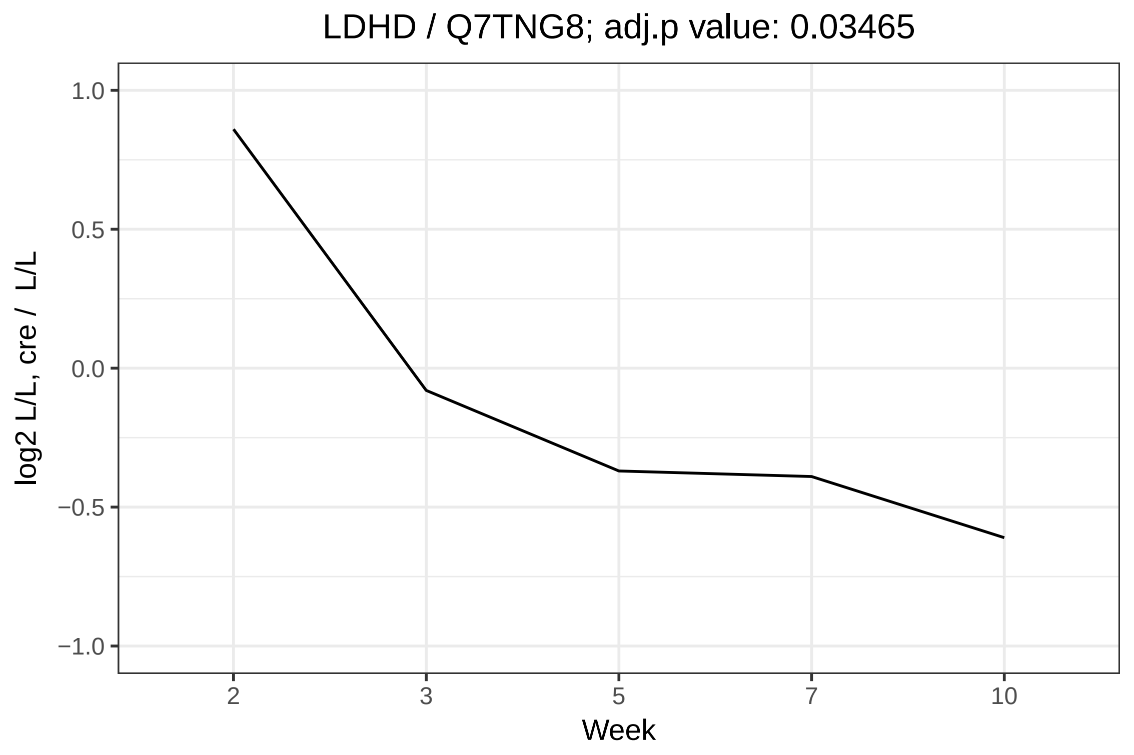

LDHD / Q7TNG8; adj.p value: 0.03465
1.0
0.5
0.0
−0.5
−1.0
log2 L/L, cre / L/L
2
3
5
7
10
Week

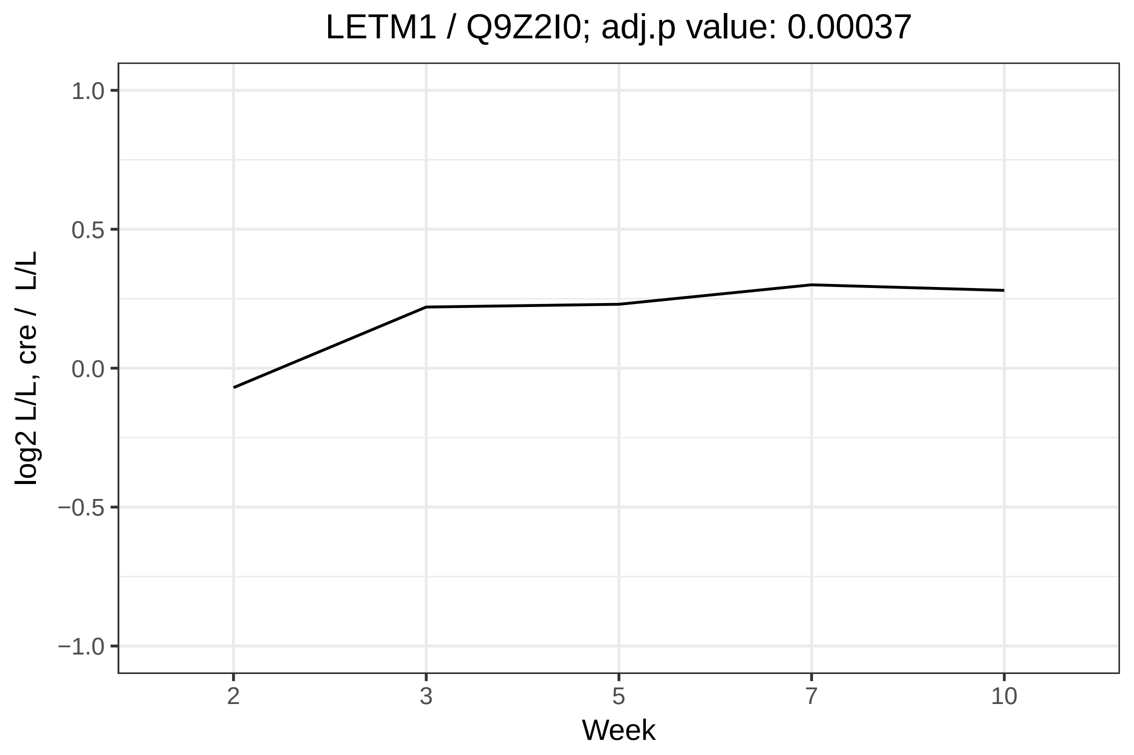
LETM1 / Q9Z2I0; adj.p value: 0.00037
log2 L/L, cre / L/L
1.0
0.5
0.0
−0.5
−1.0
2
3
5
7
10
Week

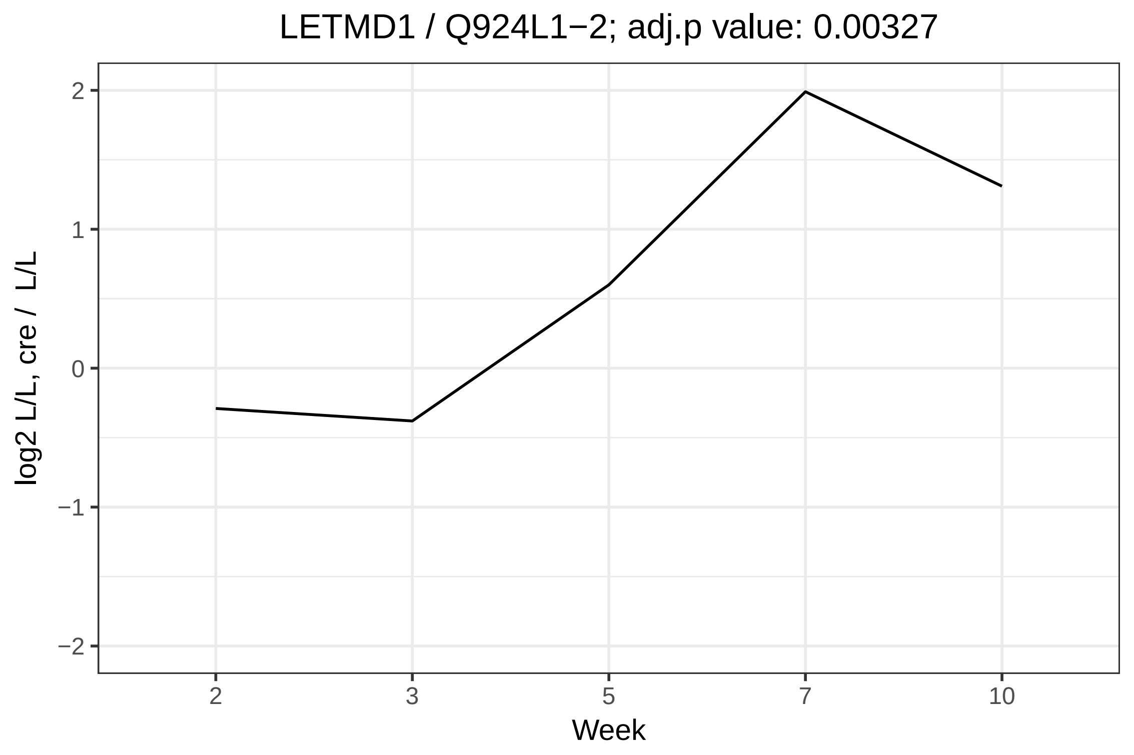
LETMD1 / Q924L1−2; adj.p value: 0.00327
log2 L/L, cre / L/L
2
1
0
−1
−2
2
3
5
7
10
Week

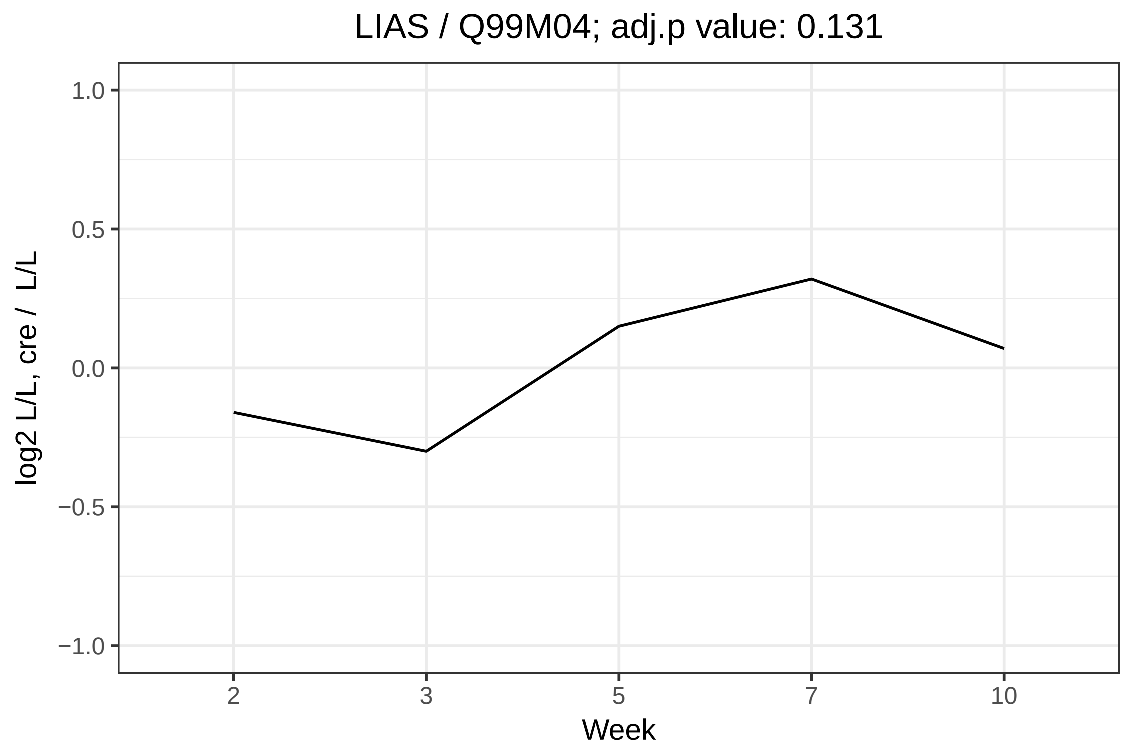
LIAS / Q99M04; adj.p value: 0.131
log2 L/L, cre / L/L
1.0
0.5
0.0
−0.5
−1.0
2
3
5
7
10
Week

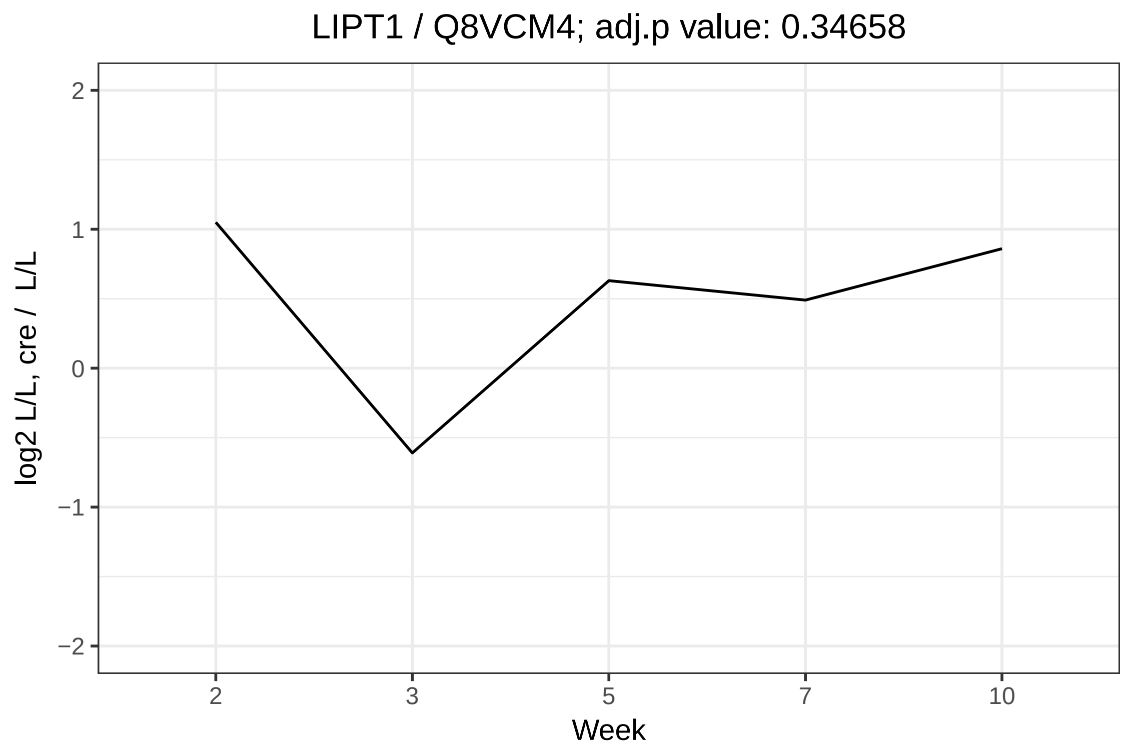

LIPT1 / Q8VCM4; adj.p value: 0.34658
log2 L/L, cre / L/L
2
1
0
−1
−2
2
3
5
7
10
Week

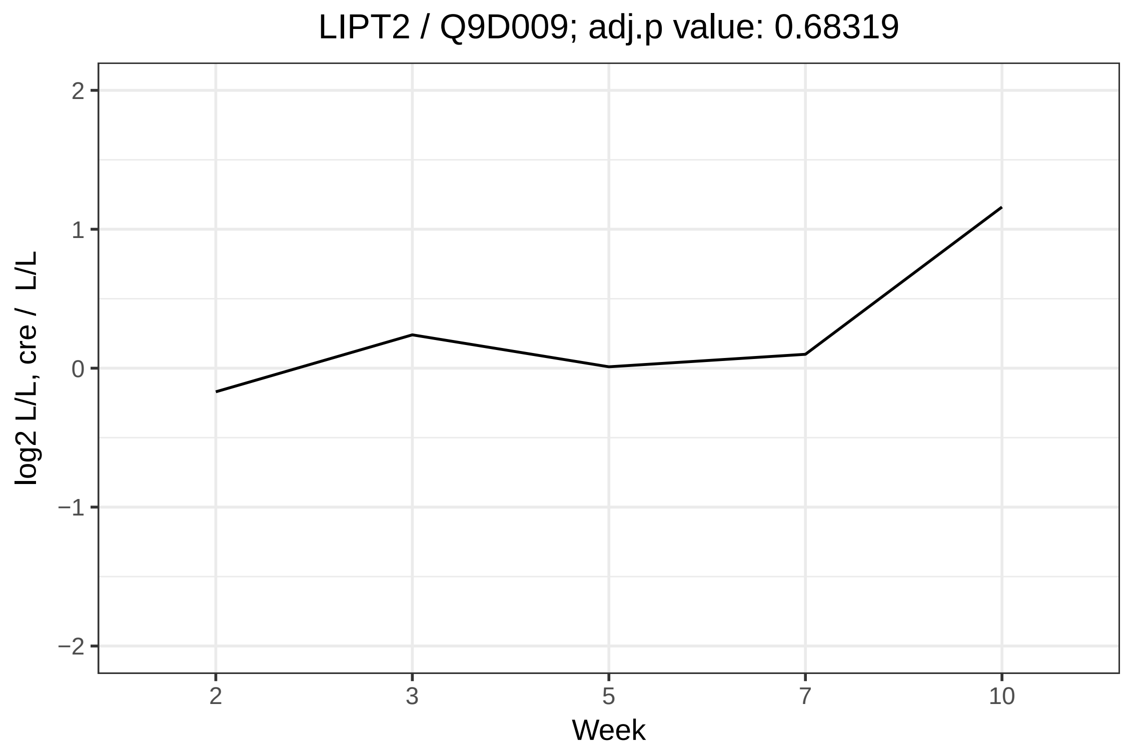
LIPT2 / Q9D009; adj.p value: 0.68319
log2 L/L, cre / L/L
Week
2
1
0
−1
−2
2
3
5
7
10

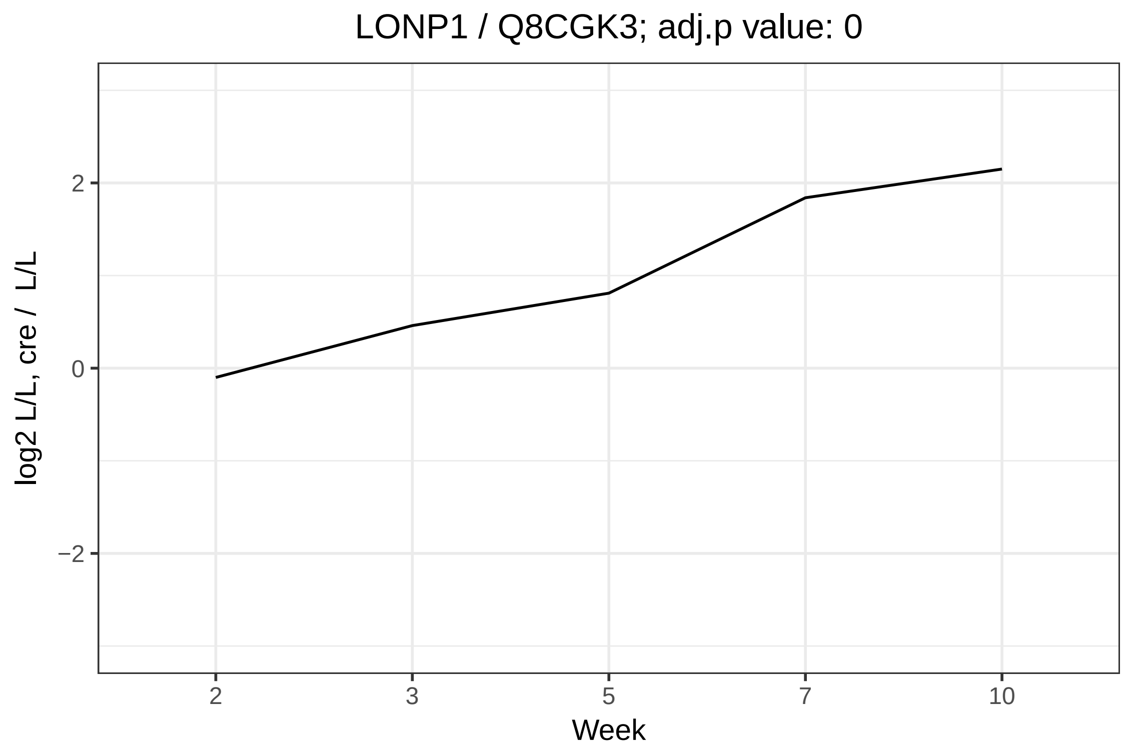

LONP1 / Q8CGK3; adj.p value: 0
log2 L/L, cre / L/L
2
0
−2
2
3
5
7
10
Week

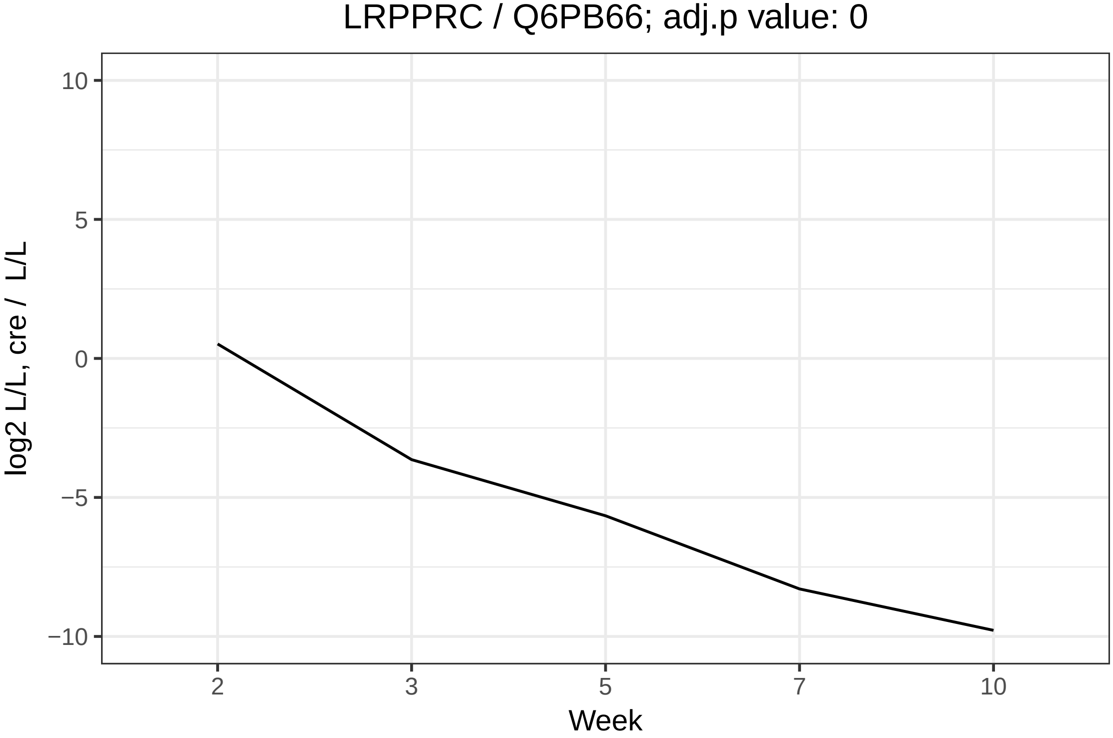

LRPPRC / Q6PB66; adj.p value: 0
10
5
0
−5
−10
log2 L/L, cre / L/L
2
3
5
7
10
Week

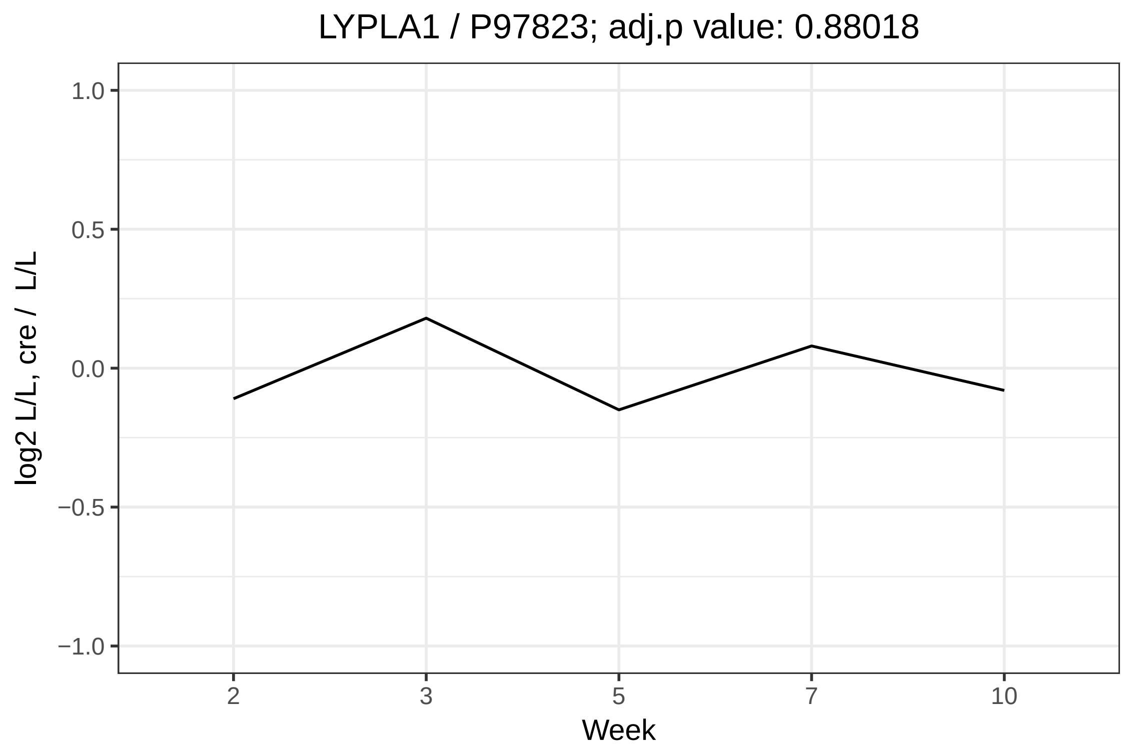
LYPLA1 / P97823; adj.p value: 0.88018
log2 L/L, cre / L/L
1.0
0.5
0.0
−0.5
−1.0
2
3
5
7
10
Week

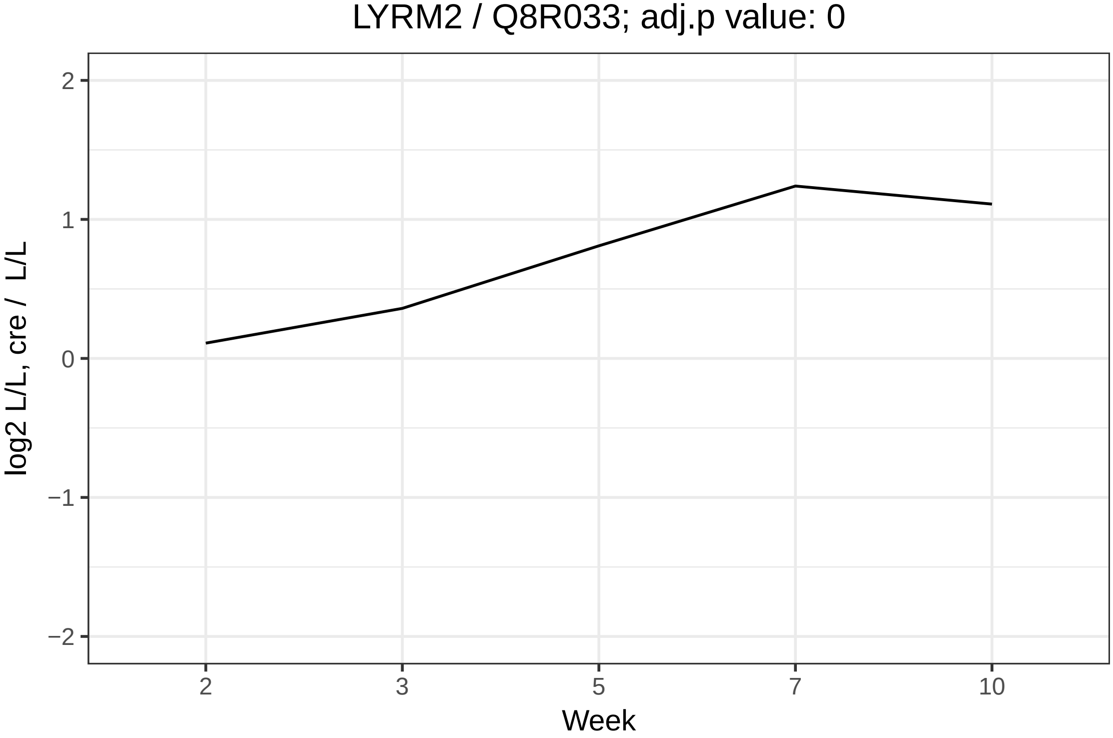
LYRM2 / Q8R033; adj.p value: 0
log2 L/L, cre / L/L
2
1
0
−1
−2
2
3
5
7
10
Week

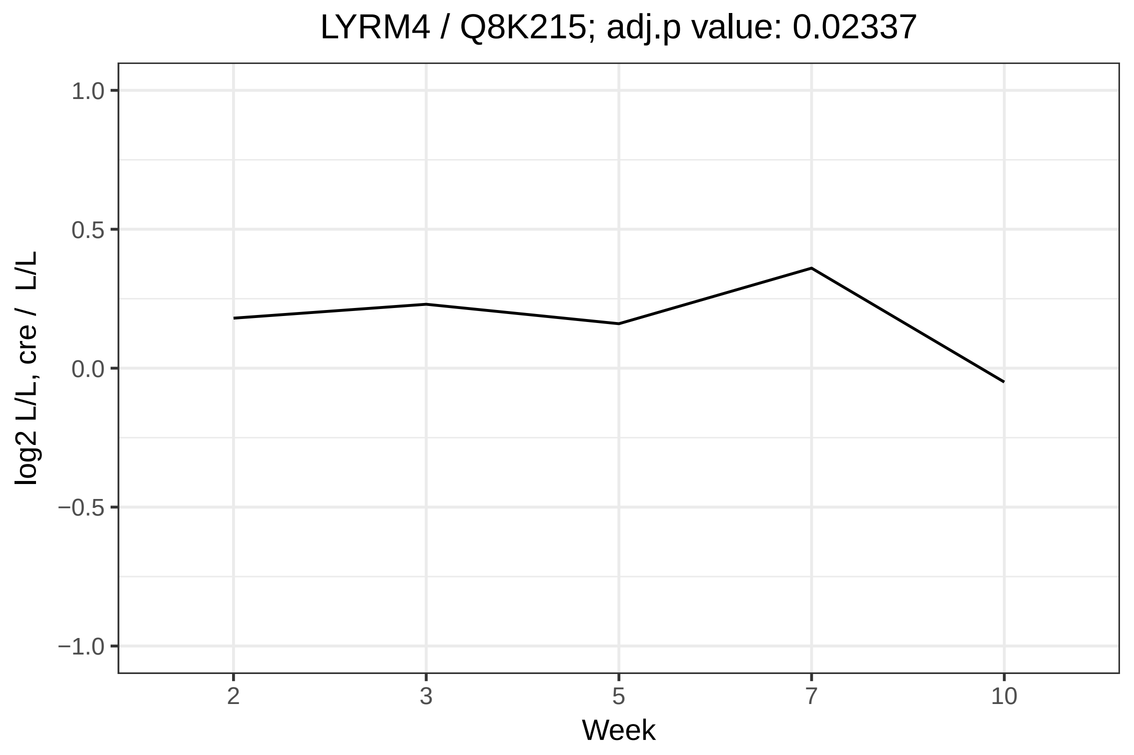
LYRM4 / Q8K215; adj.p value: 0.02337
log2 L/L, cre / L/L
1.0
0.5
0.0
−0.5
−1.0
2
3
5
7
10
Week

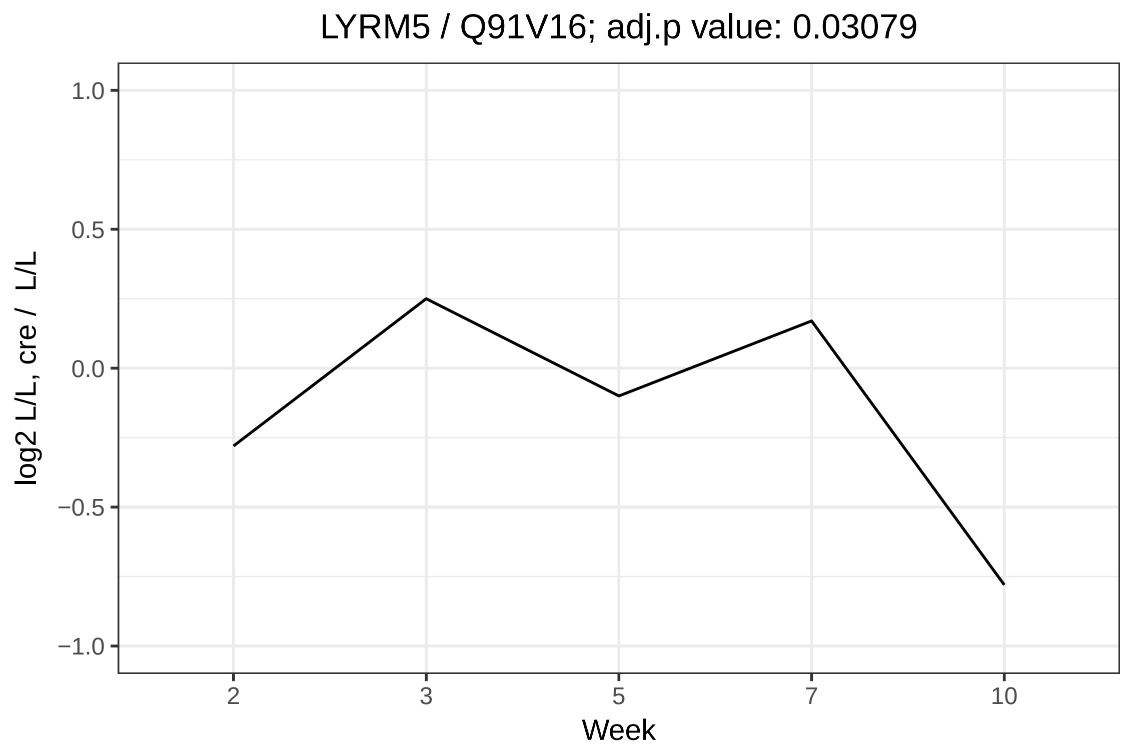

LYRM5 / Q91V16; adj.p value: 0.03079
1.0
0.5
0.0
−0.5
−1.0
log2 L/L, cre / L/L
2
3
5
7
10
Week

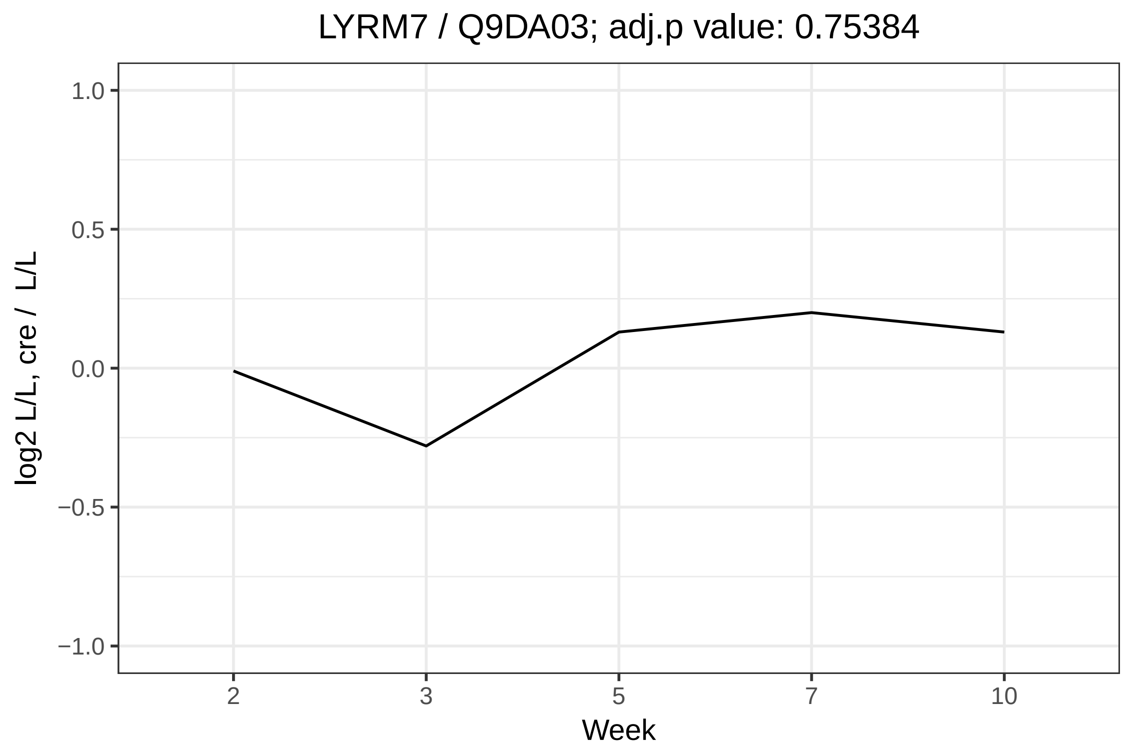
LYRM7 / Q9DA03; adj.p value: 0.75384
log2 L/L, cre / L/L
1.0
0.5
0.0
−0.5
−1.0
2
3
5
7
10
Week

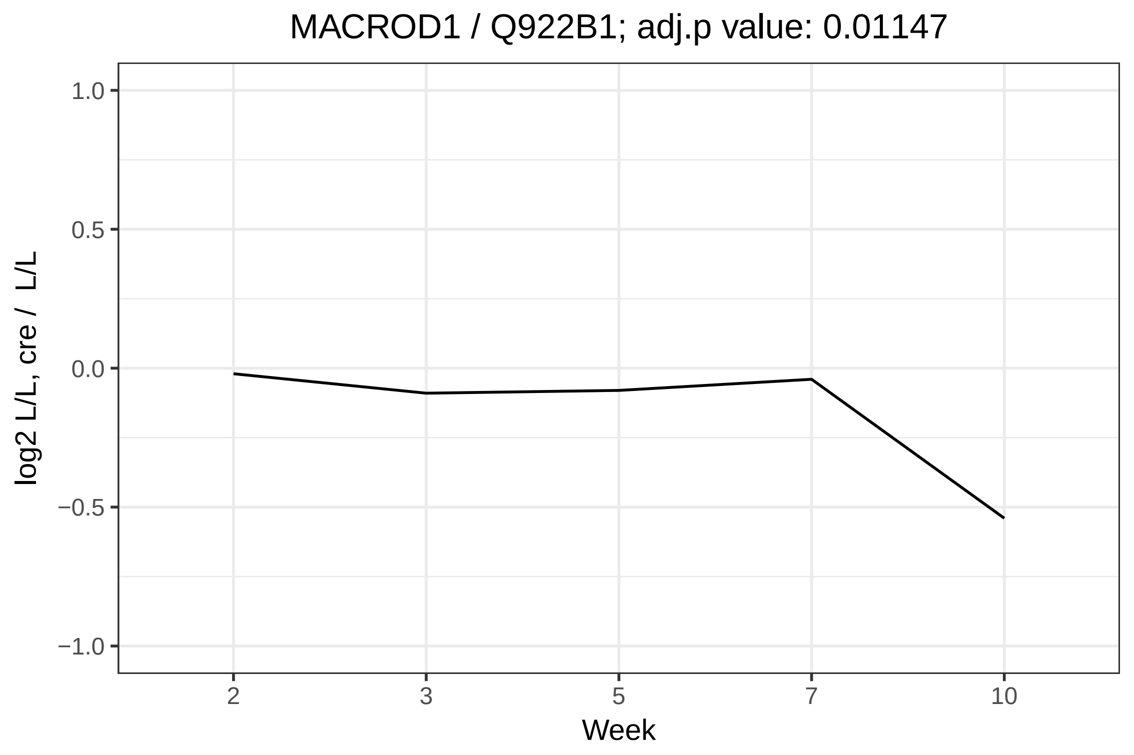

MACROD1 / Q922B1; adj.p value: 0.01147
1.0
0.5
0.0
−0.5
−1.0
log2 L/L, cre / L/L
2
3
5
7
10
Week

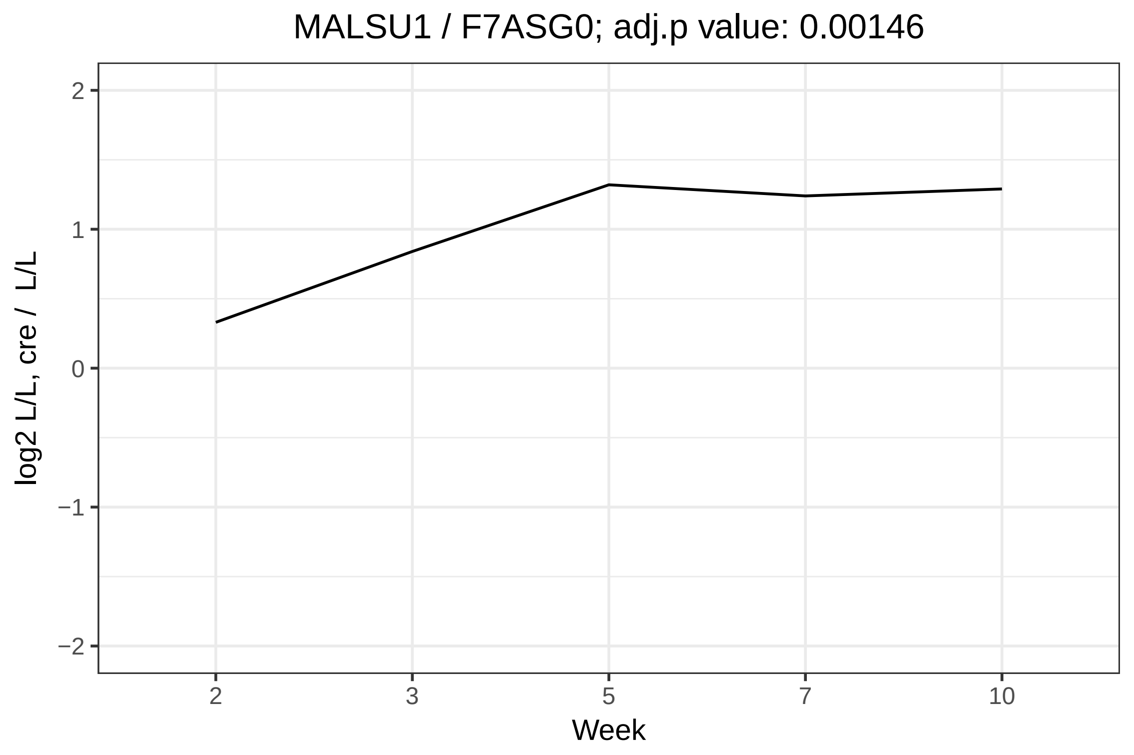
MALSU1 / F7ASG0; adj.p value: 0.00146
log2 L/L, cre / L/L
2
1
0
−1
−2
2
3
5
7
10
Week

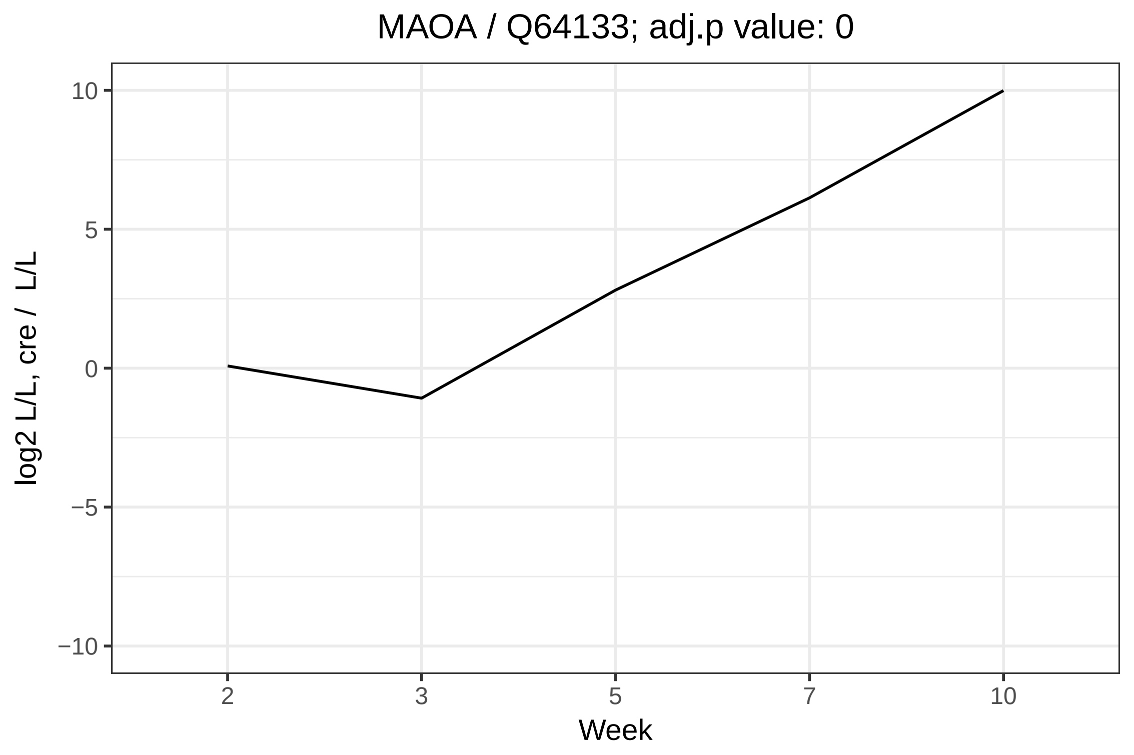
MAOA / Q64133; adj.p value: 0
log2 L/L, cre / L/L
10
5
0
−5
−10
2
3
5
7
10
Week

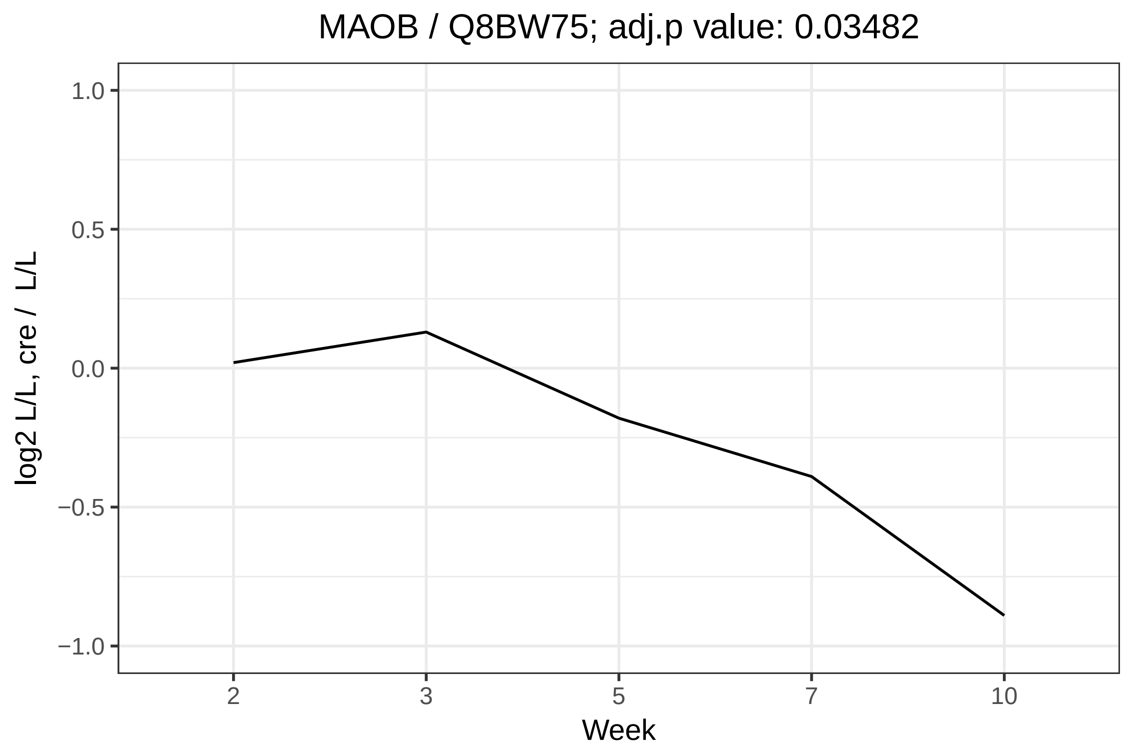
MAOB / Q8BW75; adj.p value: 0.03482
log2 L/L, cre / L/L
1.0
0.5
0.0
−0.5
−1.0
2
3
5
7
10
Week

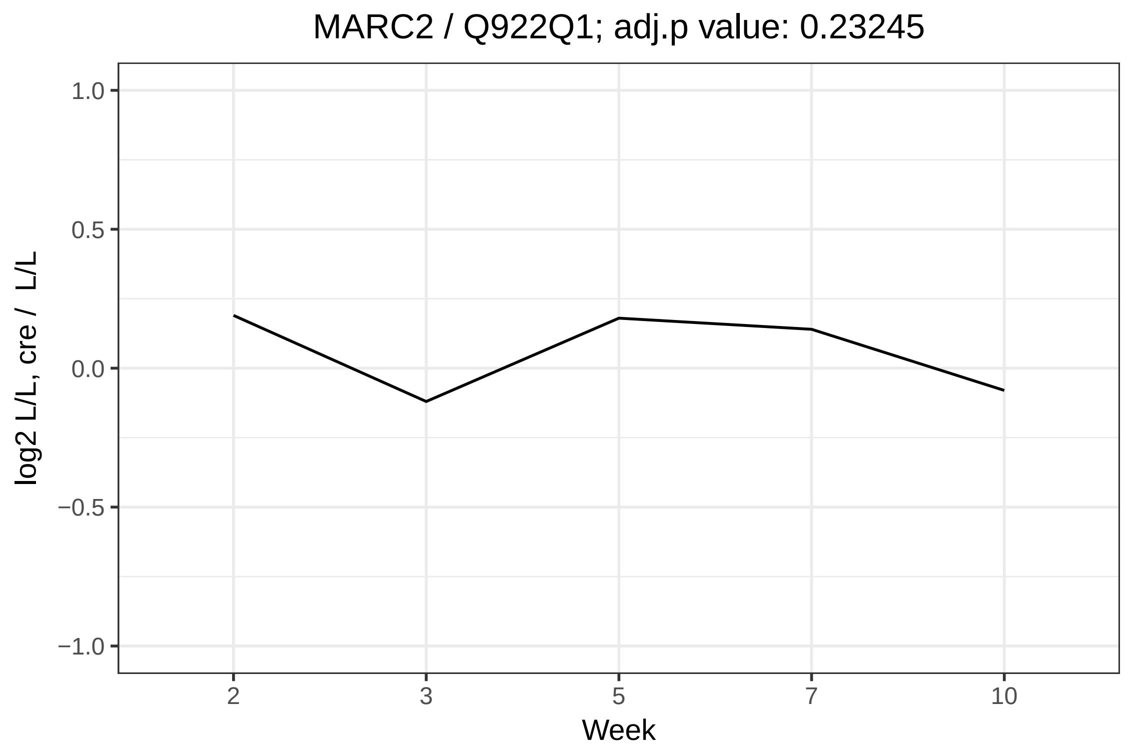
MARC2 / Q922Q1; adj.p value: 0.23245
1.0
0.5
0.0
−0.5
−1.0
log2 L/L, cre / L/L
2
3
5
7
10
Week

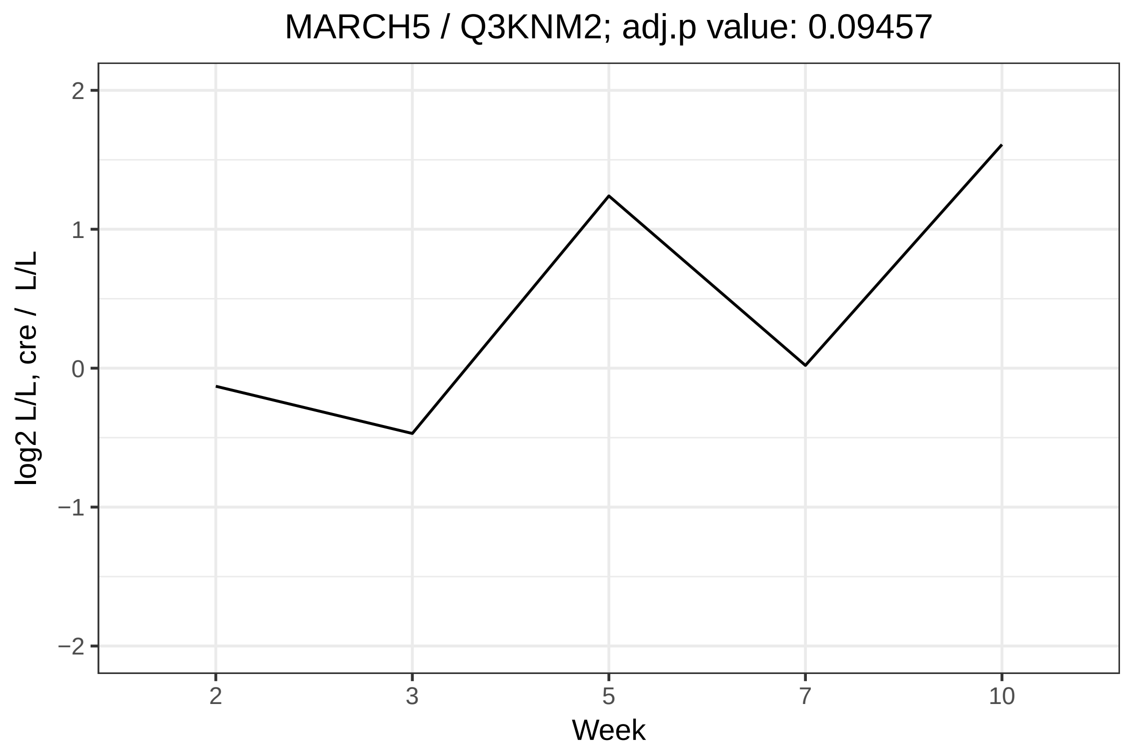
MARCH5 / Q3KNM2; adj.p value: 0.09457
log2 L/L, cre / L/L
2
1
0
−1
−2
2
3
5
7
10
Week

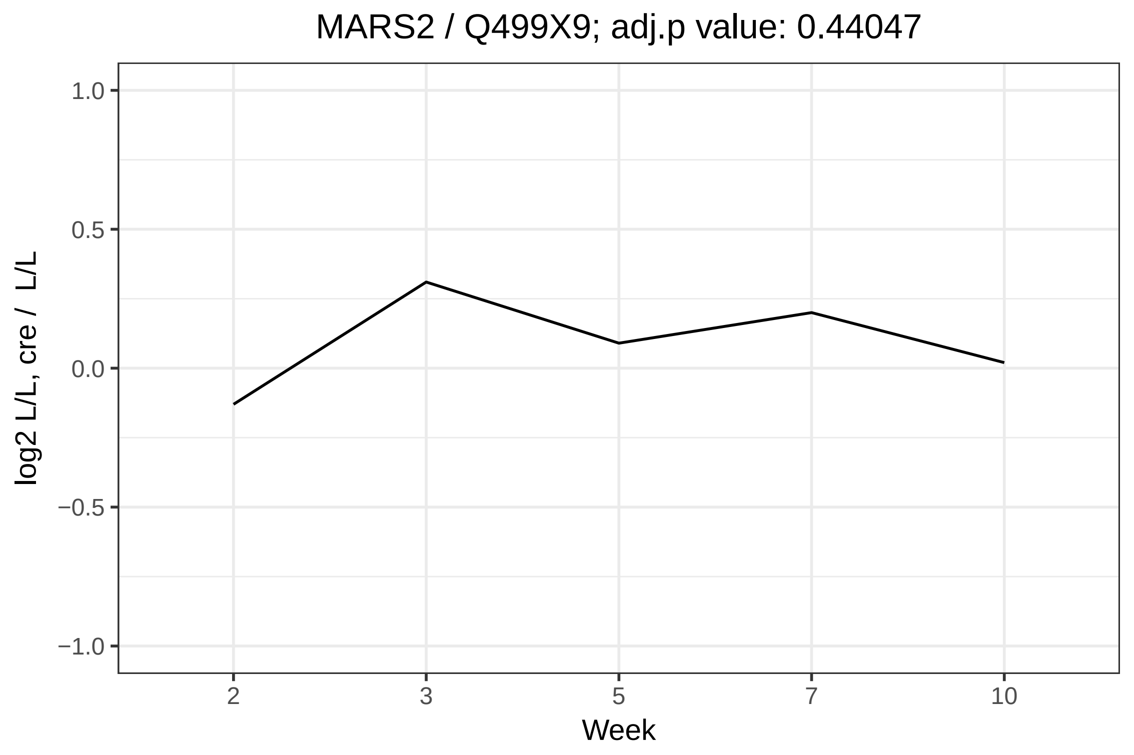

MARS2 / Q499X9; adj.p value: 0.44047
log2 L/L, cre / L/L
1.0
0.5
0.0
−0.5
−1.0
2
3
5
7
10
Week

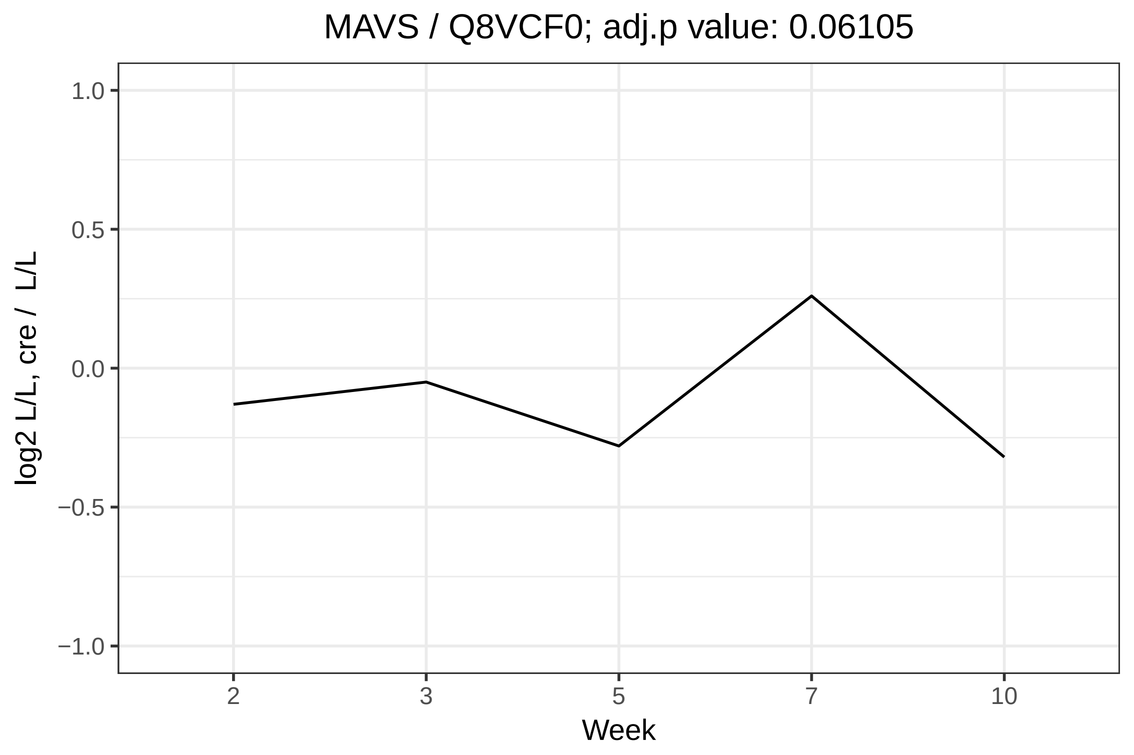
MAVS / Q8VCF0; adj.p value: 0.06105
log2 L/L, cre / L/L
1.0
0.5
0.0
−0.5
−1.0
2
3
5
7
10
Week

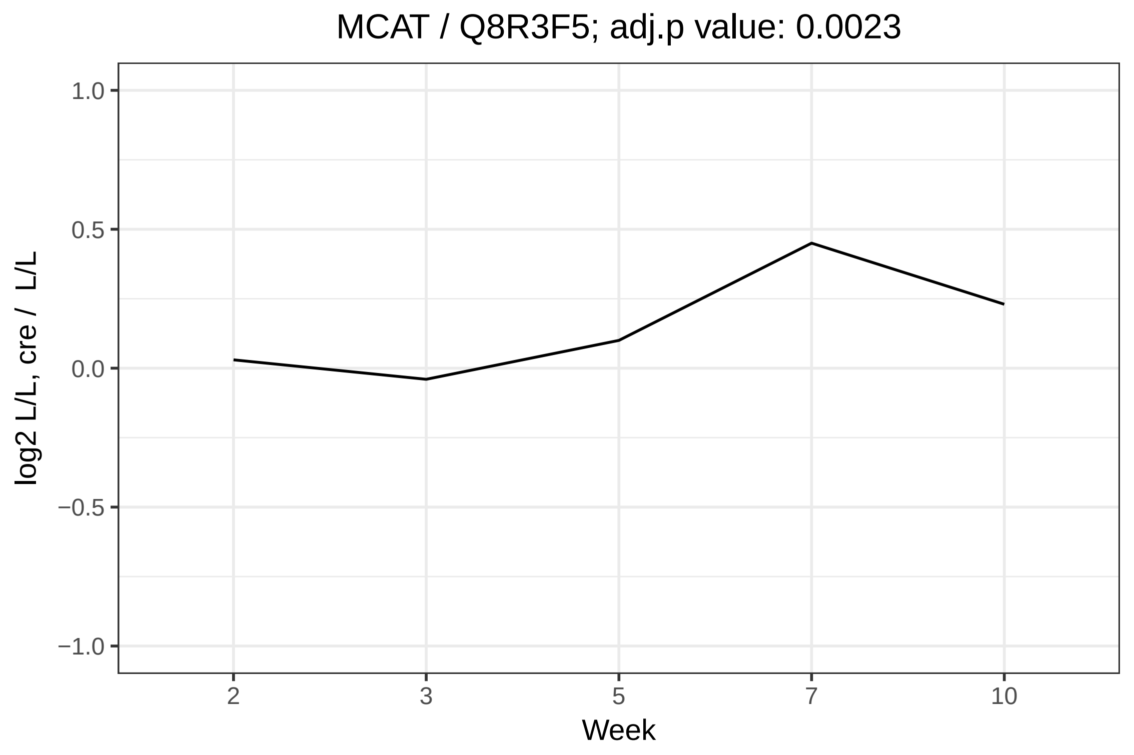
MCAT / Q8R3F5; adj.p value: 0.0023
log2 L/L, cre / L/L
1.0
0.5
0.0
−0.5
−1.0
2
3
5
7
10
Week

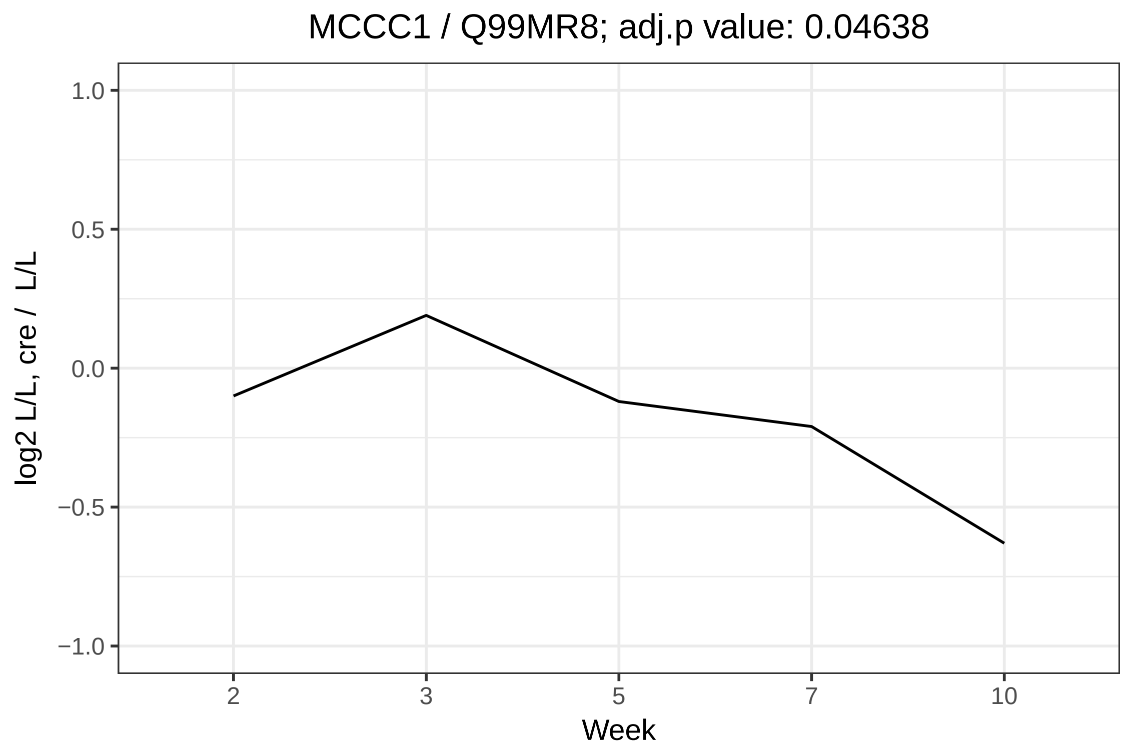
MCCC1 / Q99MR8; adj.p value: 0.04638
log2 L/L, cre / L/L
1.0
0.5
0.0
−0.5
−1.0
2
3
5
7
10
Week

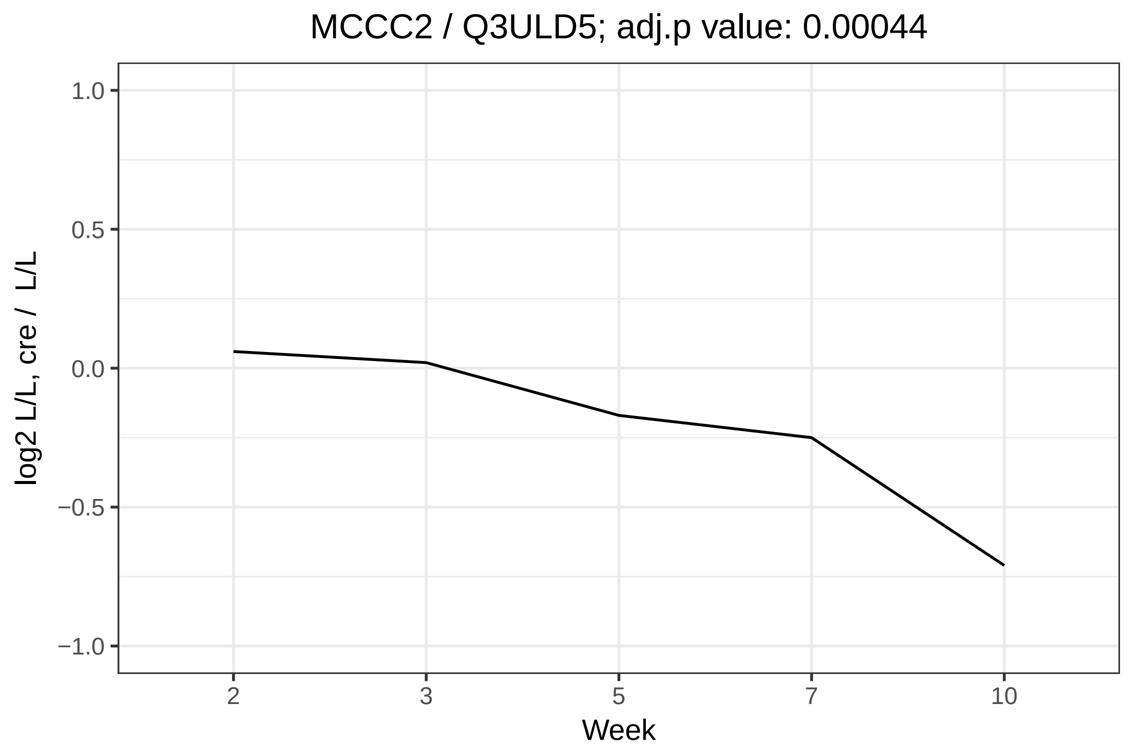

MCCC2 / Q3ULD5; adj.p value: 0.00044
1.0
0.5
0.0
−0.5
−1.0
log2 L/L, cre / L/L
2
3
5
7
10
Week

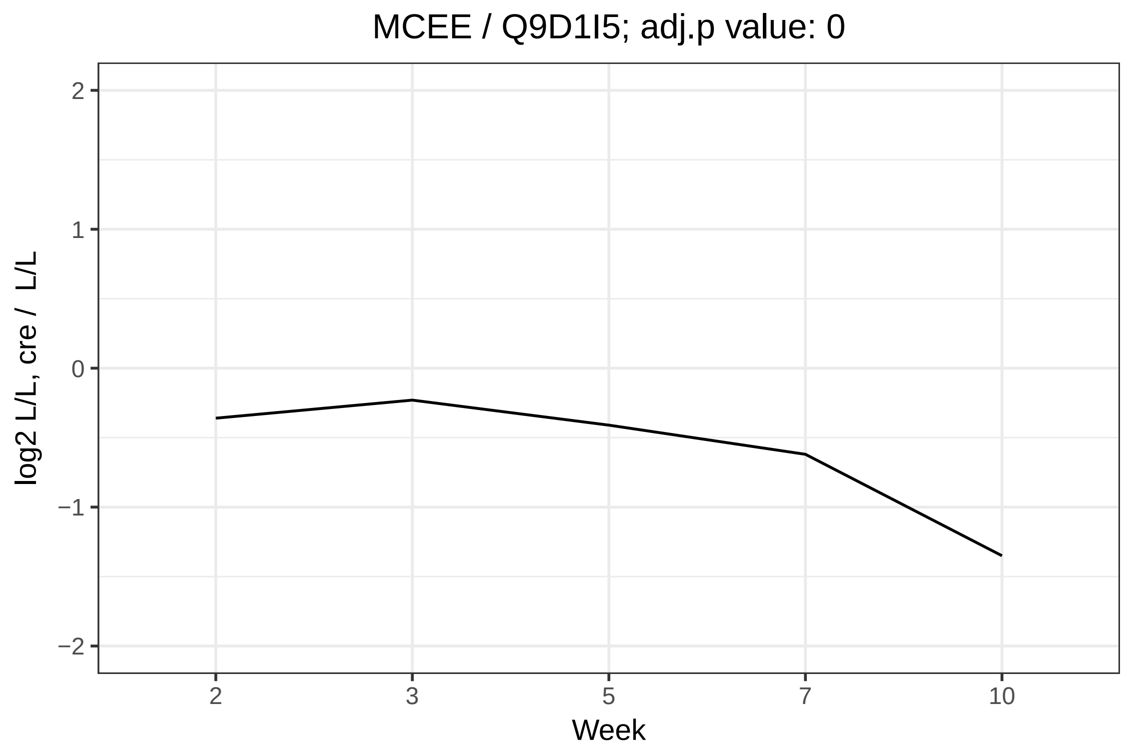

MCEE / Q9D1I5; adj.p value: 0
log2 L/L, cre / L/L
Week
2
1
0
−1
−2
2
3
5
7
10

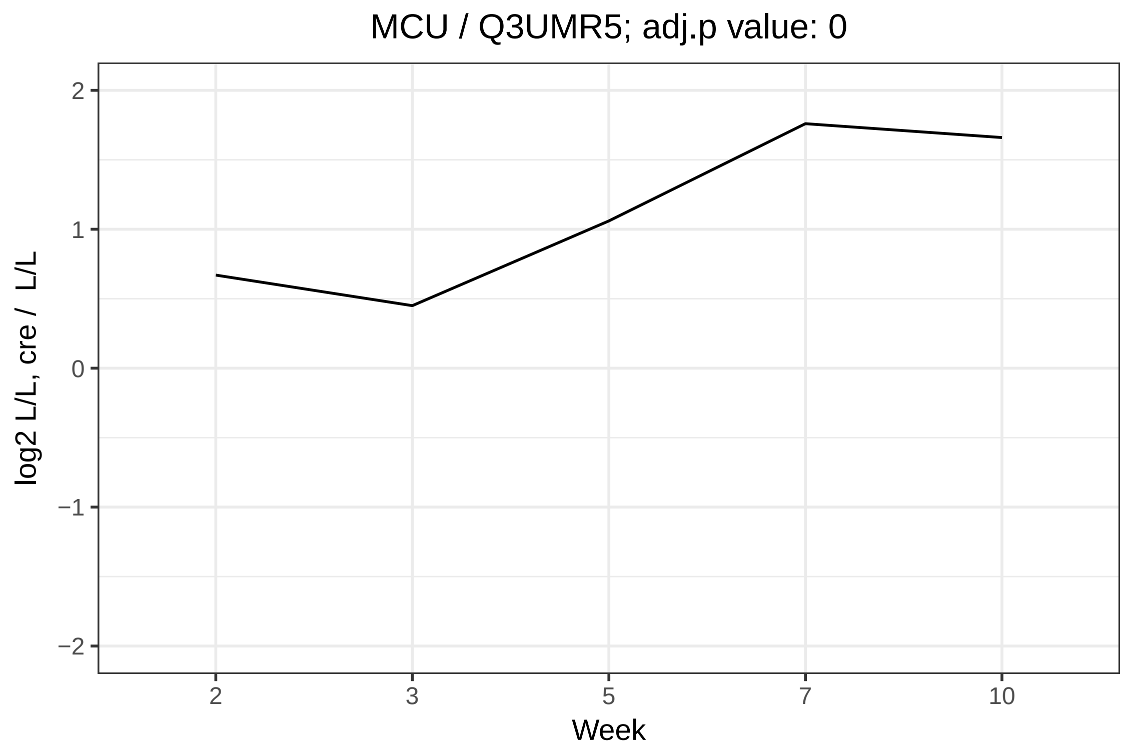

MCU / Q3UMR5; adj.p value: 0
log2 L/L, cre / L/L
2
1
0
−1
−2
2
3
5
7
10
Week

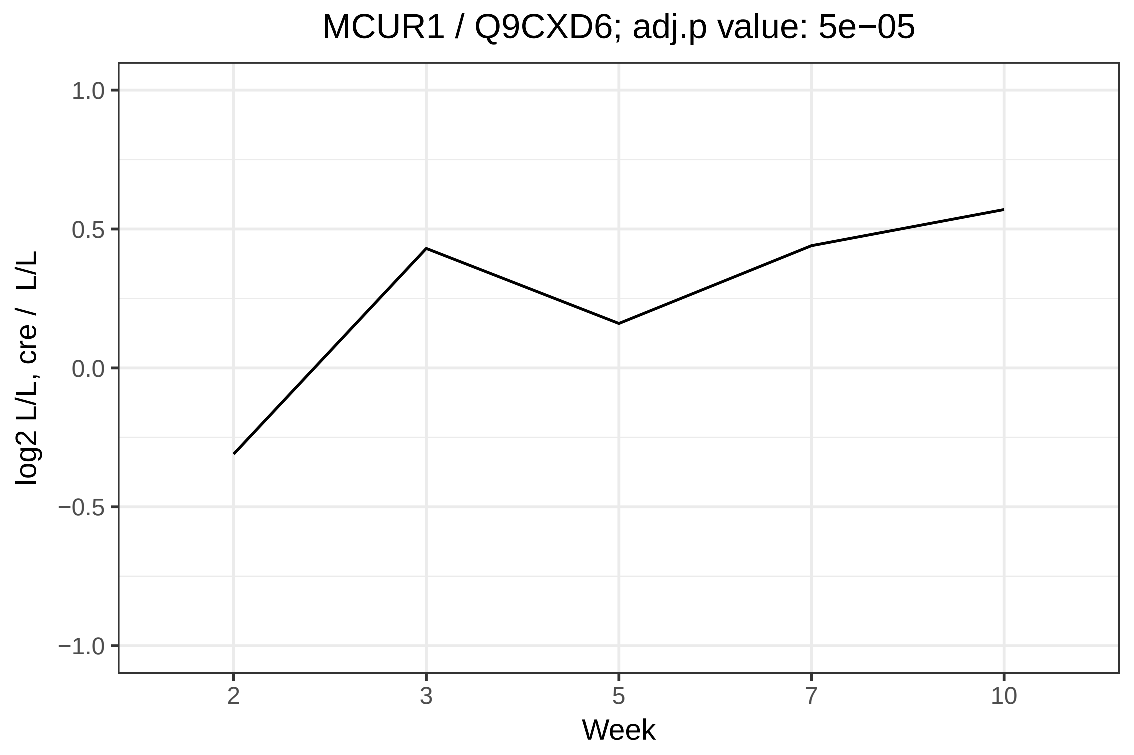
MCUR1 / Q9CXD6; adj.p value: 5e−05
1.0
0.5
0.0
−0.5
−1.0
log2 L/L, cre / L/L
2
3
5
7
10
Week

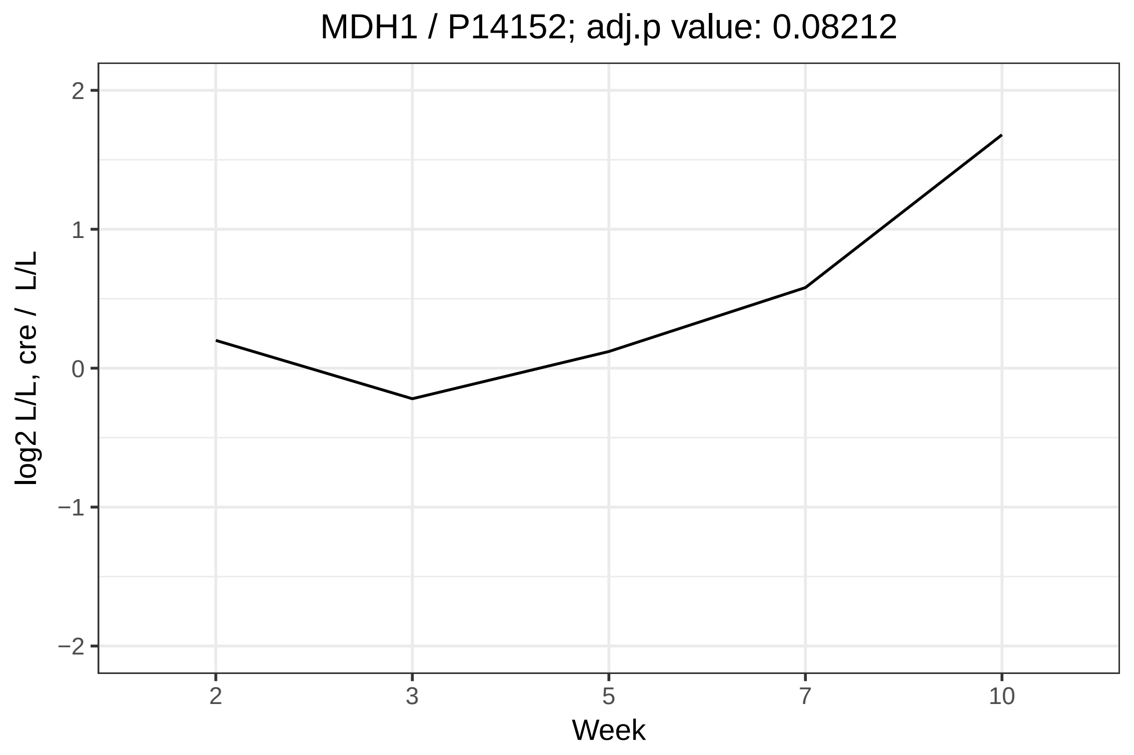
MDH1 / P14152; adj.p value: 0.08212
log2 L/L, cre / L/L
2
1
0
−1
−2
2
3
5
7
10
Week

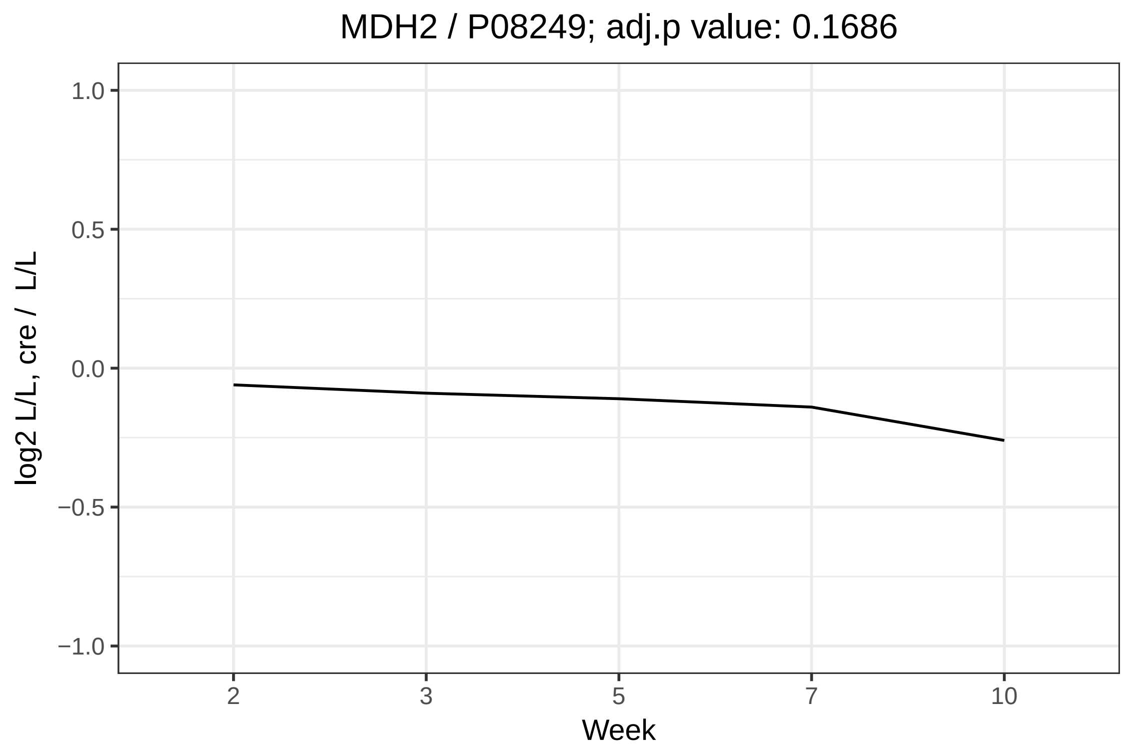

MDH2 / P08249; adj.p value: 0.1686
log2 L/L, cre / L/L
1.0
0.5
0.0
−0.5
−1.0
2
3
5
7
10
Week

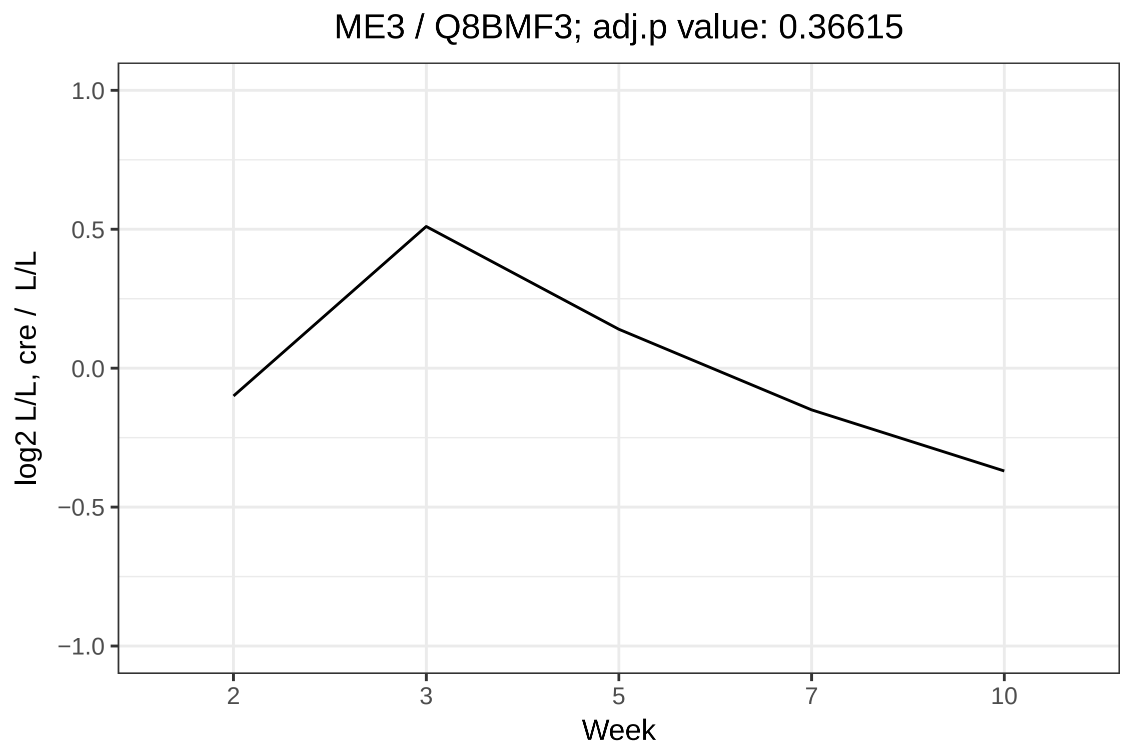
ME3 / Q8BMF3; adj.p value: 0.36615
1.0
0.5
0.0
−0.5
−1.0
log2 L/L, cre / L/L
2
3
5
7
10
Week

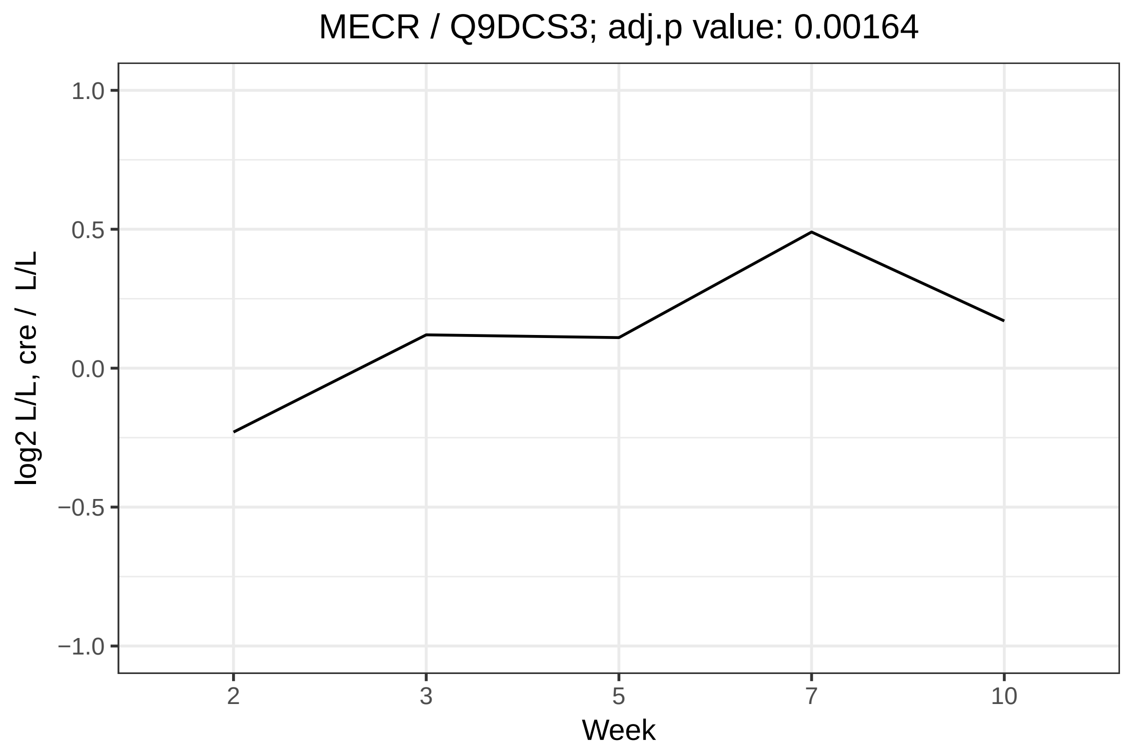

MECR / Q9DCS3; adj.p value: 0.00164
log2 L/L, cre / L/L
1.0
0.5
0.0
−0.5
−1.0
2
3
5
7
10
Week

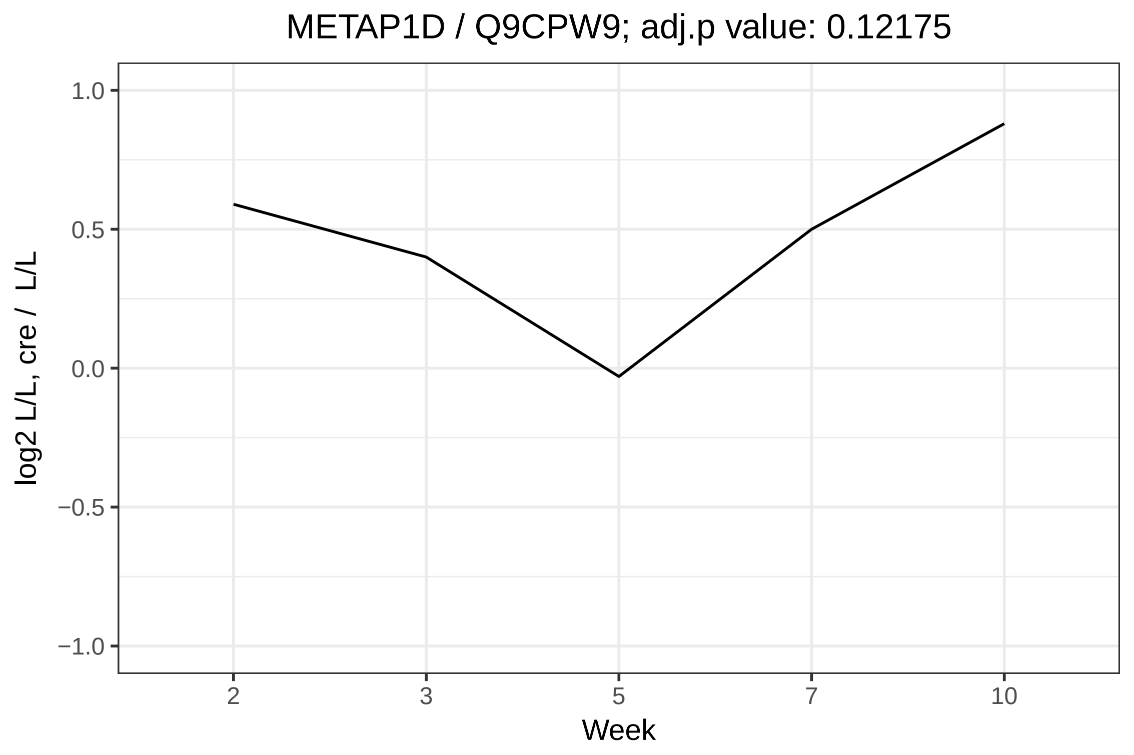

METAP1D / Q9CPW9; adj.p value: 0.12175
log2 L/L, cre / L/L
1.0
0.5
0.0
−0.5
−1.0
2
3
5
7
10
Week

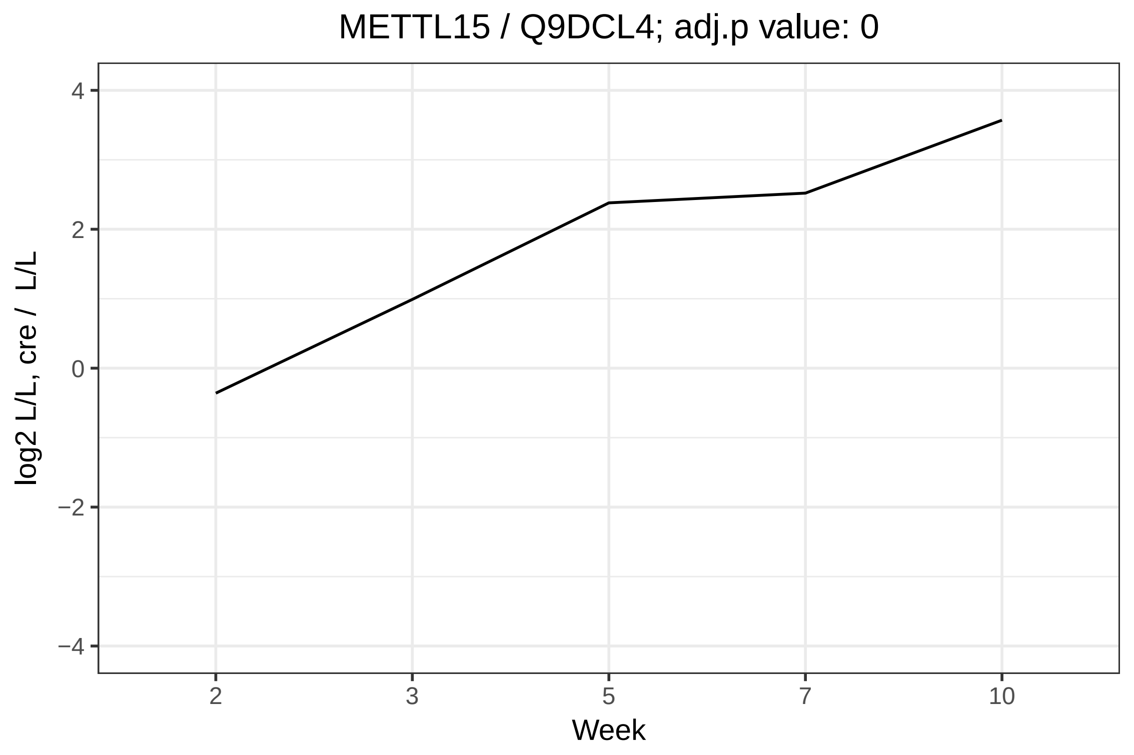
METTL15 / Q9DCL4; adj.p value: 0
log2 L/L, cre / L/L
4
2
0
−2
−4
2
3
5
7
10
Week

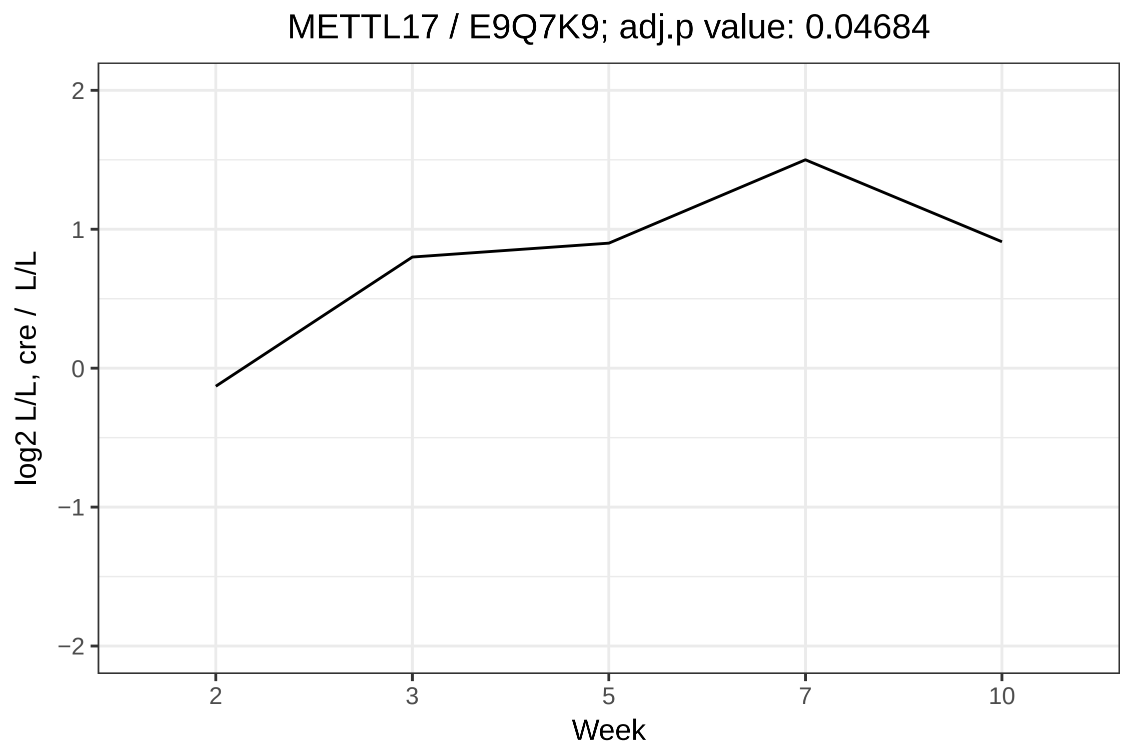

METTL17 / E9Q7K9; adj.p value: 0.04684
log2 L/L, cre / L/L
2
1
0
−1
−2
2
3
5
7
10
Week

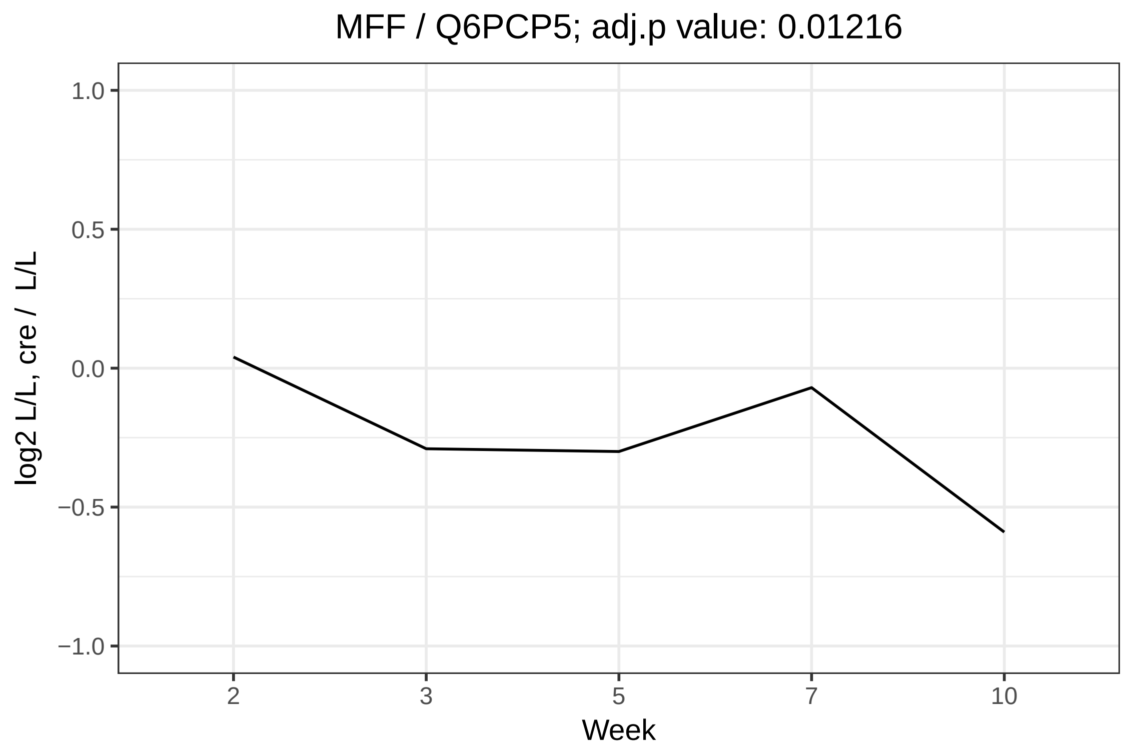
MFF / Q6PCP5; adj.p value: 0.01216
1.0
0.5
0.0
−0.5
−1.0
log2 L/L, cre / L/L
2
3
5
7
10
Week

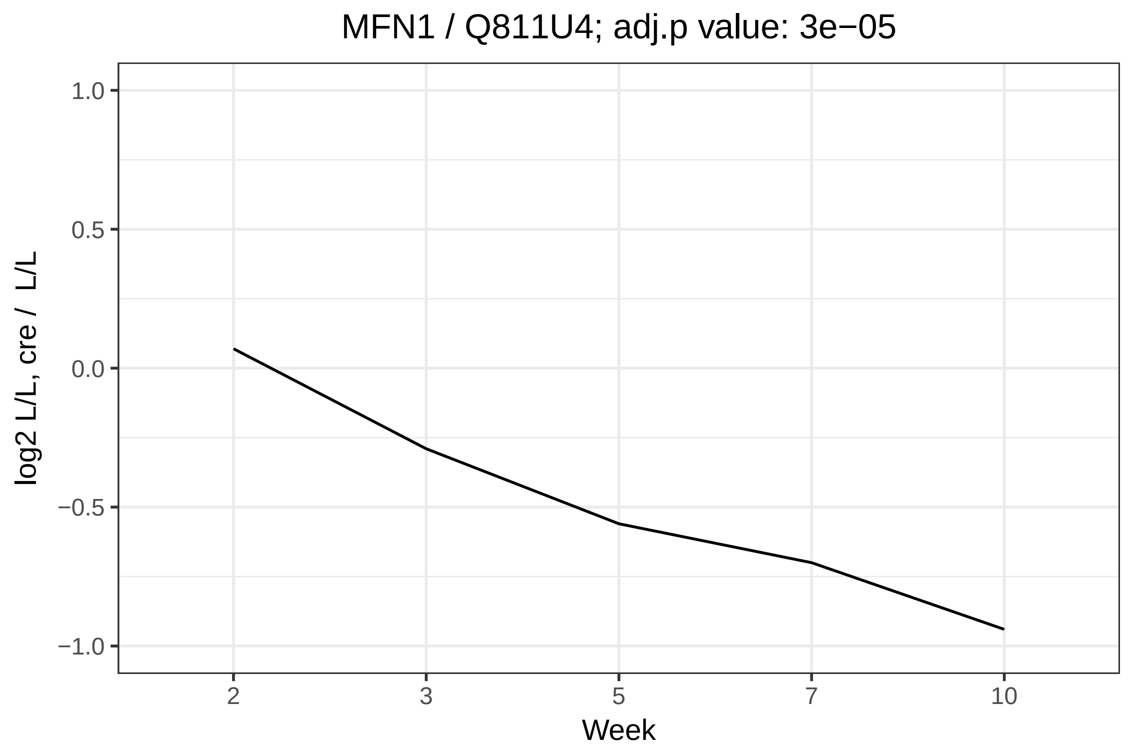
MFN1 / Q811U4; adj.p value: 3e−05
log2 L/L, cre / L/L
1.0
0.5
0.0
−0.5
−1.0
2
3
5
7
10
Week

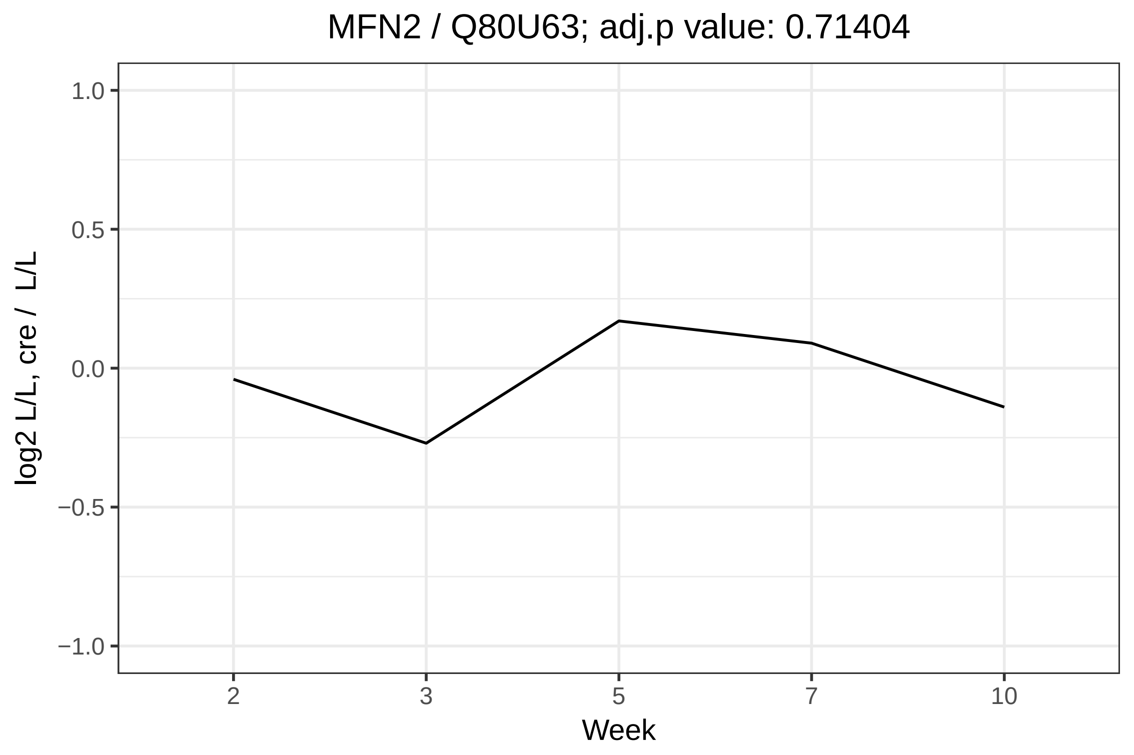
MFN2 / Q80U63; adj.p value: 0.71404
log2 L/L, cre / L/L
1.0
0.5
0.0
−0.5
−1.0
2
3
5
7
10
Week

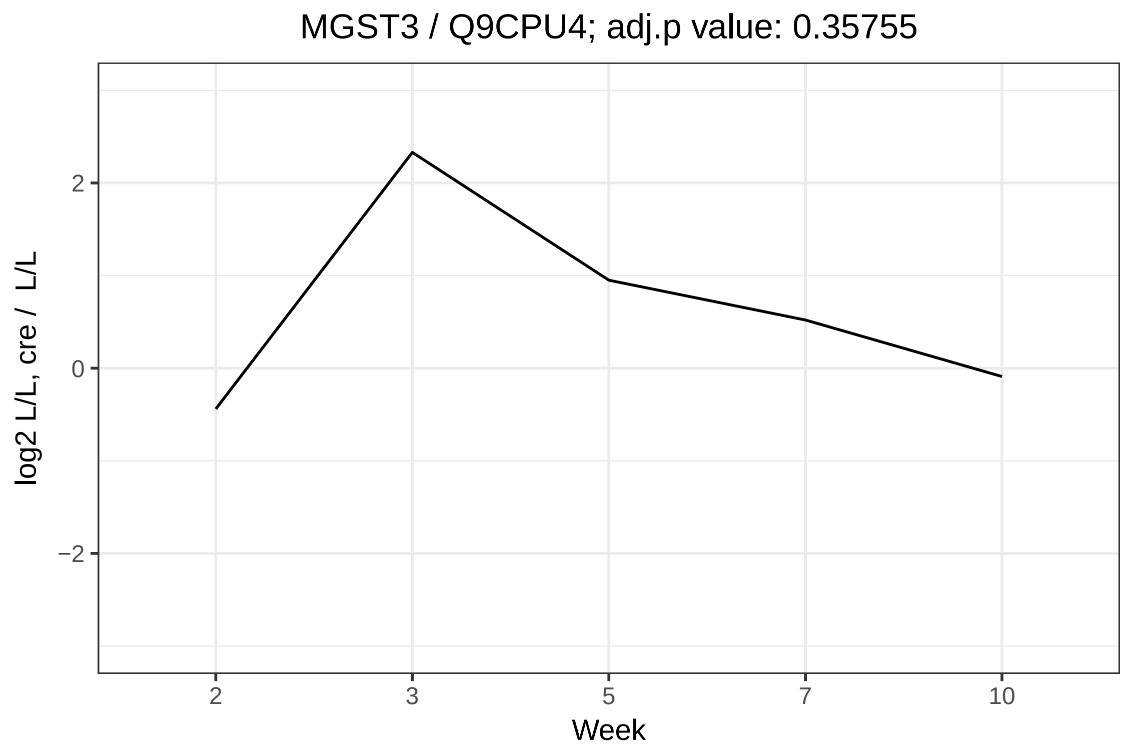
MGST3 / Q9CPU4; adj.p value: 0.35755
log2 L/L, cre / L/L
2
0
−2
2
3
5
7
10
Week

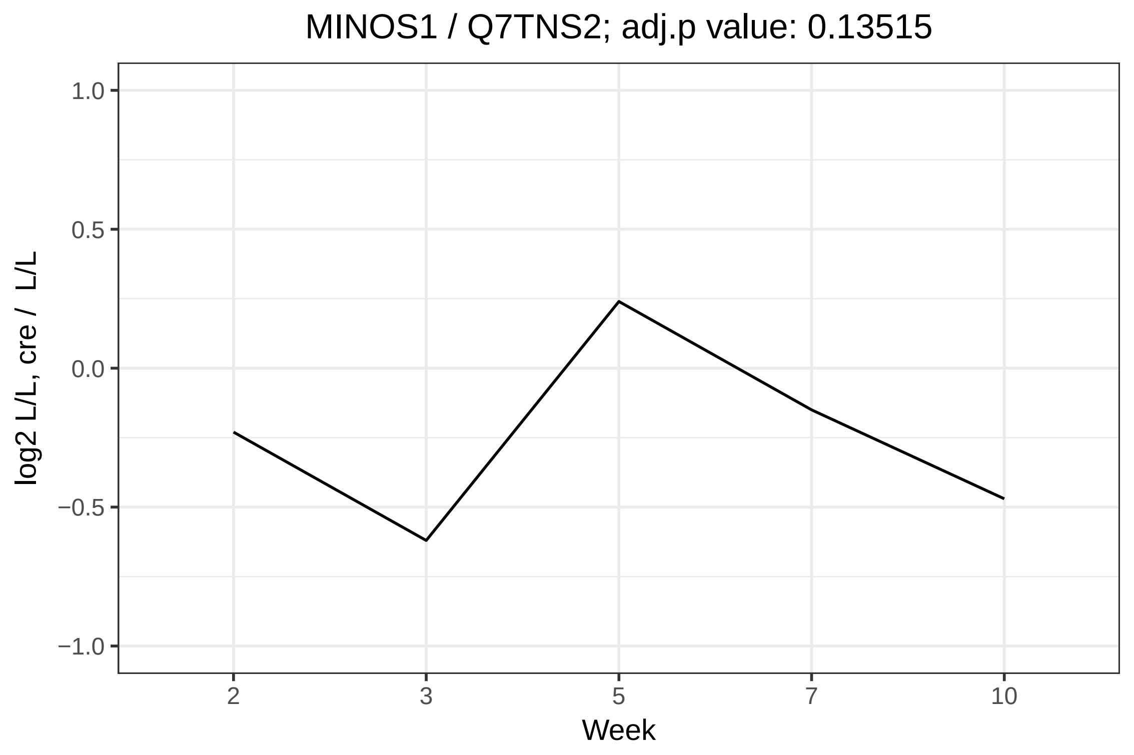

MINOS1 / Q7TNS2; adj.p value: 0.13515
1.0
0.5
0.0
−0.5
−1.0
log2 L/L, cre / L/L
2
3
5
7
10
Week

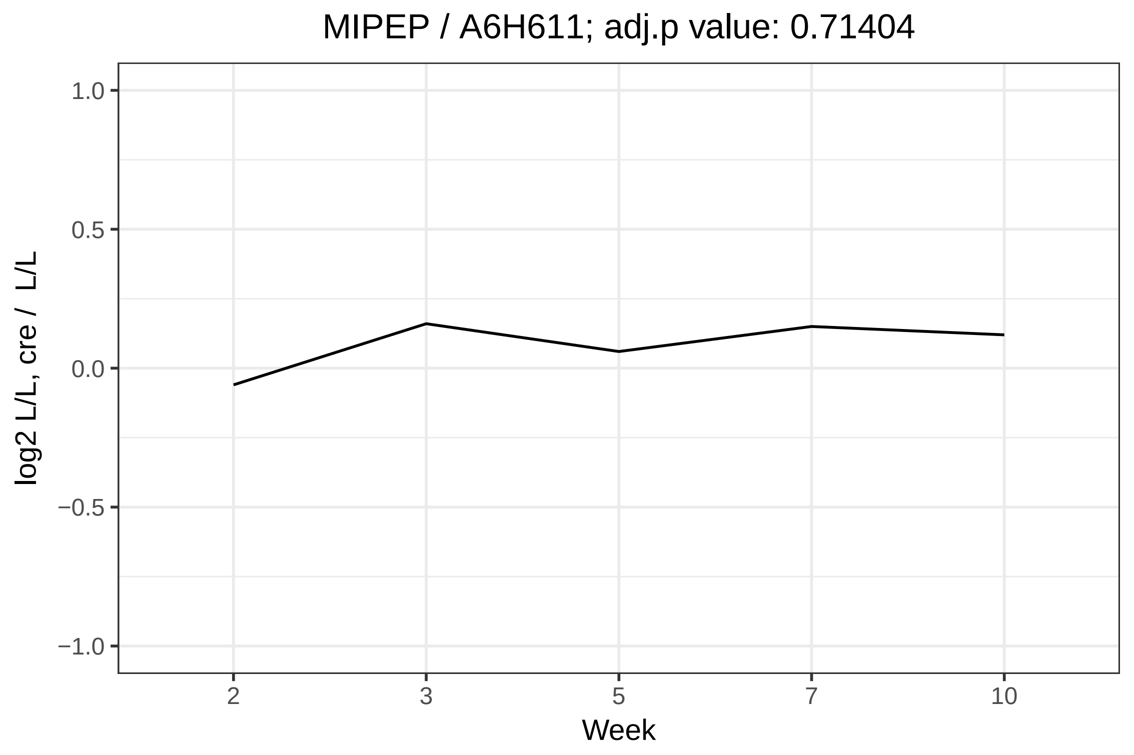
MIPEP / A6H611; adj.p value: 0.71404
log2 L/L, cre / L/L
1.0
0.5
0.0
−0.5
−1.0
2
3
5
7
10
Week

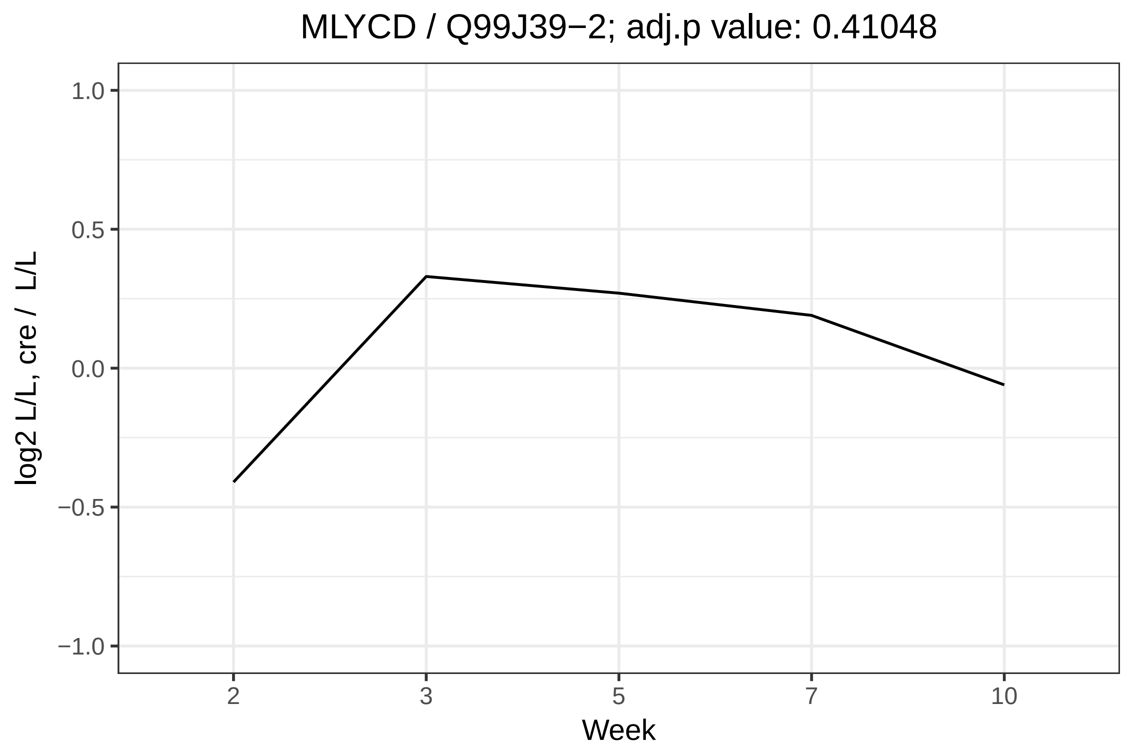

MLYCD / Q99J39−2; adj.p value: 0.41048
1.0
0.5
0.0
−0.5
−1.0
log2 L/L, cre / L/L
2
3
5
7
10
Week

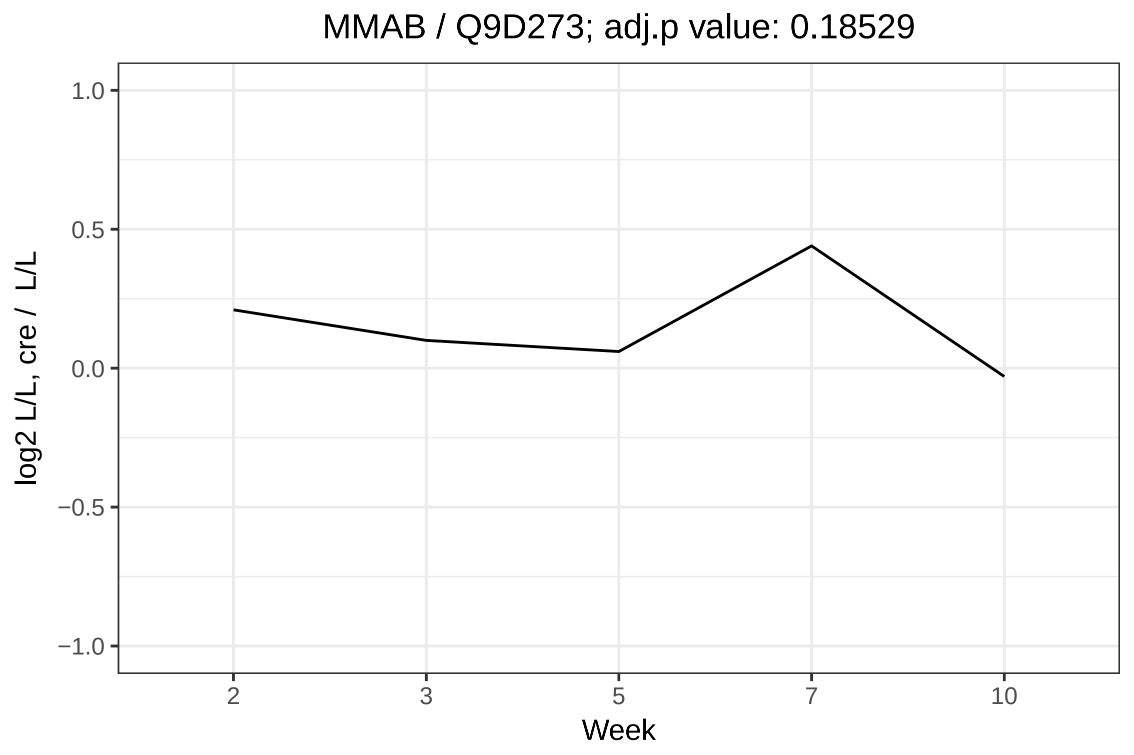

MMAB / Q9D273; adj.p value: 0.18529
log2 L/L, cre / L/L
1.0
0.5
0.0
−0.5
−1.0
2
3
5
7
10
Week

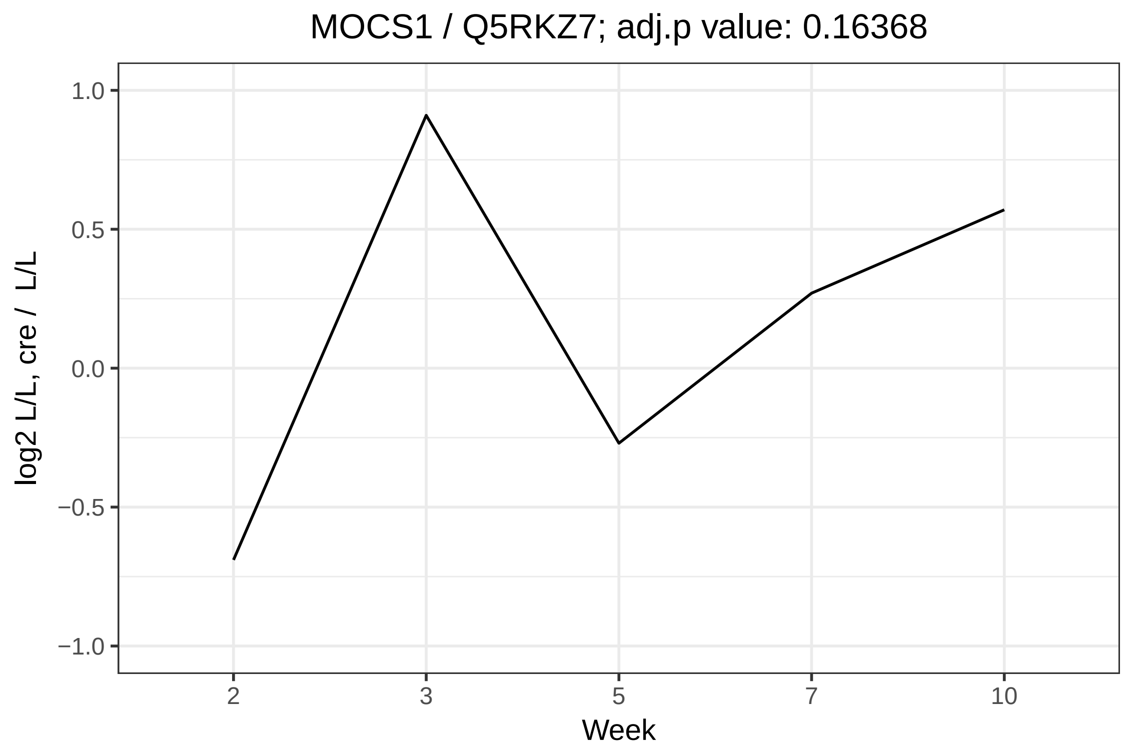

MOCS1 / Q5RKZ7; adj.p value: 0.16368
log2 L/L, cre / L/L
1.0
0.5
0.0
−0.5
−1.0
2
3
5
7
10
Week

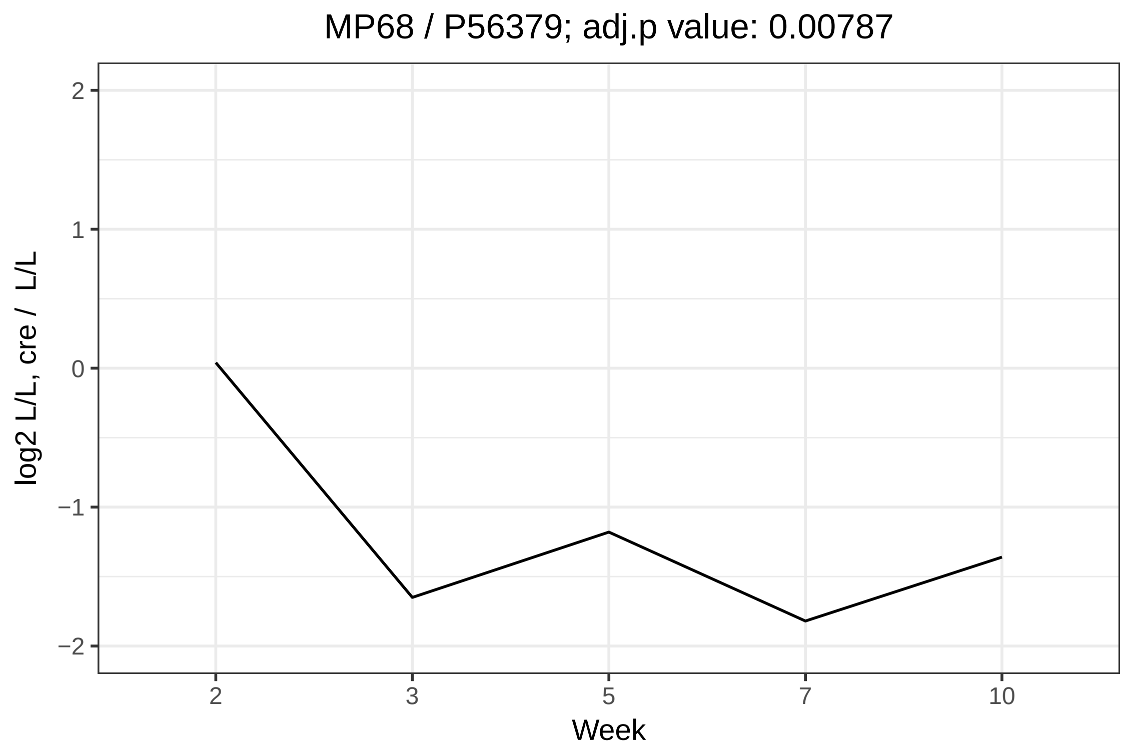

MP68 / P56379; adj.p value: 0.00787
log2 L/L, cre / L/L
2
1
0
−1
−2
2
3
5
7
10
Week

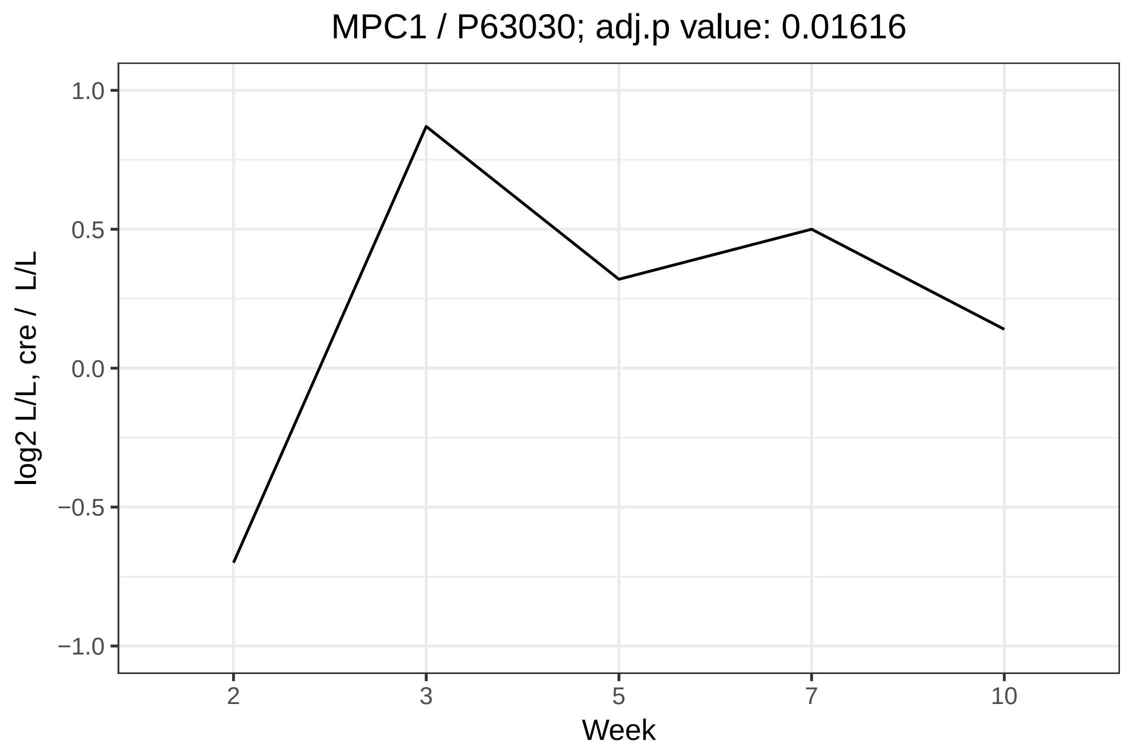
MPC1 / P63030; adj.p value: 0.01616
1.0
0.5
0.0
−0.5
−1.0
log2 L/L, cre / L/L
2
3
5
7
10
Week

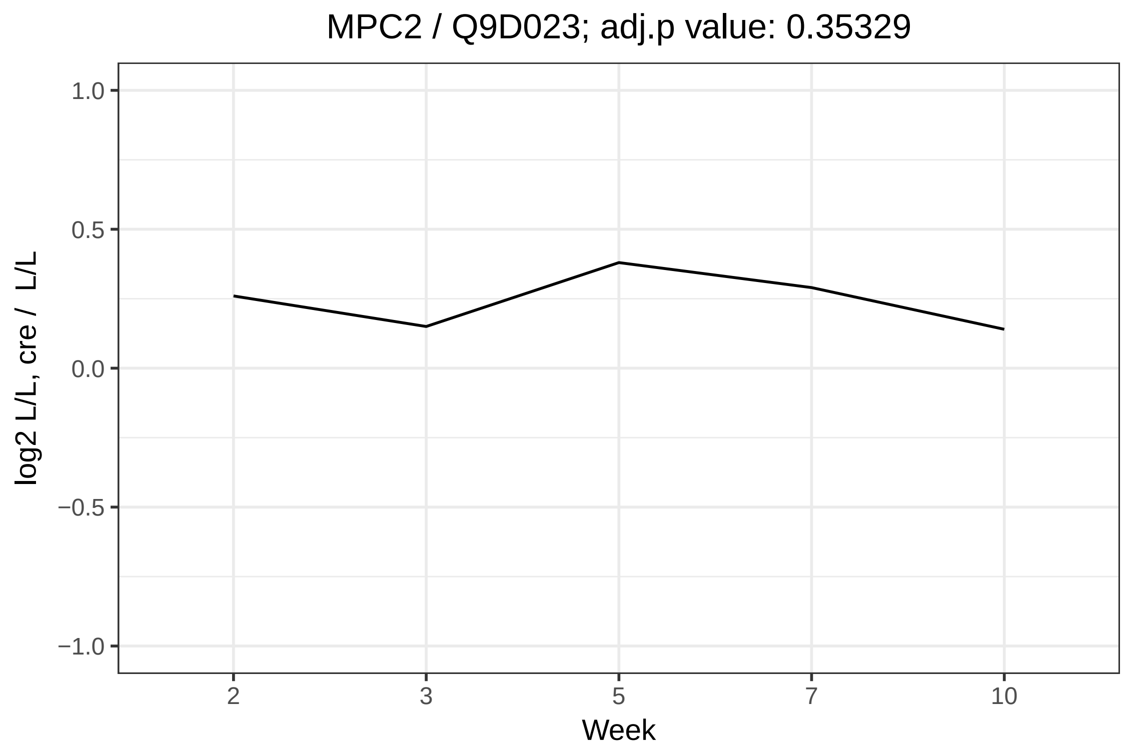
MPC2 / Q9D023; adj.p value: 0.35329
1.0
0.5
0.0
−0.5
−1.0
log2 L/L, cre / L/L
2
3
5
7
10
Week

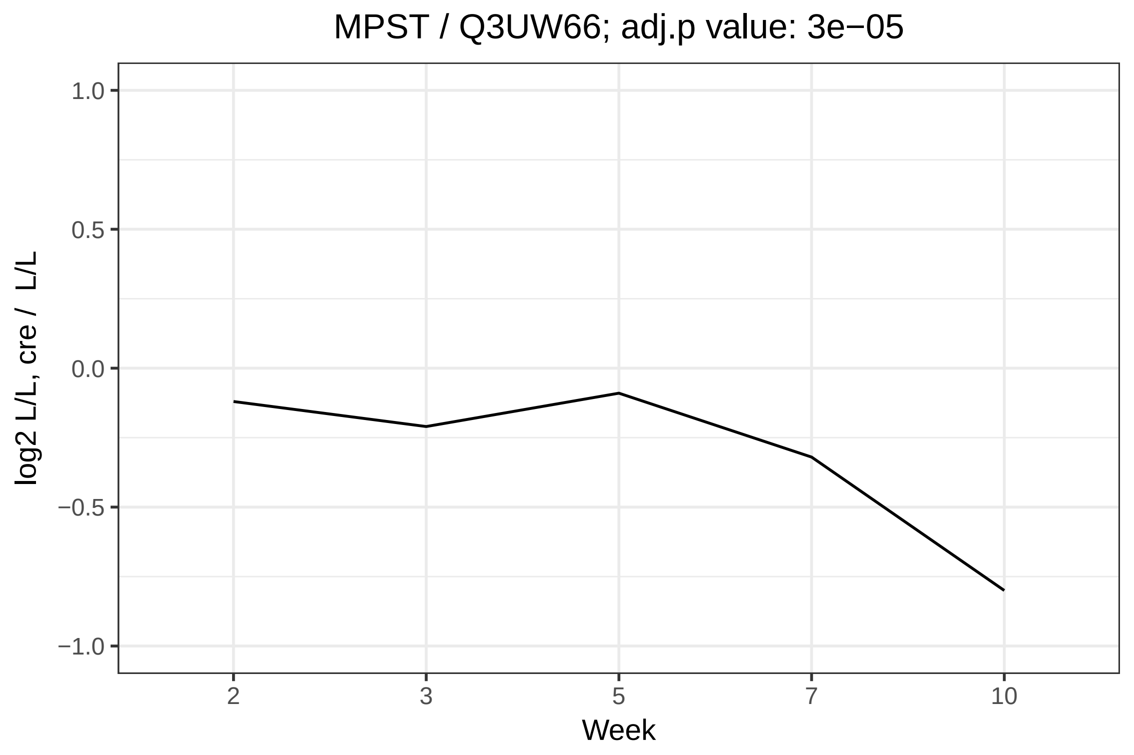
MPST / Q3UW66; adj.p value: 3e−05
log2 L/L, cre / L/L
1.0
0.5
0.0
−0.5
−1.0
2
3
5
7
10
Week

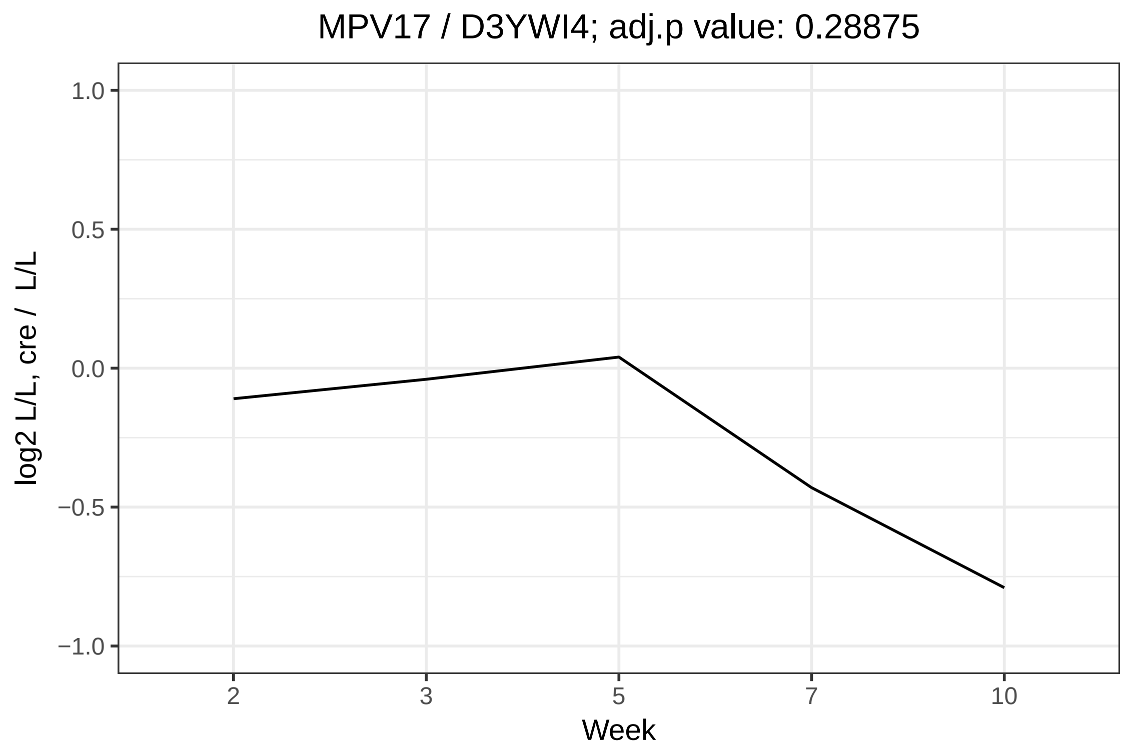

MPV17 / D3YWI4; adj.p value: 0.28875
log2 L/L, cre / L/L
1.0
0.5
0.0
−0.5
−1.0
2
3
5
7
10
Week

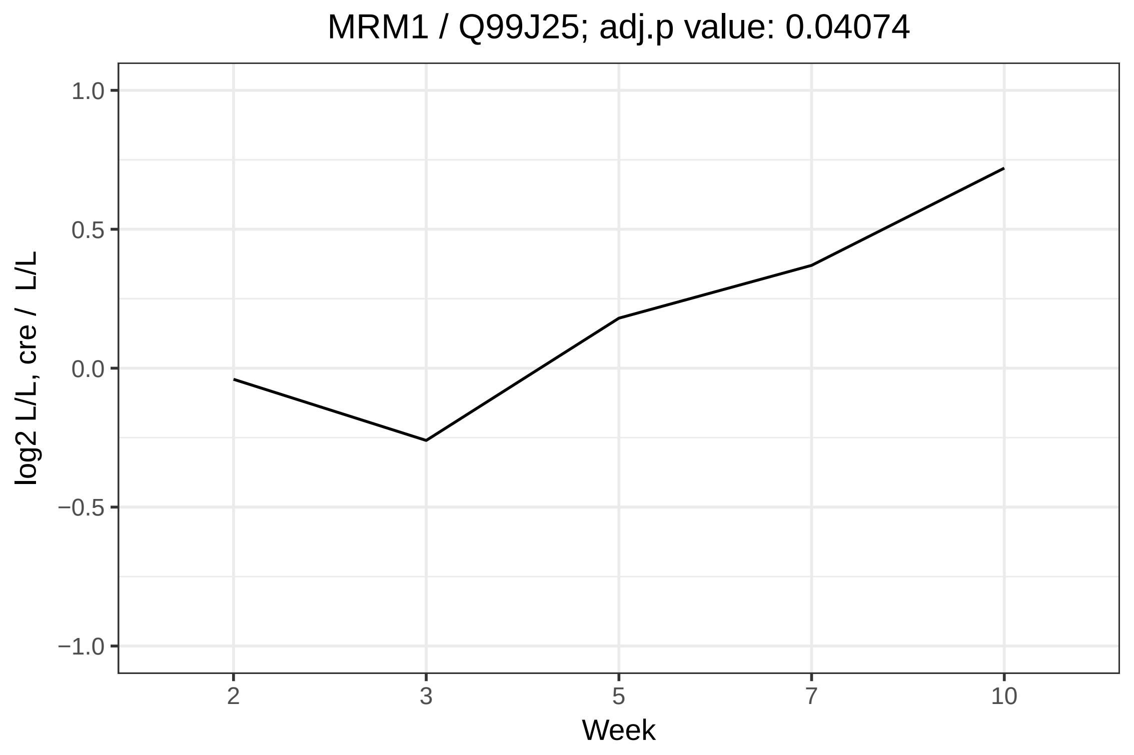

MRM1 / Q99J25; adj.p value: 0.04074
log2 L/L, cre / L/L
1.0
0.5
0.0
−0.5
−1.0
2
3
5
7
10
Week

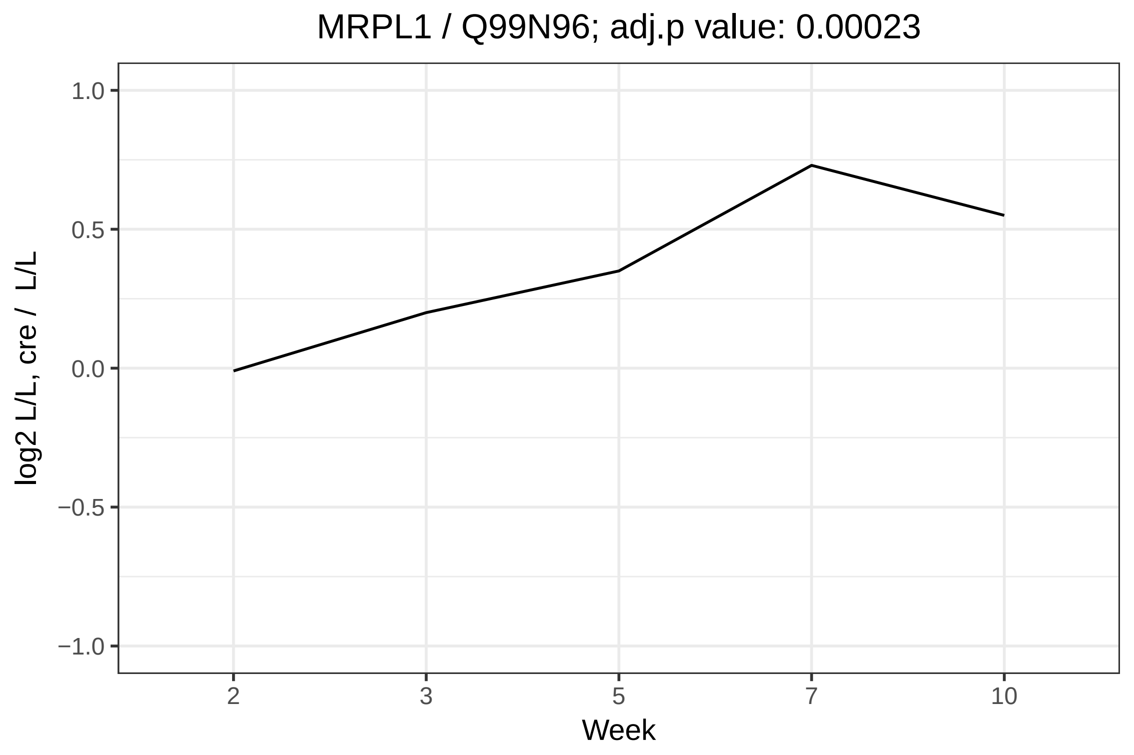
MRPL1 / Q99N96; adj.p value: 0.00023
log2 L/L, cre / L/L
1.0
0.5
0.0
−0.5
−1.0
2
3
5
7
10
Week

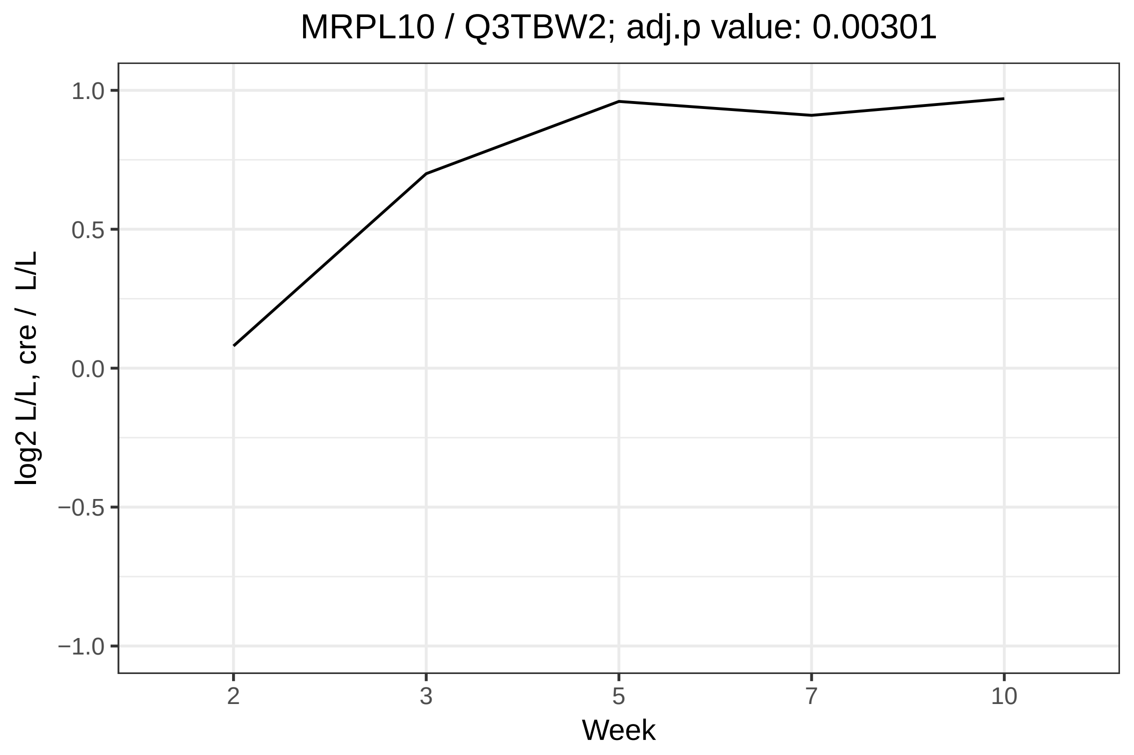

MRPL10 / Q3TBW2; adj.p value: 0.00301
log2 L/L, cre / L/L
1.0
0.5
0.0
−0.5
−1.0
2
3
5
7
10
Week

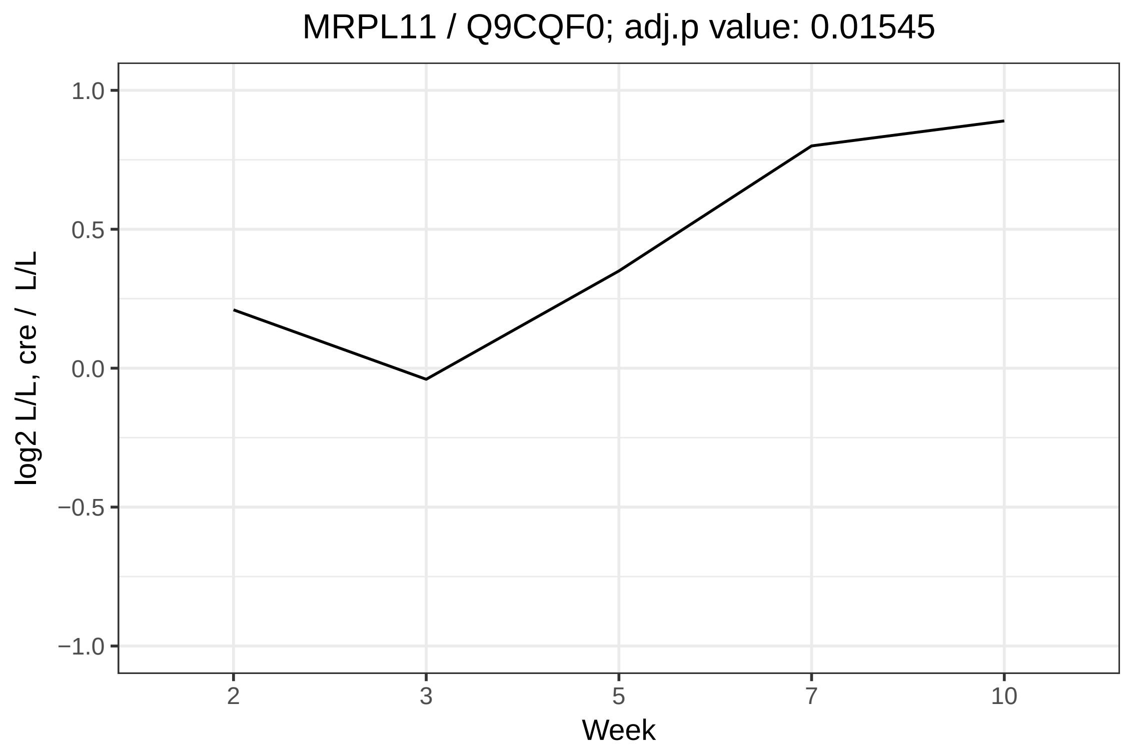

MRPL11 / Q9CQF0; adj.p value: 0.01545
1.0
0.5
0.0
−0.5
−1.0
log2 L/L, cre / L/L
2
3
5
7
10
Week

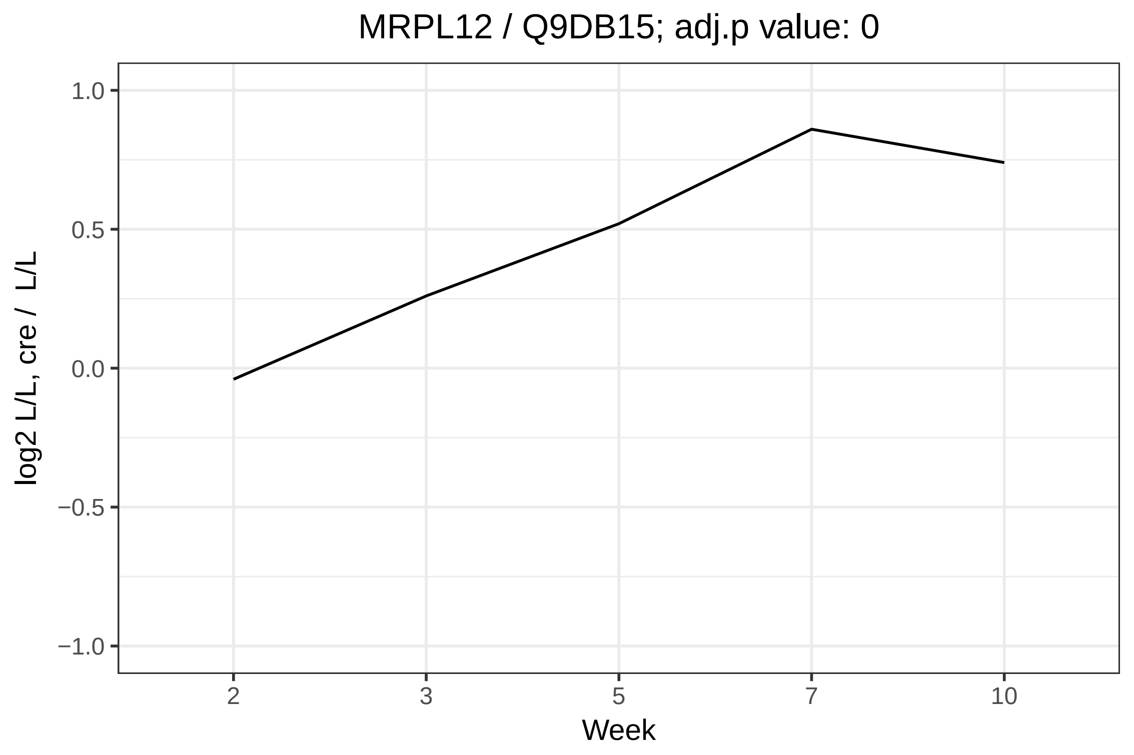
MRPL12 / Q9DB15; adj.p value: 0
log2 L/L, cre / L/L
1.0
0.5
0.0
−0.5
−1.0
2
3
5
7
10
Week

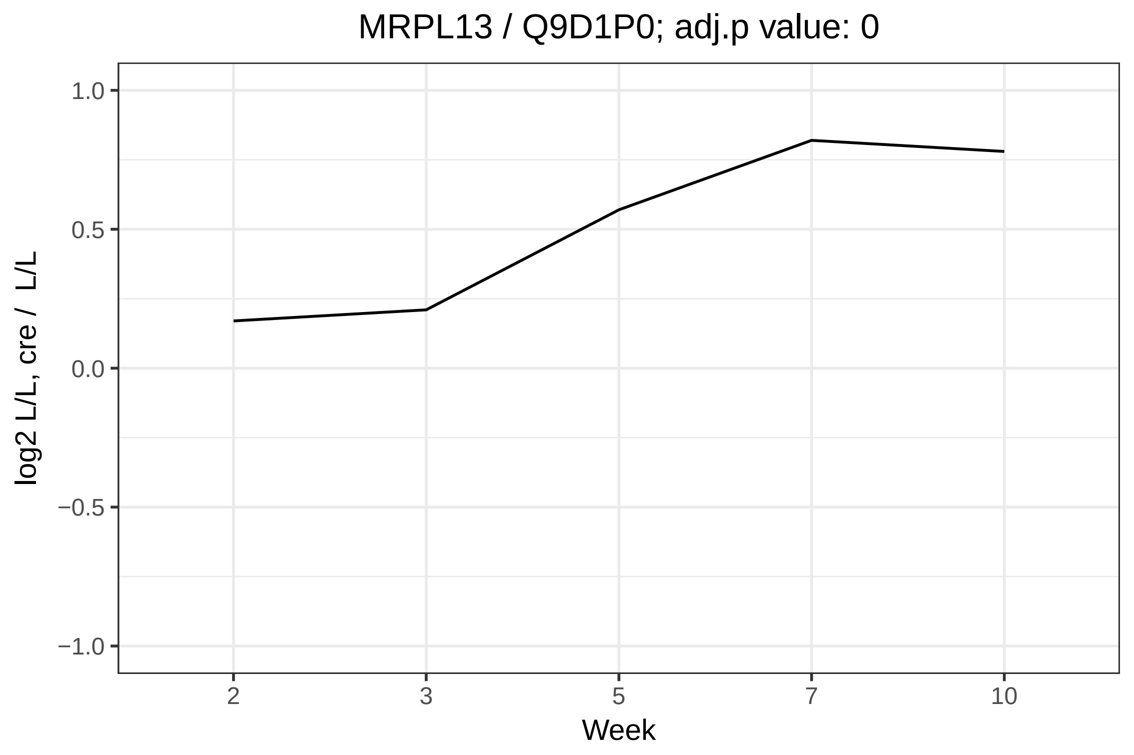
MRPL13 / Q9D1P0; adj.p value: 0
log2 L/L, cre / L/L
1.0
0.5
0.0
−0.5
−1.0
2
3
5
7
10
Week

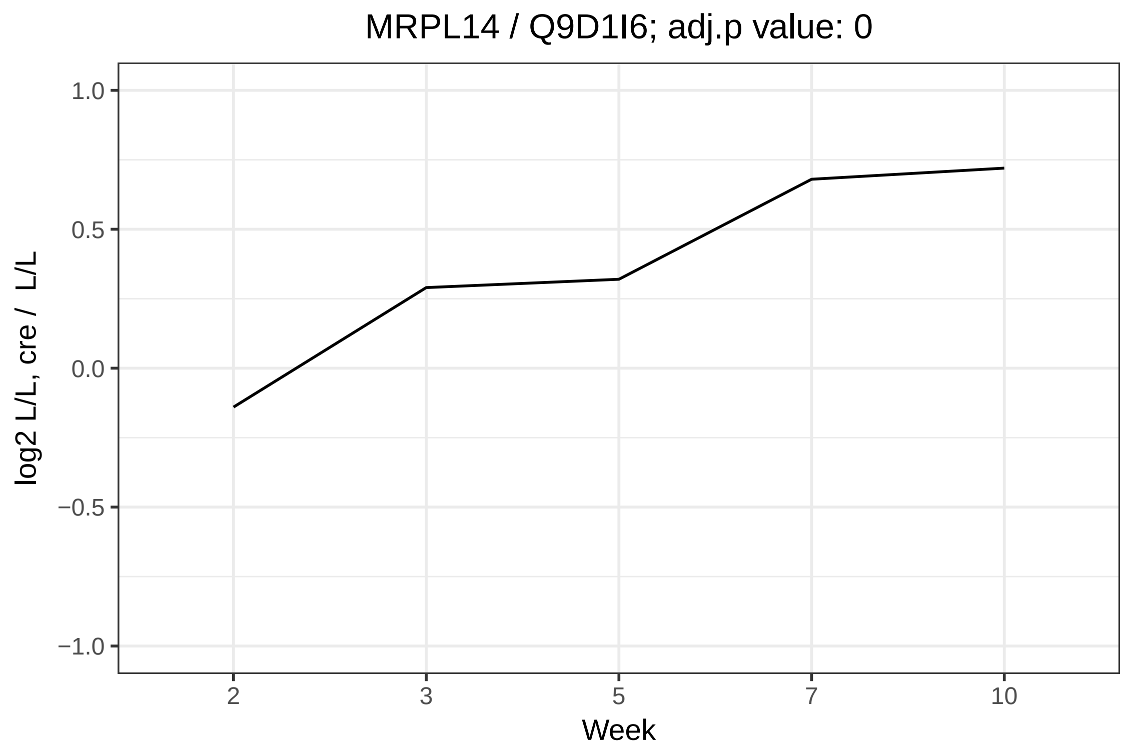

MRPL14 / Q9D1I6; adj.p value: 0
log2 L/L, cre / L/L
1.0
0.5
0.0
−0.5
−1.0
2
3
5
7
10
Week

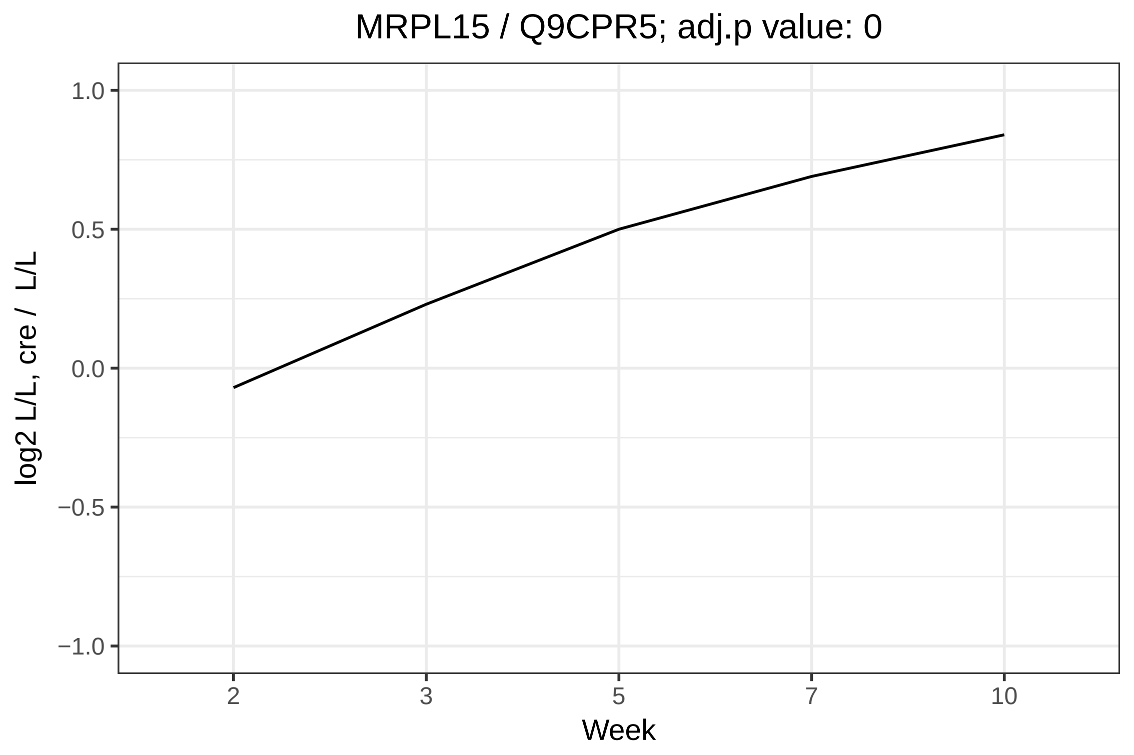
MRPL15 / Q9CPR5; adj.p value: 0
1.0
0.5
0.0
−0.5
−1.0
log2 L/L, cre / L/L
2
3
5
7
10
Week

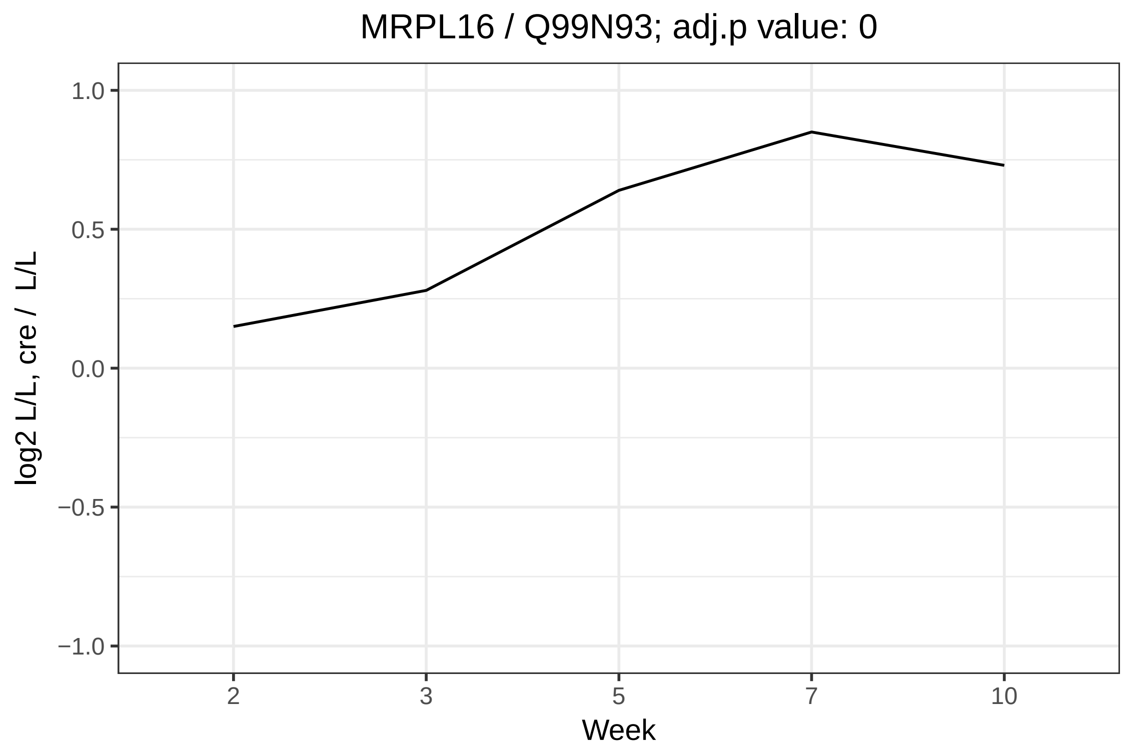

MRPL16 / Q99N93; adj.p value: 0
1.0
0.5
0.0
−0.5
−1.0
log2 L/L, cre / L/L
2
3
5
7
10
Week

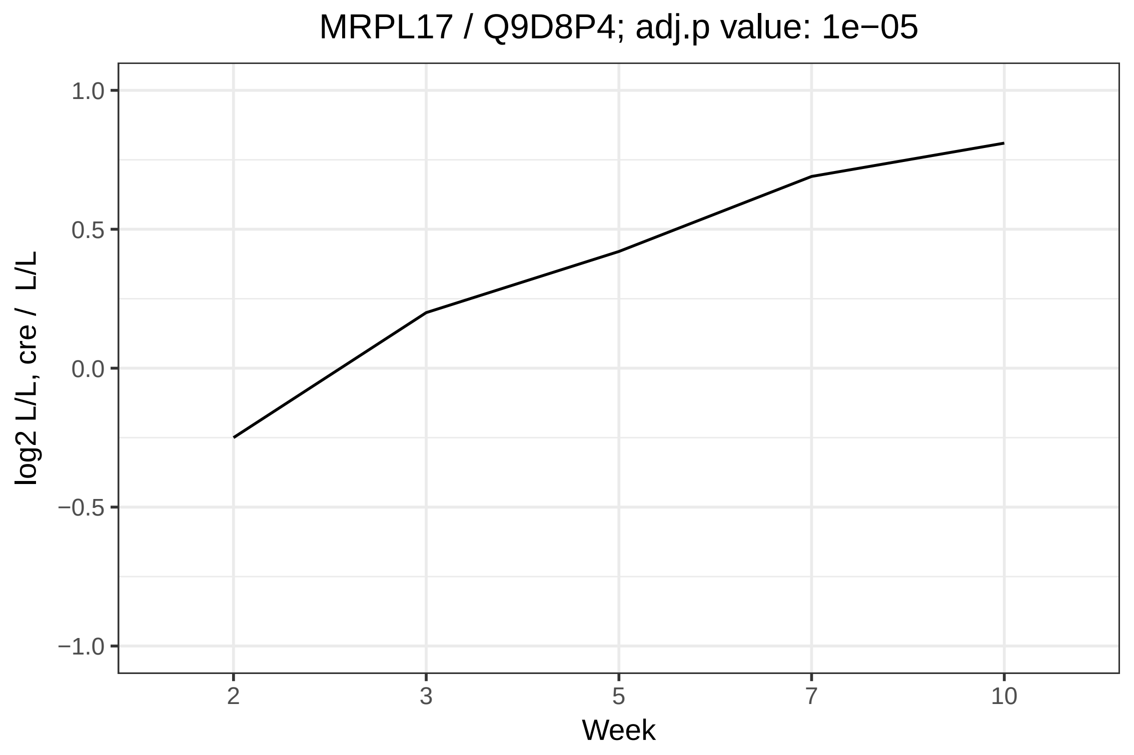
MRPL17 / Q9D8P4; adj.p value: 1e−05
log2 L/L, cre / L/L
1.0
0.5
0.0
−0.5
−1.0
2
3
5
7
10
Week

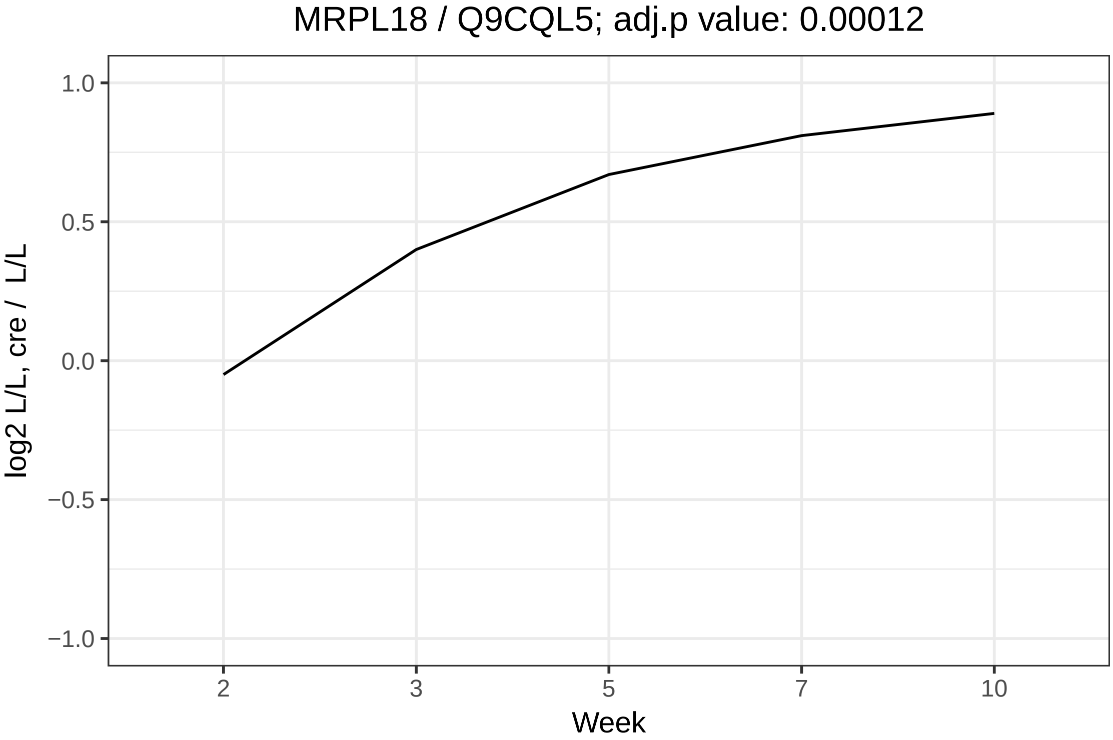
MRPL18 / Q9CQL5; adj.p value: 0.00012
1.0
0.5
0.0
−0.5
−1.0
log2 L/L, cre / L/L
2
3
5
7
10
Week

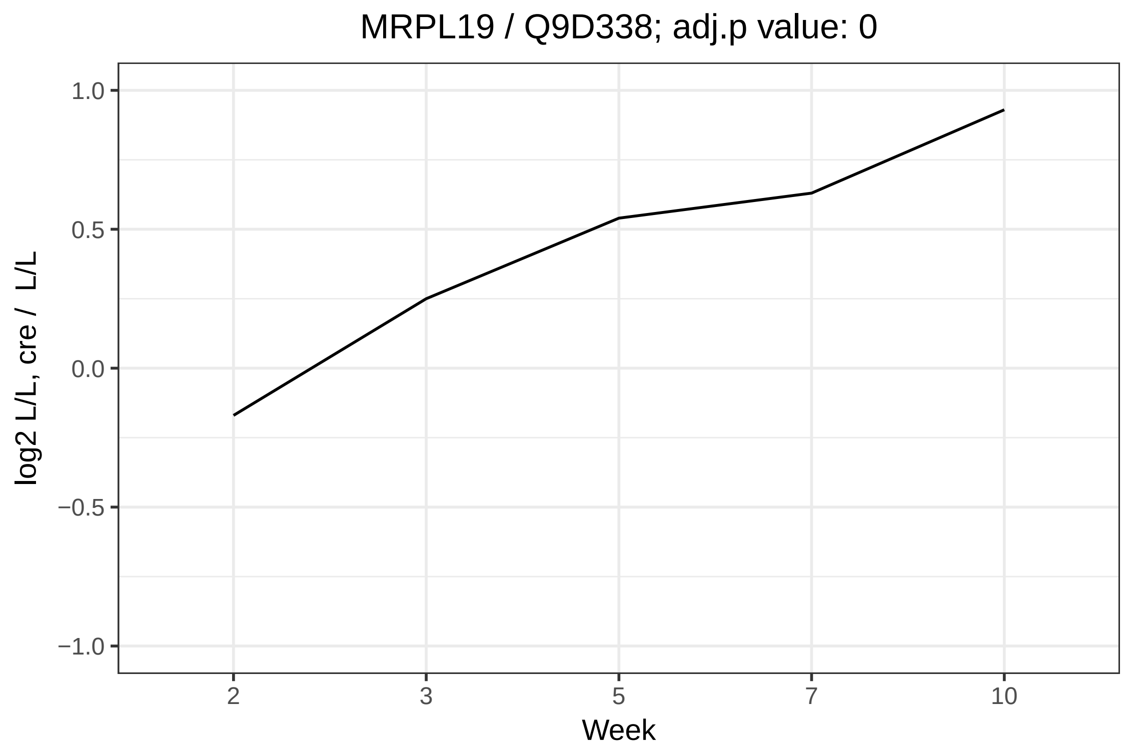

MRPL19 / Q9D338; adj.p value: 0
log2 L/L, cre / L/L
1.0
0.5
0.0
−0.5
−1.0
2
3
5
7
10
Week

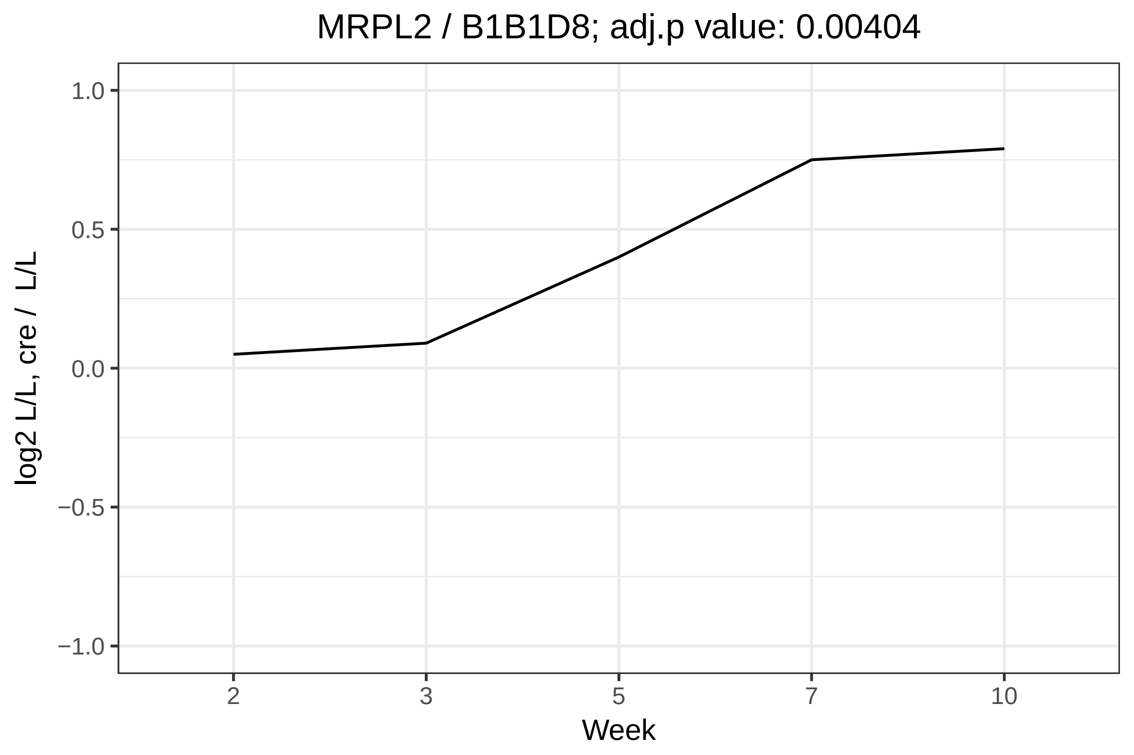

MRPL2 / B1B1D8; adj.p value: 0.00404
log2 L/L, cre / L/L
1.0
0.5
0.0
−0.5
−1.0
2
3
5
7
10
Week

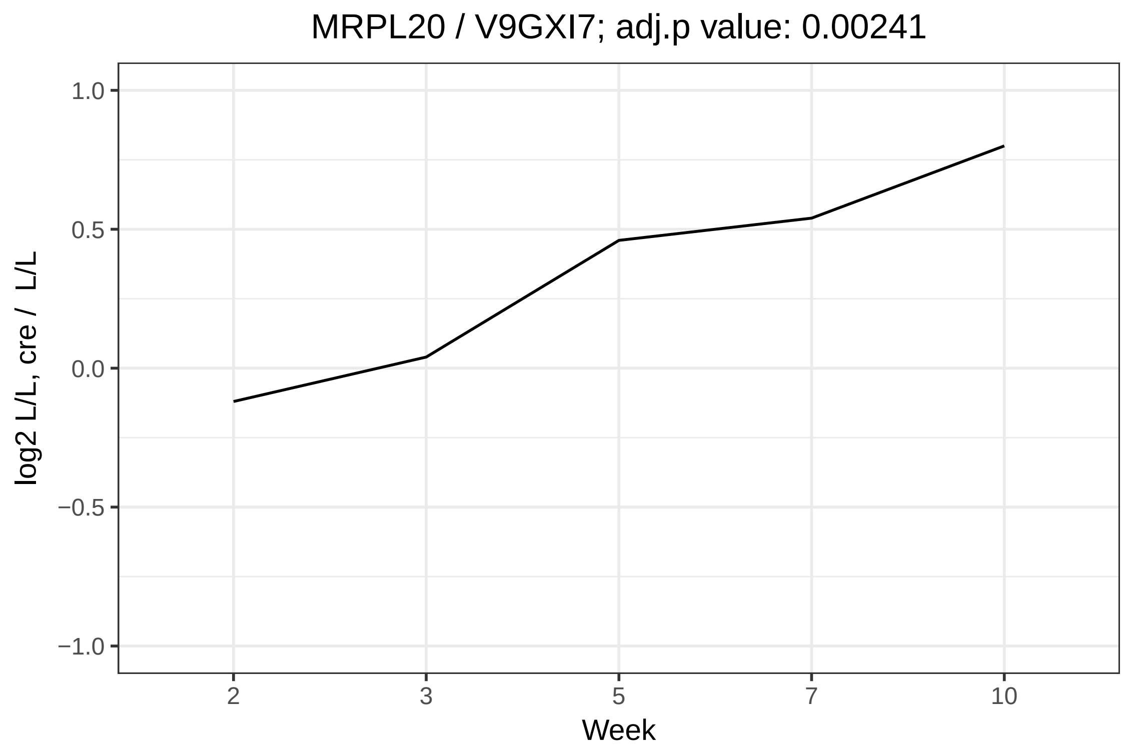

MRPL20 / V9GXI7; adj.p value: 0.00241
log2 L/L, cre / L/L
1.0
0.5
0.0
−0.5
−1.0
2
3
5
7
10
Week

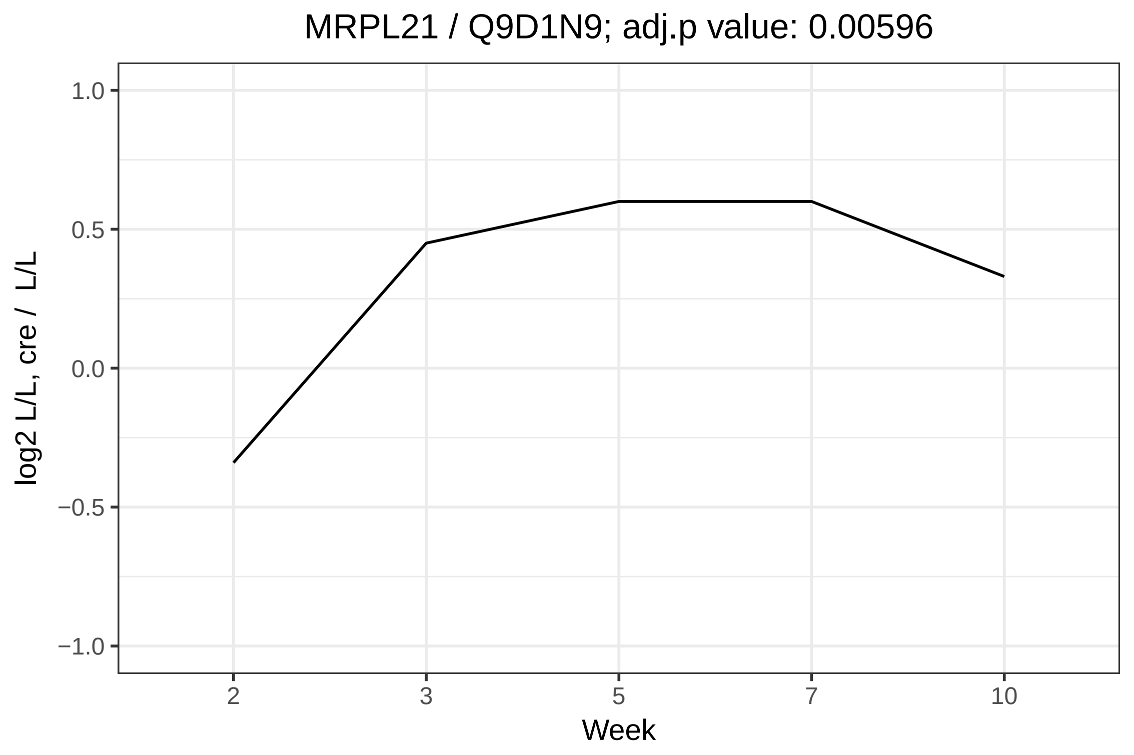

MRPL21 / Q9D1N9; adj.p value: 0.00596
1.0
0.5
0.0
−0.5
−1.0
log2 L/L, cre / L/L
2
3
5
7
10
Week

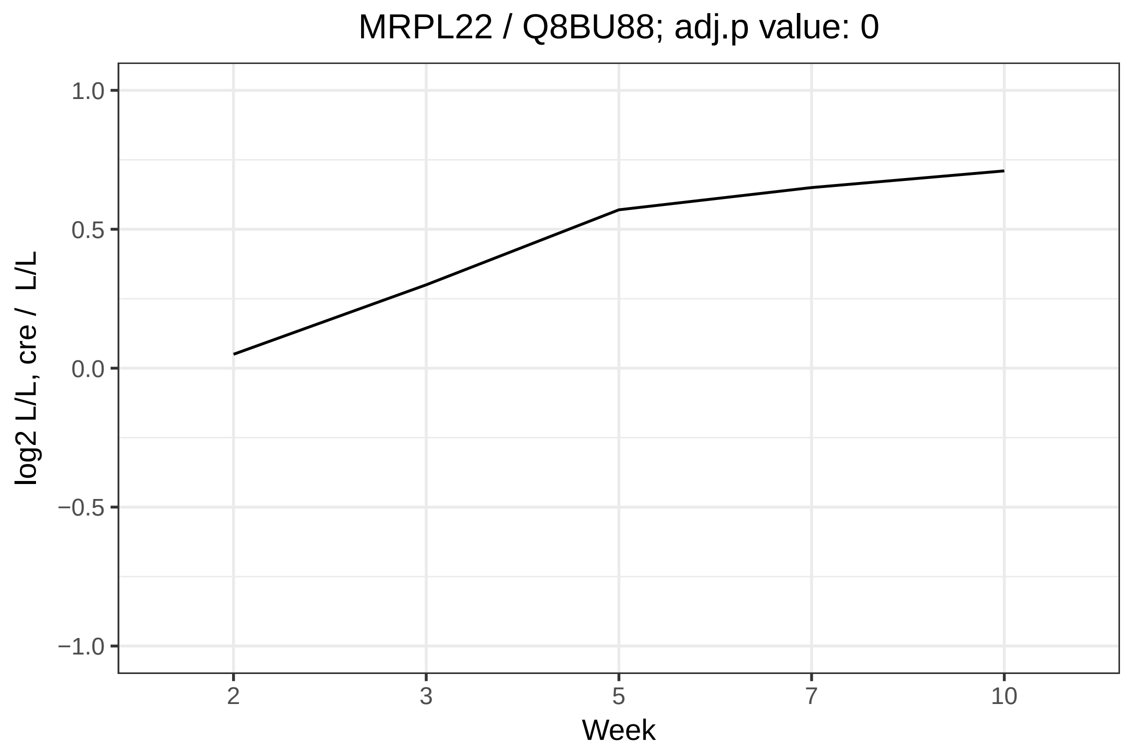

MRPL22 / Q8BU88; adj.p value: 0
1.0
0.5
0.0
−0.5
−1.0
log2 L/L, cre / L/L
2
3
5
7
10
Week

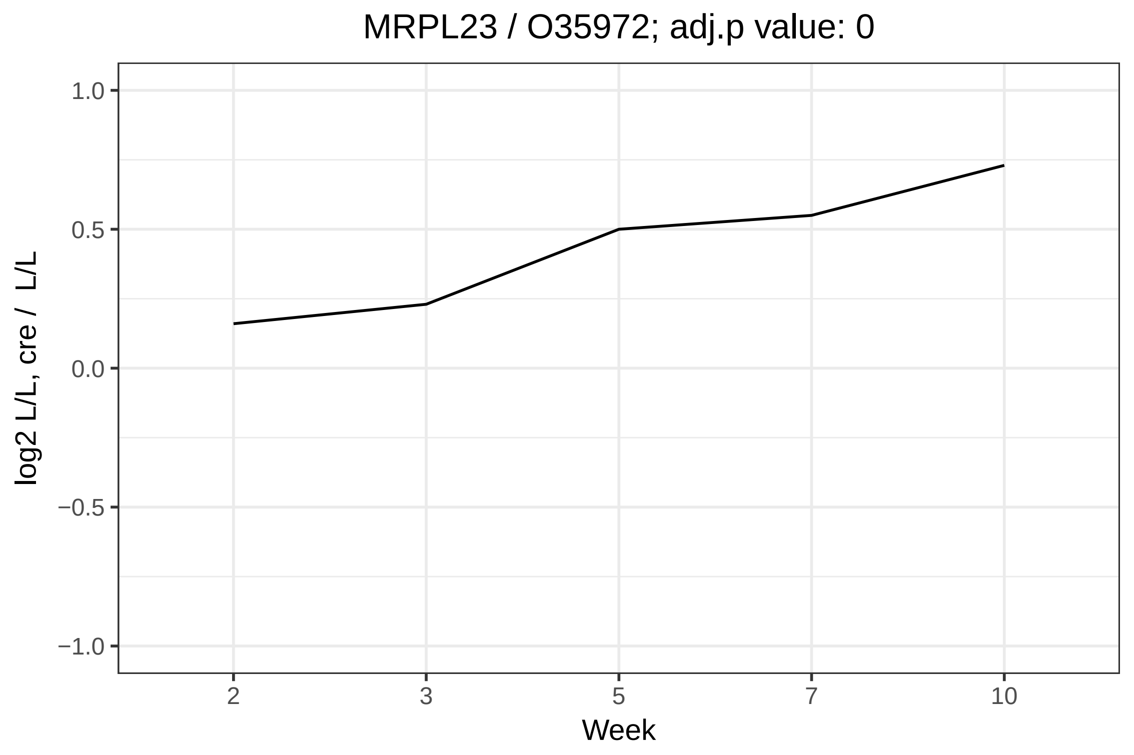
MRPL23 / O35972; adj.p value: 0
1.0
0.5
0.0
−0.5
−1.0
log2 L/L, cre / L/L
2
3
5
7
10
Week

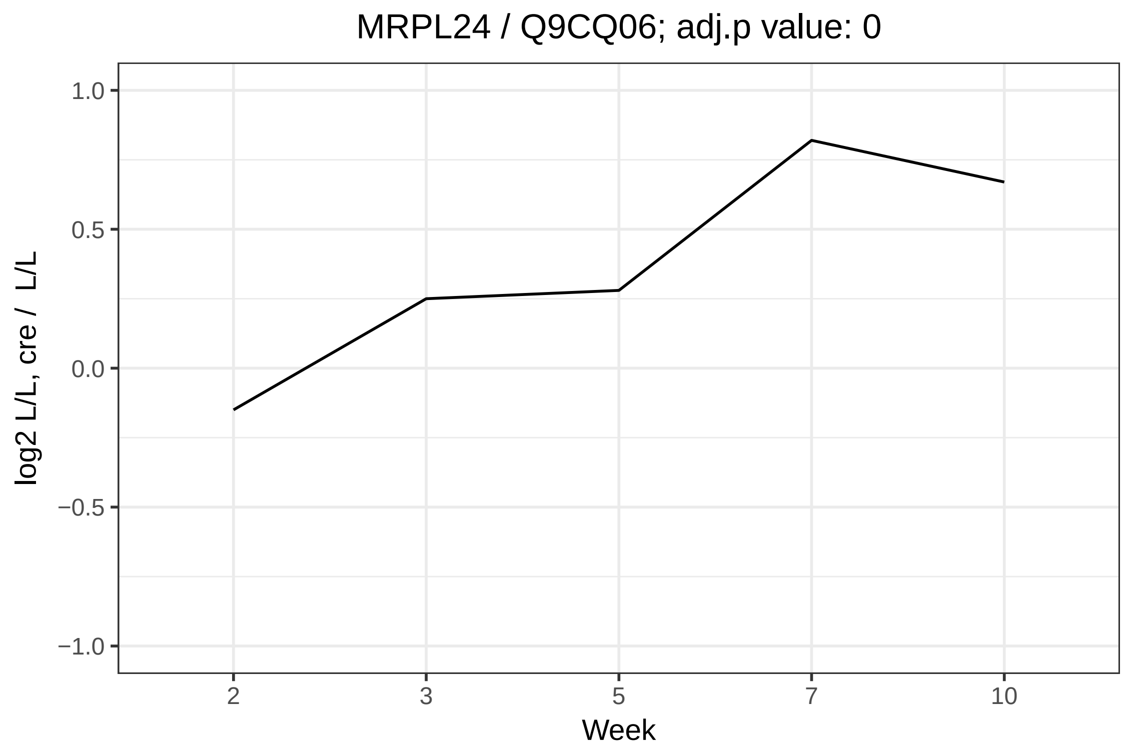

MRPL24 / Q9CQ06; adj.p value: 0
1.0
0.5
0.0
−0.5
−1.0
log2 L/L, cre / L/L
2
3
5
7
10
Week

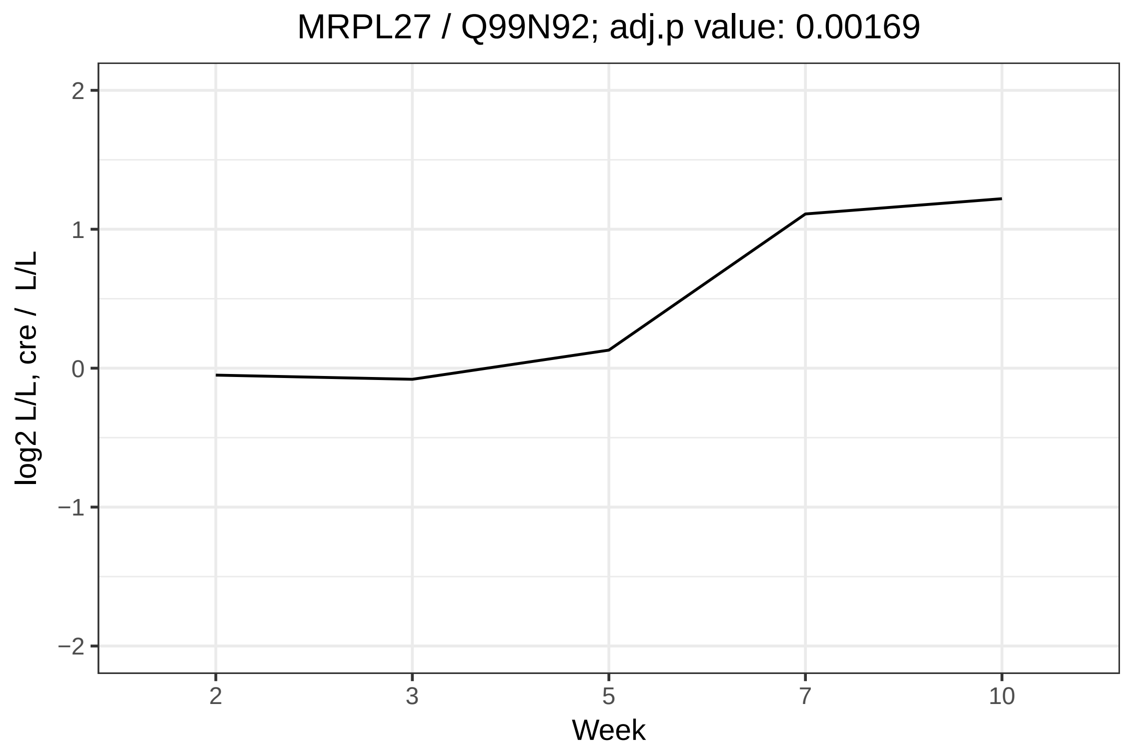
MRPL27 / Q99N92; adj.p value: 0.00169
log2 L/L, cre / L/L
2
1
0
−1
−2
2
3
5
7
10
Week

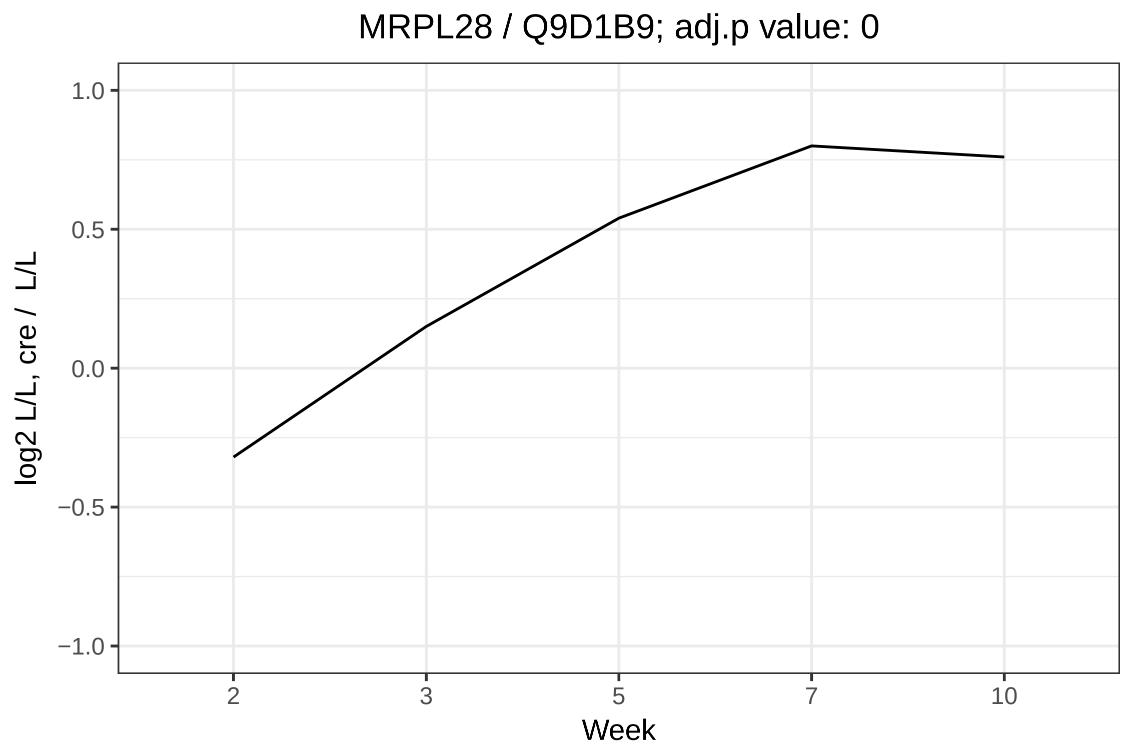

MRPL28 / Q9D1B9; adj.p value: 0
1.0
0.5
0.0
−0.5
−1.0
log2 L/L, cre / L/L
2
3
5
7
10
Week

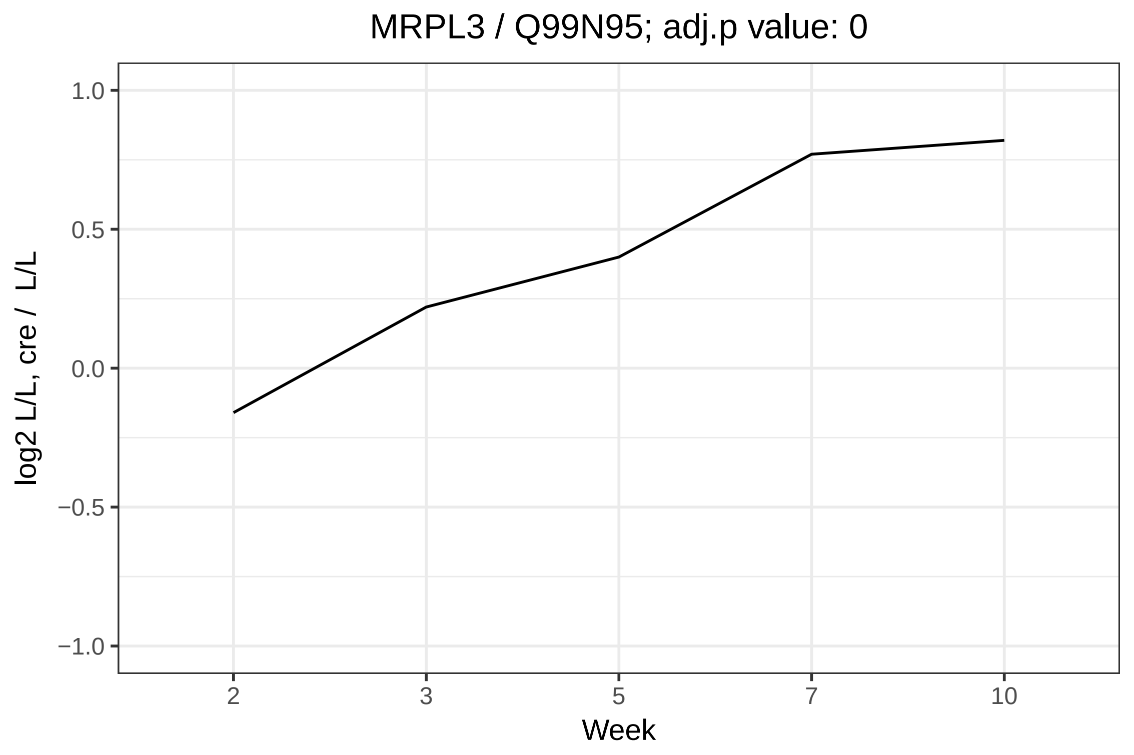

MRPL3 / Q99N95; adj.p value: 0
log2 L/L, cre / L/L
1.0
0.5
0.0
−0.5
−1.0
2
3
5
7
10
Week

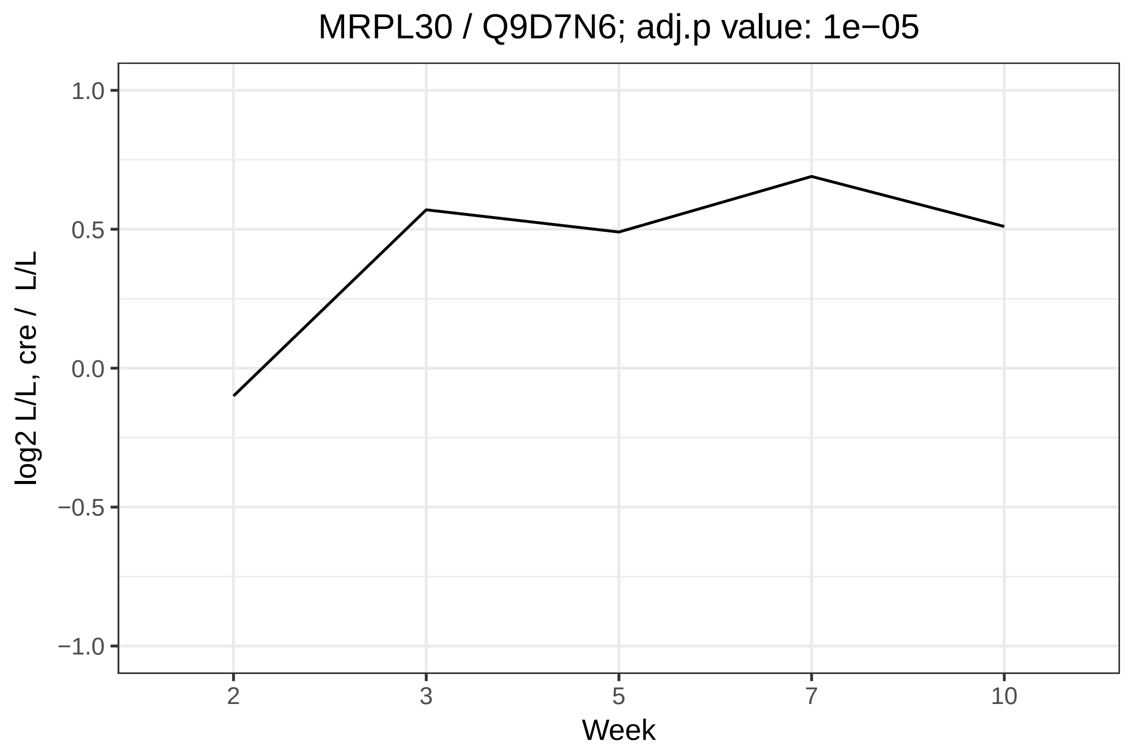
MRPL30 / Q9D7N6; adj.p value: 1e−05
log2 L/L, cre / L/L
1.0
0.5
0.0
−0.5
−1.0
2
3
5
7
10
Week

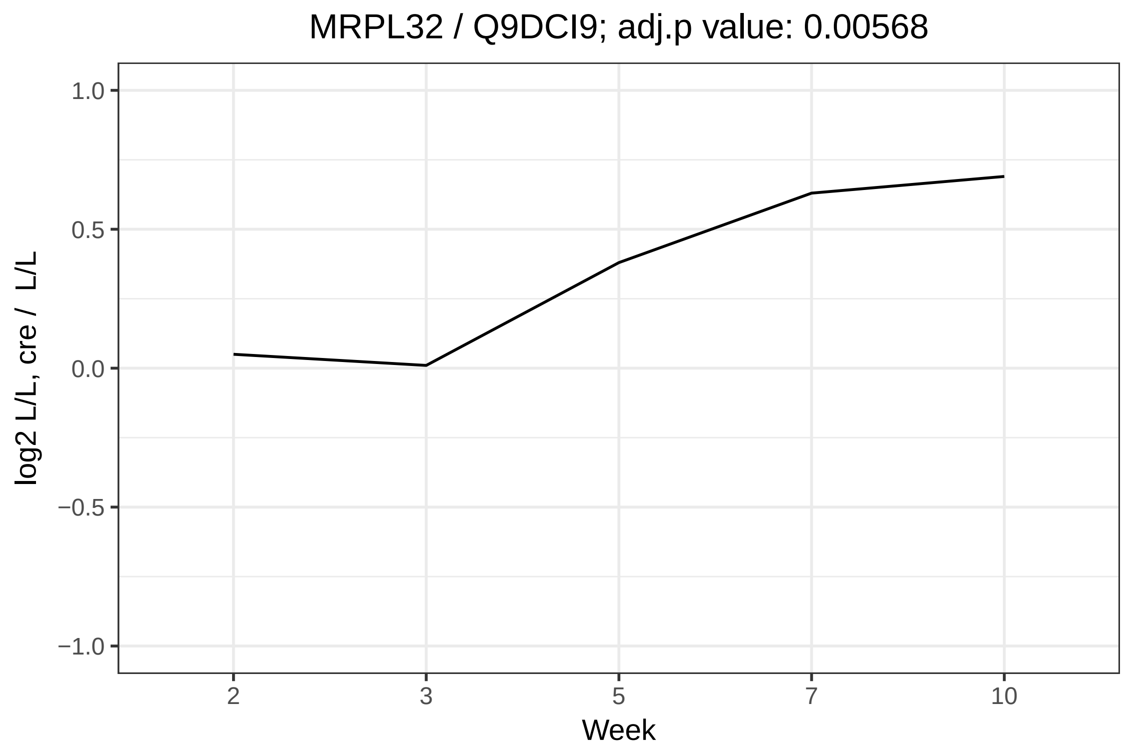
MRPL32 / Q9DCI9; adj.p value: 0.00568
log2 L/L, cre / L/L
1.0
0.5
0.0
−0.5
−1.0
2
3
5
7
10
Week

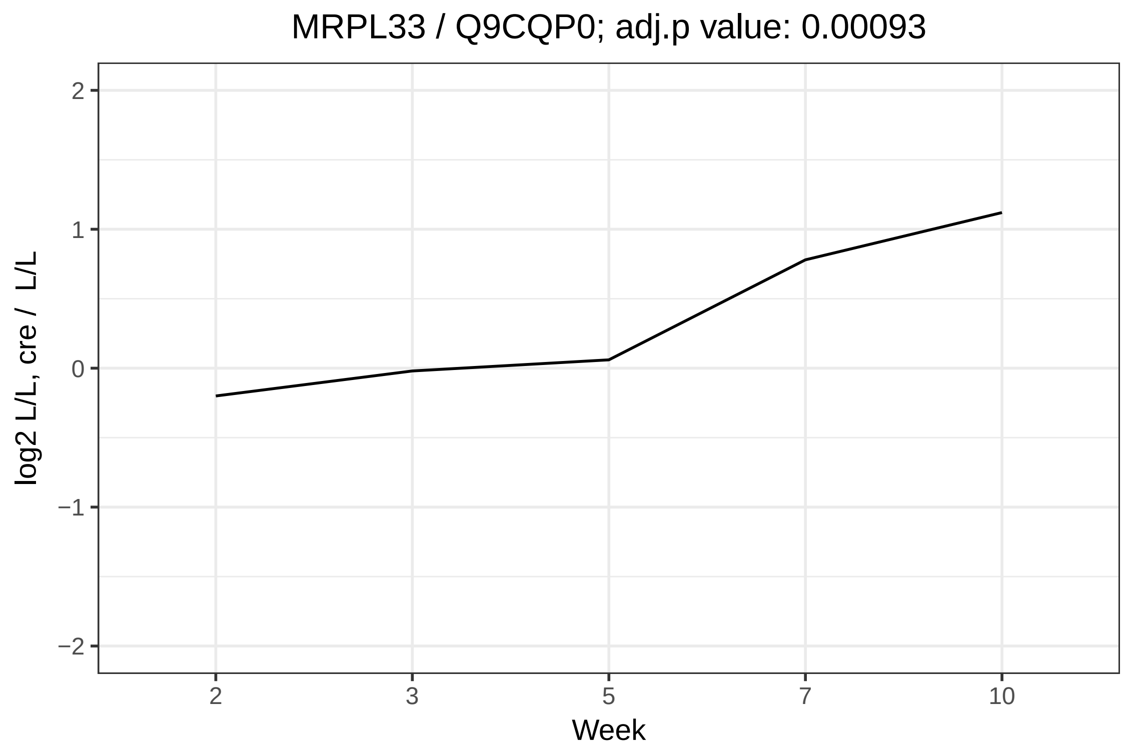
MRPL33 / Q9CQP0; adj.p value: 0.00093
log2 L/L, cre / L/L
Week
2
1
0
−1
−2
2
3
5
7
10

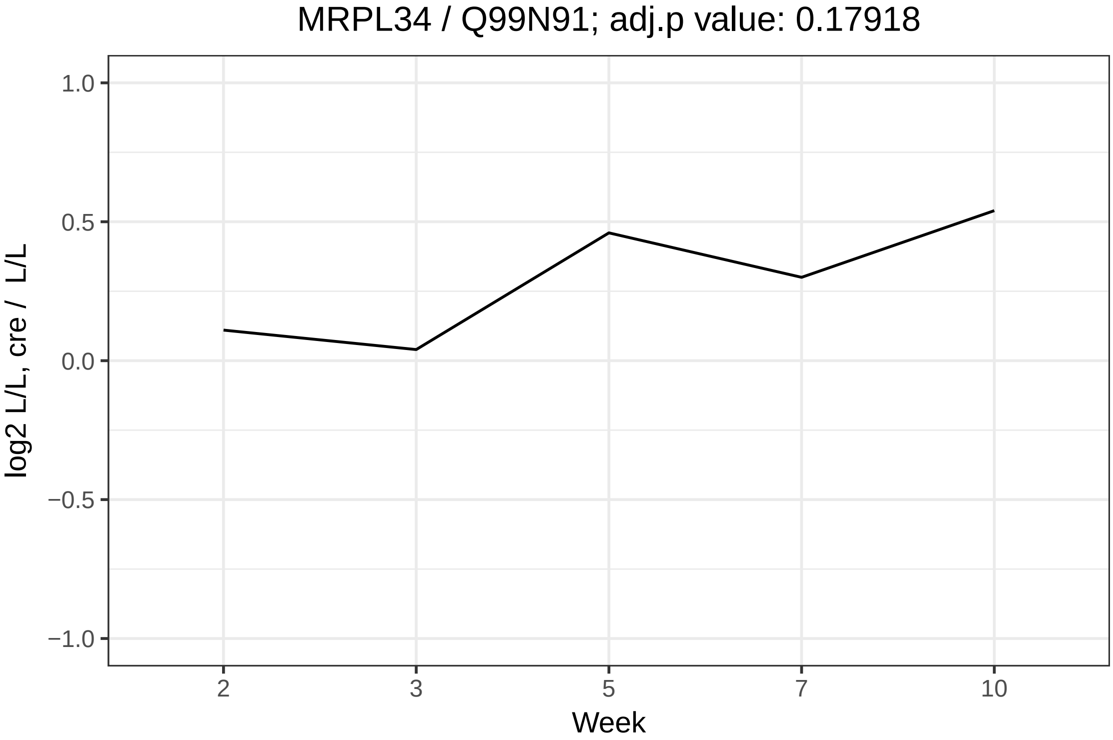
MRPL34 / Q99N91; adj.p value: 0.17918
1.0
0.5
0.0
−0.5
−1.0
log2 L/L, cre / L/L
2
3
5
7
10
Week

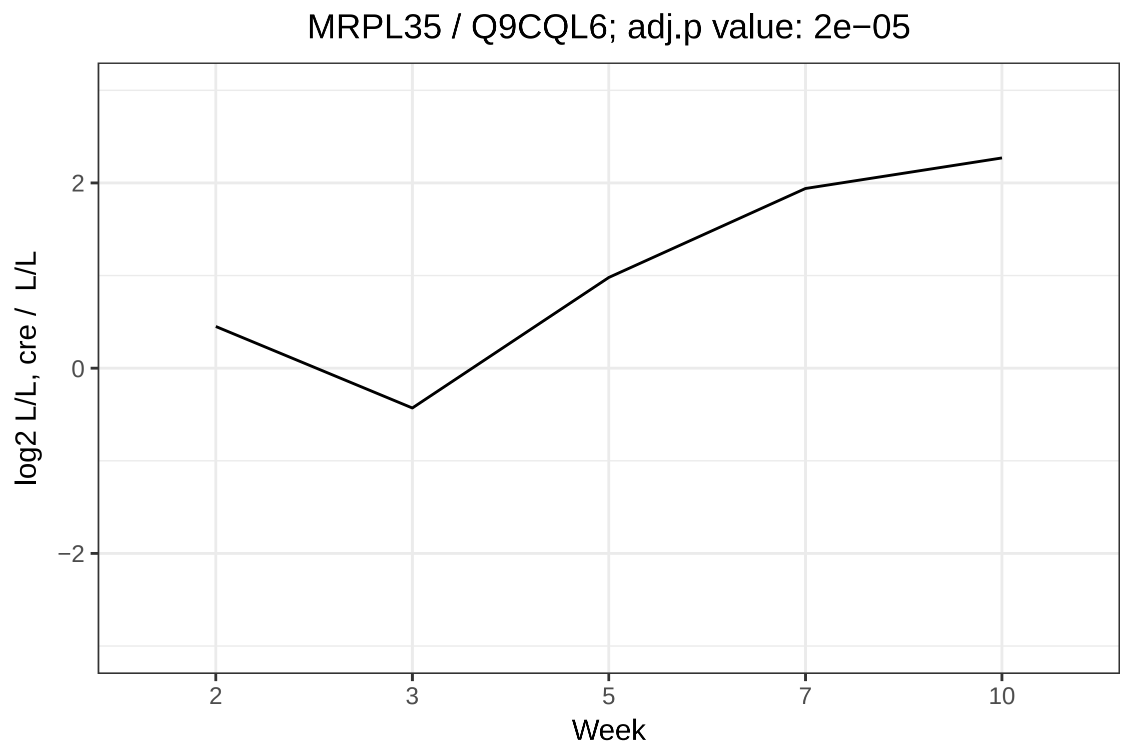

MRPL35 / Q9CQL6; adj.p value: 2e−05
log2 L/L, cre / L/L
2
0
−2
2
3
5
7
10
Week

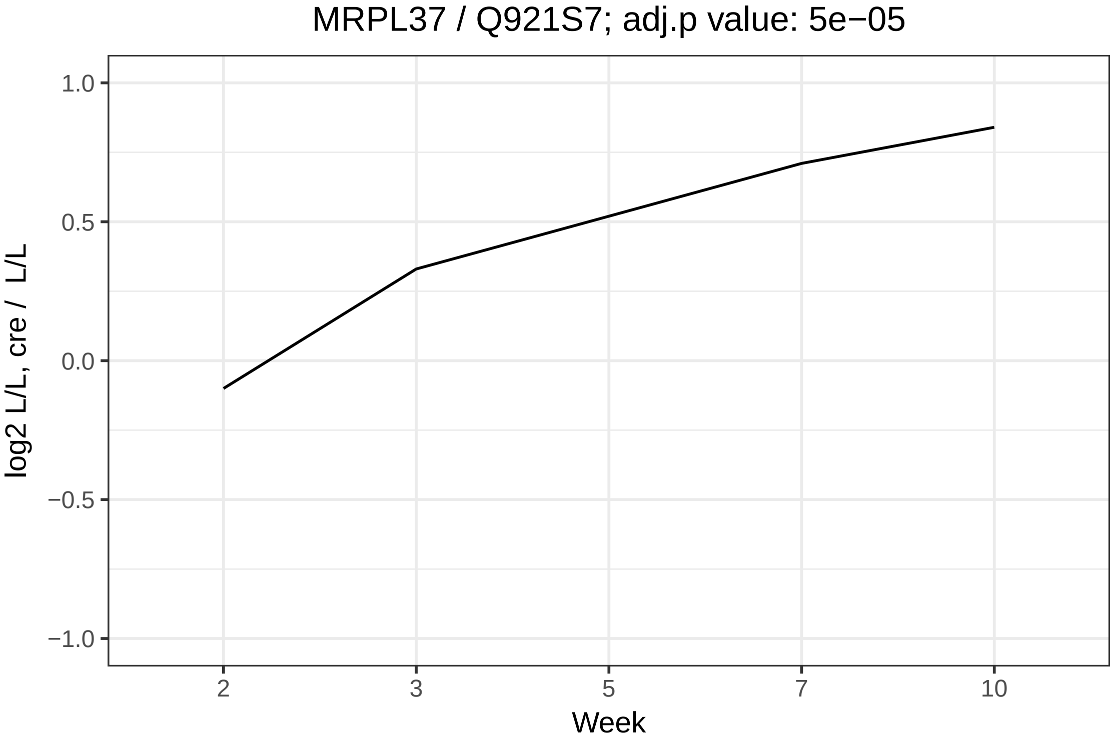

MRPL37 / Q921S7; adj.p value: 5e−05
log2 L/L, cre / L/L
1.0
0.5
0.0
−0.5
−1.0
2
3
5
7
10
Week

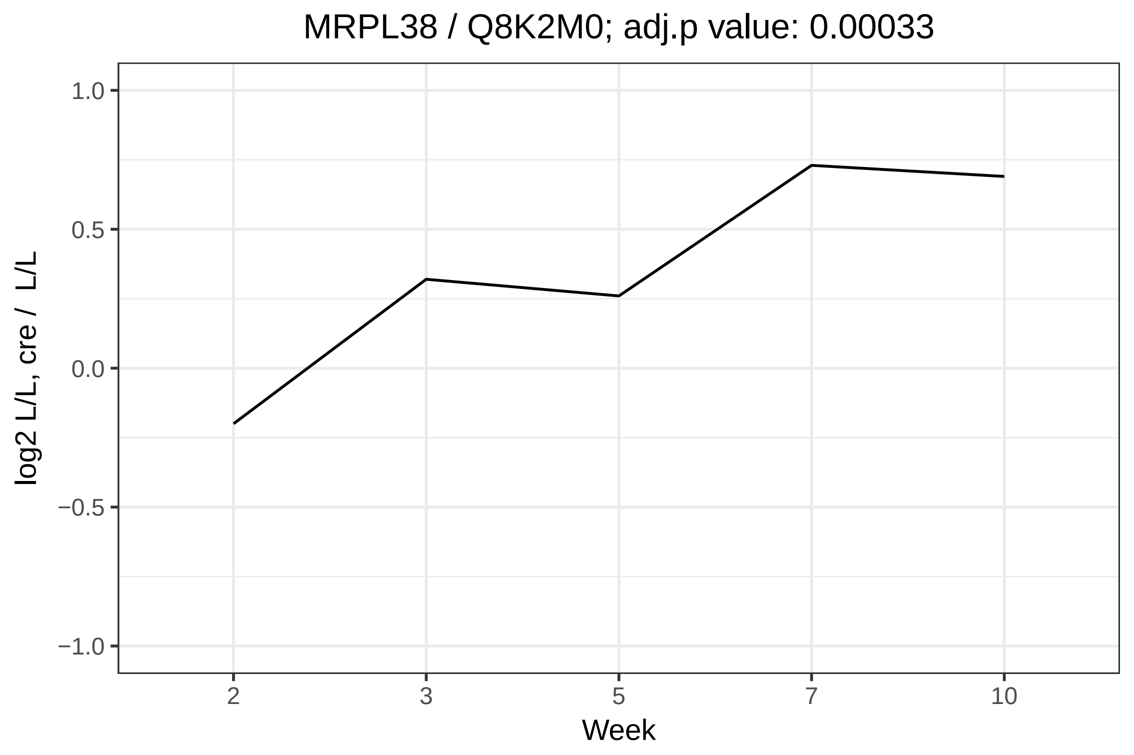

MRPL38 / Q8K2M0; adj.p value: 0.00033
log2 L/L, cre / L/L
1.0
0.5
0.0
−0.5
−1.0
2
3
5
7
10
Week

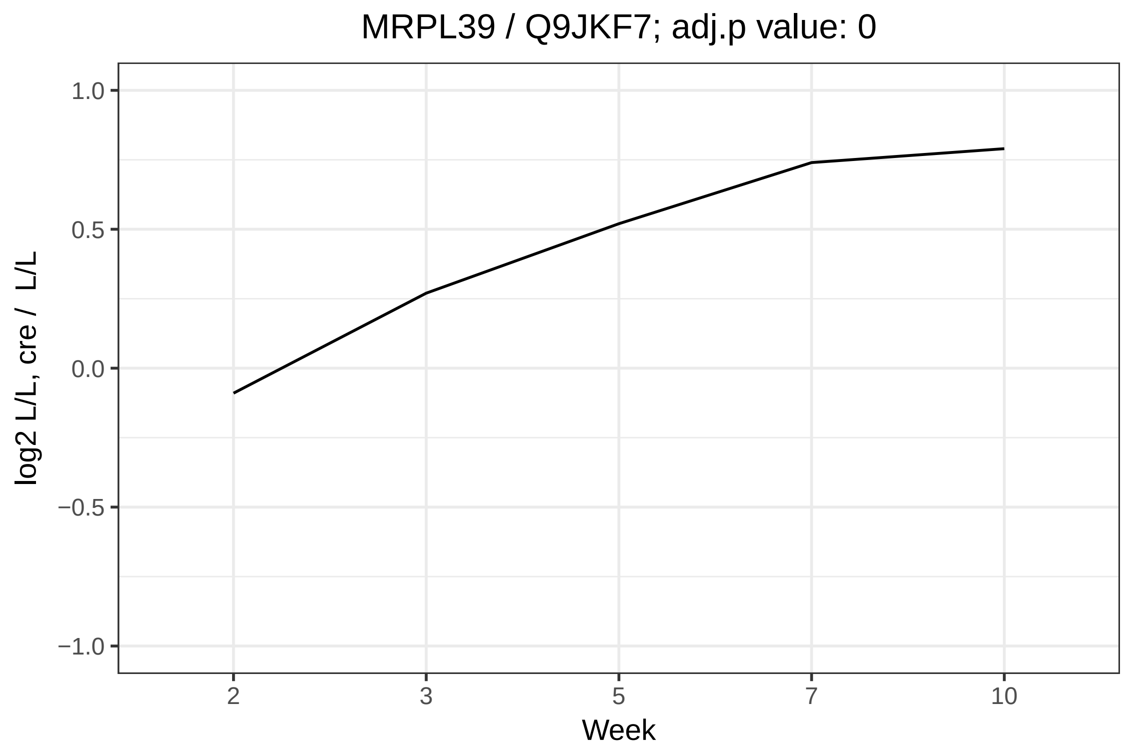

MRPL39 / Q9JKF7; adj.p value: 0
1.0
0.5
0.0
−0.5
−1.0
log2 L/L, cre / L/L
2
3
5
7
10
Week

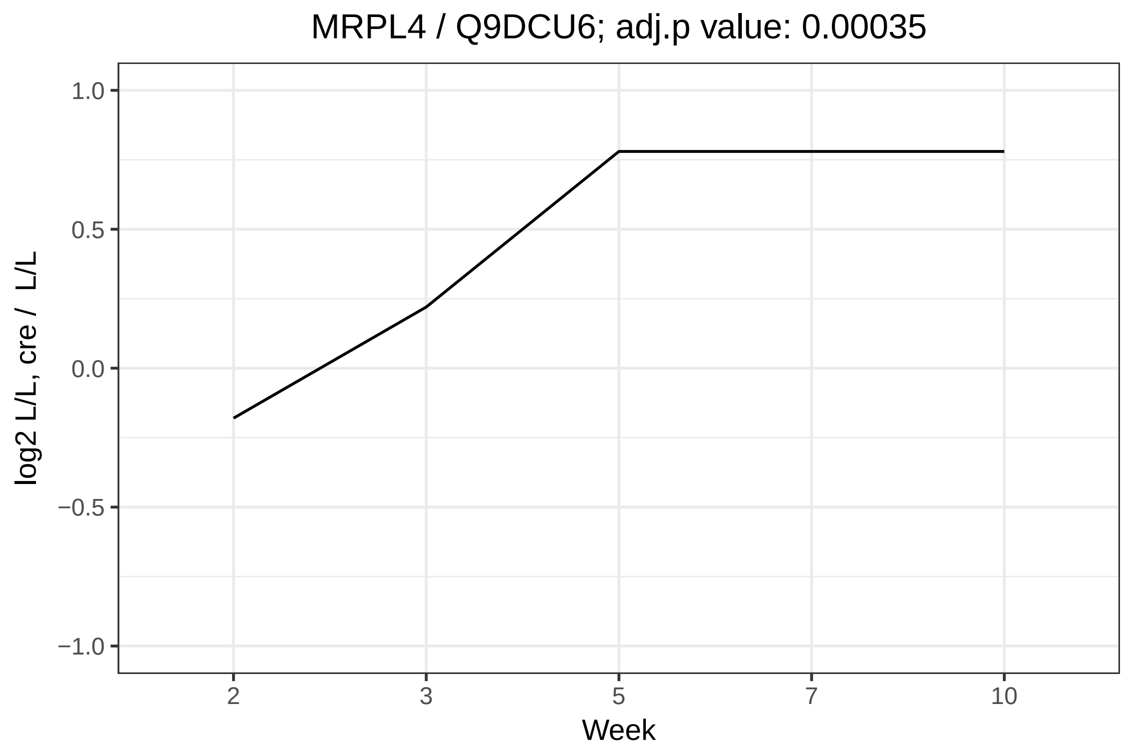
MRPL4 / Q9DCU6; adj.p value: 0.00035
log2 L/L, cre / L/L
1.0
0.5
0.0
−0.5
−1.0
2
3
5
7
10
Week

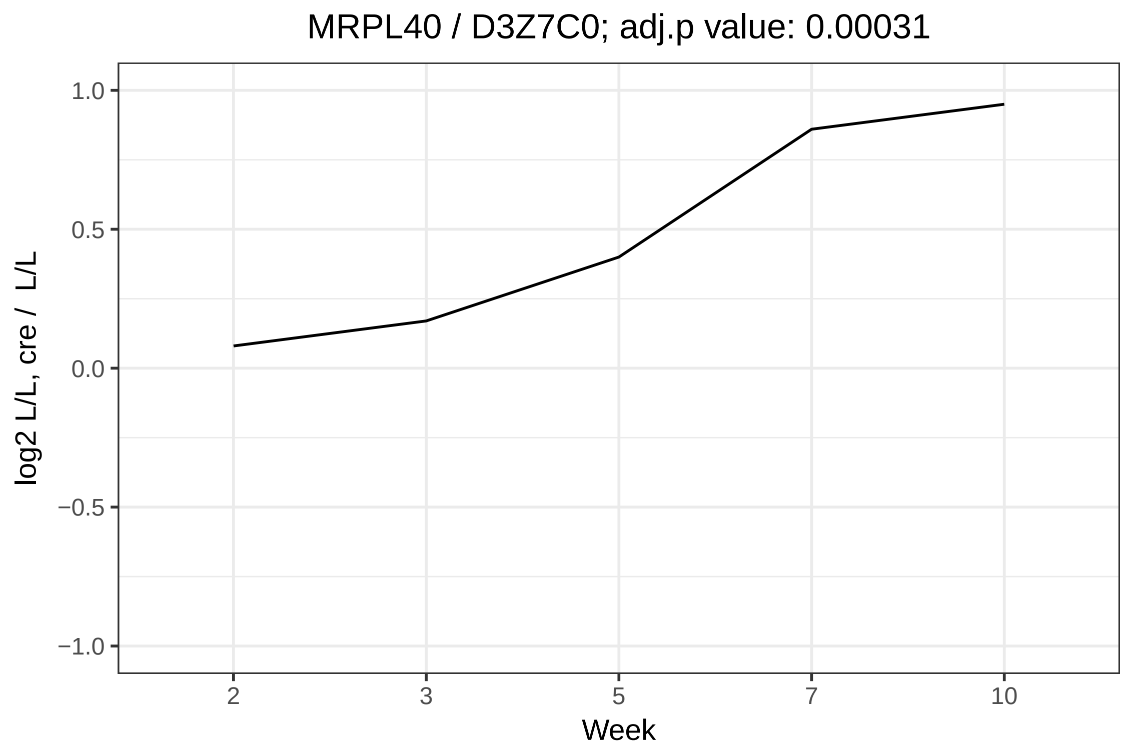
MRPL40 / D3Z7C0; adj.p value: 0.00031
log2 L/L, cre / L/L
1.0
0.5
0.0
−0.5
−1.0
2
3
5
7
10
Week

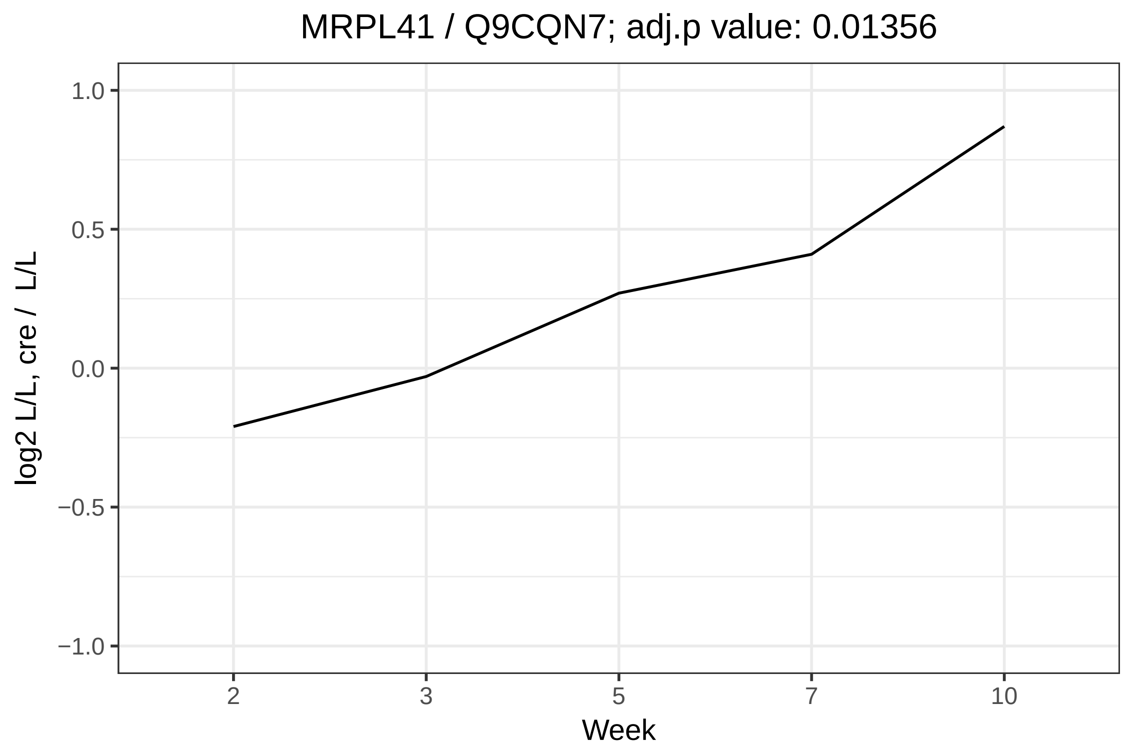
MRPL41 / Q9CQN7; adj.p value: 0.01356
1.0
0.5
0.0
−0.5
−1.0
log2 L/L, cre / L/L
2
3
5
7
10
Week

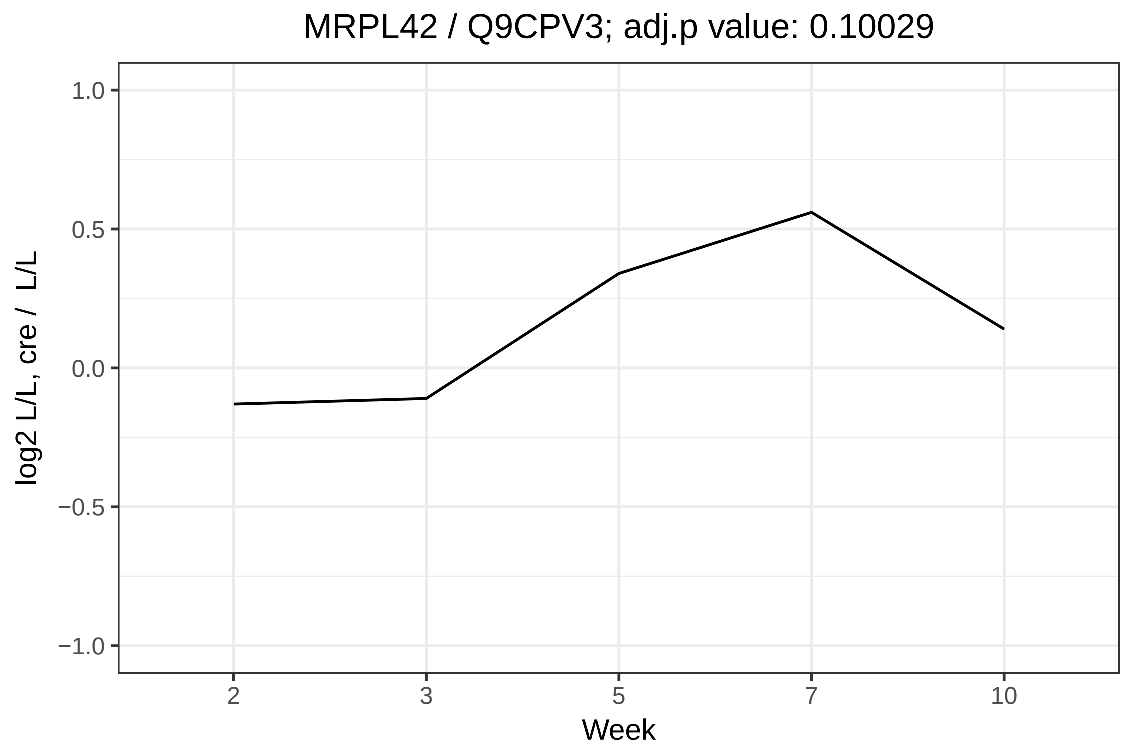

MRPL42 / Q9CPV3; adj.p value: 0.10029
log2 L/L, cre / L/L
1.0
0.5
0.0
−0.5
−1.0
2
3
5
7
10
Week

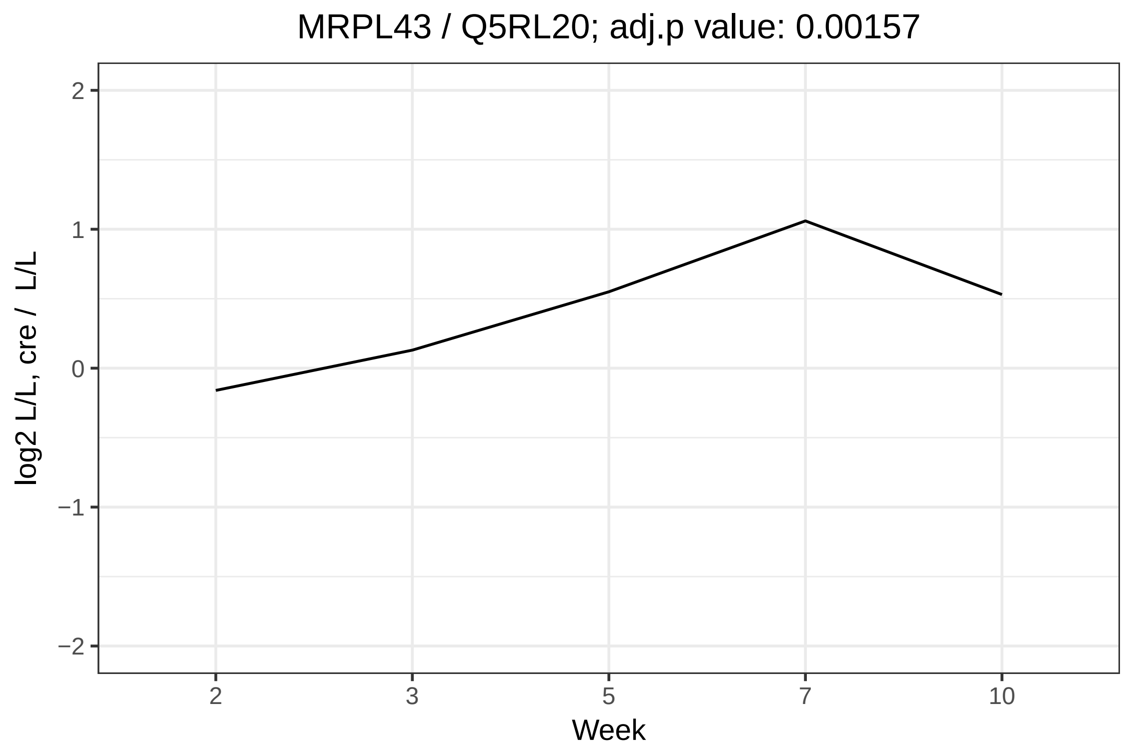
MRPL43 / Q5RL20; adj.p value: 0.00157
log2 L/L, cre / L/L
2
1
0
−1
−2
2
3
5
7
10
Week

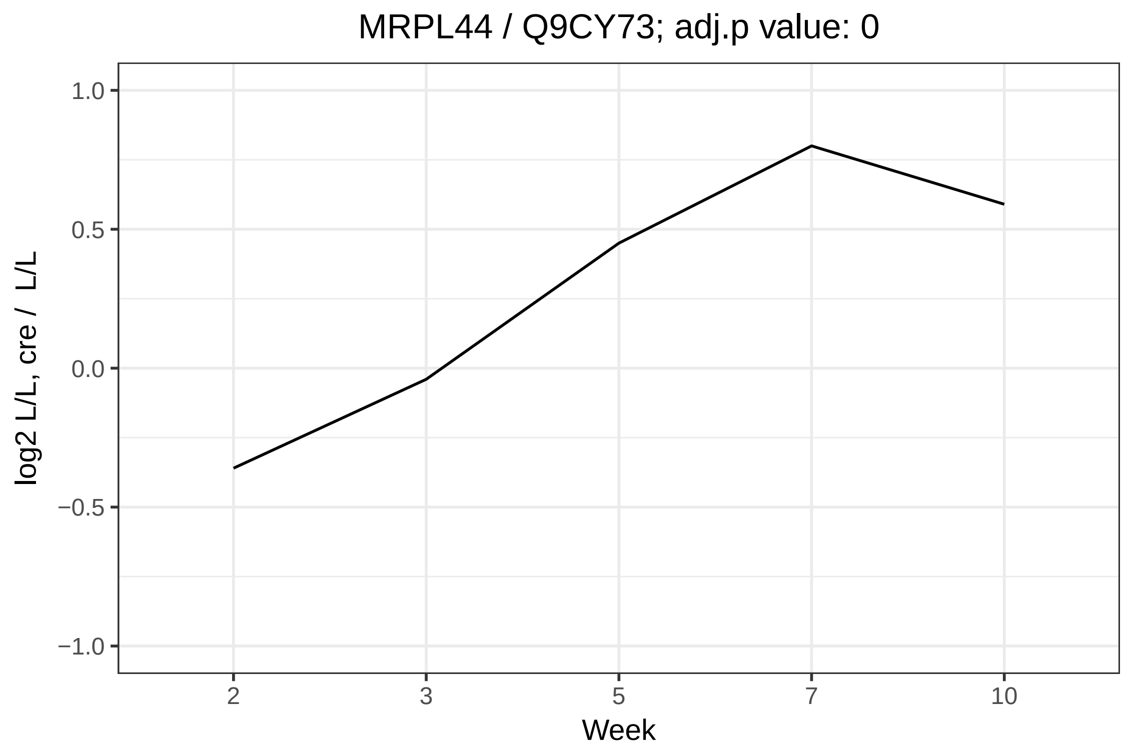
MRPL44 / Q9CY73; adj.p value: 0
1.0
0.5
0.0
−0.5
−1.0
log2 L/L, cre / L/L
2
3
5
7
10
Week

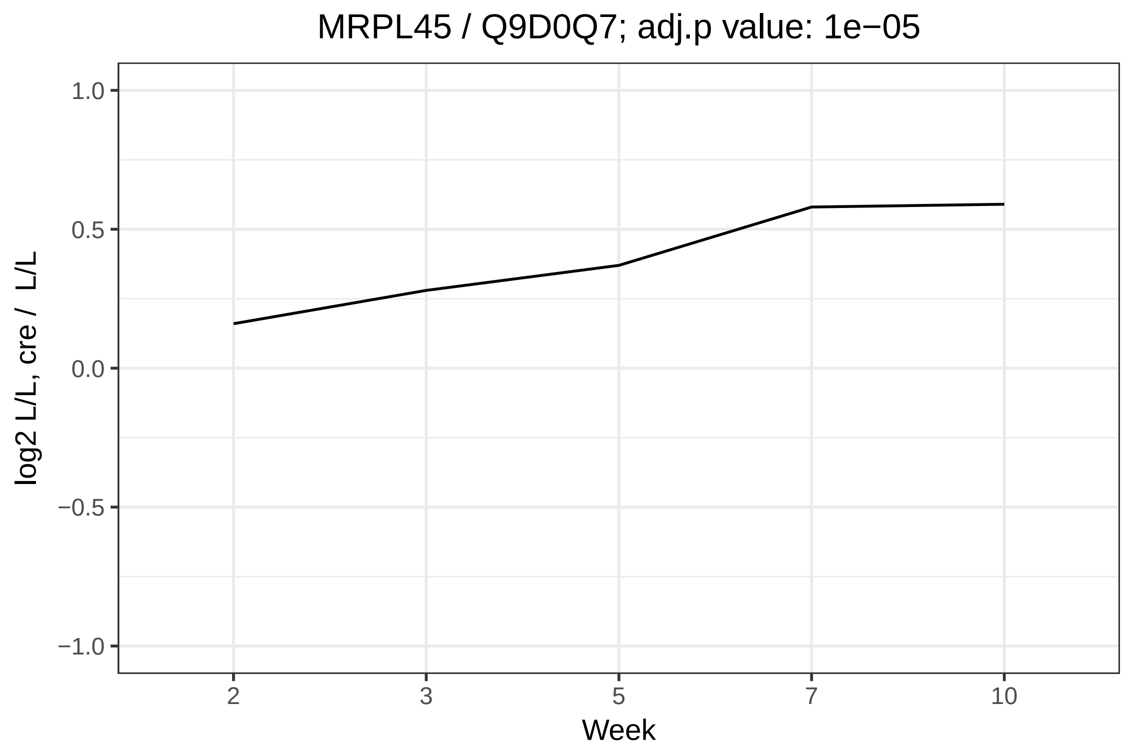

MRPL45 / Q9D0Q7; adj.p value: 1e−05
log2 L/L, cre / L/L
1.0
0.5
0.0
−0.5
−1.0
2
3
5
7
10
Week

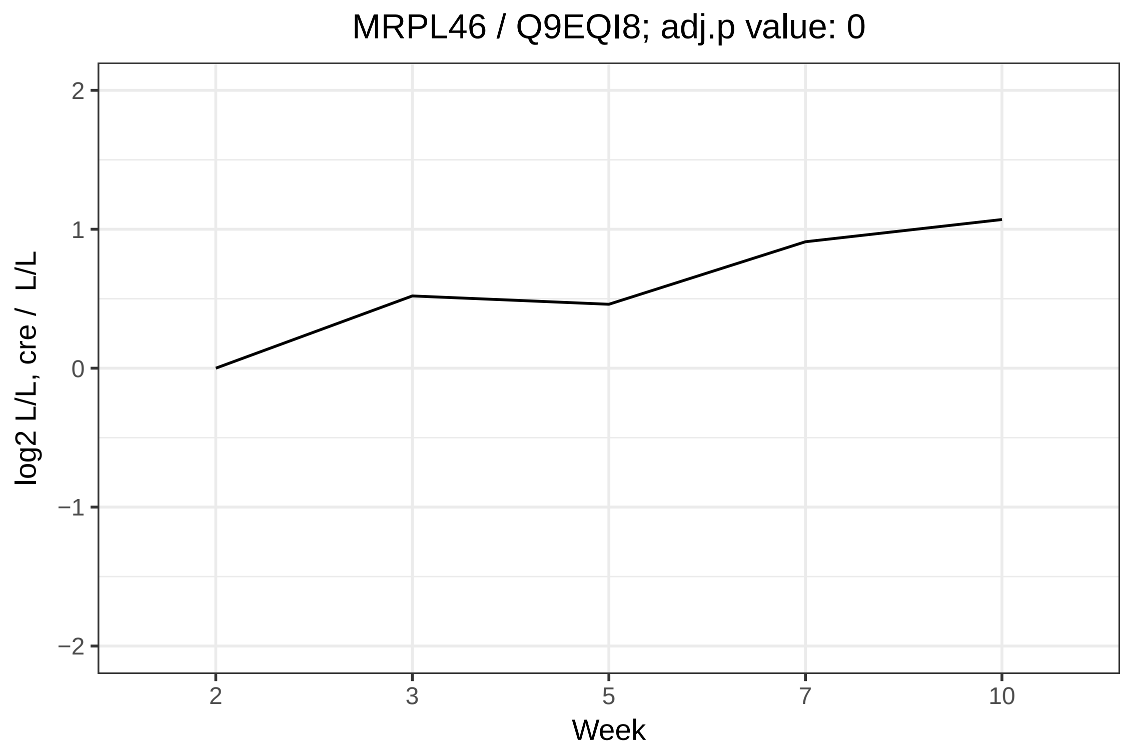
MRPL46 / Q9EQI8; adj.p value: 0
log2 L/L, cre / L/L
2
1
0
−1
−2
2
3
5
7
10
Week

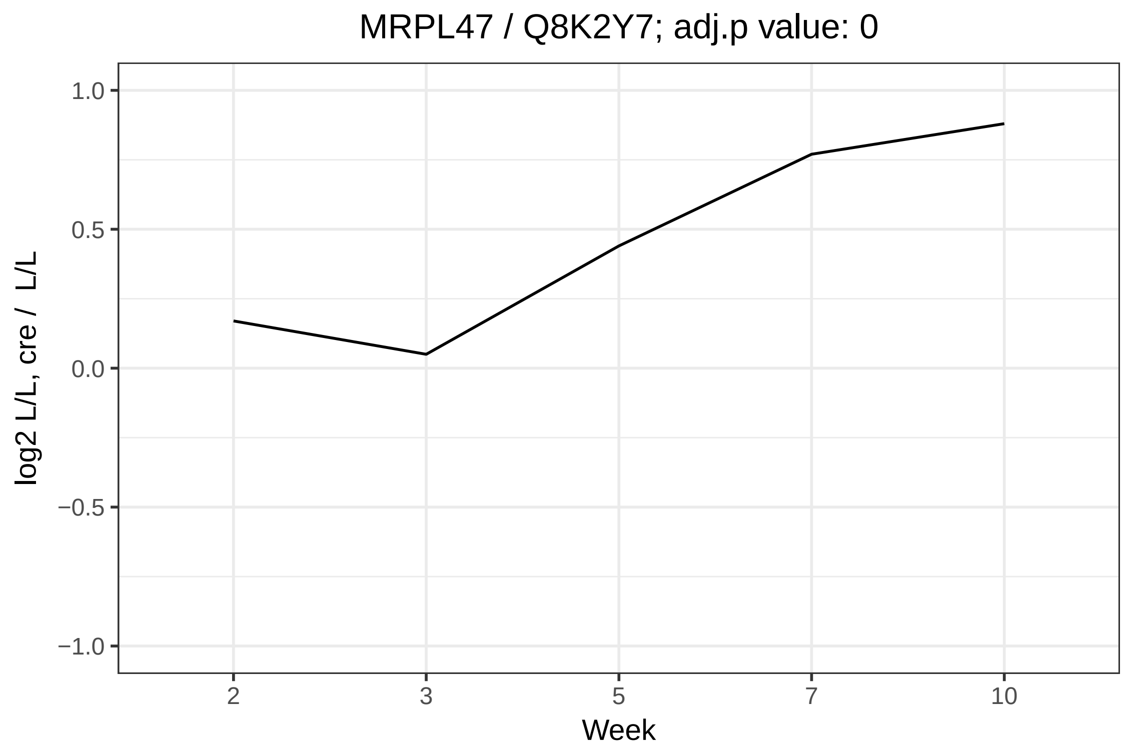

MRPL47 / Q8K2Y7; adj.p value: 0
log2 L/L, cre / L/L
1.0
0.5
0.0
−0.5
−1.0
2
3
5
7
10
Week

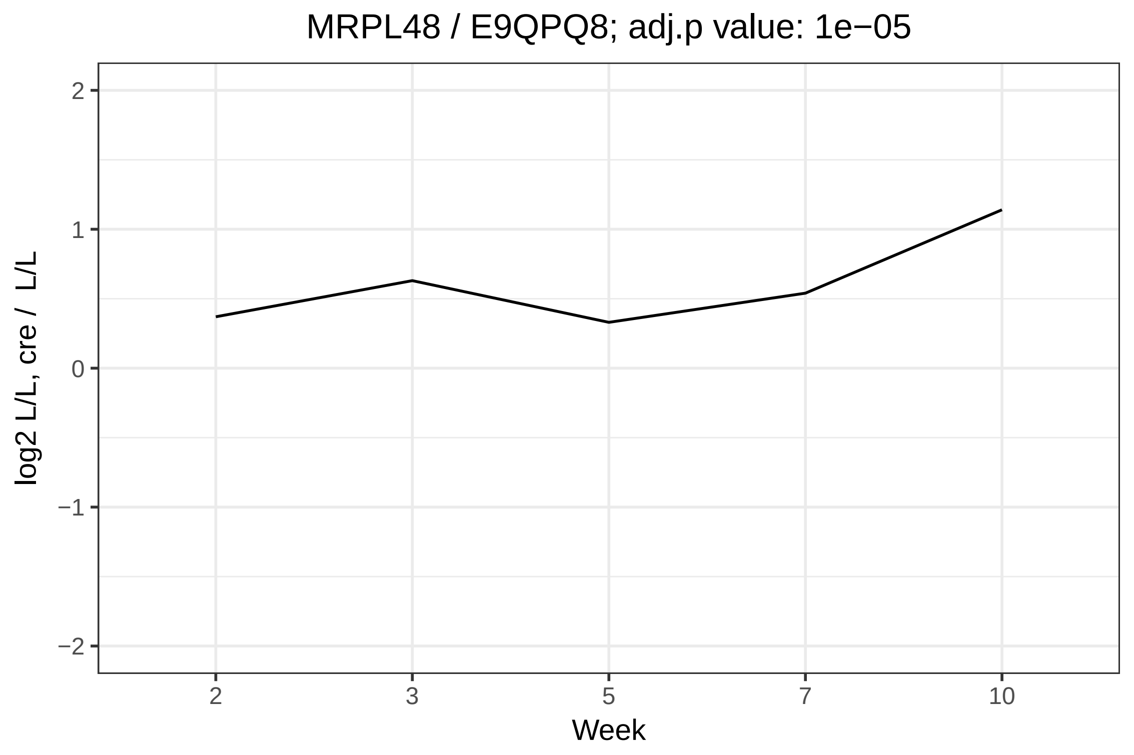
MRPL48 / E9QPQ8; adj.p value: 1e−05
log2 L/L, cre / L/L
2
1
0
−1
−2
2
3
5
7
10
Week

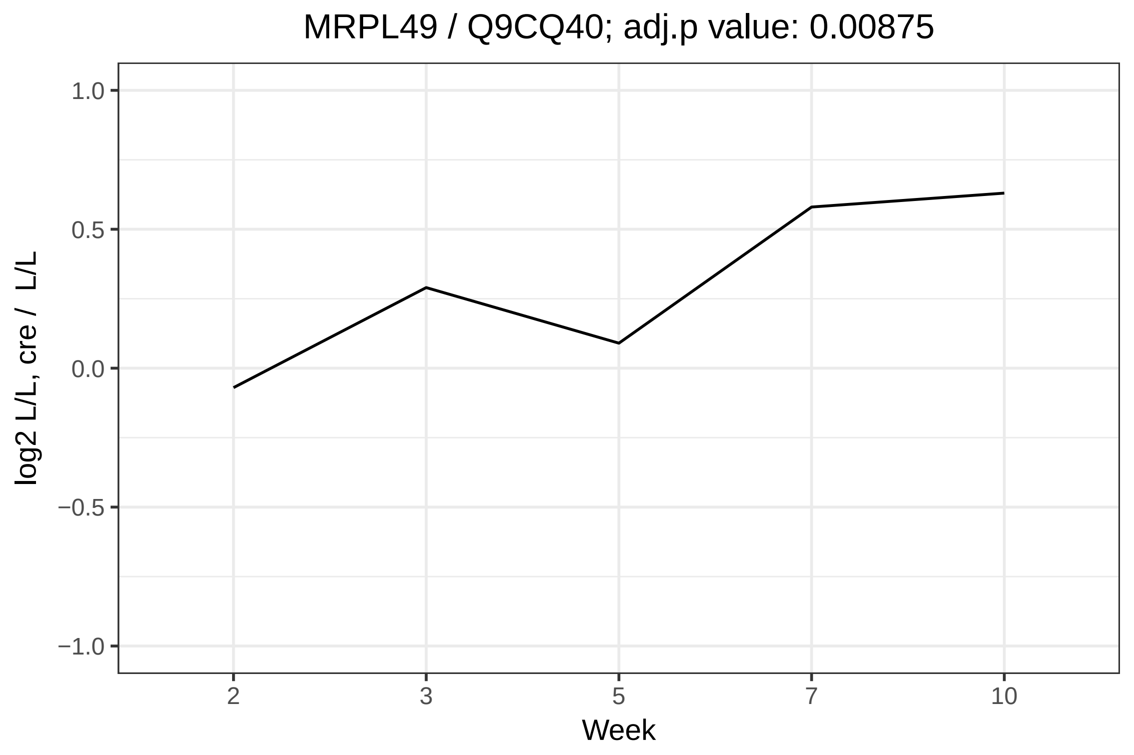

MRPL49 / Q9CQ40; adj.p value: 0.00875
log2 L/L, cre / L/L
1.0
0.5
0.0
−0.5
−1.0
2
3
5
7
10
Week

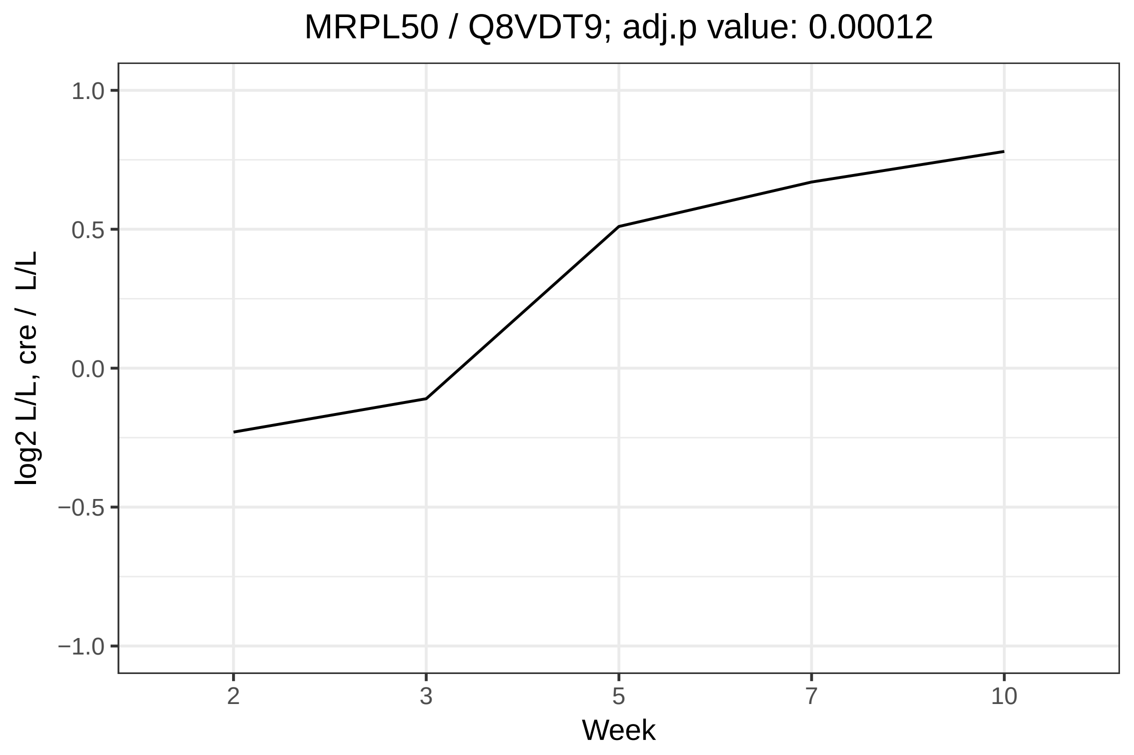
MRPL50 / Q8VDT9; adj.p value: 0.00012
log2 L/L, cre / L/L
1.0
0.5
0.0
−0.5
−1.0
2
3
5
7
10
Week

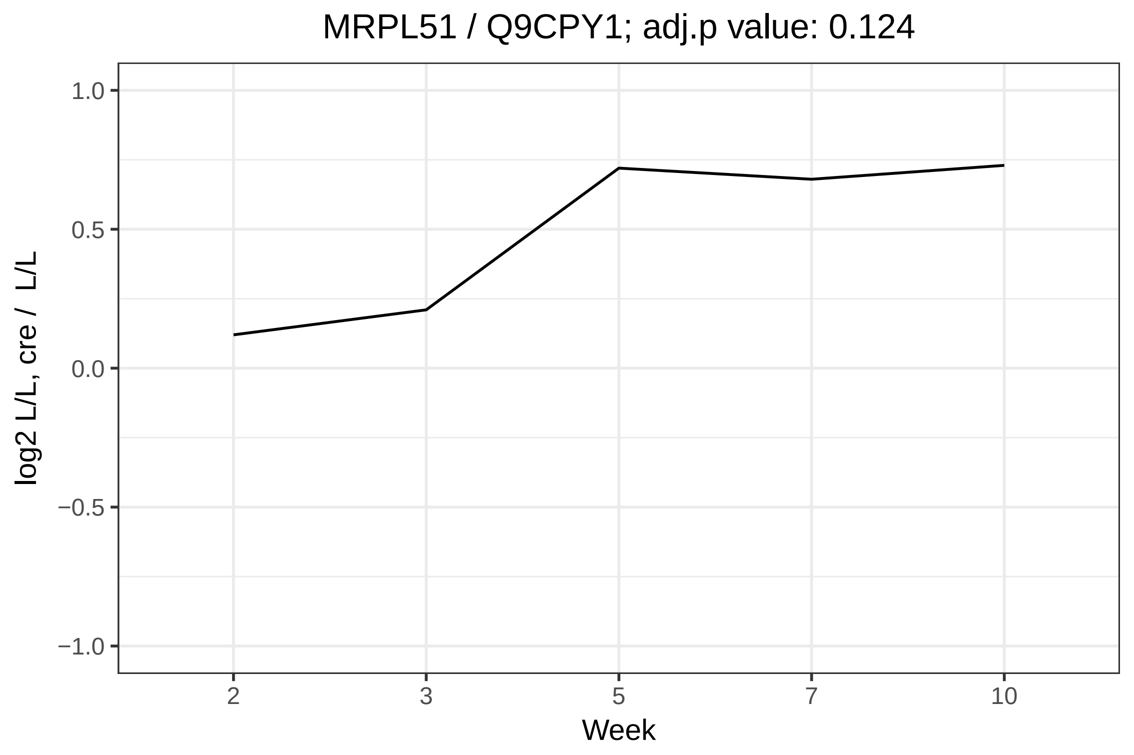

MRPL51 / Q9CPY1; adj.p value: 0.124
1.0
0.5
0.0
−0.5
−1.0
log2 L/L, cre / L/L
2
3
5
7
10
Week

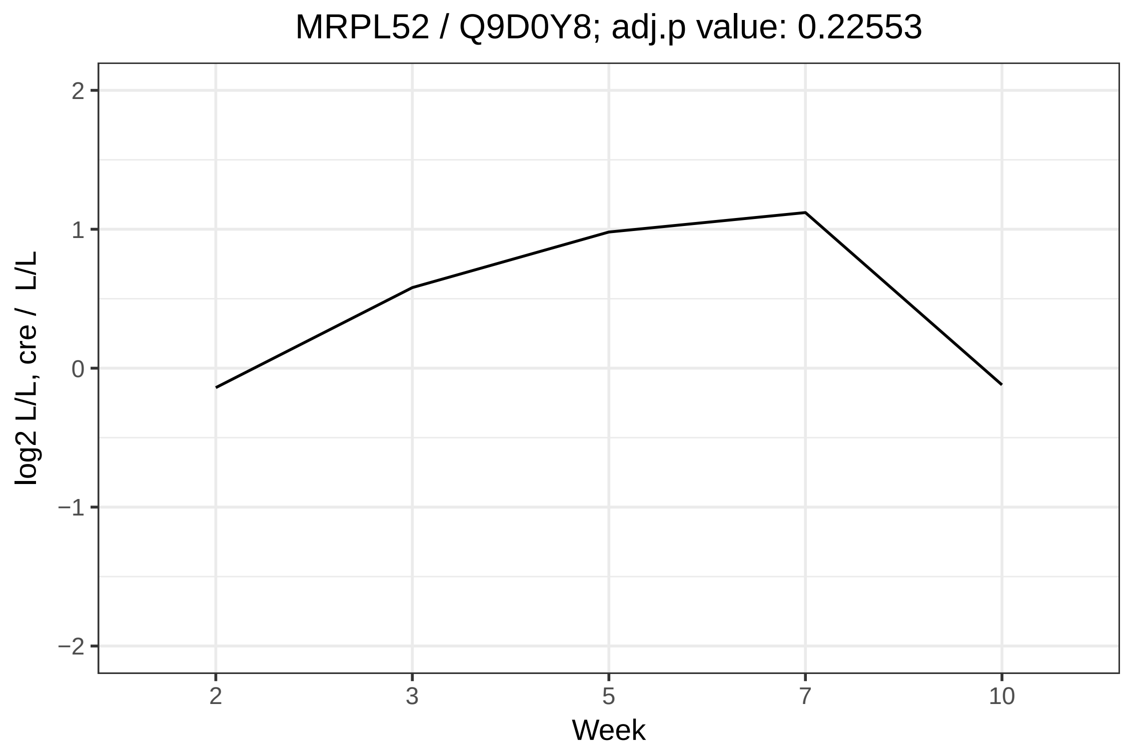

MRPL52 / Q9D0Y8; adj.p value: 0.22553
log2 L/L, cre / L/L
2
1
0
−1
−2
2
3
5
7
10
Week

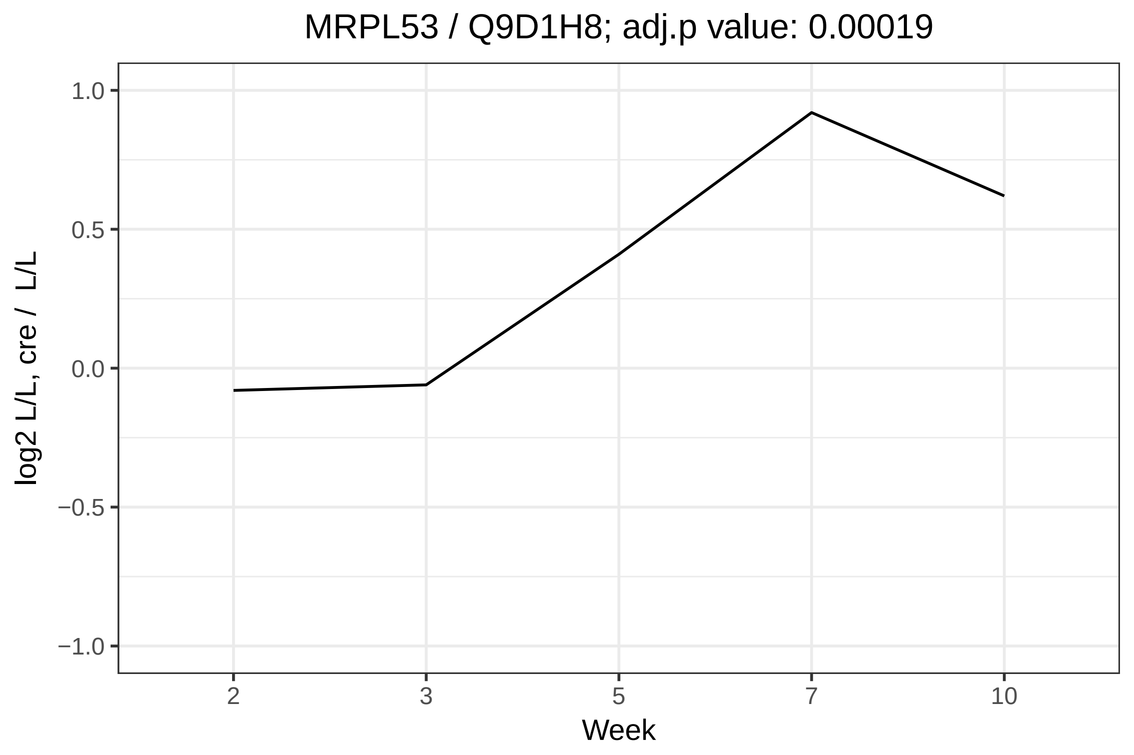
MRPL53 / Q9D1H8; adj.p value: 0.00019
log2 L/L, cre / L/L
1.0
0.5
0.0
−0.5
−1.0
2
3
5
7
10
Week

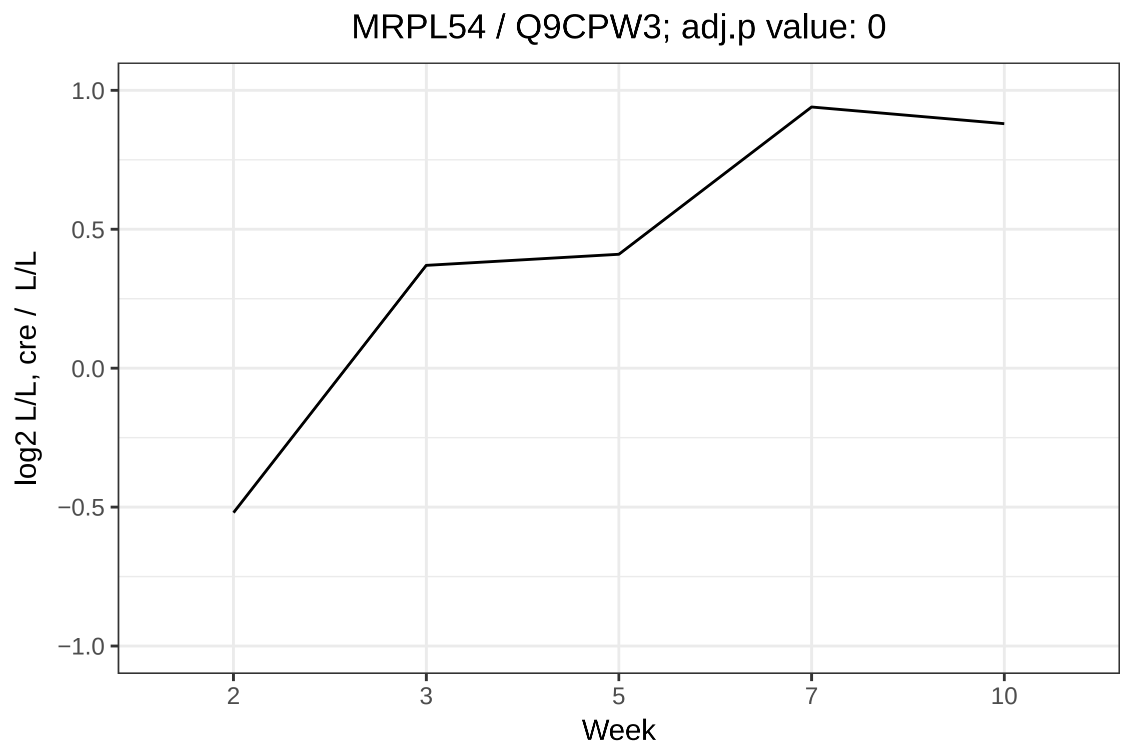

MRPL54 / Q9CPW3; adj.p value: 0
log2 L/L, cre / L/L
Week
1.0
0.5
0.0
−0.5
−1.0
2
3
5
7
10

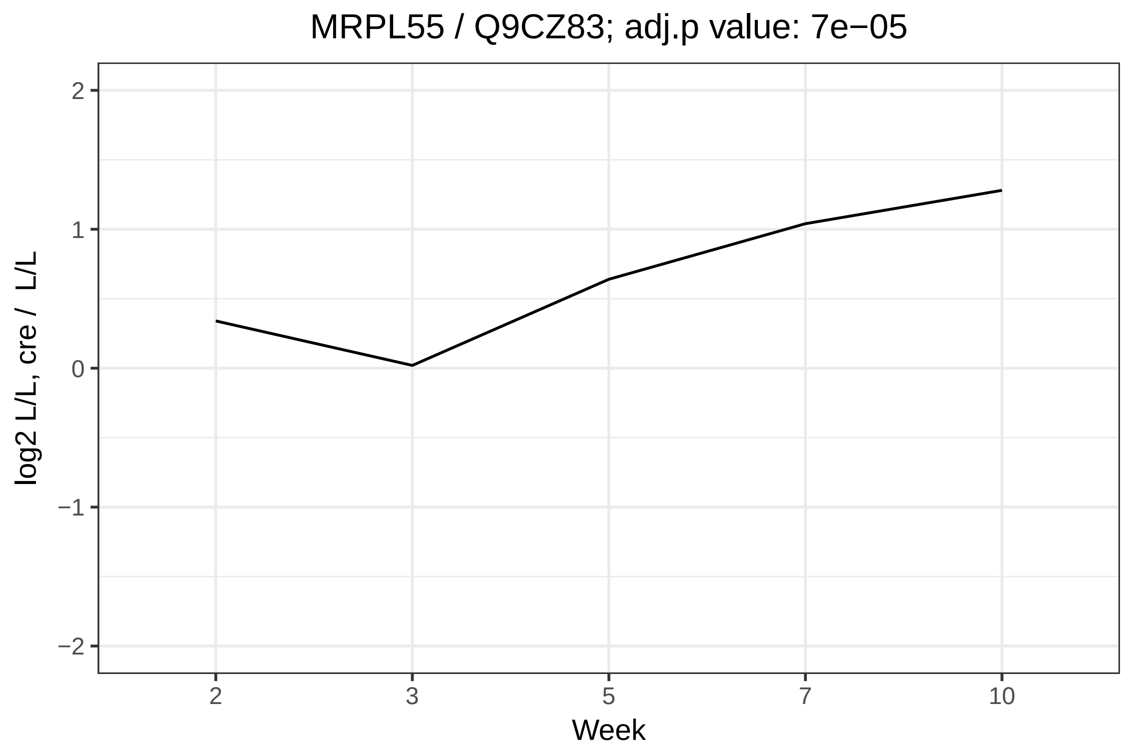

MRPL55 / Q9CZ83; adj.p value: 7e−05
log2 L/L, cre / L/L
2
1
0
−1
−2
2
3
5
7
10
Week

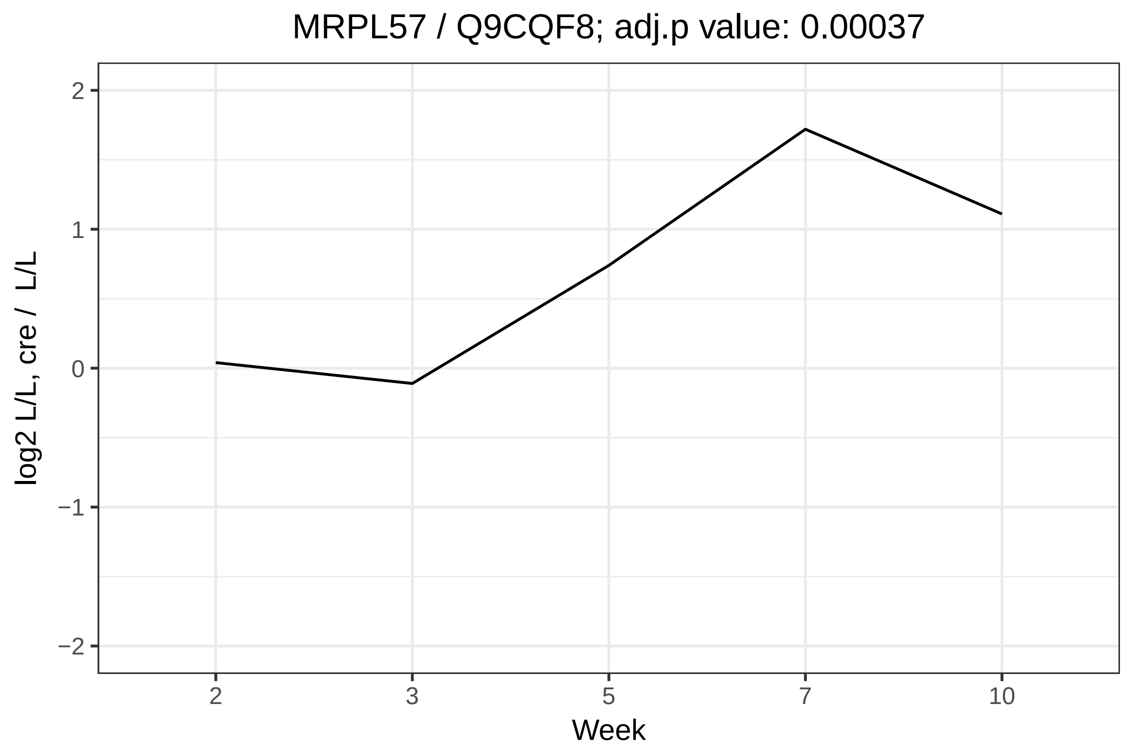

MRPL57 / Q9CQF8; adj.p value: 0.00037
2
1
0
−1
−2
log2 L/L, cre / L/L
2
3
5
7
10
Week

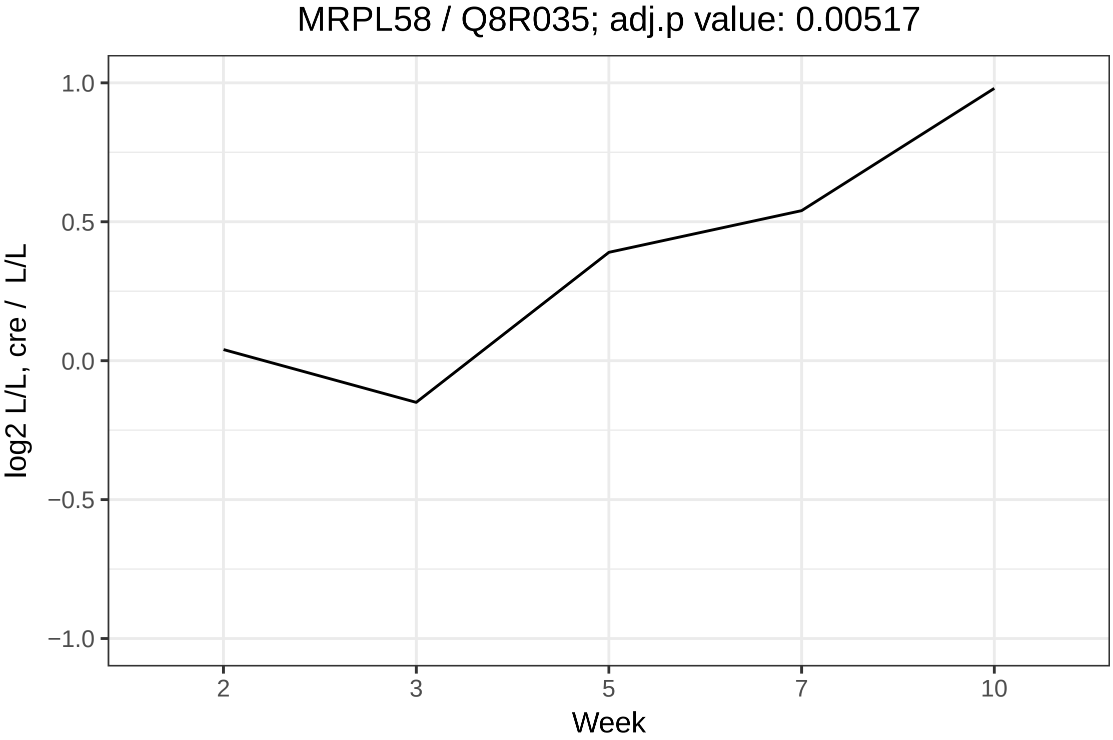

MRPL58 / Q8R035; adj.p value: 0.00517
1.0
0.5
0.0
−0.5
−1.0
log2 L/L, cre / L/L
2
3
5
7
10
Week

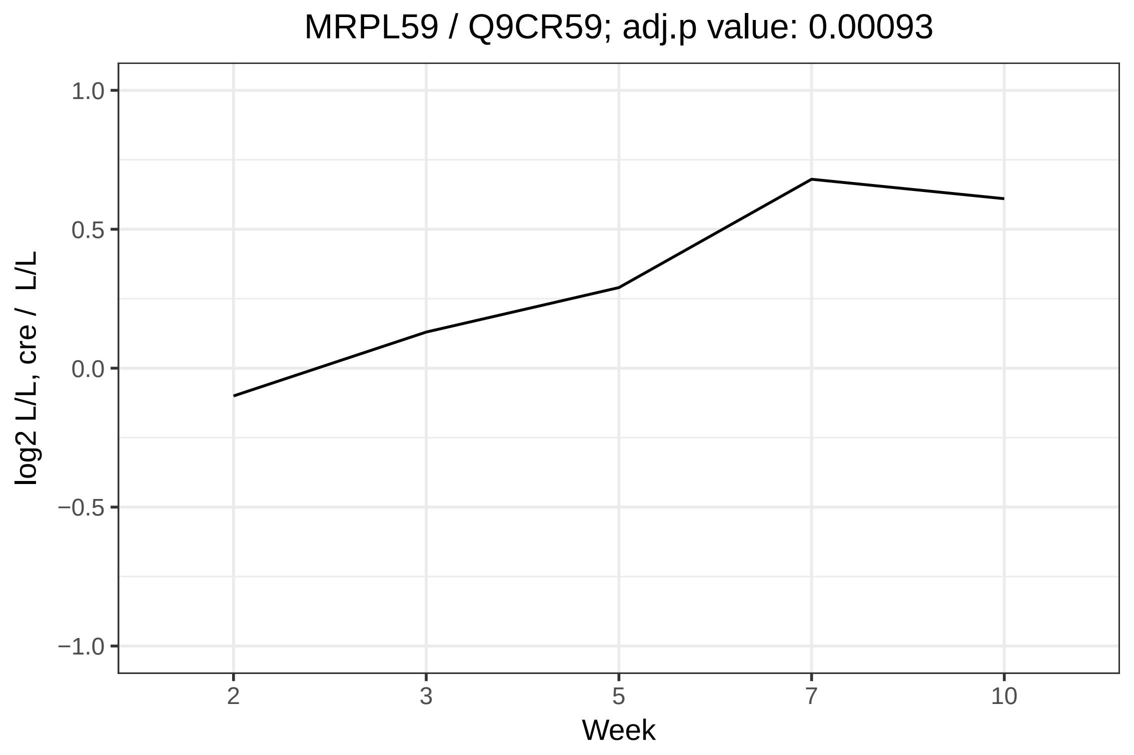
MRPL59 / Q9CR59; adj.p value: 0.00093
log2 L/L, cre / L/L
1.0
0.5
0.0
−0.5
−1.0
2
3
5
7
10
Week

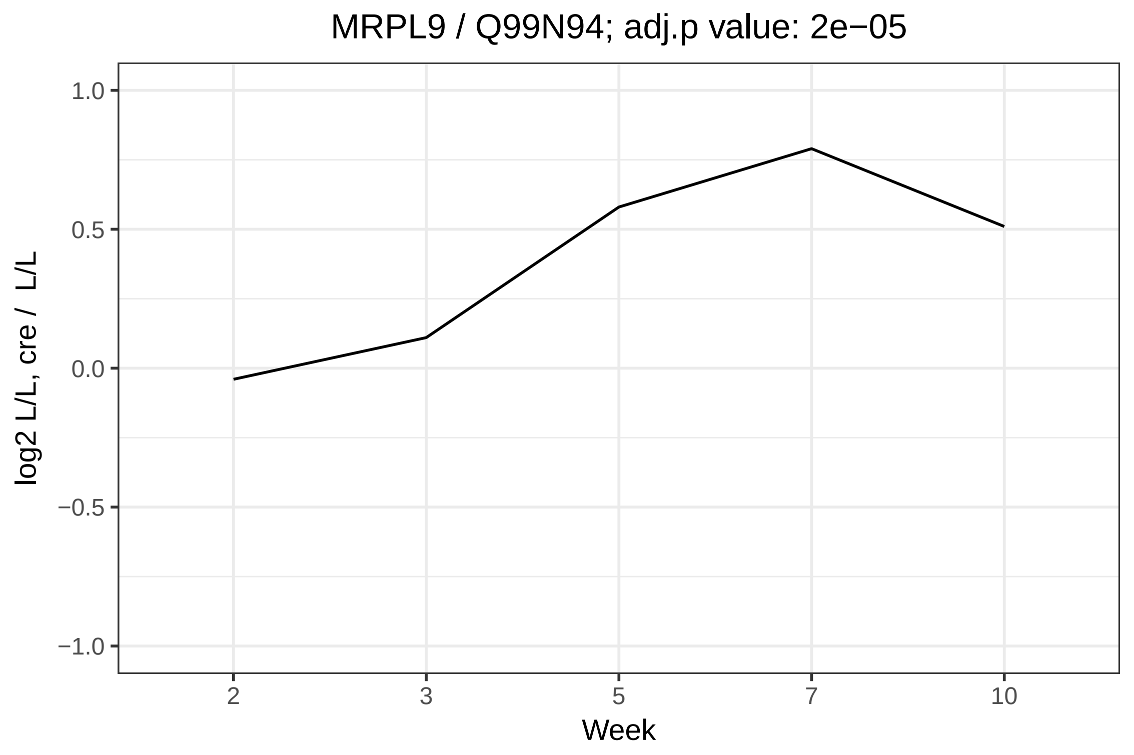

MRPL9 / Q99N94; adj.p value: 2e−05
1.0
0.5
0.0
−0.5
−1.0
log2 L/L, cre / L/L
2
3
5
7
10
Week

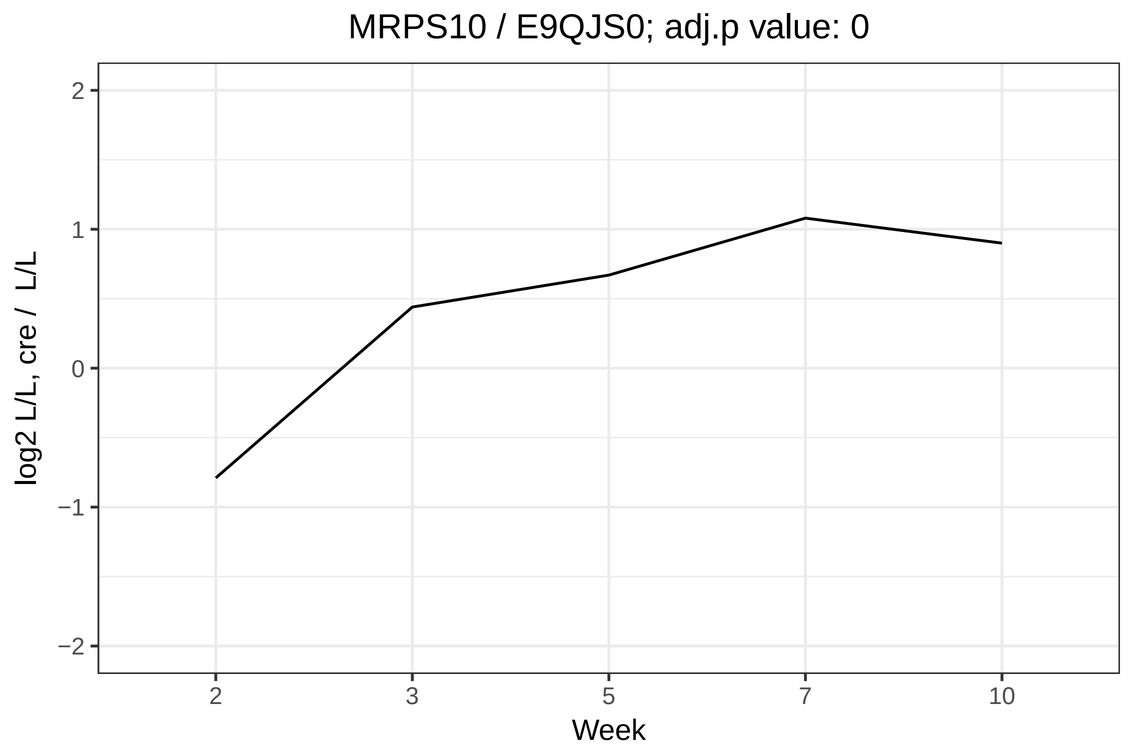

MRPS10 / E9QJS0; adj.p value: 0
log2 L/L, cre / L/L
2
1
0
−1
−2
2
3
5
7
10
Week

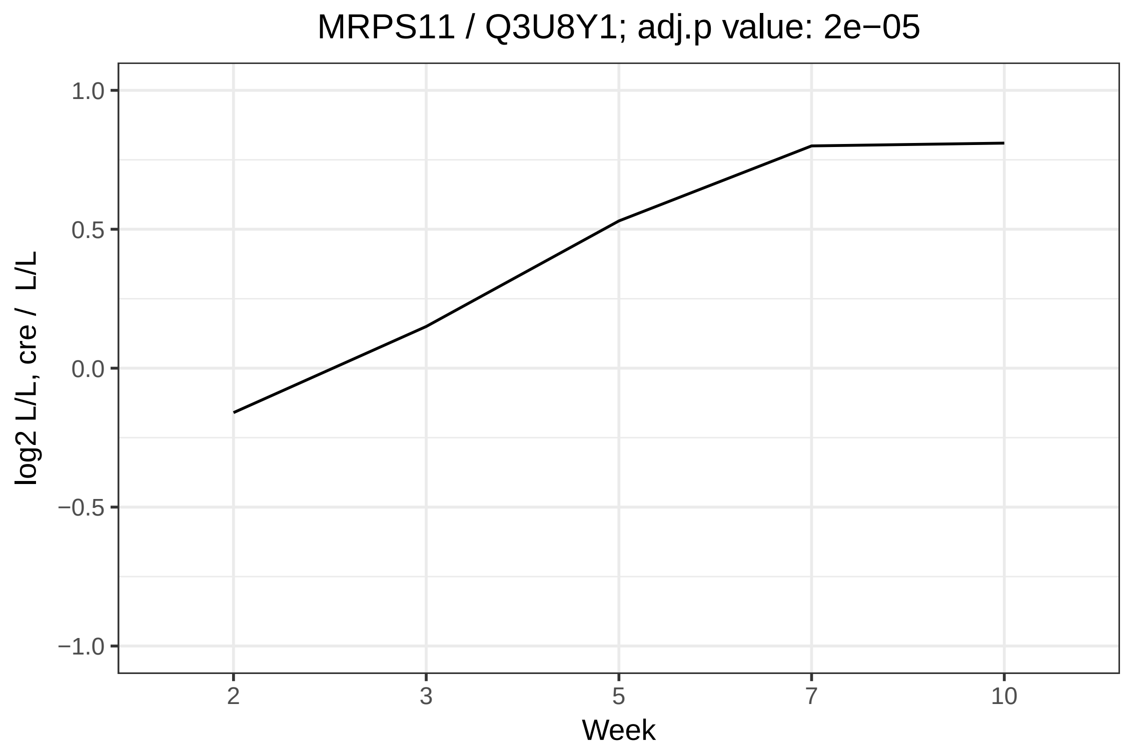
MRPS11 / Q3U8Y1; adj.p value: 2e−05
log2 L/L, cre / L/L
1.0
0.5
0.0
−0.5
−1.0
2
3
5
7
10
Week

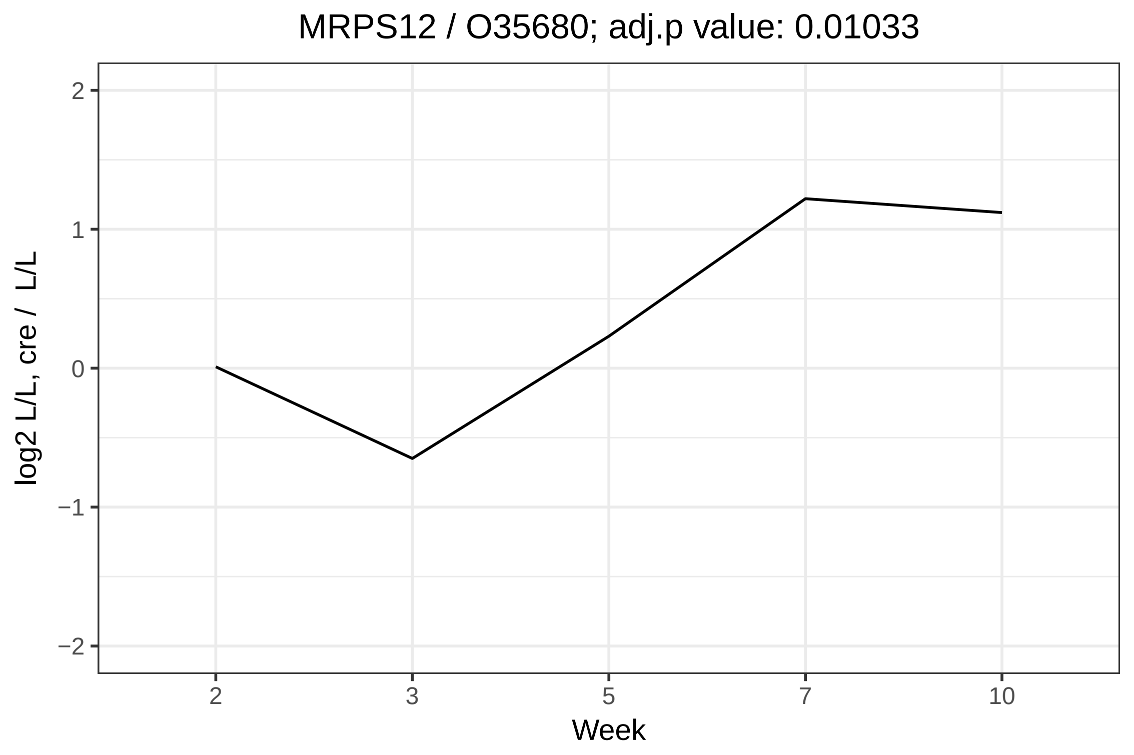
MRPS12 / O35680; adj.p value: 0.01033
log2 L/L, cre / L/L
2
1
0
−1
−2
2
3
5
7
10
Week

MRPS14 / D3Z2K2; adj.p value: 0

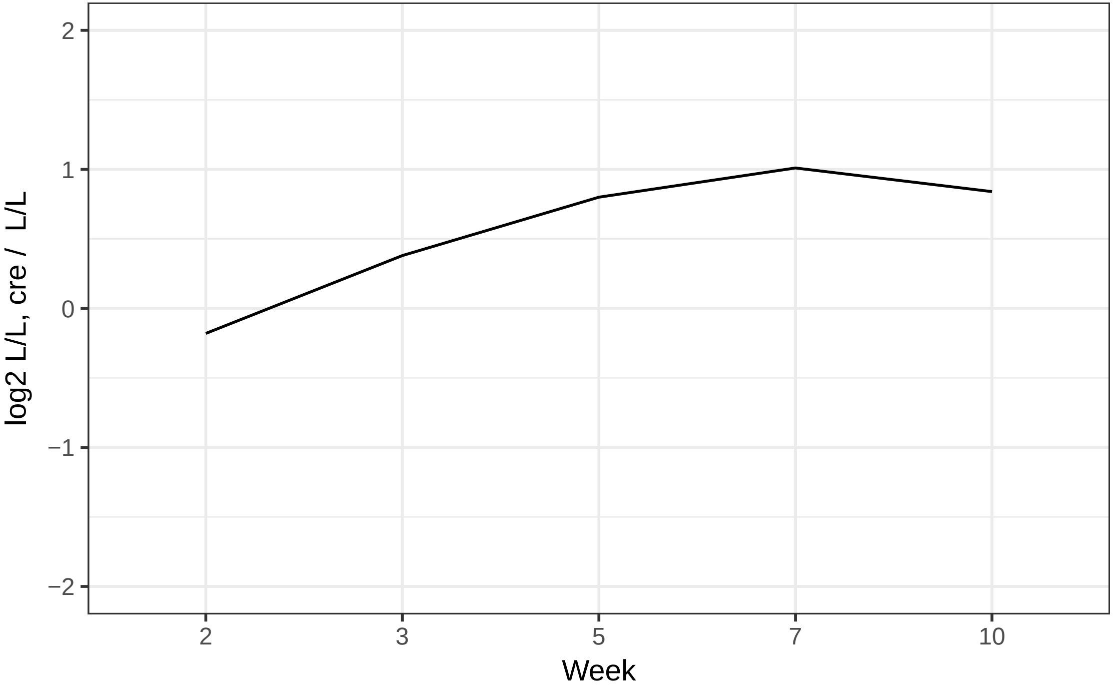

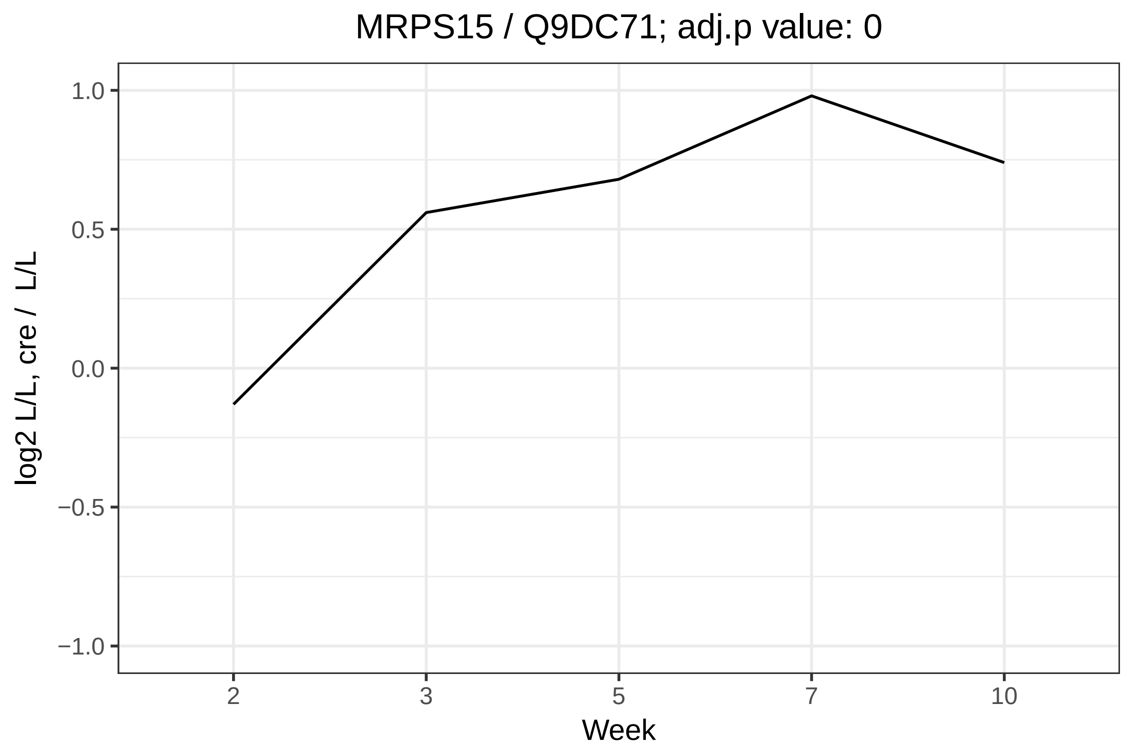

MRPS15 / Q9DC71; adj.p value: 0
1.0
0.5
0.0
−0.5
−1.0
log2 L/L, cre / L/L
2
3
5
7
10
Week

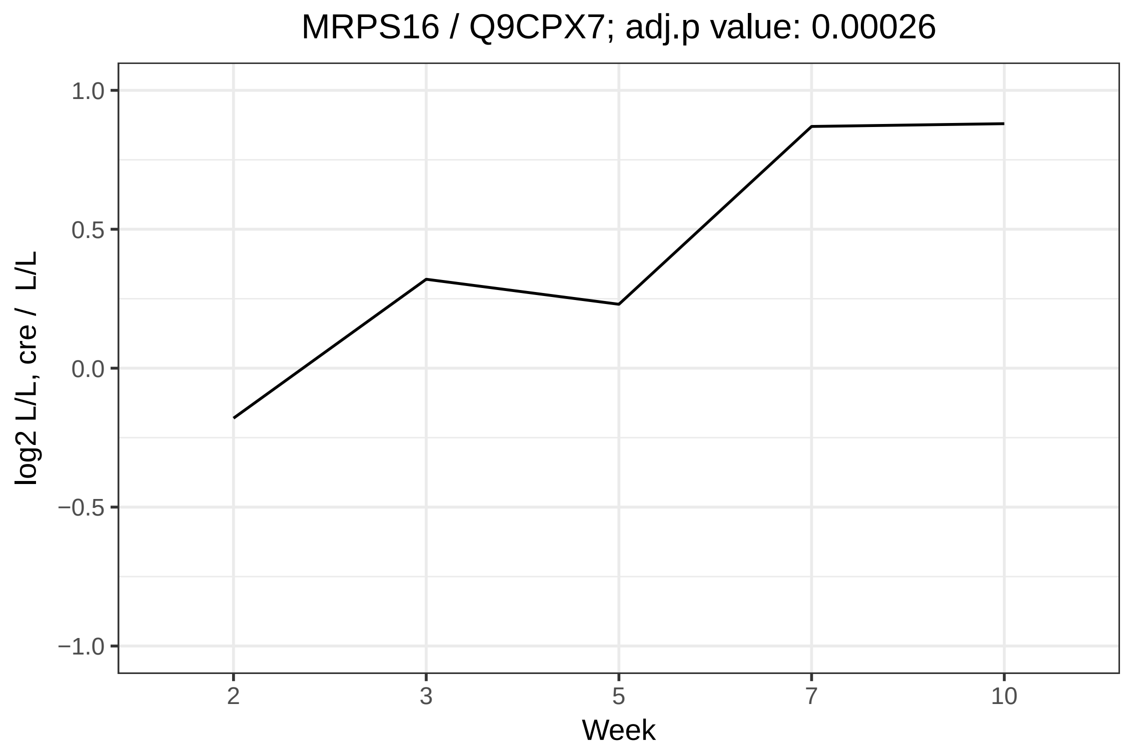
MRPS16 / Q9CPX7; adj.p value: 0.00026
1.0
0.5
0.0
−0.5
−1.0
log2 L/L, cre / L/L
2
3
5
7
10
Week

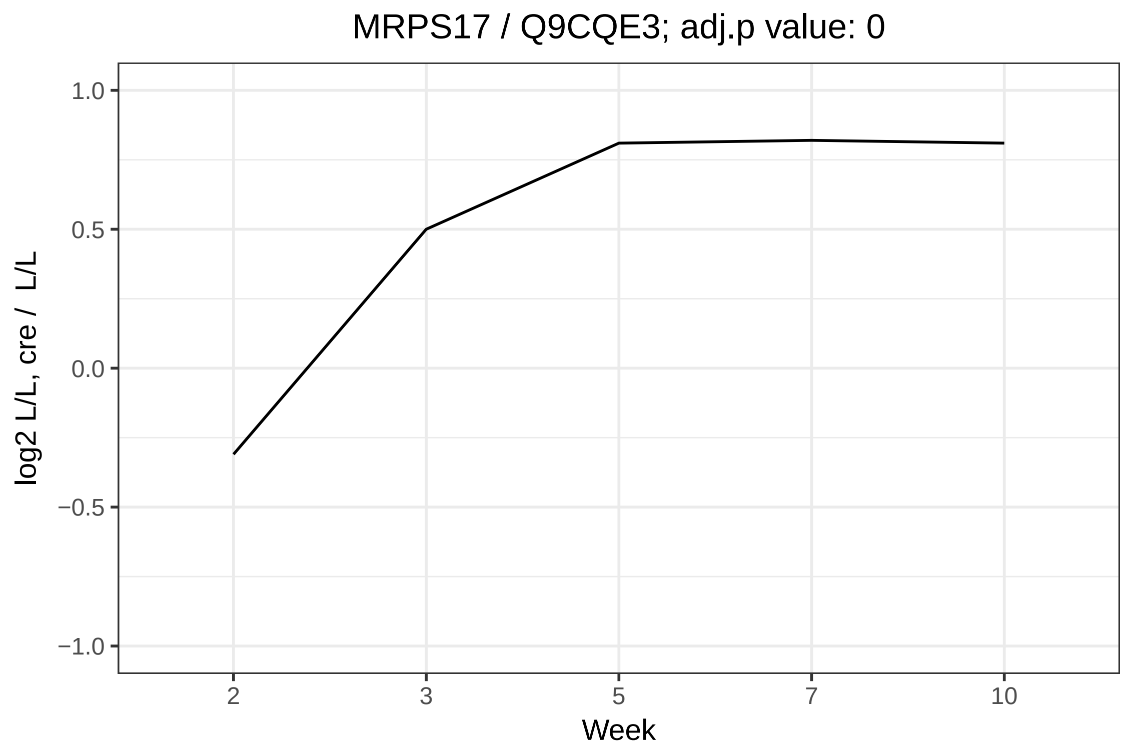
MRPS17 / Q9CQE3; adj.p value: 0
log2 L/L, cre / L/L
1.0
0.5
0.0
−0.5
−1.0
2
3
5
7
10
Week

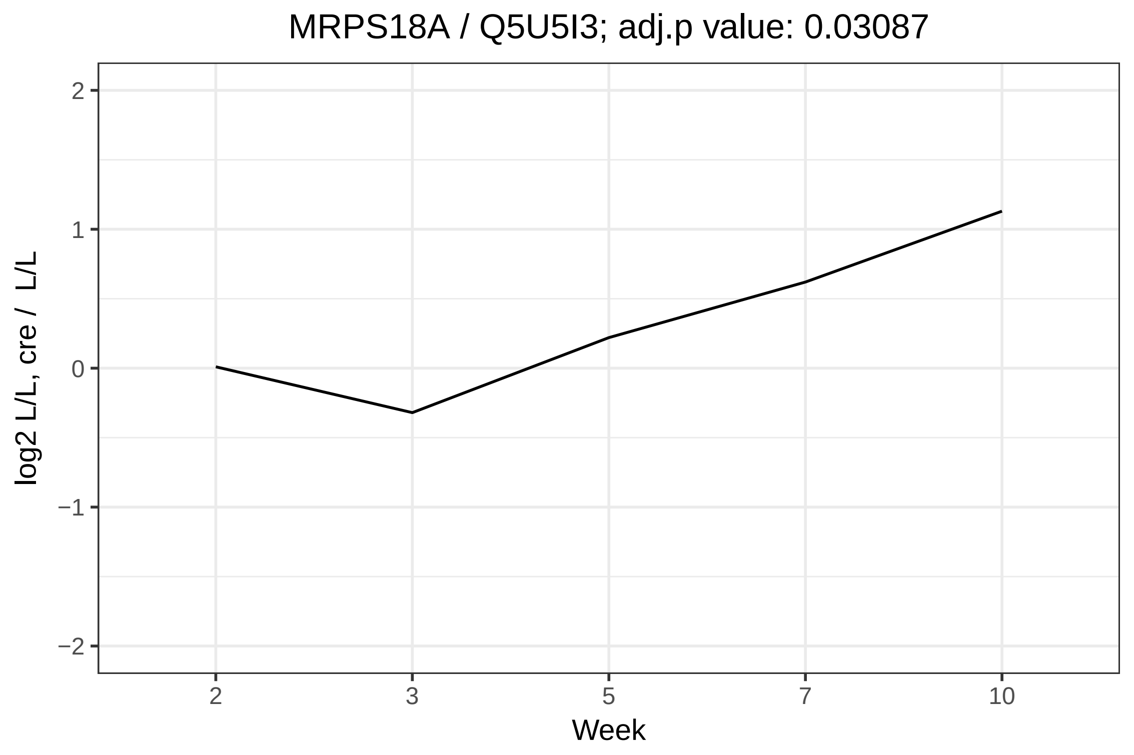
MRPS18A / Q5U5I3; adj.p value: 0.03087
log2 L/L, cre / L/L
2
1
0
−1
−2
2
3
5
7
10
Week

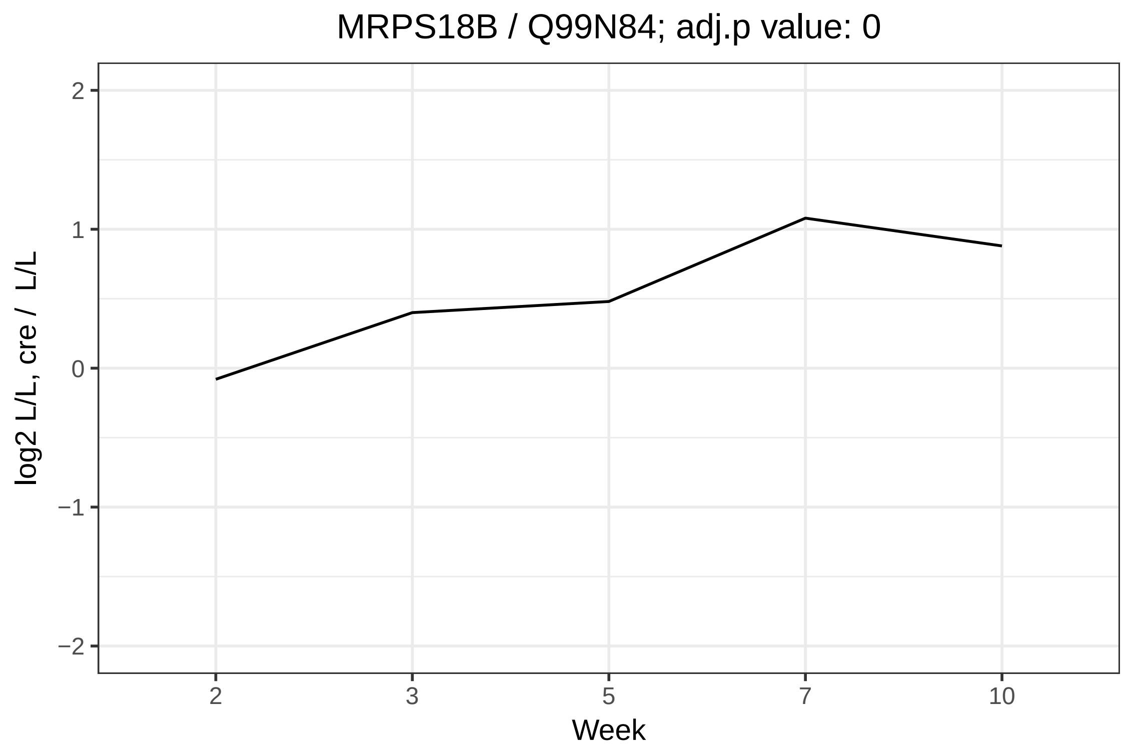
MRPS18B / Q99N84; adj.p value: 0
log2 L/L, cre / L/L
2
1
0
−1
−2
2
3
5
7
10
Week

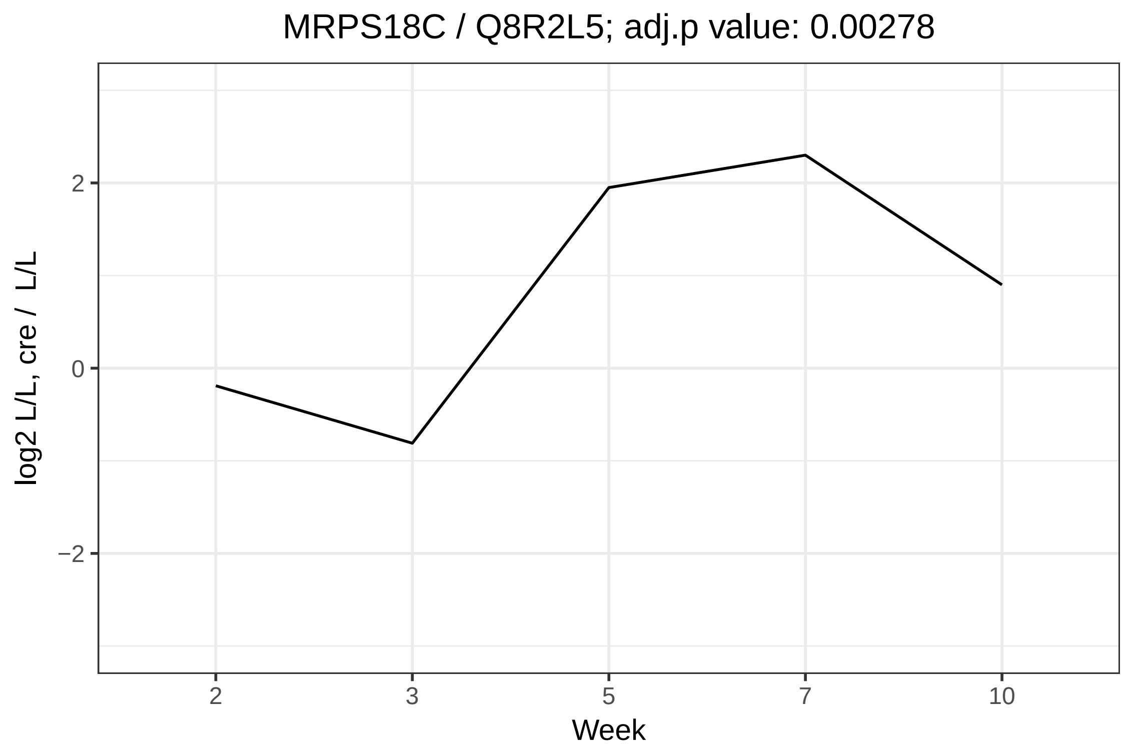
MRPS18C / Q8R2L5; adj.p value: 0.00278
log2 L/L, cre / L/L
2
0
−2
2
3
5
7
10
Week

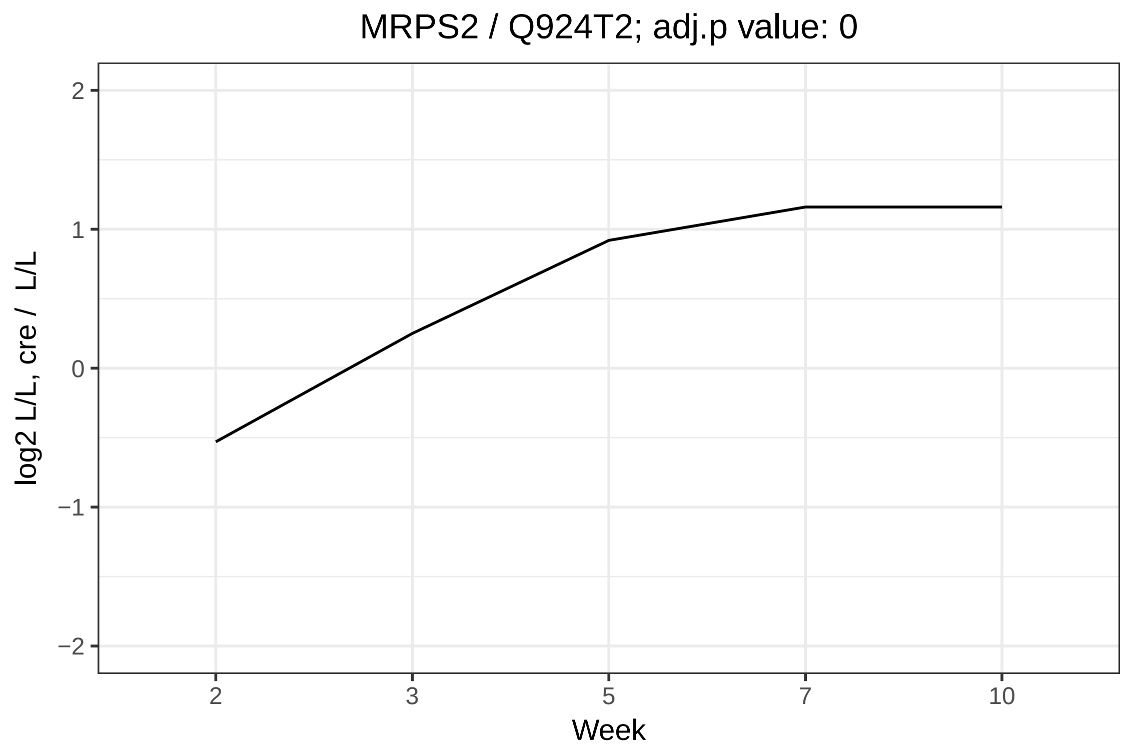

MRPS2 / Q924T2; adj.p value: 0
log2 L/L, cre / L/L
2
1
0
−1
−2
2
3
5
7
10
Week

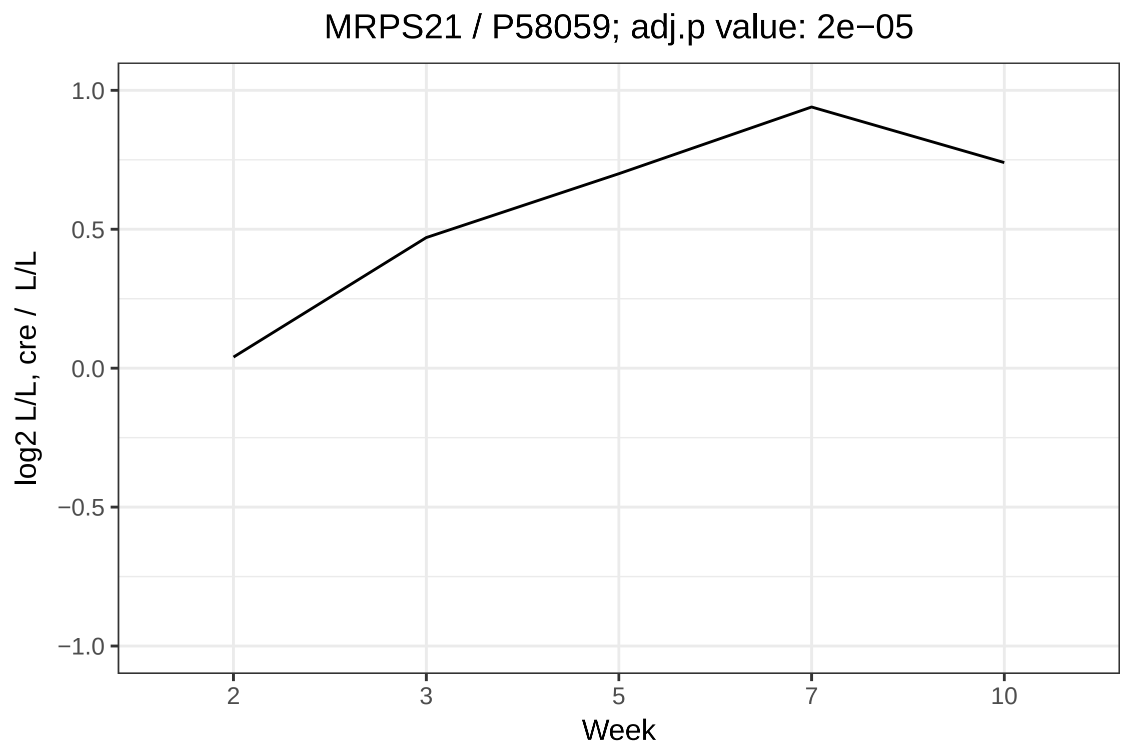

MRPS21 / P58059; adj.p value: 2e−05
1.0
0.5
0.0
−0.5
−1.0
log2 L/L, cre / L/L
2
3
5
7
10
Week

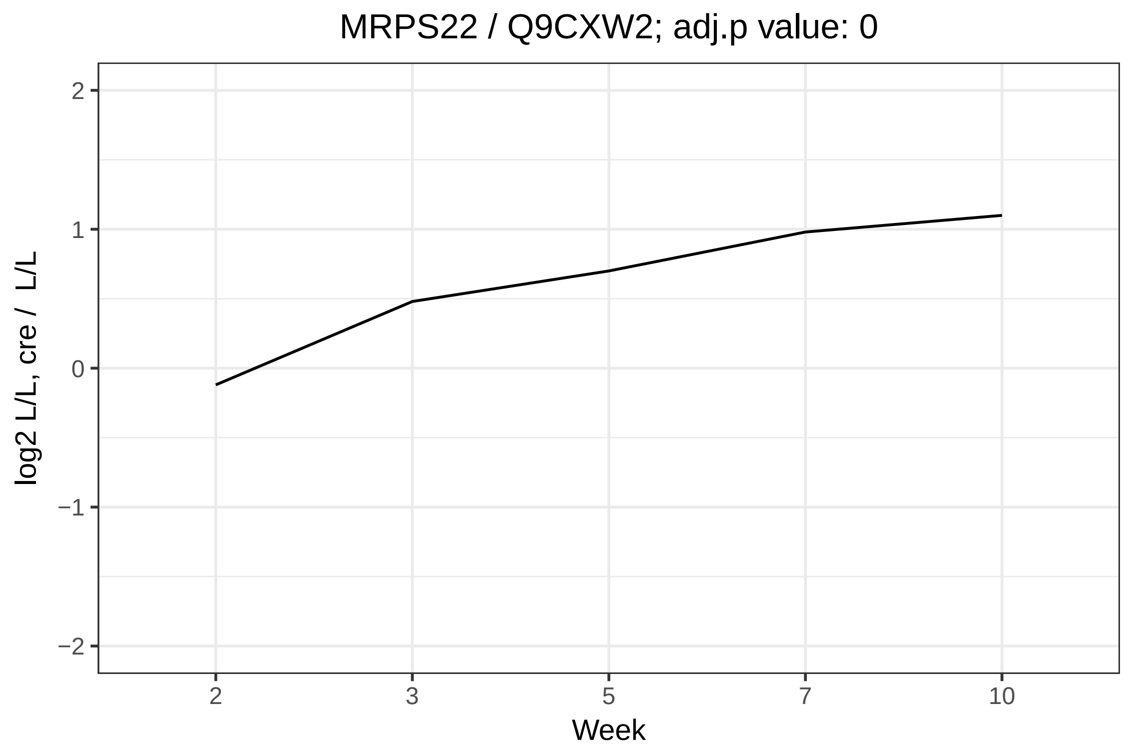

MRPS22 / Q9CXW2; adj.p value: 0
log2 L/L, cre / L/L
2
1
0
−1
−2
2
3
5
7
10
Week

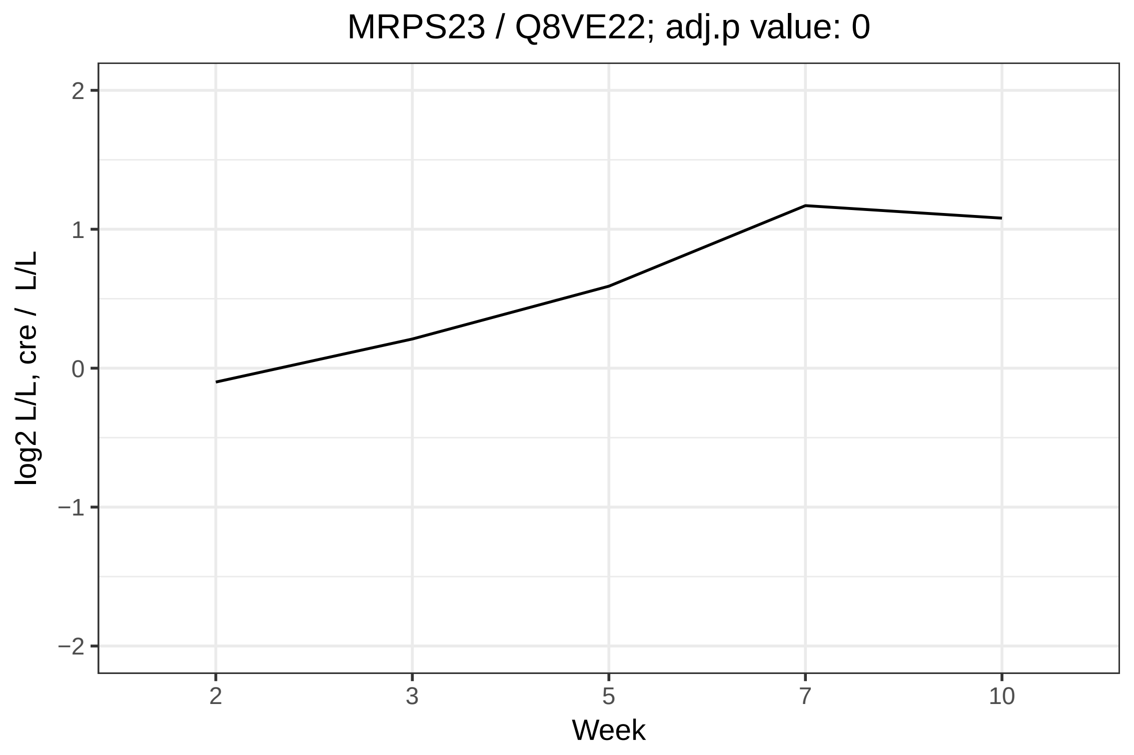
MRPS23 / Q8VE22; adj.p value: 0
log2 L/L, cre / L/L
2
1
0
−1
−2
2
3
5
7
10
Week

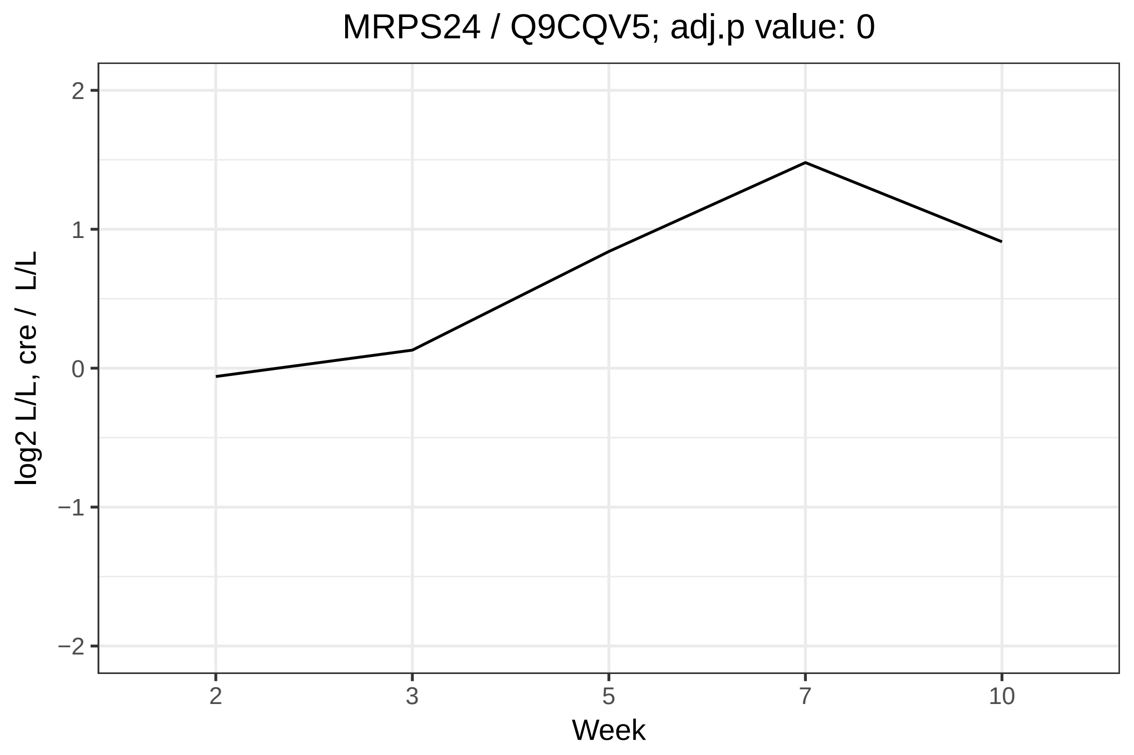

MRPS24 / Q9CQV5; adj.p value: 0
log2 L/L, cre / L/L
2
1
0
−1
−2
2
3
5
7
10
Week

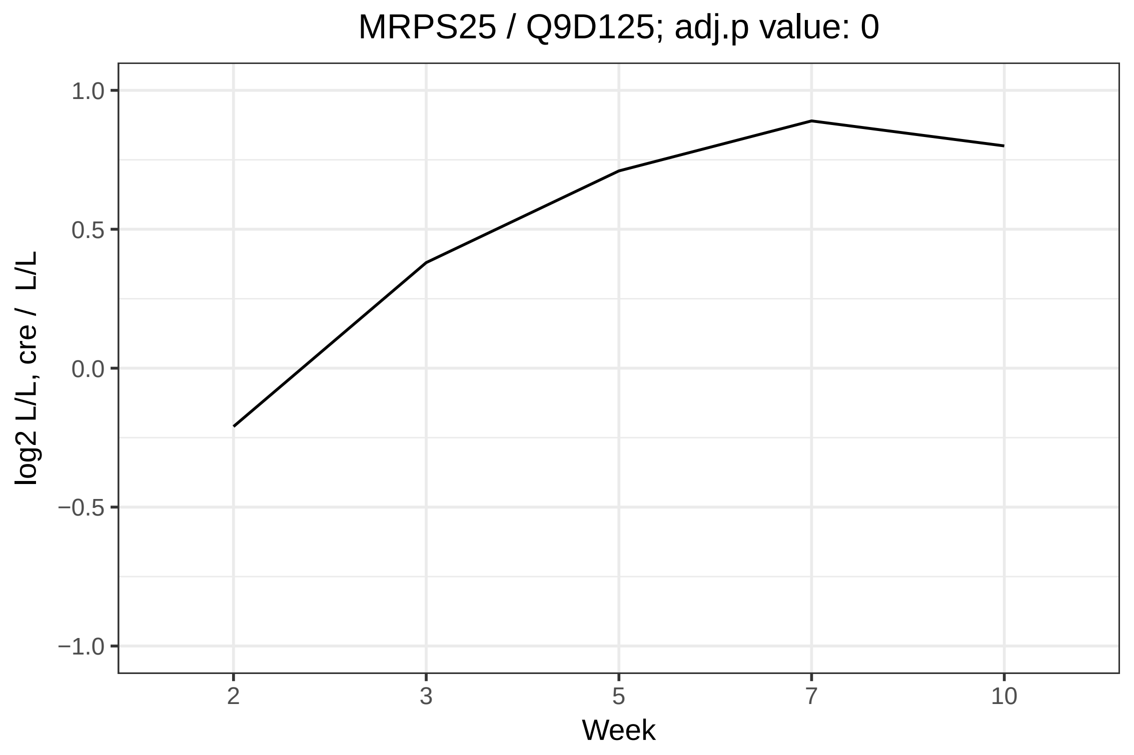
MRPS25 / Q9D125; adj.p value: 0
log2 L/L, cre / L/L
1.0
0.5
0.0
−0.5
−1.0
2
3
5
7
10
Week

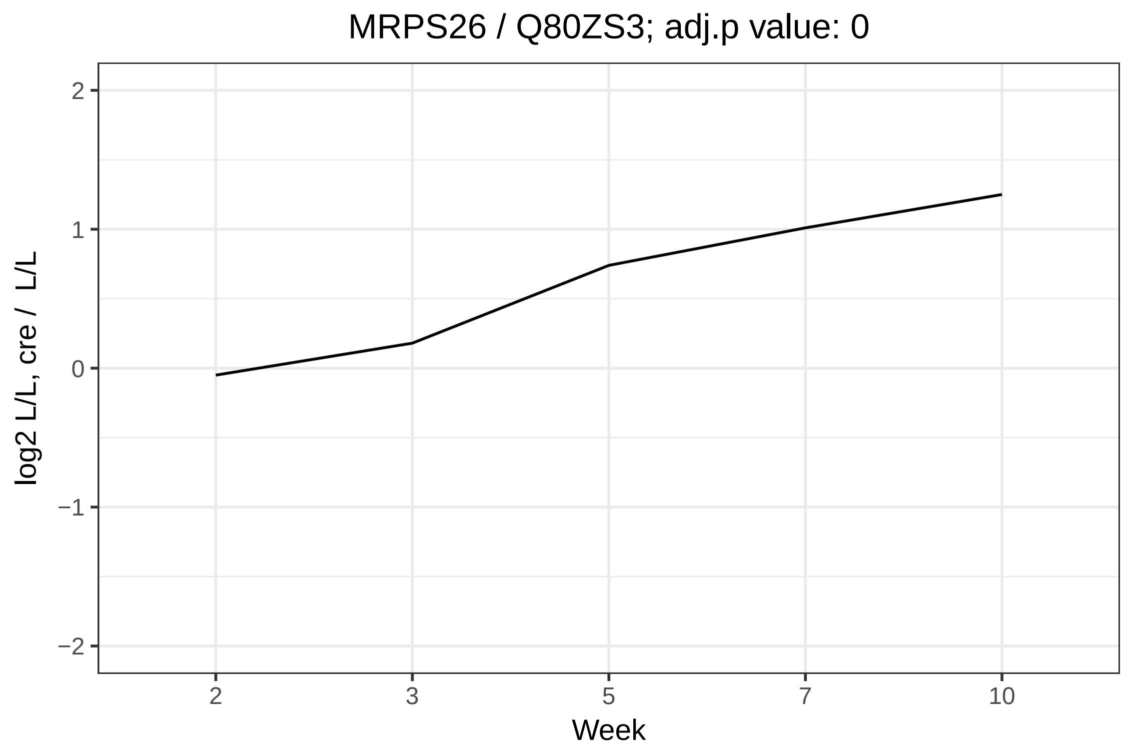
MRPS26 / Q80ZS3; adj.p value: 0
log2 L/L, cre / L/L
2
1
0
−1
−2
2
3
5
7
10
Week

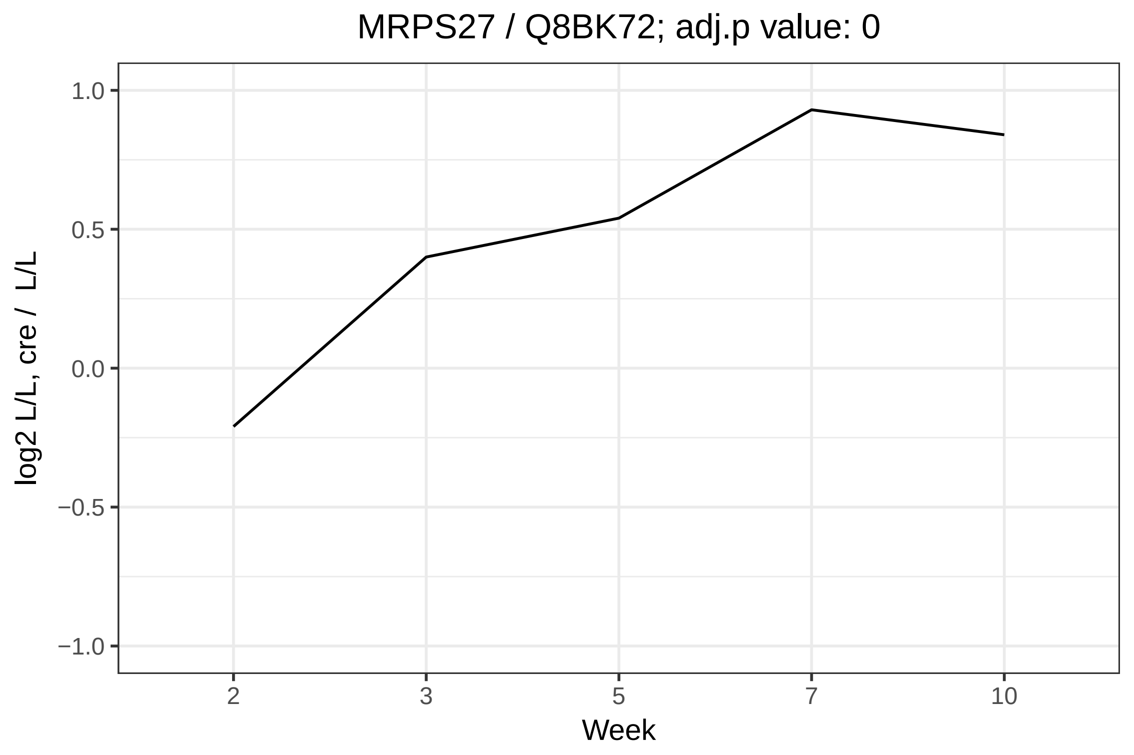
MRPS27 / Q8BK72; adj.p value: 0
log2 L/L, cre / L/L
1.0
0.5
0.0
−0.5
−1.0
2
3
5
7
10
Week

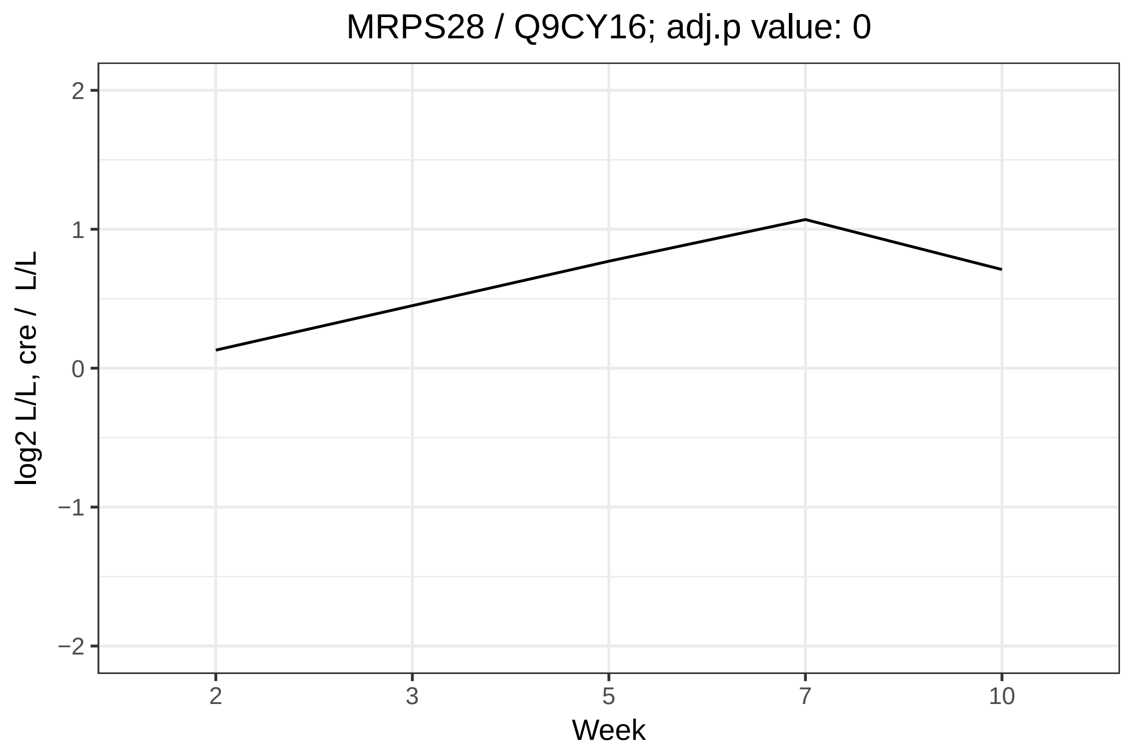
MRPS28 / Q9CY16; adj.p value: 0
log2 L/L, cre / L/L
2
1
0
−1
−2
2
3
5
7
10
Week

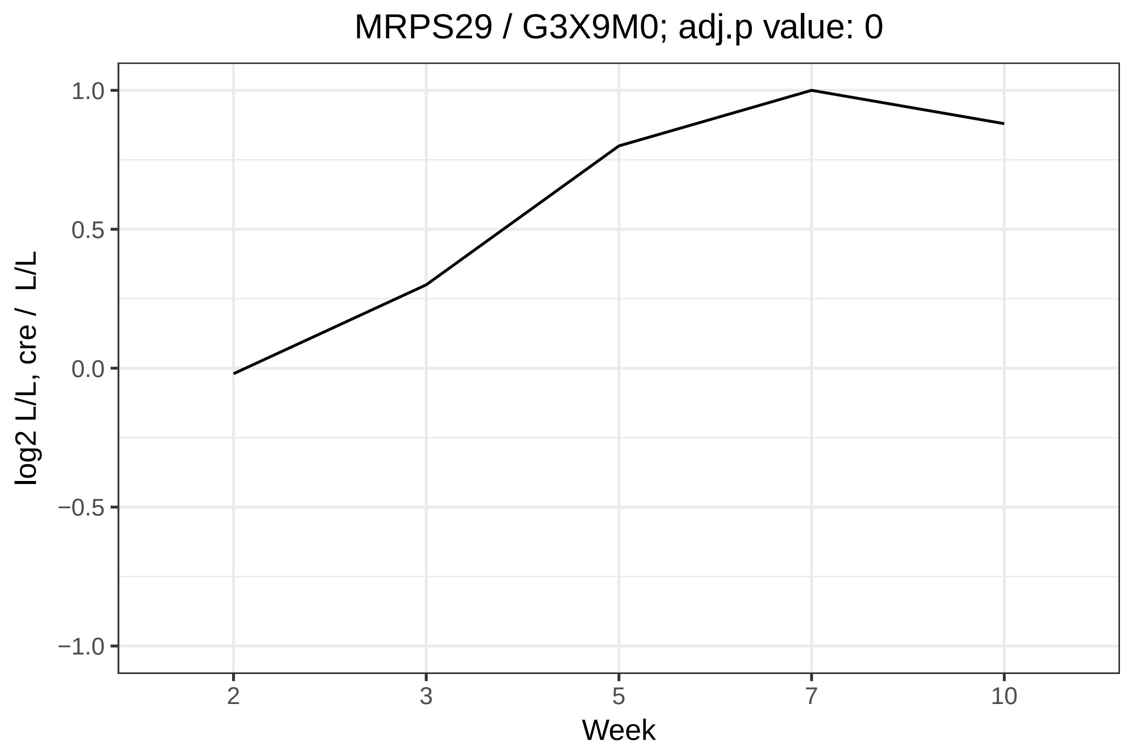

MRPS29 / G3X9M0; adj.p value: 0
log2 L/L, cre / L/L
1.0
0.5
0.0
−0.5
−1.0
2
3
5
7
10
Week

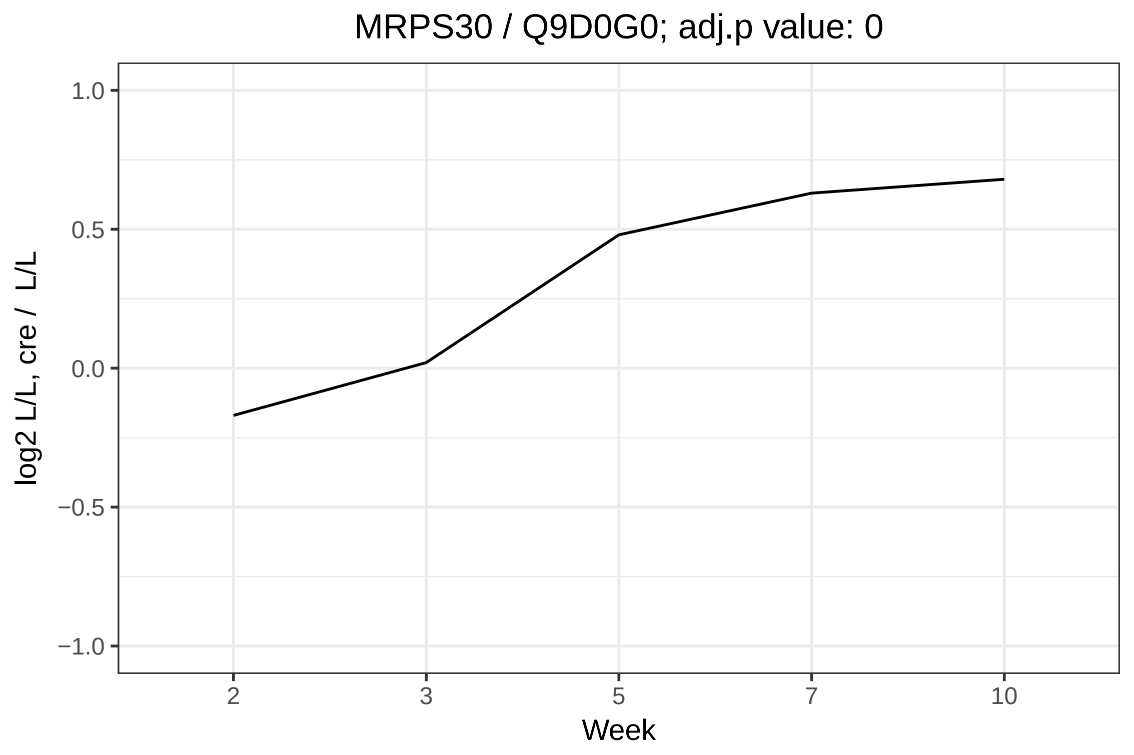
MRPS30 / Q9D0G0; adj.p value: 0
log2 L/L, cre / L/L
1.0
0.5
0.0
−0.5
−1.0
2
3
5
7
10
Week

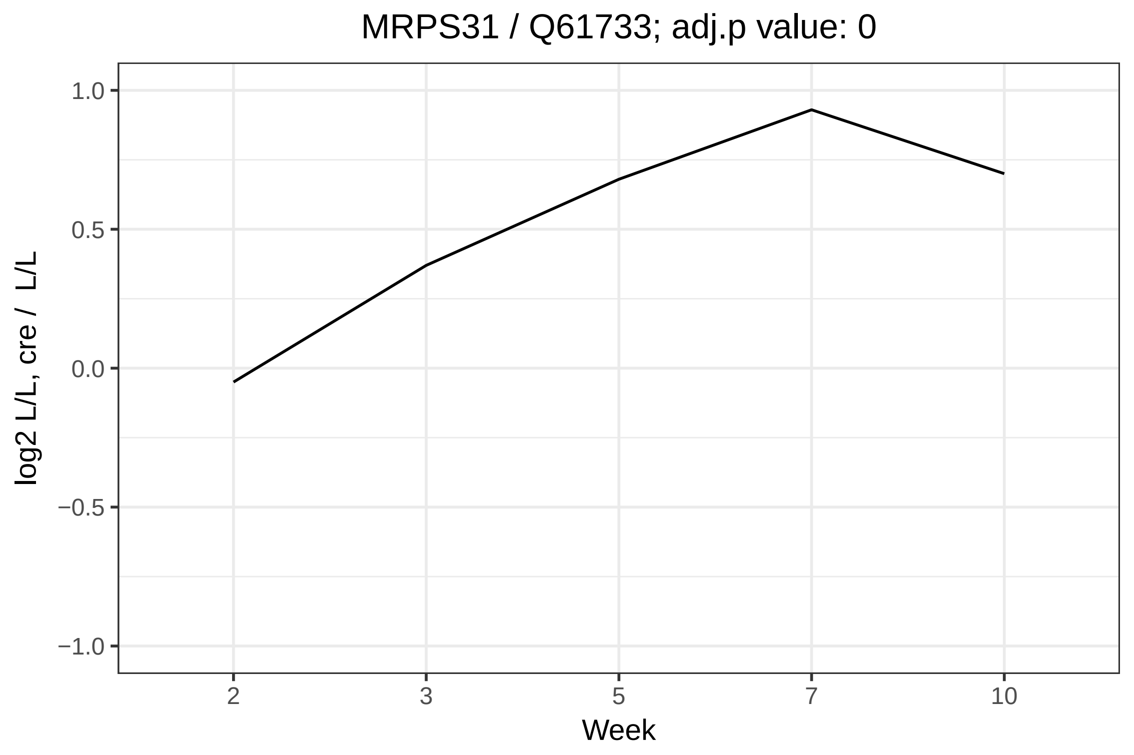

MRPS31 / Q61733; adj.p value: 0
1.0
0.5
0.0
−0.5
−1.0
log2 L/L, cre / L/L
2
3
5
7
10
Week

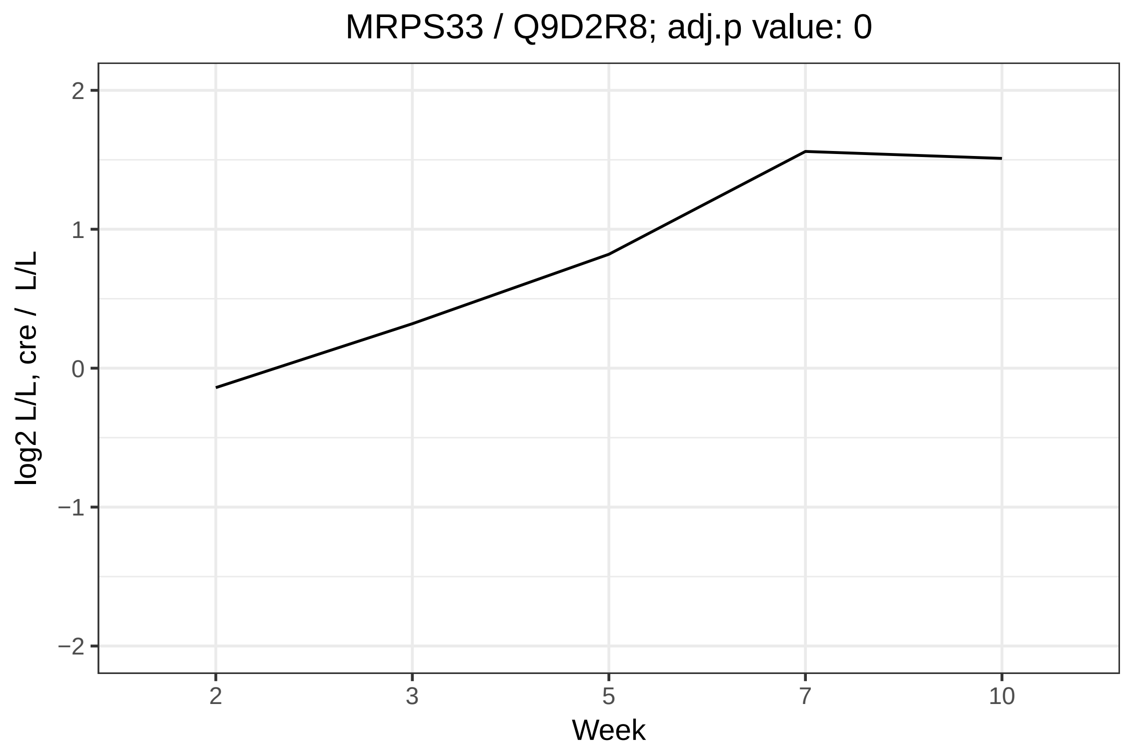
MRPS33 / Q9D2R8; adj.p value: 0
log2 L/L, cre / L/L
2
1
0
−1
−2
2
3
5
7
10
Week

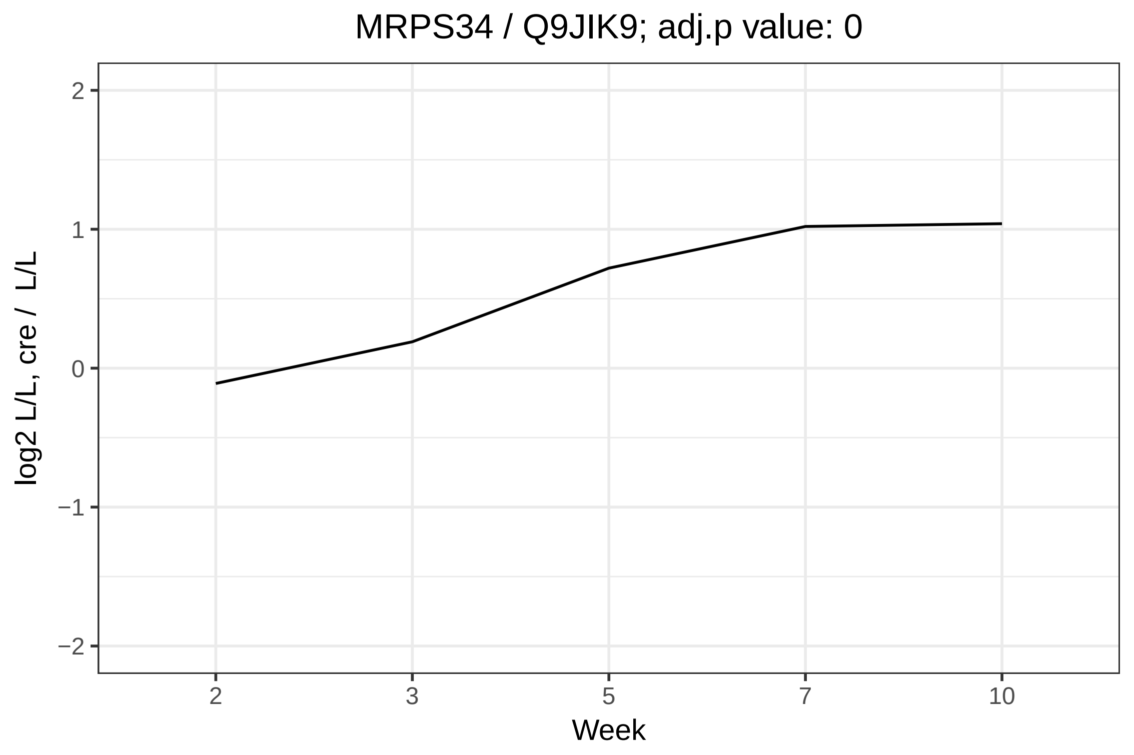
MRPS34 / Q9JIK9; adj.p value: 0
log2 L/L, cre / L/L
2
1
0
−1
−2
2
3
5
7
10
Week

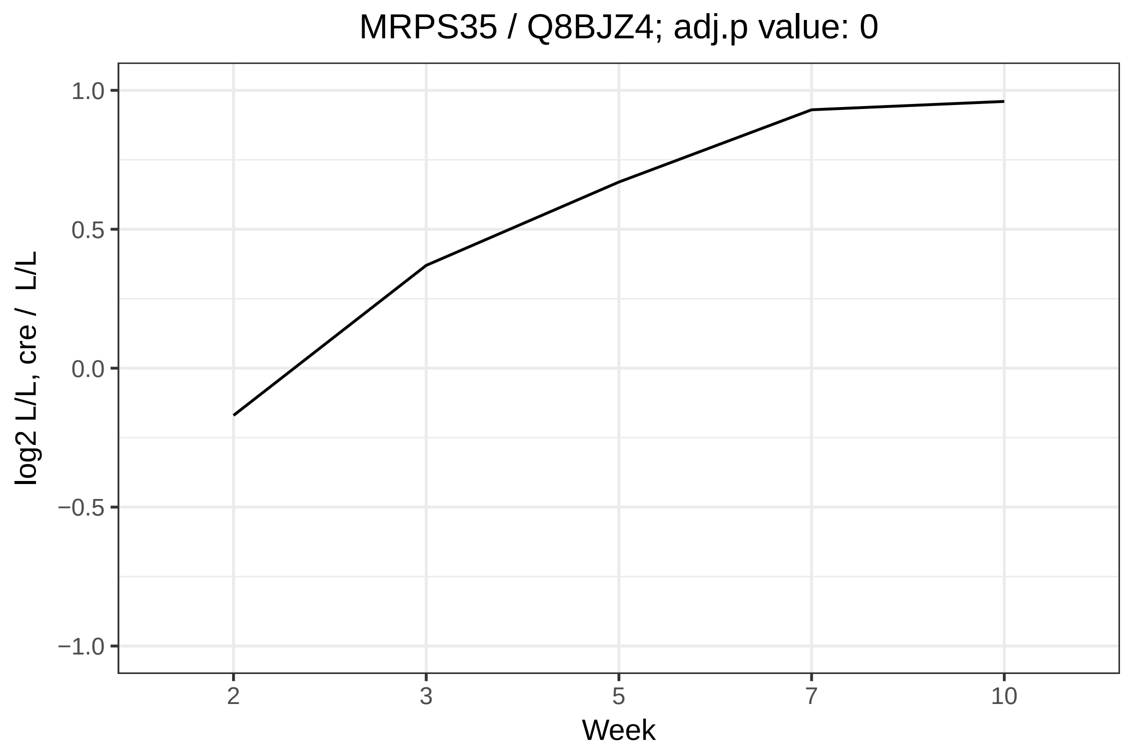
MRPS35 / Q8BJZ4; adj.p value: 0
log2 L/L, cre / L/L
1.0
0.5
0.0
−0.5
−1.0
2
3
5
7
10
Week

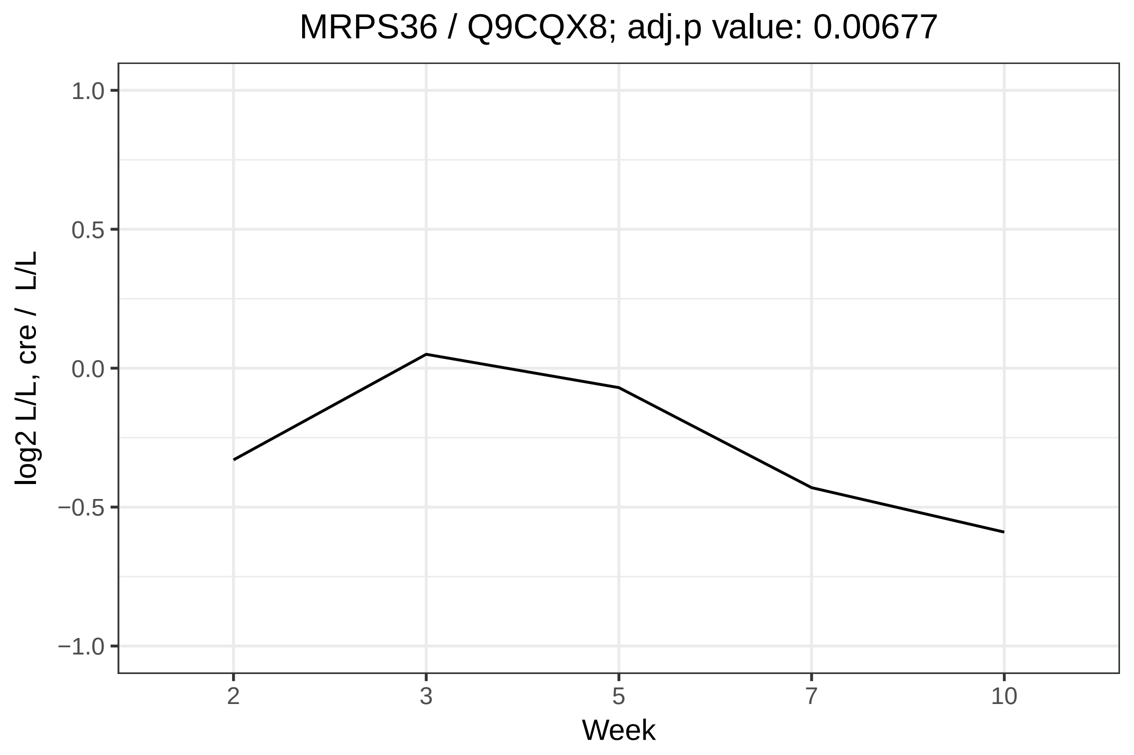
MRPS36 / Q9CQX8; adj.p value: 0.00677
log2 L/L, cre / L/L
1.0
0.5
0.0
−0.5
−1.0
2
3
5
7
10
Week

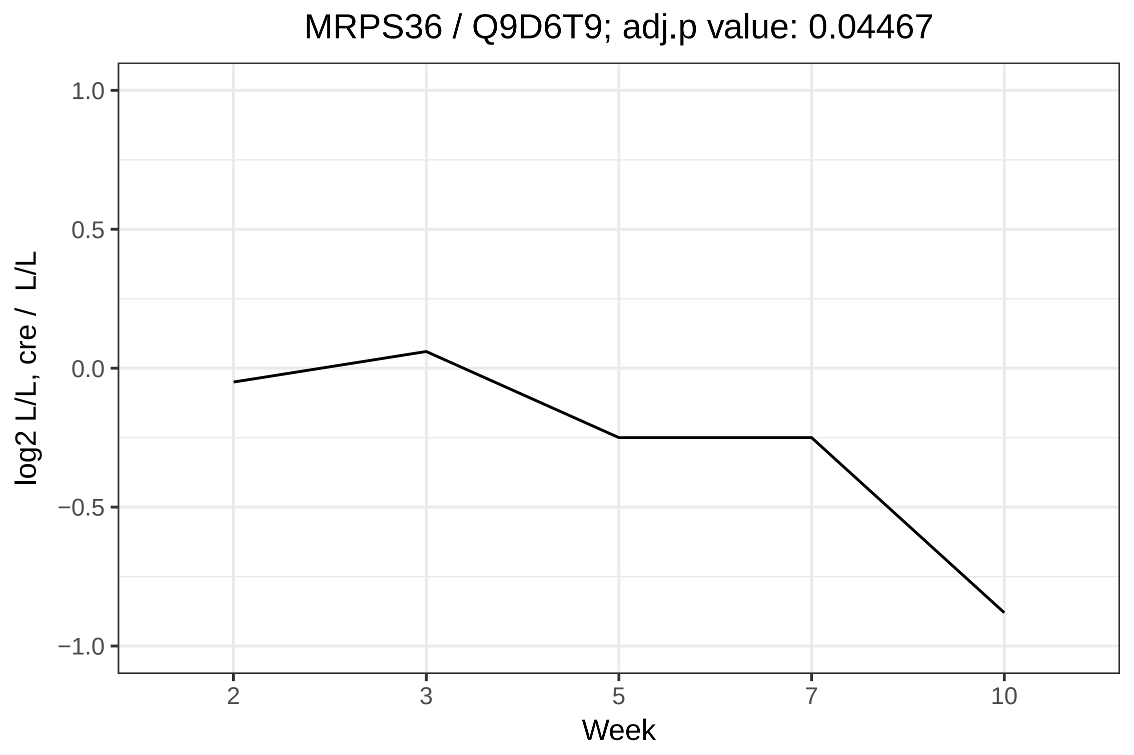
MRPS36 / Q9D6T9; adj.p value: 0.04467
log2 L/L, cre / L/L
1.0
0.5
0.0
−0.5
−1.0
2
3
5
7
10
Week

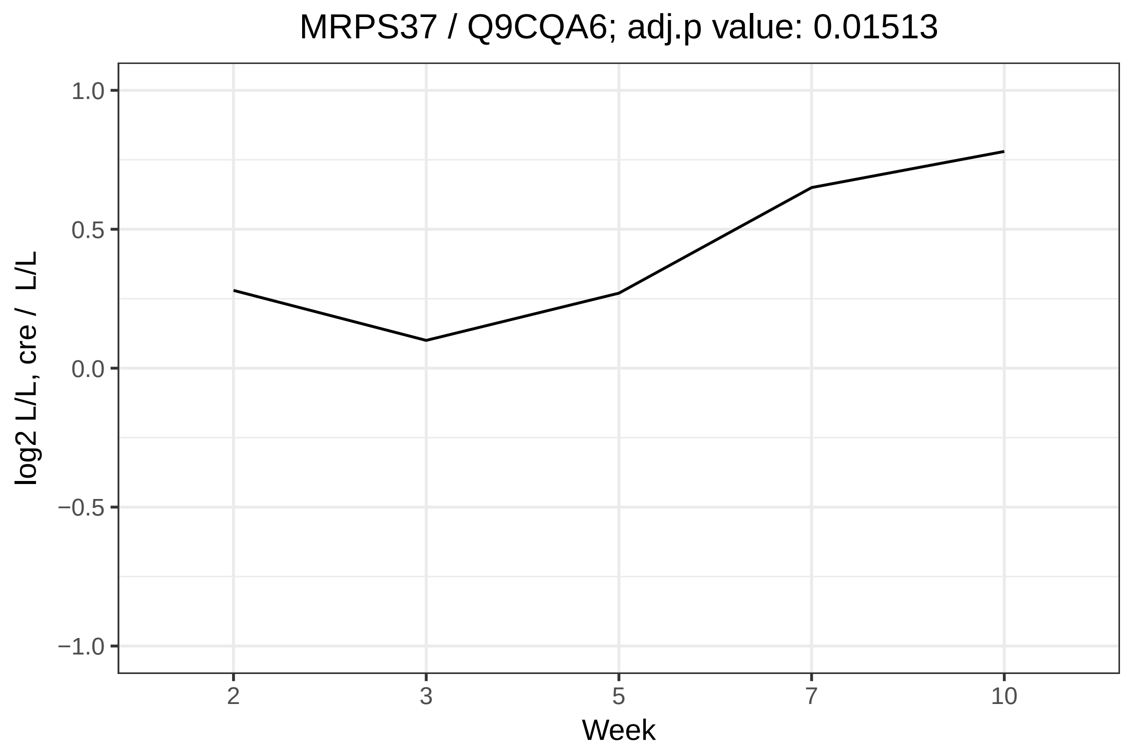
MRPS37 / Q9CQA6; adj.p value: 0.01513
1.0
0.5
0.0
−0.5
−1.0
log2 L/L, cre / L/L
2
3
5
7
10
Week

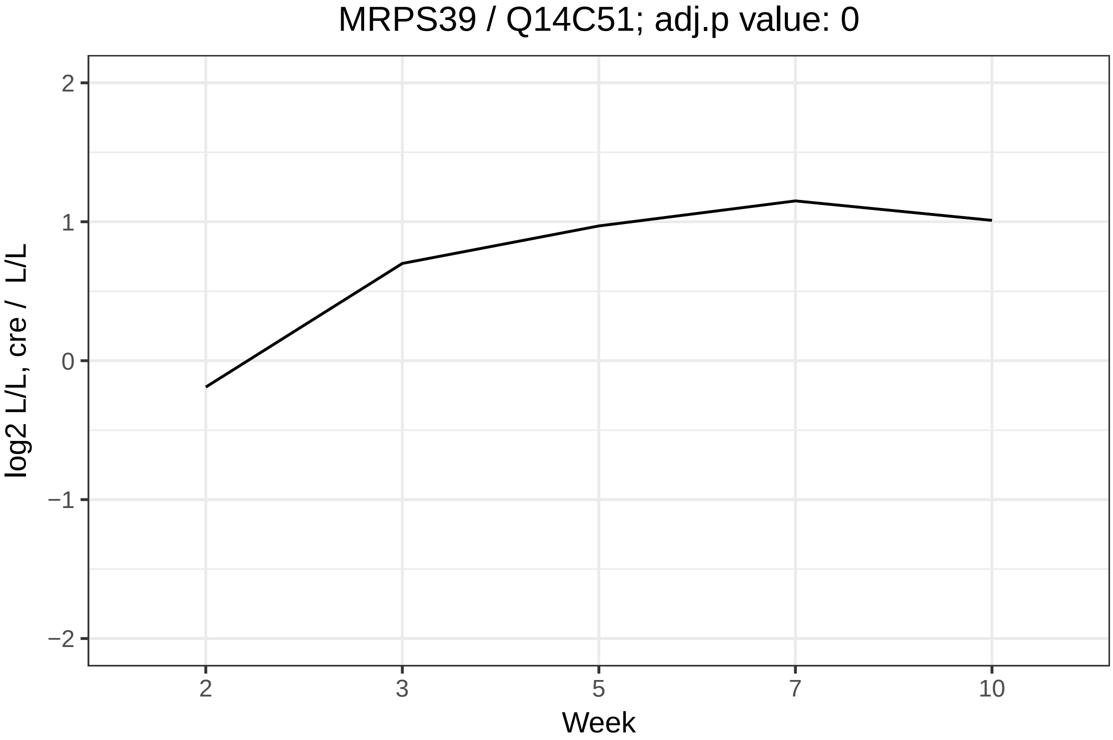
MRPS39 / Q14C51; adj.p value: 0
log2 L/L, cre / L/L
2
1
0
−1
−2
2
3
5
7
10
Week

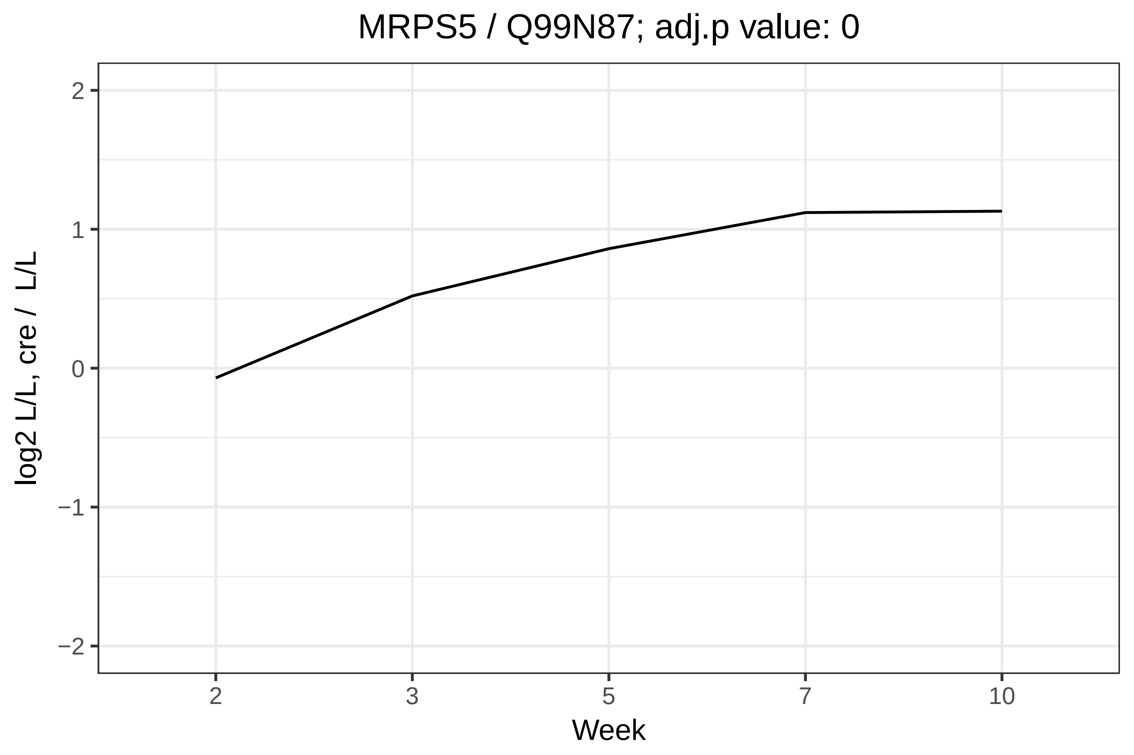

MRPS5 / Q99N87; adj.p value: 0
log2 L/L, cre / L/L
Week
2
1
0
−1
−2
2
3
5
7
10

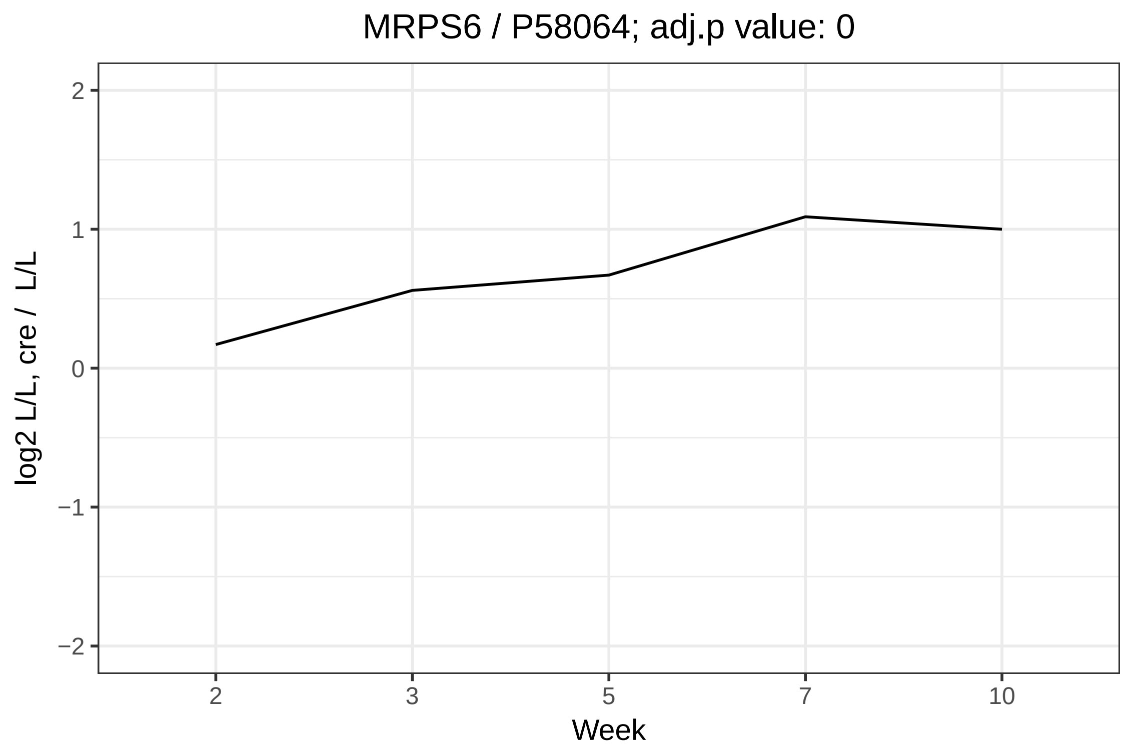
MRPS6 / P58064; adj.p value: 0
2
1
0
−1
−2
log2 L/L, cre / L/L
2
3
5
7
10
Week

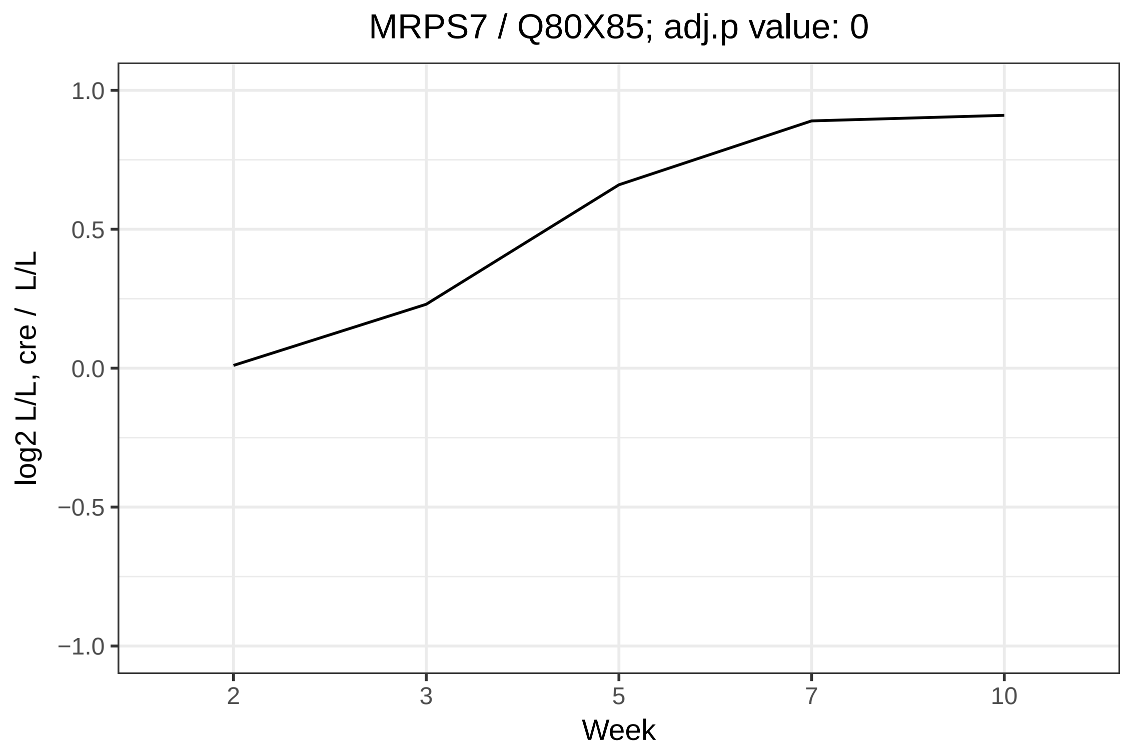
MRPS7 / Q80X85; adj.p value: 0
1.0
0.5
0.0
−0.5
−1.0
log2 L/L, cre / L/L
2
3
5
7
10
Week

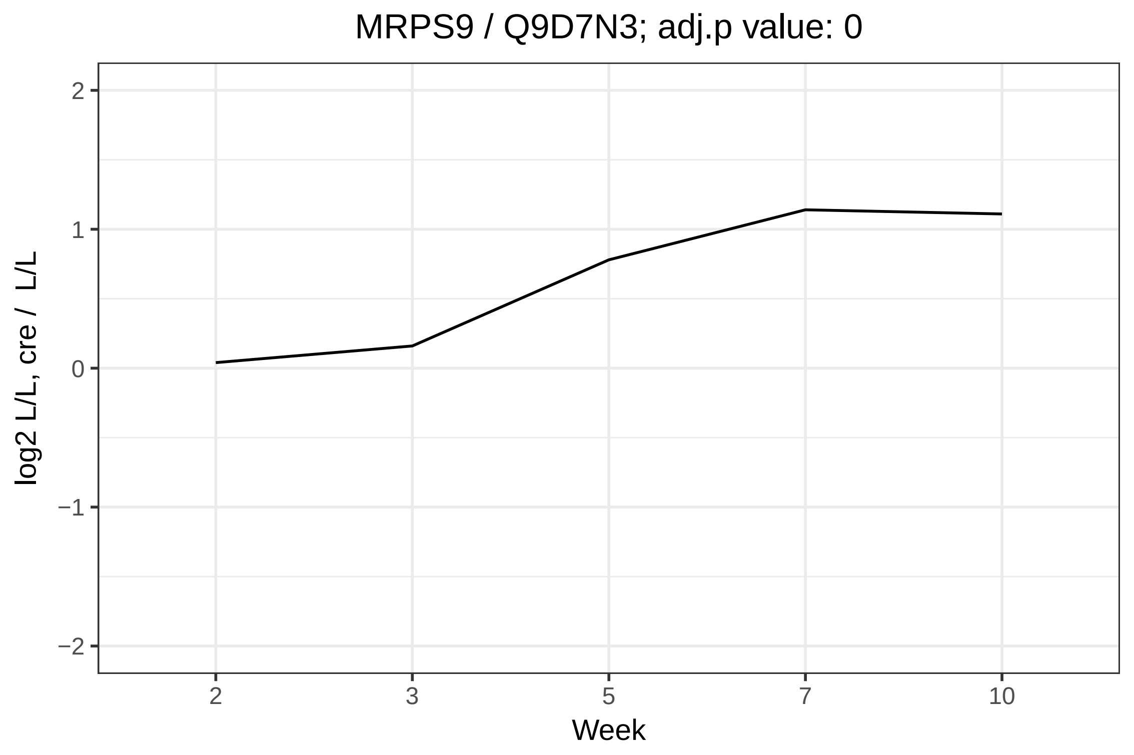

MRPS9 / Q9D7N3; adj.p value: 0
log2 L/L, cre / L/L
2
1
0
−1
−2
2
3
5
7
10
Week

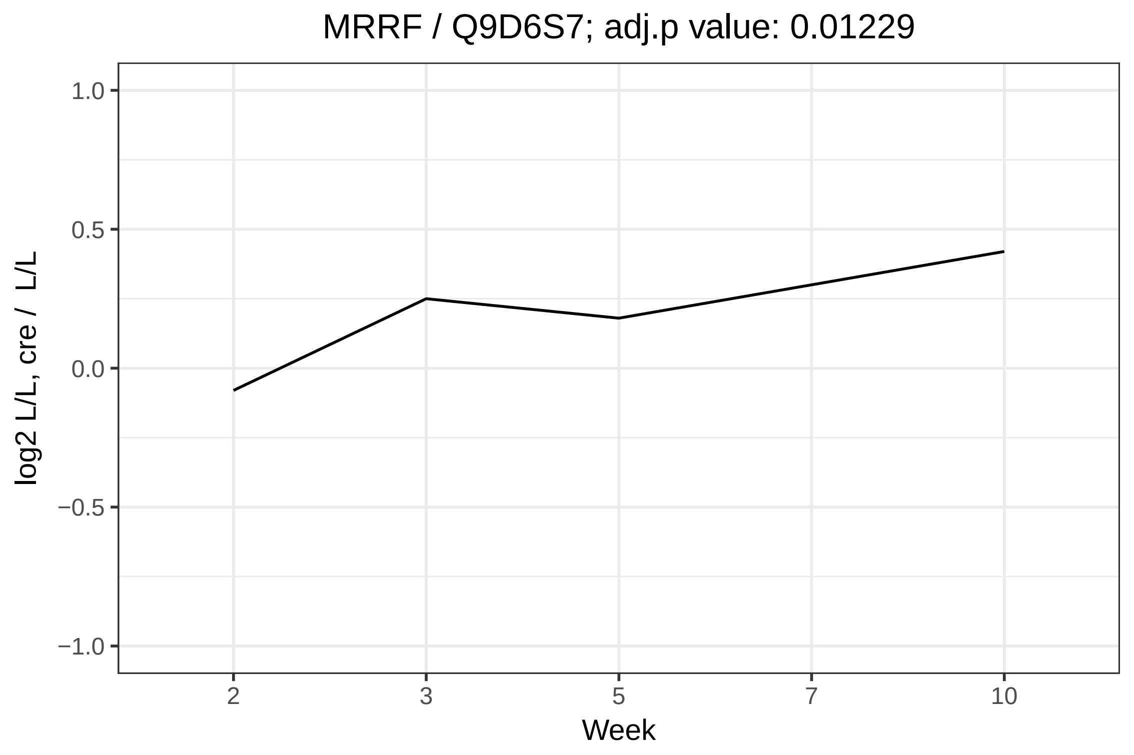
MRRF / Q9D6S7; adj.p value: 0.01229
log2 L/L, cre / L/L
1.0
0.5
0.0
−0.5
−1.0
2
3
5
7
10
Week

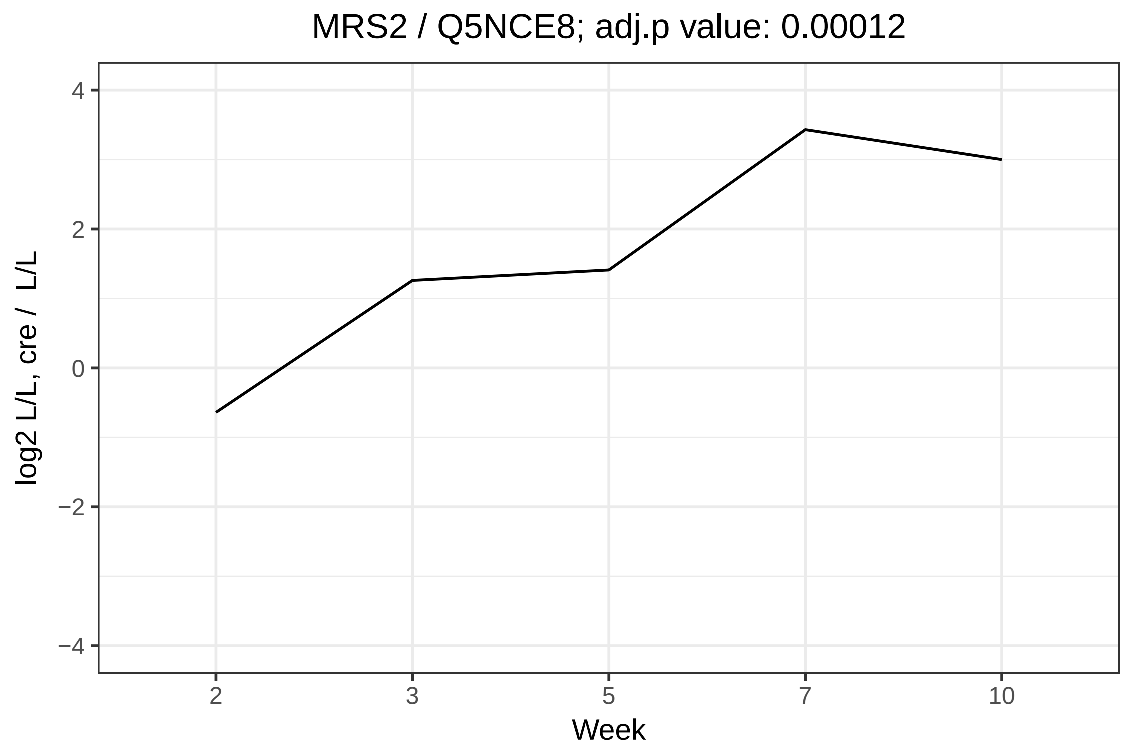

MRS2 / Q5NCE8; adj.p value: 0.00012
log2 L/L, cre / L/L
4
2
0
−2
−4
2
3
5
7
10
Week

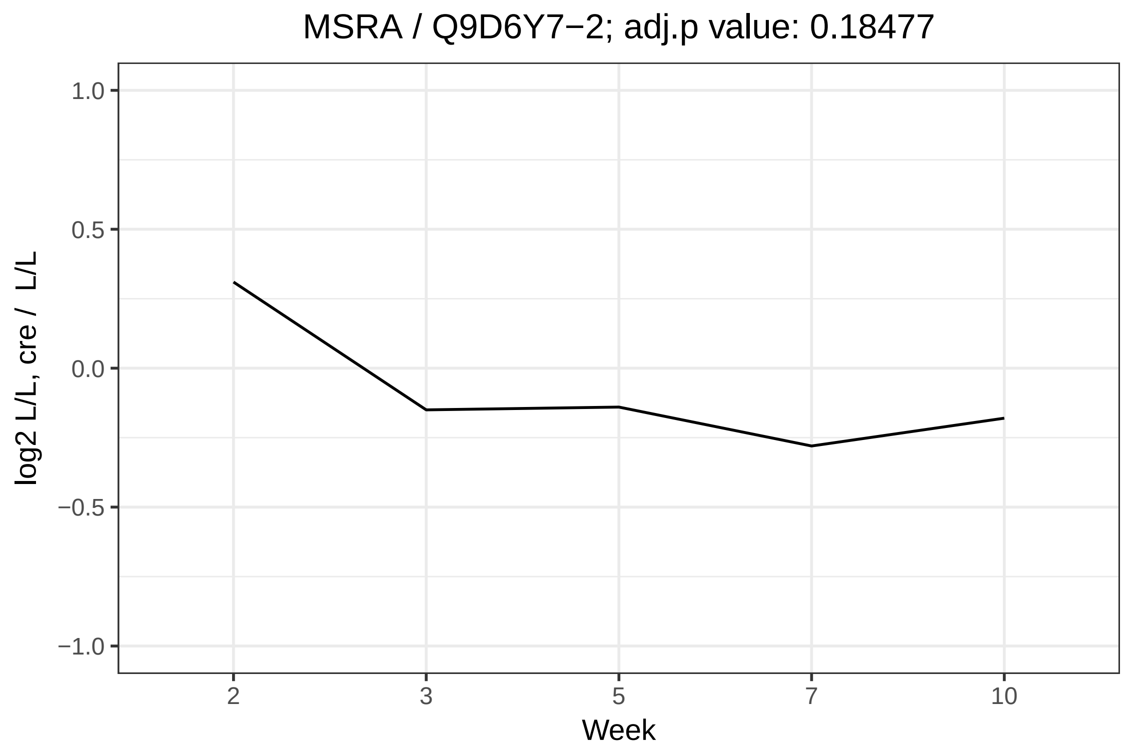
MSRA / Q9D6Y7−2; adj.p value: 0.18477
log2 L/L, cre / L/L
1.0
0.5
0.0
−0.5
−1.0
2
3
5
7
10
Week

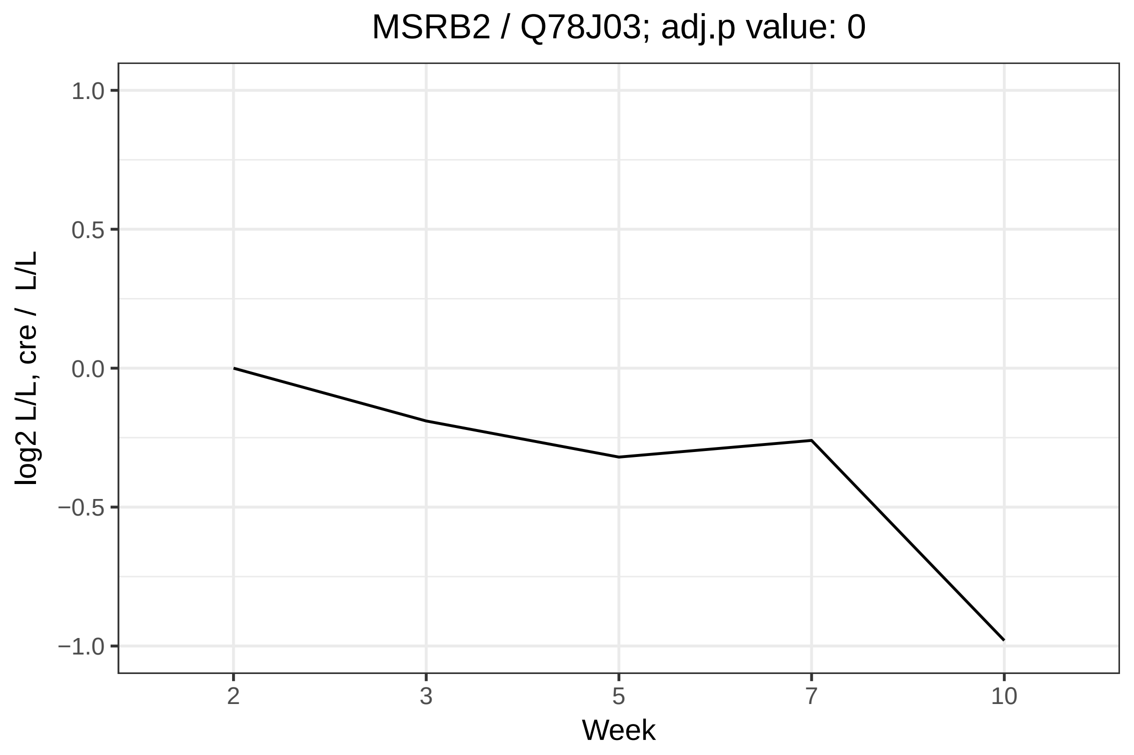

MSRB2 / Q78J03; adj.p value: 0
log2 L/L, cre / L/L
1.0
0.5
0.0
−0.5
−1.0
2
3
5
7
10
Week

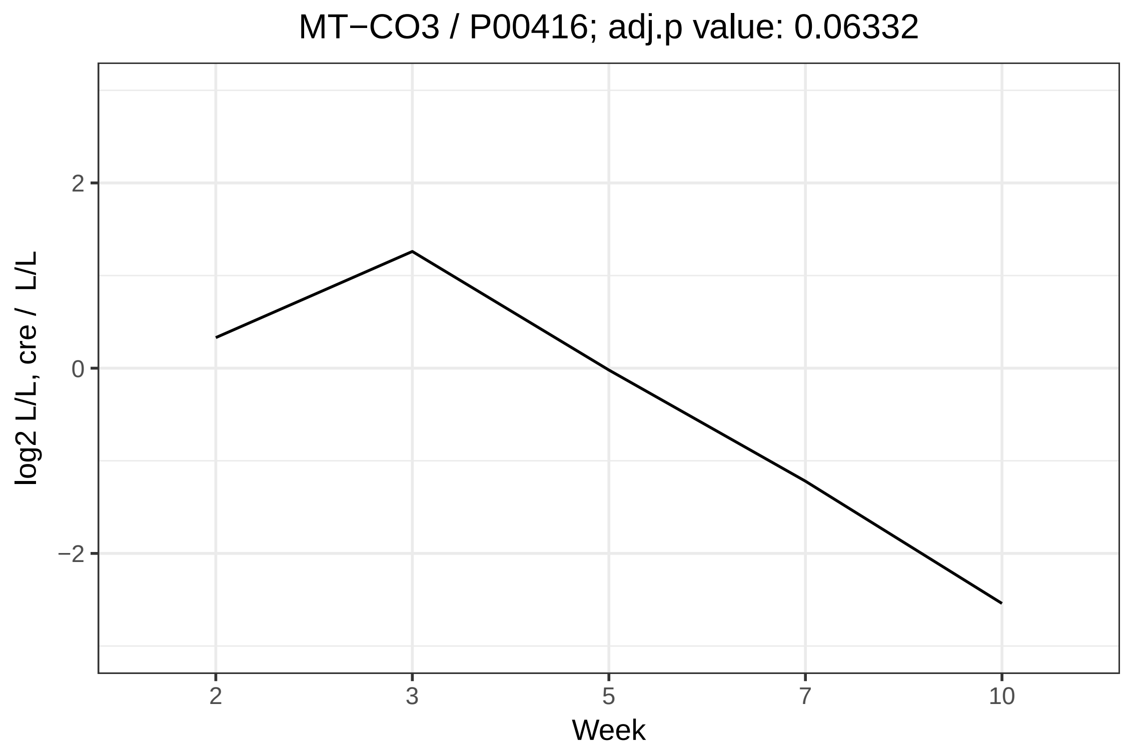

MT−CO3 / P00416; adj.p value: 0.06332
log2 L/L, cre / L/L
2
0
−2
2
3
5
7
10
Week

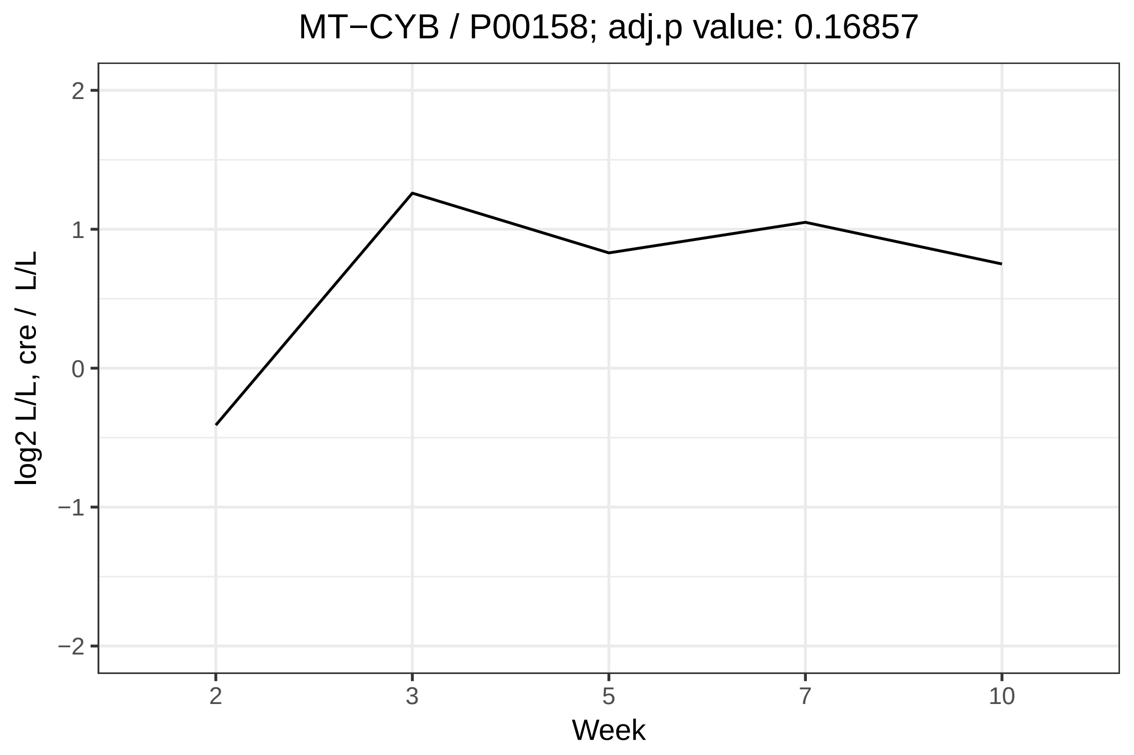
MT−CYB / P00158; adj.p value: 0.16857
log2 L/L, cre / L/L
Week
2
1
0
−1
−2
2
3
5
7
10

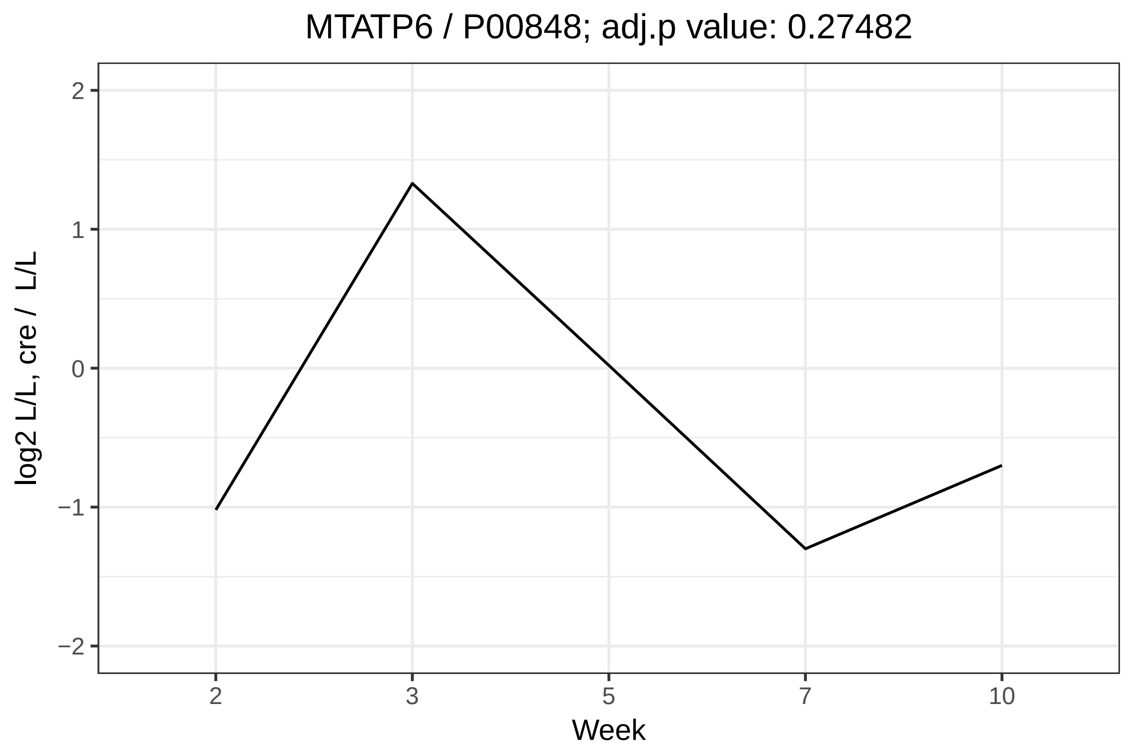

MTATP6 / P00848; adj.p value: 0.27482
log2 L/L, cre / L/L
2
1
0
−1
−2
2
3
5
7
10
Week

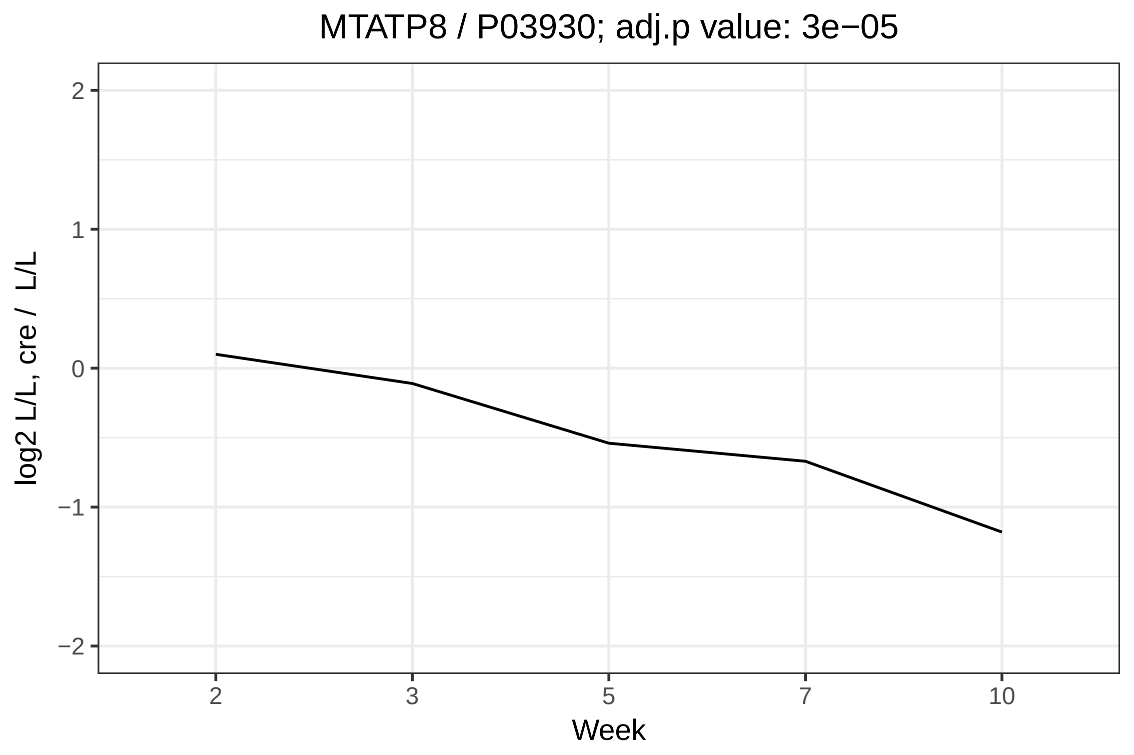
MTATP8 / P03930; adj.p value: 3e−05
log2 L/L, cre / L/L
2
1
0
−1
−2
2
3
5
7
10
Week

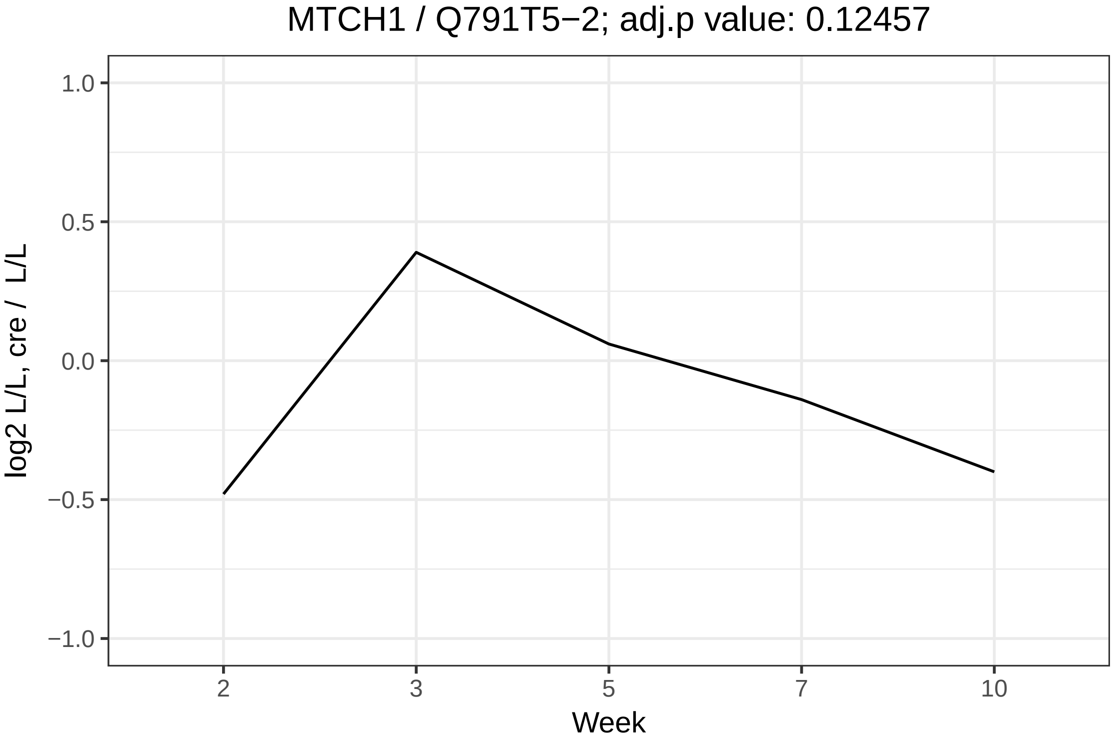
MTCH1 / Q791T5−2; adj.p value: 0.12457
log2 L/L, cre / L/L
1.0
0.5
0.0
−0.5
−1.0
2
3
5
7
10
Week

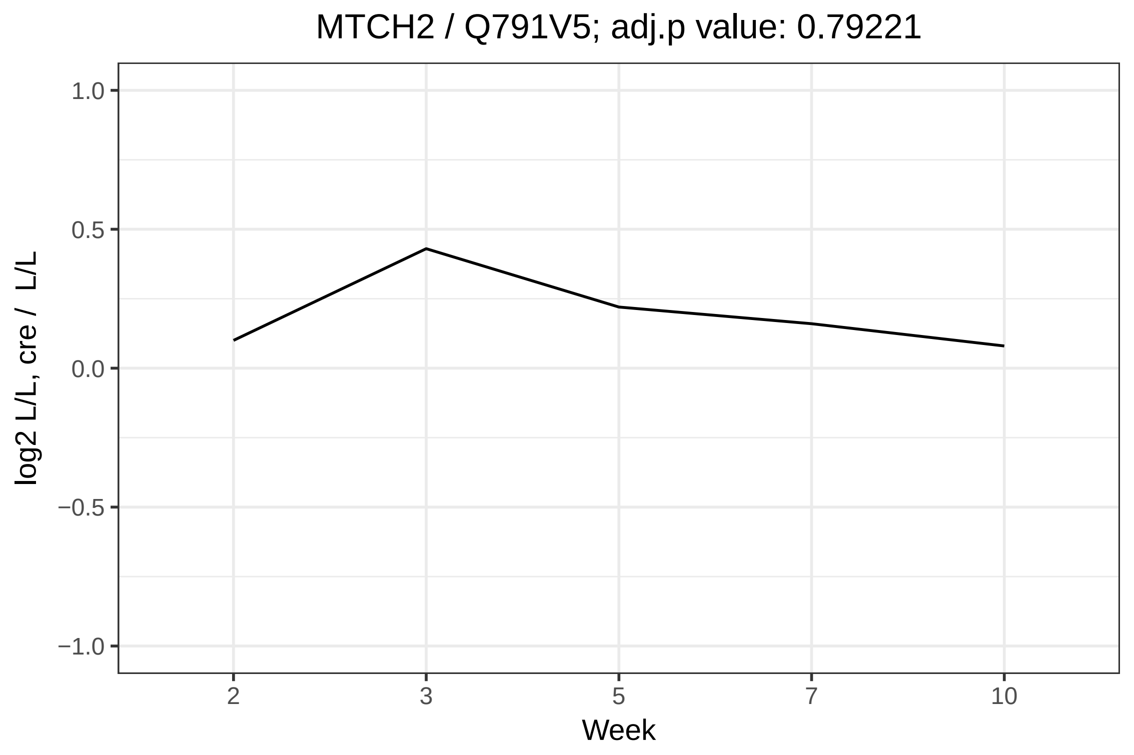
MTCH2 / Q791V5; adj.p value: 0.79221
1.0
0.5
0.0
−0.5
−1.0
log2 L/L, cre / L/L
2
3
5
7
10
Week

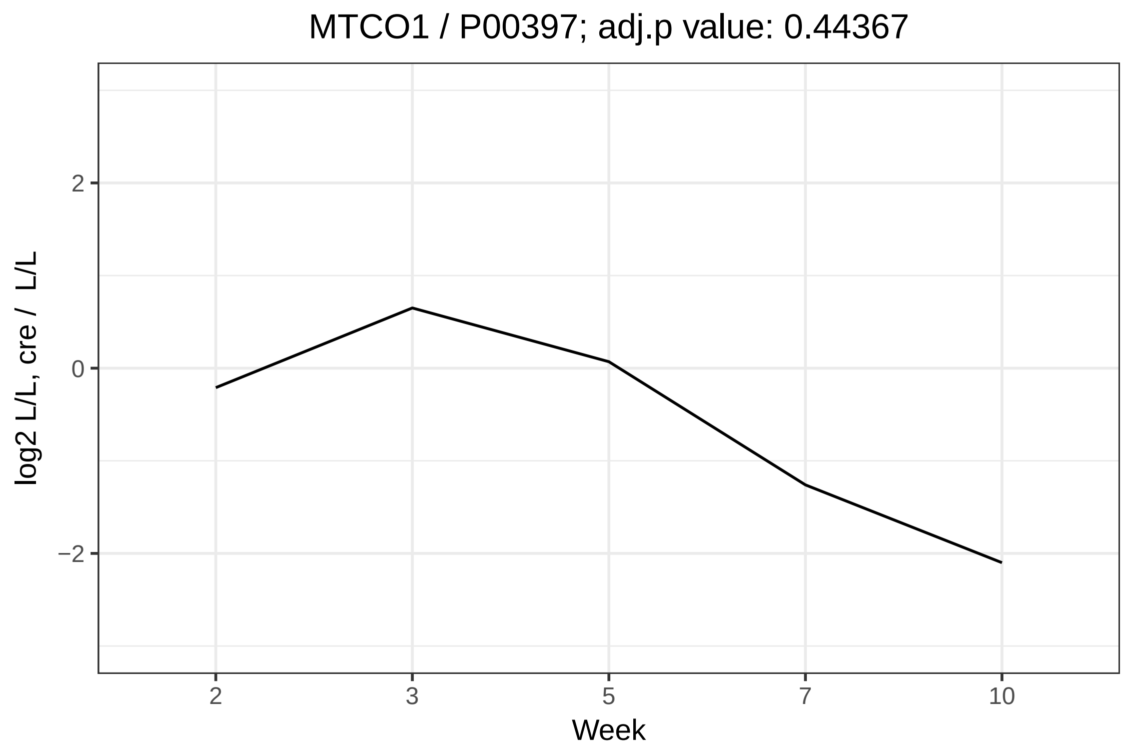

MTCO1 / P00397; adj.p value: 0.44367
log2 L/L, cre / L/L
2
0
−2
2
3
5
7
10
Week

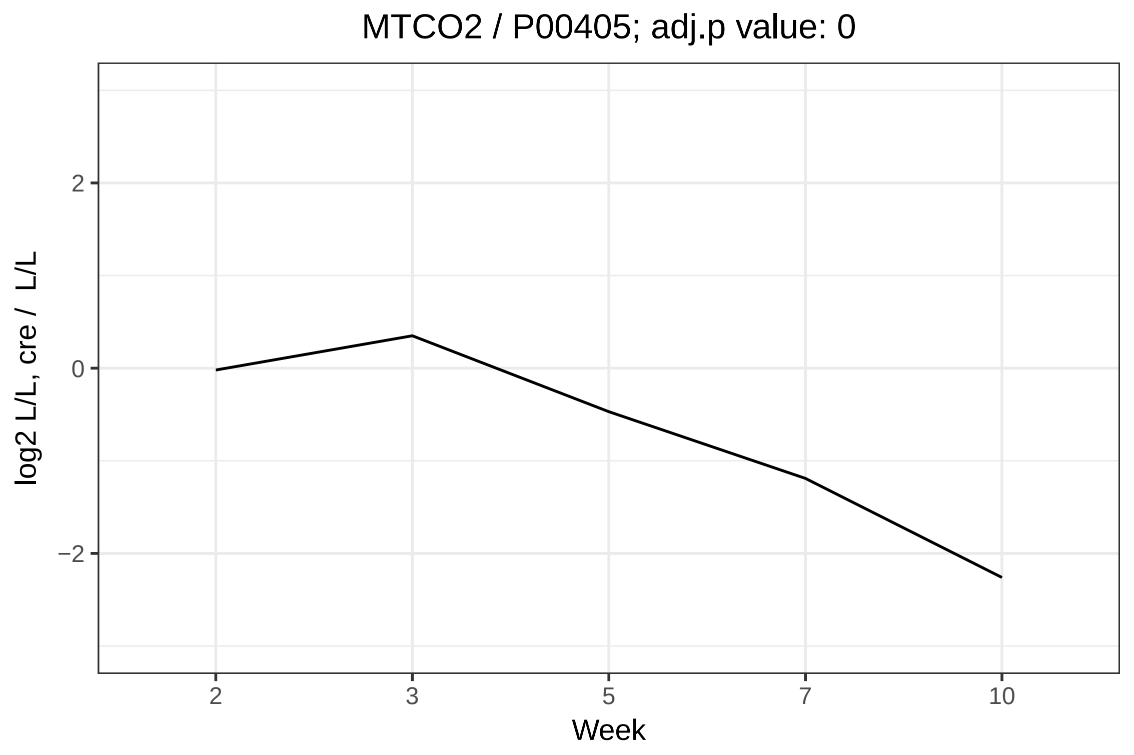
MTCO2 / P00405; adj.p value: 0
log2 L/L, cre / L/L
2
0
−2
2
3
5
7
10
Week

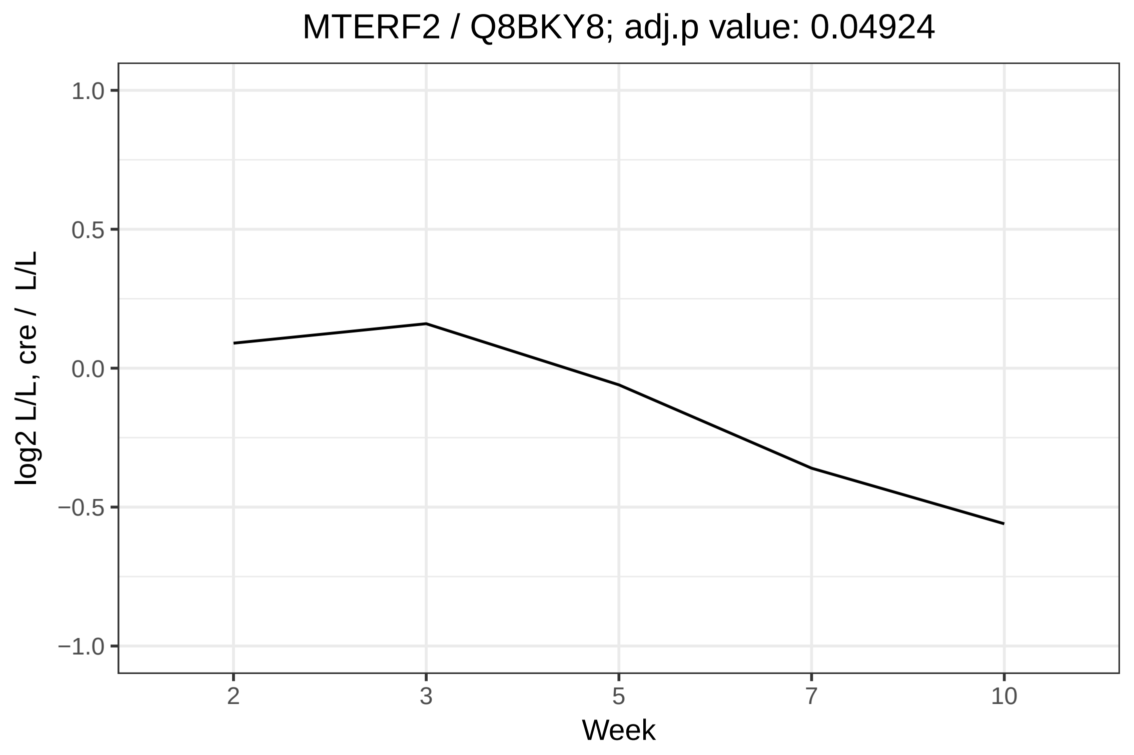
MTERF2 / Q8BKY8; adj.p value: 0.04924
log2 L/L, cre / L/L
1.0
0.5
0.0
−0.5
−1.0
2
3
5
7
10
Week

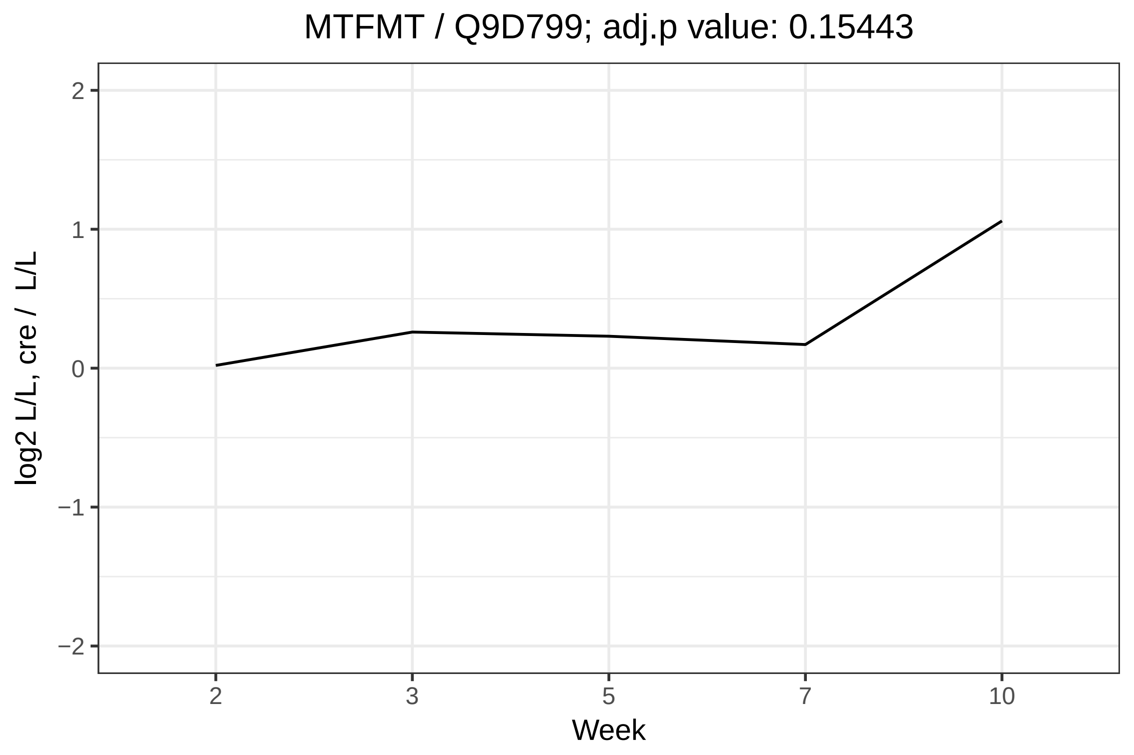

MTFMT / Q9D799; adj.p value: 0.15443
log2 L/L, cre / L/L
2
1
0
−1
−2
2
3
5
7
10
Week

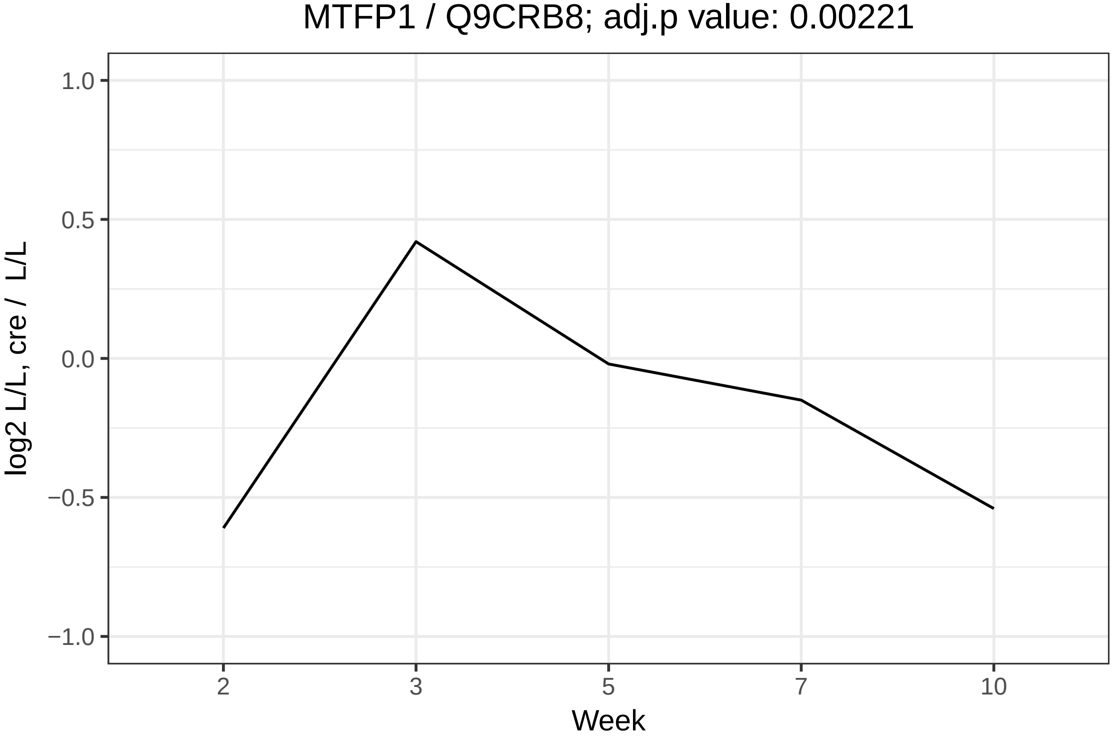
MTFP1 / Q9CRB8; adj.p value: 0.00221
1.0
0.5
0.0
−0.5
−1.0
log2 L/L, cre / L/L
2
3
5
7
10
Week

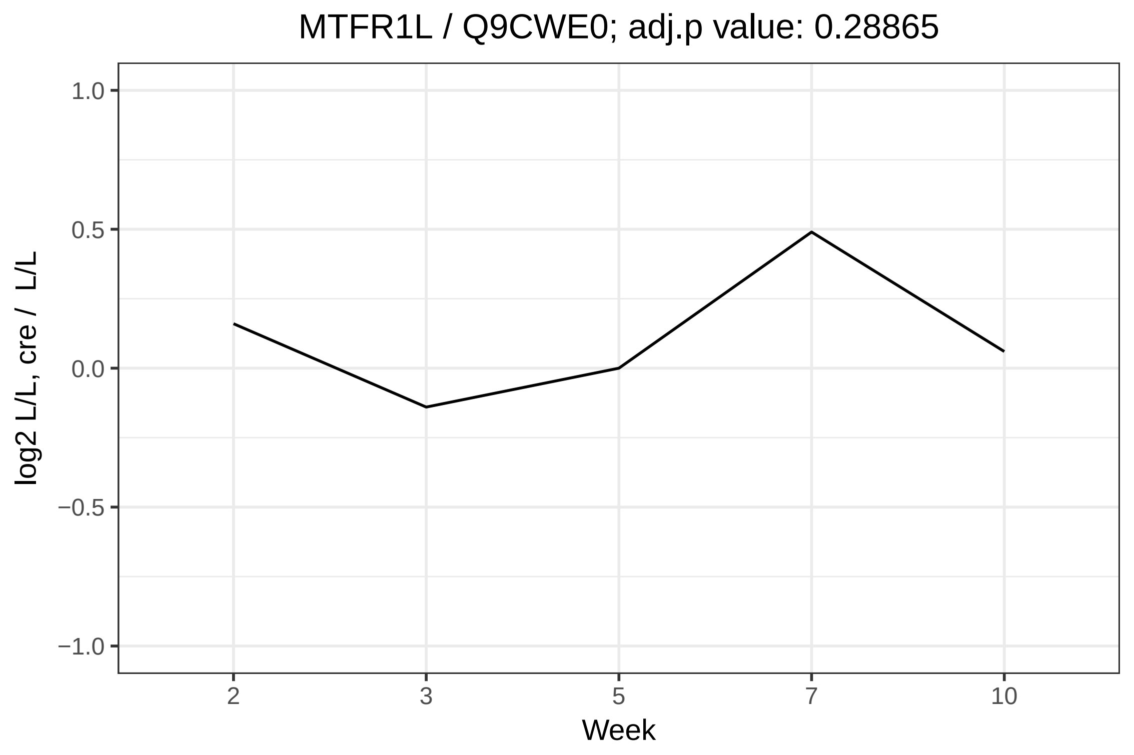
MTFR1L / Q9CWE0; adj.p value: 0.28865
log2 L/L, cre / L/L
1.0
0.5
0.0
−0.5
−1.0
2
3
5
7
10
Week

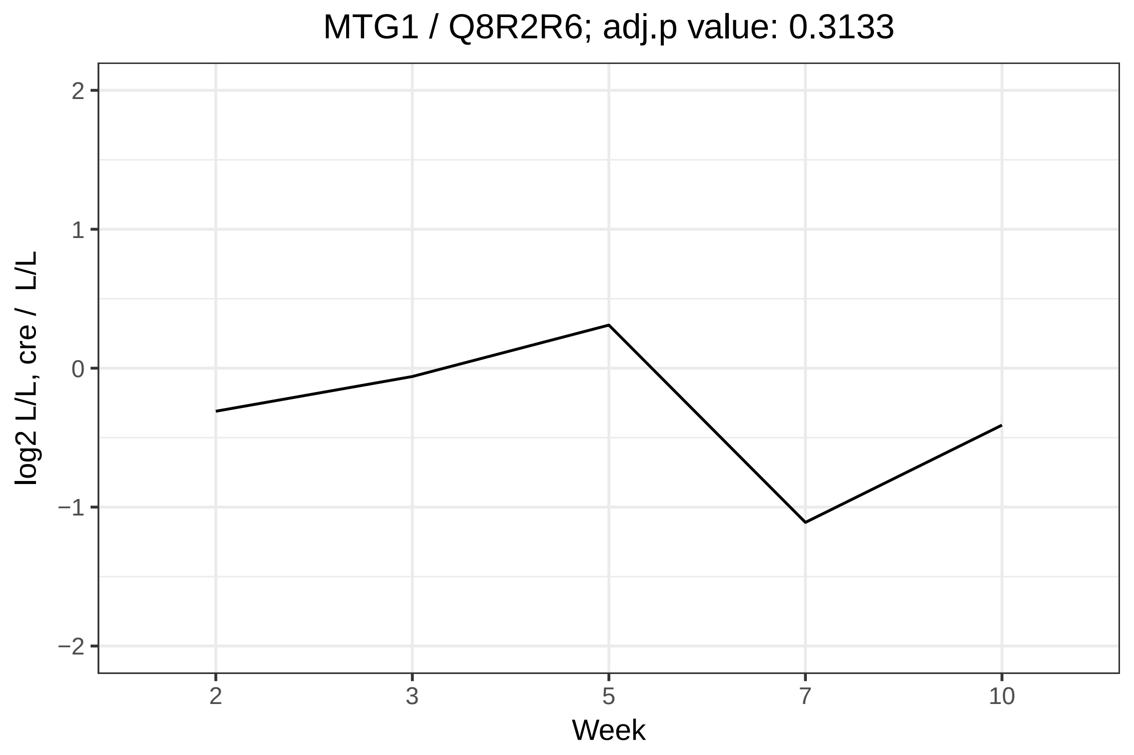

MTG1 / Q8R2R6; adj.p value: 0.3133
log2 L/L, cre / L/L
2
1
0
−1
−2
2
3
5
7
10
Week

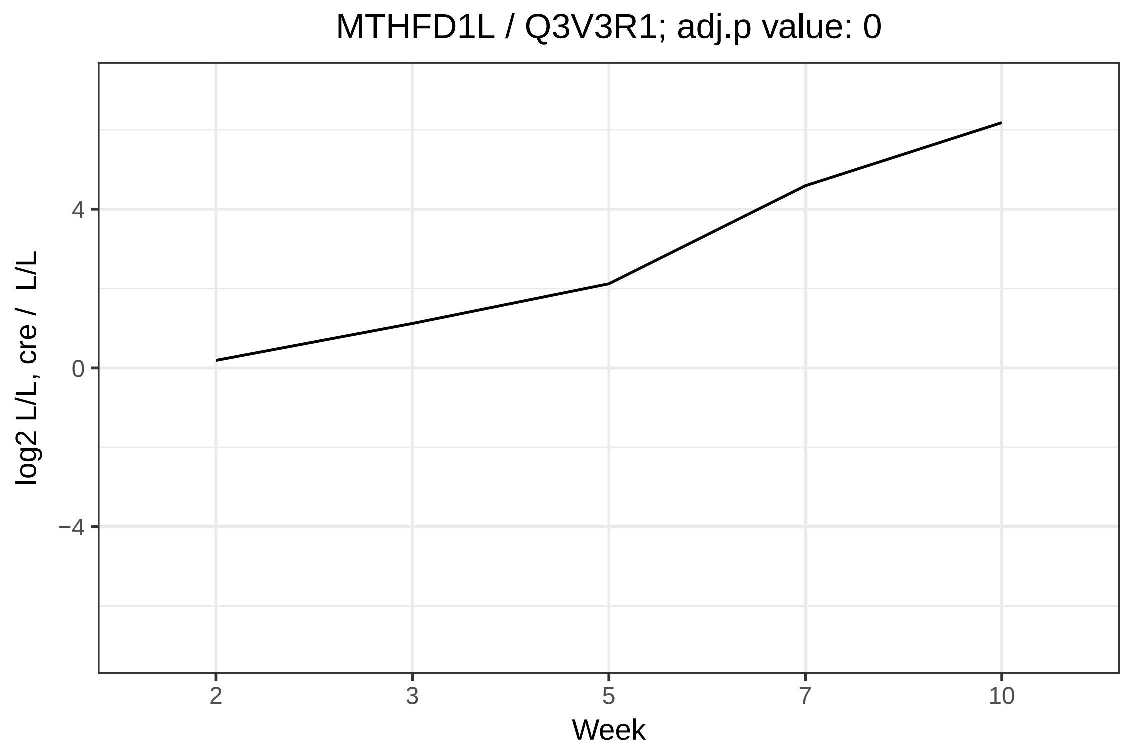
MTHFD1L / Q3V3R1; adj.p value: 0
log2 L/L, cre / L/L
4
0
−4
2
3
5
7
10
Week

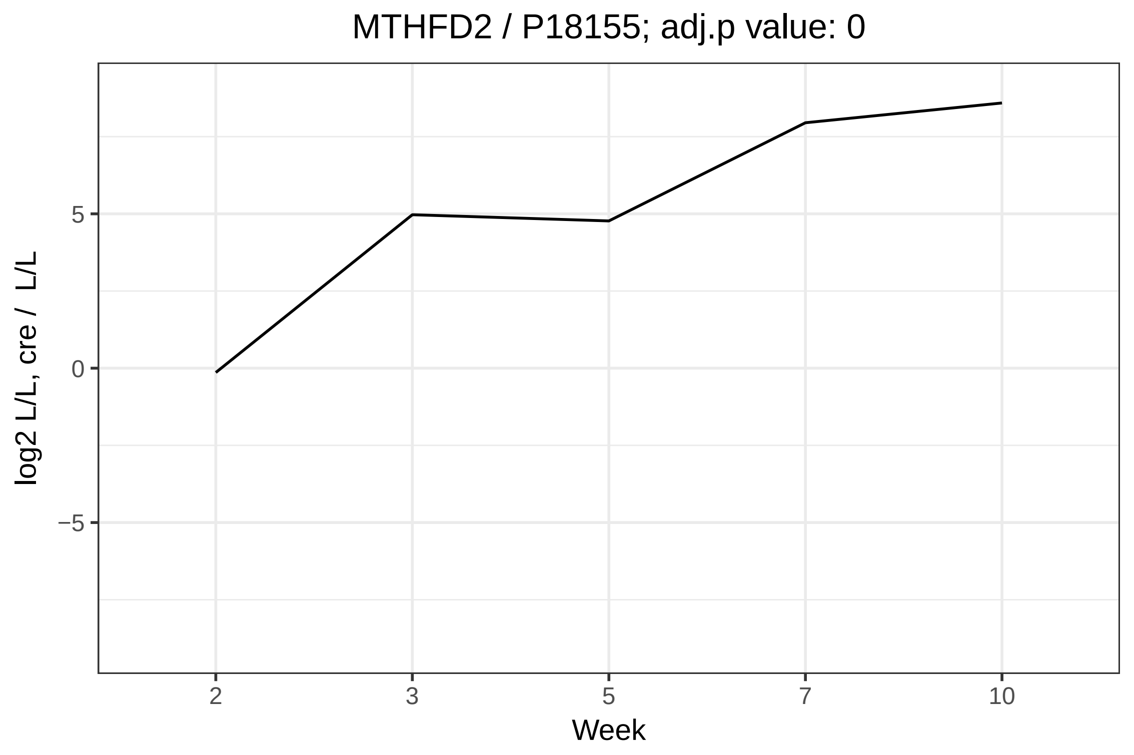

MTHFD2 / P18155; adj.p value: 0
log2 L/L, cre / L/L
5
0
−5
2
3
5
7
10
Week

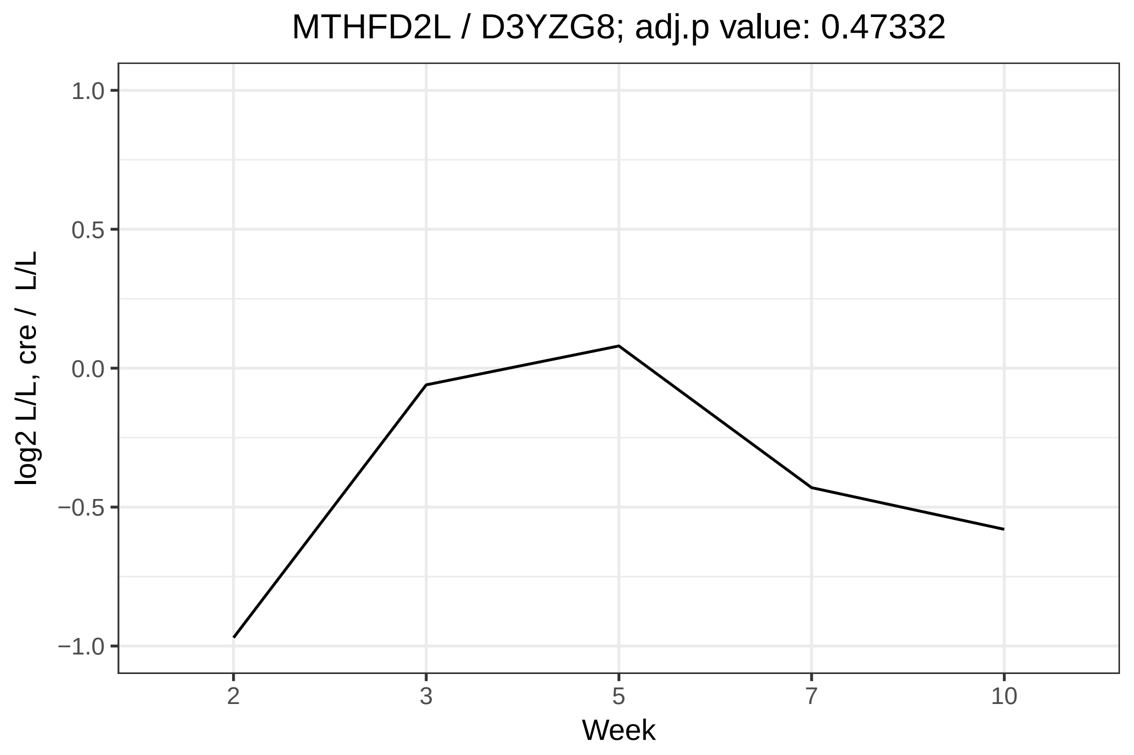
MTHFD2L / D3YZG8; adj.p value: 0.47332
log2 L/L, cre / L/L
1.0
0.5
0.0
−0.5
−1.0
2
3
5
7
10
Week

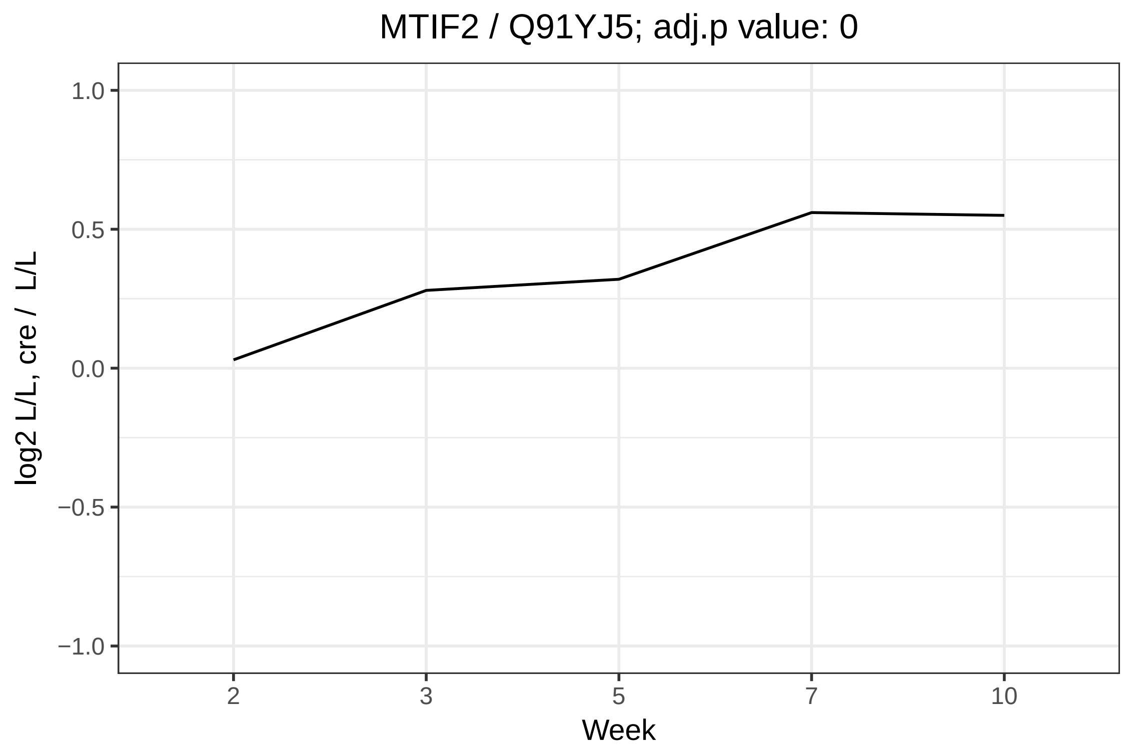
MTIF2 / Q91YJ5; adj.p value: 0
log2 L/L, cre / L/L
1.0
0.5
0.0
−0.5
−1.0
2
3
5
7
10
Week

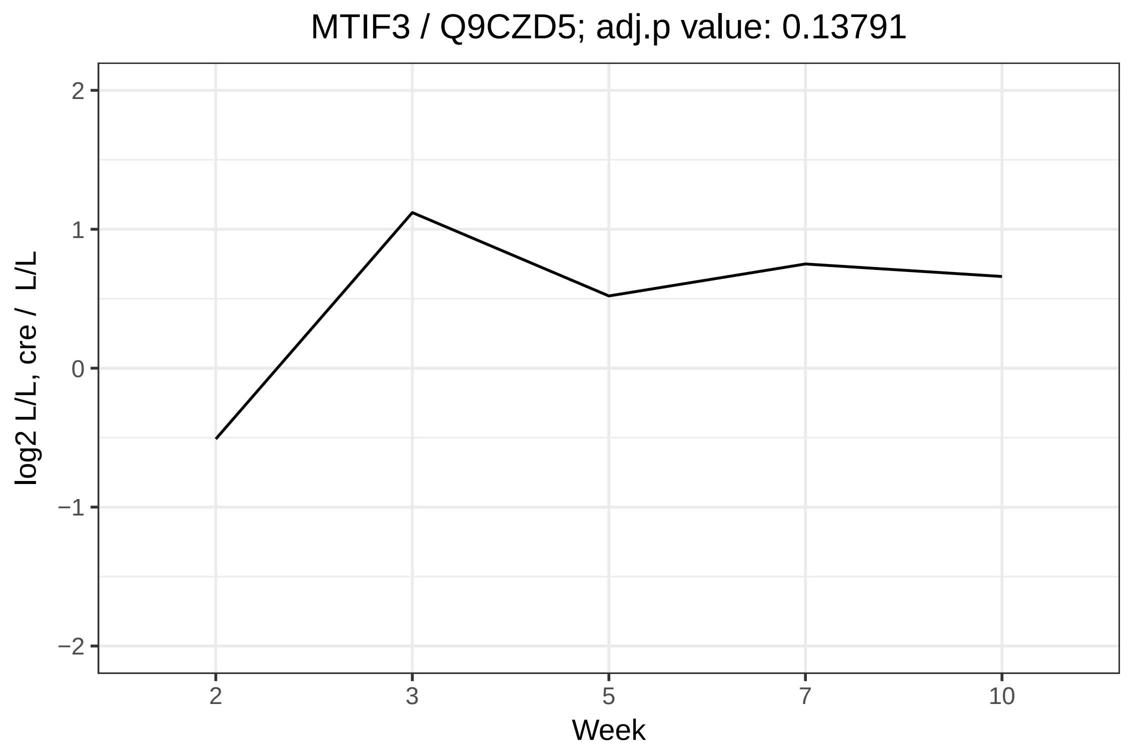
MTIF3 / Q9CZD5; adj.p value: 0.13791
log2 L/L, cre / L/L
2
1
0
−1
−2
2
3
5
7
10
Week

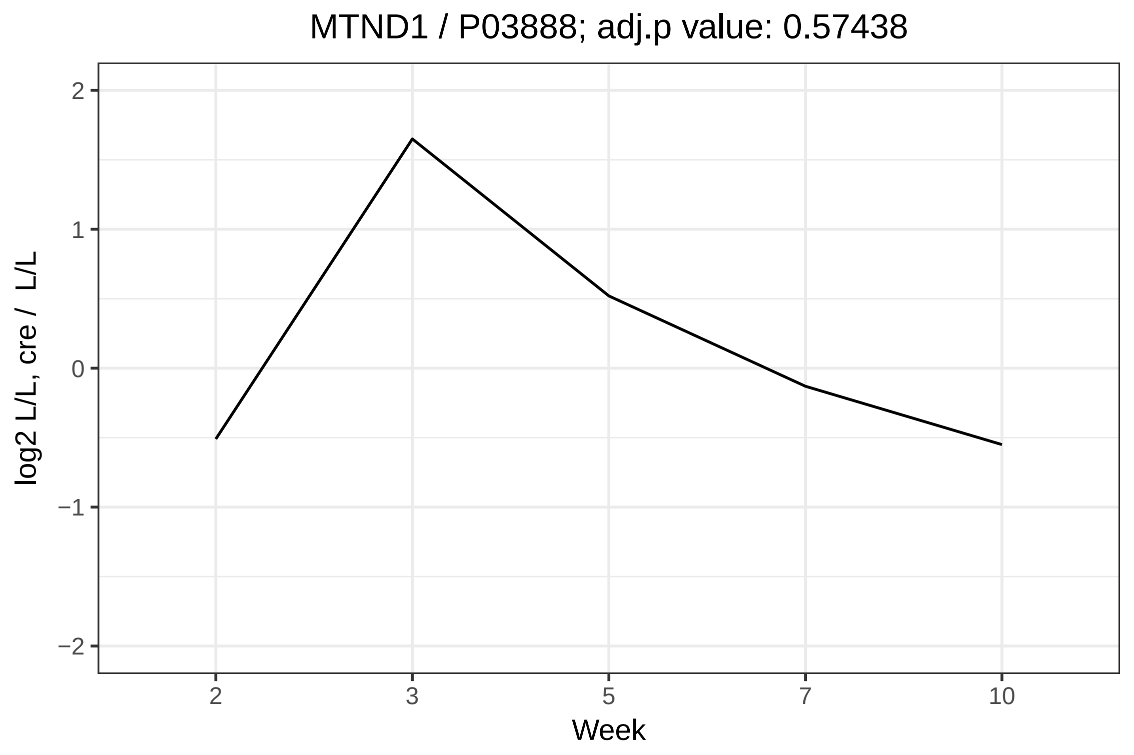
MTND1 / P03888; adj.p value: 0.57438
log2 L/L, cre / L/L
2
1
0
−1
−2
2
3
5
7
10
Week

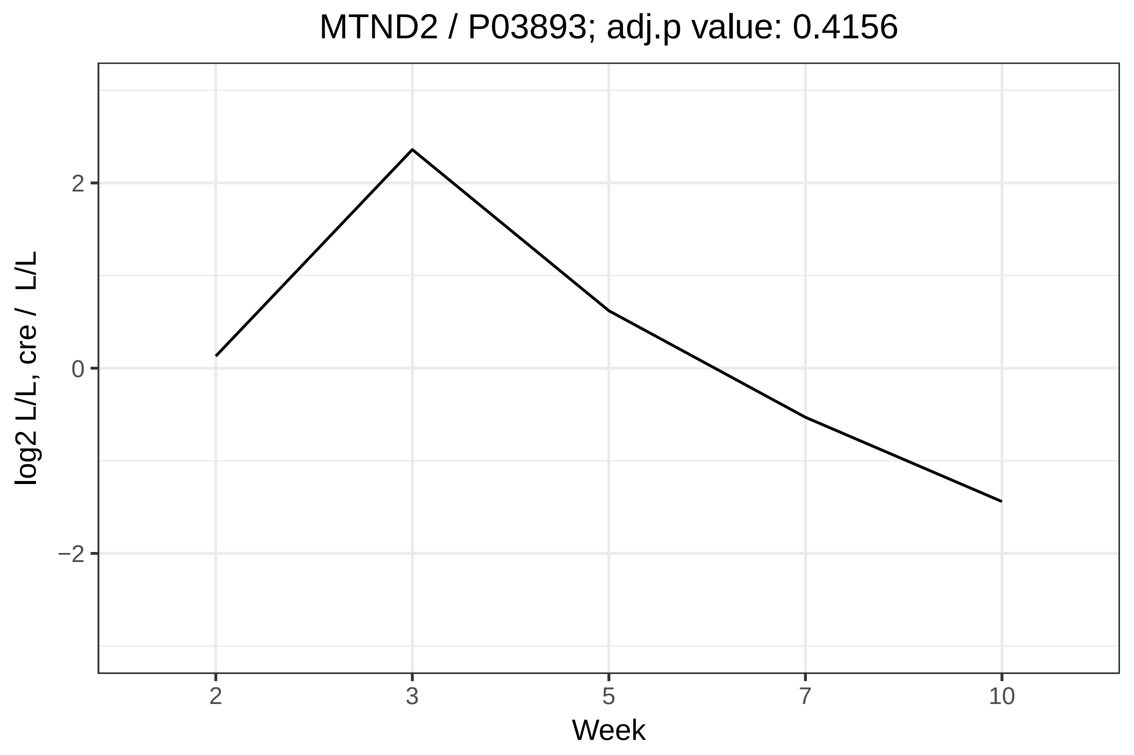
MTND2 / P03893; adj.p value: 0.4156
log2 L/L, cre / L/L
2
0
−2
2
3
5
7
10
Week

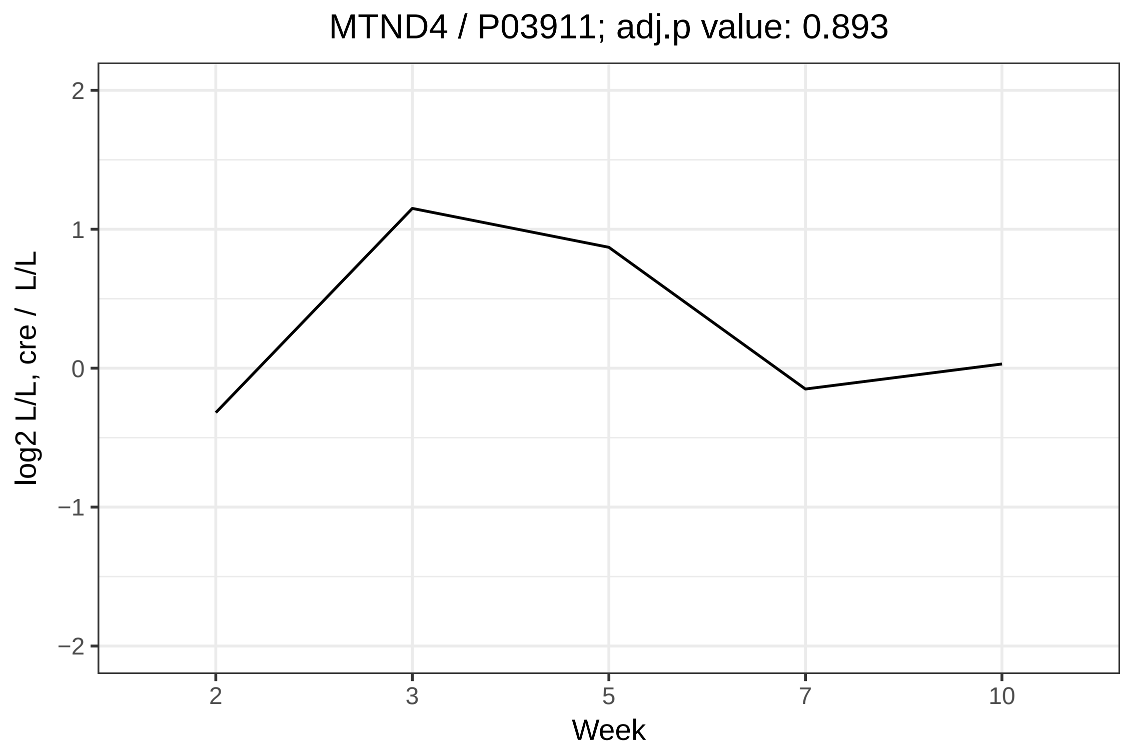
MTND4 / P03911; adj.p value: 0.893
log2 L/L, cre / L/L
2
1
0
−1
−2
2
3
5
7
10
Week

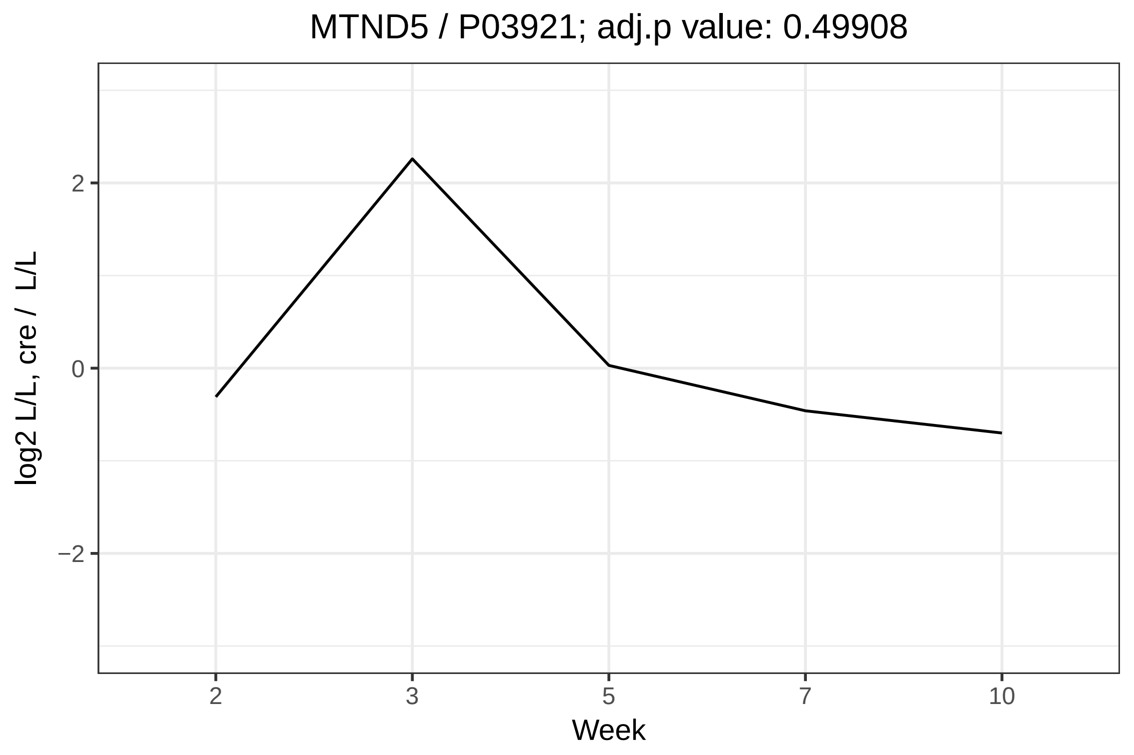

MTND5 / P03921; adj.p value: 0.49908
log2 L/L, cre / L/L
2
0
−2
2
3
5
7
10
Week

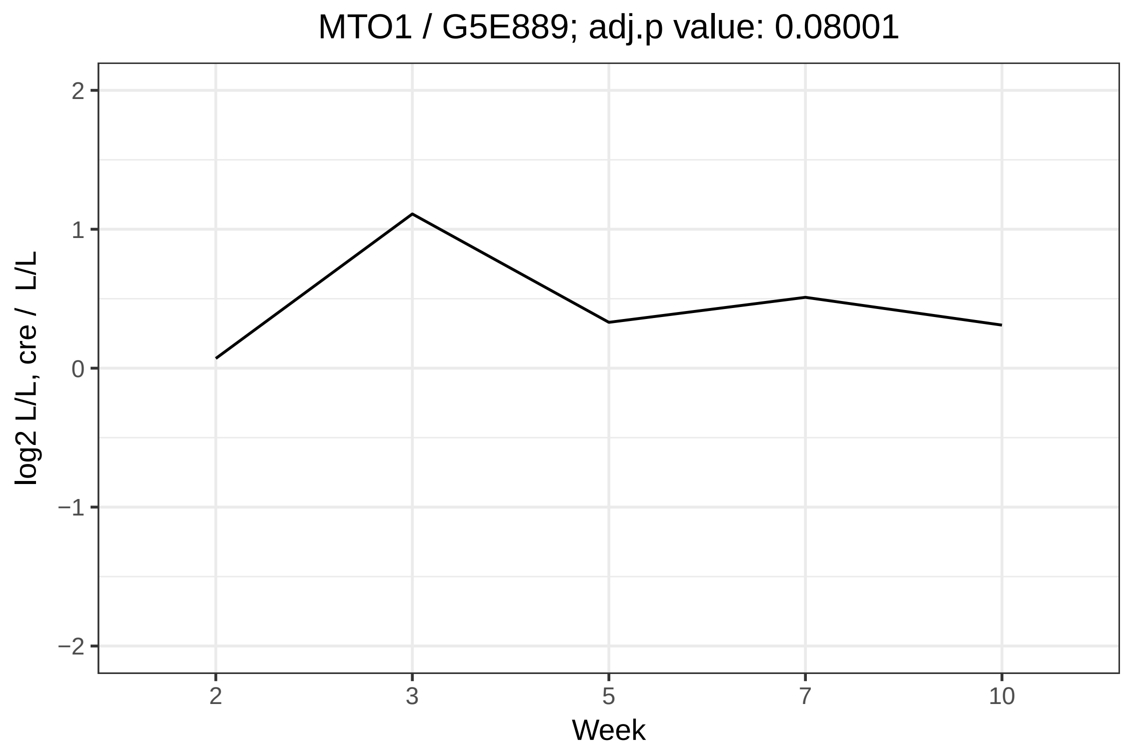
MTO1 / G5E889; adj.p value: 0.08001
log2 L/L, cre / L/L
2
1
0
−1
−2
2
3
5
7
10
Week

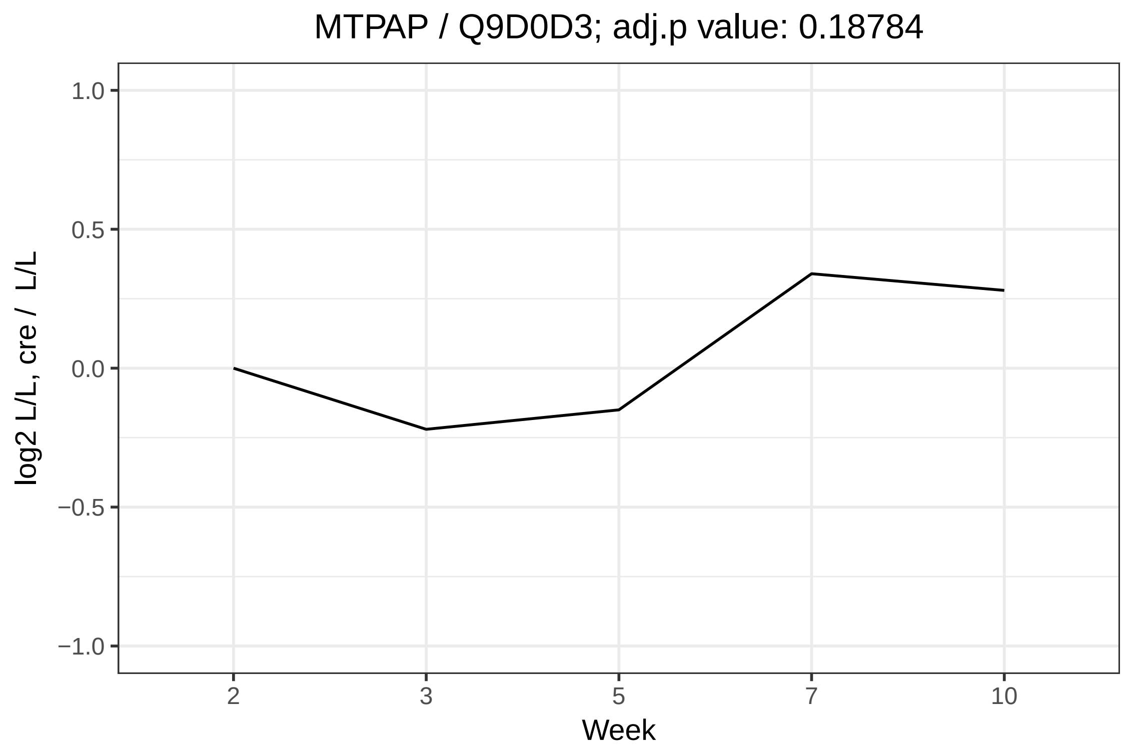
MTPAP / Q9D0D3; adj.p value: 0.18784
log2 L/L, cre / L/L
1.0
0.5
0.0
−0.5
−1.0
2
3
5
7
10
Week

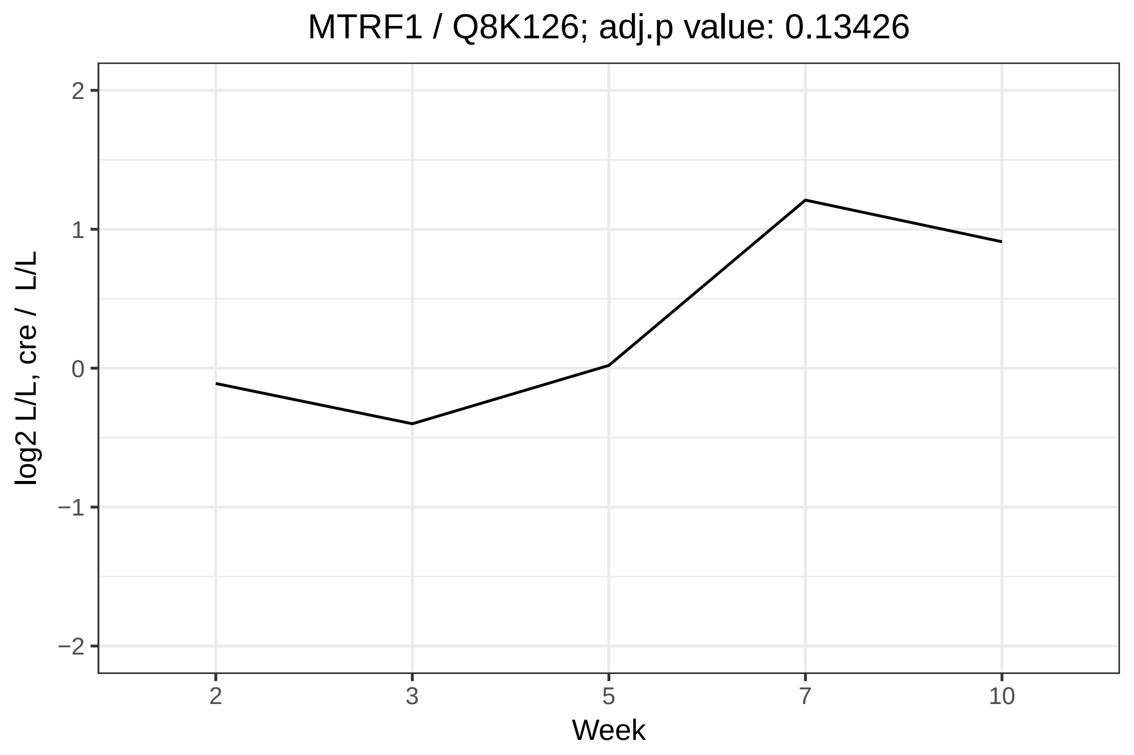
MTRF1 / Q8K126; adj.p value: 0.13426
log2 L/L, cre / L/L
Week
2
1
0
−1
−2
2
3
5
7
10

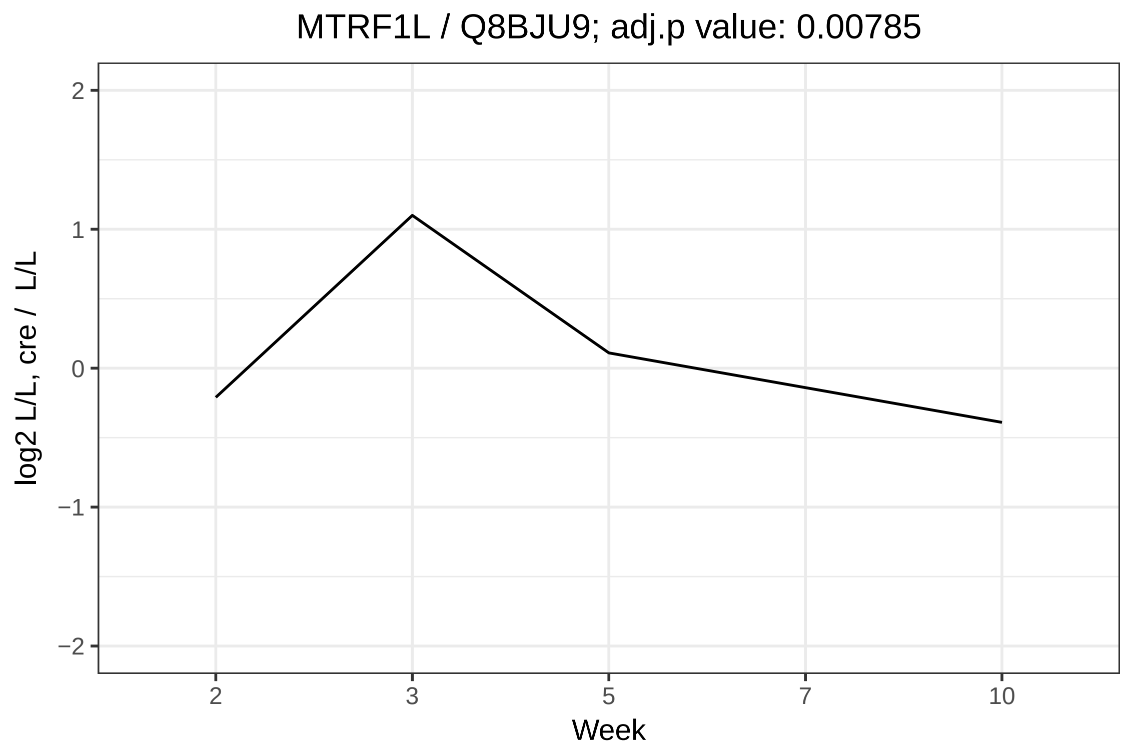

MTRF1L / Q8BJU9; adj.p value: 0.00785
log2 L/L, cre / L/L
2
1
0
−1
−2
2
3
5
7
10
Week

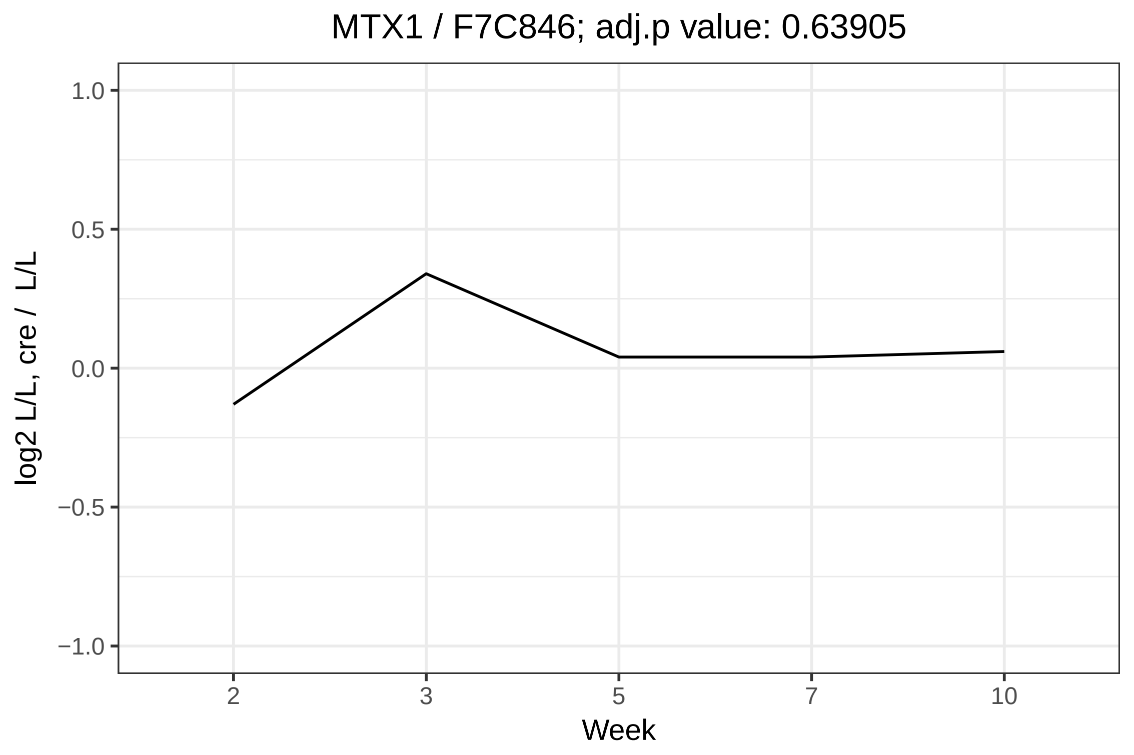
MTX1 / F7C846; adj.p value: 0.63905
log2 L/L, cre / L/L
1.0
0.5
0.0
−0.5
−1.0
2
3
5
7
10
Week

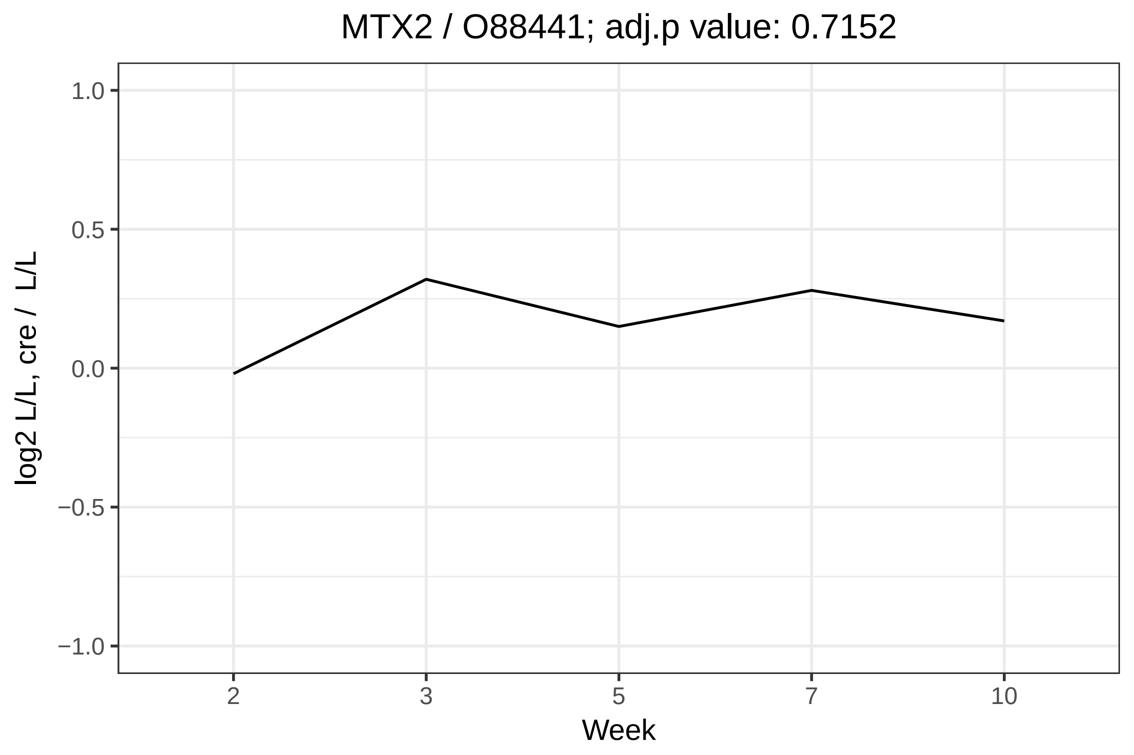
MTX2 / O88441; adj.p value: 0.7152
1.0
0.5
0.0
−0.5
−1.0
log2 L/L, cre / L/L
2
3
5
7
10
Week

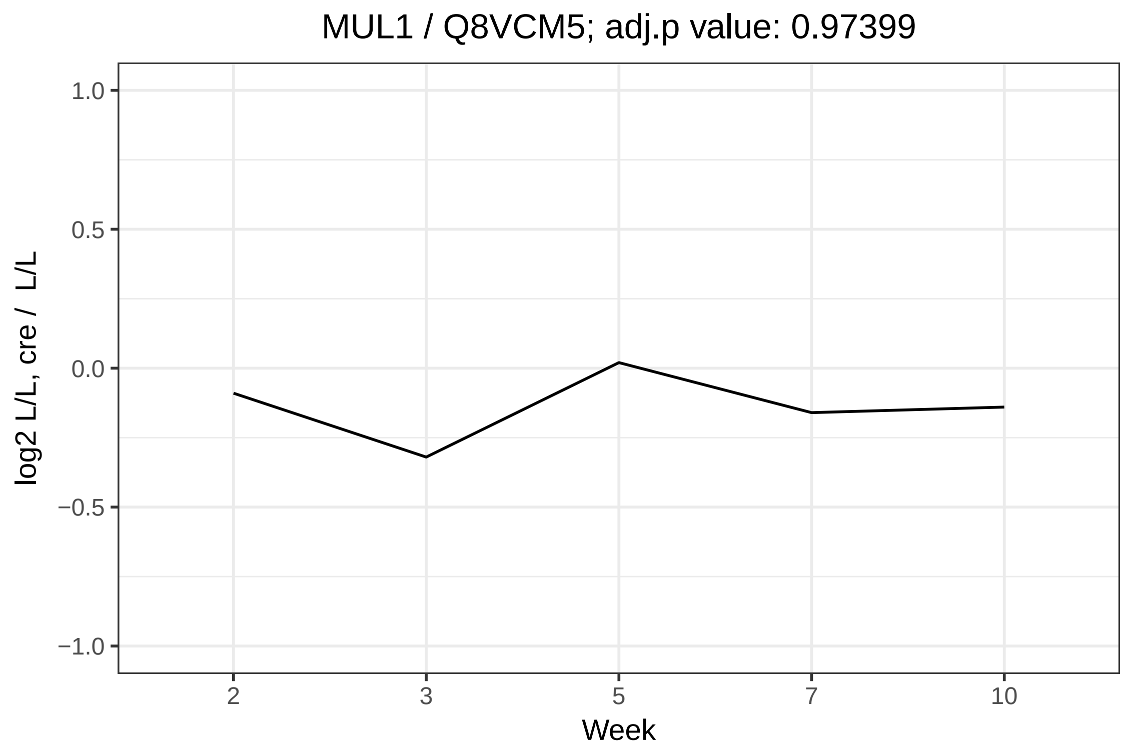

MUL1 / Q8VCM5; adj.p value: 0.97399
log2 L/L, cre / L/L
1.0
0.5
0.0
−0.5
−1.0
2
3
5
7
10
Week

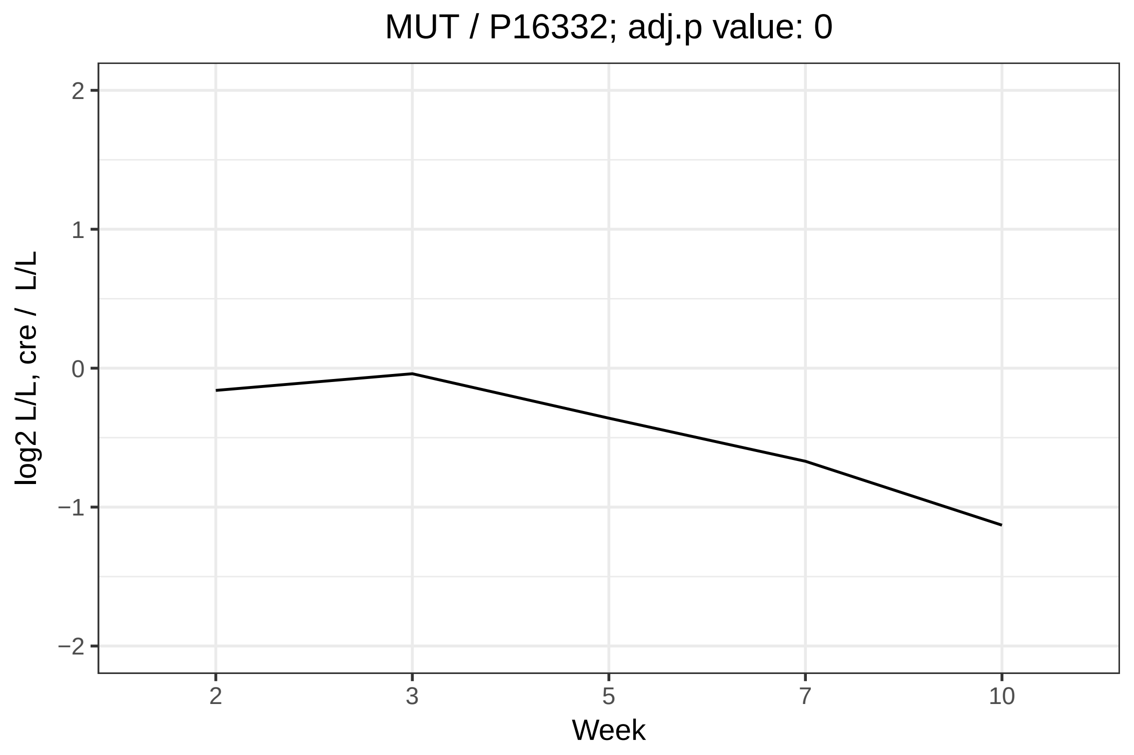
MUT / P16332; adj.p value: 0
log2 L/L, cre / L/L
2
1
0
−1
−2
2
3
5
7
10
Week

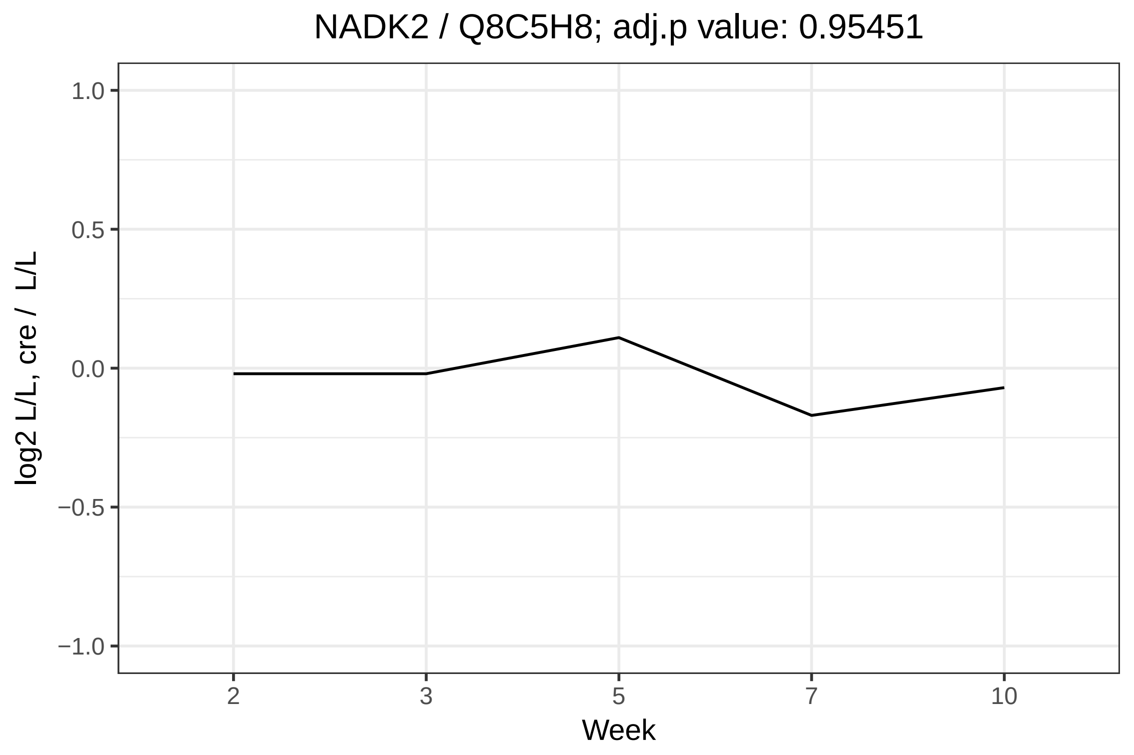
NADK2 / Q8C5H8; adj.p value: 0.95451
log2 L/L, cre / L/L
1.0
0.5
0.0
−0.5
−1.0
2
3
5
7
10
Week

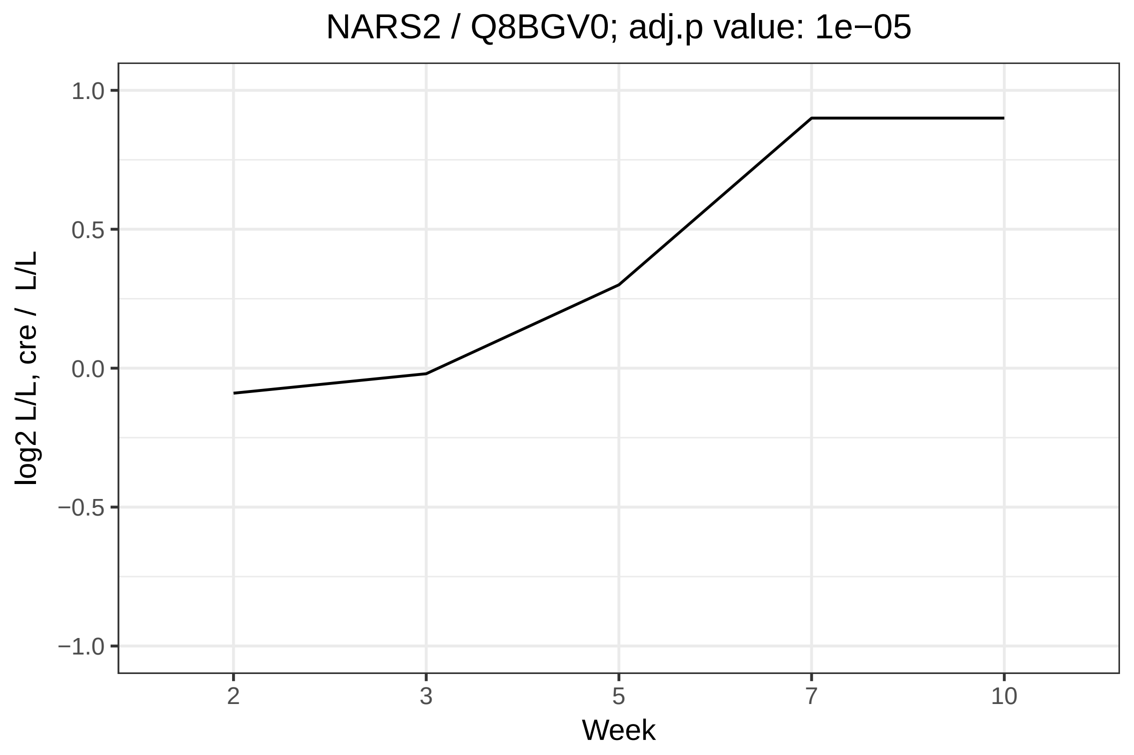

NARS2 / Q8BGV0; adj.p value: 1e−05
1.0
0.5
0.0
−0.5
−1.0
log2 L/L, cre / L/L
2
3
5
7
10
Week

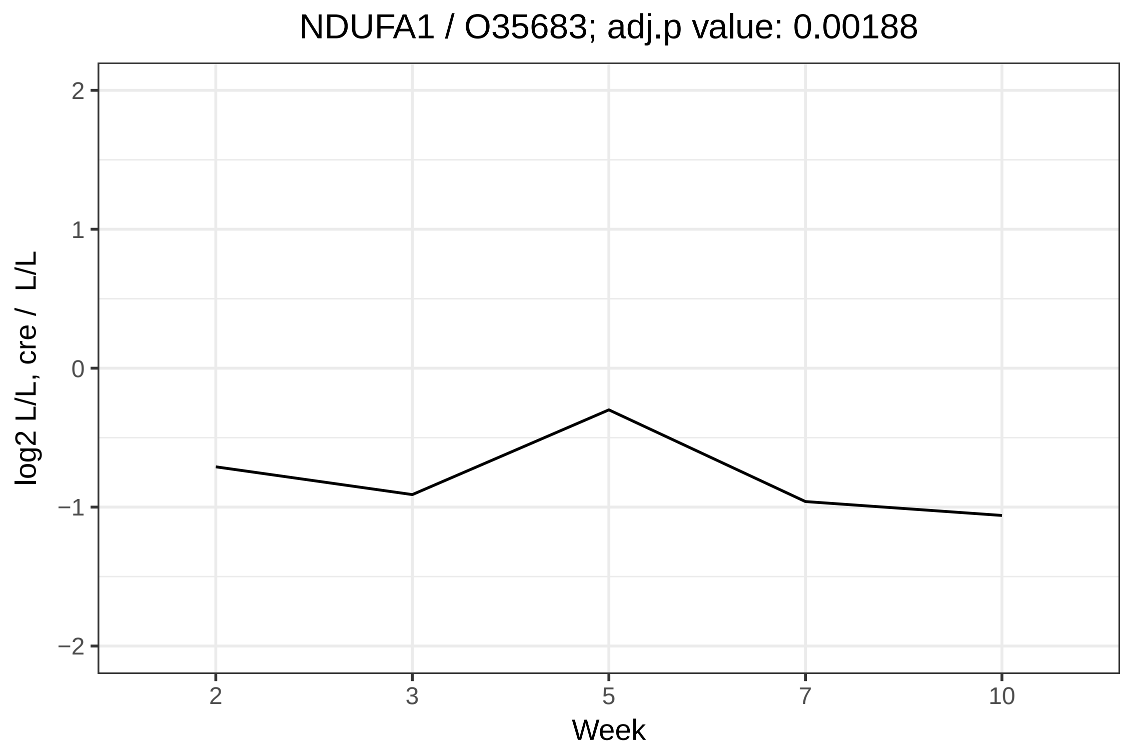

NDUFA1 / O35683; adj.p value: 0.00188
log2 L/L, cre / L/L
2
1
0
−1
−2
2
3
5
7
10
Week

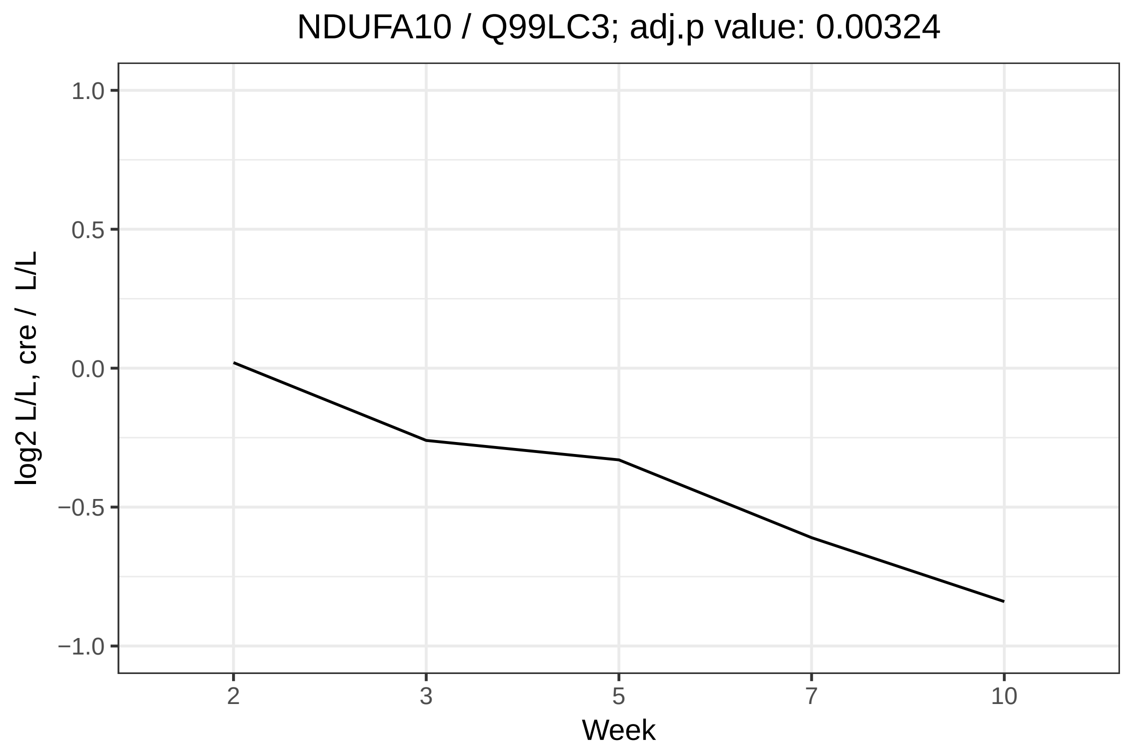
NDUFA10 / Q99LC3; adj.p value: 0.00324
log2 L/L, cre / L/L
1.0
0.5
0.0
−0.5
−1.0
2
3
5
7
10
Week

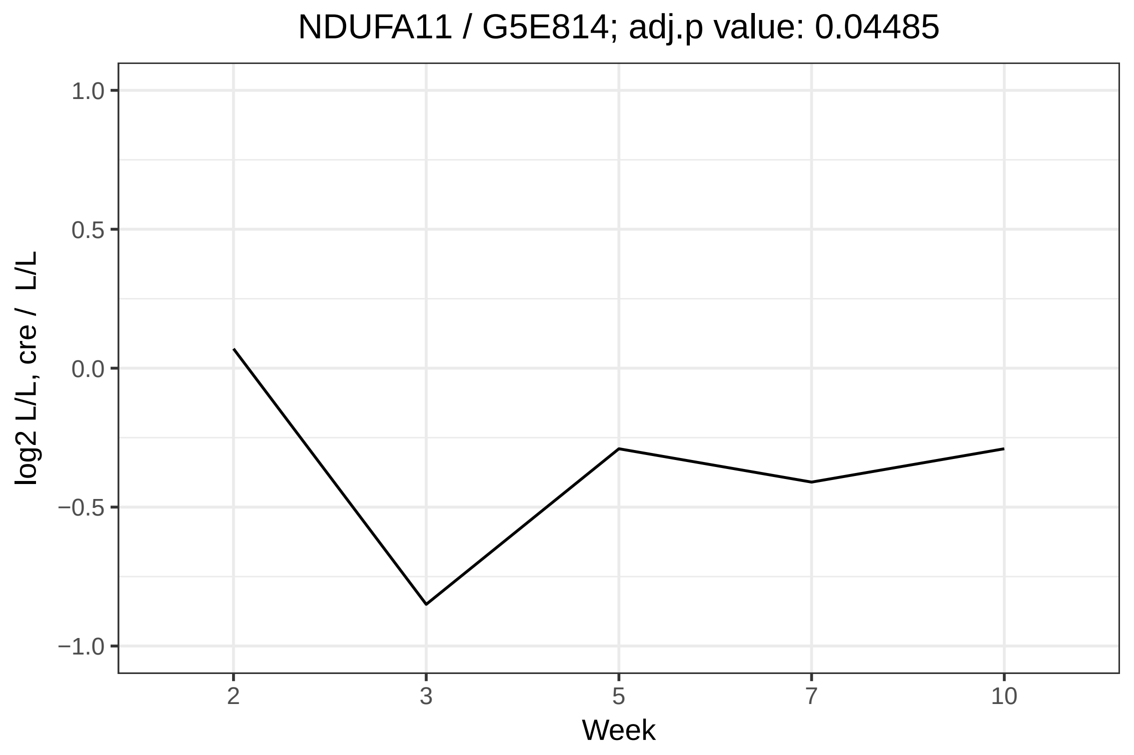
NDUFA11 / G5E814; adj.p value: 0.04485
log2 L/L, cre / L/L
1.0
0.5
0.0
−0.5
−1.0
2
3
5
7
10
Week

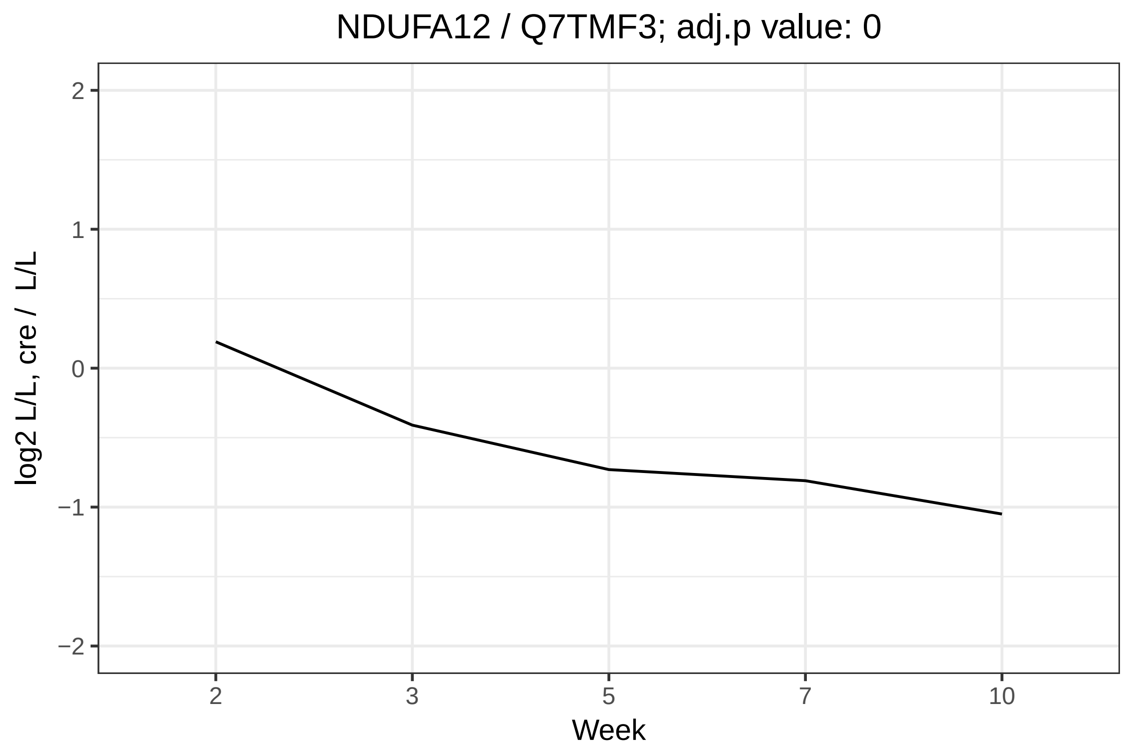
NDUFA12 / Q7TMF3; adj.p value: 0
log2 L/L, cre / L/L
2
1
0
−1
−2
2
3
5
7
10
Week

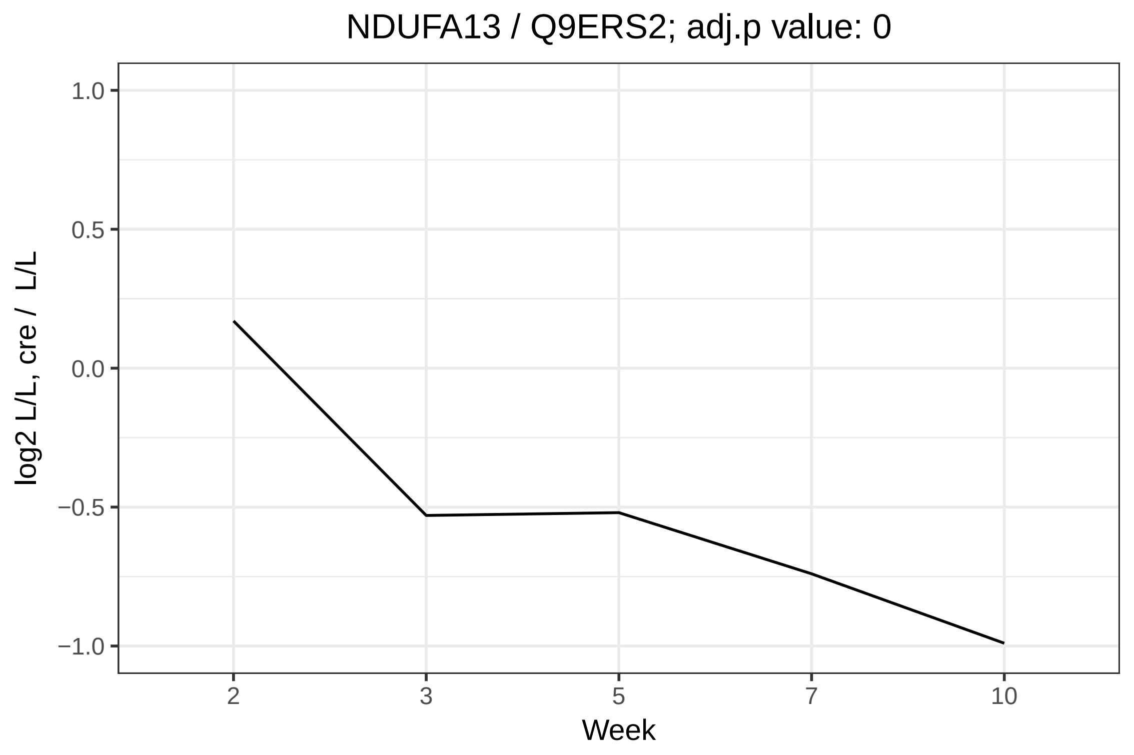
NDUFA13 / Q9ERS2; adj.p value: 0
log2 L/L, cre / L/L
1.0
0.5
0.0
−0.5
−1.0
2
3
5
7
10
Week

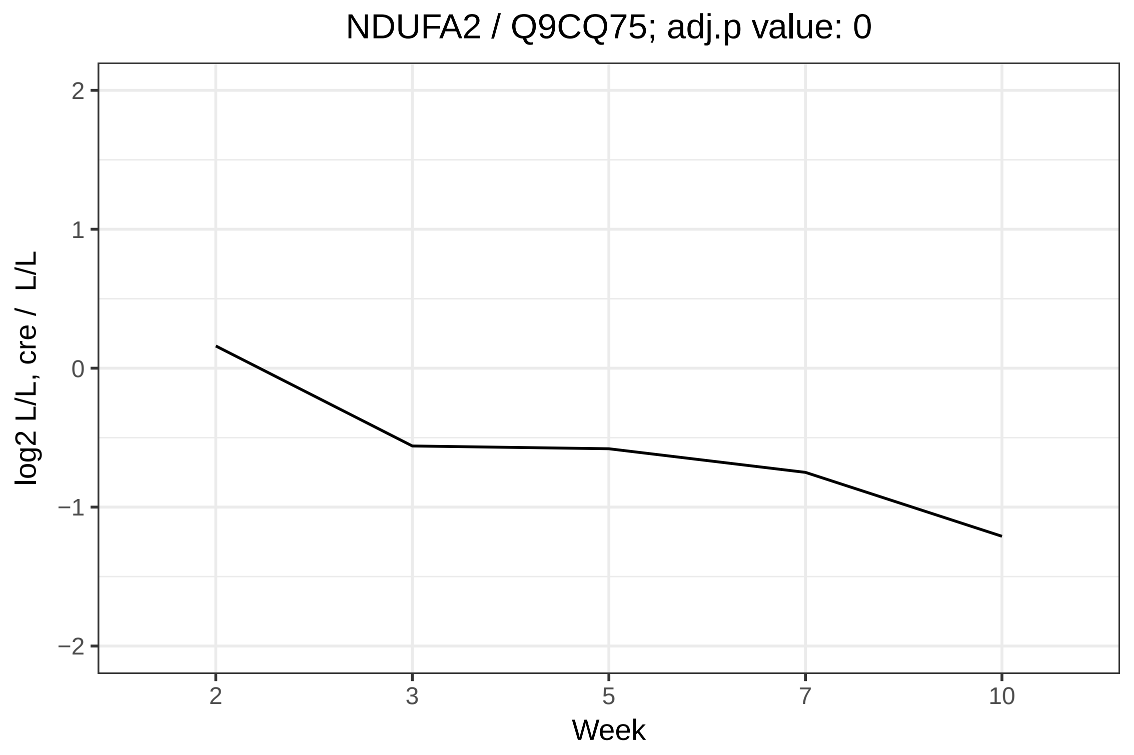
NDUFA2 / Q9CQ75; adj.p value: 0
log2 L/L, cre / L/L
2
1
0
−1
−2
2
3
5
7
10
Week

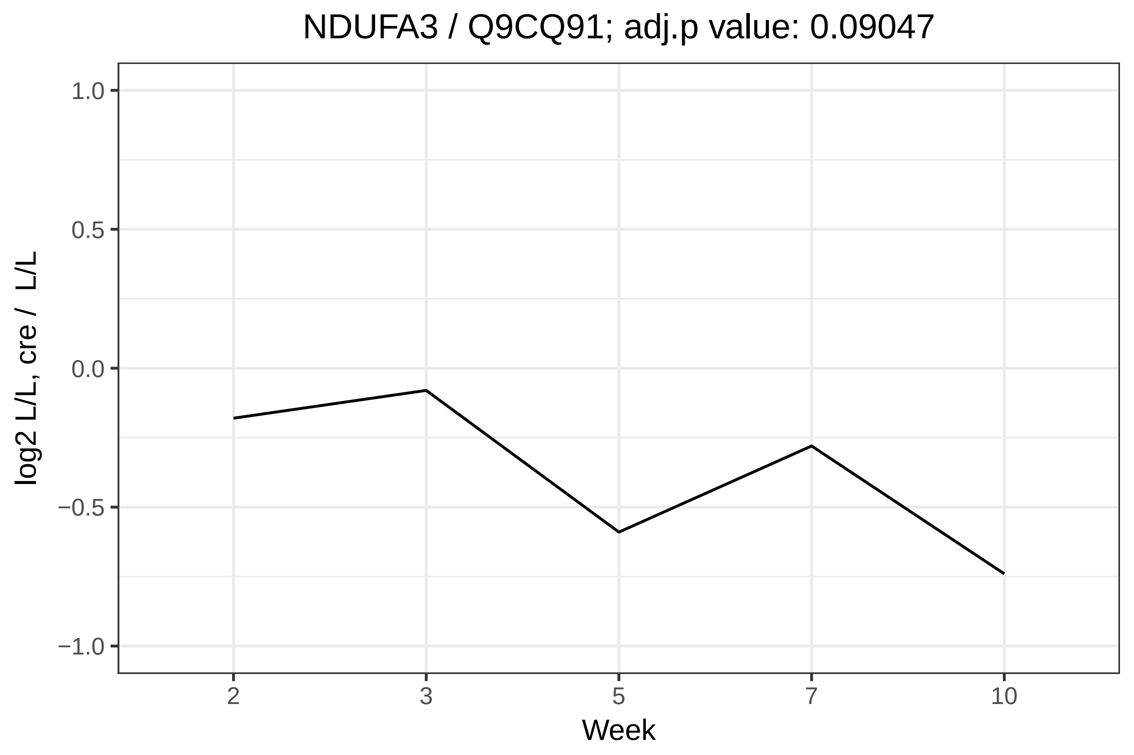
NDUFA3 / Q9CQ91; adj.p value: 0.09047
log2 L/L, cre / L/L
1.0
0.5
0.0
−0.5
−1.0
2
3
5
7
10
Week

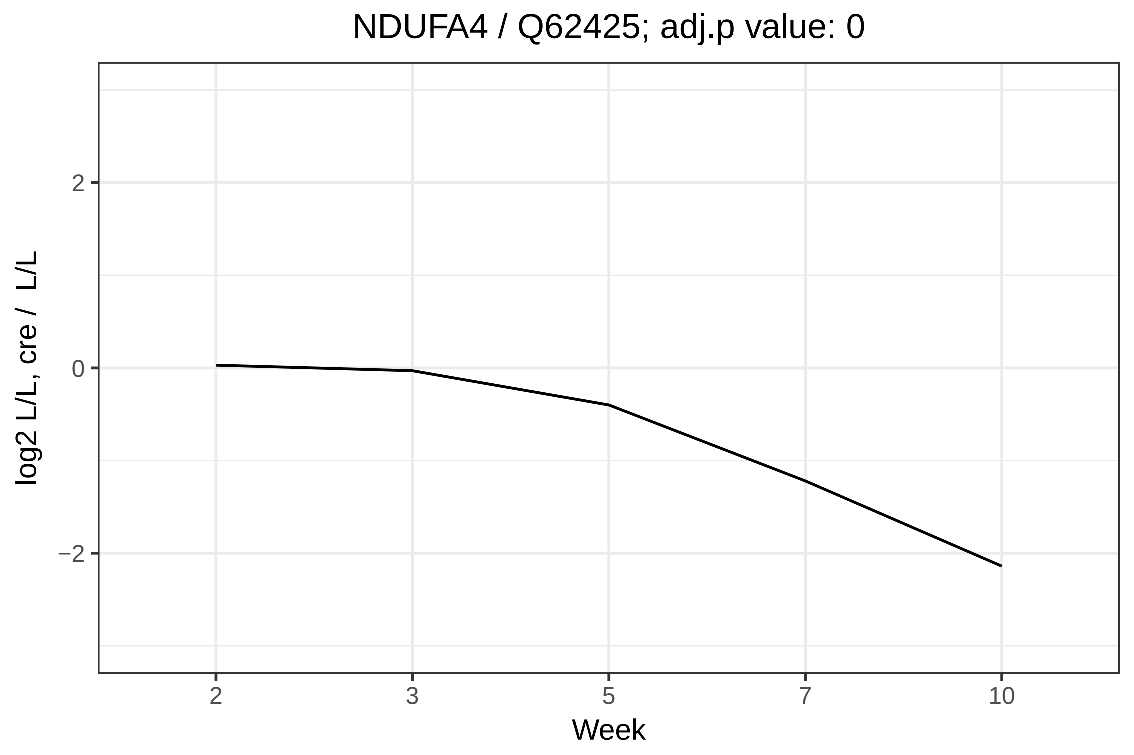

NDUFA4 / Q62425; adj.p value: 0
log2 L/L, cre / L/L
2
0
−2
2
3
5
7
10
Week

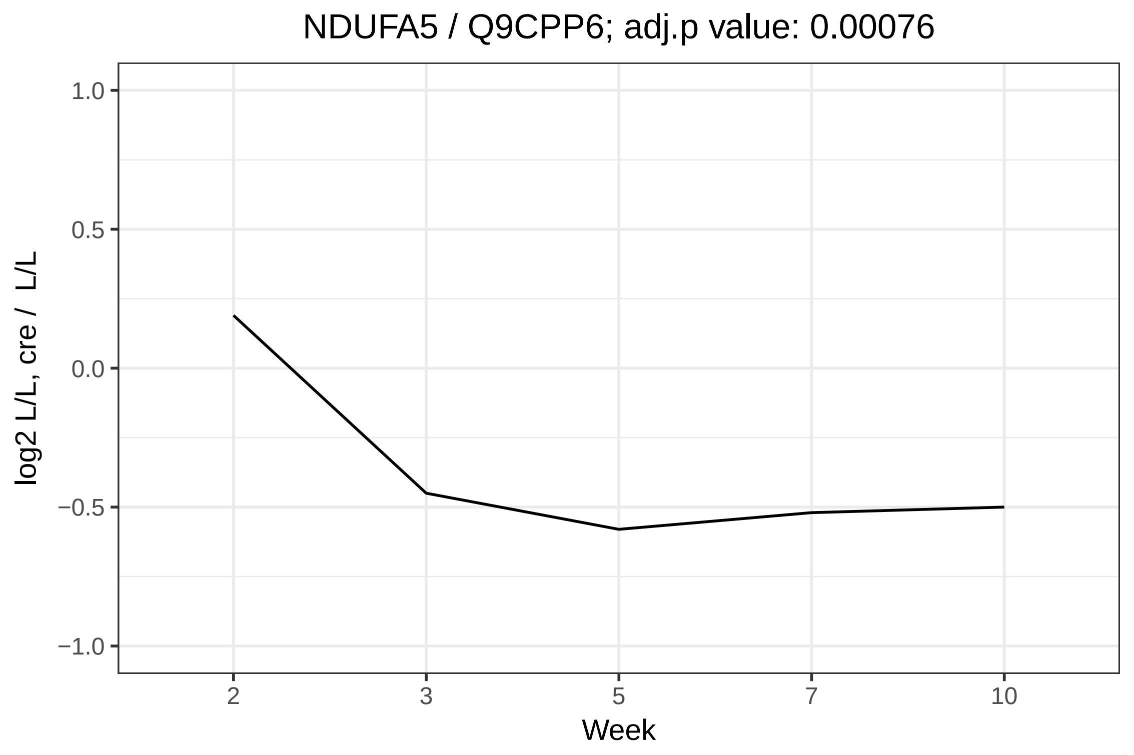
NDUFA5 / Q9CPP6; adj.p value: 0.00076
1.0
0.5
0.0
−0.5
−1.0
log2 L/L, cre / L/L
2
3
5
7
10
Week

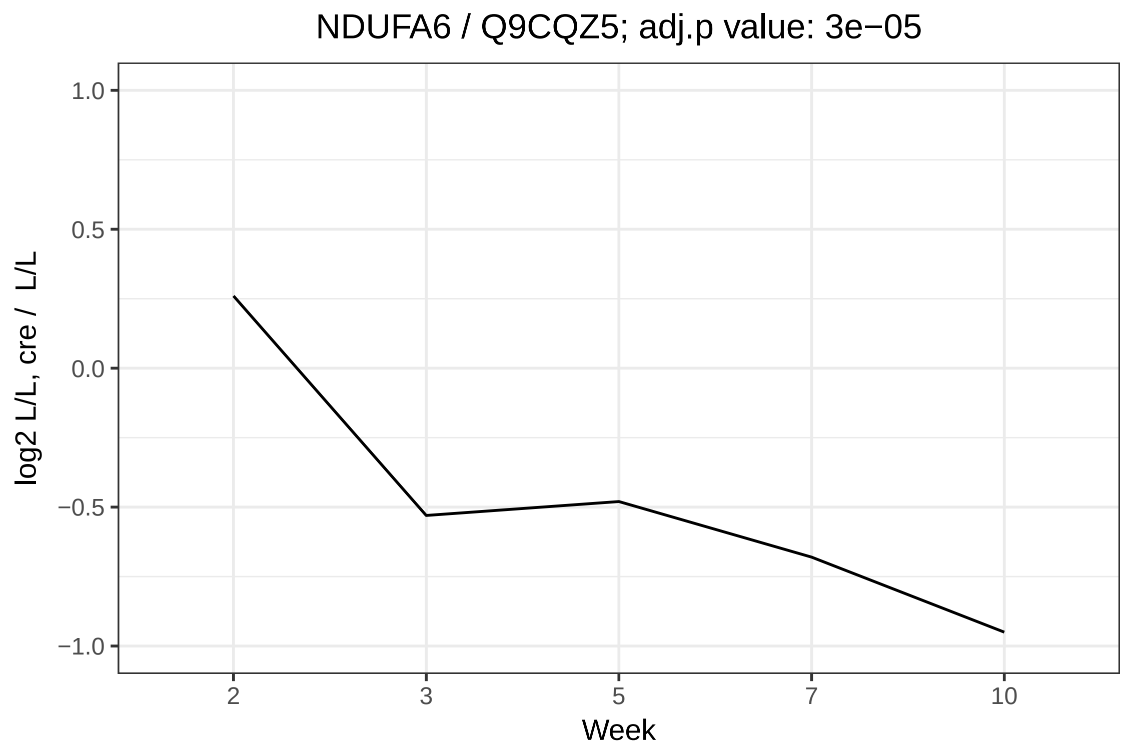

NDUFA6 / Q9CQZ5; adj.p value: 3e−05
log2 L/L, cre / L/L
1.0
0.5
0.0
−0.5
−1.0
2
3
5
7
10
Week

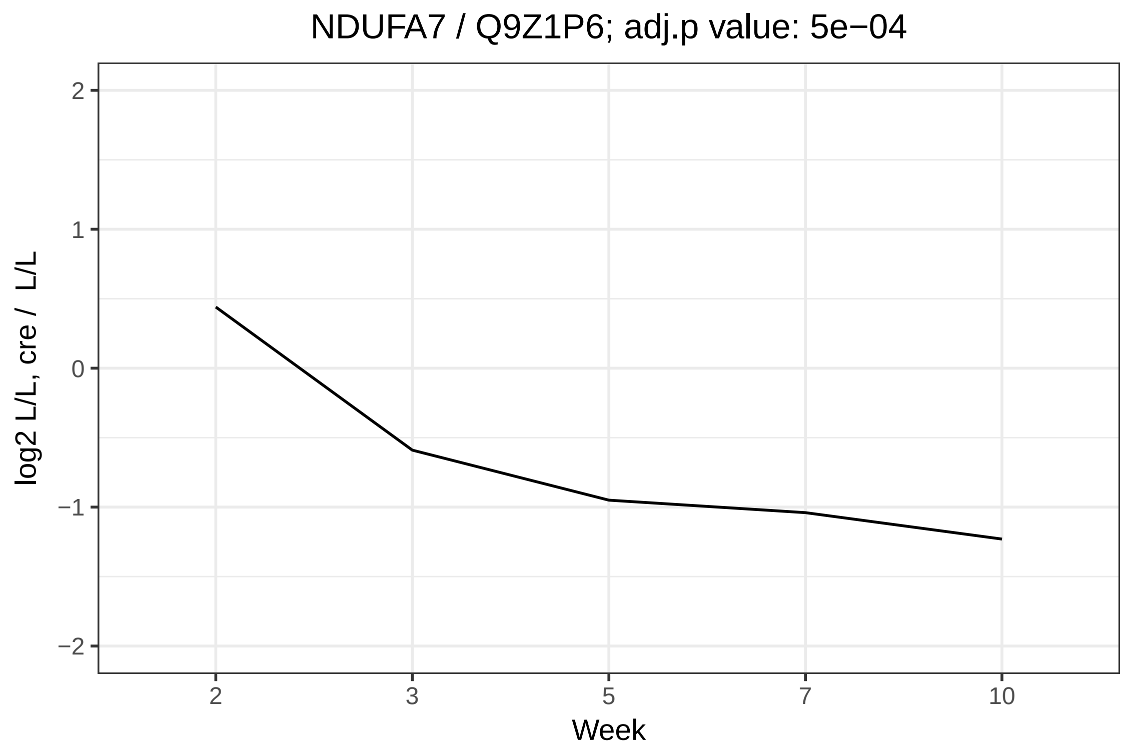
NDUFA7 / Q9Z1P6; adj.p value: 5e−04
log2 L/L, cre / L/L
2
1
0
−1
−2
2
3
5
7
10
Week

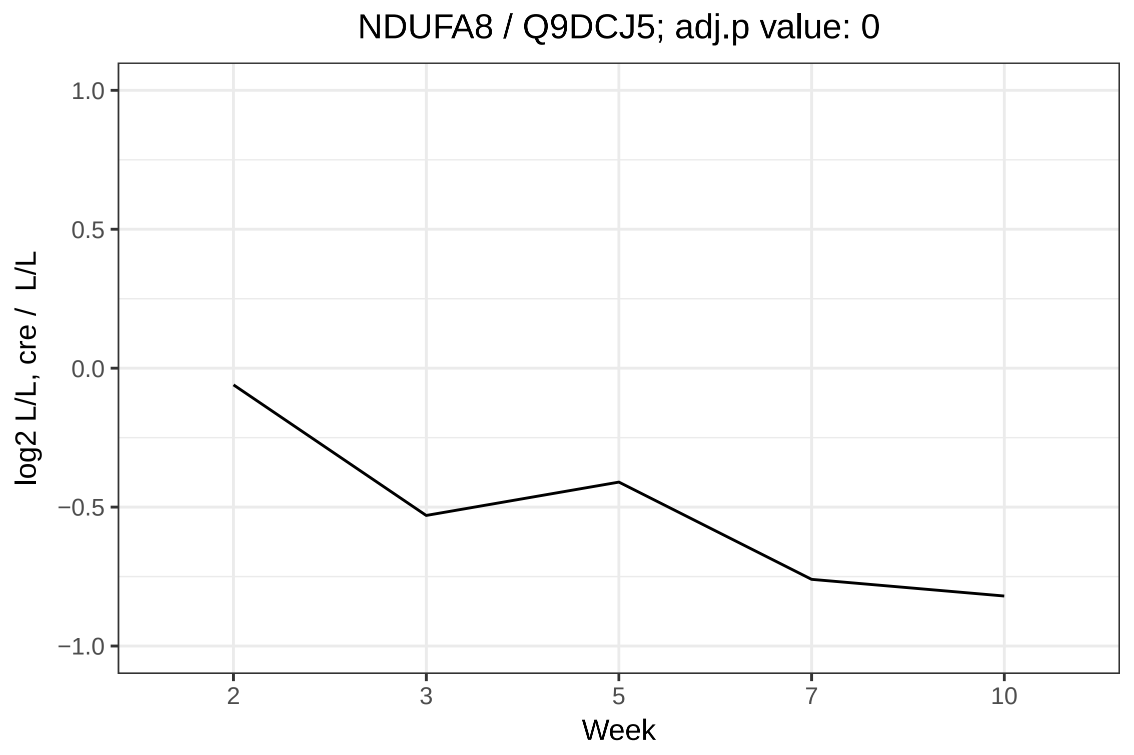

NDUFA8 / Q9DCJ5; adj.p value: 0
log2 L/L, cre / L/L
1.0
0.5
0.0
−0.5
−1.0
2
3
5
7
10
Week

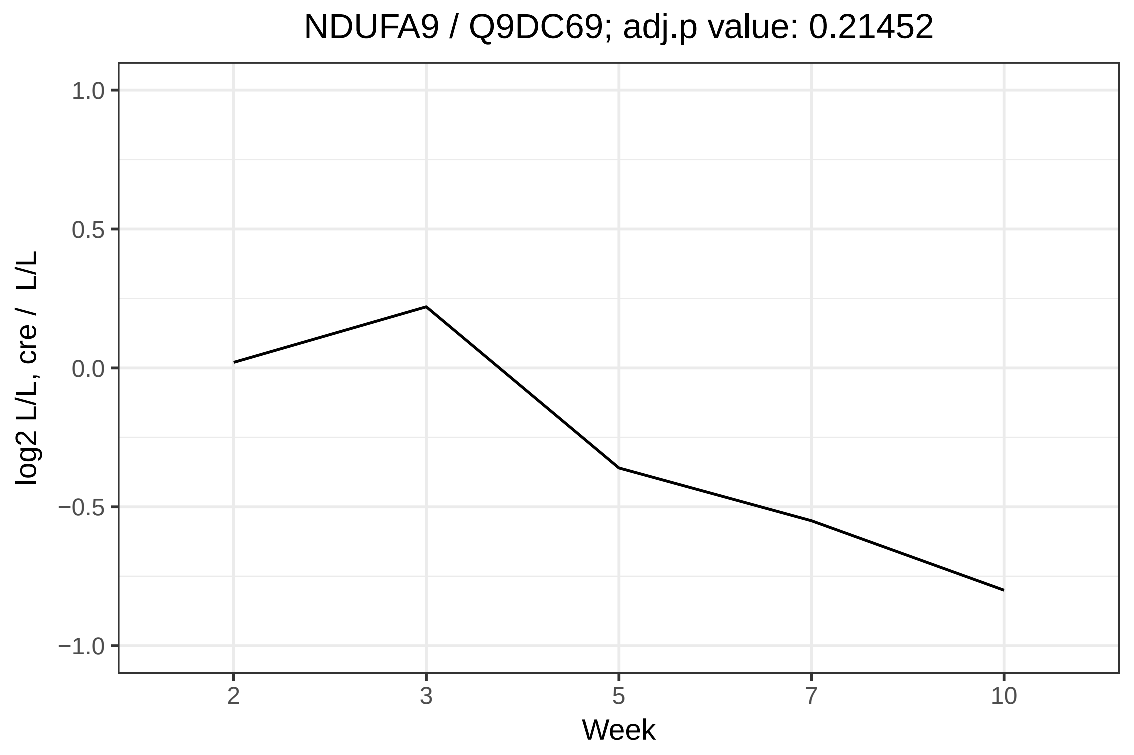
NDUFA9 / Q9DC69; adj.p value: 0.21452
log2 L/L, cre / L/L
1.0
0.5
0.0
−0.5
−1.0
2
3
5
7
10
Week

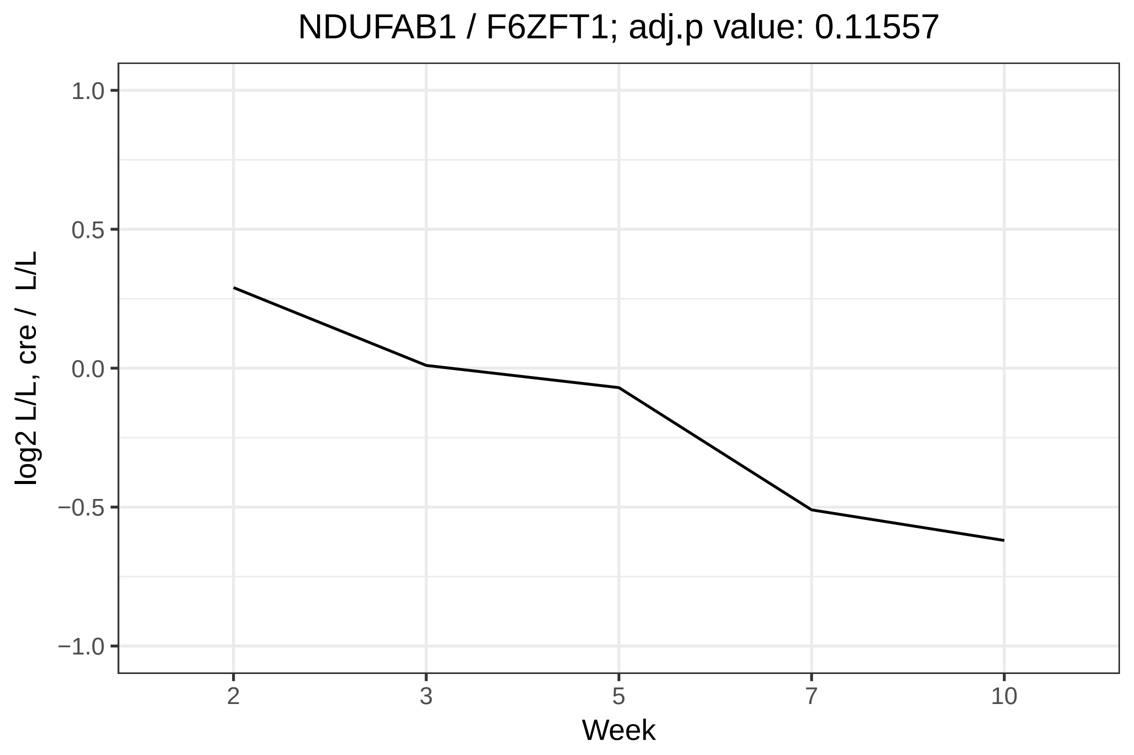
NDUFAB1 / F6ZFT1; adj.p value: 0.11557
log2 L/L, cre / L/L
1.0
0.5
0.0
−0.5
−1.0
2
3
5
7
10
Week

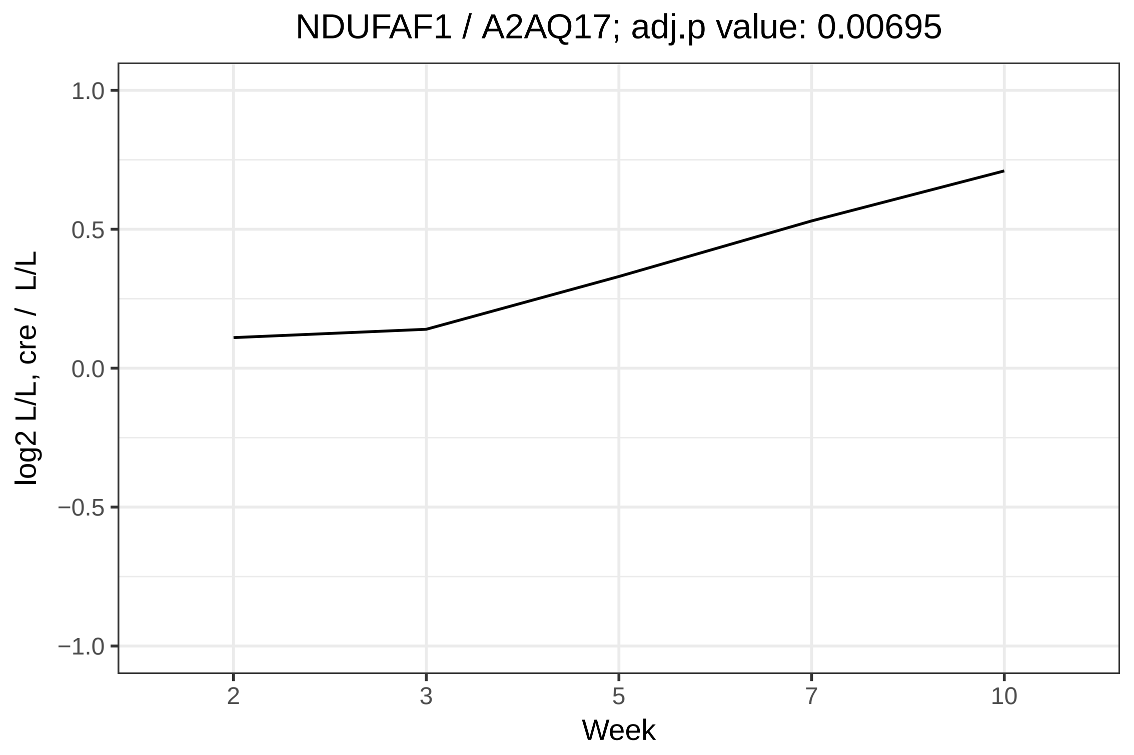
NDUFAF1 / A2AQ17; adj.p value: 0.00695
1.0
0.5
0.0
−0.5
−1.0
log2 L/L, cre / L/L
2
3
5
7
10
Week

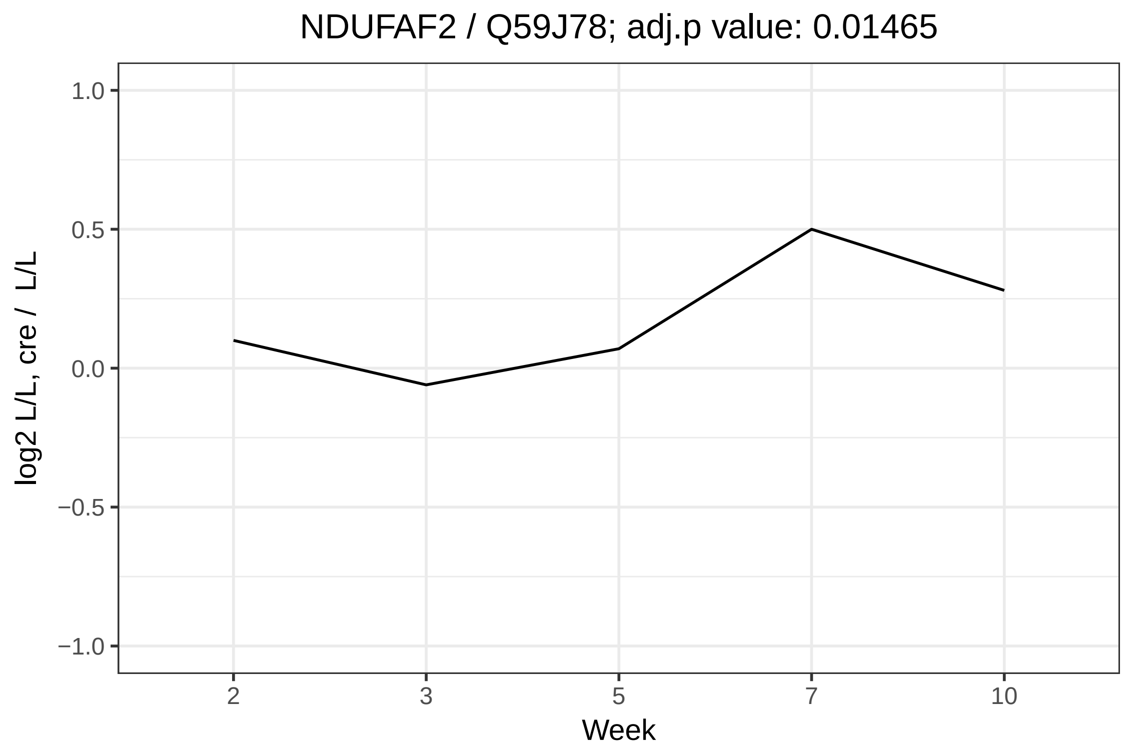

NDUFAF2 / Q59J78; adj.p value: 0.01465
log2 L/L, cre / L/L
1.0
0.5
0.0
−0.5
−1.0
2
3
5
7
10
Week

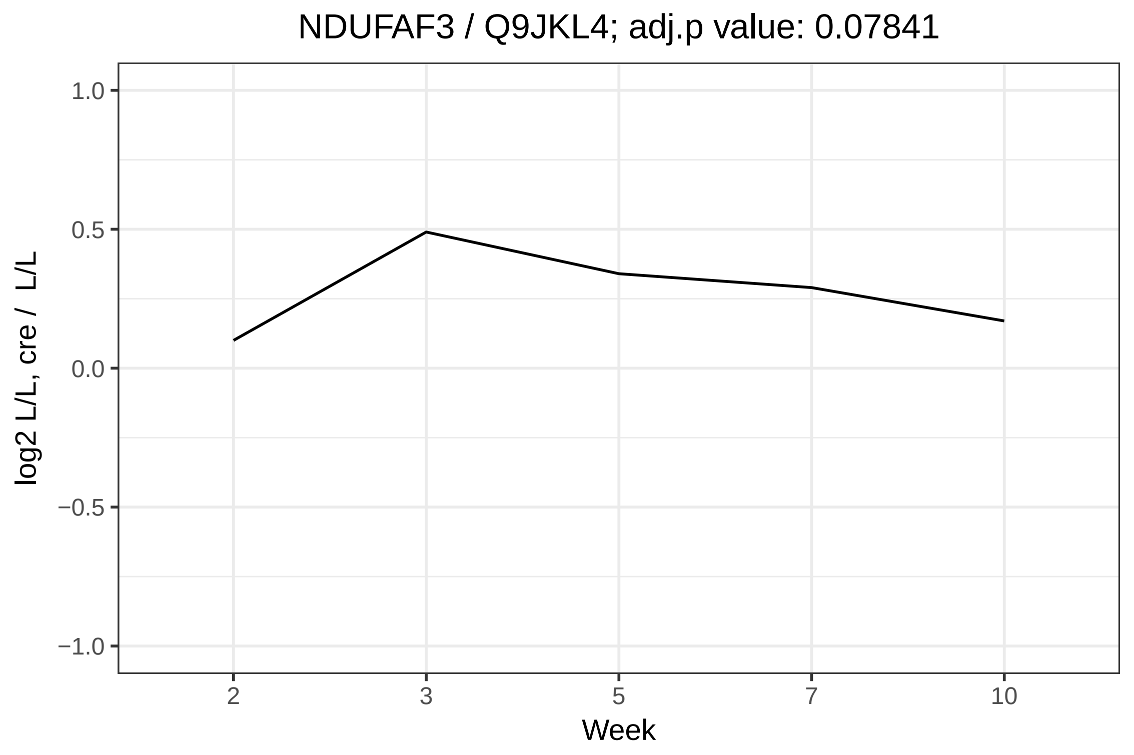
NDUFAF3 / Q9JKL4; adj.p value: 0.07841
log2 L/L, cre / L/L
1.0
0.5
0.0
−0.5
−1.0
2
3
5
7
10
Week

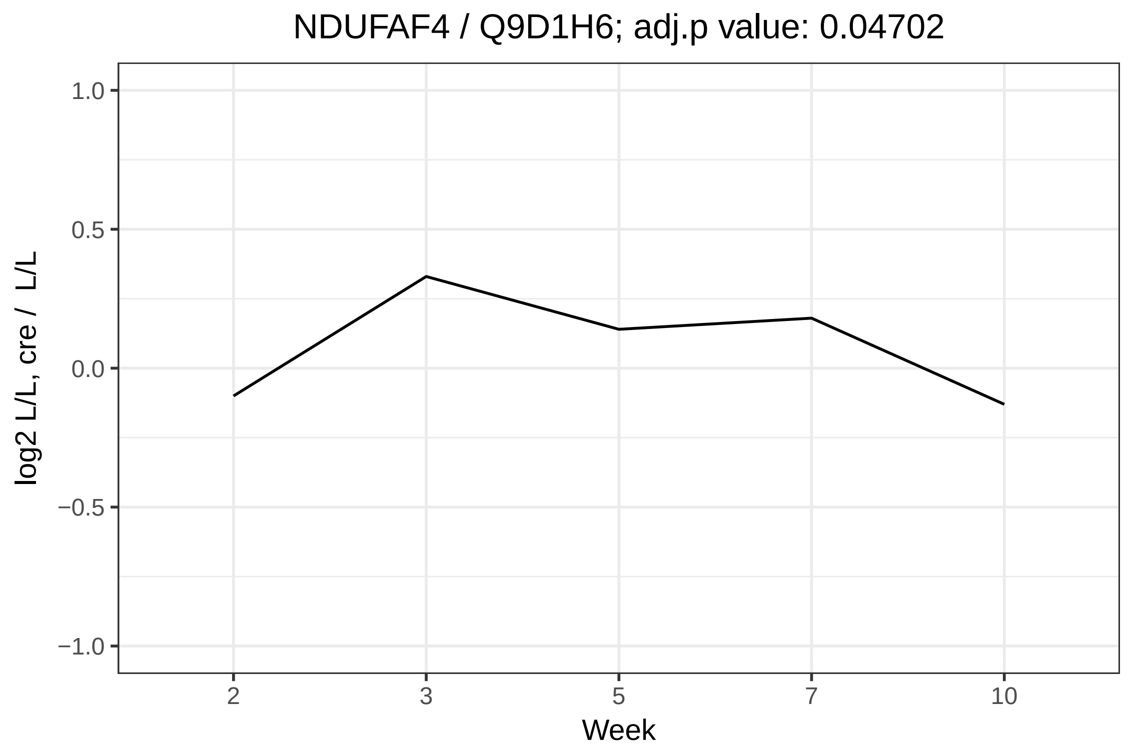

NDUFAF4 / Q9D1H6; adj.p value: 0.04702
1.0
0.5
0.0
−0.5
−1.0
log2 L/L, cre / L/L
2
3
5
7
10
Week

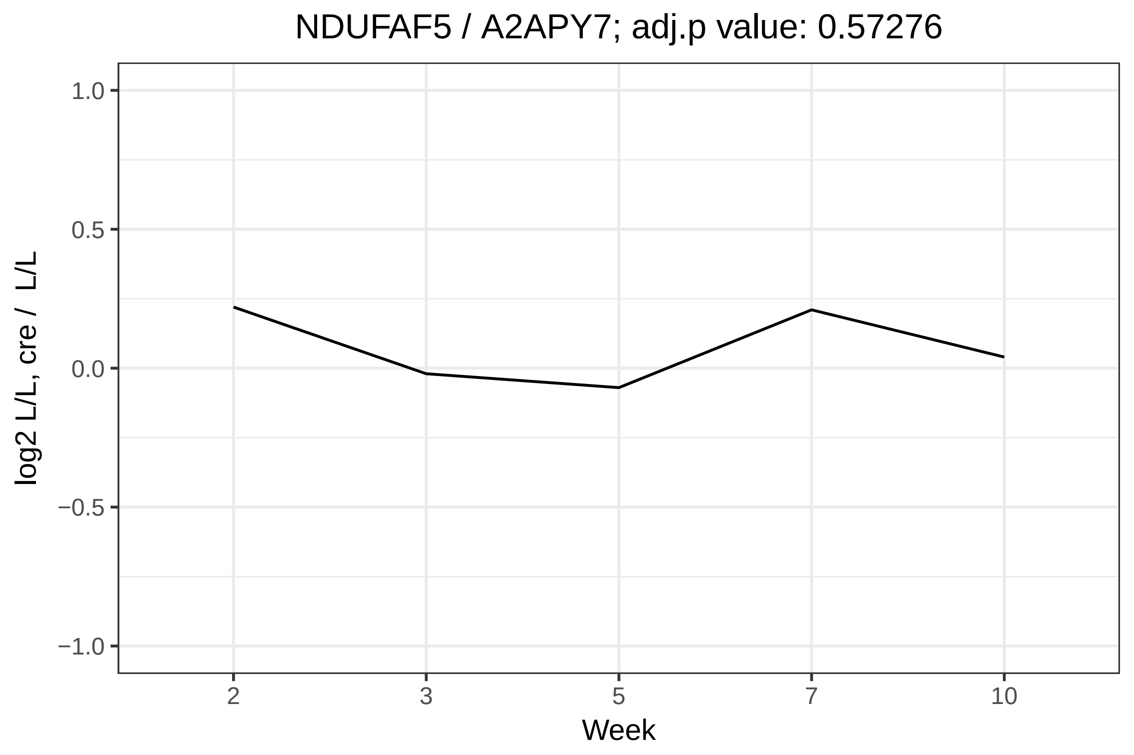
NDUFAF5 / A2APY7; adj.p value: 0.57276
log2 L/L, cre / L/L
1.0
0.5
0.0
−0.5
−1.0
2
3
5
7
10
Week

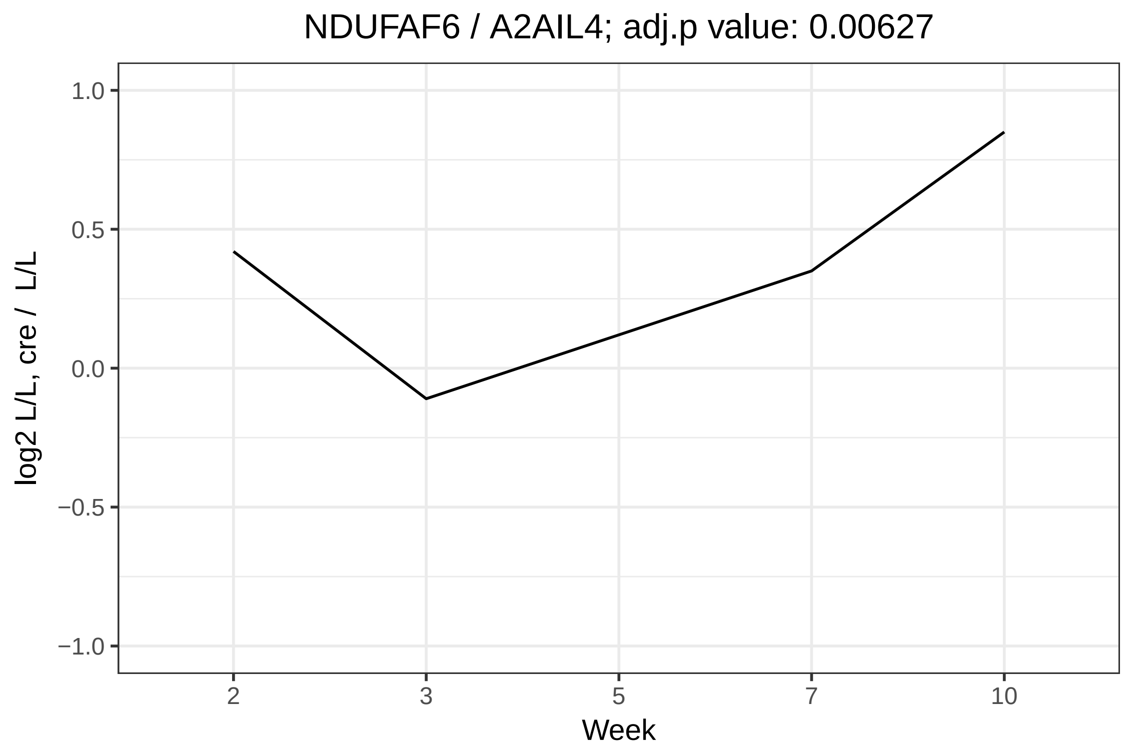
NDUFAF6 / A2AIL4; adj.p value: 0.00627
log2 L/L, cre / L/L
1.0
0.5
0.0
−0.5
−1.0
2
3
5
7
10
Week

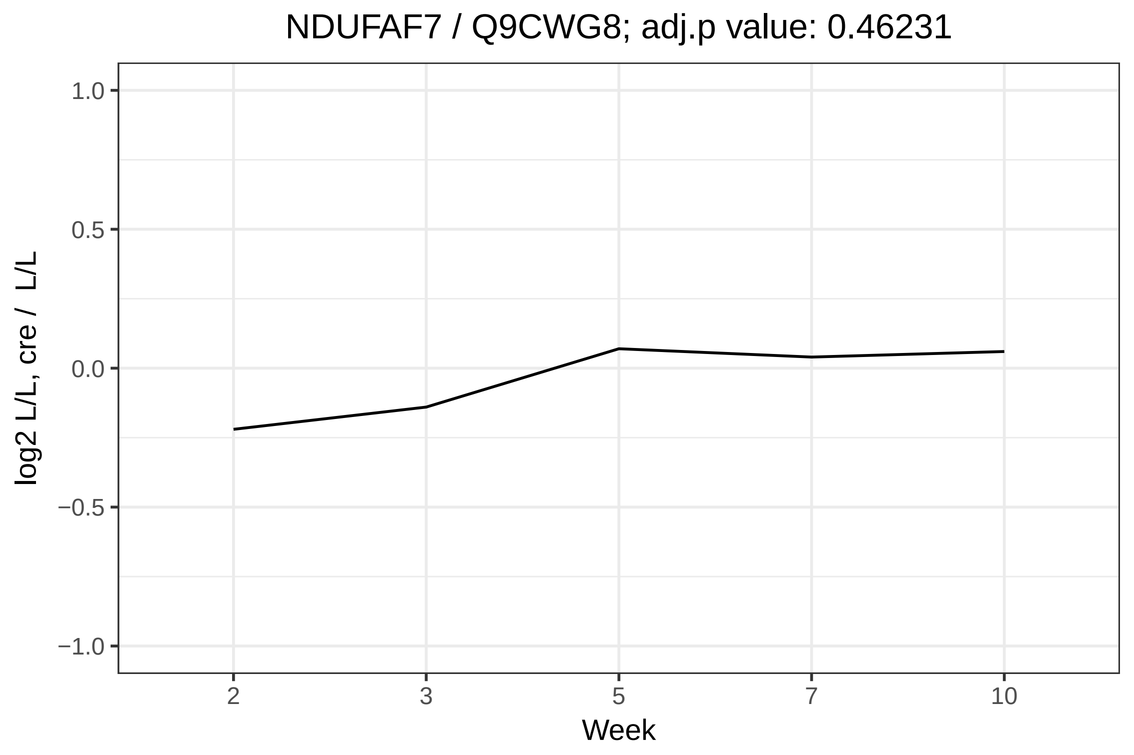
NDUFAF7 / Q9CWG8; adj.p value: 0.46231
log2 L/L, cre / L/L
1.0
0.5
0.0
−0.5
−1.0
2
3
5
7
10
Week

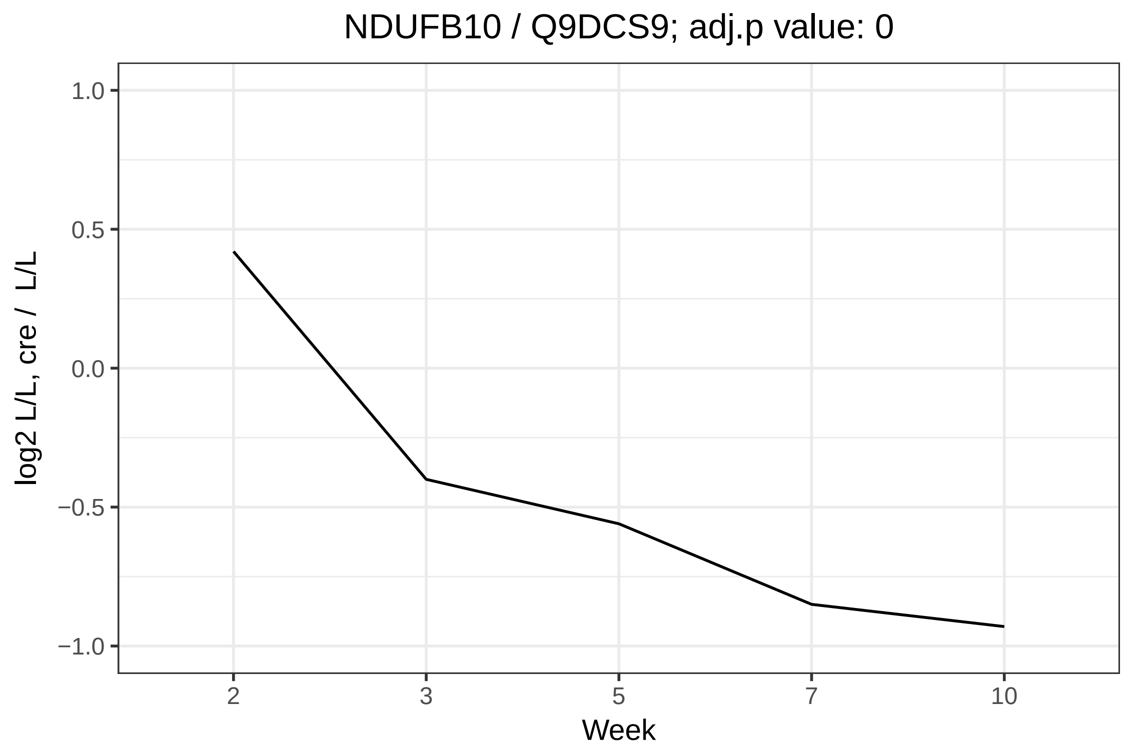

NDUFB10 / Q9DCS9; adj.p value: 0
log2 L/L, cre / L/L
1.0
0.5
0.0
−0.5
−1.0
2
3
5
7
10
Week

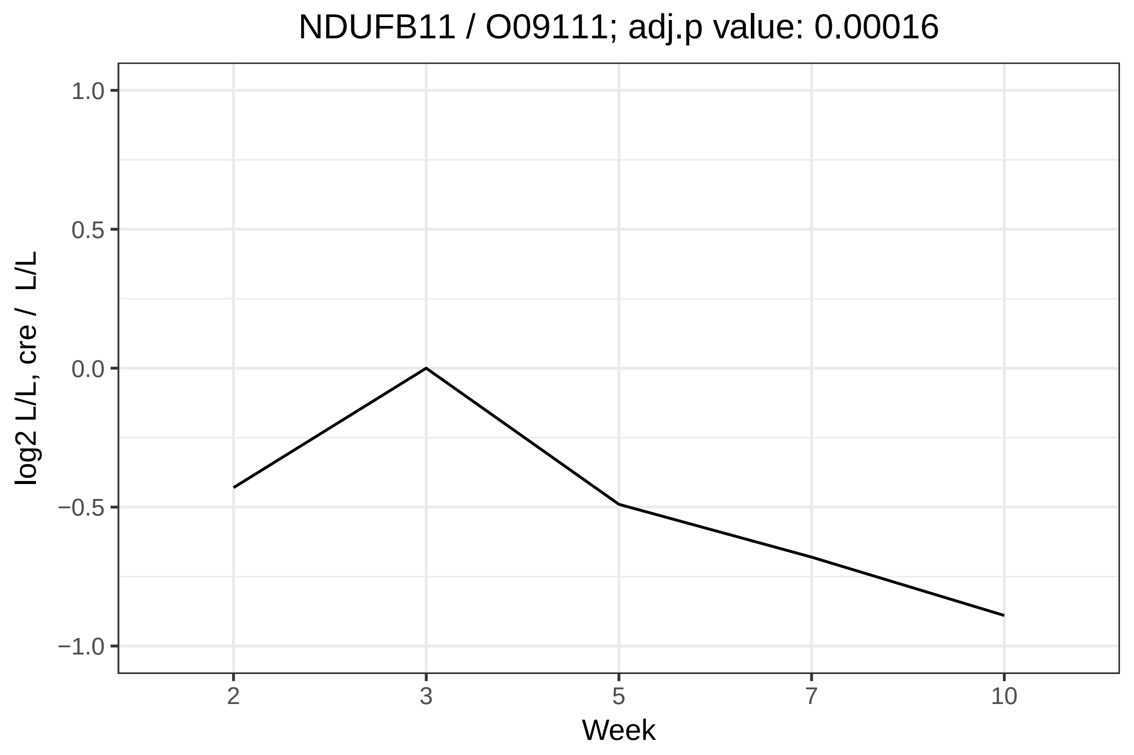
NDUFB11 / O09111; adj.p value: 0.00016
log2 L/L, cre / L/L
1.0
0.5
0.0
−0.5
−1.0
2
3
5
7
10
Week

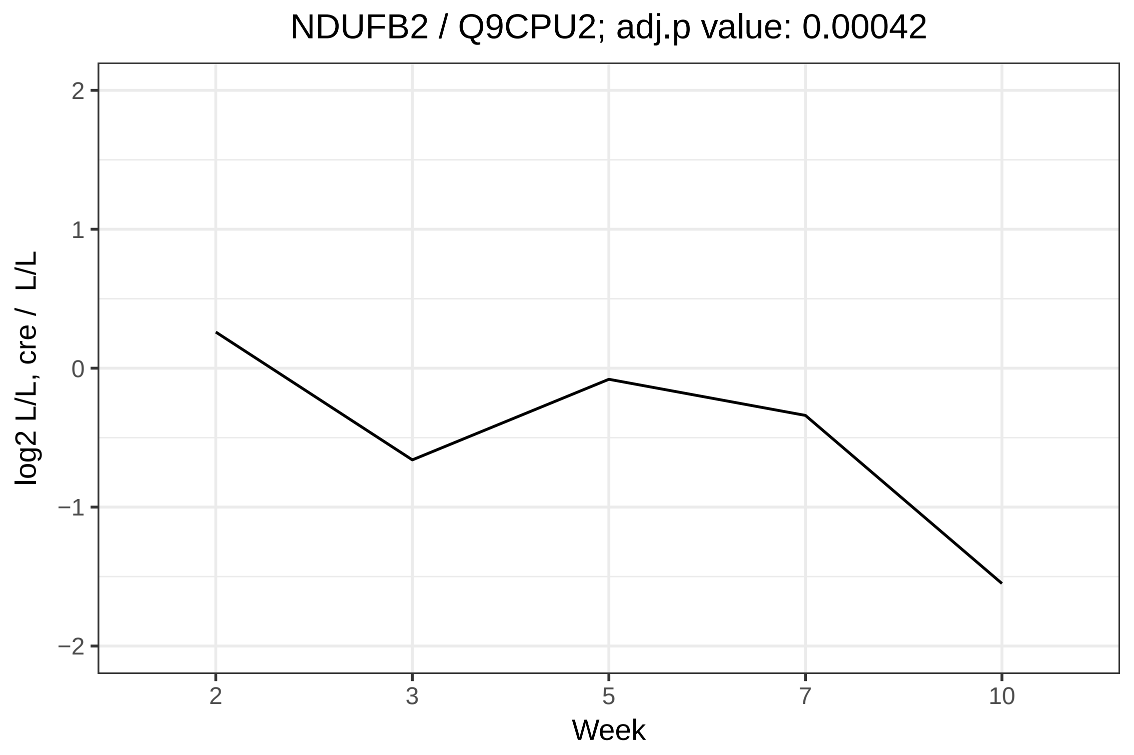

NDUFB2 / Q9CPU2; adj.p value: 0.00042
log2 L/L, cre / L/L
2
1
0
−1
−2
2
3
5
7
10
Week

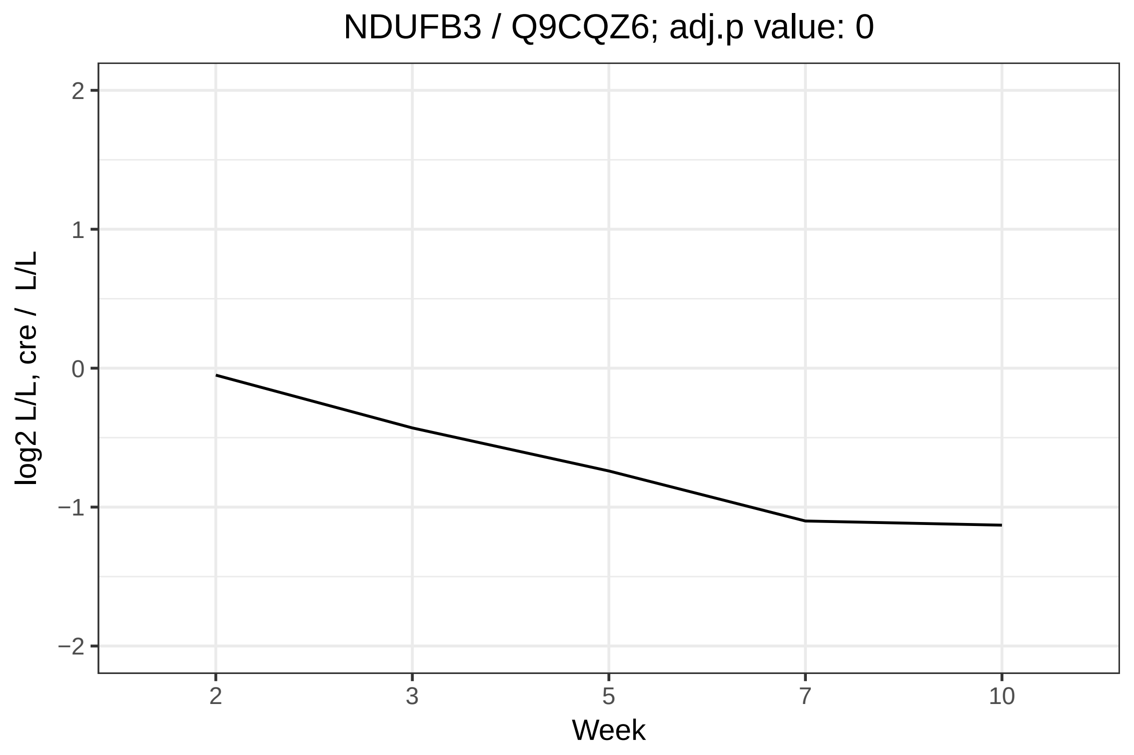
NDUFB3 / Q9CQZ6; adj.p value: 0
2
1
0
−1
−2
log2 L/L, cre / L/L
2
3
5
7
10
Week

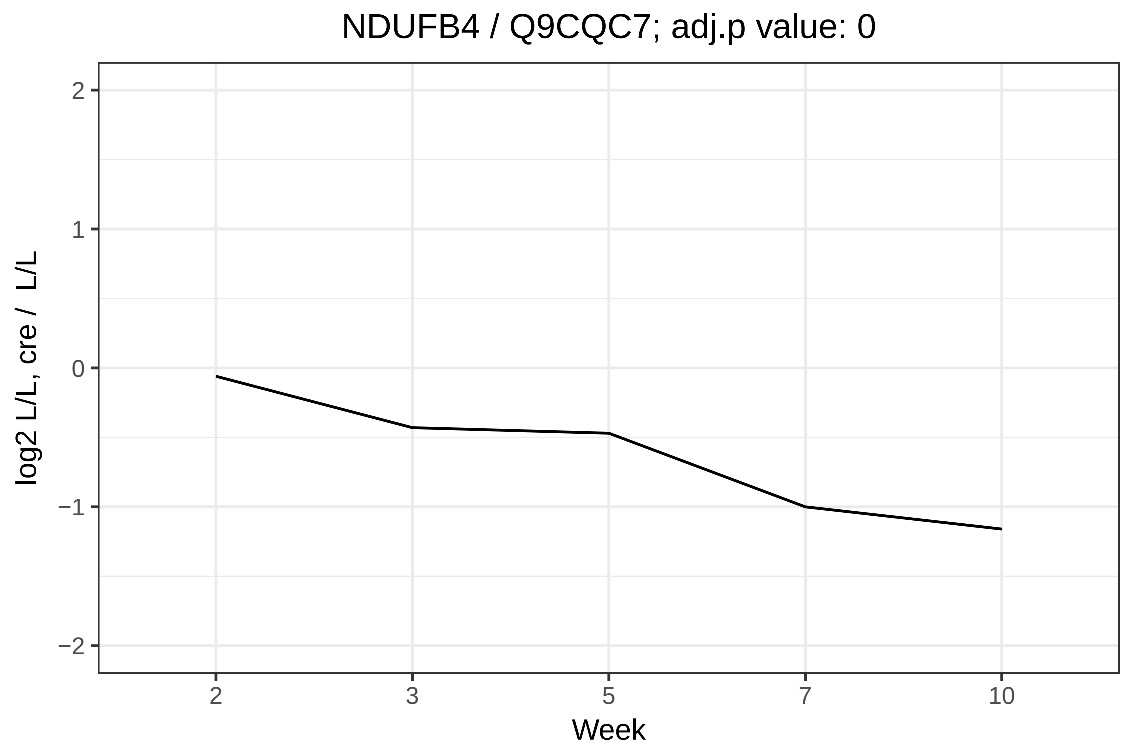

NDUFB4 / Q9CQC7; adj.p value: 0
log2 L/L, cre / L/L
Week
2
1
0
−1
−2
2
3
5
7
10

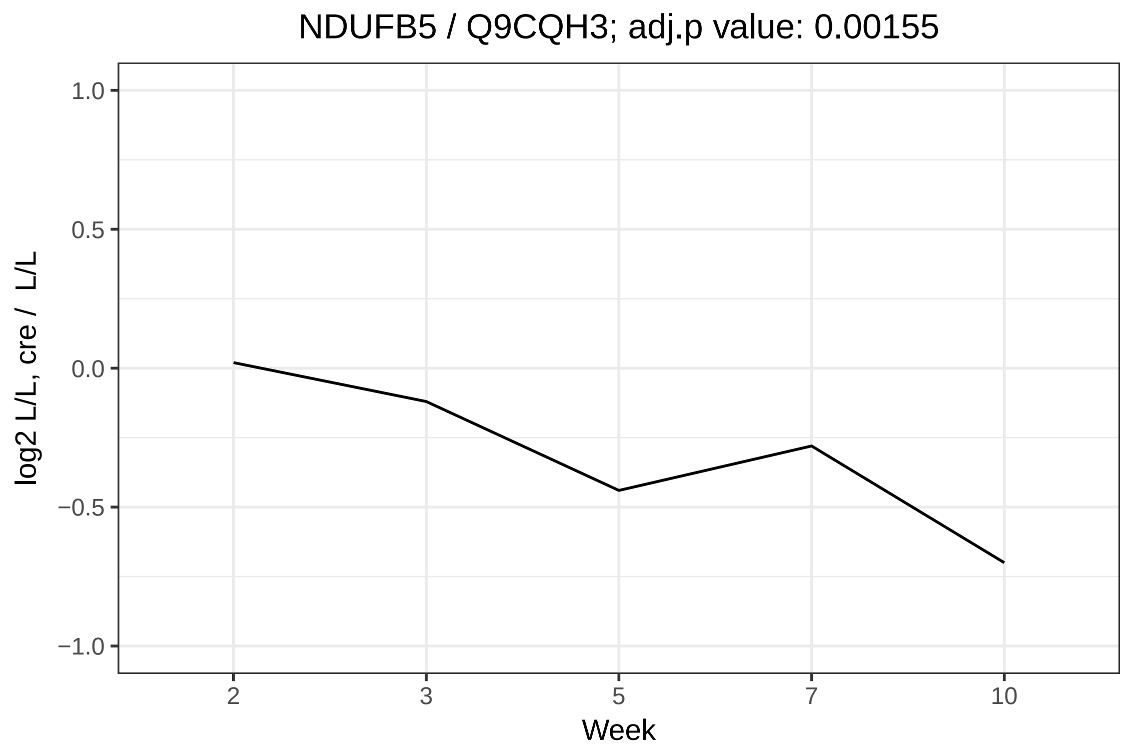
NDUFB5 / Q9CQH3; adj.p value: 0.00155
log2 L/L, cre / L/L
1.0
0.5
0.0
−0.5
−1.0
2
3
5
7
10
Week

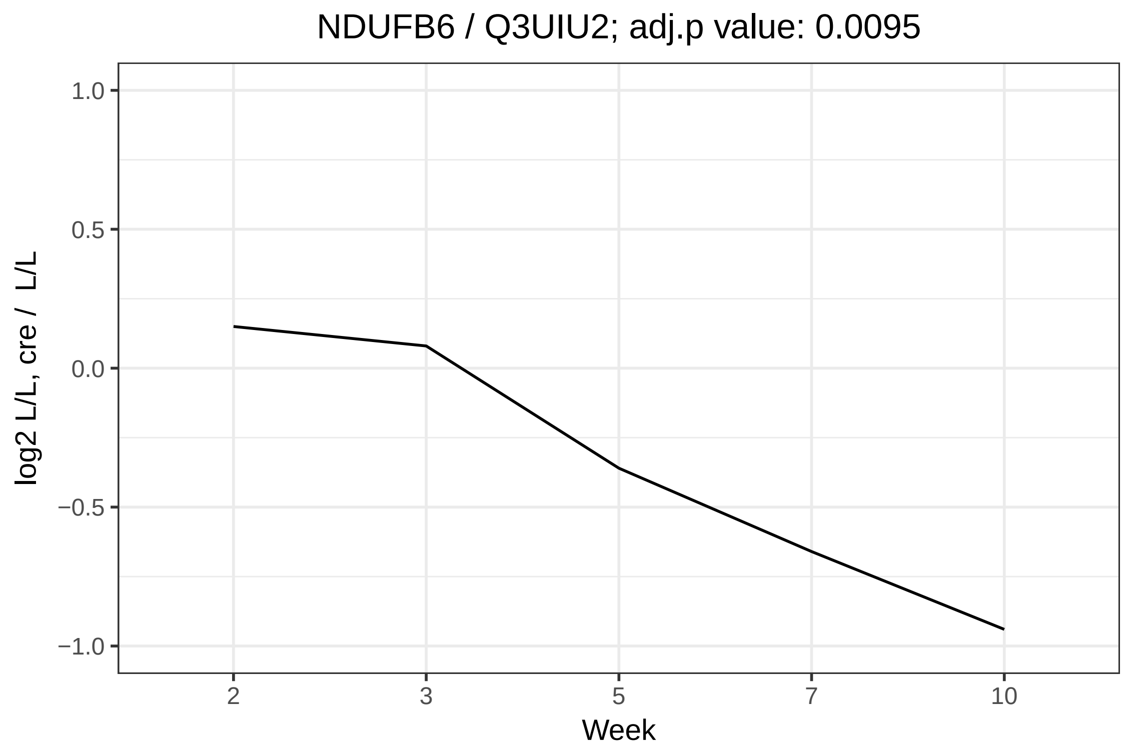
NDUFB6 / Q3UIU2; adj.p value: 0.0095
log2 L/L, cre / L/L
1.0
0.5
0.0
−0.5
−1.0
2
3
5
7
10
Week

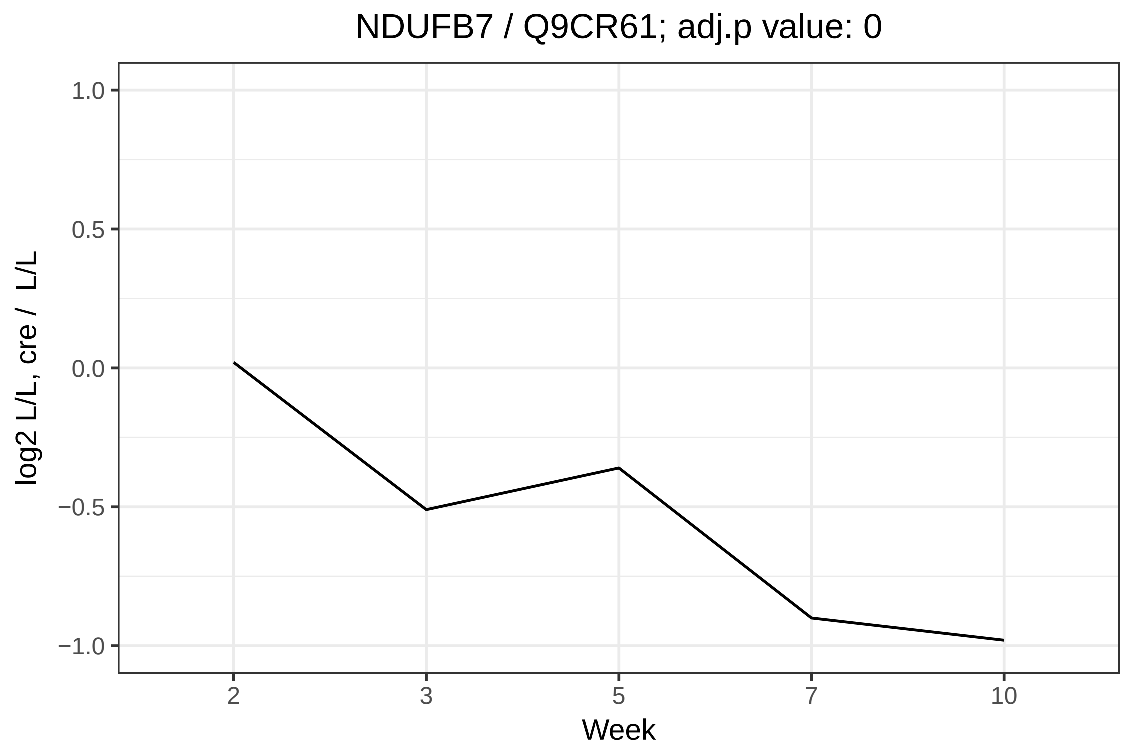

NDUFB7 / Q9CR61; adj.p value: 0
log2 L/L, cre / L/L
1.0
0.5
0.0
−0.5
−1.0
2
3
5
7
10
Week

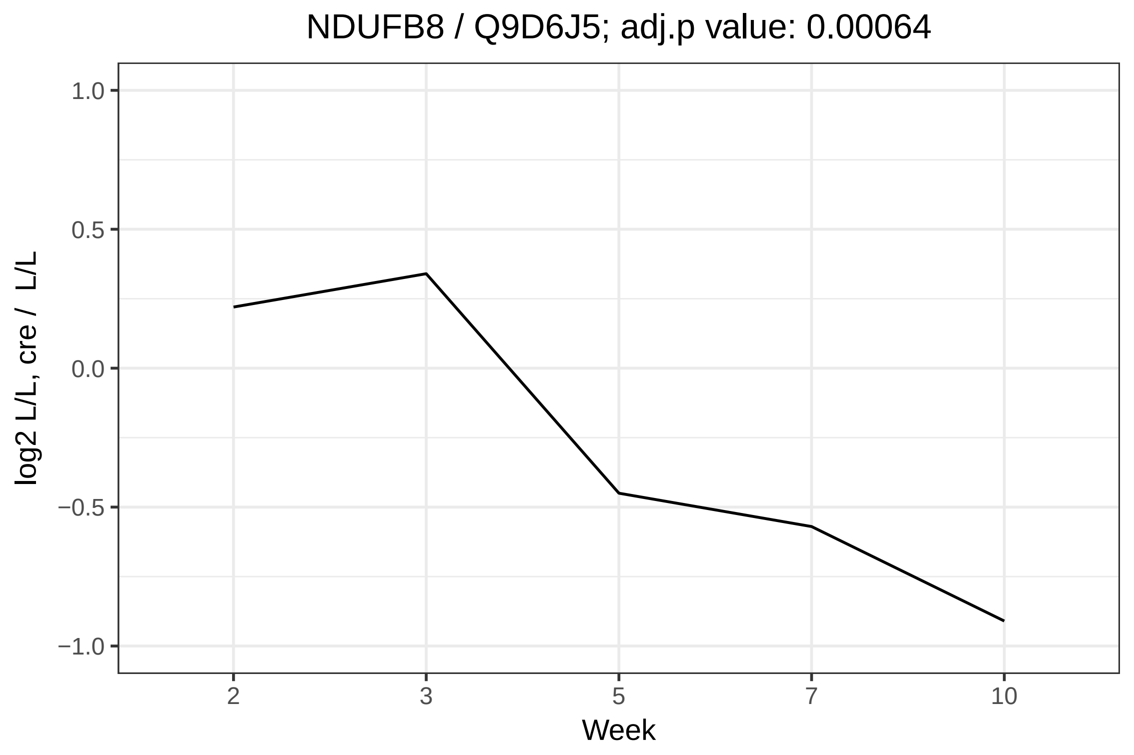
NDUFB8 / Q9D6J5; adj.p value: 0.00064
log2 L/L, cre / L/L
1.0
0.5
0.0
−0.5
−1.0
2
3
5
7
10
Week

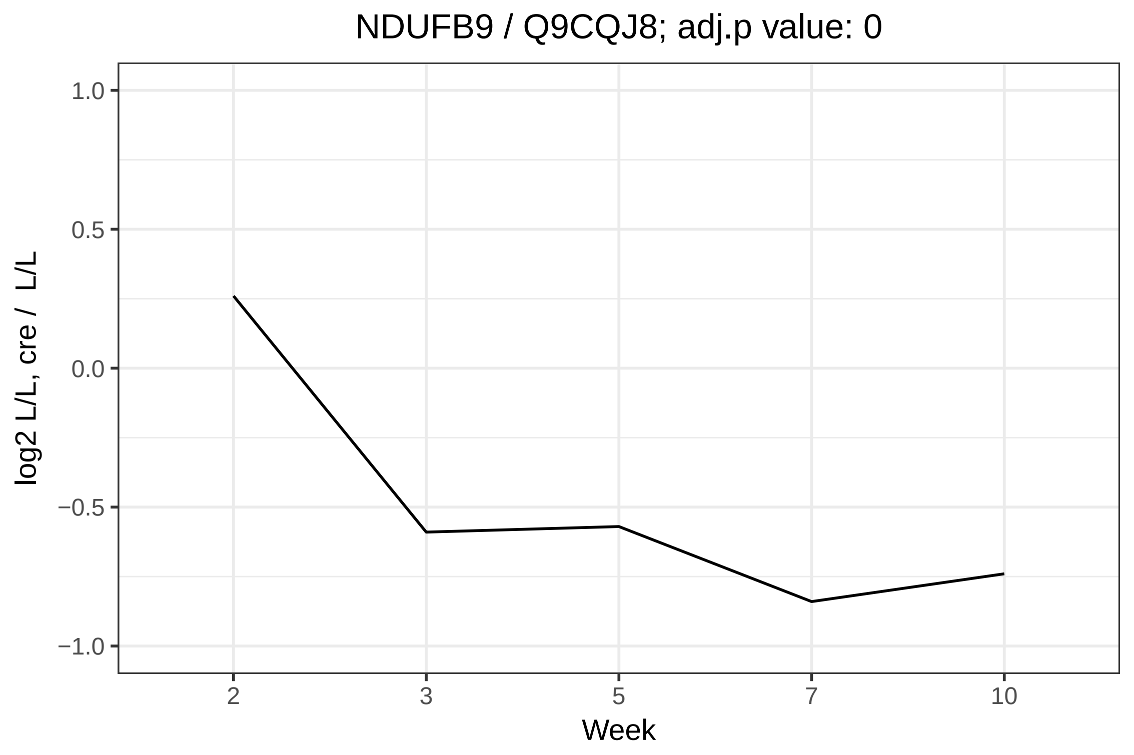
NDUFB9 / Q9CQJ8; adj.p value: 0
log2 L/L, cre / L/L
1.0
0.5
0.0
−0.5
−1.0
2
3
5
7
10
Week

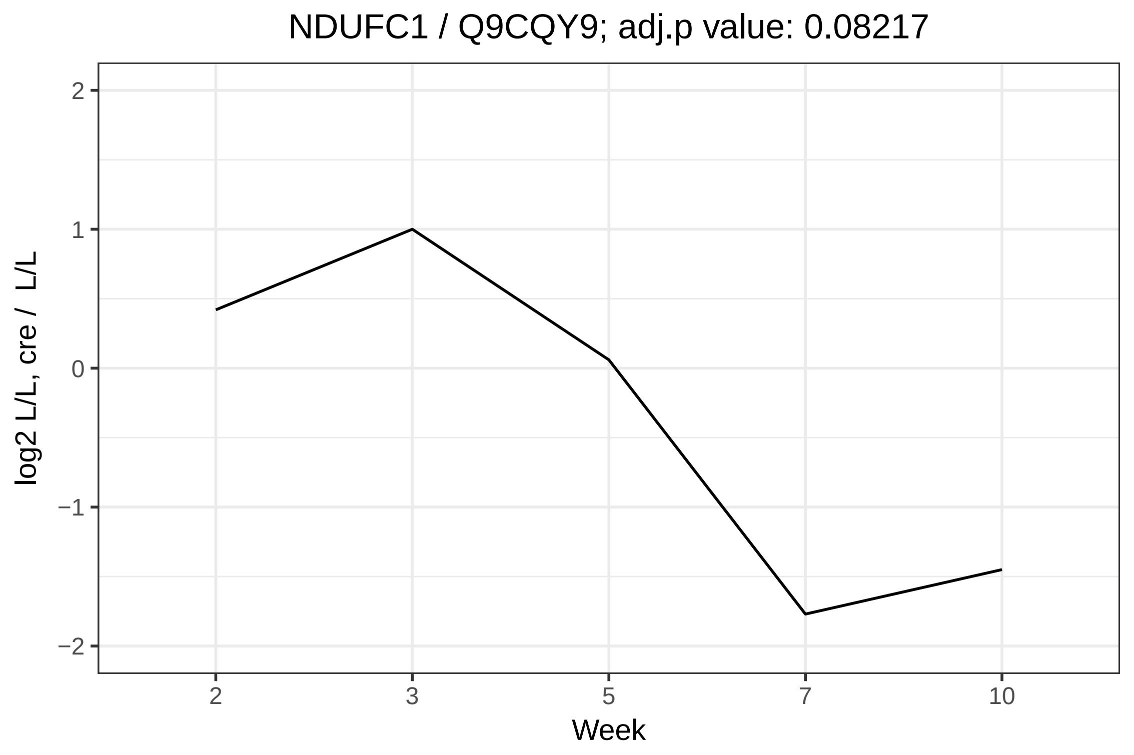
NDUFC1 / Q9CQY9; adj.p value: 0.08217
log2 L/L, cre / L/L
Week
2
1
0
−1
−2
2
3
5
7
10

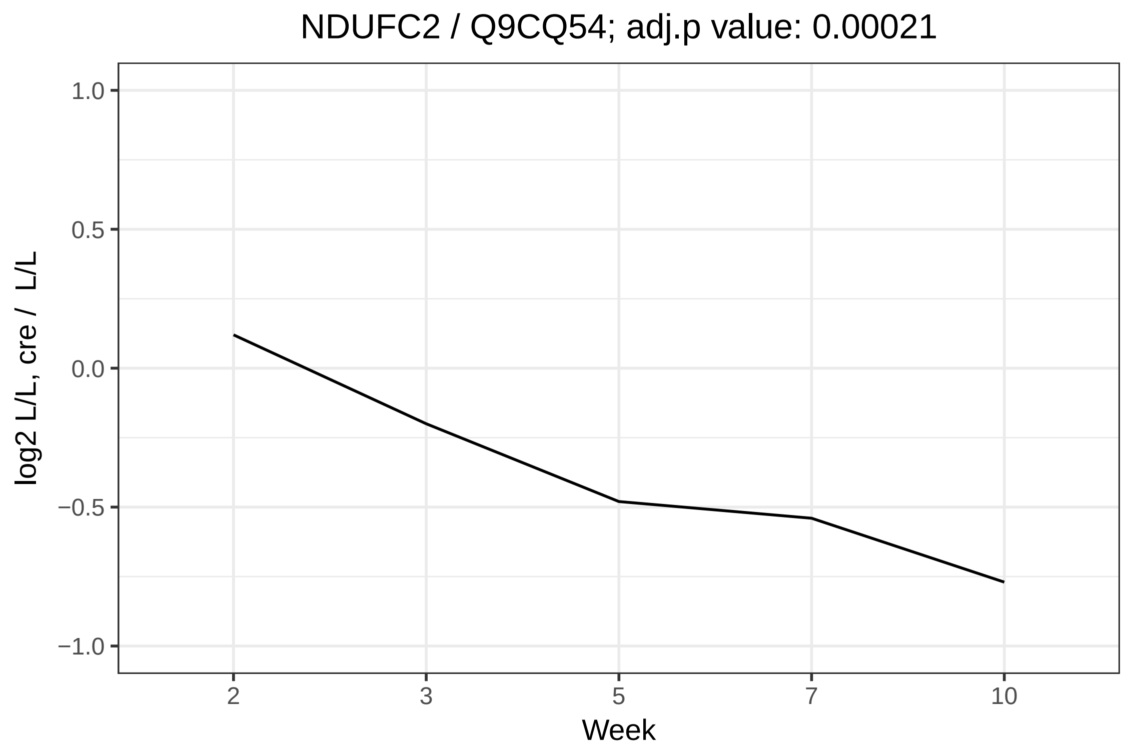

NDUFC2 / Q9CQ54; adj.p value: 0.00021
1.0
0.5
0.0
−0.5
−1.0
log2 L/L, cre / L/L
2
3
5
7
10
Week

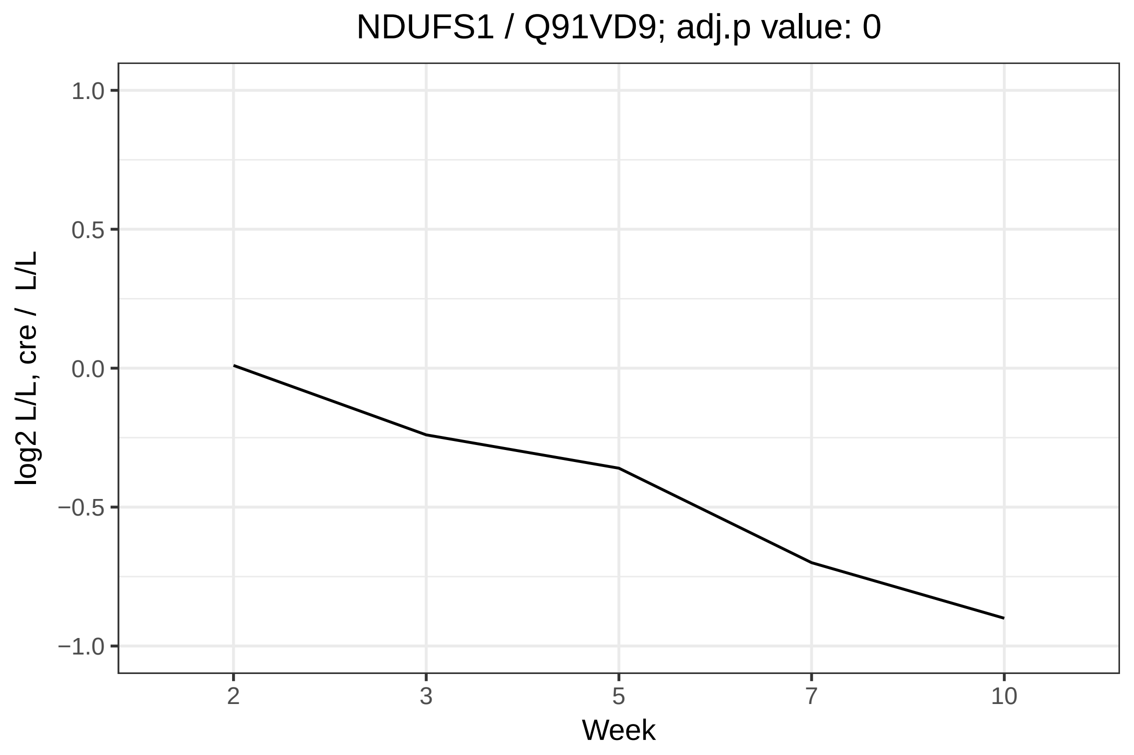

NDUFS1 / Q91VD9; adj.p value: 0
log2 L/L, cre / L/L
1.0
0.5
0.0
−0.5
−1.0
2
3
5
7
10
Week

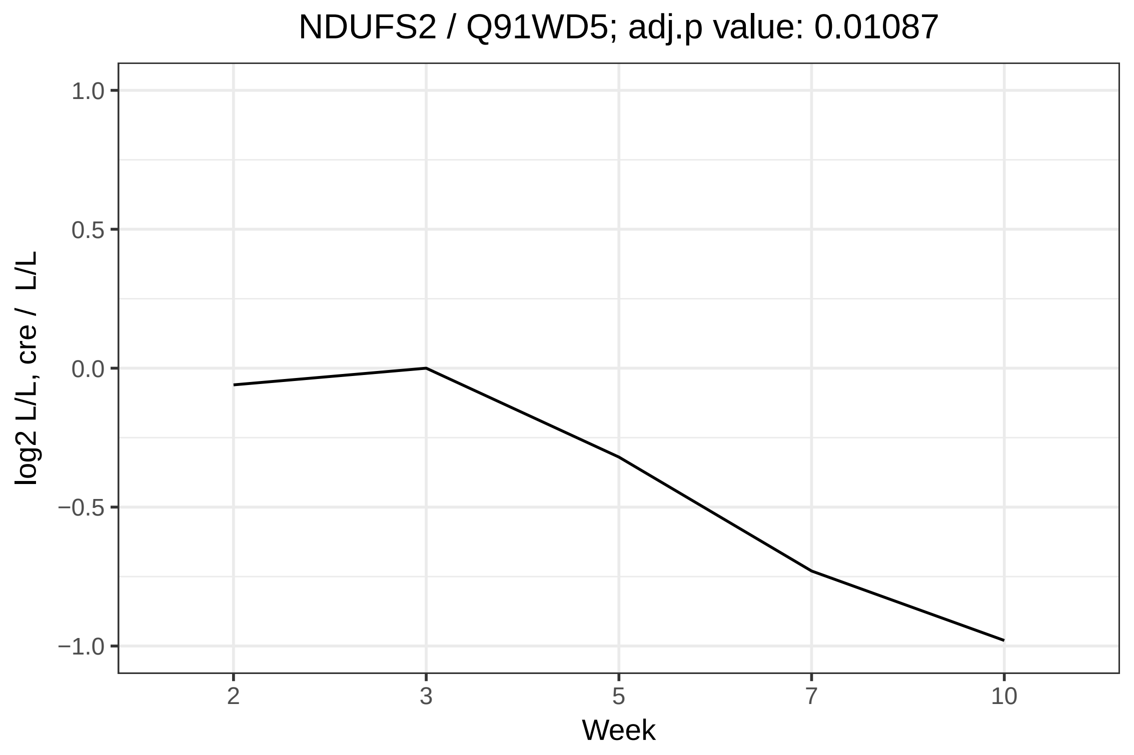
NDUFS2 / Q91WD5; adj.p value: 0.01087
1.0
0.5
0.0
−0.5
−1.0
log2 L/L, cre / L/L
2
3
5
7
10
Week

NDUFS3 / Q9DCT2; adj.p value: 0.00012

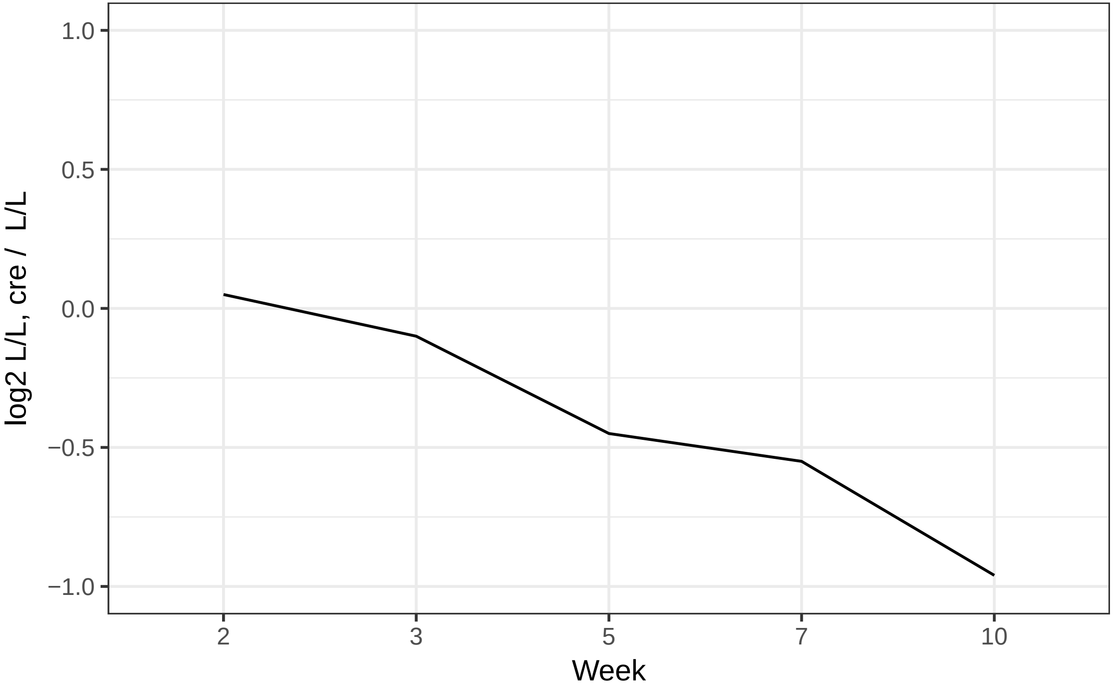

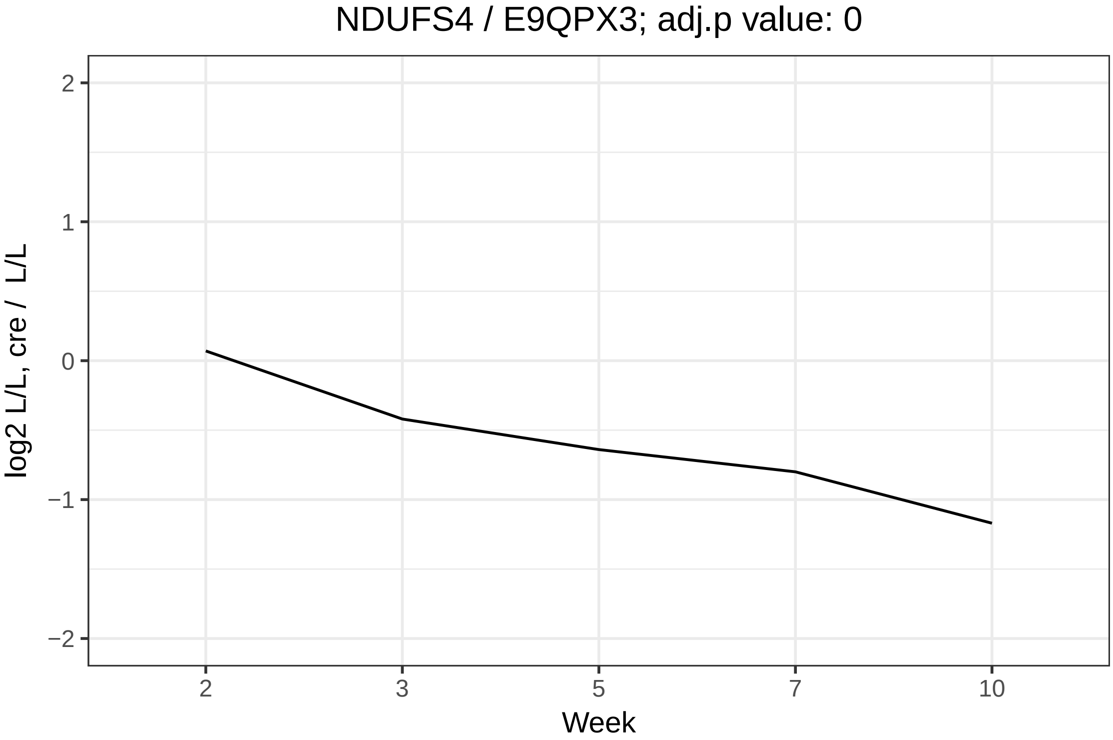
NDUFS4 / E9QPX3; adj.p value: 0
log2 L/L, cre / L/L
2
1
0
−1
−2
2
3
5
7
10
Week

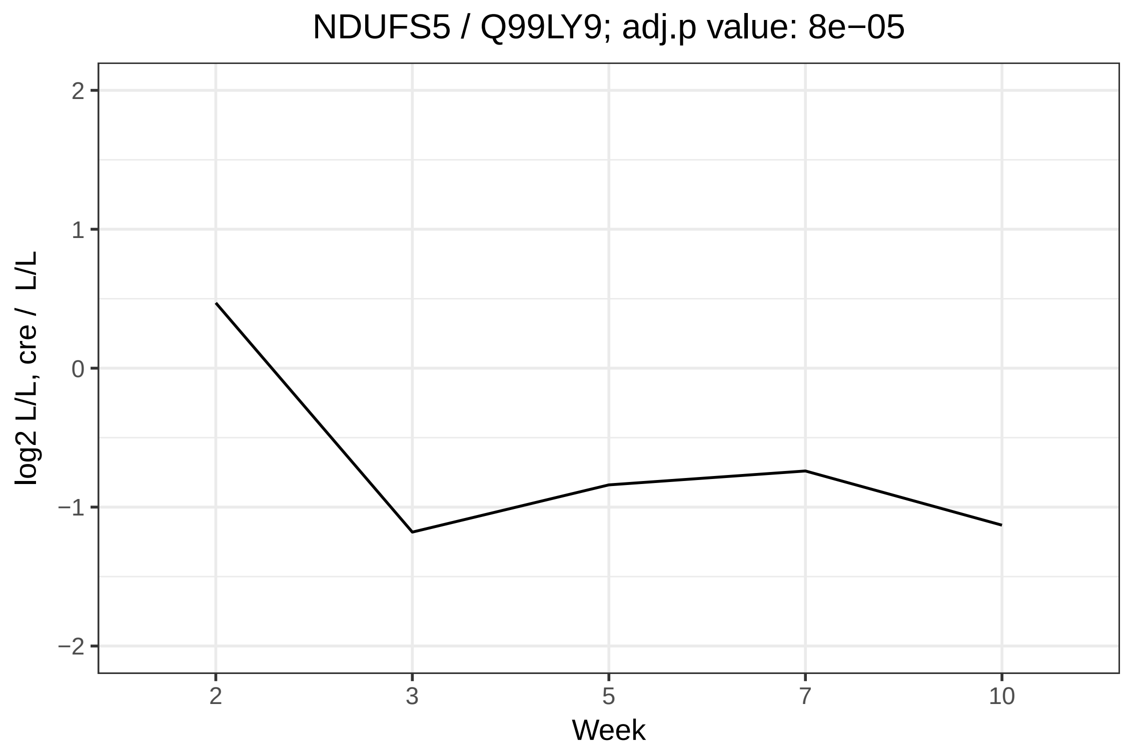
NDUFS5 / Q99LY9; adj.p value: 8e−05
log2 L/L, cre / L/L
2
1
0
−1
−2
2
3
5
7
10
Week

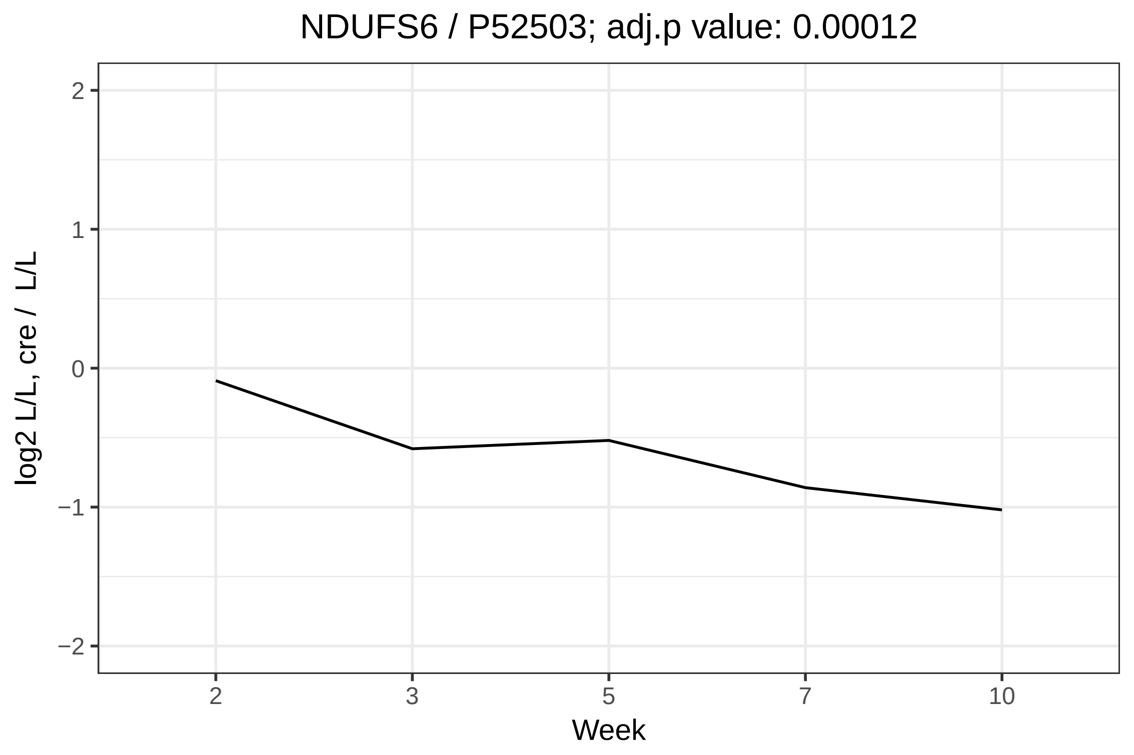
NDUFS6 / P52503; adj.p value: 0.00012
log2 L/L, cre / L/L
2
1
0
−1
−2
2
3
5
7
10
Week

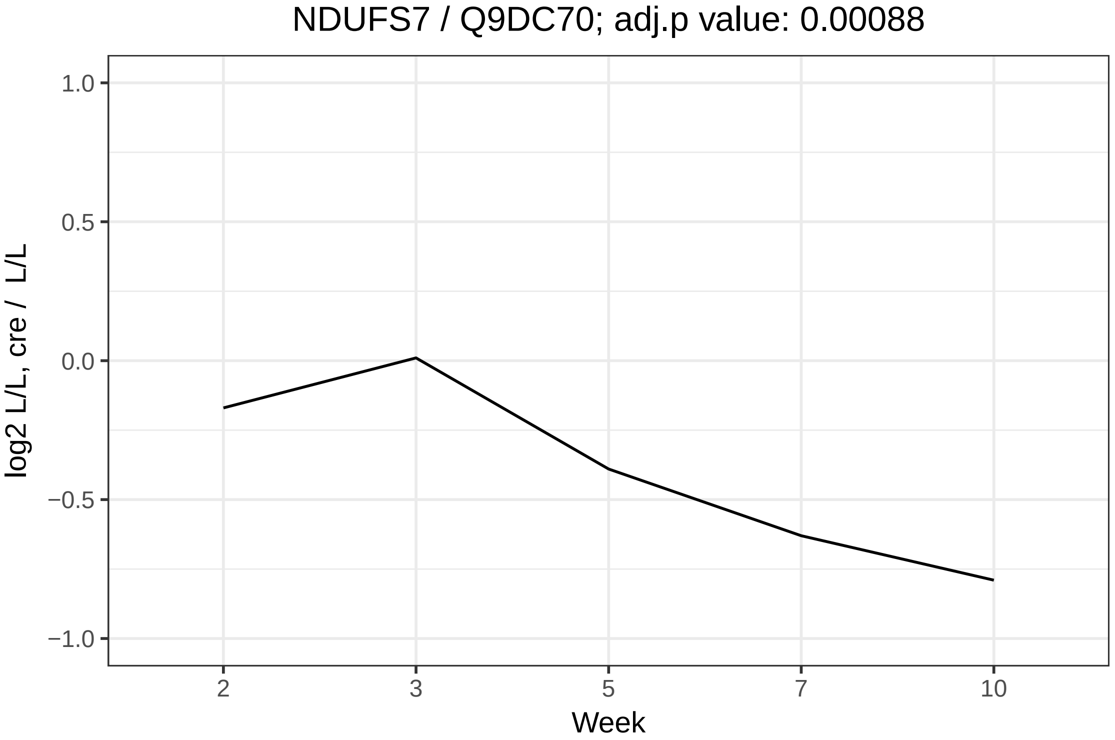
NDUFS7 / Q9DC70; adj.p value: 0.00088
log2 L/L, cre / L/L
1.0
0.5
0.0
−0.5
−1.0
2
3
5
7
10
Week

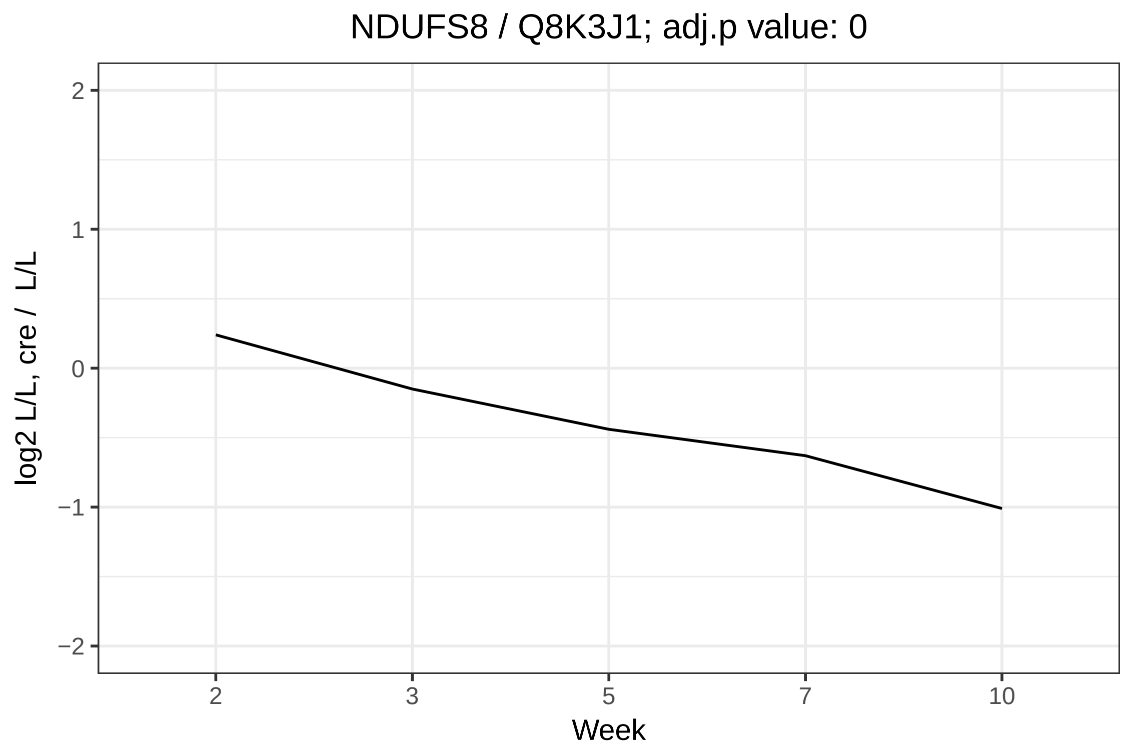

NDUFS8 / Q8K3J1; adj.p value: 0
2
1
0
−1
−2
log2 L/L, cre / L/L
2
3
5
7
10
Week

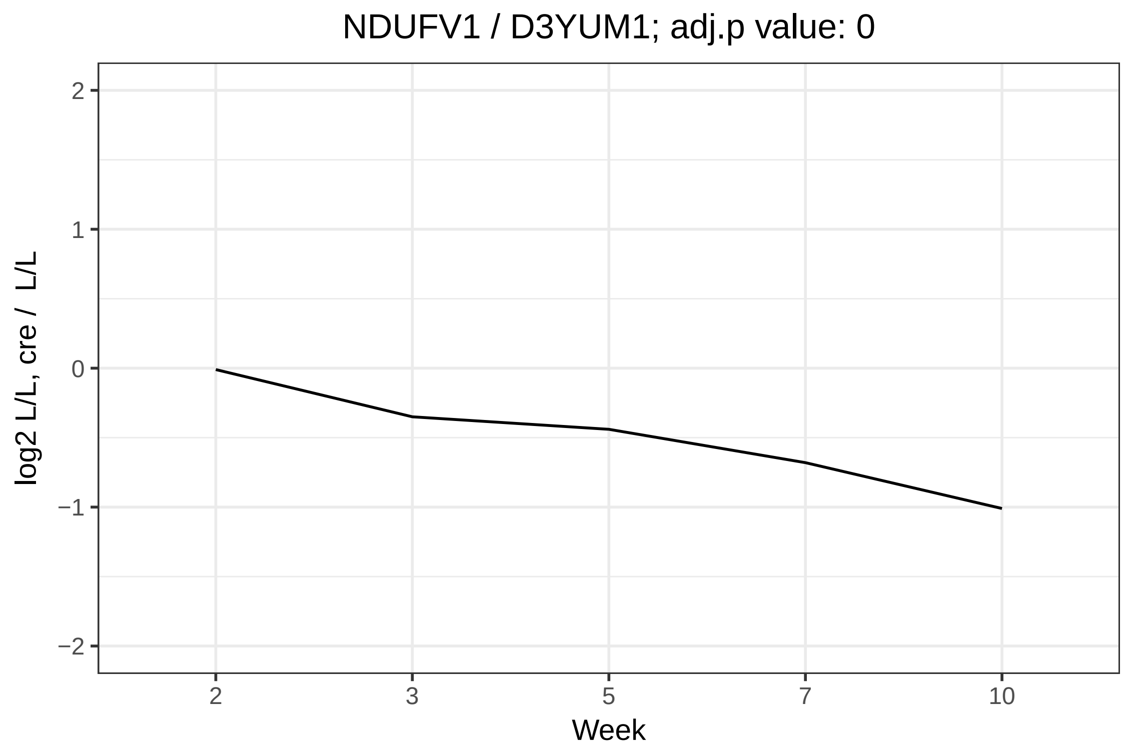
NDUFV1 / D3YUM1; adj.p value: 0
log2 L/L, cre / L/L
2
1
0
−1
−2
2
3
5
7
10
Week

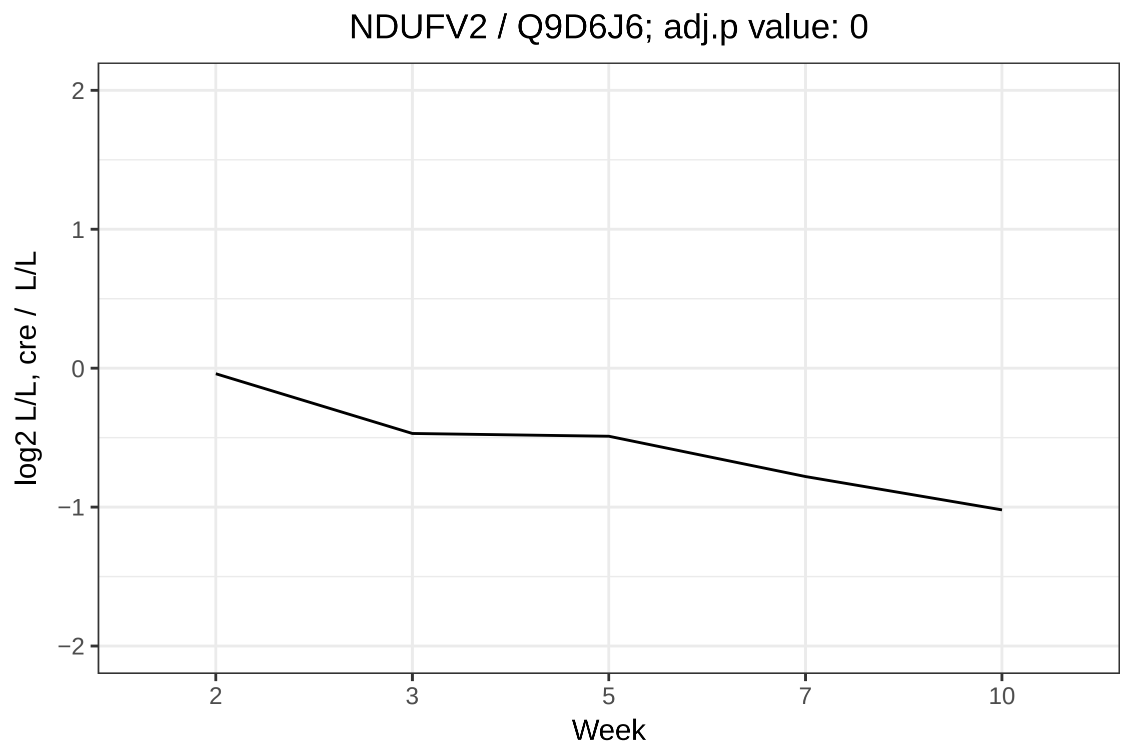
NDUFV2 / Q9D6J6; adj.p value: 0
log2 L/L, cre / L/L
2
1
0
−1
−2
2
3
5
7
10
Week

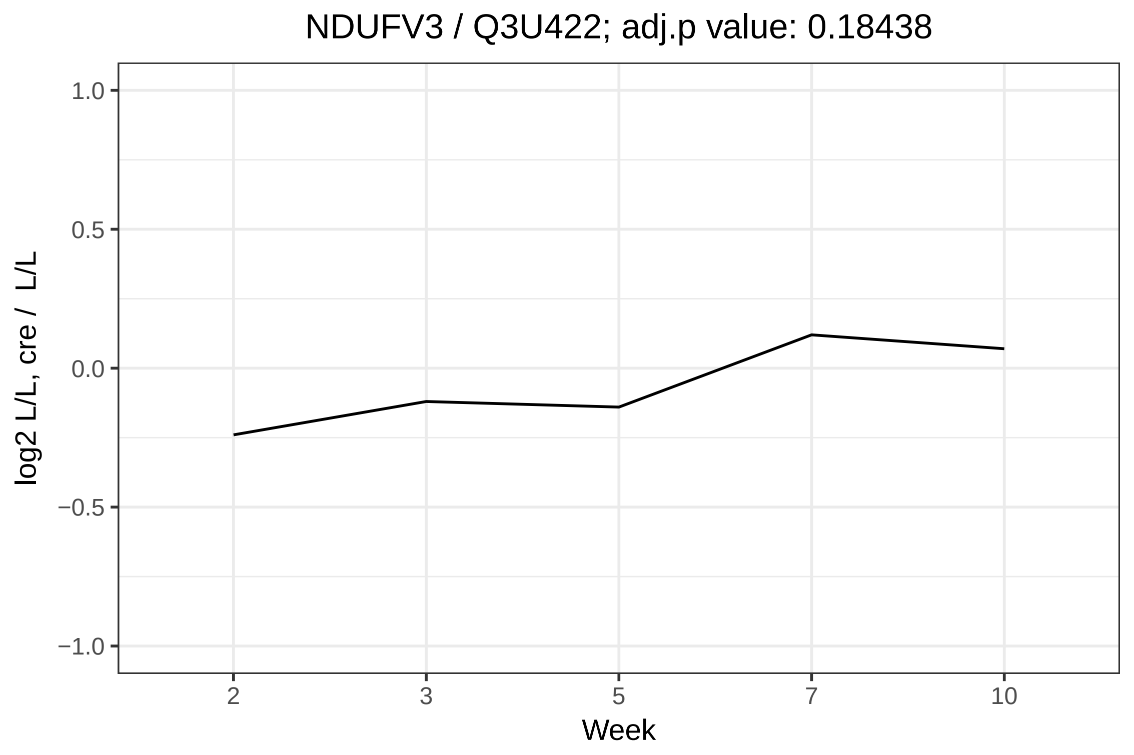
NDUFV3 / Q3U422; adj.p value: 0.18438
log2 L/L, cre / L/L
1.0
0.5
0.0
−0.5
−1.0
2
3
5
7
10
Week

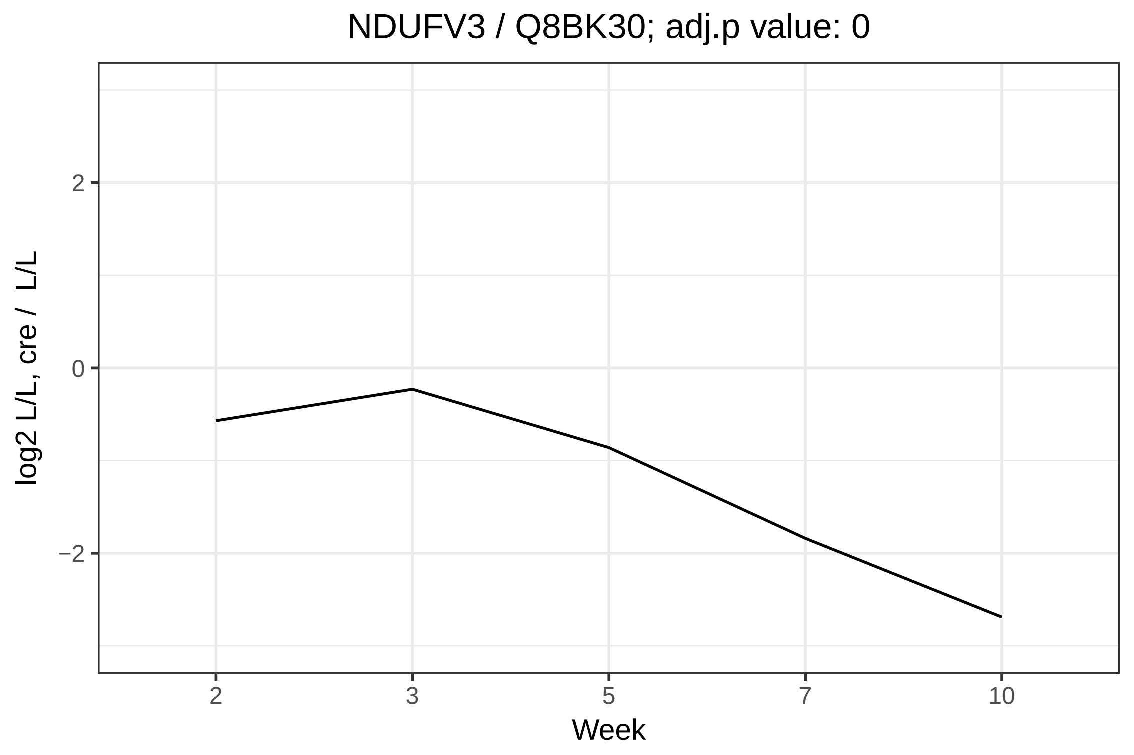

NDUFV3 / Q8BK30; adj.p value: 0
log2 L/L, cre / L/L
2
0
−2
2
3
5
7
10
Week

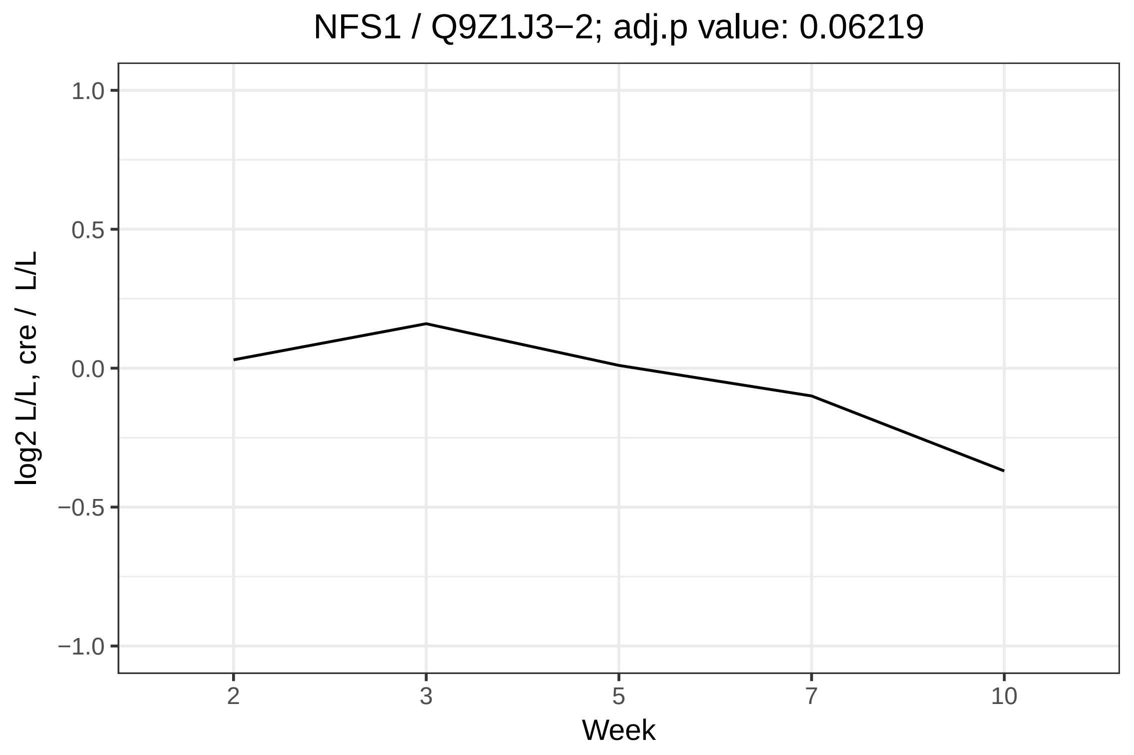

NFS1 / Q9Z1J3−2; adj.p value: 0.06219
log2 L/L, cre / L/L
1.0
0.5
0.0
−0.5
−1.0
2
3
5
7
10
Week

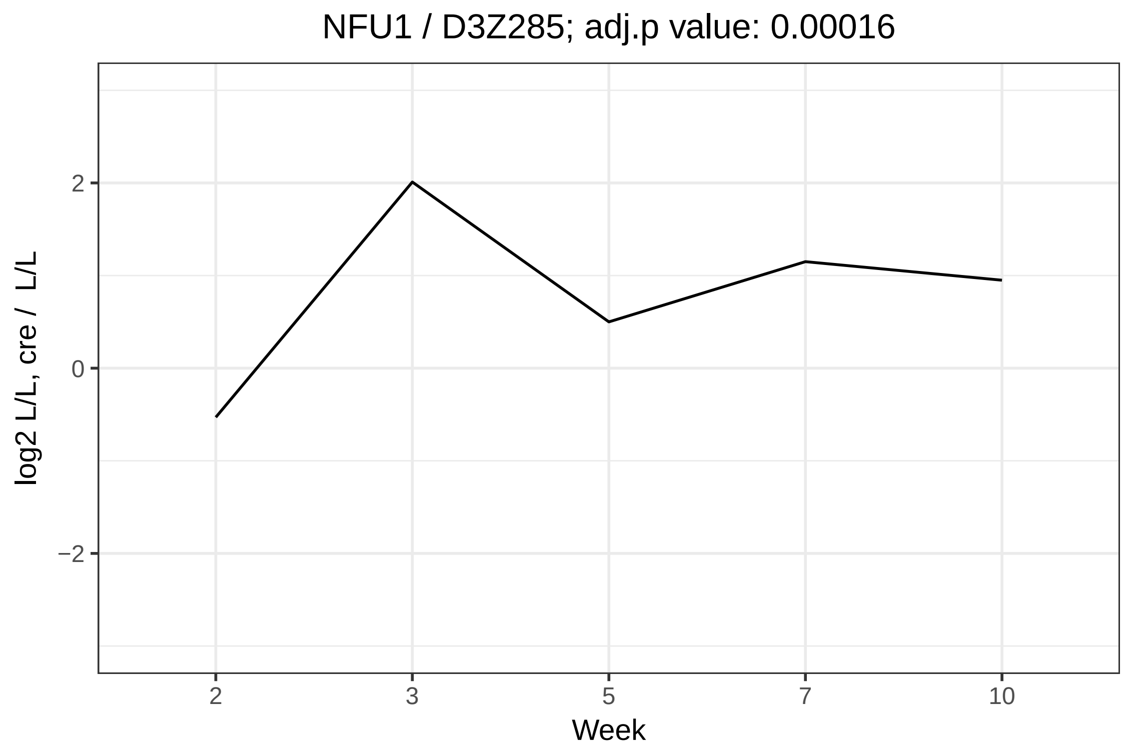

NFU1 / D3Z285; adj.p value: 0.00016
log2 L/L, cre / L/L
2
0
−2
2
3
5
7
10
Week

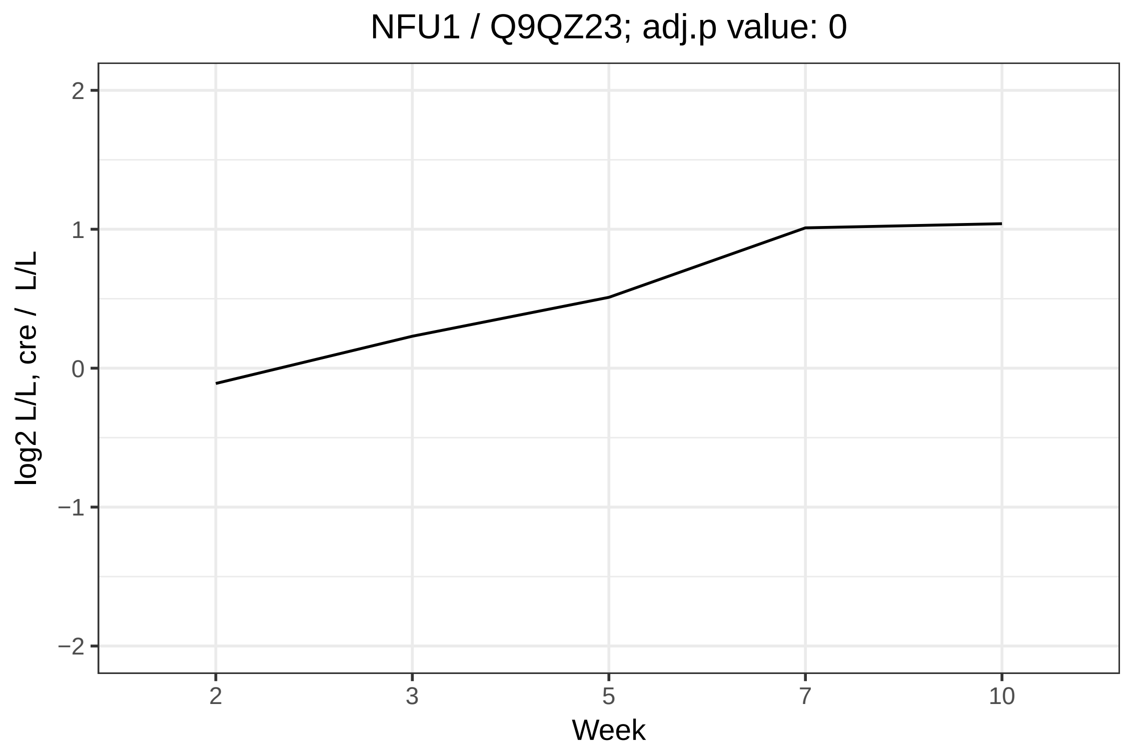
NFU1 / Q9QZ23; adj.p value: 0
log2 L/L, cre / L/L
2
1
0
−1
−2
2
3
5
7
10
Week

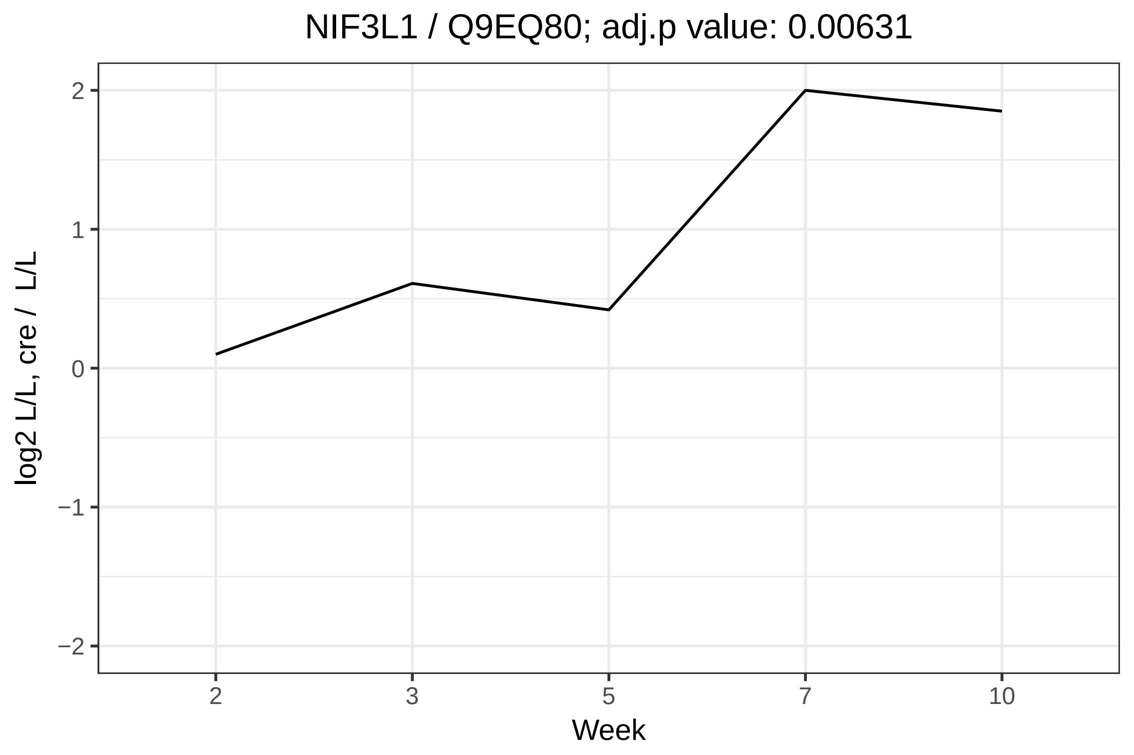

NIF3L1 / Q9EQ80; adj.p value: 0.00631
log2 L/L, cre / L/L
Week
2
1
0
−1
−2
2
3
5
7
10

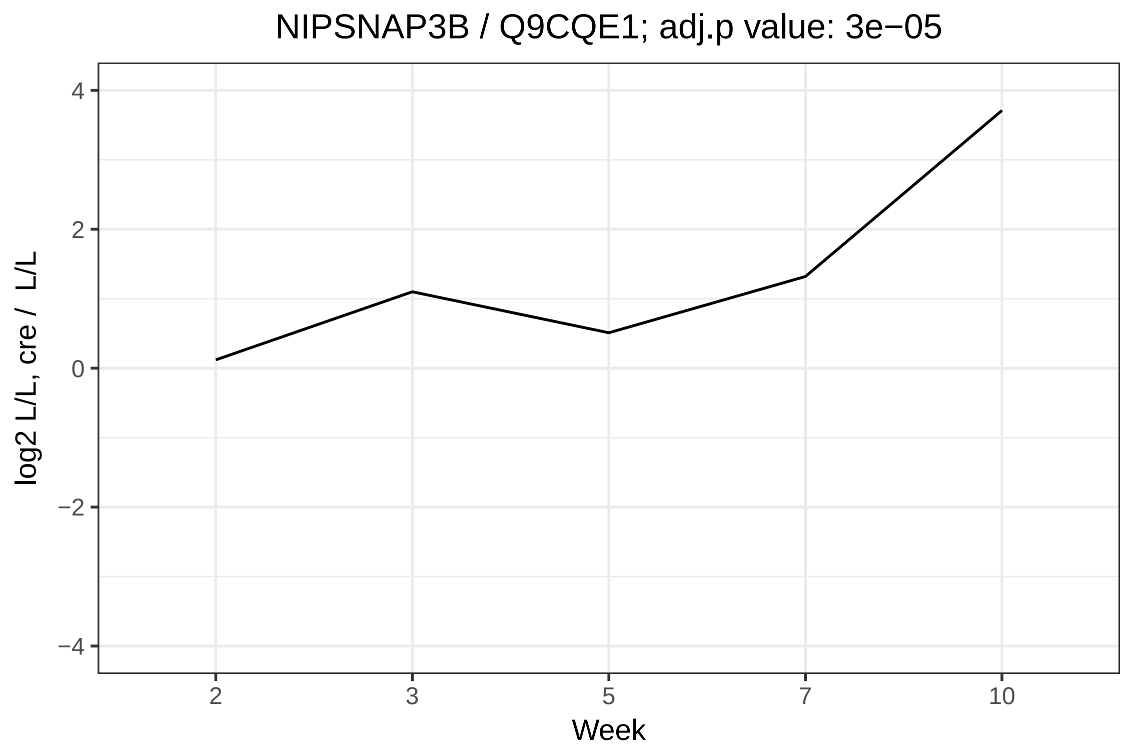
NIPSNAP3B / Q9CQE1; adj.p value: 3e−05
log2 L/L, cre / L/L
4
2
0
−2
−4
2
3
5
7
10
Week

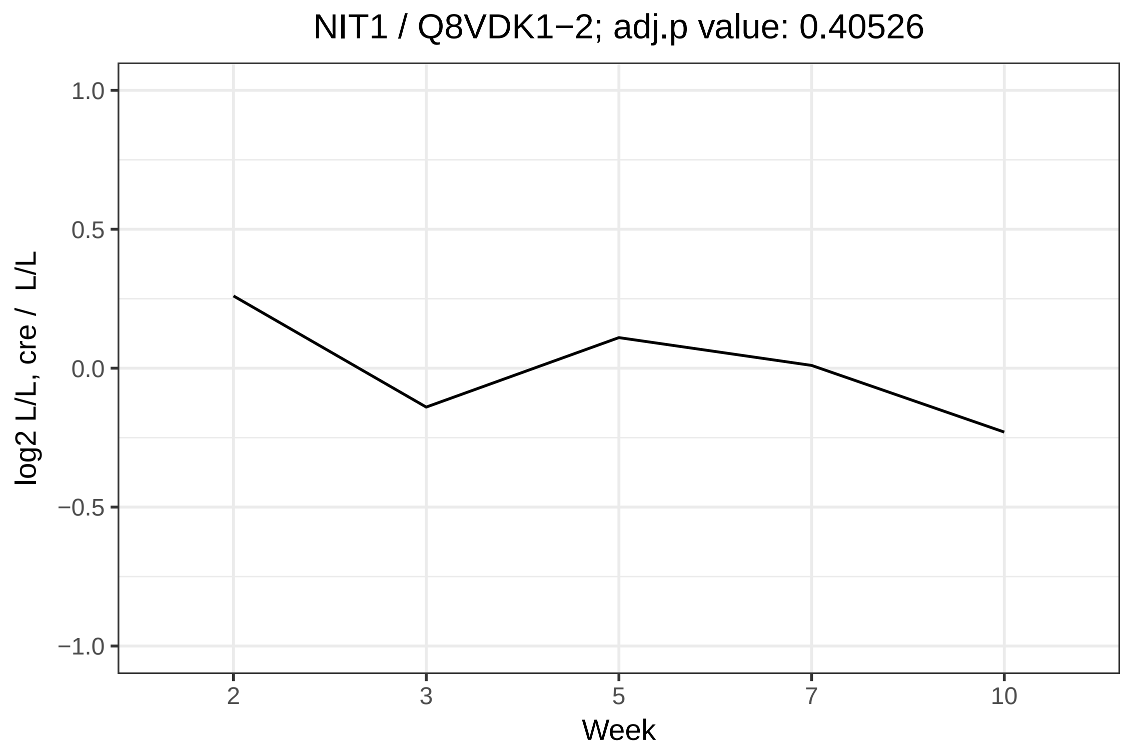

NIT1 / Q8VDK1−2; adj.p value: 0.40526
1.0
0.5
0.0
−0.5
−1.0
log2 L/L, cre / L/L
2
3
5
7
10
Week

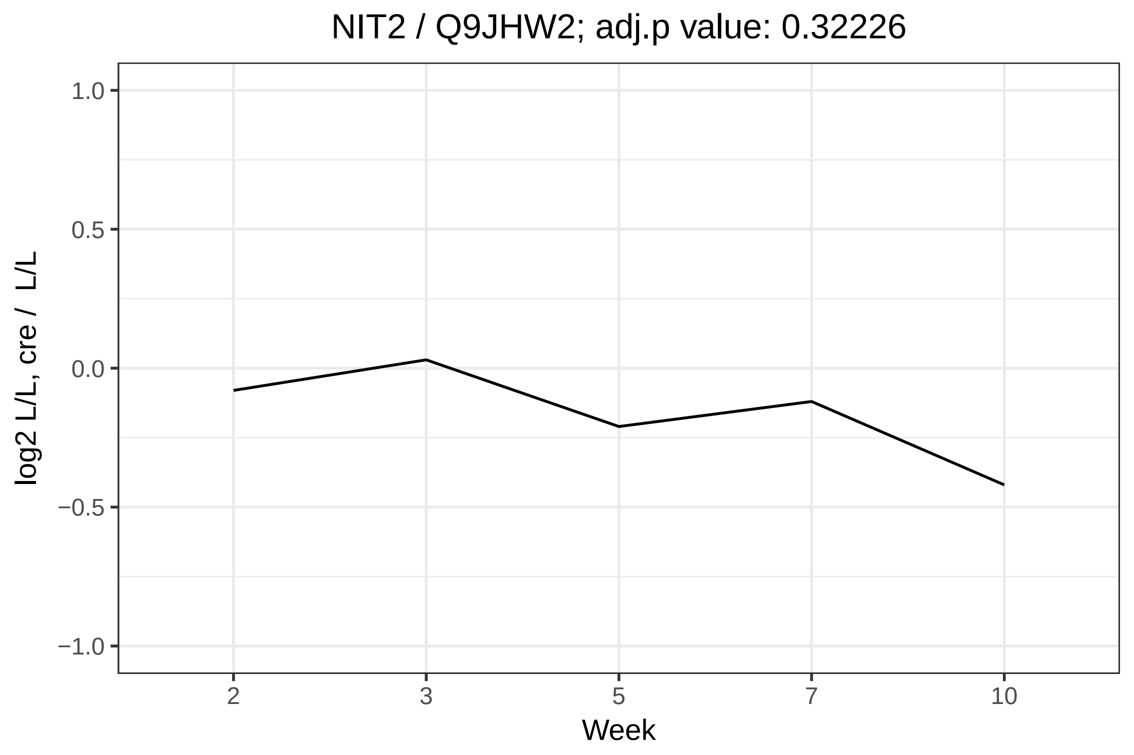
NIT2 / Q9JHW2; adj.p value: 0.32226
log2 L/L, cre / L/L
1.0
0.5
0.0
−0.5
−1.0
2
3
5
7
10
Week

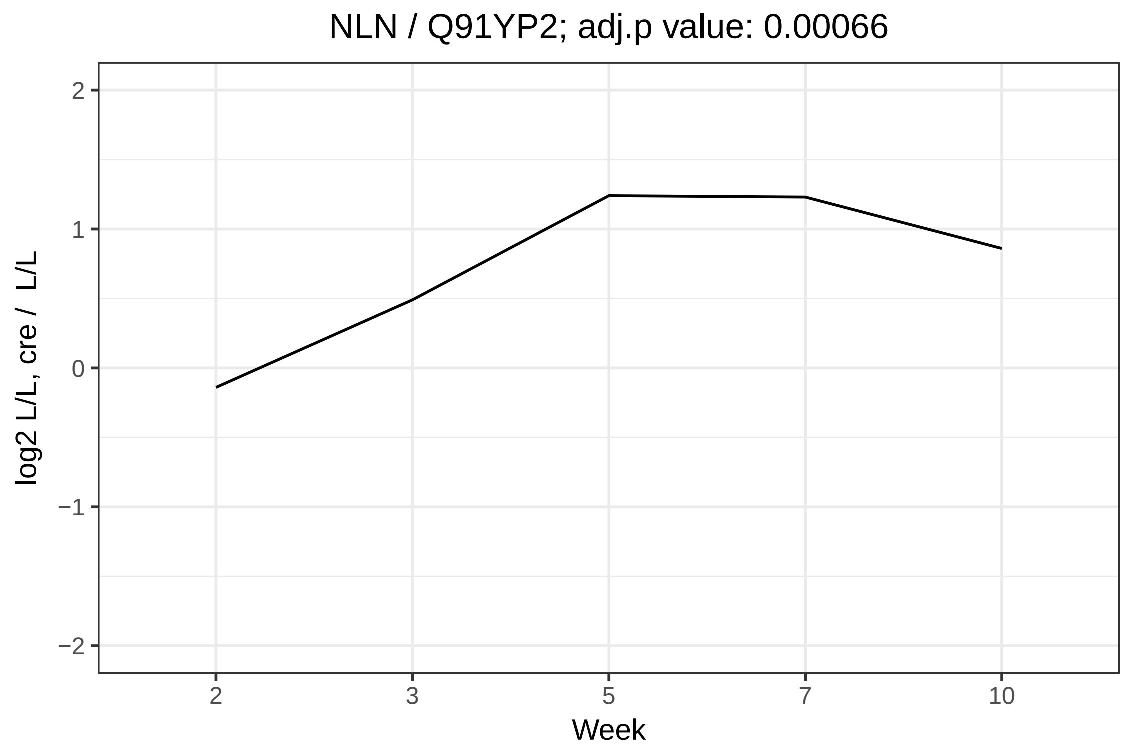
NLN / Q91YP2; adj.p value: 0.00066
log2 L/L, cre / L/L
2
1
0
−1
−2
2
3
5
7
10
Week

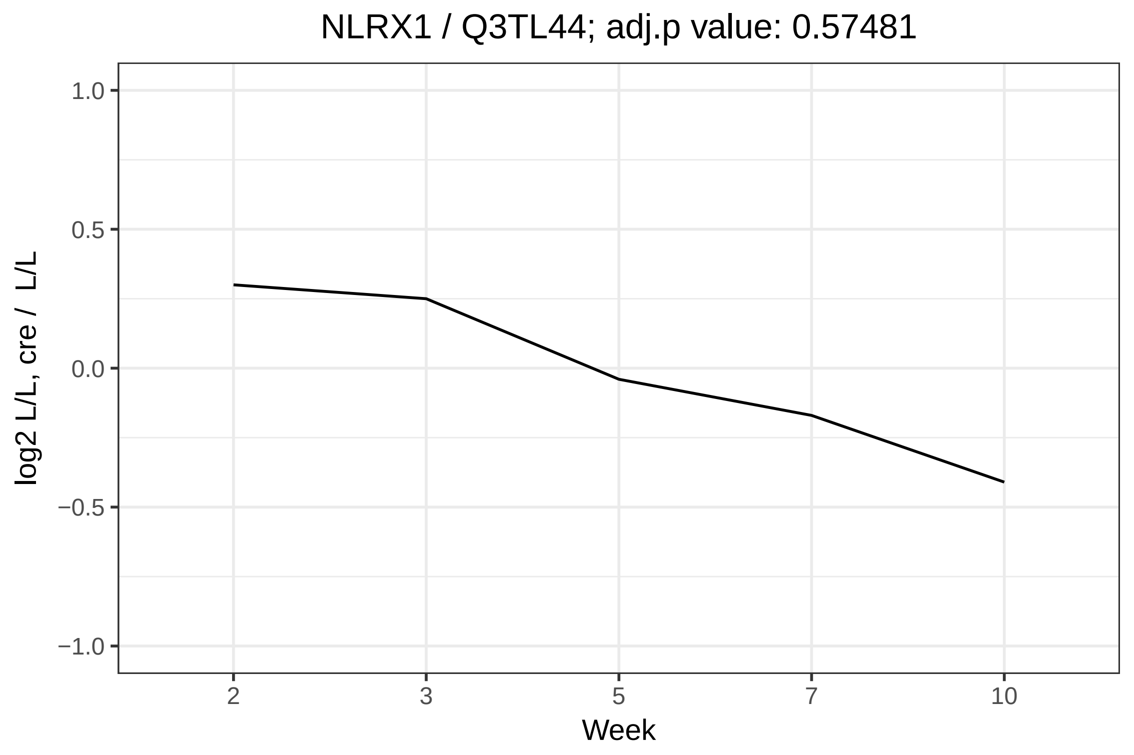

NLRX1 / Q3TL44; adj.p value: 0.57481
log2 L/L, cre / L/L
1.0
0.5
0.0
−0.5
−1.0
2
3
5
7
10
Week

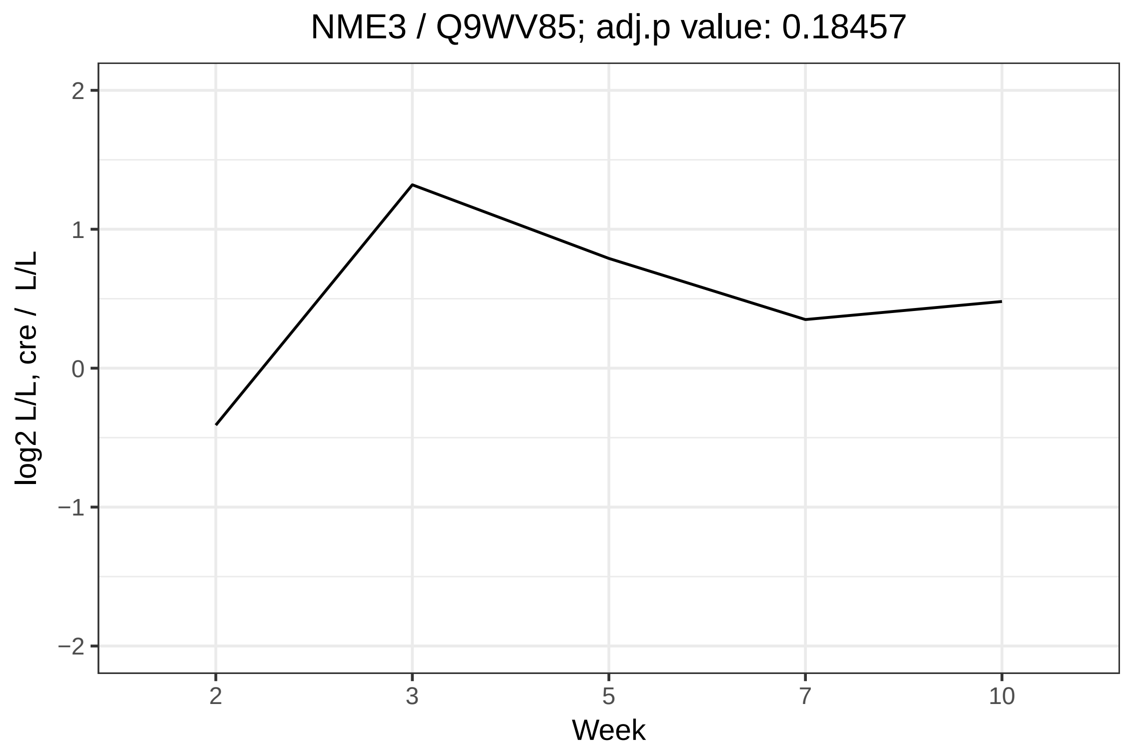
NME3 / Q9WV85; adj.p value: 0.18457
log2 L/L, cre / L/L
2
1
0
−1
−2
2
3
5
7
10
Week

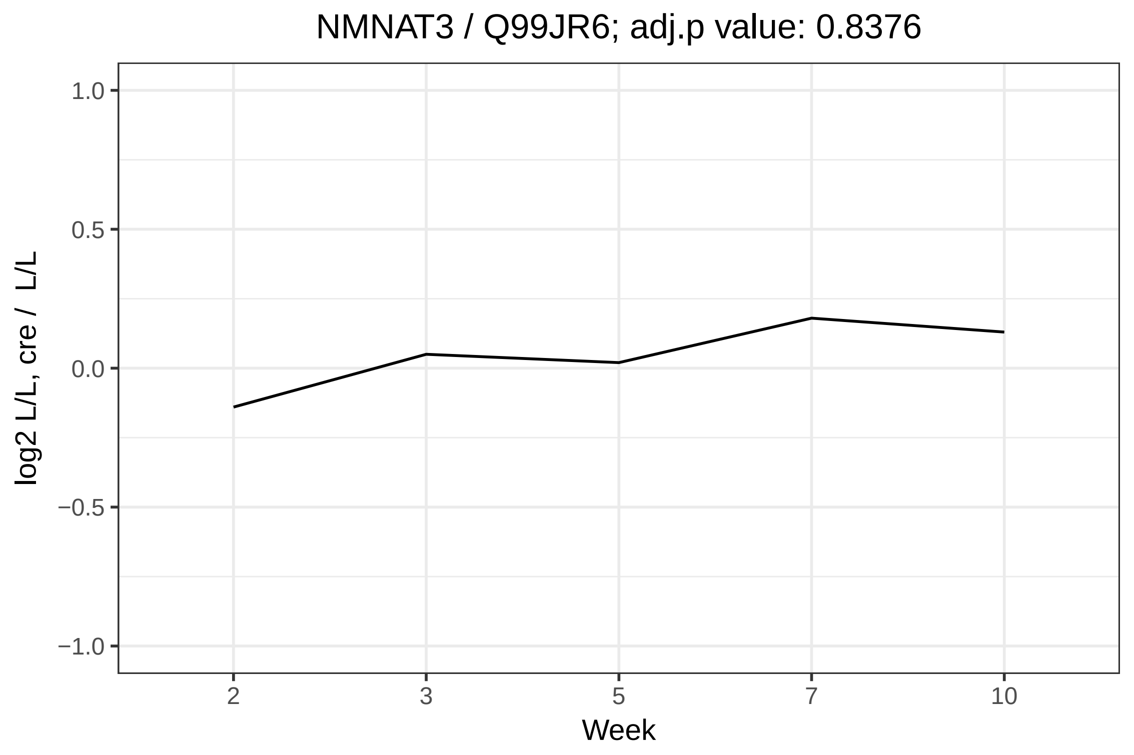

NMNAT3 / Q99JR6; adj.p value: 0.8376
log2 L/L, cre / L/L
1.0
0.5
0.0
−0.5
−1.0
2
3
5
7
10
Week

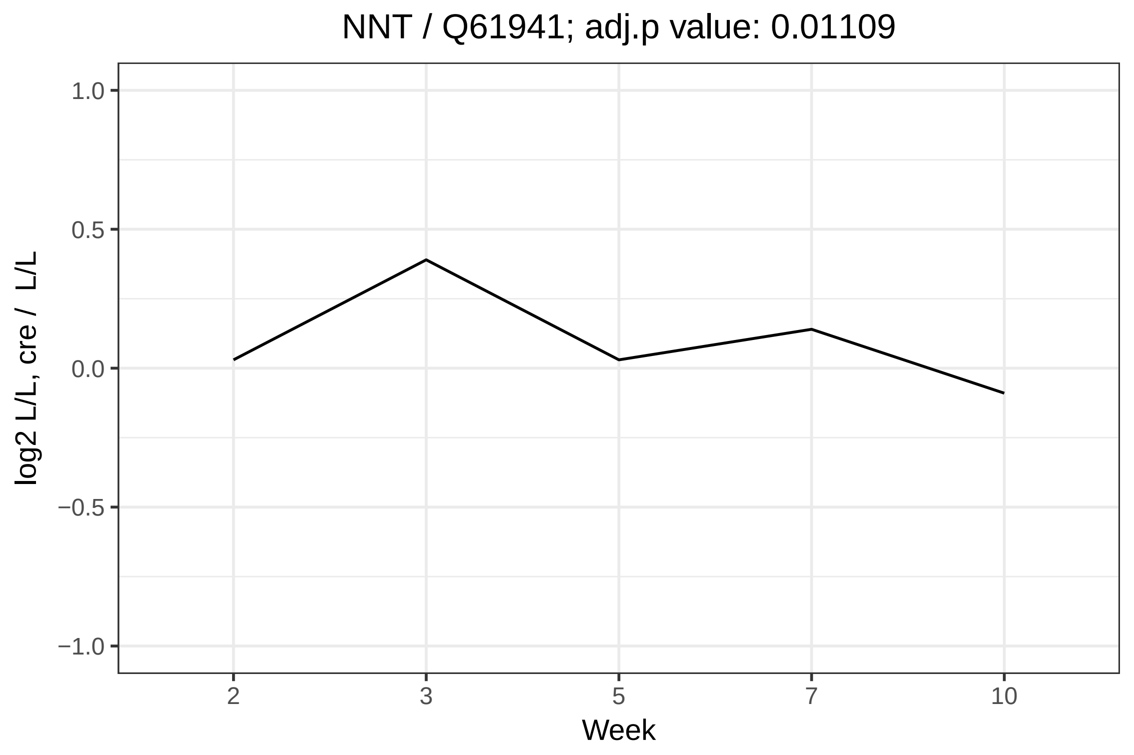

NNT / Q61941; adj.p value: 0.01109
log2 L/L, cre / L/L
1.0
0.5
0.0
−0.5
−1.0
2
3
5
7
10
Week

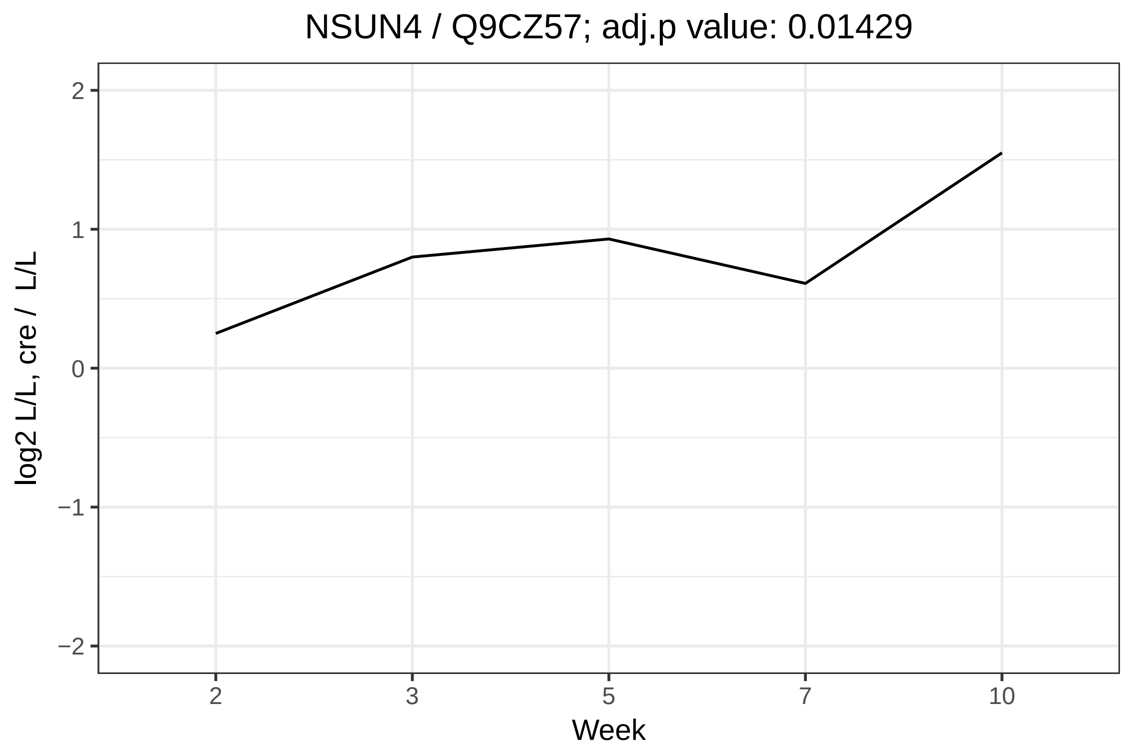
NSUN4 / Q9CZ57; adj.p value: 0.01429
log2 L/L, cre / L/L
2
1
0
−1
−2
2
3
5
7
10
Week

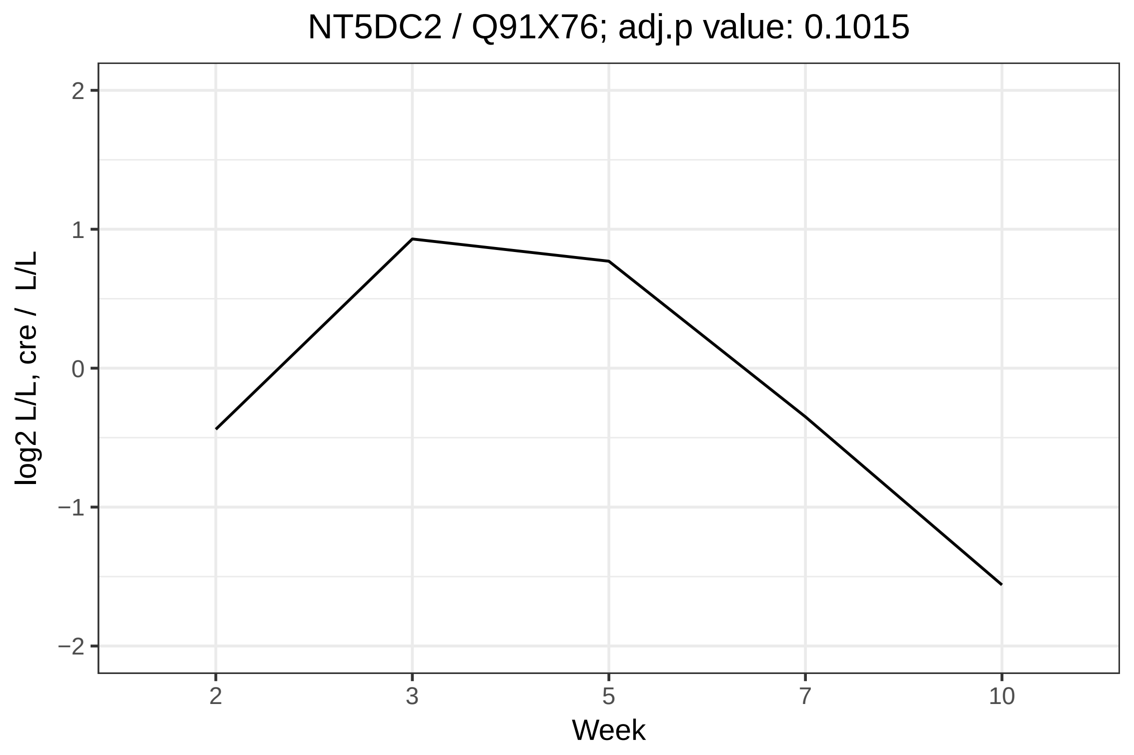
NT5DC2 / Q91X76; adj.p value: 0.1015
log2 L/L, cre / L/L
2
1
0
−1
−2
2
3
5
7
10
Week

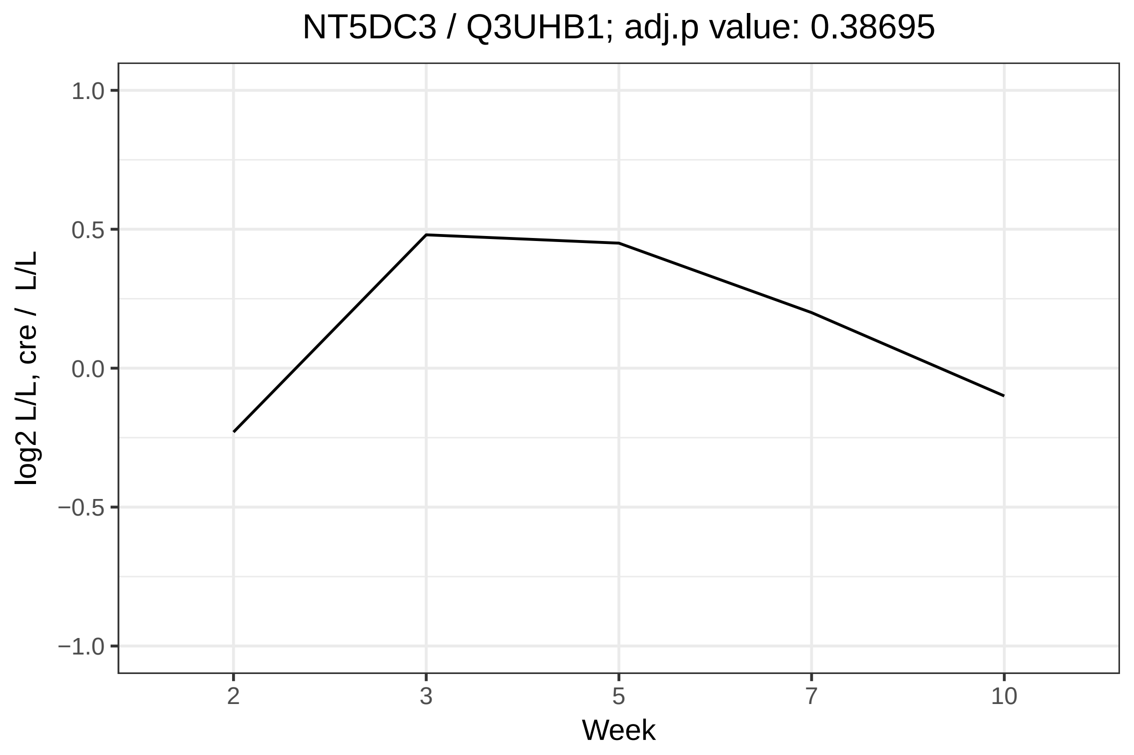
NT5DC3 / Q3UHB1; adj.p value: 0.38695
log2 L/L, cre / L/L
1.0
0.5
0.0
−0.5
−1.0
2
3
5
7
10
Week

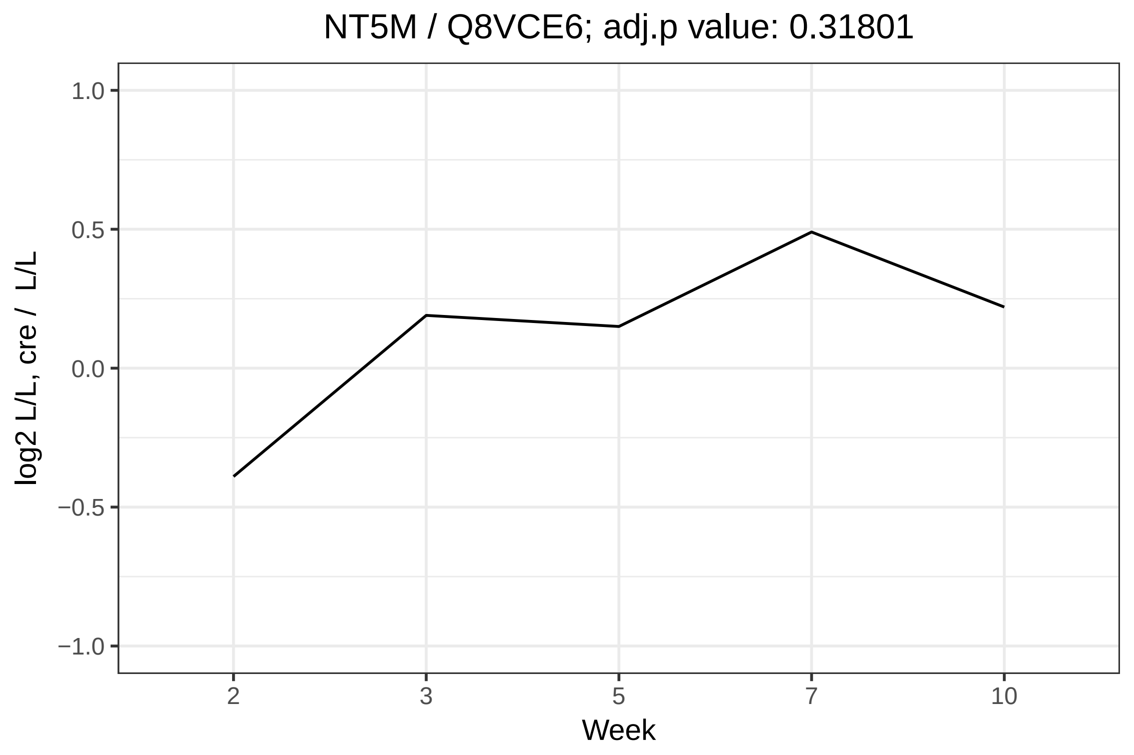

NT5M / Q8VCE6; adj.p value: 0.31801
1.0
0.5
0.0
−0.5
−1.0
log2 L/L, cre / L/L
2
3
5
7
10
Week

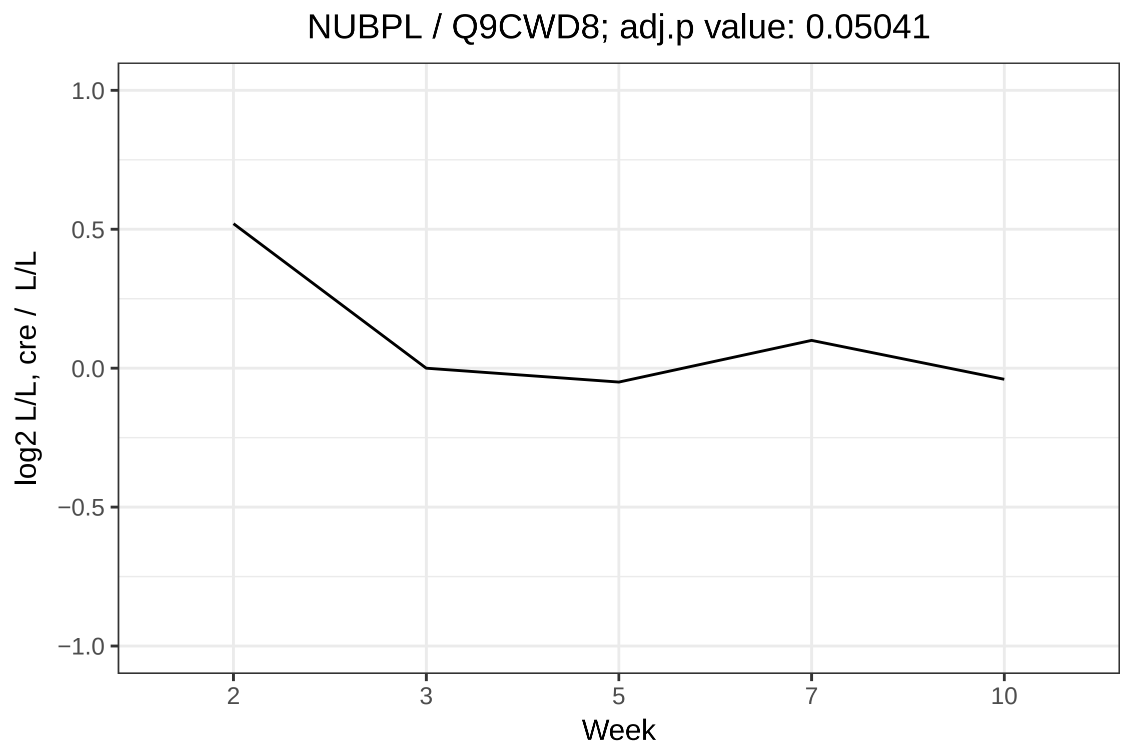
NUBPL / Q9CWD8; adj.p value: 0.05041
log2 L/L, cre / L/L
1.0
0.5
0.0
−0.5
−1.0
2
3
5
7
10
Week

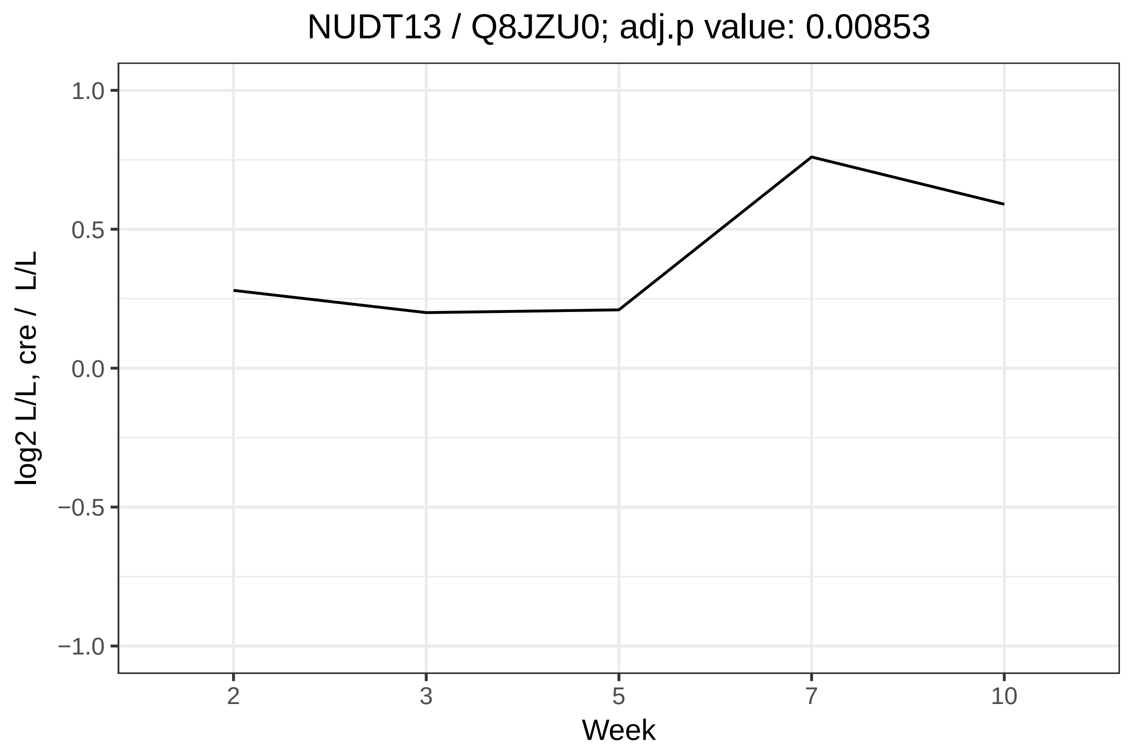
NUDT13 / Q8JZU0; adj.p value: 0.00853
1.0
0.5
0.0
−0.5
−1.0
log2 L/L, cre / L/L
2
3
5
7
10
Week

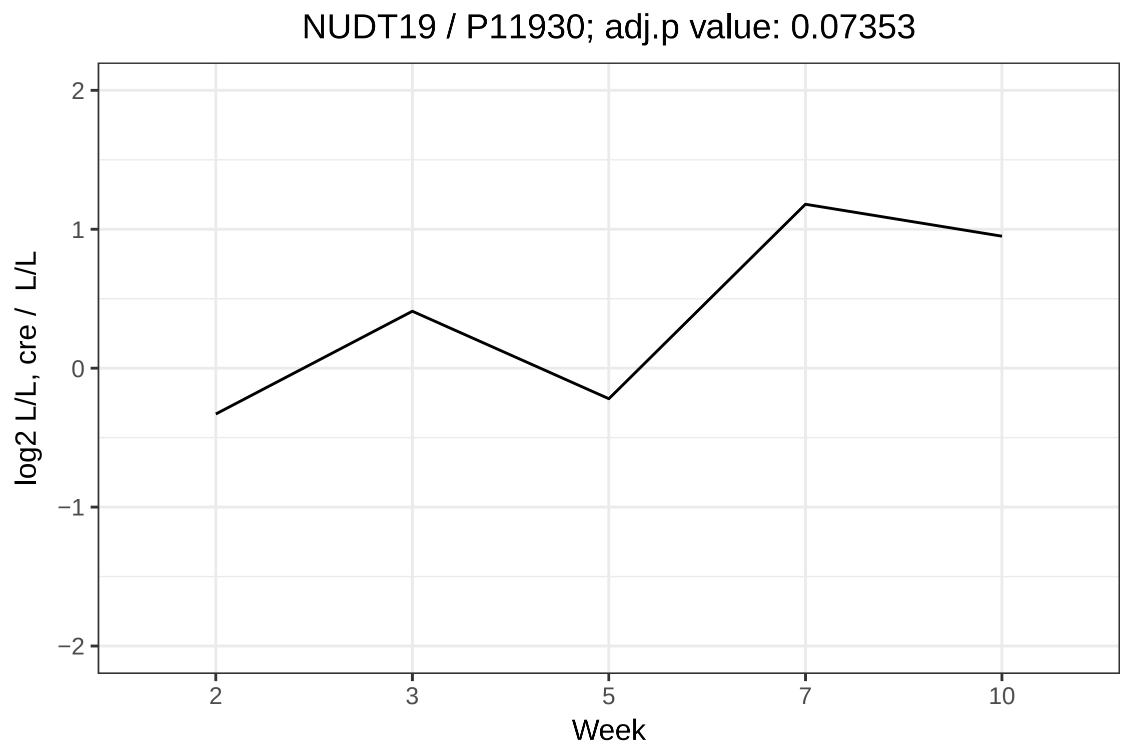
NUDT19 / P11930; adj.p value: 0.07353
log2 L/L, cre / L/L
2
1
0
−1
−2
2
3
5
7
10
Week

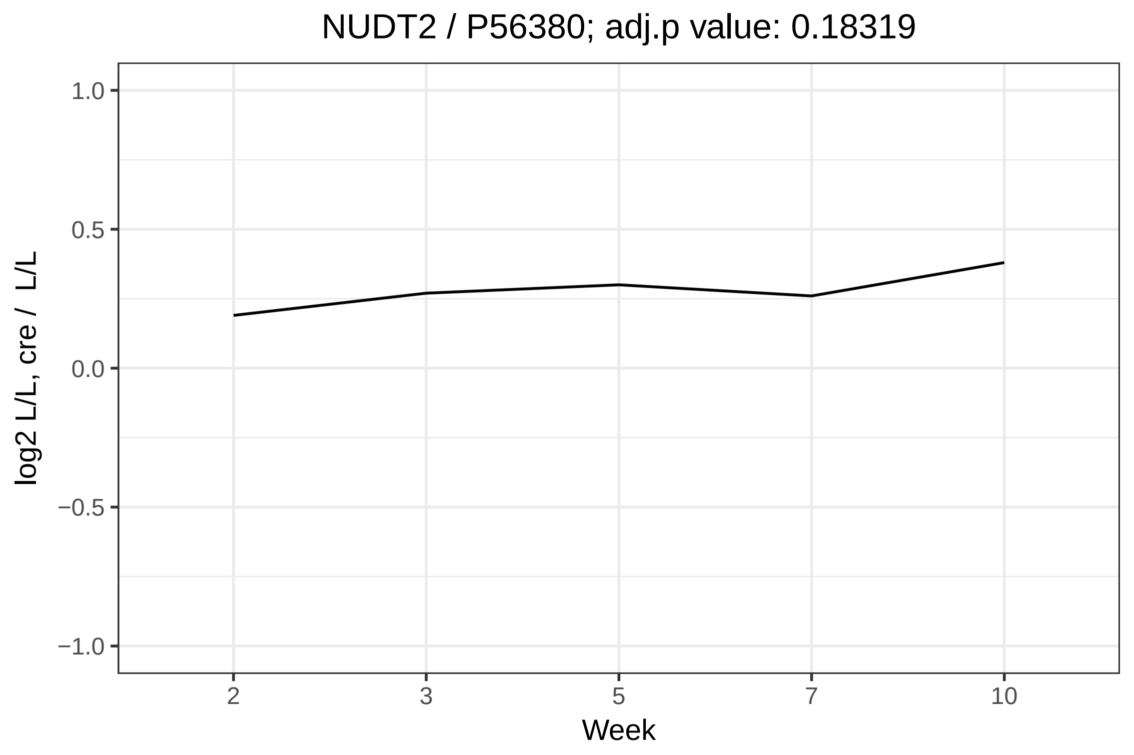

NUDT2 / P56380; adj.p value: 0.18319
1.0
0.5
0.0
−0.5
−1.0
log2 L/L, cre / L/L
2
3
5
7
10
Week

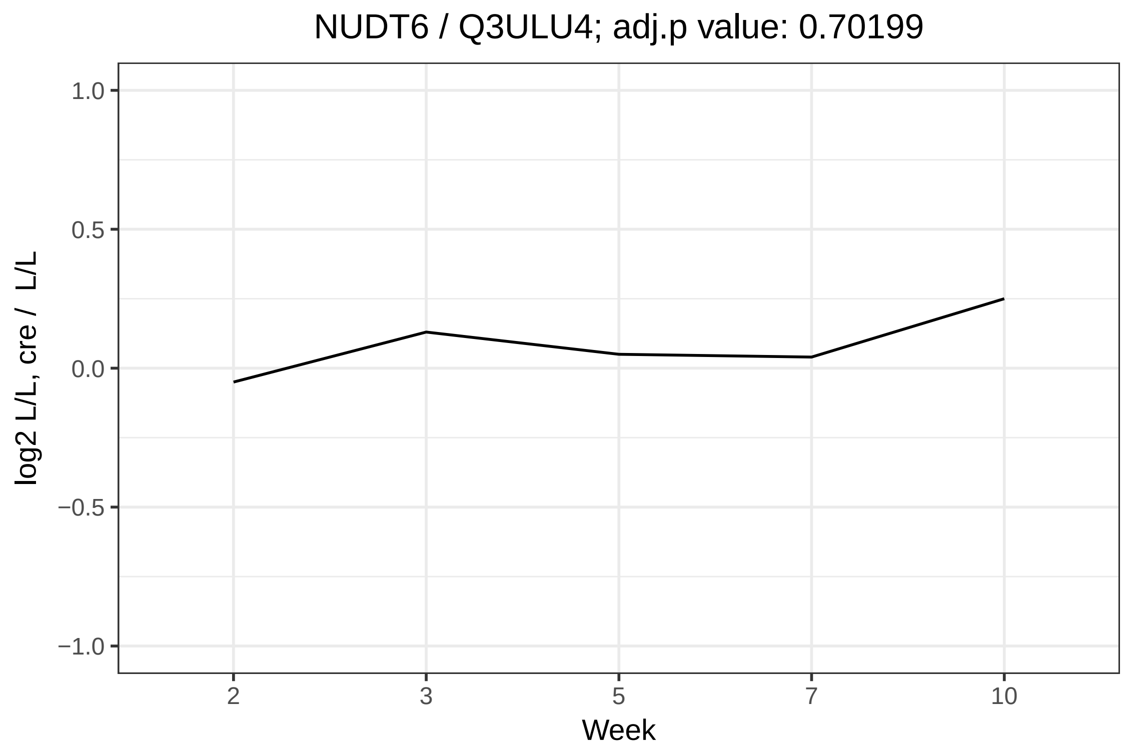
NUDT6 / Q3ULU4; adj.p value: 0.70199
log2 L/L, cre / L/L
1.0
0.5
0.0
−0.5
−1.0
2
3
5
7
10
Week

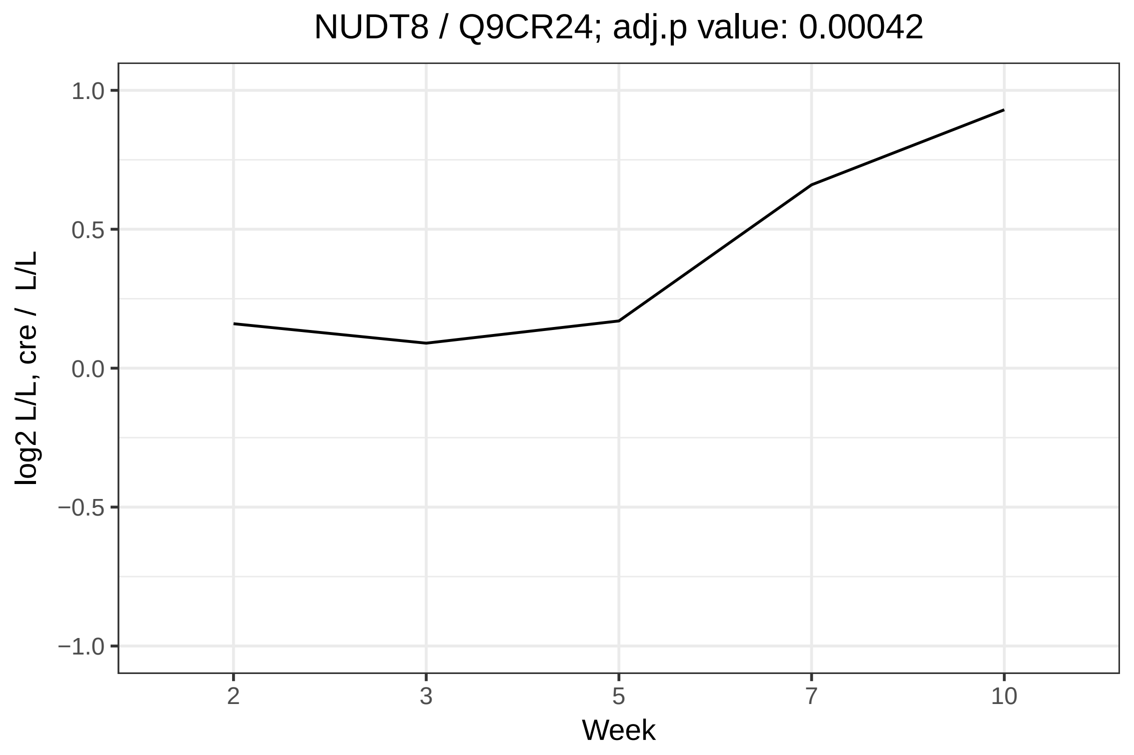

NUDT8 / Q9CR24; adj.p value: 0.00042
1.0
0.5
0.0
−0.5
−1.0
log2 L/L, cre / L/L
2
3
5
7
10
Week

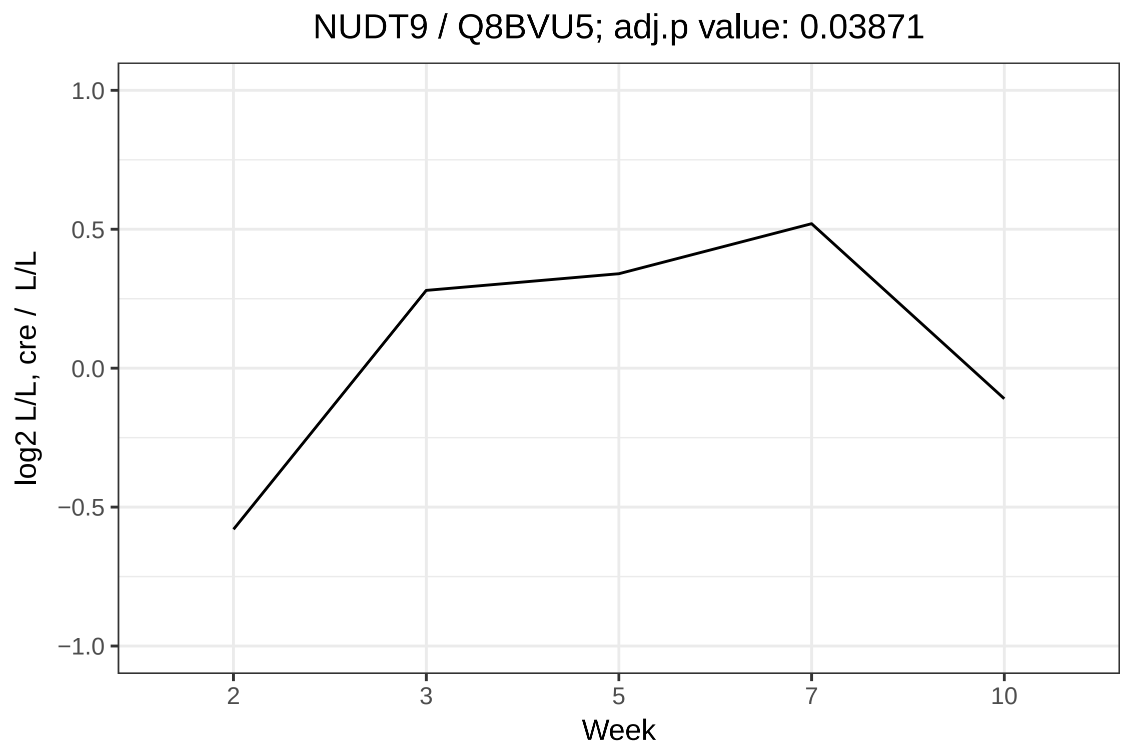
NUDT9 / Q8BVU5; adj.p value: 0.03871
log2 L/L, cre / L/L
1.0
0.5
0.0
−0.5
−1.0
2
3
5
7
10
Week

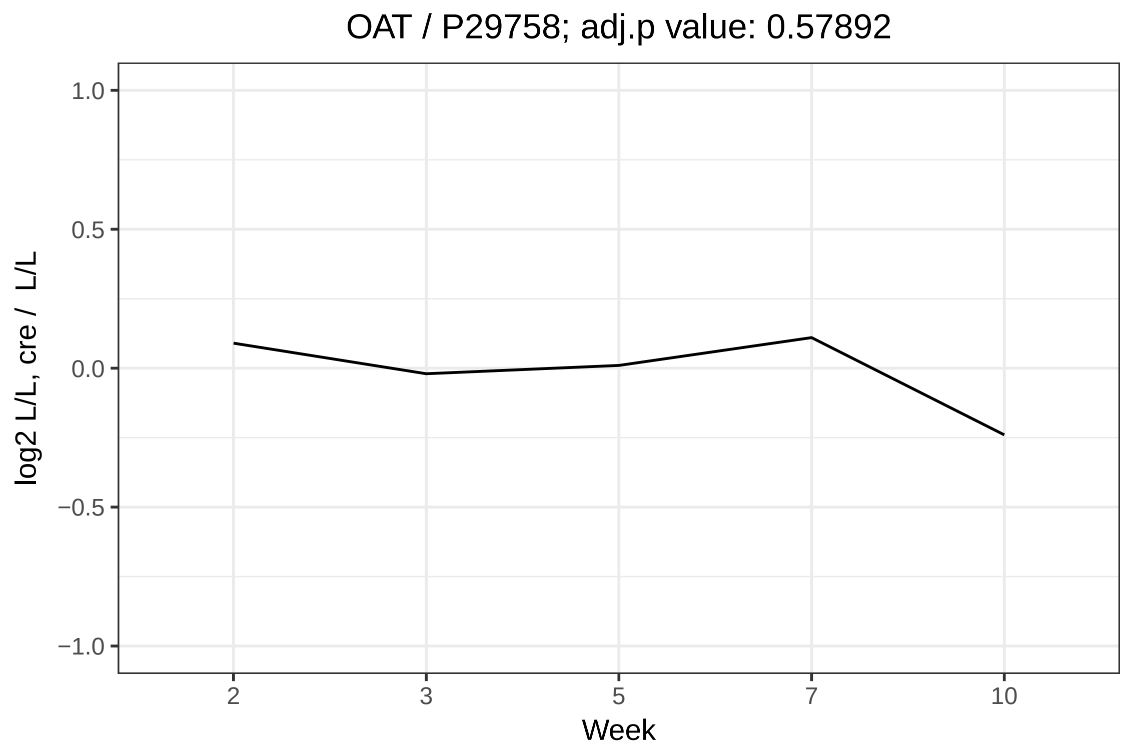

OAT / P29758; adj.p value: 0.57892
log2 L/L, cre / L/L
1.0
0.5
0.0
−0.5
−1.0
2
3
5
7
10
Week

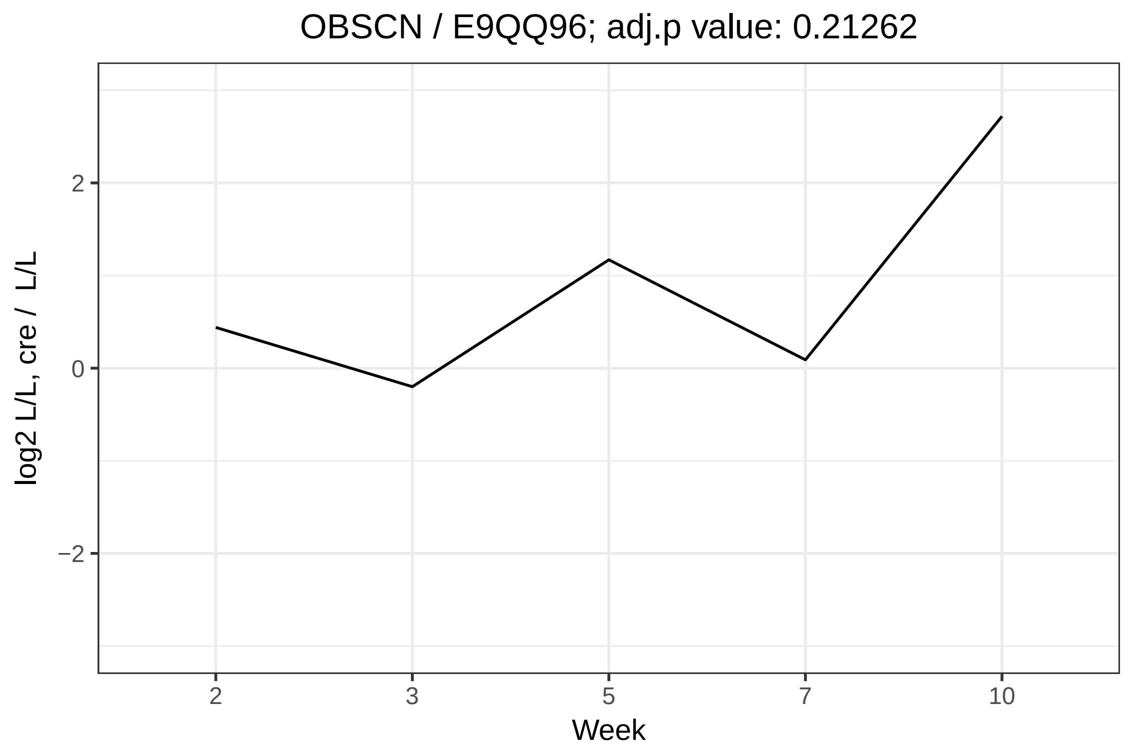

OBSCN / E9QQ96; adj.p value: 0.21262
log2 L/L, cre / L/L
2
0
−2
2
3
5
7
10
Week

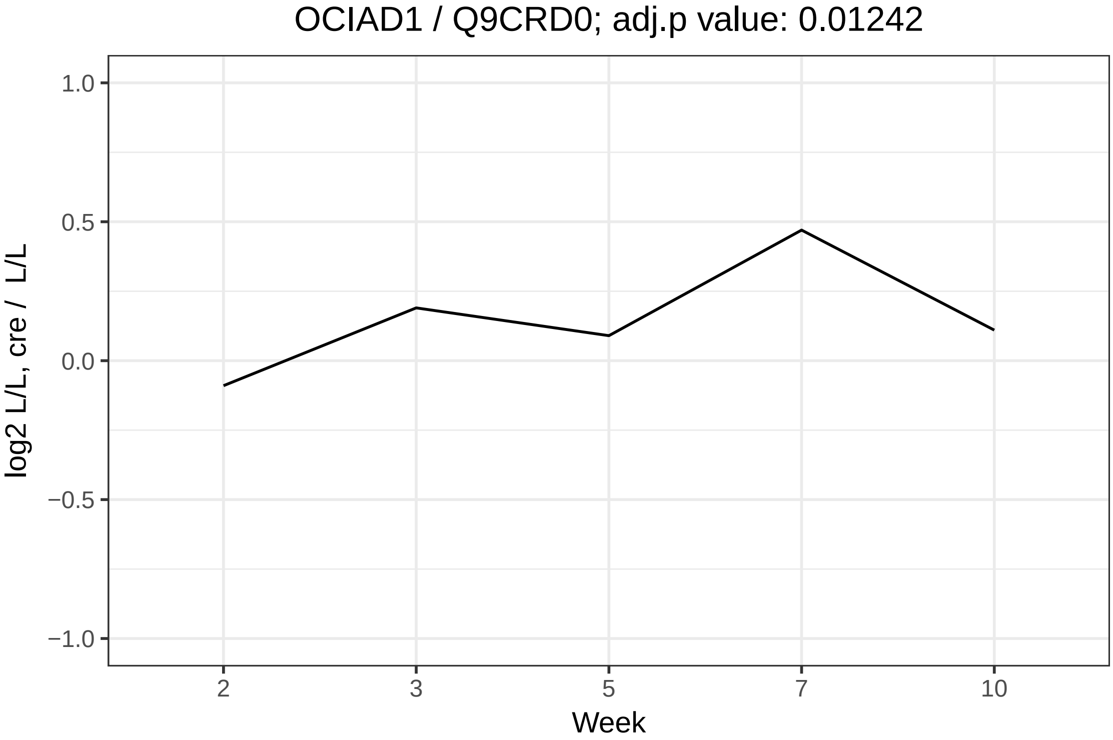
OCIAD1 / Q9CRD0; adj.p value: 0.01242
log2 L/L, cre / L/L
1.0
0.5
0.0
−0.5
−1.0
2
3
5
7
10
Week

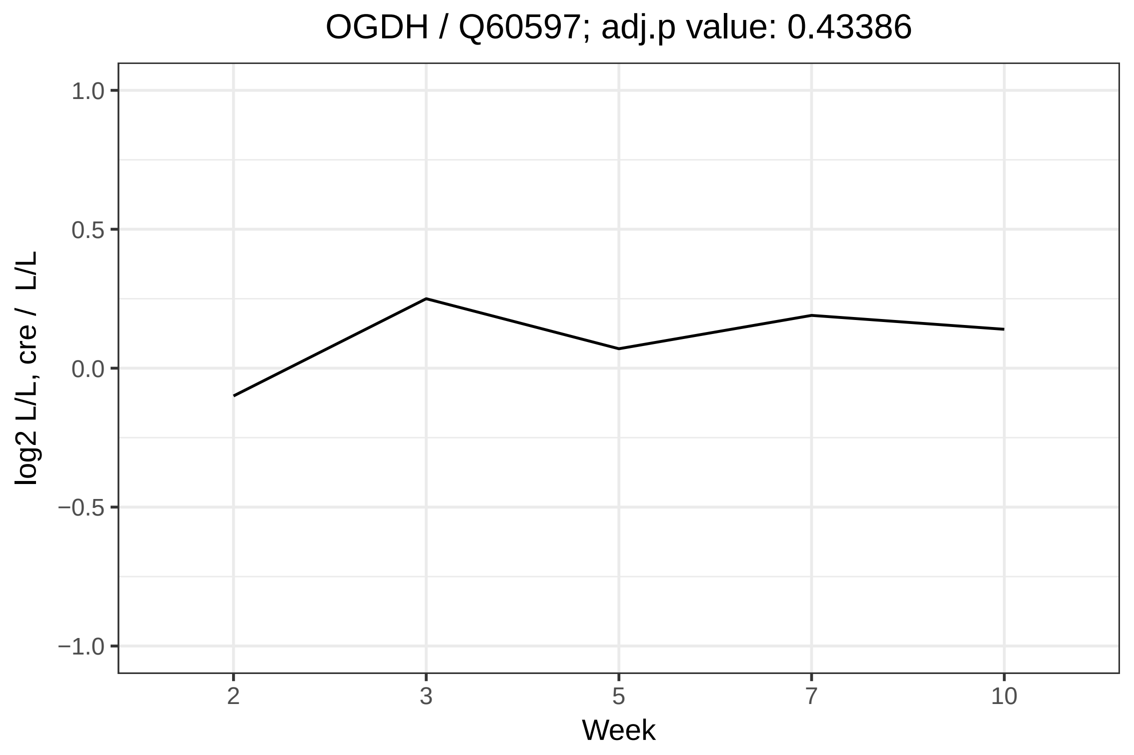
OGDH / Q60597; adj.p value: 0.43386
log2 L/L, cre / L/L
1.0
0.5
0.0
−0.5
−1.0
2
3
5
7
10
Week

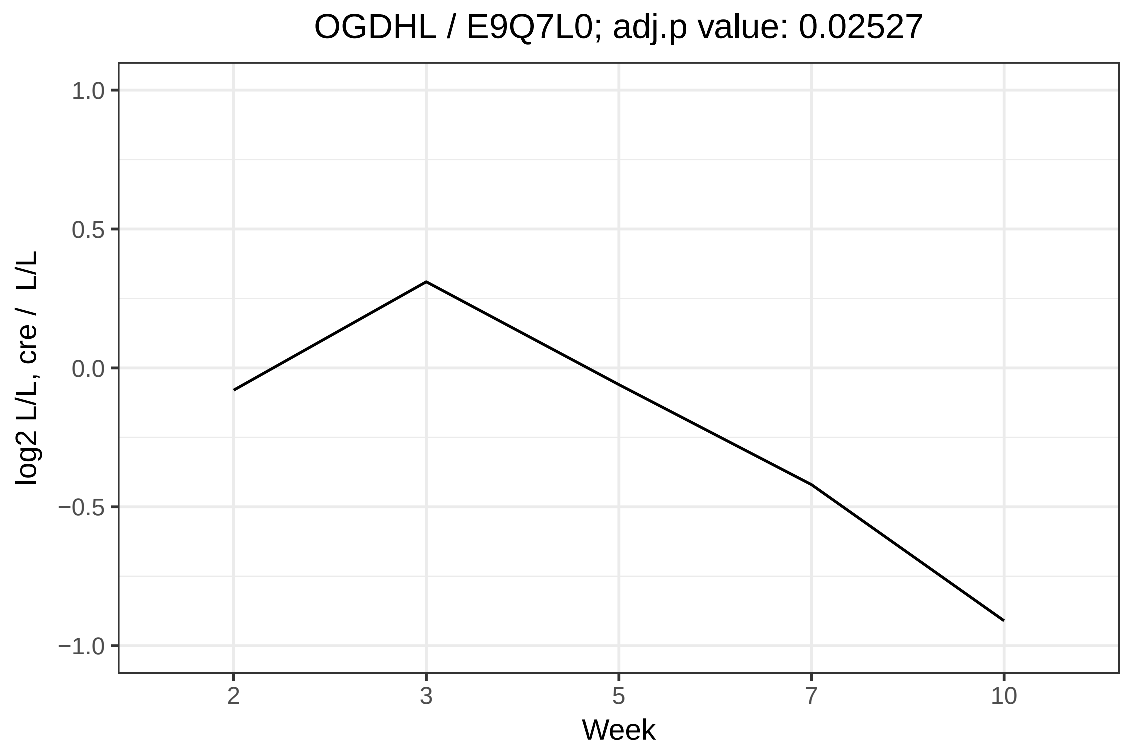
OGDHL / E9Q7L0; adj.p value: 0.02527
log2 L/L, cre / L/L
1.0
0.5
0.0
−0.5
−1.0
2
3
5
7
10
Week

OMA1 / Q9D8H7; adj.p value: 0.63457

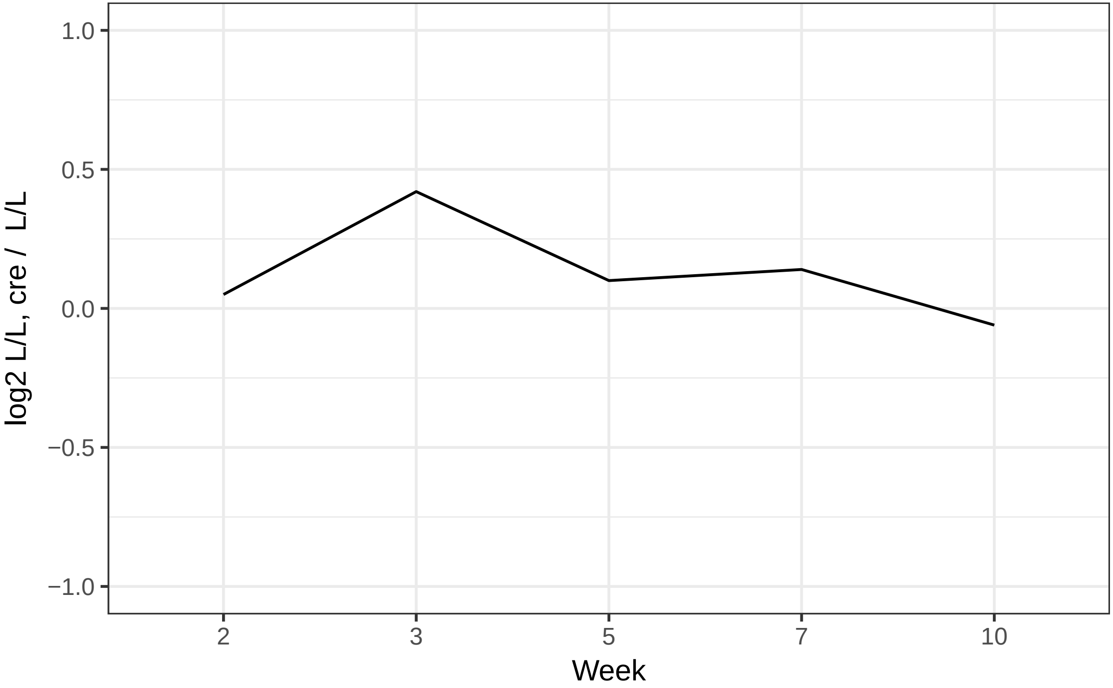

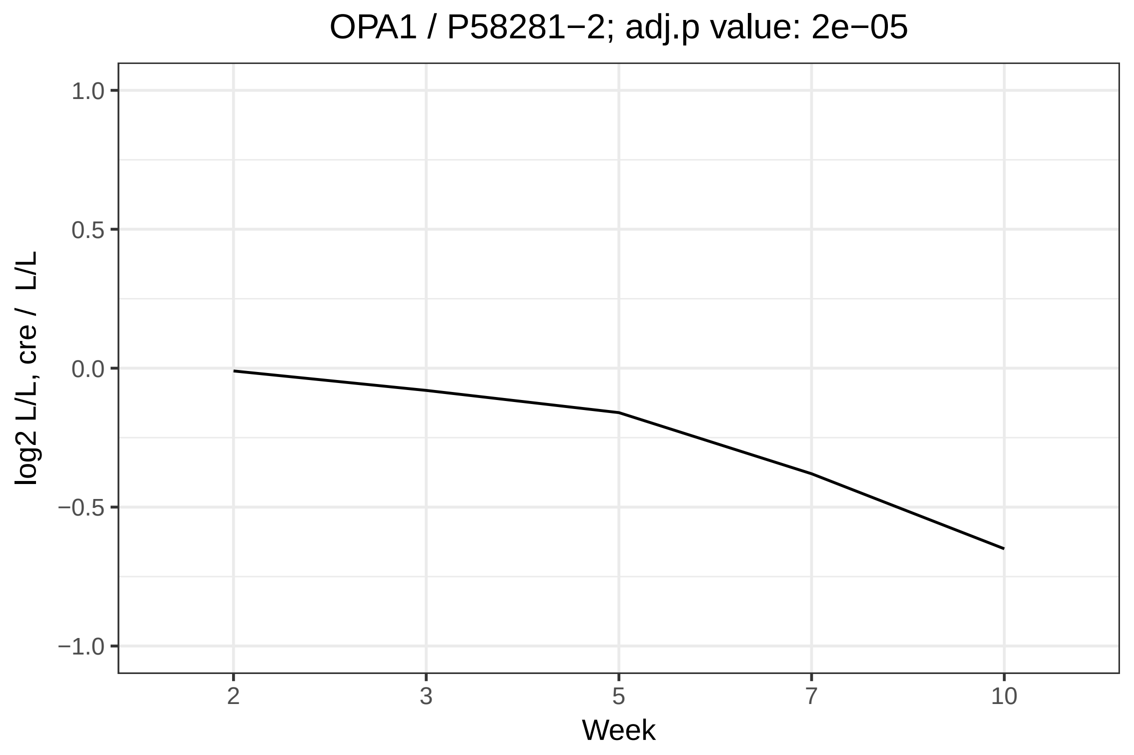

OPA1 / P58281−2; adj.p value: 2e−05
log2 L/L, cre / L/L
1.0
0.5
0.0
−0.5
−1.0
2
3
5
7
10
Week

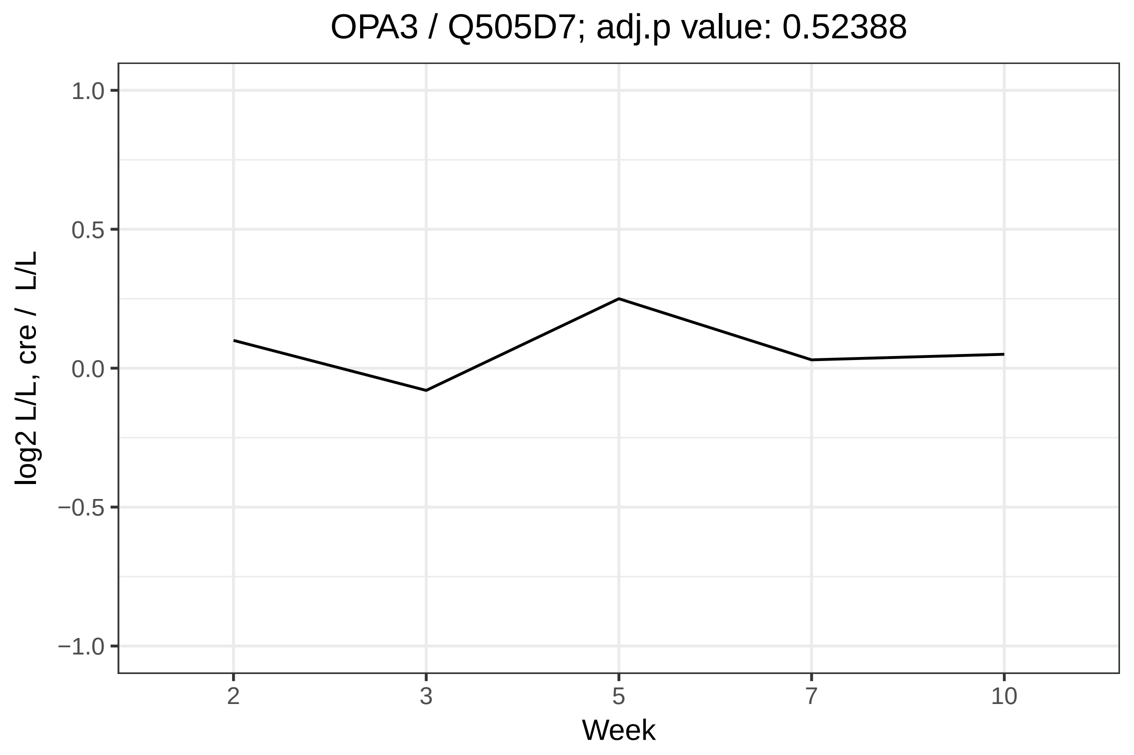
OPA3 / Q505D7; adj.p value: 0.52388
log2 L/L, cre / L/L
1.0
0.5
0.0
−0.5
−1.0
2
3
5
7
10
Week

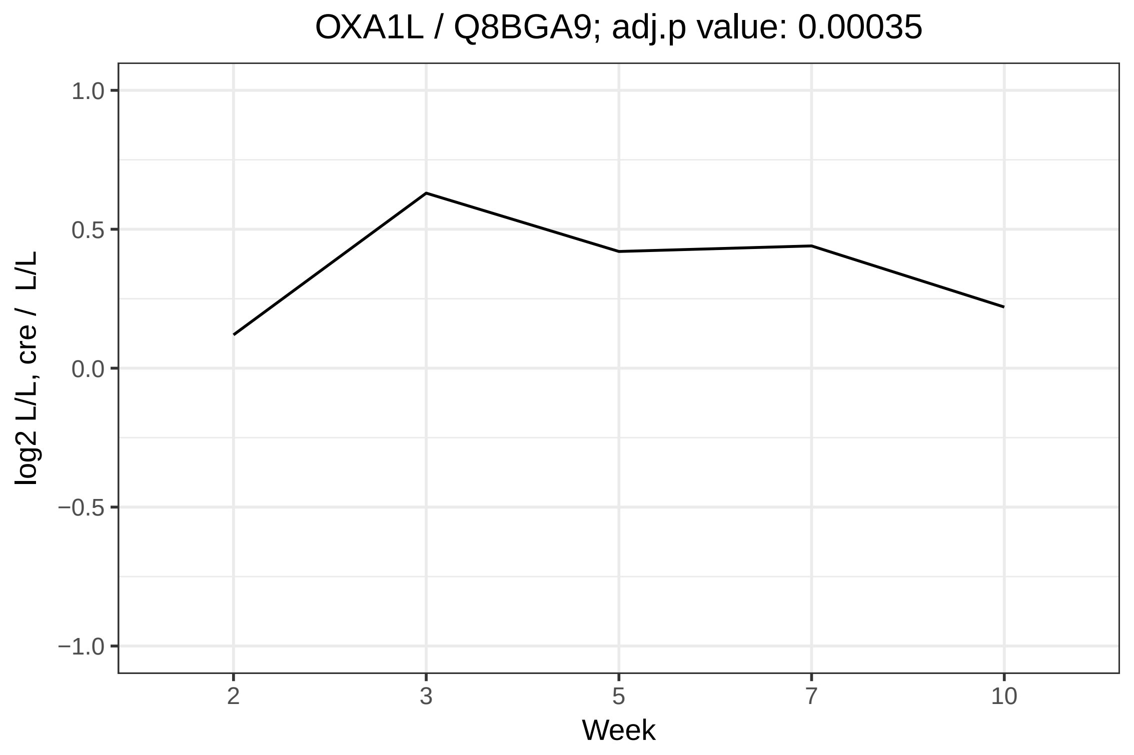
OXA1L / Q8BGA9; adj.p value: 0.00035
1.0
0.5
0.0
−0.5
−1.0
log2 L/L, cre / L/L
2
3
5
7
10
Week

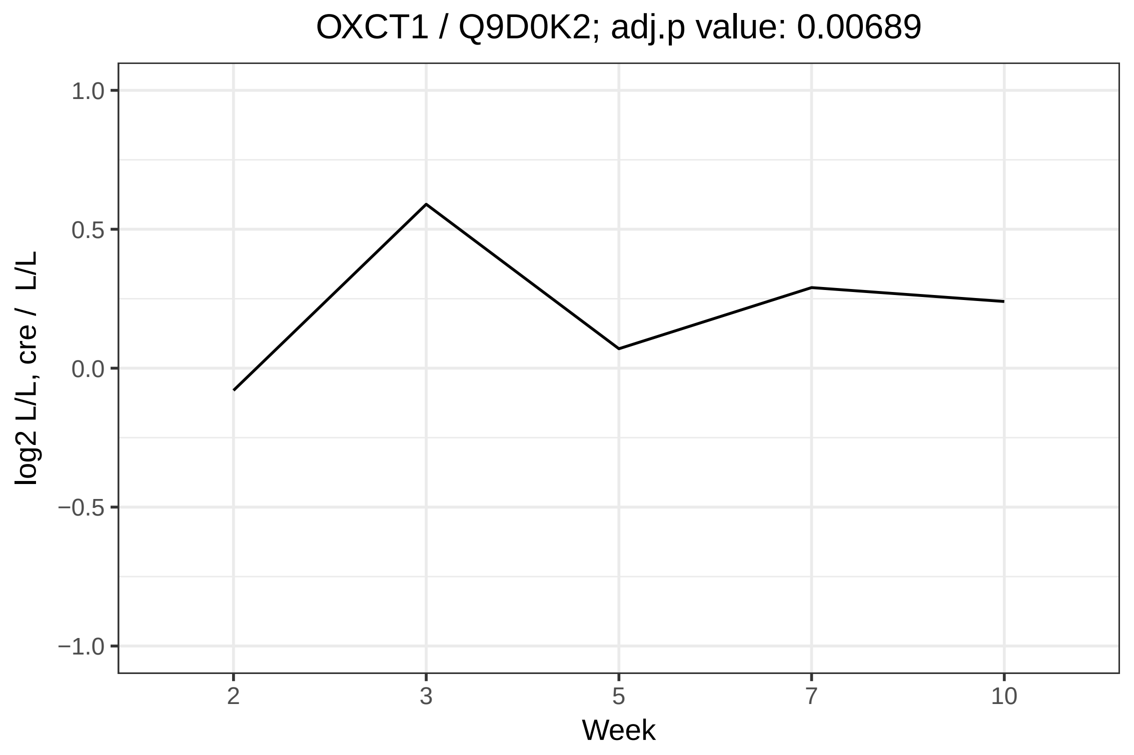
OXCT1 / Q9D0K2; adj.p value: 0.00689
log2 L/L, cre / L/L
1.0
0.5
0.0
−0.5
−1.0
2
3
5
7
10
Week

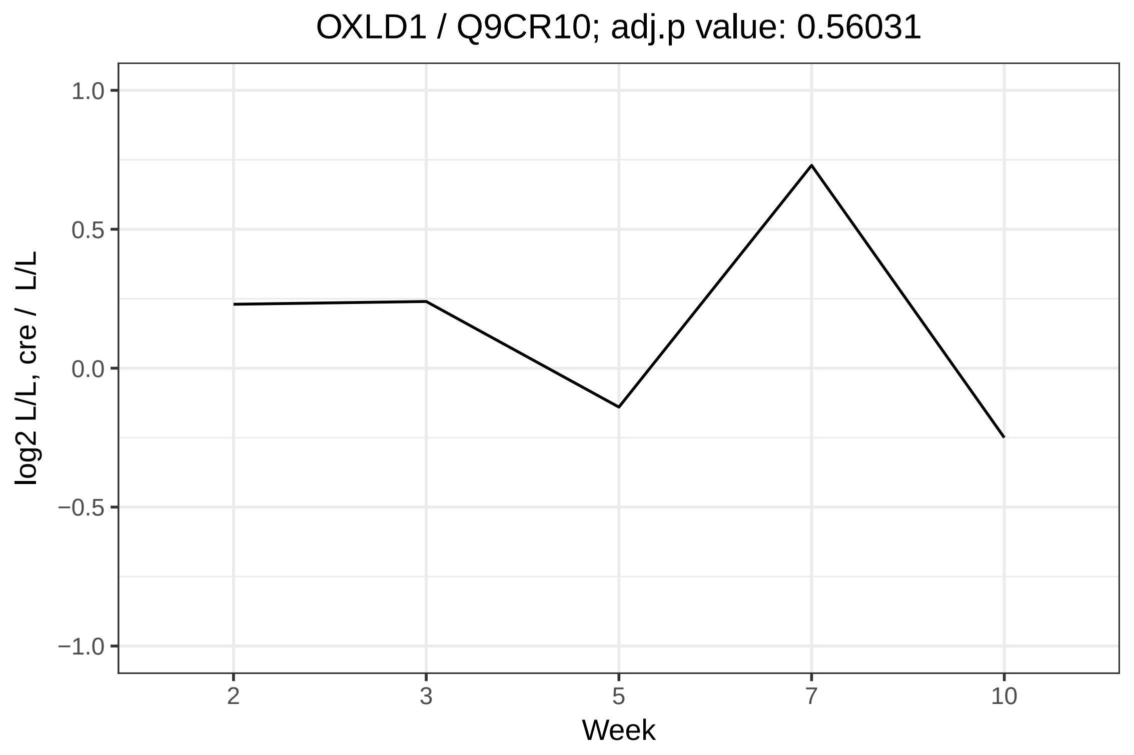

OXLD1 / Q9CR10; adj.p value: 0.56031
1.0
0.5
0.0
−0.5
−1.0
log2 L/L, cre / L/L
2
3
5
7
10
Week

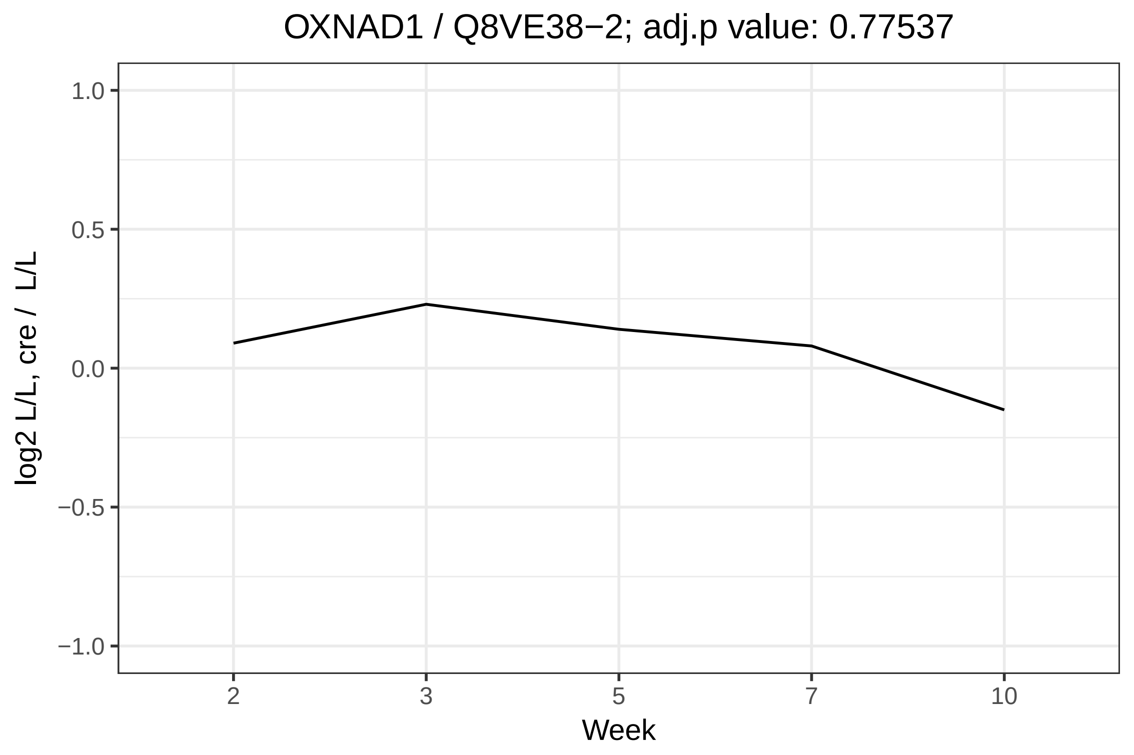
OXNAD1 / Q8VE38−2; adj.p value: 0.77537
log2 L/L, cre / L/L
1.0
0.5
0.0
−0.5
−1.0
2
3
5
7
10
Week

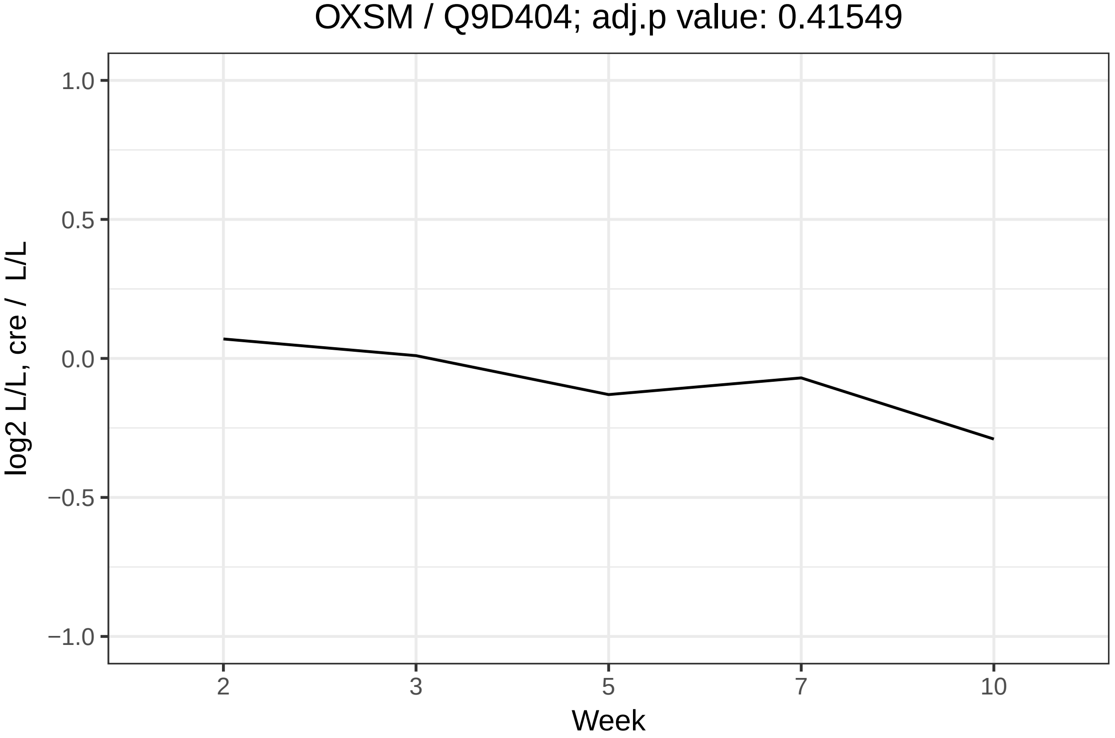

OXSM / Q9D404; adj.p value: 0.41549
1.0
0.5
0.0
−0.5
−1.0
log2 L/L, cre / L/L
2
3
5
7
10
Week

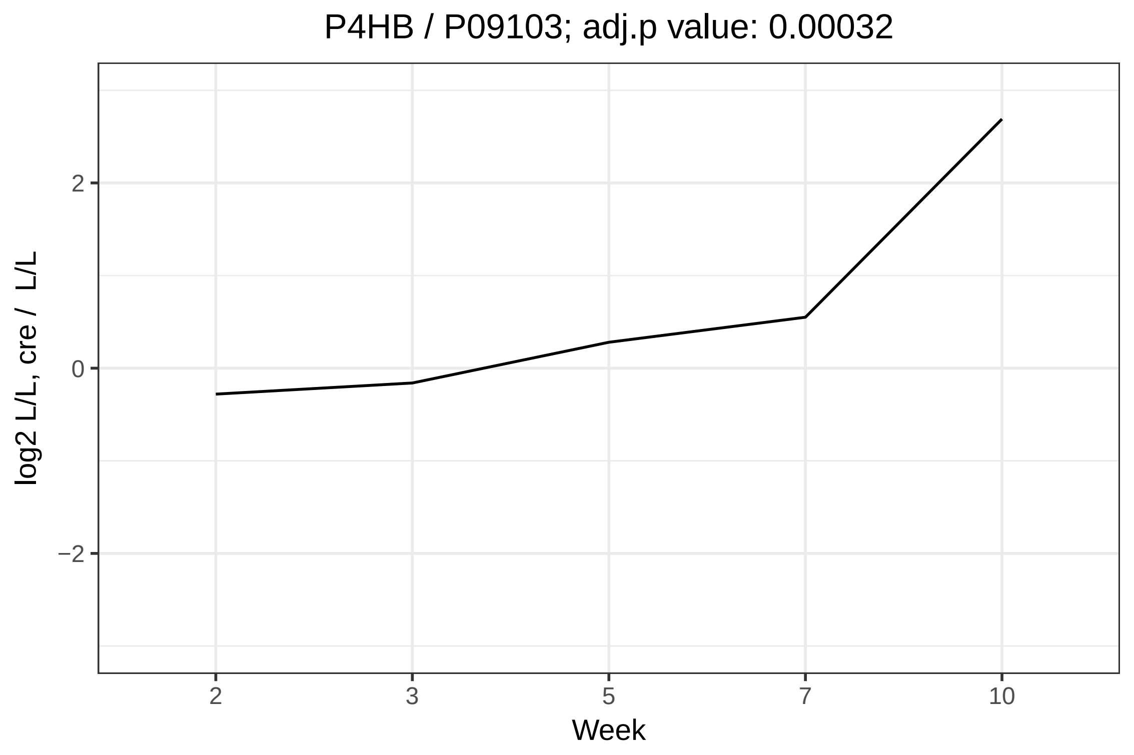
P4HB / P09103; adj.p value: 0.00032
log2 L/L, cre / L/L
2
0
−2
2
3
5
7
10
Week

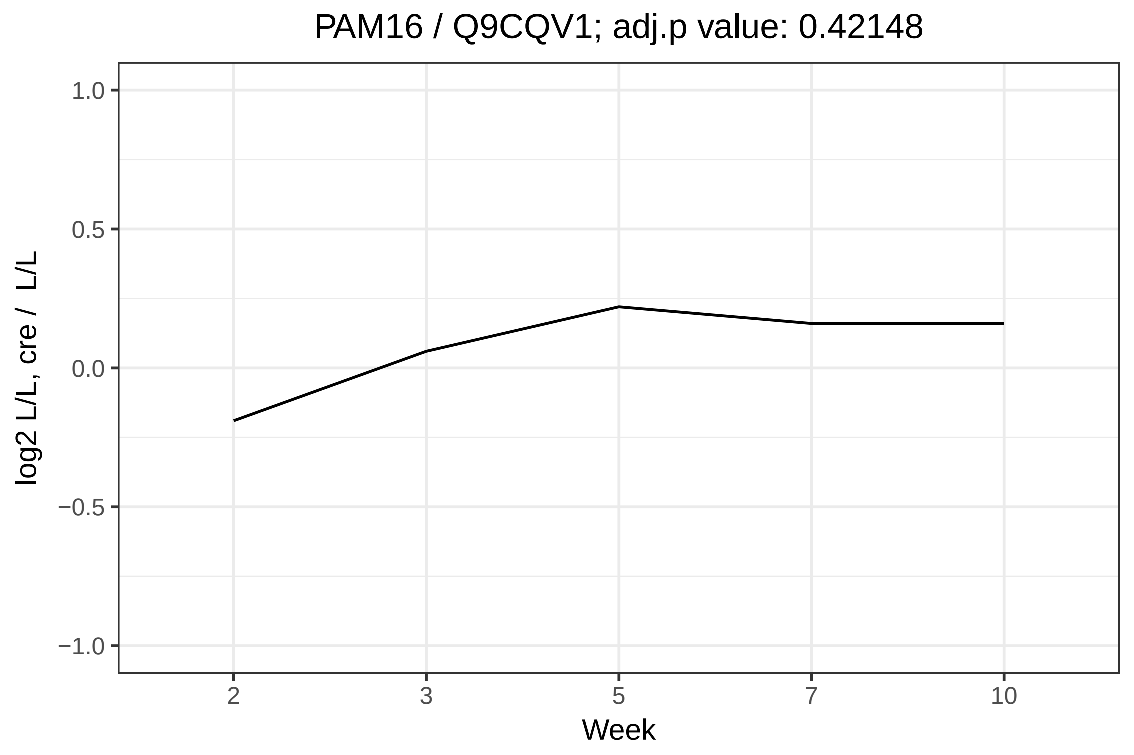
PAM16 / Q9CQV1; adj.p value: 0.42148
1.0
0.5
0.0
−0.5
−1.0
log2 L/L, cre / L/L
2
3
5
7
10
Week

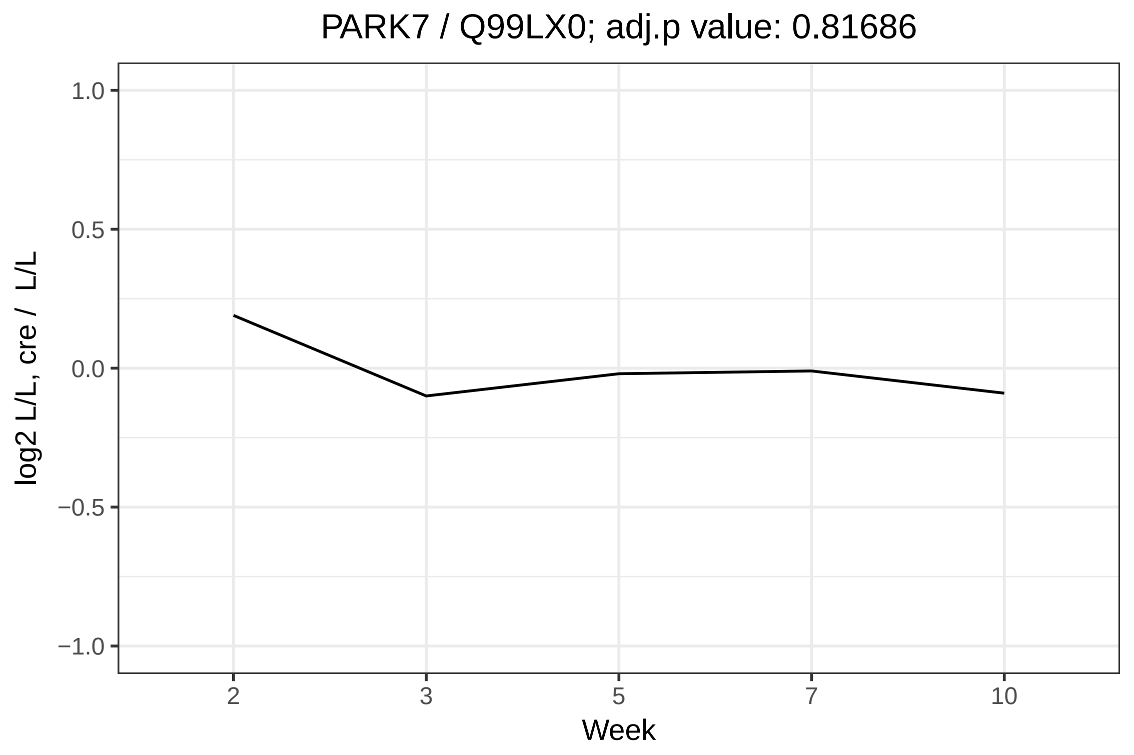
PARK7 / Q99LX0; adj.p value: 0.81686
log2 L/L, cre / L/L
1.0
0.5
0.0
−0.5
−1.0
2
3
5
7
10
Week

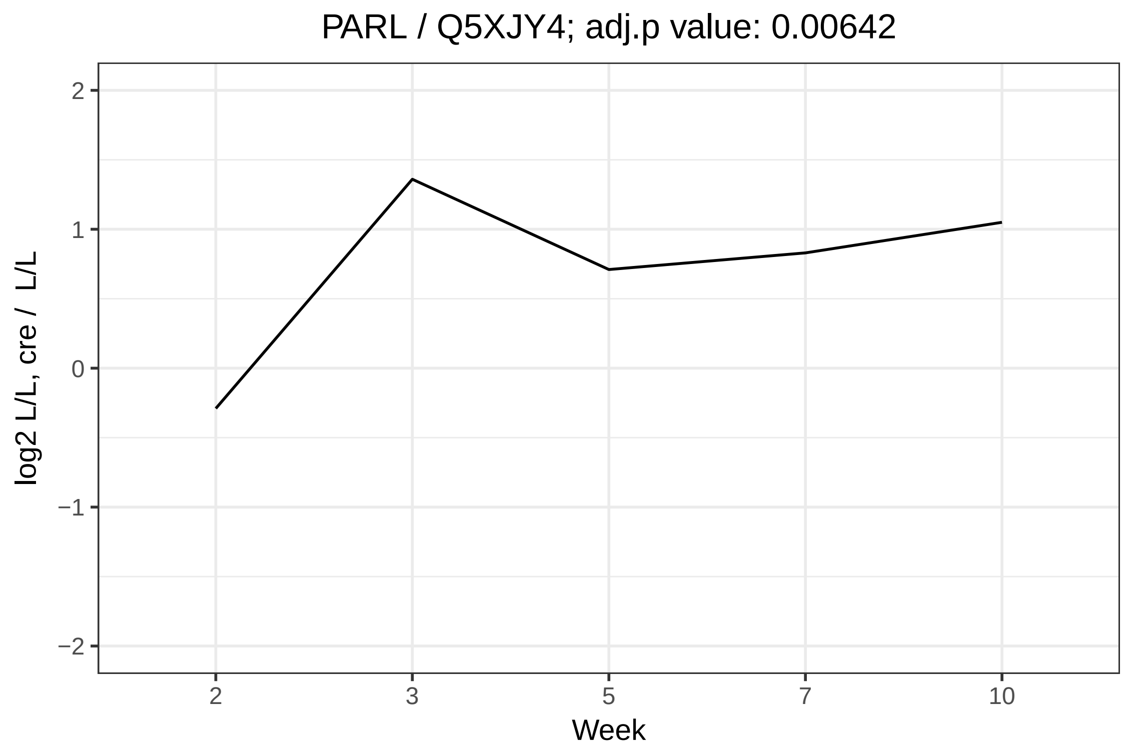
PARL / Q5XJY4; adj.p value: 0.00642
2
1
0
−1
−2
log2 L/L, cre / L/L
2
3
5
7
10
Week

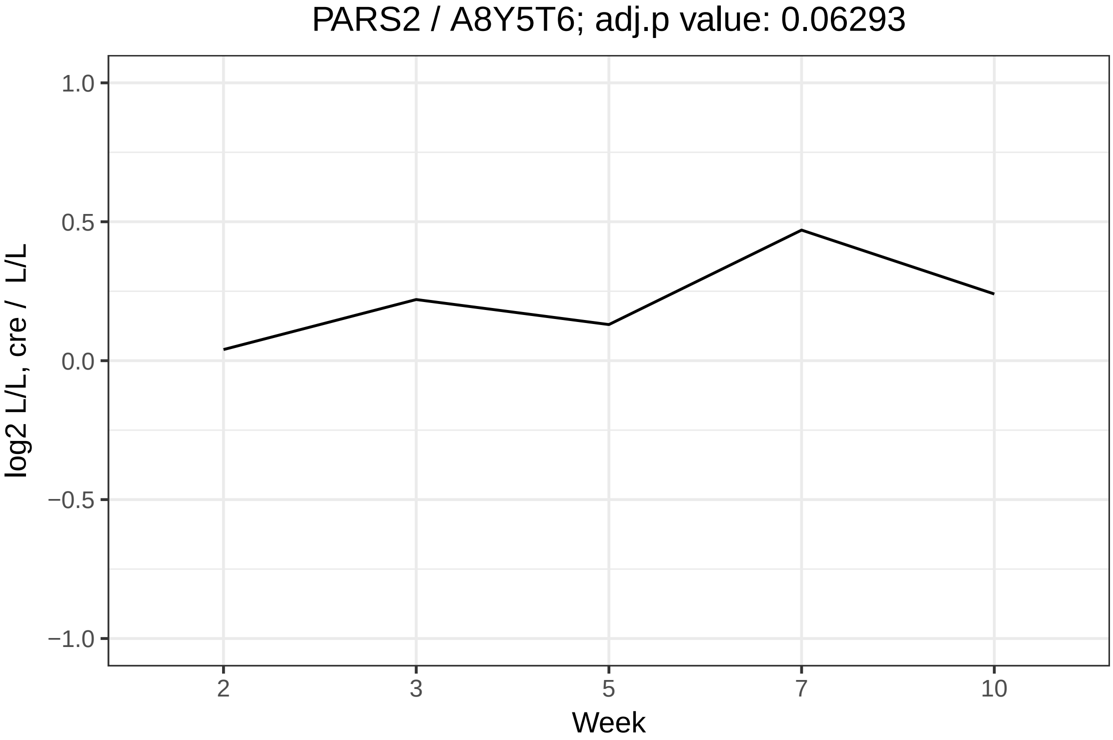
PARS2 / A8Y5T6; adj.p value: 0.06293
1.0
0.5
0.0
−0.5
−1.0
log2 L/L, cre / L/L
2
3
5
7
10
Week

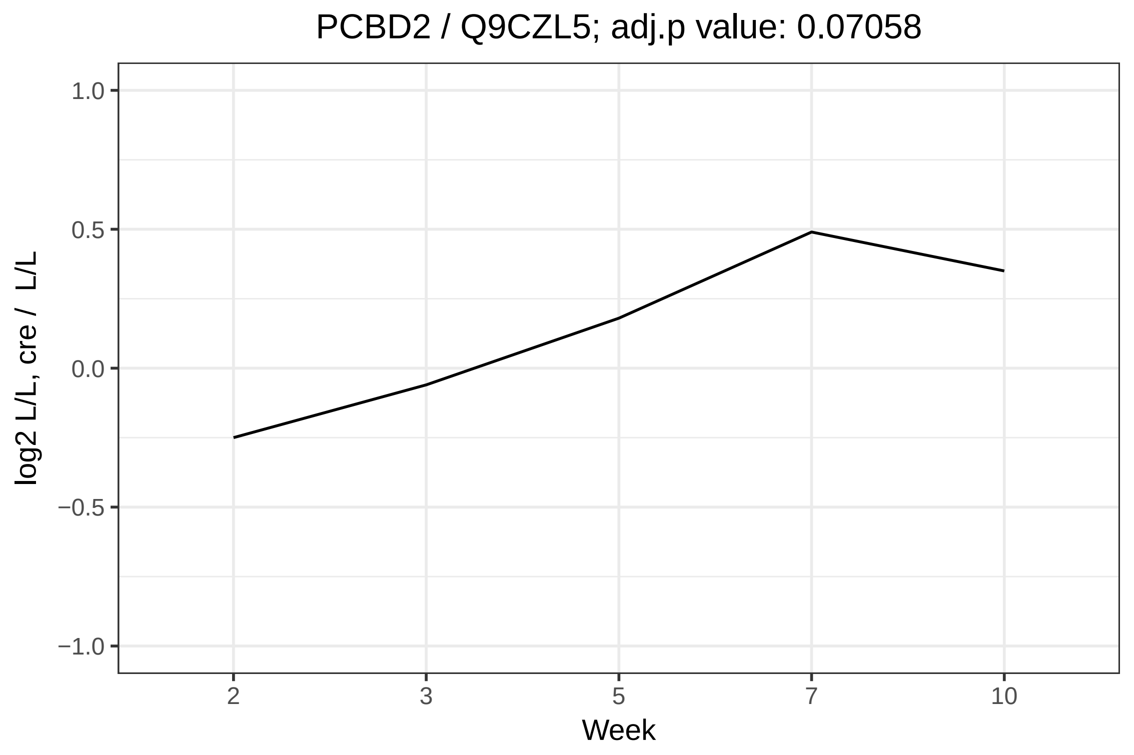
PCBD2 / Q9CZL5; adj.p value: 0.07058
log2 L/L, cre / L/L
1.0
0.5
0.0
−0.5
−1.0
2
3
5
7
10
Week

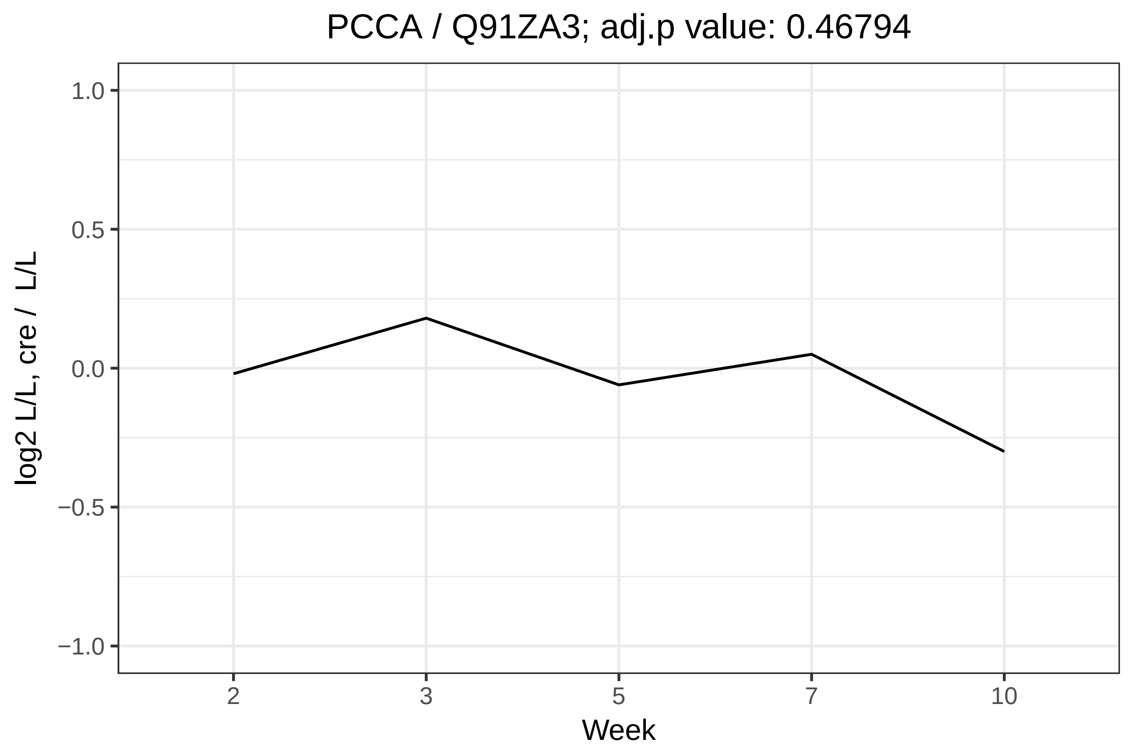
PCCA / Q91ZA3; adj.p value: 0.46794
log2 L/L, cre / L/L
1.0
0.5
0.0
−0.5
−1.0
2
3
5
7
10
Week

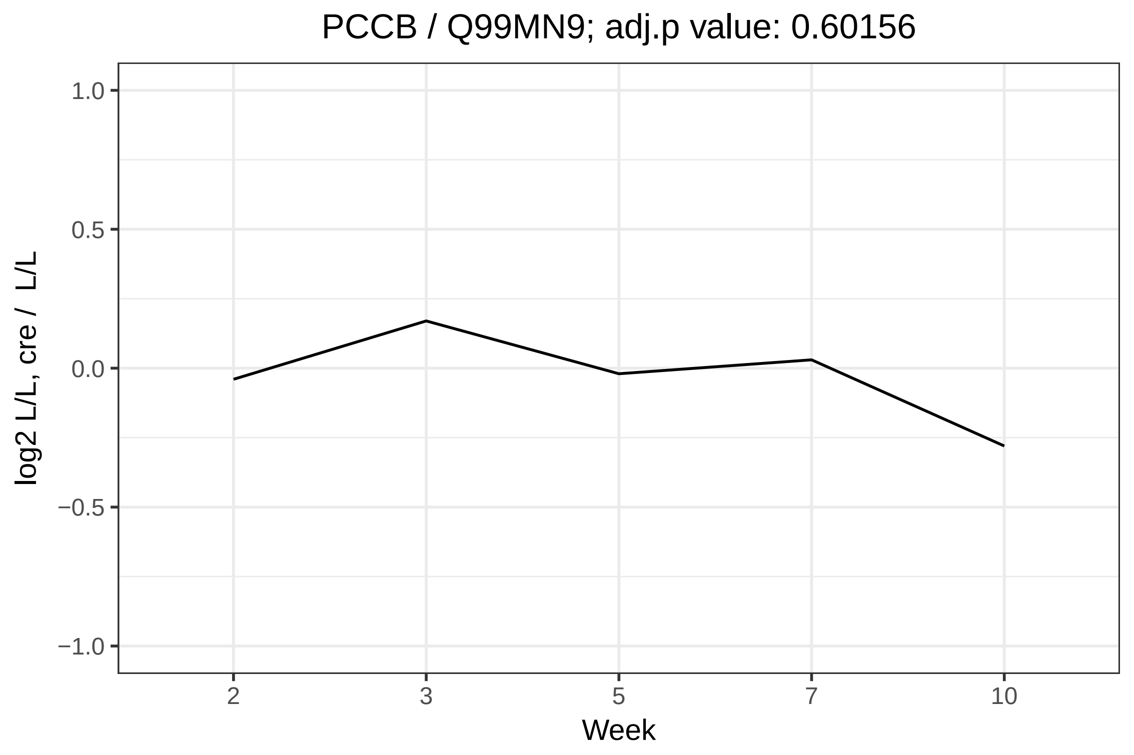

PCCB / Q99MN9; adj.p value: 0.60156
1.0
0.5
0.0
−0.5
−1.0
log2 L/L, cre / L/L
2
3
5
7
10
Week

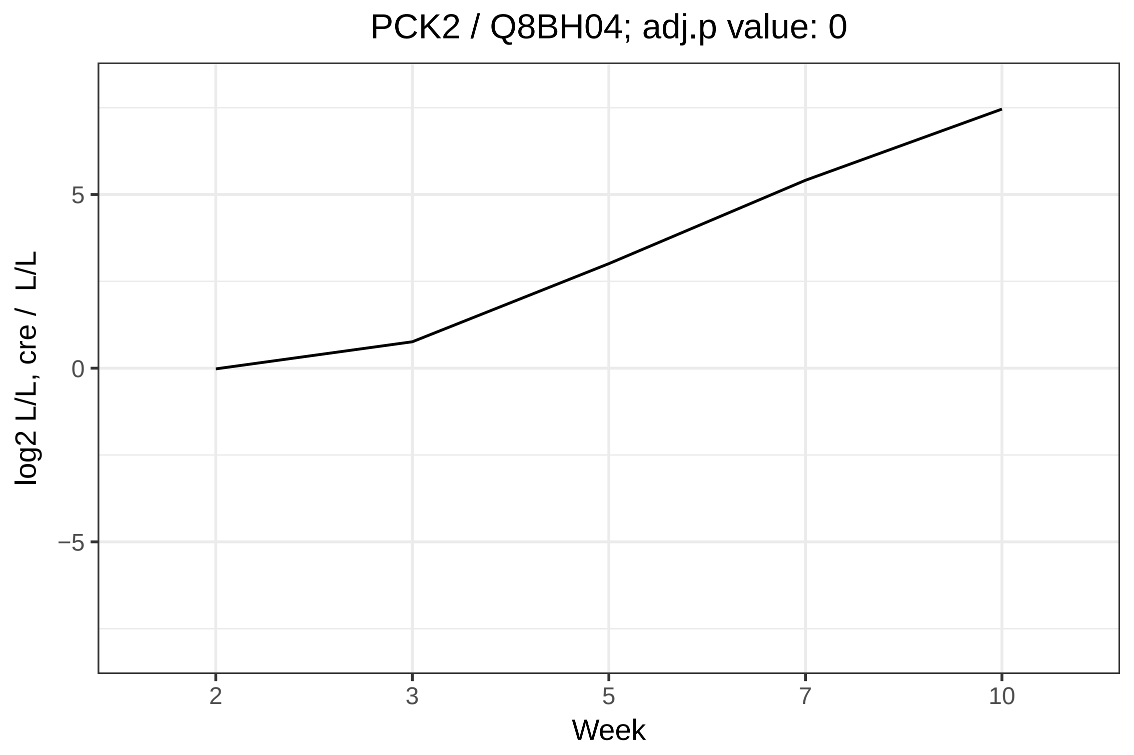

PCK2 / Q8BH04; adj.p value: 0
log2 L/L, cre / L/L
5
0
−5
2
3
5
7
10
Week

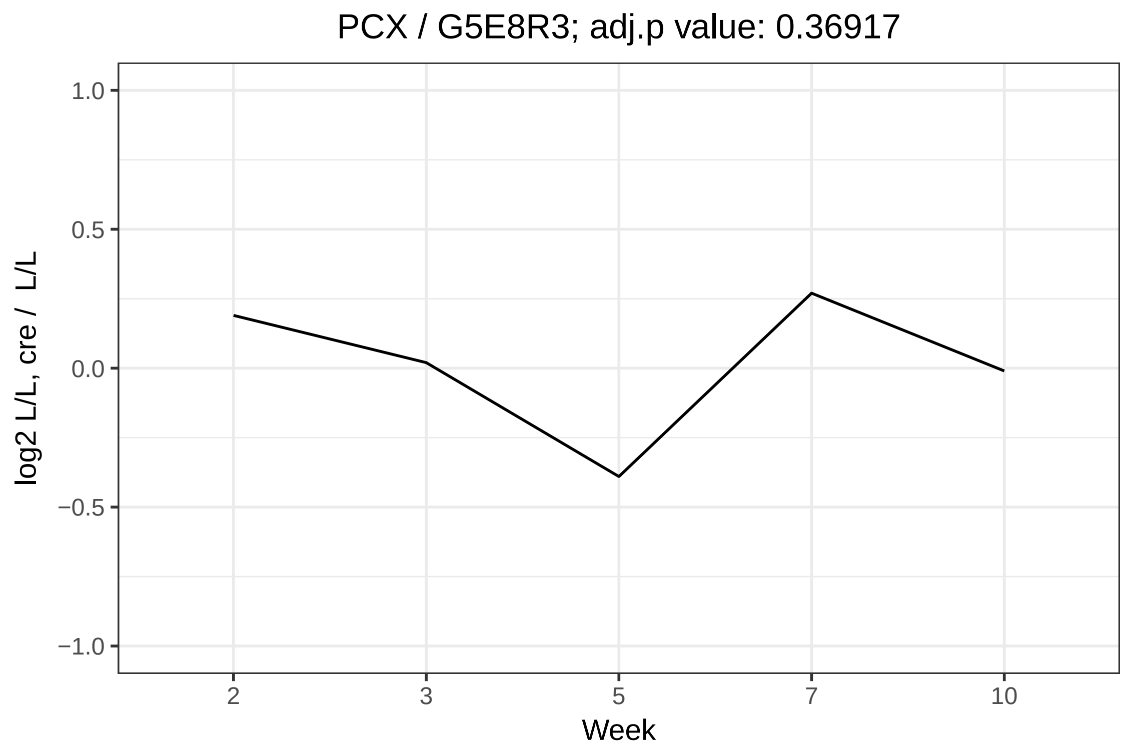

PCX / G5E8R3; adj.p value: 0.36917
1.0
0.5
0.0
−0.5
−1.0
log2 L/L, cre / L/L
2
3
5
7
10
Week

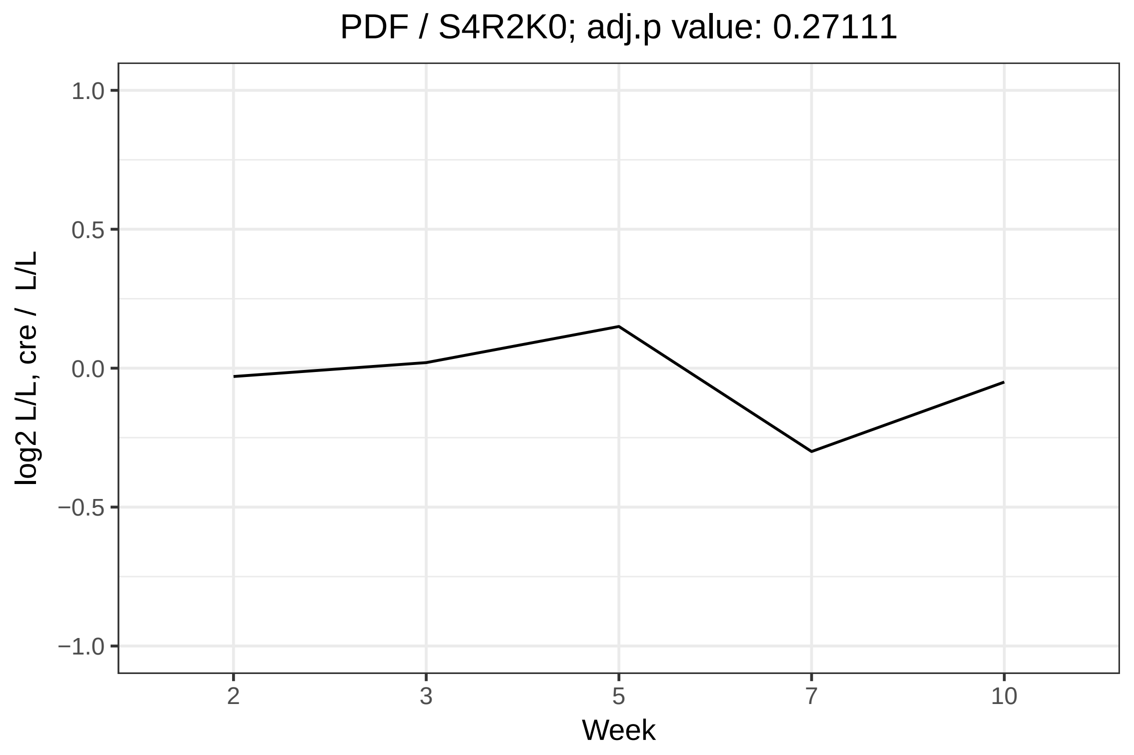
PDF / S4R2K0; adj.p value: 0.27111
log2 L/L, cre / L/L
1.0
0.5
0.0
−0.5
−1.0
2
3
5
7
10
Week

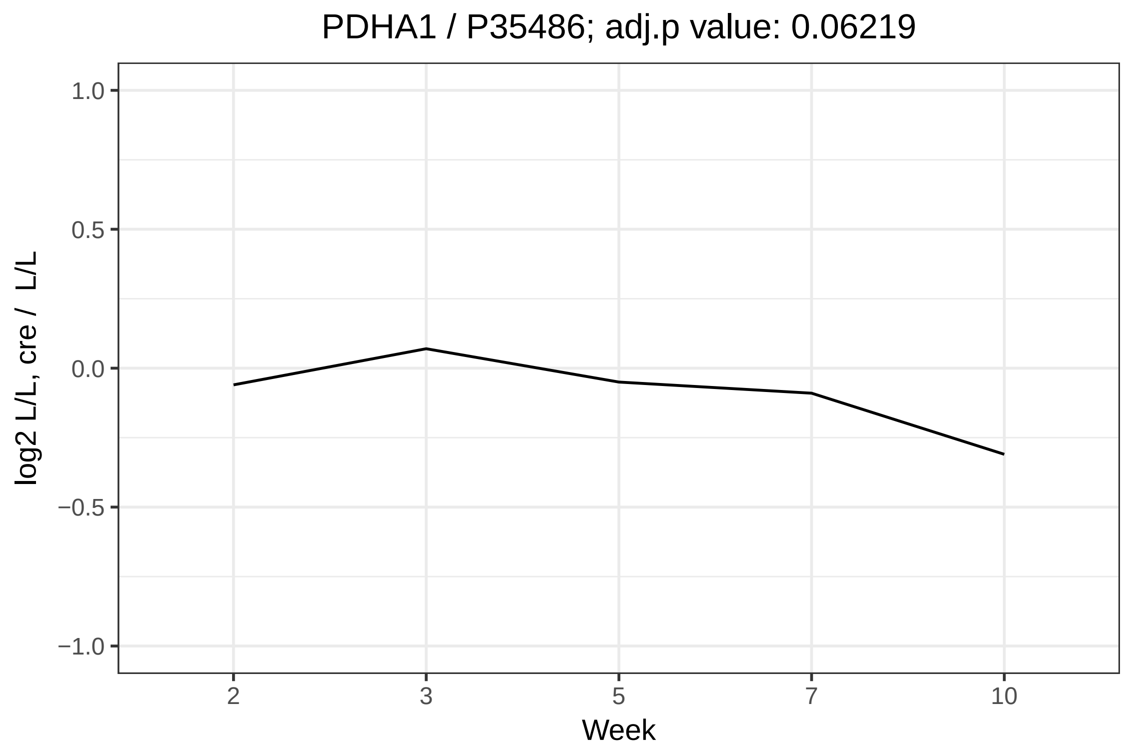
PDHA1 / P35486; adj.p value: 0.06219
log2 L/L, cre / L/L
1.0
0.5
0.0
−0.5
−1.0
2
3
5
7
10
Week

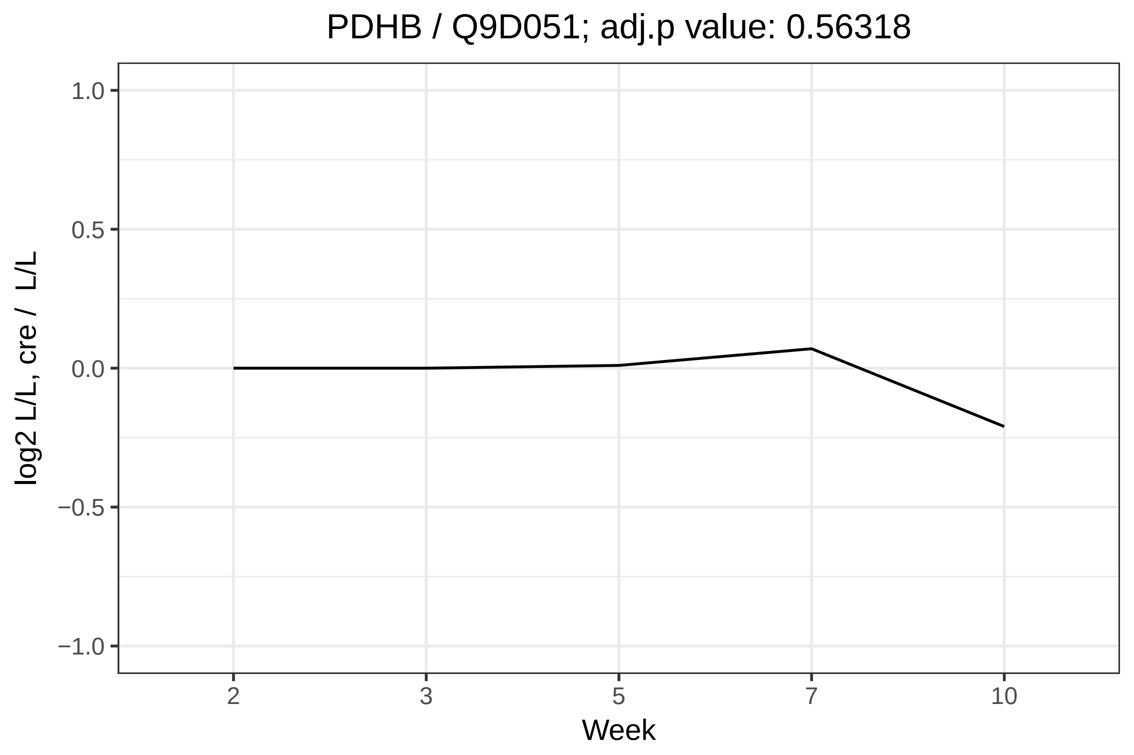
PDHB / Q9D051; adj.p value: 0.56318
1.0
0.5
0.0
−0.5
−1.0
log2 L/L, cre / L/L
2
3
5
7
10
Week

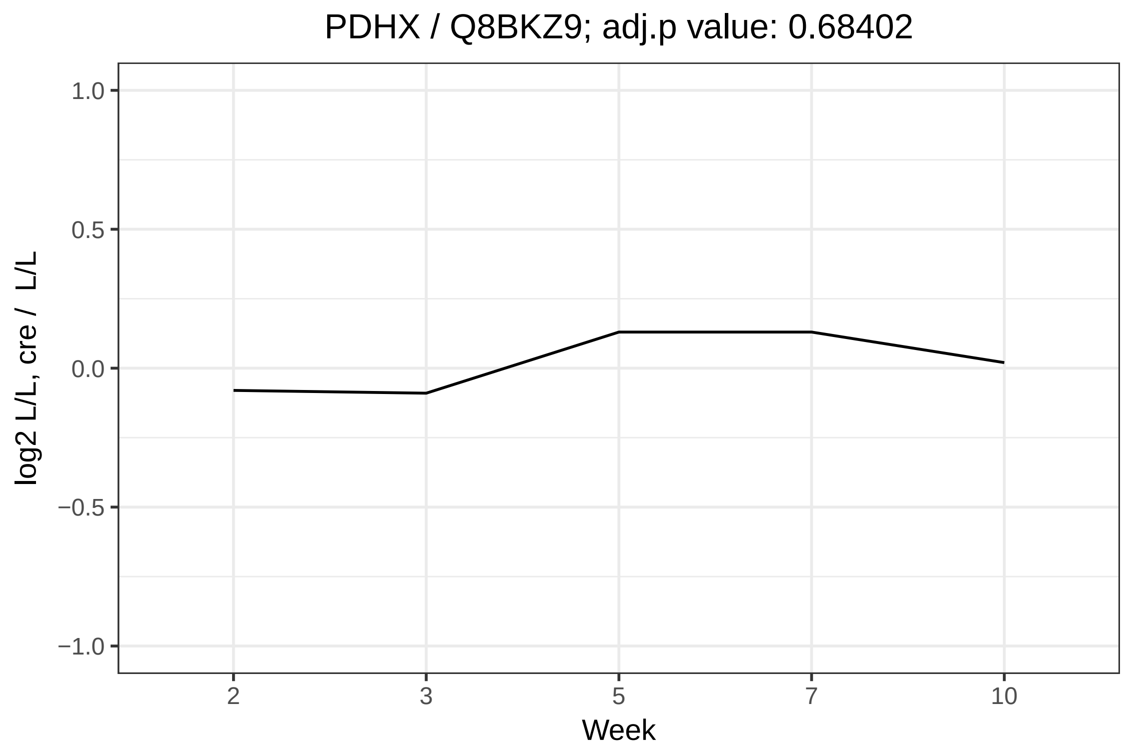
PDHX / Q8BKZ9; adj.p value: 0.68402
log2 L/L, cre / L/L
1.0
0.5
0.0
−0.5
−1.0
2
3
5
7
10
Week

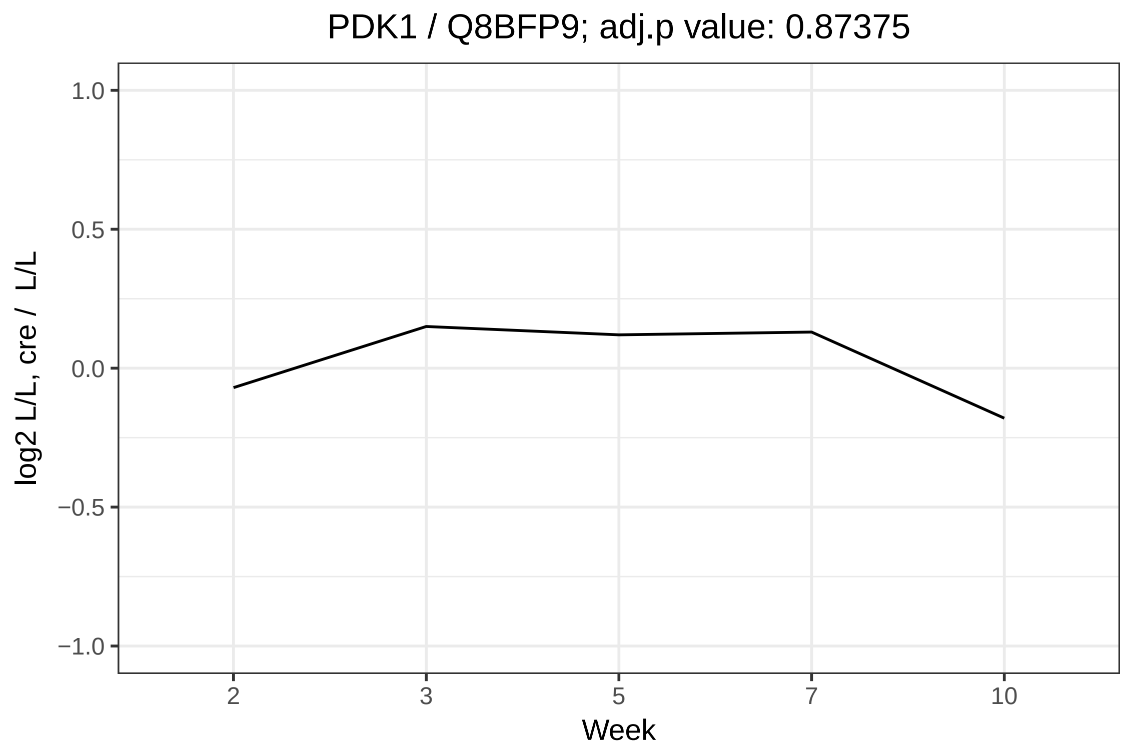

PDK1 / Q8BFP9; adj.p value: 0.87375
log2 L/L, cre / L/L
1.0
0.5
0.0
−0.5
−1.0
2
3
5
7
10
Week

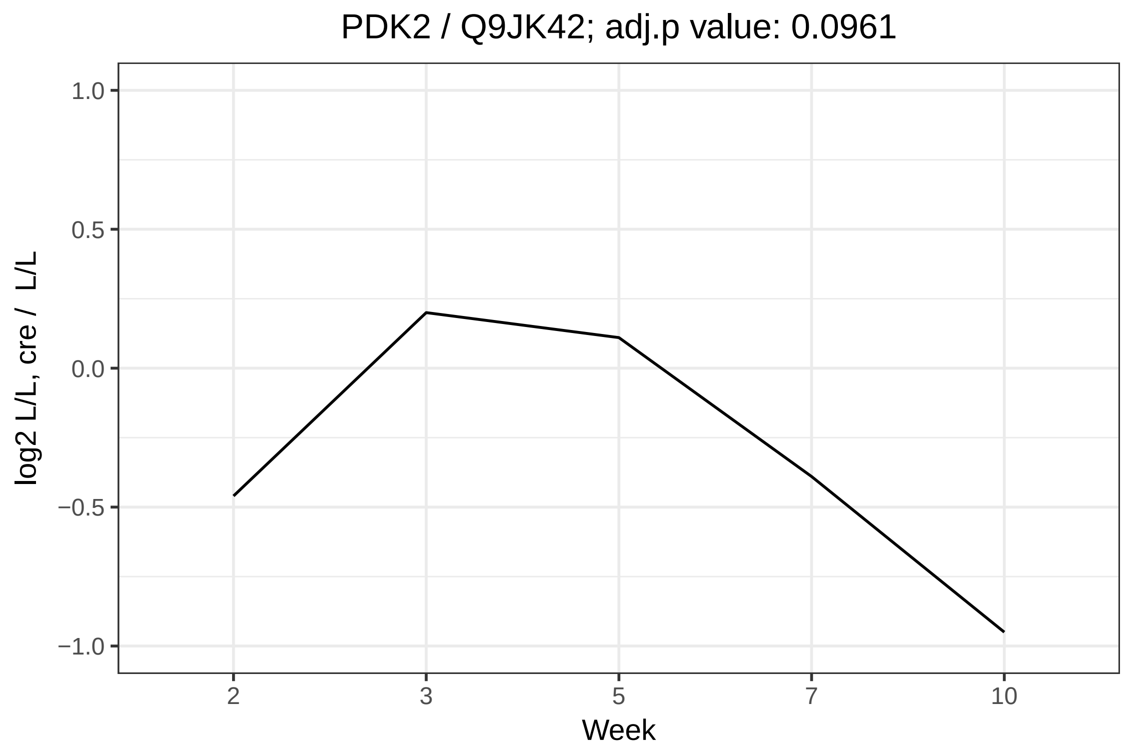
PDK2 / Q9JK42; adj.p value: 0.0961
1.0
0.5
0.0
−0.5
−1.0
log2 L/L, cre / L/L
2
3
5
7
10
Week

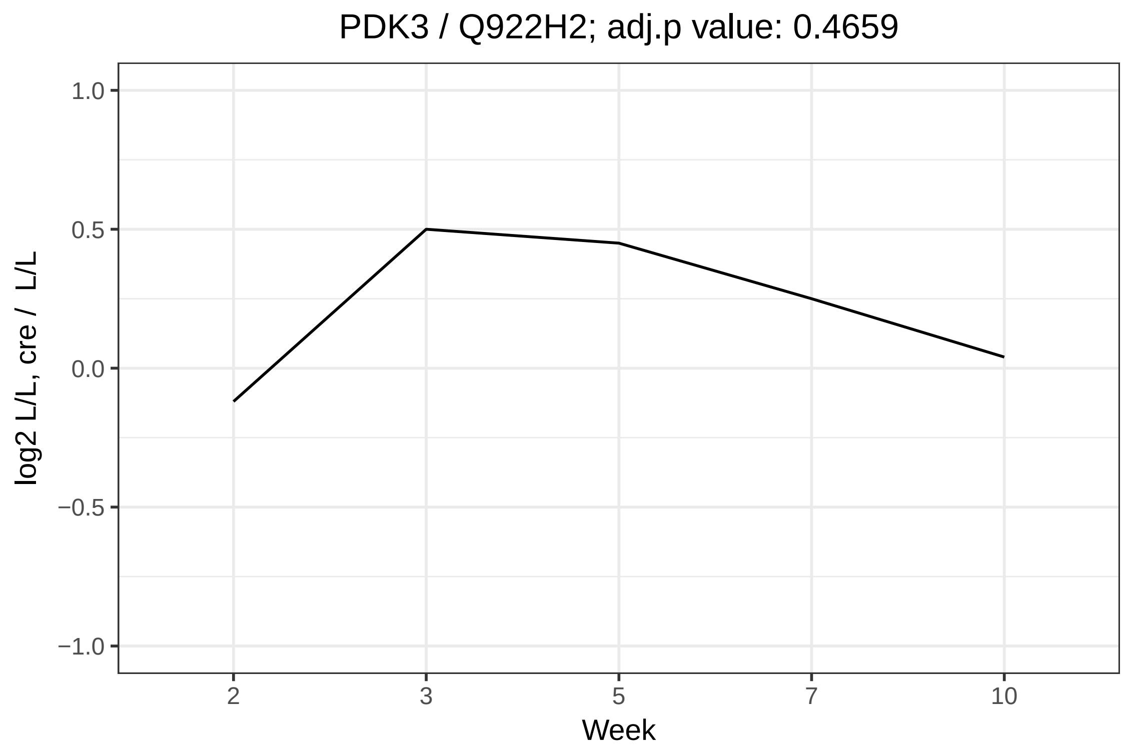
PDK3 / Q922H2; adj.p value: 0.4659
log2 L/L, cre / L/L
1.0
0.5
0.0
−0.5
−1.0
2
3
5
7
10
Week

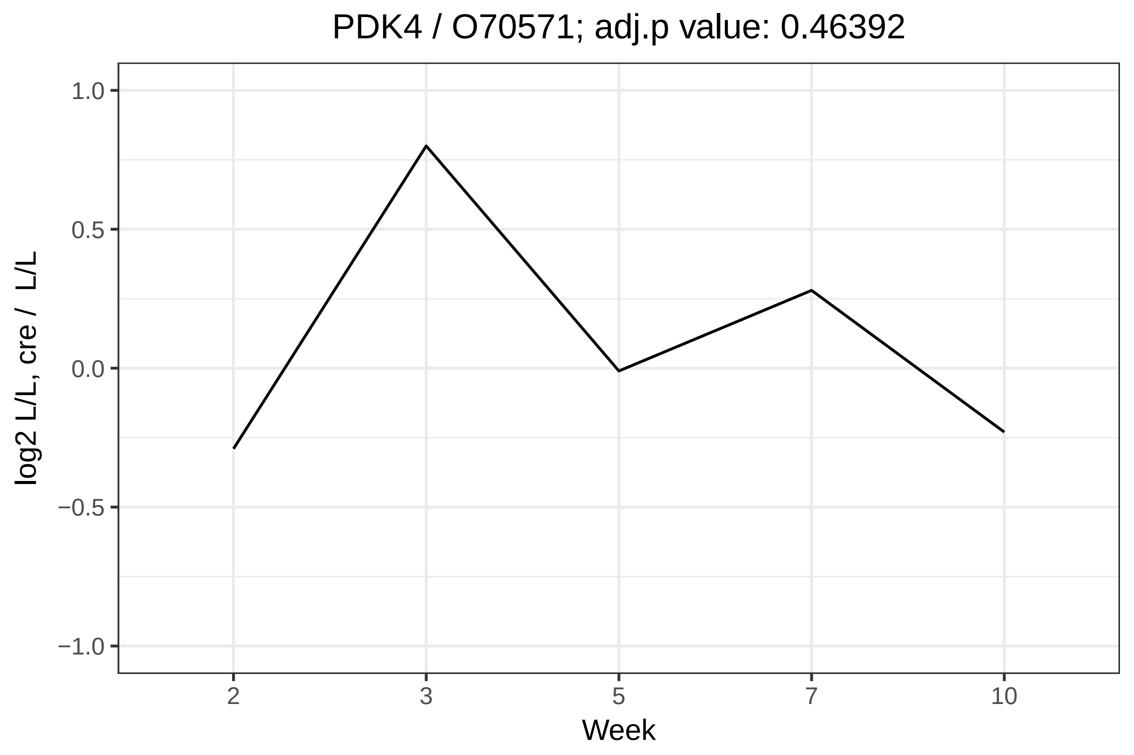

PDK4 / O70571; adj.p value: 0.46392
1.0
0.5
0.0
−0.5
−1.0
log2 L/L, cre / L/L
2
3
5
7
10
Week

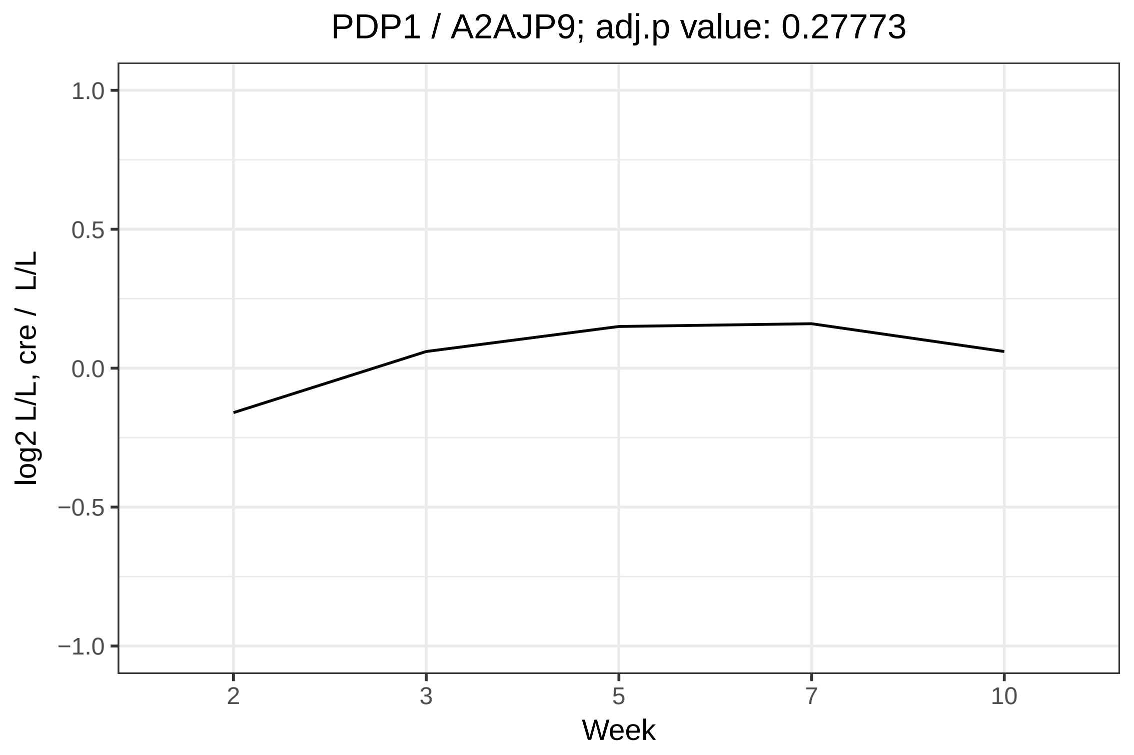
PDP1 / A2AJP9; adj.p value: 0.27773
log2 L/L, cre / L/L
1.0
0.5
0.0
−0.5
−1.0
2
3
5
7
10
Week

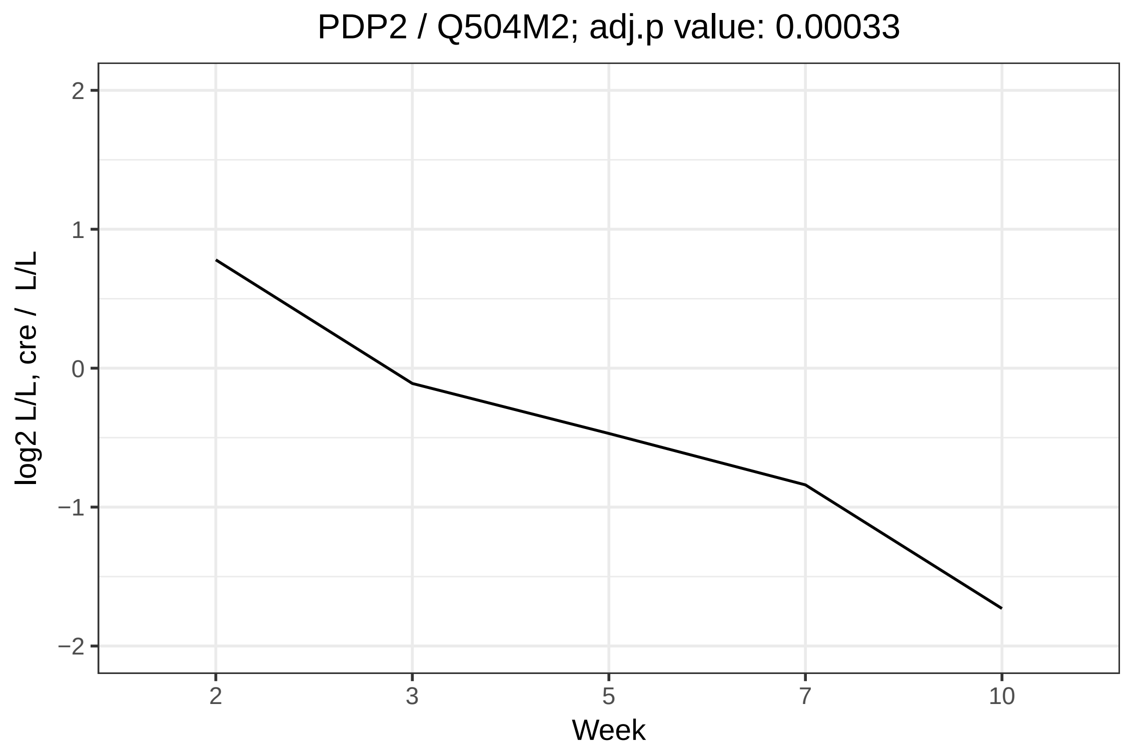
PDP2 / Q504M2; adj.p value: 0.00033
log2 L/L, cre / L/L
2
1
0
−1
−2
2
3
5
7
10
Week

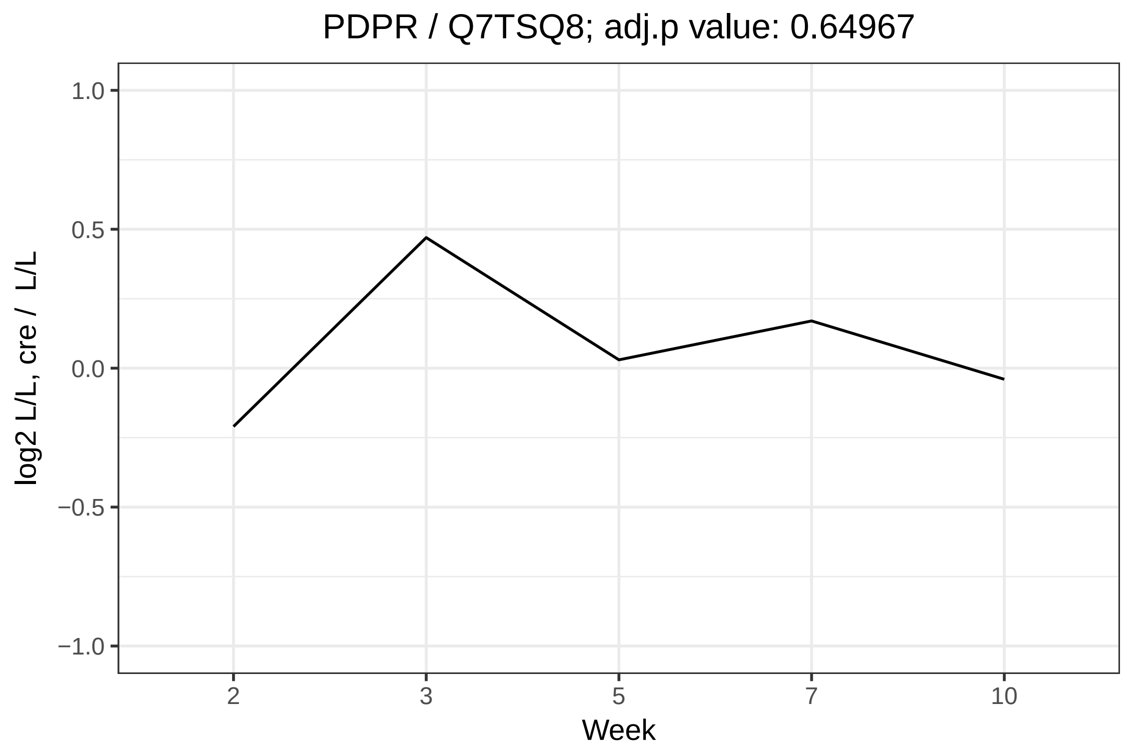
PDPR / Q7TSQ8; adj.p value: 0.64967
log2 L/L, cre / L/L
1.0
0.5
0.0
−0.5
−1.0
2
3
5
7
10
Week

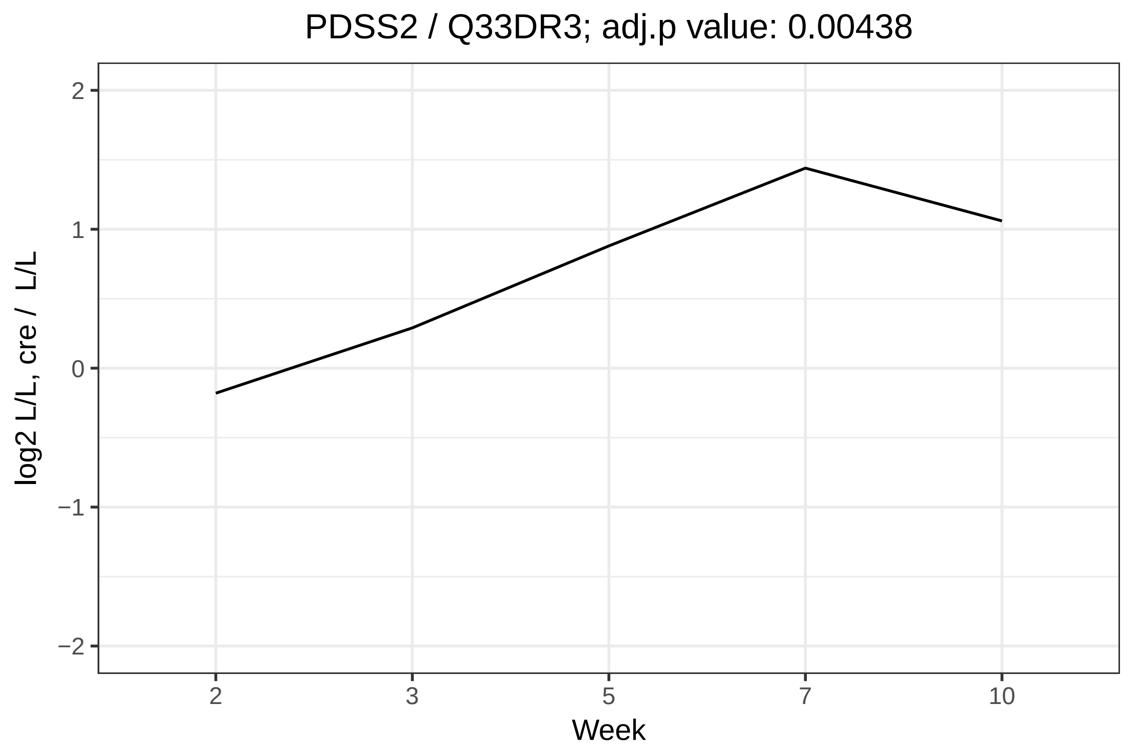
PDSS2 / Q33DR3; adj.p value: 0.00438
log2 L/L, cre / L/L
Week
2
1
0
−1
−2
2
3
5
7
10

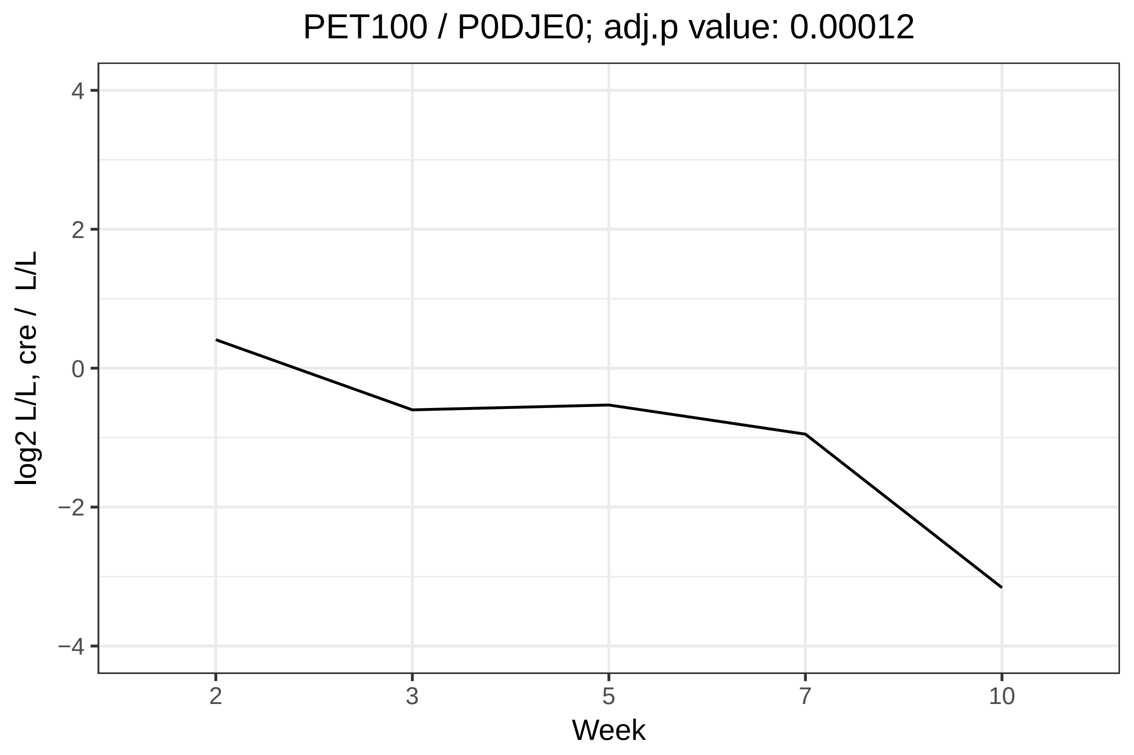
PET100 / P0DJE0; adj.p value: 0.00012
log2 L/L, cre / L/L
4
2
0
−2
−4
2
3
5
7
10
Week

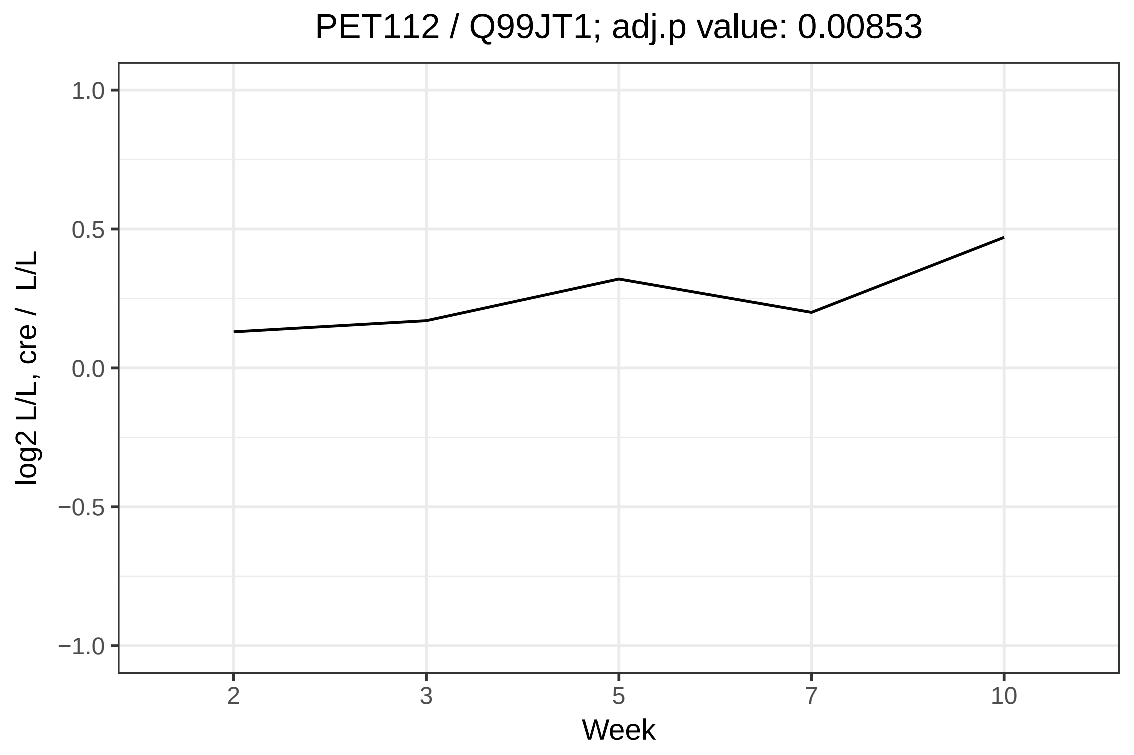
PET112 / Q99JT1; adj.p value: 0.00853
log2 L/L, cre / L/L
1.0
0.5
0.0
−0.5
−1.0
2
3
5
7
10
Week

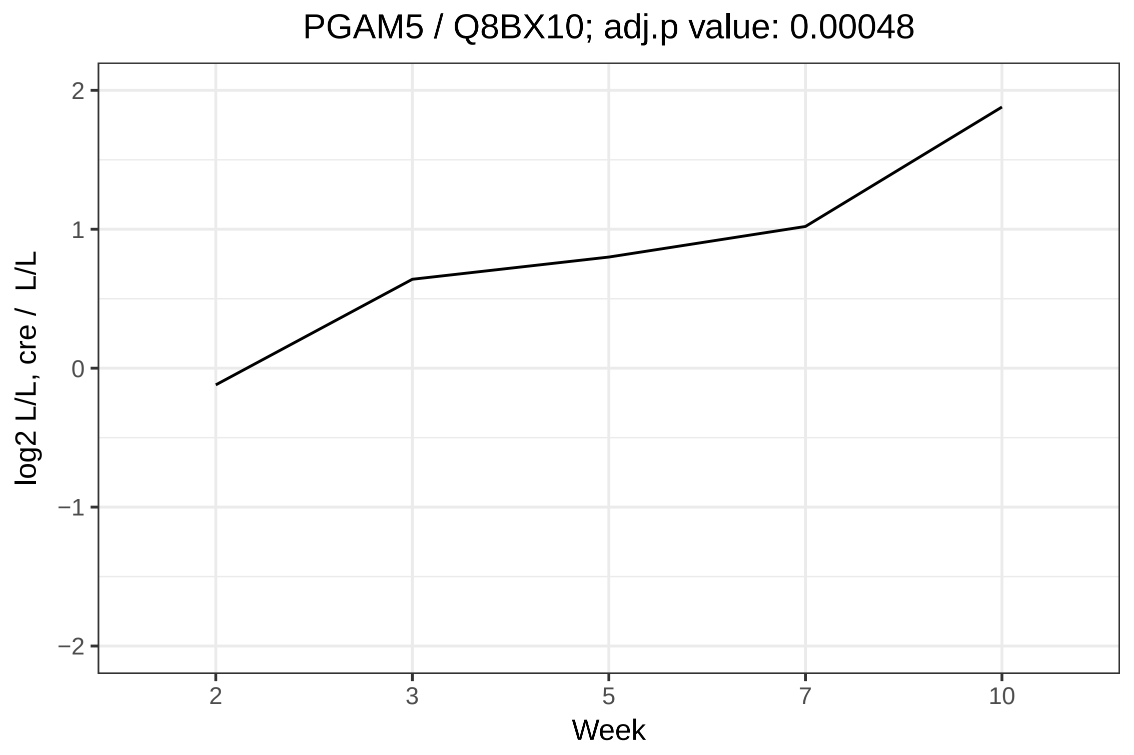
PGAM5 / Q8BX10; adj.p value: 0.00048
log2 L/L, cre / L/L
2
1
0
−1
−2
2
3
5
7
10
Week

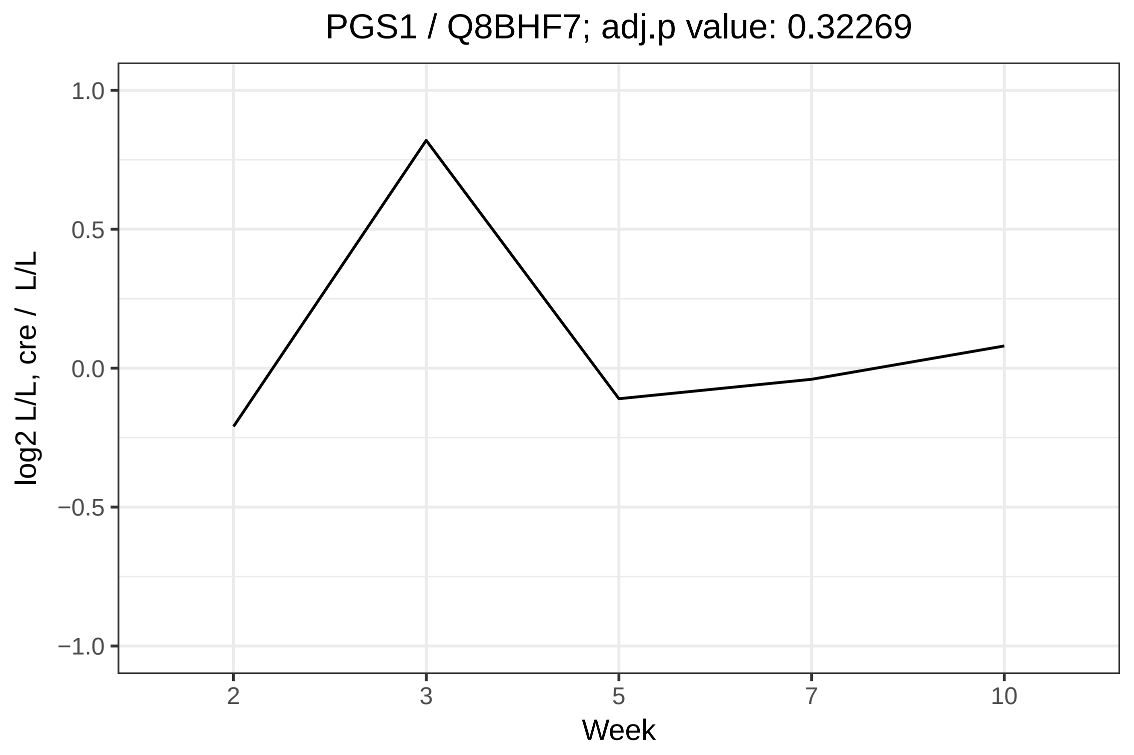
PGS1 / Q8BHF7; adj.p value: 0.32269
log2 L/L, cre / L/L
1.0
0.5
0.0
−0.5
−1.0
2
3
5
7
10
Week

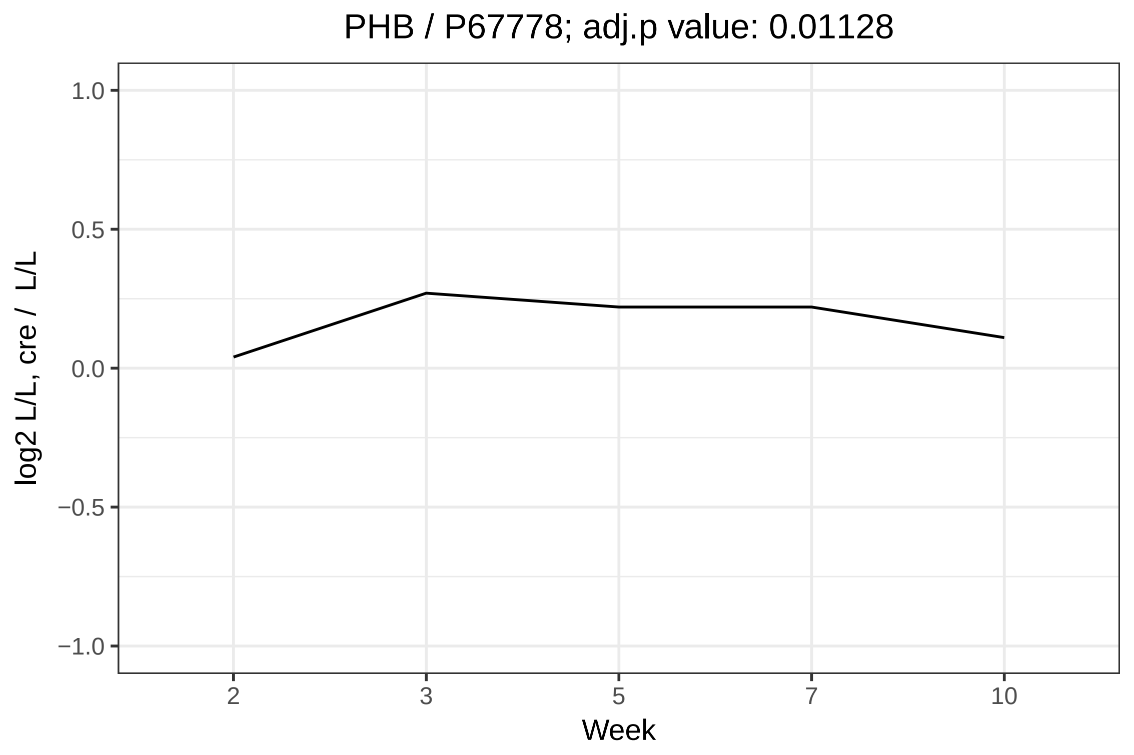
PHB / P67778; adj.p value: 0.01128
1.0
0.5
0.0
−0.5
−1.0
log2 L/L, cre / L/L
2
3
5
7
10
Week

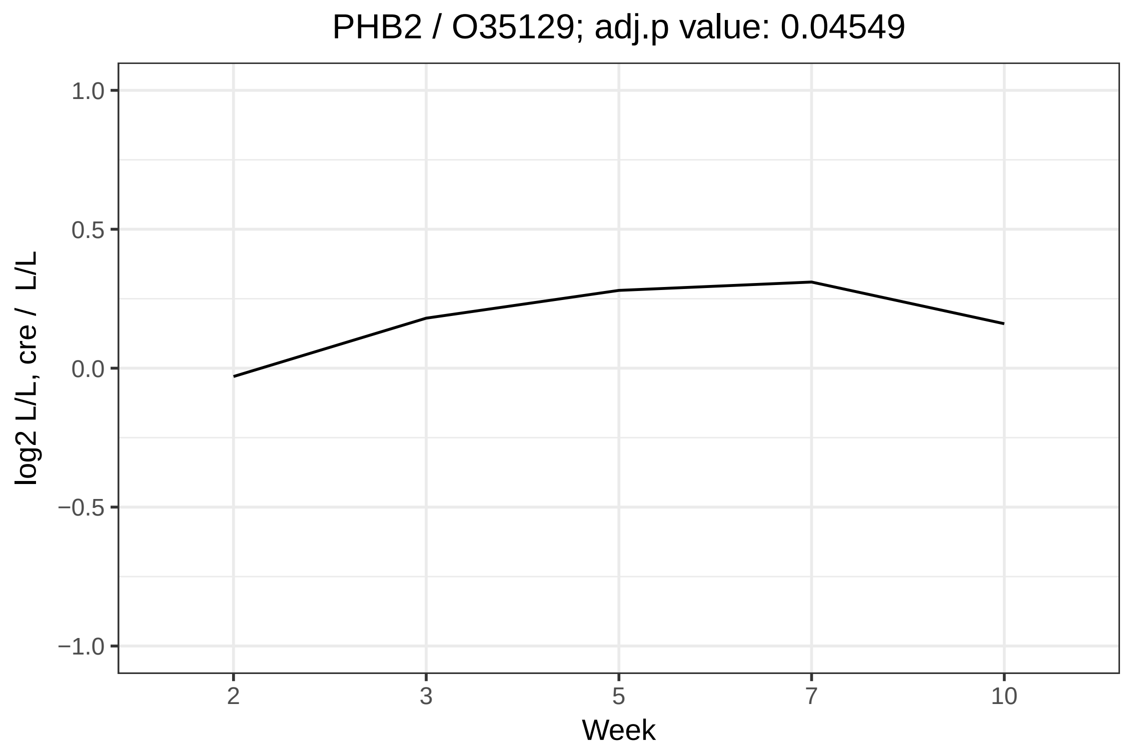

PHB2 / O35129; adj.p value: 0.04549
log2 L/L, cre / L/L
1.0
0.5
0.0
−0.5
−1.0
2
3
5
7
10
Week

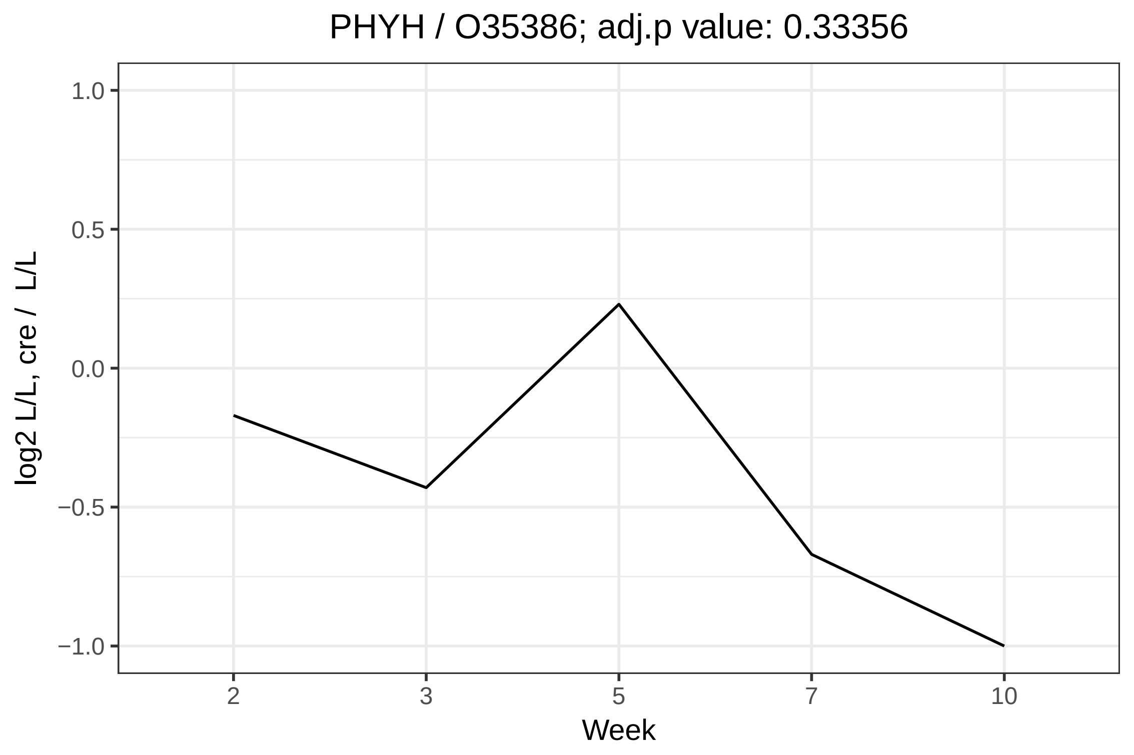
PHYH / O35386; adj.p value: 0.33356
log2 L/L, cre / L/L
1.0
0.5
0.0
−0.5
−1.0
2
3
5
7
10
Week

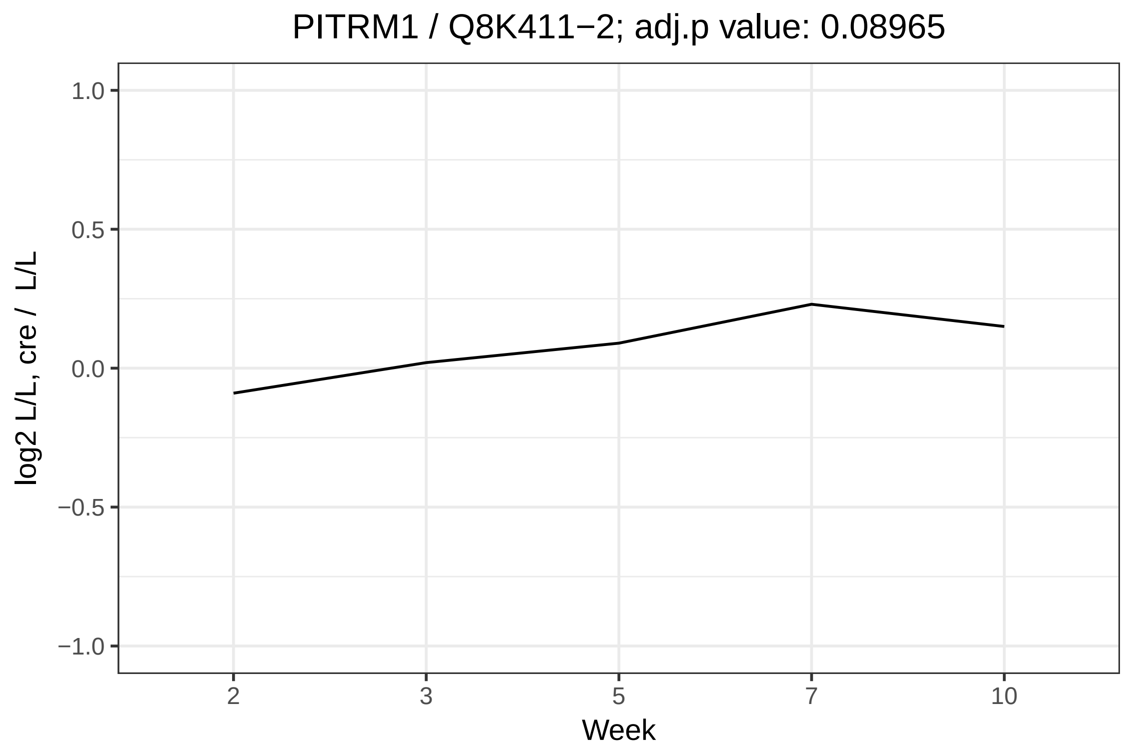
PITRM1 / Q8K411−2; adj.p value: 0.08965
log2 L/L, cre / L/L
1.0
0.5
0.0
−0.5
−1.0
2
3
5
7
10
Week

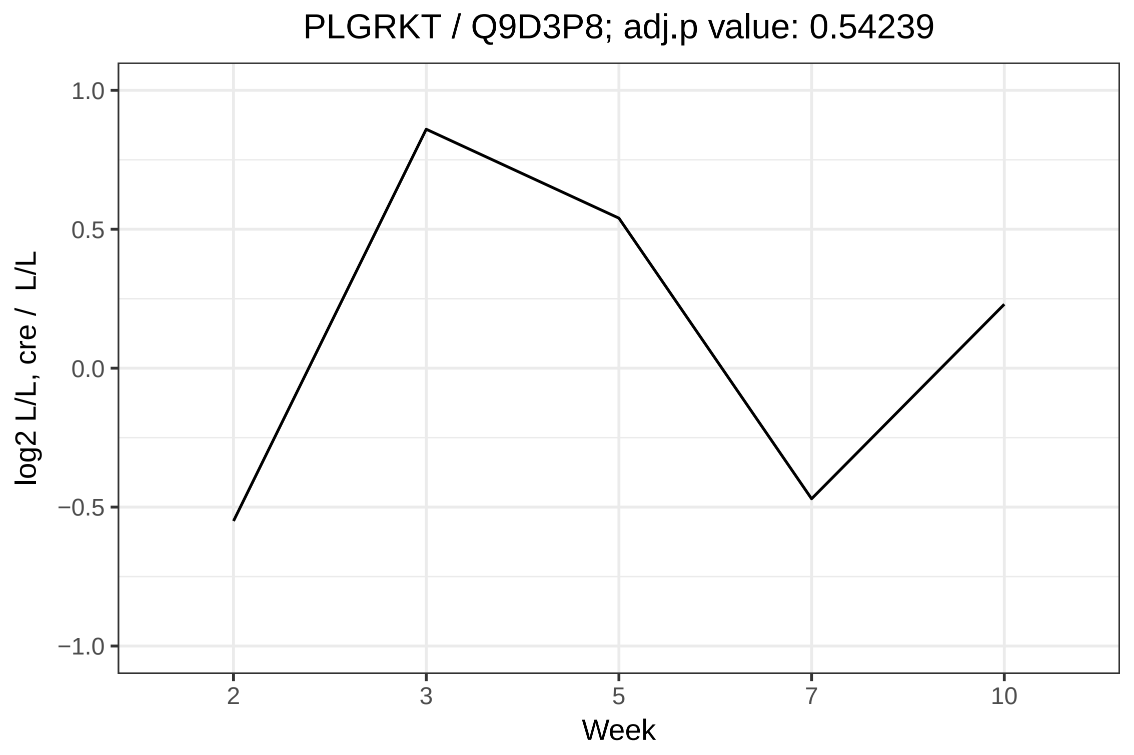
PLGRKT / Q9D3P8; adj.p value: 0.54239
1.0
0.5
0.0
−0.5
−1.0
log2 L/L, cre / L/L
2
3
5
7
10
Week

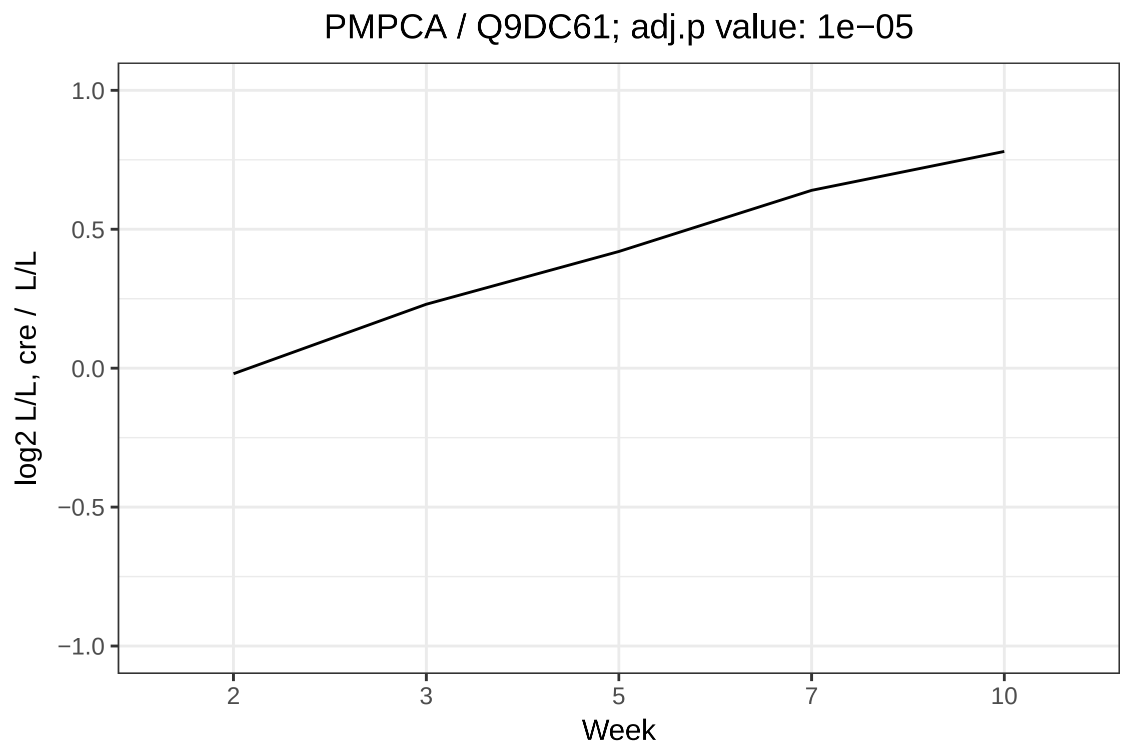

PMPCA / Q9DC61; adj.p value: 1e−05
1.0
0.5
0.0
−0.5
−1.0
log2 L/L, cre / L/L
2
3
5
7
10
Week

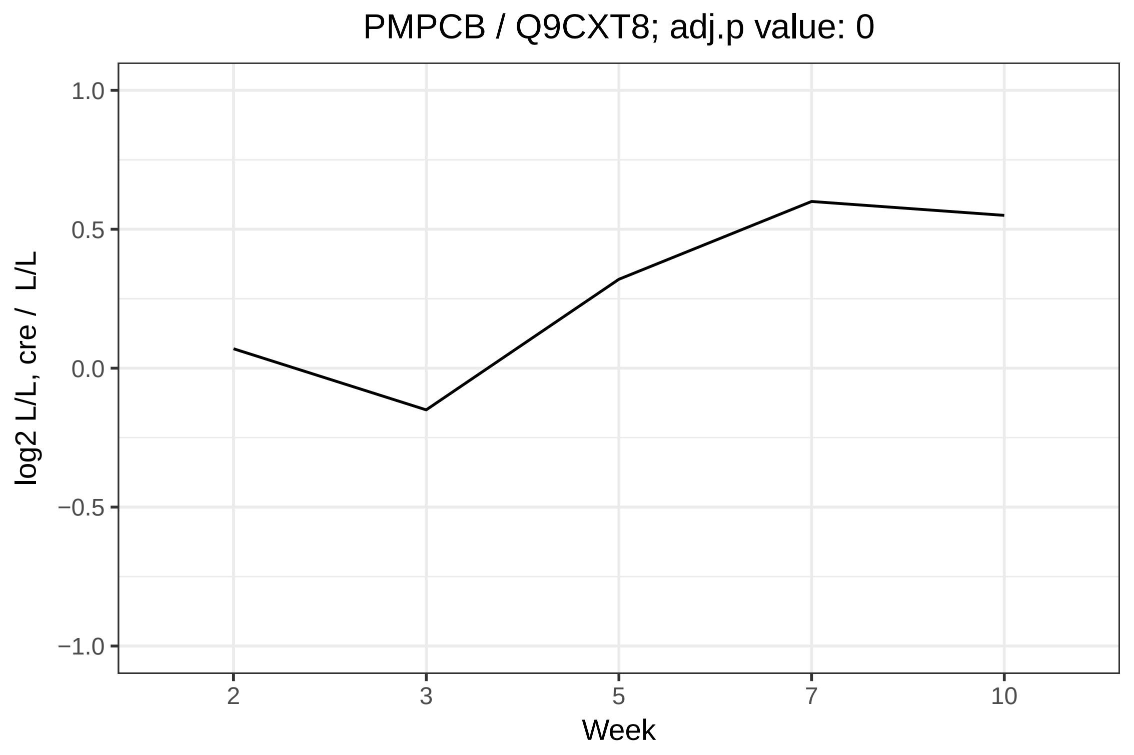
PMPCB / Q9CXT8; adj.p value: 0
1.0
0.5
0.0
−0.5
−1.0
log2 L/L, cre / L/L
2
3
5
7
10
Week

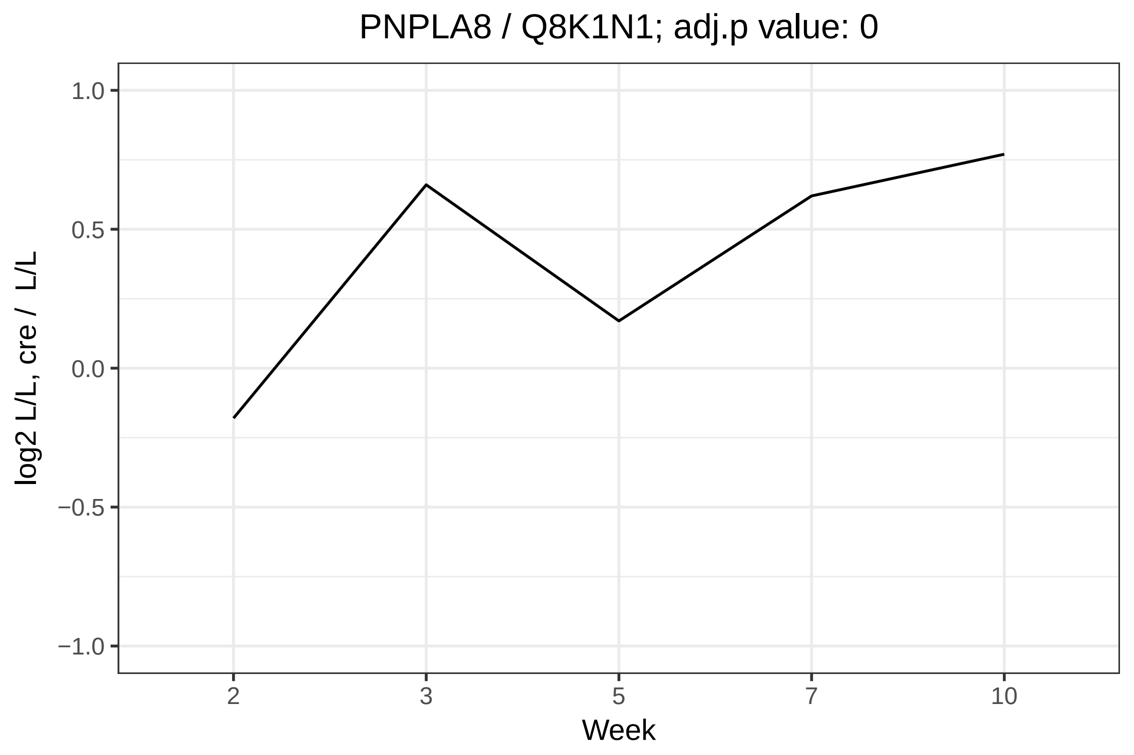
PNPLA8 / Q8K1N1; adj.p value: 0
1.0
0.5
0.0
−0.5
−1.0
log2 L/L, cre / L/L
2
3
5
7
10
Week

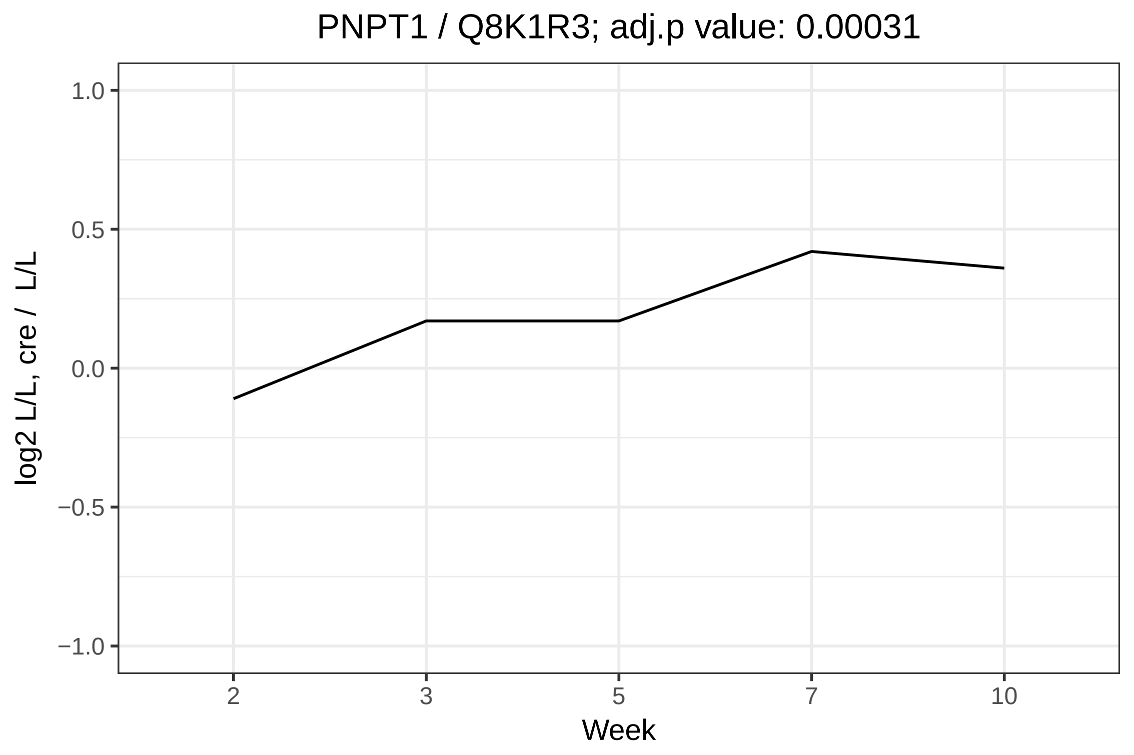

PNPT1 / Q8K1R3; adj.p value: 0.00031
1.0
0.5
0.0
−0.5
−1.0
log2 L/L, cre / L/L
2
3
5
7
10
Week

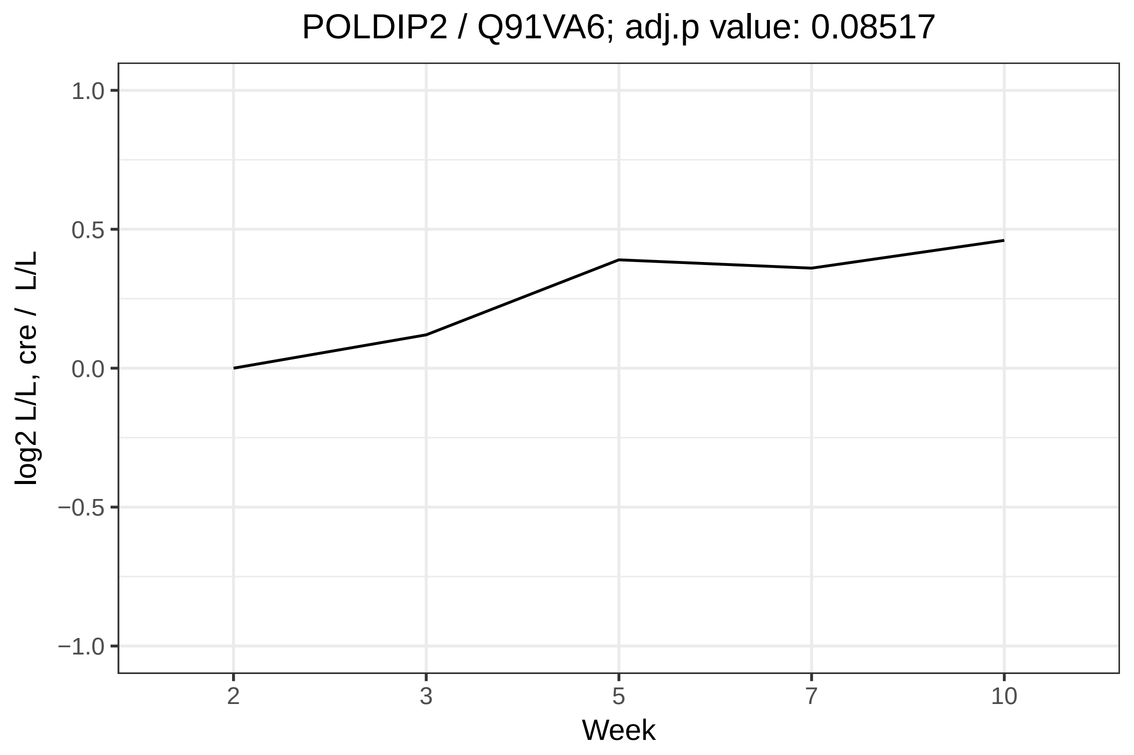

POLDIP2 / Q91VA6; adj.p value: 0.08517
log2 L/L, cre / L/L
1.0
0.5
0.0
−0.5
−1.0
2
3
5
7
10
Week

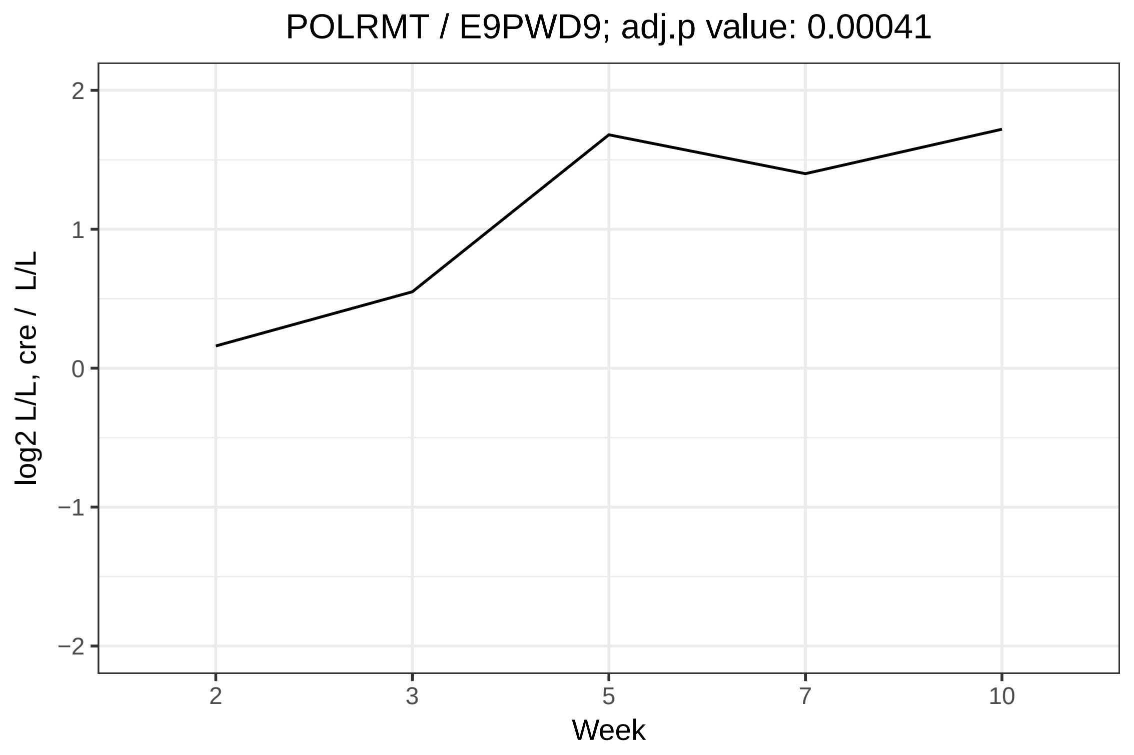

POLRMT / E9PWD9; adj.p value: 0.00041
log2 L/L, cre / L/L
Week
2
1
0
−1
−2
2
3
5
7
10

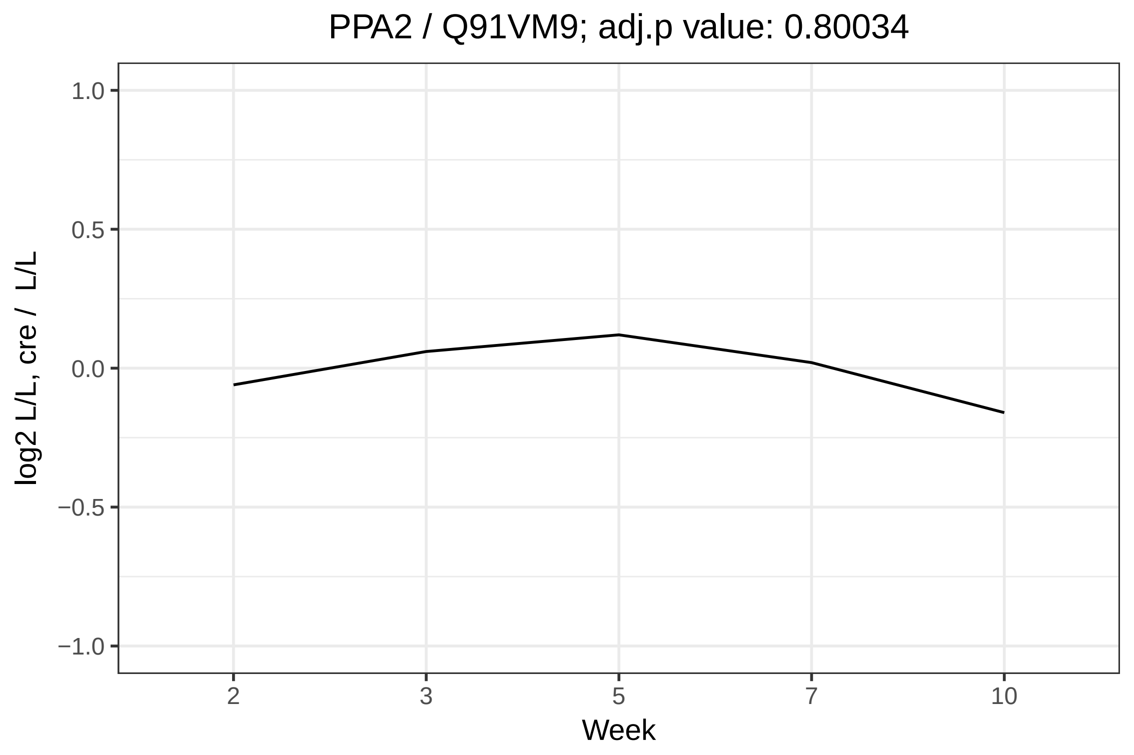
PPA2 / Q91VM9; adj.p value: 0.80034
log2 L/L, cre / L/L
1.0
0.5
0.0
−0.5
−1.0
2
3
5
7
10
Week

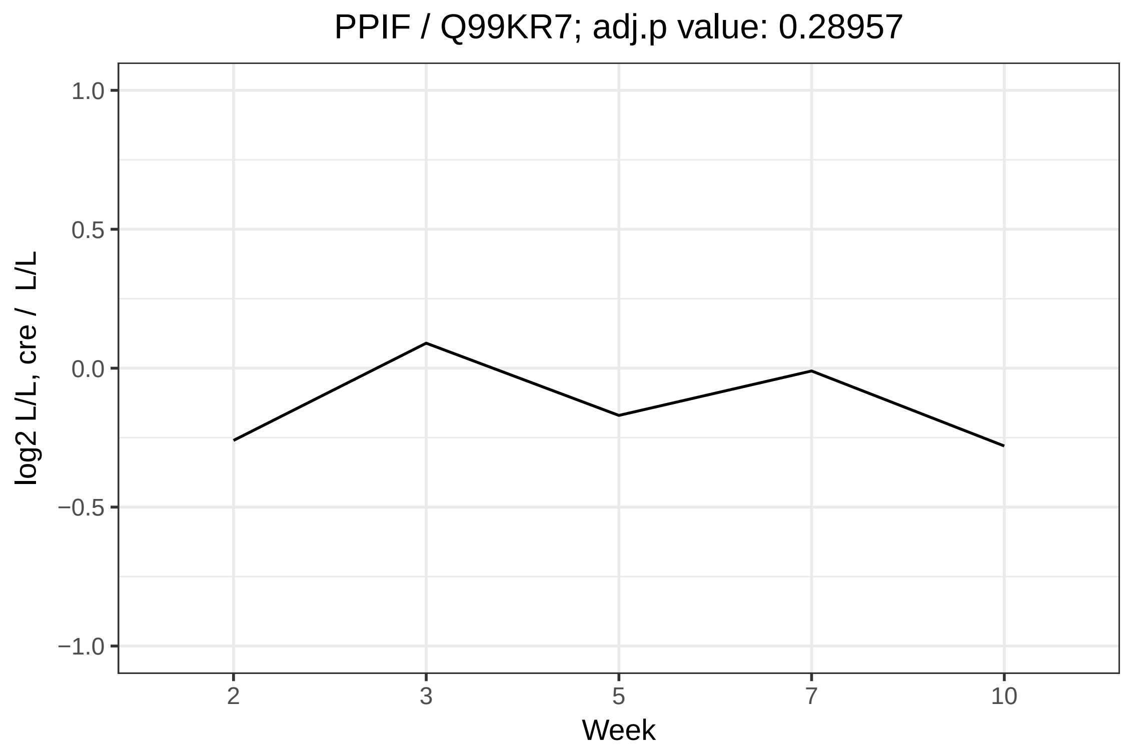

PPIF / Q99KR7; adj.p value: 0.28957
1.0
0.5
0.0
−0.5
−1.0
log2 L/L, cre / L/L
2
3
5
7
10
Week

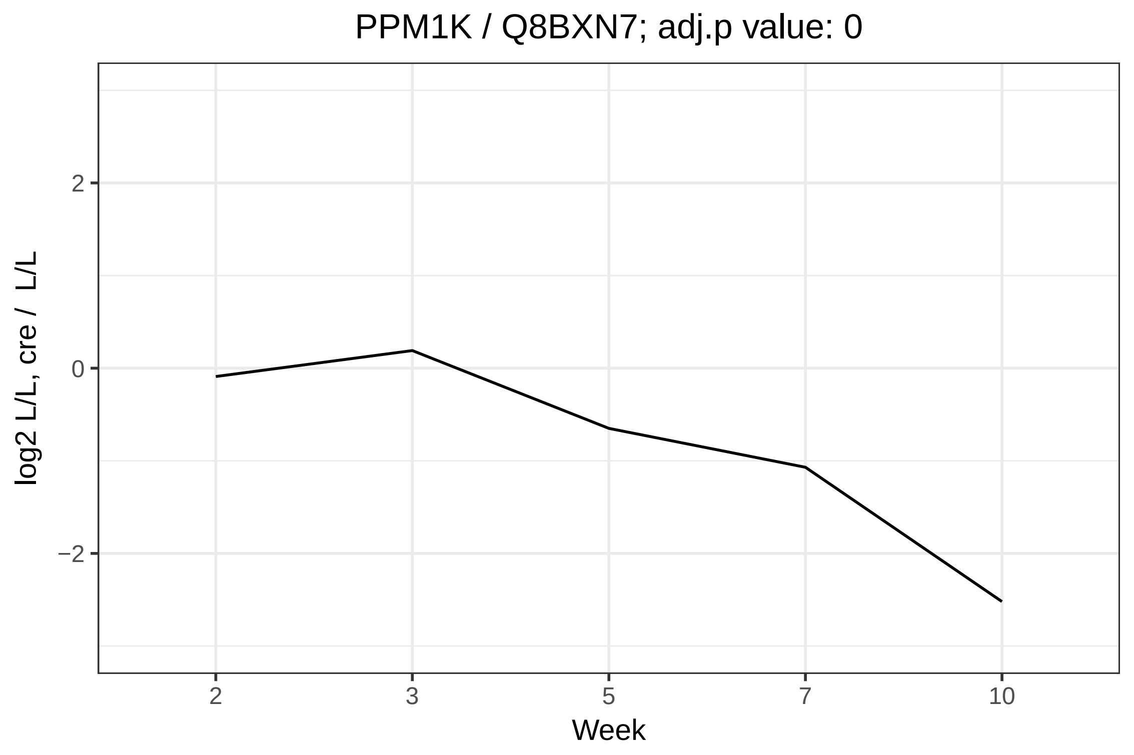

PPM1K / Q8BXN7; adj.p value: 0
log2 L/L, cre / L/L
2
0
−2
2
3
5
7
10
Week

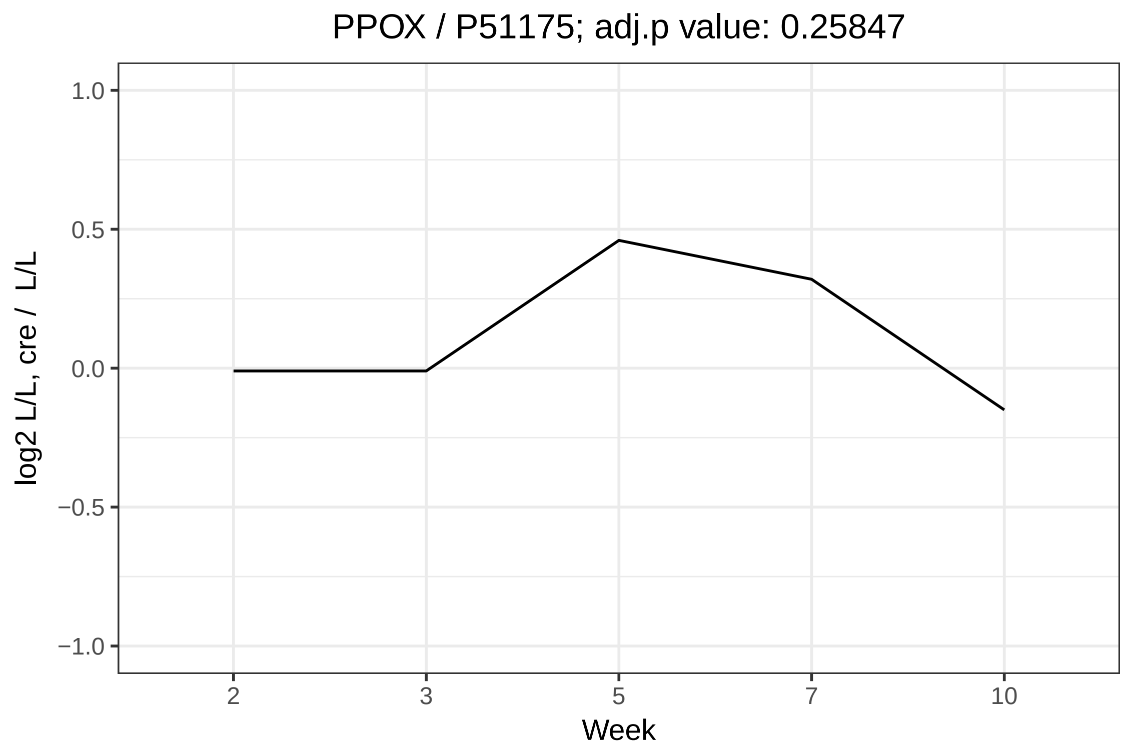

PPOX / P51175; adj.p value: 0.25847
log2 L/L, cre / L/L
1.0
0.5
0.0
−0.5
−1.0
2
3
5
7
10
Week

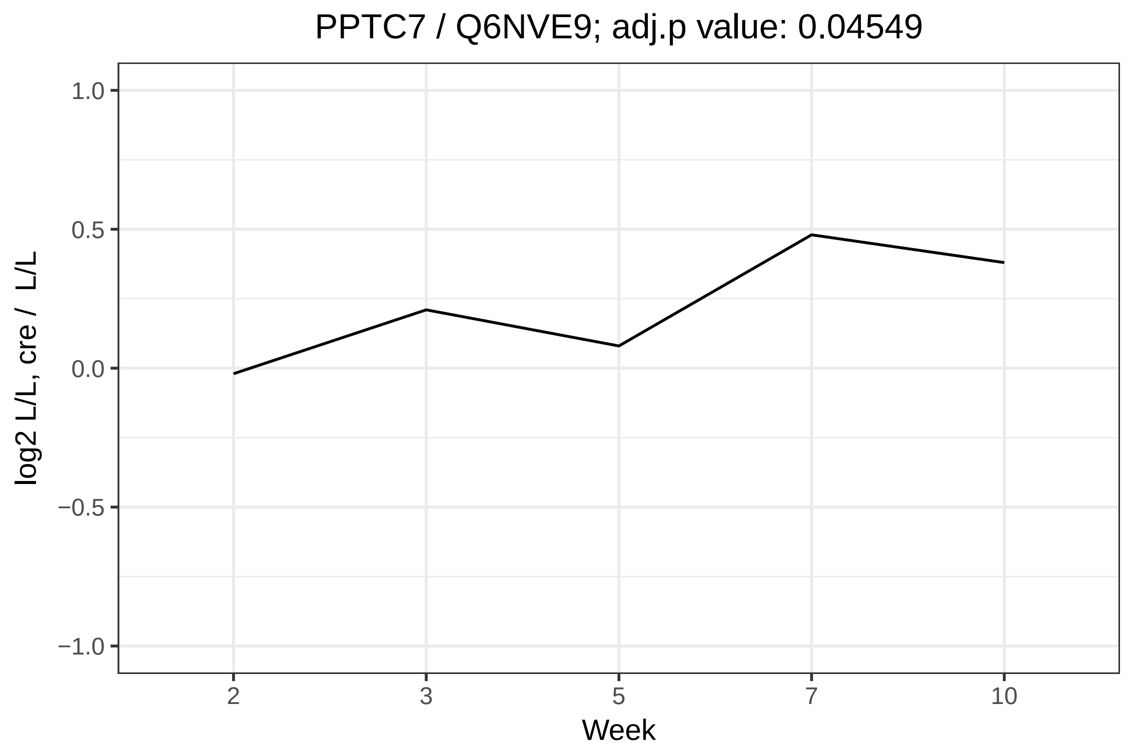
PPTC7 / Q6NVE9; adj.p value: 0.04549
1.0
0.5
0.0
−0.5
−1.0
log2 L/L, cre / L/L
2
3
5
7
10
Week

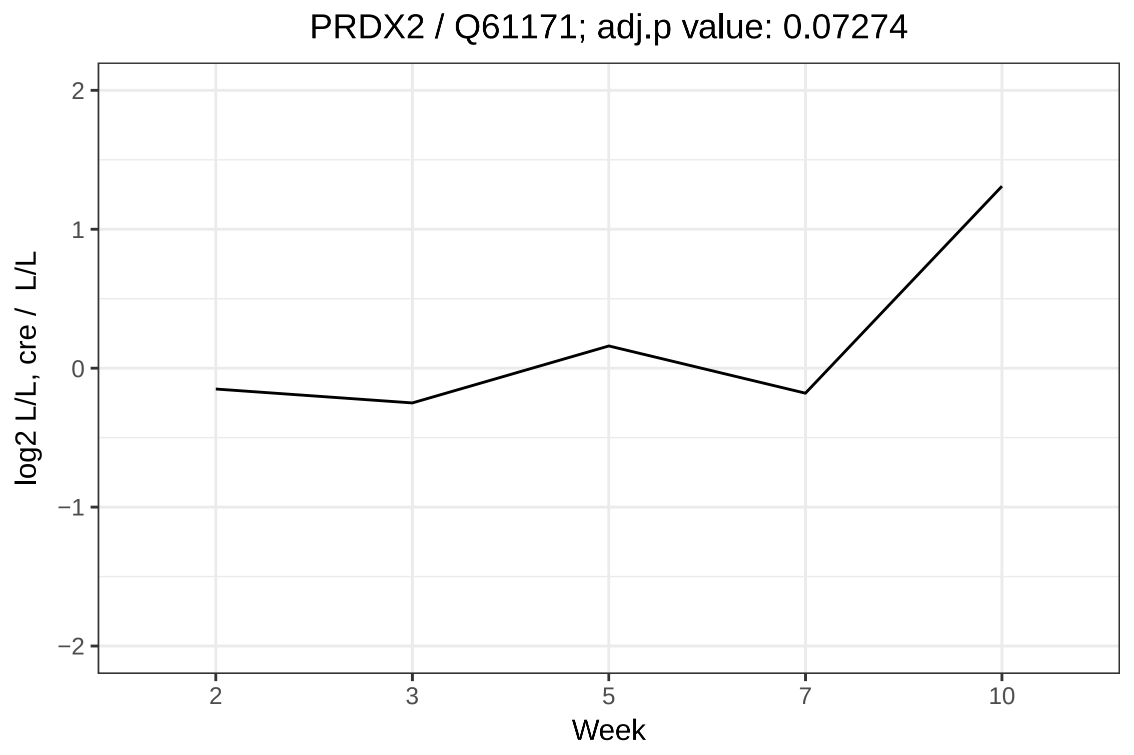
PRDX2 / Q61171; adj.p value: 0.07274
log2 L/L, cre / L/L
2
1
0
−1
−2
2
3
5
7
10
Week

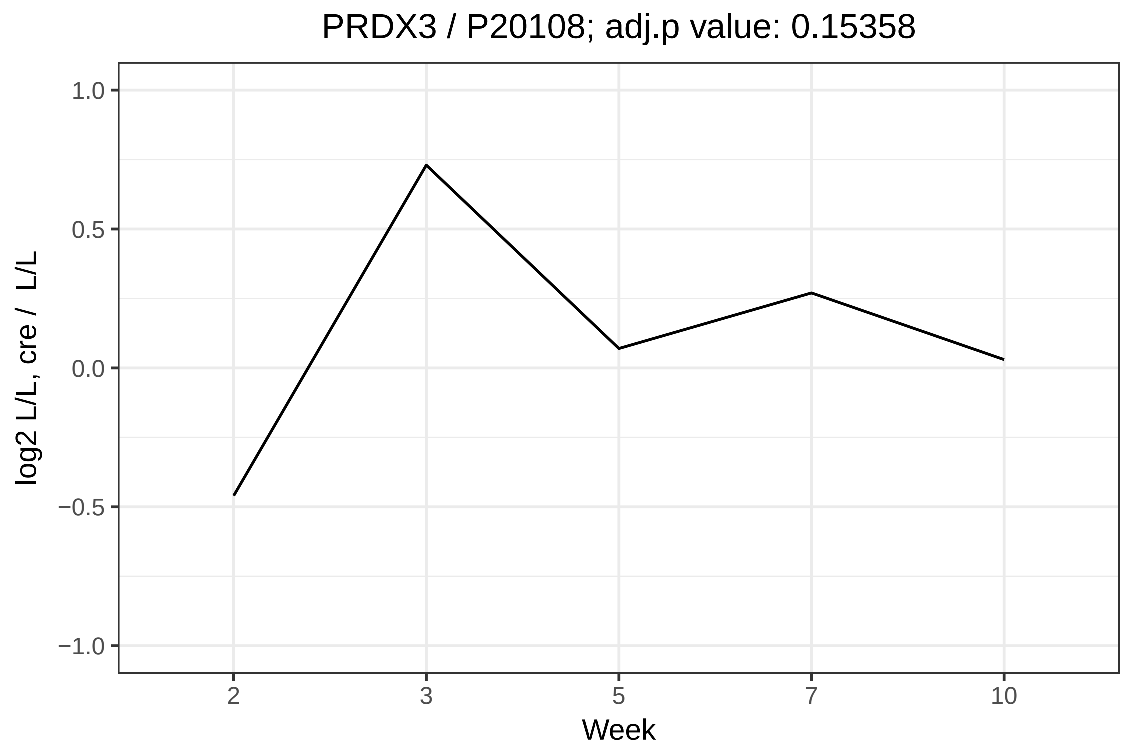
PRDX3 / P20108; adj.p value: 0.15358
log2 L/L, cre / L/L
1.0
0.5
0.0
−0.5
−1.0
2
3
5
7
10
Week

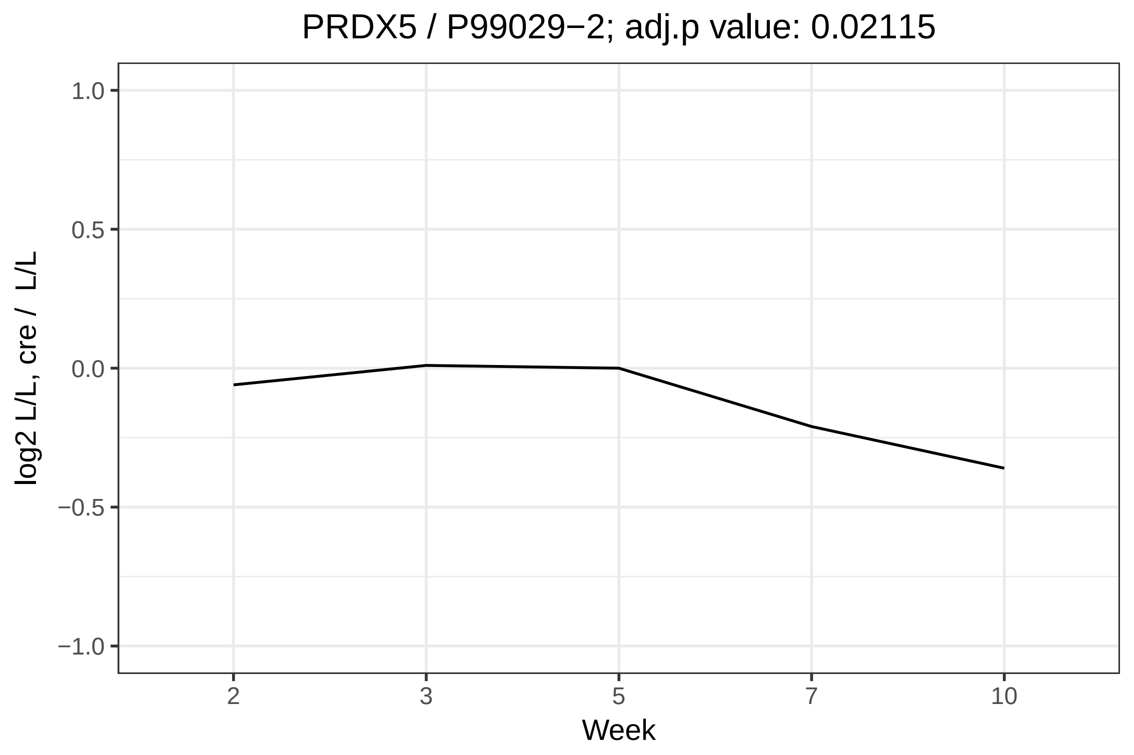

PRDX5 / P99029−2; adj.p value: 0.02115
1.0
0.5
0.0
−0.5
−1.0
log2 L/L, cre / L/L
2
3
5
7
10
Week

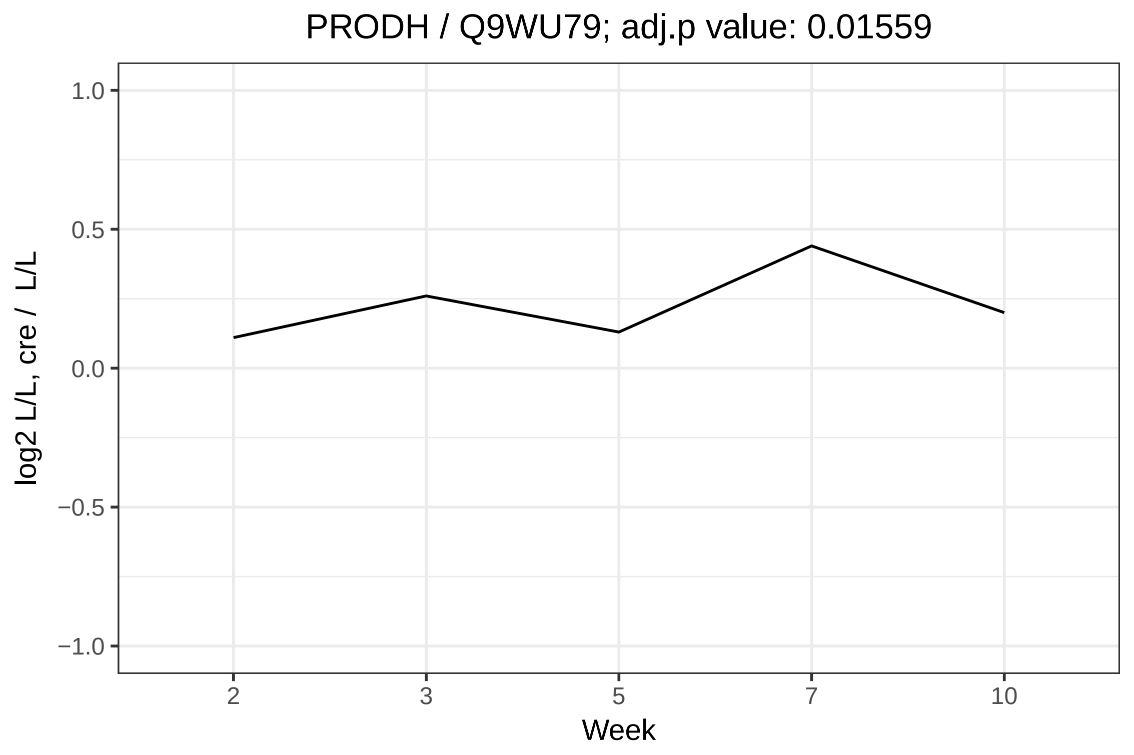

PRODH / Q9WU79; adj.p value: 0.01559
1.0
0.5
0.0
−0.5
−1.0
log2 L/L, cre / L/L
2
3
5
7
10
Week

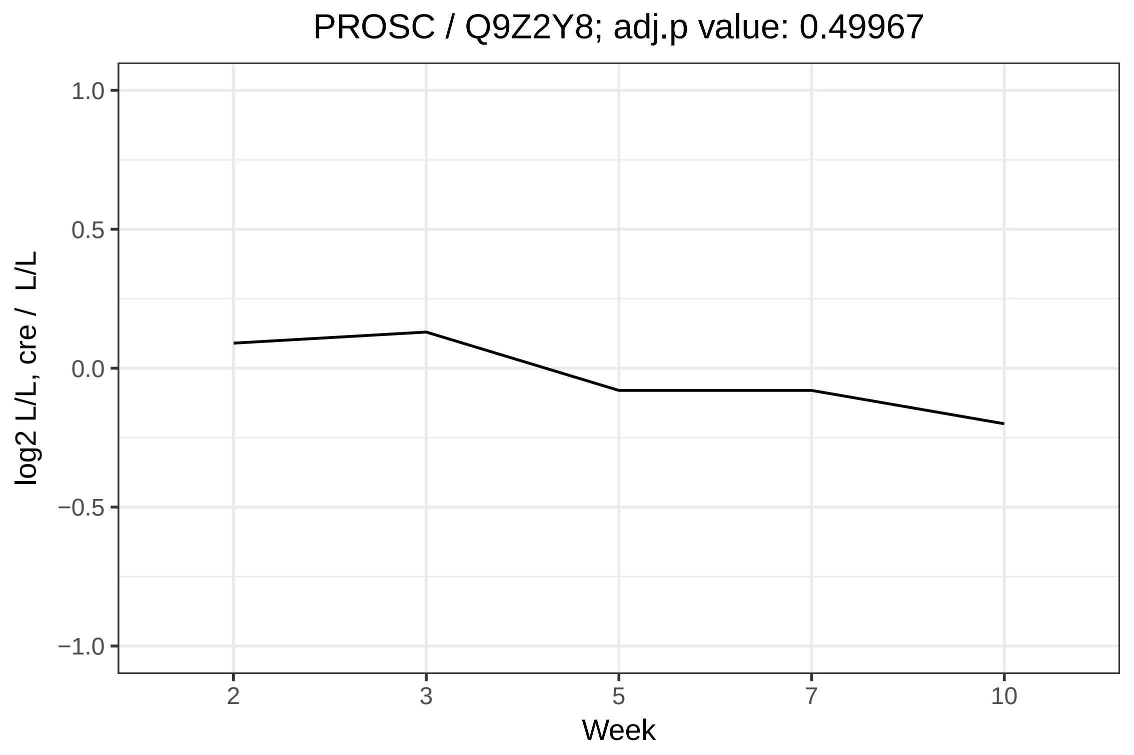
PROSC / Q9Z2Y8; adj.p value: 0.49967
log2 L/L, cre / L/L
1.0
0.5
0.0
−0.5
−1.0
2
3
5
7
10
Week

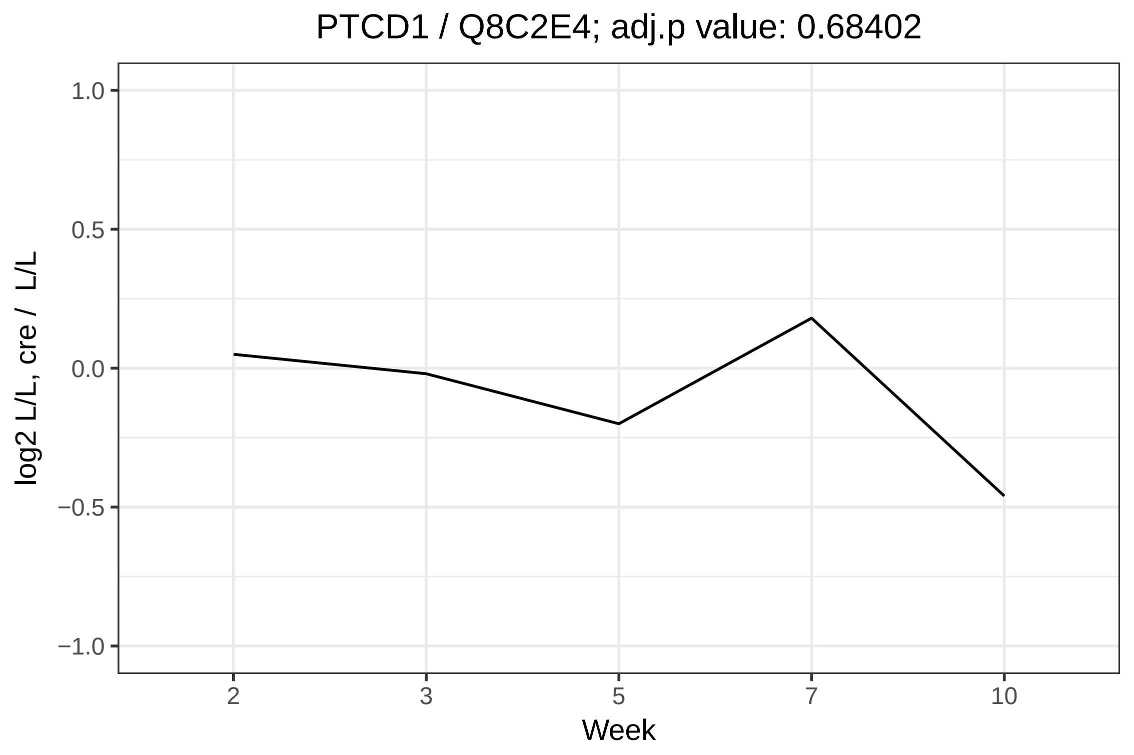

PTCD1 / Q8C2E4; adj.p value: 0.68402
log2 L/L, cre / L/L
1.0
0.5
0.0
−0.5
−1.0
2
3
5
7
10
Week

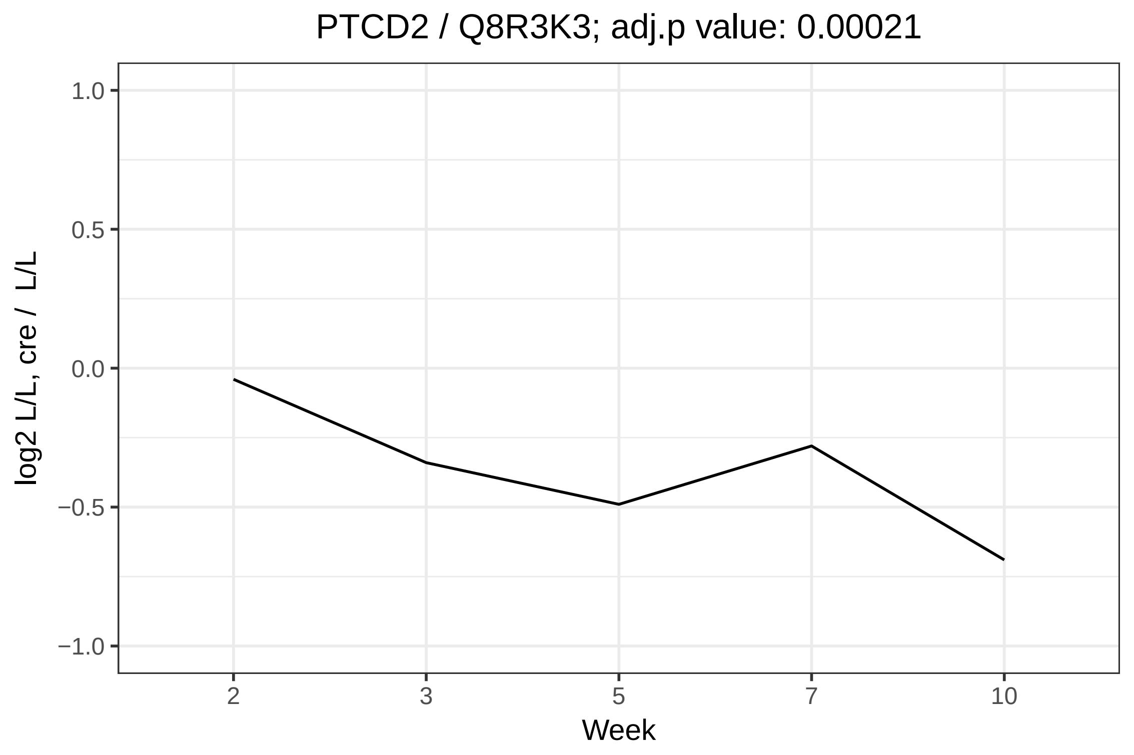
PTCD2 / Q8R3K3; adj.p value: 0.00021
log2 L/L, cre / L/L
1.0
0.5
0.0
−0.5
−1.0
2
3
5
7
10
Week

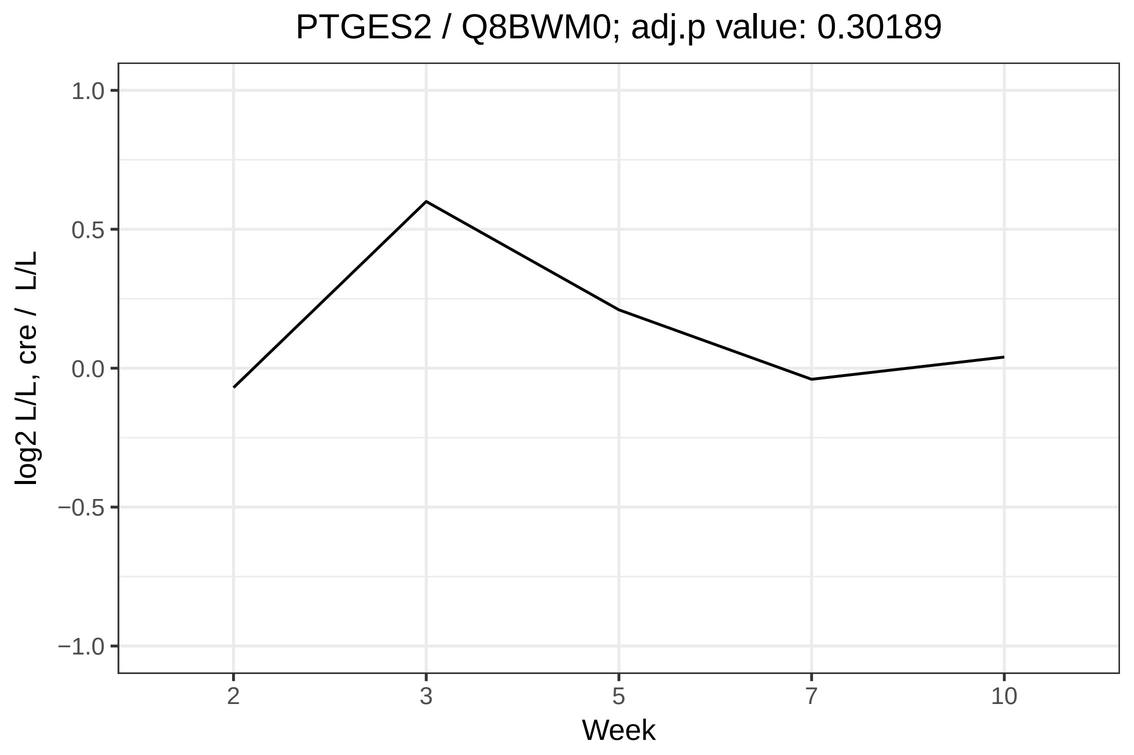
PTGES2 / Q8BWM0; adj.p value: 0.30189
log2 L/L, cre / L/L
1.0
0.5
0.0
−0.5
−1.0
2
3
5
7
10
Week

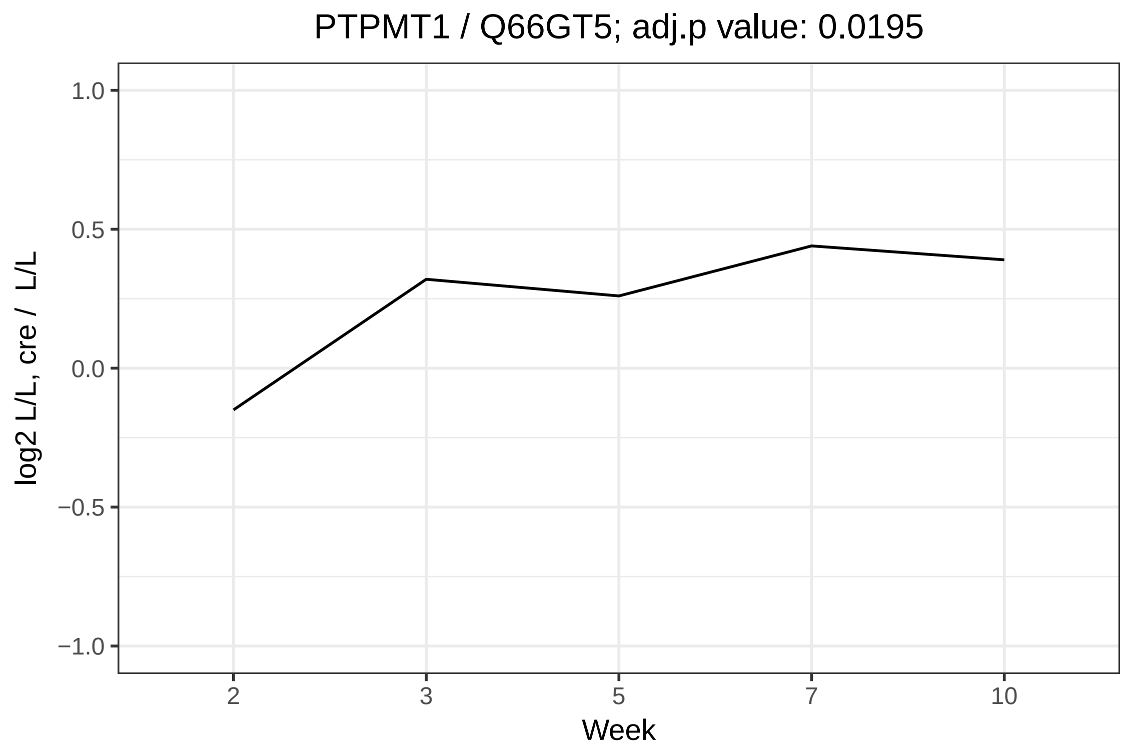
PTPMT1 / Q66GT5; adj.p value: 0.0195
1.0
0.5
0.0
−0.5
−1.0
log2 L/L, cre / L/L
2
3
5
7
10
Week

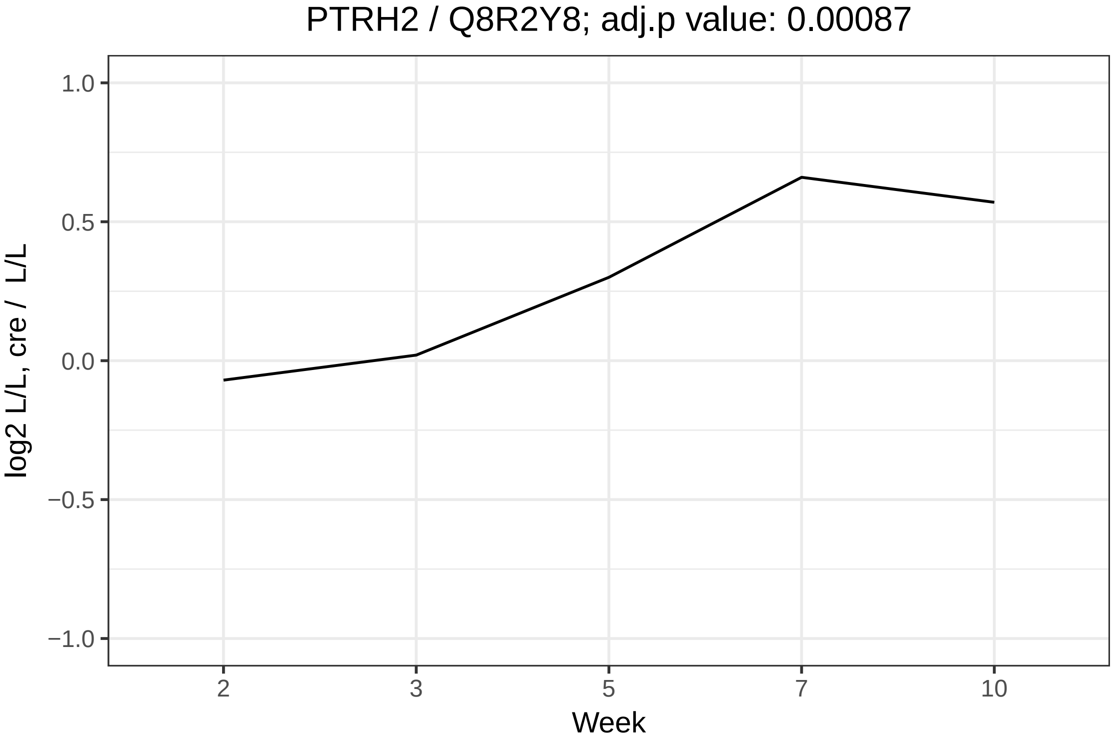
PTRH2 / Q8R2Y8; adj.p value: 0.00087
1.0
0.5
0.0
−0.5
−1.0
log2 L/L, cre / L/L
2
3
5
7
10
Week

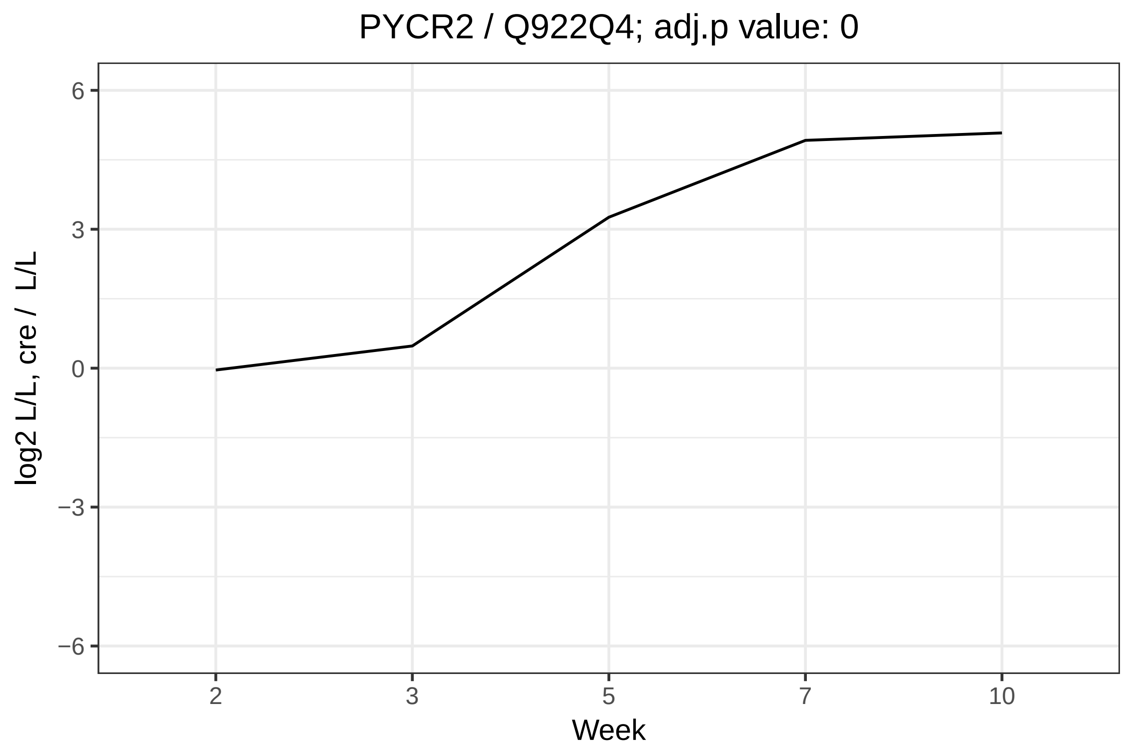

PYCR2 / Q922Q4; adj.p value: 0
log2 L/L, cre / L/L
6
3
0
−3
−6
2
3
5
7
10
Week

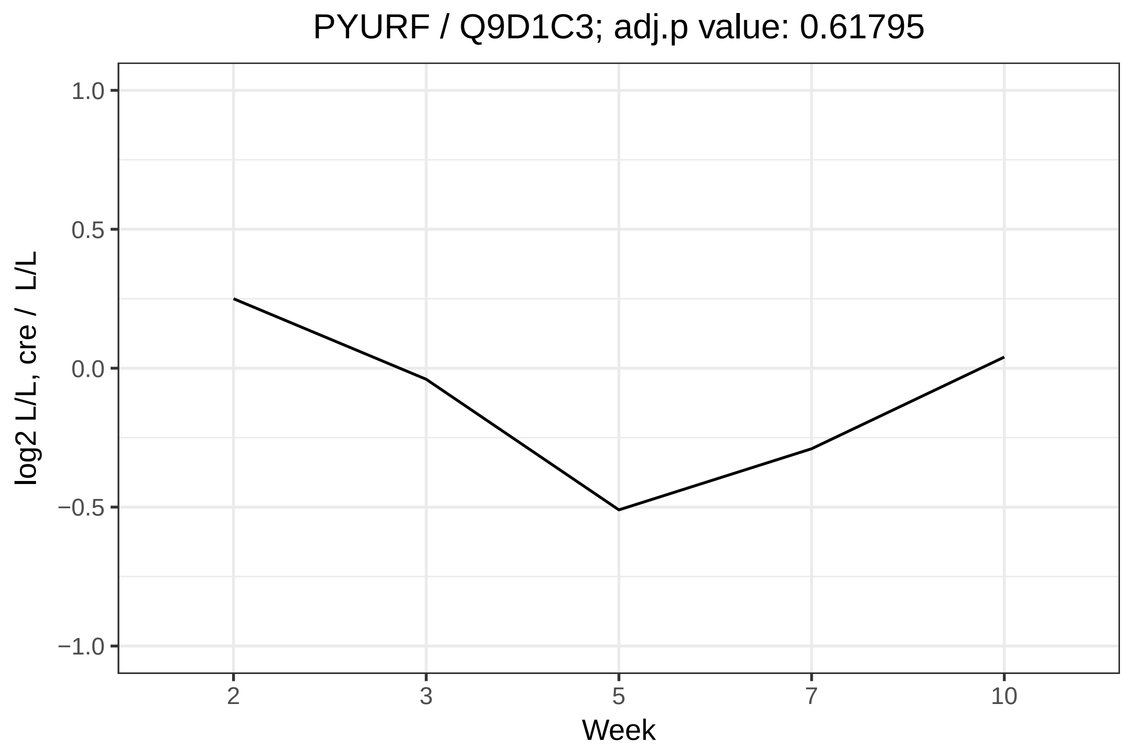
PYURF / Q9D1C3; adj.p value: 0.61795
log2 L/L, cre / L/L
1.0
0.5
0.0
−0.5
−1.0
2
3
5
7
10
Week

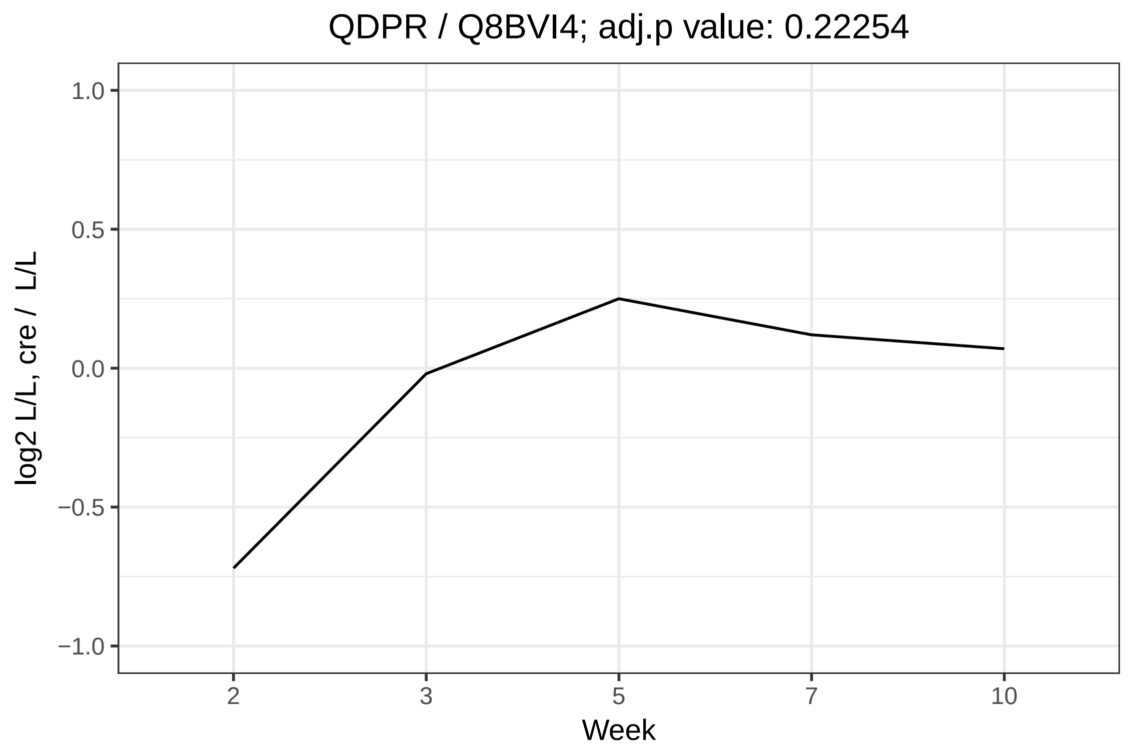
QDPR / Q8BVI4; adj.p value: 0.22254
log2 L/L, cre / L/L
1.0
0.5
0.0
−0.5
−1.0
2
3
5
7
10
Week

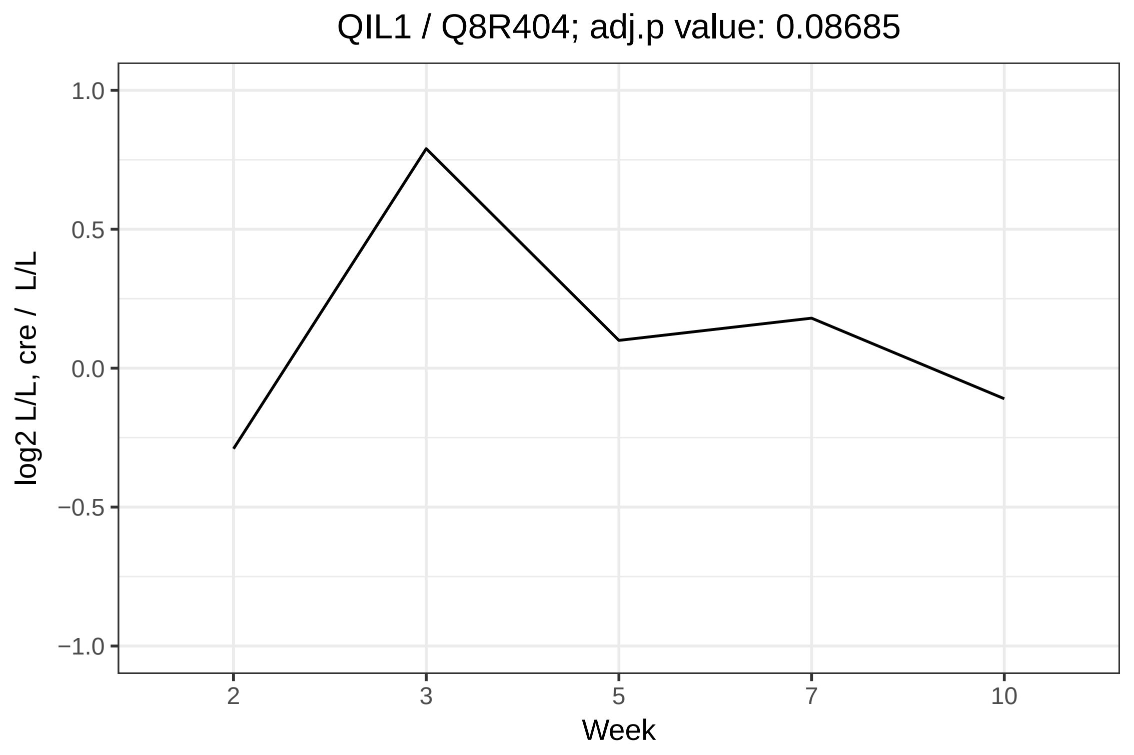

QIL1 / Q8R404; adj.p value: 0.08685
1.0
0.5
0.0
−0.5
−1.0
log2 L/L, cre / L/L
2
3
5
7
10
Week

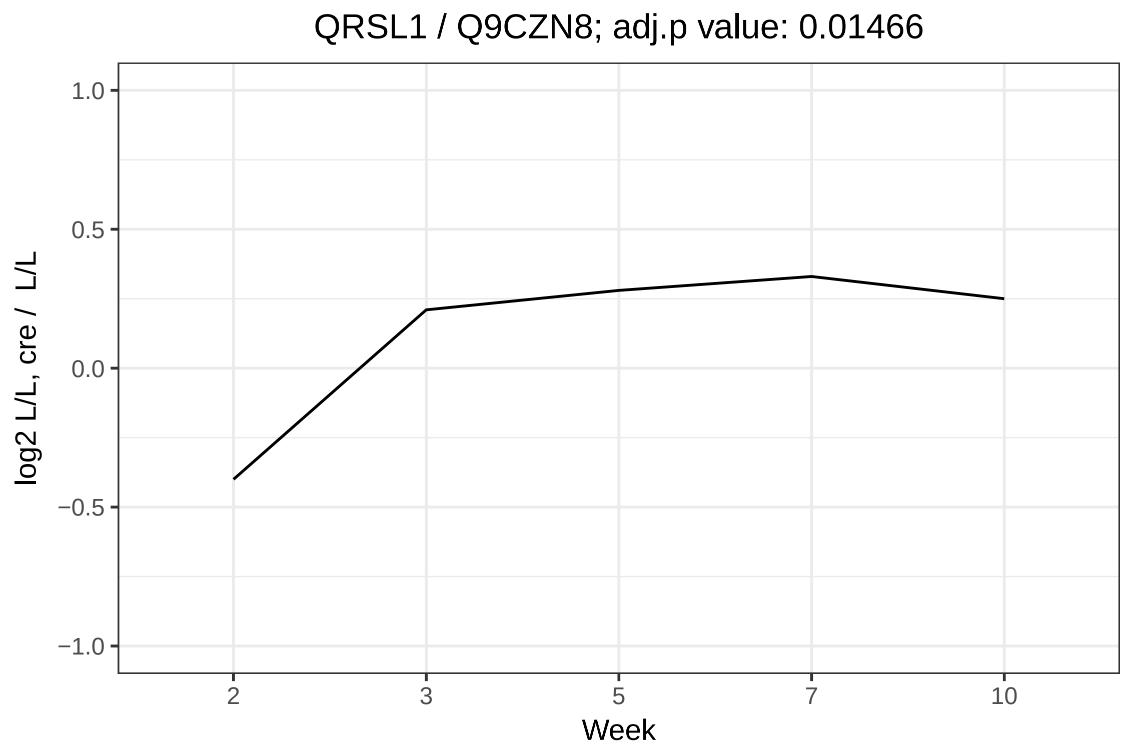
QRSL1 / Q9CZN8; adj.p value: 0.01466
log2 L/L, cre / L/L
1.0
0.5
0.0
−0.5
−1.0
2
3
5
7
10
Week

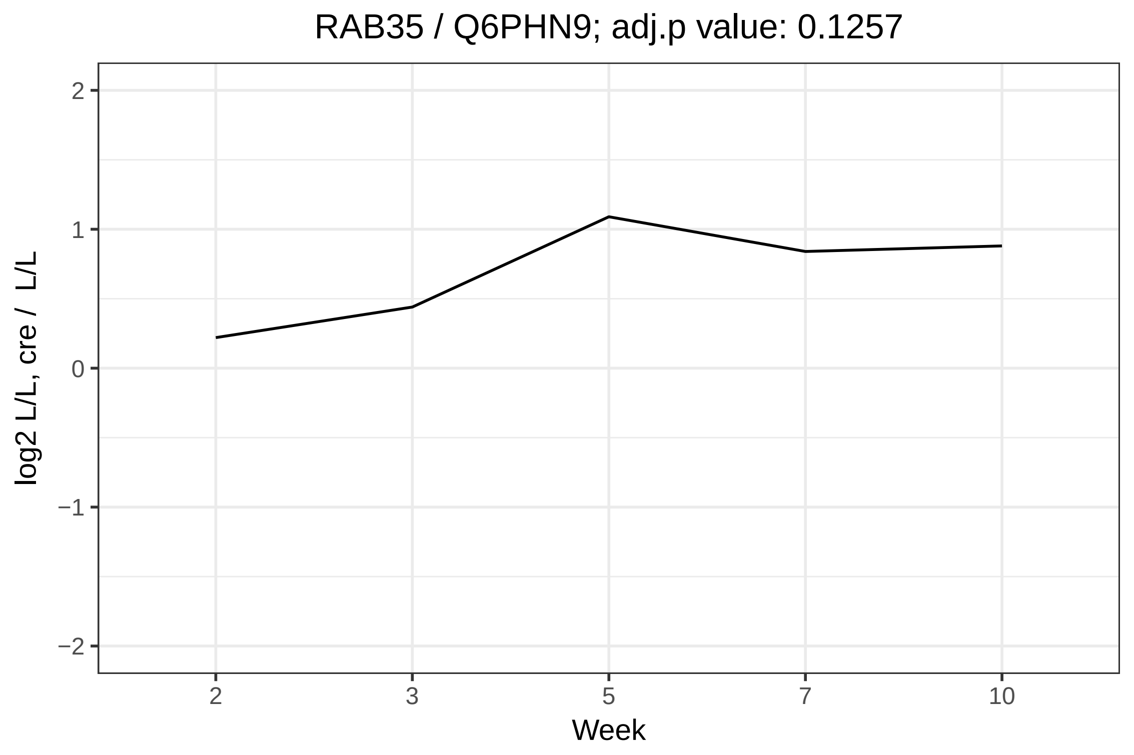
RAB35 / Q6PHN9; adj.p value: 0.1257
log2 L/L, cre / L/L
2
1
0
−1
−2
2
3
5
7
10
Week

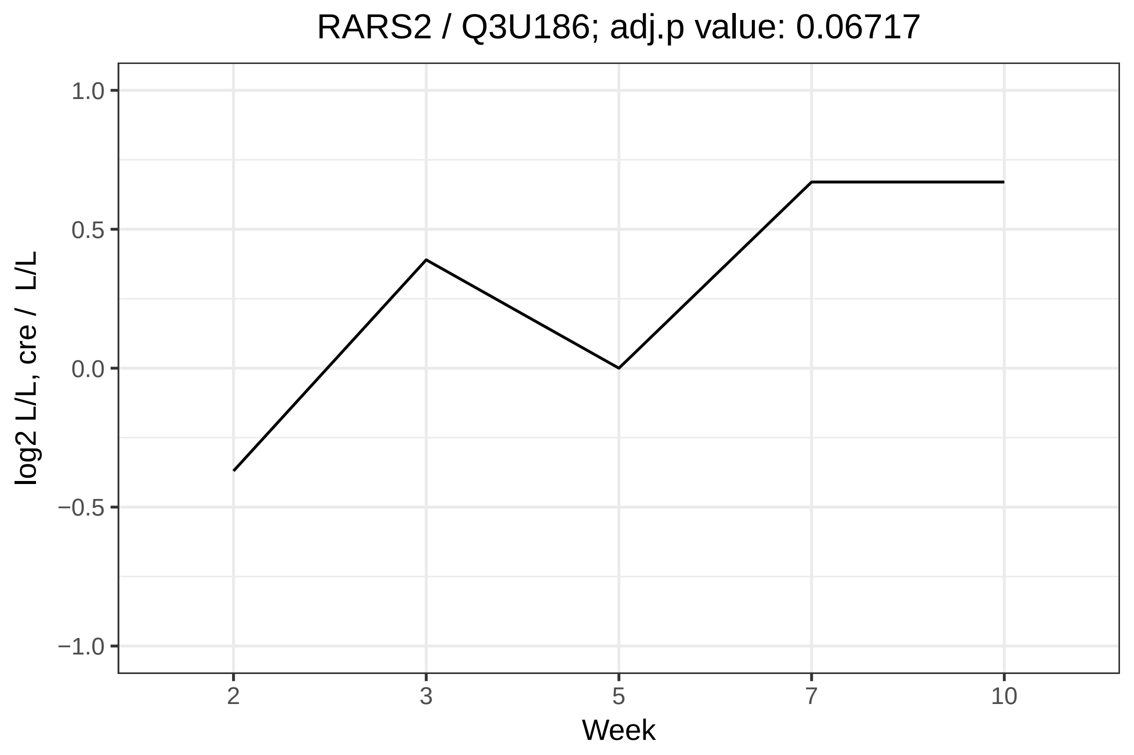

RARS2 / Q3U186; adj.p value: 0.06717
1.0
0.5
0.0
−0.5
−1.0
log2 L/L, cre / L/L
2
3
5
7
10
Week

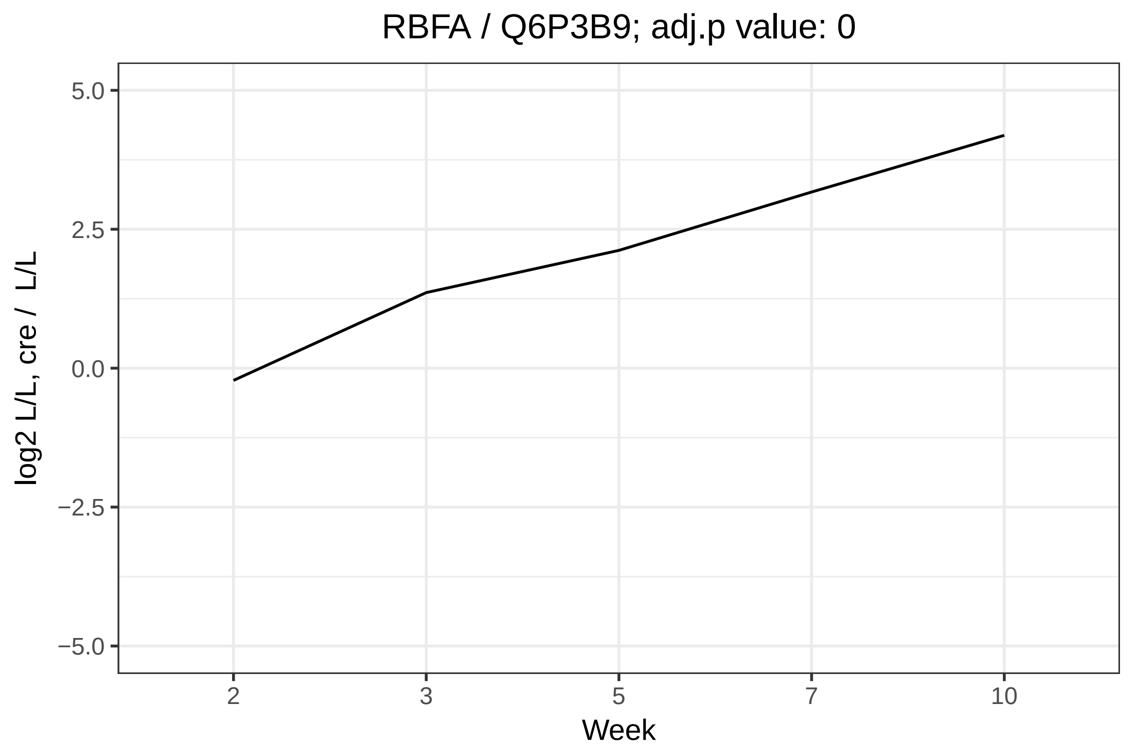
RBFA / Q6P3B9; adj.p value: 0
5.0
2.5
0.0
−2.5
−5.0
log2 L/L, cre / L/L
2
3
5
7
10
Week

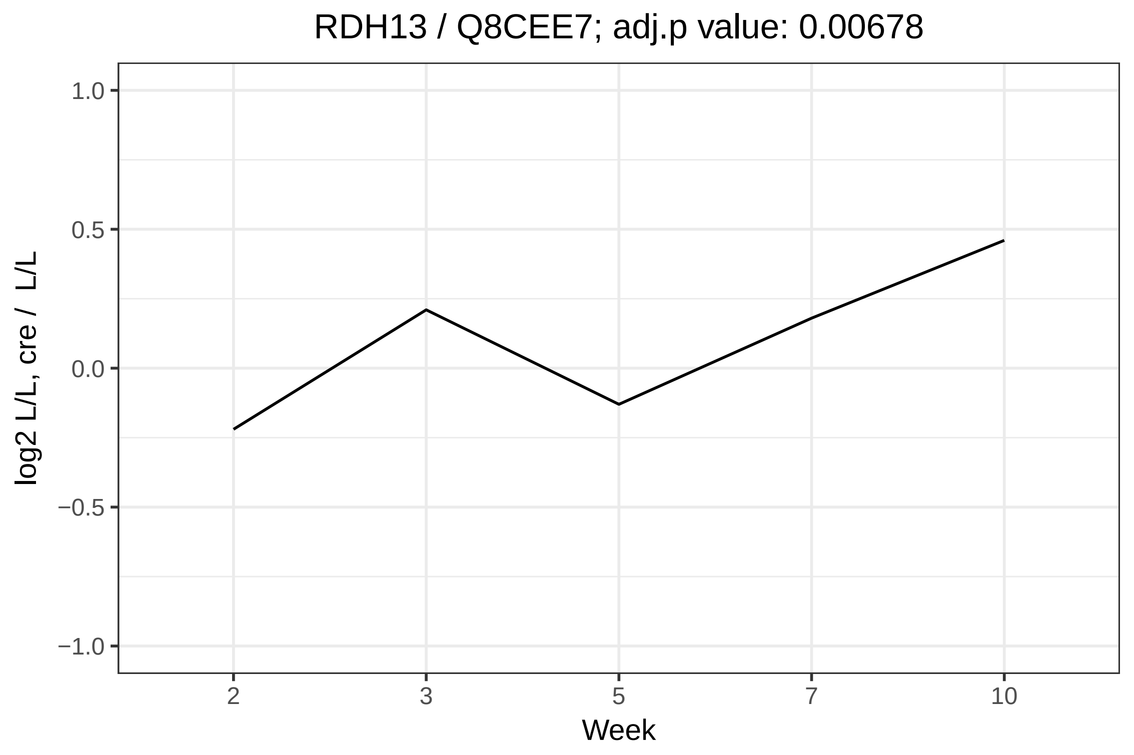
RDH13 / Q8CEE7; adj.p value: 0.00678
1.0
0.5
0.0
−0.5
−1.0
log2 L/L, cre / L/L
2
3
5
7
10
Week

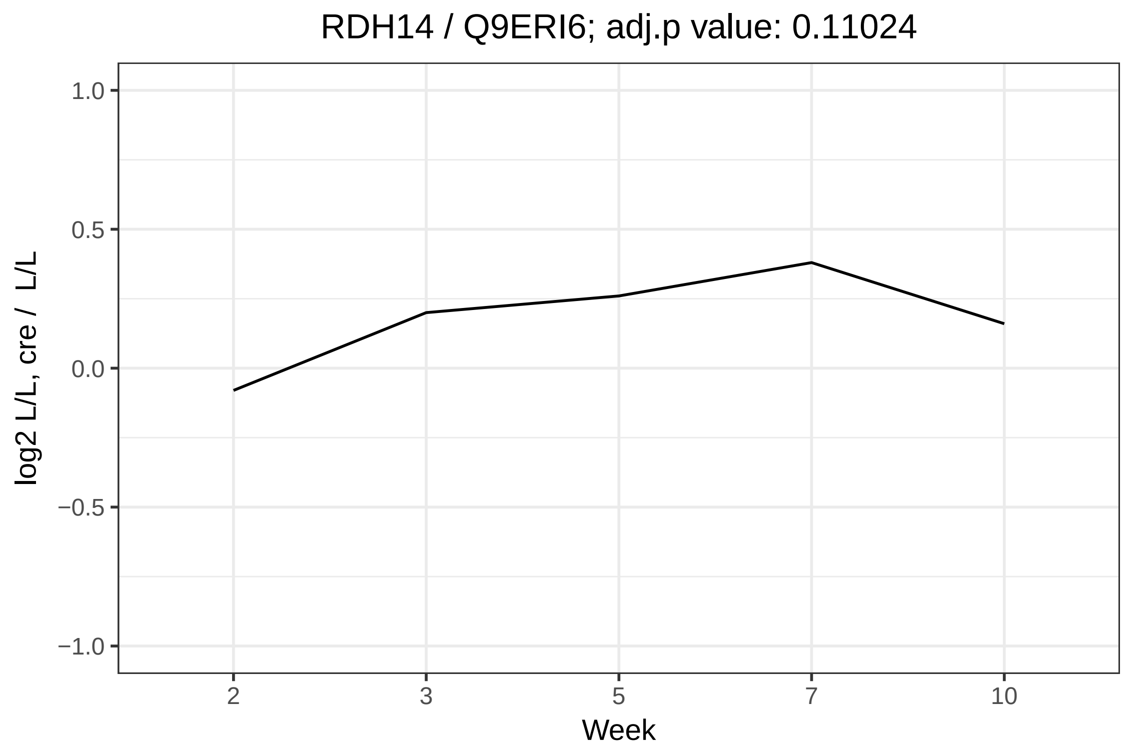
RDH14 / Q9ERI6; adj.p value: 0.11024
1.0
0.5
0.0
−0.5
−1.0
log2 L/L, cre / L/L
2
3
5
7
10
Week

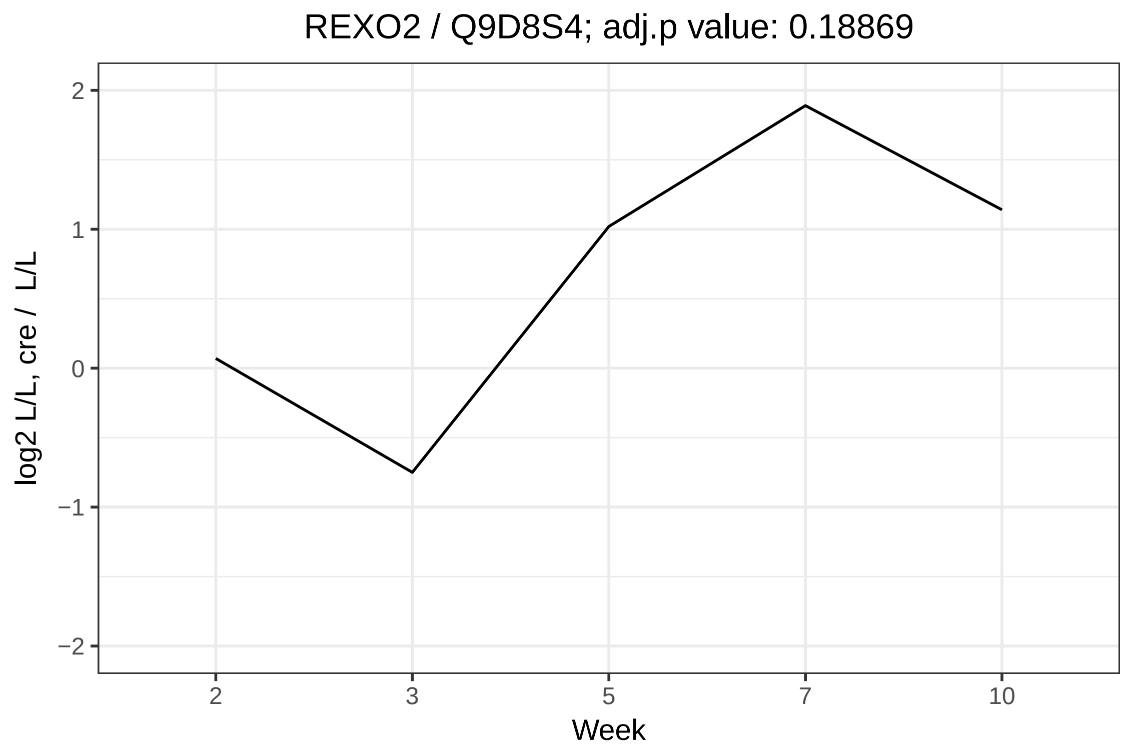
REXO2 / Q9D8S4; adj.p value: 0.18869
log2 L/L, cre / L/L
2
1
0
−1
−2
2
3
5
7
10
Week

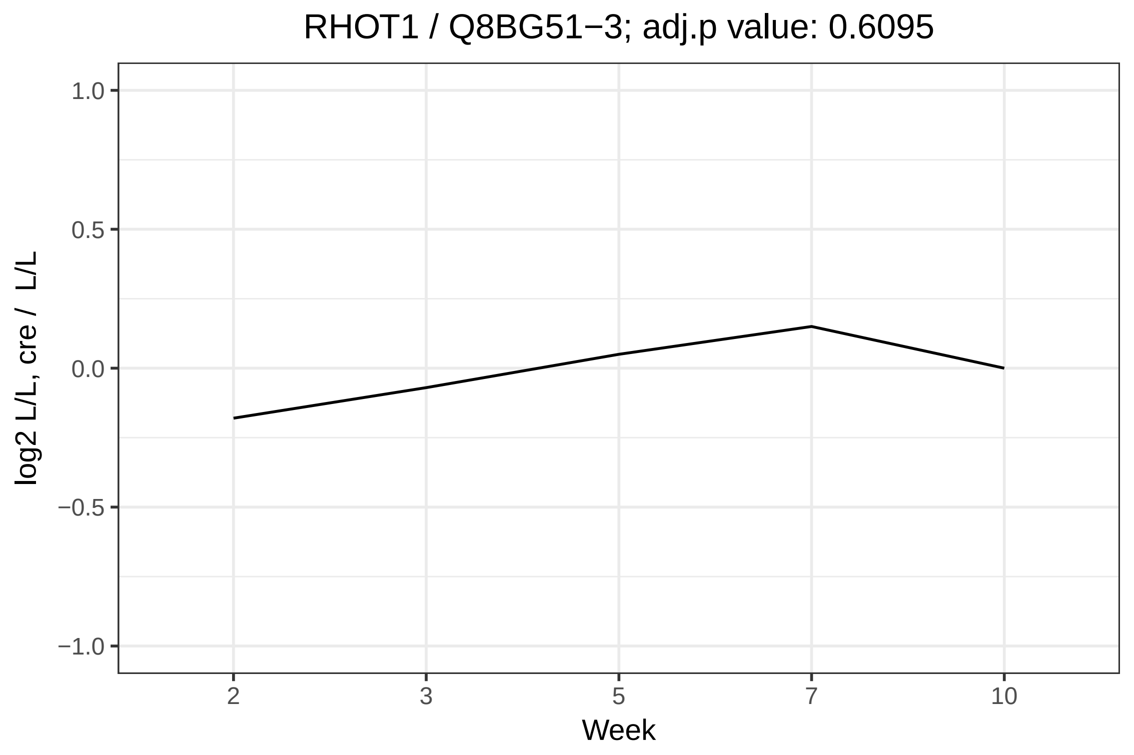

RHOT1 / Q8BG51−3; adj.p value: 0.6095
log2 L/L, cre / L/L
1.0
0.5
0.0
−0.5
−1.0
2
3
5
7
10
Week

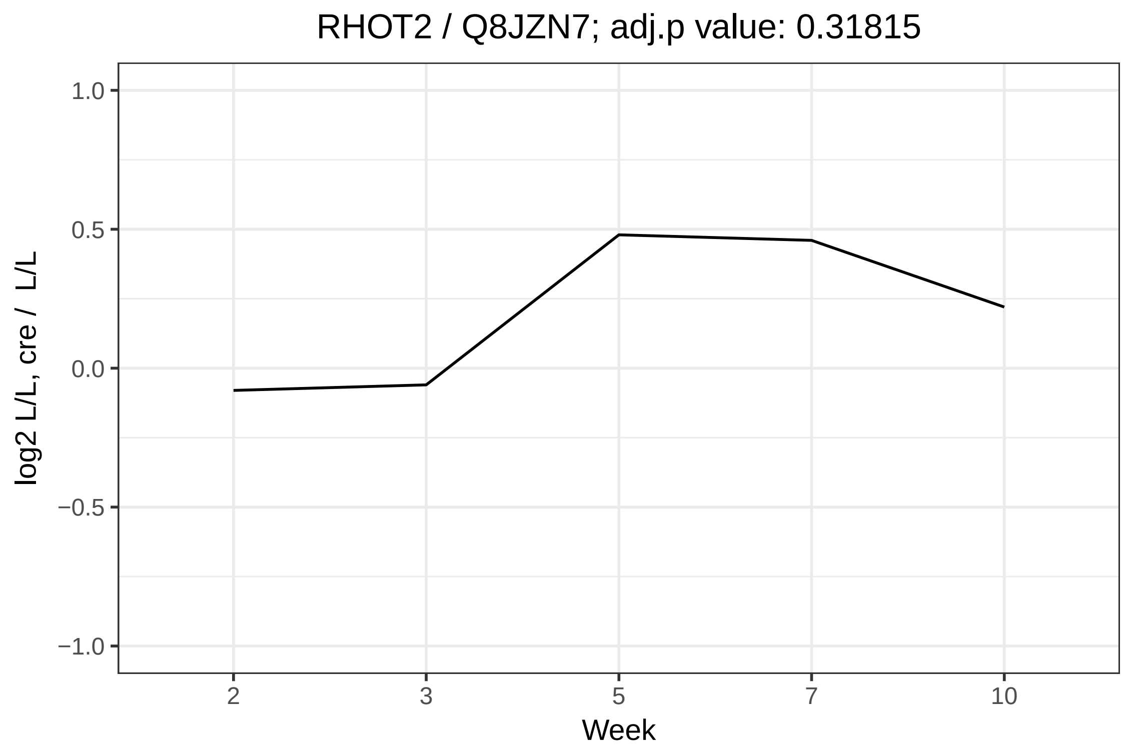
RHOT2 / Q8JZN7; adj.p value: 0.31815
log2 L/L, cre / L/L
1.0
0.5
0.0
−0.5
−1.0
2
3
5
7
10
Week

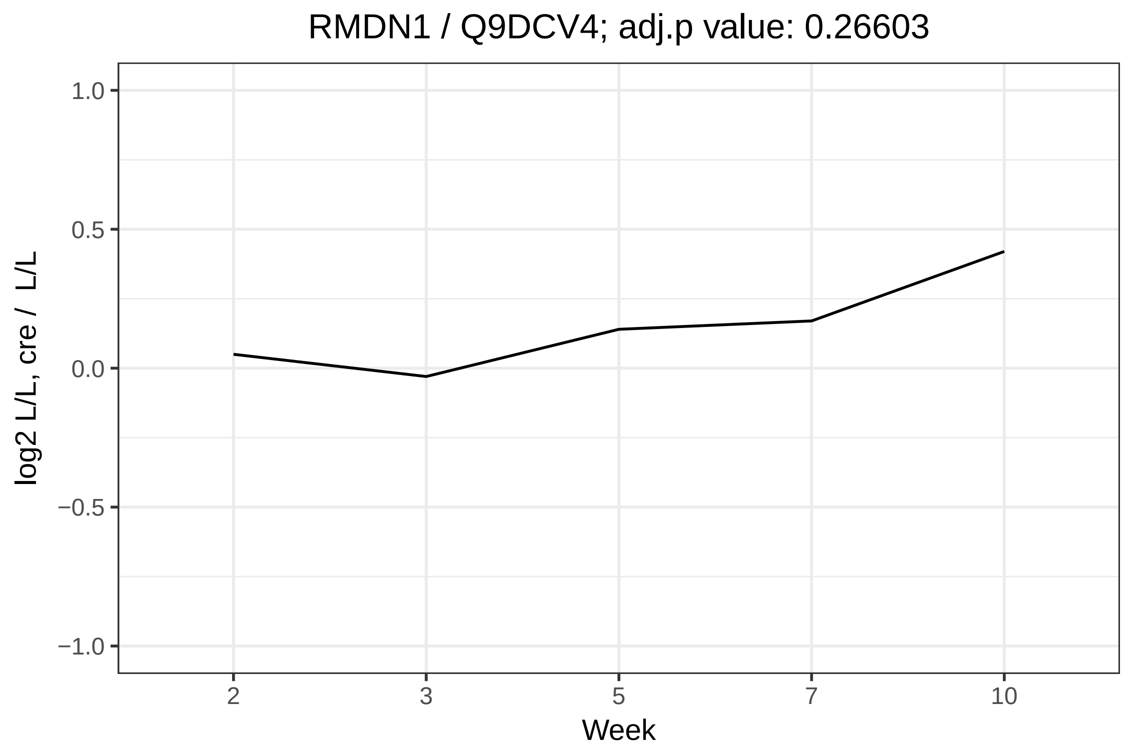

RMDN1 / Q9DCV4; adj.p value: 0.26603
log2 L/L, cre / L/L
1.0
0.5
0.0
−0.5
−1.0
2
3
5
7
10
Week

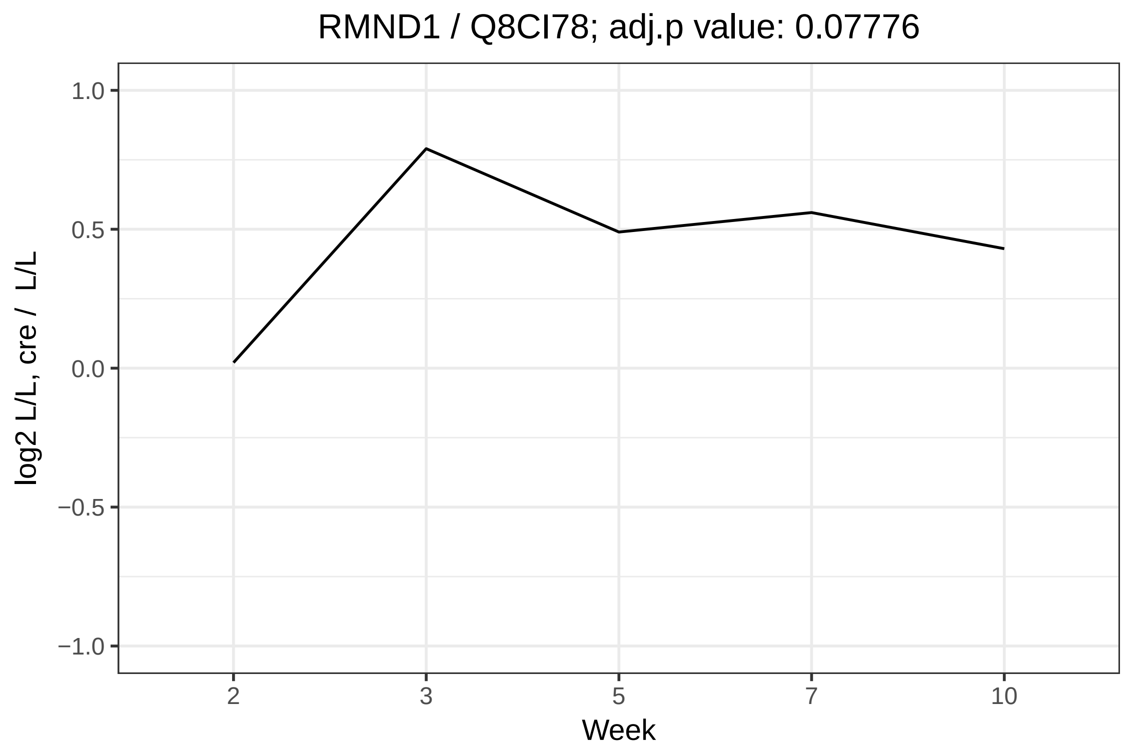

RMND1 / Q8CI78; adj.p value: 0.07776
1.0
0.5
0.0
−0.5
−1.0
log2 L/L, cre / L/L
2
3
5
7
10
Week

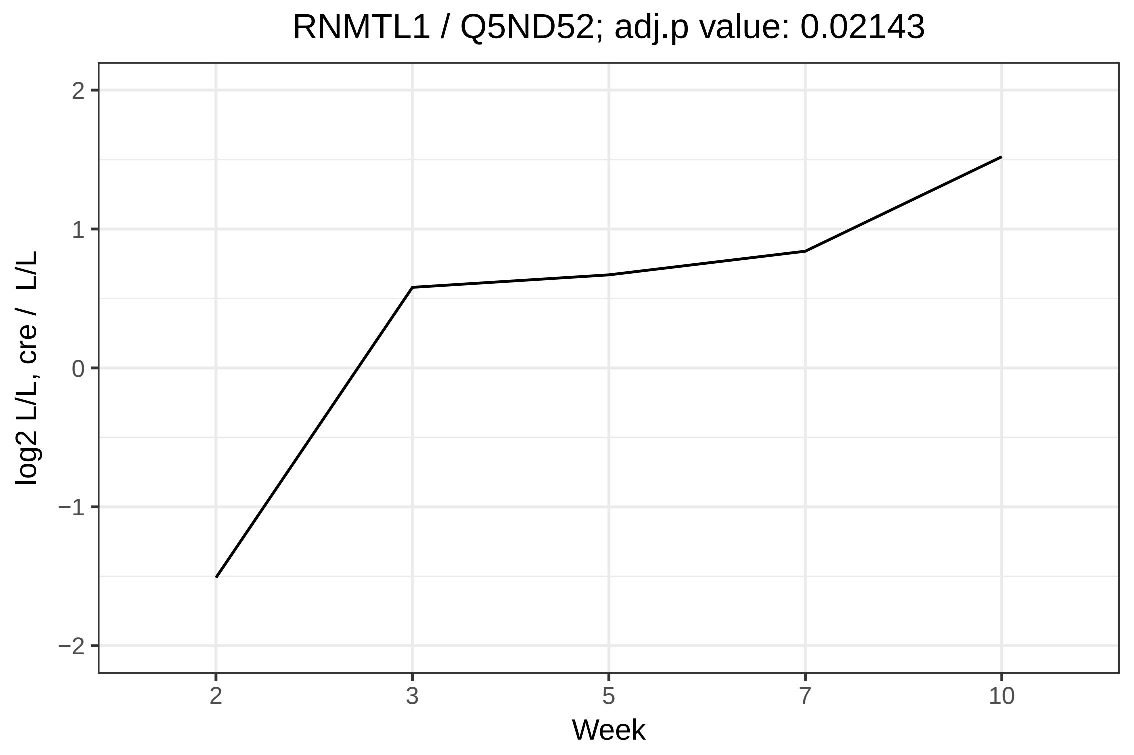
RNMTL1 / Q5ND52; adj.p value: 0.02143
log2 L/L, cre / L/L
2
1
0
−1
−2
2
3
5
7
10
Week

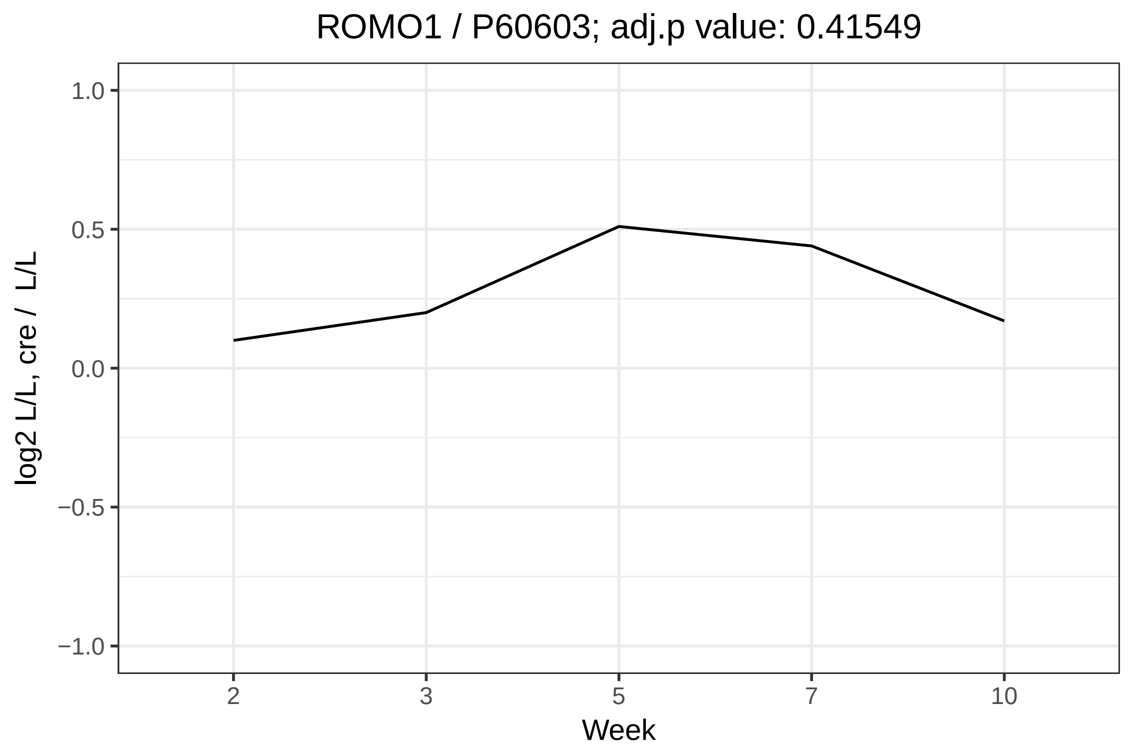

ROMO1 / P60603; adj.p value: 0.41549
log2 L/L, cre / L/L
1.0
0.5
0.0
−0.5
−1.0
2
3
5
7
10
Week

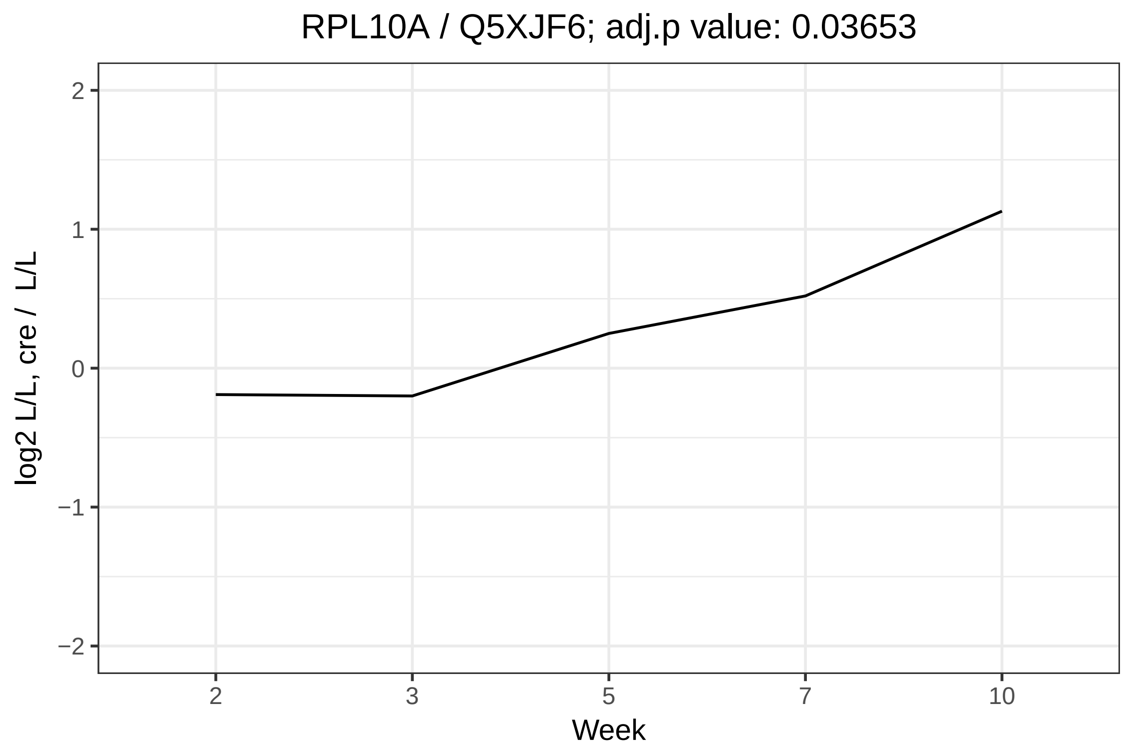
RPL10A / Q5XJF6; adj.p value: 0.03653
log2 L/L, cre / L/L
2
1
0
−1
−2
2
3
5
7
10
Week

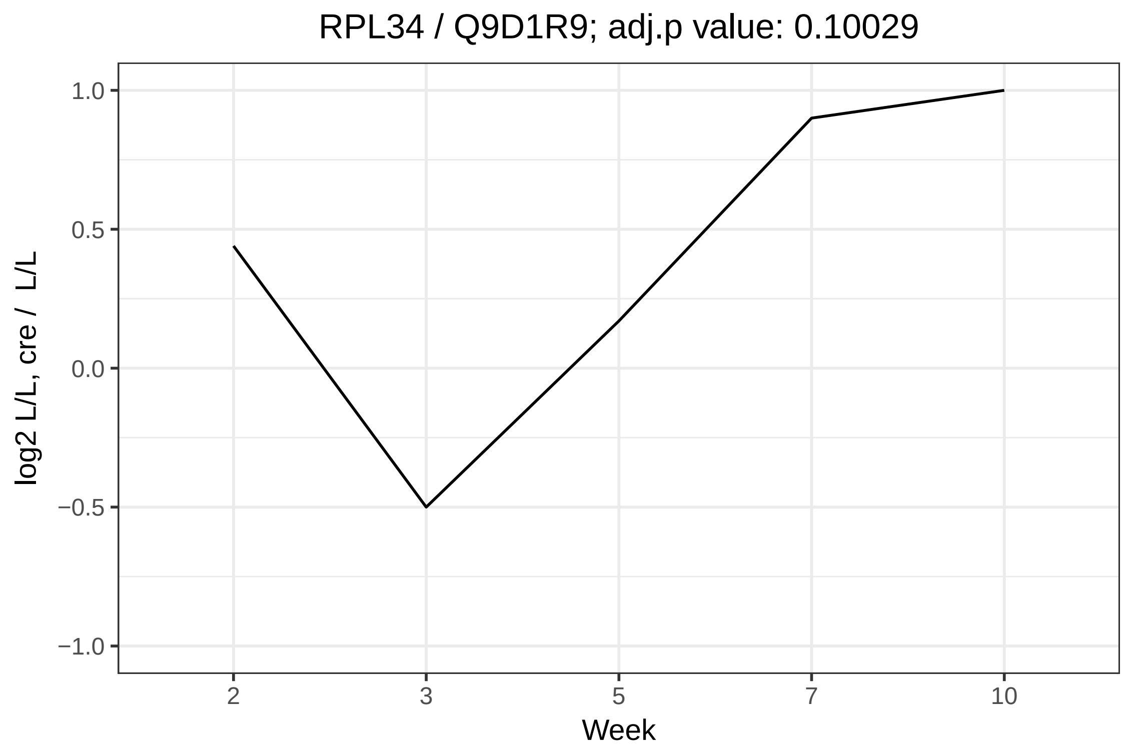

RPL34 / Q9D1R9; adj.p value: 0.10029
log2 L/L, cre / L/L
1.0
0.5
0.0
−0.5
−1.0
2
3
5
7
10
Week

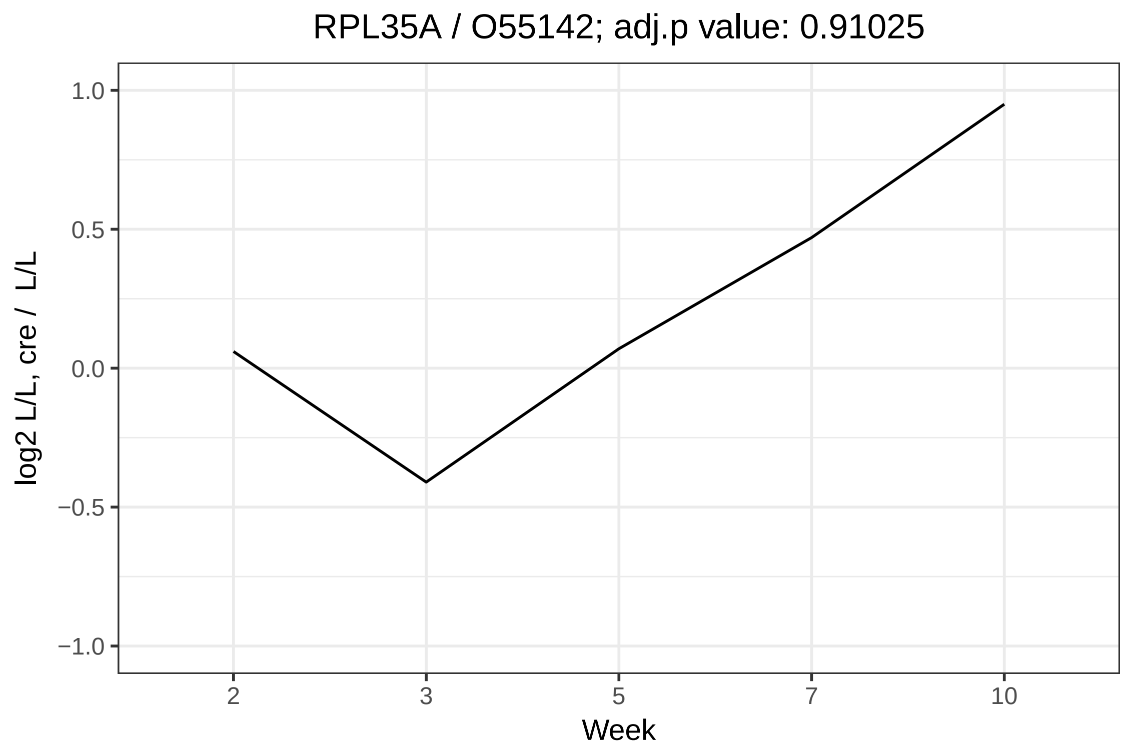
RPL35A / O55142; adj.p value: 0.91025
1.0
0.5
0.0
−0.5
−1.0
log2 L/L, cre / L/L
2
3
5
7
10
Week

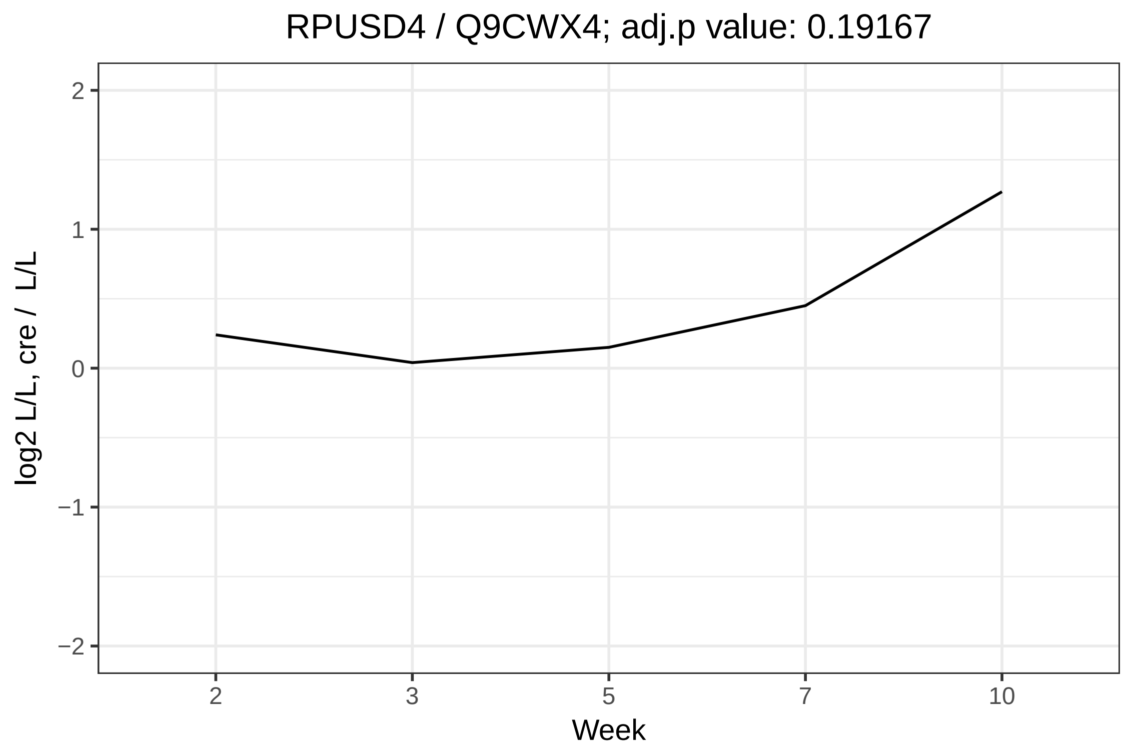

RPUSD4 / Q9CWX4; adj.p value: 0.19167
2
1
0
−1
−2
log2 L/L, cre / L/L
2
3
5
7
10
Week

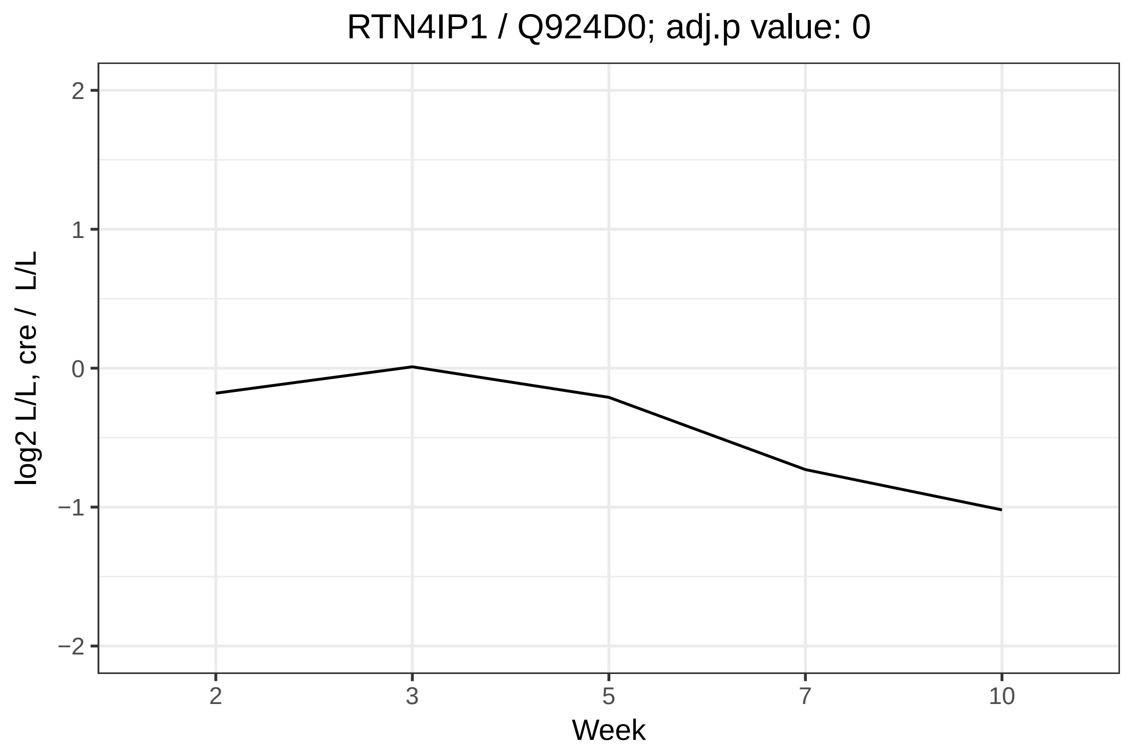
RTN4IP1 / Q924D0; adj.p value: 0
2
1
0
−1
−2
log2 L/L, cre / L/L
2
3
5
7
10
Week

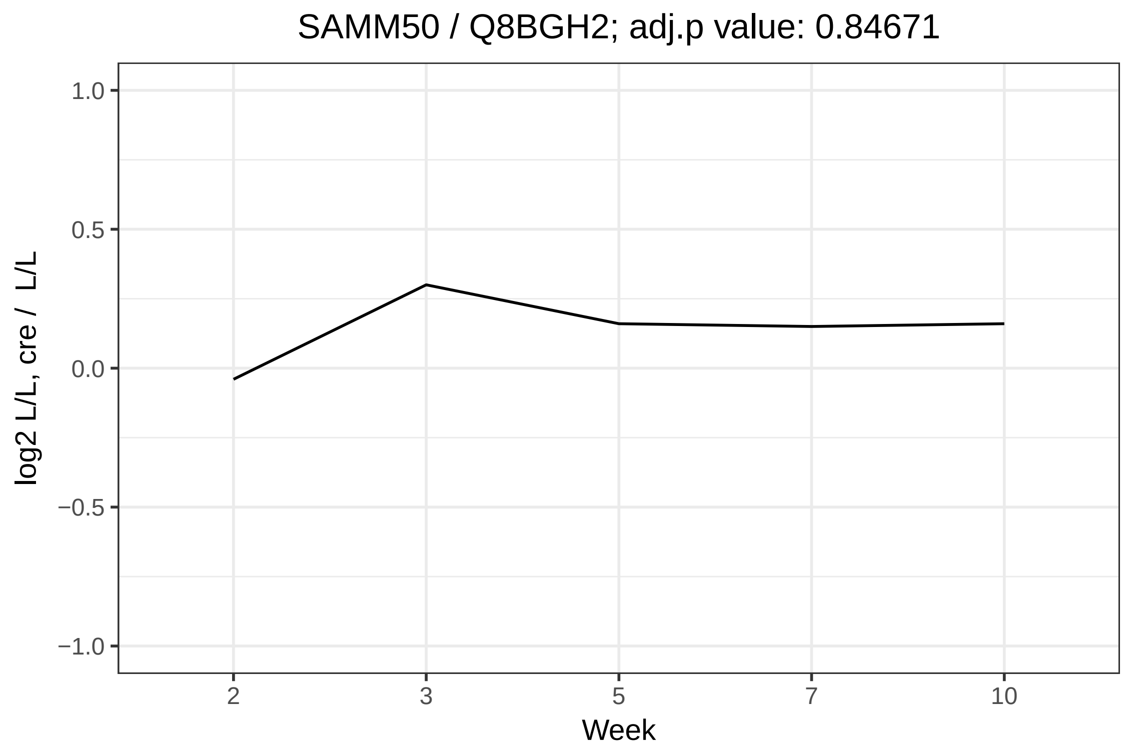
SAMM50 / Q8BGH2; adj.p value: 0.84671
log2 L/L, cre / L/L
1.0
0.5
0.0
−0.5
−1.0
2
3
5
7
10
Week

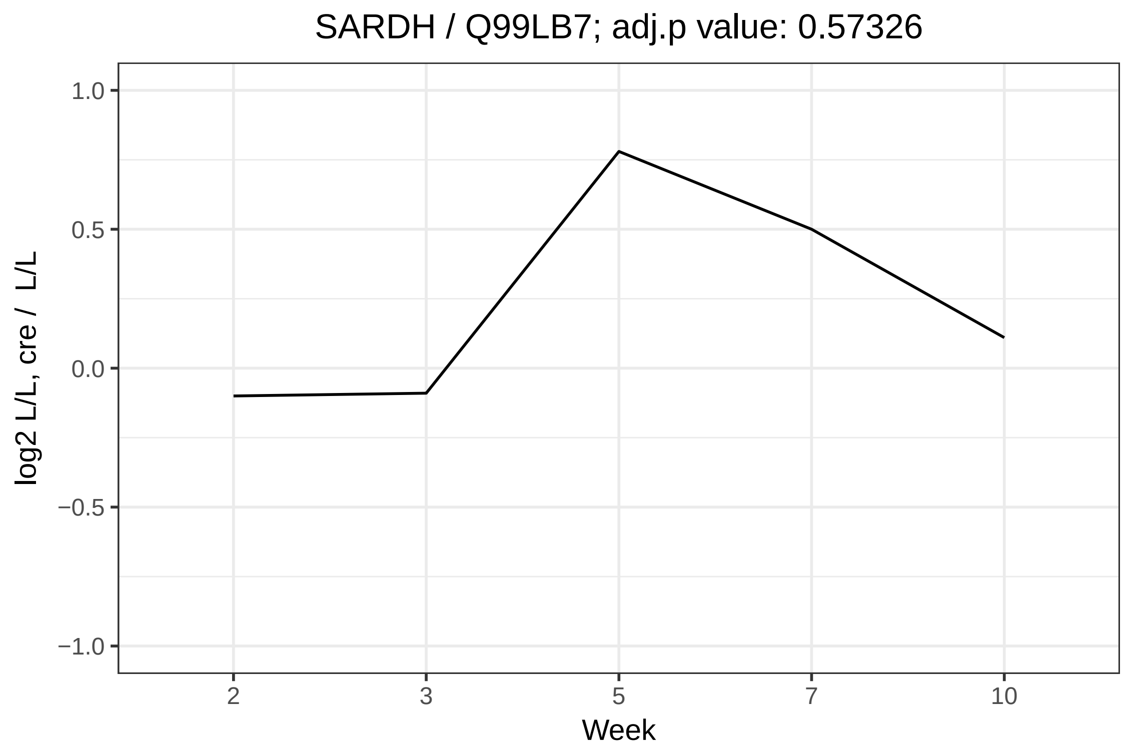
SARDH / Q99LB7; adj.p value: 0.57326
log2 L/L, cre / L/L
1.0
0.5
0.0
−0.5
−1.0
2
3
5
7
10
Week

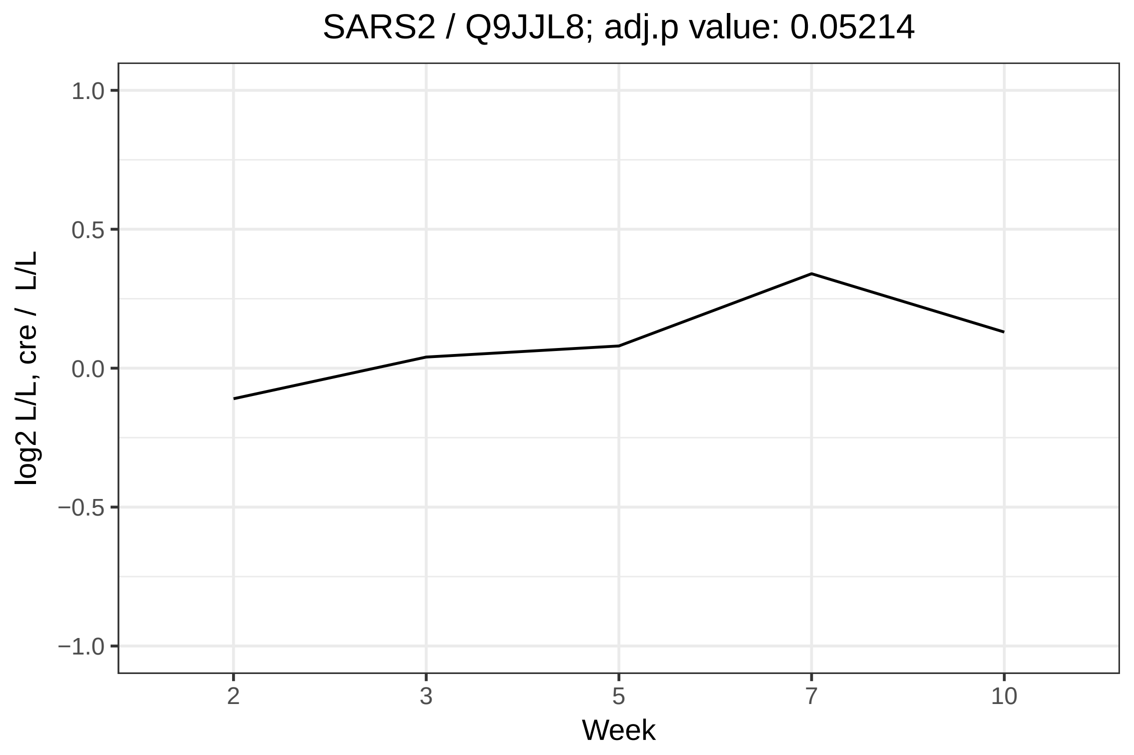

SARS2 / Q9JJL8; adj.p value: 0.05214
log2 L/L, cre / L/L
1.0
0.5
0.0
−0.5
−1.0
2
3
5
7
10
Week

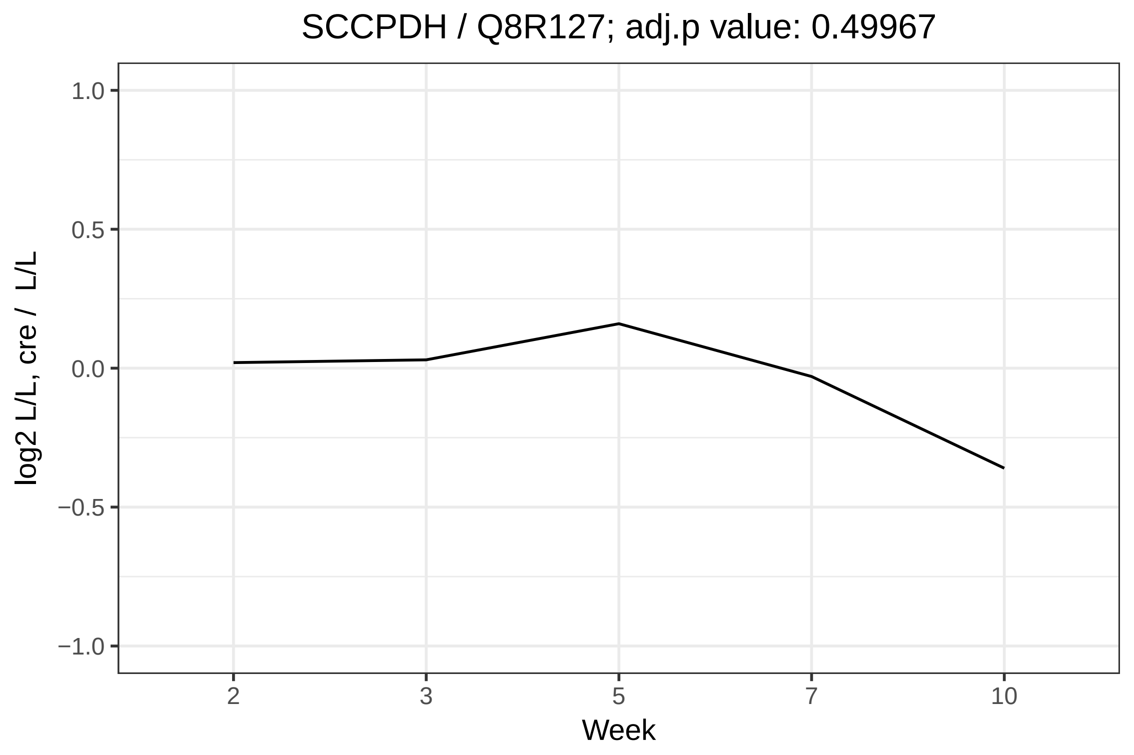

SCCPDH / Q8R127; adj.p value: 0.49967
log2 L/L, cre / L/L
1.0
0.5
0.0
−0.5
−1.0
2
3
5
7
10
Week

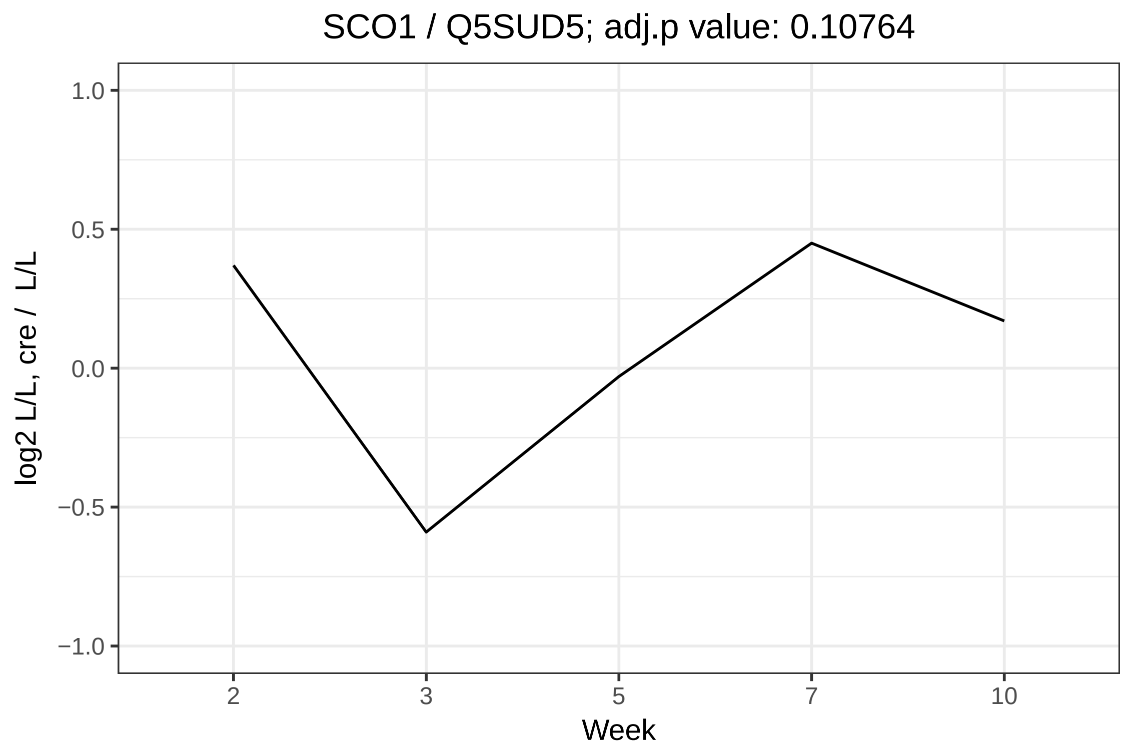
SCO1 / Q5SUD5; adj.p value: 0.10764
1.0
0.5
0.0
−0.5
−1.0
log2 L/L, cre / L/L
2
3
5
7
10
Week

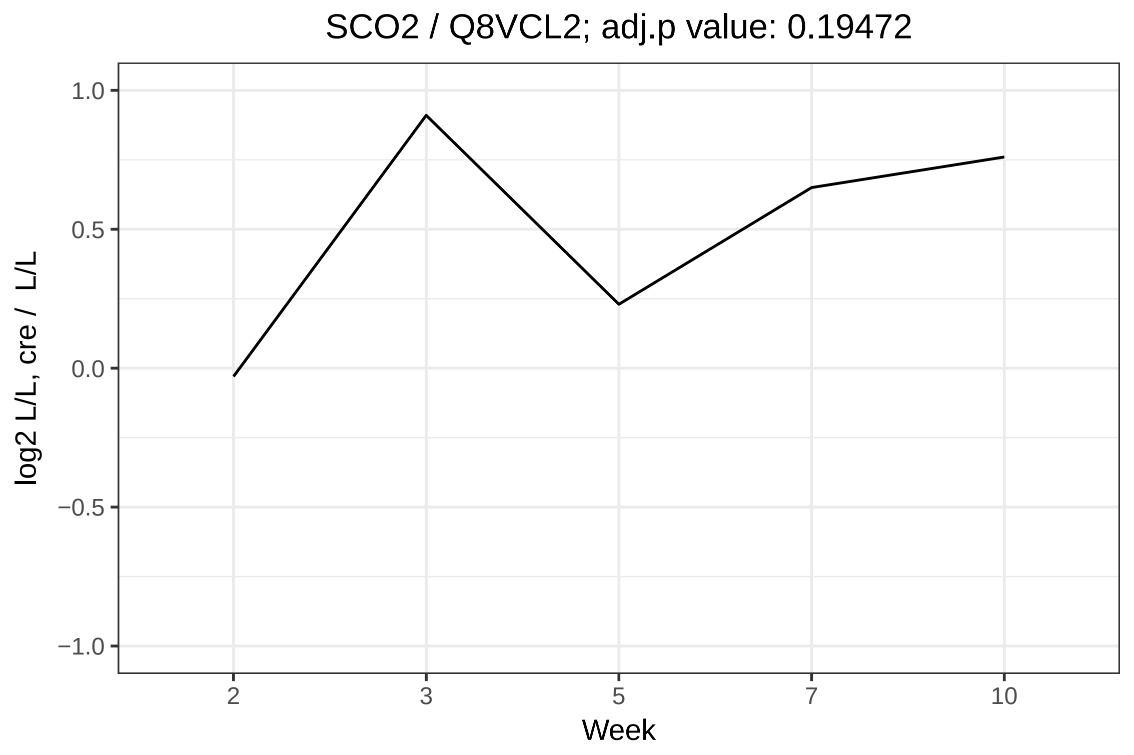
SCO2 / Q8VCL2; adj.p value: 0.19472
1.0
0.5
0.0
−0.5
−1.0
log2 L/L, cre / L/L
2
3
5
7
10
Week

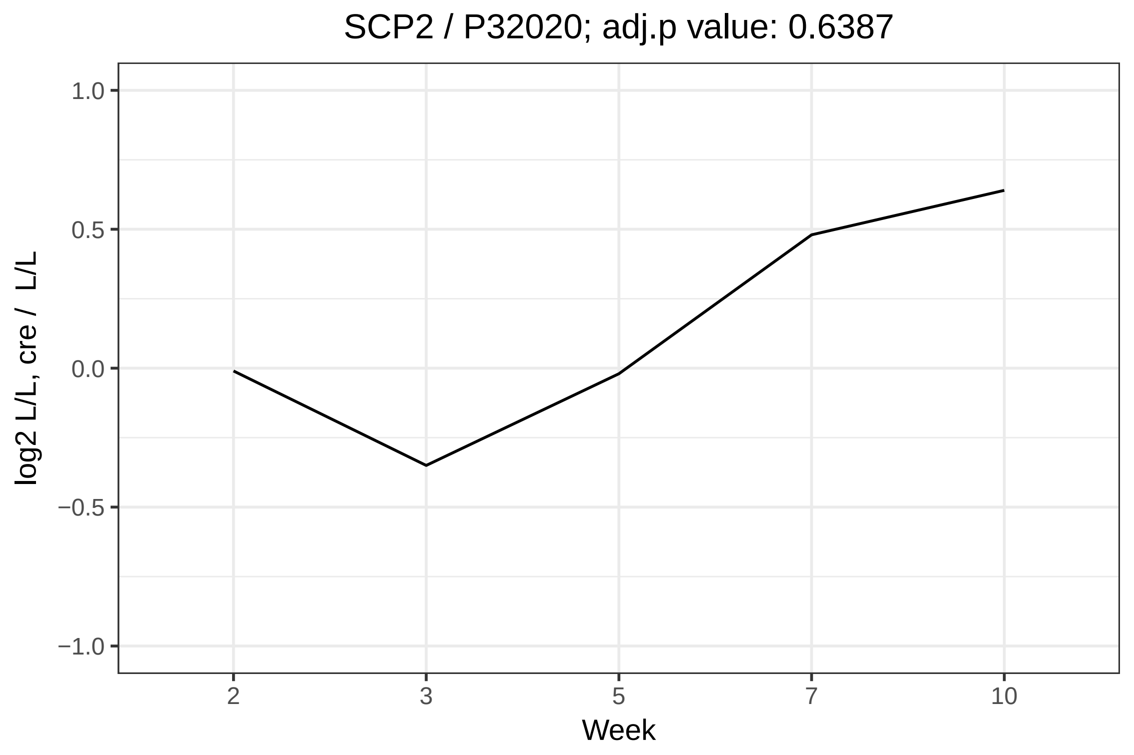
SCP2 / P32020; adj.p value: 0.6387
1.0
0.5
0.0
−0.5
−1.0
log2 L/L, cre / L/L
2
3
5
7
10
Week

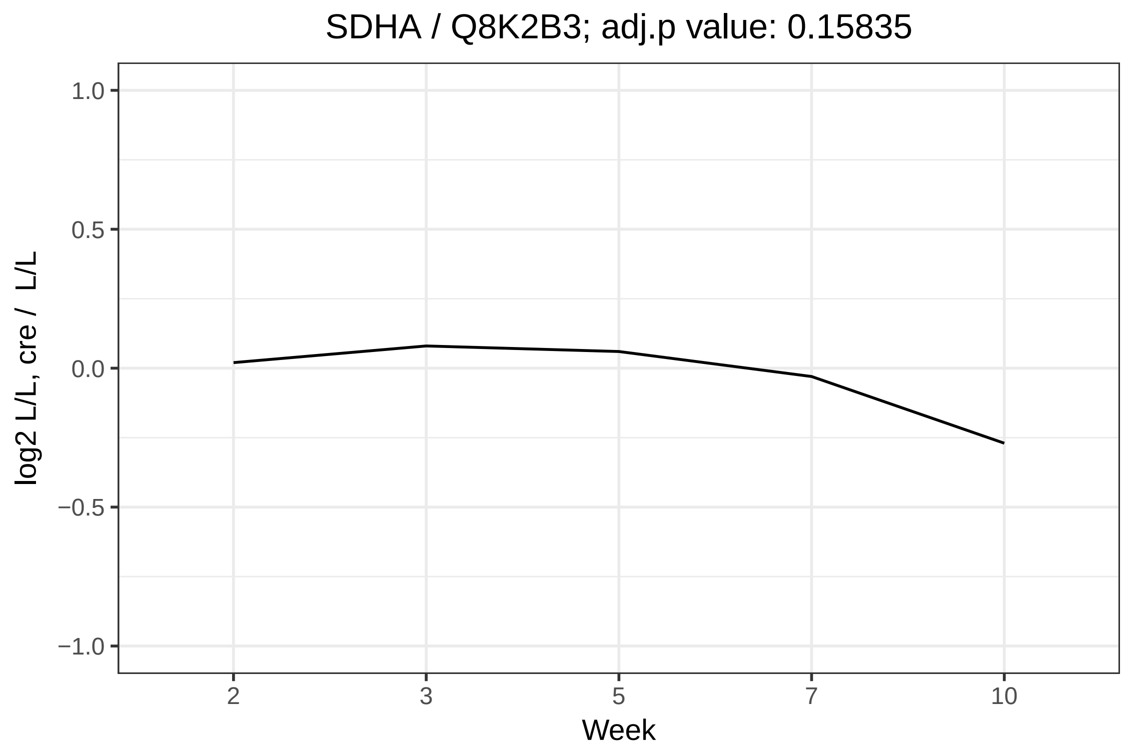

SDHA / Q8K2B3; adj.p value: 0.15835
1.0
0.5
0.0
−0.5
−1.0
log2 L/L, cre / L/L
2
3
5
7
10
Week

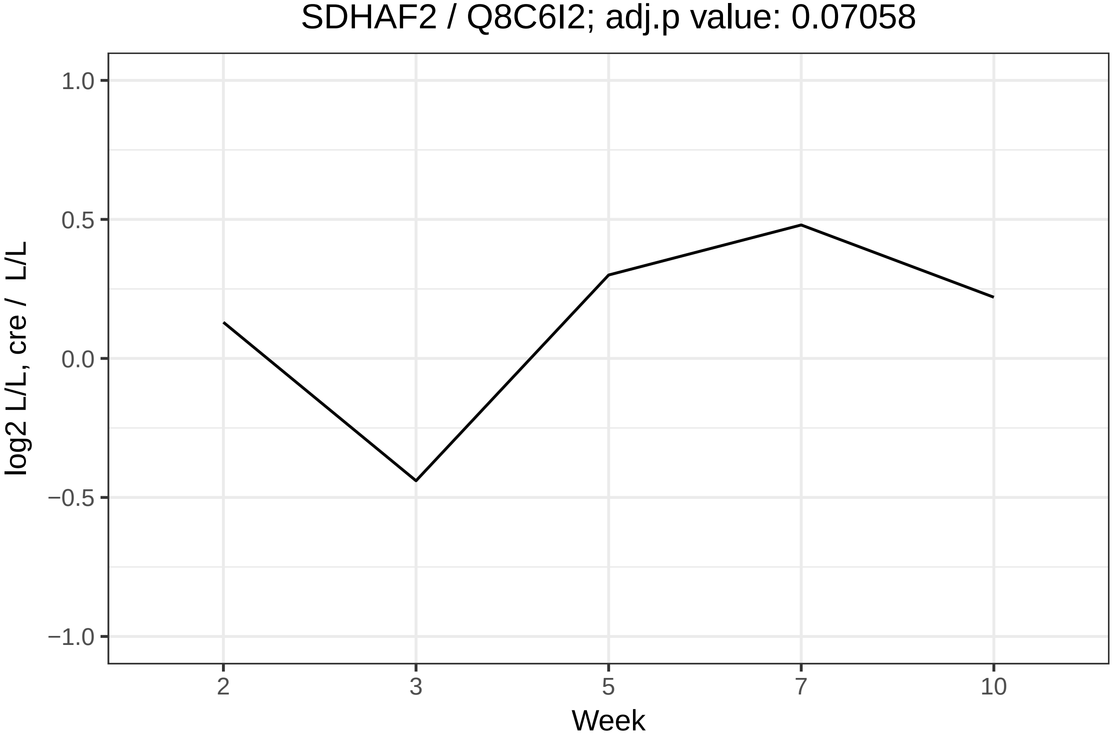

SDHAF2 / Q8C6I2; adj.p value: 0.07058
log2 L/L, cre / L/L
1.0
0.5
0.0
−0.5
−1.0
2
3
5
7
10
Week

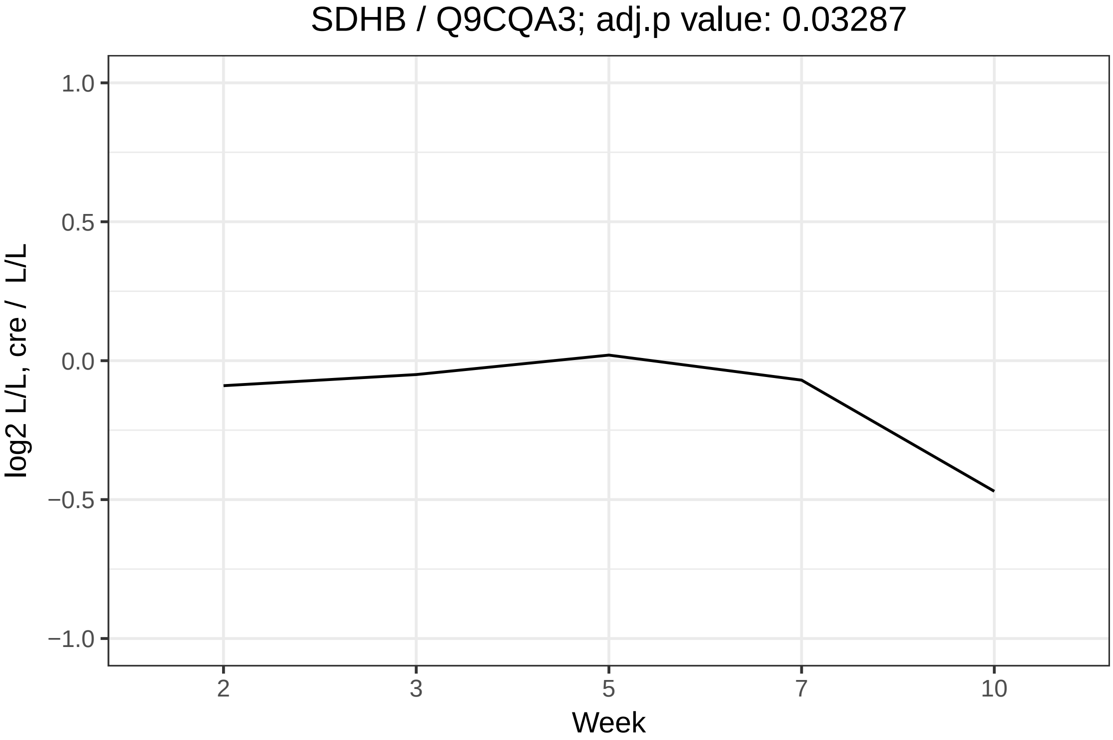
SDHB / Q9CQA3; adj.p value: 0.03287
1.0
0.5
0.0
−0.5
−1.0
log2 L/L, cre / L/L
2
3
5
7
10
Week

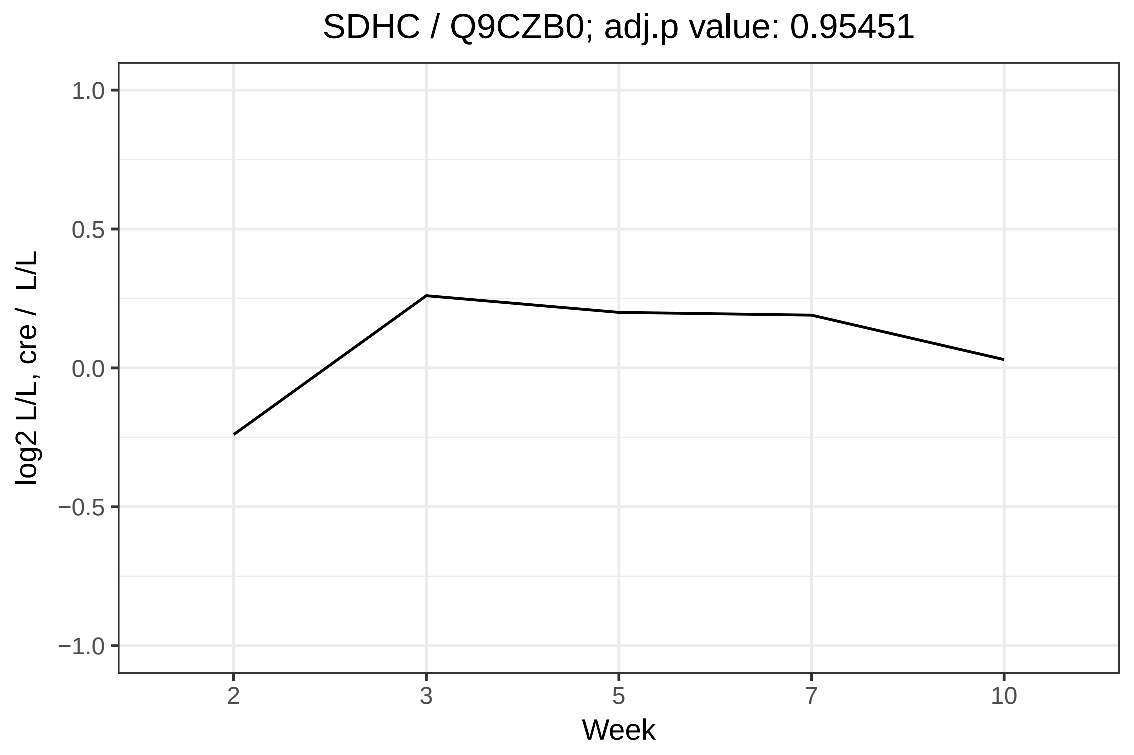

SDHC / Q9CZB0; adj.p value: 0.95451
log2 L/L, cre / L/L
1.0
0.5
0.0
−0.5
−1.0
2
3
5
7
10
Week

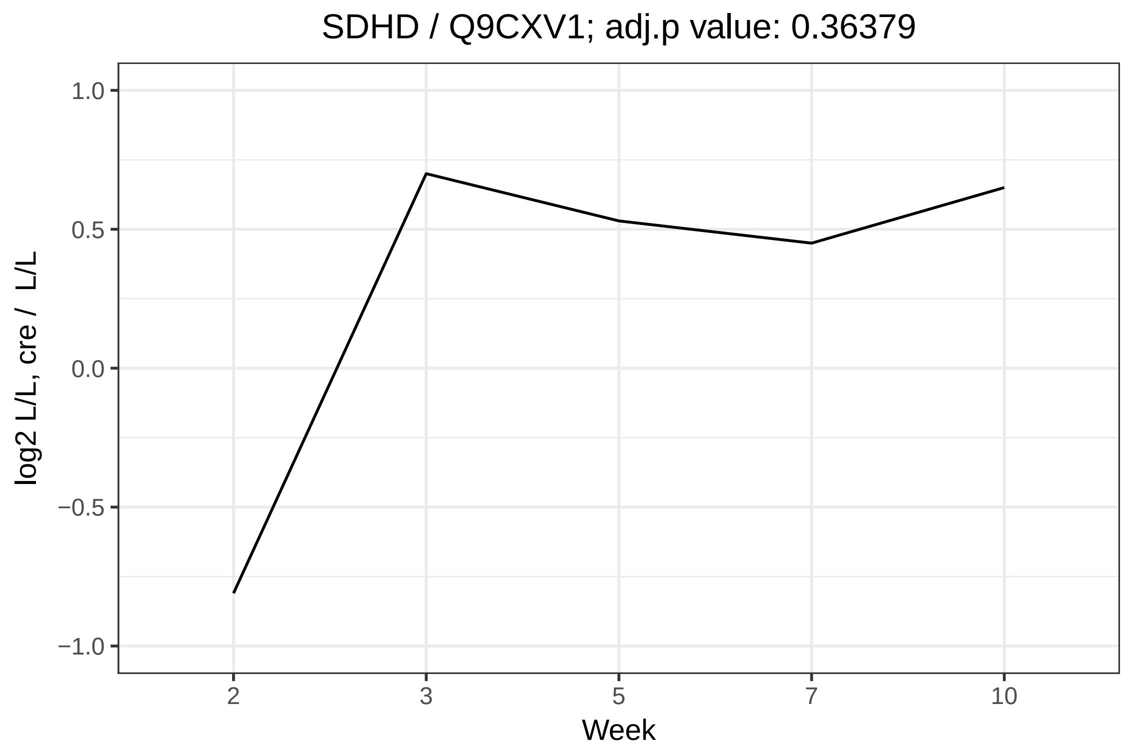

SDHD / Q9CXV1; adj.p value: 0.36379
1.0
0.5
0.0
−0.5
−1.0
log2 L/L, cre / L/L
2
3
5
7
10
Week

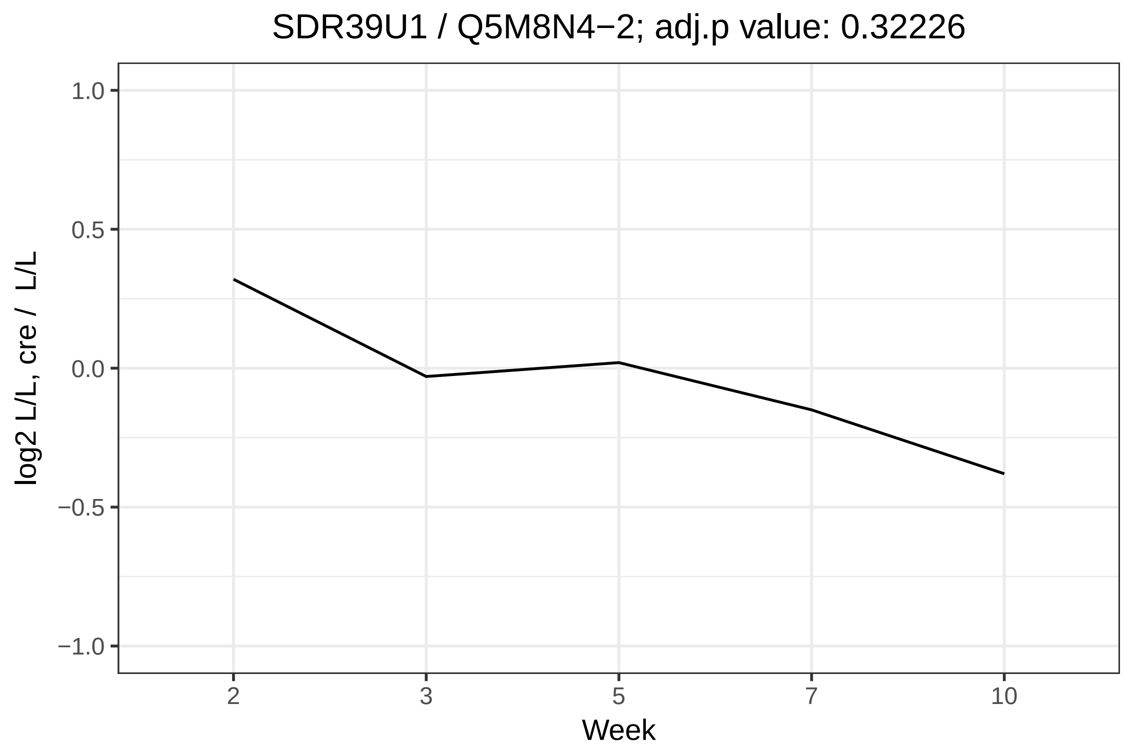

SDR39U1 / Q5M8N4−2; adj.p value: 0.32226
1.0
0.5
0.0
−0.5
−1.0
log2 L/L, cre / L/L
2
3
5
7
10
Week

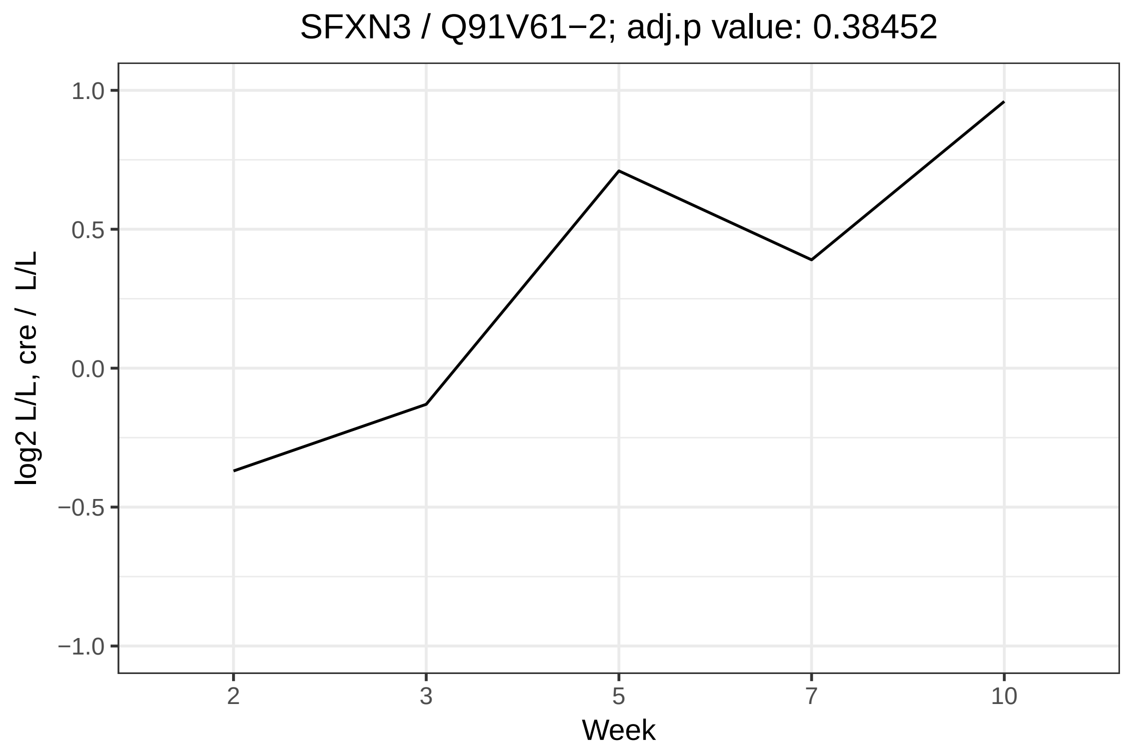
SFXN3 / Q91V61−2; adj.p value: 0.38452
log2 L/L, cre / L/L
1.0
0.5
0.0
−0.5
−1.0
2
3
5
7
10
Week

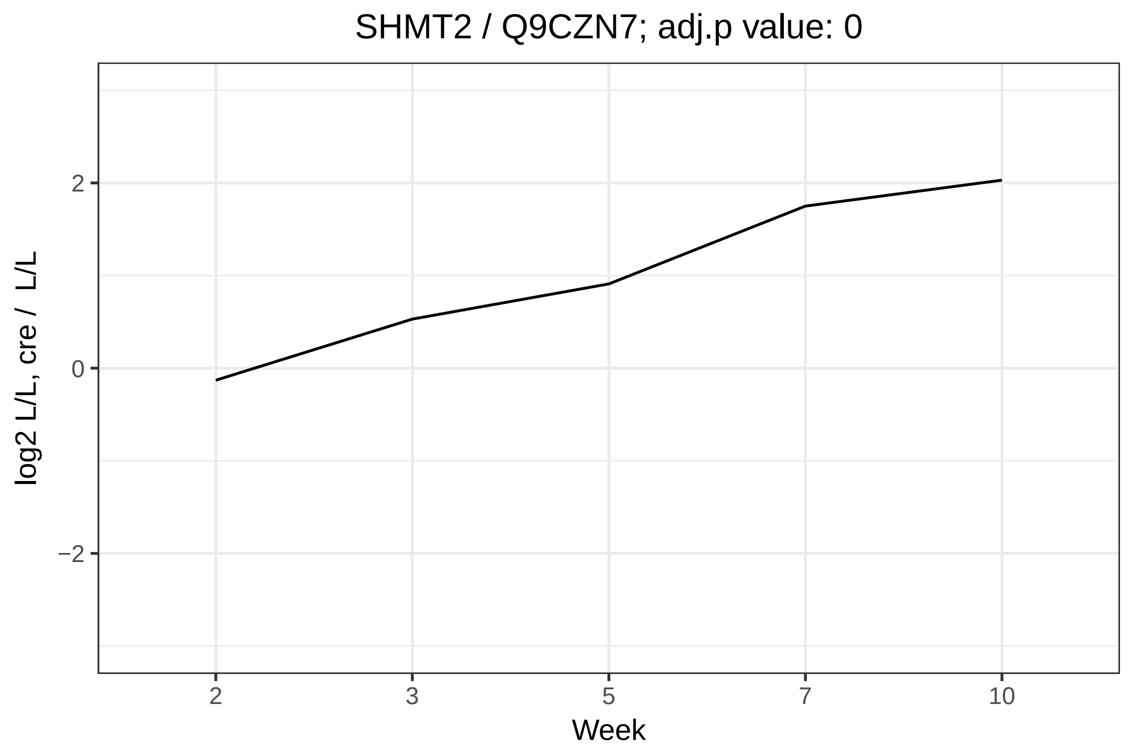

SHMT2 / Q9CZN7; adj.p value: 0
log2 L/L, cre / L/L
2
0
−2
2
3
5
7
10
Week

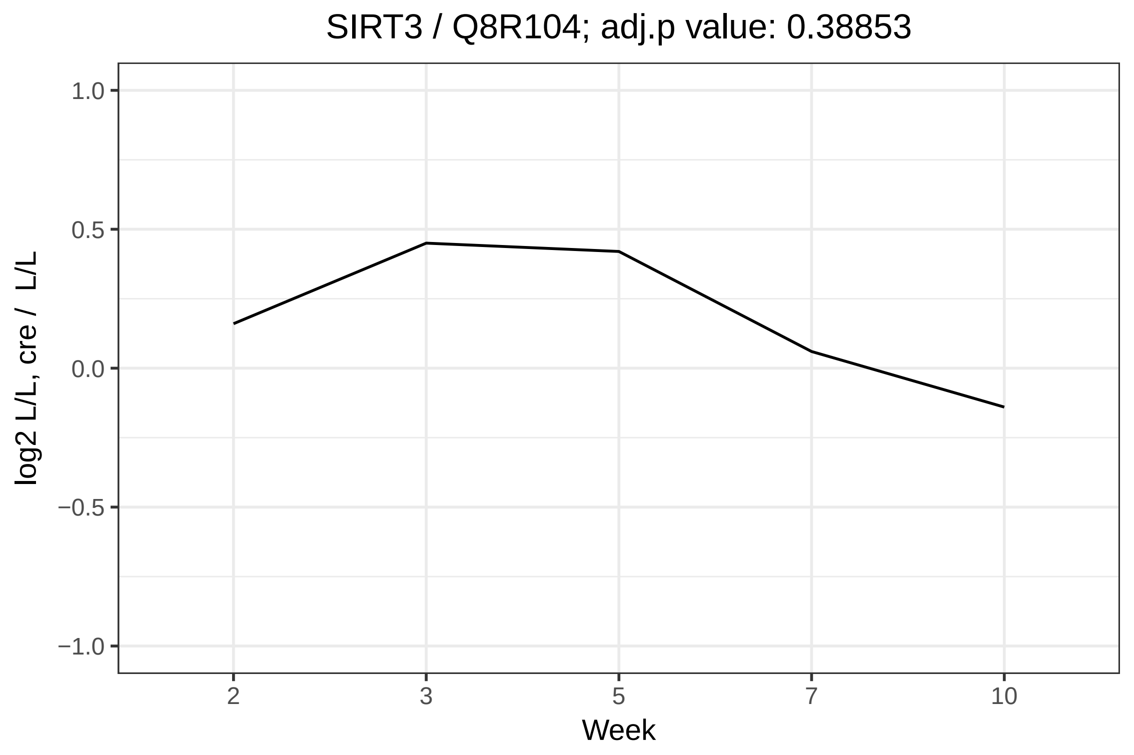
SIRT3 / Q8R104; adj.p value: 0.38853
1.0
0.5
0.0
−0.5
−1.0
log2 L/L, cre / L/L
2
3
5
7
10
Week

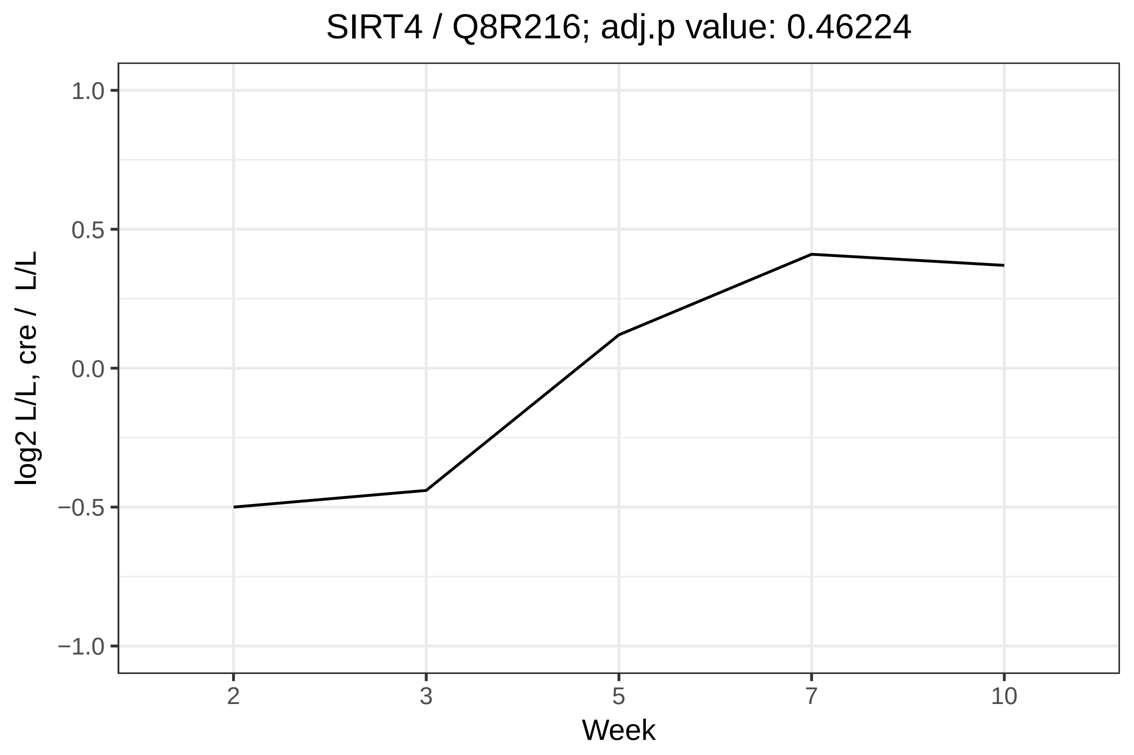

SIRT4 / Q8R216; adj.p value: 0.46224
1.0
0.5
0.0
−0.5
−1.0
log2 L/L, cre / L/L
2
3
5
7
10
Week

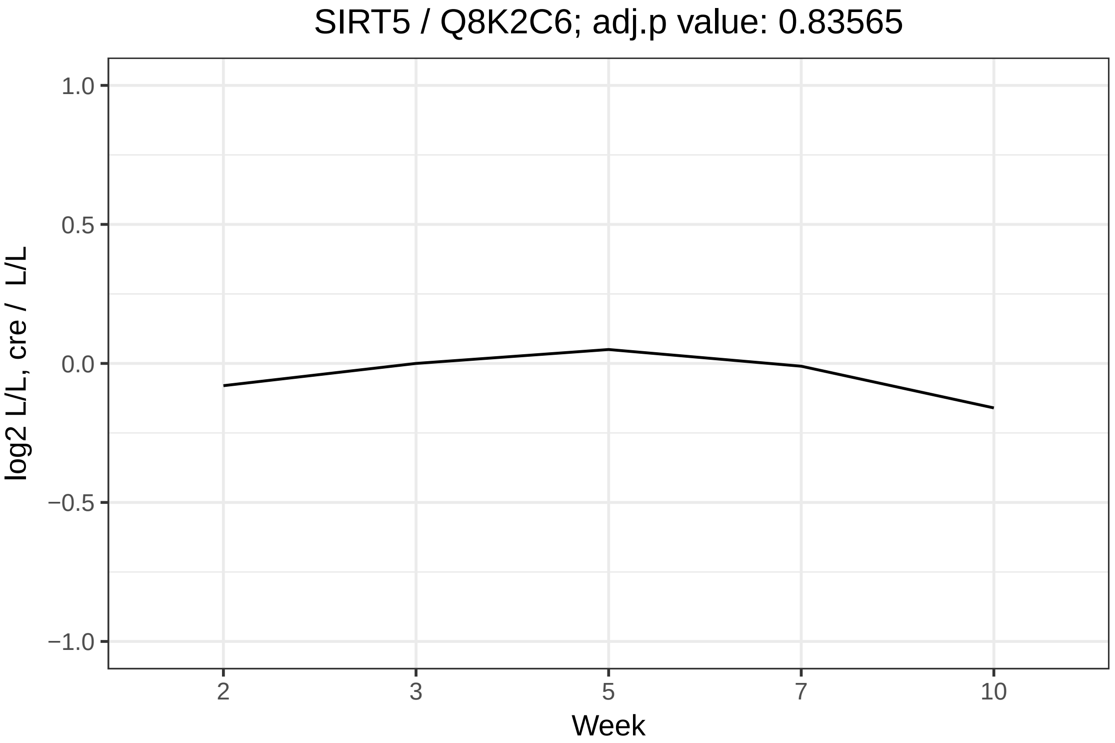
SIRT5 / Q8K2C6; adj.p value: 0.83565
log2 L/L, cre / L/L
1.0
0.5
0.0
−0.5
−1.0
2
3
5
7
10
Week

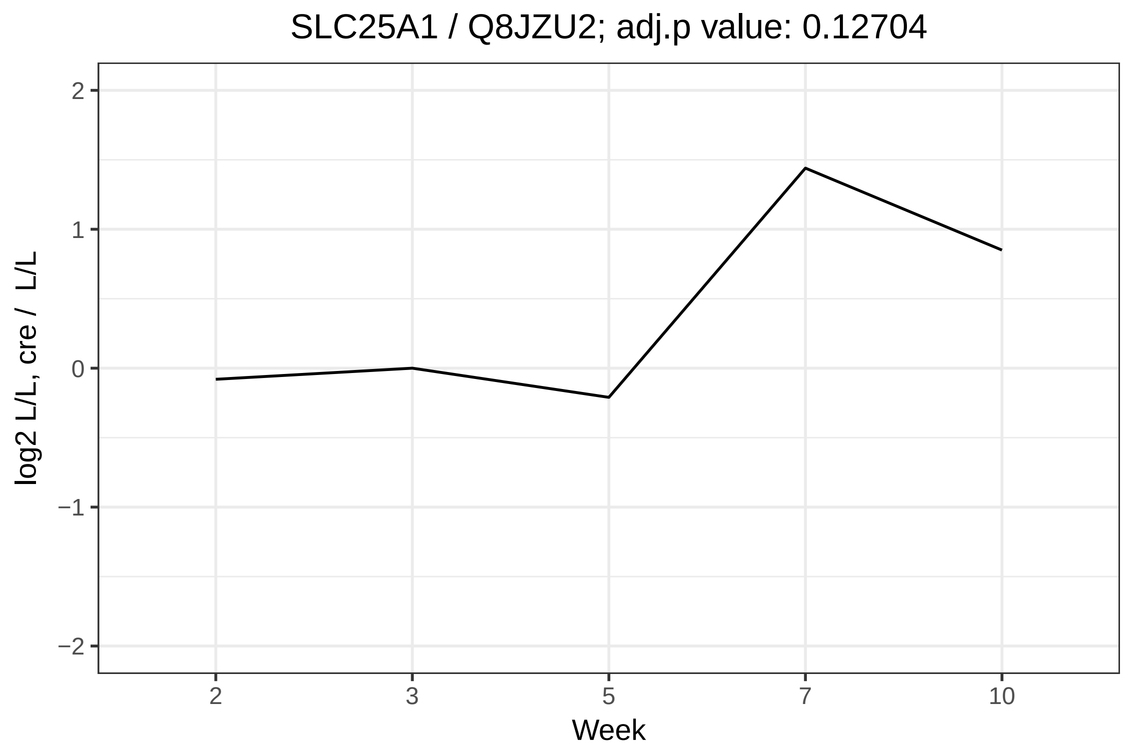

SLC25A1 / Q8JZU2; adj.p value: 0.12704
log2 L/L, cre / L/L
2
1
0
−1
−2
2
3
5
7
10
Week

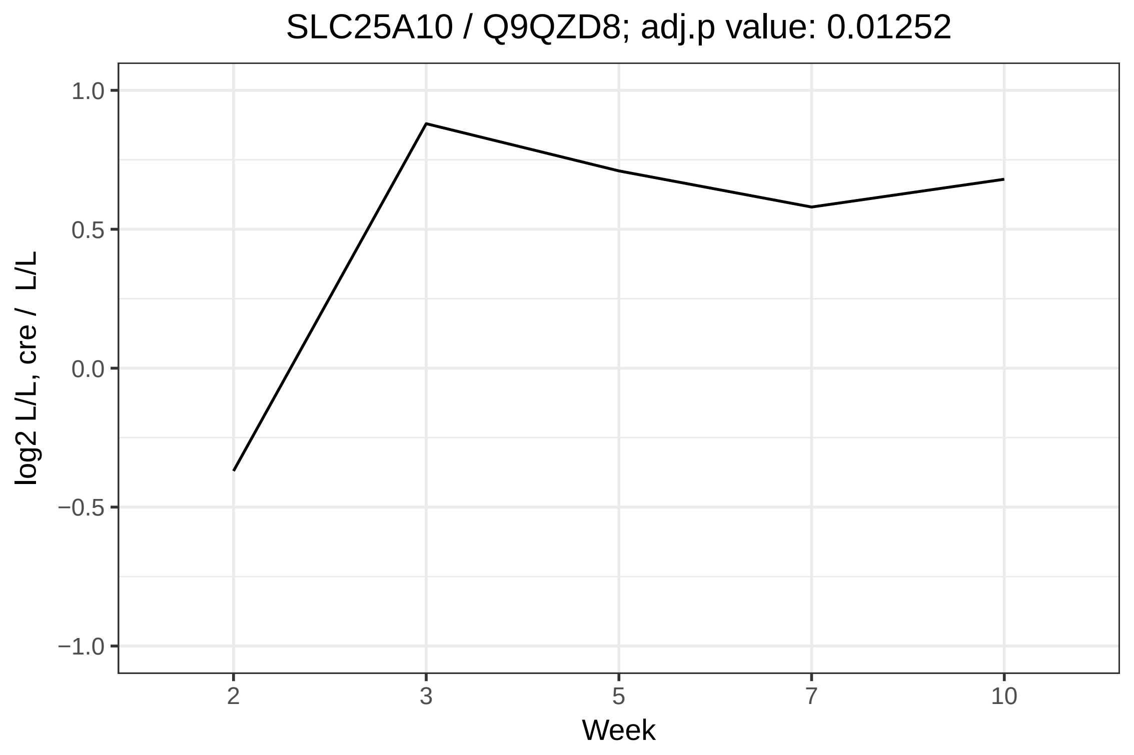
SLC25A10 / Q9QZD8; adj.p value: 0.01252
1.0
0.5
0.0
−0.5
−1.0
log2 L/L, cre / L/L
2
3
5
7
10
Week

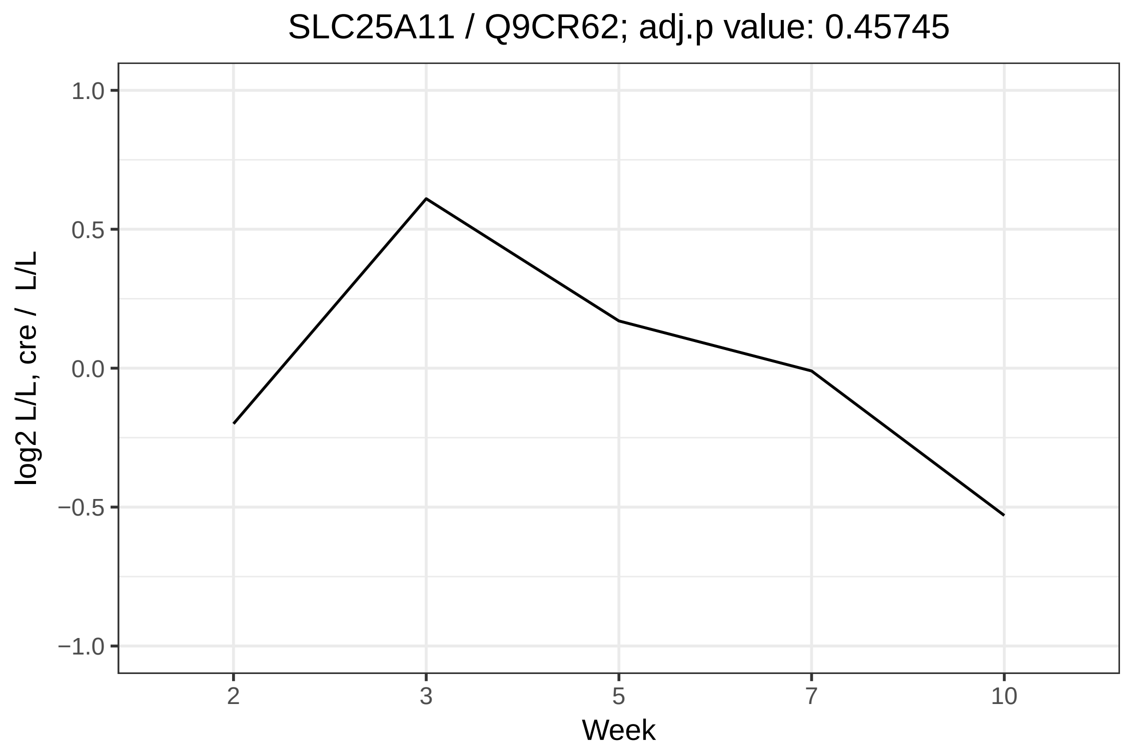
SLC25A11 / Q9CR62; adj.p value: 0.45745
log2 L/L, cre / L/L
1.0
0.5
0.0
−0.5
−1.0
2
3
5
7
10
Week

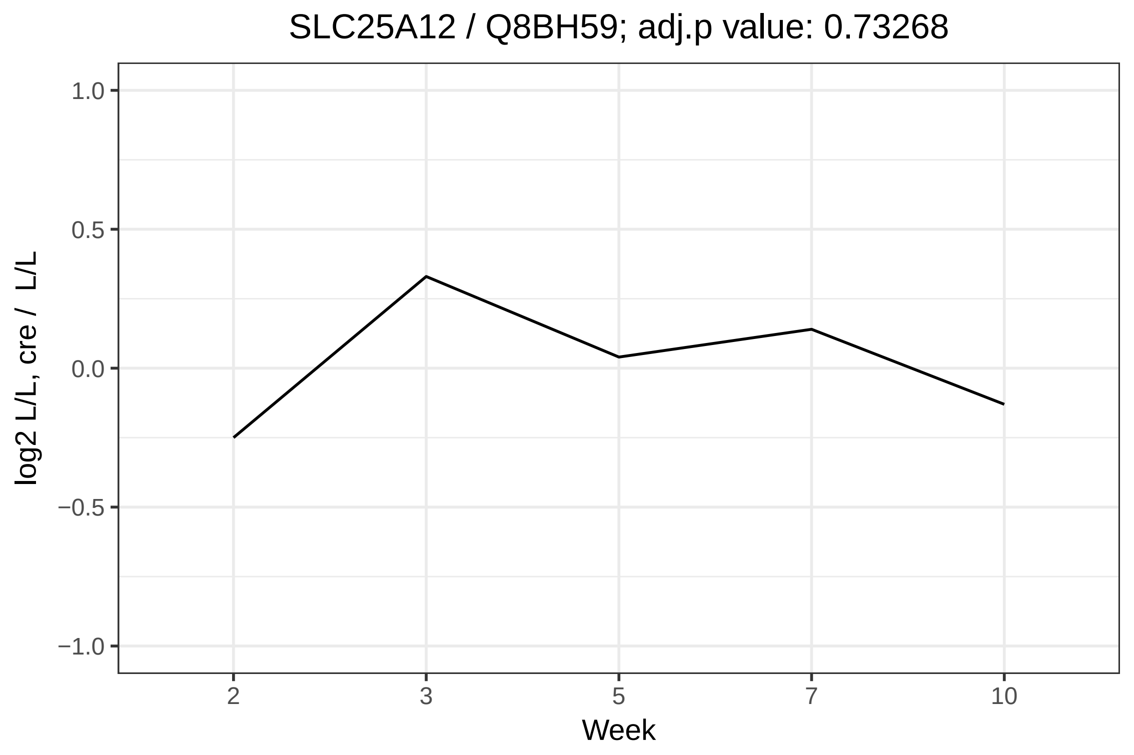

SLC25A12 / Q8BH59; adj.p value: 0.73268
1.0
0.5
0.0
−0.5
−1.0
log2 L/L, cre / L/L
2
3
5
7
10
Week

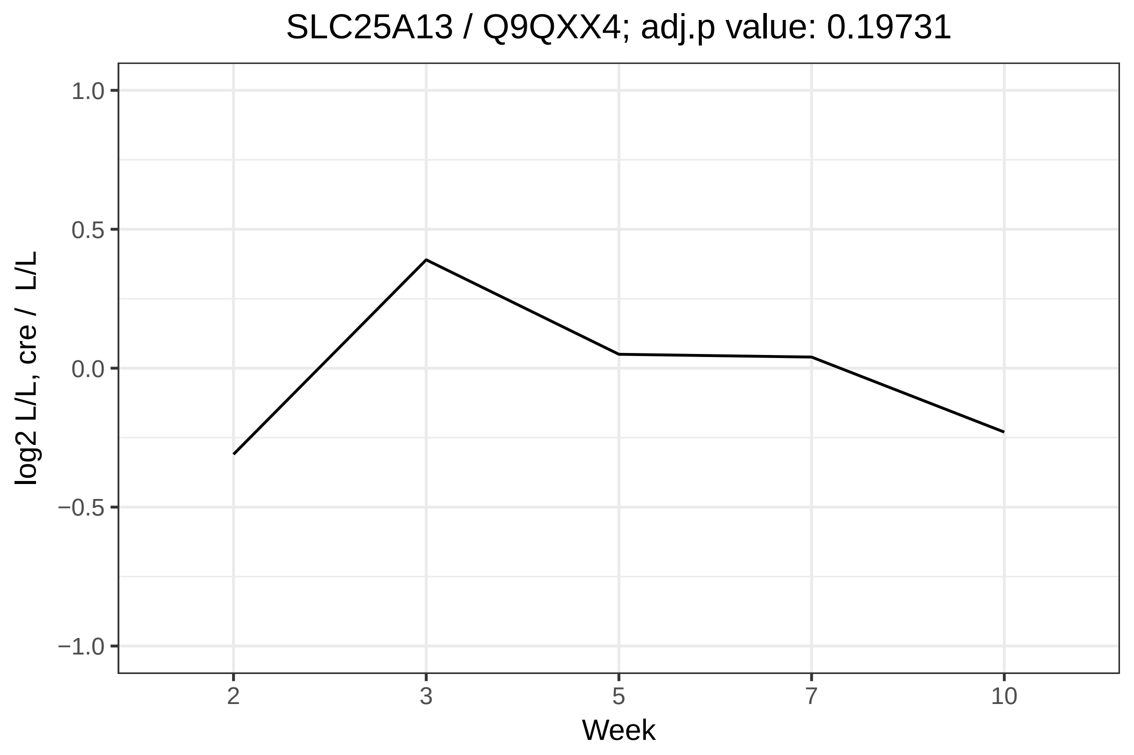

SLC25A13 / Q9QXX4; adj.p value: 0.19731
1.0
0.5
0.0
−0.5
−1.0
log2 L/L, cre / L/L
2
3
5
7
10
Week

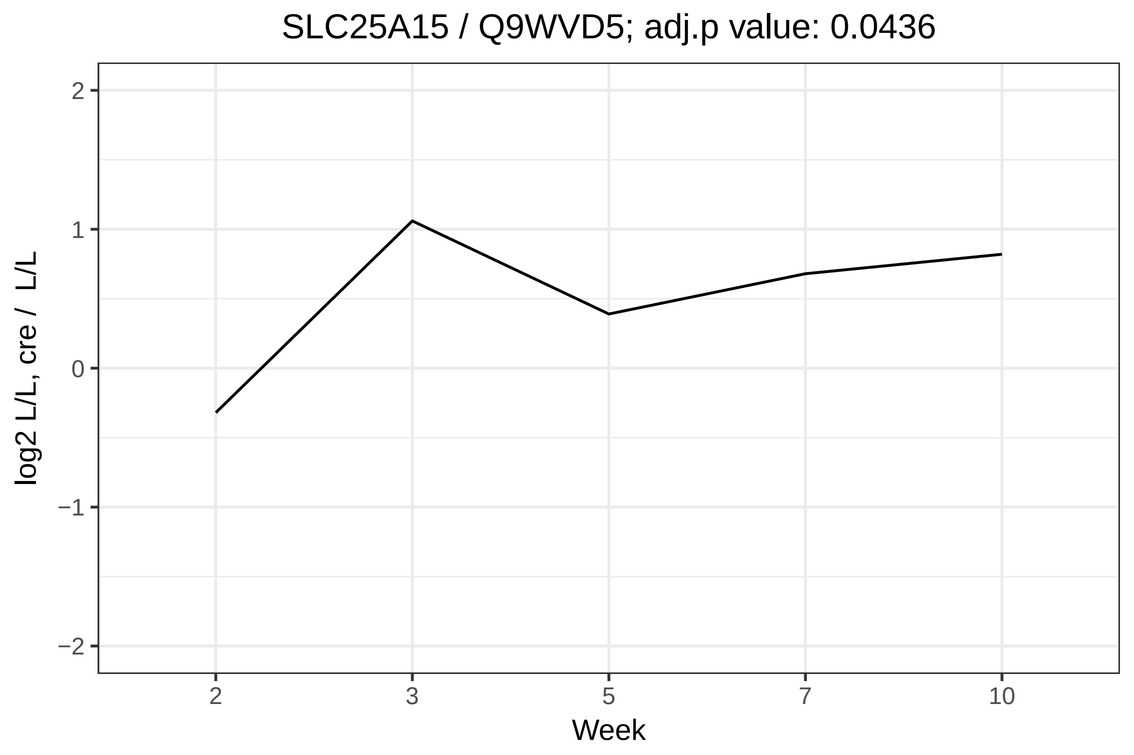

SLC25A15 / Q9WVD5; adj.p value: 0.0436
log2 L/L, cre / L/L
2
1
0
−1
−2
2
3
5
7
10
Week

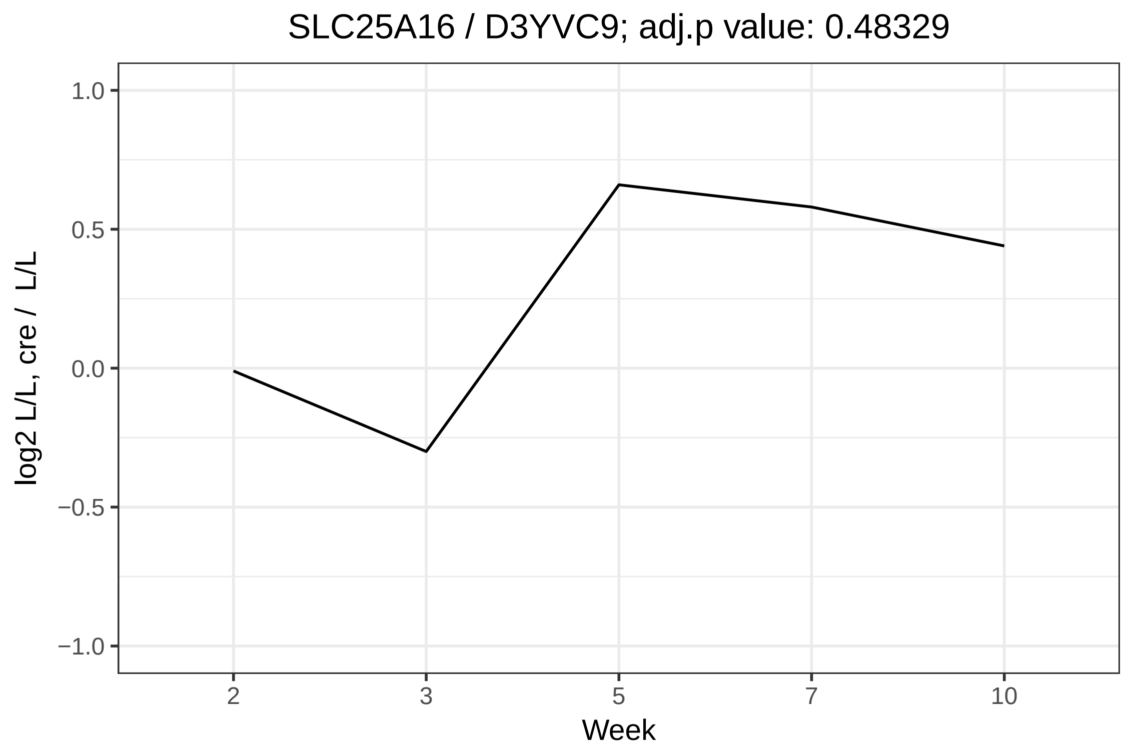
SLC25A16 / D3YVC9; adj.p value: 0.48329
log2 L/L, cre / L/L
1.0
0.5
0.0
−0.5
−1.0
2
3
5
7
10
Week

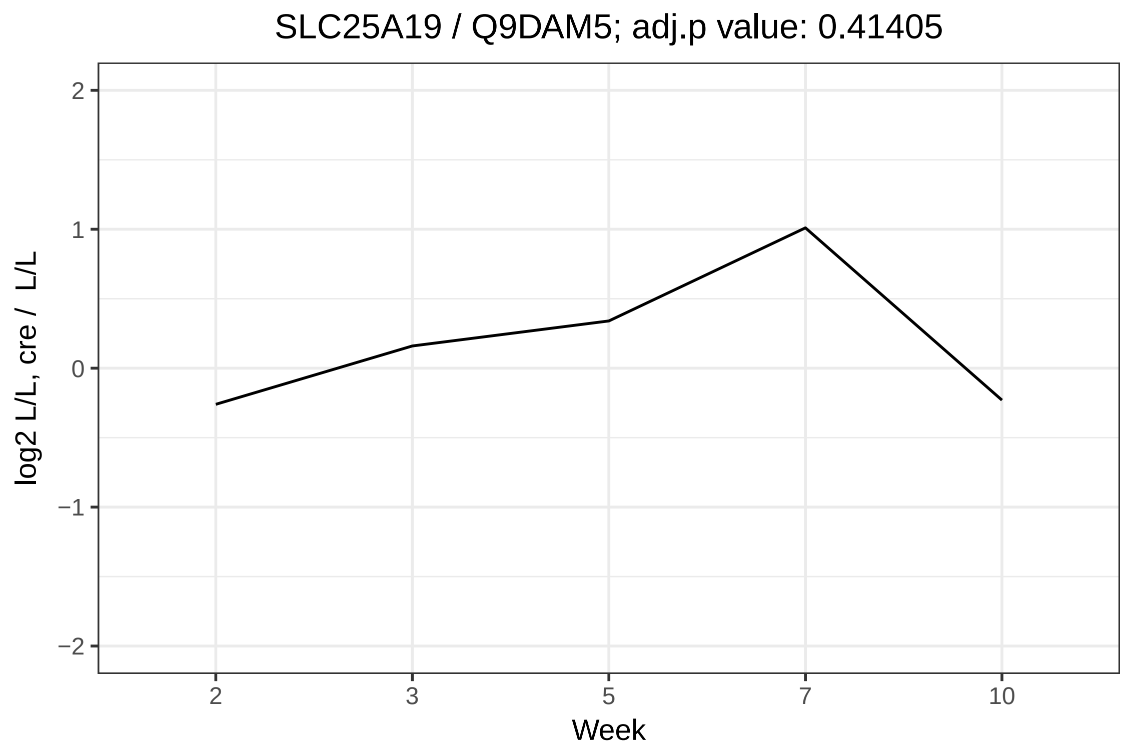
SLC25A19 / Q9DAM5; adj.p value: 0.41405
log2 L/L, cre / L/L
2
1
0
−1
−2
2
3
5
7
10
Week

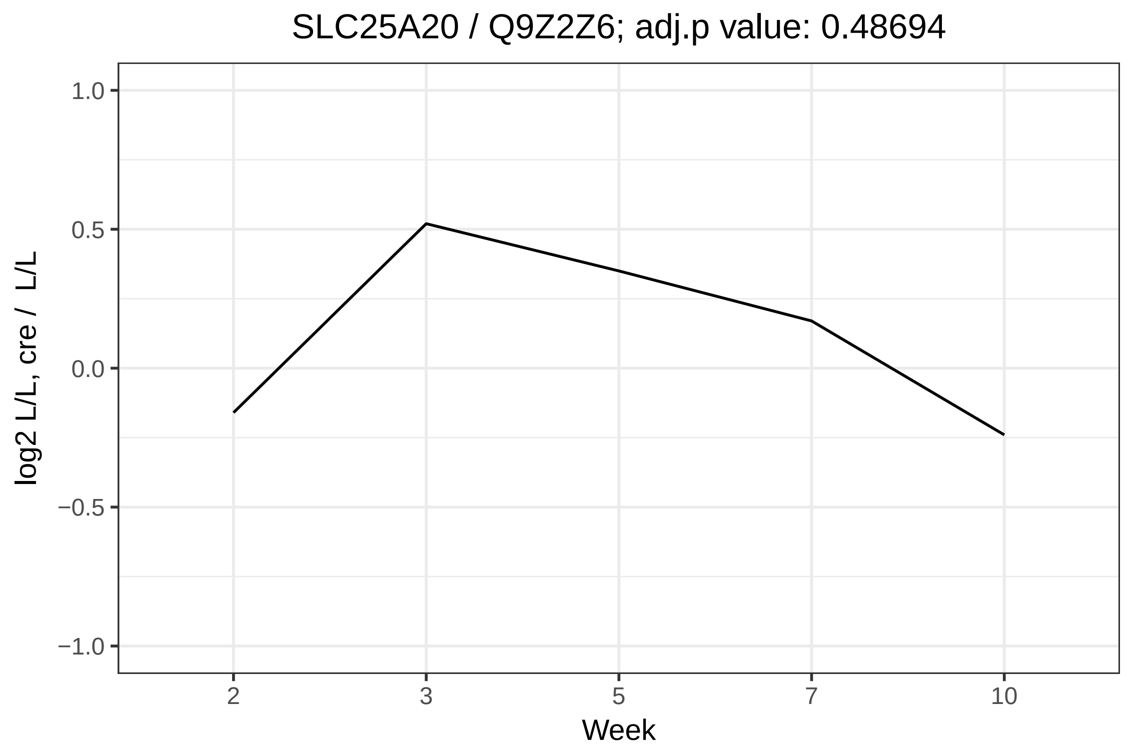
SLC25A20 / Q9Z2Z6; adj.p value: 0.48694
log2 L/L, cre / L/L
1.0
0.5
0.0
−0.5
−1.0
2
3
5
7
10
Week

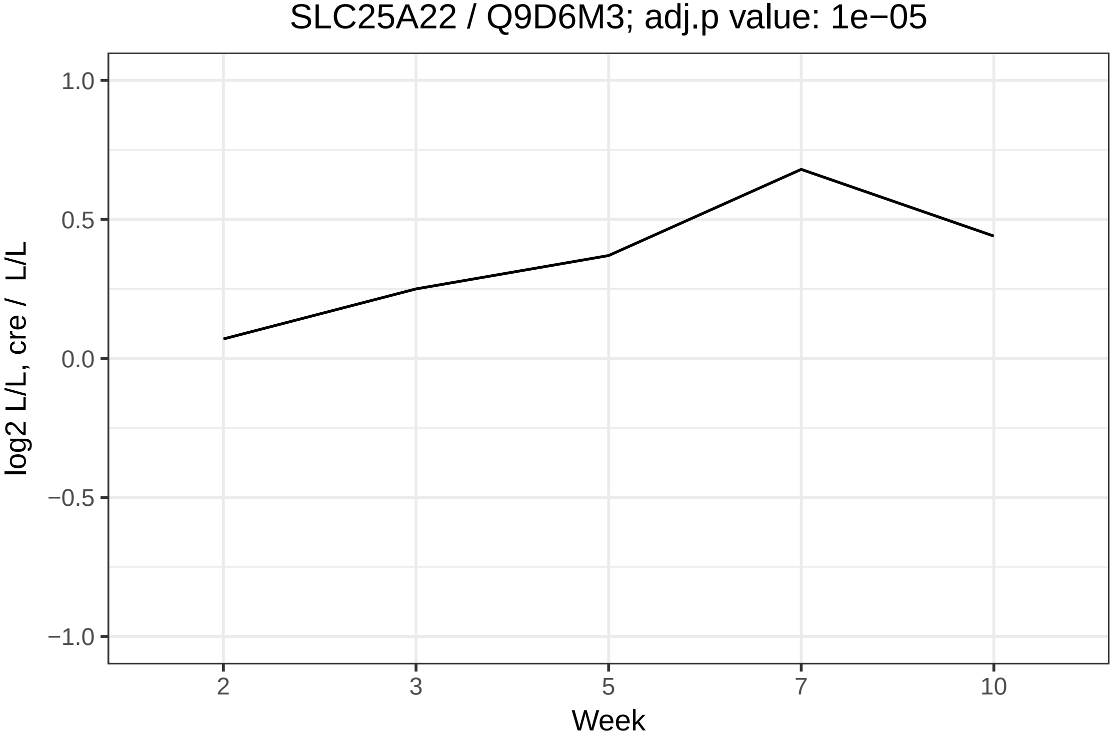

SLC25A22 / Q9D6M3; adj.p value: 1e−05
log2 L/L, cre / L/L
1.0
0.5
0.0
−0.5
−1.0
2
3
5
7
10
Week

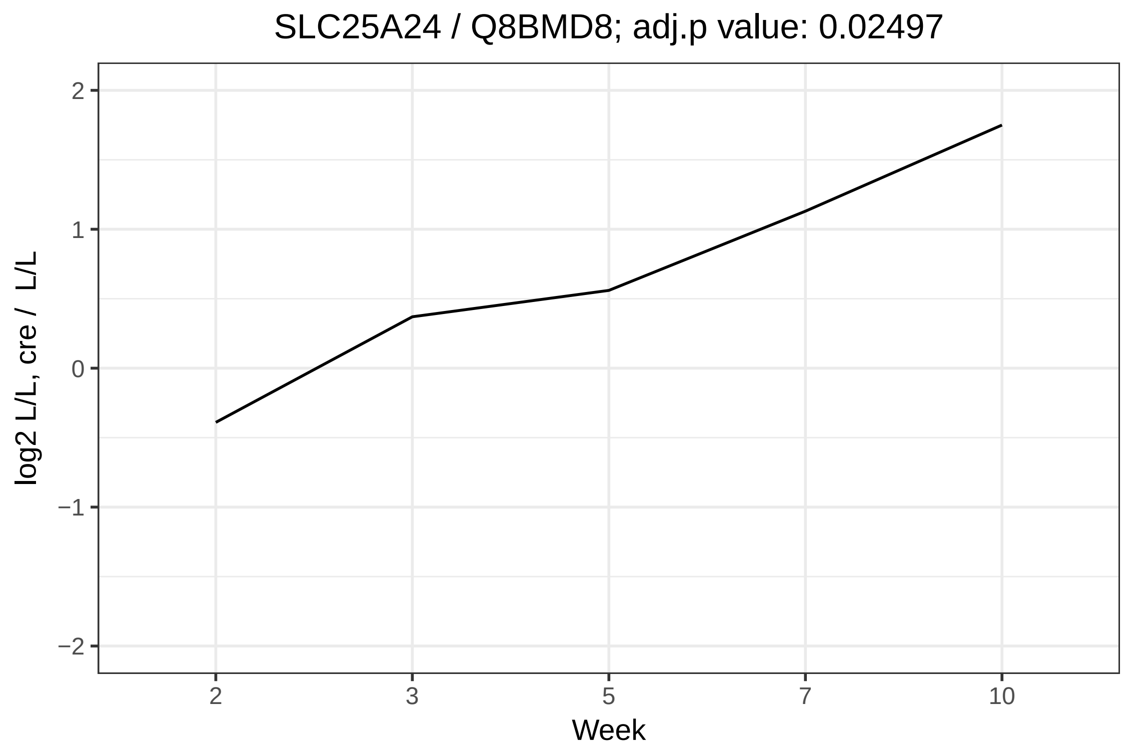

SLC25A24 / Q8BMD8; adj.p value: 0.02497
log2 L/L, cre / L/L
2
1
0
−1
−2
2
3
5
7
10
Week

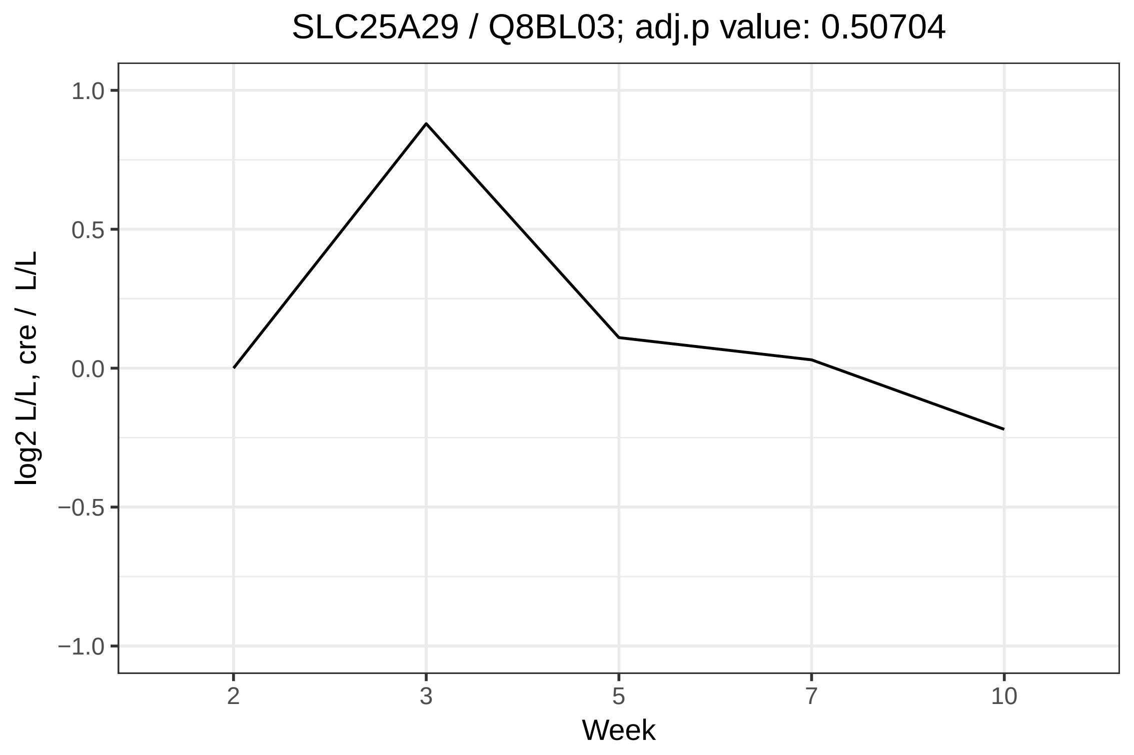
SLC25A29 / Q8BL03; adj.p value: 0.50704
log2 L/L, cre / L/L
1.0
0.5
0.0
−0.5
−1.0
2
3
5
7
10
Week

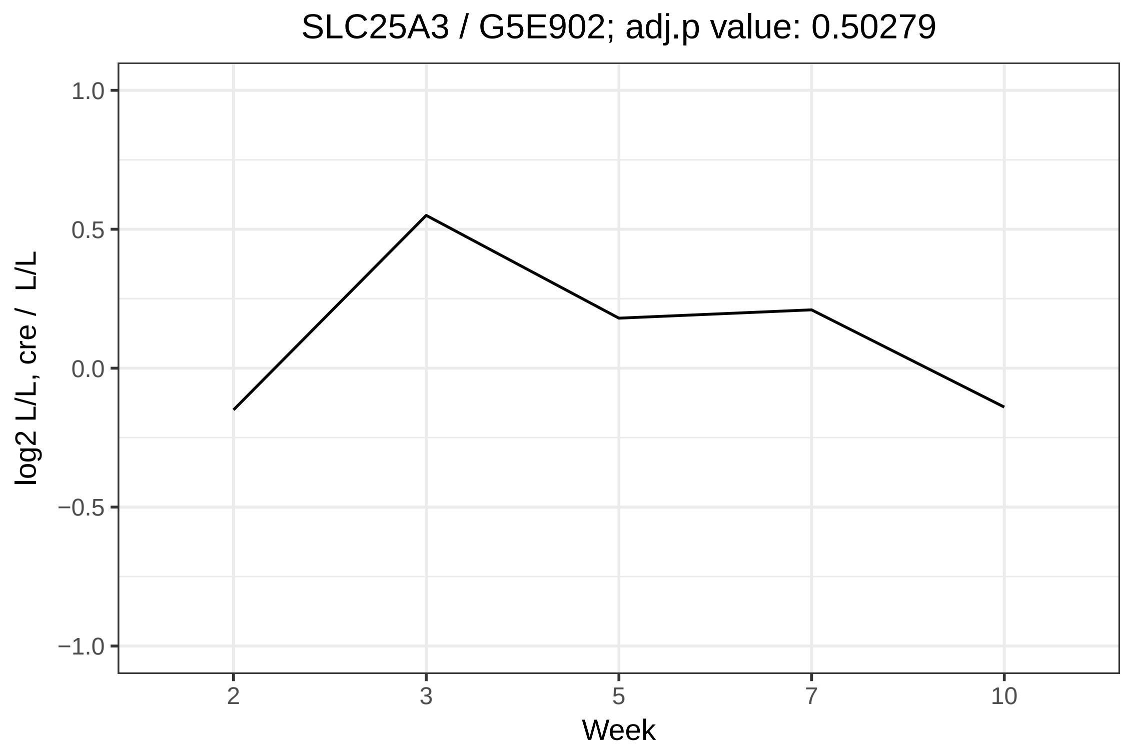

SLC25A3 / G5E902; adj.p value: 0.50279
1.0
0.5
0.0
−0.5
−1.0
log2 L/L, cre / L/L
2
3
5
7
10
Week

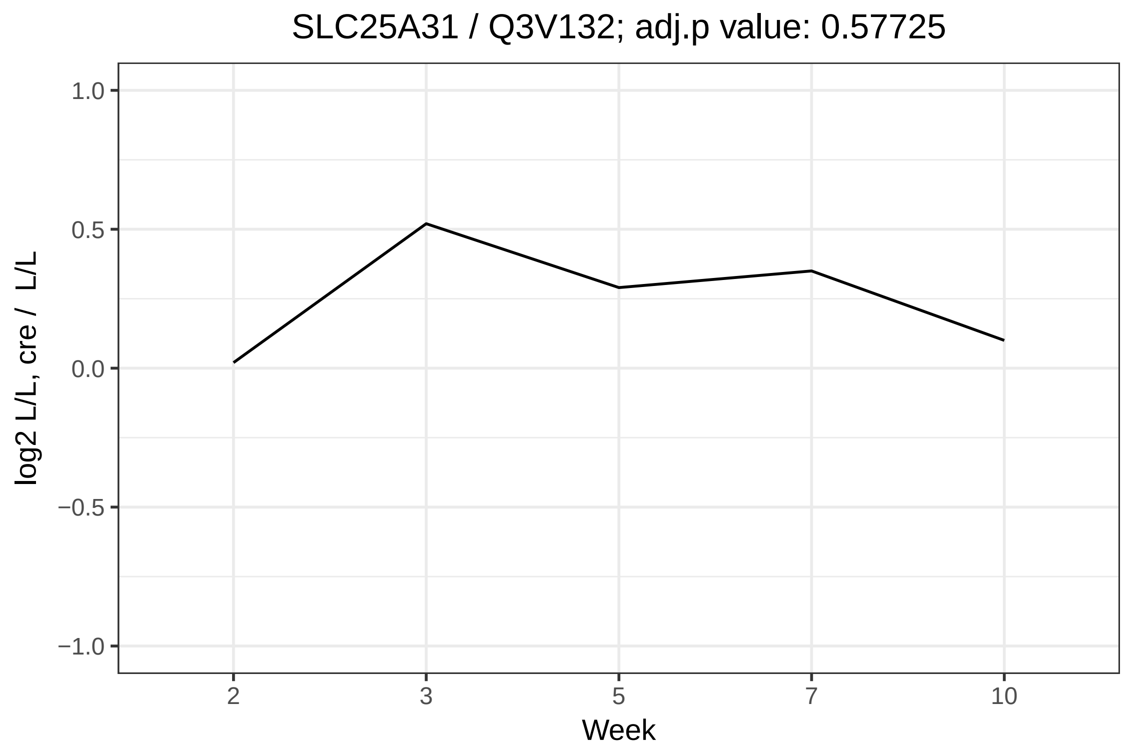

SLC25A31 / Q3V132; adj.p value: 0.57725
log2 L/L, cre / L/L
1.0
0.5
0.0
−0.5
−1.0
2
3
5
7
10
Week

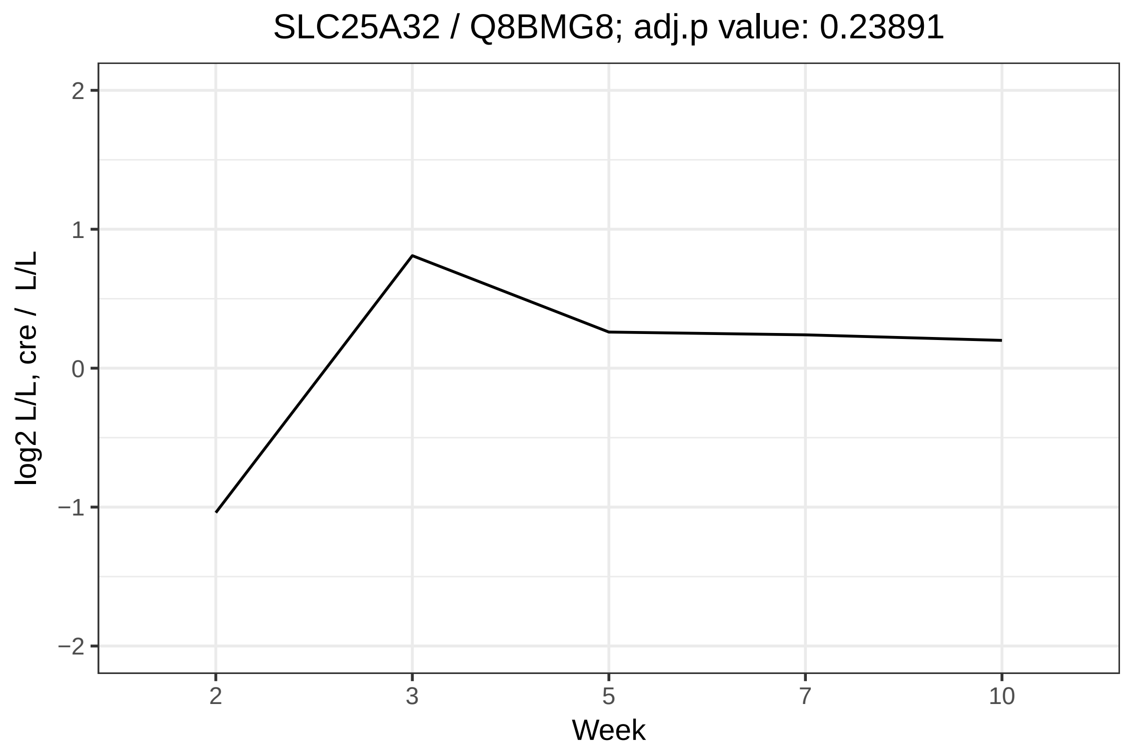
SLC25A32 / Q8BMG8; adj.p value: 0.23891
log2 L/L, cre / L/L
2
1
0
−1
−2
2
3
5
7
10
Week

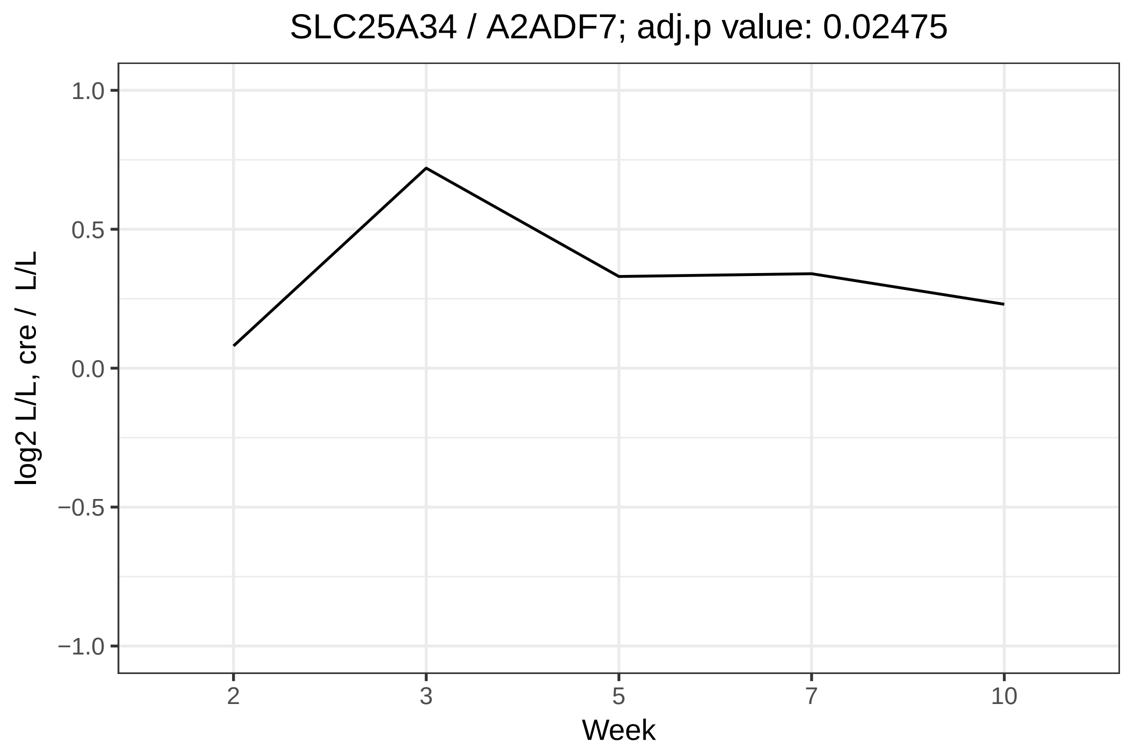
SLC25A34 / A2ADF7; adj.p value: 0.02475
log2 L/L, cre / L/L
1.0
0.5
0.0
−0.5
−1.0
2
3
5
7
10
Week

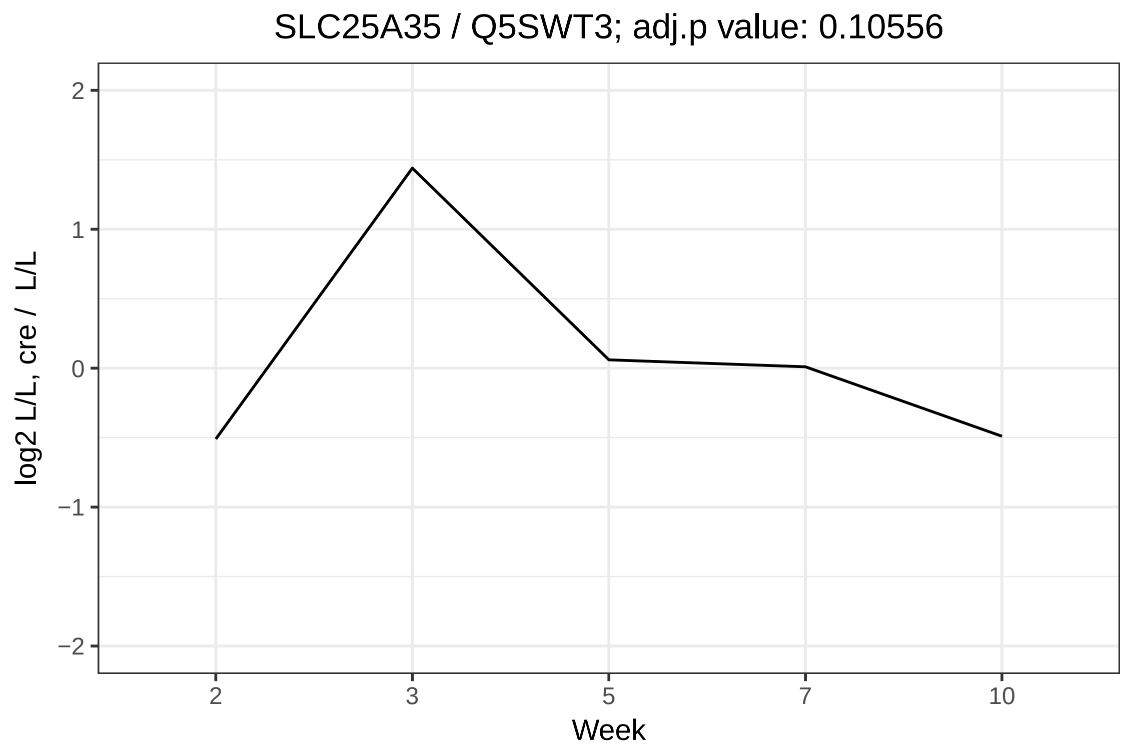
SLC25A35 / Q5SWT3; adj.p value: 0.10556
log2 L/L, cre / L/L
2
1
0
−1
−2
2
3
5
7
10
Week

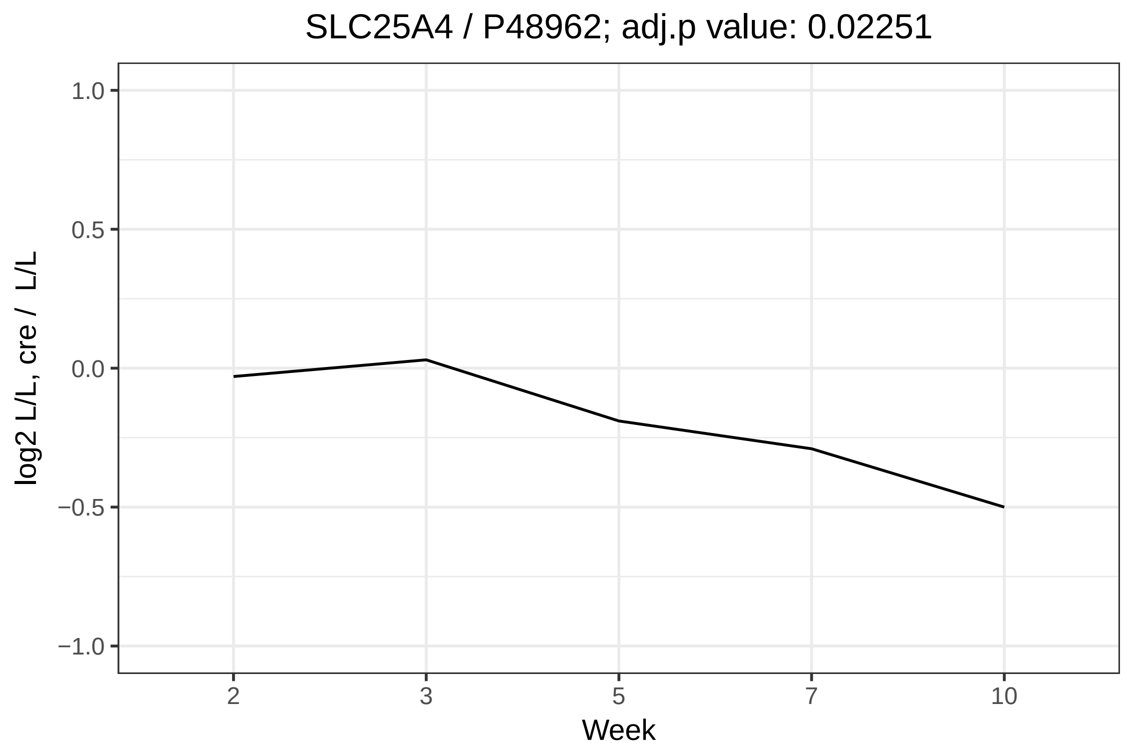
SLC25A4 / P48962; adj.p value: 0.02251
log2 L/L, cre / L/L
1.0
0.5
0.0
−0.5
−1.0
2
3
5
7
10
Week

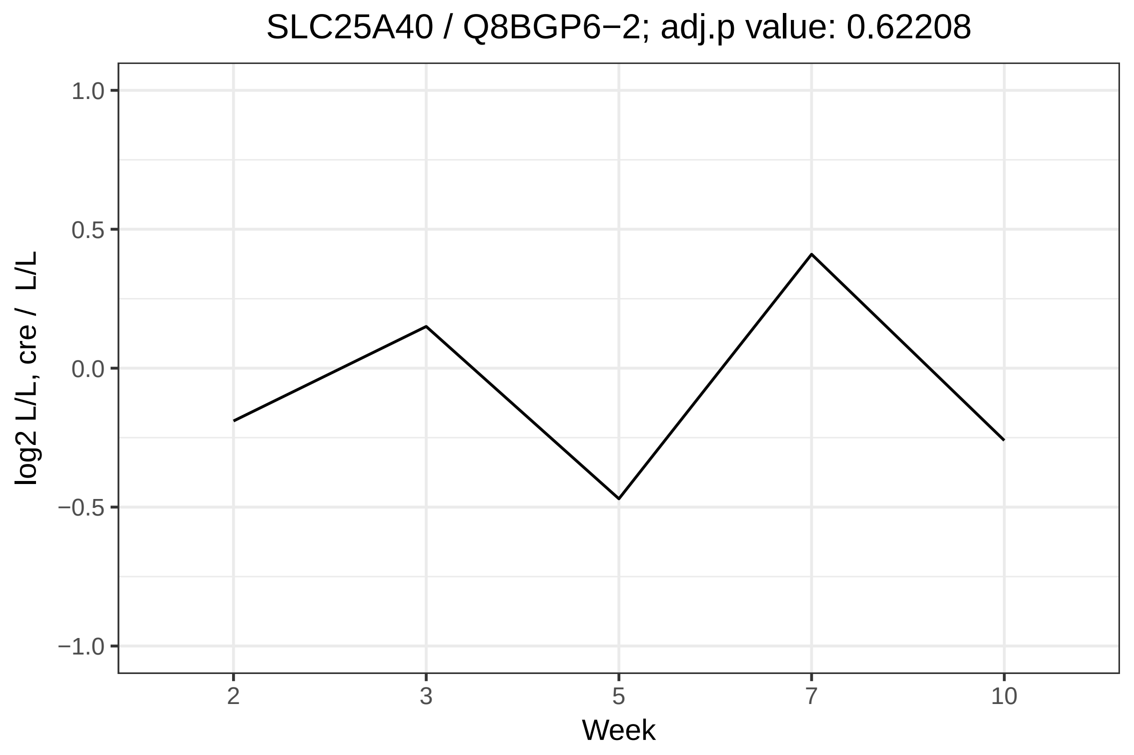
SLC25A40 / Q8BGP6−2; adj.p value: 0.62208
log2 L/L, cre / L/L
1.0
0.5
0.0
−0.5
−1.0
2
3
5
7
10
Week

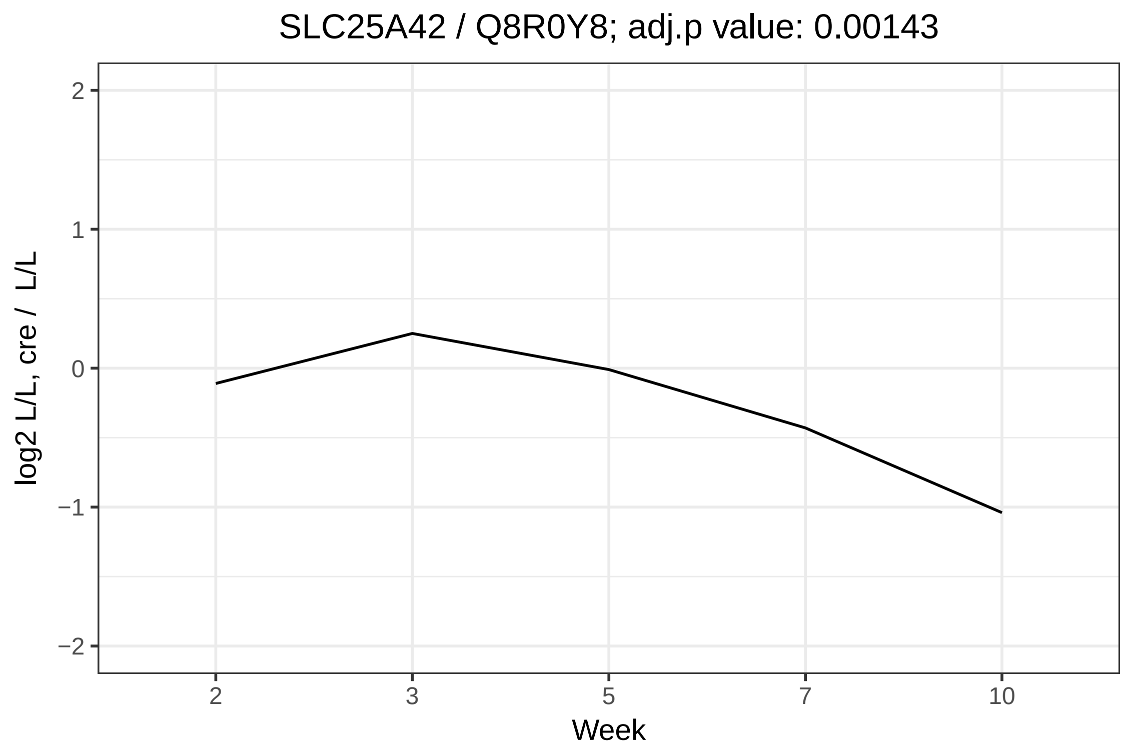
SLC25A42 / Q8R0Y8; adj.p value: 0.00143
log2 L/L, cre / L/L
2
1
0
−1
−2
2
3
5
7
10
Week

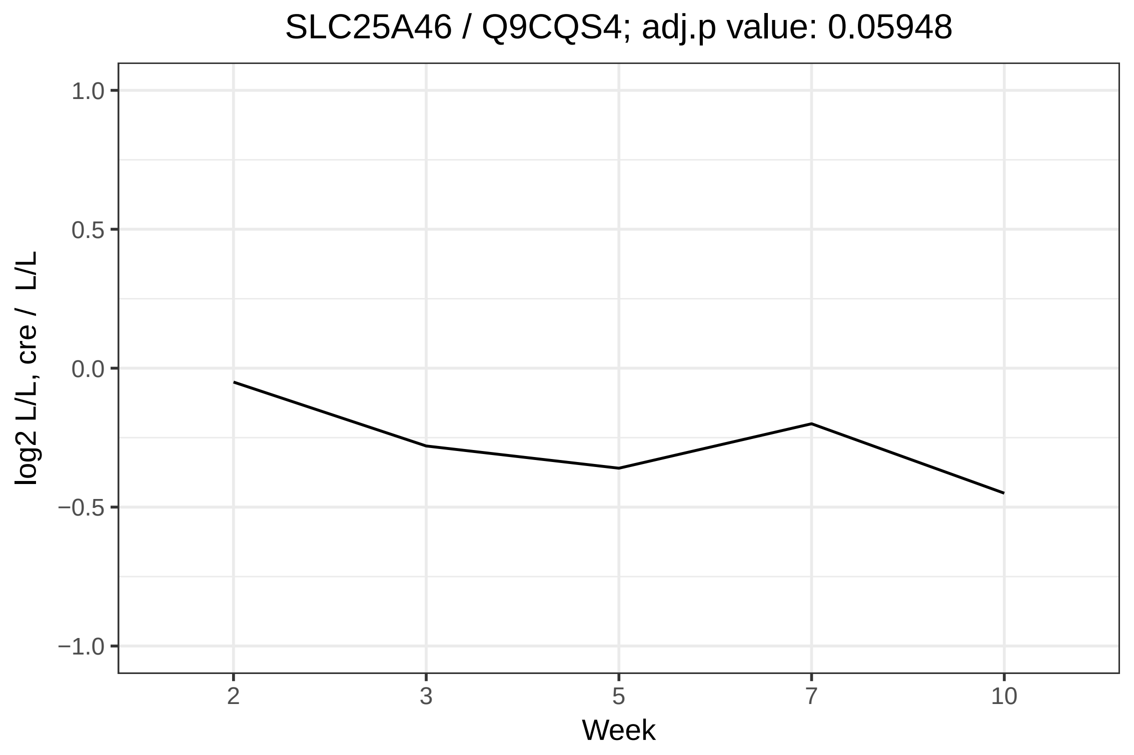
SLC25A46 / Q9CQS4; adj.p value: 0.05948
log2 L/L, cre / L/L
1.0
0.5
0.0
−0.5
−1.0
2
3
5
7
10
Week

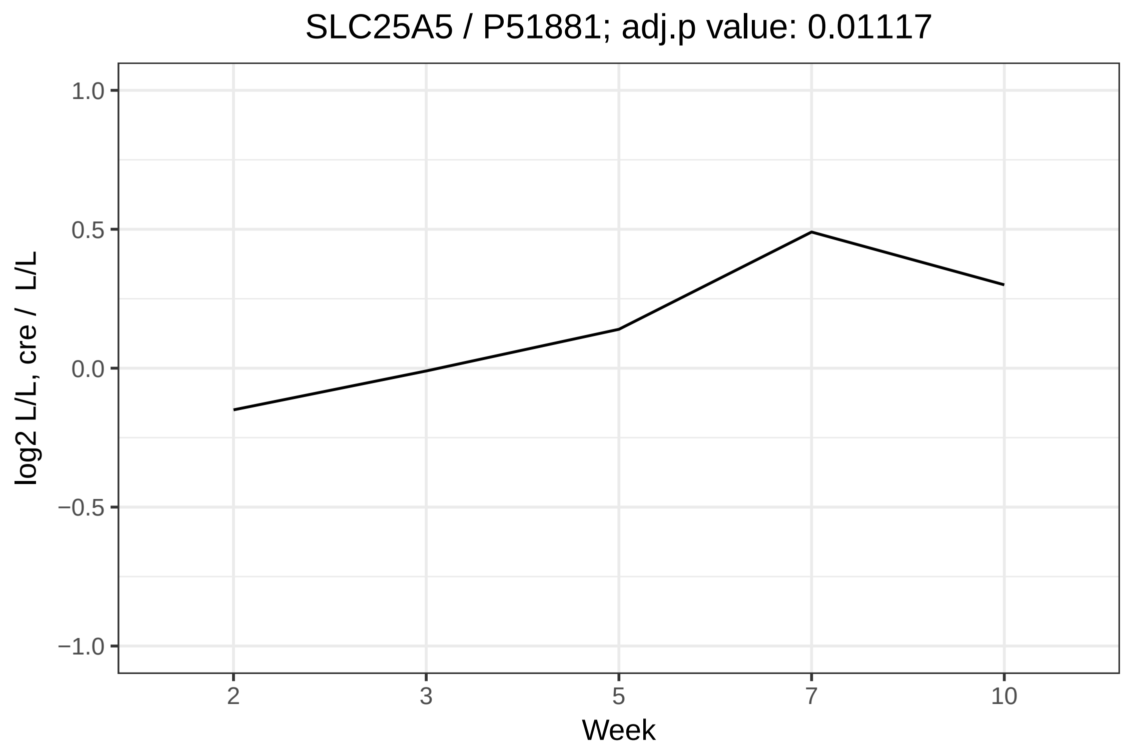

SLC25A5 / P51881; adj.p value: 0.01117
1.0
0.5
0.0
−0.5
−1.0
log2 L/L, cre / L/L
2
3
5
7
10
Week

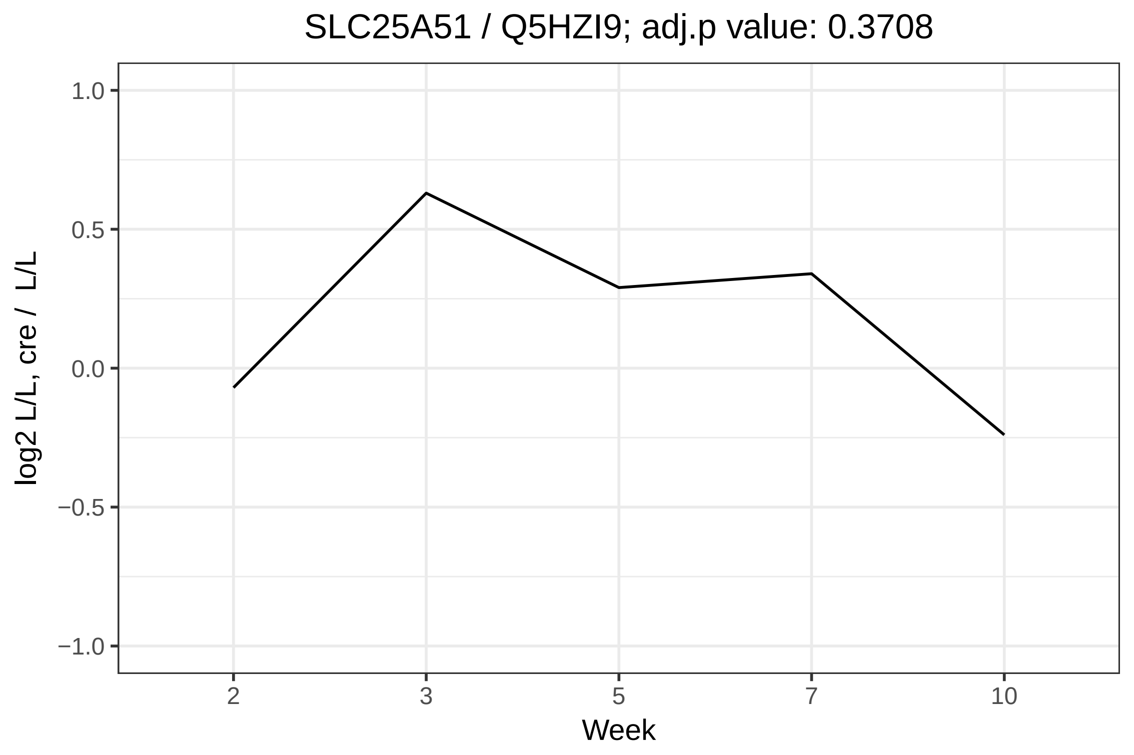

SLC25A51 / Q5HZI9; adj.p value: 0.3708
1.0
0.5
0.0
−0.5
−1.0
log2 L/L, cre / L/L
2
3
5
7
10
Week

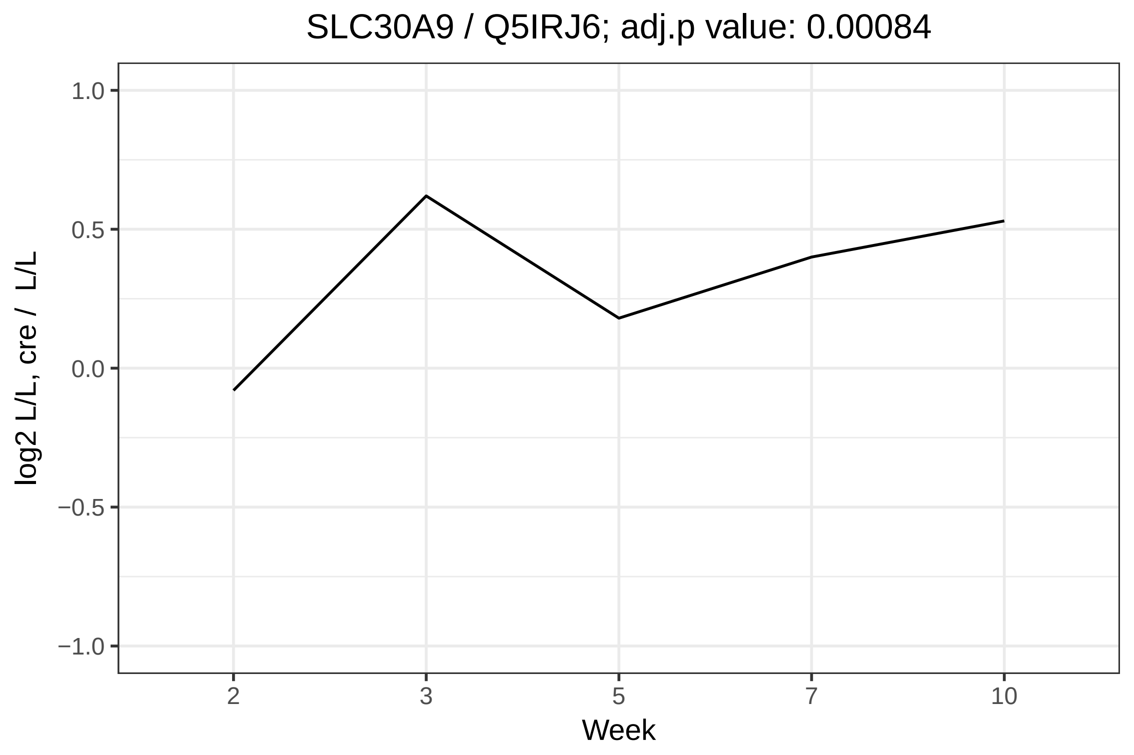

SLC30A9 / Q5IRJ6; adj.p value: 0.00084
1.0
0.5
0.0
−0.5
−1.0
log2 L/L, cre / L/L
2
3
5
7
10
Week

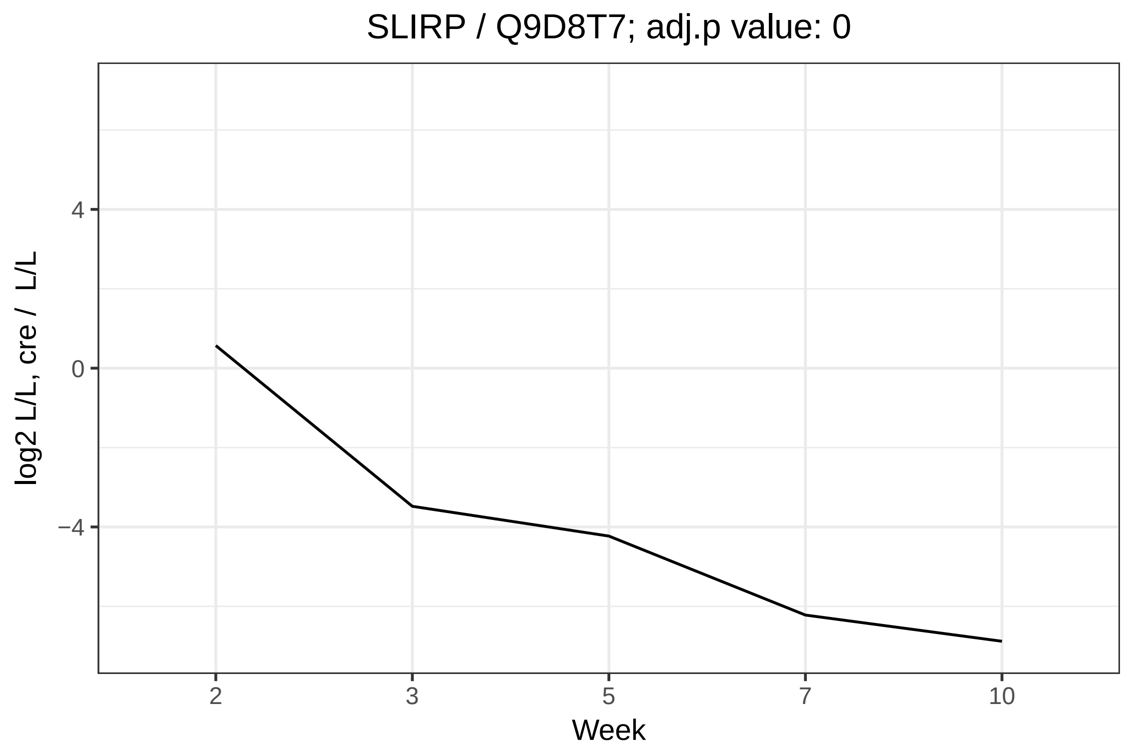

SLIRP / Q9D8T7; adj.p value: 0
log2 L/L, cre / L/L
4
0
−4
2
3
5
7
10
Week

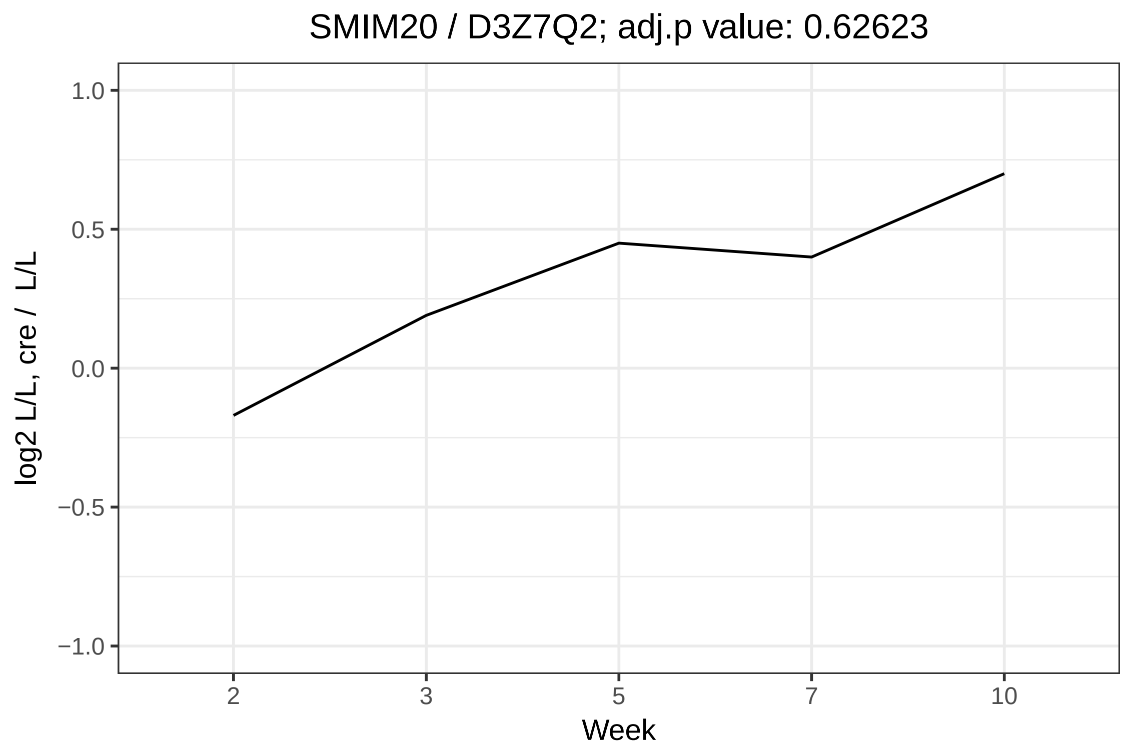

SMIM20 / D3Z7Q2; adj.p value: 0.62623
1.0
0.5
0.0
−0.5
−1.0
log2 L/L, cre / L/L
2
3
5
7
10
Week

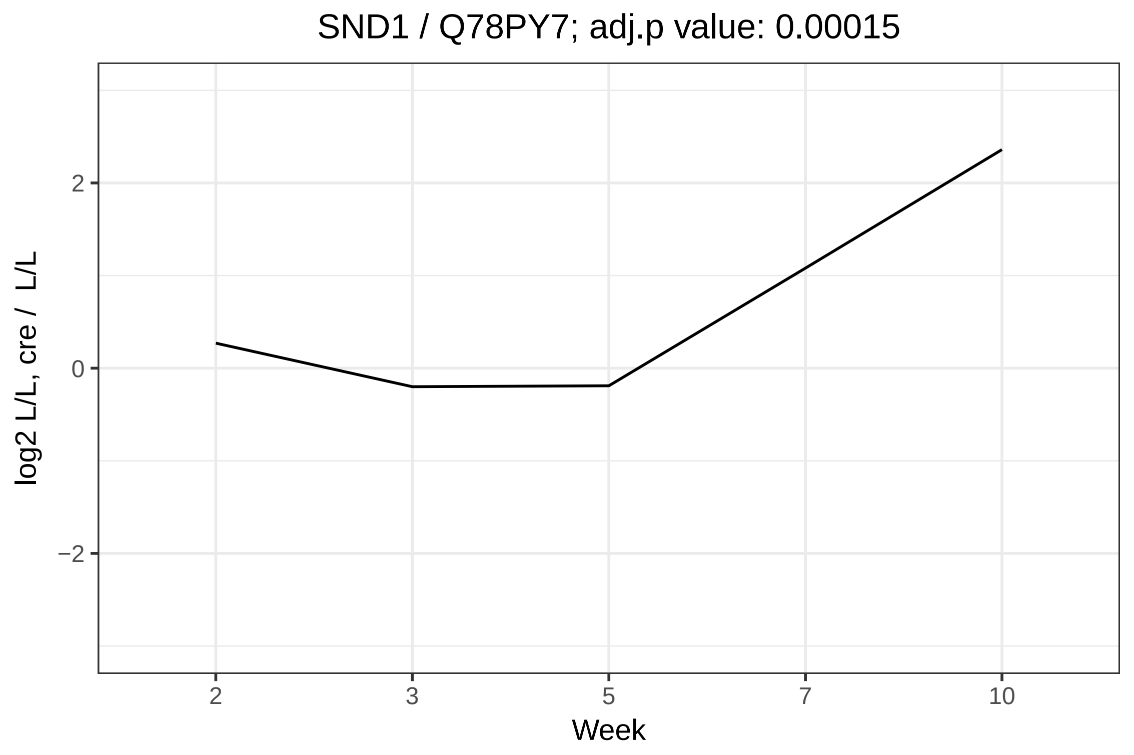
SND1 / Q78PY7; adj.p value: 0.00015
log2 L/L, cre / L/L
2
0
−2
2
3
5
7
10
Week

SOD1 / P08228; adj.p value: 0.00195

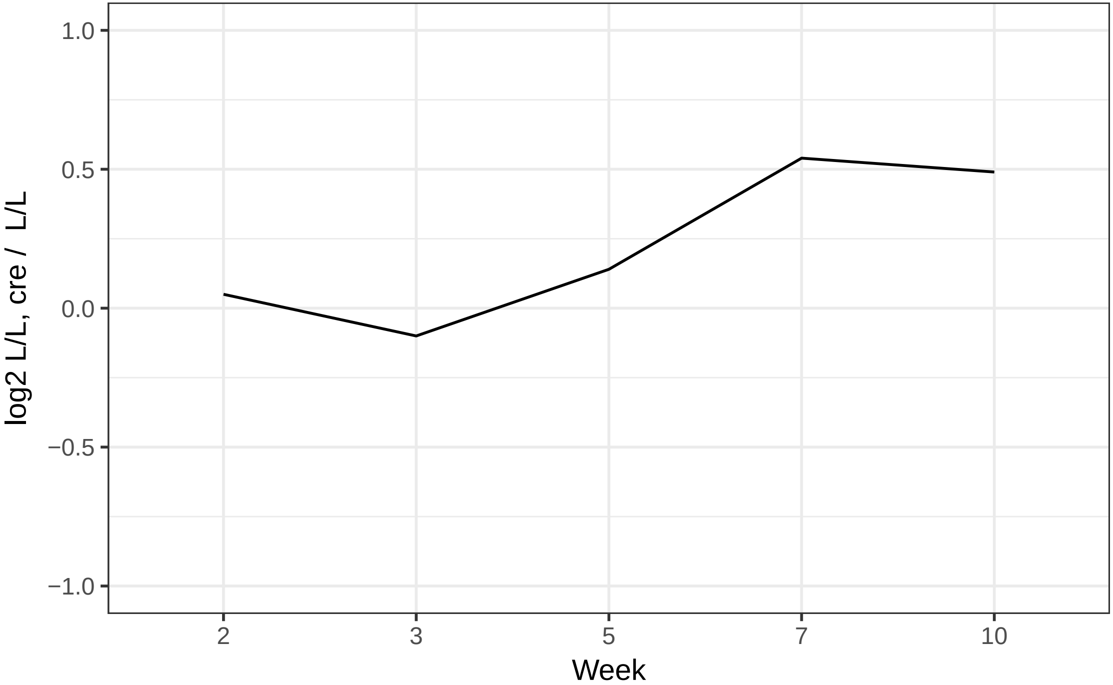

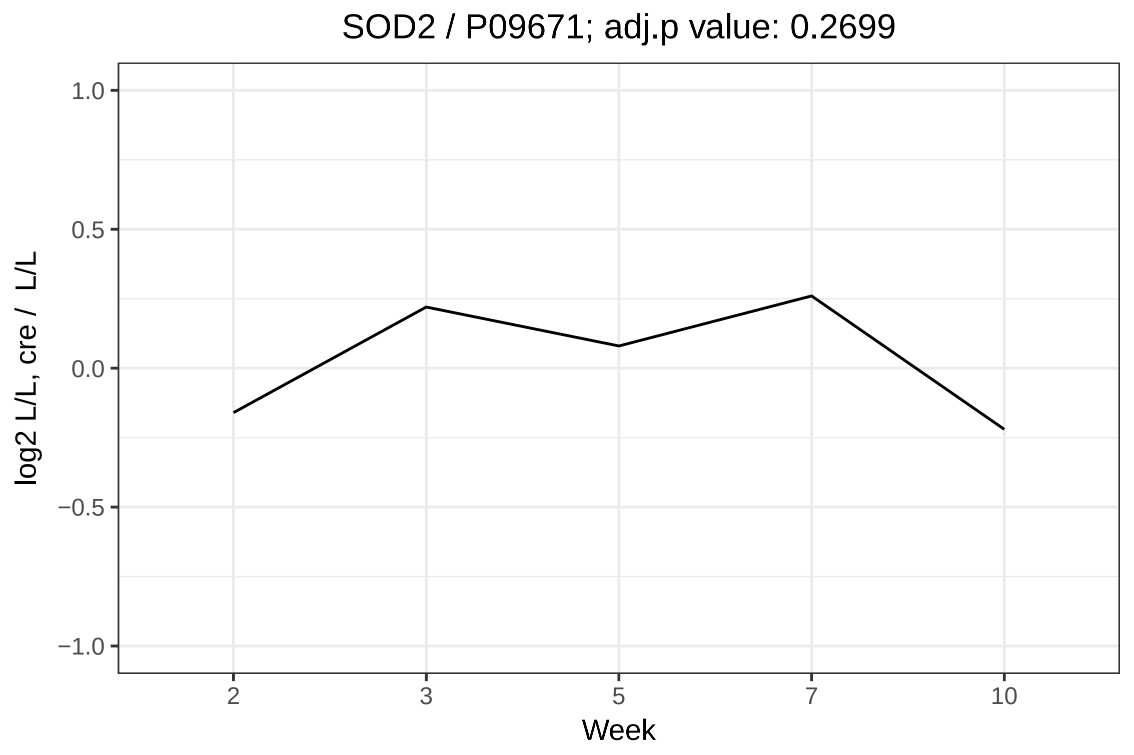

SOD2 / P09671; adj.p value: 0.2699
log2 L/L, cre / L/L
1.0
0.5
0.0
−0.5
−1.0
2
3
5
7
10
Week

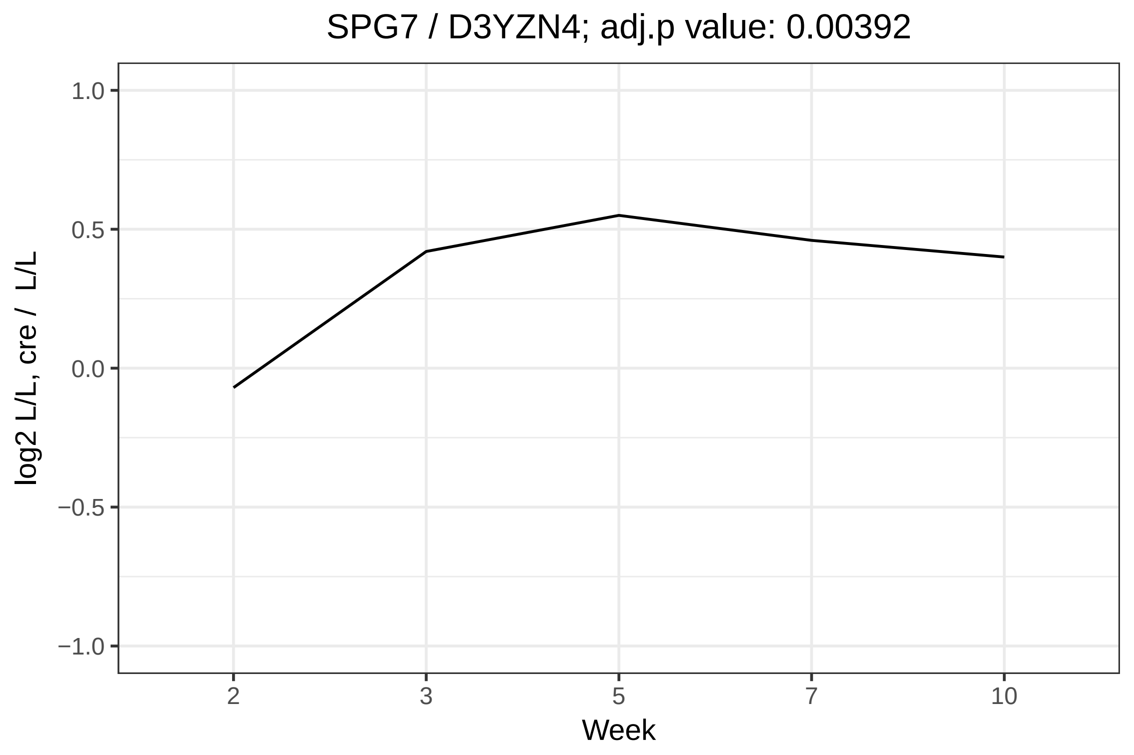
SPG7 / D3YZN4; adj.p value: 0.00392
log2 L/L, cre / L/L
1.0
0.5
0.0
−0.5
−1.0
2
3
5
7
10
Week

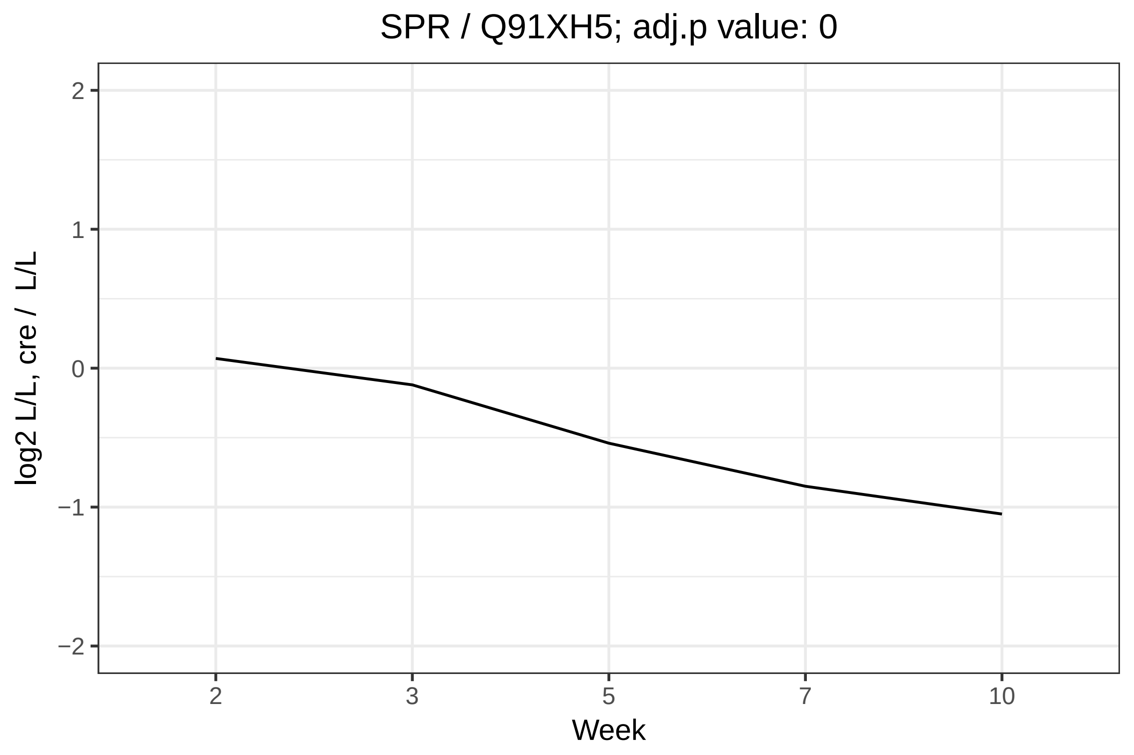

SPR / Q91XH5; adj.p value: 0
log2 L/L, cre / L/L
2
1
0
−1
−2
2
3
5
7
10
Week

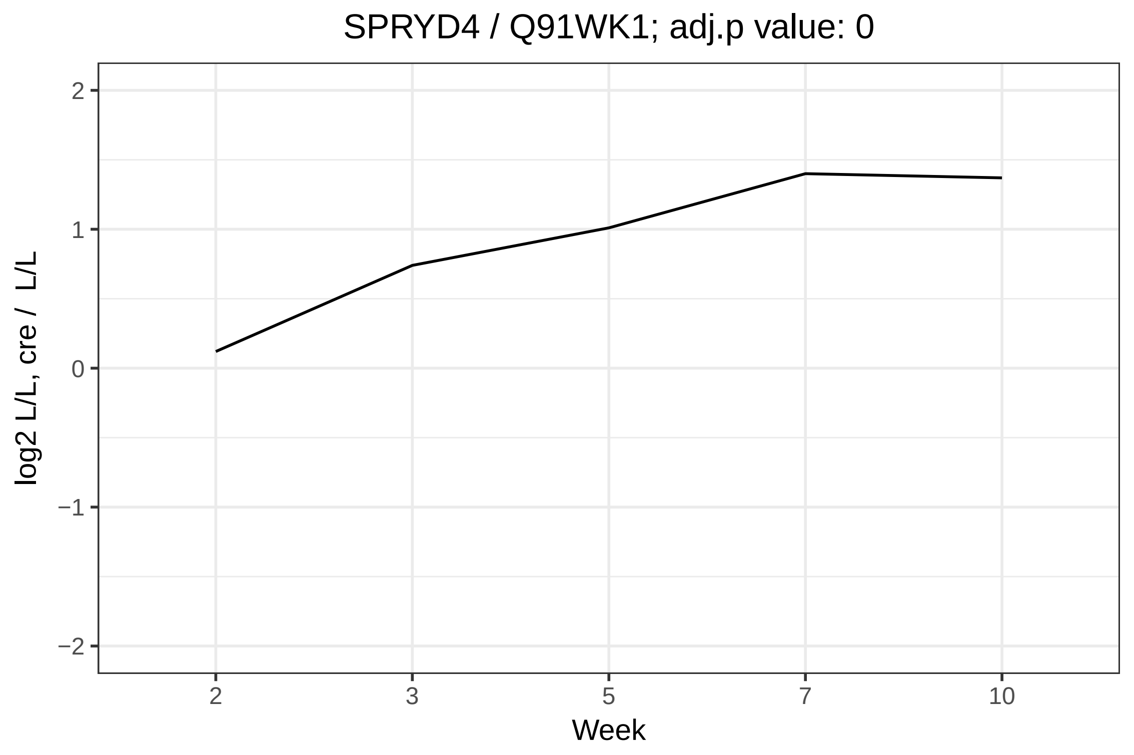
SPRYD4 / Q91WK1; adj.p value: 0
log2 L/L, cre / L/L
Week
2
1
0
−1
−2
2
3
5
7
10

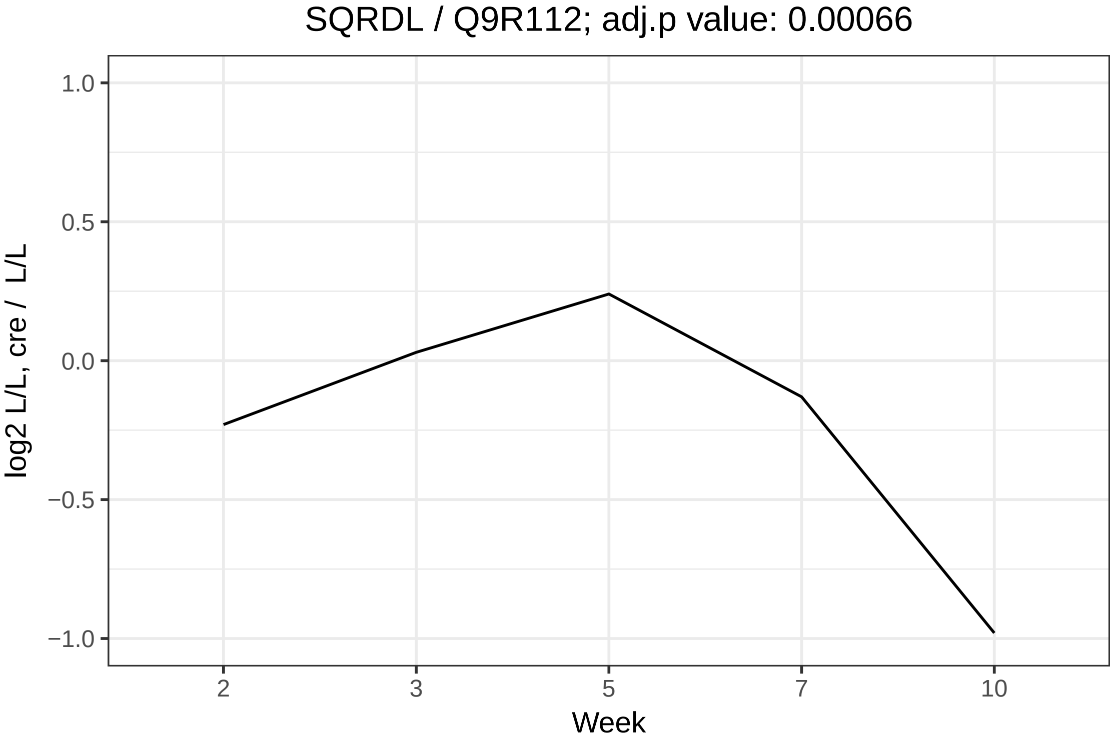

SQRDL / Q9R112; adj.p value: 0.00066
1.0
0.5
0.0
−0.5
−1.0
log2 L/L, cre / L/L
2
3
5
7
10
Week

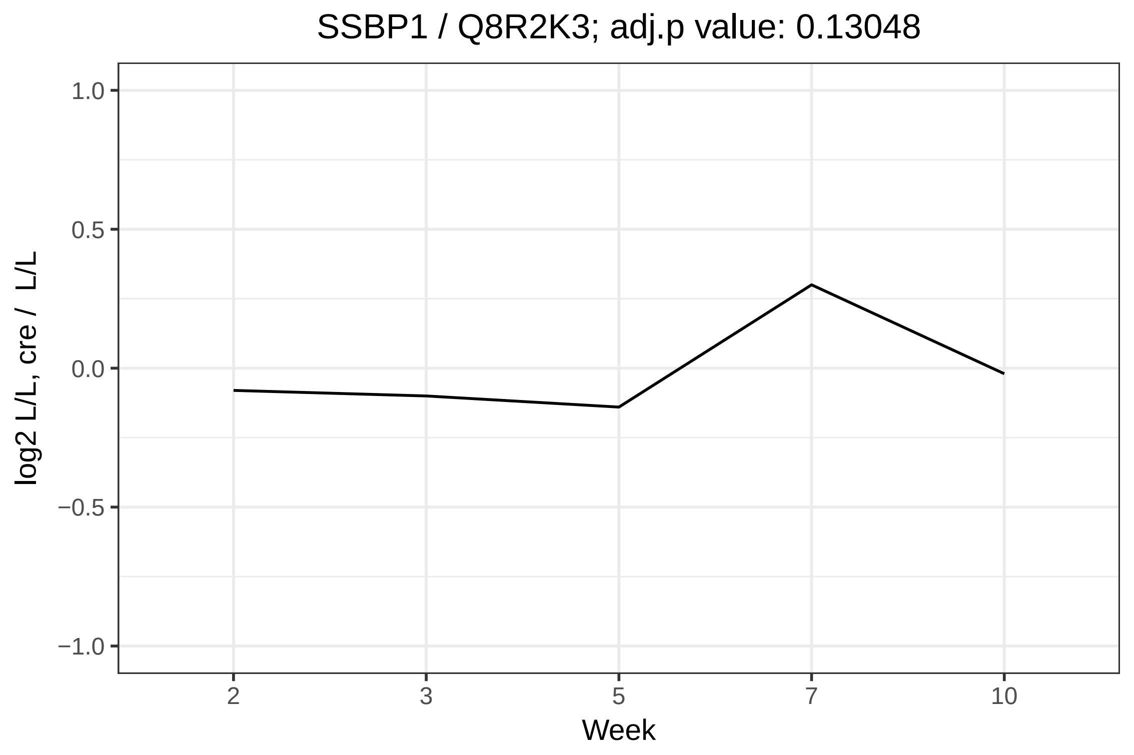
SSBP1 / Q8R2K3; adj.p value: 0.13048
log2 L/L, cre / L/L
1.0
0.5
0.0
−0.5
−1.0
2
3
5
7
10
Week

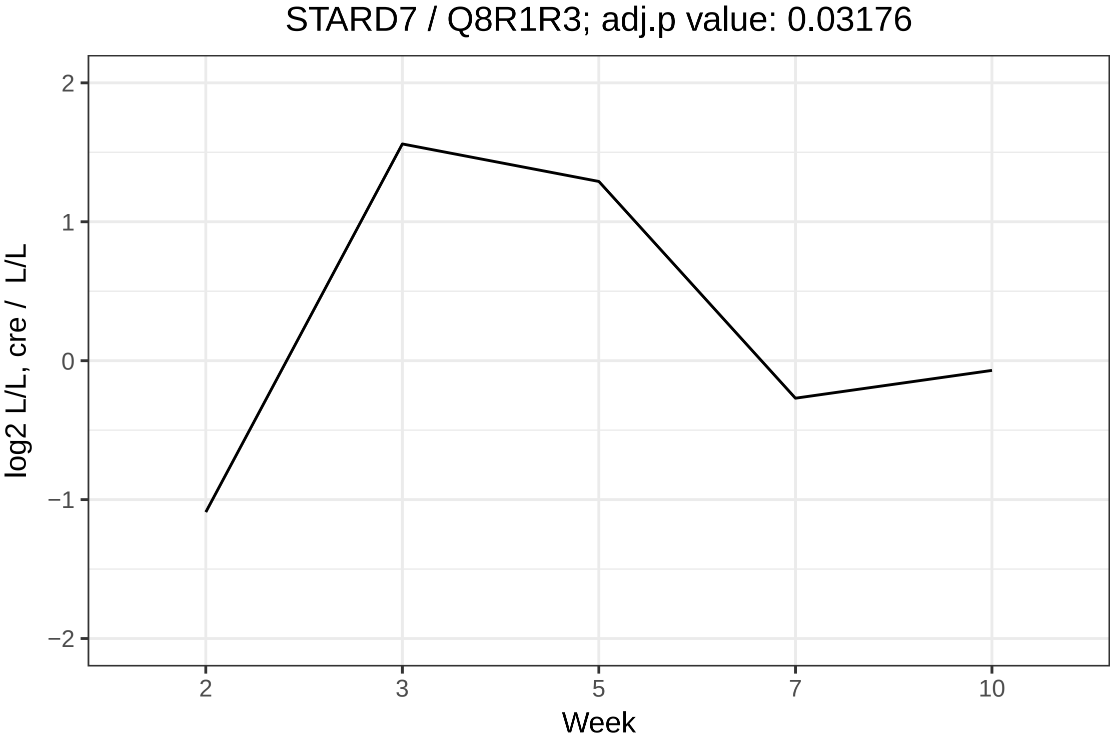
STARD7 / Q8R1R3; adj.p value: 0.03176
log2 L/L, cre / L/L
2
1
0
−1
−2
2
3
5
7
10
Week

STOML2 / Q99JB2; adj.p value: 0

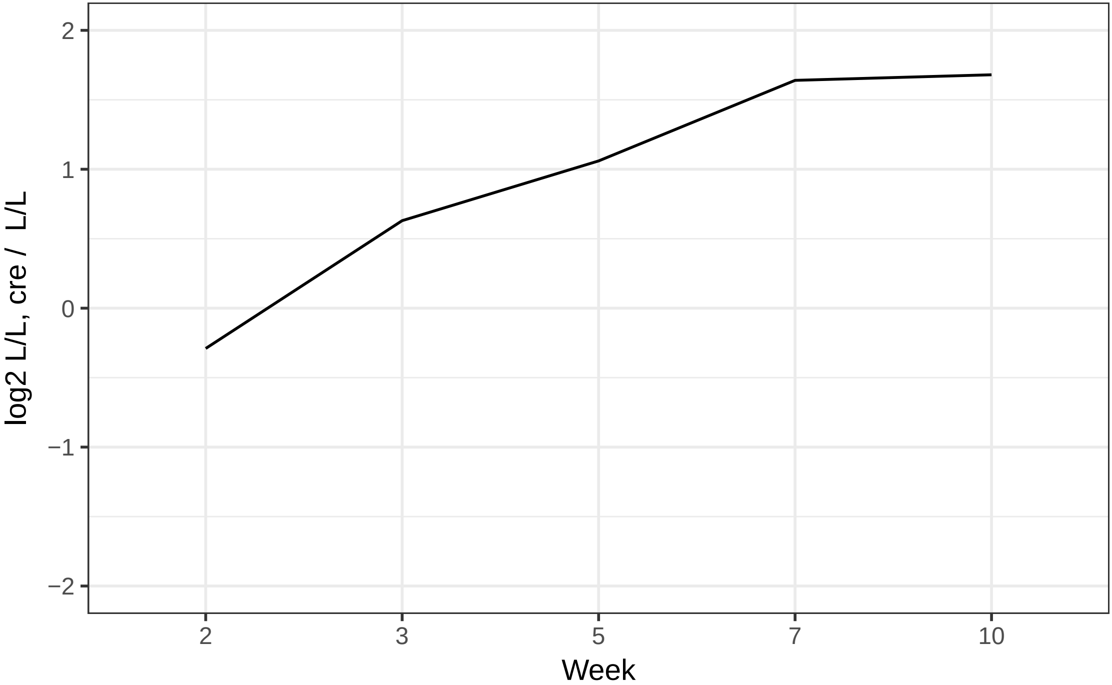

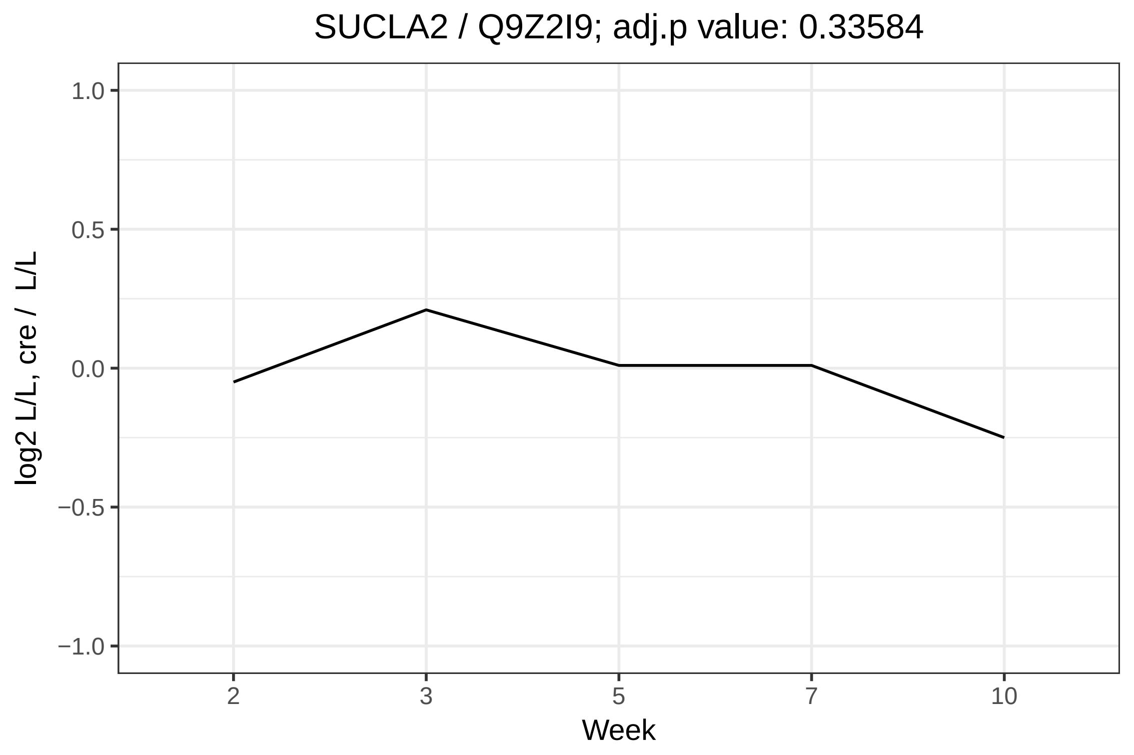

SUCLA2 / Q9Z2I9; adj.p value: 0.33584
1.0
0.5
0.0
−0.5
−1.0
log2 L/L, cre / L/L
2
3
5
7
10
Week

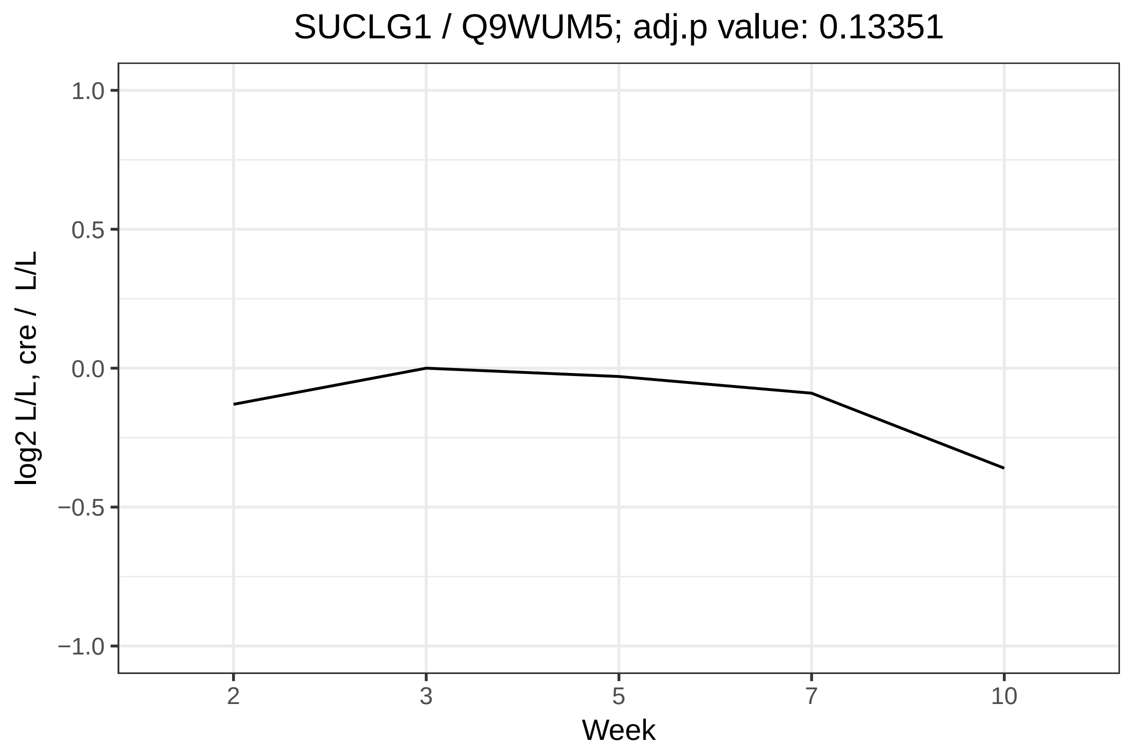
SUCLG1 / Q9WUM5; adj.p value: 0.13351
log2 L/L, cre / L/L
1.0
0.5
0.0
−0.5
−1.0
2
3
5
7
10
Week

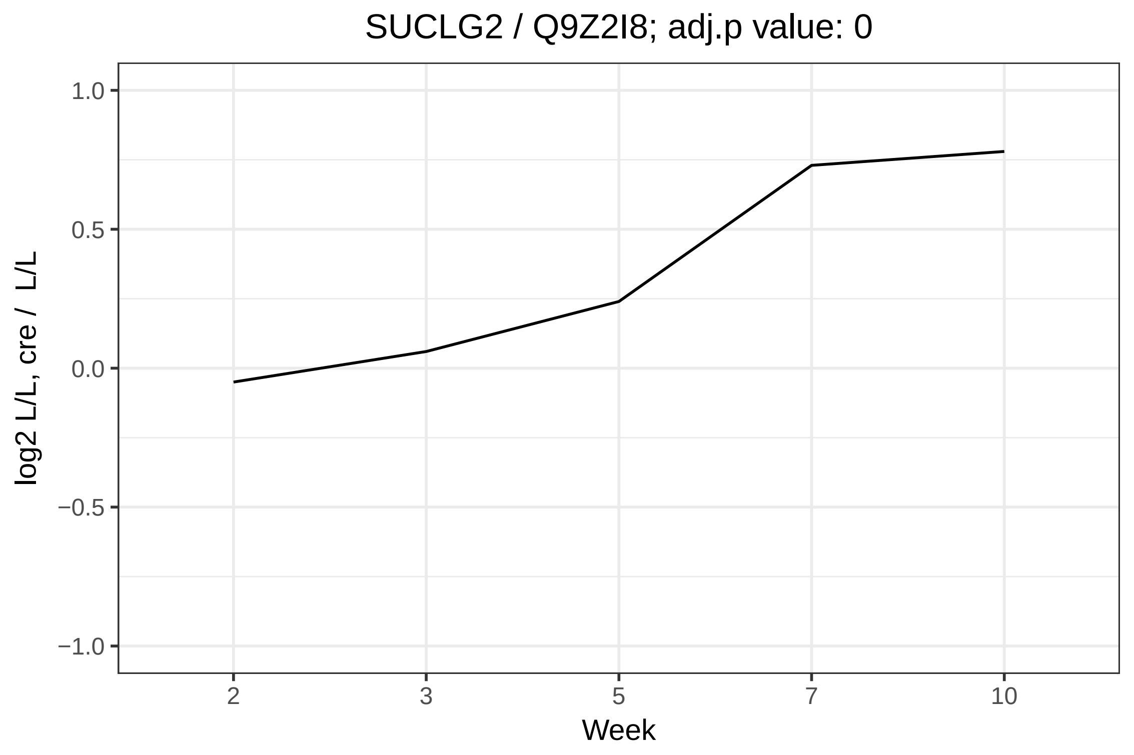

SUCLG2 / Q9Z2I8; adj.p value: 0
log2 L/L, cre / L/L
1.0
0.5
0.0
−0.5
−1.0
2
3
5
7
10
Week

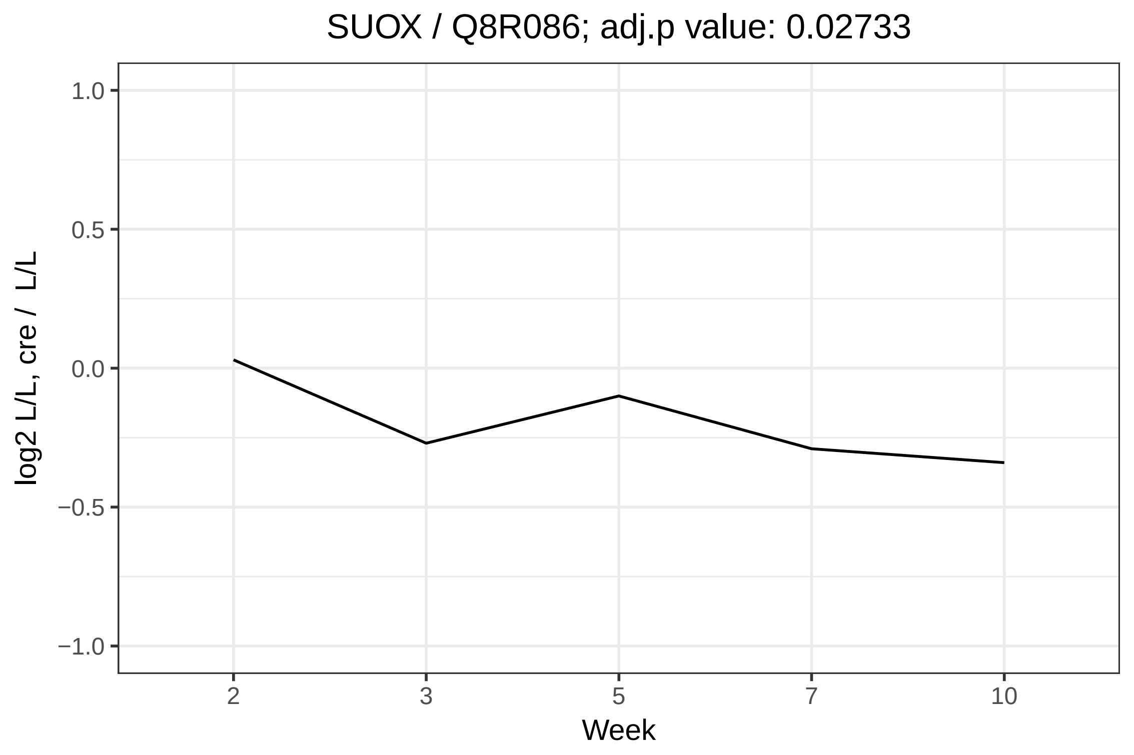

SUOX / Q8R086; adj.p value: 0.02733
1.0
0.5
0.0
−0.5
−1.0
log2 L/L, cre / L/L
2
3
5
7
10
Week

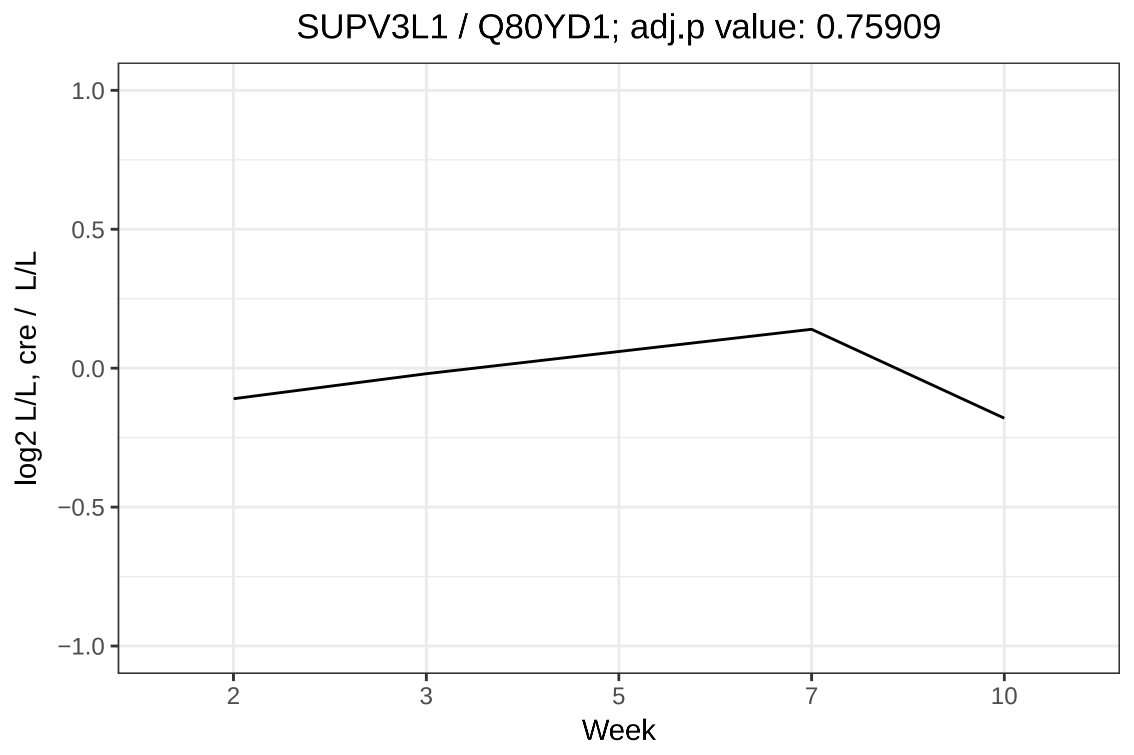
SUPV3L1 / Q80YD1; adj.p value: 0.75909
log2 L/L, cre / L/L
1.0
0.5
0.0
−0.5
−1.0
2
3
5
7
10
Week

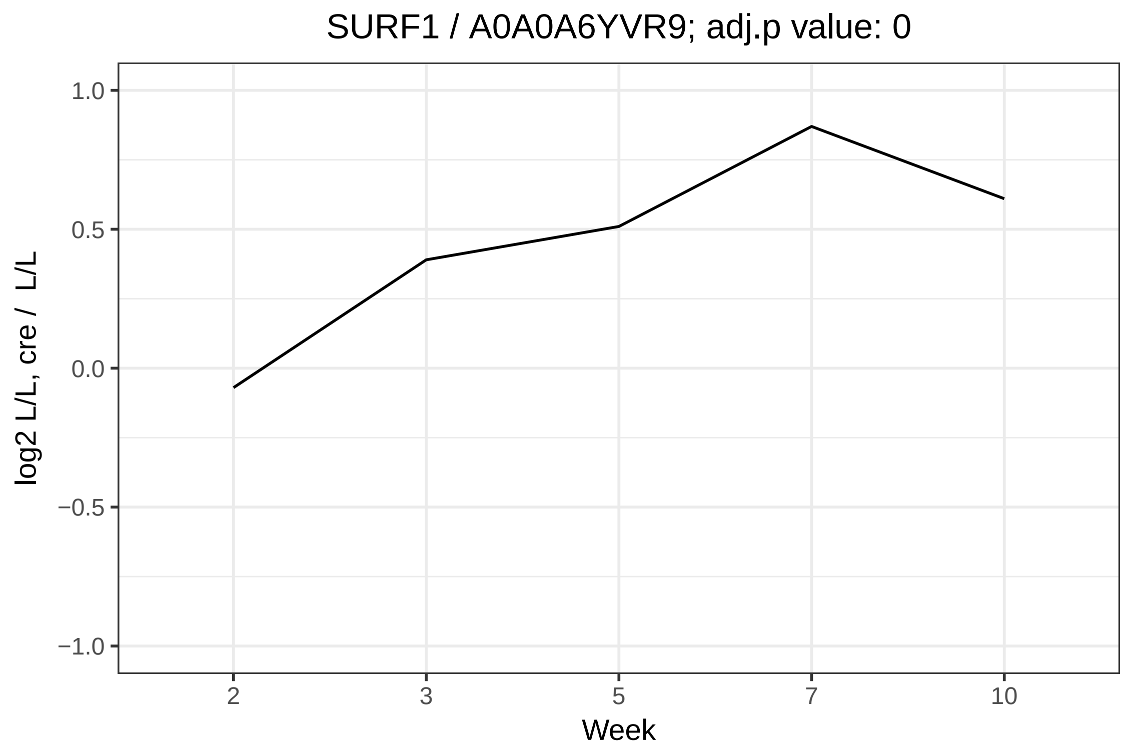

SURF1 / A0A0A6YVR9; adj.p value: 0
1.0
0.5
0.0
−0.5
−1.0
log2 L/L, cre / L/L
2
3
5
7
10
Week

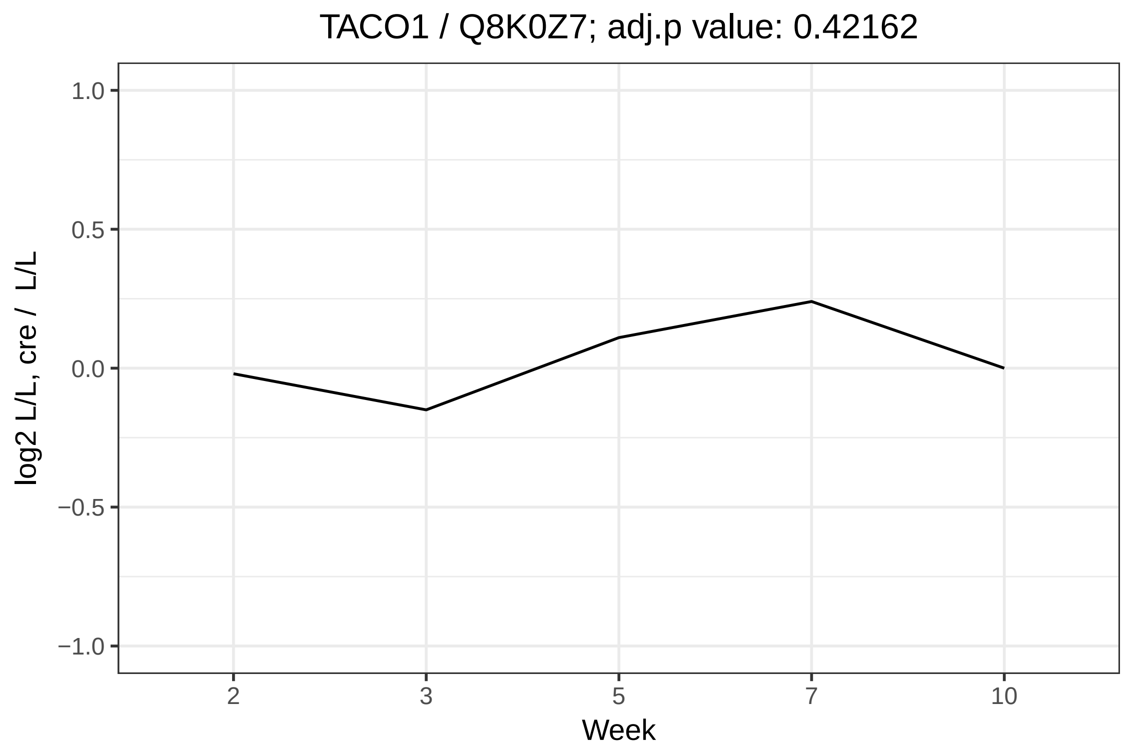
TACO1 / Q8K0Z7; adj.p value: 0.42162
log2 L/L, cre / L/L
1.0
0.5
0.0
−0.5
−1.0
2
3
5
7
10
Week

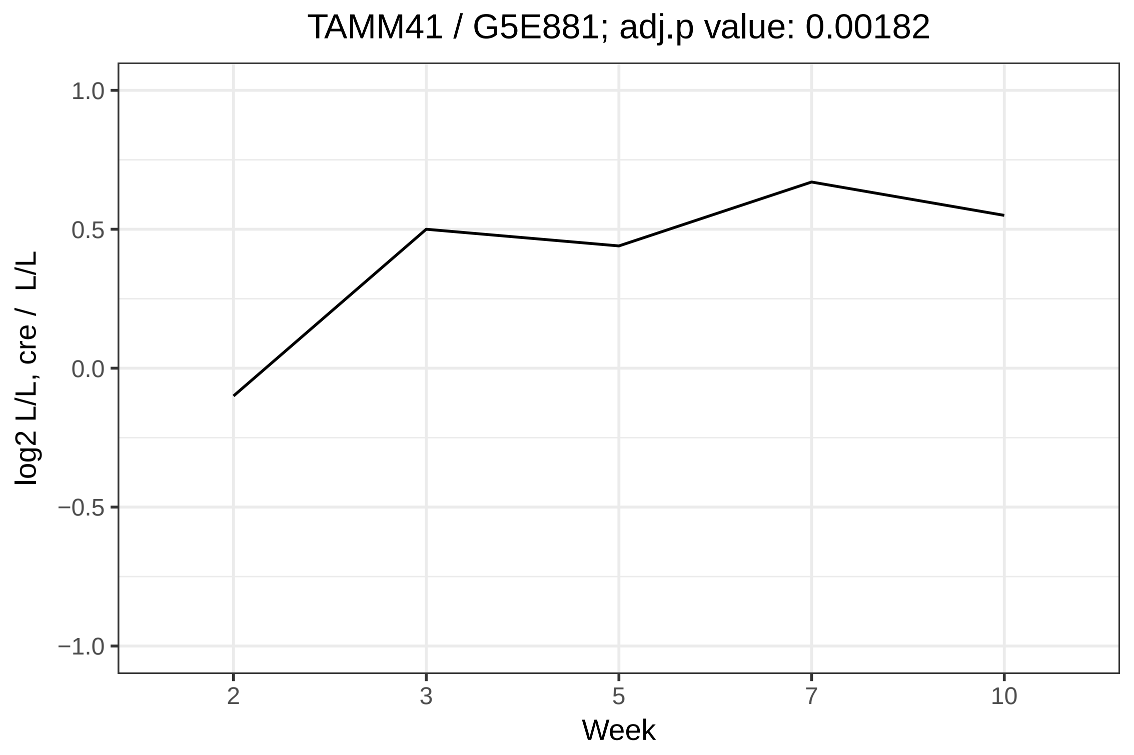
TAMM41 / G5E881; adj.p value: 0.00182
log2 L/L, cre / L/L
1.0
0.5
0.0
−0.5
−1.0
2
3
5
7
10
Week

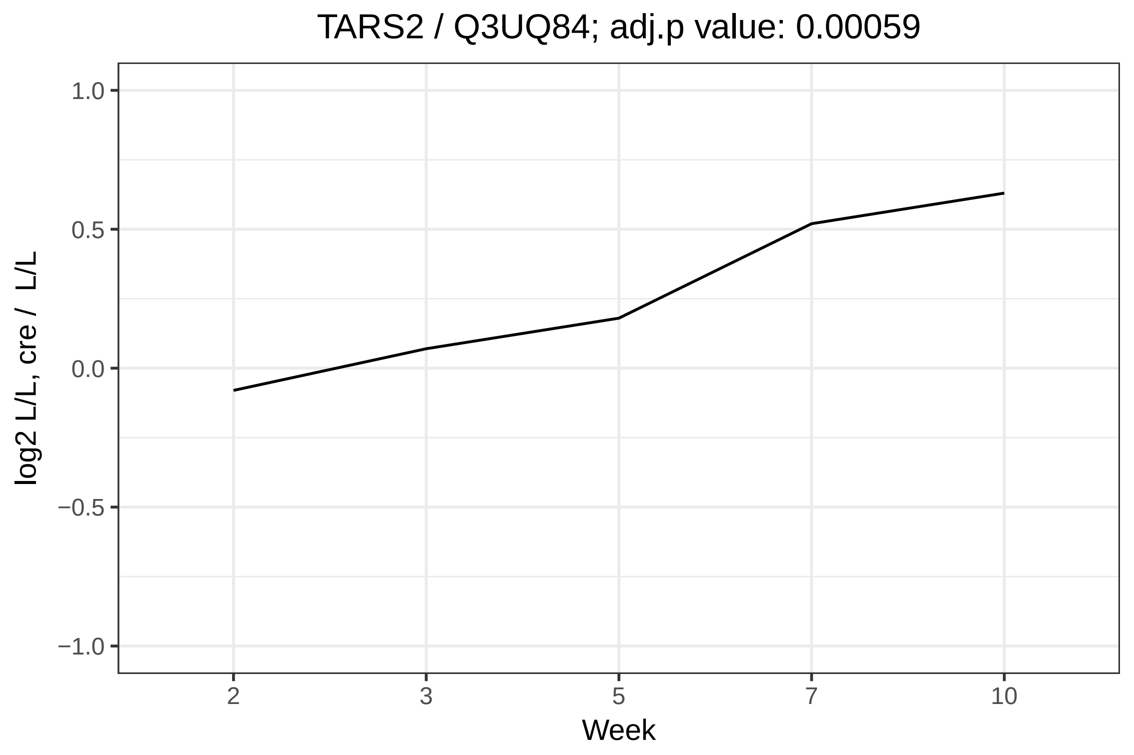

TARS2 / Q3UQ84; adj.p value: 0.00059
1.0
0.5
0.0
−0.5
−1.0
log2 L/L, cre / L/L
2
3
5
7
10
Week

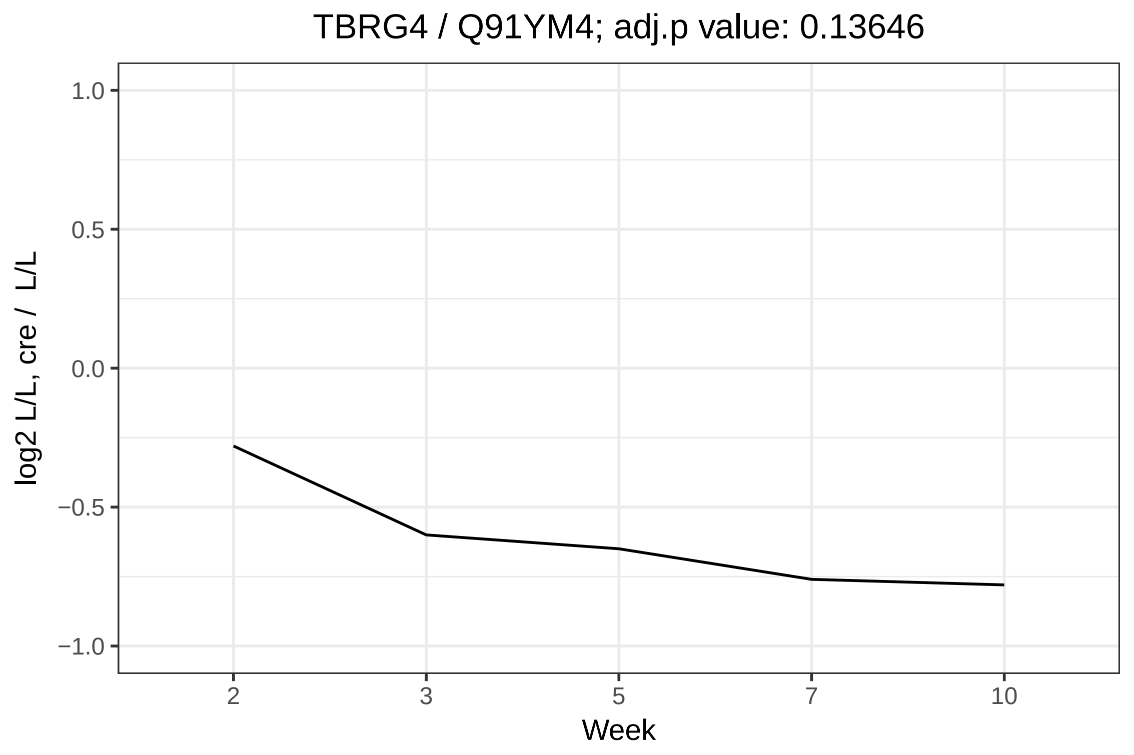
TBRG4 / Q91YM4; adj.p value: 0.13646
1.0
0.5
0.0
−0.5
−1.0
log2 L/L, cre / L/L
2
3
5
7
10
Week

TCAIM / G3X983; adj.p value: 0.18759

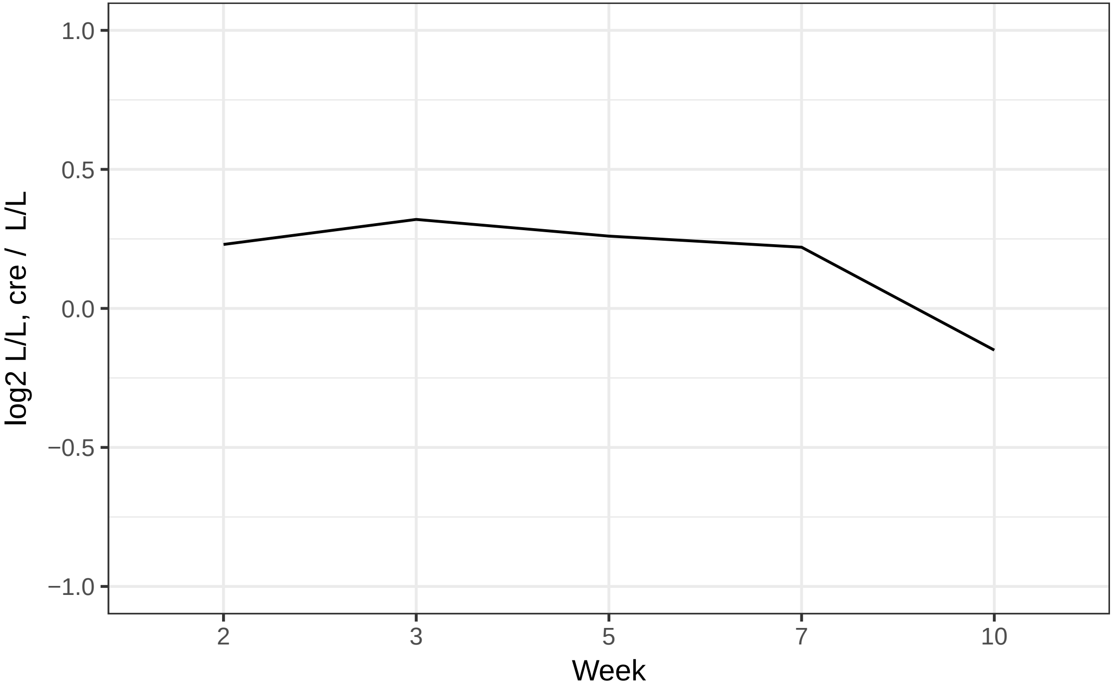

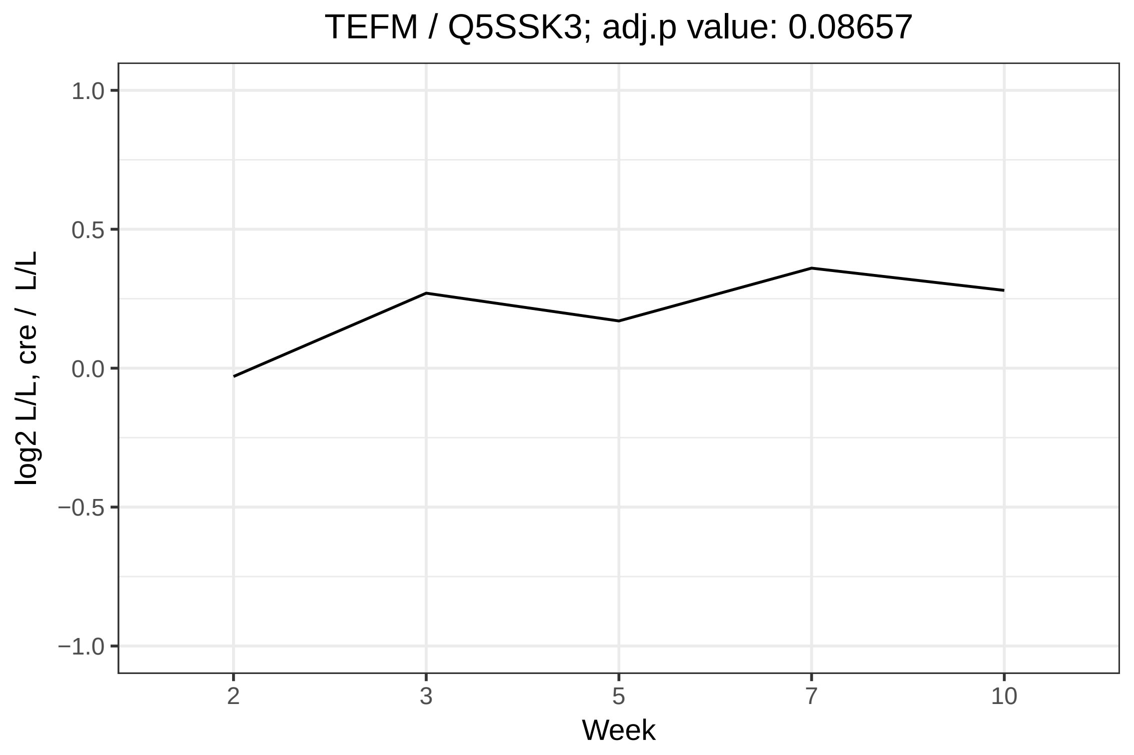
TEFM / Q5SSK3; adj.p value: 0.08657
log2 L/L, cre / L/L
1.0
0.5
0.0
−0.5
−1.0
2
3
5
7
10
Week

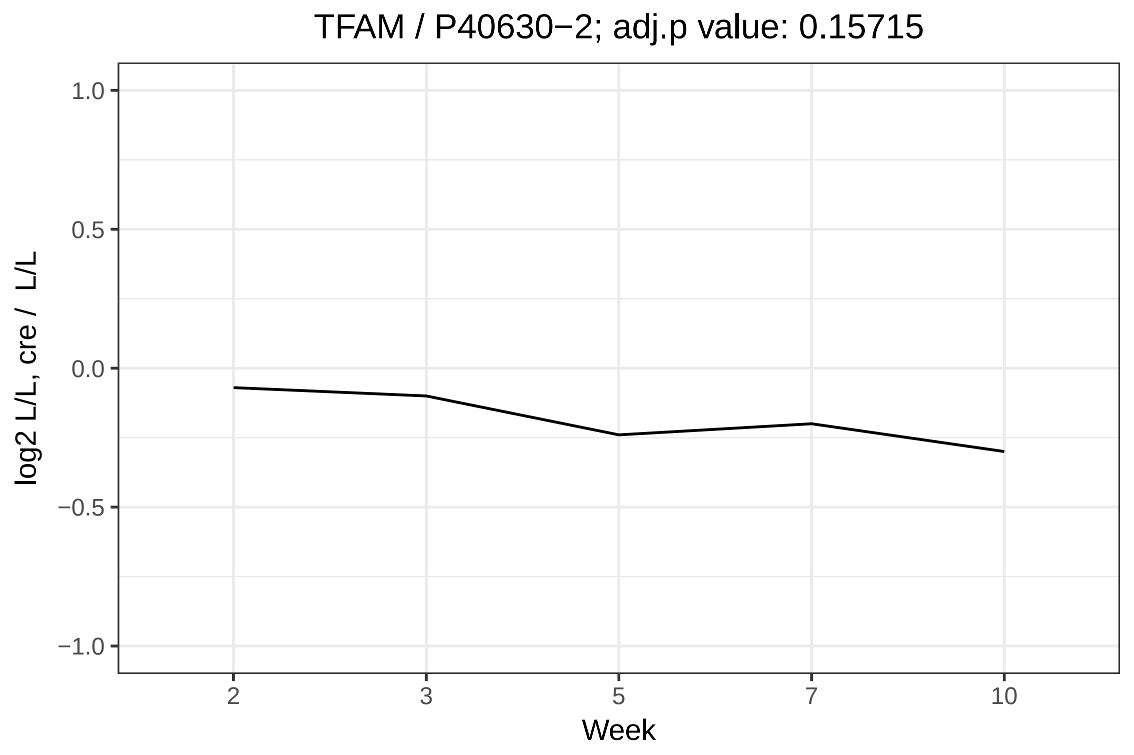
TFAM / P40630−2; adj.p value: 0.15716
1.0
0.5
0.0
−0.5
−1.0
log2 L/L, cre / L/L
2
3
5
7
10
Week

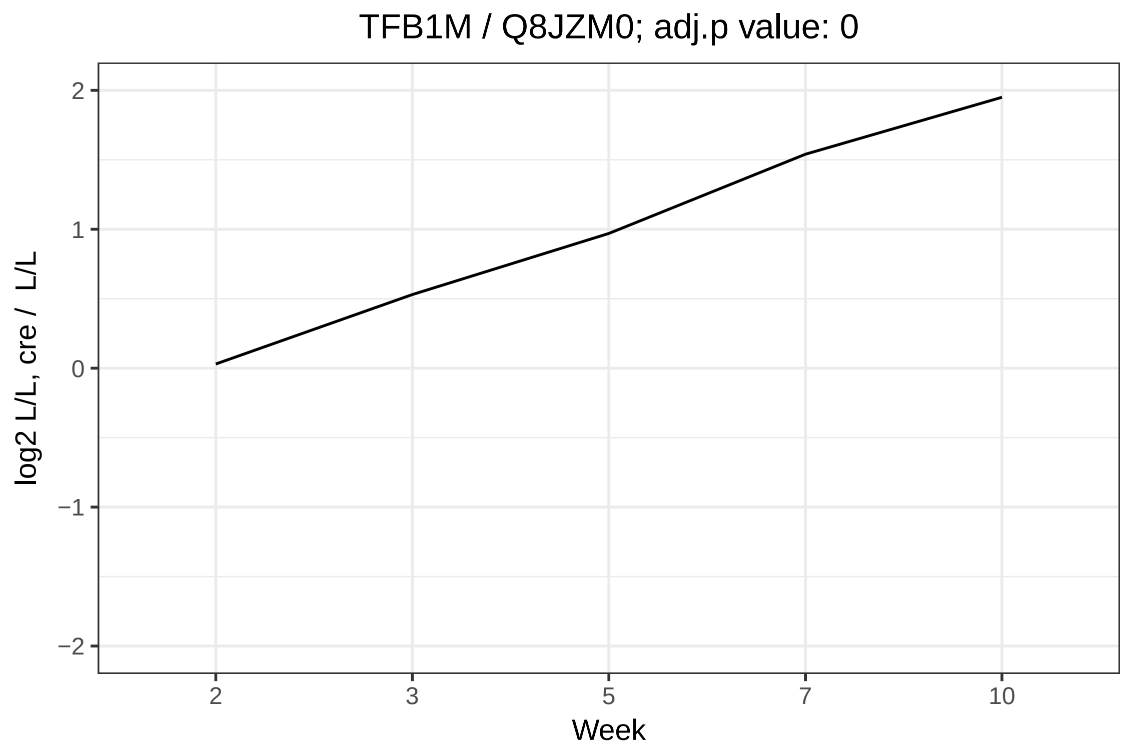

TFB1M / Q8JZM0; adj.p value: 0
2
1
0
−1
−2
log2 L/L, cre / L/L
2
3
5
7
10
Week

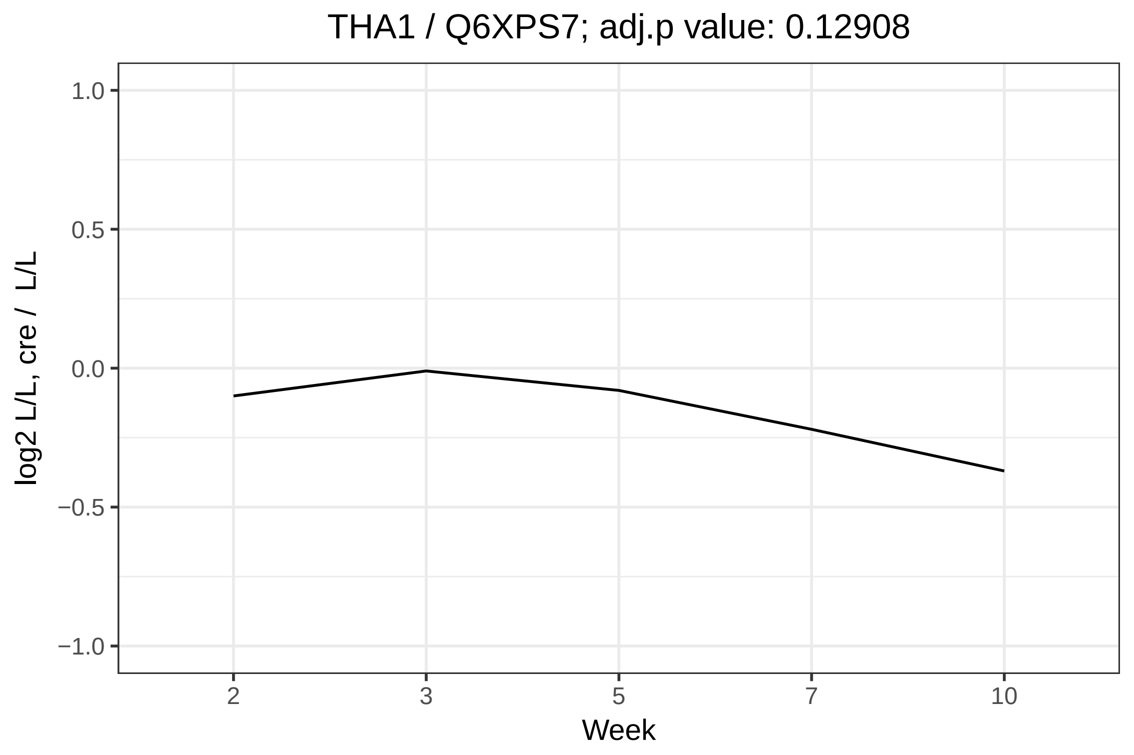

THA1 / Q6XPS7; adj.p value: 0.12908
log2 L/L, cre / L/L
1.0
0.5
0.0
−0.5
−1.0
2
3
5
7
10
Week

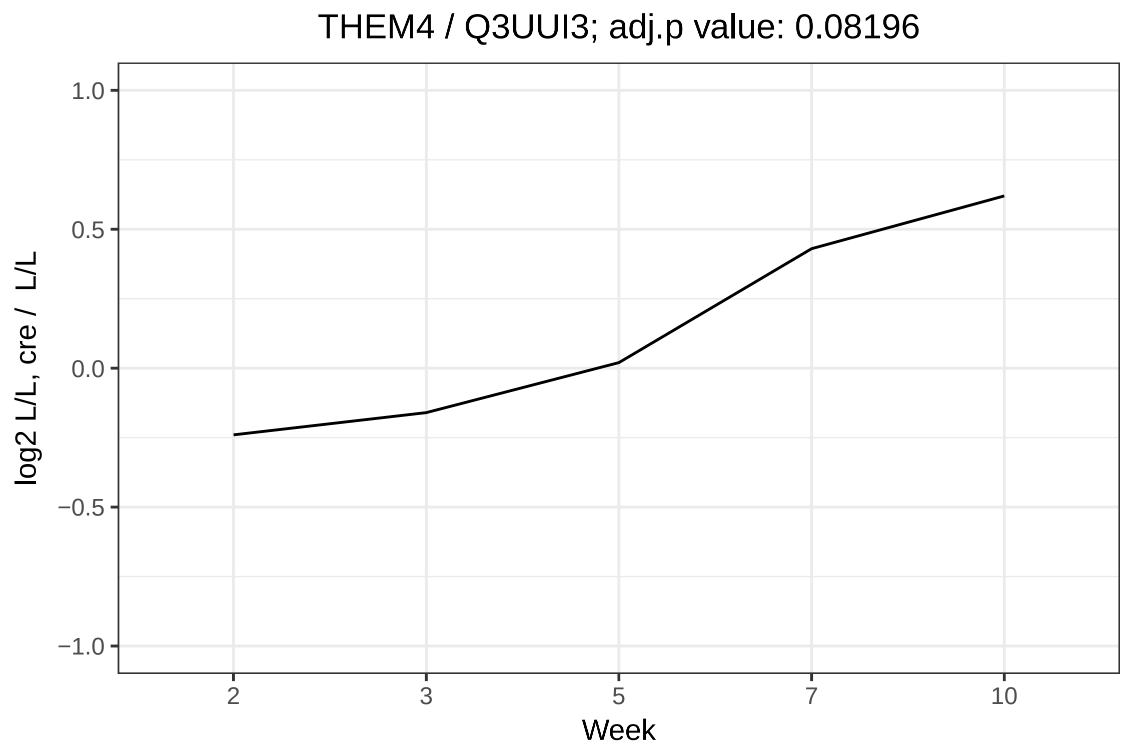
THEM4 / Q3UUI3; adj.p value: 0.08196
log2 L/L, cre / L/L
1.0
0.5
0.0
−0.5
−1.0
2
3
5
7
10
Week

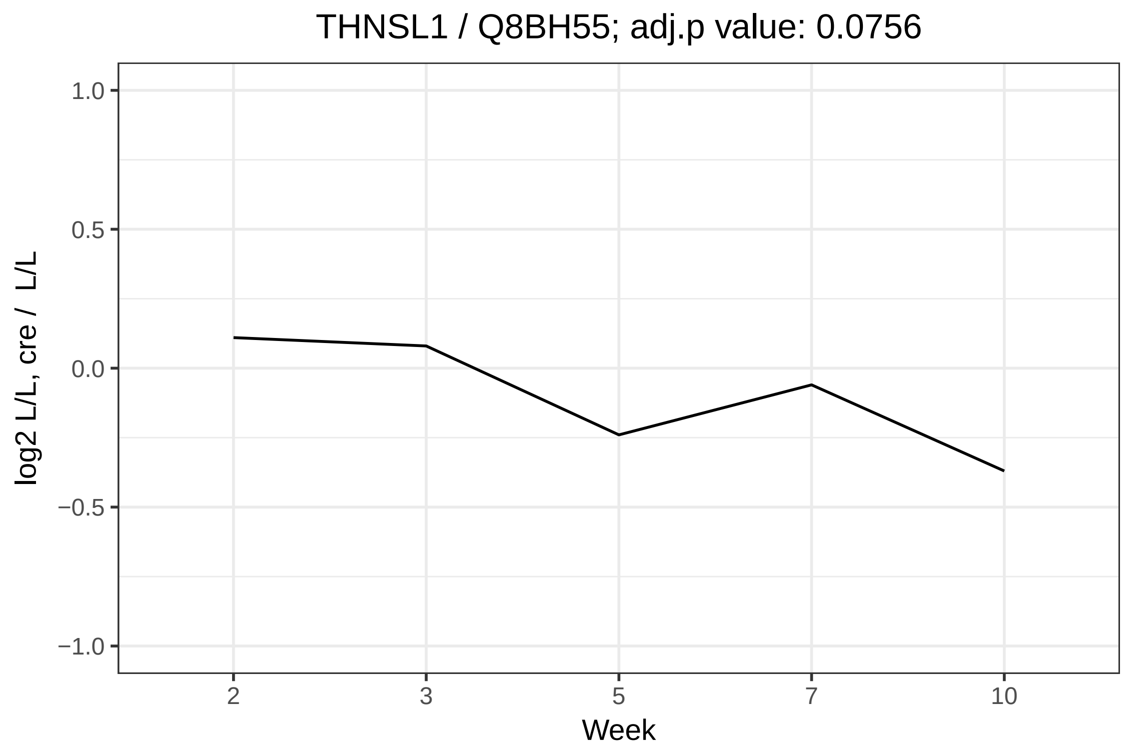

THNSL1 / Q8BH55; adj.p value: 0.0756
1.0
0.5
0.0
−0.5
−1.0
log2 L/L, cre / L/L
2
3
5
7
10
Week

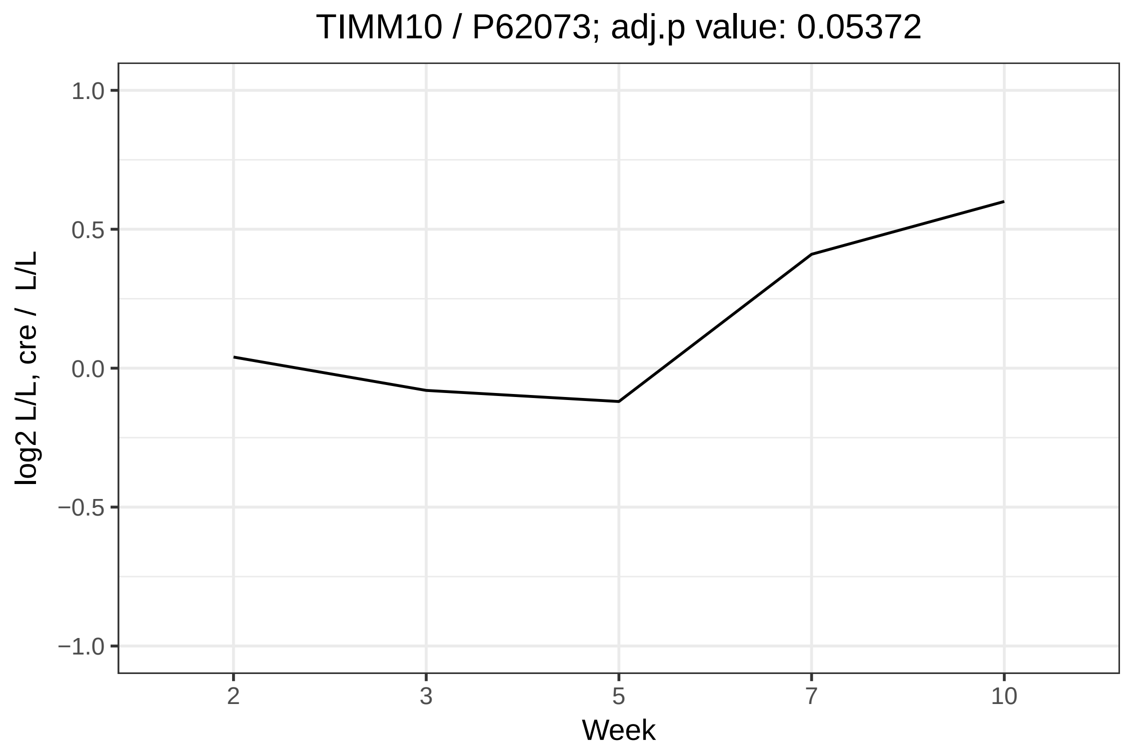

TIMM10 / P62073; adj.p value: 0.05372
log2 L/L, cre / L/L
1.0
0.5
0.0
−0.5
−1.0
2
3
5
7
10
Week

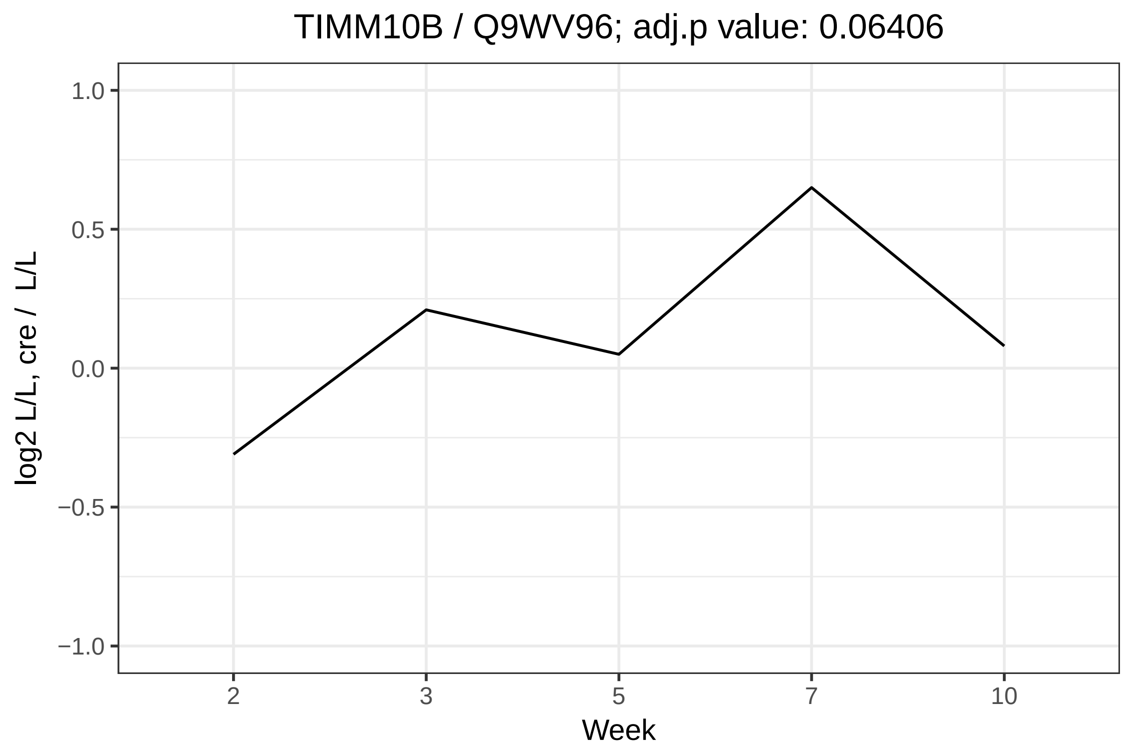

TIMM10B / Q9WV96; adj.p value: 0.06406
log2 L/L, cre / L/L
1.0
0.5
0.0
−0.5
−1.0
2
3
5
7
10
Week

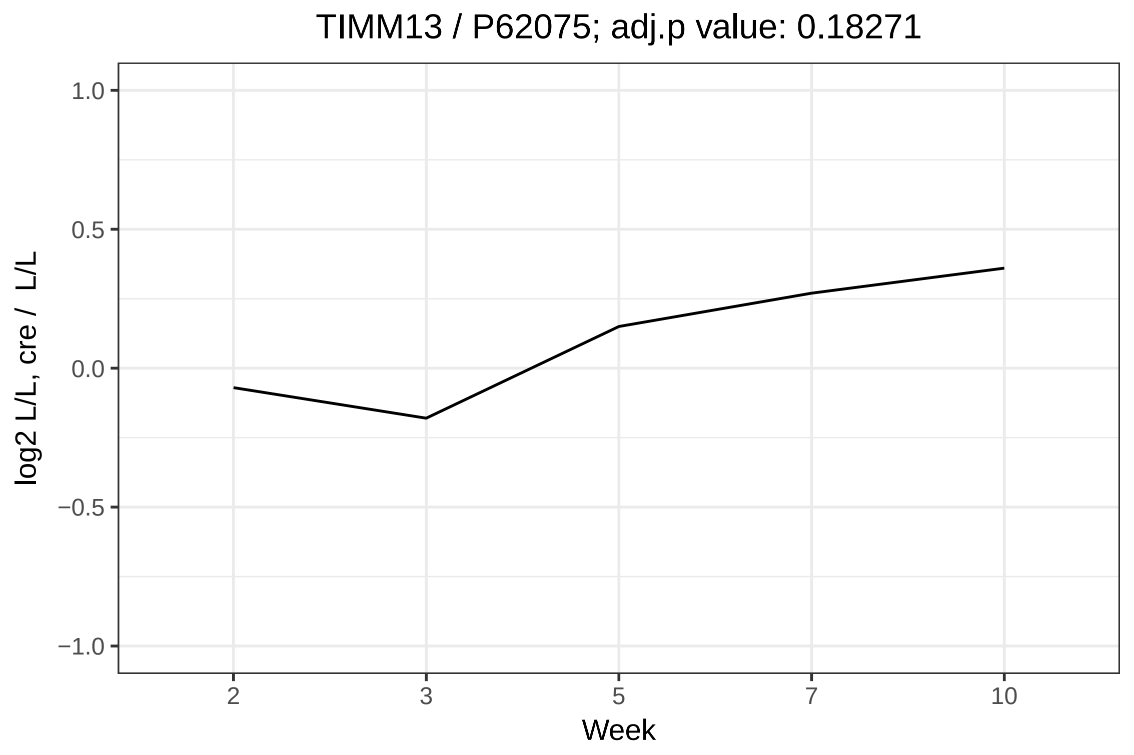
TIMM13 / P62075; adj.p value: 0.18271
1.0
0.5
0.0
−0.5
−1.0
log2 L/L, cre / L/L
2
3
5
7
10
Week

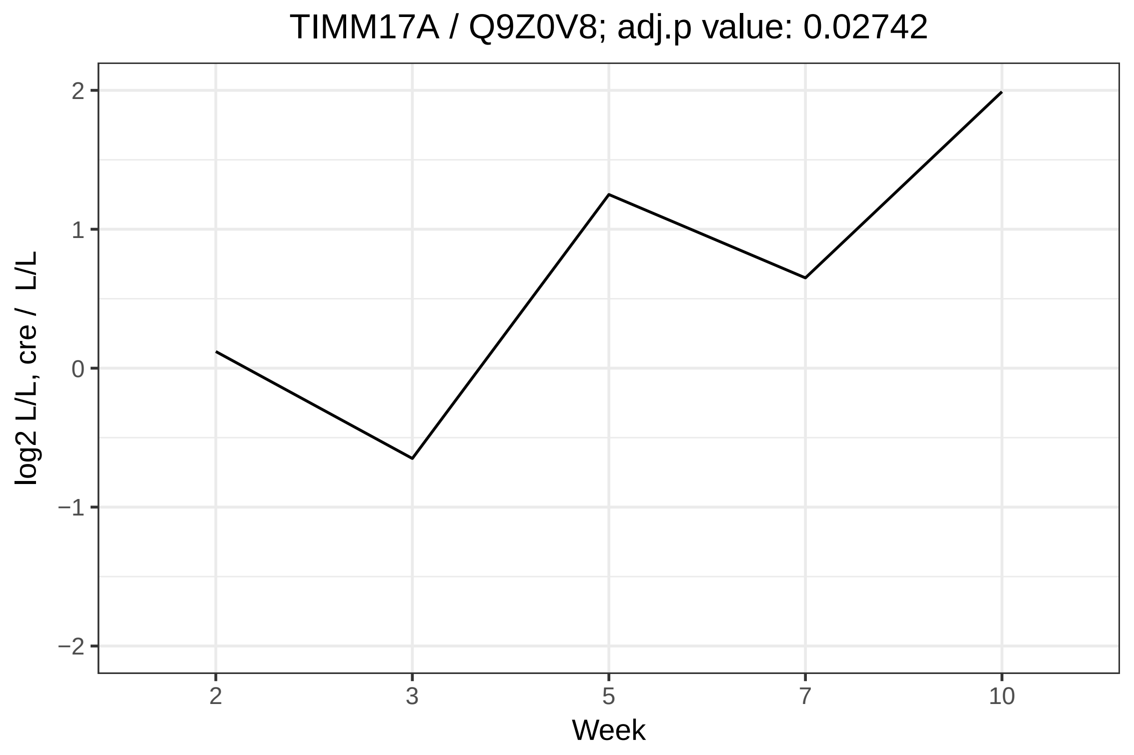
TIMM17A / Q9Z0V8; adj.p value: 0.02742
log2 L/L, cre / L/L
2
1
0
−1
−2
2
3
5
7
10
Week

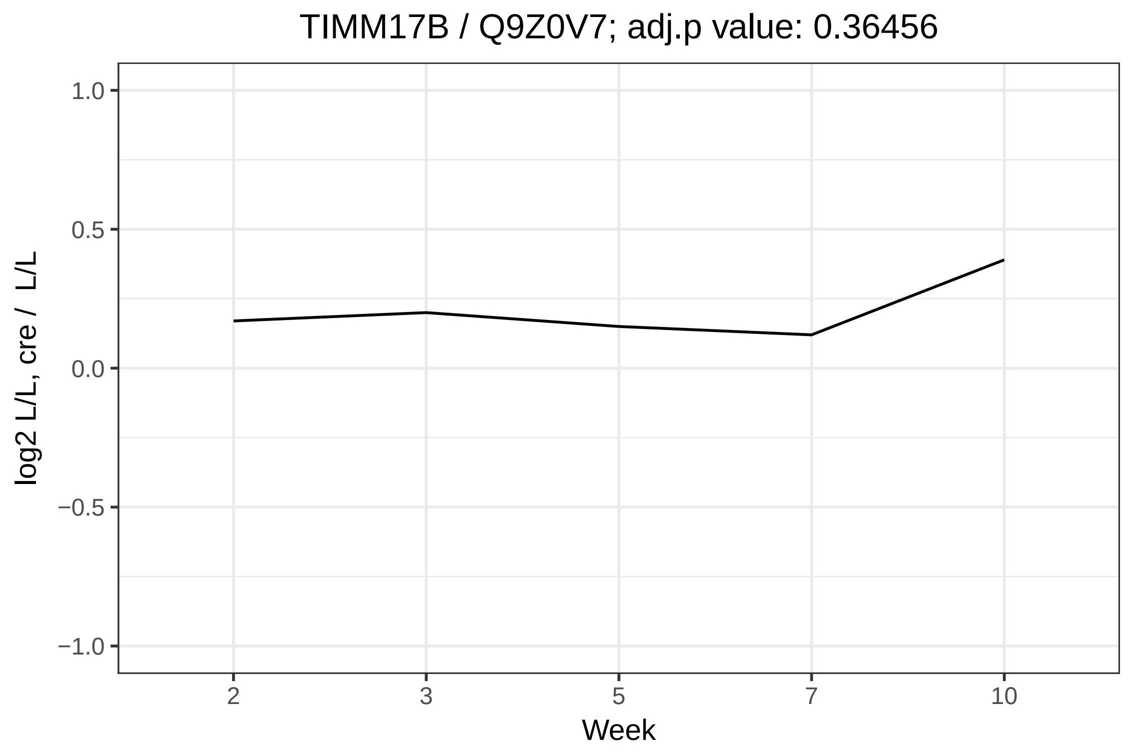
TIMM17B / Q9Z0V7; adj.p value: 0.36456
1.0
0.5
0.0
−0.5
−1.0
log2 L/L, cre / L/L
2
3
5
7
10
Week

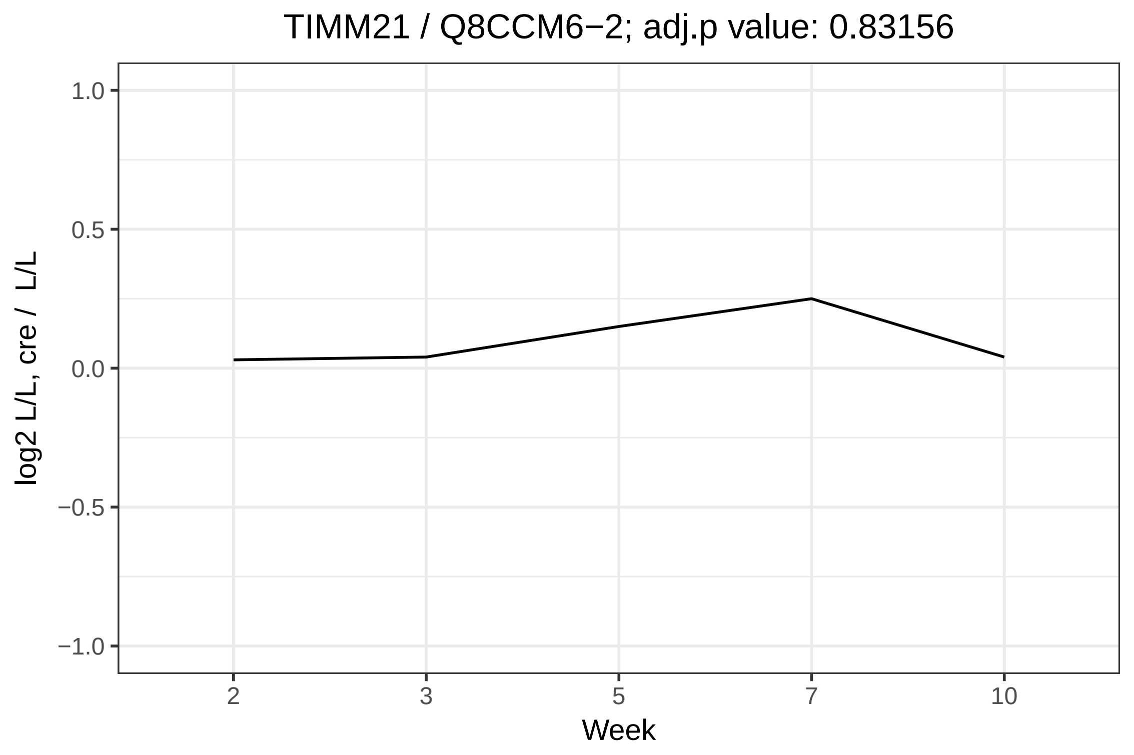

TIMM21 / Q8CCM6−2; adj.p value: 0.83156
log2 L/L, cre / L/L
1.0
0.5
0.0
−0.5
−1.0
2
3
5
7
10
Week

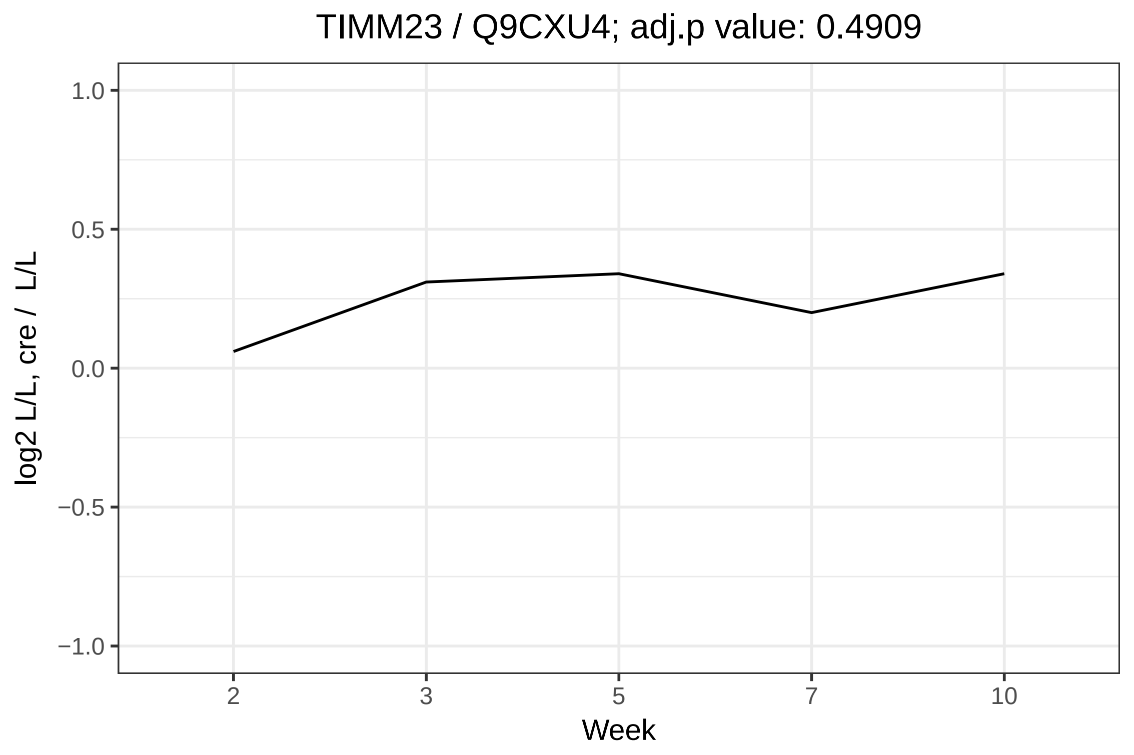

TIMM23 / Q9CXU4; adj.p value: 0.4909
1.0
0.5
0.0
−0.5
−1.0
log2 L/L, cre / L/L
2
3
5
7
10
Week

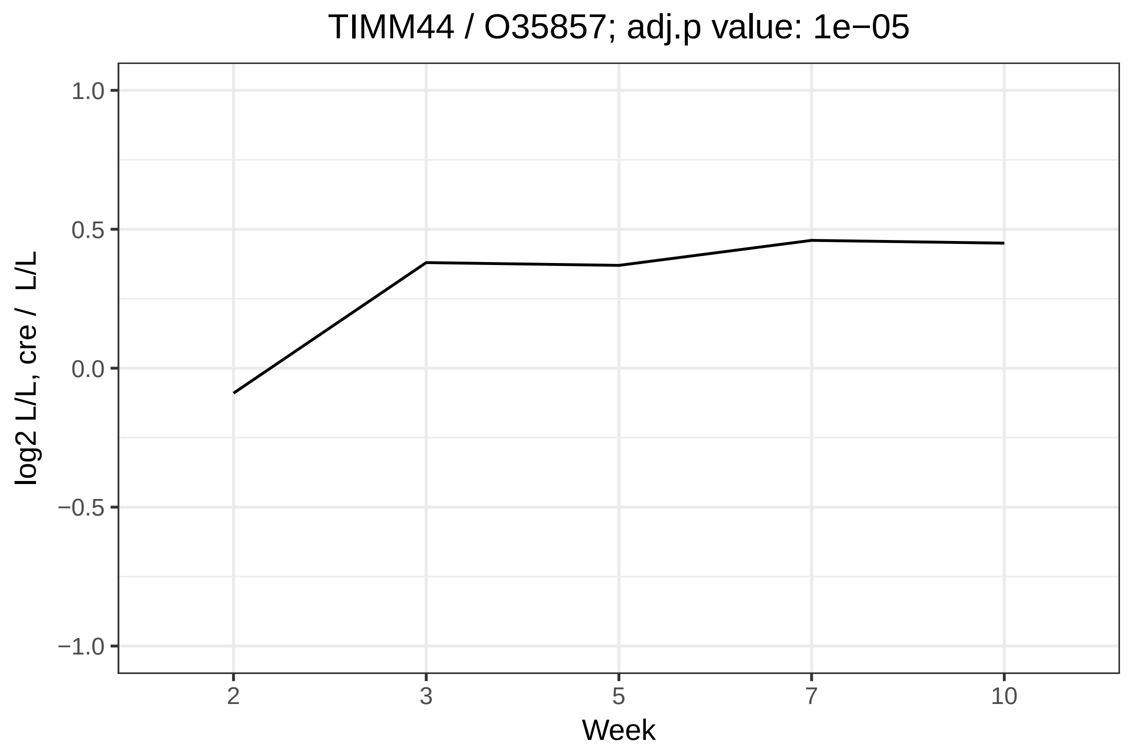

TIMM44 / O35857; adj.p value: 1e−05
1.0
0.5
0.0
−0.5
−1.0
log2 L/L, cre / L/L
2
3
5
7
10
Week

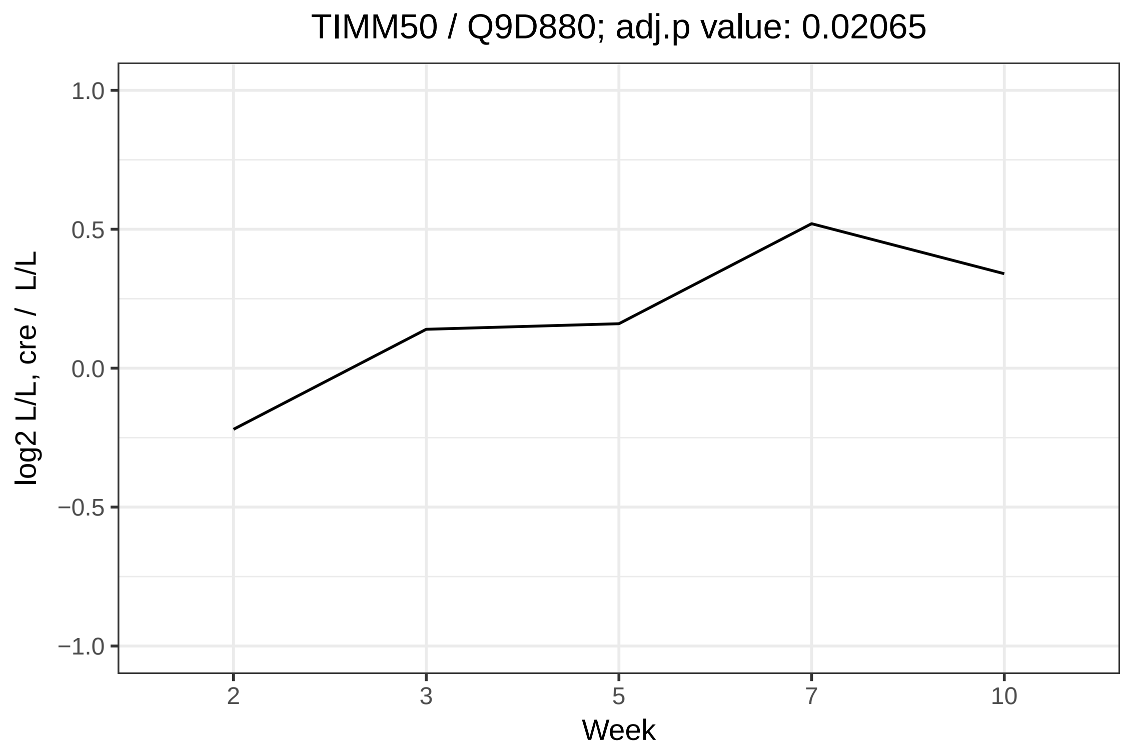
TIMM50 / Q9D880; adj.p value: 0.02065
log2 L/L, cre / L/L
1.0
0.5
0.0
−0.5
−1.0
2
3
5
7
10
Week

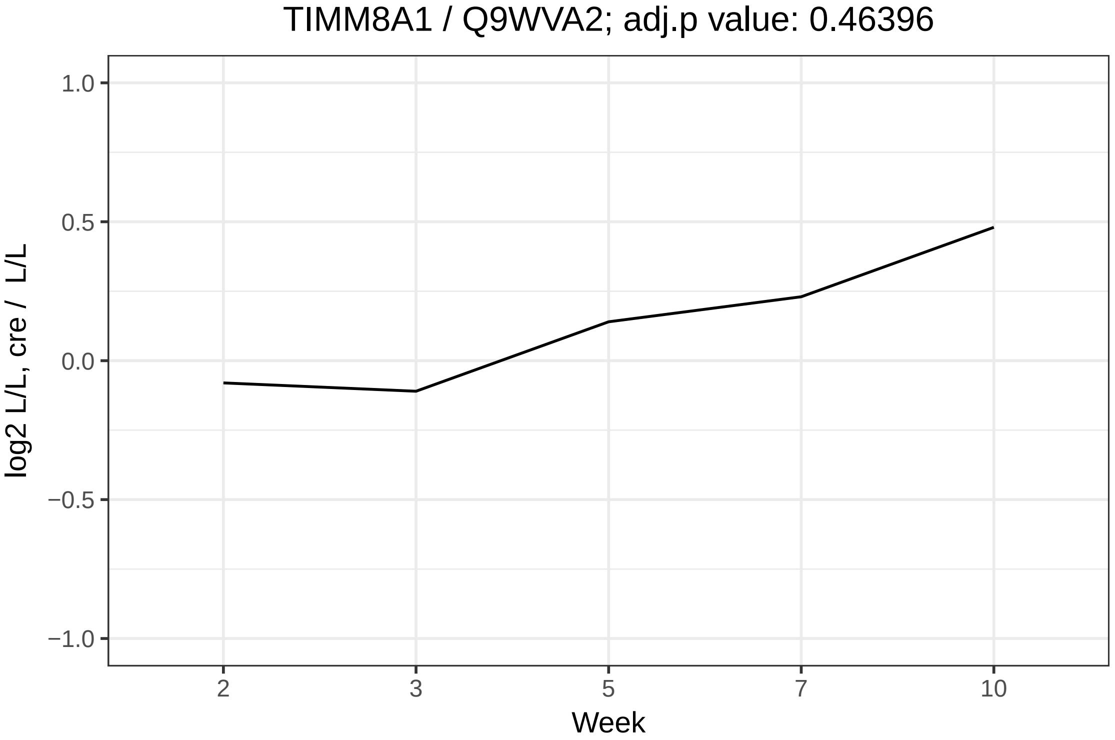

TIMM8A1 / Q9WVA2; adj.p value: 0.46396
1.0
0.5
0.0
−0.5
−1.0
log2 L/L, cre / L/L
2
3
5
7
10
Week

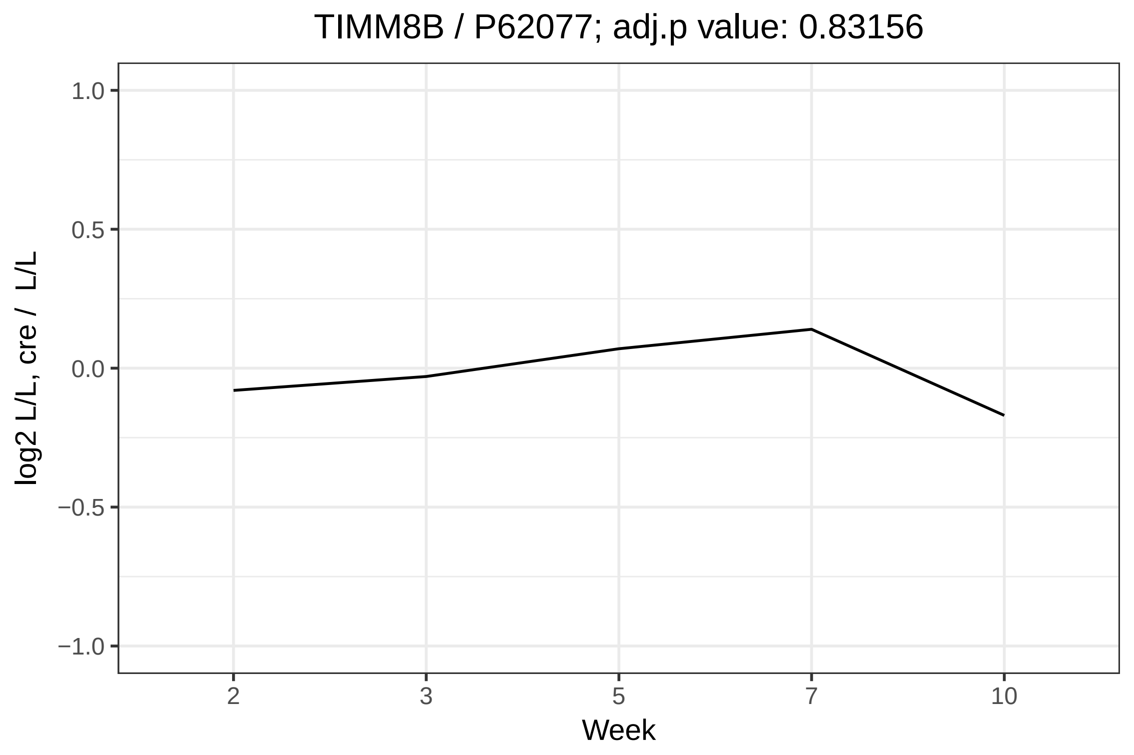
TIMM8B / P62077; adj.p value: 0.83156
1.0
0.5
0.0
−0.5
−1.0
log2 L/L, cre / L/L
2
3
5
7
10
Week

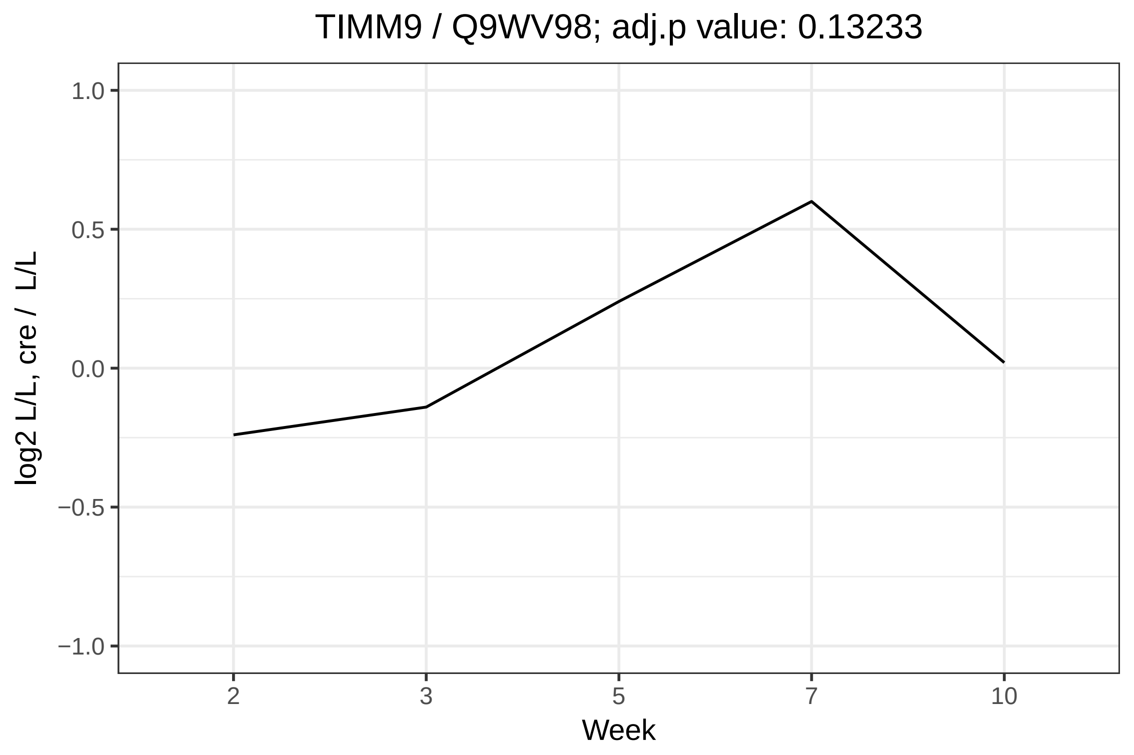

TIMM9 / Q9WV98; adj.p value: 0.13233
log2 L/L, cre / L/L
1.0
0.5
0.0
−0.5
−1.0
2
3
5
7
10
Week

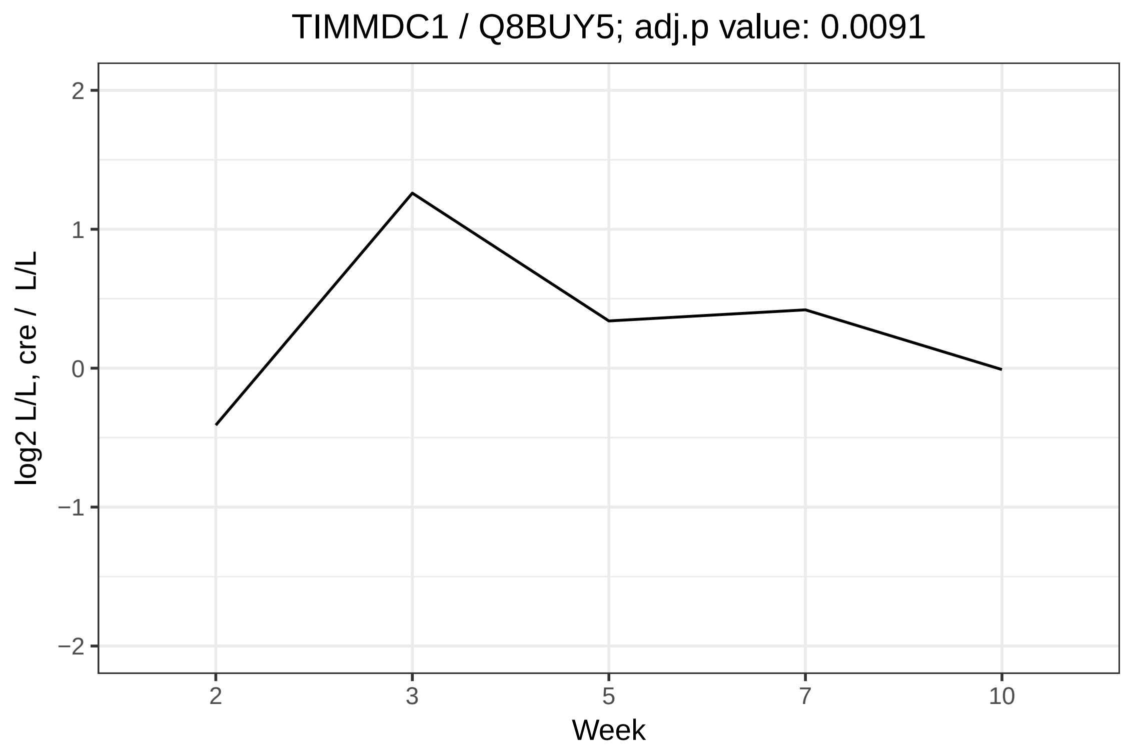
TIMMDC1 / Q8BUY5; adj.p value: 0.0091
log2 L/L, cre / L/L
2
1
0
−1
−2
2
3
5
7
10
Week

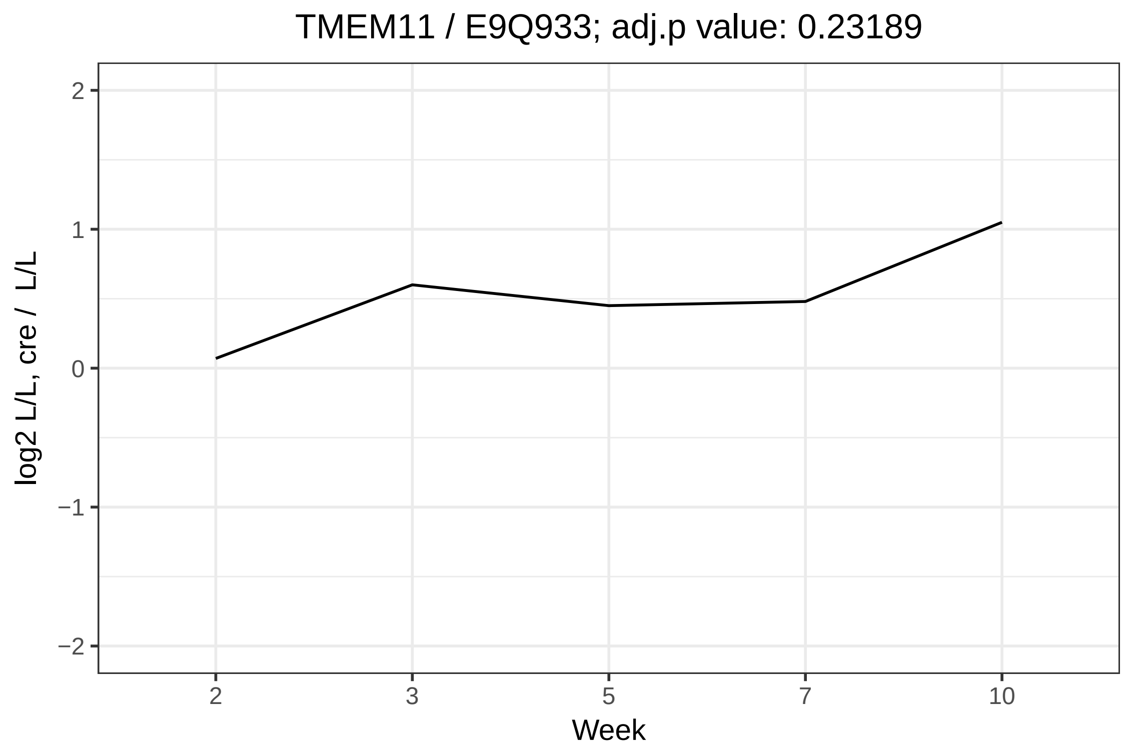

TMEM11 / E9Q933; adj.p value: 0.23189
log2 L/L, cre / L/L
2
1
0
−1
−2
2
3
5
7
10
Week

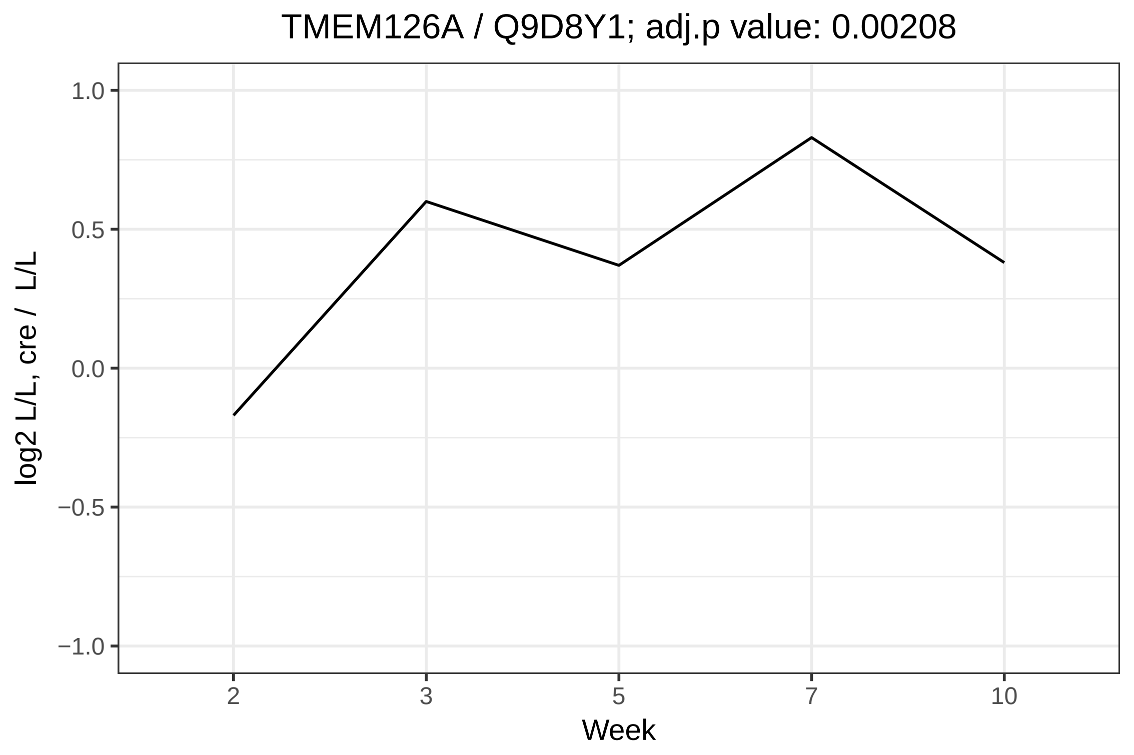
TMEM126A / Q9D8Y1; adj.p value: 0.00208
log2 L/L, cre / L/L
1.0
0.5
0.0
−0.5
−1.0
2
3
5
7
10
Week

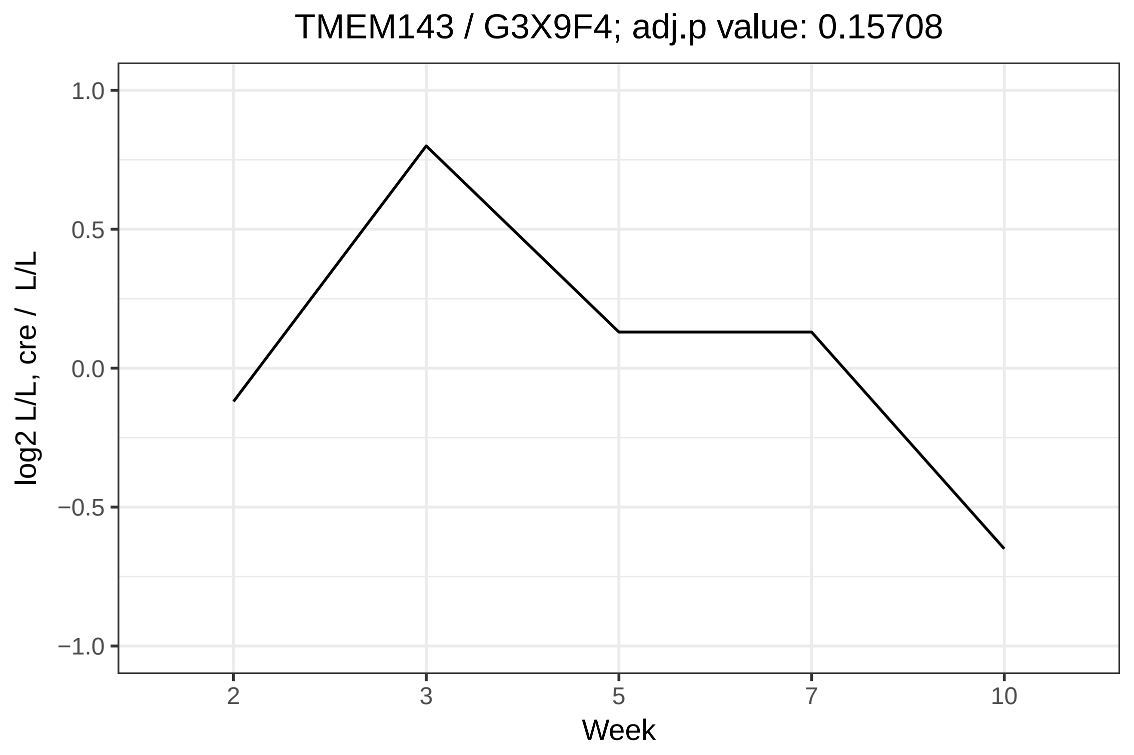
TMEM143 / G3X9F4; adj.p value: 0.15708
1.0
0.5
0.0
−0.5
−1.0
log2 L/L, cre / L/L
2
3
5
7
10
Week

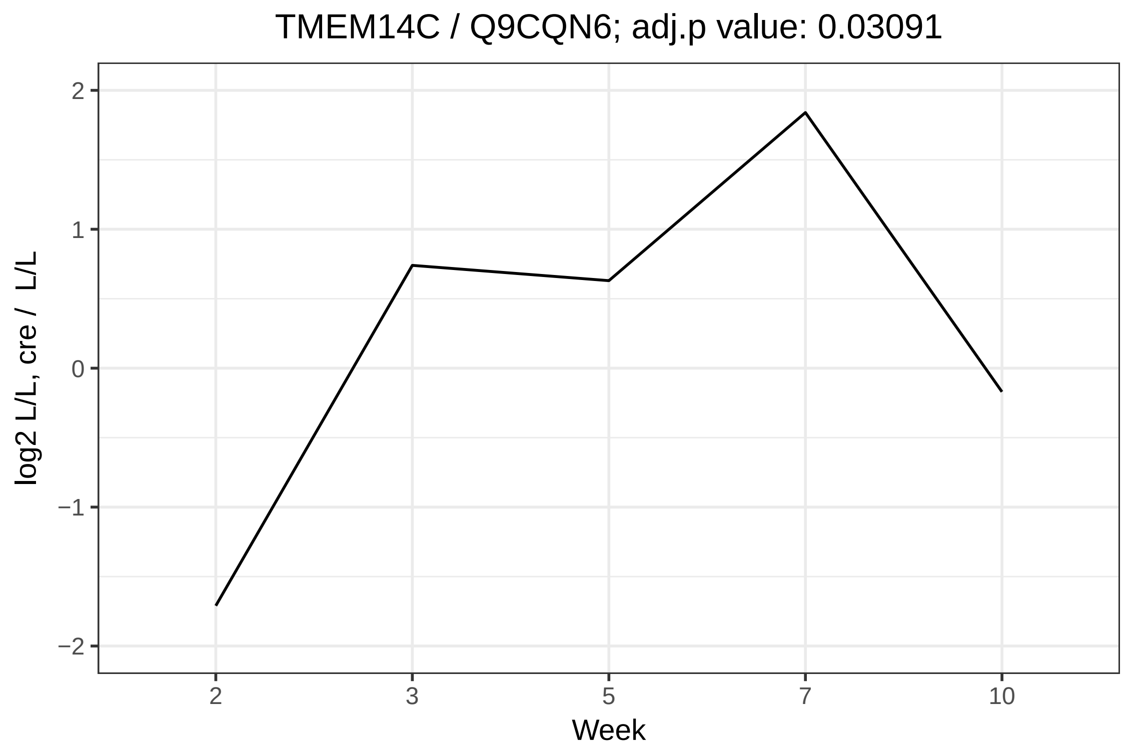

TMEM14C / Q9CQN6; adj.p value: 0.03091
log2 L/L, cre / L/L
2
1
0
−1
−2
2
3
5
7
10
Week

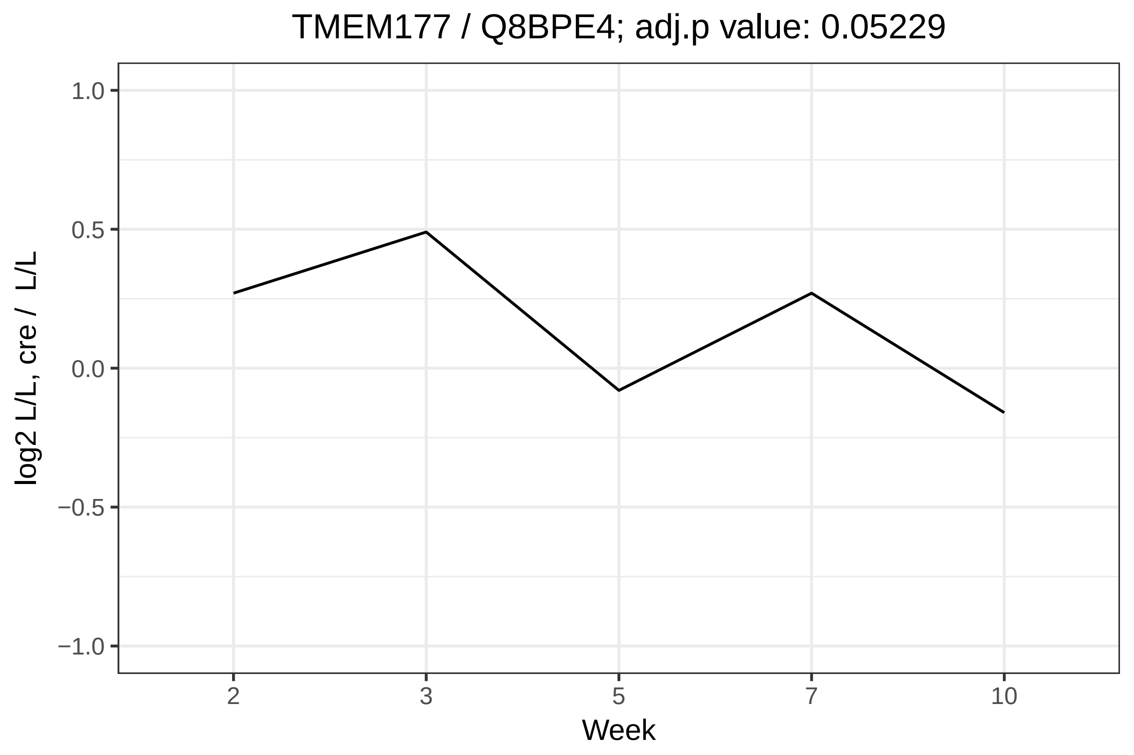

TMEM177 / Q8BPE4; adj.p value: 0.05229
log2 L/L, cre / L/L
1.0
0.5
0.0
−0.5
−1.0
2
3
5
7
10
Week

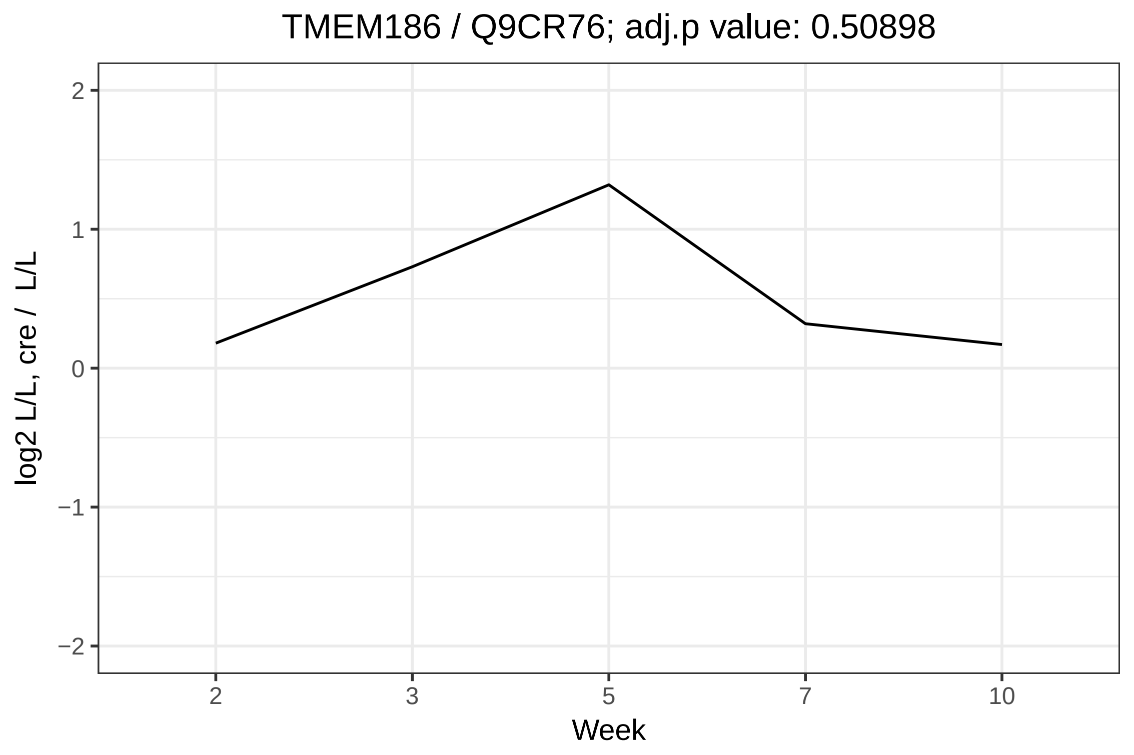

TMEM186 / Q9CR76; adj.p value: 0.50898
log2 L/L, cre / L/L
2
1
0
−1
−2
2
3
5
7
10
Week

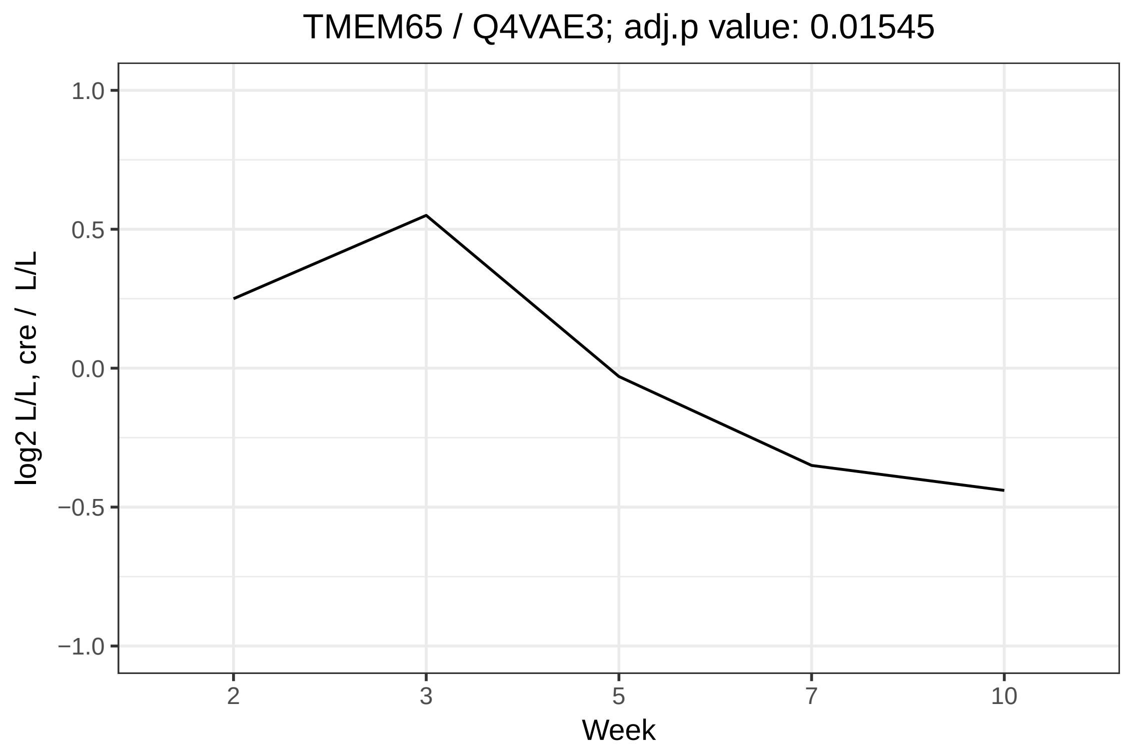
TMEM65 / Q4VAE3; adj.p value: 0.01545
log2 L/L, cre / L/L
1.0
0.5
0.0
−0.5
−1.0
2
3
5
7
10
Week

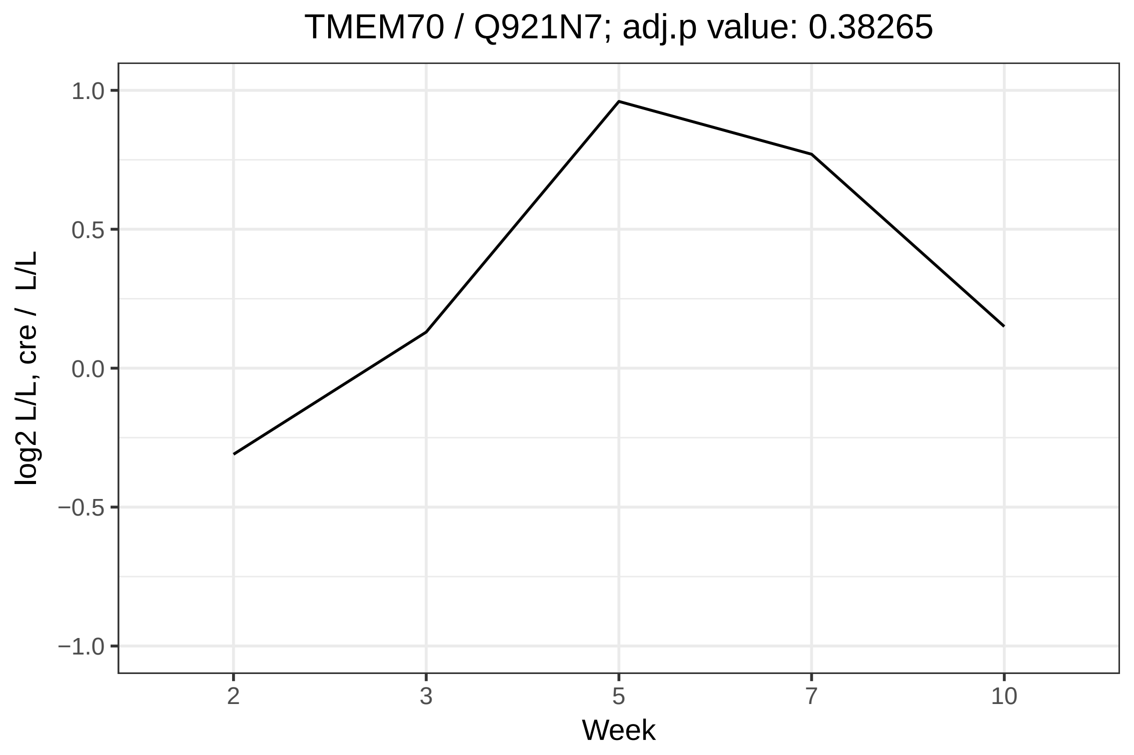
TMEM70 / Q921N7; adj.p value: 0.38265
log2 L/L, cre / L/L
1.0
0.5
0.0
−0.5
−1.0
2
3
5
7
10
Week

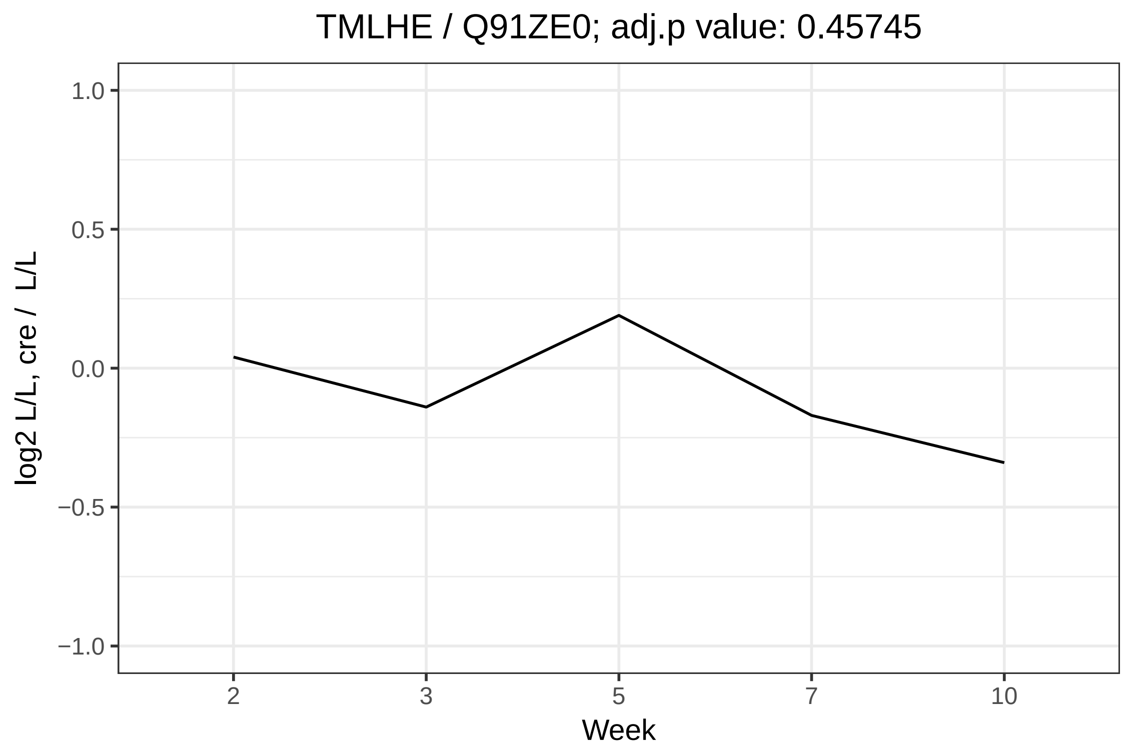
TMLHE / Q91ZE0; adj.p value: 0.45745
log2 L/L, cre / L/L
1.0
0.5
0.0
−0.5
−1.0
2
3
5
7
10
Week

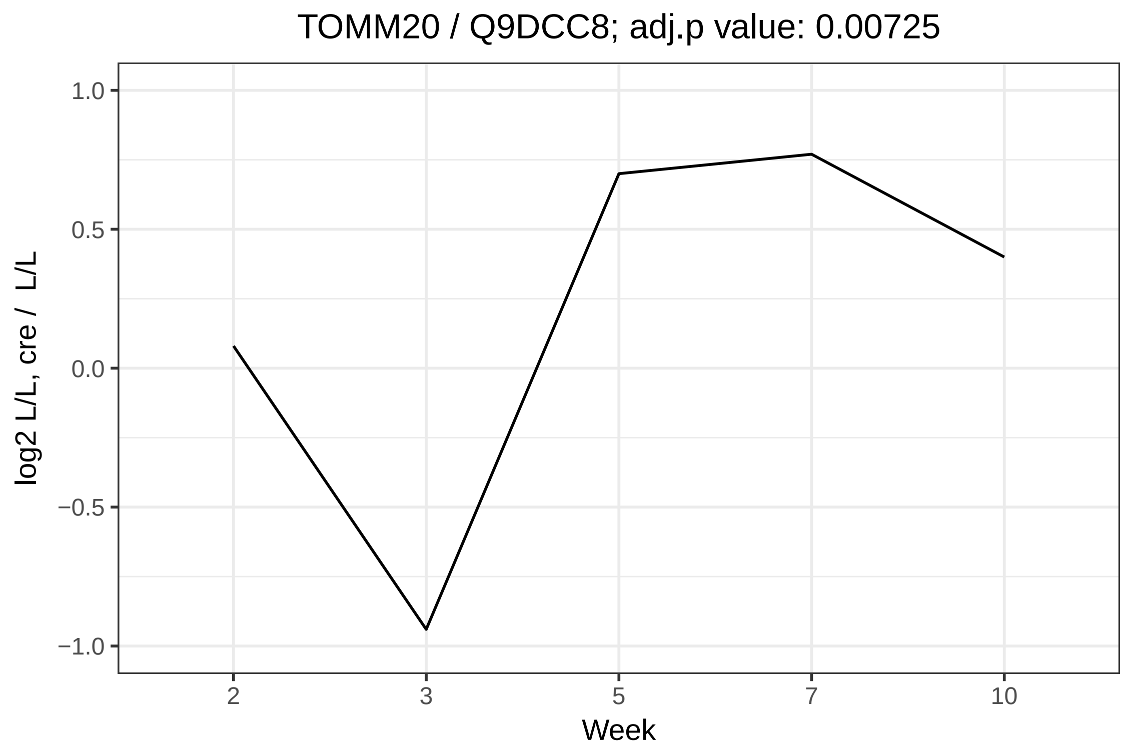

TOMM20 / Q9DCC8; adj.p value: 0.00725
1.0
0.5
0.0
−0.5
−1.0
log2 L/L, cre / L/L
2
3
5
7
10
Week

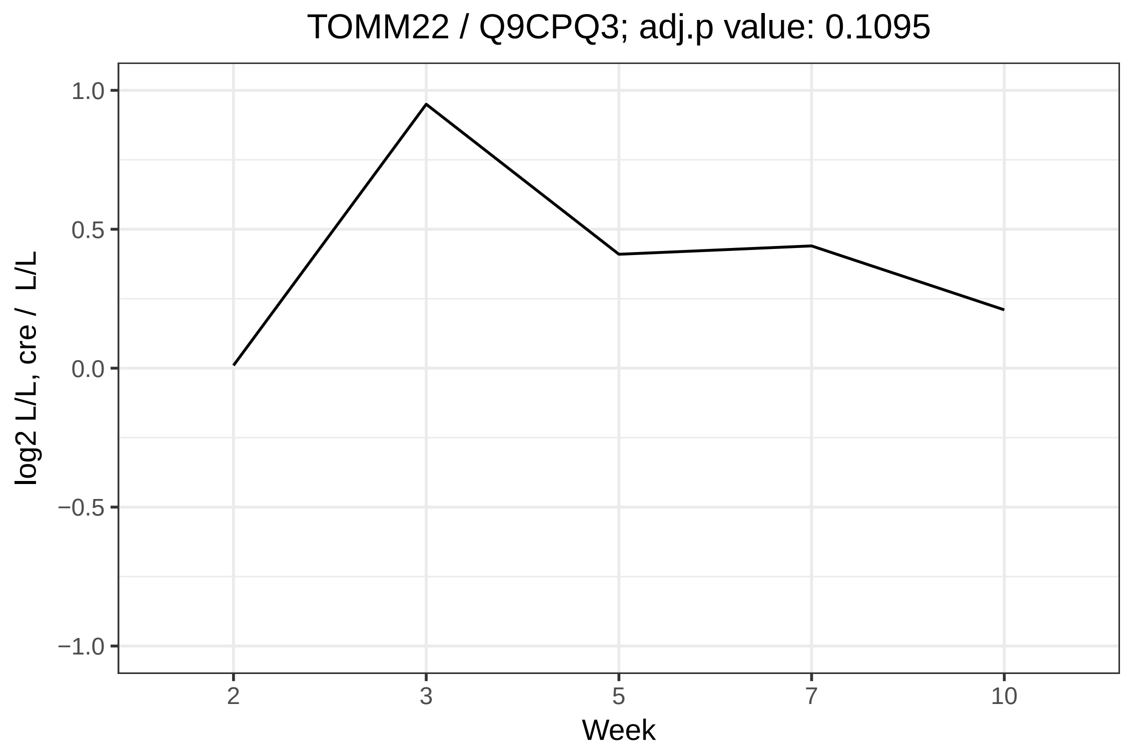
TOMM22 / Q9CPQ3; adj.p value: 0.1095
log2 L/L, cre / L/L
1.0
0.5
0.0
−0.5
−1.0
2
3
5
7
10
Week

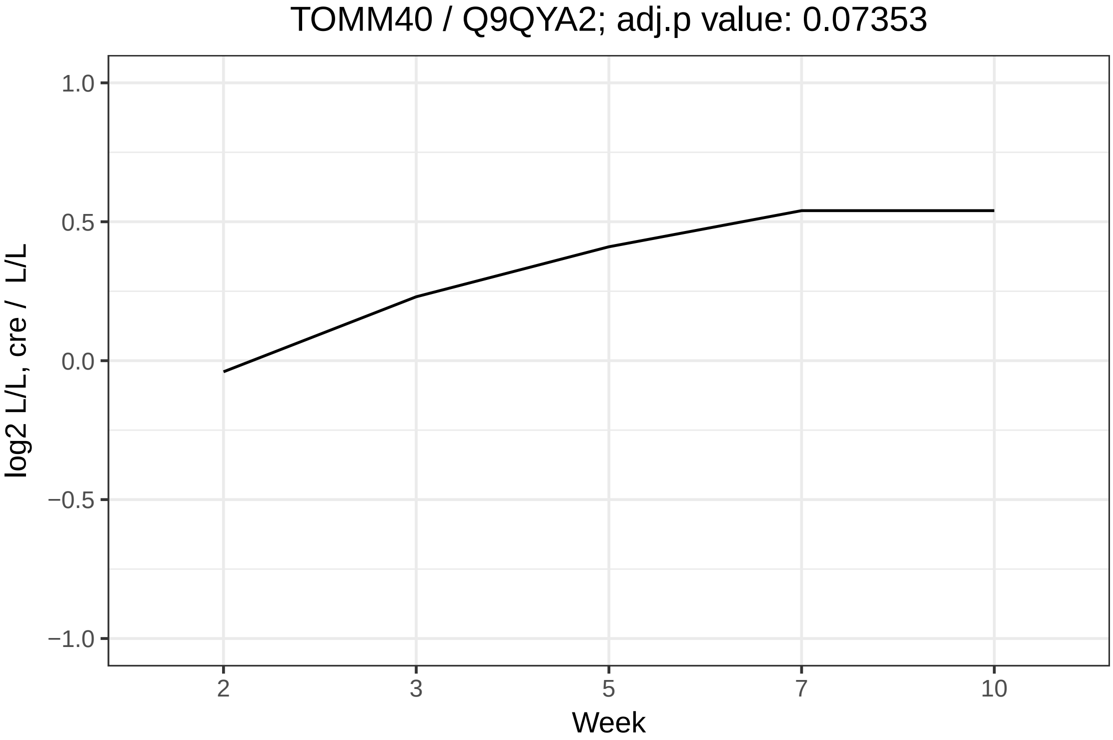

TOMM40 / Q9QYA2; adj.p value: 0.07353
log2 L/L, cre / L/L
1.0
0.5
0.0
−0.5
−1.0
2
3
5
7
10
Week

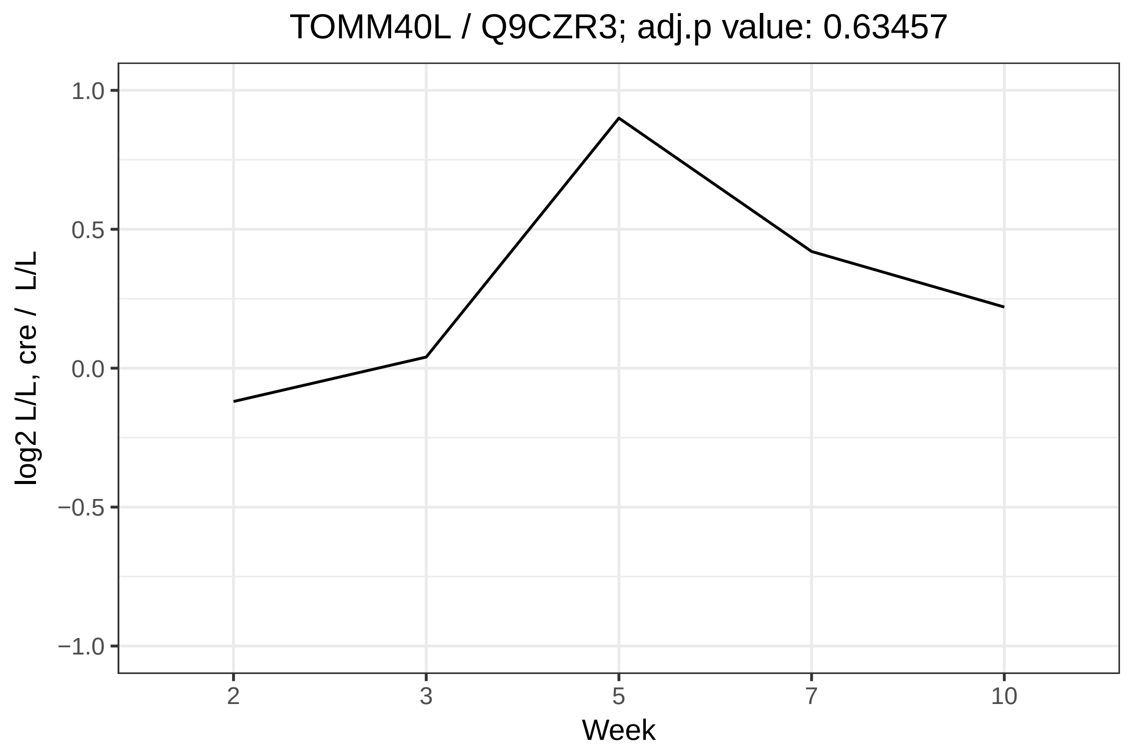

TOMM40L / Q9CZR3; adj.p value: 0.63457
log2 L/L, cre / L/L
1.0
0.5
0.0
−0.5
−1.0
2
3
5
7
10
Week

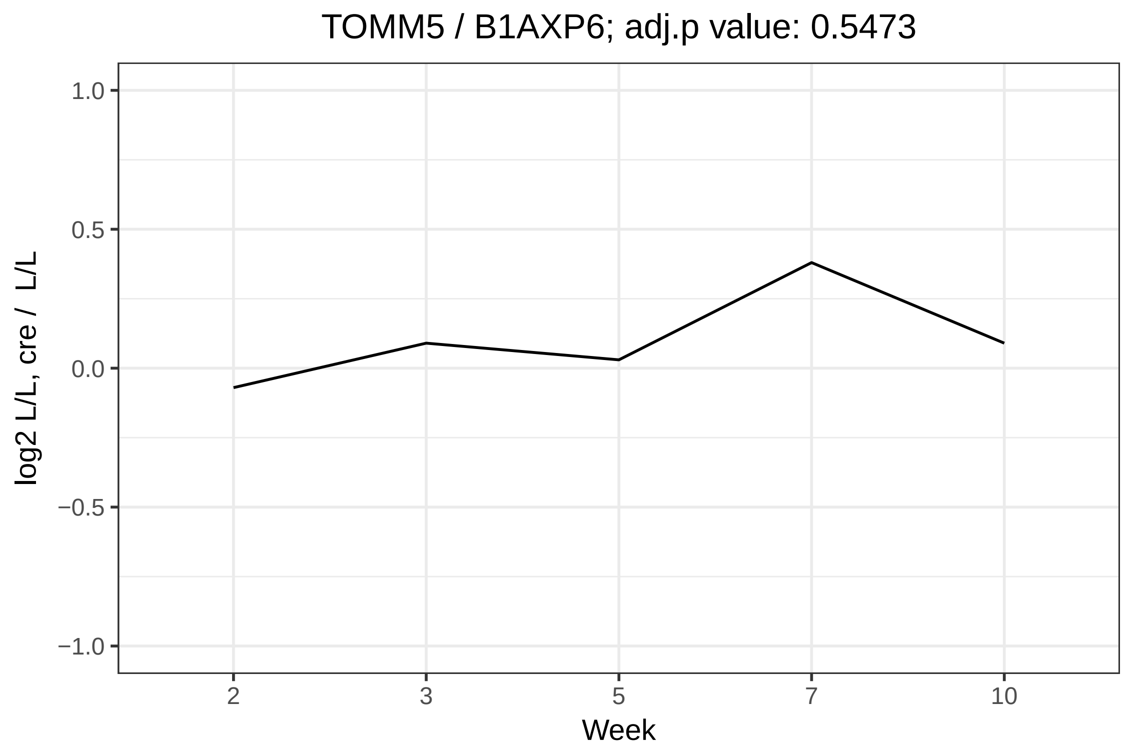

TOMM5 / B1AXP6; adj.p value: 0.5473
1.0
0.5
0.0
−0.5
−1.0
log2 L/L, cre / L/L
2
3
5
7
10
Week

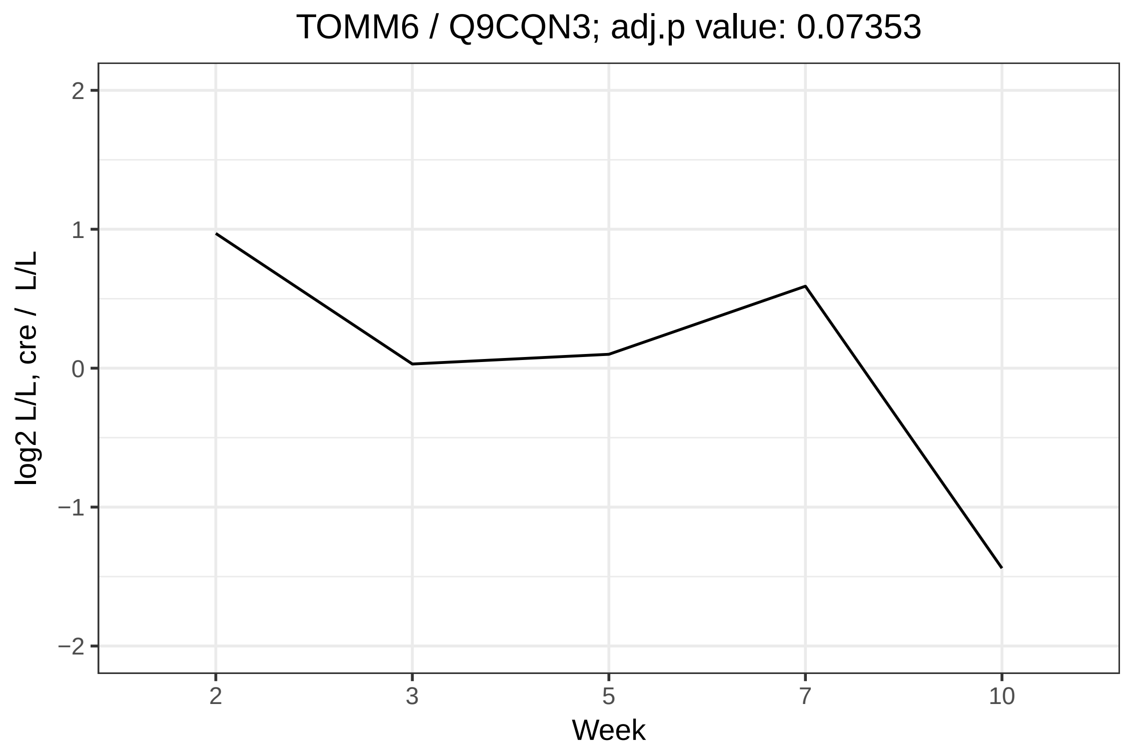

TOMM6 / Q9CQN3; adj.p value: 0.07353
log2 L/L, cre / L/L
2
1
0
−1
−2
2
3
5
7
10
Week

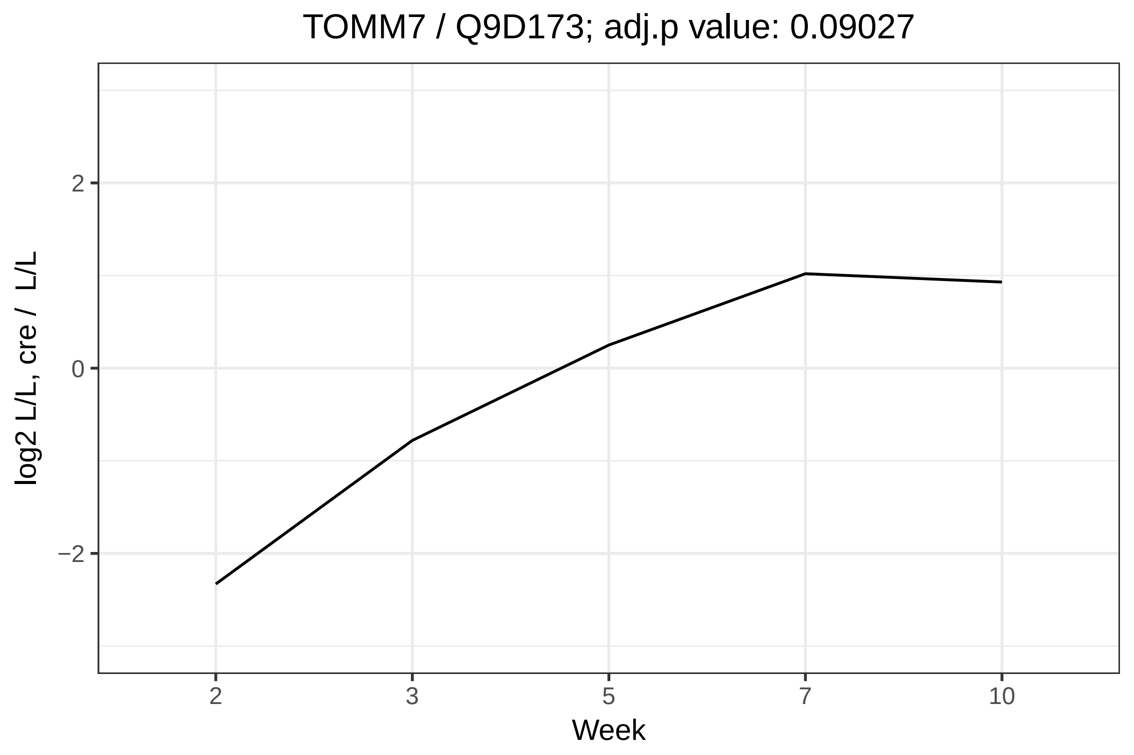
TOMM7 / Q9D173; adj.p value: 0.09027
log2 L/L, cre / L/L
2
0
−2
2
3
5
7
10
Week

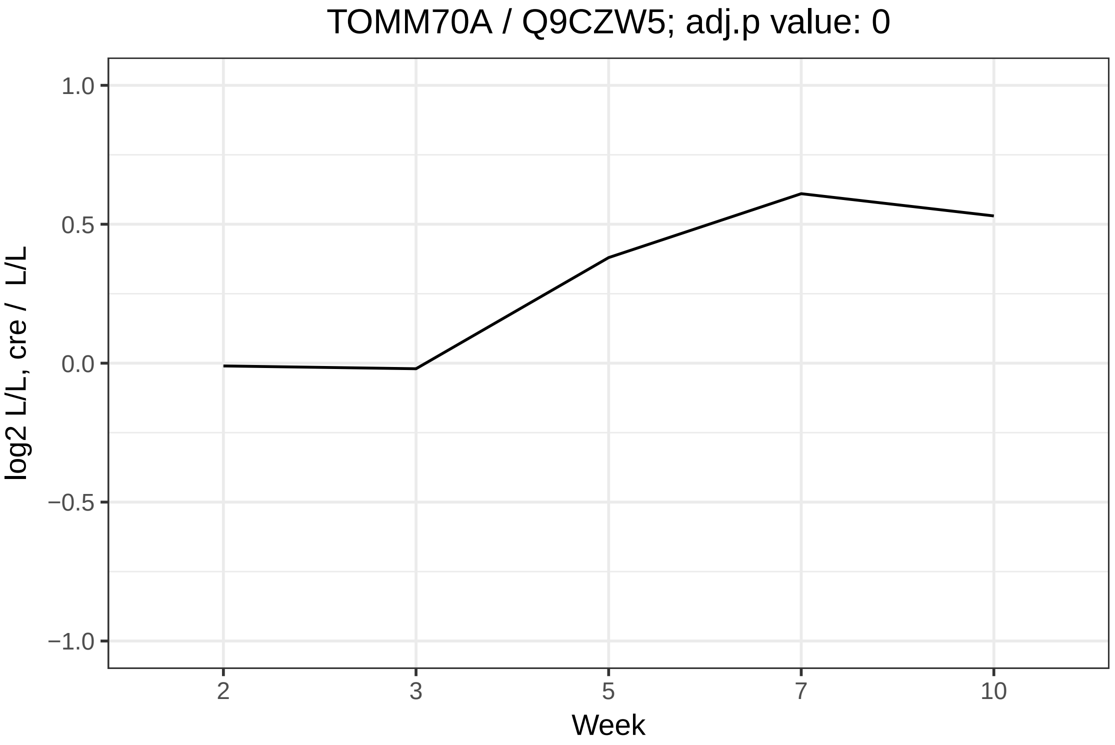

TOMM70A / Q9CZW5; adj.p value: 0
1.0
0.5
0.0
−0.5
−1.0
log2 L/L, cre / L/L
2
3
5
7
10
Week

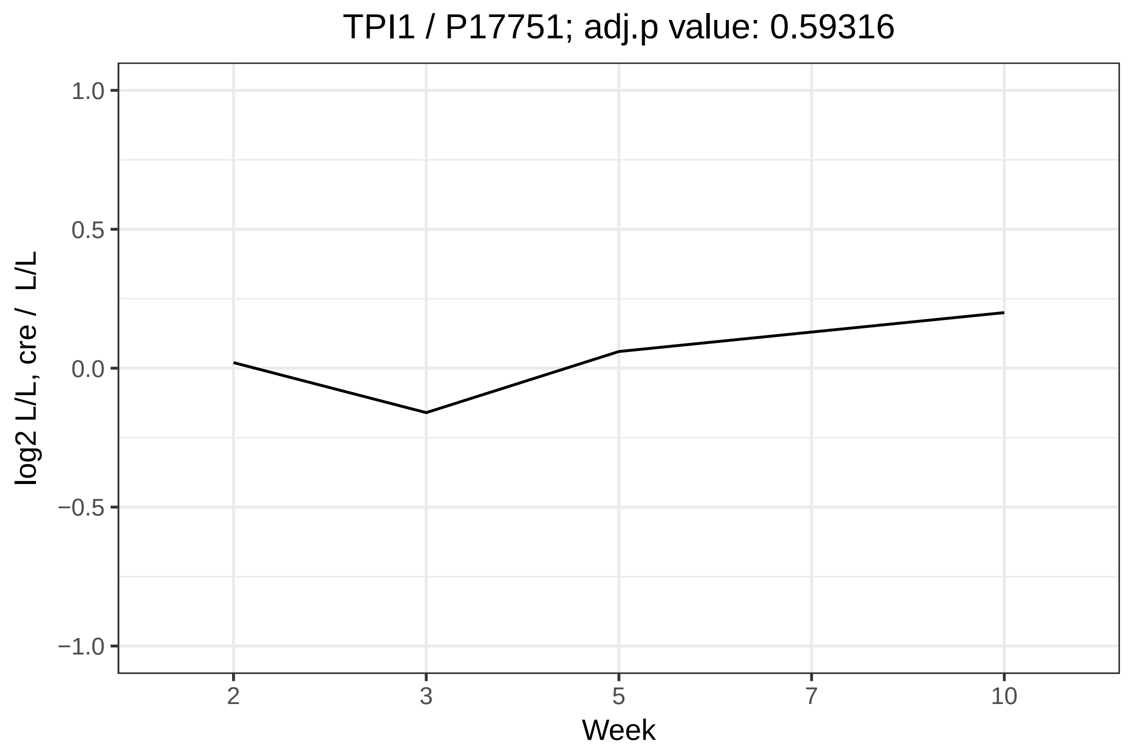
TPI1 / P17751; adj.p value: 0.59316
log2 L/L, cre / L/L
1.0
0.5
0.0
−0.5
−1.0
2
3
5
7
10
Week

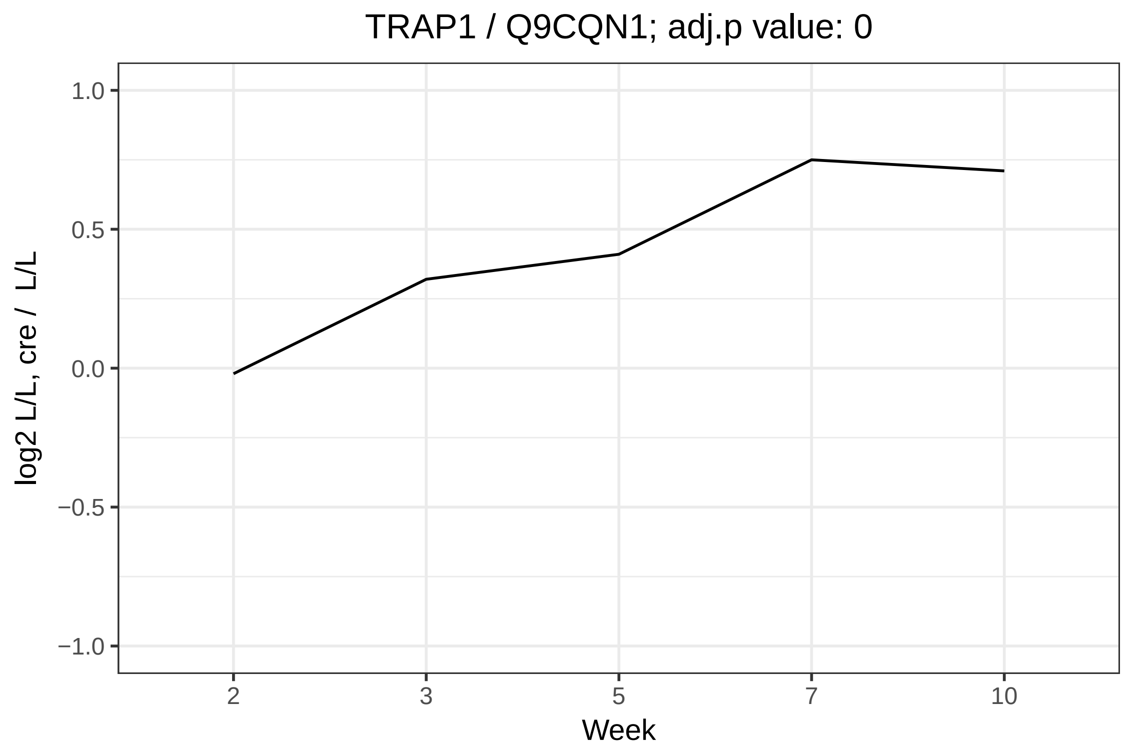
TRAP1 / Q9CQN1; adj.p value: 0
1.0
0.5
0.0
−0.5
−1.0
log2 L/L, cre / L/L
2
3
5
7
10
Week

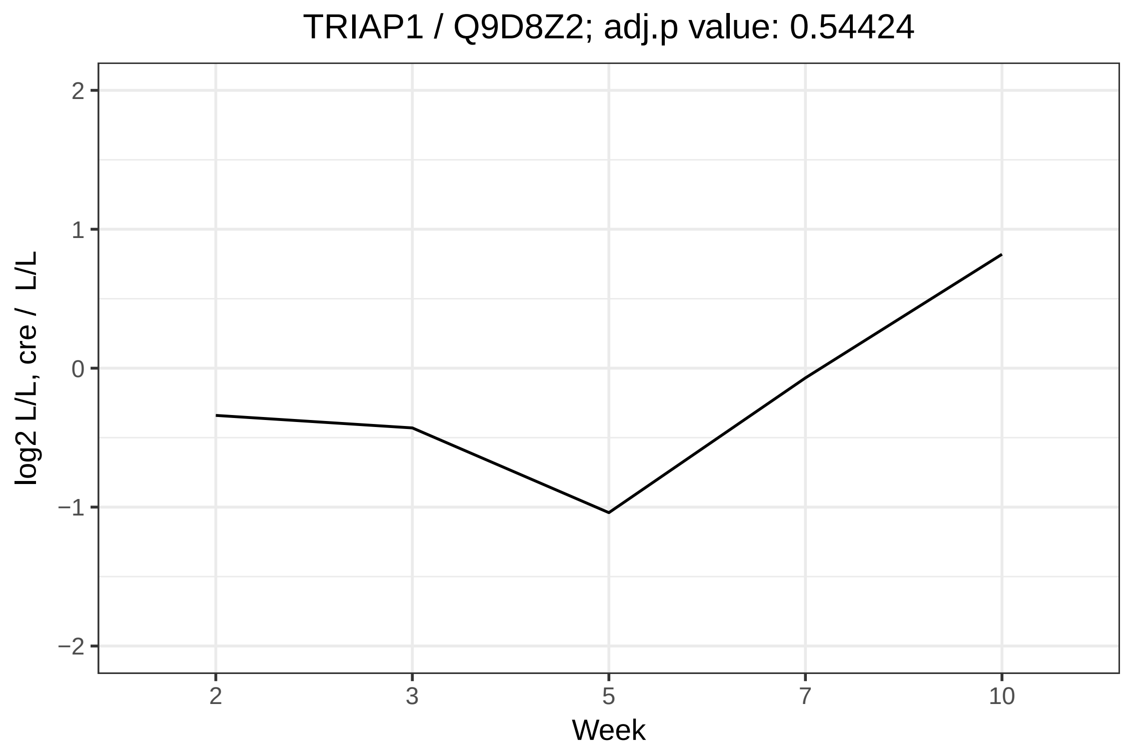

TRIAP1 / Q9D8Z2; adj.p value: 0.54424
log2 L/L, cre / L/L
2
1
0
−1
−2
2
3
5
7
10
Week

TRMT10C / Q3UFY8; adj.p value: 0.00583

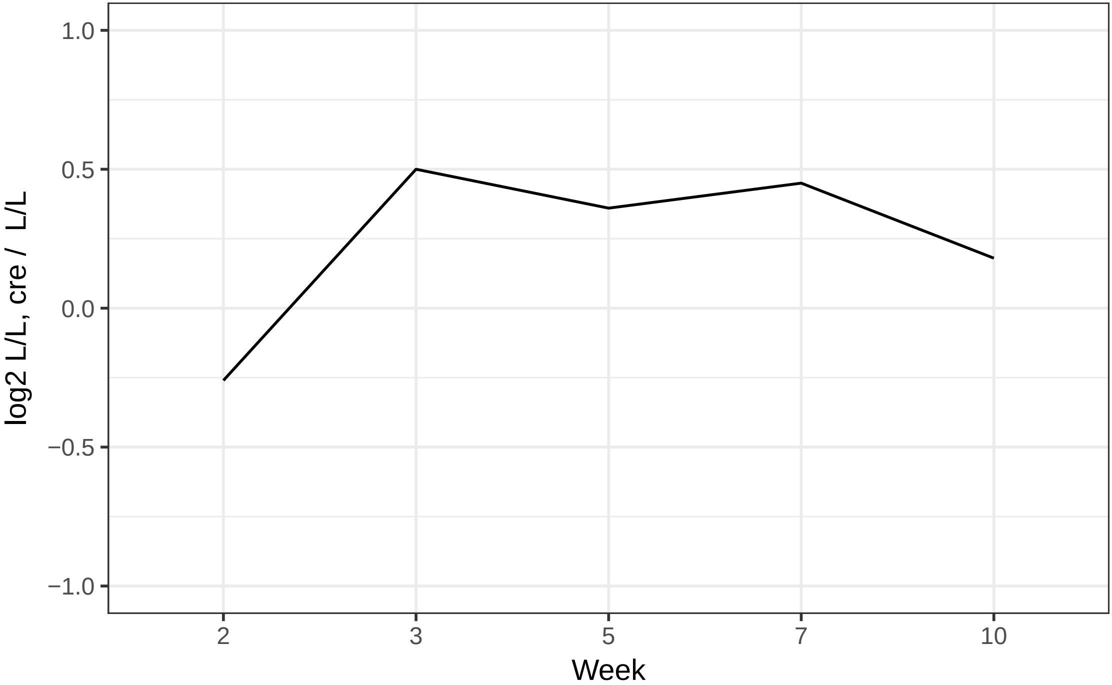

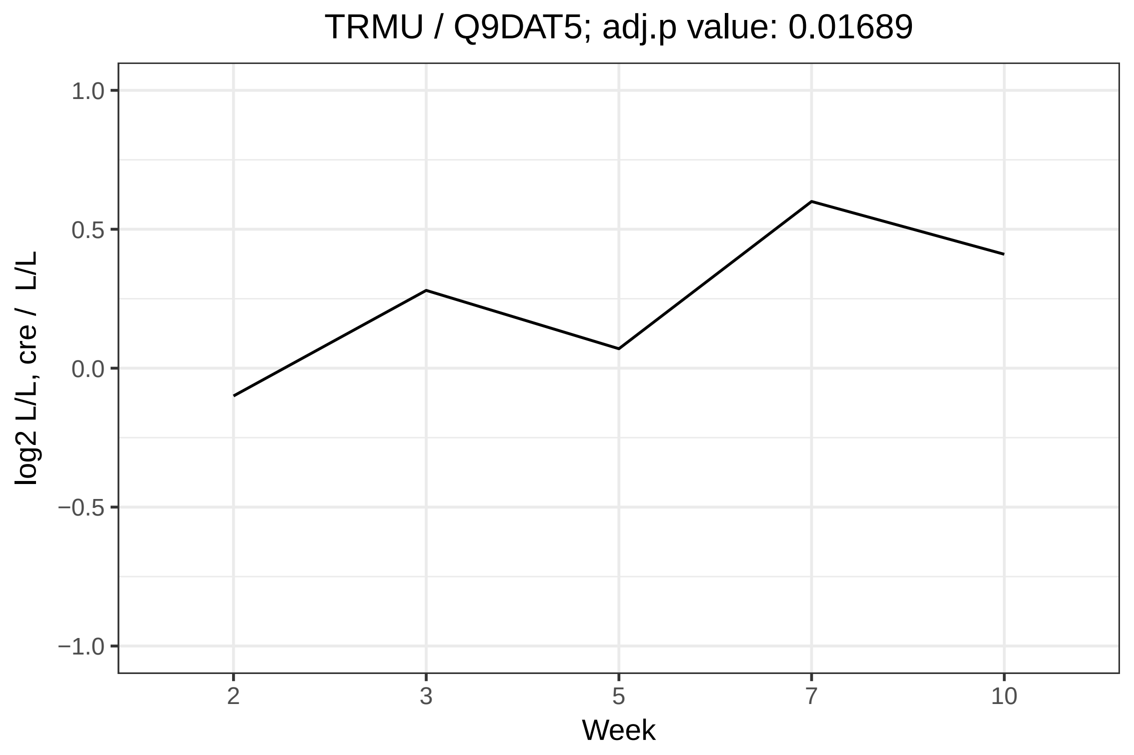

TRMU / Q9DAT5; adj.p value: 0.01689
1.0
0.5
0.0
−0.5
−1.0
log2 L/L, cre / L/L
2
3
5
7
10
Week

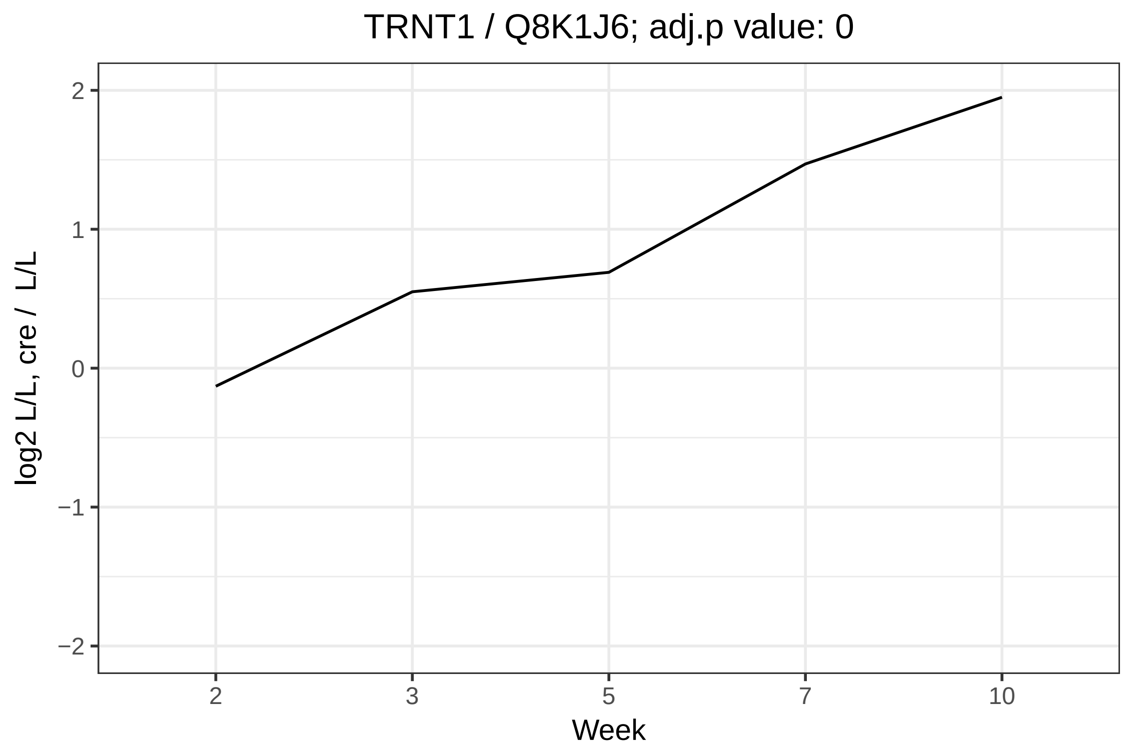
TRNT1 / Q8K1J6; adj.p value: 0
log2 L/L, cre / L/L
2
1
0
−1
−2
2
3
5
7
10
Week

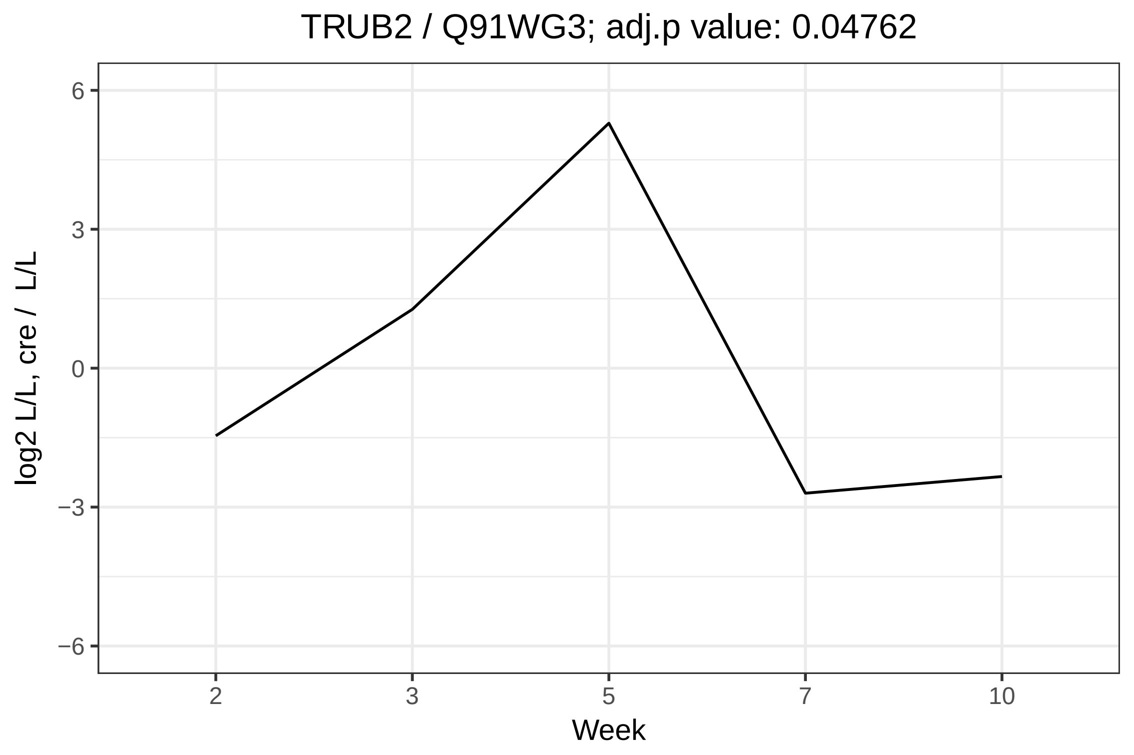

TRUB2 / Q91WG3; adj.p value: 0.04762
log2 L/L, cre / L/L
6
3
0
−3
−6
2
3
5
7
10
Week

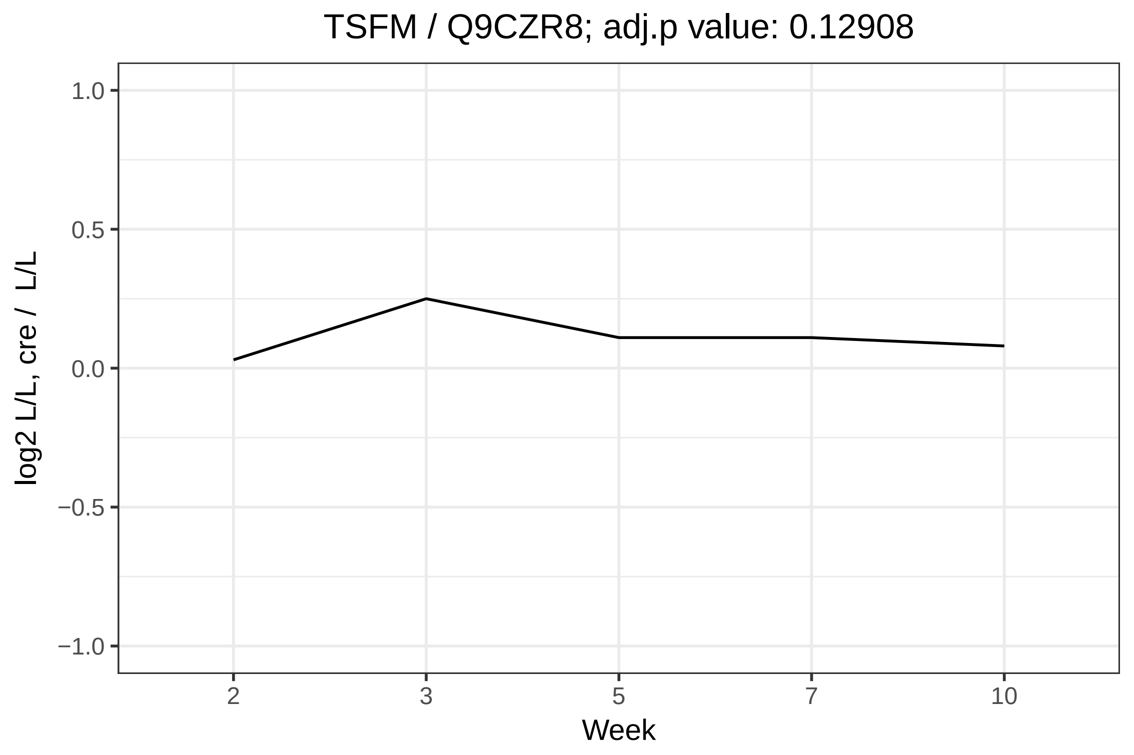
TSFM / Q9CZR8; adj.p value: 0.12908
log2 L/L, cre / L/L
1.0
0.5
0.0
−0.5
−1.0
2
3
5
7
10
Week

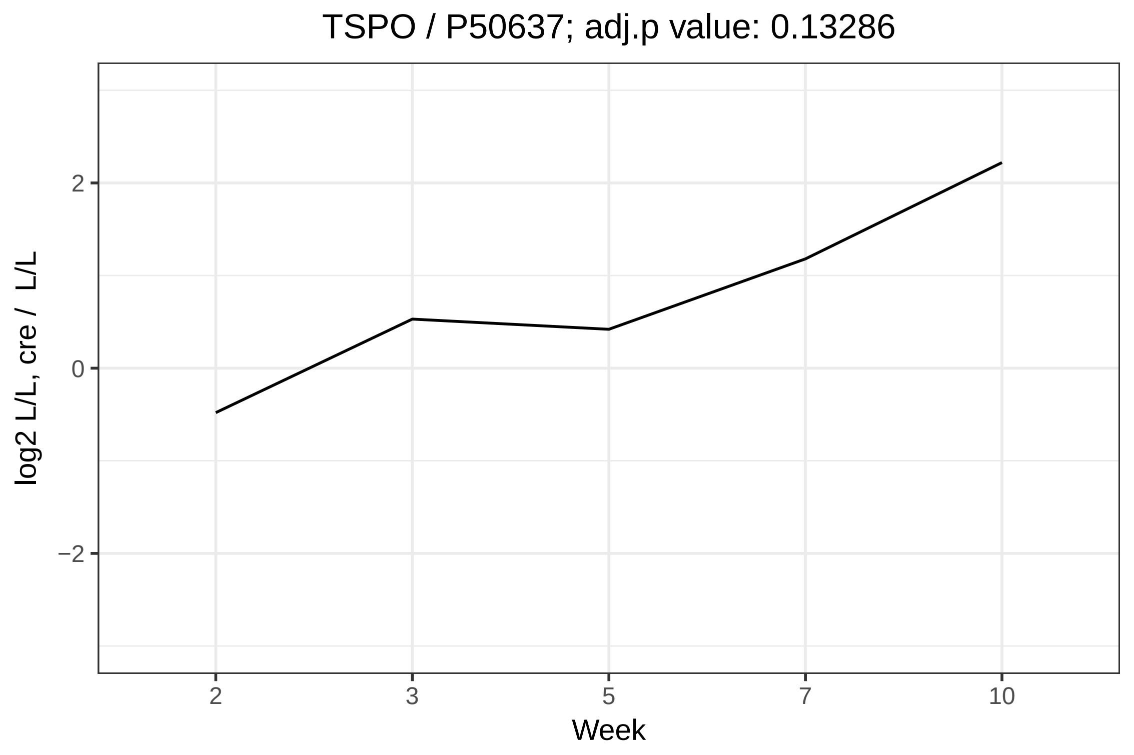
TSPO / P50637; adj.p value: 0.13286
log2 L/L, cre / L/L
2
0
−2
2
3
5
7
10
Week

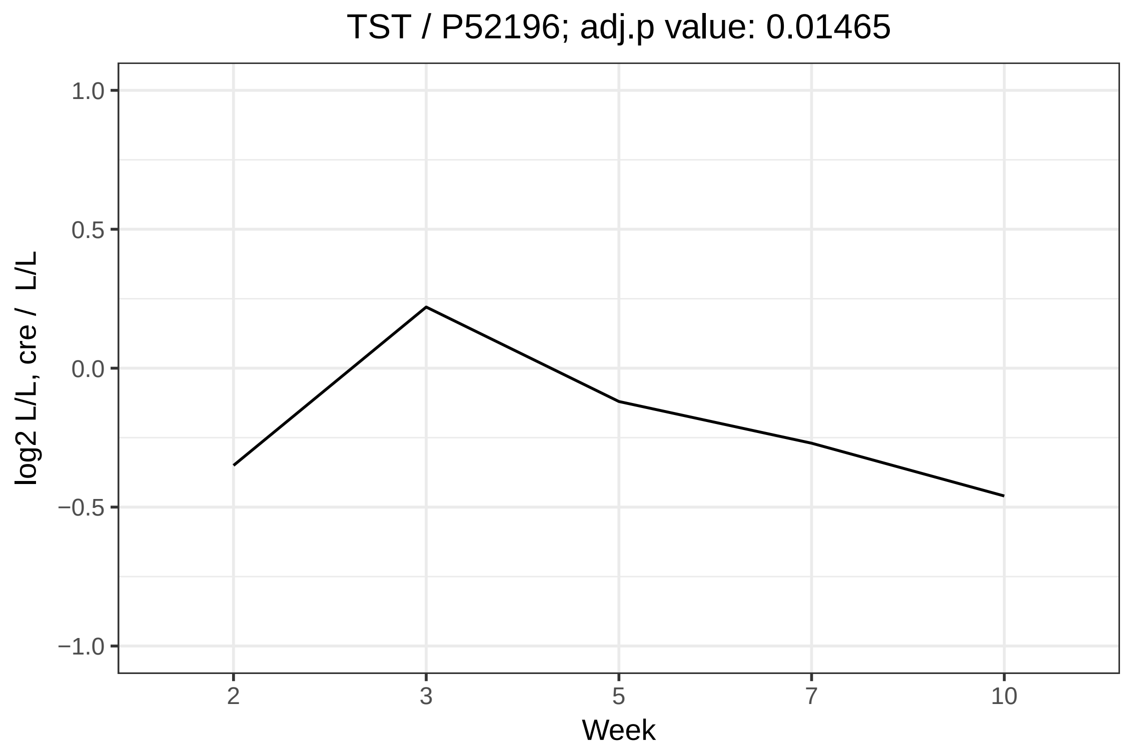
TST / P52196; adj.p value: 0.01465
log2 L/L, cre / L/L
1.0
0.5
0.0
−0.5
−1.0
2
3
5
7
10
Week

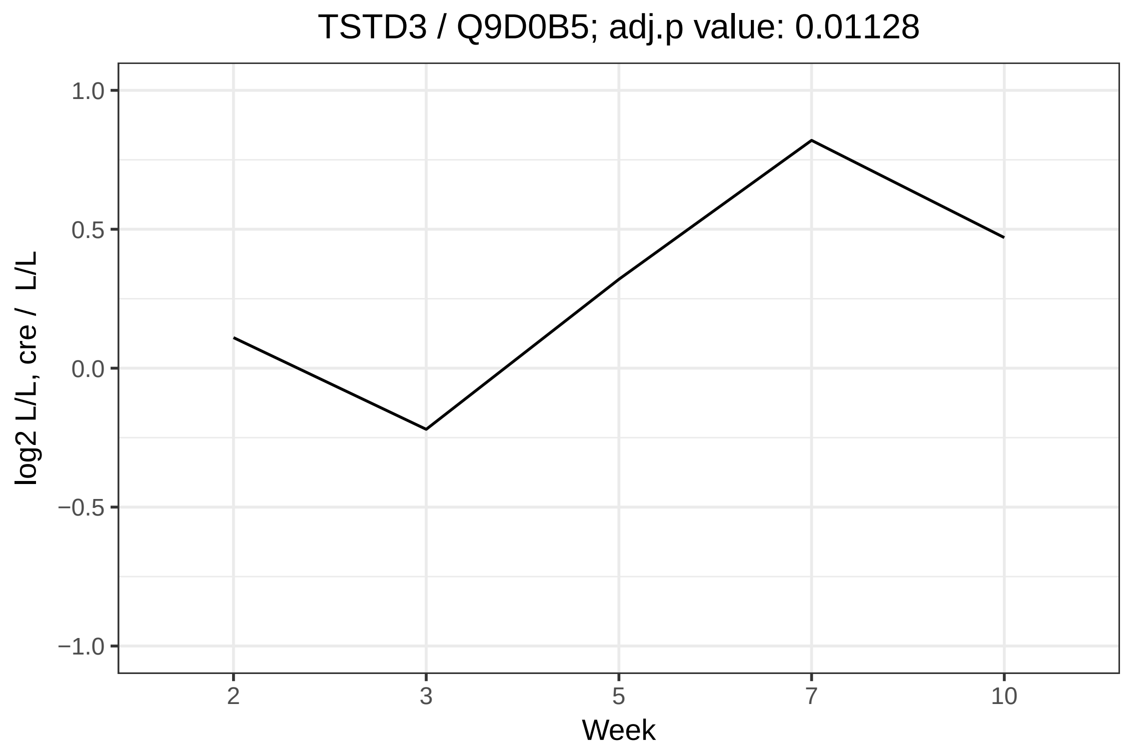
TSTD3 / Q9D0B5; adj.p value: 0.01128
log2 L/L, cre / L/L
1.0
0.5
0.0
−0.5
−1.0
2
3
5
7
10
Week

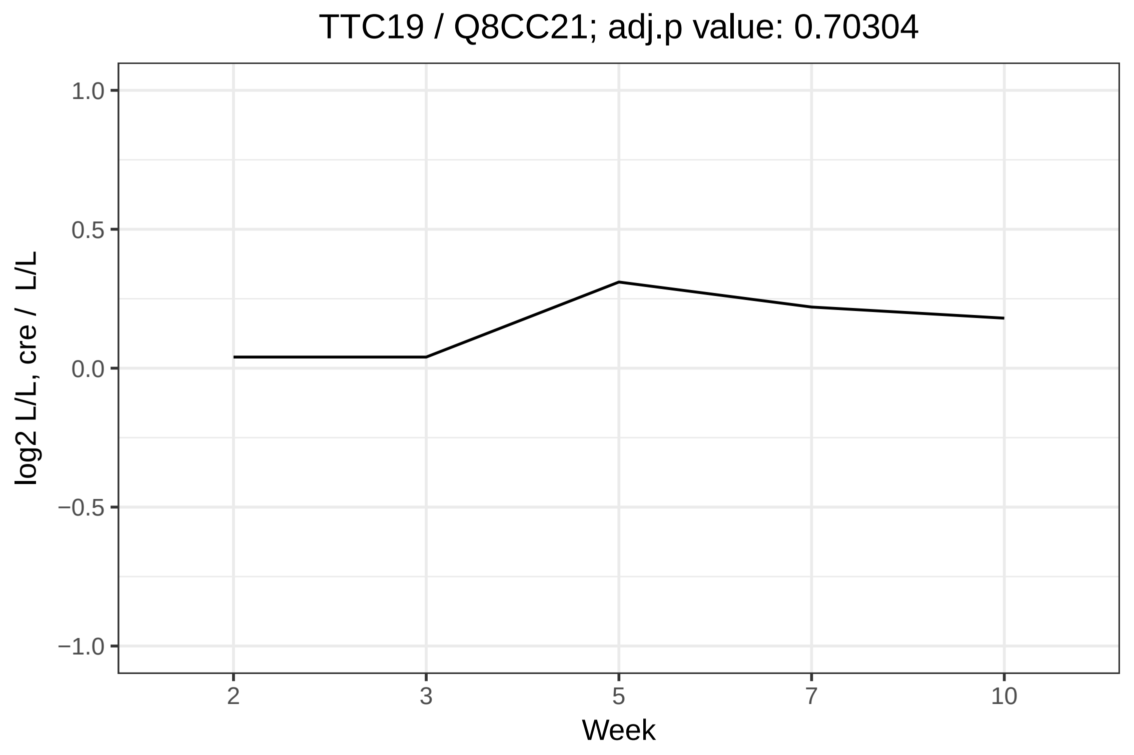

TTC19 / Q8CC21; adj.p value: 0.70304
1.0
0.5
0.0
−0.5
−1.0
log2 L/L, cre / L/L
2
3
5
7
10
Week

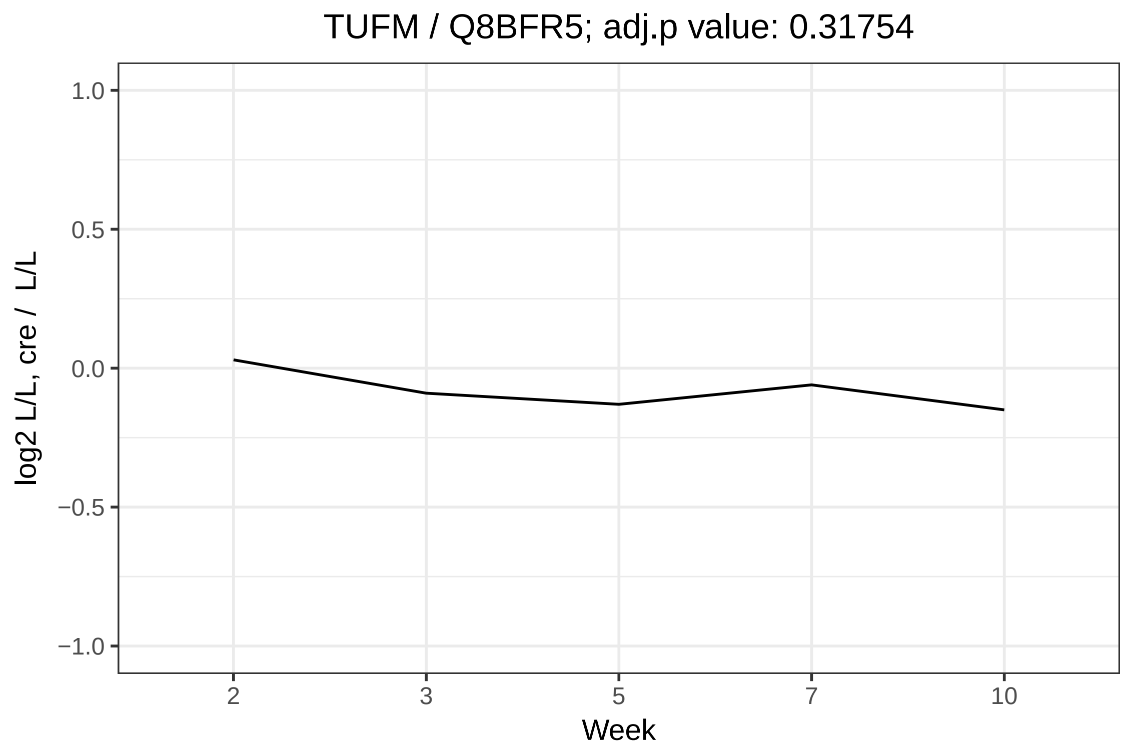

TUFM / Q8BFR5; adj.p value: 0.31754
log2 L/L, cre / L/L
1.0
0.5
0.0
−0.5
−1.0
2
3
5
7
10
Week

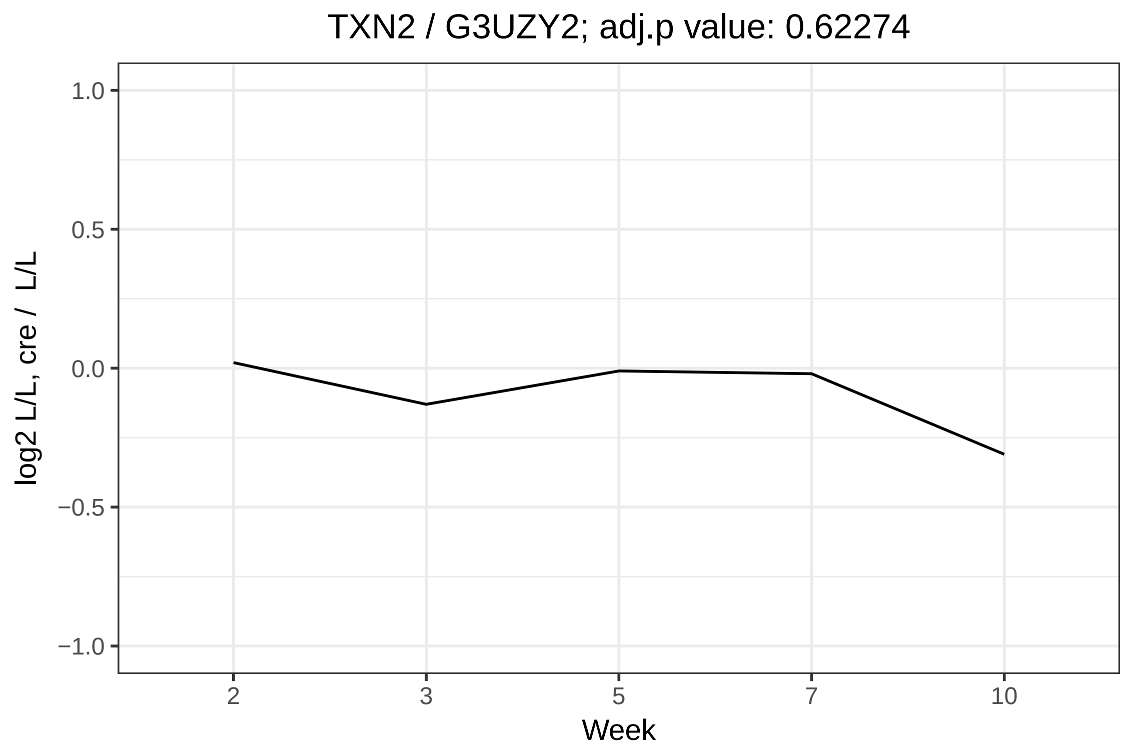
TXN2 / G3UZY2; adj.p value: 0.62274
log2 L/L, cre / L/L
1.0
0.5
0.0
−0.5
−1.0
2
3
5
7
10
Week

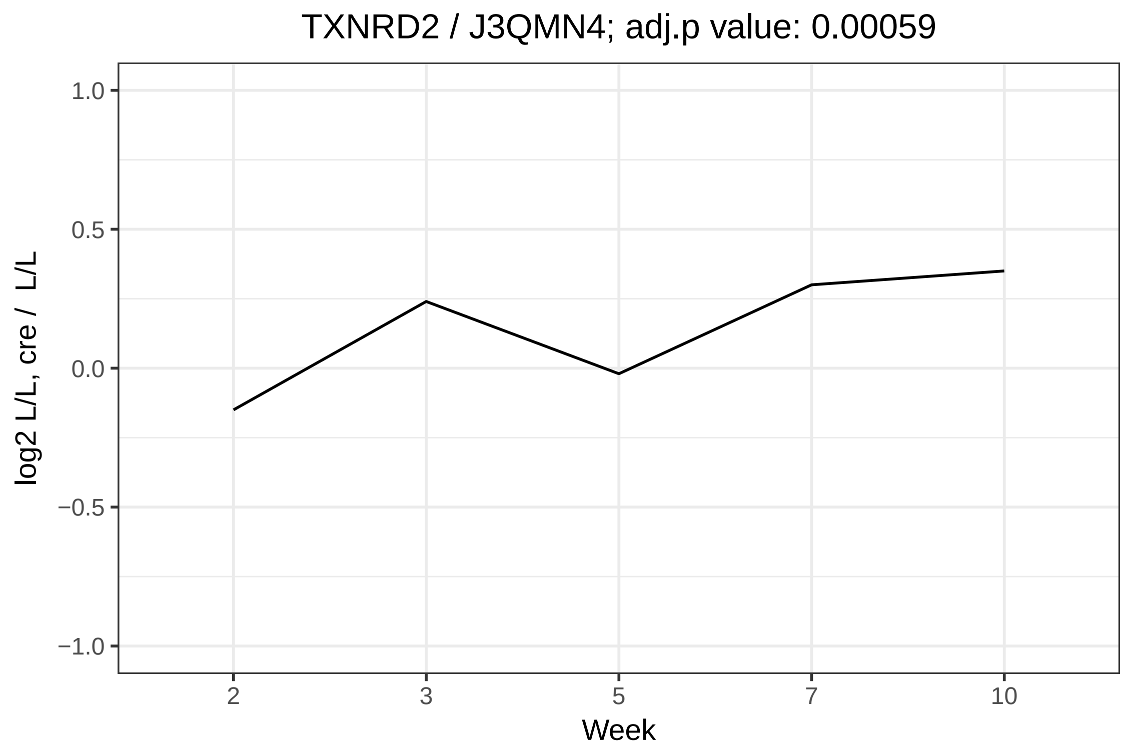
TXNRD2 / J3QMN4; adj.p value: 0.00059
log2 L/L, cre / L/L
1.0
0.5
0.0
−0.5
−1.0
2
3
5
7
10
Week

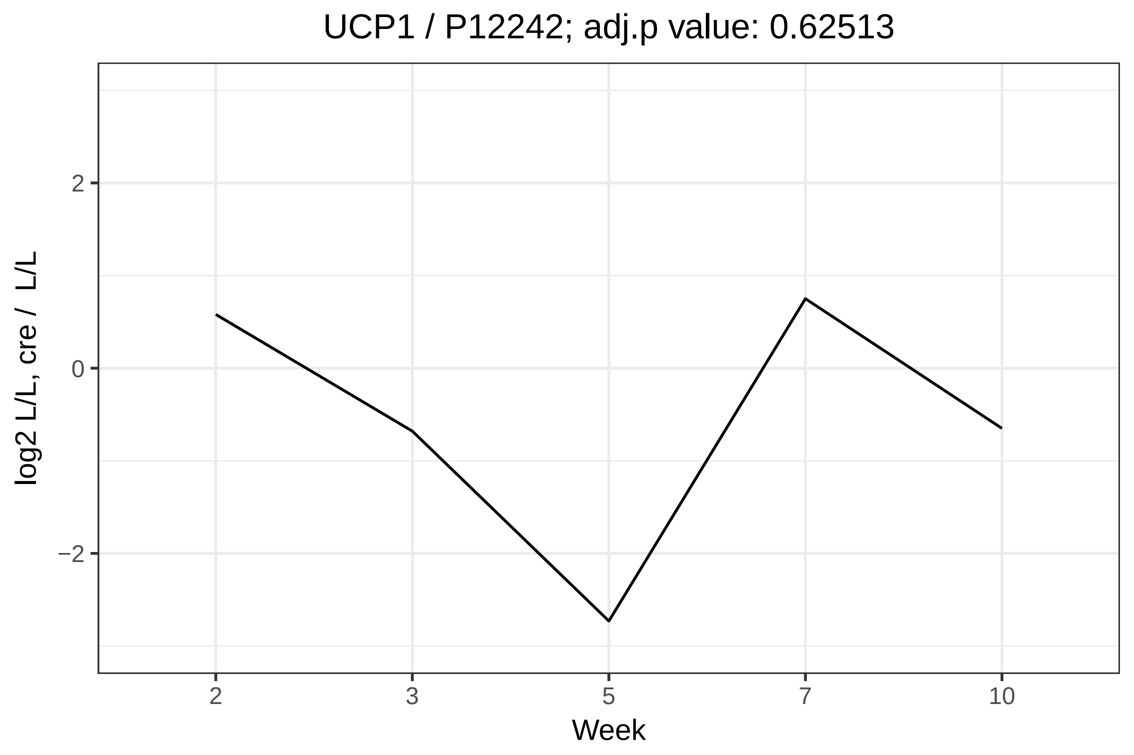
UCP1 / P12242; adj.p value: 0.62513
log2 L/L, cre / L/L
2
0
−2
2
3
5
7
10
Week

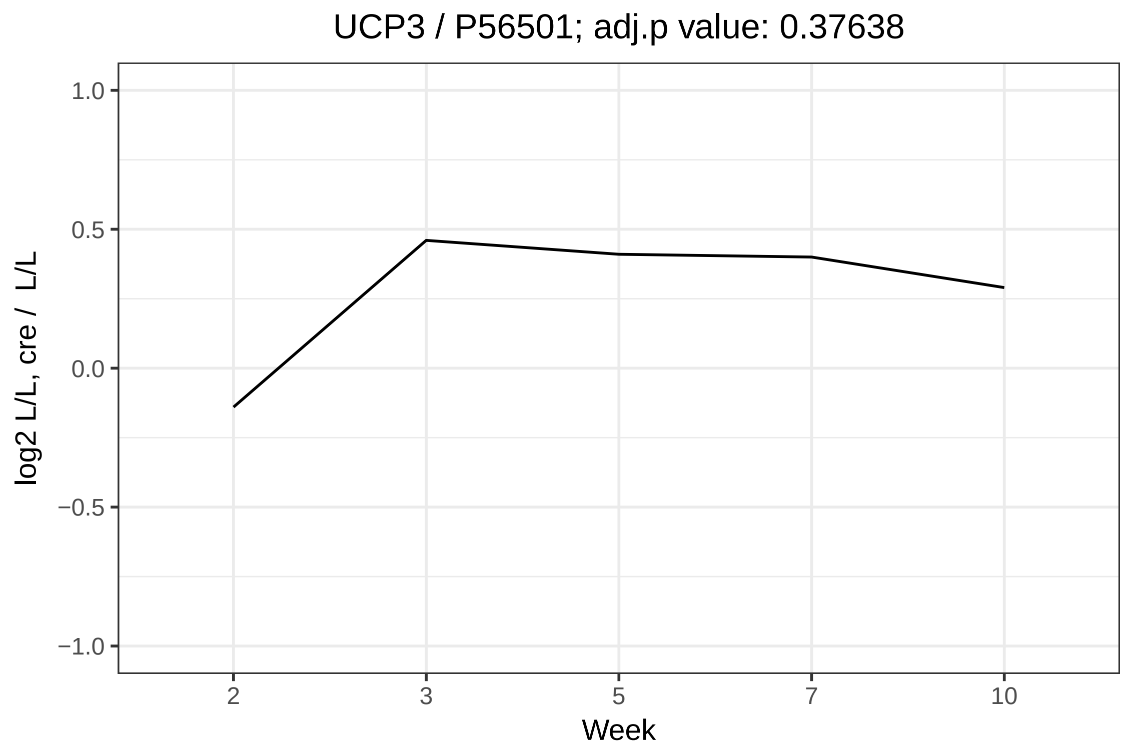

UCP3 / P56501; adj.p value: 0.37638
log2 L/L, cre / L/L
1.0
0.5
0.0
−0.5
−1.0
2
3
5
7
10
Week

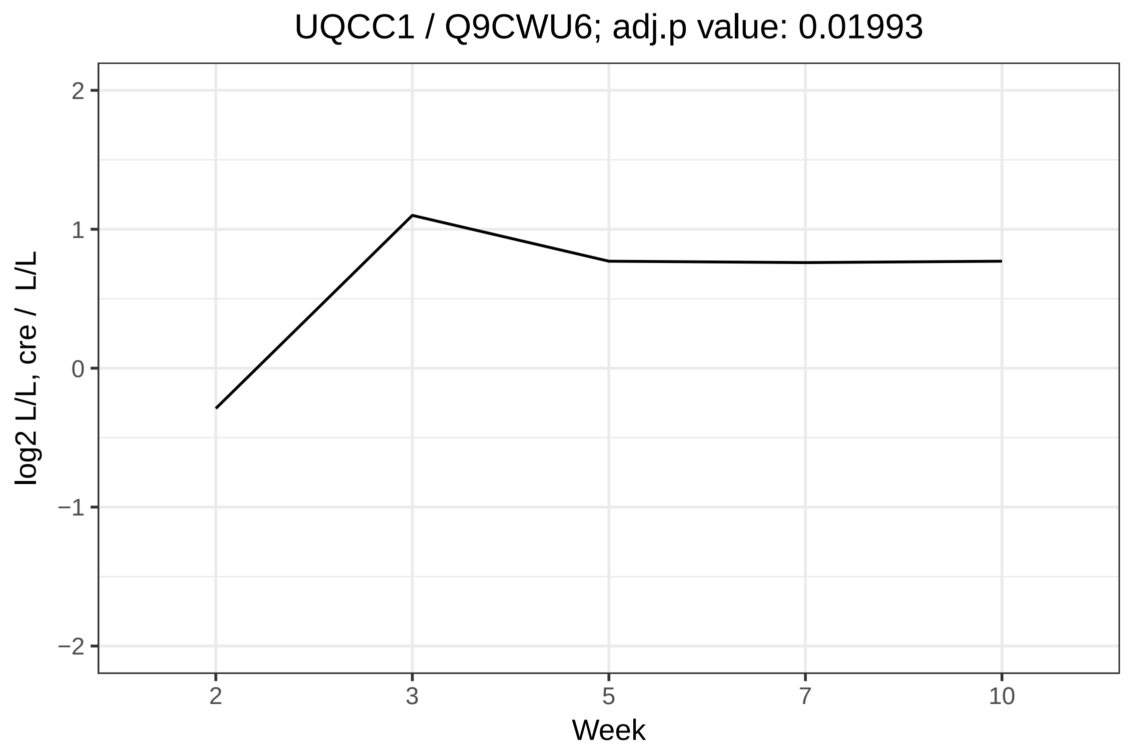

UQCC1 / Q9CWU6; adj.p value: 0.01993
log2 L/L, cre / L/L
2
1
0
−1
−2
2
3
5
7
10
Week

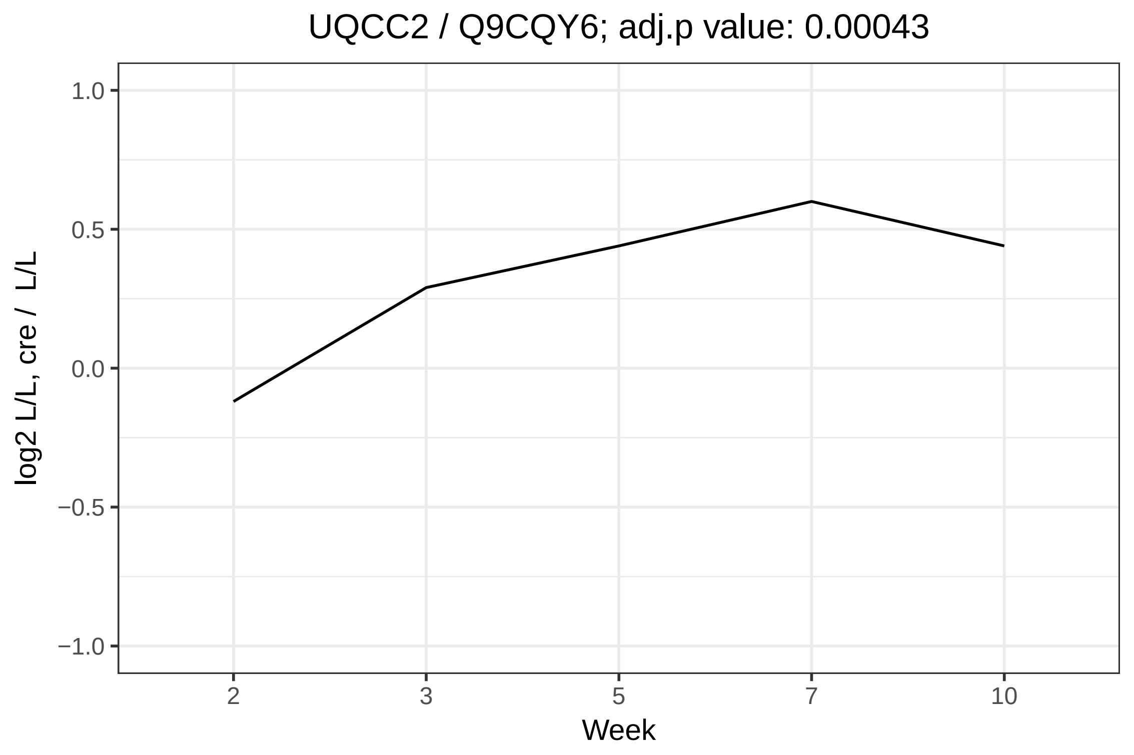
UQCC2 / Q9CQY6; adj.p value: 0.00043
log2 L/L, cre / L/L
1.0
0.5
0.0
−0.5
−1.0
2
3
5
7
10
Week

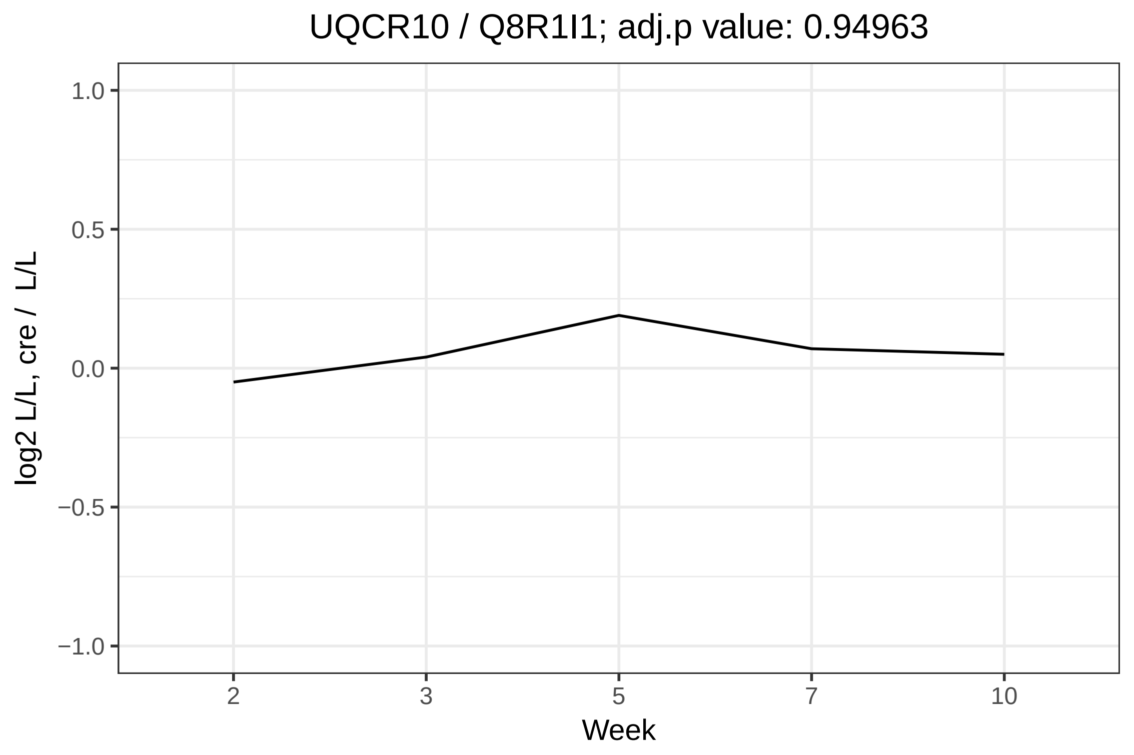
UQCR10 / Q8R1I1; adj.p value: 0.94963
log2 L/L, cre / L/L
1.0
0.5
0.0
−0.5
−1.0
2
3
5
7
10
Week

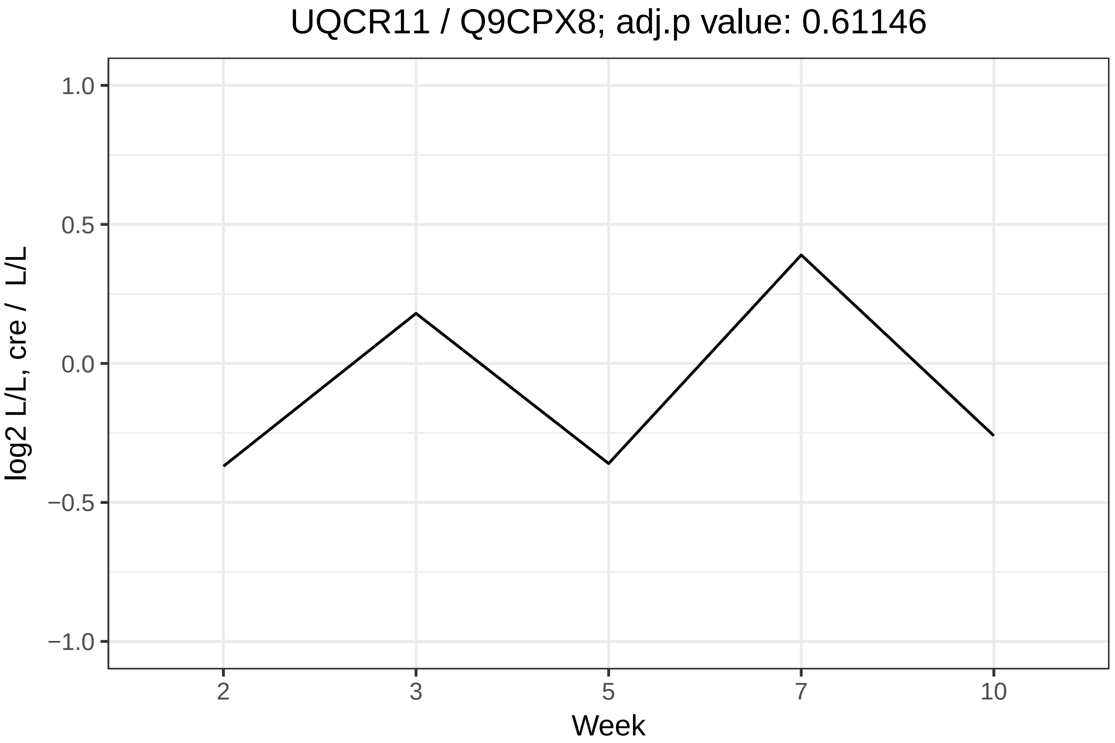
UQCR11 / Q9CPX8; adj.p value: 0.61146
log2 L/L, cre / L/L
1.0
0.5
0.0
−0.5
−1.0
2
3
5
7
10
Week

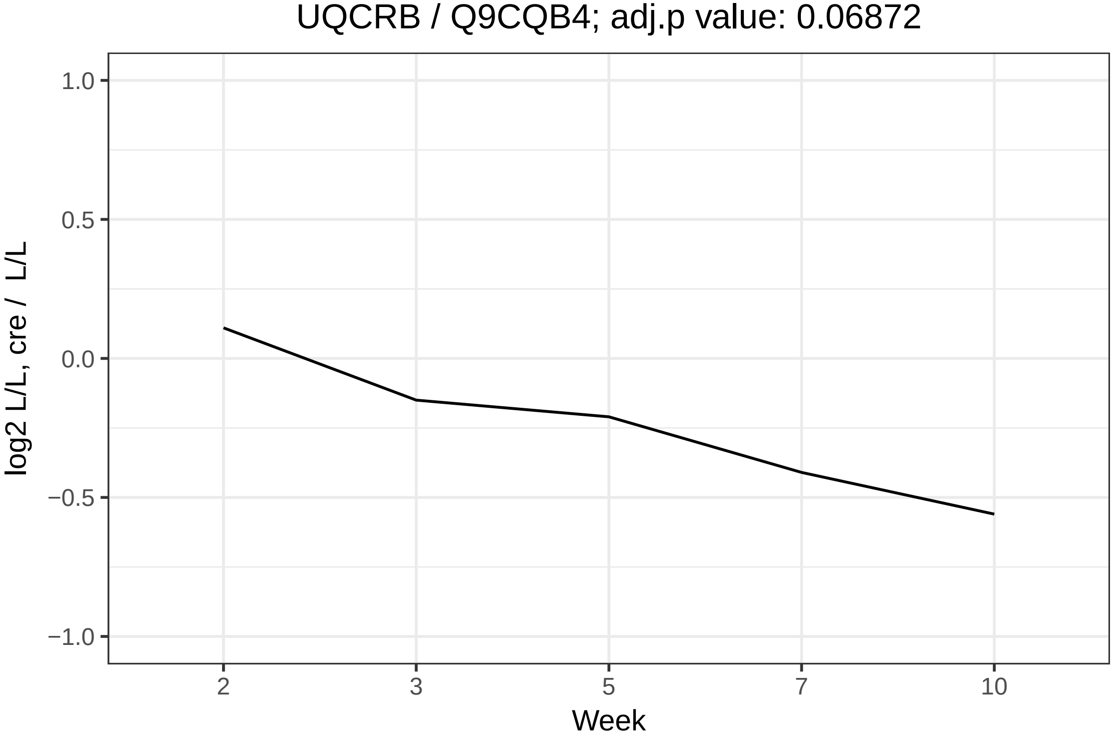

UQCRB / Q9CQB4; adj.p value: 0.06872
1.0
0.5
0.0
−0.5
−1.0
log2 L/L, cre / L/L
2
3
5
7
10
Week

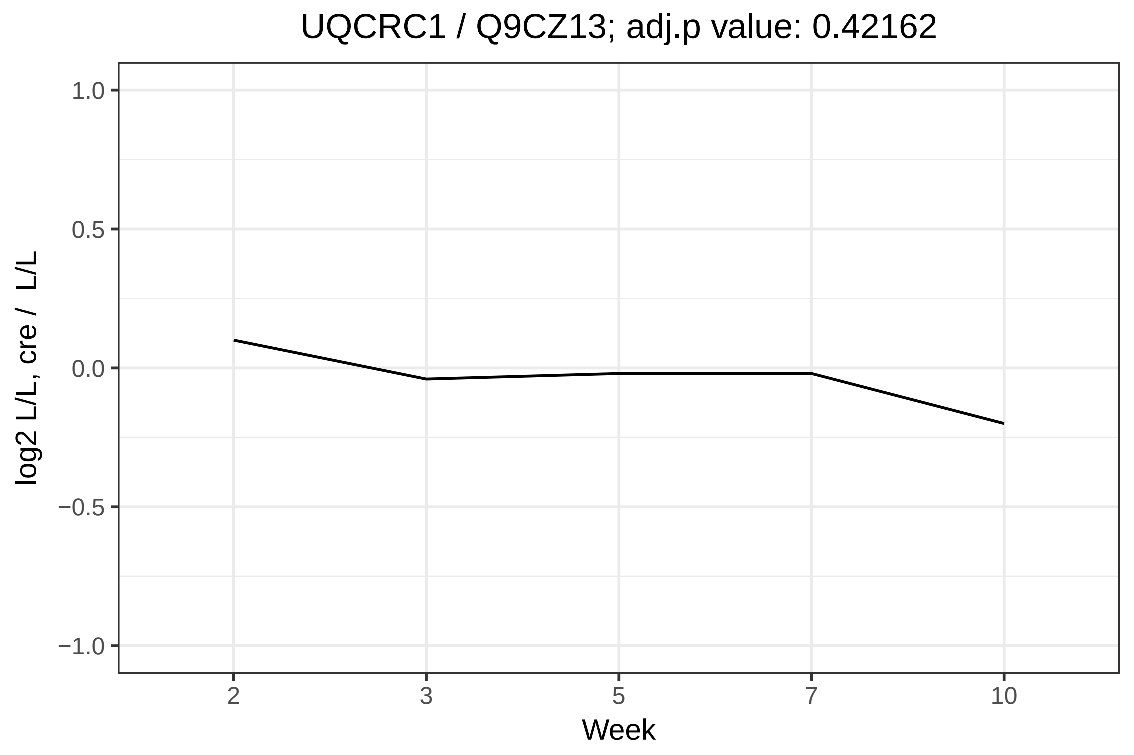

UQCRC1 / Q9CZ13; adj.p value: 0.42162
1.0
0.5
0.0
−0.5
−1.0
log2 L/L, cre / L/L
2
3
5
7
10
Week

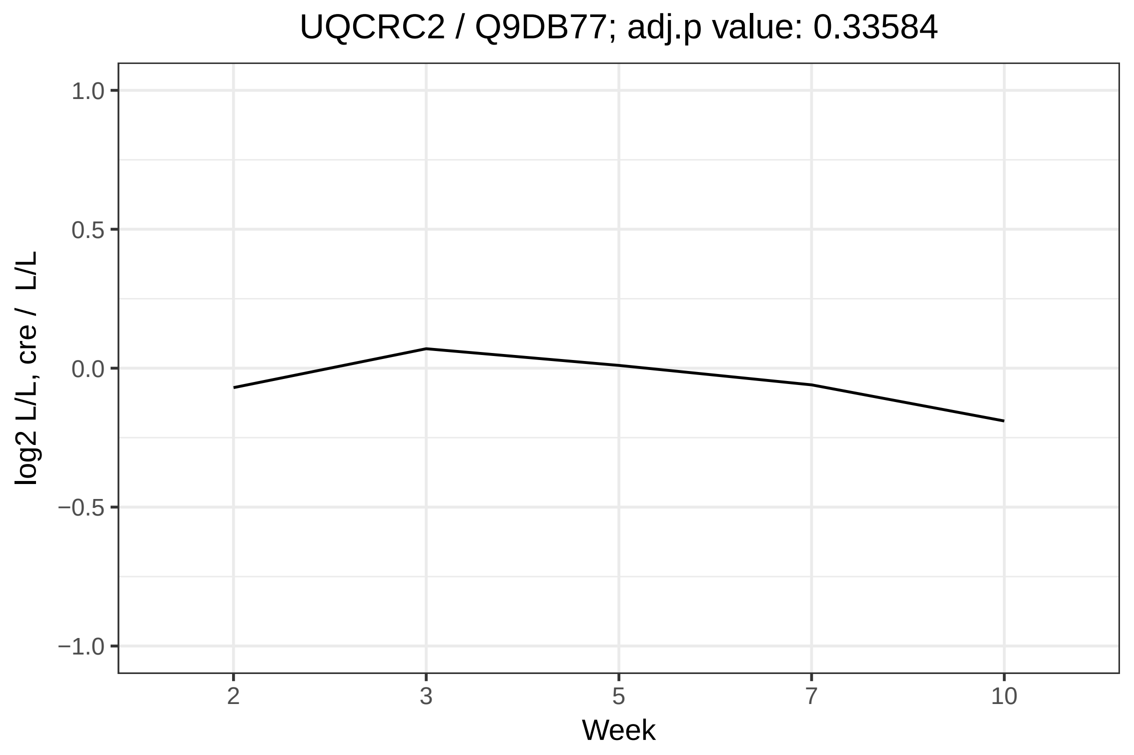

UQCRC2 / Q9DB77; adj.p value: 0.33584
1.0
0.5
0.0
−0.5
−1.0
log2 L/L, cre / L/L
2
3
5
7
10
Week

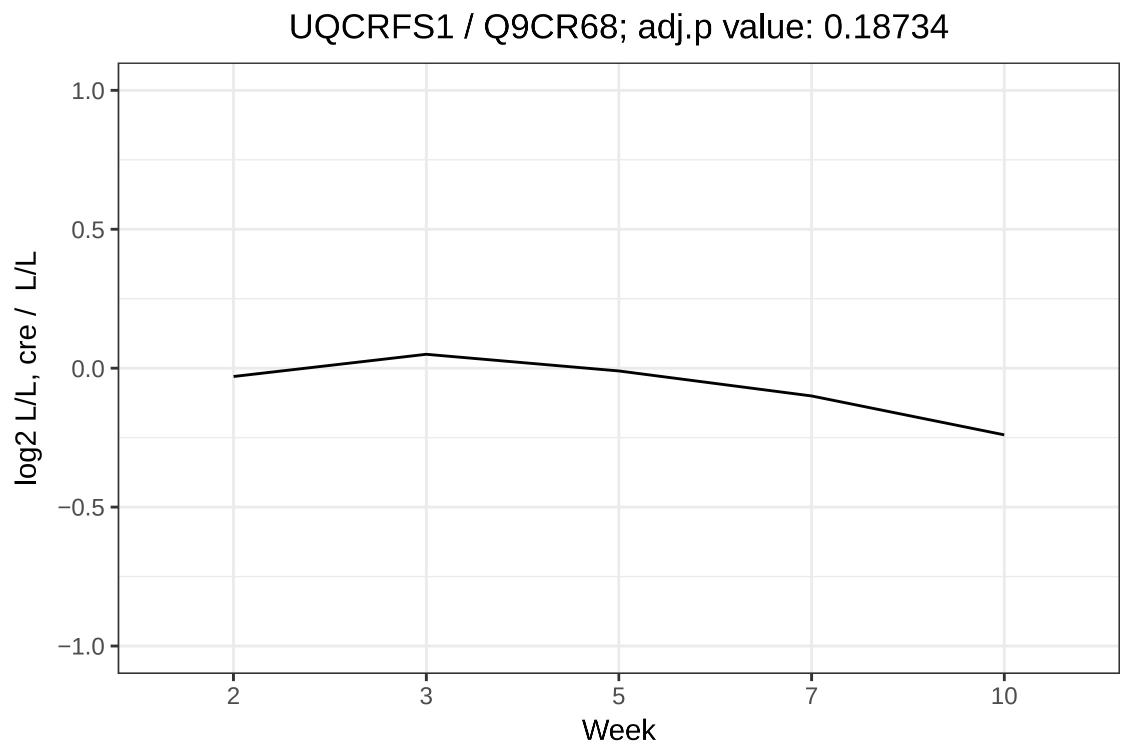

UQCRFS1 / Q9CR68; adj.p value: 0.18734
log2 L/L, cre / L/L
1.0
0.5
0.0
−0.5
−1.0
2
3
5
7
10
Week

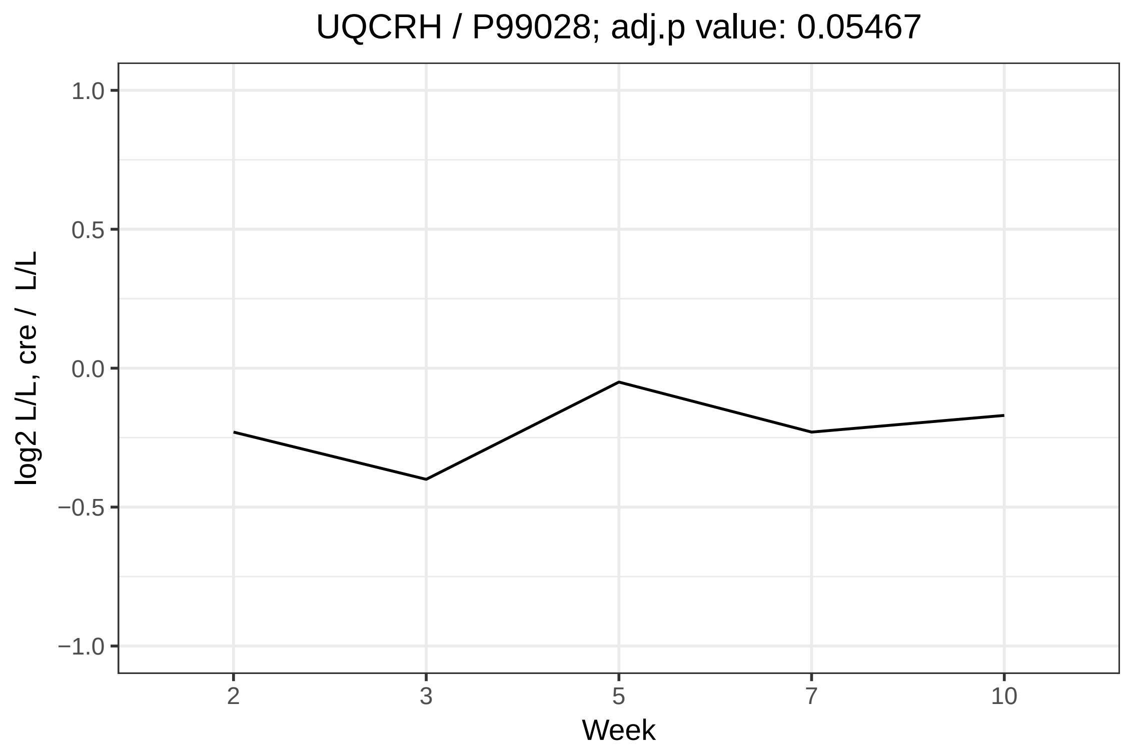
UQCRH / P99028; adj.p value: 0.05467
log2 L/L, cre / L/L
1.0
0.5
0.0
−0.5
−1.0
2
3
5
7
10
Week

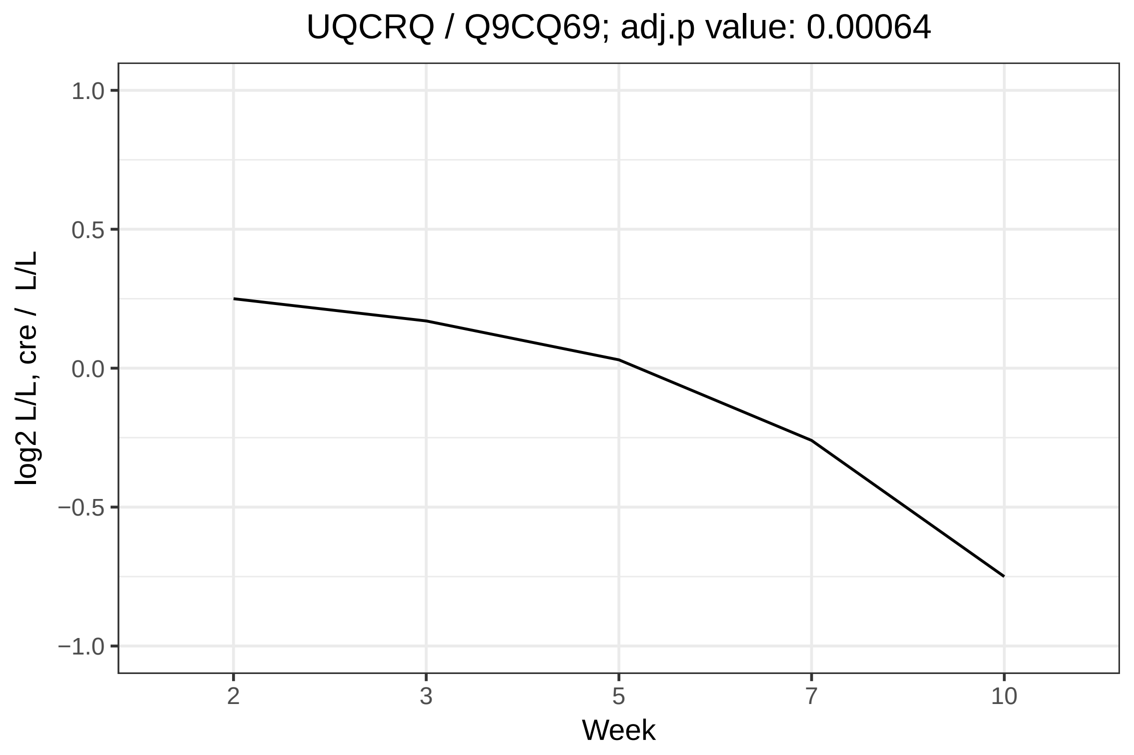

UQCRQ / Q9CQ69; adj.p value: 0.00064
1.0
0.5
0.0
−0.5
−1.0
log2 L/L, cre / L/L
2
3
5
7
10
Week

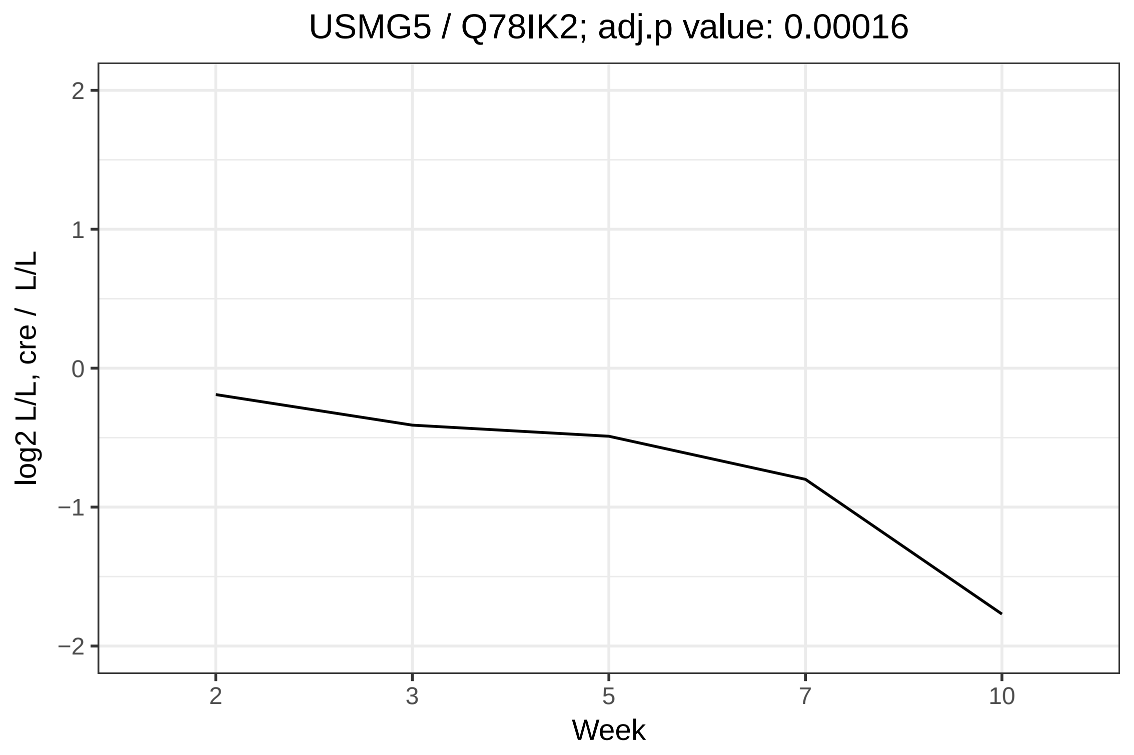
USMG5 / Q78IK2; adj.p value: 0.00016
log2 L/L, cre / L/L
2
1
0
−1
−2
2
3
5
7
10
Week

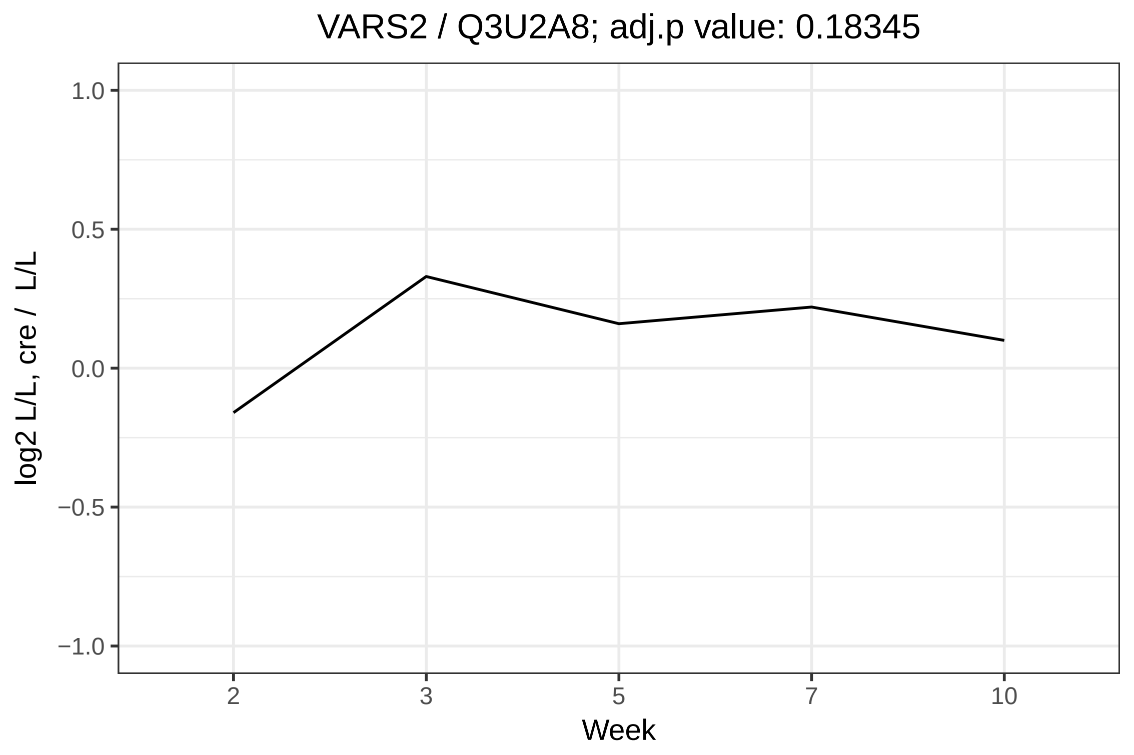
VARS2 / Q3U2A8; adj.p value: 0.18345
1.0
0.5
0.0
−0.5
−1.0
log2 L/L, cre / L/L
2
3
5
7
10
Week

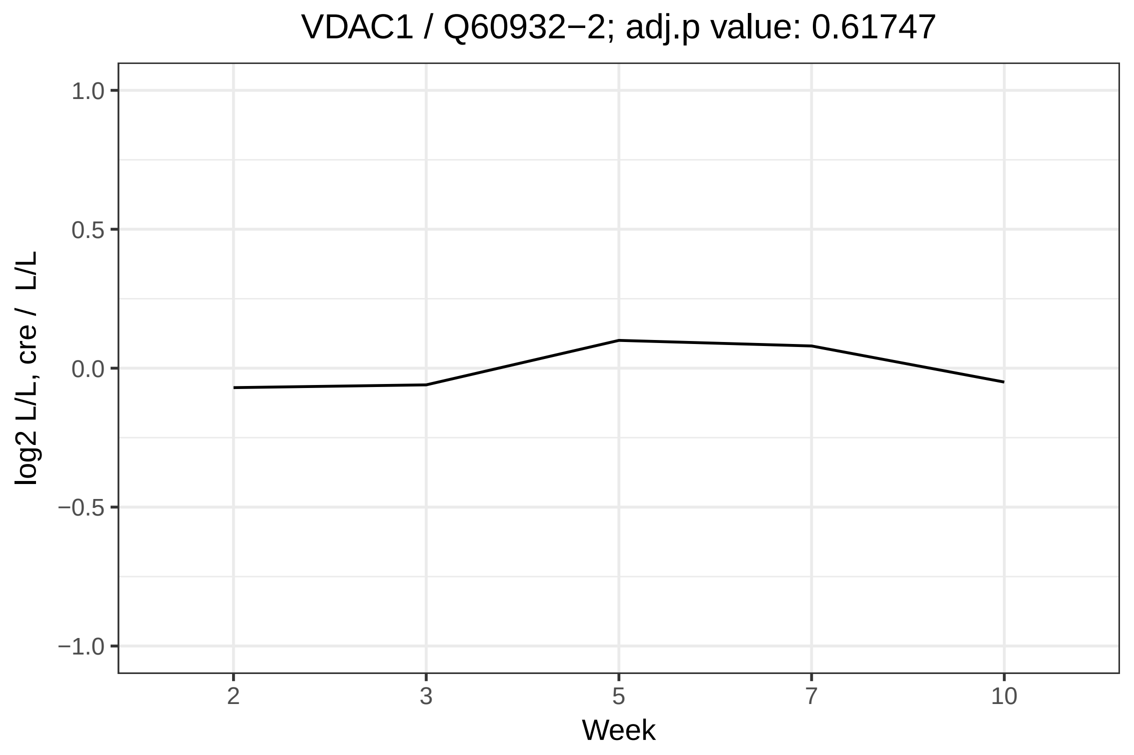
VDAC1 / Q60932−2; adj.p value: 0.61747
1.0
0.5
0.0
−0.5
−1.0
log2 L/L, cre / L/L
2
3
5
7
10
Week

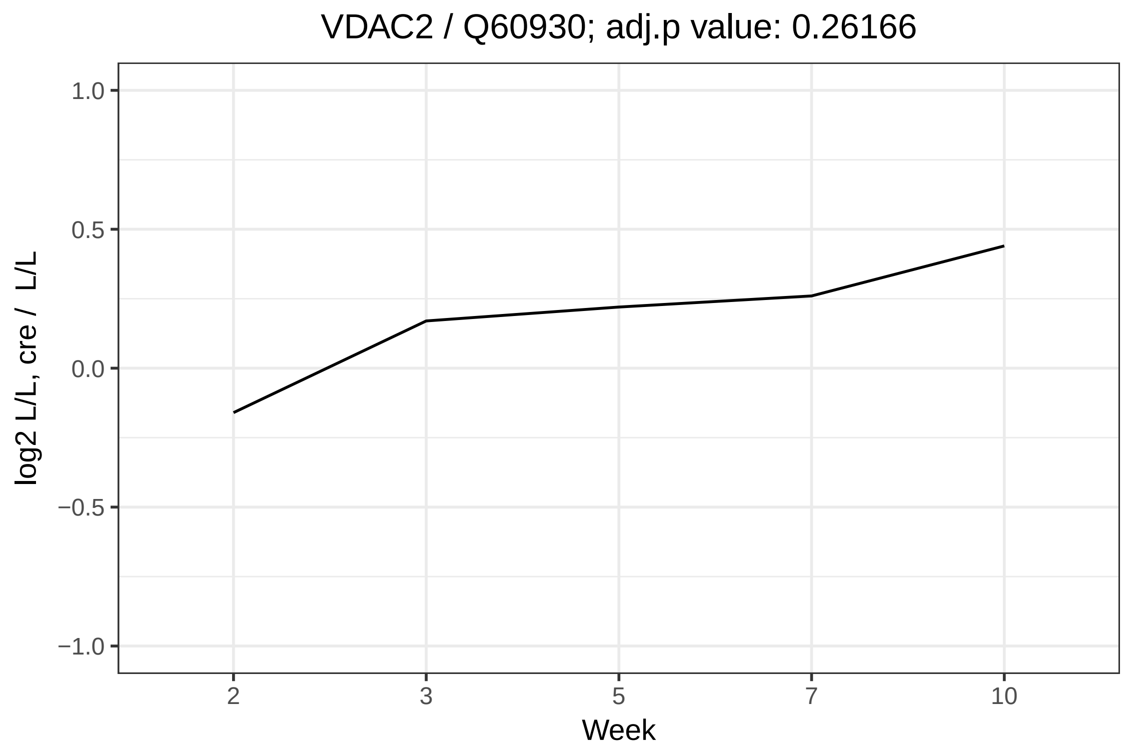

VDAC2 / Q60930; adj.p value: 0.26166
1.0
0.5
0.0
−0.5
−1.0
log2 L/L, cre / L/L
2
3
5
7
10
Week

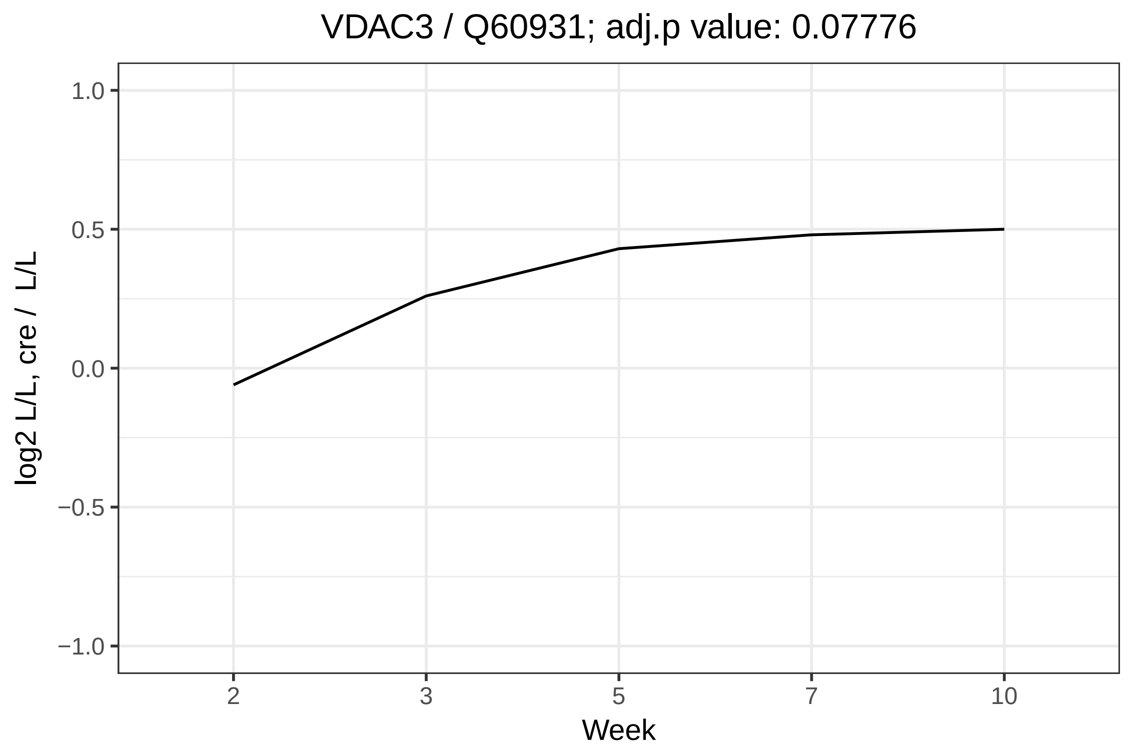

VDAC3 / Q60931; adj.p value: 0.07776
1.0
0.5
0.0
−0.5
−1.0
log2 L/L, cre / L/L
2
3
5
7
10
Week

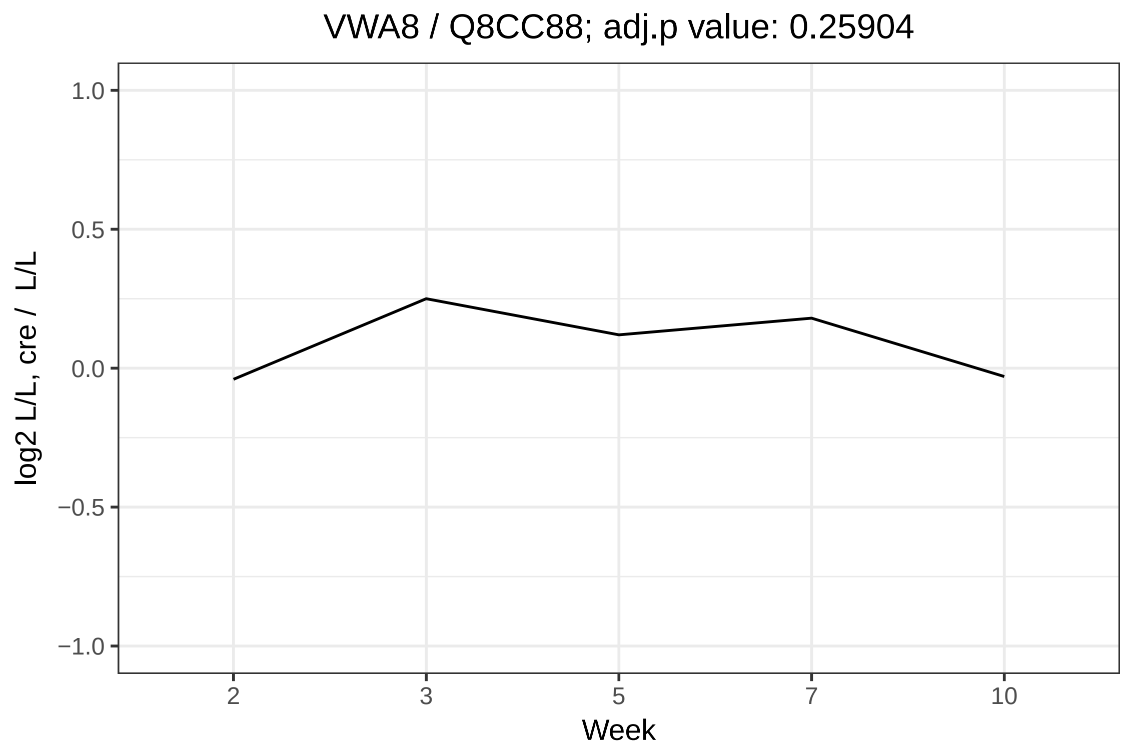

VWA8 / Q8CC88; adj.p value: 0.25904
1.0
0.5
0.0
−0.5
−1.0
log2 L/L, cre / L/L
2
3
5
7
10
Week

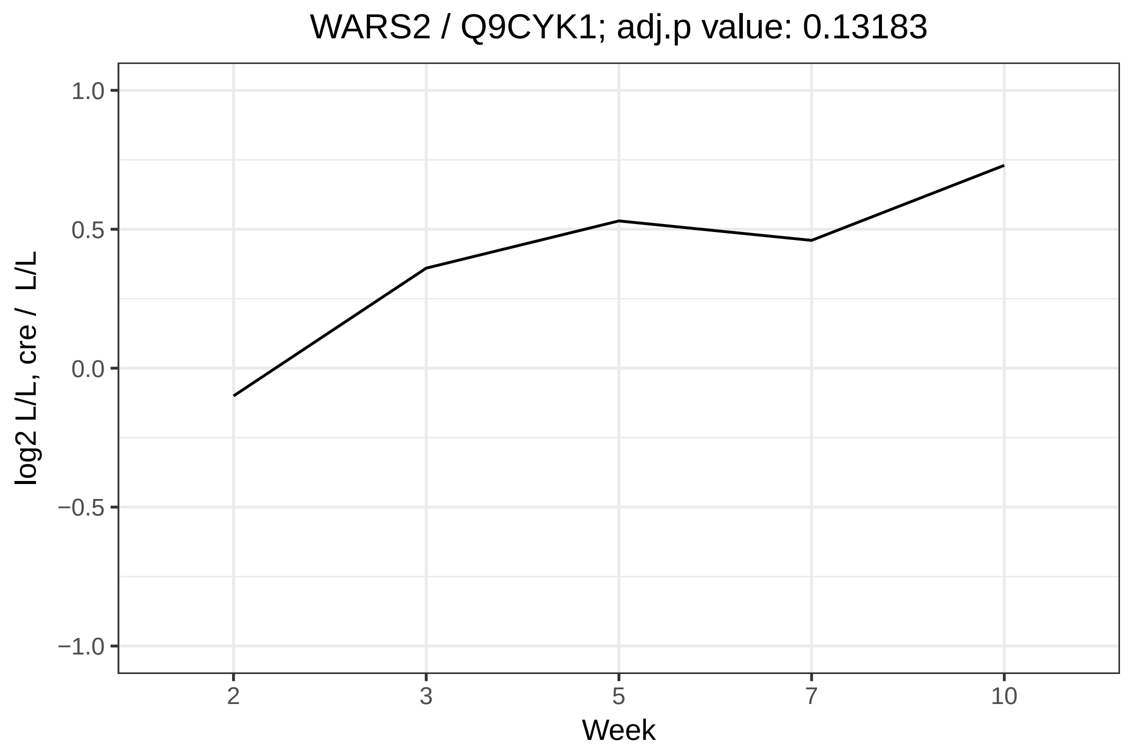
WARS2 / Q9CYK1; adj.p value: 0.13183
1.0
0.5
0.0
−0.5
−1.0
log2 L/L, cre / L/L
2
3
5
7
10
Week

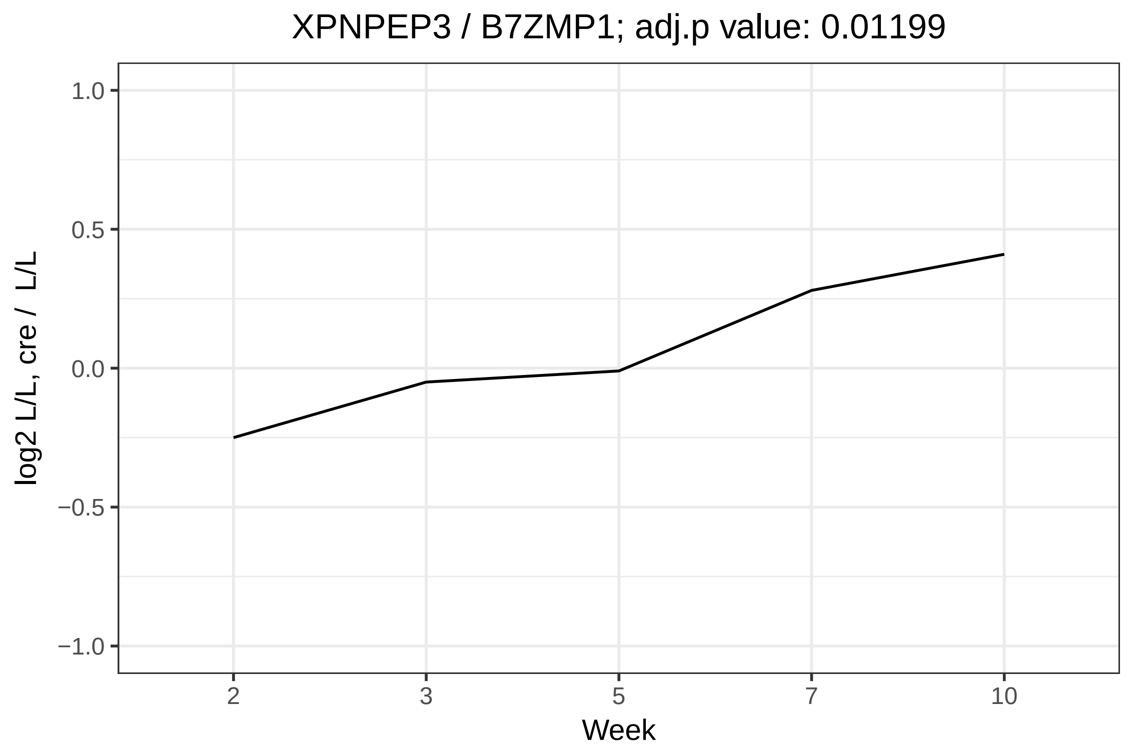
XPNPEP3 / B7ZMP1; adj.p value: 0.01199
1.0
0.5
0.0
−0.5
−1.0
log2 L/L, cre / L/L
2
3
5
7
10
Week

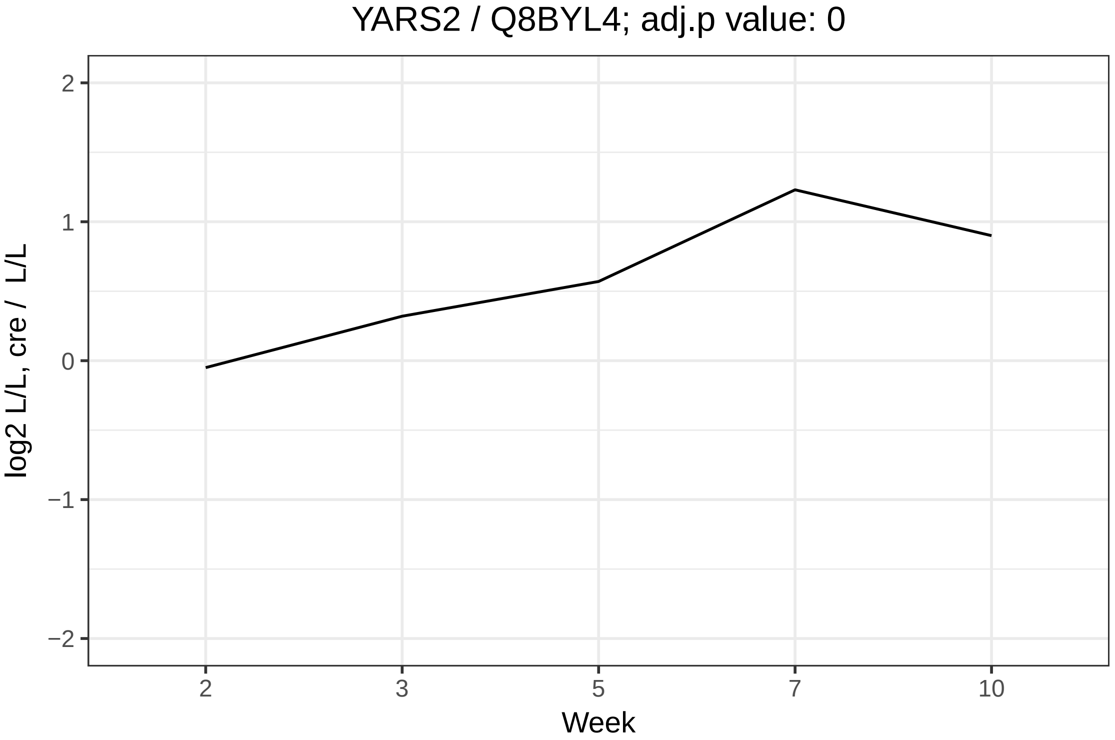
YARS2 / Q8BYL4; adj.p value: 0
log2 L/L, cre / L/L
2
1
0
−1
−2
2
3
5
7
10
Week

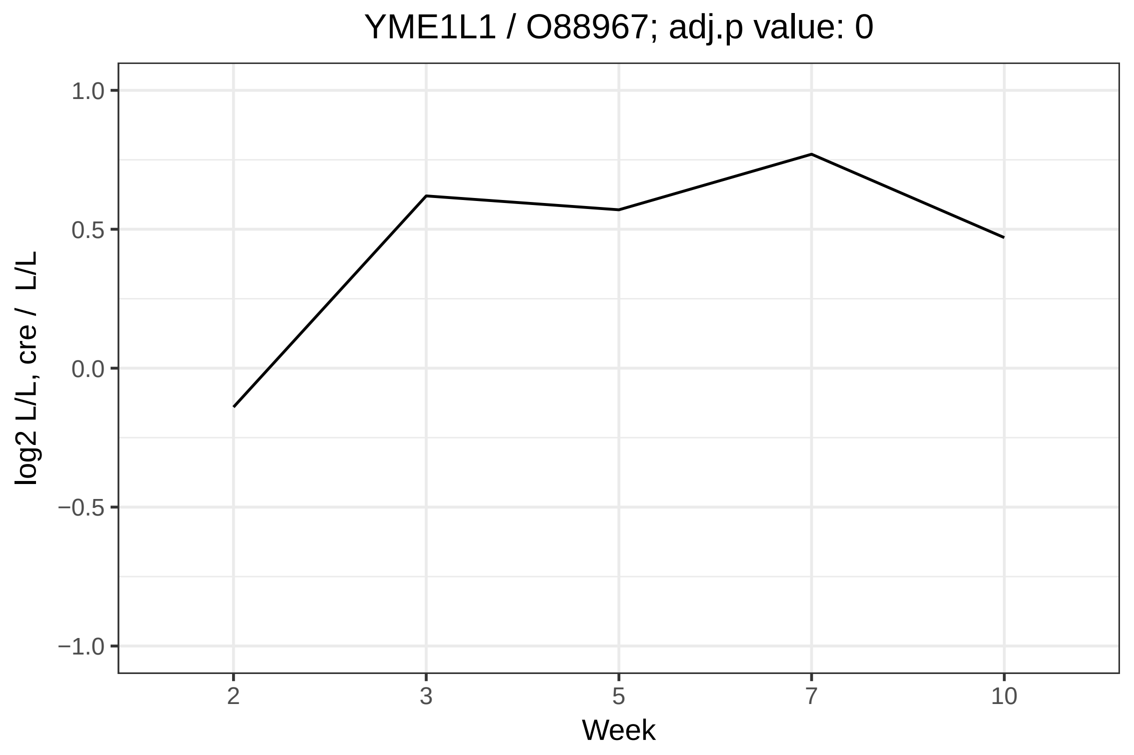
YME1L1 / O88967; adj.p value: 0
log2 L/L, cre / L/L
1.0
0.5
0.0
−0.5
−1.0
2
3
5
7
10
Week

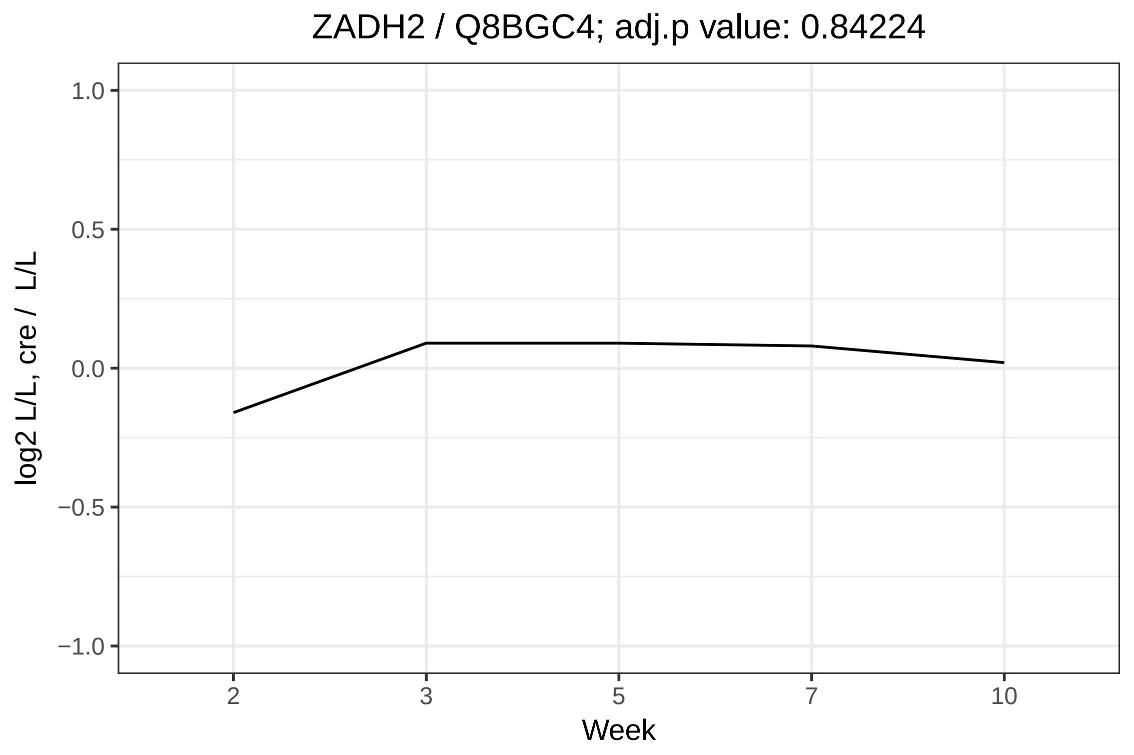
ZADH2 / Q8BGC4; adj.p value: 0.84224
log2 L/L, cre / L/L
1.0
0.5
0.0
−0.5
−1.0
2
3
5
7
10
Week
