# Supplementary material for: Transcriptomic and proteomic landscape of mitochondrial dysfunction reveals secondary coenzyme Q deficiency in mammals
Source: eLife. 2017 Nov 14;6:e30952. doi: 10.7554/eLife.30952 (PMC5703644; doi:10.7554/eLife.30952)
Supplement: Supplementary file 8. [file elife-30952-supp8.pdf]

Supplementary File 8: Differential expression proteomic analysis all knockouts Kühl *et al.*

Boxplots of the intensity detected by mass spectrometry per protein.

Boxplots represent median ± 25% quantile. The whiskers extend from the hinge to the highest and lowest value within 1.5x IQR, respectively. Red dot: mean.

Each mouse strain is labeled in the boxplots with the protein name of the gene they are knockout for:

Twnk: TWINKLE
Tfam: TFAM
Polrmt: POLRMT
Lrpprc: LRPPRC
Mterf4: MTERF4

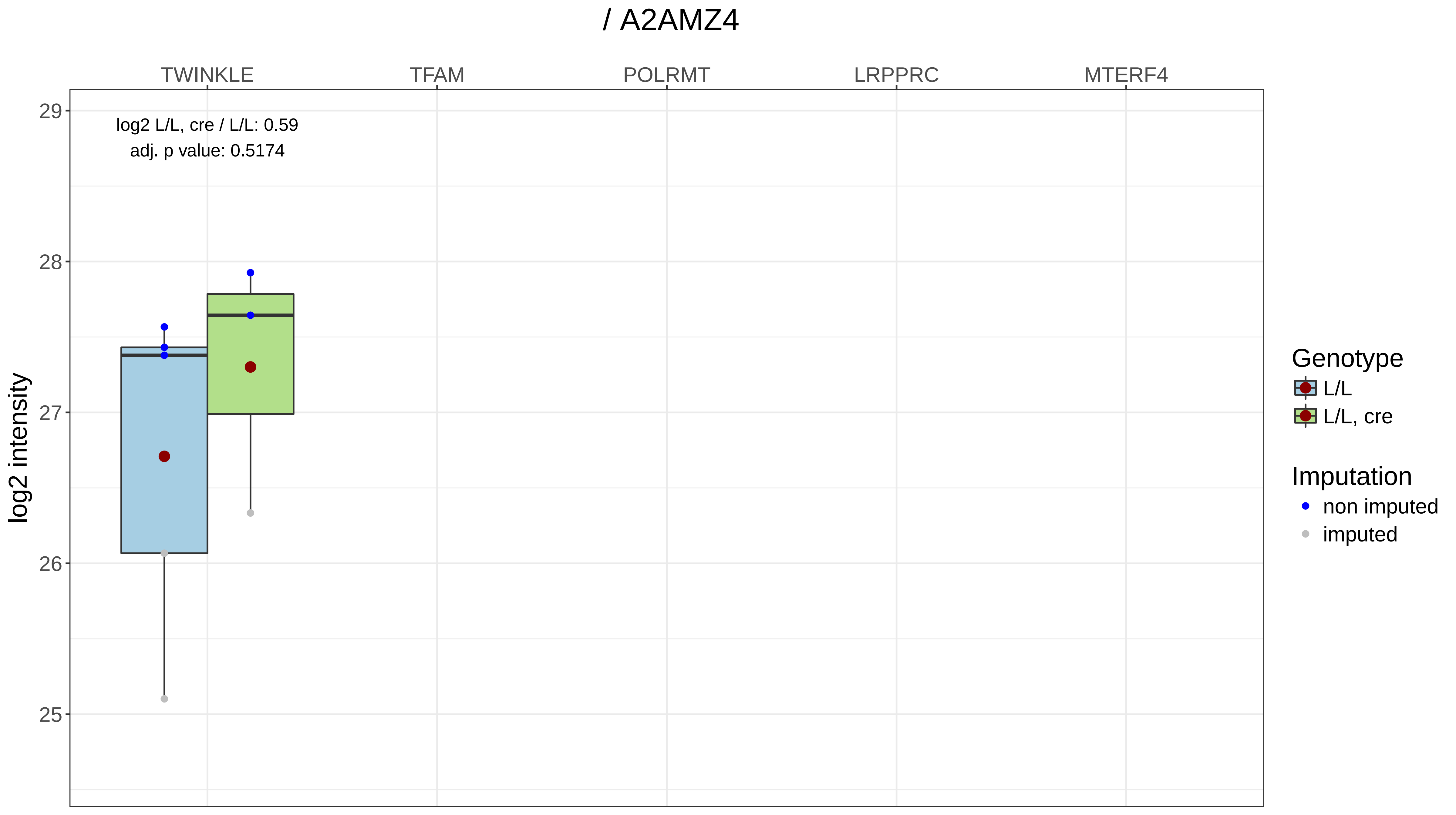
/ A2AMZ4
TWINKLE
TFAM
POLRMT
LRPPRC
MTERF4
log2 L/L, cre / L/L: 0.59
adj. p value: 0.5174
log2 intensity
29
28
27
26
25
Genotype
L/L
L/L, cre
Imputation
non imputed
imputed

/ Q3UNZ8

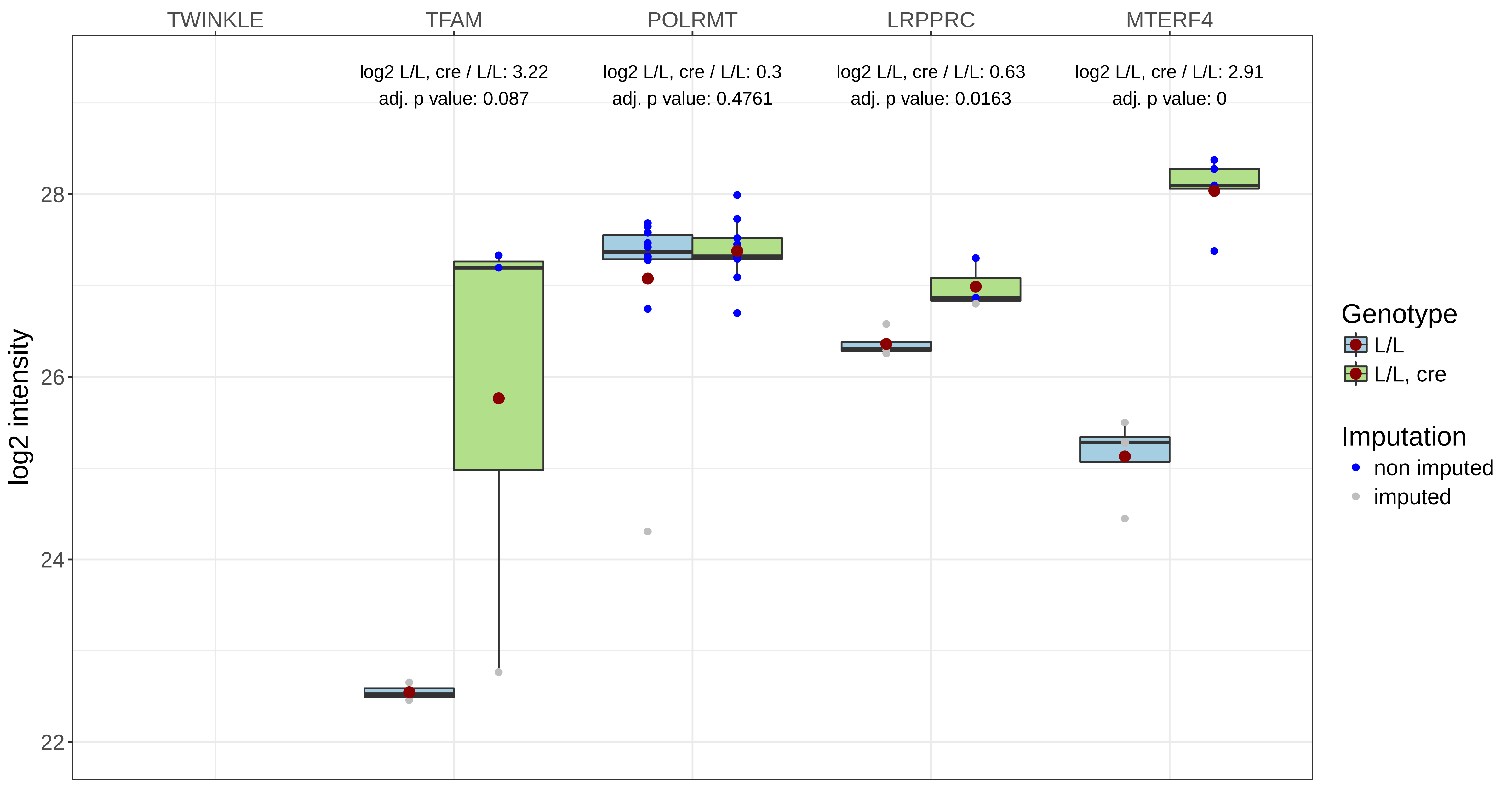

TWINKLE
TFAM
POLRMT
LRPPRC
MTERF4
log2 L/L, cre / L/L: 3.22
adj. p value: 0.087
log2 L/L, cre / L/L: 0.3
adj. p value: 0.4761
log2 L/L, cre / L/L: 0.63
adj. p value: 0.0163
log2 L/L, cre / L/L: 2.91
adj. p value: 0
log2 intensity
28
26
24
22
Genotype
L/L
L/L, cre
Imputation
non imputed
imputed

/ Q80VP5

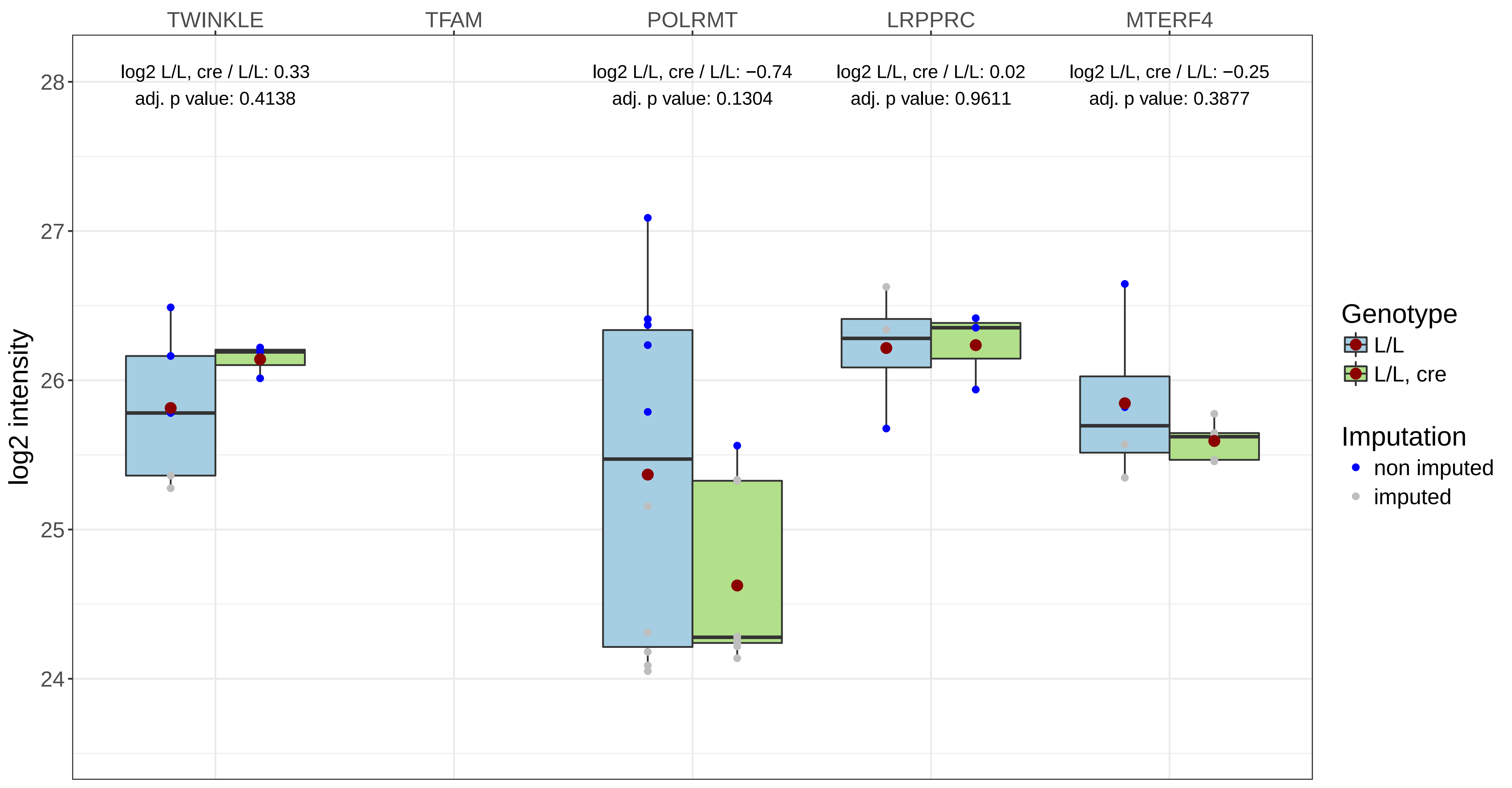
TWINKLE
TFAM
POLRMT
LRPPRC
MTERF4
log2 L/L, cre / L/L: 0.33
adj. p value: 0.4138
log2 L/L, cre / L/L: −0.74
adj. p value: 0.1304
log2 L/L, cre / L/L: 0.02
adj. p value: 0.9611
log2 L/L, cre / L/L: −0.25
adj. p value: 0.3877
log2 intensity
28
27
26
25
24
Genotype
L/L
L/L, cre
Imputation
non imputed
imputed

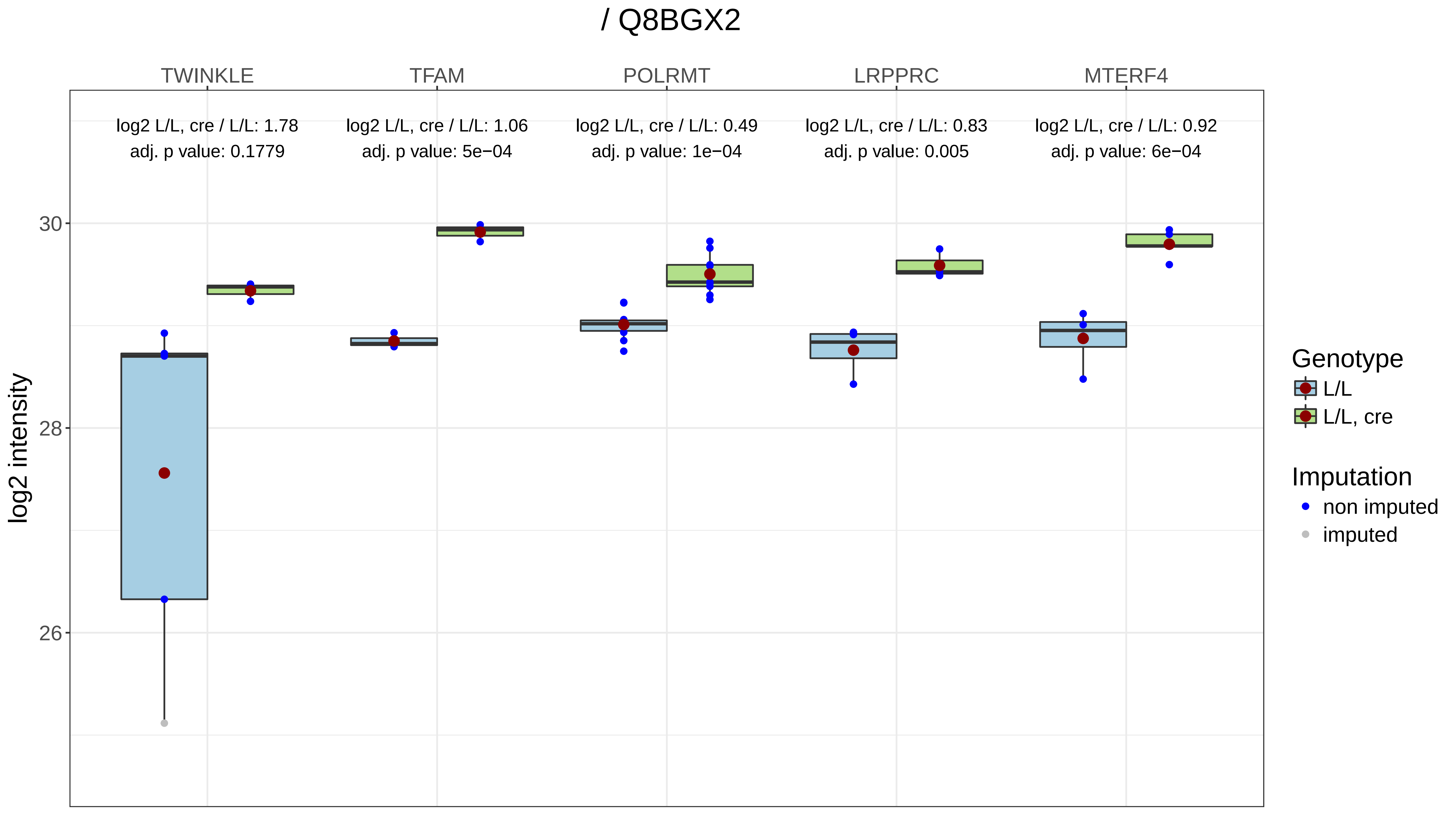
/ Q8BGX2
TWINKLE
TFAM
POLRMT
LRPPRC
MTERF4
log2 L/L, cre / L/L: 1.78
adj. p value: 0.1779
log2 L/L, cre / L/L: 1.06
adj. p value: 5e−04
log2 L/L, cre / L/L: 0.49
adj. p value: 1e−04
log2 L/L, cre / L/L: 0.83
adj. p value: 0.005
log2 L/L, cre / L/L: 0.92
adj. p value: 6e−04
30
28
26
log2 intensity
Genotype
L/L
L/L, cre
Imputation
non imputed
imputed

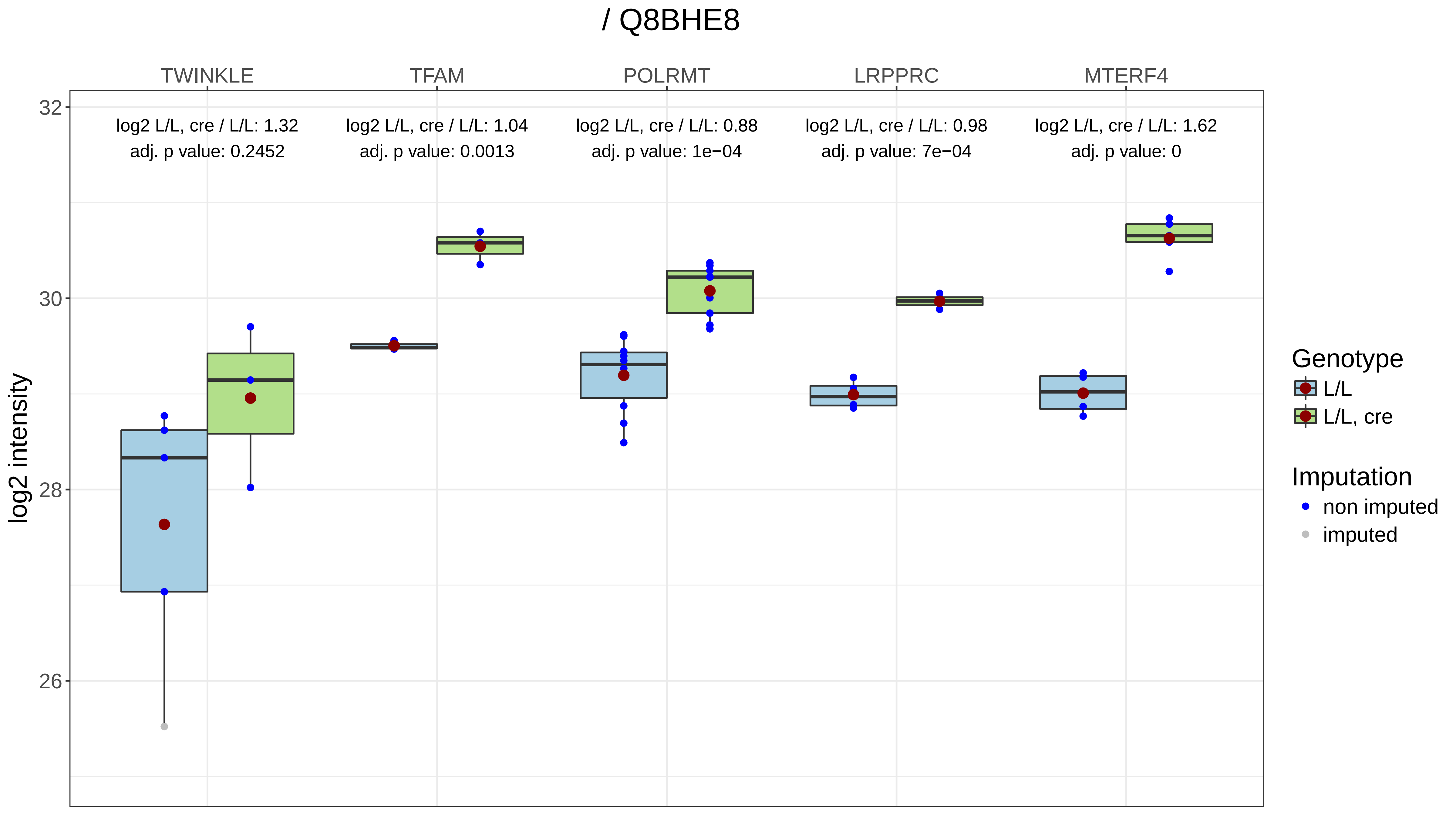
/ Q8BHE8
TWINKLE
TFAM
POLRMT
LRPPRC
MTERF4
log2 L/L, cre / L/L: 1.32
adj. p value: 0.2452
log2 L/L, cre / L/L: 1.04
adj. p value: 0.0013
log2 L/L, cre / L/L: 0.88
adj. p value: 1e−04
log2 L/L, cre / L/L: 0.98
adj. p value: 7e−04
log2 L/L, cre / L/L: 1.62
adj. p value: 0
32
30
28
26
log2 intensity
Genotype
L/L
L/L, cre
Imputation
non imputed
imputed

/ Q8BTC1

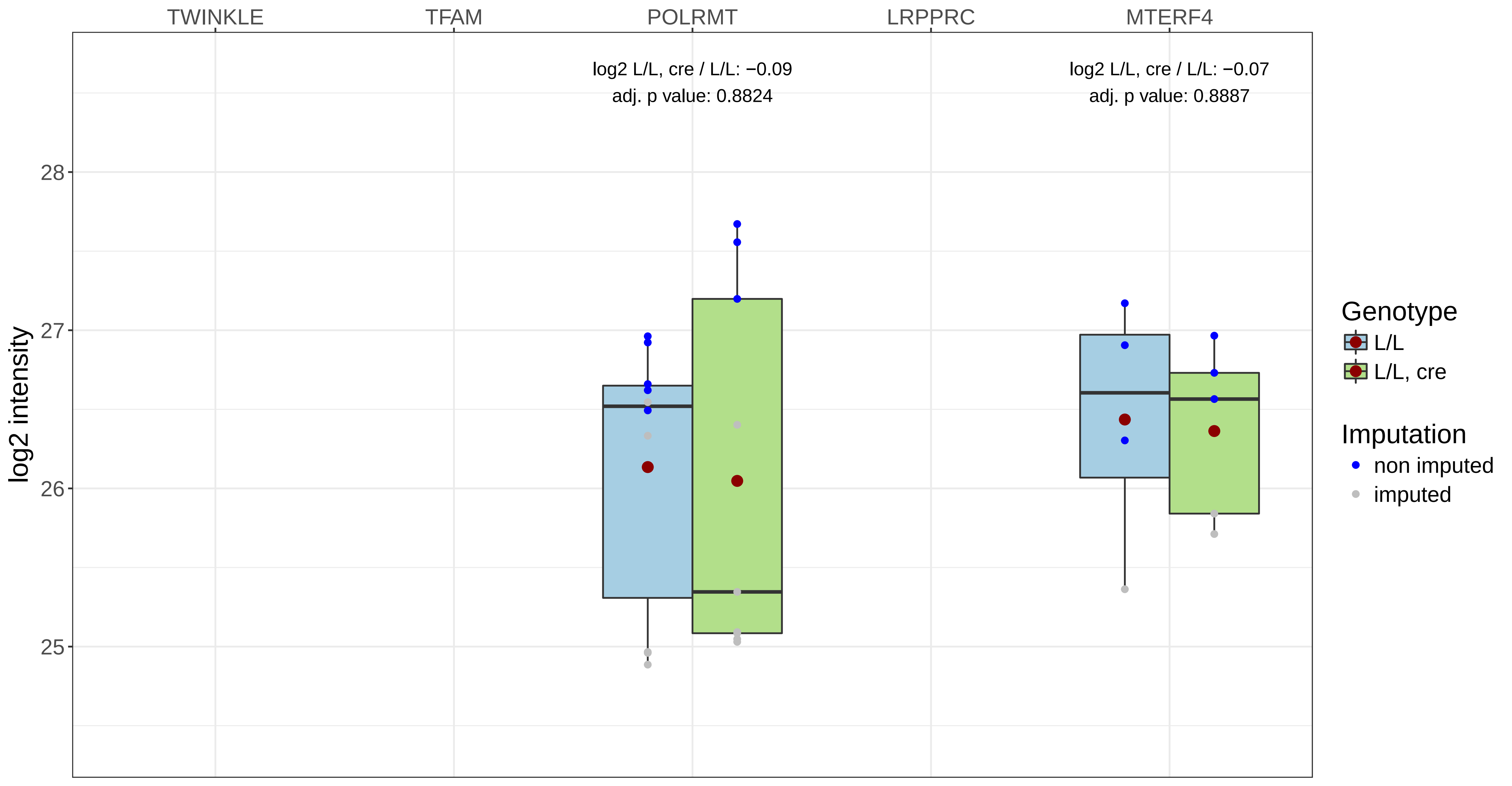
TWINKLE
TFAM
POLRMT
LRPPRC
MTERF4
log2 L/L, cre / L/L: −0.09
adj. p value: 0.8824
log2 L/L, cre / L/L: −0.07
adj. p value: 0.8887
log2 intensity
28
27
26
25
Genotype
L/L
L/L, cre
Imputation
non imputed
imputed

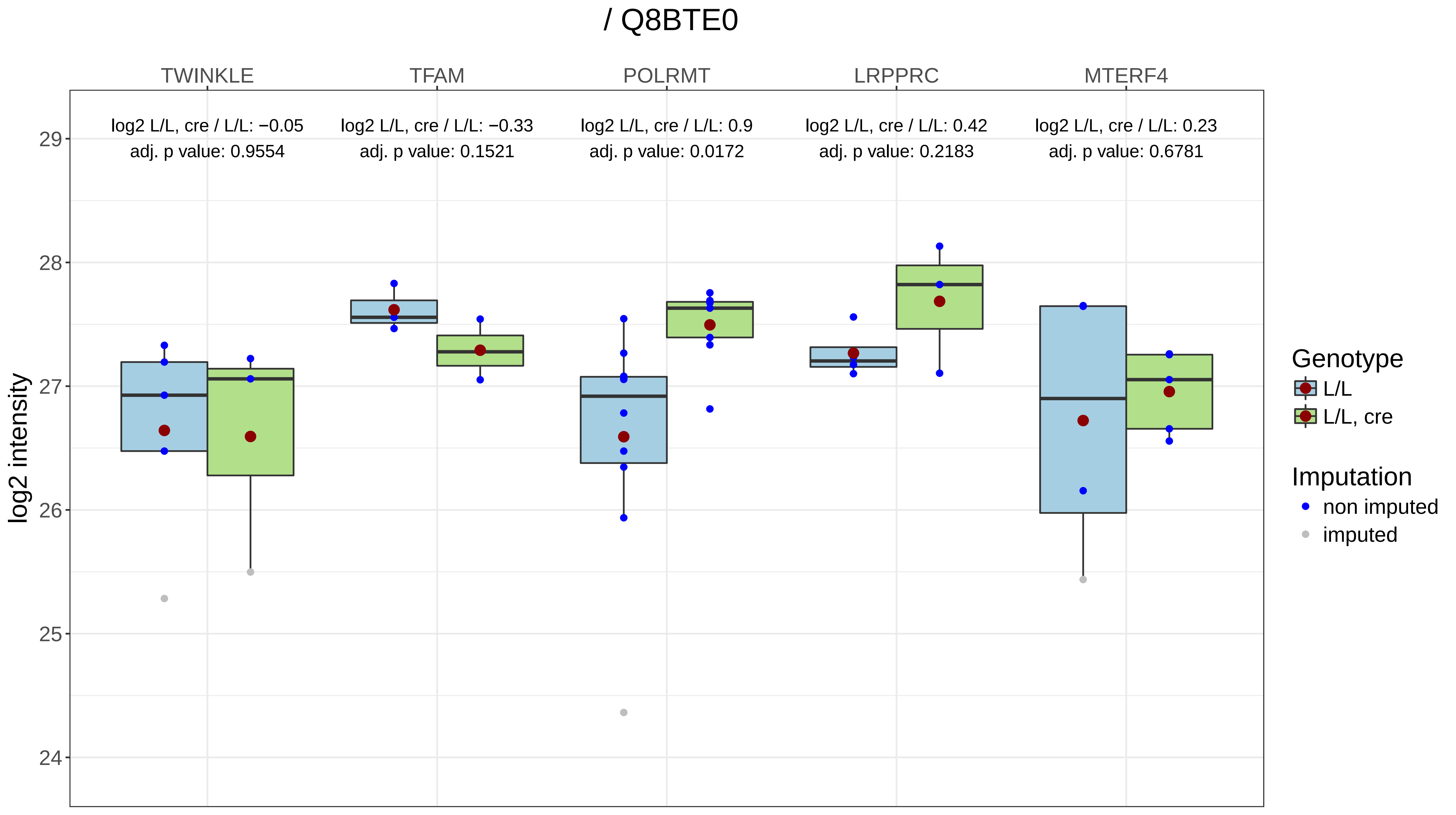
/ Q8BTE0
TWINKLE
TFAM
POLRMT
LRPPRC
MTERF4
log2 L/L, cre / L/L: −0.05
adj. p value: 0.9554
log2 L/L, cre / L/L: −0.33
adj. p value: 0.1521
log2 L/L, cre / L/L: 0.9
adj. p value: 0.0172
log2 L/L, cre / L/L: 0.42
adj. p value: 0.2183
log2 L/L, cre / L/L: 0.23
adj. p value: 0.6781
log2 intensity
29
28
27
26
25
24
Genotype
L/L
L/L, cre
Imputation
non imputed
imputed

/ Q8K2T4

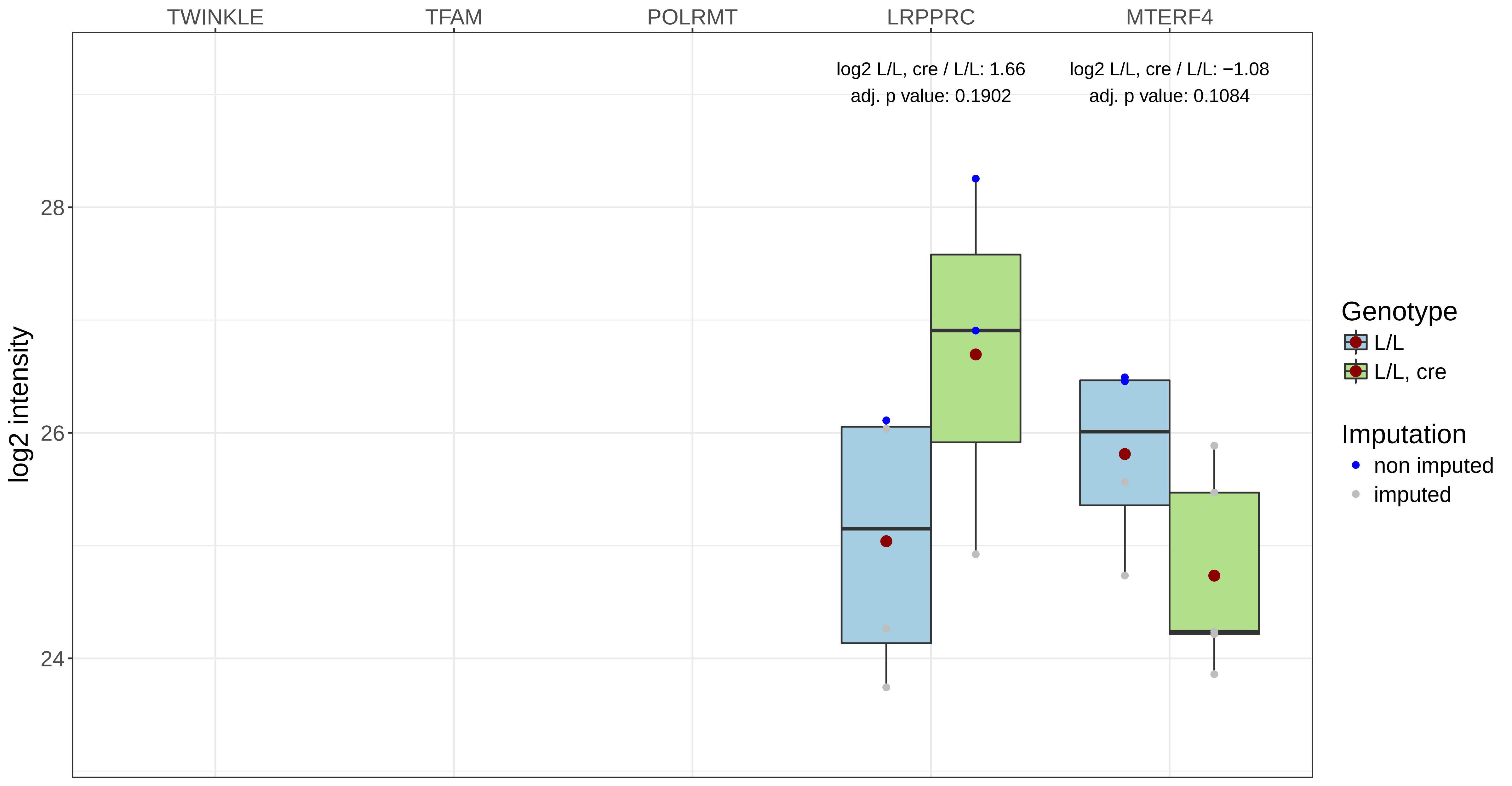
TWINKLE
TFAM
POLRMT
LRPPRC
MTERF4
log2 L/L, cre / L/L: 1.66
adj. p value: 0.1902
log2 L/L, cre / L/L: −1.08
adj. p value: 0.1084
28
26
24
log2 intensity
Genotype
L/L
L/L, cre
Imputation
non imputed
imputed

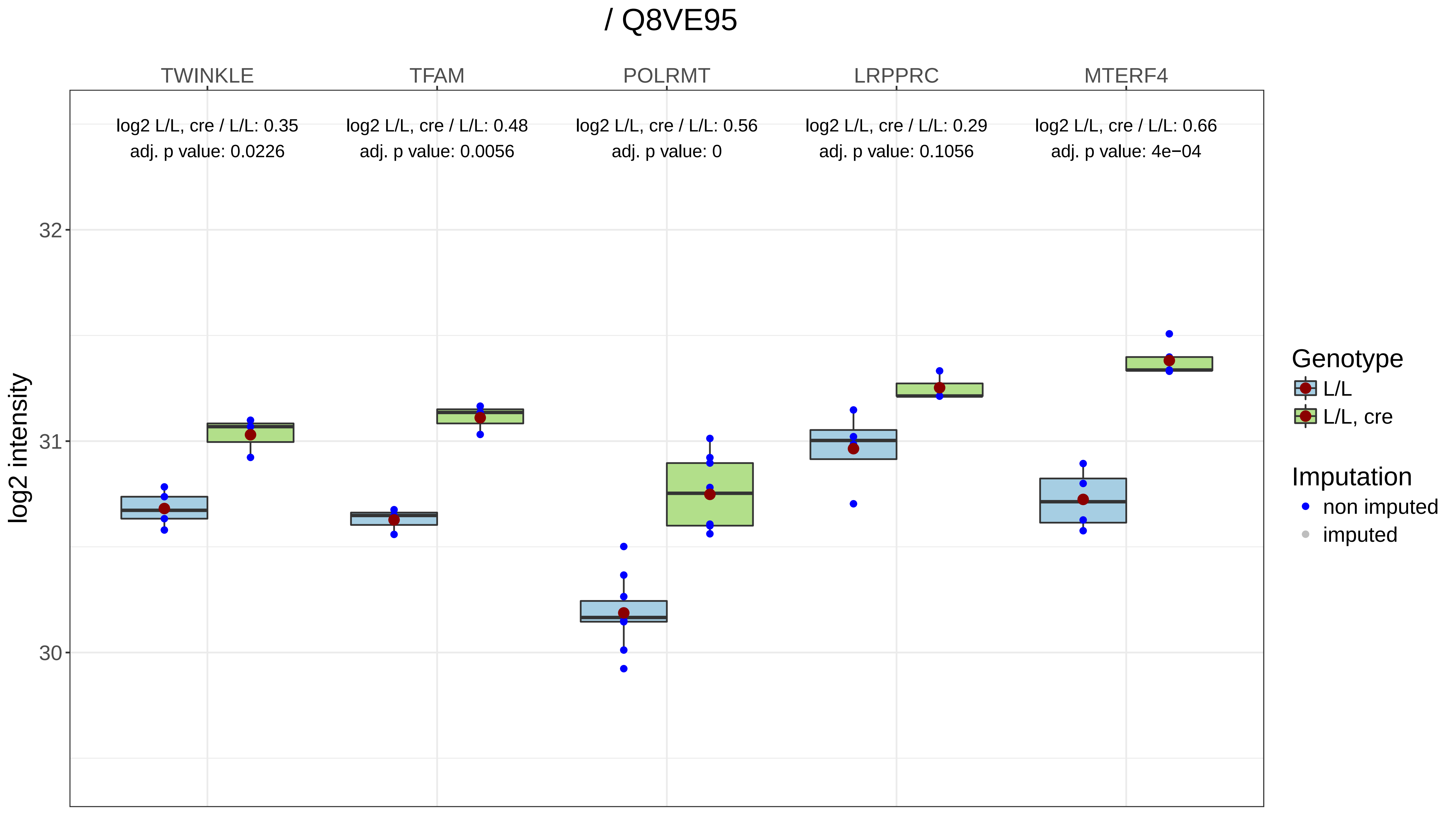
/ Q8VE95
TWINKLE
TFAM
POLRMT
LRPPRC
MTERF4
log2 L/L, cre / L/L: 0.35
adj. p value: 0.0226
log2 L/L, cre / L/L: 0.48
adj. p value: 0.0056
log2 L/L, cre / L/L: 0.56
adj. p value: 0
log2 L/L, cre / L/L: 0.29
adj. p value: 0.1056
log2 L/L, cre / L/L: 0.66
adj. p value: 4e−04
32
31
30
log2 intensity
Genotype
L/L
L/L, cre
Imputation
non imputed
imputed

/ Q9CR13

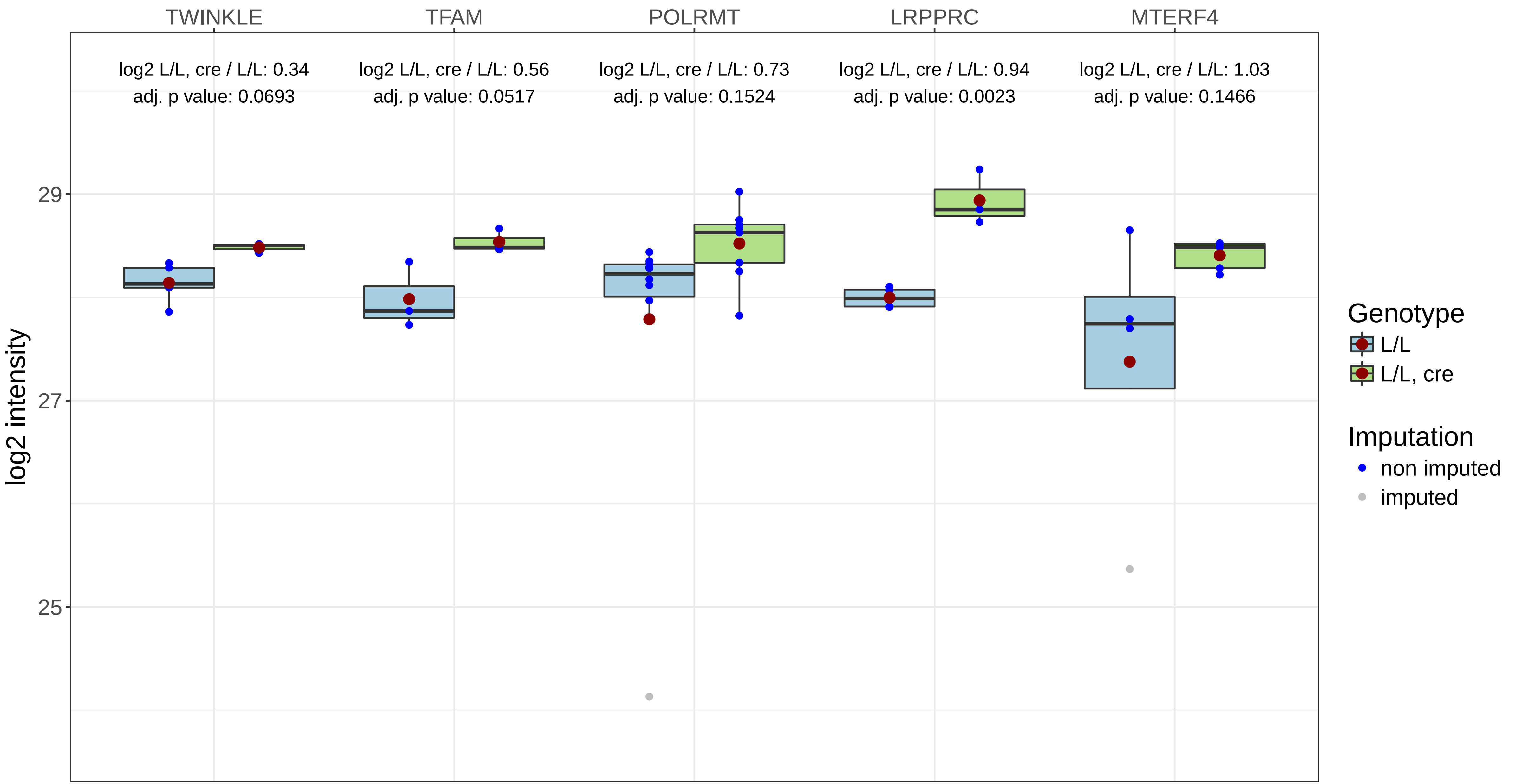
TWINKLE
TFAM
POLRMT
LRPPRC
MTERF4
log2 L/L, cre / L/L: 0.34
adj. p value: 0.0693
log2 L/L, cre / L/L: 0.56
adj. p value: 0.0517
log2 L/L, cre / L/L: 0.73
adj. p value: 0.1524
log2 L/L, cre / L/L: 0.94
adj. p value: 0.0023
log2 L/L, cre / L/L: 1.03
adj. p value: 0.1466
29
27
25
log2 intensity
Genotype
L/L
L/L, cre
Imputation
non imputed
imputed

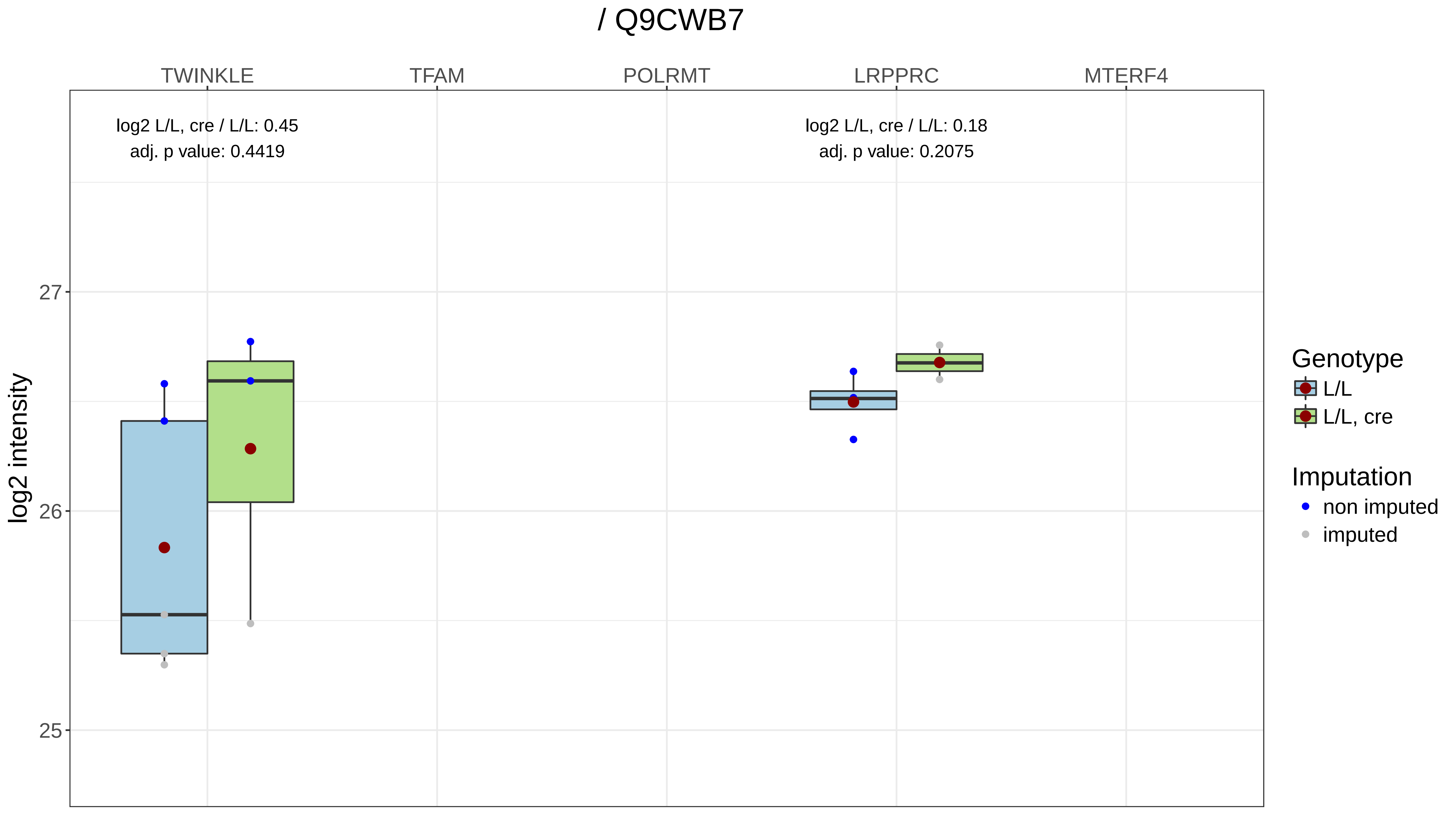
/ Q9CWB7
TWINKLE
TFAM
POLRMT
LRPPRC
MTERF4
log2 L/L, cre / L/L: 0.45
adj. p value: 0.4419
log2 L/L, cre / L/L: 0.18
adj. p value: 0.2075
log2 intensity
27
26
25
Genotype
L/L
L/L, cre
Imputation
non imputed
imputed

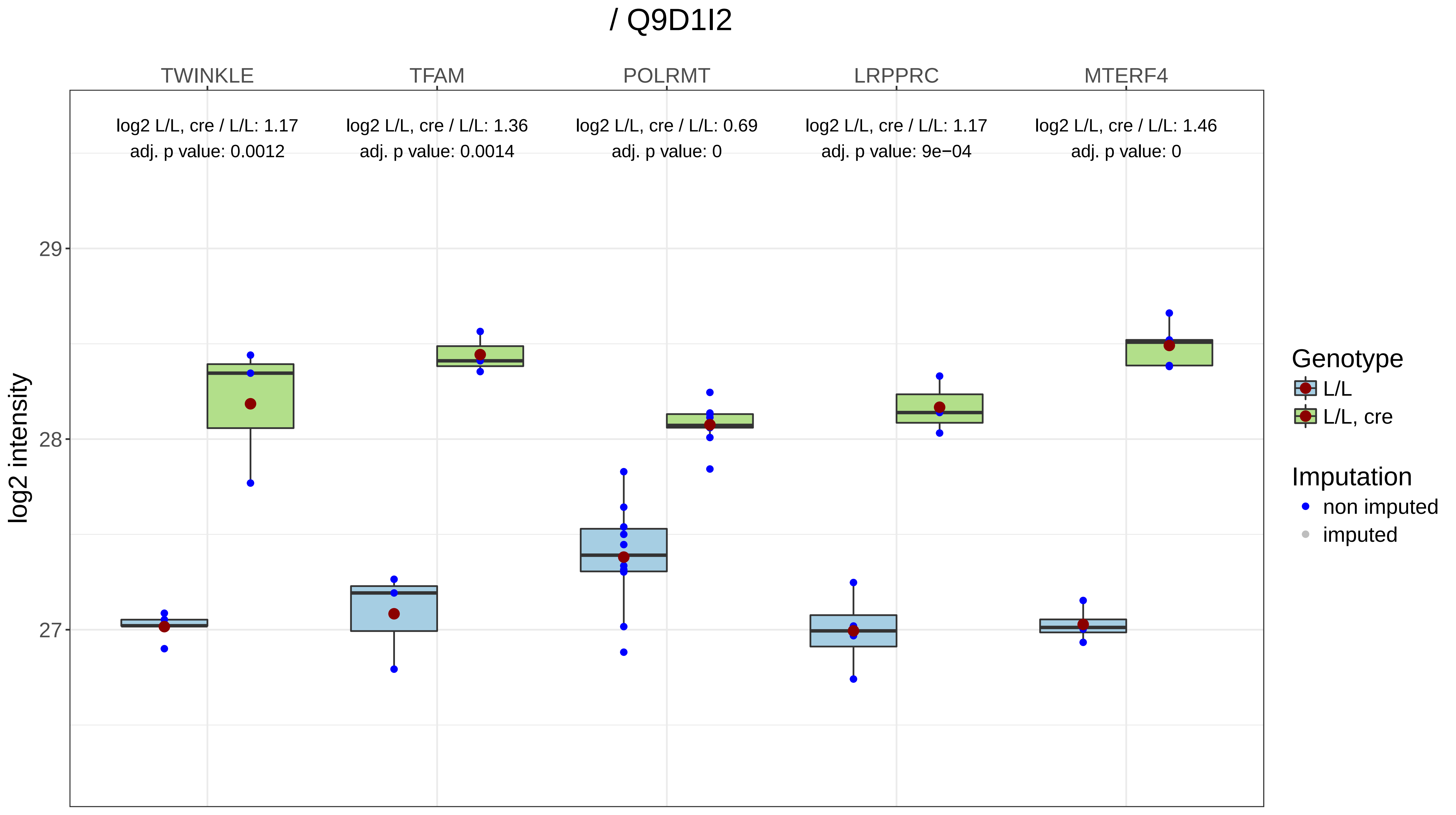
/ Q9D1I2
TWINKLE
TFAM
POLRMT
LRPPRC
MTERF4
log2 L/L, cre / L/L: 1.17
adj. p value: 0.0012
log2 L/L, cre / L/L: 1.36
adj. p value: 0.0014
log2 L/L, cre / L/L: 0.69
adj. p value: 0
log2 L/L, cre / L/L: 1.17
adj. p value: 9e−04
log2 L/L, cre / L/L: 1.46
adj. p value: 0
log2 intensity
29
28
27
Genotype
L/L
L/L, cre
Imputation
non imputed
imputed

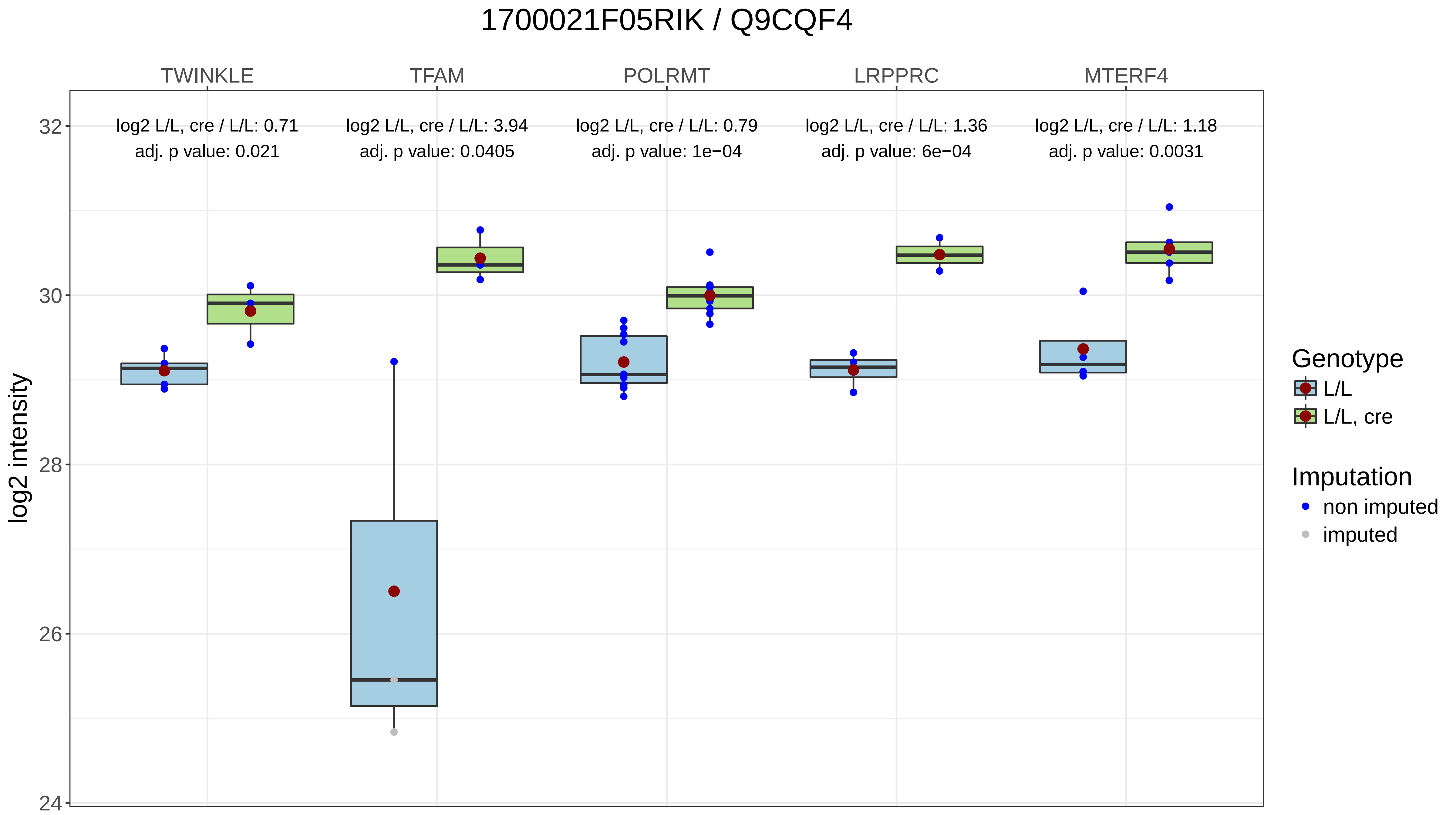
1700021F05RIK / Q9CQF4
TWINKLE
TFAM
POLRMT
LRPPRC
MTERF4
log2 L/L, cre / L/L: 0.71
adj. p value: 0.021
log2 L/L, cre / L/L: 3.94
adj. p value: 0.0405
log2 L/L, cre / L/L: 0.79
adj. p value: 1e−04
log2 L/L, cre / L/L: 1.36
adj. p value: 6e−04
log2 L/L, cre / L/L: 1.18
adj. p value: 0.0031
log2 intensity
32
30
28
26
24
Genotype
L/L
L/L, cre
Imputation
non imputed
imputed

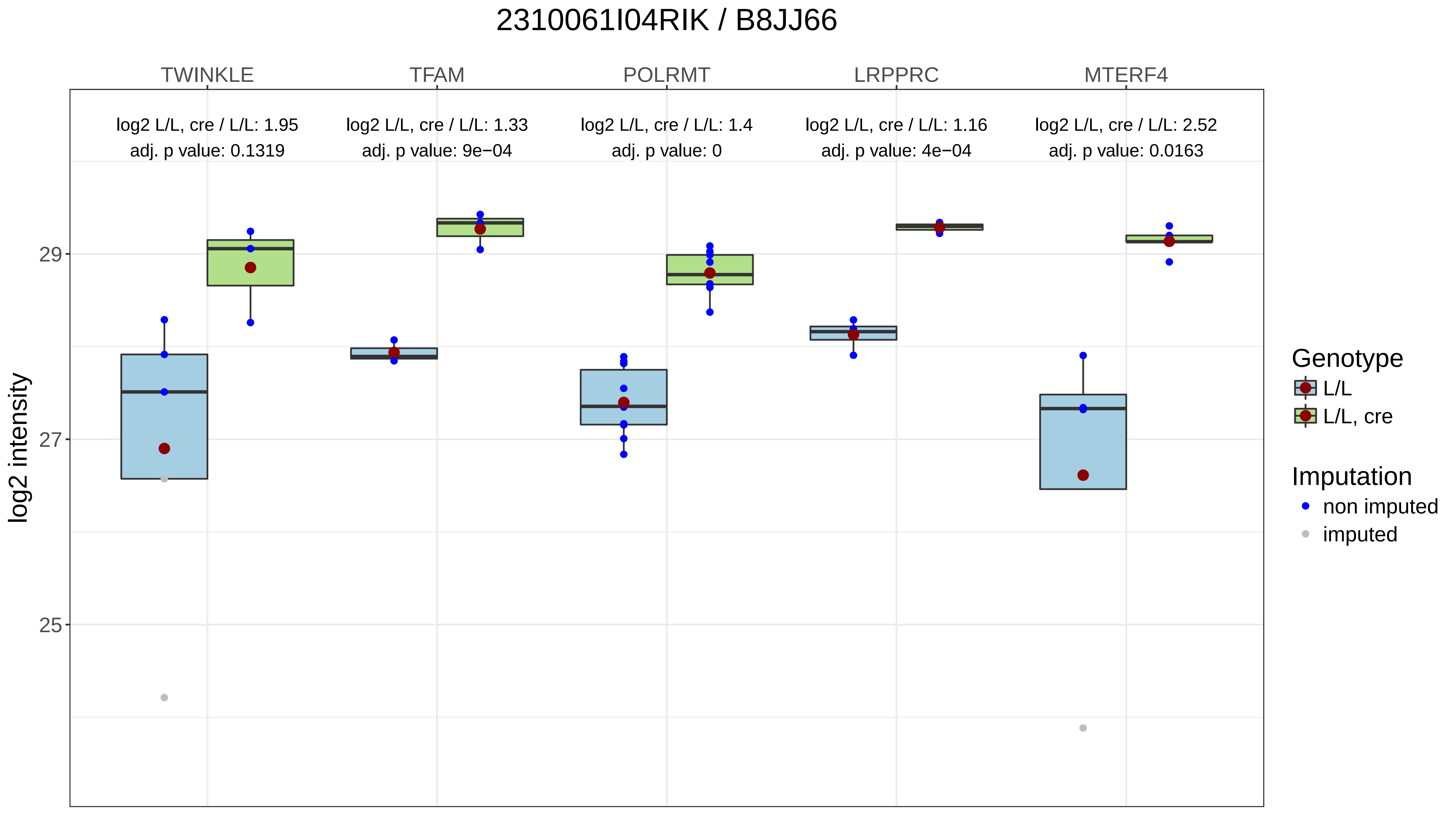
2310061I04RIK / B8JJ66
TWINKLE
TFAM
POLRMT
LRPPRC
MTERF4
log2 L/L, cre / L/L: 1.95
adj. p value: 0.1319
log2 L/L, cre / L/L: 1.33
adj. p value: 9e−04
log2 L/L, cre / L/L: 1.4
adj. p value: 0
log2 L/L, cre / L/L: 1.16
adj. p value: 4e−04
log2 L/L, cre / L/L: 2.52
adj. p value: 0.0163
log2 intensity
29
27
25
Genotype
L/L
L/L, cre
Imputation
non imputed
imputed

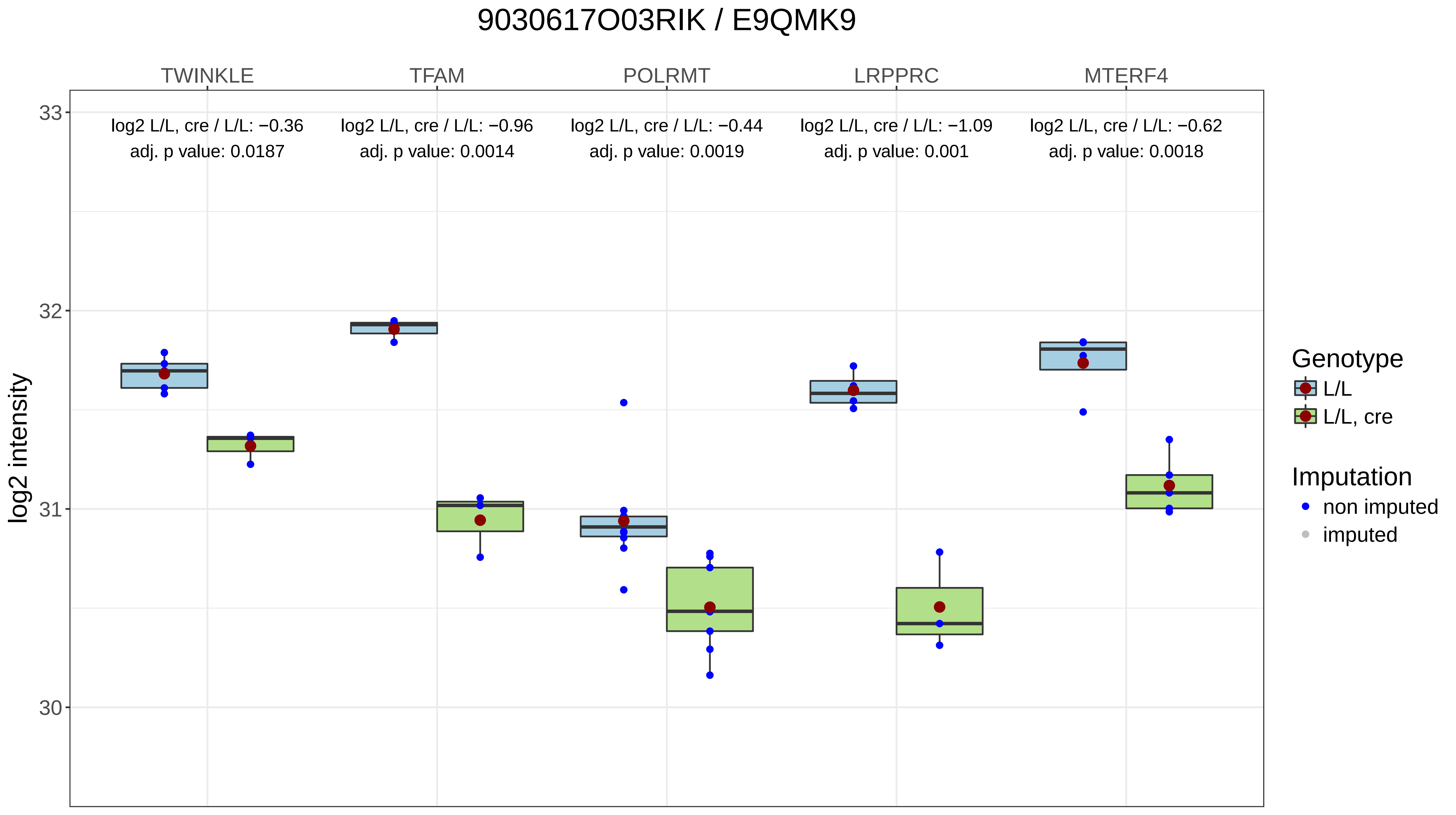
9030617O03RIK / E9QMK9
TWINKLE
TFAM
POLRMT
LRPPRC
MTERF4
log2 L/L, cre / L/L: −0.36
adj. p value: 0.0187
log2 L/L, cre / L/L: −0.96
adj. p value: 0.0014
log2 L/L, cre / L/L: −0.44
adj. p value: 0.0019
log2 L/L, cre / L/L: −1.09
adj. p value: 0.001
log2 L/L, cre / L/L: −0.62
adj. p value: 0.0018
log2 intensity
33
32
31
30
Genotype
L/L
L/L, cre
Imputation
non imputed
imputed

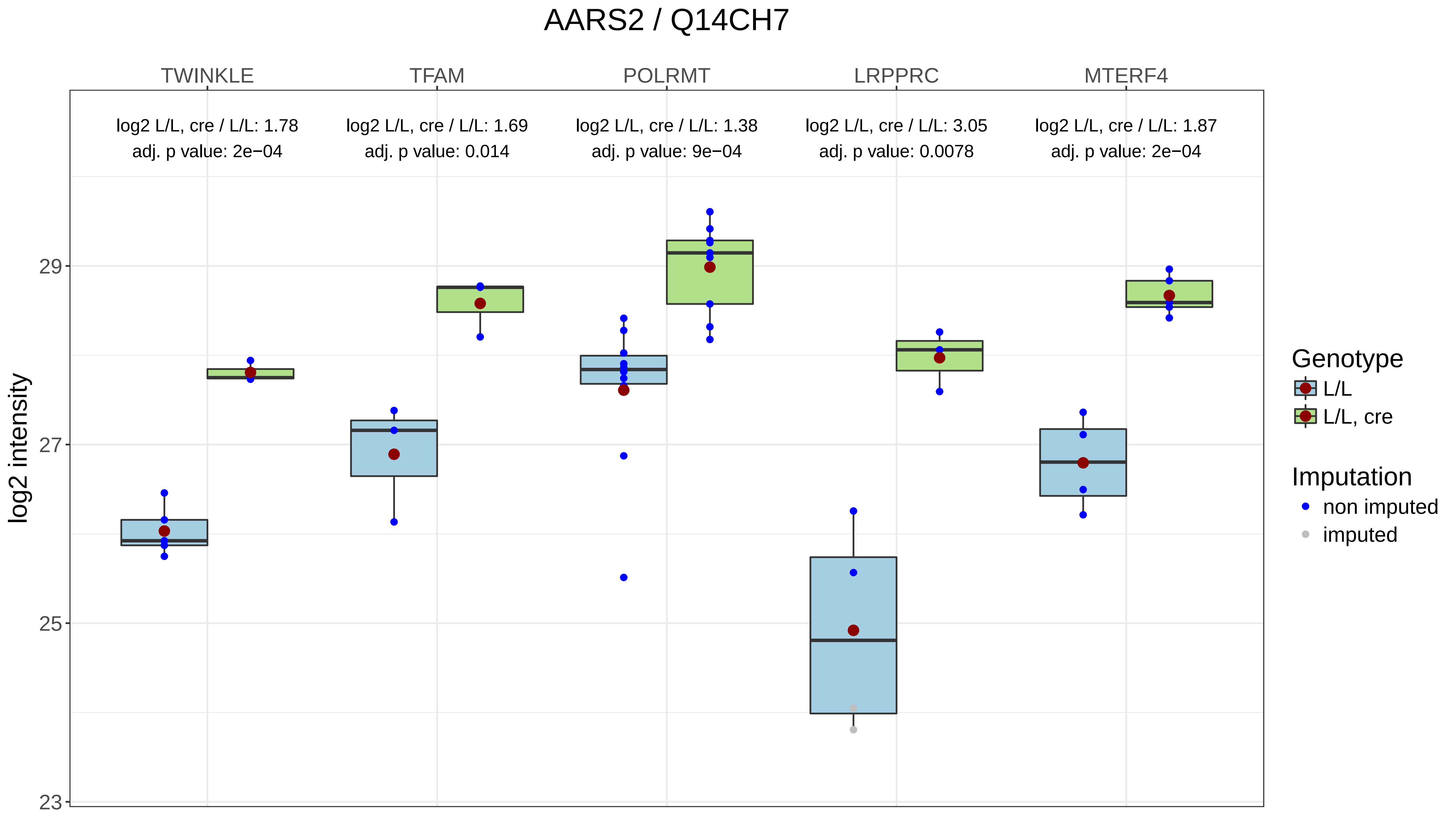
AARS2 / Q14CH7
TWINKLE
TFAM
POLRMT
LRPPRC
MTERF4
log2 L/L, cre / L/L: 1.78
adj. p value: 2e−04
log2 L/L, cre / L/L: 1.69
adj. p value: 0.014
log2 L/L, cre / L/L: 1.38
adj. p value: 9e−04
log2 L/L, cre / L/L: 3.05
adj. p value: 0.0078
log2 L/L, cre / L/L: 1.87
adj. p value: 2e−04
log2 intensity
29
27
25
23
Genotype
L/L
L/L, cre
Imputation
non imputed
imputed

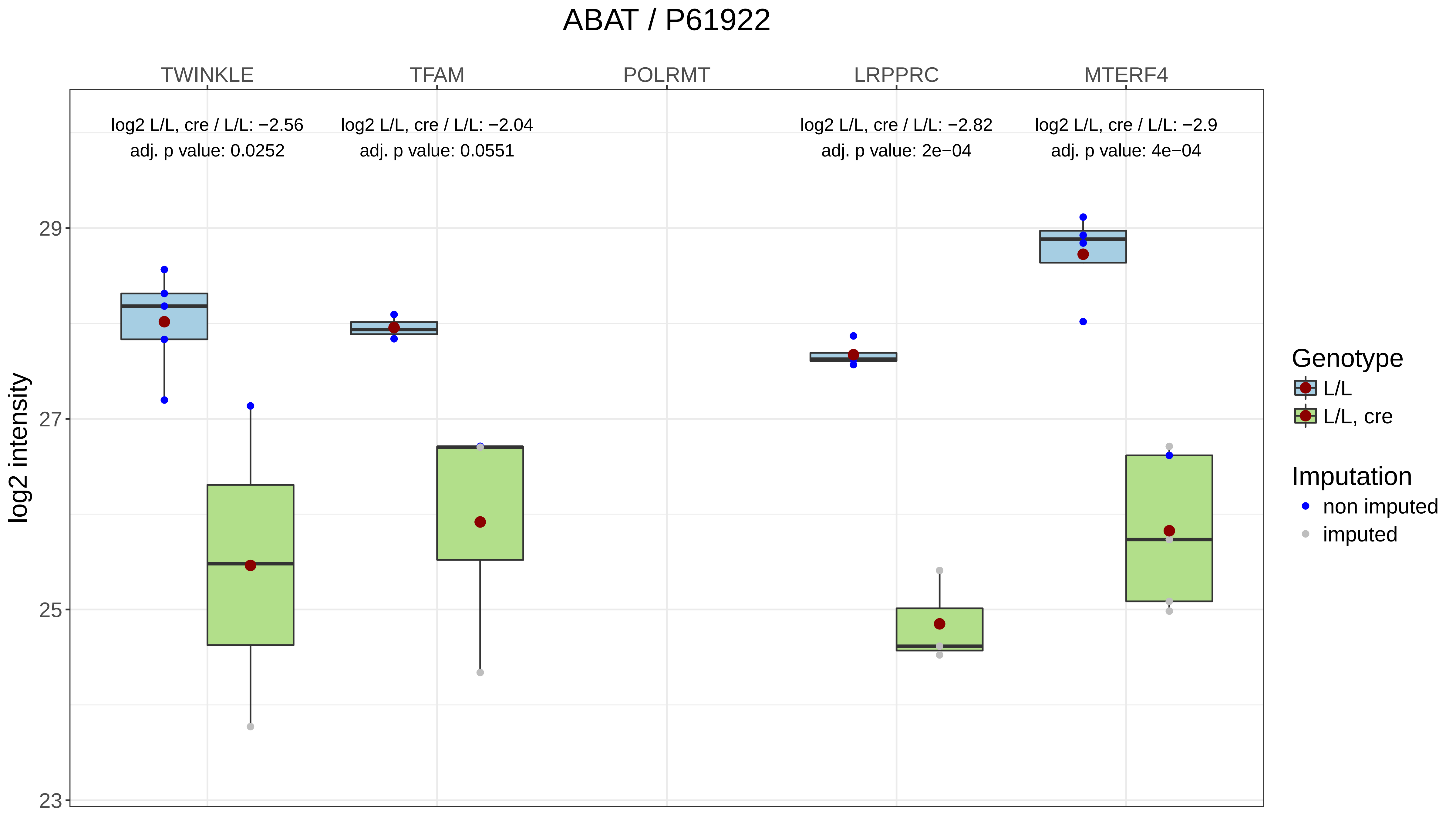
ABAT / P61922
TWINKLE
TFAM
POLRMT
LRPPRC
MTERF4
log2 L/L, cre / L/L: −2.56
adj. p value: 0.0252
log2 L/L, cre / L/L: −2.04
adj. p value: 0.0551
log2 L/L, cre / L/L: −2.82
adj. p value: 2e−04
log2 L/L, cre / L/L: −2.9
adj. p value: 4e−04
log2 intensity
29
27
25
23
Genotype
L/L
L/L, cre
Imputation
non imputed
imputed

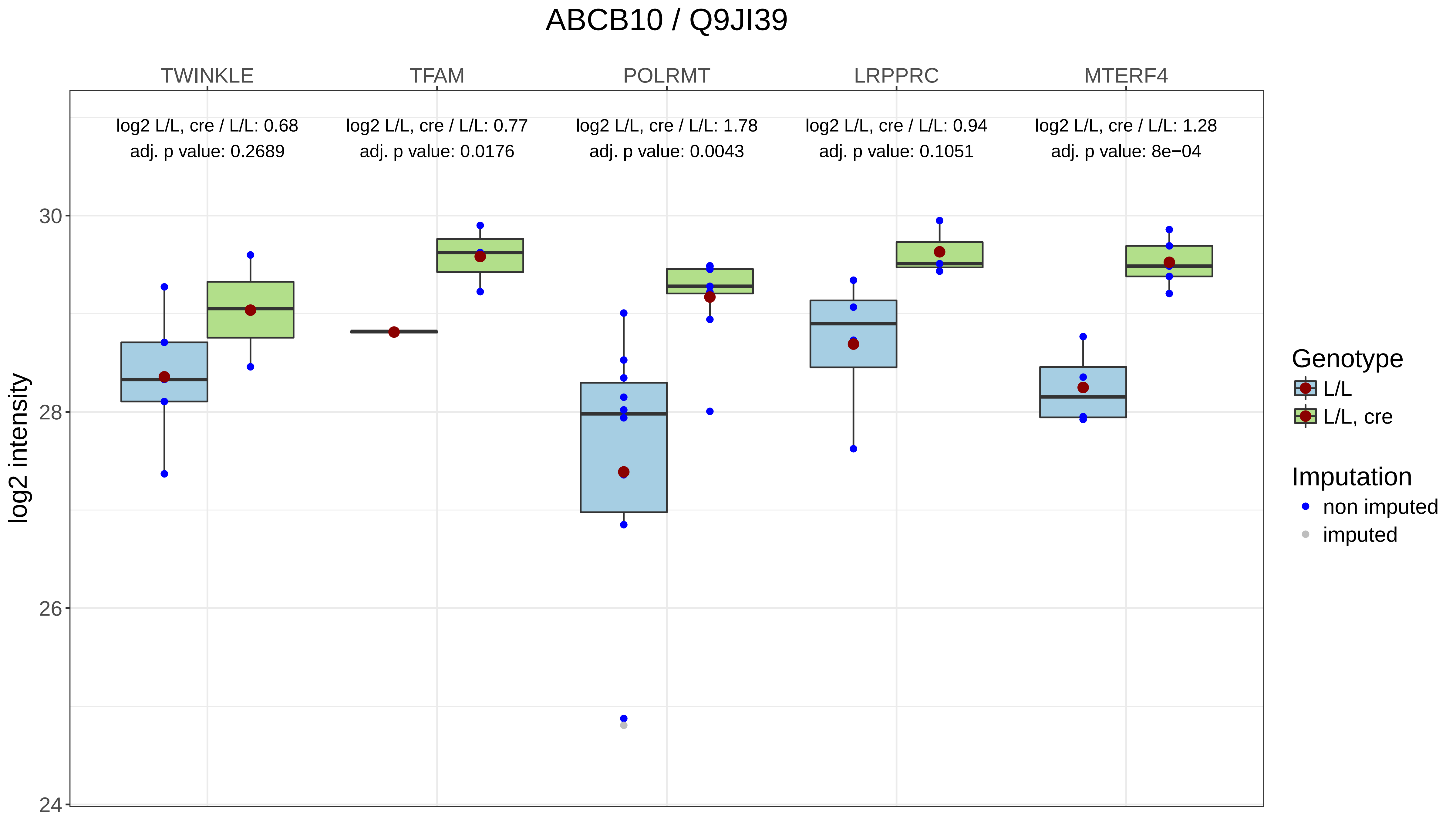
ABCB10 / Q9JI39
TWINKLE
TFAM
POLRMT
LRPPRC
MTERF4
log2 L/L, cre / L/L: 0.68
adj. p value: 0.2689
log2 L/L, cre / L/L: 0.77
adj. p value: 0.0176
log2 L/L, cre / L/L: 1.78
adj. p value: 0.0043
log2 L/L, cre / L/L: 0.94
adj. p value: 0.1051
log2 L/L, cre / L/L: 1.28
adj. p value: 8e−04
log2 intensity
30
28
26
24
Genotype
L/L
L/L, cre
Imputation
non imputed
imputed

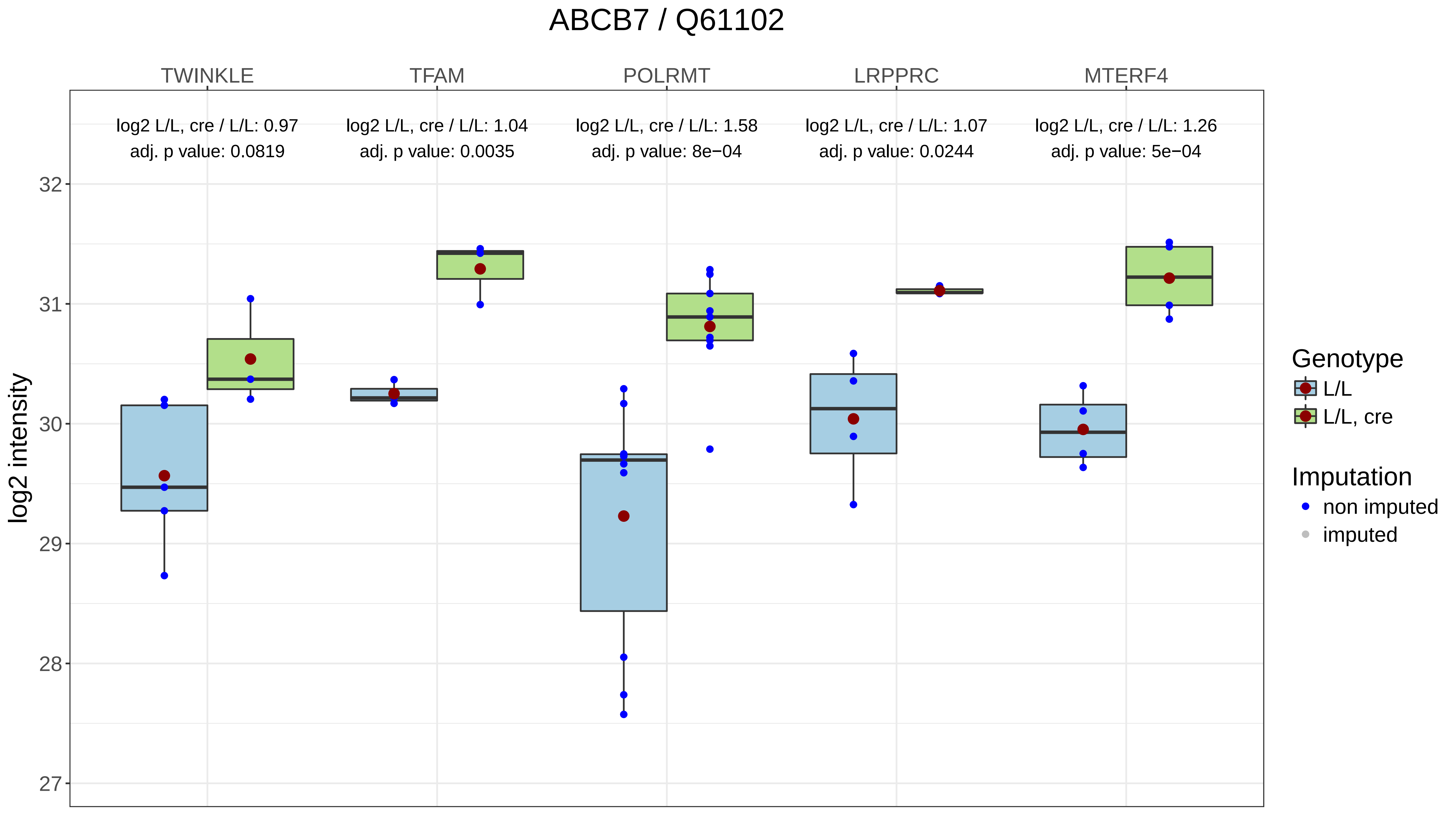
ABCB7 / Q61102
TWINKLE
TFAM
POLRMT
LRPPRC
MTERF4
log2 L/L, cre / L/L: 0.97
adj. p value: 0.0819
log2 L/L, cre / L/L: 1.04
adj. p value: 0.0035
log2 L/L, cre / L/L: 1.58
adj. p value: 8e−04
log2 L/L, cre / L/L: 1.07
adj. p value: 0.0244
log2 L/L, cre / L/L: 1.26
adj. p value: 5e−04
log2 intensity
32
31
30
29
28
27
Genotype
L/L
L/L, cre
Imputation
non imputed
imputed

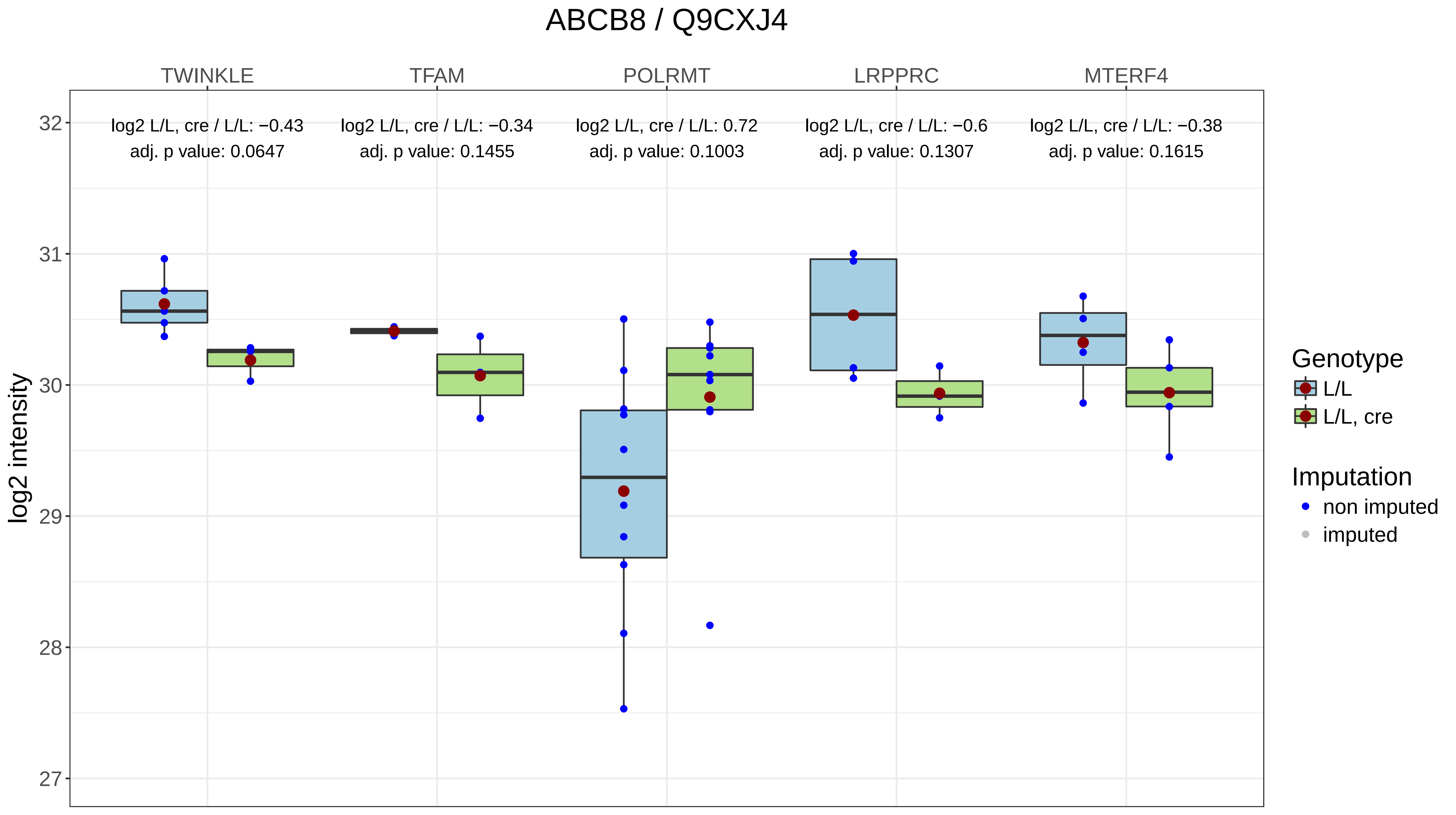
ABCB8 / Q9CXJ4
TWINKLE
TFAM
POLRMT
LRPPRC
MTERF4
log2 L/L, cre / L/L: −0.43
adj. p value: 0.0647
log2 L/L, cre / L/L: −0.34
adj. p value: 0.1455
log2 L/L, cre / L/L: 0.72
adj. p value: 0.1003
log2 L/L, cre / L/L: −0.6
adj. p value: 0.1307
log2 L/L, cre / L/L: −0.38
adj. p value: 0.1615
log2 intensity
32
31
30
29
28
27
Genotype
L/L
L/L, cre
Imputation
non imputed
imputed

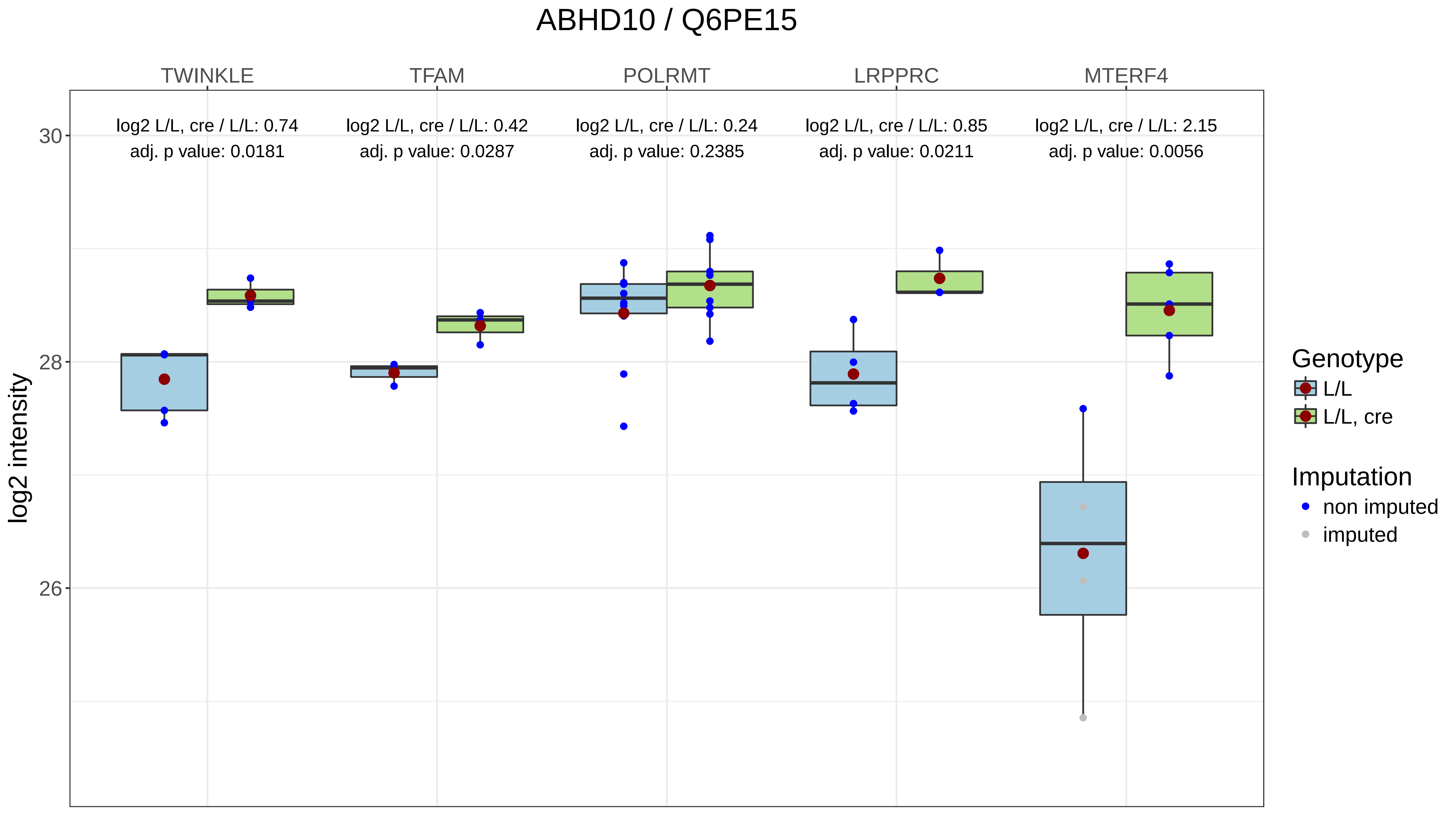
ABHD10 / Q6PE15
TWINKLE
TFAM
POLRMT
LRPPRC
MTERF4
log2 L/L, cre / L/L: 0.74
adj. p value: 0.0181
log2 L/L, cre / L/L: 0.42
adj. p value: 0.0287
log2 L/L, cre / L/L: 0.24
adj. p value: 0.2385
log2 L/L, cre / L/L: 0.85
adj. p value: 0.0211
log2 L/L, cre / L/L: 2.15
adj. p value: 0.0056
log2 intensity
30
28
26
Genotype
L/L
L/L, cre
Imputation
non imputed
imputed

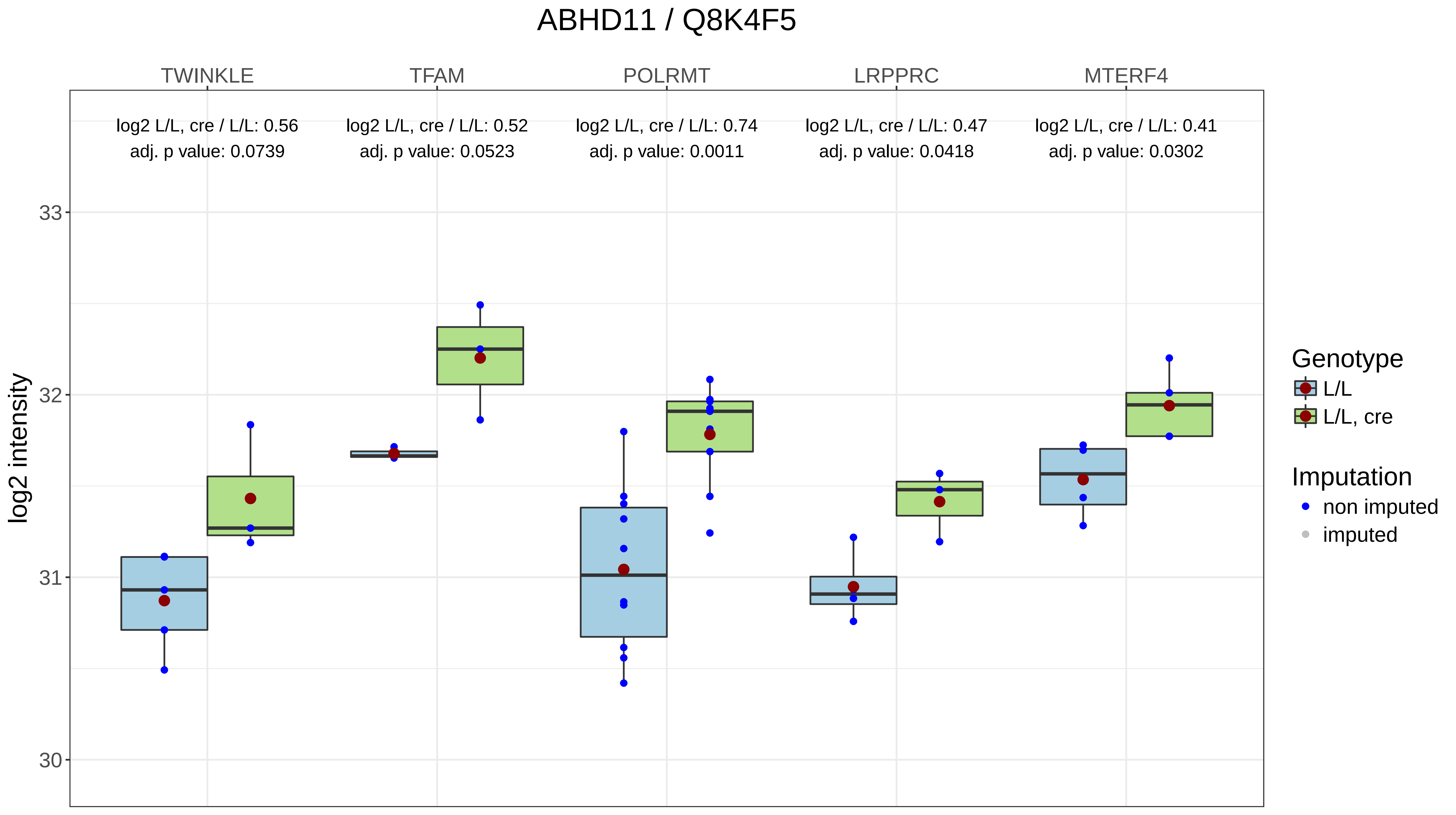

ABHD11 / Q8K4F5
TWINKLE
TFAM
POLRMT
LRPPRC
MTERF4
log2 L/L, cre / L/L: 0.56
adj. p value: 0.0739
log2 L/L, cre / L/L: 0.52
adj. p value: 0.0523
log2 L/L, cre / L/L: 0.74
adj. p value: 0.0011
log2 L/L, cre / L/L: 0.47
adj. p value: 0.0418
log2 L/L, cre / L/L: 0.41
adj. p value: 0.0302
log2 intensity
33
32
31
30
Genotype
L/L
L/L, cre
Imputation
non imputed
imputed

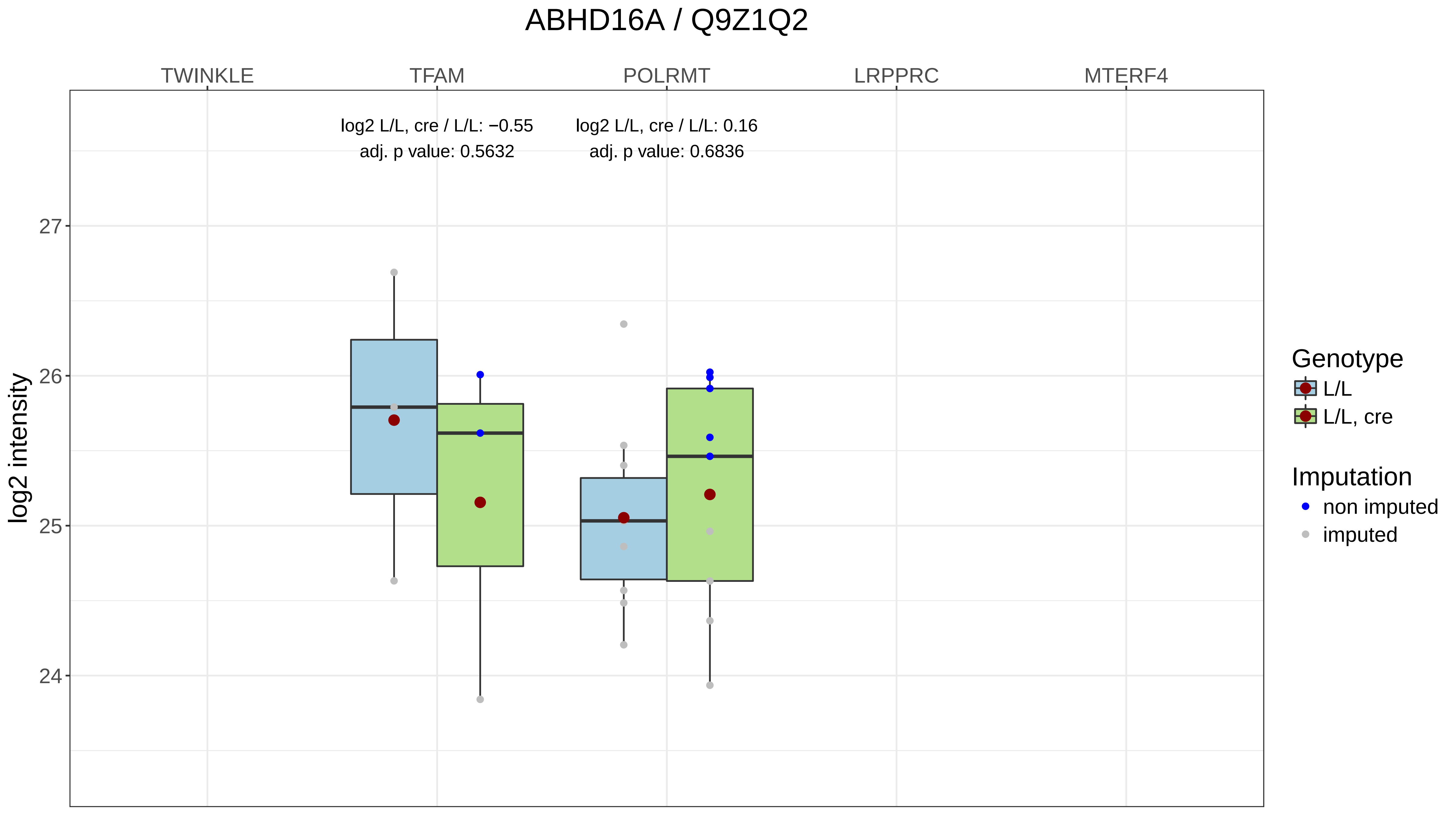
ABHD16A / Q9Z1Q2
TWINKLE
TFAM
POLRMT
LRPPRC
MTERF4
log2 L/L, cre / L/L: −0.55
adj. p value: 0.5632
log2 L/L, cre / L/L: 0.16
adj. p value: 0.6836
log2 intensity
27
26
25
24
Genotype
L/L
L/L, cre
Imputation
non imputed
imputed

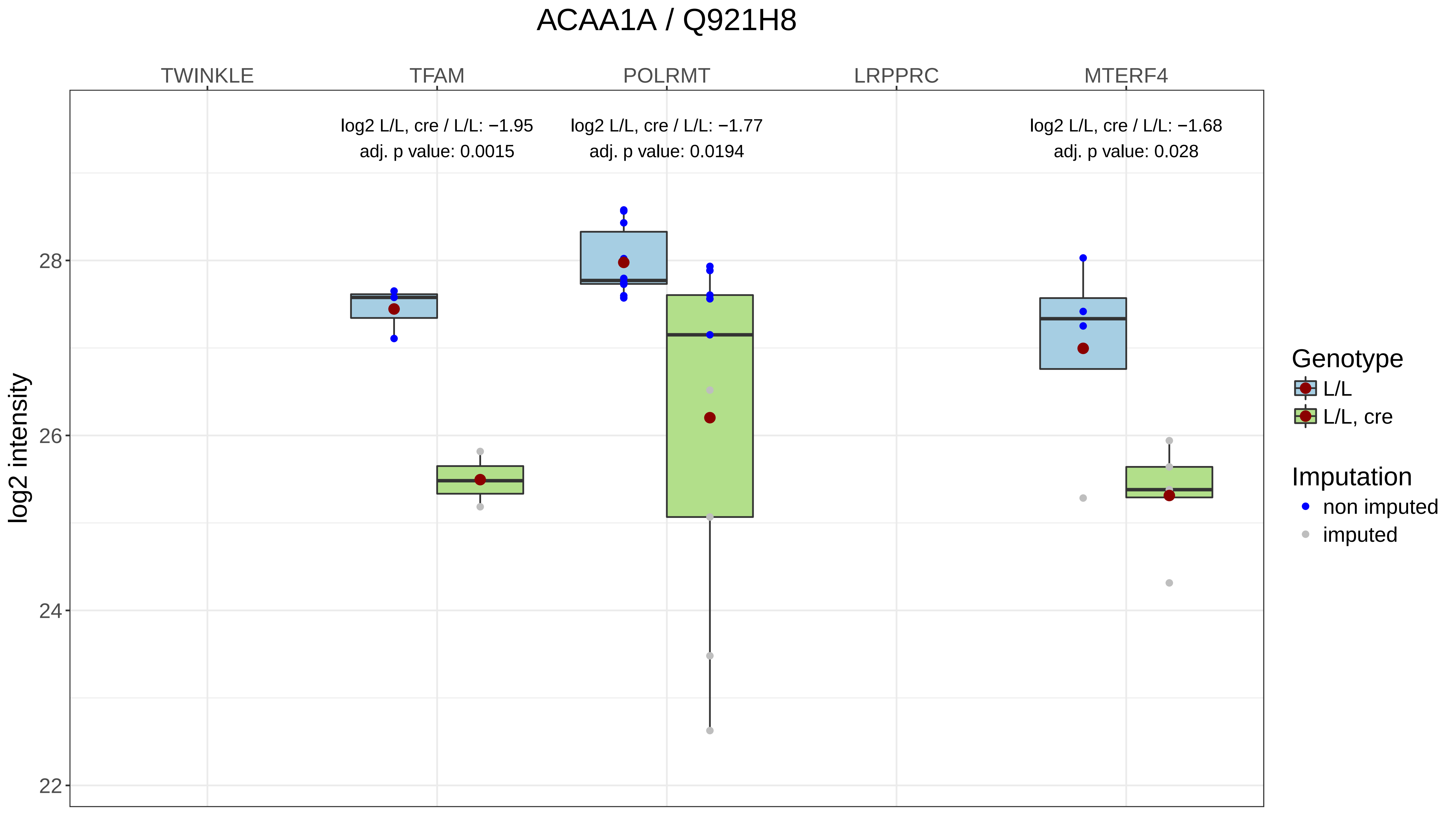

ACAA1A / Q921H8
TWINKLE
TFAM
POLRMT
LRPPRC
MTERF4
log2 L/L, cre / L/L: −1.95
adj. p value: 0.0015
log2 L/L, cre / L/L: −1.77
adj. p value: 0.0194
log2 L/L, cre / L/L: −1.68
adj. p value: 0.028
log2 intensity
28
26
24
22
Genotype
L/L
L/L, cre
Imputation
non imputed
imputed

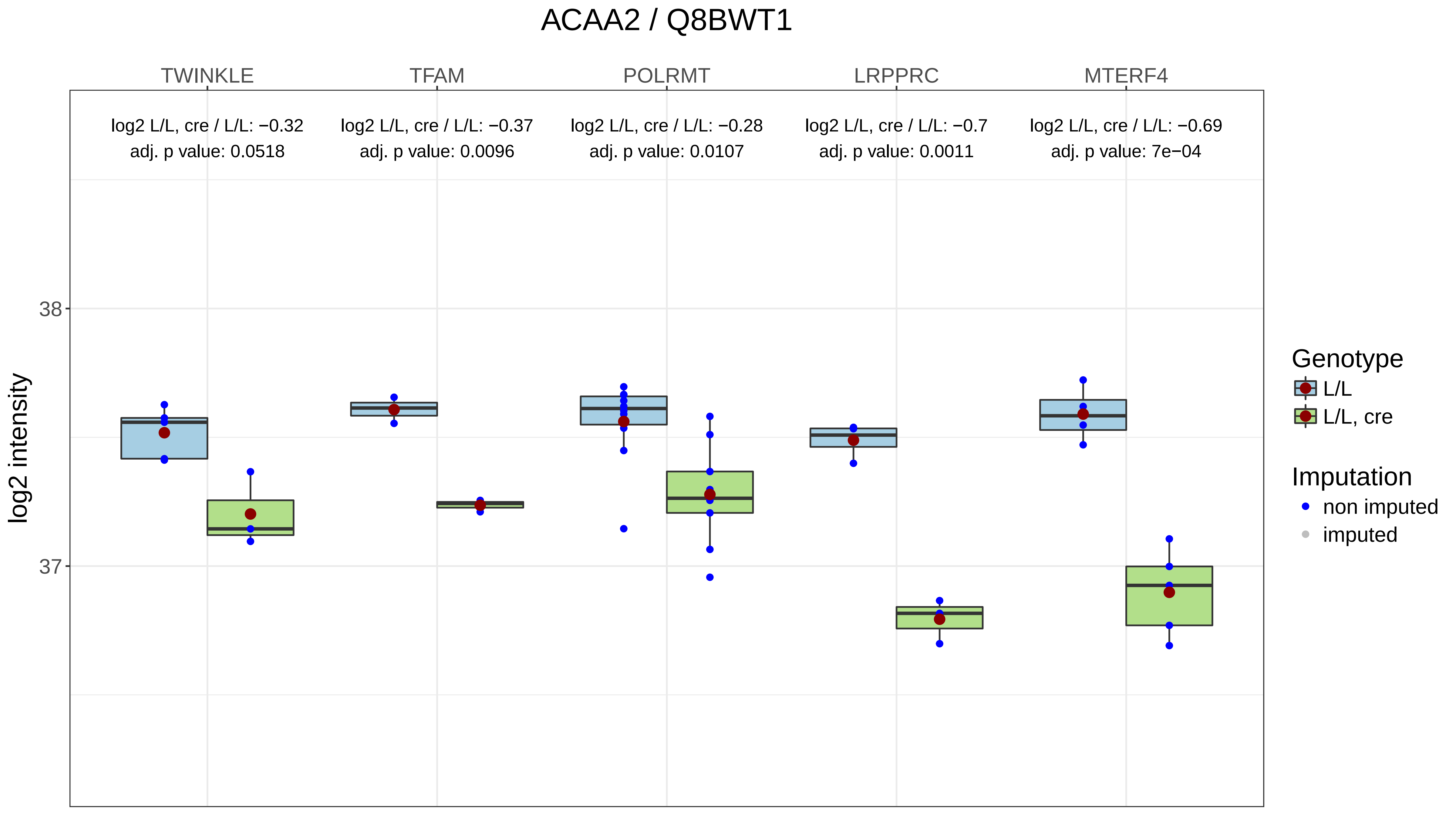
ACAA2 / Q8BWT1
TWINKLE
TFAM
POLRMT
LRPPRC
MTERF4
log2 L/L, cre / L/L: −0.32
adj. p value: 0.0518
log2 L/L, cre / L/L: −0.37
adj. p value: 0.0096
log2 L/L, cre / L/L: −0.28
adj. p value: 0.0107
log2 L/L, cre / L/L: −0.7
adj. p value: 0.0011
log2 L/L, cre / L/L: −0.69
adj. p value: 7e−04
log2 intensity
38
37
Genotype
L/L
L/L, cre
Imputation
non imputed
imputed

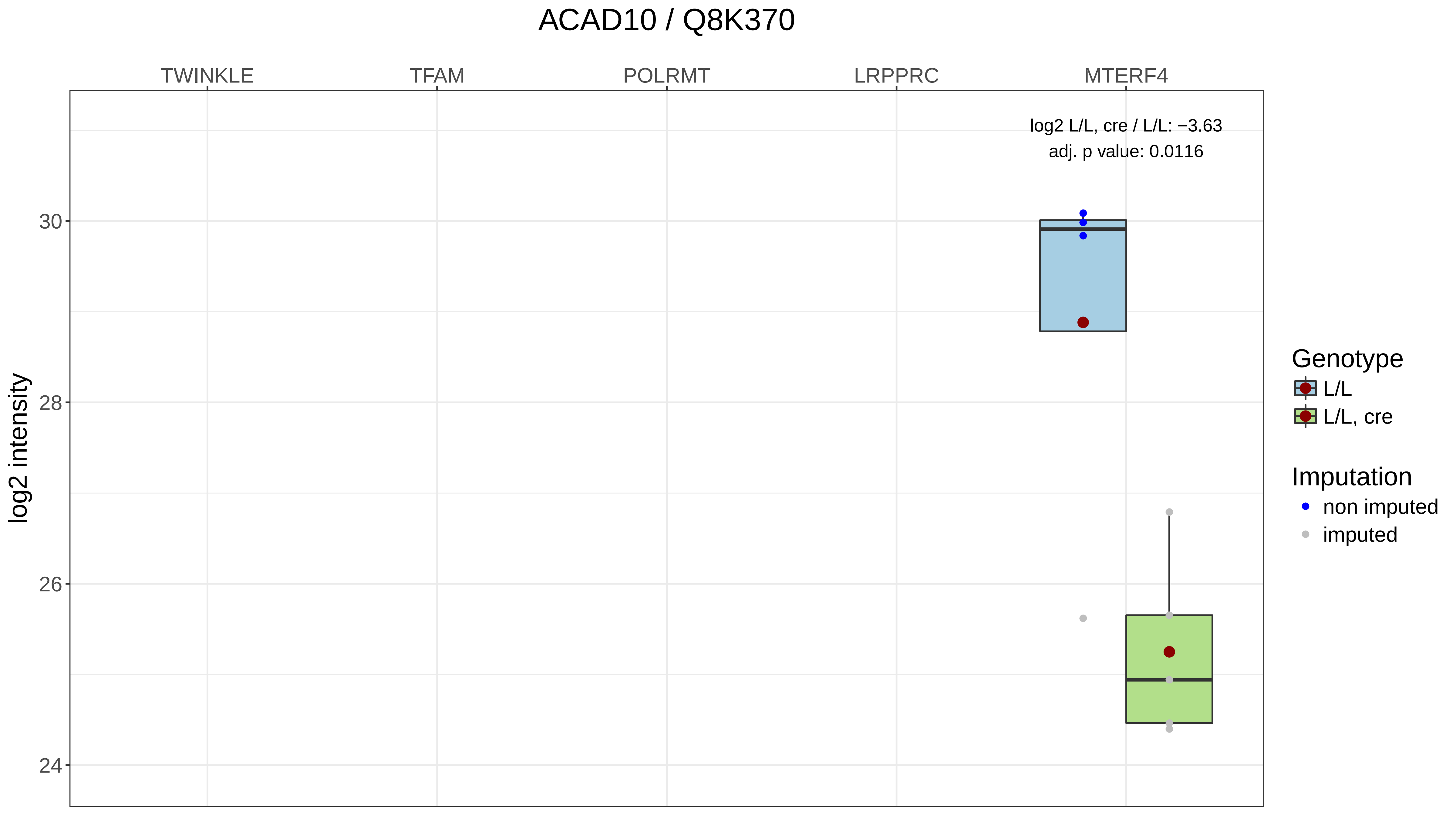

ACAD10 / Q8K370
TWINKLE
TFAM
POLRMT
LRPPRC
MTERF4
log2 L/L, cre / L/L: −3.63
adj. p value: 0.0116
log2 intensity
30
28
26
24
Genotype
L/L
L/L, cre
Imputation
non imputed
imputed

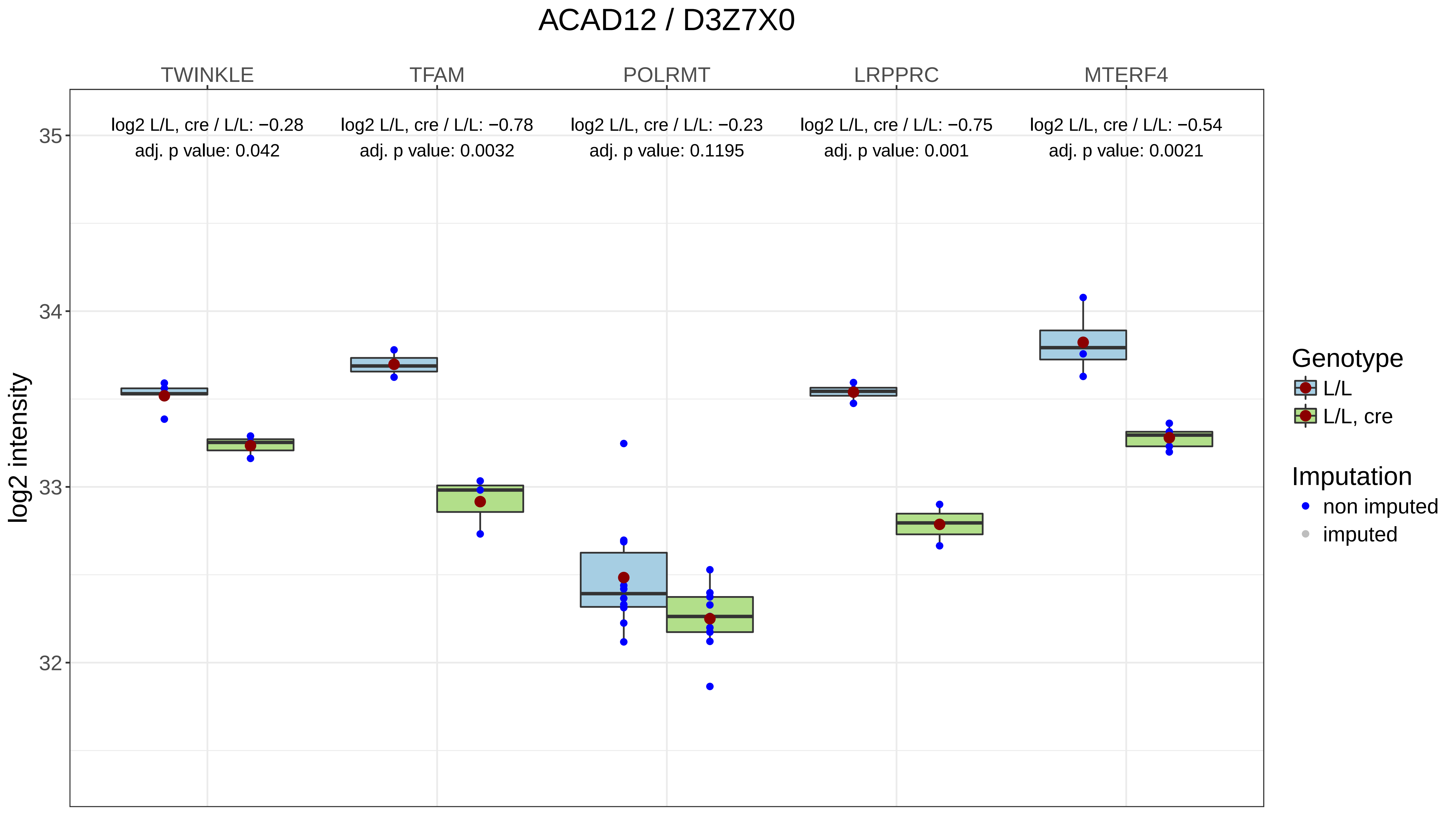
ACAD12 / D3Z7X0
TWINKLE
TFAM
POLRMT
LRPPRC
MTERF4
log2 L/L, cre / L/L: −0.28
adj. p value: 0.042
log2 L/L, cre / L/L: −0.78
adj. p value: 0.0032
log2 L/L, cre / L/L: −0.23
adj. p value: 0.1195
log2 L/L, cre / L/L: −0.75
adj. p value: 0.001
log2 L/L, cre / L/L: −0.54
adj. p value: 0.0021
log2 intensity
35
34
33
32
Genotype
L/L
L/L, cre
Imputation
non imputed
imputed

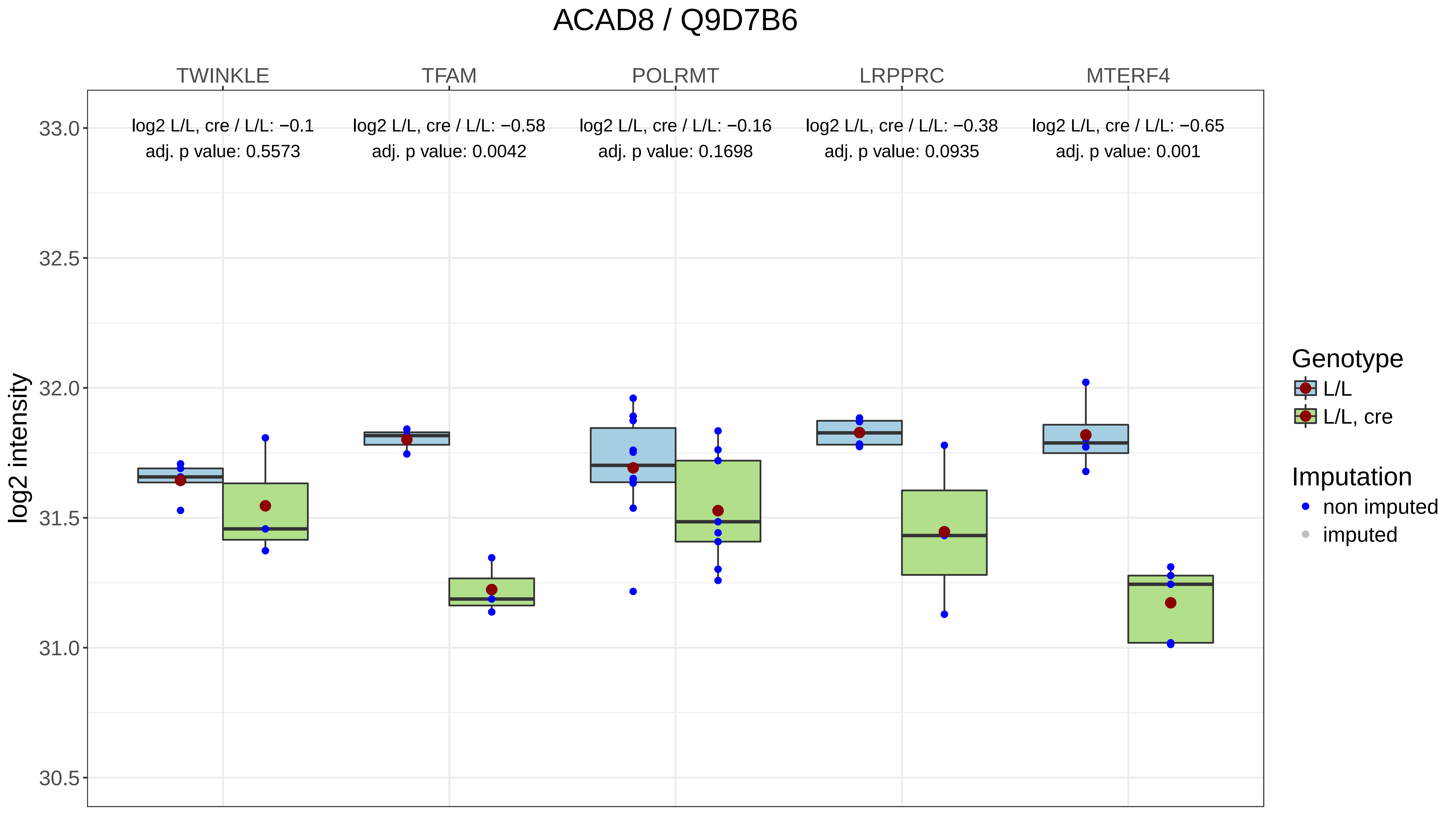
ACAD8 / Q9D7B6
TWINKLE
TFAM
POLRMT
LRPPRC
MTERF4
log2 L/L, cre / L/L: −0.1
adj. p value: 0.5573
log2 L/L, cre / L/L: −0.58
adj. p value: 0.0042
log2 L/L, cre / L/L: −0.16
adj. p value: 0.1698
log2 L/L, cre / L/L: −0.38
adj. p value: 0.0935
log2 L/L, cre / L/L: −0.65
adj. p value: 0.001
log2 intensity
33.0
32.5
32.0
31.5
31.0
30.5
Genotype
L/L
L/L, cre
Imputation
non imputed
imputed

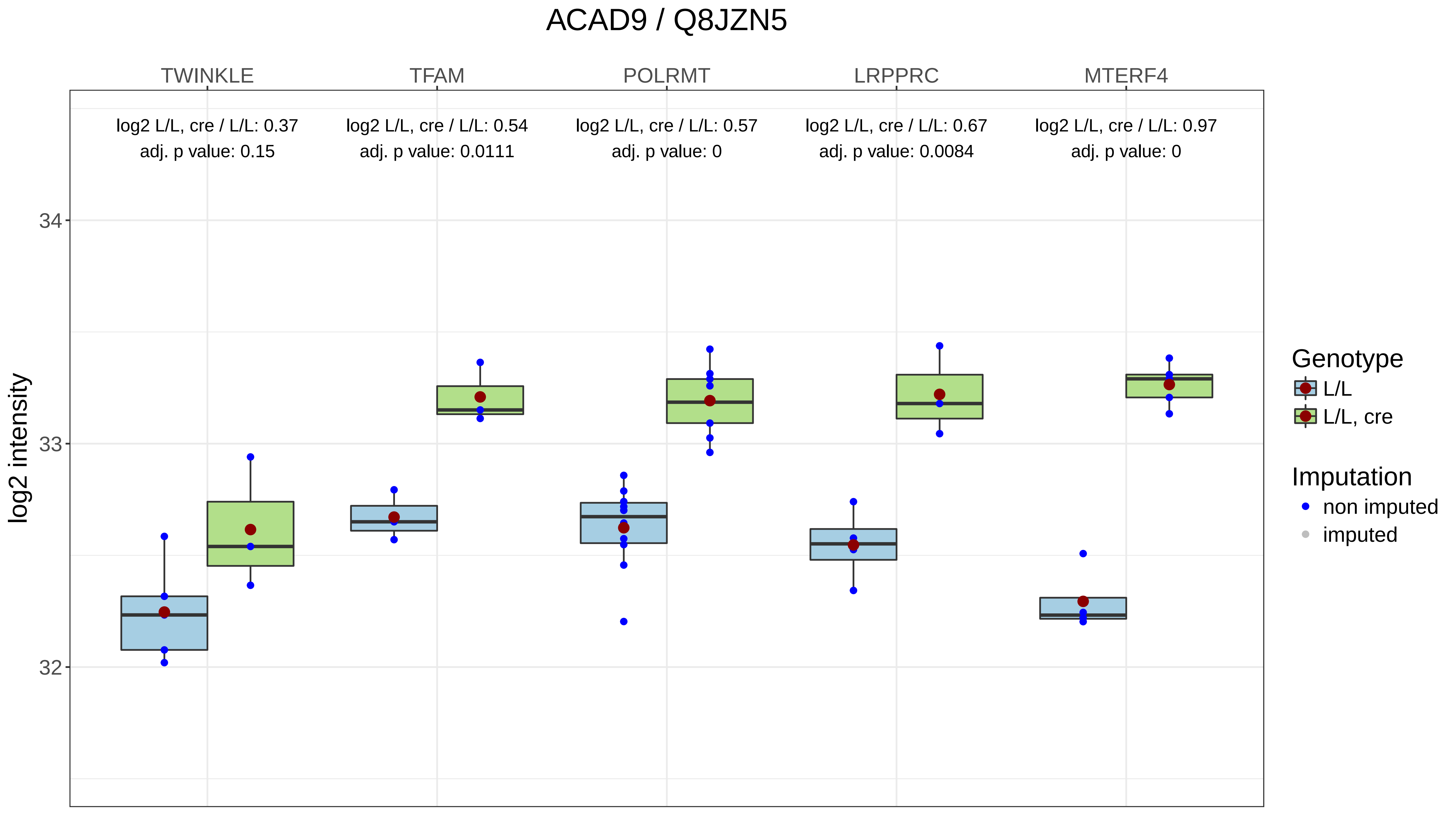

ACAD9 / Q8JZN5
TWINKLE
TFAM
POLRMT
LRPPRC
MTERF4
log2 L/L, cre / L/L: 0.37
adj. p value: 0.15
log2 L/L, cre / L/L: 0.54
adj. p value: 0.0111
log2 L/L, cre / L/L: 0.57
adj. p value: 0
log2 L/L, cre / L/L: 0.67
adj. p value: 0.0084
log2 L/L, cre / L/L: 0.97
adj. p value: 0
34
33
32
log2 intensity
Genotype
L/L
L/L, cre
Imputation
non imputed
imputed

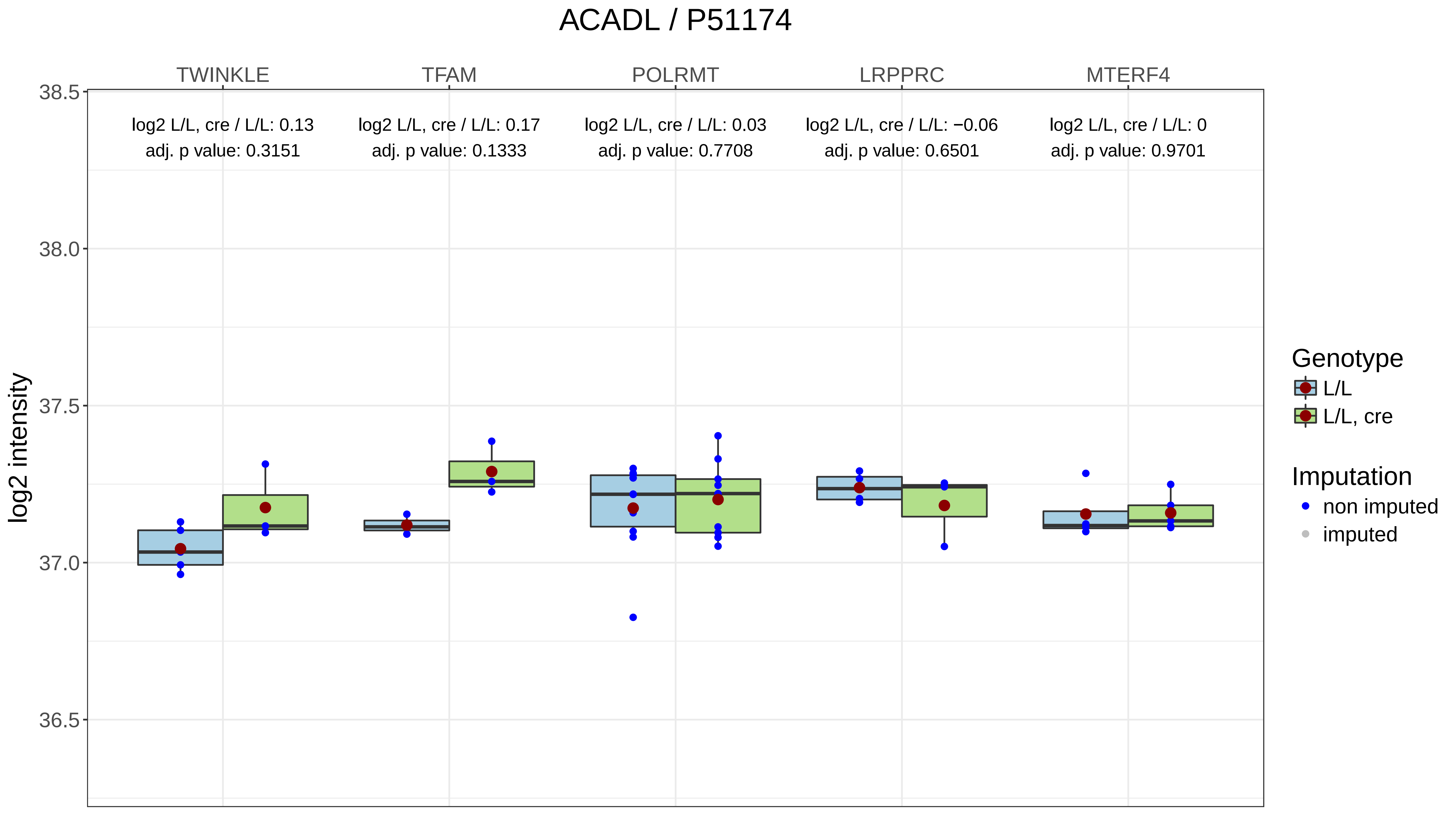
ACADL / P51174
TWINKLE
TFAM
POLRMT
LRPPRC
MTERF4
log2 L/L, cre / L/L: 0.13
adj. p value: 0.3151
log2 L/L, cre / L/L: 0.17
adj. p value: 0.1333
log2 L/L, cre / L/L: 0.03
adj. p value: 0.7708
log2 L/L, cre / L/L: −0.06
adj. p value: 0.6501
log2 L/L, cre / L/L: 0
adj. p value: 0.9701
log2 intensity
38.5
38.0
37.5
37.0
36.5
Genotype
L/L
L/L, cre
Imputation
non imputed
imputed

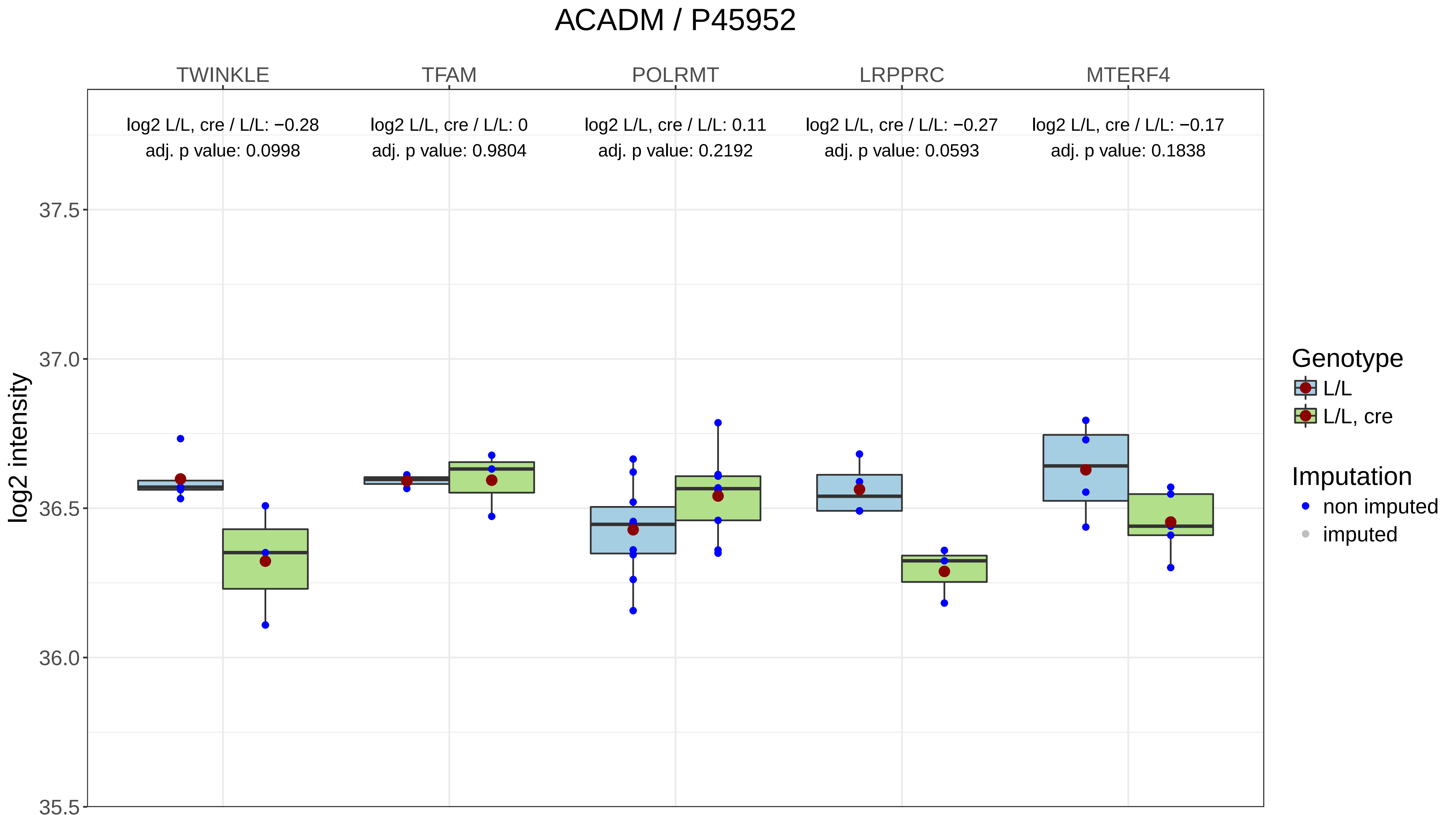
ACADM / P45952
TWINKLE
TFAM
POLRMT
LRPPRC
MTERF4
log2 L/L, cre / L/L: −0.28
adj. p value: 0.0998
log2 L/L, cre / L/L: 0
adj. p value: 0.9804
log2 L/L, cre / L/L: 0.11
adj. p value: 0.2192
log2 L/L, cre / L/L: −0.27
adj. p value: 0.0593
log2 L/L, cre / L/L: −0.17
adj. p value: 0.1838
log2 intensity
37.5
37.0
36.5
36.0
35.5
Genotype
L/L
L/L, cre
Imputation
non imputed
imputed

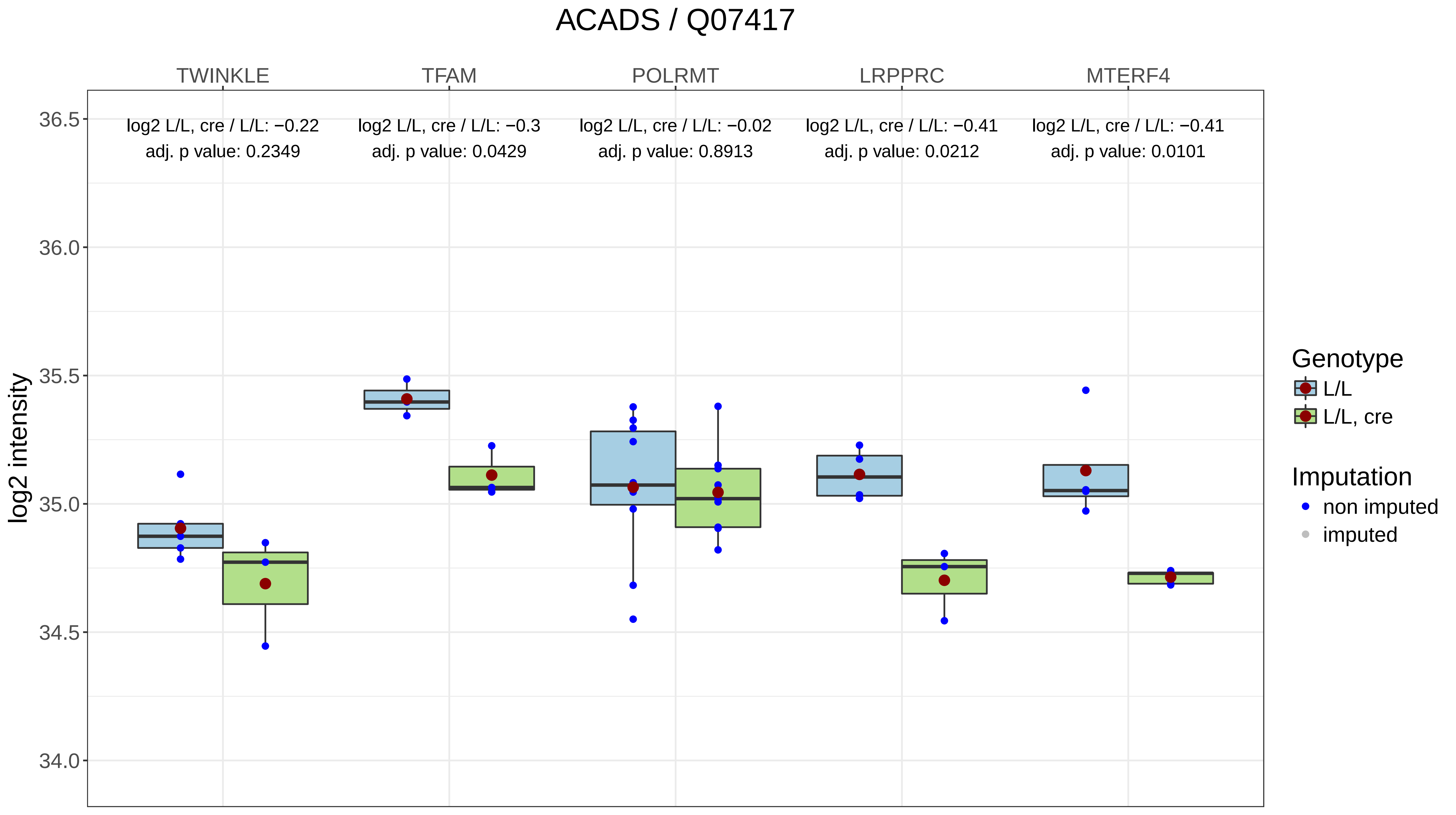
ACADS / Q07417
TWINKLE
TFAM
POLRMT
LRPPRC
MTERF4
log2 L/L, cre / L/L: −0.22
adj. p value: 0.2349
log2 L/L, cre / L/L: −0.3
adj. p value: 0.0429
log2 L/L, cre / L/L: −0.02
adj. p value: 0.8913
log2 L/L, cre / L/L: −0.41
adj. p value: 0.0212
log2 L/L, cre / L/L: −0.41
adj. p value: 0.0101
log2 intensity
36.5
36.0
35.5
35.0
34.5
34.0
Genotype
L/L
L/L, cre
Imputation
non imputed
imputed

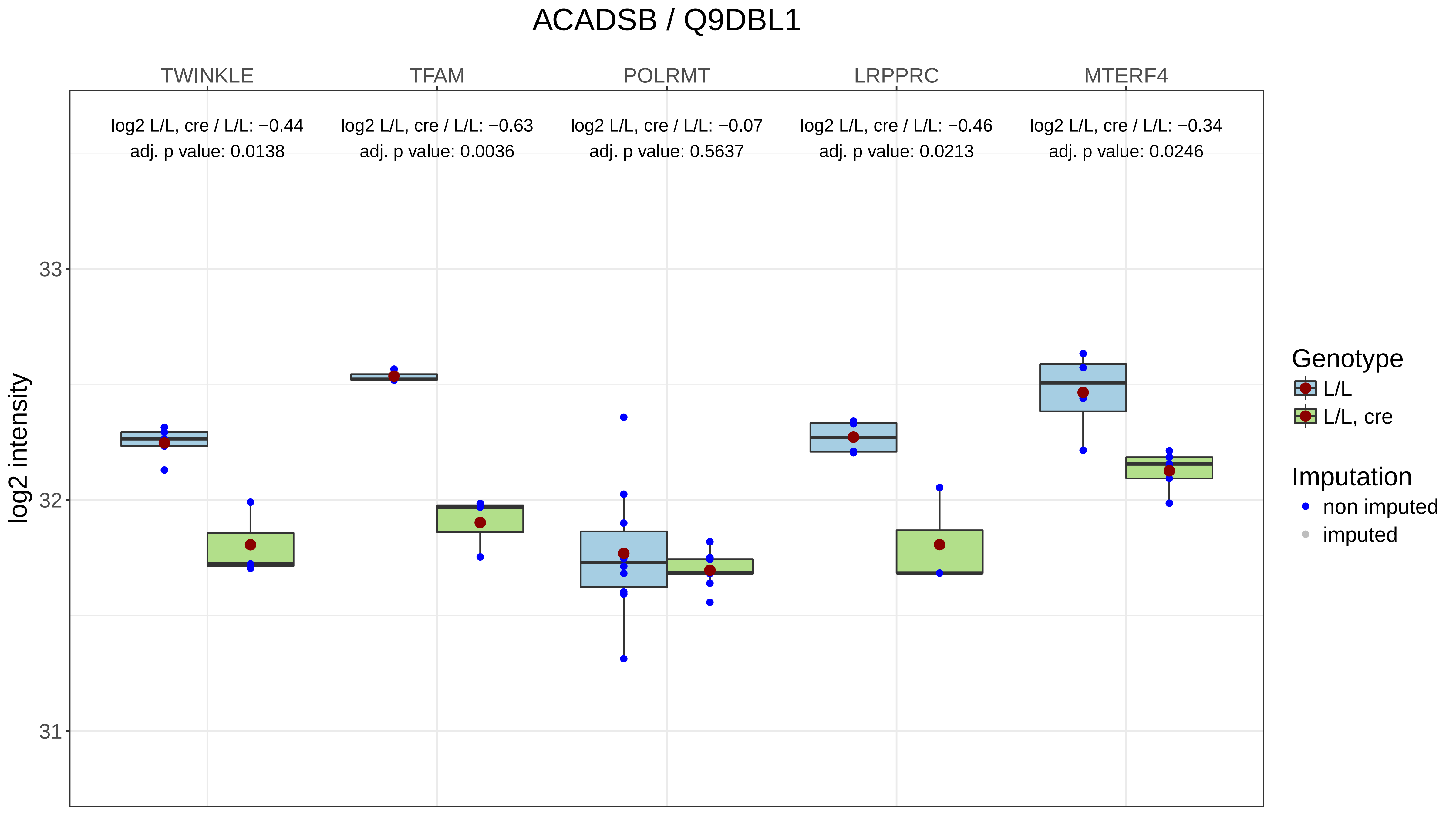
ACADSB / Q9DBL1
TWINKLE
TFAM
POLRMT
LRPPRC
MTERF4
log2 L/L, cre / L/L: −0.44
adj. p value: 0.0138
log2 L/L, cre / L/L: −0.63
adj. p value: 0.0036
log2 L/L, cre / L/L: −0.07
adj. p value: 0.5637
log2 L/L, cre / L/L: −0.46
adj. p value: 0.0213
log2 L/L, cre / L/L: −0.34
adj. p value: 0.0246
log2 intensity
33
32
31
Genotype
L/L
L/L, cre
Imputation
non imputed
imputed

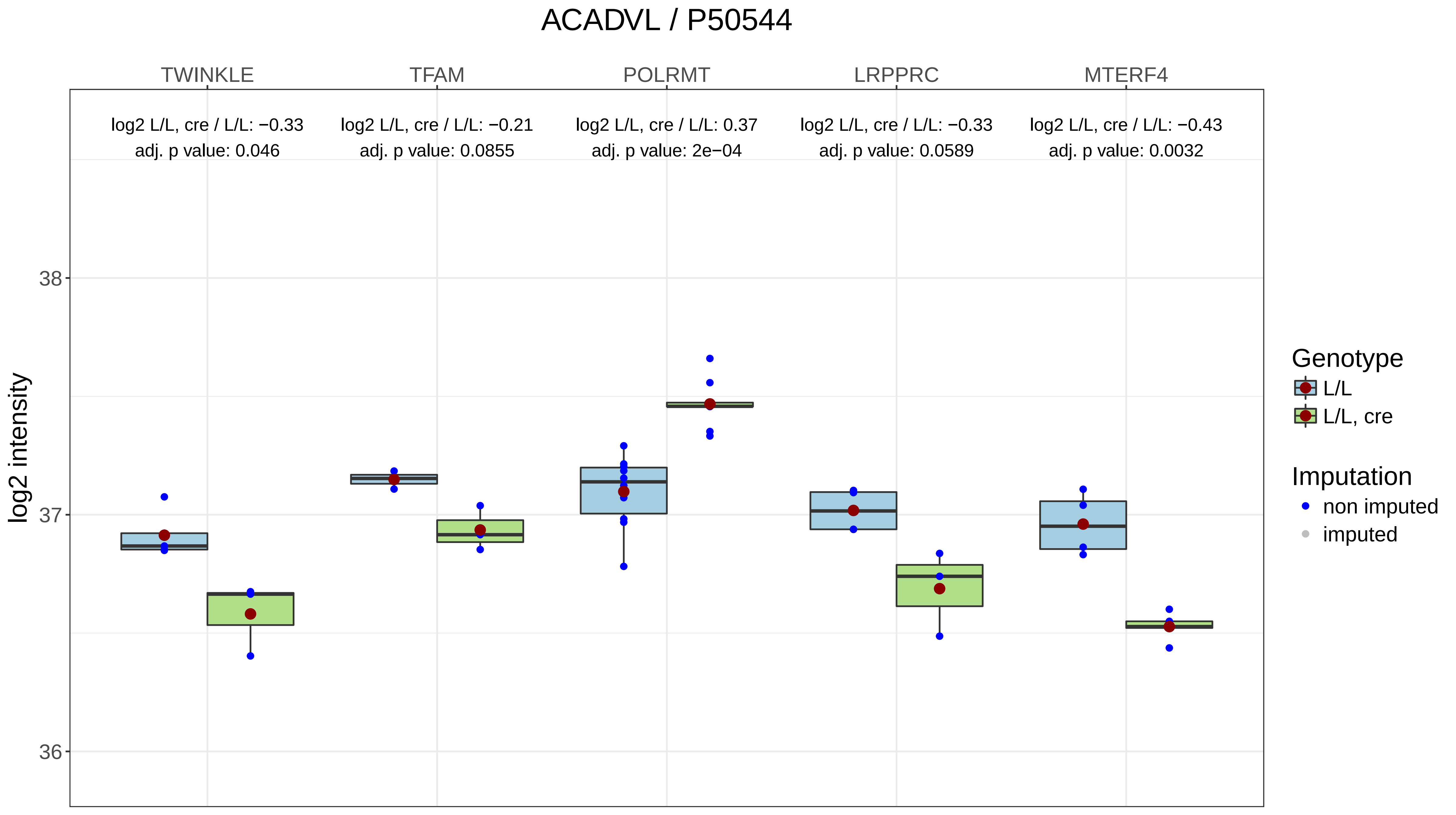
ACADVL / P50544
TWINKLE
TFAM
POLRMT
LRPPRC
MTERF4
log2 L/L, cre / L/L: −0.33
adj. p value: 0.046
log2 L/L, cre / L/L: −0.21
adj. p value: 0.0855
log2 L/L, cre / L/L: 0.37
adj. p value: 2e−04
log2 L/L, cre / L/L: −0.33
adj. p value: 0.0589
log2 L/L, cre / L/L: −0.43
adj. p value: 0.0032
log2 intensity
38
37
36
Genotype
L/L
L/L, cre
Imputation
non imputed
imputed

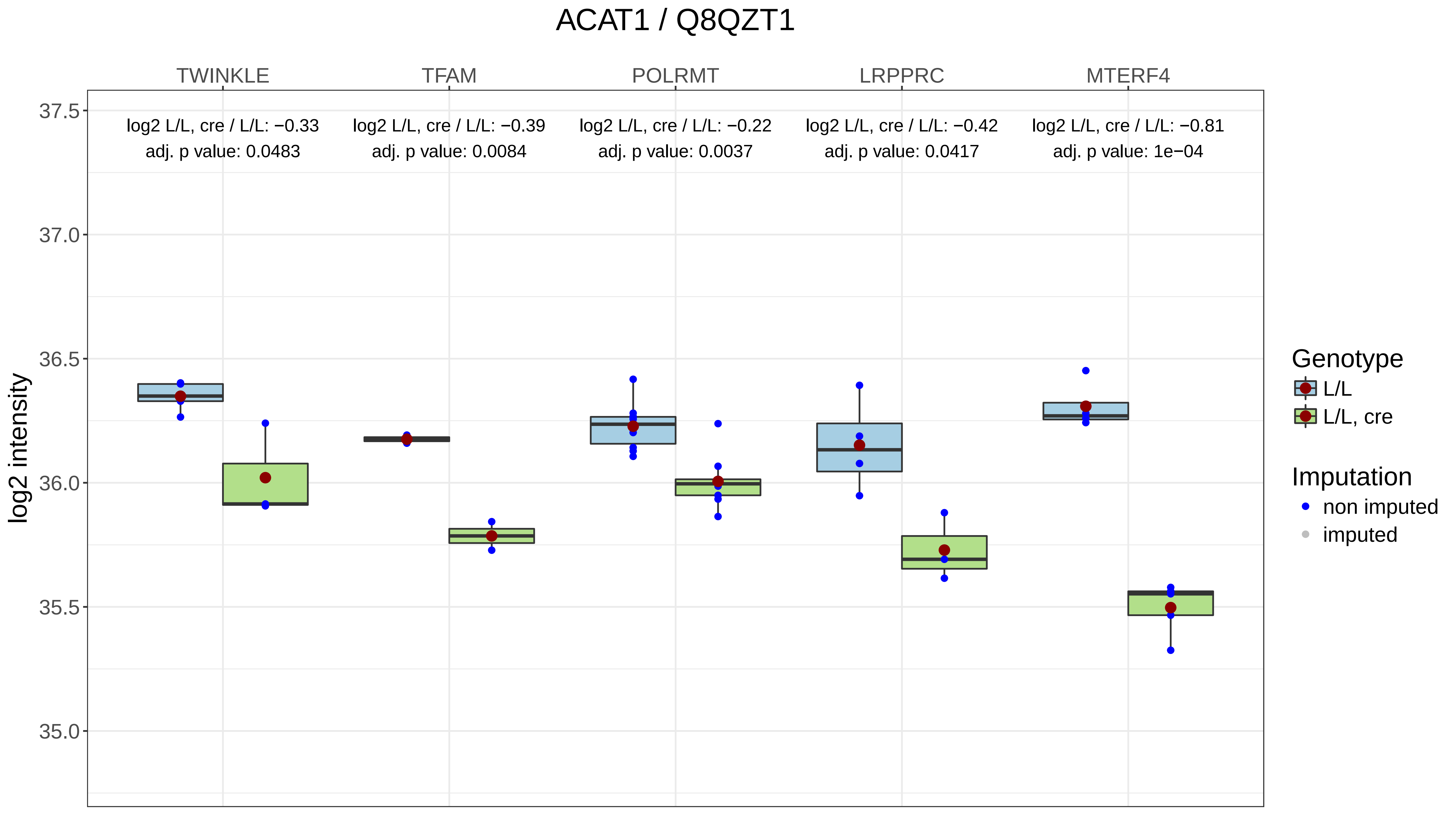
ACAT1 / Q8QZT1
TWINKLE
TFAM
POLRMT
LRPPRC
MTERF4
log2 L/L, cre / L/L: −0.33
adj. p value: 0.0483
log2 L/L, cre / L/L: −0.39
adj. p value: 0.0084
log2 L/L, cre / L/L: −0.22
adj. p value: 0.0037
log2 L/L, cre / L/L: −0.42
adj. p value: 0.0417
log2 L/L, cre / L/L: −0.81
adj. p value: 1e−04
log2 intensity
37.5
37.0
36.5
36.0
35.5
35.0
Genotype
L/L
L/L, cre
Imputation
non imputed
imputed

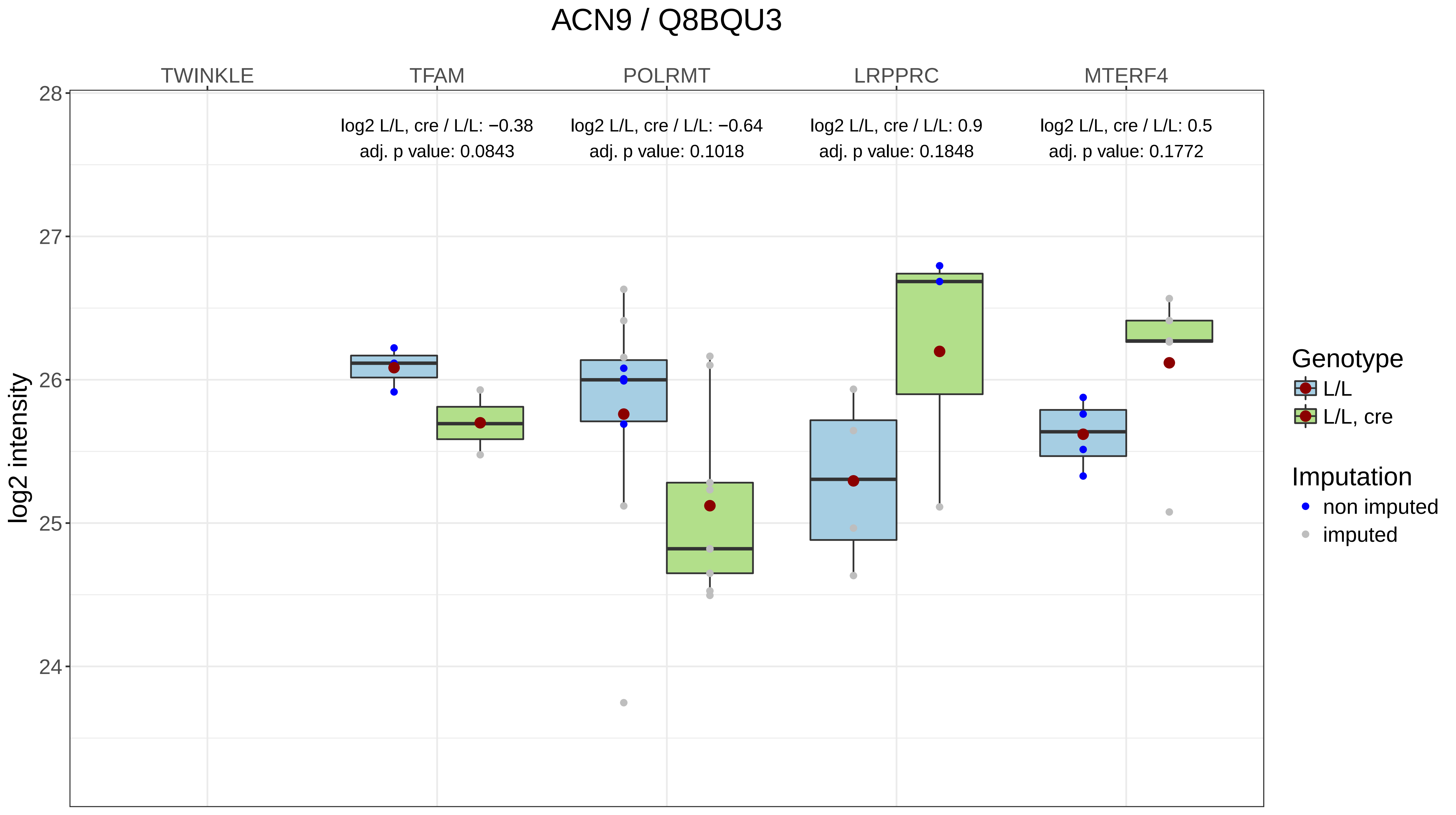
ACN9 / Q8BQU3
TWINKLE
TFAM
POLRMT
LRPPRC
MTERF4
log2 L/L, cre / L/L: −0.38
adj. p value: 0.0843
log2 L/L, cre / L/L: −0.64
adj. p value: 0.1018
log2 L/L, cre / L/L: 0.9
adj. p value: 0.1848
log2 L/L, cre / L/L: 0.5
adj. p value: 0.1772
28
27
26
25
24
log2 intensity
Genotype
L/L
L/L, cre
Imputation
non imputed
imputed

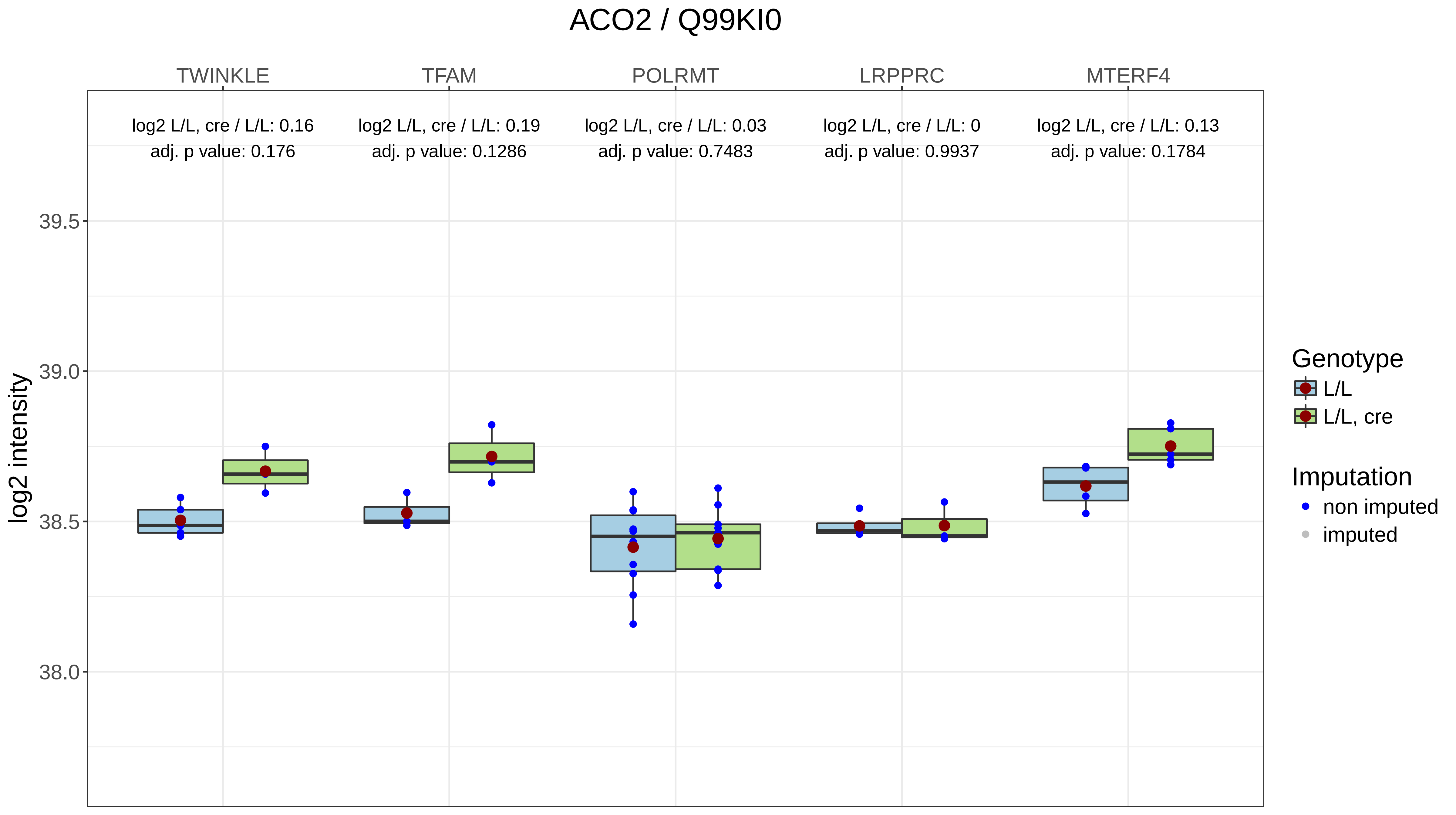

ACO2 / Q99KI0
TWINKLE
TFAM
POLRMT
LRPPRC
MTERF4
log2 L/L, cre / L/L: 0.16
adj. p value: 0.176
log2 L/L, cre / L/L: 0.19
adj. p value: 0.1286
log2 L/L, cre / L/L: 0.03
adj. p value: 0.7483
log2 L/L, cre / L/L: 0
adj. p value: 0.9937
log2 L/L, cre / L/L: 0.13
adj. p value: 0.1784
log2 intensity
39.5
39.0
38.5
38.0
Genotype
L/L
L/L, cre
Imputation
non imputed
imputed

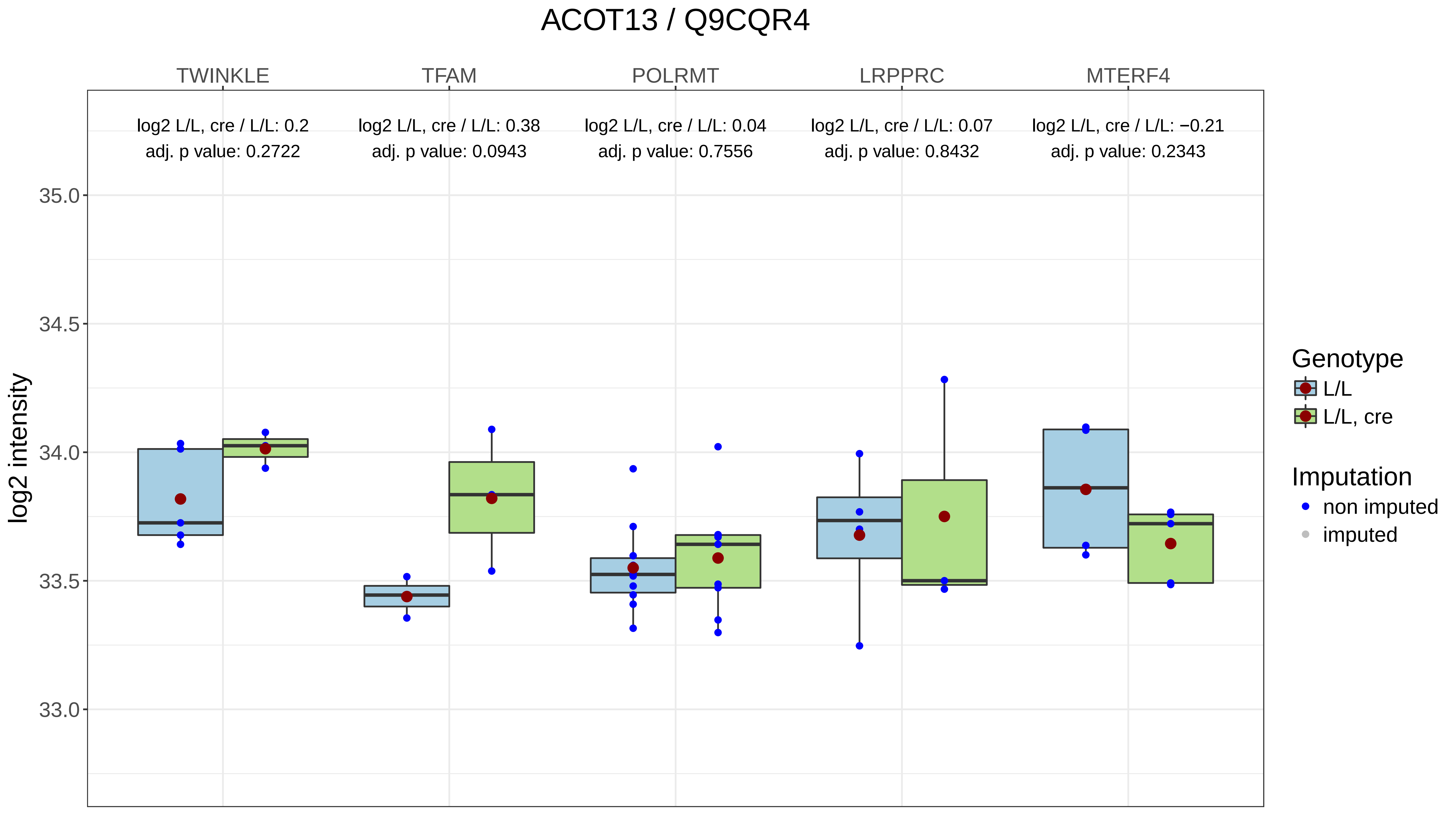

ACOT13 / Q9CQR4
TWINKLE
TFAM
POLRMT
LRPPRC
MTERF4
log2 L/L, cre / L/L: 0.2
adj. p value: 0.2722
log2 L/L, cre / L/L: 0.38
adj. p value: 0.0943
log2 L/L, cre / L/L: 0.04
adj. p value: 0.7556
log2 L/L, cre / L/L: 0.07
adj. p value: 0.8432
log2 L/L, cre / L/L: −0.21
adj. p value: 0.2343
log2 intensity
35.0
34.5
34.0
33.5
33.0
Genotype
L/L
L/L, cre
Imputation
non imputed
imputed

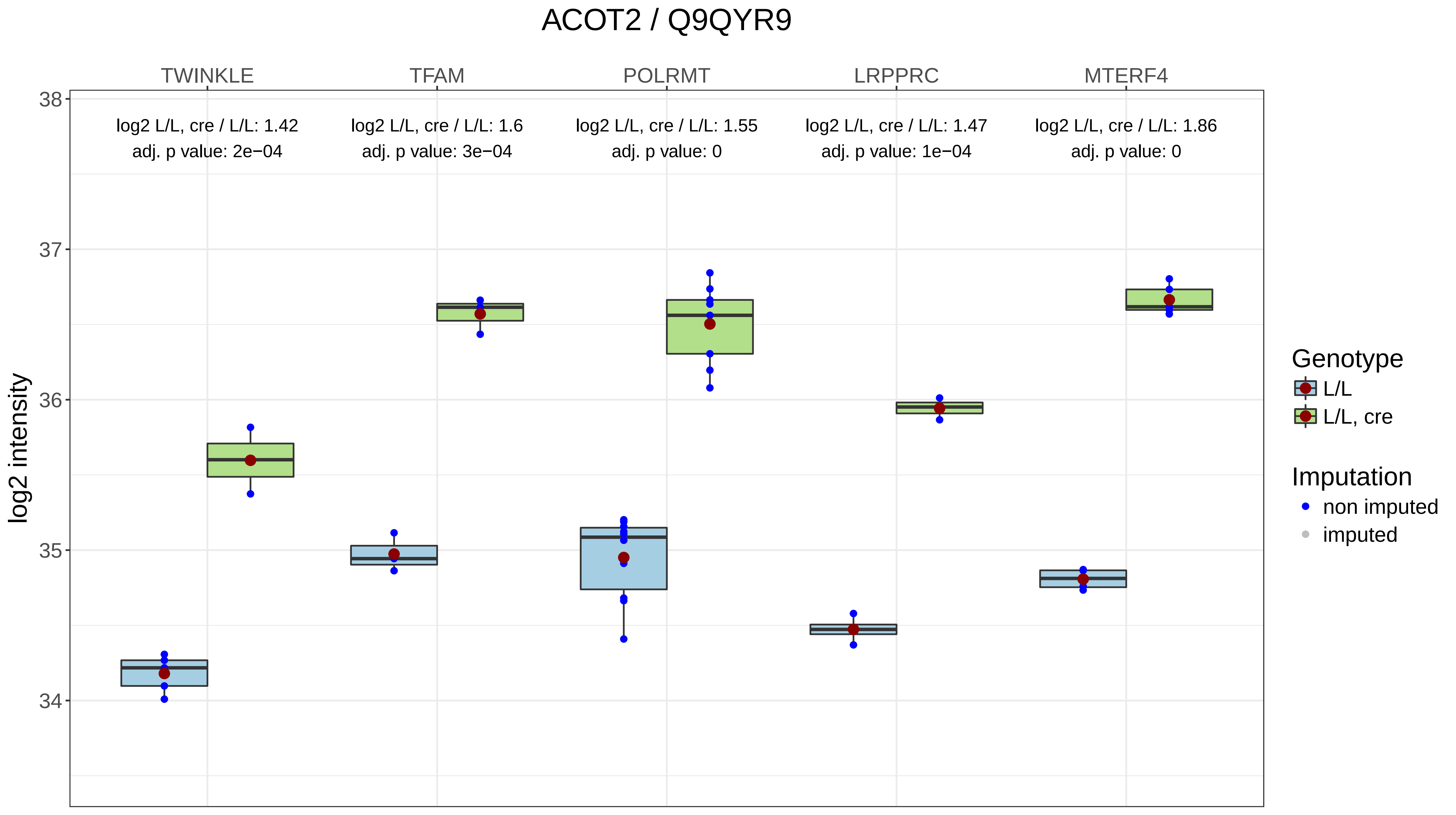

ACOT2 / Q9QYR9
TWINKLE
TFAM
POLRMT
LRPPRC
MTERF4
log2 L/L, cre / L/L: 1.42
adj. p value: 2e−04
log2 L/L, cre / L/L: 1.6
adj. p value: 3e−04
log2 L/L, cre / L/L: 1.55
adj. p value: 0
log2 L/L, cre / L/L: 1.47
adj. p value: 1e−04
log2 L/L, cre / L/L: 1.86
adj. p value: 0
38
37
36
35
34
log2 intensity
Genotype
L/L
L/L, cre
Imputation
non imputed
imputed

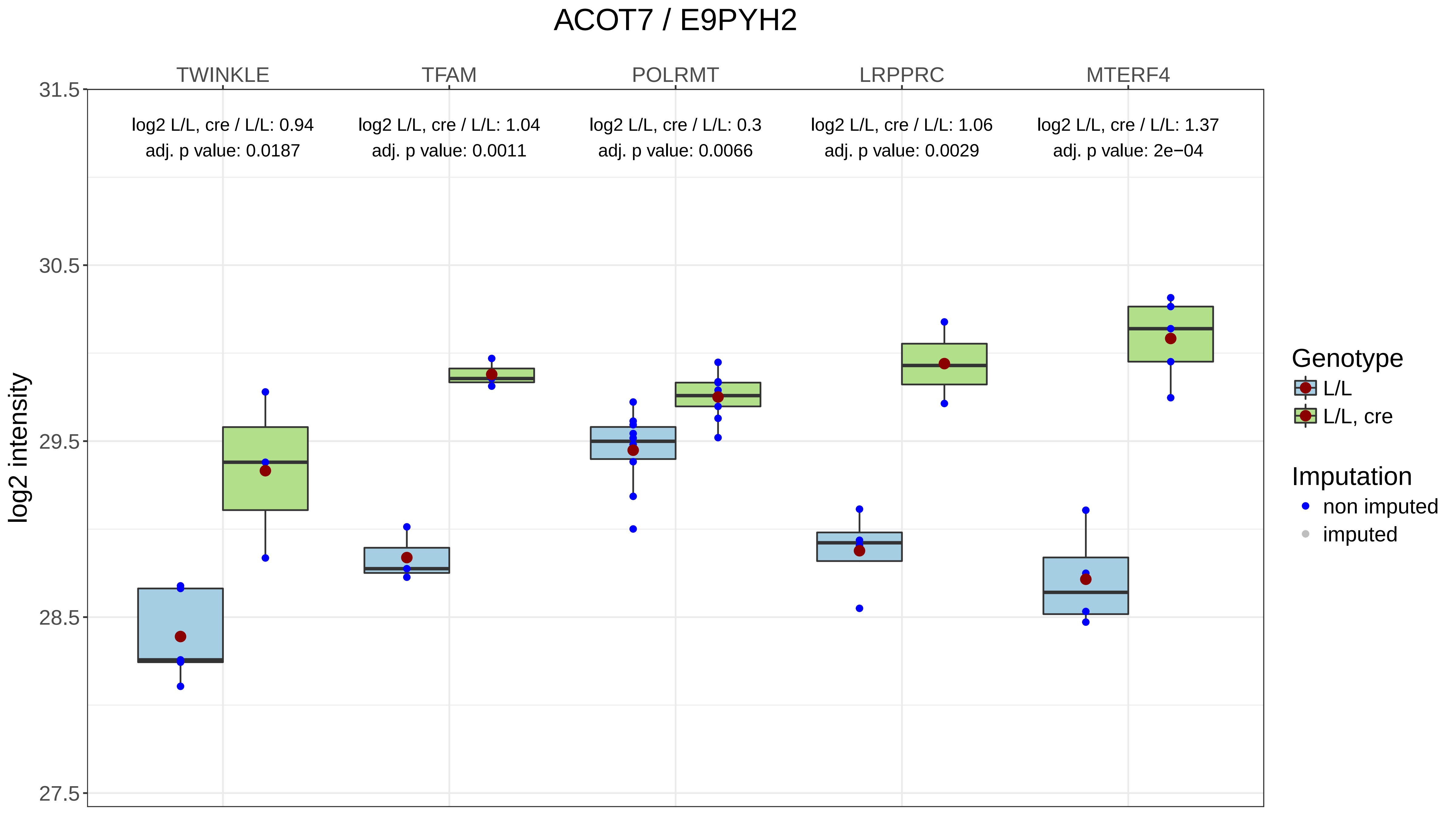
ACOT7 / E9PYH2
TWINKLE
TFAM
POLRMT
LRPPRC
MTERF4
log2 L/L, cre / L/L: 0.94
adj. p value: 0.0187
log2 L/L, cre / L/L: 1.04
adj. p value: 0.0011
log2 L/L, cre / L/L: 0.3
adj. p value: 0.0066
log2 L/L, cre / L/L: 1.06
adj. p value: 0.0029
log2 L/L, cre / L/L: 1.37
adj. p value: 2e−04
31.5
30.5
29.5
28.5
27.5
log2 intensity
Genotype
L/L
L/L, cre
Imputation
non imputed
imputed

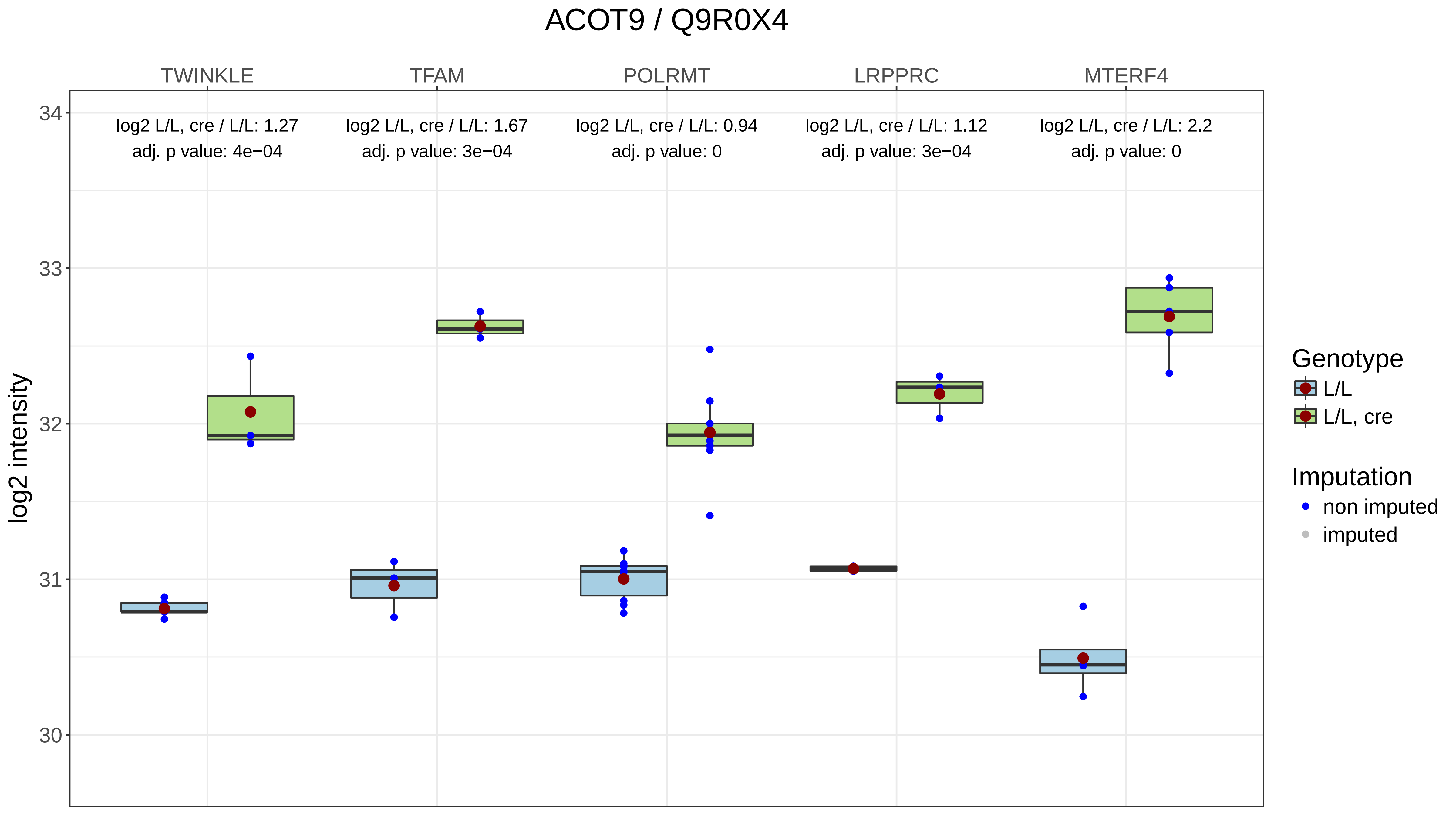
ACOT9 / Q9R0X4
TWINKLE
TFAM
POLRMT
LRPPRC
MTERF4
log2 L/L, cre / L/L: 1.27
adj. p value: 4e−04
log2 L/L, cre / L/L: 1.67
adj. p value: 3e−04
log2 L/L, cre / L/L: 0.94
adj. p value: 0
log2 L/L, cre / L/L: 1.12
adj. p value: 3e−04
log2 L/L, cre / L/L: 2.2
adj. p value: 0
log2 intensity
34
33
32
31
30
Genotype
L/L
L/L, cre
Imputation
non imputed
imputed

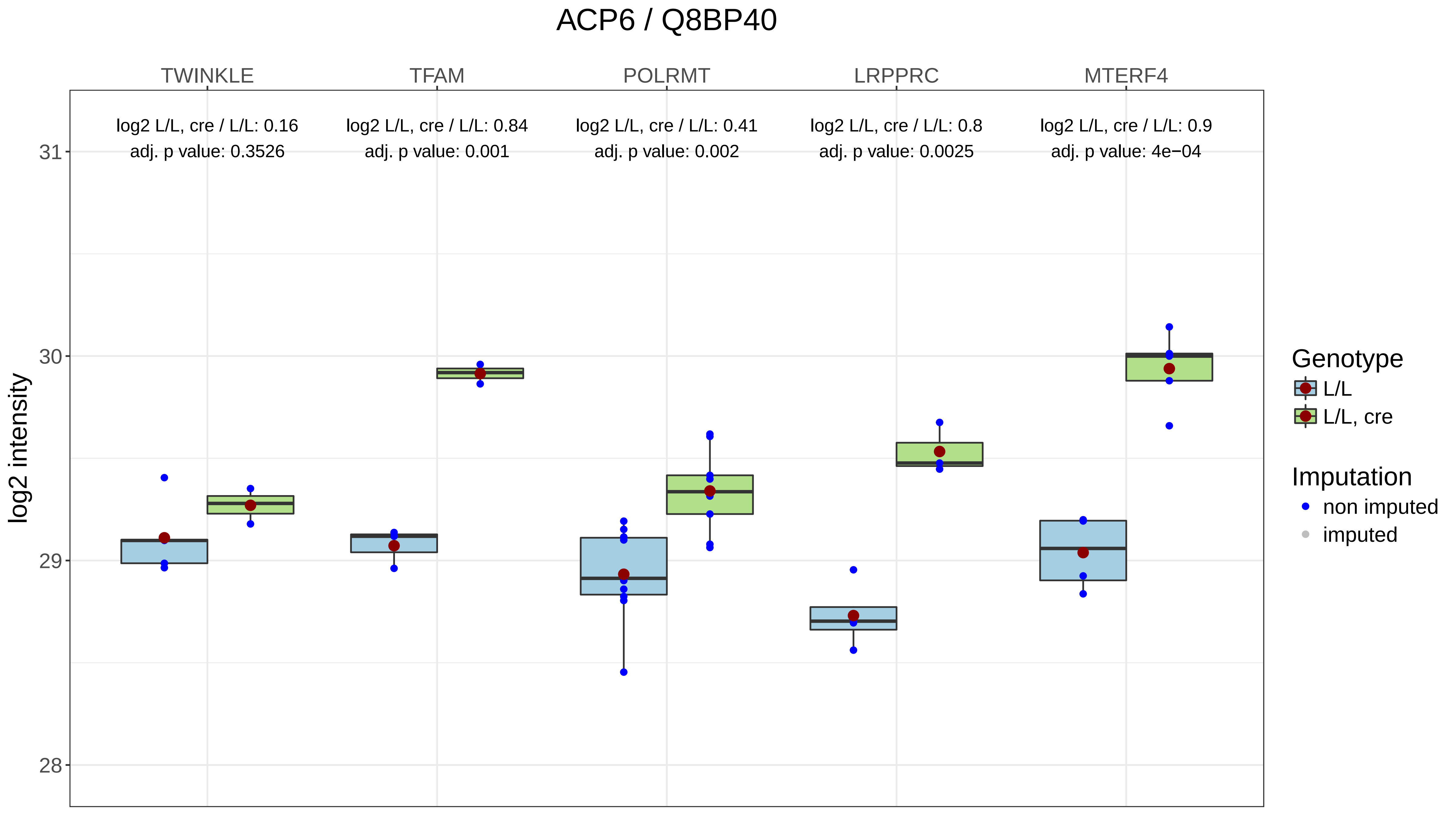

ACP6 / Q8BP40
TWINKLE
TFAM
POLRMT
LRPPRC
MTERF4
log2 L/L, cre / L/L: 0.16
adj. p value: 0.3526
log2 L/L, cre / L/L: 0.84
adj. p value: 0.001
log2 L/L, cre / L/L: 0.41
adj. p value: 0.002
log2 L/L, cre / L/L: 0.8
adj. p value: 0.0025
log2 L/L, cre / L/L: 0.9
adj. p value: 4e−04
log2 intensity
31
30
29
28
Genotype
L/L
L/L, cre
Imputation
non imputed
imputed

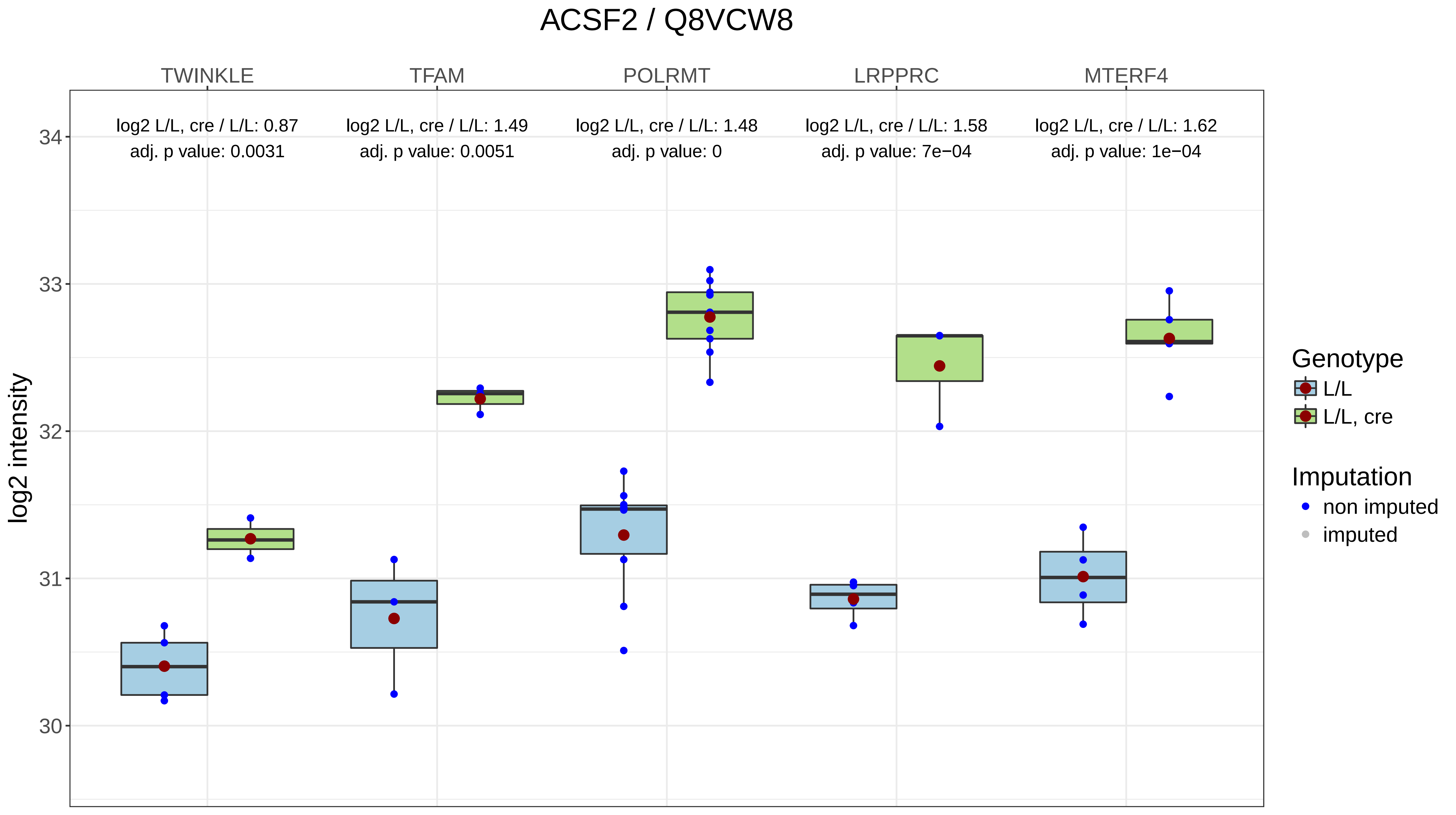
ACSF2 / Q8VCW8
TWINKLE
TFAM
POLRMT
LRPPRC
MTERF4
log2 L/L, cre / L/L: 0.87
adj. p value: 0.0031
log2 L/L, cre / L/L: 1.49
adj. p value: 0.0051
log2 L/L, cre / L/L: 1.48
adj. p value: 0
log2 L/L, cre / L/L: 1.58
adj. p value: 7e−04
log2 L/L, cre / L/L: 1.62
adj. p value: 1e−04
log2 intensity
34
33
32
31
30
Genotype
L/L
L/L, cre
Imputation
non imputed
imputed

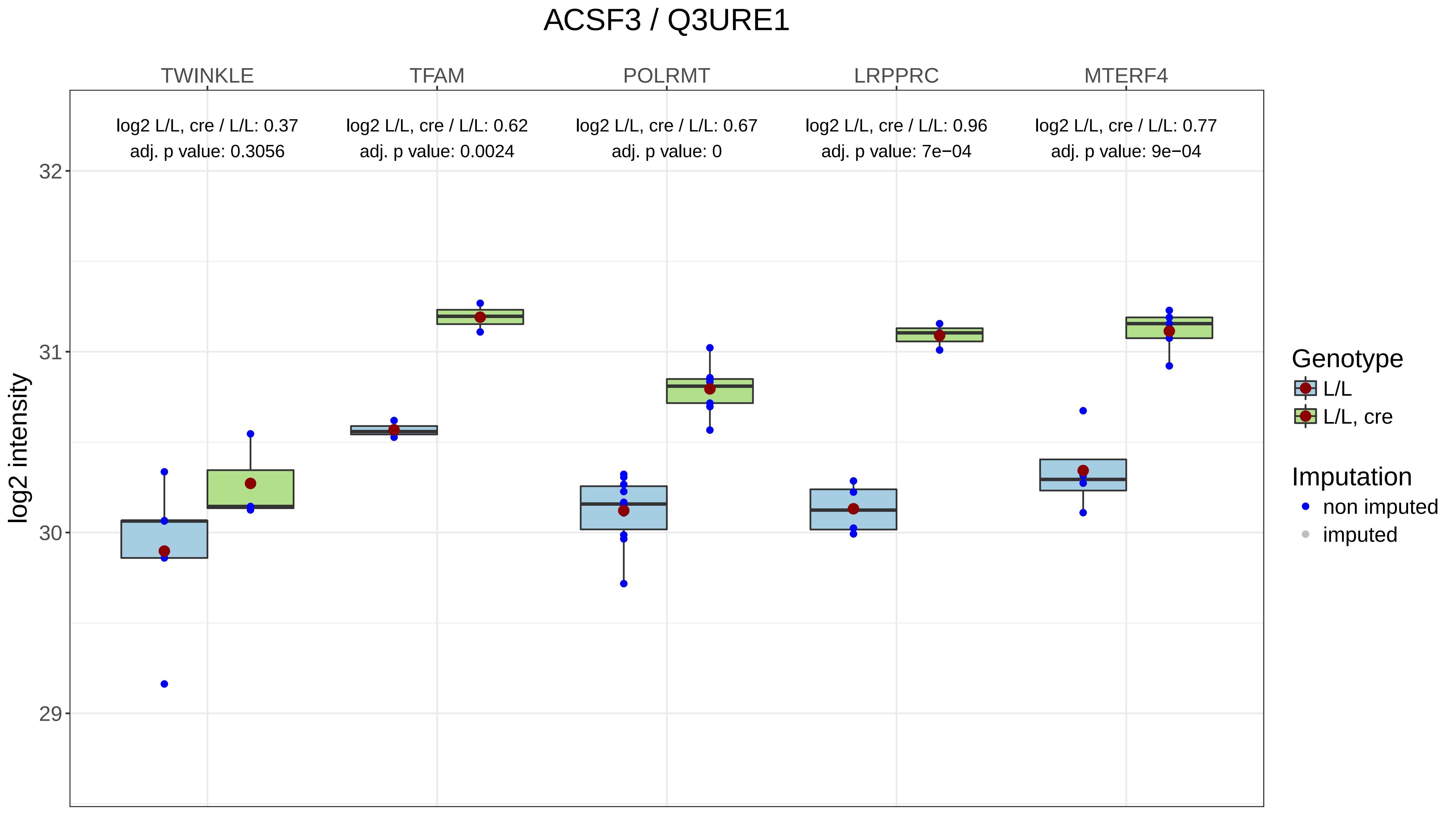
ACSF3 / Q3URE1
TWINKLE
TFAM
POLRMT
LRPPRC
MTERF4
log2 L/L, cre / L/L: 0.37
adj. p value: 0.3056
log2 L/L, cre / L/L: 0.62
adj. p value: 0.0024
log2 L/L, cre / L/L: 0.67
adj. p value: 0
log2 L/L, cre / L/L: 0.96
adj. p value: 7e−04
log2 L/L, cre / L/L: 0.77
adj. p value: 9e−04
log2 intensity
32
31
30
29
Genotype
L/L
L/L, cre
Imputation
non imputed
imputed

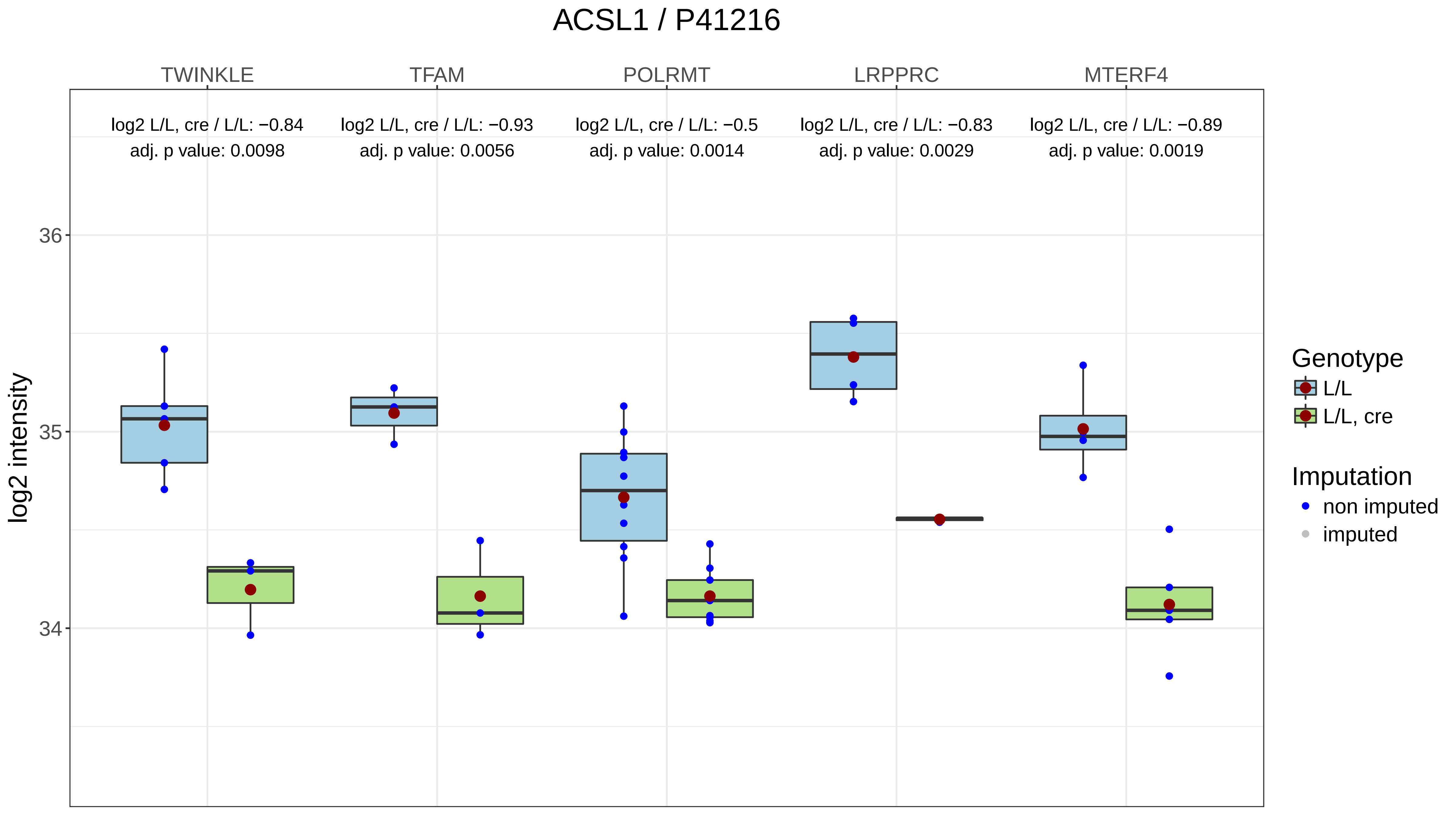
ACSL1 / P41216
TWINKLE
TFAM
POLRMT
LRPPRC
MTERF4
log2 L/L, cre / L/L: −0.84
adj. p value: 0.0098
log2 L/L, cre / L/L: −0.93
adj. p value: 0.0056
log2 L/L, cre / L/L: −0.5
adj. p value: 0.0014
log2 L/L, cre / L/L: −0.83
adj. p value: 0.0029
log2 L/L, cre / L/L: −0.89
adj. p value: 0.0019
log2 intensity
36
35
34
Genotype
L/L
L/L, cre
Imputation
non imputed
imputed

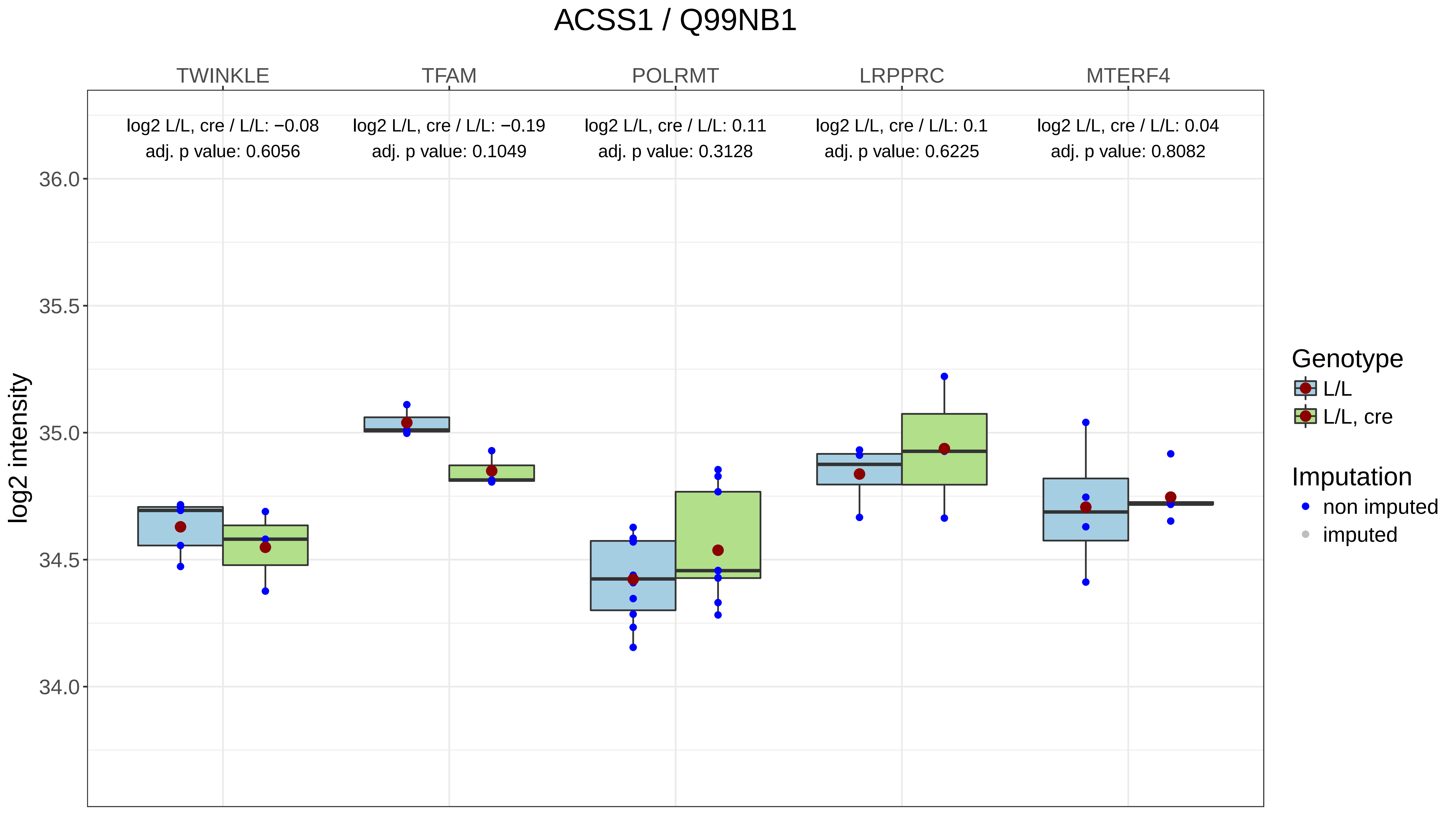

ACSS1 / Q99NB1
TWINKLE
TFAM
POLRMT
LRPPRC
MTERF4
log2 L/L, cre / L/L: −0.08
adj. p value: 0.6056
log2 L/L, cre / L/L: −0.19
adj. p value: 0.1049
log2 L/L, cre / L/L: 0.11
adj. p value: 0.3128
log2 L/L, cre / L/L: 0.1
adj. p value: 0.6225
log2 L/L, cre / L/L: 0.04
adj. p value: 0.8082
log2 intensity
36.0
35.5
35.0
34.5
34.0
Genotype
L/L
L/L, cre
Imputation
non imputed
imputed

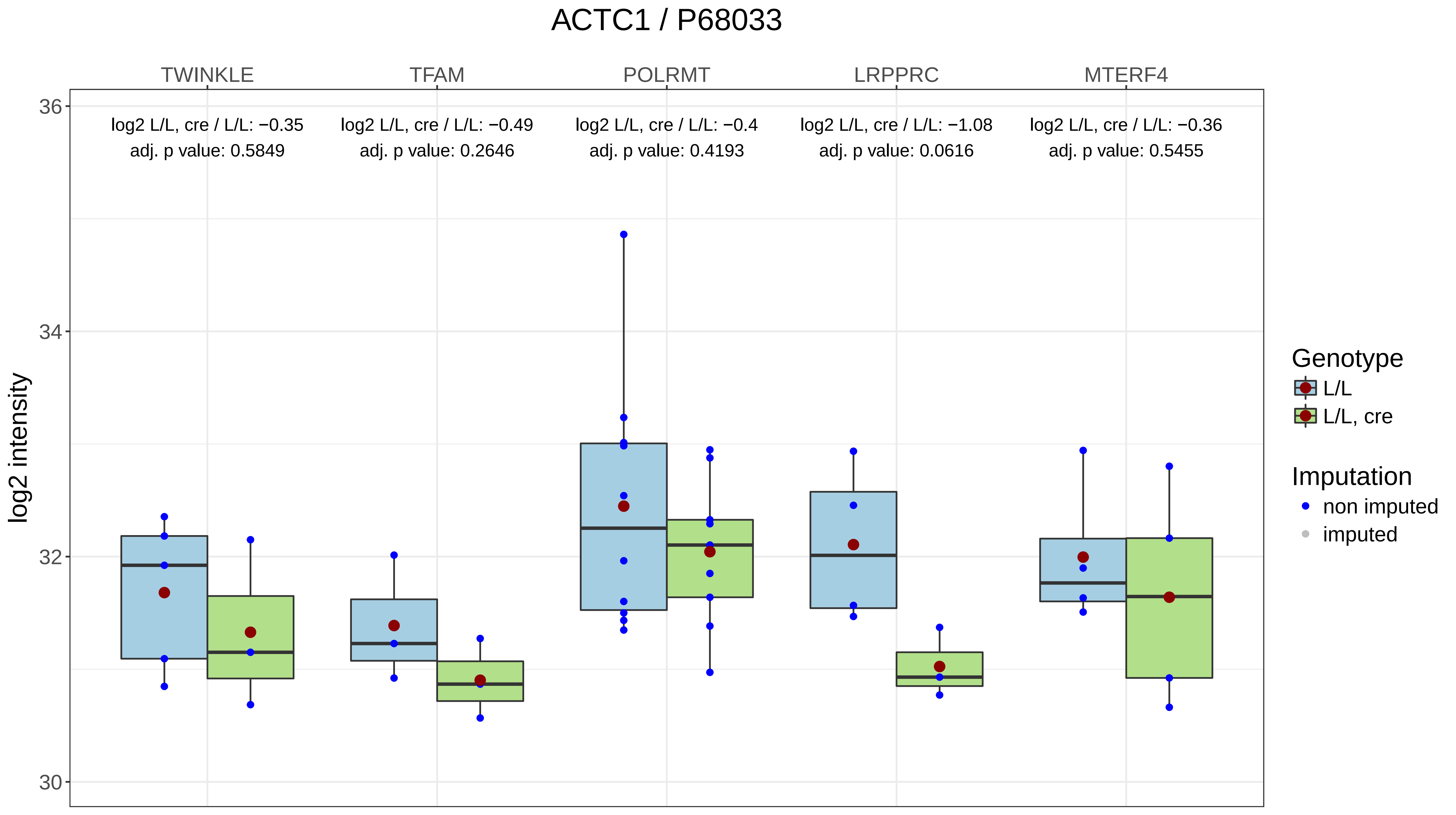
ACTC1 / P68033
TWINKLE
TFAM
POLRMT
LRPPRC
MTERF4
log2 L/L, cre / L/L: −0.35
adj. p value: 0.5849
log2 L/L, cre / L/L: −0.49
adj. p value: 0.2646
log2 L/L, cre / L/L: −0.4
adj. p value: 0.4193
log2 L/L, cre / L/L: −1.08
adj. p value: 0.0616
log2 L/L, cre / L/L: −0.36
adj. p value: 0.5455
log2 intensity
36
34
32
30
Genotype
L/L
L/L, cre
Imputation
non imputed
imputed

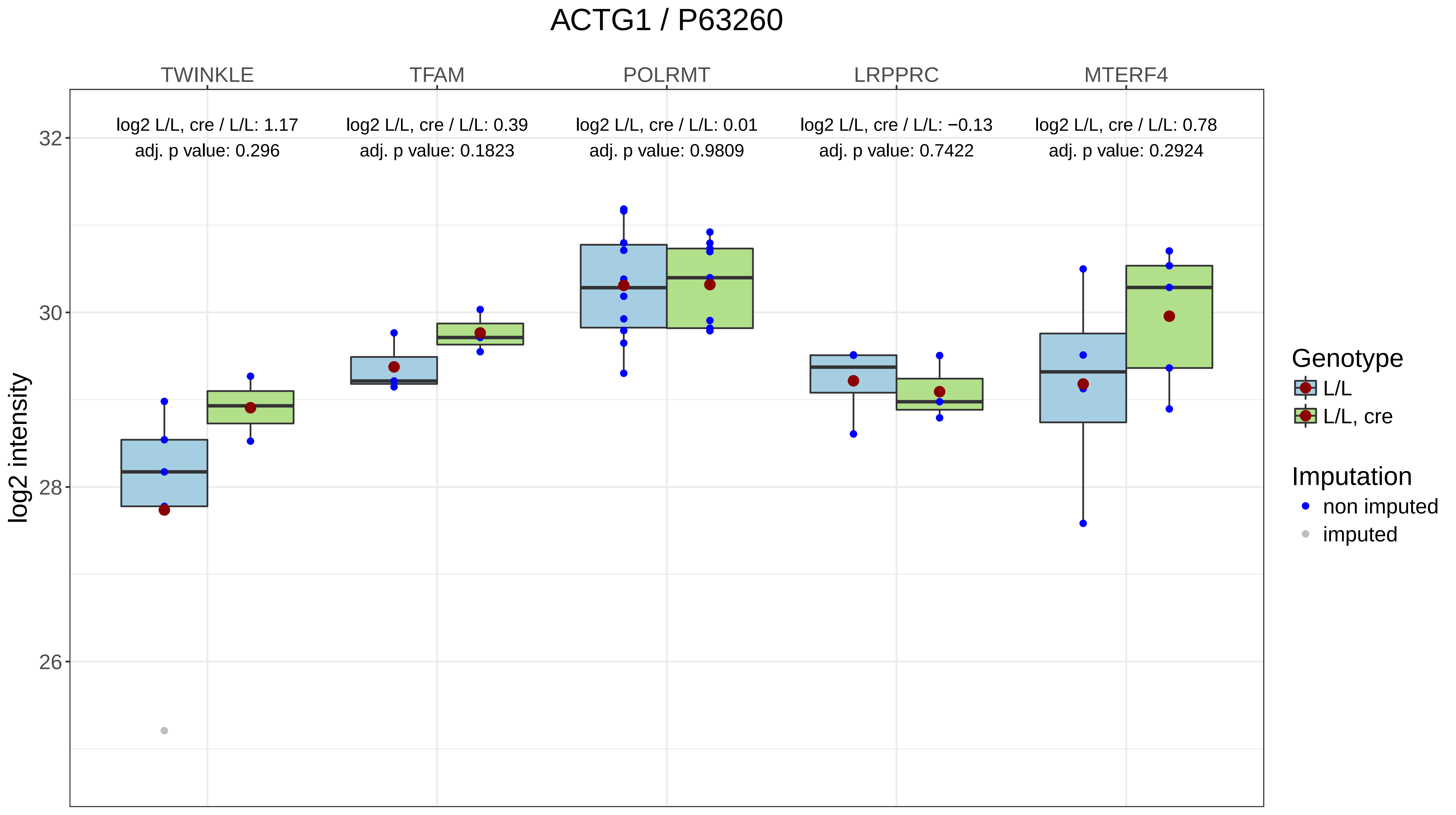
ACTG1 / P63260
TWINKLE
TFAM
POLRMT
LRPPRC
MTERF4
log2 L/L, cre / L/L: 1.17
adj. p value: 0.296
log2 L/L, cre / L/L: 0.39
adj. p value: 0.1823
log2 L/L, cre / L/L: 0.01
adj. p value: 0.9809
log2 L/L, cre / L/L: −0.13
adj. p value: 0.7422
log2 L/L, cre / L/L: 0.78
adj. p value: 0.2924
log2 intensity
32
30
28
26
Genotype
L/L
L/L, cre
Imputation
non imputed
imputed

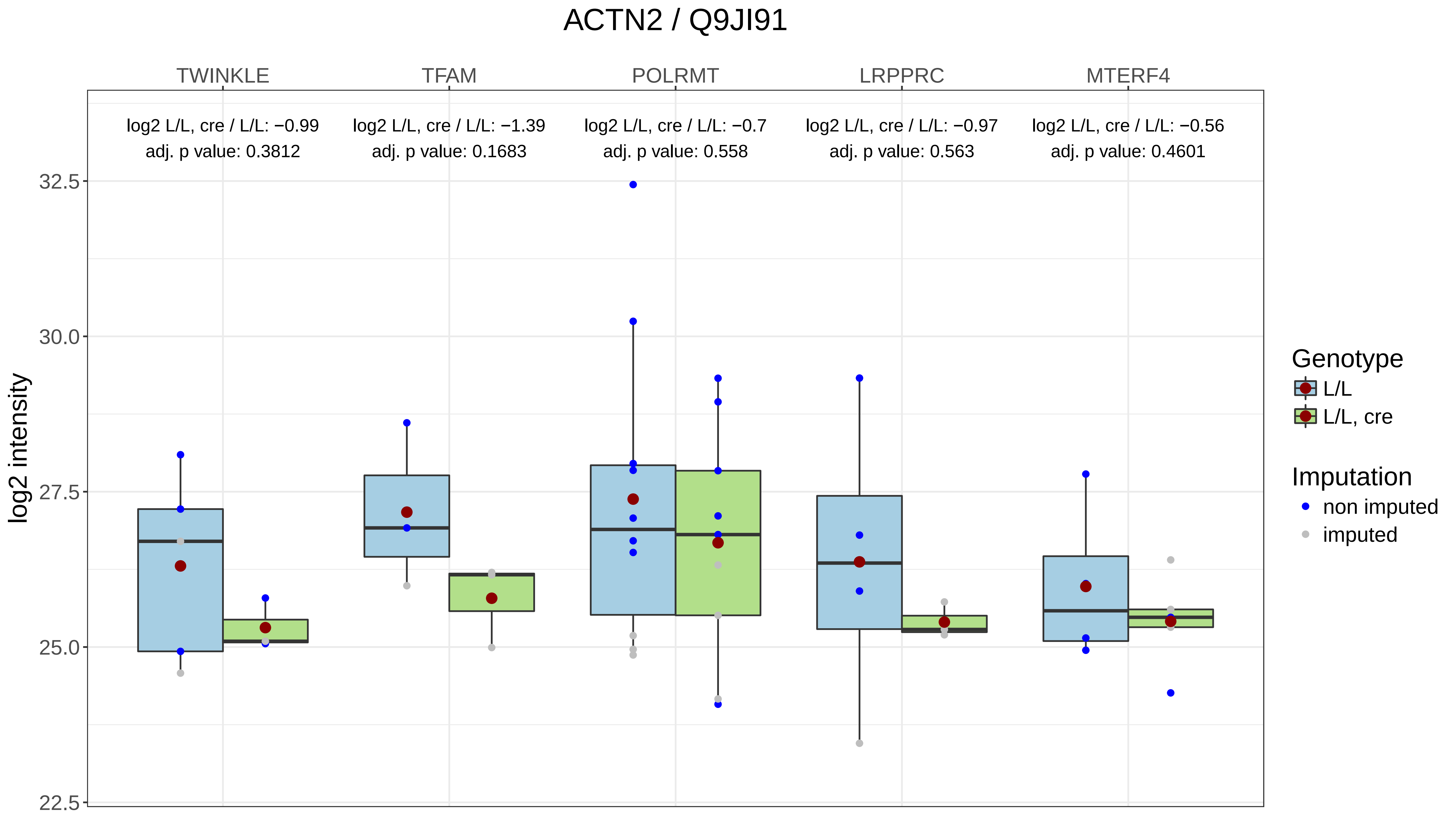
ACTN2 / Q9JI91
TWINKLE
TFAM
POLRMT
LRPPRC
MTERF4
log2 L/L, cre / L/L: −0.99
adj. p value: 0.3812
log2 L/L, cre / L/L: −1.39
adj. p value: 0.1683
log2 L/L, cre / L/L: −0.7
adj. p value: 0.558
log2 L/L, cre / L/L: −0.97
adj. p value: 0.563
log2 L/L, cre / L/L: −0.56
adj. p value: 0.4601
log2 intensity
32.5
30.0
27.5
25.0
22.5
Genotype
L/L
L/L, cre
Imputation
non imputed
imputed

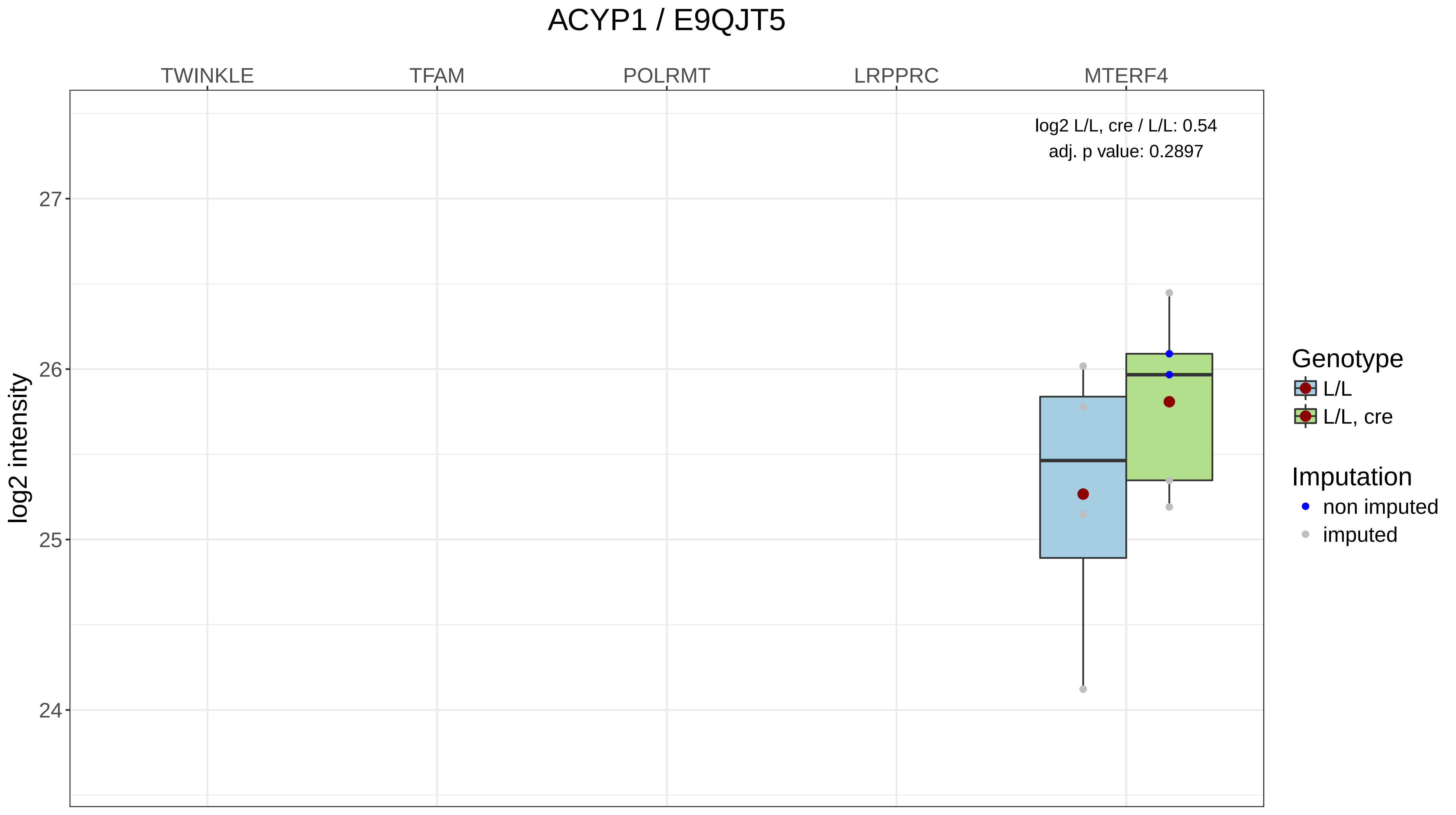
ACYP1 / E9QJT5
TWINKLE
TFAM
POLRMT
LRPPRC
MTERF4
log2 L/L, cre / L/L: 0.54
adj. p value: 0.2897
log2 intensity
27
26
25
24
Genotype
L/L
L/L, cre
Imputation
non imputed
imputed

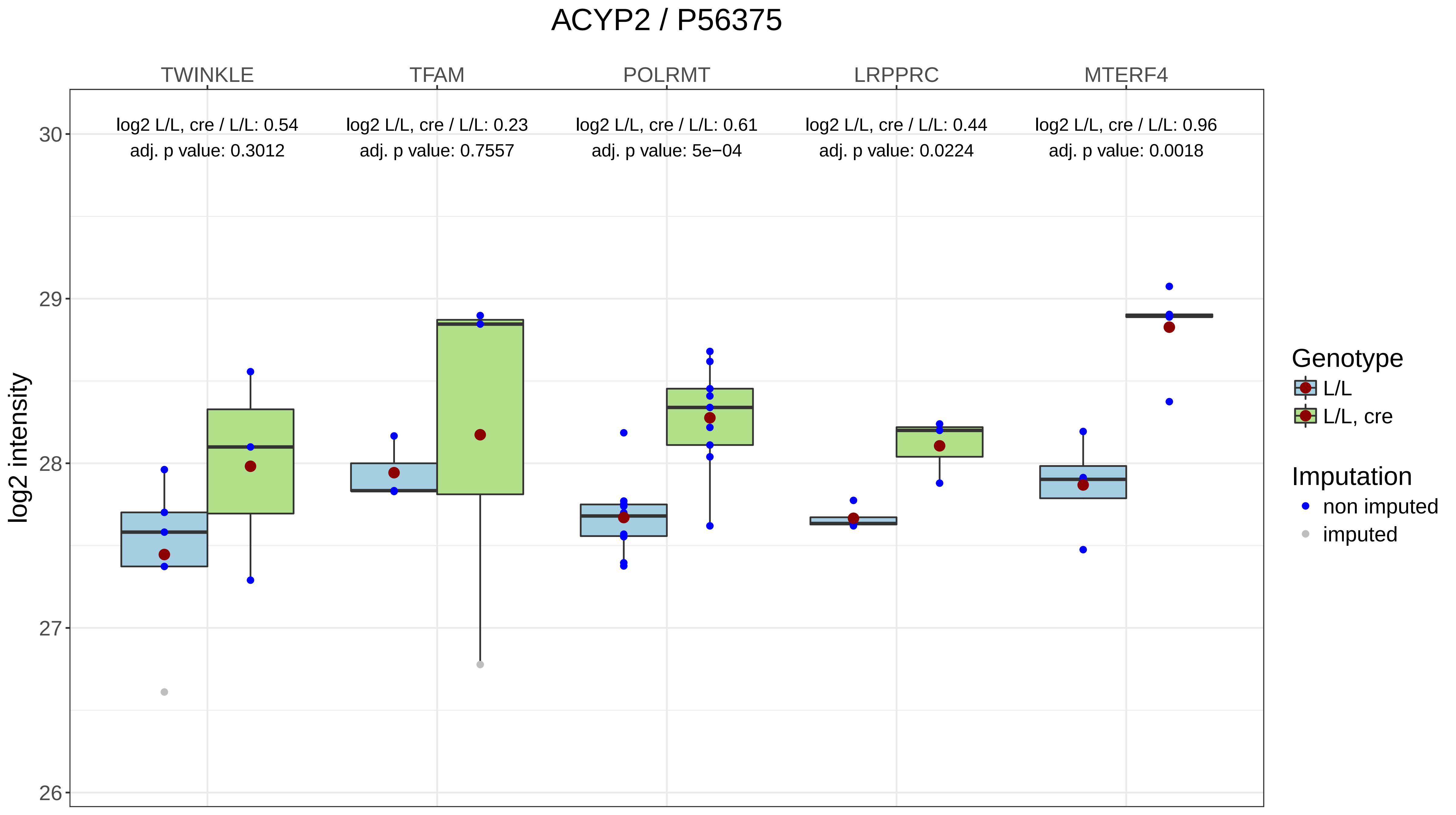
ACYP2 / P56375
TWINKLE
TFAM
POLRMT
LRPPRC
MTERF4
log2 L/L, cre / L/L: 0.54
adj. p value: 0.3012
log2 L/L, cre / L/L: 0.23
adj. p value: 0.7557
log2 L/L, cre / L/L: 0.61
adj. p value: 5e−04
log2 L/L, cre / L/L: 0.44
adj. p value: 0.0224
log2 L/L, cre / L/L: 0.96
adj. p value: 0.0018
log2 intensity
30
29
28
27
26
Genotype
L/L
L/L, cre
Imputation
non imputed
imputed

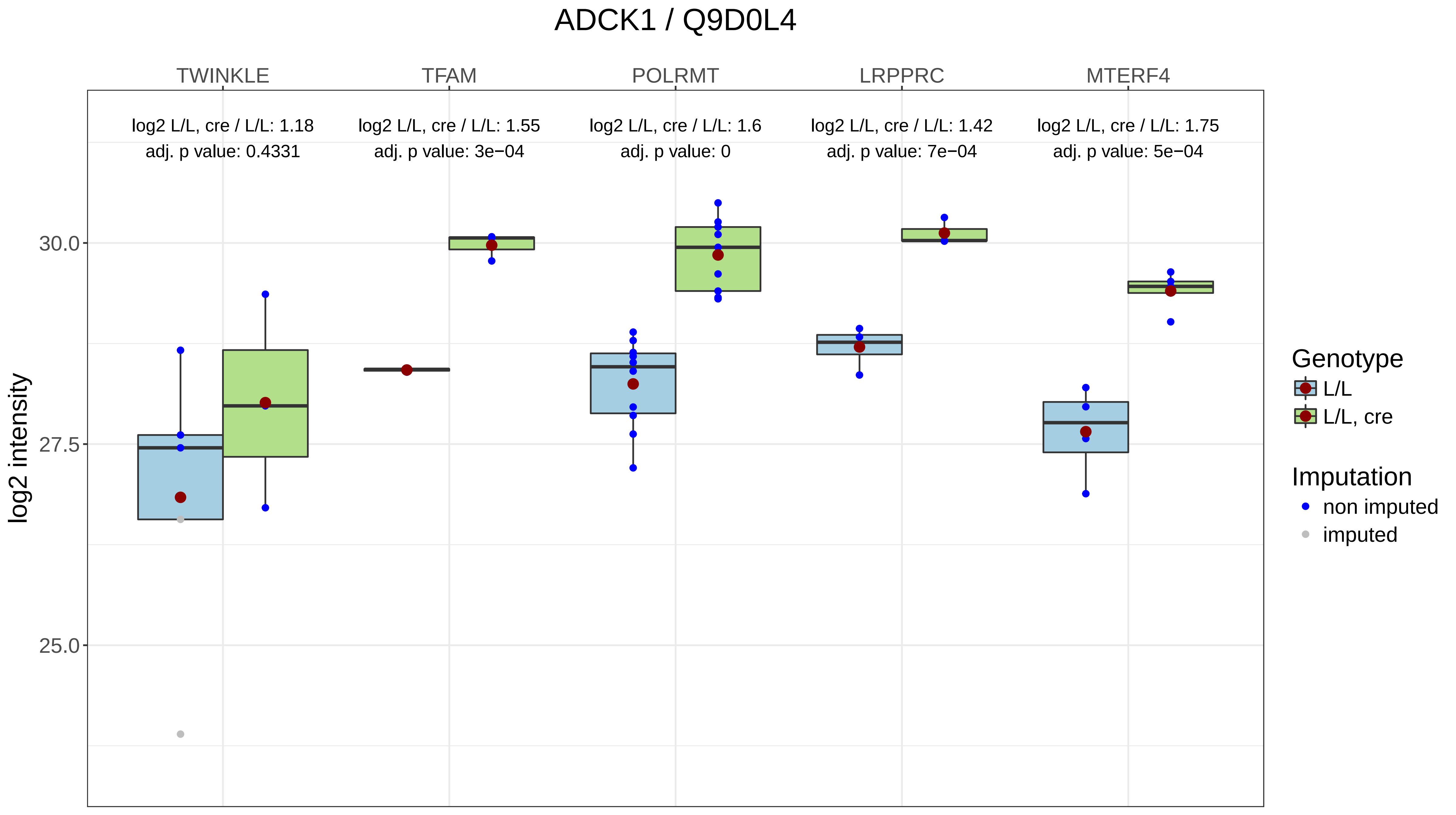
ADCK1 / Q9D0L4
TWINKLE
TFAM
POLRMT
LRPPRC
MTERF4
log2 L/L, cre / L/L: 1.18
adj. p value: 0.4331
log2 L/L, cre / L/L: 1.55
adj. p value: 3e−04
log2 L/L, cre / L/L: 1.6
adj. p value: 0
log2 L/L, cre / L/L: 1.42
adj. p value: 7e−04
log2 L/L, cre / L/L: 1.75
adj. p value: 5e−04
30.0
27.5
25.0
log2 intensity
Genotype
L/L
L/L, cre
Imputation
non imputed
imputed

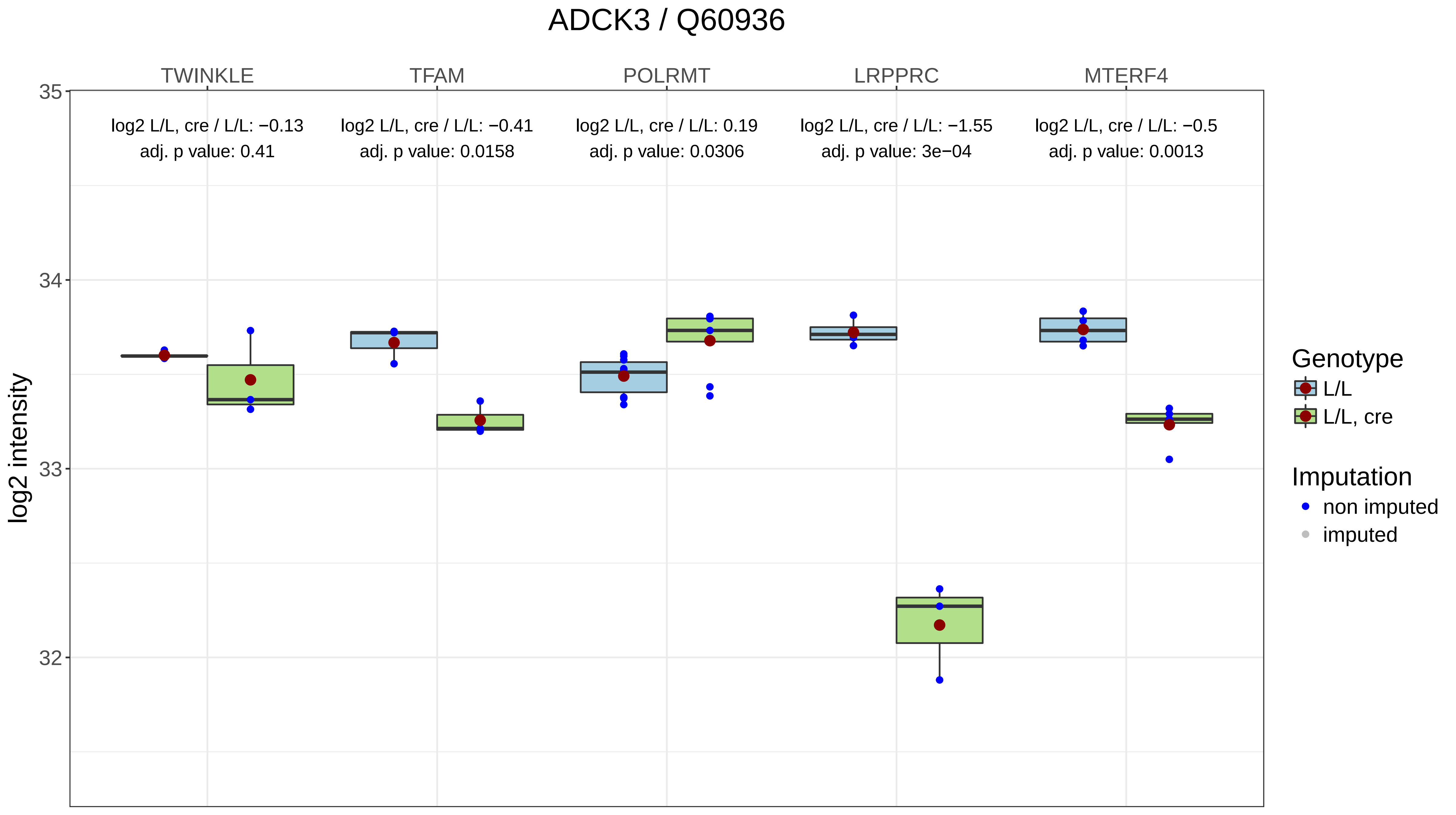

ADCK3 / Q60936
TWINKLE
TFAM
POLRMT
LRPPRC
MTERF4
log2 L/L, cre / L/L: −0.13
adj. p value: 0.41
log2 L/L, cre / L/L: −0.41
adj. p value: 0.0158
log2 L/L, cre / L/L: 0.19
adj. p value: 0.0306
log2 L/L, cre / L/L: −1.55
adj. p value: 3e−04
log2 L/L, cre / L/L: −0.5
adj. p value: 0.0013
35
34
33
32
log2 intensity
Genotype
L/L
L/L, cre
Imputation
non imputed
imputed

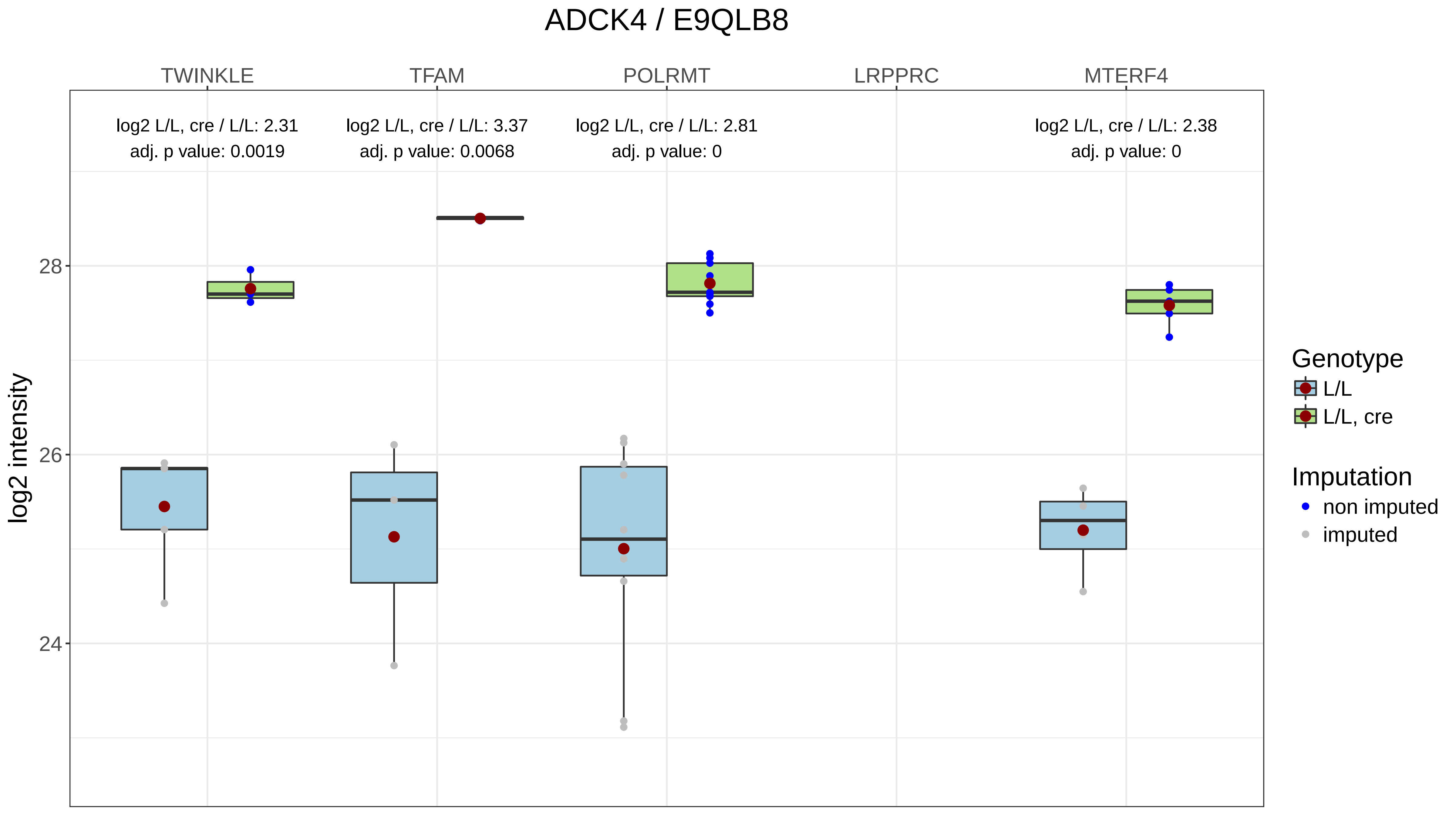

ADCK4 / E9QLB8
TWINKLE
TFAM
POLRMT
LRPPRC
MTERF4
log2 L/L, cre / L/L: 2.31
adj. p value: 0.0019
log2 L/L, cre / L/L: 3.37
adj. p value: 0.0068
log2 L/L, cre / L/L: 2.81
adj. p value: 0
log2 L/L, cre / L/L: 2.38
adj. p value: 0
log2 intensity
28
26
24
Genotype
L/L
L/L, cre
Imputation
non imputed
imputed

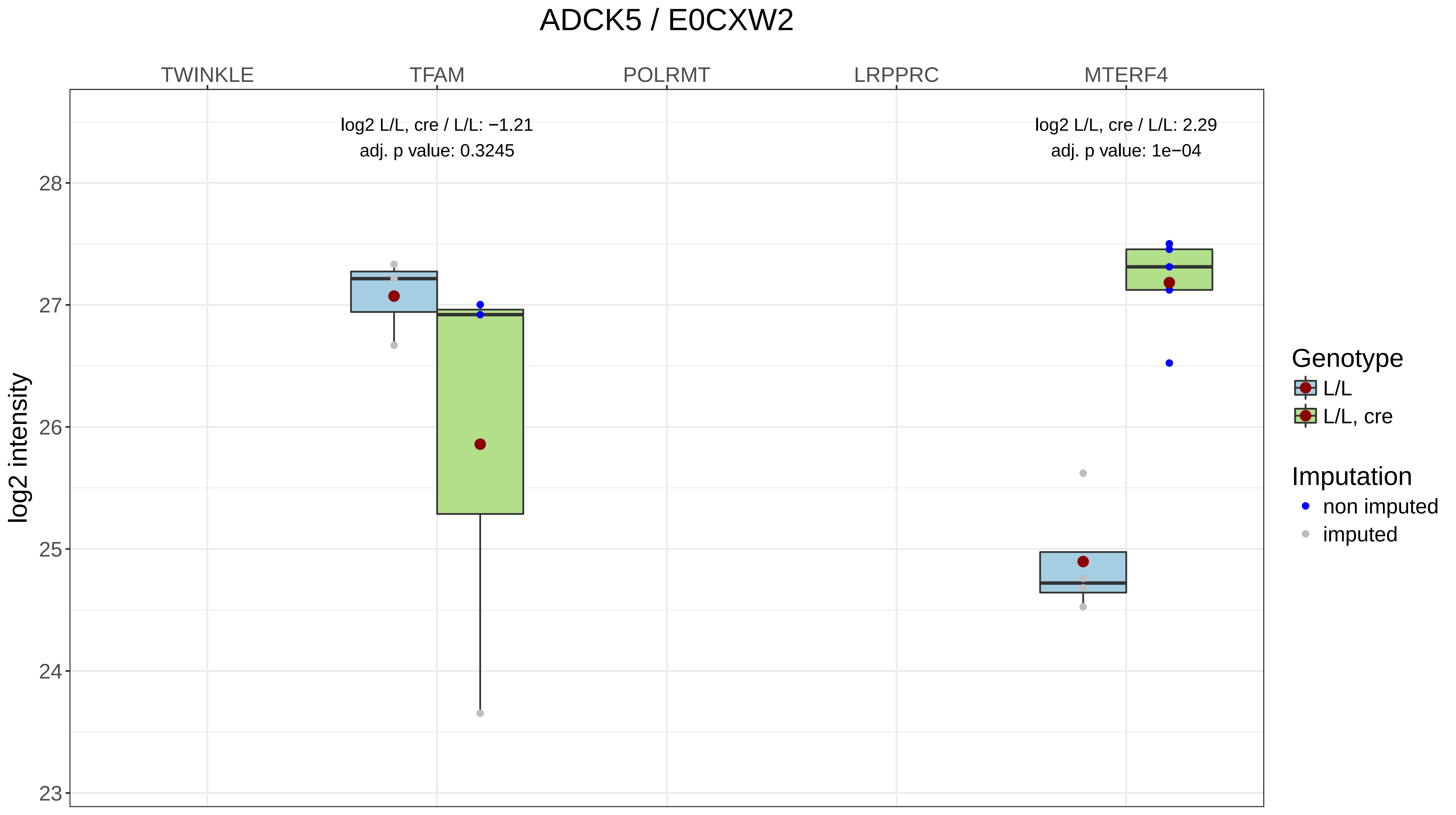
ADCK5 / E0CXW2
TWINKLE
TFAM
POLRMT
LRPPRC
MTERF4
log2 L/L, cre / L/L: −1.21
adj. p value: 0.3245
log2 L/L, cre / L/L: 2.29
adj. p value: 1e−04
log2 intensity
28
27
26
25
24
23
Genotype
L/L
L/L, cre
Imputation
non imputed
imputed

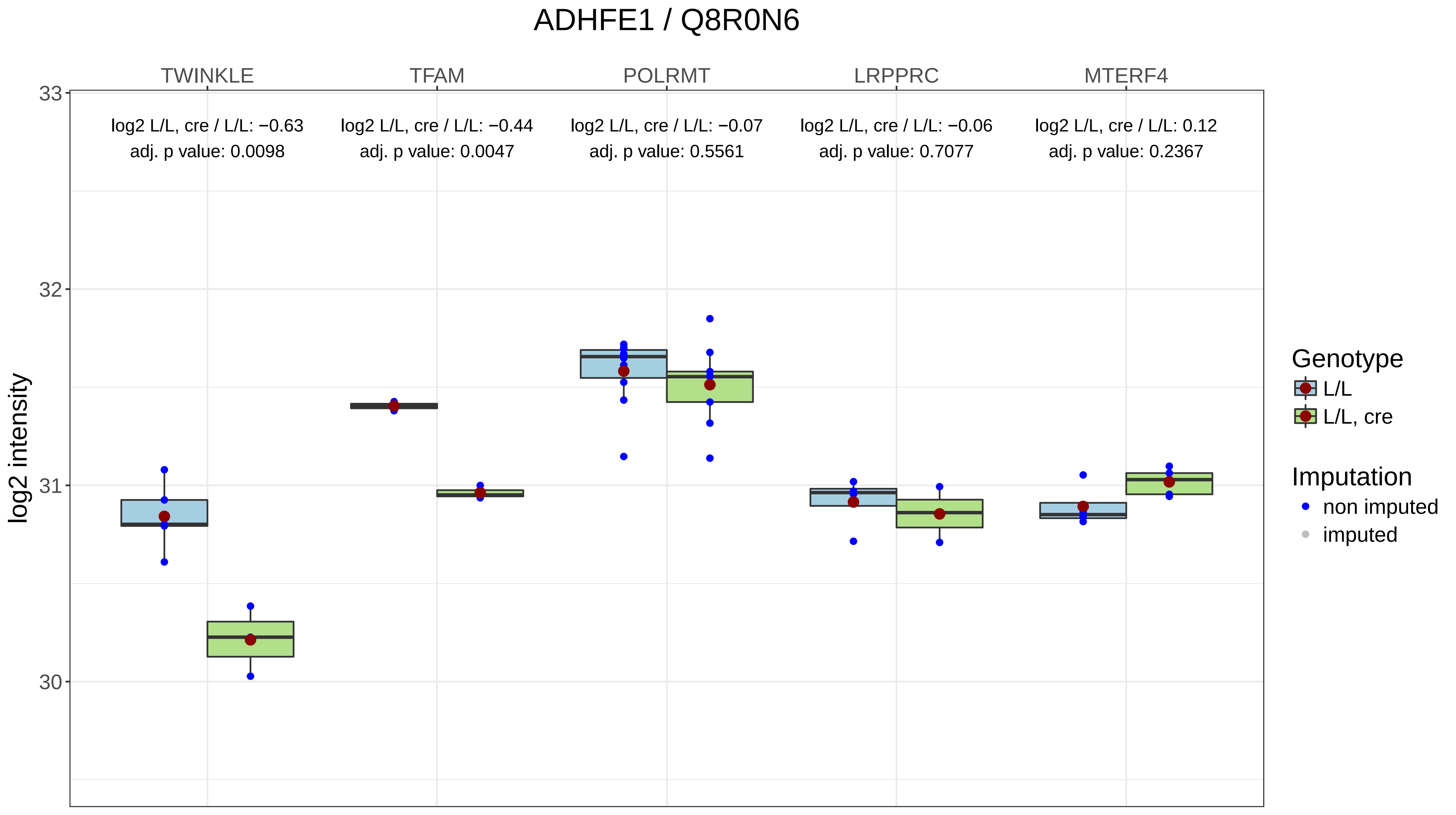
ADHFE1 / Q8R0N6
TWINKLE
TFAM
POLRMT
LRPPRC
MTERF4
log2 L/L, cre / L/L: −0.63
adj. p value: 0.0098
log2 L/L, cre / L/L: −0.44
adj. p value: 0.0047
log2 L/L, cre / L/L: −0.07
adj. p value: 0.5561
log2 L/L, cre / L/L: −0.06
adj. p value: 0.7077
log2 L/L, cre / L/L: 0.12
adj. p value: 0.2367
log2 intensity
33
32
31
30
Genotype
L/L
L/L, cre
Imputation
non imputed
imputed

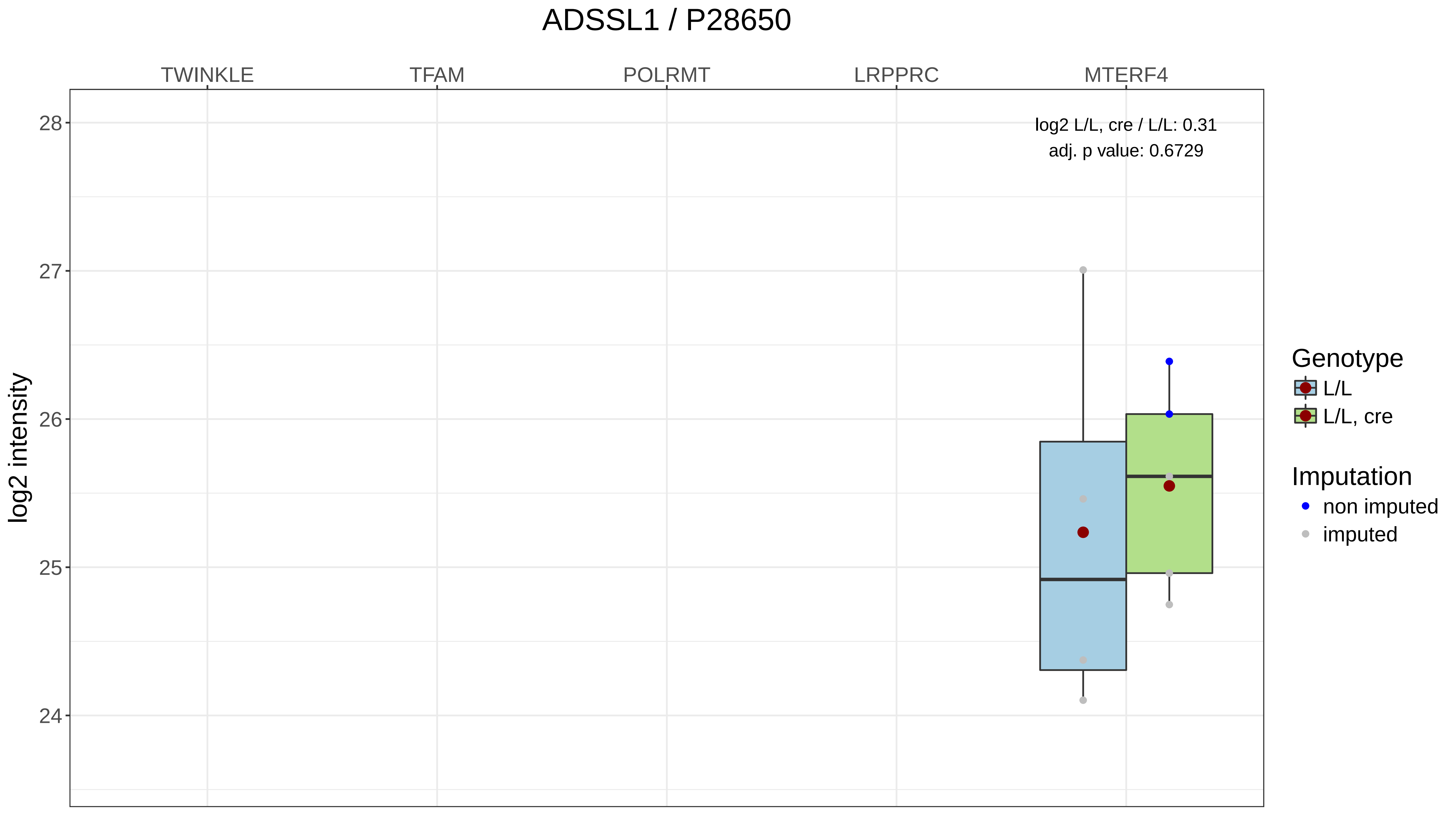
ADSSL1 / P28650
TWINKLE
TFAM
POLRMT
LRPPRC
MTERF4
log2 L/L, cre / L/L: 0.31
adj. p value: 0.6729
log2 intensity
28
27
26
25
24
Genotype
L/L
L/L, cre
Imputation
non imputed
imputed

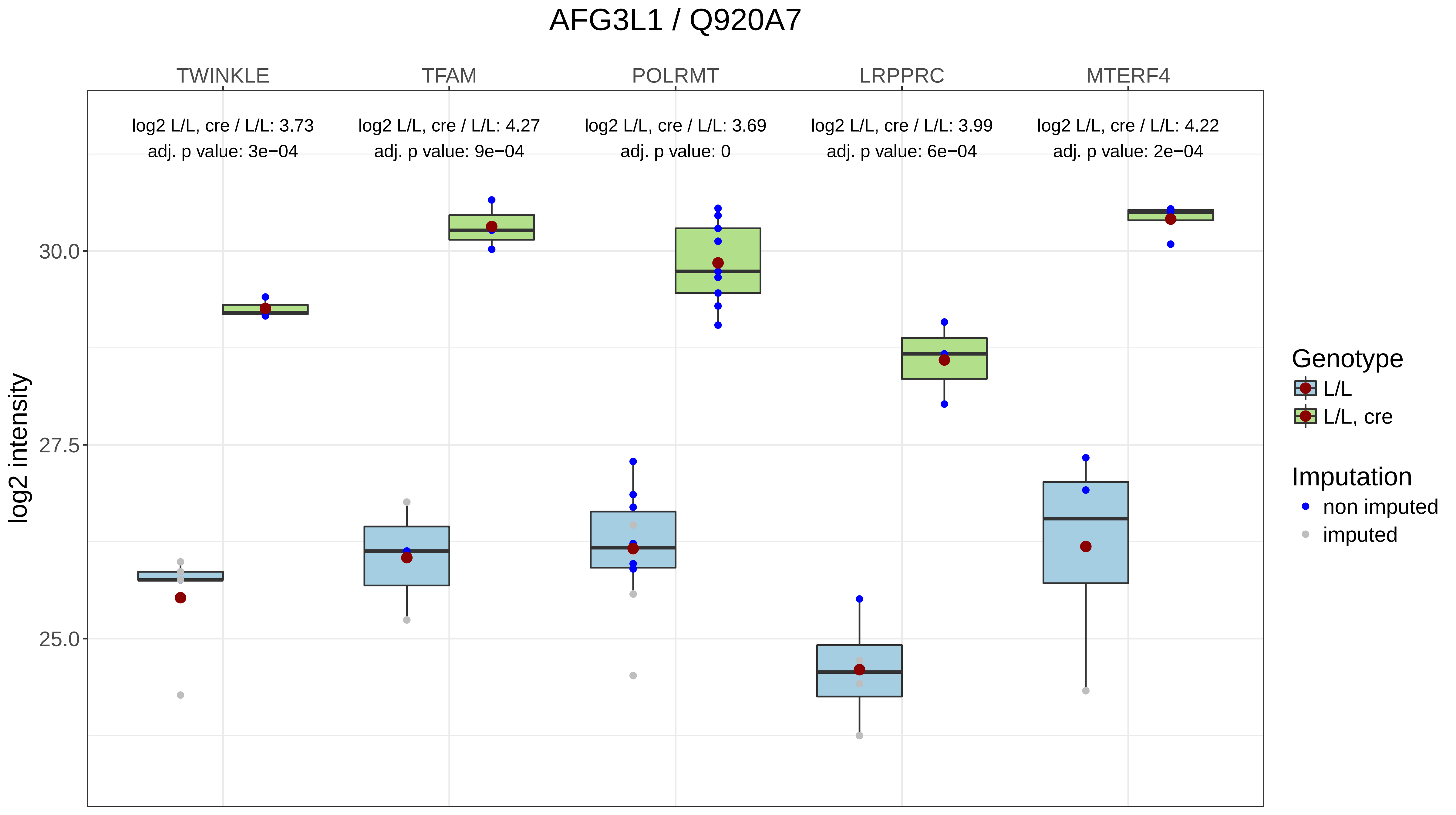
AFG3L1 / Q920A7
TWINKLE
TFAM
POLRMT
LRPPRC
MTERF4
log2 L/L, cre / L/L: 3.73
adj. p value: 3e−04
log2 L/L, cre / L/L: 4.27
adj. p value: 9e−04
log2 L/L, cre / L/L: 3.69
adj. p value: 0
log2 L/L, cre / L/L: 3.99
adj. p value: 6e−04
log2 L/L, cre / L/L: 4.22
adj. p value: 2e−04
log2 intensity
30.0
27.5
25.0
Genotype
L/L
L/L, cre
Imputation
non imputed
imputed

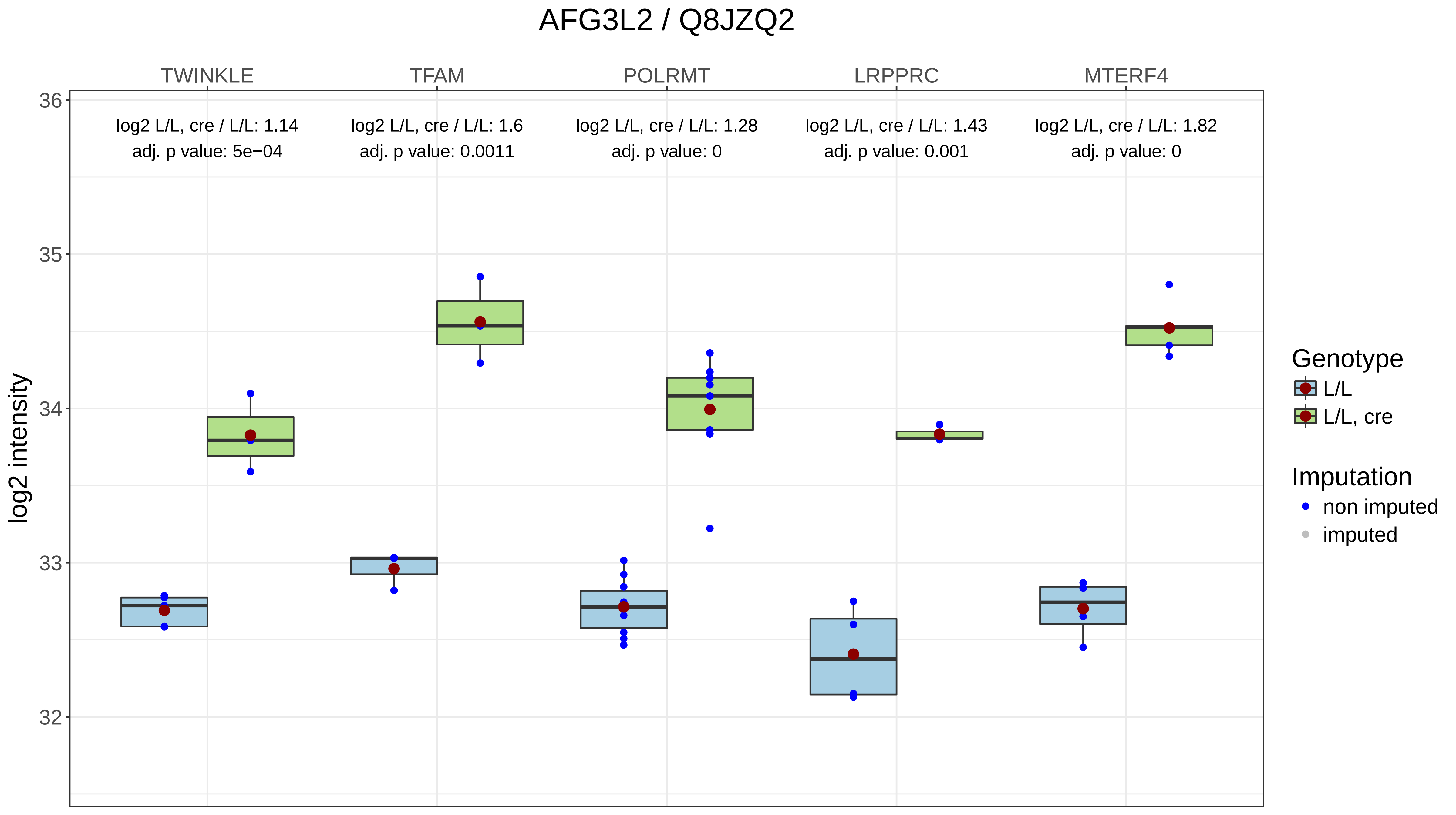
AFG3L2 / Q8JZQ2
TWINKLE
TFAM
POLRMT
LRPPRC
MTERF4
log2 L/L, cre / L/L: 1.14
adj. p value: 5e−04
log2 L/L, cre / L/L: 1.6
adj. p value: 0.0011
log2 L/L, cre / L/L: 1.28
adj. p value: 0
log2 L/L, cre / L/L: 1.43
adj. p value: 0.001
log2 L/L, cre / L/L: 1.82
adj. p value: 0
log2 intensity
36
35
34
33
32
Genotype
L/L
L/L, cre
Imputation
non imputed
imputed

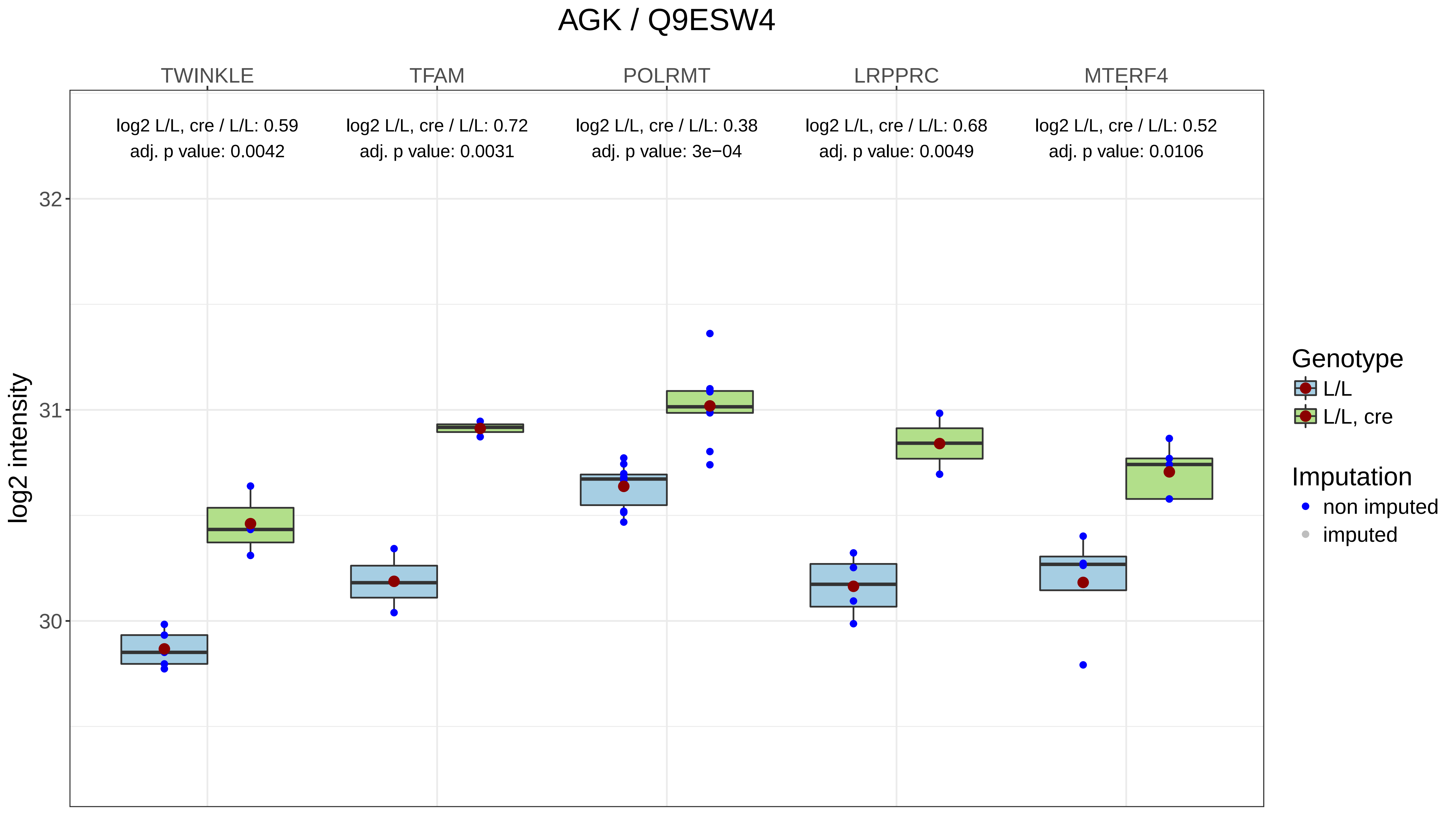

AGK / Q9ESW4
TWINKLE
TFAM
POLRMT
LRPPRC
MTERF4
log2 L/L, cre / L/L: 0.59
adj. p value: 0.0042
log2 L/L, cre / L/L: 0.72
adj. p value: 0.0031
log2 L/L, cre / L/L: 0.38
adj. p value: 3e−04
log2 L/L, cre / L/L: 0.68
adj. p value: 0.0049
log2 L/L, cre / L/L: 0.52
adj. p value: 0.0106
log2 intensity
32
31
30
Genotype
L/L
L/L, cre
Imputation
non imputed
imputed

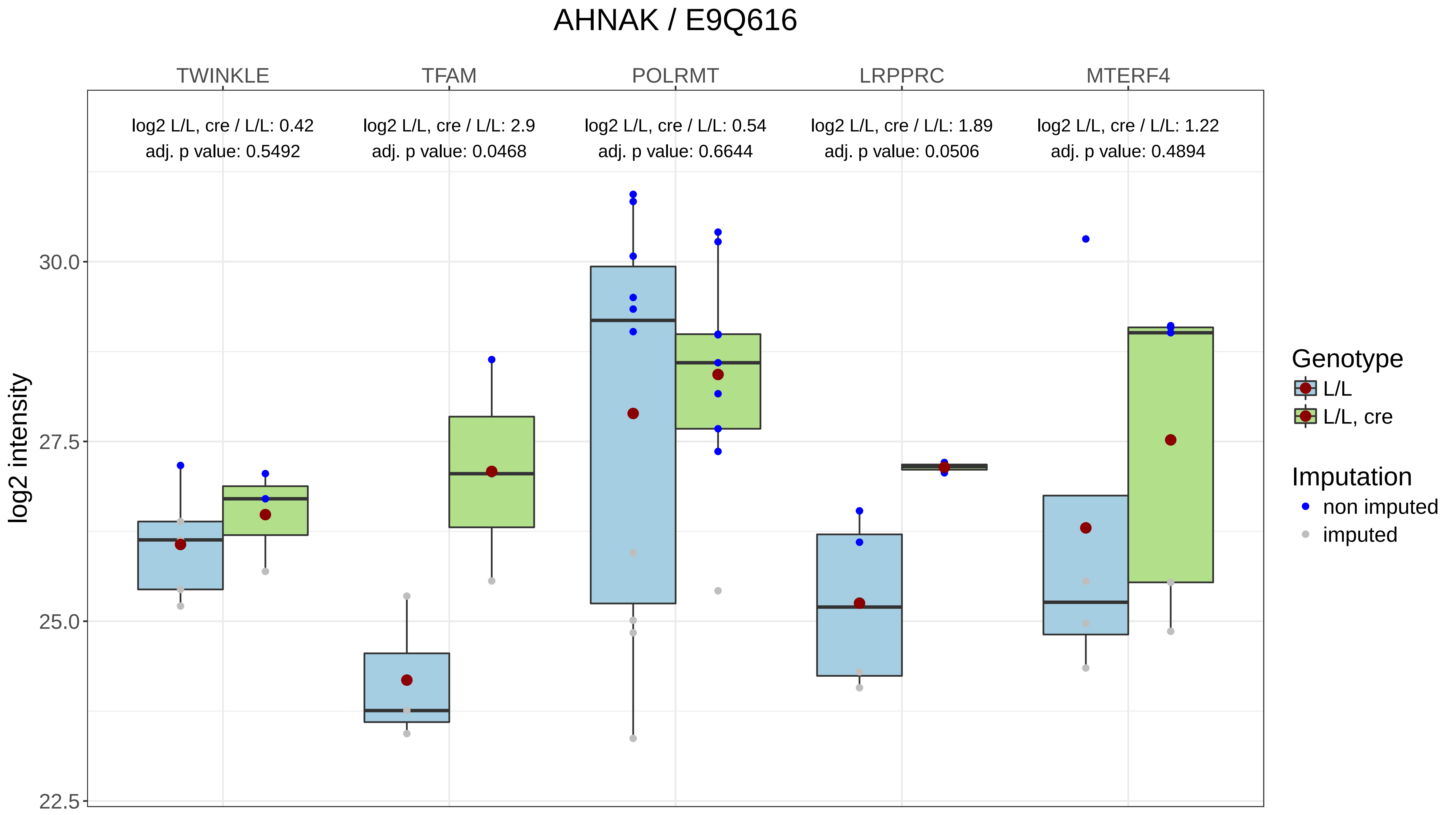
AHNAK / E9Q616
TWINKLE
TFAM
POLRMT
LRPPRC
MTERF4
log2 L/L, cre / L/L: 0.42
adj. p value: 0.5492
log2 L/L, cre / L/L: 2.9
adj. p value: 0.0468
log2 L/L, cre / L/L: 0.54
adj. p value: 0.6644
log2 L/L, cre / L/L: 1.89
adj. p value: 0.0506
log2 L/L, cre / L/L: 1.22
adj. p value: 0.4894
log2 intensity
30.0
27.5
25.0
22.5
Genotype
L/L
L/L, cre
Imputation
non imputed
imputed

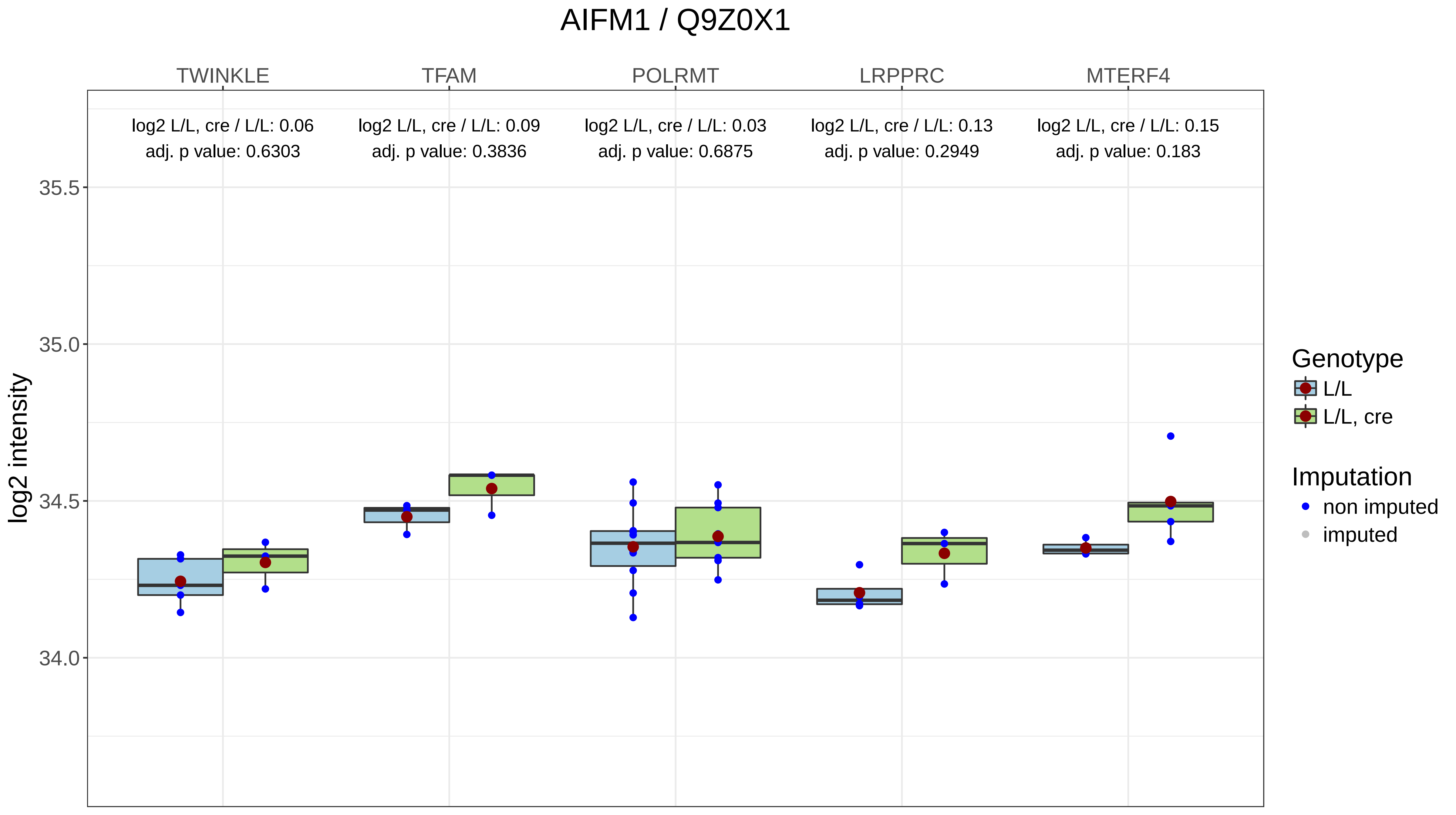

AIFM1 / Q9Z0X1
TWINKLE
TFAM
POLRMT
LRPPRC
MTERF4
log2 L/L, cre / L/L: 0.06
adj. p value: 0.6303
log2 L/L, cre / L/L: 0.09
adj. p value: 0.3836
log2 L/L, cre / L/L: 0.03
adj. p value: 0.6875
log2 L/L, cre / L/L: 0.13
adj. p value: 0.2949
log2 L/L, cre / L/L: 0.15
adj. p value: 0.183
log2 intensity
35.5
35.0
34.5
34.0
Genotype
L/L
L/L, cre
Imputation
non imputed
imputed

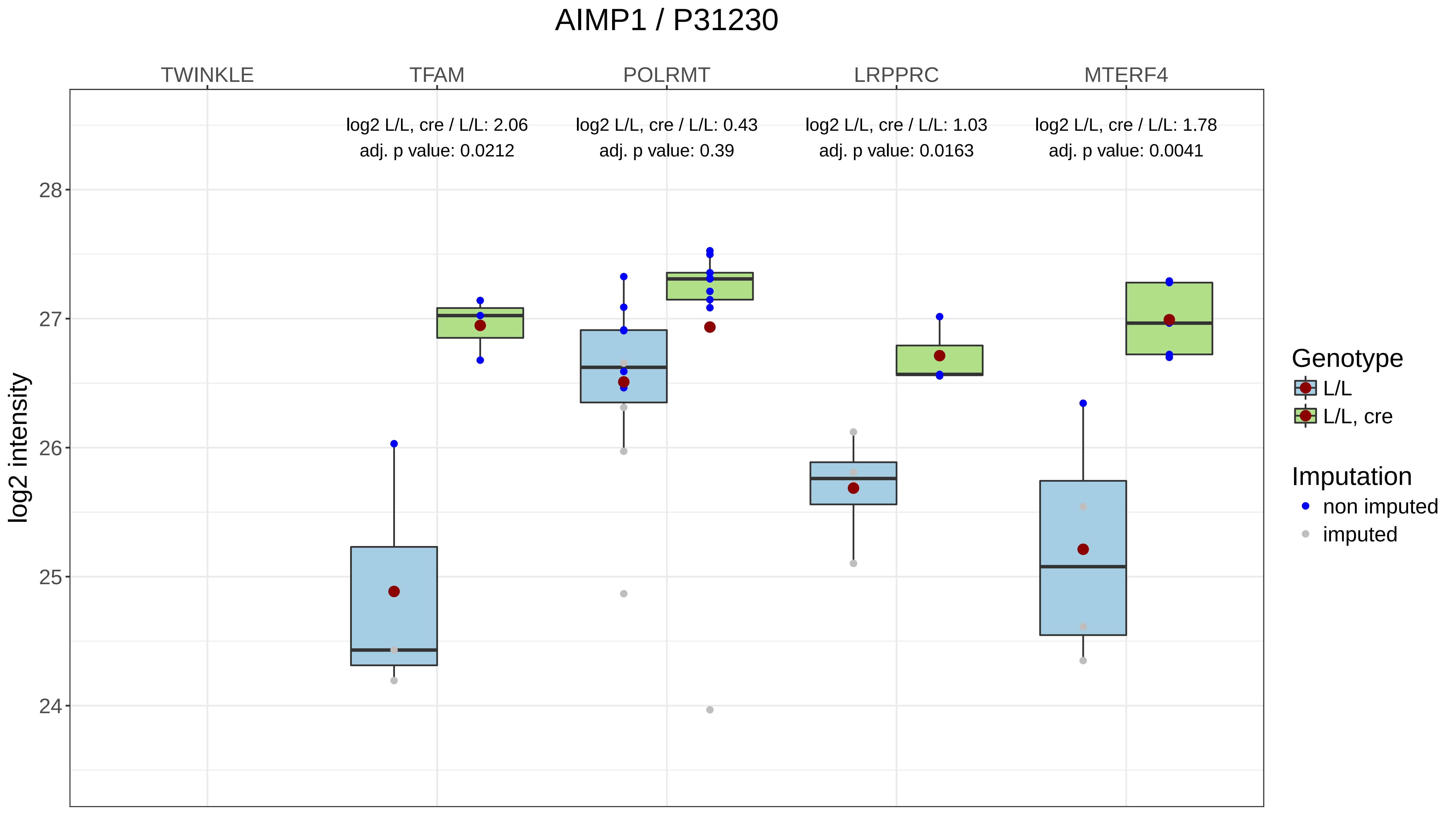

AIMP1 / P31230
TWINKLE
TFAM
POLRMT
LRPPRC
MTERF4
log2 L/L, cre / L/L: 2.06
adj. p value: 0.0212
log2 L/L, cre / L/L: 0.43
adj. p value: 0.39
log2 L/L, cre / L/L: 1.03
adj. p value: 0.0163
log2 L/L, cre / L/L: 1.78
adj. p value: 0.0041
28
27
26
25
24
log2 intensity
Genotype
L/L
L/L, cre
Imputation
non imputed
imputed

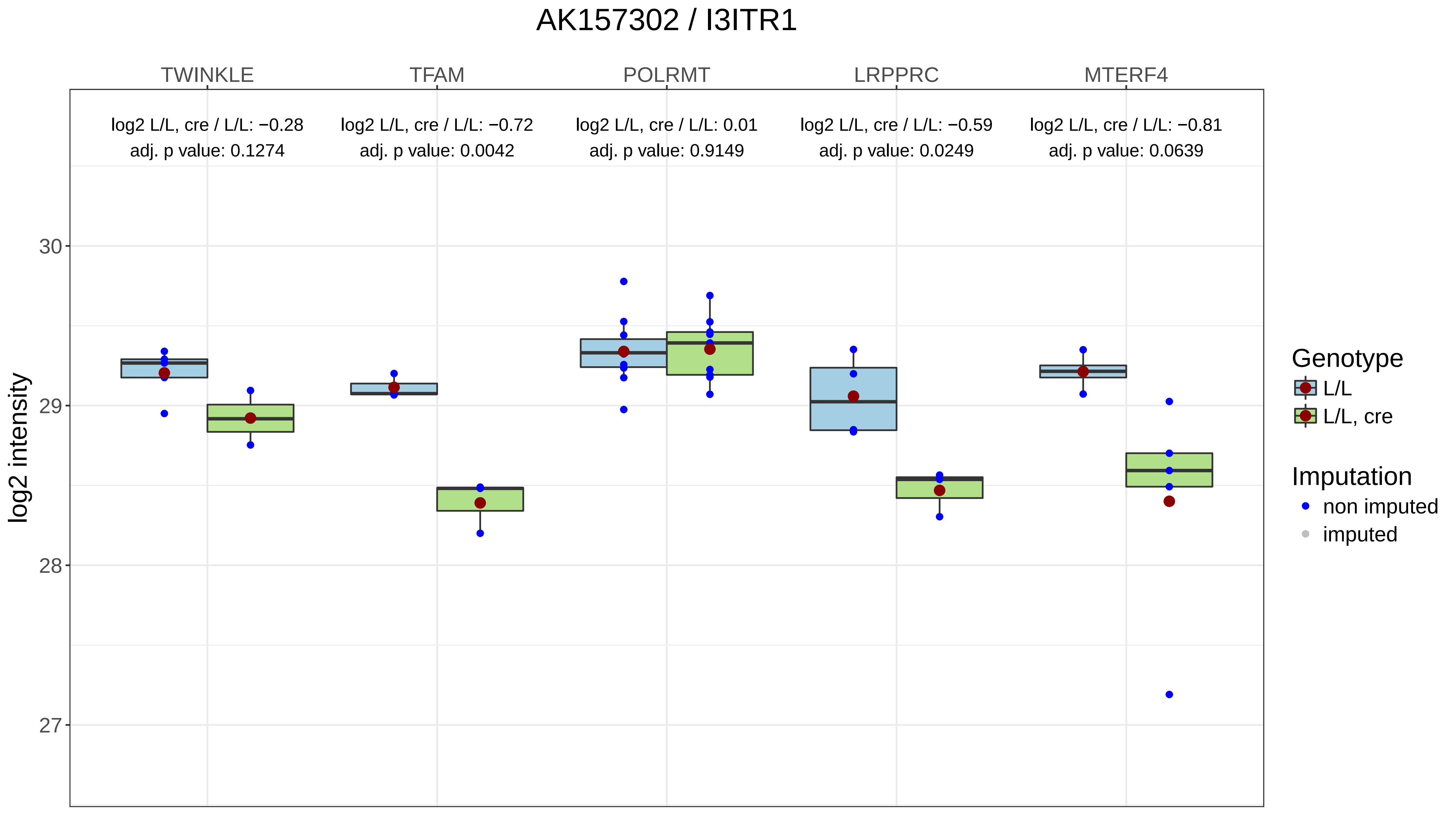
AK157302 / I3ITR1
TWINKLE
TFAM
POLRMT
LRPPRC
MTERF4
log2 L/L, cre / L/L: −0.28
adj. p value: 0.1274
log2 L/L, cre / L/L: −0.72
adj. p value: 0.0042
log2 L/L, cre / L/L: 0.01
adj. p value: 0.9149
log2 L/L, cre / L/L: −0.59
adj. p value: 0.0249
log2 L/L, cre / L/L: −0.81
adj. p value: 0.0639
log2 intensity
30
29
28
27
Genotype
L/L
L/L, cre
Imputation
non imputed
imputed

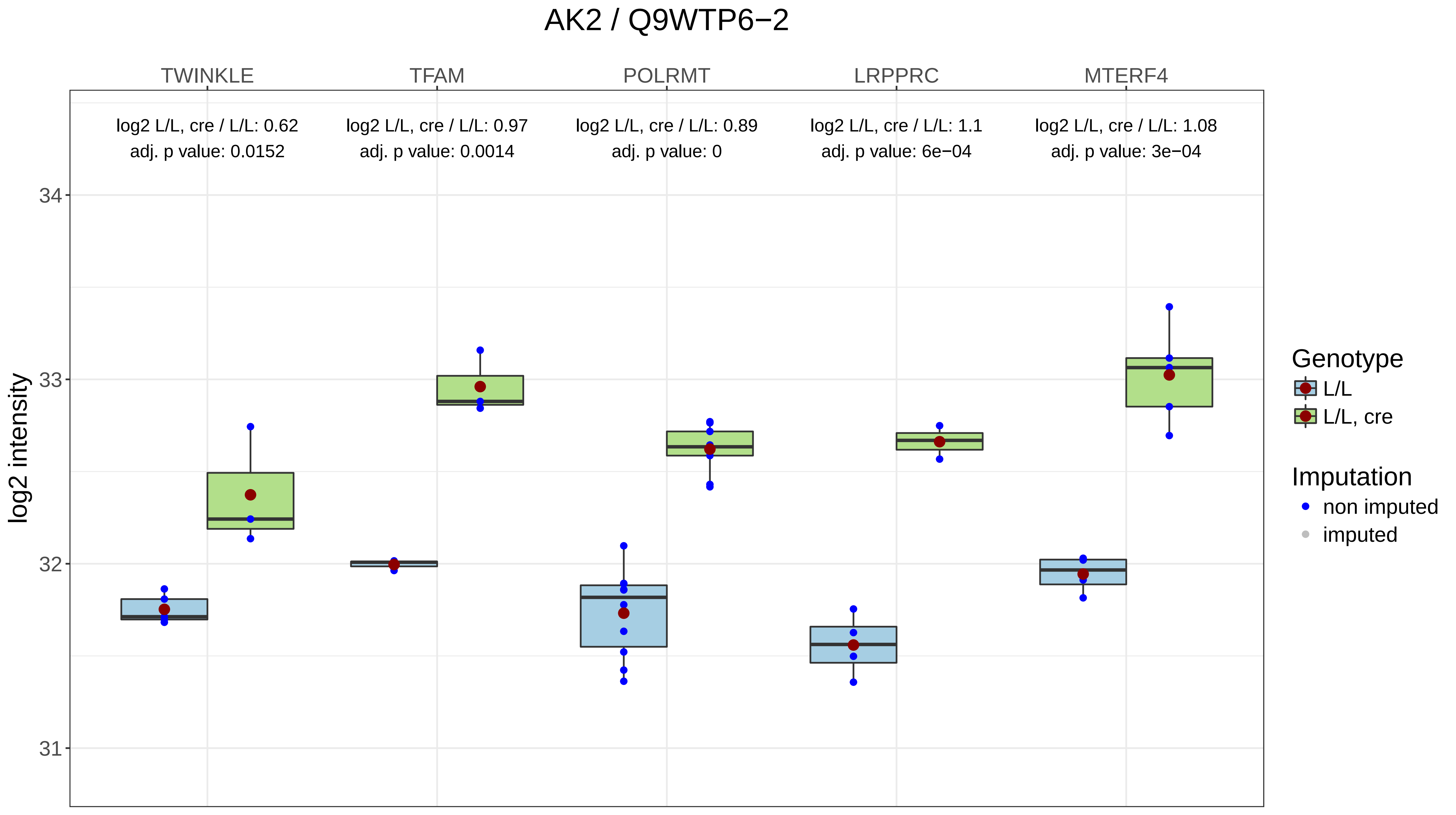
AK2 / Q9WTP6−2
TWINKLE
TFAM
POLRMT
LRPPRC
MTERF4
log2 L/L, cre / L/L: 0.62
adj. p value: 0.0152
log2 L/L, cre / L/L: 0.97
adj. p value: 0.0014
log2 L/L, cre / L/L: 0.89
adj. p value: 0
log2 L/L, cre / L/L: 1.1
adj. p value: 6e−04
log2 L/L, cre / L/L: 1.08
adj. p value: 3e−04
log2 intensity
34
33
32
31
Genotype
L/L
L/L, cre
Imputation
non imputed
imputed

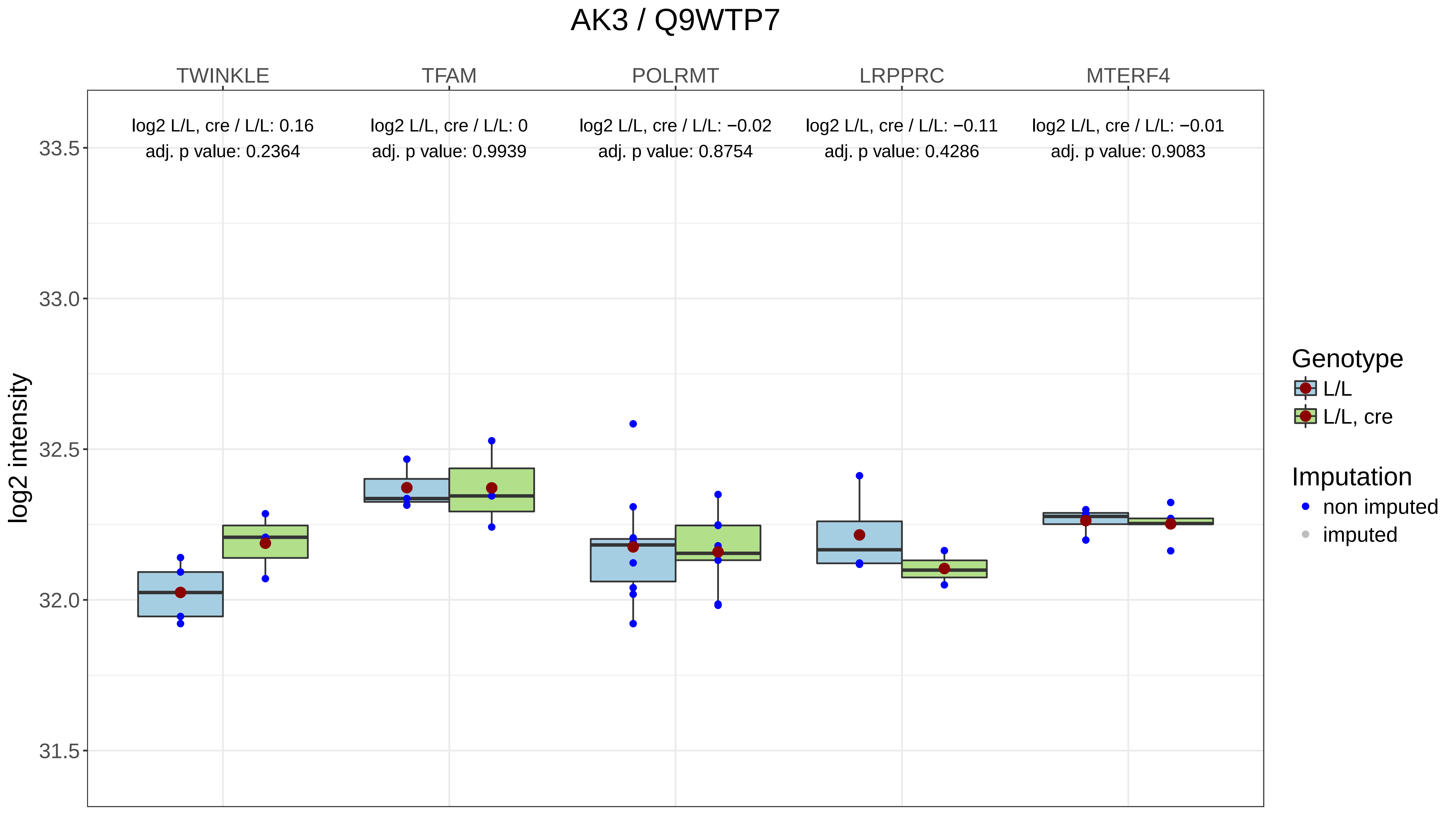
AK3 / Q9WTP7
TWINKLE
TFAM
POLRMT
LRPPRC
MTERF4
log2 L/L, cre / L/L: 0.16
adj. p value: 0.2364
log2 L/L, cre / L/L: 0
adj. p value: 0.9939
log2 L/L, cre / L/L: −0.02
adj. p value: 0.8754
log2 L/L, cre / L/L: −0.11
adj. p value: 0.4286
log2 L/L, cre / L/L: −0.01
adj. p value: 0.9083
log2 intensity
33.5
33.0
32.5
32.0
31.5
Genotype
L/L
L/L, cre
Imputation
non imputed
imputed

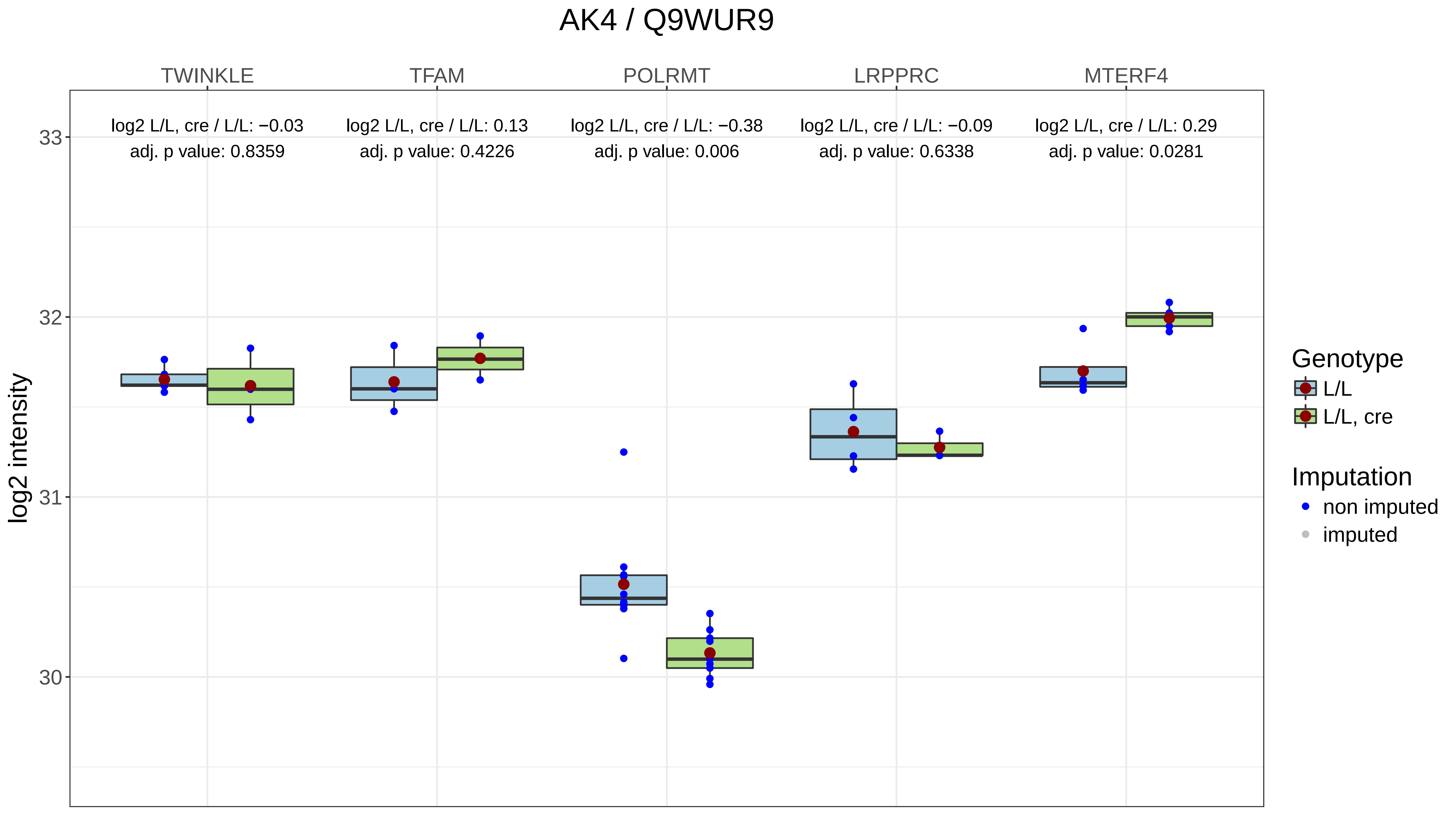
AK4 / Q9WUR9
TWINKLE
TFAM
POLRMT
LRPPRC
MTERF4
log2 L/L, cre / L/L: −0.03
adj. p value: 0.8359
log2 L/L, cre / L/L: 0.13
adj. p value: 0.4226
log2 L/L, cre / L/L: −0.38
adj. p value: 0.006
log2 L/L, cre / L/L: −0.09
adj. p value: 0.6338
log2 L/L, cre / L/L: 0.29
adj. p value: 0.0281
log2 intensity
33
32
31
30
Genotype
L/L
L/L, cre
Imputation
non imputed
imputed

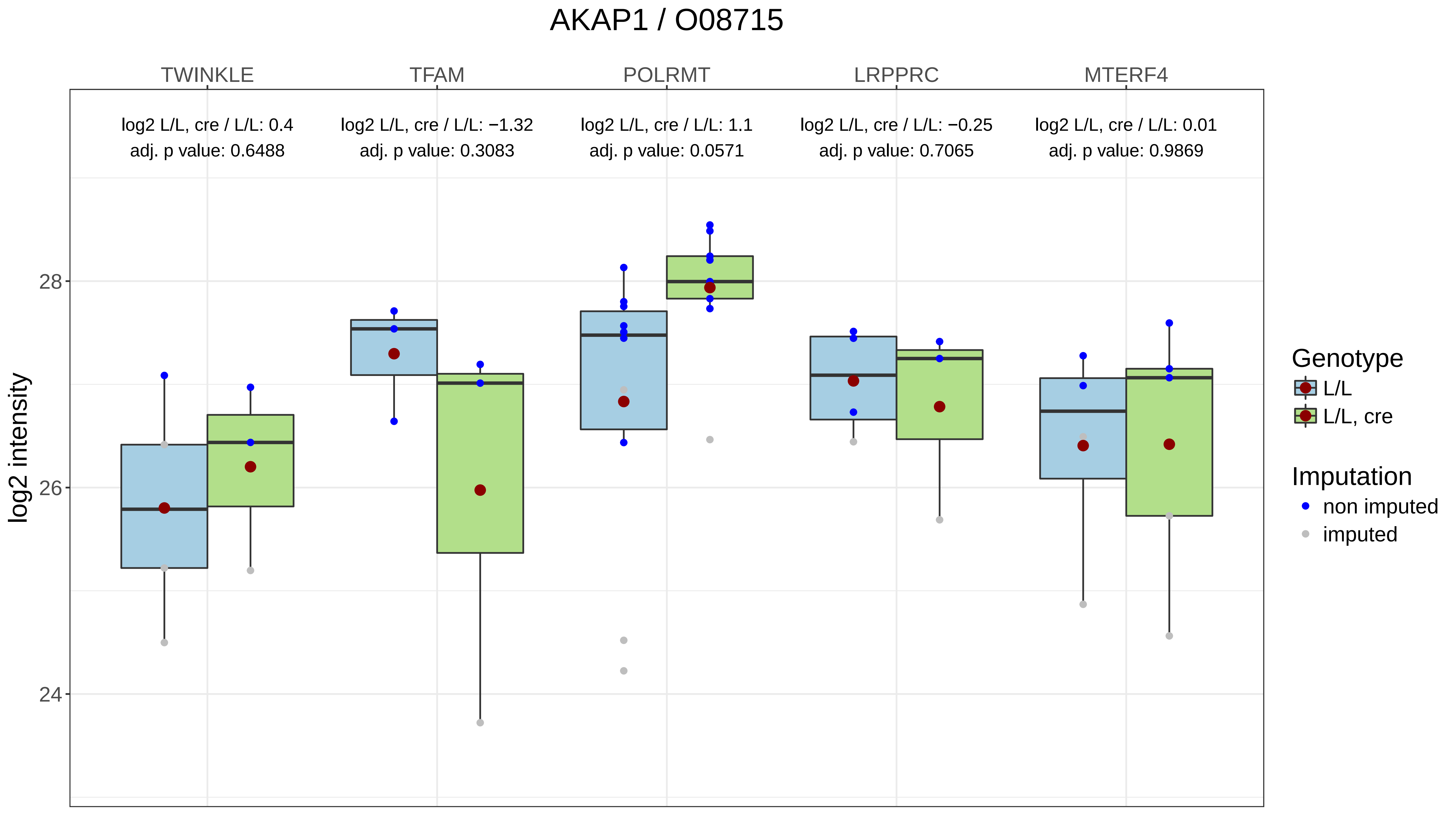
AKAP1 / O08715
TWINKLE
TFAM
POLRMT
LRPPRC
MTERF4
log2 L/L, cre / L/L: 0.4
adj. p value: 0.6488
log2 L/L, cre / L/L: −1.32
adj. p value: 0.3083
log2 L/L, cre / L/L: 1.1
adj. p value: 0.0571
log2 L/L, cre / L/L: −0.25
adj. p value: 0.7065
log2 L/L, cre / L/L: 0.01
adj. p value: 0.9869
log2 intensity
28
26
24
Genotype
L/L
L/L, cre
Imputation
non imputed
imputed

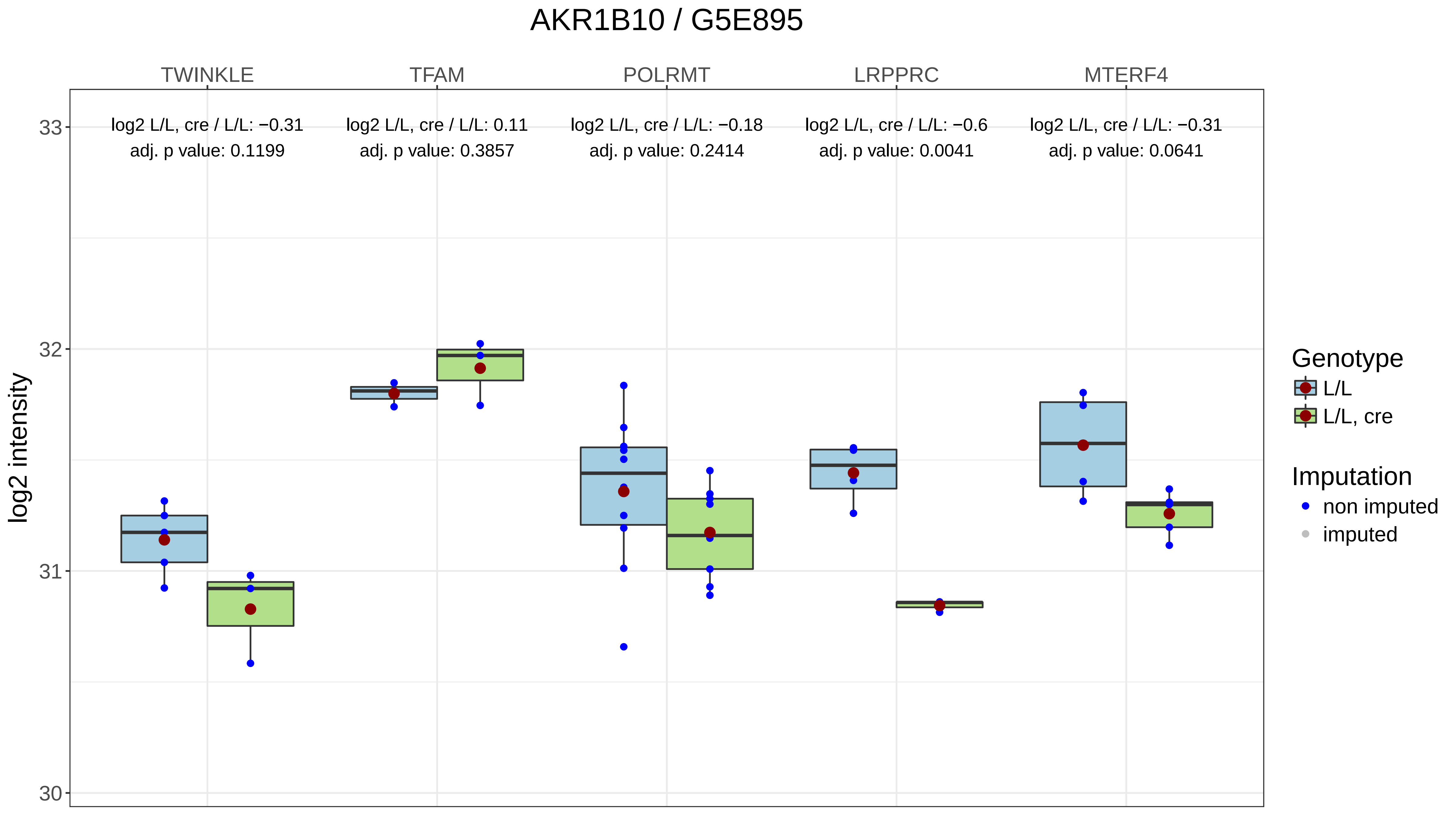
AKR1B10 / G5E895
TWINKLE
TFAM
POLRMT
LRPPRC
MTERF4
log2 L/L, cre / L/L: −0.31
adj. p value: 0.1199
log2 L/L, cre / L/L: 0.11
adj. p value: 0.3857
log2 L/L, cre / L/L: −0.18
adj. p value: 0.2414
log2 L/L, cre / L/L: −0.6
adj. p value: 0.0041
log2 L/L, cre / L/L: −0.31
adj. p value: 0.0641
log2 intensity
33
32
31
30
Genotype
L/L
L/L, cre
Imputation
non imputed
imputed

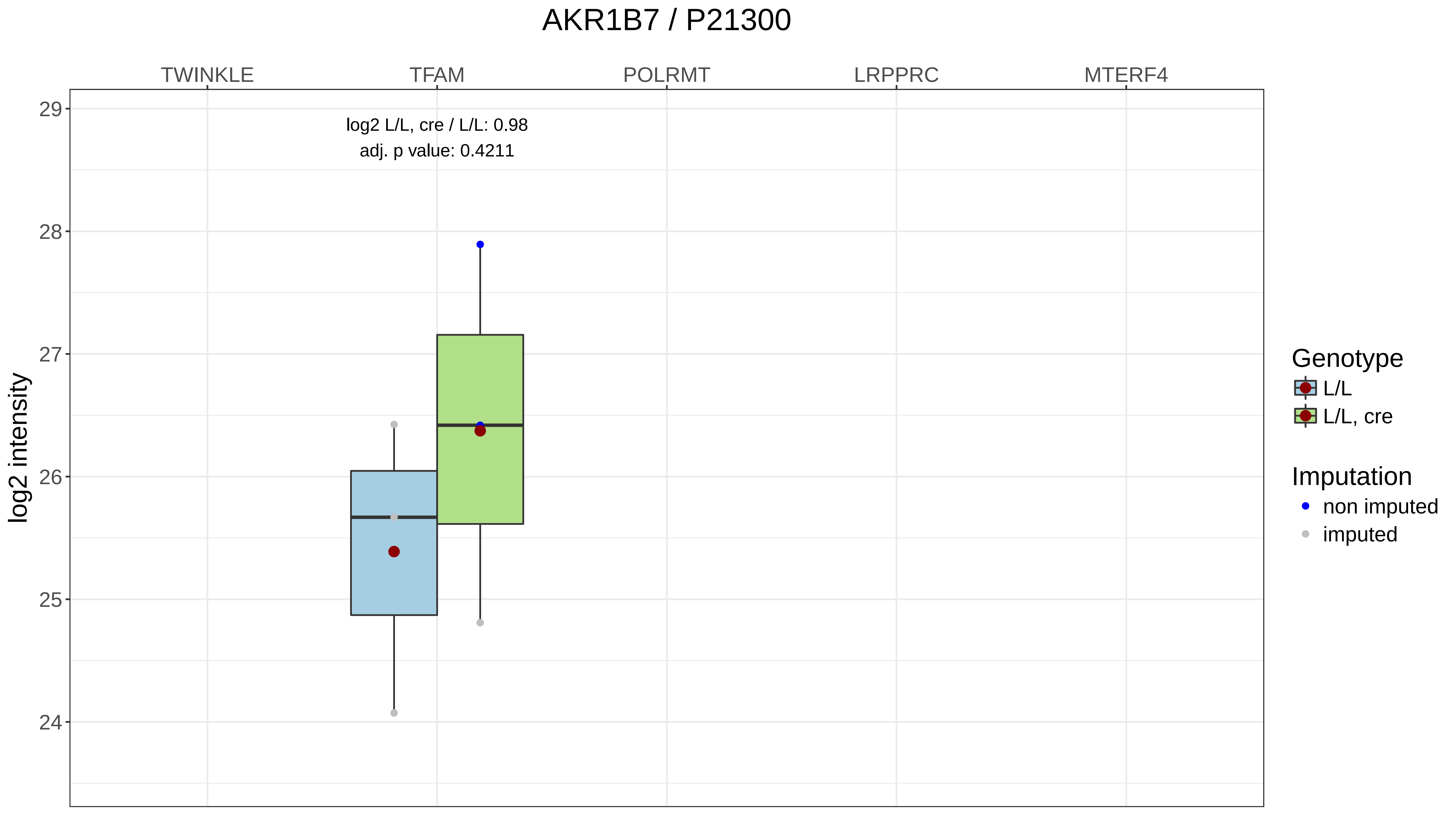

AKR1B7 / P21300
TWINKLE
TFAM
POLRMT
LRPPRC
MTERF4
log2 L/L, cre / L/L: 0.98
adj. p value: 0.4211
log2 intensity
29
28
27
26
25
24
Genotype
L/L
L/L, cre
Imputation
non imputed
imputed

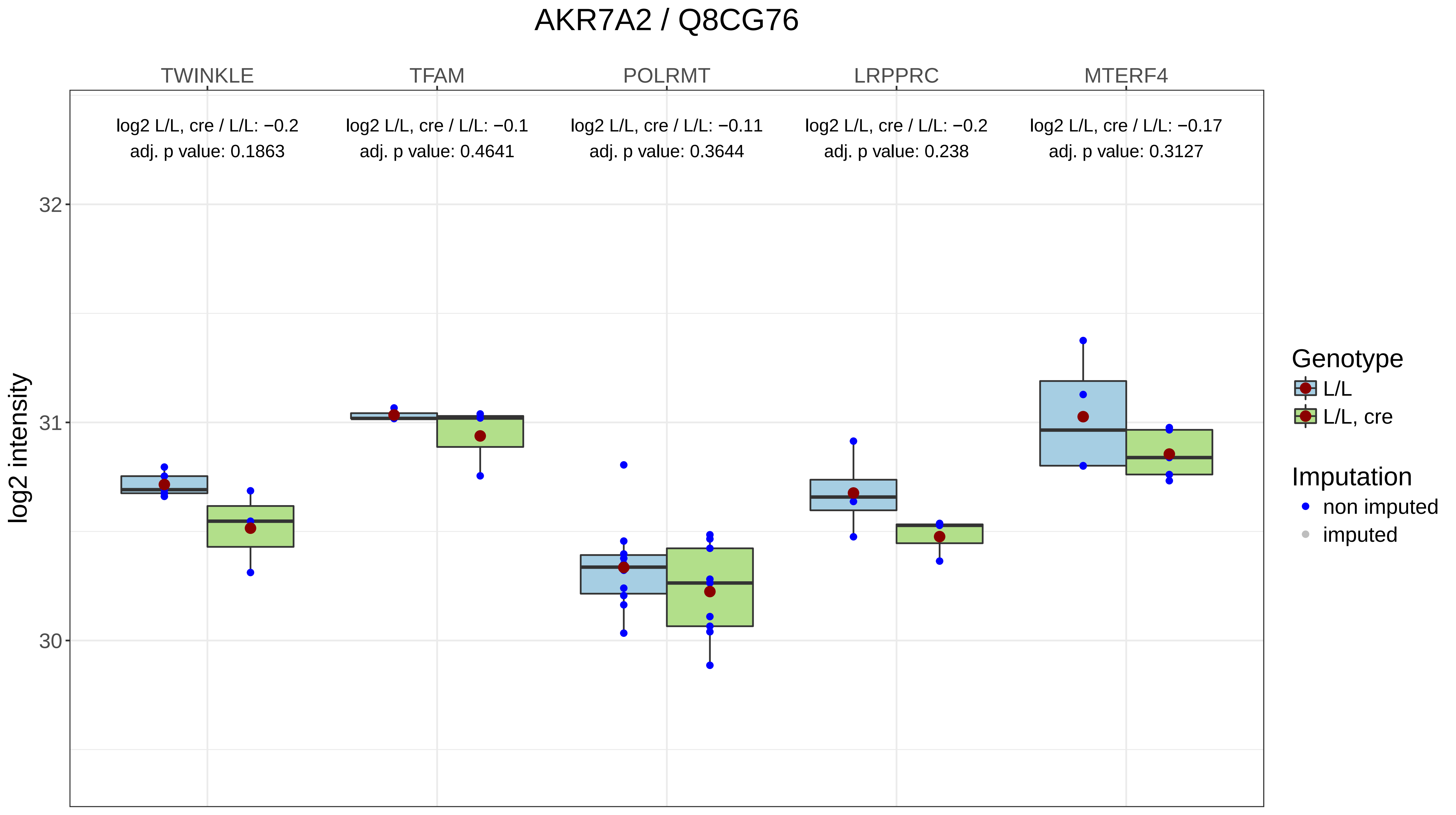
AKR7A2 / Q8CG76
TWINKLE
TFAM
POLRMT
LRPPRC
MTERF4
log2 L/L, cre / L/L: −0.2
adj. p value: 0.1863
log2 L/L, cre / L/L: −0.1
adj. p value: 0.4641
log2 L/L, cre / L/L: −0.11
adj. p value: 0.3644
log2 L/L, cre / L/L: −0.2
adj. p value: 0.238
log2 L/L, cre / L/L: −0.17
adj. p value: 0.3127
log2 intensity
32
31
30
Genotype
L/L
L/L, cre
Imputation
non imputed
imputed

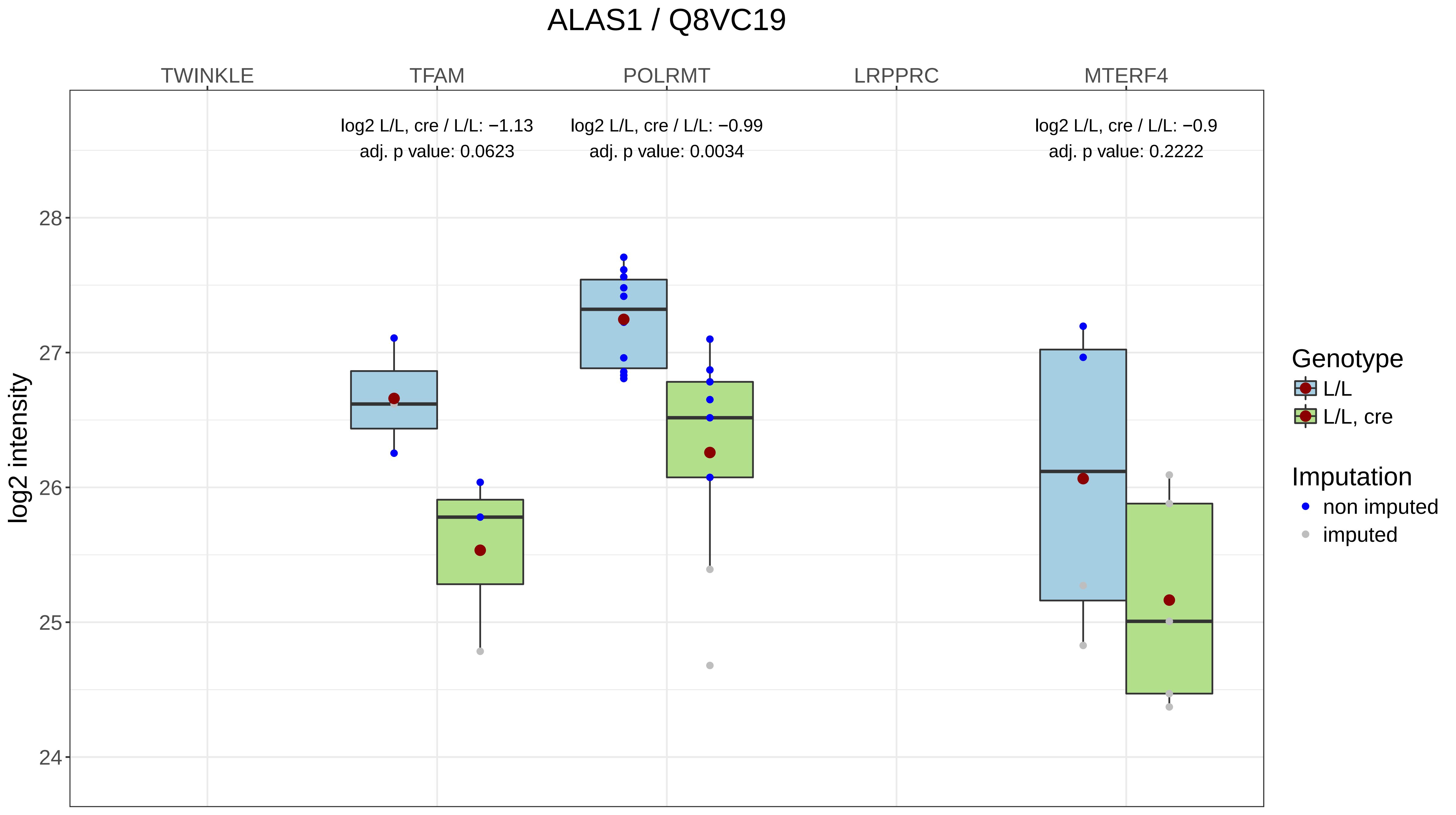
ALAS1 / Q8VC19
TWINKLE
TFAM
POLRMT
LRPPRC
MTERF4
log2 L/L, cre / L/L: −1.13
adj. p value: 0.0623
log2 L/L, cre / L/L: −0.99
adj. p value: 0.0034
log2 L/L, cre / L/L: −0.9
adj. p value: 0.2222
log2 intensity
28
27
26
25
24
Genotype
L/L
L/L, cre
Imputation
non imputed
imputed

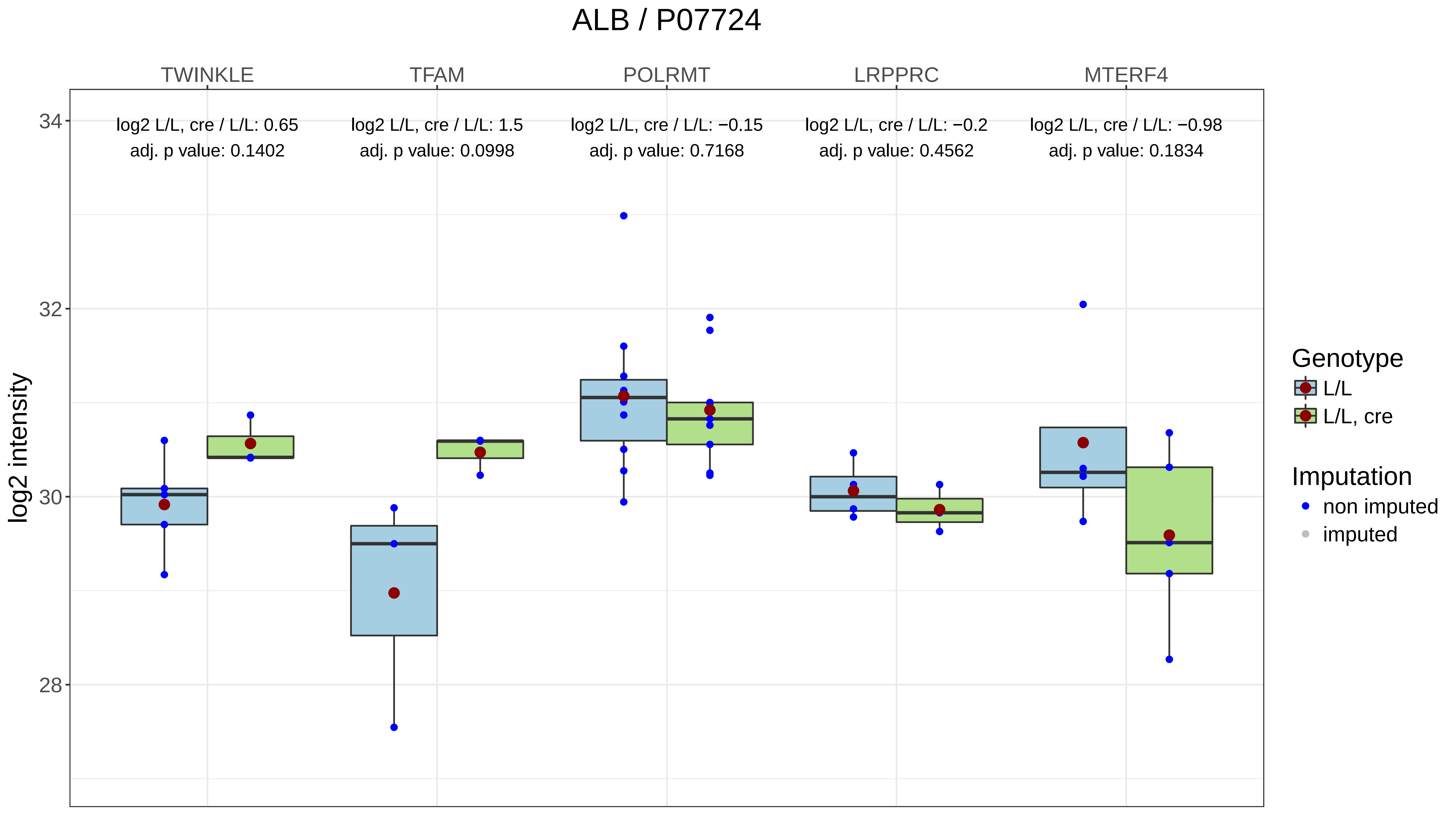
ALB / P07724
TWINKLE
TFAM
POLRMT
LRPPRC
MTERF4
log2 L/L, cre / L/L: 0.65
adj. p value: 0.1402
log2 L/L, cre / L/L: 1.5
adj. p value: 0.0998
log2 L/L, cre / L/L: −0.15
adj. p value: 0.7168
log2 L/L, cre / L/L: −0.2
adj. p value: 0.4562
log2 L/L, cre / L/L: −0.98
adj. p value: 0.1834
log2 intensity
34
32
30
28
Genotype
L/L
L/L, cre
Imputation
non imputed
imputed

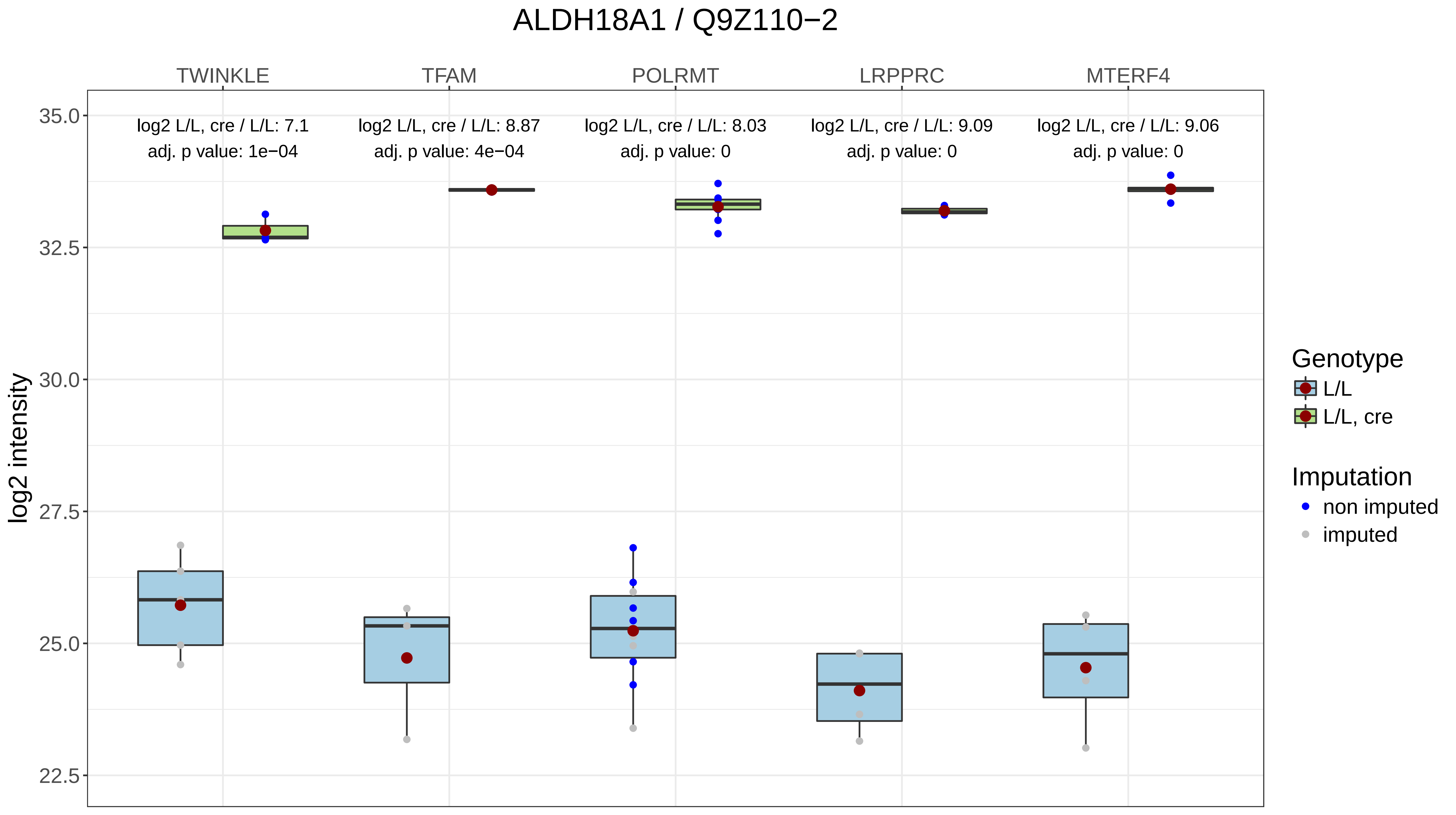
ALDH18A1 / Q9Z110−2
TWINKLE
TFAM
POLRMT
LRPPRC
MTERF4
log2 L/L, cre / L/L: 7.1
adj. p value: 1e−04
log2 L/L, cre / L/L: 8.87
adj. p value: 4e−04
log2 L/L, cre / L/L: 8.03
adj. p value: 0
log2 L/L, cre / L/L: 9.09
adj. p value: 0
log2 L/L, cre / L/L: 9.06
adj. p value: 0
log2 intensity
35.0
32.5
30.0
27.5
25.0
22.5
Genotype
L/L
L/L, cre
Imputation
non imputed
imputed

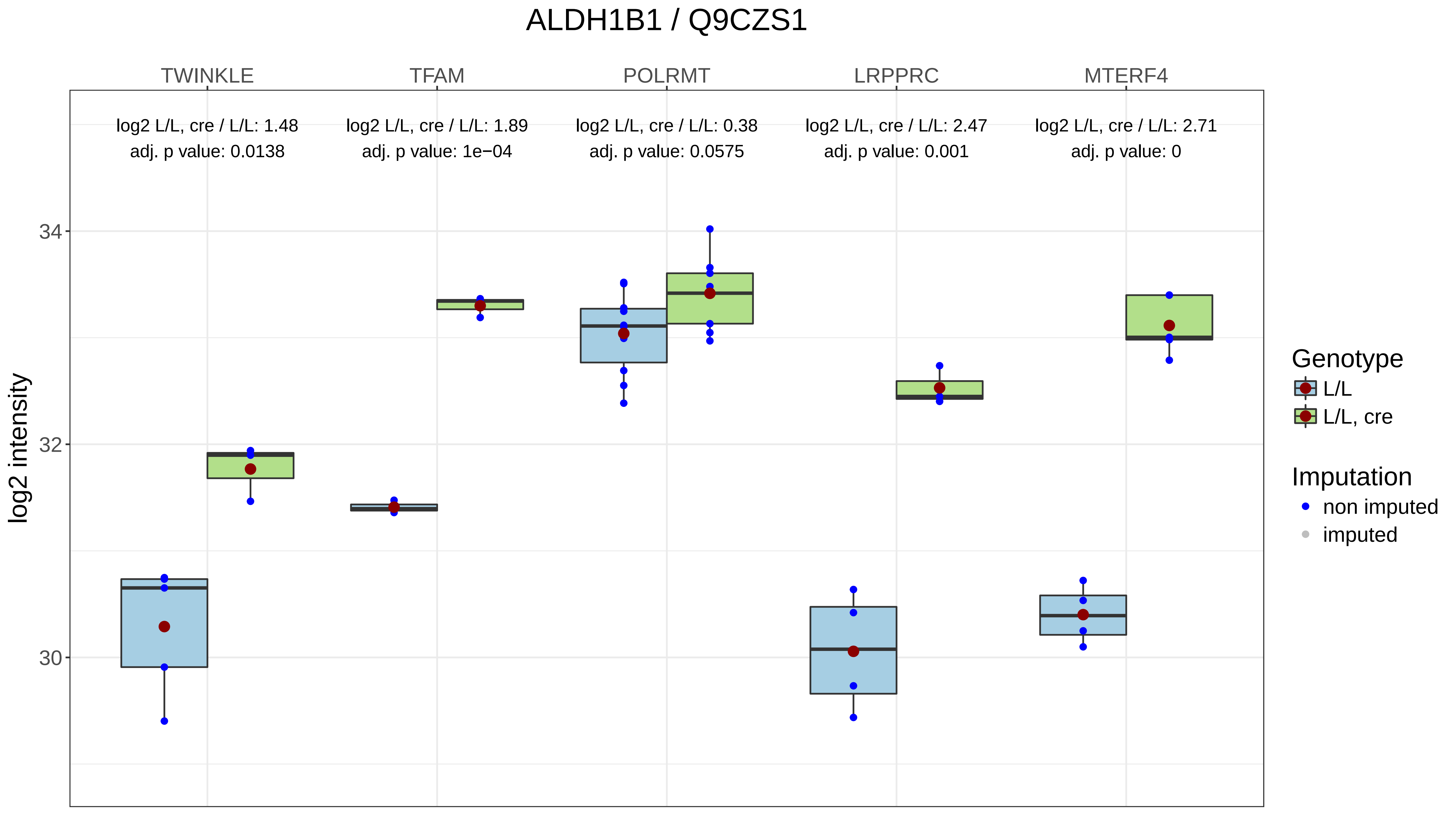
ALDH1B1 / Q9CZS1
TWINKLE
TFAM
POLRMT
LRPPRC
MTERF4
log2 L/L, cre / L/L: 1.48
adj. p value: 0.0138
log2 L/L, cre / L/L: 1.89
adj. p value: 1e−04
log2 L/L, cre / L/L: 0.38
adj. p value: 0.0575
log2 L/L, cre / L/L: 2.47
adj. p value: 0.001
log2 L/L, cre / L/L: 2.71
adj. p value: 0
34
32
30
log2 intensity
Genotype
L/L
L/L, cre
Imputation
non imputed
imputed

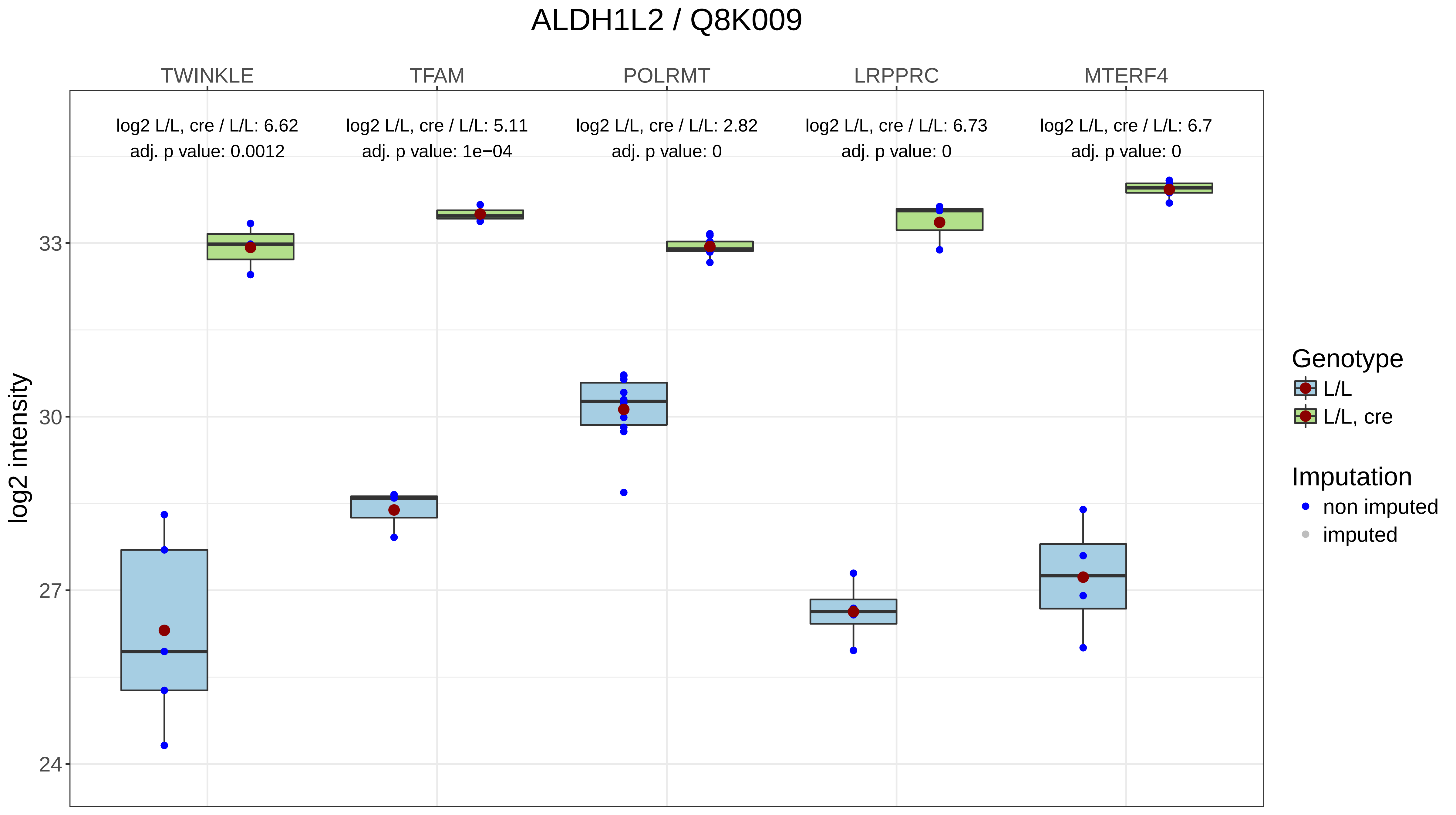
ALDH1L2 / Q8K009
TWINKLE
TFAM
POLRMT
LRPPRC
MTERF4
log2 L/L, cre / L/L: 6.62
adj. p value: 0.0012
log2 L/L, cre / L/L: 5.11
adj. p value: 1e−04
log2 L/L, cre / L/L: 2.82
adj. p value: 0
log2 L/L, cre / L/L: 6.73
adj. p value: 0
log2 L/L, cre / L/L: 6.7
adj. p value: 0
log2 intensity
33
30
27
24
Genotype
L/L
L/L, cre
Imputation
non imputed
imputed

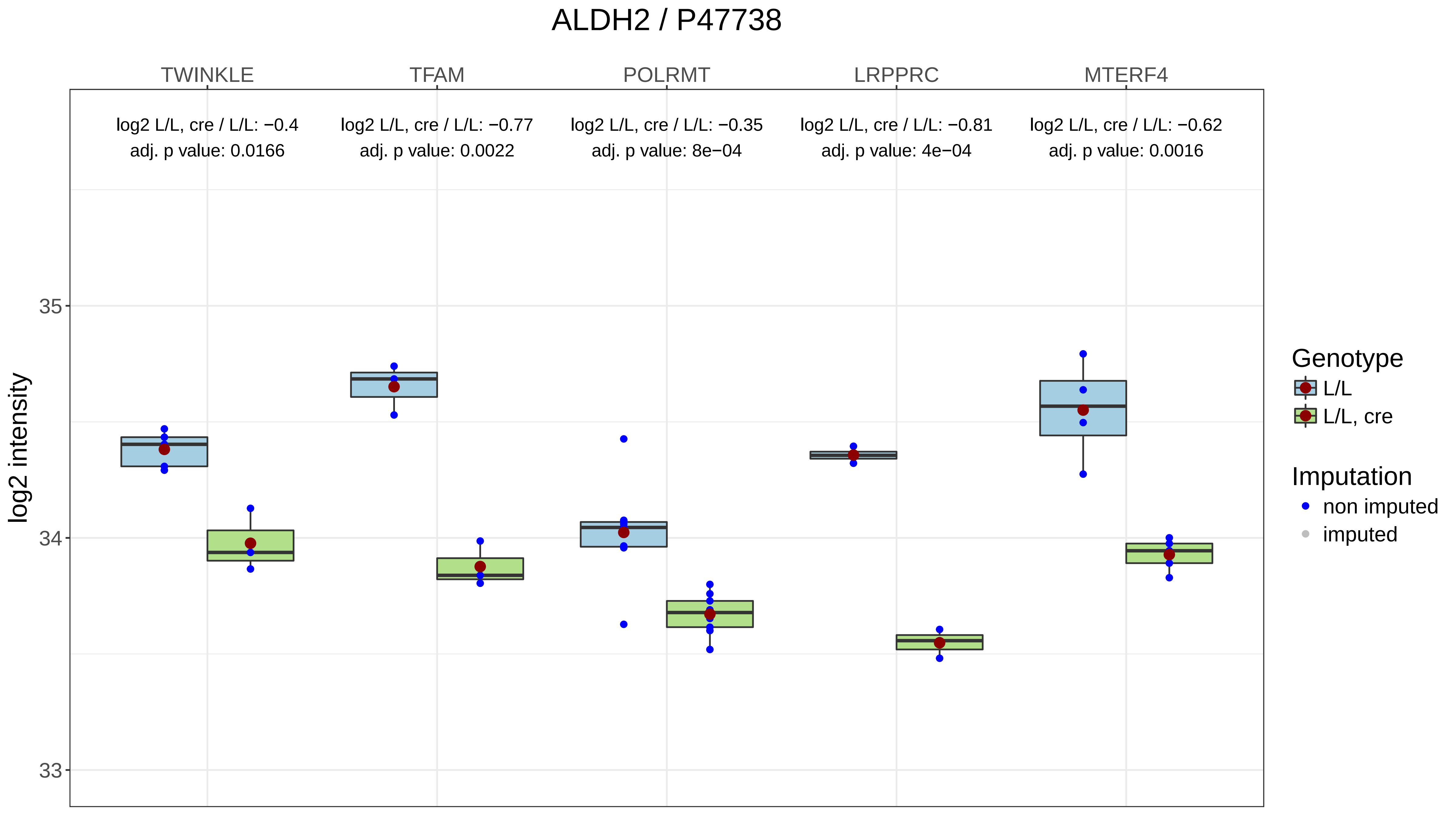

ALDH2 / P47738
TWINKLE
TFAM
POLRMT
LRPPRC
MTERF4
log2 L/L, cre / L/L: −0.4
adj. p value: 0.0166
log2 L/L, cre / L/L: −0.77
adj. p value: 0.0022
log2 L/L, cre / L/L: −0.35
adj. p value: 8e−04
log2 L/L, cre / L/L: −0.81
adj. p value: 4e−04
log2 L/L, cre / L/L: −0.62
adj. p value: 0.0016
log2 intensity
35
34
33
Genotype
L/L
L/L, cre
Imputation
non imputed
imputed

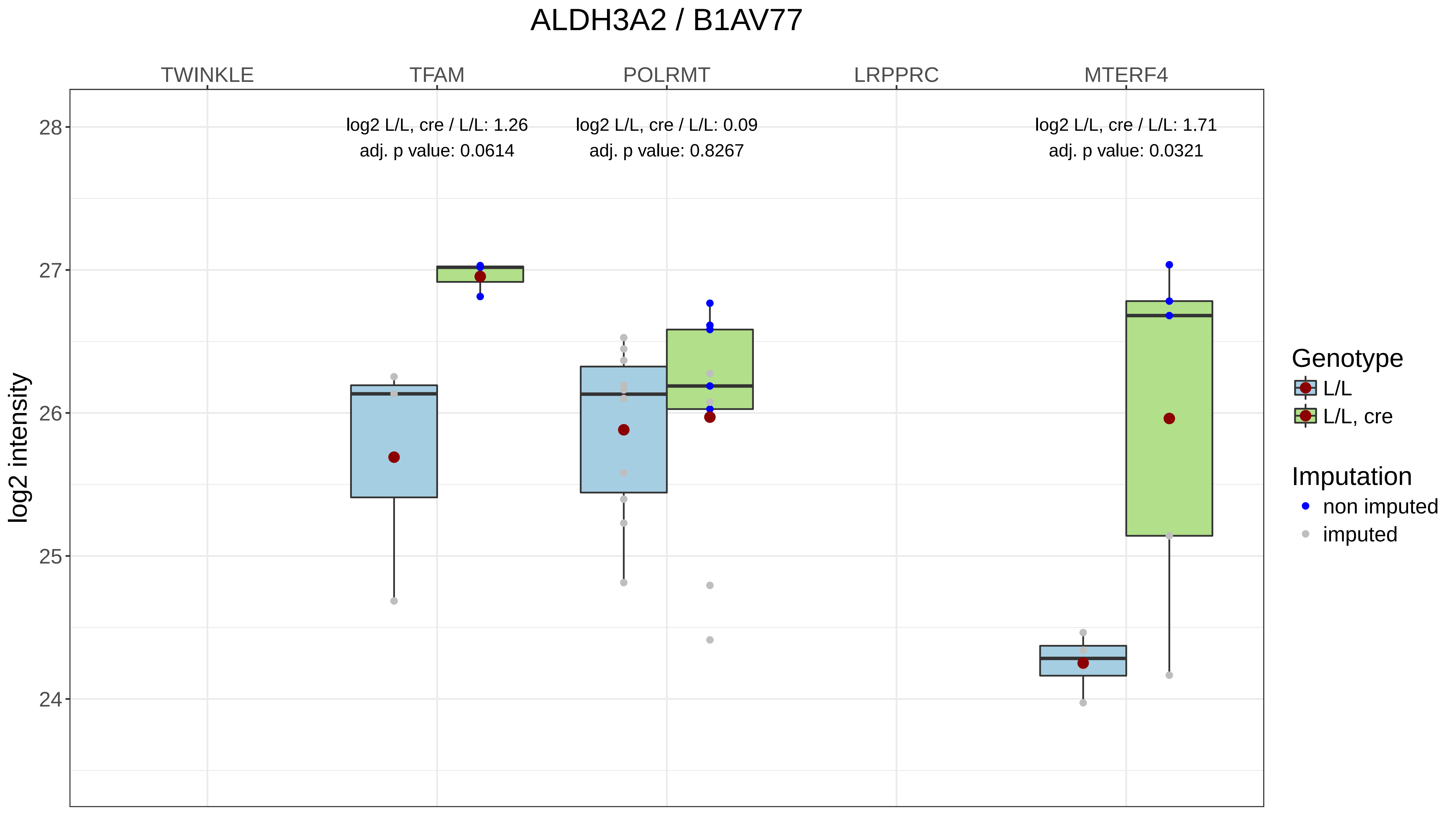

ALDH3A2 / B1AV77
TWINKLE
TFAM
POLRMT
LRPPRC
MTERF4
log2 L/L, cre / L/L: 1.26
adj. p value: 0.0614
log2 L/L, cre / L/L: 0.09
adj. p value: 0.8267
log2 L/L, cre / L/L: 1.71
adj. p value: 0.0321
28
27
26
25
24
log2 intensity
Genotype
L/L
L/L, cre
Imputation
non imputed
imputed

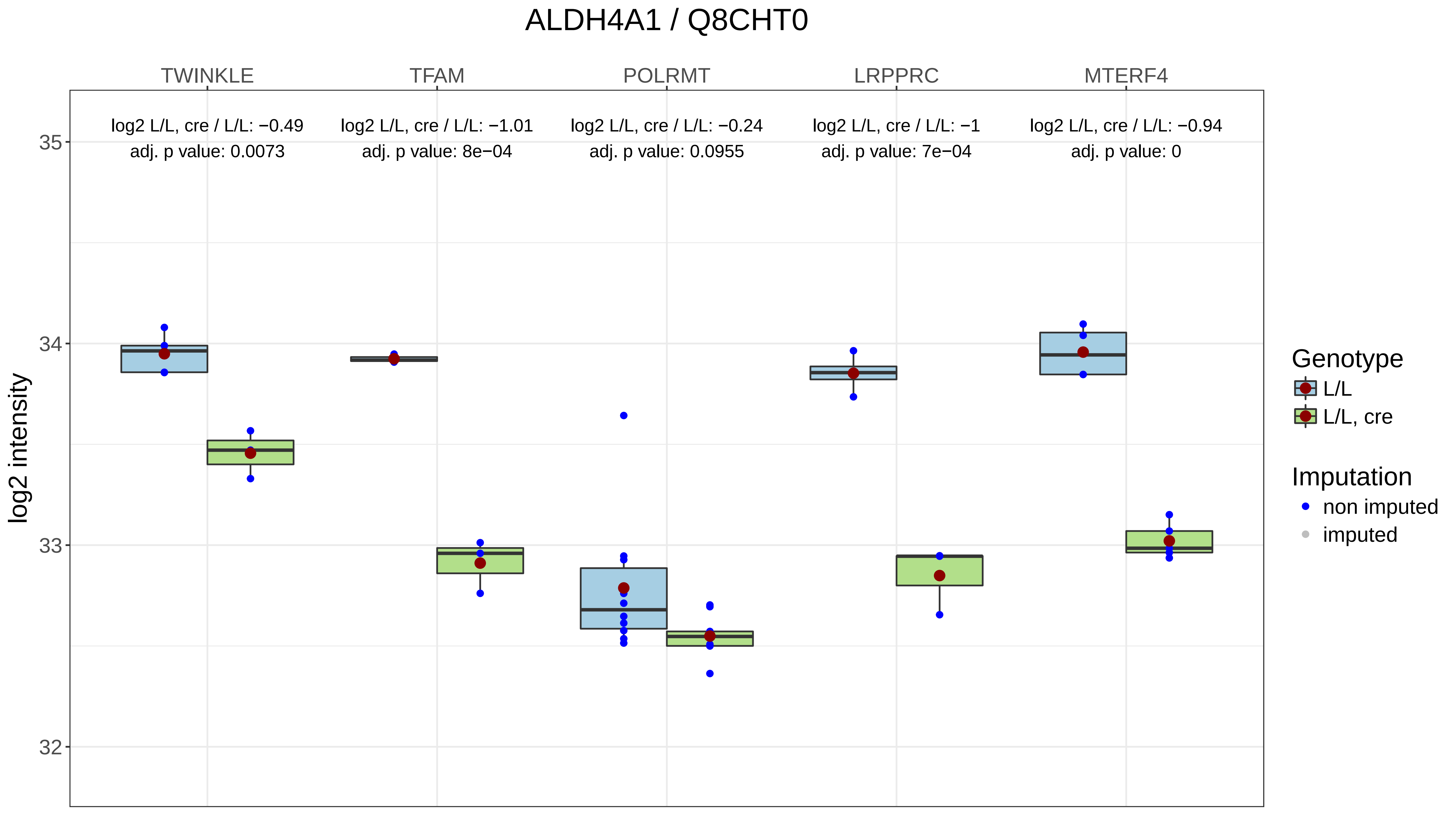

ALDH4A1 / Q8CHT0
TWINKLE
TFAM
POLRMT
LRPPRC
MTERF4
log2 L/L, cre / L/L: −0.49
adj. p value: 0.0073
log2 L/L, cre / L/L: −1.01
adj. p value: 8e−04
log2 L/L, cre / L/L: −0.24
adj. p value: 0.0955
log2 L/L, cre / L/L: −1
adj. p value: 7e−04
log2 L/L, cre / L/L: −0.94
adj. p value: 0
log2 intensity
35
34
33
32
Genotype
L/L
L/L, cre
Imputation
non imputed
imputed

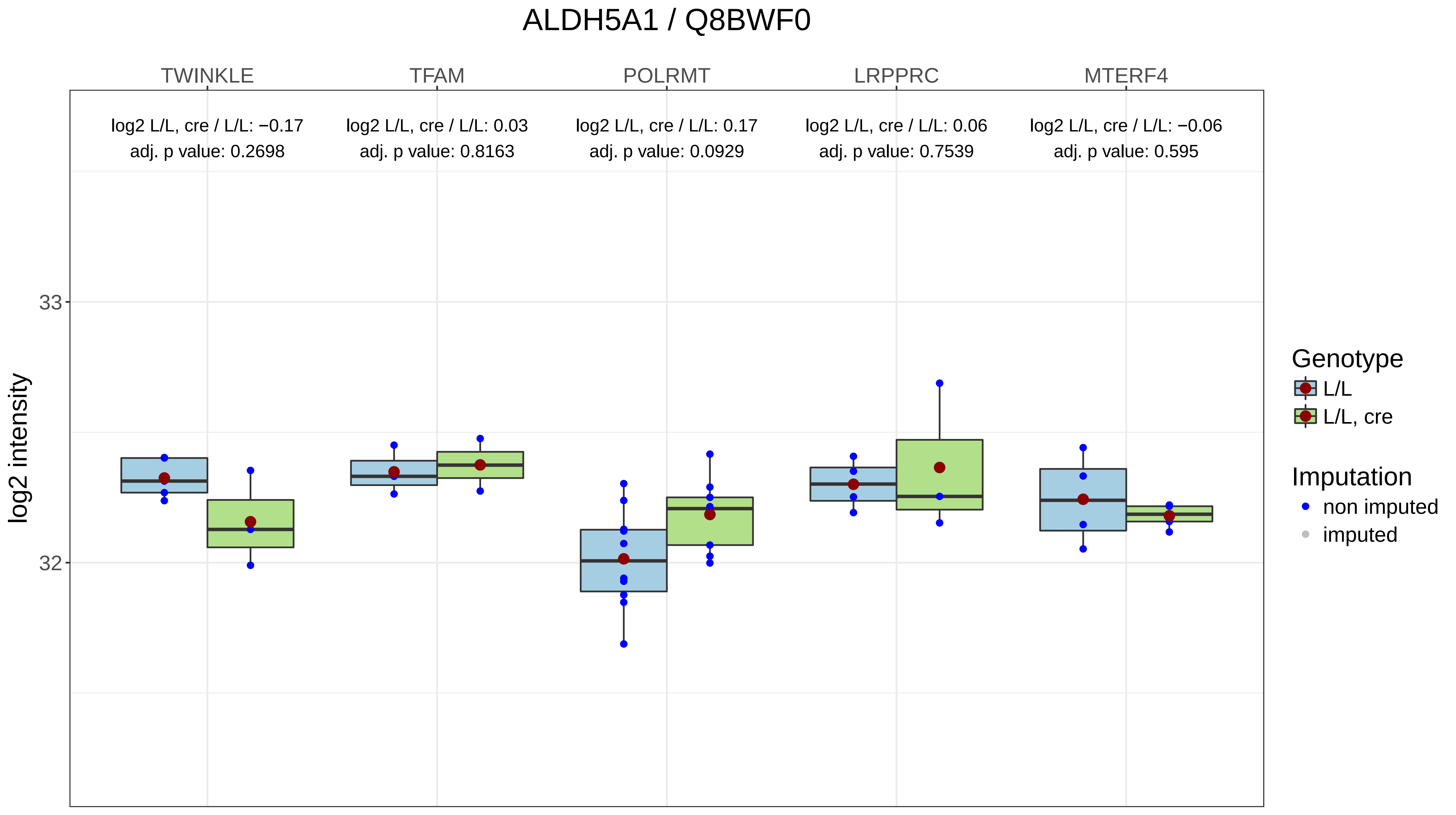
ALDH5A1 / Q8BWF0
TWINKLE
TFAM
POLRMT
LRPPRC
MTERF4
log2 L/L, cre / L/L: −0.17
adj. p value: 0.2698
log2 L/L, cre / L/L: 0.03
adj. p value: 0.8163
log2 L/L, cre / L/L: 0.17
adj. p value: 0.0929
log2 L/L, cre / L/L: 0.06
adj. p value: 0.7539
log2 L/L, cre / L/L: −0.06
adj. p value: 0.595
log2 intensity
33
32
Genotype
L/L
L/L, cre
Imputation
non imputed
imputed

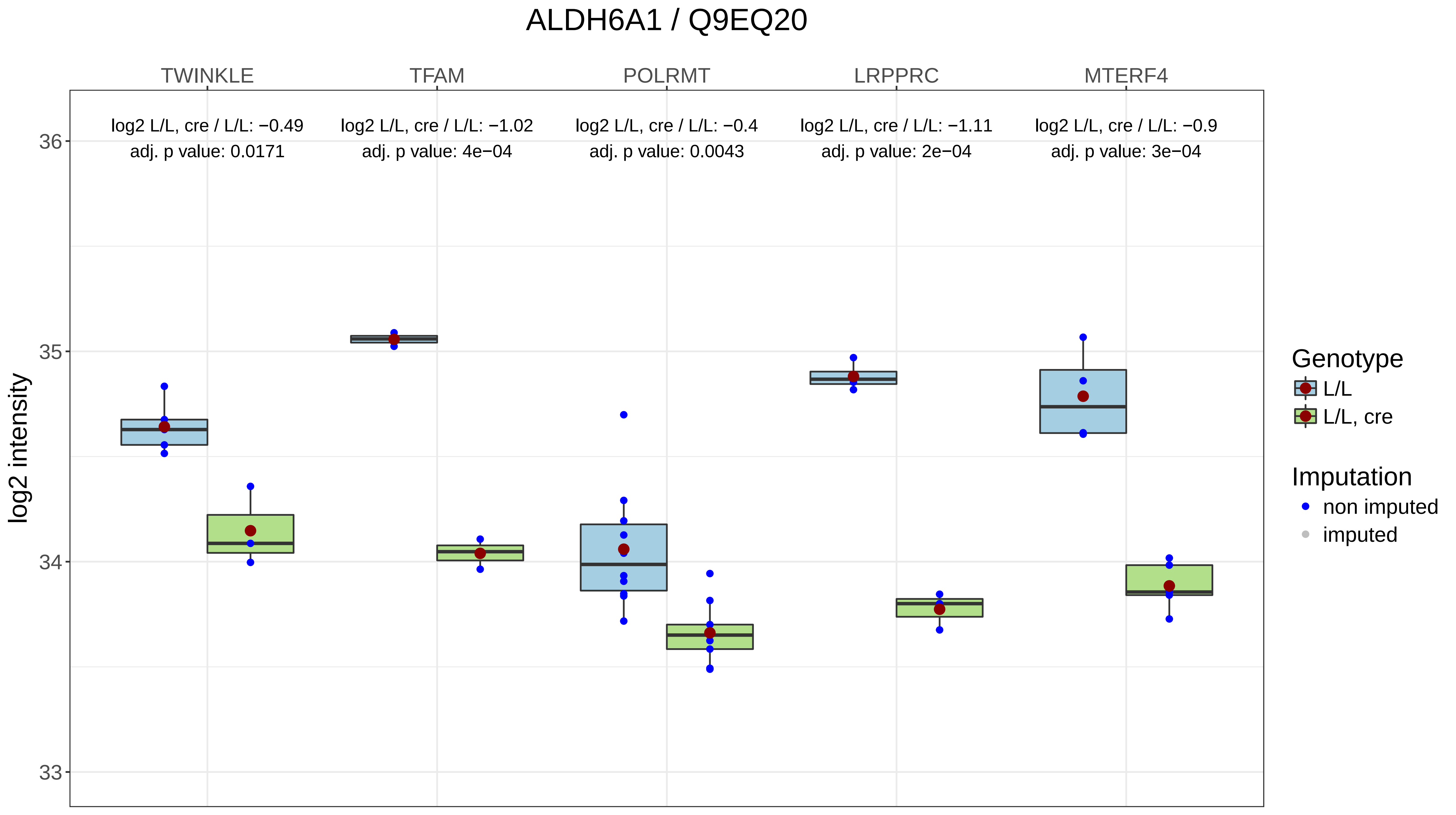

ALDH6A1 / Q9EQ20
TWINKLE
TFAM
POLRMT
LRPPRC
MTERF4
log2 L/L, cre / L/L: −0.49
adj. p value: 0.0171
log2 L/L, cre / L/L: −1.02
adj. p value: 4e−04
log2 L/L, cre / L/L: −0.4
adj. p value: 0.0043
log2 L/L, cre / L/L: −1.11
adj. p value: 2e−04
log2 L/L, cre / L/L: −0.9
adj. p value: 3e−04
log2 intensity
36
35
34
33
Genotype
L/L
L/L, cre
Imputation
non imputed
imputed

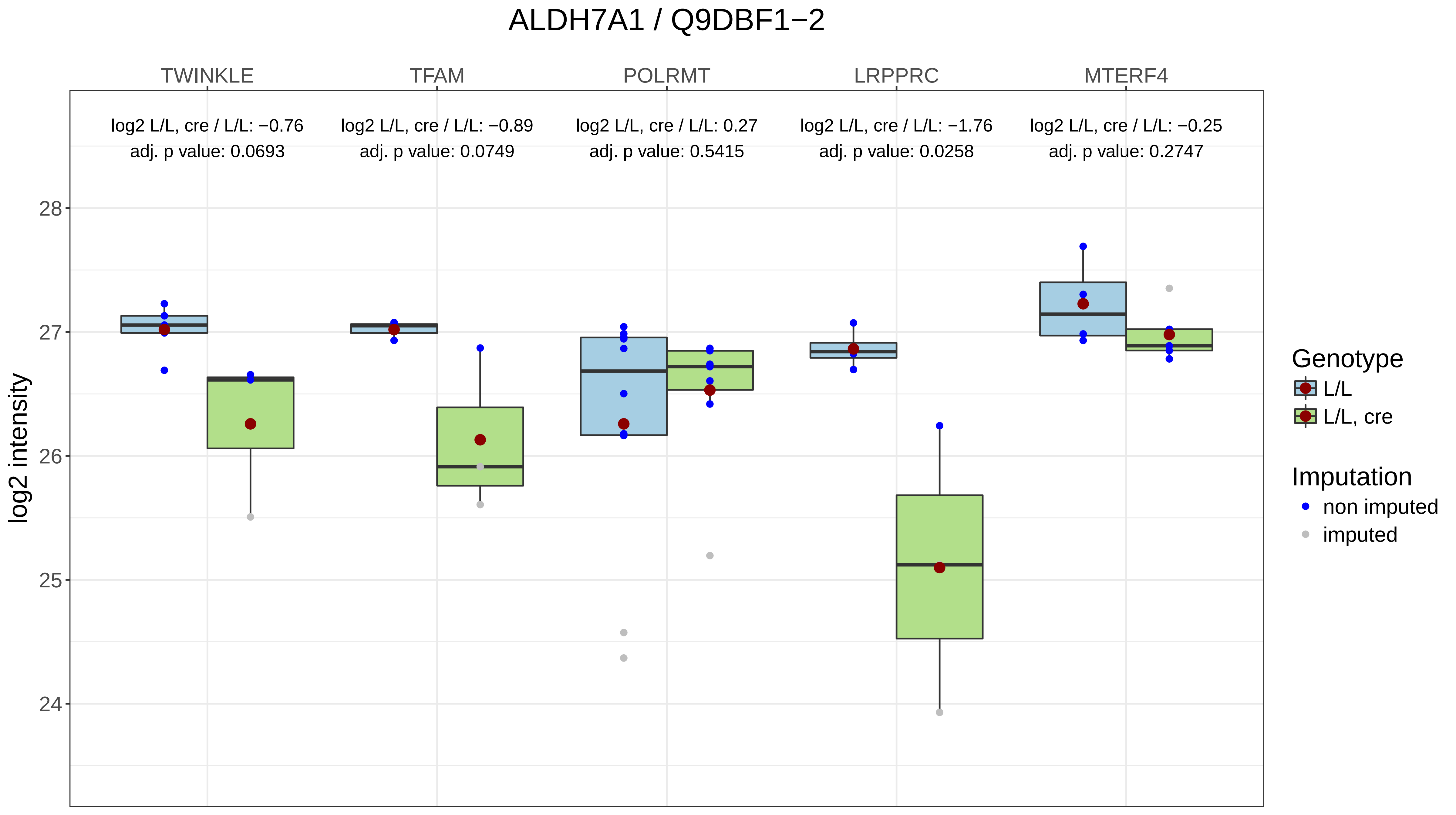
ALDH7A1 / Q9DBF1−2
TWINKLE
TFAM
POLRMT
LRPPRC
MTERF4
log2 L/L, cre / L/L: −0.76
adj. p value: 0.0693
log2 L/L, cre / L/L: −0.89
adj. p value: 0.0749
log2 L/L, cre / L/L: 0.27
adj. p value: 0.5415
log2 L/L, cre / L/L: −1.76
adj. p value: 0.0258
log2 L/L, cre / L/L: −0.25
adj. p value: 0.2747
log2 intensity
28
27
26
25
24
Genotype
L/L
L/L, cre
Imputation
non imputed
imputed

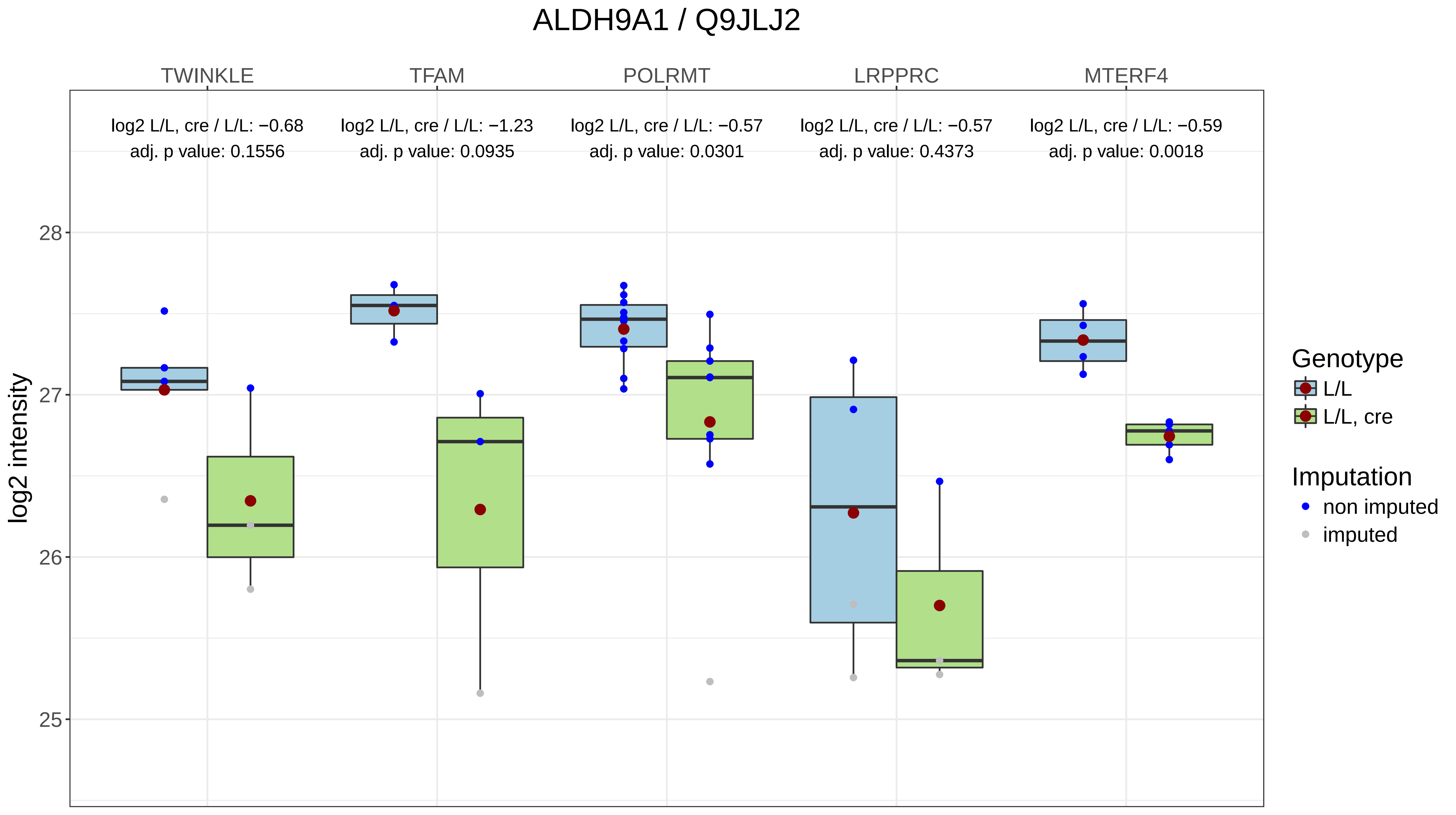
ALDH9A1 / Q9JLJ2
TWINKLE
TFAM
POLRMT
LRPPRC
MTERF4
log2 L/L, cre / L/L: −0.68
adj. p value: 0.1556
log2 L/L, cre / L/L: −1.23
adj. p value: 0.0935
log2 L/L, cre / L/L: −0.57
adj. p value: 0.0301
log2 L/L, cre / L/L: −0.57
adj. p value: 0.4373
log2 L/L, cre / L/L: −0.59
adj. p value: 0.0018
log2 intensity
28
27
26
25
Genotype
L/L
L/L, cre
Imputation
non imputed
imputed

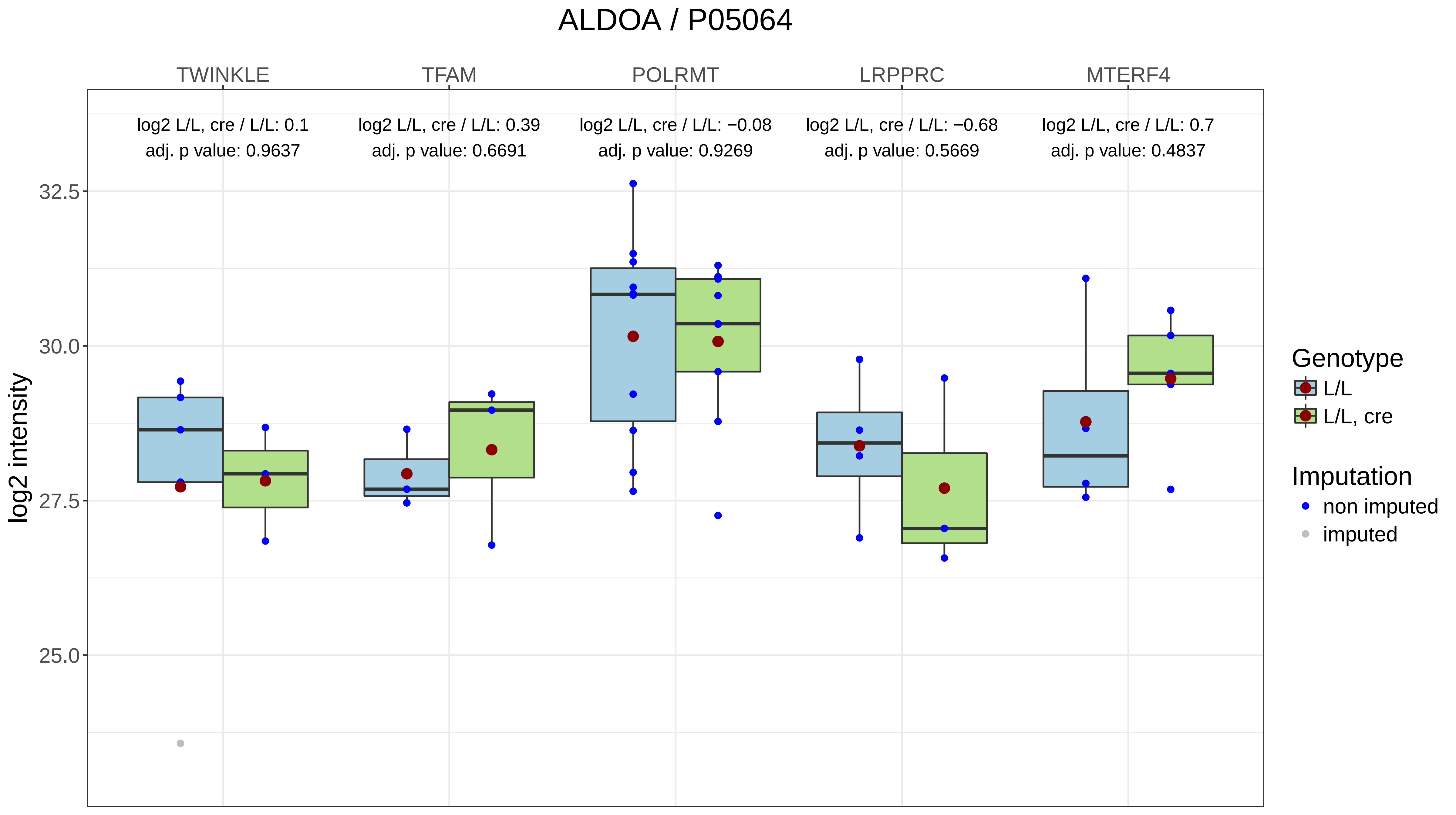
ALDOA / P05064
TWINKLE
TFAM
POLRMT
LRPPRC
MTERF4
log2 L/L, cre / L/L: 0.1
adj. p value: 0.9637
log2 L/L, cre / L/L: 0.39
adj. p value: 0.6691
log2 L/L, cre / L/L: −0.08
adj. p value: 0.9269
log2 L/L, cre / L/L: −0.68
adj. p value: 0.5669
log2 L/L, cre / L/L: 0.7
adj. p value: 0.4837
32.5
30.0
27.5
25.0
log2 intensity
Genotype
L/L
L/L, cre
Imputation
non imputed
imputed

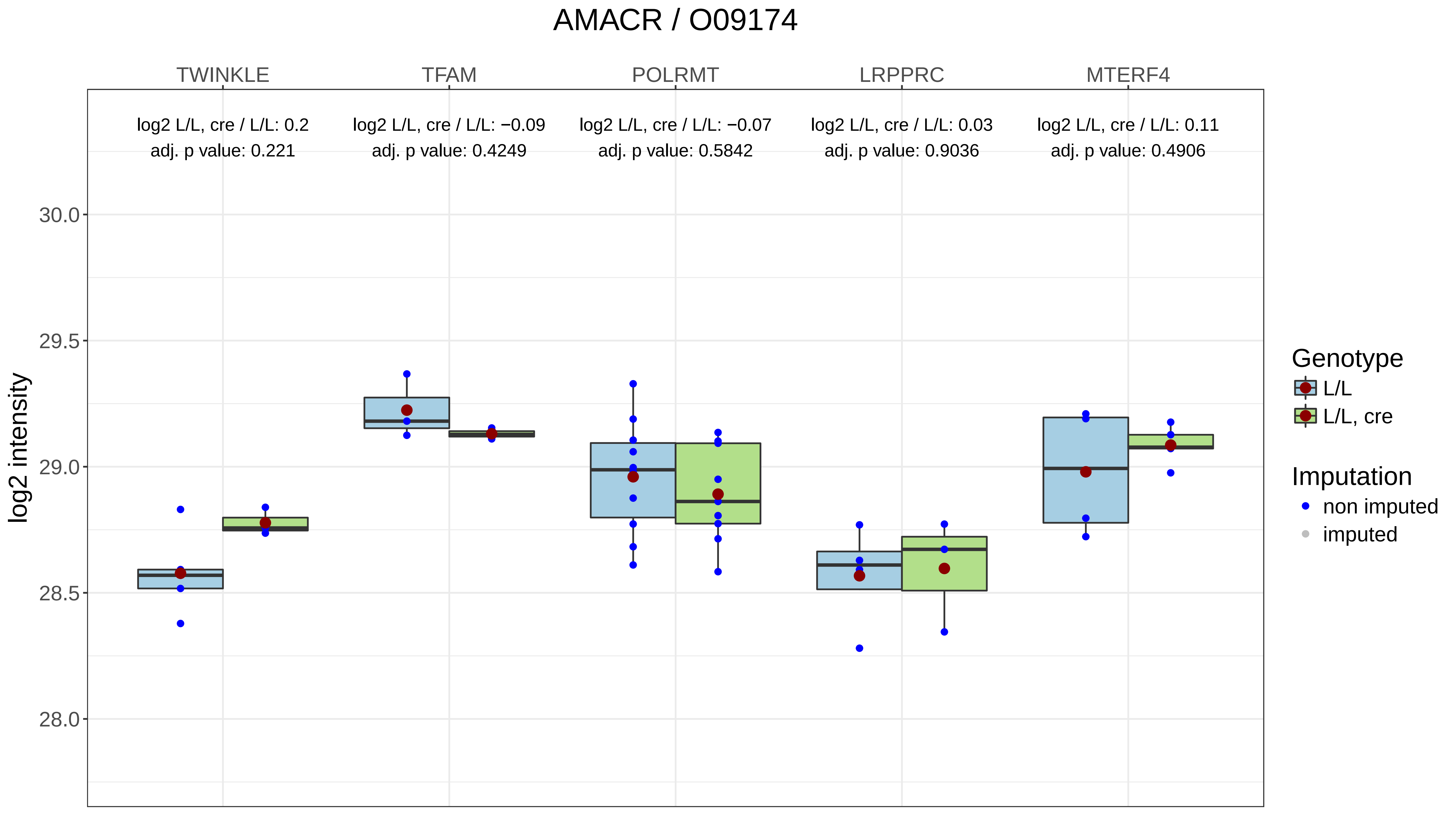
AMACR / O09174
TWINKLE
TFAM
POLRMT
LRPPRC
MTERF4
log2 L/L, cre / L/L: 0.2
adj. p value: 0.221
log2 L/L, cre / L/L: −0.09
adj. p value: 0.4249
log2 L/L, cre / L/L: −0.07
adj. p value: 0.5842
log2 L/L, cre / L/L: 0.03
adj. p value: 0.9036
log2 L/L, cre / L/L: 0.11
adj. p value: 0.4906
30.0
29.5
29.0
28.5
28.0
log2 intensity
Genotype
L/L
L/L, cre
Imputation
non imputed
imputed

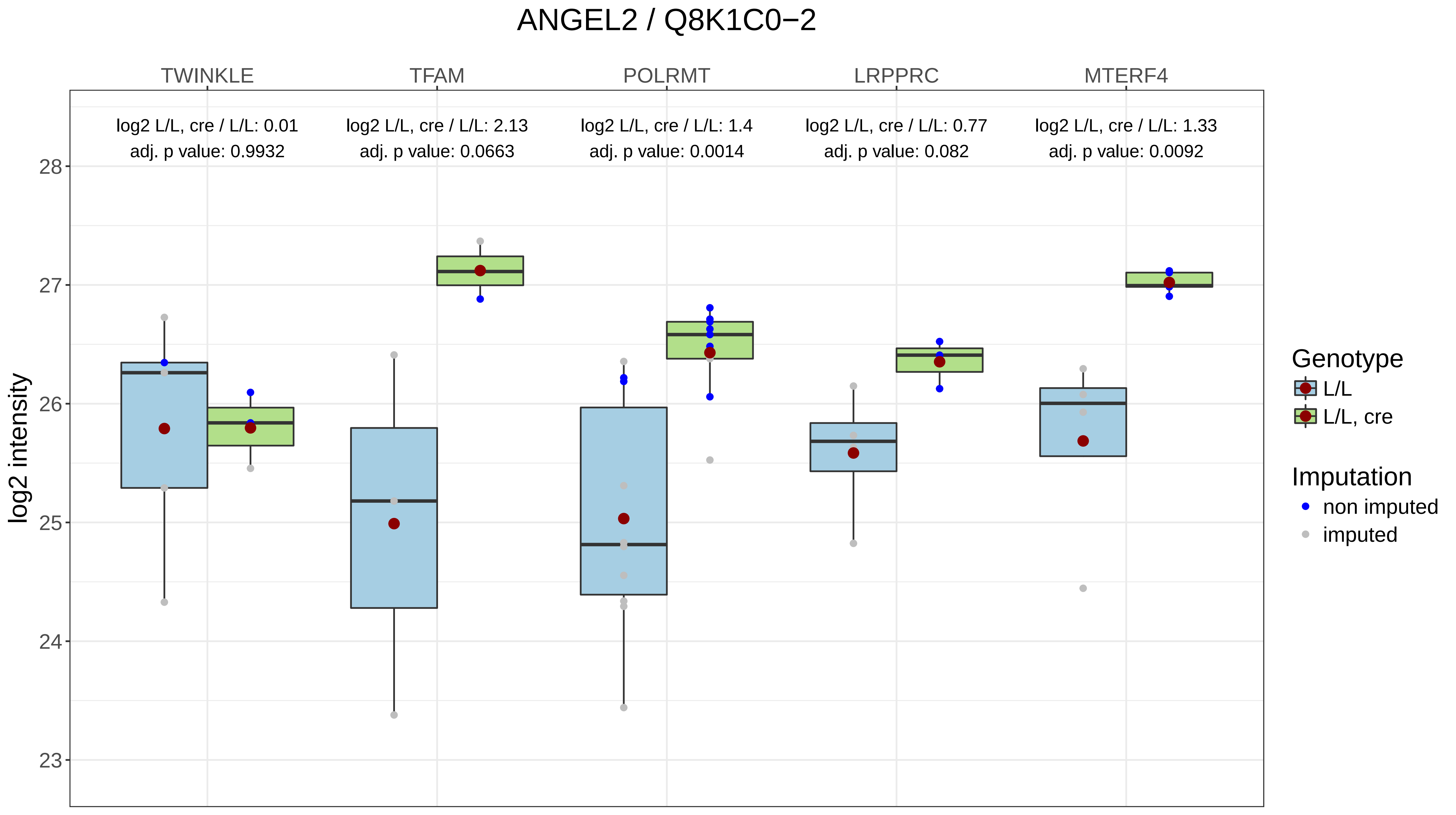
ANGEL2 / Q8K1C0−2
TWINKLE
TFAM
POLRMT
LRPPRC
MTERF4
log2 L/L, cre / L/L: 0.01
adj. p value: 0.9932
log2 L/L, cre / L/L: 2.13
adj. p value: 0.0663
log2 L/L, cre / L/L: 1.4
adj. p value: 0.0014
log2 L/L, cre / L/L: 0.77
adj. p value: 0.082
log2 L/L, cre / L/L: 1.33
adj. p value: 0.0092
log2 intensity
28
27
26
25
24
23
Genotype
L/L
L/L, cre
Imputation
non imputed
imputed

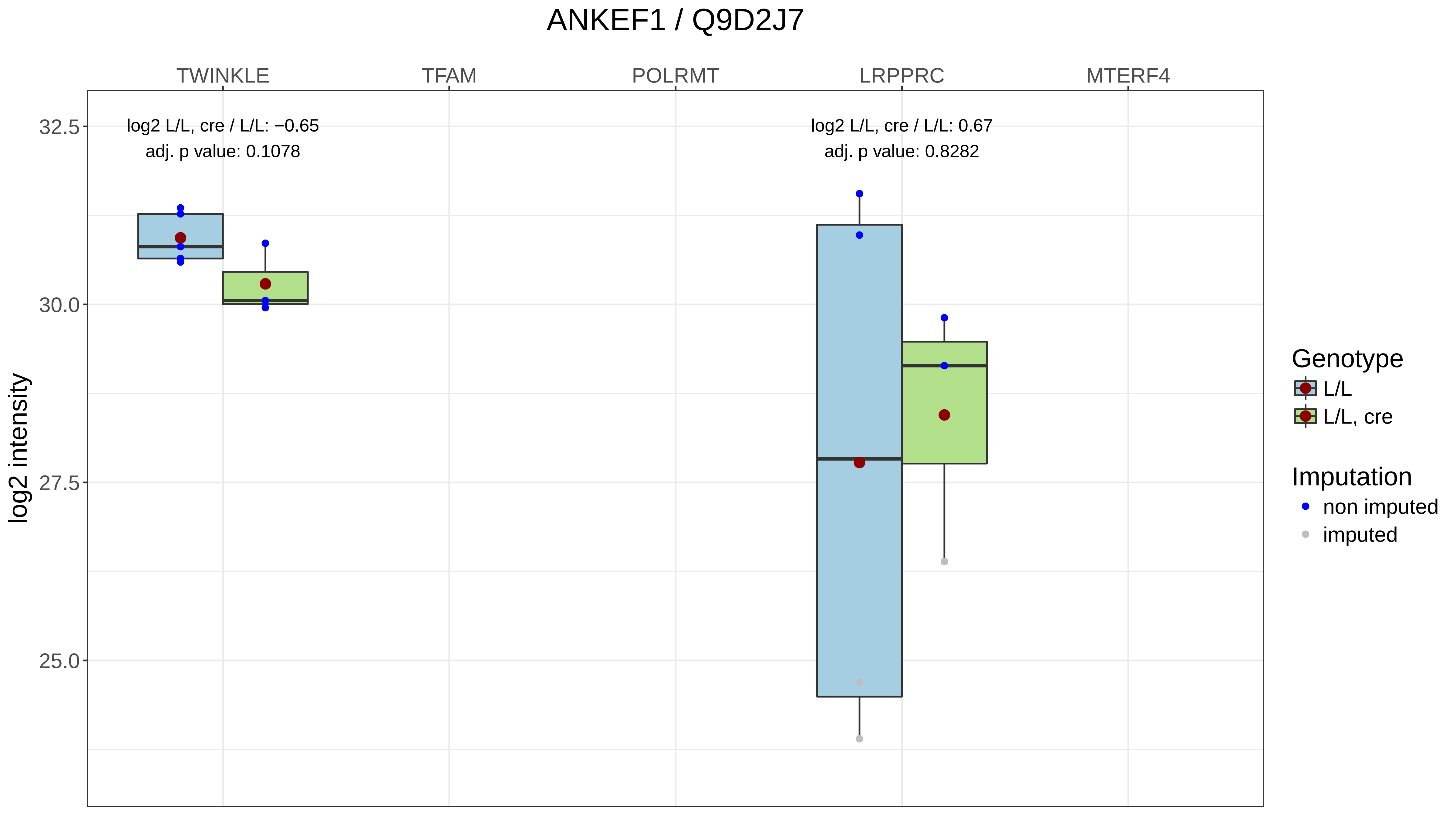
ANKEF1 / Q9D2J7
TWINKLE
TFAM
POLRMT
LRPPRC
MTERF4
log2 L/L, cre / L/L: −0.65
adj. p value: 0.1078
log2 L/L, cre / L/L: 0.67
adj. p value: 0.8282
32.5
30.0
27.5
25.0
log2 intensity
Genotype
L/L
L/L, cre
Imputation
non imputed
imputed

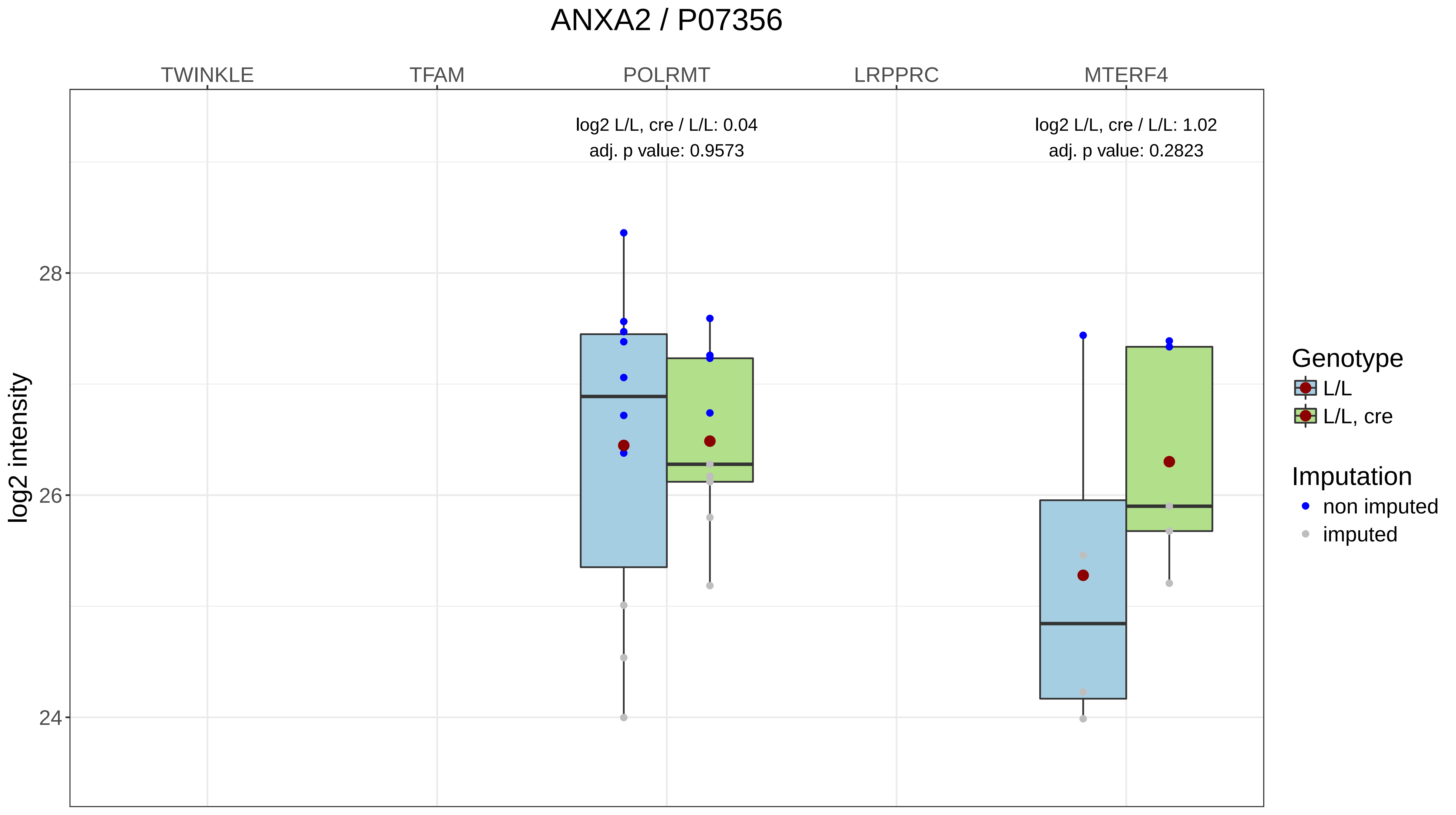

ANXA2 / P07356
TWINKLE
TFAM
POLRMT
LRPPRC
MTERF4
log2 L/L, cre / L/L: 0.04
adj. p value: 0.9573
log2 L/L, cre / L/L: 1.02
adj. p value: 0.2823
log2 intensity
28
26
24
Genotype
L/L
L/L, cre
Imputation
non imputed
imputed

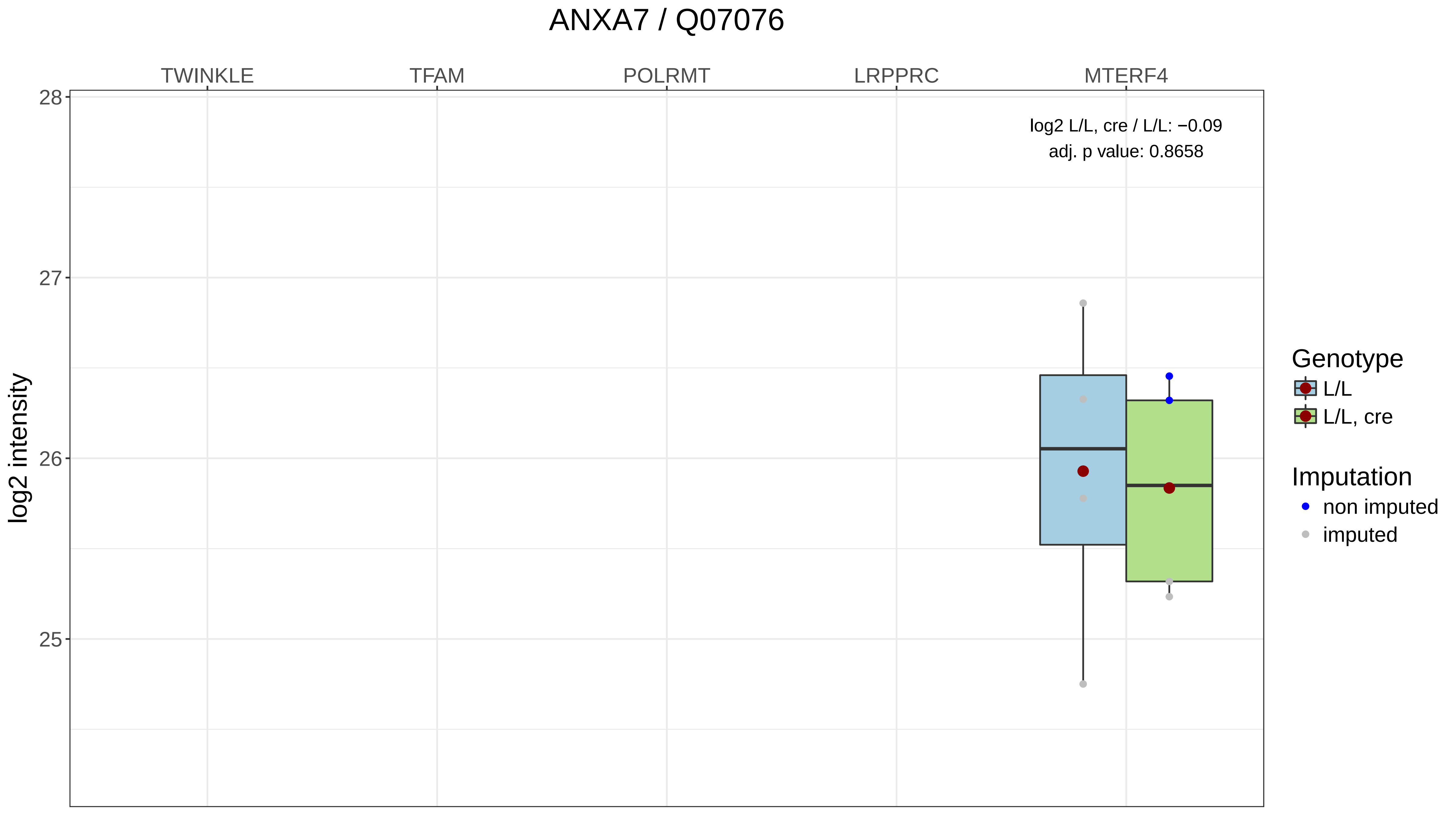

ANXA7 / Q07076
TWINKLE
TFAM
POLRMT
LRPPRC
MTERF4
log2 L/L, cre / L/L: −0.09
adj. p value: 0.8658
log2 intensity
28
27
26
25
Genotype
L/L
L/L, cre
Imputation
non imputed
imputed

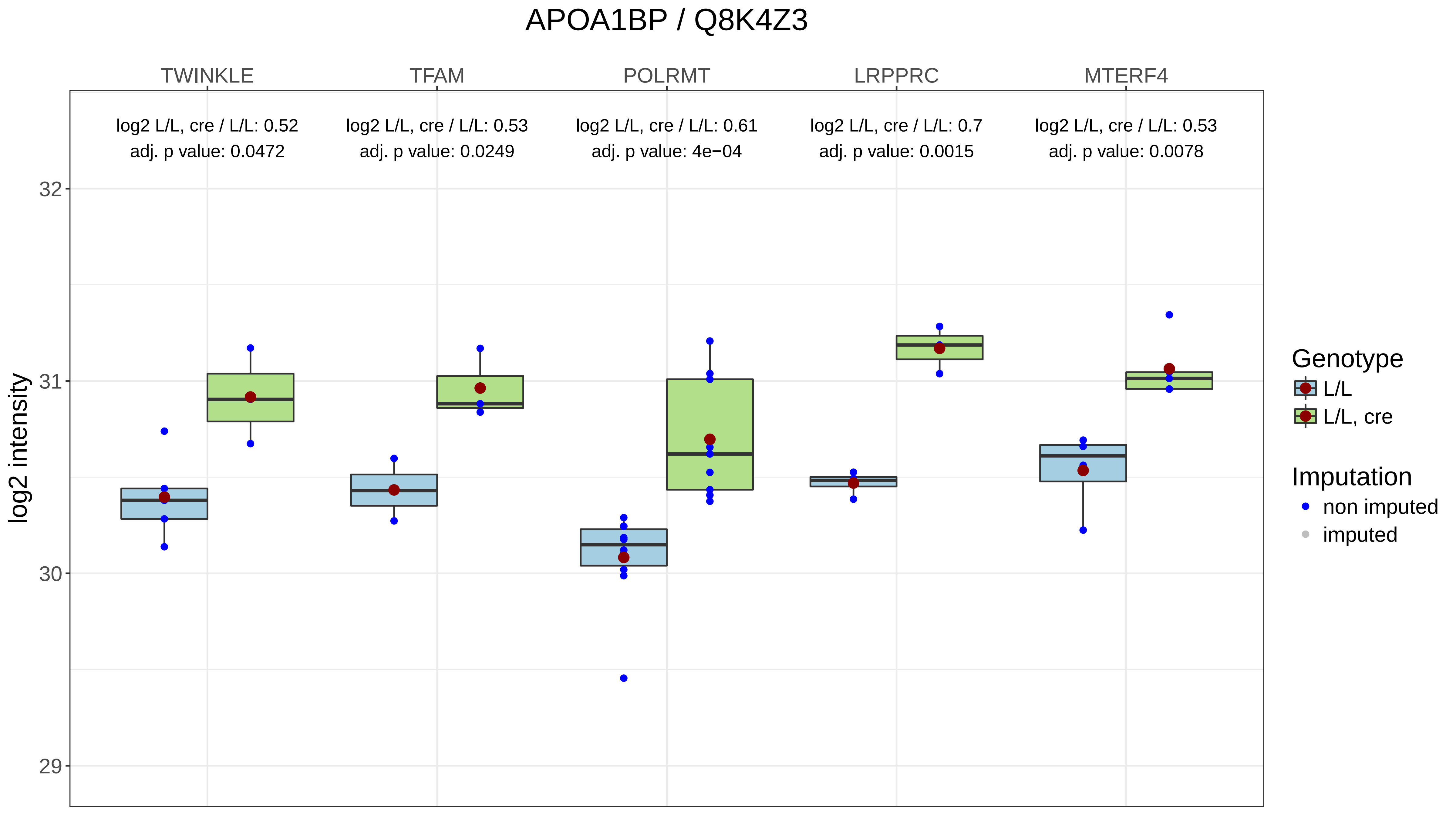
APOA1BP / Q8K4Z3
TWINKLE
TFAM
POLRMT
LRPPRC
MTERF4
log2 L/L, cre / L/L: 0.52
adj. p value: 0.0472
log2 L/L, cre / L/L: 0.53
adj. p value: 0.0249
log2 L/L, cre / L/L: 0.61
adj. p value: 4e−04
log2 L/L, cre / L/L: 0.7
adj. p value: 0.0015
log2 L/L, cre / L/L: 0.53
adj. p value: 0.0078
log2 intensity
32
31
30
29
Genotype
L/L
L/L, cre
Imputation
non imputed
imputed

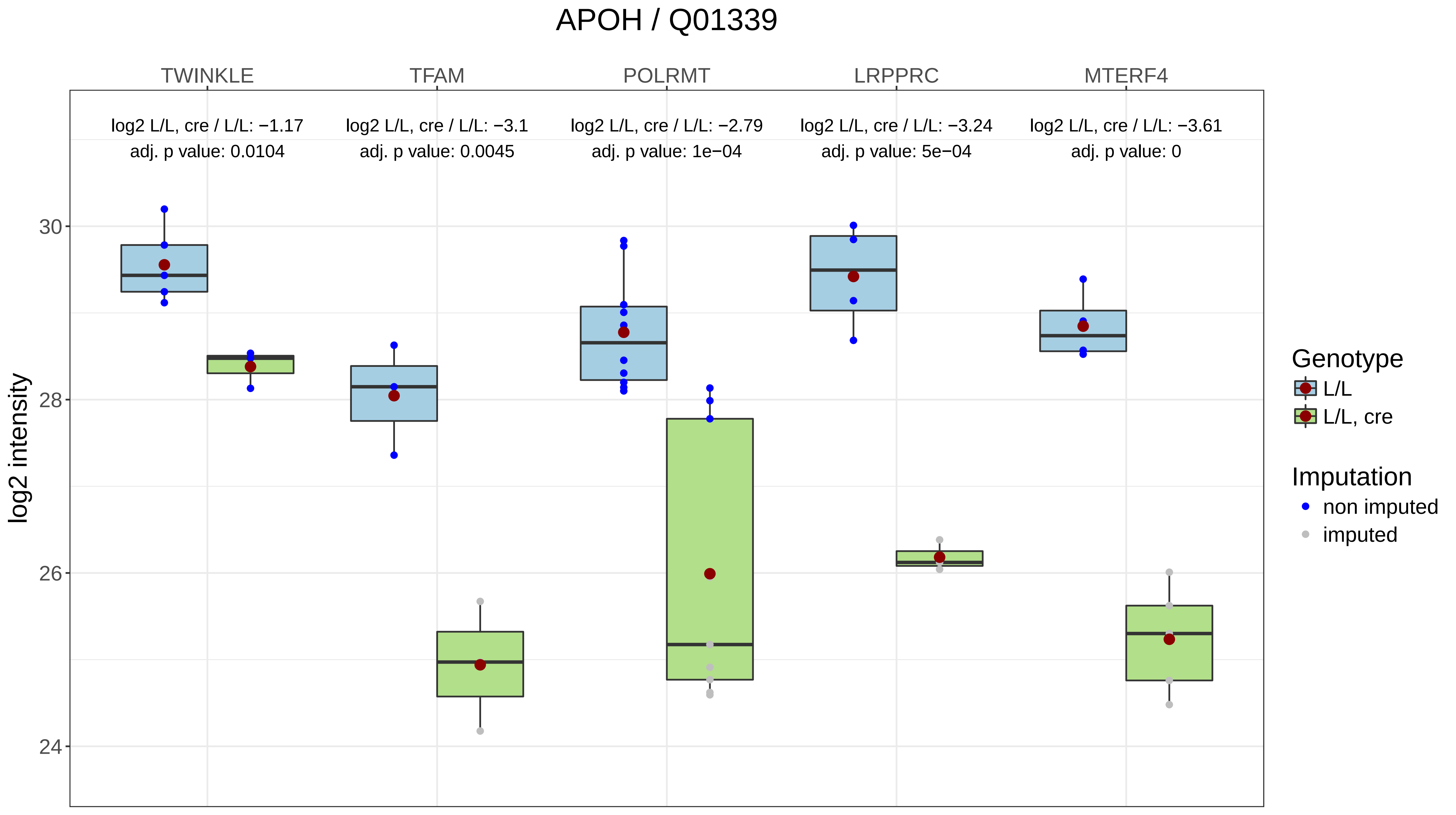
APOH / Q01339
TWINKLE
TFAM
POLRMT
LRPPRC
MTERF4
log2 L/L, cre / L/L: −1.17
adj. p value: 0.0104
log2 L/L, cre / L/L: −3.1
adj. p value: 0.0045
log2 L/L, cre / L/L: −2.79
adj. p value: 1e−04
log2 L/L, cre / L/L: −3.24
adj. p value: 5e−04
log2 L/L, cre / L/L: −3.61
adj. p value: 0
log2 intensity
30
28
26
24
Genotype
L/L
L/L, cre
Imputation
non imputed
imputed

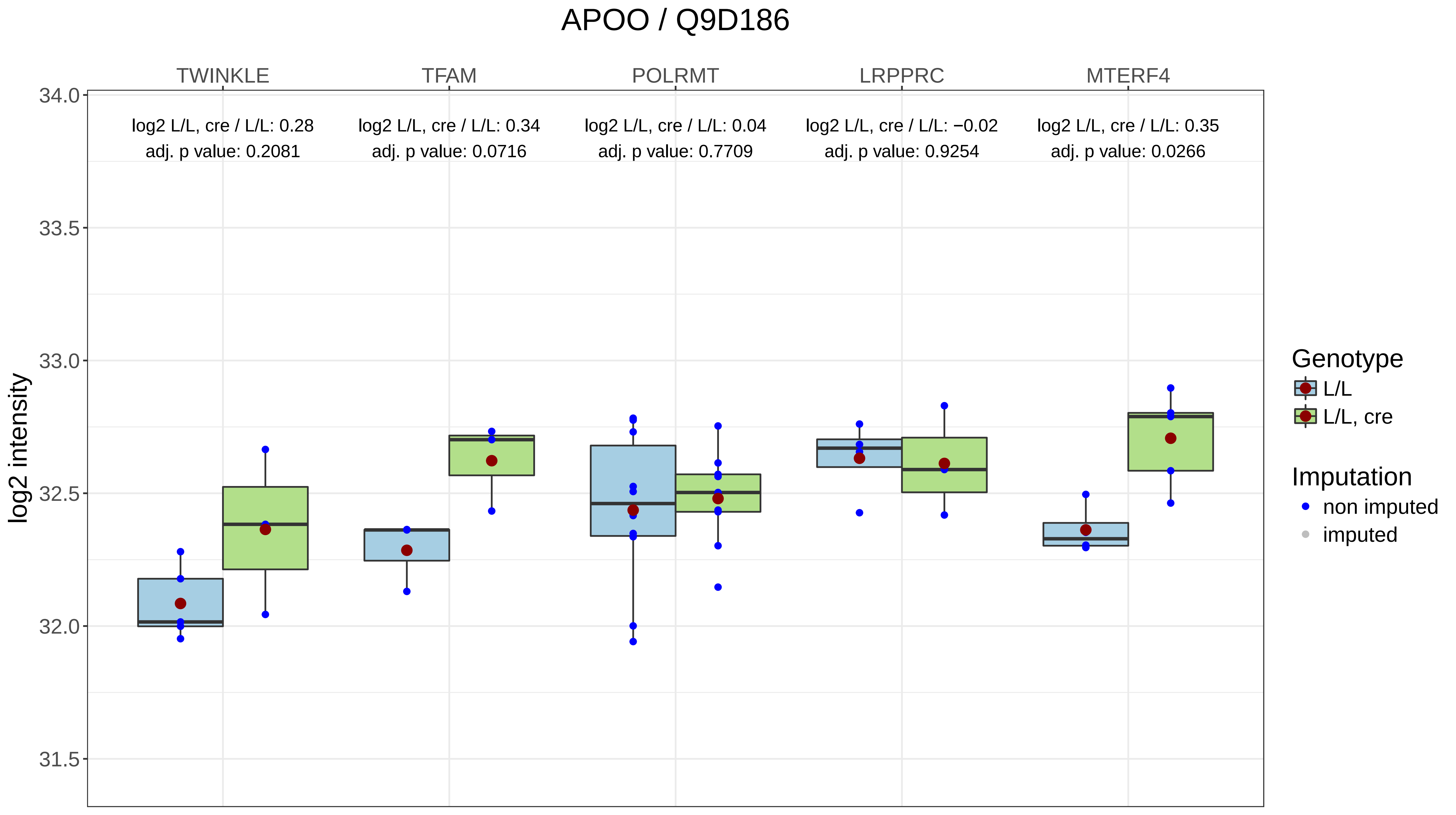

APOO / Q9D186
TWINKLE
TFAM
POLRMT
LRPPRC
MTERF4
log2 L/L, cre / L/L: 0.28
adj. p value: 0.2081
log2 L/L, cre / L/L: 0.34
adj. p value: 0.0716
log2 L/L, cre / L/L: 0.04
adj. p value: 0.7709
log2 L/L, cre / L/L: −0.02
adj. p value: 0.9254
log2 L/L, cre / L/L: 0.35
adj. p value: 0.0266
log2 intensity
34.0
33.5
33.0
32.5
32.0
31.5
Genotype
L/L
L/L, cre
Imputation
non imputed
imputed

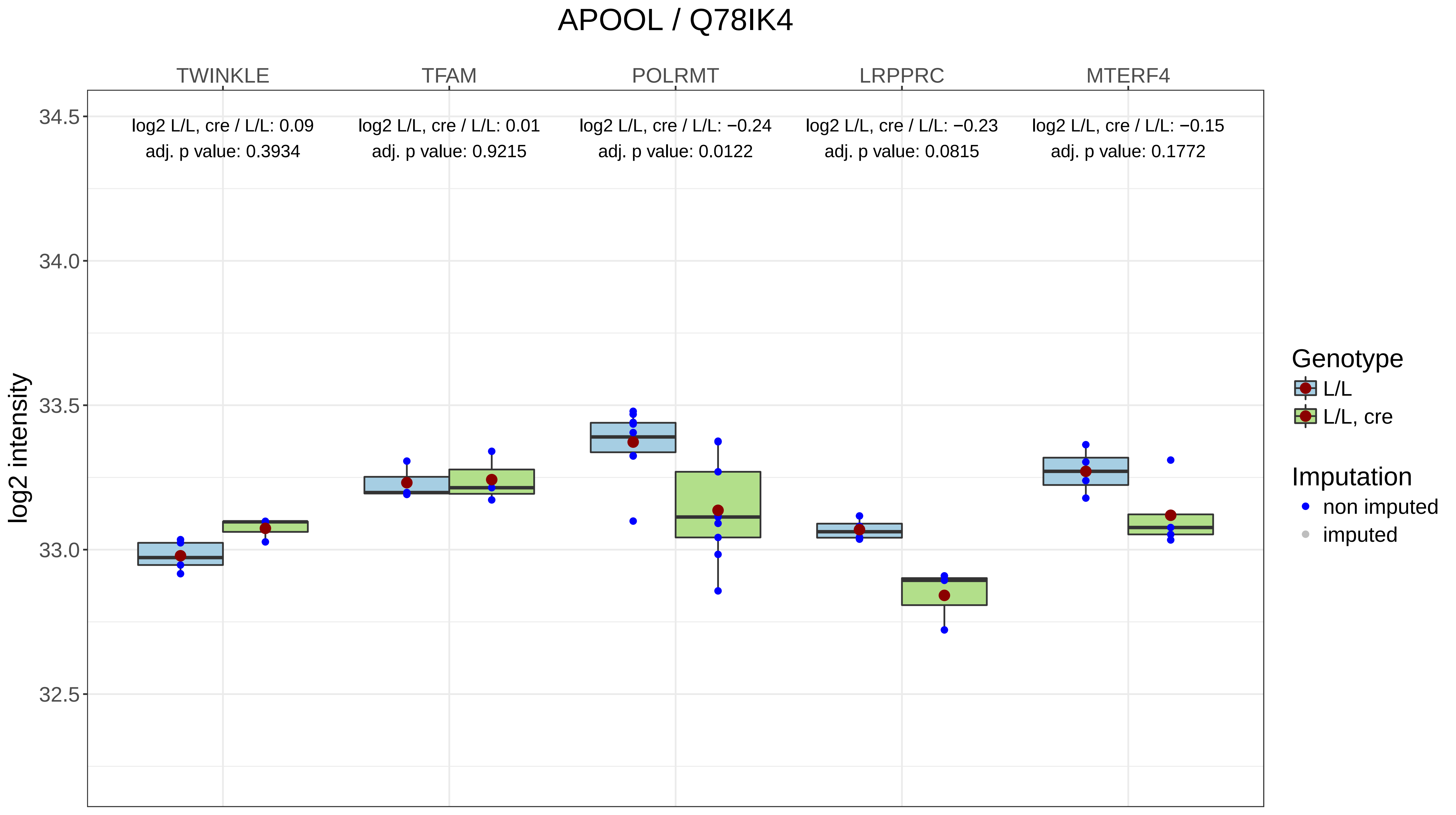

APOOL / Q78IK4
TWINKLE
TFAM
POLRMT
LRPPRC
MTERF4
log2 L/L, cre / L/L: 0.09
adj. p value: 0.3934
log2 L/L, cre / L/L: 0.01
adj. p value: 0.9215
log2 L/L, cre / L/L: −0.24
adj. p value: 0.0122
log2 L/L, cre / L/L: −0.23
adj. p value: 0.0815
log2 L/L, cre / L/L: −0.15
adj. p value: 0.1772
34.5
34.0
33.5
33.0
32.5
log2 intensity
Genotype
L/L
L/L, cre
Imputation
non imputed
imputed

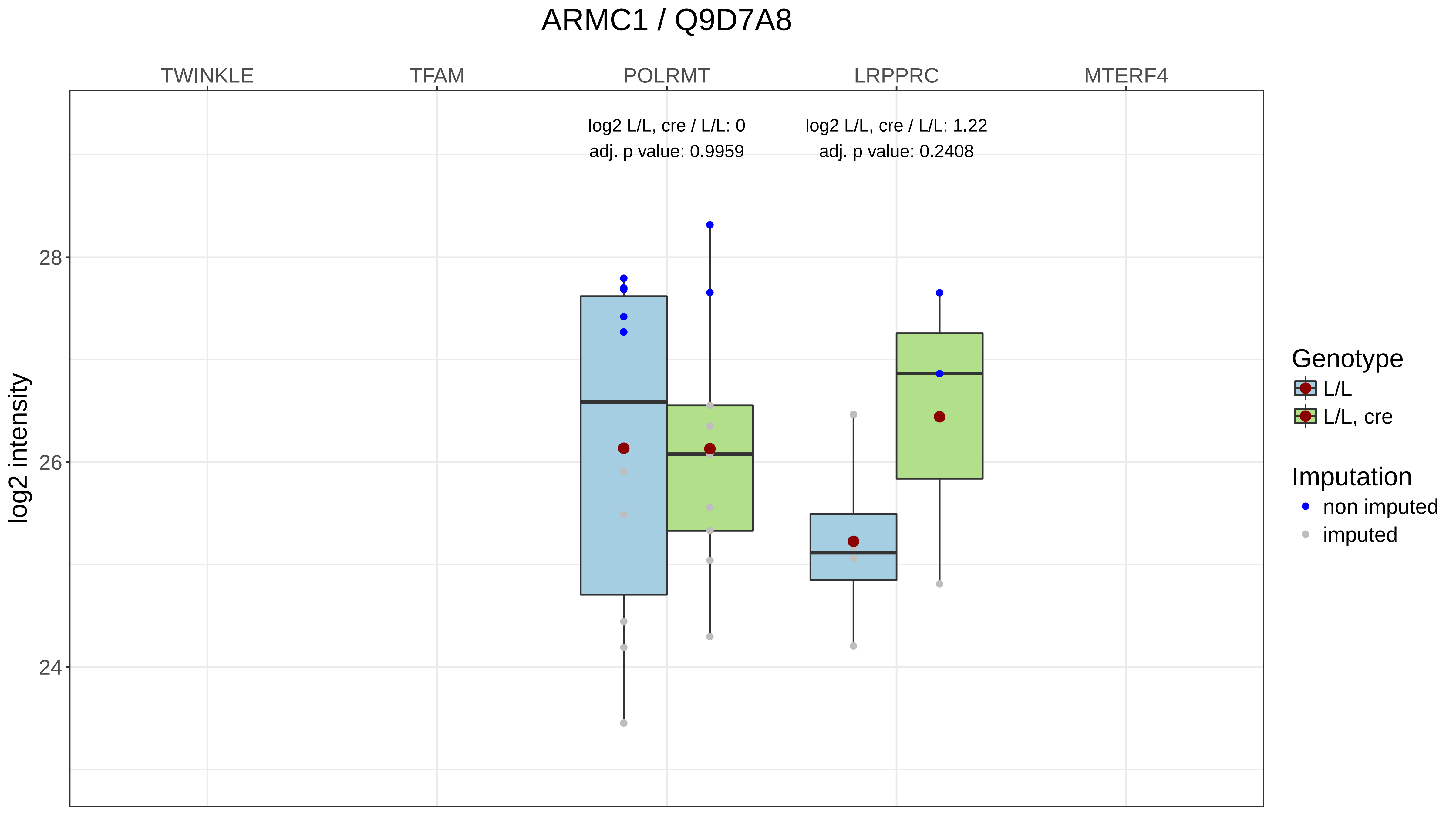

ARMC1 / Q9D7A8
TWINKLE
TFAM
POLRMT
LRPPRC
MTERF4
log2 L/L, cre / L/L: 0
adj. p value: 0.9959
log2 L/L, cre / L/L: 1.22
adj. p value: 0.2408
log2 intensity
28
26
24
Genotype
L/L
L/L, cre
Imputation
non imputed
imputed

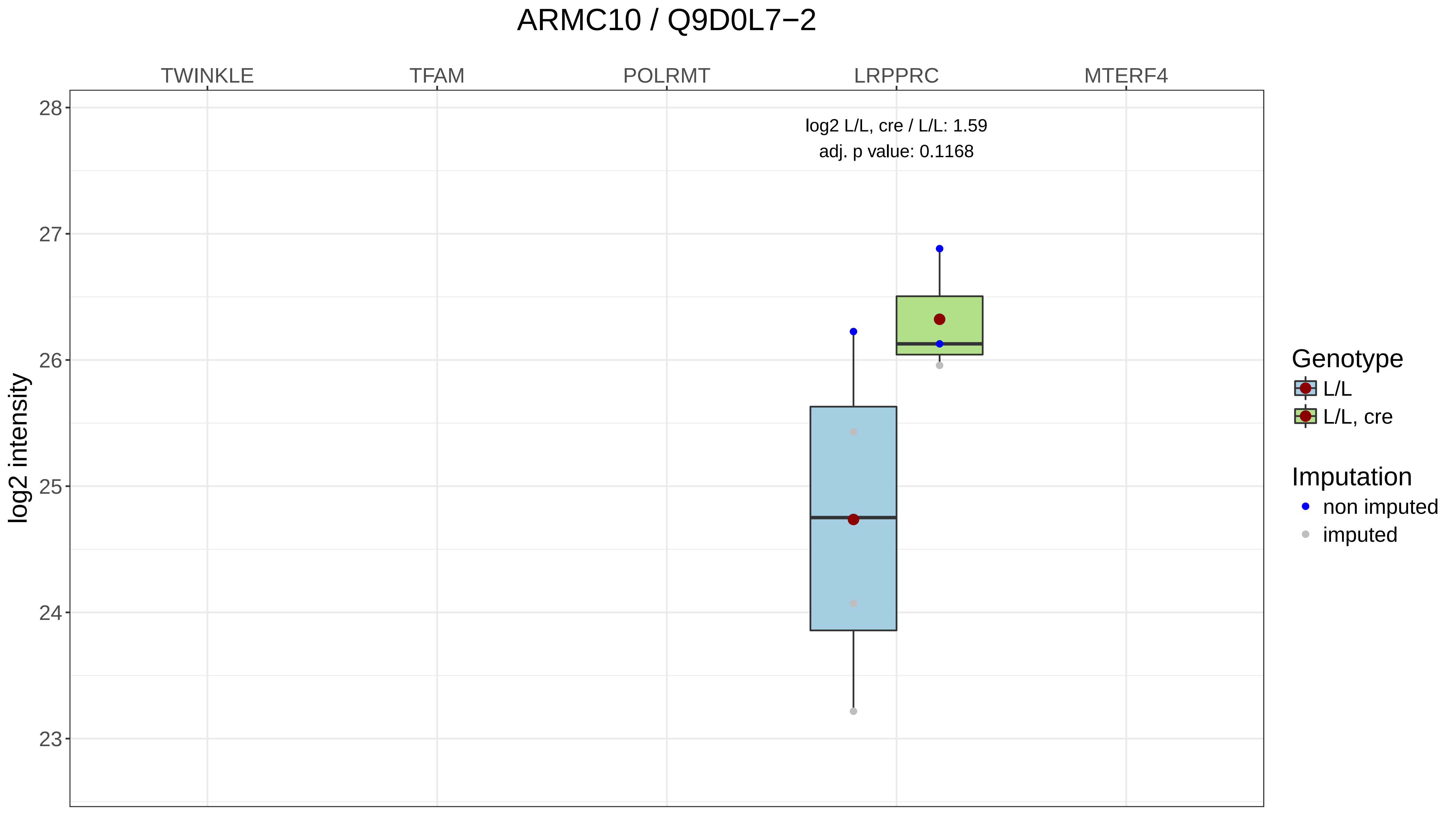

ARMC10 / Q9D0L7−2
TWINKLE
TFAM
POLRMT
LRPPRC
MTERF4
log2 L/L, cre / L/L: 1.59
adj. p value: 0.1168
log2 intensity
28
27
26
25
24
23
Genotype
L/L
L/L, cre
Imputation
non imputed
imputed

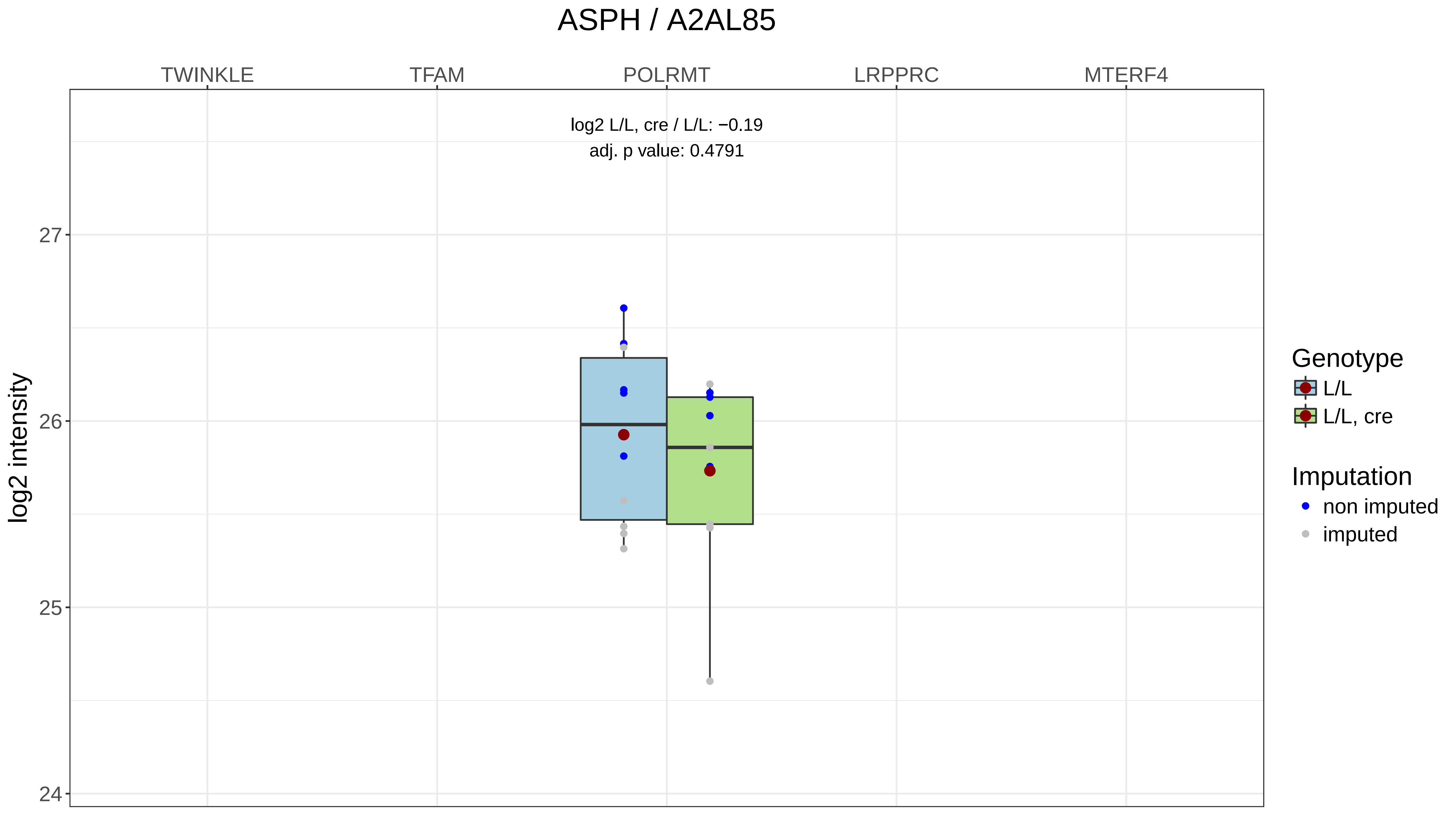
ASPH / A2AL85
TWINKLE
TFAM
POLRMT
LRPPRC
MTERF4
log2 L/L, cre / L/L: −0.19
adj. p value: 0.4791
log2 intensity
27
26
25
24
Genotype
L/L
L/L, cre
Imputation
non imputed
imputed

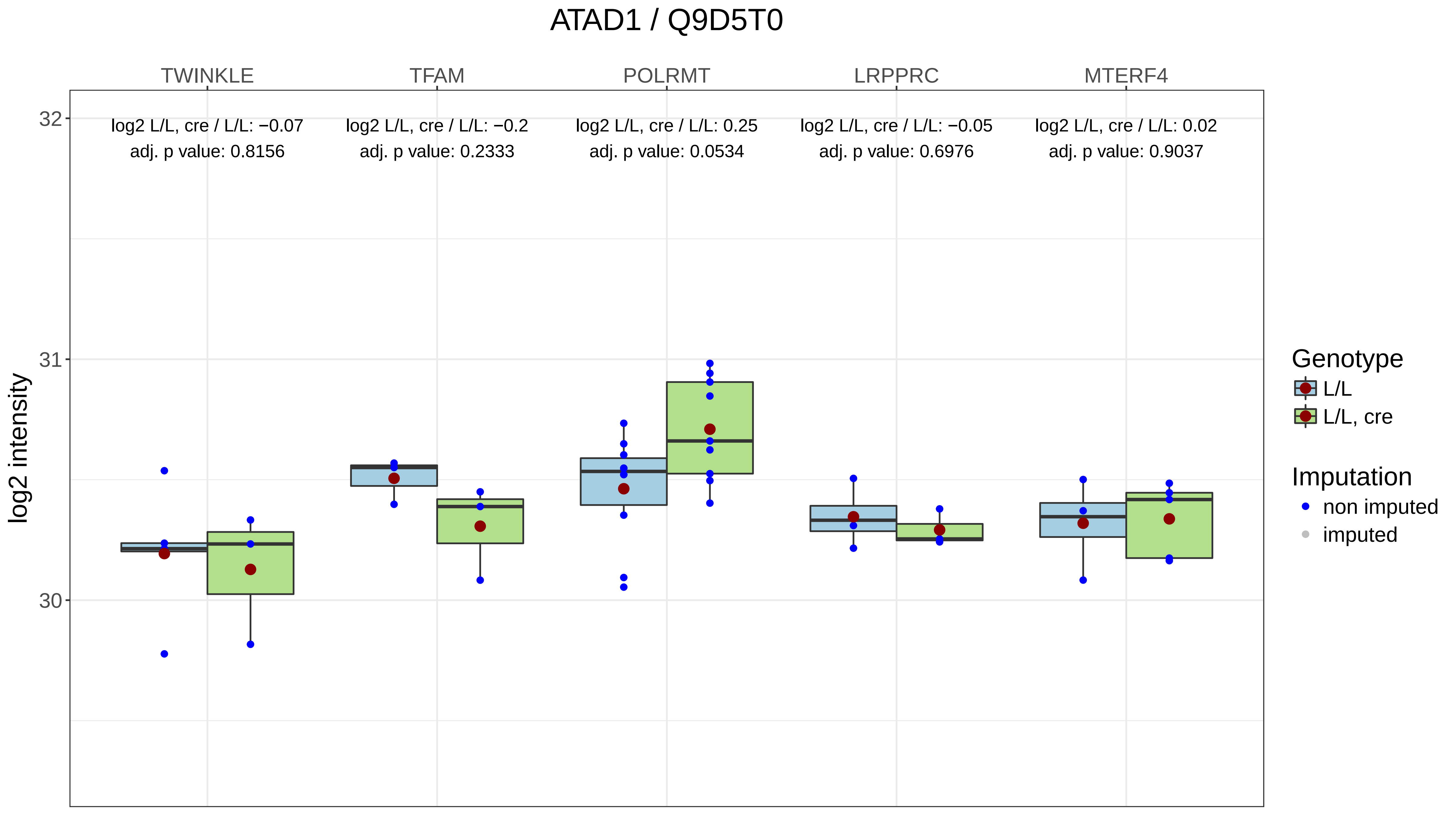
ATAD1 / Q9D5T0
TWINKLE
TFAM
POLRMT
LRPPRC
MTERF4
log2 L/L, cre / L/L: −0.07
adj. p value: 0.8156
log2 L/L, cre / L/L: −0.2
adj. p value: 0.2333
log2 L/L, cre / L/L: 0.25
adj. p value: 0.0534
log2 L/L, cre / L/L: −0.05
adj. p value: 0.6976
log2 L/L, cre / L/L: 0.02
adj. p value: 0.9037
32
31
30
log2 intensity
Genotype
L/L
L/L, cre
Imputation
non imputed
imputed

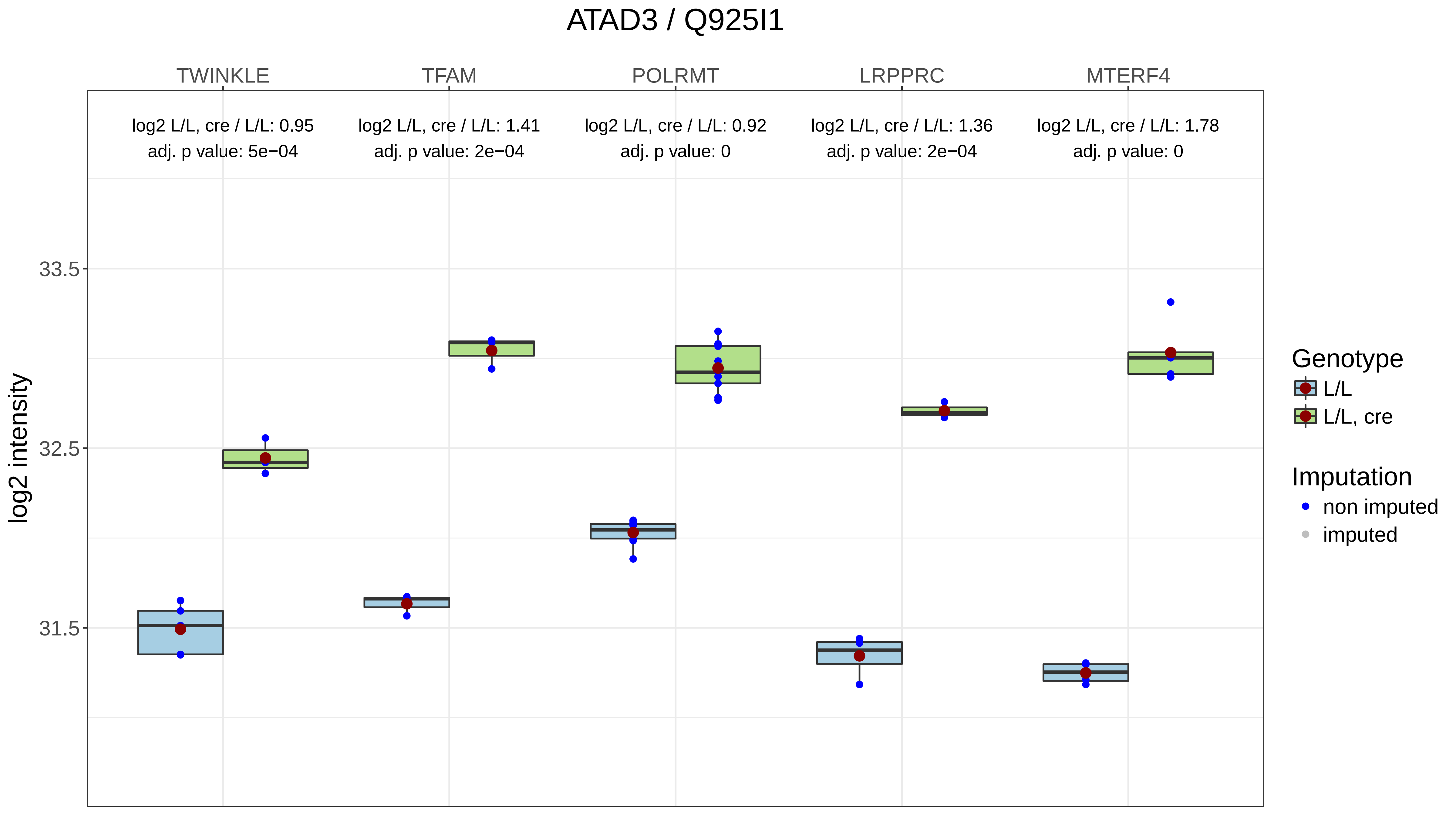

ATAD3 / Q925I1
TWINKLE
TFAM
POLRMT
LRPPRC
MTERF4
log2 L/L, cre / L/L: 0.95
adj. p value: 5e−04
log2 L/L, cre / L/L: 1.41
adj. p value: 2e−04
log2 L/L, cre / L/L: 0.92
adj. p value: 0
log2 L/L, cre / L/L: 1.36
adj. p value: 2e−04
log2 L/L, cre / L/L: 1.78
adj. p value: 0
33.5
32.5
31.5
log2 intensity
Genotype
L/L
L/L, cre
Imputation
non imputed
imputed

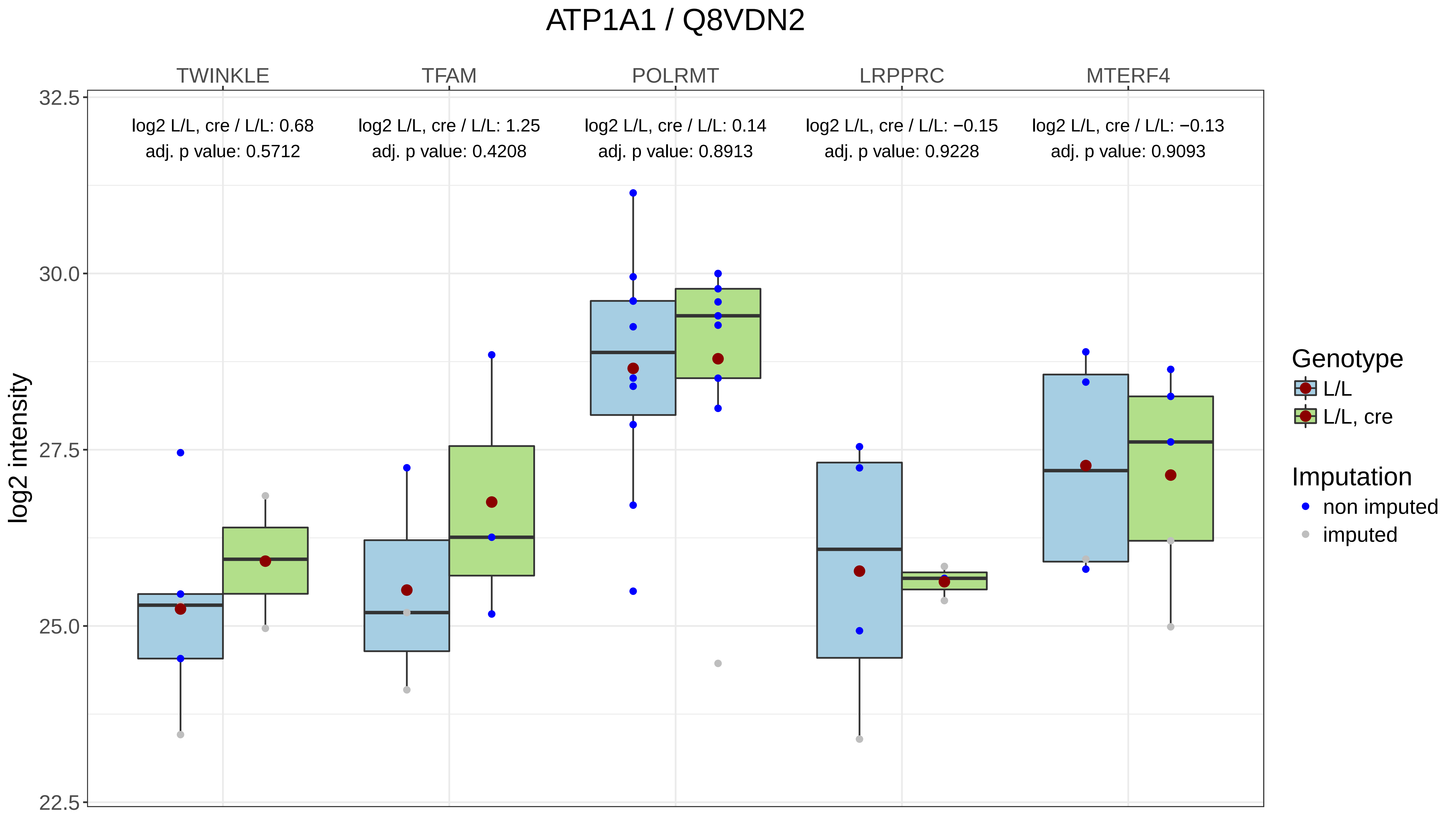

ATP1A1 / Q8VDN2
TWINKLE
TFAM
POLRMT
LRPPRC
MTERF4
log2 L/L, cre / L/L: 0.68
adj. p value: 0.5712
log2 L/L, cre / L/L: 1.25
adj. p value: 0.4208
log2 L/L, cre / L/L: 0.14
adj. p value: 0.8913
log2 L/L, cre / L/L: −0.15
adj. p value: 0.9228
log2 L/L, cre / L/L: −0.13
adj. p value: 0.9093
log2 intensity
32.5
30.0
27.5
25.0
22.5
Genotype
L/L
L/L, cre
Imputation
non imputed
imputed

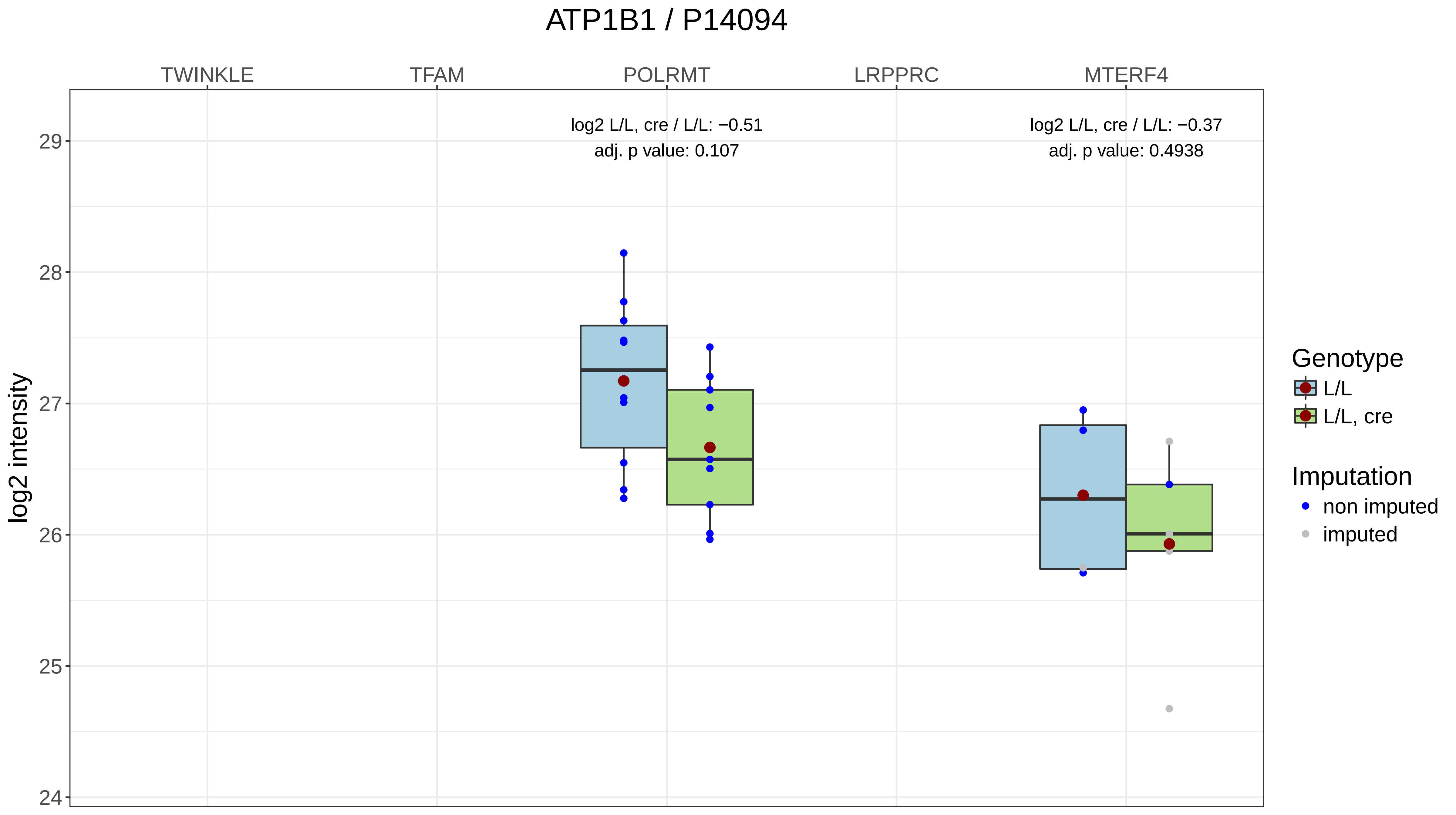
ATP1B1 / P14094
TWINKLE
TFAM
POLRMT
LRPPRC
MTERF4
log2 L/L, cre / L/L: −0.51
adj. p value: 0.107
log2 L/L, cre / L/L: −0.37
adj. p value: 0.4938
log2 intensity
29
28
27
26
25
24
Genotype
L/L
L/L, cre
Imputation
non imputed
imputed

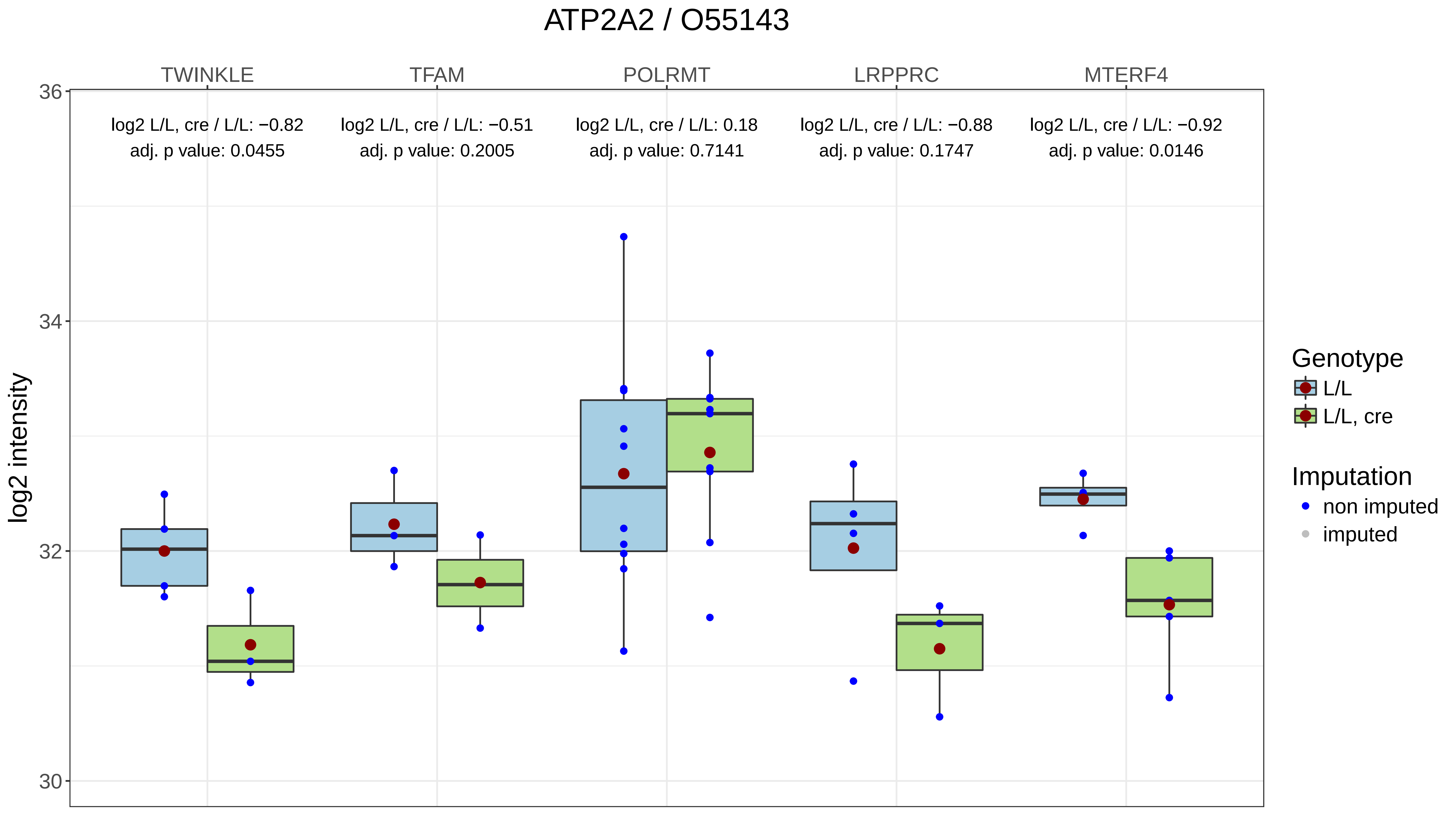
ATP2A2 / O55143
TWINKLE
TFAM
POLRMT
LRPPRC
MTERF4
log2 L/L, cre / L/L: −0.82
adj. p value: 0.0455
log2 L/L, cre / L/L: −0.51
adj. p value: 0.2005
log2 L/L, cre / L/L: 0.18
adj. p value: 0.7141
log2 L/L, cre / L/L: −0.88
adj. p value: 0.1747
log2 L/L, cre / L/L: −0.92
adj. p value: 0.0146
log2 intensity
36
34
32
30
Genotype
L/L
L/L, cre
Imputation
non imputed
imputed

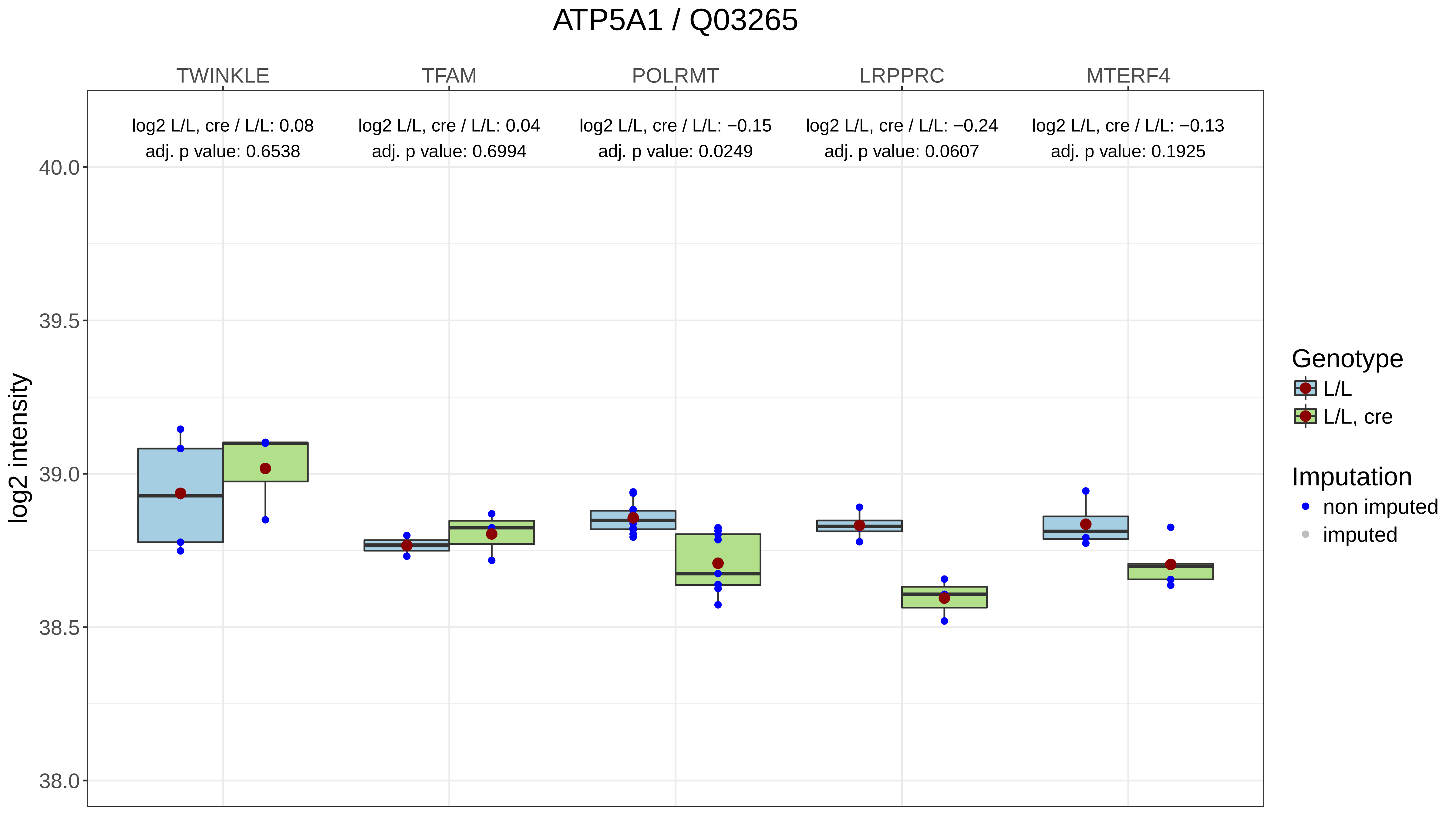
ATP5A1 / Q03265
TWINKLE
TFAM
POLRMT
LRPPRC
MTERF4
log2 L/L, cre / L/L: 0.08
adj. p value: 0.6538
log2 L/L, cre / L/L: 0.04
adj. p value: 0.6994
log2 L/L, cre / L/L: −0.15
adj. p value: 0.0249
log2 L/L, cre / L/L: −0.24
adj. p value: 0.0607
log2 L/L, cre / L/L: −0.13
adj. p value: 0.1925
log2 intensity
40.0
39.5
39.0
38.5
38.0
Genotype
L/L
L/L, cre
Imputation
non imputed
imputed

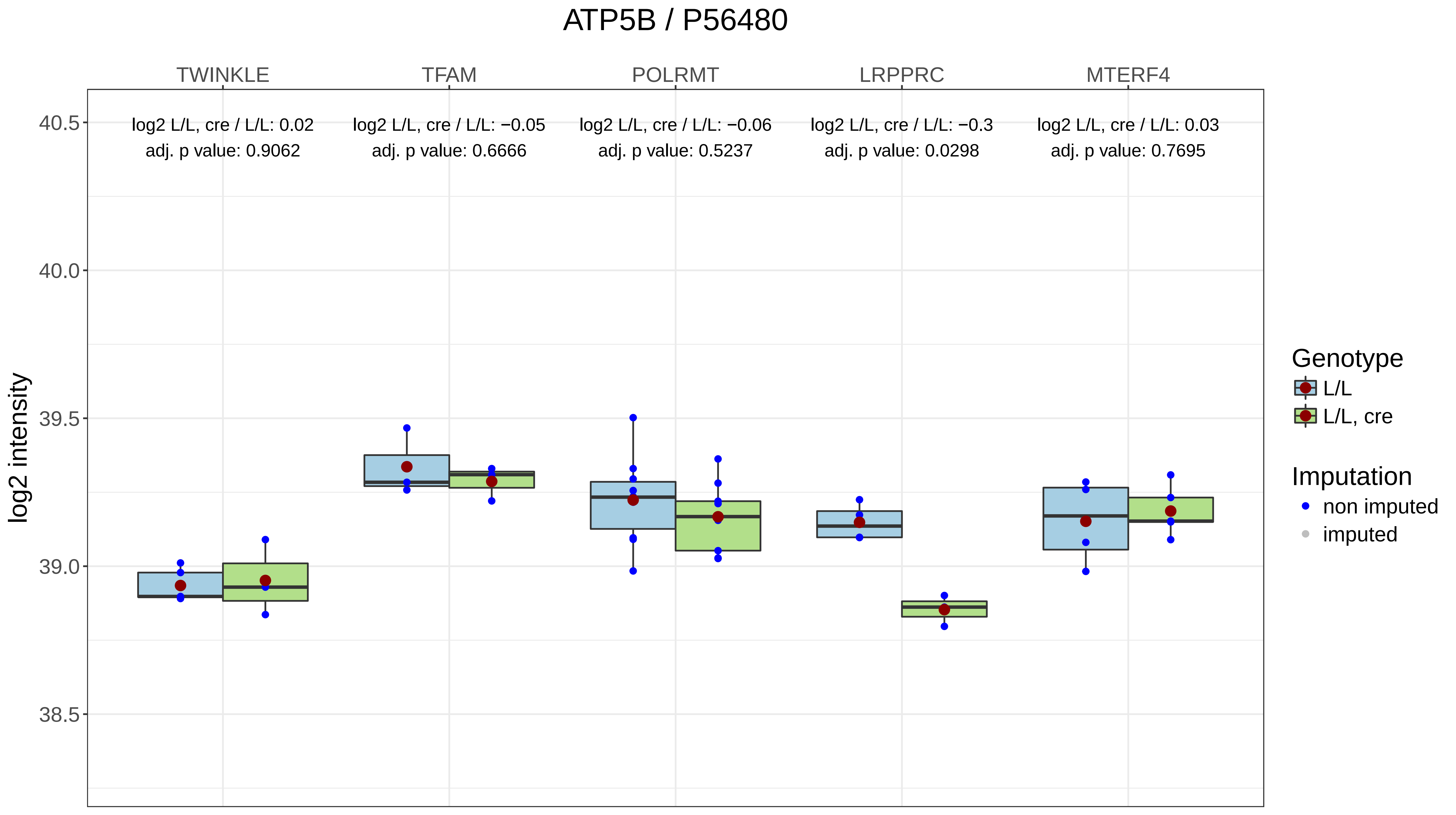
ATP5B / P56480
TWINKLE
TFAM
POLRMT
LRPPRC
MTERF4
log2 L/L, cre / L/L: 0.02
adj. p value: 0.9062
log2 L/L, cre / L/L: −0.05
adj. p value: 0.6666
log2 L/L, cre / L/L: −0.06
adj. p value: 0.5237
log2 L/L, cre / L/L: −0.3
adj. p value: 0.0298
log2 L/L, cre / L/L: 0.03
adj. p value: 0.7695
log2 intensity
40.5
40.0
39.5
39.0
38.5
Genotype
L/L
L/L, cre
Imputation
non imputed
imputed

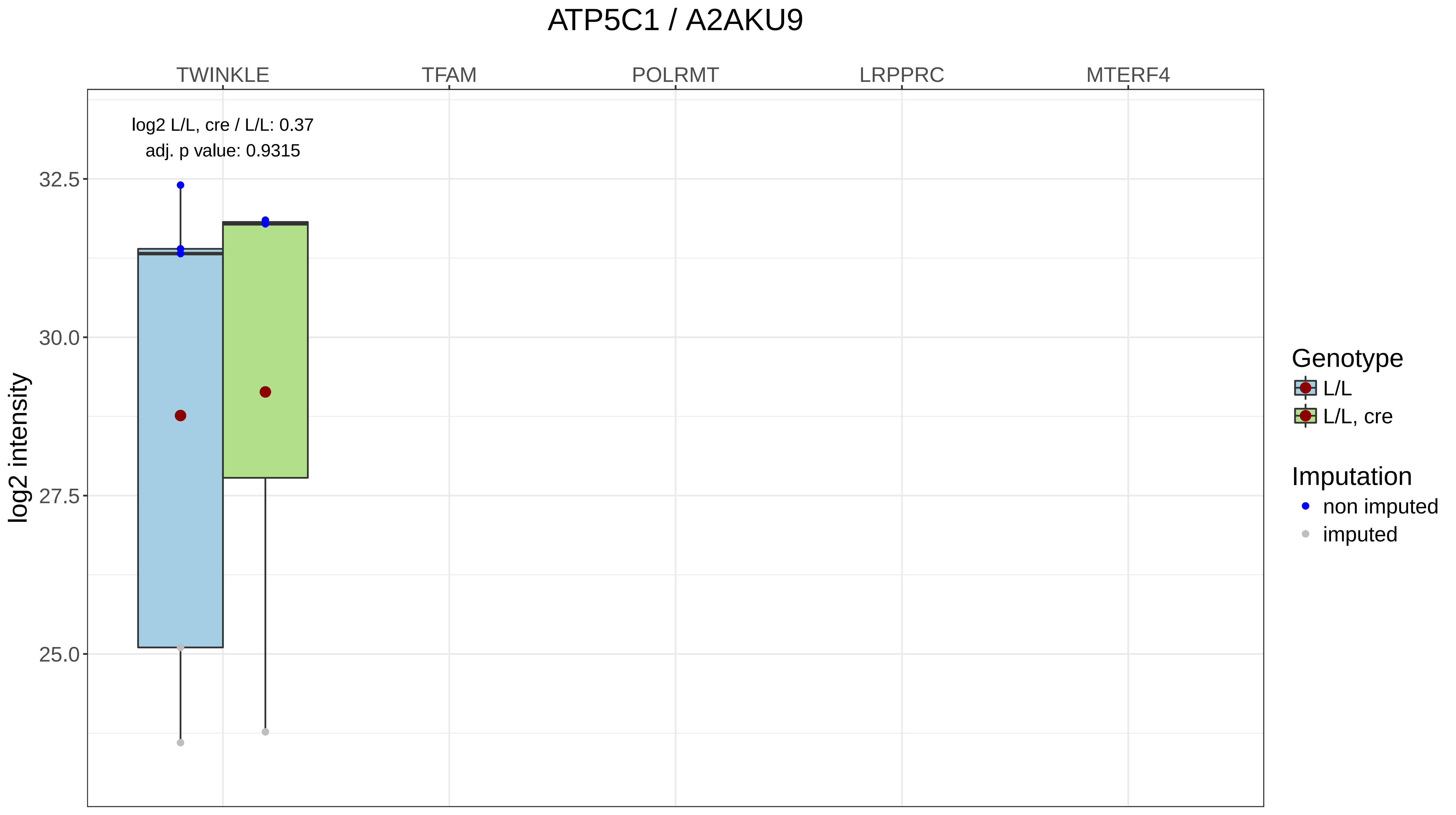
ATP5C1 / A2AKU9
TWINKLE
TFAM
POLRMT
LRPPRC
MTERF4
log2 L/L, cre / L/L: 0.37
adj. p value: 0.9315
32.5
30.0
27.5
25.0
log2 intensity
Genotype
L/L
L/L, cre
Imputation
non imputed
imputed

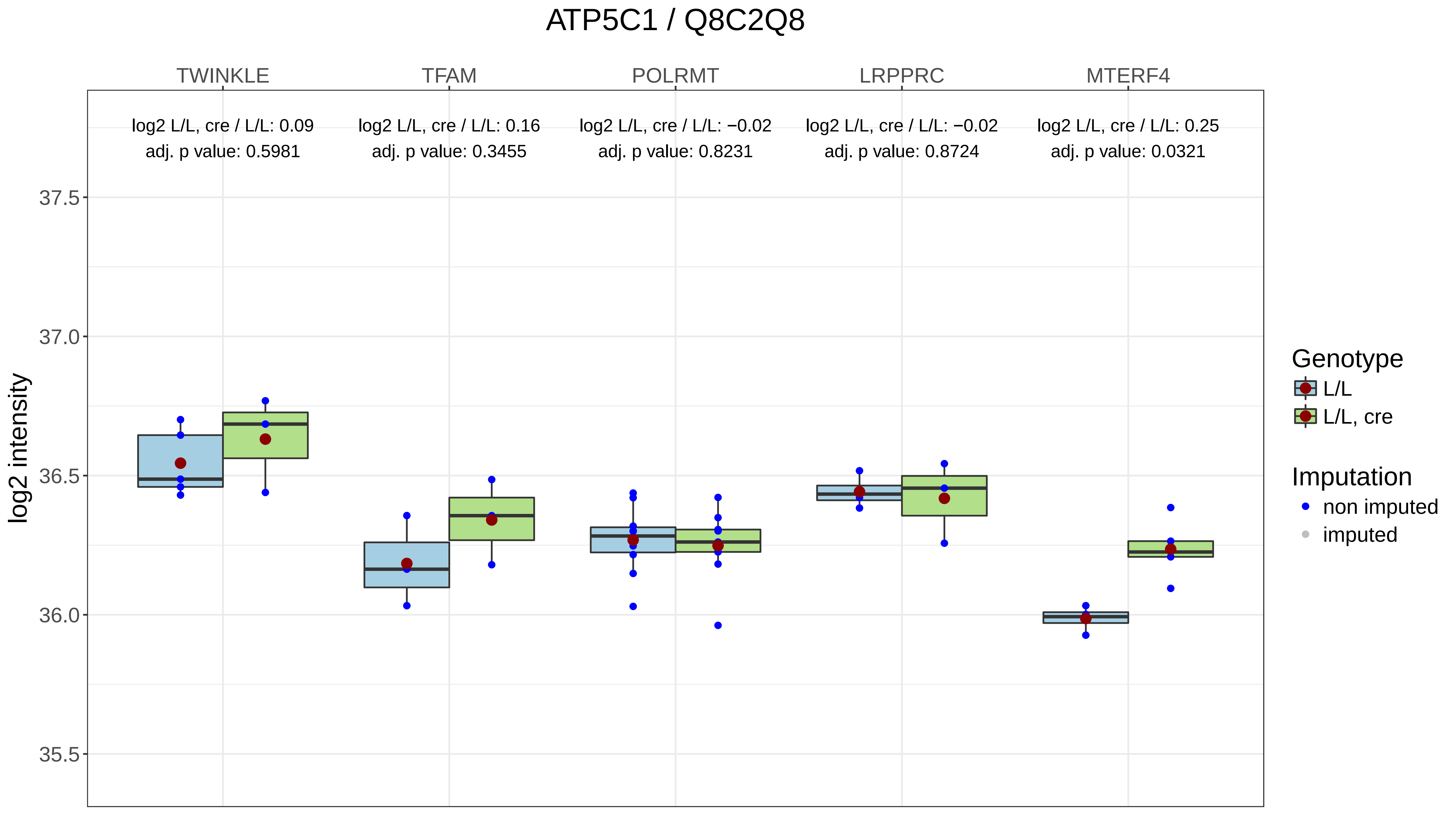
ATP5C1 / Q8C2Q8
TWINKLE
TFAM
POLRMT
LRPPRC
MTERF4
log2 L/L, cre / L/L: 0.09
adj. p value: 0.5981
log2 L/L, cre / L/L: 0.16
adj. p value: 0.3455
log2 L/L, cre / L/L: −0.02
adj. p value: 0.8231
log2 L/L, cre / L/L: −0.02
adj. p value: 0.8724
log2 L/L, cre / L/L: 0.25
adj. p value: 0.0321
log2 intensity
37.5
37.0
36.5
36.0
35.5
Genotype
L/L
L/L, cre
Imputation
non imputed
imputed

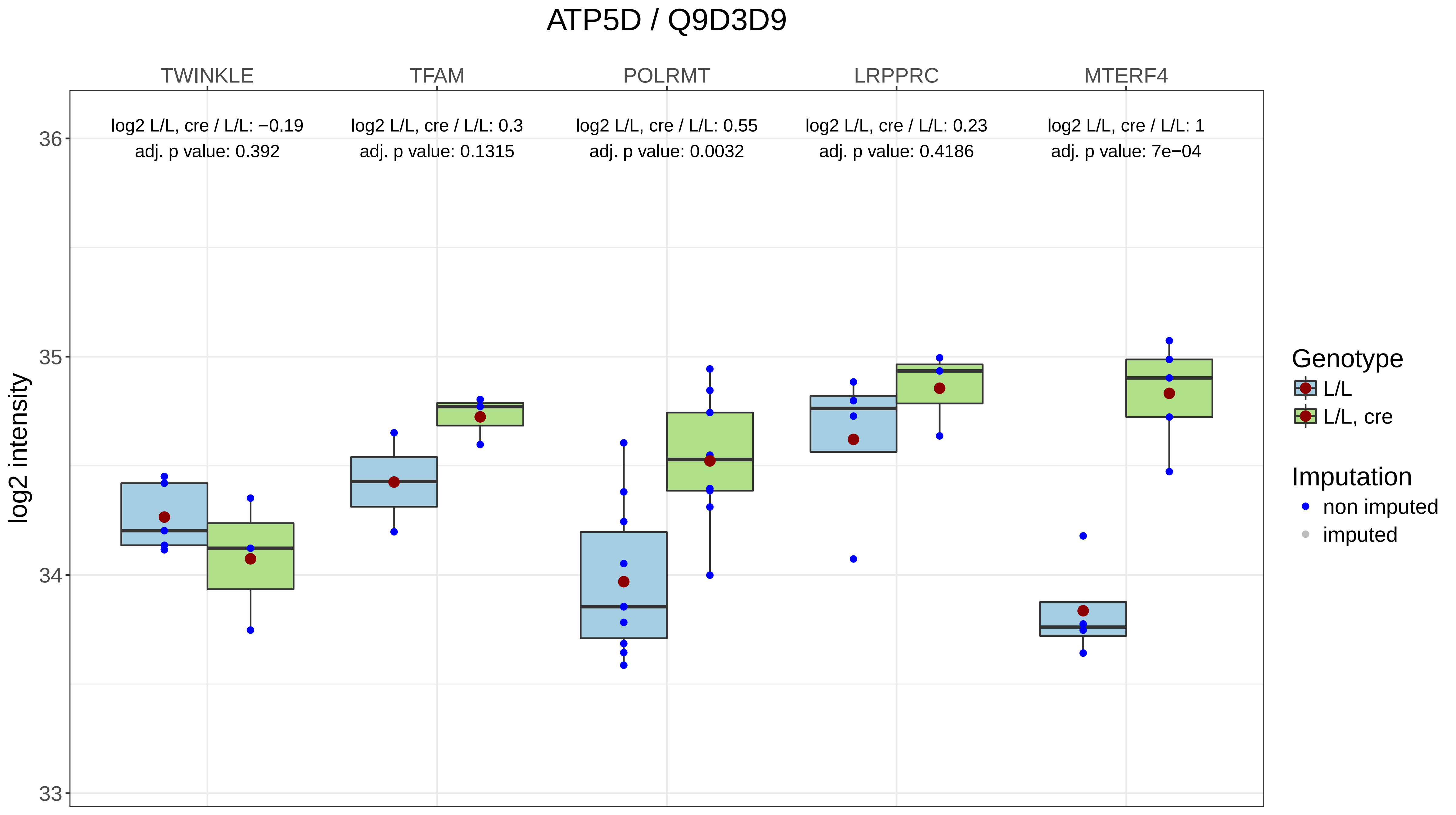
ATP5D / Q9D3D9
TWINKLE
TFAM
POLRMT
LRPPRC
MTERF4
log2 L/L, cre / L/L: −0.19
adj. p value: 0.392
log2 L/L, cre / L/L: 0.3
adj. p value: 0.1315
log2 L/L, cre / L/L: 0.55
adj. p value: 0.0032
log2 L/L, cre / L/L: 0.23
adj. p value: 0.4186
log2 L/L, cre / L/L: 1
adj. p value: 7e−04
log2 intensity
36
35
34
33
Genotype
L/L
L/L, cre
Imputation
non imputed
imputed

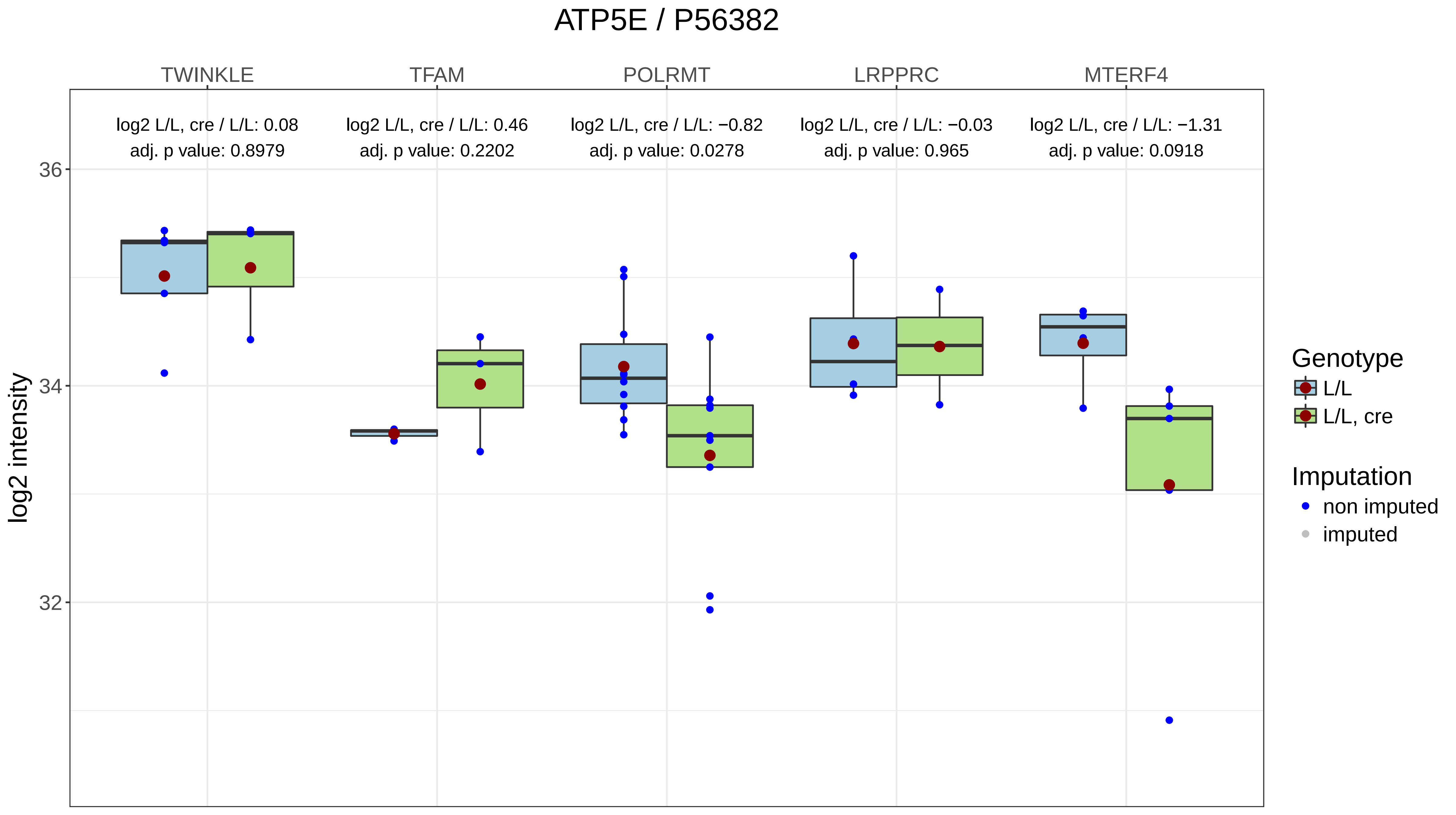
ATP5E / P56382
TWINKLE
TFAM
POLRMT
LRPPRC
MTERF4
log2 L/L, cre / L/L: 0.08
adj. p value: 0.8979
log2 L/L, cre / L/L: 0.46
adj. p value: 0.2202
log2 L/L, cre / L/L: −0.82
adj. p value: 0.0278
log2 L/L, cre / L/L: −0.03
adj. p value: 0.965
log2 L/L, cre / L/L: −1.31
adj. p value: 0.0918
log2 intensity
36
34
32
Genotype
L/L
L/L, cre
Imputation
non imputed
imputed

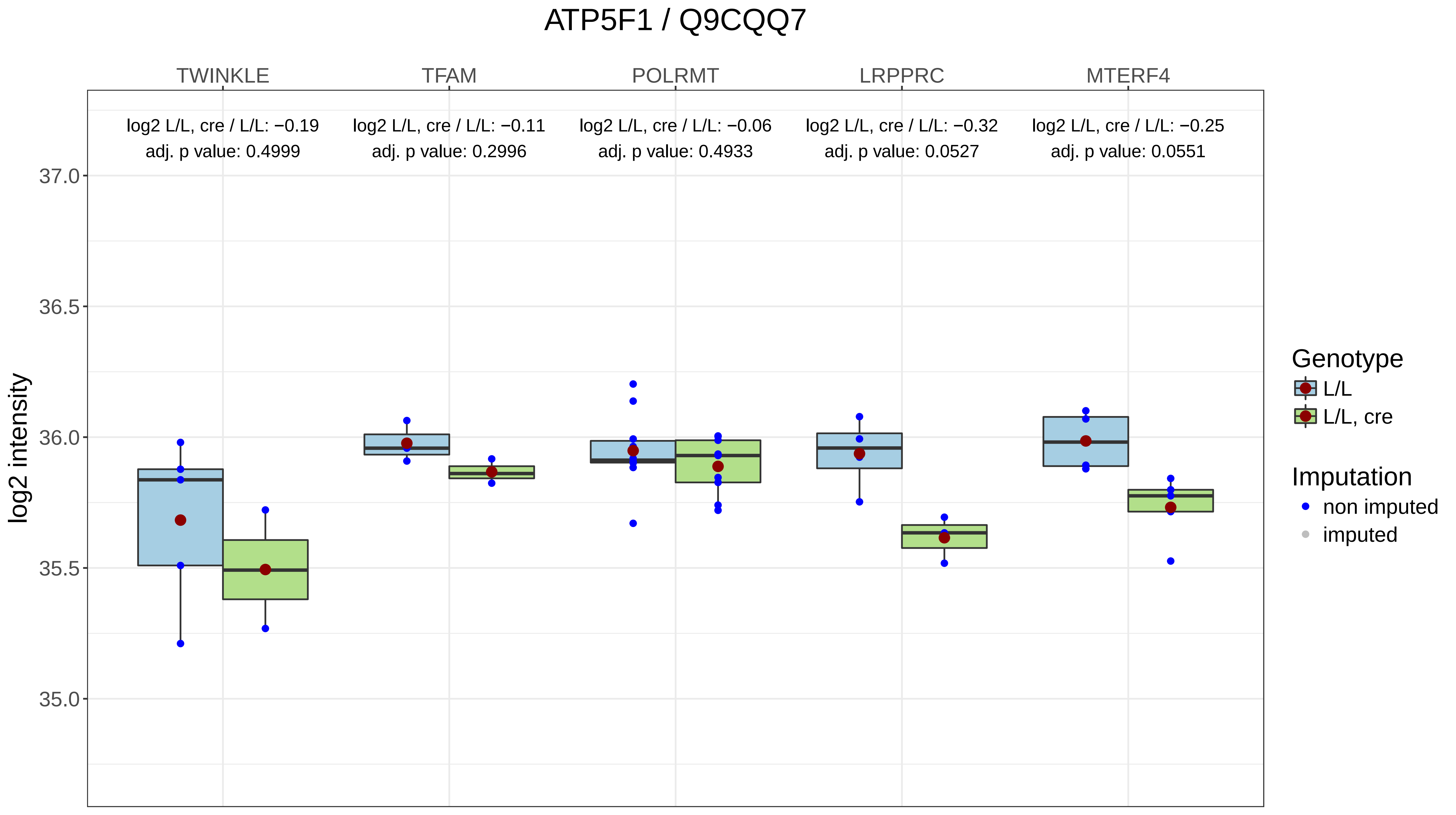
ATP5F1 / Q9CQQ7
TWINKLE
TFAM
POLRMT
LRPPRC
MTERF4
log2 L/L, cre / L/L: −0.19
adj. p value: 0.4999
log2 L/L, cre / L/L: −0.11
adj. p value: 0.2996
log2 L/L, cre / L/L: −0.06
adj. p value: 0.4933
log2 L/L, cre / L/L: −0.32
adj. p value: 0.0527
log2 L/L, cre / L/L: −0.25
adj. p value: 0.0551
37.0
36.5
36.0
35.5
35.0
log2 intensity
Genotype
L/L
L/L, cre
Imputation
non imputed
imputed

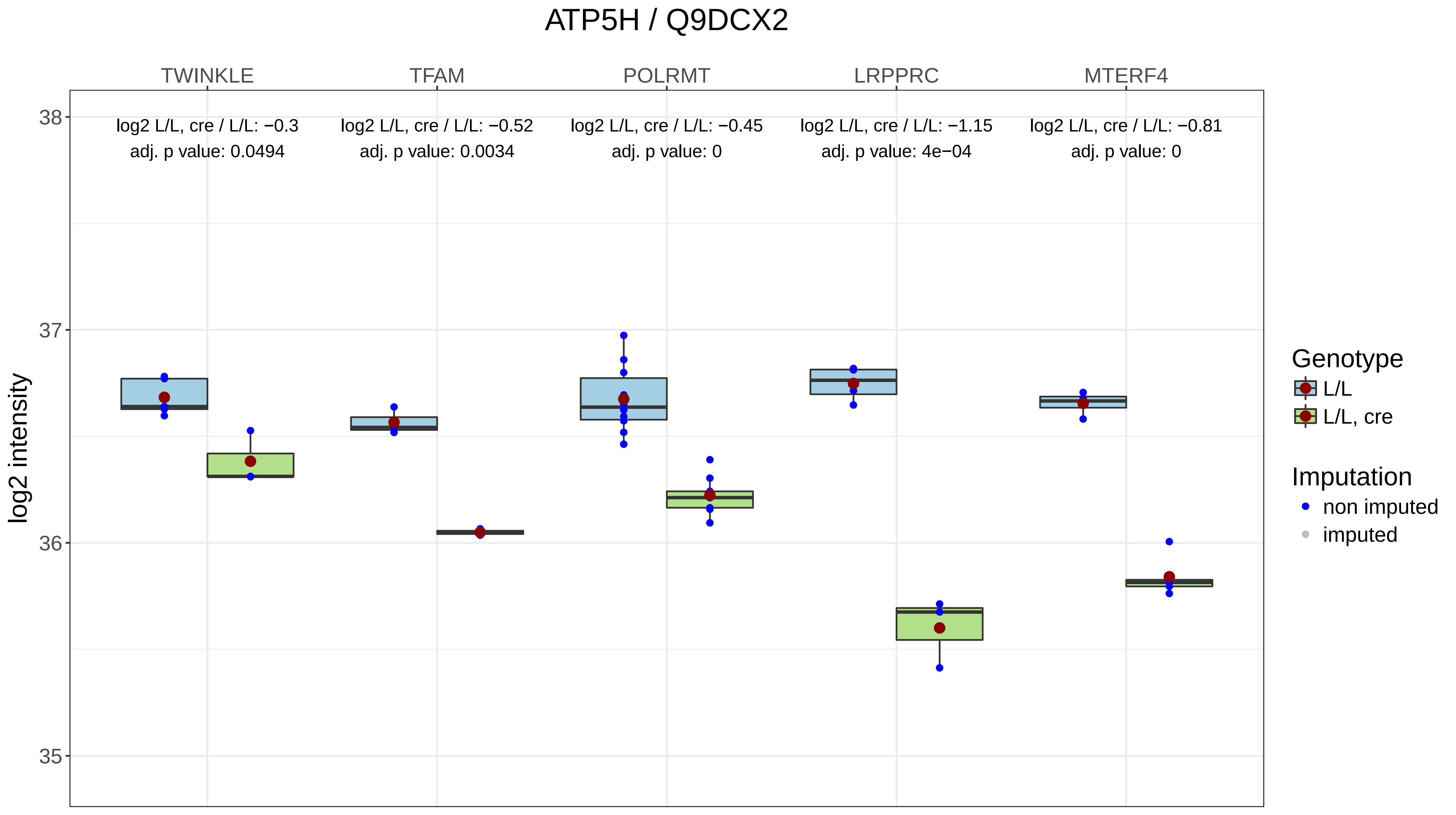
ATP5H / Q9DCX2
TWINKLE
TFAM
POLRMT
LRPPRC
MTERF4
log2 L/L, cre / L/L: −0.3
adj. p value: 0.0494
log2 L/L, cre / L/L: −0.52
adj. p value: 0.0034
log2 L/L, cre / L/L: −0.45
adj. p value: 0
log2 L/L, cre / L/L: −1.15
adj. p value: 4e−04
log2 L/L, cre / L/L: −0.81
adj. p value: 0
log2 intensity
38
37
36
35
Genotype
L/L
L/L, cre
Imputation
non imputed
imputed

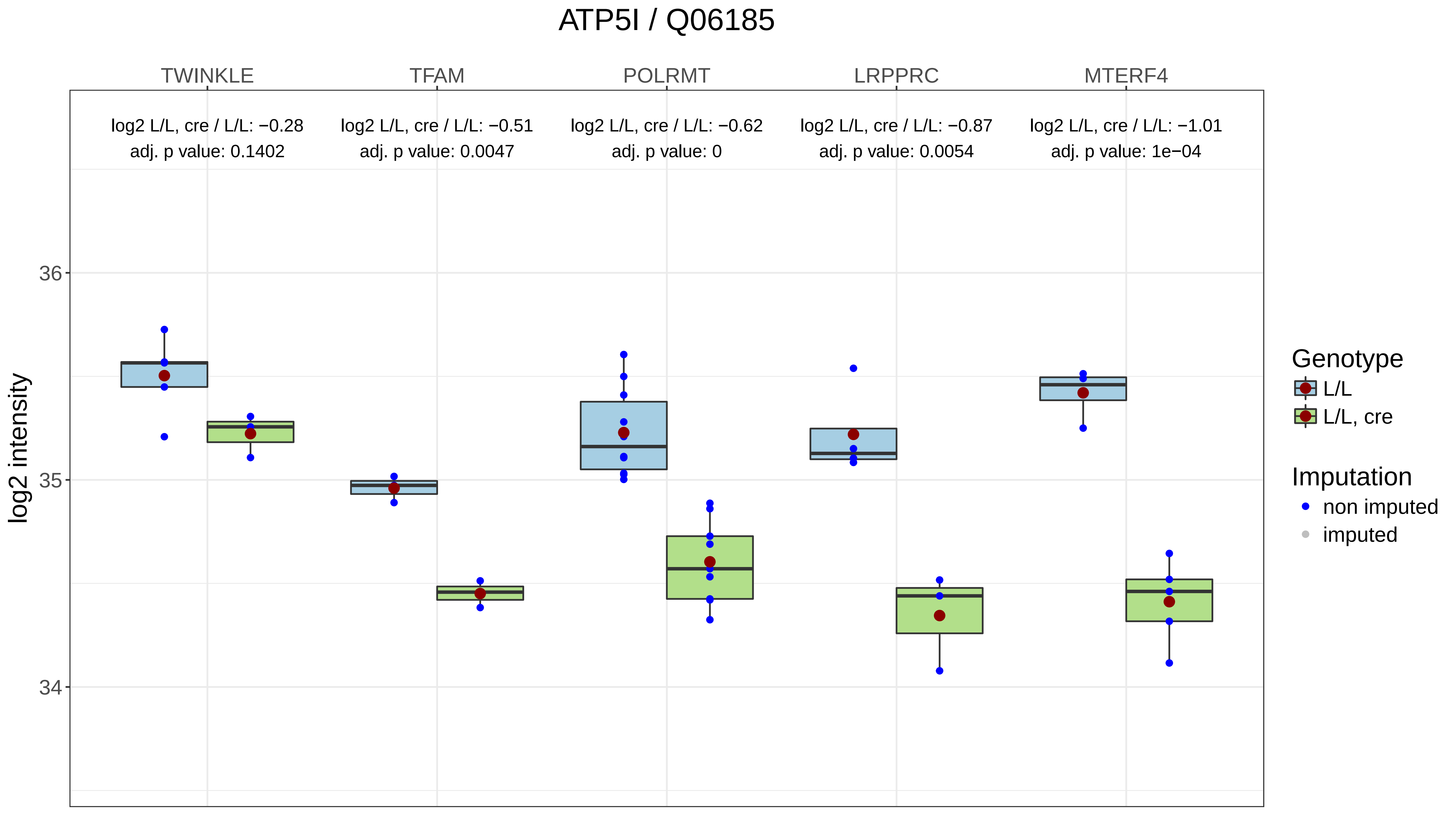
ATP5I / Q06185
TWINKLE
TFAM
POLRMT
LRPPRC
MTERF4
log2 L/L, cre / L/L: −0.28
adj. p value: 0.1402
log2 L/L, cre / L/L: −0.51
adj. p value: 0.0047
log2 L/L, cre / L/L: −0.62
adj. p value: 0
log2 L/L, cre / L/L: −0.87
adj. p value: 0.0054
log2 L/L, cre / L/L: −1.01
adj. p value: 1e−04
log2 intensity
36
35
34
Genotype
L/L
L/L, cre
Imputation
non imputed
imputed

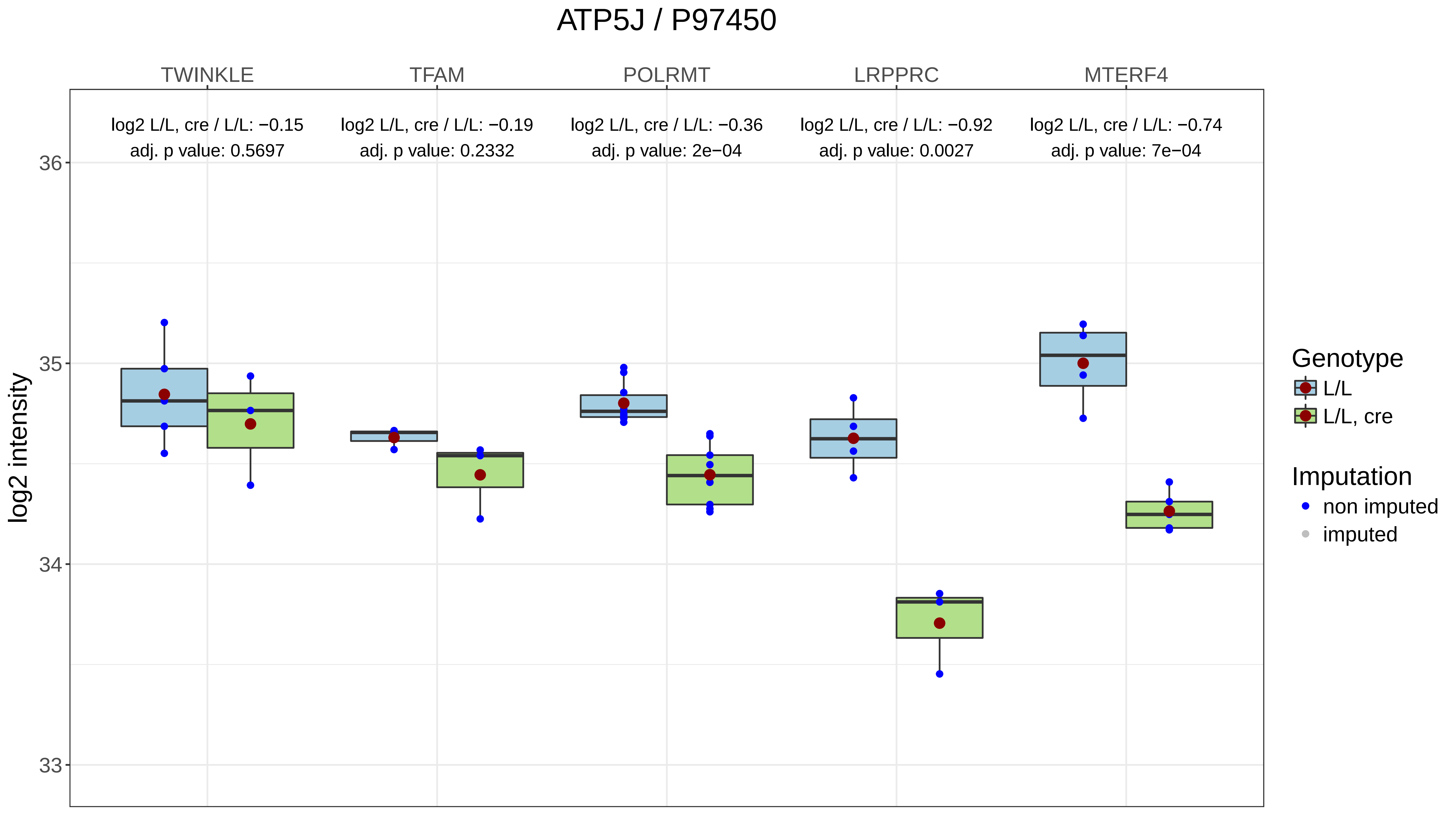
ATP5J / P97450
TWINKLE
TFAM
POLRMT
LRPPRC
MTERF4
log2 L/L, cre / L/L: −0.15
adj. p value: 0.5697
log2 L/L, cre / L/L: −0.19
adj. p value: 0.2332
log2 L/L, cre / L/L: −0.36
adj. p value: 2e−04
log2 L/L, cre / L/L: −0.92
adj. p value: 0.0027
log2 L/L, cre / L/L: −0.74
adj. p value: 7e−04
log2 intensity
36
35
34
33
Genotype
L/L
L/L, cre
Imputation
non imputed
imputed

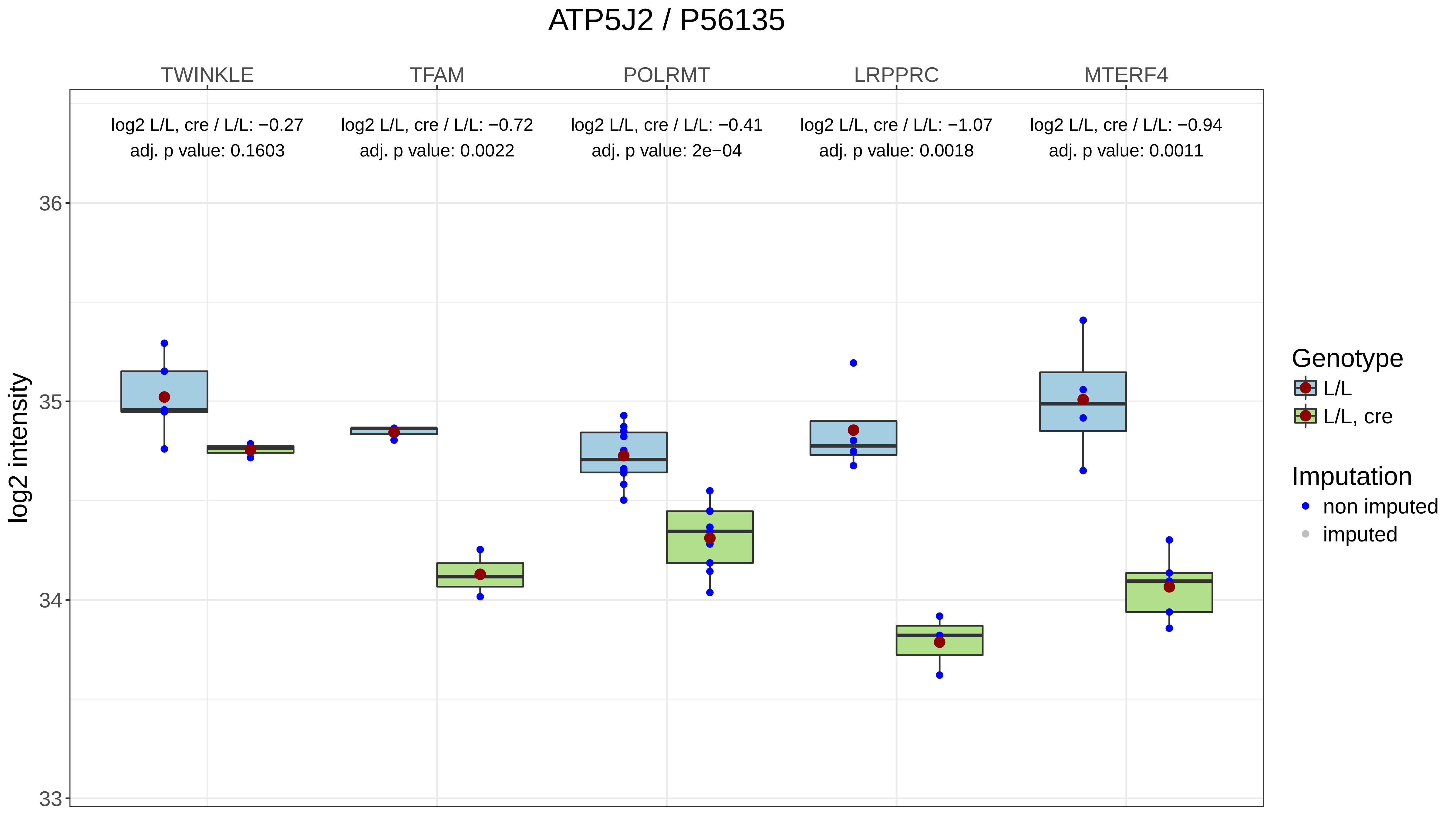
ATP5J2 / P56135
TWINKLE
TFAM
POLRMT
LRPPRC
MTERF4
log2 L/L, cre / L/L: −0.27
adj. p value: 0.1603
log2 L/L, cre / L/L: −0.72
adj. p value: 0.0022
log2 L/L, cre / L/L: −0.41
adj. p value: 2e−04
log2 L/L, cre / L/L: −1.07
adj. p value: 0.0018
log2 L/L, cre / L/L: −0.94
adj. p value: 0.0011
log2 intensity
36
35
34
33
Genotype
L/L
L/L, cre
Imputation
non imputed
imputed

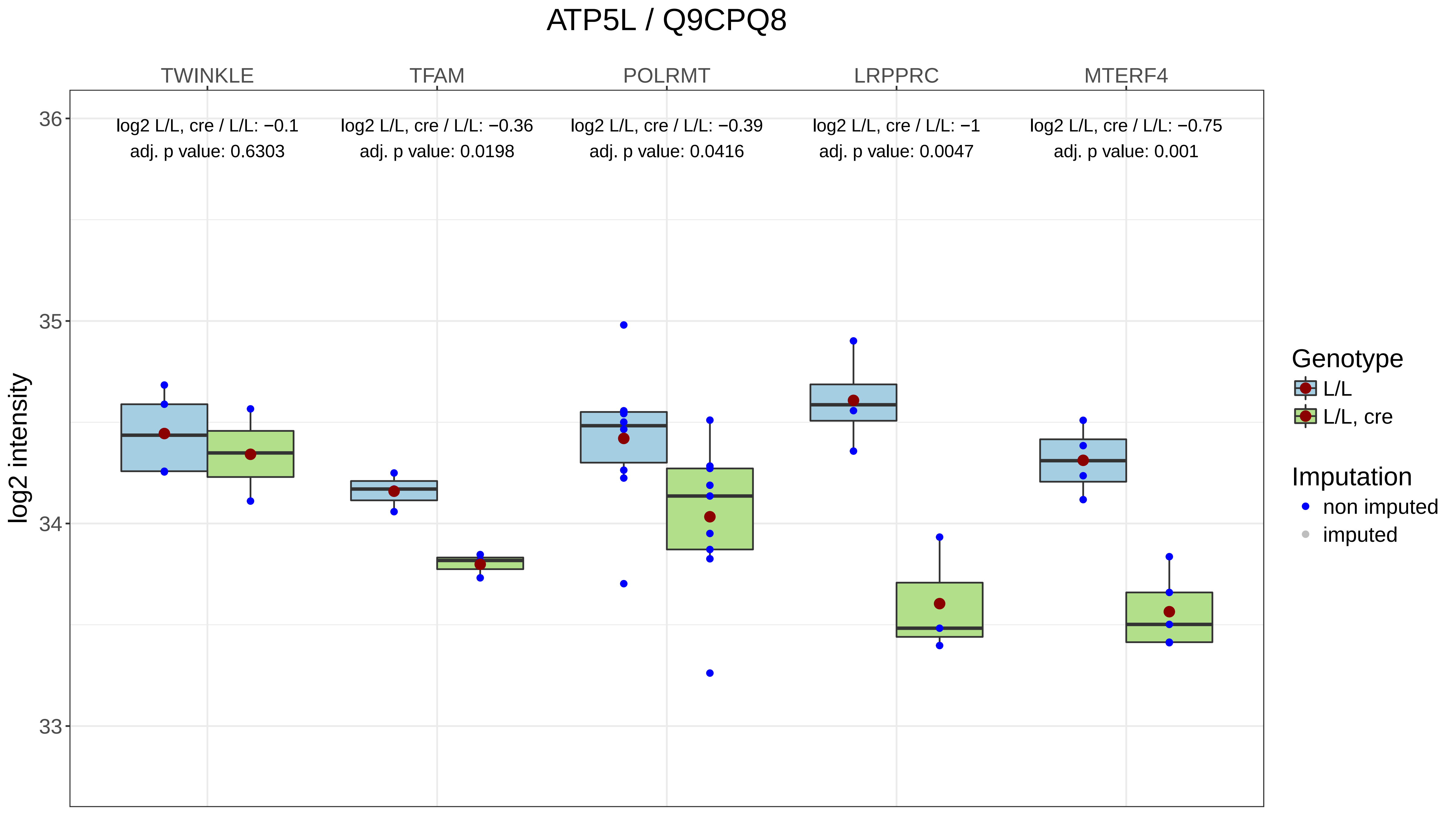
ATP5L / Q9CPQ8
TWINKLE
TFAM
POLRMT
LRPPRC
MTERF4
log2 L/L, cre / L/L: −0.1
adj. p value: 0.6303
log2 L/L, cre / L/L: −0.36
adj. p value: 0.0198
log2 L/L, cre / L/L: −0.39
adj. p value: 0.0416
log2 L/L, cre / L/L: −1
adj. p value: 0.0047
log2 L/L, cre / L/L: −0.75
adj. p value: 0.001
log2 intensity
36
35
34
33
Genotype
L/L
L/L, cre
Imputation
non imputed
imputed

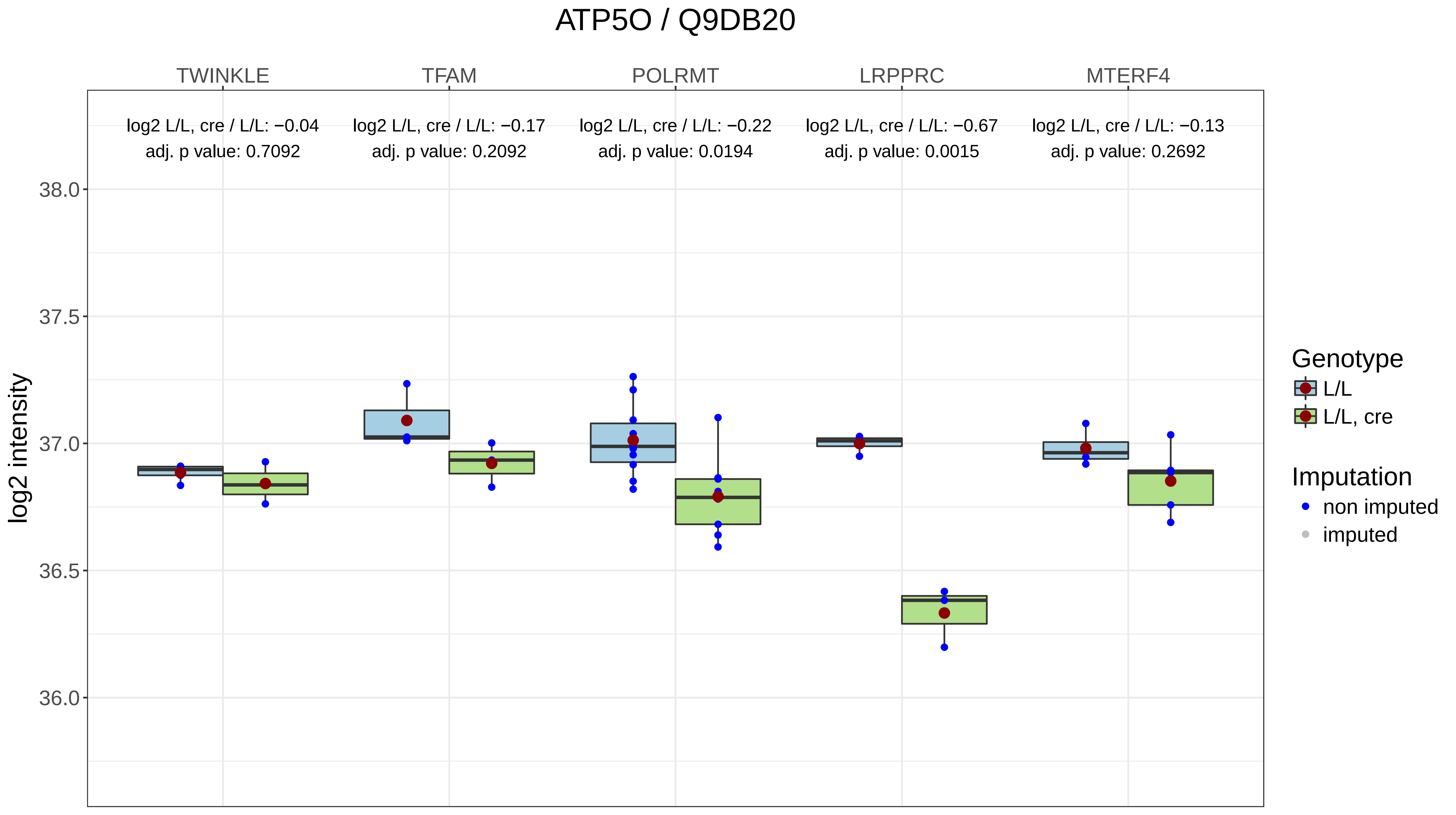

ATP5O / Q9DB20
TWINKLE
TFAM
POLRMT
LRPPRC
MTERF4
log2 L/L, cre / L/L: −0.04
adj. p value: 0.7092
log2 L/L, cre / L/L: −0.17
adj. p value: 0.2092
log2 L/L, cre / L/L: −0.22
adj. p value: 0.0194
log2 L/L, cre / L/L: −0.67
adj. p value: 0.0015
log2 L/L, cre / L/L: −0.13
adj. p value: 0.2692
38.0
37.5
37.0
36.5
36.0
log2 intensity
Genotype
L/L
L/L, cre
Imputation
non imputed
imputed

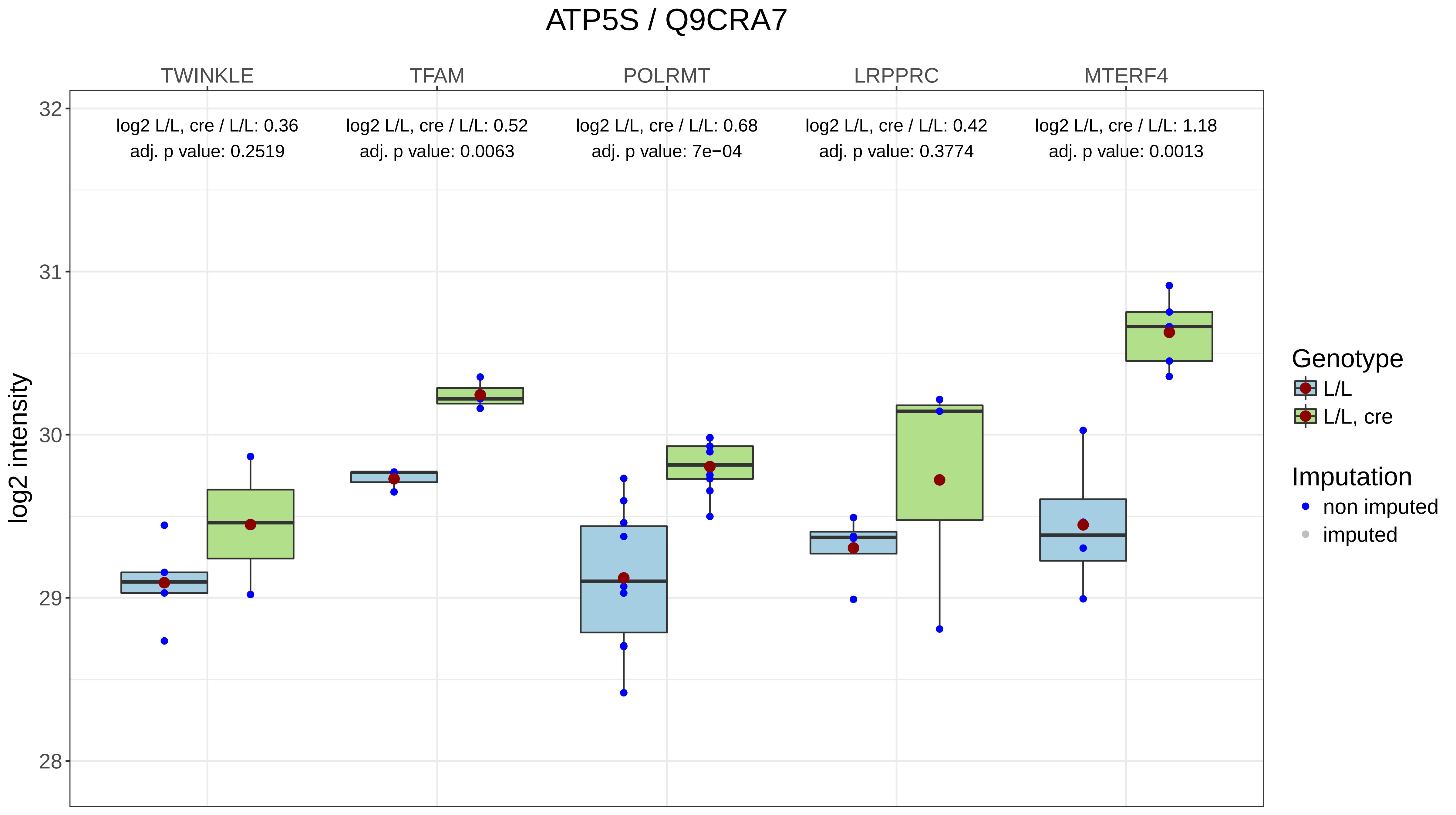
ATP5S / Q9CRA7
TWINKLE
TFAM
POLRMT
LRPPRC
MTERF4
log2 L/L, cre / L/L: 0.36
adj. p value: 0.2519
log2 L/L, cre / L/L: 0.52
adj. p value: 0.0063
log2 L/L, cre / L/L: 0.68
adj. p value: 7e−04
log2 L/L, cre / L/L: 0.42
adj. p value: 0.3774
log2 L/L, cre / L/L: 1.18
adj. p value: 0.0013
log2 intensity
32
31
30
29
28
Genotype
L/L
L/L, cre
Imputation
non imputed
imputed

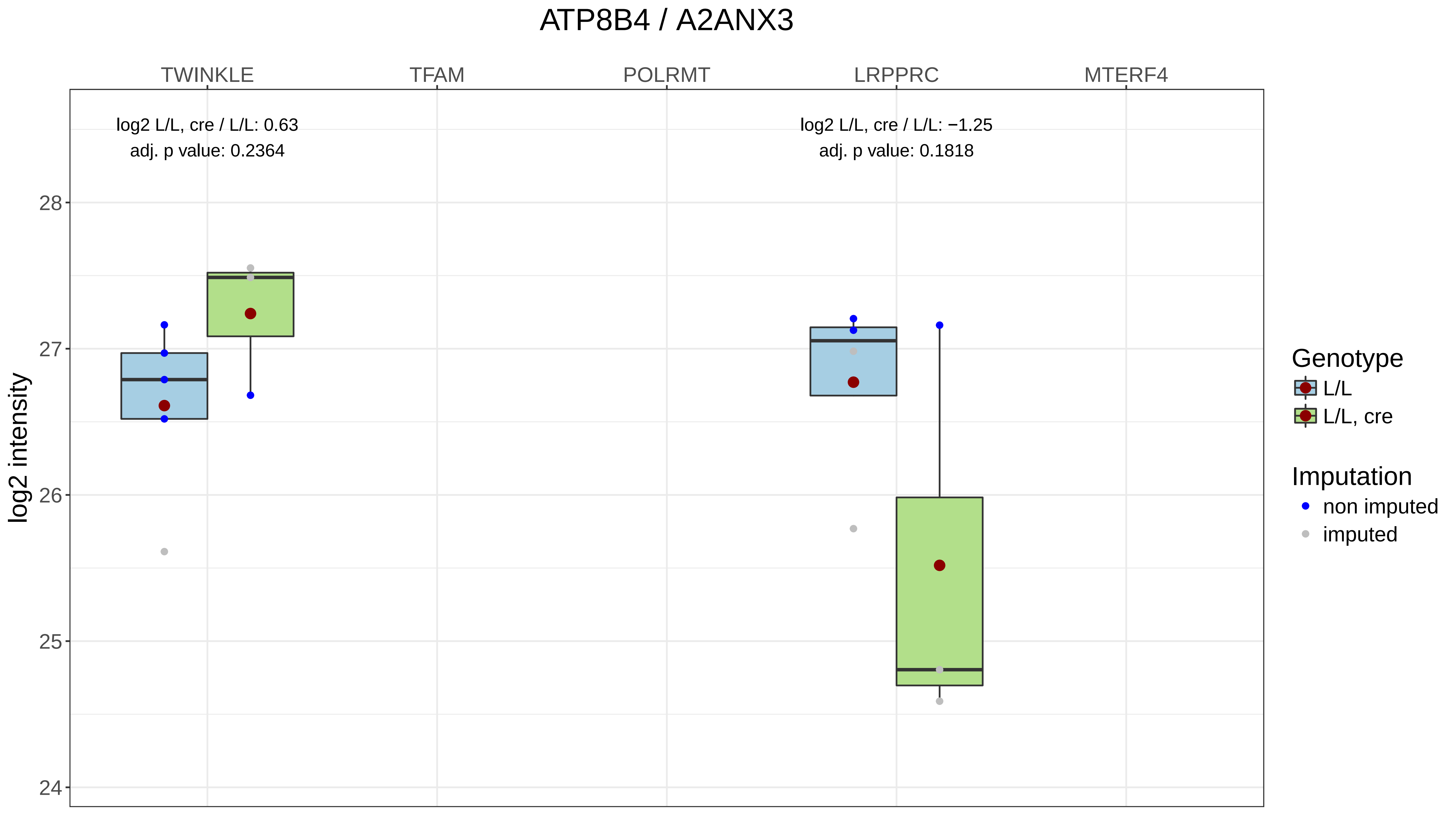
ATP8B4 / A2ANX3
TWINKLE
TFAM
POLRMT
LRPPRC
MTERF4
log2 L/L, cre / L/L: 0.63
adj. p value: 0.2364
log2 L/L, cre / L/L: −1.25
adj. p value: 0.1818
log2 intensity
28
27
26
25
24
Genotype
L/L
L/L, cre
Imputation
non imputed
imputed

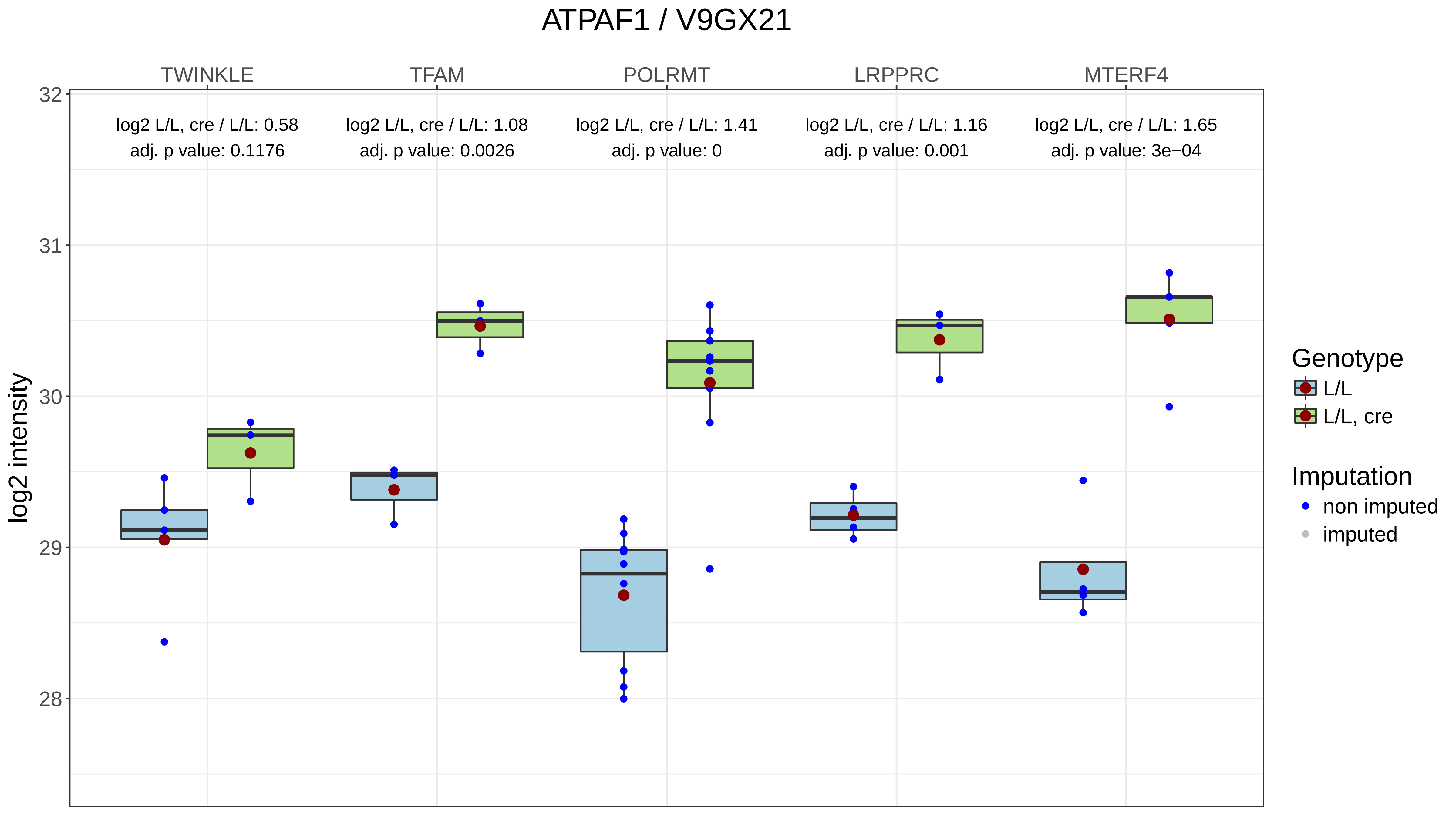
ATPAF1 / V9GX21
TWINKLE
TFAM
POLRMT
LRPPRC
MTERF4
log2 L/L, cre / L/L: 0.58
adj. p value: 0.1176
log2 L/L, cre / L/L: 1.08
adj. p value: 0.0026
log2 L/L, cre / L/L: 1.41
adj. p value: 0
log2 L/L, cre / L/L: 1.16
adj. p value: 0.001
log2 L/L, cre / L/L: 1.65
adj. p value: 3e−04
log2 intensity
32
31
30
29
28
Genotype
L/L
L/L, cre
Imputation
non imputed
imputed

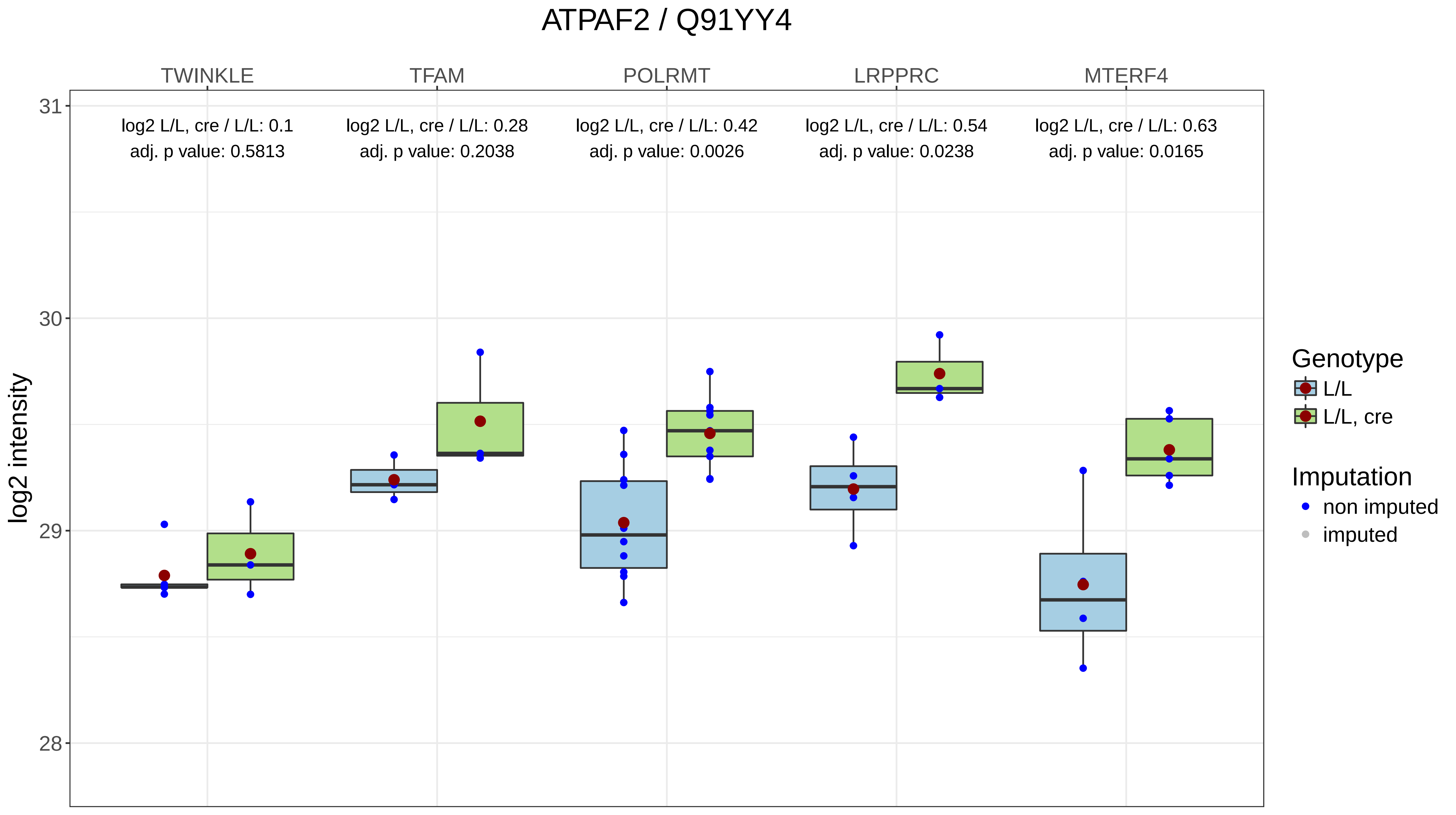

ATPAF2 / Q91YY4
TWINKLE
TFAM
POLRMT
LRPPRC
MTERF4
log2 L/L, cre / L/L: 0.1
adj. p value: 0.5813
log2 L/L, cre / L/L: 0.28
adj. p value: 0.2038
log2 L/L, cre / L/L: 0.42
adj. p value: 0.0026
log2 L/L, cre / L/L: 0.54
adj. p value: 0.0238
log2 L/L, cre / L/L: 0.63
adj. p value: 0.0165
31
30
29
28
log2 intensity
Genotype
L/L
L/L, cre
Imputation
non imputed
imputed

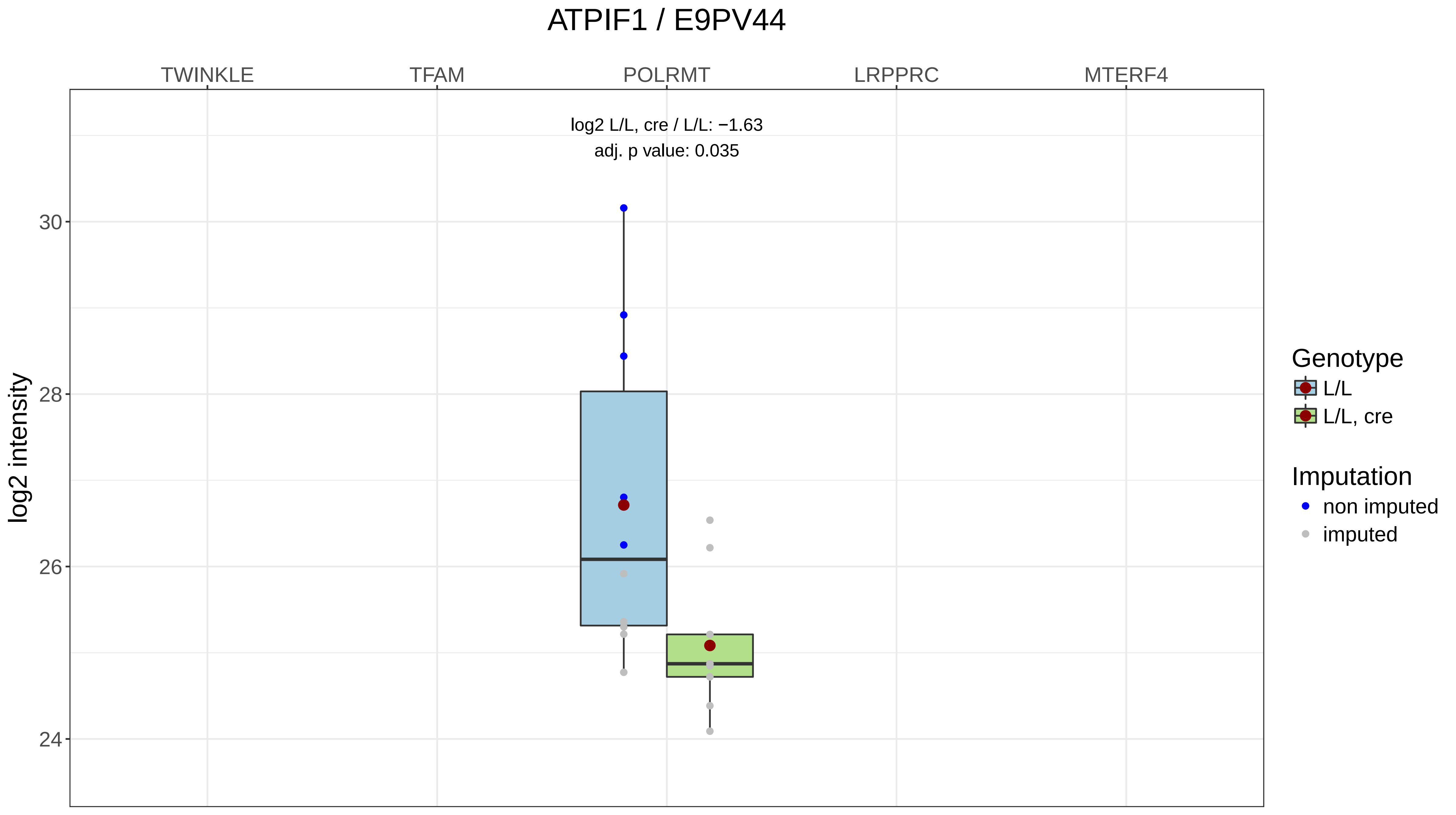
ATPIF1 / E9PV44
TWINKLE
TFAM
POLRMT
LRPPRC
MTERF4
log2 L/L, cre / L/L: −1.63
adj. p value: 0.035
log2 intensity
30
28
26
24
Genotype
L/L
L/L, cre
Imputation
non imputed
imputed

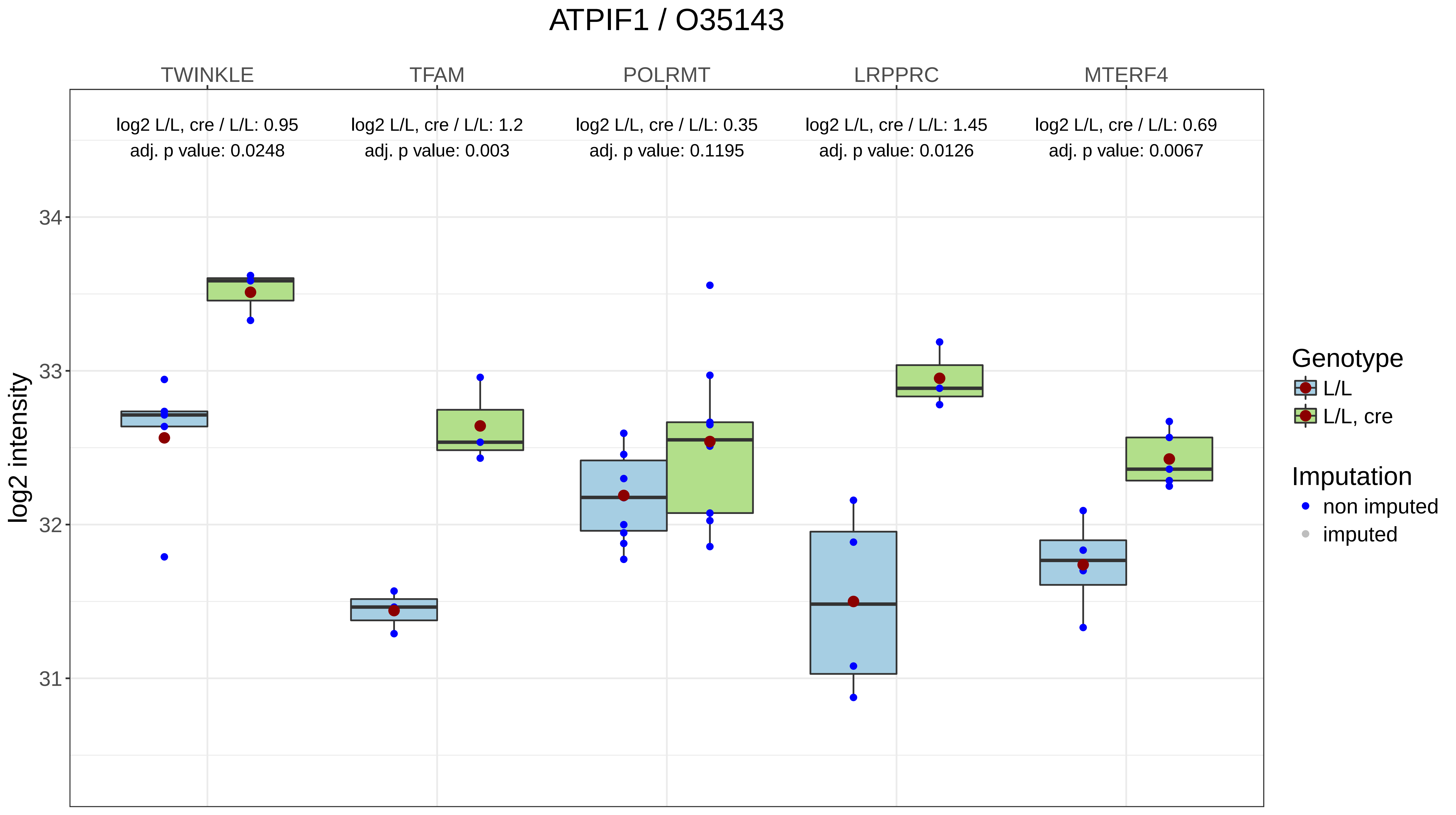
ATPIF1 / O35143
TWINKLE
TFAM
POLRMT
LRPPRC
MTERF4
log2 L/L, cre / L/L: 0.95
adj. p value: 0.0248
log2 L/L, cre / L/L: 1.2
adj. p value: 0.003
log2 L/L, cre / L/L: 0.35
adj. p value: 0.1195
log2 L/L, cre / L/L: 1.45
adj. p value: 0.0126
log2 L/L, cre / L/L: 0.69
adj. p value: 0.0067
log2 intensity
34
33
32
31
Genotype
L/L
L/L, cre
Imputation
non imputed
imputed

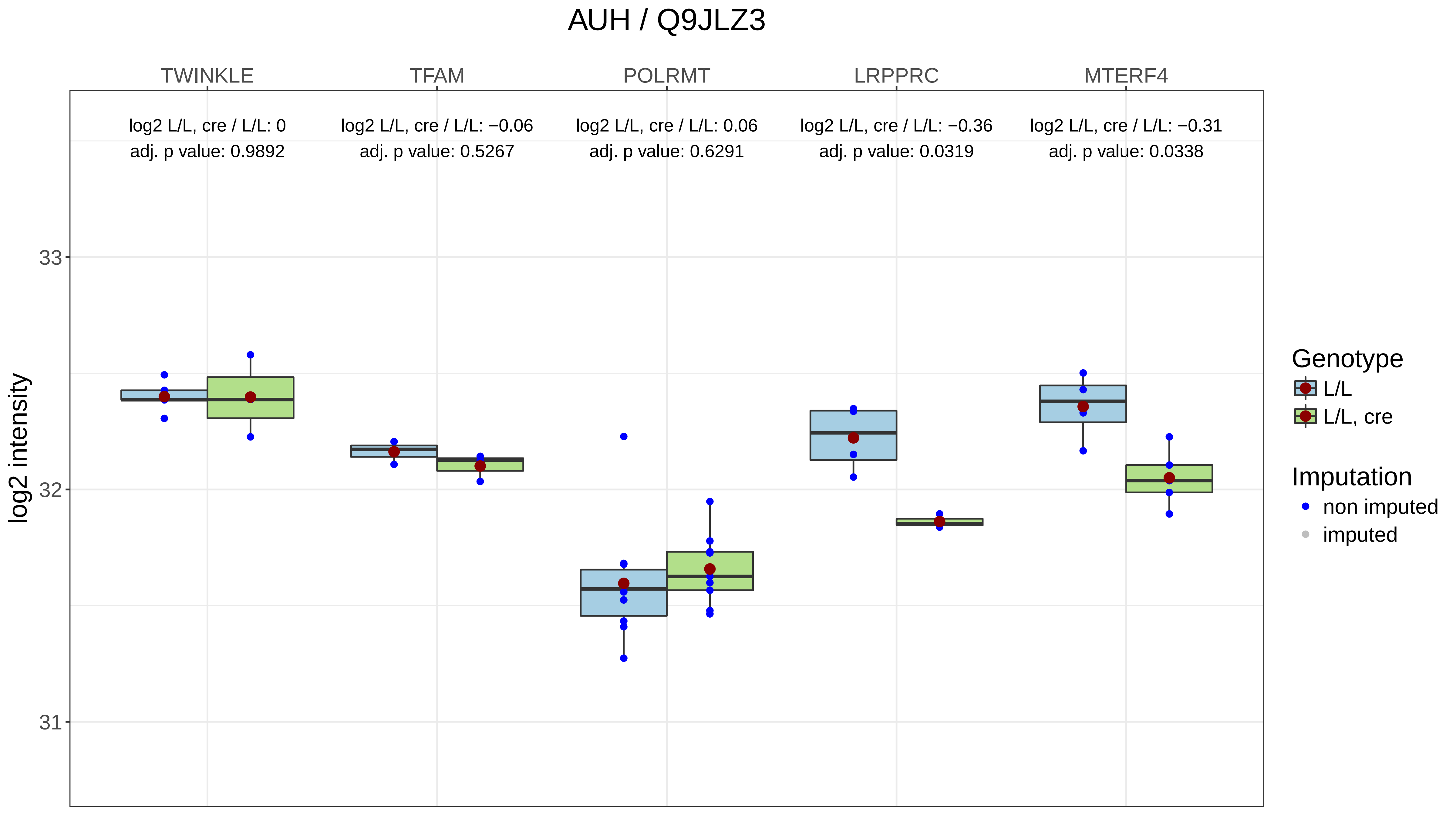
AUH / Q9JLZ3
TWINKLE
TFAM
POLRMT
LRPPRC
MTERF4
log2 L/L, cre / L/L: 0
adj. p value: 0.9892
log2 L/L, cre / L/L: −0.06
adj. p value: 0.5267
log2 L/L, cre / L/L: 0.06
adj. p value: 0.6291
log2 L/L, cre / L/L: −0.36
adj. p value: 0.0319
log2 L/L, cre / L/L: −0.31
adj. p value: 0.0338
33
32
31
log2 intensity
Genotype
L/L
L/L, cre
Imputation
non imputed
imputed

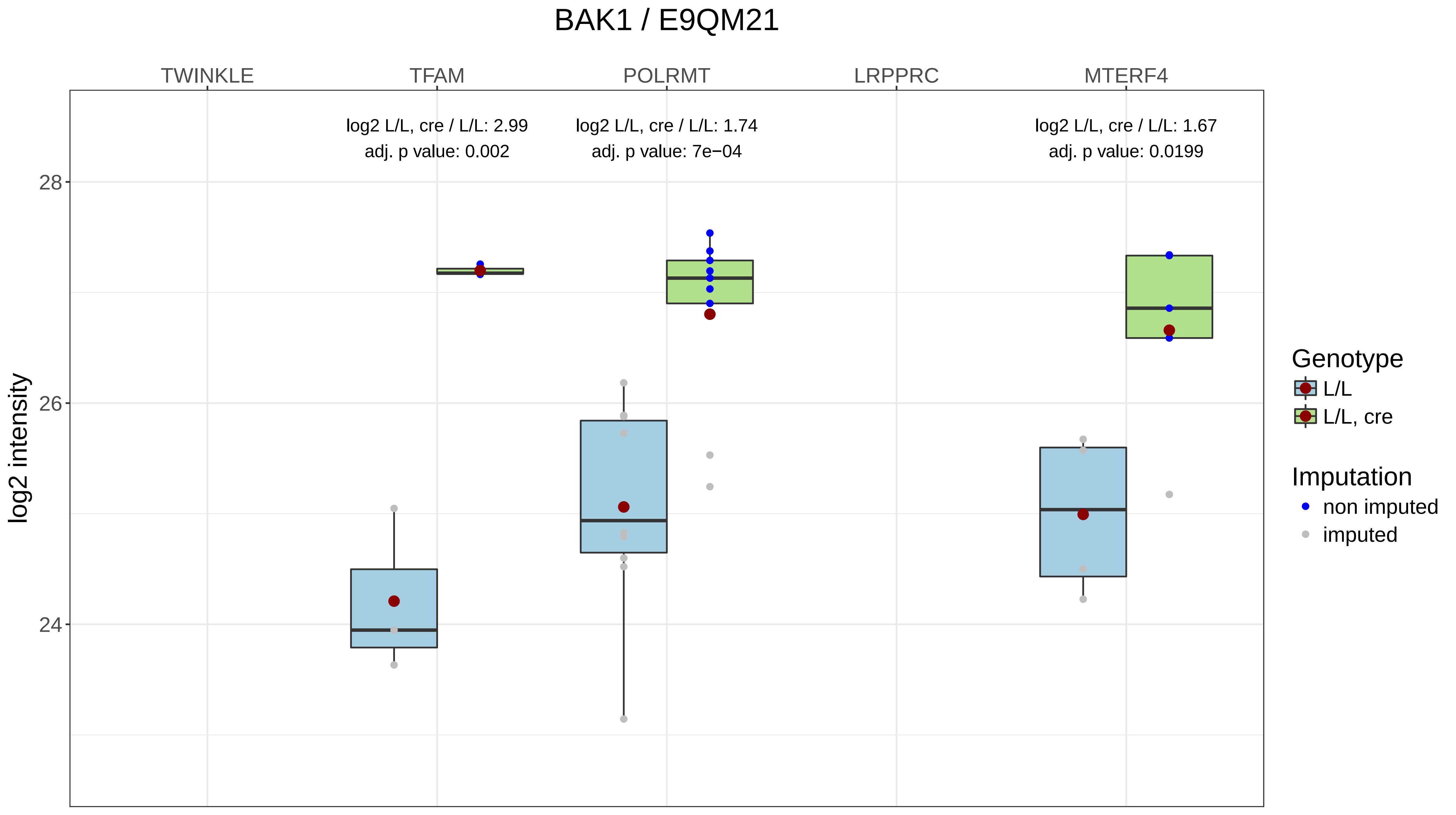
BAK1 / E9QM21
TWINKLE
TFAM
POLRMT
LRPPRC
MTERF4
log2 L/L, cre / L/L: 2.99
adj. p value: 0.002
log2 L/L, cre / L/L: 1.74
adj. p value: 7e−04
log2 L/L, cre / L/L: 1.67
adj. p value: 0.0199
28
26
24
log2 intensity
Genotype
L/L
L/L, cre
Imputation
non imputed
imputed

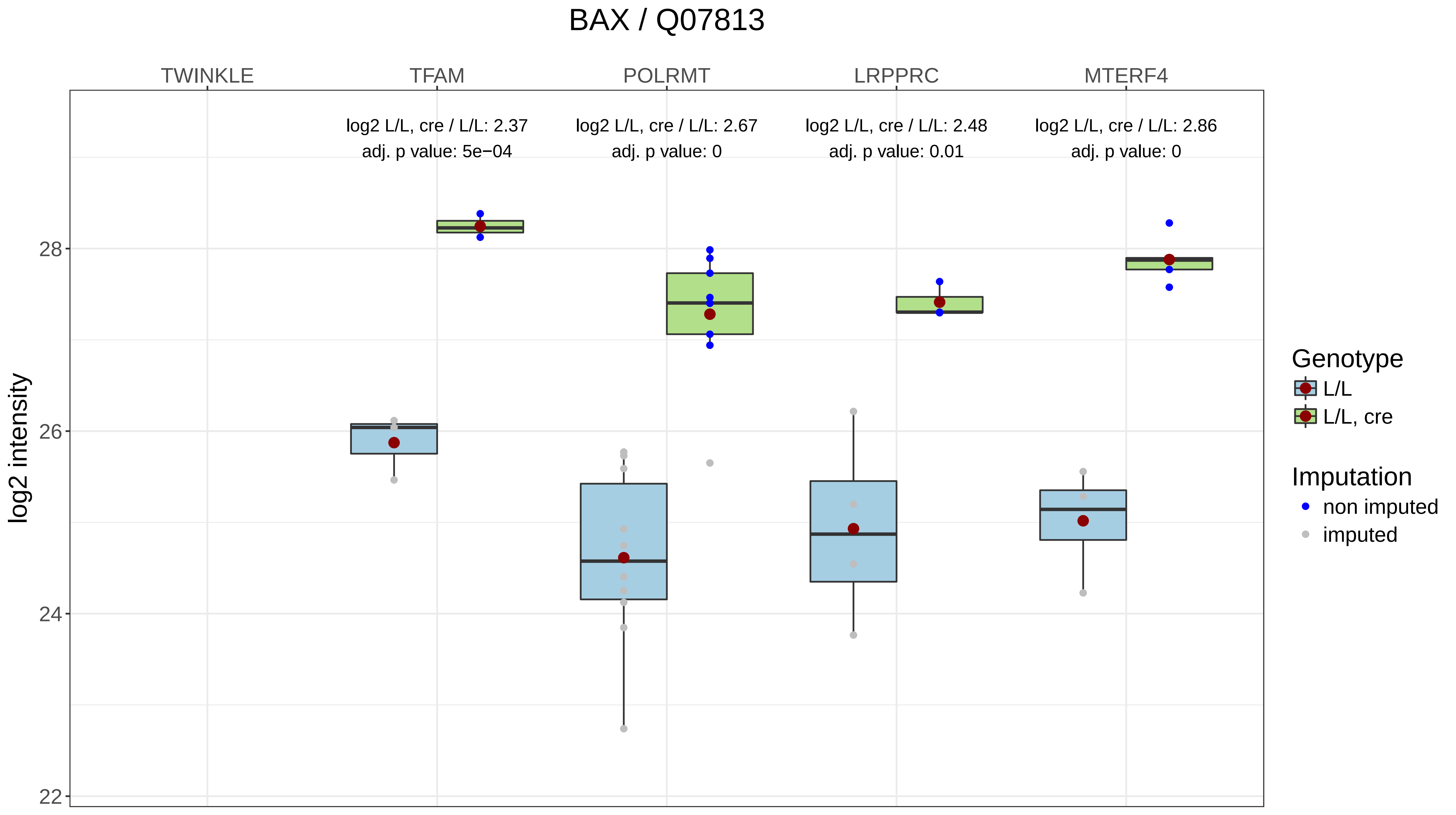
BAX / Q07813
TWINKLE
TFAM
POLRMT
LRPPRC
MTERF4
log2 L/L, cre / L/L: 2.37
adj. p value: 5e−04
log2 L/L, cre / L/L: 2.67
adj. p value: 0
log2 L/L, cre / L/L: 2.48
adj. p value: 0.01
log2 L/L, cre / L/L: 2.86
adj. p value: 0
log2 intensity
28
26
24
22
Genotype
L/L
L/L, cre
Imputation
non imputed
imputed

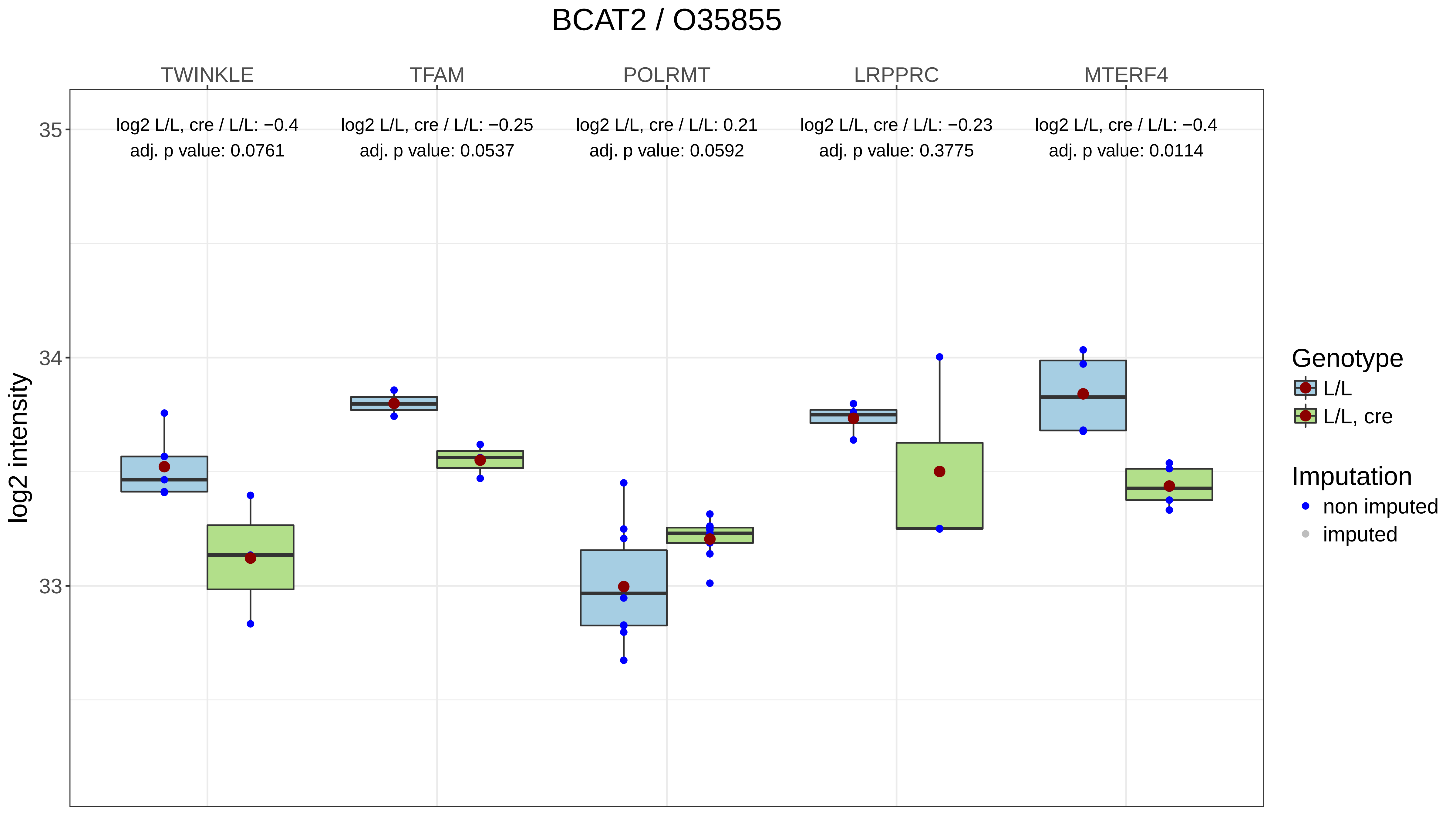
BCAT2 / O35855
TWINKLE
TFAM
POLRMT
LRPPRC
MTERF4
log2 L/L, cre / L/L: −0.4
adj. p value: 0.0761
log2 L/L, cre / L/L: −0.25
adj. p value: 0.0537
log2 L/L, cre / L/L: 0.21
adj. p value: 0.0592
log2 L/L, cre / L/L: −0.23
adj. p value: 0.3775
log2 L/L, cre / L/L: −0.4
adj. p value: 0.0114
35
34
33
log2 intensity
Genotype
L/L
L/L, cre
Imputation
non imputed
imputed

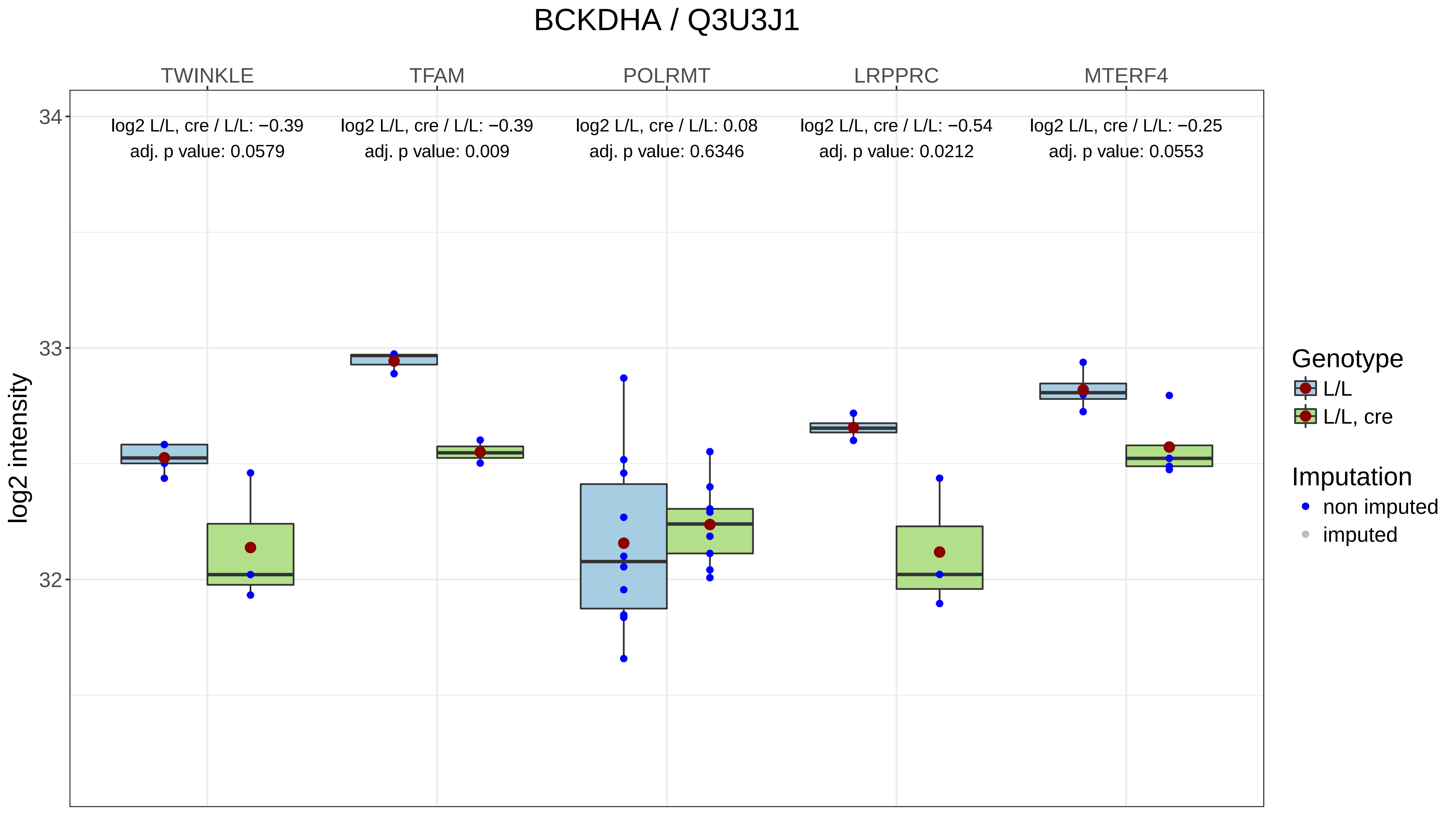
BCKDHA / Q3U3J1
TWINKLE
TFAM
POLRMT
LRPPRC
MTERF4
log2 L/L, cre / L/L: −0.39
adj. p value: 0.0579
log2 L/L, cre / L/L: −0.39
adj. p value: 0.009
log2 L/L, cre / L/L: 0.08
adj. p value: 0.6346
log2 L/L, cre / L/L: −0.54
adj. p value: 0.0212
log2 L/L, cre / L/L: −0.25
adj. p value: 0.0553
34
33
32
log2 intensity
Genotype
L/L
L/L, cre
Imputation
non imputed
imputed

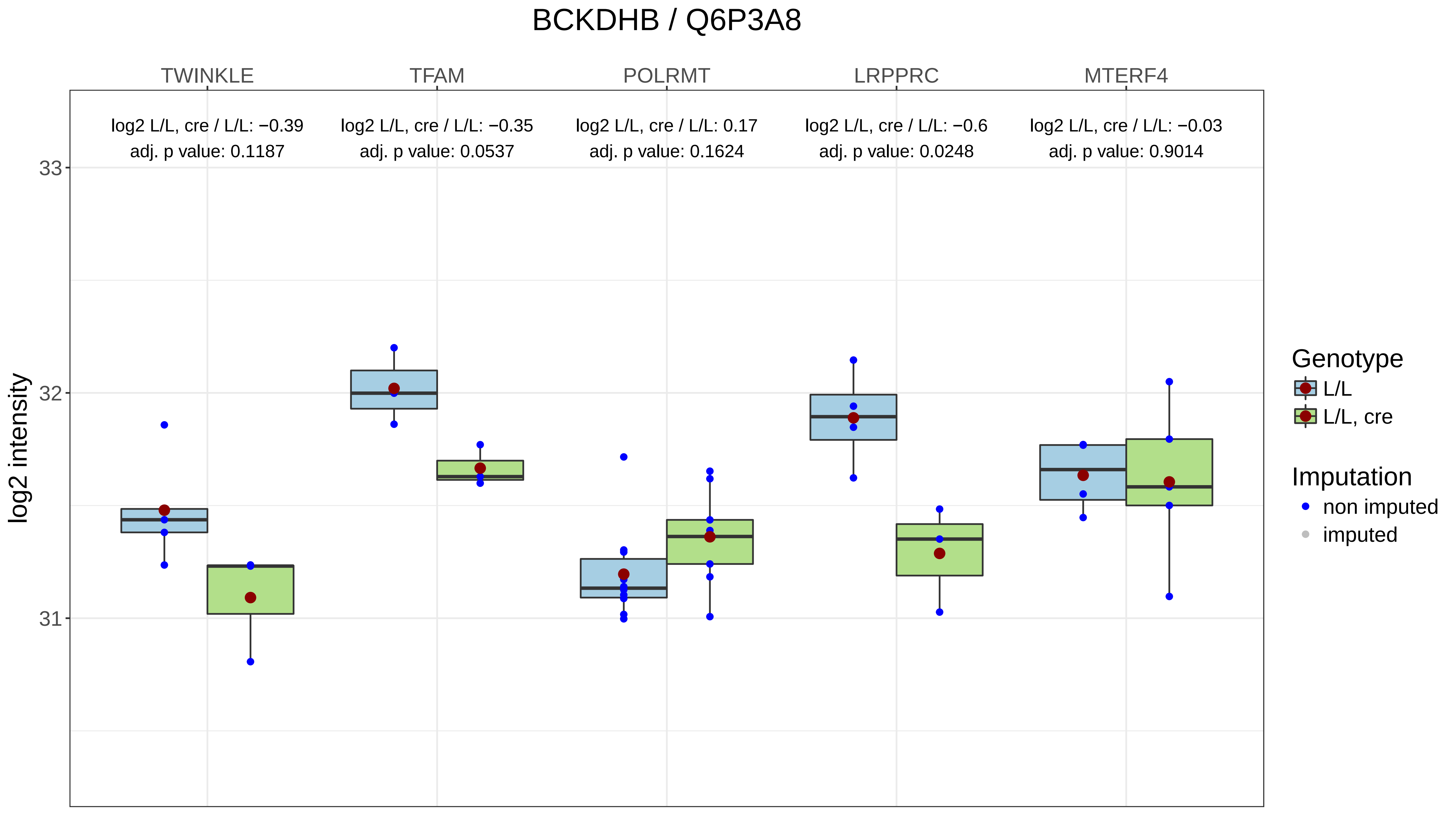
BCKDHB / Q6P3A8
TWINKLE
TFAM
POLRMT
LRPPRC
MTERF4
log2 L/L, cre / L/L: −0.39
adj. p value: 0.1187
log2 L/L, cre / L/L: −0.35
adj. p value: 0.0537
log2 L/L, cre / L/L: 0.17
adj. p value: 0.1624
log2 L/L, cre / L/L: −0.6
adj. p value: 0.0248
log2 L/L, cre / L/L: −0.03
adj. p value: 0.9014
log2 intensity
33
32
31
Genotype
L/L
L/L, cre
Imputation
non imputed
imputed

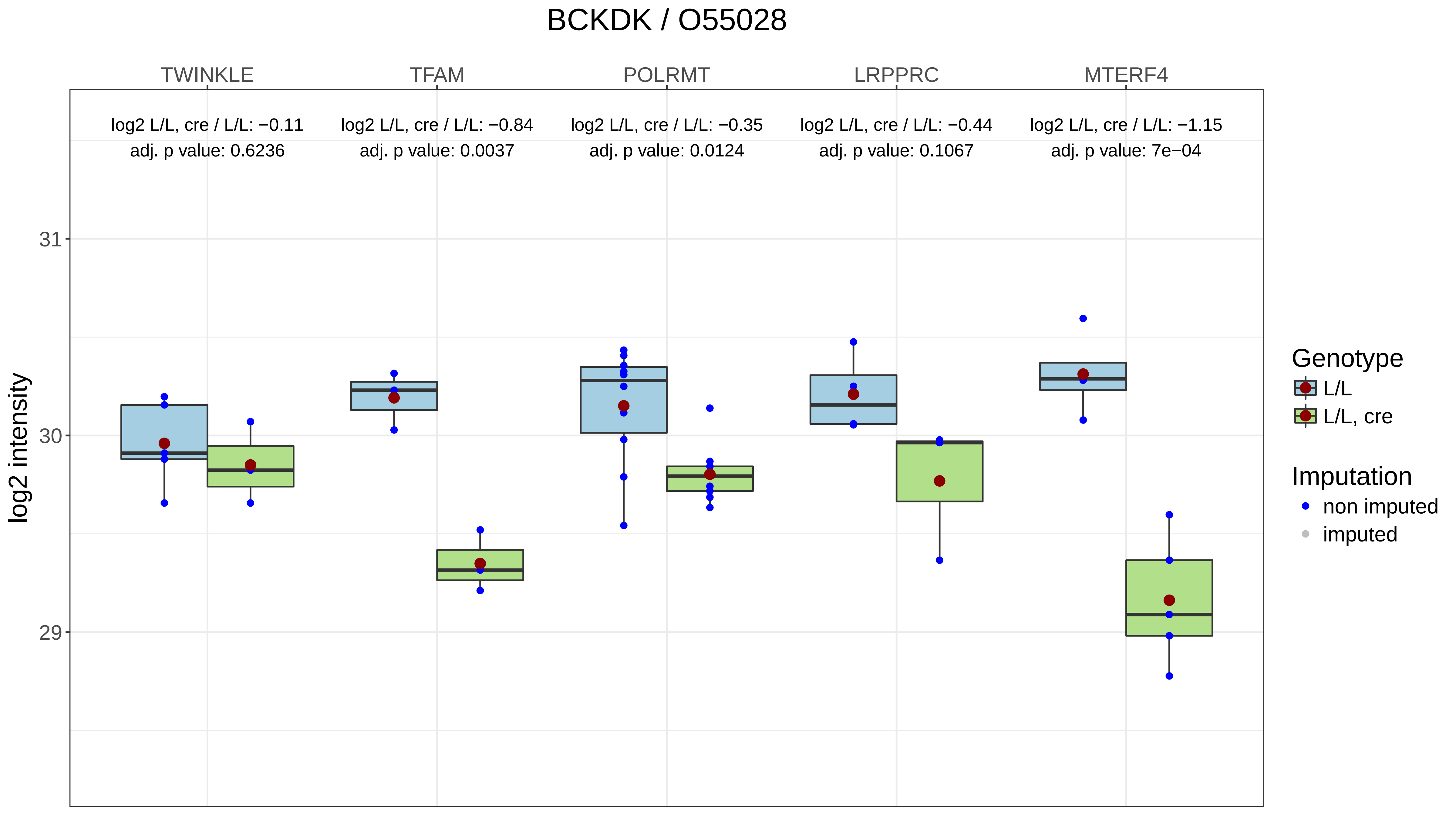
BCKDK / O55028
TWINKLE
TFAM
POLRMT
LRPPRC
MTERF4
log2 L/L, cre / L/L: −0.11
adj. p value: 0.6236
log2 L/L, cre / L/L: −0.84
adj. p value: 0.0037
log2 L/L, cre / L/L: −0.35
adj. p value: 0.0124
log2 L/L, cre / L/L: −0.44
adj. p value: 0.1067
log2 L/L, cre / L/L: −1.15
adj. p value: 7e−04
log2 intensity
31
30
29
Genotype
L/L
L/L, cre
Imputation
non imputed
imputed

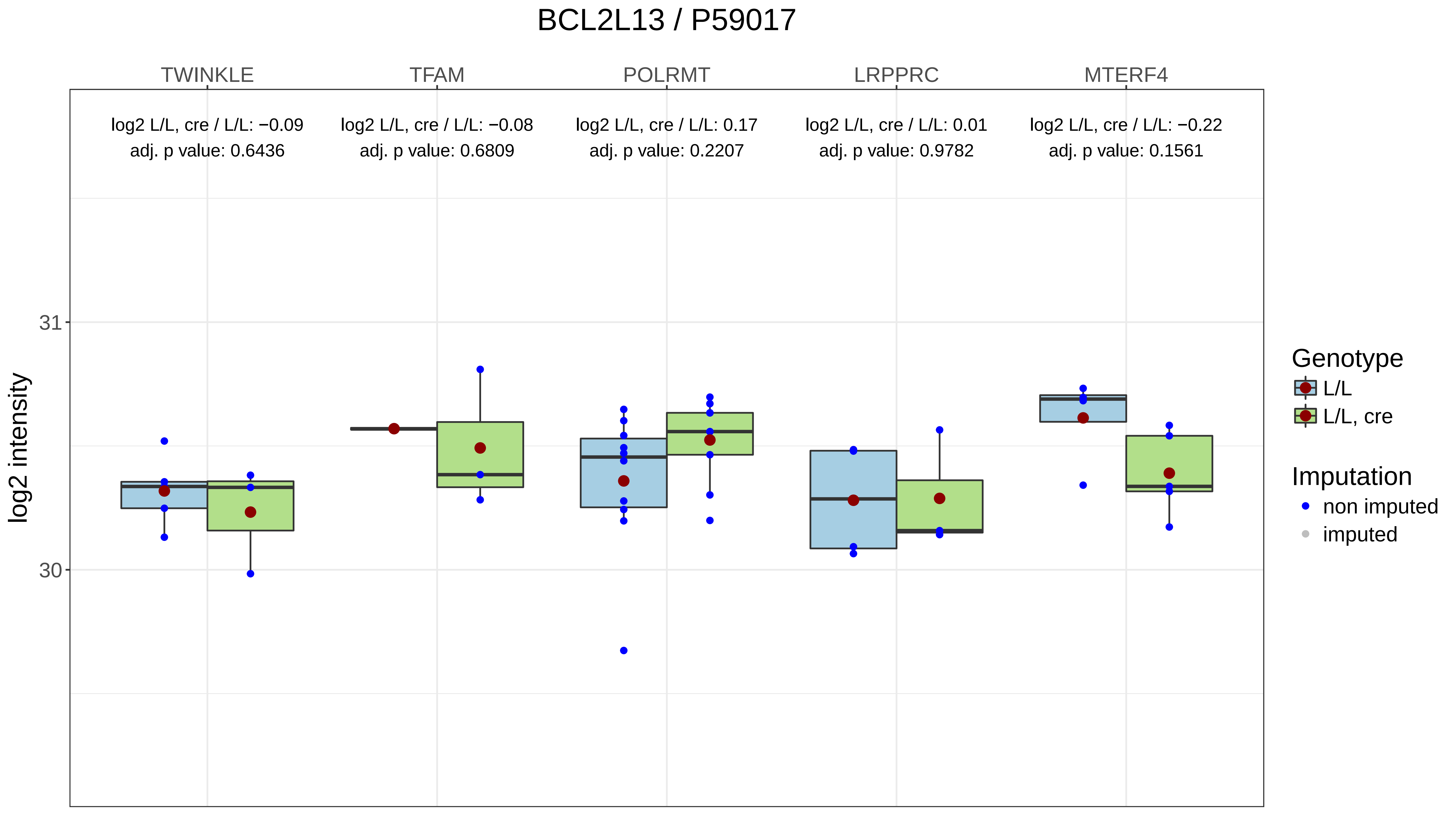
BCL2L13 / P59017
TWINKLE
TFAM
POLRMT
LRPPRC
MTERF4
log2 L/L, cre / L/L: −0.09
adj. p value: 0.6436
log2 L/L, cre / L/L: −0.08
adj. p value: 0.6809
log2 L/L, cre / L/L: 0.17
adj. p value: 0.2207
log2 L/L, cre / L/L: 0.01
adj. p value: 0.9782
log2 L/L, cre / L/L: −0.22
adj. p value: 0.1561
log2 intensity
31
30
Genotype
L/L
L/L, cre
Imputation
non imputed
imputed

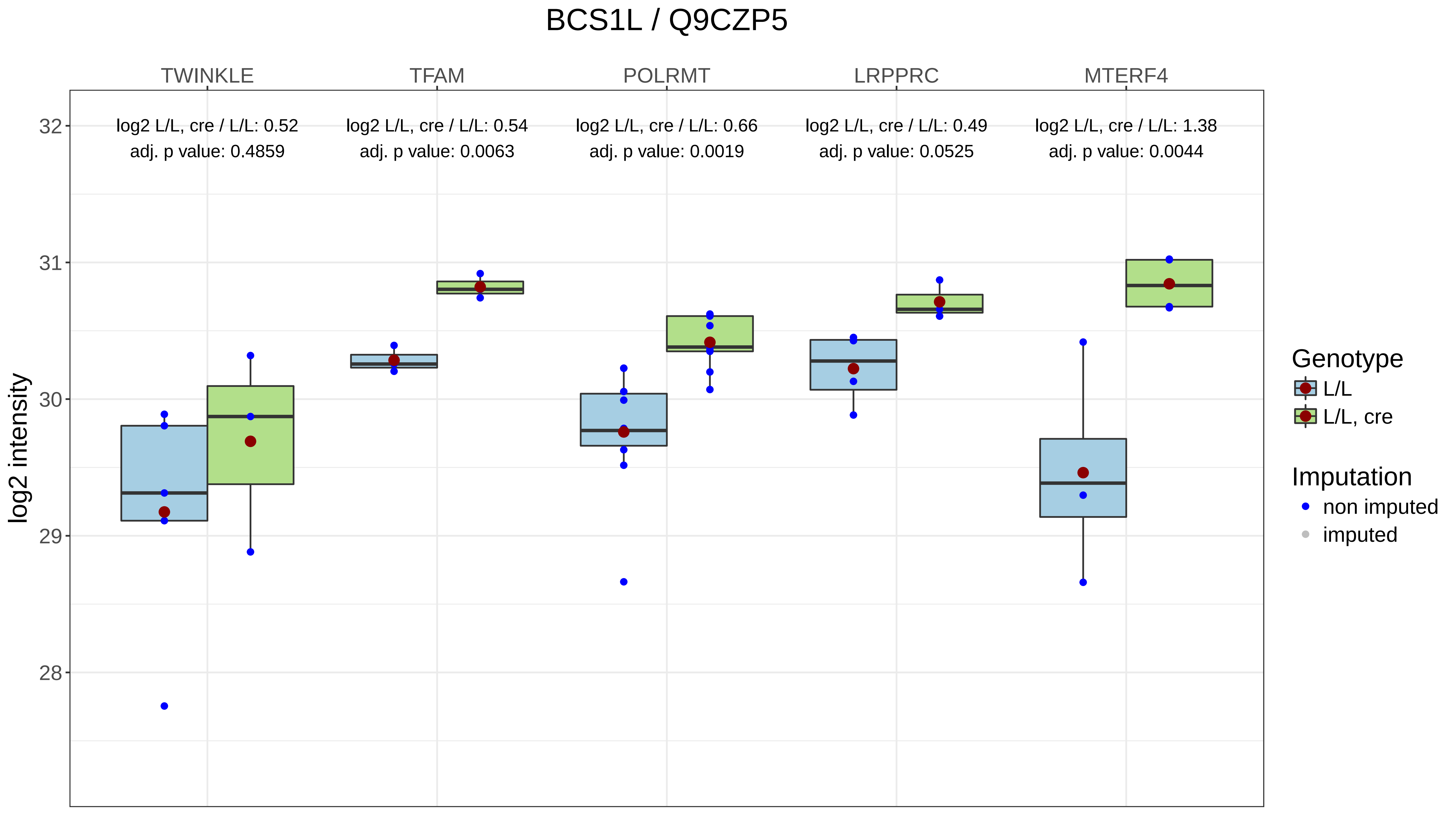
BCS1L / Q9CZP5
TWINKLE
TFAM
POLRMT
LRPPRC
MTERF4
log2 L/L, cre / L/L: 0.52
adj. p value: 0.4859
log2 L/L, cre / L/L: 0.54
adj. p value: 0.0063
log2 L/L, cre / L/L: 0.66
adj. p value: 0.0019
log2 L/L, cre / L/L: 0.49
adj. p value: 0.0525
log2 L/L, cre / L/L: 1.38
adj. p value: 0.0044
log2 intensity
32
31
30
29
28
Genotype
L/L
L/L, cre
Imputation
non imputed
imputed

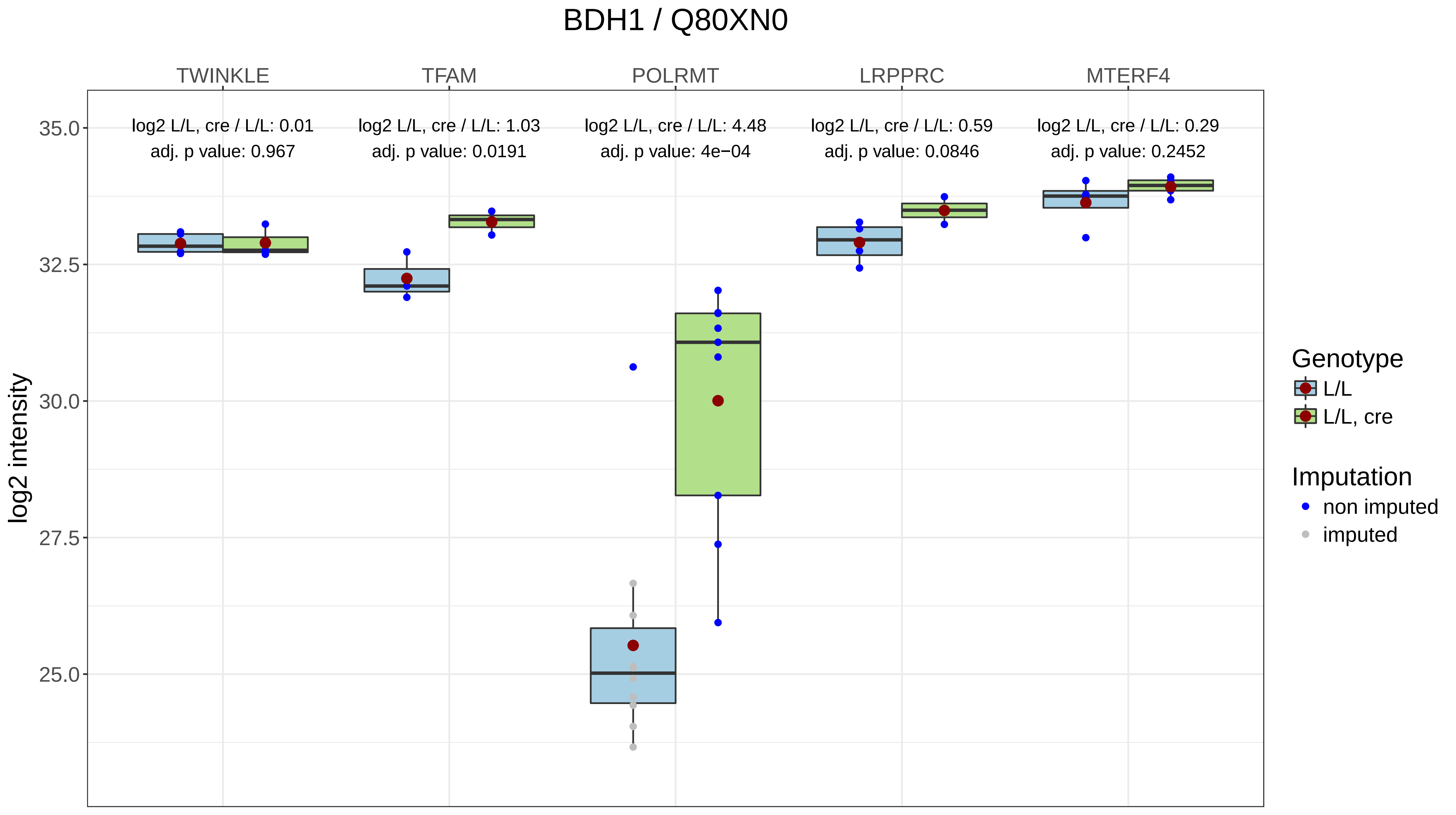
BDH1 / Q80XN0
TWINKLE
TFAM
POLRMT
LRPPRC
MTERF4
log2 L/L, cre / L/L: 0.01
adj. p value: 0.967
log2 L/L, cre / L/L: 1.03
adj. p value: 0.0191
log2 L/L, cre / L/L: 4.48
adj. p value: 4e−04
log2 L/L, cre / L/L: 0.59
adj. p value: 0.0846
log2 L/L, cre / L/L: 0.29
adj. p value: 0.2452
log2 intensity
35.0
32.5
30.0
27.5
25.0
Genotype
L/L
L/L, cre
Imputation
non imputed
imputed

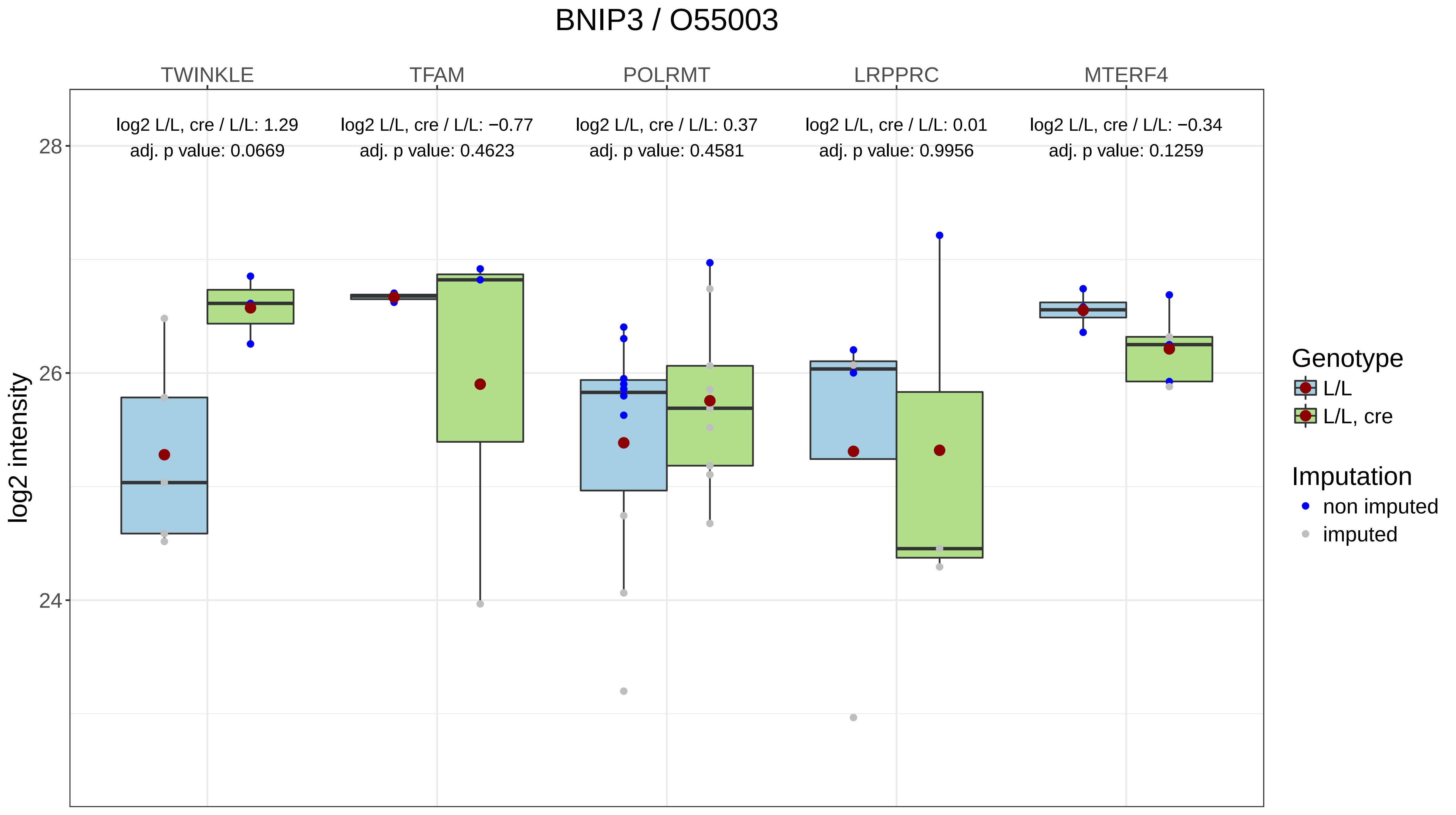
BNIP3 / O55003
TWINKLE
TFAM
POLRMT
LRPPRC
MTERF4
log2 L/L, cre / L/L: 1.29
adj. p value: 0.0669
log2 L/L, cre / L/L: −0.77
adj. p value: 0.4623
log2 L/L, cre / L/L: 0.37
adj. p value: 0.4581
log2 L/L, cre / L/L: 0.01
adj. p value: 0.9956
log2 L/L, cre / L/L: −0.34
adj. p value: 0.1259
28
26
24
log2 intensity
Genotype
L/L
L/L, cre
Imputation
non imputed
imputed

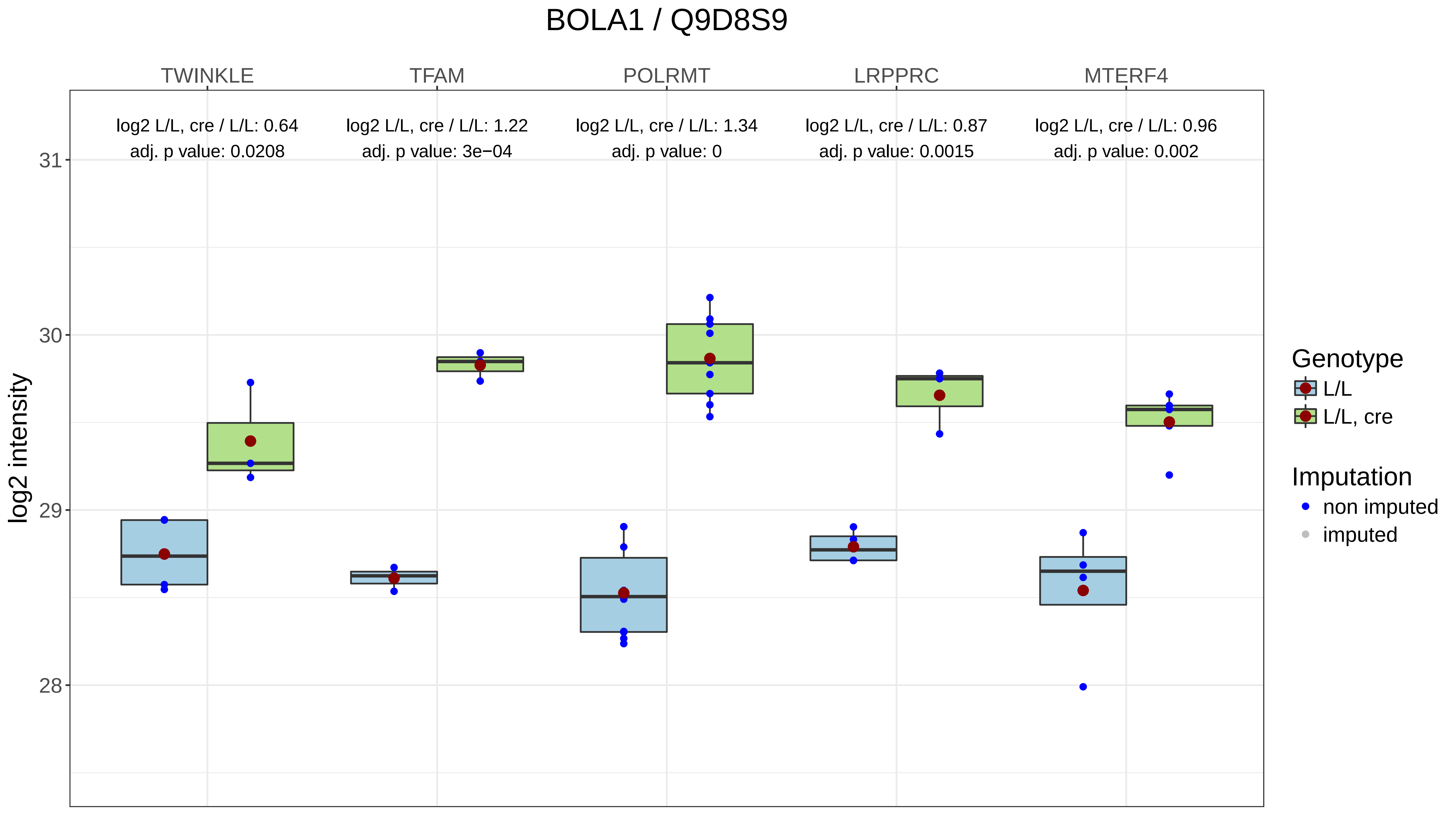
BOLA1 / Q9D8S9
TWINKLE
TFAM
POLRMT
LRPPRC
MTERF4
log2 L/L, cre / L/L: 0.64
adj. p value: 0.0208
log2 L/L, cre / L/L: 1.22
adj. p value: 3e−04
log2 L/L, cre / L/L: 1.34
adj. p value: 0
log2 L/L, cre / L/L: 0.87
adj. p value: 0.0015
log2 L/L, cre / L/L: 0.96
adj. p value: 0.002
log2 intensity
31
30
29
28
Genotype
L/L
L/L, cre
Imputation
non imputed
imputed

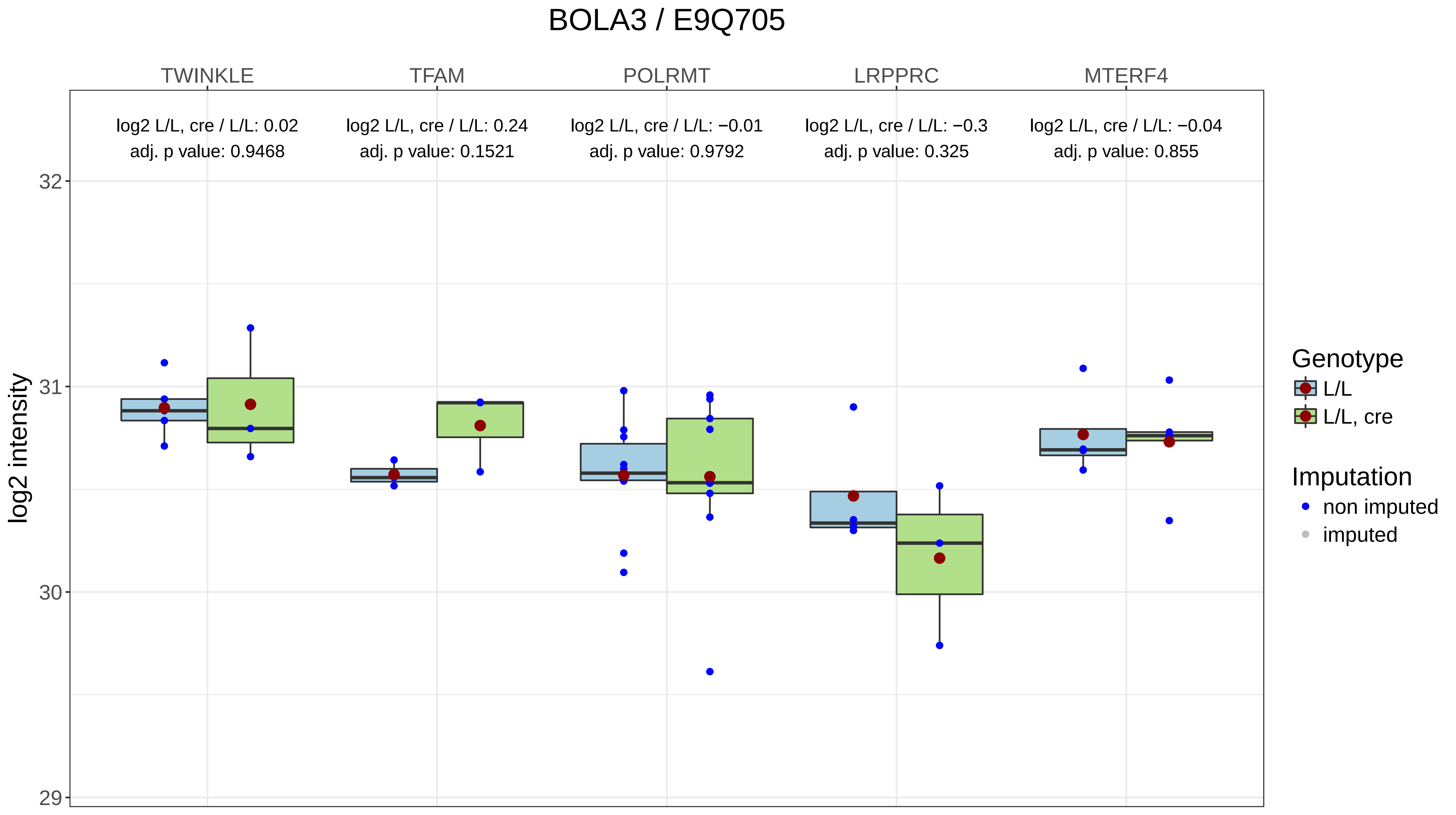
BOLA3 / E9Q705
TWINKLE
TFAM
POLRMT
LRPPRC
MTERF4
log2 L/L, cre / L/L: 0.02
adj. p value: 0.9468
log2 L/L, cre / L/L: 0.24
adj. p value: 0.1521
log2 L/L, cre / L/L: −0.01
adj. p value: 0.9792
log2 L/L, cre / L/L: −0.3
adj. p value: 0.325
log2 L/L, cre / L/L: −0.04
adj. p value: 0.855
log2 intensity
32
31
30
29
Genotype
L/L
L/L, cre
Imputation
non imputed
imputed

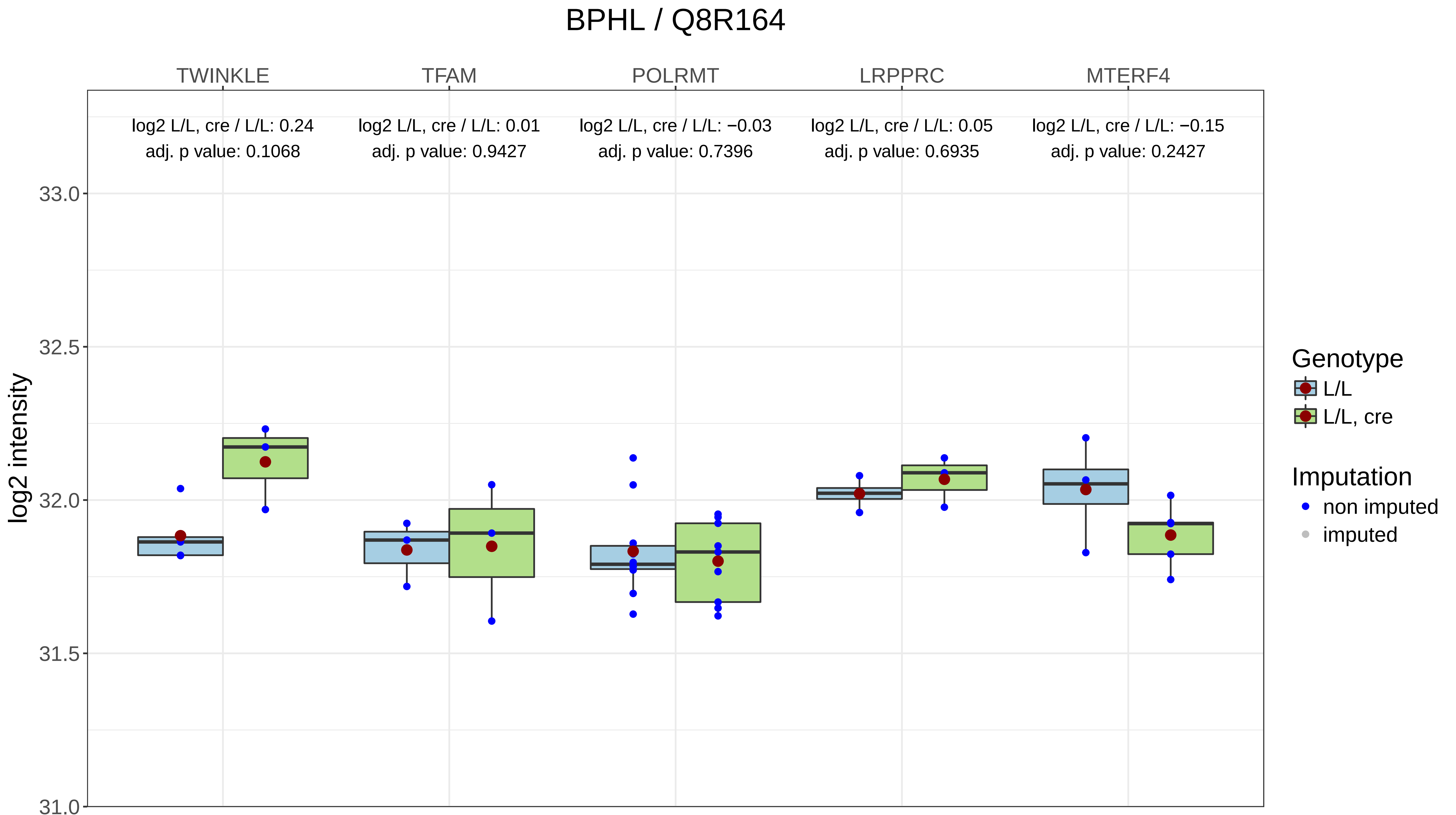
BPHL / Q8R164
TWINKLE
TFAM
POLRMT
LRPPRC
MTERF4
log2 L/L, cre / L/L: 0.24
adj. p value: 0.1068
log2 L/L, cre / L/L: 0.01
adj. p value: 0.9427
log2 L/L, cre / L/L: −0.03
adj. p value: 0.7396
log2 L/L, cre / L/L: 0.05
adj. p value: 0.6935
log2 L/L, cre / L/L: −0.15
adj. p value: 0.2427
log2 intensity
33.0
32.5
32.0
31.5
31.0
Genotype
L/L
L/L, cre
Imputation
non imputed
imputed

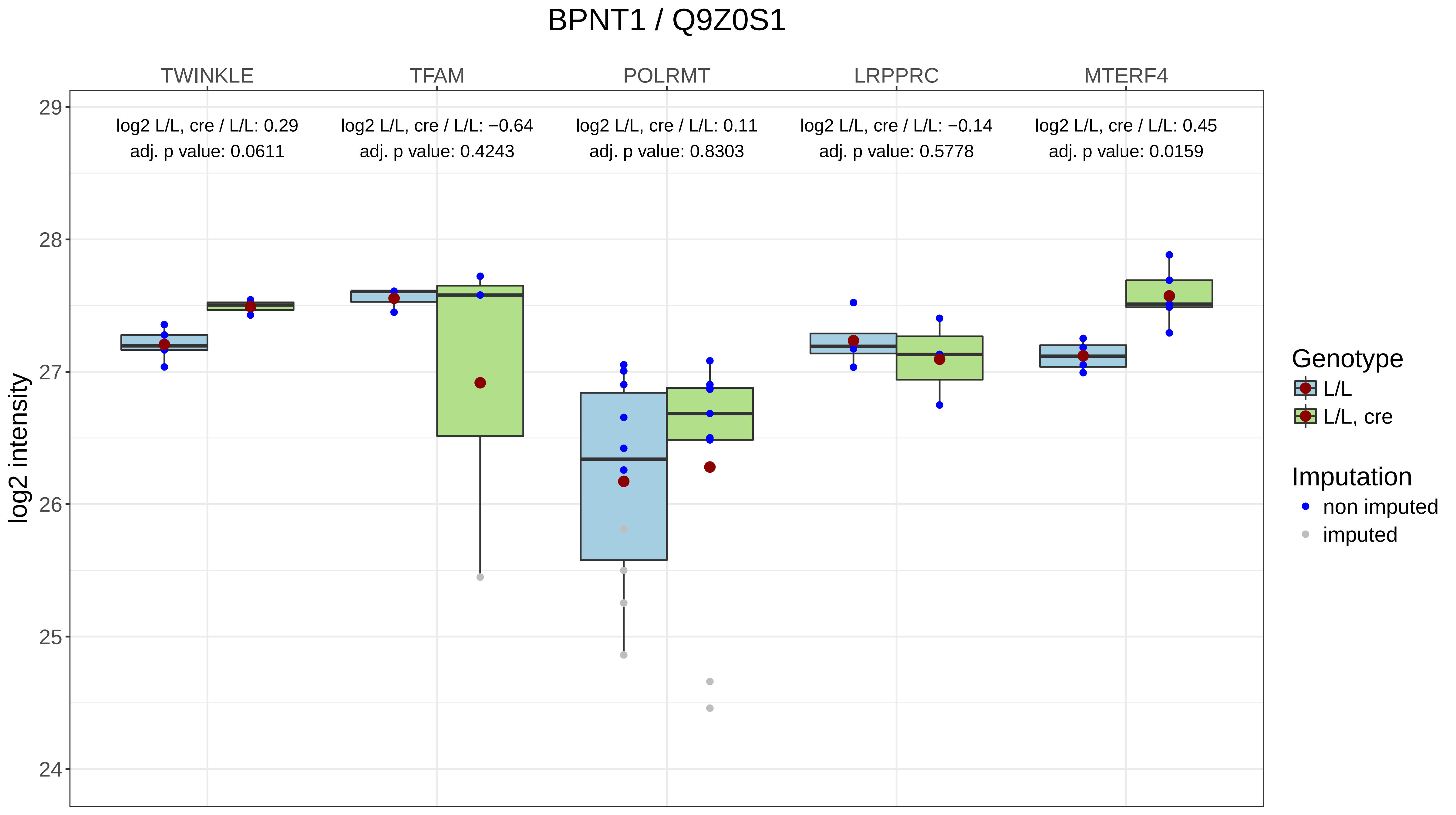
BPNT1 / Q9Z0S1
TWINKLE
TFAM
POLRMT
LRPPRC
MTERF4
log2 L/L, cre / L/L: 0.29
adj. p value: 0.0611
log2 L/L, cre / L/L: −0.64
adj. p value: 0.4243
log2 L/L, cre / L/L: 0.11
adj. p value: 0.8303
log2 L/L, cre / L/L: −0.14
adj. p value: 0.5778
log2 L/L, cre / L/L: 0.45
adj. p value: 0.0159
log2 intensity
29
28
27
26
25
24
Genotype
L/L
L/L, cre
Imputation
non imputed
imputed

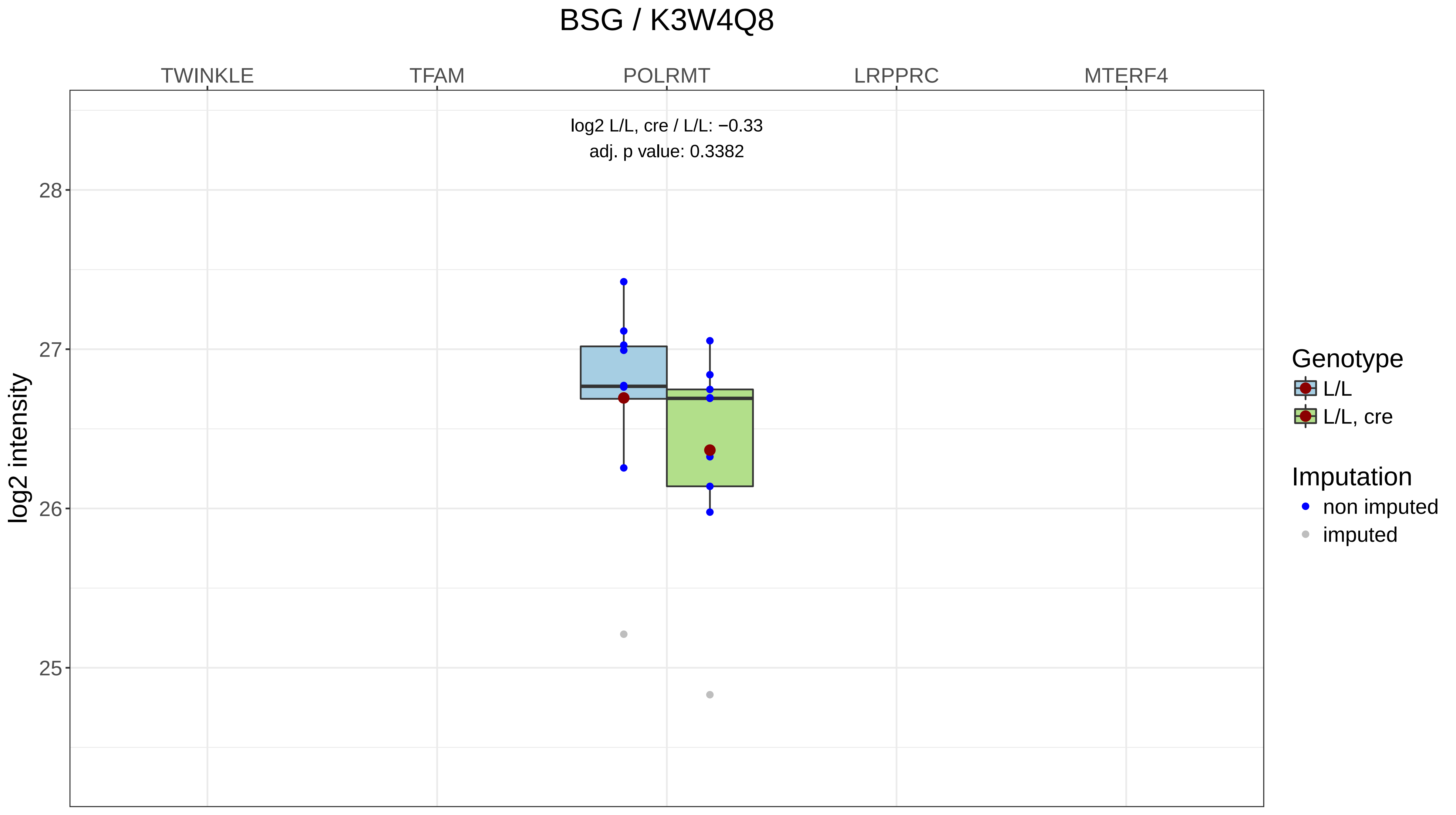
BSG / K3W4Q8
TWINKLE
TFAM
POLRMT
LRPPRC
MTERF4
log2 L/L, cre / L/L: −0.33
adj. p value: 0.3382
28
27
26
25
log2 intensity
Genotype
L/L
L/L, cre
Imputation
non imputed
imputed

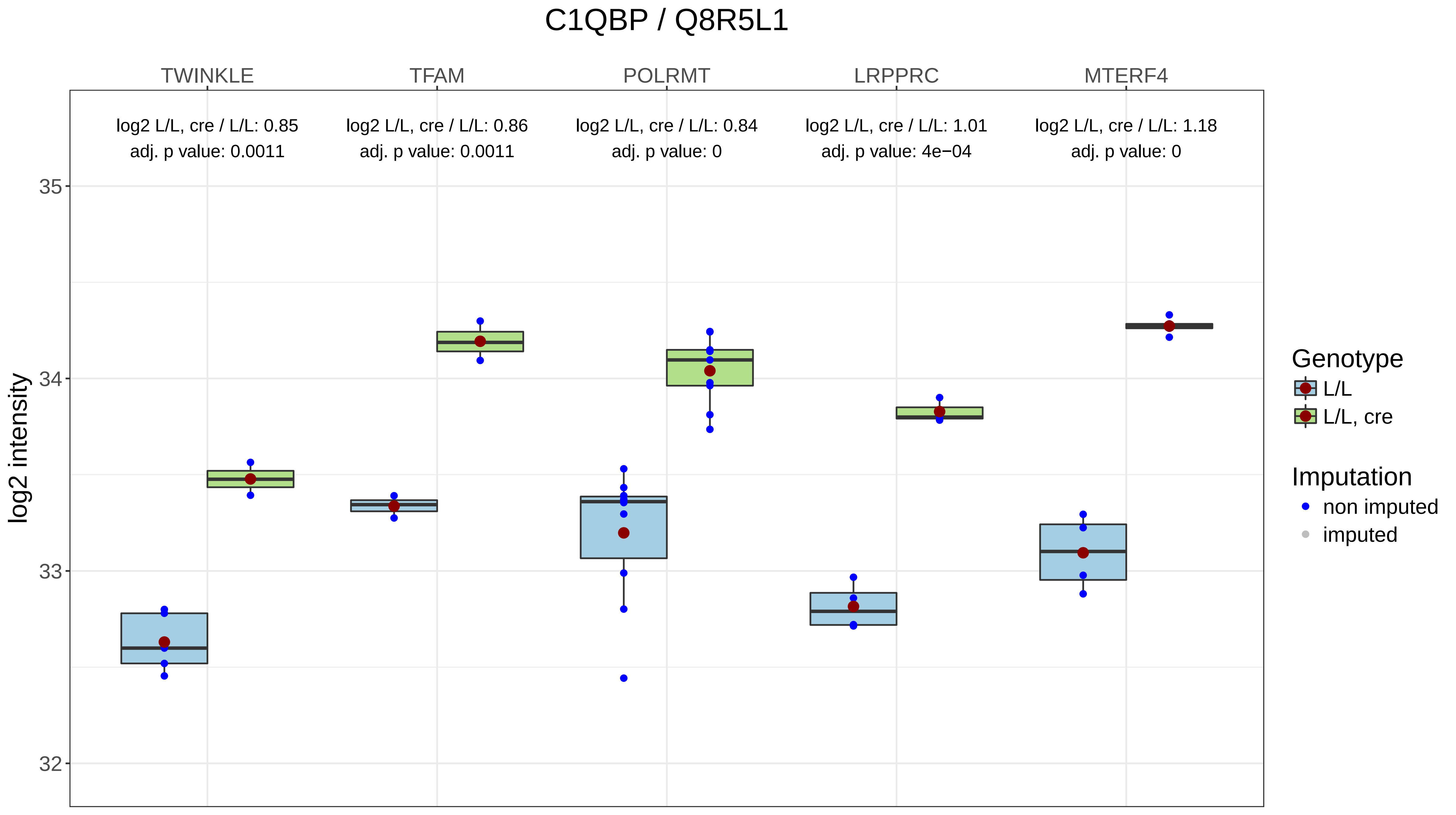

C1QBP / Q8R5L1
TWINKLE
TFAM
POLRMT
LRPPRC
MTERF4
log2 L/L, cre / L/L: 0.85
adj. p value: 0.0011
log2 L/L, cre / L/L: 0.86
adj. p value: 0.0011
log2 L/L, cre / L/L: 0.84
adj. p value: 0
log2 L/L, cre / L/L: 1.01
adj. p value: 4e−04
log2 L/L, cre / L/L: 1.18
adj. p value: 0
log2 intensity
35
34
33
32
Genotype
L/L
L/L, cre
Imputation
non imputed
imputed

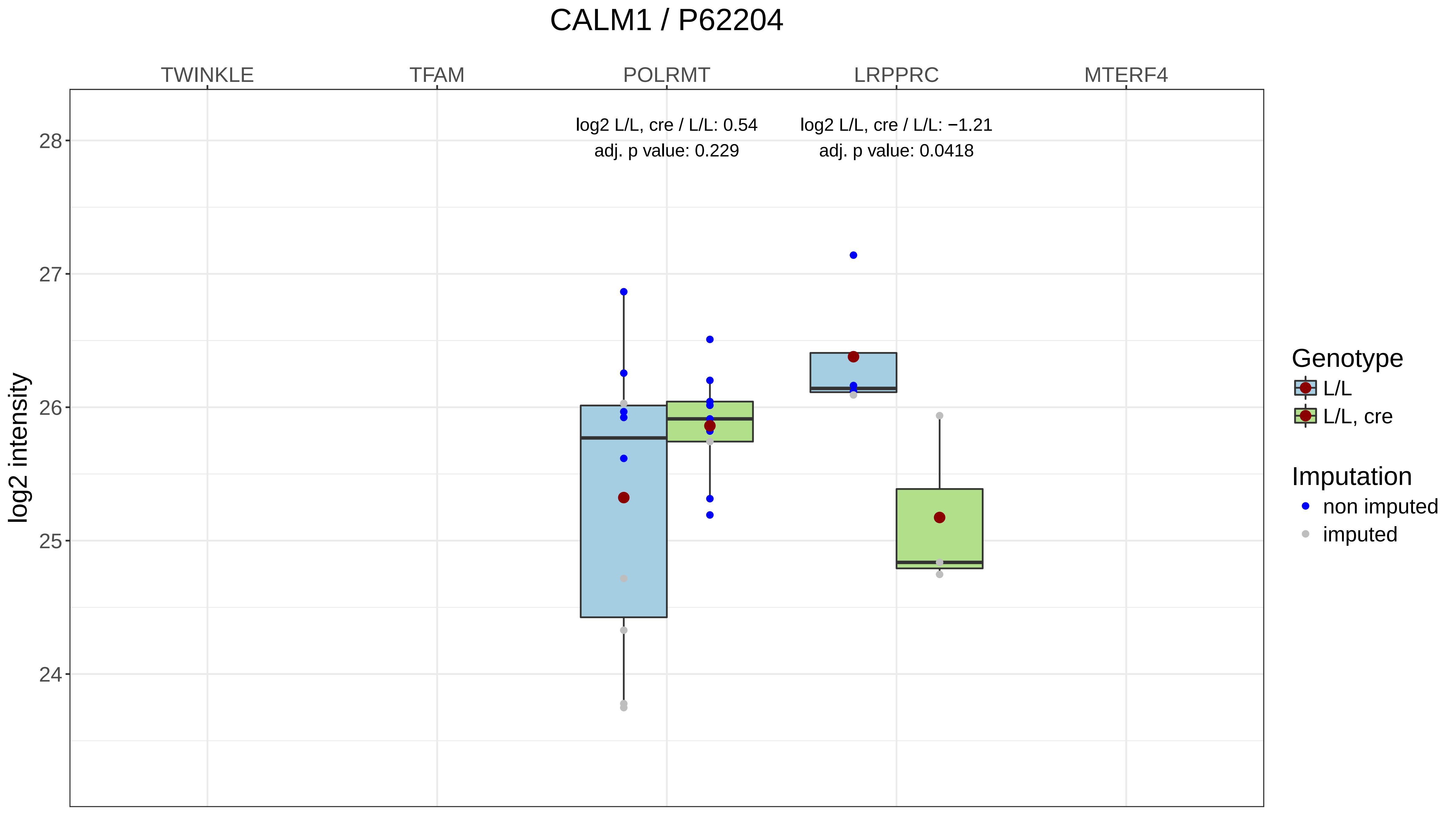

CALM1 / P62204
TWINKLE
TFAM
POLRMT
LRPPRC
MTERF4
log2 L/L, cre / L/L: 0.54
adj. p value: 0.229
log2 L/L, cre / L/L: −1.21
adj. p value: 0.0418
28
27
26
25
24
log2 intensity
Genotype
L/L
L/L, cre
Imputation
non imputed
imputed

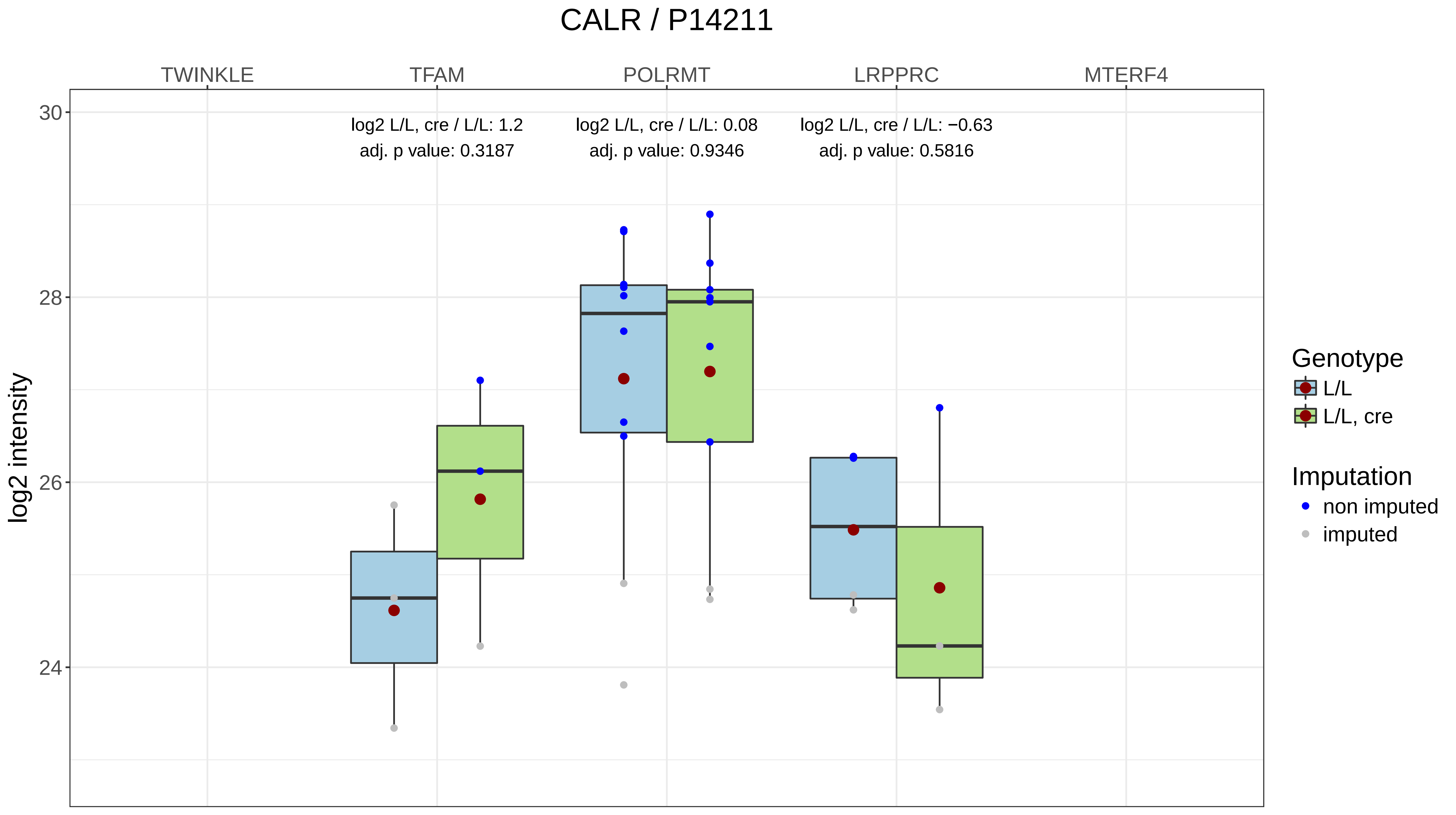

CALR / P14211
TWINKLE
TFAM
POLRMT
LRPPRC
MTERF4
log2 L/L, cre / L/L: 1.2
adj. p value: 0.3187
log2 L/L, cre / L/L: 0.08
adj. p value: 0.9346
log2 L/L, cre / L/L: −0.63
adj. p value: 0.5816
30
28
26
24
log2 intensity
Genotype
L/L
L/L, cre
Imputation
non imputed
imputed

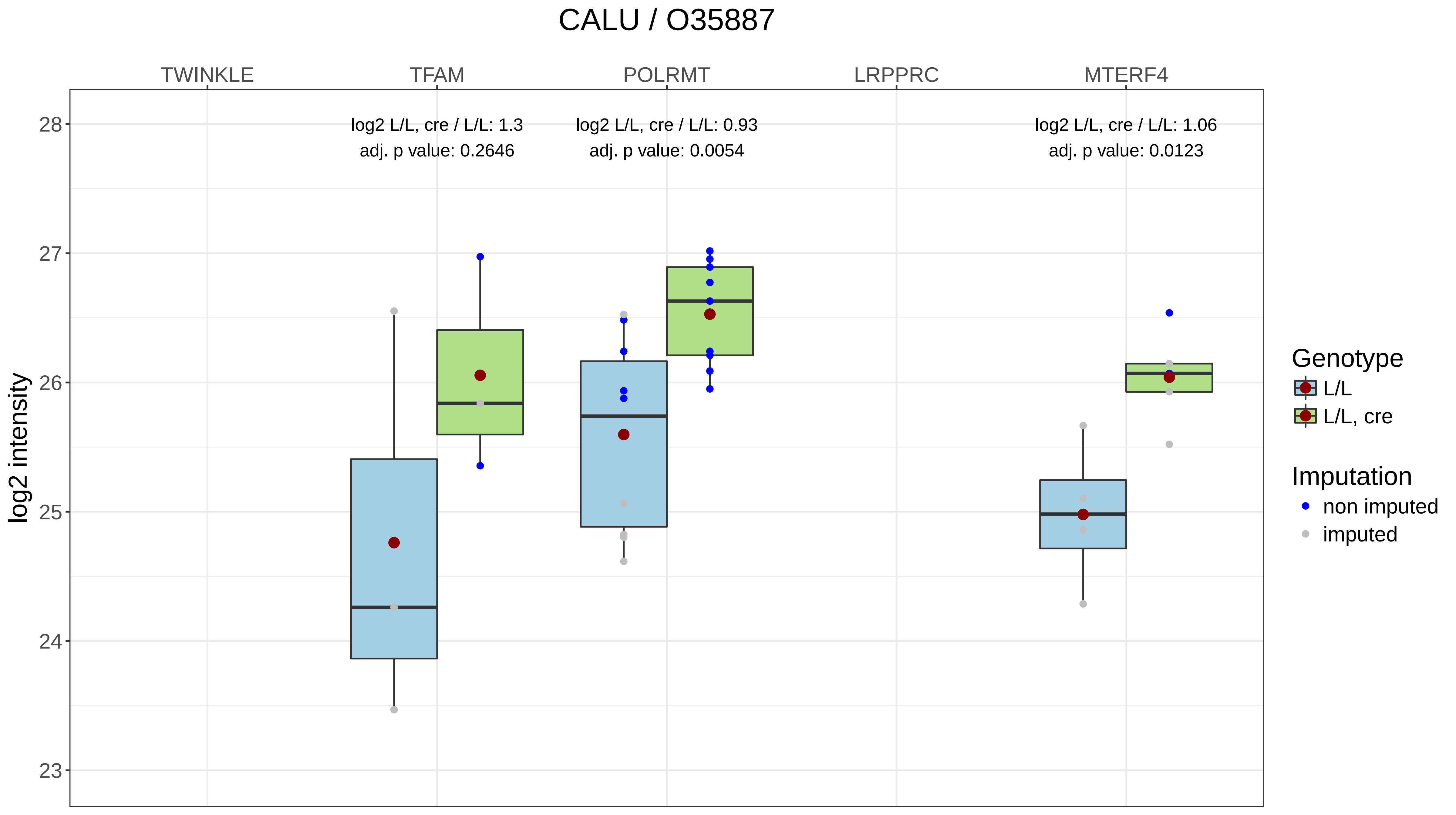
CALU / O35887
TWINKLE
TFAM
POLRMT
LRPPRC
MTERF4
log2 L/L, cre / L/L: 1.3
adj. p value: 0.2646
log2 L/L, cre / L/L: 0.93
adj. p value: 0.0054
log2 L/L, cre / L/L: 1.06
adj. p value: 0.0123
log2 intensity
28
27
26
25
24
23
Genotype
L/L
L/L, cre
Imputation
non imputed
imputed

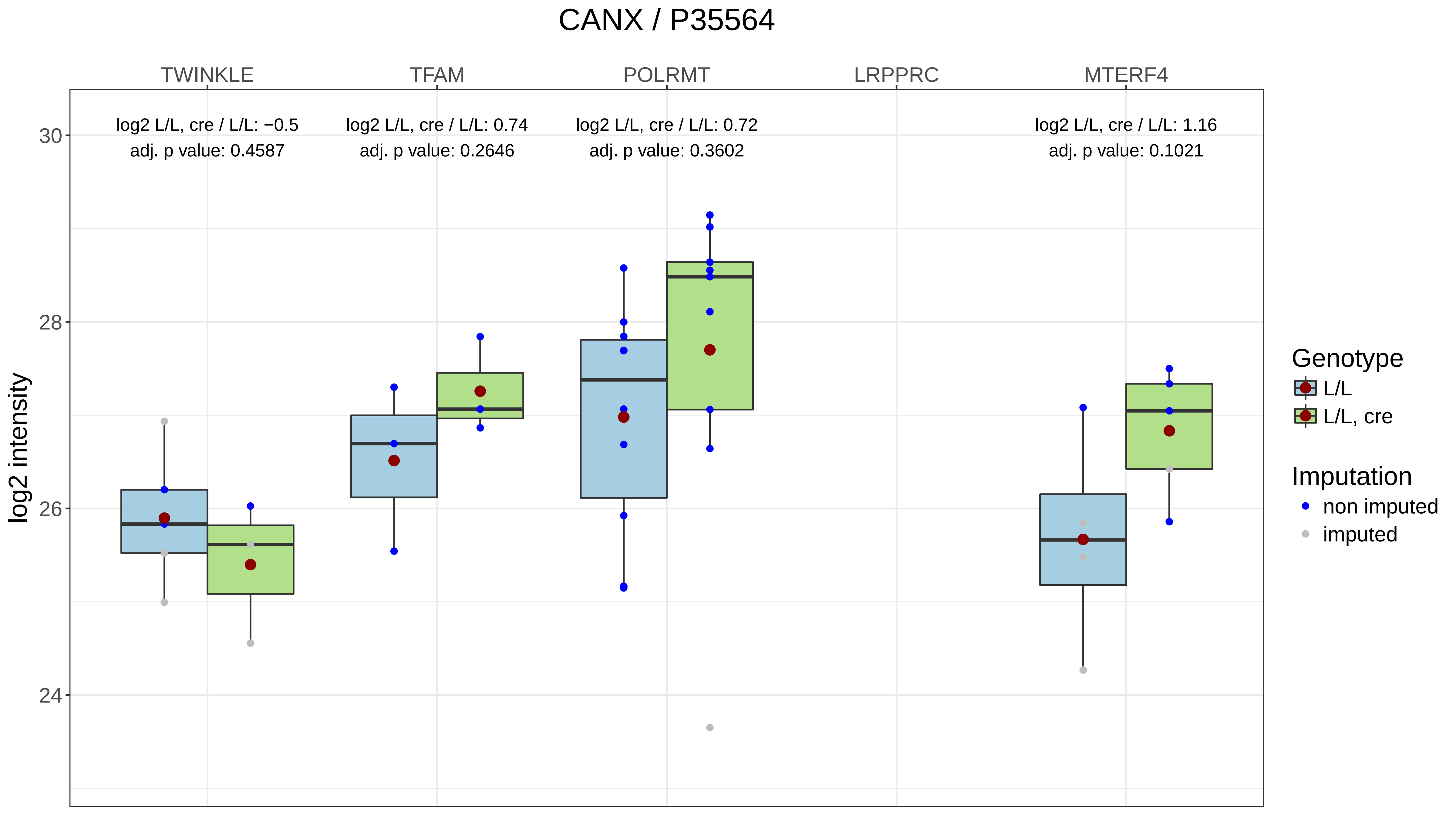
CANX / P35564
TWINKLE
TFAM
POLRMT
LRPPRC
MTERF4
log2 L/L, cre / L/L: −0.5
adj. p value: 0.4587
log2 L/L, cre / L/L: 0.74
adj. p value: 0.2646
log2 L/L, cre / L/L: 0.72
adj. p value: 0.3602
log2 L/L, cre / L/L: 1.16
adj. p value: 0.1021
log2 intensity
30
28
26
24
Genotype
L/L
L/L, cre
Imputation
non imputed
imputed

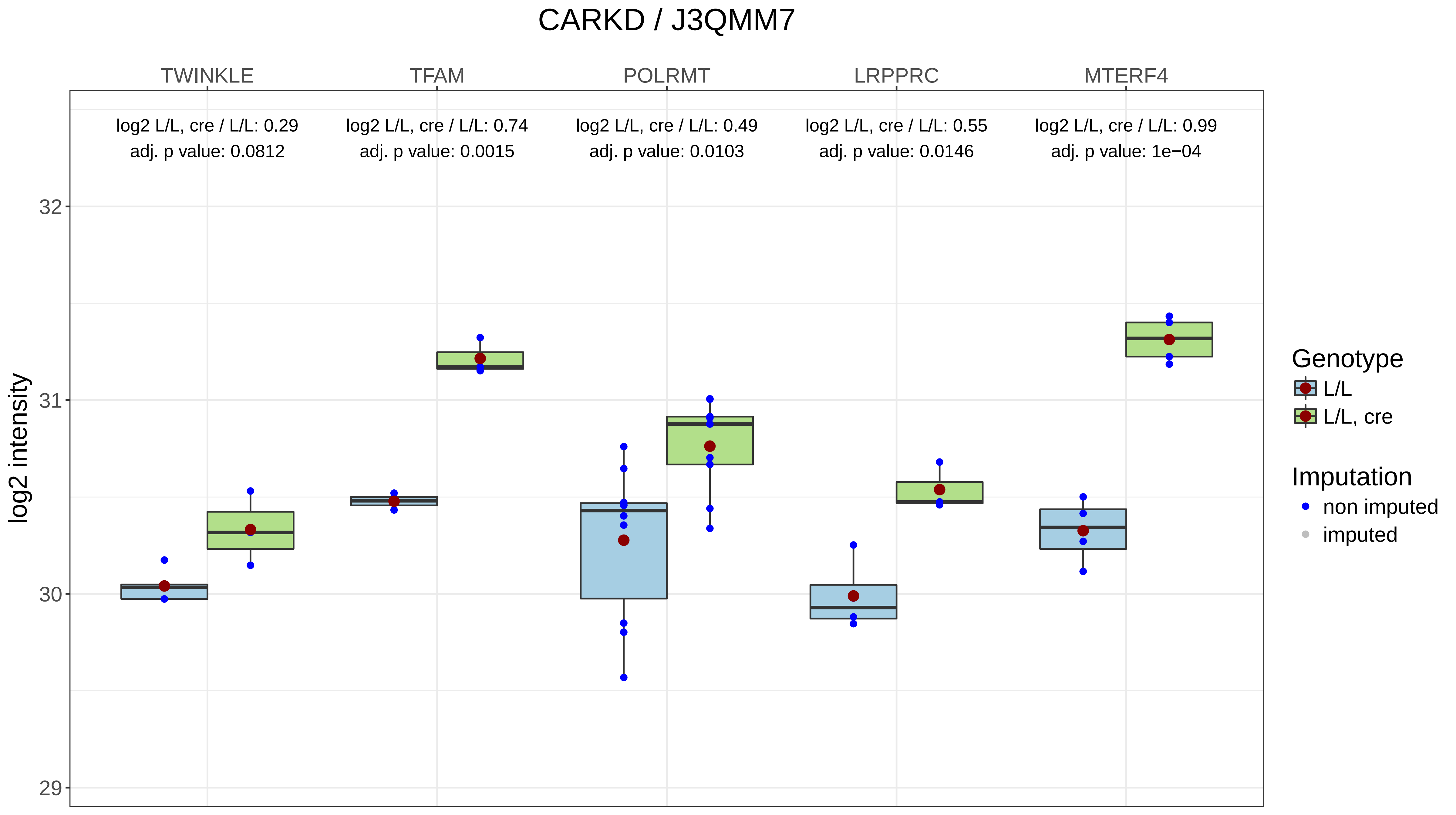
CARKD / J3QMM7
TWINKLE
TFAM
POLRMT
LRPPRC
MTERF4
log2 L/L, cre / L/L: 0.29
adj. p value: 0.0812
log2 L/L, cre / L/L: 0.74
adj. p value: 0.0015
log2 L/L, cre / L/L: 0.49
adj. p value: 0.0103
log2 L/L, cre / L/L: 0.55
adj. p value: 0.0146
log2 L/L, cre / L/L: 0.99
adj. p value: 1e−04
log2 intensity
32
31
30
29
Genotype
L/L
L/L, cre
Imputation
non imputed
imputed

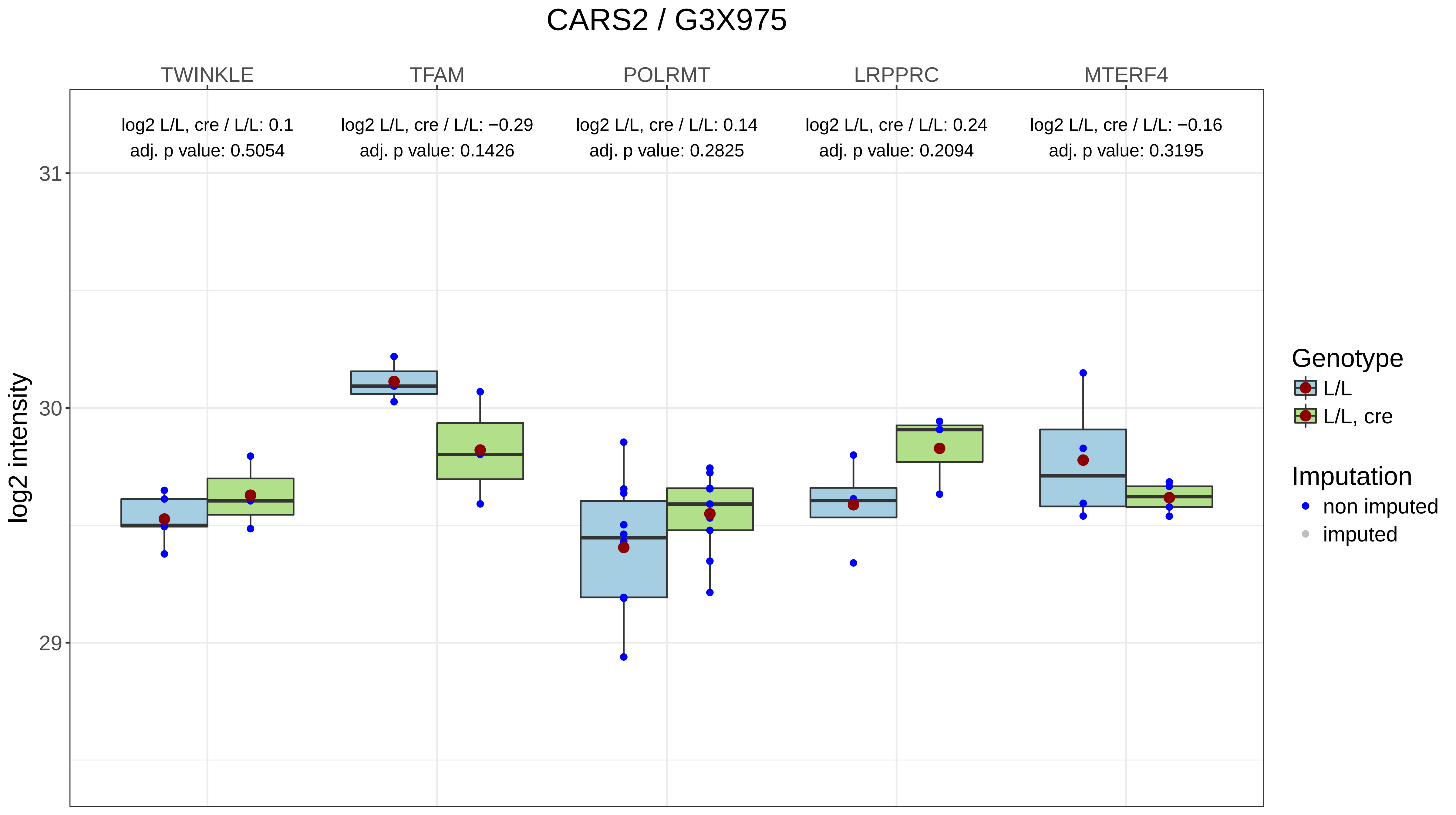
CARS2 / G3X975
TWINKLE
TFAM
POLRMT
LRPPRC
MTERF4
log2 L/L, cre / L/L: 0.1
adj. p value: 0.5054
log2 L/L, cre / L/L: −0.29
adj. p value: 0.1426
log2 L/L, cre / L/L: 0.14
adj. p value: 0.2825
log2 L/L, cre / L/L: 0.24
adj. p value: 0.2094
log2 L/L, cre / L/L: −0.16
adj. p value: 0.3195
log2 intensity
31
30
29
Genotype
L/L
L/L, cre
Imputation
non imputed
imputed

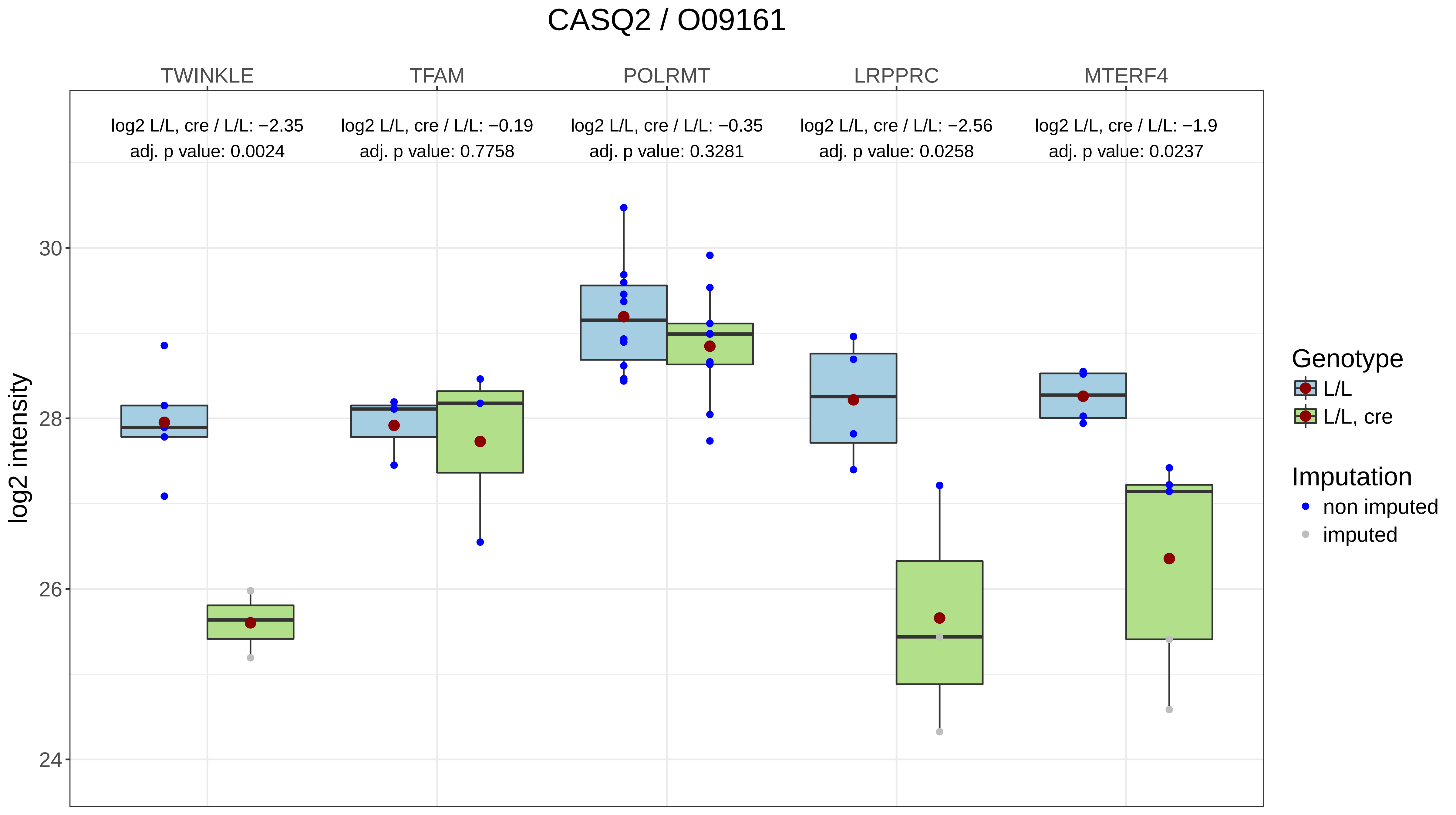
CASQ2 / O09161
TWINKLE
TFAM
POLRMT
LRPPRC
MTERF4
log2 L/L, cre / L/L: −2.35
adj. p value: 0.0024
log2 L/L, cre / L/L: −0.19
adj. p value: 0.7758
log2 L/L, cre / L/L: −0.35
adj. p value: 0.3281
log2 L/L, cre / L/L: −2.56
adj. p value: 0.0258
log2 L/L, cre / L/L: −1.9
adj. p value: 0.0237
log2 intensity
30
28
26
24
Genotype
L/L
L/L, cre
Imputation
non imputed
imputed

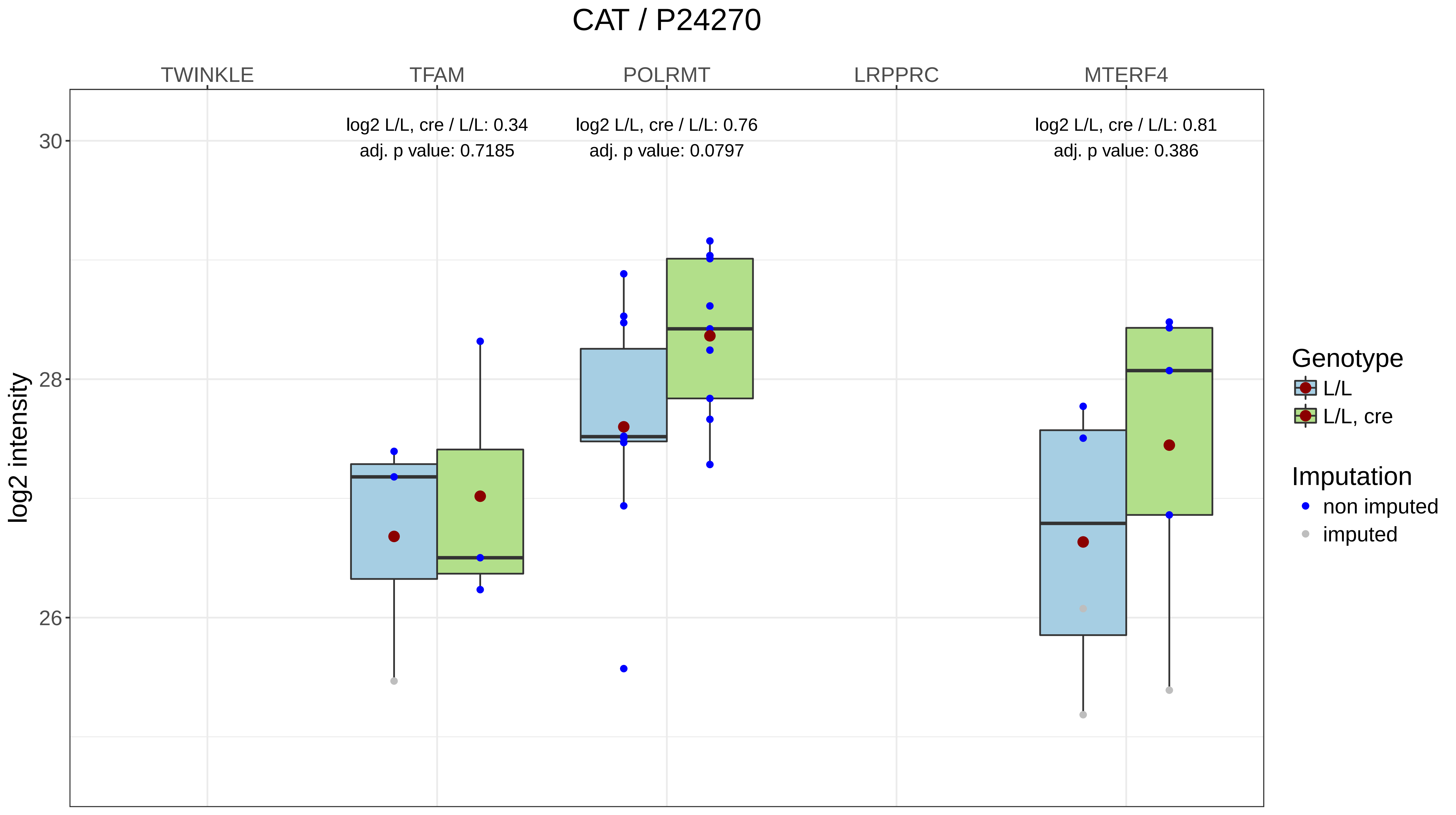
CAT / P24270
TWINKLE
TFAM
POLRMT
LRPPRC
MTERF4
log2 L/L, cre / L/L: 0.34
adj. p value: 0.7185
log2 L/L, cre / L/L: 0.76
adj. p value: 0.0797
log2 L/L, cre / L/L: 0.81
adj. p value: 0.386
30
28
26
log2 intensity
Genotype
L/L
L/L, cre
Imputation
non imputed
imputed

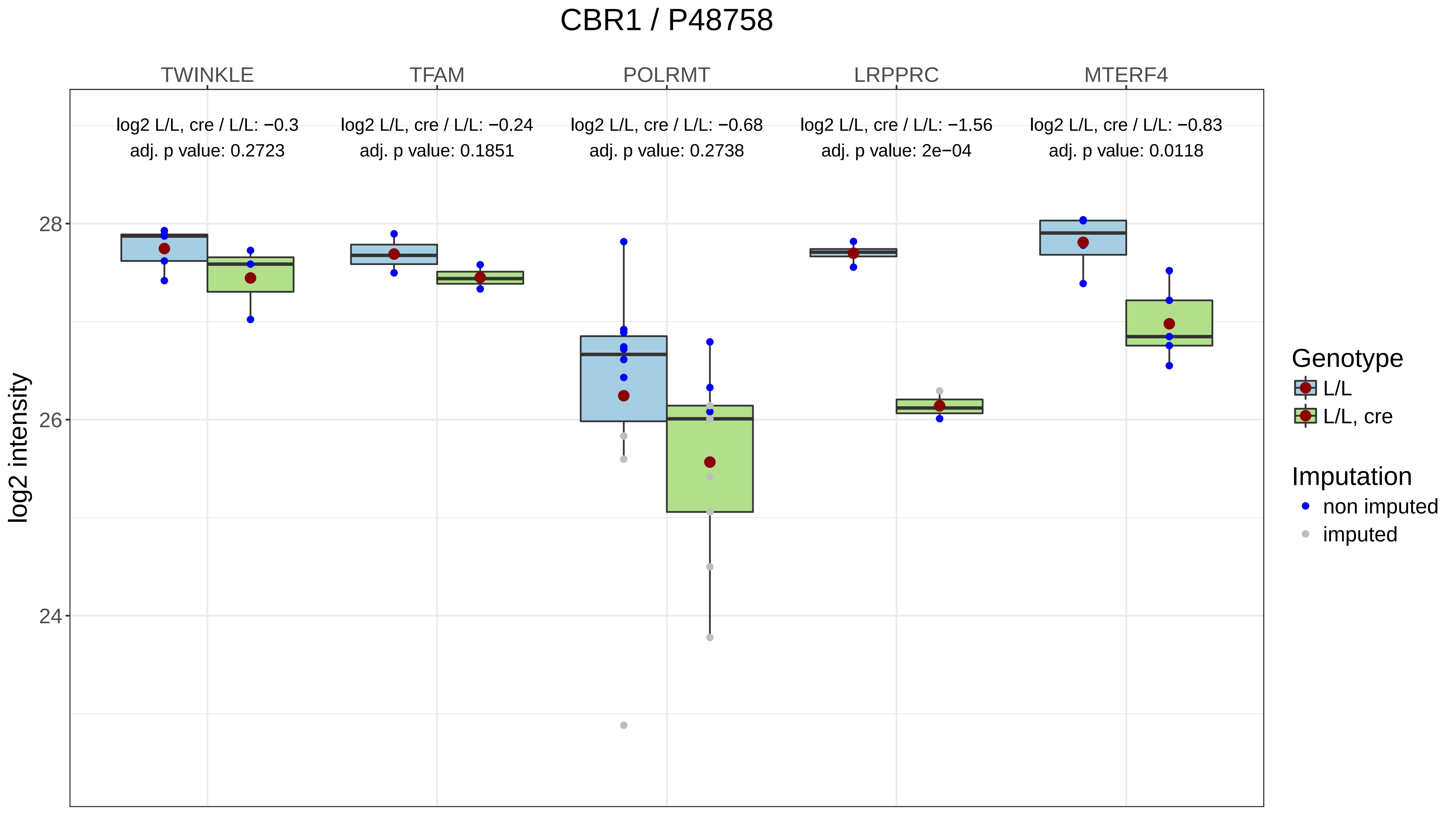

CBR1 / P48758
TWINKLE
TFAM
POLRMT
LRPPRC
MTERF4
log2 L/L, cre / L/L: −0.3
adj. p value: 0.2723
log2 L/L, cre / L/L: −0.24
adj. p value: 0.1851
log2 L/L, cre / L/L: −0.68
adj. p value: 0.2738
log2 L/L, cre / L/L: −1.56
adj. p value: 2e−04
log2 L/L, cre / L/L: −0.83
adj. p value: 0.0118
28
26
24
log2 intensity
Genotype
L/L
L/L, cre
Imputation
non imputed
imputed

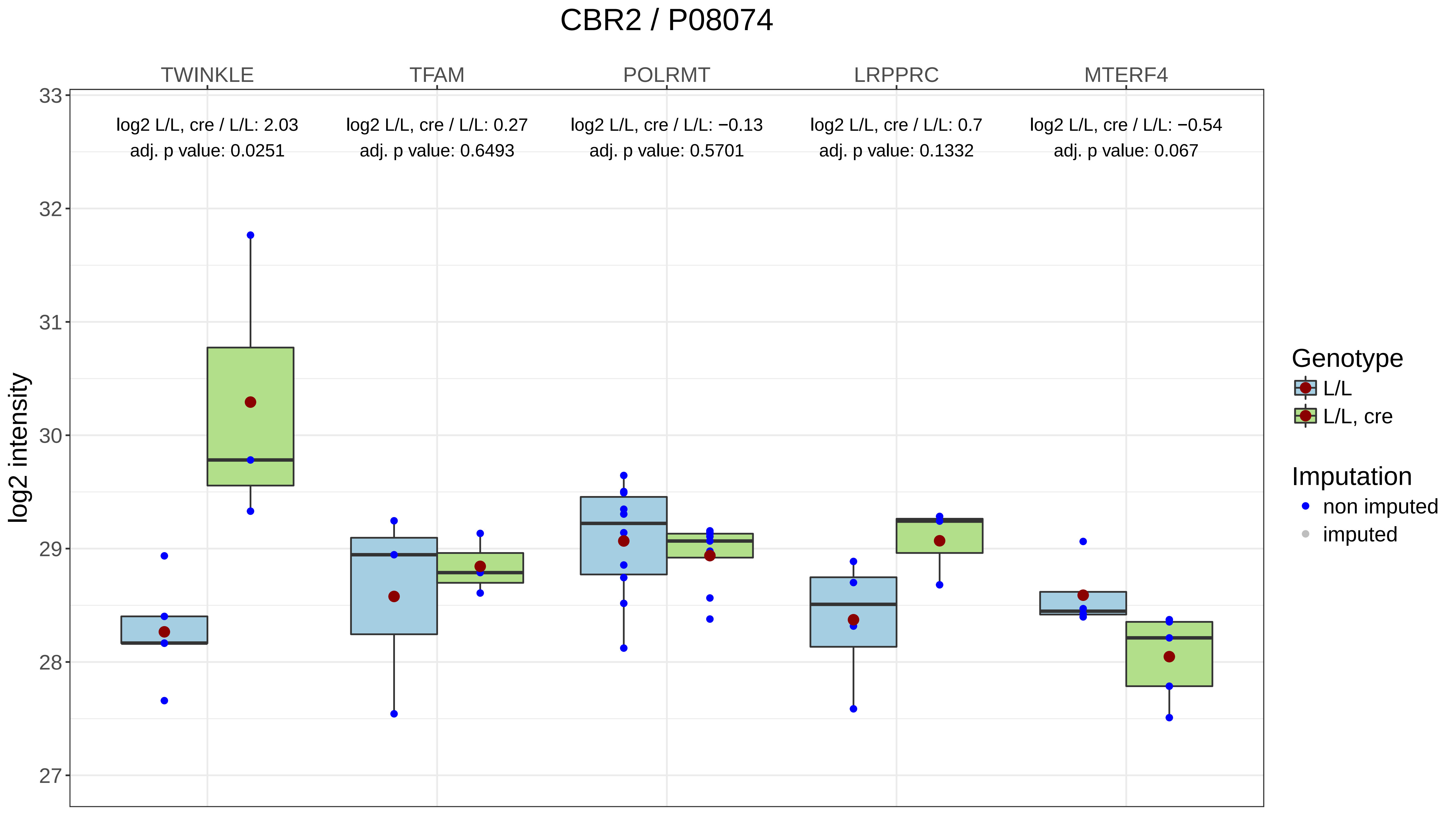
CBR2 / P08074
TWINKLE
TFAM
POLRMT
LRPPRC
MTERF4
log2 L/L, cre / L/L: 2.03
adj. p value: 0.0251
log2 L/L, cre / L/L: 0.27
adj. p value: 0.6493
log2 L/L, cre / L/L: −0.13
adj. p value: 0.5701
log2 L/L, cre / L/L: 0.7
adj. p value: 0.1332
log2 L/L, cre / L/L: −0.54
adj. p value: 0.067
log2 intensity
33
32
31
30
29
28
27
Genotype
L/L
L/L, cre
Imputation
non imputed
imputed

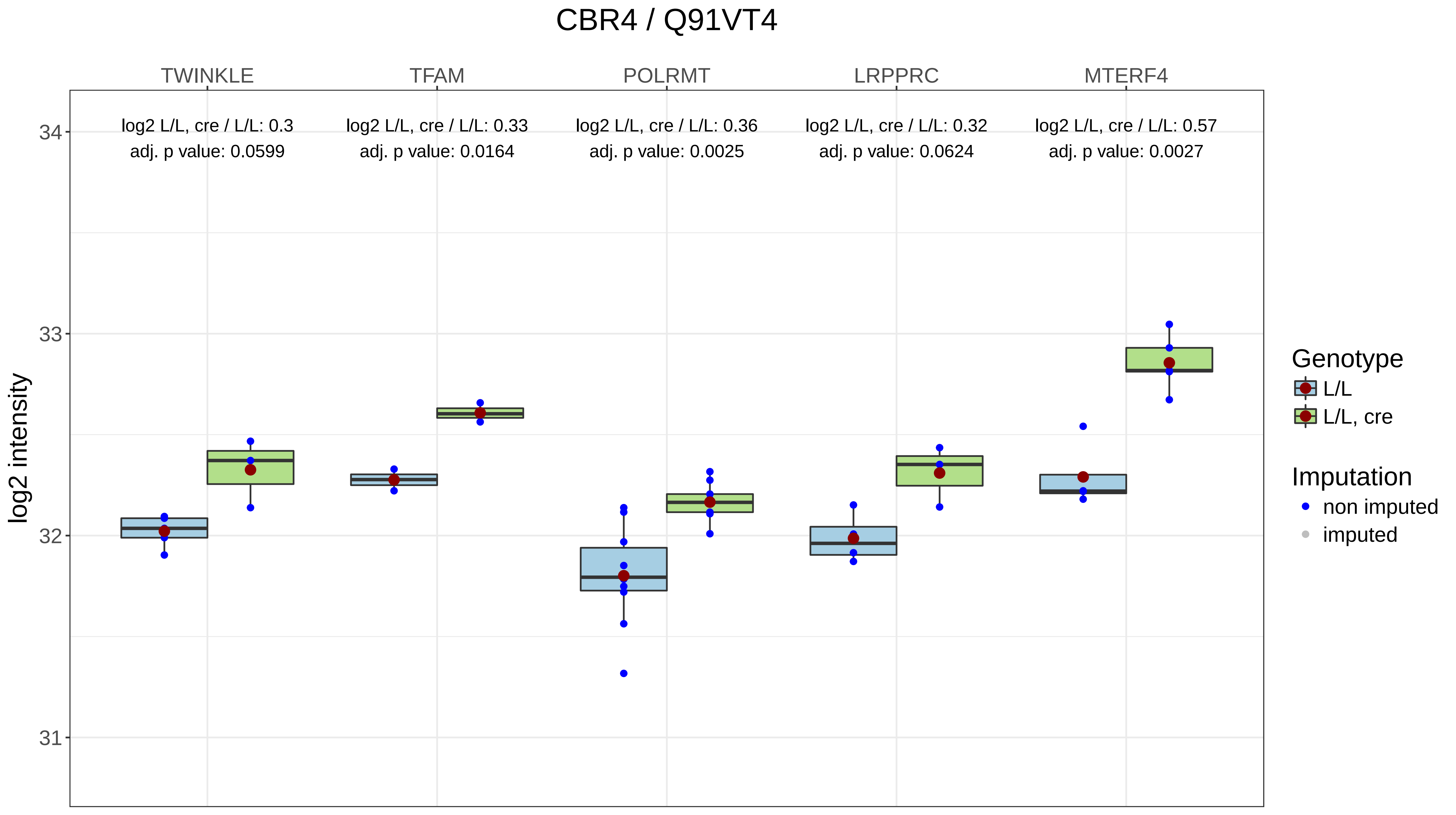
CBR4 / Q91VT4
TWINKLE
TFAM
POLRMT
LRPPRC
MTERF4
log2 L/L, cre / L/L: 0.3
adj. p value: 0.0599
log2 L/L, cre / L/L: 0.33
adj. p value: 0.0164
log2 L/L, cre / L/L: 0.36
adj. p value: 0.0025
log2 L/L, cre / L/L: 0.32
adj. p value: 0.0624
log2 L/L, cre / L/L: 0.57
adj. p value: 0.0027
log2 intensity
34
33
32
31
Genotype
L/L
L/L, cre
Imputation
non imputed
imputed

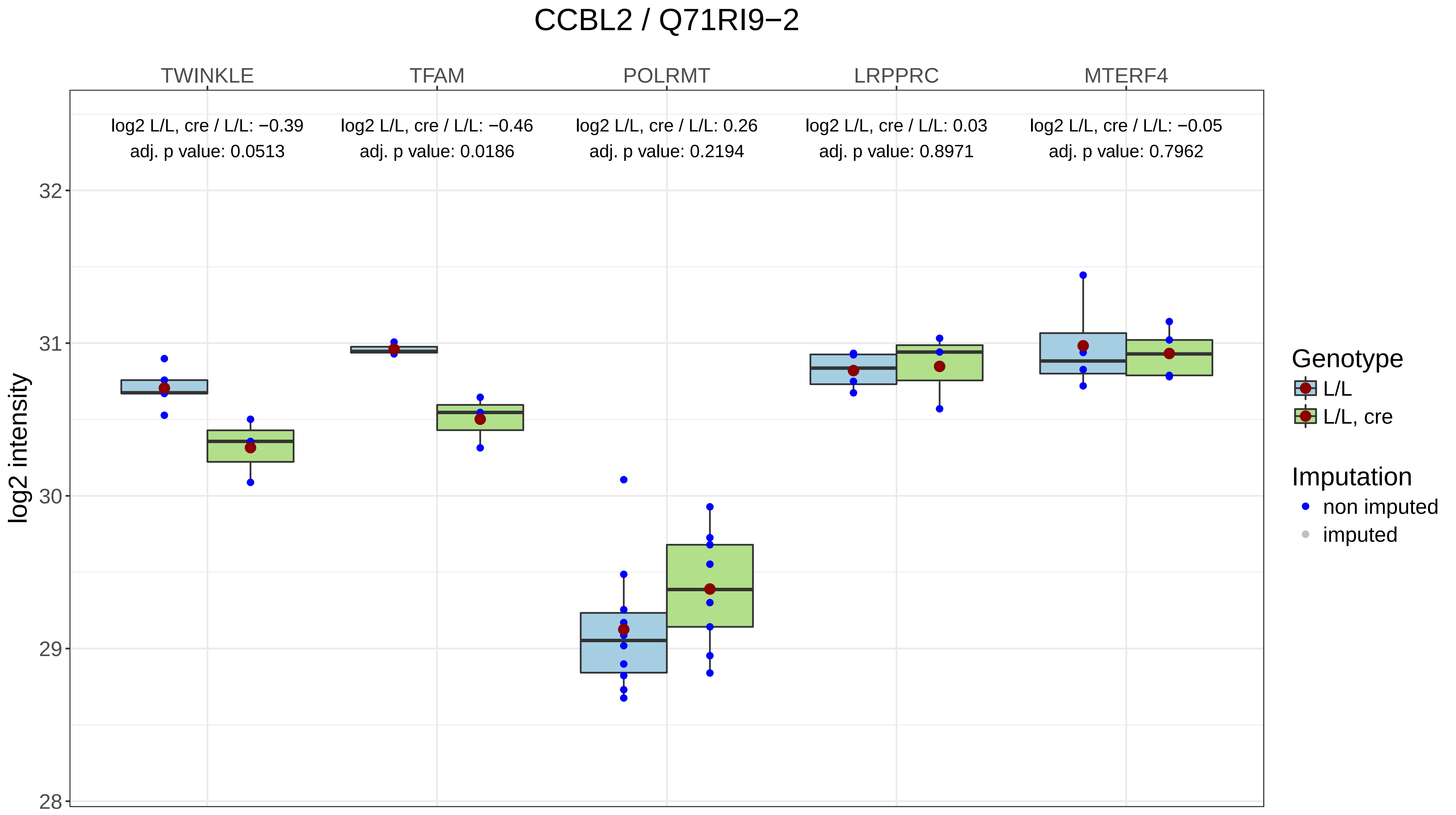
CCBL2 / Q71RI9−2
TWINKLE
TFAM
POLRMT
LRPPRC
MTERF4
log2 L/L, cre / L/L: −0.39
adj. p value: 0.0513
log2 L/L, cre / L/L: −0.46
adj. p value: 0.0186
log2 L/L, cre / L/L: 0.26
adj. p value: 0.2194
log2 L/L, cre / L/L: 0.03
adj. p value: 0.8971
log2 L/L, cre / L/L: −0.05
adj. p value: 0.7962
log2 intensity
32
31
30
29
28
Genotype
L/L
L/L, cre
Imputation
non imputed
imputed

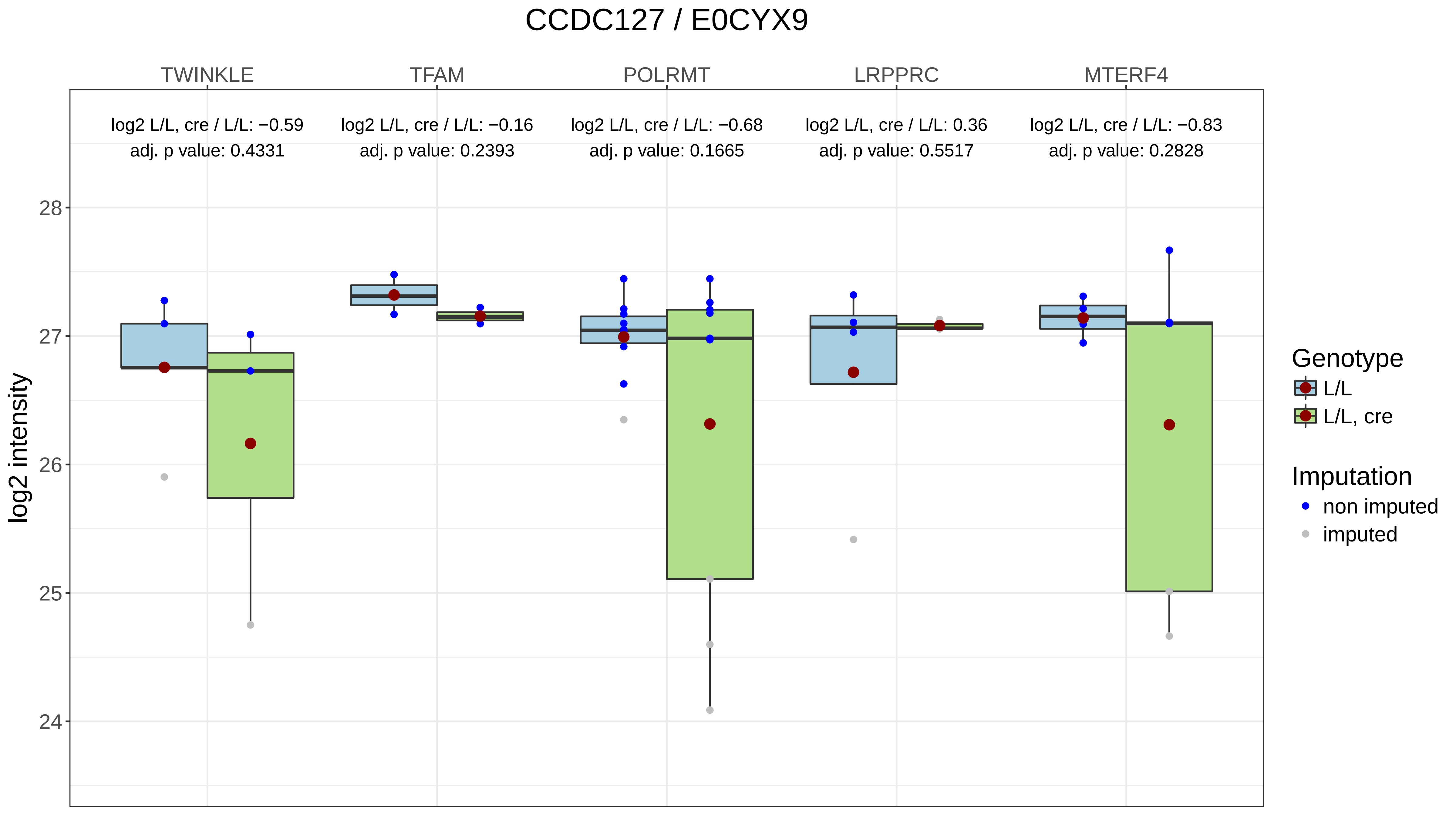
CCDC127 / E0CYX9
TWINKLE
TFAM
POLRMT
LRPPRC
MTERF4
log2 L/L, cre / L/L: −0.59
adj. p value: 0.4331
log2 L/L, cre / L/L: −0.16
adj. p value: 0.2393
log2 L/L, cre / L/L: −0.68
adj. p value: 0.1665
log2 L/L, cre / L/L: 0.36
adj. p value: 0.5517
log2 L/L, cre / L/L: −0.83
adj. p value: 0.2828
log2 intensity
28
27
26
25
24
Genotype
L/L
L/L, cre
Imputation
non imputed
imputed

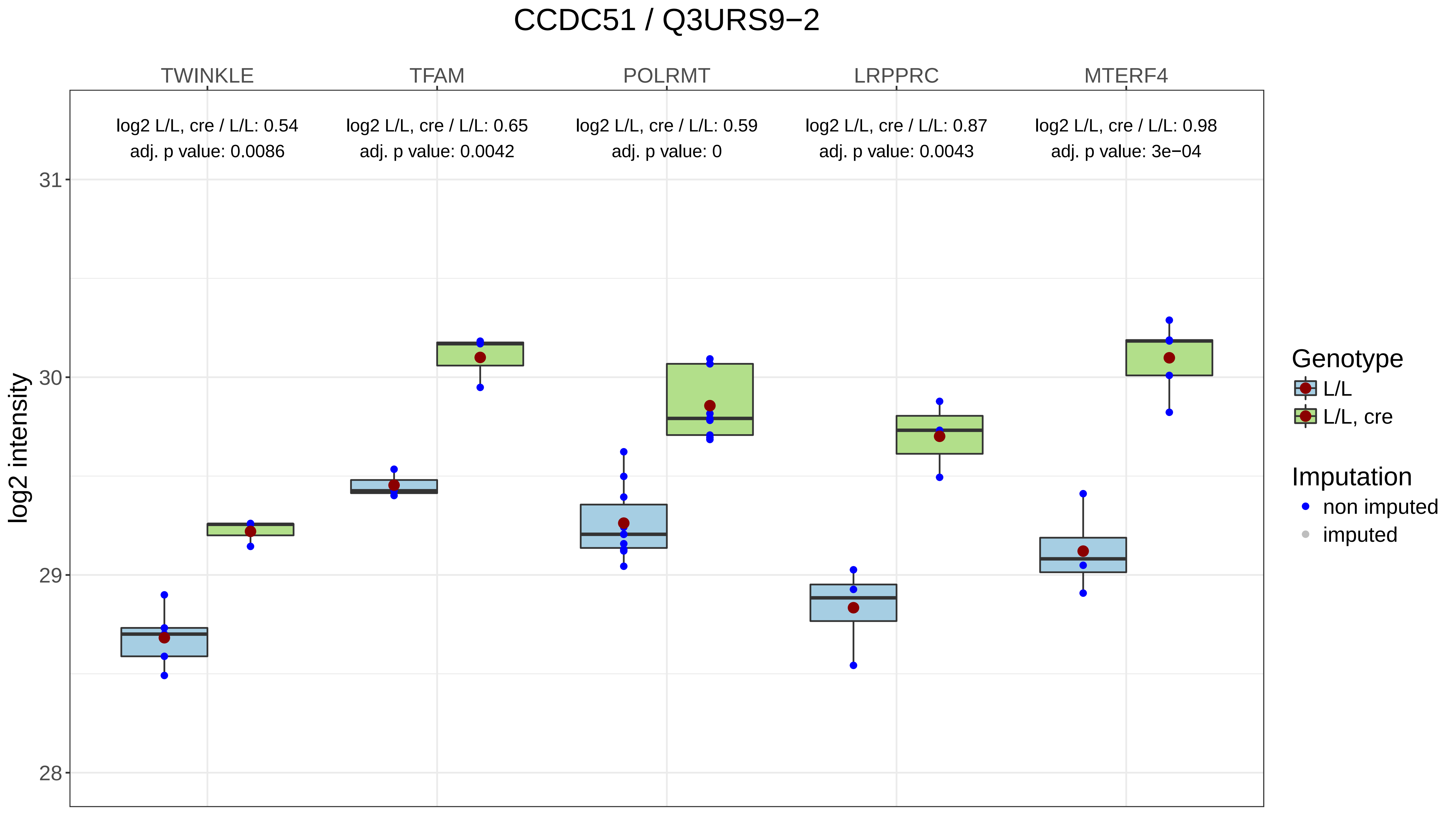
CCDC51 / Q3URS9−2
TWINKLE
TFAM
POLRMT
LRPPRC
MTERF4
log2 L/L, cre / L/L: 0.54
adj. p value: 0.0086
log2 L/L, cre / L/L: 0.65
adj. p value: 0.0042
log2 L/L, cre / L/L: 0.59
adj. p value: 0
log2 L/L, cre / L/L: 0.87
adj. p value: 0.0043
log2 L/L, cre / L/L: 0.98
adj. p value: 3e−04
log2 intensity
31
30
29
28
Genotype
L/L
L/L, cre
Imputation
non imputed
imputed

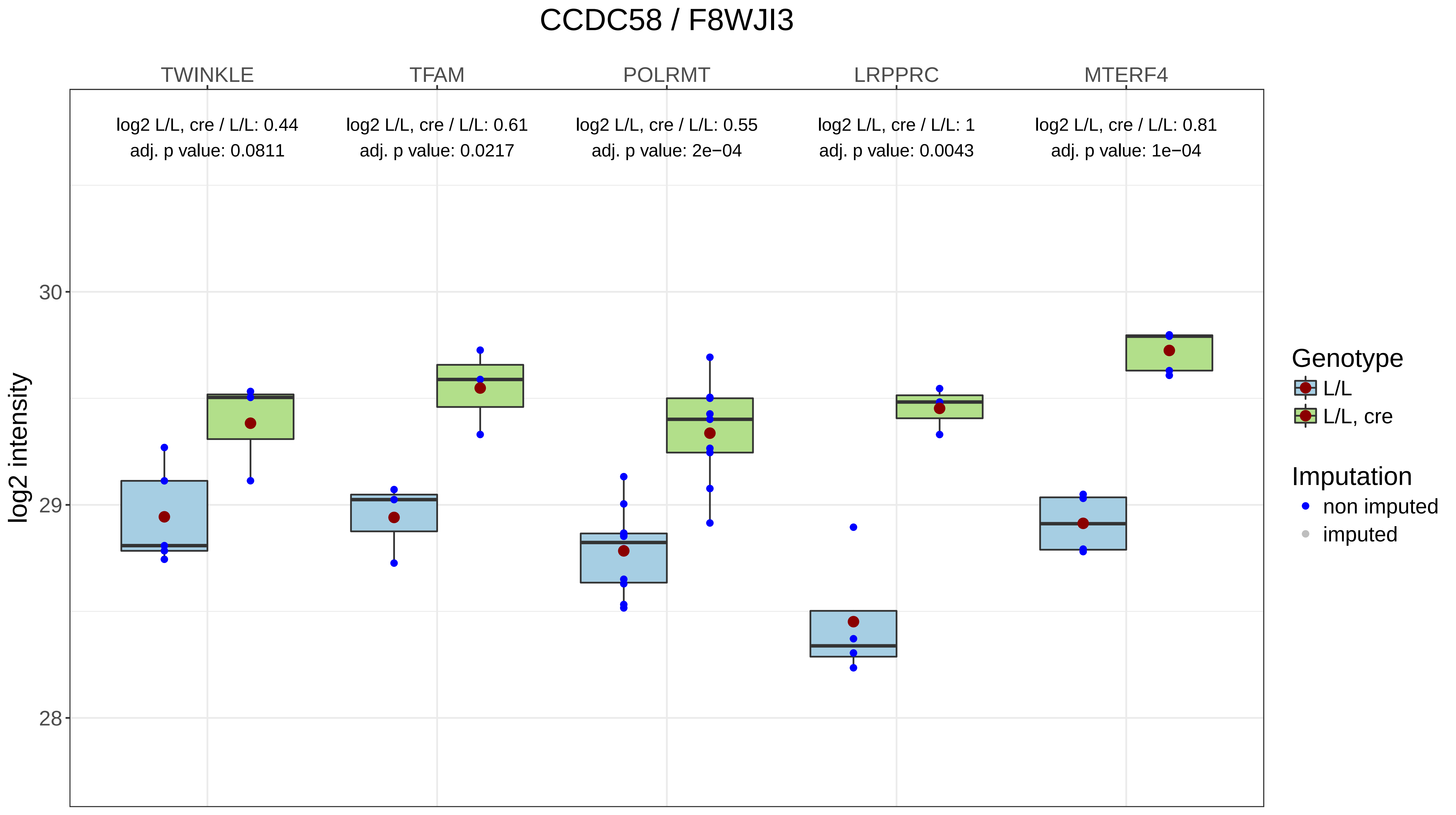
CCDC58 / F8WJI3
TWINKLE
TFAM
POLRMT
LRPPRC
MTERF4
log2 L/L, cre / L/L: 0.44
adj. p value: 0.0811
log2 L/L, cre / L/L: 0.61
adj. p value: 0.0217
log2 L/L, cre / L/L: 0.55
adj. p value: 2e−04
log2 L/L, cre / L/L: 1
adj. p value: 0.0043
log2 L/L, cre / L/L: 0.81
adj. p value: 1e−04
log2 intensity
30
29
28
Genotype
L/L
L/L, cre
Imputation
non imputed
imputed

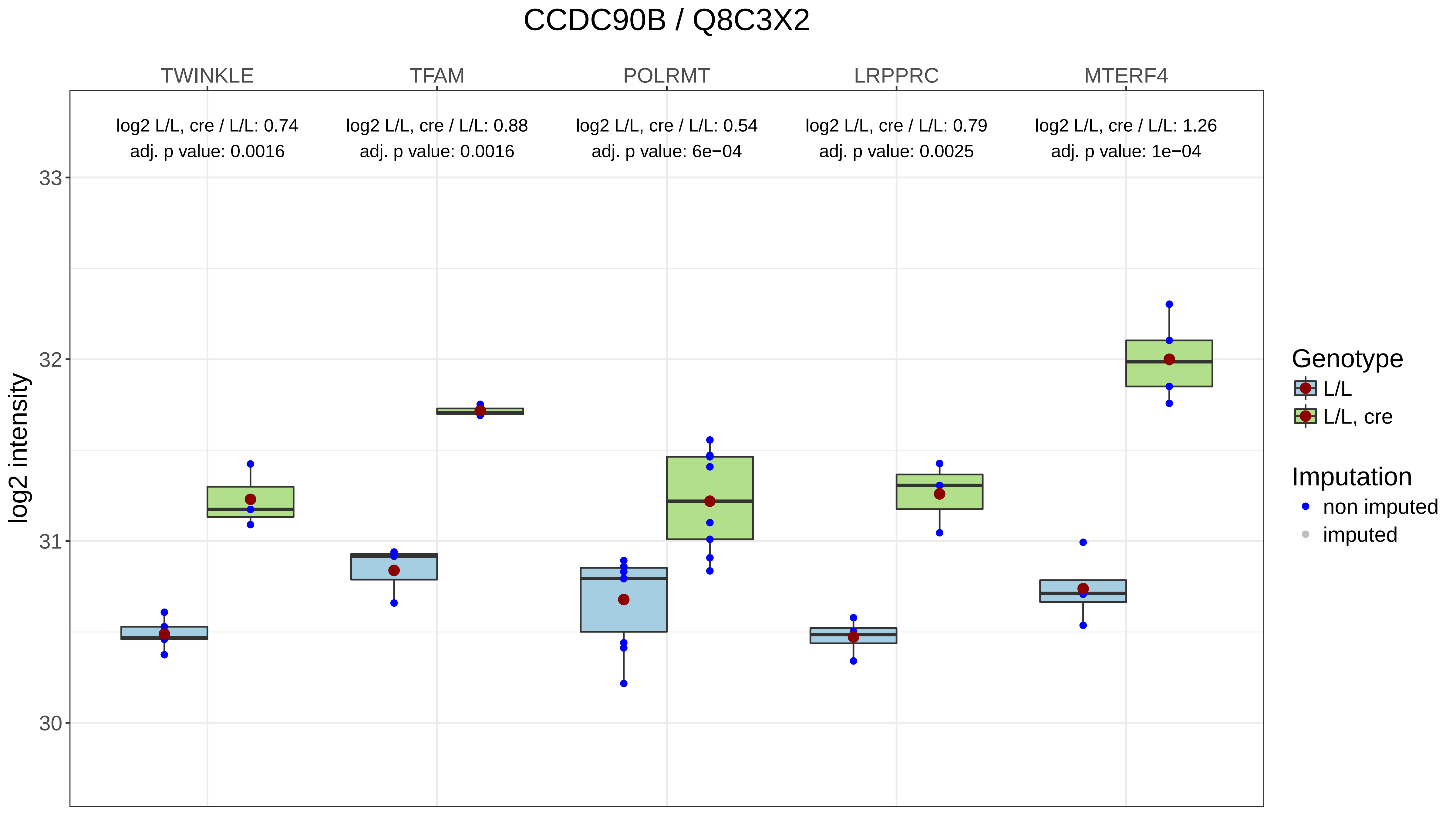
CCDC90B / Q8C3X2
TWINKLE
TFAM
POLRMT
LRPPRC
MTERF4
log2 L/L, cre / L/L: 0.74
adj. p value: 0.0016
log2 L/L, cre / L/L: 0.88
adj. p value: 0.0016
log2 L/L, cre / L/L: 0.54
adj. p value: 6e−04
log2 L/L, cre / L/L: 0.79
adj. p value: 0.0025
log2 L/L, cre / L/L: 1.26
adj. p value: 1e−04
log2 intensity
33
32
31
30
Genotype
L/L
L/L, cre
Imputation
non imputed
imputed

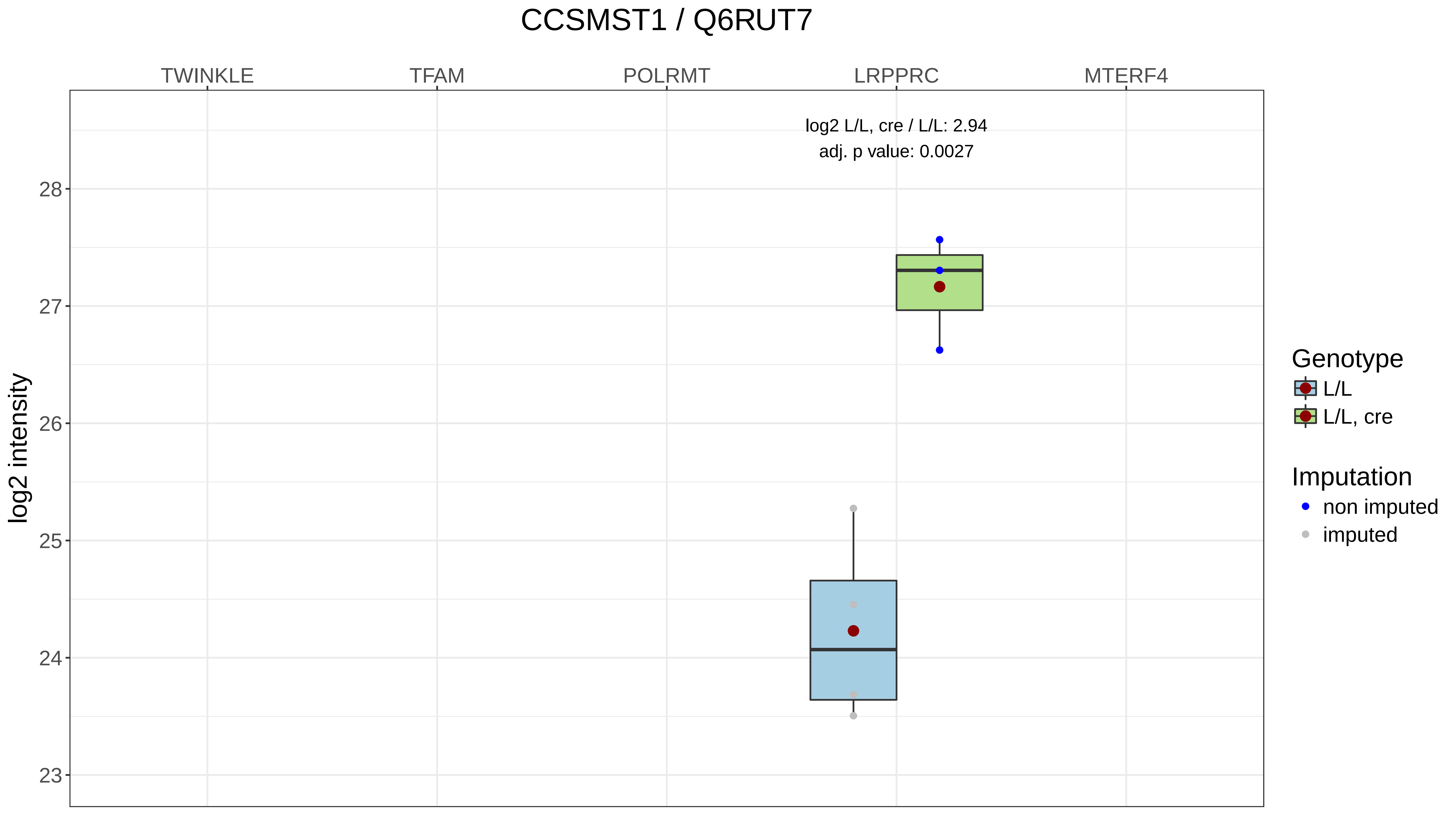
CCSMST1 / Q6RUT7
TWINKLE
TFAM
POLRMT
LRPPRC
MTERF4
log2 L/L, cre / L/L: 2.94
adj. p value: 0.0027
28
27
26
25
24
23
log2 intensity
Genotype
L/L
L/L, cre
Imputation
non imputed
imputed

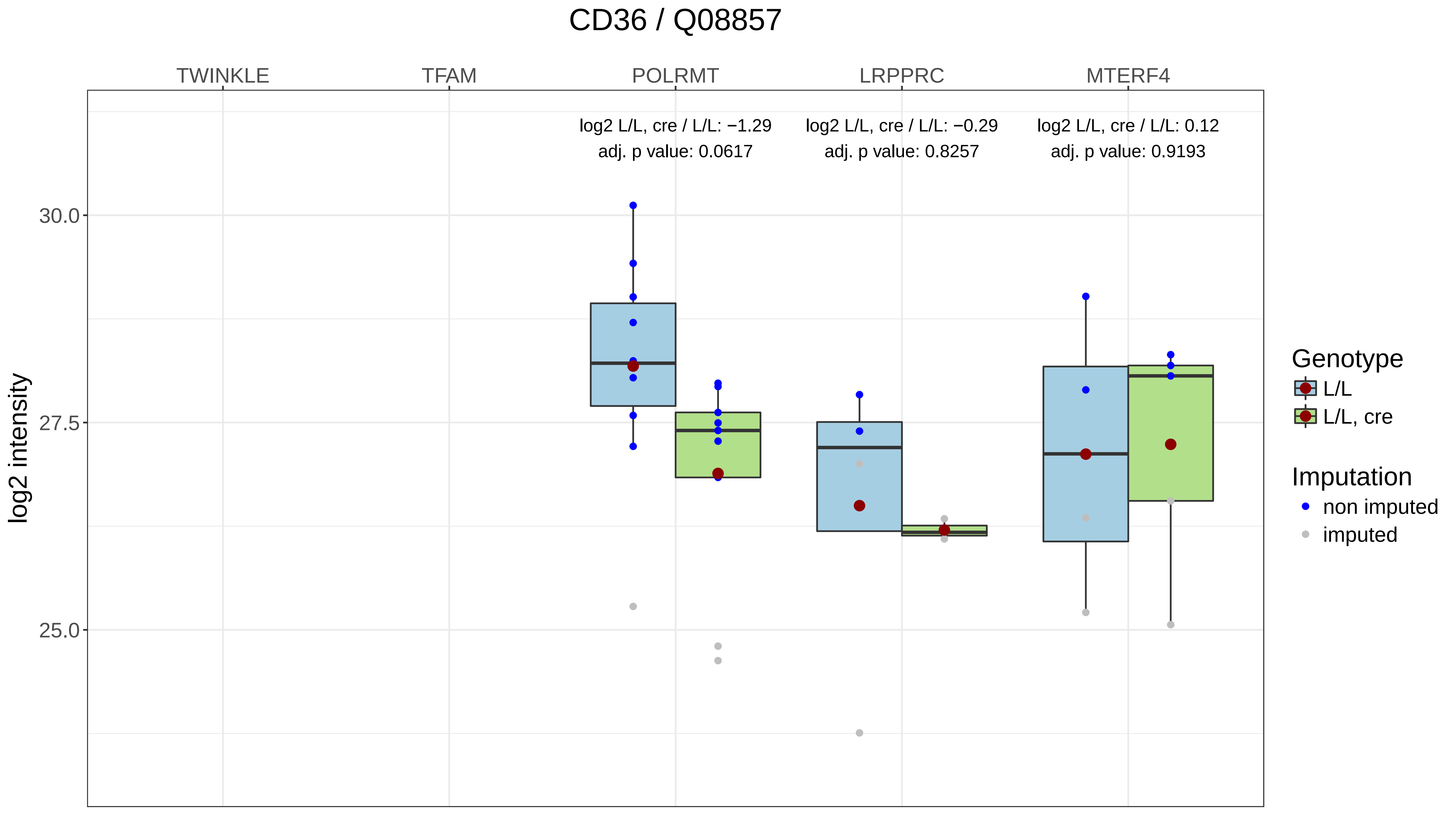
CD36 / Q08857
TWINKLE
TFAM
POLRMT
LRPPRC
MTERF4
log2 L/L, cre / L/L: −1.29
adj. p value: 0.0617
log2 L/L, cre / L/L: −0.29
adj. p value: 0.8257
log2 L/L, cre / L/L: 0.12
adj. p value: 0.9193
30.0
27.5
25.0
log2 intensity
Genotype
L/L
L/L, cre
Imputation
non imputed
imputed

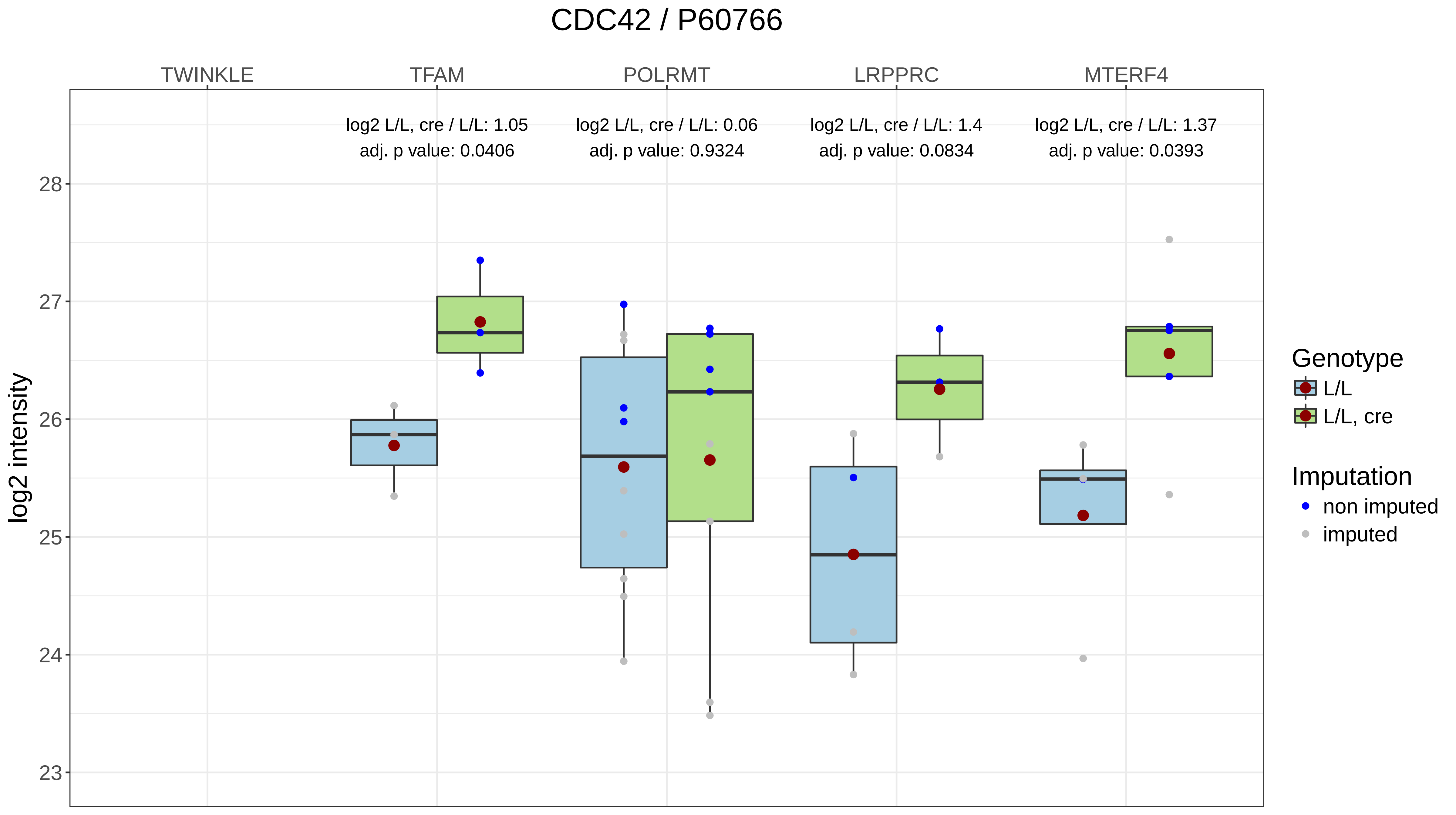
CDC42 / P60766
TWINKLE
TFAM
POLRMT
LRPPRC
MTERF4
log2 L/L, cre / L/L: 1.05
adj. p value: 0.0406
log2 L/L, cre / L/L: 0.06
adj. p value: 0.9324
log2 L/L, cre / L/L: 1.4
adj. p value: 0.0834
log2 L/L, cre / L/L: 1.37
adj. p value: 0.0393
log2 intensity
28
27
26
25
24
23
Genotype
L/L
L/L, cre
Imputation
non imputed
imputed

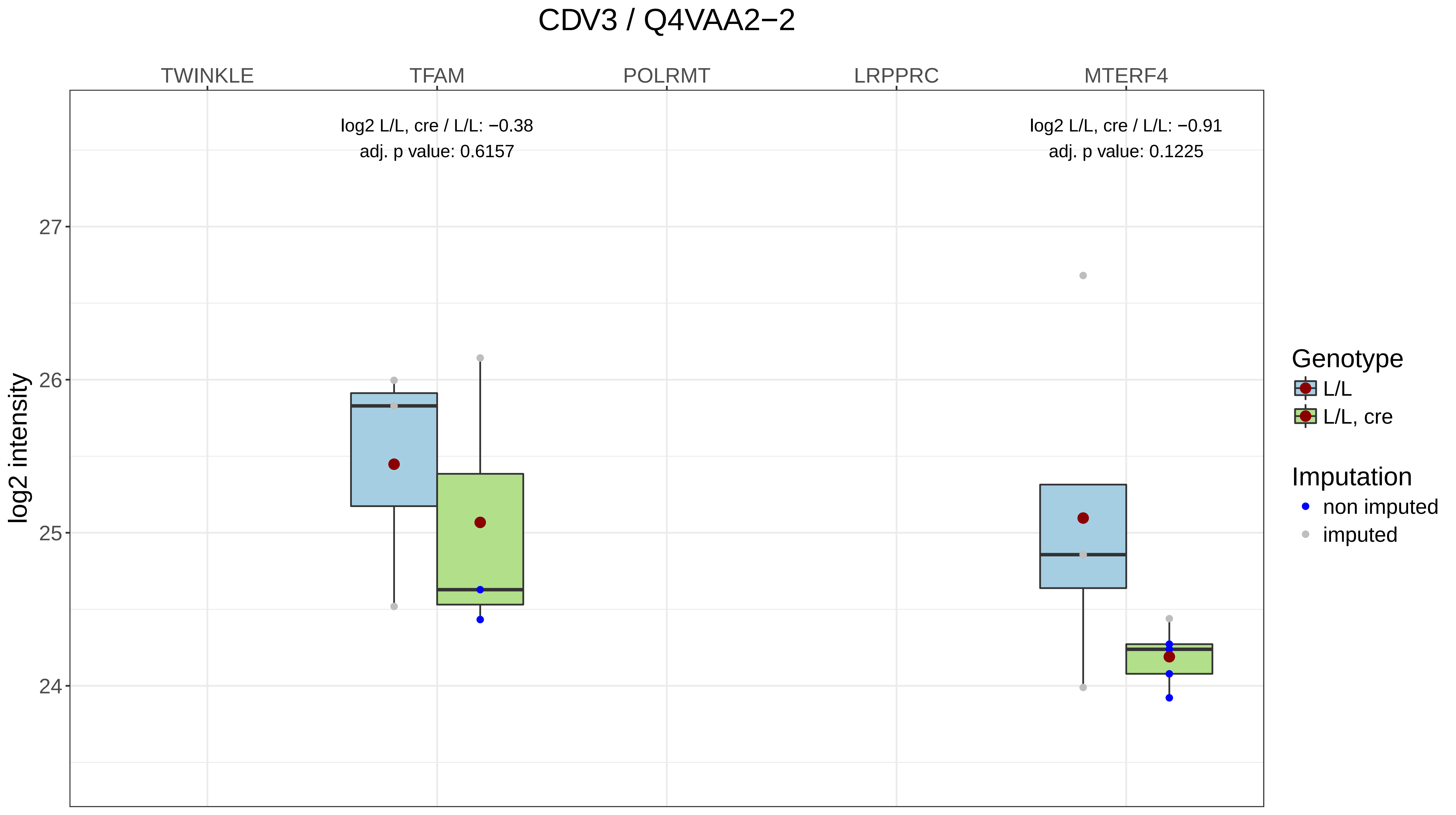

CDV3 / Q4VAA2−2
TWINKLE
TFAM
POLRMT
LRPPRC
MTERF4
log2 L/L, cre / L/L: −0.38
adj. p value: 0.6157
log2 L/L, cre / L/L: −0.91
adj. p value: 0.1225
log2 intensity
27
26
25
24
Genotype
L/L
L/L, cre
Imputation
non imputed
imputed

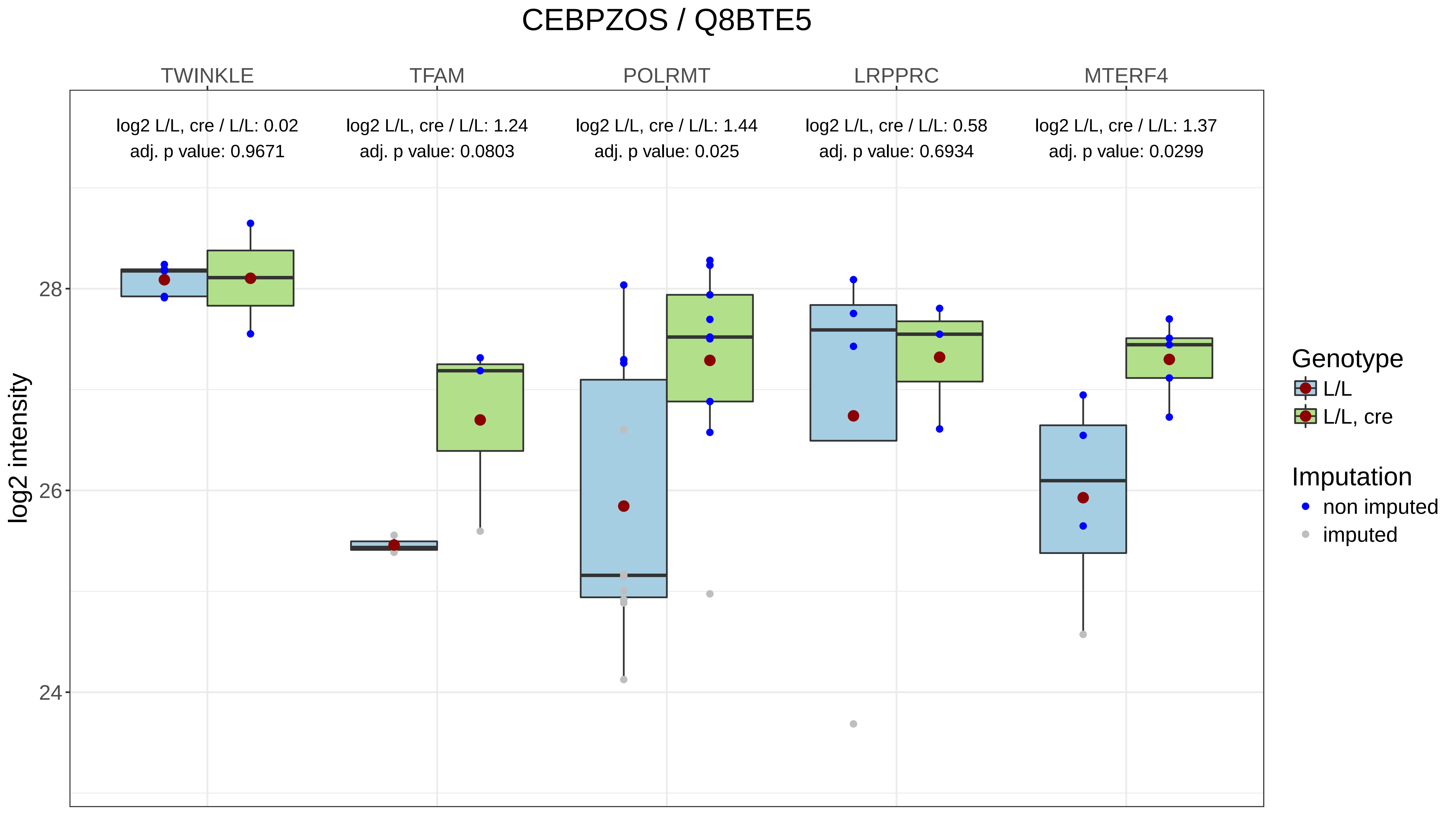

CEBPZOS / Q8BTE5
TWINKLE
TFAM
POLRMT
LRPPRC
MTERF4
log2 L/L, cre / L/L: 0.02
adj. p value: 0.9671
log2 L/L, cre / L/L: 1.24
adj. p value: 0.0803
log2 L/L, cre / L/L: 1.44
adj. p value: 0.025
log2 L/L, cre / L/L: 0.58
adj. p value: 0.6934
log2 L/L, cre / L/L: 1.37
adj. p value: 0.0299
log2 intensity
28
26
24
Genotype
L/L
L/L, cre
Imputation
non imputed
imputed

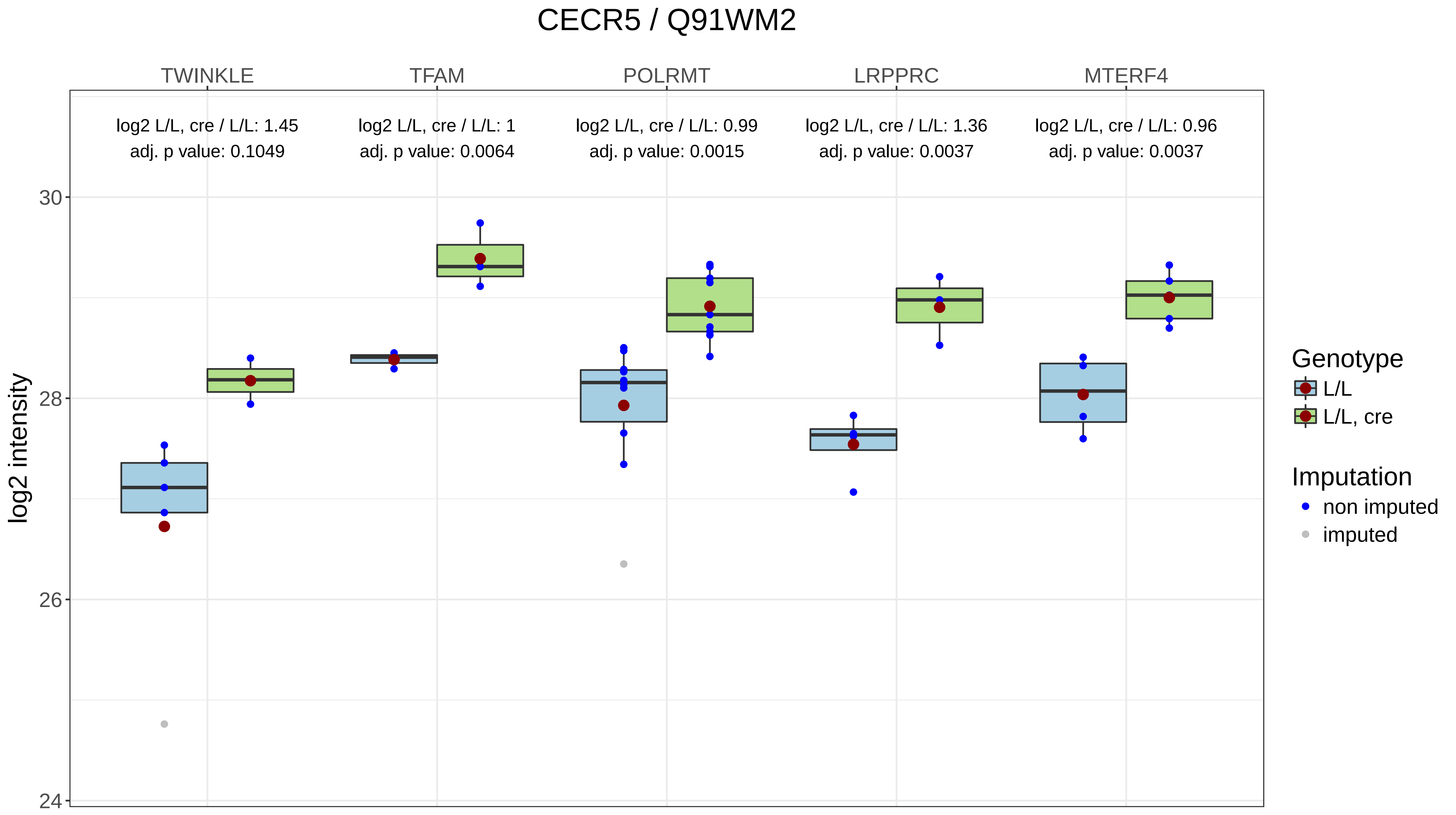
CECR5 / Q91WM2
TWINKLE
TFAM
POLRMT
LRPPRC
MTERF4
log2 L/L, cre / L/L: 1.45
adj. p value: 0.1049
log2 L/L, cre / L/L: 1
adj. p value: 0.0064
log2 L/L, cre / L/L: 0.99
adj. p value: 0.0015
log2 L/L, cre / L/L: 1.36
adj. p value: 0.0037
log2 L/L, cre / L/L: 0.96
adj. p value: 0.0037
log2 intensity
30
28
26
24
Genotype
L/L
L/L, cre
Imputation
non imputed
imputed

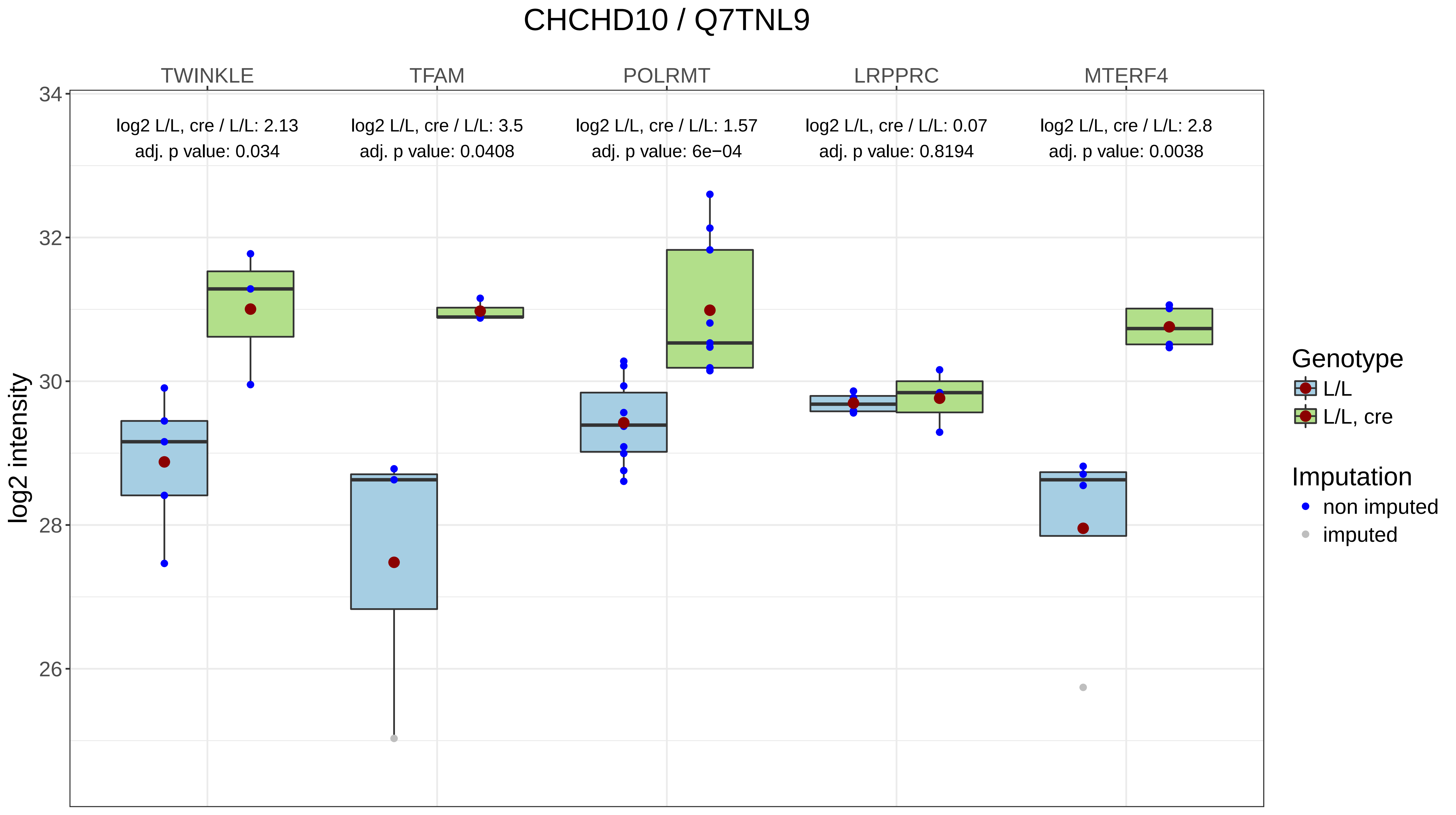
CHCHD10 / Q7TNL9
TWINKLE
TFAM
POLRMT
LRPPRC
MTERF4
log2 L/L, cre / L/L: 2.13
adj. p value: 0.034
log2 L/L, cre / L/L: 3.5
adj. p value: 0.0408
log2 L/L, cre / L/L: 1.57
adj. p value: 6e−04
log2 L/L, cre / L/L: 0.07
adj. p value: 0.8194
log2 L/L, cre / L/L: 2.8
adj. p value: 0.0038
log2 intensity
34
32
30
28
26
Genotype
L/L
L/L, cre
Imputation
non imputed
imputed

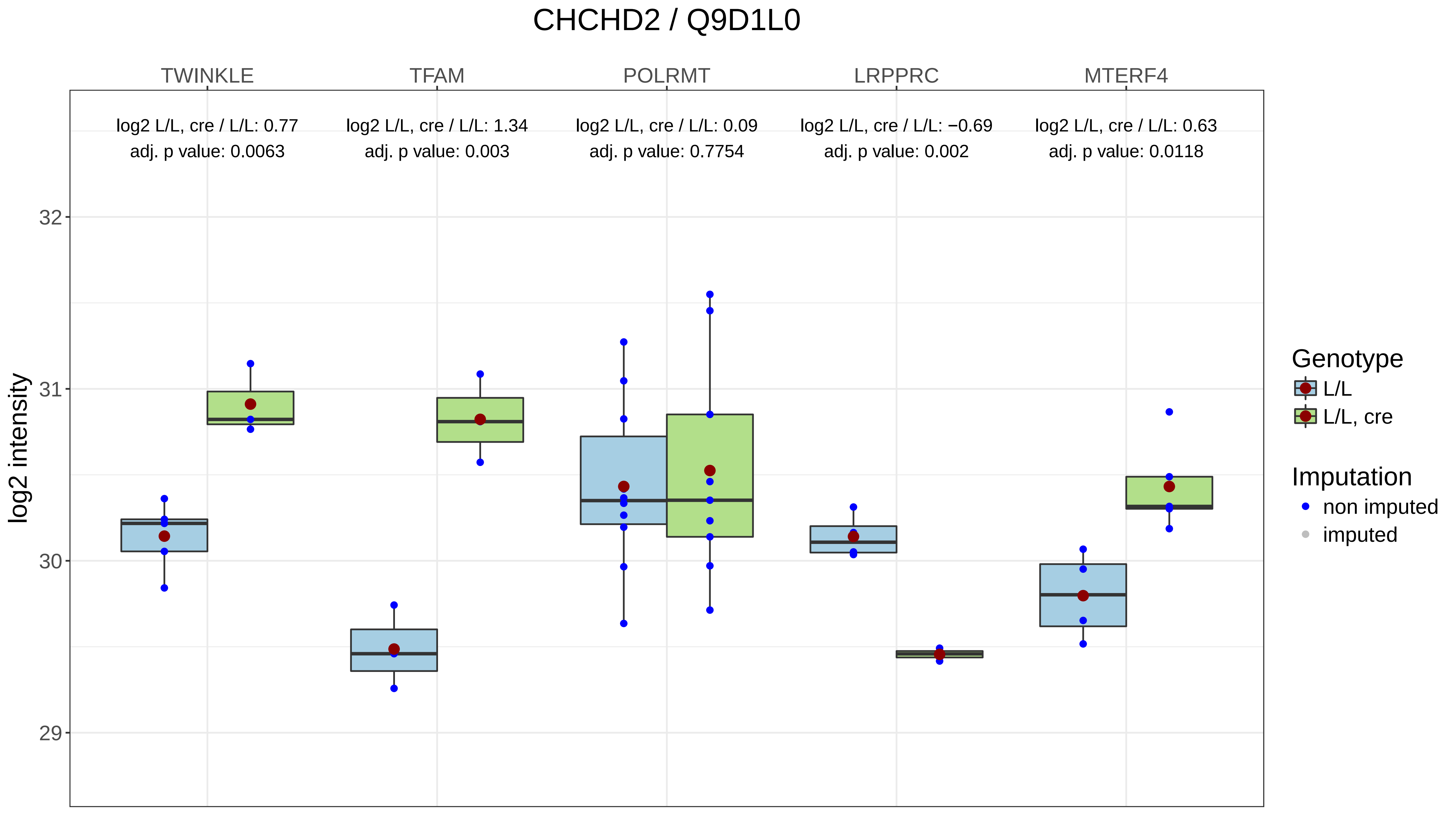
CHCHD2 / Q9D1L0
TWINKLE
TFAM
POLRMT
LRPPRC
MTERF4
log2 L/L, cre / L/L: 0.77
adj. p value: 0.0063
log2 L/L, cre / L/L: 1.34
adj. p value: 0.003
log2 L/L, cre / L/L: 0.09
adj. p value: 0.7754
log2 L/L, cre / L/L: −0.69
adj. p value: 0.002
log2 L/L, cre / L/L: 0.63
adj. p value: 0.0118
log2 intensity
32
31
30
29
Genotype
L/L
L/L, cre
Imputation
non imputed
imputed

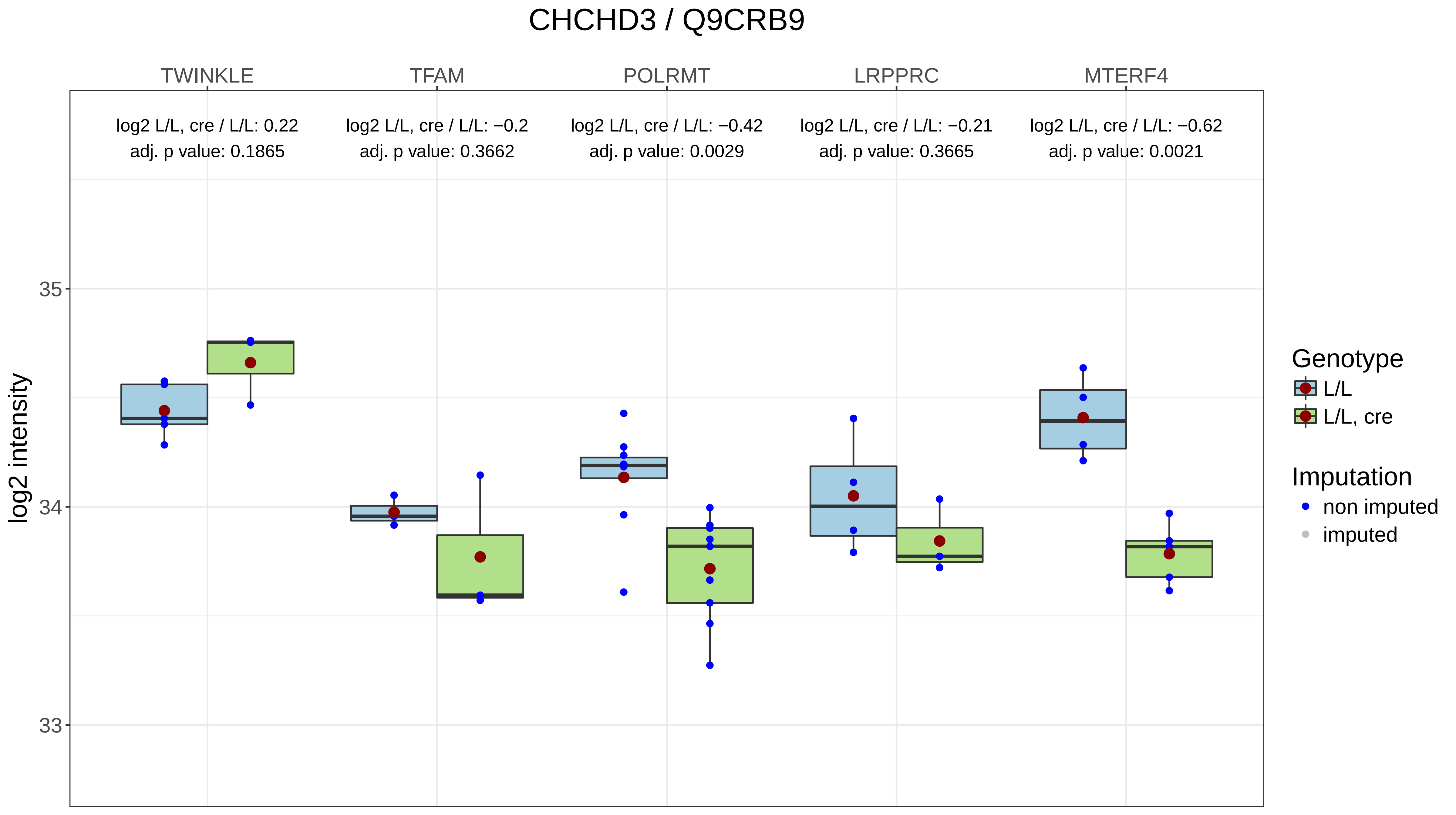
CHCHD3 / Q9CRB9
TWINKLE
TFAM
POLRMT
LRPPRC
MTERF4
log2 L/L, cre / L/L: 0.22
adj. p value: 0.1865
log2 L/L, cre / L/L: −0.2
adj. p value: 0.3662
log2 L/L, cre / L/L: −0.42
adj. p value: 0.0029
log2 L/L, cre / L/L: −0.21
adj. p value: 0.3665
log2 L/L, cre / L/L: −0.62
adj. p value: 0.0021
log2 intensity
35
34
33
Genotype
L/L
L/L, cre
Imputation
non imputed
imputed

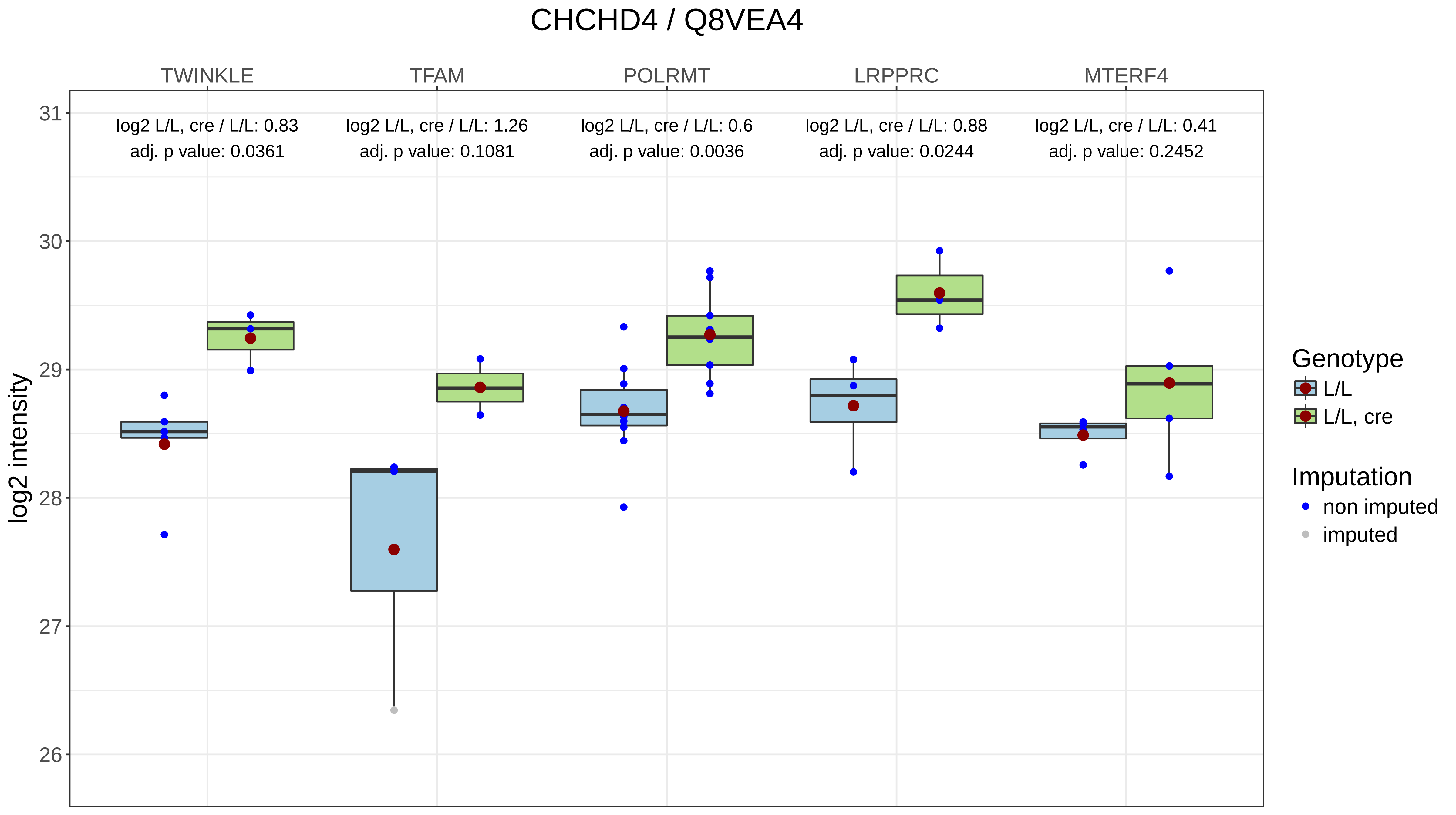

CHCHD4 / Q8VEA4
TWINKLE
TFAM
POLRMT
LRPPRC
MTERF4
log2 L/L, cre / L/L: 0.83
adj. p value: 0.0361
log2 L/L, cre / L/L: 1.26
adj. p value: 0.1081
log2 L/L, cre / L/L: 0.6
adj. p value: 0.0036
log2 L/L, cre / L/L: 0.88
adj. p value: 0.0244
log2 L/L, cre / L/L: 0.41
adj. p value: 0.2452
log2 intensity
31
30
29
28
27
26
Genotype
L/L
L/L, cre
Imputation
non imputed
imputed

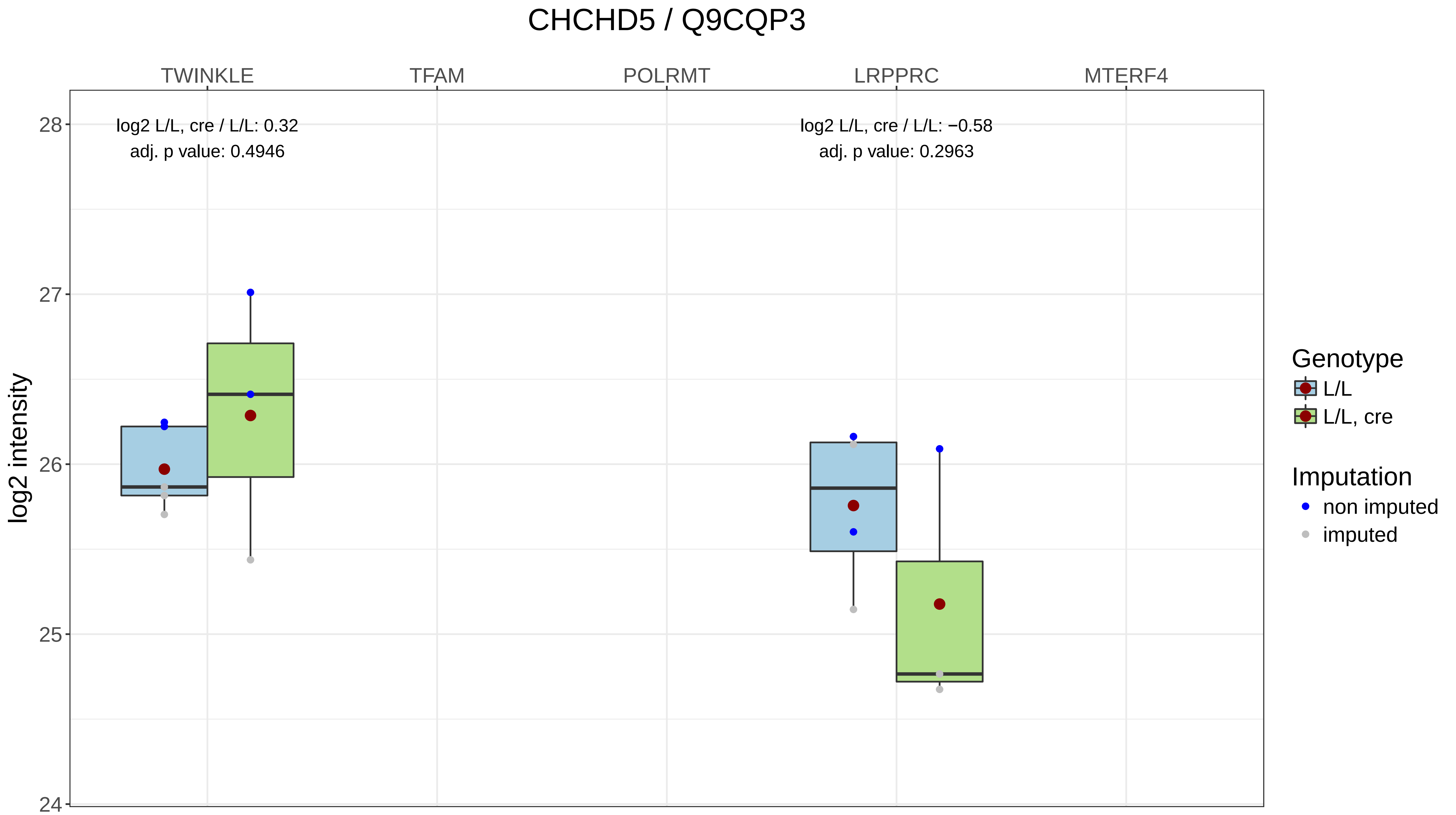
CHCHD5 / Q9CQP3
TWINKLE
TFAM
POLRMT
LRPPRC
MTERF4
log2 L/L, cre / L/L: 0.32
adj. p value: 0.4946
log2 L/L, cre / L/L: −0.58
adj. p value: 0.2963
log2 intensity
28
27
26
25
24
Genotype
L/L
L/L, cre
Imputation
non imputed
imputed

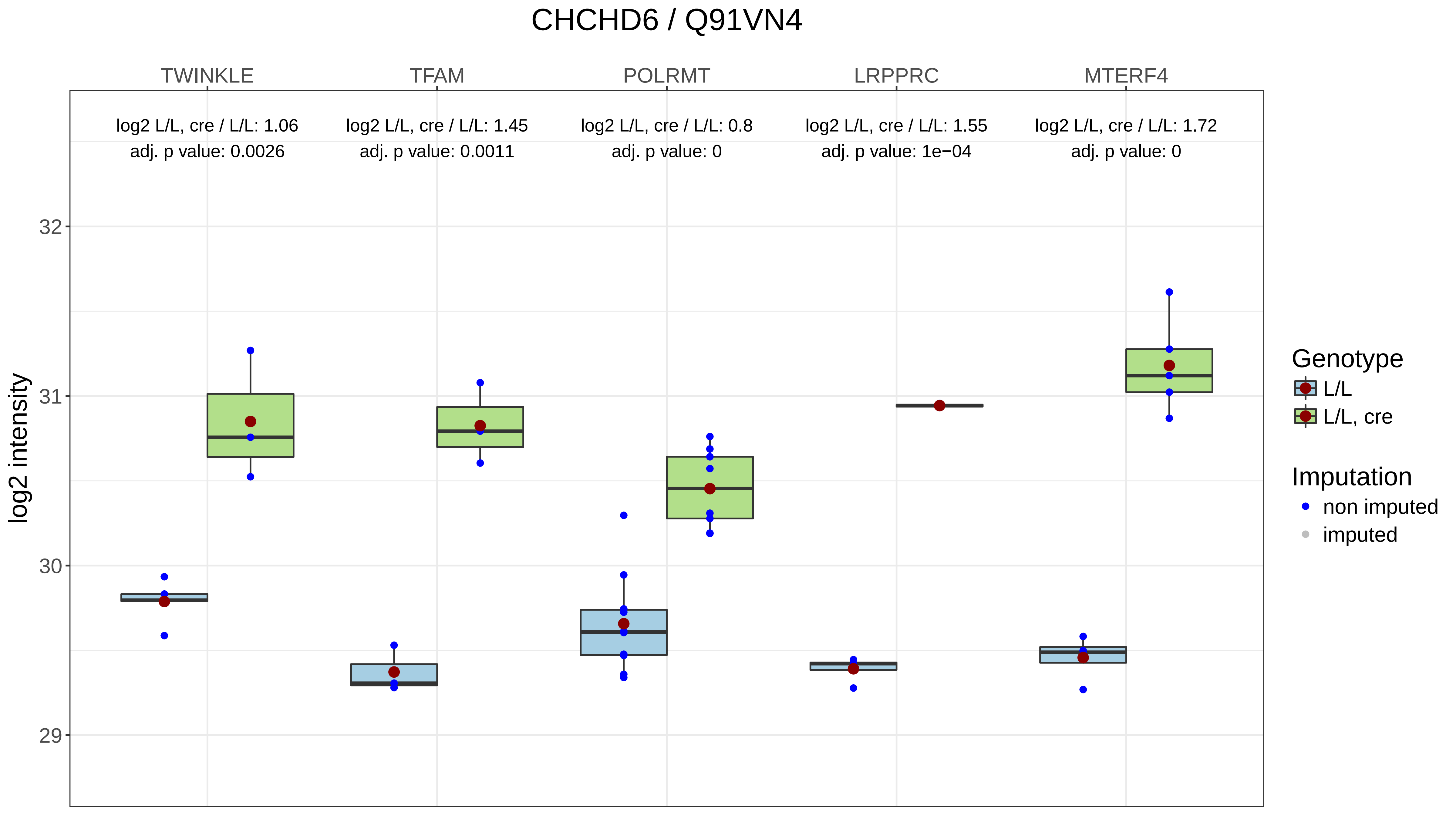
CHCHD6 / Q91VN4
TWINKLE
TFAM
POLRMT
LRPPRC
MTERF4
log2 L/L, cre / L/L: 1.06
adj. p value: 0.0026
log2 L/L, cre / L/L: 1.45
adj. p value: 0.0011
log2 L/L, cre / L/L: 0.8
adj. p value: 0
log2 L/L, cre / L/L: 1.55
adj. p value: 1e−04
log2 L/L, cre / L/L: 1.72
adj. p value: 0
log2 intensity
32
31
30
29
Genotype
L/L
L/L, cre
Imputation
non imputed
imputed

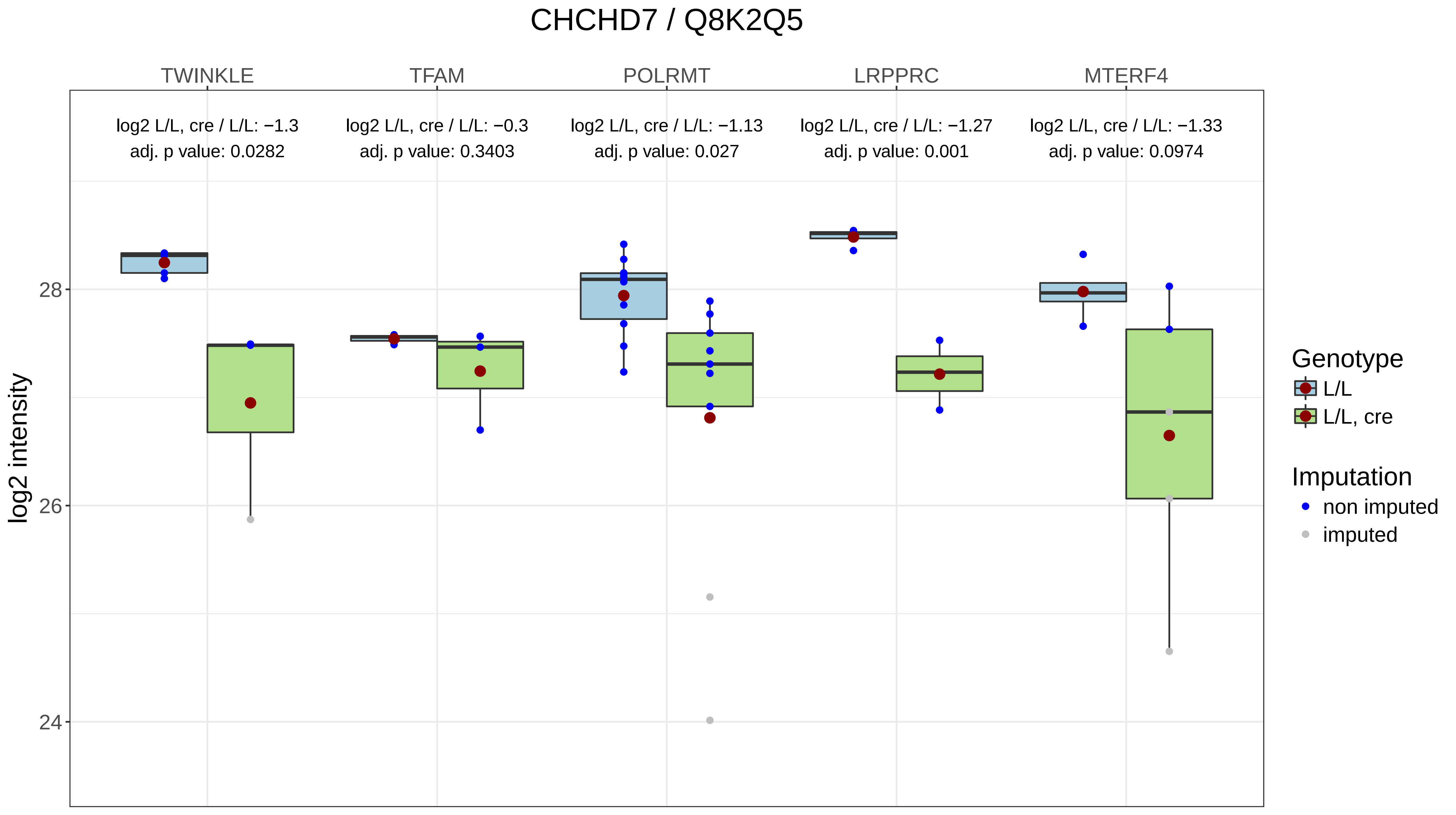
CHCHD7 / Q8K2Q5
TWINKLE
TFAM
POLRMT
LRPPRC
MTERF4
log2 L/L, cre / L/L: −1.3
adj. p value: 0.0282
log2 L/L, cre / L/L: −0.3
adj. p value: 0.3403
log2 L/L, cre / L/L: −1.13
adj. p value: 0.027
log2 L/L, cre / L/L: −1.27
adj. p value: 0.001
log2 L/L, cre / L/L: −1.33
adj. p value: 0.0974
log2 intensity
28
26
24
Genotype
L/L
L/L, cre
Imputation
non imputed
imputed

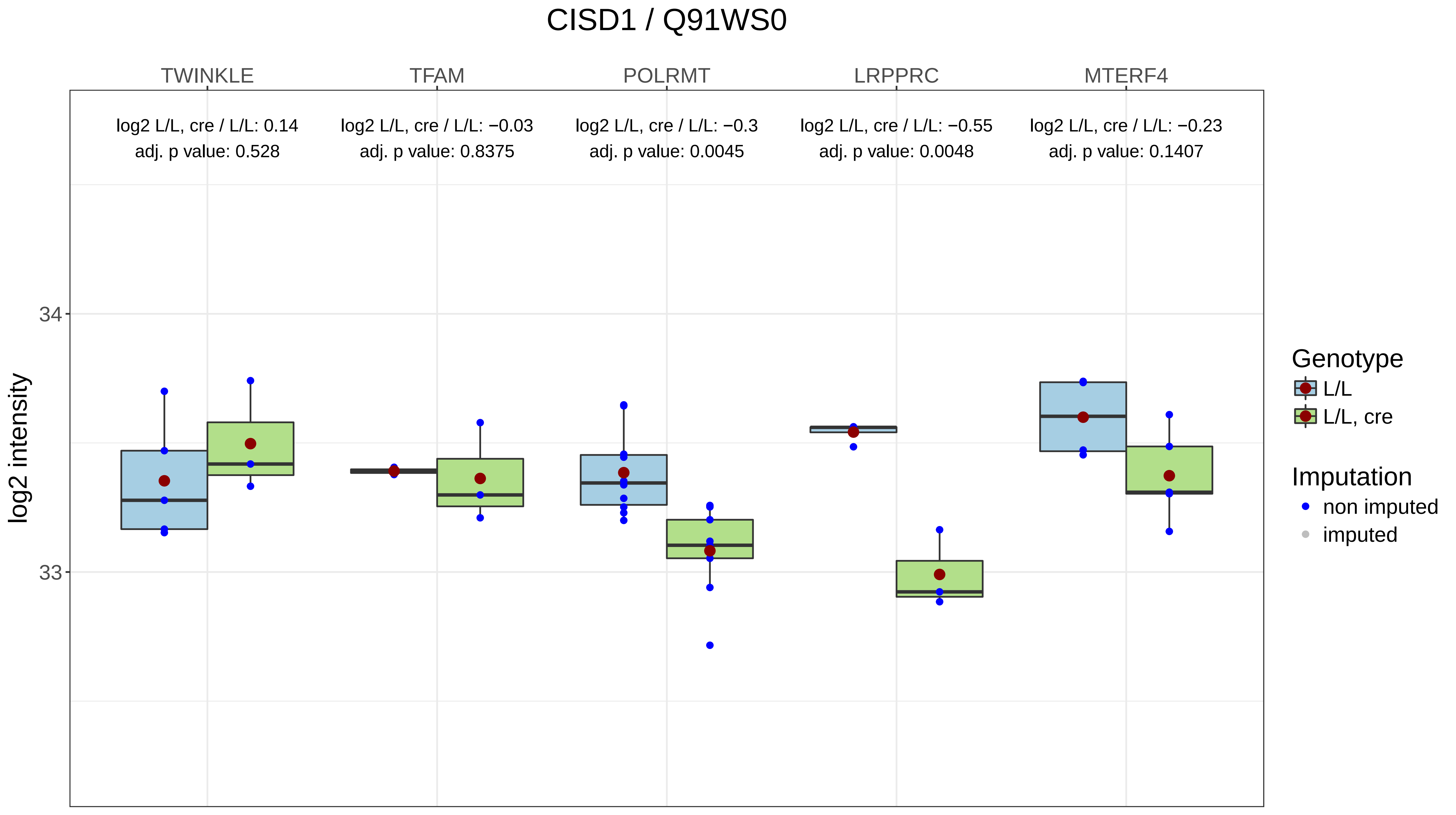
CISD1 / Q91WS0
TWINKLE
TFAM
POLRMT
LRPPRC
MTERF4
log2 L/L, cre / L/L: 0.14
adj. p value: 0.528
log2 L/L, cre / L/L: −0.03
adj. p value: 0.8375
log2 L/L, cre / L/L: −0.3
adj. p value: 0.0045
log2 L/L, cre / L/L: −0.55
adj. p value: 0.0048
log2 L/L, cre / L/L: −0.23
adj. p value: 0.1407
log2 intensity
34
33
Genotype
L/L
L/L, cre
Imputation
non imputed
imputed

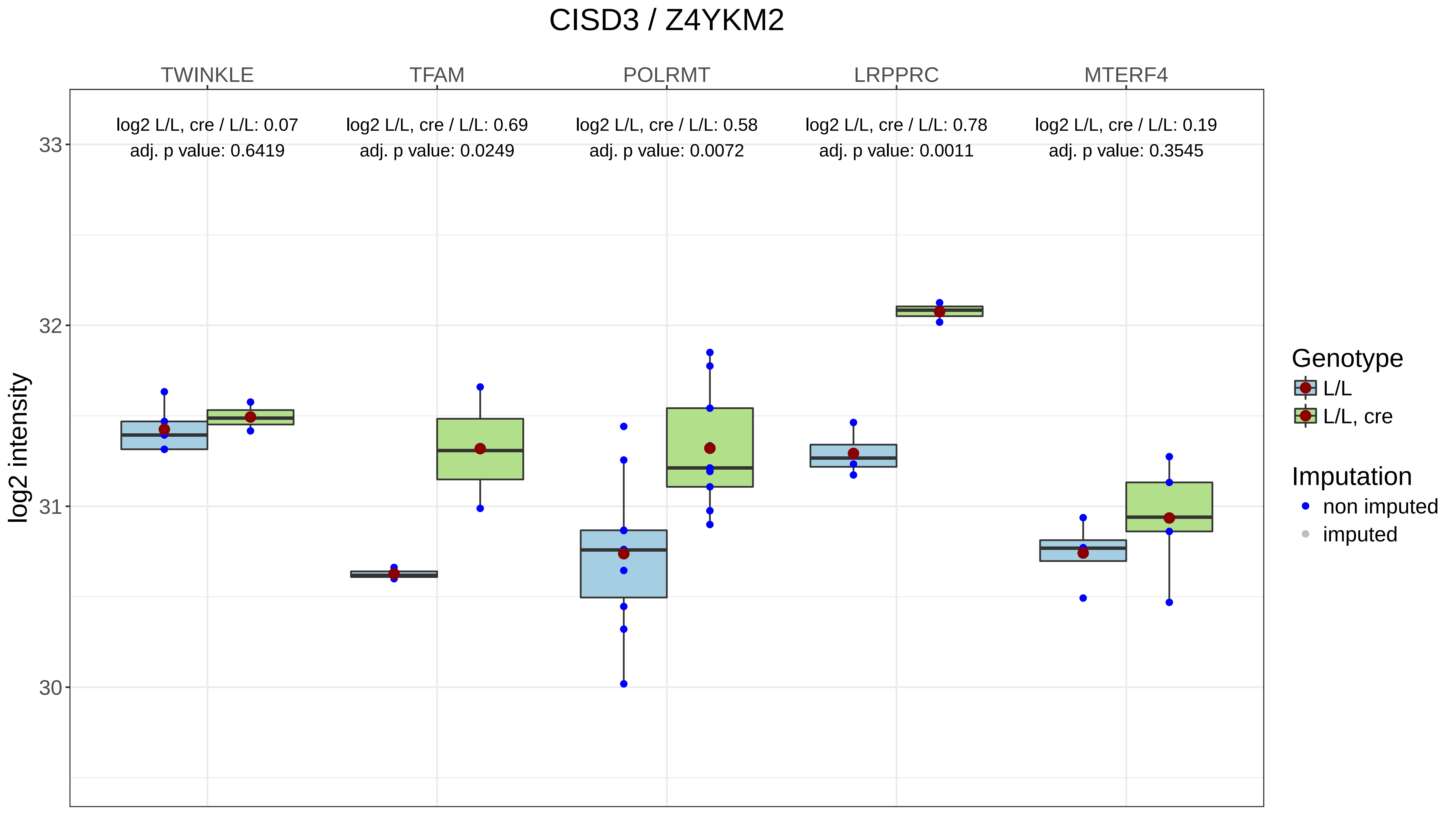
CISD3 / Z4YKM2
TWINKLE
TFAM
POLRMT
LRPPRC
MTERF4
log2 L/L, cre / L/L: 0.07
adj. p value: 0.6419
log2 L/L, cre / L/L: 0.69
adj. p value: 0.0249
log2 L/L, cre / L/L: 0.58
adj. p value: 0.0072
log2 L/L, cre / L/L: 0.78
adj. p value: 0.0011
log2 L/L, cre / L/L: 0.19
adj. p value: 0.3545
log2 intensity
33
32
31
30
Genotype
L/L
L/L, cre
Imputation
non imputed
imputed

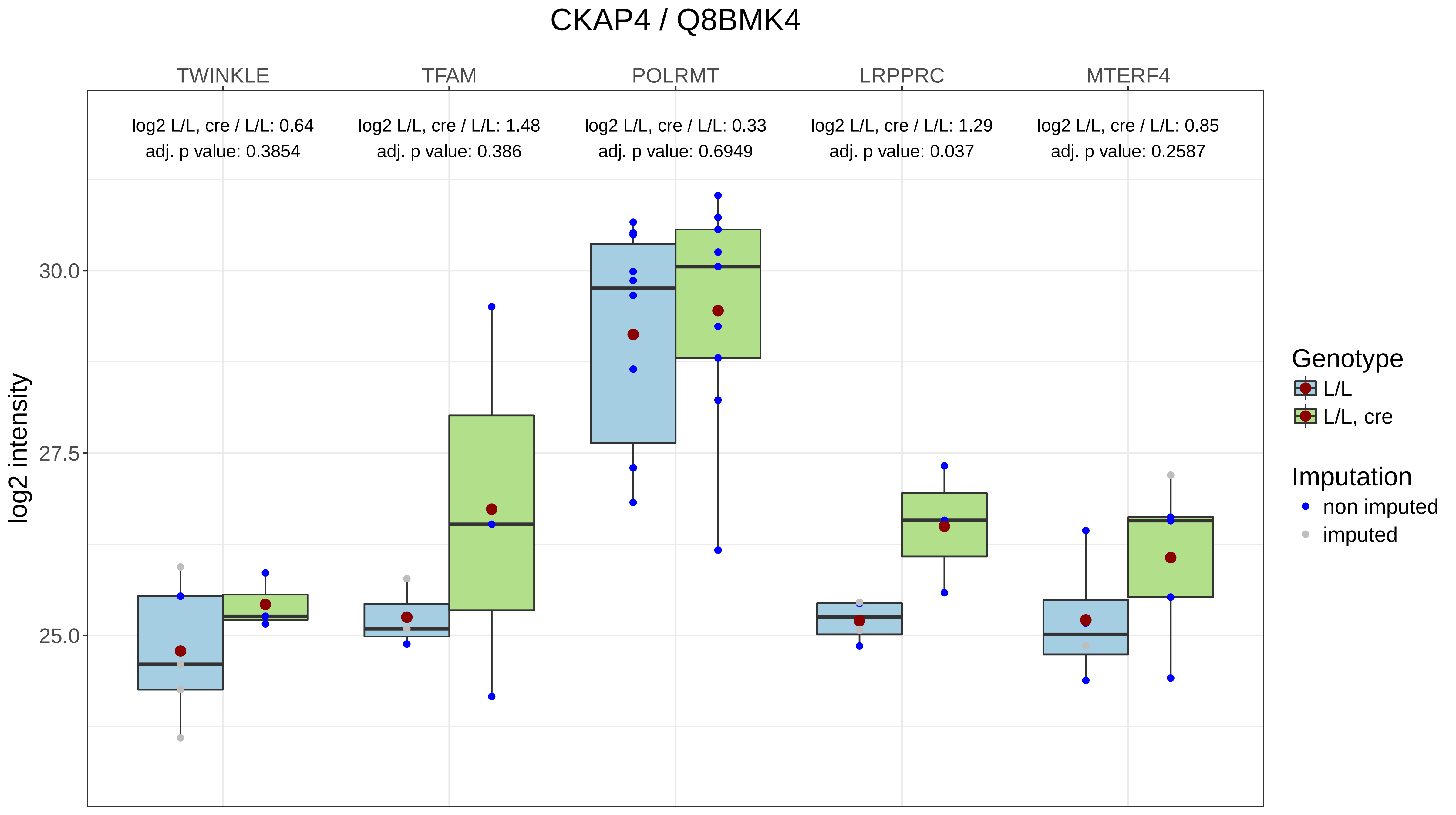

CKAP4 / Q8BMK4
TWINKLE
TFAM
POLRMT
LRPPRC
MTERF4
log2 L/L, cre / L/L: 0.64
adj. p value: 0.3854
log2 L/L, cre / L/L: 1.48
adj. p value: 0.386
log2 L/L, cre / L/L: 0.33
adj. p value: 0.6949
log2 L/L, cre / L/L: 1.29
adj. p value: 0.037
log2 L/L, cre / L/L: 0.85
adj. p value: 0.2587
log2 intensity
30.0
27.5
25.0
Genotype
L/L
L/L, cre
Imputation
non imputed
imputed

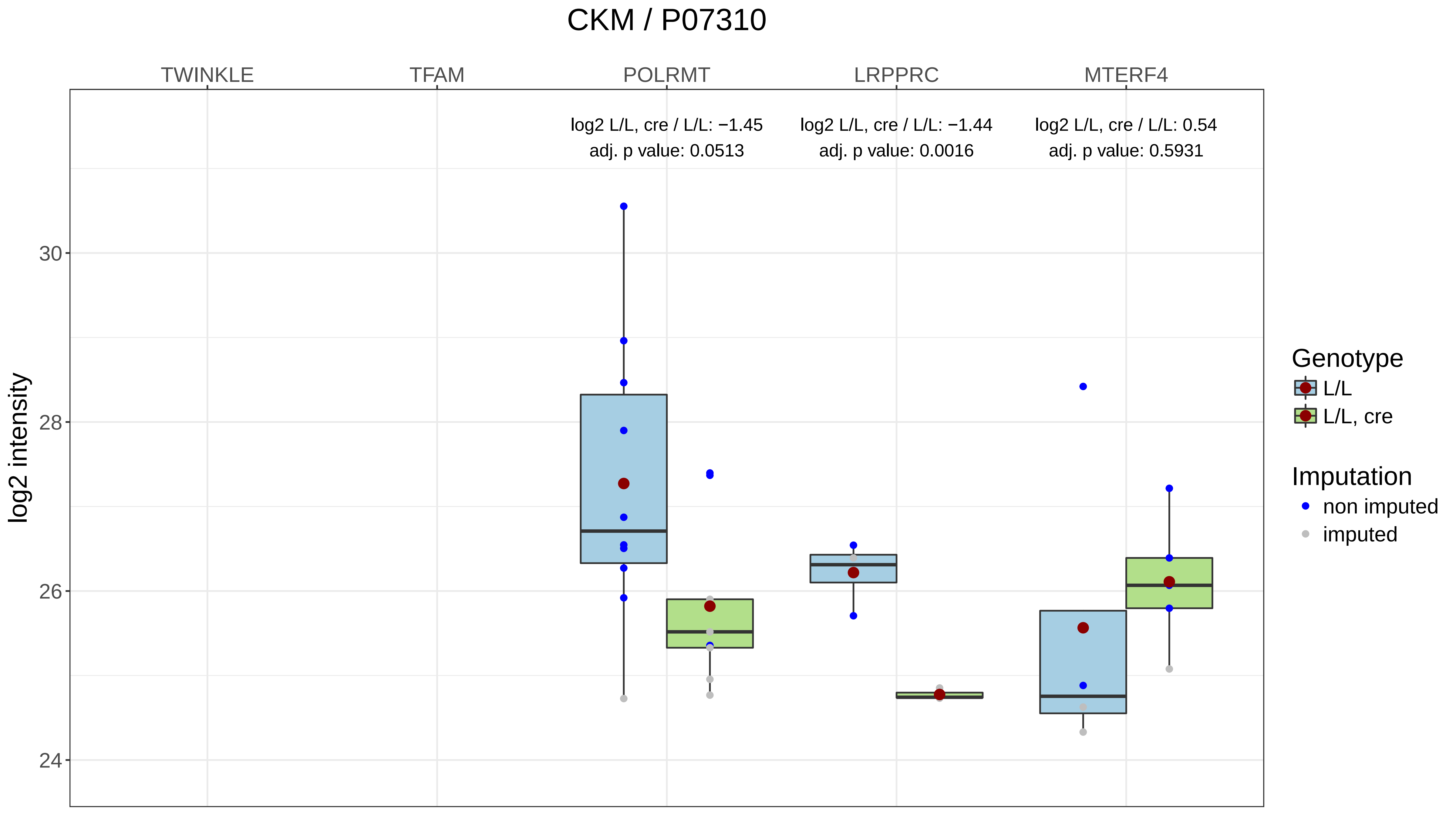
CKM / P07310
TWINKLE
TFAM
POLRMT
LRPPRC
MTERF4
log2 L/L, cre / L/L: −1.45
adj. p value: 0.0513
log2 L/L, cre / L/L: −1.44
adj. p value: 0.0016
log2 L/L, cre / L/L: 0.54
adj. p value: 0.5931
log2 intensity
30
28
26
24
Genotype
L/L
L/L, cre
Imputation
non imputed
imputed

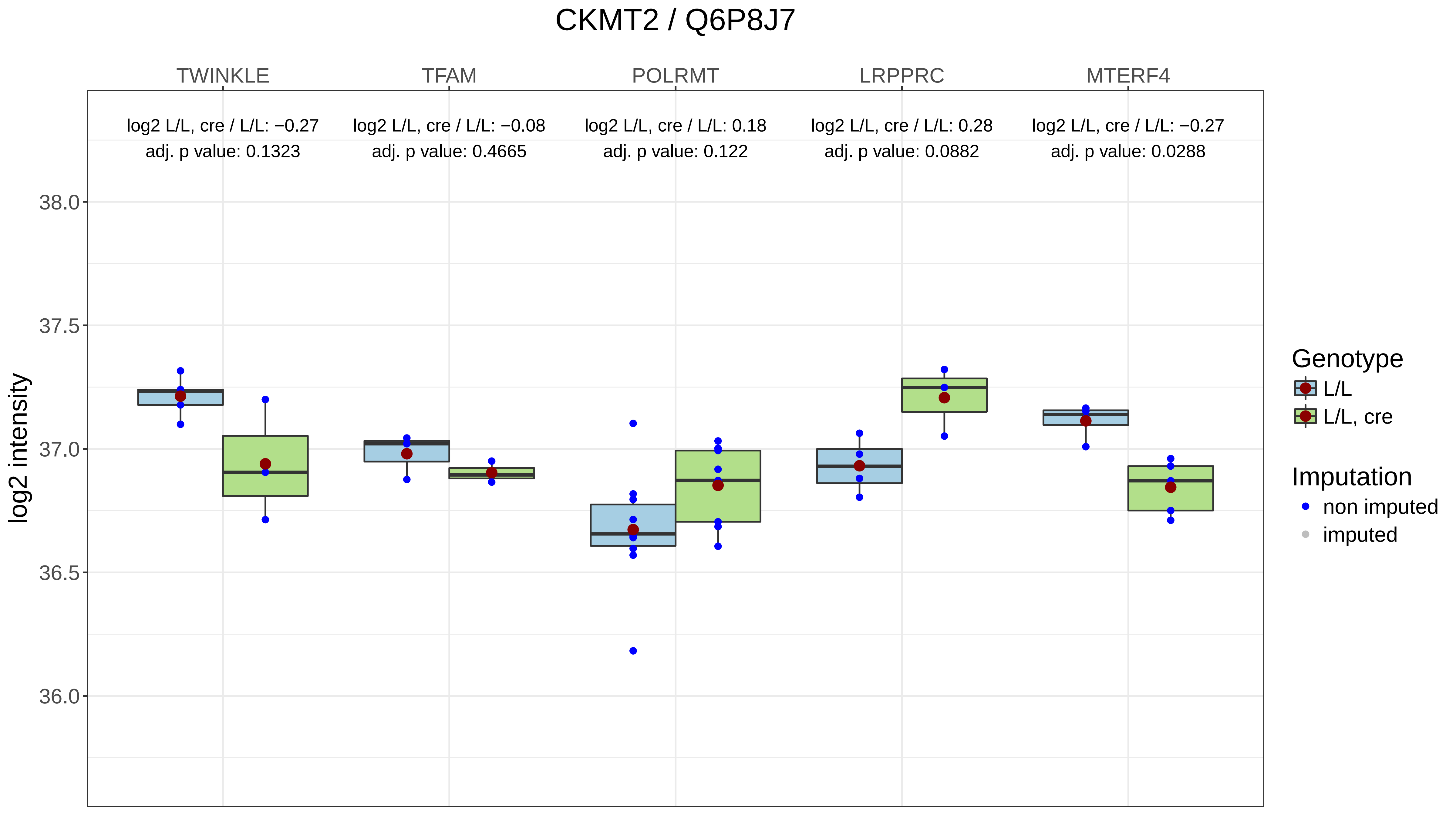
CKMT2 / Q6P8J7
TWINKLE
TFAM
POLRMT
LRPPRC
MTERF4
log2 L/L, cre / L/L: −0.27
adj. p value: 0.1323
log2 L/L, cre / L/L: −0.08
adj. p value: 0.4665
log2 L/L, cre / L/L: 0.18
adj. p value: 0.122
log2 L/L, cre / L/L: 0.28
adj. p value: 0.0882
log2 L/L, cre / L/L: −0.27
adj. p value: 0.0288
log2 intensity
38.0
37.5
37.0
36.5
36.0
Genotype
L/L
L/L, cre
Imputation
non imputed
imputed

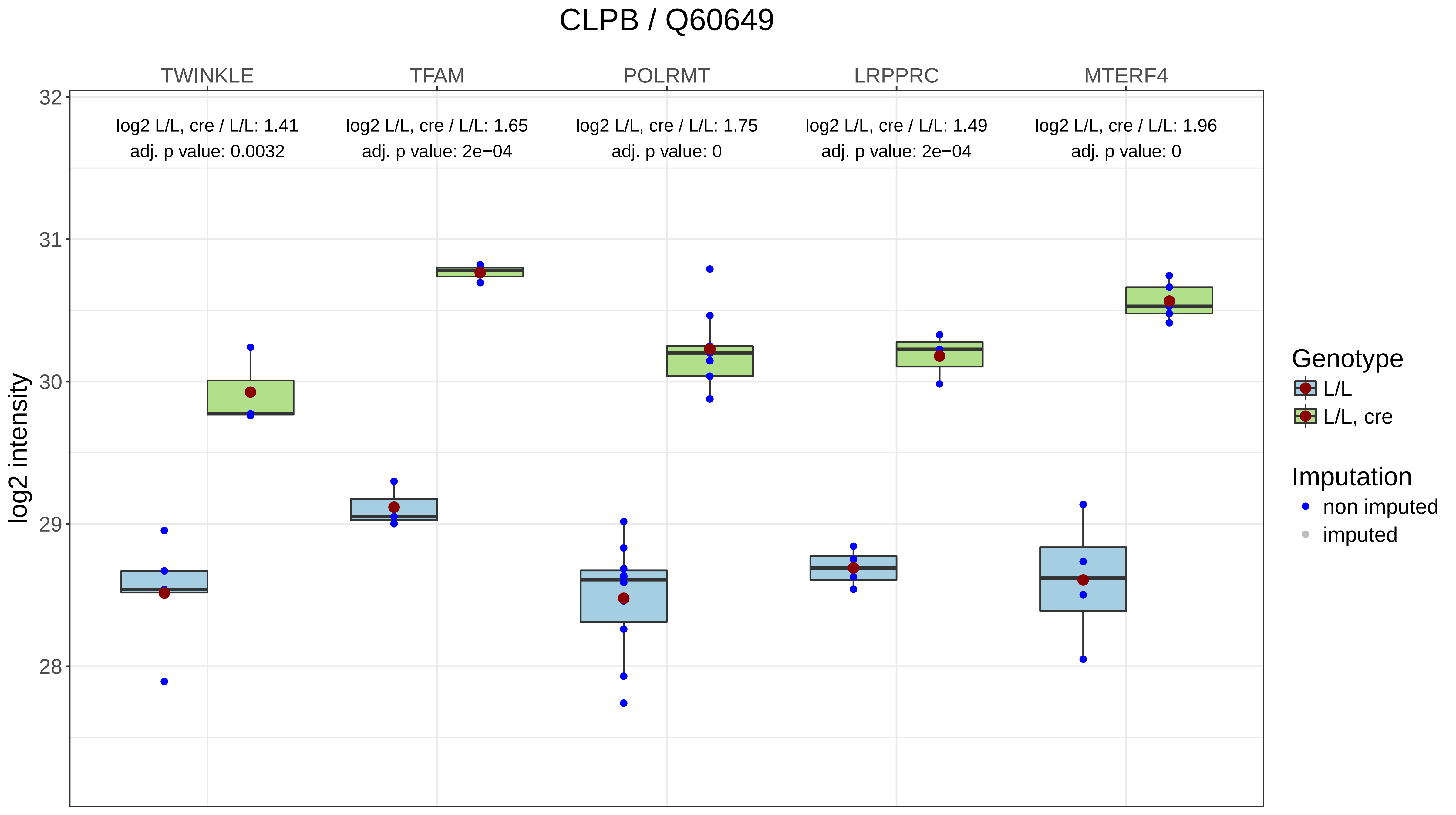
CLPB / Q60649
TWINKLE
TFAM
POLRMT
LRPPRC
MTERF4
log2 L/L, cre / L/L: 1.41
adj. p value: 0.0032
log2 L/L, cre / L/L: 1.65
adj. p value: 2e−04
log2 L/L, cre / L/L: 1.75
adj. p value: 0
log2 L/L, cre / L/L: 1.49
adj. p value: 2e−04
log2 L/L, cre / L/L: 1.96
adj. p value: 0
log2 intensity
32
31
30
29
28
Genotype
L/L
L/L, cre
Imputation
non imputed
imputed

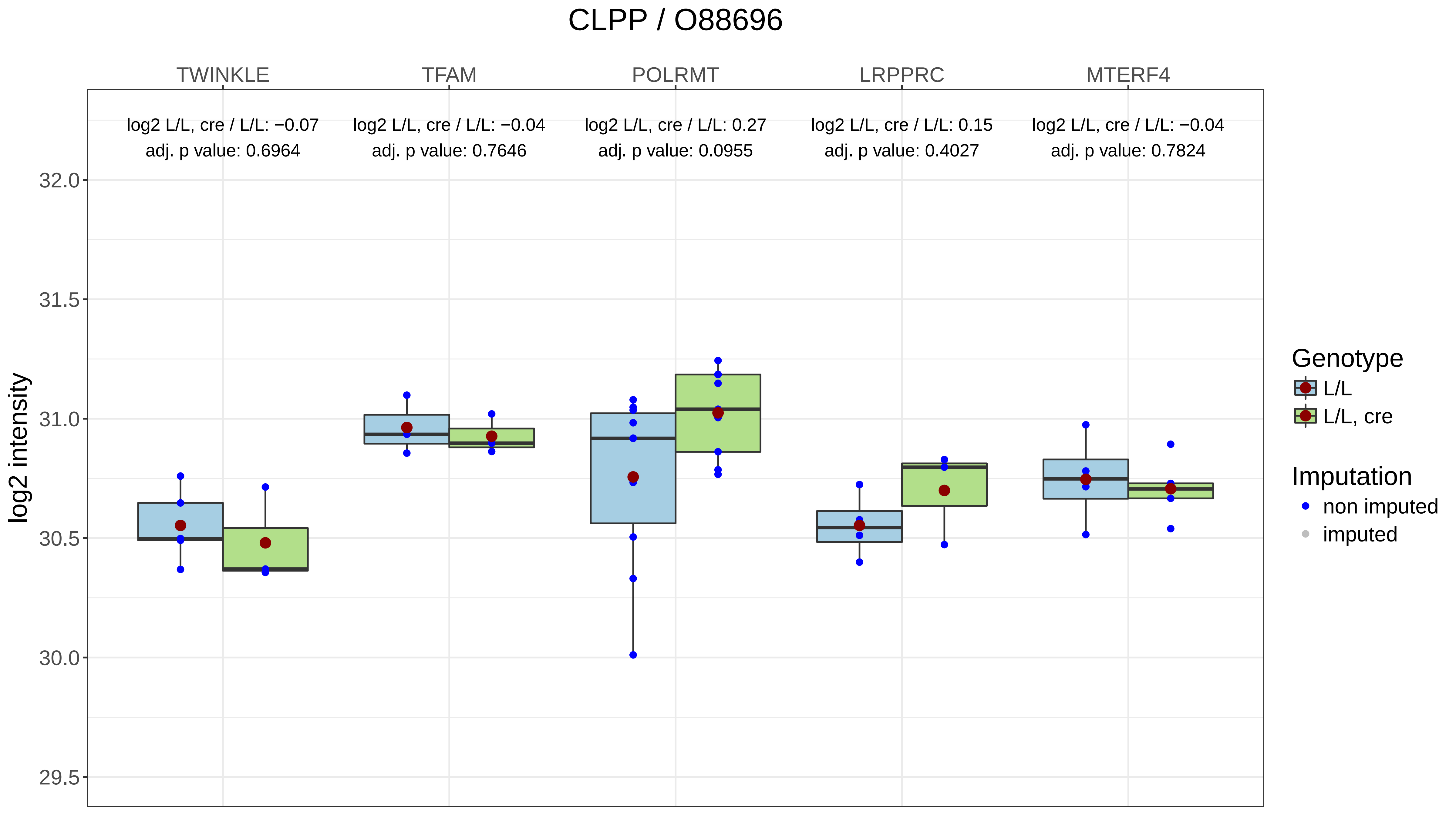

CLPP / O88696
TWINKLE
TFAM
POLRMT
LRPPRC
MTERF4
log2 L/L, cre / L/L: −0.07
adj. p value: 0.6964
log2 L/L, cre / L/L: −0.04
adj. p value: 0.7646
log2 L/L, cre / L/L: 0.27
adj. p value: 0.0955
log2 L/L, cre / L/L: 0.15
adj. p value: 0.4027
log2 L/L, cre / L/L: −0.04
adj. p value: 0.7824
log2 intensity
32.0
31.5
31.0
30.5
30.0
29.5
Genotype
L/L
L/L, cre
Imputation
non imputed
imputed

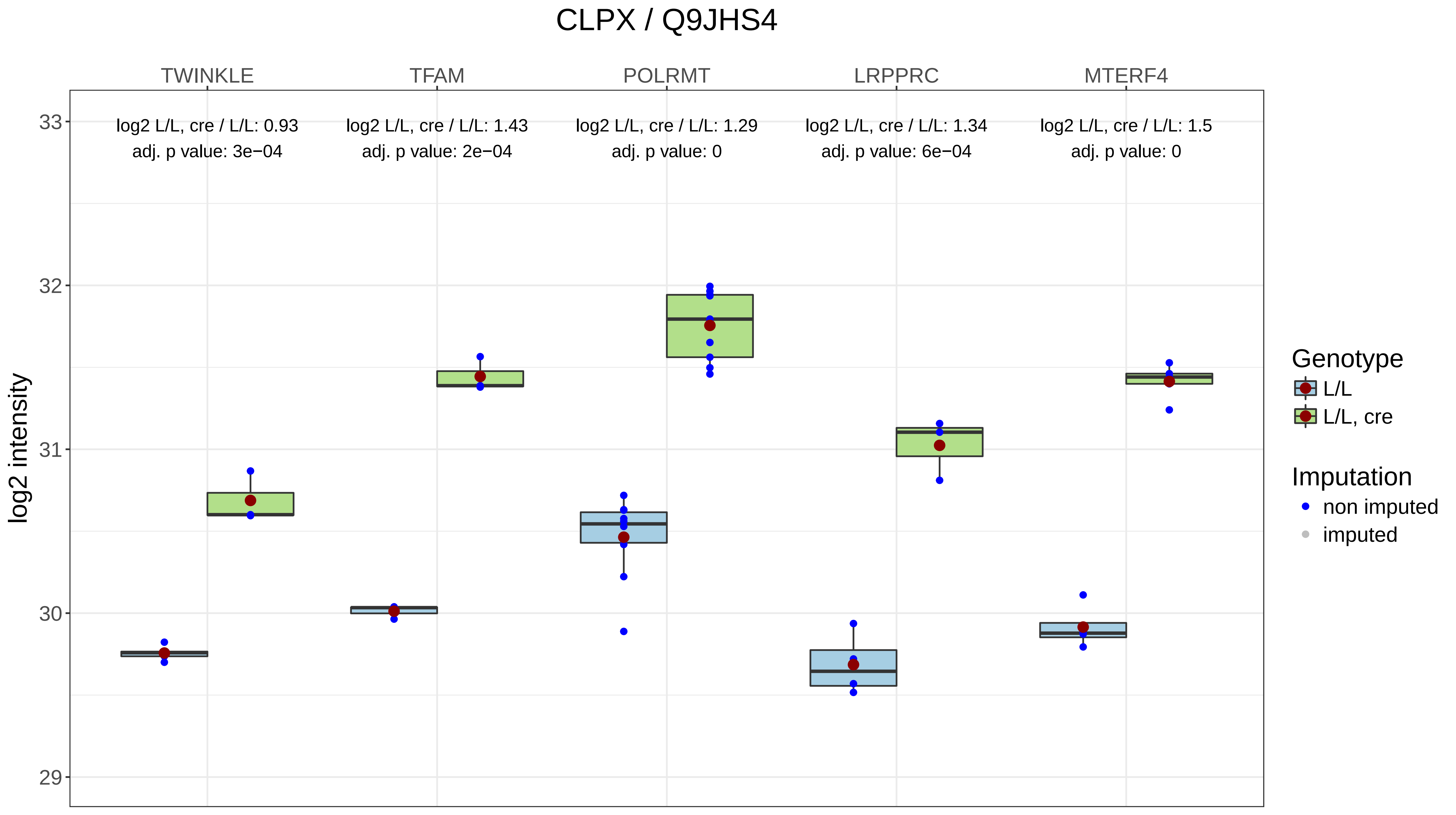
CLPX / Q9JHS4
TWINKLE
TFAM
POLRMT
LRPPRC
MTERF4
log2 L/L, cre / L/L: 0.93
adj. p value: 3e−04
log2 L/L, cre / L/L: 1.43
adj. p value: 2e−04
log2 L/L, cre / L/L: 1.29
adj. p value: 0
log2 L/L, cre / L/L: 1.34
adj. p value: 6e−04
log2 L/L, cre / L/L: 1.5
adj. p value: 0
log2 intensity
33
32
31
30
29
Genotype
L/L
L/L, cre
Imputation
non imputed
imputed

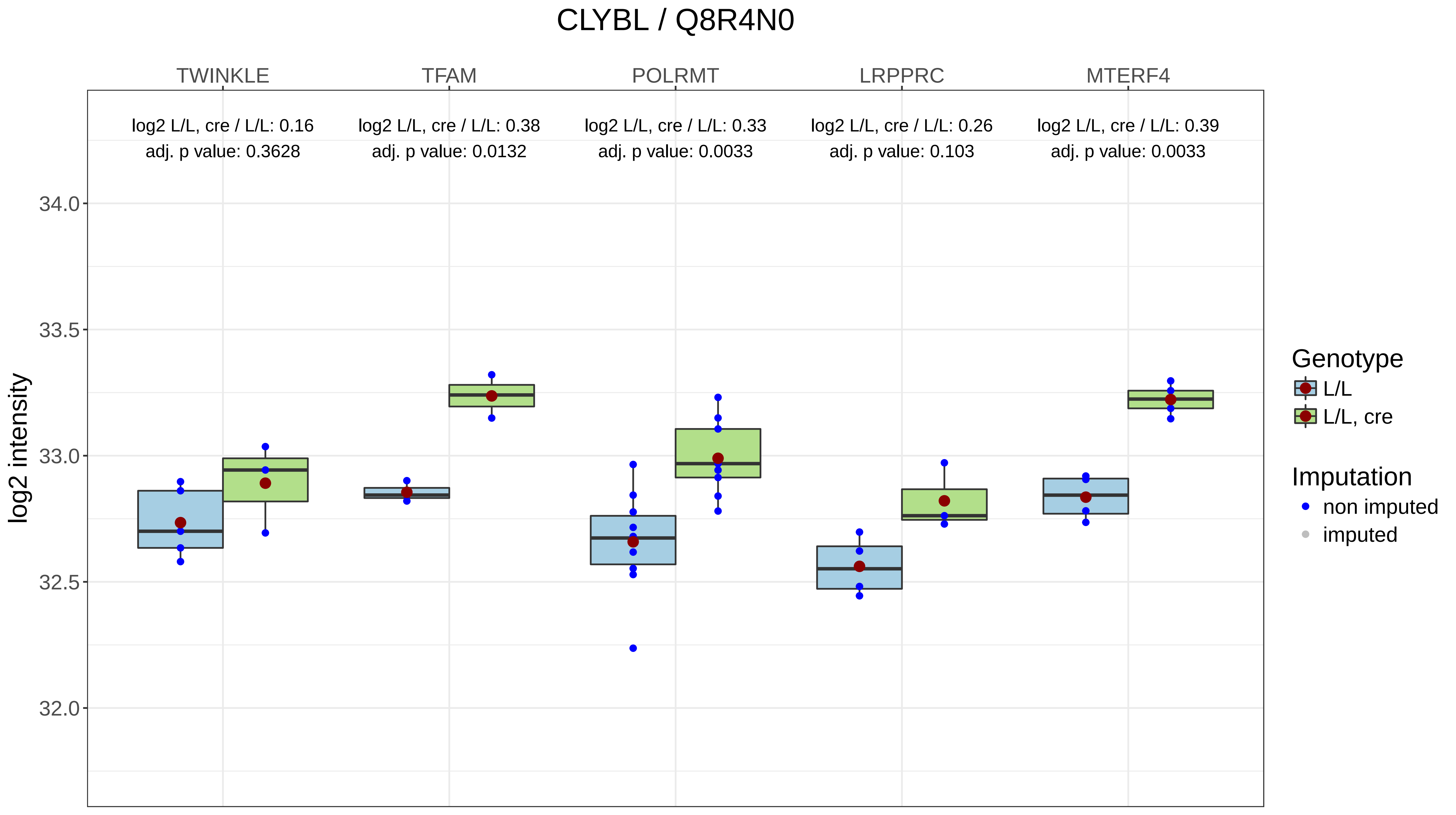
CLYBL / Q8R4N0
TWINKLE
TFAM
POLRMT
LRPPRC
MTERF4
log2 L/L, cre / L/L: 0.16
adj. p value: 0.3628
log2 L/L, cre / L/L: 0.38
adj. p value: 0.0132
log2 L/L, cre / L/L: 0.33
adj. p value: 0.0033
log2 L/L, cre / L/L: 0.26
adj. p value: 0.103
log2 L/L, cre / L/L: 0.39
adj. p value: 0.0033
log2 intensity
34.0
33.5
33.0
32.5
32.0
Genotype
L/L
L/L, cre
Imputation
non imputed
imputed

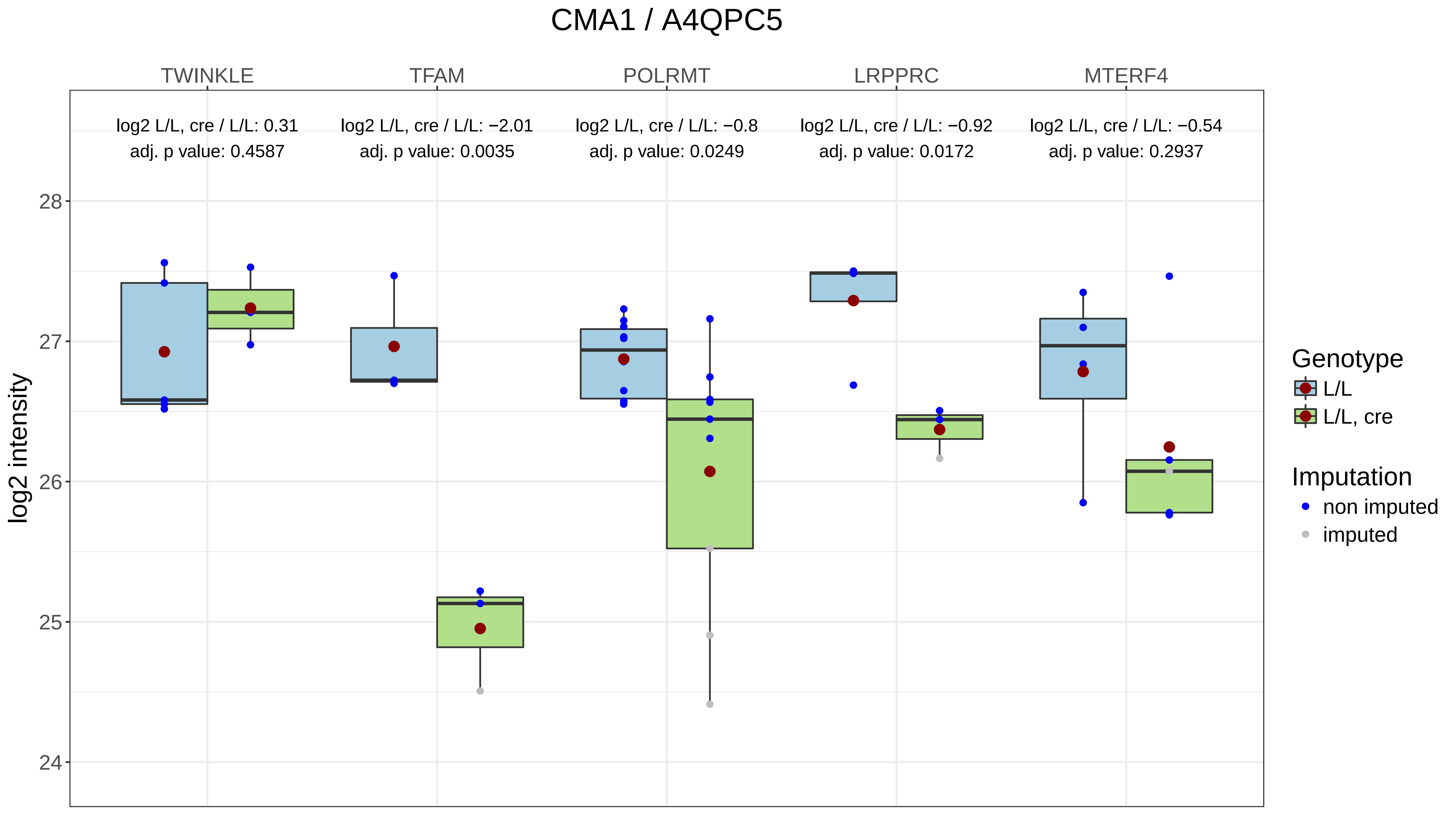

CMA1 / A4QPC5
TWINKLE
TFAM
POLRMT
LRPPRC
MTERF4
log2 L/L, cre / L/L: 0.31
adj. p value: 0.4587
log2 L/L, cre / L/L: −2.01
adj. p value: 0.0035
log2 L/L, cre / L/L: −0.8
adj. p value: 0.0249
log2 L/L, cre / L/L: −0.92
adj. p value: 0.0172
log2 L/L, cre / L/L: −0.54
adj. p value: 0.2937
log2 intensity
28
27
26
25
24
Genotype
L/L
L/L, cre
Imputation
non imputed
imputed

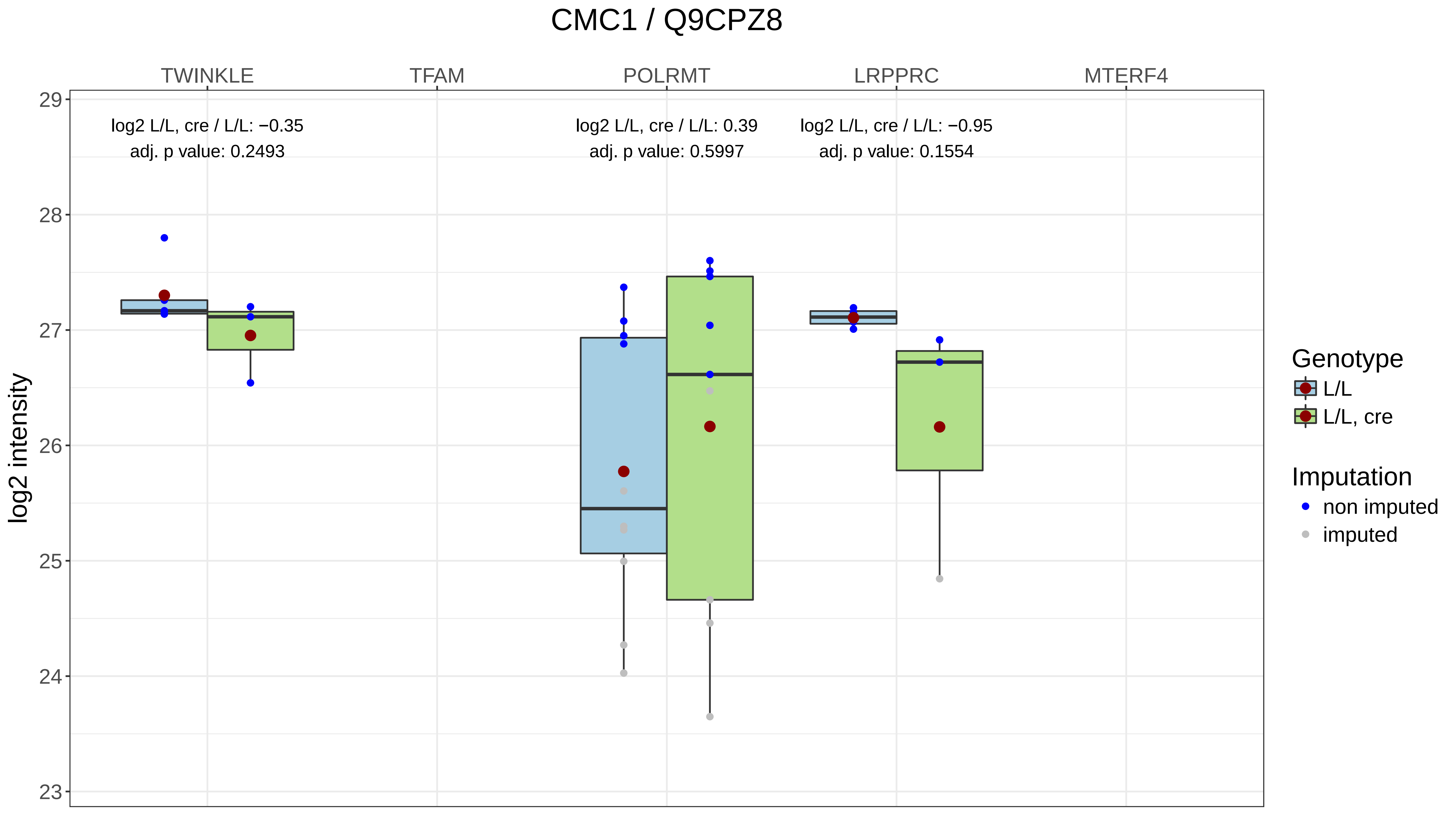
CMC1 / Q9CPZ8
TWINKLE
TFAM
POLRMT
LRPPRC
MTERF4
log2 L/L, cre / L/L: −0.35
adj. p value: 0.2493
log2 L/L, cre / L/L: 0.39
adj. p value: 0.5997
log2 L/L, cre / L/L: −0.95
adj. p value: 0.1554
log2 intensity
29
28
27
26
25
24
23
Genotype
L/L
L/L, cre
Imputation
non imputed
imputed

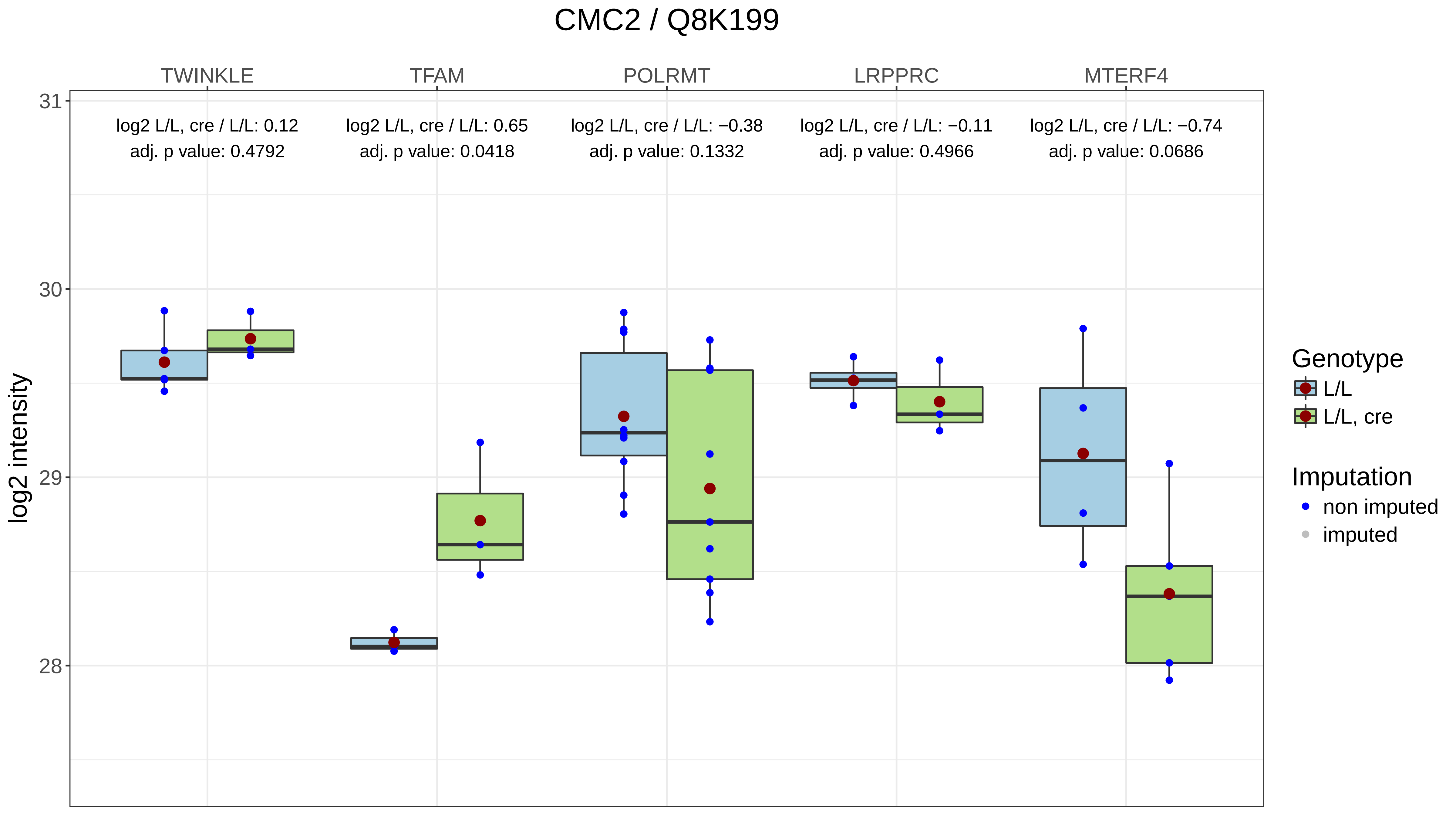
CMC2 / Q8K199
TWINKLE
TFAM
POLRMT
LRPPRC
MTERF4
log2 L/L, cre / L/L: 0.12
adj. p value: 0.4792
log2 L/L, cre / L/L: 0.65
adj. p value: 0.0418
log2 L/L, cre / L/L: −0.38
adj. p value: 0.1332
log2 L/L, cre / L/L: −0.11
adj. p value: 0.4966
log2 L/L, cre / L/L: −0.74
adj. p value: 0.0686
log2 intensity
31
30
29
28
Genotype
L/L
L/L, cre
Imputation
non imputed
imputed

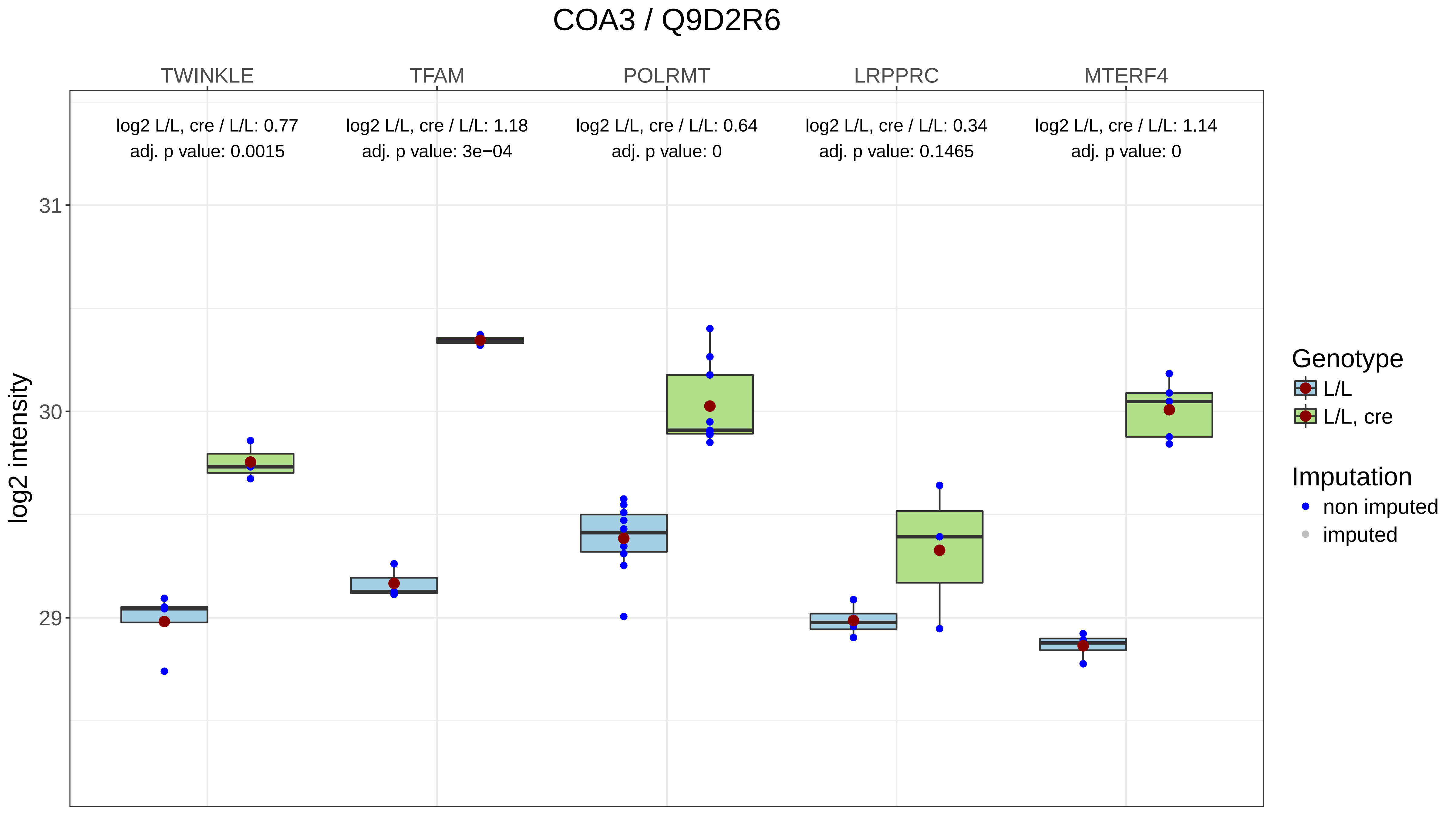
COA3 / Q9D2R6
TWINKLE
TFAM
POLRMT
LRPPRC
MTERF4
log2 L/L, cre / L/L: 0.77
adj. p value: 0.0015
log2 L/L, cre / L/L: 1.18
adj. p value: 3e−04
log2 L/L, cre / L/L: 0.64
adj. p value: 0
log2 L/L, cre / L/L: 0.34
adj. p value: 0.1465
log2 L/L, cre / L/L: 1.14
adj. p value: 0
log2 intensity
31
30
29
Genotype
L/L
L/L, cre
Imputation
non imputed
imputed

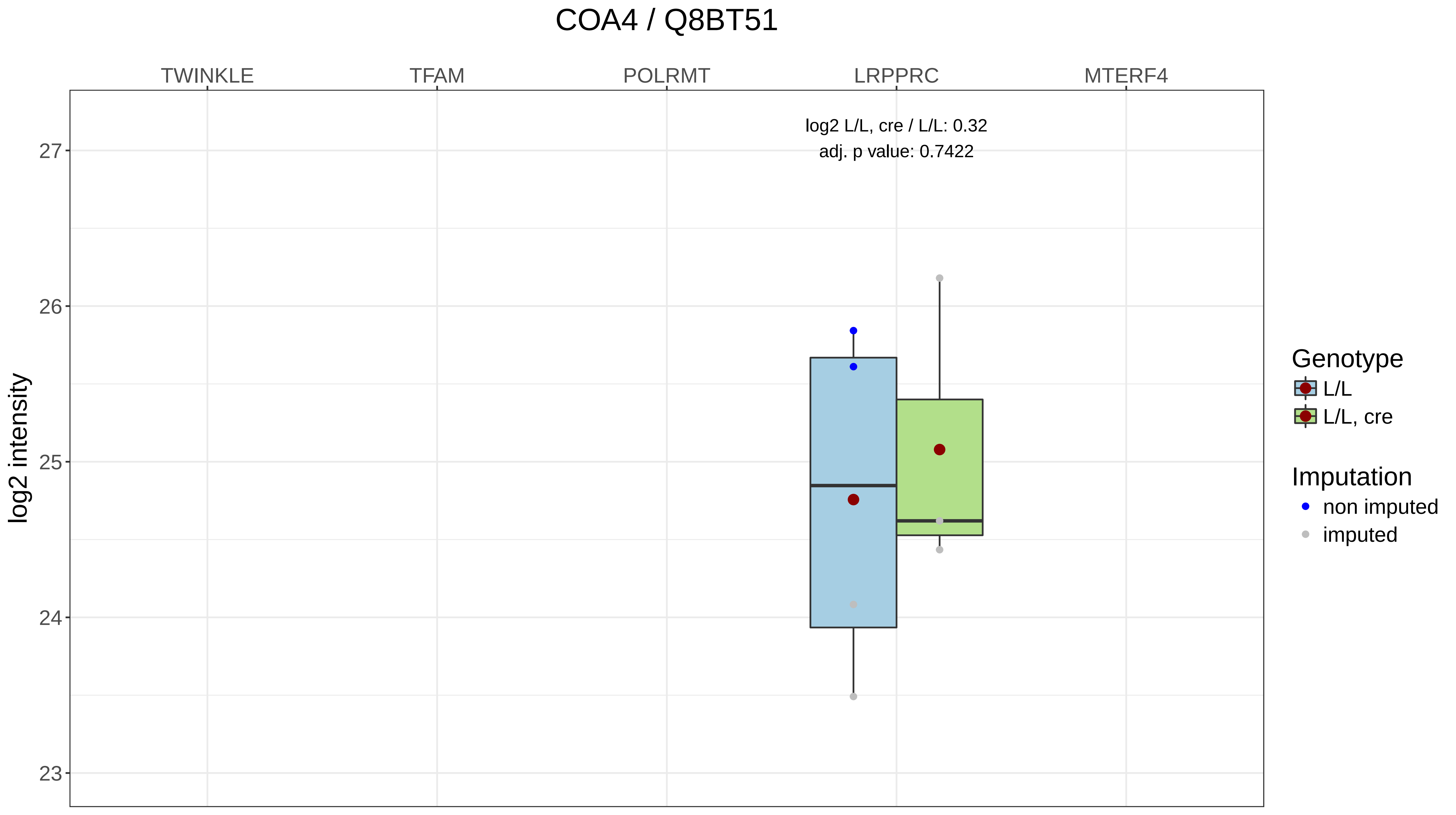

COA4 / Q8BT51
TWINKLE
TFAM
POLRMT
LRPPRC
MTERF4
log2 L/L, cre / L/L: 0.32
adj. p value: 0.7422
log2 intensity
27
26
25
24
23
Genotype
L/L
L/L, cre
Imputation
non imputed
imputed

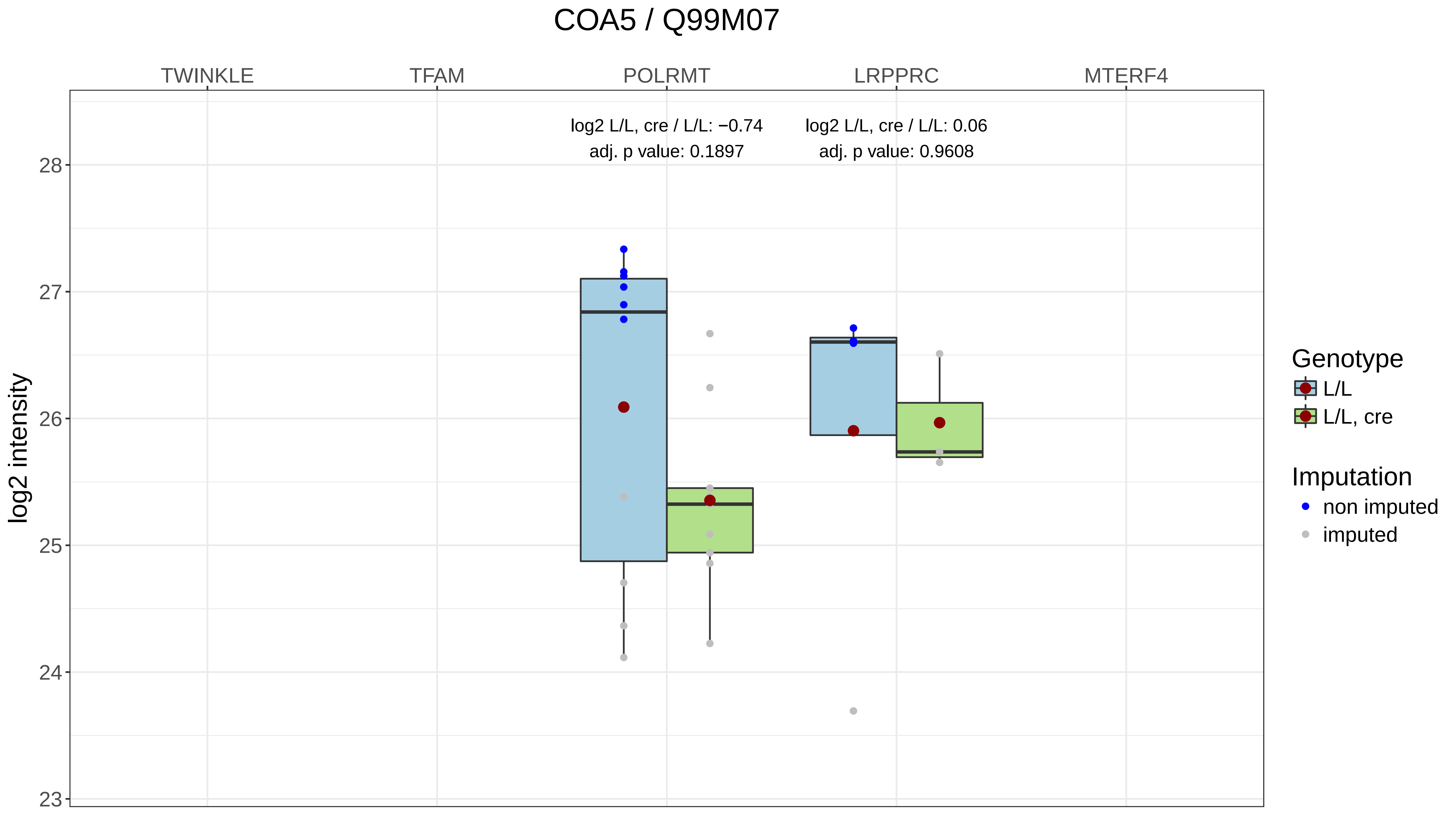
COA5 / Q99M07
TWINKLE
TFAM
POLRMT
LRPPRC
MTERF4
log2 L/L, cre / L/L: −0.74
adj. p value: 0.1897
log2 L/L, cre / L/L: 0.06
adj. p value: 0.9608
log2 intensity
28
27
26
25
24
23
Genotype
L/L
L/L, cre
Imputation
non imputed
imputed

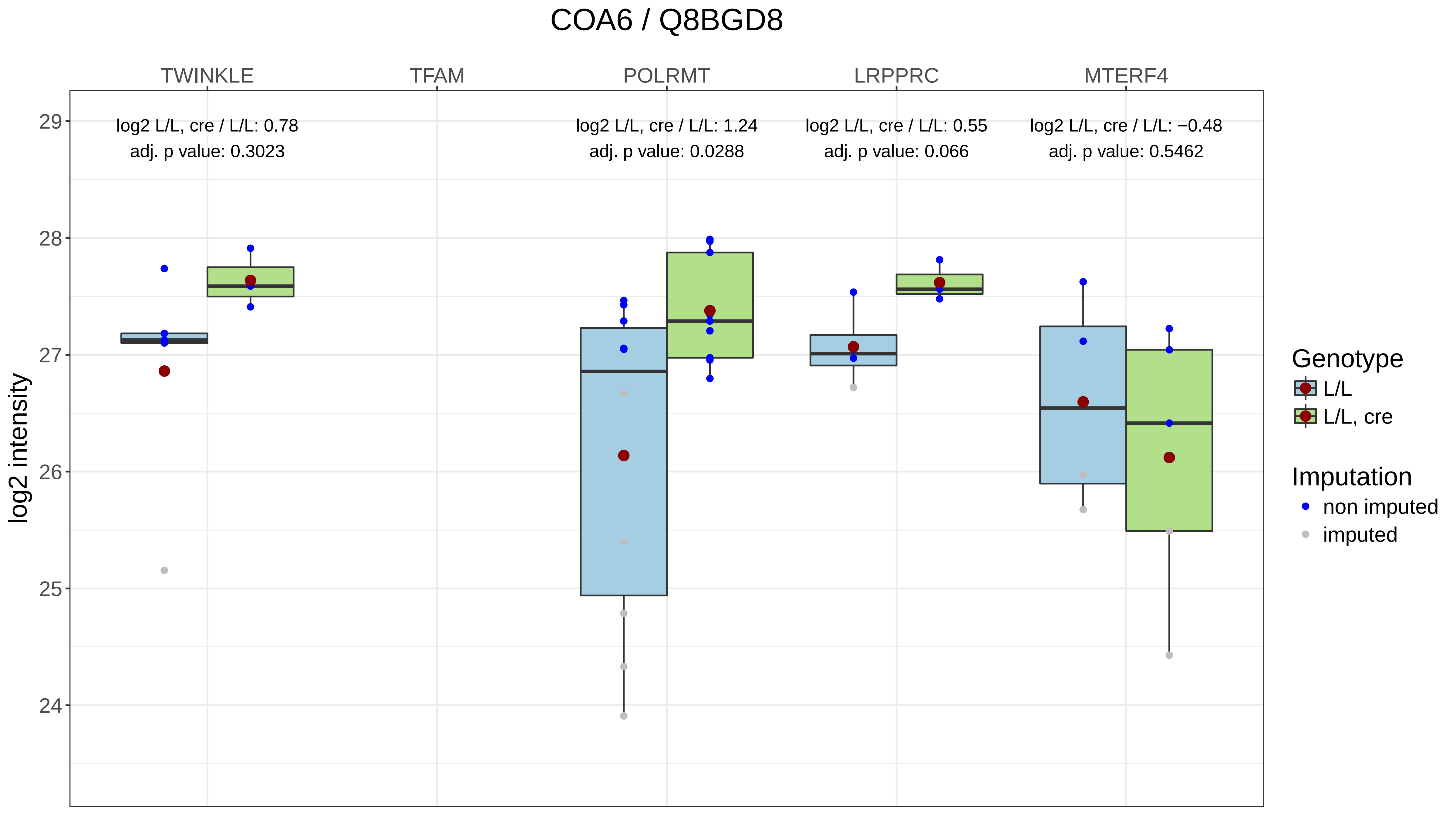

COA6 / Q8BGD8
TWINKLE
TFAM
POLRMT
LRPPRC
MTERF4
log2 L/L, cre / L/L: 0.78
adj. p value: 0.3023
log2 L/L, cre / L/L: 1.24
adj. p value: 0.0288
log2 L/L, cre / L/L: 0.55
adj. p value: 0.066
log2 L/L, cre / L/L: −0.48
adj. p value: 0.5462
log2 intensity
29
28
27
26
25
24
Genotype
L/L
L/L, cre
Imputation
non imputed
imputed

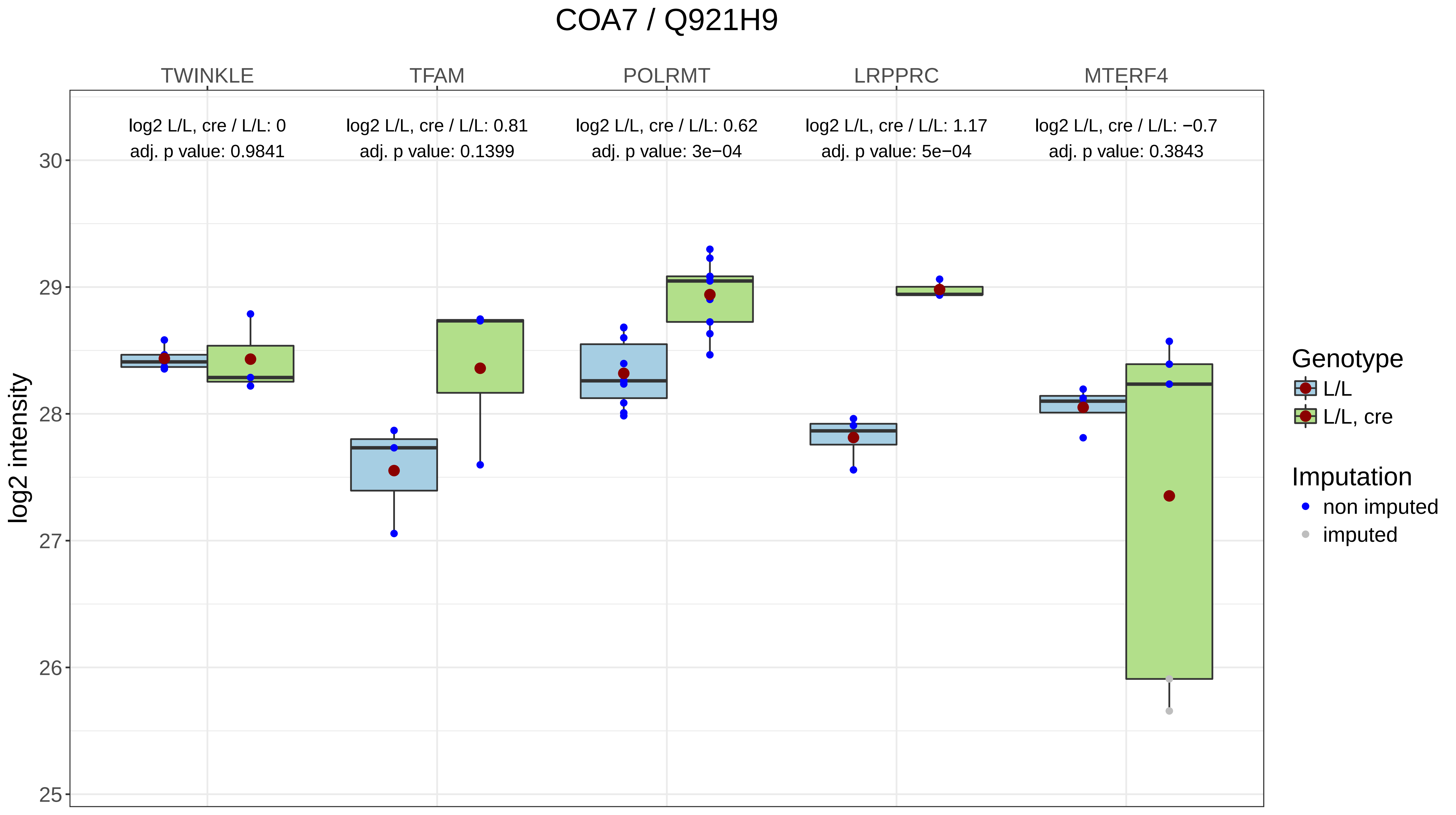
COA7 / Q921H9
TWINKLE
TFAM
POLRMT
LRPPRC
MTERF4
log2 L/L, cre / L/L: 0
adj. p value: 0.9841
log2 L/L, cre / L/L: 0.81
adj. p value: 0.1399
log2 L/L, cre / L/L: 0.62
adj. p value: 3e−04
log2 L/L, cre / L/L: 1.17
adj. p value: 5e−04
log2 L/L, cre / L/L: −0.7
adj. p value: 0.3843
log2 intensity
30
29
28
27
26
25
Genotype
L/L
L/L, cre
Imputation
non imputed
imputed

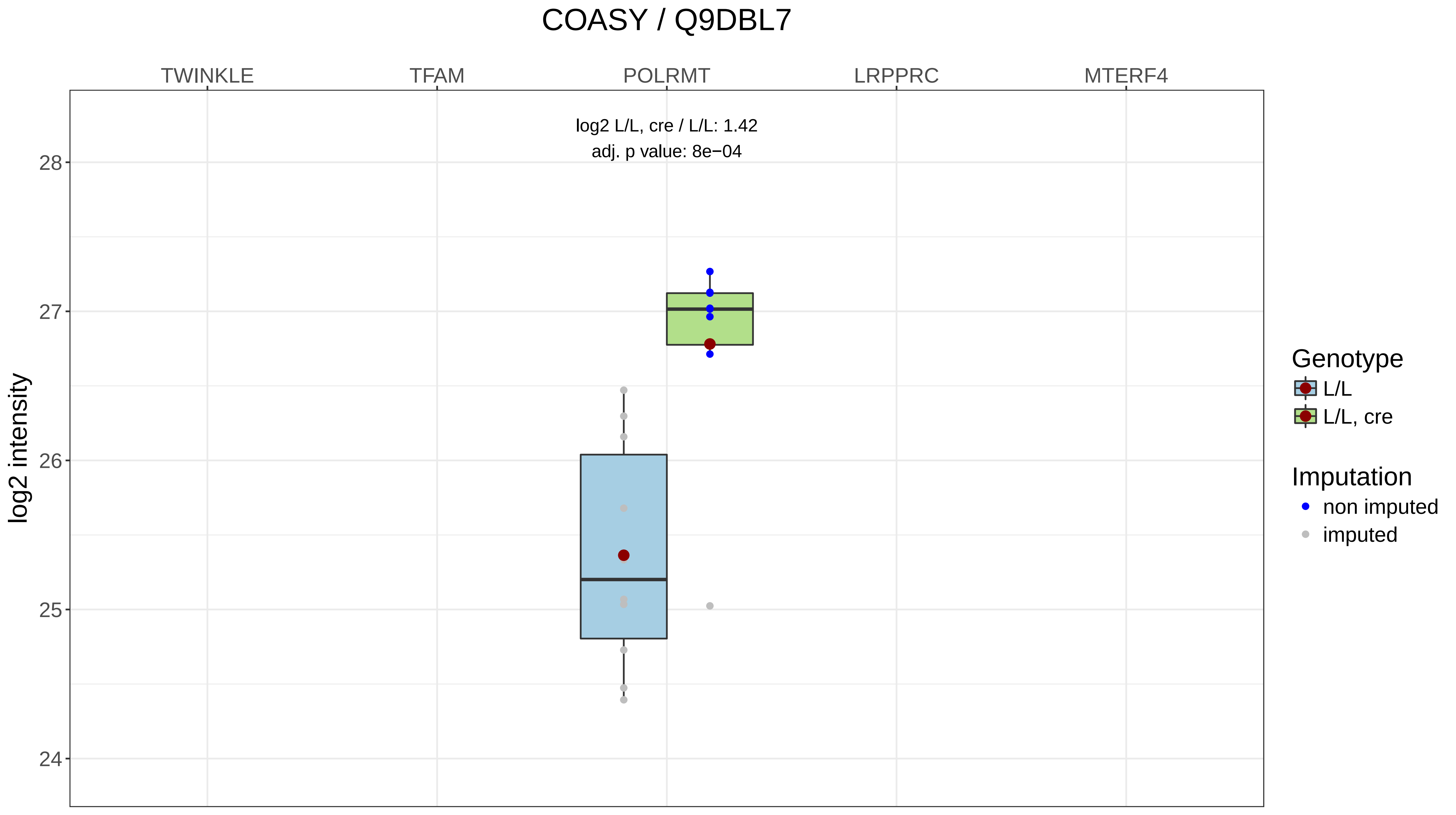
COASY / Q9DBL7
TWINKLE
TFAM
POLRMT
LRPPRC
MTERF4
log2 L/L, cre / L/L: 1.42
adj. p value: 8e−04
log2 intensity
28
27
26
25
24
Genotype
L/L
L/L, cre
Imputation
non imputed
imputed

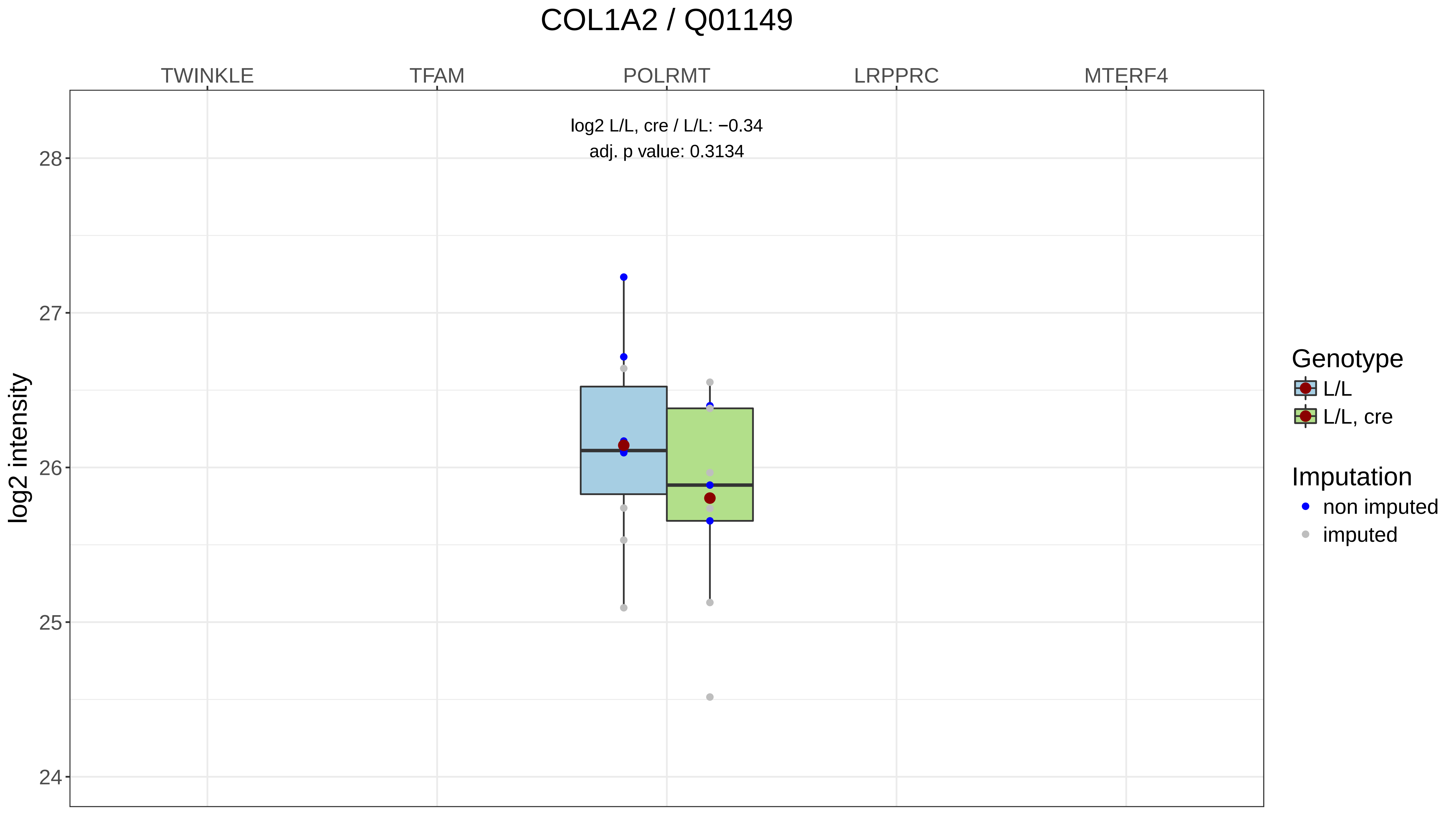
COL1A2 / Q01149
TWINKLE
TFAM
POLRMT
LRPPRC
MTERF4
log2 L/L, cre / L/L: −0.34
adj. p value: 0.3134
log2 intensity
28
27
26
25
24
Genotype
L/L
L/L, cre
Imputation
non imputed
imputed

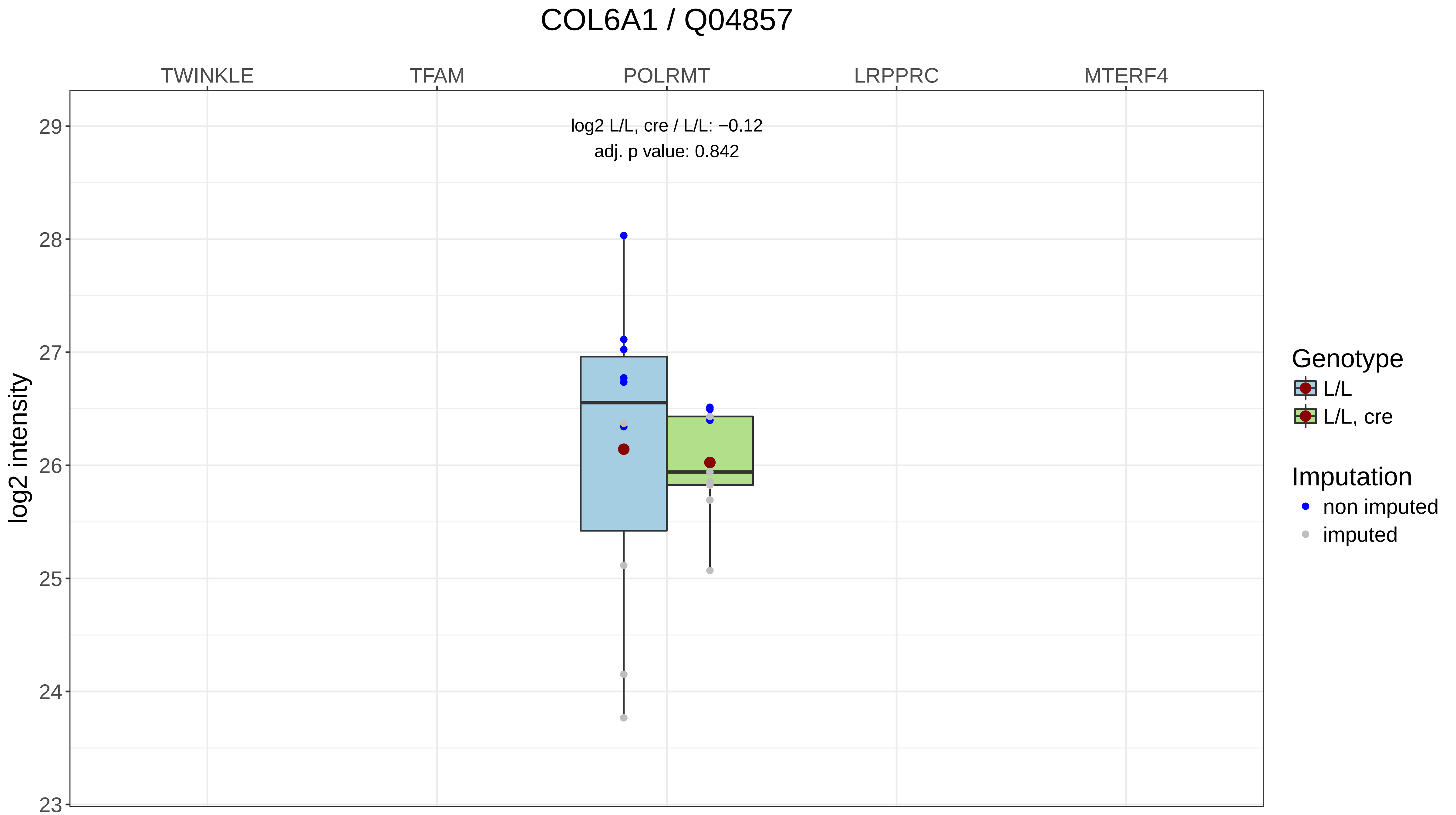
COL6A1 / Q04857
TWINKLE
TFAM
POLRMT
LRPPRC
MTERF4
log2 L/L, cre / L/L: −0.12
adj. p value: 0.842
log2 intensity
29
28
27
26
25
24
23
Genotype
L/L
L/L, cre
Imputation
non imputed
imputed

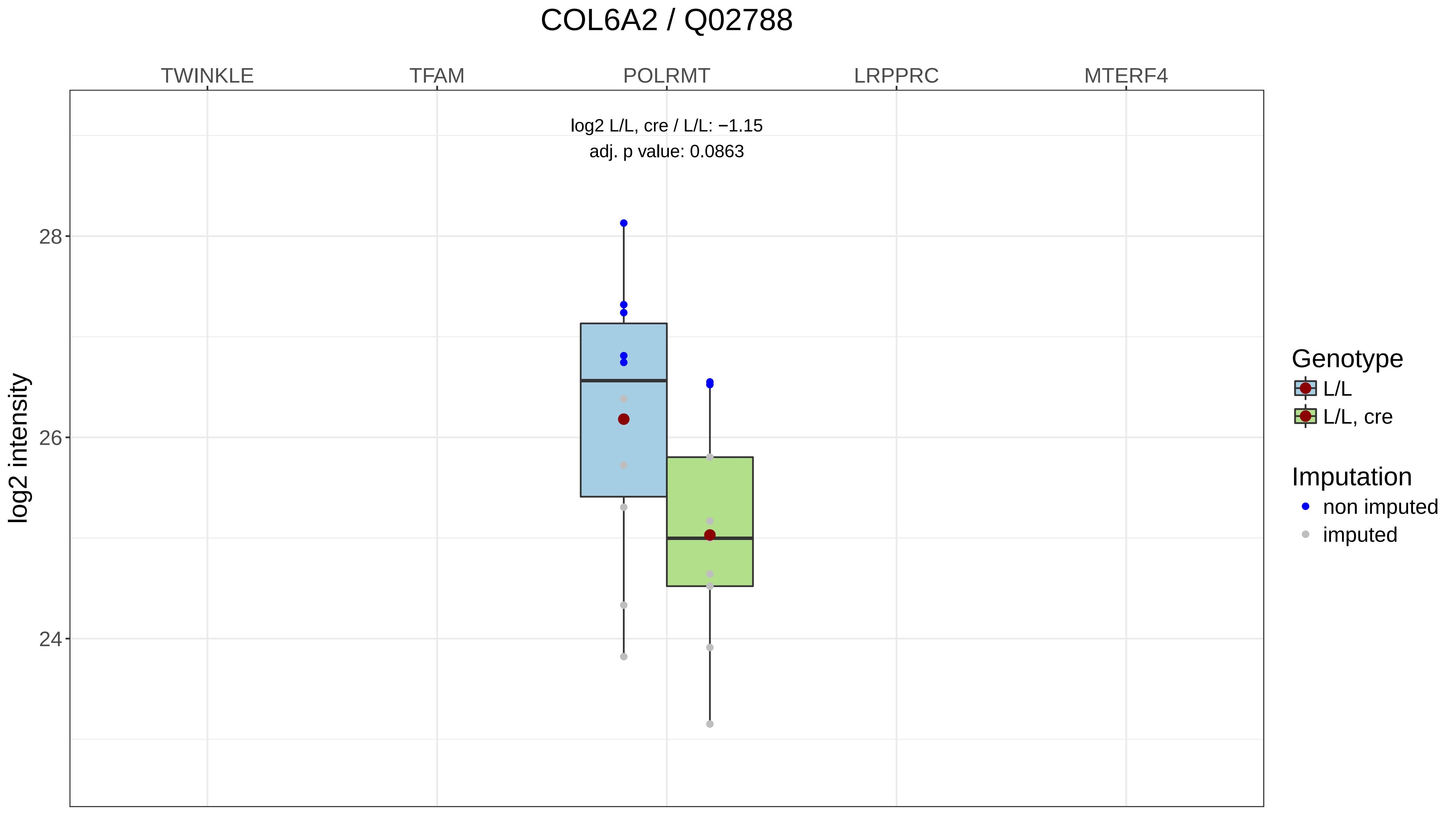
COL6A2 / Q02788
TWINKLE
TFAM
POLRMT
LRPPRC
MTERF4
log2 L/L, cre / L/L: −1.15
adj. p value: 0.0863
28
26
24
log2 intensity
Genotype
L/L
L/L, cre
Imputation
non imputed
imputed

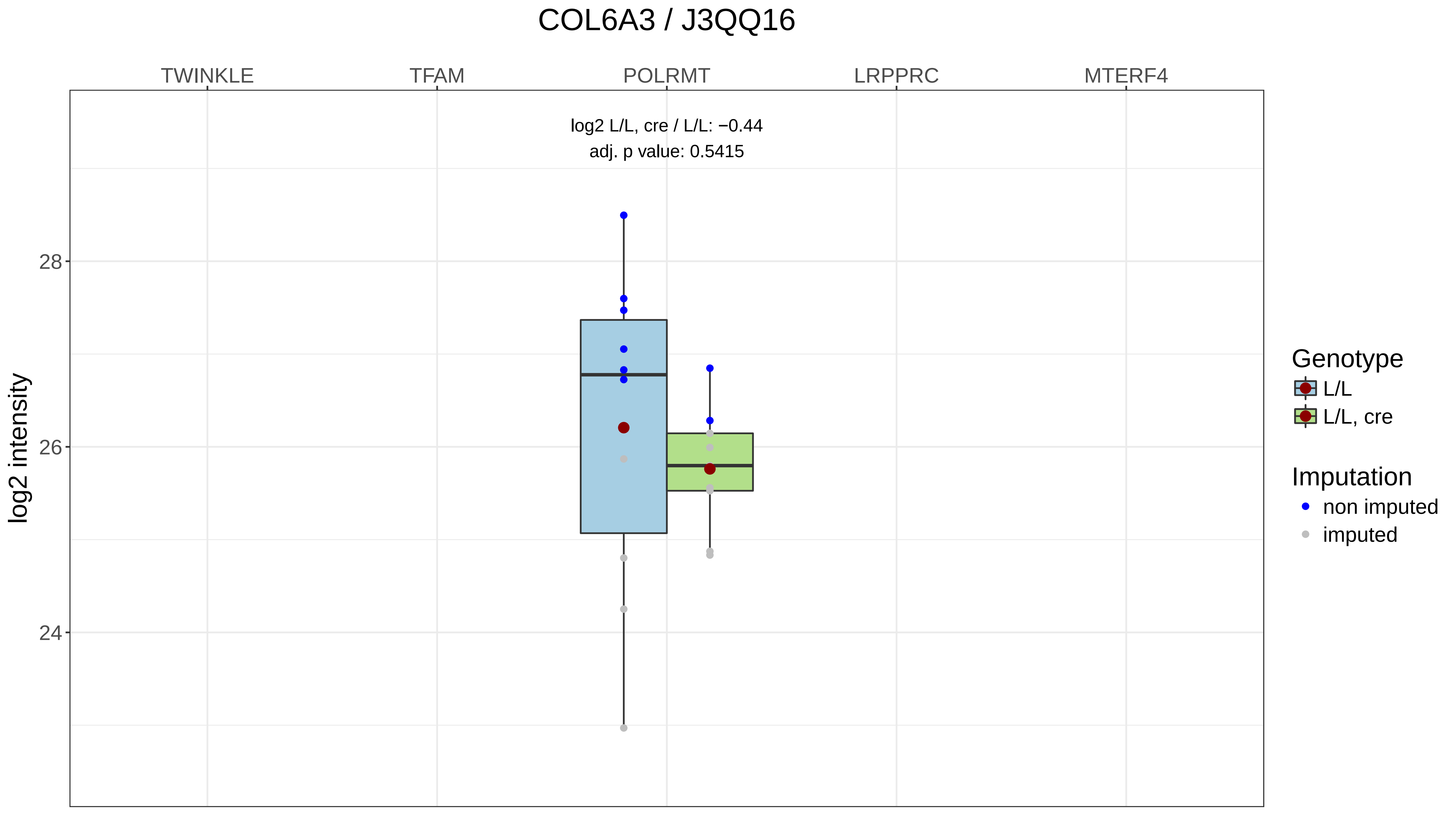
COL6A3 / J3QQ16
TWINKLE
TFAM
POLRMT
LRPPRC
MTERF4
log2 L/L, cre / L/L: −0.44
adj. p value: 0.5415
log2 intensity
28
26
24
Genotype
L/L
L/L, cre
Imputation
non imputed
imputed

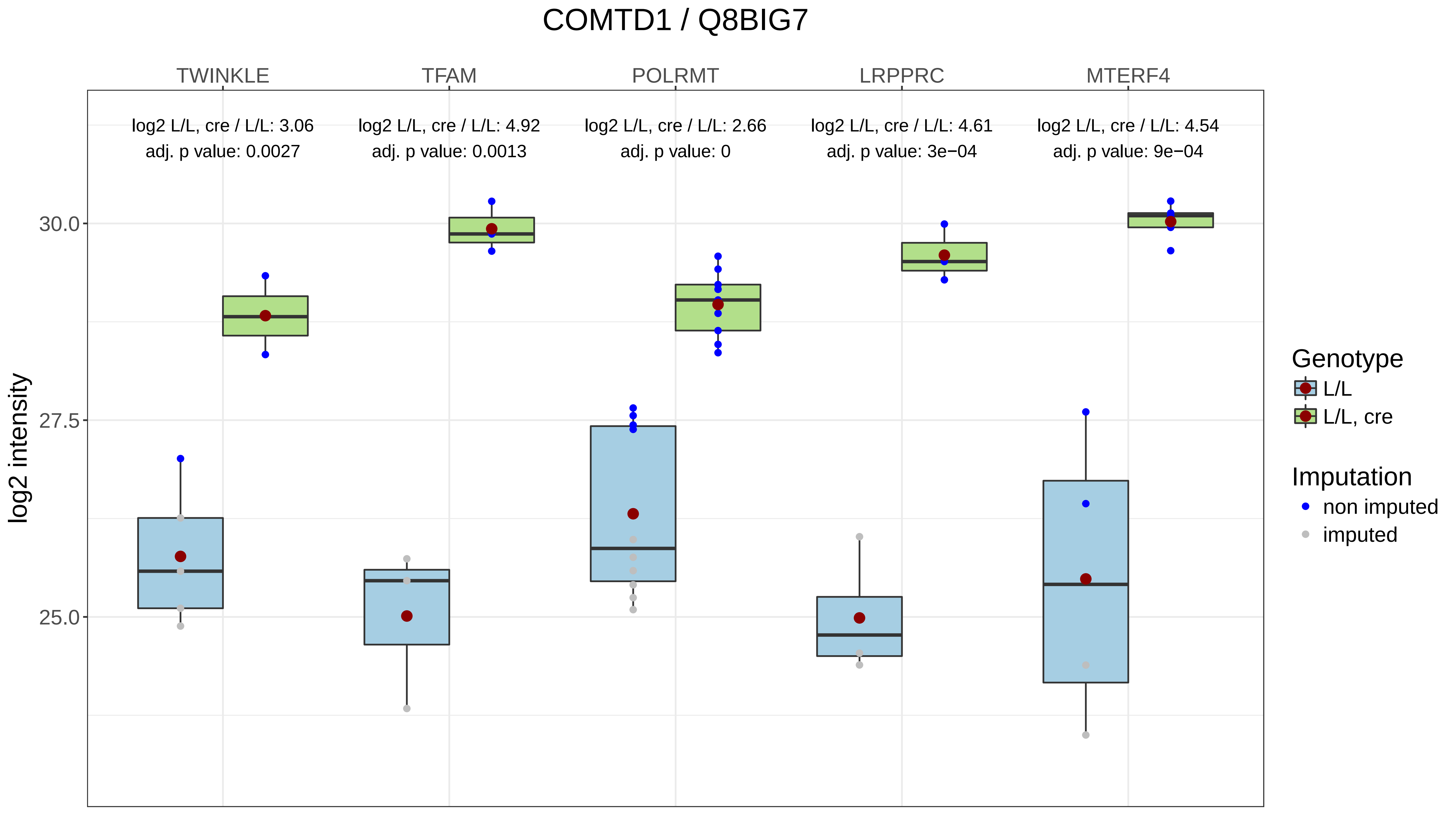

COMTD1 / Q8BIG7
TWINKLE
TFAM
POLRMT
LRPPRC
MTERF4
log2 L/L, cre / L/L: 3.06
adj. p value: 0.0027
log2 L/L, cre / L/L: 4.92
adj. p value: 0.0013
log2 L/L, cre / L/L: 2.66
adj. p value: 0
log2 L/L, cre / L/L: 4.61
adj. p value: 3e−04
log2 L/L, cre / L/L: 4.54
adj. p value: 9e−04
log2 intensity
30.0
27.5
25.0
Genotype
L/L
L/L, cre
Imputation
non imputed
imputed

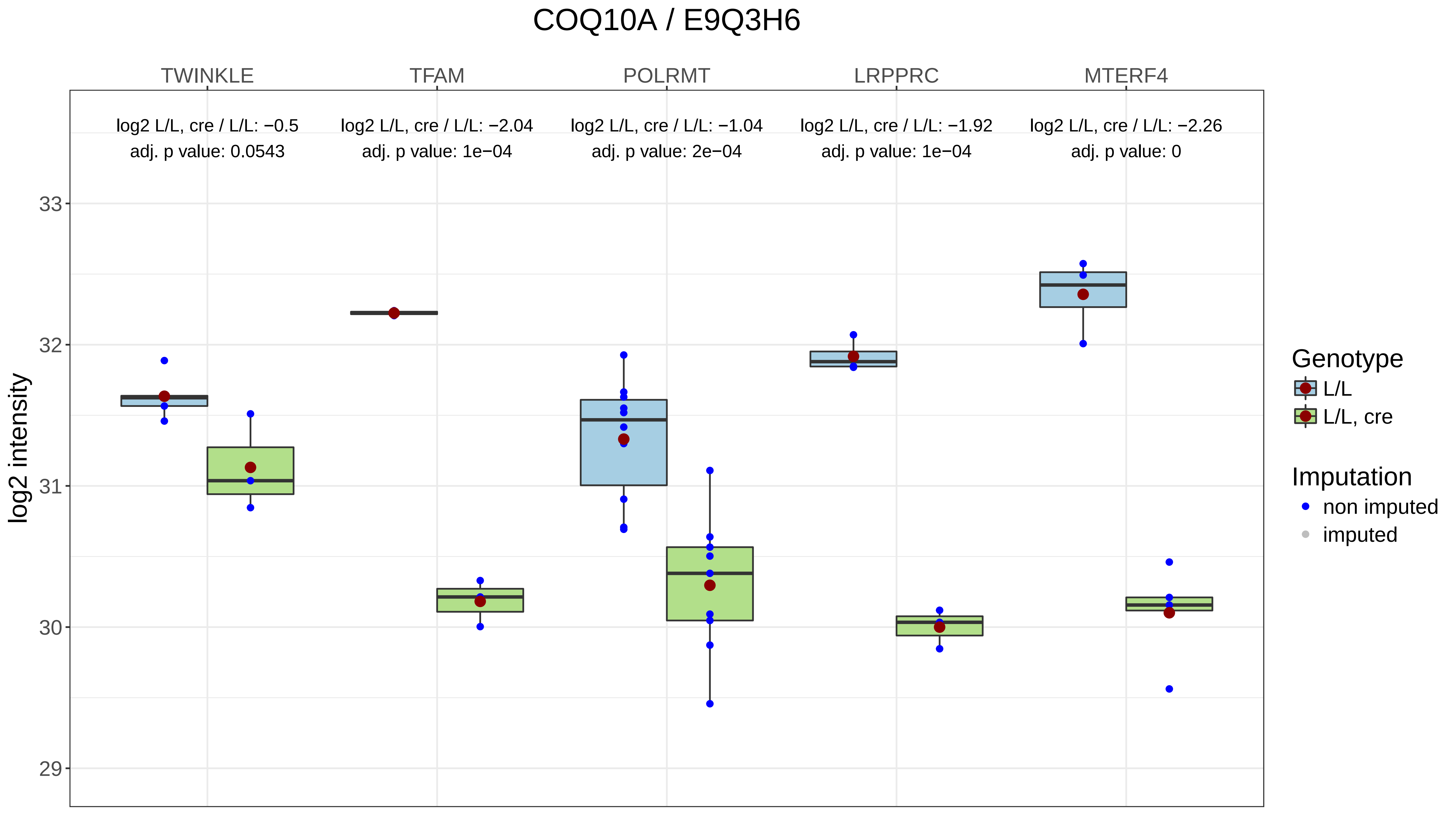
COQ10A / E9Q3H6
TWINKLE
TFAM
POLRMT
LRPPRC
MTERF4
log2 L/L, cre / L/L: −0.5
adj. p value: 0.0543
log2 L/L, cre / L/L: −2.04
adj. p value: 1e−04
log2 L/L, cre / L/L: −1.04
adj. p value: 2e−04
log2 L/L, cre / L/L: −1.92
adj. p value: 1e−04
log2 L/L, cre / L/L: −2.26
adj. p value: 0
log2 intensity
33
32
31
30
29
Genotype
L/L
L/L, cre
Imputation
non imputed
imputed

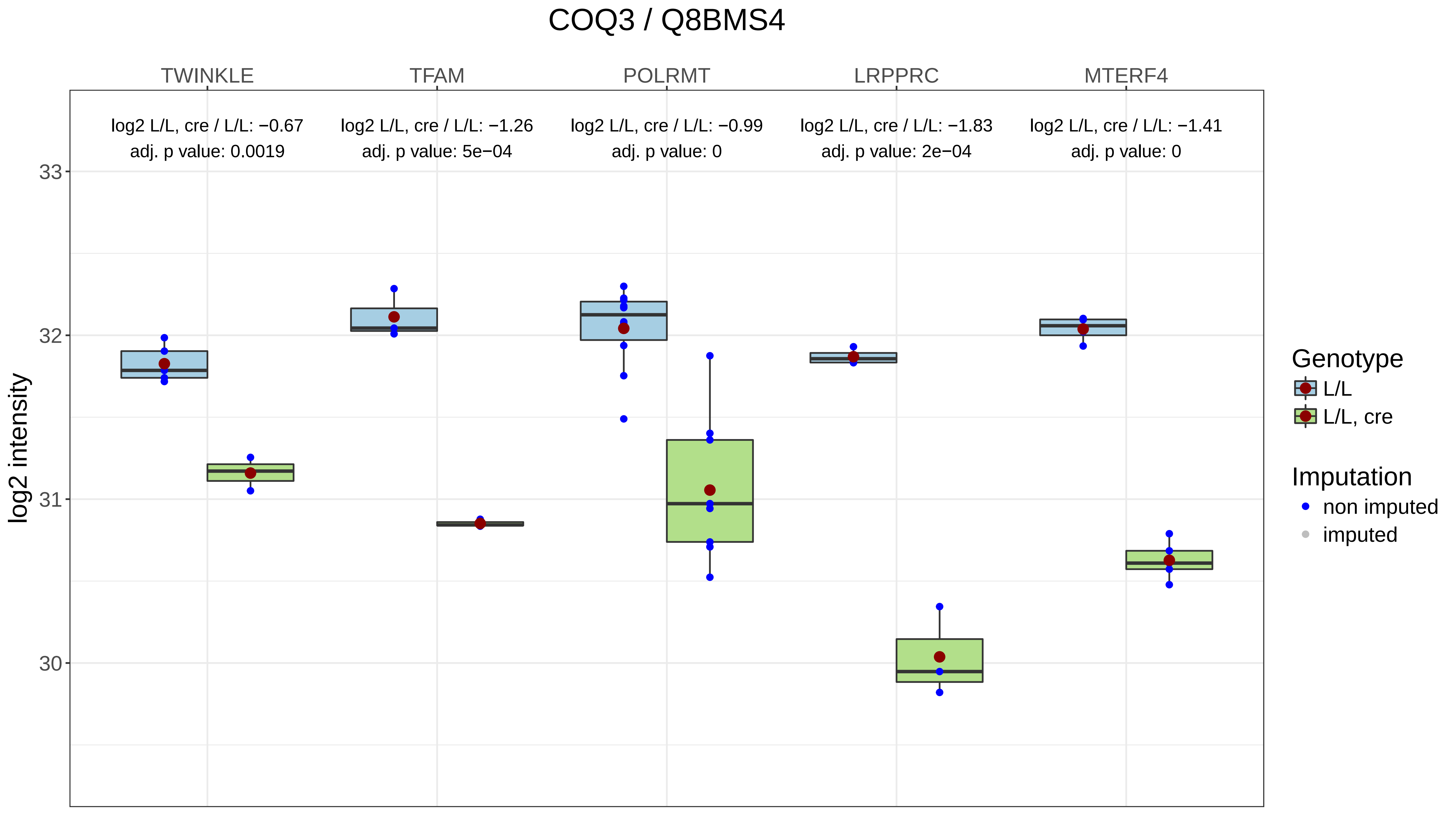
COQ3 / Q8BMS4
TWINKLE
TFAM
POLRMT
LRPPRC
MTERF4
log2 L/L, cre / L/L: −0.67
adj. p value: 0.0019
log2 L/L, cre / L/L: −1.26
adj. p value: 5e−04
log2 L/L, cre / L/L: −0.99
adj. p value: 0
log2 L/L, cre / L/L: −1.83
adj. p value: 2e−04
log2 L/L, cre / L/L: −1.41
adj. p value: 0
log2 intensity
33
32
31
30
Genotype
L/L
L/L, cre
Imputation
non imputed
imputed

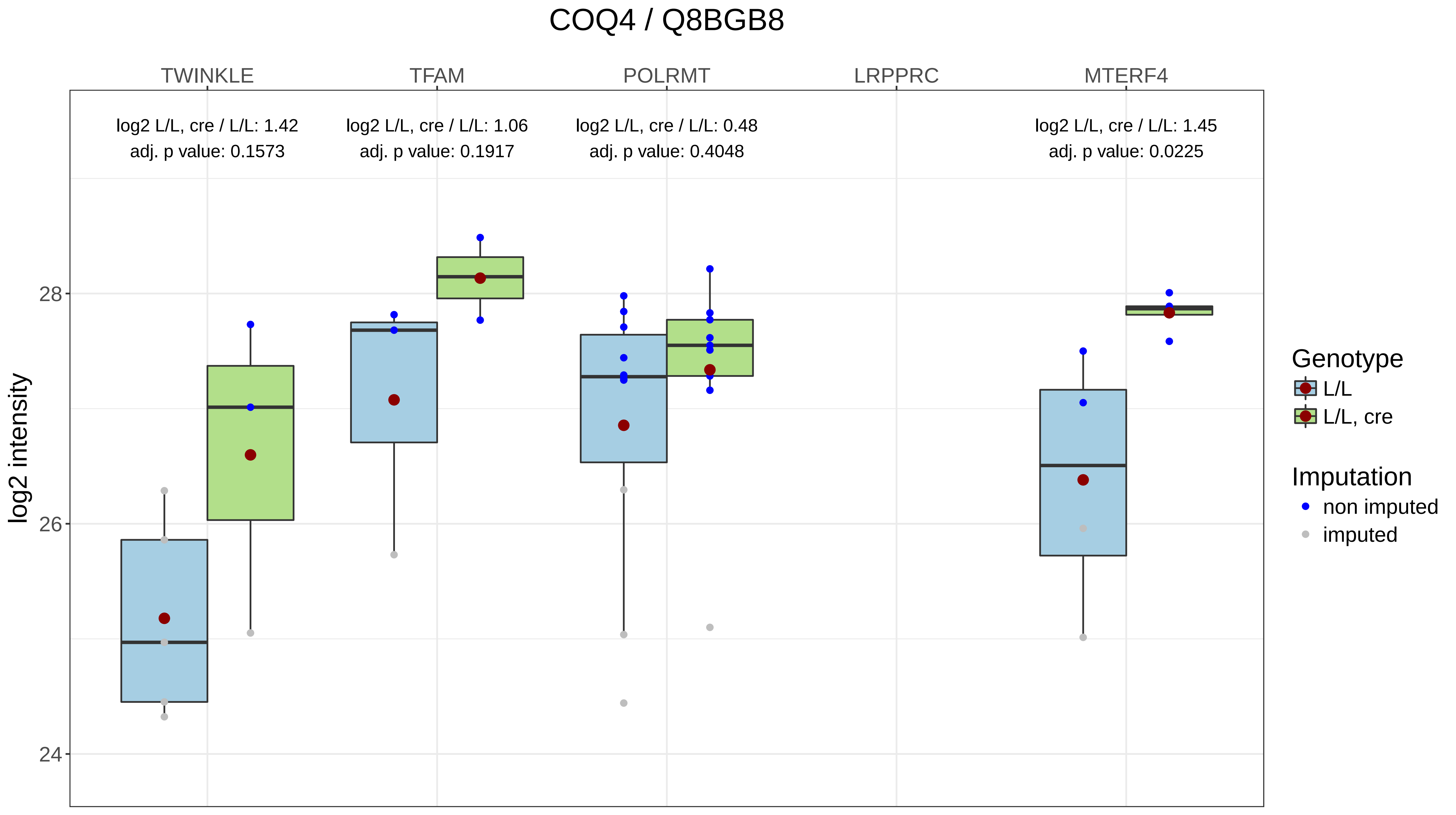
COQ4 / Q8BGB8
TWINKLE
TFAM
POLRMT
LRPPRC
MTERF4
log2 L/L, cre / L/L: 1.42
adj. p value: 0.1573
log2 L/L, cre / L/L: 1.06
adj. p value: 0.1917
log2 L/L, cre / L/L: 0.48
adj. p value: 0.4048
log2 L/L, cre / L/L: 1.45
adj. p value: 0.0225
log2 intensity
28
26
24
Genotype
L/L
L/L, cre
Imputation
non imputed
imputed

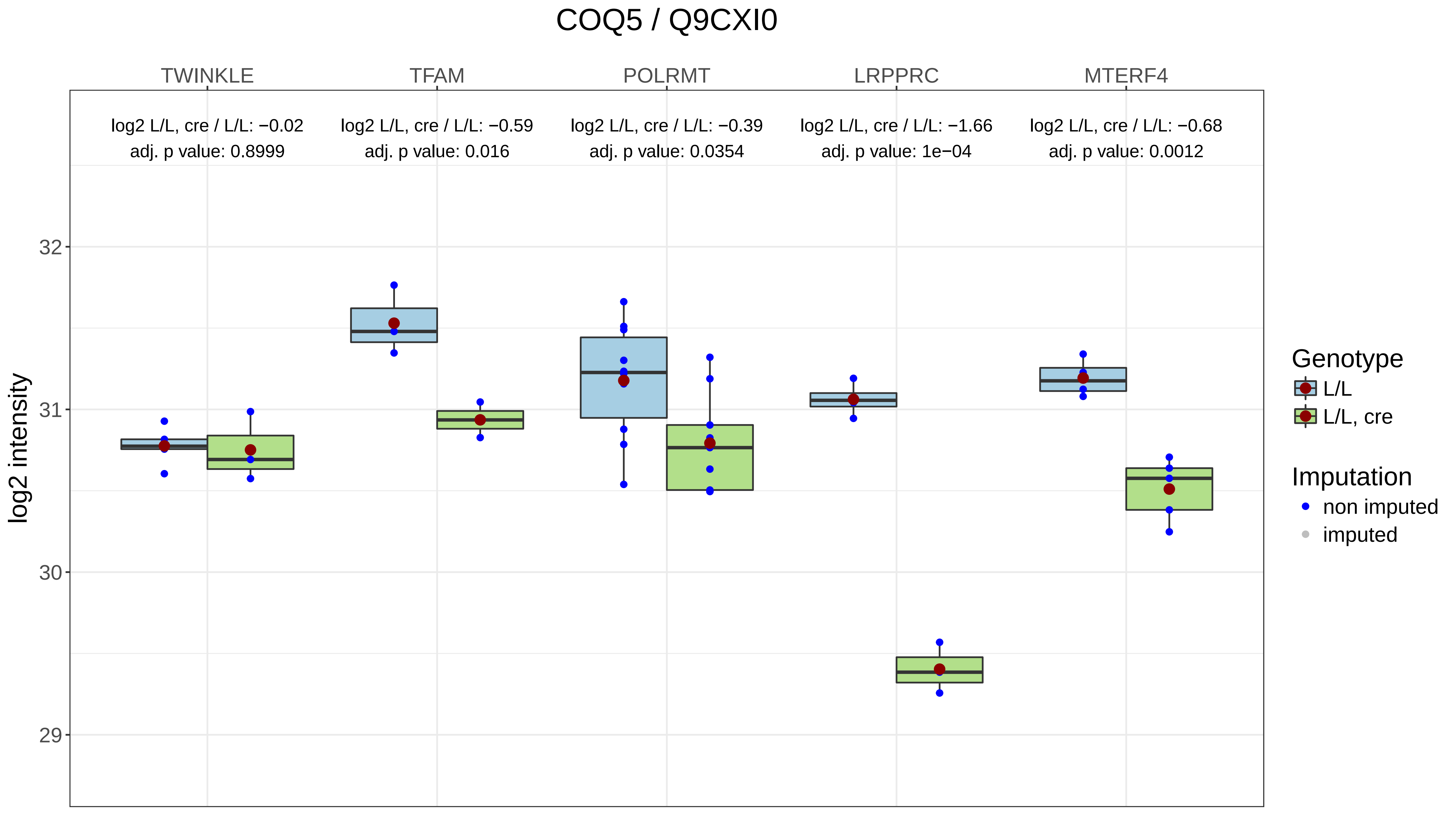
COQ5 / Q9CXI0
TWINKLE
TFAM
POLRMT
LRPPRC
MTERF4
log2 L/L, cre / L/L: −0.02
adj. p value: 0.8999
log2 L/L, cre / L/L: −0.59
adj. p value: 0.016
log2 L/L, cre / L/L: −0.39
adj. p value: 0.0354
log2 L/L, cre / L/L: −1.66
adj. p value: 1e−04
log2 L/L, cre / L/L: −0.68
adj. p value: 0.0012
log2 intensity
32
31
30
29
Genotype
L/L
L/L, cre
Imputation
non imputed
imputed

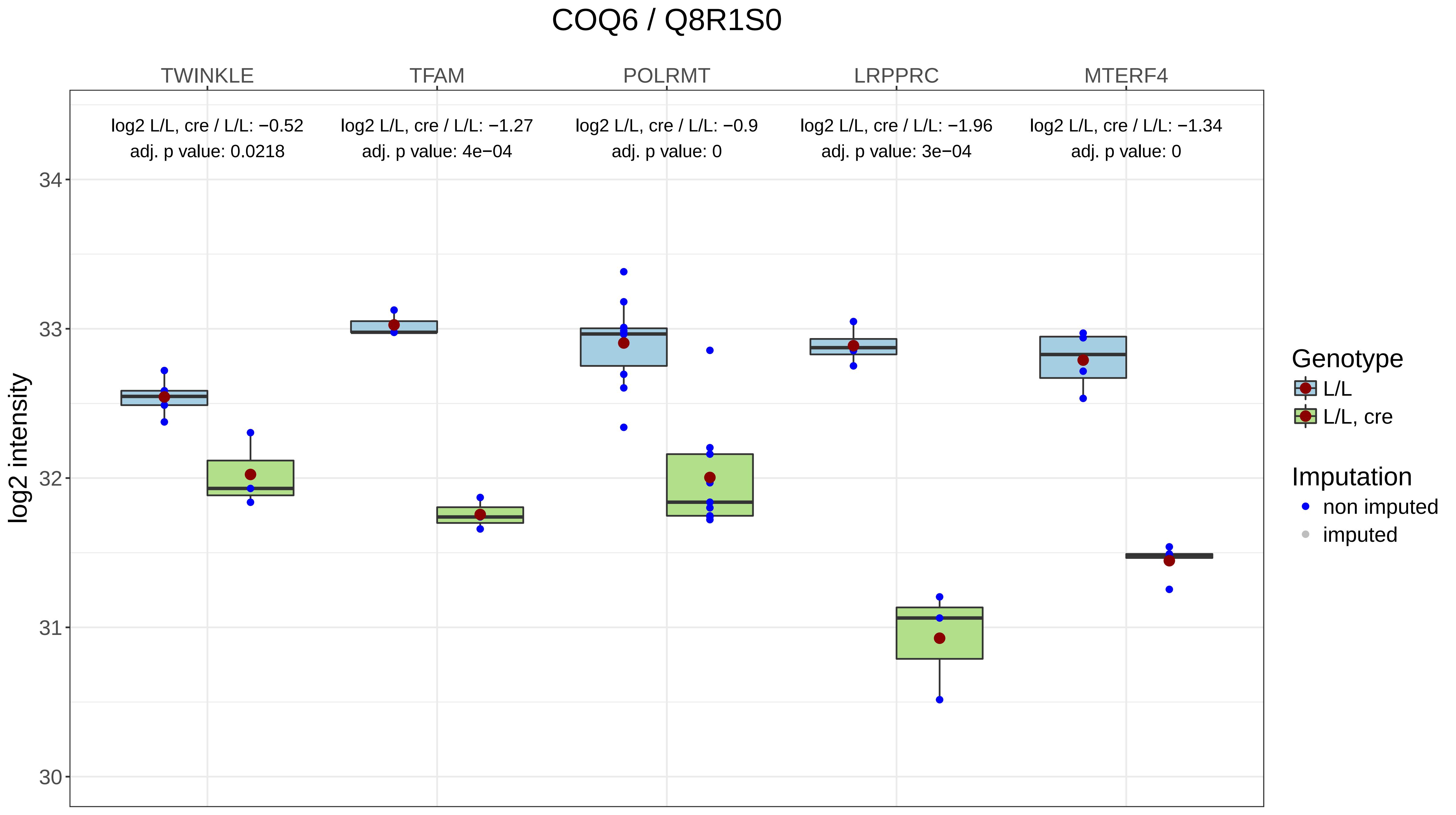
COQ6 / Q8R1S0
TWINKLE
TFAM
POLRMT
LRPPRC
MTERF4
log2 L/L, cre / L/L: −0.52
adj. p value: 0.0218
log2 L/L, cre / L/L: −1.27
adj. p value: 4e−04
log2 L/L, cre / L/L: −0.9
adj. p value: 0
log2 L/L, cre / L/L: −1.96
adj. p value: 3e−04
log2 L/L, cre / L/L: −1.34
adj. p value: 0
log2 intensity
34
33
32
31
30
Genotype
L/L
L/L, cre
Imputation
non imputed
imputed

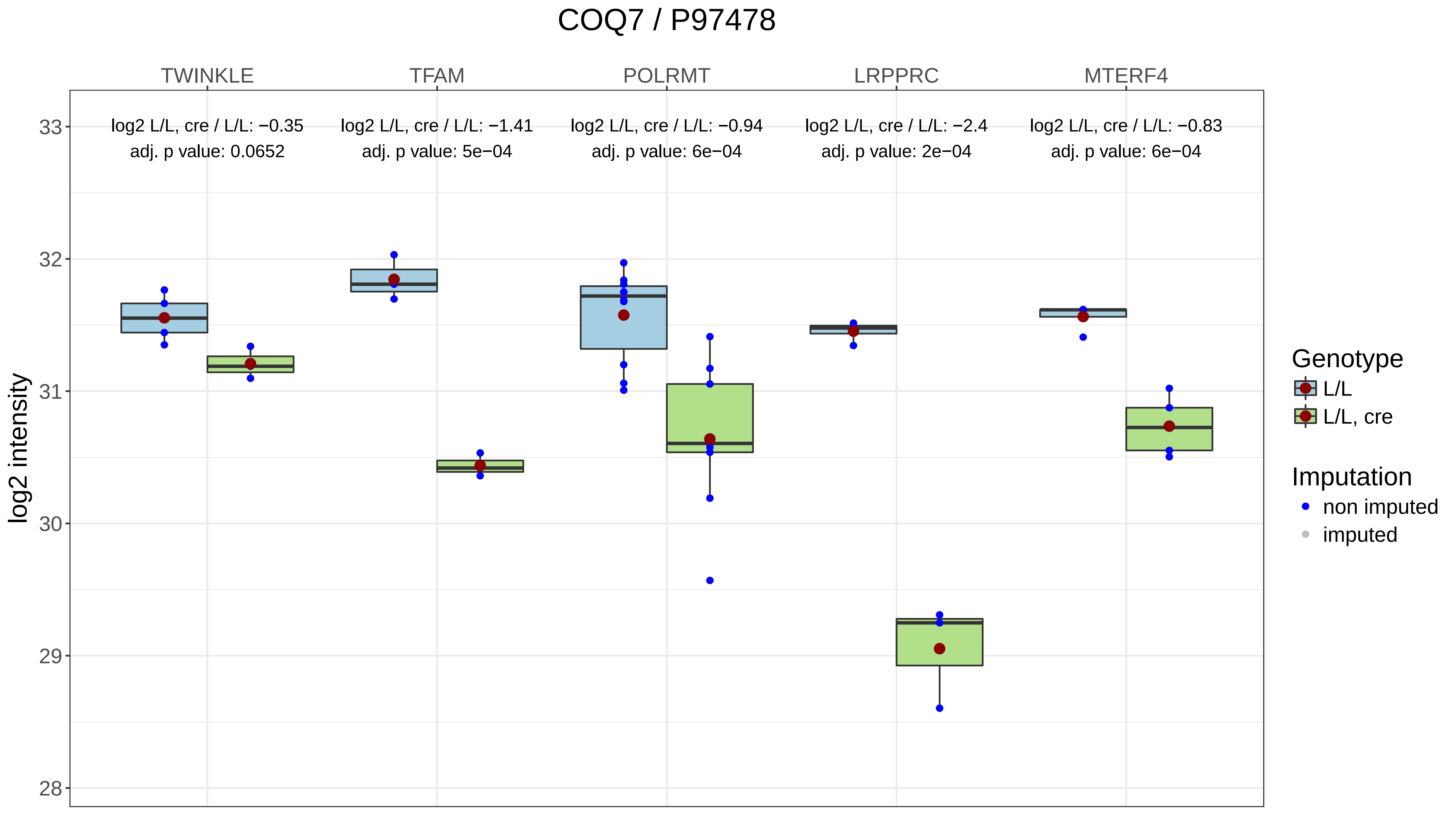
COQ7 / P97478
TWINKLE
TFAM
POLRMT
LRPPRC
MTERF4
log2 L/L, cre / L/L: −0.35
adj. p value: 0.0652
log2 L/L, cre / L/L: −1.41
adj. p value: 5e−04
log2 L/L, cre / L/L: −0.94
adj. p value: 6e−04
log2 L/L, cre / L/L: −2.4
adj. p value: 2e−04
log2 L/L, cre / L/L: −0.83
adj. p value: 6e−04
log2 intensity
33
32
31
30
29
28
Genotype
L/L
L/L, cre
Imputation
non imputed
imputed

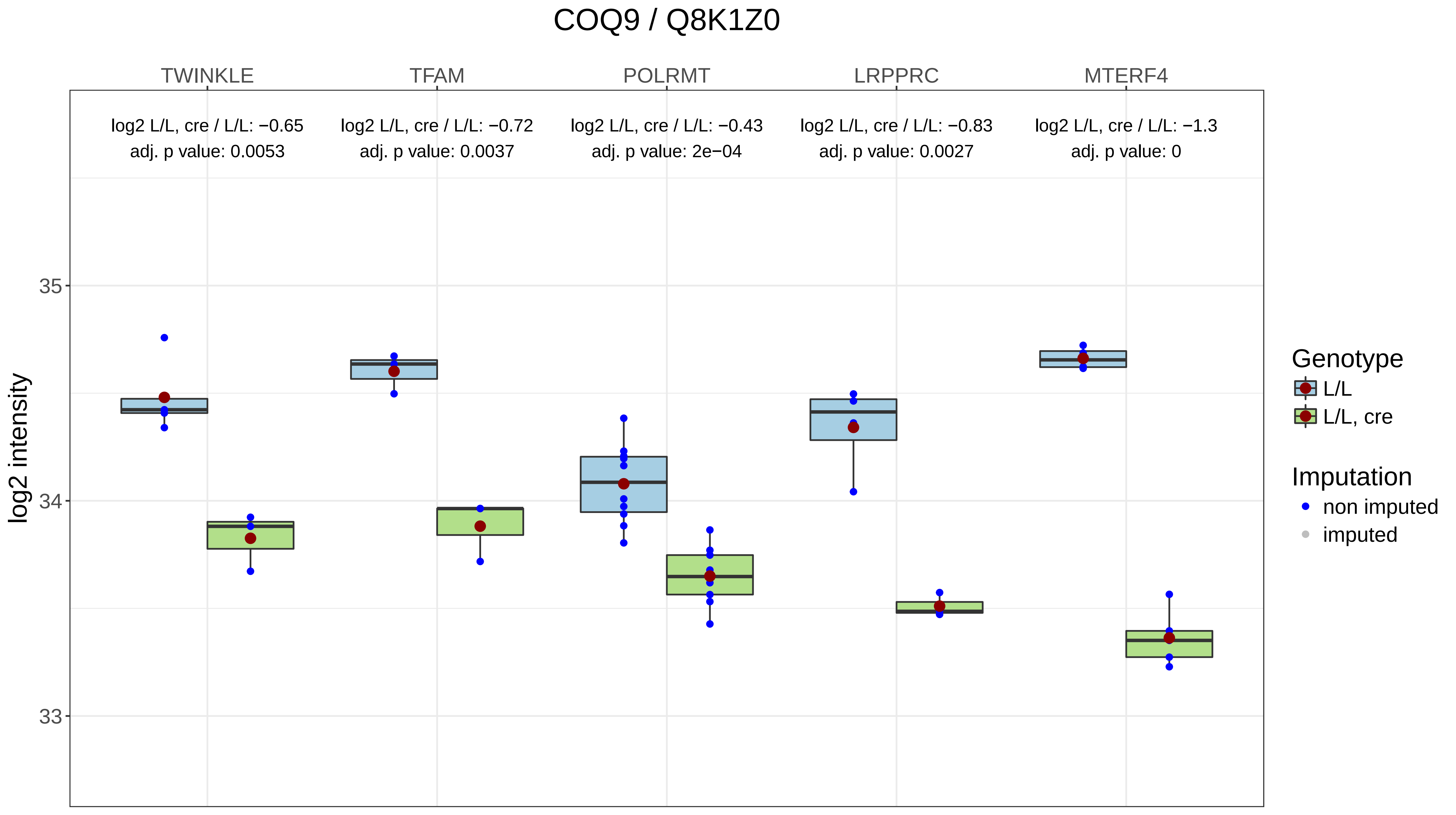
COQ9 / Q8K1Z0
TWINKLE
TFAM
POLRMT
LRPPRC
MTERF4
log2 L/L, cre / L/L: −0.65
adj. p value: 0.0053
log2 L/L, cre / L/L: −0.72
adj. p value: 0.0037
log2 L/L, cre / L/L: −0.43
adj. p value: 2e−04
log2 L/L, cre / L/L: −0.83
adj. p value: 0.0027
log2 L/L, cre / L/L: −1.3
adj. p value: 0
log2 intensity
35
34
33
Genotype
L/L
L/L, cre
Imputation
non imputed
imputed

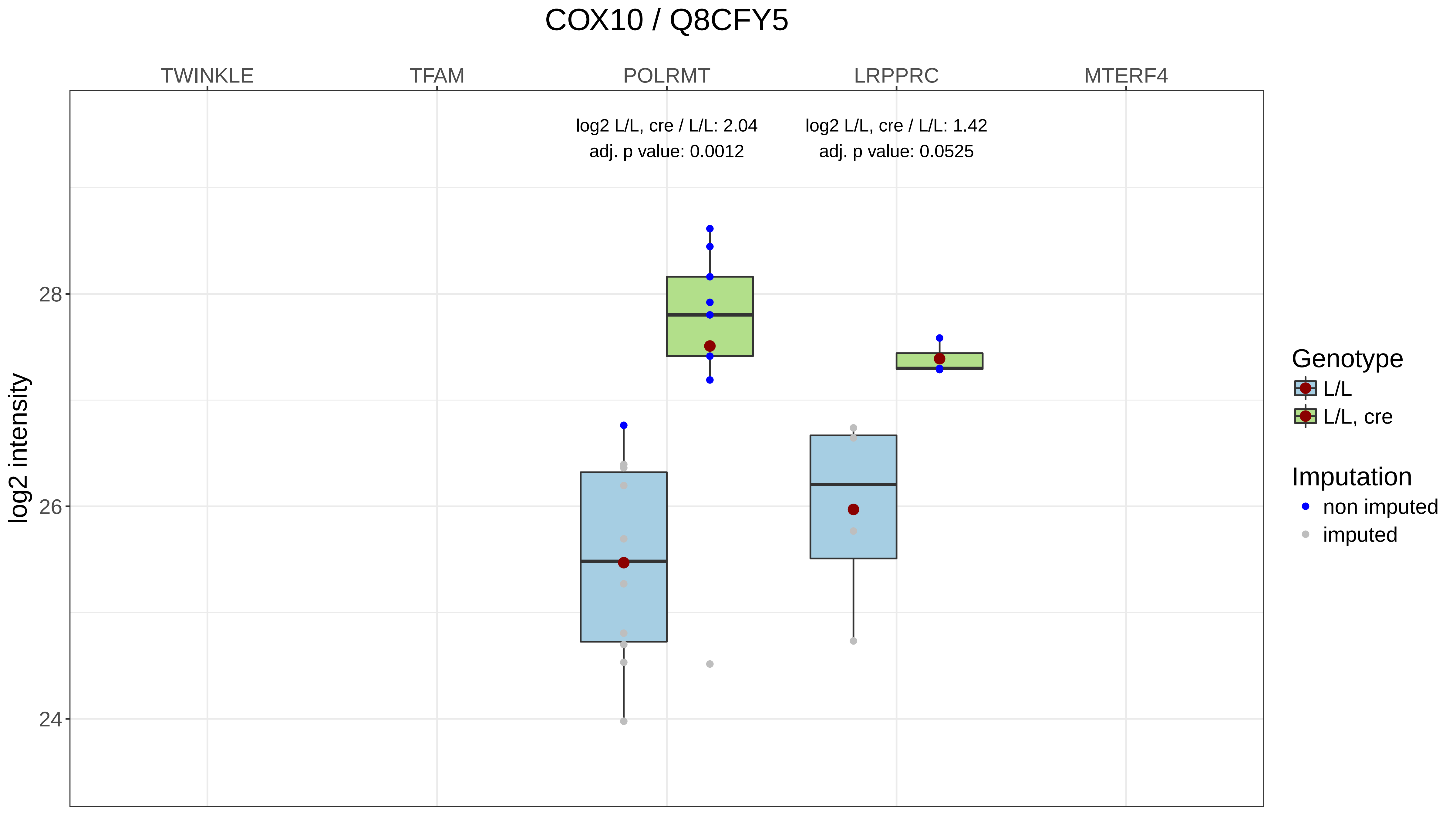
COX10 / Q8CFY5
TWINKLE
TFAM
POLRMT
LRPPRC
MTERF4
log2 L/L, cre / L/L: 2.04
adj. p value: 0.0012
log2 L/L, cre / L/L: 1.42
adj. p value: 0.0525
log2 intensity
28
26
24
Genotype
L/L
L/L, cre
Imputation
non imputed
imputed

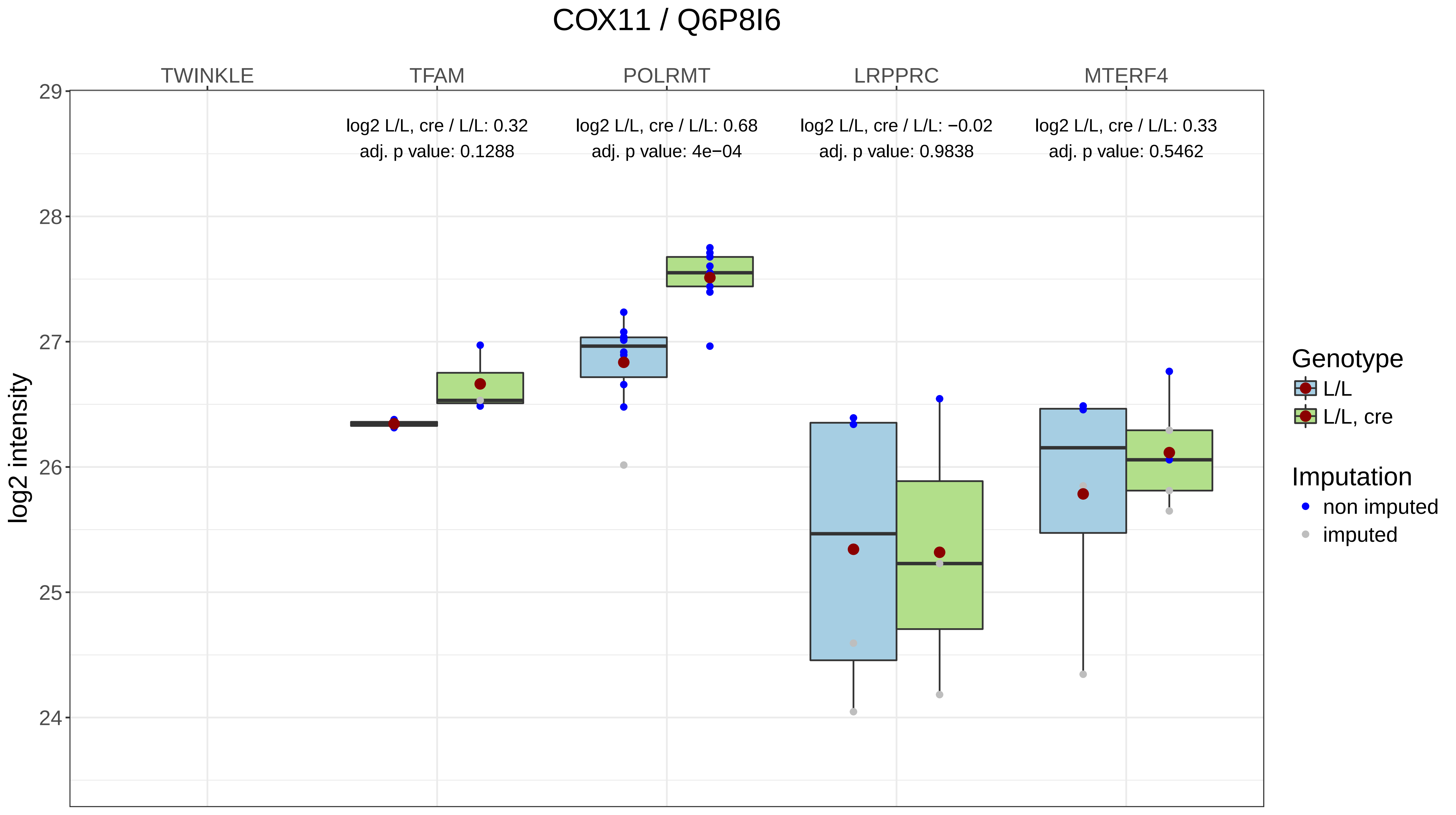
COX11 / Q6P8I6
TWINKLE
TFAM
POLRMT
LRPPRC
MTERF4
log2 L/L, cre / L/L: 0.32
adj. p value: 0.1288
log2 L/L, cre / L/L: 0.68
adj. p value: 4e−04
log2 L/L, cre / L/L: −0.02
adj. p value: 0.9838
log2 L/L, cre / L/L: 0.33
adj. p value: 0.5462
log2 intensity
29
28
27
26
25
24
Genotype
L/L
L/L, cre
Imputation
non imputed
imputed

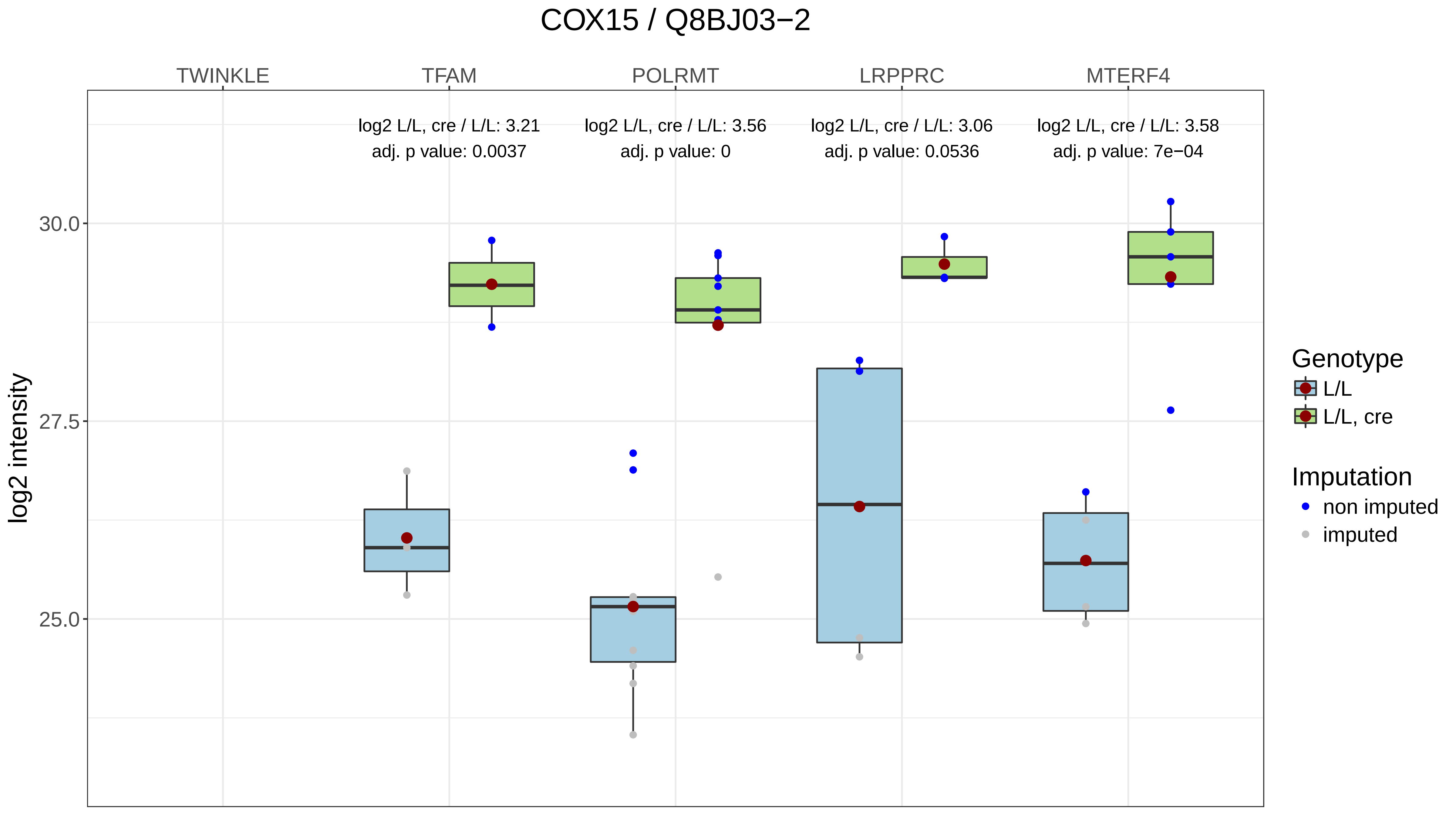
COX15 / Q8BJ03−2
TWINKLE
TFAM
POLRMT
LRPPRC
MTERF4
log2 L/L, cre / L/L: 3.21
adj. p value: 0.0037
log2 L/L, cre / L/L: 3.56
adj. p value: 0
log2 L/L, cre / L/L: 3.06
adj. p value: 0.0536
log2 L/L, cre / L/L: 3.58
adj. p value: 7e−04
30.0
27.5
25.0
log2 intensity
Genotype
L/L
L/L, cre
Imputation
non imputed
imputed

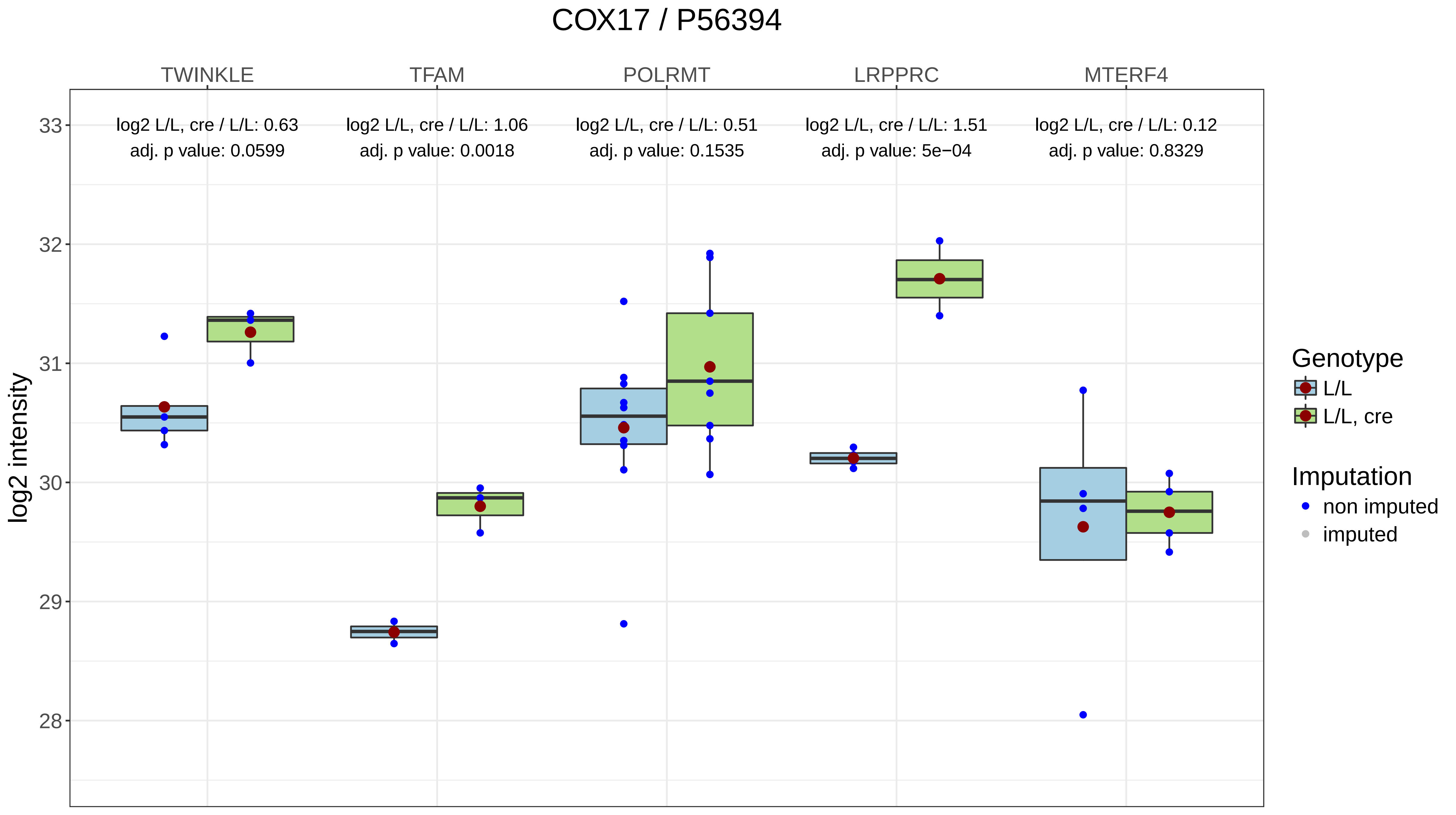

COX17 / P56394
TWINKLE
TFAM
POLRMT
LRPPRC
MTERF4
log2 L/L, cre / L/L: 0.63
adj. p value: 0.0599
log2 L/L, cre / L/L: 1.06
adj. p value: 0.0018
log2 L/L, cre / L/L: 0.51
adj. p value: 0.1535
log2 L/L, cre / L/L: 1.51
adj. p value: 5e−04
log2 L/L, cre / L/L: 0.12
adj. p value: 0.8329
log2 intensity
33
32
31
30
29
28
Genotype
L/L
L/L, cre
Imputation
non imputed
imputed

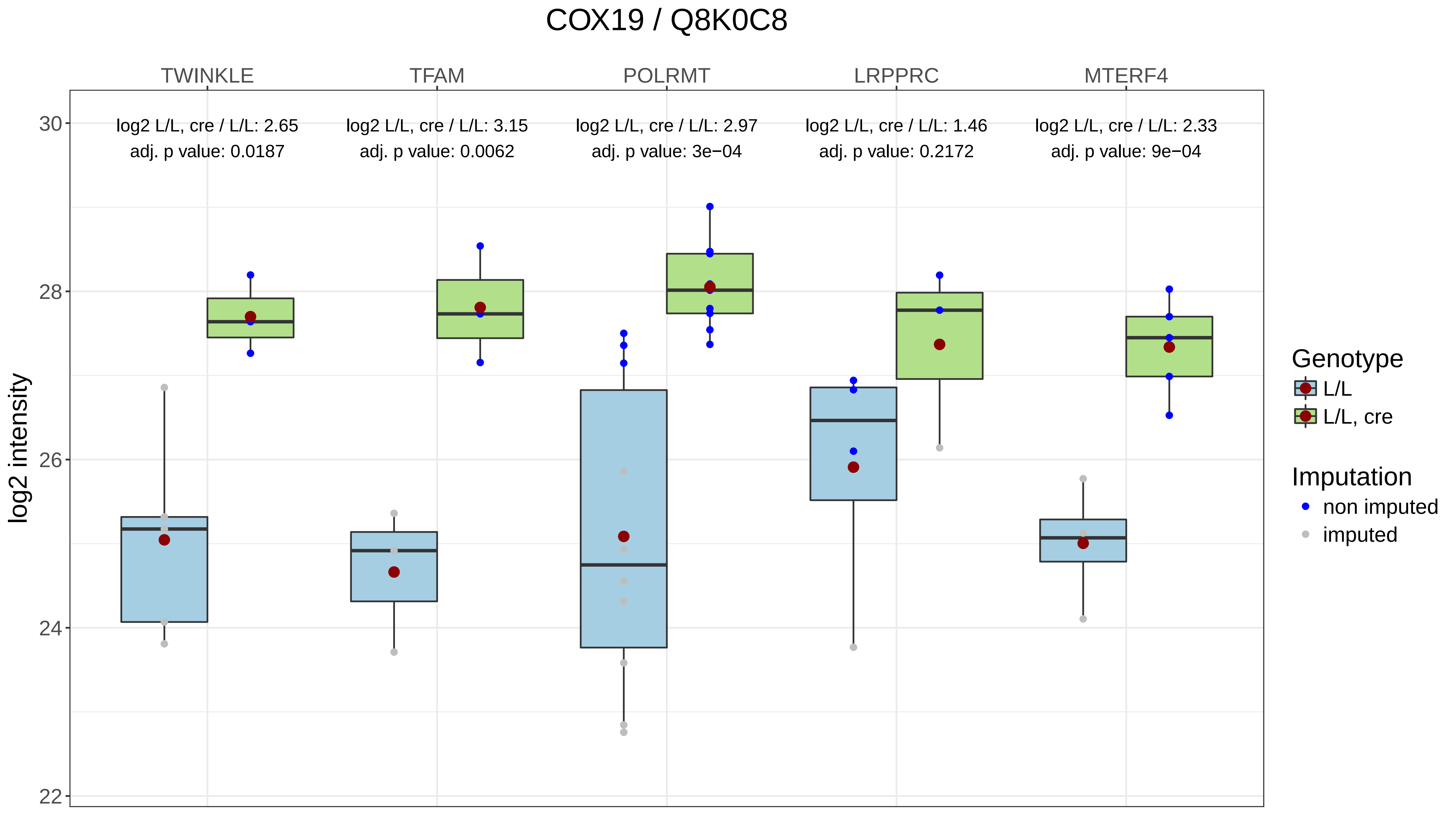

COX19 / Q8K0C8
TWINKLE
TFAM
POLRMT
LRPPRC
MTERF4
log2 L/L, cre / L/L: 2.65
adj. p value: 0.0187
log2 L/L, cre / L/L: 3.15
adj. p value: 0.0062
log2 L/L, cre / L/L: 2.97
adj. p value: 3e−04
log2 L/L, cre / L/L: 1.46
adj. p value: 0.2172
log2 L/L, cre / L/L: 2.33
adj. p value: 9e−04
log2 intensity
30
28
26
24
22
Genotype
L/L
L/L, cre
Imputation
non imputed
imputed

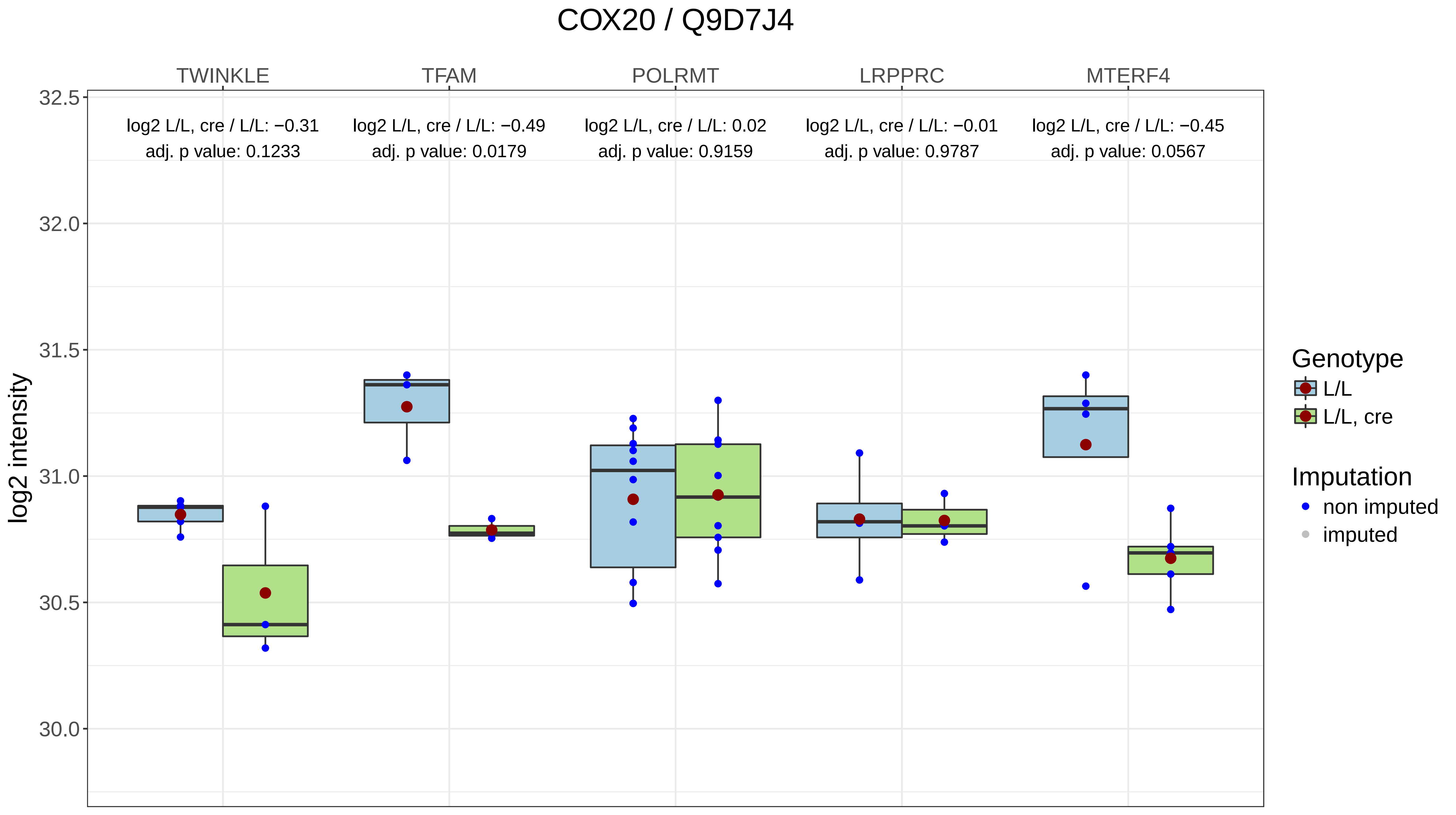
COX20 / Q9D7J4
TWINKLE
TFAM
POLRMT
LRPPRC
MTERF4
log2 L/L, cre / L/L: −0.31
adj. p value: 0.1233
log2 L/L, cre / L/L: −0.49
adj. p value: 0.0179
log2 L/L, cre / L/L: 0.02
adj. p value: 0.9159
log2 L/L, cre / L/L: −0.01
adj. p value: 0.9787
log2 L/L, cre / L/L: −0.45
adj. p value: 0.0567
log2 intensity
32.5
32.0
31.5
31.0
30.5
30.0
Genotype
L/L
L/L, cre
Imputation
non imputed
imputed

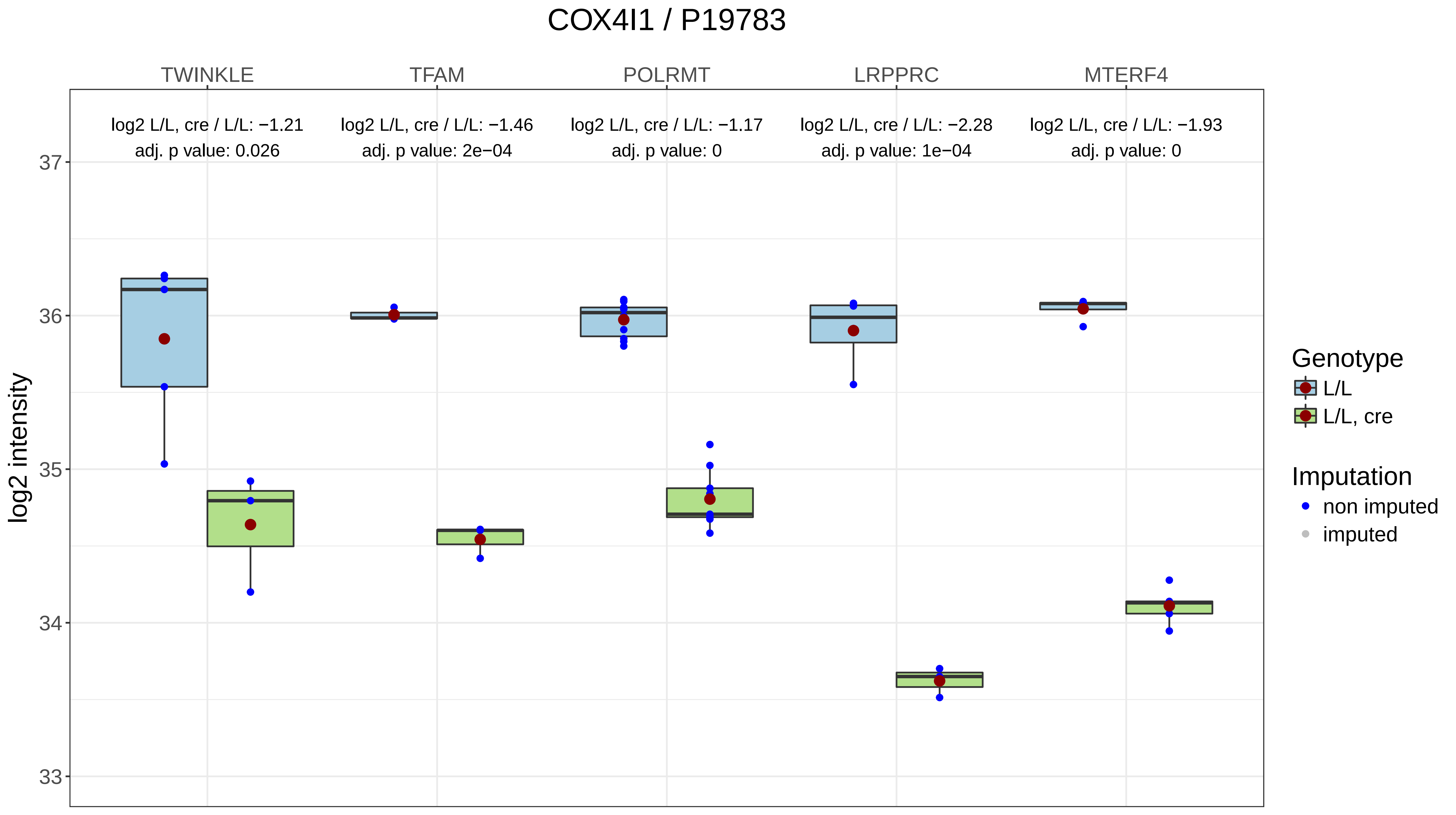
COX4I1 / P19783
TWINKLE
TFAM
POLRMT
LRPPRC
MTERF4
log2 L/L, cre / L/L: −1.21
adj. p value: 0.026
log2 L/L, cre / L/L: −1.46
adj. p value: 2e−04
log2 L/L, cre / L/L: −1.17
adj. p value: 0
log2 L/L, cre / L/L: −2.28
adj. p value: 1e−04
log2 L/L, cre / L/L: −1.93
adj. p value: 0
log2 intensity
37
36
35
34
33
Genotype
L/L
L/L, cre
Imputation
non imputed
imputed

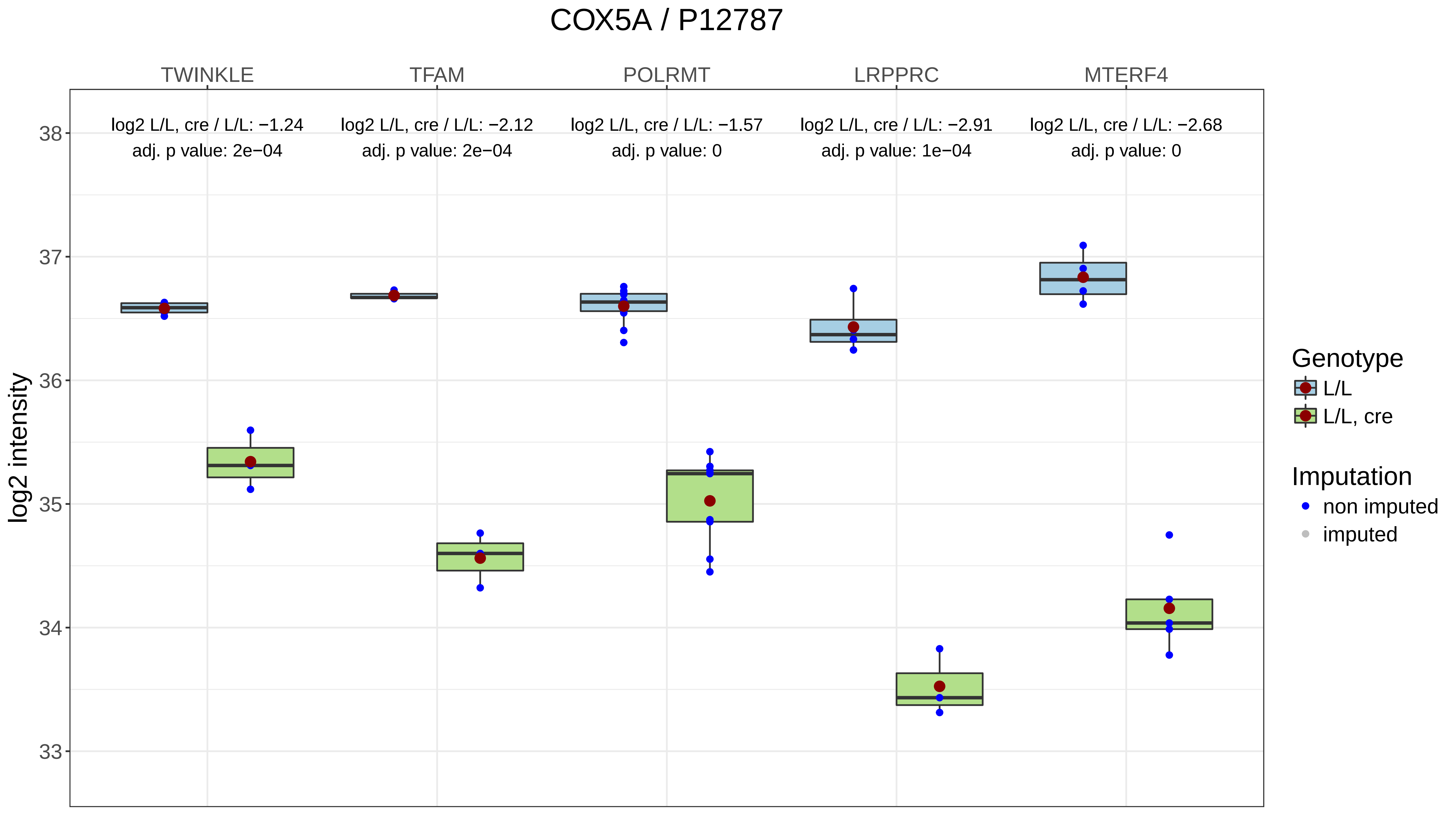

COX5A / P12787
TWINKLE
TFAM
POLRMT
LRPPRC
MTERF4
log2 L/L, cre / L/L: −1.24
adj. p value: 2e−04
log2 L/L, cre / L/L: −2.12
adj. p value: 2e−04
log2 L/L, cre / L/L: −1.57
adj. p value: 0
log2 L/L, cre / L/L: −2.91
adj. p value: 1e−04
log2 L/L, cre / L/L: −2.68
adj. p value: 0
log2 intensity
38
37
36
35
34
33
Genotype
L/L
L/L, cre
Imputation
non imputed
imputed

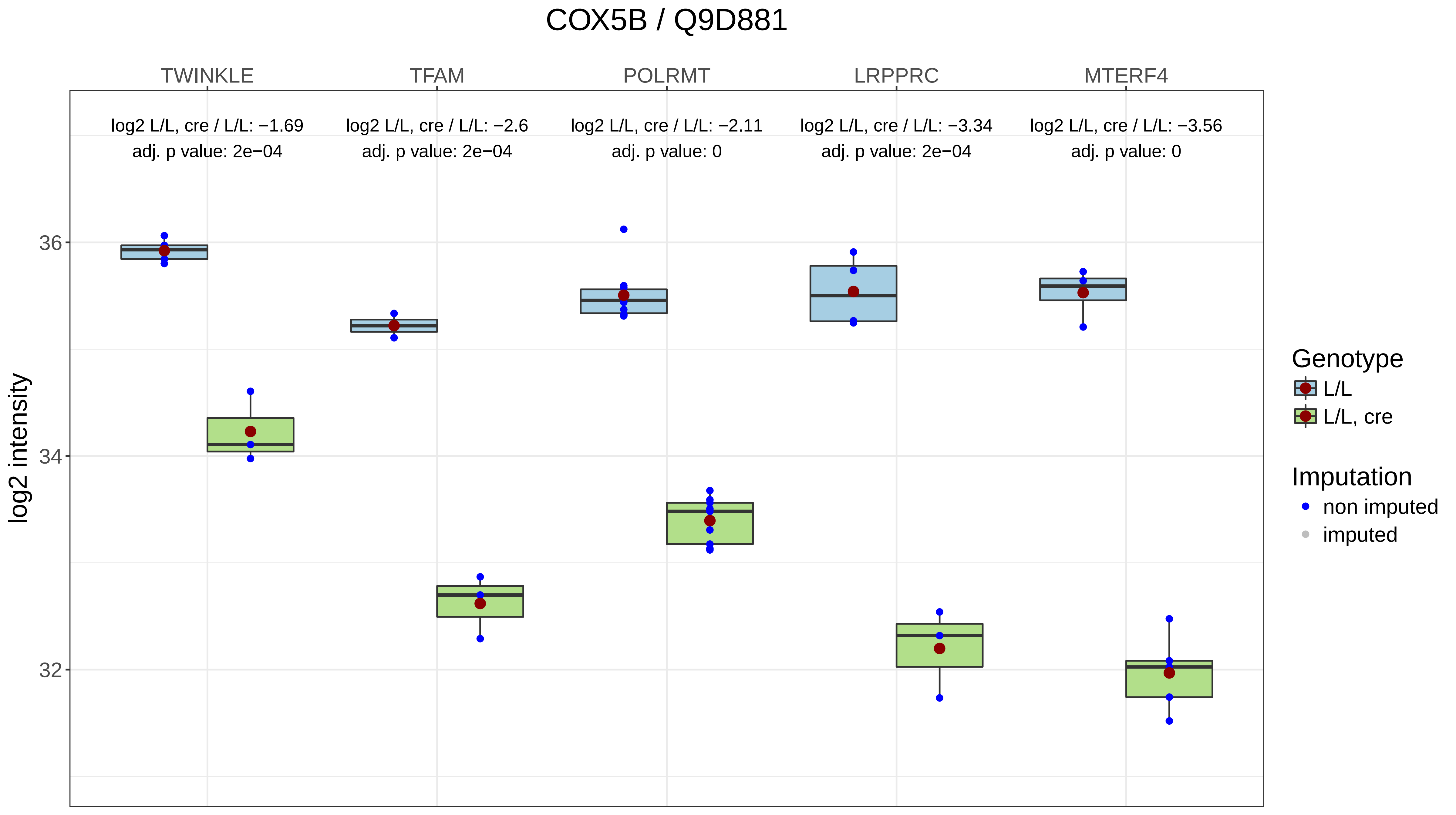
COX5B / Q9D881
TWINKLE
TFAM
POLRMT
LRPPRC
MTERF4
log2 L/L, cre / L/L: −1.69
adj. p value: 2e−04
log2 L/L, cre / L/L: −2.6
adj. p value: 2e−04
log2 L/L, cre / L/L: −2.11
adj. p value: 0
log2 L/L, cre / L/L: −3.34
adj. p value: 2e−04
log2 L/L, cre / L/L: −3.56
adj. p value: 0
log2 intensity
36
34
32
Genotype
L/L
L/L, cre
Imputation
non imputed
imputed

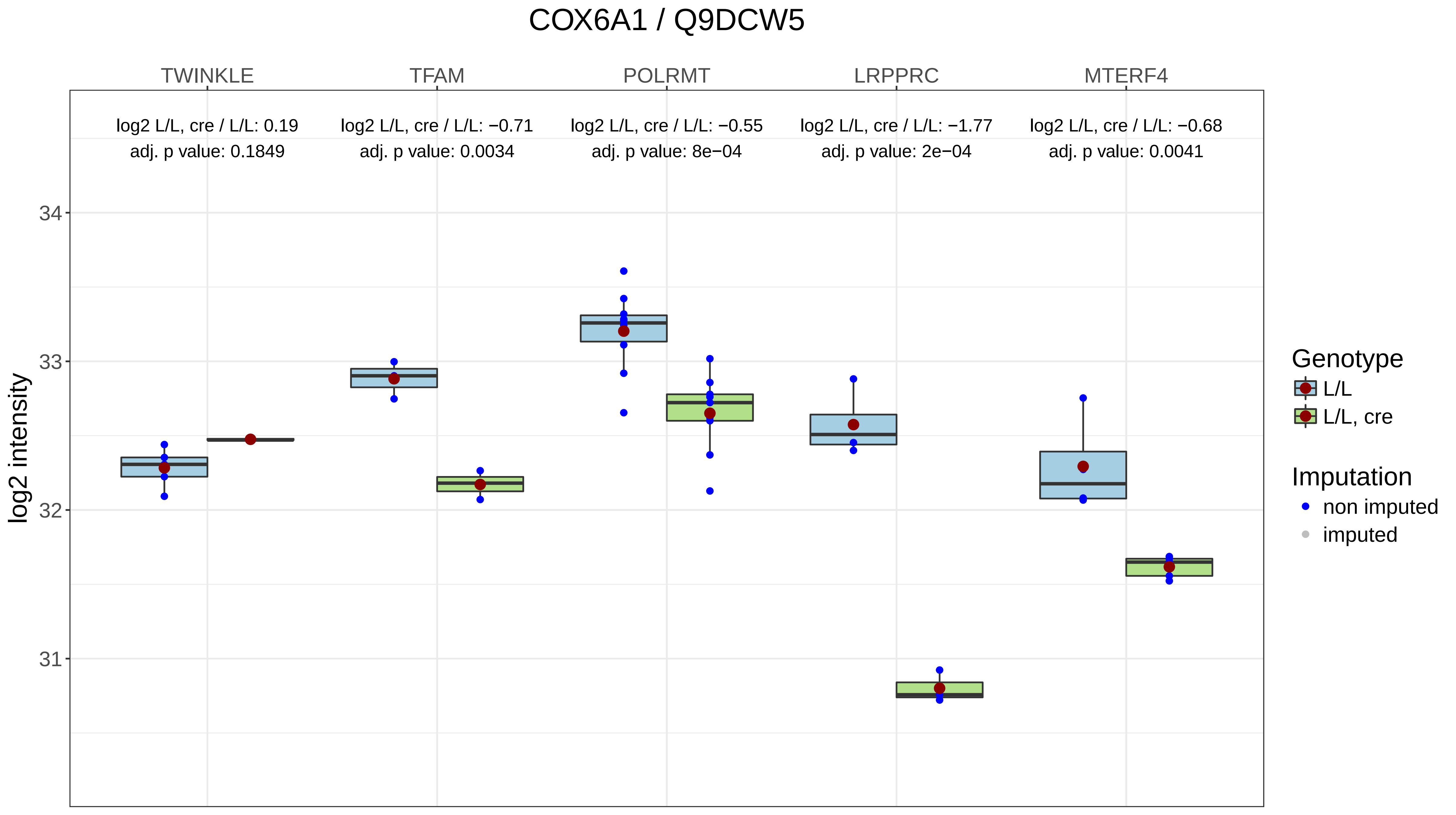
COX6A1 / Q9DCW5
TWINKLE
TFAM
POLRMT
LRPPRC
MTERF4
log2 L/L, cre / L/L: 0.19
adj. p value: 0.1849
log2 L/L, cre / L/L: −0.71
adj. p value: 0.0034
log2 L/L, cre / L/L: −0.55
adj. p value: 8e−04
log2 L/L, cre / L/L: −1.77
adj. p value: 2e−04
log2 L/L, cre / L/L: −0.68
adj. p value: 0.0041
log2 intensity
34
33
32
31
Genotype
L/L
L/L, cre
Imputation
non imputed
imputed

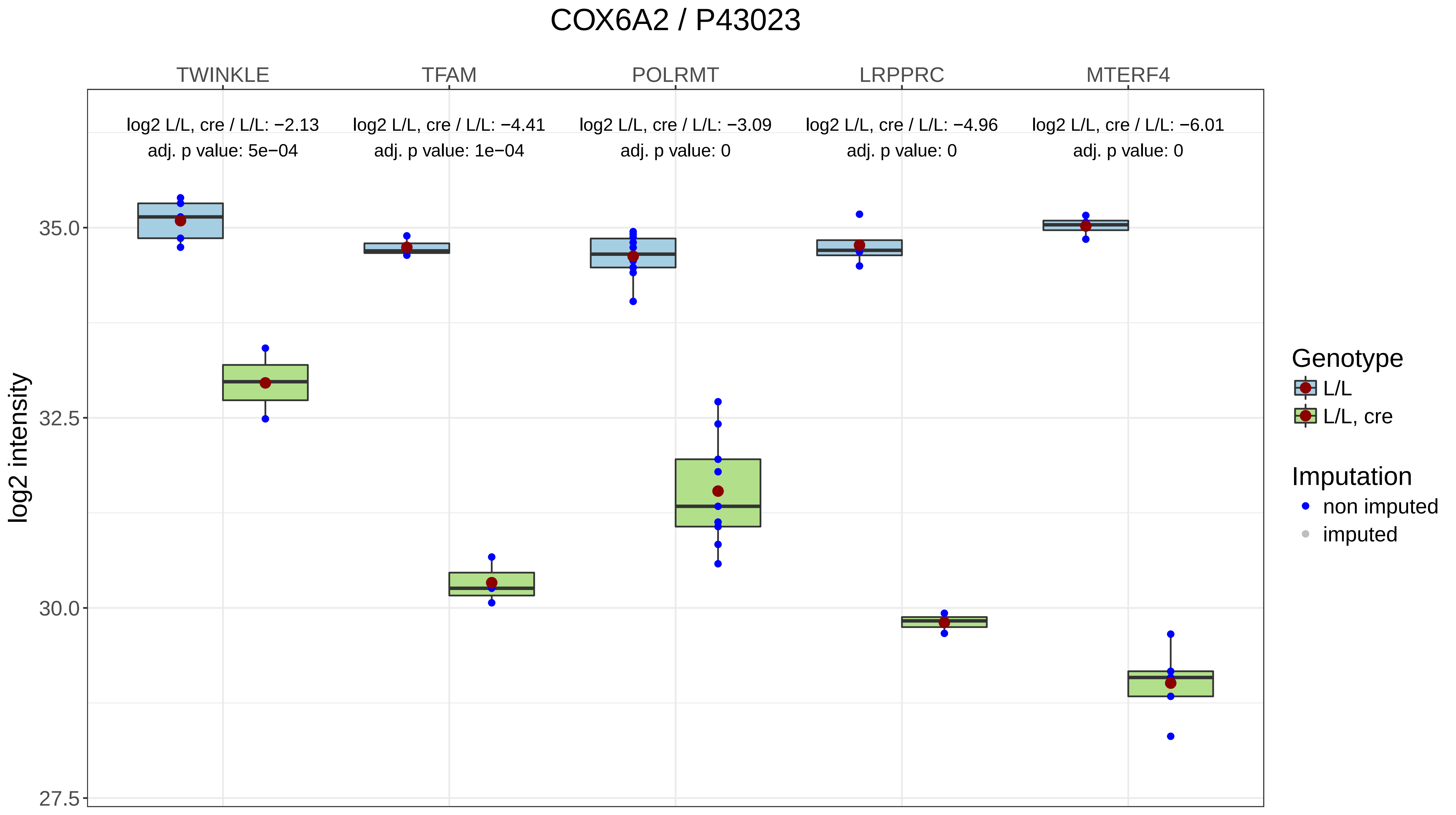
COX6A2 / P43023
TWINKLE
TFAM
POLRMT
LRPPRC
MTERF4
log2 L/L, cre / L/L: −2.13
adj. p value: 5e−04
log2 L/L, cre / L/L: −4.41
adj. p value: 1e−04
log2 L/L, cre / L/L: −3.09
adj. p value: 0
log2 L/L, cre / L/L: −4.96
adj. p value: 0
log2 L/L, cre / L/L: −6.01
adj. p value: 0
log2 intensity
35.0
32.5
30.0
27.5
Genotype
L/L
L/L, cre
Imputation
non imputed
imputed

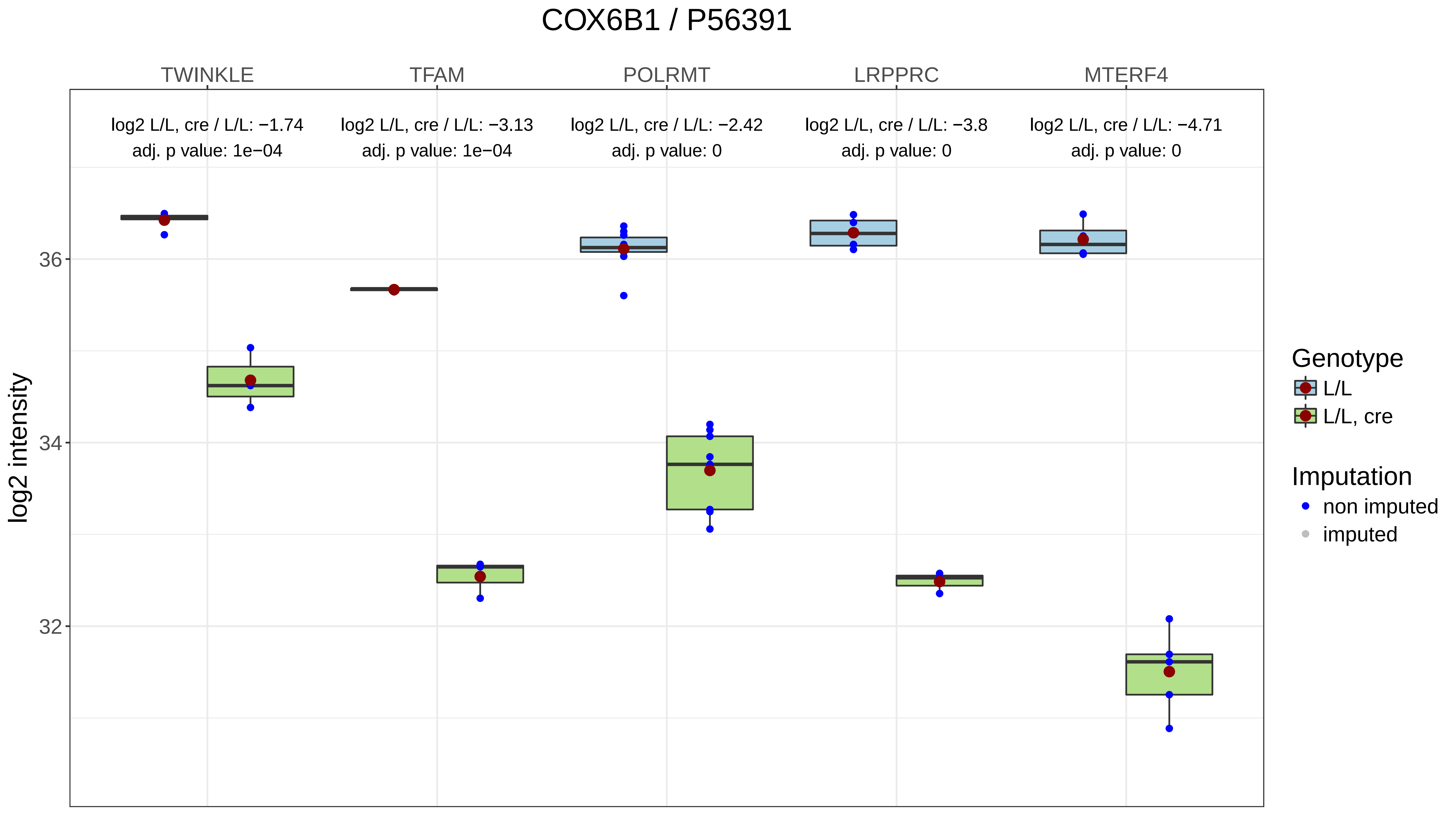
COX6B1 / P56391
TWINKLE
TFAM
POLRMT
LRPPRC
MTERF4
log2 L/L, cre / L/L: −1.74
adj. p value: 1e−04
log2 L/L, cre / L/L: −3.13
adj. p value: 1e−04
log2 L/L, cre / L/L: −2.42
adj. p value: 0
log2 L/L, cre / L/L: −3.8
adj. p value: 0
log2 L/L, cre / L/L: −4.71
adj. p value: 0
log2 intensity
36
34
32
Genotype
L/L
L/L, cre
Imputation
non imputed
imputed

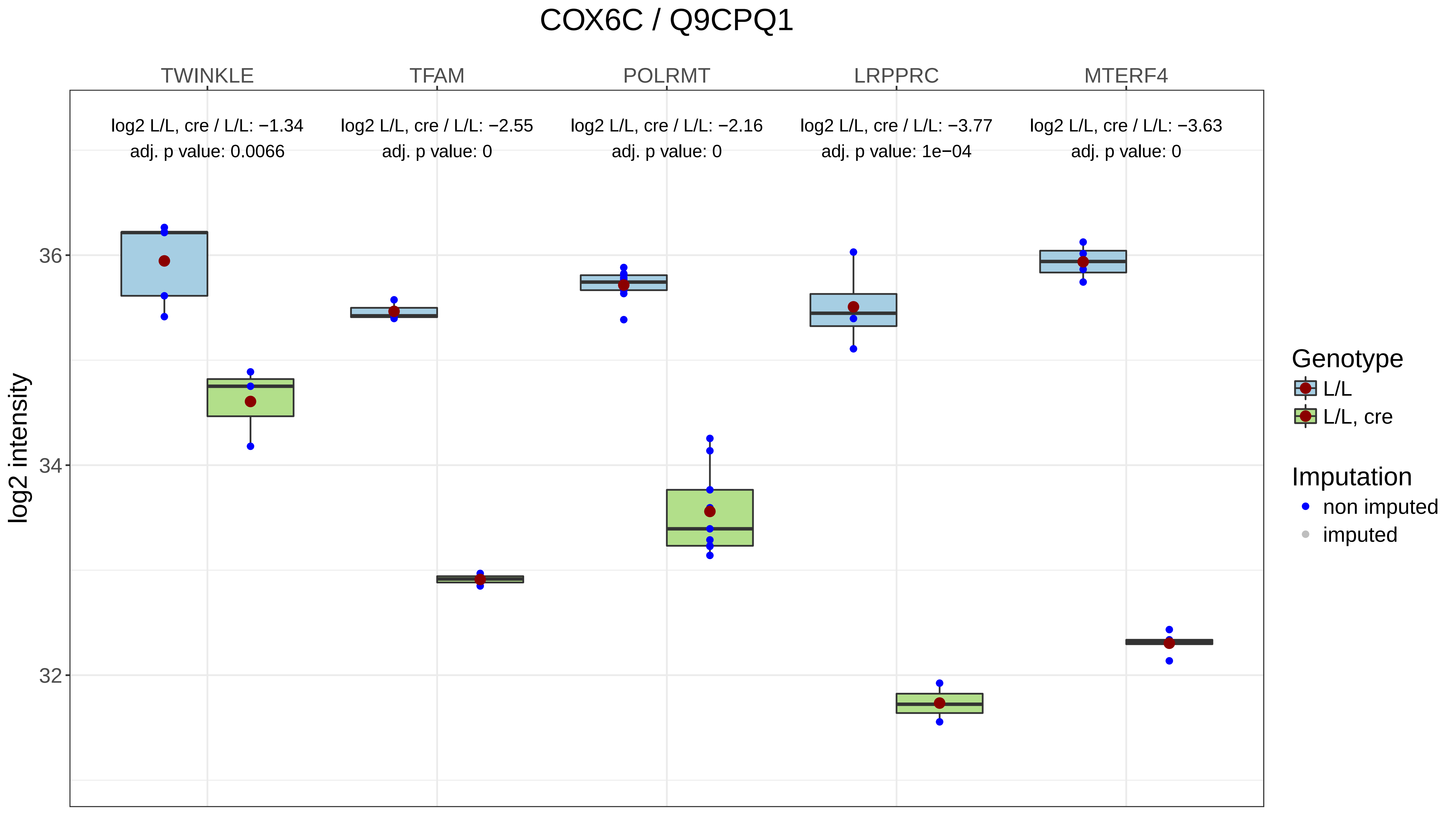

COX6C / Q9CPQ1
TWINKLE
TFAM
POLRMT
LRPPRC
MTERF4
log2 L/L, cre / L/L: −1.34
adj. p value: 0.0066
log2 L/L, cre / L/L: −2.55
adj. p value: 0
log2 L/L, cre / L/L: −2.16
adj. p value: 0
log2 L/L, cre / L/L: −3.77
adj. p value: 1e−04
log2 L/L, cre / L/L: −3.63
adj. p value: 0
log2 intensity
36
34
32
Genotype
L/L
L/L, cre
Imputation
non imputed
imputed

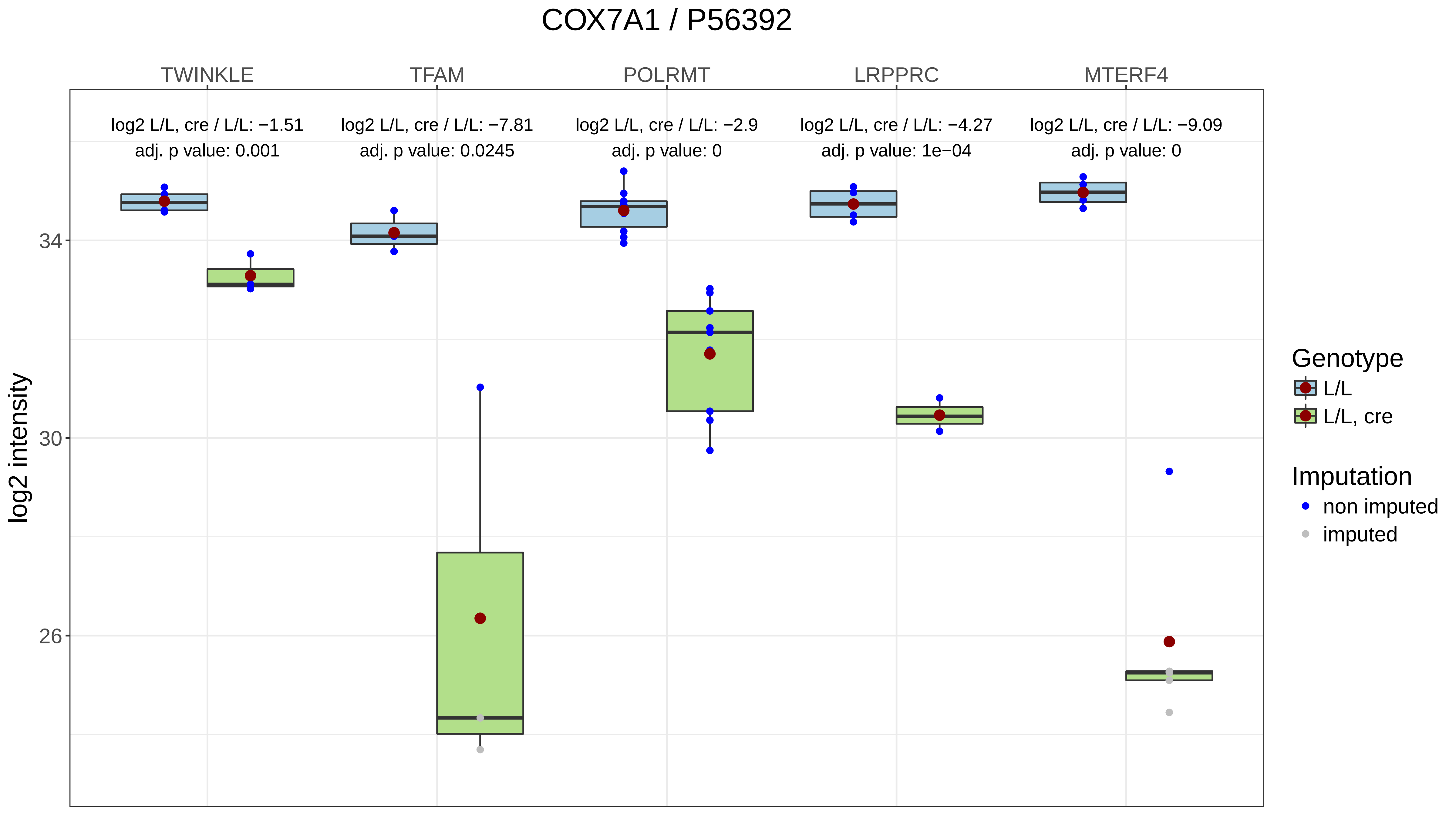
COX7A1 / P56392
TWINKLE
TFAM
POLRMT
LRPPRC
MTERF4
log2 L/L, cre / L/L: −1.51
adj. p value: 0.001
log2 L/L, cre / L/L: −7.81
adj. p value: 0.0245
log2 L/L, cre / L/L: −2.9
adj. p value: 0
log2 L/L, cre / L/L: −4.27
adj. p value: 1e−04
log2 L/L, cre / L/L: −9.09
adj. p value: 0
34
30
26
log2 intensity
Genotype
L/L
L/L, cre
Imputation
non imputed
imputed

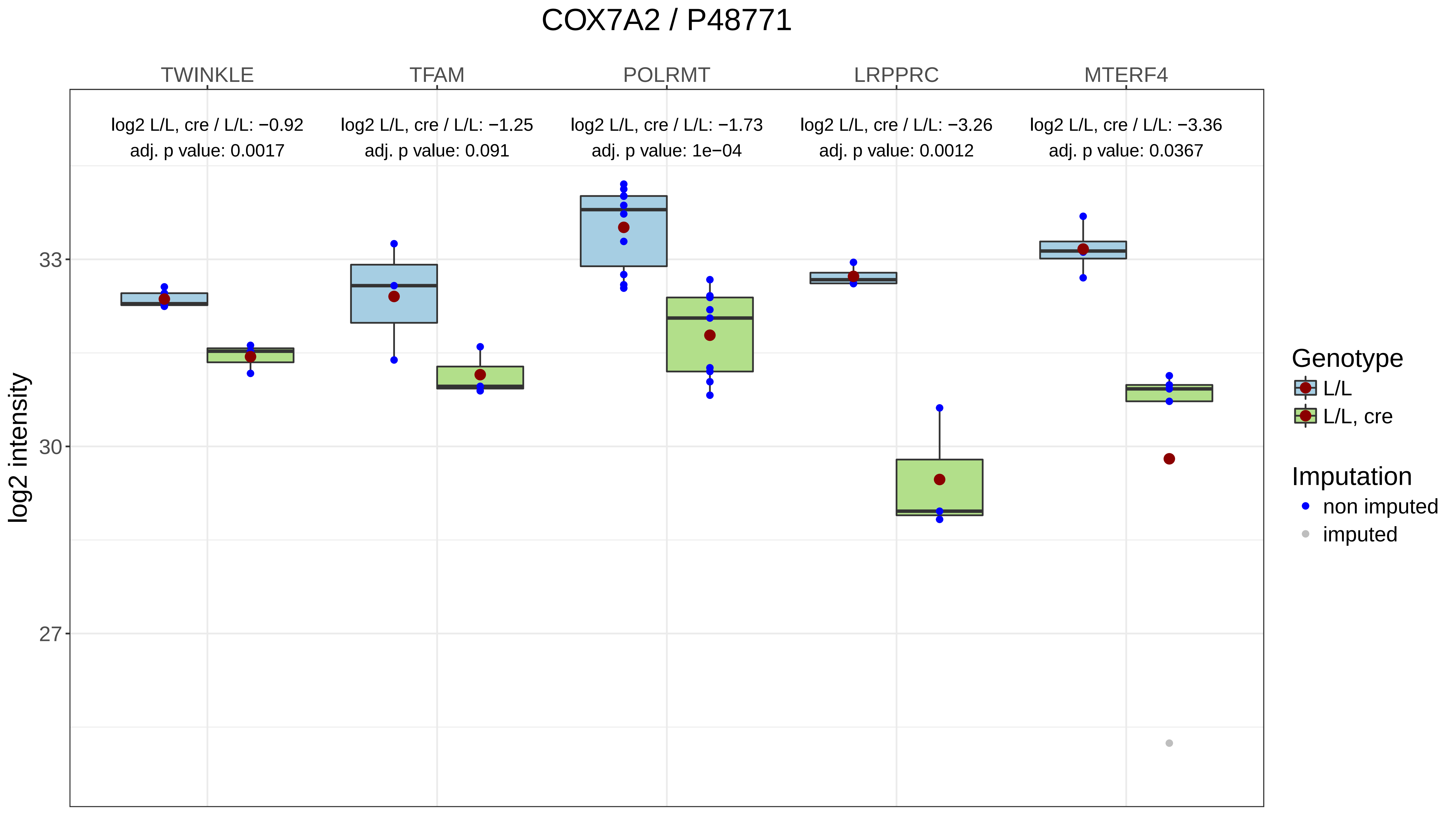

COX7A2 / P48771
TWINKLE
TFAM
POLRMT
LRPPRC
MTERF4
log2 L/L, cre / L/L: −0.92
adj. p value: 0.0017
log2 L/L, cre / L/L: −1.25
adj. p value: 0.091
log2 L/L, cre / L/L: −1.73
adj. p value: 1e−04
log2 L/L, cre / L/L: −3.26
adj. p value: 0.0012
log2 L/L, cre / L/L: −3.36
adj. p value: 0.0367
33
30
27
log2 intensity
Genotype
L/L
L/L, cre
Imputation
non imputed
imputed

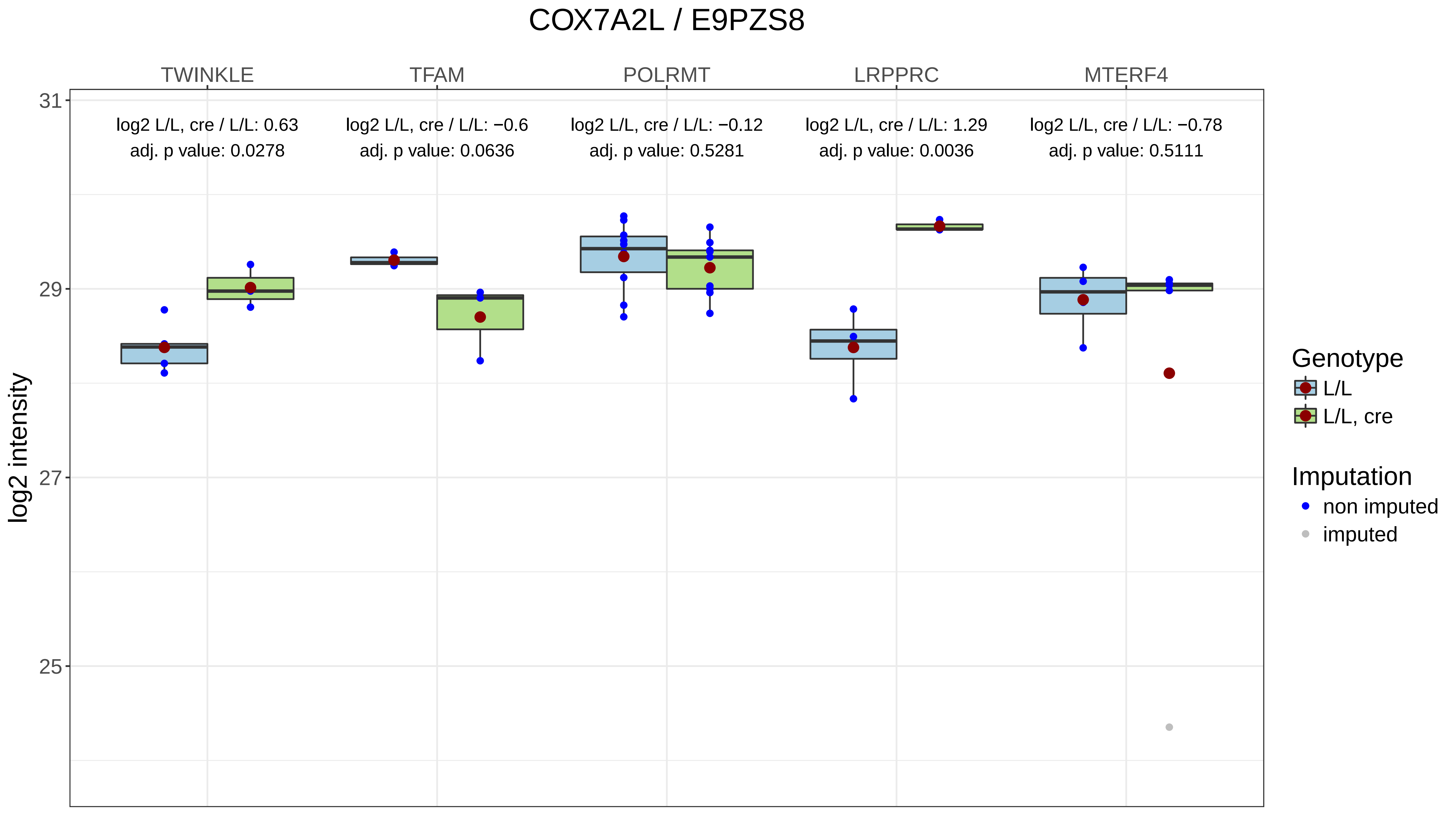
COX7A2L / E9PZS8
TWINKLE
TFAM
POLRMT
LRPPRC
MTERF4
log2 L/L, cre / L/L: 0.63
adj. p value: 0.0278
log2 L/L, cre / L/L: −0.6
adj. p value: 0.0636
log2 L/L, cre / L/L: −0.12
adj. p value: 0.5281
log2 L/L, cre / L/L: 1.29
adj. p value: 0.0036
log2 L/L, cre / L/L: −0.78
adj. p value: 0.5111
31
29
27
25
log2 intensity
Genotype
L/L
L/L, cre
Imputation
non imputed
imputed

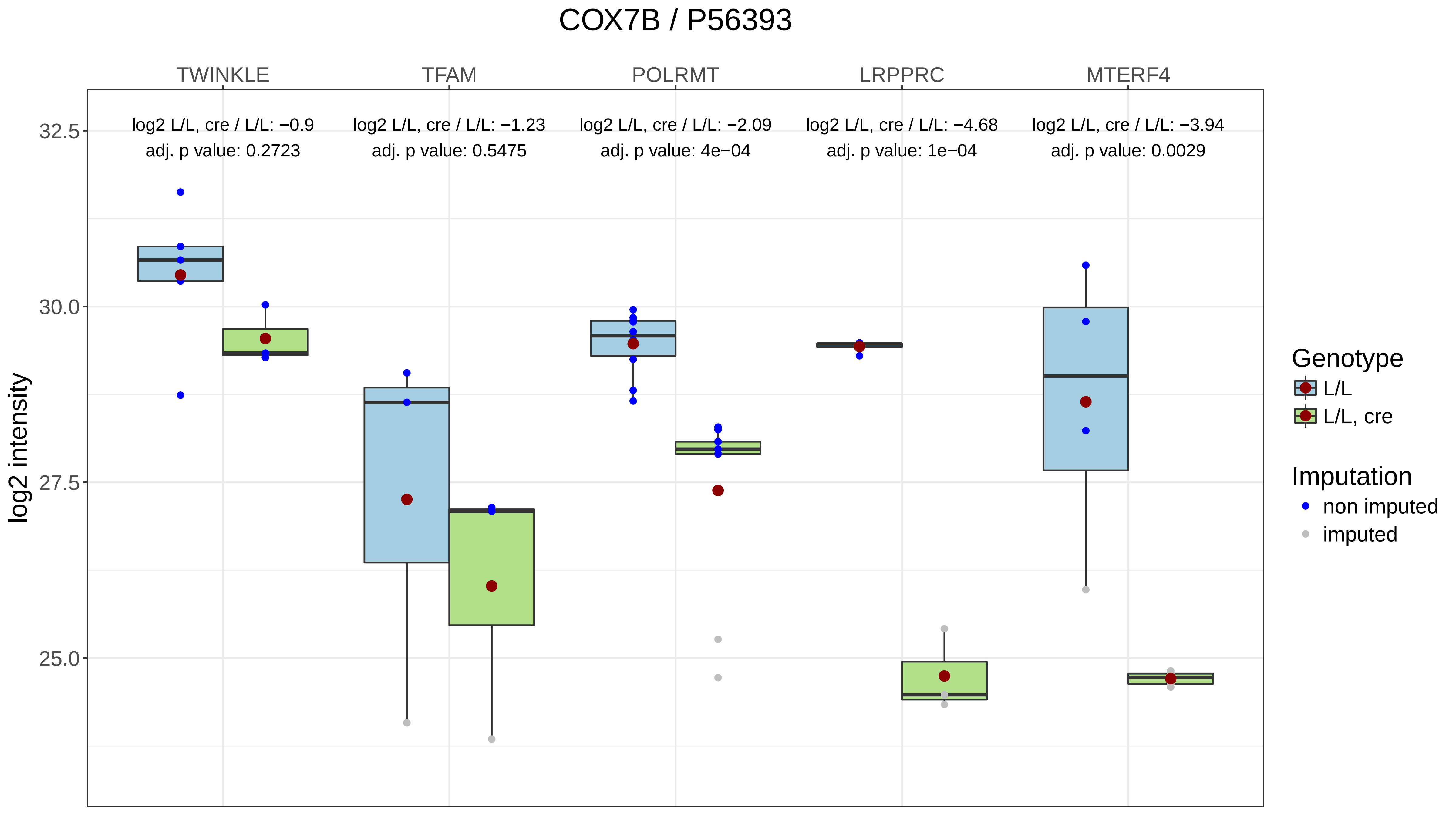
COX7B / P56393
TWINKLE
TFAM
POLRMT
LRPPRC
MTERF4
log2 L/L, cre / L/L: −0.9
adj. p value: 0.2723
log2 L/L, cre / L/L: −1.23
adj. p value: 0.5475
log2 L/L, cre / L/L: −2.09
adj. p value: 4e−04
log2 L/L, cre / L/L: −4.68
adj. p value: 1e−04
log2 L/L, cre / L/L: −3.94
adj. p value: 0.0029
log2 intensity
32.5
30.0
27.5
25.0
Genotype
L/L
L/L, cre
Imputation
non imputed
imputed

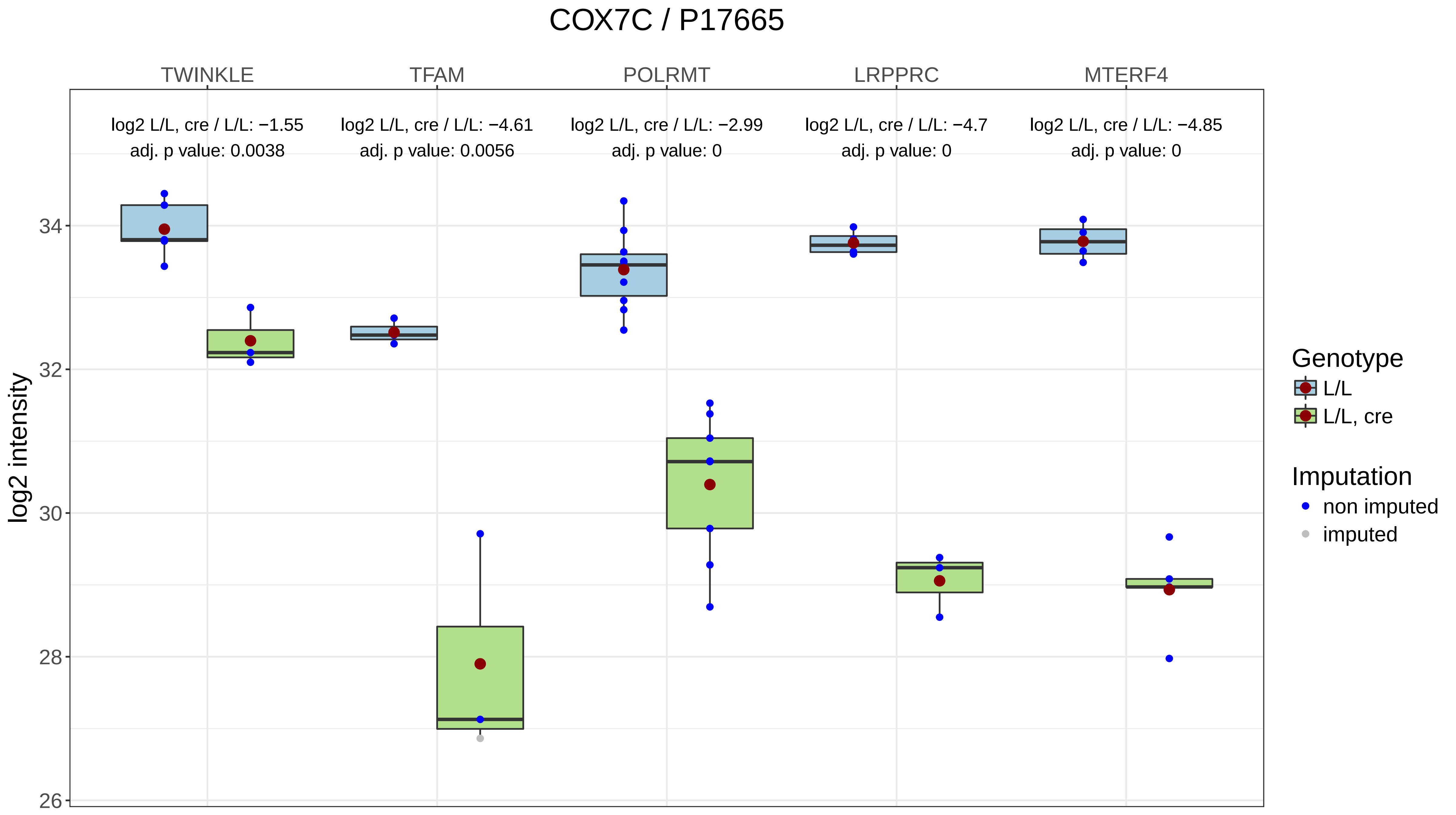
COX7C / P17665
TWINKLE
TFAM
POLRMT
LRPPRC
MTERF4
log2 L/L, cre / L/L: −1.55
adj. p value: 0.0038
log2 L/L, cre / L/L: −4.61
adj. p value: 0.0056
log2 L/L, cre / L/L: −2.99
adj. p value: 0
log2 L/L, cre / L/L: −4.7
adj. p value: 0
log2 L/L, cre / L/L: −4.85
adj. p value: 0
log2 intensity
34
32
30
28
26
Genotype
L/L
L/L, cre
Imputation
non imputed
imputed

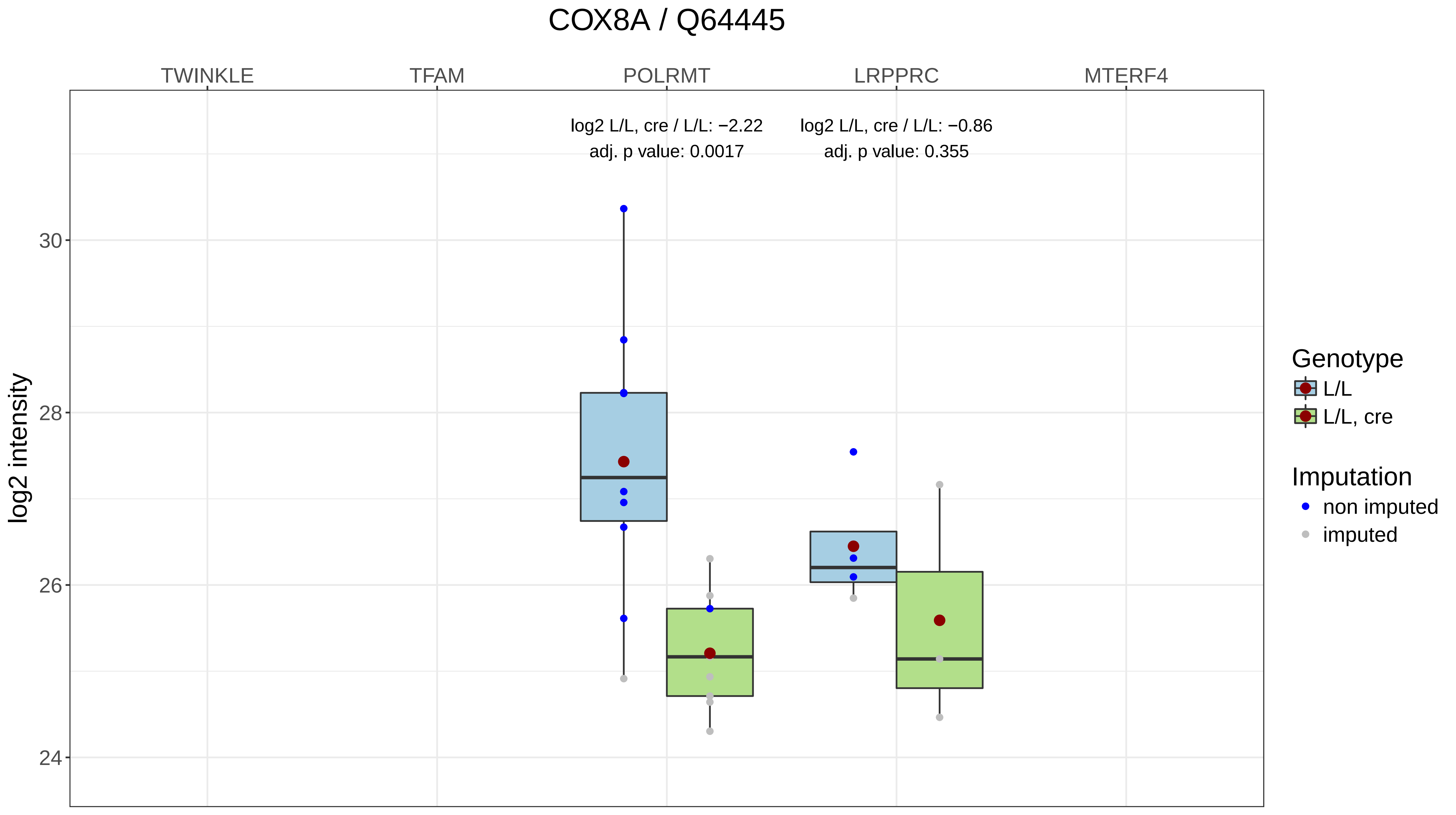
COX8A / Q64445
TWINKLE
TFAM
POLRMT
LRPPRC
MTERF4
log2 L/L, cre / L/L: −2.22
adj. p value: 0.0017
log2 L/L, cre / L/L: −0.86
adj. p value: 0.355
log2 intensity
30
28
26
24
Genotype
L/L
L/L, cre
Imputation
non imputed
imputed

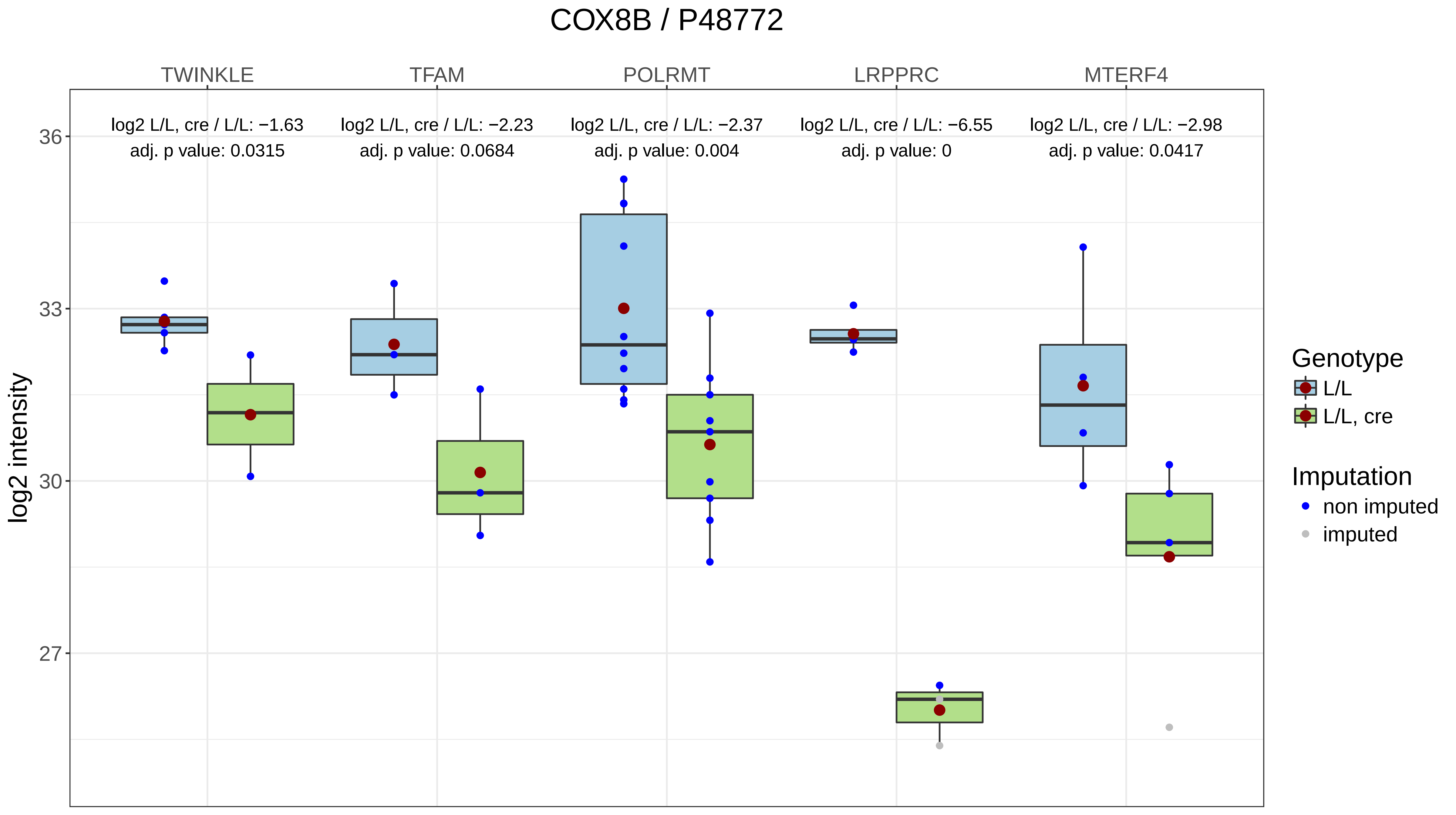
COX8B / P48772
TWINKLE
TFAM
POLRMT
LRPPRC
MTERF4
log2 L/L, cre / L/L: −1.63
adj. p value: 0.0315
log2 L/L, cre / L/L: −2.23
adj. p value: 0.0684
log2 L/L, cre / L/L: −2.37
adj. p value: 0.004
log2 L/L, cre / L/L: −6.55
adj. p value: 0
log2 L/L, cre / L/L: −2.98
adj. p value: 0.0417
log2 intensity
36
33
30
27
Genotype
L/L
L/L, cre
Imputation
non imputed
imputed

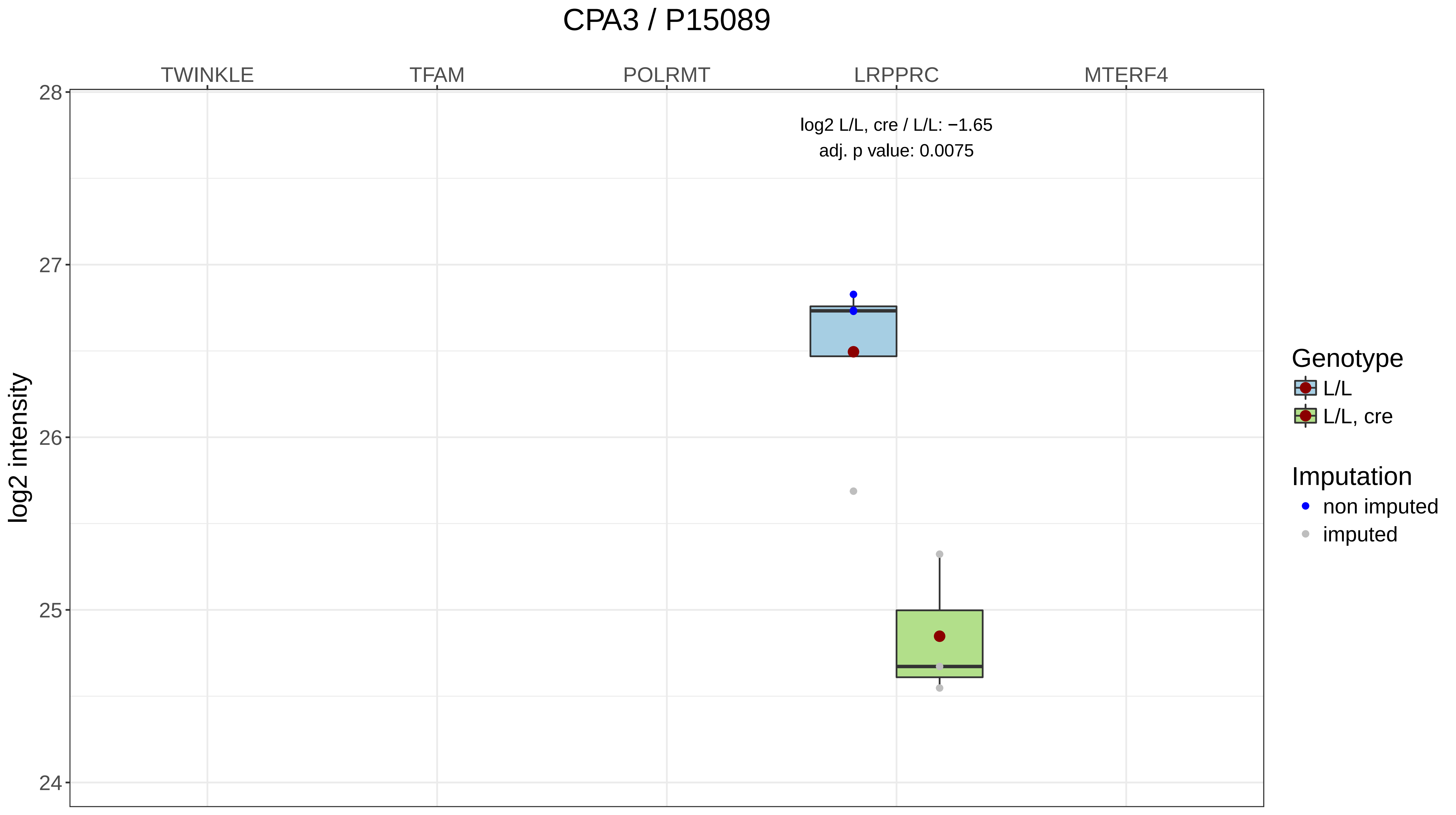

CPA3 / P15089
TWINKLE
TFAM
POLRMT
LRPPRC
MTERF4
log2 L/L, cre / L/L: −1.65
adj. p value: 0.0075
log2 intensity
28
27
26
25
24
Genotype
L/L
L/L, cre
Imputation
non imputed
imputed

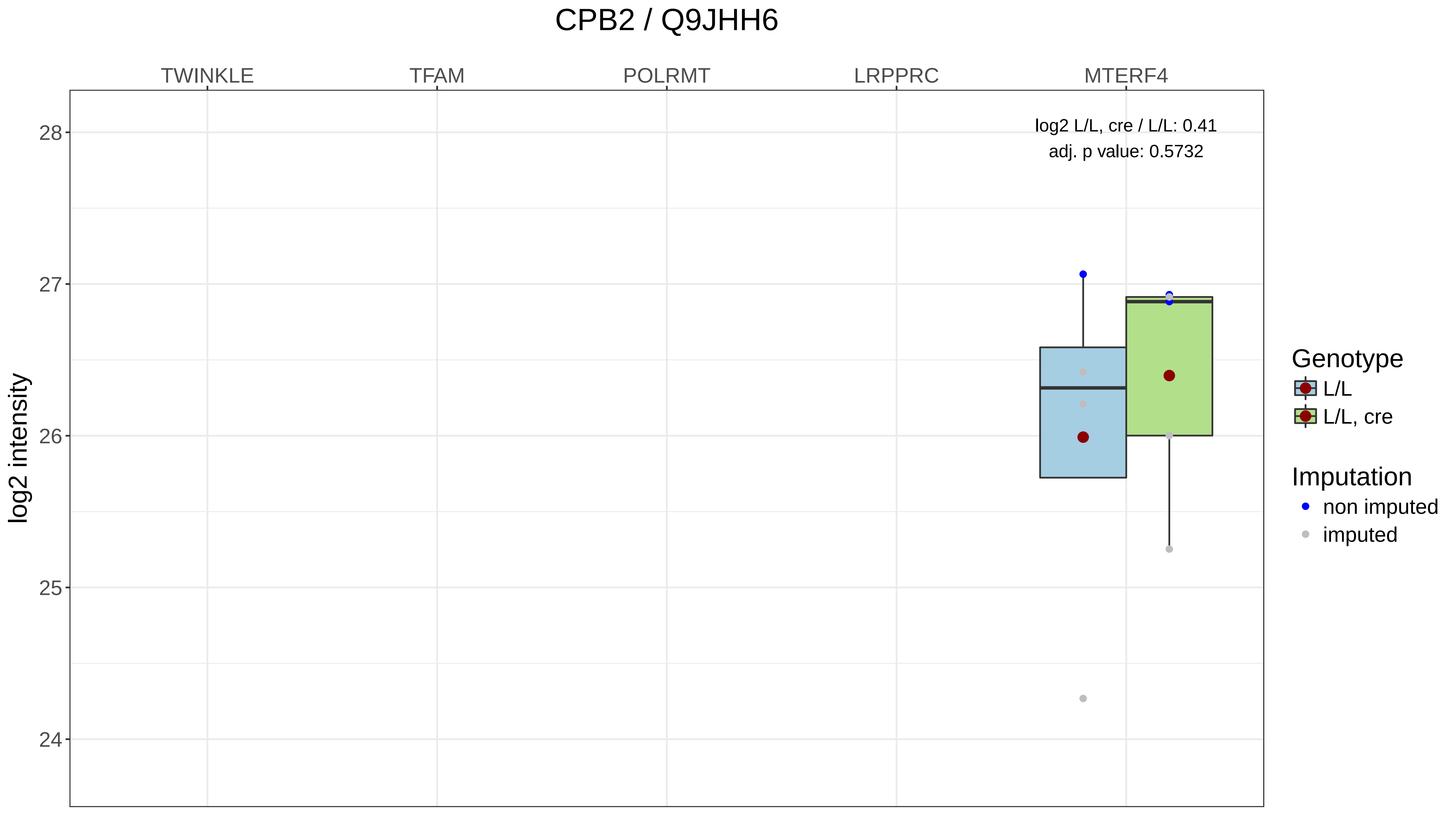
CPB2 / Q9JHH6
TWINKLE
TFAM
POLRMT
LRPPRC
MTERF4
log2 L/L, cre / L/L: 0.41
adj. p value: 0.5732
log2 intensity
28
27
26
25
24
Genotype
L/L
L/L, cre
Imputation
non imputed
imputed

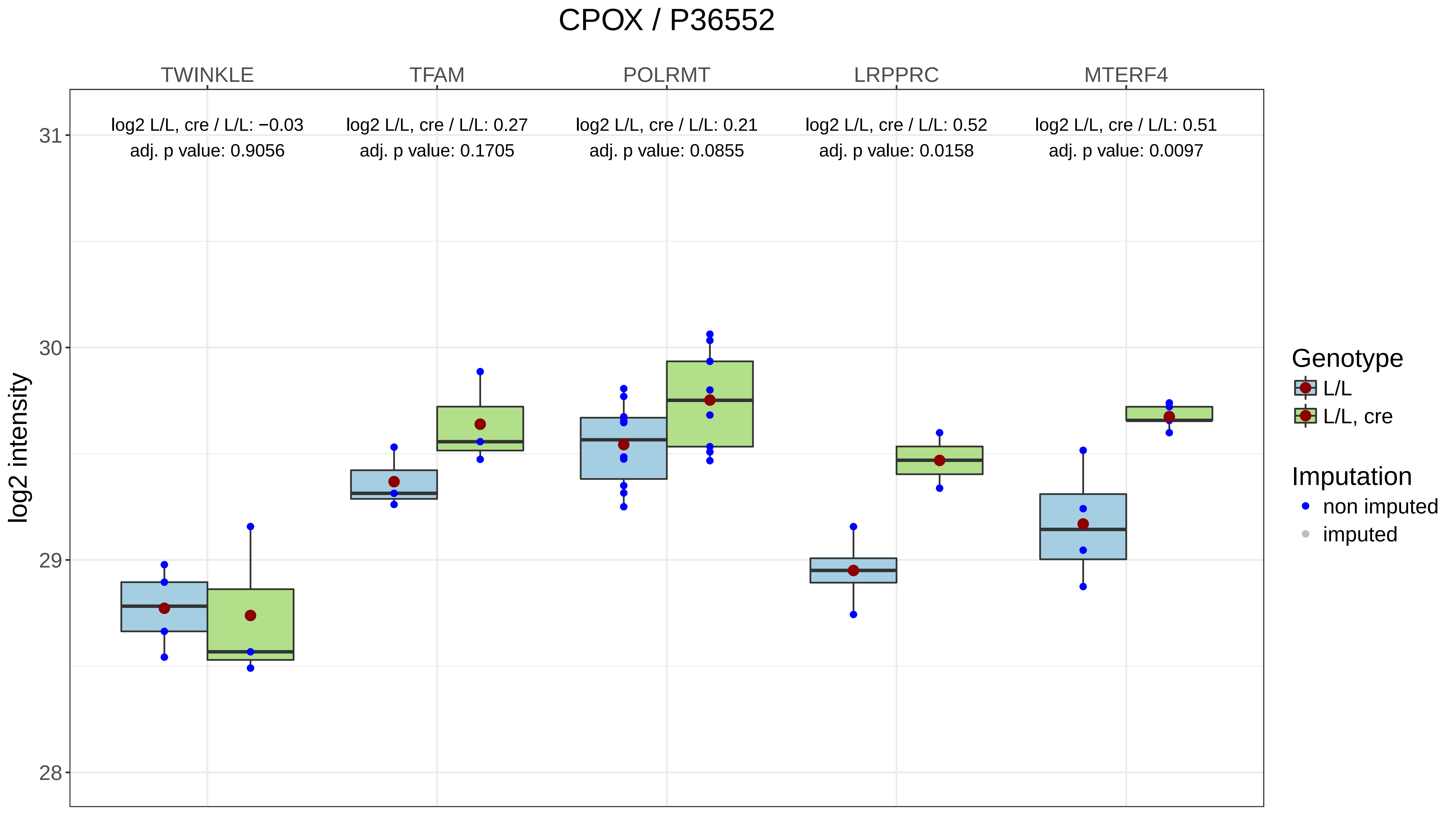
CPOX / P36552
TWINKLE
TFAM
POLRMT
LRPPRC
MTERF4
log2 L/L, cre / L/L: −0.03
adj. p value: 0.9056
log2 L/L, cre / L/L: 0.27
adj. p value: 0.1705
log2 L/L, cre / L/L: 0.21
adj. p value: 0.0855
log2 L/L, cre / L/L: 0.52
adj. p value: 0.0158
log2 L/L, cre / L/L: 0.51
adj. p value: 0.0097
log2 intensity
31
30
29
28
Genotype
L/L
L/L, cre
Imputation
non imputed
imputed

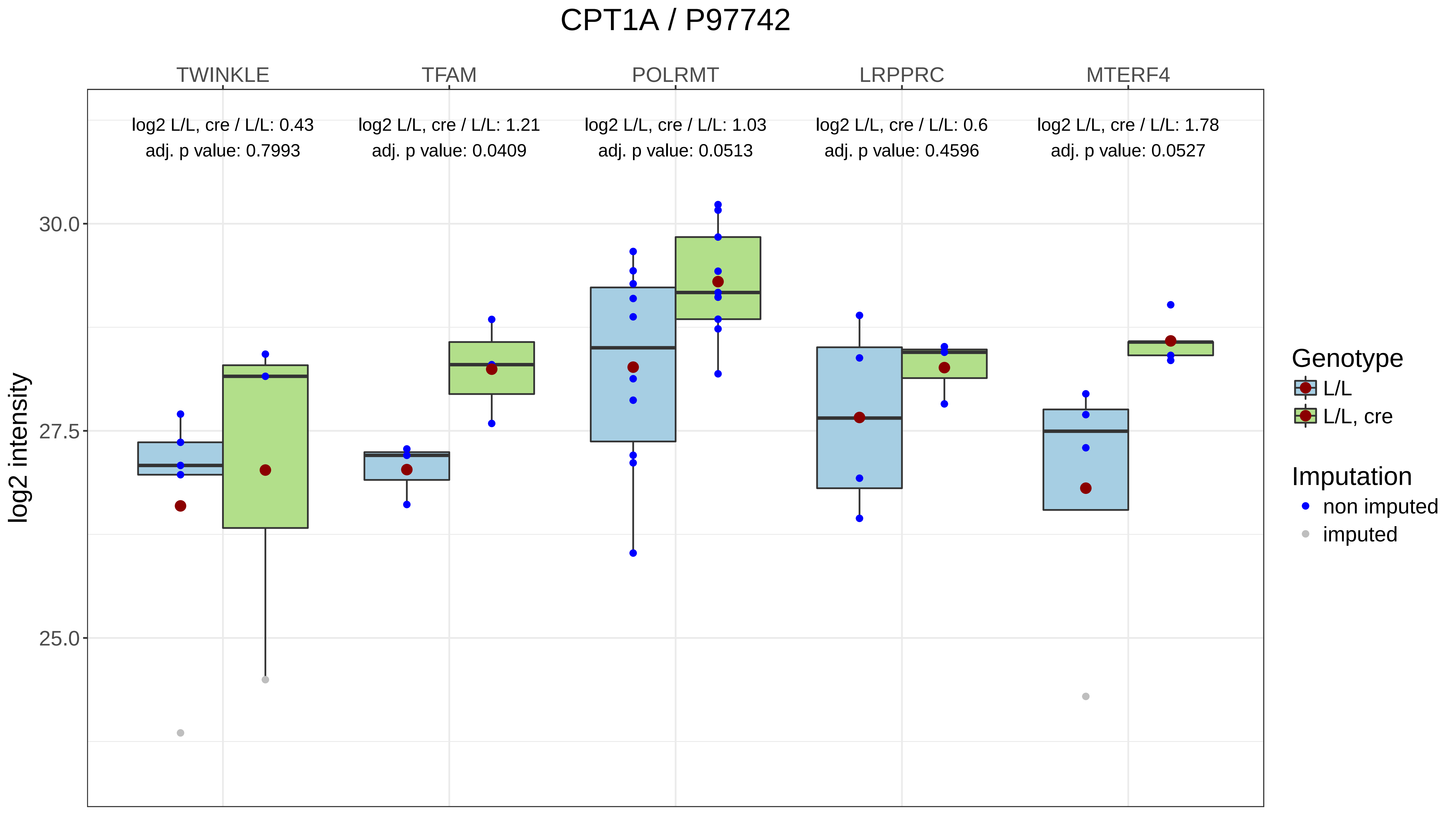
CPT1A / P97742
TWINKLE
TFAM
POLRMT
LRPPRC
MTERF4
log2 L/L, cre / L/L: 0.43
adj. p value: 0.7993
log2 L/L, cre / L/L: 1.21
adj. p value: 0.0409
log2 L/L, cre / L/L: 1.03
adj. p value: 0.0513
log2 L/L, cre / L/L: 0.6
adj. p value: 0.4596
log2 L/L, cre / L/L: 1.78
adj. p value: 0.0527
30.0
27.5
25.0
log2 intensity
Genotype
L/L
L/L, cre
Imputation
non imputed
imputed

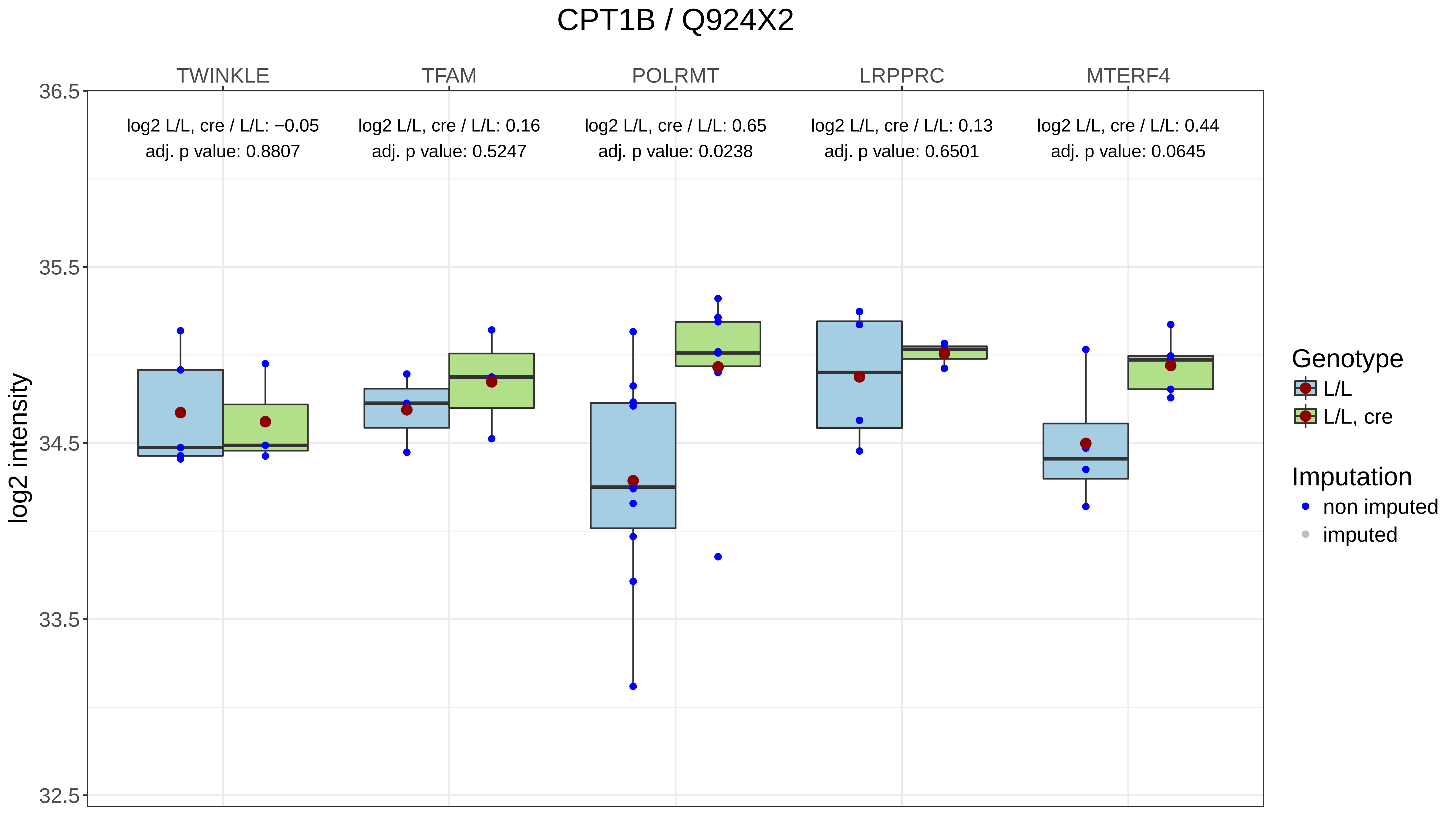
CPT1B / Q924X2
TWINKLE
TFAM
POLRMT
LRPPRC
MTERF4
log2 L/L, cre / L/L: −0.05
adj. p value: 0.8807
log2 L/L, cre / L/L: 0.16
adj. p value: 0.5247
log2 L/L, cre / L/L: 0.65
adj. p value: 0.0238
log2 L/L, cre / L/L: 0.13
adj. p value: 0.6501
log2 L/L, cre / L/L: 0.44
adj. p value: 0.0645
log2 intensity
36.5
35.5
34.5
33.5
32.5
Genotype
L/L
L/L, cre
Imputation
non imputed
imputed

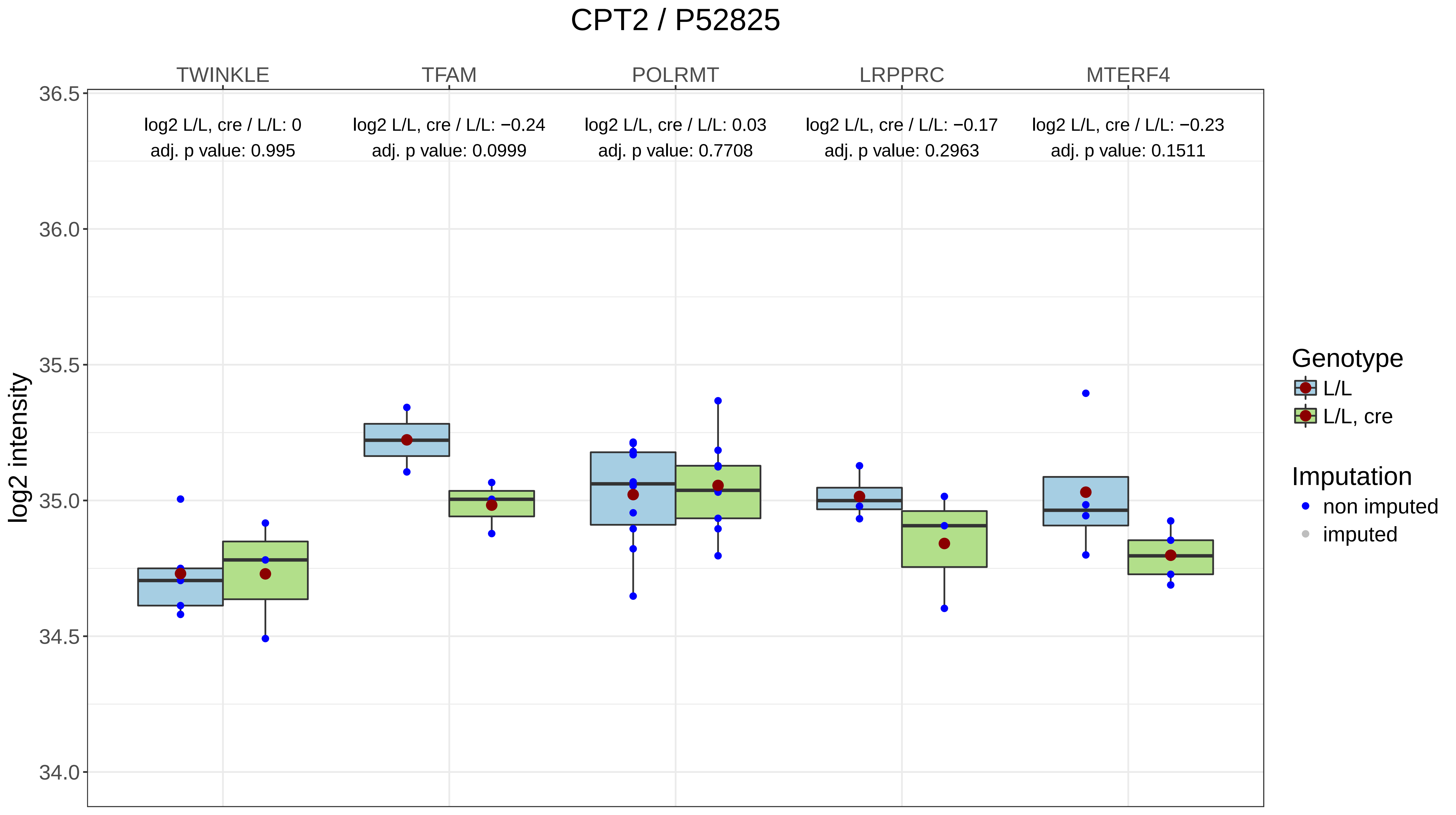
CPT2 / P52825
TWINKLE
TFAM
POLRMT
LRPPRC
MTERF4
log2 L/L, cre / L/L: 0
adj. p value: 0.995
log2 L/L, cre / L/L: −0.24
adj. p value: 0.0999
log2 L/L, cre / L/L: 0.03
adj. p value: 0.7708
log2 L/L, cre / L/L: −0.17
adj. p value: 0.2963
log2 L/L, cre / L/L: −0.23
adj. p value: 0.1511
log2 intensity
36.5
36.0
35.5
35.0
34.5
34.0
Genotype
L/L
L/L, cre
Imputation
non imputed
imputed

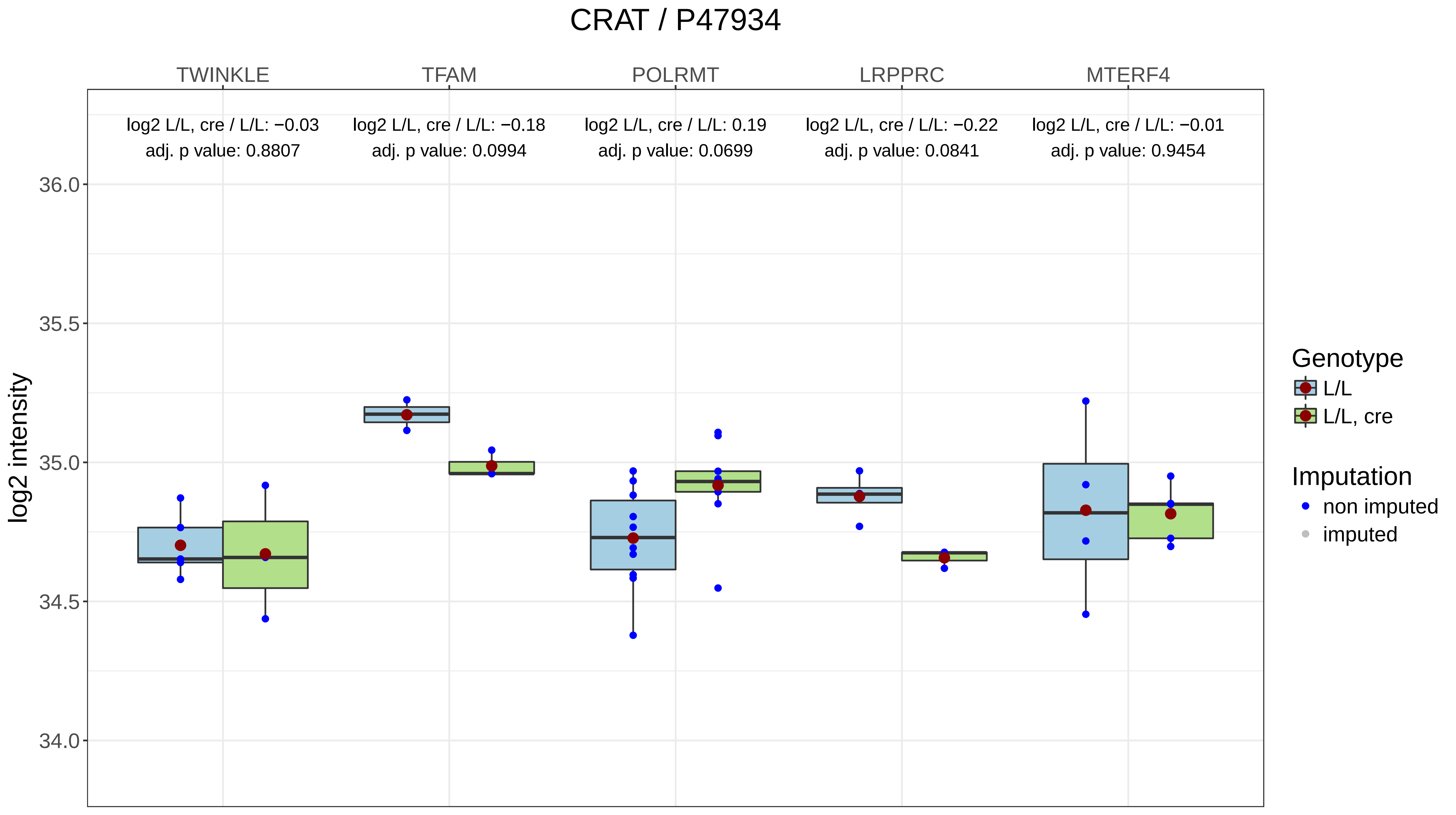
CRAT / P47934
TWINKLE
TFAM
POLRMT
LRPPRC
MTERF4
log2 L/L, cre / L/L: −0.03
adj. p value: 0.8807
log2 L/L, cre / L/L: −0.18
adj. p value: 0.0994
log2 L/L, cre / L/L: 0.19
adj. p value: 0.0699
log2 L/L, cre / L/L: −0.22
adj. p value: 0.0841
log2 L/L, cre / L/L: −0.01
adj. p value: 0.9454
log2 intensity
36.0
35.5
35.0
34.5
34.0
Genotype
L/L
L/L, cre
Imputation
non imputed
imputed

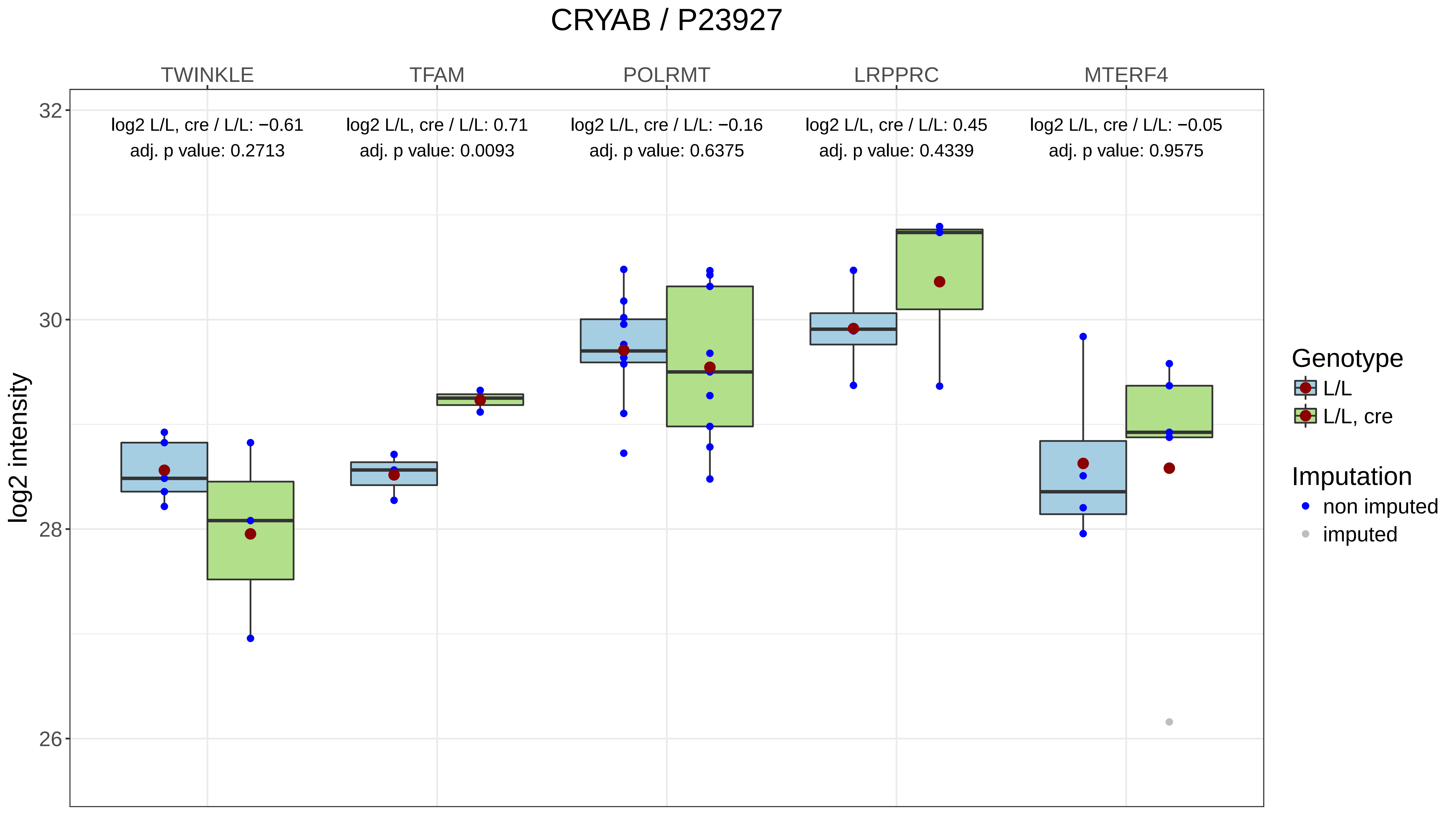
CRYAB / P23927
TWINKLE
TFAM
POLRMT
LRPPRC
MTERF4
log2 L/L, cre / L/L: −0.61
adj. p value: 0.2713
log2 L/L, cre / L/L: 0.71
adj. p value: 0.0093
log2 L/L, cre / L/L: −0.16
adj. p value: 0.6375
log2 L/L, cre / L/L: 0.45
adj. p value: 0.4339
log2 L/L, cre / L/L: −0.05
adj. p value: 0.9575
log2 intensity
32
30
28
26
Genotype
L/L
L/L, cre
Imputation
non imputed
imputed

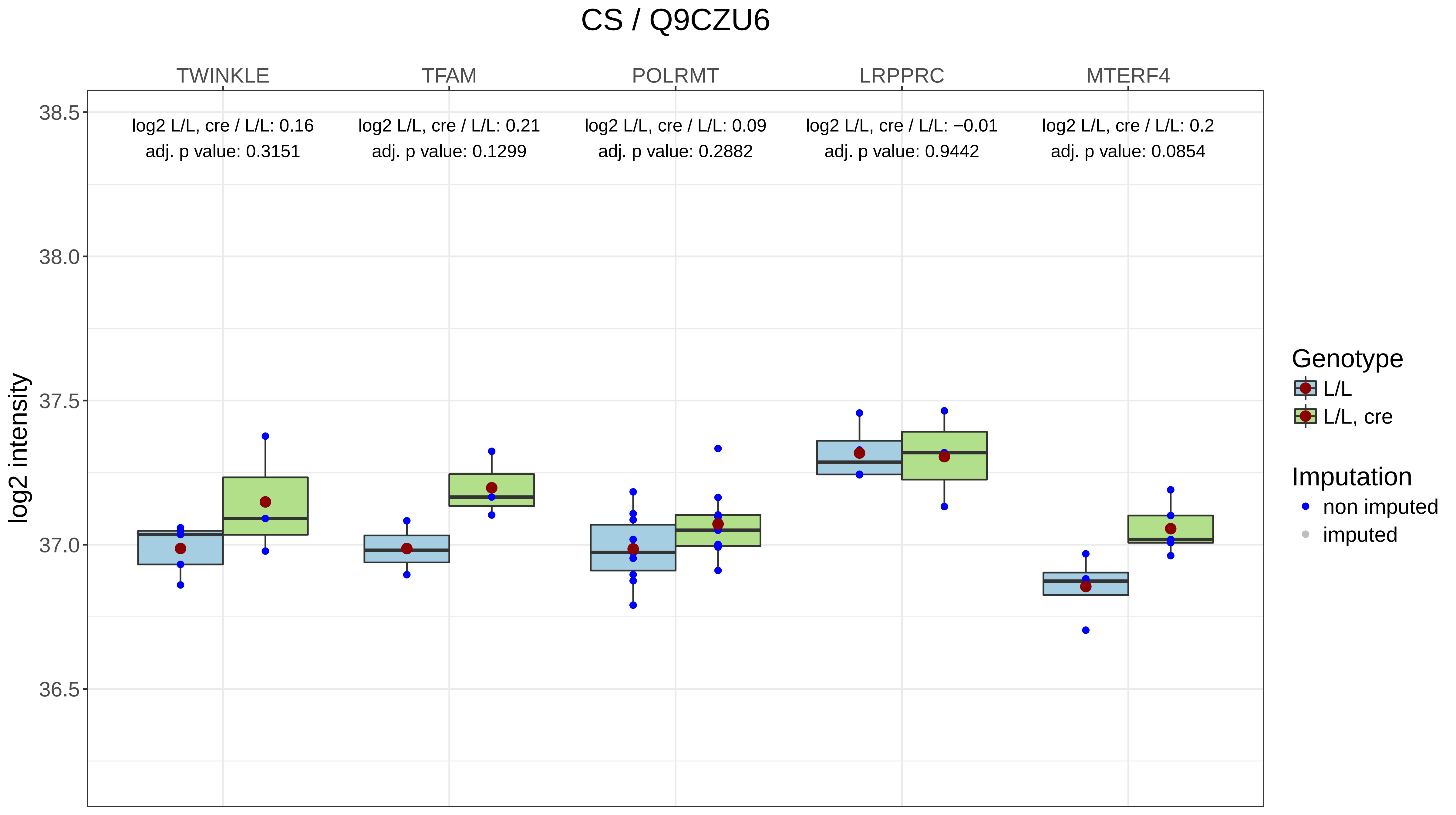
CS / Q9CZU6
TWINKLE
TFAM
POLRMT
LRPPRC
MTERF4
log2 L/L, cre / L/L: 0.16
adj. p value: 0.3151
log2 L/L, cre / L/L: 0.21
adj. p value: 0.1299
log2 L/L, cre / L/L: 0.09
adj. p value: 0.2882
log2 L/L, cre / L/L: −0.01
adj. p value: 0.9442
log2 L/L, cre / L/L: 0.2
adj. p value: 0.0854
log2 intensity
38.5
38.0
37.5
37.0
36.5
Genotype
L/L
L/L, cre
Imputation
non imputed
imputed

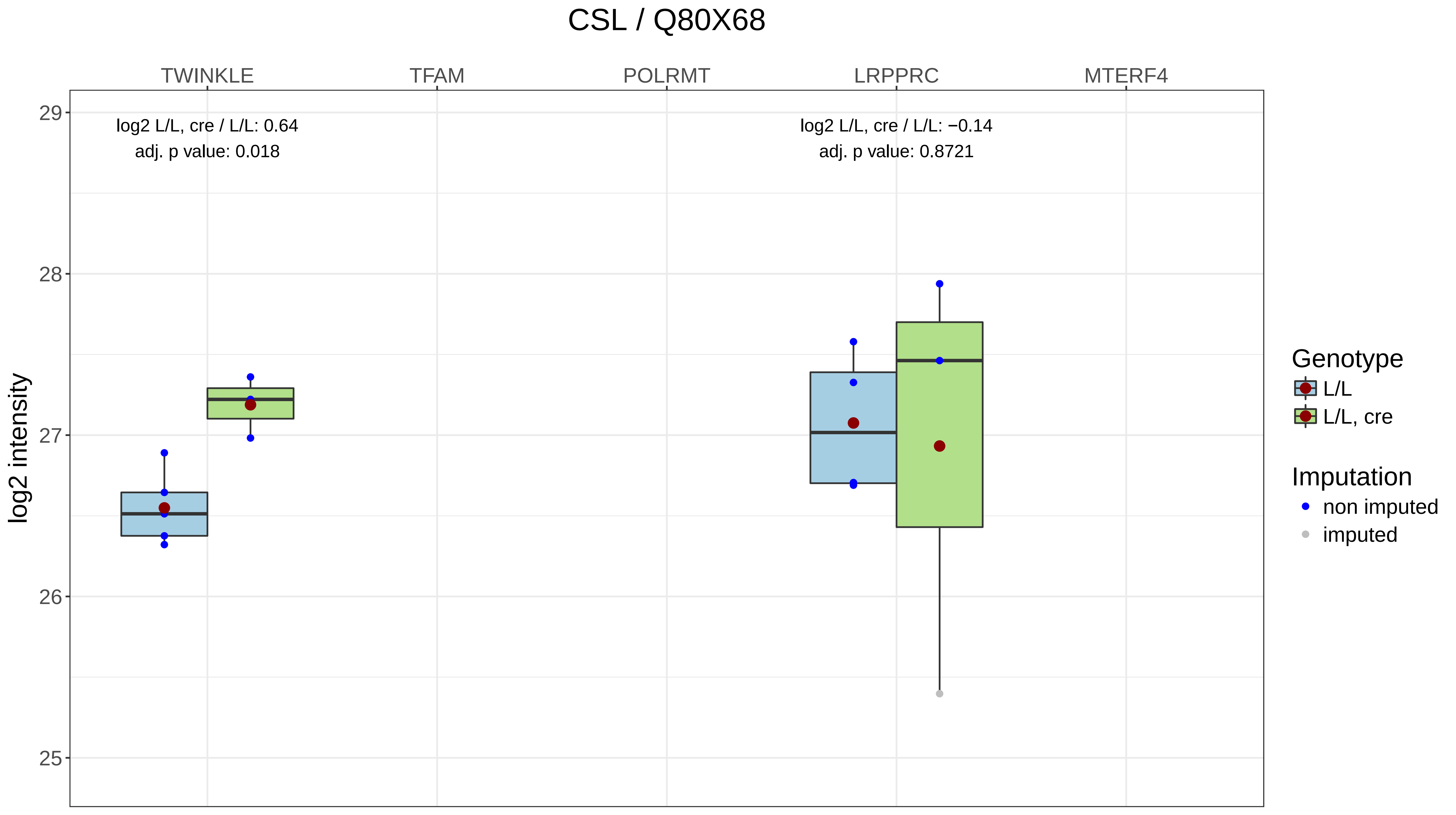
CSL / Q80X68
TWINKLE
TFAM
POLRMT
LRPPRC
MTERF4
log2 L/L, cre / L/L: 0.64
adj. p value: 0.018
log2 L/L, cre / L/L: −0.14
adj. p value: 0.8721
29
28
27
26
25
log2 intensity
Genotype
L/L
L/L, cre
Imputation
non imputed
imputed

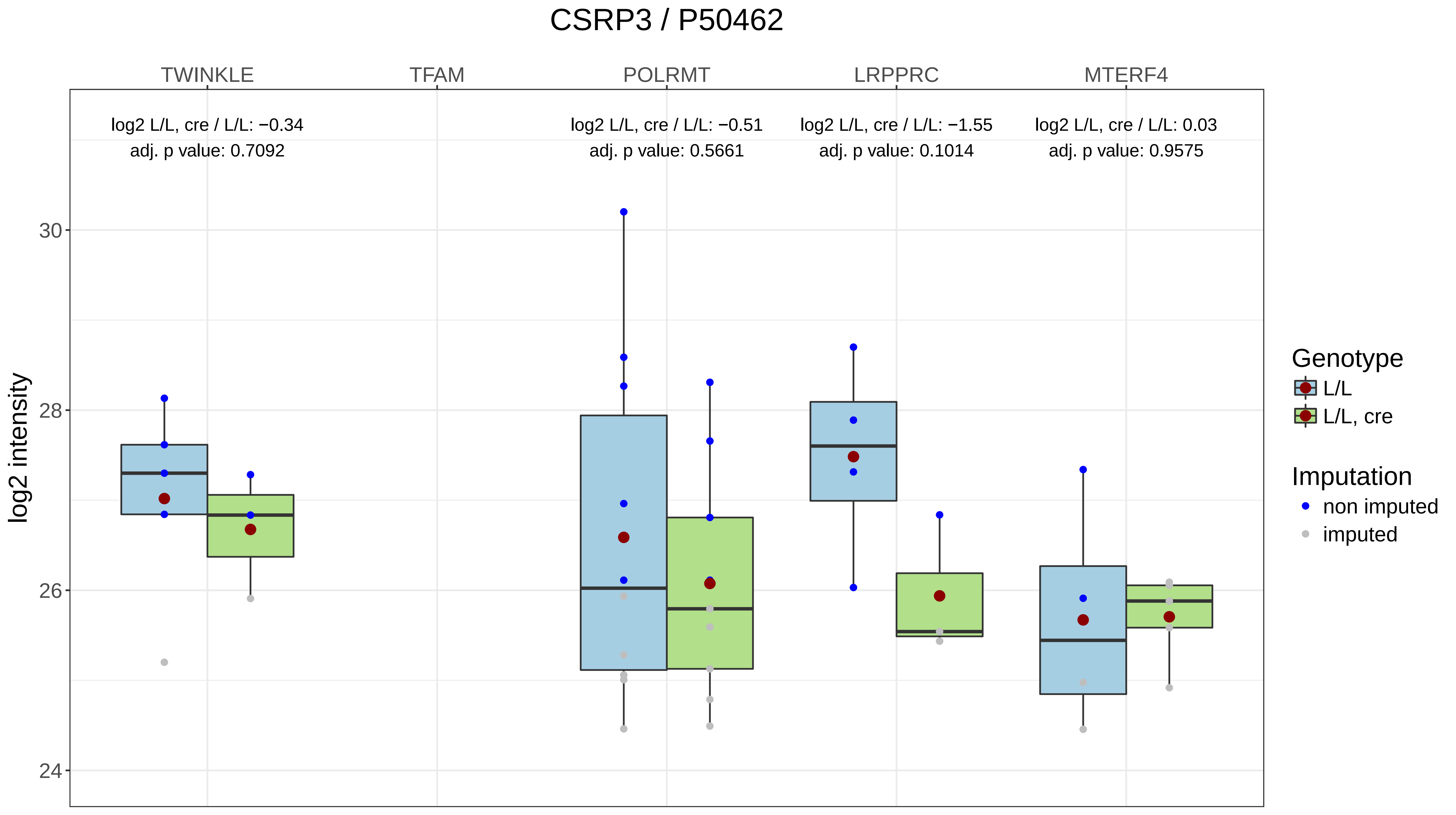
CSRP3 / P50462
TWINKLE
TFAM
POLRMT
LRPPRC
MTERF4
log2 L/L, cre / L/L: −0.34
adj. p value: 0.7092
log2 L/L, cre / L/L: −0.51
adj. p value: 0.5661
log2 L/L, cre / L/L: −1.55
adj. p value: 0.1014
log2 L/L, cre / L/L: 0.03
adj. p value: 0.9575
log2 intensity
30
28
26
24
Genotype
L/L
L/L, cre
Imputation
non imputed
imputed

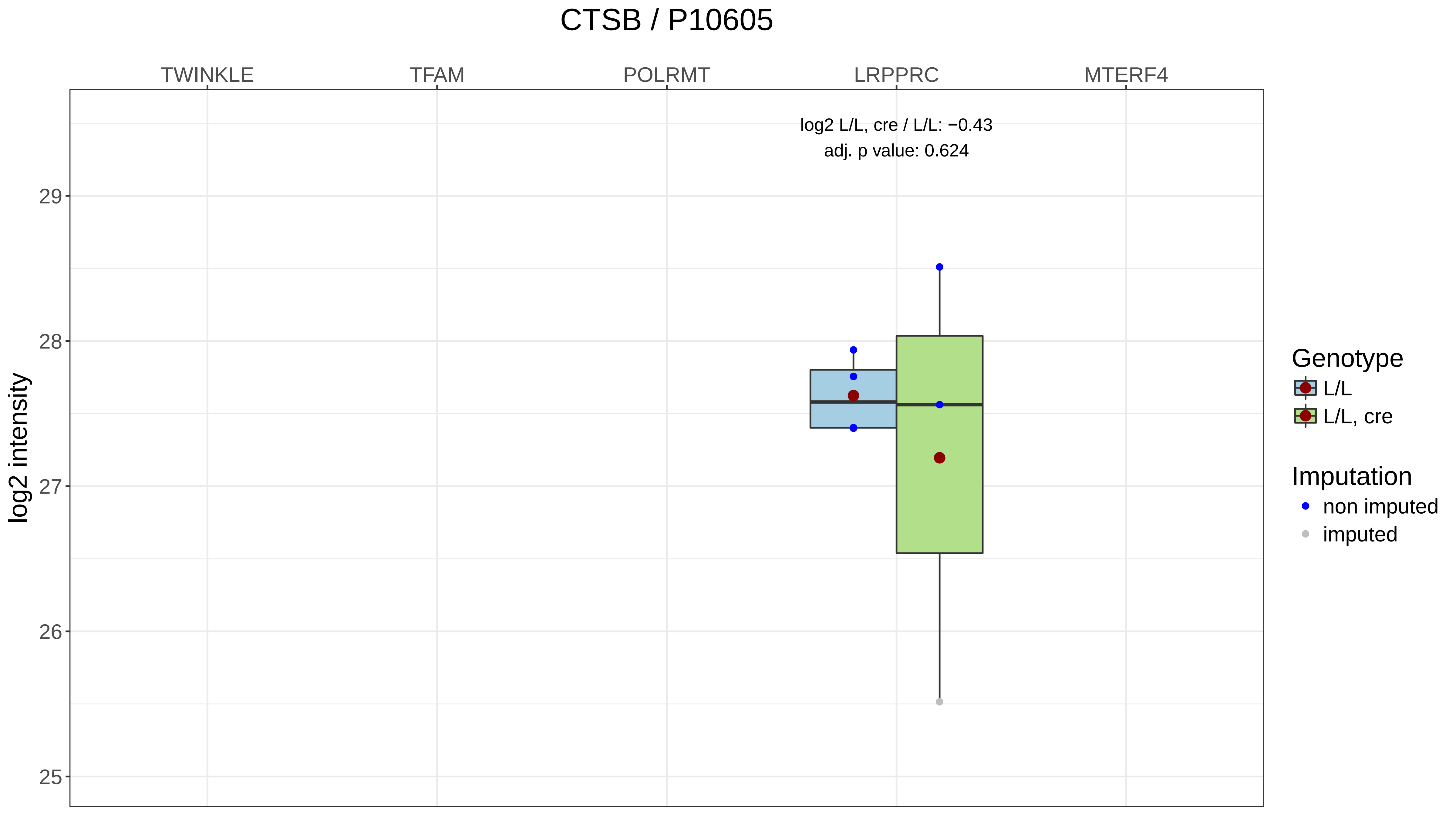

CTSB / P10605
TWINKLE
TFAM
POLRMT
LRPPRC
MTERF4
log2 L/L, cre / L/L: −0.43
adj. p value: 0.624
log2 intensity
29
28
27
26
25
Genotype
L/L
L/L, cre
Imputation
non imputed
imputed

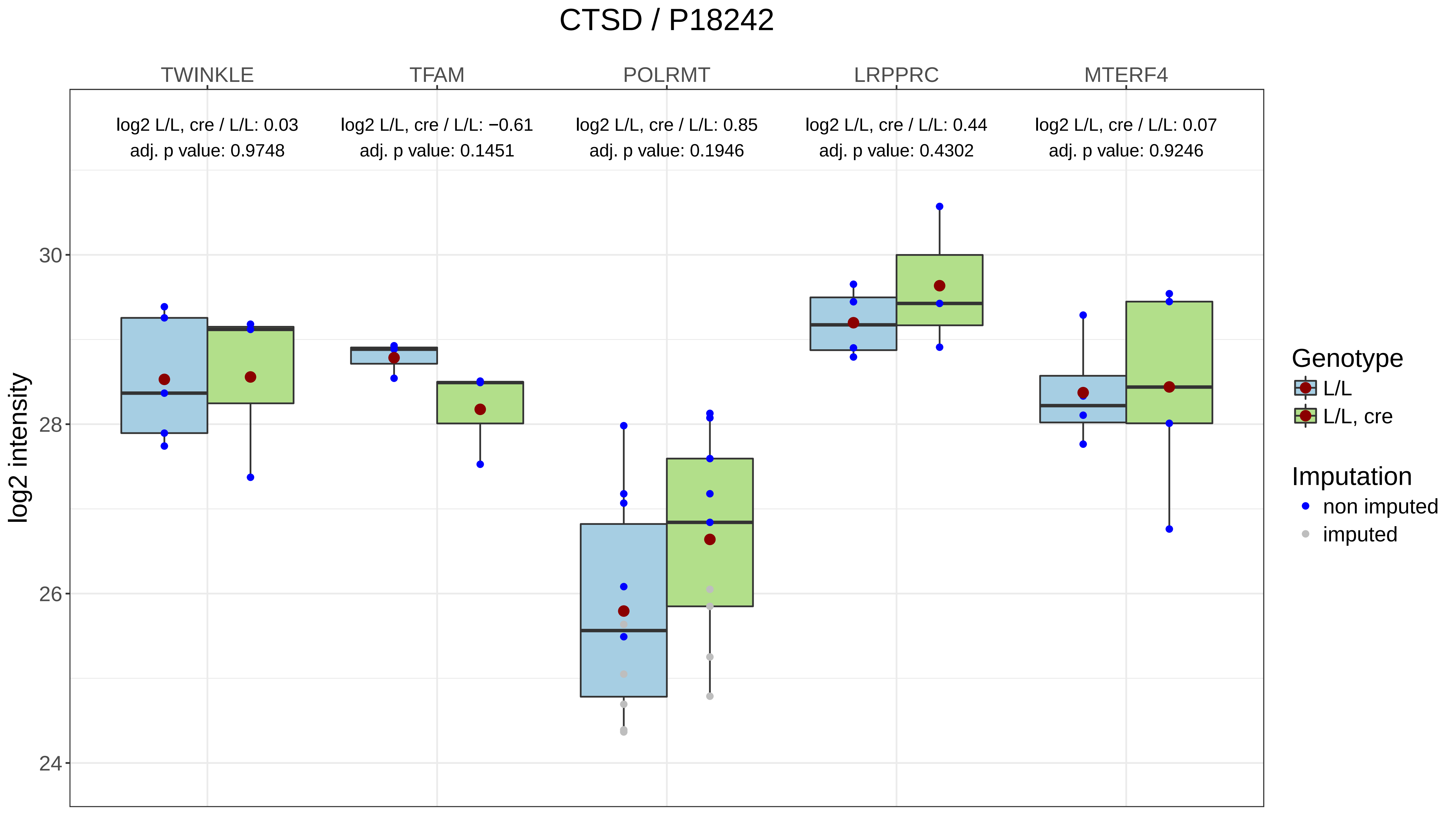

CTSD / P18242
TWINKLE
TFAM
POLRMT
LRPPRC
MTERF4
log2 L/L, cre / L/L: 0.03
adj. p value: 0.9748
log2 L/L, cre / L/L: −0.61
adj. p value: 0.1451
log2 L/L, cre / L/L: 0.85
adj. p value: 0.1946
log2 L/L, cre / L/L: 0.44
adj. p value: 0.4302
log2 L/L, cre / L/L: 0.07
adj. p value: 0.9246
log2 intensity
30
28
26
24
Genotype
L/L
L/L, cre
Imputation
non imputed
imputed

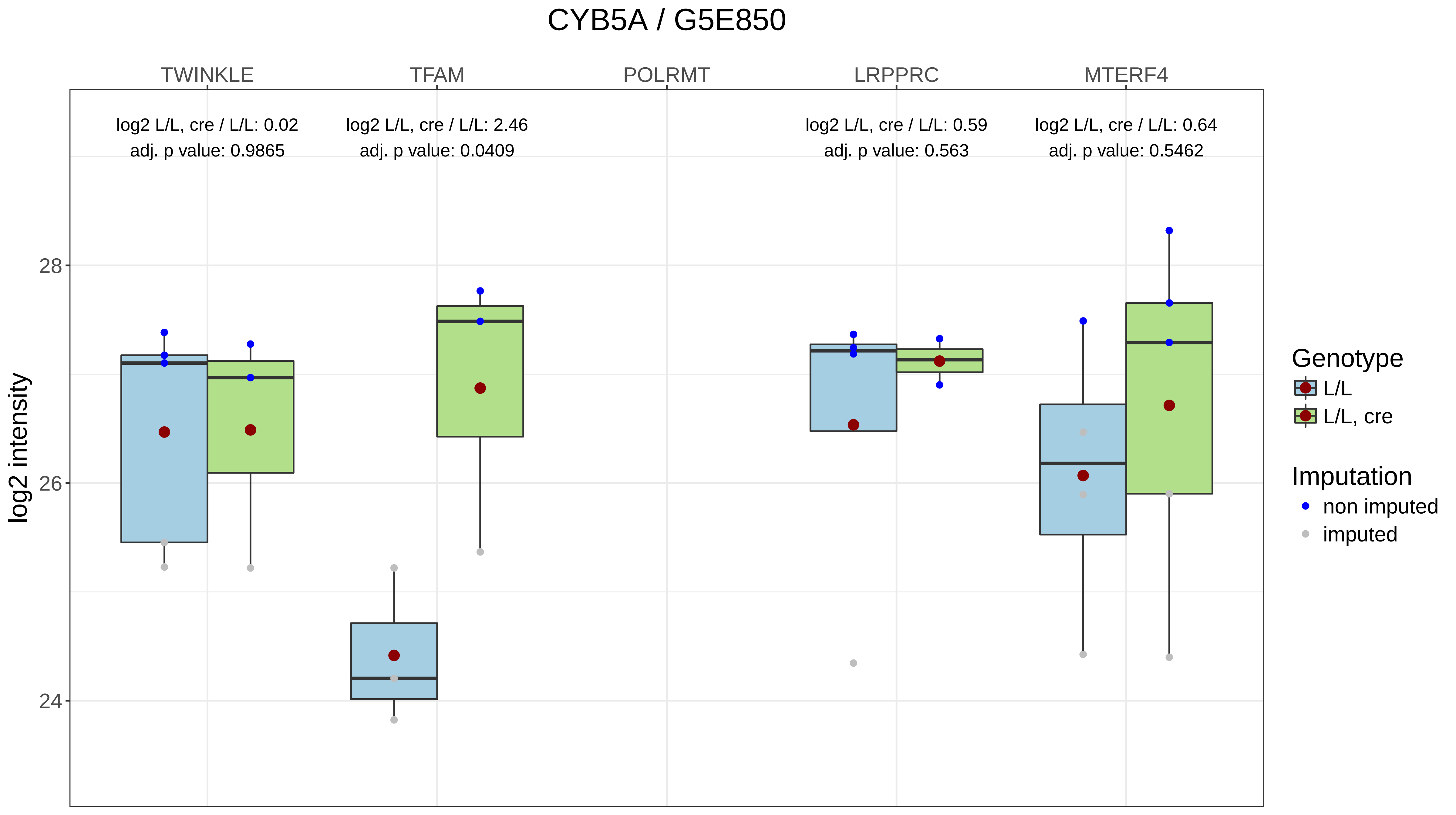
CYB5A / G5E850
TWINKLE
TFAM
POLRMT
LRPPRC
MTERF4
log2 L/L, cre / L/L: 0.02
adj. p value: 0.9865
log2 L/L, cre / L/L: 2.46
adj. p value: 0.0409
log2 L/L, cre / L/L: 0.59
adj. p value: 0.563
log2 L/L, cre / L/L: 0.64
adj. p value: 0.5462
log2 intensity
28
26
24
Genotype
L/L
L/L, cre
Imputation
non imputed
imputed

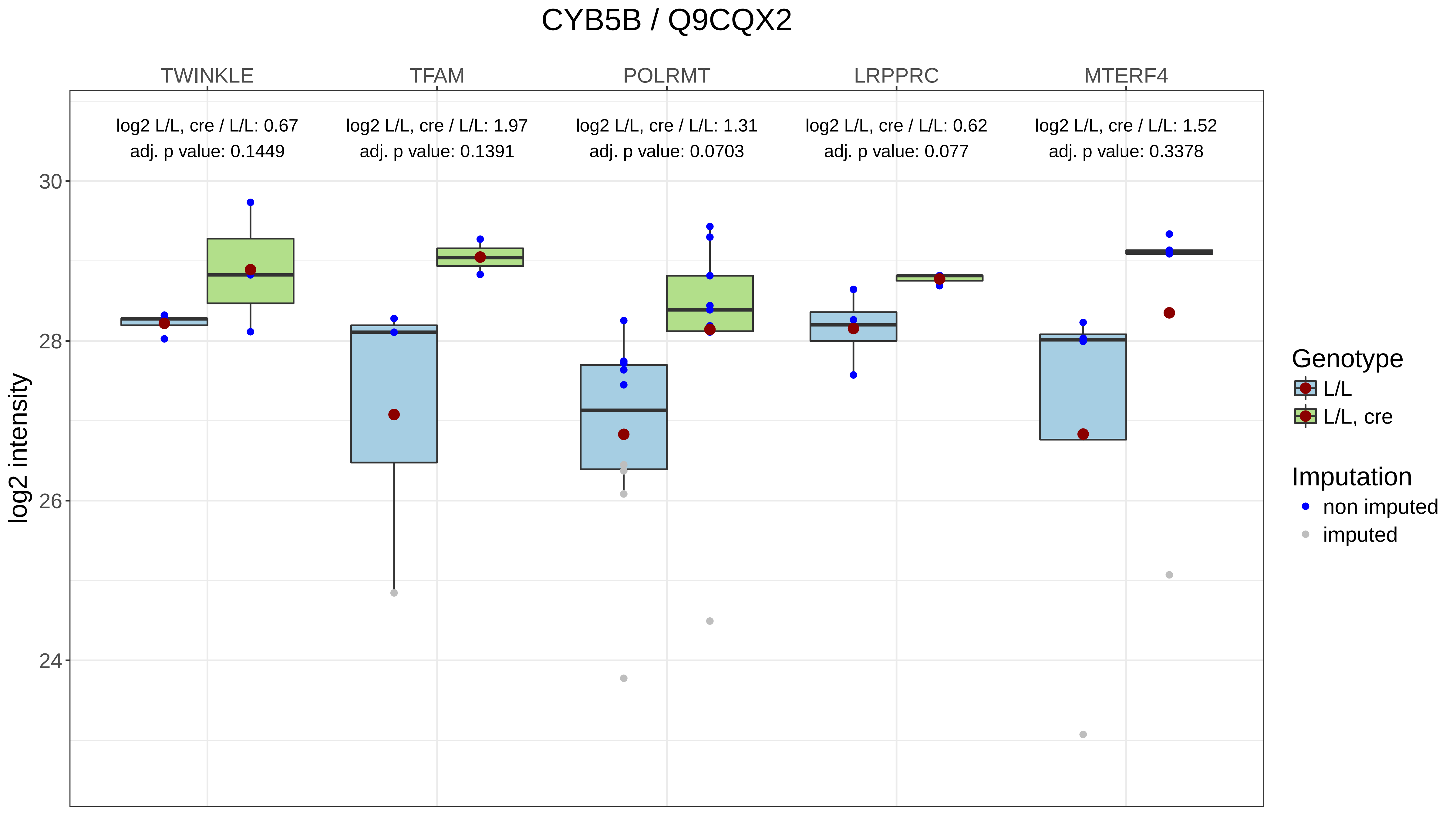
CYB5B / Q9CQX2
TWINKLE
TFAM
POLRMT
LRPPRC
MTERF4
log2 L/L, cre / L/L: 0.67
adj. p value: 0.1449
log2 L/L, cre / L/L: 1.97
adj. p value: 0.1391
log2 L/L, cre / L/L: 1.31
adj. p value: 0.0703
log2 L/L, cre / L/L: 0.62
adj. p value: 0.077
log2 L/L, cre / L/L: 1.52
adj. p value: 0.3378
log2 intensity
30
28
26
24
Genotype
L/L
L/L, cre
Imputation
non imputed
imputed

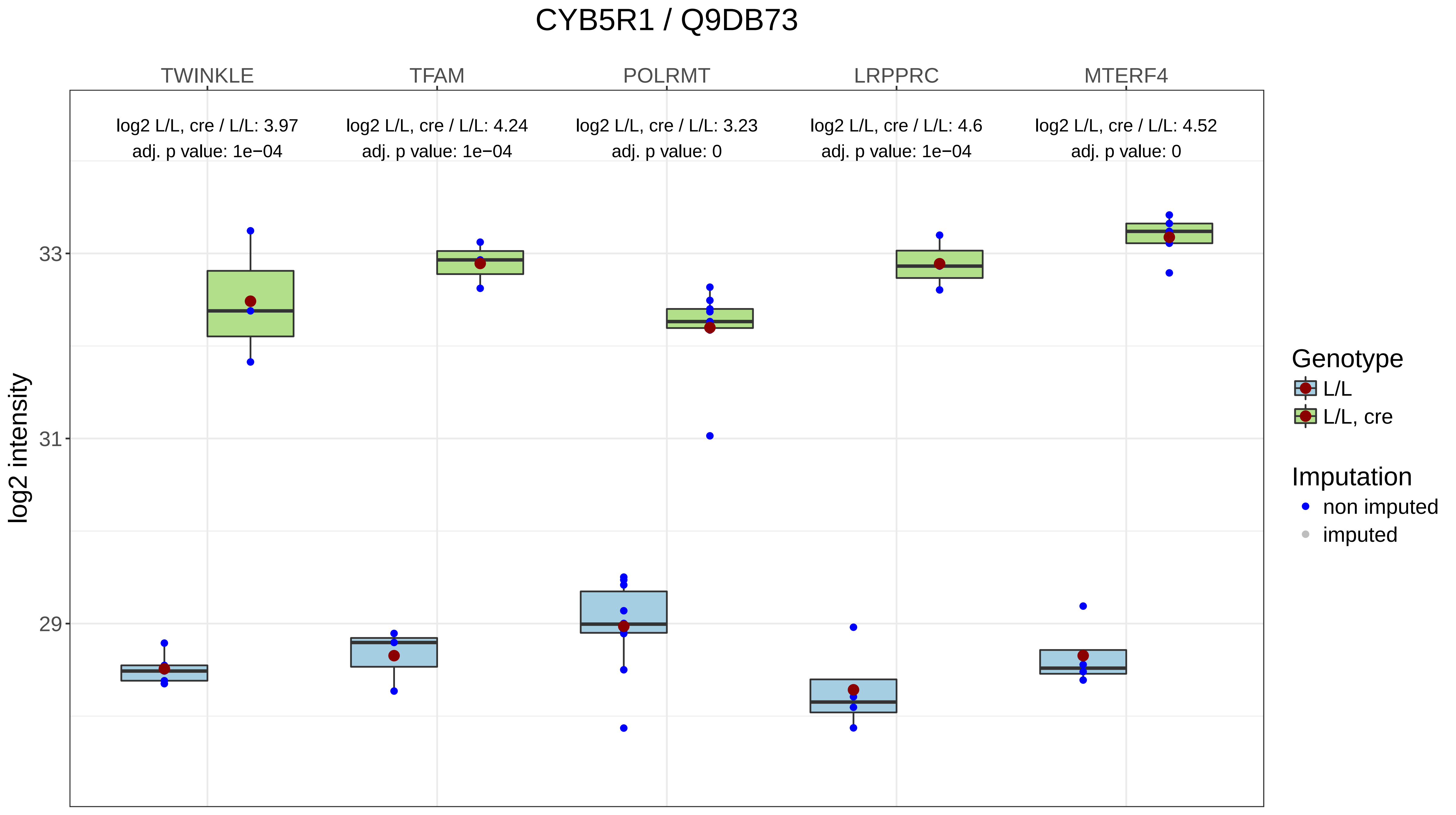
CYB5R1 / Q9DB73
TWINKLE
TFAM
POLRMT
LRPPRC
MTERF4
log2 L/L, cre / L/L: 3.97
adj. p value: 1e−04
log2 L/L, cre / L/L: 4.24
adj. p value: 1e−04
log2 L/L, cre / L/L: 3.23
adj. p value: 0
log2 L/L, cre / L/L: 4.6
adj. p value: 1e−04
log2 L/L, cre / L/L: 4.52
adj. p value: 0
33
31
29
log2 intensity
Genotype
L/L
L/L, cre
Imputation
non imputed
imputed

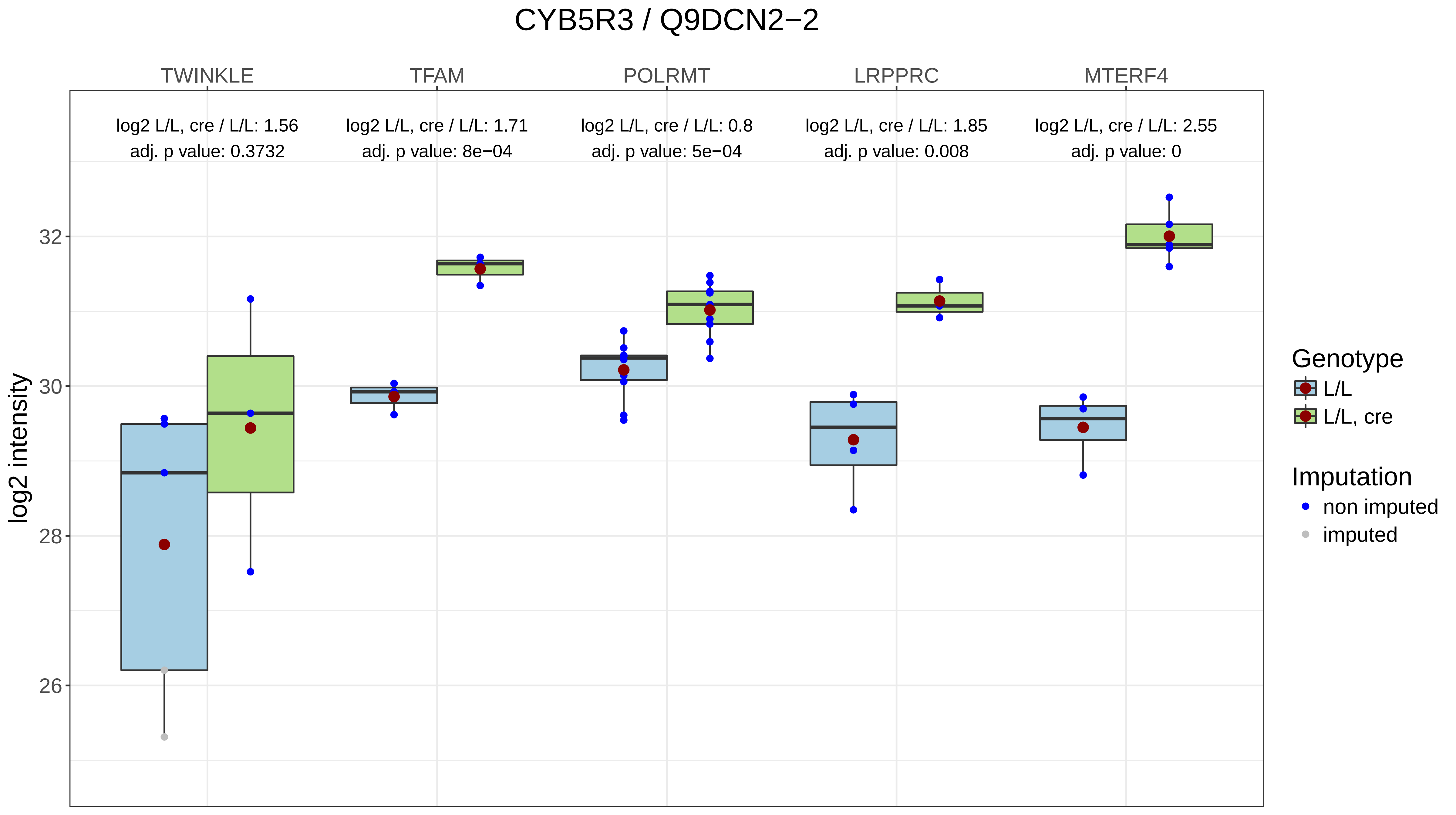
CYB5R3 / Q9DCN2−2
TWINKLE
TFAM
POLRMT
LRPPRC
MTERF4
log2 L/L, cre / L/L: 1.56
adj. p value: 0.3732
log2 L/L, cre / L/L: 1.71
adj. p value: 8e−04
log2 L/L, cre / L/L: 0.8
adj. p value: 5e−04
log2 L/L, cre / L/L: 1.85
adj. p value: 0.008
log2 L/L, cre / L/L: 2.55
adj. p value: 0
log2 intensity
32
30
28
26
Genotype
L/L
L/L, cre
Imputation
non imputed
imputed

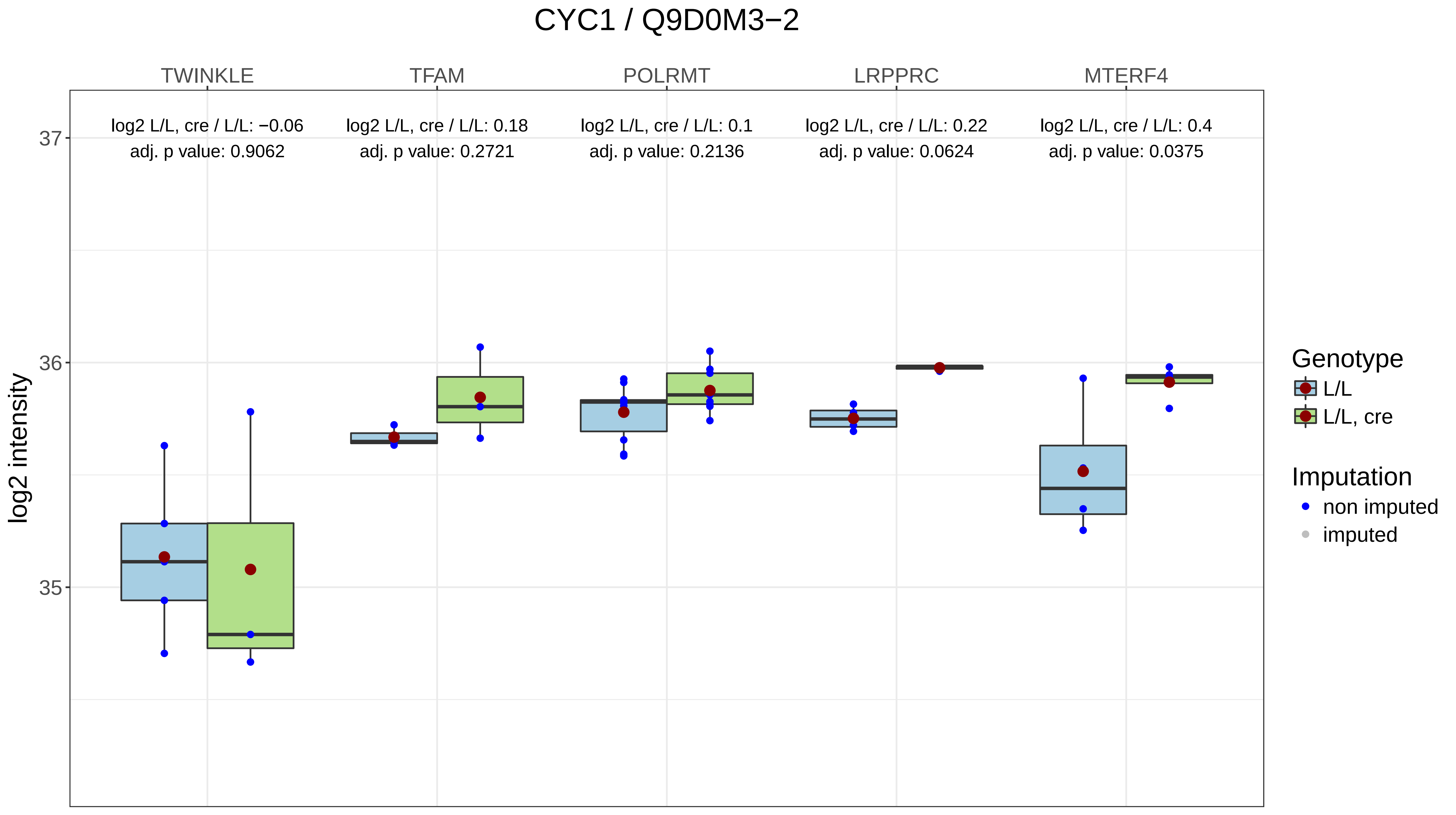
CYC1 / Q9D0M3−2
TWINKLE
TFAM
POLRMT
LRPPRC
MTERF4
log2 L/L, cre / L/L: −0.06
adj. p value: 0.9062
log2 L/L, cre / L/L: 0.18
adj. p value: 0.2721
log2 L/L, cre / L/L: 0.1
adj. p value: 0.2136
log2 L/L, cre / L/L: 0.22
adj. p value: 0.0624
log2 L/L, cre / L/L: 0.4
adj. p value: 0.0375
log2 intensity
37
36
35
Genotype
L/L
L/L, cre
Imputation
non imputed
imputed

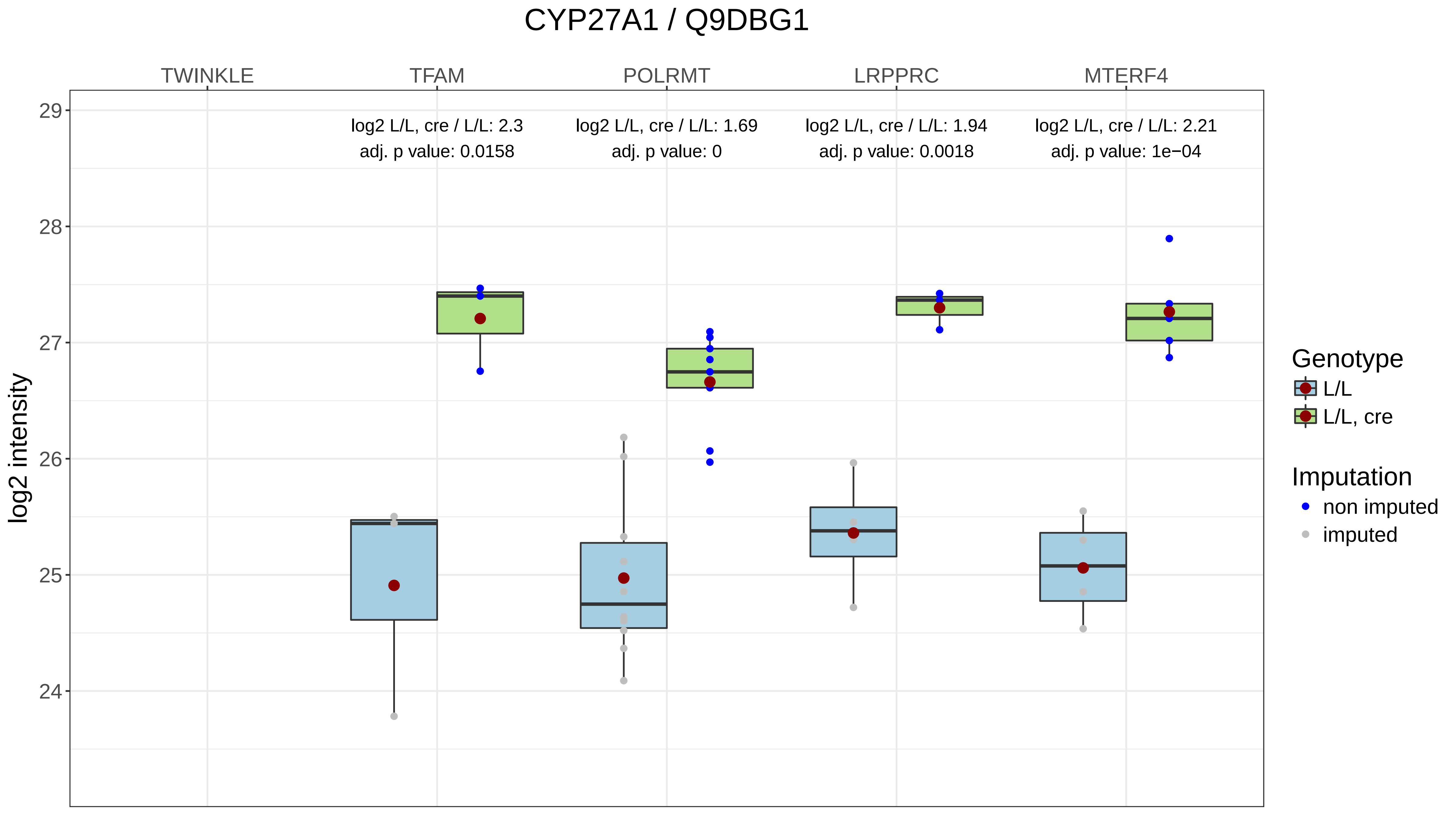
CYP27A1 / Q9DBG1
TWINKLE
TFAM
POLRMT
LRPPRC
MTERF4
log2 L/L, cre / L/L: 2.3
adj. p value: 0.0158
log2 L/L, cre / L/L: 1.69
adj. p value: 0
log2 L/L, cre / L/L: 1.94
adj. p value: 0.0018
log2 L/L, cre / L/L: 2.21
adj. p value: 1e−04
log2 intensity
29
28
27
26
25
24
Genotype
L/L
L/L, cre
Imputation
non imputed
imputed

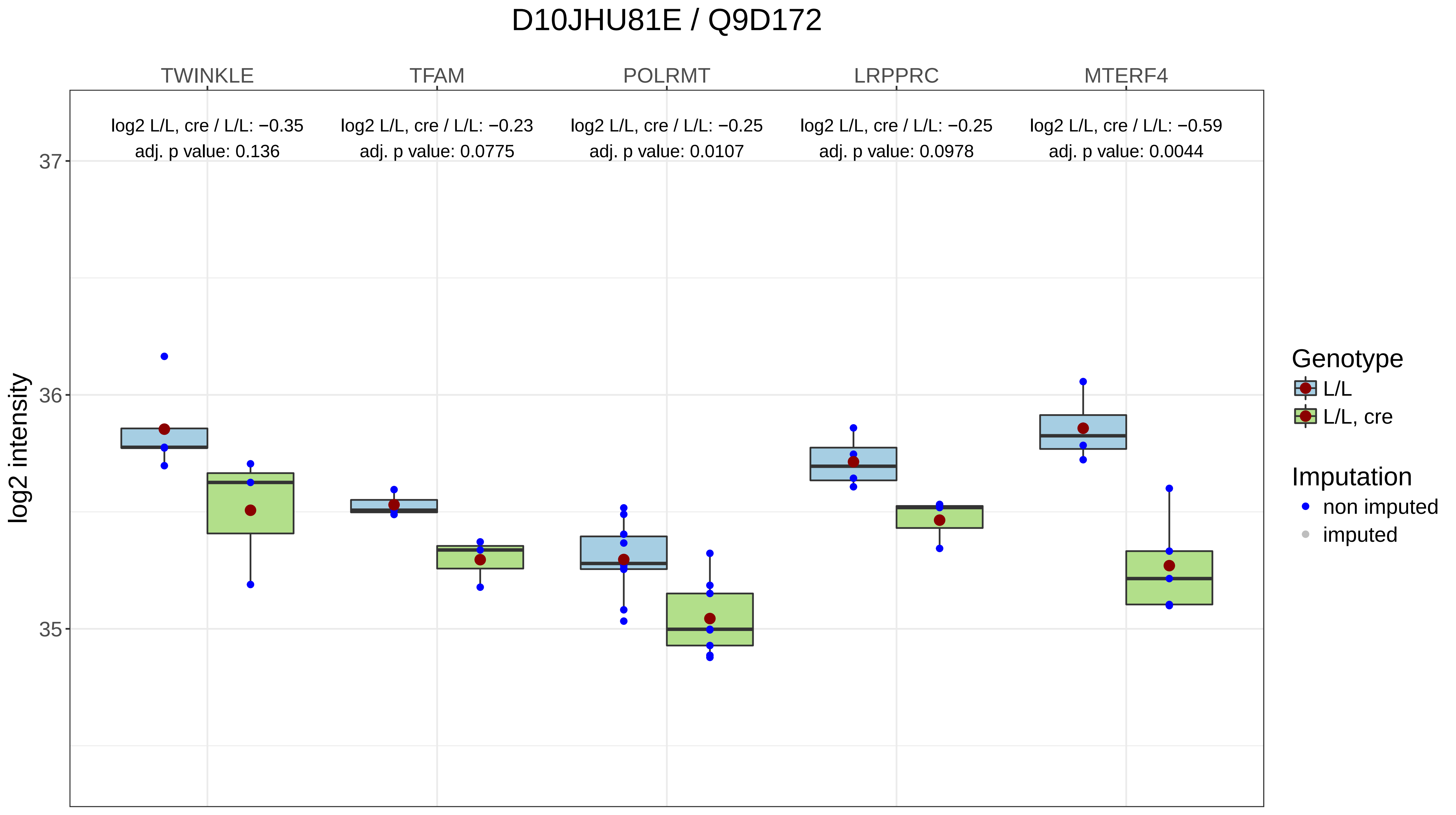

D10JHU81E / Q9D172
TWINKLE
TFAM
POLRMT
LRPPRC
MTERF4
log2 L/L, cre / L/L: −0.35
adj. p value: 0.136
log2 L/L, cre / L/L: −0.23
adj. p value: 0.0775
log2 L/L, cre / L/L: −0.25
adj. p value: 0.0107
log2 L/L, cre / L/L: −0.25
adj. p value: 0.0978
log2 L/L, cre / L/L: −0.59
adj. p value: 0.0044
37
36
35
log2 intensity
Genotype
L/L
L/L, cre
Imputation
non imputed
imputed

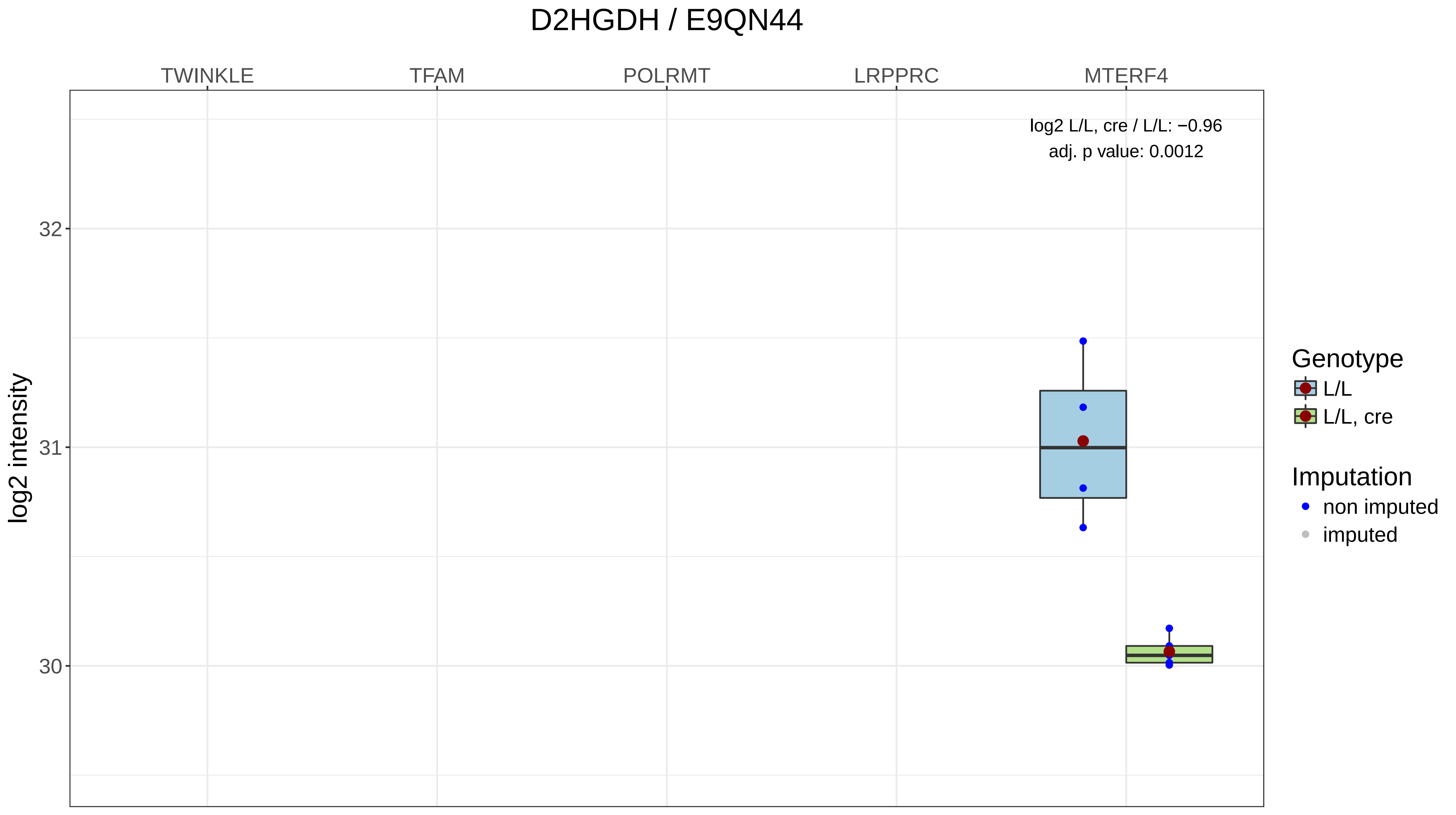
D2HGDH / E9QN44
TWINKLE
TFAM
POLRMT
LRPPRC
MTERF4
log2 L/L, cre / L/L: −0.96
adj. p value: 0.0012
log2 intensity
32
31
30
Genotype
L/L
L/L, cre
Imputation
non imputed
imputed

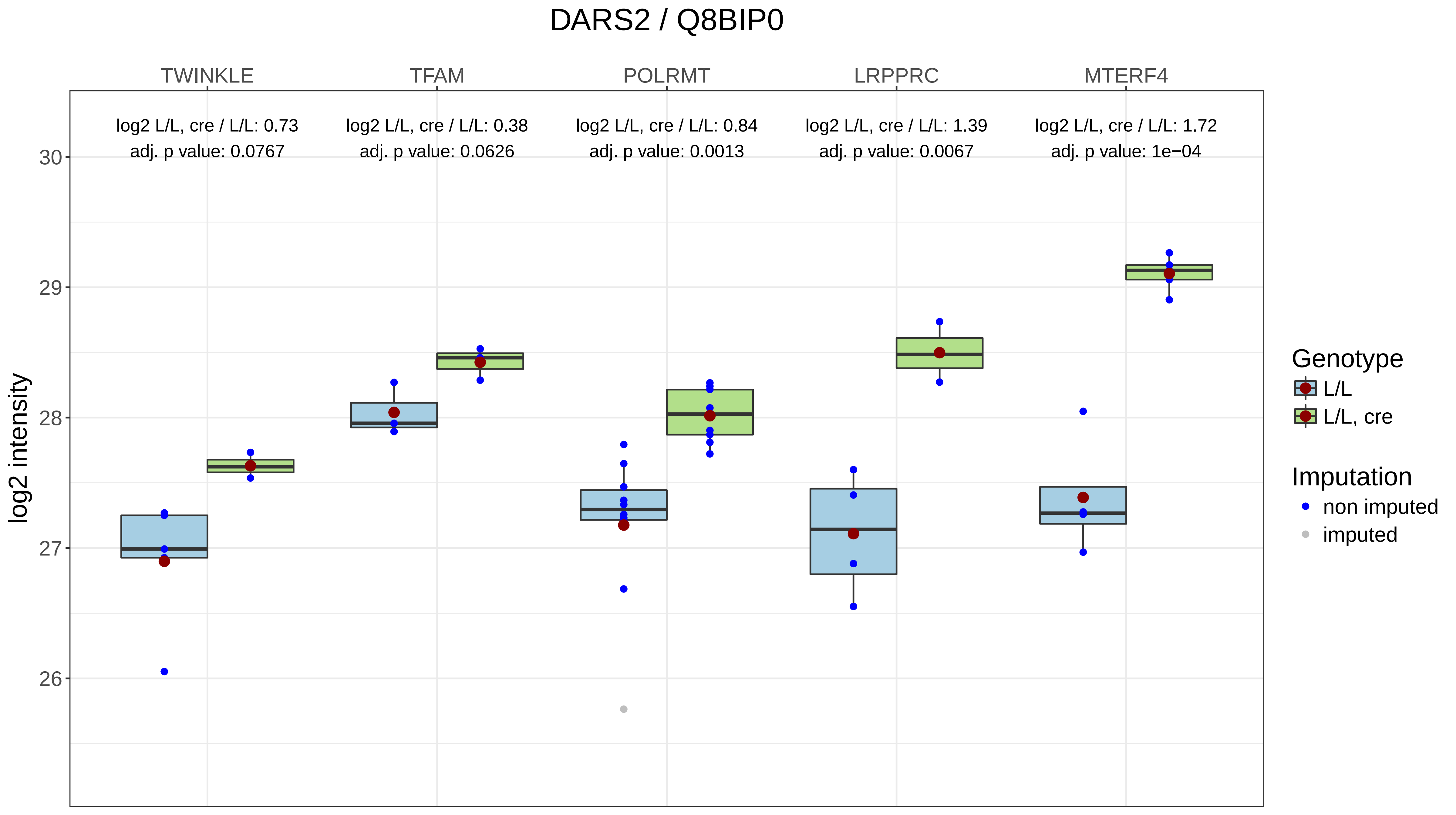
DARS2 / Q8BIP0
TWINKLE
TFAM
POLRMT
LRPPRC
MTERF4
log2 L/L, cre / L/L: 0.73
adj. p value: 0.0767
log2 L/L, cre / L/L: 0.38
adj. p value: 0.0626
log2 L/L, cre / L/L: 0.84
adj. p value: 0.0013
log2 L/L, cre / L/L: 1.39
adj. p value: 0.0067
log2 L/L, cre / L/L: 1.72
adj. p value: 1e−04
log2 intensity
30
29
28
27
26
Genotype
L/L
L/L, cre
Imputation
non imputed
imputed

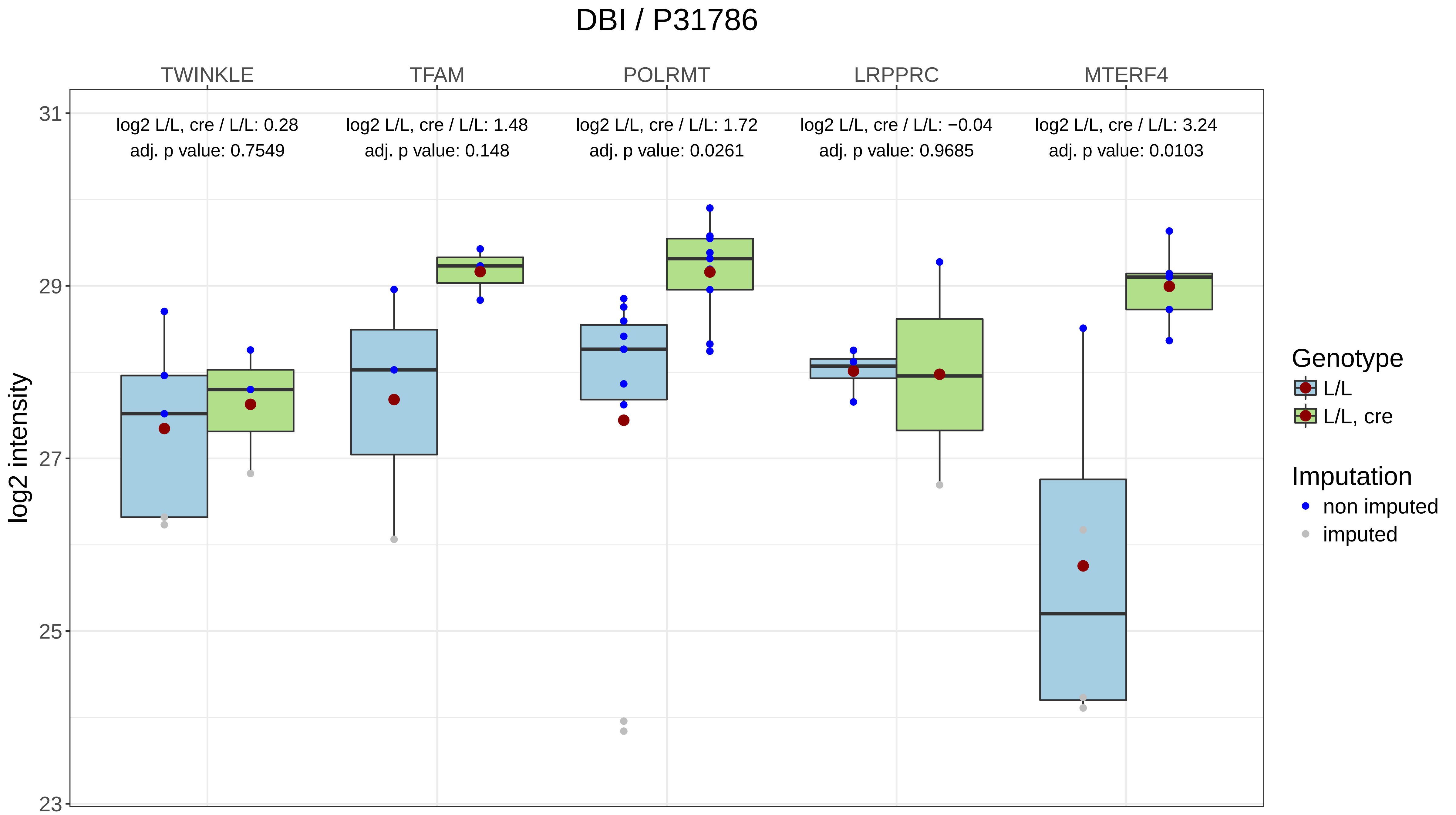
DBI / P31786
TWINKLE
TFAM
POLRMT
LRPPRC
MTERF4
log2 L/L, cre / L/L: 0.28
adj. p value: 0.7549
log2 L/L, cre / L/L: 1.48
adj. p value: 0.148
log2 L/L, cre / L/L: 1.72
adj. p value: 0.0261
log2 L/L, cre / L/L: −0.04
adj. p value: 0.9685
log2 L/L, cre / L/L: 3.24
adj. p value: 0.0103
log2 intensity
31
29
27
25
23
Genotype
L/L
L/L, cre
Imputation
non imputed
imputed

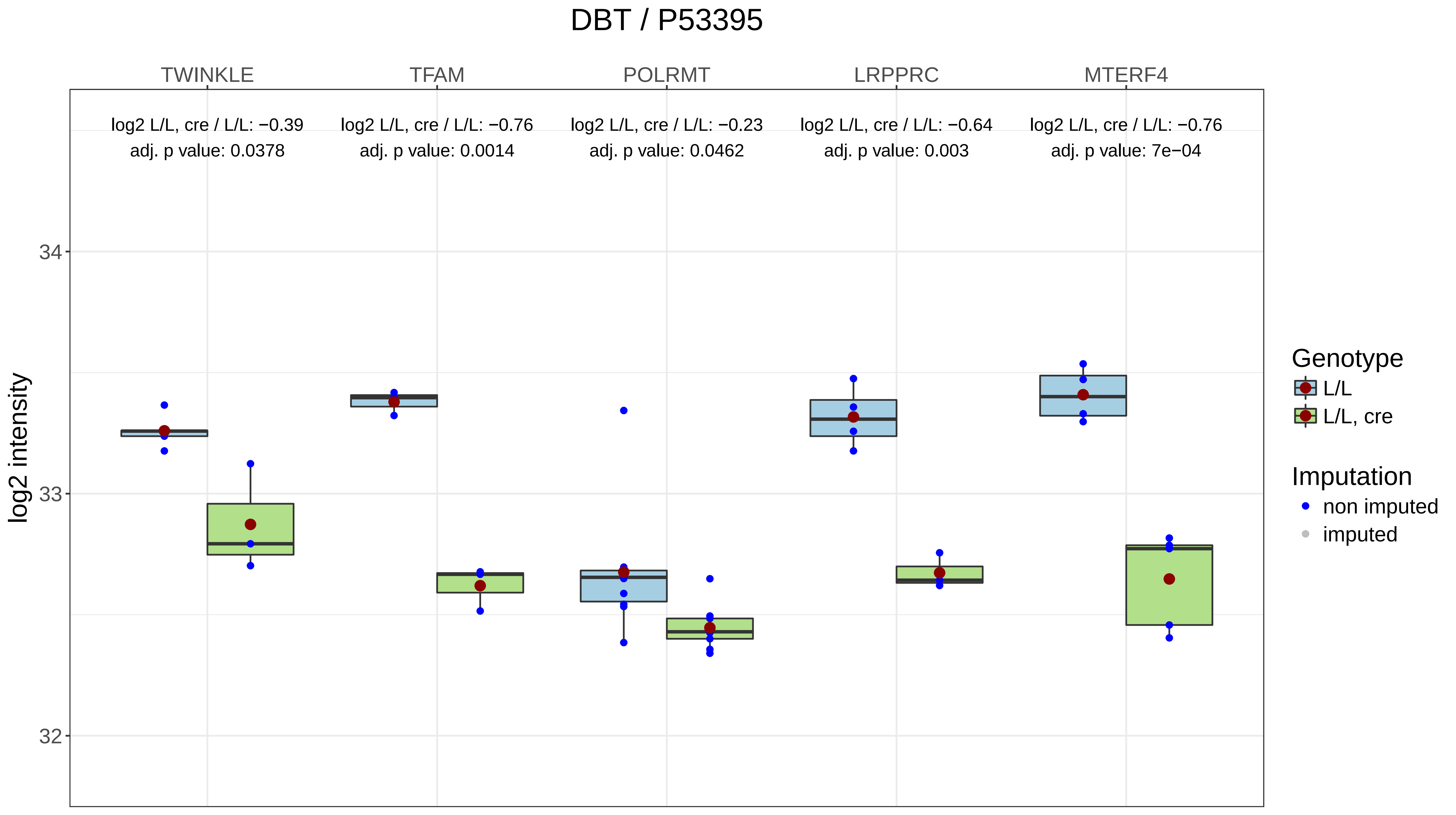

DBT / P53395
TWINKLE
TFAM
POLRMT
LRPPRC
MTERF4
log2 L/L, cre / L/L: −0.39
adj. p value: 0.0378
log2 L/L, cre / L/L: −0.76
adj. p value: 0.0014
log2 L/L, cre / L/L: −0.23
adj. p value: 0.0462
log2 L/L, cre / L/L: −0.64
adj. p value: 0.003
log2 L/L, cre / L/L: −0.76
adj. p value: 7e−04
log2 intensity
34
33
32
Genotype
L/L
L/L, cre
Imputation
non imputed
imputed

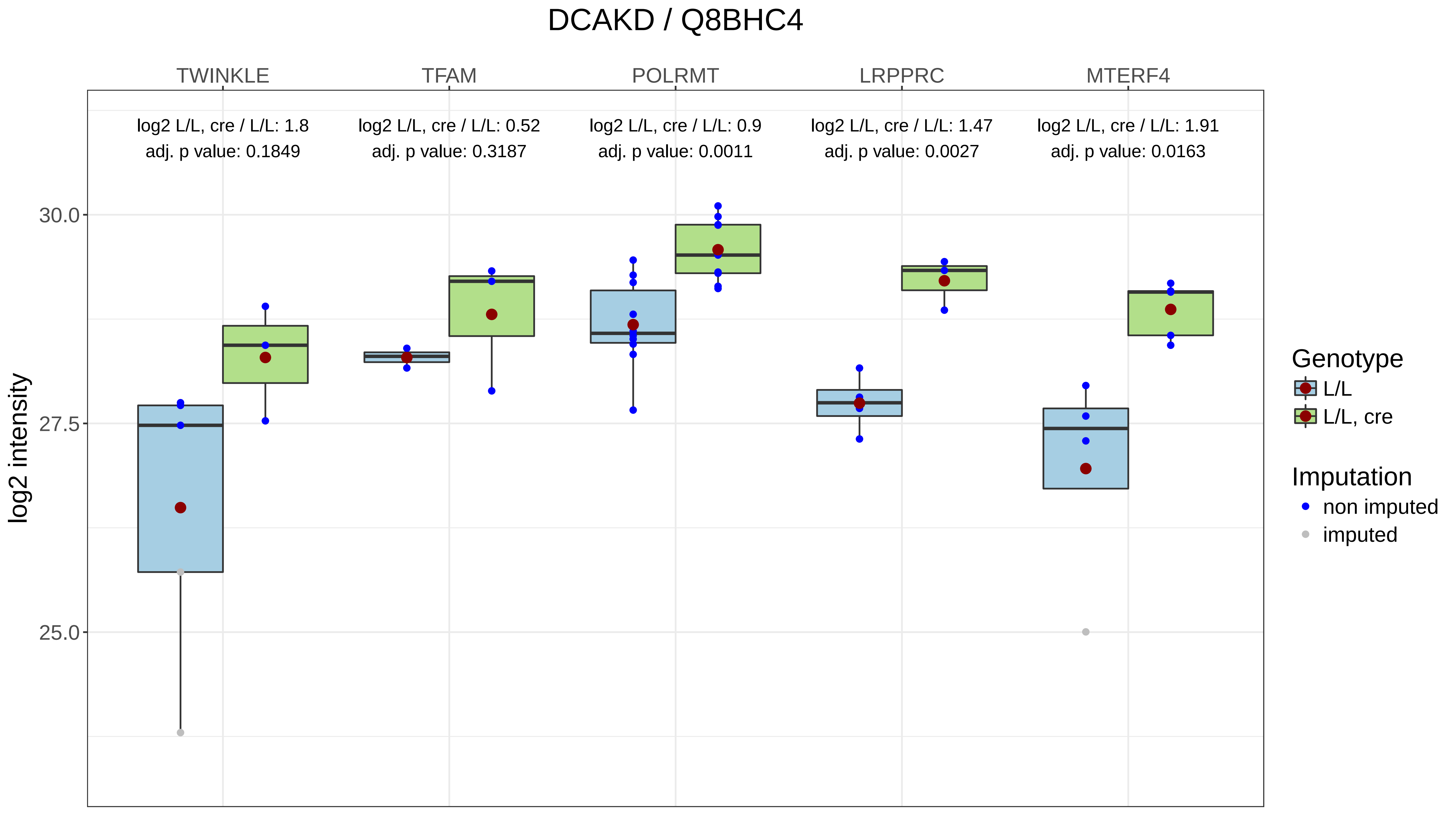
DCAKD / Q8BHC4
TWINKLE
TFAM
POLRMT
LRPPRC
MTERF4
log2 L/L, cre / L/L: 1.8
adj. p value: 0.1849
log2 L/L, cre / L/L: 0.52
adj. p value: 0.3187
log2 L/L, cre / L/L: 0.9
adj. p value: 0.0011
log2 L/L, cre / L/L: 1.47
adj. p value: 0.0027
log2 L/L, cre / L/L: 1.91
adj. p value: 0.0163
30.0
27.5
25.0
log2 intensity
Genotype
L/L
L/L, cre
Imputation
non imputed
imputed

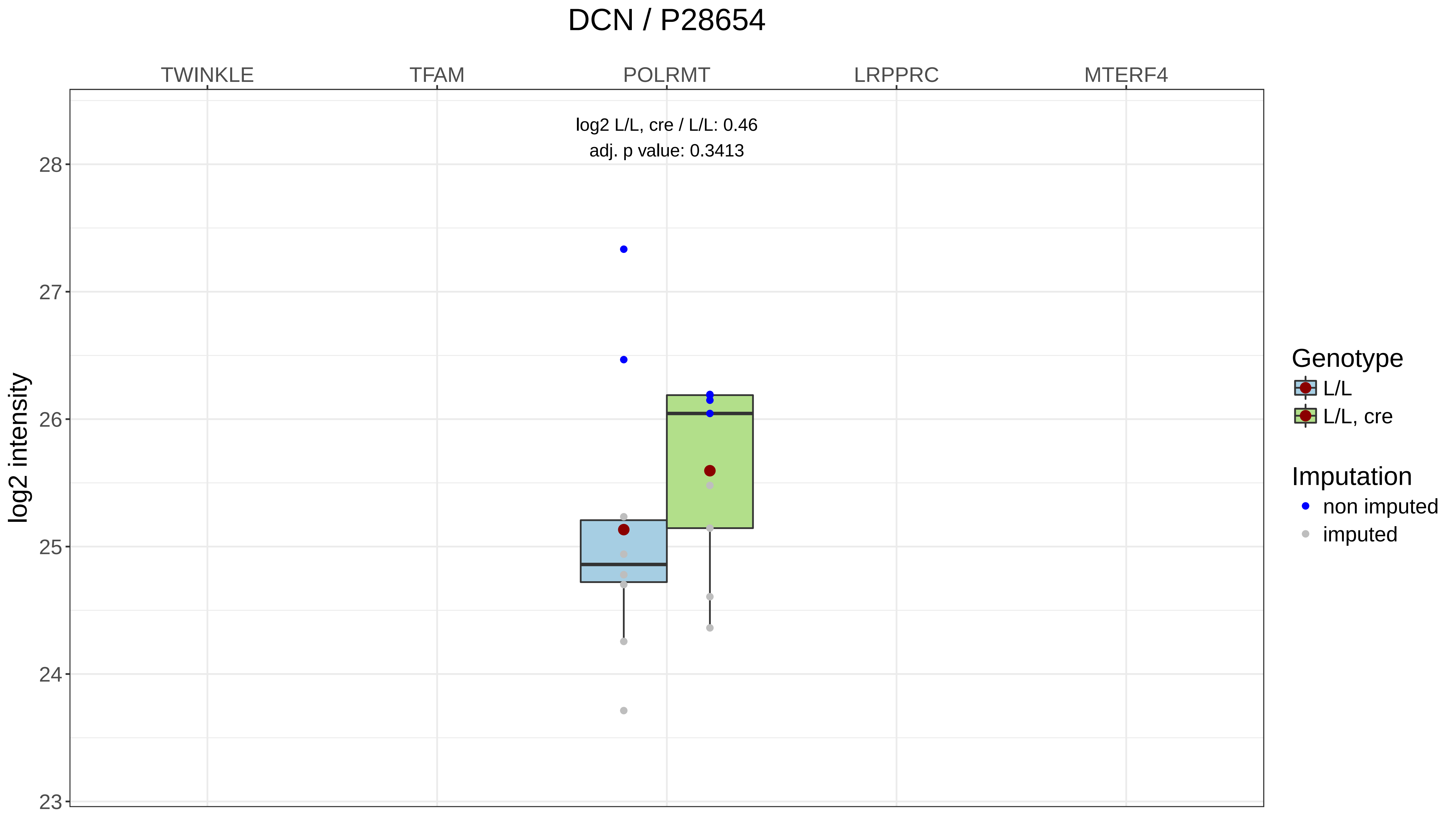
DCN / P28654
TWINKLE
TFAM
POLRMT
LRPPRC
MTERF4
log2 L/L, cre / L/L: 0.46
adj. p value: 0.3413
log2 intensity
23
24
25
26
27
28
Genotype
L/L
L/L, cre
Imputation
non imputed
imputed

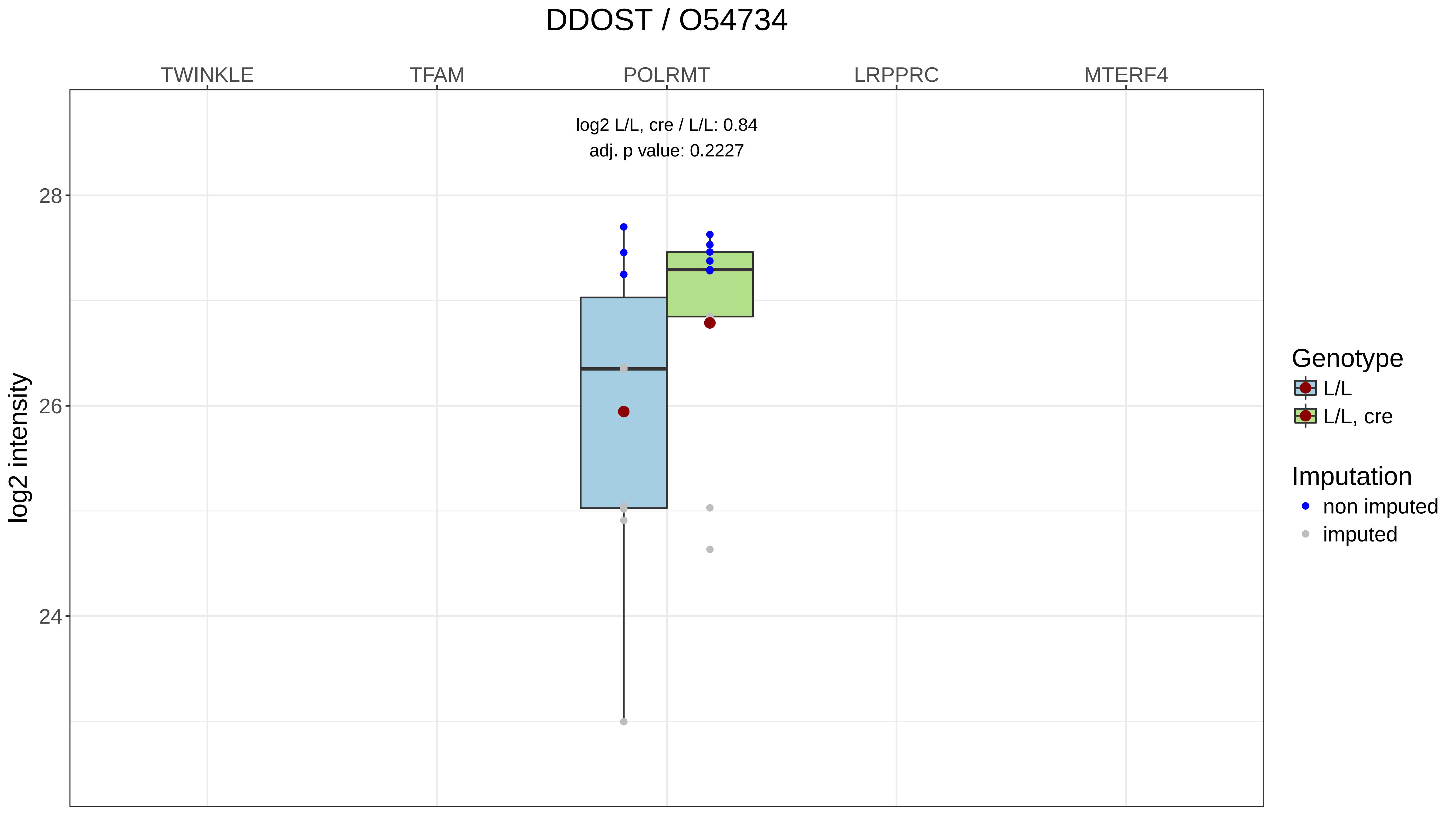

DDOST / O54734
TWINKLE
TFAM
POLRMT
LRPPRC
MTERF4
log2 L/L, cre / L/L: 0.84
adj. p value: 0.2227
28
26
24
log2 intensity
Genotype
L/L
L/L, cre
Imputation
non imputed
imputed

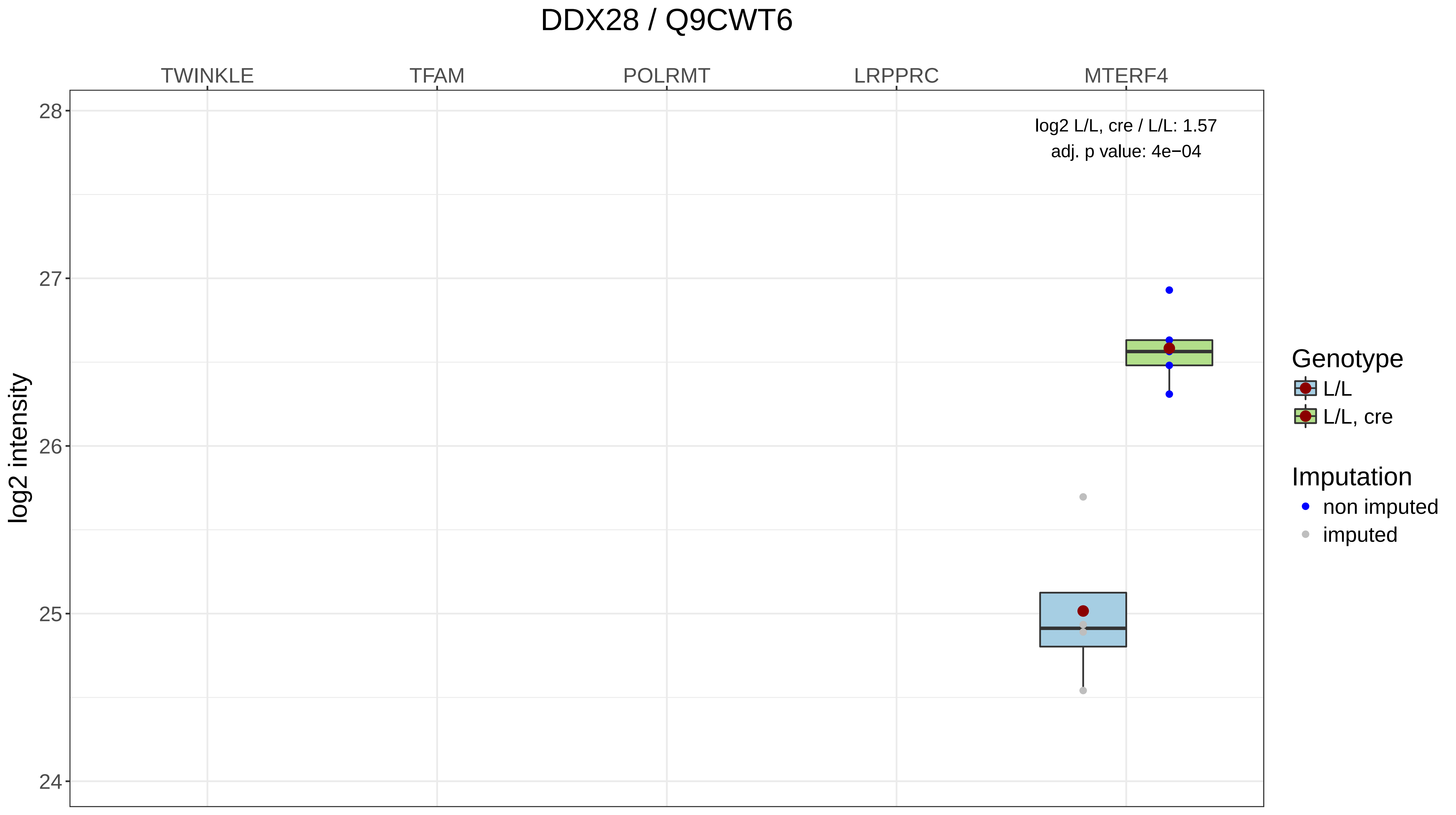
DDX28 / Q9CWT6
TWINKLE
TFAM
POLRMT
LRPPRC
MTERF4
log2 L/L, cre / L/L: 1.57
adj. p value: 4e−04
28
27
26
25
24
log2 intensity
Genotype
L/L
L/L, cre
Imputation
non imputed
imputed

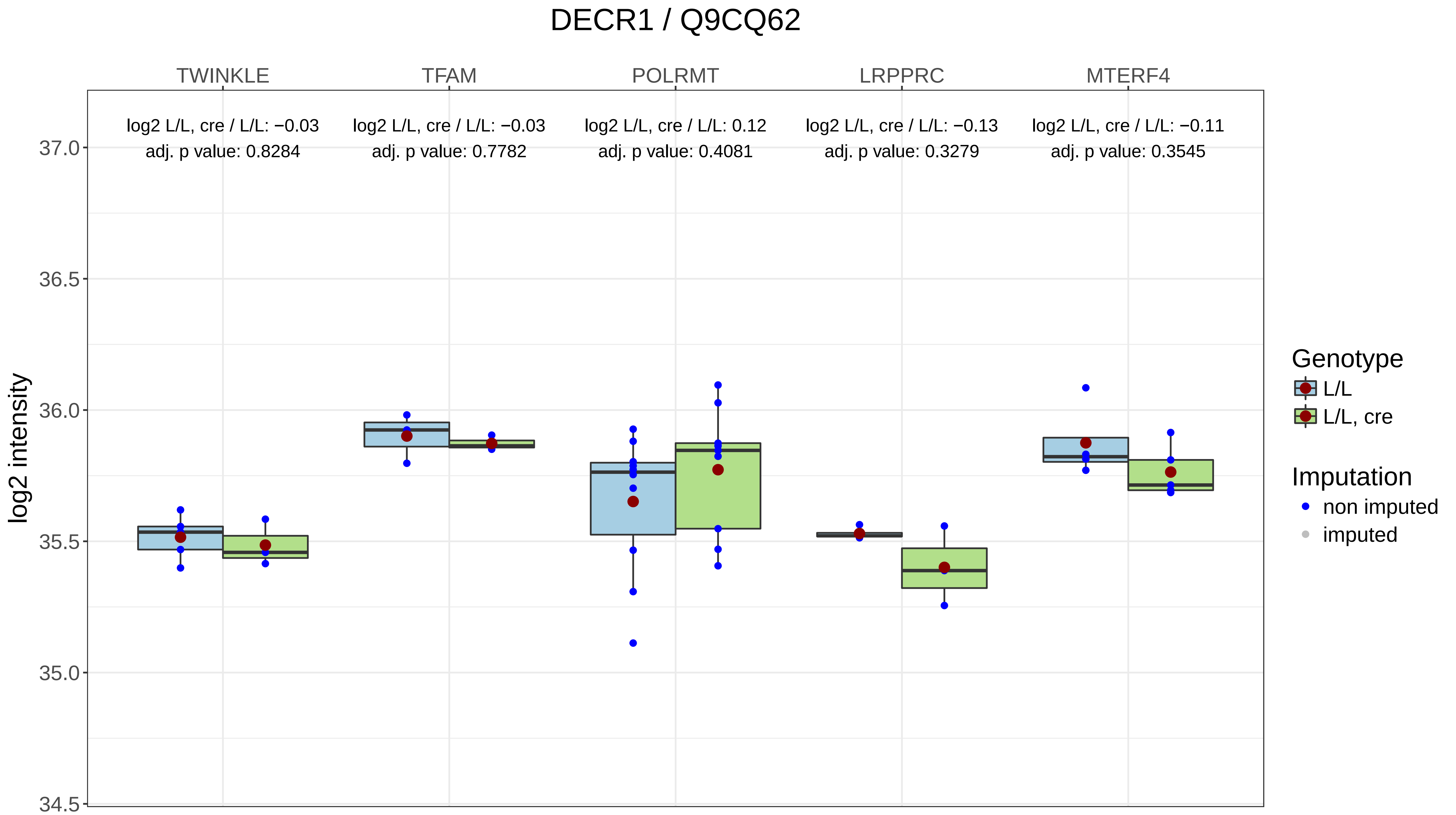
DECR1 / Q9CQ62
TWINKLE
TFAM
POLRMT
LRPPRC
MTERF4
log2 L/L, cre / L/L: −0.03
adj. p value: 0.8284
log2 L/L, cre / L/L: −0.03
adj. p value: 0.7782
log2 L/L, cre / L/L: 0.12
adj. p value: 0.4081
log2 L/L, cre / L/L: −0.13
adj. p value: 0.3279
log2 L/L, cre / L/L: −0.11
adj. p value: 0.3545
log2 intensity
37.0
36.5
36.0
35.5
35.0
34.5
Genotype
L/L
L/L, cre
Imputation
non imputed
imputed

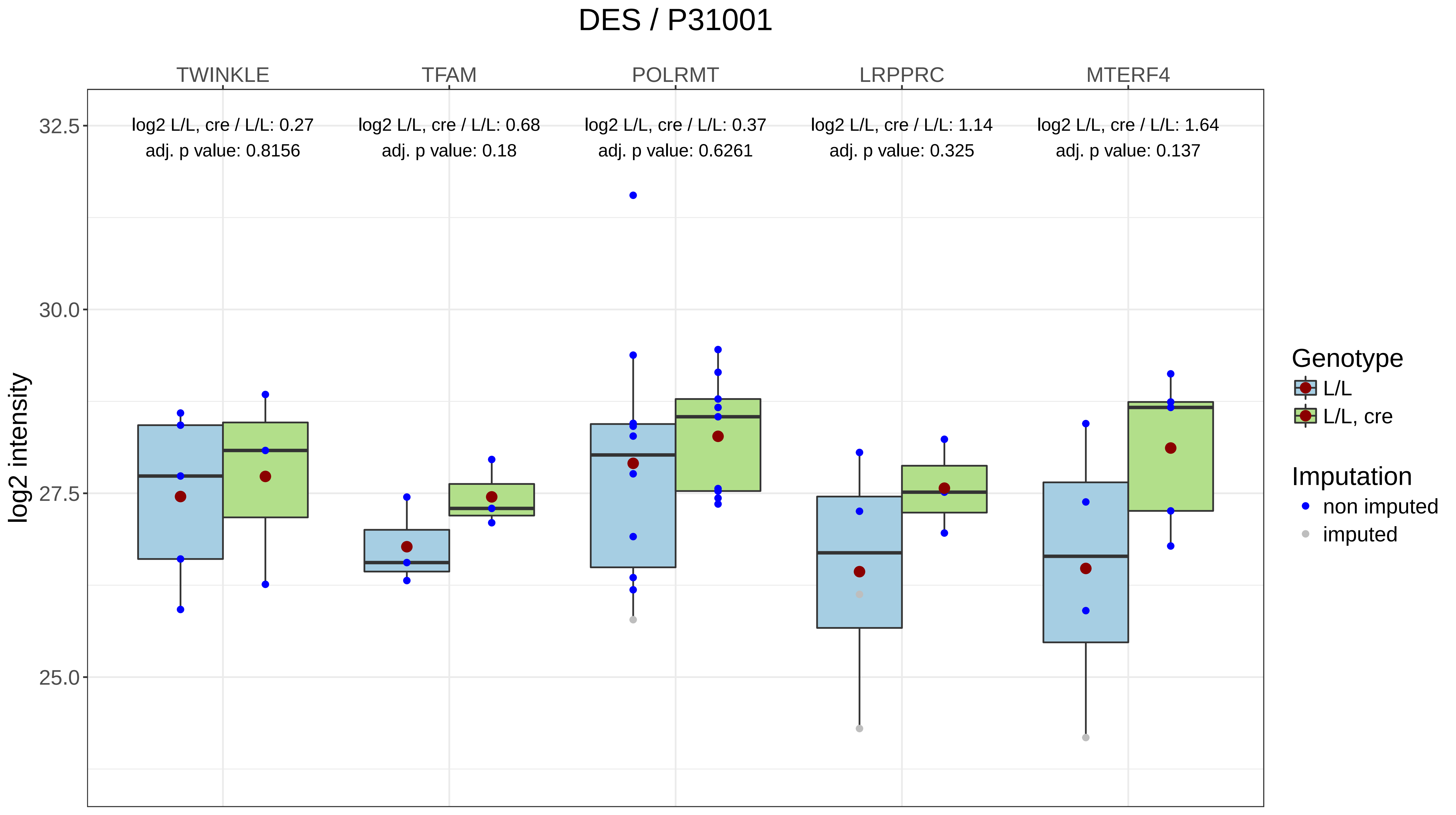
DES / P31001
TWINKLE
TFAM
POLRMT
LRPPRC
MTERF4
log2 L/L, cre / L/L: 0.27
adj. p value: 0.8156
log2 L/L, cre / L/L: 0.68
adj. p value: 0.18
log2 L/L, cre / L/L: 0.37
adj. p value: 0.6261
log2 L/L, cre / L/L: 1.14
adj. p value: 0.325
log2 L/L, cre / L/L: 1.64
adj. p value: 0.137
32.5
30.0
27.5
25.0
log2 intensity
Genotype
L/L
L/L, cre
Imputation
non imputed
imputed

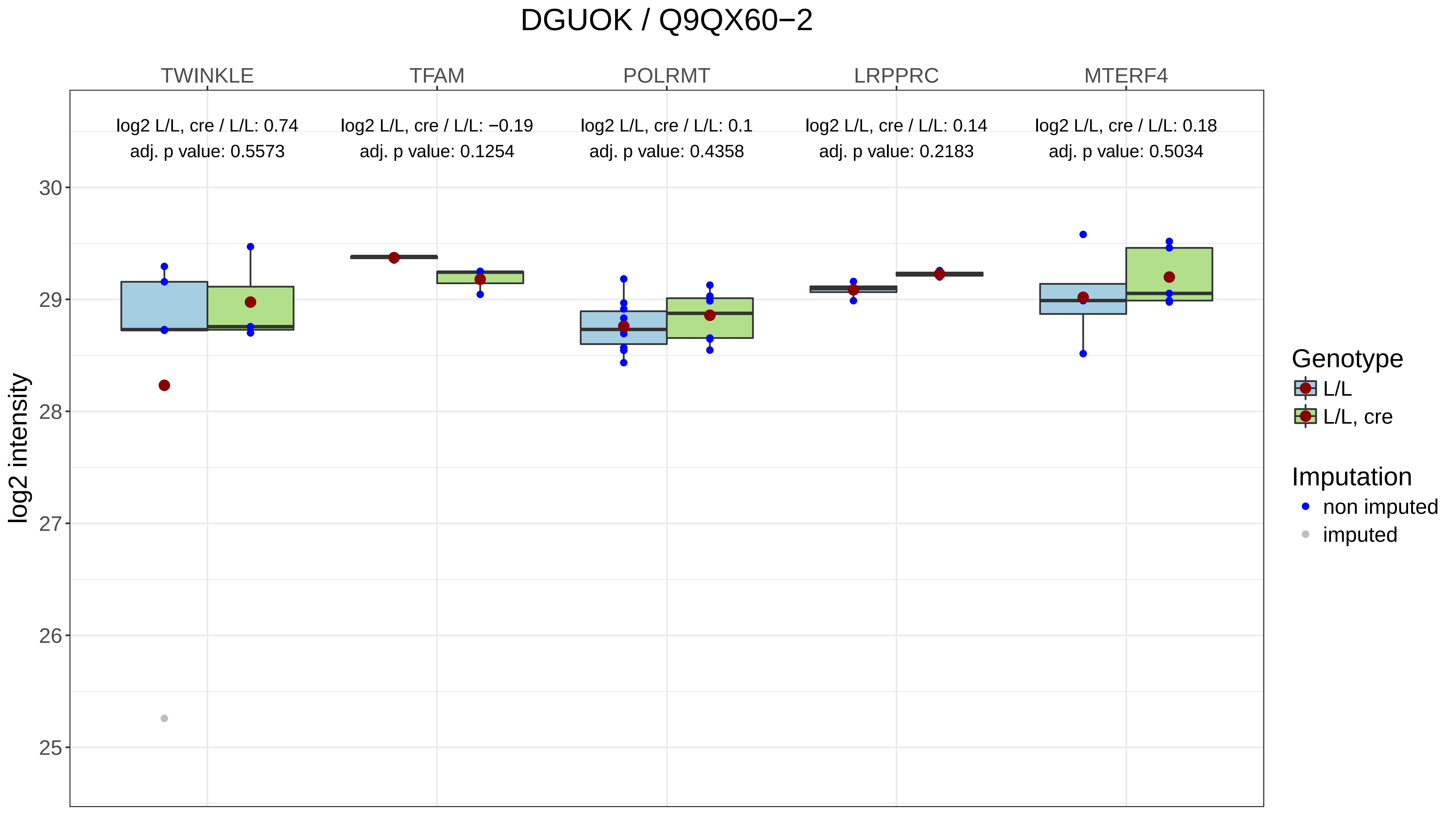

DGUOK / Q9QX60−2
TWINKLE
TFAM
POLRMT
LRPPRC
MTERF4
log2 L/L, cre / L/L: 0.74
adj. p value: 0.5573
log2 L/L, cre / L/L: −0.19
adj. p value: 0.1254
log2 L/L, cre / L/L: 0.1
adj. p value: 0.4358
log2 L/L, cre / L/L: 0.14
adj. p value: 0.2183
log2 L/L, cre / L/L: 0.18
adj. p value: 0.5034
log2 intensity
30
29
28
27
26
25
Genotype
L/L
L/L, cre
Imputation
non imputed
imputed

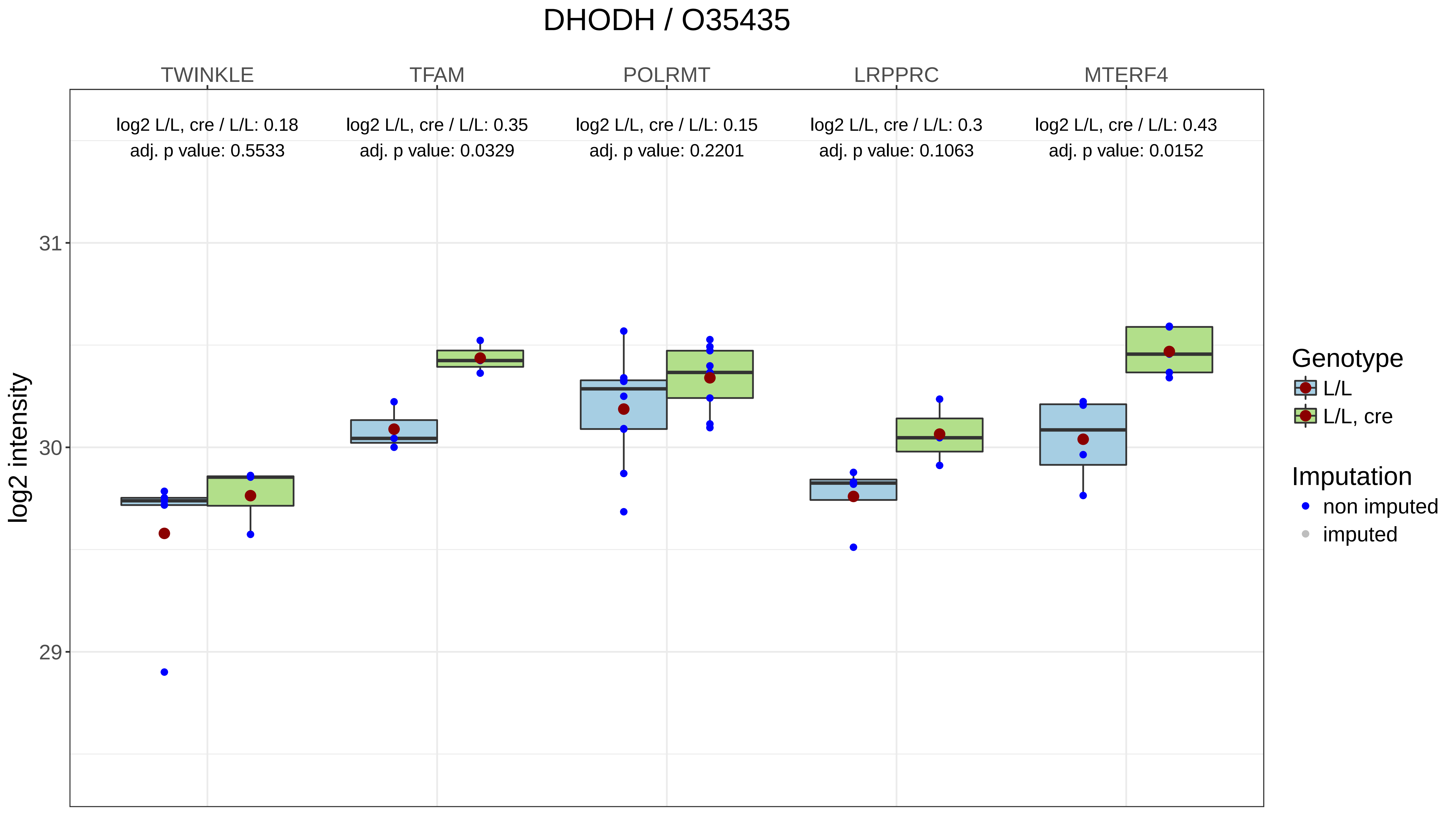
DHODH / O35435
TWINKLE
TFAM
POLRMT
LRPPRC
MTERF4
log2 L/L, cre / L/L: 0.18
adj. p value: 0.5533
log2 L/L, cre / L/L: 0.35
adj. p value: 0.0329
log2 L/L, cre / L/L: 0.15
adj. p value: 0.2201
log2 L/L, cre / L/L: 0.3
adj. p value: 0.1063
log2 L/L, cre / L/L: 0.43
adj. p value: 0.0152
log2 intensity
31
30
29
Genotype
L/L
L/L, cre
Imputation
non imputed
imputed

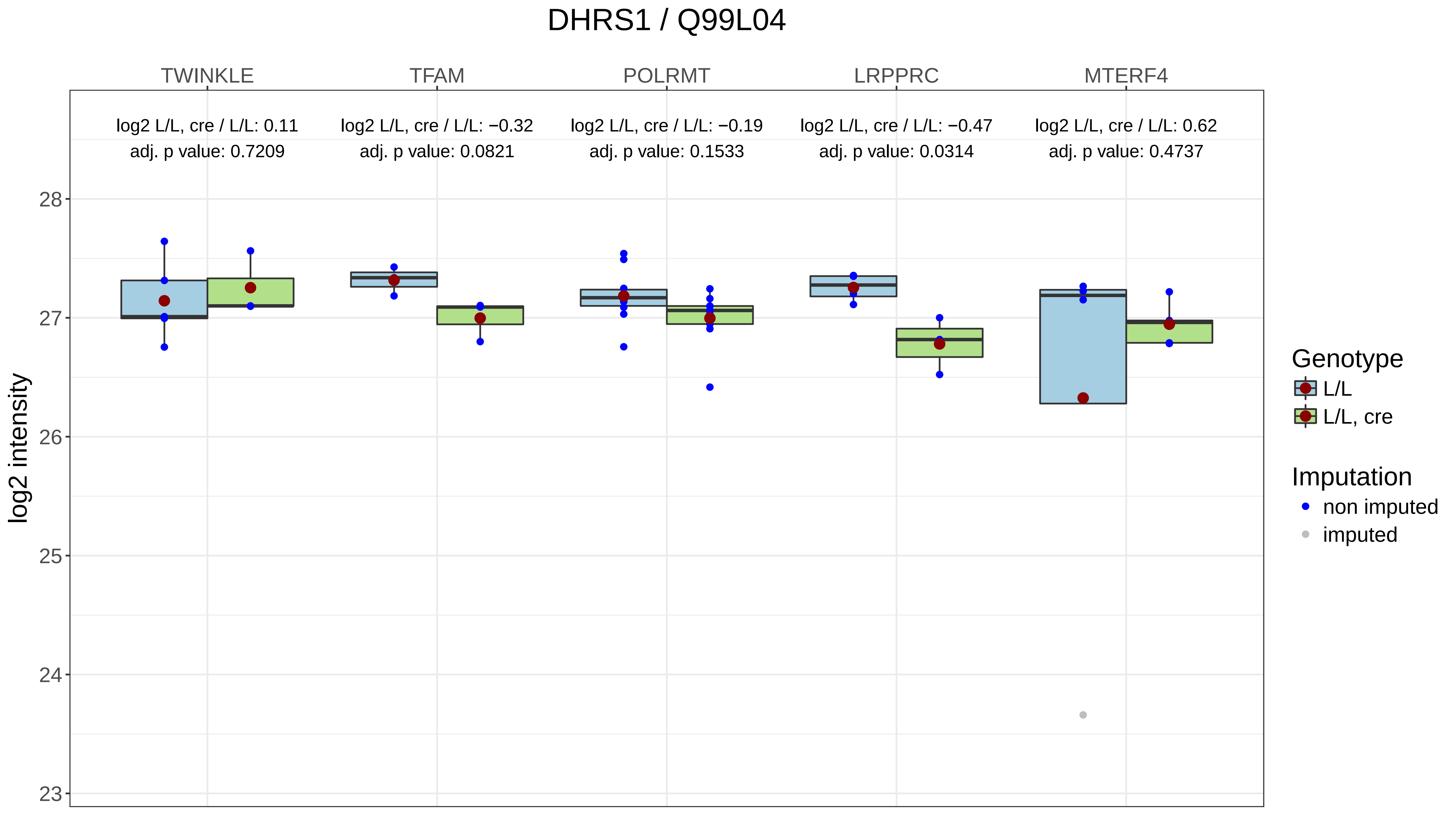
DHRS1 / Q99L04
TWINKLE
TFAM
POLRMT
LRPPRC
MTERF4
log2 L/L, cre / L/L: 0.11
adj. p value: 0.7209
log2 L/L, cre / L/L: −0.32
adj. p value: 0.0821
log2 L/L, cre / L/L: −0.19
adj. p value: 0.1533
log2 L/L, cre / L/L: −0.47
adj. p value: 0.0314
log2 L/L, cre / L/L: 0.62
adj. p value: 0.4737
log2 intensity
28
27
26
25
24
23
Genotype
L/L
L/L, cre
Imputation
non imputed
imputed

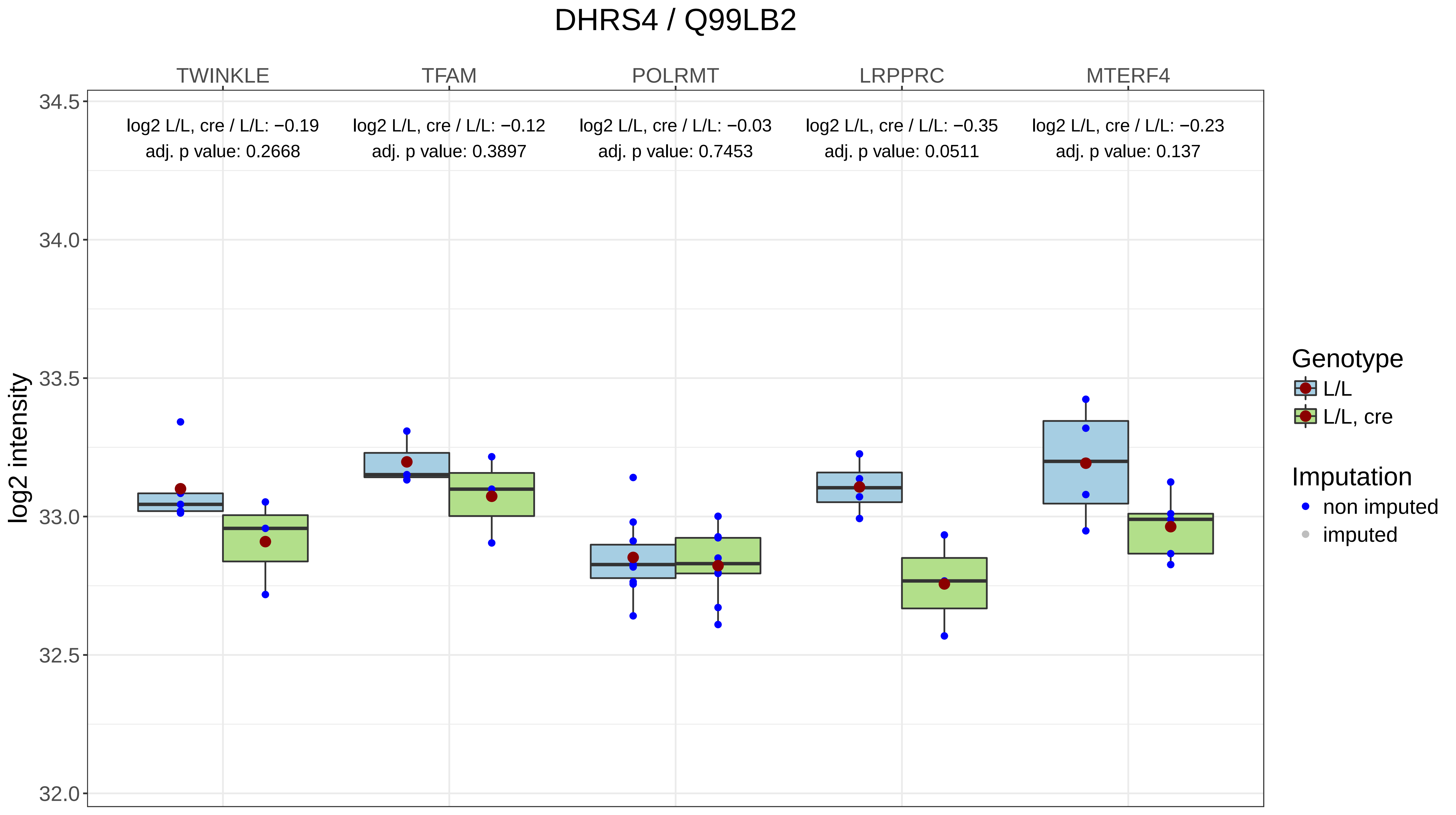
DHRS4 / Q99LB2
TWINKLE
TFAM
POLRMT
LRPPRC
MTERF4
log2 L/L, cre / L/L: −0.19
adj. p value: 0.2668
log2 L/L, cre / L/L: −0.12
adj. p value: 0.3897
log2 L/L, cre / L/L: −0.03
adj. p value: 0.7453
log2 L/L, cre / L/L: −0.35
adj. p value: 0.0511
log2 L/L, cre / L/L: −0.23
adj. p value: 0.137
log2 intensity
34.5
34.0
33.5
33.0
32.5
32.0
Genotype
L/L
L/L, cre
Imputation
non imputed
imputed

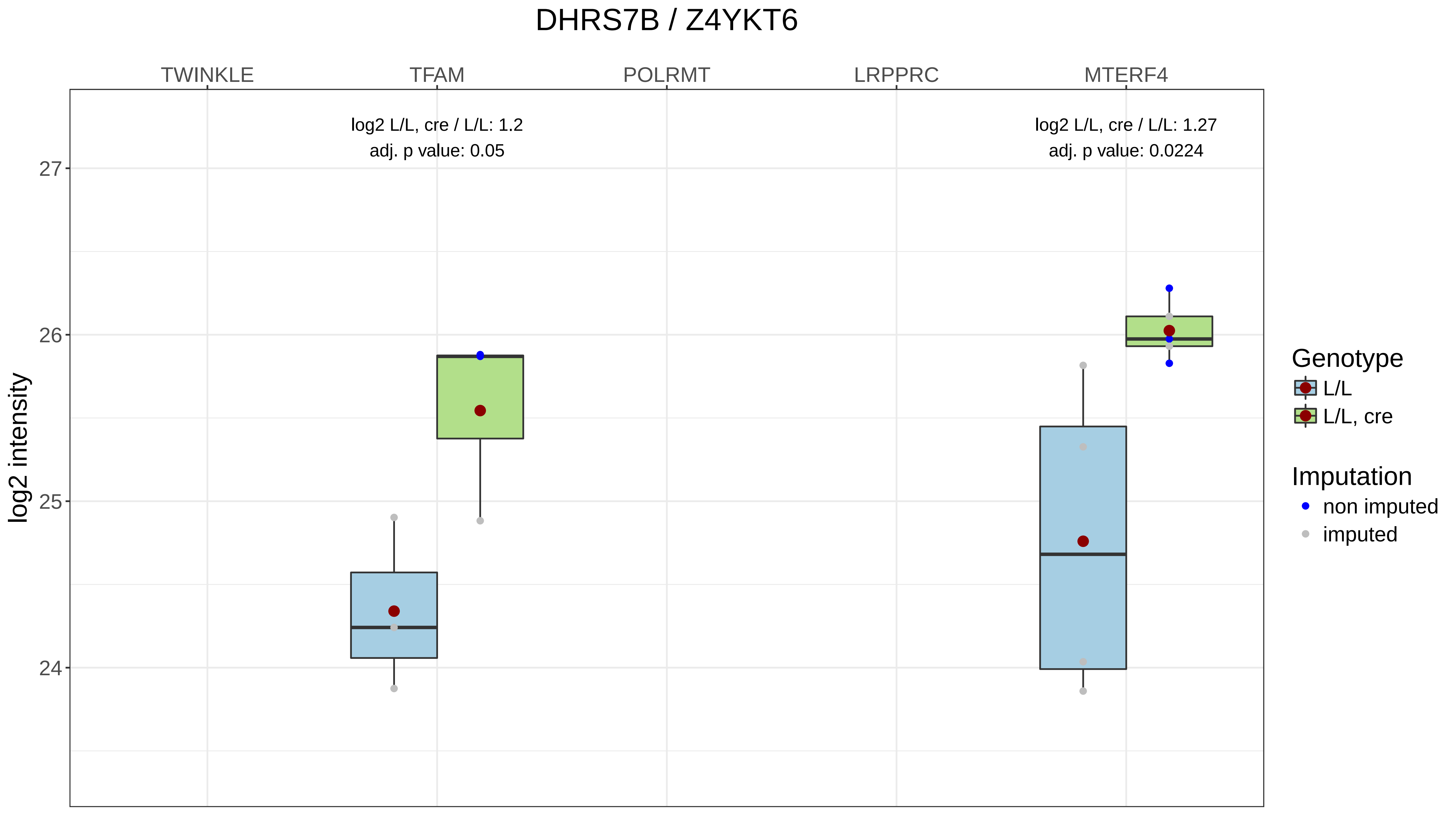
DHRS7B / Z4YKT6
TWINKLE
TFAM
POLRMT
LRPPRC
MTERF4
log2 L/L, cre / L/L: 1.2
adj. p value: 0.05
log2 L/L, cre / L/L: 1.27
adj. p value: 0.0224
log2 intensity
27
26
25
24
Genotype
L/L
L/L, cre
Imputation
non imputed
imputed

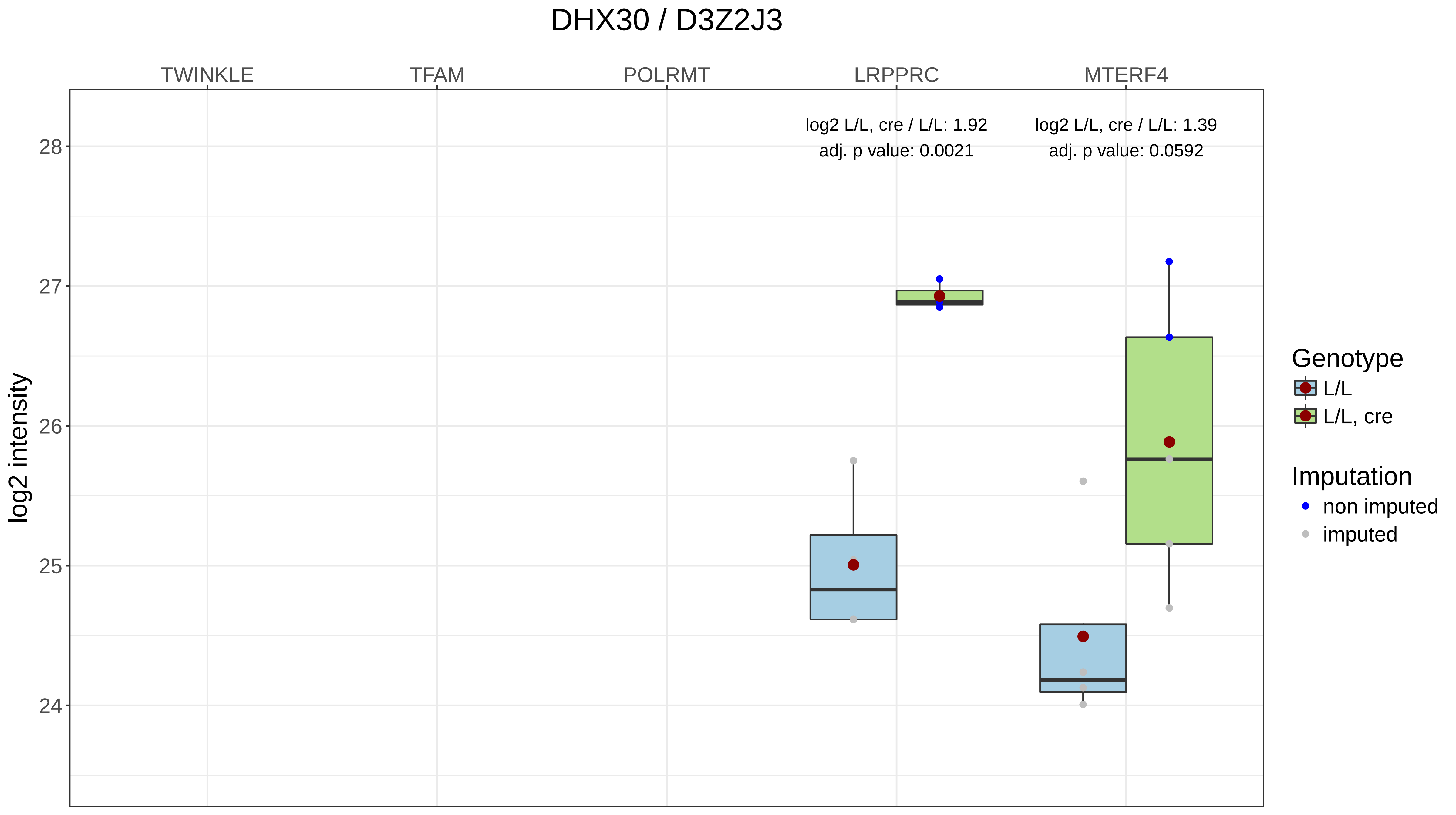
DHX30 / D3Z2J3
TWINKLE
TFAM
POLRMT
LRPPRC
MTERF4
log2 L/L, cre / L/L: 1.92
adj. p value: 0.0021
log2 L/L, cre / L/L: 1.39
adj. p value: 0.0592
log2 intensity
28
27
26
25
24
Genotype
L/L
L/L, cre
Imputation
non imputed
imputed

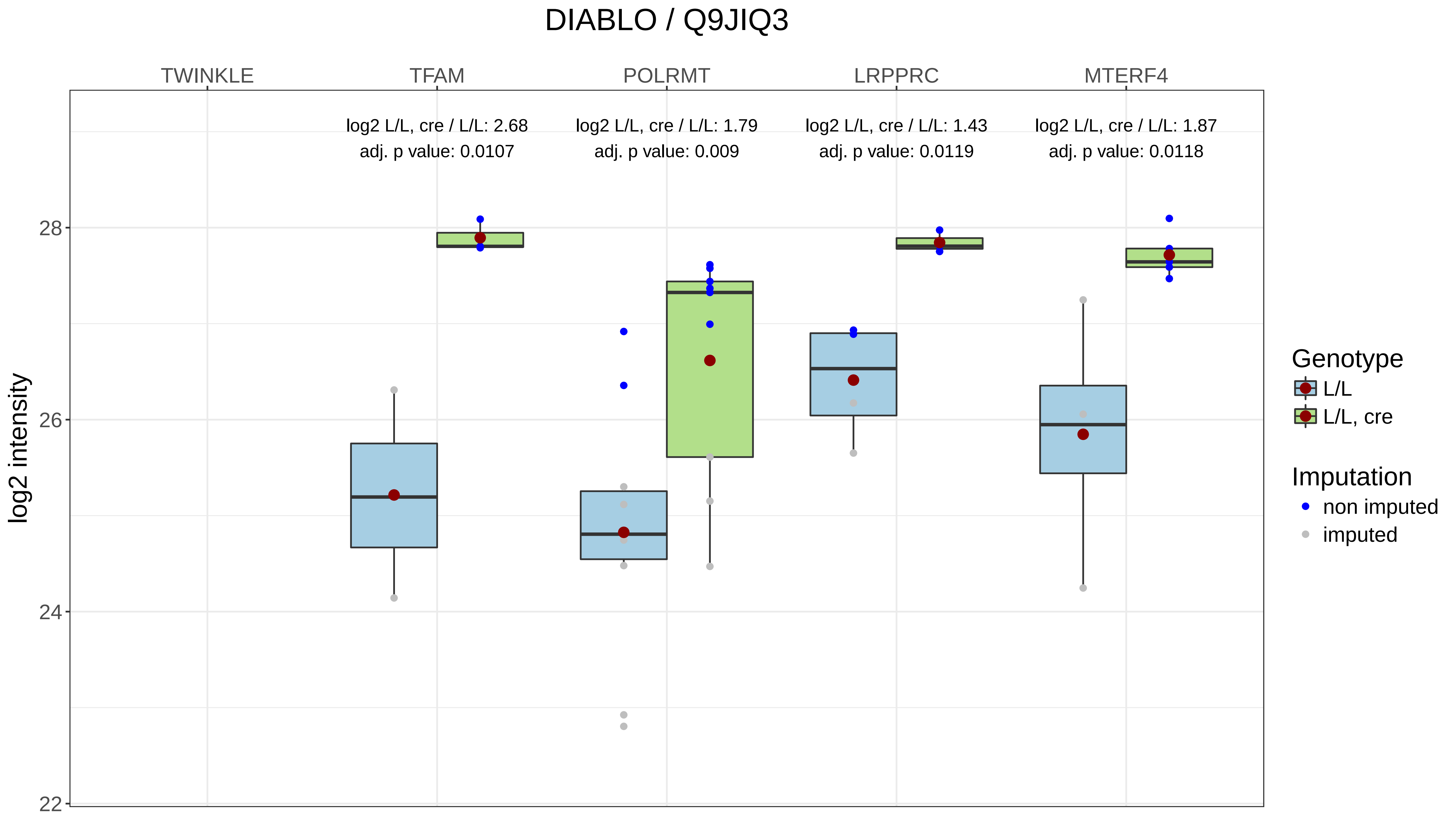

DIABLO / Q9JIQ3
TWINKLE
TFAM
POLRMT
LRPPRC
MTERF4
log2 L/L, cre / L/L: 2.68
adj. p value: 0.0107
log2 L/L, cre / L/L: 1.79
adj. p value: 0.009
log2 L/L, cre / L/L: 1.43
adj. p value: 0.0119
log2 L/L, cre / L/L: 1.87
adj. p value: 0.0118
log2 intensity
28
26
24
22
Genotype
L/L
L/L, cre
Imputation
non imputed
imputed

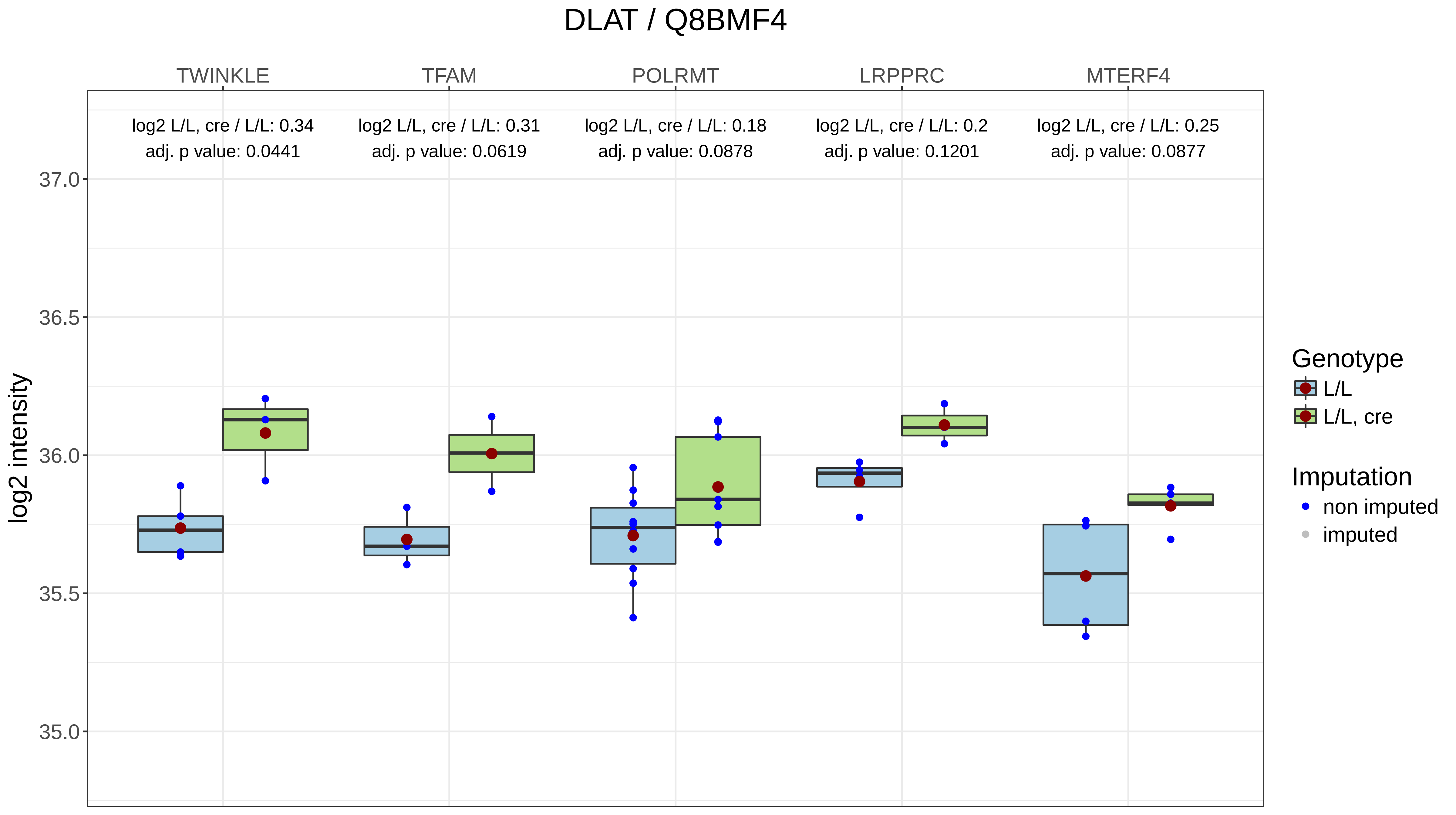

DLAT / Q8BMF4
TWINKLE
TFAM
POLRMT
LRPPRC
MTERF4
log2 L/L, cre / L/L: 0.34
adj. p value: 0.0441
log2 L/L, cre / L/L: 0.31
adj. p value: 0.0619
log2 L/L, cre / L/L: 0.18
adj. p value: 0.0878
log2 L/L, cre / L/L: 0.2
adj. p value: 0.1201
log2 L/L, cre / L/L: 0.25
adj. p value: 0.0877
log2 intensity
37.0
36.5
36.0
35.5
35.0
Genotype
L/L
L/L, cre
Imputation
non imputed
imputed

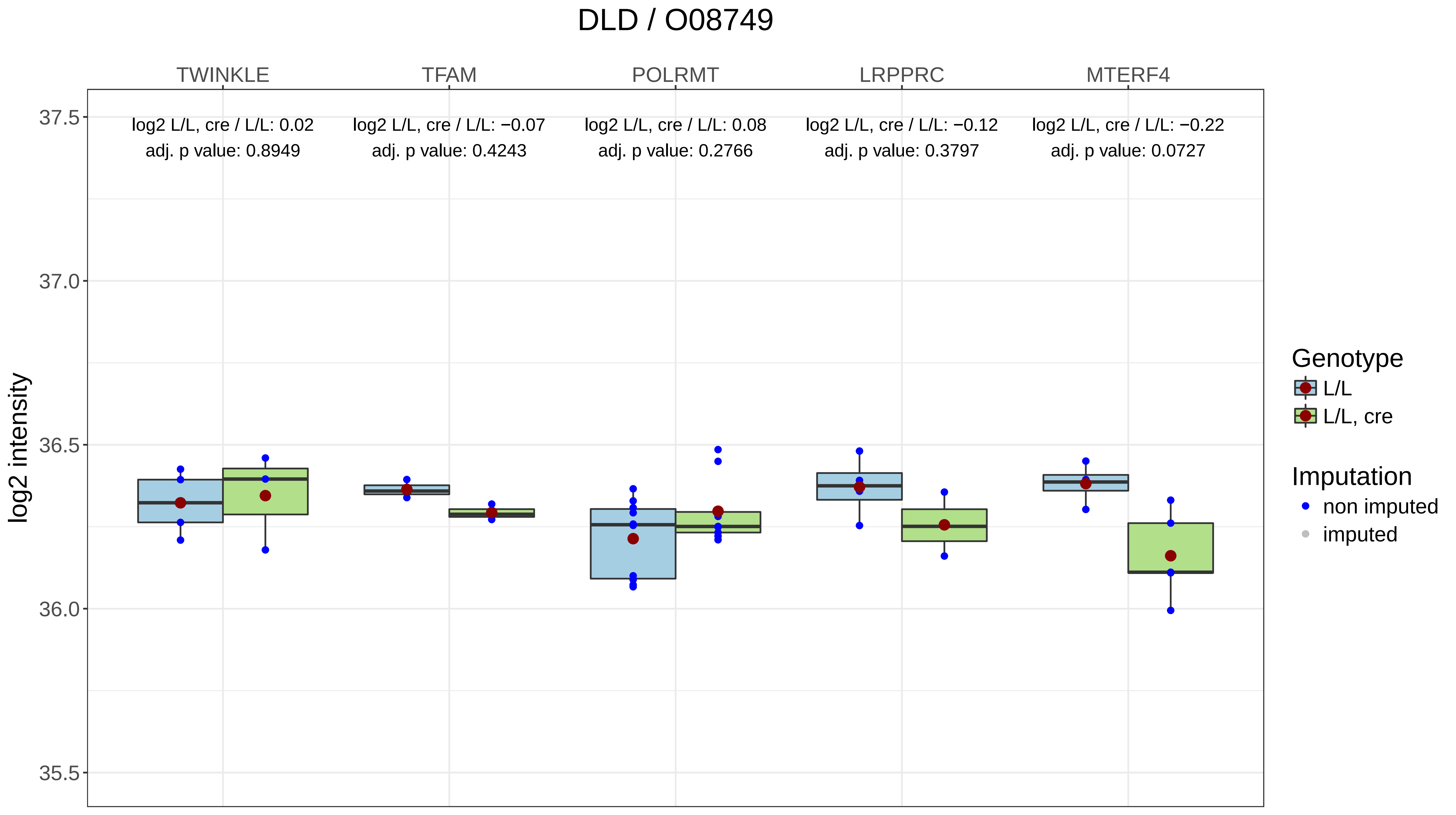
DLD / O08749
TWINKLE
TFAM
POLRMT
LRPPRC
MTERF4
log2 L/L, cre / L/L: 0.02
adj. p value: 0.8949
log2 L/L, cre / L/L: −0.07
adj. p value: 0.4243
log2 L/L, cre / L/L: 0.08
adj. p value: 0.2766
log2 L/L, cre / L/L: −0.12
adj. p value: 0.3797
log2 L/L, cre / L/L: −0.22
adj. p value: 0.0727
log2 intensity
37.5
37.0
36.5
36.0
35.5
Genotype
L/L
L/L, cre
Imputation
non imputed
imputed

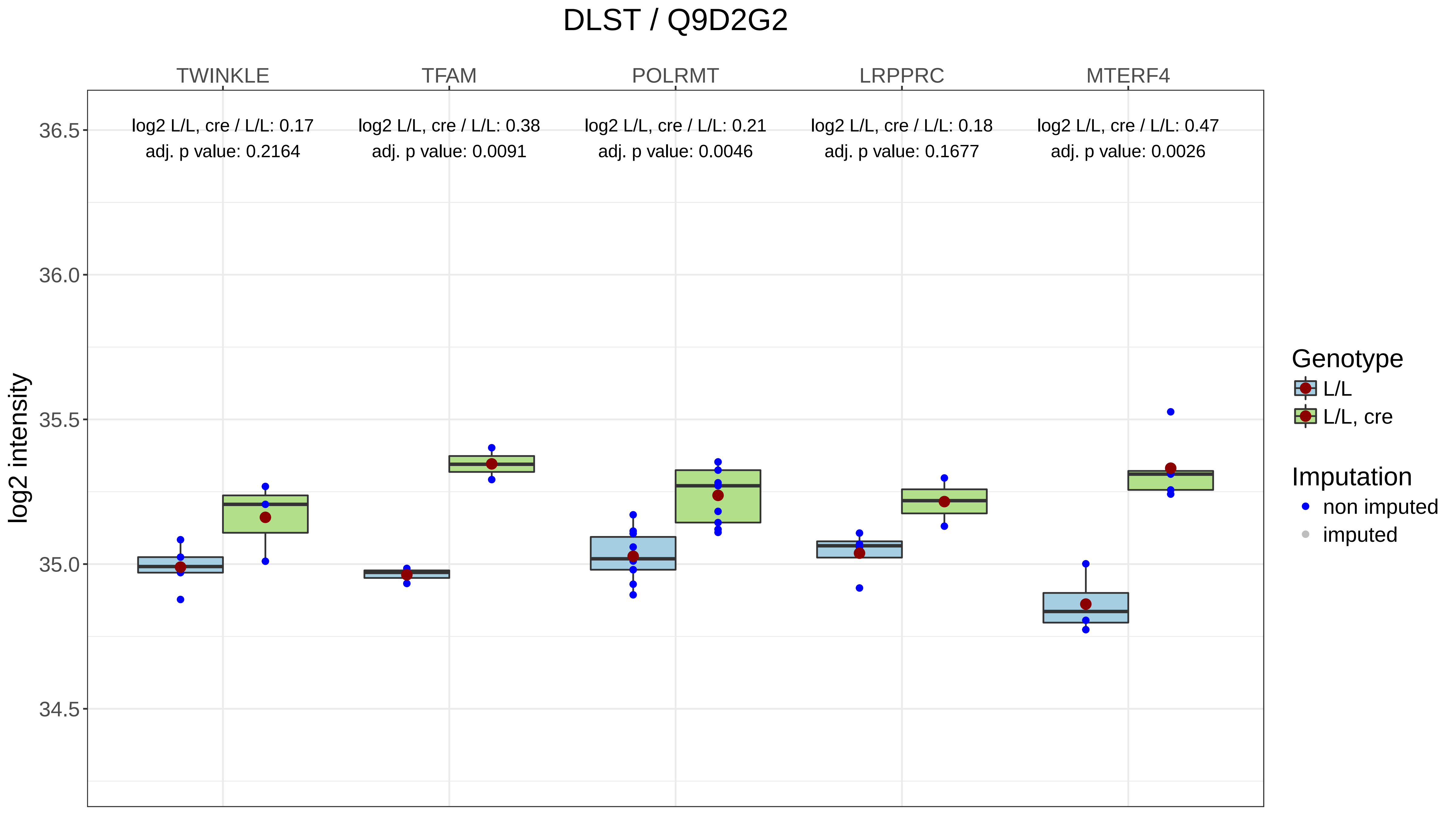
DLST / Q9D2G2
TWINKLE
TFAM
POLRMT
LRPPRC
MTERF4
log2 L/L, cre / L/L: 0.17
adj. p value: 0.2164
log2 L/L, cre / L/L: 0.38
adj. p value: 0.0091
log2 L/L, cre / L/L: 0.21
adj. p value: 0.0046
log2 L/L, cre / L/L: 0.18
adj. p value: 0.1677
log2 L/L, cre / L/L: 0.47
adj. p value: 0.0026
log2 intensity
36.5
36.0
35.5
35.0
34.5
Genotype
L/L
L/L, cre
Imputation
non imputed
imputed

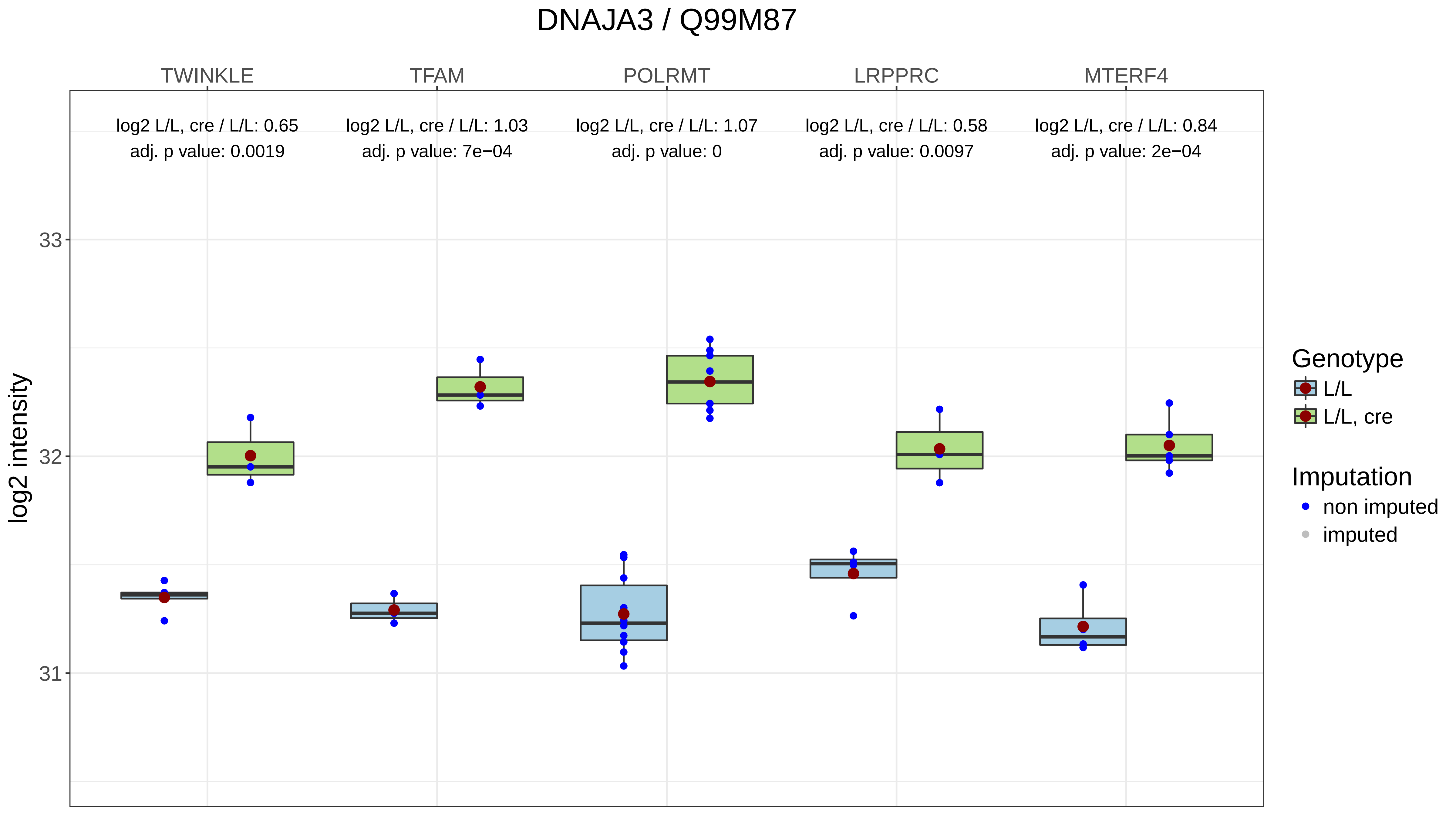
DNAJA3 / Q99M87
TWINKLE
TFAM
POLRMT
LRPPRC
MTERF4
log2 L/L, cre / L/L: 0.65
adj. p value: 0.0019
log2 L/L, cre / L/L: 1.03
adj. p value: 7e−04
log2 L/L, cre / L/L: 1.07
adj. p value: 0
log2 L/L, cre / L/L: 0.58
adj. p value: 0.0097
log2 L/L, cre / L/L: 0.84
adj. p value: 2e−04
33
32
31
log2 intensity
Genotype
L/L
L/L, cre
Imputation
non imputed
imputed

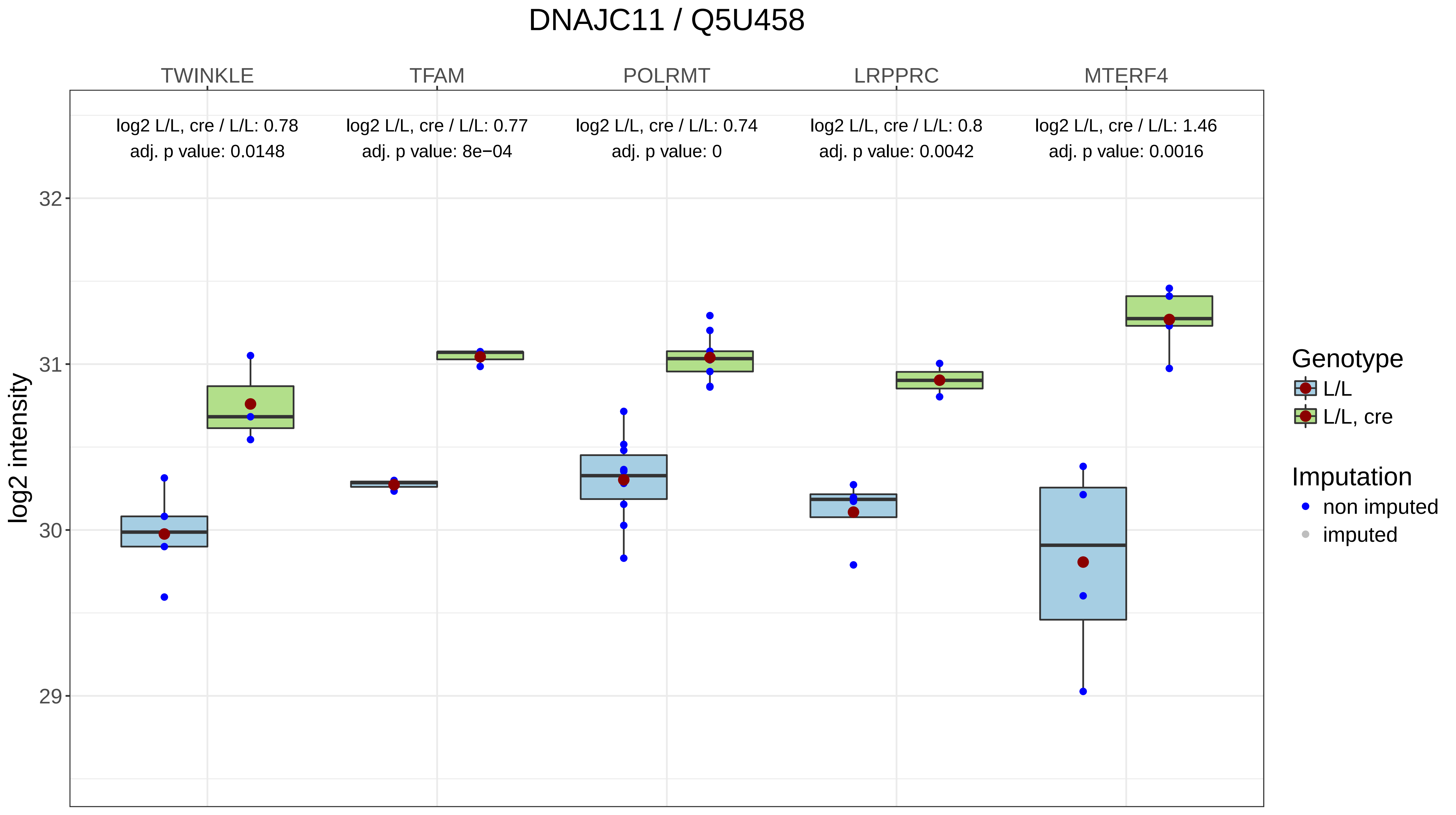

DNAJC11 / Q5U458
TWINKLE
TFAM
POLRMT
LRPPRC
MTERF4
log2 L/L, cre / L/L: 0.78
adj. p value: 0.0148
log2 L/L, cre / L/L: 0.77
adj. p value: 8e−04
log2 L/L, cre / L/L: 0.74
adj. p value: 0
log2 L/L, cre / L/L: 0.8
adj. p value: 0.0042
log2 L/L, cre / L/L: 1.46
adj. p value: 0.0016
log2 intensity
32
31
30
29
Genotype
L/L
L/L, cre
Imputation
non imputed
imputed

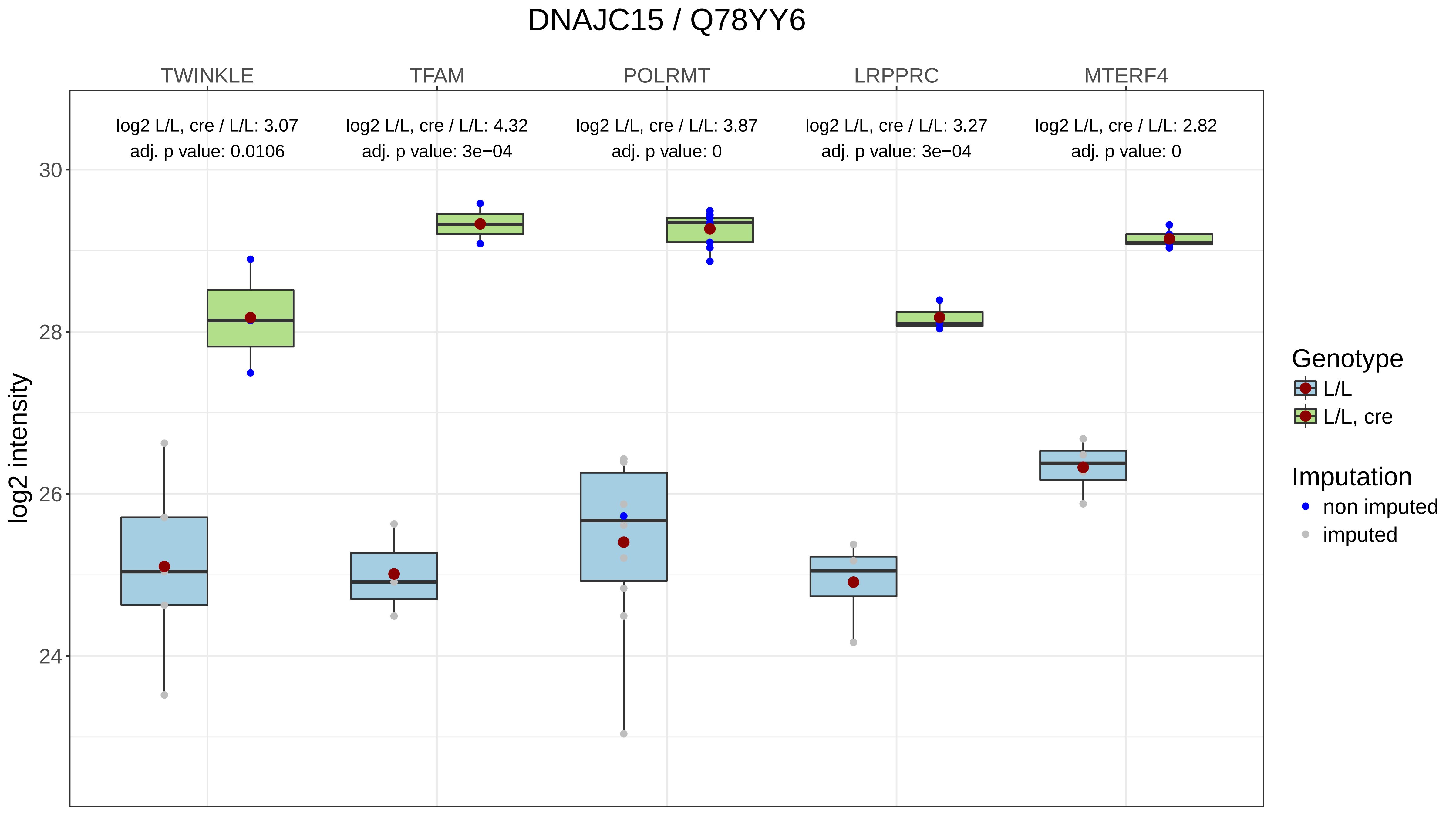

DNAJC15 / Q78YY6
TWINKLE
TFAM
POLRMT
LRPPRC
MTERF4
log2 L/L, cre / L/L: 3.07
adj. p value: 0.0106
log2 L/L, cre / L/L: 4.32
adj. p value: 3e−04
log2 L/L, cre / L/L: 3.87
adj. p value: 0
log2 L/L, cre / L/L: 3.27
adj. p value: 3e−04
log2 L/L, cre / L/L: 2.82
adj. p value: 0
log2 intensity
30
28
26
24
Genotype
L/L
L/L, cre
Imputation
non imputed
imputed

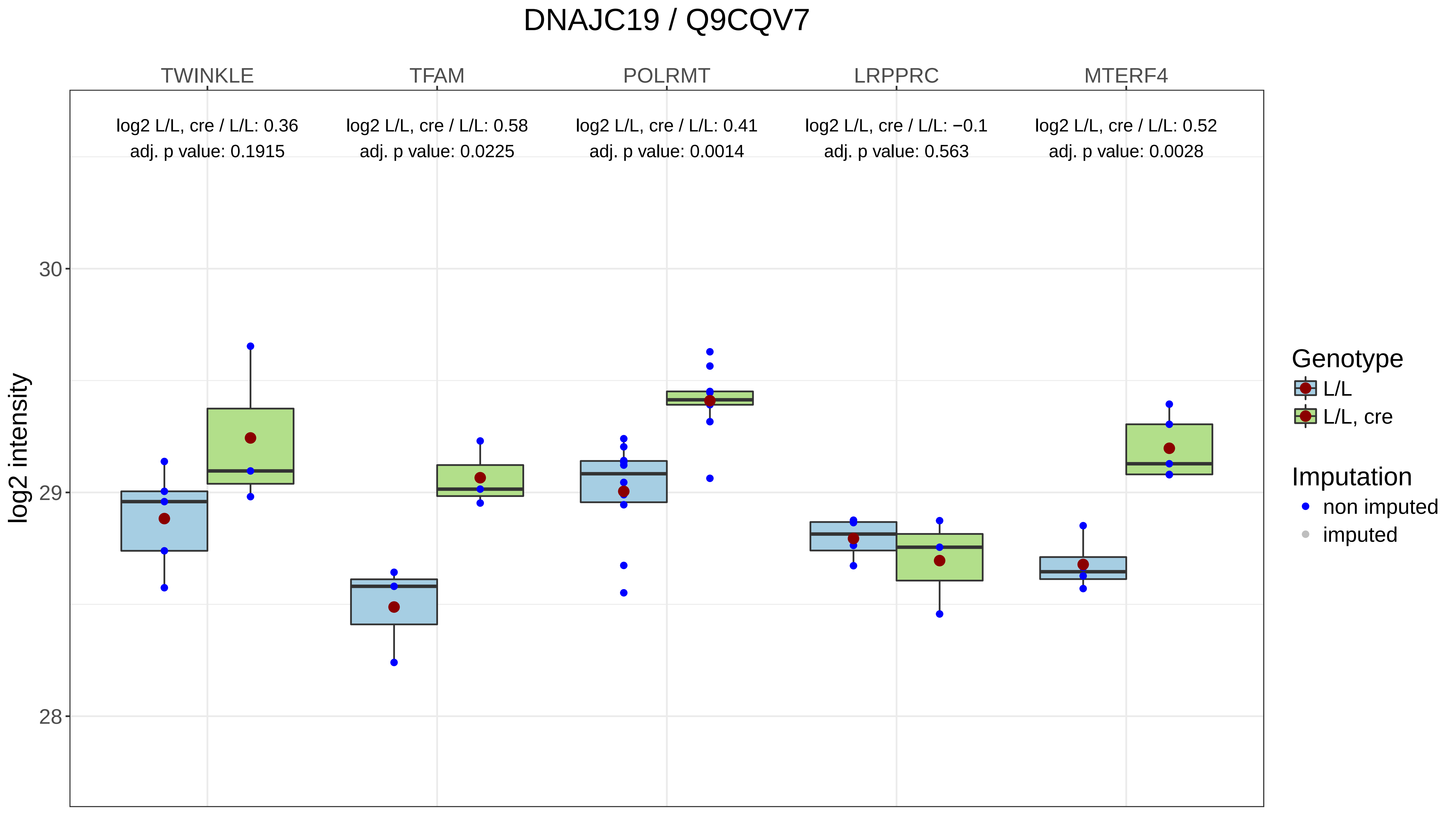

DNAJC19 / Q9CQV7
TWINKLE
TFAM
POLRMT
LRPPRC
MTERF4
log2 L/L, cre / L/L: 0.36
adj. p value: 0.1915
log2 L/L, cre / L/L: 0.58
adj. p value: 0.0225
log2 L/L, cre / L/L: 0.41
adj. p value: 0.0014
log2 L/L, cre / L/L: −0.1
adj. p value: 0.563
log2 L/L, cre / L/L: 0.52
adj. p value: 0.0028
log2 intensity
30
29
28
Genotype
L/L
L/L, cre
Imputation
non imputed
imputed

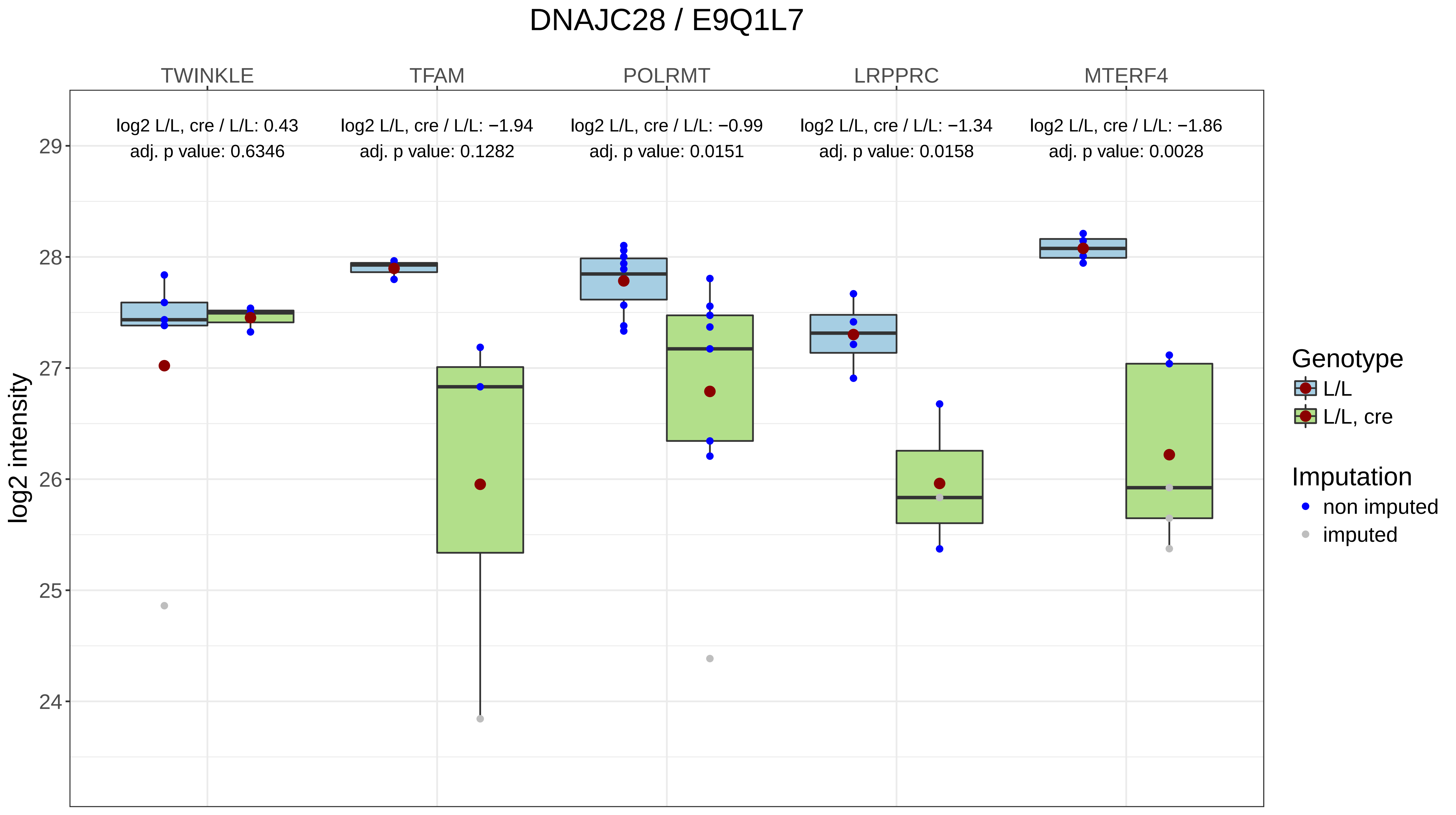
DNAJC28 / E9Q1L7
TWINKLE
TFAM
POLRMT
LRPPRC
MTERF4
log2 L/L, cre / L/L: 0.43
adj. p value: 0.6346
log2 L/L, cre / L/L: −1.94
adj. p value: 0.1282
log2 L/L, cre / L/L: −0.99
adj. p value: 0.0151
log2 L/L, cre / L/L: −1.34
adj. p value: 0.0158
log2 L/L, cre / L/L: −1.86
adj. p value: 0.0028
log2 intensity
29
28
27
26
25
24
Genotype
L/L
L/L, cre
Imputation
non imputed
imputed

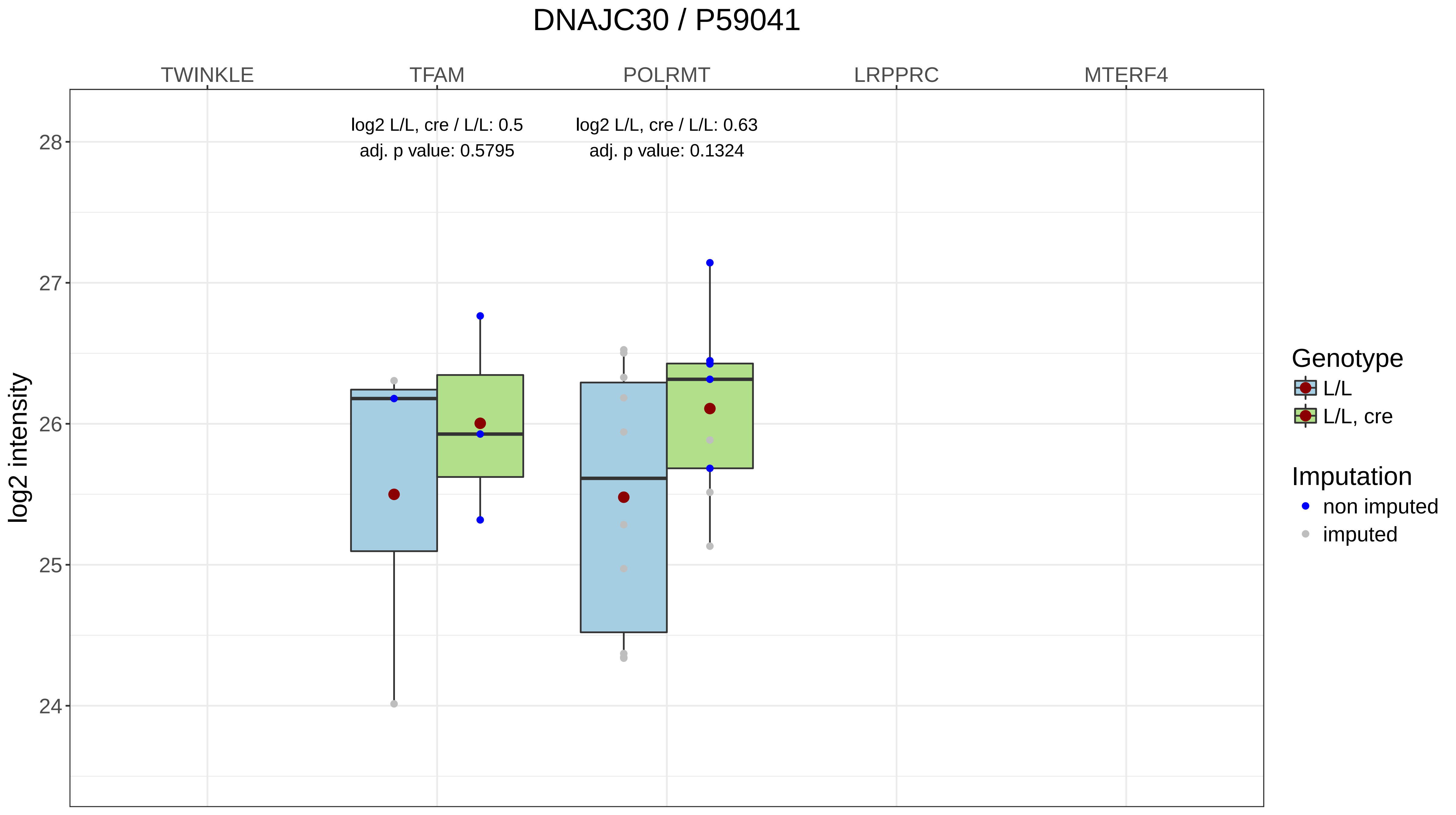

DNAJC30 / P59041
TWINKLE
TFAM
POLRMT
LRPPRC
MTERF4
log2 L/L, cre / L/L: 0.5
adj. p value: 0.5795
log2 L/L, cre / L/L: 0.63
adj. p value: 0.1324
28
27
26
25
24
log2 intensity
Genotype
L/L
L/L, cre
Imputation
non imputed
imputed

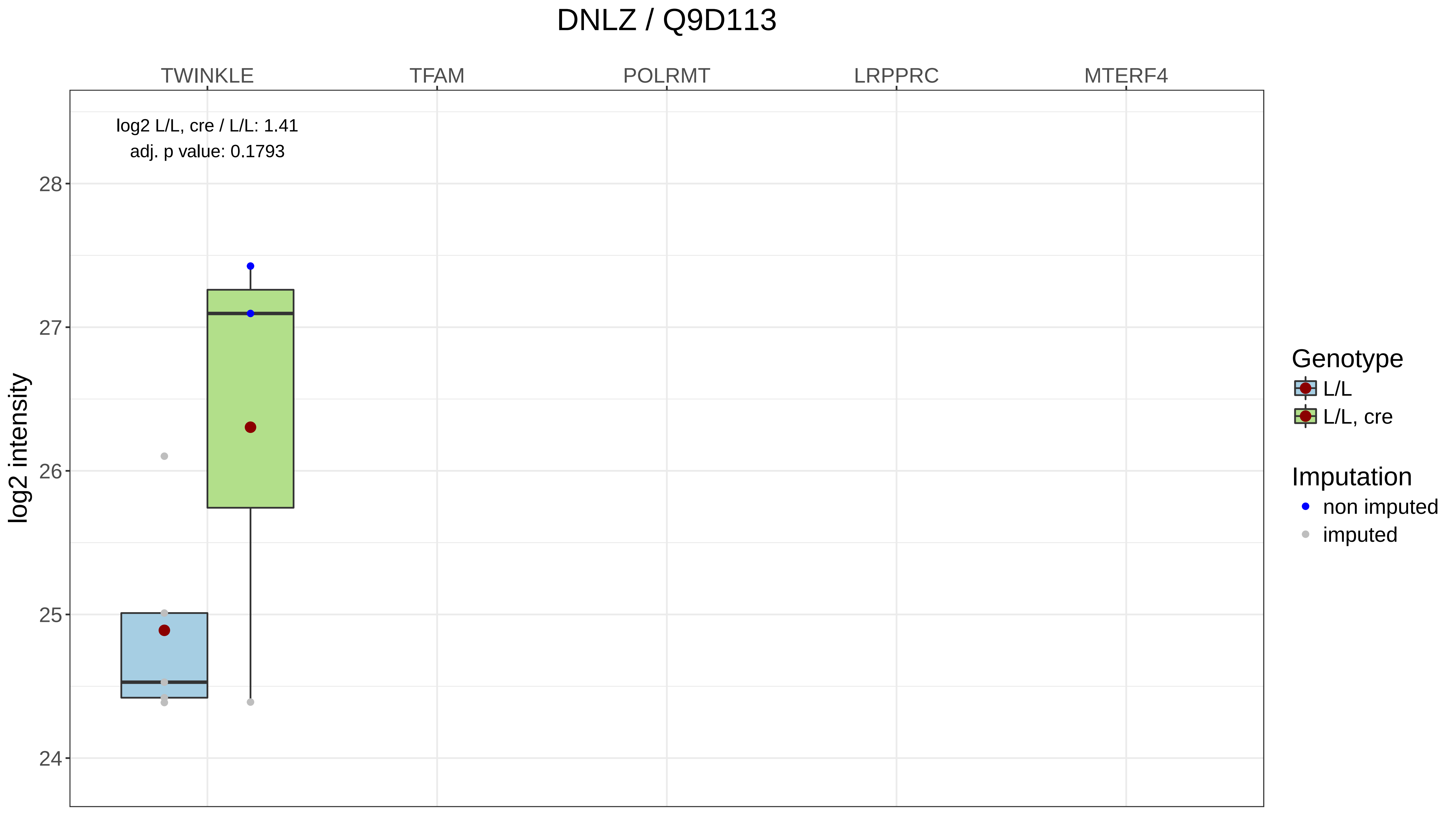
DNLZ / Q9D113
TWINKLE
TFAM
POLRMT
LRPPRC
MTERF4
log2 L/L, cre / L/L: 1.41
adj. p value: 0.1793
log2 intensity
28
27
26
25
24
Genotype
L/L
L/L, cre
Imputation
non imputed
imputed

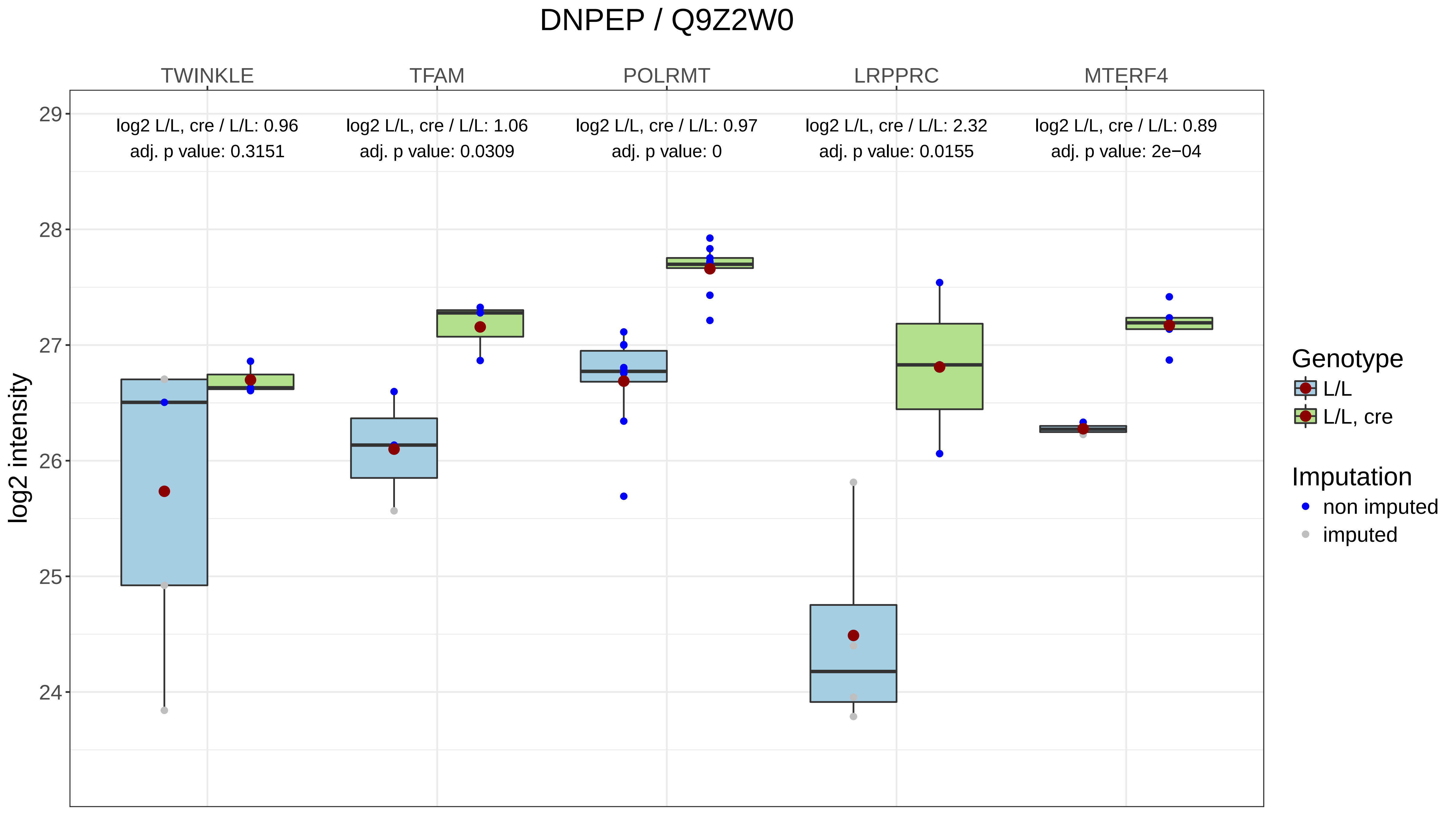

DNPEP / Q9Z2W0
TWINKLE
TFAM
POLRMT
LRPPRC
MTERF4
log2 L/L, cre / L/L: 0.96
adj. p value: 0.3151
log2 L/L, cre / L/L: 1.06
adj. p value: 0.0309
log2 L/L, cre / L/L: 0.97
adj. p value: 0
log2 L/L, cre / L/L: 2.32
adj. p value: 0.0155
log2 L/L, cre / L/L: 0.89
adj. p value: 2e−04
log2 intensity
29
28
27
26
25
24
Genotype
L/L
L/L, cre
Imputation
non imputed
imputed

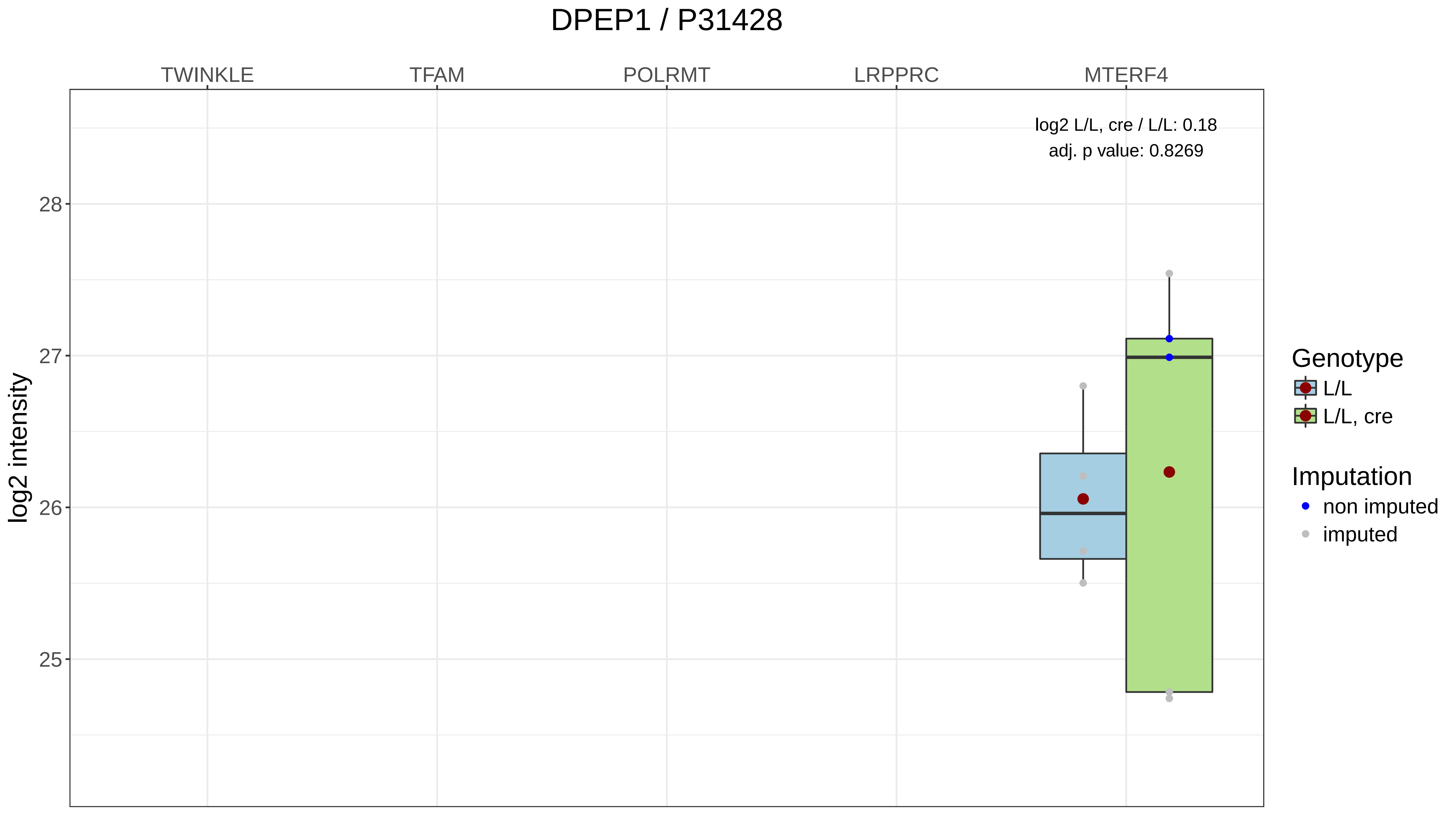
DPEP1 / P31428
TWINKLE
TFAM
POLRMT
LRPPRC
MTERF4
log2 L/L, cre / L/L: 0.18
adj. p value: 0.8269
log2 intensity
28
27
26
25
Genotype
L/L
L/L, cre
Imputation
non imputed
imputed

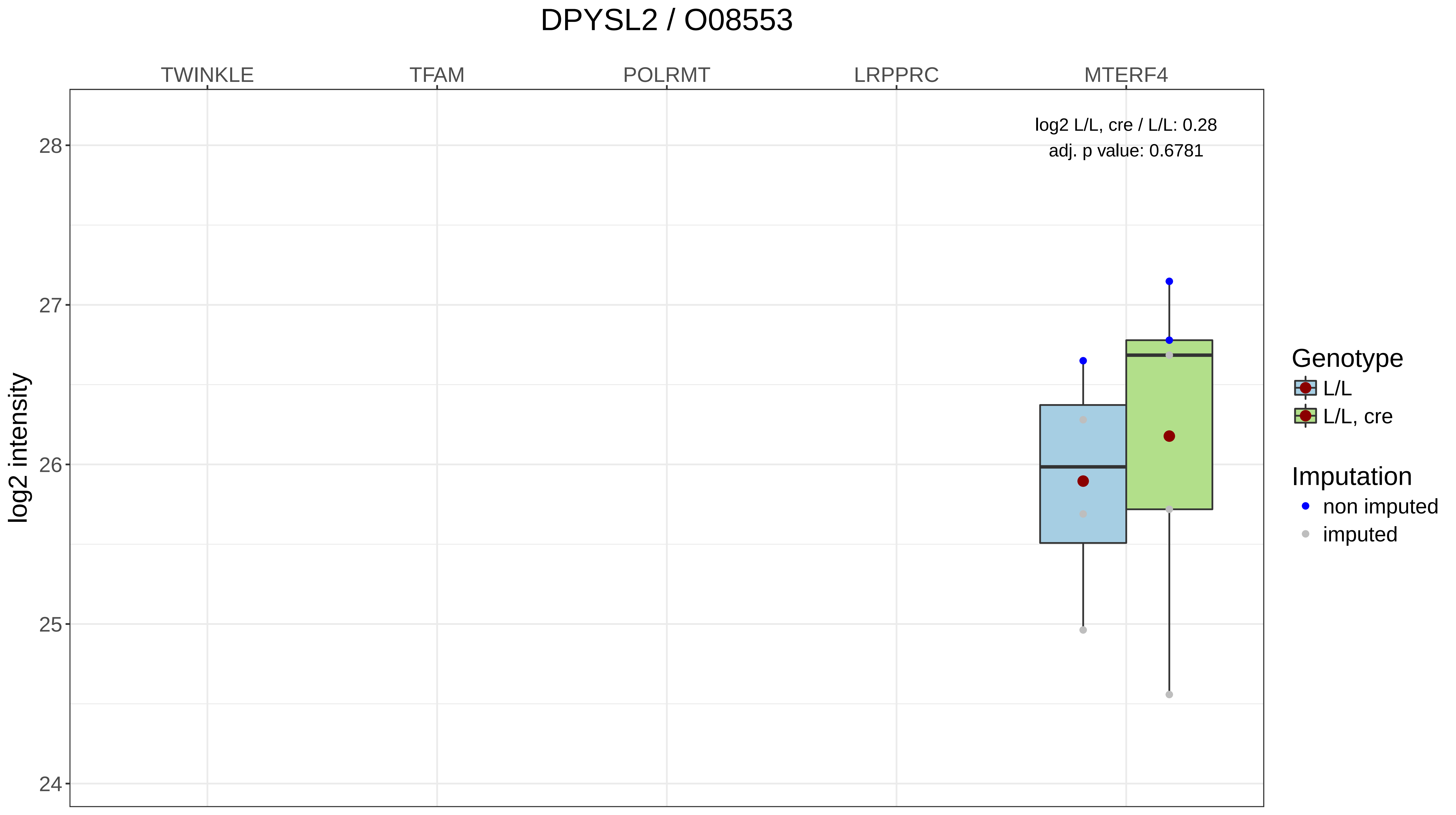

DPYSL2 / O08553
TWINKLE
TFAM
POLRMT
LRPPRC
MTERF4
log2 L/L, cre / L/L: 0.28
adj. p value: 0.6781
log2 intensity
28
27
26
25
24
Genotype
L/L
L/L, cre
Imputation
non imputed
imputed

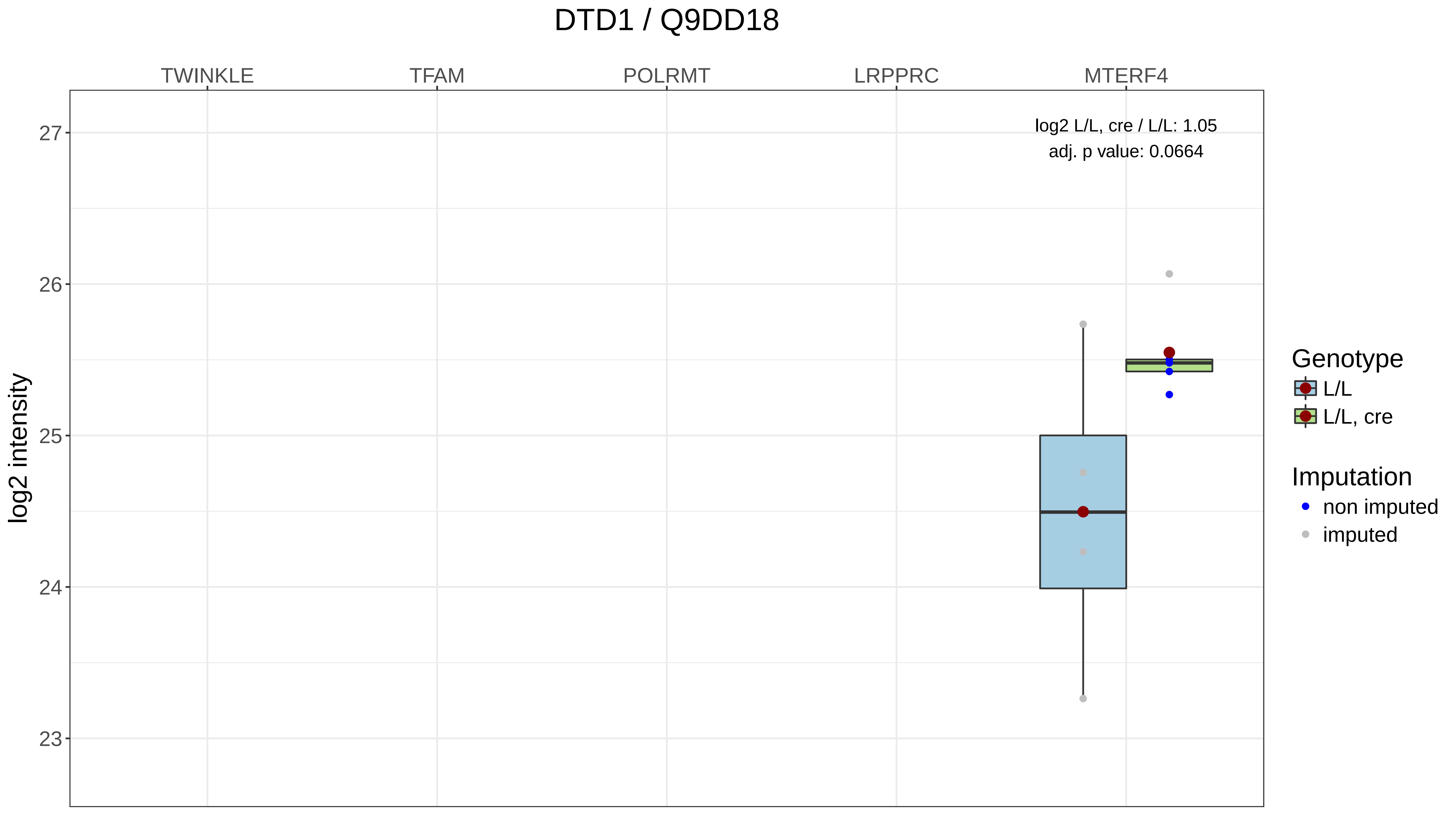

DTD1 / Q9DD18
TWINKLE
TFAM
POLRMT
LRPPRC
MTERF4
log2 L/L, cre / L/L: 1.05
adj. p value: 0.0664
27
26
25
24
23
log2 intensity
Genotype
L/L
L/L, cre
Imputation
non imputed
imputed

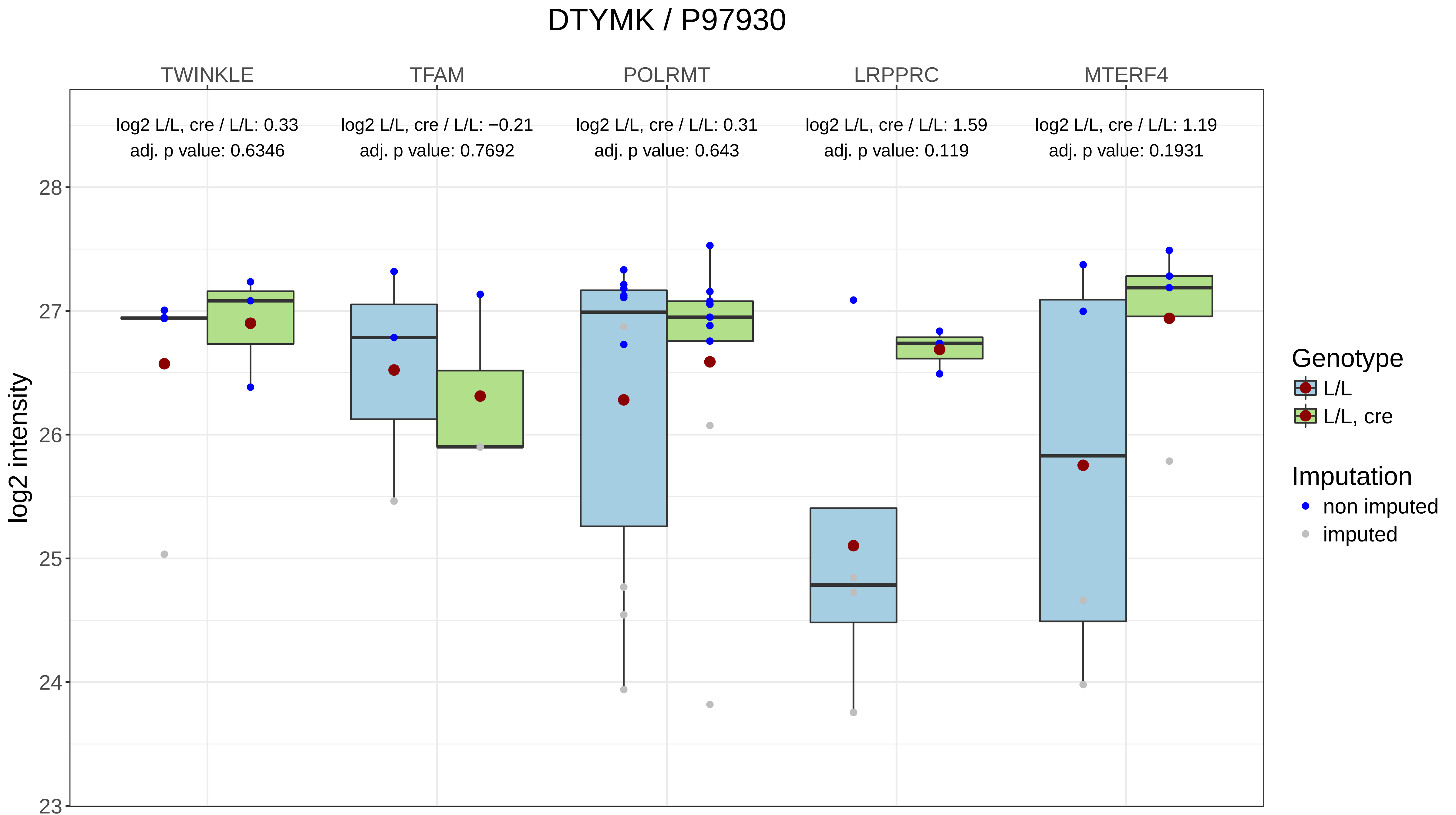
DTYMK / P97930
TWINKLE
TFAM
POLRMT
LRPPRC
MTERF4
log2 L/L, cre / L/L: 0.33
adj. p value: 0.6346
log2 L/L, cre / L/L: −0.21
adj. p value: 0.7692
log2 L/L, cre / L/L: 0.31
adj. p value: 0.643
log2 L/L, cre / L/L: 1.59
adj. p value: 0.119
log2 L/L, cre / L/L: 1.19
adj. p value: 0.1931
log2 intensity
28
27
26
25
24
23
Genotype
L/L
L/L, cre
Imputation
non imputed
imputed

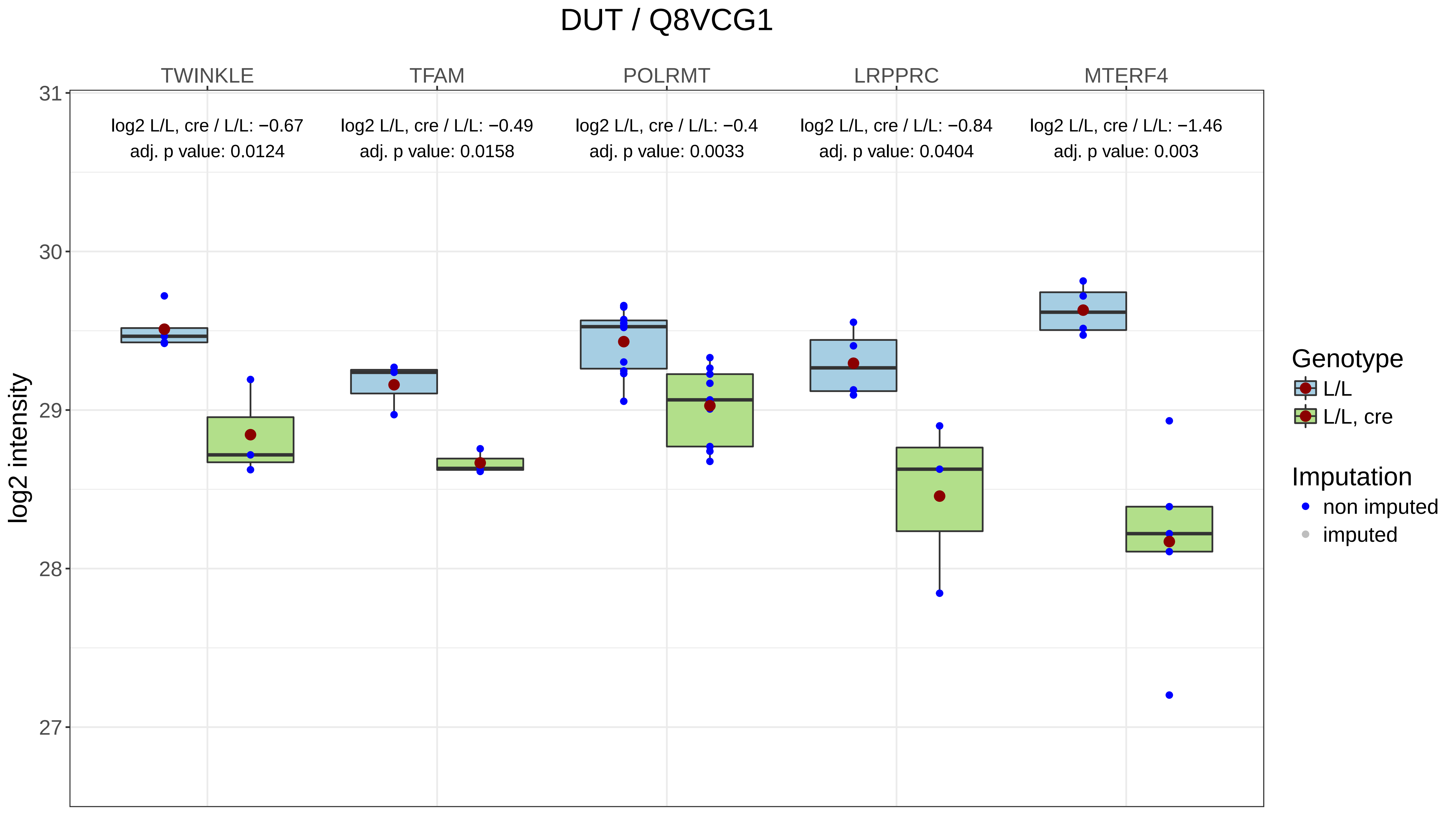
DUT / Q8VCG1
TWINKLE
TFAM
POLRMT
LRPPRC
MTERF4
log2 L/L, cre / L/L: −0.67
adj. p value: 0.0124
log2 L/L, cre / L/L: −0.49
adj. p value: 0.0158
log2 L/L, cre / L/L: −0.4
adj. p value: 0.0033
log2 L/L, cre / L/L: −0.84
adj. p value: 0.0404
log2 L/L, cre / L/L: −1.46
adj. p value: 0.003
log2 intensity
31
30
29
28
27
Genotype
L/L
L/L, cre
Imputation
non imputed
imputed

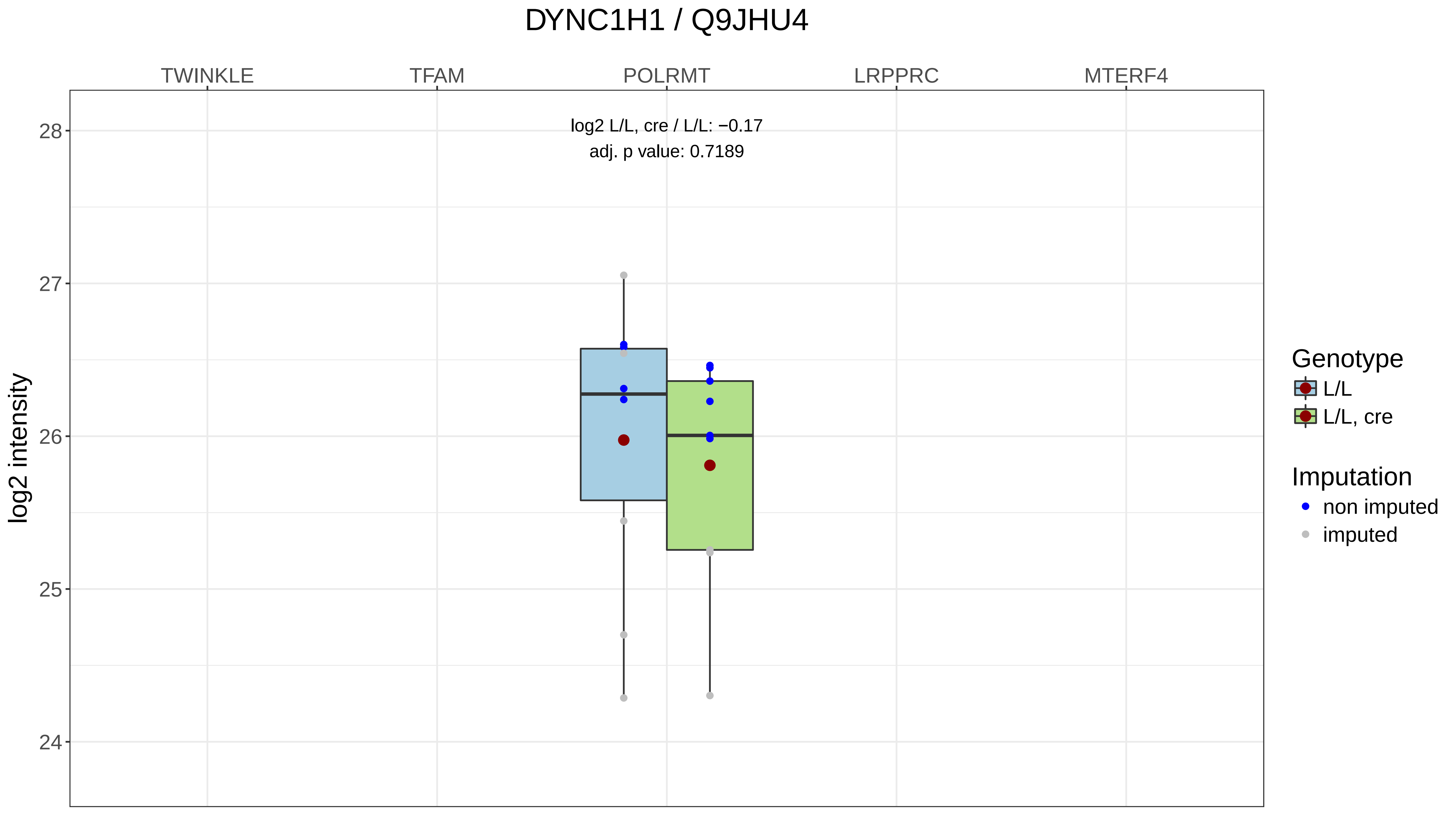
DYNC1H1 / Q9JHU4
TWINKLE
TFAM
POLRMT
LRPPRC
MTERF4
log2 L/L, cre / L/L: −0.17
adj. p value: 0.7189
log2 intensity
28
27
26
25
24
Genotype
L/L
L/L, cre
Imputation
non imputed
imputed

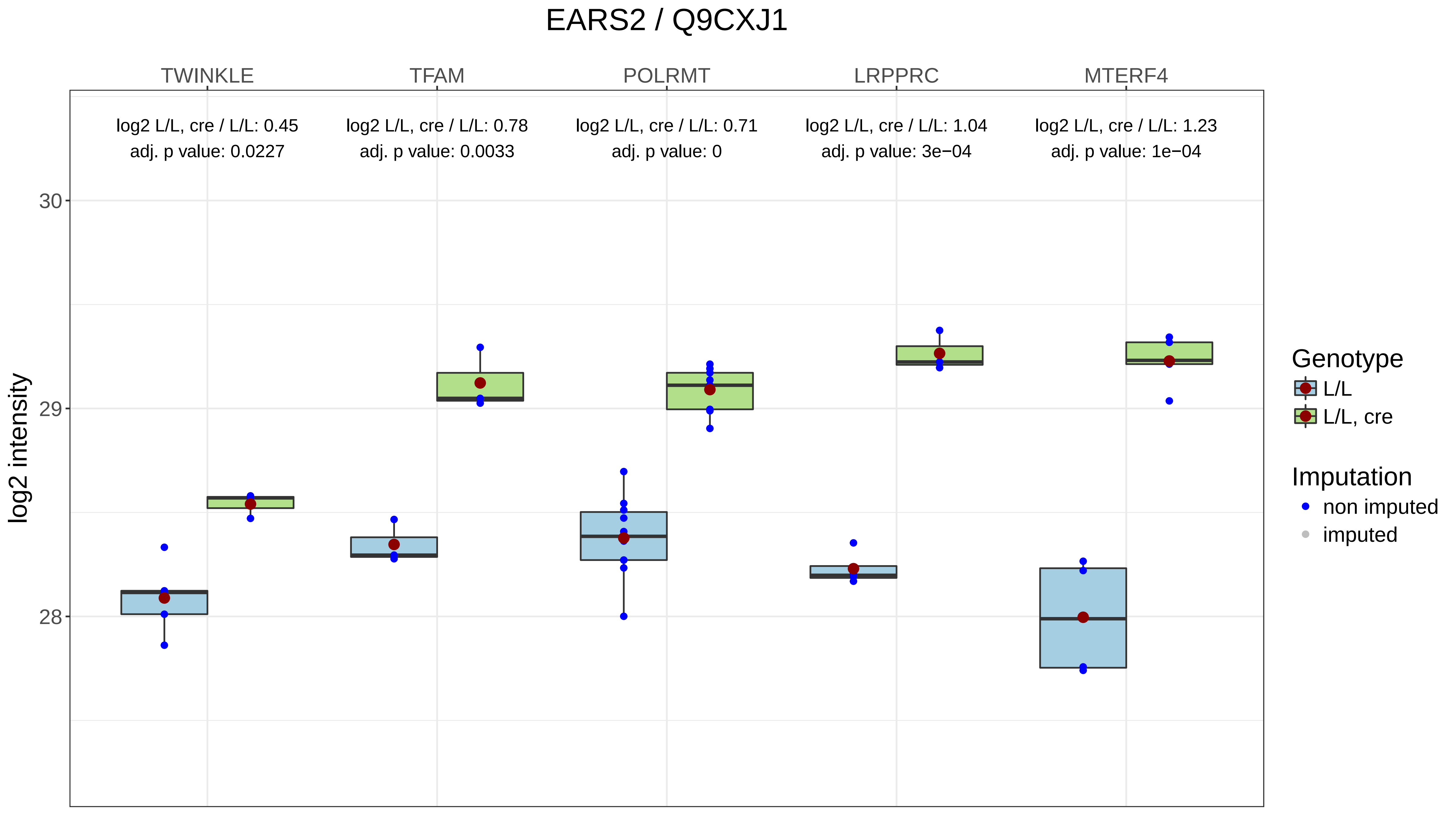
EARS2 / Q9CXJ1
TWINKLE
TFAM
POLRMT
LRPPRC
MTERF4
log2 L/L, cre / L/L: 0.45
adj. p value: 0.0227
log2 L/L, cre / L/L: 0.78
adj. p value: 0.0033
log2 L/L, cre / L/L: 0.71
adj. p value: 0
log2 L/L, cre / L/L: 1.04
adj. p value: 3e−04
log2 L/L, cre / L/L: 1.23
adj. p value: 1e−04
log2 intensity
30
29
28
Genotype
L/L
L/L, cre
Imputation
non imputed
imputed

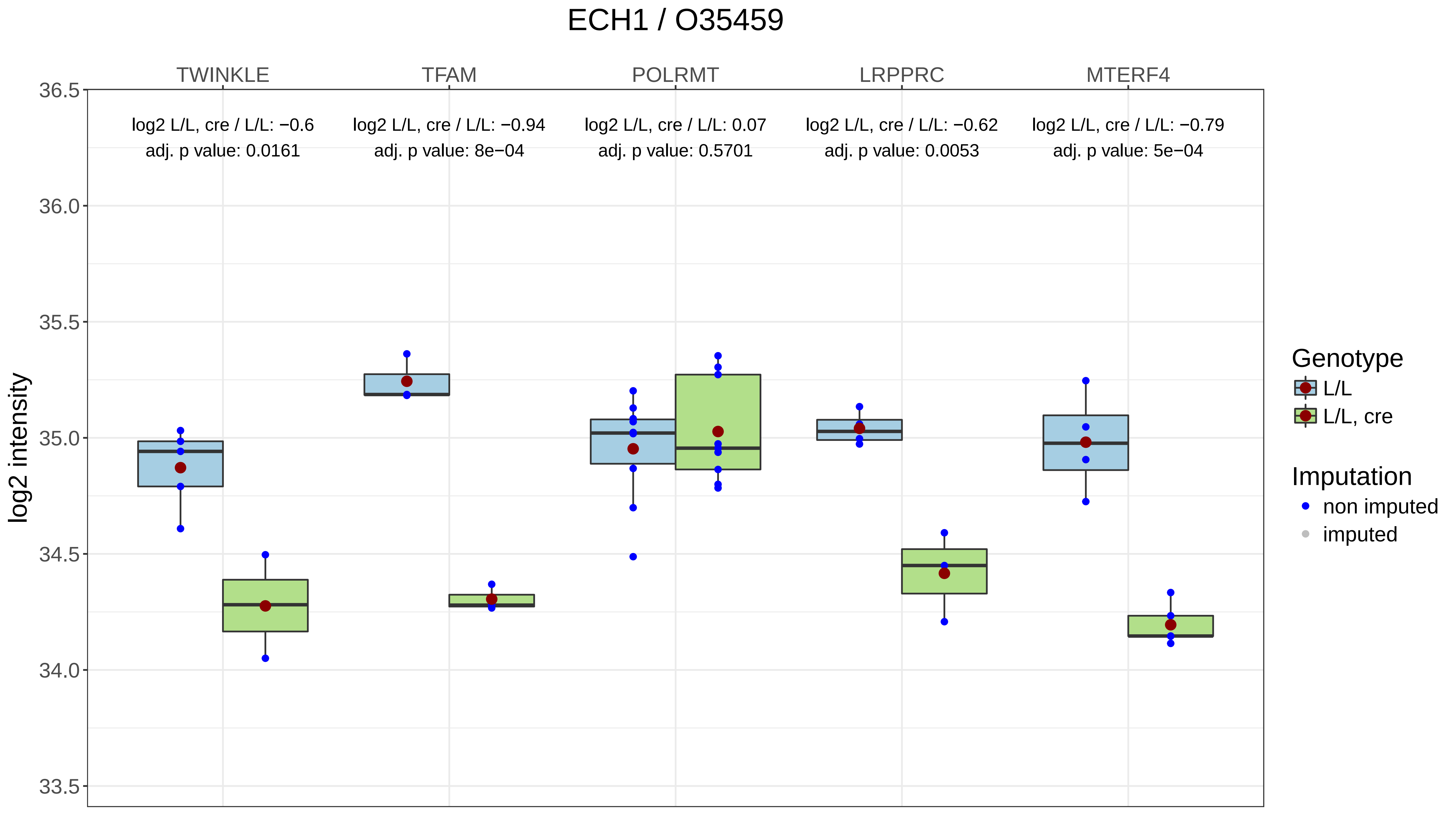
ECH1 / O35459
TWINKLE
TFAM
POLRMT
LRPPRC
MTERF4
log2 L/L, cre / L/L: −0.6
adj. p value: 0.0161
log2 L/L, cre / L/L: −0.94
adj. p value: 8e−04
log2 L/L, cre / L/L: 0.07
adj. p value: 0.5701
log2 L/L, cre / L/L: −0.62
adj. p value: 0.0053
log2 L/L, cre / L/L: −0.79
adj. p value: 5e−04
log2 intensity
36.5
36.0
35.5
35.0
34.5
34.0
33.5
Genotype
L/L
L/L, cre
Imputation
non imputed
imputed

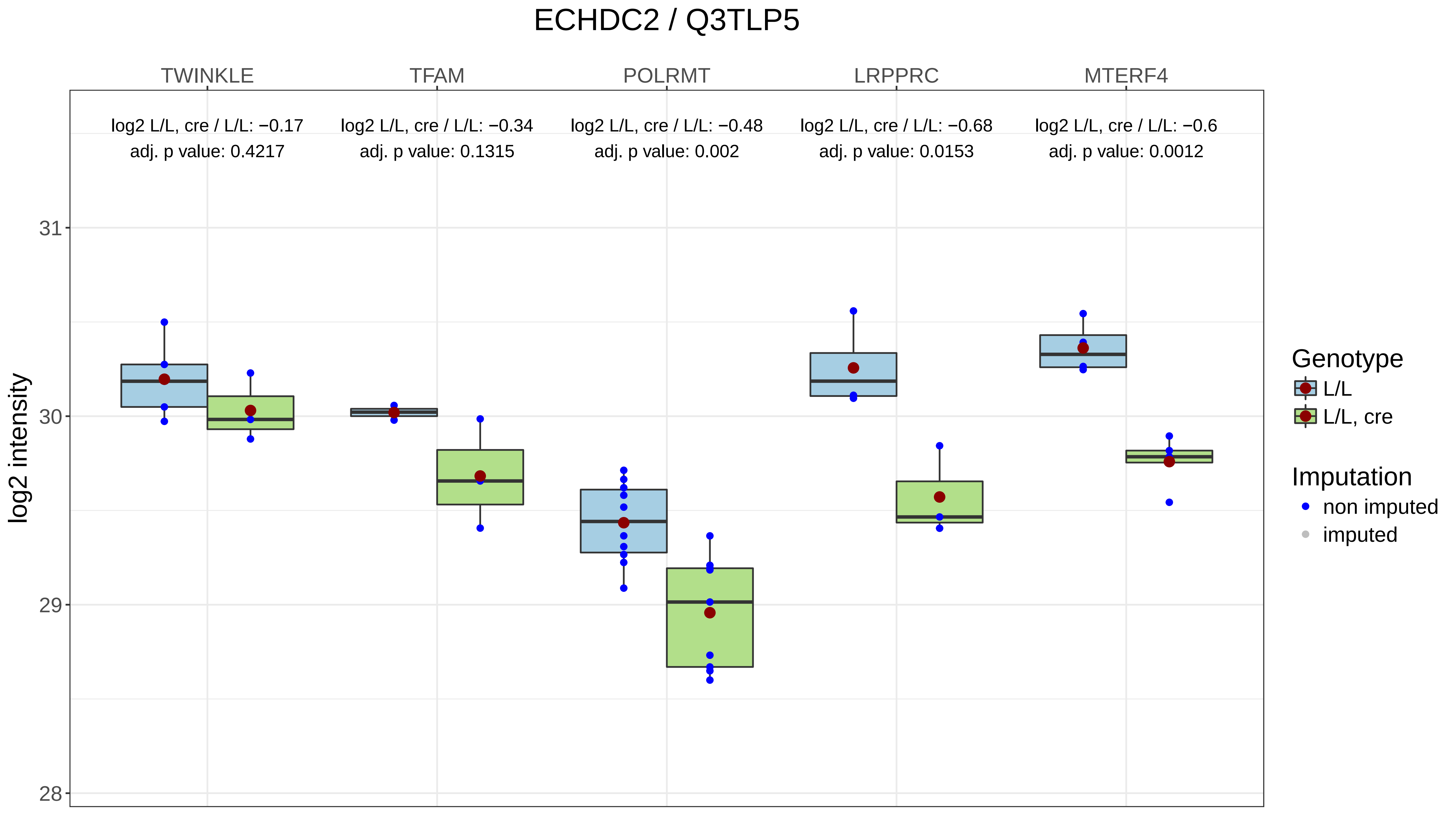

ECHDC2 / Q3TLP5
TWINKLE
TFAM
POLRMT
LRPPRC
MTERF4
log2 L/L, cre / L/L: −0.17
adj. p value: 0.4217
log2 L/L, cre / L/L: −0.34
adj. p value: 0.1315
log2 L/L, cre / L/L: −0.48
adj. p value: 0.002
log2 L/L, cre / L/L: −0.68
adj. p value: 0.0153
log2 L/L, cre / L/L: −0.6
adj. p value: 0.0012
log2 intensity
31
30
29
28
Genotype
L/L
L/L, cre
Imputation
non imputed
imputed

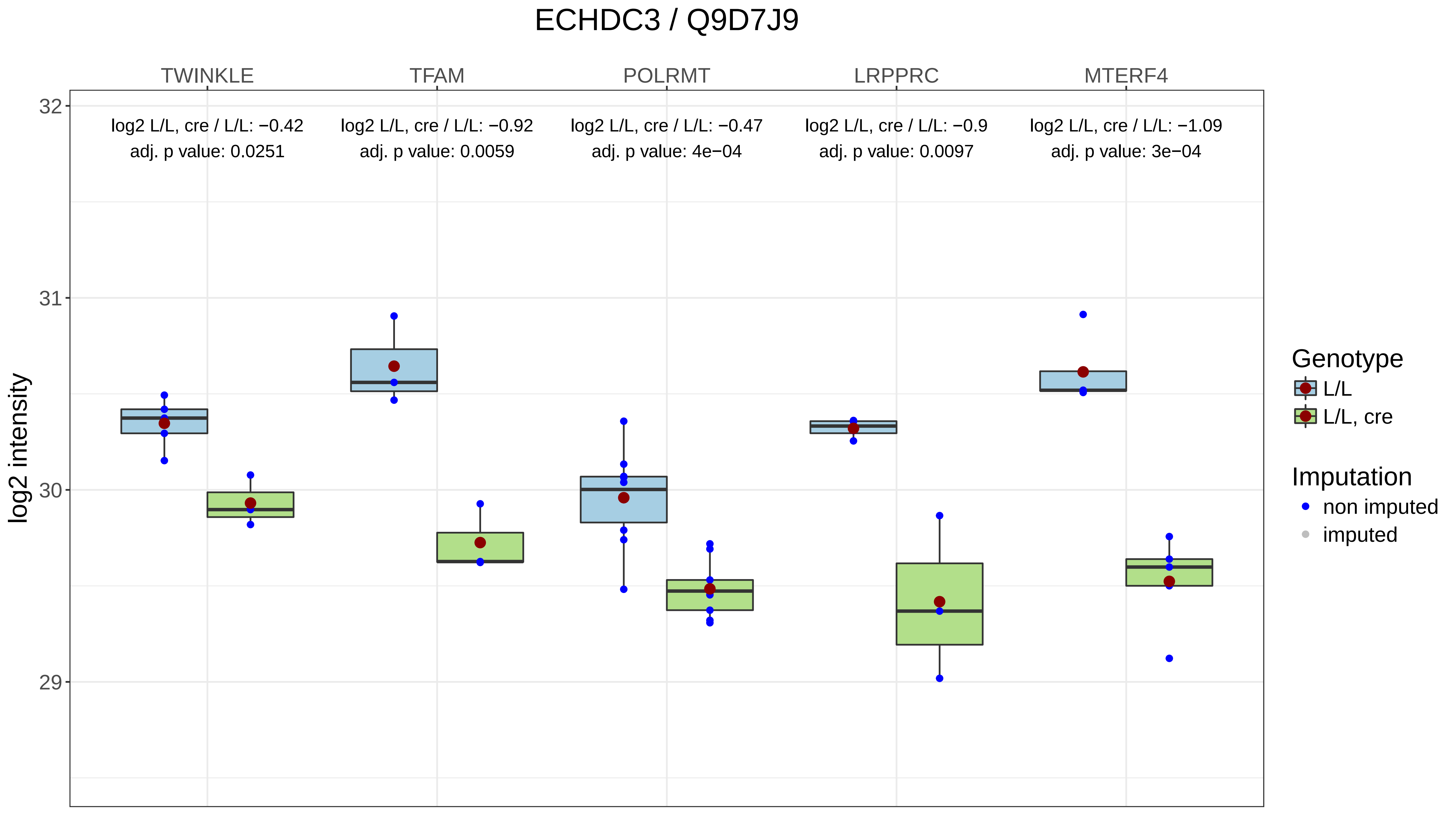
ECHDC3 / Q9D7J9
TWINKLE
TFAM
POLRMT
LRPPRC
MTERF4
log2 L/L, cre / L/L: −0.42
adj. p value: 0.0251
log2 L/L, cre / L/L: −0.92
adj. p value: 0.0059
log2 L/L, cre / L/L: −0.47
adj. p value: 4e−04
log2 L/L, cre / L/L: −0.9
adj. p value: 0.0097
log2 L/L, cre / L/L: −1.09
adj. p value: 3e−04
32
31
30
29
log2 intensity
Genotype
L/L
L/L, cre
Imputation
non imputed
imputed

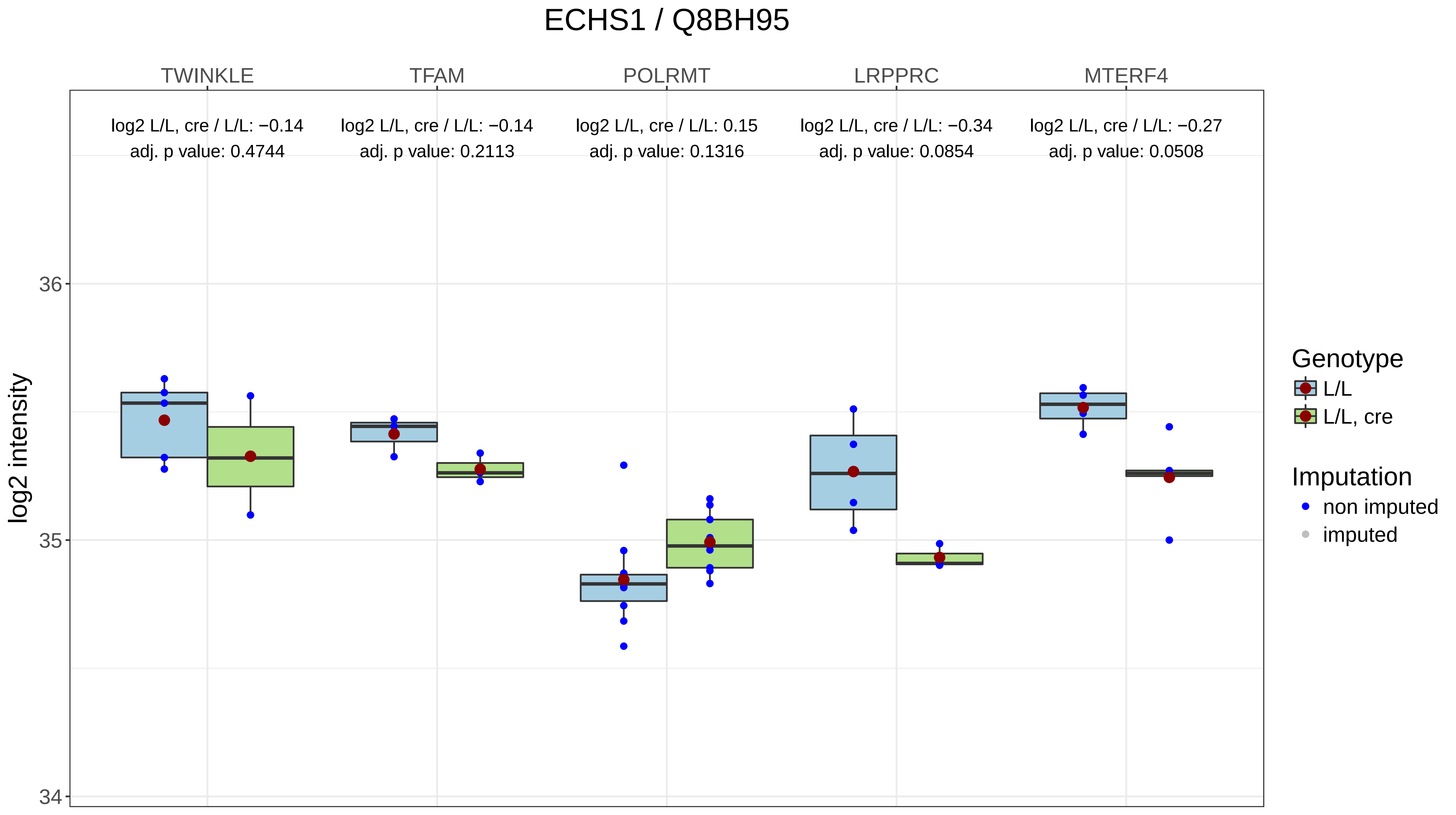
ECHS1 / Q8BH95
TWINKLE
TFAM
POLRMT
LRPPRC
MTERF4
log2 L/L, cre / L/L: −0.14
adj. p value: 0.4744
log2 L/L, cre / L/L: −0.14
adj. p value: 0.2113
log2 L/L, cre / L/L: 0.15
adj. p value: 0.1316
log2 L/L, cre / L/L: −0.34
adj. p value: 0.0854
log2 L/L, cre / L/L: −0.27
adj. p value: 0.0508
log2 intensity
36
35
34
Genotype
L/L
L/L, cre
Imputation
non imputed
imputed

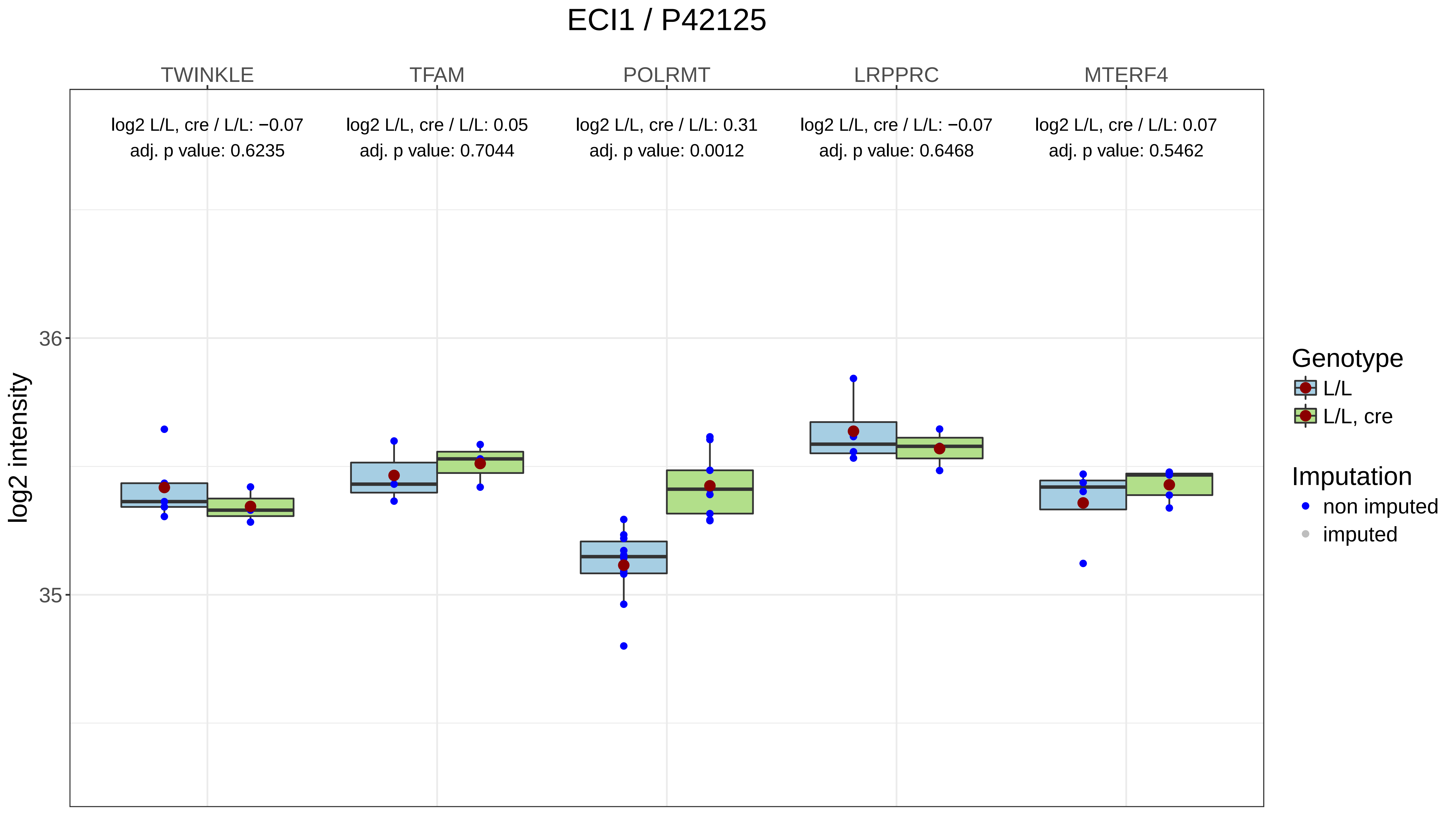

ECI1 / P42125
TWINKLE
TFAM
POLRMT
LRPPRC
MTERF4
log2 L/L, cre / L/L: −0.07
adj. p value: 0.6235
log2 L/L, cre / L/L: 0.05
adj. p value: 0.7044
log2 L/L, cre / L/L: 0.31
adj. p value: 0.0012
log2 L/L, cre / L/L: −0.07
adj. p value: 0.6468
log2 L/L, cre / L/L: 0.07
adj. p value: 0.5462
36
35
log2 intensity
Genotype
L/L
L/L, cre
Imputation
non imputed
imputed

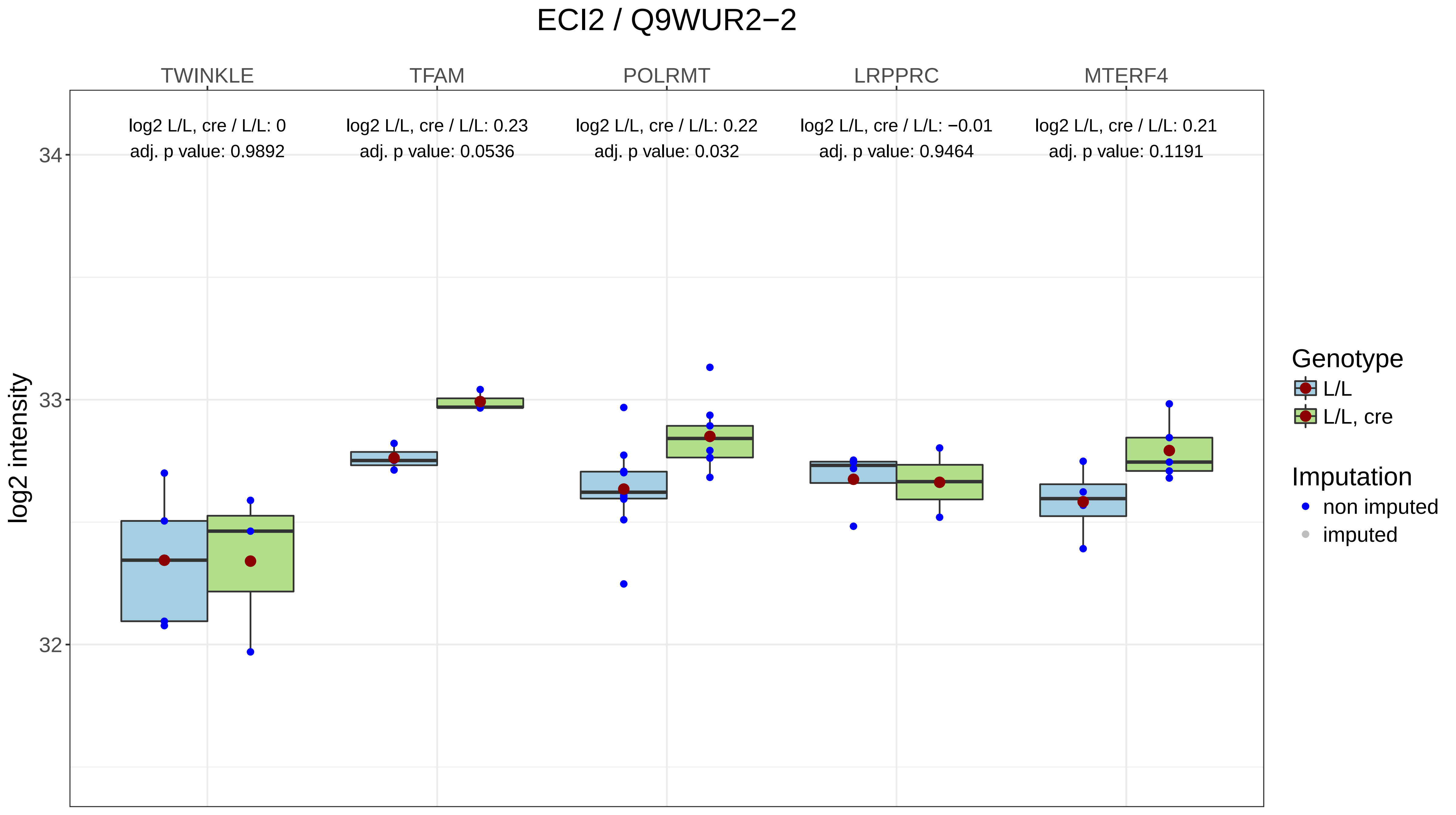
ECI2 / Q9WUR2−2
TWINKLE
TFAM
POLRMT
LRPPRC
MTERF4
log2 L/L, cre / L/L: 0
adj. p value: 0.9892
log2 L/L, cre / L/L: 0.23
adj. p value: 0.0536
log2 L/L, cre / L/L: 0.22
adj. p value: 0.032
log2 L/L, cre / L/L: −0.01
adj. p value: 0.9464
log2 L/L, cre / L/L: 0.21
adj. p value: 0.1191
log2 intensity
34
33
32
Genotype
L/L
L/L, cre
Imputation
non imputed
imputed

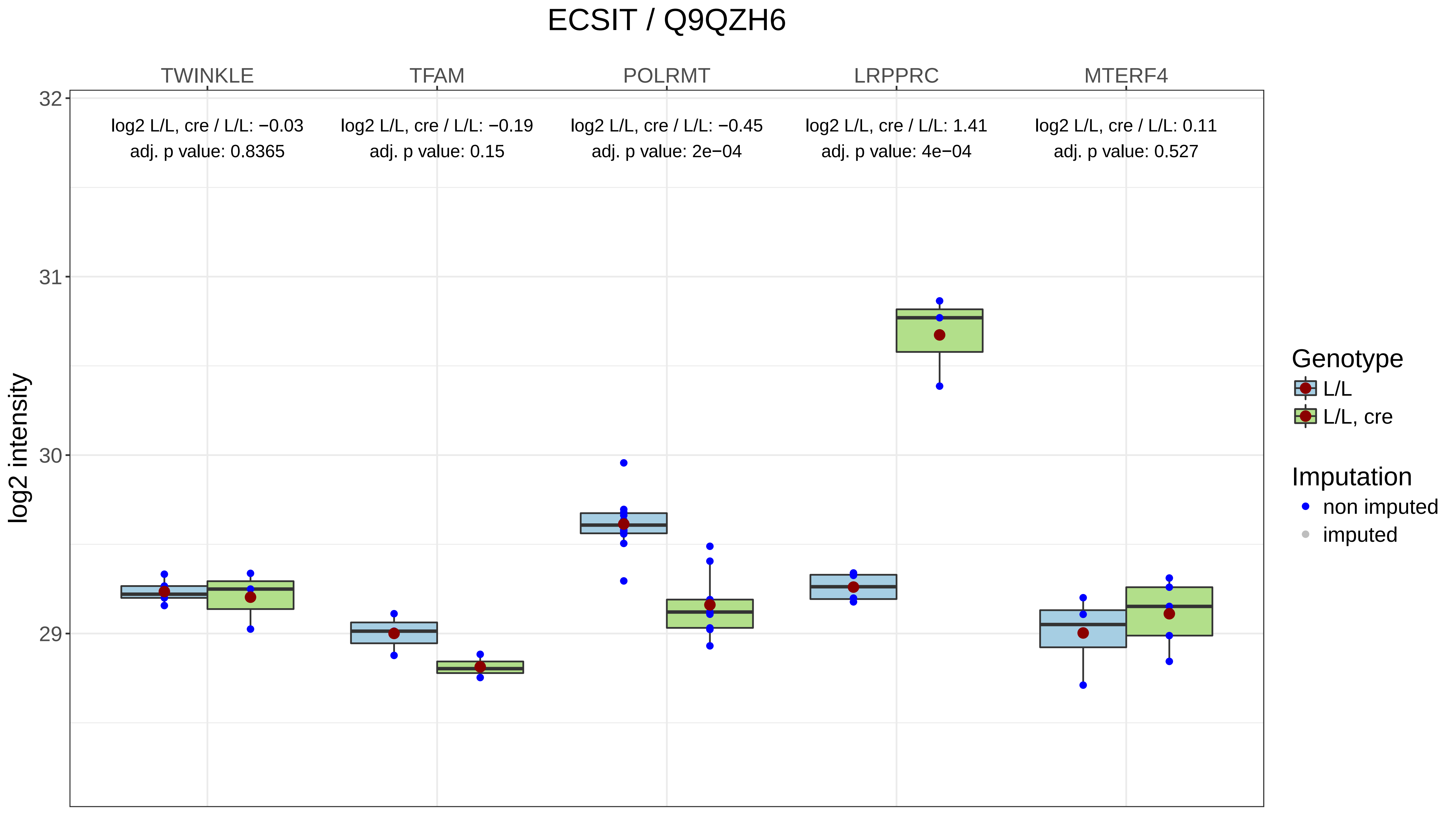

ECSIT / Q9QZH6
TWINKLE
TFAM
POLRMT
LRPPRC
MTERF4
log2 L/L, cre / L/L: −0.03
adj. p value: 0.8365
log2 L/L, cre / L/L: −0.19
adj. p value: 0.15
log2 L/L, cre / L/L: −0.45
adj. p value: 2e−04
log2 L/L, cre / L/L: 1.41
adj. p value: 4e−04
log2 L/L, cre / L/L: 0.11
adj. p value: 0.527
32
31
30
29
log2 intensity
Genotype
L/L
L/L, cre
Imputation
non imputed
imputed

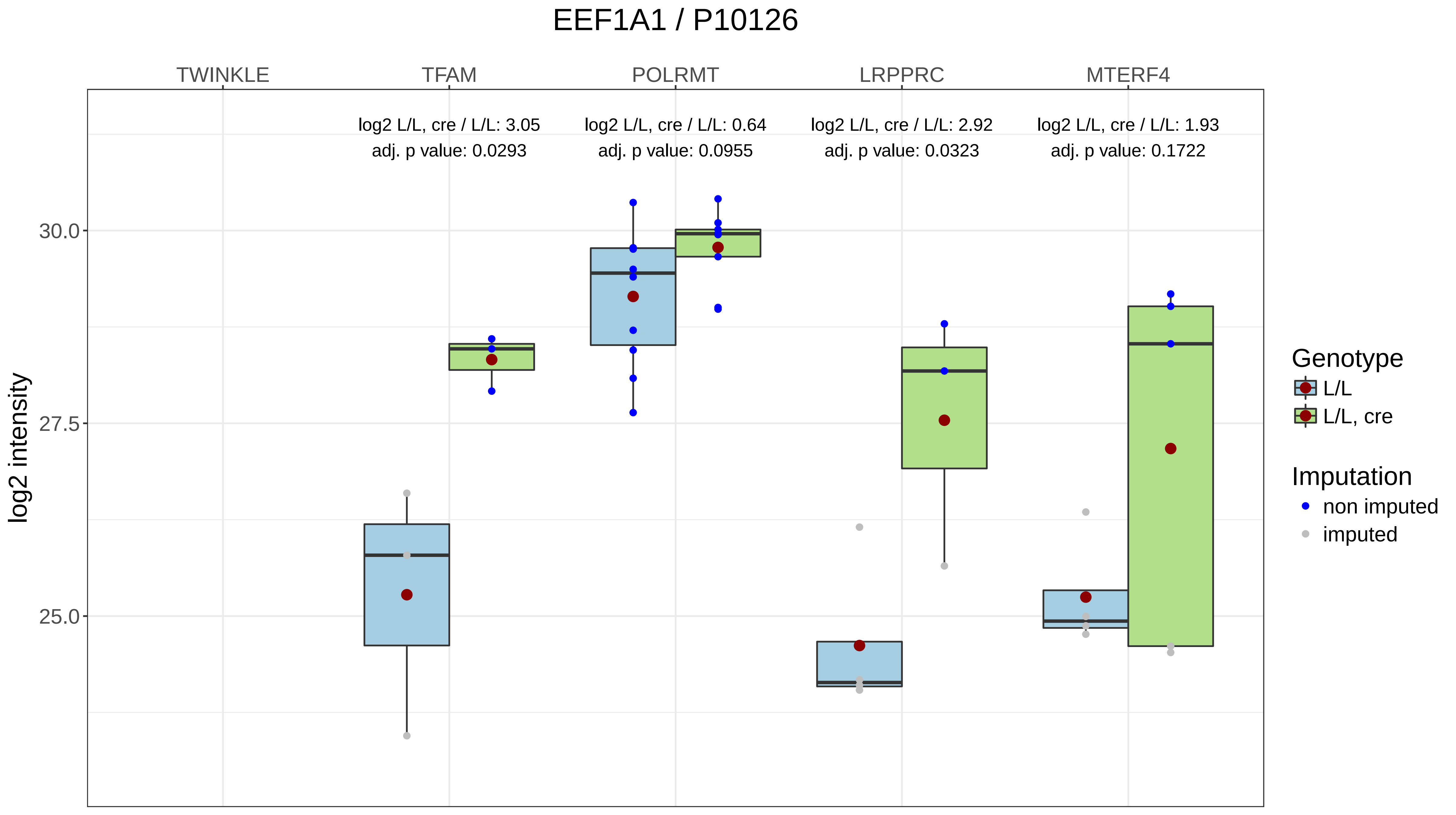
EEF1A1 / P10126
TWINKLE
TFAM
POLRMT
LRPPRC
MTERF4
log2 L/L, cre / L/L: 3.05
adj. p value: 0.0293
log2 L/L, cre / L/L: 0.64
adj. p value: 0.0955
log2 L/L, cre / L/L: 2.92
adj. p value: 0.0323
log2 L/L, cre / L/L: 1.93
adj. p value: 0.1722
log2 intensity
30.0
27.5
25.0
Genotype
L/L
L/L, cre
Imputation
non imputed
imputed

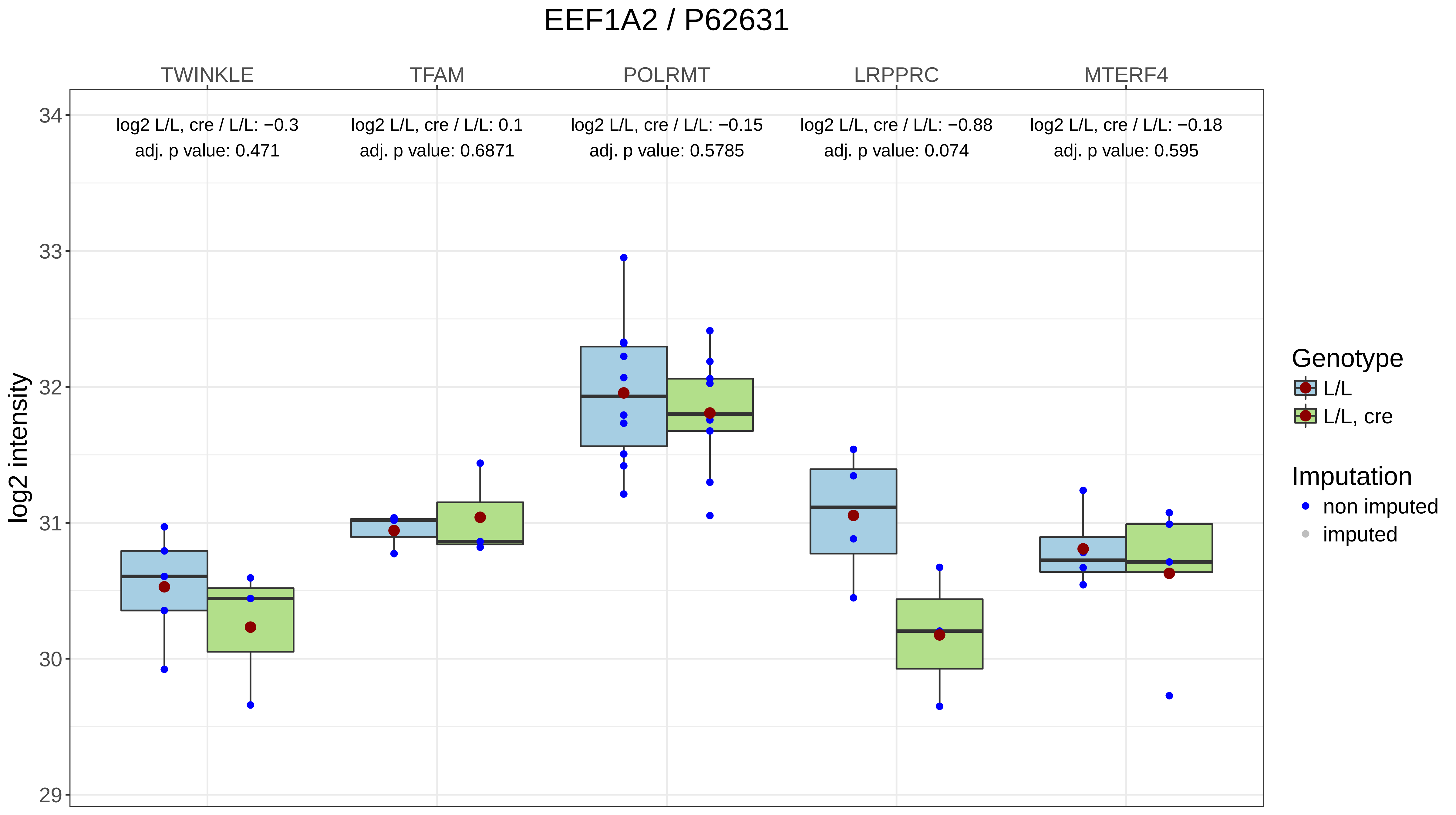
EEF1A2 / P62631
TWINKLE
TFAM
POLRMT
LRPPRC
MTERF4
log2 L/L, cre / L/L: −0.3
adj. p value: 0.471
log2 L/L, cre / L/L: 0.1
adj. p value: 0.6871
log2 L/L, cre / L/L: −0.15
adj. p value: 0.5785
log2 L/L, cre / L/L: −0.88
adj. p value: 0.074
log2 L/L, cre / L/L: −0.18
adj. p value: 0.595
log2 intensity
34
33
32
31
30
29
Genotype
L/L
L/L, cre
Imputation
non imputed
imputed

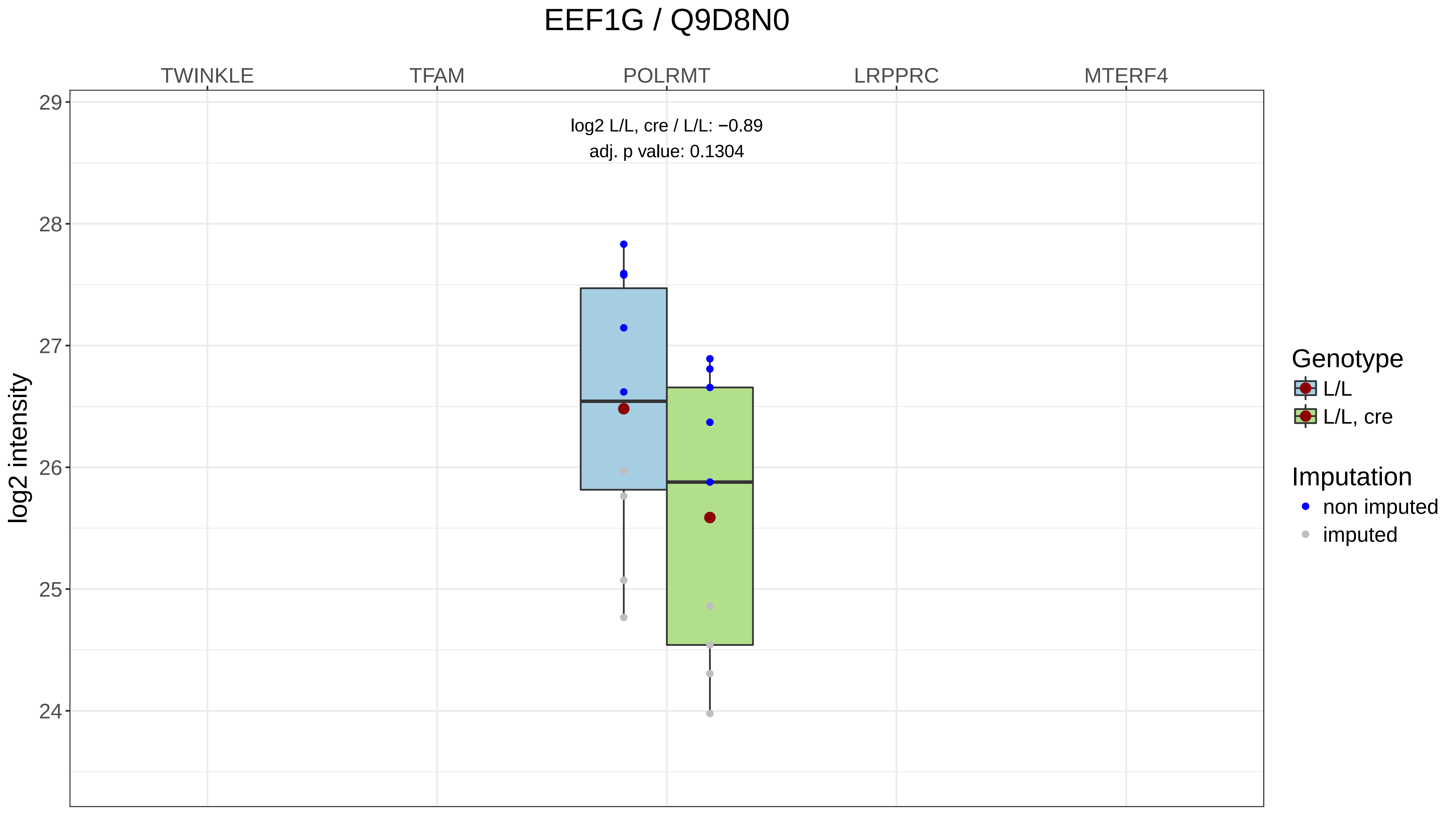
EEF1G / Q9D8N0
TWINKLE
TFAM
POLRMT
LRPPRC
MTERF4
log2 L/L, cre / L/L: −0.89
adj. p value: 0.1304
log2 intensity
29
28
27
26
25
24
Genotype
L/L
L/L, cre
Imputation
non imputed
imputed

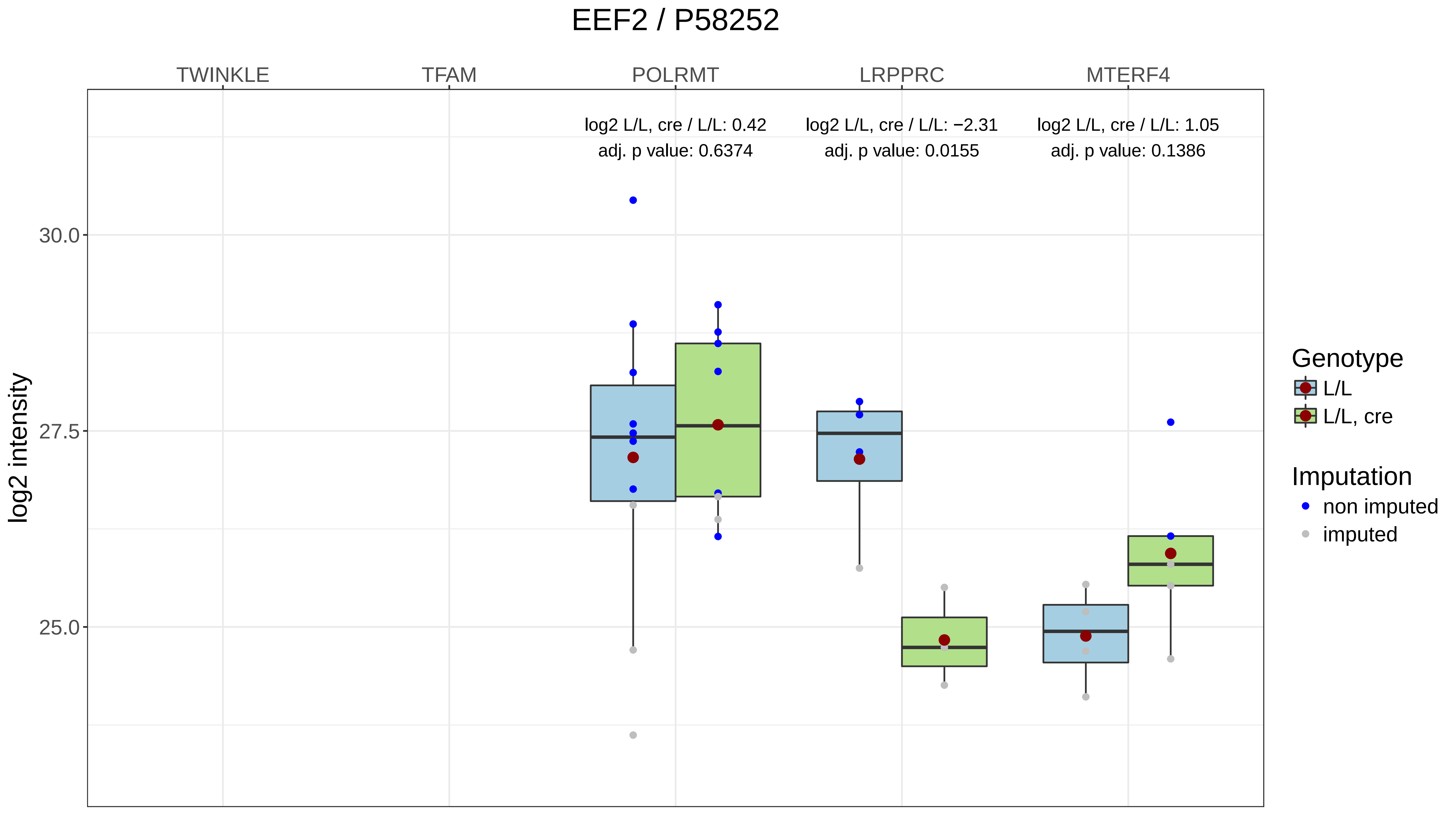
EEF2 / P58252
TWINKLE
TFAM
POLRMT
LRPPRC
MTERF4
log2 L/L, cre / L/L: 0.42
adj. p value: 0.6374
log2 L/L, cre / L/L: −2.31
adj. p value: 0.0155
log2 L/L, cre / L/L: 1.05
adj. p value: 0.1386
30.0
27.5
25.0
log2 intensity
Genotype
L/L
L/L, cre
Imputation
non imputed
imputed

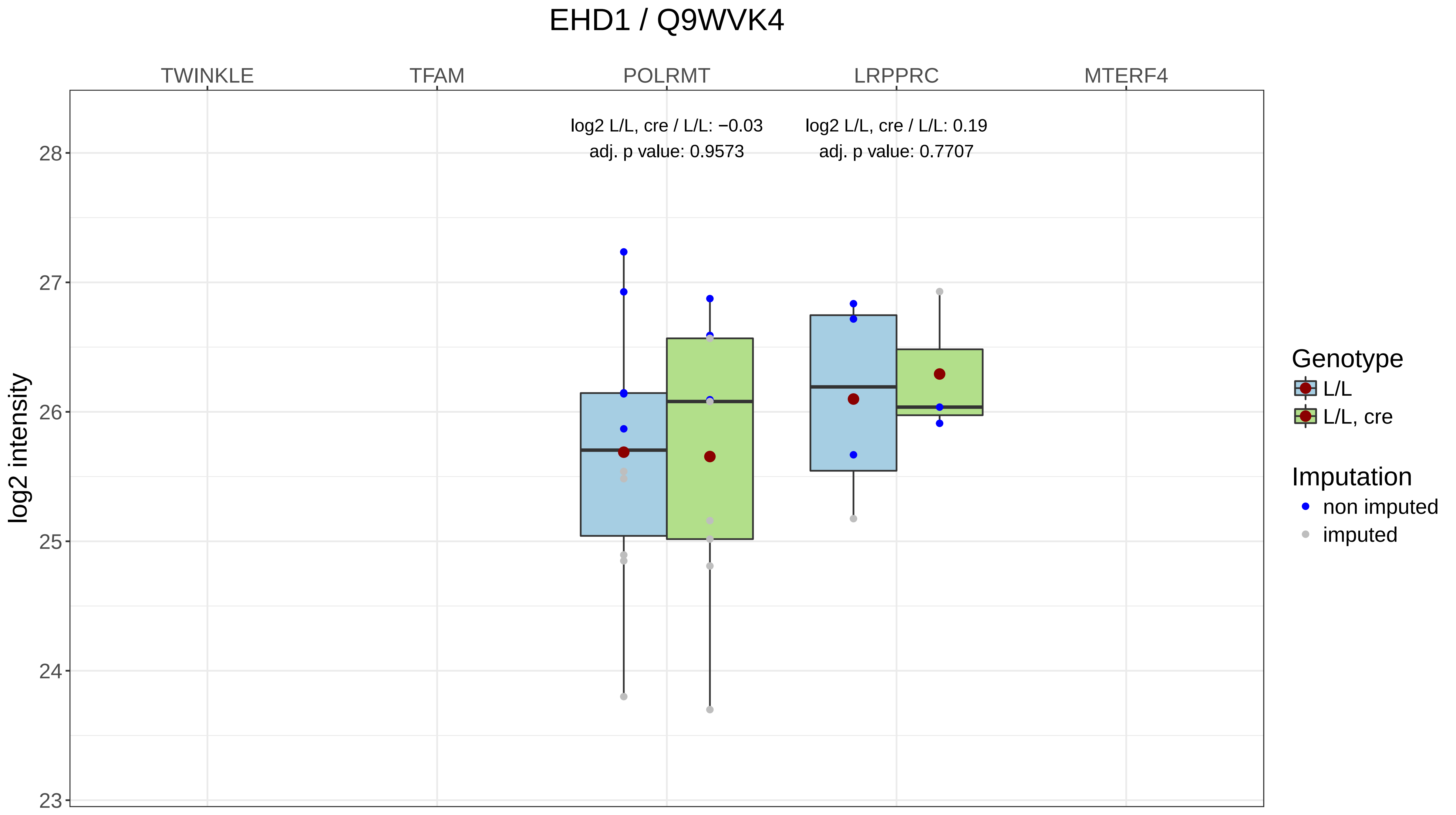
EHD1 / Q9WVK4
TWINKLE
TFAM
POLRMT
LRPPRC
MTERF4
log2 L/L, cre / L/L: −0.03
adj. p value: 0.9573
log2 L/L, cre / L/L: 0.19
adj. p value: 0.7707
log2 intensity
28
27
26
25
24
23
Genotype
L/L
L/L, cre
Imputation
non imputed
imputed

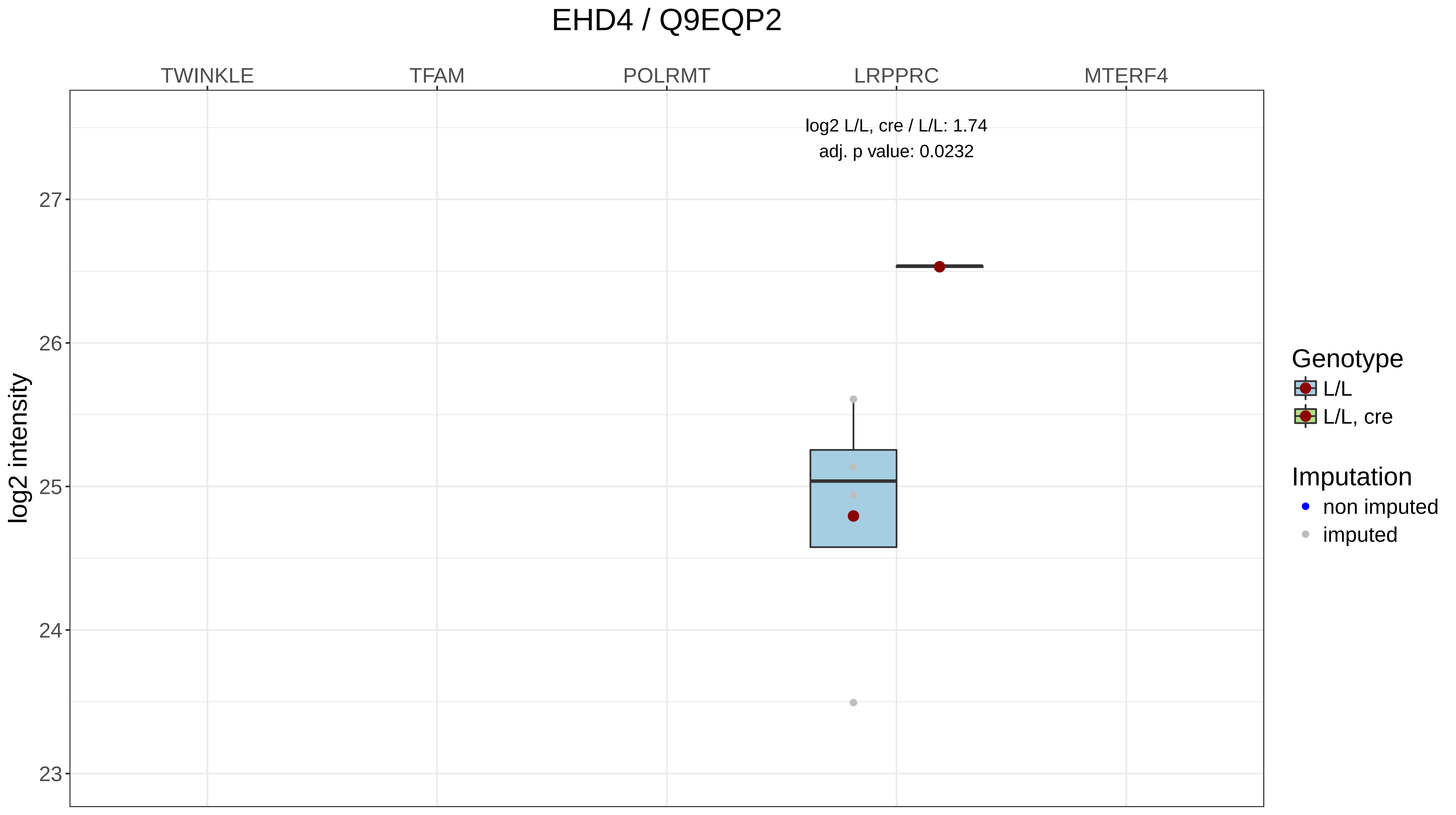
EHD4 / Q9EQP2
TWINKLE
TFAM
POLRMT
LRPPRC
MTERF4
log2 L/L, cre / L/L: 1.74
adj. p value: 0.0232
log2 intensity
27
26
25
24
23
Genotype
L/L
L/L, cre
Imputation
non imputed
imputed

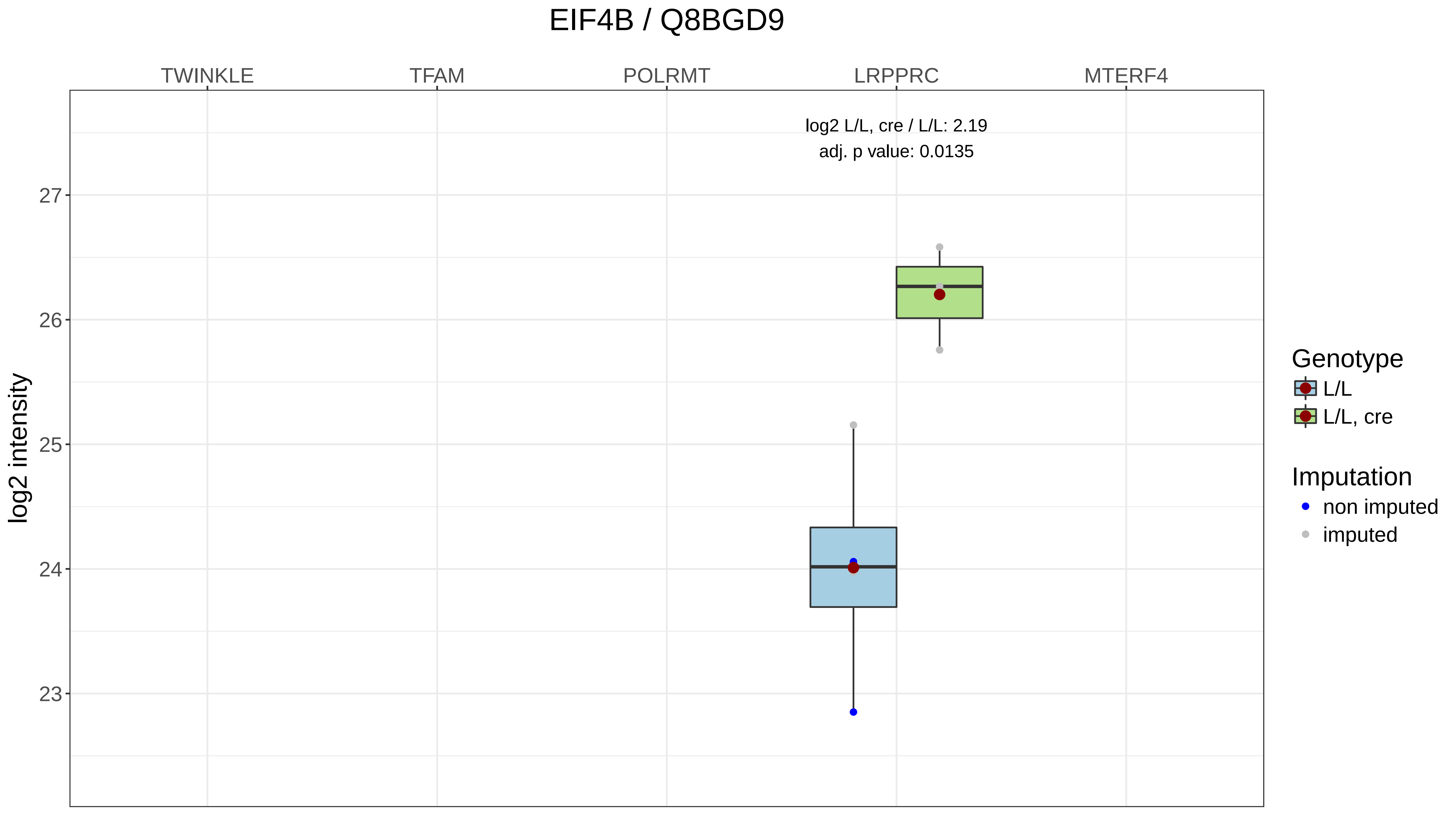
EIF4B / Q8BGD9
TWINKLE
TFAM
POLRMT
LRPPRC
MTERF4
log2 L/L, cre / L/L: 2.19
adj. p value: 0.0135
27
26
25
24
23
log2 intensity
Genotype
L/L
L/L, cre
Imputation
non imputed
imputed

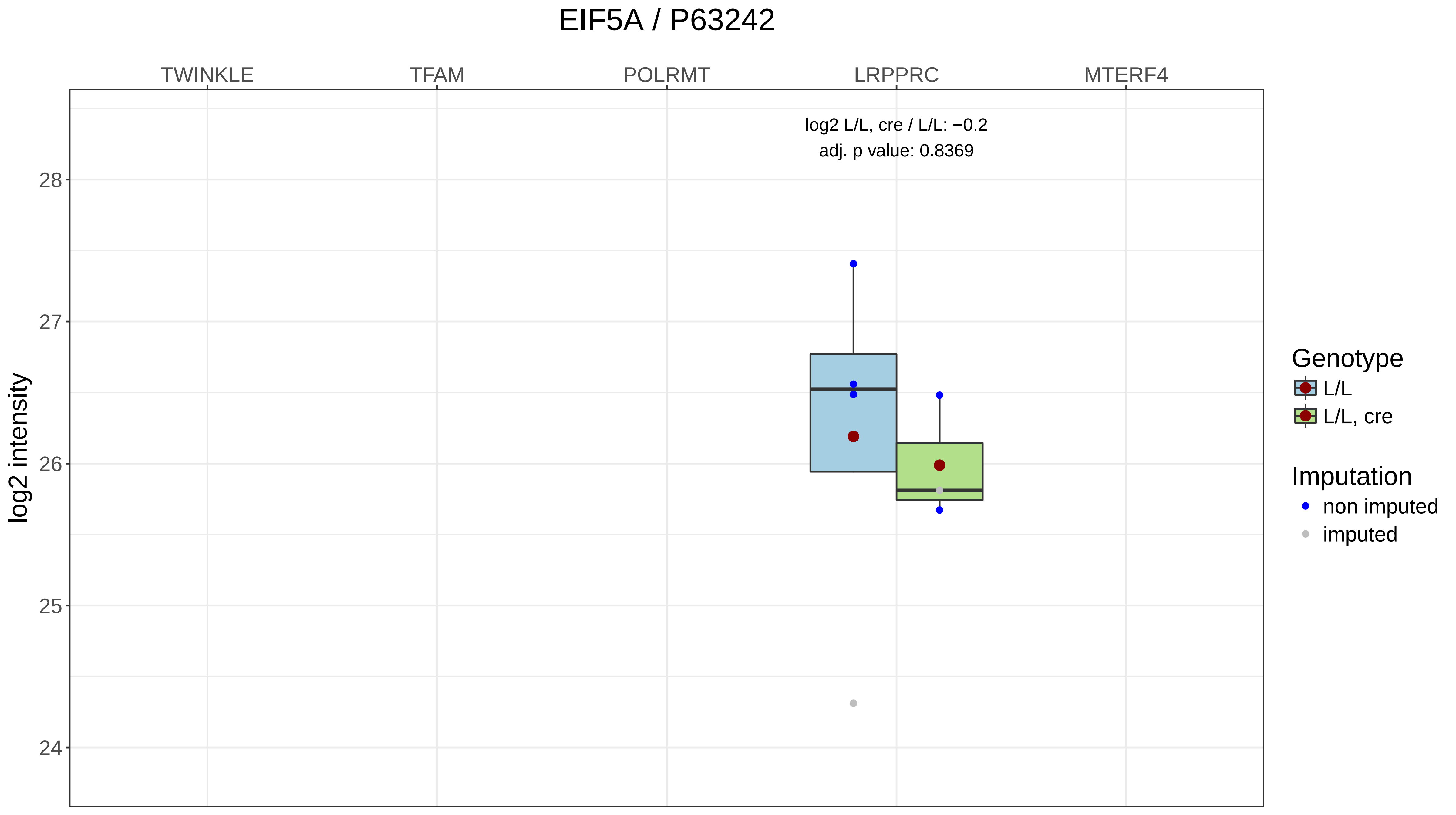

EIF5A / P63242
TWINKLE
TFAM
POLRMT
LRPPRC
MTERF4
log2 L/L, cre / L/L: −0.2
adj. p value: 0.8369
28
27
26
25
24
log2 intensity
Genotype
L/L
L/L, cre
Imputation
non imputed
imputed

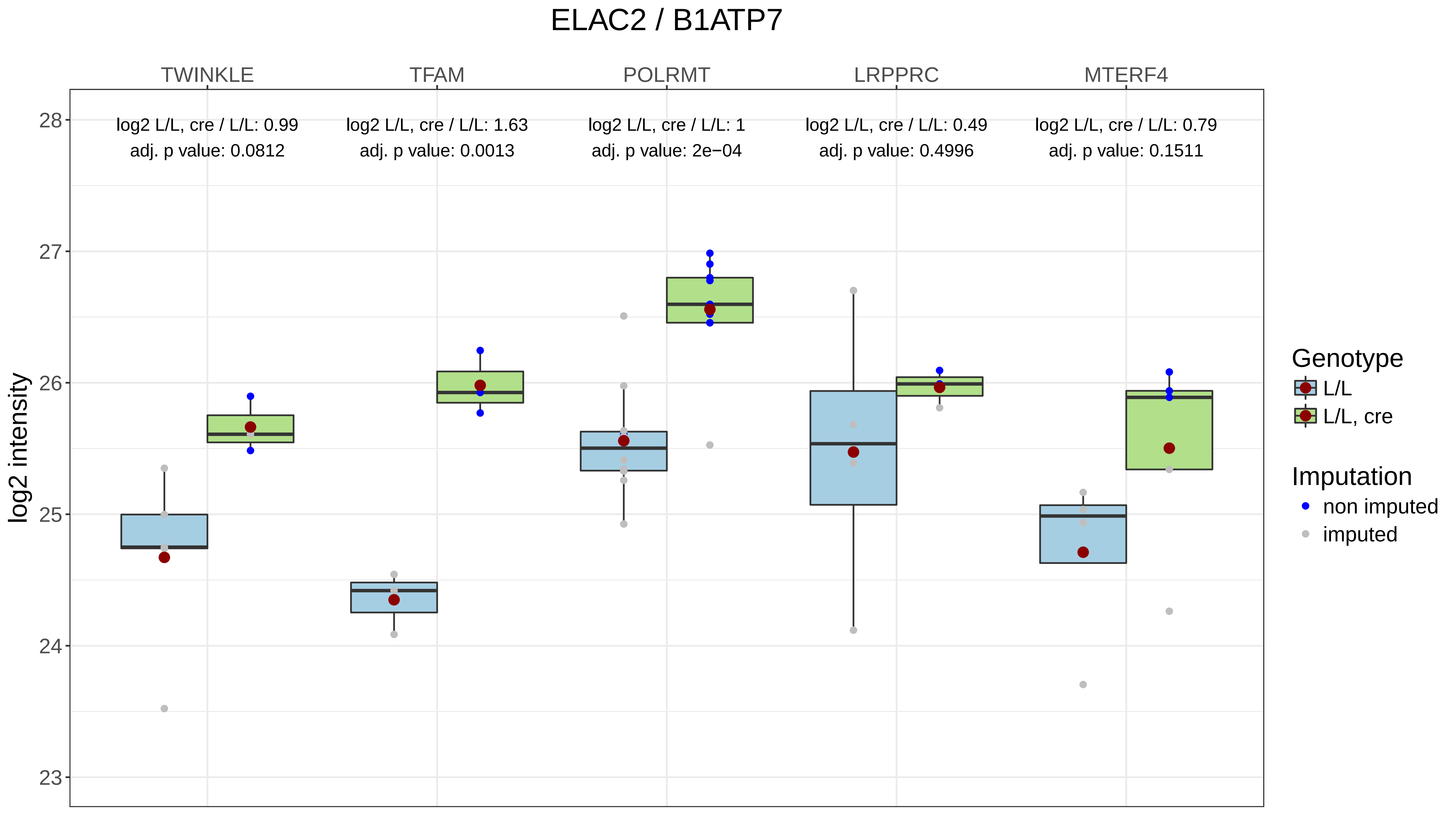
ELAC2 / B1ATP7
TWINKLE
TFAM
POLRMT
LRPPRC
MTERF4
log2 L/L, cre / L/L: 0.99
adj. p value: 0.0812
log2 L/L, cre / L/L: 1.63
adj. p value: 0.0013
log2 L/L, cre / L/L: 1
adj. p value: 2e−04
log2 L/L, cre / L/L: 0.49
adj. p value: 0.4996
log2 L/L, cre / L/L: 0.79
adj. p value: 0.1511
log2 intensity
28
27
26
25
24
23
Genotype
L/L
L/L, cre
Imputation
non imputed
imputed

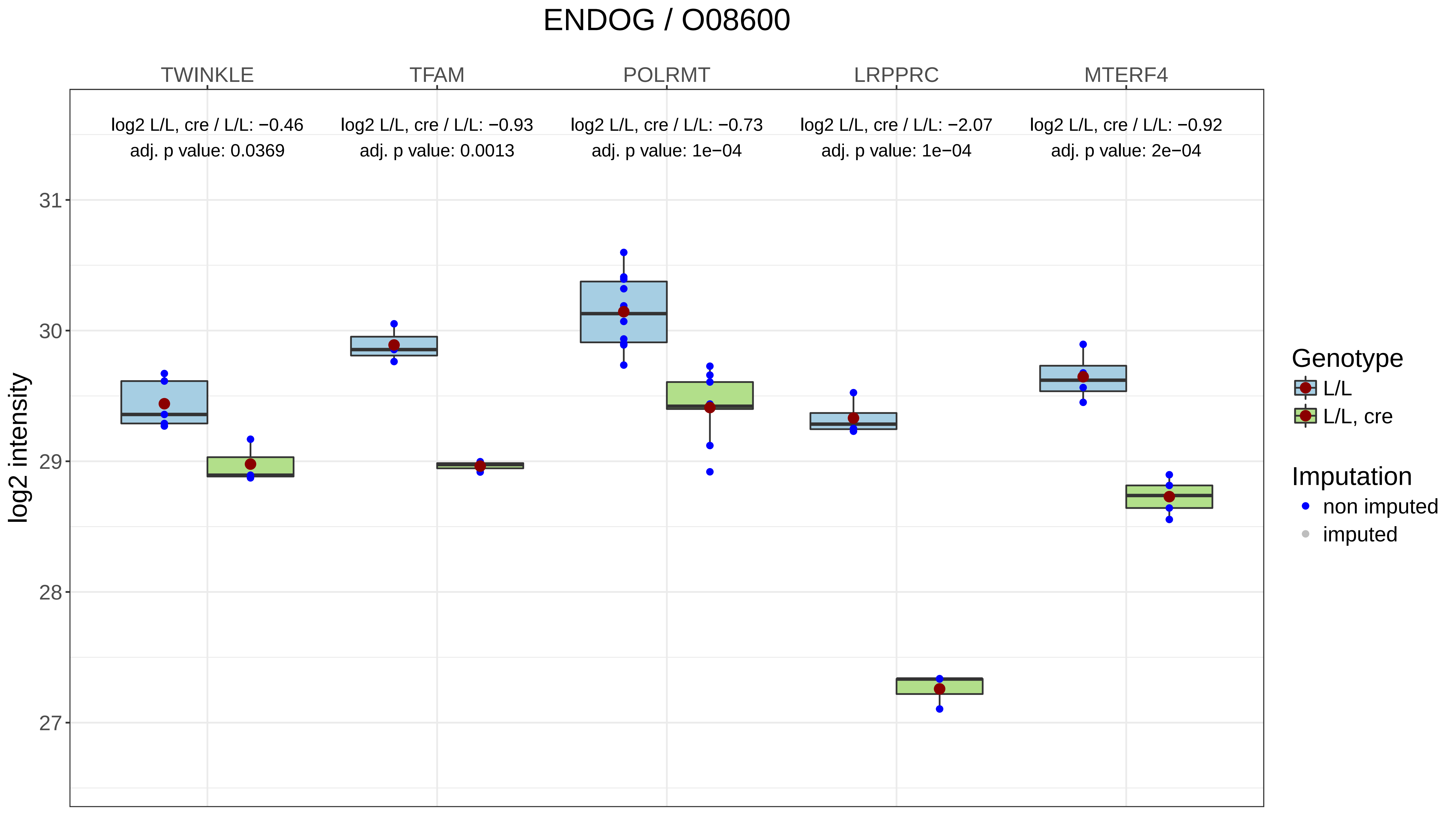
ENDOG / O08600
TWINKLE
TFAM
POLRMT
LRPPRC
MTERF4
log2 L/L, cre / L/L: −0.46
adj. p value: 0.0369
log2 L/L, cre / L/L: −0.93
adj. p value: 0.0013
log2 L/L, cre / L/L: −0.73
adj. p value: 1e−04
log2 L/L, cre / L/L: −2.07
adj. p value: 1e−04
log2 L/L, cre / L/L: −0.92
adj. p value: 2e−04
log2 intensity
31
30
29
28
27
Genotype
L/L
L/L, cre
Imputation
non imputed
imputed

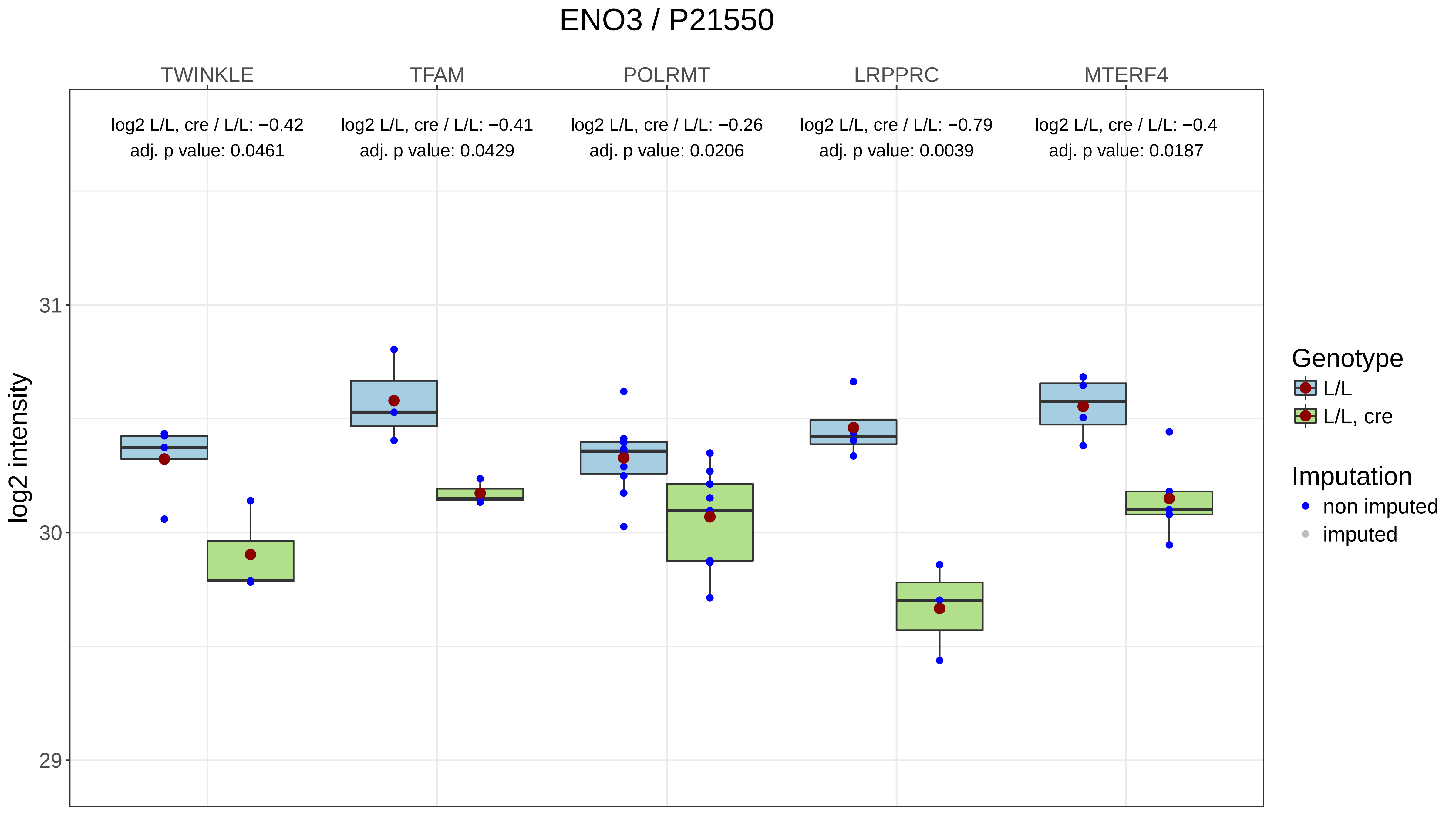
ENO3 / P21550
TWINKLE
TFAM
POLRMT
LRPPRC
MTERF4
log2 L/L, cre / L/L: −0.42
adj. p value: 0.0461
log2 L/L, cre / L/L: −0.41
adj. p value: 0.0429
log2 L/L, cre / L/L: −0.26
adj. p value: 0.0206
log2 L/L, cre / L/L: −0.79
adj. p value: 0.0039
log2 L/L, cre / L/L: −0.4
adj. p value: 0.0187
log2 intensity
31
30
29
Genotype
L/L
L/L, cre
Imputation
non imputed
imputed

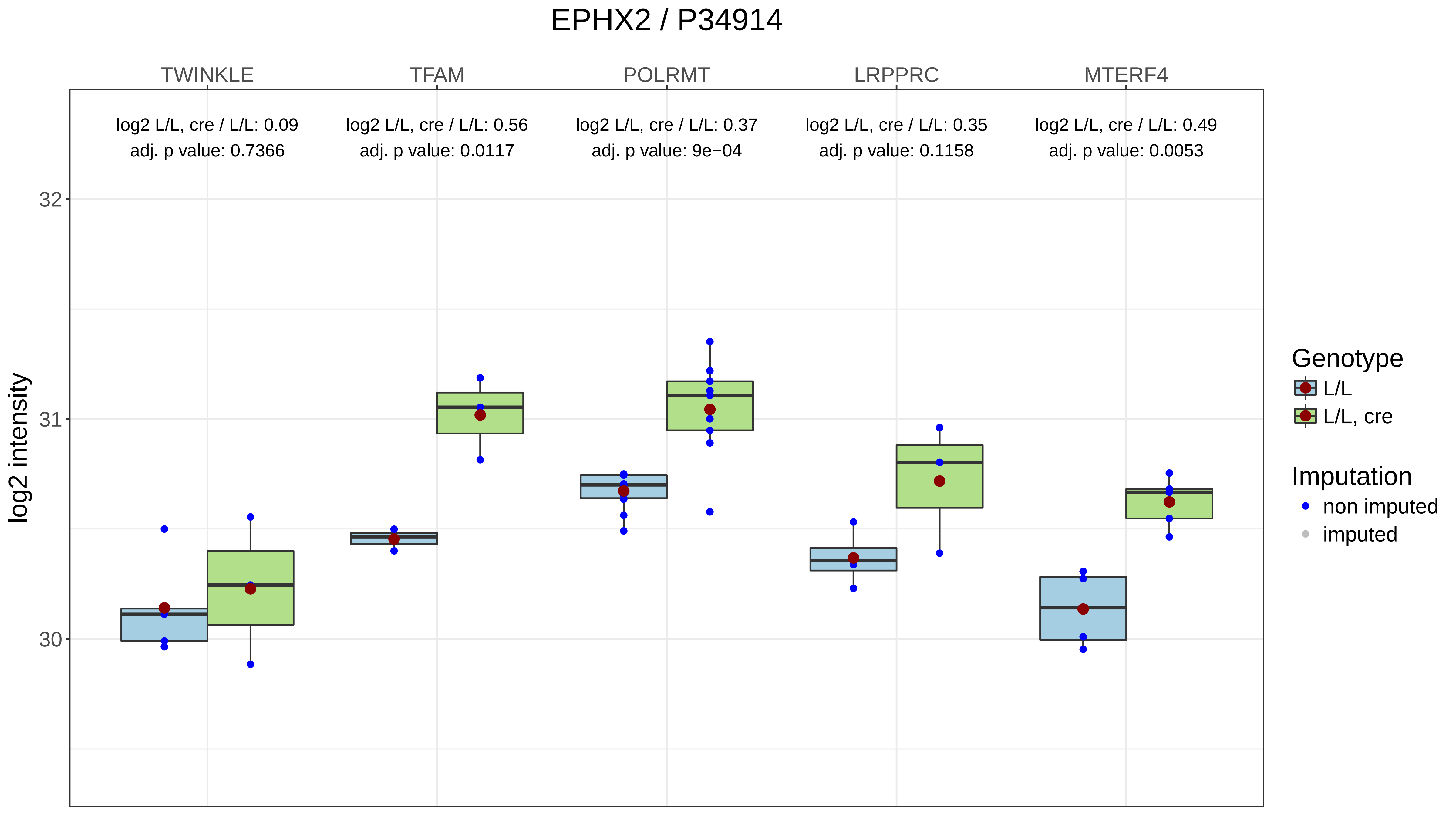

EPHX2 / P34914
TWINKLE
TFAM
POLRMT
LRPPRC
MTERF4
log2 L/L, cre / L/L: 0.09
adj. p value: 0.7366
log2 L/L, cre / L/L: 0.56
adj. p value: 0.0117
log2 L/L, cre / L/L: 0.37
adj. p value: 9e−04
log2 L/L, cre / L/L: 0.35
adj. p value: 0.1158
log2 L/L, cre / L/L: 0.49
adj. p value: 0.0053
log2 intensity
32
31
30
Genotype
L/L
L/L, cre
Imputation
non imputed
imputed

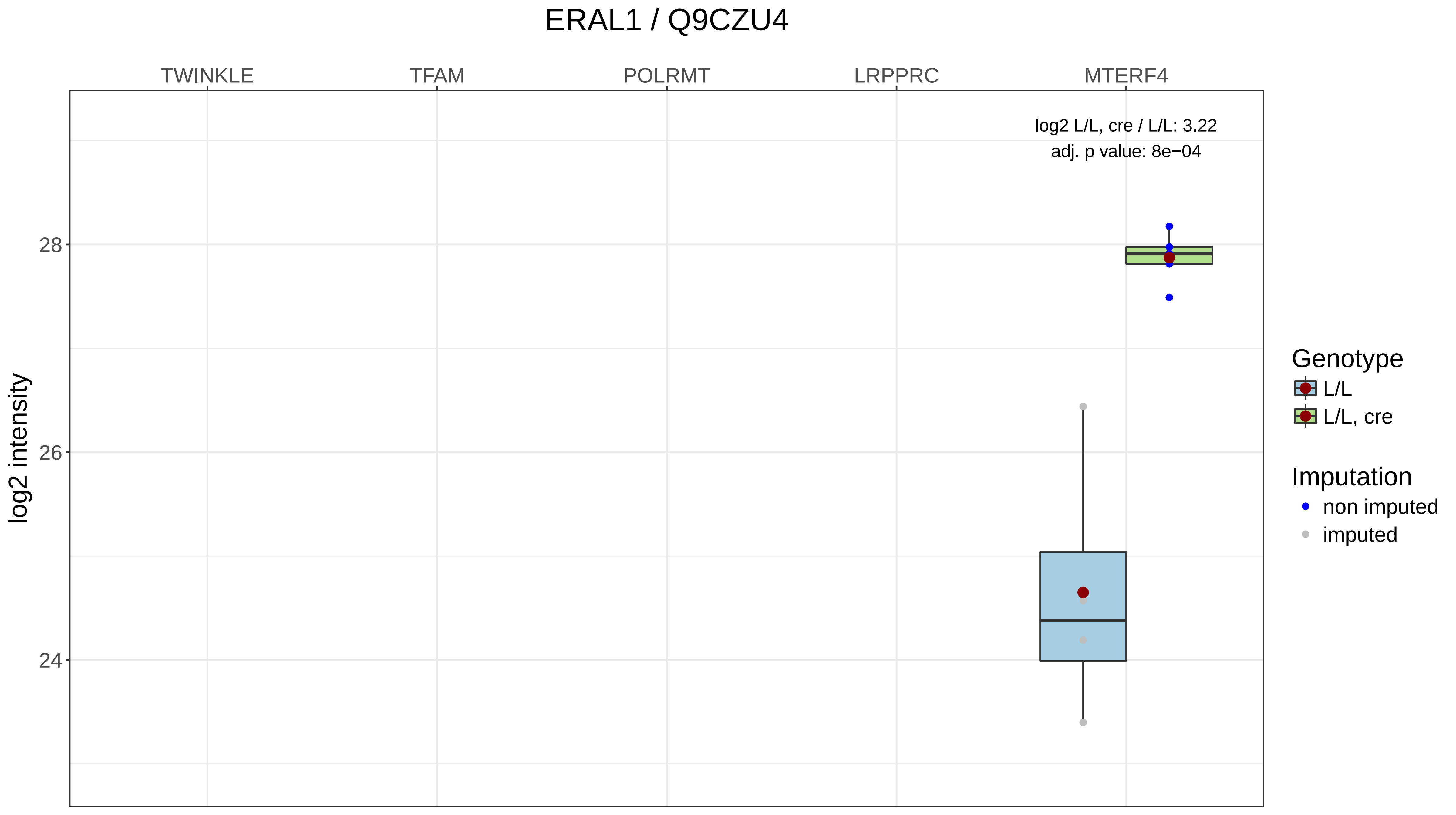
ERAL1 / Q9CZU4
TWINKLE
TFAM
POLRMT
LRPPRC
MTERF4
log2 L/L, cre / L/L: 3.22
adj. p value: 8e−04
28
26
24
log2 intensity
Genotype
L/L
L/L, cre
Imputation
non imputed
imputed

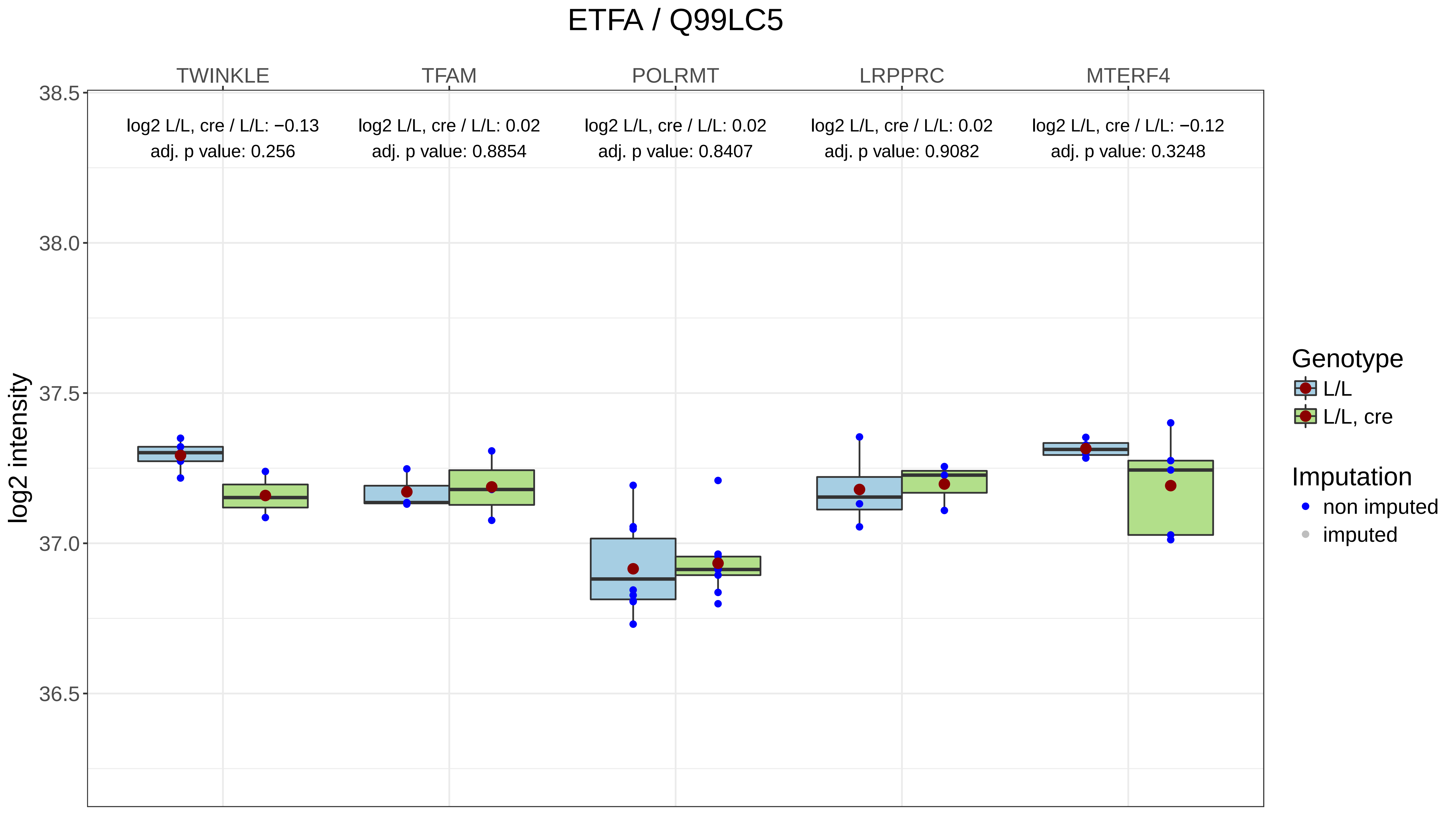

ETFA / Q99LC5
TWINKLE
TFAM
POLRMT
LRPPRC
MTERF4
log2 L/L, cre / L/L: −0.13
adj. p value: 0.256
log2 L/L, cre / L/L: 0.02
adj. p value: 0.8854
log2 L/L, cre / L/L: 0.02
adj. p value: 0.8407
log2 L/L, cre / L/L: 0.02
adj. p value: 0.9082
log2 L/L, cre / L/L: −0.12
adj. p value: 0.3248
log2 intensity
38.5
38.0
37.5
37.0
36.5
Genotype
L/L
L/L, cre
Imputation
non imputed
imputed

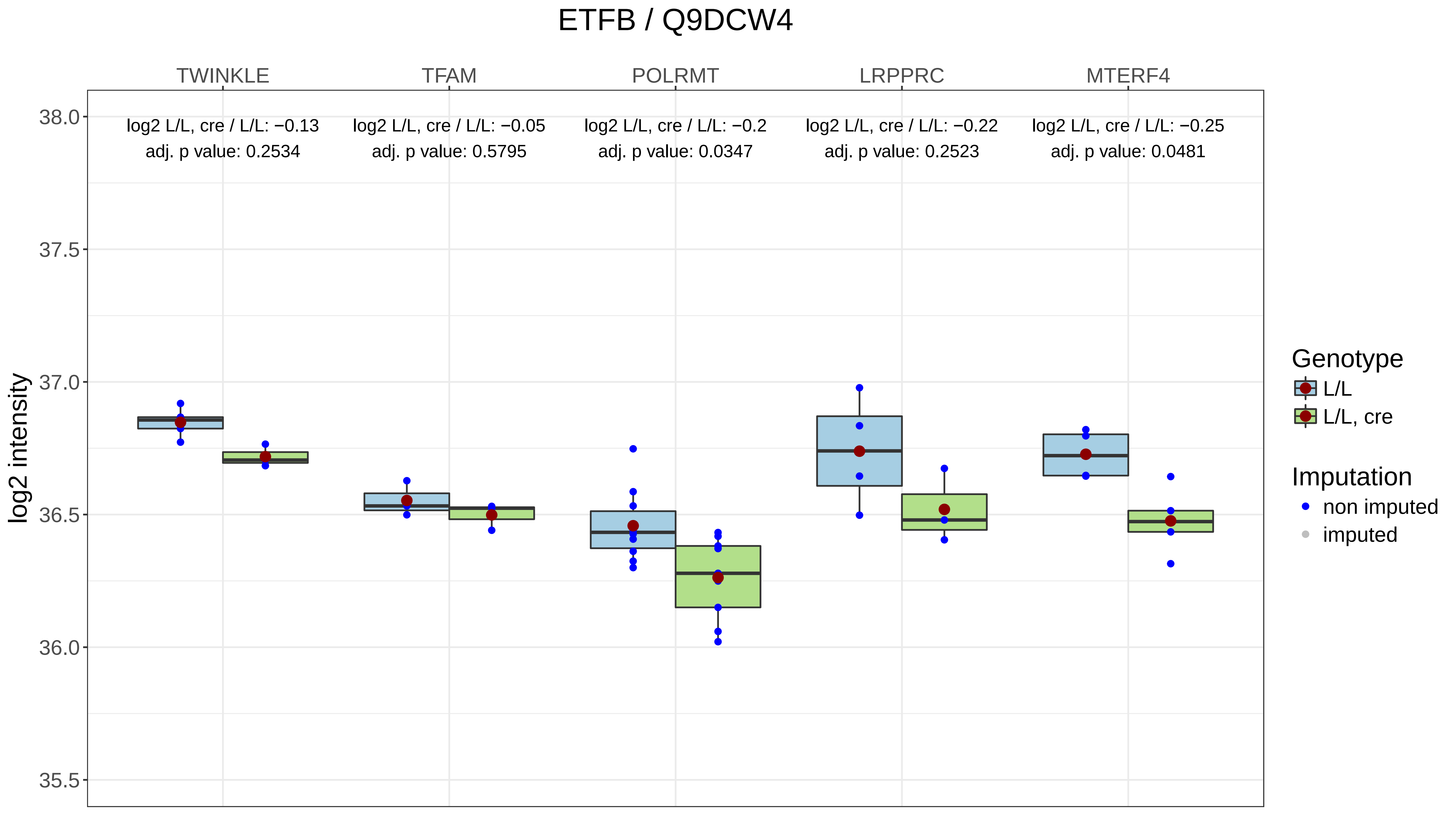
ETFB / Q9DCW4
TWINKLE
TFAM
POLRMT
LRPPRC
MTERF4
log2 L/L, cre / L/L: −0.13
adj. p value: 0.2534
log2 L/L, cre / L/L: −0.05
adj. p value: 0.5795
log2 L/L, cre / L/L: −0.2
adj. p value: 0.0347
log2 L/L, cre / L/L: −0.22
adj. p value: 0.2523
log2 L/L, cre / L/L: −0.25
adj. p value: 0.0481
log2 intensity
38.0
37.5
37.0
36.5
36.0
35.5
Genotype
L/L
L/L, cre
Imputation
non imputed
imputed

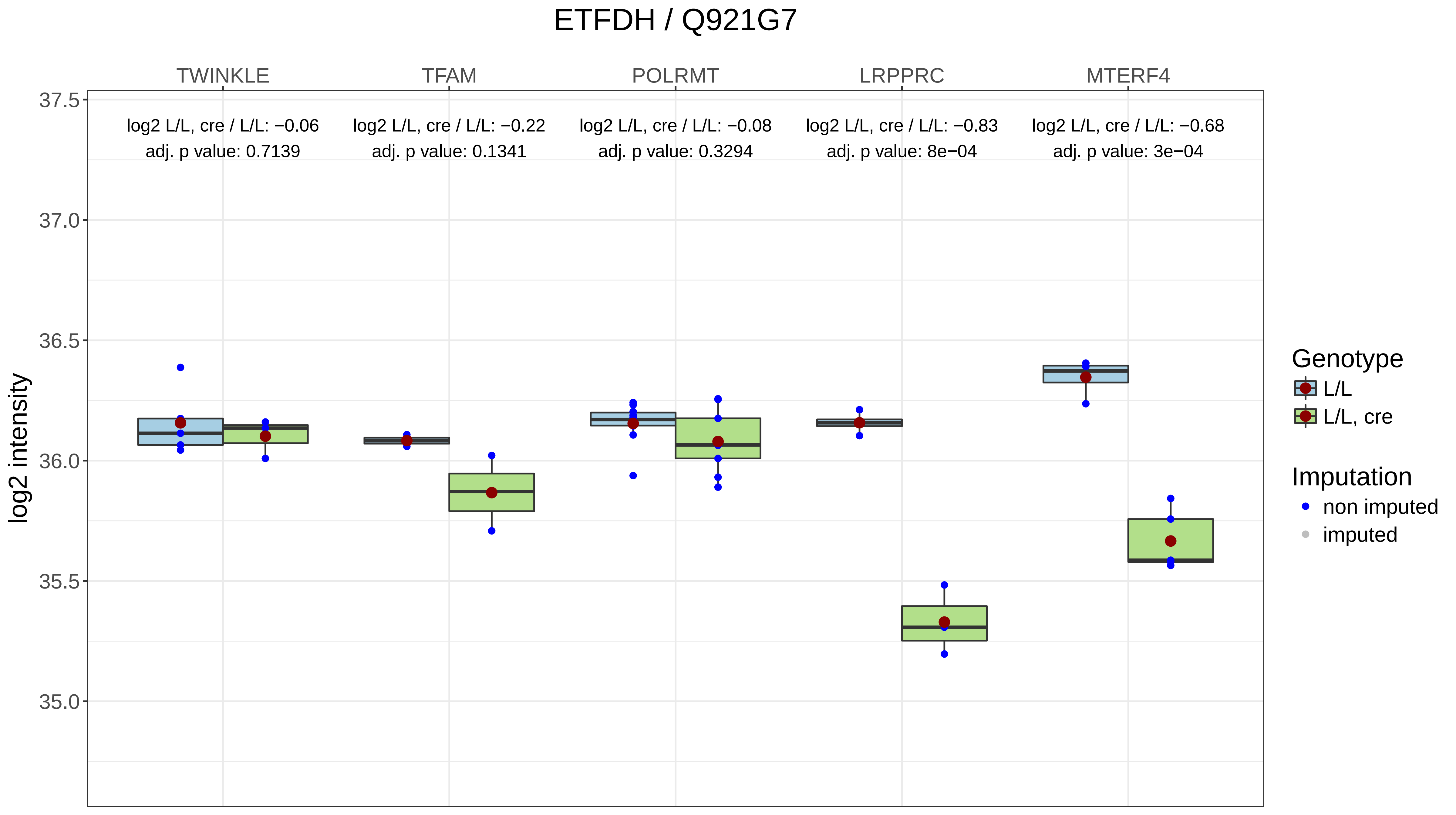

ETFDH / Q921G7
TWINKLE
TFAM
POLRMT
LRPPRC
MTERF4
log2 L/L, cre / L/L: −0.06
adj. p value: 0.7139
log2 L/L, cre / L/L: −0.22
adj. p value: 0.1341
log2 L/L, cre / L/L: −0.08
adj. p value: 0.3294
log2 L/L, cre / L/L: −0.83
adj. p value: 8e−04
log2 L/L, cre / L/L: −0.68
adj. p value: 3e−04
log2 intensity
37.5
37.0
36.5
36.0
35.5
35.0
Genotype
L/L
L/L, cre
Imputation
non imputed
imputed

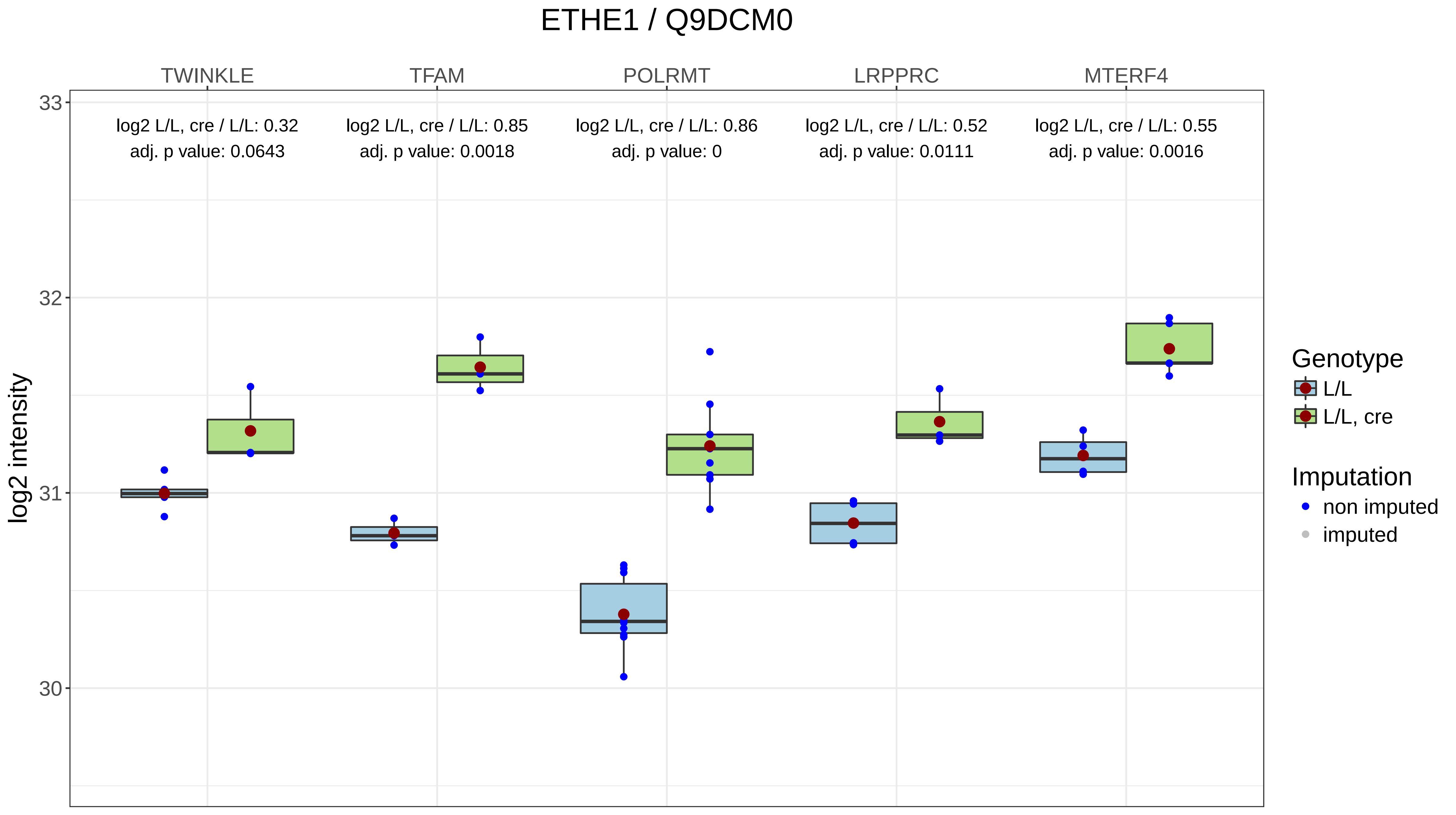
ETHE1 / Q9DCM0
TWINKLE
TFAM
POLRMT
LRPPRC
MTERF4
log2 L/L, cre / L/L: 0.32
adj. p value: 0.0643
log2 L/L, cre / L/L: 0.85
adj. p value: 0.0018
log2 L/L, cre / L/L: 0.86
adj. p value: 0
log2 L/L, cre / L/L: 0.52
adj. p value: 0.0111
log2 L/L, cre / L/L: 0.55
adj. p value: 0.0016
log2 intensity
33
32
31
30
Genotype
L/L
L/L, cre
Imputation
non imputed
imputed

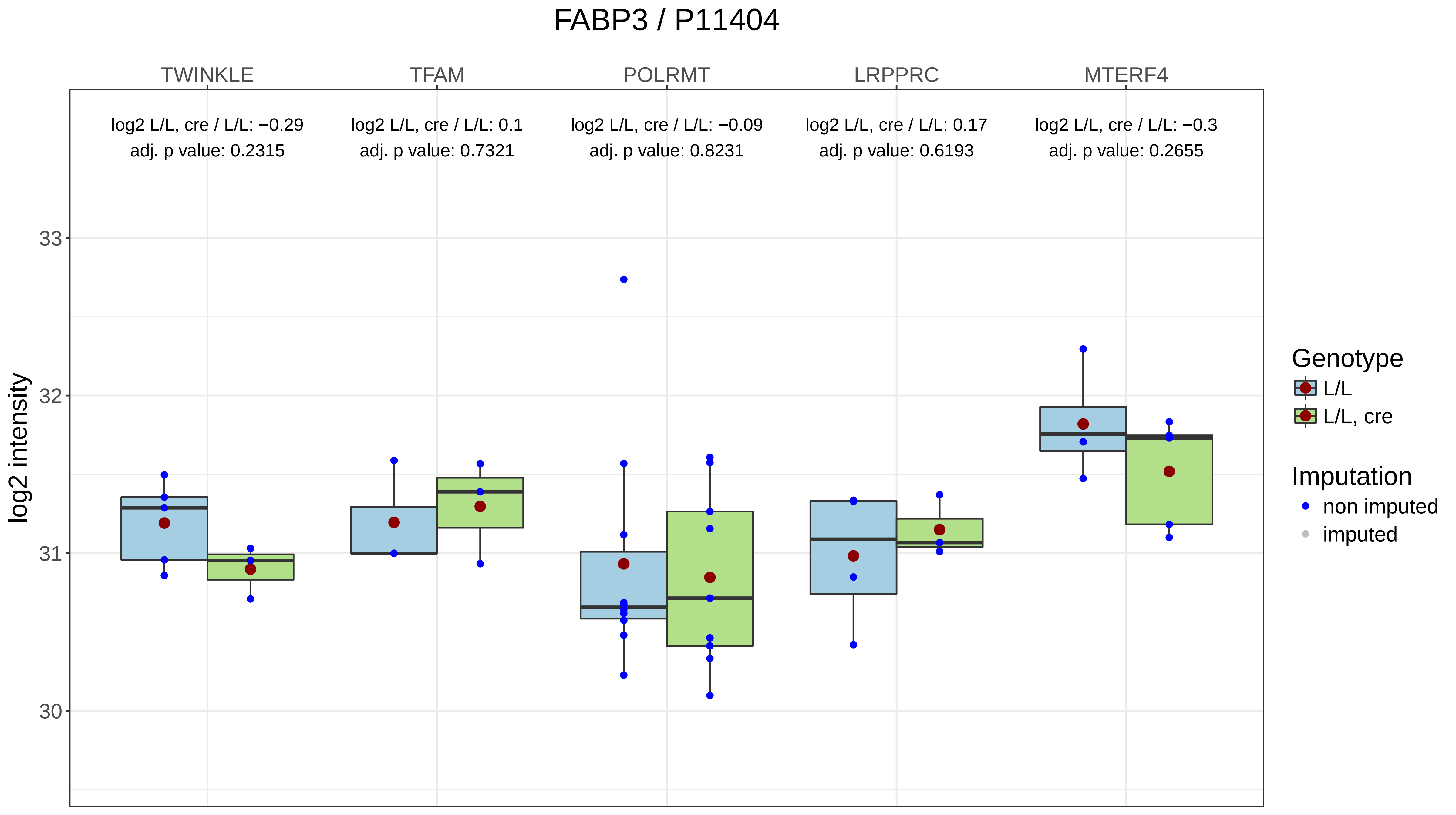
FABP3 / P11404
TWINKLE
TFAM
POLRMT
LRPPRC
MTERF4
log2 L/L, cre / L/L: −0.29
adj. p value: 0.2315
log2 L/L, cre / L/L: 0.1
adj. p value: 0.7321
log2 L/L, cre / L/L: −0.09
adj. p value: 0.8231
log2 L/L, cre / L/L: 0.17
adj. p value: 0.6193
log2 L/L, cre / L/L: −0.3
adj. p value: 0.2655
log2 intensity
33
32
31
30
Genotype
L/L
L/L, cre
Imputation
non imputed
imputed

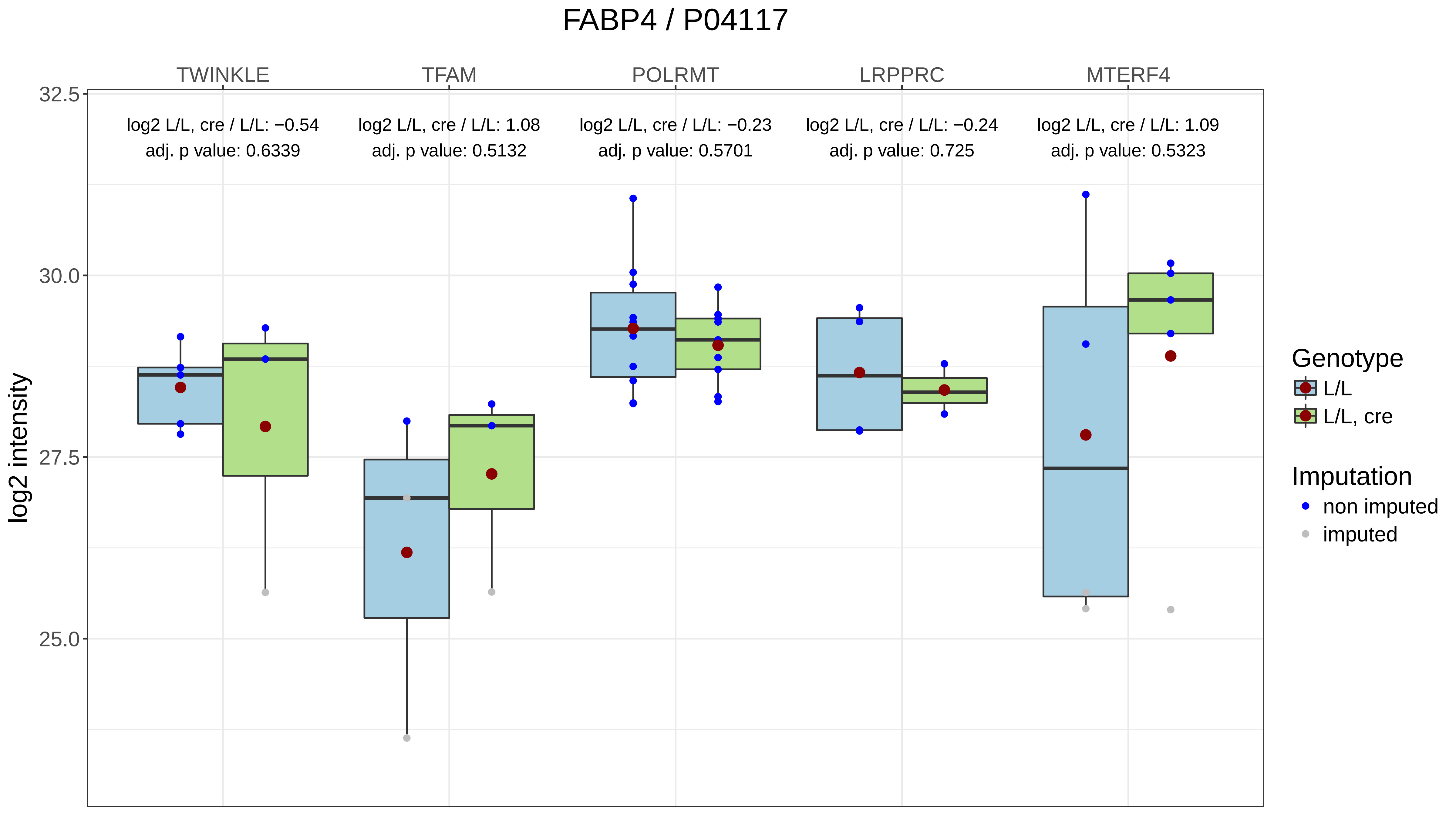
FABP4 / P04117
TWINKLE
TFAM
POLRMT
LRPPRC
MTERF4
log2 L/L, cre / L/L: −0.54
adj. p value: 0.6339
log2 L/L, cre / L/L: 1.08
adj. p value: 0.5132
log2 L/L, cre / L/L: −0.23
adj. p value: 0.5701
log2 L/L, cre / L/L: −0.24
adj. p value: 0.725
log2 L/L, cre / L/L: 1.09
adj. p value: 0.5323
32.5
30.0
27.5
25.0
log2 intensity
Genotype
L/L
L/L, cre
Imputation
non imputed
imputed

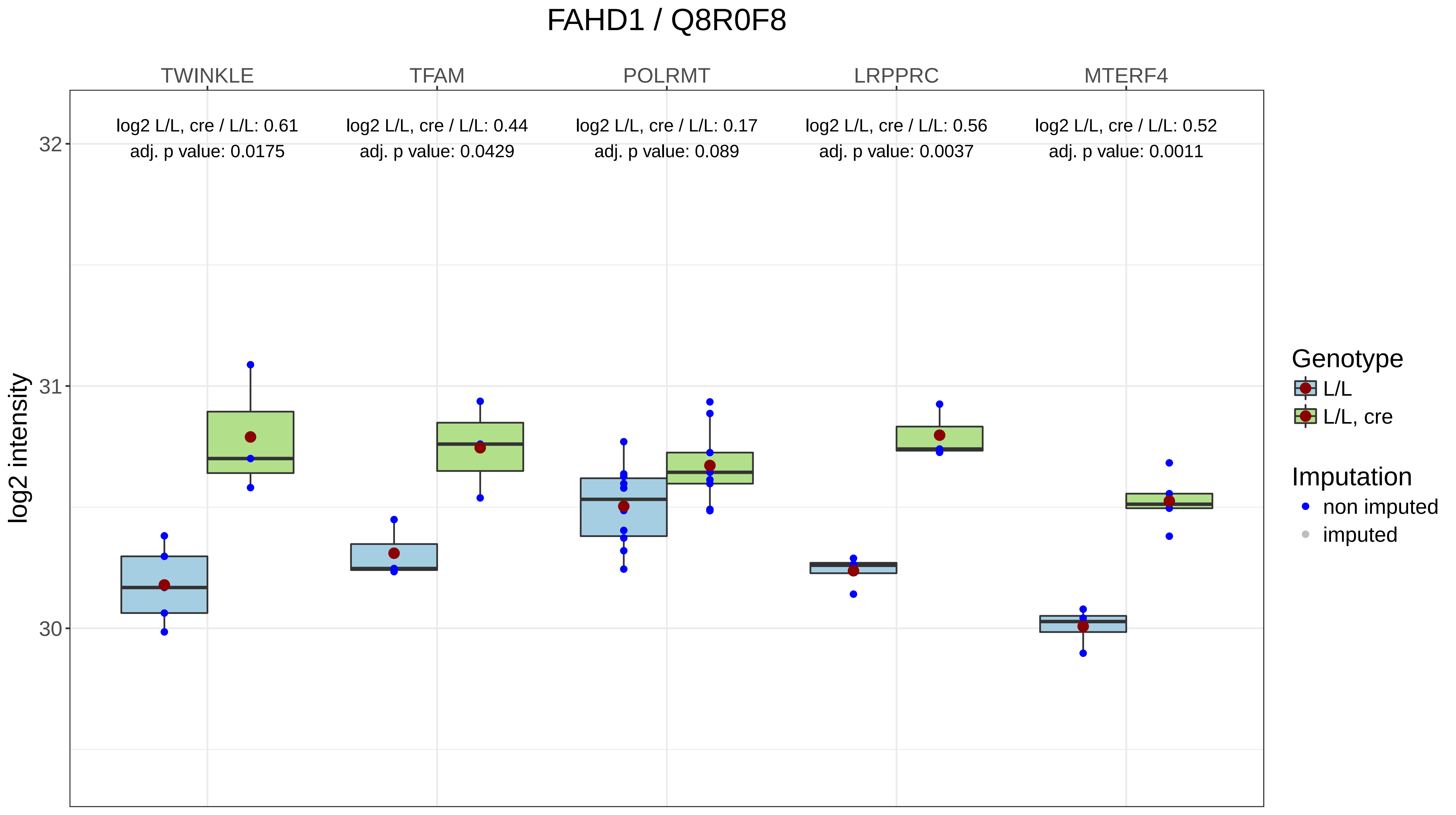
FAHD1 / Q8R0F8
TWINKLE
TFAM
POLRMT
LRPPRC
MTERF4
log2 L/L, cre / L/L: 0.61
adj. p value: 0.0175
log2 L/L, cre / L/L: 0.44
adj. p value: 0.0429
log2 L/L, cre / L/L: 0.17
adj. p value: 0.089
log2 L/L, cre / L/L: 0.56
adj. p value: 0.0037
log2 L/L, cre / L/L: 0.52
adj. p value: 0.0011
32
31
30
log2 intensity
Genotype
L/L
L/L, cre
Imputation
non imputed
imputed

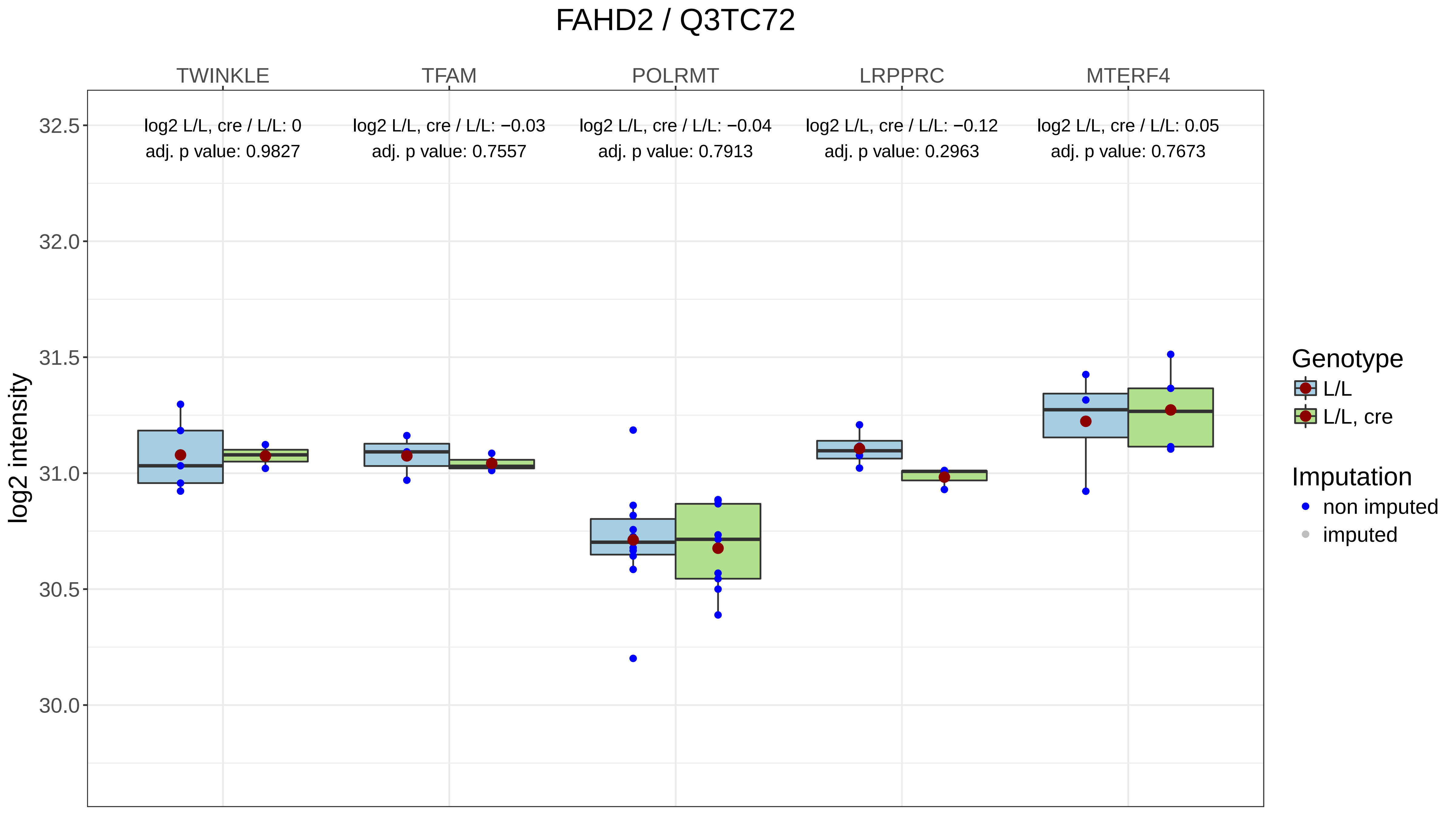
FAHD2 / Q3TC72
TWINKLE
TFAM
POLRMT
LRPPRC
MTERF4
log2 L/L, cre / L/L: 0
adj. p value: 0.9827
log2 L/L, cre / L/L: −0.03
adj. p value: 0.7557
log2 L/L, cre / L/L: −0.04
adj. p value: 0.7913
log2 L/L, cre / L/L: −0.12
adj. p value: 0.2963
log2 L/L, cre / L/L: 0.05
adj. p value: 0.7673
log2 intensity
32.5
32.0
31.5
31.0
30.5
30.0
Genotype
L/L
L/L, cre
Imputation
non imputed
imputed

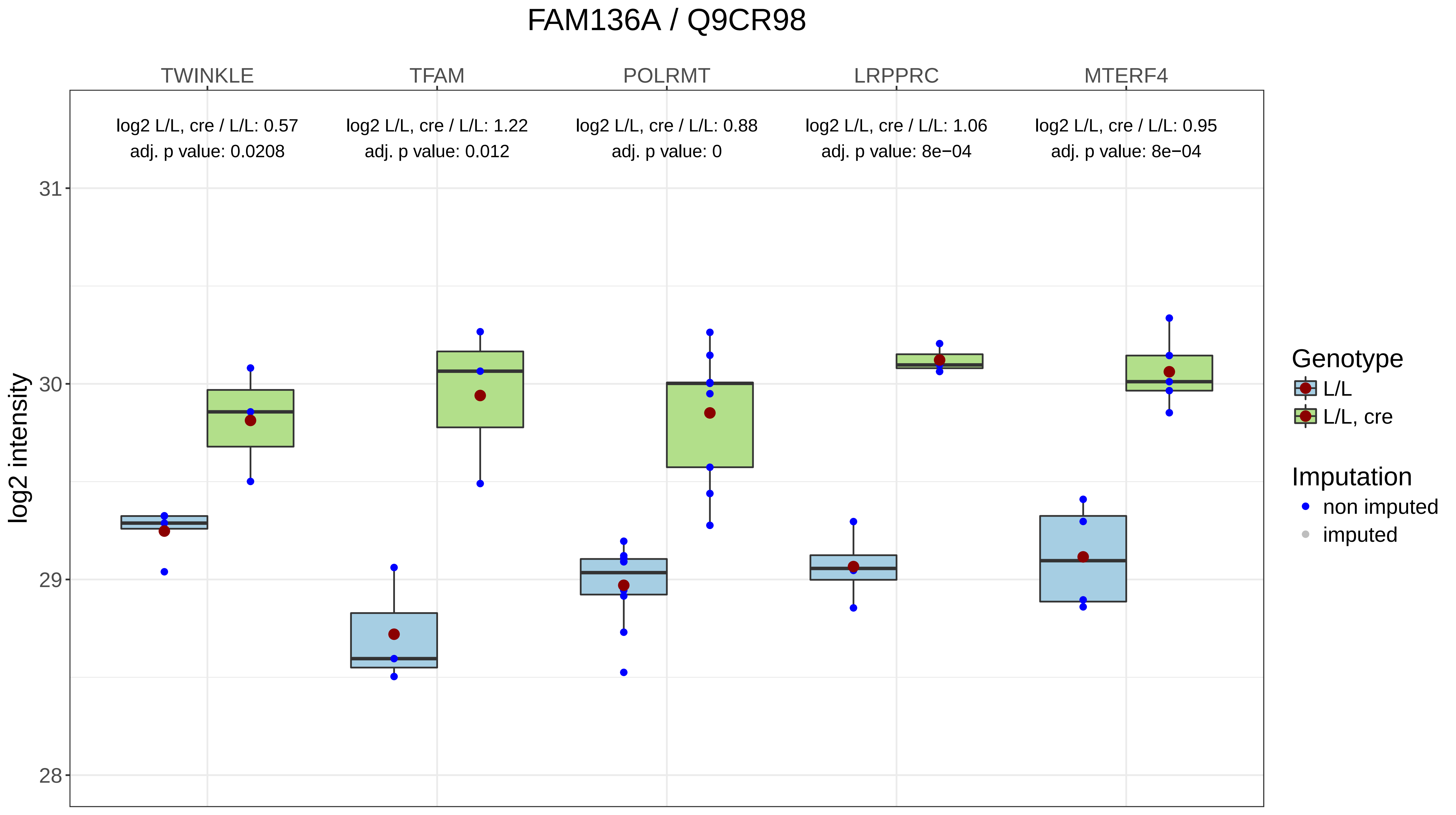
FAM136A / Q9CR98
TWINKLE
TFAM
POLRMT
LRPPRC
MTERF4
log2 L/L, cre / L/L: 0.57
adj. p value: 0.0208
log2 L/L, cre / L/L: 1.22
adj. p value: 0.012
log2 L/L, cre / L/L: 0.88
adj. p value: 0
log2 L/L, cre / L/L: 1.06
adj. p value: 8e−04
log2 L/L, cre / L/L: 0.95
adj. p value: 8e−04
log2 intensity
31
30
29
28
Genotype
L/L
L/L, cre
Imputation
non imputed
imputed

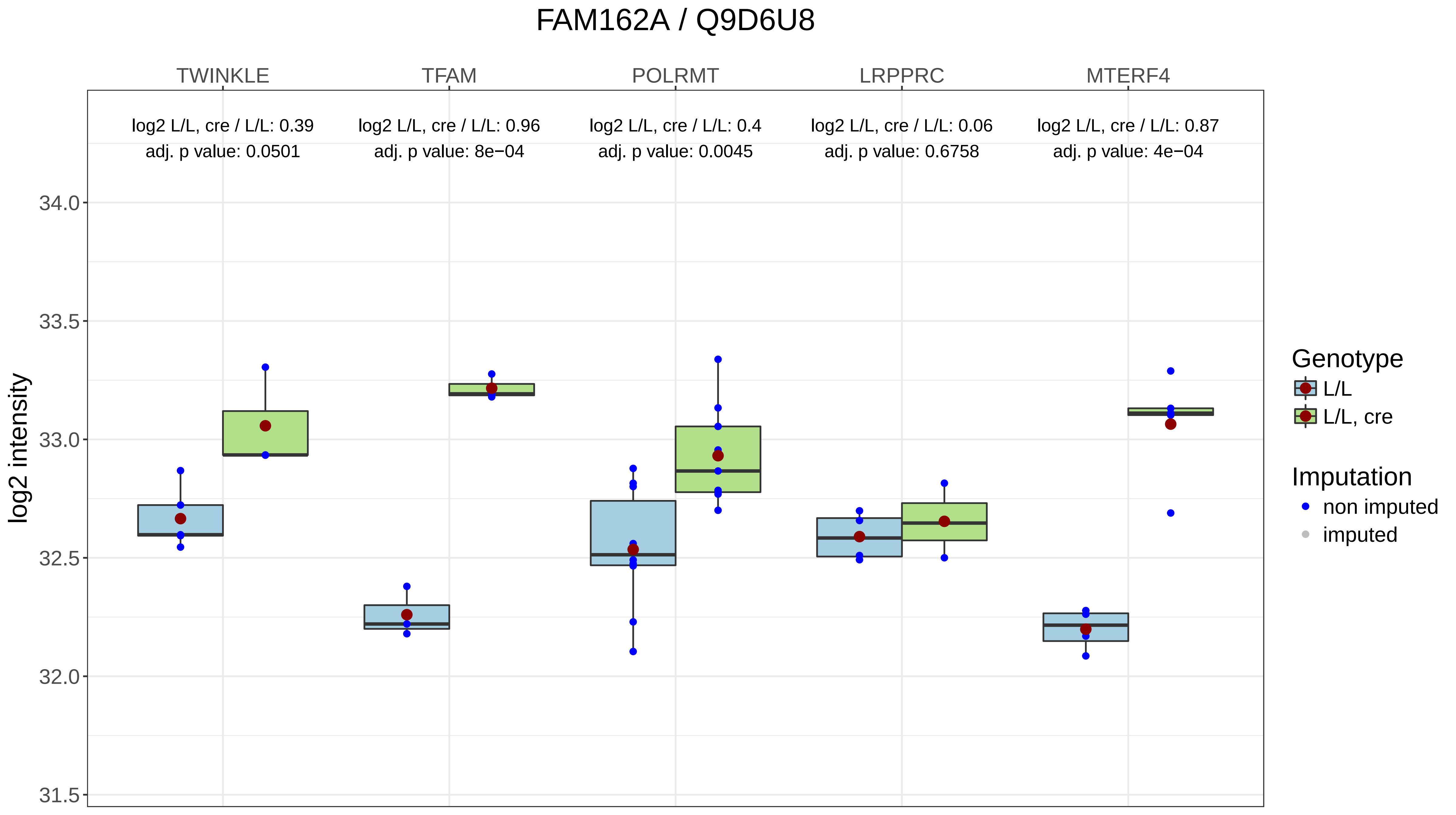
FAM162A / Q9D6U8
TWINKLE
TFAM
POLRMT
LRPPRC
MTERF4
log2 L/L, cre / L/L: 0.39
adj. p value: 0.0501
log2 L/L, cre / L/L: 0.96
adj. p value: 8e−04
log2 L/L, cre / L/L: 0.4
adj. p value: 0.0045
log2 L/L, cre / L/L: 0.06
adj. p value: 0.6758
log2 L/L, cre / L/L: 0.87
adj. p value: 4e−04
log2 intensity
34.0
33.5
33.0
32.5
32.0
31.5
Genotype
L/L
L/L, cre
Imputation
non imputed
imputed

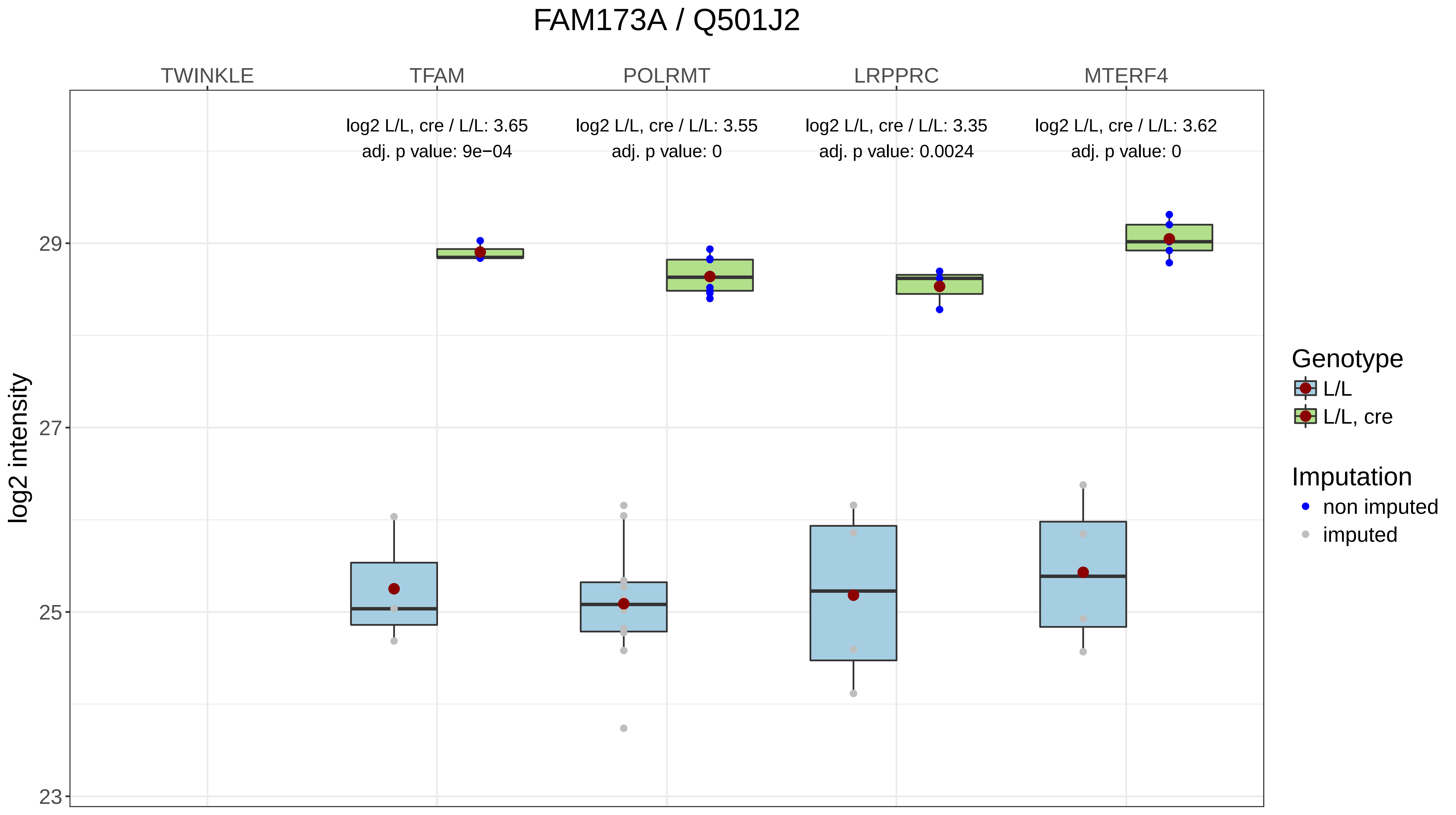
FAM173A / Q501J2
TWINKLE
TFAM
POLRMT
LRPPRC
MTERF4
log2 L/L, cre / L/L: 3.65
adj. p value: 9e−04
log2 L/L, cre / L/L: 3.55
adj. p value: 0
log2 L/L, cre / L/L: 3.35
adj. p value: 0.0024
log2 L/L, cre / L/L: 3.62
adj. p value: 0
log2 intensity
29
27
25
23
Genotype
L/L
L/L, cre
Imputation
non imputed
imputed

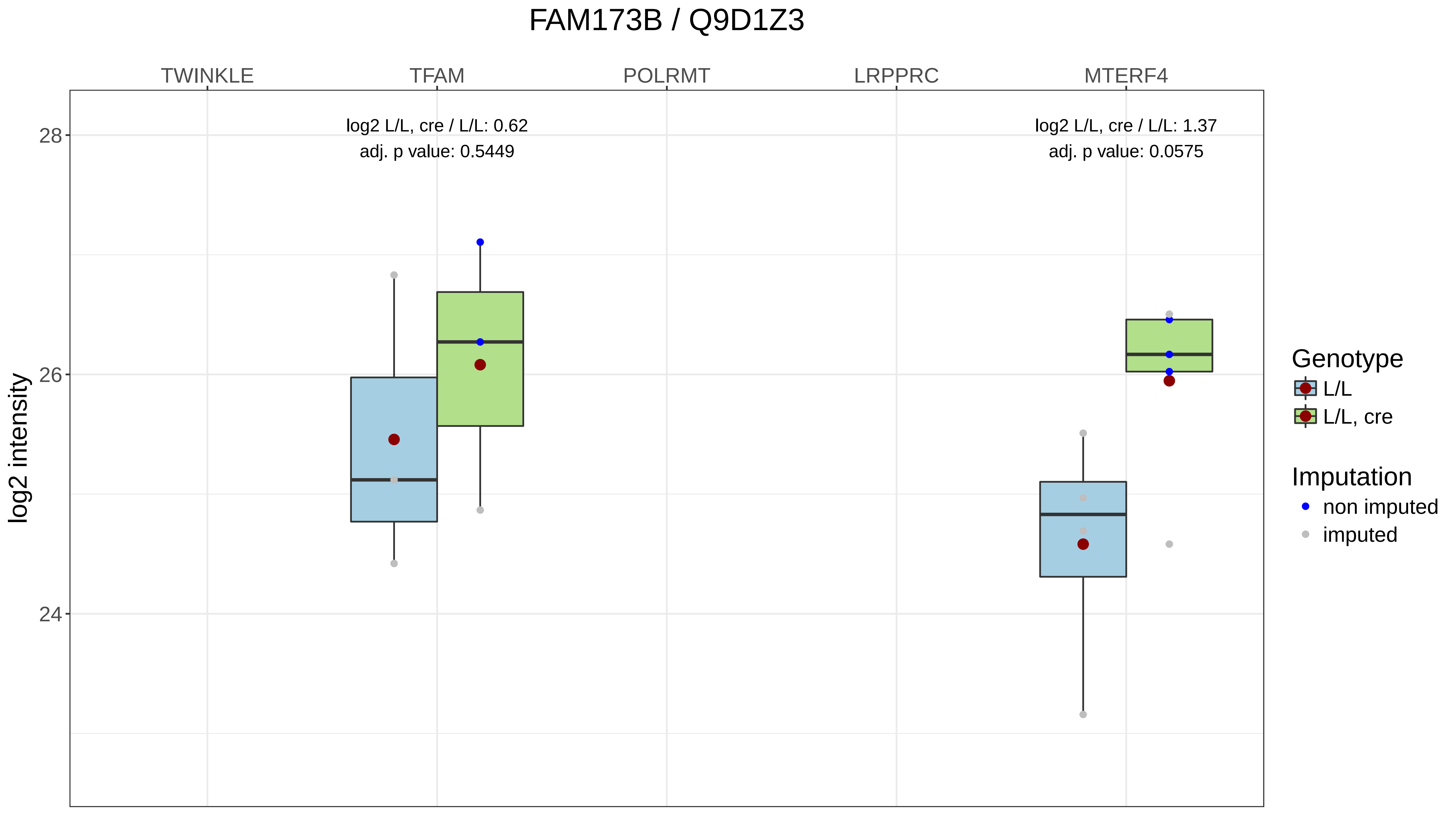
FAM173B / Q9D1Z3
TWINKLE
TFAM
POLRMT
LRPPRC
MTERF4
log2 L/L, cre / L/L: 0.62
adj. p value: 0.5449
log2 L/L, cre / L/L: 1.37
adj. p value: 0.0575
28
26
24
log2 intensity
Genotype
L/L
L/L, cre
Imputation
non imputed
imputed

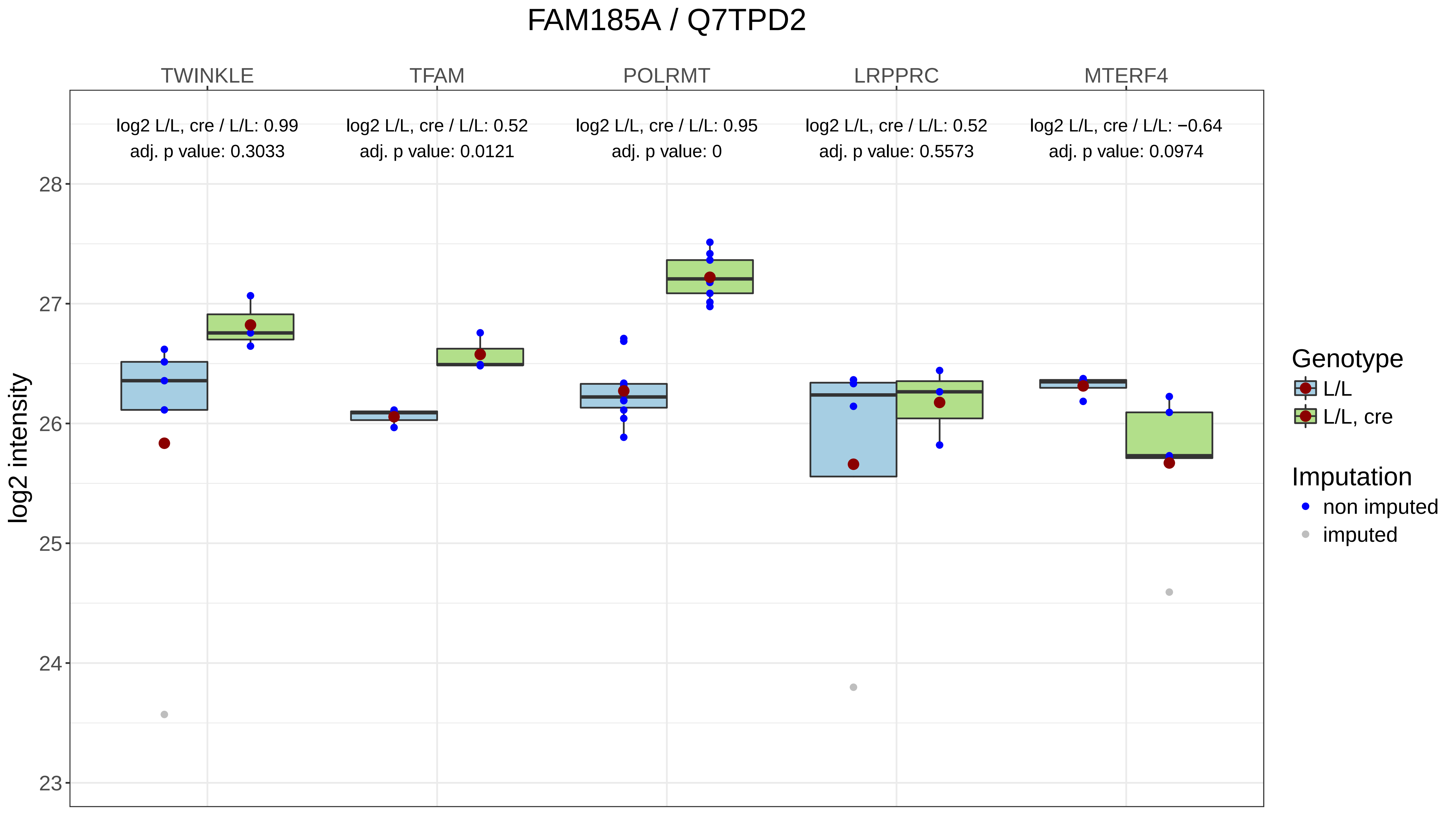
FAM185A / Q7TPD2
TWINKLE
TFAM
POLRMT
LRPPRC
MTERF4
log2 L/L, cre / L/L: 0.99
adj. p value: 0.3033
log2 L/L, cre / L/L: 0.52
adj. p value: 0.0121
log2 L/L, cre / L/L: 0.95
adj. p value: 0
log2 L/L, cre / L/L: 0.52
adj. p value: 0.5573
log2 L/L, cre / L/L: −0.64
adj. p value: 0.0974
log2 intensity
28
27
26
25
24
23
Genotype
L/L
L/L, cre
Imputation
non imputed
imputed

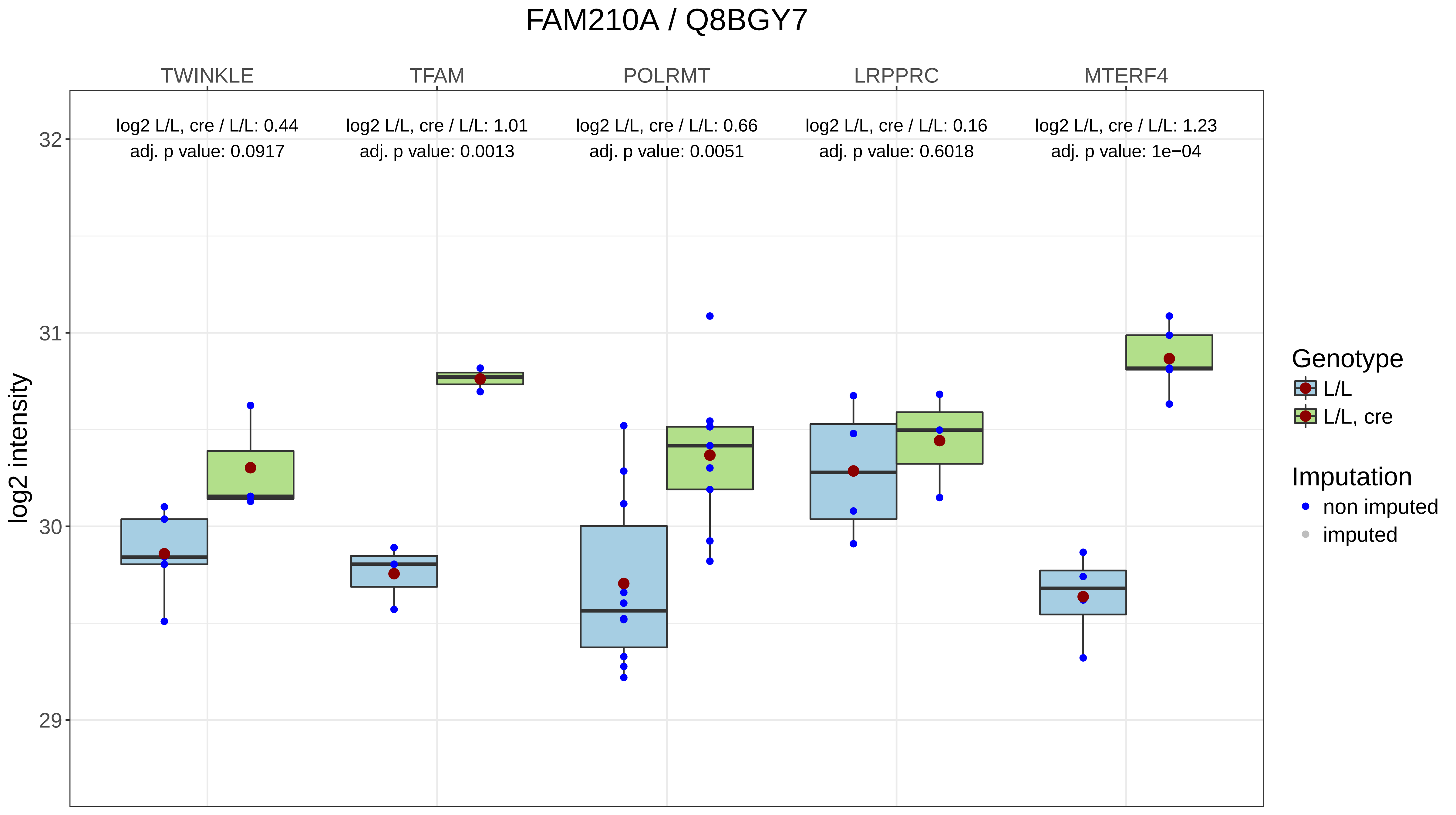
FAM210A / Q8BGY7
TWINKLE
TFAM
POLRMT
LRPPRC
MTERF4
log2 L/L, cre / L/L: 0.44
adj. p value: 0.0917
log2 L/L, cre / L/L: 1.01
adj. p value: 0.0013
log2 L/L, cre / L/L: 0.66
adj. p value: 0.0051
log2 L/L, cre / L/L: 0.16
adj. p value: 0.6018
log2 L/L, cre / L/L: 1.23
adj. p value: 1e−04
log2 intensity
32
31
30
29
Genotype
L/L
L/L, cre
Imputation
non imputed
imputed

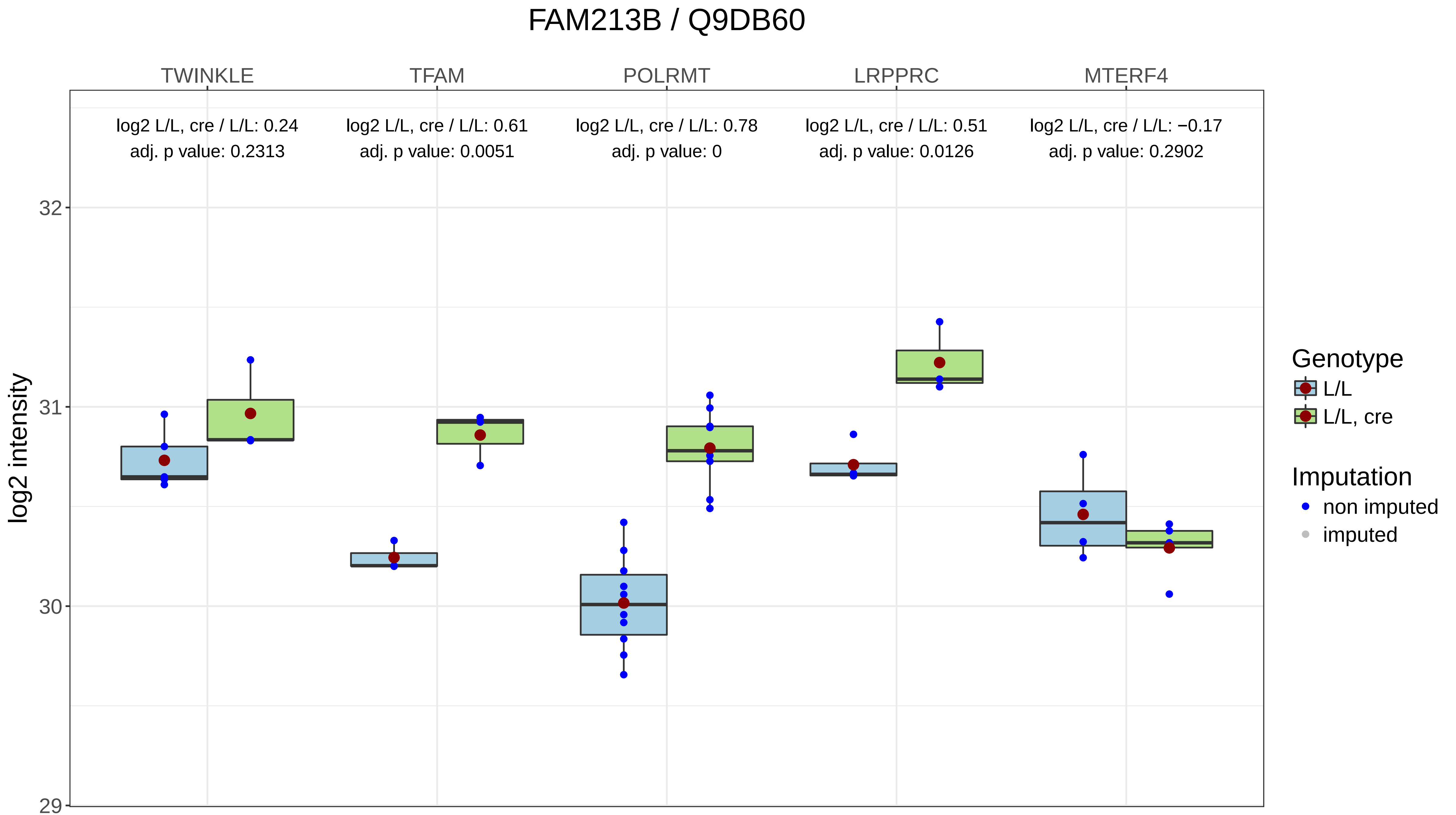
FAM213B / Q9DB60
TWINKLE
TFAM
POLRMT
LRPPRC
MTERF4
log2 L/L, cre / L/L: 0.24
adj. p value: 0.2313
log2 L/L, cre / L/L: 0.61
adj. p value: 0.0051
log2 L/L, cre / L/L: 0.78
adj. p value: 0
log2 L/L, cre / L/L: 0.51
adj. p value: 0.0126
log2 L/L, cre / L/L: −0.17
adj. p value: 0.2902
log2 intensity
32
31
30
29
Genotype
L/L
L/L, cre
Imputation
non imputed
imputed

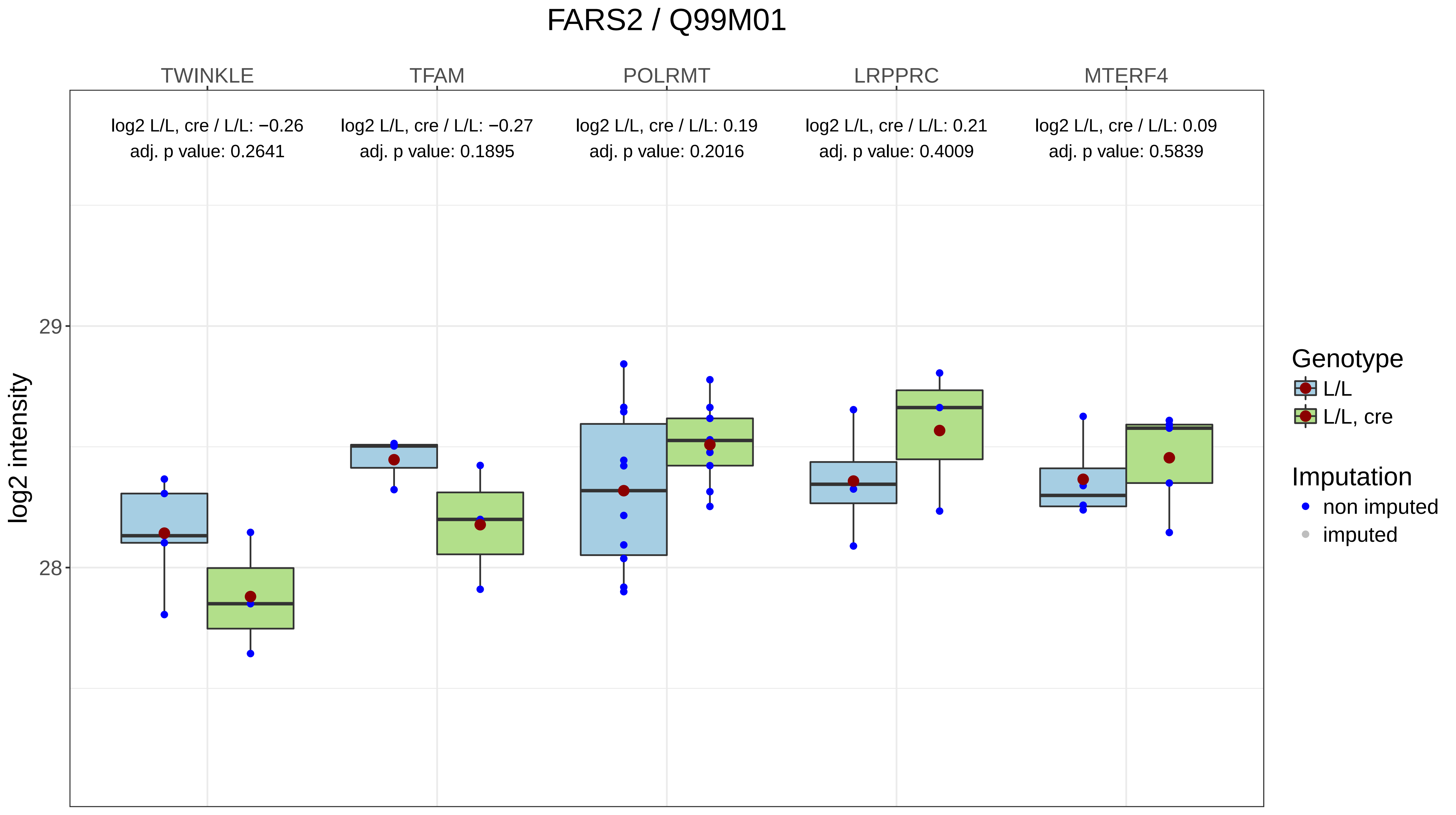

FARS2 / Q99M01
TWINKLE
TFAM
POLRMT
LRPPRC
MTERF4
log2 L/L, cre / L/L: −0.26
adj. p value: 0.2641
log2 L/L, cre / L/L: −0.27
adj. p value: 0.1895
log2 L/L, cre / L/L: 0.19
adj. p value: 0.2016
log2 L/L, cre / L/L: 0.21
adj. p value: 0.4009
log2 L/L, cre / L/L: 0.09
adj. p value: 0.5839
29
28
log2 intensity
Genotype
L/L
L/L, cre
Imputation
non imputed
imputed

FASTKD2 / Q922E6

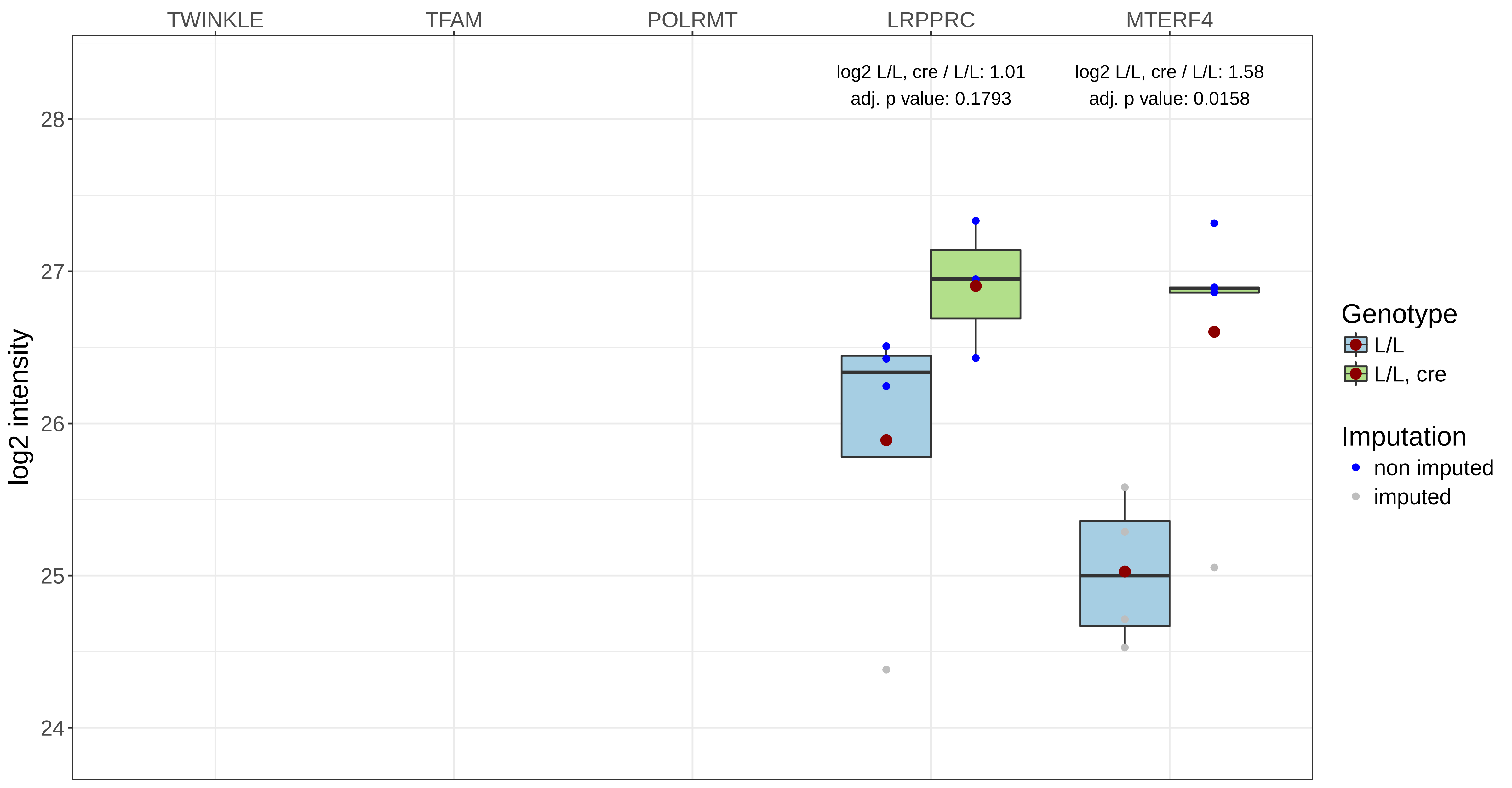

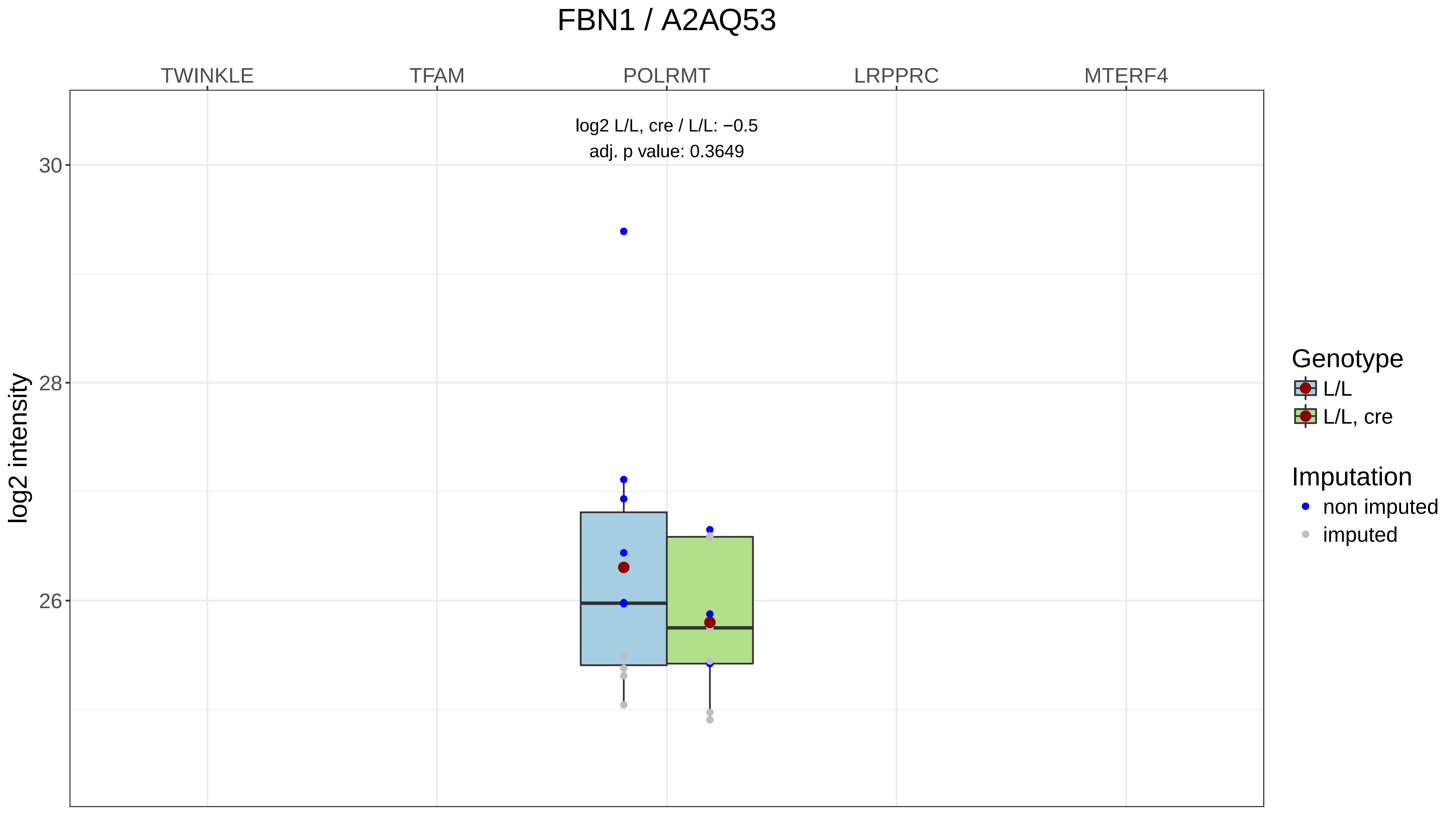
FBN1 / A2AQ53
TWINKLE
TFAM
POLRMT
LRPPRC
MTERF4
log2 L/L, cre / L/L: −0.5
adj. p value: 0.3649
30
28
26
log2 intensity
Genotype
L/L
L/L, cre
Imputation
non imputed
imputed

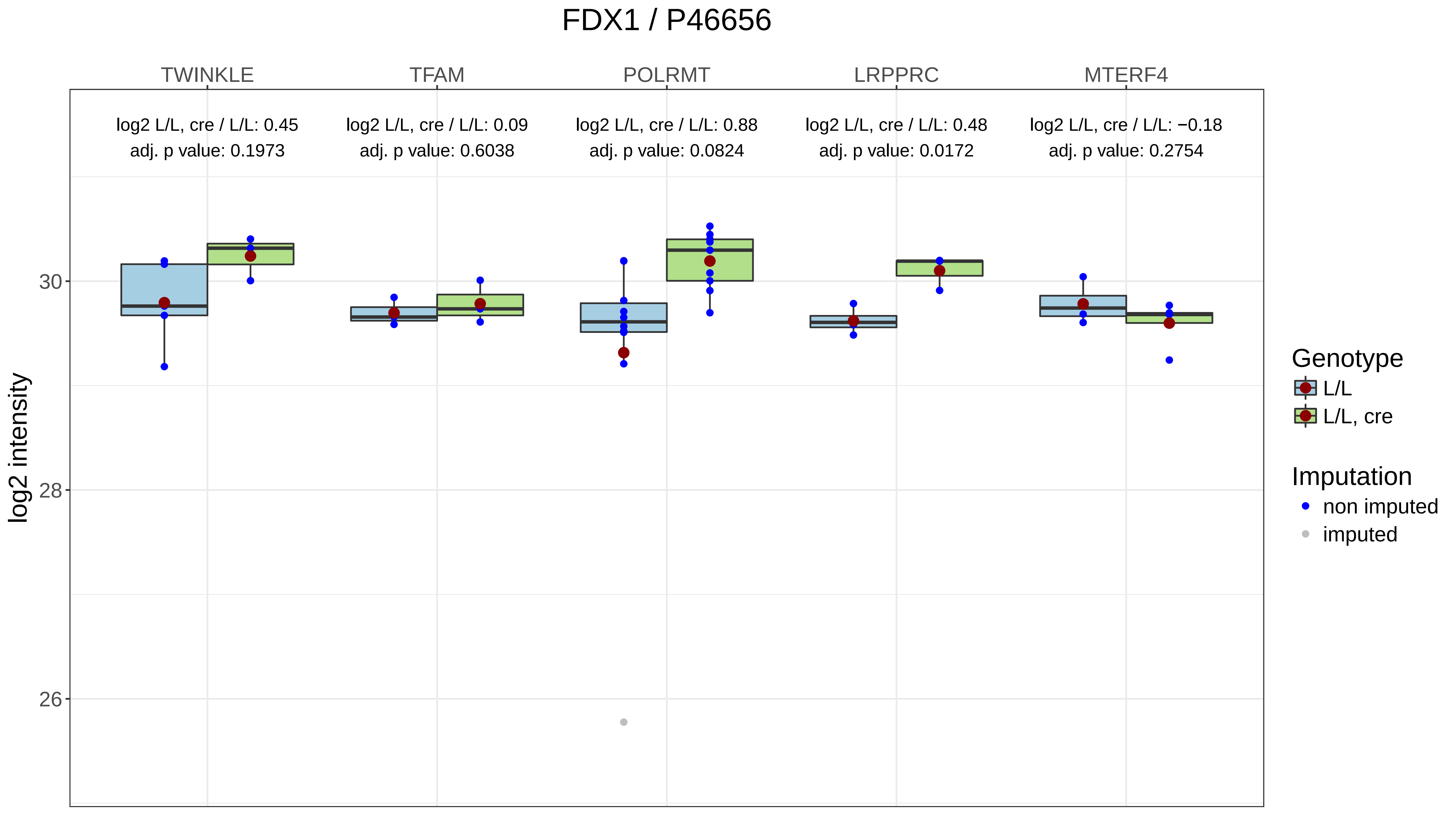
FDX1 / P46656
TWINKLE
TFAM
POLRMT
LRPPRC
MTERF4
log2 L/L, cre / L/L: 0.45
adj. p value: 0.1973
log2 L/L, cre / L/L: 0.09
adj. p value: 0.6038
log2 L/L, cre / L/L: 0.88
adj. p value: 0.0824
log2 L/L, cre / L/L: 0.48
adj. p value: 0.0172
log2 L/L, cre / L/L: −0.18
adj. p value: 0.2754
log2 intensity
30
28
26
Genotype
L/L
L/L, cre
Imputation
non imputed
imputed

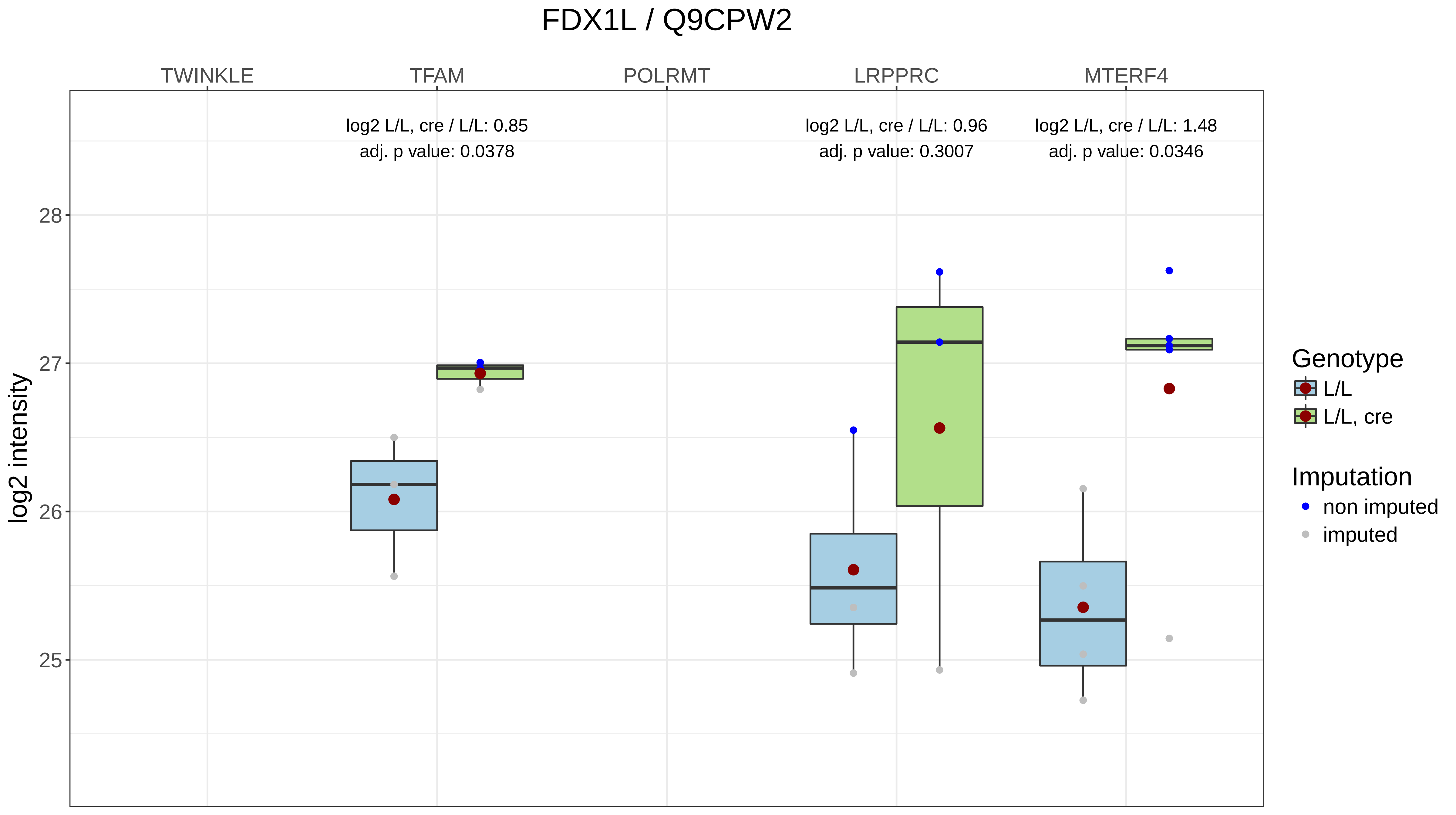
FDX1L / Q9CPW2
TWINKLE
TFAM
POLRMT
LRPPRC
MTERF4
log2 L/L, cre / L/L: 0.85
adj. p value: 0.0378
log2 L/L, cre / L/L: 0.96
adj. p value: 0.3007
log2 L/L, cre / L/L: 1.48
adj. p value: 0.0346
log2 intensity
28
27
26
25
Genotype
L/L
L/L, cre
Imputation
non imputed
imputed

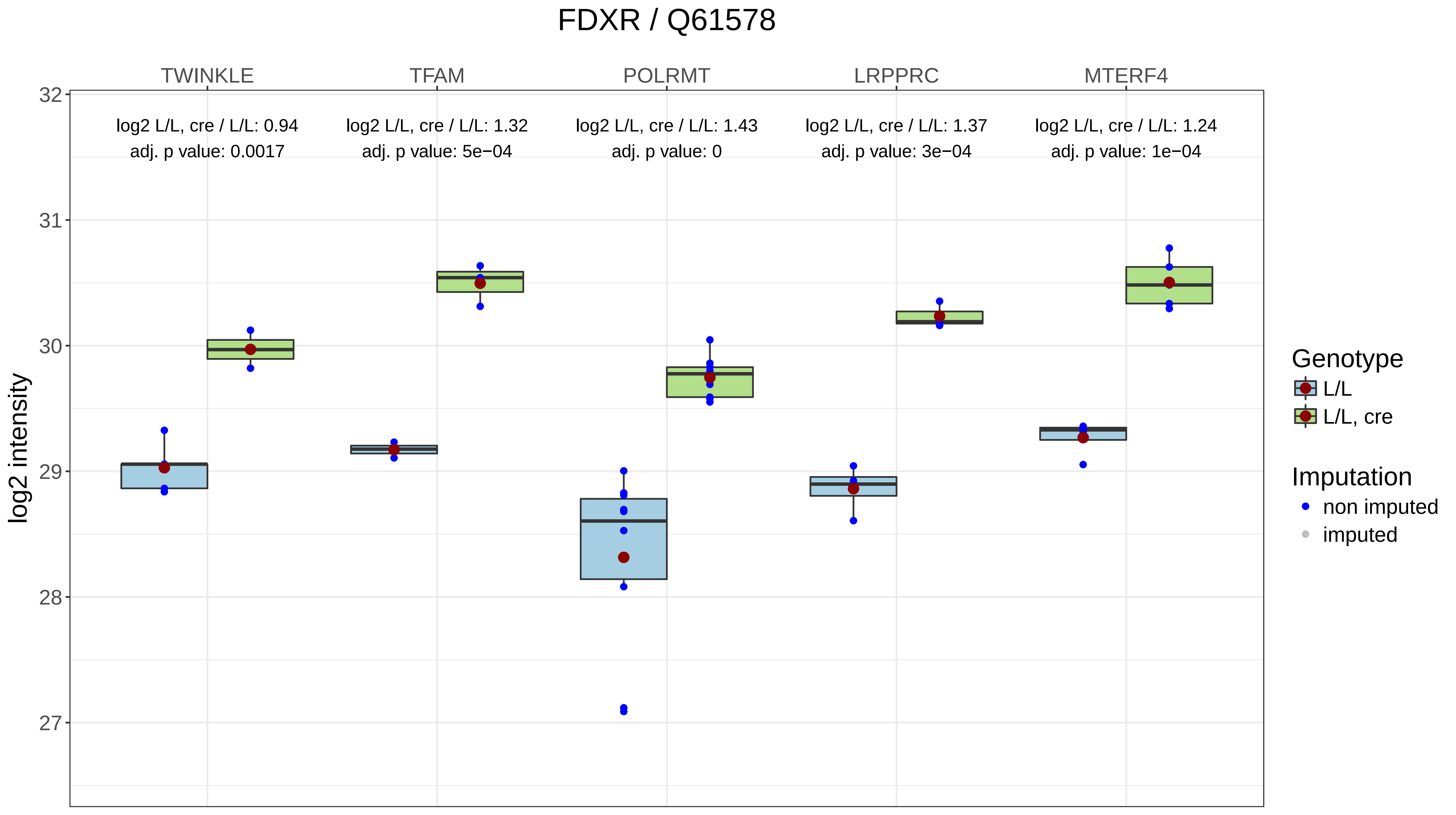
FDXR / Q61578
TWINKLE
TFAM
POLRMT
LRPPRC
MTERF4
log2 L/L, cre / L/L: 0.94
adj. p value: 0.0017
log2 L/L, cre / L/L: 1.32
adj. p value: 5e−04
log2 L/L, cre / L/L: 1.43
adj. p value: 0
log2 L/L, cre / L/L: 1.37
adj. p value: 3e−04
log2 L/L, cre / L/L: 1.24
adj. p value: 1e−04
log2 intensity
32
31
30
29
28
27
Genotype
L/L
L/L, cre
Imputation
non imputed
imputed

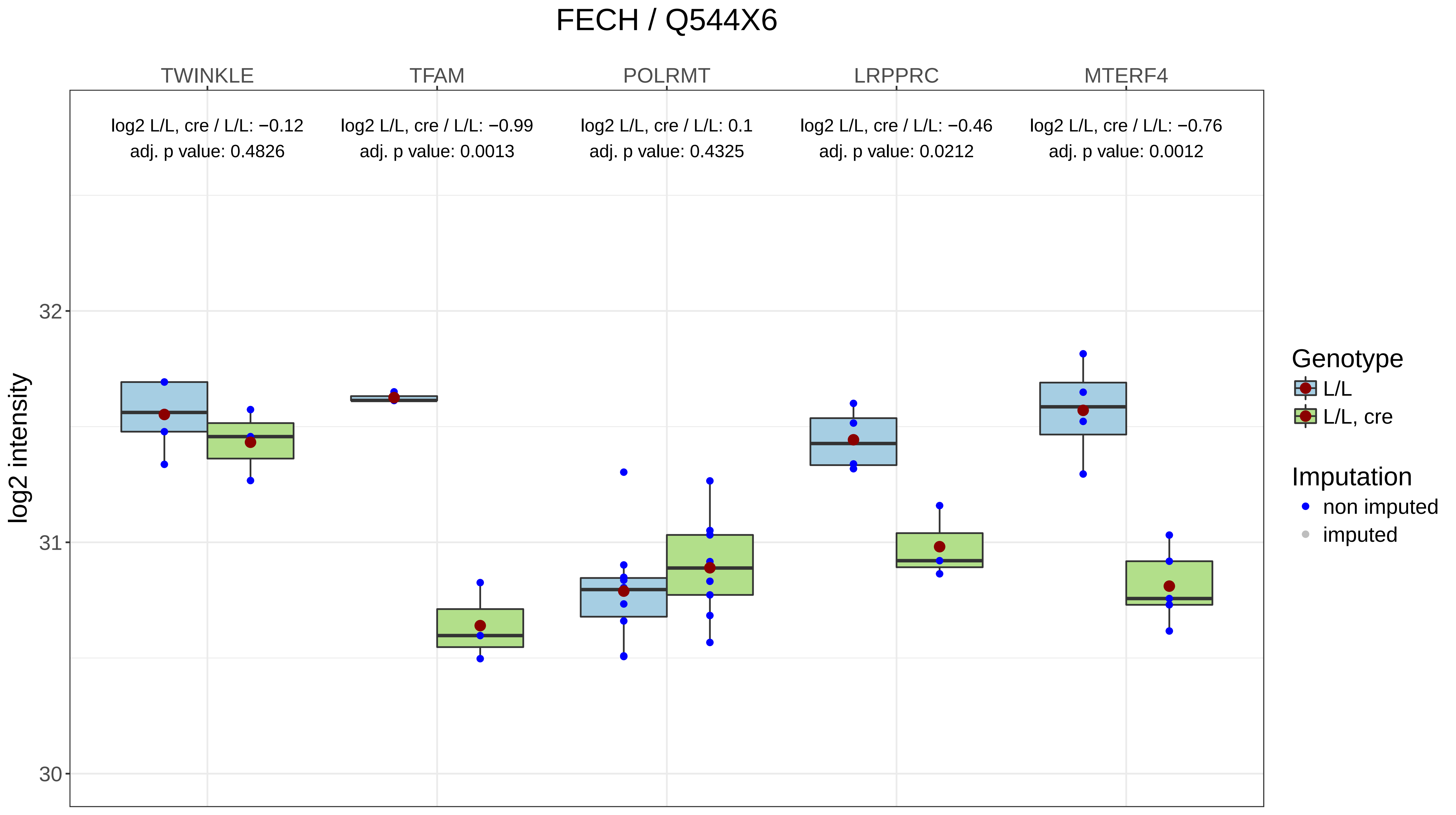
FECH / Q544X6
TWINKLE
TFAM
POLRMT
LRPPRC
MTERF4
log2 L/L, cre / L/L: −0.12
adj. p value: 0.4826
log2 L/L, cre / L/L: −0.99
adj. p value: 0.0013
log2 L/L, cre / L/L: 0.1
adj. p value: 0.4325
log2 L/L, cre / L/L: −0.46
adj. p value: 0.0212
log2 L/L, cre / L/L: −0.76
adj. p value: 0.0012
log2 intensity
32
31
30
Genotype
L/L
L/L, cre
Imputation
non imputed
imputed

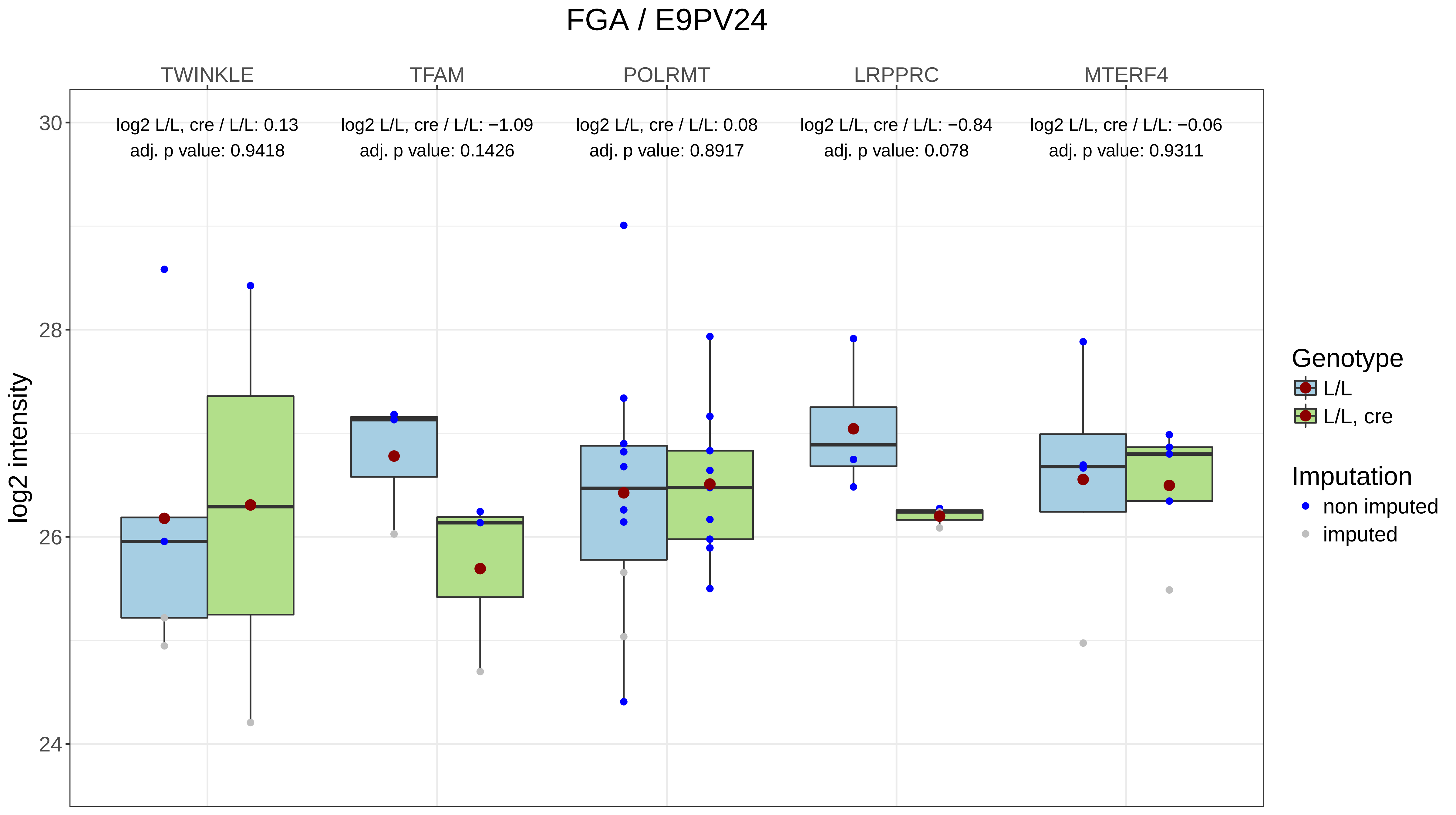

FGA / E9PV24
TWINKLE
TFAM
POLRMT
LRPPRC
MTERF4
log2 L/L, cre / L/L: 0.13
adj. p value: 0.9418
log2 L/L, cre / L/L: −1.09
adj. p value: 0.1426
log2 L/L, cre / L/L: 0.08
adj. p value: 0.8917
log2 L/L, cre / L/L: −0.84
adj. p value: 0.078
log2 L/L, cre / L/L: −0.06
adj. p value: 0.9311
30
28
26
24
log2 intensity
Genotype
L/L
L/L, cre
Imputation
non imputed
imputed

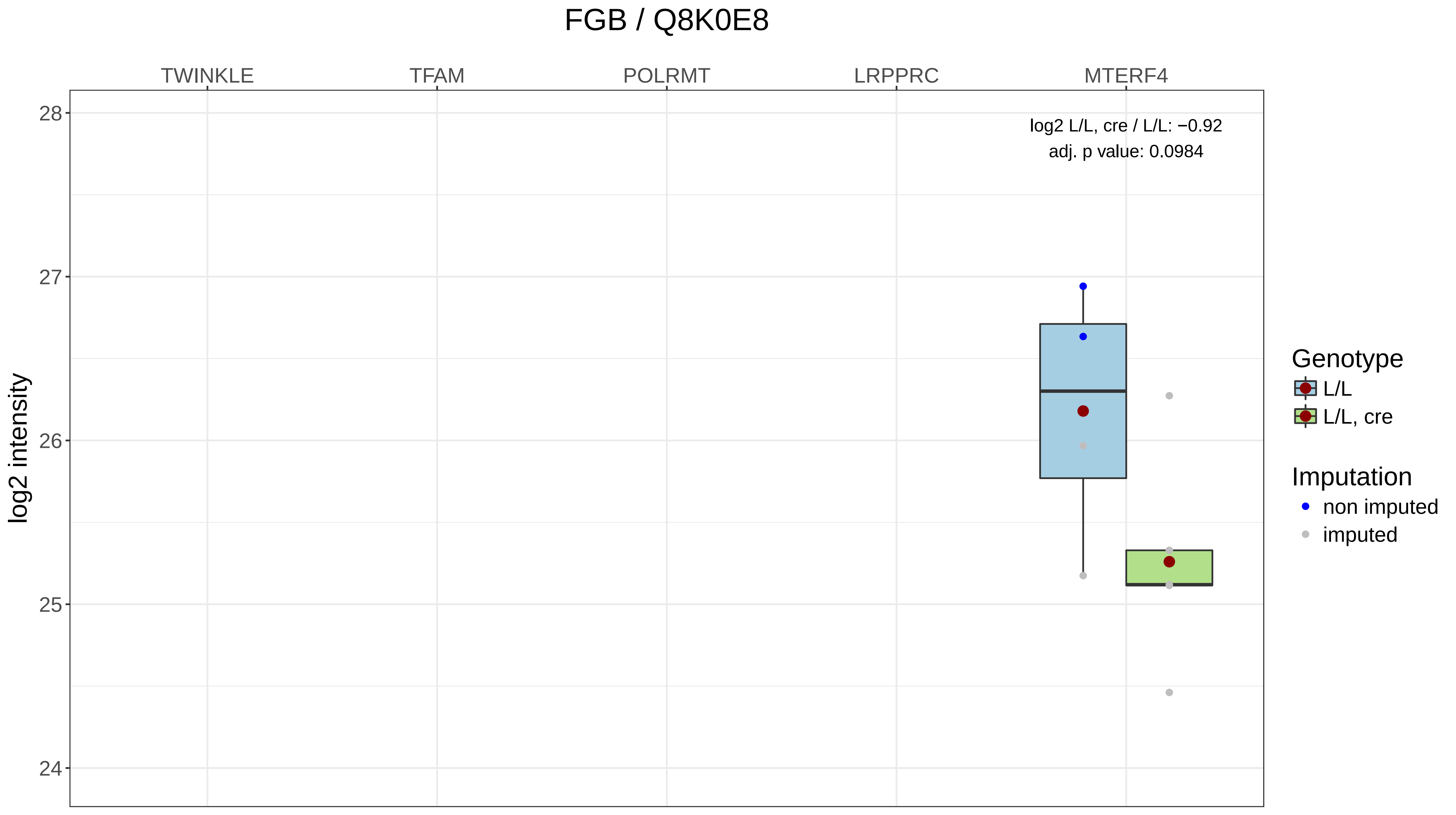
FGB / Q8K0E8
TWINKLE
TFAM
POLRMT
LRPPRC
MTERF4
log2 L/L, cre / L/L: −0.92
adj. p value: 0.0984
log2 intensity
28
27
26
25
24
Genotype
L/L
L/L, cre
Imputation
non imputed
imputed

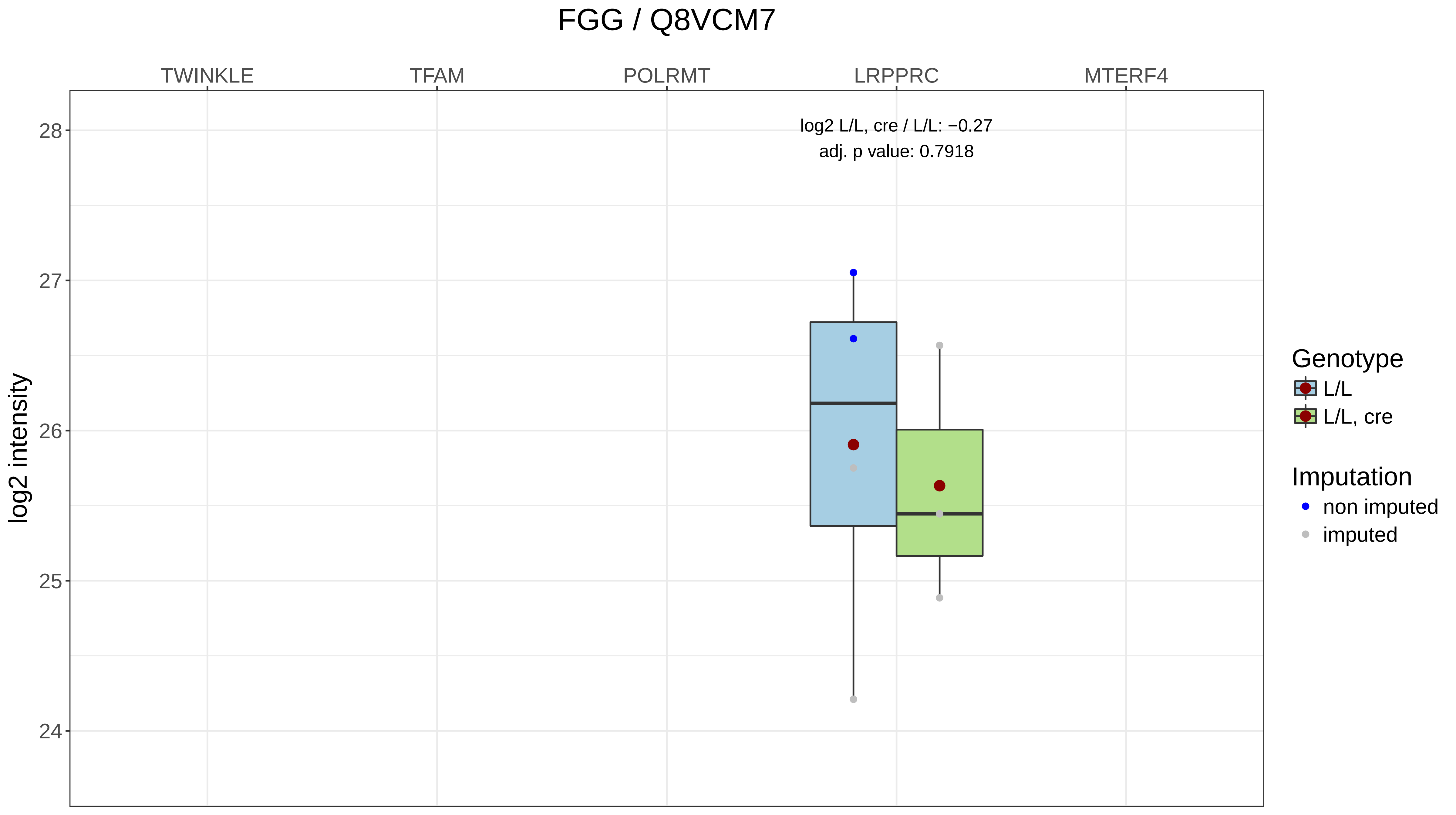
FGG / Q8VCM7
TWINKLE
TFAM
POLRMT
LRPPRC
MTERF4
log2 L/L, cre / L/L: −0.27
adj. p value: 0.7918
28
27
26
25
24
log2 intensity
Genotype
L/L
L/L, cre
Imputation
non imputed
imputed

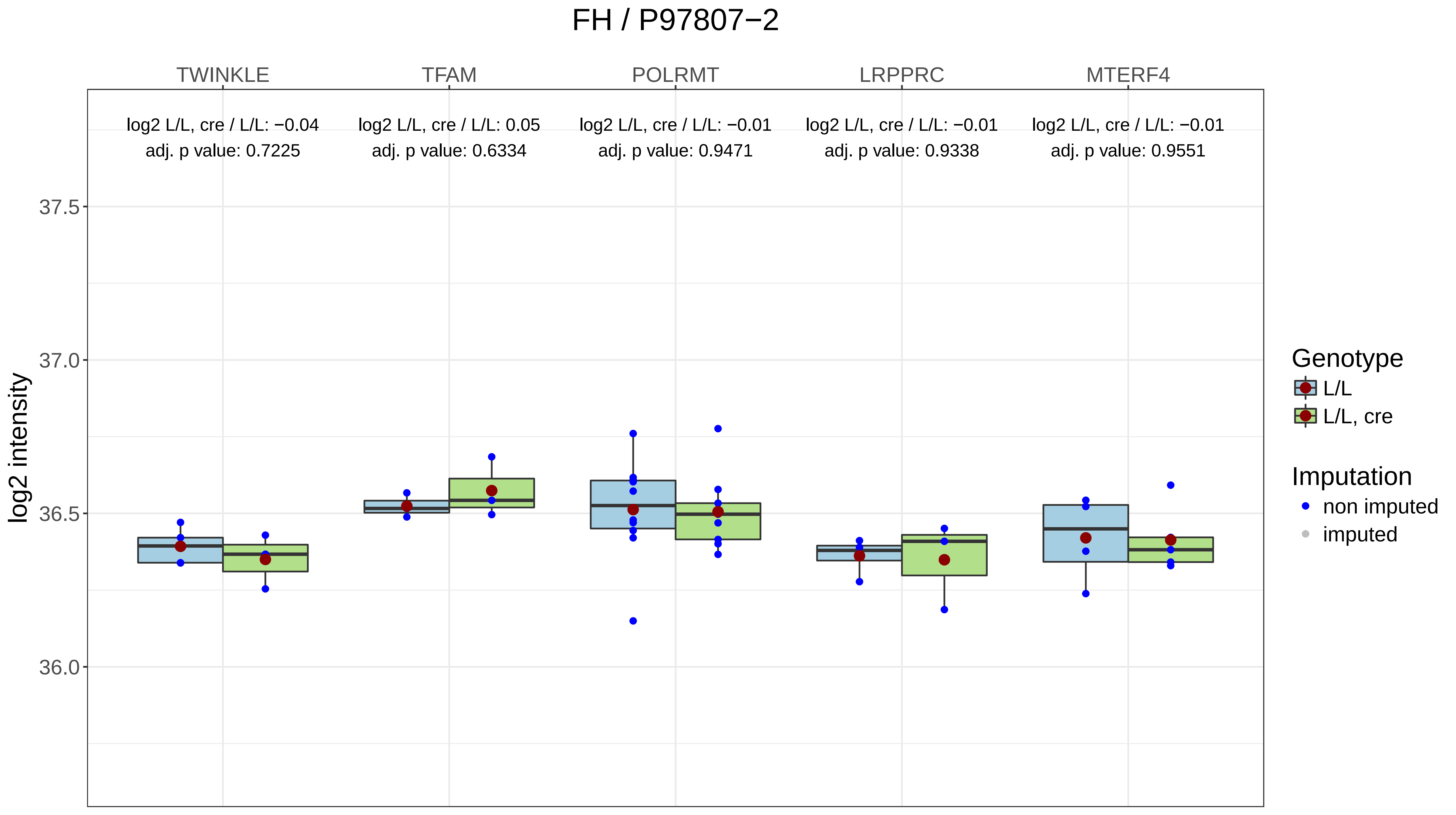
FH / P97807−2
TWINKLE
TFAM
POLRMT
LRPPRC
MTERF4
log2 L/L, cre / L/L: −0.04
adj. p value: 0.7225
log2 L/L, cre / L/L: 0.05
adj. p value: 0.6334
log2 L/L, cre / L/L: −0.01
adj. p value: 0.9471
log2 L/L, cre / L/L: −0.01
adj. p value: 0.9338
log2 L/L, cre / L/L: −0.01
adj. p value: 0.9551
log2 intensity
37.5
37.0
36.5
36.0
Genotype
L/L
L/L, cre
Imputation
non imputed
imputed

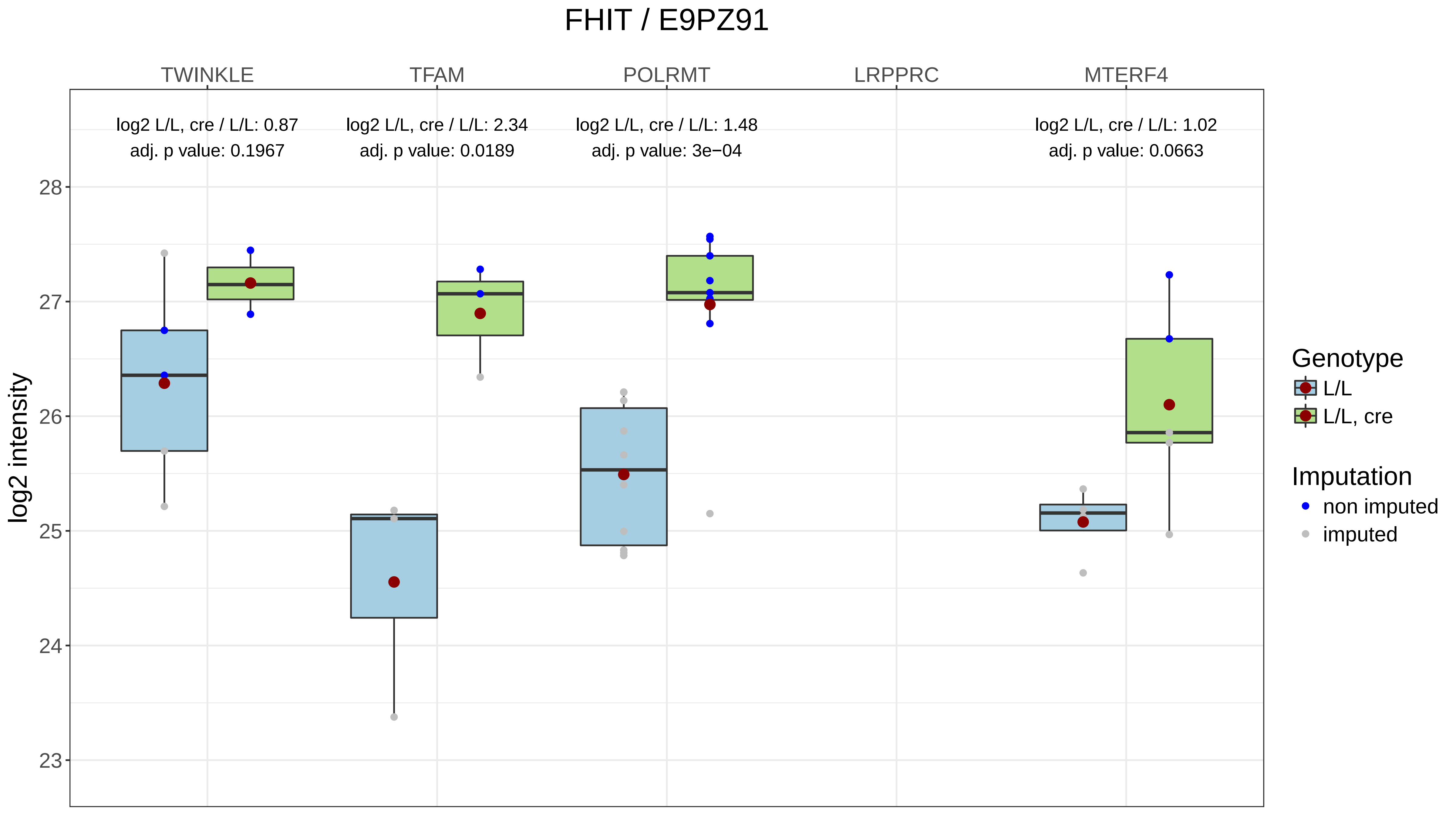
FHIT / E9PZ91
TWINKLE
TFAM
POLRMT
LRPPRC
MTERF4
log2 L/L, cre / L/L: 0.87
adj. p value: 0.1967
log2 L/L, cre / L/L: 2.34
adj. p value: 0.0189
log2 L/L, cre / L/L: 1.48
adj. p value: 3e−04
log2 L/L, cre / L/L: 1.02
adj. p value: 0.0663
log2 intensity
28
27
26
25
24
23
Genotype
L/L
L/L, cre
Imputation
non imputed
imputed

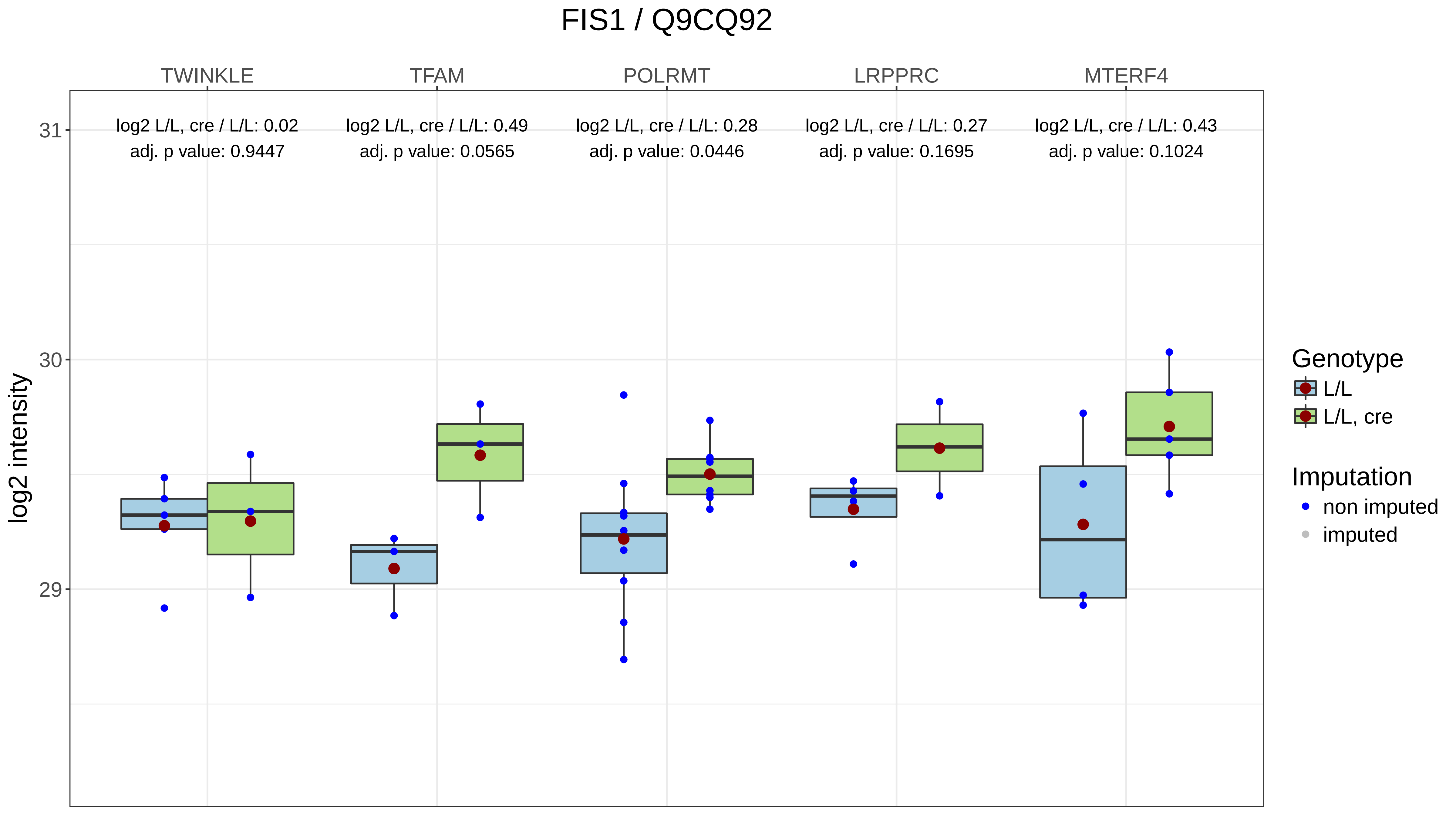
FIS1 / Q9CQ92
TWINKLE
TFAM
POLRMT
LRPPRC
MTERF4
log2 L/L, cre / L/L: 0.02
adj. p value: 0.9447
log2 L/L, cre / L/L: 0.49
adj. p value: 0.0565
log2 L/L, cre / L/L: 0.28
adj. p value: 0.0446
log2 L/L, cre / L/L: 0.27
adj. p value: 0.1695
log2 L/L, cre / L/L: 0.43
adj. p value: 0.1024
log2 intensity
31
30
29
Genotype
L/L
L/L, cre
Imputation
non imputed
imputed

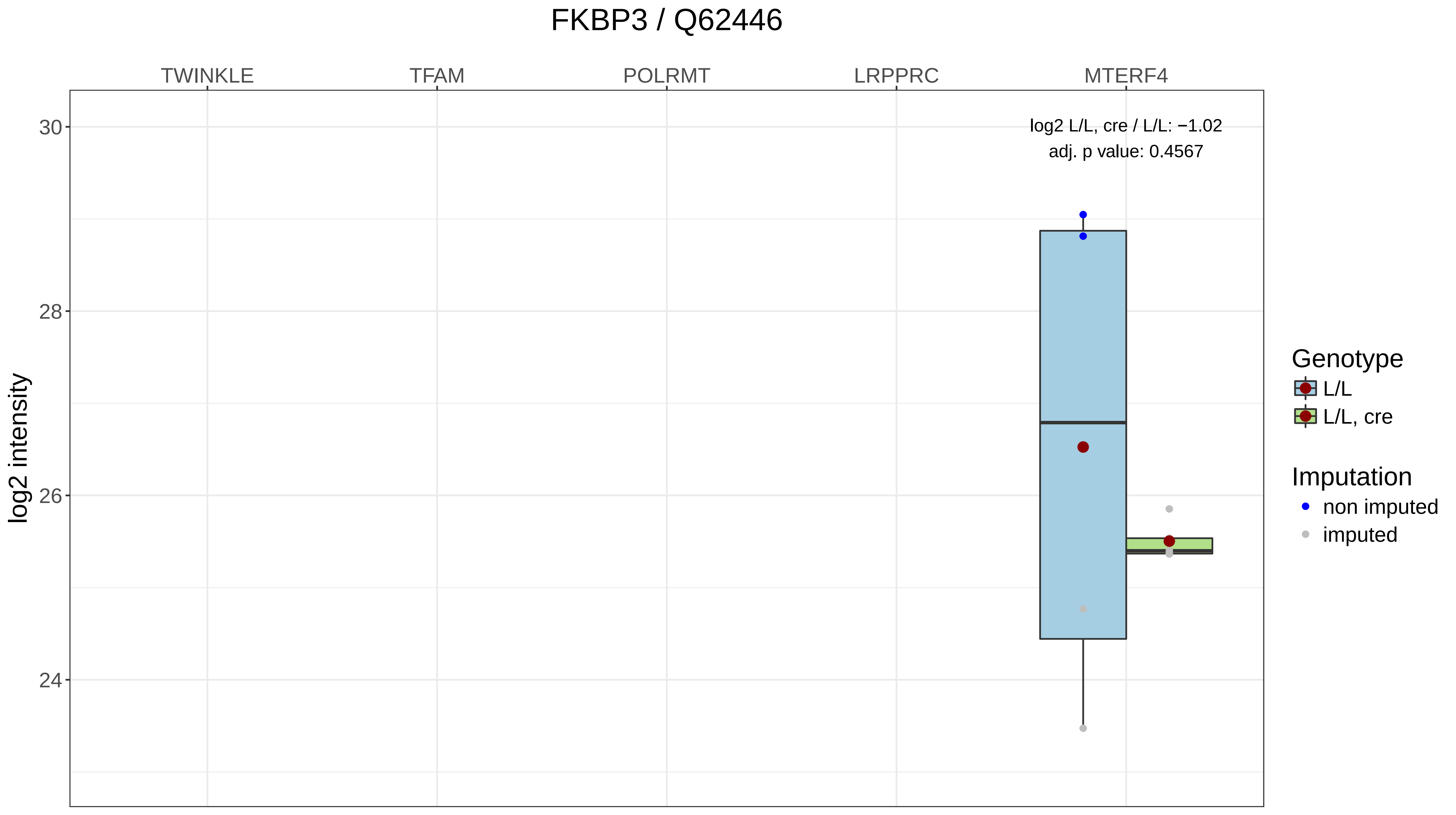
FKBP3 / Q62446
TWINKLE
TFAM
POLRMT
LRPPRC
MTERF4
log2 L/L, cre / L/L: −1.02
adj. p value: 0.4567
log2 intensity
30
28
26
24
Genotype
L/L
L/L, cre
Imputation
non imputed
imputed

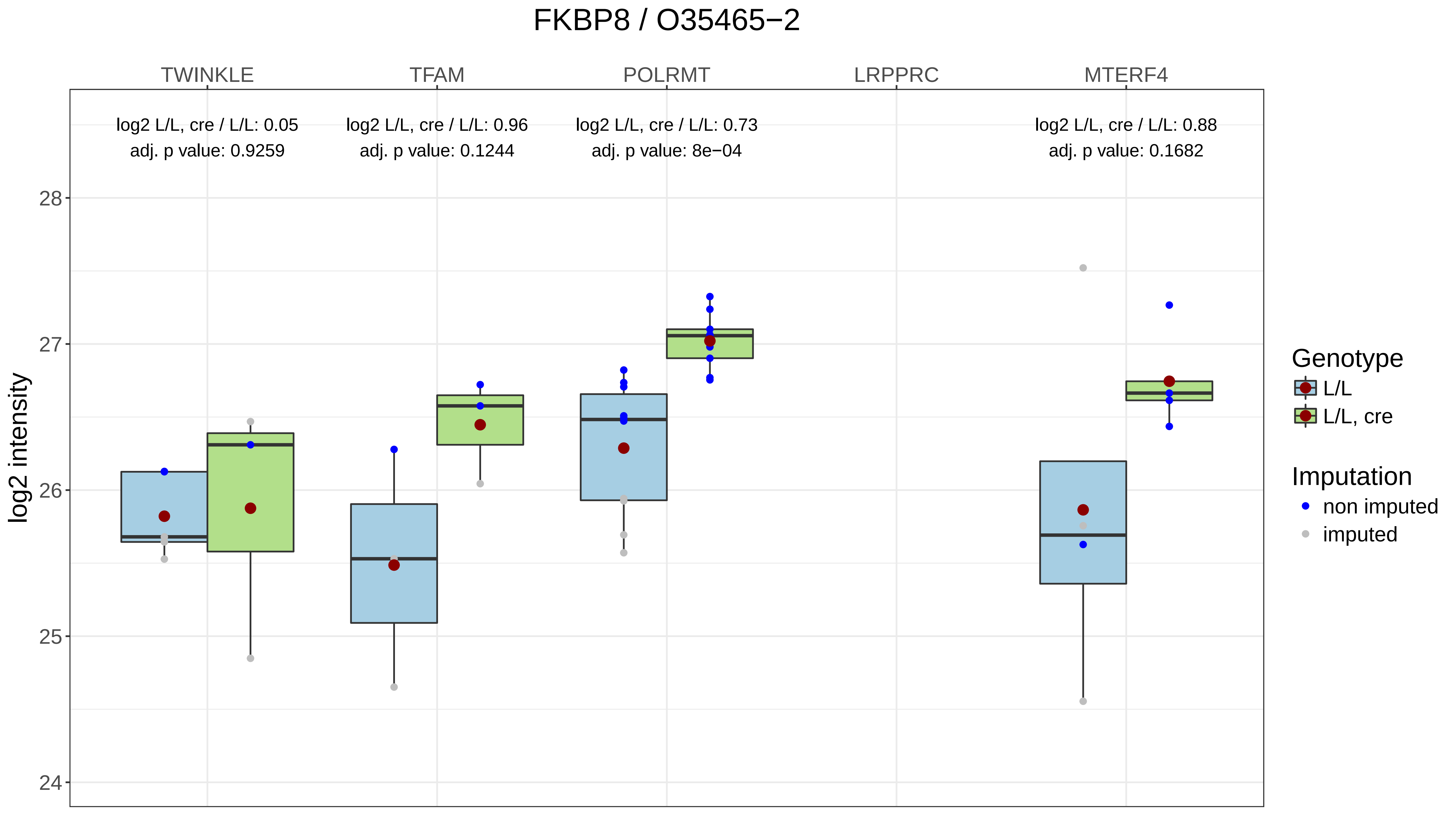

FKBP8 / O35465−2
TWINKLE
TFAM
POLRMT
LRPPRC
MTERF4
log2 L/L, cre / L/L: 0.05
adj. p value: 0.9259
log2 L/L, cre / L/L: 0.96
adj. p value: 0.1244
log2 L/L, cre / L/L: 0.73
adj. p value: 8e−04
log2 L/L, cre / L/L: 0.88
adj. p value: 0.1682
log2 intensity
28
27
26
25
24
Genotype
L/L
L/L, cre
Imputation
non imputed
imputed

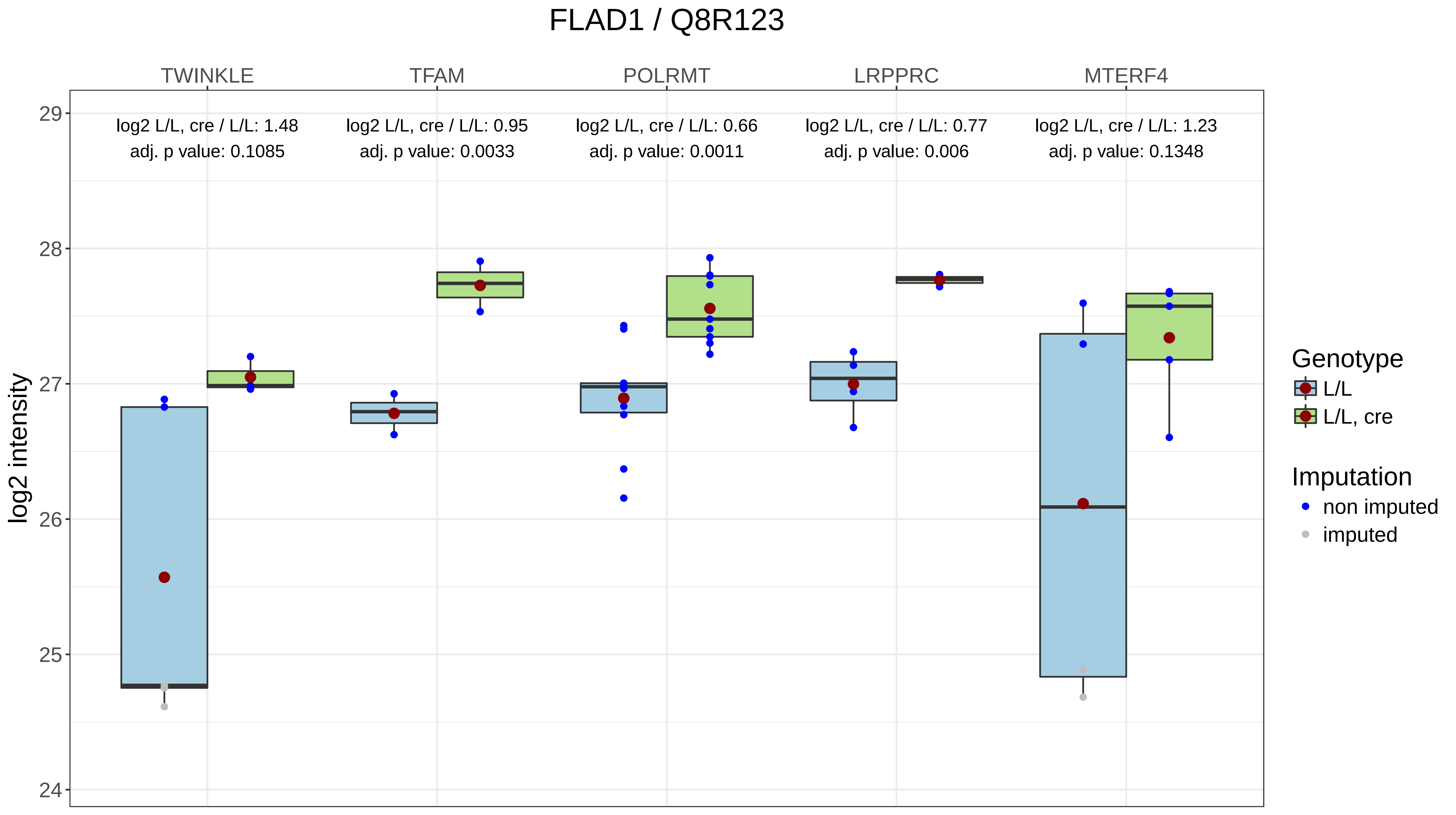

FLAD1 / Q8R123
TWINKLE
TFAM
POLRMT
LRPPRC
MTERF4
log2 L/L, cre / L/L: 1.48
adj. p value: 0.1085
log2 L/L, cre / L/L: 0.95
adj. p value: 0.0033
log2 L/L, cre / L/L: 0.66
adj. p value: 0.0011
log2 L/L, cre / L/L: 0.77
adj. p value: 0.006
log2 L/L, cre / L/L: 1.23
adj. p value: 0.1348
log2 intensity
29
28
27
26
25
24
Genotype
L/L
L/L, cre
Imputation
non imputed
imputed

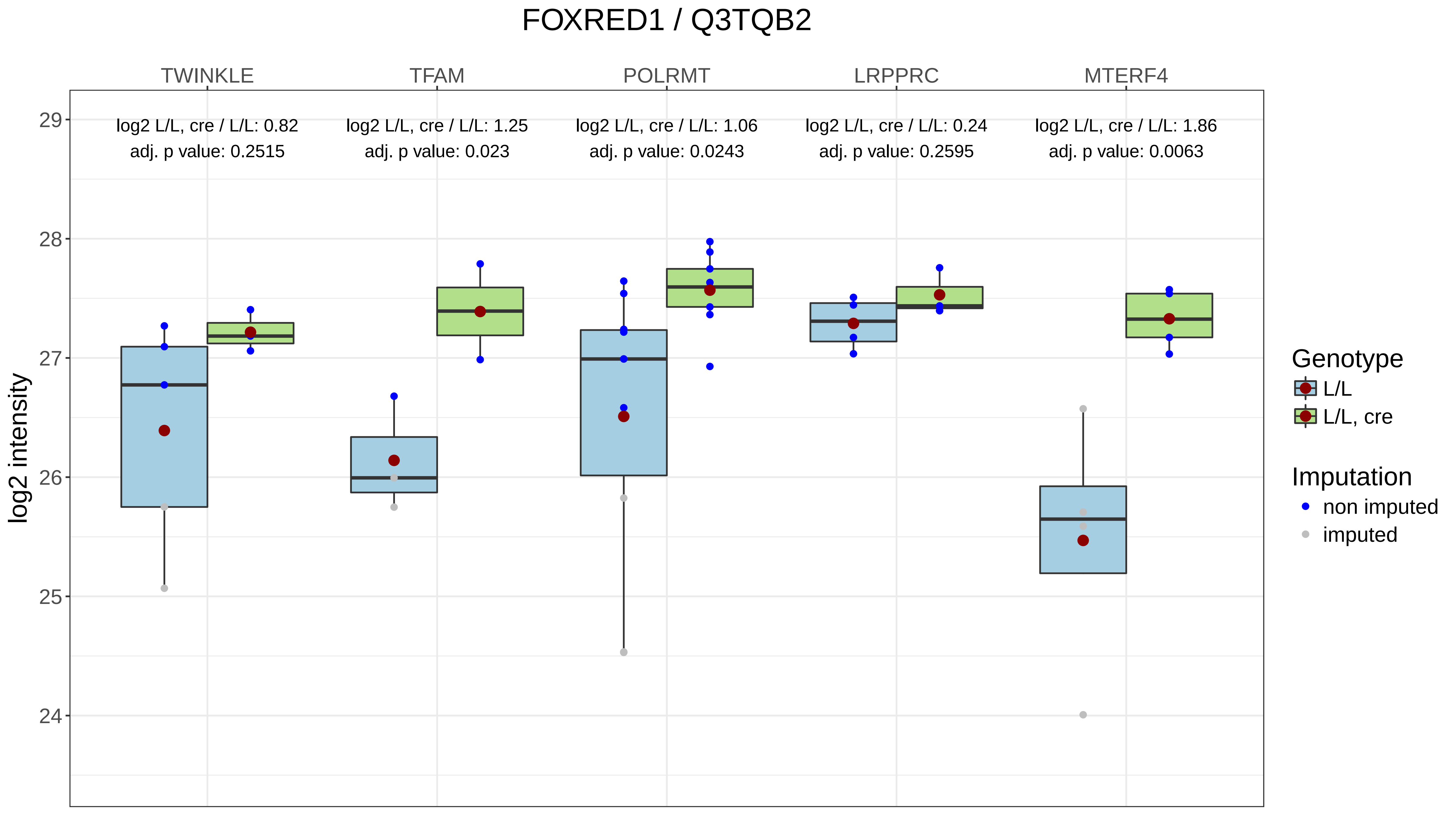
FOXRED1 / Q3TQB2
TWINKLE
TFAM
POLRMT
LRPPRC
MTERF4
log2 L/L, cre / L/L: 0.82
adj. p value: 0.2515
log2 L/L, cre / L/L: 1.25
adj. p value: 0.023
log2 L/L, cre / L/L: 1.06
adj. p value: 0.0243
log2 L/L, cre / L/L: 0.24
adj. p value: 0.2595
log2 L/L, cre / L/L: 1.86
adj. p value: 0.0063
log2 intensity
29
28
27
26
25
24
Genotype
L/L
L/L, cre
Imputation
non imputed
imputed

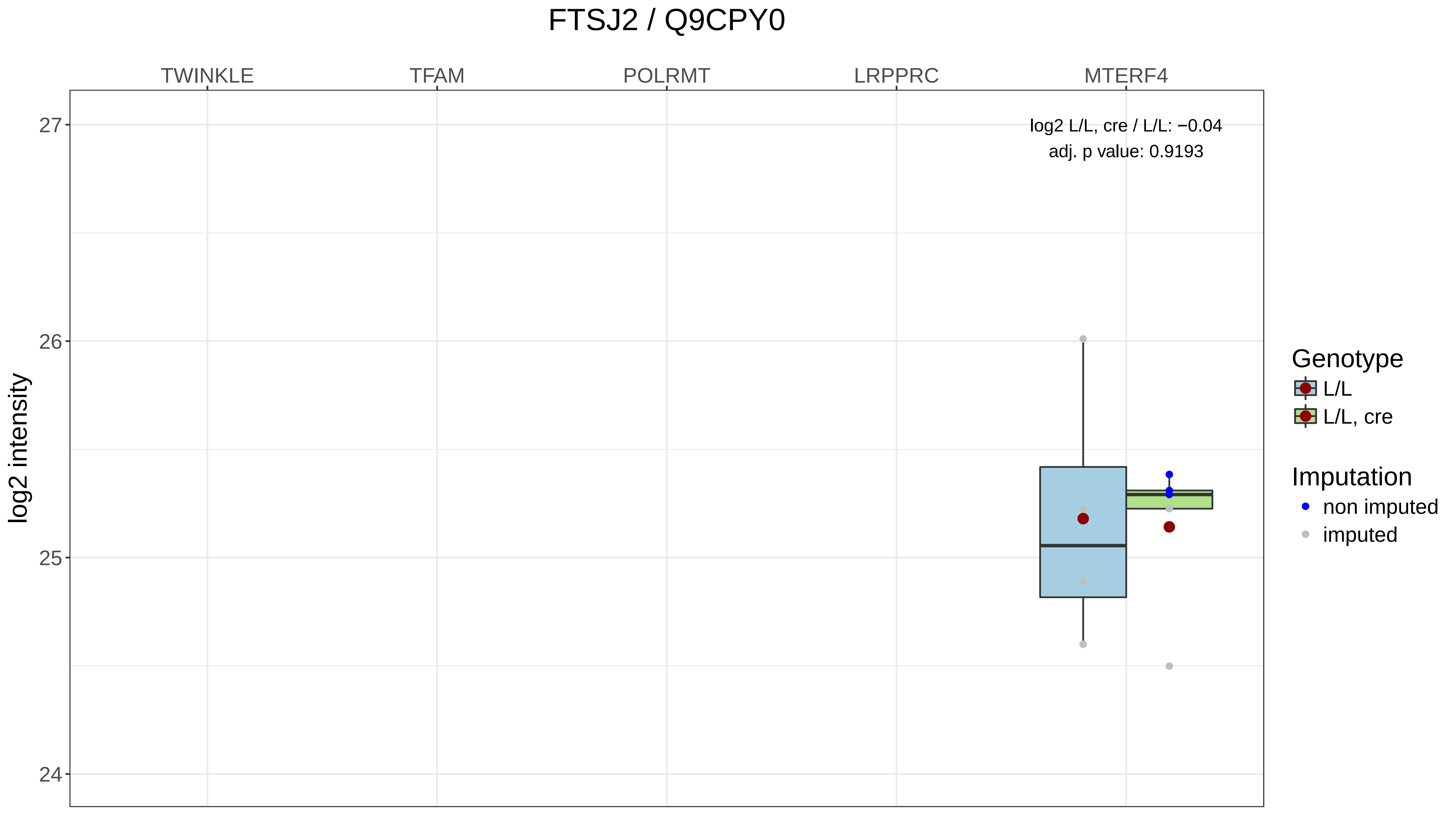

FTSJ2 / Q9CPY0
TWINKLE
TFAM
POLRMT
LRPPRC
MTERF4
log2 L/L, cre / L/L: −0.04
adj. p value: 0.9193
27
26
25
24
log2 intensity
Genotype
L/L
L/L, cre
Imputation
non imputed
imputed

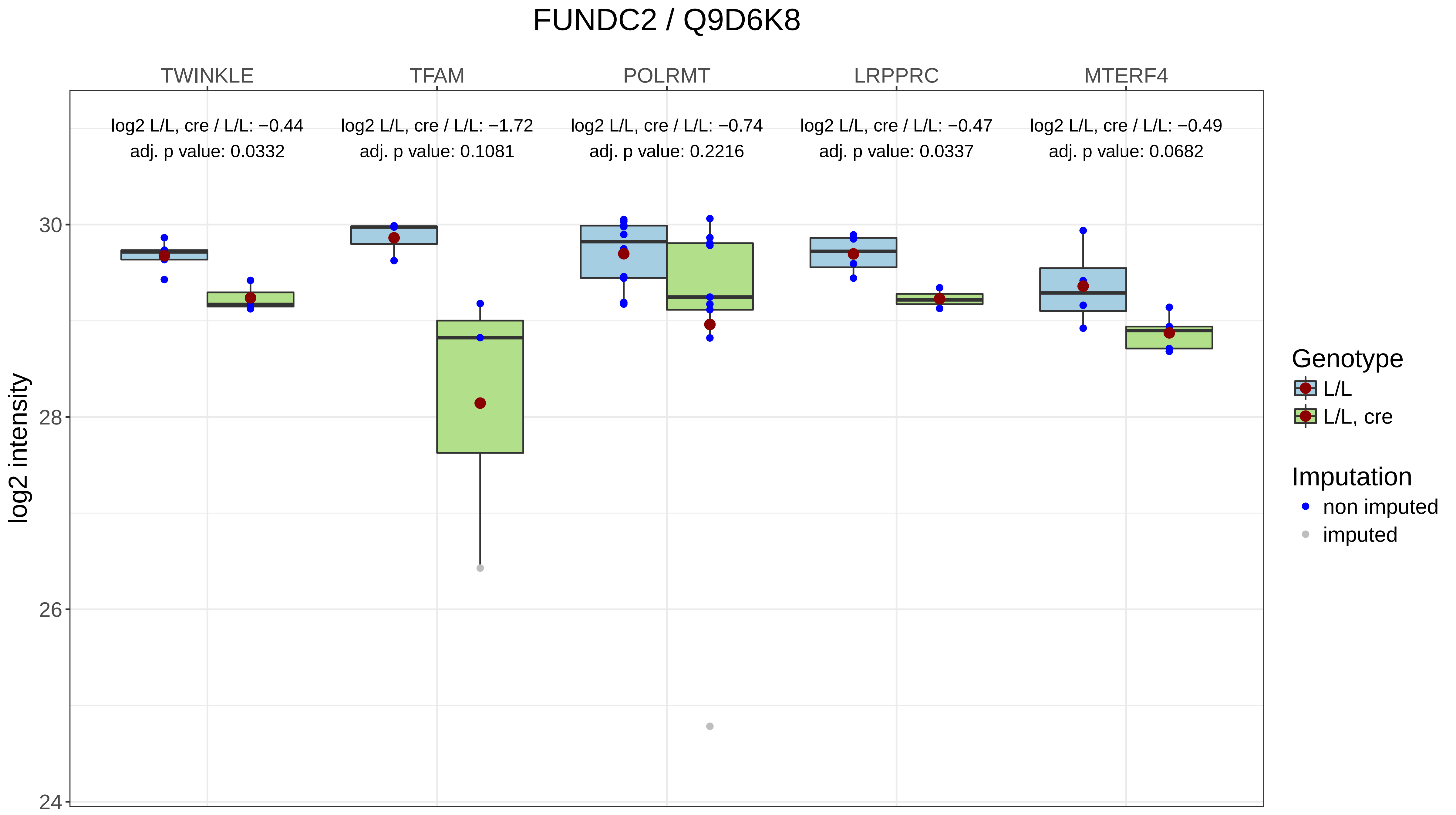
FUNDC2 / Q9D6K8
TWINKLE
TFAM
POLRMT
LRPPRC
MTERF4
log2 L/L, cre / L/L: −0.44
adj. p value: 0.0332
log2 L/L, cre / L/L: −1.72
adj. p value: 0.1081
log2 L/L, cre / L/L: −0.74
adj. p value: 0.2216
log2 L/L, cre / L/L: −0.47
adj. p value: 0.0337
log2 L/L, cre / L/L: −0.49
adj. p value: 0.0682
log2 intensity
30
28
26
24
Genotype
L/L
L/L, cre
Imputation
non imputed
imputed

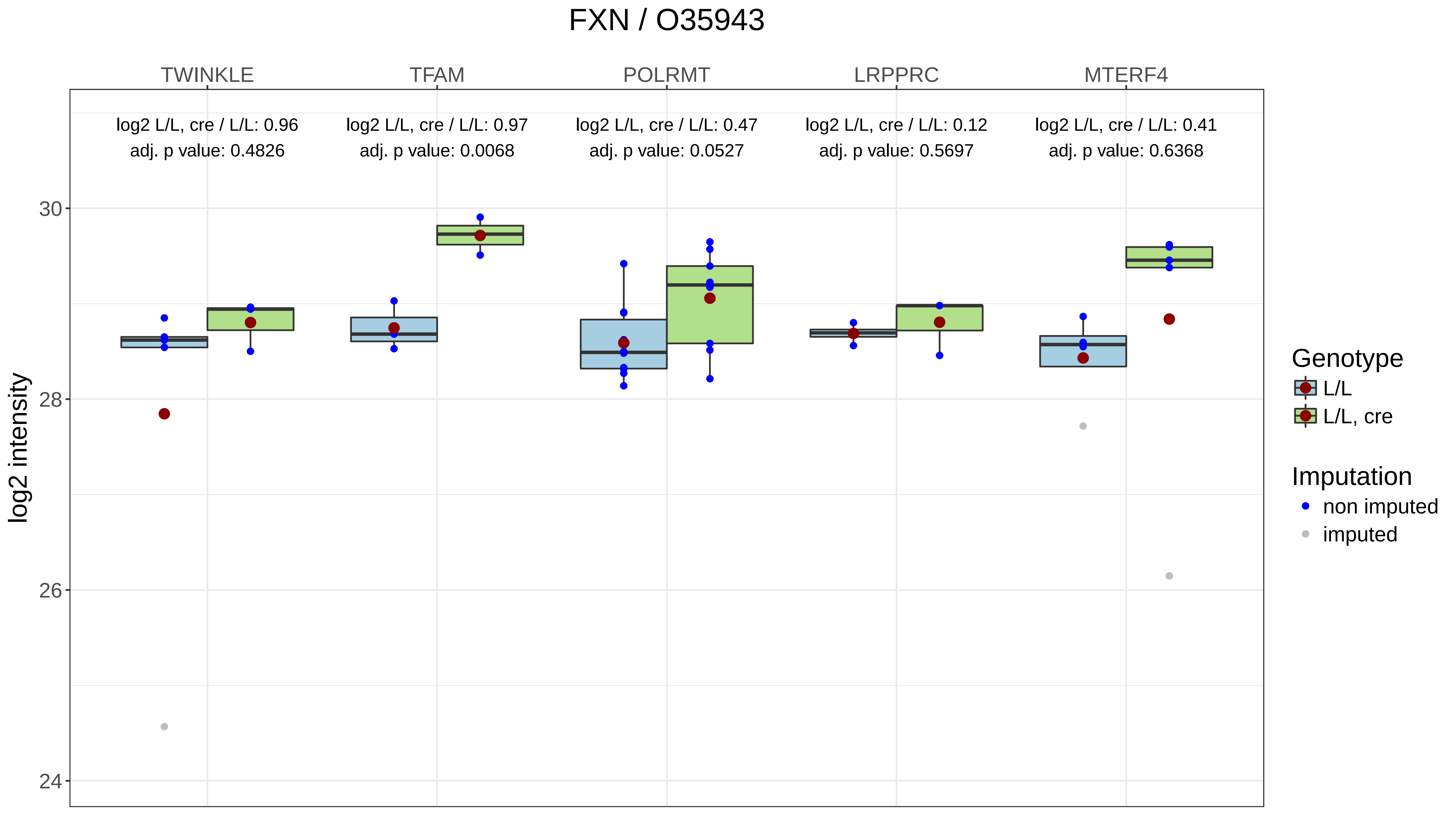
FXN / O35943
TWINKLE
TFAM
POLRMT
LRPPRC
MTERF4
log2 L/L, cre / L/L: 0.96
adj. p value: 0.4826
log2 L/L, cre / L/L: 0.97
adj. p value: 0.0068
log2 L/L, cre / L/L: 0.47
adj. p value: 0.0527
log2 L/L, cre / L/L: 0.12
adj. p value: 0.5697
log2 L/L, cre / L/L: 0.41
adj. p value: 0.6368
log2 intensity
30
28
26
24
Genotype
L/L
L/L, cre
Imputation
non imputed
imputed

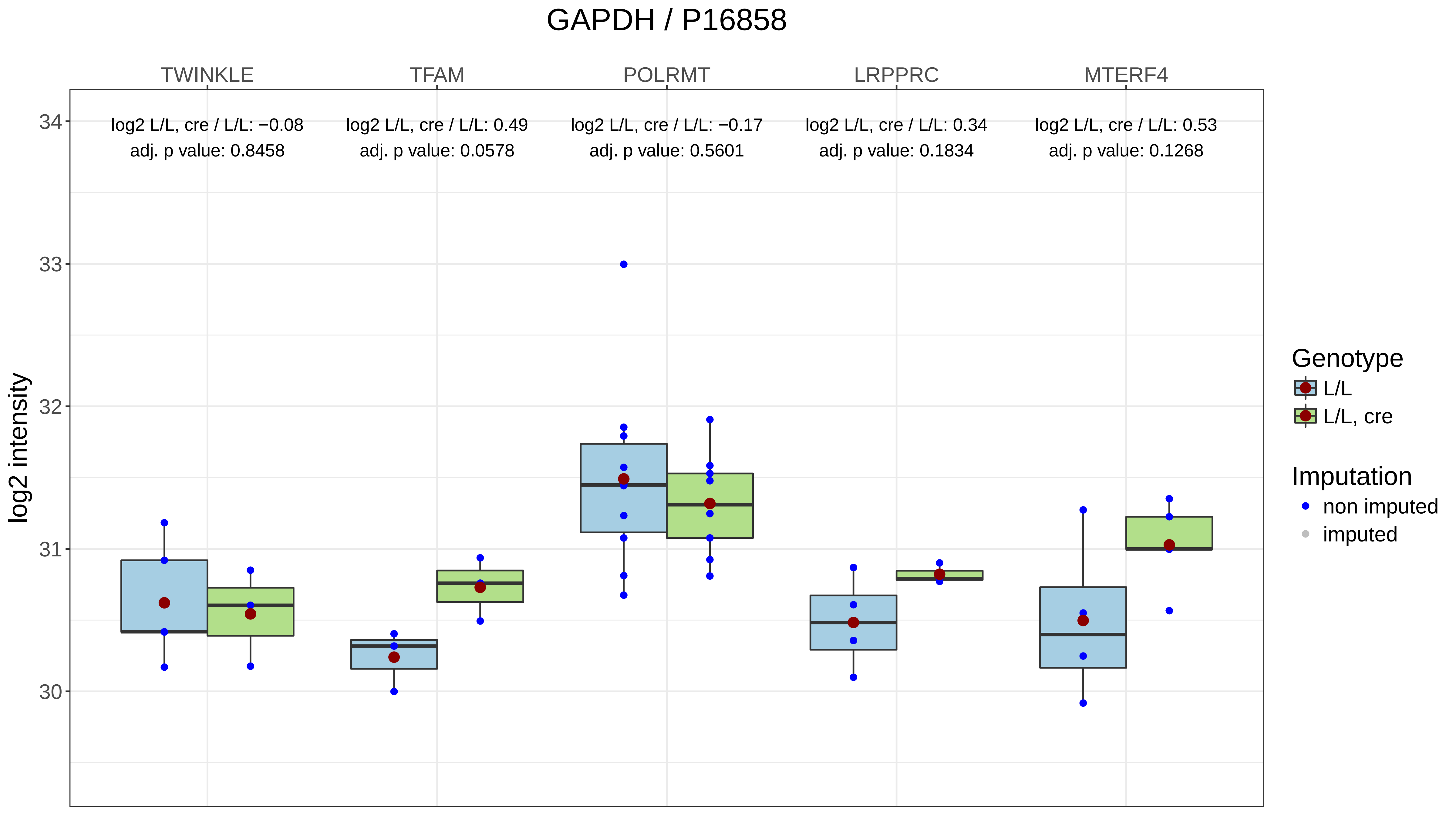
GAPDH / P16858
TWINKLE
TFAM
POLRMT
LRPPRC
MTERF4
log2 L/L, cre / L/L: −0.08
adj. p value: 0.8458
log2 L/L, cre / L/L: 0.49
adj. p value: 0.0578
log2 L/L, cre / L/L: −0.17
adj. p value: 0.5601
log2 L/L, cre / L/L: 0.34
adj. p value: 0.1834
log2 L/L, cre / L/L: 0.53
adj. p value: 0.1268
log2 intensity
34
33
32
31
30
Genotype
L/L
L/L, cre
Imputation
non imputed
imputed

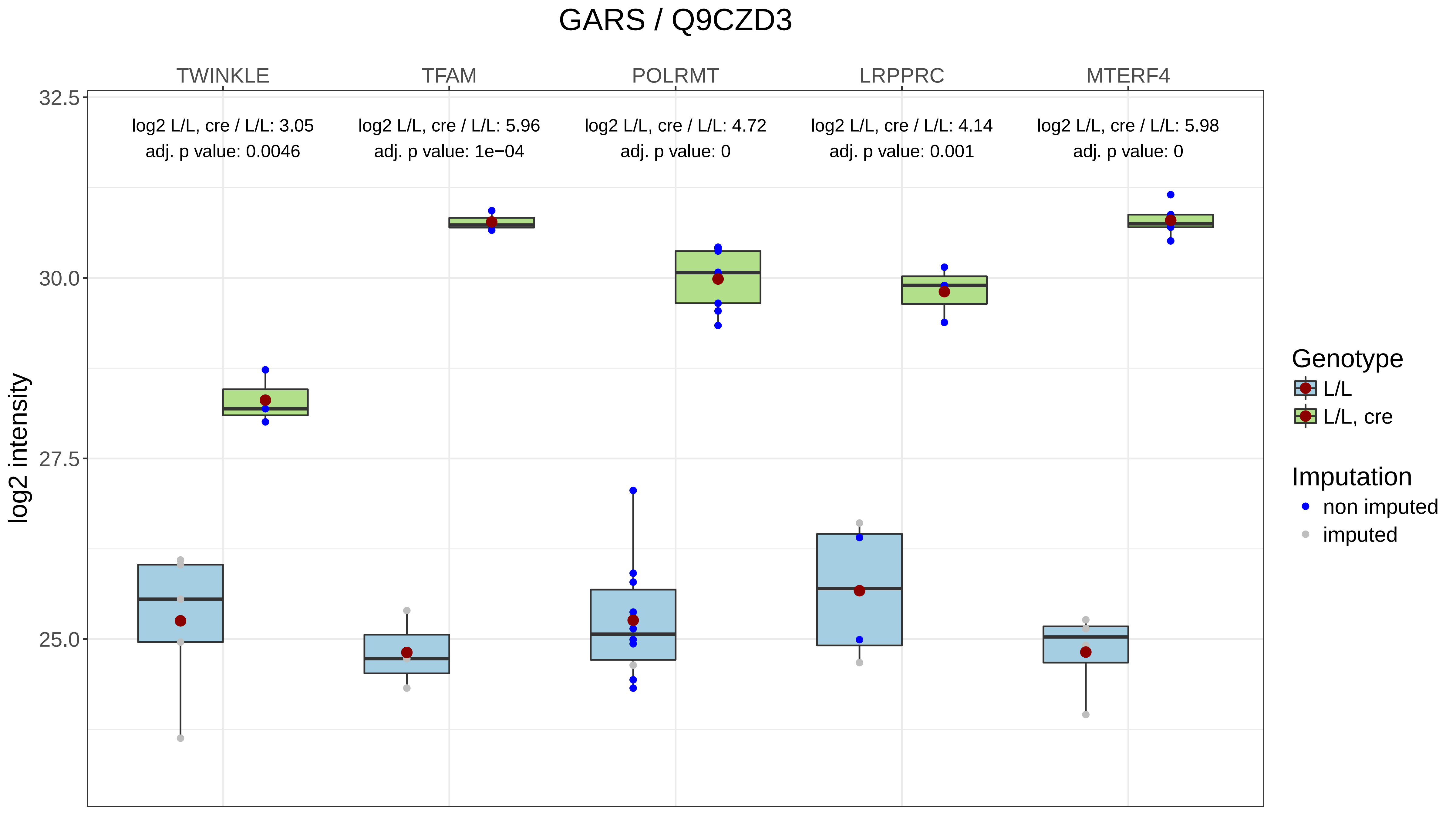
GARS / Q9CZD3
TWINKLE
TFAM
POLRMT
LRPPRC
MTERF4
log2 L/L, cre / L/L: 3.05
adj. p value: 0.0046
log2 L/L, cre / L/L: 5.96
adj. p value: 1e−04
log2 L/L, cre / L/L: 4.72
adj. p value: 0
log2 L/L, cre / L/L: 4.14
adj. p value: 0.001
log2 L/L, cre / L/L: 5.98
adj. p value: 0
32.5
30.0
27.5
25.0
log2 intensity
Genotype
L/L
L/L, cre
Imputation
non imputed
imputed

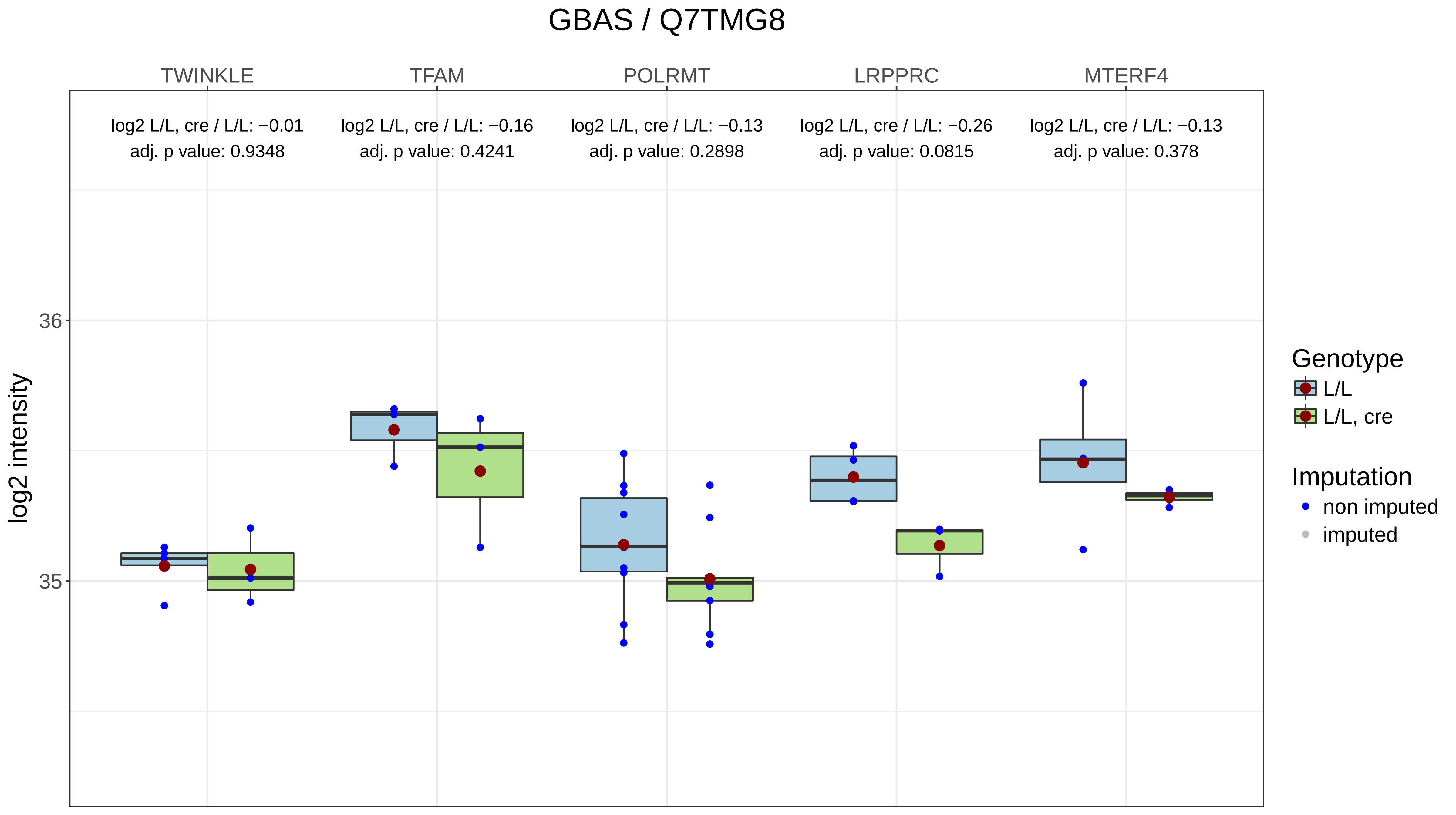
GBAS / Q7TMG8
TWINKLE
TFAM
POLRMT
LRPPRC
MTERF4
log2 L/L, cre / L/L: −0.01
adj. p value: 0.9348
log2 L/L, cre / L/L: −0.16
adj. p value: 0.4241
log2 L/L, cre / L/L: −0.13
adj. p value: 0.2898
log2 L/L, cre / L/L: −0.26
adj. p value: 0.0815
log2 L/L, cre / L/L: −0.13
adj. p value: 0.378
log2 intensity
36
35
Genotype
L/L
L/L, cre
Imputation
non imputed
imputed

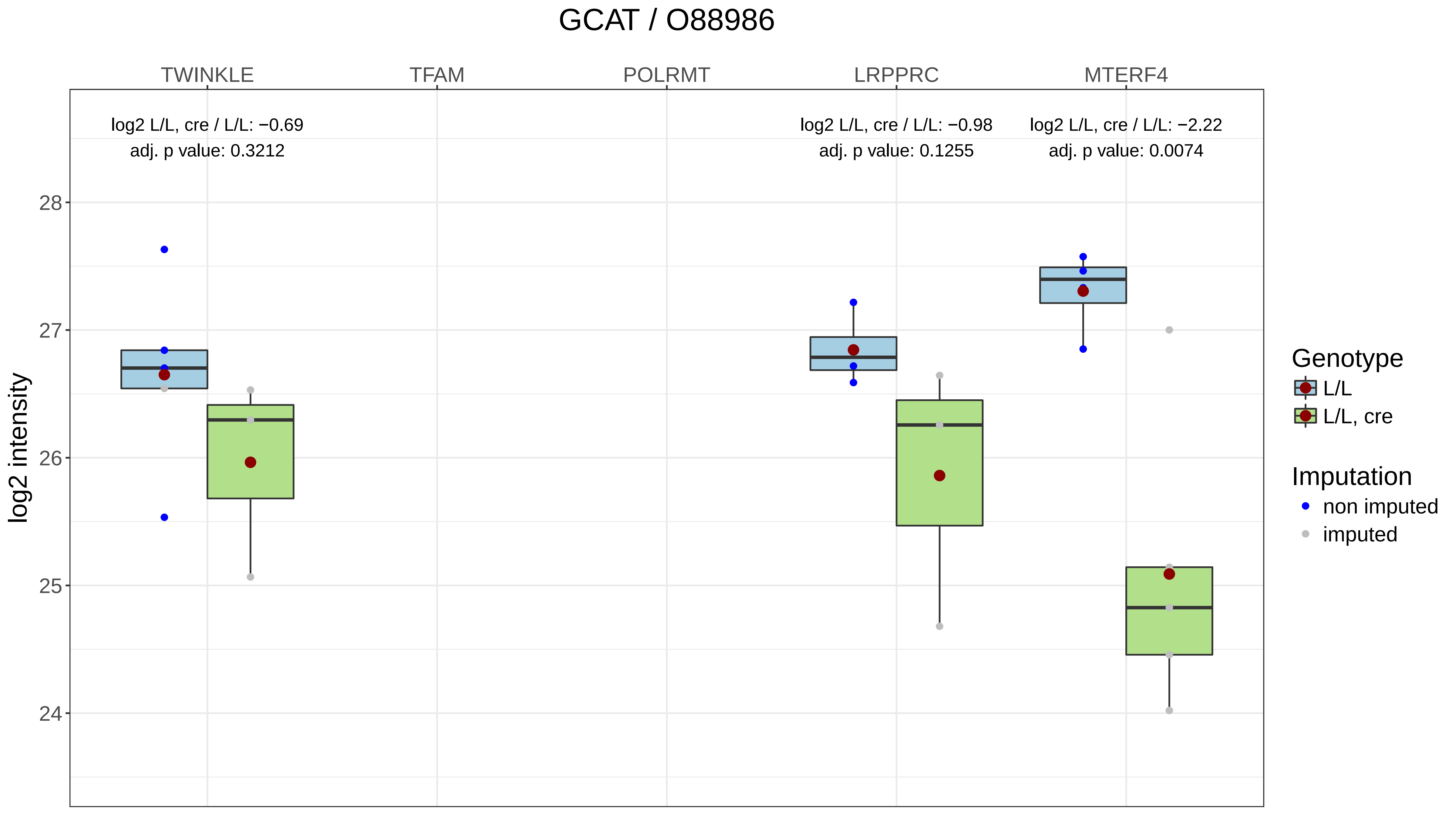
GCAT / O88986
TWINKLE
TFAM
POLRMT
LRPPRC
MTERF4
log2 L/L, cre / L/L: −0.69
adj. p value: 0.3212
log2 L/L, cre / L/L: −0.98
adj. p value: 0.1255
log2 L/L, cre / L/L: −2.22
adj. p value: 0.0074
log2 intensity
28
27
26
25
24
Genotype
L/L
L/L, cre
Imputation
non imputed
imputed

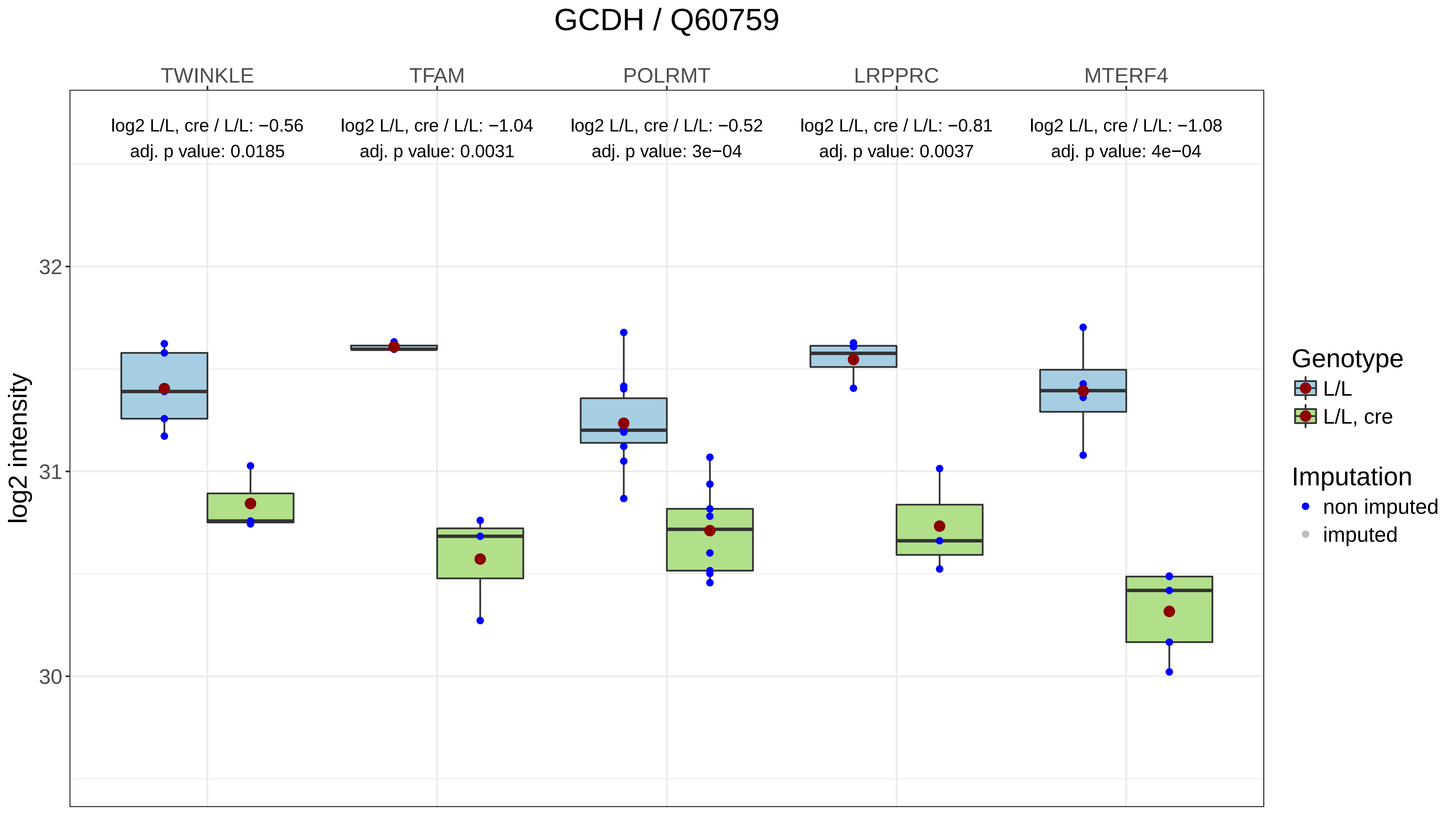
GCDH / Q60759
TWINKLE
TFAM
POLRMT
LRPPRC
MTERF4
log2 L/L, cre / L/L: −0.56
adj. p value: 0.0185
log2 L/L, cre / L/L: −1.04
adj. p value: 0.0031
log2 L/L, cre / L/L: −0.52
adj. p value: 3e−04
log2 L/L, cre / L/L: −0.81
adj. p value: 0.0037
log2 L/L, cre / L/L: −1.08
adj. p value: 4e−04
log2 intensity
32
31
30
Genotype
L/L
L/L, cre
Imputation
non imputed
imputed

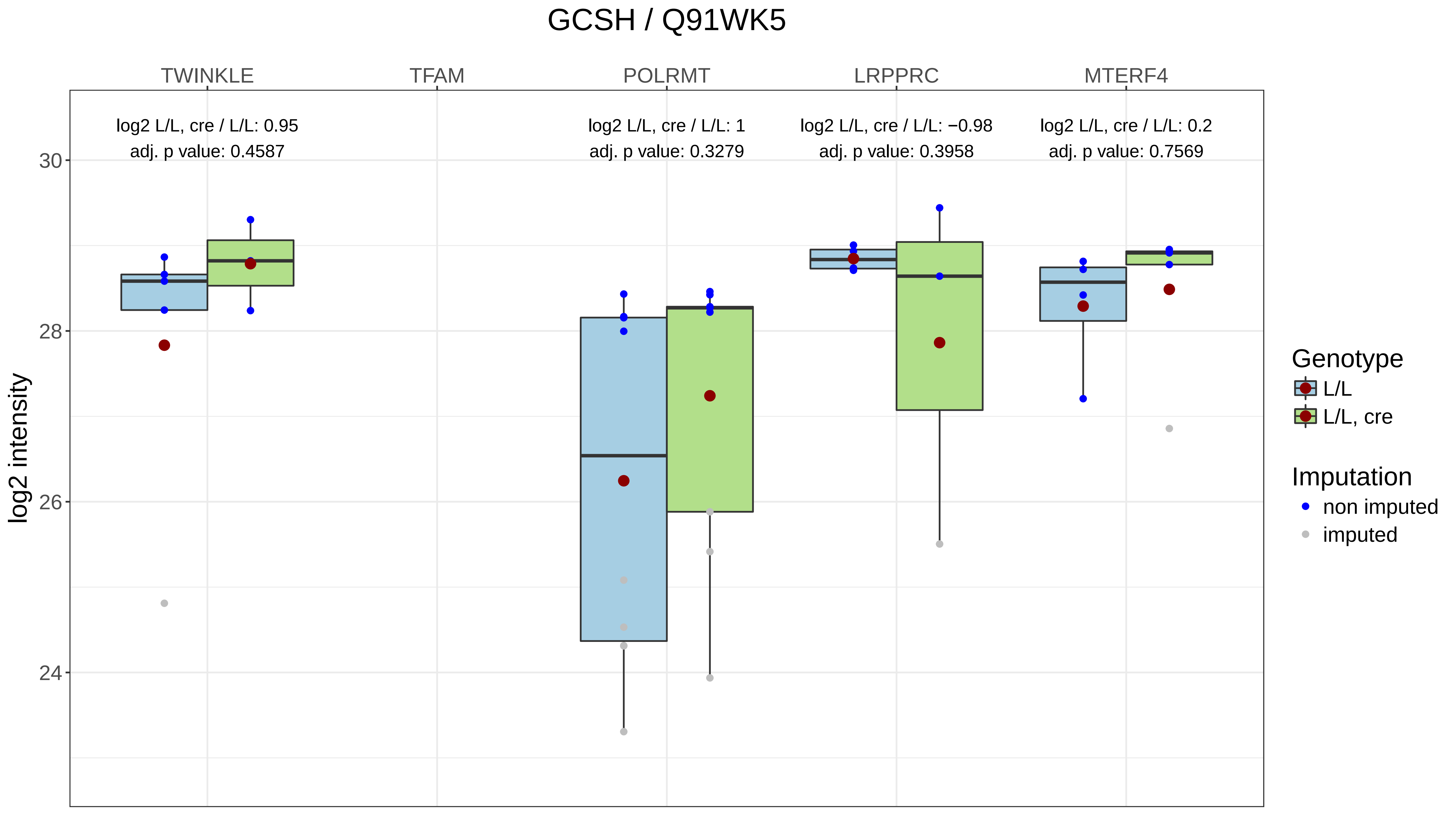
GCSH / Q91WK5
TWINKLE
TFAM
POLRMT
LRPPRC
MTERF4
log2 L/L, cre / L/L: 0.95
adj. p value: 0.4587
log2 L/L, cre / L/L: 1
adj. p value: 0.3279
log2 L/L, cre / L/L: −0.98
adj. p value: 0.3958
log2 L/L, cre / L/L: 0.2
adj. p value: 0.7569
log2 intensity
30
28
26
24
Genotype
L/L
L/L, cre
Imputation
non imputed
imputed

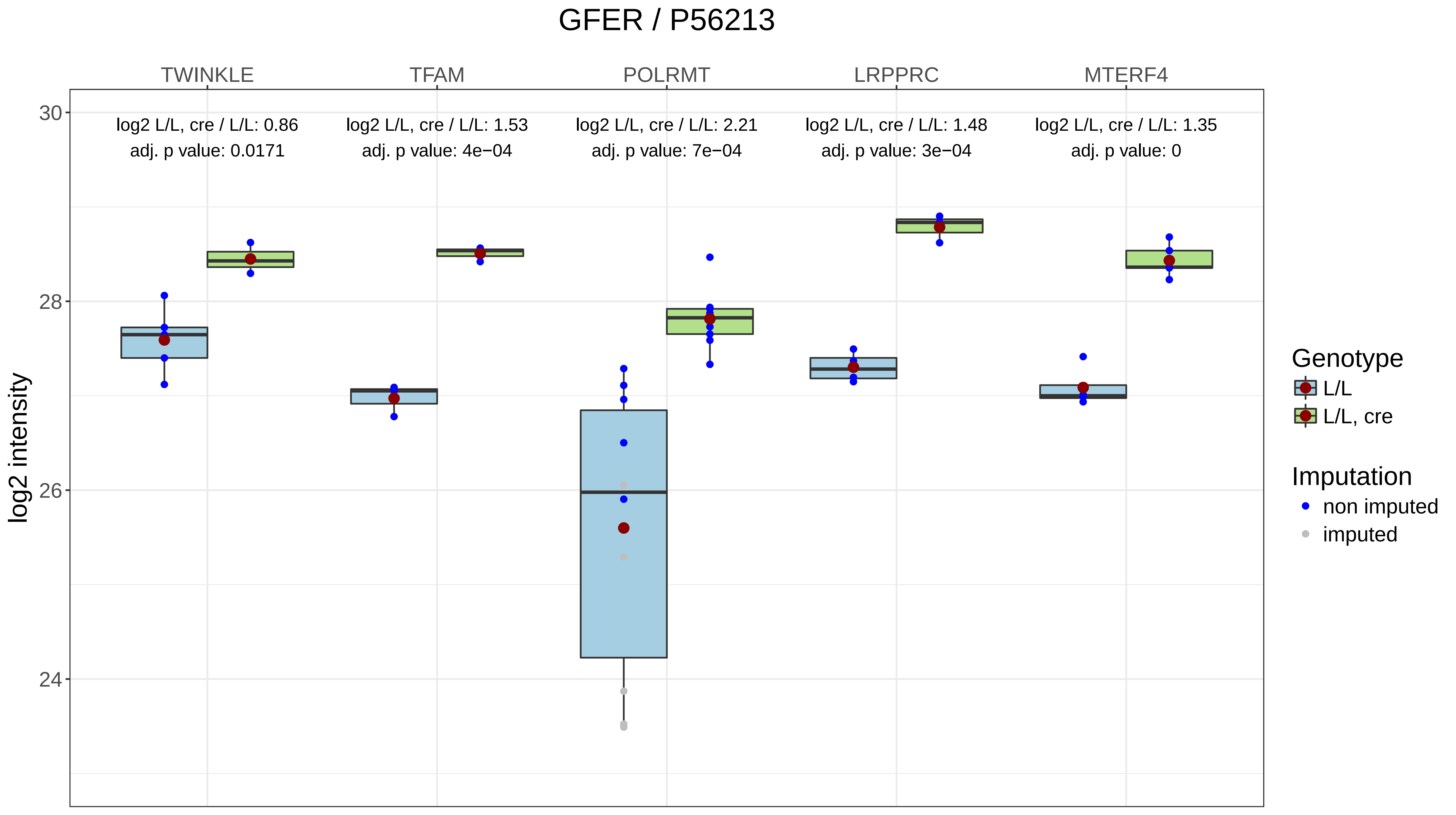
GFER / P56213
TWINKLE
TFAM
POLRMT
LRPPRC
MTERF4
log2 L/L, cre / L/L: 0.86
adj. p value: 0.0171
log2 L/L, cre / L/L: 1.53
adj. p value: 4e−04
log2 L/L, cre / L/L: 2.21
adj. p value: 7e−04
log2 L/L, cre / L/L: 1.48
adj. p value: 3e−04
log2 L/L, cre / L/L: 1.35
adj. p value: 0
30
28
26
24
log2 intensity
Genotype
L/L
L/L, cre
Imputation
non imputed
imputed

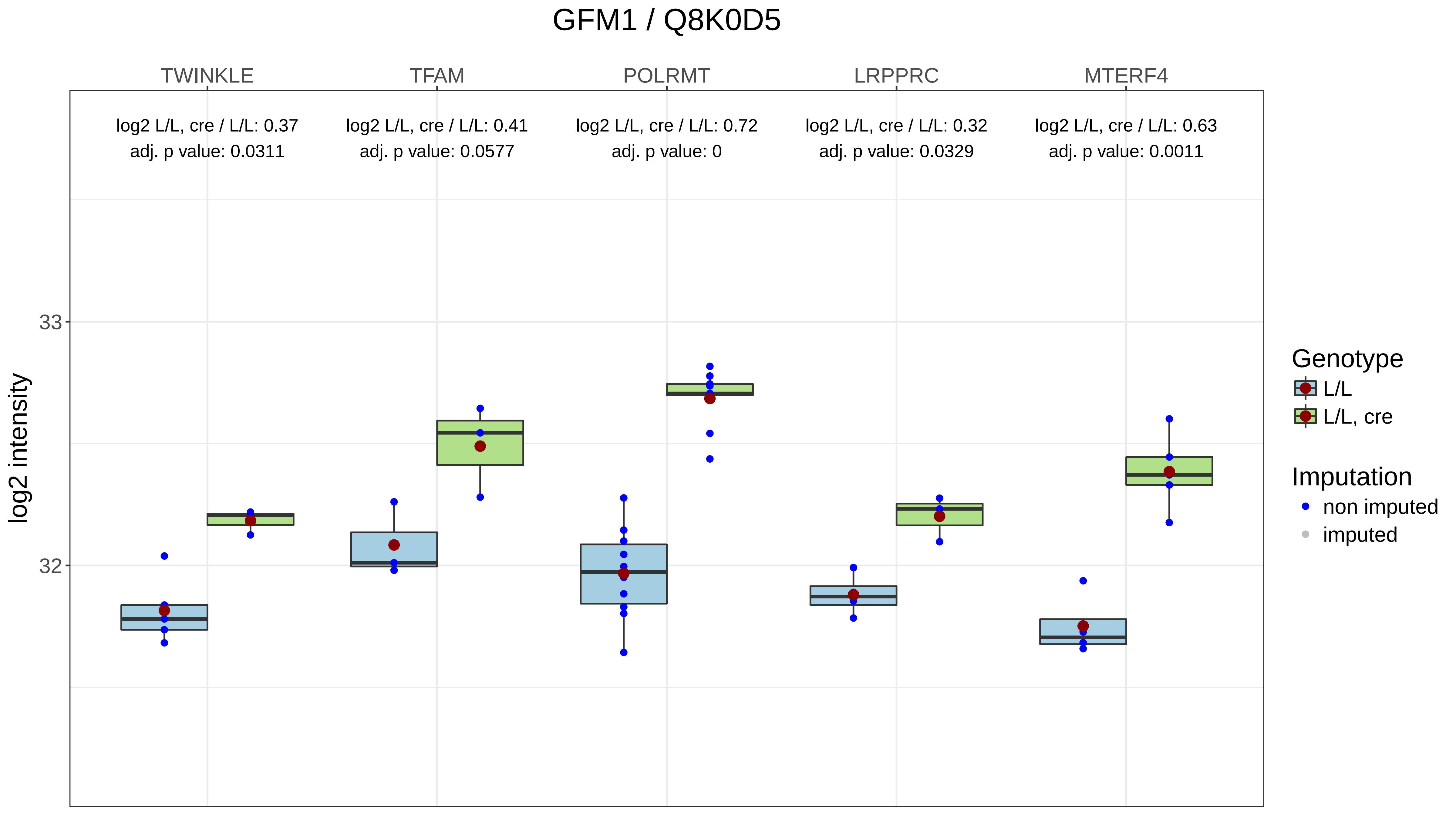

GFM1 / Q8K0D5
TWINKLE
TFAM
POLRMT
LRPPRC
MTERF4
log2 L/L, cre / L/L: 0.37
adj. p value: 0.0311
log2 L/L, cre / L/L: 0.41
adj. p value: 0.0577
log2 L/L, cre / L/L: 0.72
adj. p value: 0
log2 L/L, cre / L/L: 0.32
adj. p value: 0.0329
log2 L/L, cre / L/L: 0.63
adj. p value: 0.0011
log2 intensity
33
32
Genotype
L/L
L/L, cre
Imputation
non imputed
imputed

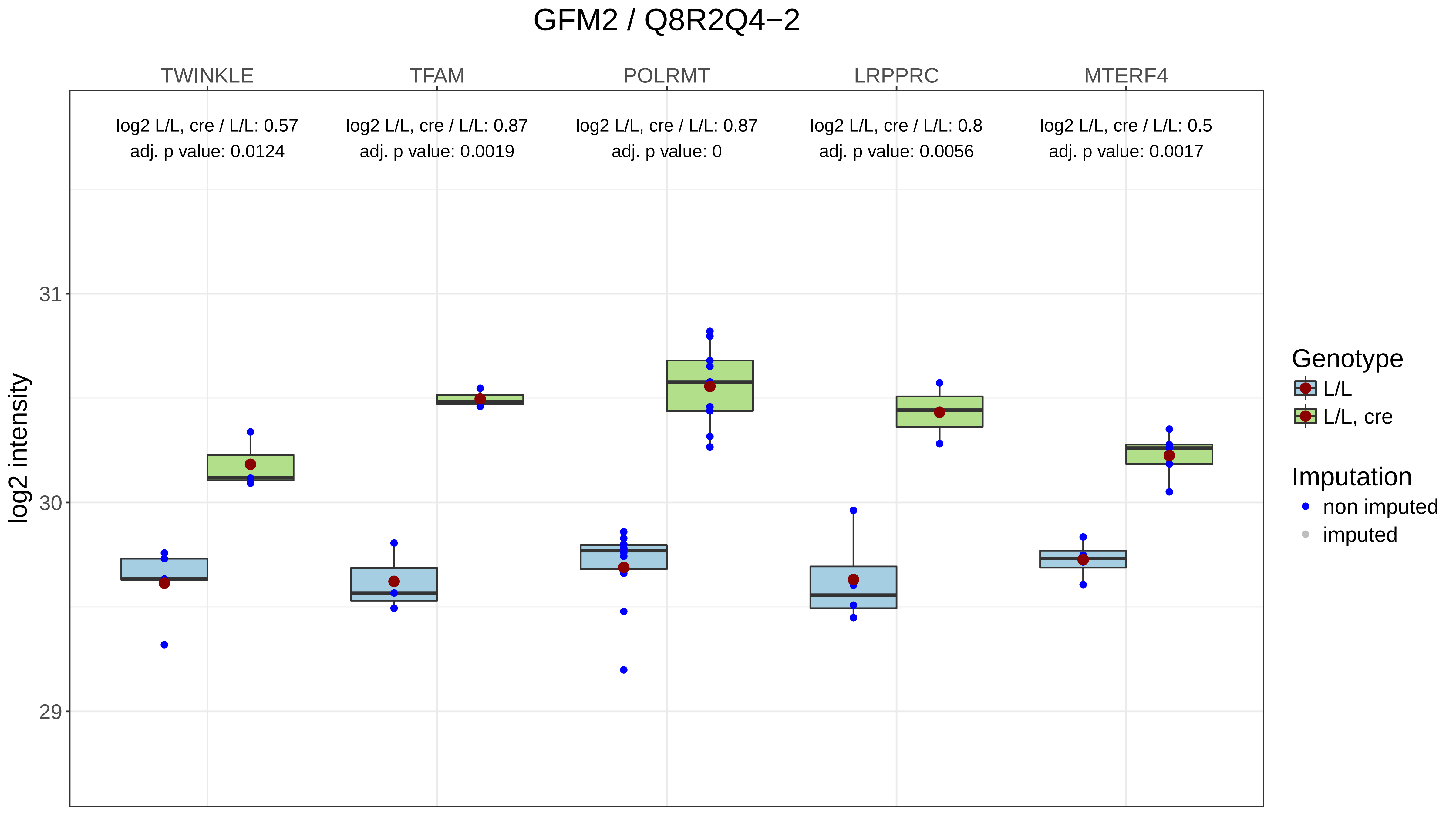

GFM2 / Q8R2Q4−2
TWINKLE
TFAM
POLRMT
LRPPRC
MTERF4
log2 L/L, cre / L/L: 0.57
adj. p value: 0.0124
log2 L/L, cre / L/L: 0.87
adj. p value: 0.0019
log2 L/L, cre / L/L: 0.87
adj. p value: 0
log2 L/L, cre / L/L: 0.8
adj. p value: 0.0056
log2 L/L, cre / L/L: 0.5
adj. p value: 0.0017
log2 intensity
31
30
29
Genotype
L/L
L/L, cre
Imputation
non imputed
imputed

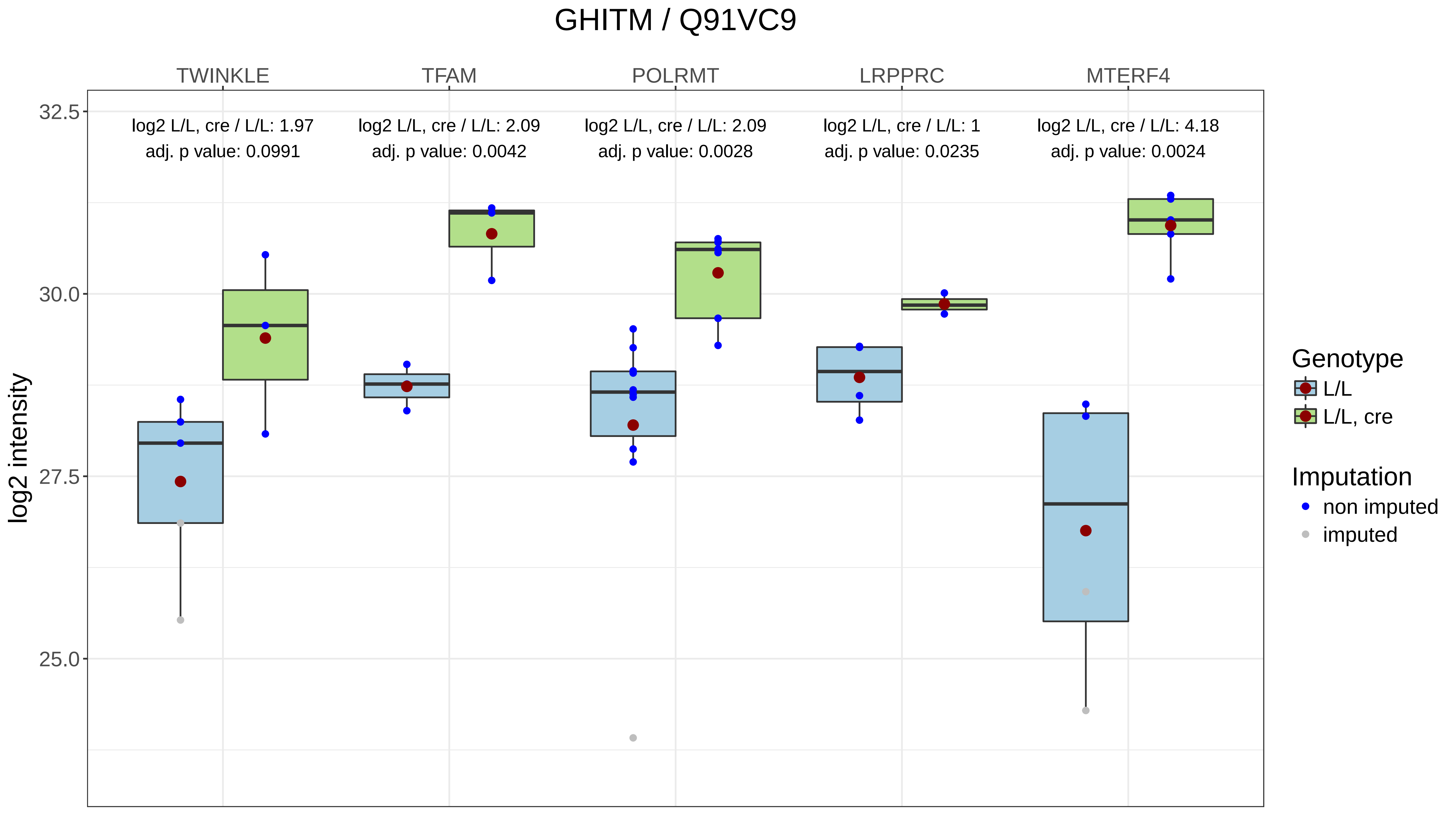
GHITM / Q91VC9
TWINKLE
TFAM
POLRMT
LRPPRC
MTERF4
log2 L/L, cre / L/L: 1.97
adj. p value: 0.0991
log2 L/L, cre / L/L: 2.09
adj. p value: 0.0042
log2 L/L, cre / L/L: 2.09
adj. p value: 0.0028
log2 L/L, cre / L/L: 1
adj. p value: 0.0235
log2 L/L, cre / L/L: 4.18
adj. p value: 0.0024
32.5
30.0
27.5
25.0
log2 intensity
Genotype
L/L
L/L, cre
Imputation
non imputed
imputed

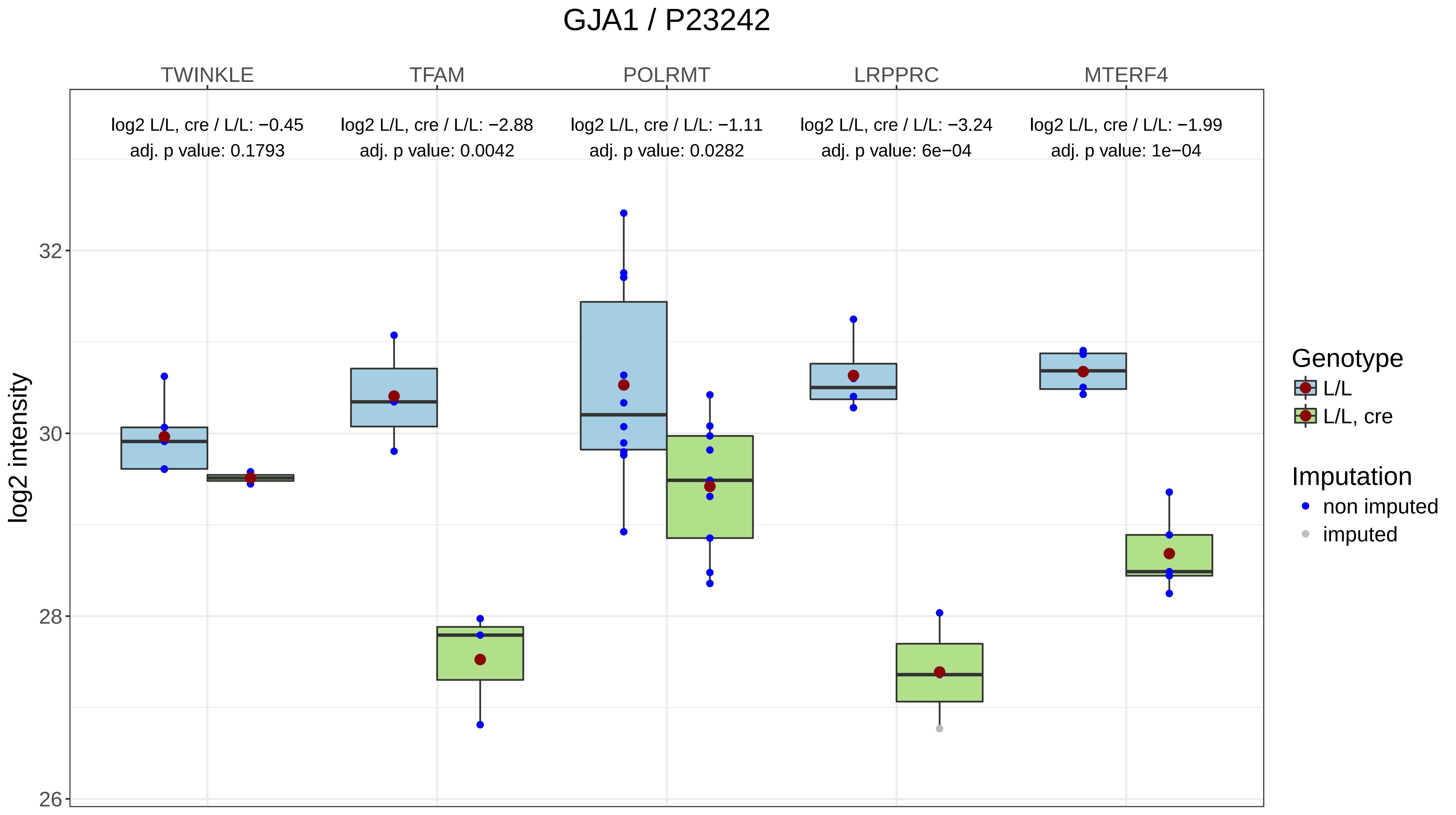

GJA1 / P23242
TWINKLE
TFAM
POLRMT
LRPPRC
MTERF4
log2 L/L, cre / L/L: −0.45
adj. p value: 0.1793
log2 L/L, cre / L/L: −2.88
adj. p value: 0.0042
log2 L/L, cre / L/L: −1.11
adj. p value: 0.0282
log2 L/L, cre / L/L: −3.24
adj. p value: 6e−04
log2 L/L, cre / L/L: −1.99
adj. p value: 1e−04
log2 intensity
32
30
28
26
Genotype
L/L
L/L, cre
Imputation
non imputed
imputed

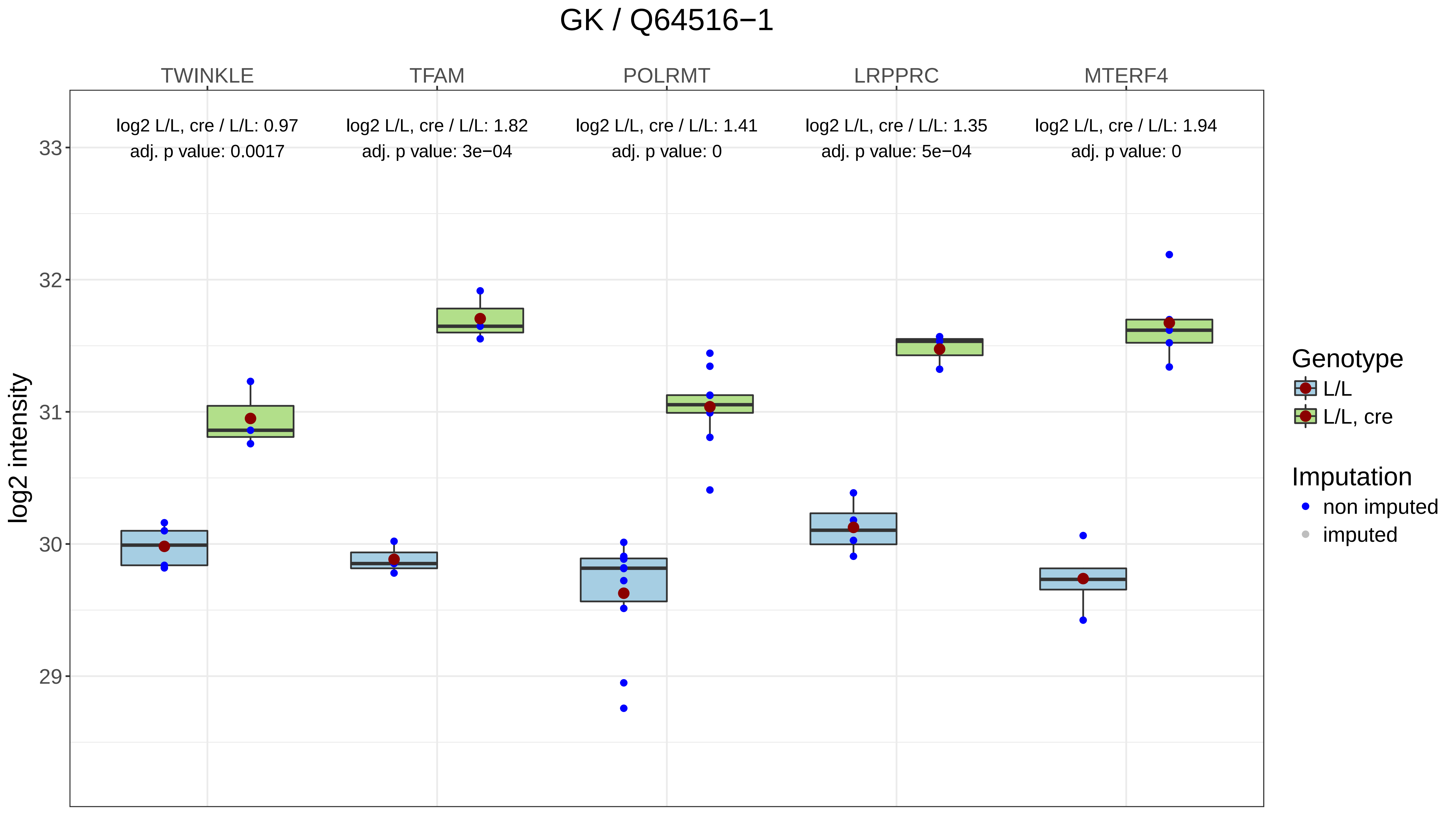
GK / Q64516−1
TWINKLE
TFAM
POLRMT
LRPPRC
MTERF4
log2 L/L, cre / L/L: 0.97
adj. p value: 0.0017
log2 L/L, cre / L/L: 1.82
adj. p value: 3e−04
log2 L/L, cre / L/L: 1.41
adj. p value: 0
log2 L/L, cre / L/L: 1.35
adj. p value: 5e−04
log2 L/L, cre / L/L: 1.94
adj. p value: 0
log2 intensity
33
32
31
30
29
Genotype
L/L
L/L, cre
Imputation
non imputed
imputed

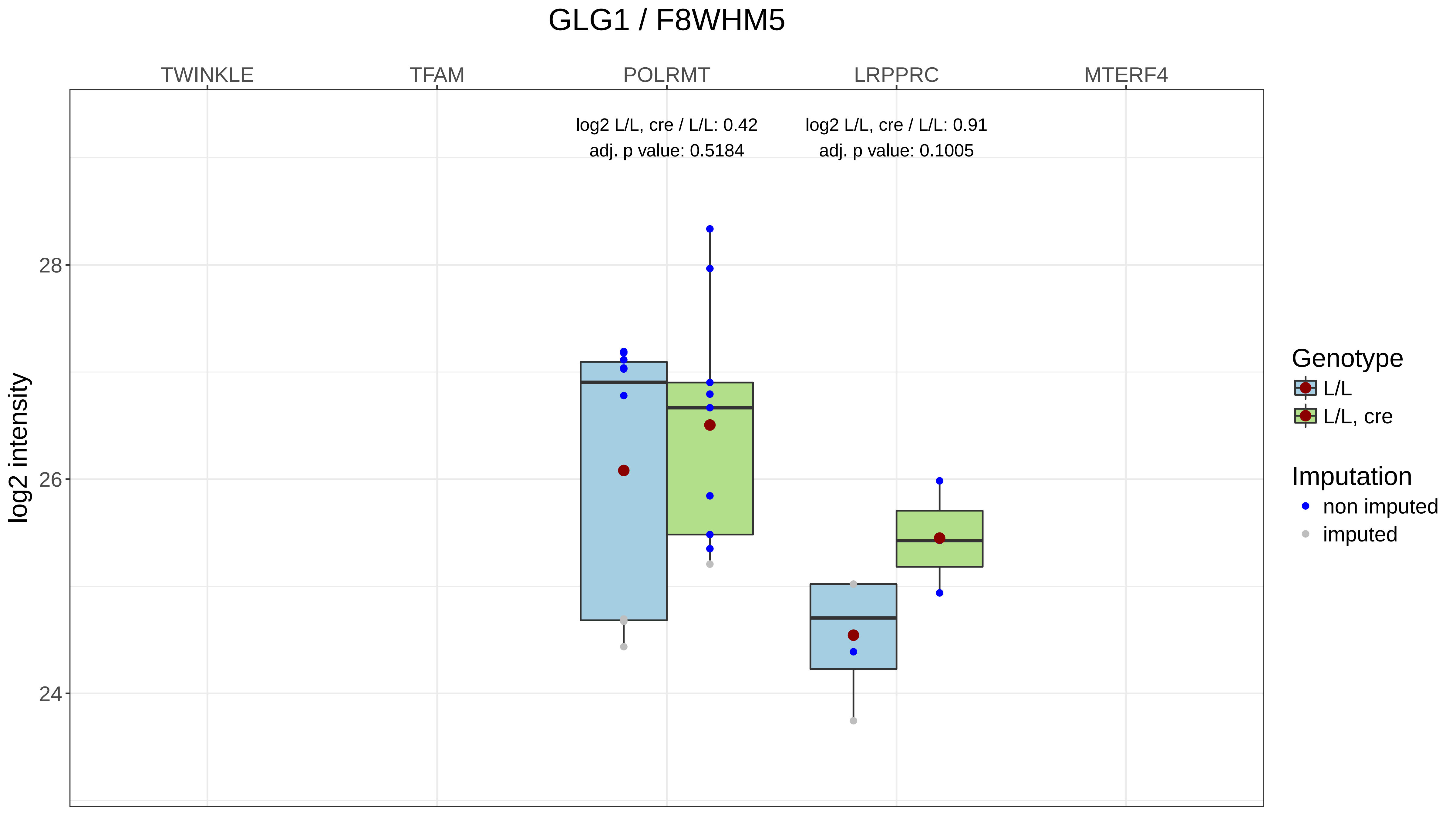
GLG1 / F8WHM5
TWINKLE
TFAM
POLRMT
LRPPRC
MTERF4
log2 L/L, cre / L/L: 0.42
adj. p value: 0.5184
log2 L/L, cre / L/L: 0.91
adj. p value: 0.1005
28
26
24
log2 intensity
Genotype
L/L
L/L, cre
Imputation
non imputed
imputed

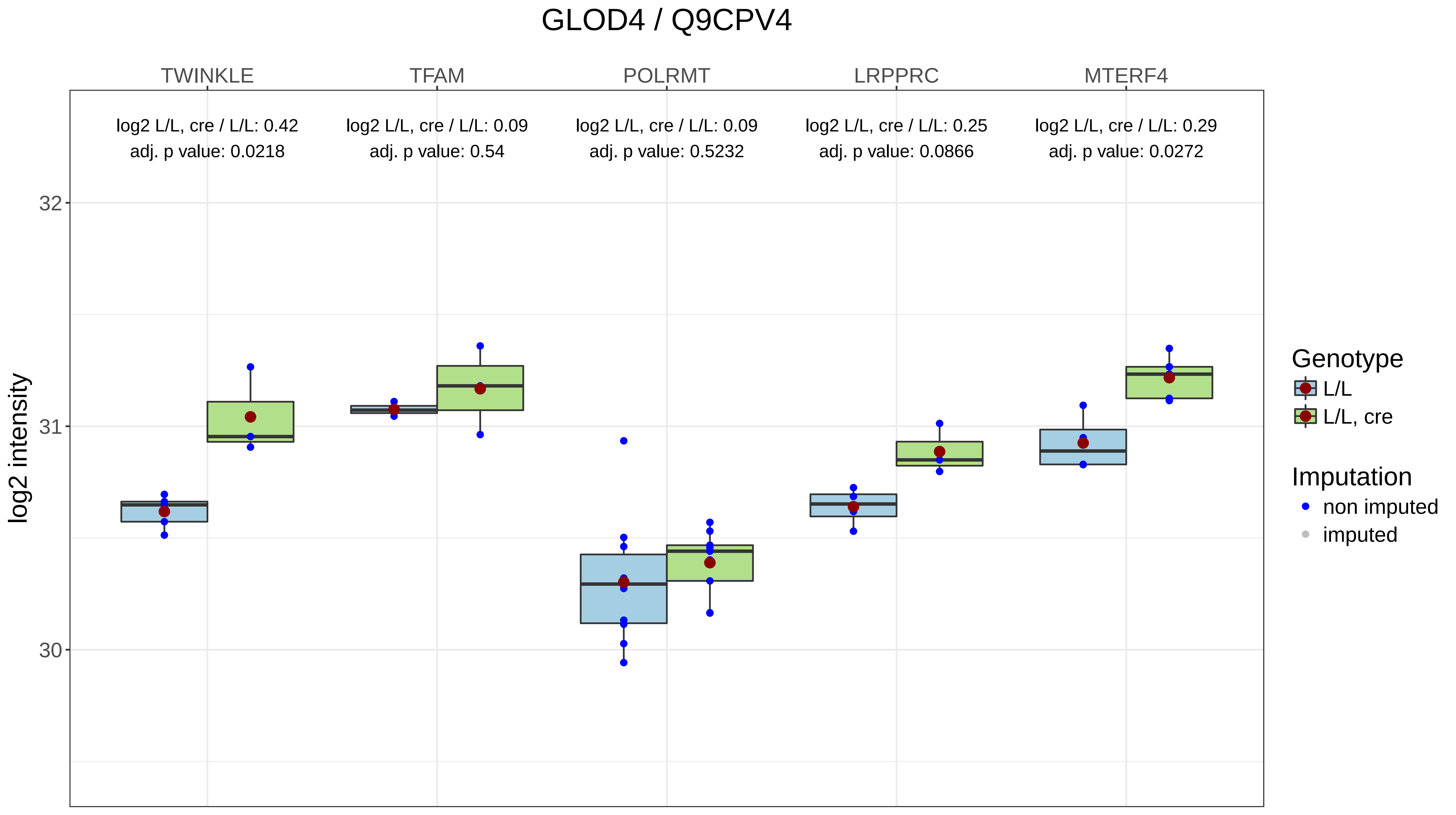
GLOD4 / Q9CPV4
TWINKLE
TFAM
POLRMT
LRPPRC
MTERF4
log2 L/L, cre / L/L: 0.42
adj. p value: 0.0218
log2 L/L, cre / L/L: 0.09
adj. p value: 0.54
log2 L/L, cre / L/L: 0.09
adj. p value: 0.5232
log2 L/L, cre / L/L: 0.25
adj. p value: 0.0866
log2 L/L, cre / L/L: 0.29
adj. p value: 0.0272
log2 intensity
32
31
30
Genotype
L/L
L/L, cre
Imputation
non imputed
imputed

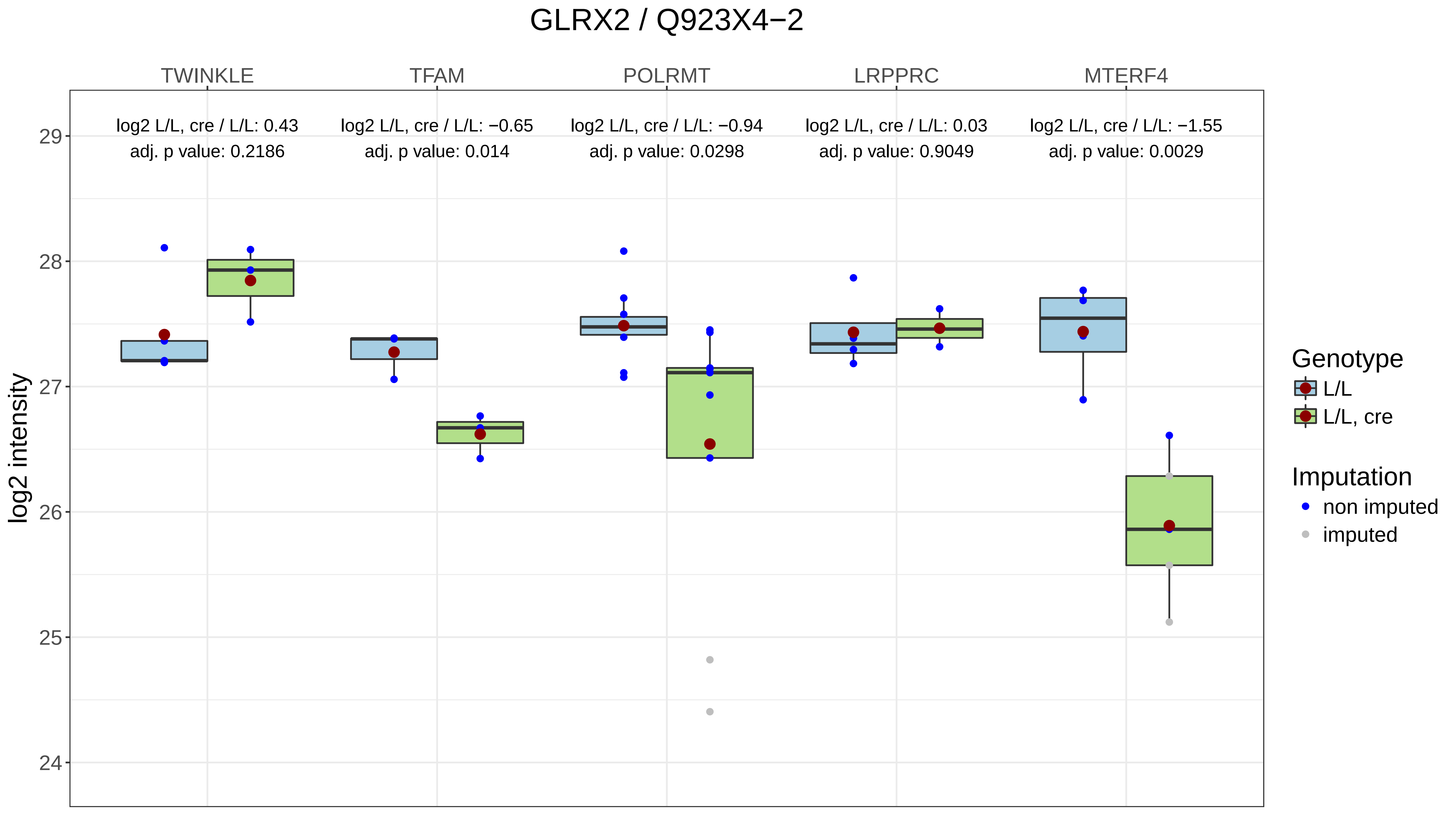
GLRX2 / Q923X4−2
TWINKLE
TFAM
POLRMT
LRPPRC
MTERF4
log2 L/L, cre / L/L: 0.43
adj. p value: 0.2186
log2 L/L, cre / L/L: −0.65
adj. p value: 0.014
log2 L/L, cre / L/L: −0.94
adj. p value: 0.0298
log2 L/L, cre / L/L: 0.03
adj. p value: 0.9049
log2 L/L, cre / L/L: −1.55
adj. p value: 0.0029
log2 intensity
29
28
27
26
25
24
Genotype
L/L
L/L, cre
Imputation
non imputed
imputed

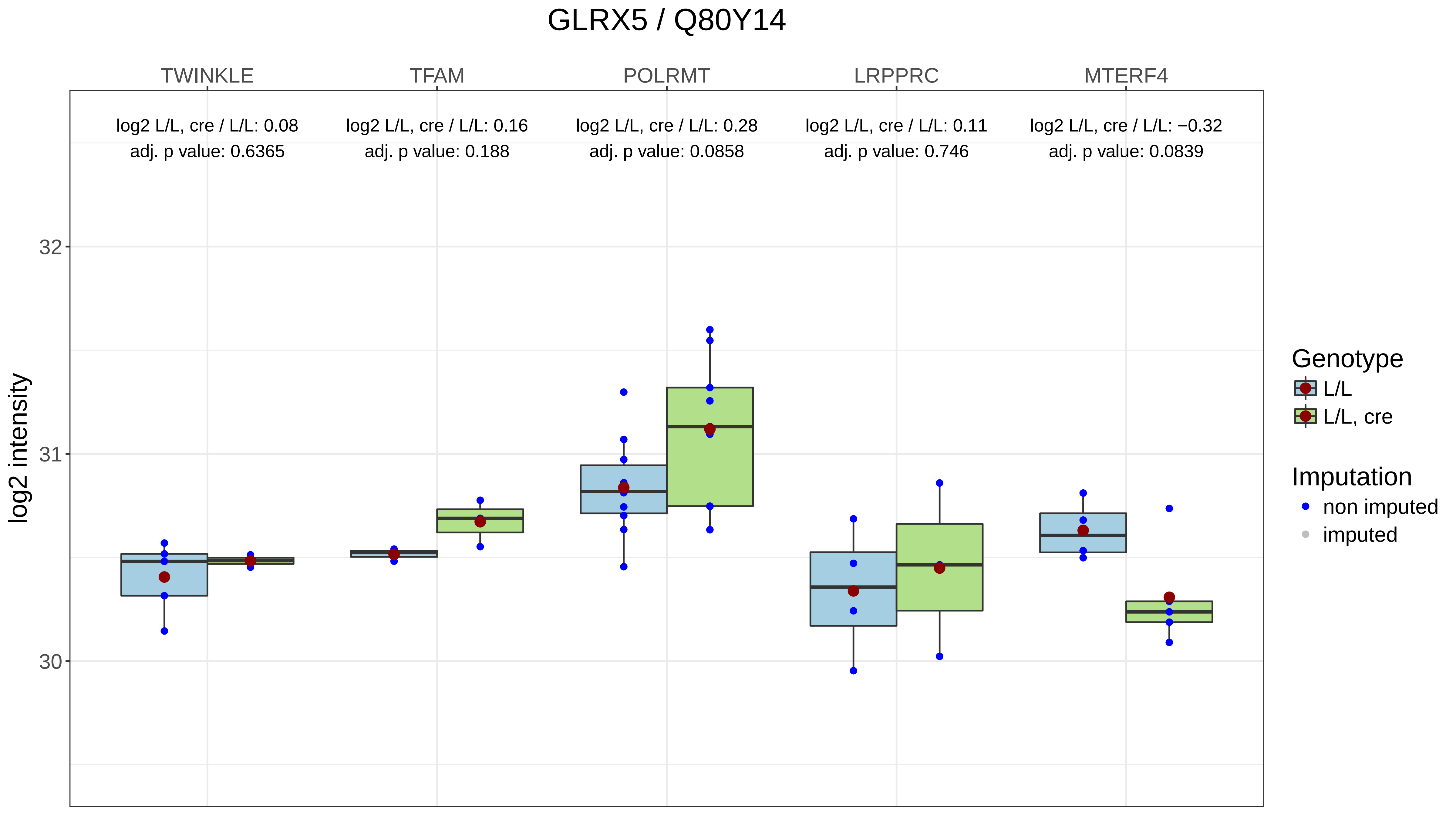
GLRX5 / Q80Y14
TWINKLE
TFAM
POLRMT
LRPPRC
MTERF4
log2 L/L, cre / L/L: 0.08
adj. p value: 0.6365
log2 L/L, cre / L/L: 0.16
adj. p value: 0.188
log2 L/L, cre / L/L: 0.28
adj. p value: 0.0858
log2 L/L, cre / L/L: 0.11
adj. p value: 0.746
log2 L/L, cre / L/L: −0.32
adj. p value: 0.0839
log2 intensity
32
31
30
Genotype
L/L
L/L, cre
Imputation
non imputed
imputed

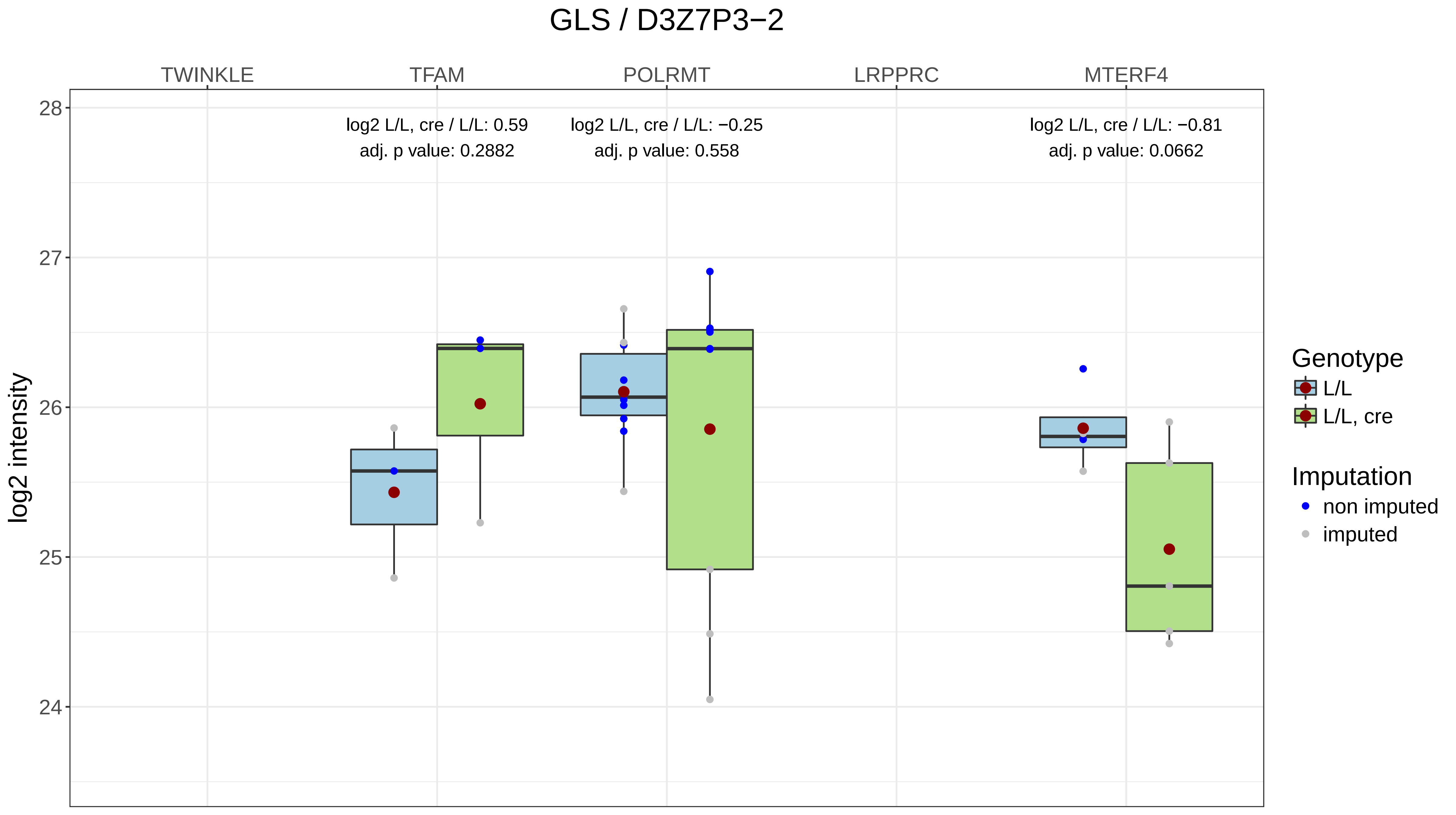

GLS / D3Z7P3−2
TWINKLE
TFAM
POLRMT
LRPPRC
MTERF4
log2 L/L, cre / L/L: 0.59
adj. p value: 0.2882
log2 L/L, cre / L/L: −0.25
adj. p value: 0.558
log2 L/L, cre / L/L: −0.81
adj. p value: 0.0662
log2 intensity
28
27
26
25
24
Genotype
L/L
L/L, cre
Imputation
non imputed
imputed

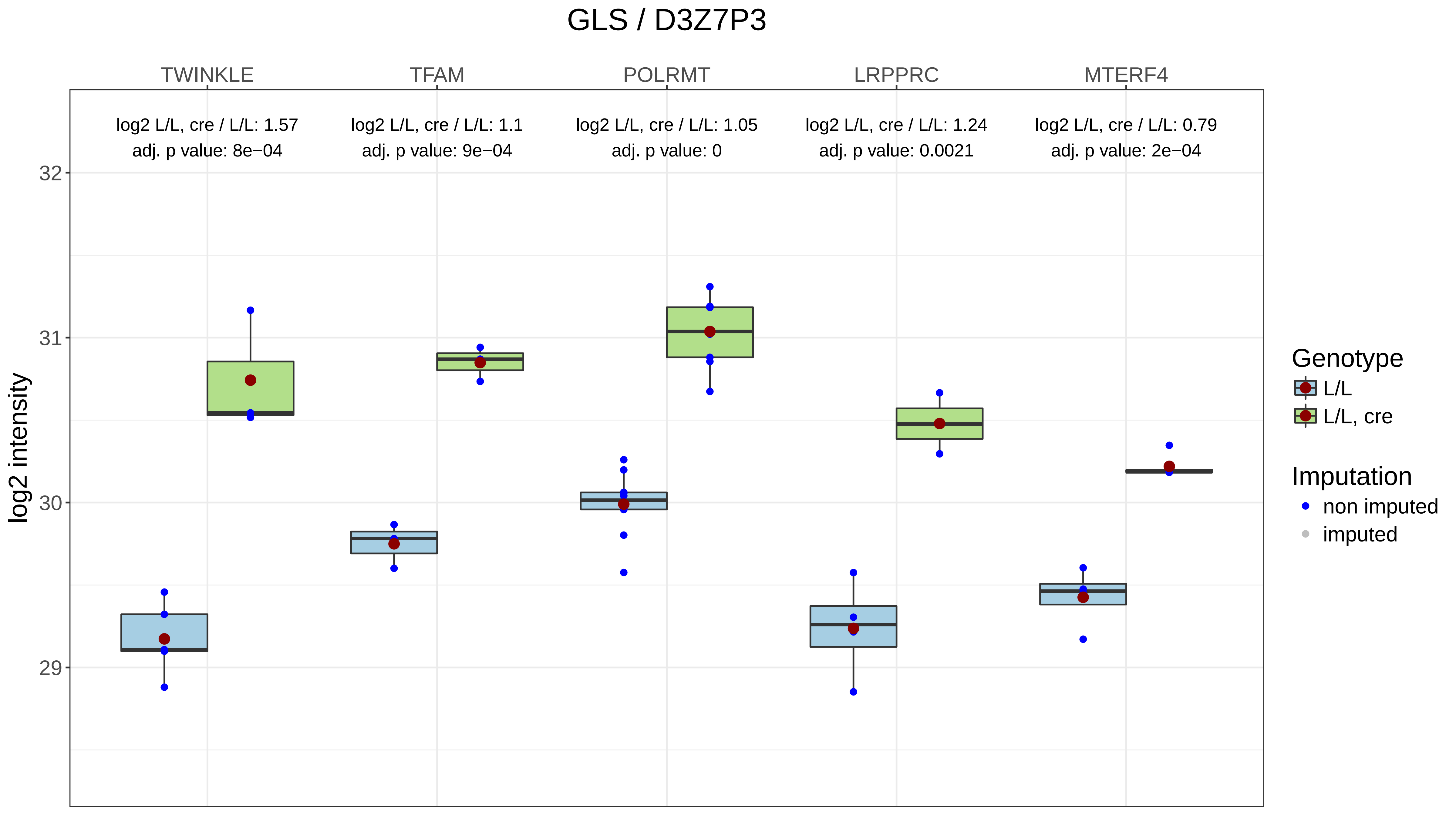

GLS / D3Z7P3
TWINKLE
TFAM
POLRMT
LRPPRC
MTERF4
log2 L/L, cre / L/L: 1.57
adj. p value: 8e−04
log2 L/L, cre / L/L: 1.1
adj. p value: 9e−04
log2 L/L, cre / L/L: 1.05
adj. p value: 0
log2 L/L, cre / L/L: 1.24
adj. p value: 0.0021
log2 L/L, cre / L/L: 0.79
adj. p value: 2e−04
log2 intensity
32
31
30
29
Genotype
L/L
L/L, cre
Imputation
non imputed
imputed

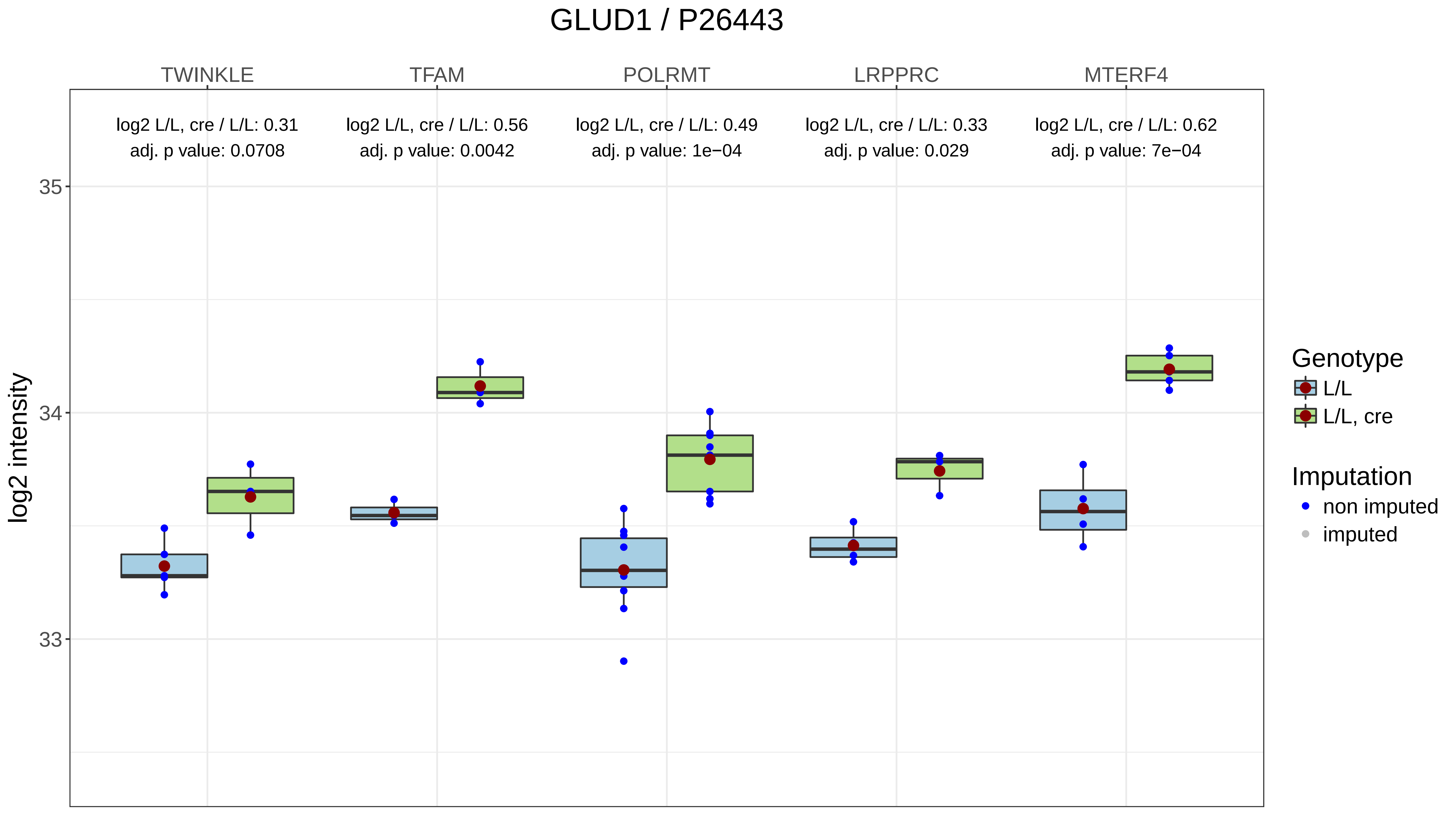
GLUD1 / P26443
TWINKLE
TFAM
POLRMT
LRPPRC
MTERF4
log2 L/L, cre / L/L: 0.31
adj. p value: 0.0708
log2 L/L, cre / L/L: 0.56
adj. p value: 0.0042
log2 L/L, cre / L/L: 0.49
adj. p value: 1e−04
log2 L/L, cre / L/L: 0.33
adj. p value: 0.029
log2 L/L, cre / L/L: 0.62
adj. p value: 7e−04
log2 intensity
35
34
33
Genotype
L/L
L/L, cre
Imputation
non imputed
imputed

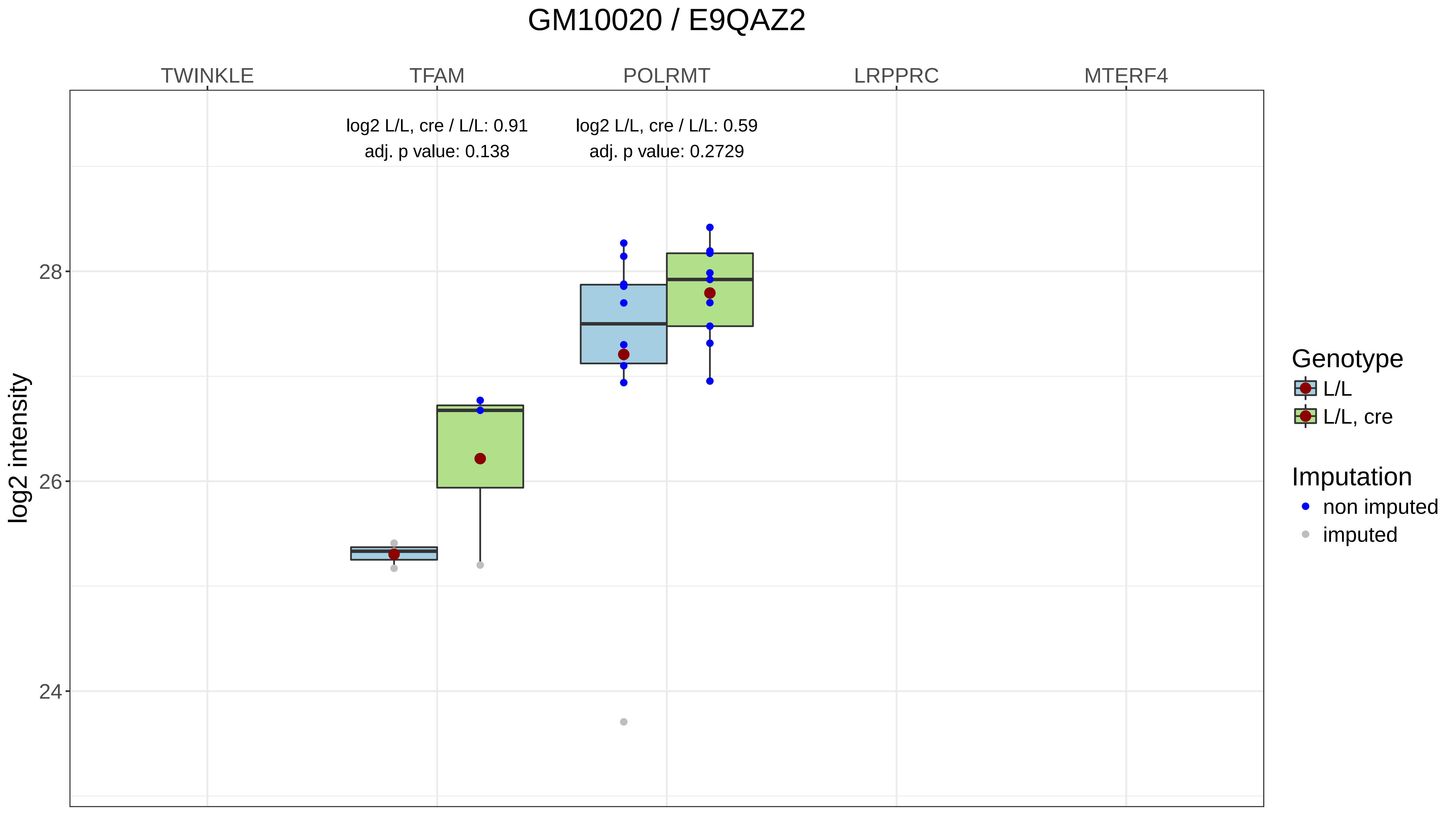

GM10020 / E9QAZ2
TWINKLE
TFAM
POLRMT
LRPPRC
MTERF4
log2 L/L, cre / L/L: 0.91
adj. p value: 0.138
log2 L/L, cre / L/L: 0.59
adj. p value: 0.2729
28
26
24
log2 intensity
Genotype
L/L
L/L, cre
Imputation
non imputed
imputed

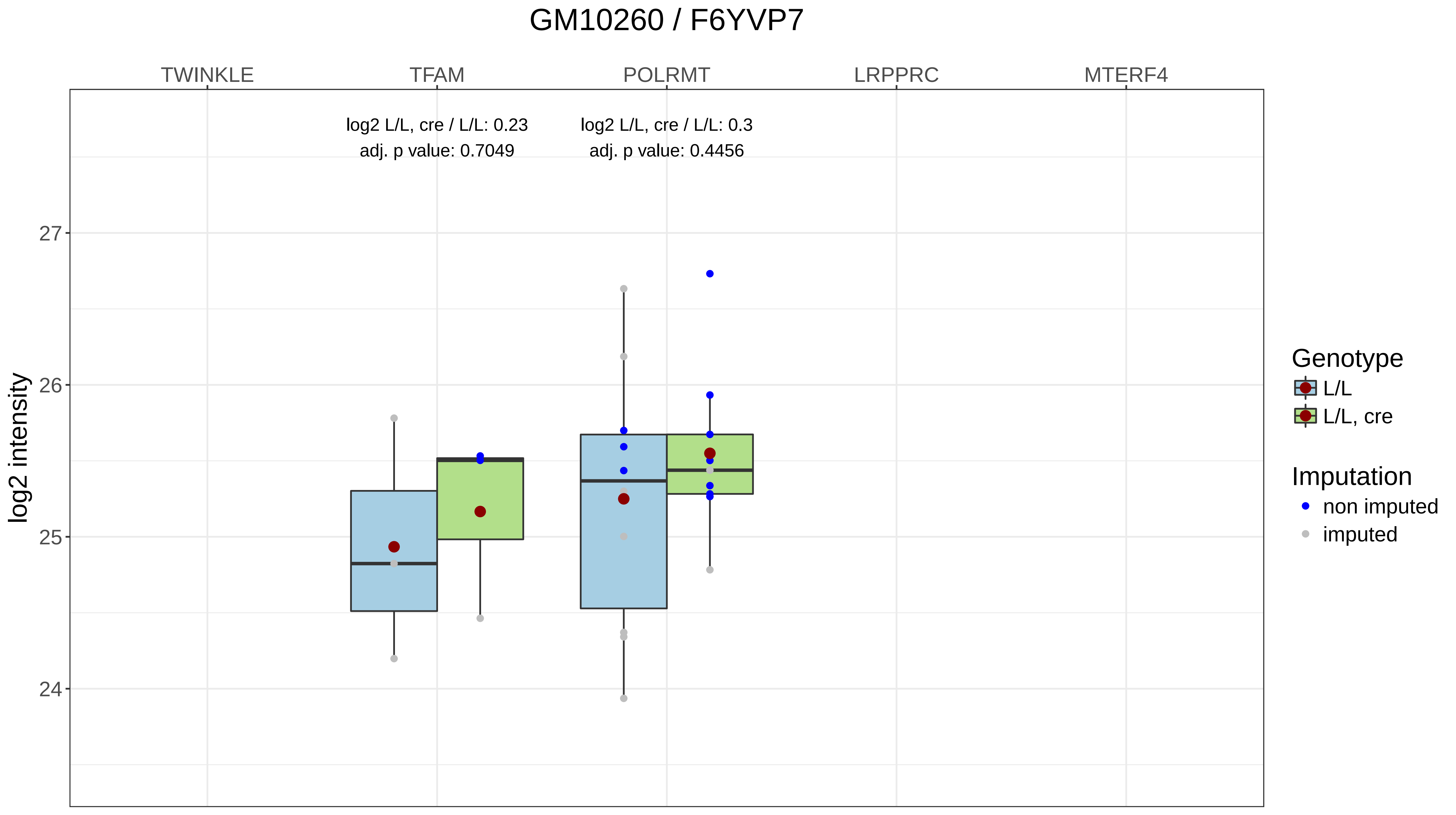
GM10260 / F6YVP7
TWINKLE
TFAM
POLRMT
LRPPRC
MTERF4
log2 L/L, cre / L/L: 0.23
adj. p value: 0.7049
log2 L/L, cre / L/L: 0.3
adj. p value: 0.4456
log2 intensity
27
26
25
24
Genotype
L/L
L/L, cre
Imputation
non imputed
imputed

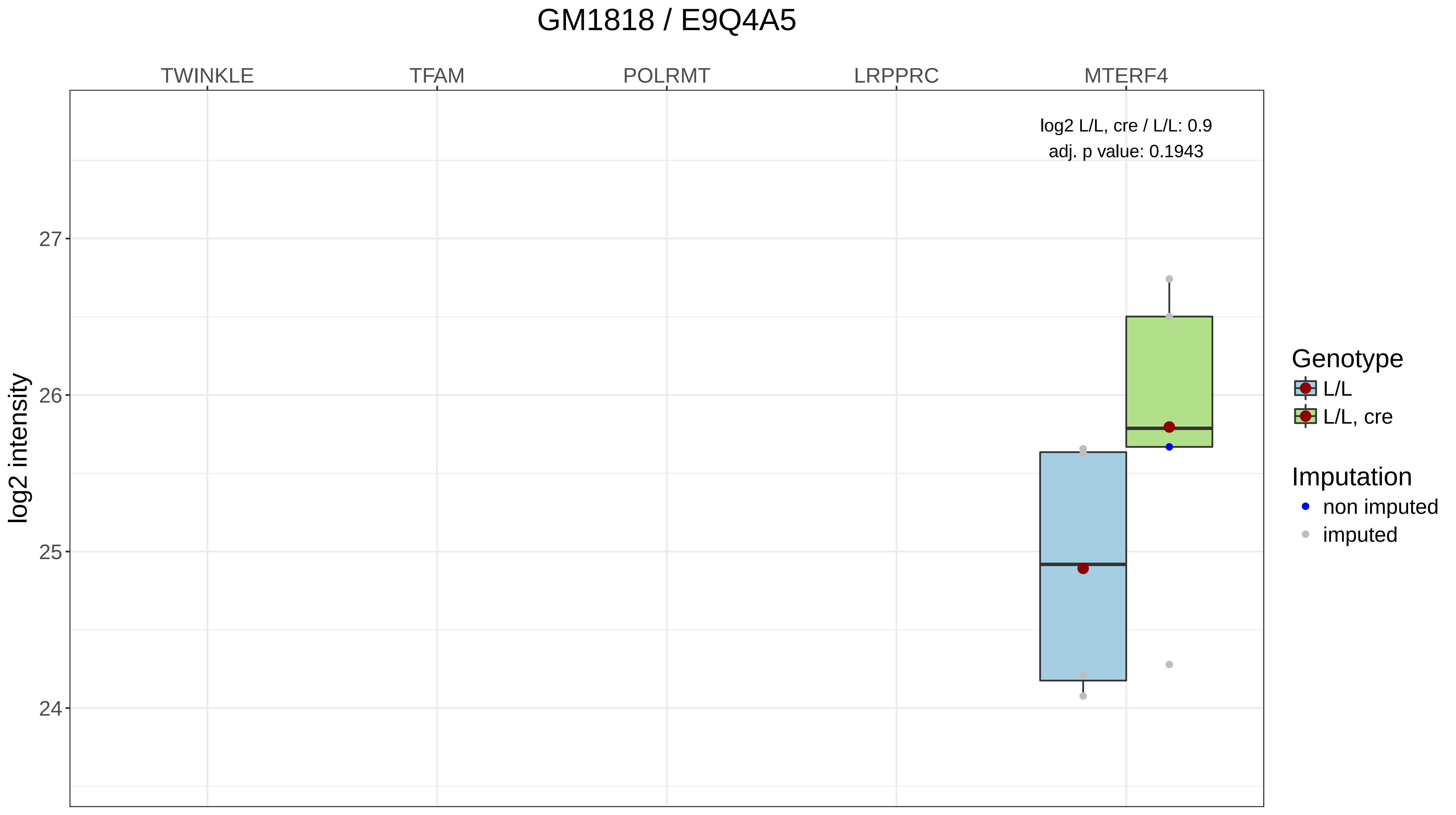
GM1818 / E9Q4A5
TWINKLE
TFAM
POLRMT
LRPPRC
MTERF4
log2 L/L, cre / L/L: 0.9
adj. p value: 0.1943
27
26
25
24
log2 intensity
Genotype
L/L
L/L, cre
Imputation
non imputed
imputed

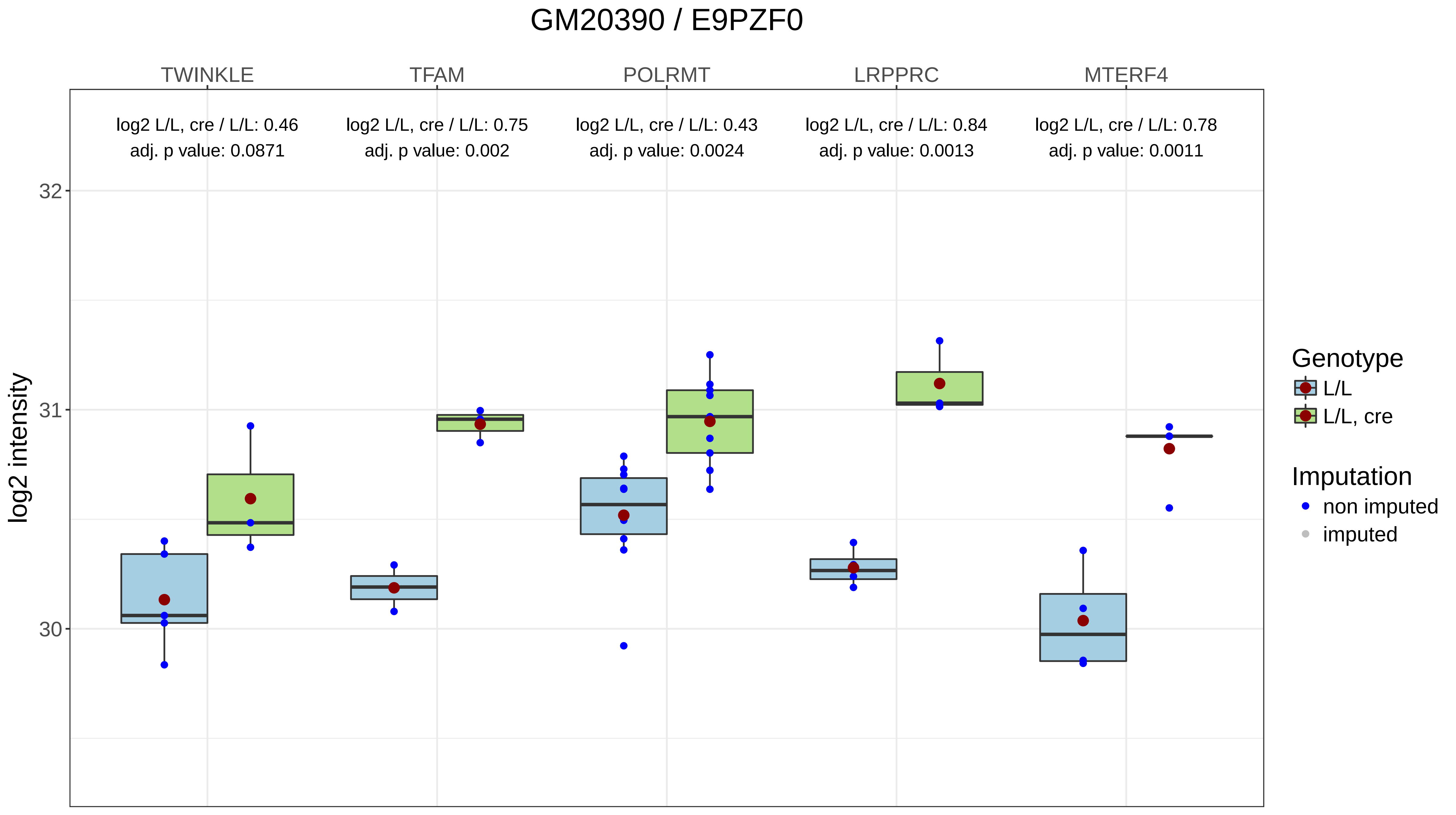

GM20390 / E9PZF0
TWINKLE
TFAM
POLRMT
LRPPRC
MTERF4
log2 L/L, cre / L/L: 0.46
adj. p value: 0.0871
log2 L/L, cre / L/L: 0.75
adj. p value: 0.002
log2 L/L, cre / L/L: 0.43
adj. p value: 0.0024
log2 L/L, cre / L/L: 0.84
adj. p value: 0.0013
log2 L/L, cre / L/L: 0.78
adj. p value: 0.0011
32
31
30
log2 intensity
Genotype
L/L
L/L, cre
Imputation
non imputed
imputed

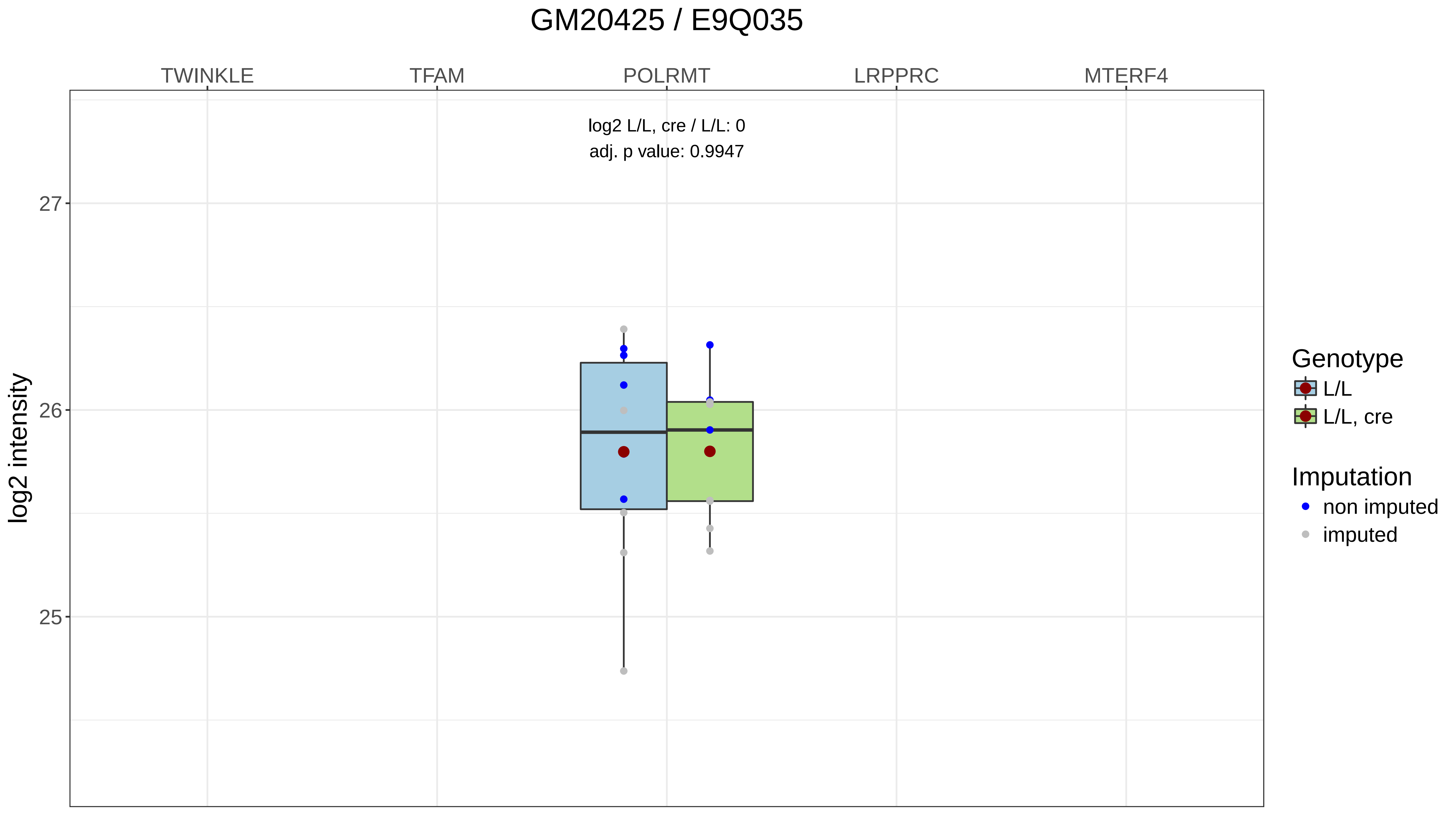
GM20425 / E9Q035
TWINKLE
TFAM
POLRMT
LRPPRC
MTERF4
log2 L/L, cre / L/L: 0
adj. p value: 0.9947
27
26
25
log2 intensity
Genotype
L/L
L/L, cre
Imputation
non imputed
imputed

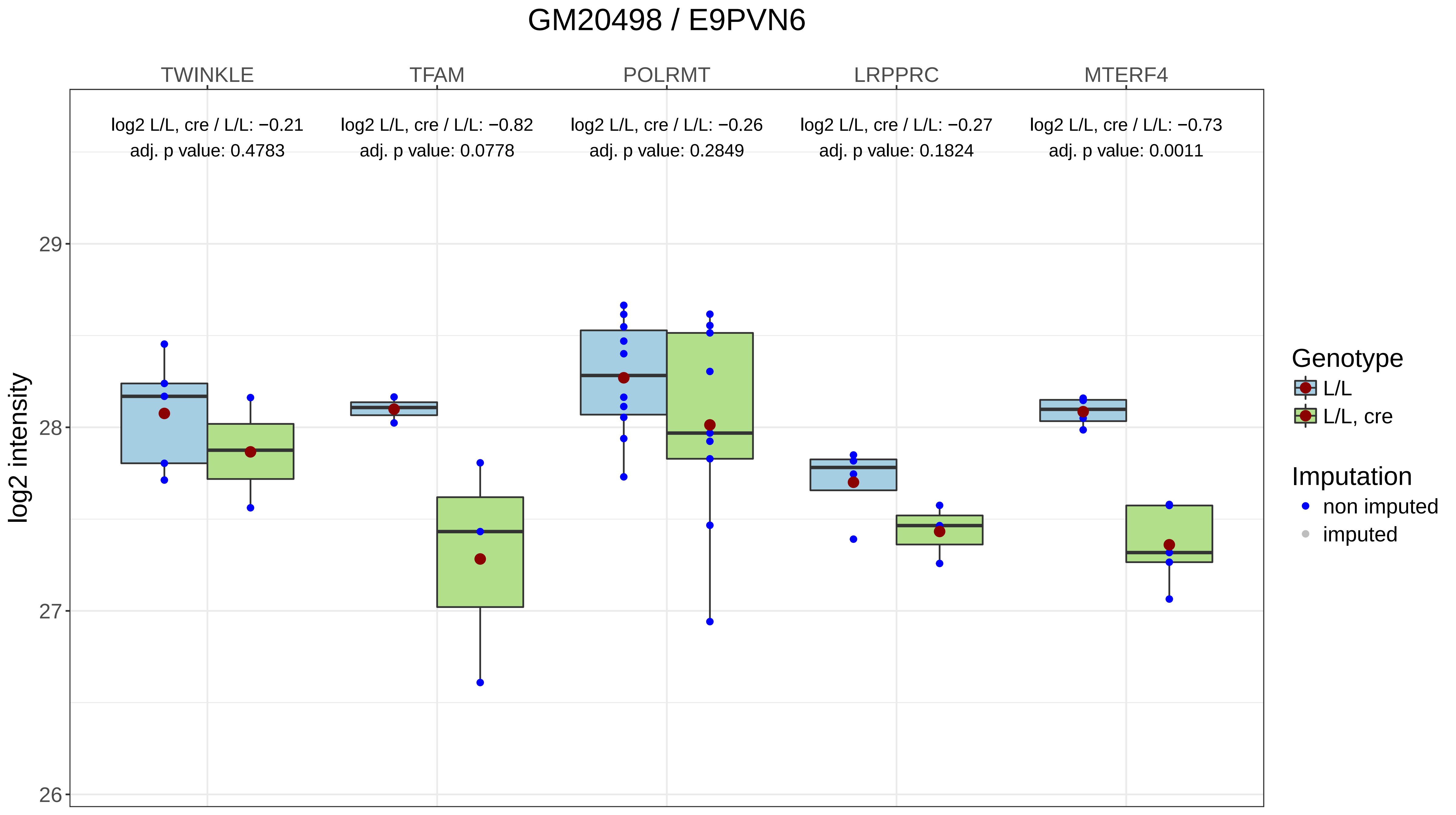
GM20498 / E9PVN6
TWINKLE
TFAM
POLRMT
LRPPRC
MTERF4
log2 L/L, cre / L/L: −0.21
adj. p value: 0.4783
log2 L/L, cre / L/L: −0.82
adj. p value: 0.0778
log2 L/L, cre / L/L: −0.26
adj. p value: 0.2849
log2 L/L, cre / L/L: −0.27
adj. p value: 0.1824
log2 L/L, cre / L/L: −0.73
adj. p value: 0.0011
log2 intensity
29
28
27
26
Genotype
L/L
L/L, cre
Imputation
non imputed
imputed

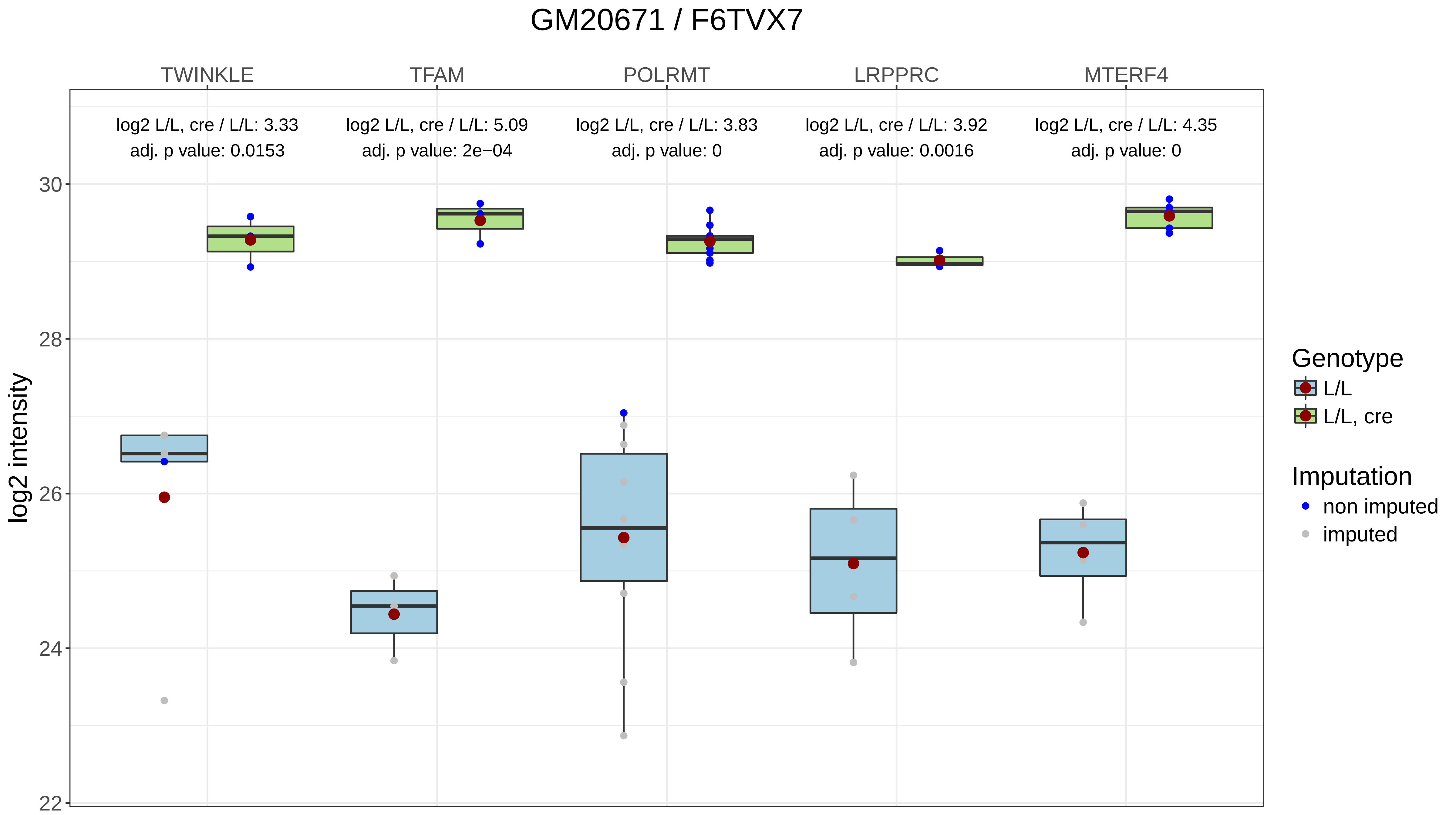
GM20671 / F6TVX7
TWINKLE
TFAM
POLRMT
LRPPRC
MTERF4
log2 L/L, cre / L/L: 3.33
adj. p value: 0.0153
log2 L/L, cre / L/L: 5.09
adj. p value: 2e−04
log2 L/L, cre / L/L: 3.83
adj. p value: 0
log2 L/L, cre / L/L: 3.92
adj. p value: 0.0016
log2 L/L, cre / L/L: 4.35
adj. p value: 0
log2 intensity
30
28
26
24
22
Genotype
L/L
L/L, cre
Imputation
non imputed
imputed

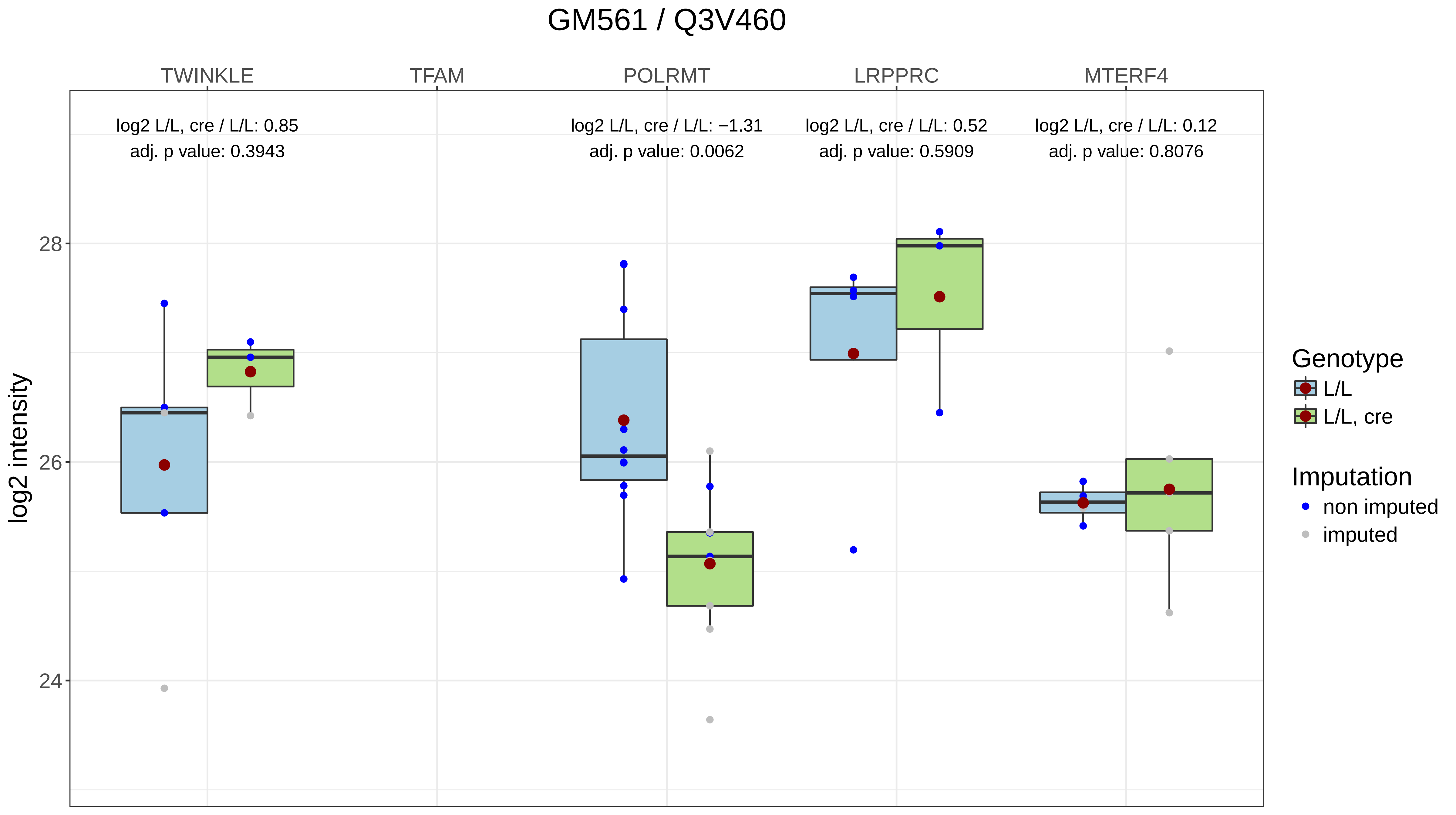
GM561 / Q3V460
TWINKLE
TFAM
POLRMT
LRPPRC
MTERF4
log2 L/L, cre / L/L: 0.85
adj. p value: 0.3943
log2 L/L, cre / L/L: −1.31
adj. p value: 0.0062
log2 L/L, cre / L/L: 0.52
adj. p value: 0.5909
log2 L/L, cre / L/L: 0.12
adj. p value: 0.8076
log2 intensity
28
26
24
Genotype
L/L
L/L, cre
Imputation
non imputed
imputed

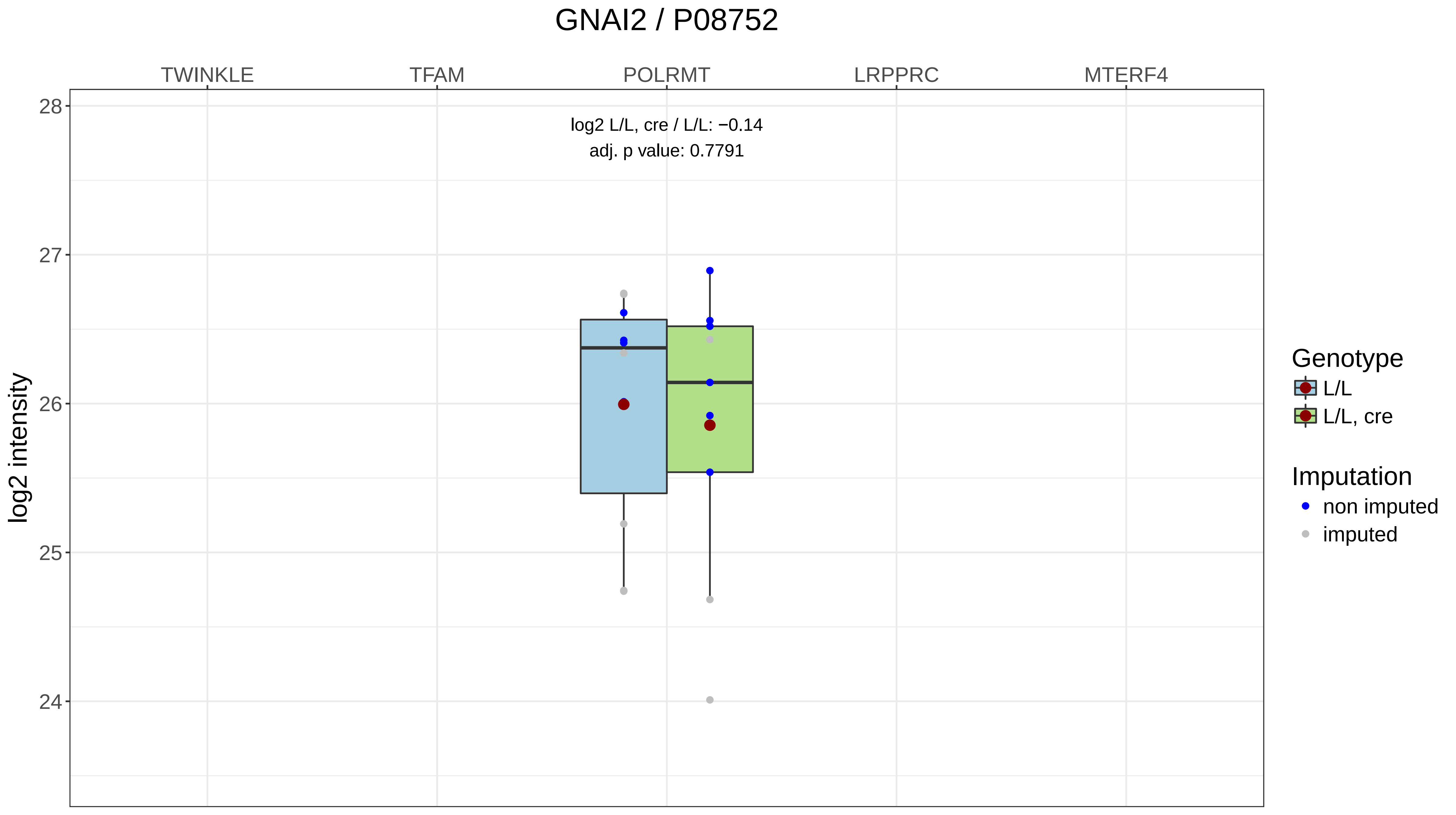
GNAI2 / P08752
TWINKLE
TFAM
POLRMT
LRPPRC
MTERF4
log2 L/L, cre / L/L: −0.14
adj. p value: 0.7791
log2 intensity
28
27
26
25
24
Genotype
L/L
L/L, cre
Imputation
non imputed
imputed

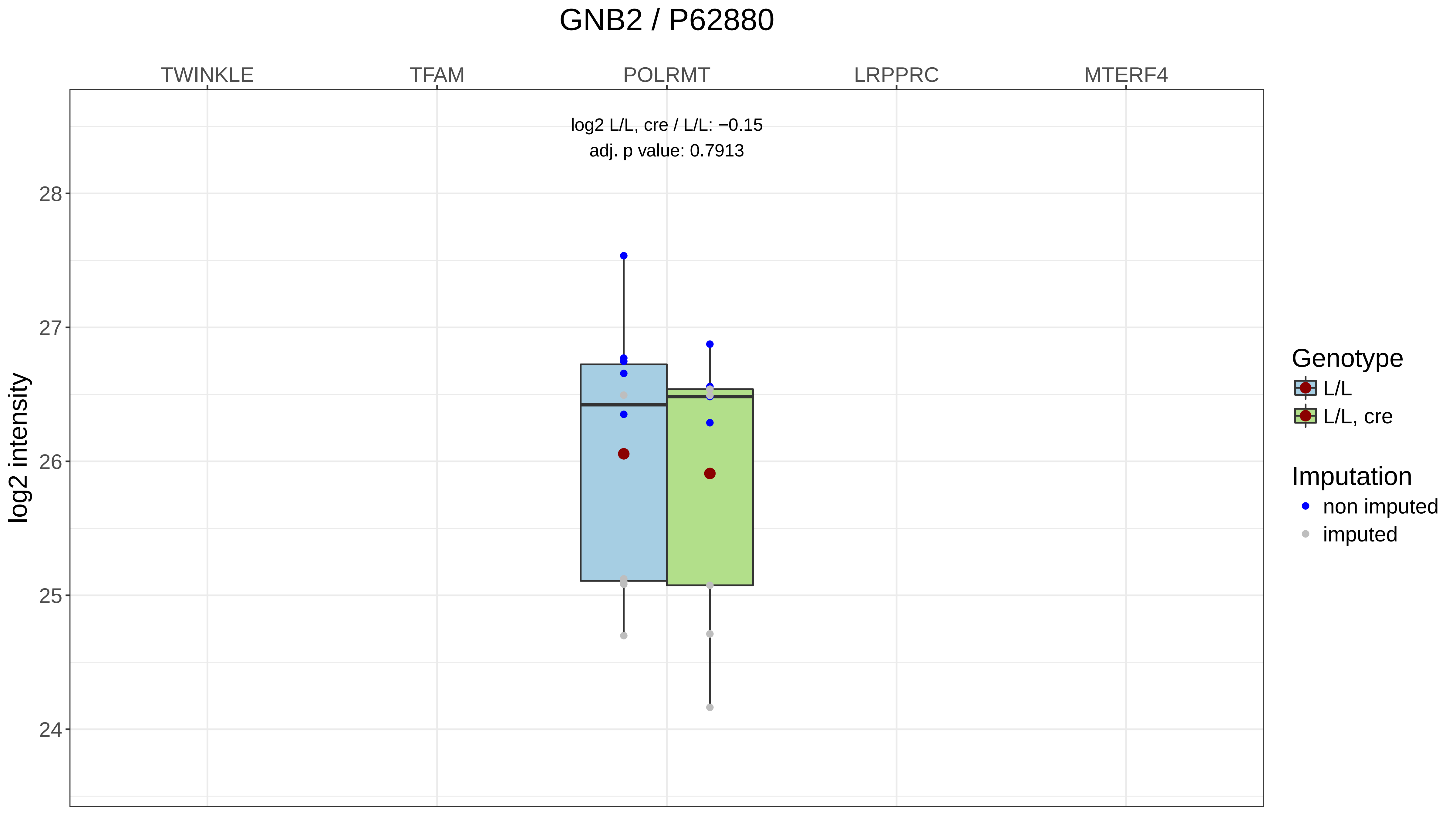
GNB2 / P62880
TWINKLE
TFAM
POLRMT
LRPPRC
MTERF4
log2 L/L, cre / L/L: −0.15
adj. p value: 0.7913
28
27
26
25
24
log2 intensity
Genotype
L/L
L/L, cre
Imputation
non imputed
imputed

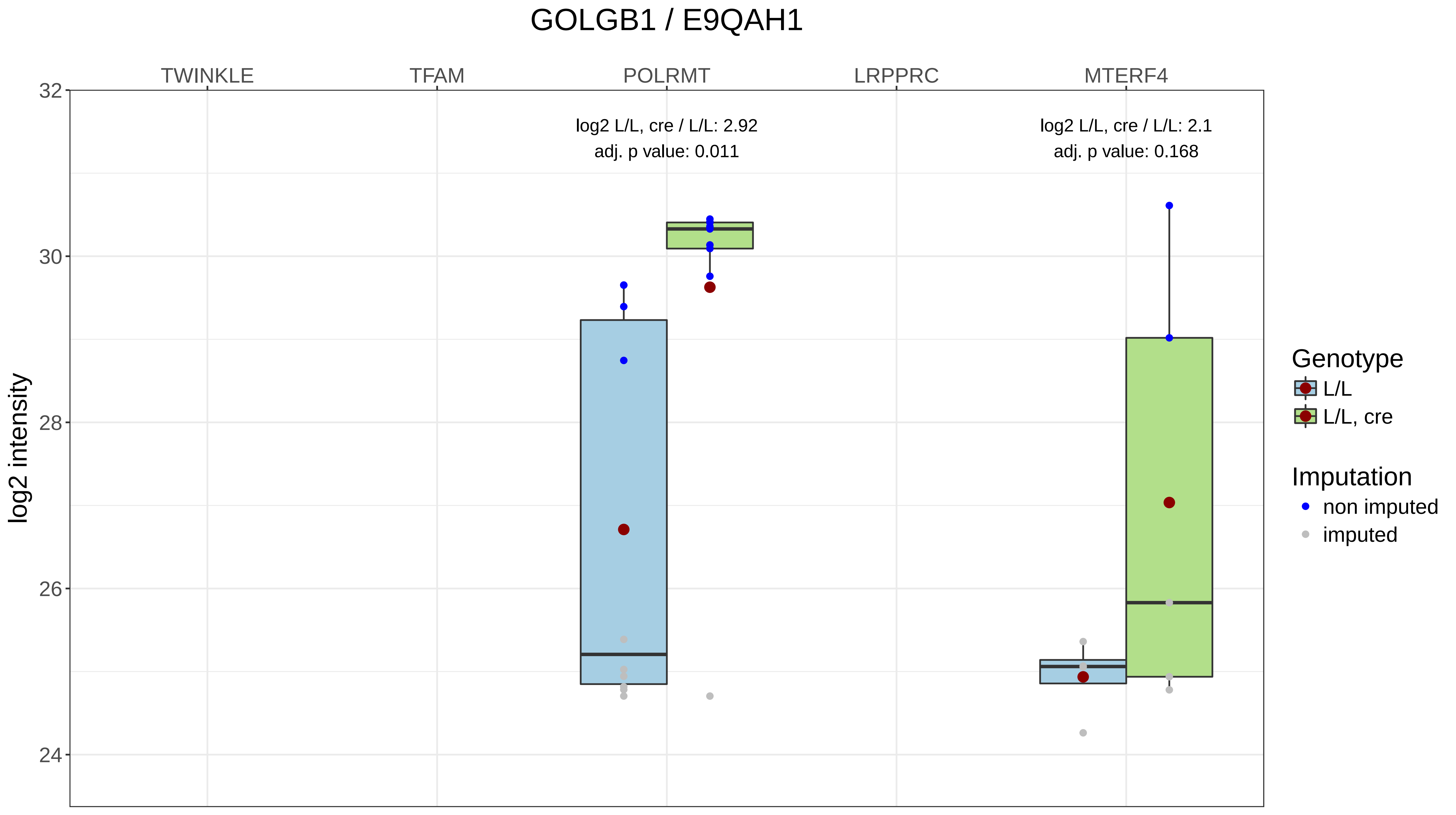
GOLGB1 / E9QAH1
TWINKLE
TFAM
POLRMT
LRPPRC
MTERF4
log2 L/L, cre / L/L: 2.92
adj. p value: 0.011
log2 L/L, cre / L/L: 2.1
adj. p value: 0.168
log2 intensity
32
30
28
26
24
Genotype
L/L
L/L, cre
Imputation
non imputed
imputed

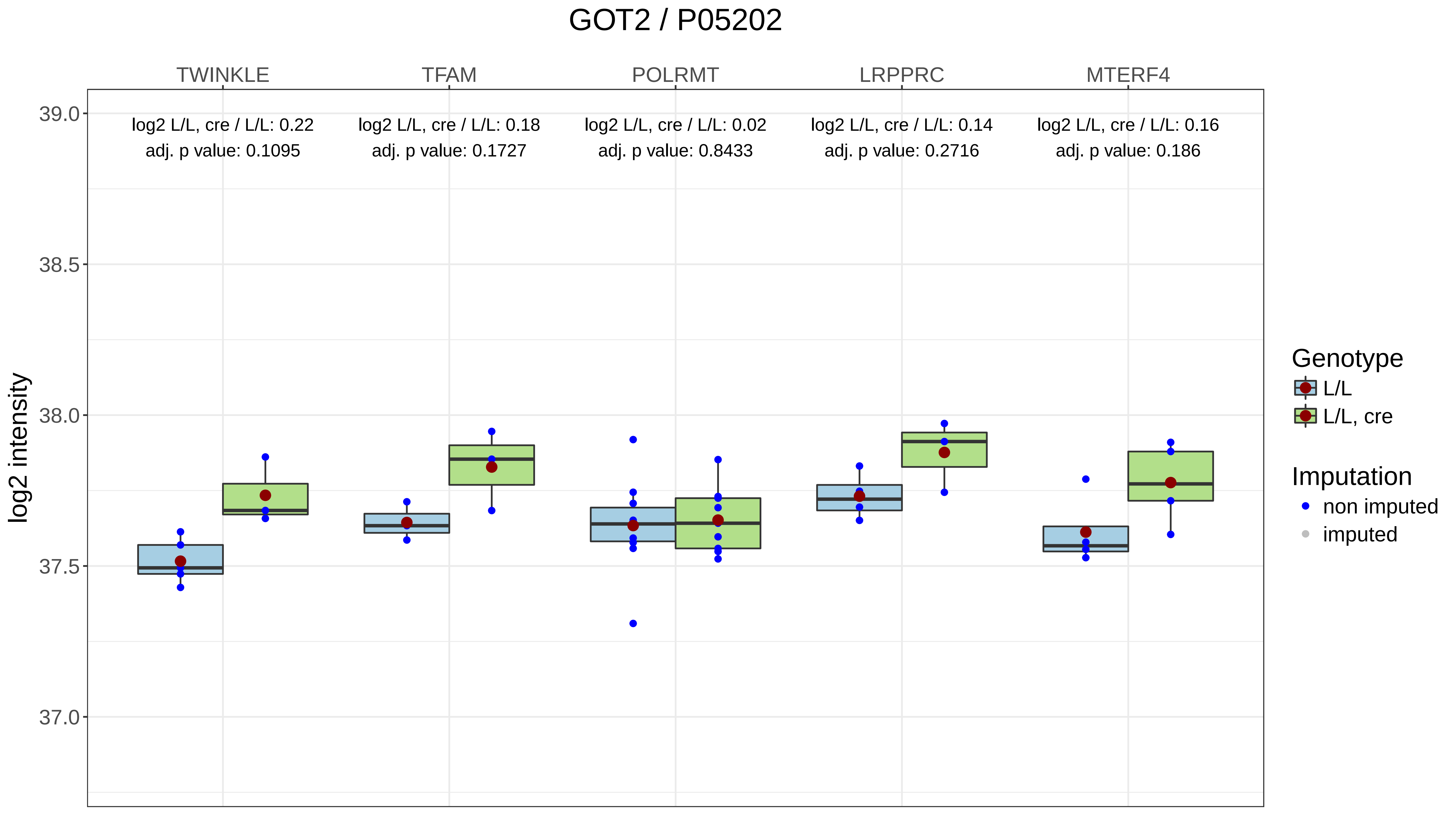
GOT2 / P05202
TWINKLE
TFAM
POLRMT
LRPPRC
MTERF4
log2 L/L, cre / L/L: 0.22
adj. p value: 0.1095
log2 L/L, cre / L/L: 0.18
adj. p value: 0.1727
log2 L/L, cre / L/L: 0.02
adj. p value: 0.8433
log2 L/L, cre / L/L: 0.14
adj. p value: 0.2716
log2 L/L, cre / L/L: 0.16
adj. p value: 0.186
log2 intensity
39.0
38.5
38.0
37.5
37.0
Genotype
L/L
L/L, cre
Imputation
non imputed
imputed

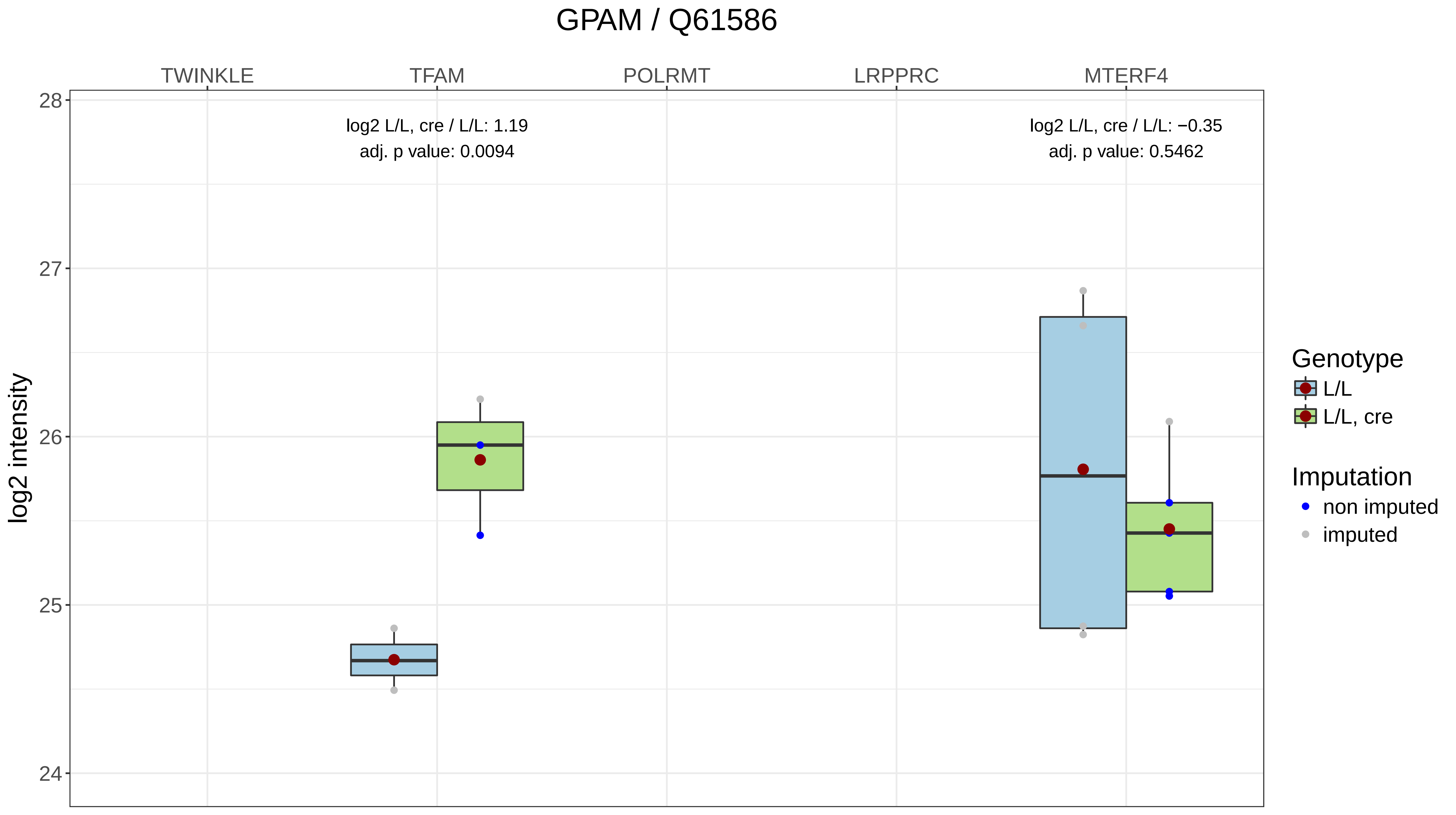
GPAM / Q61586
TWINKLE
TFAM
POLRMT
LRPPRC
MTERF4
log2 L/L, cre / L/L: 1.19
adj. p value: 0.0094
log2 L/L, cre / L/L: −0.35
adj. p value: 0.5462
28
27
26
25
24
log2 intensity
Genotype
L/L
L/L, cre
Imputation
non imputed
imputed

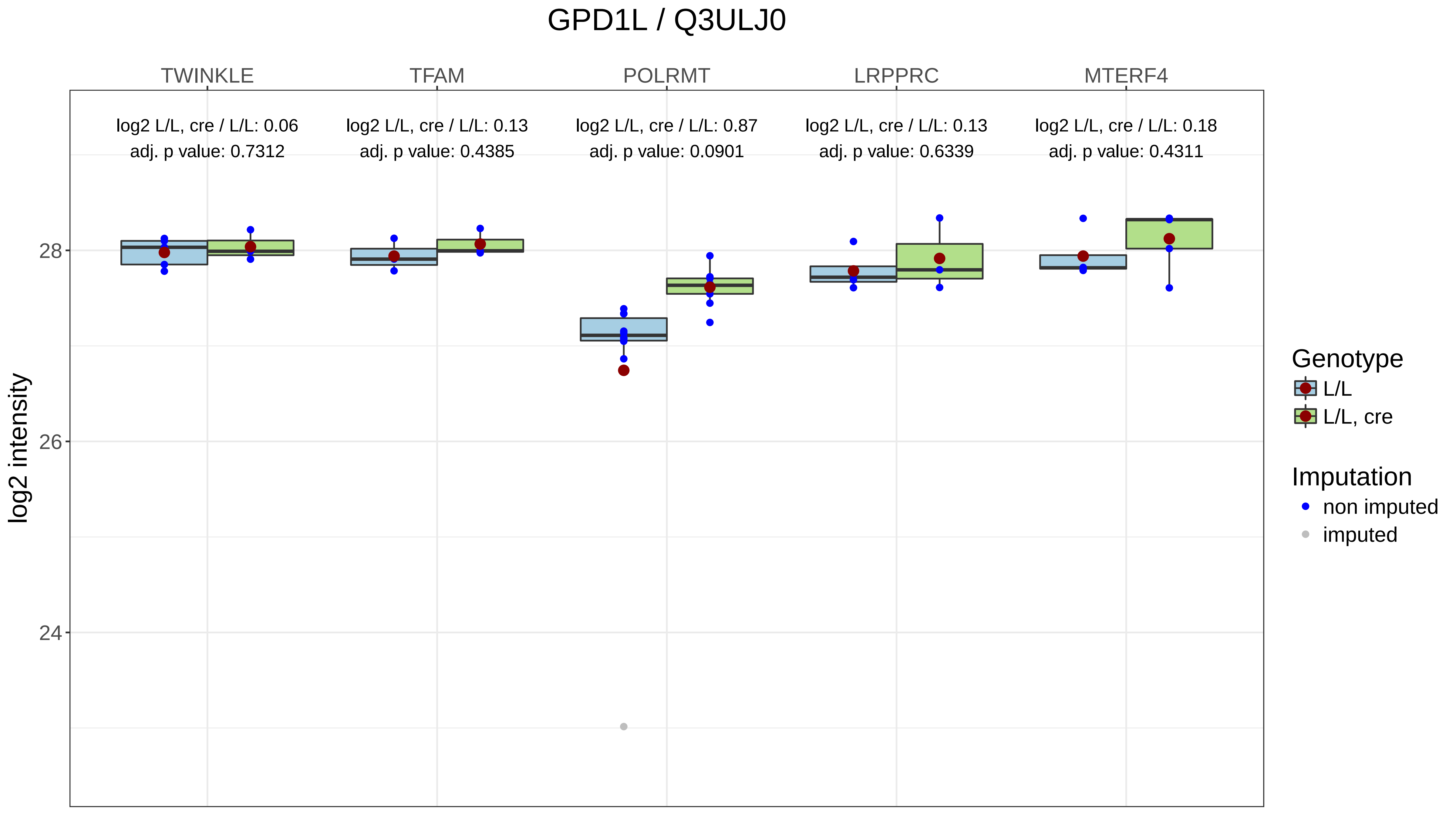
GPD1L / Q3ULJ0
TWINKLE
TFAM
POLRMT
LRPPRC
MTERF4
log2 L/L, cre / L/L: 0.06
adj. p value: 0.7312
log2 L/L, cre / L/L: 0.13
adj. p value: 0.4385
log2 L/L, cre / L/L: 0.87
adj. p value: 0.0901
log2 L/L, cre / L/L: 0.13
adj. p value: 0.6339
log2 L/L, cre / L/L: 0.18
adj. p value: 0.4311
log2 intensity
28
26
24
Genotype
L/L
L/L, cre
Imputation
non imputed
imputed

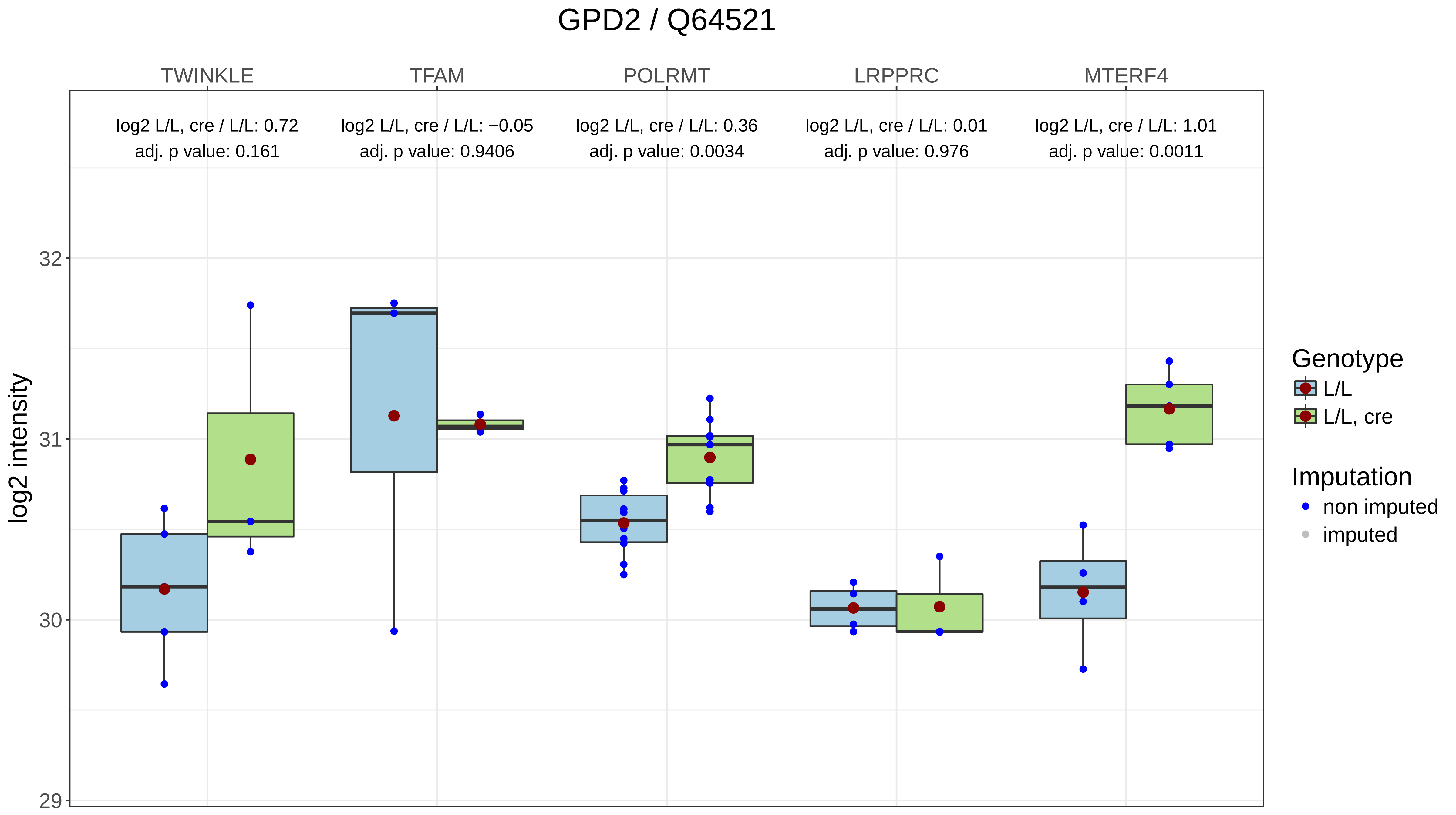
GPD2 / Q64521
TWINKLE
TFAM
POLRMT
LRPPRC
MTERF4
log2 L/L, cre / L/L: 0.72
adj. p value: 0.161
log2 L/L, cre / L/L: −0.05
adj. p value: 0.9406
log2 L/L, cre / L/L: 0.36
adj. p value: 0.0034
log2 L/L, cre / L/L: 0.01
adj. p value: 0.976
log2 L/L, cre / L/L: 1.01
adj. p value: 0.0011
log2 intensity
32
31
30
29
Genotype
L/L
L/L, cre
Imputation
non imputed
imputed

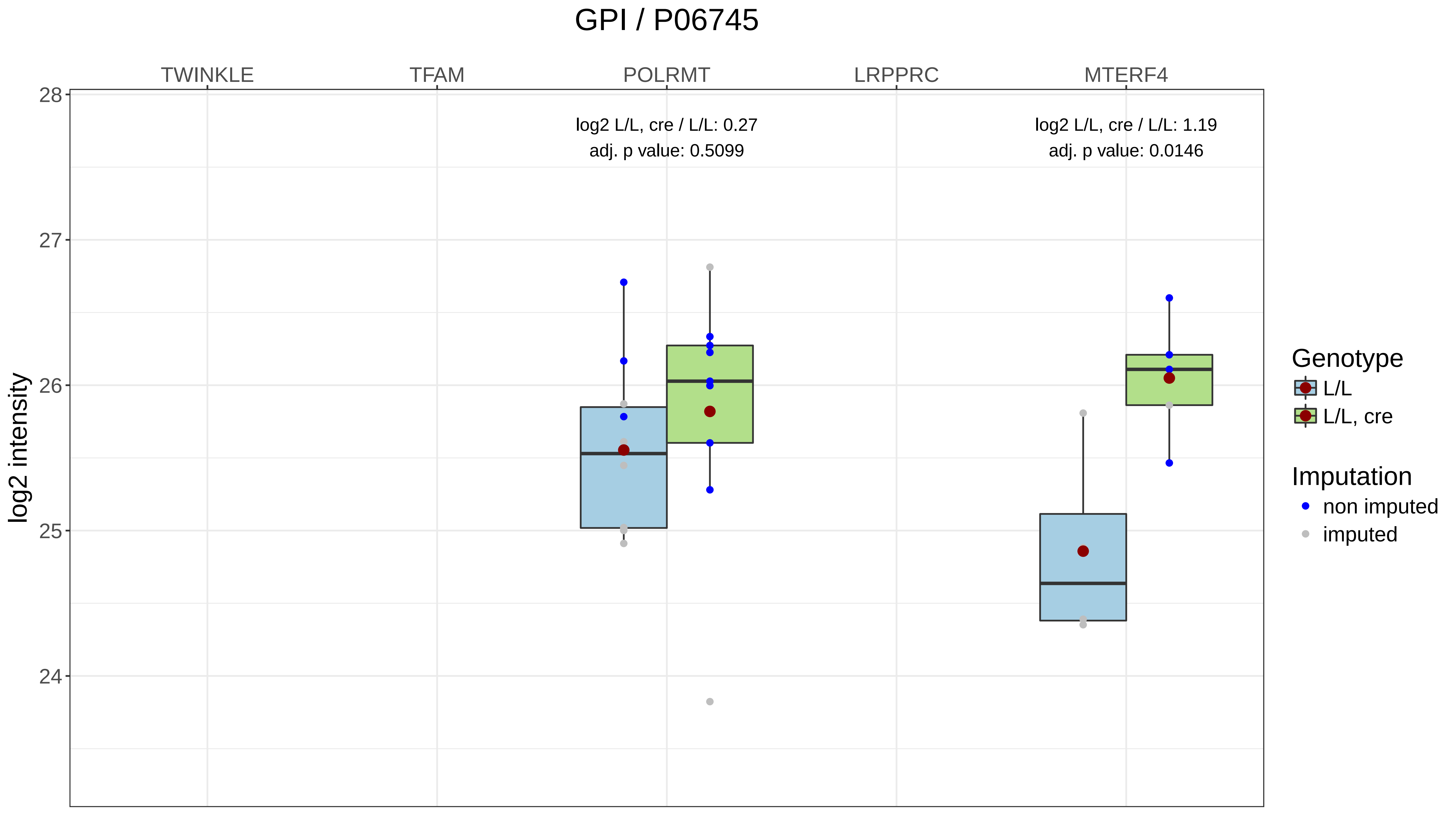
GPI / P06745
TWINKLE
TFAM
POLRMT
LRPPRC
MTERF4
log2 L/L, cre / L/L: 0.27
adj. p value: 0.5099
log2 L/L, cre / L/L: 1.19
adj. p value: 0.0146
log2 intensity
28
27
26
25
24
Genotype
L/L
L/L, cre
Imputation
non imputed
imputed

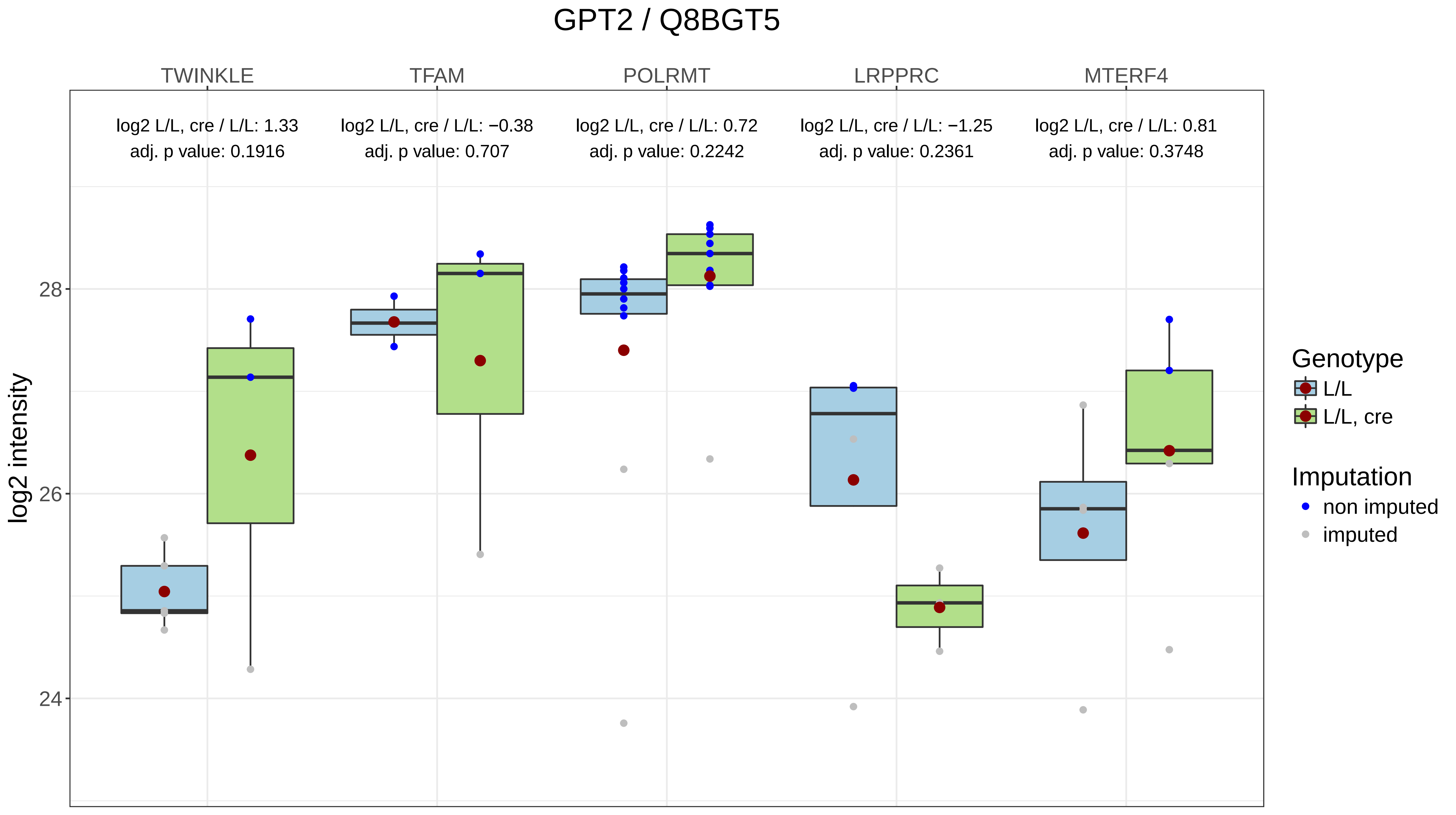

GPT2 / Q8BGT5
TWINKLE
TFAM
POLRMT
LRPPRC
MTERF4
log2 L/L, cre / L/L: 1.33
adj. p value: 0.1916
log2 L/L, cre / L/L: −0.38
adj. p value: 0.707
log2 L/L, cre / L/L: 0.72
adj. p value: 0.2242
log2 L/L, cre / L/L: −1.25
adj. p value: 0.2361
log2 L/L, cre / L/L: 0.81
adj. p value: 0.3748
log2 intensity
28
26
24
Genotype
L/L
L/L, cre
Imputation
non imputed
imputed

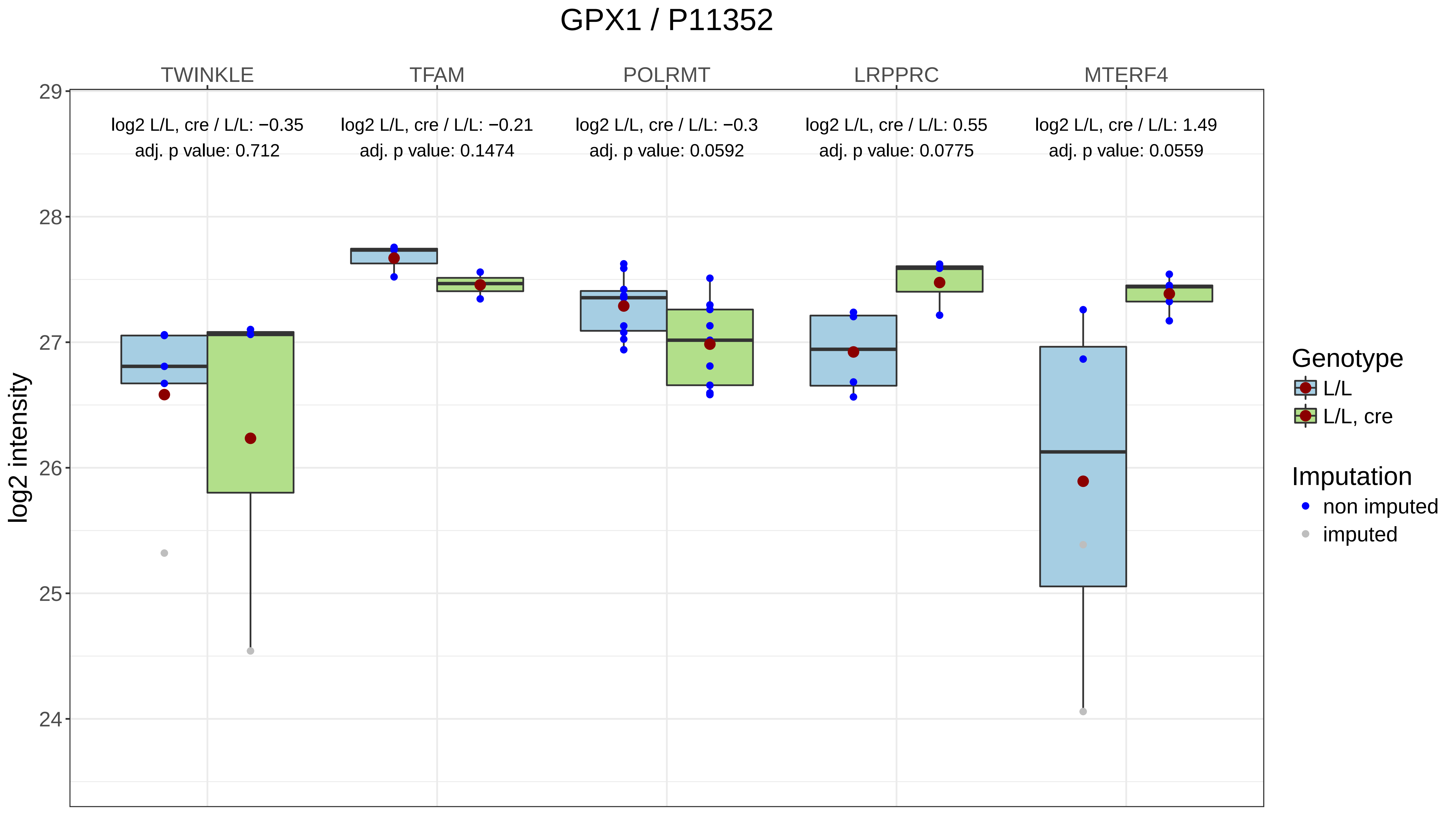
GPX1 / P11352
TWINKLE
TFAM
POLRMT
LRPPRC
MTERF4
log2 L/L, cre / L/L: −0.35
adj. p value: 0.712
log2 L/L, cre / L/L: −0.21
adj. p value: 0.1474
log2 L/L, cre / L/L: −0.3
adj. p value: 0.0592
log2 L/L, cre / L/L: 0.55
adj. p value: 0.0775
log2 L/L, cre / L/L: 1.49
adj. p value: 0.0559
log2 intensity
29
28
27
26
25
24
Genotype
L/L
L/L, cre
Imputation
non imputed
imputed

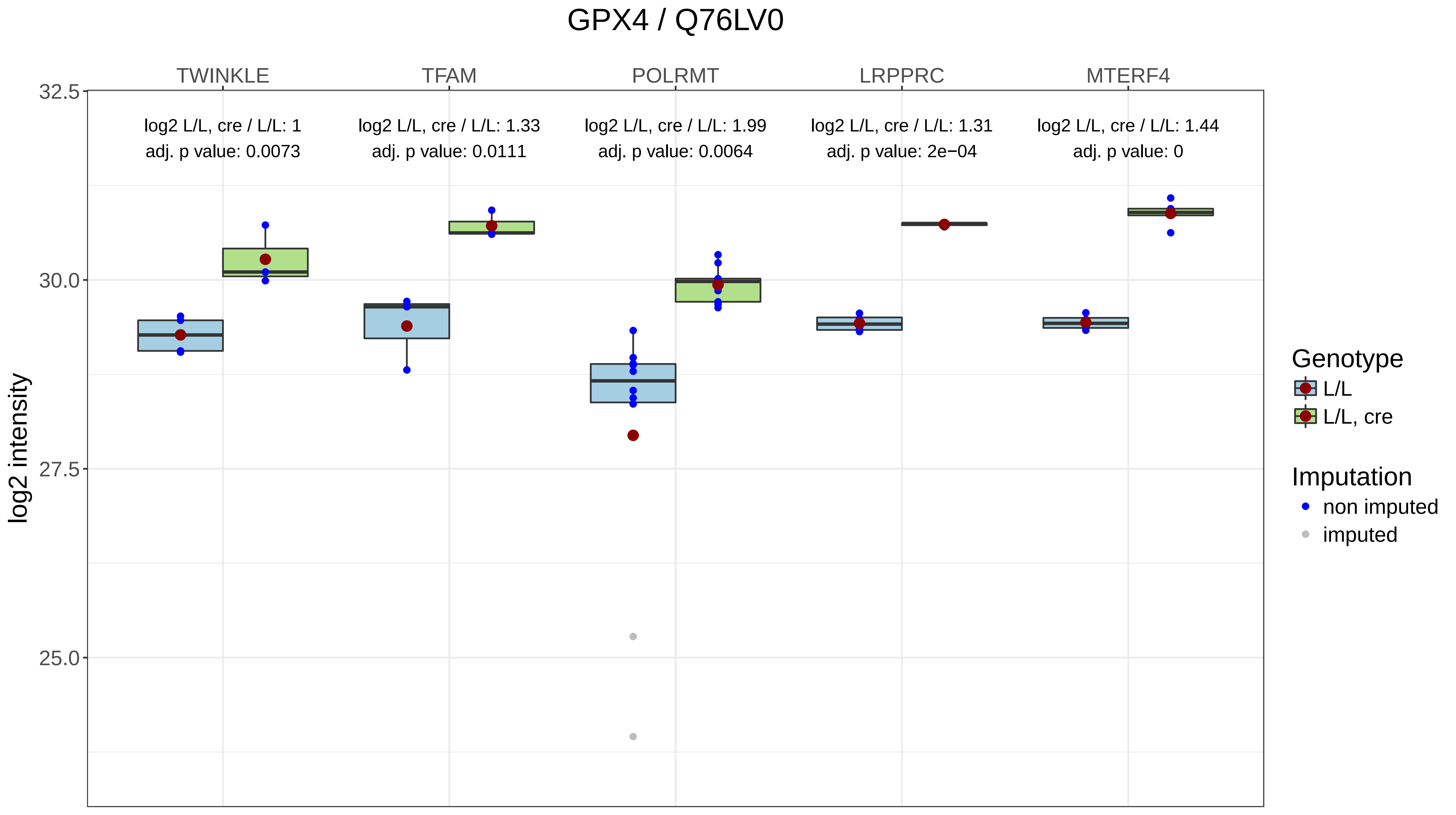
GPX4 / Q76LV0
TWINKLE
TFAM
POLRMT
LRPPRC
MTERF4
log2 L/L, cre / L/L: 1
adj. p value: 0.0073
log2 L/L, cre / L/L: 1.33
adj. p value: 0.0111
log2 L/L, cre / L/L: 1.99
adj. p value: 0.0064
log2 L/L, cre / L/L: 1.31
adj. p value: 2e−04
log2 L/L, cre / L/L: 1.44
adj. p value: 0
32.5
30.0
27.5
25.0
log2 intensity
Genotype
L/L
L/L, cre
Imputation
non imputed
imputed

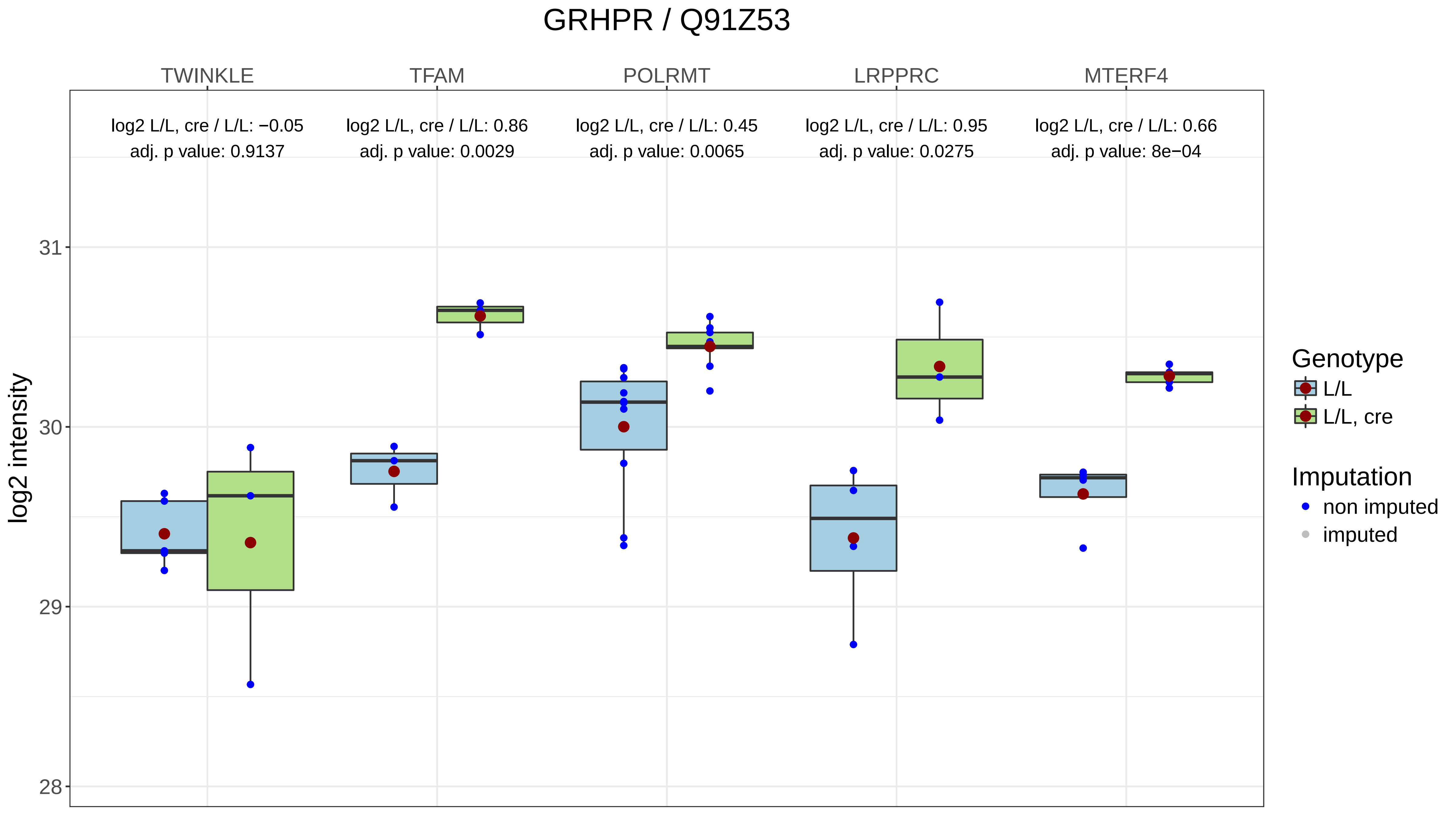
GRHPR / Q91Z53
TWINKLE
TFAM
POLRMT
LRPPRC
MTERF4
log2 L/L, cre / L/L: −0.05
adj. p value: 0.9137
log2 L/L, cre / L/L: 0.86
adj. p value: 0.0029
log2 L/L, cre / L/L: 0.45
adj. p value: 0.0065
log2 L/L, cre / L/L: 0.95
adj. p value: 0.0275
log2 L/L, cre / L/L: 0.66
adj. p value: 8e−04
log2 intensity
31
30
29
28
Genotype
L/L
L/L, cre
Imputation
non imputed
imputed

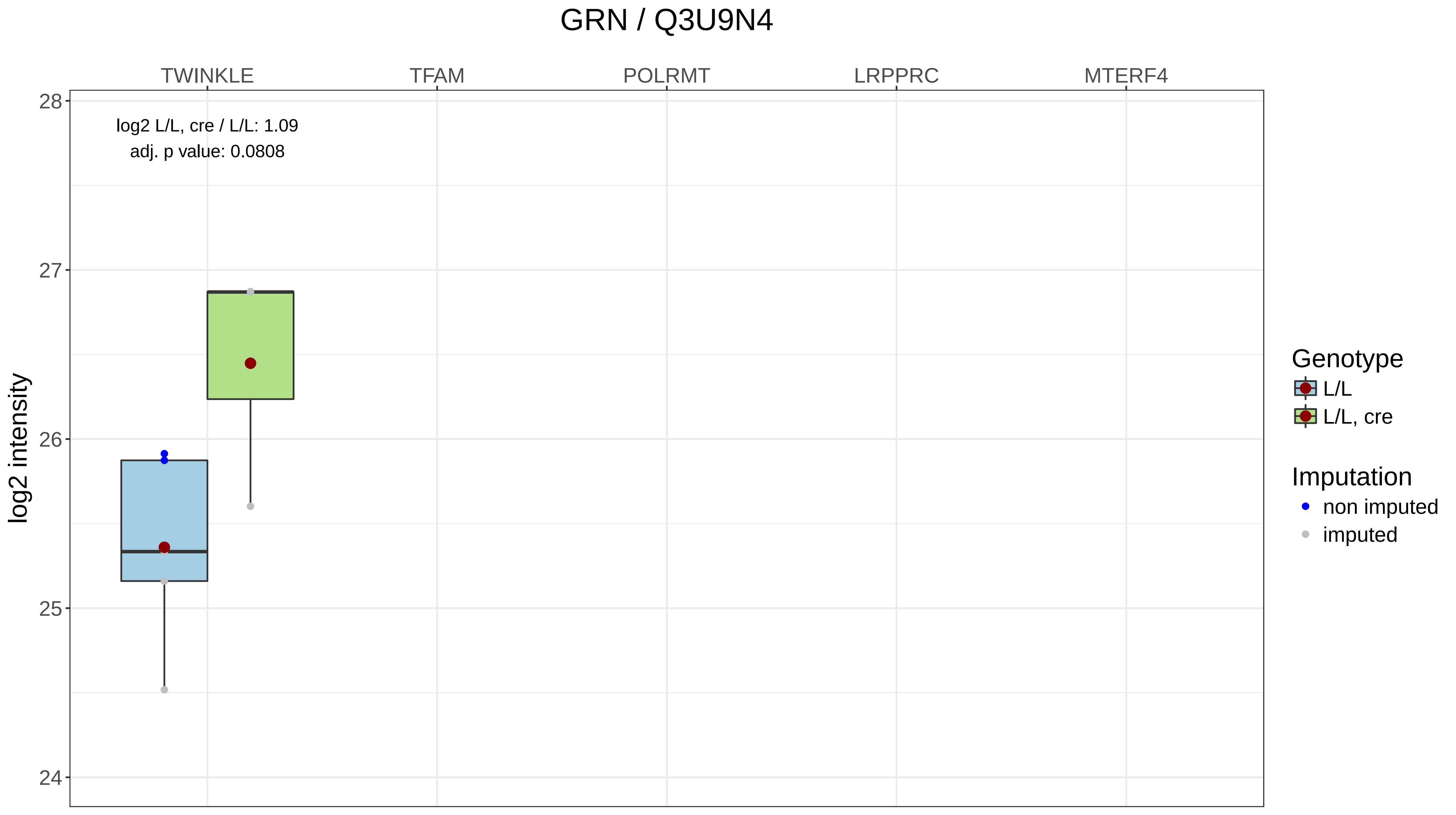
GRN / Q3U9N4
TWINKLE
TFAM
POLRMT
LRPPRC
MTERF4
log2 L/L, cre / L/L: 1.09
adj. p value: 0.0808
log2 intensity
28
27
26
25
24
Genotype
L/L
L/L, cre
Imputation
non imputed
imputed

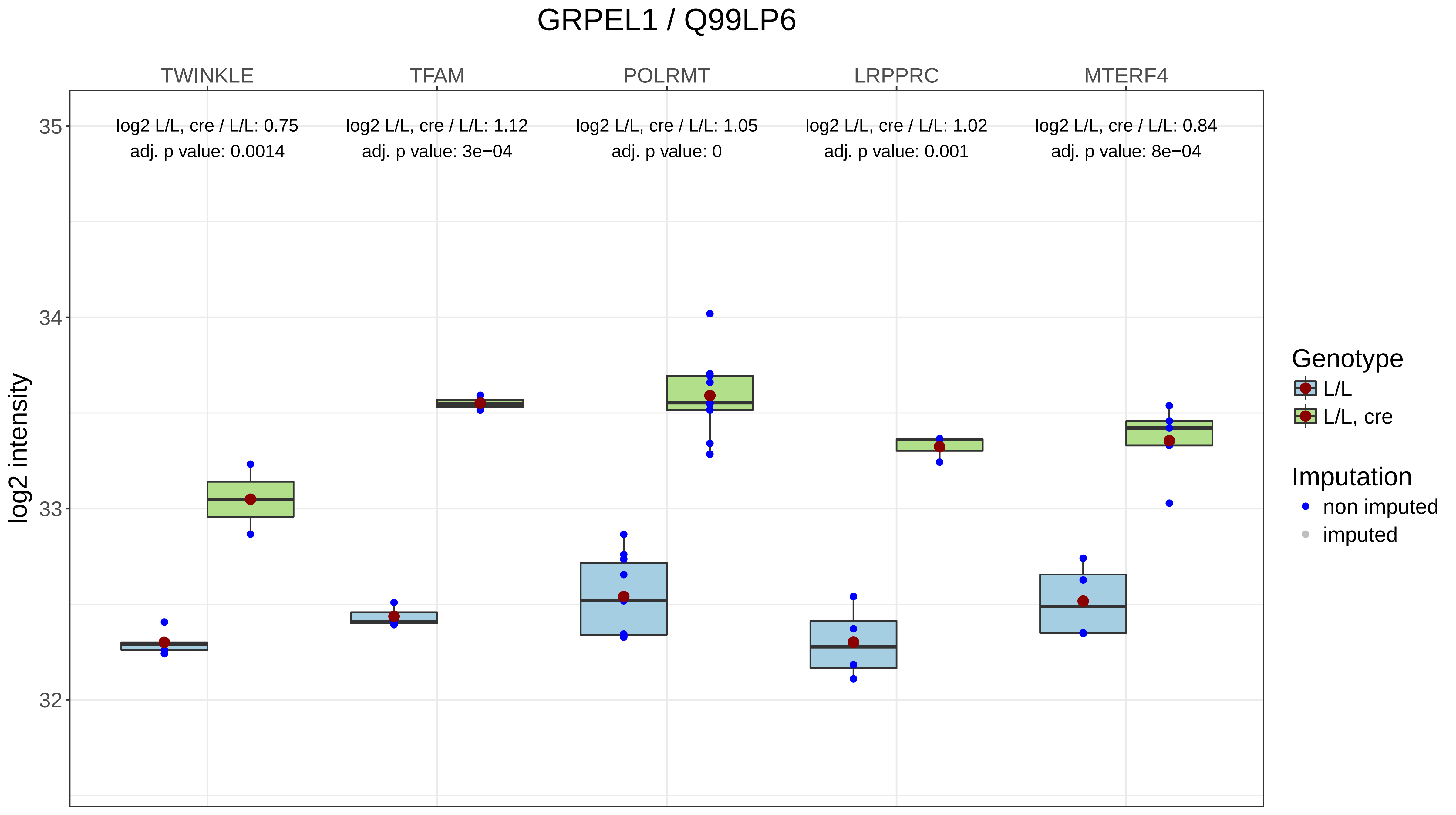
GRPEL1 / Q99LP6
TWINKLE
TFAM
POLRMT
LRPPRC
MTERF4
log2 L/L, cre / L/L: 0.75
adj. p value: 0.0014
log2 L/L, cre / L/L: 1.12
adj. p value: 3e−04
log2 L/L, cre / L/L: 1.05
adj. p value: 0
log2 L/L, cre / L/L: 1.02
adj. p value: 0.001
log2 L/L, cre / L/L: 0.84
adj. p value: 8e−04
log2 intensity
35
34
33
32
Genotype
L/L
L/L, cre
Imputation
non imputed
imputed

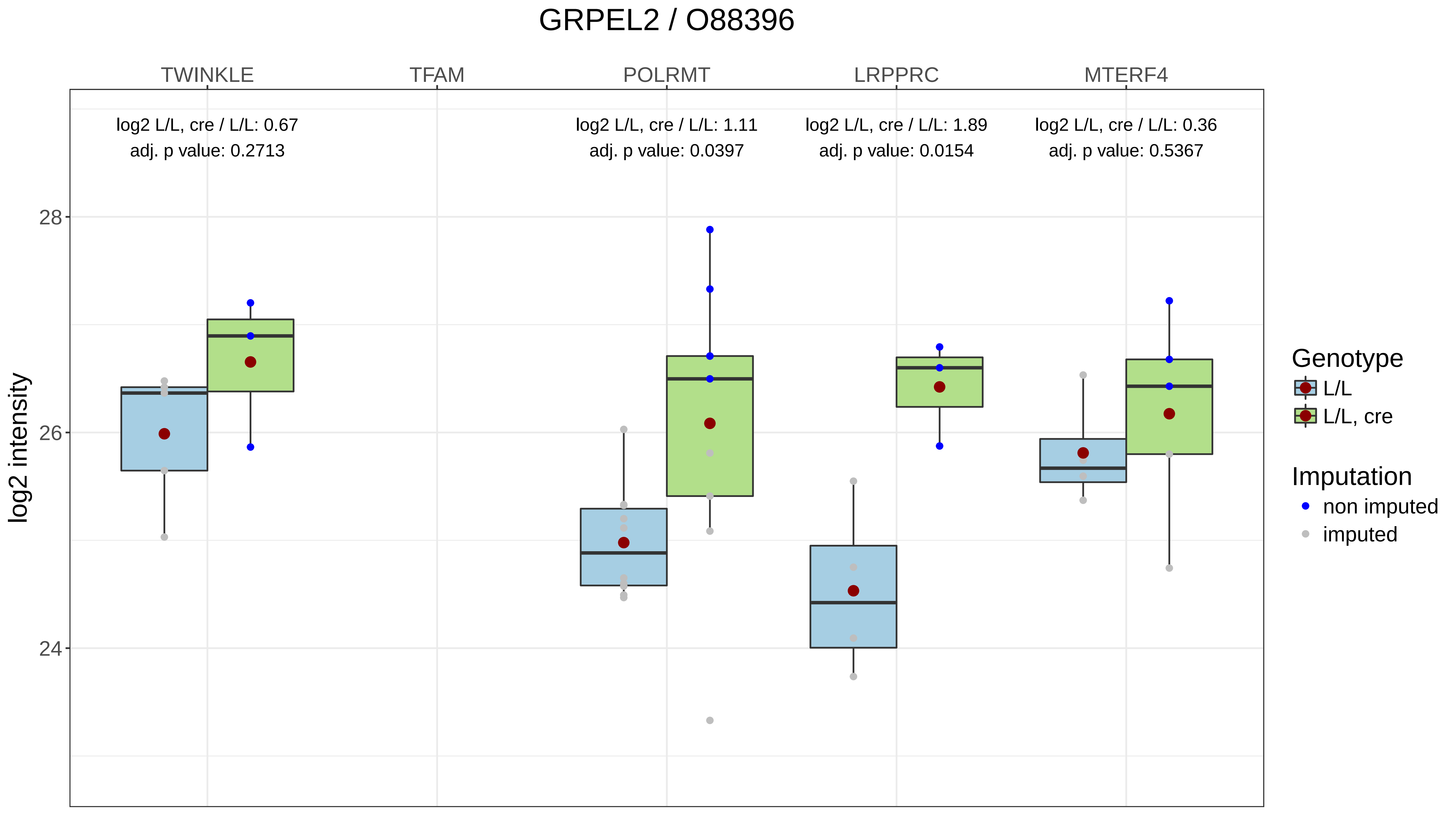
GRPEL2 / O88396
TWINKLE
TFAM
POLRMT
LRPPRC
MTERF4
log2 L/L, cre / L/L: 0.67
adj. p value: 0.2713
log2 L/L, cre / L/L: 1.11
adj. p value: 0.0397
log2 L/L, cre / L/L: 1.89
adj. p value: 0.0154
log2 L/L, cre / L/L: 0.36
adj. p value: 0.5367
28
26
24
log2 intensity
Genotype
L/L
L/L, cre
Imputation
non imputed
imputed

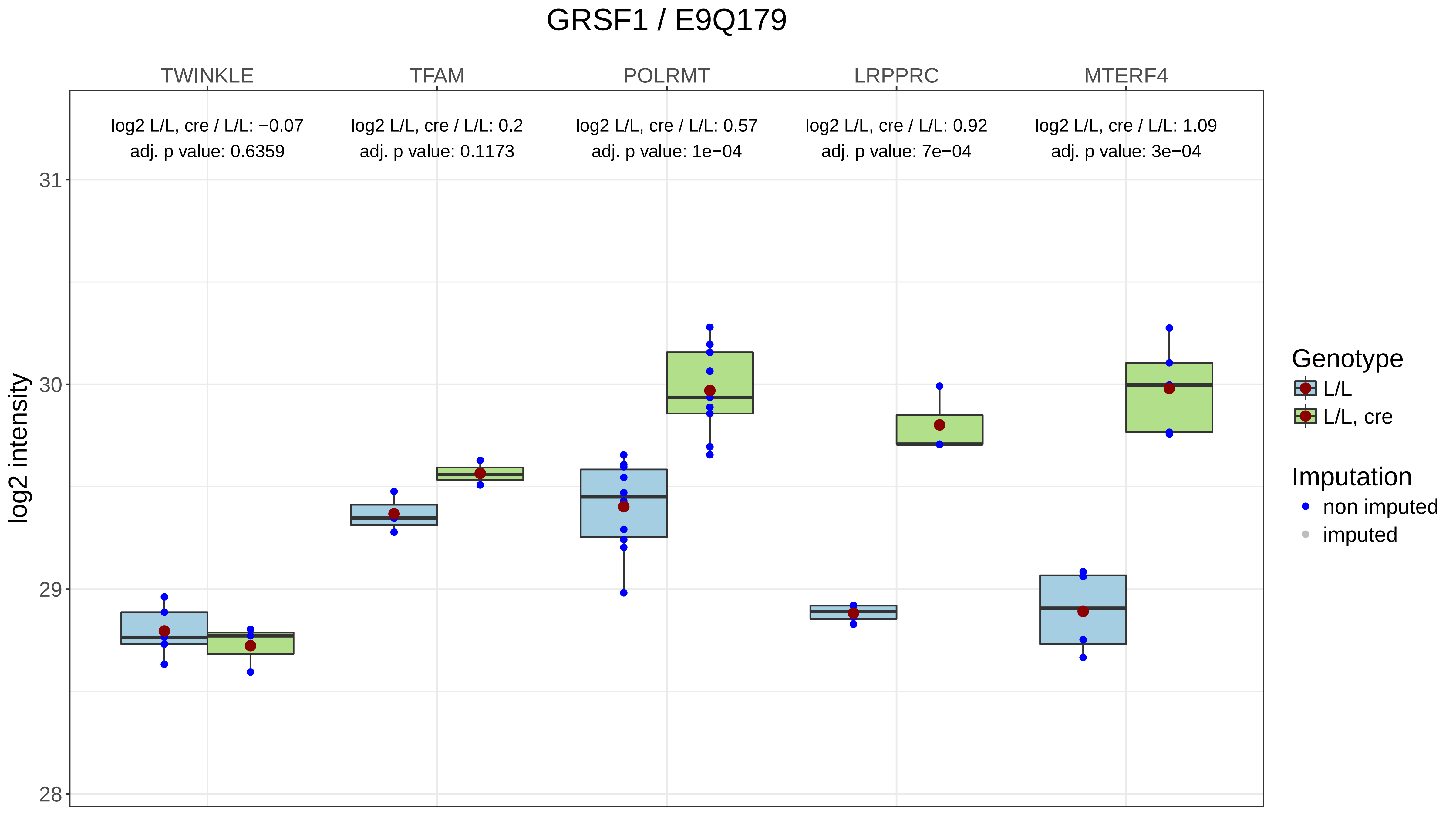
GRSF1 / E9Q179
TWINKLE
TFAM
POLRMT
LRPPRC
MTERF4
log2 L/L, cre / L/L: −0.07
adj. p value: 0.6359
log2 L/L, cre / L/L: 0.2
adj. p value: 0.1173
log2 L/L, cre / L/L: 0.57
adj. p value: 1e−04
log2 L/L, cre / L/L: 0.92
adj. p value: 7e−04
log2 L/L, cre / L/L: 1.09
adj. p value: 3e−04
log2 intensity
31
30
29
28
Genotype
L/L
L/L, cre
Imputation
non imputed
imputed

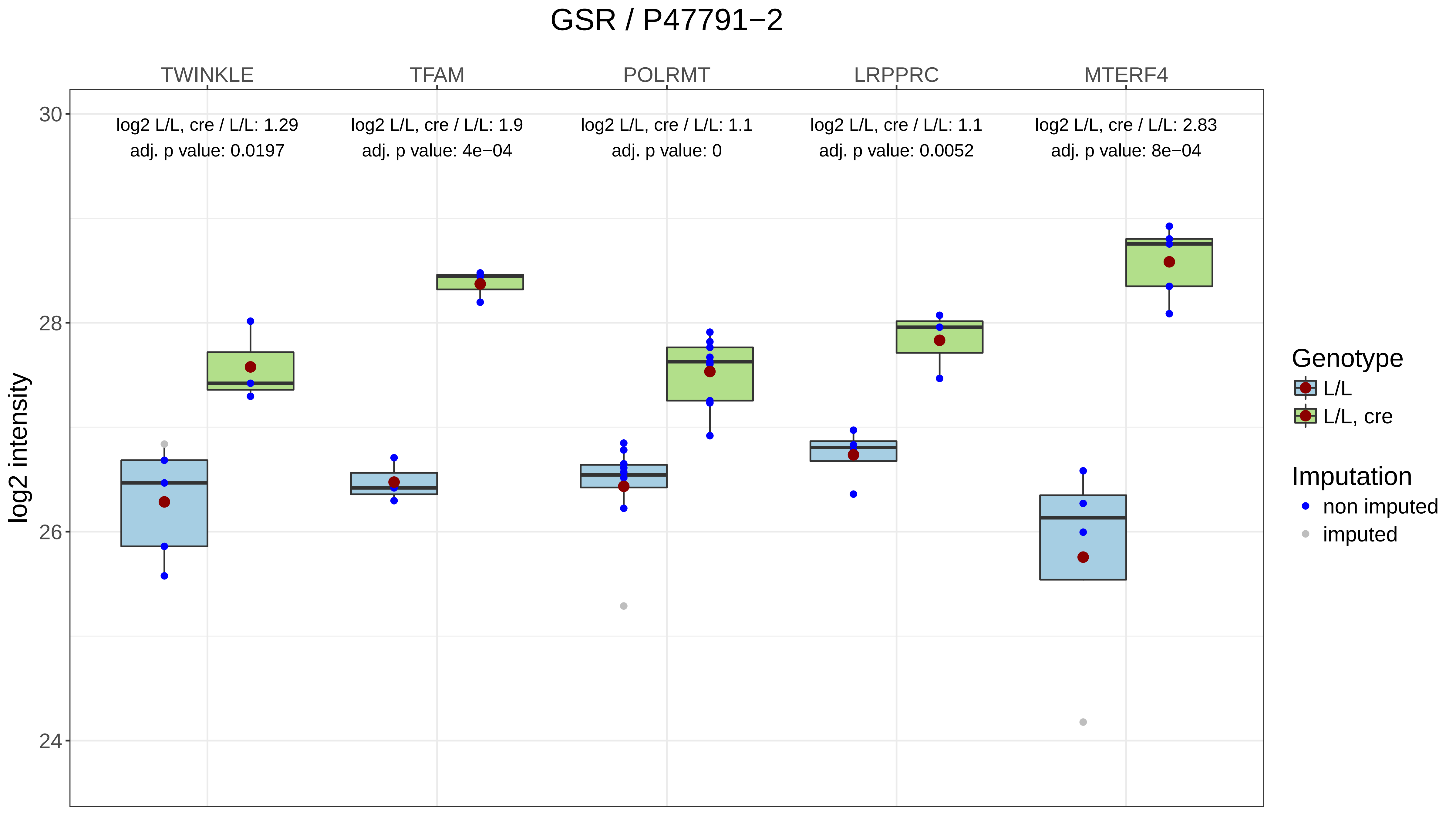
GSR / P47791−2
TWINKLE
TFAM
POLRMT
LRPPRC
MTERF4
log2 L/L, cre / L/L: 1.29
adj. p value: 0.0197
log2 L/L, cre / L/L: 1.9
adj. p value: 4e−04
log2 L/L, cre / L/L: 1.1
adj. p value: 0
log2 L/L, cre / L/L: 1.1
adj. p value: 0.0052
log2 L/L, cre / L/L: 2.83
adj. p value: 8e−04
log2 intensity
30
28
26
24
Genotype
L/L
L/L, cre
Imputation
non imputed
imputed

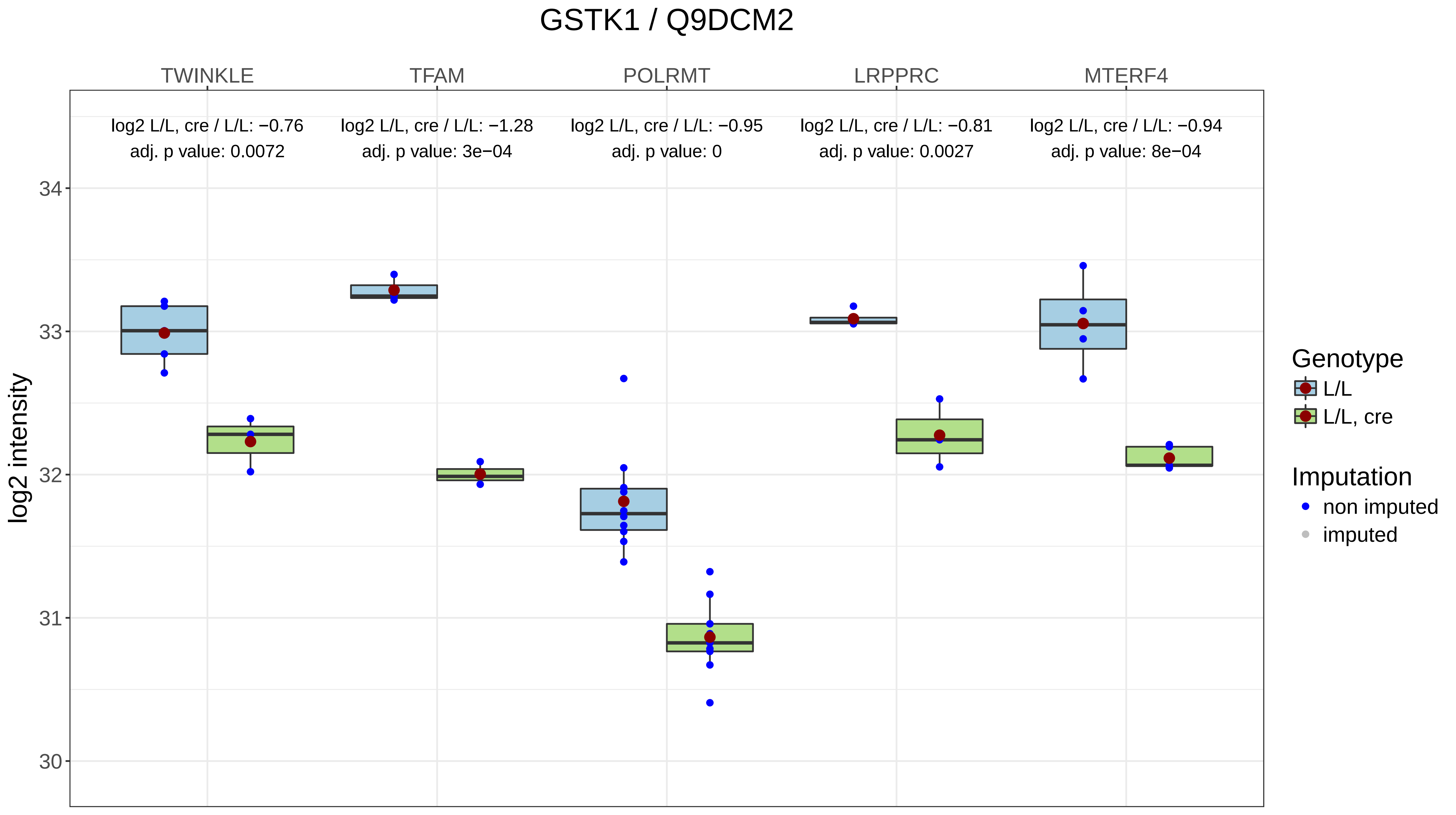

GSTK1 / Q9DCM2
TWINKLE
TFAM
POLRMT
LRPPRC
MTERF4
log2 L/L, cre / L/L: −0.76
adj. p value: 0.0072
log2 L/L, cre / L/L: −1.28
adj. p value: 3e−04
log2 L/L, cre / L/L: −0.95
adj. p value: 0
log2 L/L, cre / L/L: −0.81
adj. p value: 0.0027
log2 L/L, cre / L/L: −0.94
adj. p value: 8e−04
34
33
32
31
30
log2 intensity
Genotype
L/L
L/L, cre
Imputation
non imputed
imputed

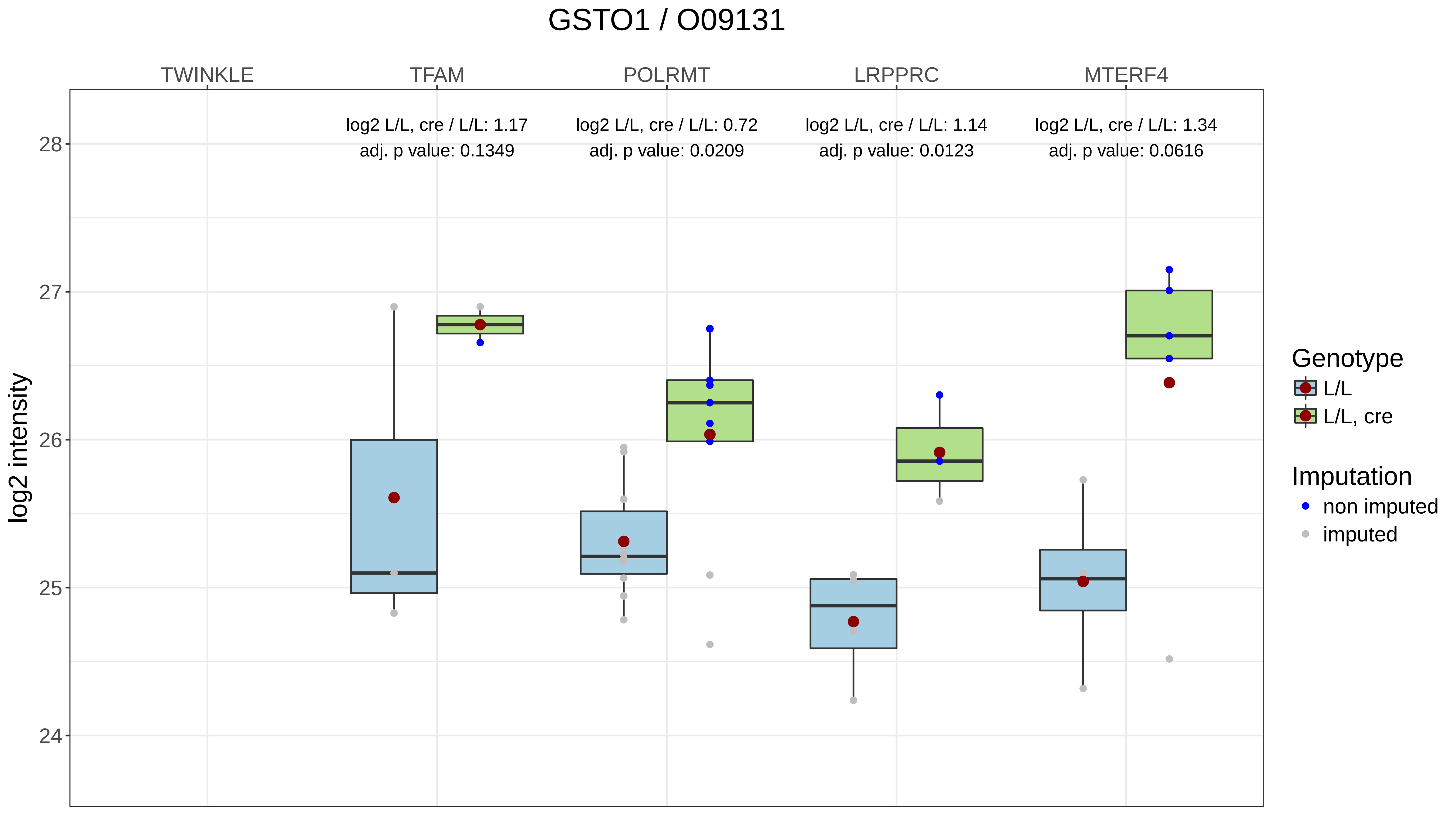
GSTO1 / O09131
TWINKLE
TFAM
POLRMT
LRPPRC
MTERF4
log2 L/L, cre / L/L: 1.17
adj. p value: 0.1349
log2 L/L, cre / L/L: 0.72
adj. p value: 0.0209
log2 L/L, cre / L/L: 1.14
adj. p value: 0.0123
log2 L/L, cre / L/L: 1.34
adj. p value: 0.0616
log2 intensity
28
27
26
25
24
Genotype
L/L
L/L, cre
Imputation
non imputed
imputed

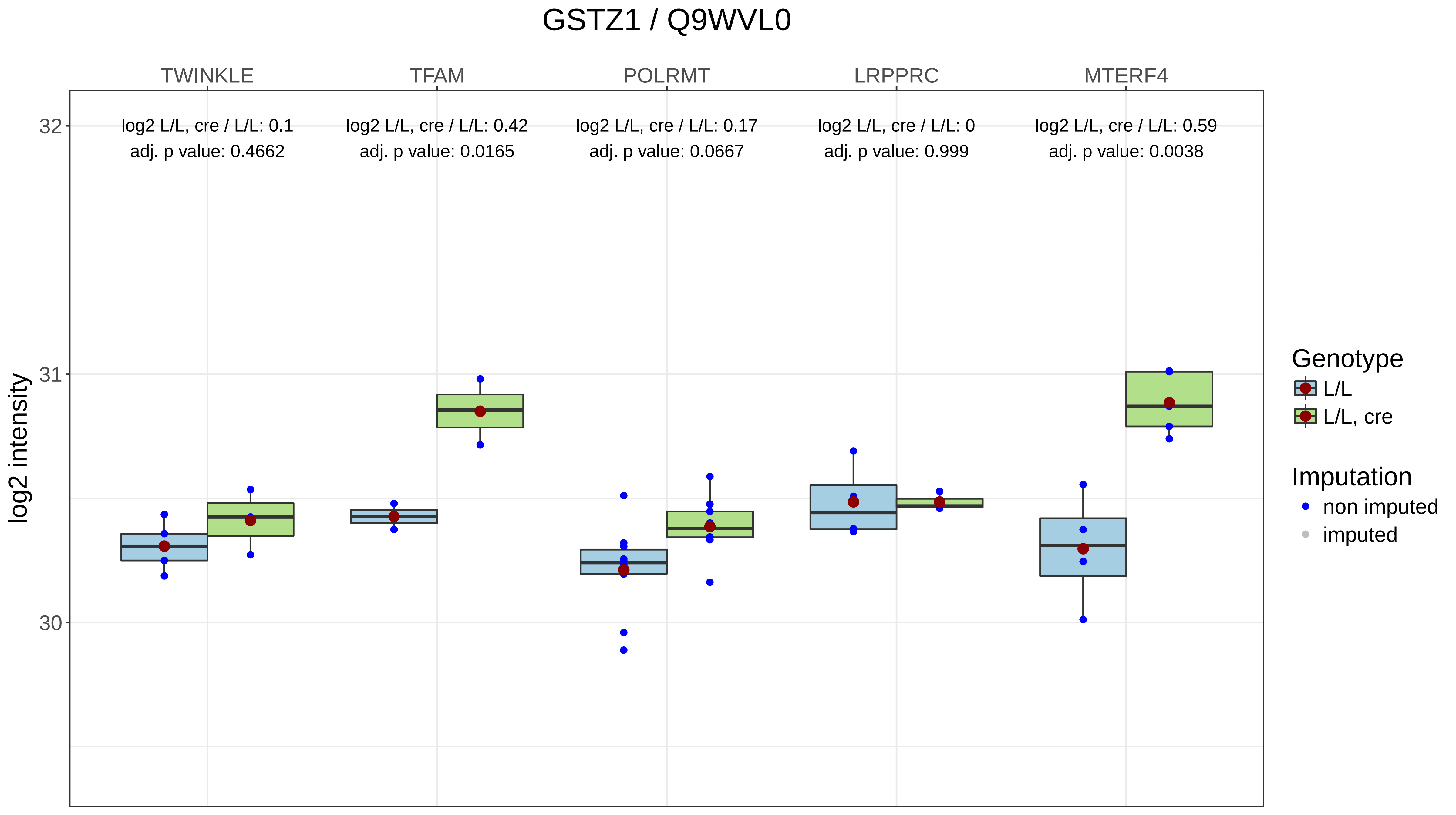
GSTZ1 / Q9WVL0
TWINKLE
TFAM
POLRMT
LRPPRC
MTERF4
log2 L/L, cre / L/L: 0.1
adj. p value: 0.4662
log2 L/L, cre / L/L: 0.42
adj. p value: 0.0165
log2 L/L, cre / L/L: 0.17
adj. p value: 0.0667
log2 L/L, cre / L/L: 0
adj. p value: 0.999
log2 L/L, cre / L/L: 0.59
adj. p value: 0.0038
32
31
30
log2 intensity
Genotype
L/L
L/L, cre
Imputation
non imputed
imputed

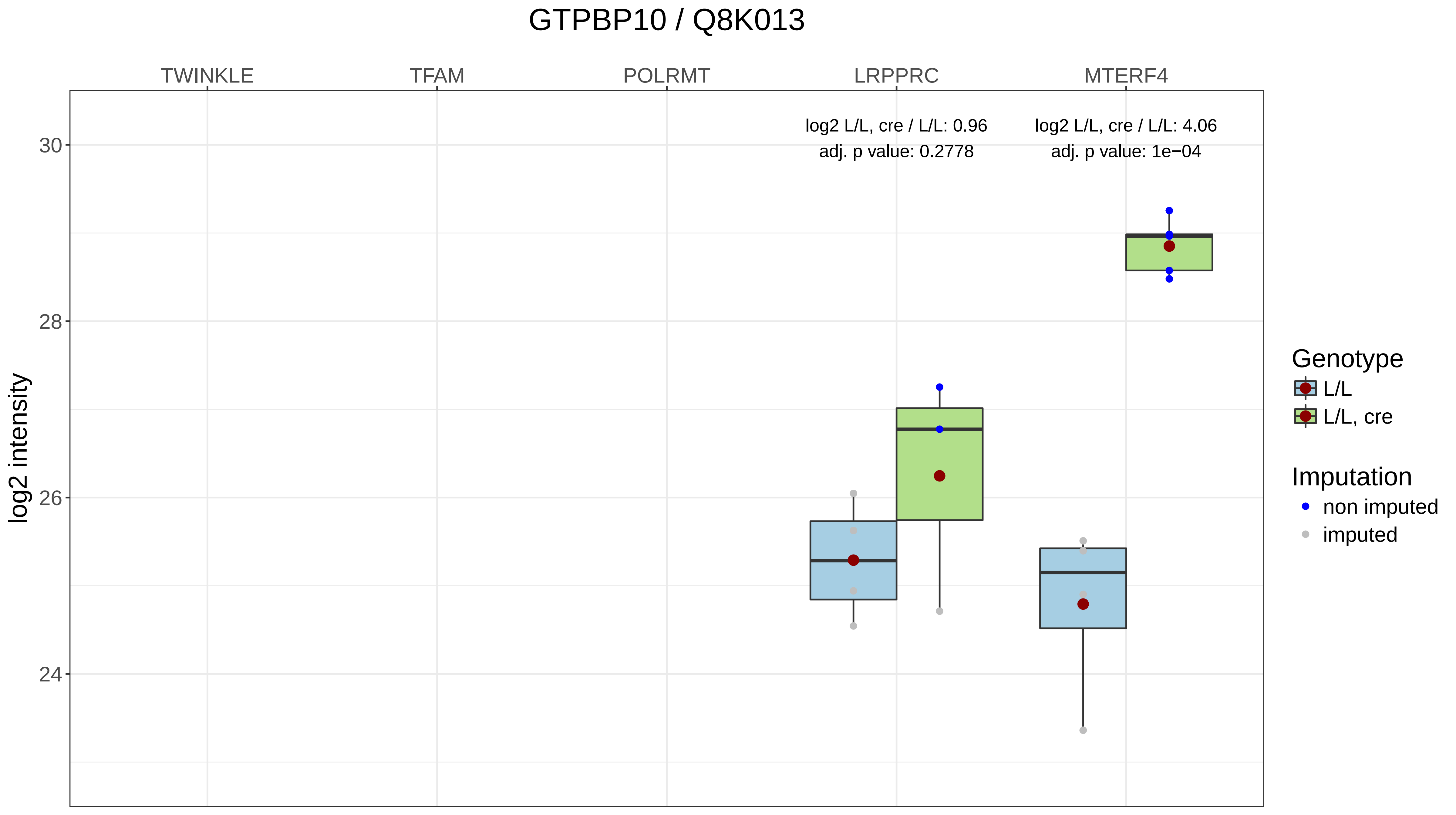
GTPBP10 / Q8K013
TWINKLE
TFAM
POLRMT
LRPPRC
MTERF4
log2 L/L, cre / L/L: 0.96
adj. p value: 0.2778
log2 L/L, cre / L/L: 4.06
adj. p value: 1e−04
log2 intensity
30
28
26
24
Genotype
L/L
L/L, cre
Imputation
non imputed
imputed

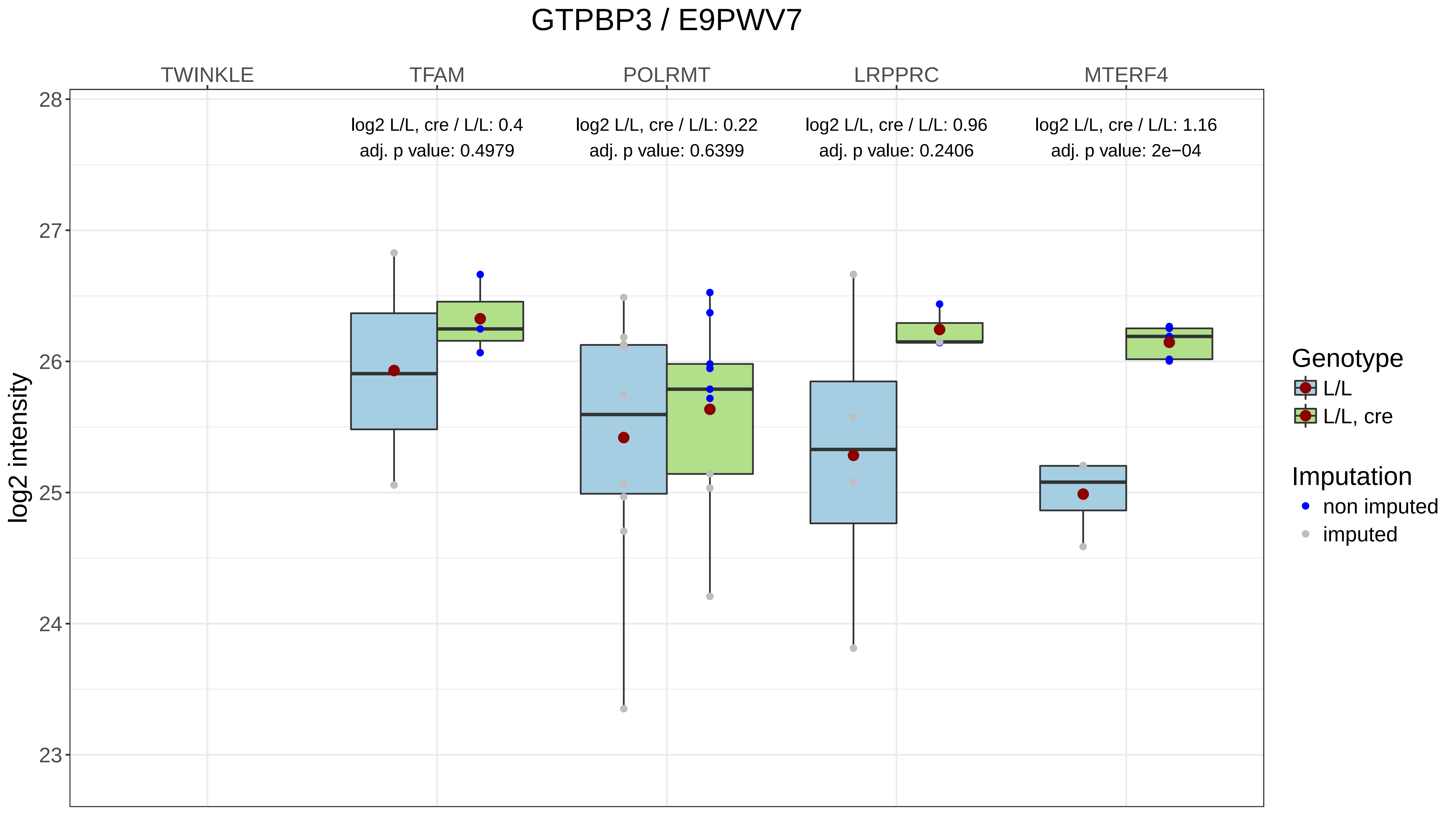
GTPBP3 / E9PWV7
TWINKLE
TFAM
POLRMT
LRPPRC
MTERF4
log2 L/L, cre / L/L: 0.4
adj. p value: 0.4979
log2 L/L, cre / L/L: 0.22
adj. p value: 0.6399
log2 L/L, cre / L/L: 0.96
adj. p value: 0.2406
log2 L/L, cre / L/L: 1.16
adj. p value: 2e−04
log2 intensity
28
27
26
25
24
23
Genotype
L/L
L/L, cre
Imputation
non imputed
imputed

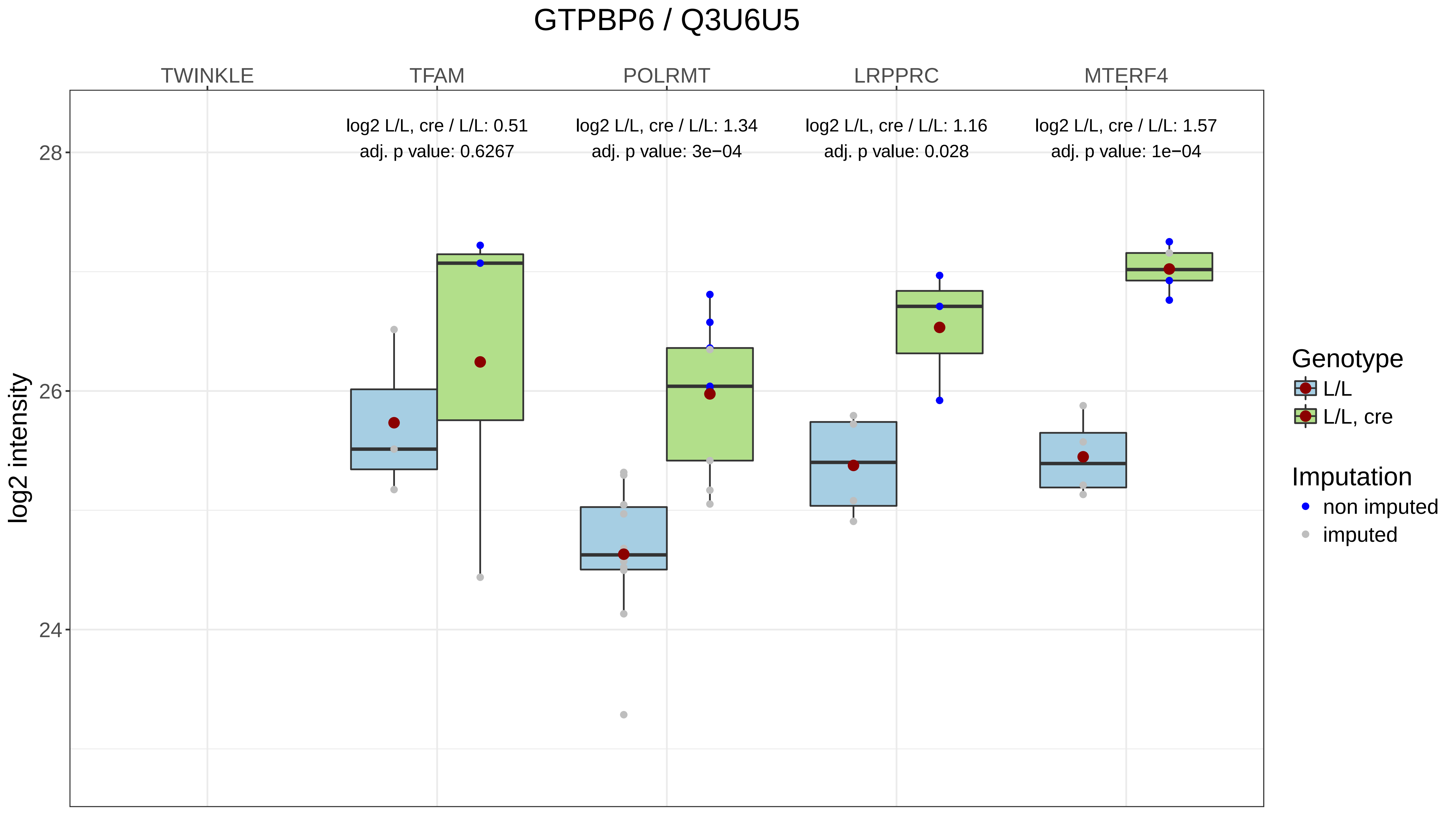

GTPBP6 / Q3U6U5
TWINKLE
TFAM
POLRMT
LRPPRC
MTERF4
log2 L/L, cre / L/L: 0.51
adj. p value: 0.6267
log2 L/L, cre / L/L: 1.34
adj. p value: 3e−04
log2 L/L, cre / L/L: 1.16
adj. p value: 0.028
log2 L/L, cre / L/L: 1.57
adj. p value: 1e−04
28
26
24
log2 intensity
Genotype
L/L
L/L, cre
Imputation
non imputed
imputed

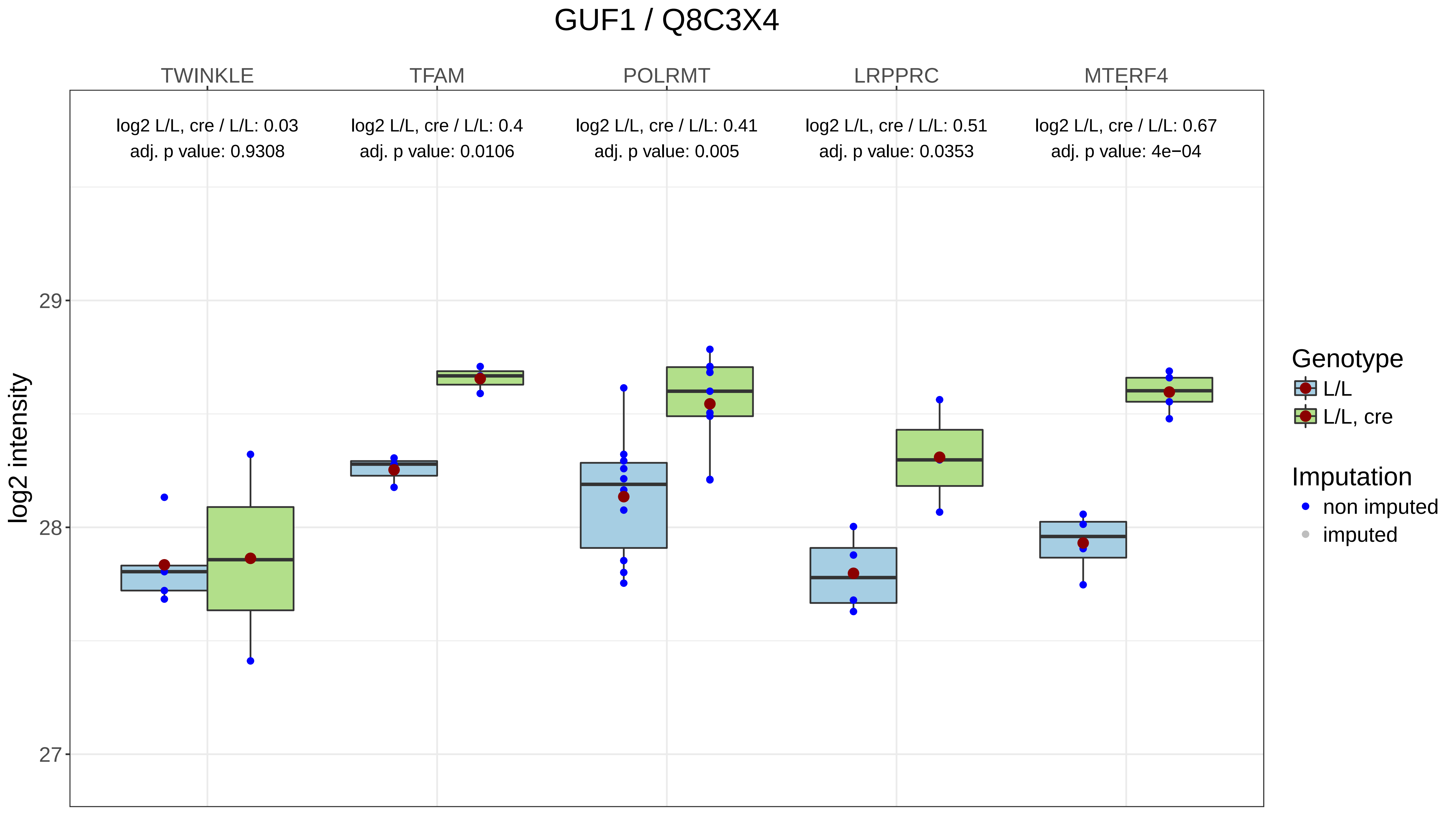
GUF1 / Q8C3X4
TWINKLE
TFAM
POLRMT
LRPPRC
MTERF4
log2 L/L, cre / L/L: 0.03
adj. p value: 0.9308
log2 L/L, cre / L/L: 0.4
adj. p value: 0.0106
log2 L/L, cre / L/L: 0.41
adj. p value: 0.005
log2 L/L, cre / L/L: 0.51
adj. p value: 0.0353
log2 L/L, cre / L/L: 0.67
adj. p value: 4e−04
log2 intensity
29
28
27
Genotype
L/L
L/L, cre
Imputation
non imputed
imputed

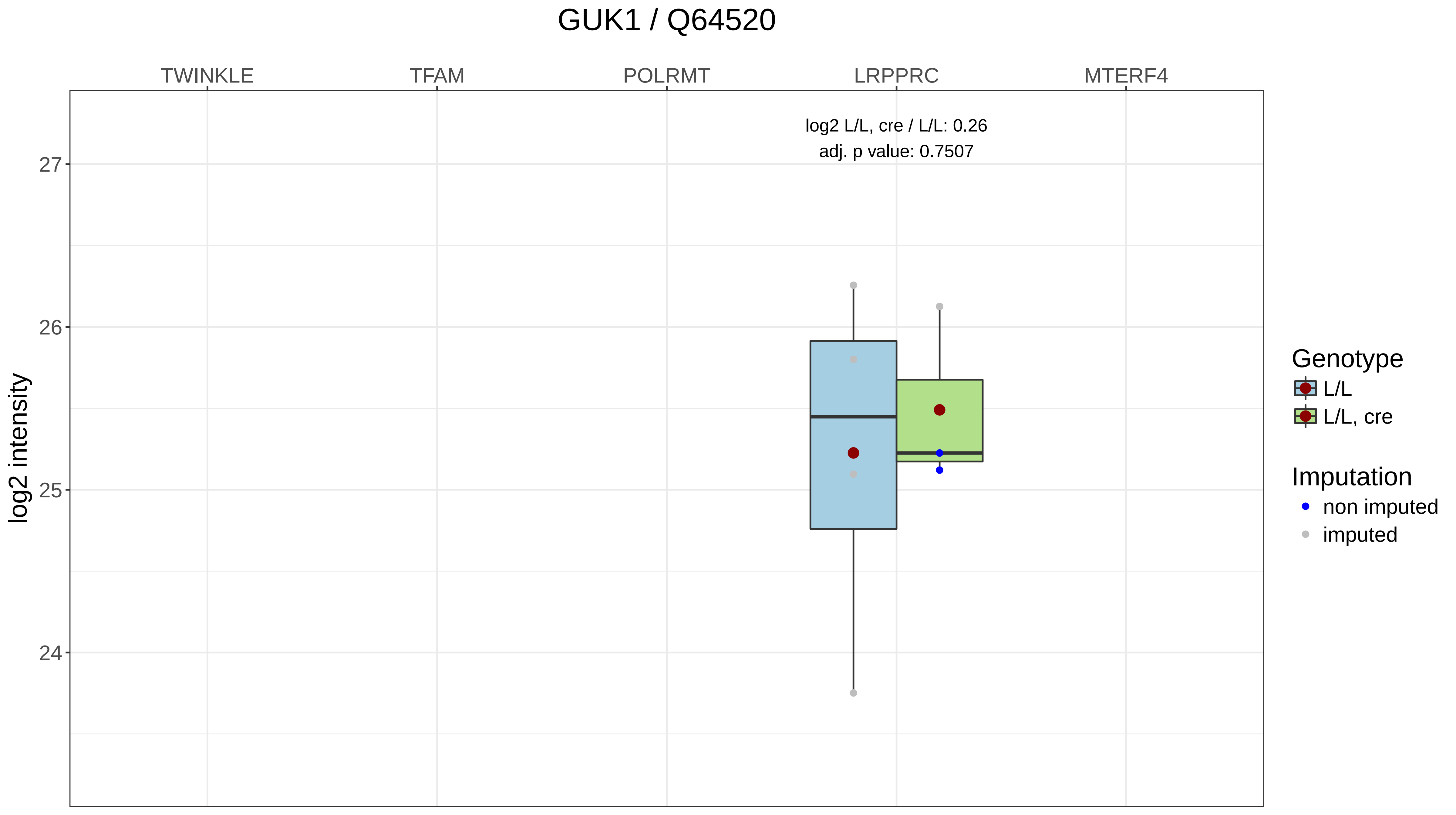
GUK1 / Q64520
TWINKLE
TFAM
POLRMT
LRPPRC
MTERF4
log2 L/L, cre / L/L: 0.26
adj. p value: 0.7507
log2 intensity
27
26
25
24
Genotype
L/L
L/L, cre
Imputation
non imputed
imputed

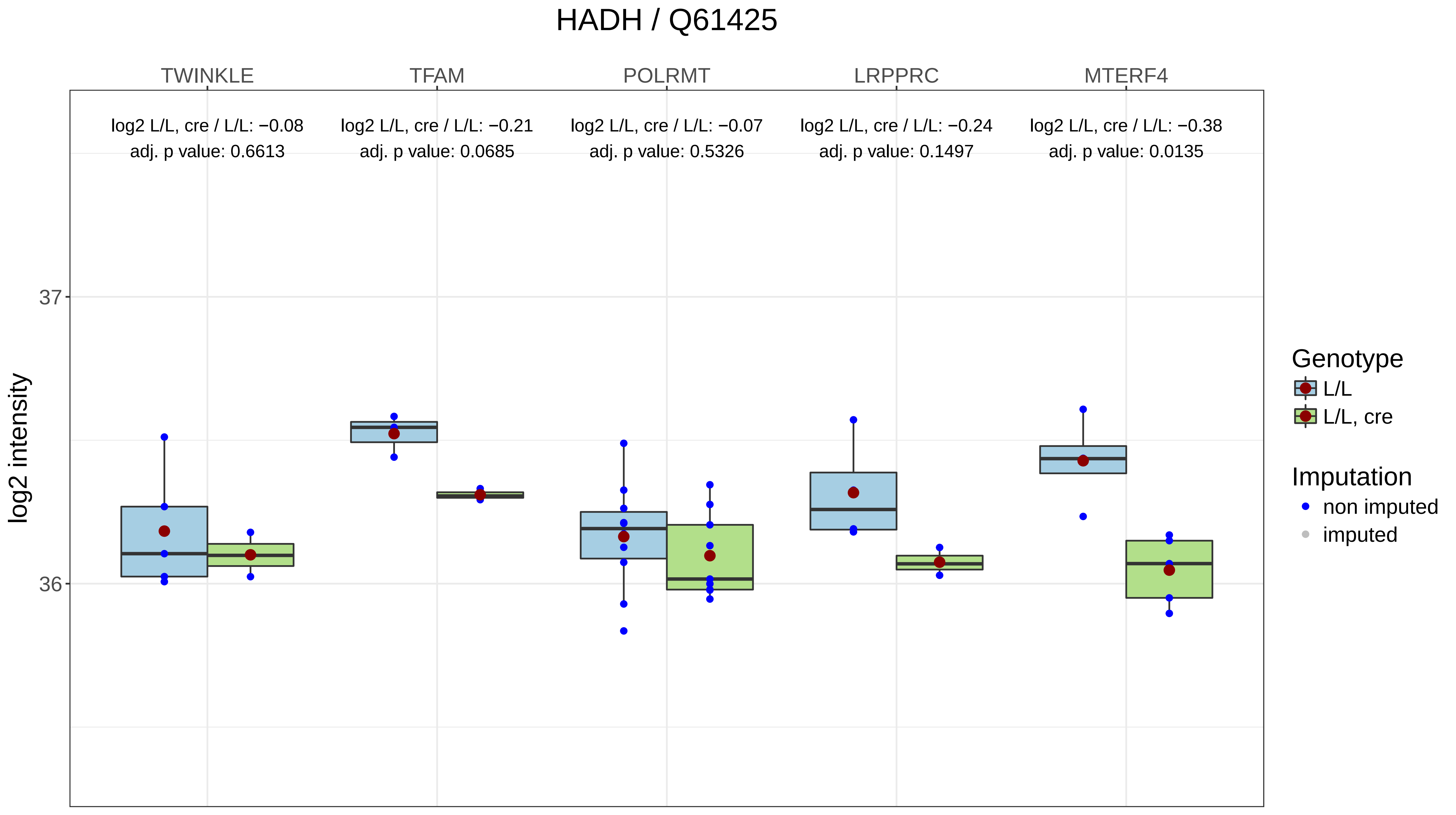

HADH / Q61425
TWINKLE
TFAM
POLRMT
LRPPRC
MTERF4
log2 L/L, cre / L/L: −0.08
adj. p value: 0.6613
log2 L/L, cre / L/L: −0.21
adj. p value: 0.0685
log2 L/L, cre / L/L: −0.07
adj. p value: 0.5326
log2 L/L, cre / L/L: −0.24
adj. p value: 0.1497
log2 L/L, cre / L/L: −0.38
adj. p value: 0.0135
log2 intensity
37
36
Genotype
L/L
L/L, cre
Imputation
non imputed
imputed

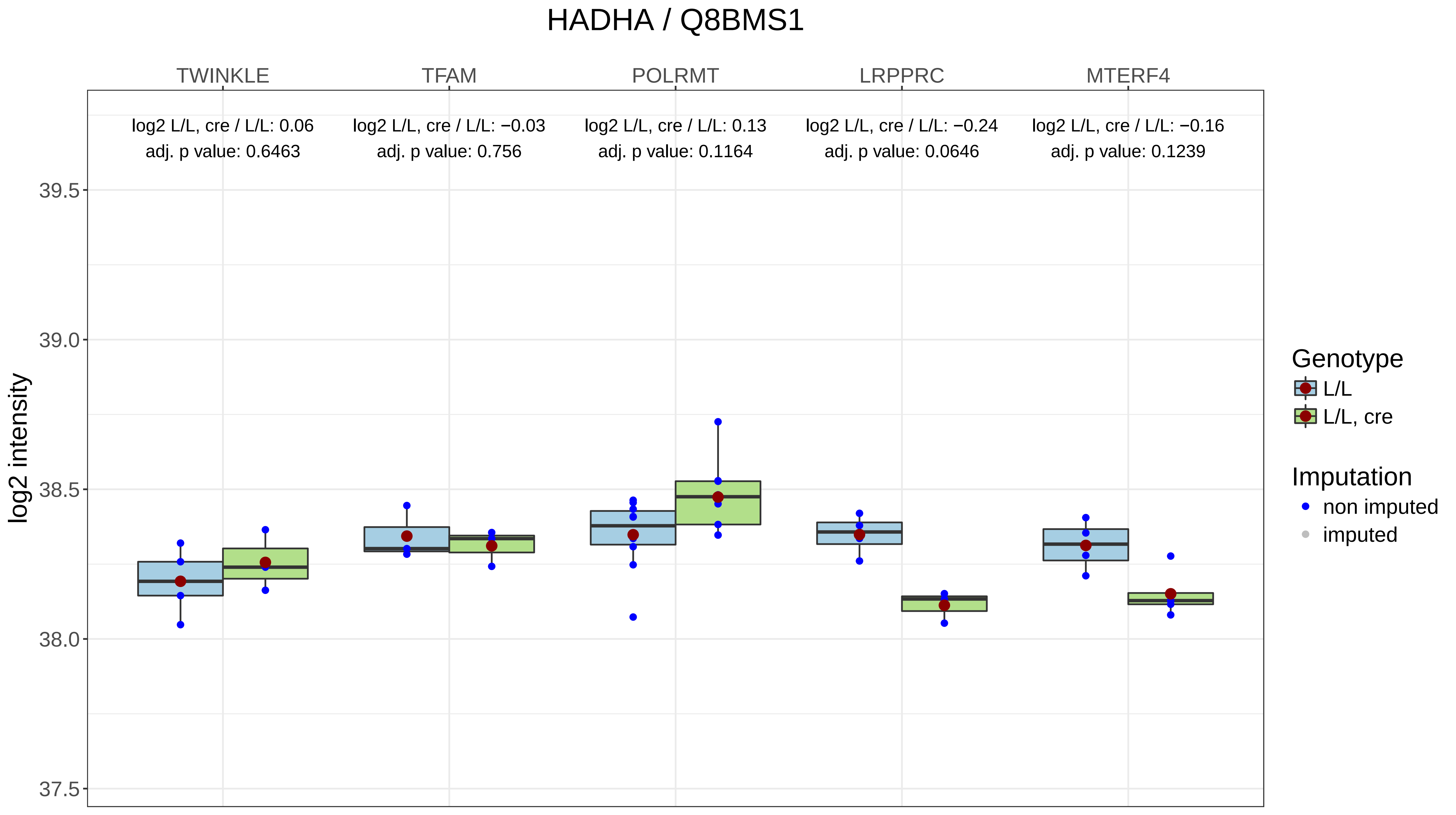
HADHA / Q8BMS1
TWINKLE
TFAM
POLRMT
LRPPRC
MTERF4
log2 L/L, cre / L/L: 0.06
adj. p value: 0.6463
log2 L/L, cre / L/L: −0.03
adj. p value: 0.756
log2 L/L, cre / L/L: 0.13
adj. p value: 0.1164
log2 L/L, cre / L/L: −0.24
adj. p value: 0.0646
log2 L/L, cre / L/L: −0.16
adj. p value: 0.1239
log2 intensity
39.5
39.0
38.5
38.0
37.5
Genotype
L/L
L/L, cre
Imputation
non imputed
imputed

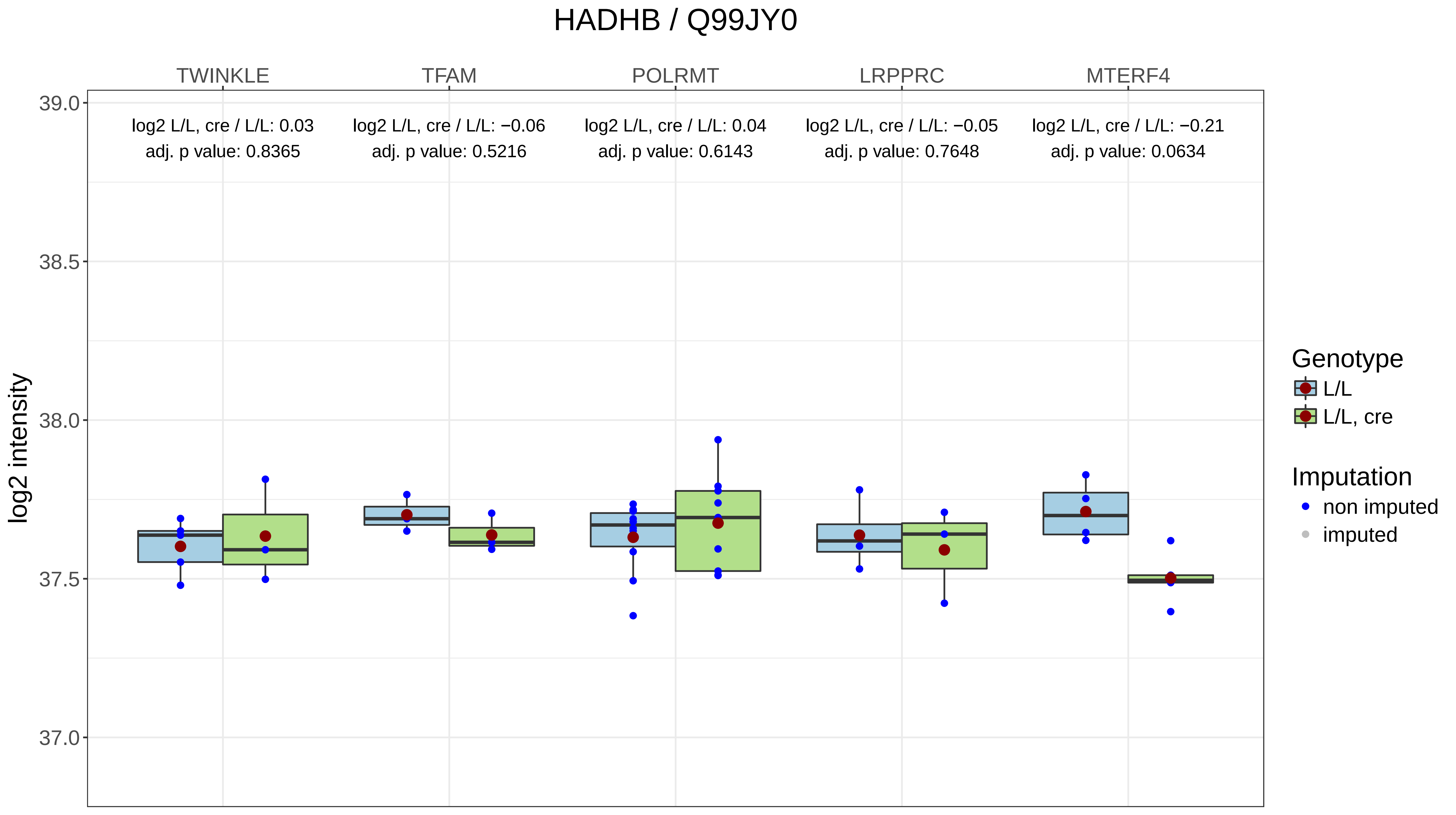
HADHB / Q99JY0
TWINKLE
TFAM
POLRMT
LRPPRC
MTERF4
log2 L/L, cre / L/L: 0.03
adj. p value: 0.8365
log2 L/L, cre / L/L: −0.06
adj. p value: 0.5216
log2 L/L, cre / L/L: 0.04
adj. p value: 0.6143
log2 L/L, cre / L/L: −0.05
adj. p value: 0.7648
log2 L/L, cre / L/L: −0.21
adj. p value: 0.0634
log2 intensity
39.0
38.5
38.0
37.5
37.0
Genotype
L/L
L/L, cre
Imputation
non imputed
imputed

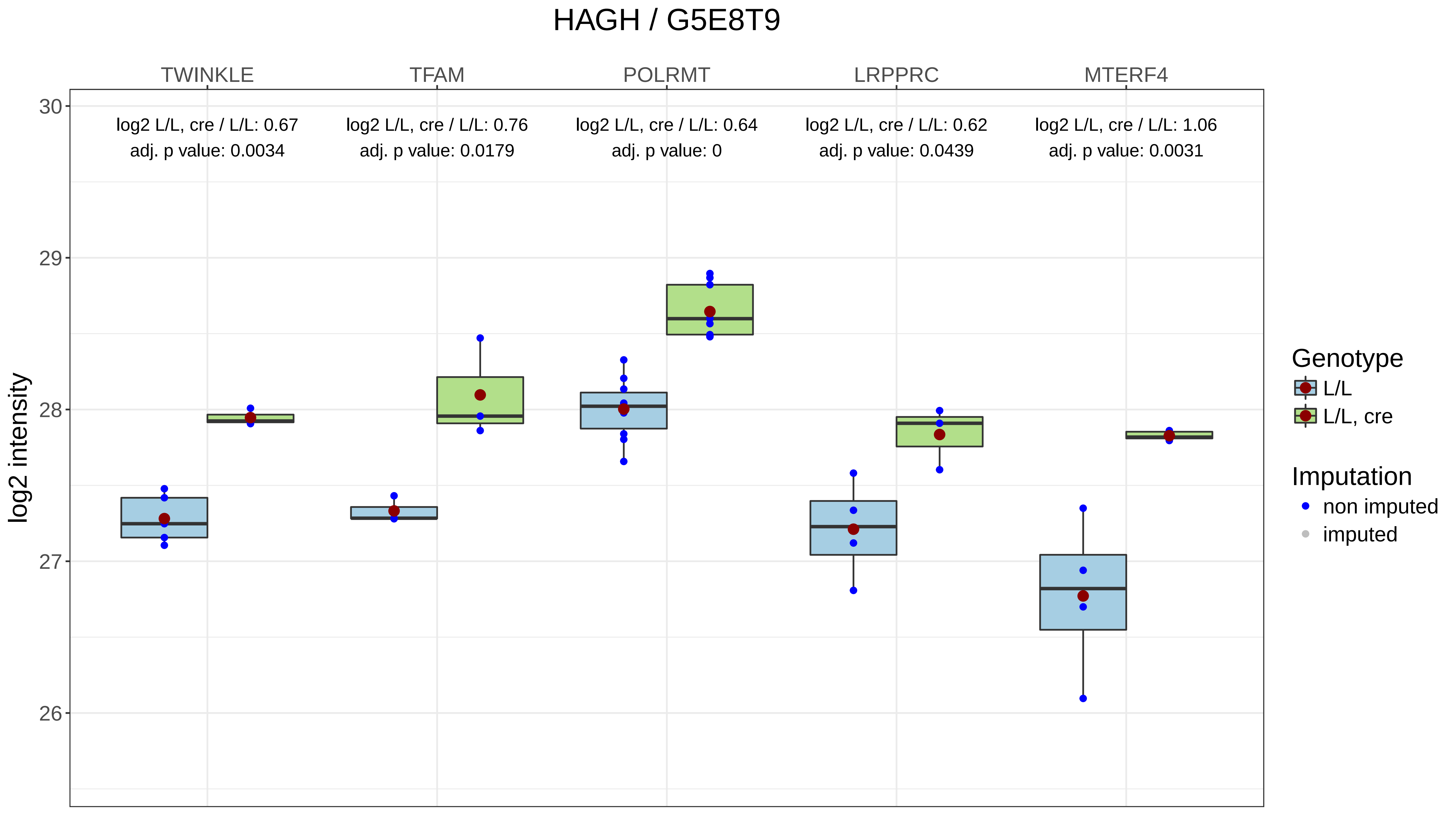
HAGH / G5E8T9
TWINKLE
TFAM
POLRMT
LRPPRC
MTERF4
log2 L/L, cre / L/L: 0.67
adj. p value: 0.0034
log2 L/L, cre / L/L: 0.76
adj. p value: 0.0179
log2 L/L, cre / L/L: 0.64
adj. p value: 0
log2 L/L, cre / L/L: 0.62
adj. p value: 0.0439
log2 L/L, cre / L/L: 1.06
adj. p value: 0.0031
30
29
28
27
26
log2 intensity
Genotype
L/L
L/L, cre
Imputation
non imputed
imputed

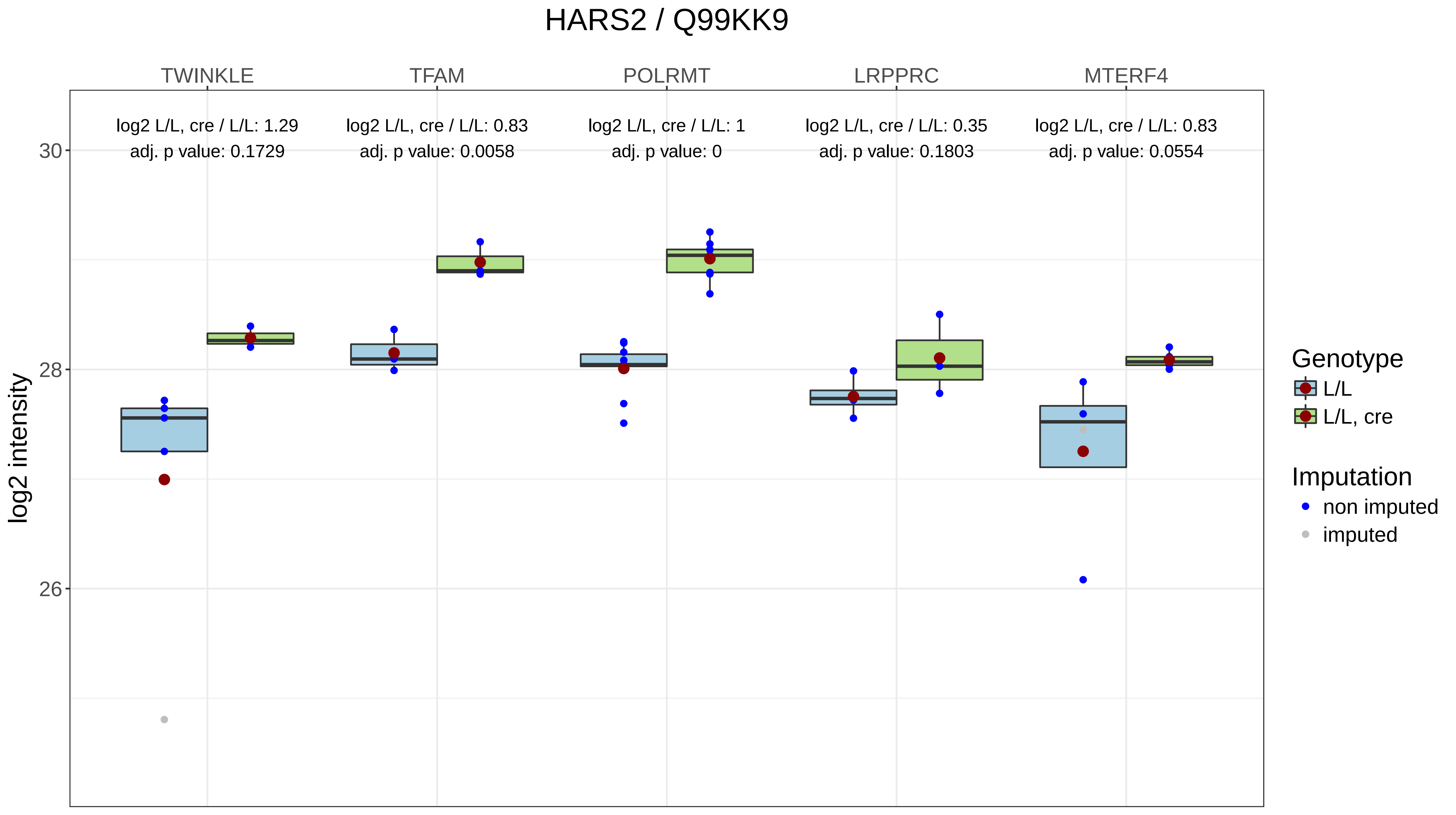
HARS2 / Q99KK9
TWINKLE
TFAM
POLRMT
LRPPRC
MTERF4
log2 L/L, cre / L/L: 1.29
adj. p value: 0.1729
log2 L/L, cre / L/L: 0.83
adj. p value: 0.0058
log2 L/L, cre / L/L: 1
adj. p value: 0
log2 L/L, cre / L/L: 0.35
adj. p value: 0.1803
log2 L/L, cre / L/L: 0.83
adj. p value: 0.0554
30
28
26
log2 intensity
Genotype
L/L
L/L, cre
Imputation
non imputed
imputed

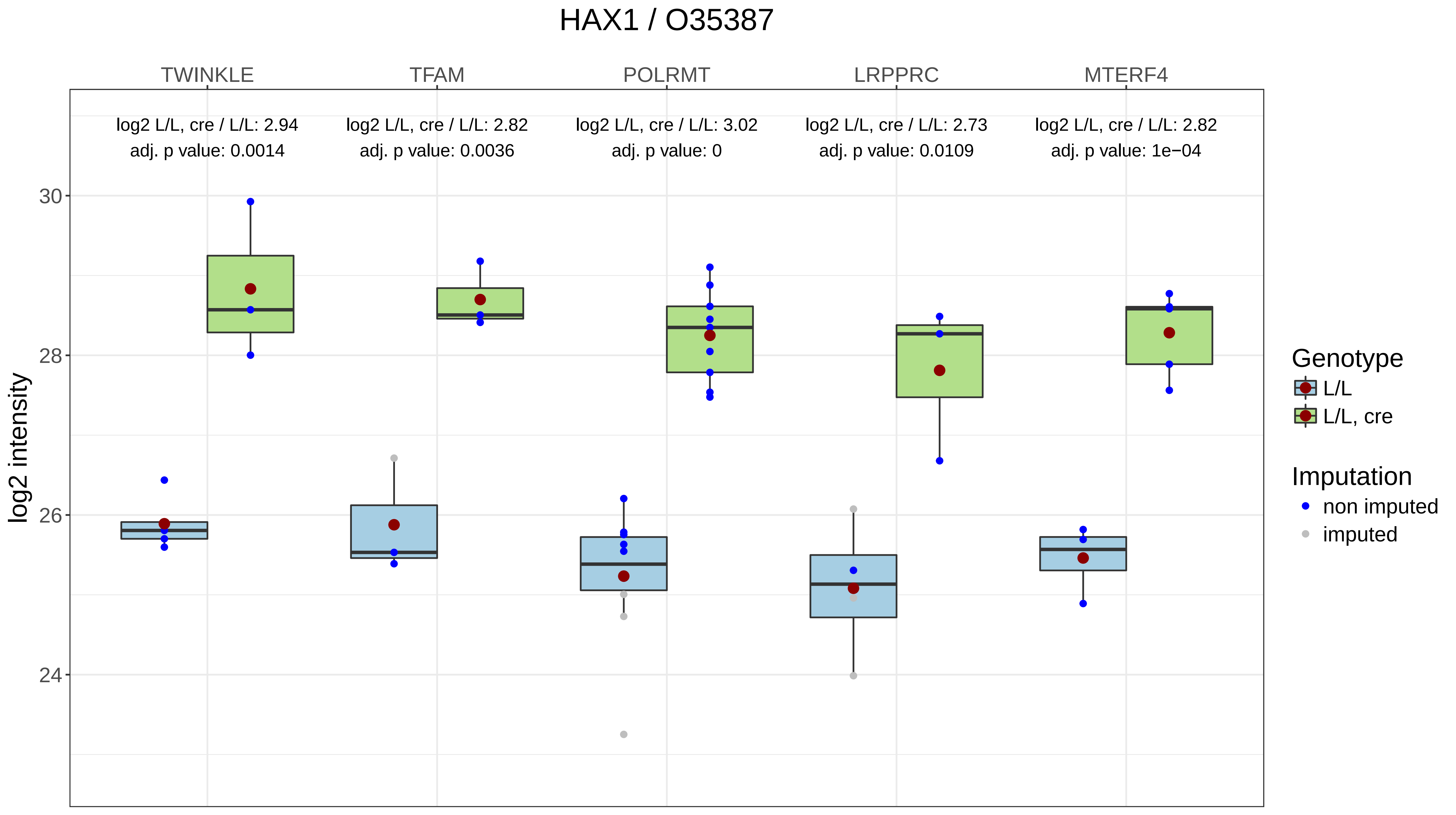

HAX1 / O35387
TWINKLE
TFAM
POLRMT
LRPPRC
MTERF4
log2 L/L, cre / L/L: 2.94
adj. p value: 0.0014
log2 L/L, cre / L/L: 2.82
adj. p value: 0.0036
log2 L/L, cre / L/L: 3.02
adj. p value: 0
log2 L/L, cre / L/L: 2.73
adj. p value: 0.0109
log2 L/L, cre / L/L: 2.82
adj. p value: 1e−04
log2 intensity
30
28
26
24
Genotype
L/L
L/L, cre
Imputation
non imputed
imputed

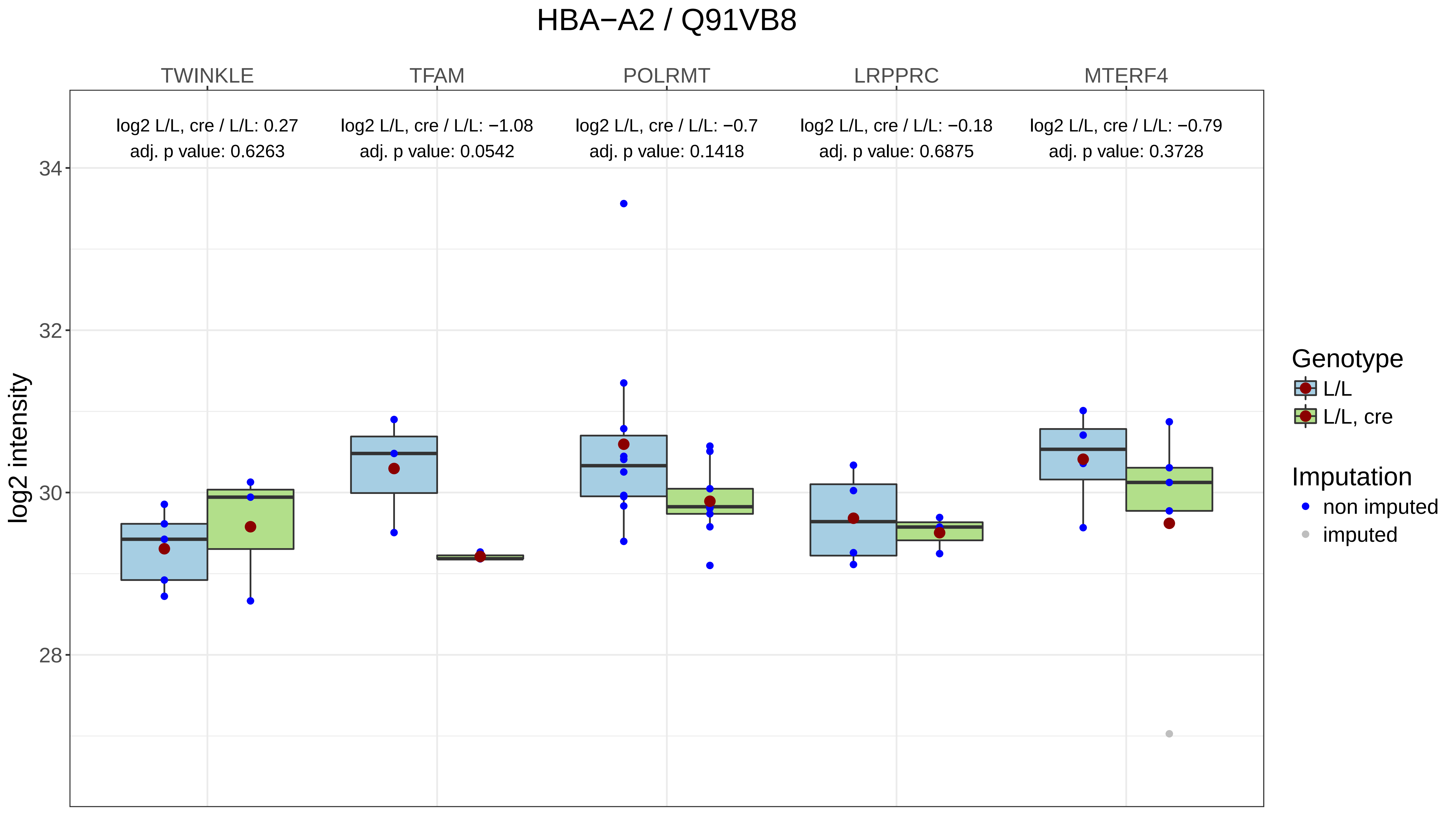

HBA−A2 / Q91VB8
TWINKLE
TFAM
POLRMT
LRPPRC
MTERF4
log2 L/L, cre / L/L: 0.27
adj. p value: 0.6263
log2 L/L, cre / L/L: −1.08
adj. p value: 0.0542
log2 L/L, cre / L/L: −0.7
adj. p value: 0.1418
log2 L/L, cre / L/L: −0.18
adj. p value: 0.6875
log2 L/L, cre / L/L: −0.79
adj. p value: 0.3728
log2 intensity
34
32
30
28
Genotype
L/L
L/L, cre
Imputation
non imputed
imputed

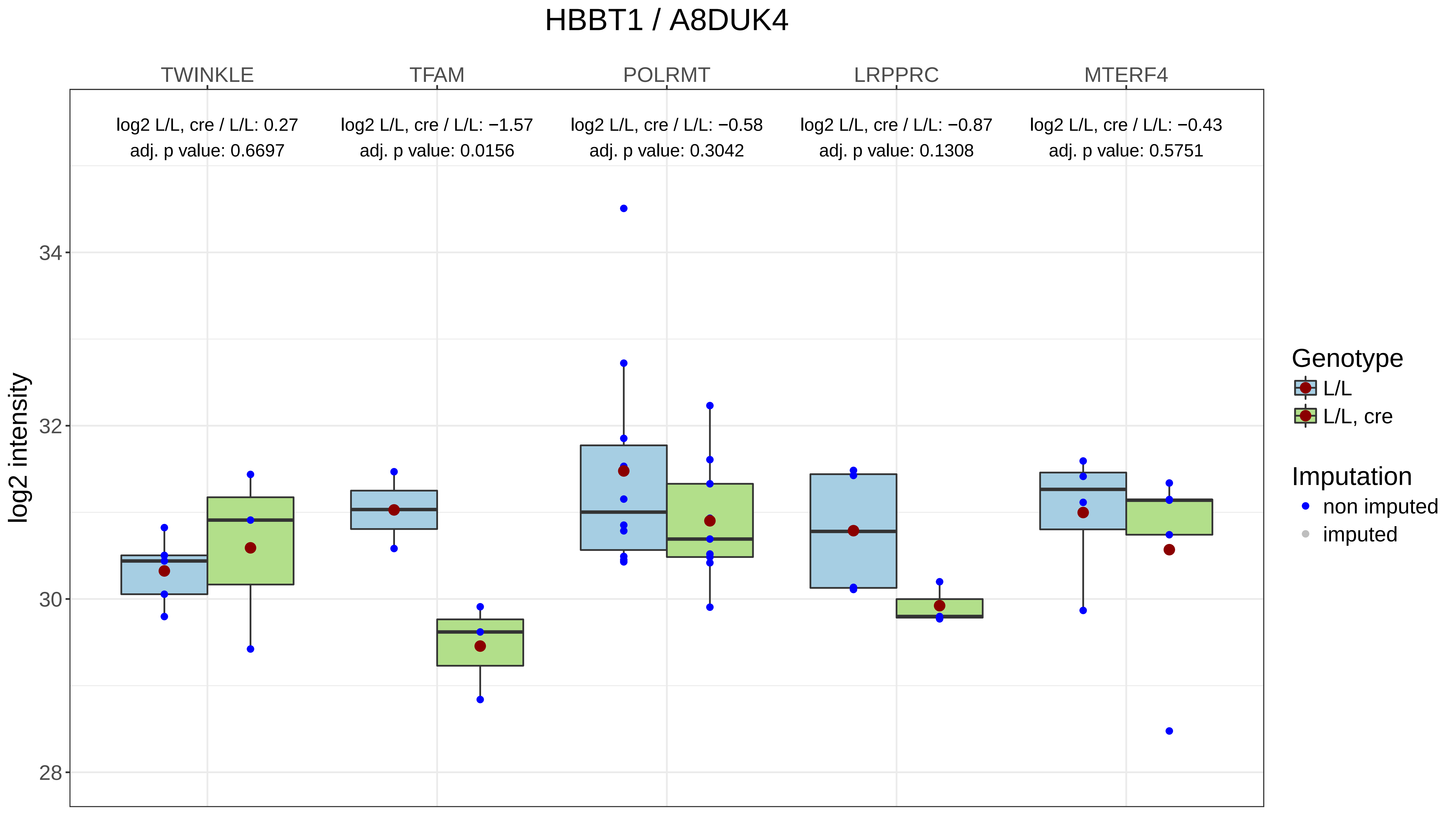
HBBT1 / A8DUK4
TWINKLE
TFAM
POLRMT
LRPPRC
MTERF4
log2 L/L, cre / L/L: 0.27
adj. p value: 0.6697
log2 L/L, cre / L/L: −1.57
adj. p value: 0.0156
log2 L/L, cre / L/L: −0.58
adj. p value: 0.3042
log2 L/L, cre / L/L: −0.87
adj. p value: 0.1308
log2 L/L, cre / L/L: −0.43
adj. p value: 0.5751
log2 intensity
34
32
30
28
Genotype
L/L
L/L, cre
Imputation
non imputed
imputed

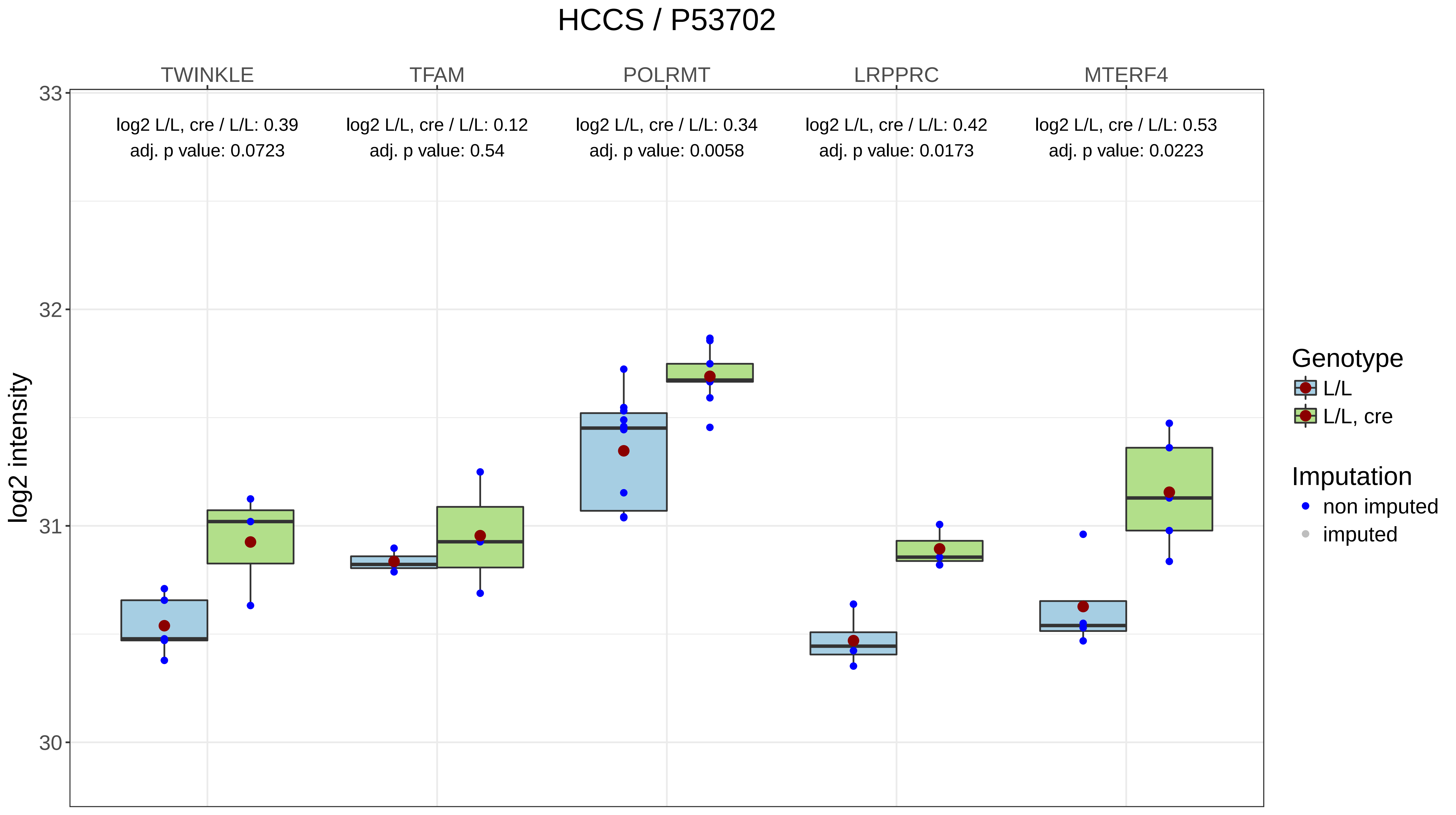

HCCS / P53702
TWINKLE
TFAM
POLRMT
LRPPRC
MTERF4
log2 L/L, cre / L/L: 0.39
adj. p value: 0.0723
log2 L/L, cre / L/L: 0.12
adj. p value: 0.54
log2 L/L, cre / L/L: 0.34
adj. p value: 0.0058
log2 L/L, cre / L/L: 0.42
adj. p value: 0.0173
log2 L/L, cre / L/L: 0.53
adj. p value: 0.0223
log2 intensity
33
32
31
30
Genotype
L/L
L/L, cre
Imputation
non imputed
imputed

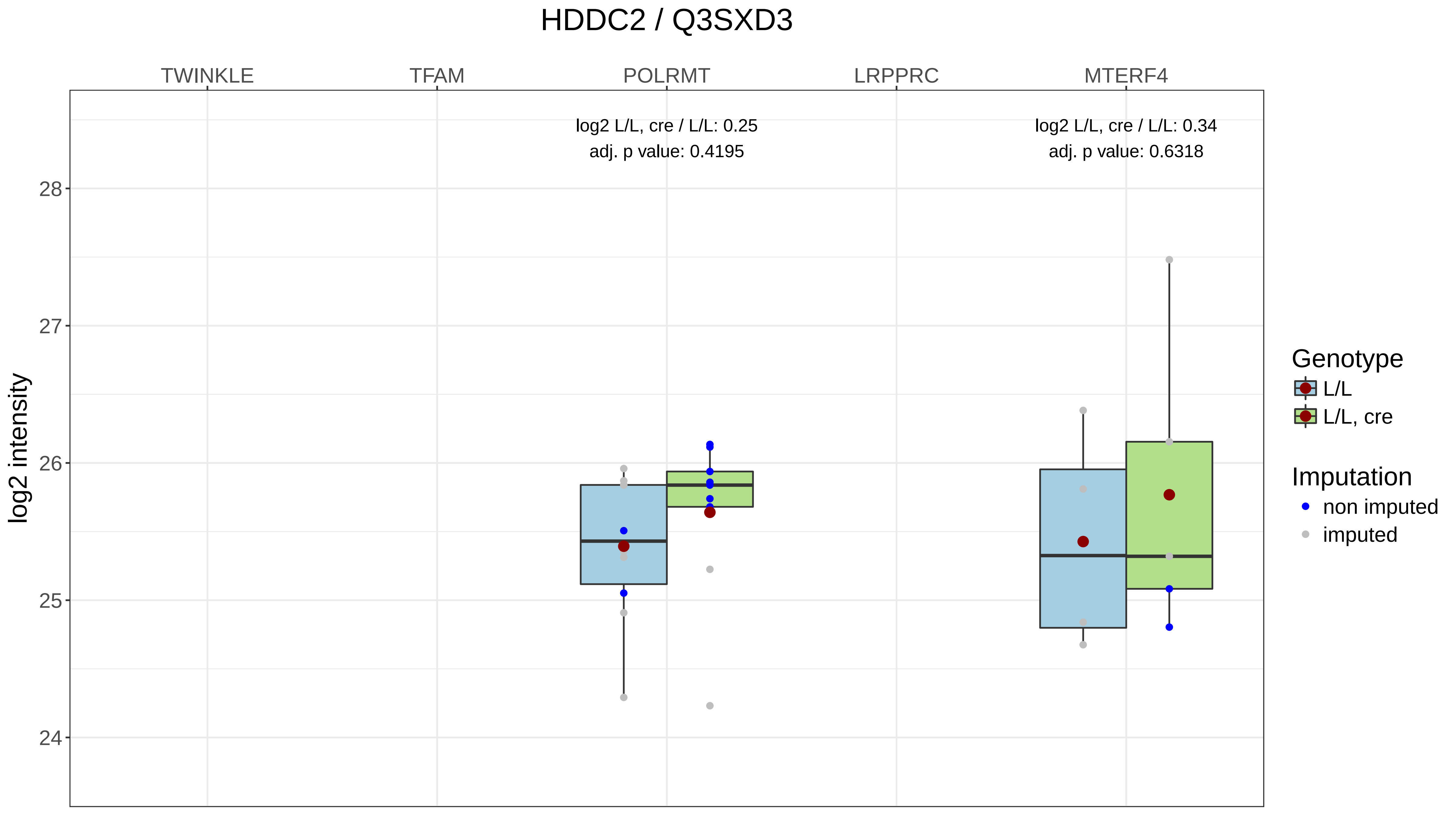
HDDC2 / Q3SXD3
TWINKLE
TFAM
POLRMT
LRPPRC
MTERF4
log2 L/L, cre / L/L: 0.25
adj. p value: 0.4195
log2 L/L, cre / L/L: 0.34
adj. p value: 0.6318
log2 intensity
28
27
26
25
24
Genotype
L/L
L/L, cre
Imputation
non imputed
imputed

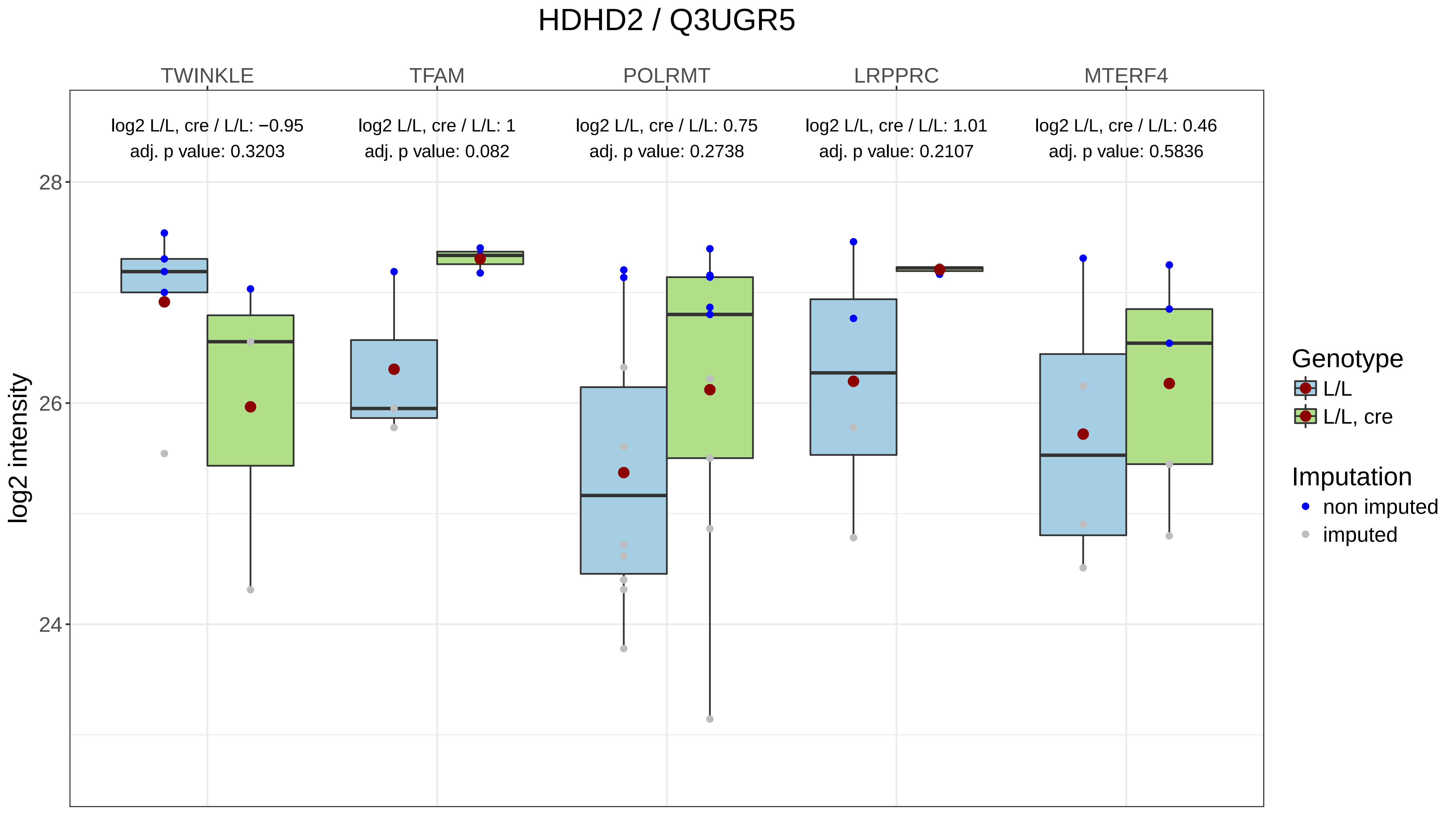

HDHD2 / Q3UGR5
TWINKLE
TFAM
POLRMT
LRPPRC
MTERF4
log2 L/L, cre / L/L: −0.95
adj. p value: 0.3203
log2 L/L, cre / L/L: 1
adj. p value: 0.082
log2 L/L, cre / L/L: 0.75
adj. p value: 0.2738
log2 L/L, cre / L/L: 1.01
adj. p value: 0.2107
log2 L/L, cre / L/L: 0.46
adj. p value: 0.5836
28
26
24
log2 intensity
Genotype
L/L
L/L, cre
Imputation
non imputed
imputed

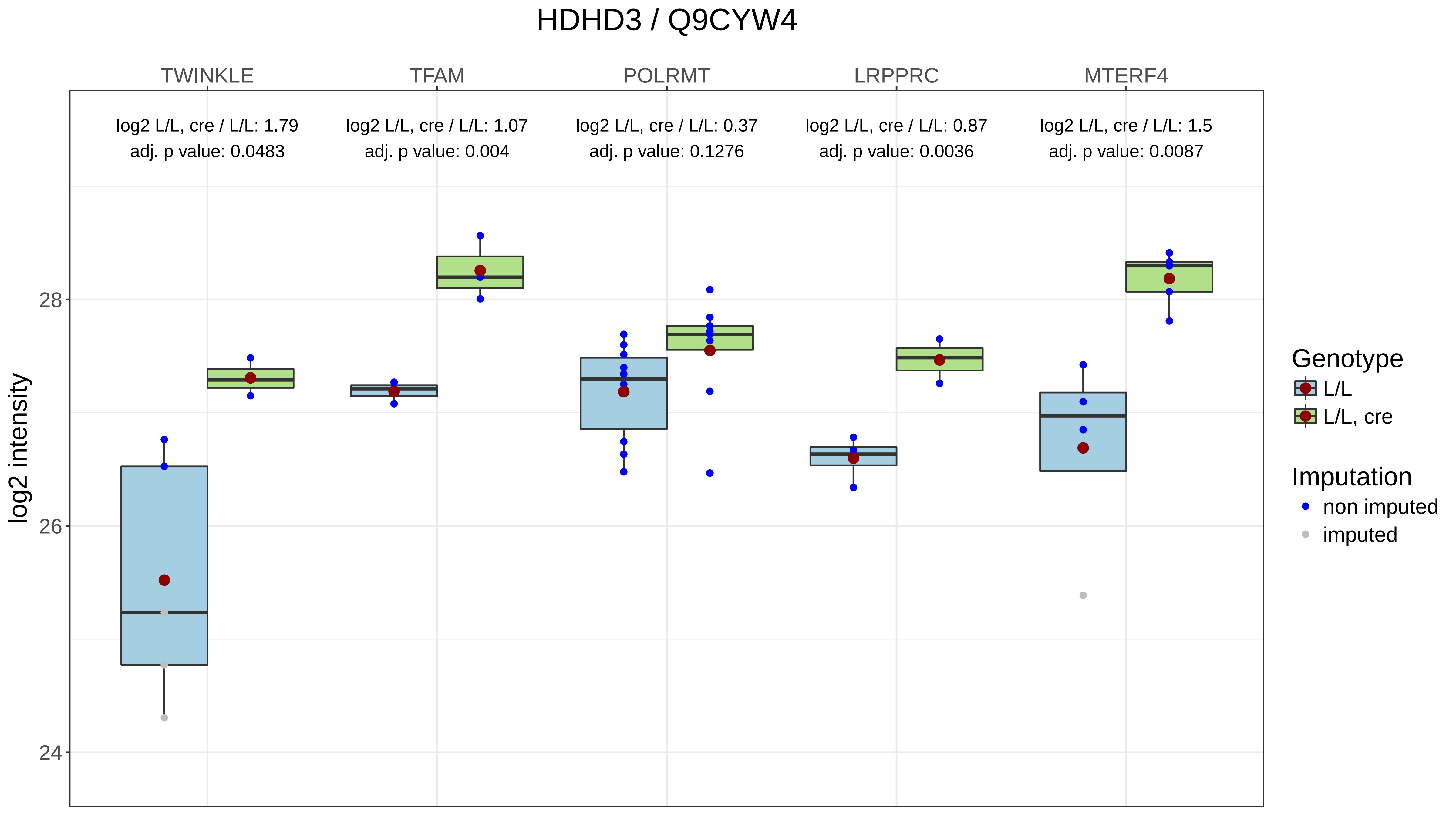

HDHD3 / Q9CYW4
TWINKLE
TFAM
POLRMT
LRPPRC
MTERF4
log2 L/L, cre / L/L: 1.79
adj. p value: 0.0483
log2 L/L, cre / L/L: 1.07
adj. p value: 0.004
log2 L/L, cre / L/L: 0.37
adj. p value: 0.1276
log2 L/L, cre / L/L: 0.87
adj. p value: 0.0036
log2 L/L, cre / L/L: 1.5
adj. p value: 0.0087
log2 intensity
28
26
24
Genotype
L/L
L/L, cre
Imputation
non imputed
imputed

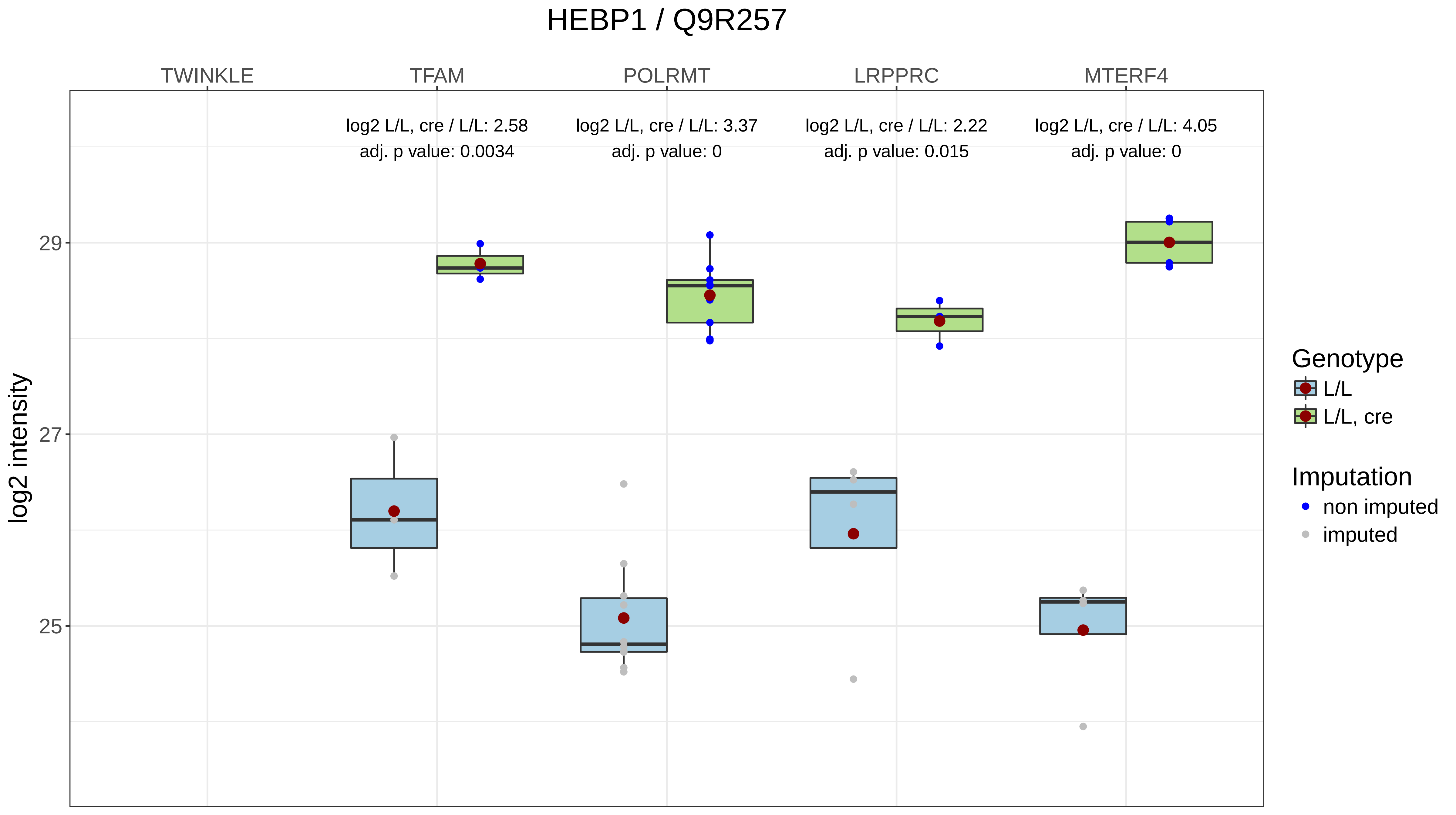
HEBP1 / Q9R257
TWINKLE
TFAM
POLRMT
LRPPRC
MTERF4
log2 L/L, cre / L/L: 2.58
adj. p value: 0.0034
log2 L/L, cre / L/L: 3.37
adj. p value: 0
log2 L/L, cre / L/L: 2.22
adj. p value: 0.015
log2 L/L, cre / L/L: 4.05
adj. p value: 0
29
27
25
log2 intensity
Genotype
L/L
L/L, cre
Imputation
non imputed
imputed

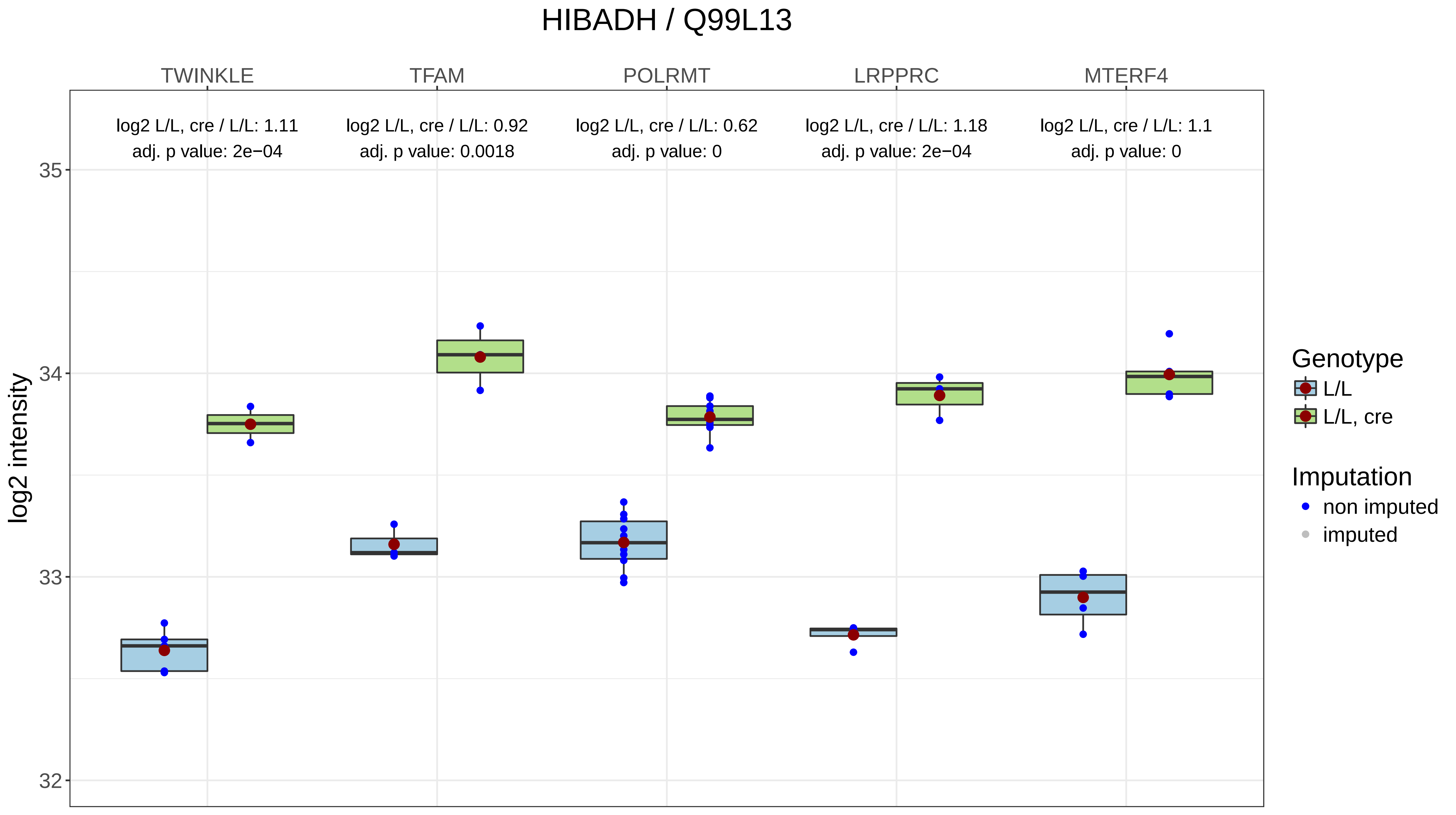
HIBADH / Q99L13
TWINKLE
TFAM
POLRMT
LRPPRC
MTERF4
log2 L/L, cre / L/L: 1.11
adj. p value: 2e−04
log2 L/L, cre / L/L: 0.92
adj. p value: 0.0018
log2 L/L, cre / L/L: 0.62
adj. p value: 0
log2 L/L, cre / L/L: 1.18
adj. p value: 2e−04
log2 L/L, cre / L/L: 1.1
adj. p value: 0
log2 intensity
35
34
33
32
Genotype
L/L
L/L, cre
Imputation
non imputed
imputed

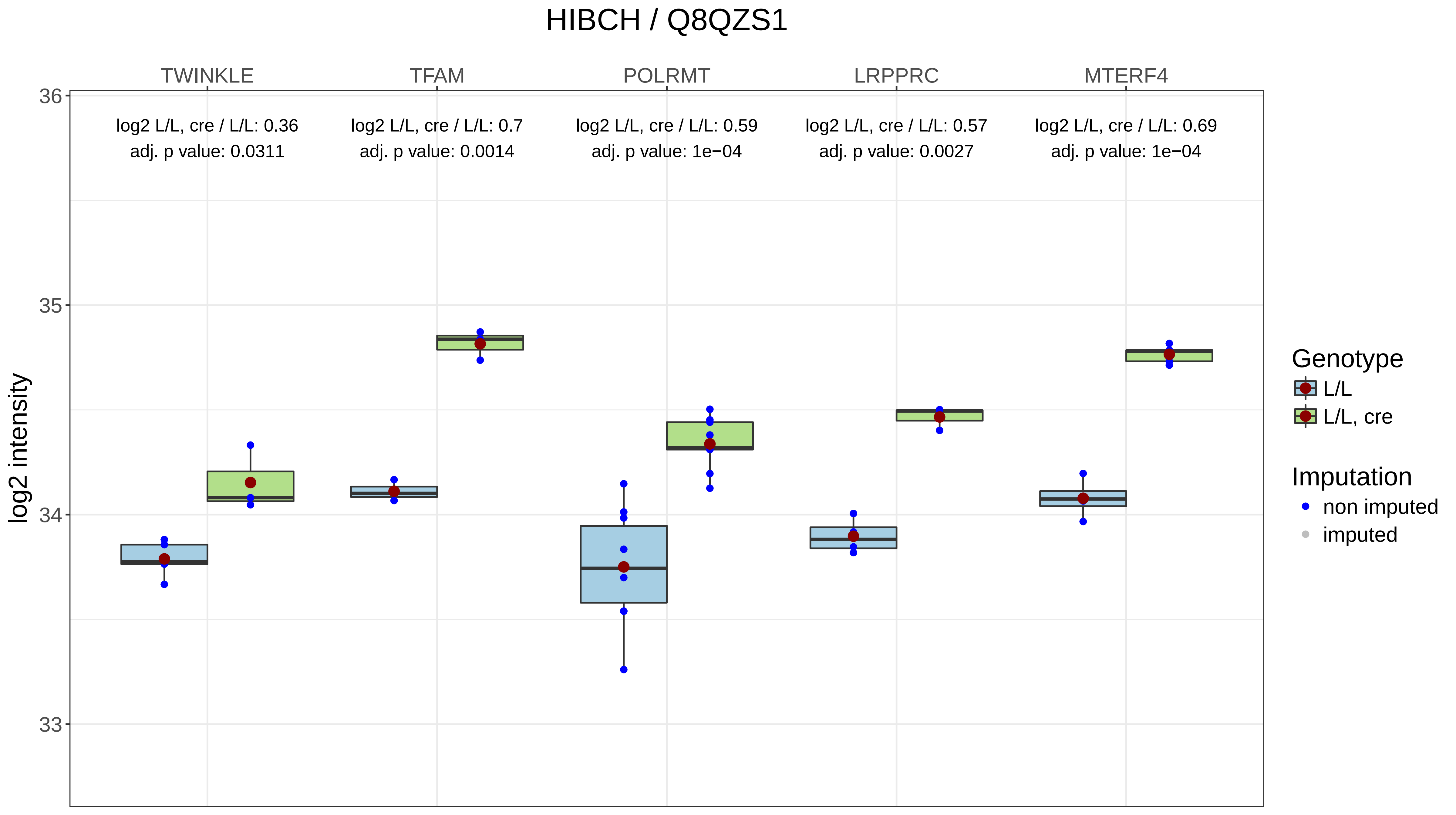

HIBCH / Q8QZS1
TWINKLE
TFAM
POLRMT
LRPPRC
MTERF4
log2 L/L, cre / L/L: 0.36
adj. p value: 0.0311
log2 L/L, cre / L/L: 0.7
adj. p value: 0.0014
log2 L/L, cre / L/L: 0.59
adj. p value: 1e−04
log2 L/L, cre / L/L: 0.57
adj. p value: 0.0027
log2 L/L, cre / L/L: 0.69
adj. p value: 1e−04
log2 intensity
36
35
34
33
Genotype
L/L
L/L, cre
Imputation
non imputed
imputed

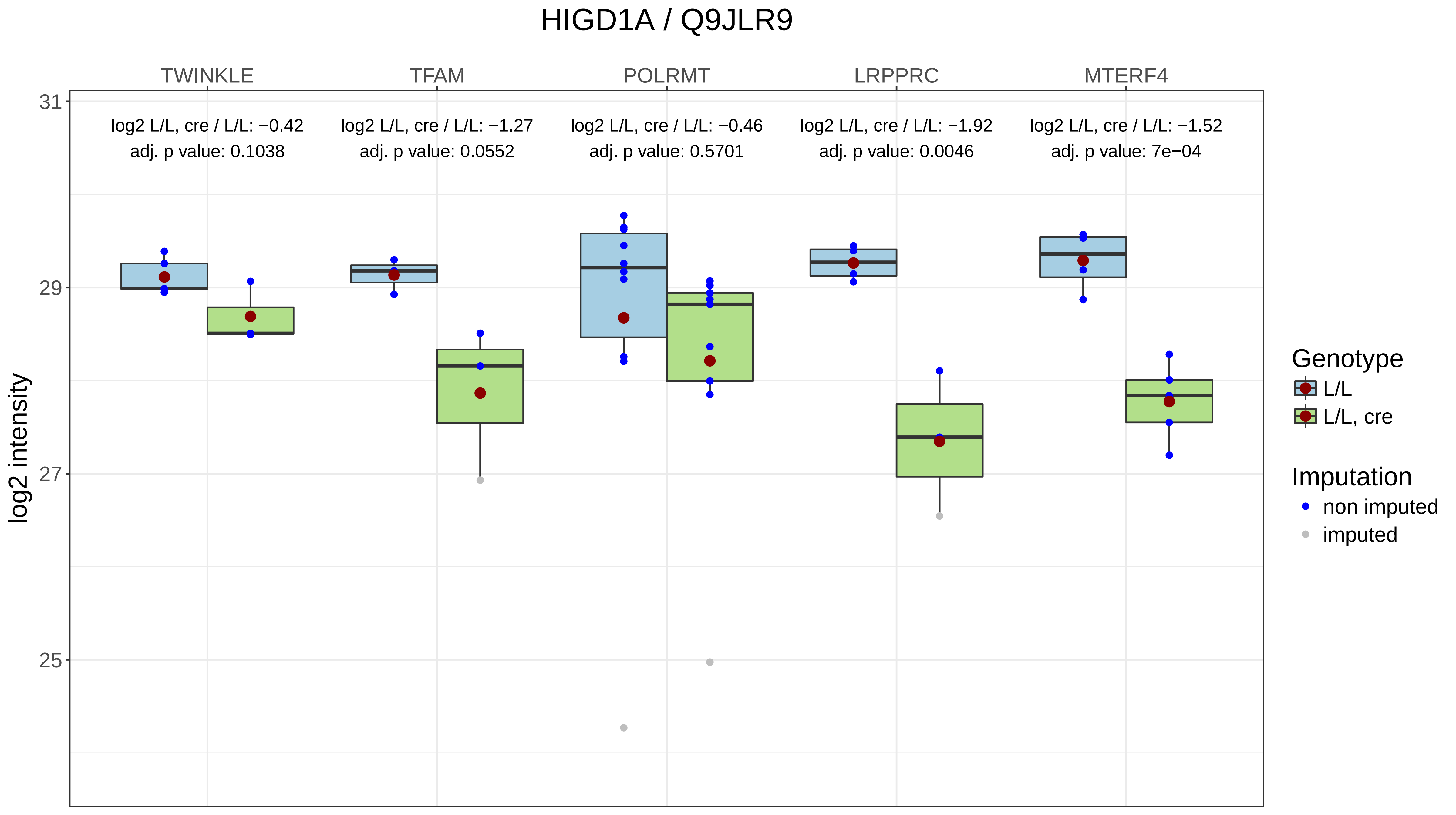
HIGD1A / Q9JLR9
TWINKLE
TFAM
POLRMT
LRPPRC
MTERF4
log2 L/L, cre / L/L: −0.42
adj. p value: 0.1038
log2 L/L, cre / L/L: −1.27
adj. p value: 0.0552
log2 L/L, cre / L/L: −0.46
adj. p value: 0.5701
log2 L/L, cre / L/L: −1.92
adj. p value: 0.0046
log2 L/L, cre / L/L: −1.52
adj. p value: 7e−04
31
29
27
25
log2 intensity
Genotype
L/L
L/L, cre
Imputation
non imputed
imputed

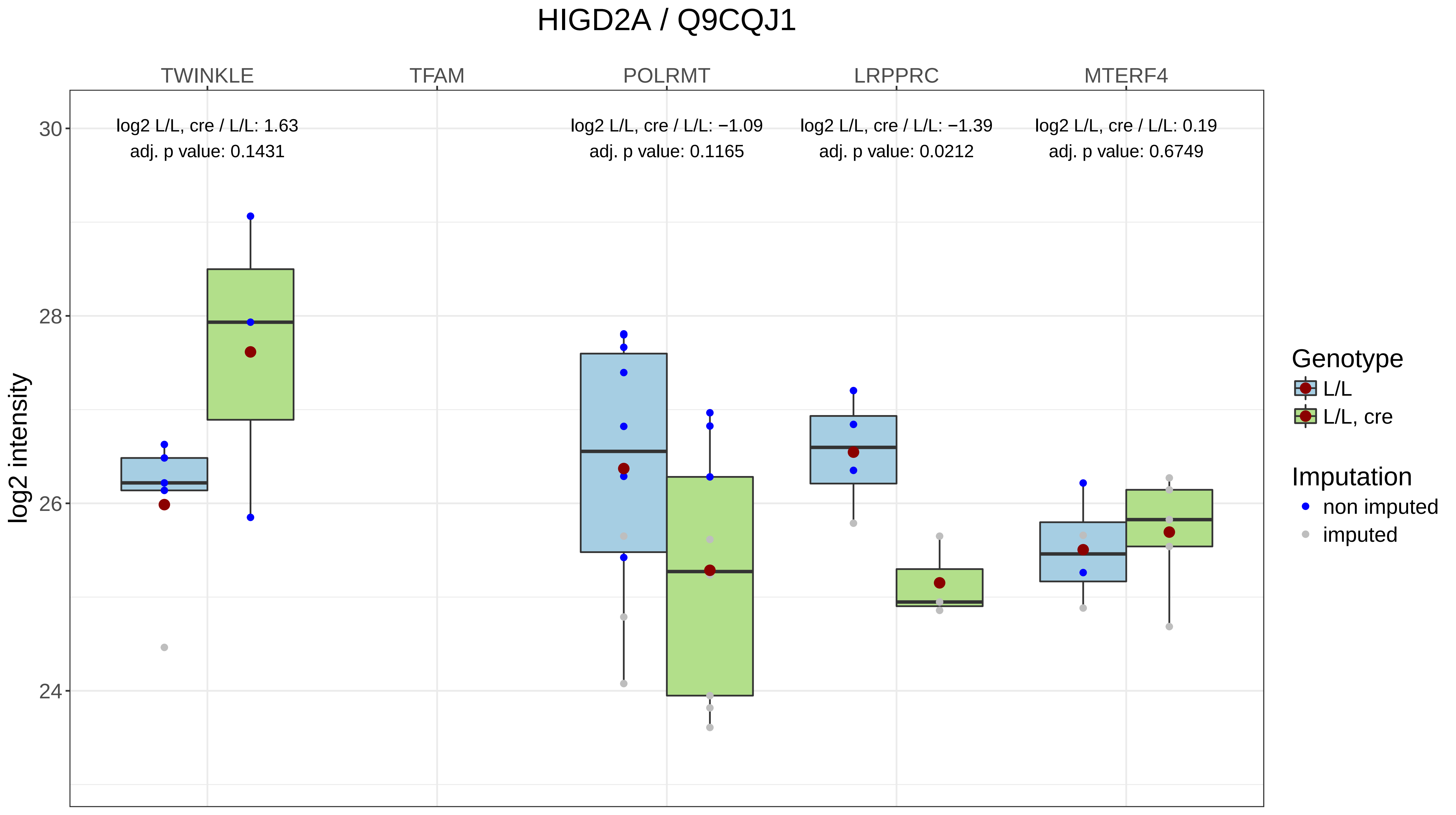
HIGD2A / Q9CQJ1
TWINKLE
TFAM
POLRMT
LRPPRC
MTERF4
log2 L/L, cre / L/L: 1.63
adj. p value: 0.1431
log2 L/L, cre / L/L: −1.09
adj. p value: 0.1165
log2 L/L, cre / L/L: −1.39
adj. p value: 0.0212
log2 L/L, cre / L/L: 0.19
adj. p value: 0.6749
30
28
26
24
log2 intensity
Genotype
L/L
L/L, cre
Imputation
non imputed
imputed

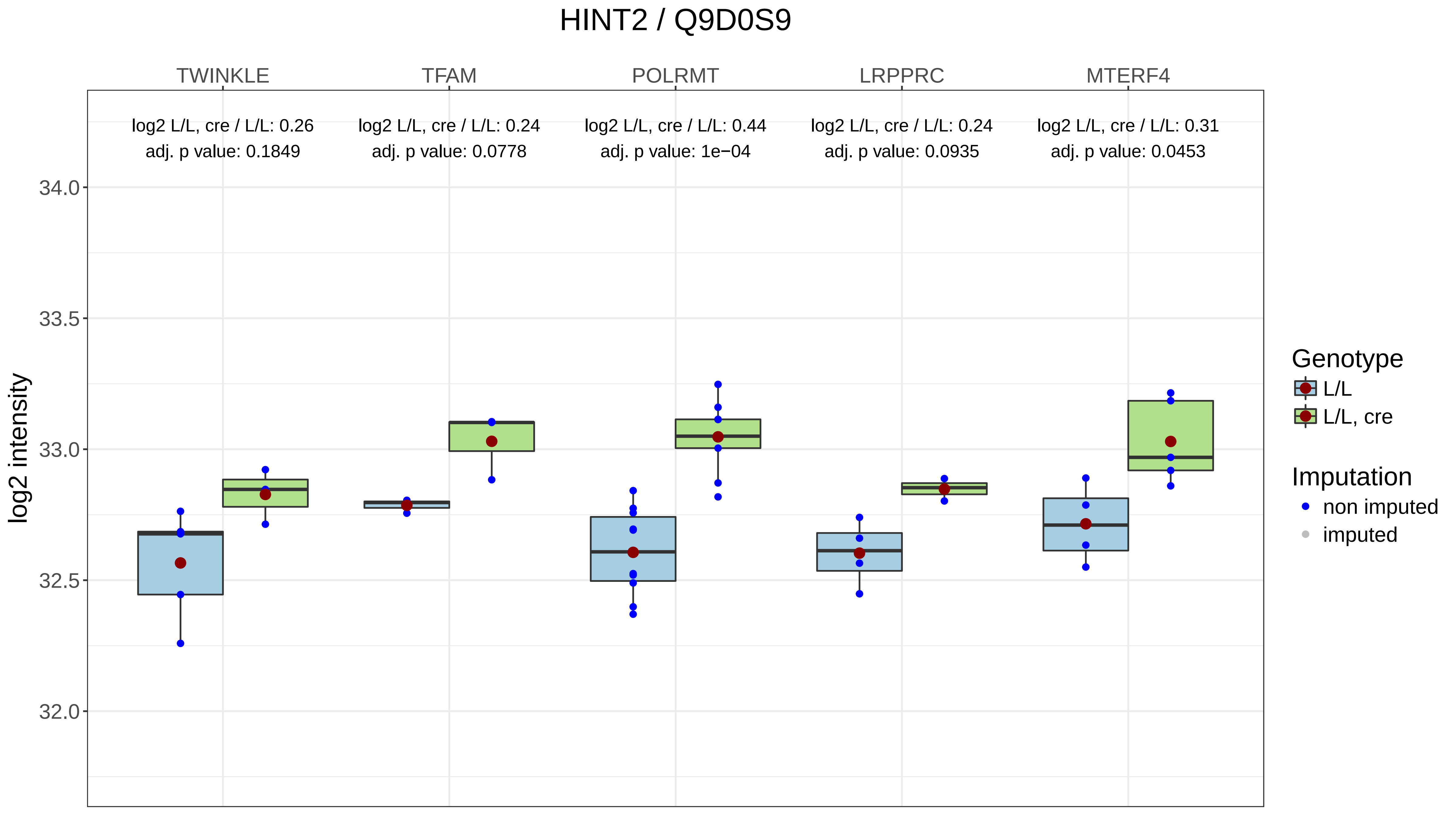

HINT2 / Q9D0S9
TWINKLE
TFAM
POLRMT
LRPPRC
MTERF4
log2 L/L, cre / L/L: 0.26
adj. p value: 0.1849
log2 L/L, cre / L/L: 0.24
adj. p value: 0.0778
log2 L/L, cre / L/L: 0.44
adj. p value: 1e−04
log2 L/L, cre / L/L: 0.24
adj. p value: 0.0935
log2 L/L, cre / L/L: 0.31
adj. p value: 0.0453
log2 intensity
34.0
33.5
33.0
32.5
32.0
Genotype
L/L
L/L, cre
Imputation
non imputed
imputed

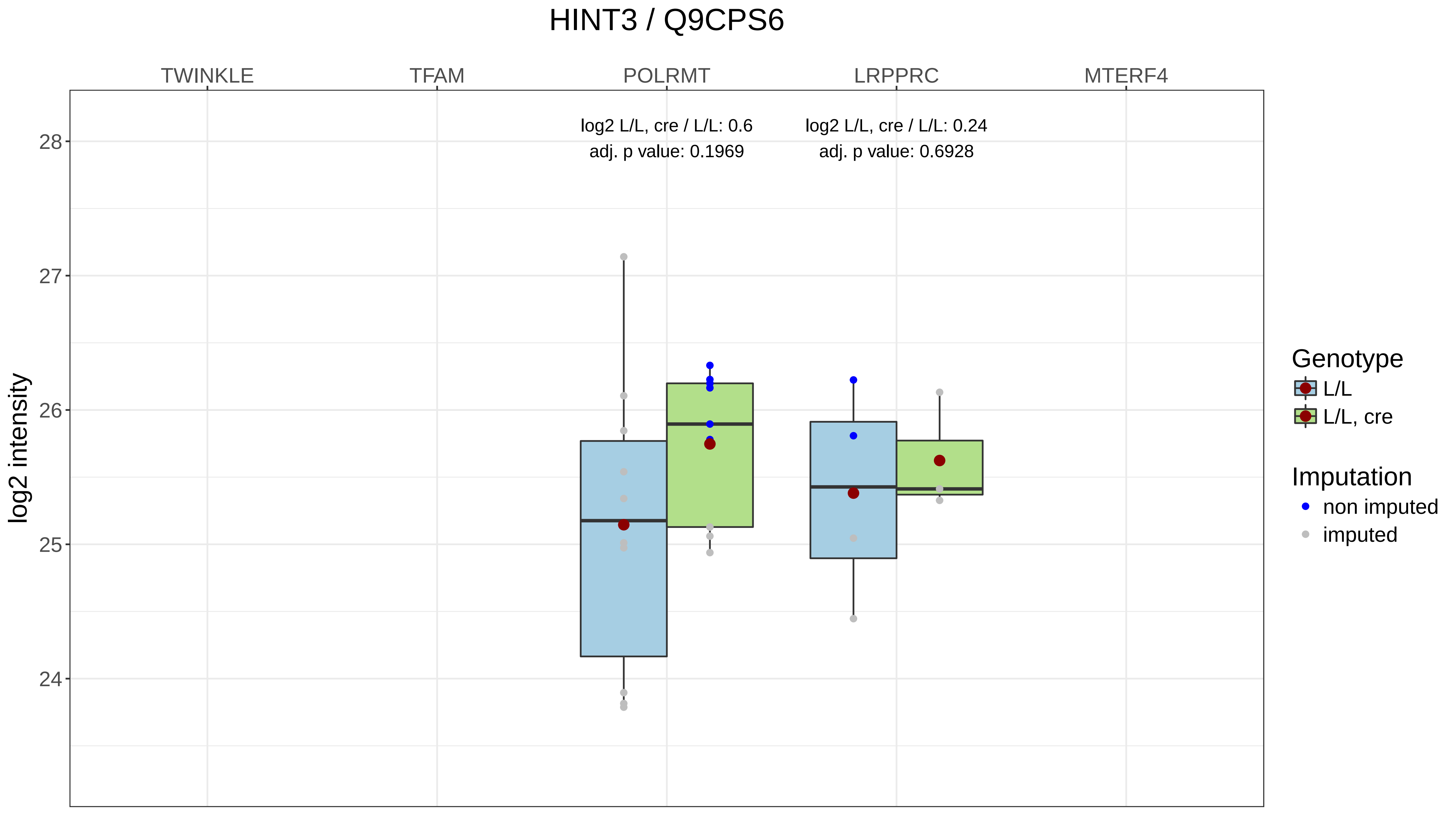
HINT3 / Q9CPS6
TWINKLE
TFAM
POLRMT
LRPPRC
MTERF4
log2 L/L, cre / L/L: 0.6
adj. p value: 0.1969
log2 L/L, cre / L/L: 0.24
adj. p value: 0.6928
log2 intensity
28
27
26
25
24
Genotype
L/L
L/L, cre
Imputation
non imputed
imputed

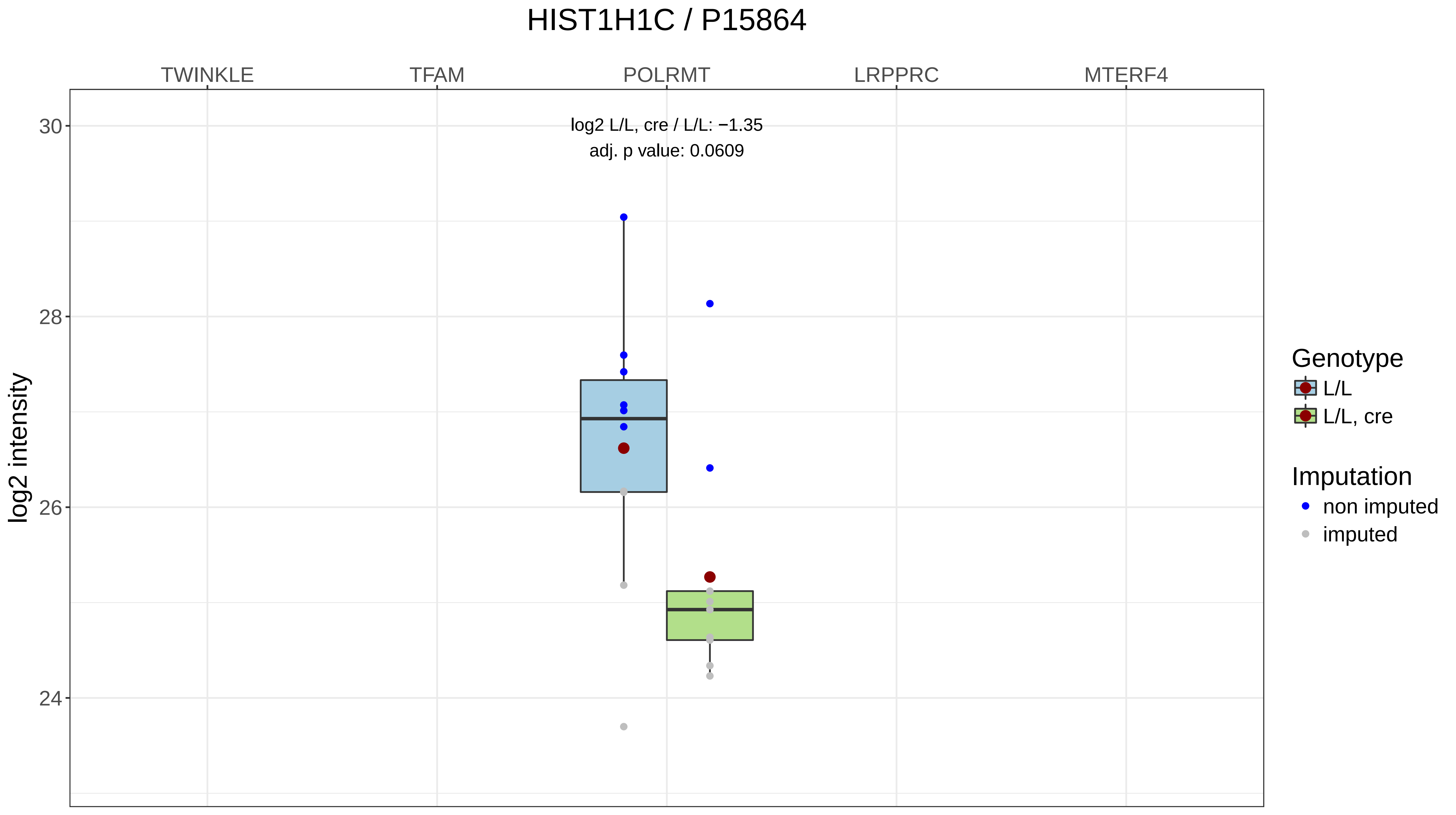
HIST1H1C / P15864
TWINKLE
TFAM
POLRMT
LRPPRC
MTERF4
log2 L/L, cre / L/L: −1.35
adj. p value: 0.0609
30
28
26
24
log2 intensity
Genotype
L/L
L/L, cre
Imputation
non imputed
imputed

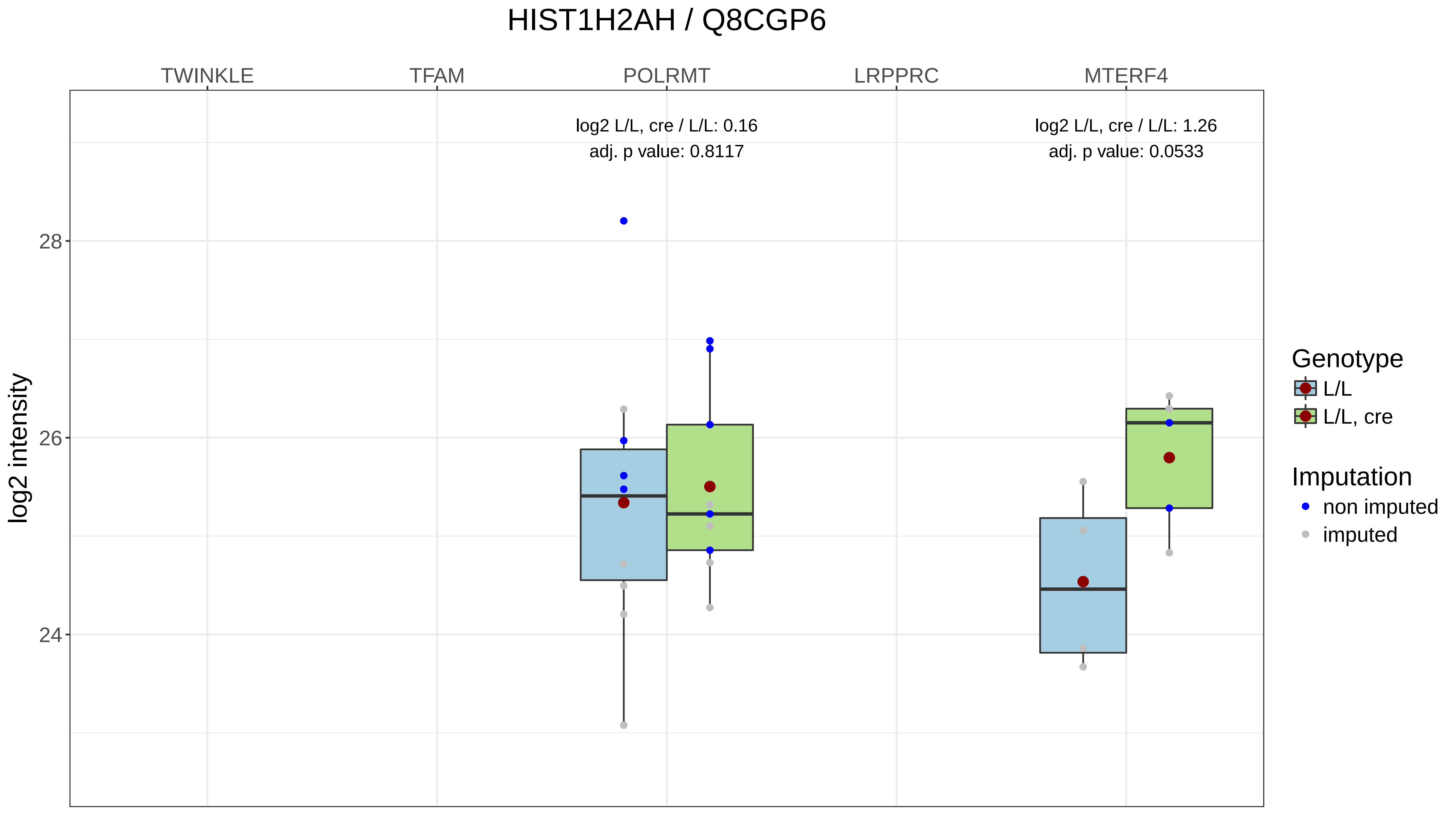

HIST1H2AH / Q8CGP6
TWINKLE
TFAM
POLRMT
LRPPRC
MTERF4
log2 L/L, cre / L/L: 0.16
adj. p value: 0.8117
log2 L/L, cre / L/L: 1.26
adj. p value: 0.0533
28
26
24
log2 intensity
Genotype
L/L
L/L, cre
Imputation
non imputed
imputed

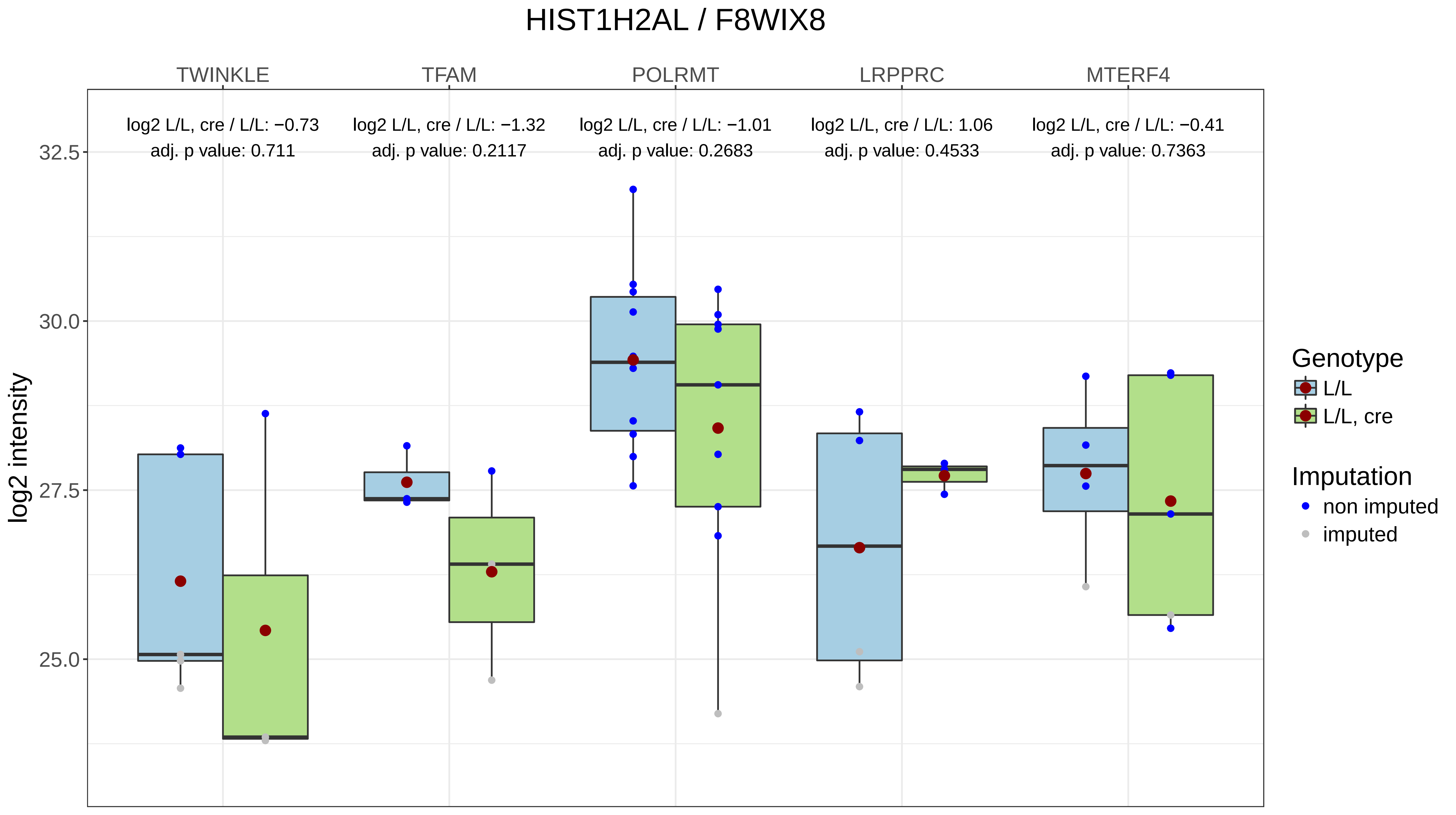

HIST1H2AL / F8WIX8
TWINKLE
TFAM
POLRMT
LRPPRC
MTERF4
log2 L/L, cre / L/L: −0.73
adj. p value: 0.711
log2 L/L, cre / L/L: −1.32
adj. p value: 0.2117
log2 L/L, cre / L/L: −1.01
adj. p value: 0.2683
log2 L/L, cre / L/L: 1.06
adj. p value: 0.4533
log2 L/L, cre / L/L: −0.41
adj. p value: 0.7363
log2 intensity
32.5
30.0
27.5
25.0
Genotype
L/L
L/L, cre
Imputation
non imputed
imputed

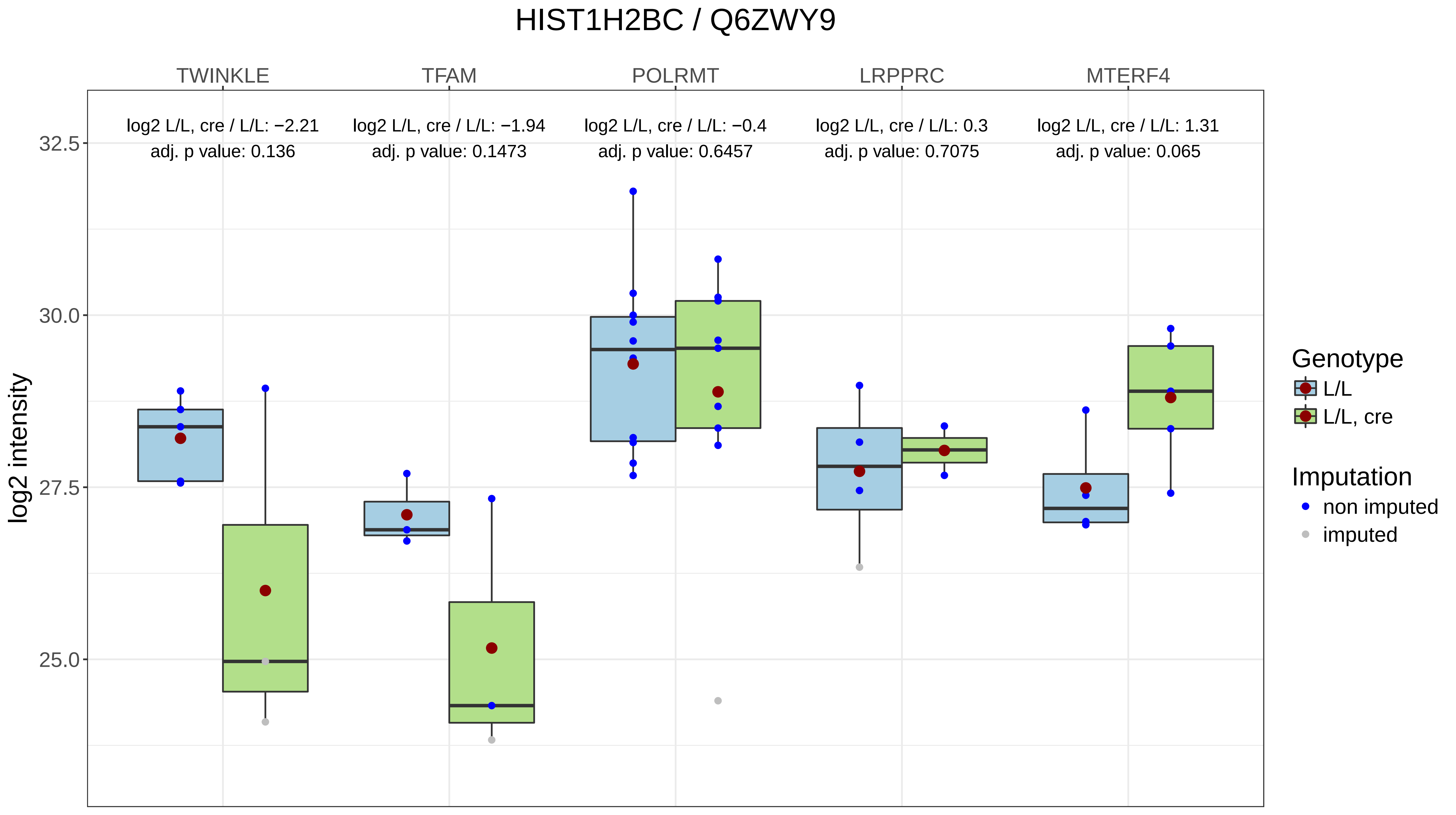
HIST1H2BC / Q6ZWY9
TWINKLE
TFAM
POLRMT
LRPPRC
MTERF4
log2 L/L, cre / L/L: −2.21
adj. p value: 0.136
log2 L/L, cre / L/L: −1.94
adj. p value: 0.1473
log2 L/L, cre / L/L: −0.4
adj. p value: 0.6457
log2 L/L, cre / L/L: 0.3
adj. p value: 0.7075
log2 L/L, cre / L/L: 1.31
adj. p value: 0.065
32.5
30.0
27.5
25.0
log2 intensity
Genotype
L/L
L/L, cre
Imputation
non imputed
imputed

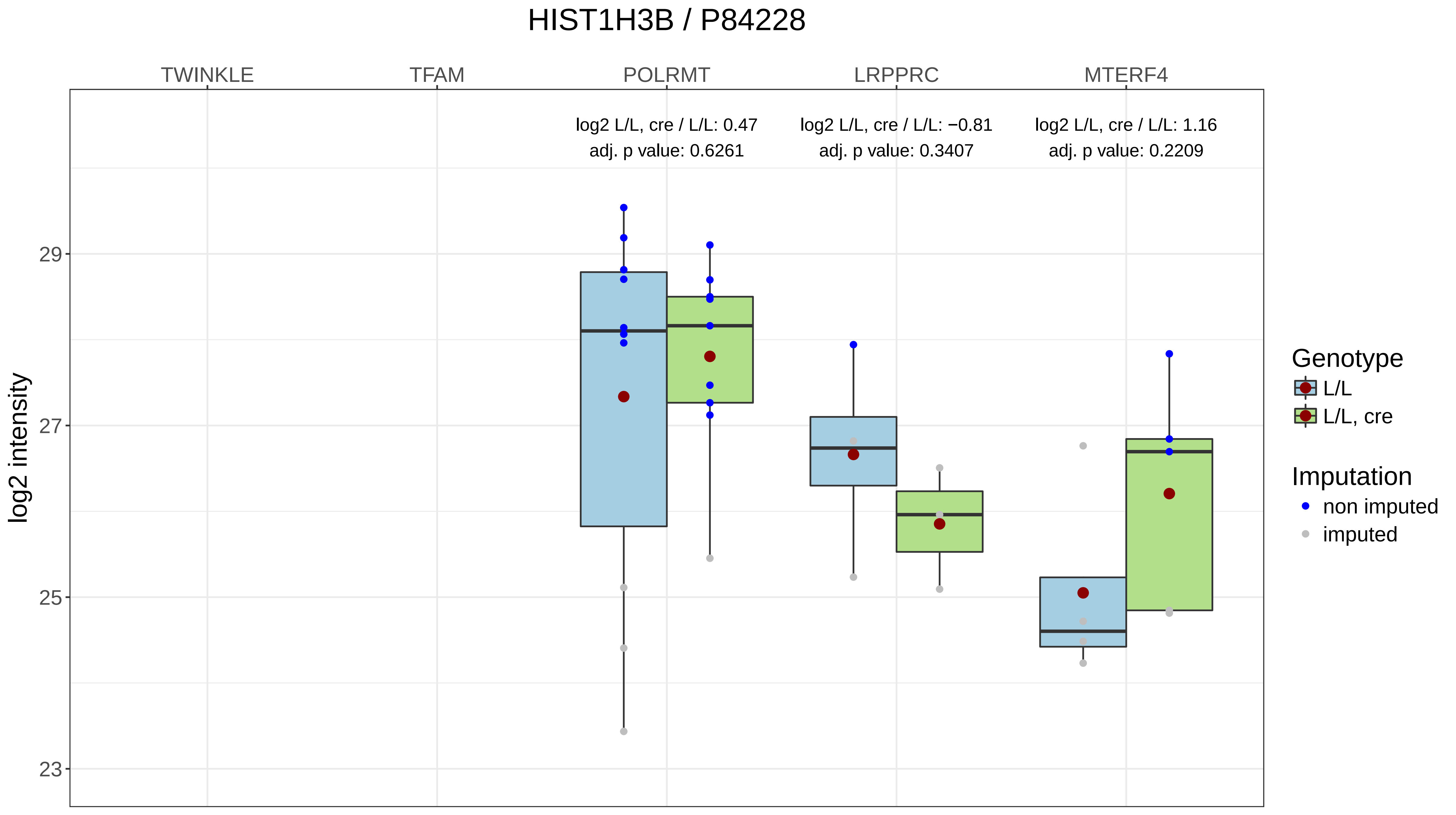
HIST1H3B / P84228
TWINKLE
TFAM
POLRMT
LRPPRC
MTERF4
log2 L/L, cre / L/L: 0.47
adj. p value: 0.6261
log2 L/L, cre / L/L: −0.81
adj. p value: 0.3407
log2 L/L, cre / L/L: 1.16
adj. p value: 0.2209
log2 intensity
29
27
25
23
Genotype
L/L
L/L, cre
Imputation
non imputed
imputed

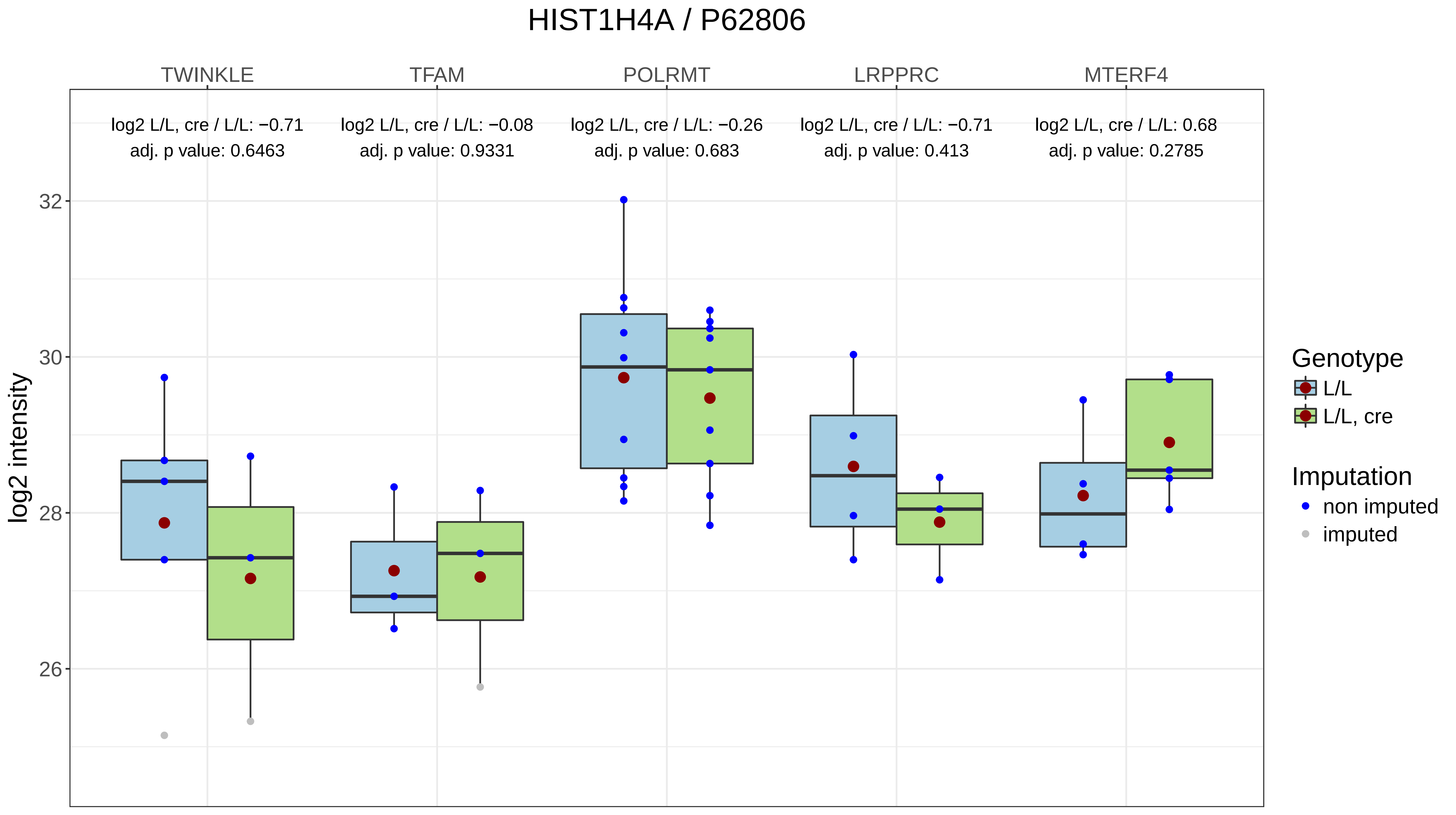
HIST1H4A / P62806
TWINKLE
TFAM
POLRMT
LRPPRC
MTERF4
log2 L/L, cre / L/L: −0.71
adj. p value: 0.6463
log2 L/L, cre / L/L: −0.08
adj. p value: 0.9331
log2 L/L, cre / L/L: −0.26
adj. p value: 0.683
log2 L/L, cre / L/L: −0.71
adj. p value: 0.413
log2 L/L, cre / L/L: 0.68
adj. p value: 0.2785
log2 intensity
32
30
28
26
Genotype
L/L
L/L, cre
Imputation
non imputed
imputed

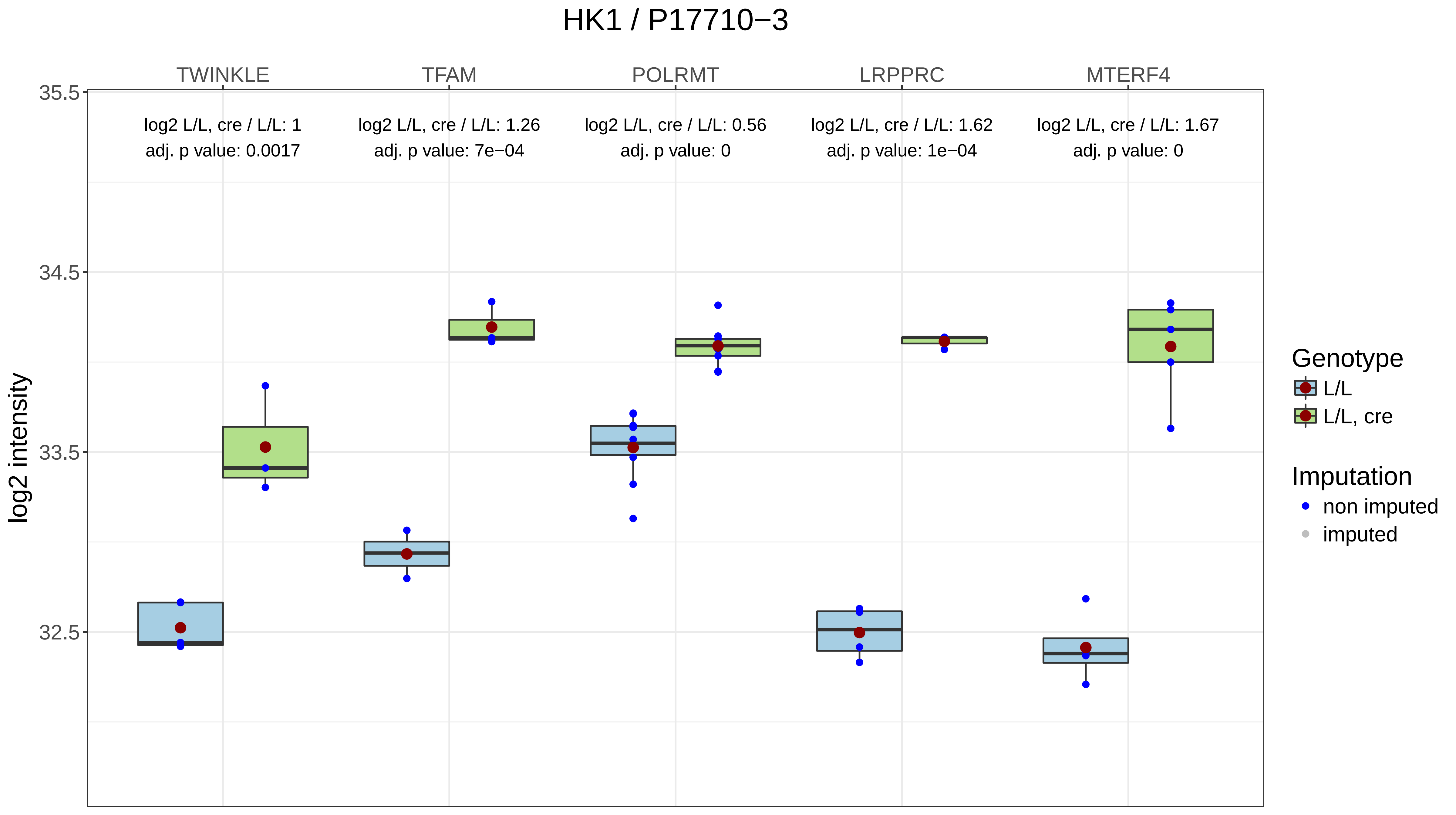
HK1 / P17710−3
TWINKLE
TFAM
POLRMT
LRPPRC
MTERF4
log2 L/L, cre / L/L: 1
adj. p value: 0.0017
log2 L/L, cre / L/L: 1.26
adj. p value: 7e−04
log2 L/L, cre / L/L: 0.56
adj. p value: 0
log2 L/L, cre / L/L: 1.62
adj. p value: 1e−04
log2 L/L, cre / L/L: 1.67
adj. p value: 0
35.5
34.5
33.5
32.5
log2 intensity
Genotype
L/L
L/L, cre
Imputation
non imputed
imputed

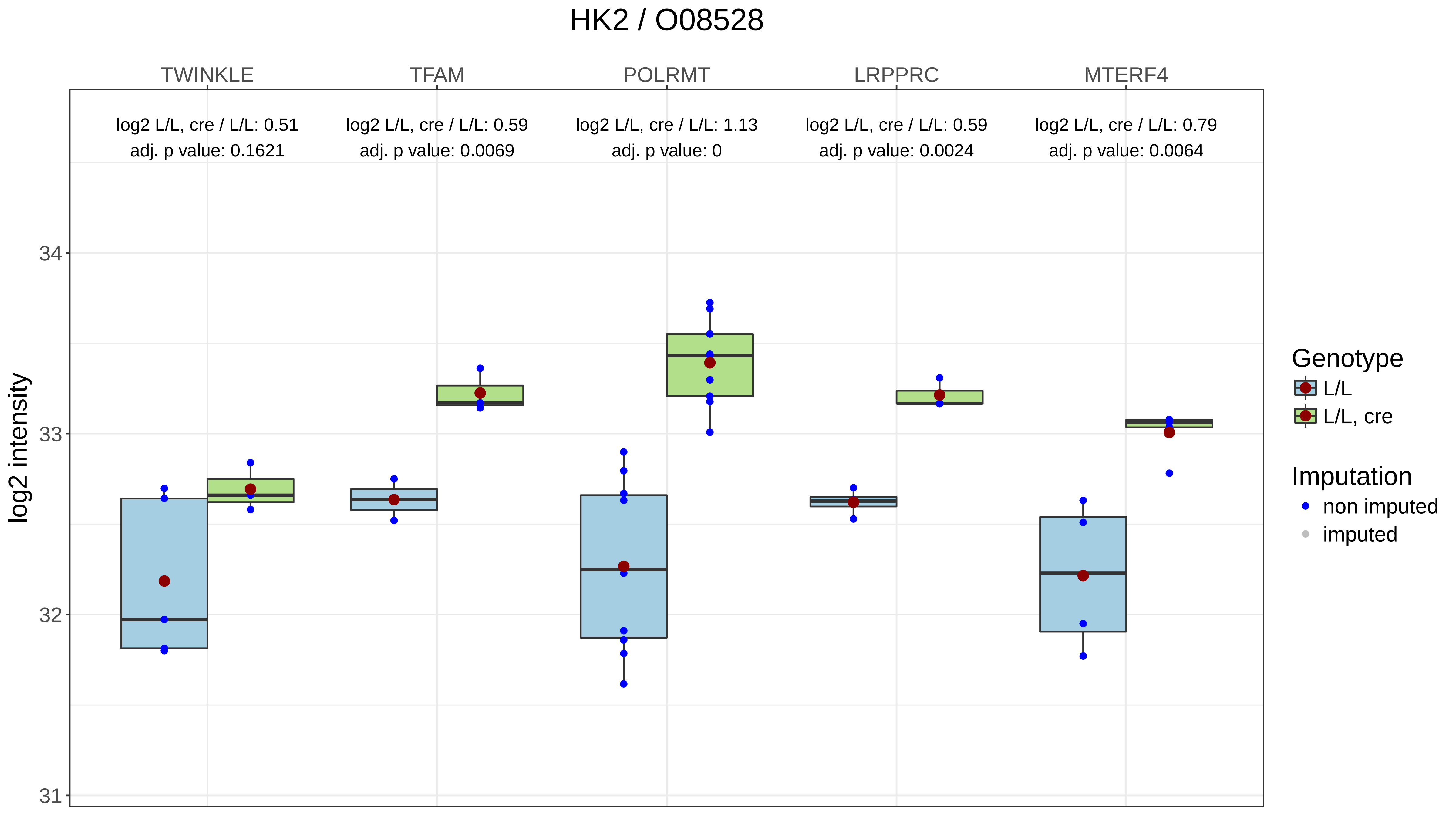
HK2 / O08528
TWINKLE
TFAM
POLRMT
LRPPRC
MTERF4
log2 L/L, cre / L/L: 0.51
adj. p value: 0.1621
log2 L/L, cre / L/L: 0.59
adj. p value: 0.0069
log2 L/L, cre / L/L: 1.13
adj. p value: 0
log2 L/L, cre / L/L: 0.59
adj. p value: 0.0024
log2 L/L, cre / L/L: 0.79
adj. p value: 0.0064
log2 intensity
34
33
32
31
Genotype
L/L
L/L, cre
Imputation
non imputed
imputed

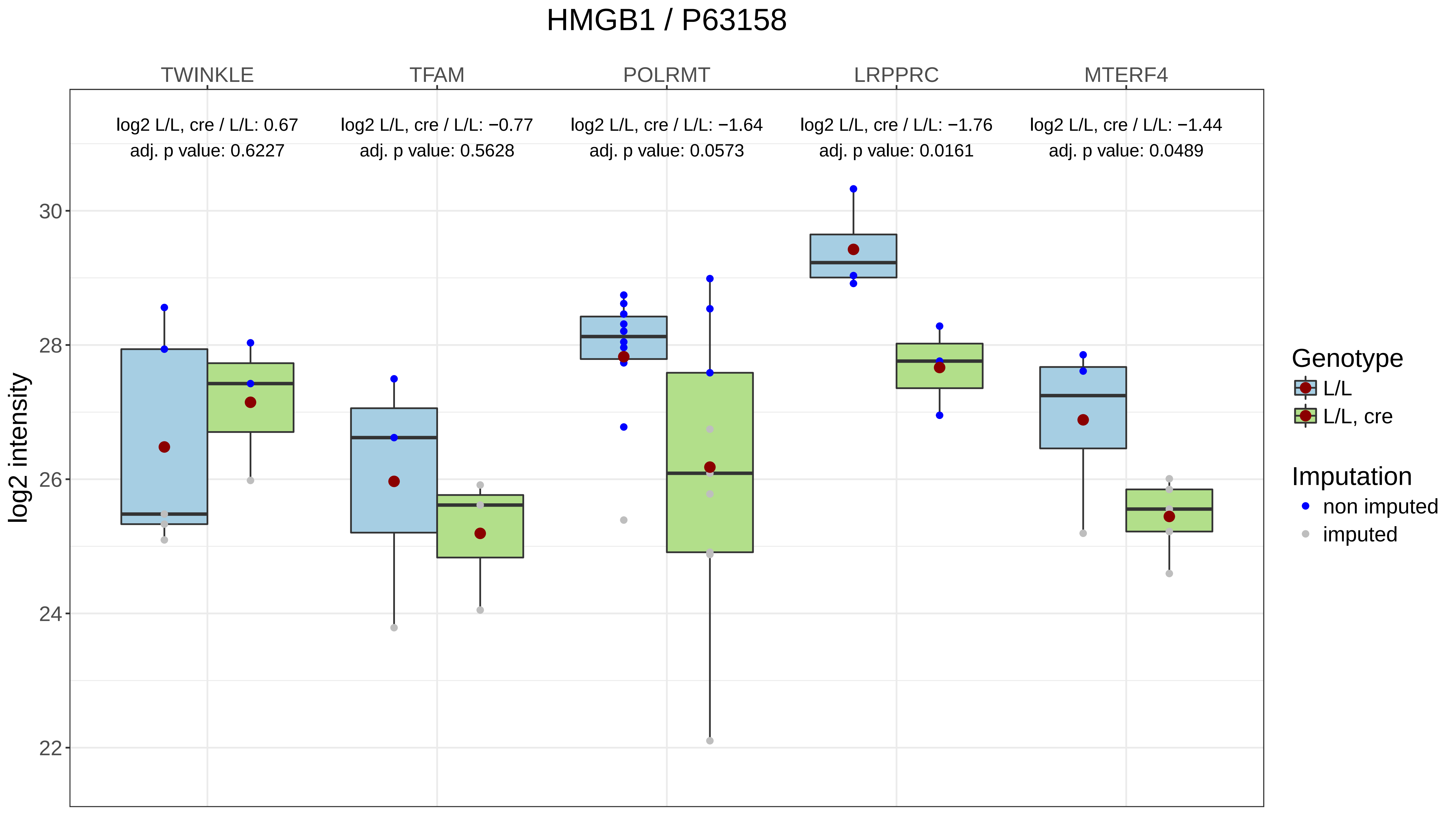
HMGB1 / P63158
TWINKLE
TFAM
POLRMT
LRPPRC
MTERF4
log2 L/L, cre / L/L: 0.67
adj. p value: 0.6227
log2 L/L, cre / L/L: −0.77
adj. p value: 0.5628
log2 L/L, cre / L/L: −1.64
adj. p value: 0.0573
log2 L/L, cre / L/L: −1.76
adj. p value: 0.0161
log2 L/L, cre / L/L: −1.44
adj. p value: 0.0489
log2 intensity
30
28
26
24
22
Genotype
L/L
L/L, cre
Imputation
non imputed
imputed

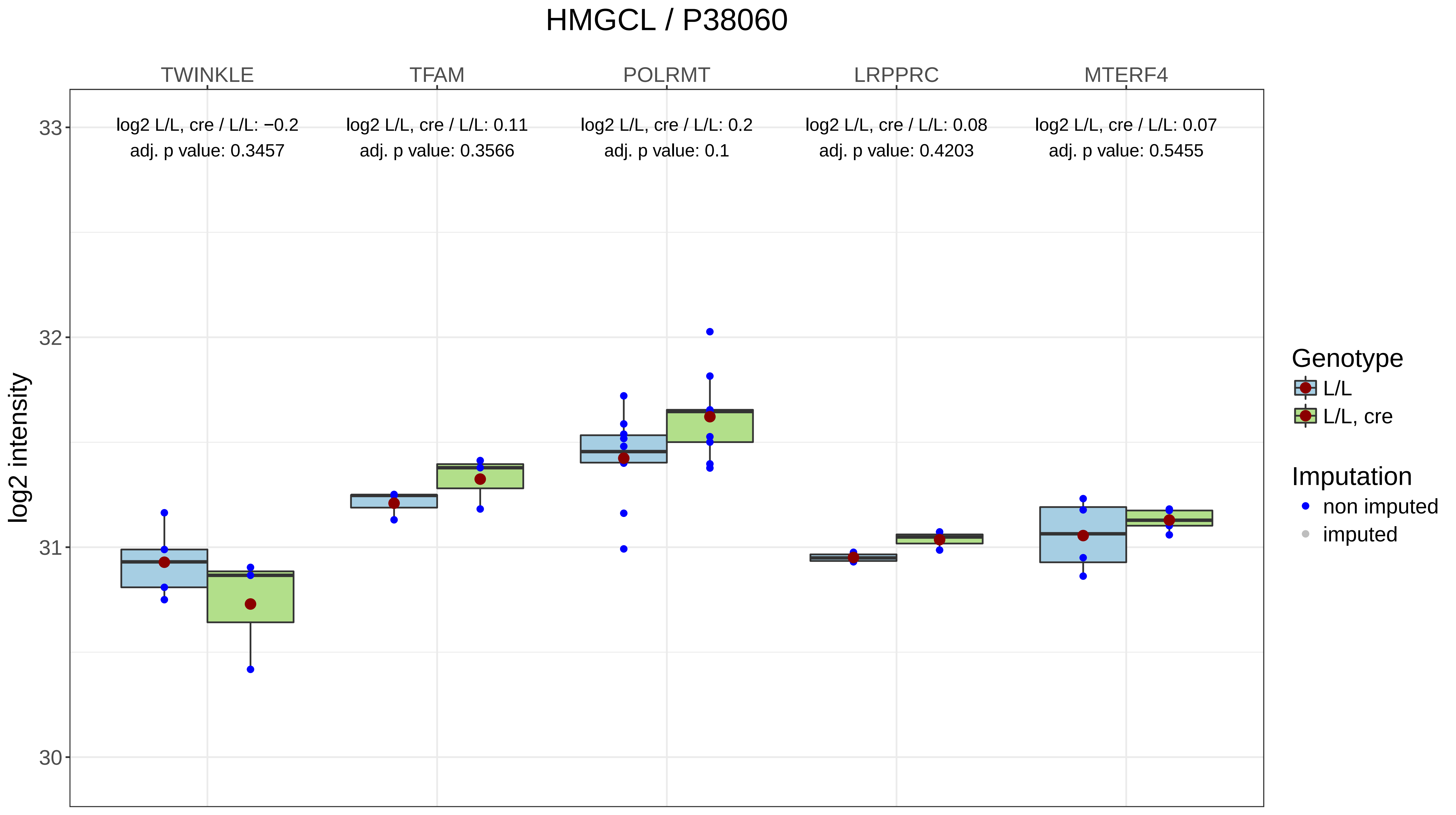

HMGCL / P38060
TWINKLE
TFAM
POLRMT
LRPPRC
MTERF4
log2 L/L, cre / L/L: −0.2
adj. p value: 0.3457
log2 L/L, cre / L/L: 0.11
adj. p value: 0.3566
log2 L/L, cre / L/L: 0.2
adj. p value: 0.1
log2 L/L, cre / L/L: 0.08
adj. p value: 0.4203
log2 L/L, cre / L/L: 0.07
adj. p value: 0.5455
log2 intensity
33
32
31
30
Genotype
L/L
L/L, cre
Imputation
non imputed
imputed

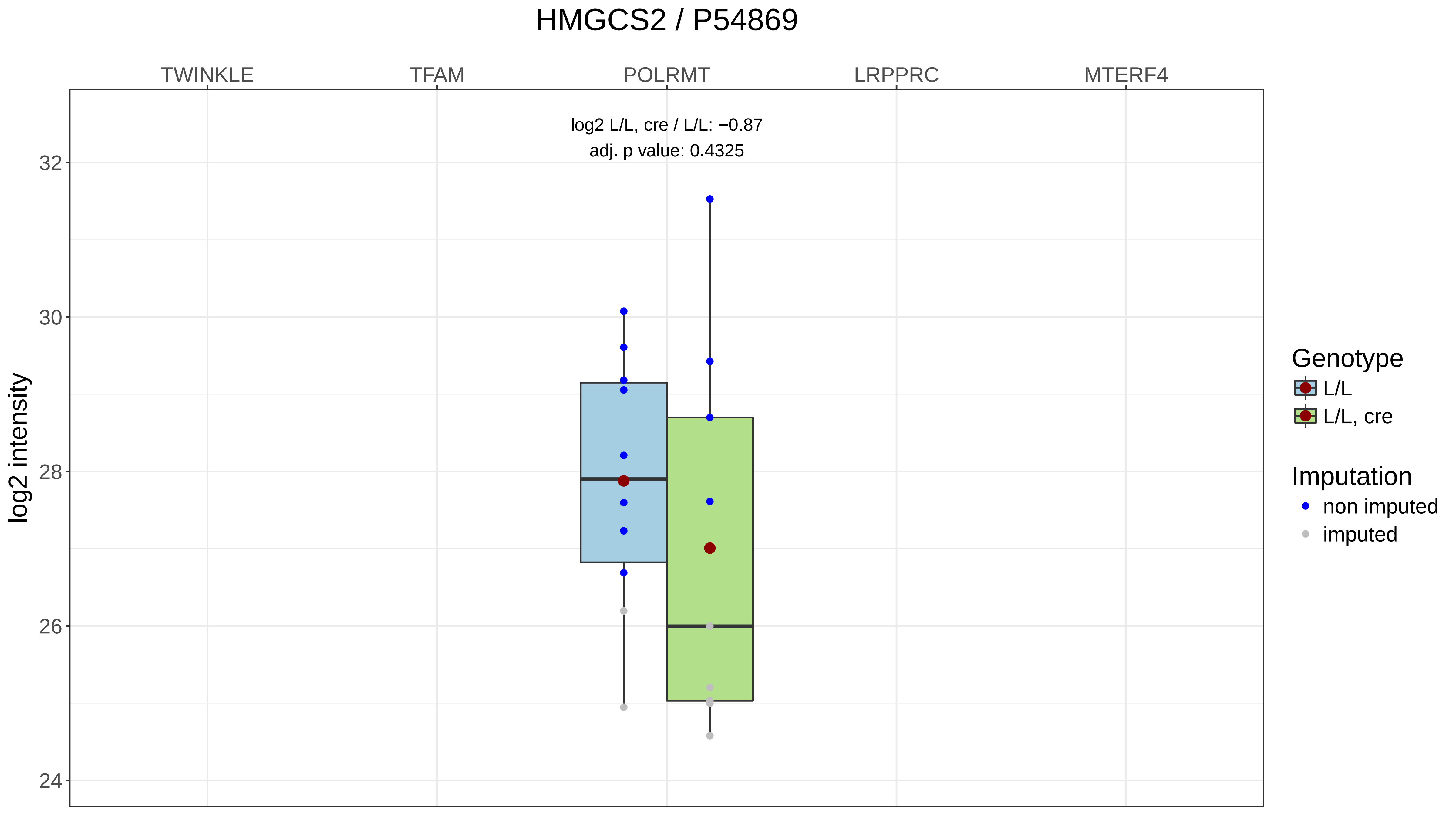
HMGCS2 / P54869
TWINKLE
TFAM
POLRMT
LRPPRC
MTERF4
log2 L/L, cre / L/L: −0.87
adj. p value: 0.4325
log2 intensity
32
30
28
26
24
Genotype
L/L
L/L, cre
Imputation
non imputed
imputed

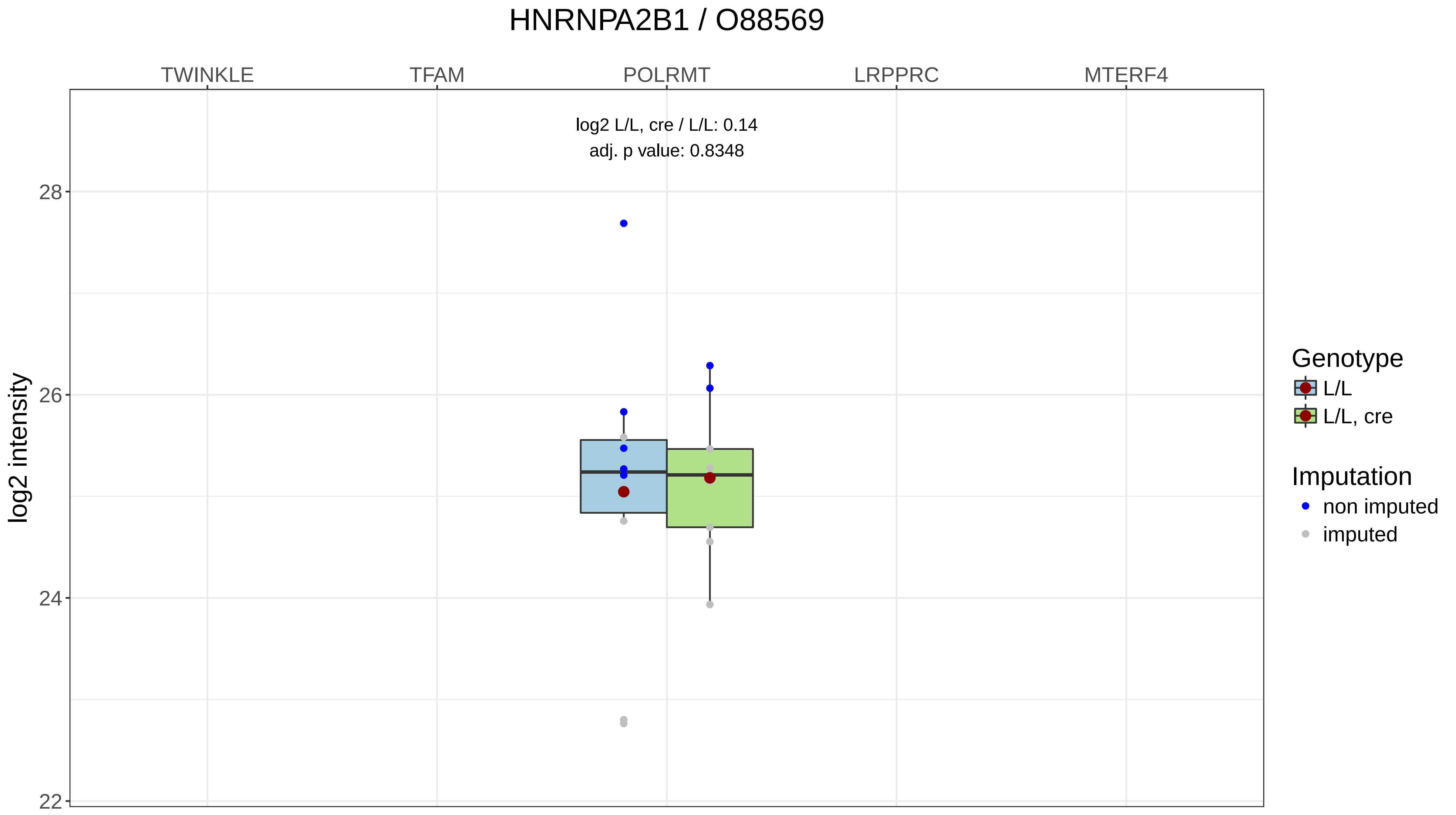
HNRNPA2B1 / O88569
TWINKLE
TFAM
POLRMT
LRPPRC
MTERF4
log2 L/L, cre / L/L: 0.14
adj. p value: 0.8348
28
26
24
22
log2 intensity
Genotype
L/L
L/L, cre
Imputation
non imputed
imputed

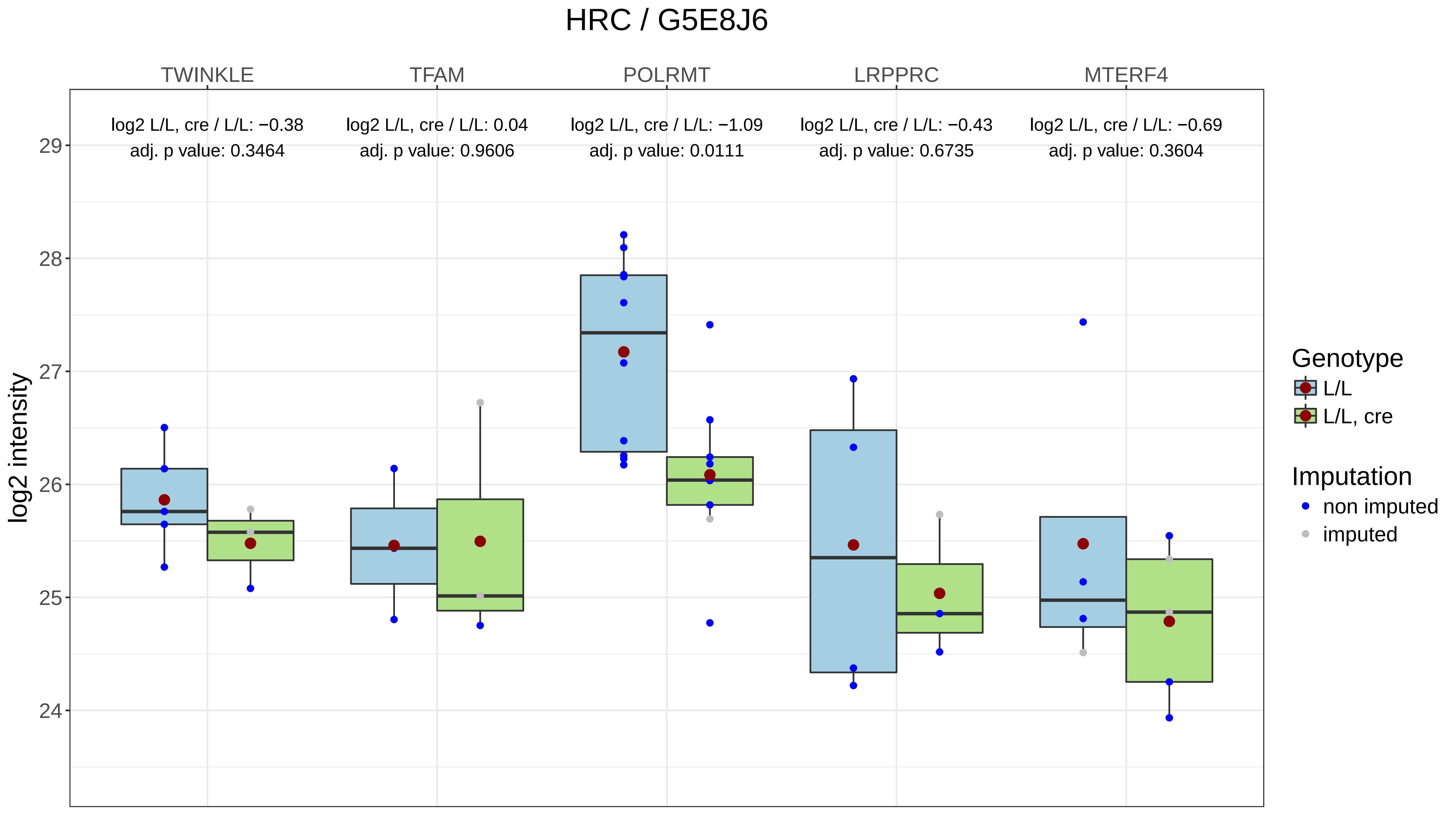
HRC / G5E8J6
TWINKLE
TFAM
POLRMT
LRPPRC
MTERF4
log2 L/L, cre / L/L: −0.38
adj. p value: 0.3464
log2 L/L, cre / L/L: 0.04
adj. p value: 0.9606
log2 L/L, cre / L/L: −1.09
adj. p value: 0.0111
log2 L/L, cre / L/L: −0.43
adj. p value: 0.6735
log2 L/L, cre / L/L: −0.69
adj. p value: 0.3604
log2 intensity
29
28
27
26
25
24
Genotype
L/L
L/L, cre
Imputation
non imputed
imputed

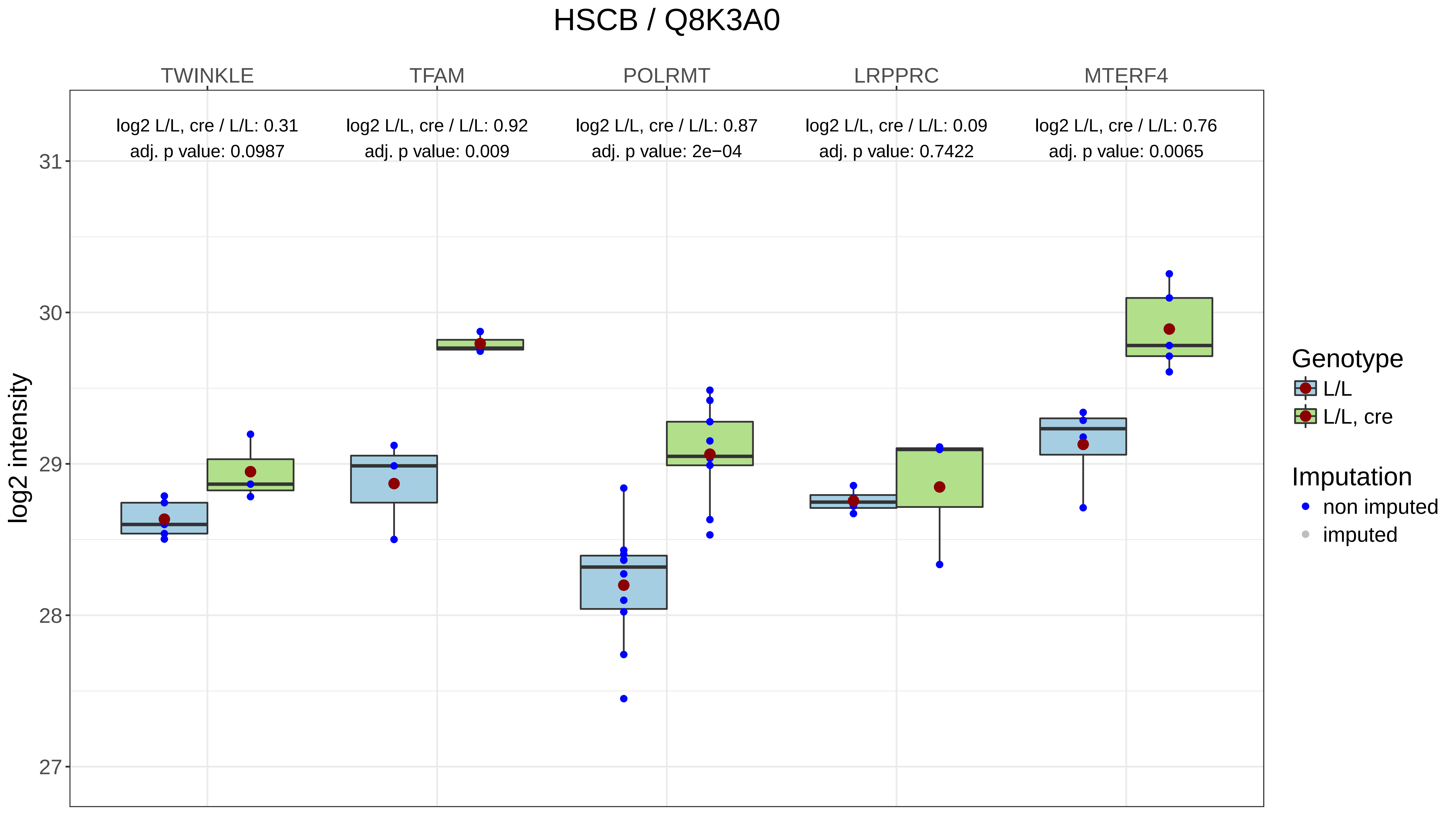
HSCB / Q8K3A0
TWINKLE
TFAM
POLRMT
LRPPRC
MTERF4
log2 L/L, cre / L/L: 0.31
adj. p value: 0.0987
log2 L/L, cre / L/L: 0.92
adj. p value: 0.009
log2 L/L, cre / L/L: 0.87
adj. p value: 2e−04
log2 L/L, cre / L/L: 0.09
adj. p value: 0.7422
log2 L/L, cre / L/L: 0.76
adj. p value: 0.0065
log2 intensity
31
30
29
28
27
Genotype
L/L
L/L, cre
Imputation
non imputed
imputed

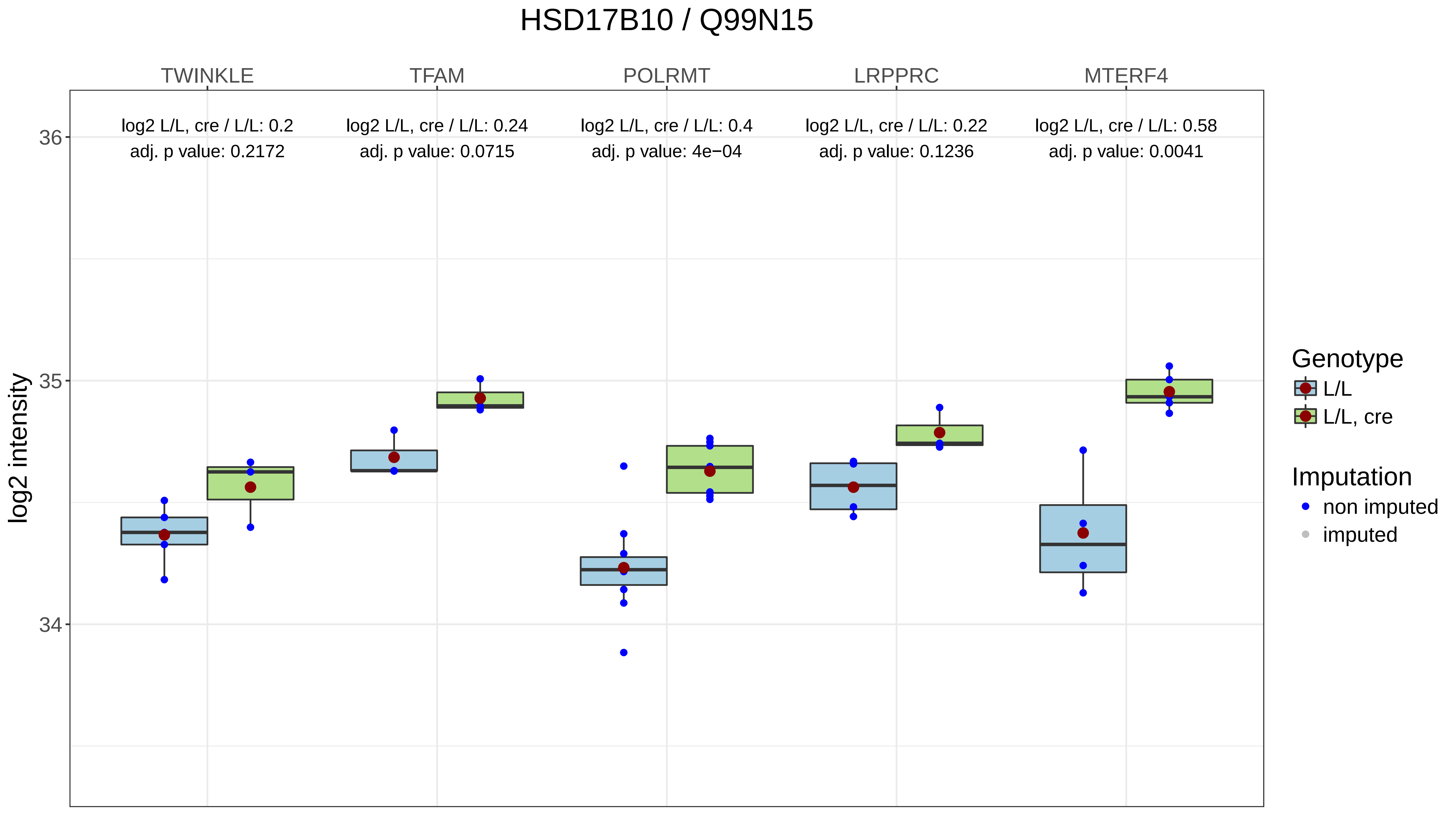
HSD17B10 / Q99N15
TWINKLE
TFAM
POLRMT
LRPPRC
MTERF4
log2 L/L, cre / L/L: 0.2
adj. p value: 0.2172
log2 L/L, cre / L/L: 0.24
adj. p value: 0.0715
log2 L/L, cre / L/L: 0.4
adj. p value: 4e−04
log2 L/L, cre / L/L: 0.22
adj. p value: 0.1236
log2 L/L, cre / L/L: 0.58
adj. p value: 0.0041
36
35
34
log2 intensity
Genotype
L/L
L/L, cre
Imputation
non imputed
imputed

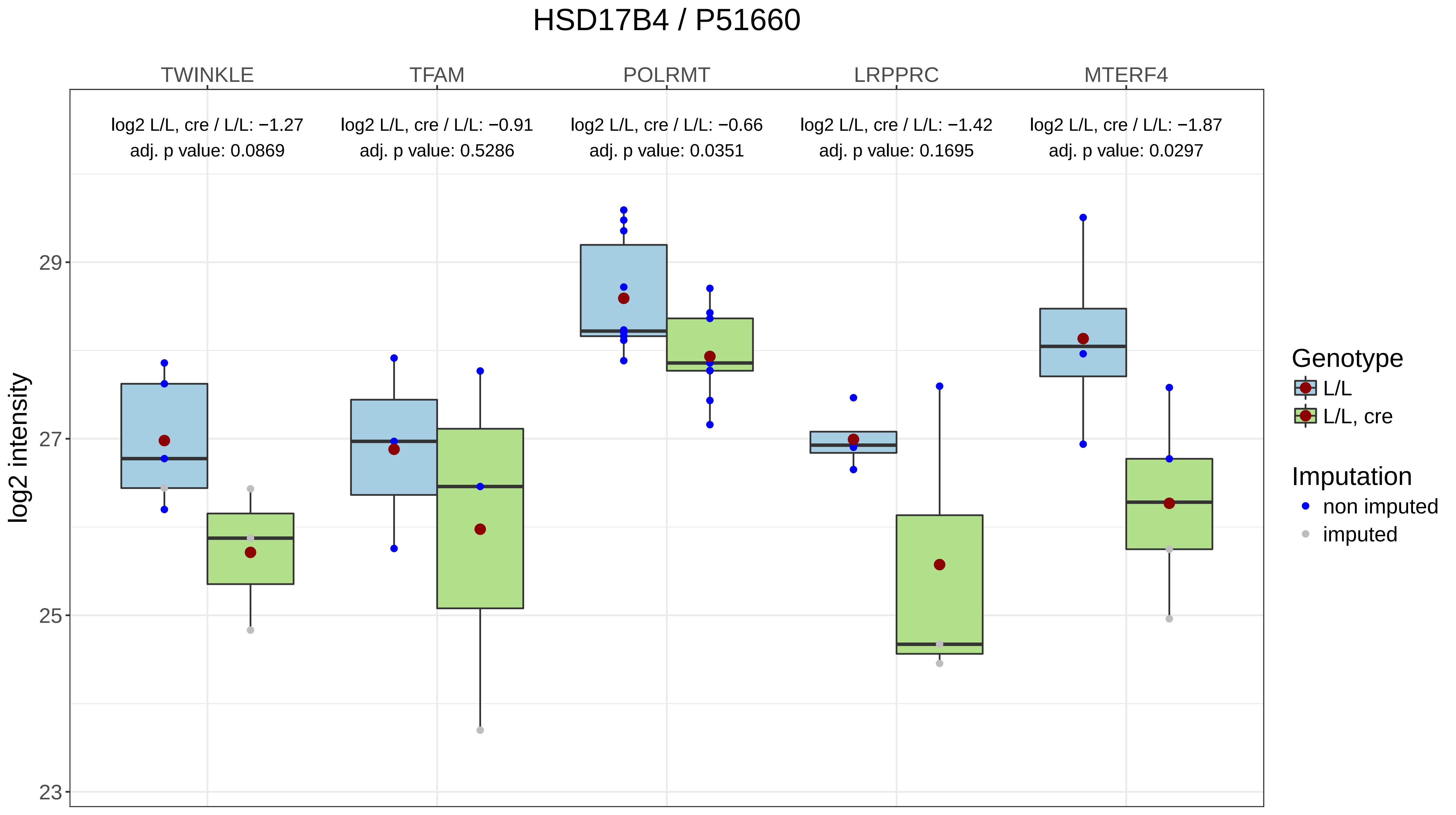
HSD17B4 / P51660
TWINKLE
TFAM
POLRMT
LRPPRC
MTERF4
log2 L/L, cre / L/L: −1.27
adj. p value: 0.0869
log2 L/L, cre / L/L: −0.91
adj. p value: 0.5286
log2 L/L, cre / L/L: −0.66
adj. p value: 0.0351
log2 L/L, cre / L/L: −1.42
adj. p value: 0.1695
log2 L/L, cre / L/L: −1.87
adj. p value: 0.0297
log2 intensity
29
27
25
23
Genotype
L/L
L/L, cre
Imputation
non imputed
imputed

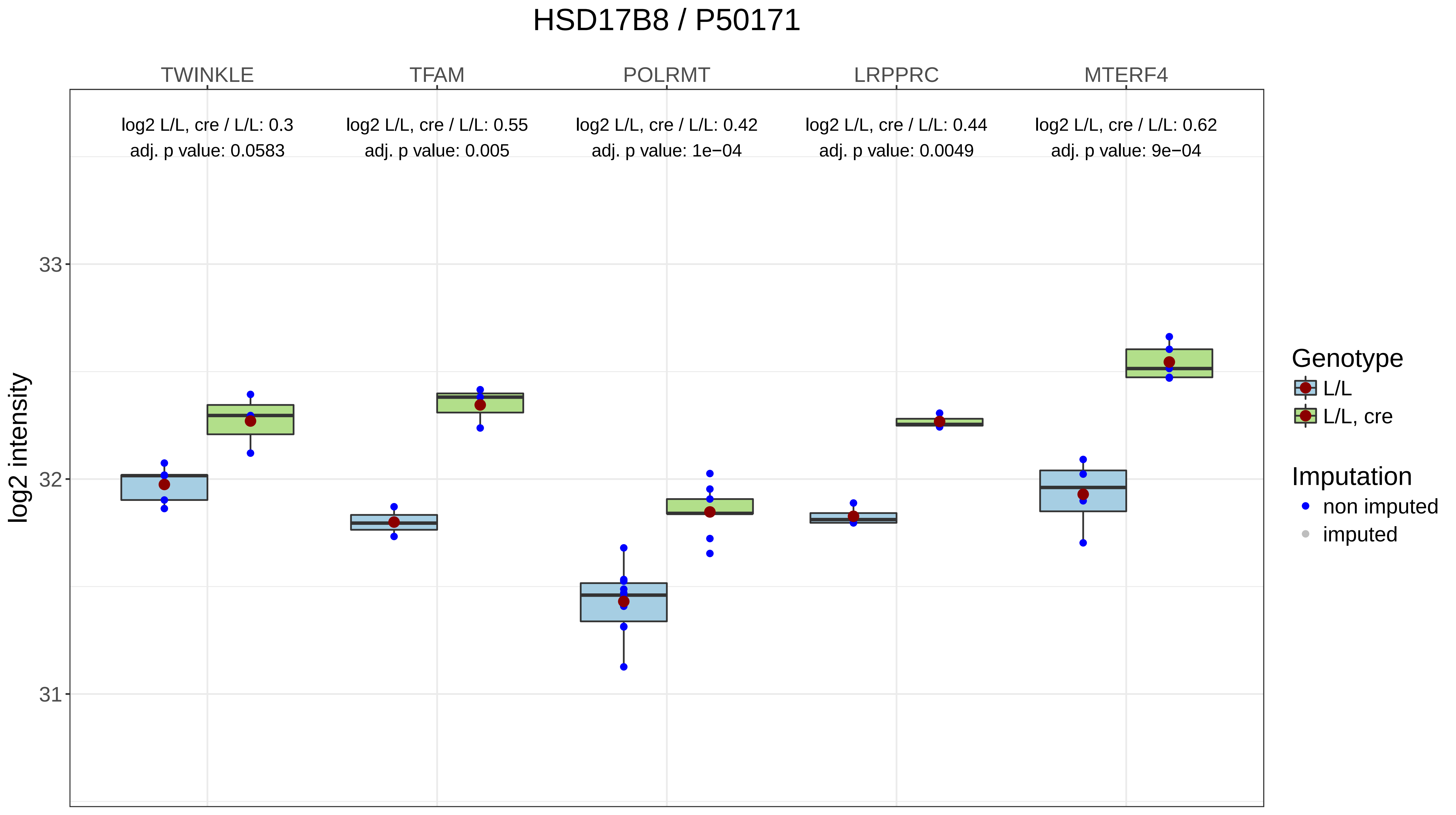
HSD17B8 / P50171
TWINKLE
TFAM
POLRMT
LRPPRC
MTERF4
log2 L/L, cre / L/L: 0.3
adj. p value: 0.0583
log2 L/L, cre / L/L: 0.55
adj. p value: 0.005
log2 L/L, cre / L/L: 0.42
adj. p value: 1e−04
log2 L/L, cre / L/L: 0.44
adj. p value: 0.0049
log2 L/L, cre / L/L: 0.62
adj. p value: 9e−04
log2 intensity
33
32
31
Genotype
L/L
L/L, cre
Imputation
non imputed
imputed

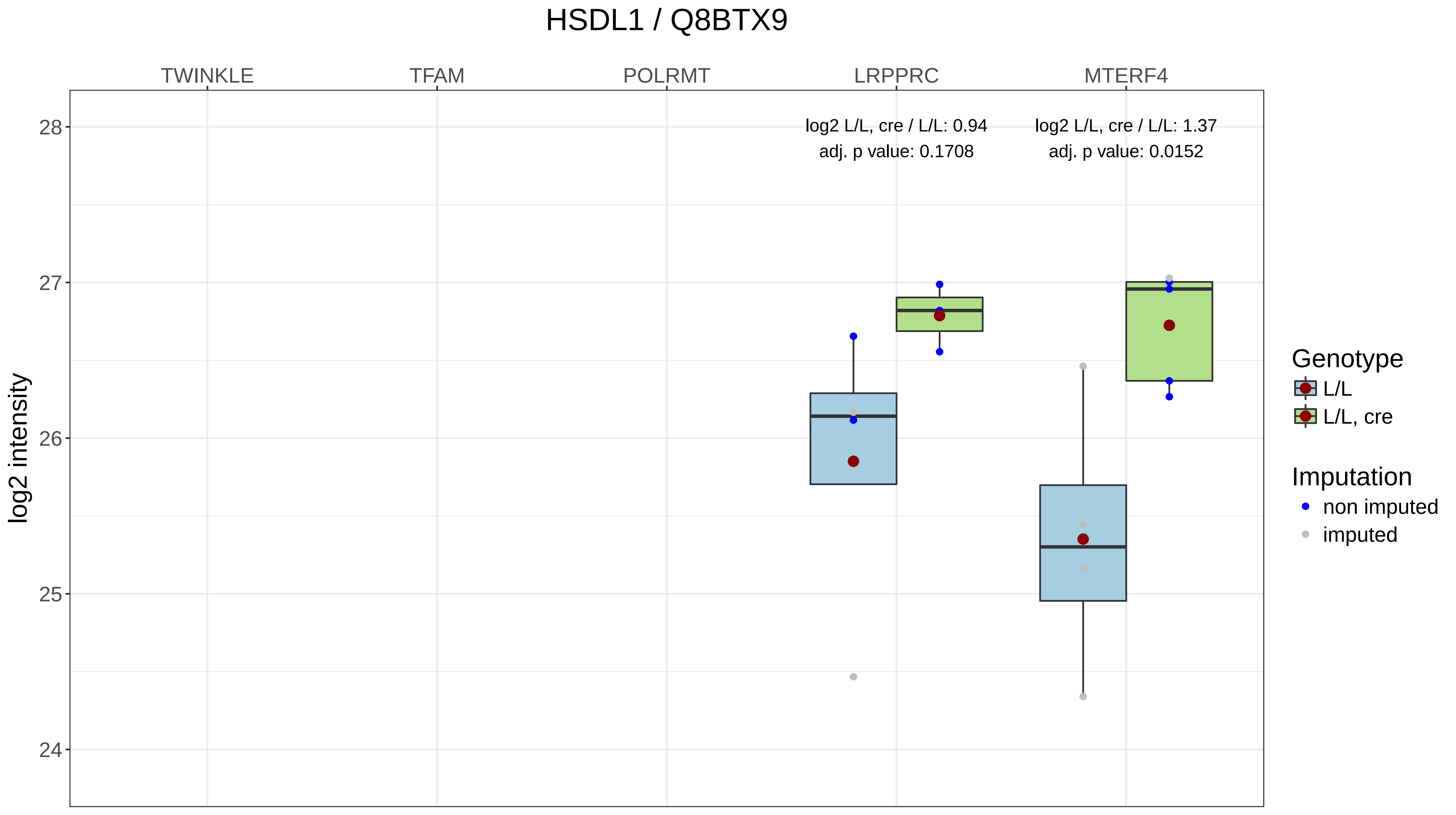
HSDL1 / Q8BTX9
TWINKLE
TFAM
POLRMT
LRPPRC
MTERF4
log2 L/L, cre / L/L: 0.94
adj. p value: 0.1708
log2 L/L, cre / L/L: 1.37
adj. p value: 0.0152
28
27
26
25
24
log2 intensity
Genotype
L/L
L/L, cre
Imputation
non imputed
imputed

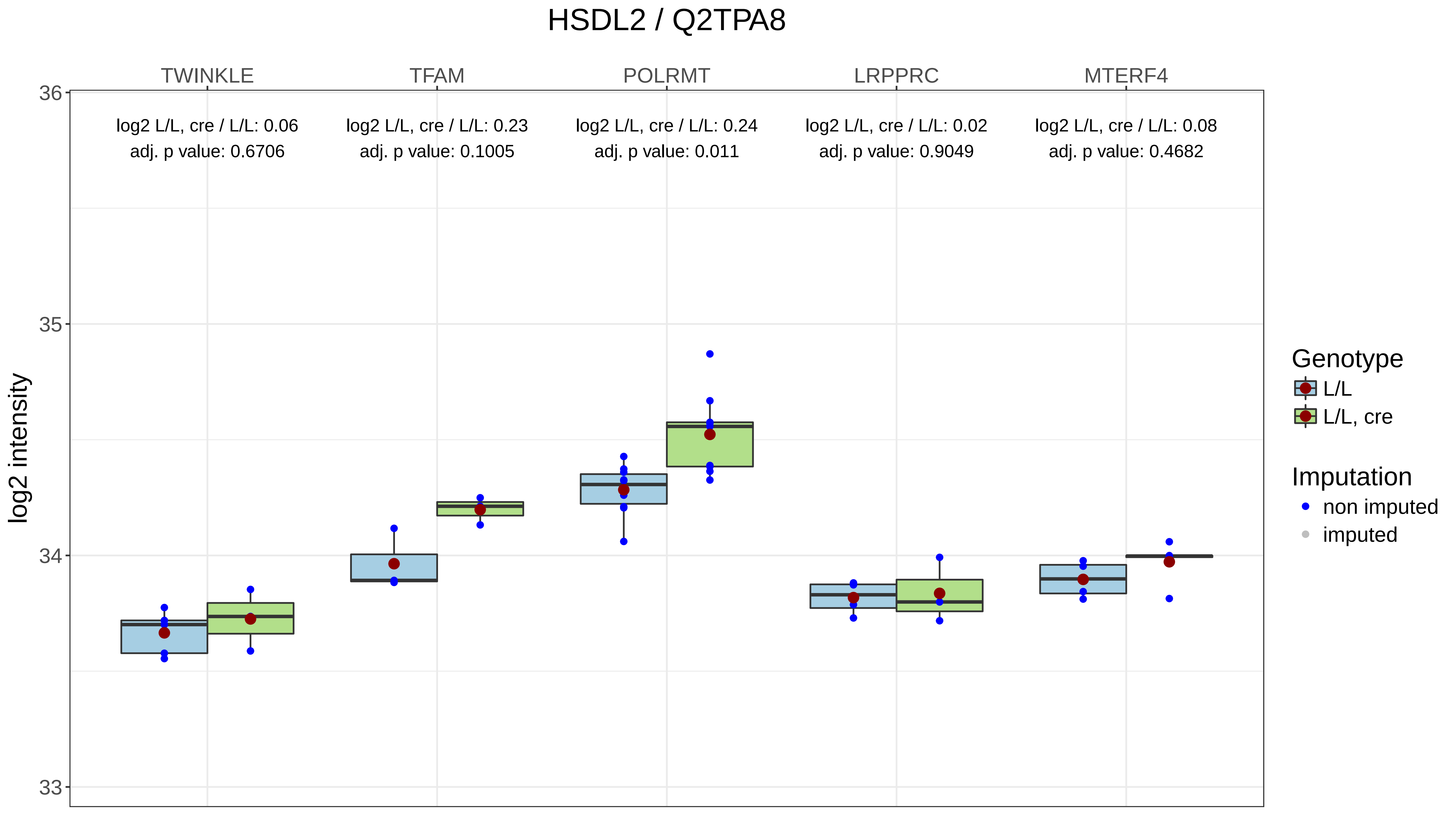
HSDL2 / Q2TPA8
TWINKLE
TFAM
POLRMT
LRPPRC
MTERF4
log2 L/L, cre / L/L: 0.06
adj. p value: 0.6706
log2 L/L, cre / L/L: 0.23
adj. p value: 0.1005
log2 L/L, cre / L/L: 0.24
adj. p value: 0.011
log2 L/L, cre / L/L: 0.02
adj. p value: 0.9049
log2 L/L, cre / L/L: 0.08
adj. p value: 0.4682
36
35
34
33
log2 intensity
Genotype
L/L
L/L, cre
Imputation
non imputed
imputed

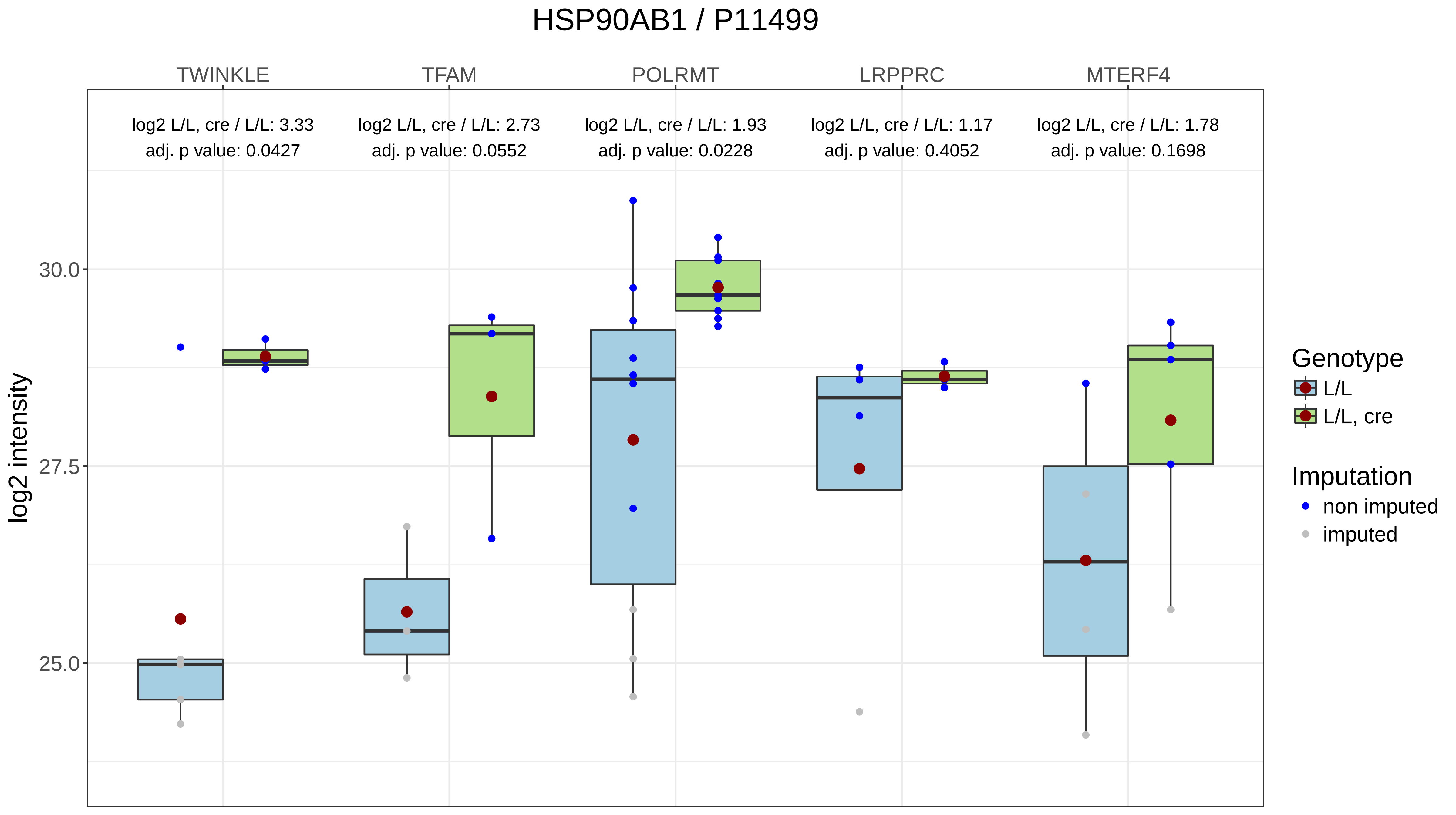
HSP90AB1 / P11499
TWINKLE
TFAM
POLRMT
LRPPRC
MTERF4
log2 L/L, cre / L/L: 3.33
adj. p value: 0.0427
log2 L/L, cre / L/L: 2.73
adj. p value: 0.0552
log2 L/L, cre / L/L: 1.93
adj. p value: 0.0228
log2 L/L, cre / L/L: 1.17
adj. p value: 0.4052
log2 L/L, cre / L/L: 1.78
adj. p value: 0.1698
log2 intensity
30.0
27.5
25.0
Genotype
L/L
L/L, cre
Imputation
non imputed
imputed

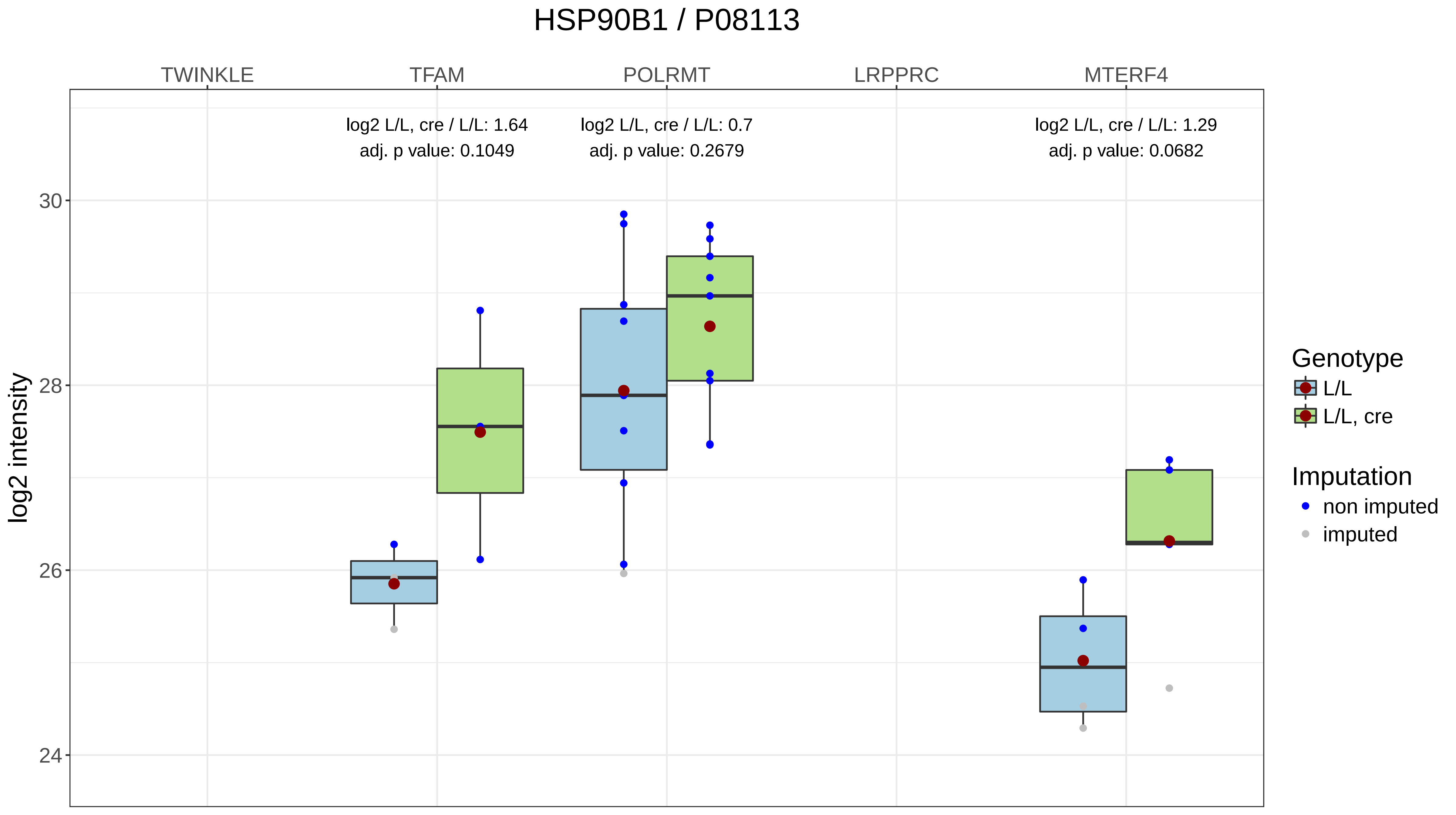
HSP90B1 / P08113
TWINKLE
TFAM
POLRMT
LRPPRC
MTERF4
log2 L/L, cre / L/L: 1.64
adj. p value: 0.1049
log2 L/L, cre / L/L: 0.7
adj. p value: 0.2679
log2 L/L, cre / L/L: 1.29
adj. p value: 0.0682
log2 intensity
30
28
26
24
Genotype
L/L
L/L, cre
Imputation
non imputed
imputed

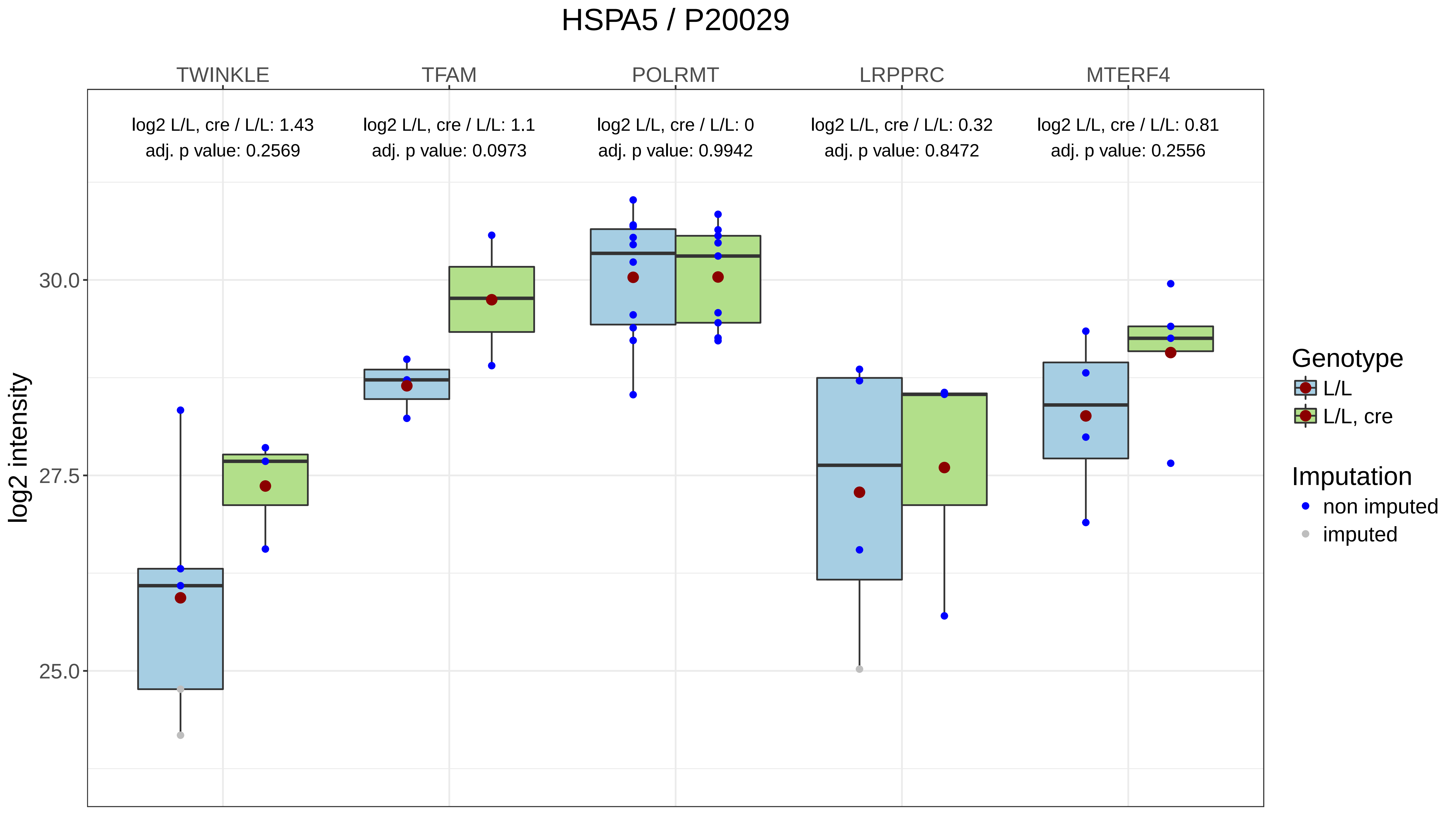

HSPA5 / P20029
TWINKLE
TFAM
POLRMT
LRPPRC
MTERF4
log2 L/L, cre / L/L: 1.43
adj. p value: 0.2569
log2 L/L, cre / L/L: 1.1
adj. p value: 0.0973
log2 L/L, cre / L/L: 0
adj. p value: 0.9942
log2 L/L, cre / L/L: 0.32
adj. p value: 0.8472
log2 L/L, cre / L/L: 0.81
adj. p value: 0.2556
30.0
27.5
25.0
log2 intensity
Genotype
L/L
L/L, cre
Imputation
non imputed
imputed

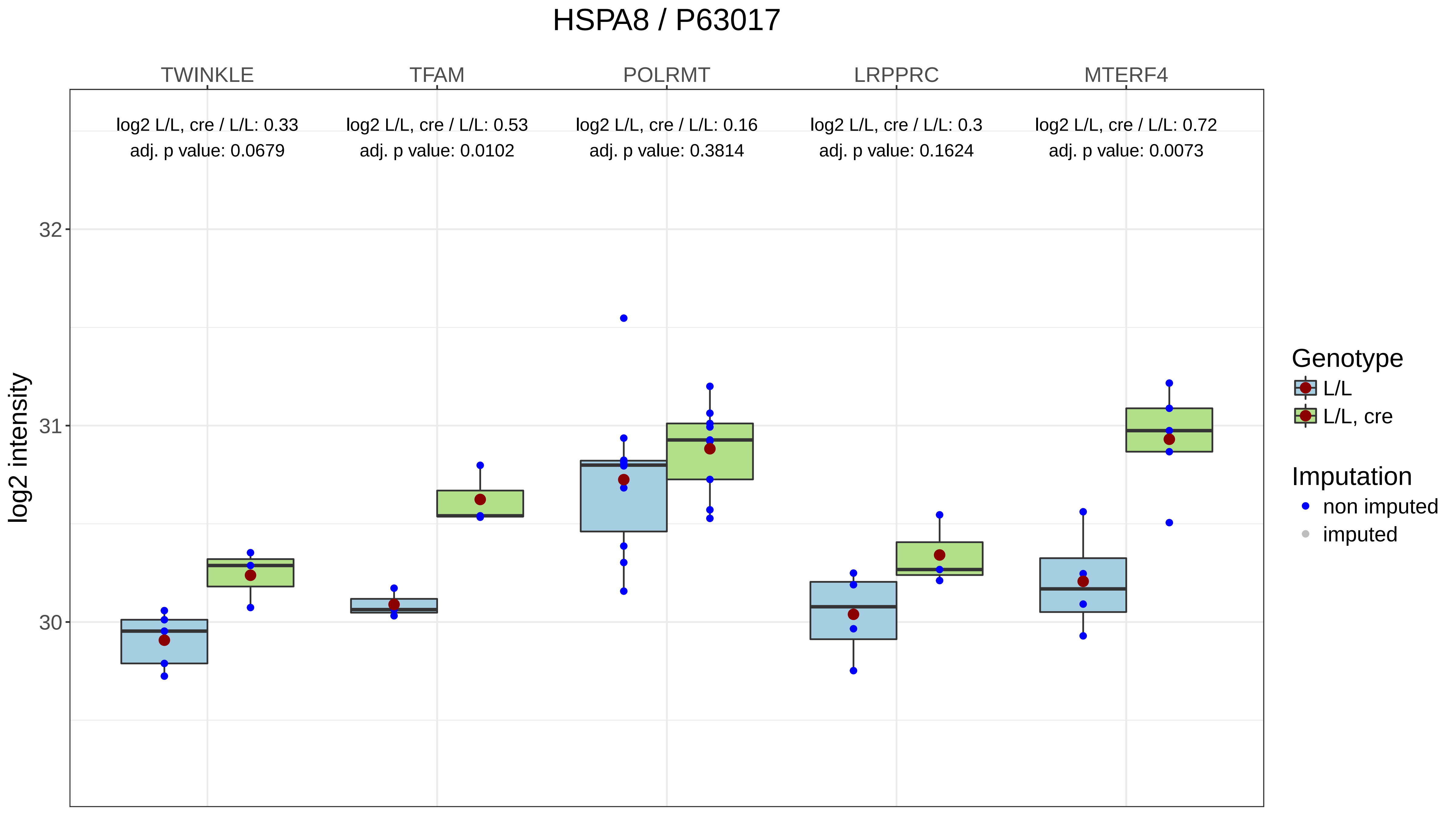
HSPA8 / P63017
TWINKLE
TFAM
POLRMT
LRPPRC
MTERF4
log2 L/L, cre / L/L: 0.33
adj. p value: 0.0679
log2 L/L, cre / L/L: 0.53
adj. p value: 0.0102
log2 L/L, cre / L/L: 0.16
adj. p value: 0.3814
log2 L/L, cre / L/L: 0.3
adj. p value: 0.1624
log2 L/L, cre / L/L: 0.72
adj. p value: 0.0073
log2 intensity
32
31
30
Genotype
L/L
L/L, cre
Imputation
non imputed
imputed

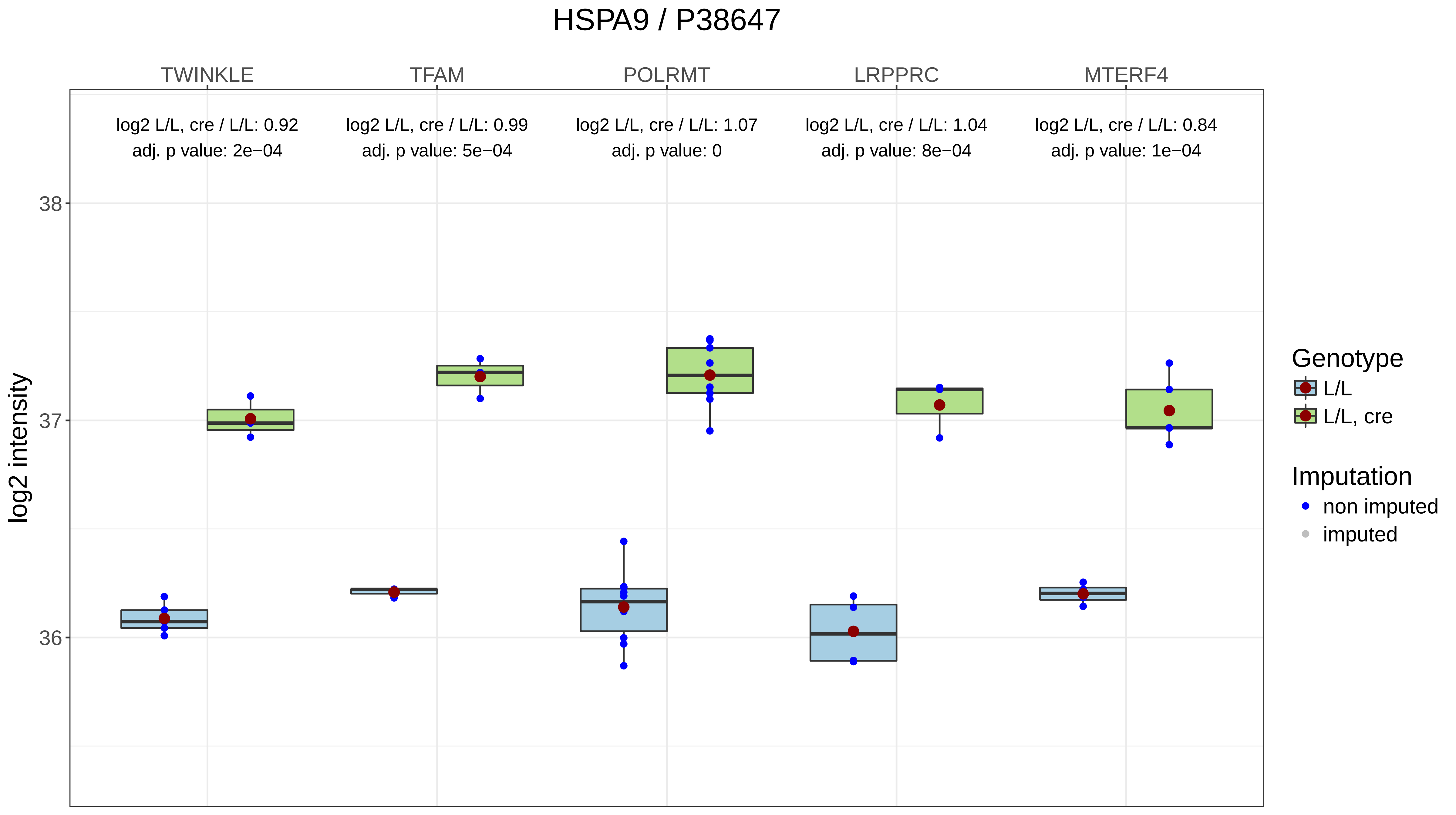
HSPA9 / P38647
TWINKLE
TFAM
POLRMT
LRPPRC
MTERF4
log2 L/L, cre / L/L: 0.92
adj. p value: 2e−04
log2 L/L, cre / L/L: 0.99
adj. p value: 5e−04
log2 L/L, cre / L/L: 1.07
adj. p value: 0
log2 L/L, cre / L/L: 1.04
adj. p value: 8e−04
log2 L/L, cre / L/L: 0.84
adj. p value: 1e−04
log2 intensity
38
37
36
Genotype
L/L
L/L, cre
Imputation
non imputed
imputed

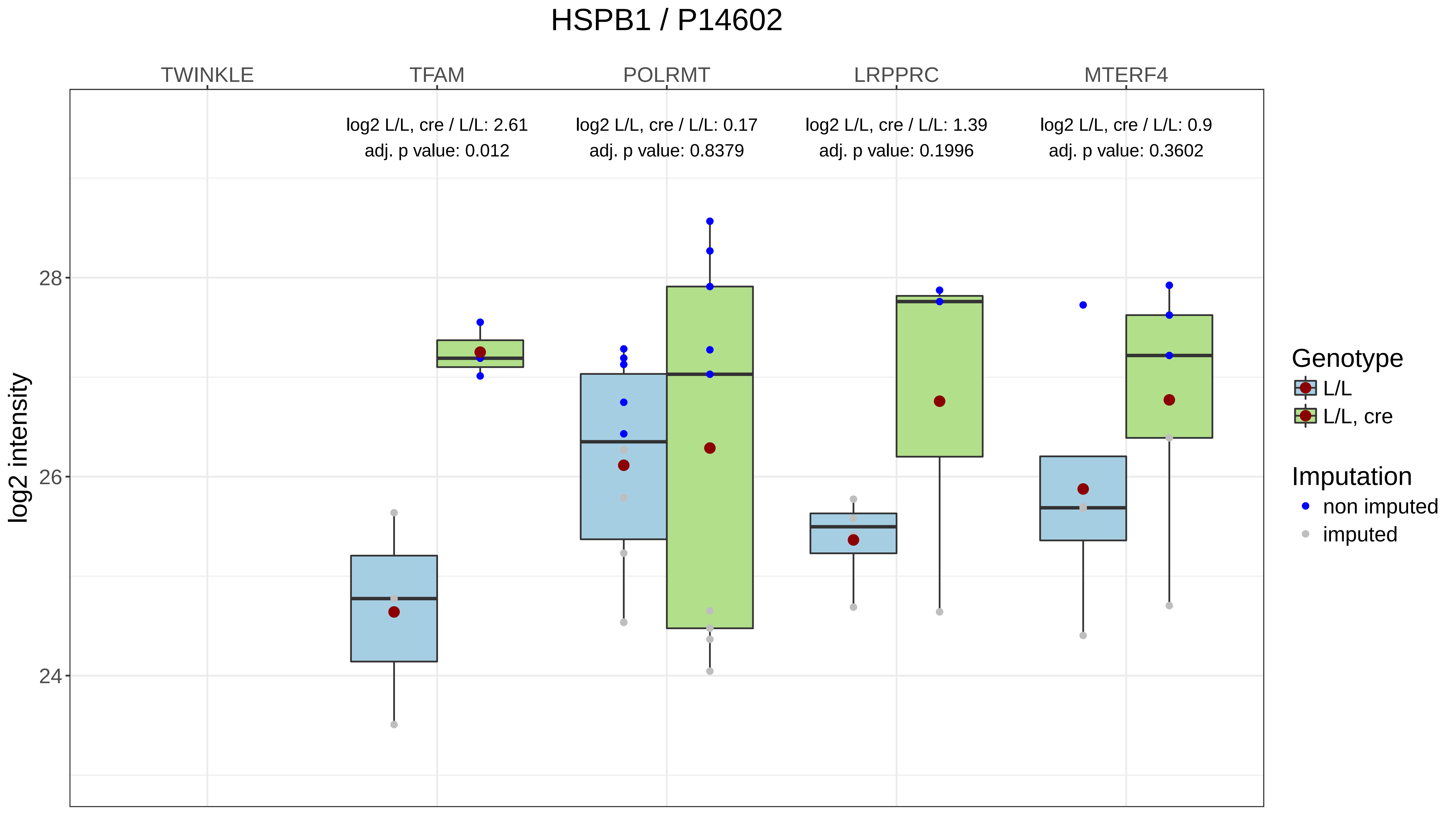
HSPB1 / P14602
TWINKLE
TFAM
POLRMT
LRPPRC
MTERF4
log2 L/L, cre / L/L: 2.61
adj. p value: 0.012
log2 L/L, cre / L/L: 0.17
adj. p value: 0.8379
log2 L/L, cre / L/L: 1.39
adj. p value: 0.1996
log2 L/L, cre / L/L: 0.9
adj. p value: 0.3602
log2 intensity
28
26
24
Genotype
L/L
L/L, cre
Imputation
non imputed
imputed

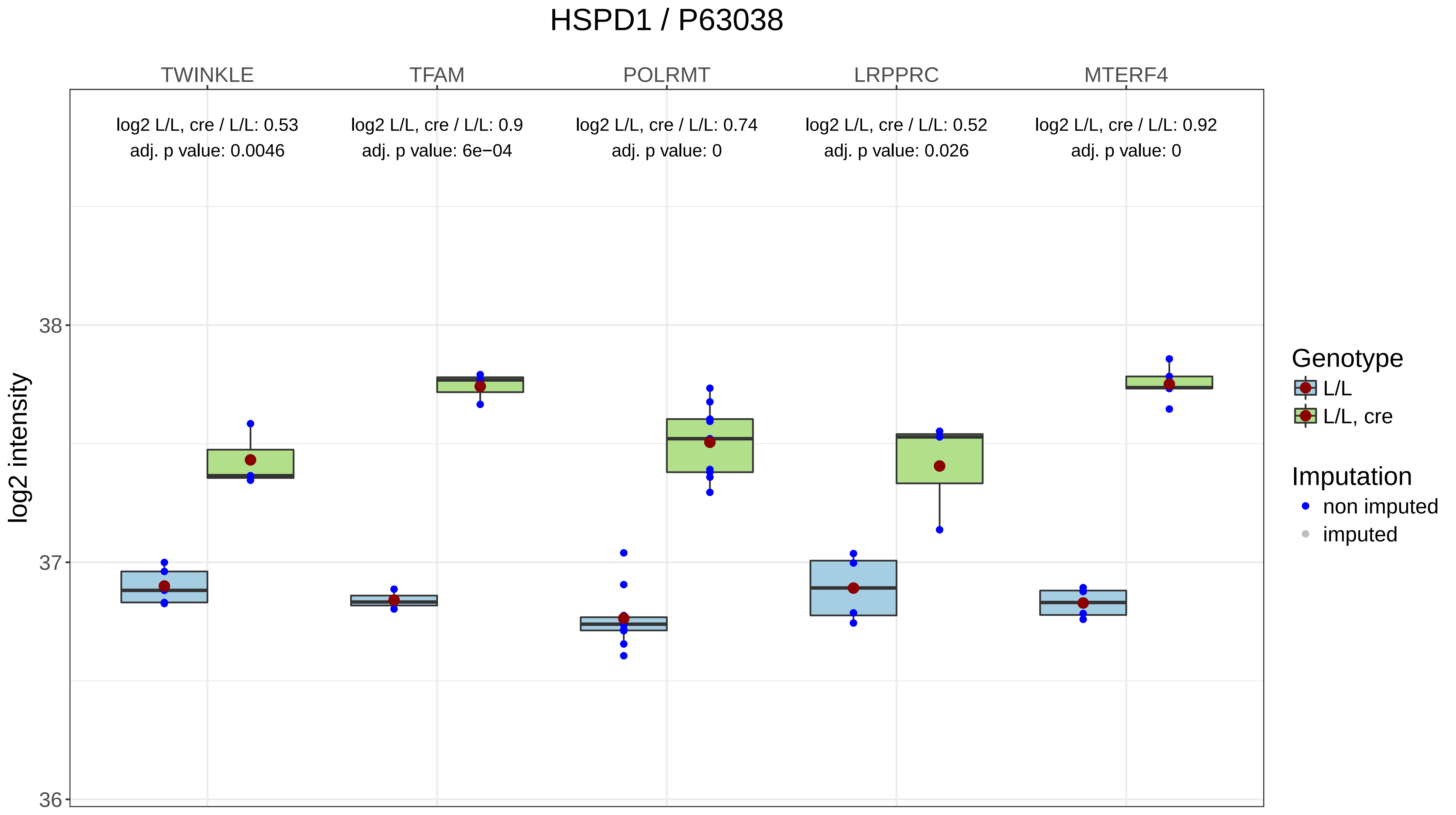
HSPD1 / P63038
TWINKLE
TFAM
POLRMT
LRPPRC
MTERF4
log2 L/L, cre / L/L: 0.53
adj. p value: 0.0046
log2 L/L, cre / L/L: 0.9
adj. p value: 6e−04
log2 L/L, cre / L/L: 0.74
adj. p value: 0
log2 L/L, cre / L/L: 0.52
adj. p value: 0.026
log2 L/L, cre / L/L: 0.92
adj. p value: 0
log2 intensity
38
37
36
Genotype
L/L
L/L, cre
Imputation
non imputed
imputed

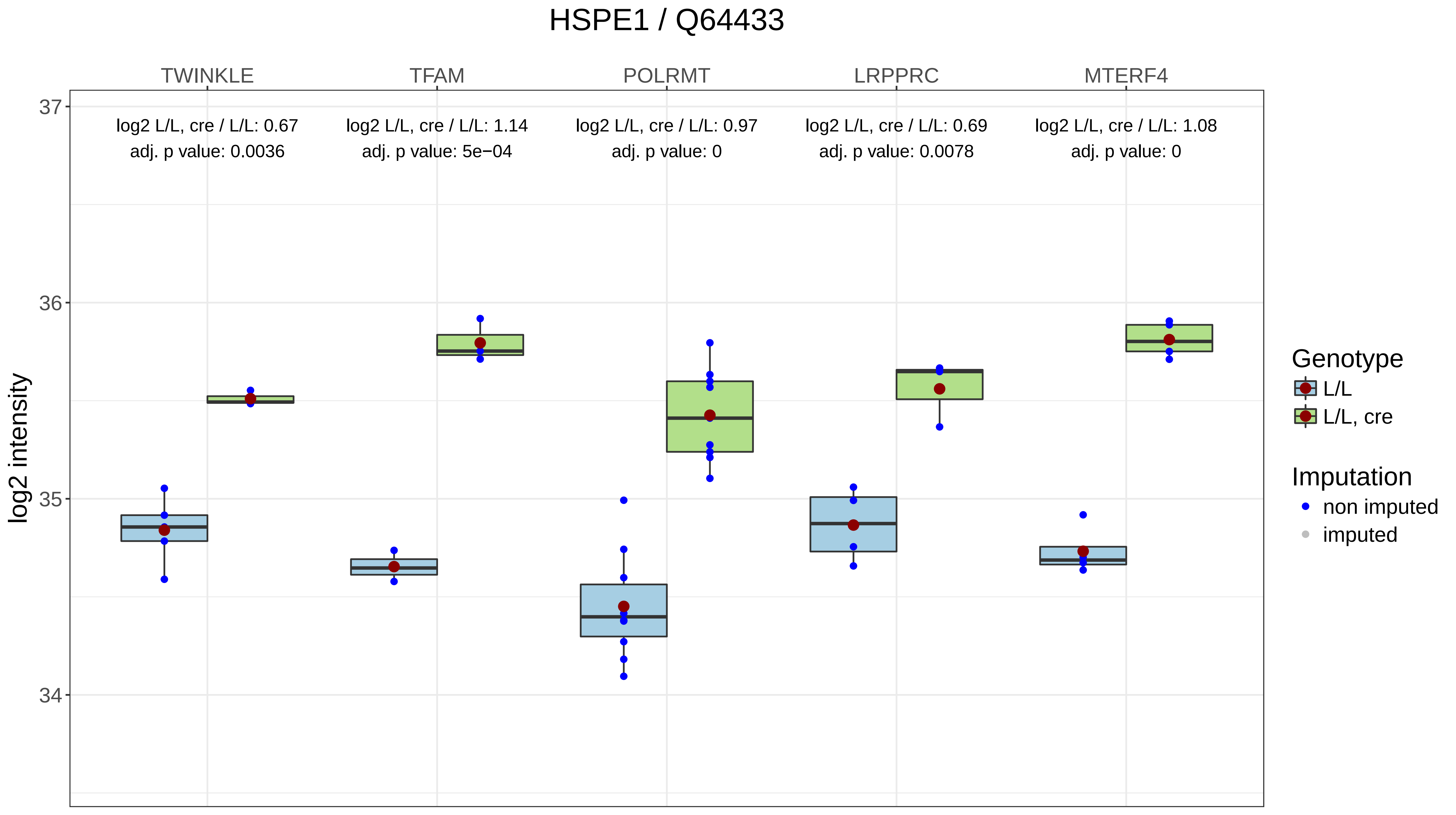
HSPE1 / Q64433
TWINKLE
TFAM
POLRMT
LRPPRC
MTERF4
log2 L/L, cre / L/L: 0.67
adj. p value: 0.0036
log2 L/L, cre / L/L: 1.14
adj. p value: 5e−04
log2 L/L, cre / L/L: 0.97
adj. p value: 0
log2 L/L, cre / L/L: 0.69
adj. p value: 0.0078
log2 L/L, cre / L/L: 1.08
adj. p value: 0
log2 intensity
37
36
35
34
Genotype
L/L
L/L, cre
Imputation
non imputed
imputed

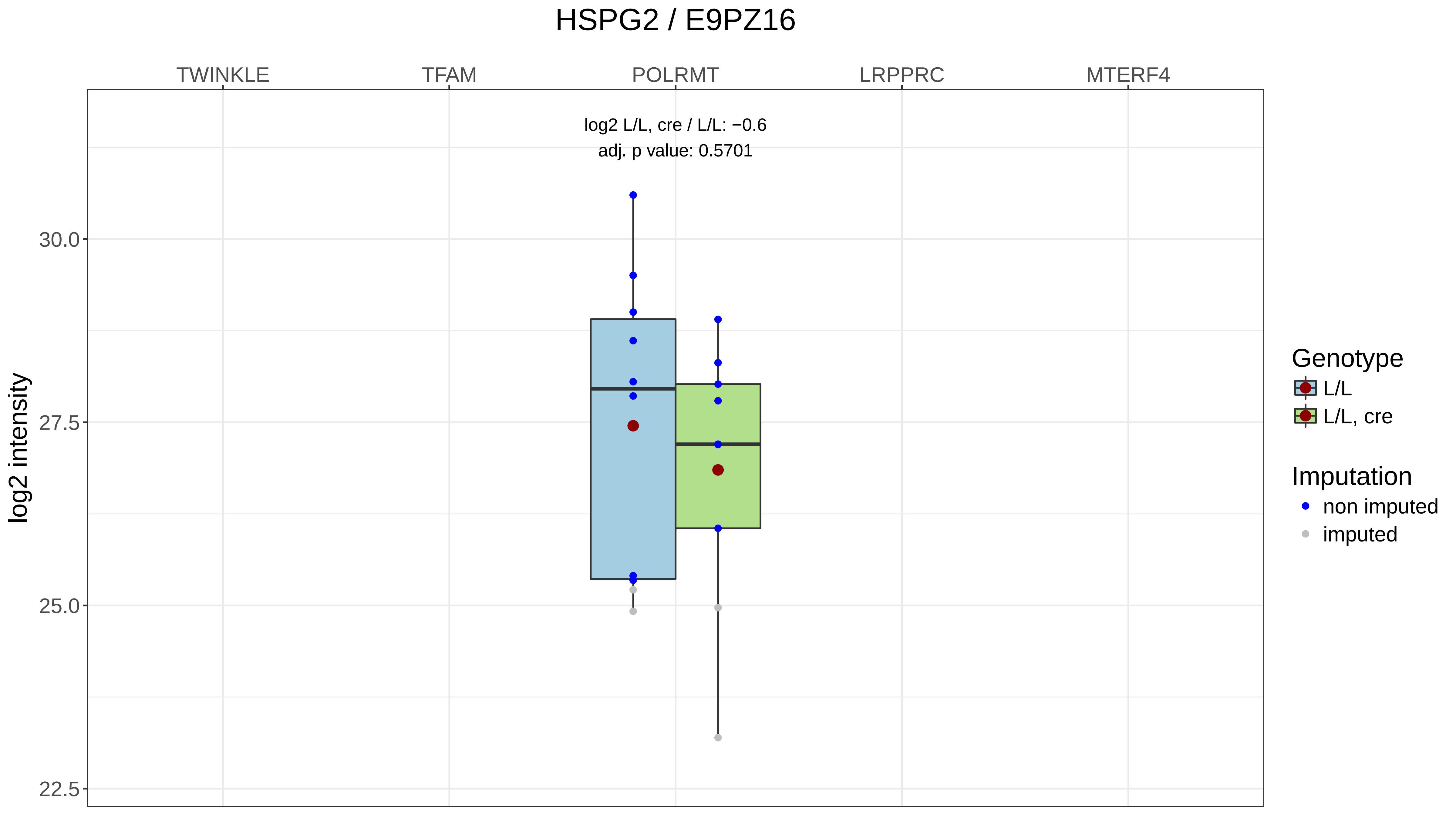

HSPG2 / E9PZ16
TWINKLE
TFAM
POLRMT
LRPPRC
MTERF4
log2 L/L, cre / L/L: −0.6
adj. p value: 0.5701
30.0
27.5
25.0
22.5
log2 intensity
Genotype
L/L
L/L, cre
Imputation
non imputed
imputed

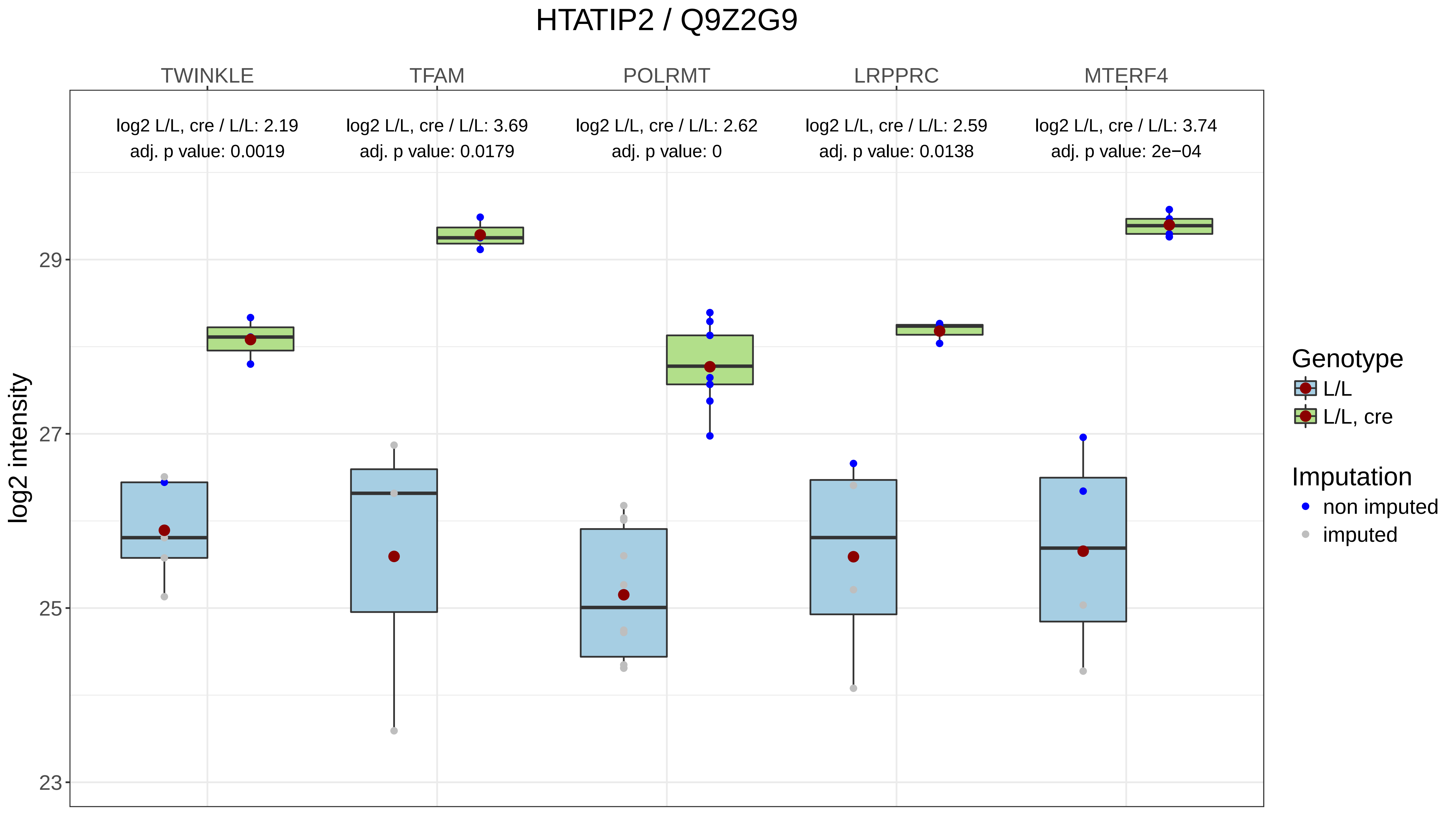
HTATIP2 / Q9Z2G9
TWINKLE
TFAM
POLRMT
LRPPRC
MTERF4
log2 L/L, cre / L/L: 2.19
adj. p value: 0.0019
log2 L/L, cre / L/L: 3.69
adj. p value: 0.0179
log2 L/L, cre / L/L: 2.62
adj. p value: 0
log2 L/L, cre / L/L: 2.59
adj. p value: 0.0138
log2 L/L, cre / L/L: 3.74
adj. p value: 2e−04
log2 intensity
29
27
25
23
Genotype
L/L
L/L, cre
Imputation
non imputed
imputed

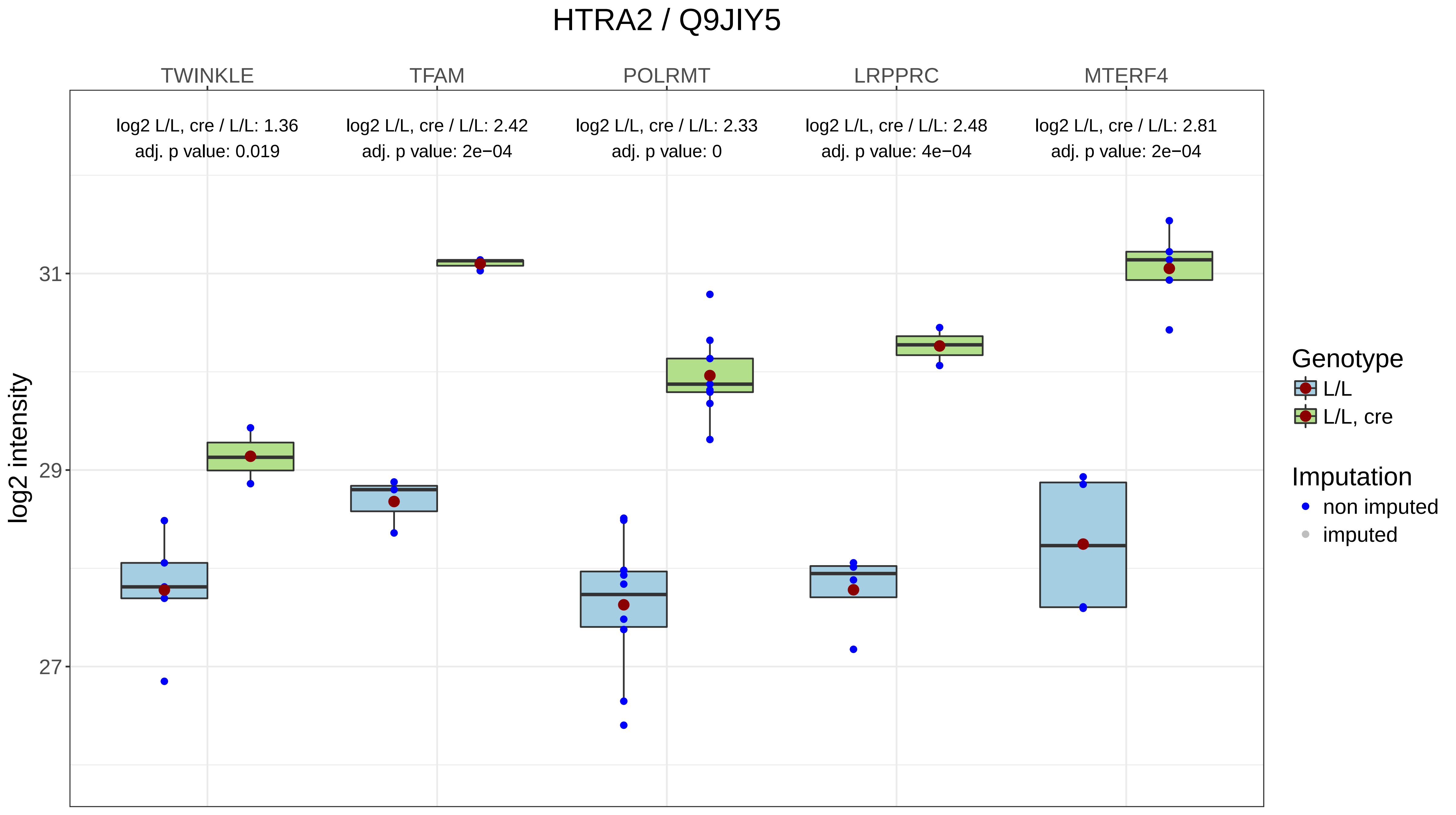
HTRA2 / Q9JIY5
TWINKLE
TFAM
POLRMT
LRPPRC
MTERF4
log2 L/L, cre / L/L: 1.36
adj. p value: 0.019
log2 L/L, cre / L/L: 2.42
adj. p value: 2e−04
log2 L/L, cre / L/L: 2.33
adj. p value: 0
log2 L/L, cre / L/L: 2.48
adj. p value: 4e−04
log2 L/L, cre / L/L: 2.81
adj. p value: 2e−04
log2 intensity
31
29
27
Genotype
L/L
L/L, cre
Imputation
non imputed
imputed

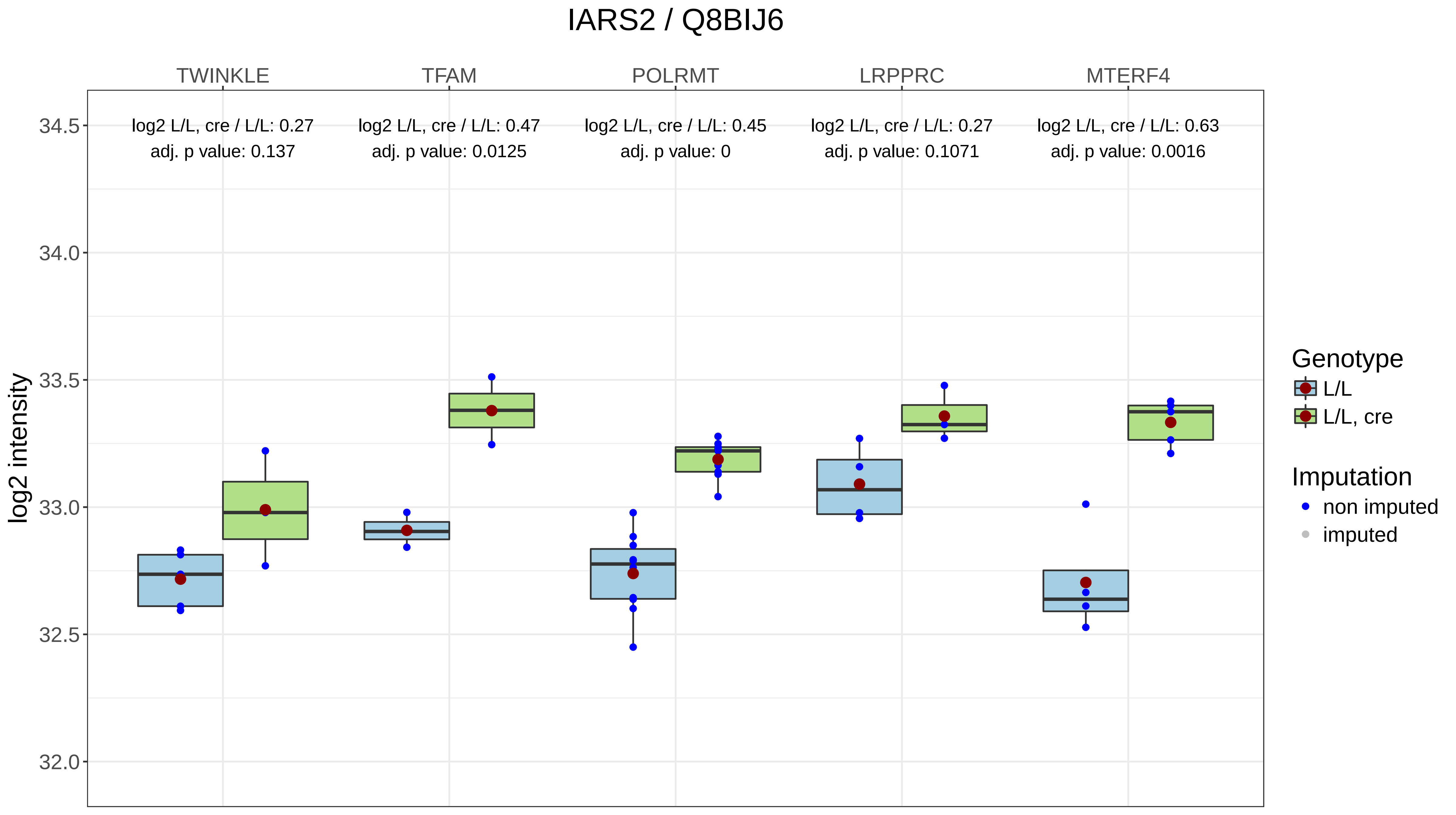
IARS2 / Q8BIJ6
TWINKLE
TFAM
POLRMT
LRPPRC
MTERF4
log2 L/L, cre / L/L: 0.27
adj. p value: 0.137
log2 L/L, cre / L/L: 0.47
adj. p value: 0.0125
log2 L/L, cre / L/L: 0.45
adj. p value: 0
log2 L/L, cre / L/L: 0.27
adj. p value: 0.1071
log2 L/L, cre / L/L: 0.63
adj. p value: 0.0016
log2 intensity
34.5
34.0
33.5
33.0
32.5
32.0
Genotype
L/L
L/L, cre
Imputation
non imputed
imputed

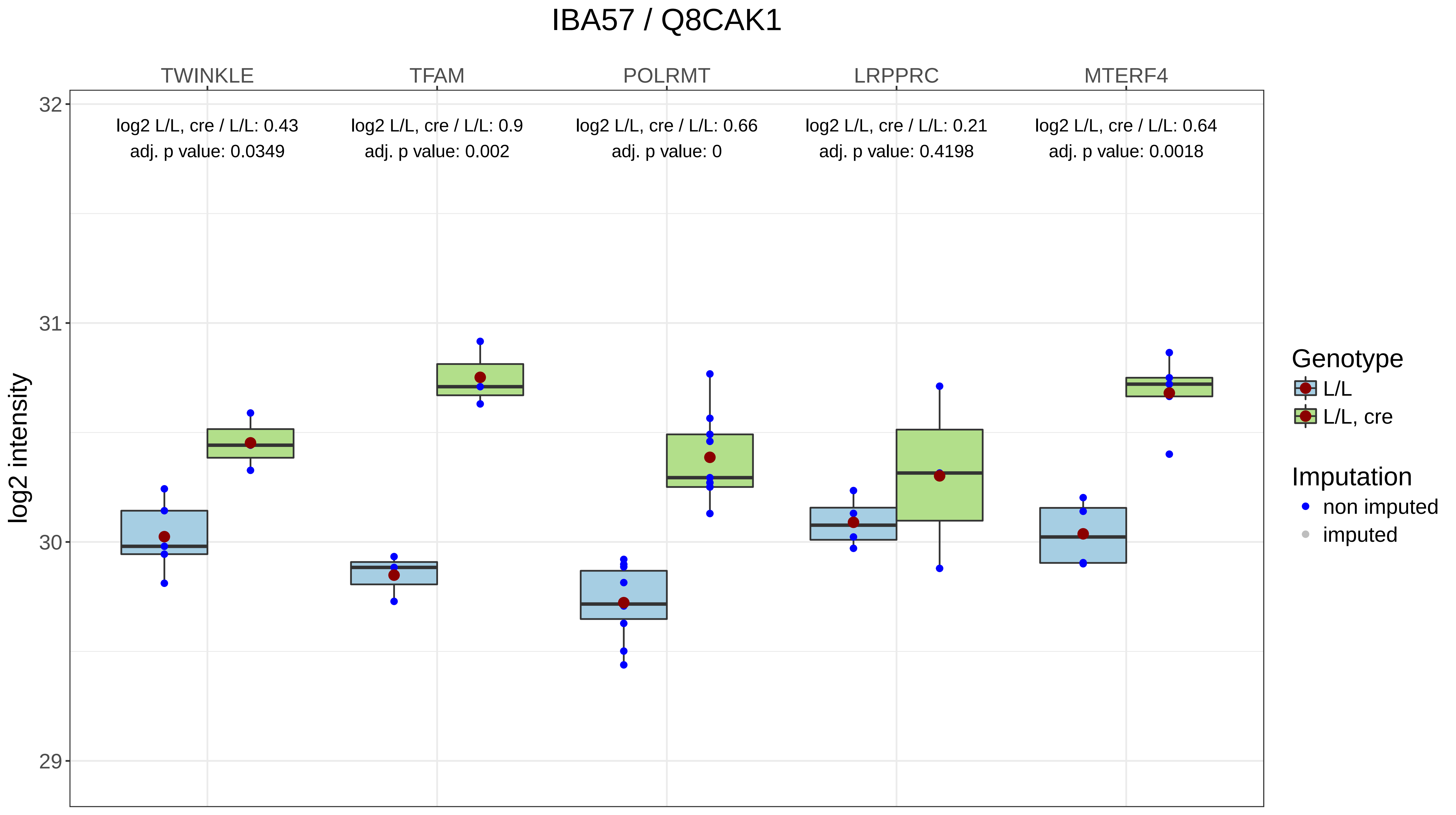
IBA57 / Q8CAK1
TWINKLE
TFAM
POLRMT
LRPPRC
MTERF4
log2 L/L, cre / L/L: 0.43
adj. p value: 0.0349
log2 L/L, cre / L/L: 0.9
adj. p value: 0.002
log2 L/L, cre / L/L: 0.66
adj. p value: 0
log2 L/L, cre / L/L: 0.21
adj. p value: 0.4198
log2 L/L, cre / L/L: 0.64
adj. p value: 0.0018
log2 intensity
32
31
30
29
Genotype
L/L
L/L, cre
Imputation
non imputed
imputed

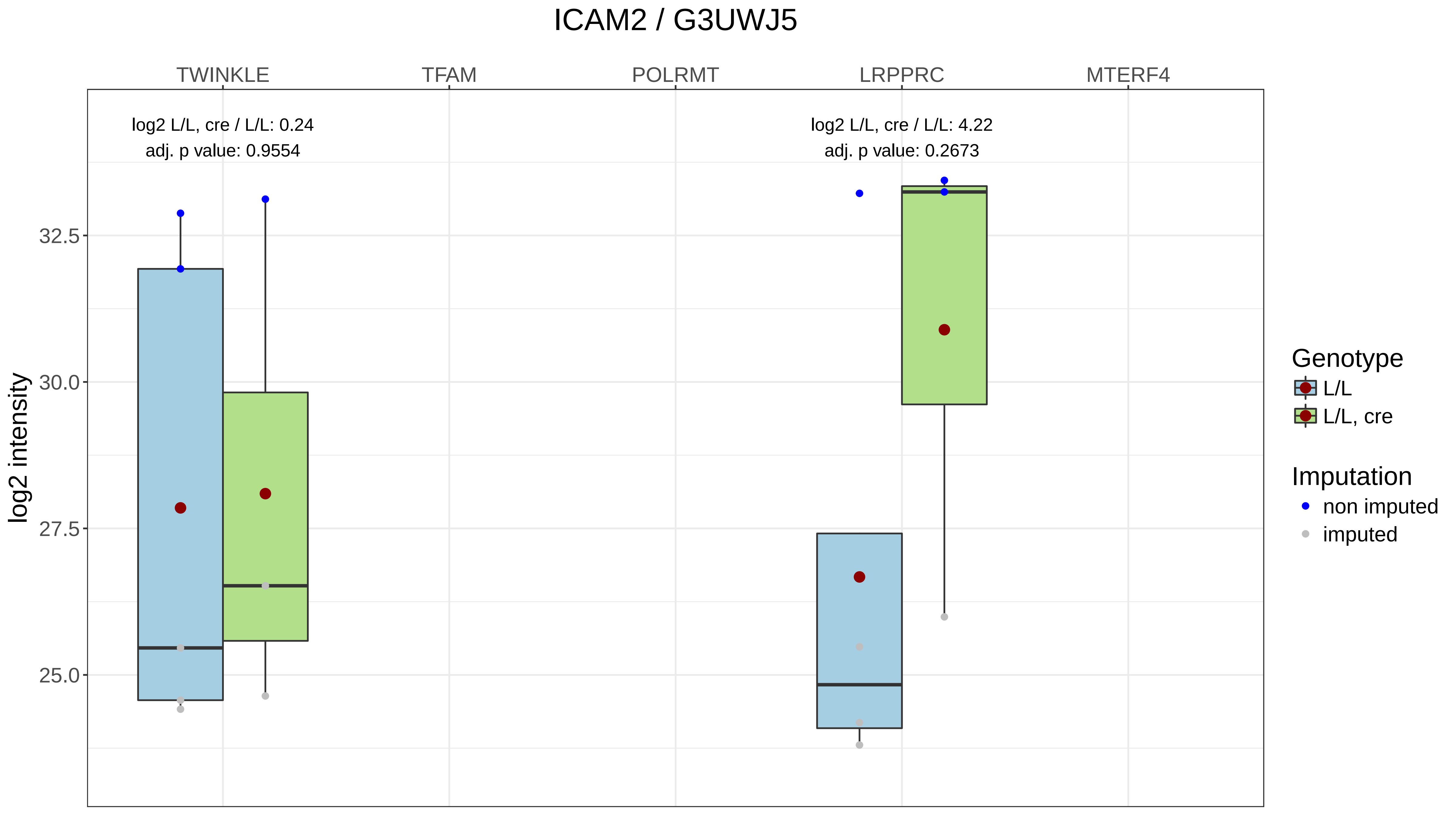
ICAM2 / G3UWJ5
TWINKLE
TFAM
POLRMT
LRPPRC
MTERF4
log2 L/L, cre / L/L: 0.24
adj. p value: 0.9554
log2 L/L, cre / L/L: 4.22
adj. p value: 0.2673
log2 intensity
32.5
30.0
27.5
25.0
Genotype
L/L
L/L, cre
Imputation
non imputed
imputed

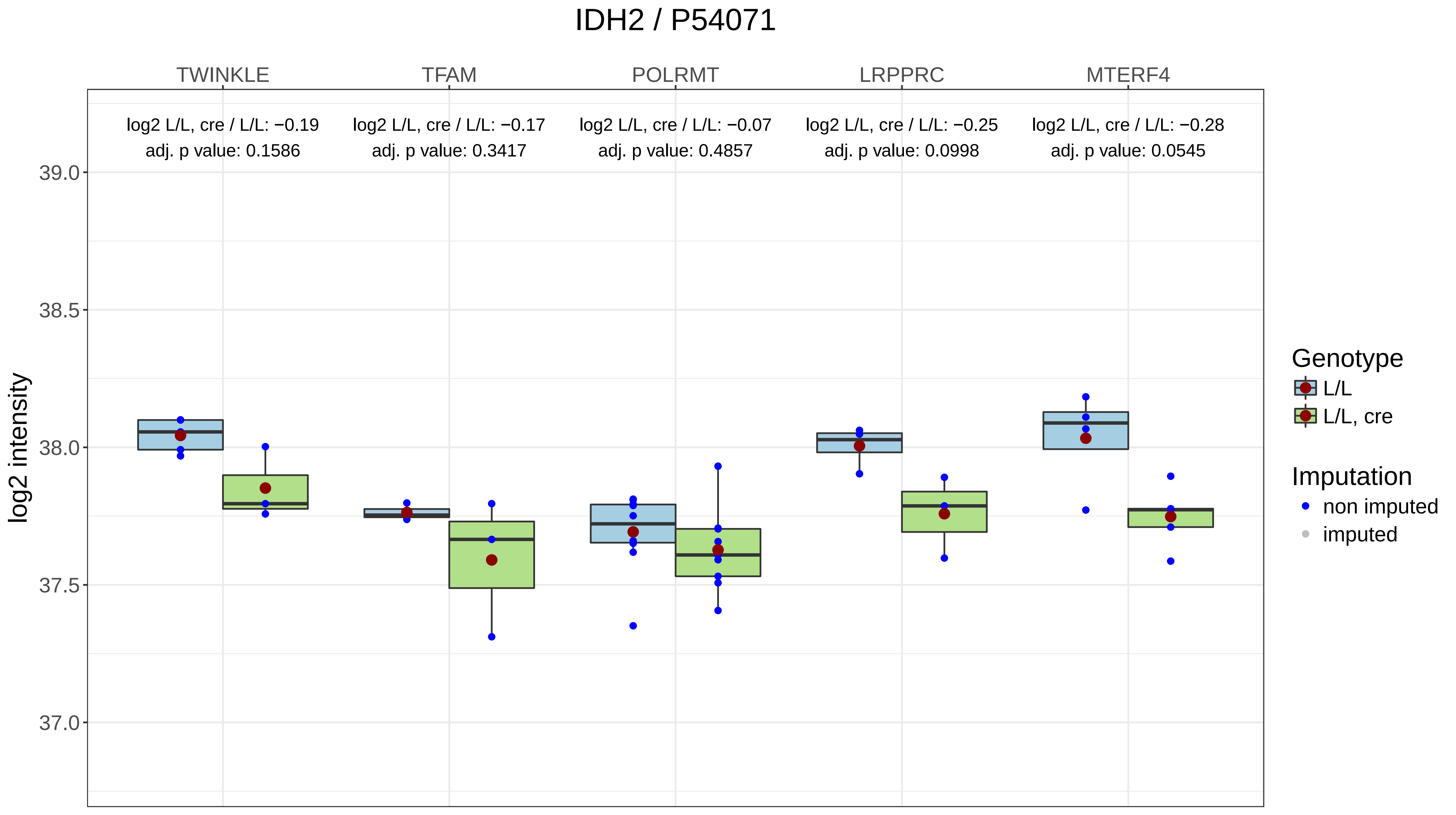
IDH2 / P54071
TWINKLE
TFAM
POLRMT
LRPPRC
MTERF4
log2 L/L, cre / L/L: −0.19
adj. p value: 0.1586
log2 L/L, cre / L/L: −0.17
adj. p value: 0.3417
log2 L/L, cre / L/L: −0.07
adj. p value: 0.4857
log2 L/L, cre / L/L: −0.25
adj. p value: 0.0998
log2 L/L, cre / L/L: −0.28
adj. p value: 0.0545
log2 intensity
39.0
38.5
38.0
37.5
37.0
Genotype
L/L
L/L, cre
Imputation
non imputed
imputed

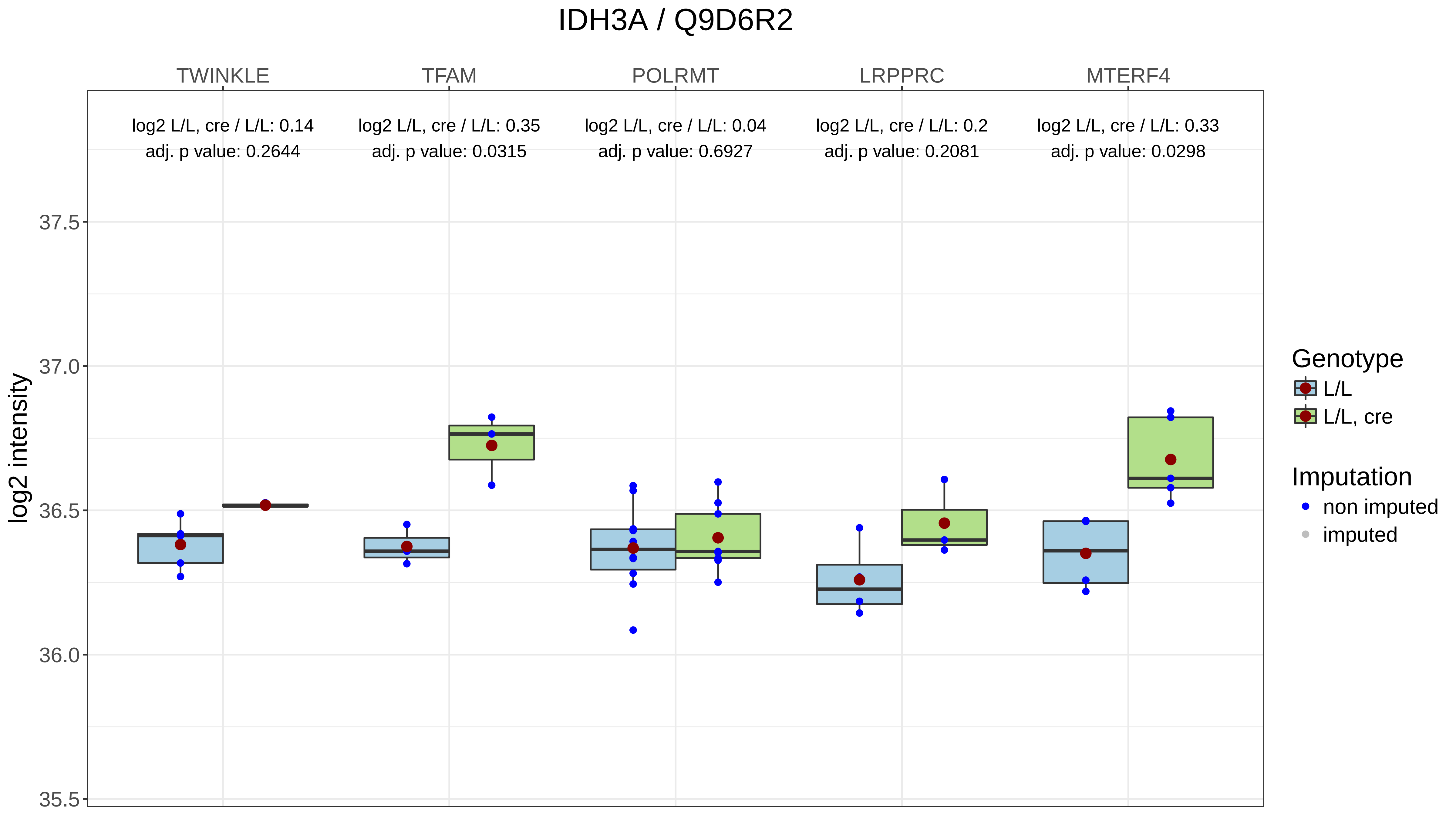
IDH3A / Q9D6R2
TWINKLE
TFAM
POLRMT
LRPPRC
MTERF4
log2 L/L, cre / L/L: 0.14
adj. p value: 0.2644
log2 L/L, cre / L/L: 0.35
adj. p value: 0.0315
log2 L/L, cre / L/L: 0.04
adj. p value: 0.6927
log2 L/L, cre / L/L: 0.2
adj. p value: 0.2081
log2 L/L, cre / L/L: 0.33
adj. p value: 0.0298
log2 intensity
37.5
37.0
36.5
36.0
35.5
Genotype
L/L
L/L, cre
Imputation
non imputed
imputed

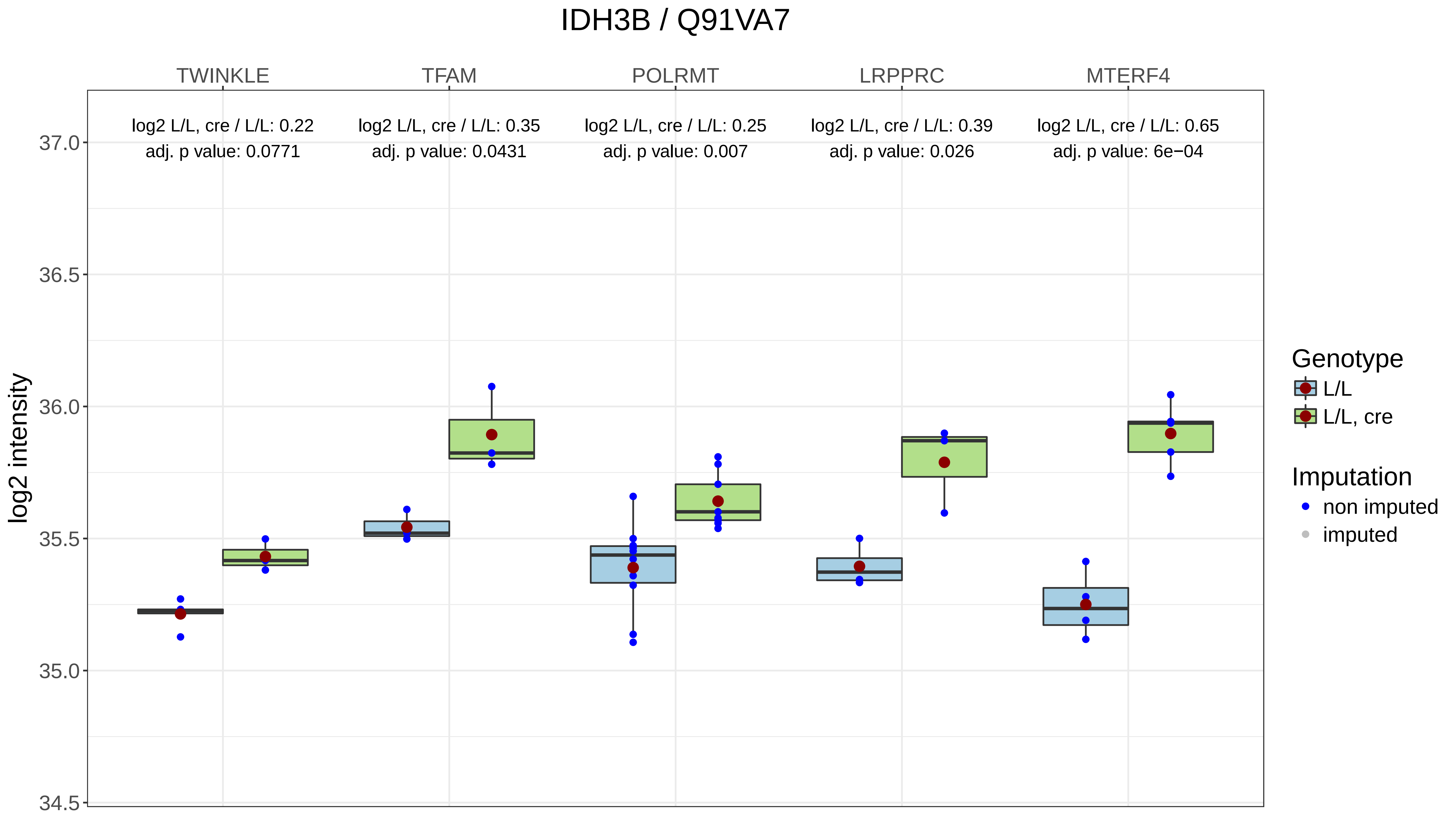
IDH3B / Q91VA7
TWINKLE
TFAM
POLRMT
LRPPRC
MTERF4
log2 L/L, cre / L/L: 0.22
adj. p value: 0.0771
log2 L/L, cre / L/L: 0.35
adj. p value: 0.0431
log2 L/L, cre / L/L: 0.25
adj. p value: 0.007
log2 L/L, cre / L/L: 0.39
adj. p value: 0.026
log2 L/L, cre / L/L: 0.65
adj. p value: 6e−04
log2 intensity
37.0
36.5
36.0
35.5
35.0
34.5
Genotype
L/L
L/L, cre
Imputation
non imputed
imputed

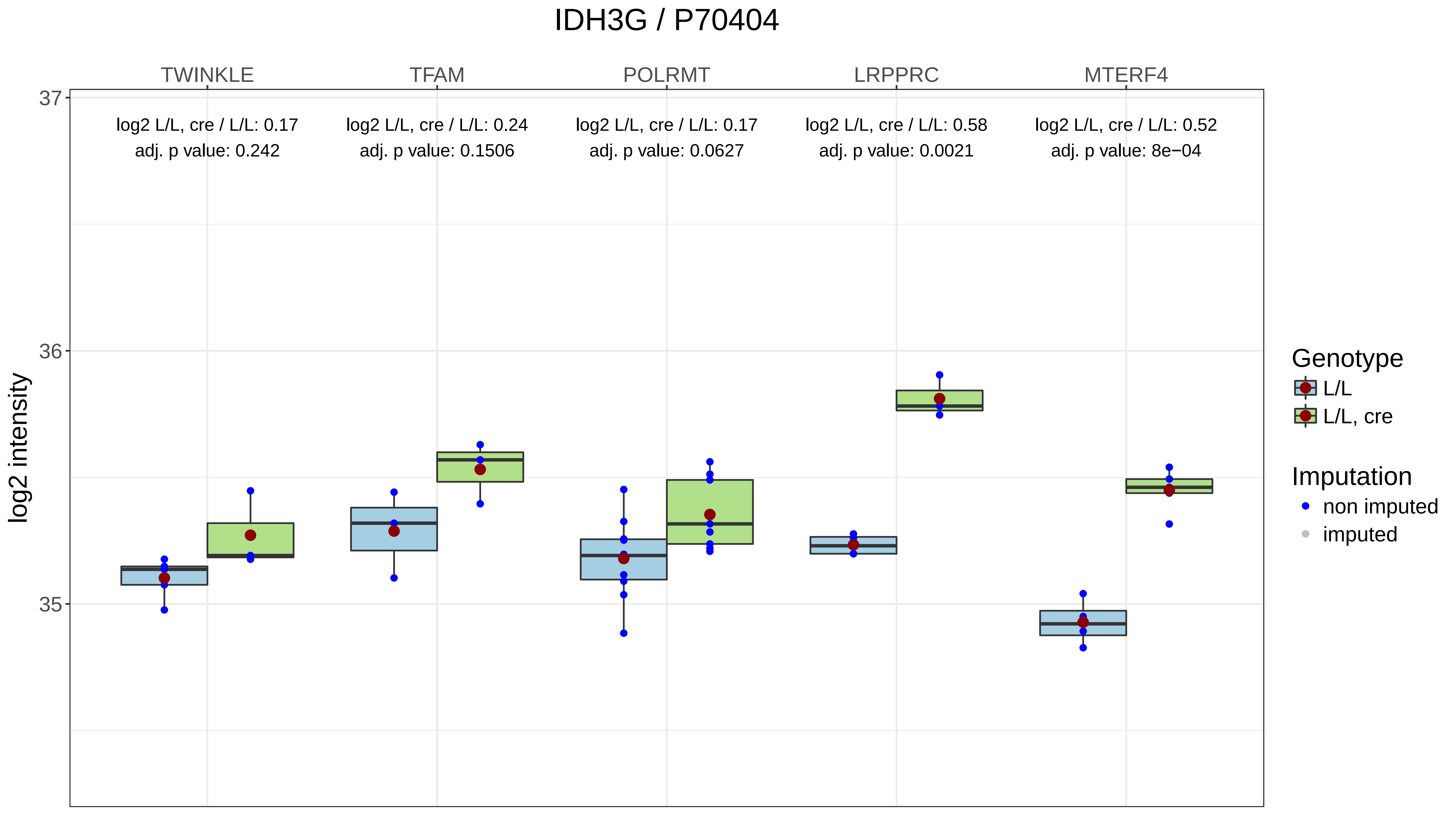
IDH3G / P70404
TWINKLE
TFAM
POLRMT
LRPPRC
MTERF4
log2 L/L, cre / L/L: 0.17
adj. p value: 0.242
log2 L/L, cre / L/L: 0.24
adj. p value: 0.1506
log2 L/L, cre / L/L: 0.17
adj. p value: 0.0627
log2 L/L, cre / L/L: 0.58
adj. p value: 0.0021
log2 L/L, cre / L/L: 0.52
adj. p value: 8e−04
37
36
35
log2 intensity
Genotype
L/L
L/L, cre
Imputation
non imputed
imputed

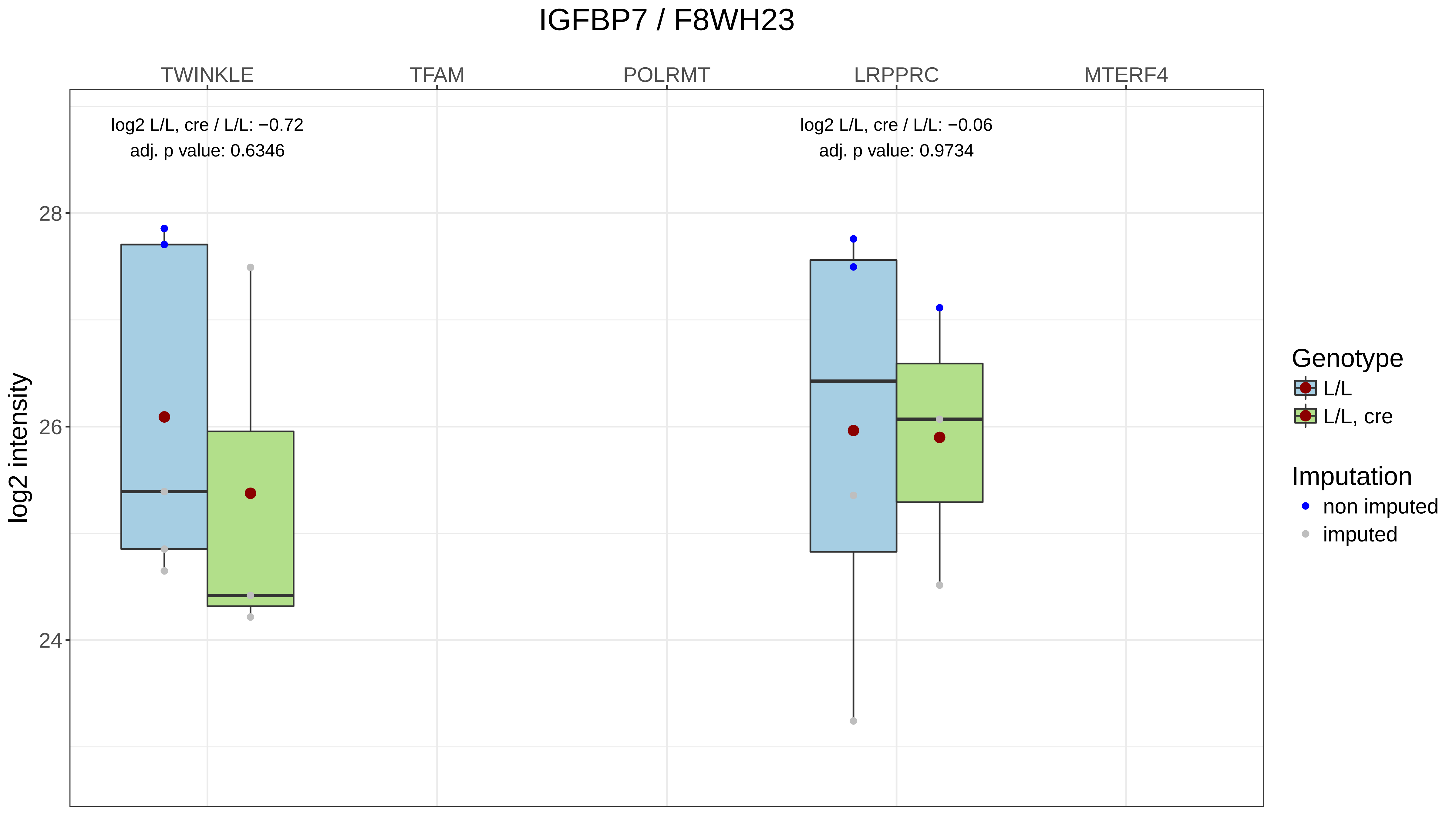

IGFBP7 / F8WH23
TWINKLE
TFAM
POLRMT
LRPPRC
MTERF4
log2 L/L, cre / L/L: −0.72
adj. p value: 0.6346
log2 L/L, cre / L/L: −0.06
adj. p value: 0.9734
28
26
24
log2 intensity
Genotype
L/L
L/L, cre
Imputation
non imputed
imputed

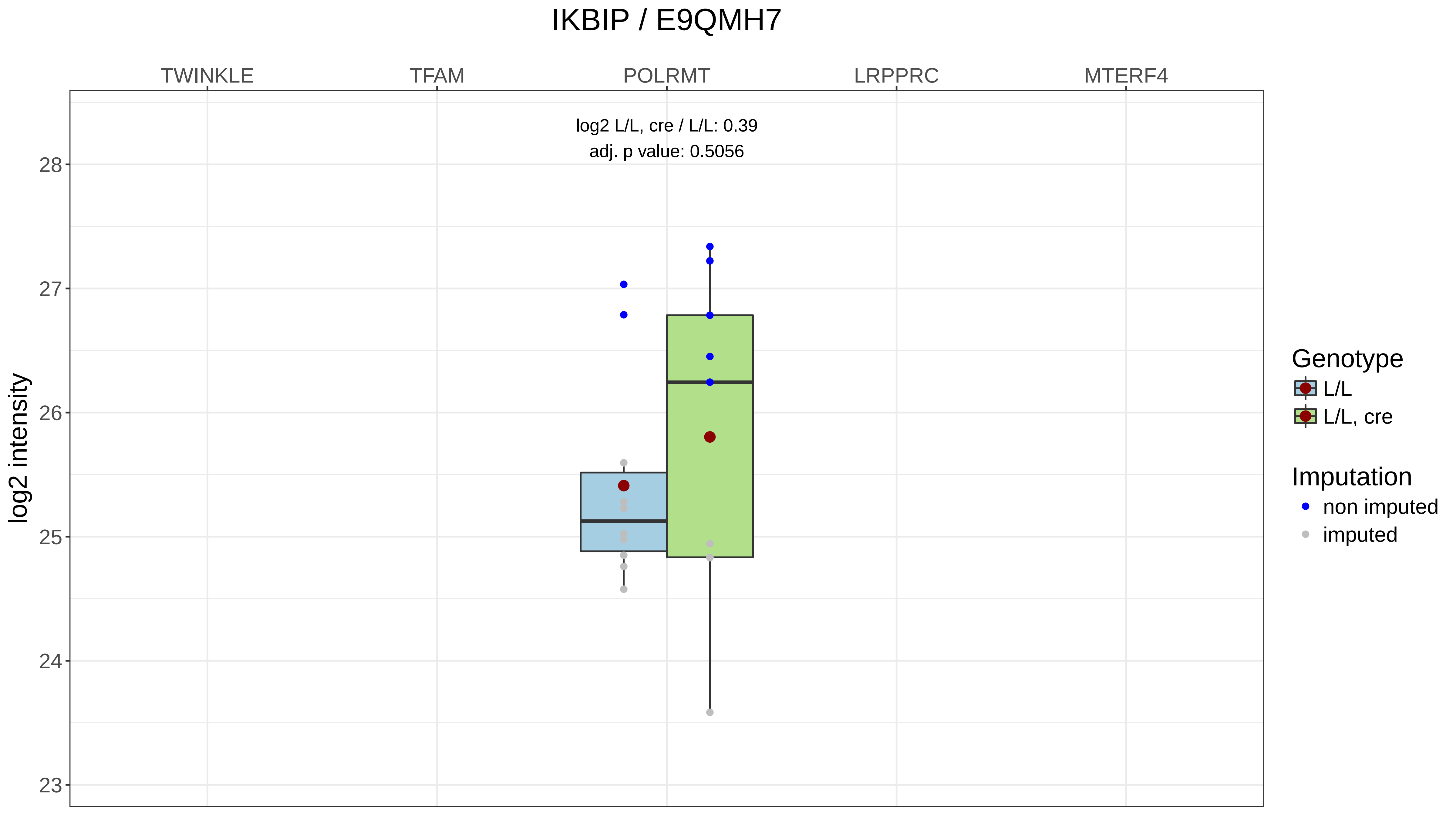
IKBIP / E9QMH7
TWINKLE
TFAM
POLRMT
LRPPRC
MTERF4
log2 L/L, cre / L/L: 0.39
adj. p value: 0.5056
log2 intensity
28
27
26
25
24
23
Genotype
L/L
L/L, cre
Imputation
non imputed
imputed

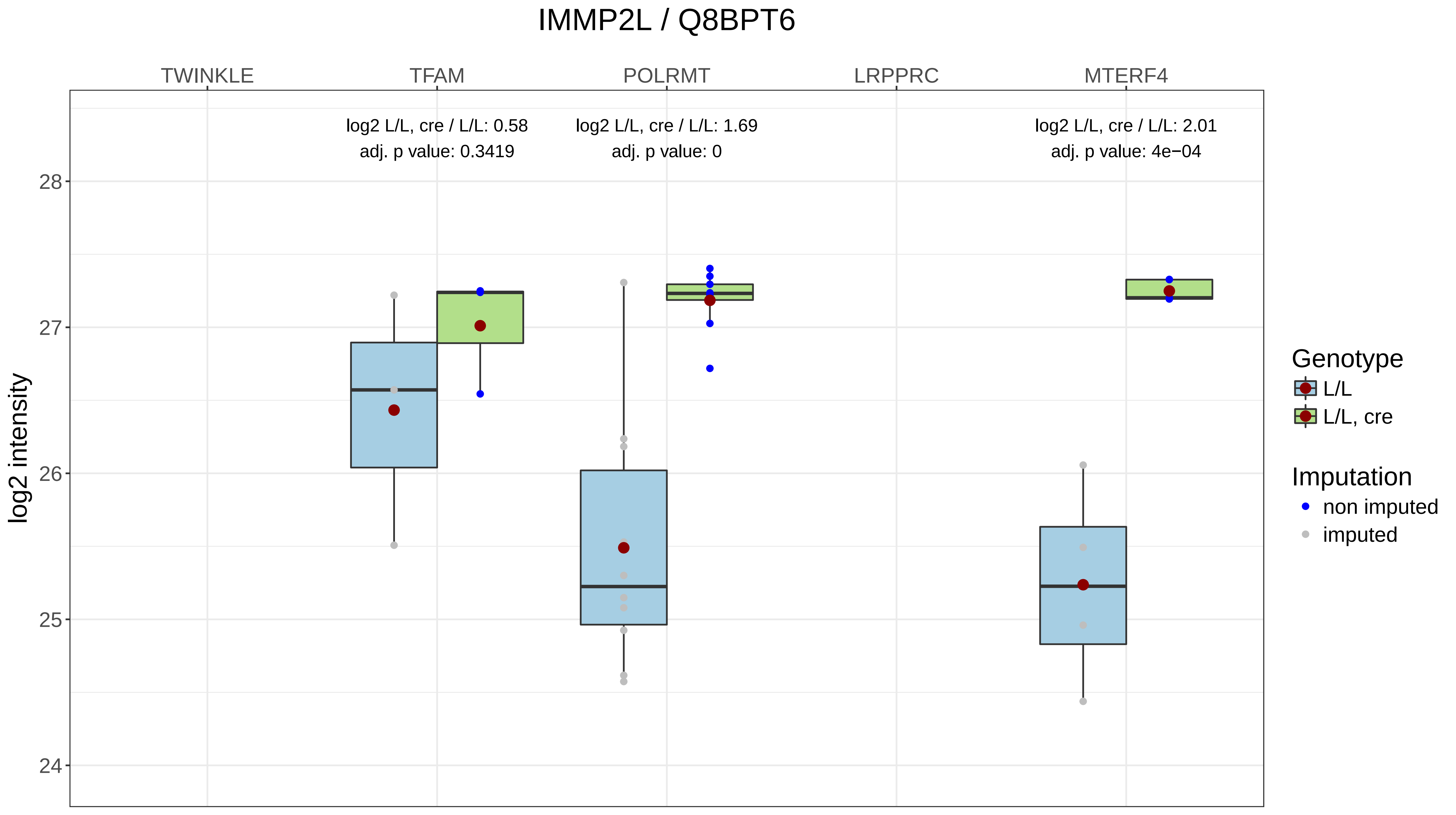

IMMP2L / Q8BPT6
TWINKLE
TFAM
POLRMT
LRPPRC
MTERF4
log2 L/L, cre / L/L: 0.58
adj. p value: 0.3419
log2 L/L, cre / L/L: 1.69
adj. p value: 0
log2 L/L, cre / L/L: 2.01
adj. p value: 4e−04
log2 intensity
28
27
26
25
24
Genotype
L/L
L/L, cre
Imputation
non imputed
imputed

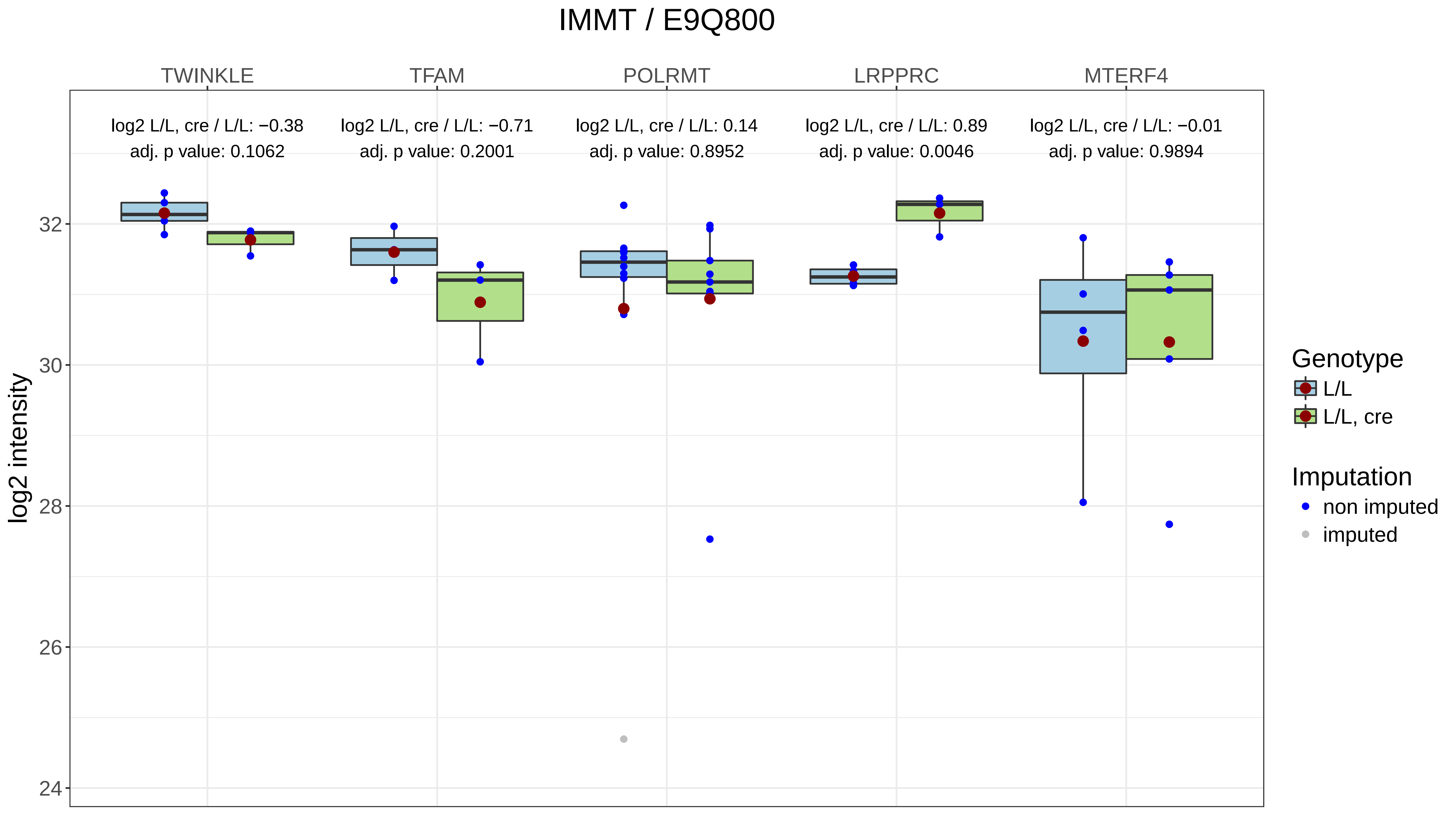
IMMT / E9Q800
TWINKLE
TFAM
POLRMT
LRPPRC
MTERF4
log2 L/L, cre / L/L: −0.38
adj. p value: 0.1062
log2 L/L, cre / L/L: −0.71
adj. p value: 0.2001
log2 L/L, cre / L/L: 0.14
adj. p value: 0.8952
log2 L/L, cre / L/L: 0.89
adj. p value: 0.0046
log2 L/L, cre / L/L: −0.01
adj. p value: 0.9894
log2 intensity
32
30
28
26
24
Genotype
L/L
L/L, cre
Imputation
non imputed
imputed

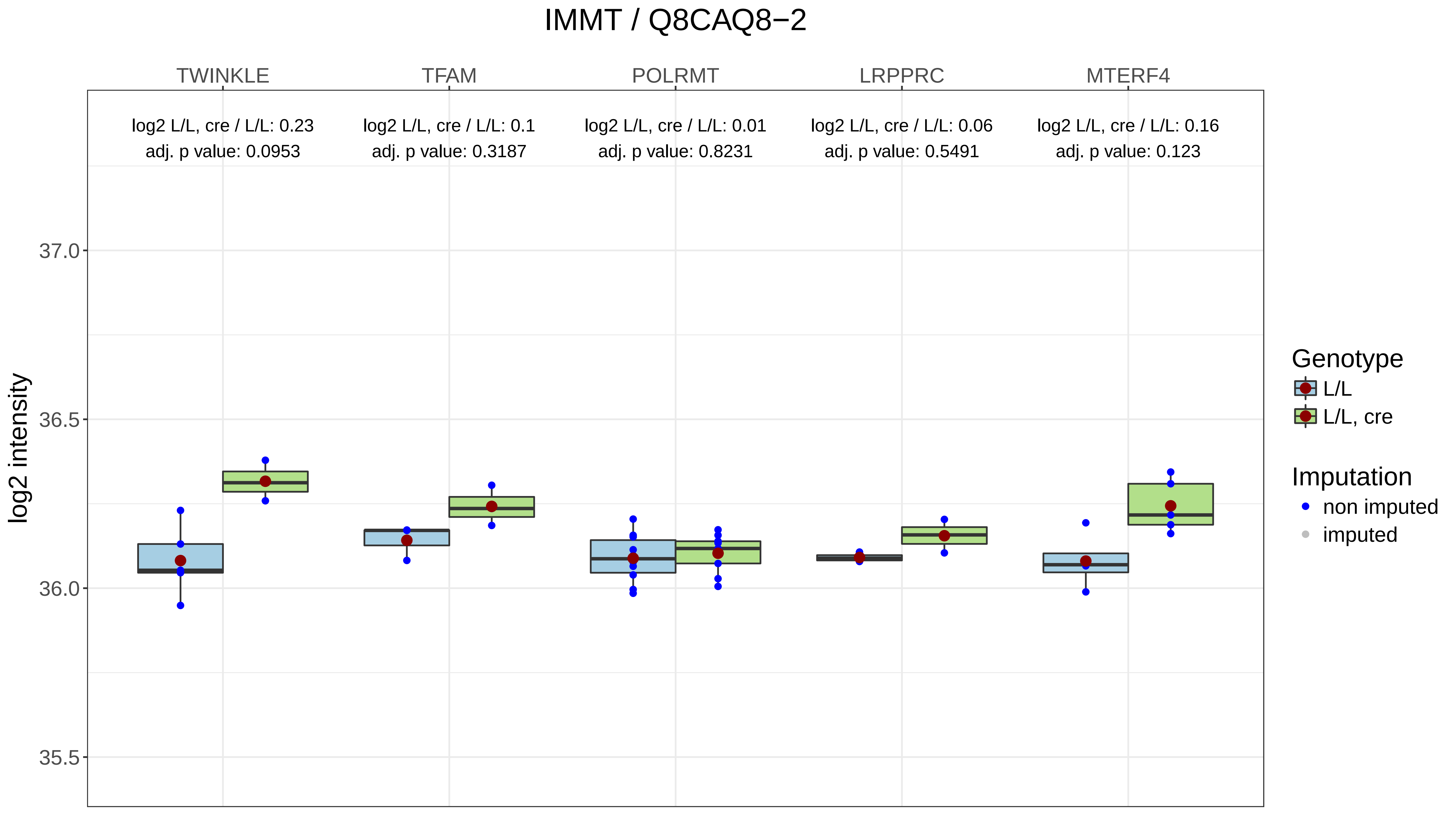
IMMT / Q8CAQ8−2
TWINKLE
TFAM
POLRMT
LRPPRC
MTERF4
log2 L/L, cre / L/L: 0.23
adj. p value: 0.0953
log2 L/L, cre / L/L: 0.1
adj. p value: 0.3187
log2 L/L, cre / L/L: 0.01
adj. p value: 0.8231
log2 L/L, cre / L/L: 0.06
adj. p value: 0.5491
log2 L/L, cre / L/L: 0.16
adj. p value: 0.123
log2 intensity
37.0
36.5
36.0
35.5
Genotype
L/L
L/L, cre
Imputation
non imputed
imputed

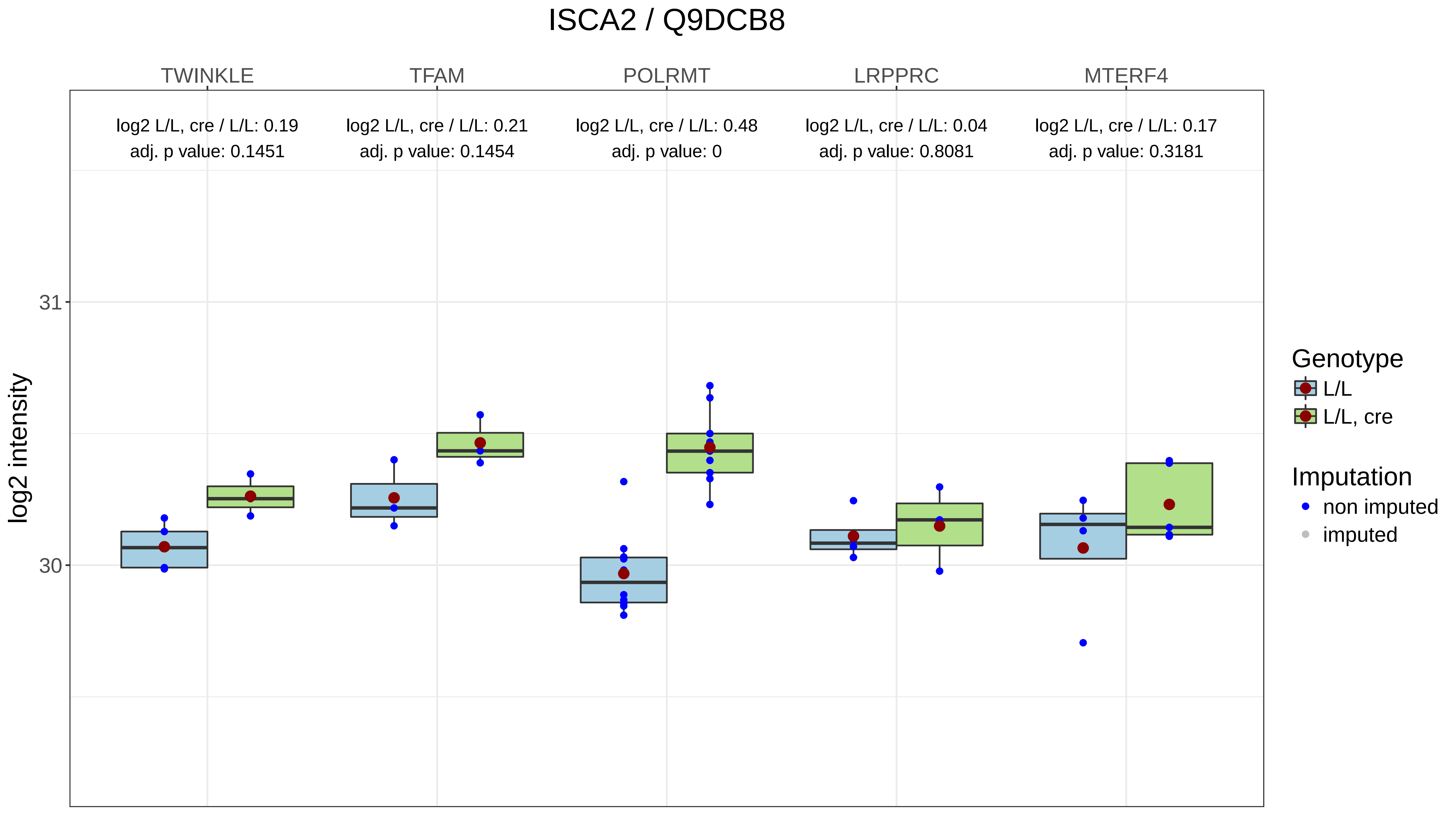
ISCA2 / Q9DCB8
TWINKLE
TFAM
POLRMT
LRPPRC
MTERF4
log2 L/L, cre / L/L: 0.19
adj. p value: 0.1451
log2 L/L, cre / L/L: 0.21
adj. p value: 0.1454
log2 L/L, cre / L/L: 0.48
adj. p value: 0
log2 L/L, cre / L/L: 0.04
adj. p value: 0.8081
log2 L/L, cre / L/L: 0.17
adj. p value: 0.3181
log2 intensity
31
30
Genotype
L/L
L/L, cre
Imputation
non imputed
imputed

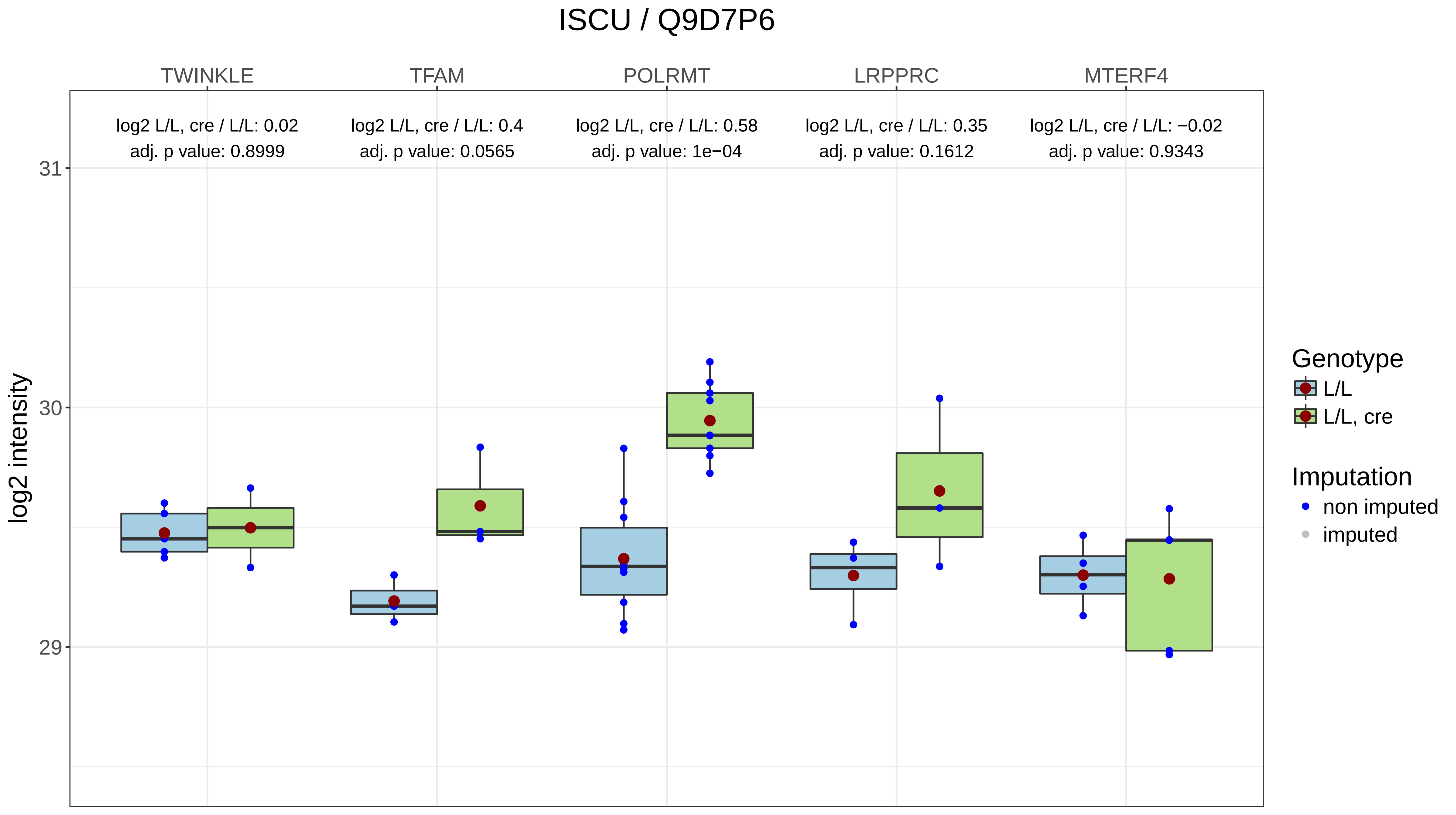
ISCU / Q9D7P6
TWINKLE
TFAM
POLRMT
LRPPRC
MTERF4
log2 L/L, cre / L/L: 0.02
adj. p value: 0.8999
log2 L/L, cre / L/L: 0.4
adj. p value: 0.0565
log2 L/L, cre / L/L: 0.58
adj. p value: 1e−04
log2 L/L, cre / L/L: 0.35
adj. p value: 0.1612
log2 L/L, cre / L/L: −0.02
adj. p value: 0.9343
log2 intensity
31
30
29
Genotype
L/L
L/L, cre
Imputation
non imputed
imputed

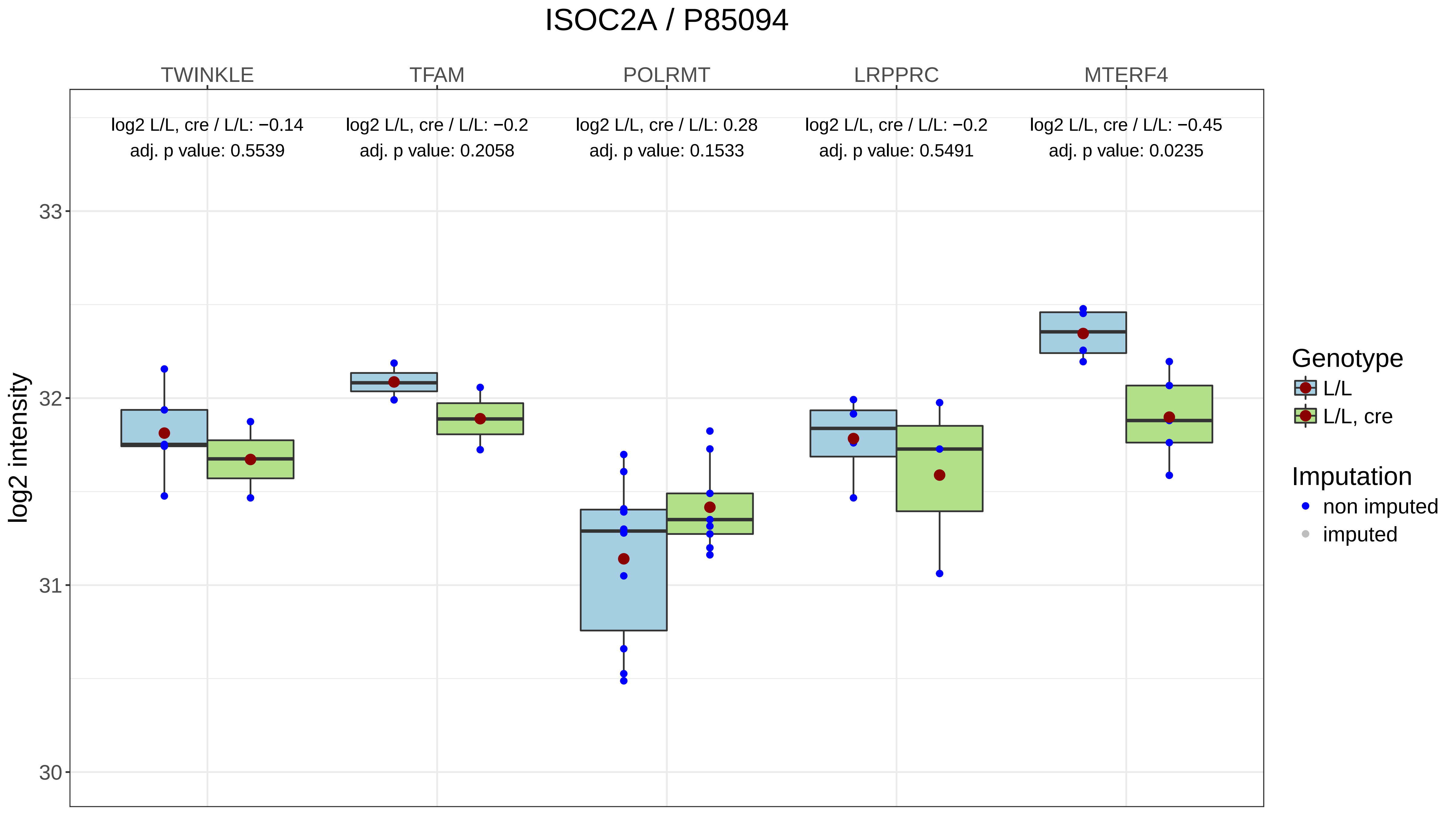
ISOC2A / P85094
TWINKLE
TFAM
POLRMT
LRPPRC
MTERF4
log2 L/L, cre / L/L: −0.14
adj. p value: 0.5539
log2 L/L, cre / L/L: −0.2
adj. p value: 0.2058
log2 L/L, cre / L/L: 0.28
adj. p value: 0.1533
log2 L/L, cre / L/L: −0.2
adj. p value: 0.5491
log2 L/L, cre / L/L: −0.45
adj. p value: 0.0235
log2 intensity
33
32
31
30
Genotype
L/L
L/L, cre
Imputation
non imputed
imputed

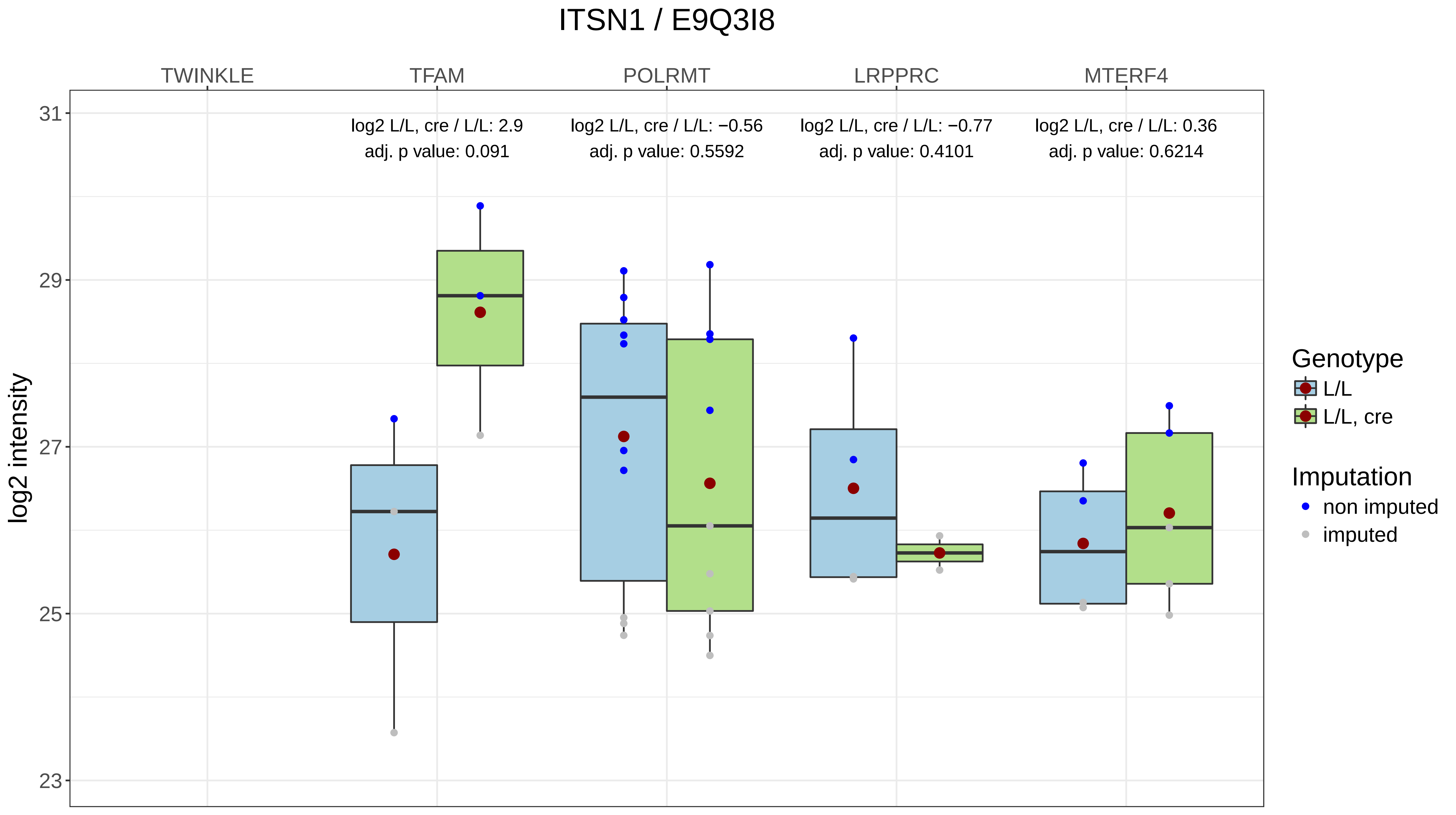

ITSN1 / E9Q3I8
TWINKLE
TFAM
POLRMT
LRPPRC
MTERF4
log2 L/L, cre / L/L: 2.9
adj. p value: 0.091
log2 L/L, cre / L/L: −0.56
adj. p value: 0.5592
log2 L/L, cre / L/L: −0.77
adj. p value: 0.4101
log2 L/L, cre / L/L: 0.36
adj. p value: 0.6214
log2 intensity
31
29
27
25
23
Genotype
L/L
L/L, cre
Imputation
non imputed
imputed

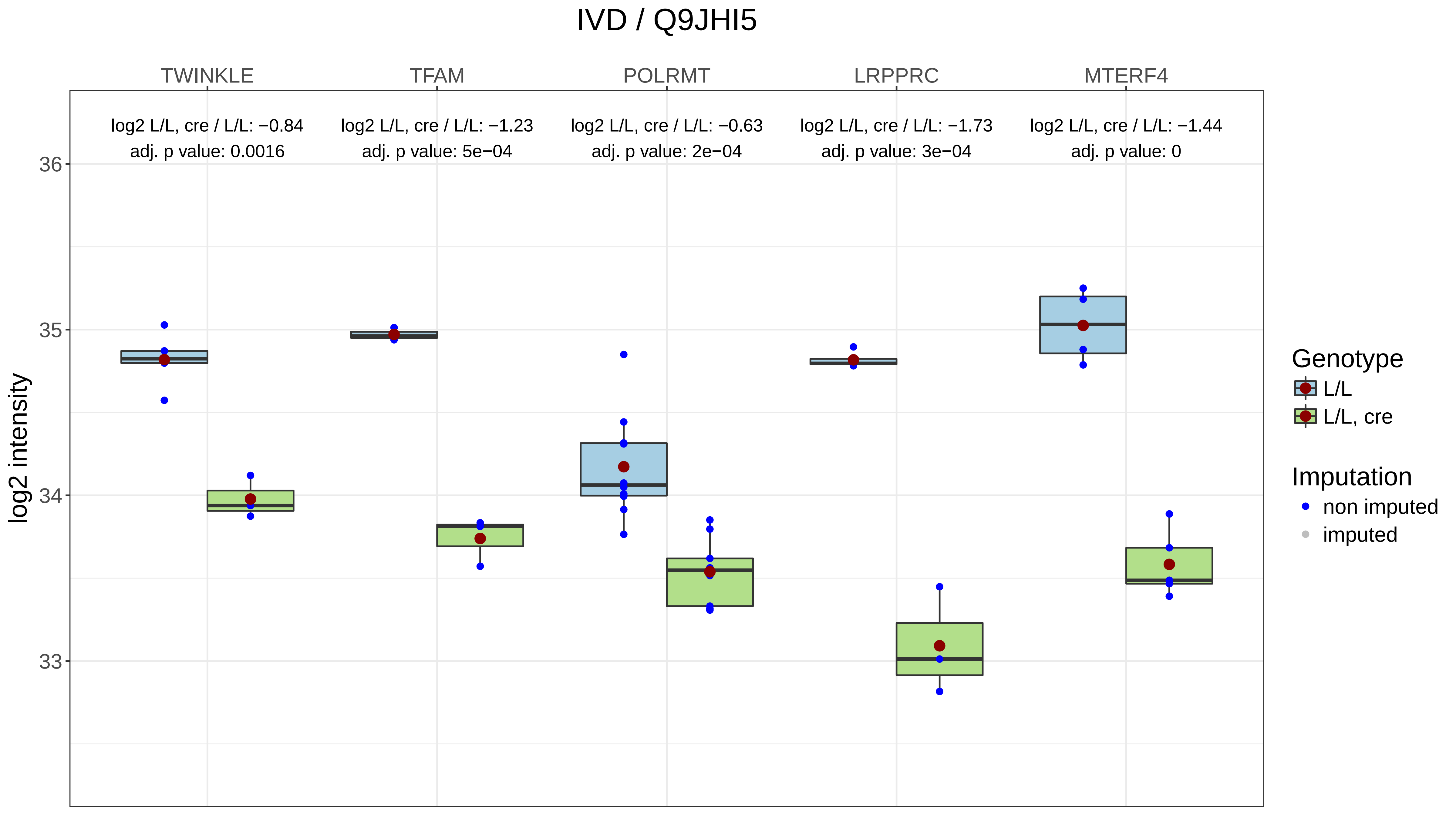

IVD / Q9JHI5
TWINKLE
TFAM
POLRMT
LRPPRC
MTERF4
log2 L/L, cre / L/L: −0.84
adj. p value: 0.0016
log2 L/L, cre / L/L: −1.23
adj. p value: 5e−04
log2 L/L, cre / L/L: −0.63
adj. p value: 2e−04
log2 L/L, cre / L/L: −1.73
adj. p value: 3e−04
log2 L/L, cre / L/L: −1.44
adj. p value: 0
36
35
34
33
log2 intensity
Genotype
L/L
L/L, cre
Imputation
non imputed
imputed

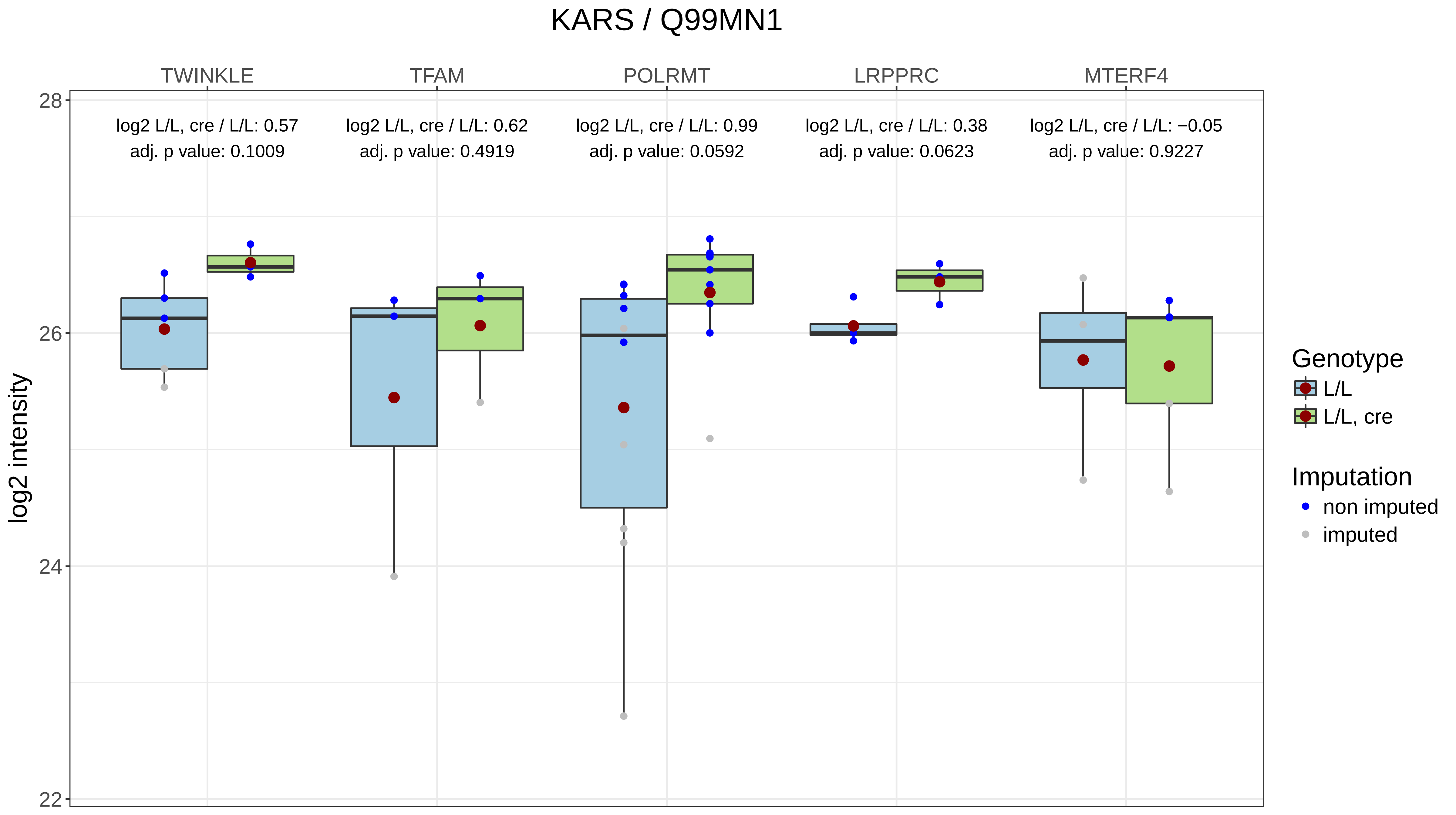
KARS / Q99MN1
TWINKLE
TFAM
POLRMT
LRPPRC
MTERF4
log2 L/L, cre / L/L: 0.57
adj. p value: 0.1009
log2 L/L, cre / L/L: 0.62
adj. p value: 0.4919
log2 L/L, cre / L/L: 0.99
adj. p value: 0.0592
log2 L/L, cre / L/L: 0.38
adj. p value: 0.0623
log2 L/L, cre / L/L: −0.05
adj. p value: 0.9227
log2 intensity
28
26
24
22
Genotype
L/L
L/L, cre
Imputation
non imputed
imputed

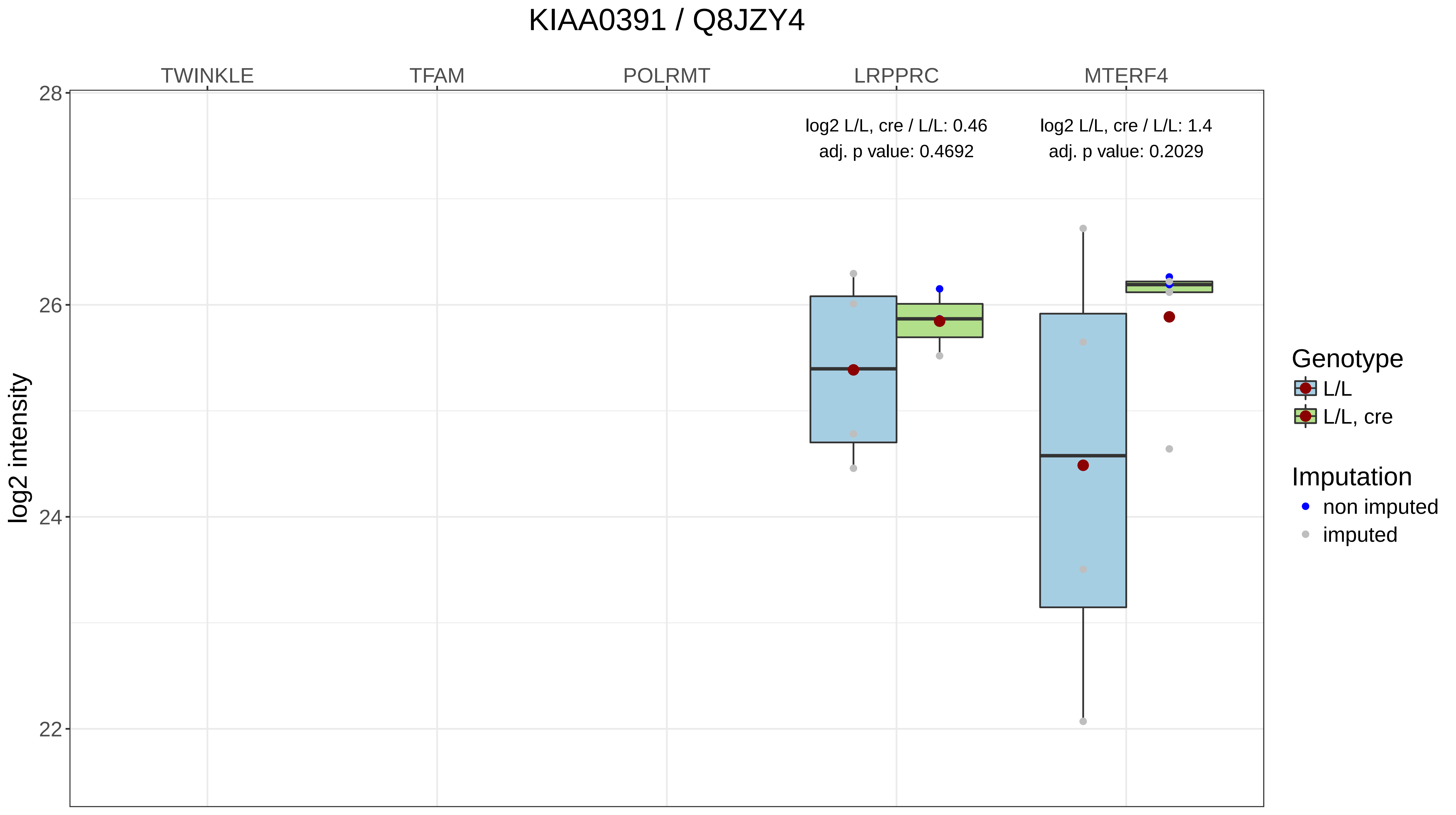

KIAA0391 / Q8JZY4
TWINKLE
TFAM
POLRMT
LRPPRC
MTERF4
log2 L/L, cre / L/L: 0.46
adj. p value: 0.4692
log2 L/L, cre / L/L: 1.4
adj. p value: 0.2029
28
26
24
22
log2 intensity
Genotype
L/L
L/L, cre
Imputation
non imputed
imputed

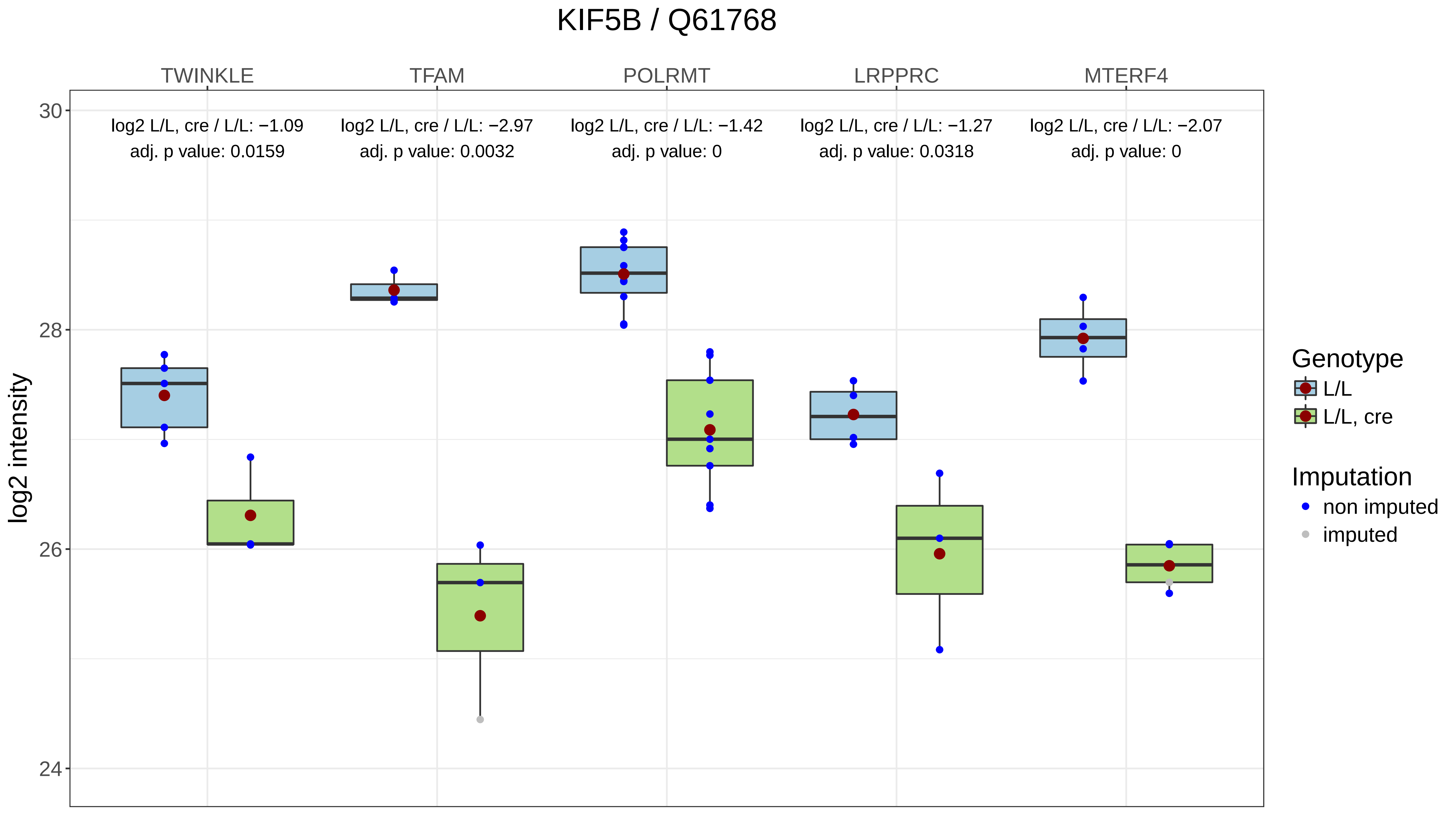

KIF5B / Q61768
TWINKLE
TFAM
POLRMT
LRPPRC
MTERF4
log2 L/L, cre / L/L: −1.09
adj. p value: 0.0159
log2 L/L, cre / L/L: −2.97
adj. p value: 0.0032
log2 L/L, cre / L/L: −1.42
adj. p value: 0
log2 L/L, cre / L/L: −1.27
adj. p value: 0.0318
log2 L/L, cre / L/L: −2.07
adj. p value: 0
log2 intensity
30
28
26
24
Genotype
L/L
L/L, cre
Imputation
non imputed
imputed

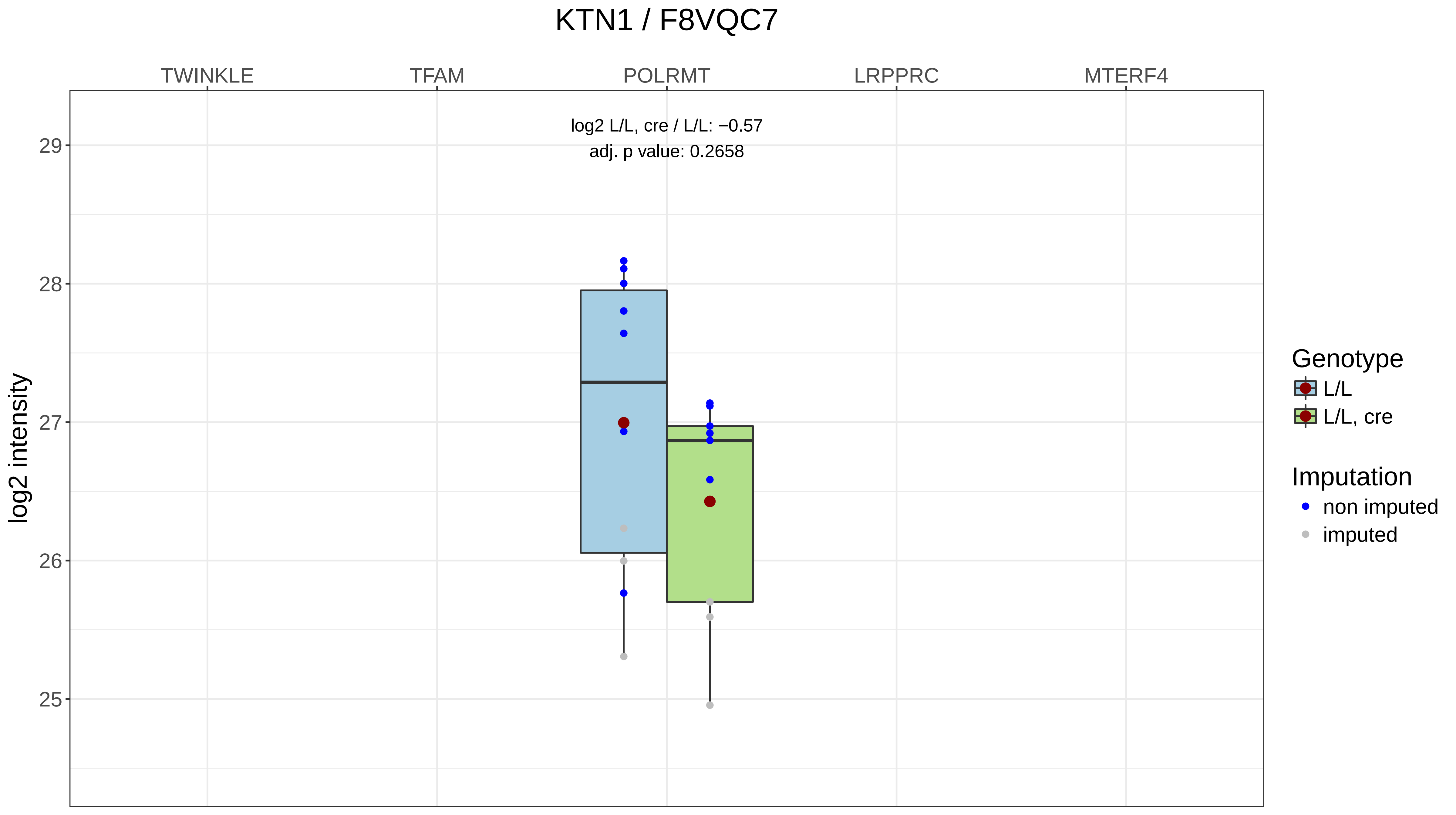
KTN1 / F8VQC7
TWINKLE
TFAM
POLRMT
LRPPRC
MTERF4
log2 L/L, cre / L/L: −0.57
adj. p value: 0.2658
log2 intensity
29
28
27
26
25
Genotype
L/L
L/L, cre
Imputation
non imputed
imputed

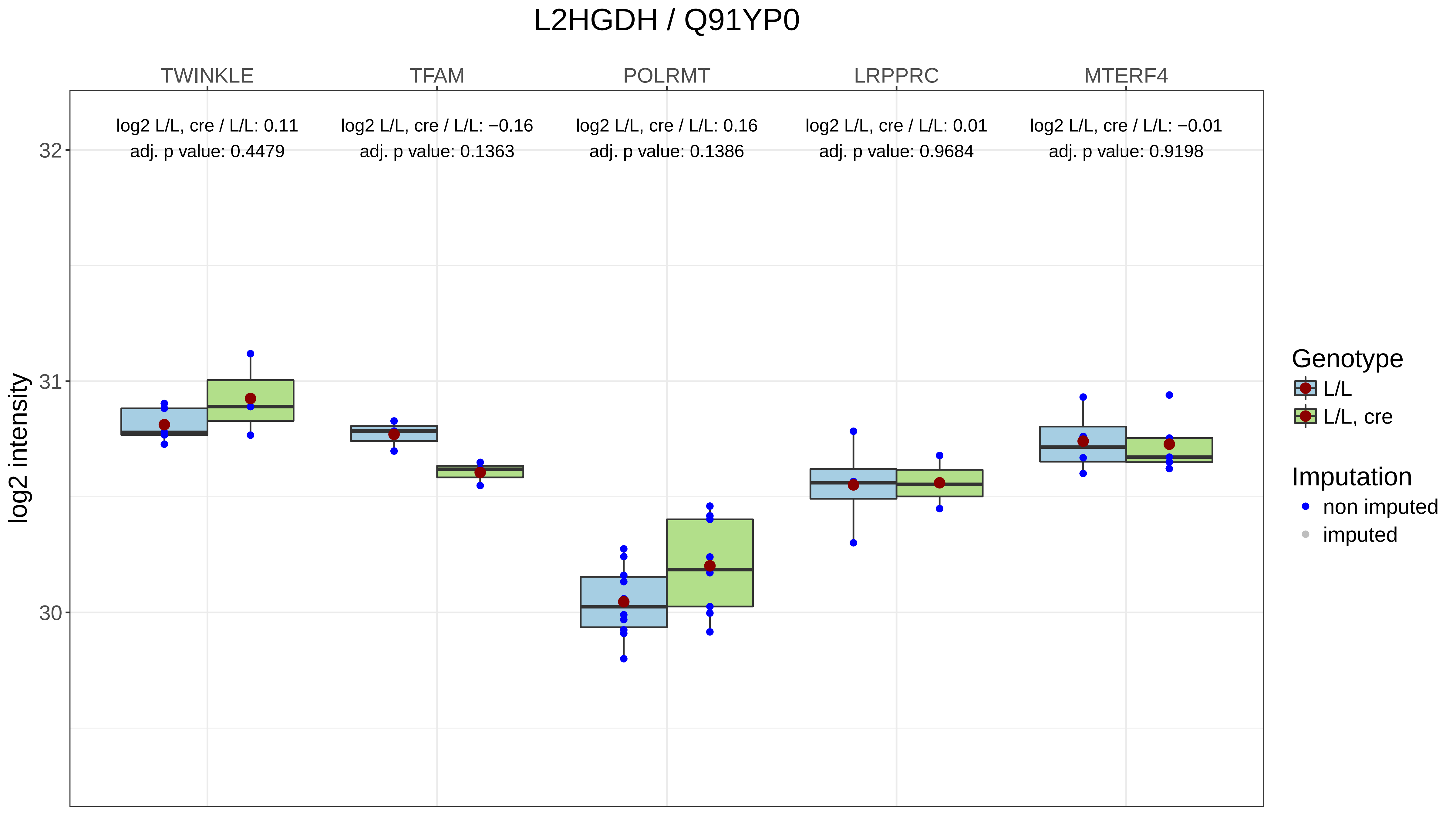

L2HGDH / Q91YP0
TWINKLE
TFAM
POLRMT
LRPPRC
MTERF4
log2 L/L, cre / L/L: 0.11
adj. p value: 0.4479
log2 L/L, cre / L/L: −0.16
adj. p value: 0.1363
log2 L/L, cre / L/L: 0.16
adj. p value: 0.1386
log2 L/L, cre / L/L: 0.01
adj. p value: 0.9684
log2 L/L, cre / L/L: −0.01
adj. p value: 0.9198
32
31
30
log2 intensity
Genotype
L/L
L/L, cre
Imputation
non imputed
imputed

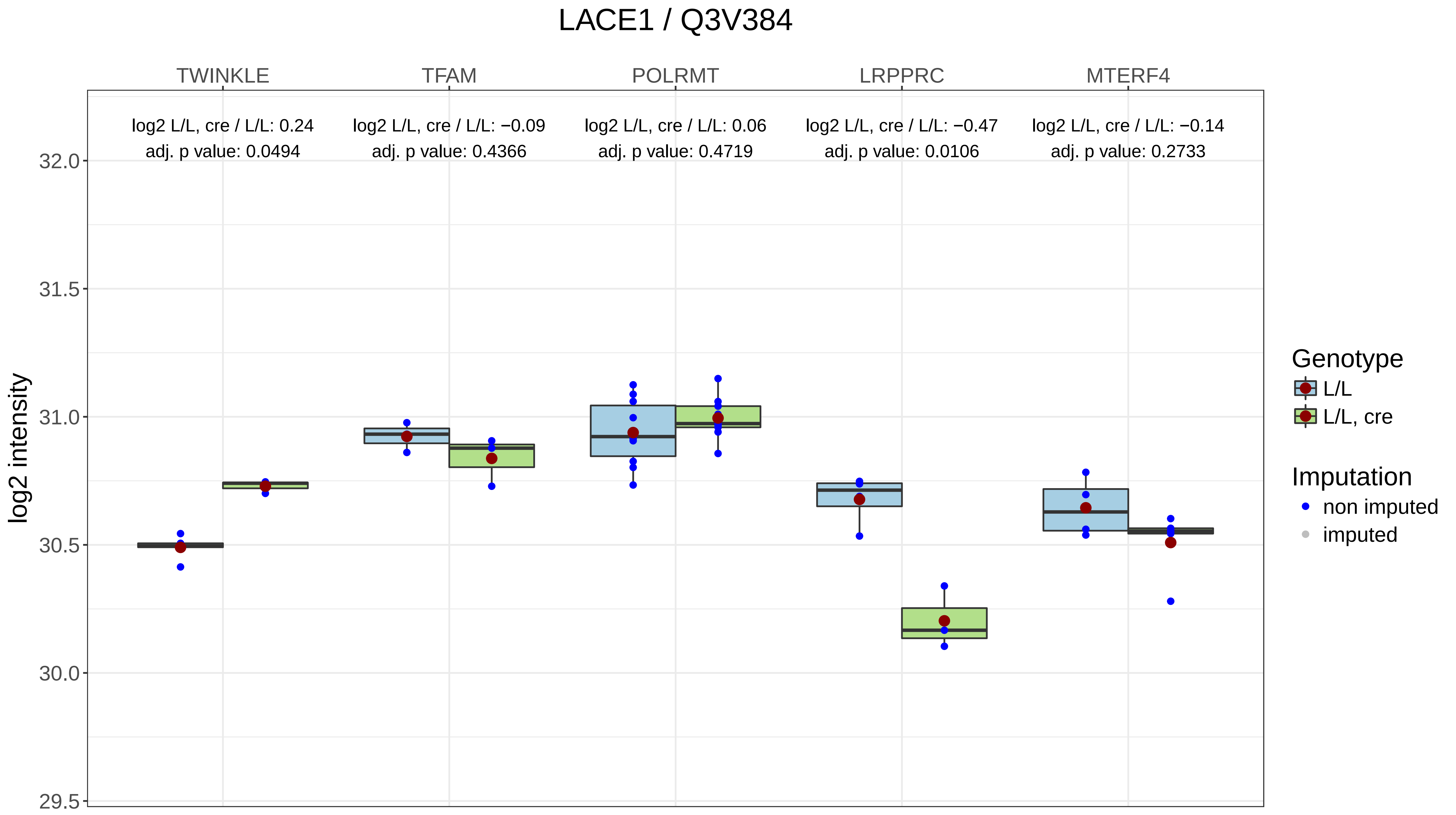

LACE1 / Q3V384
TWINKLE
TFAM
POLRMT
LRPPRC
MTERF4
log2 L/L, cre / L/L: 0.24
adj. p value: 0.0494
log2 L/L, cre / L/L: −0.09
adj. p value: 0.4366
log2 L/L, cre / L/L: 0.06
adj. p value: 0.4719
log2 L/L, cre / L/L: −0.47
adj. p value: 0.0106
log2 L/L, cre / L/L: −0.14
adj. p value: 0.2733
log2 intensity
32.0
31.5
31.0
30.5
30.0
29.5
Genotype
L/L
L/L, cre
Imputation
non imputed
imputed

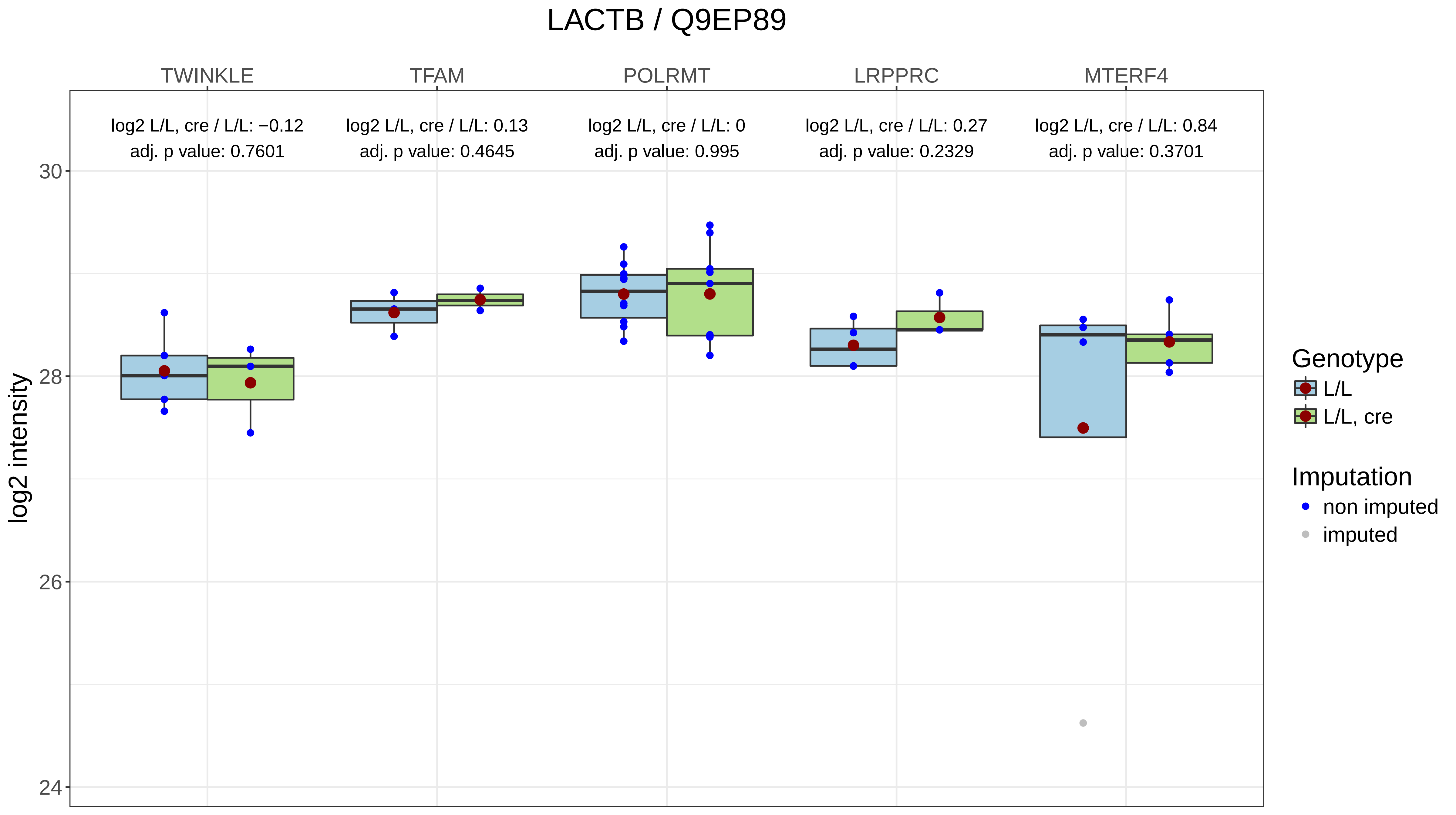
LACTB / Q9EP89
TWINKLE
TFAM
POLRMT
LRPPRC
MTERF4
log2 L/L, cre / L/L: −0.12
adj. p value: 0.7601
log2 L/L, cre / L/L: 0.13
adj. p value: 0.4645
log2 L/L, cre / L/L: 0
adj. p value: 0.995
log2 L/L, cre / L/L: 0.27
adj. p value: 0.2329
log2 L/L, cre / L/L: 0.84
adj. p value: 0.3701
log2 intensity
30
28
26
24
Genotype
L/L
L/L, cre
Imputation
non imputed
imputed

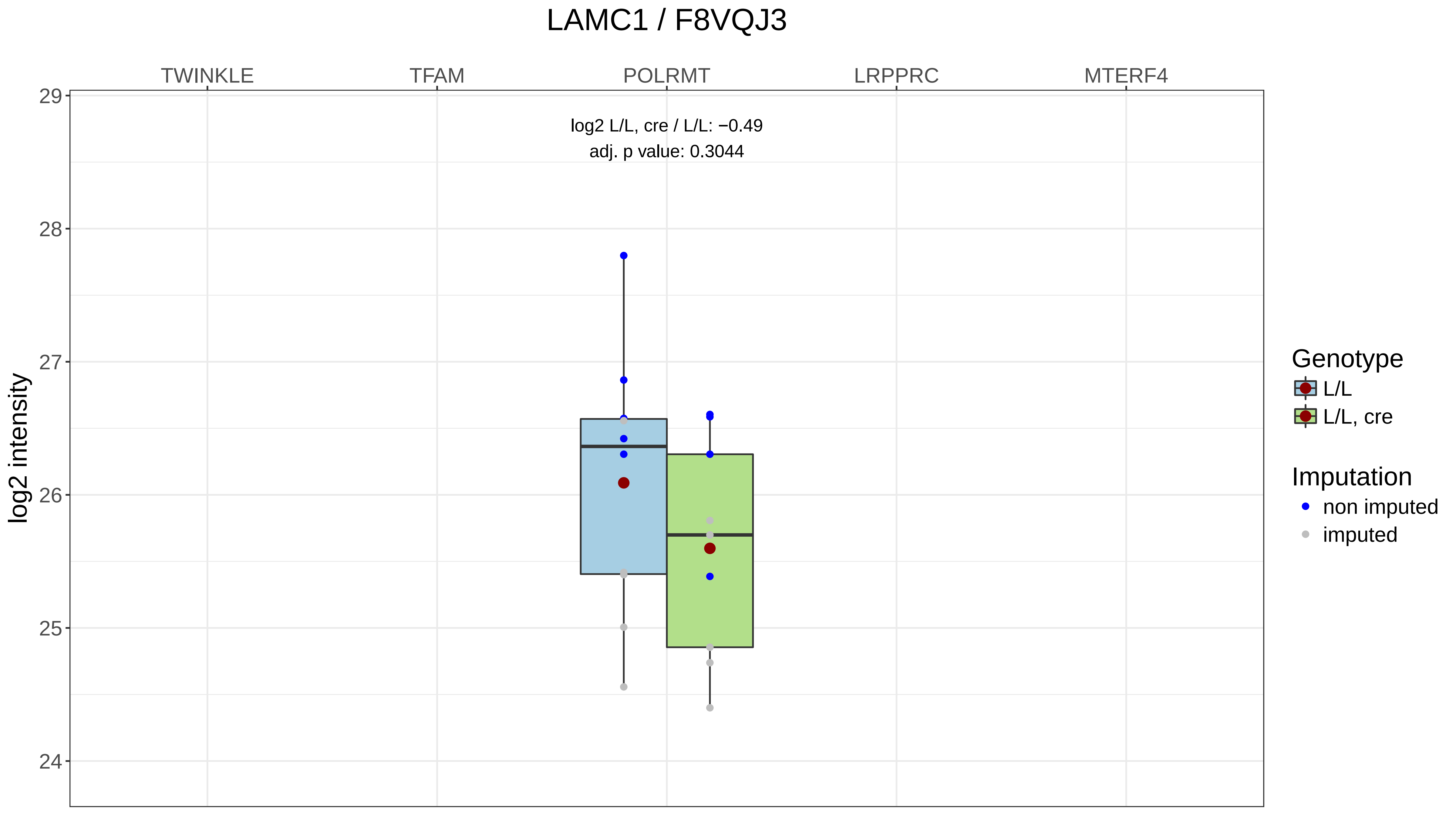
LAMC1 / F8VQJ3
TWINKLE
TFAM
POLRMT
LRPPRC
MTERF4
log2 L/L, cre / L/L: −0.49
adj. p value: 0.3044
log2 intensity
29
28
27
26
25
24
Genotype
L/L
L/L, cre
Imputation
non imputed
imputed

LAMP1 / P11438

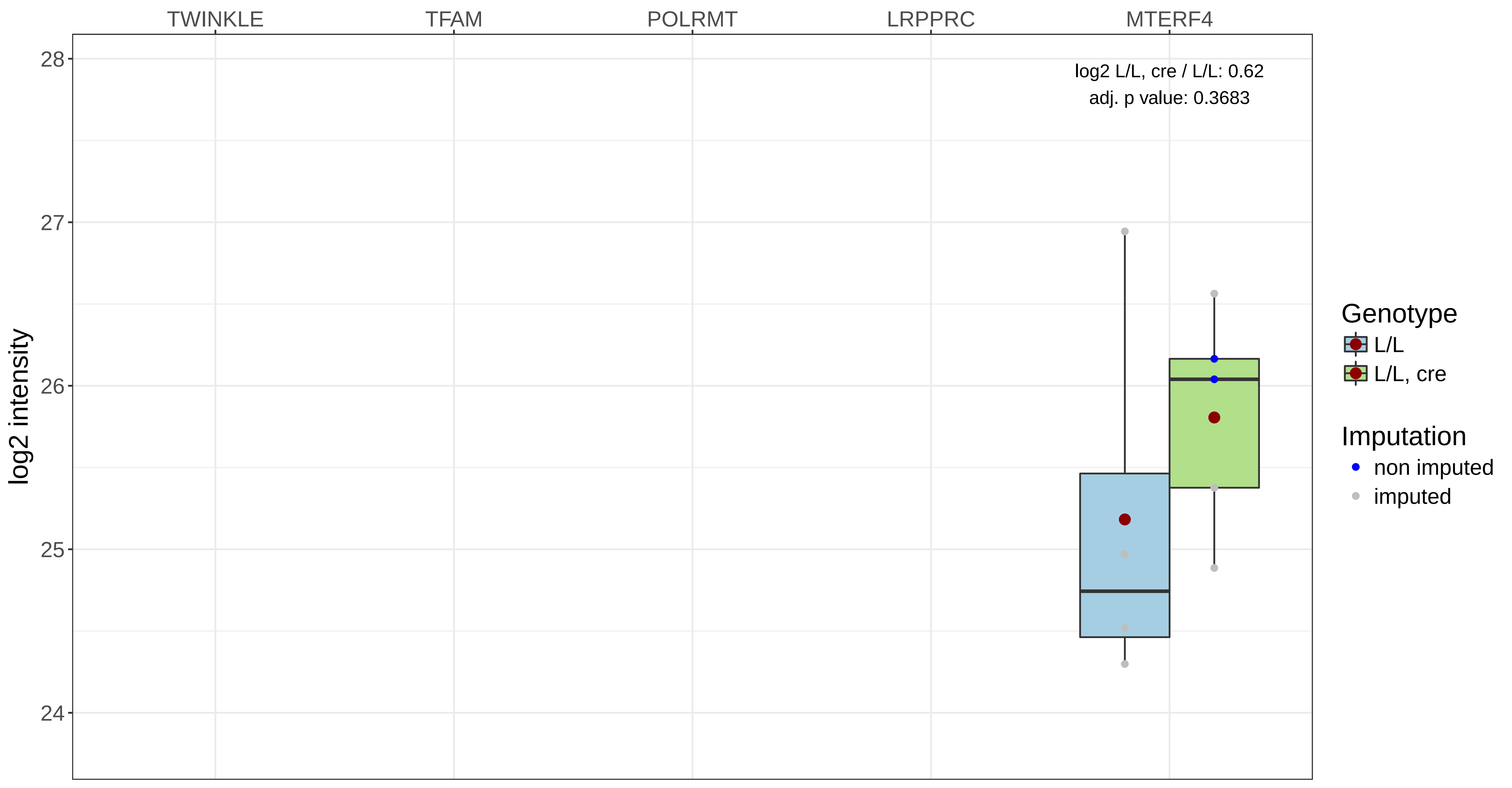

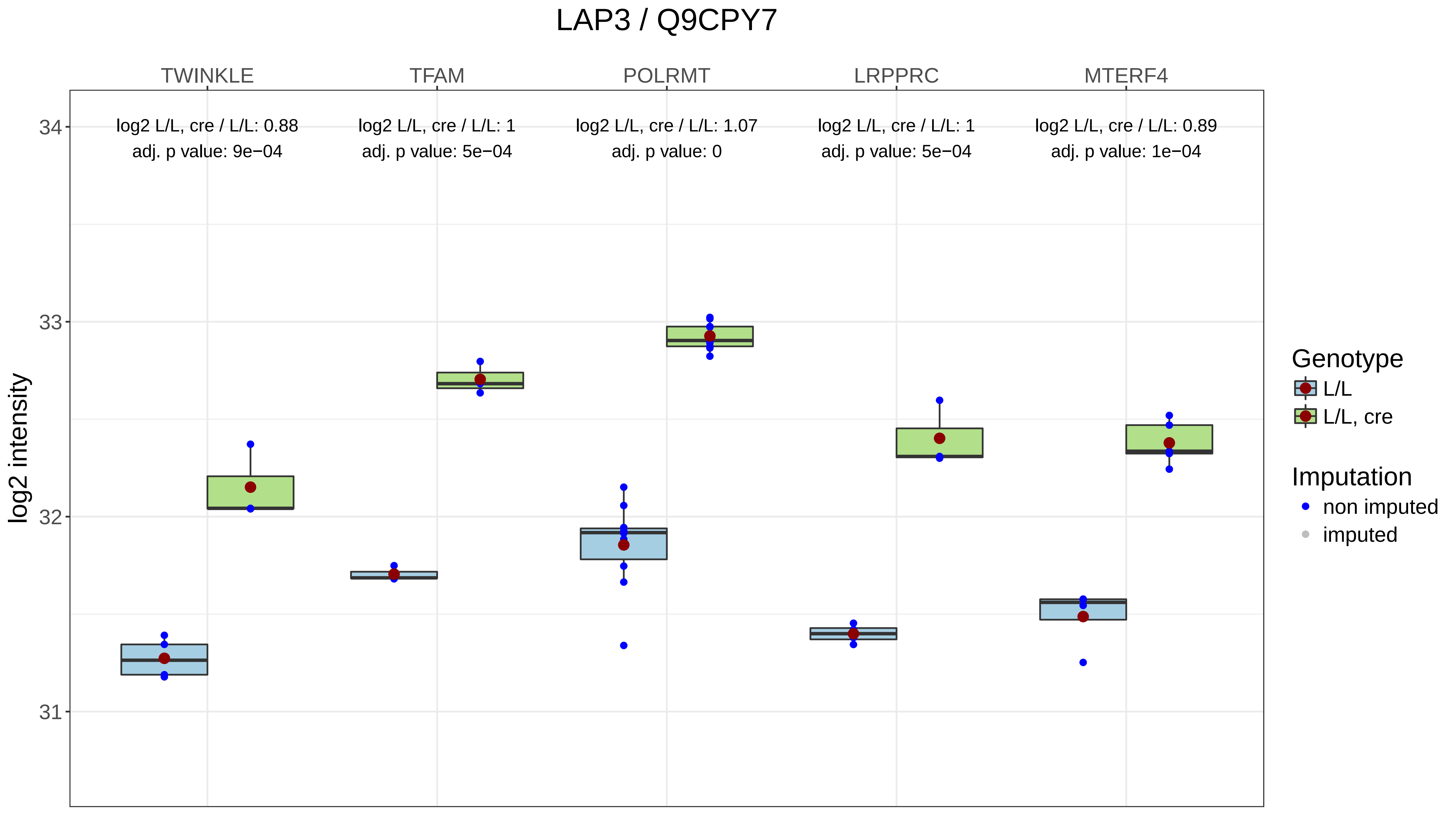

LAP3 / Q9CPY7
TWINKLE
TFAM
POLRMT
LRPPRC
MTERF4
log2 L/L, cre / L/L: 0.88
adj. p value: 9e−04
log2 L/L, cre / L/L: 1
adj. p value: 5e−04
log2 L/L, cre / L/L: 1.07
adj. p value: 0
log2 L/L, cre / L/L: 1
adj. p value: 5e−04
log2 L/L, cre / L/L: 0.89
adj. p value: 1e−04
log2 intensity
34
33
32
31
Genotype
L/L
L/L, cre
Imputation
non imputed
imputed

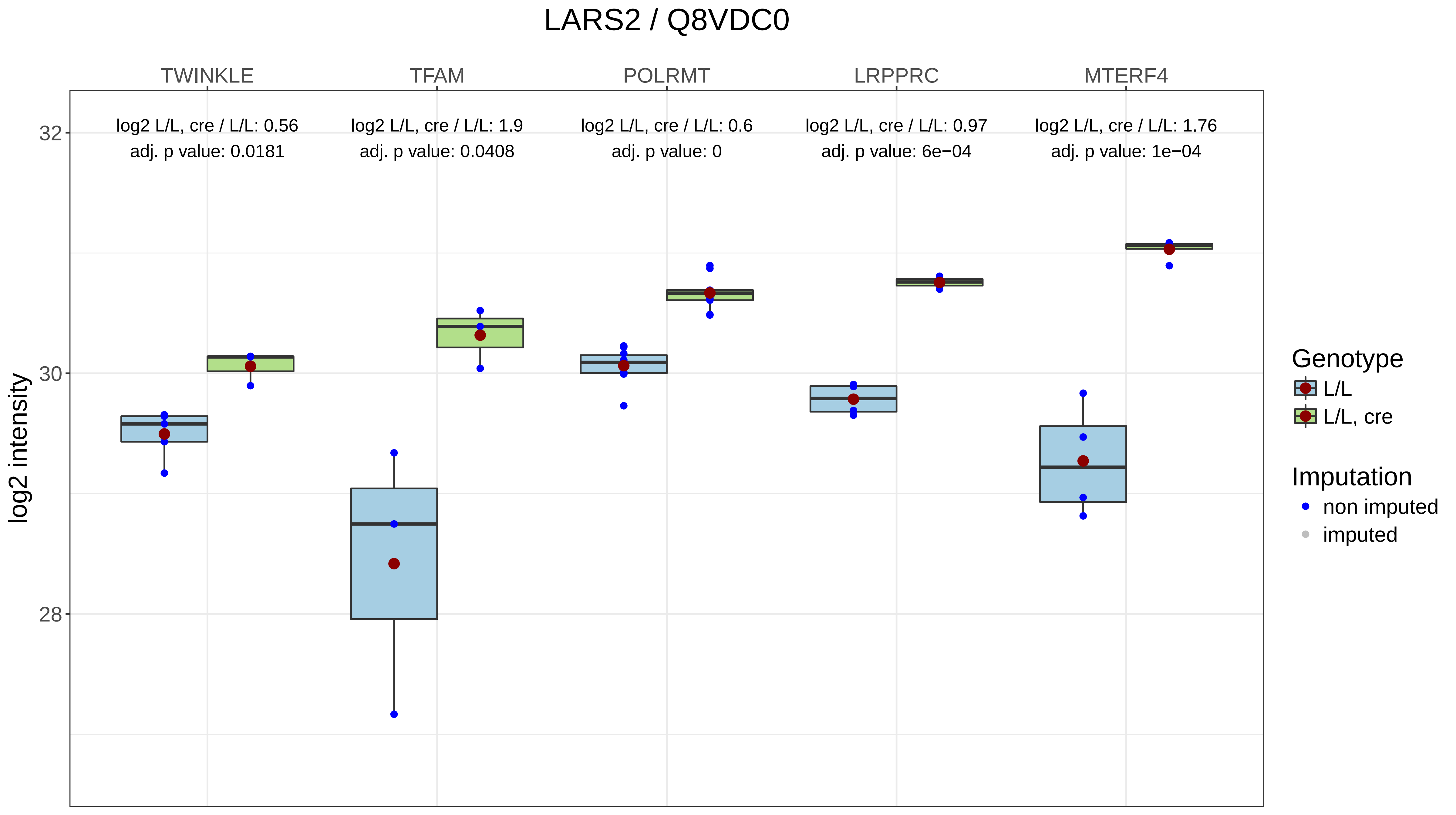
LARS2 / Q8VDC0
TWINKLE
TFAM
POLRMT
LRPPRC
MTERF4
log2 L/L, cre / L/L: 0.56
adj. p value: 0.0181
log2 L/L, cre / L/L: 1.9
adj. p value: 0.0408
log2 L/L, cre / L/L: 0.6
adj. p value: 0
log2 L/L, cre / L/L: 0.97
adj. p value: 6e−04
log2 L/L, cre / L/L: 1.76
adj. p value: 1e−04
32
30
28
log2 intensity
Genotype
L/L
L/L, cre
Imputation
non imputed
imputed

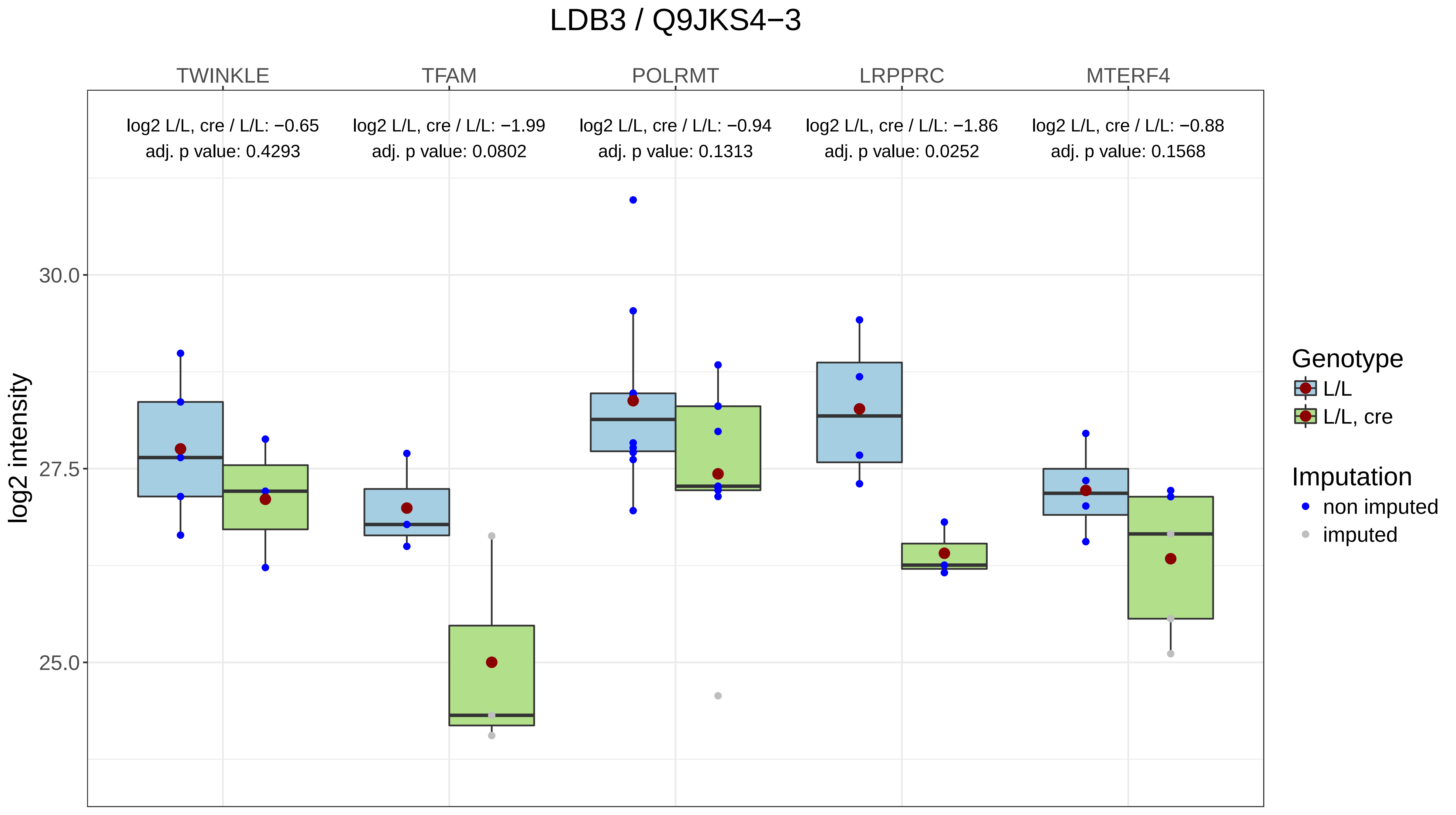
LDB3 / Q9JKS4−3
TWINKLE
TFAM
POLRMT
LRPPRC
MTERF4
log2 L/L, cre / L/L: −0.65
adj. p value: 0.4293
log2 L/L, cre / L/L: −1.99
adj. p value: 0.0802
log2 L/L, cre / L/L: −0.94
adj. p value: 0.1313
log2 L/L, cre / L/L: −1.86
adj. p value: 0.0252
log2 L/L, cre / L/L: −0.88
adj. p value: 0.1568
log2 intensity
30.0
27.5
25.0
Genotype
L/L
L/L, cre
Imputation
non imputed
imputed

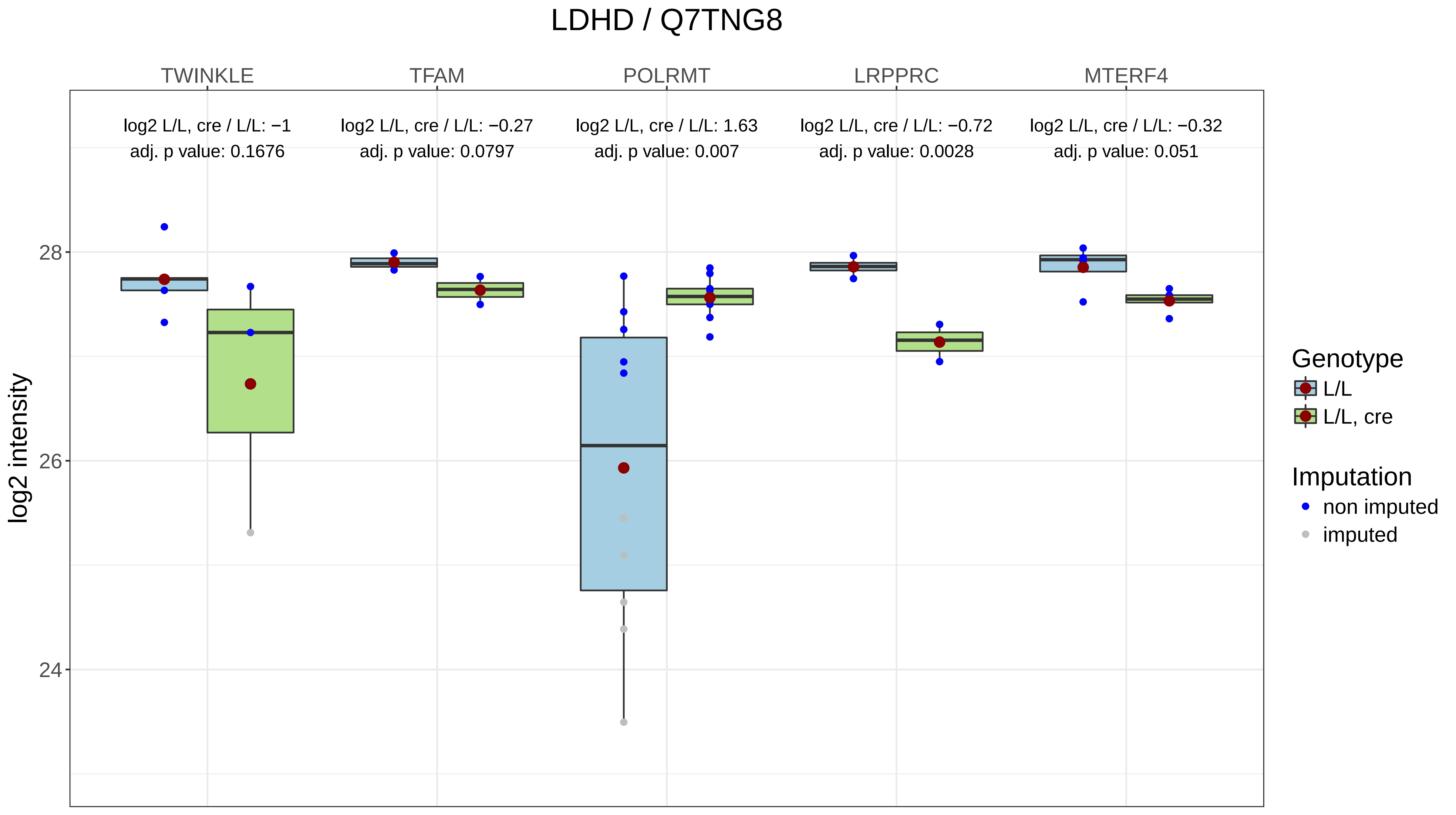

LDHD / Q7TNG8
TWINKLE
TFAM
POLRMT
LRPPRC
MTERF4
log2 L/L, cre / L/L: −1
adj. p value: 0.1676
log2 L/L, cre / L/L: −0.27
adj. p value: 0.0797
log2 L/L, cre / L/L: 1.63
adj. p value: 0.007
log2 L/L, cre / L/L: −0.72
adj. p value: 0.0028
log2 L/L, cre / L/L: −0.32
adj. p value: 0.051
log2 intensity
28
26
24
Genotype
L/L
L/L, cre
Imputation
non imputed
imputed

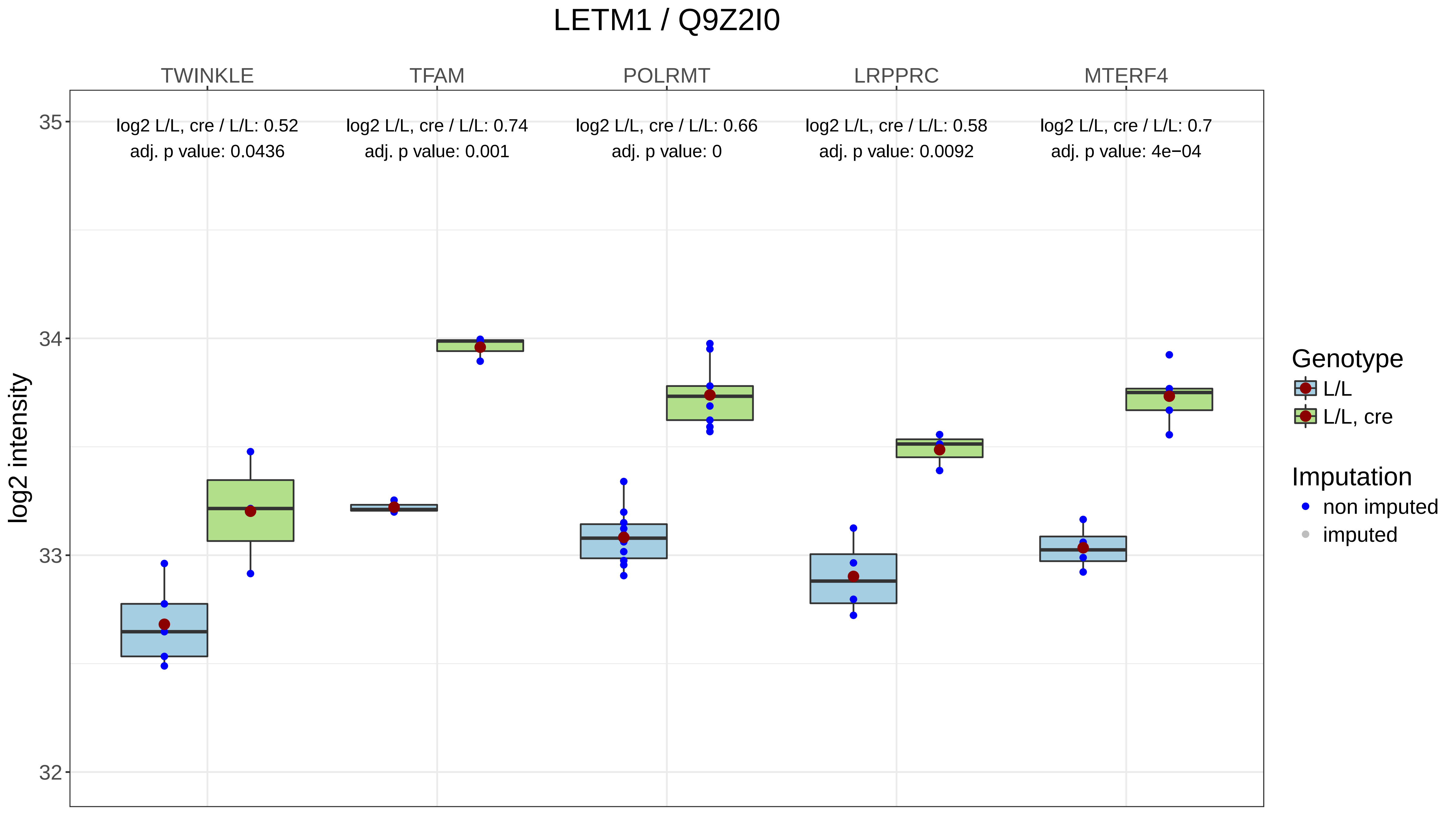

LETM1 / Q9Z2I0
TWINKLE
TFAM
POLRMT
LRPPRC
MTERF4
log2 L/L, cre / L/L: 0.52
adj. p value: 0.0436
log2 L/L, cre / L/L: 0.74
adj. p value: 0.001
log2 L/L, cre / L/L: 0.66
adj. p value: 0
log2 L/L, cre / L/L: 0.58
adj. p value: 0.0092
log2 L/L, cre / L/L: 0.7
adj. p value: 4e−04
log2 intensity
35
34
33
32
Genotype
L/L
L/L, cre
Imputation
non imputed
imputed

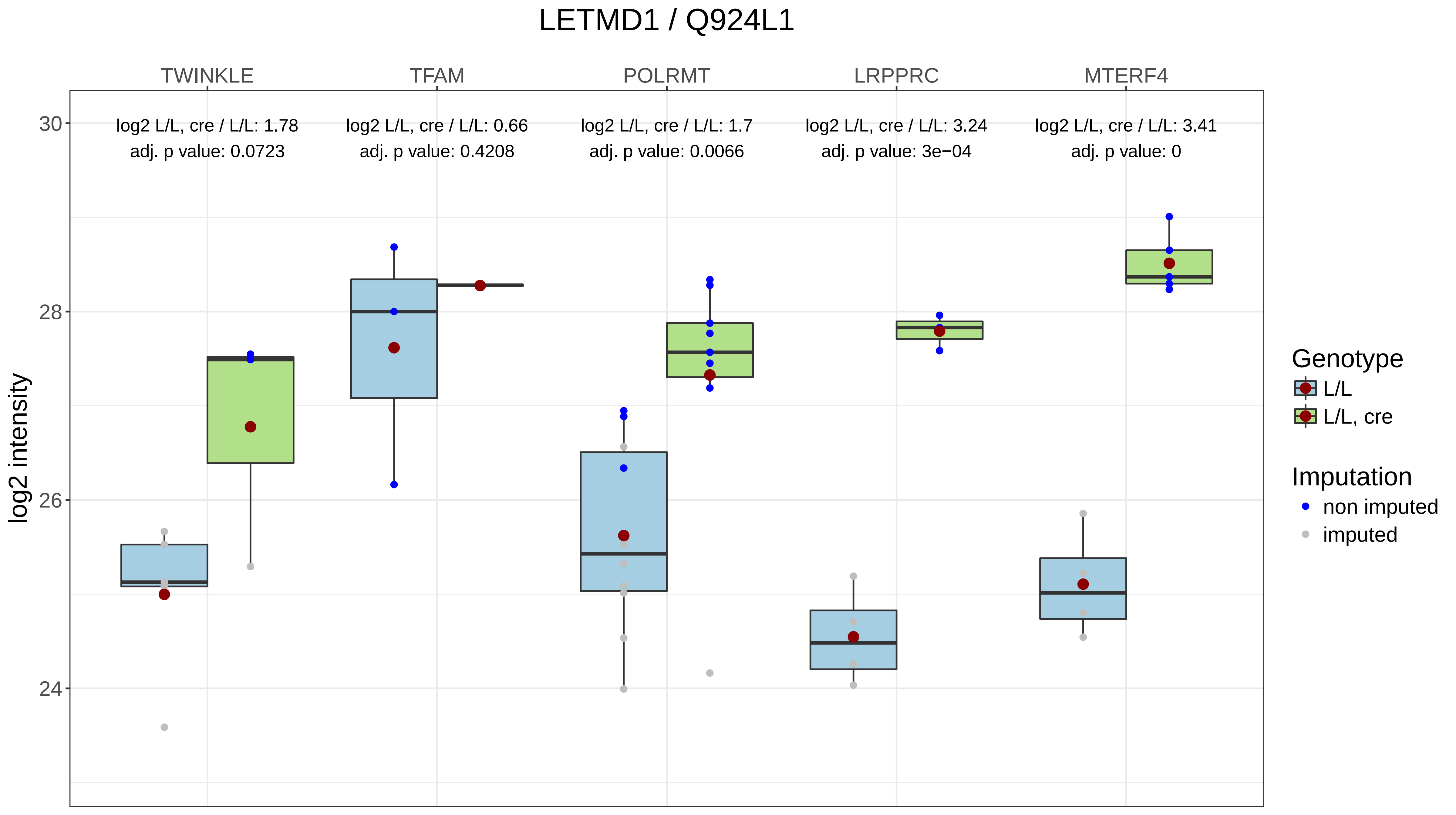
LETMD1 / Q924L1
TWINKLE
TFAM
POLRMT
LRPPRC
MTERF4
log2 L/L, cre / L/L: 1.78
adj. p value: 0.0723
log2 L/L, cre / L/L: 0.66
adj. p value: 0.4208
log2 L/L, cre / L/L: 1.7
adj. p value: 0.0066
log2 L/L, cre / L/L: 3.24
adj. p value: 3e−04
log2 L/L, cre / L/L: 3.41
adj. p value: 0
30
28
26
24
log2 intensity
Genotype
L/L
L/L, cre
Imputation
non imputed
imputed

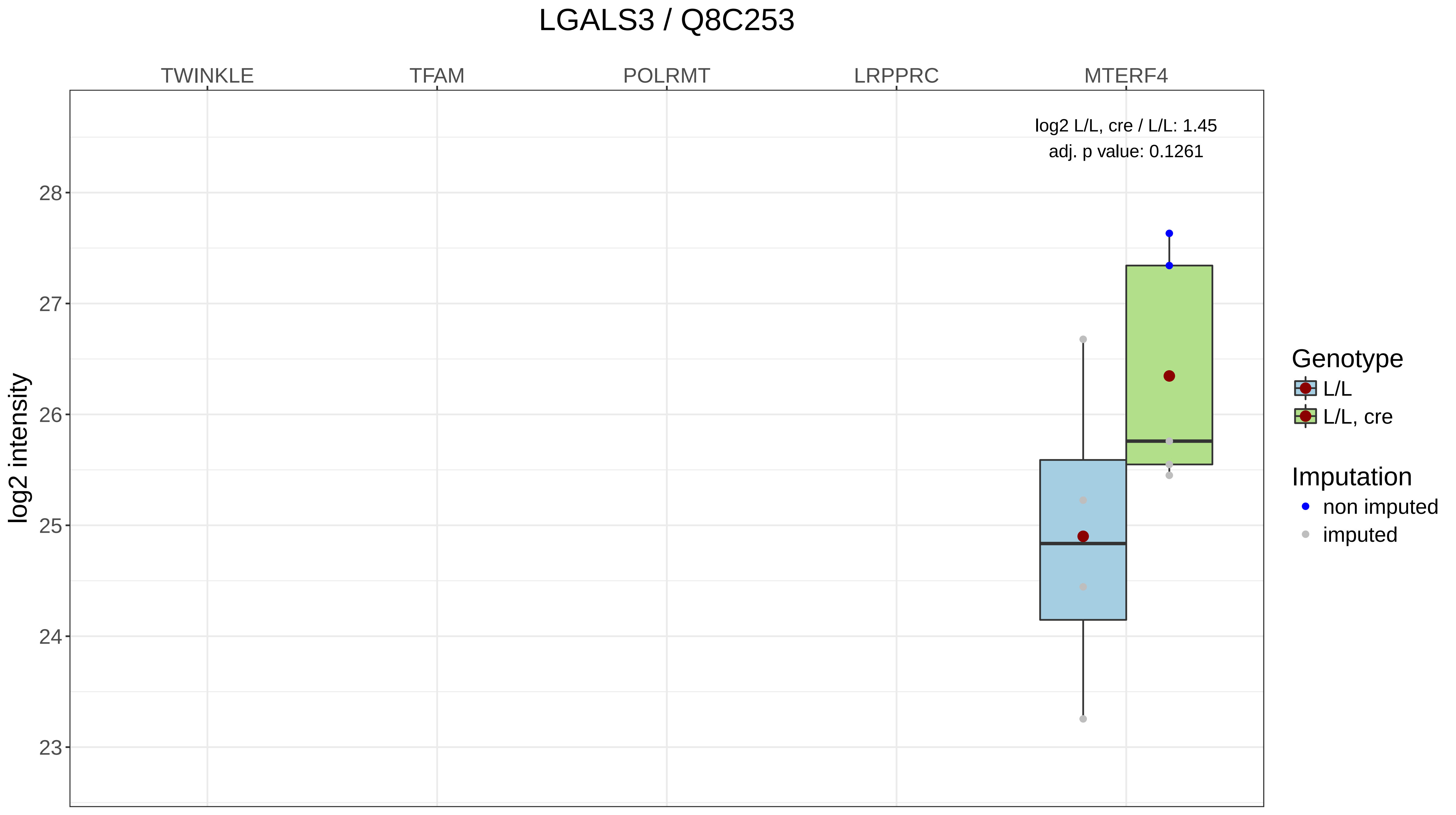

LGALS3 / Q8C253
TWINKLE
TFAM
POLRMT
LRPPRC
MTERF4
log2 L/L, cre / L/L: 1.45
adj. p value: 0.1261
log2 intensity
28
27
26
25
24
23
Genotype
L/L
L/L, cre
Imputation
non imputed
imputed

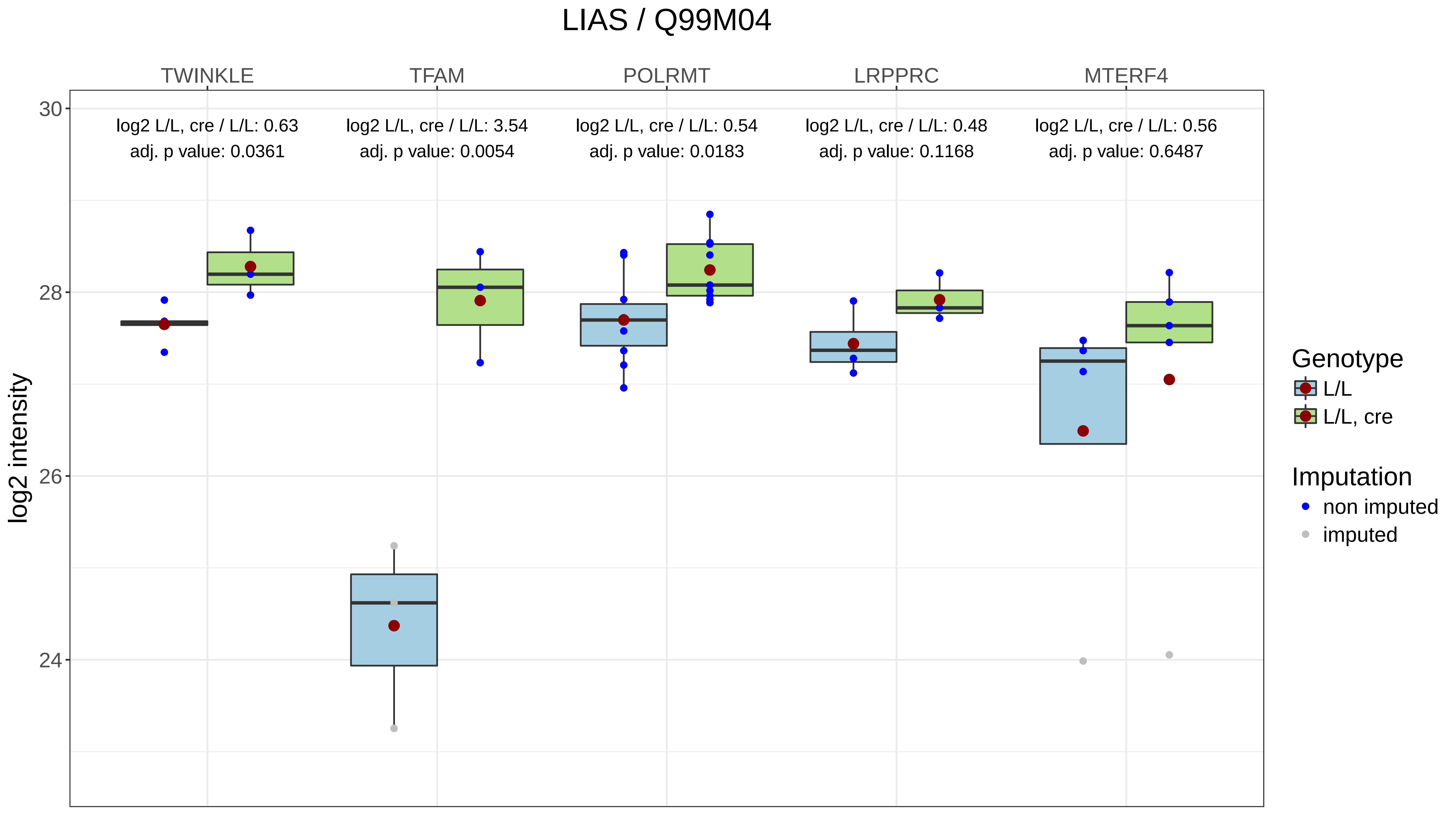
LIAS / Q99M04
TWINKLE
TFAM
POLRMT
LRPPRC
MTERF4
log2 L/L, cre / L/L: 0.63
adj. p value: 0.0361
log2 L/L, cre / L/L: 3.54
adj. p value: 0.0054
log2 L/L, cre / L/L: 0.54
adj. p value: 0.0183
log2 L/L, cre / L/L: 0.48
adj. p value: 0.1168
log2 L/L, cre / L/L: 0.56
adj. p value: 0.6487
log2 intensity
30
28
26
24
Genotype
L/L
L/L, cre
Imputation
non imputed
imputed

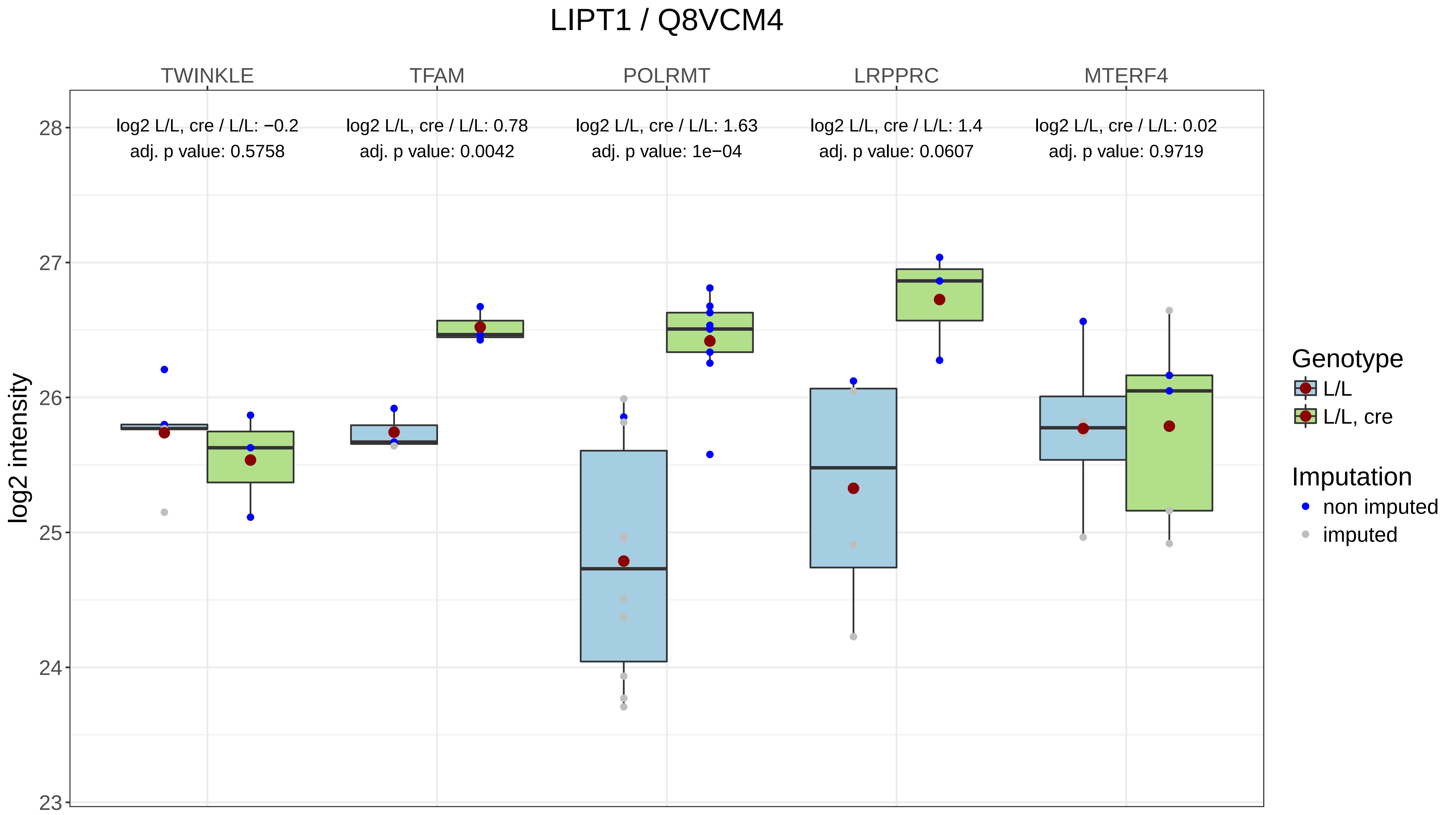
LIPT1 / Q8VCM4
TWINKLE
TFAM
POLRMT
LRPPRC
MTERF4
log2 L/L, cre / L/L: −0.2
adj. p value: 0.5758
log2 L/L, cre / L/L: 0.78
adj. p value: 0.0042
log2 L/L, cre / L/L: 1.63
adj. p value: 1e−04
log2 L/L, cre / L/L: 1.4
adj. p value: 0.0607
log2 L/L, cre / L/L: 0.02
adj. p value: 0.9719
log2 intensity
28
27
26
25
24
23
Genotype
L/L
L/L, cre
Imputation
non imputed
imputed

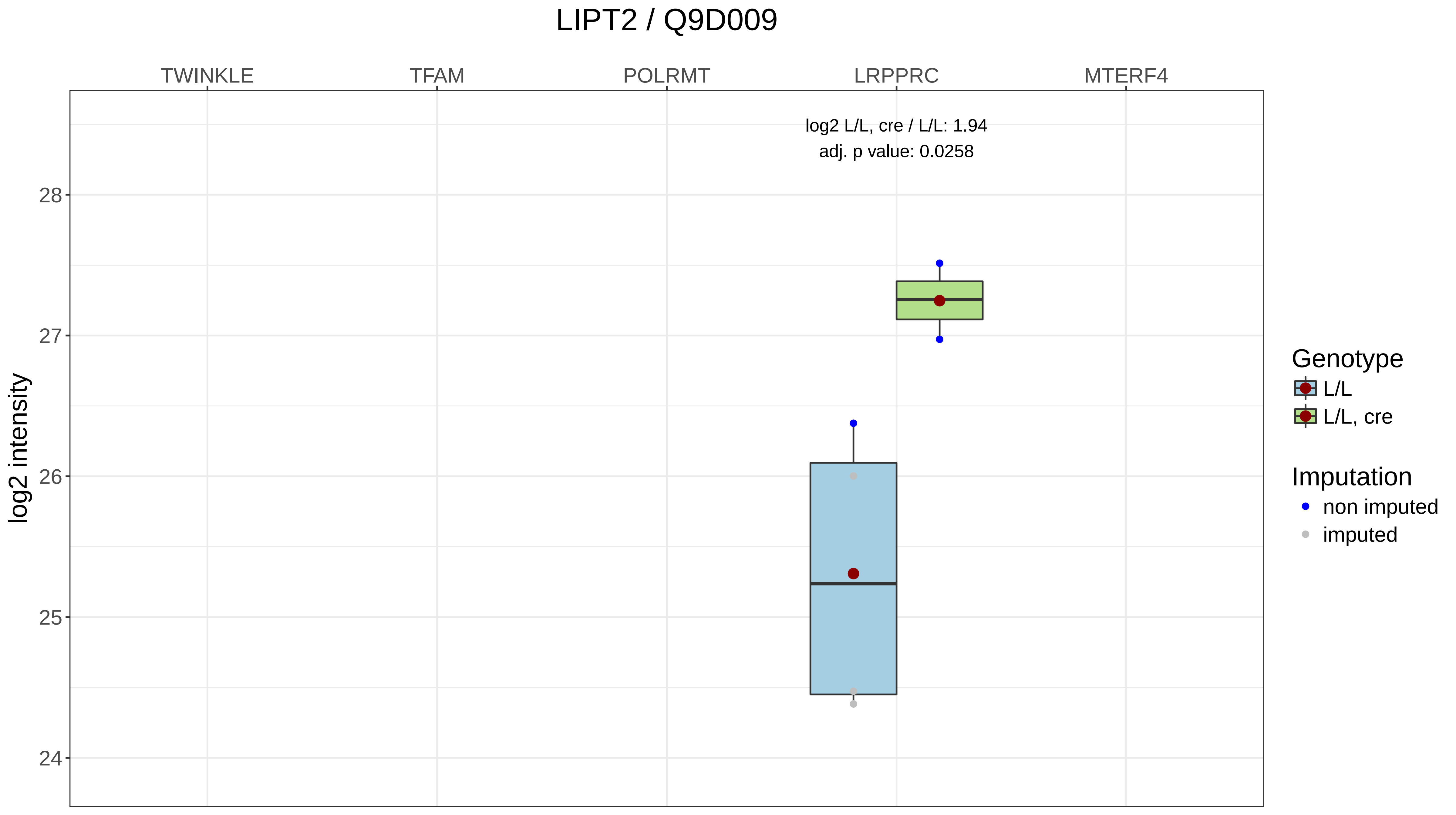

LIPT2 / Q9D009
TWINKLE
TFAM
POLRMT
LRPPRC
MTERF4
log2 L/L, cre / L/L: 1.94
adj. p value: 0.0258
28
27
26
25
24
log2 intensity
Genotype
L/L
L/L, cre
Imputation
non imputed
imputed

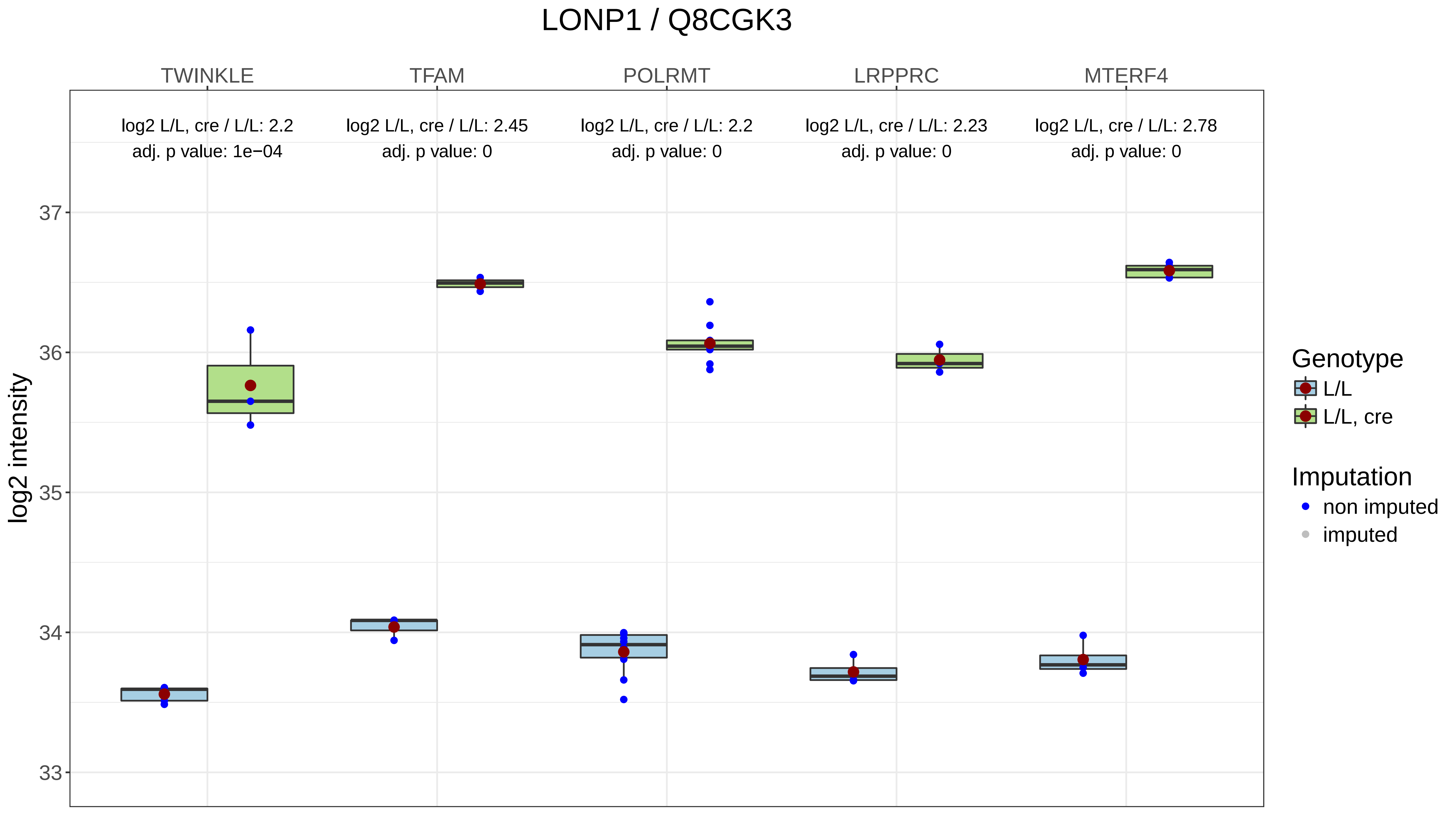

LONP1 / Q8CGK3
TWINKLE
TFAM
POLRMT
LRPPRC
MTERF4
log2 L/L, cre / L/L: 2.2
adj. p value: 1e−04
log2 L/L, cre / L/L: 2.45
adj. p value: 0
log2 L/L, cre / L/L: 2.2
adj. p value: 0
log2 L/L, cre / L/L: 2.23
adj. p value: 0
log2 L/L, cre / L/L: 2.78
adj. p value: 0
log2 intensity
37
36
35
34
33
Genotype
L/L
L/L, cre
Imputation
non imputed
imputed

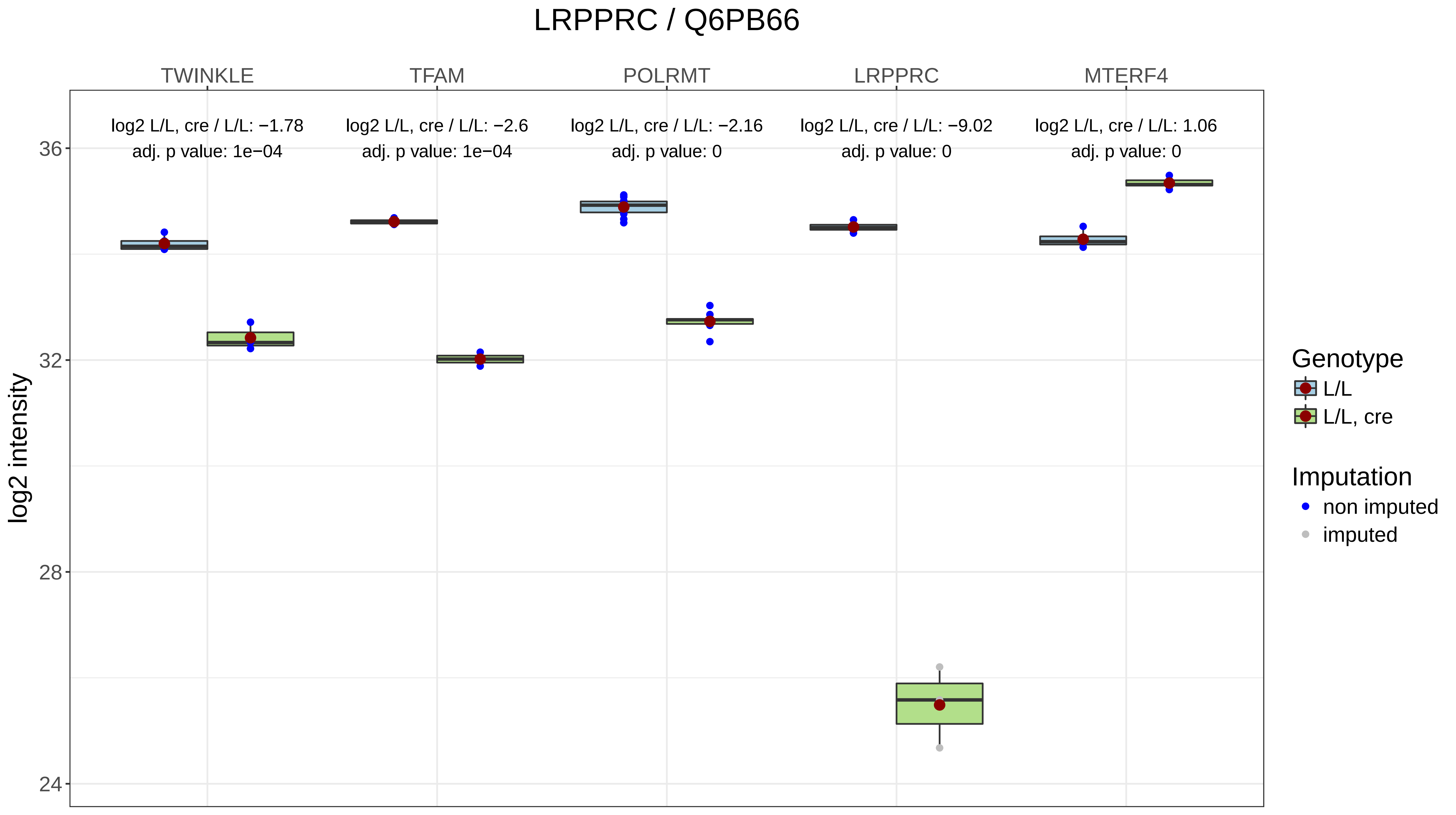
LRPPRC / Q6PB66
TWINKLE
TFAM
POLRMT
LRPPRC
MTERF4
log2 L/L, cre / L/L: −1.78
adj. p value: 1e−04
log2 L/L, cre / L/L: −2.6
adj. p value: 1e−04
log2 L/L, cre / L/L: −2.16
adj. p value: 0
log2 L/L, cre / L/L: −9.02
adj. p value: 0
log2 L/L, cre / L/L: 1.06
adj. p value: 0
36
32
28
24
log2 intensity
Genotype
L/L
L/L, cre
Imputation
non imputed
imputed

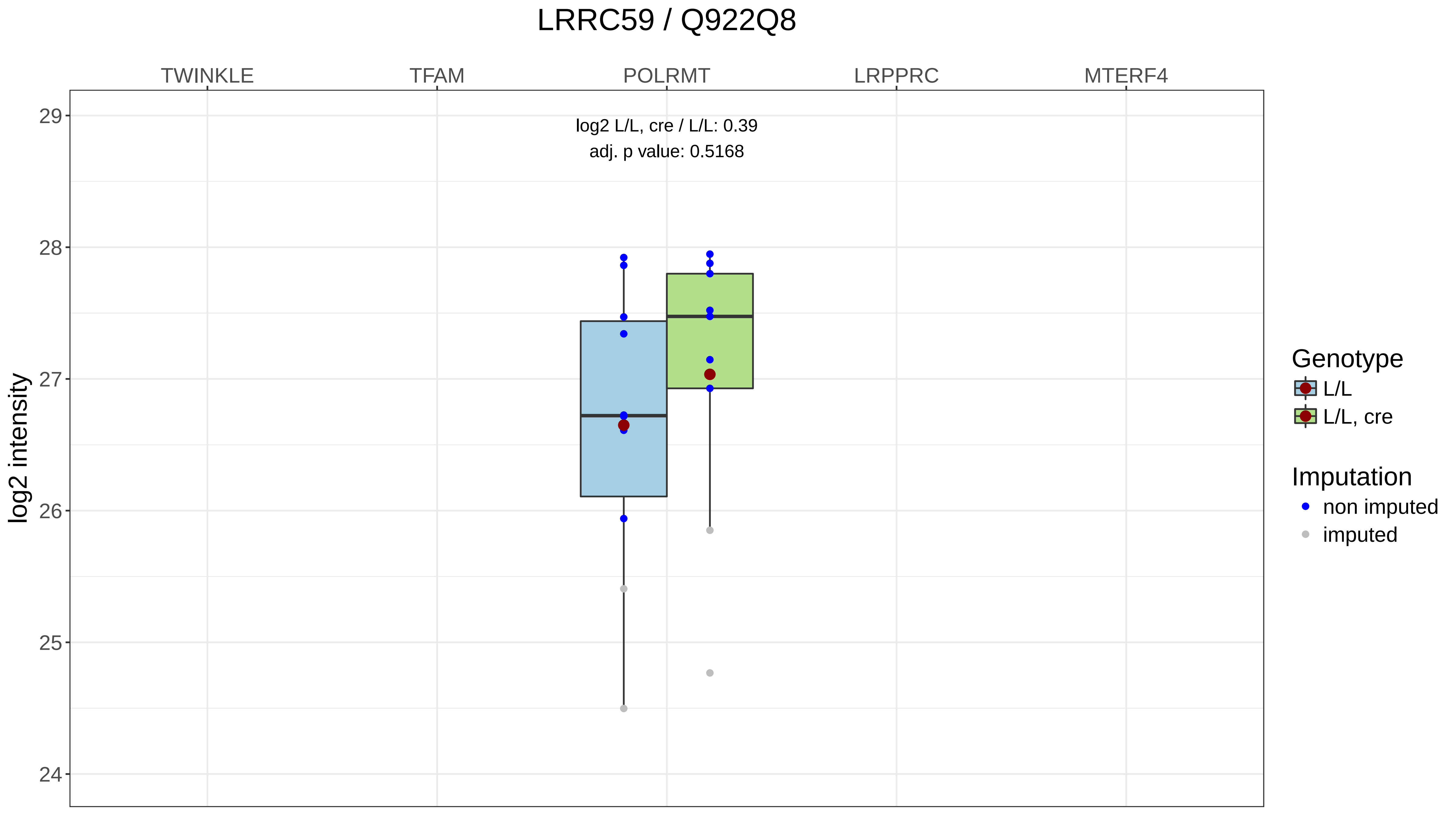
LRRC59 / Q922Q8
TWINKLE
TFAM
POLRMT
LRPPRC
MTERF4
log2 L/L, cre / L/L: 0.39
adj. p value: 0.5168
log2 intensity
29
28
27
26
25
24
Genotype
L/L
L/L, cre
Imputation
non imputed
imputed

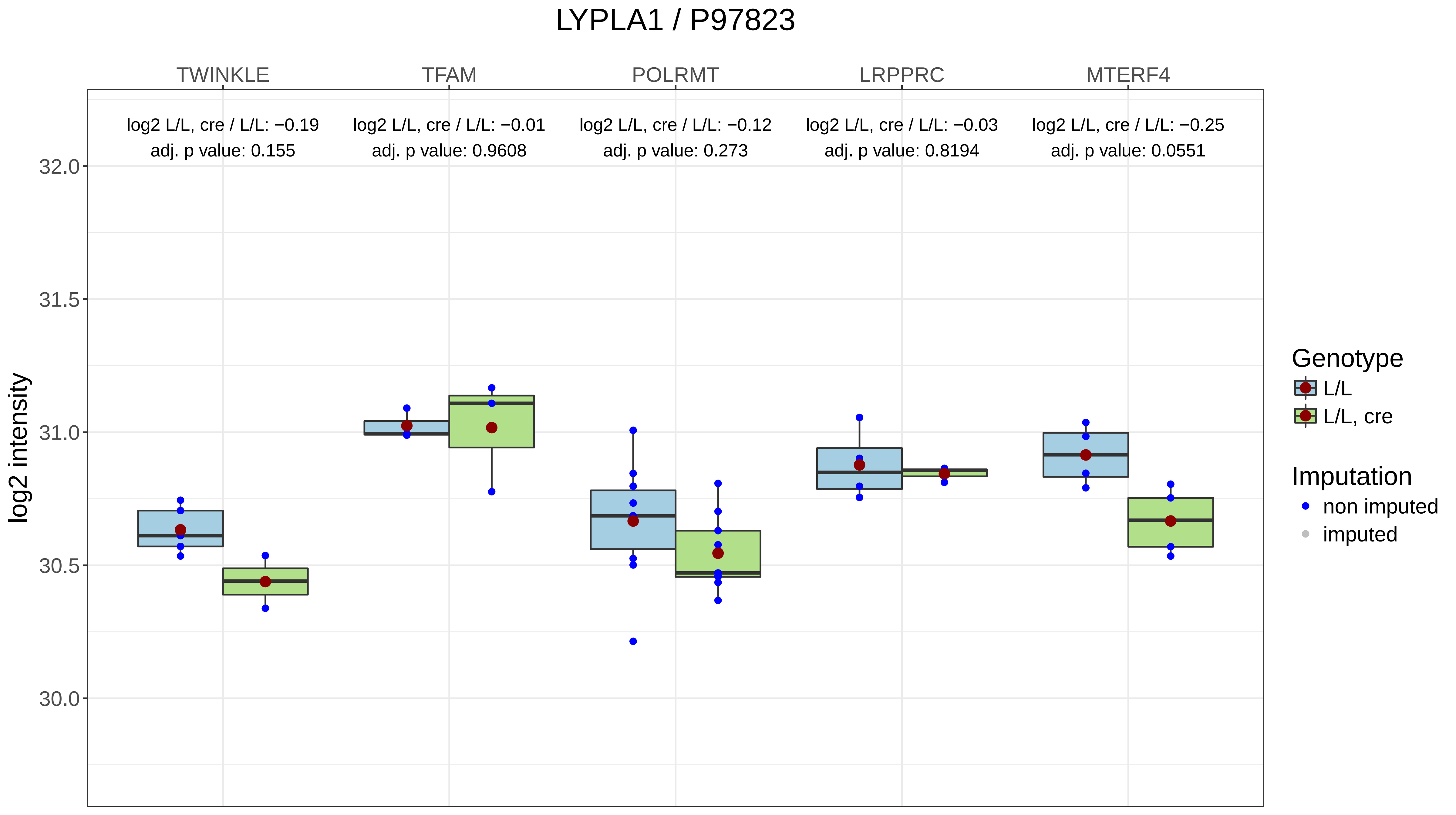

LYPLA1 / P97823
TWINKLE
TFAM
POLRMT
LRPPRC
MTERF4
log2 L/L, cre / L/L: −0.19
adj. p value: 0.155
log2 L/L, cre / L/L: −0.01
adj. p value: 0.9608
log2 L/L, cre / L/L: −0.12
adj. p value: 0.273
log2 L/L, cre / L/L: −0.03
adj. p value: 0.8194
log2 L/L, cre / L/L: −0.25
adj. p value: 0.0551
log2 intensity
32.0
31.5
31.0
30.5
30.0
Genotype
L/L
L/L, cre
Imputation
non imputed
imputed

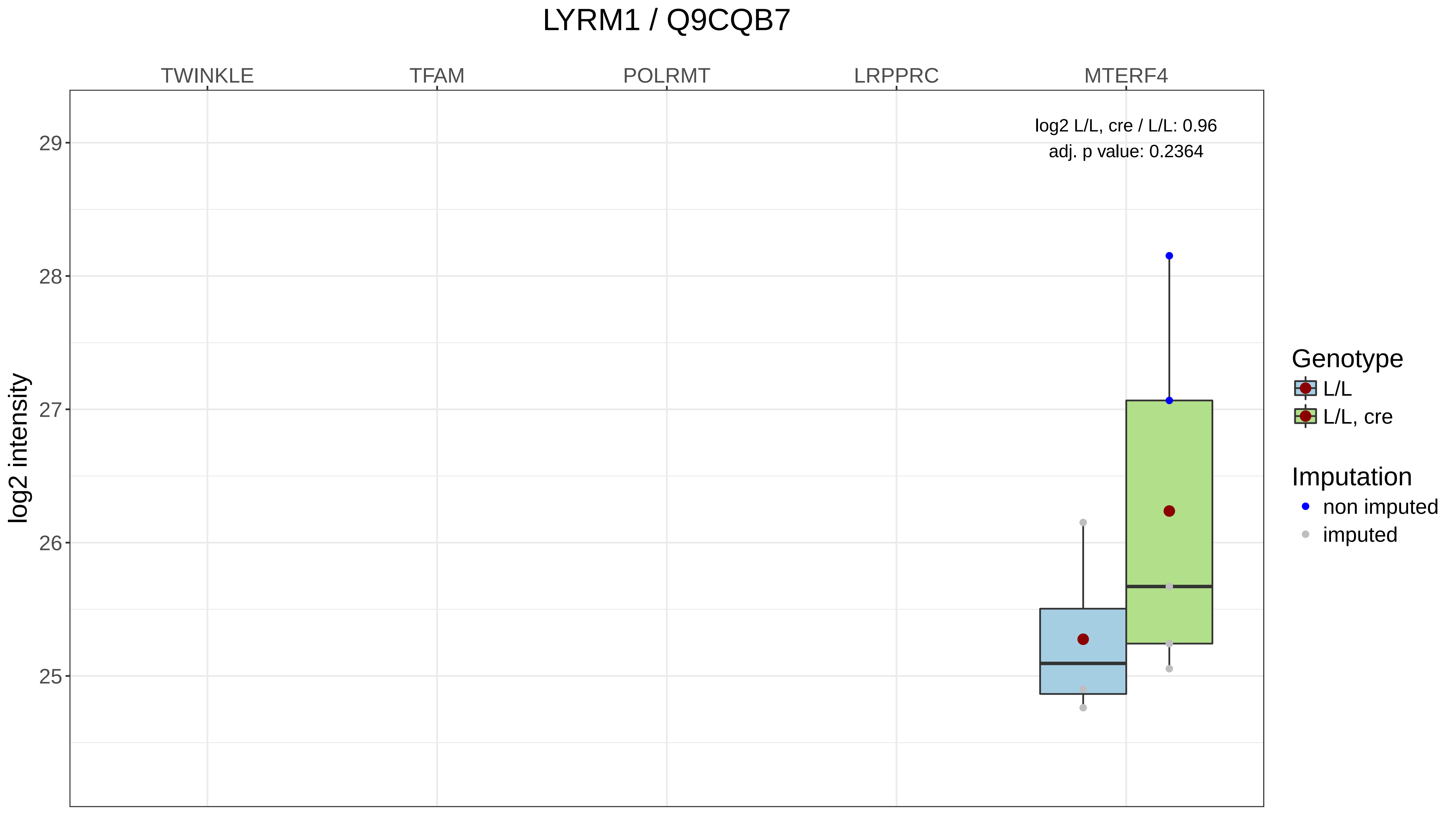

LYRM1 / Q9CQB7
TWINKLE
TFAM
POLRMT
LRPPRC
MTERF4
log2 L/L, cre / L/L: 0.96
adj. p value: 0.2364
log2 intensity
29
28
27
26
25
Genotype
L/L
L/L, cre
Imputation
non imputed
imputed

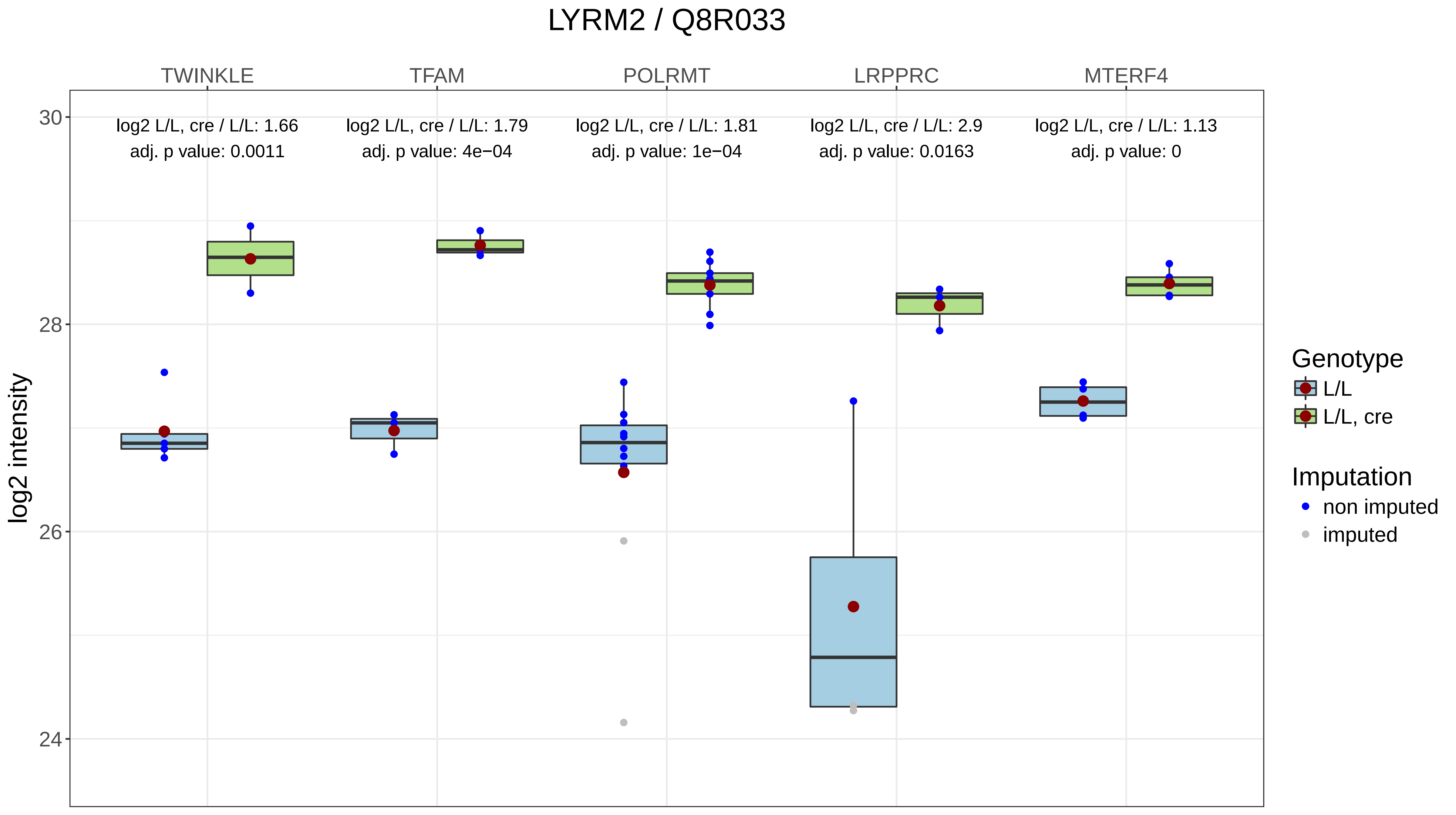
LYRM2 / Q8R033
TWINKLE
TFAM
POLRMT
LRPPRC
MTERF4
log2 L/L, cre / L/L: 1.66
adj. p value: 0.0011
log2 L/L, cre / L/L: 1.79
adj. p value: 4e−04
log2 L/L, cre / L/L: 1.81
adj. p value: 1e−04
log2 L/L, cre / L/L: 2.9
adj. p value: 0.0163
log2 L/L, cre / L/L: 1.13
adj. p value: 0
log2 intensity
30
28
26
24
Genotype
L/L
L/L, cre
Imputation
non imputed
imputed

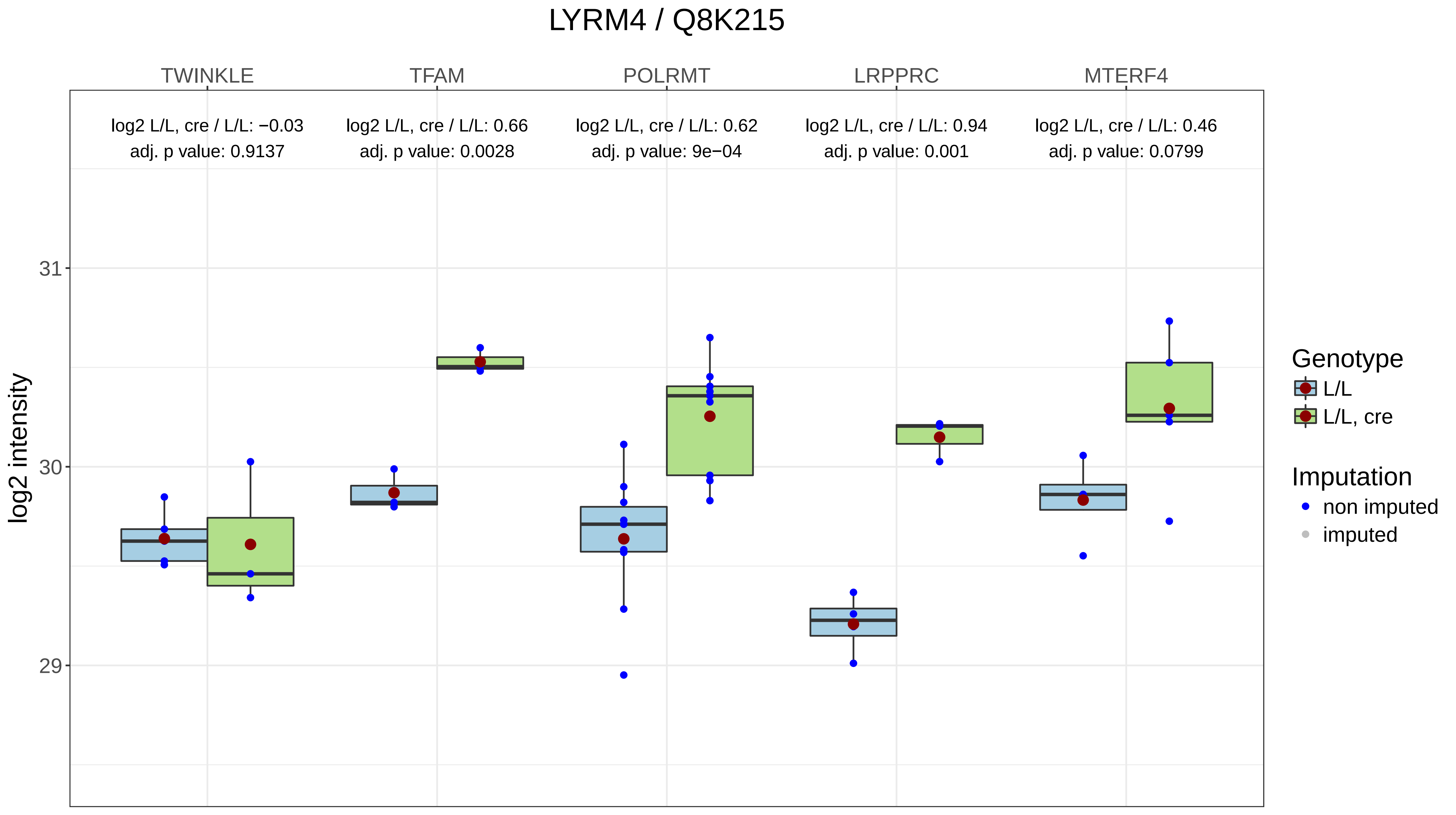
LYRM4 / Q8K215
TWINKLE
TFAM
POLRMT
LRPPRC
MTERF4
log2 L/L, cre / L/L: −0.03
adj. p value: 0.9137
log2 L/L, cre / L/L: 0.66
adj. p value: 0.0028
log2 L/L, cre / L/L: 0.62
adj. p value: 9e−04
log2 L/L, cre / L/L: 0.94
adj. p value: 0.001
log2 L/L, cre / L/L: 0.46
adj. p value: 0.0799
log2 intensity
31
30
29
Genotype
L/L
L/L, cre
Imputation
non imputed
imputed

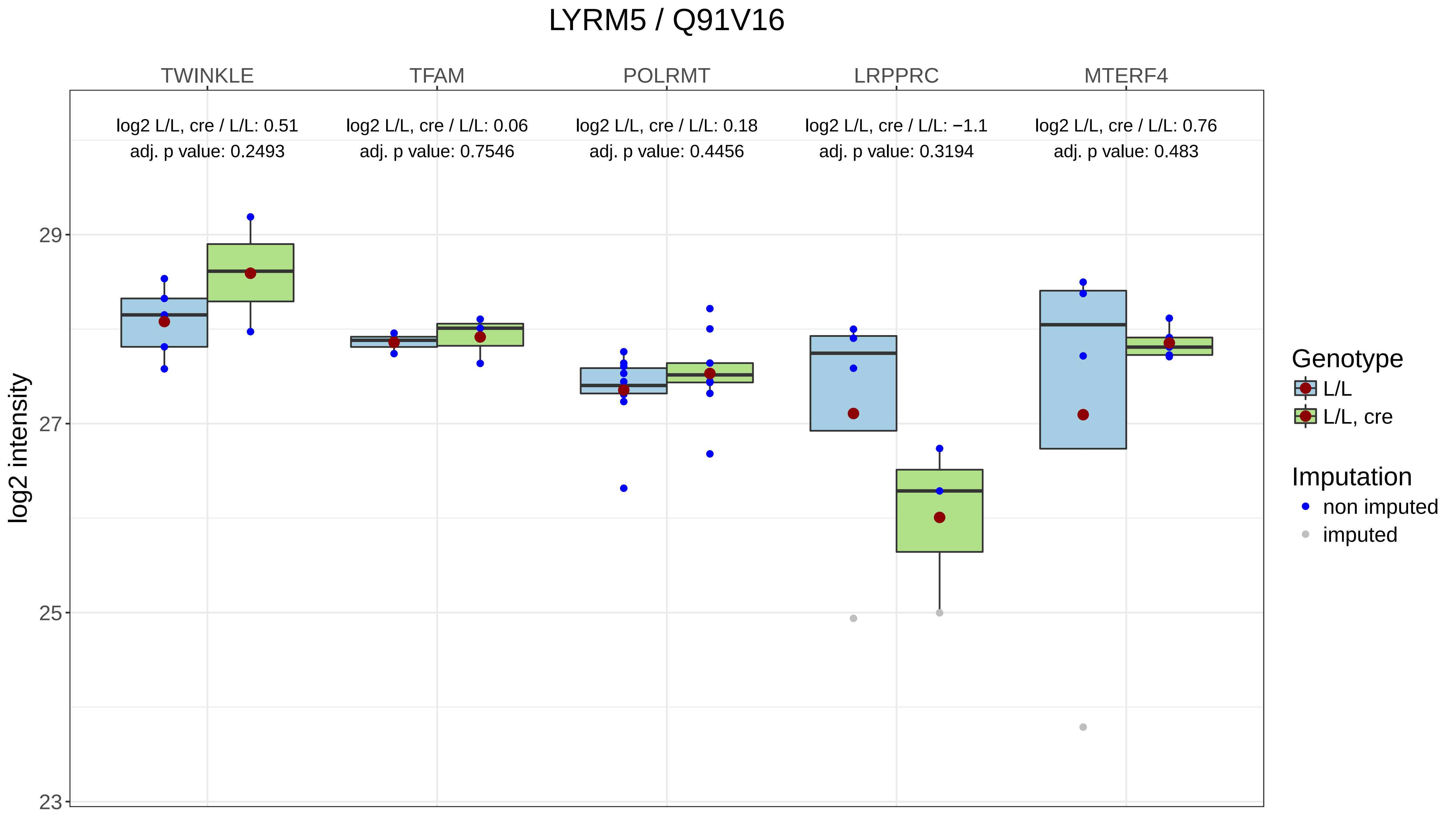
LYRM5 / Q91V16
TWINKLE
TFAM
POLRMT
LRPPRC
MTERF4
log2 L/L, cre / L/L: 0.51
adj. p value: 0.2493
log2 L/L, cre / L/L: 0.06
adj. p value: 0.7546
log2 L/L, cre / L/L: 0.18
adj. p value: 0.4456
log2 L/L, cre / L/L: −1.1
adj. p value: 0.3194
log2 L/L, cre / L/L: 0.76
adj. p value: 0.483
log2 intensity
29
27
25
23
Genotype
L/L
L/L, cre
Imputation
non imputed
imputed

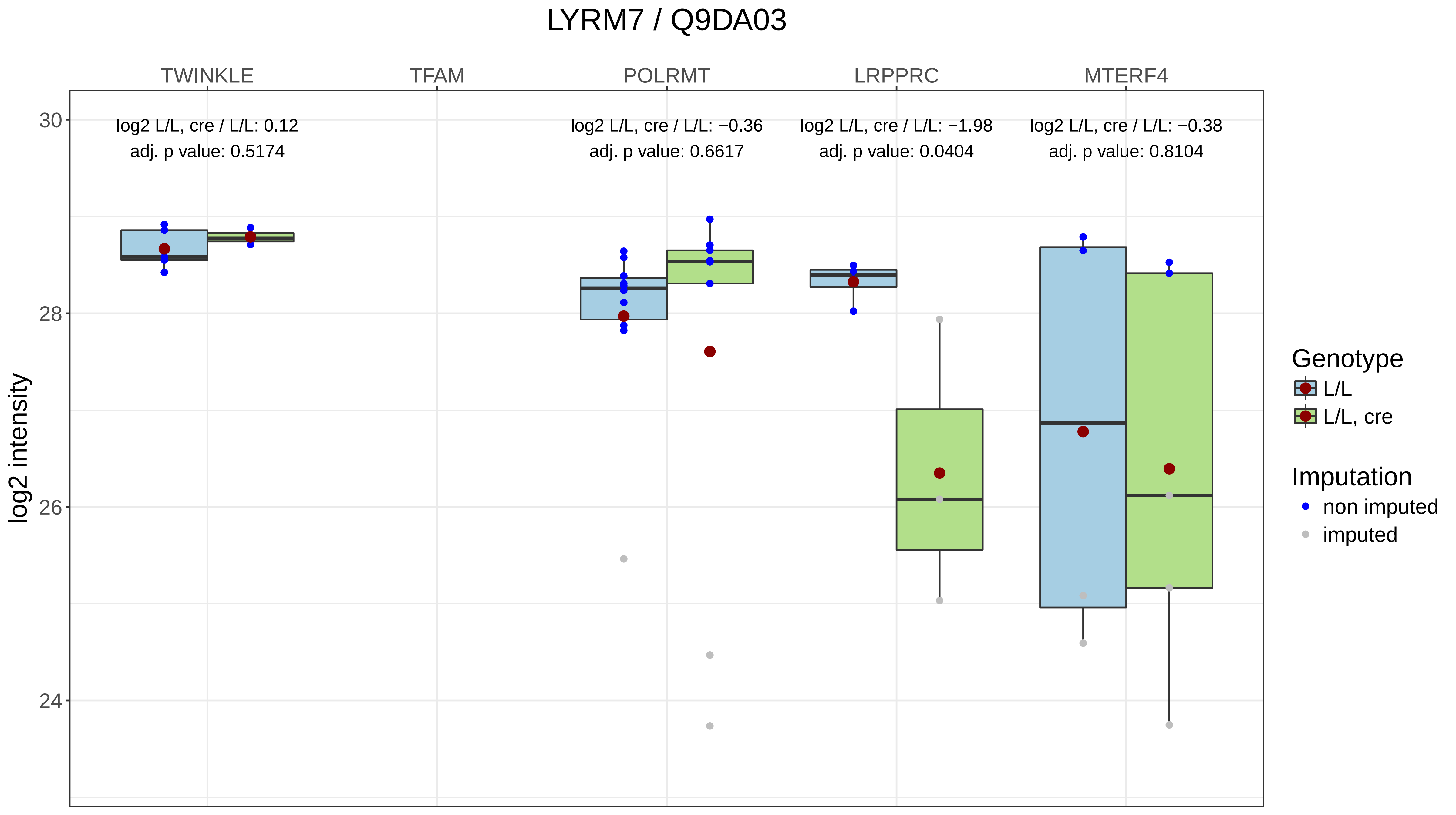

LYRM7 / Q9DA03
TWINKLE
TFAM
POLRMT
LRPPRC
MTERF4
log2 L/L, cre / L/L: 0.12
adj. p value: 0.5174
log2 L/L, cre / L/L: −0.36
adj. p value: 0.6617
log2 L/L, cre / L/L: −1.98
adj. p value: 0.0404
log2 L/L, cre / L/L: −0.38
adj. p value: 0.8104
log2 intensity
30
28
26
24
Genotype
L/L
L/L, cre
Imputation
non imputed
imputed

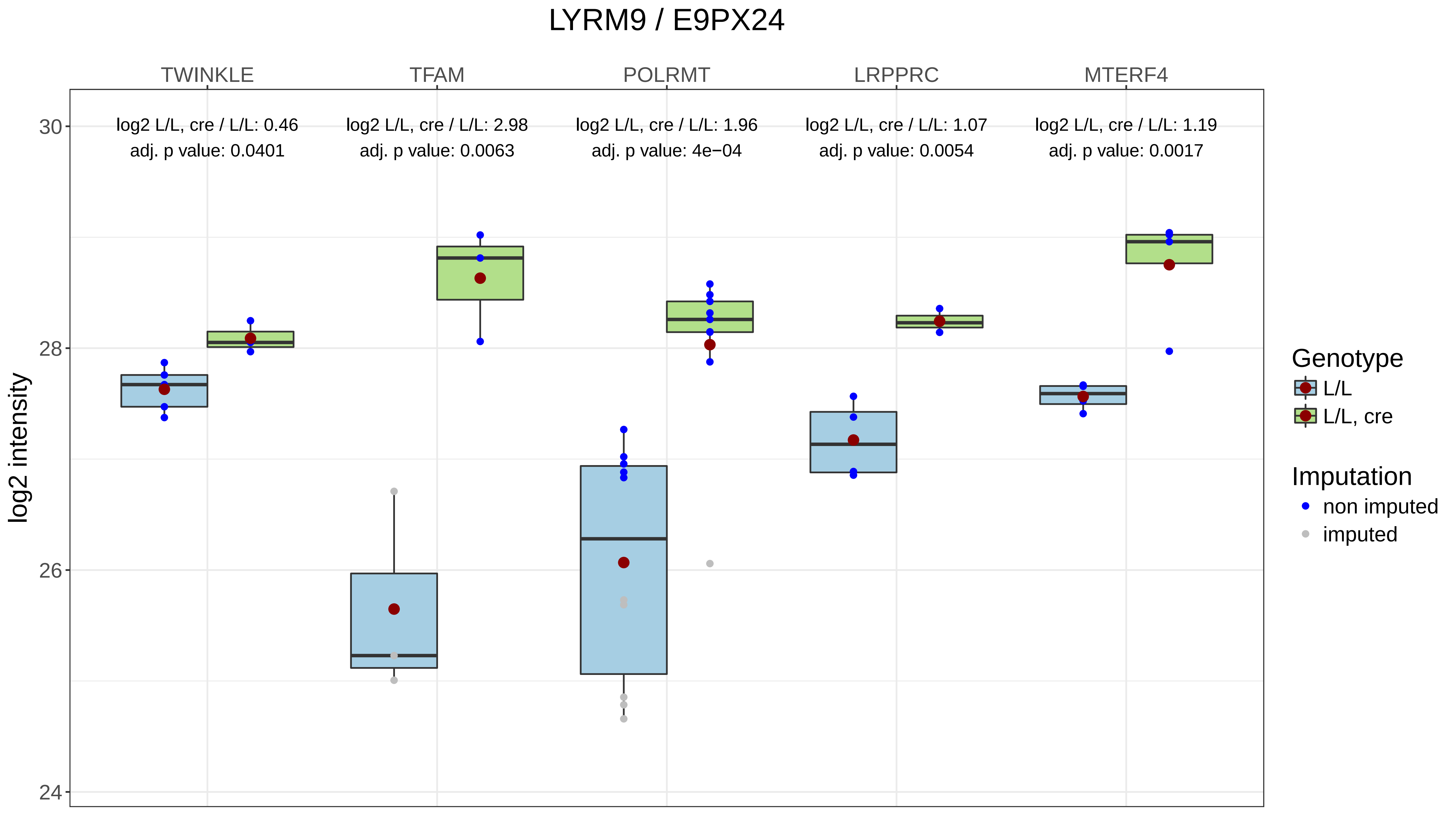
LYRM9 / E9PX24
TWINKLE
TFAM
POLRMT
LRPPRC
MTERF4
log2 L/L, cre / L/L: 0.46
adj. p value: 0.0401
log2 L/L, cre / L/L: 2.98
adj. p value: 0.0063
log2 L/L, cre / L/L: 1.96
adj. p value: 4e−04
log2 L/L, cre / L/L: 1.07
adj. p value: 0.0054
log2 L/L, cre / L/L: 1.19
adj. p value: 0.0017
log2 intensity
30
28
26
24
Genotype
L/L
L/L, cre
Imputation
non imputed
imputed

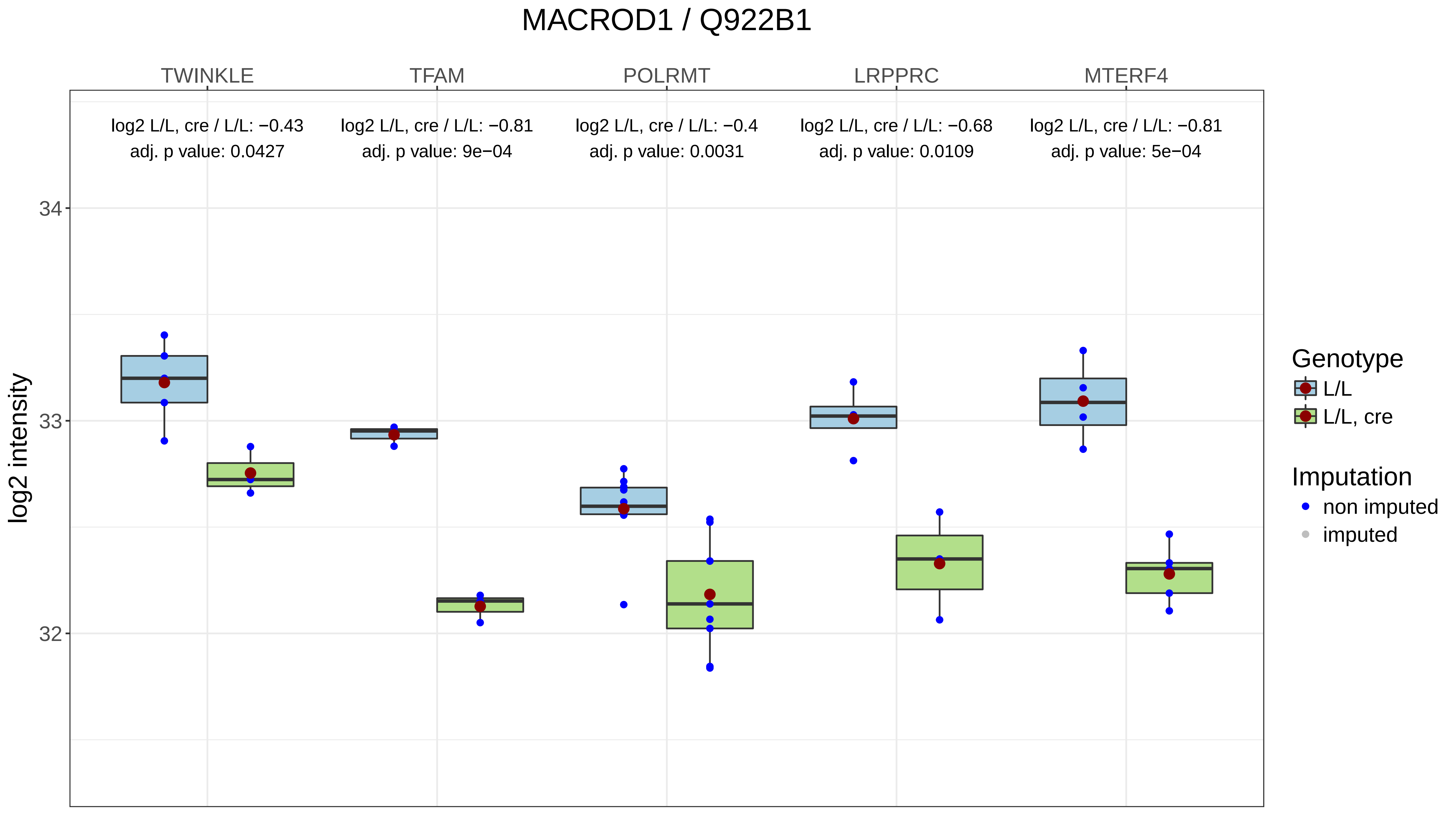
MACROD1 / Q922B1
TWINKLE
TFAM
POLRMT
LRPPRC
MTERF4
log2 L/L, cre / L/L: −0.43
adj. p value: 0.0427
log2 L/L, cre / L/L: −0.81
adj. p value: 9e−04
log2 L/L, cre / L/L: −0.4
adj. p value: 0.0031
log2 L/L, cre / L/L: −0.68
adj. p value: 0.0109
log2 L/L, cre / L/L: −0.81
adj. p value: 5e−04
34
33
32
log2 intensity
Genotype
L/L
L/L, cre
Imputation
non imputed
imputed

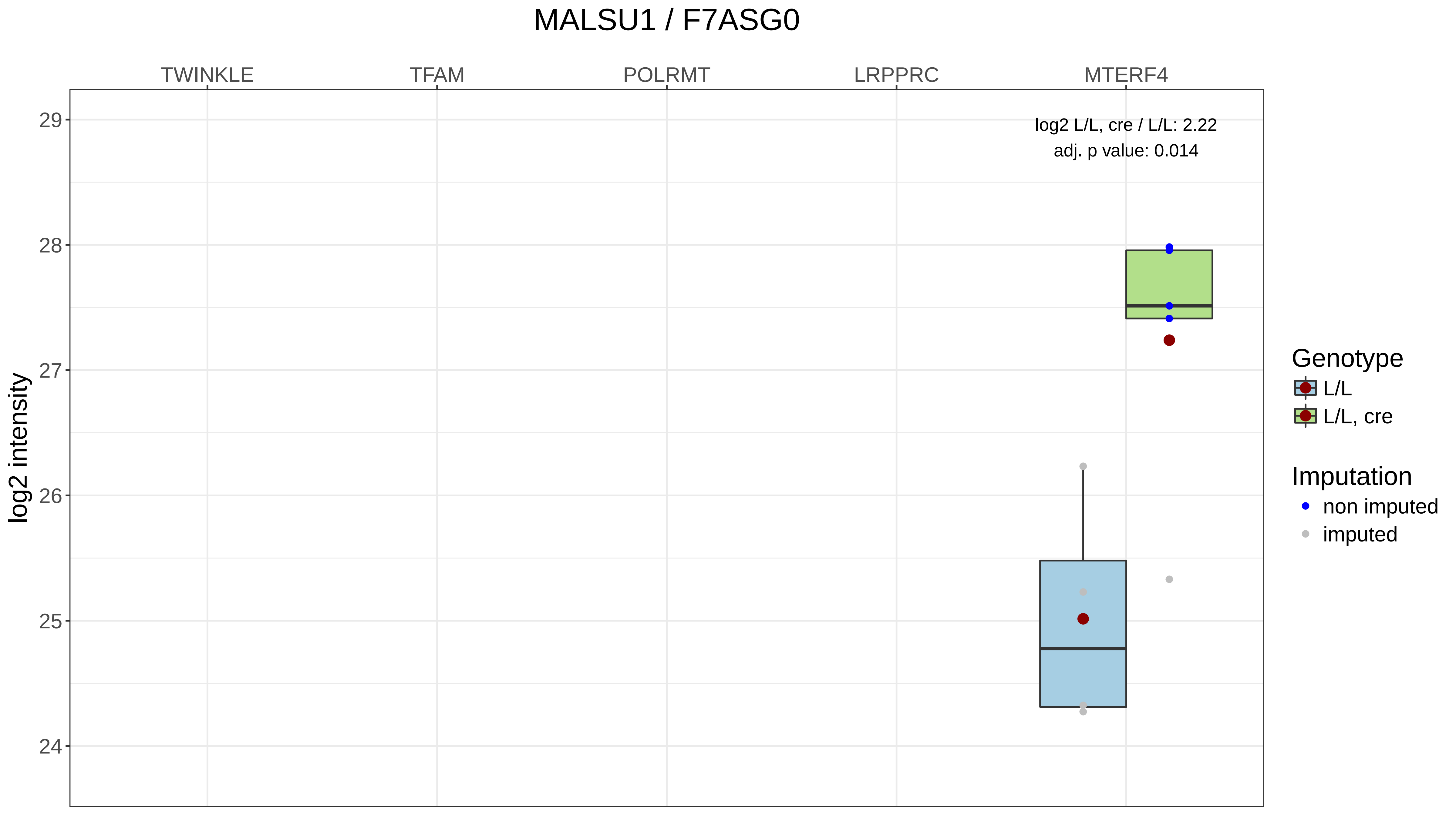
MALSU1 / F7ASG0
TWINKLE
TFAM
POLRMT
LRPPRC
MTERF4
log2 L/L, cre / L/L: 2.22
adj. p value: 0.014
log2 intensity
29
28
27
26
25
24
Genotype
L/L
L/L, cre
Imputation
non imputed
imputed

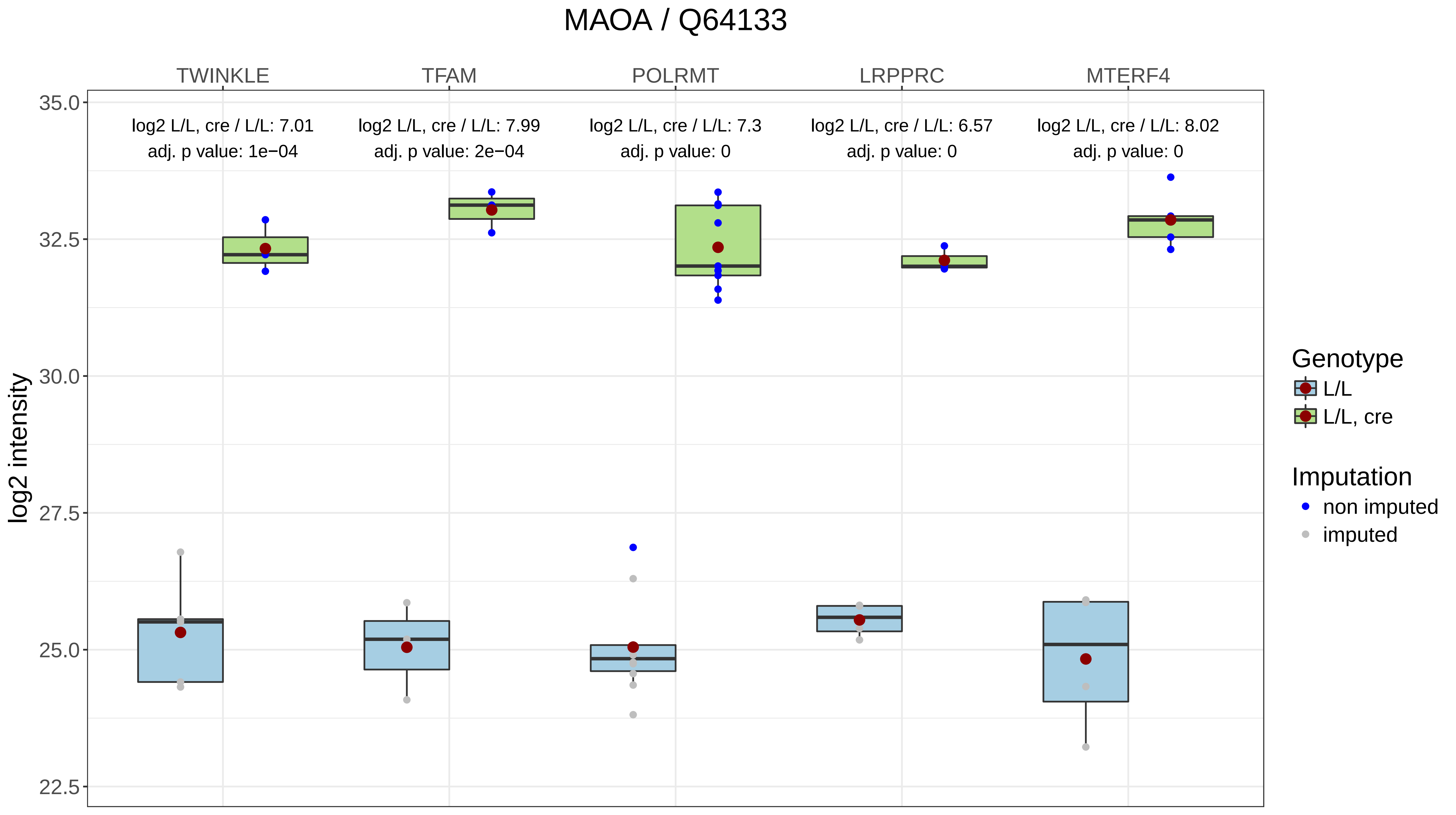
MAOA / Q64133
TWINKLE
TFAM
POLRMT
LRPPRC
MTERF4
log2 L/L, cre / L/L: 7.01
adj. p value: 1e−04
log2 L/L, cre / L/L: 7.99
adj. p value: 2e−04
log2 L/L, cre / L/L: 7.3
adj. p value: 0
log2 L/L, cre / L/L: 6.57
adj. p value: 0
log2 L/L, cre / L/L: 8.02
adj. p value: 0
log2 intensity
35.0
32.5
30.0
27.5
25.0
22.5
Genotype
L/L
L/L, cre
Imputation
non imputed
imputed

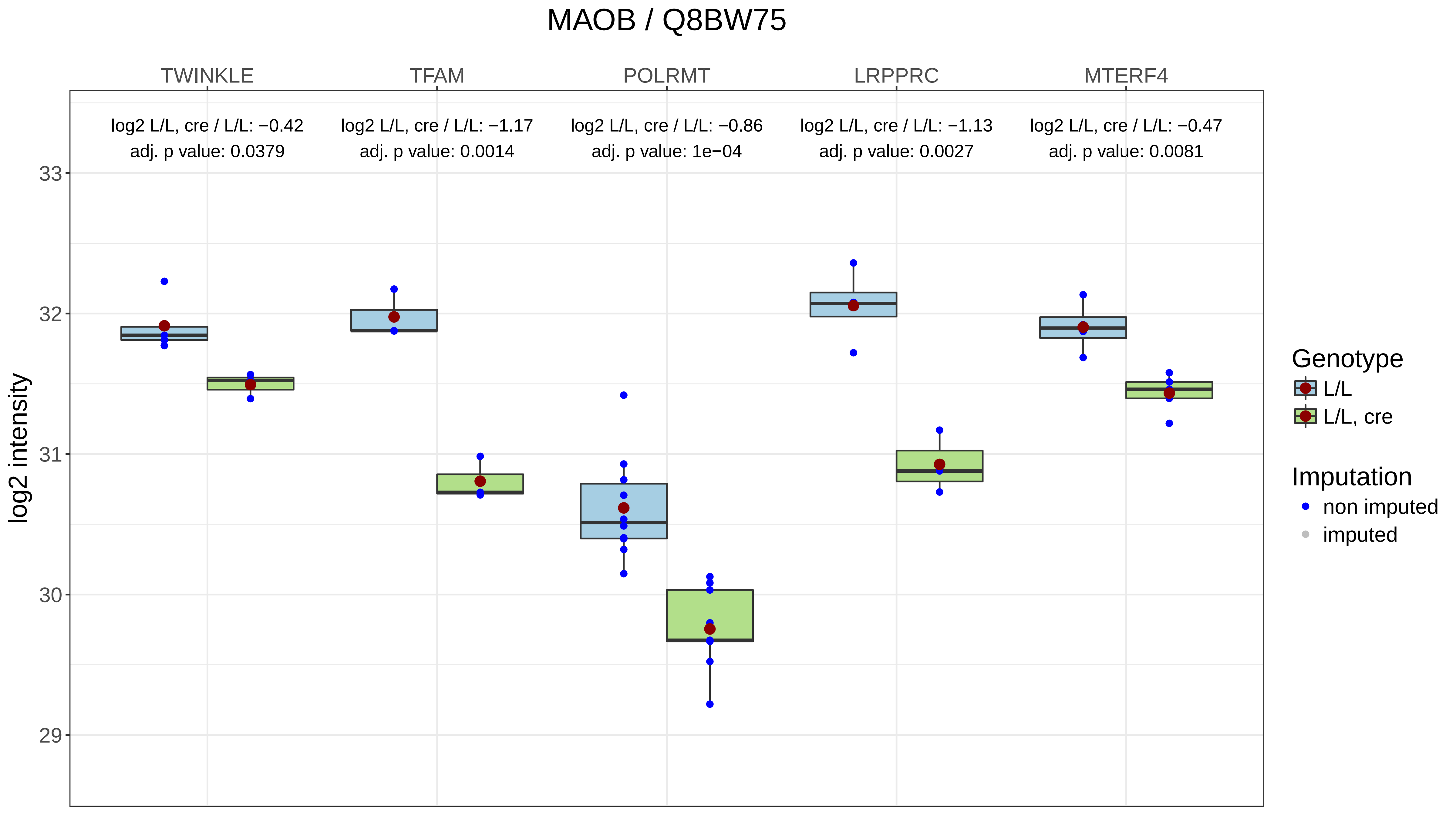
MAOB / Q8BW75
TWINKLE
TFAM
POLRMT
LRPPRC
MTERF4
log2 L/L, cre / L/L: −0.42
adj. p value: 0.0379
log2 L/L, cre / L/L: −1.17
adj. p value: 0.0014
log2 L/L, cre / L/L: −0.86
adj. p value: 1e−04
log2 L/L, cre / L/L: −1.13
adj. p value: 0.0027
log2 L/L, cre / L/L: −0.47
adj. p value: 0.0081
log2 intensity
33
32
31
30
29
Genotype
L/L
L/L, cre
Imputation
non imputed
imputed

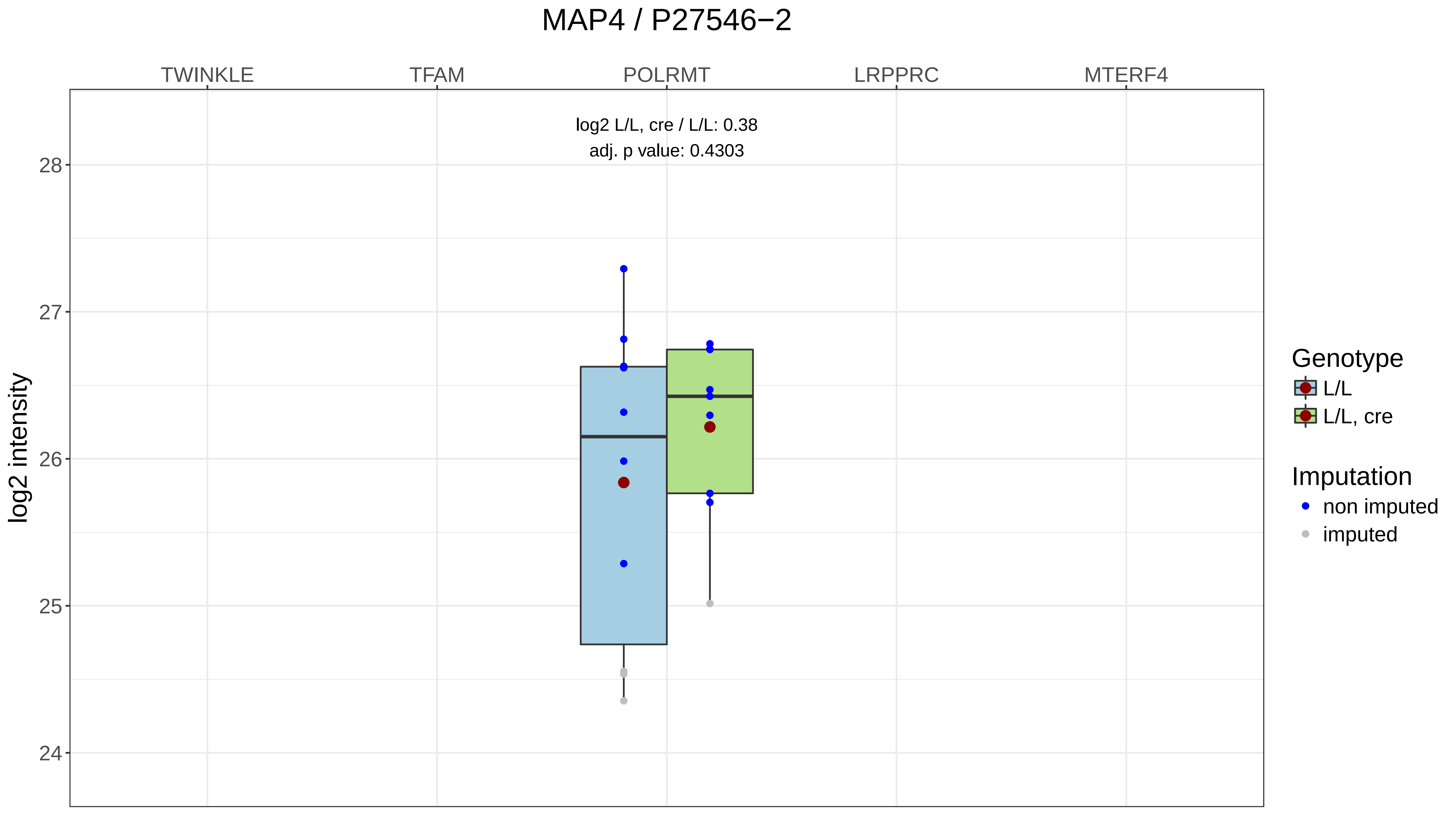
MAP4 / P27546−2
TWINKLE
TFAM
POLRMT
LRPPRC
MTERF4
log2 L/L, cre / L/L: 0.38
adj. p value: 0.4303
28
27
26
25
24
log2 intensity
Genotype
L/L
L/L, cre
Imputation
non imputed
imputed

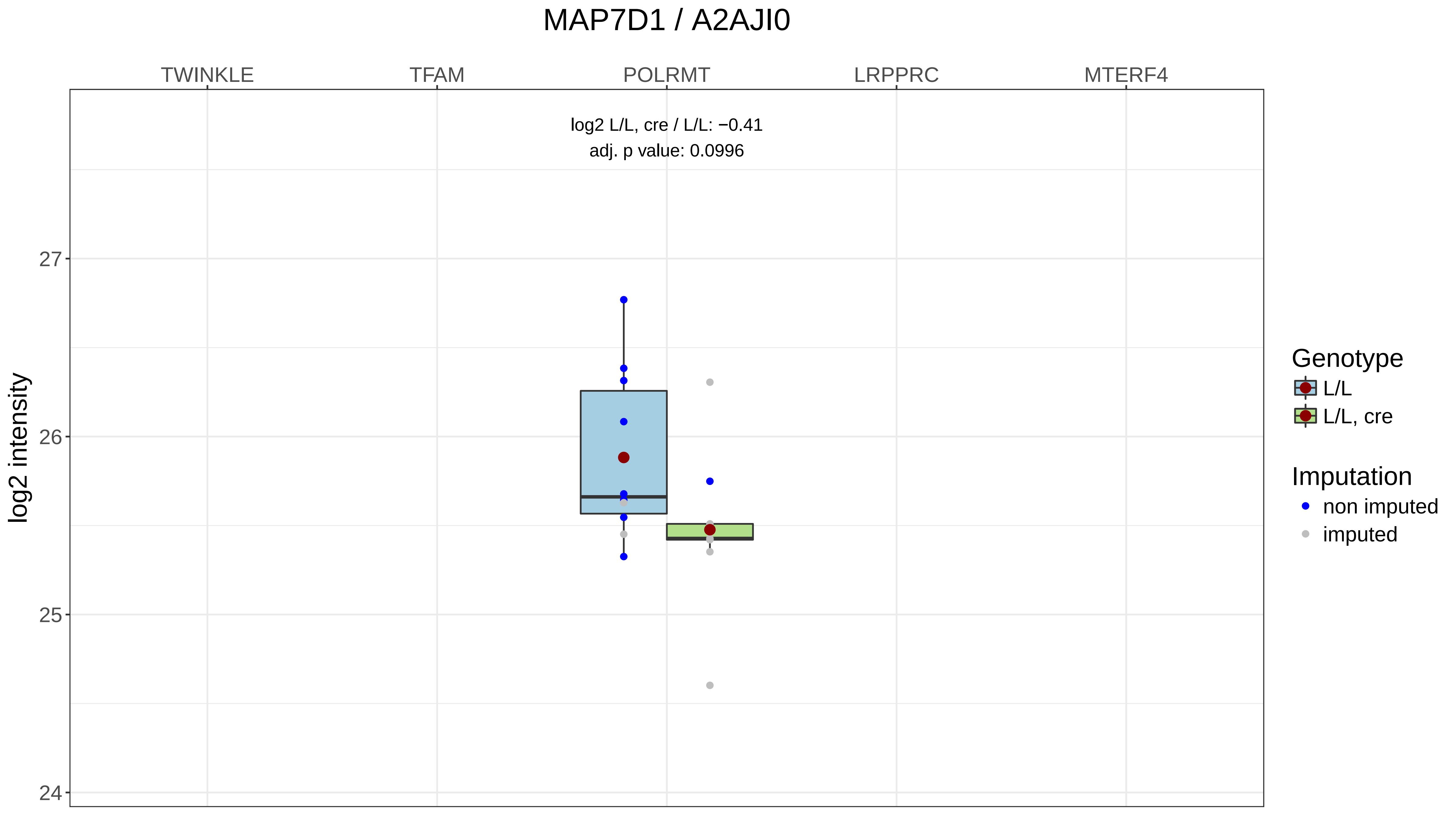
MAP7D1 / A2AJI0
TWINKLE
TFAM
POLRMT
LRPPRC
MTERF4
log2 L/L, cre / L/L: −0.41
adj. p value: 0.0996
27
26
25
24
log2 intensity
Genotype
L/L
L/L, cre
Imputation
non imputed
imputed

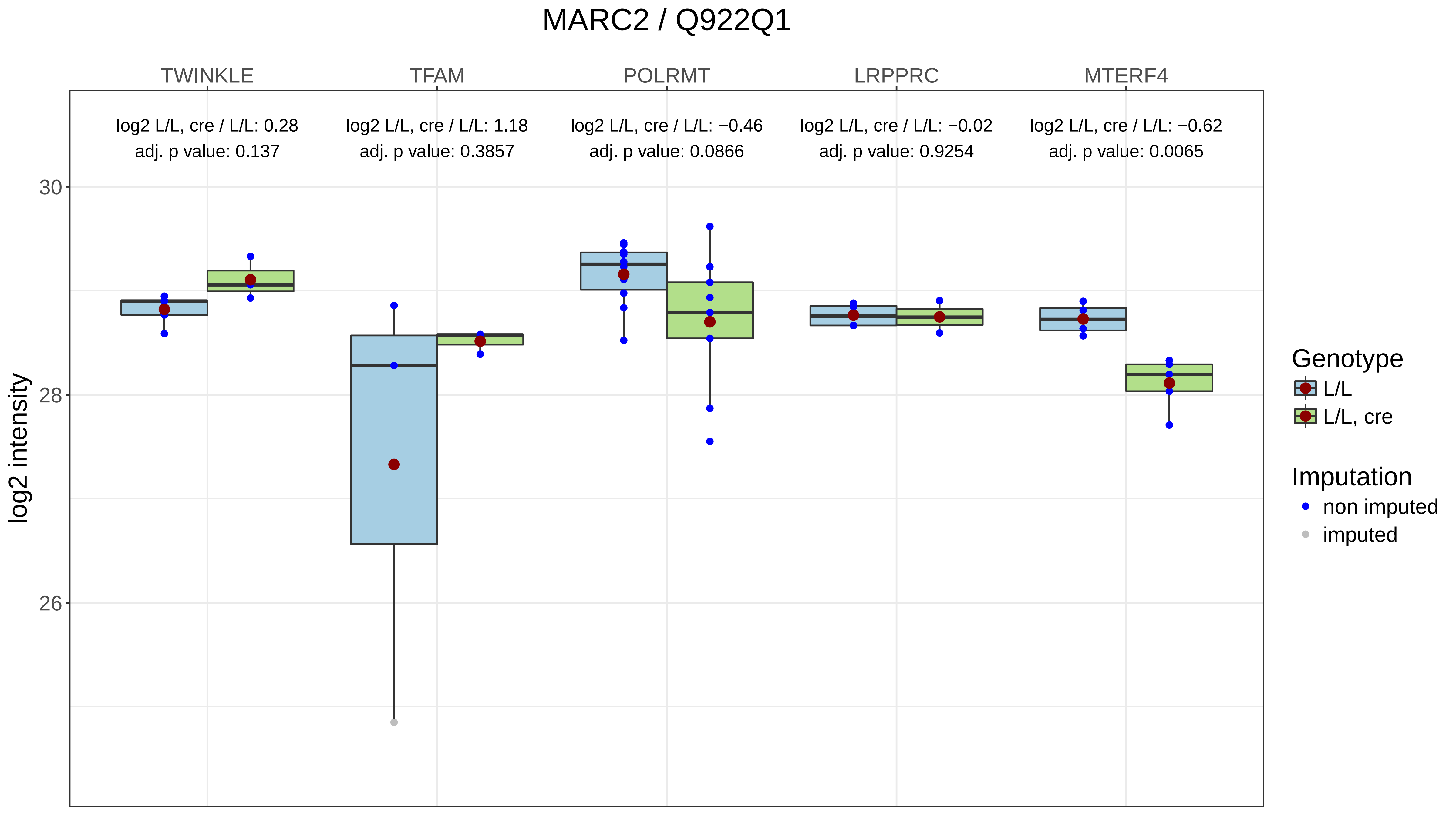
MARC2 / Q922Q1
TWINKLE
TFAM
POLRMT
LRPPRC
MTERF4
log2 L/L, cre / L/L: 0.28
adj. p value: 0.137
log2 L/L, cre / L/L: 1.18
adj. p value: 0.3857
log2 L/L, cre / L/L: −0.46
adj. p value: 0.0866
log2 L/L, cre / L/L: −0.02
adj. p value: 0.9254
log2 L/L, cre / L/L: −0.62
adj. p value: 0.0065
30
28
26
log2 intensity
Genotype
L/L
L/L, cre
Imputation
non imputed
imputed

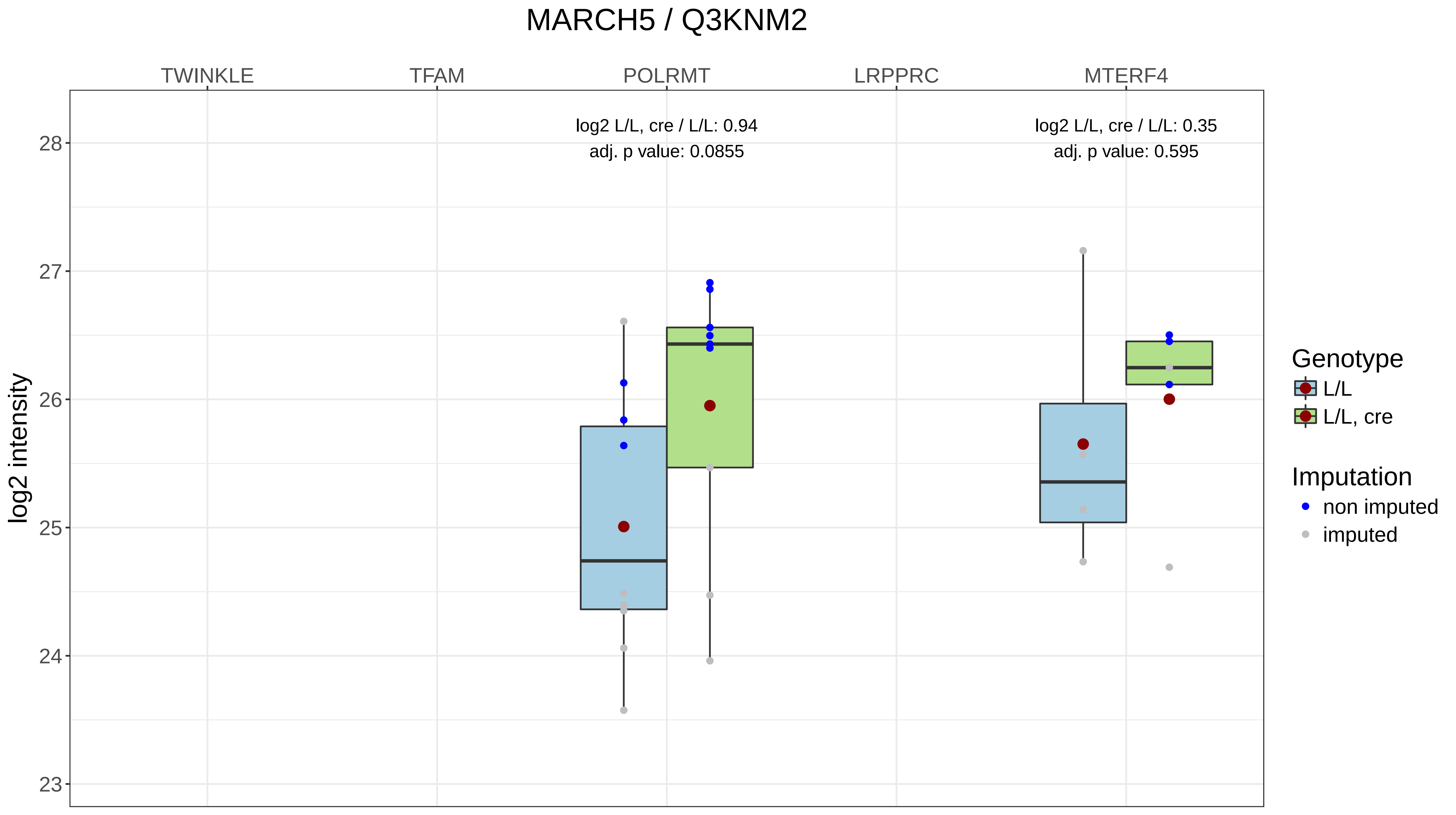

MARCH5 / Q3KNM2
TWINKLE
TFAM
POLRMT
LRPPRC
MTERF4
log2 L/L, cre / L/L: 0.94
adj. p value: 0.0855
log2 L/L, cre / L/L: 0.35
adj. p value: 0.595
log2 intensity
28
27
26
25
24
23
Genotype
L/L
L/L, cre
Imputation
non imputed
imputed

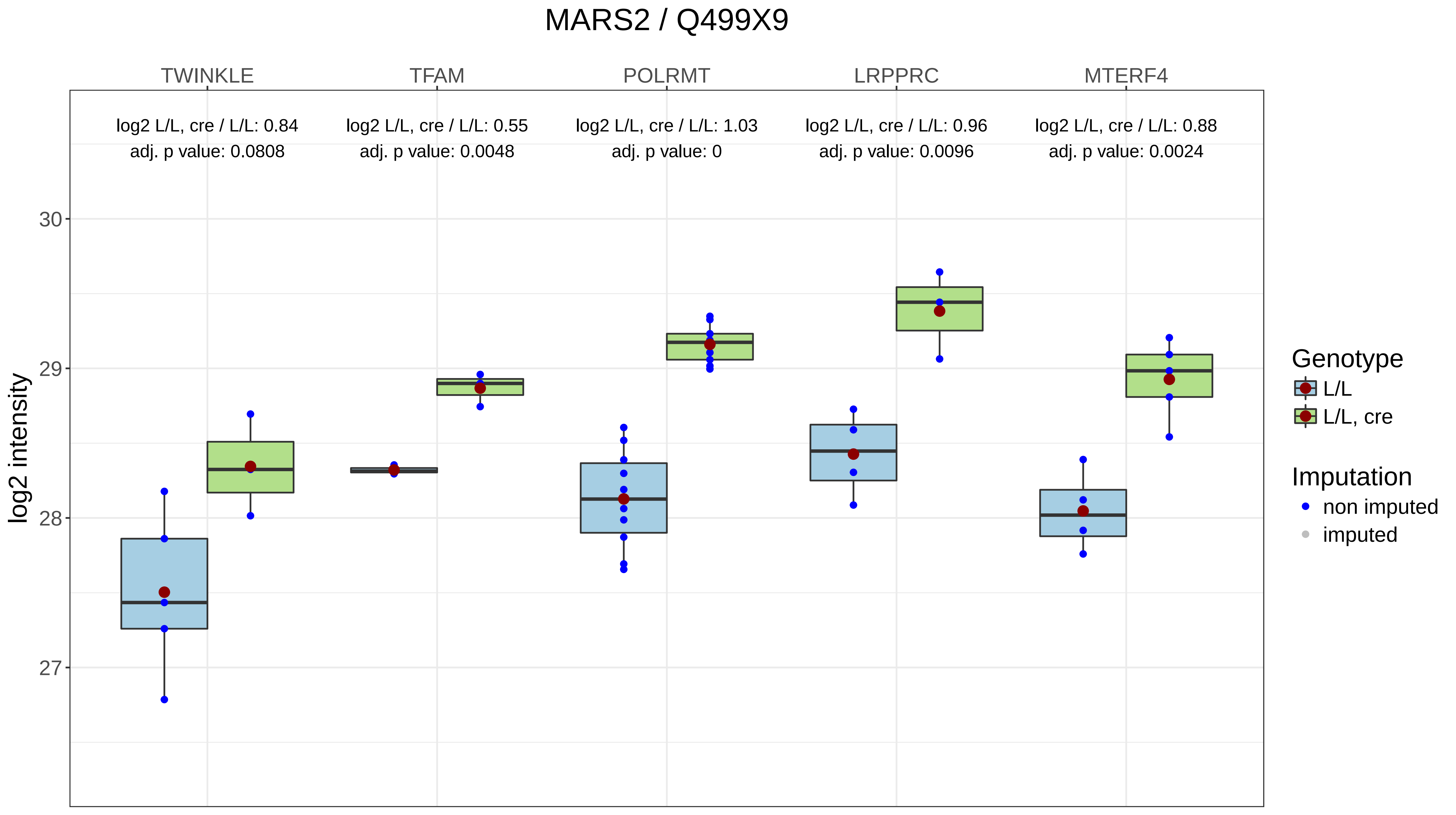

MARS2 / Q499X9
TWINKLE
TFAM
POLRMT
LRPPRC
MTERF4
log2 L/L, cre / L/L: 0.84
adj. p value: 0.0808
log2 L/L, cre / L/L: 0.55
adj. p value: 0.0048
log2 L/L, cre / L/L: 1.03
adj. p value: 0
log2 L/L, cre / L/L: 0.96
adj. p value: 0.0096
log2 L/L, cre / L/L: 0.88
adj. p value: 0.0024
30
29
28
27
log2 intensity
Genotype
L/L
L/L, cre
Imputation
non imputed
imputed

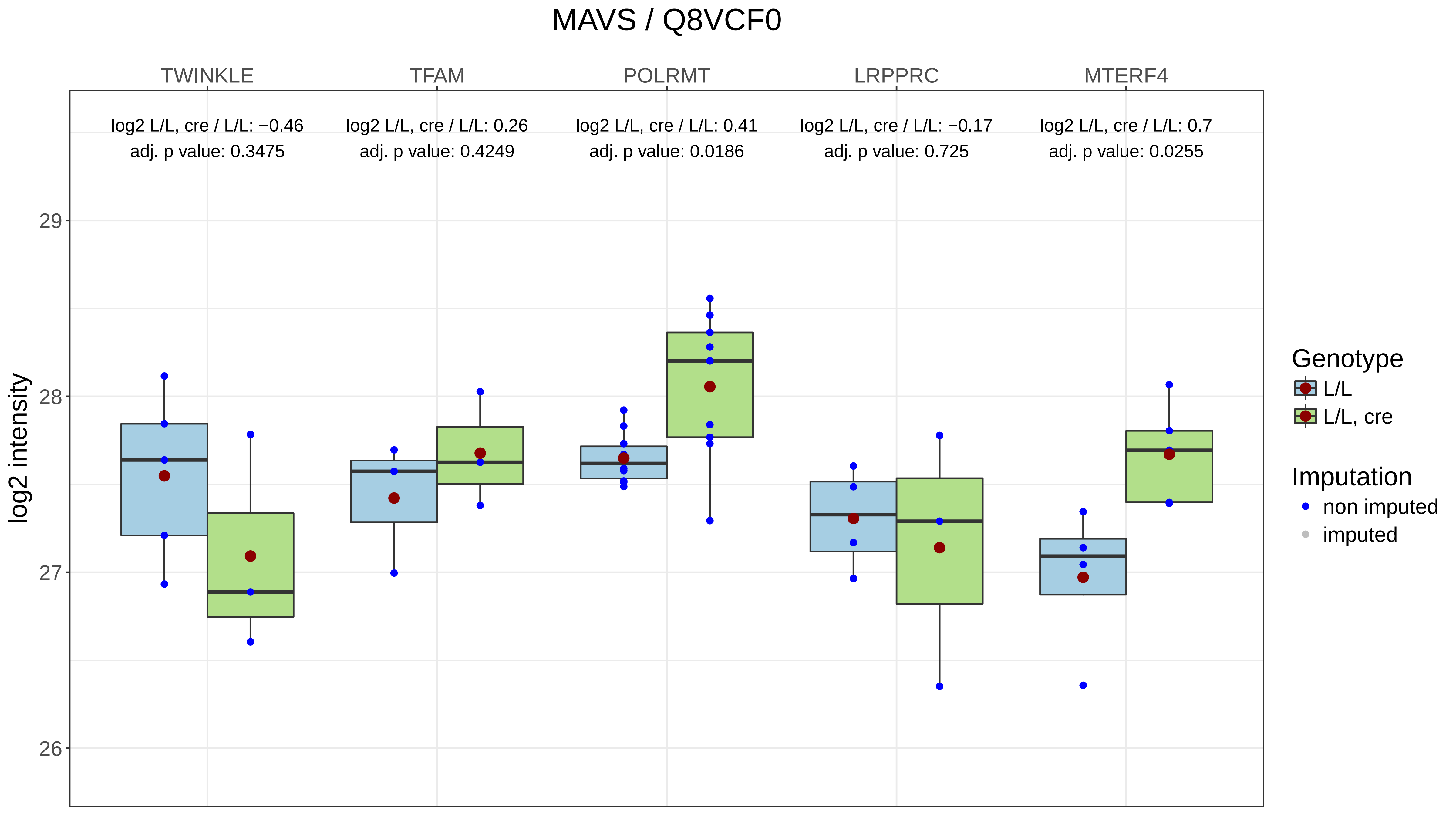
MAVS / Q8VCF0
TWINKLE
TFAM
POLRMT
LRPPRC
MTERF4
log2 L/L, cre / L/L: −0.46
adj. p value: 0.3475
log2 L/L, cre / L/L: 0.26
adj. p value: 0.4249
log2 L/L, cre / L/L: 0.41
adj. p value: 0.0186
log2 L/L, cre / L/L: −0.17
adj. p value: 0.725
log2 L/L, cre / L/L: 0.7
adj. p value: 0.0255
log2 intensity
29
28
27
26
Genotype
L/L
L/L, cre
Imputation
non imputed
imputed

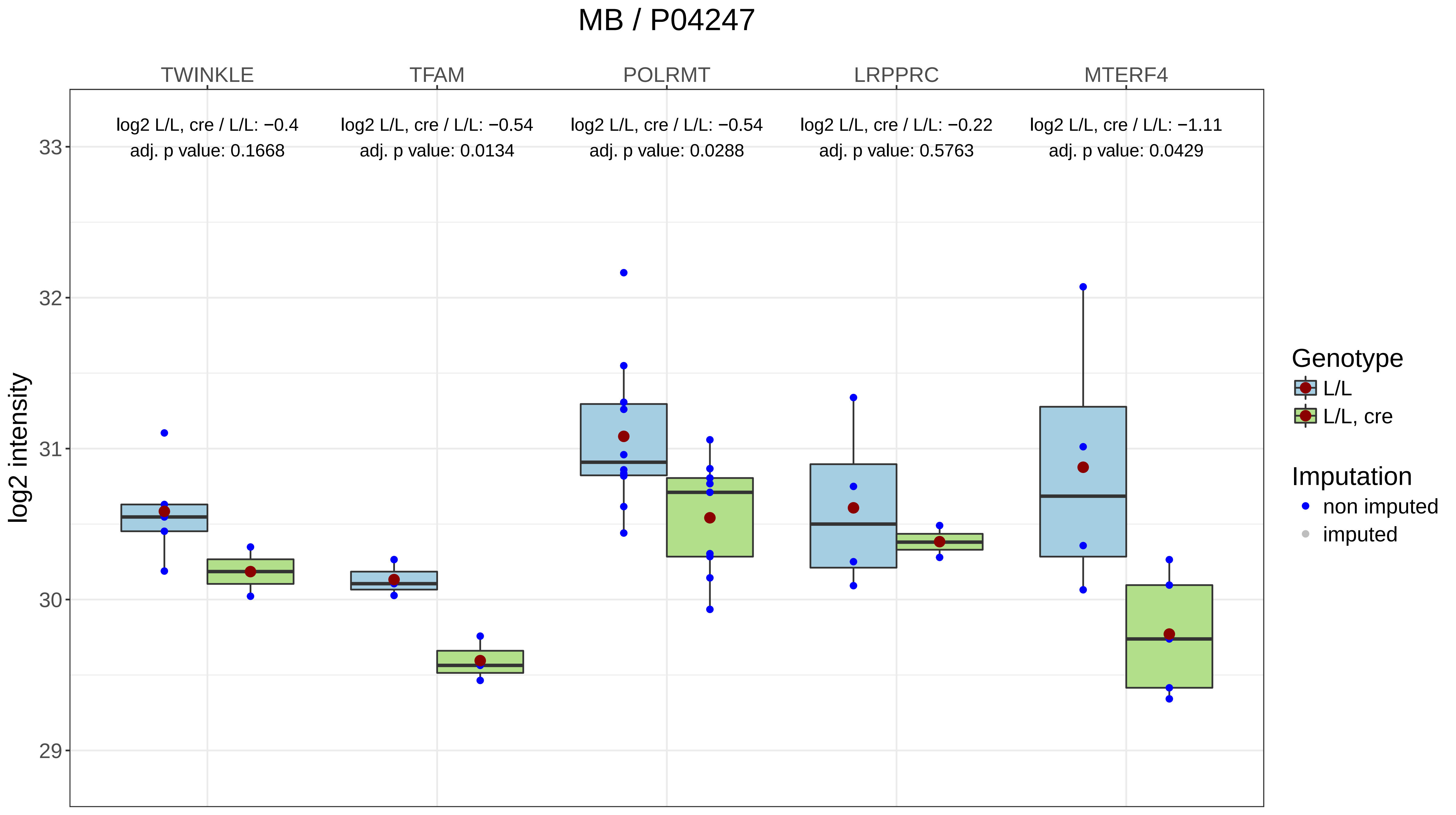
MB / P04247
TWINKLE
TFAM
POLRMT
LRPPRC
MTERF4
log2 L/L, cre / L/L: −0.4
adj. p value: 0.1668
log2 L/L, cre / L/L: −0.54
adj. p value: 0.0134
log2 L/L, cre / L/L: −0.54
adj. p value: 0.0288
log2 L/L, cre / L/L: −0.22
adj. p value: 0.5763
log2 L/L, cre / L/L: −1.11
adj. p value: 0.0429
log2 intensity
33
32
31
30
29
Genotype
L/L
L/L, cre
Imputation
non imputed
imputed

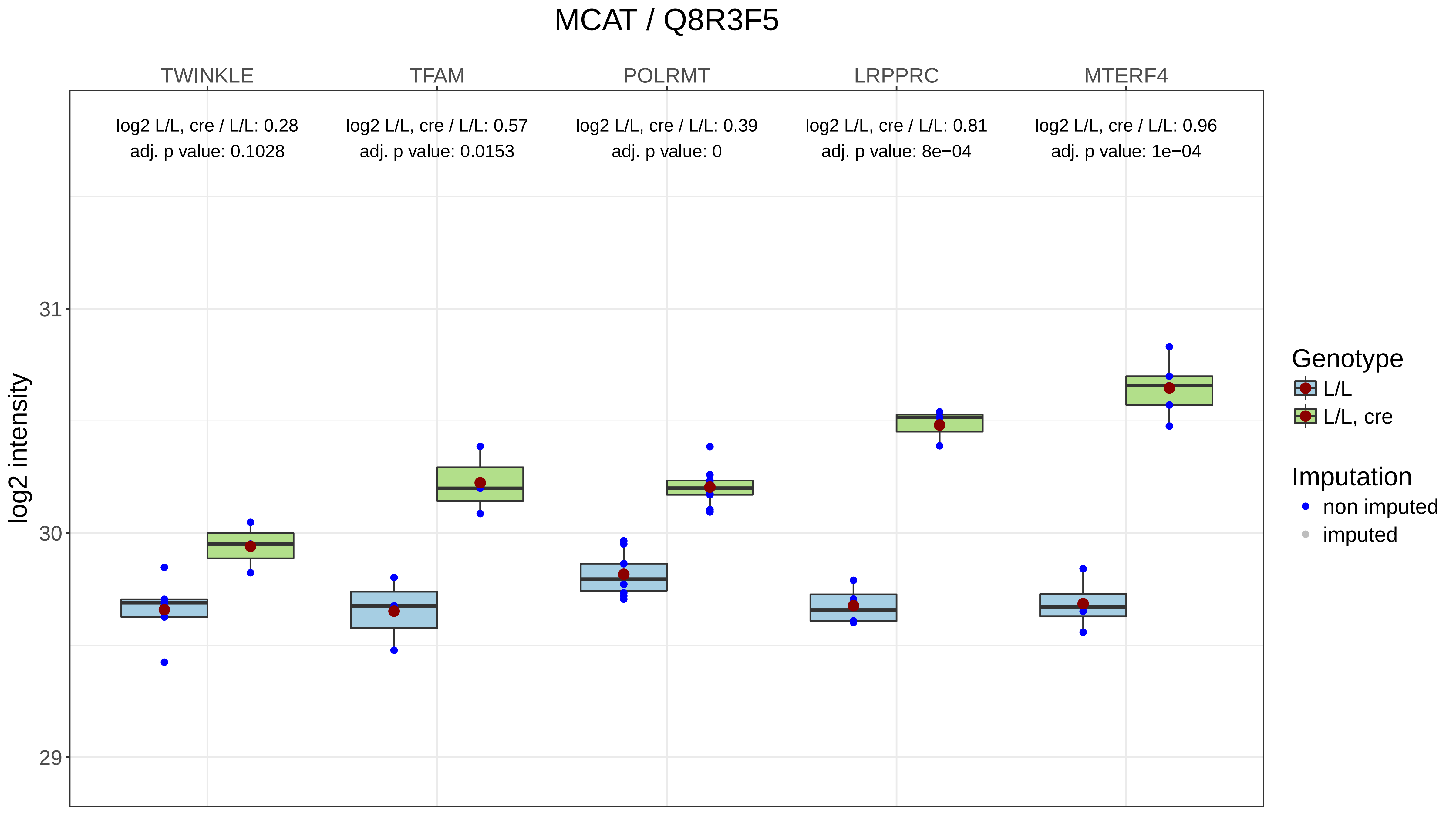

MCAT / Q8R3F5
TWINKLE
TFAM
POLRMT
LRPPRC
MTERF4
log2 L/L, cre / L/L: 0.28
adj. p value: 0.1028
log2 L/L, cre / L/L: 0.57
adj. p value: 0.0153
log2 L/L, cre / L/L: 0.39
adj. p value: 0
log2 L/L, cre / L/L: 0.81
adj. p value: 8e−04
log2 L/L, cre / L/L: 0.96
adj. p value: 1e−04
log2 intensity
31
30
29
Genotype
L/L
L/L, cre
Imputation
non imputed
imputed

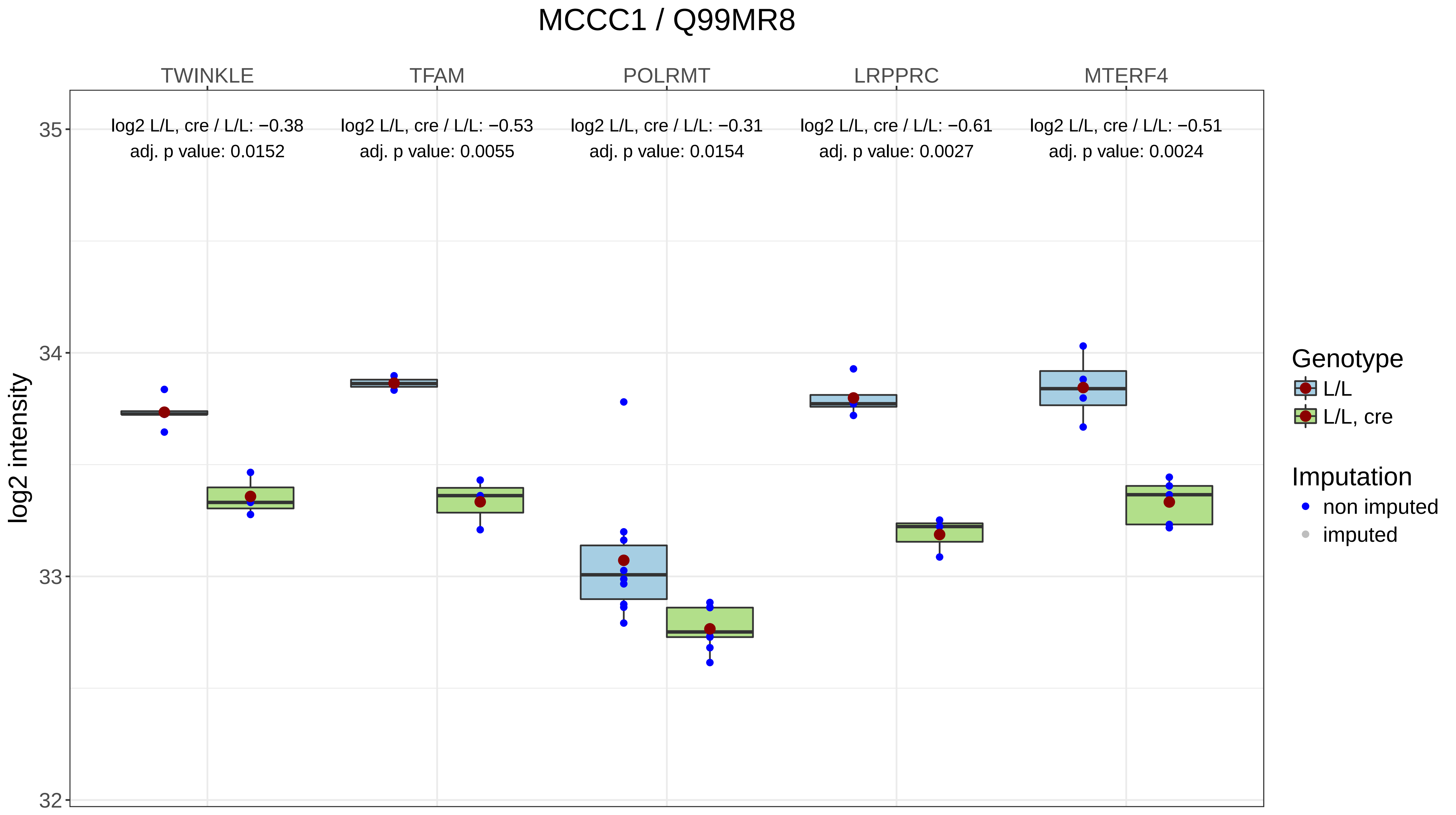
MCCC1 / Q99MR8
TWINKLE
TFAM
POLRMT
LRPPRC
MTERF4
log2 L/L, cre / L/L: −0.38
adj. p value: 0.0152
log2 L/L, cre / L/L: −0.53
adj. p value: 0.0055
log2 L/L, cre / L/L: −0.31
adj. p value: 0.0154
log2 L/L, cre / L/L: −0.61
adj. p value: 0.0027
log2 L/L, cre / L/L: −0.51
adj. p value: 0.0024
log2 intensity
35
34
33
32
Genotype
L/L
L/L, cre
Imputation
non imputed
imputed

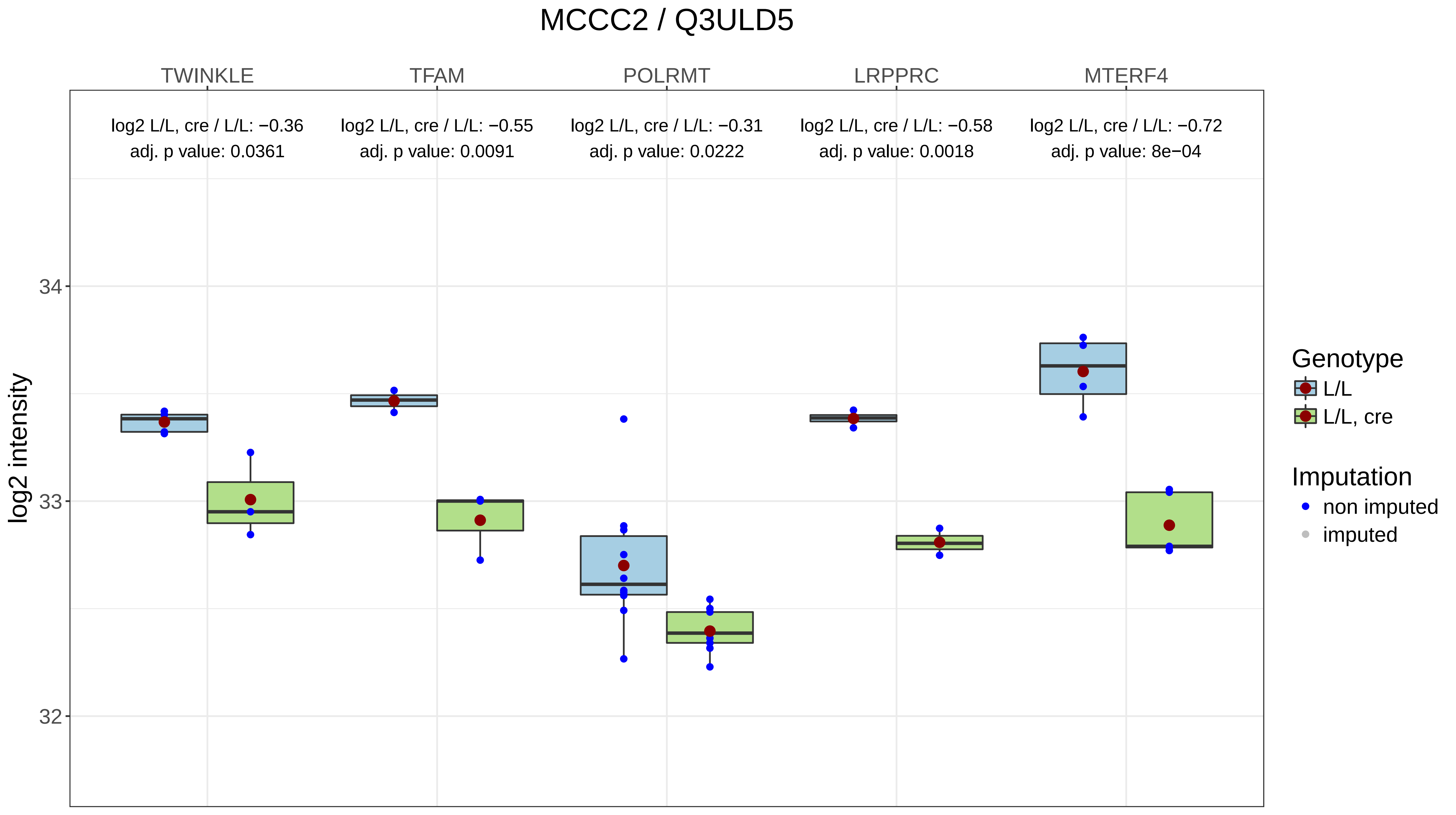
MCCC2 / Q3ULD5
TWINKLE
TFAM
POLRMT
LRPPRC
MTERF4
log2 L/L, cre / L/L: −0.36
adj. p value: 0.0361
log2 L/L, cre / L/L: −0.55
adj. p value: 0.0091
log2 L/L, cre / L/L: −0.31
adj. p value: 0.0222
log2 L/L, cre / L/L: −0.58
adj. p value: 0.0018
log2 L/L, cre / L/L: −0.72
adj. p value: 8e−04
log2 intensity
34
33
32
Genotype
L/L
L/L, cre
Imputation
non imputed
imputed

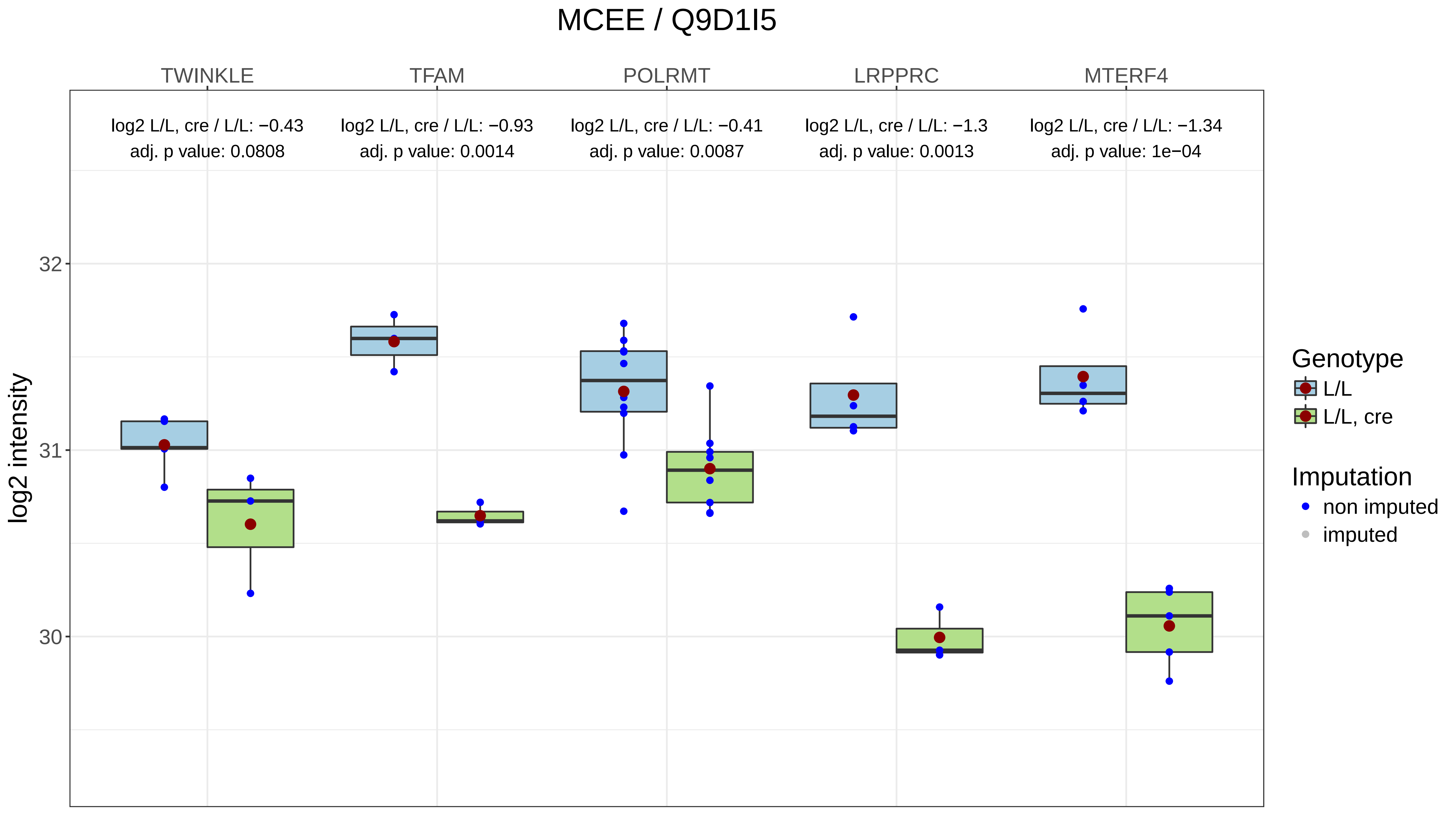
MCEE / Q9D1I5
TWINKLE
TFAM
POLRMT
LRPPRC
MTERF4
log2 L/L, cre / L/L: −0.43
adj. p value: 0.0808
log2 L/L, cre / L/L: −0.93
adj. p value: 0.0014
log2 L/L, cre / L/L: −0.41
adj. p value: 0.0087
log2 L/L, cre / L/L: −1.3
adj. p value: 0.0013
log2 L/L, cre / L/L: −1.34
adj. p value: 1e−04
log2 intensity
32
31
30
Genotype
L/L
L/L, cre
Imputation
non imputed
imputed

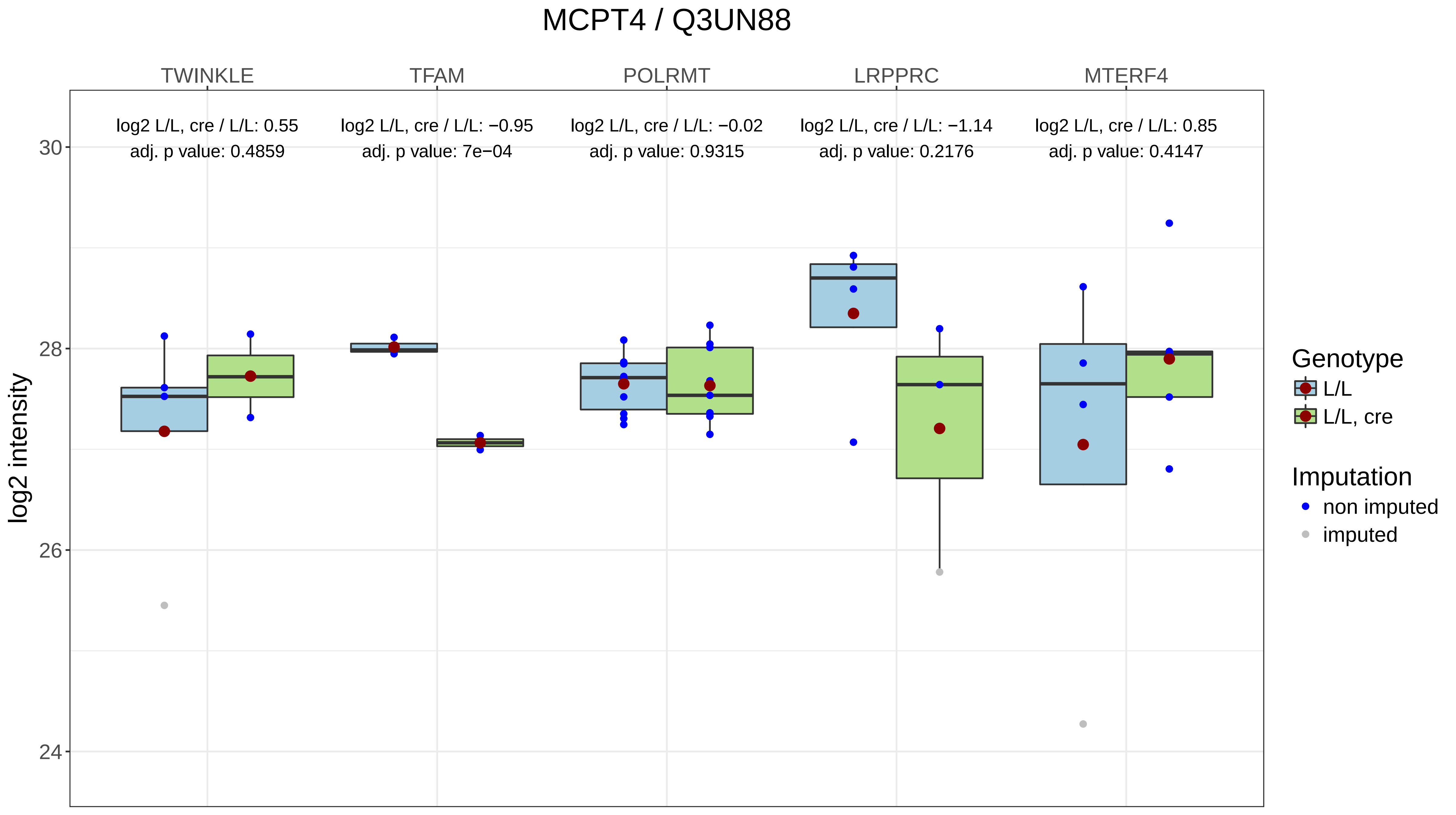
MCPT4 / Q3UN88
TWINKLE
TFAM
POLRMT
LRPPRC
MTERF4
log2 L/L, cre / L/L: 0.55
adj. p value: 0.4859
log2 L/L, cre / L/L: −0.95
adj. p value: 7e−04
log2 L/L, cre / L/L: −0.02
adj. p value: 0.9315
log2 L/L, cre / L/L: −1.14
adj. p value: 0.2176
log2 L/L, cre / L/L: 0.85
adj. p value: 0.4147
log2 intensity
30
28
26
24
Genotype
L/L
L/L, cre
Imputation
non imputed
imputed

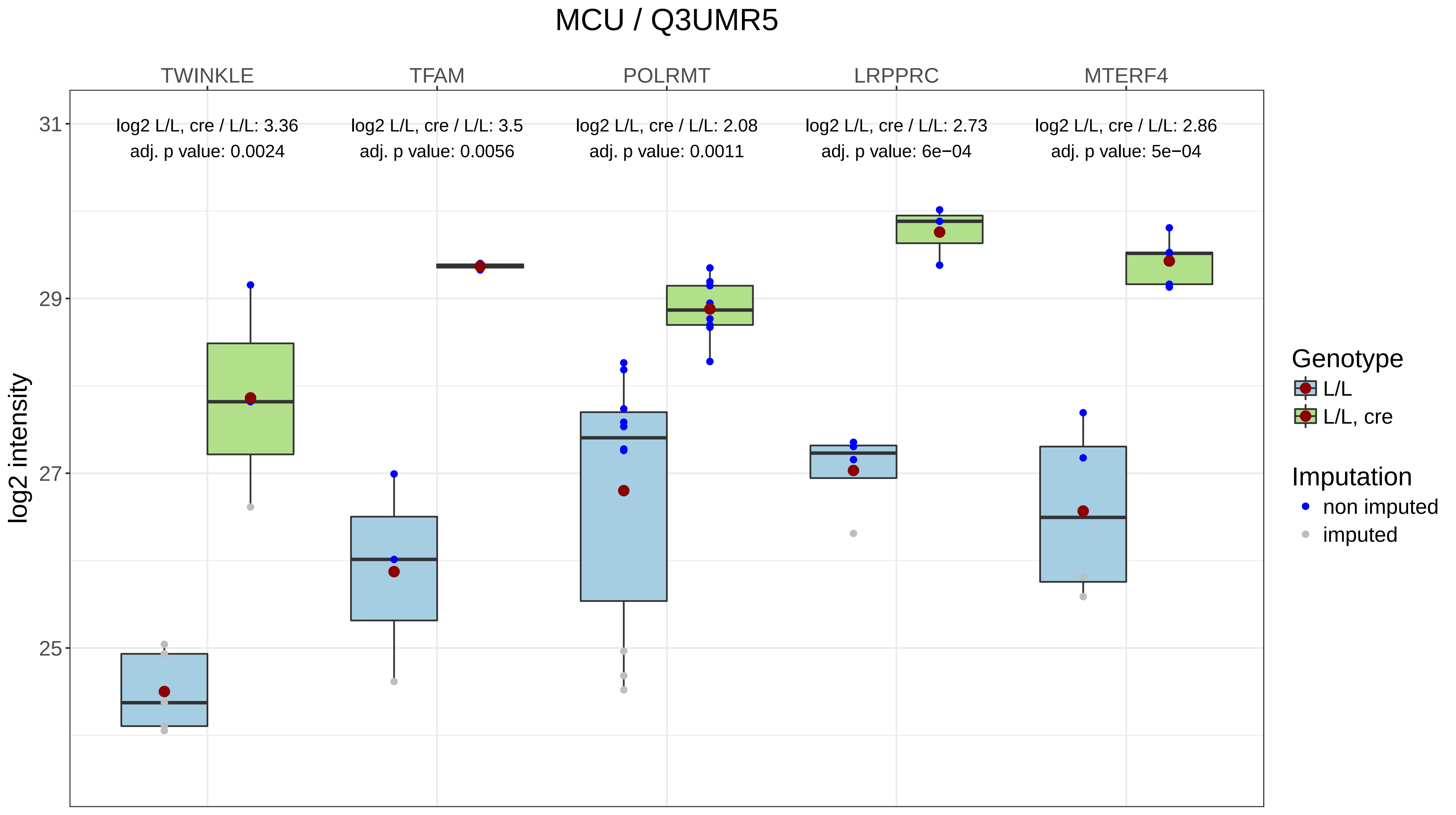
MCU / Q3UMR5
TWINKLE
TFAM
POLRMT
LRPPRC
MTERF4
log2 L/L, cre / L/L: 3.36
adj. p value: 0.0024
log2 L/L, cre / L/L: 3.5
adj. p value: 0.0056
log2 L/L, cre / L/L: 2.08
adj. p value: 0.0011
log2 L/L, cre / L/L: 2.73
adj. p value: 6e−04
log2 L/L, cre / L/L: 2.86
adj. p value: 5e−04
31
29
27
25
log2 intensity
Genotype
L/L
L/L, cre
Imputation
non imputed
imputed

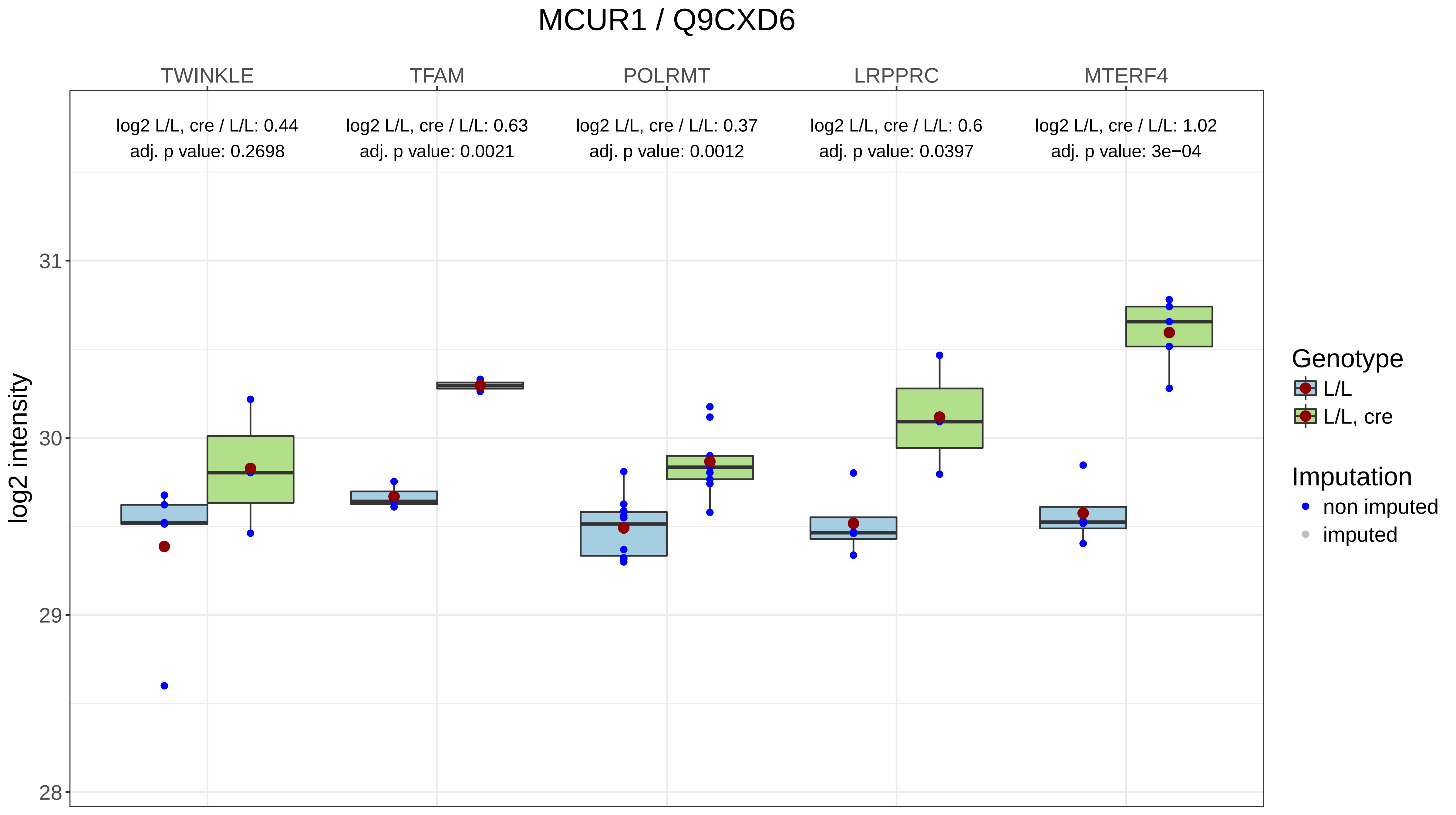
MCUR1 / Q9CXD6
TWINKLE
TFAM
POLRMT
LRPPRC
MTERF4
log2 L/L, cre / L/L: 0.44
adj. p value: 0.2698
log2 L/L, cre / L/L: 0.63
adj. p value: 0.0021
log2 L/L, cre / L/L: 0.37
adj. p value: 0.0012
log2 L/L, cre / L/L: 0.6
adj. p value: 0.0397
log2 L/L, cre / L/L: 1.02
adj. p value: 3e−04
log2 intensity
31
30
29
28
Genotype
L/L
L/L, cre
Imputation
non imputed
imputed

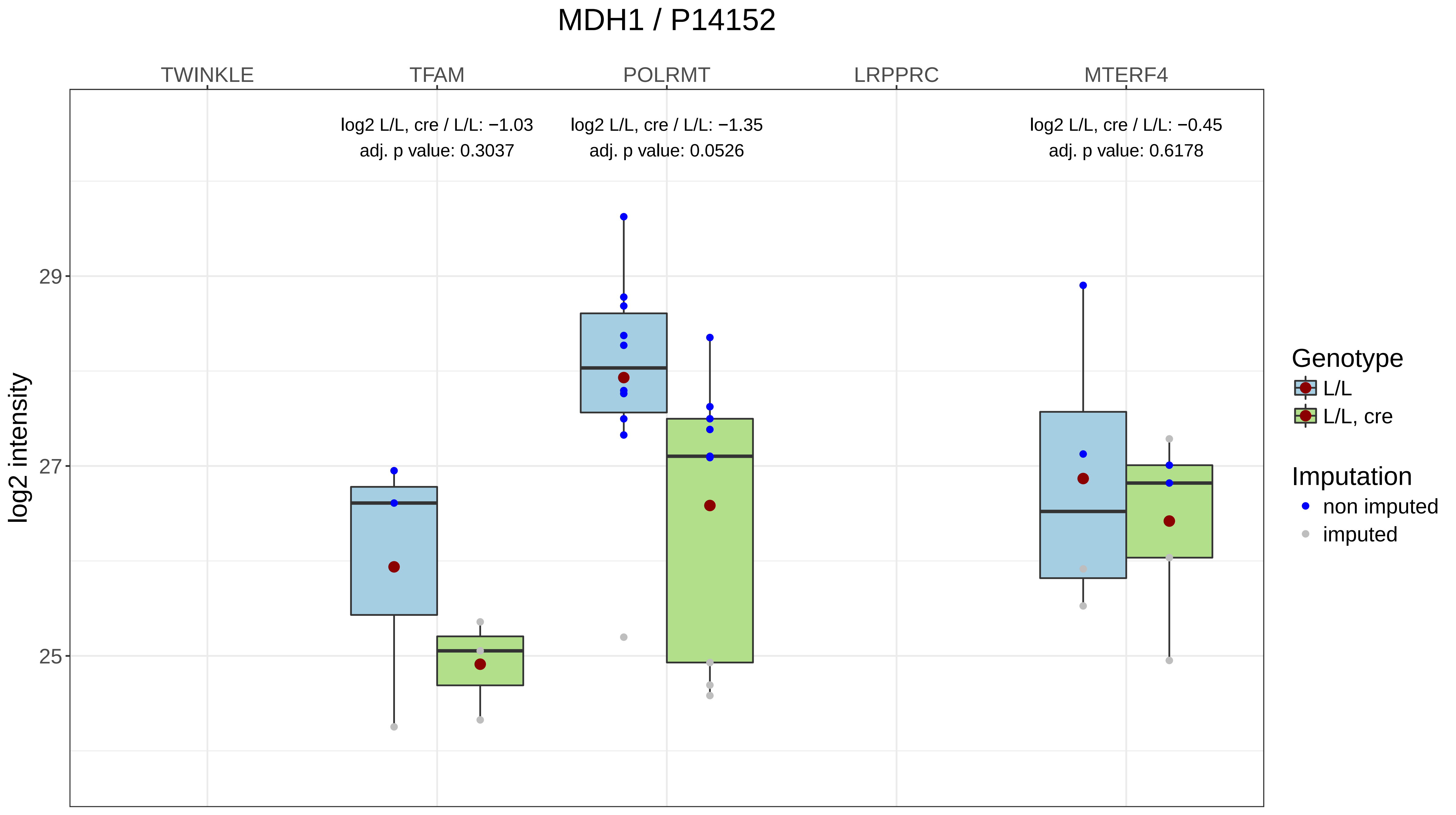
MDH1 / P14152
TWINKLE
TFAM
POLRMT
LRPPRC
MTERF4
log2 L/L, cre / L/L: −1.03
adj. p value: 0.3037
log2 L/L, cre / L/L: −1.35
adj. p value: 0.0526
log2 L/L, cre / L/L: −0.45
adj. p value: 0.6178
log2 intensity
29
27
25
Genotype
L/L
L/L, cre
Imputation
non imputed
imputed

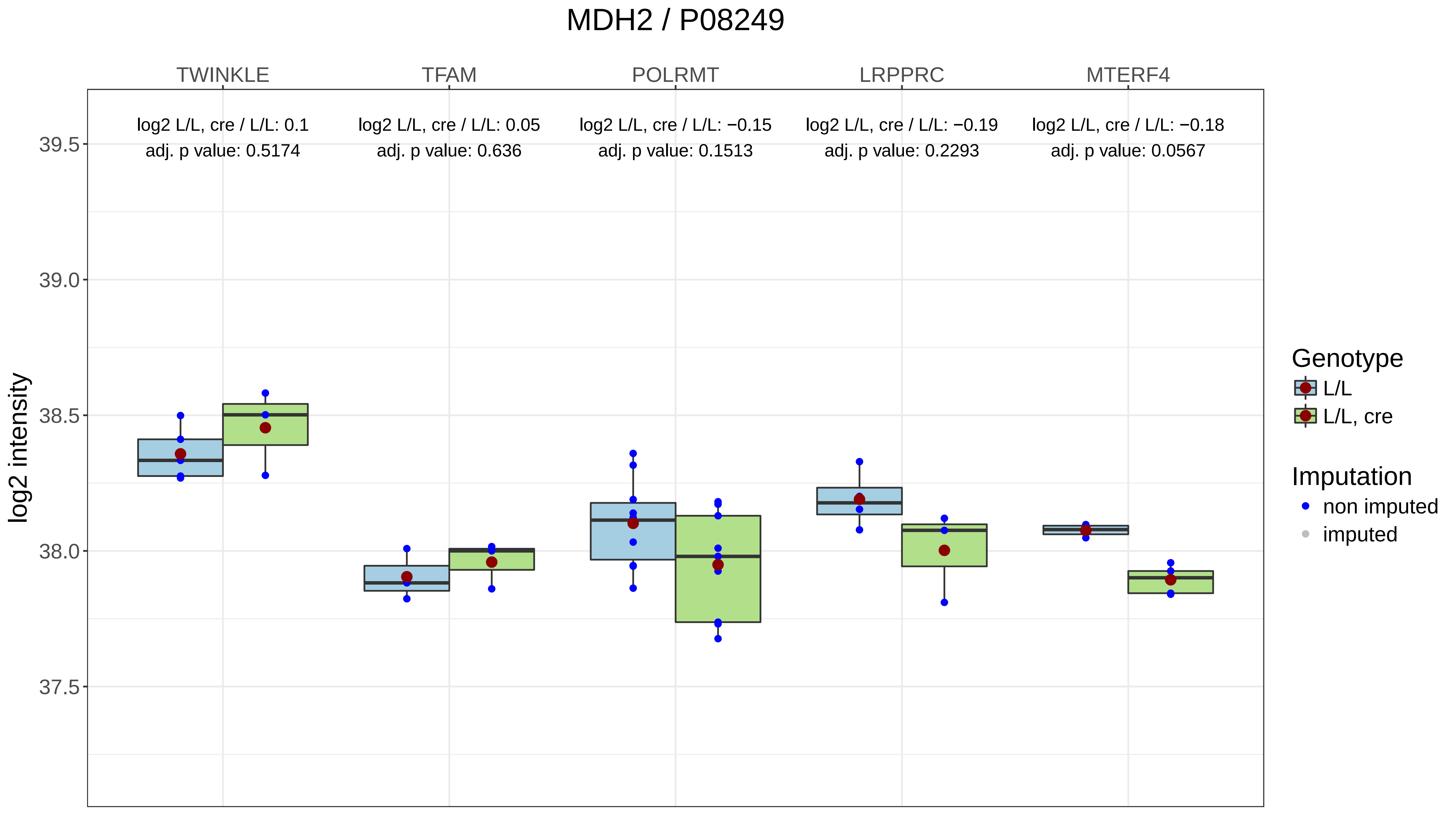
MDH2 / P08249
TWINKLE
TFAM
POLRMT
LRPPRC
MTERF4
log2 L/L, cre / L/L: 0.1
adj. p value: 0.5174
log2 L/L, cre / L/L: 0.05
adj. p value: 0.636
log2 L/L, cre / L/L: −0.15
adj. p value: 0.1513
log2 L/L, cre / L/L: −0.19
adj. p value: 0.2293
log2 L/L, cre / L/L: −0.18
adj. p value: 0.0567
log2 intensity
39.5
39.0
38.5
38.0
37.5
Genotype
L/L
L/L, cre
Imputation
non imputed
imputed

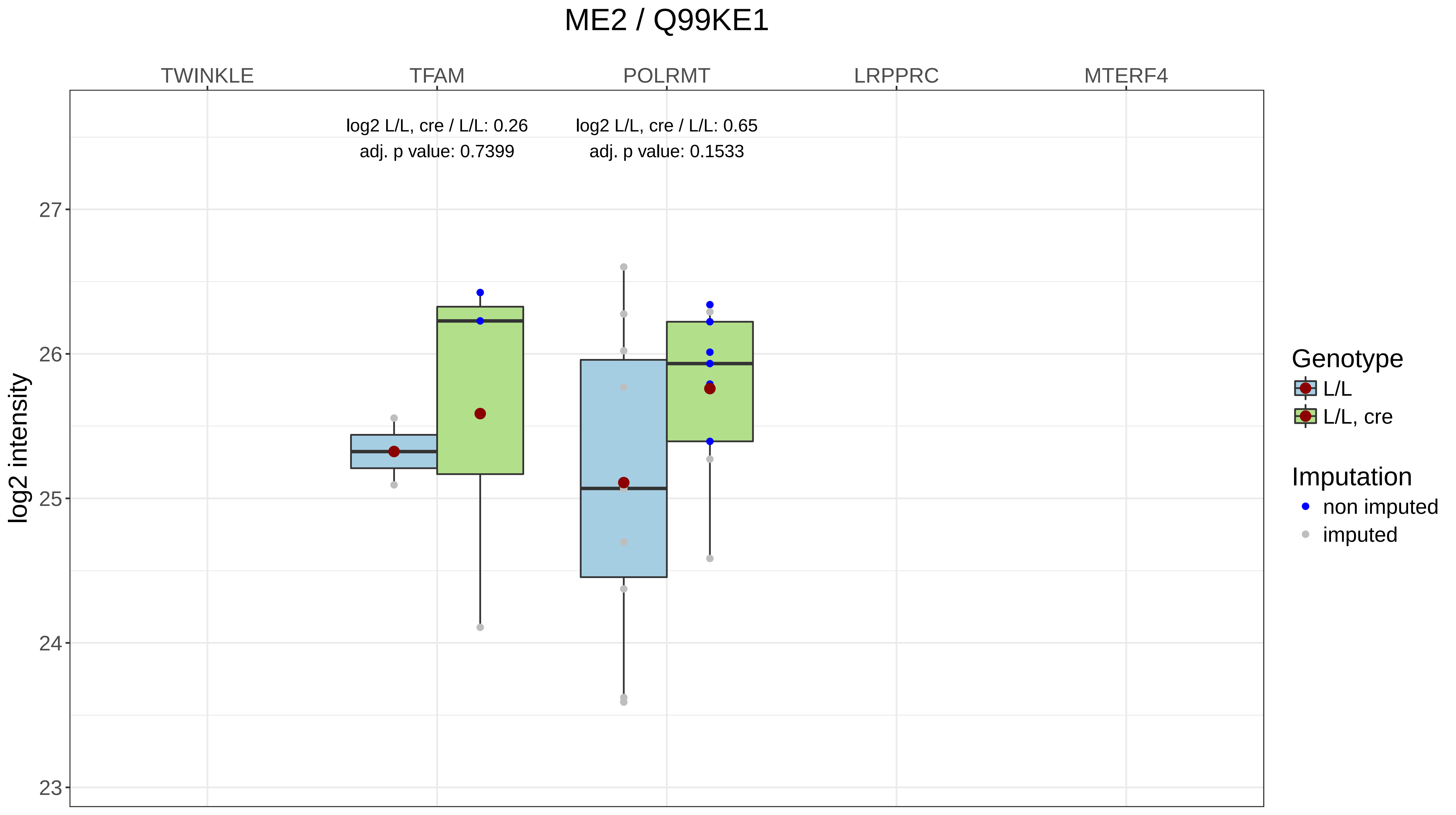

ME2 / Q99KE1
TWINKLE
TFAM
POLRMT
LRPPRC
MTERF4
log2 L/L, cre / L/L: 0.26
adj. p value: 0.7399
log2 L/L, cre / L/L: 0.65
adj. p value: 0.1533
log2 intensity
27
26
25
24
23
Genotype
L/L
L/L, cre
Imputation
non imputed
imputed

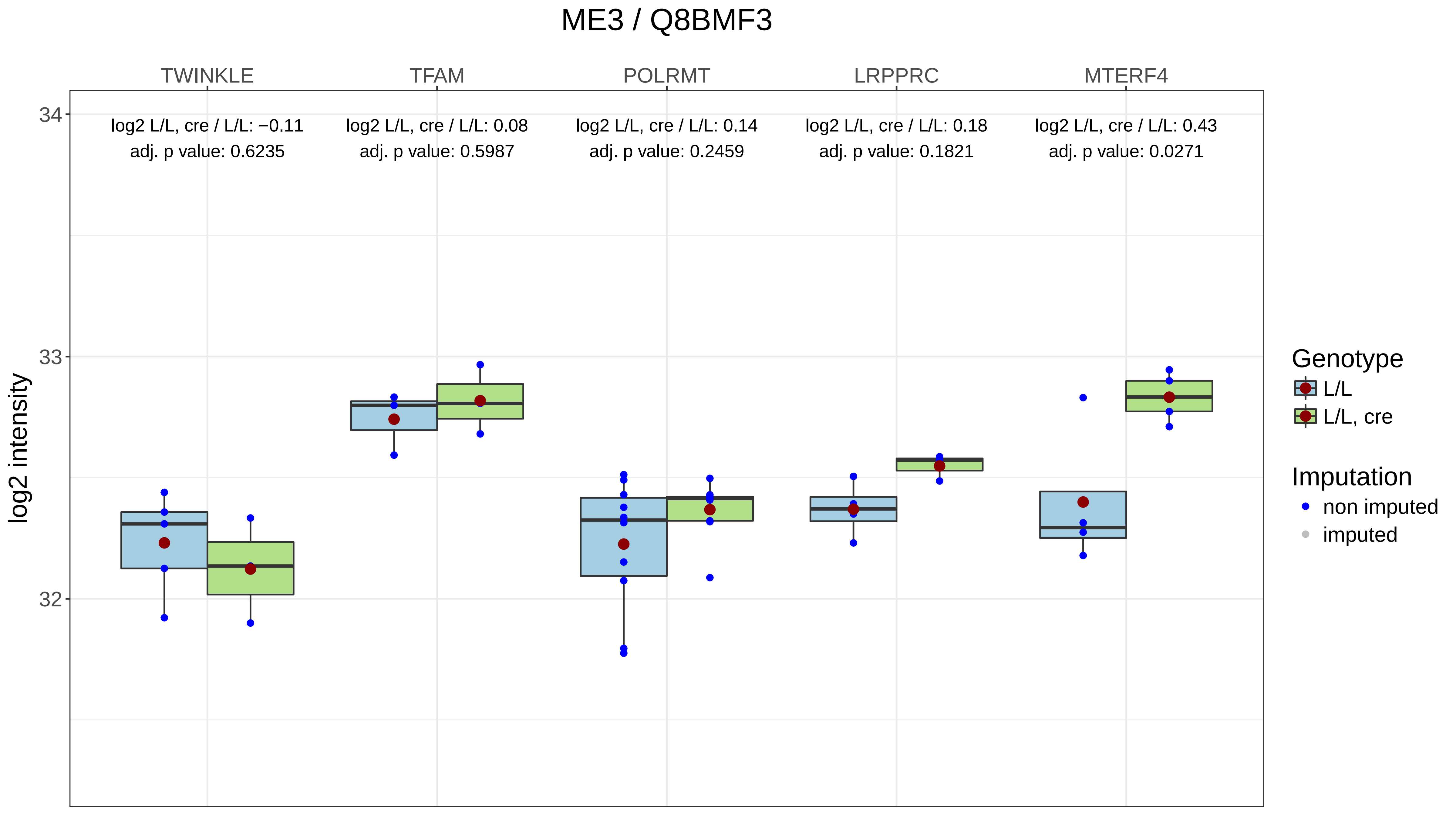

ME3 / Q8BMF3
TWINKLE
TFAM
POLRMT
LRPPRC
MTERF4
log2 L/L, cre / L/L: −0.11
adj. p value: 0.6235
log2 L/L, cre / L/L: 0.08
adj. p value: 0.5987
log2 L/L, cre / L/L: 0.14
adj. p value: 0.2459
log2 L/L, cre / L/L: 0.18
adj. p value: 0.1821
log2 L/L, cre / L/L: 0.43
adj. p value: 0.0271
34
33
32
log2 intensity
Genotype
L/L
L/L, cre
Imputation
non imputed
imputed

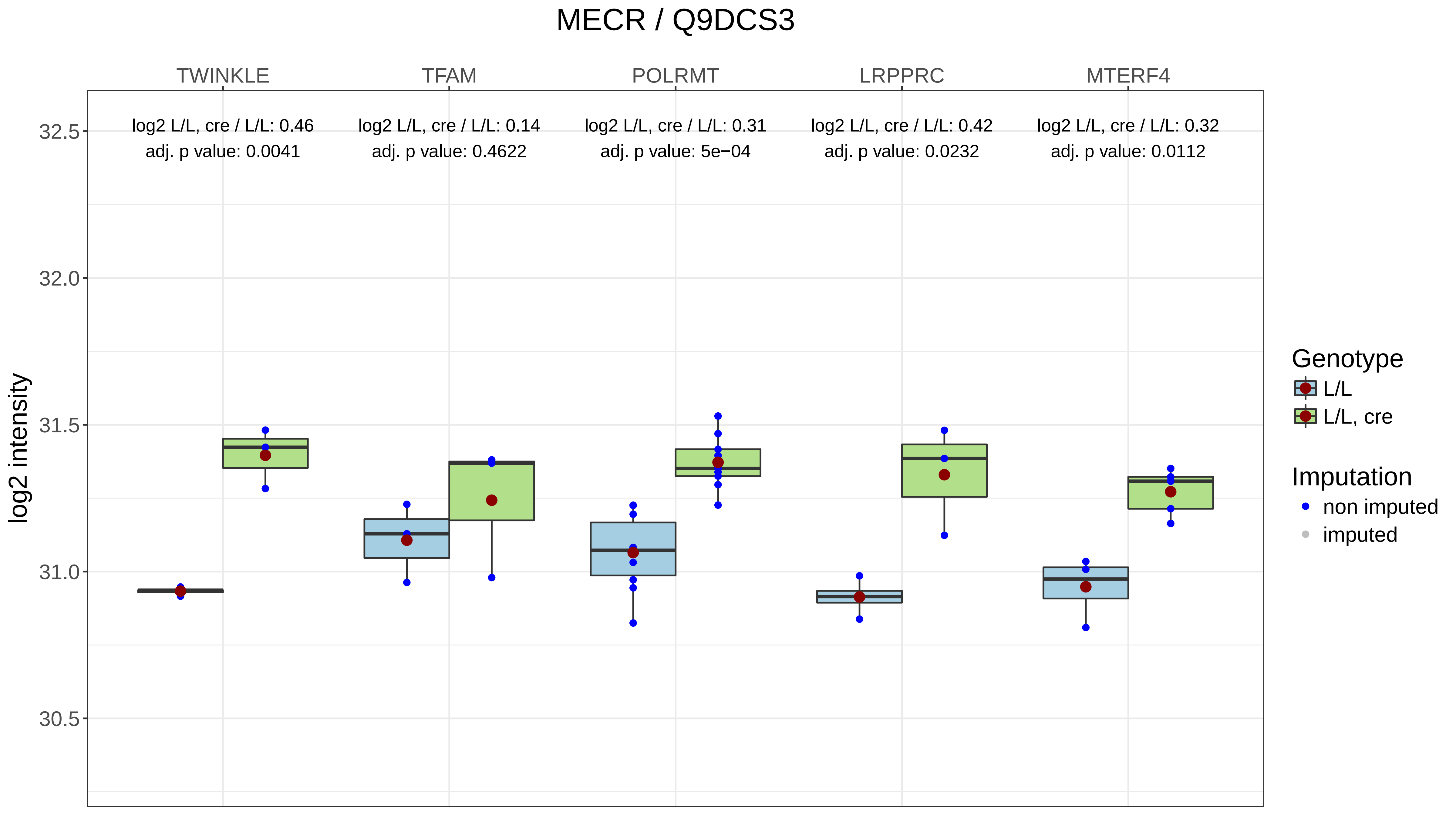
MECR / Q9DCS3
TWINKLE
TFAM
POLRMT
LRPPRC
MTERF4
log2 L/L, cre / L/L: 0.46
adj. p value: 0.0041
log2 L/L, cre / L/L: 0.14
adj. p value: 0.4622
log2 L/L, cre / L/L: 0.31
adj. p value: 5e−04
log2 L/L, cre / L/L: 0.42
adj. p value: 0.0232
log2 L/L, cre / L/L: 0.32
adj. p value: 0.0112
32.5
32.0
31.5
31.0
30.5
log2 intensity
Genotype
L/L
L/L, cre
Imputation
non imputed
imputed

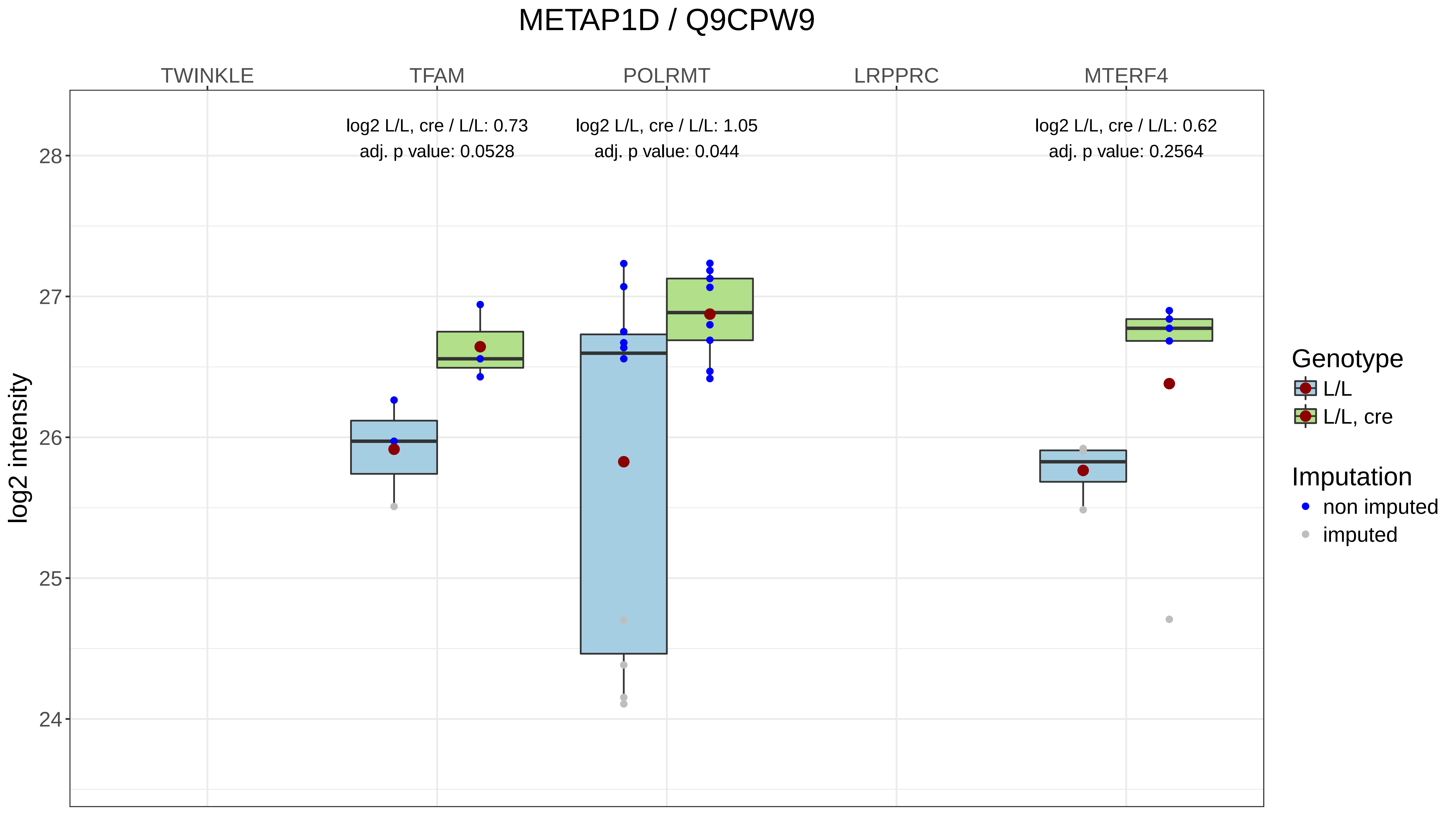

METAP1D / Q9CPW9
TWINKLE
TFAM
POLRMT
LRPPRC
MTERF4
log2 L/L, cre / L/L: 0.73
adj. p value: 0.0528
log2 L/L, cre / L/L: 1.05
adj. p value: 0.044
log2 L/L, cre / L/L: 0.62
adj. p value: 0.2564
log2 intensity
28
27
26
25
24
Genotype
L/L
L/L, cre
Imputation
non imputed
imputed

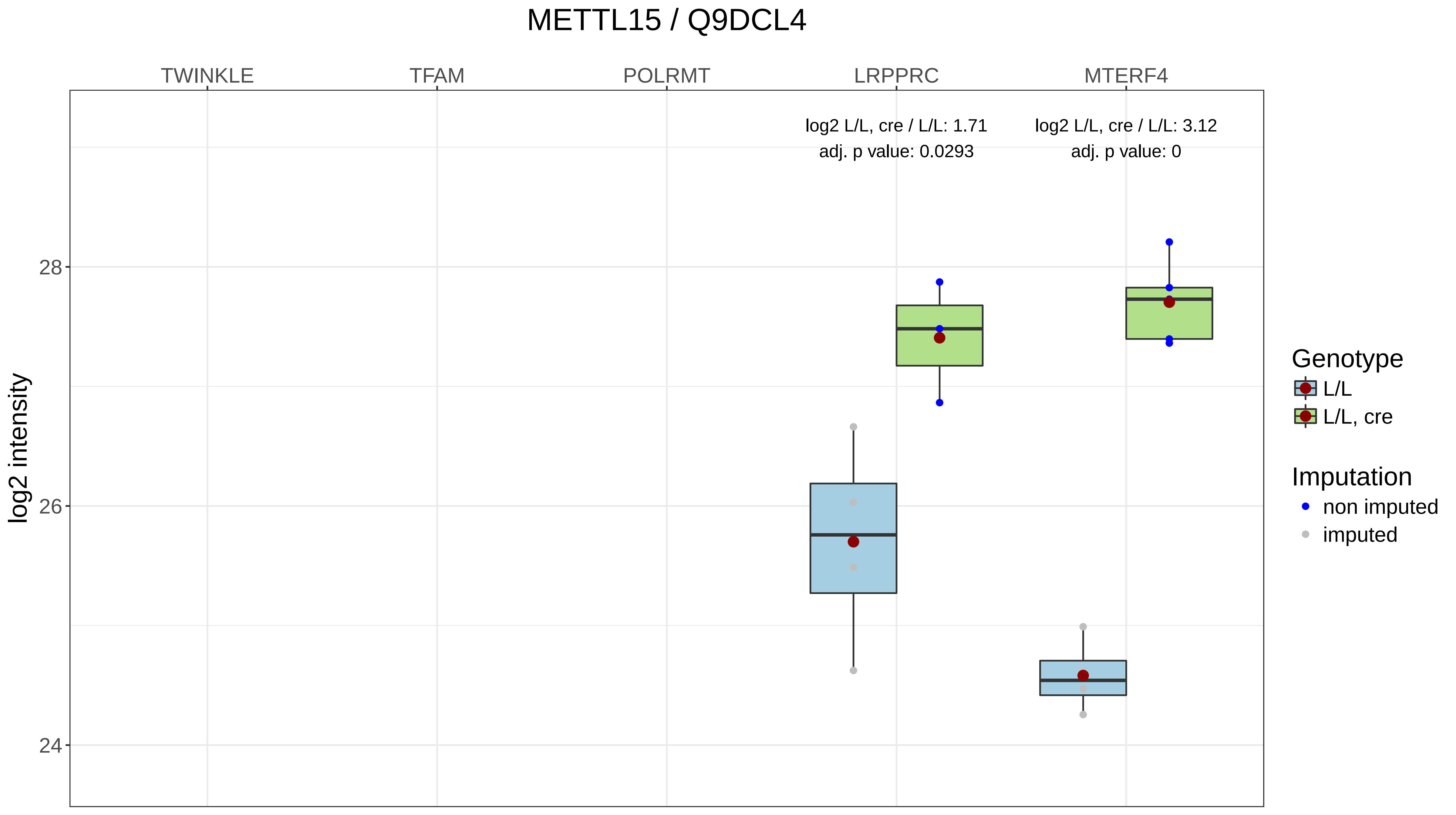
METTL15 / Q9DCL4
TWINKLE
TFAM
POLRMT
LRPPRC
MTERF4
log2 L/L, cre / L/L: 1.71
adj. p value: 0.0293
log2 L/L, cre / L/L: 3.12
adj. p value: 0
log2 intensity
28
26
24
Genotype
L/L
L/L, cre
Imputation
non imputed
imputed

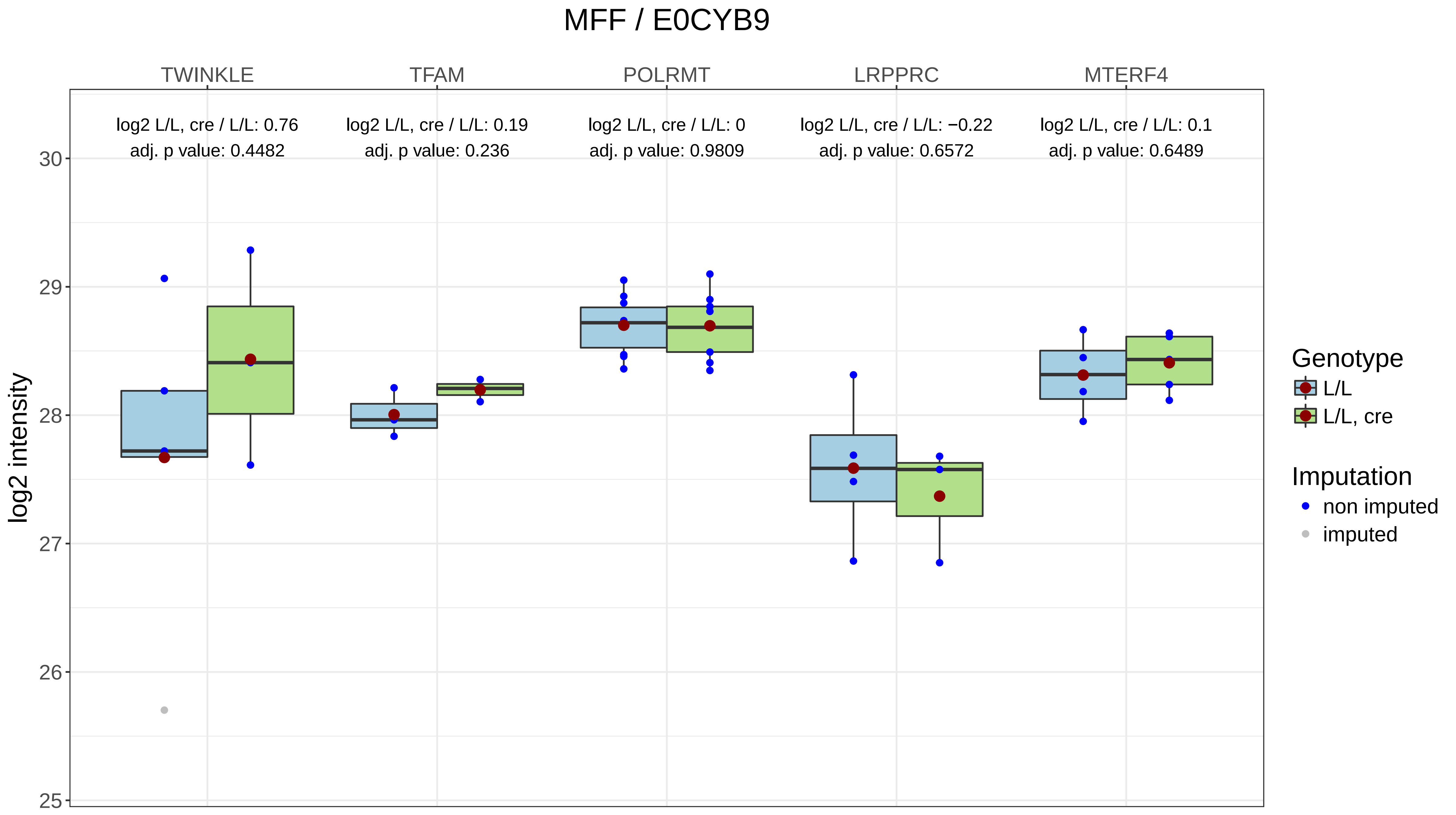
MFF / E0CYB9
TWINKLE
TFAM
POLRMT
LRPPRC
MTERF4
log2 L/L, cre / L/L: 0.76
adj. p value: 0.4482
log2 L/L, cre / L/L: 0.19
adj. p value: 0.236
log2 L/L, cre / L/L: 0
adj. p value: 0.9809
log2 L/L, cre / L/L: −0.22
adj. p value: 0.6572
log2 L/L, cre / L/L: 0.1
adj. p value: 0.6489
log2 intensity
30
29
28
27
26
25
Genotype
L/L
L/L, cre
Imputation
non imputed
imputed

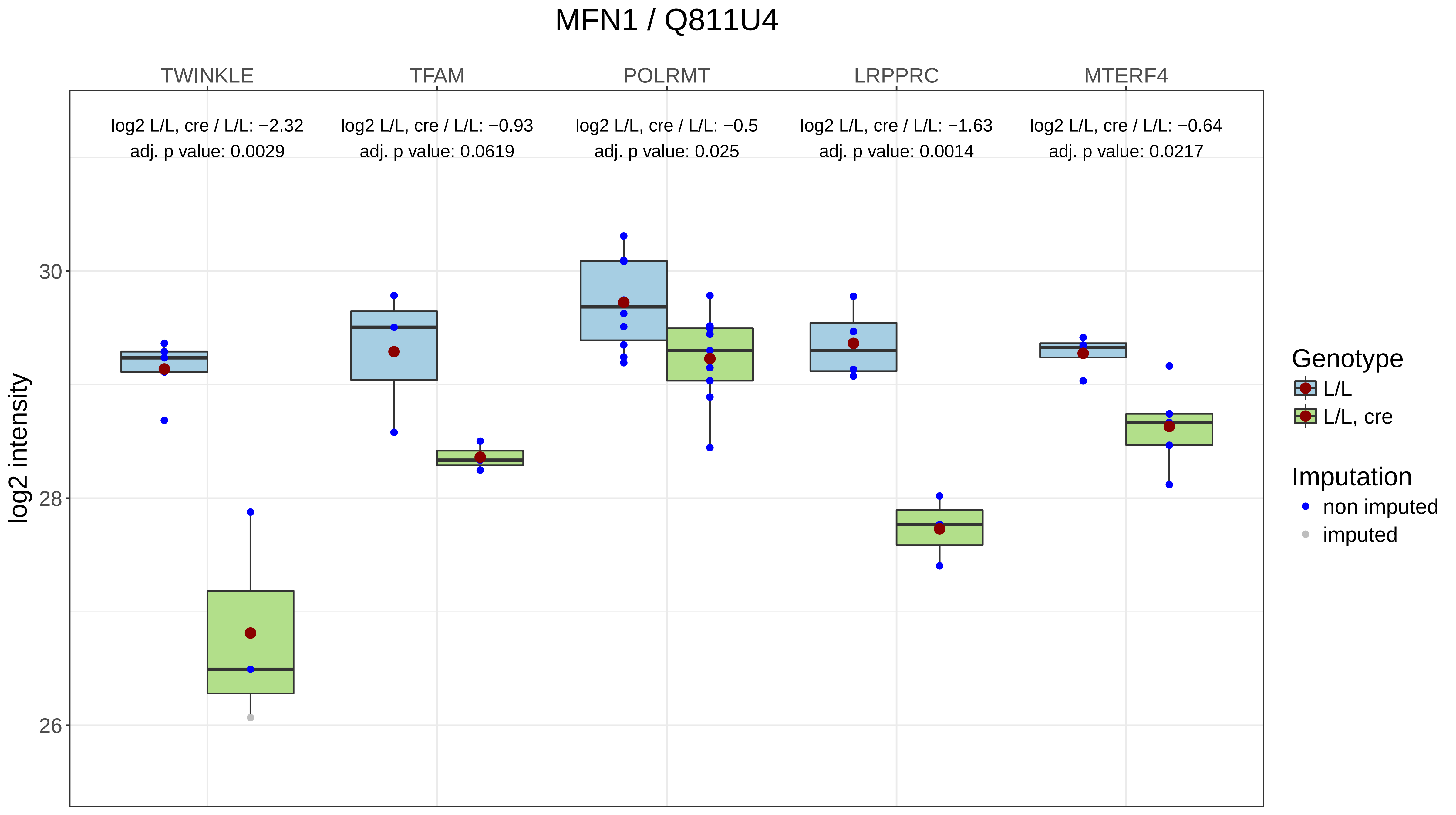

MFN1 / Q811U4
TWINKLE
TFAM
POLRMT
LRPPRC
MTERF4
log2 L/L, cre / L/L: −2.32
adj. p value: 0.0029
log2 L/L, cre / L/L: −0.93
adj. p value: 0.0619
log2 L/L, cre / L/L: −0.5
adj. p value: 0.025
log2 L/L, cre / L/L: −1.63
adj. p value: 0.0014
log2 L/L, cre / L/L: −0.64
adj. p value: 0.0217
30
28
26
log2 intensity
Genotype
L/L
L/L, cre
Imputation
non imputed
imputed

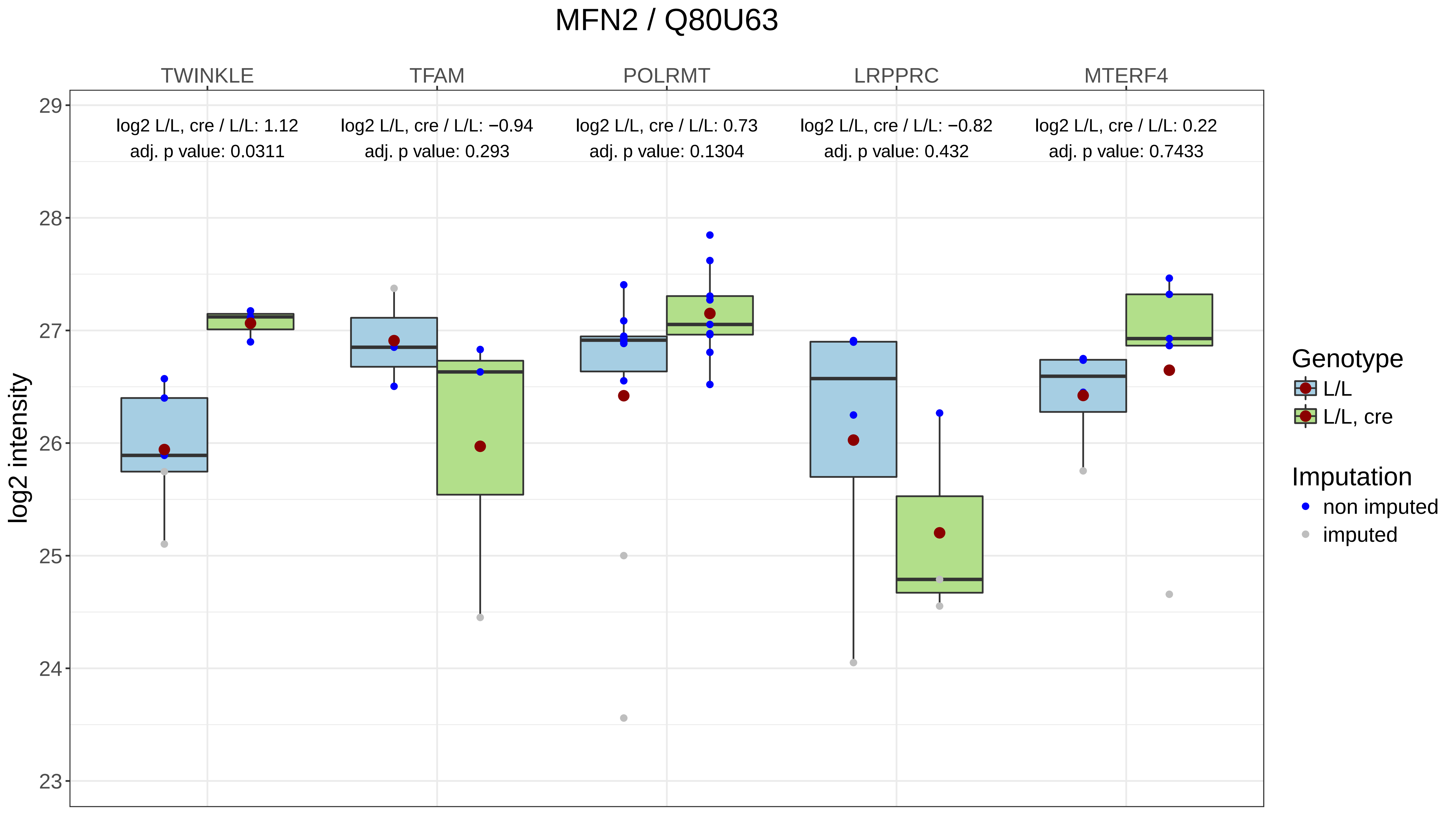

MFN2 / Q80U63
TWINKLE
TFAM
POLRMT
LRPPRC
MTERF4
log2 L/L, cre / L/L: 1.12
adj. p value: 0.0311
log2 L/L, cre / L/L: −0.94
adj. p value: 0.293
log2 L/L, cre / L/L: 0.73
adj. p value: 0.1304
log2 L/L, cre / L/L: −0.82
adj. p value: 0.432
log2 L/L, cre / L/L: 0.22
adj. p value: 0.7433
log2 intensity
29
28
27
26
25
24
23
Genotype
L/L
L/L, cre
Imputation
non imputed
imputed

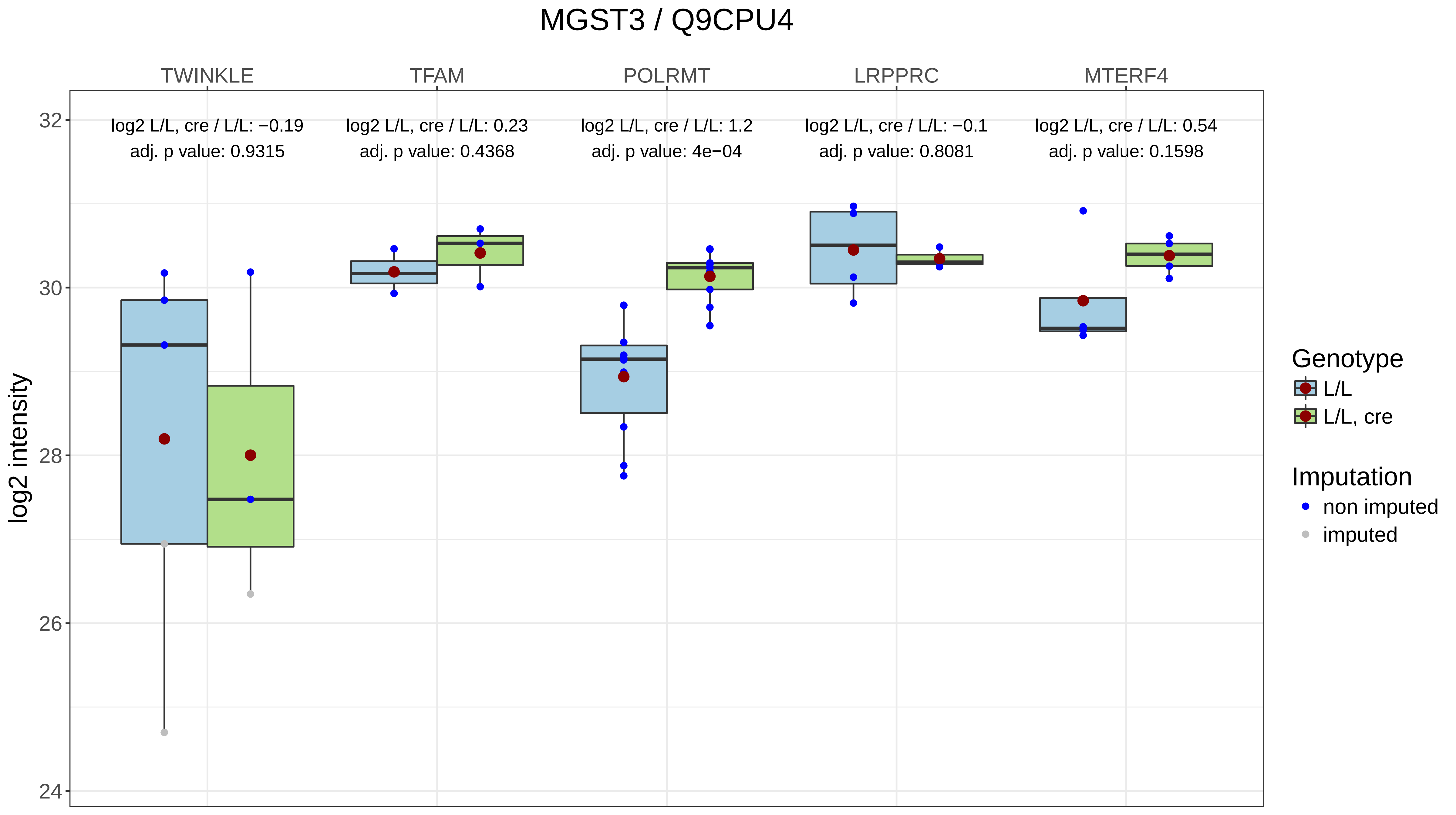
MGST3 / Q9CPU4
TWINKLE
TFAM
POLRMT
LRPPRC
MTERF4
log2 L/L, cre / L/L: −0.19
adj. p value: 0.9315
log2 L/L, cre / L/L: 0.23
adj. p value: 0.4368
log2 L/L, cre / L/L: 1.2
adj. p value: 4e−04
log2 L/L, cre / L/L: −0.1
adj. p value: 0.8081
log2 L/L, cre / L/L: 0.54
adj. p value: 0.1598
log2 intensity
32
30
28
26
24
Genotype
L/L
L/L, cre
Imputation
non imputed
imputed

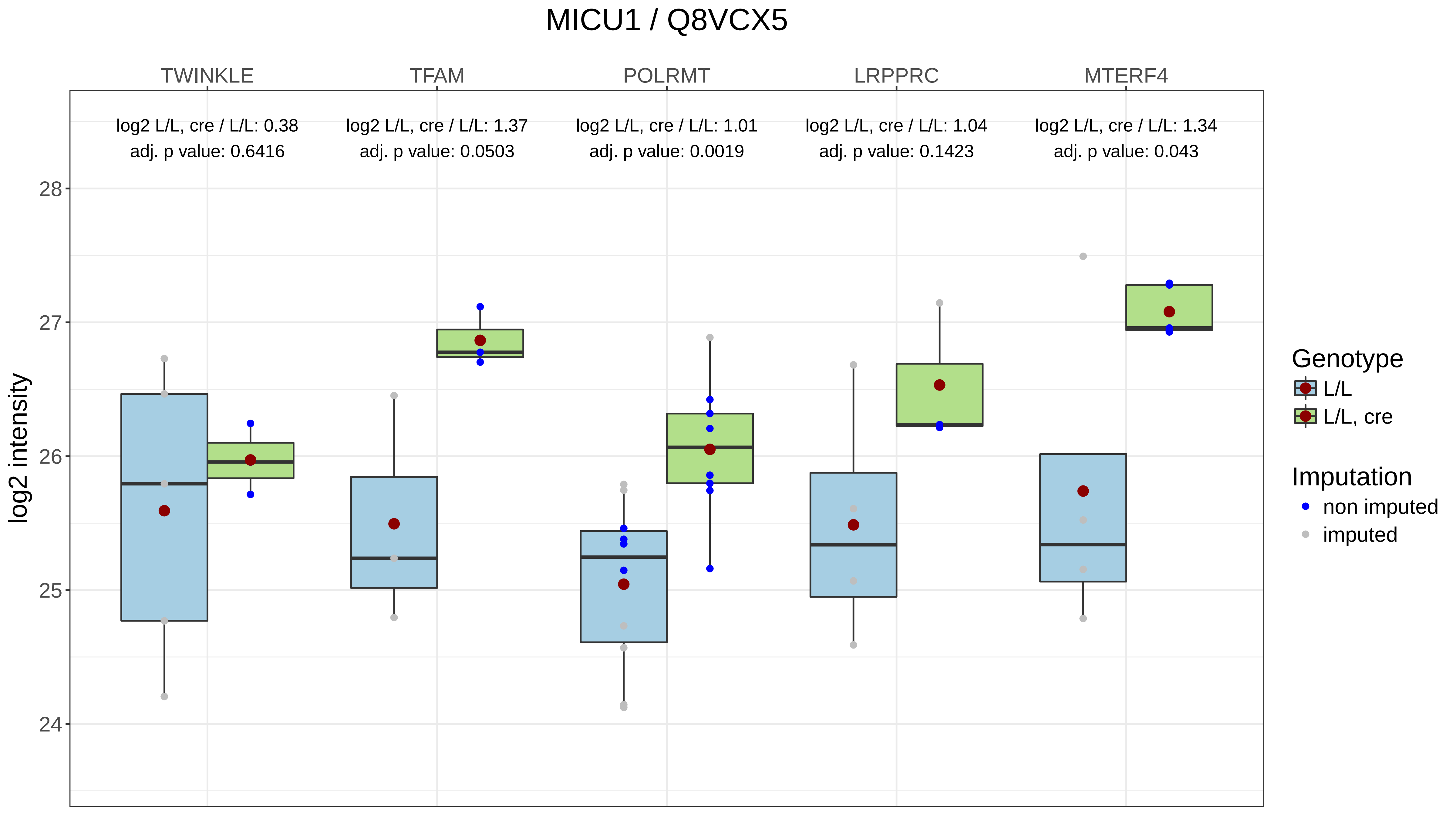

MICU1 / Q8VCX5
TWINKLE
TFAM
POLRMT
LRPPRC
MTERF4
log2 L/L, cre / L/L: 0.38
adj. p value: 0.6416
log2 L/L, cre / L/L: 1.37
adj. p value: 0.0503
log2 L/L, cre / L/L: 1.01
adj. p value: 0.0019
log2 L/L, cre / L/L: 1.04
adj. p value: 0.1423
log2 L/L, cre / L/L: 1.34
adj. p value: 0.043
log2 intensity
28
27
26
25
24
Genotype
L/L
L/L, cre
Imputation
non imputed
imputed

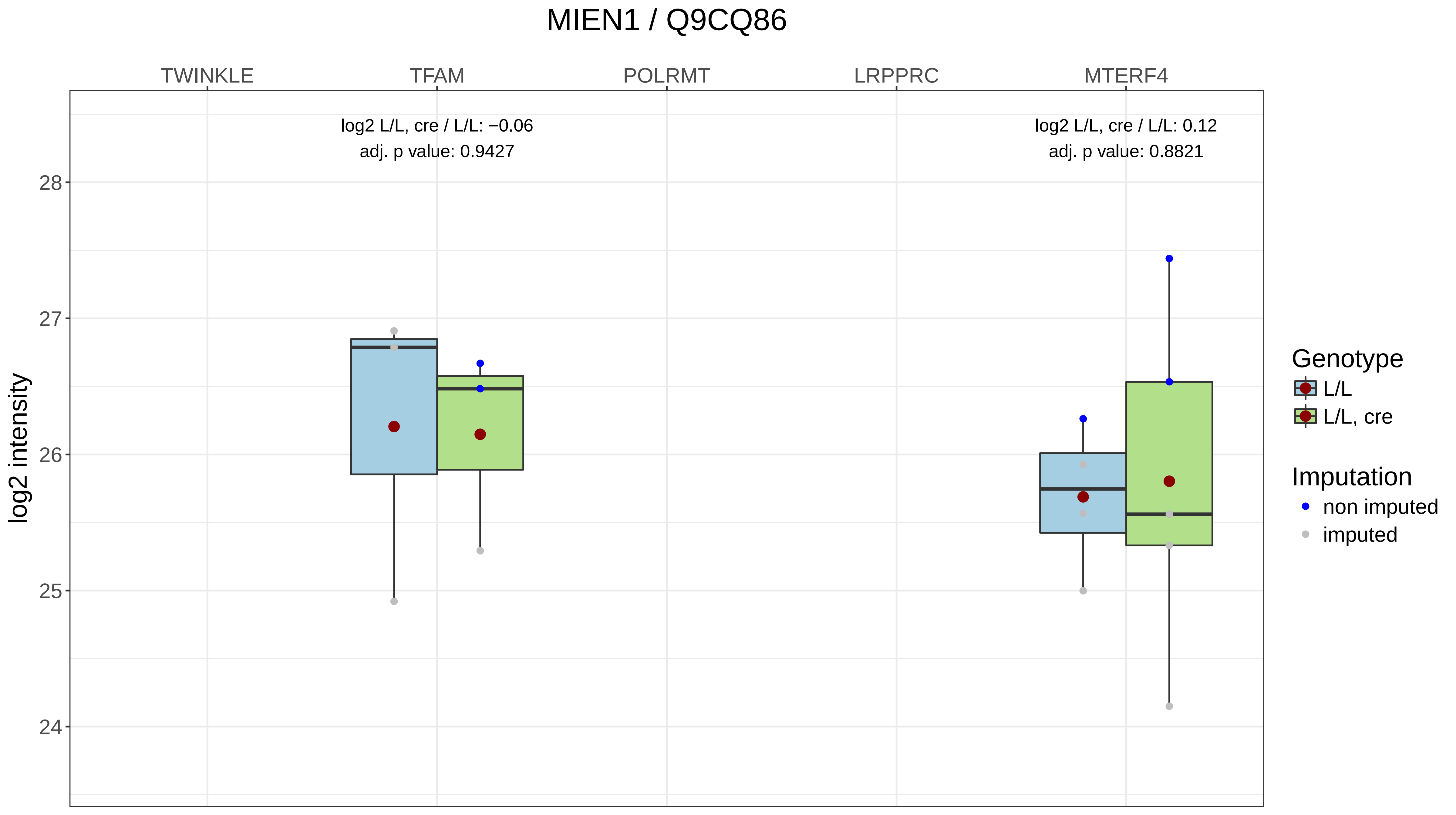

MIEN1 / Q9CQ86
TWINKLE
TFAM
POLRMT
LRPPRC
MTERF4
log2 L/L, cre / L/L: −0.06
adj. p value: 0.9427
log2 L/L, cre / L/L: 0.12
adj. p value: 0.8821
log2 intensity
28
27
26
25
24
Genotype
L/L
L/L, cre
Imputation
non imputed
imputed

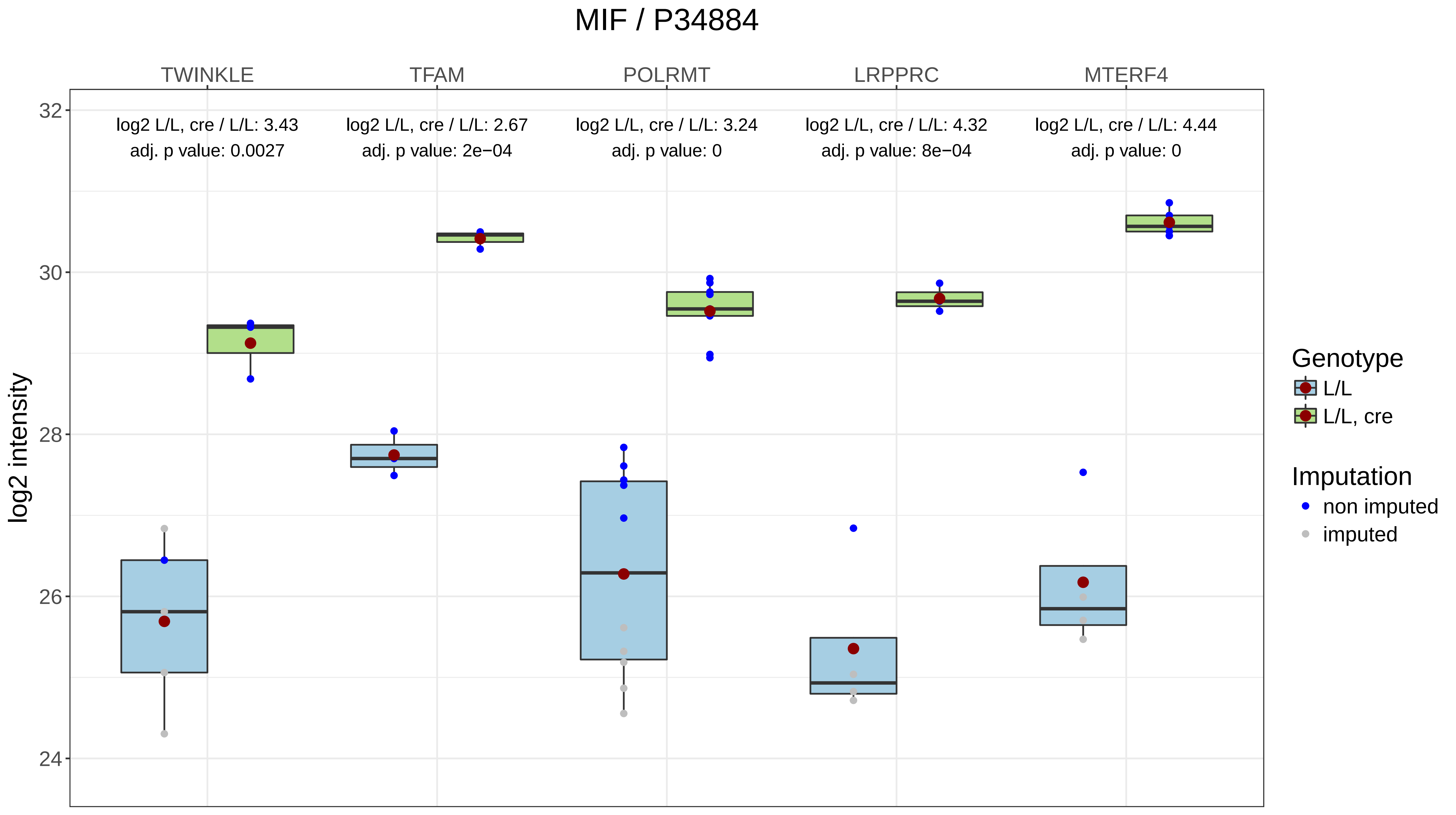
MIF / P34884
TWINKLE
TFAM
POLRMT
LRPPRC
MTERF4
log2 L/L, cre / L/L: 3.43
adj. p value: 0.0027
log2 L/L, cre / L/L: 2.67
adj. p value: 2e−04
log2 L/L, cre / L/L: 3.24
adj. p value: 0
log2 L/L, cre / L/L: 4.32
adj. p value: 8e−04
log2 L/L, cre / L/L: 4.44
adj. p value: 0
log2 intensity
32
30
28
26
24
Genotype
L/L
L/L, cre
Imputation
non imputed
imputed

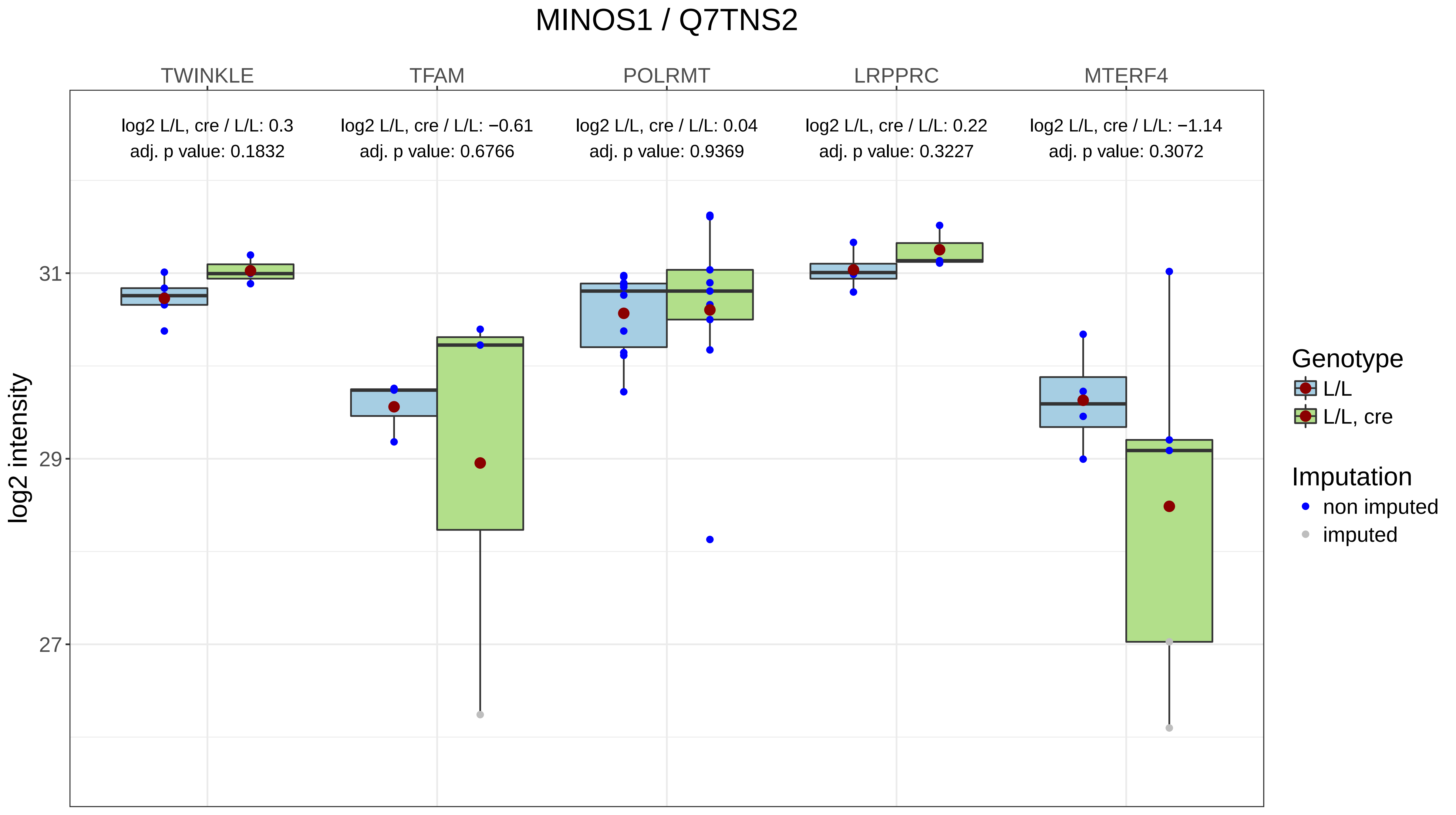
MINOS1 / Q7TNS2
TWINKLE
TFAM
POLRMT
LRPPRC
MTERF4
log2 L/L, cre / L/L: 0.3
adj. p value: 0.1832
log2 L/L, cre / L/L: −0.61
adj. p value: 0.6766
log2 L/L, cre / L/L: 0.04
adj. p value: 0.9369
log2 L/L, cre / L/L: 0.22
adj. p value: 0.3227
log2 L/L, cre / L/L: −1.14
adj. p value: 0.3072
log2 intensity
31
29
27
Genotype
L/L
L/L, cre
Imputation
non imputed
imputed

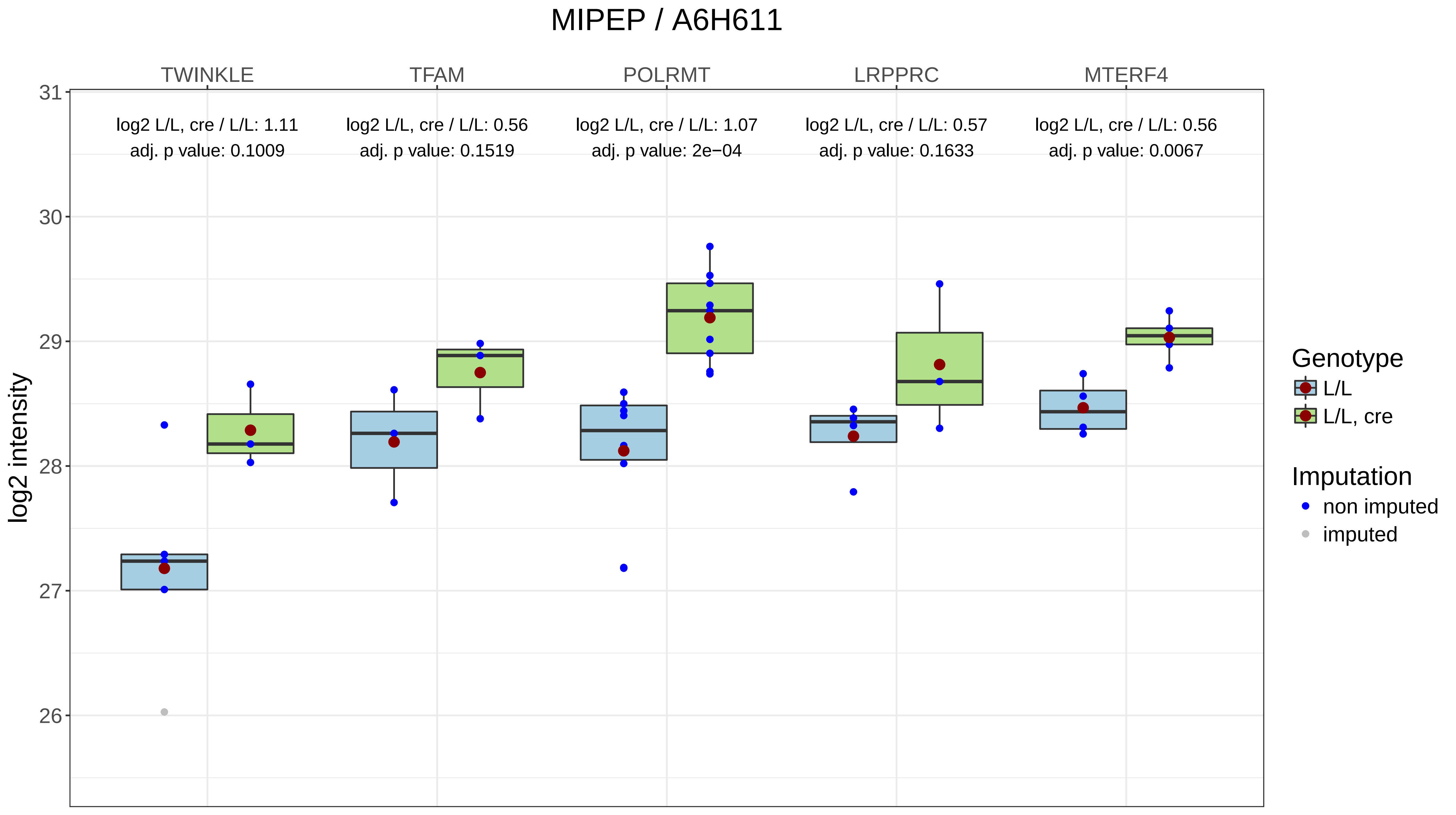
MIPEP / A6H611
TWINKLE
TFAM
POLRMT
LRPPRC
MTERF4
log2 L/L, cre / L/L: 1.11
adj. p value: 0.1009
log2 L/L, cre / L/L: 0.56
adj. p value: 0.1519
log2 L/L, cre / L/L: 1.07
adj. p value: 2e−04
log2 L/L, cre / L/L: 0.57
adj. p value: 0.1633
log2 L/L, cre / L/L: 0.56
adj. p value: 0.0067
log2 intensity
31
30
29
28
27
26
Genotype
L/L
L/L, cre
Imputation
non imputed
imputed

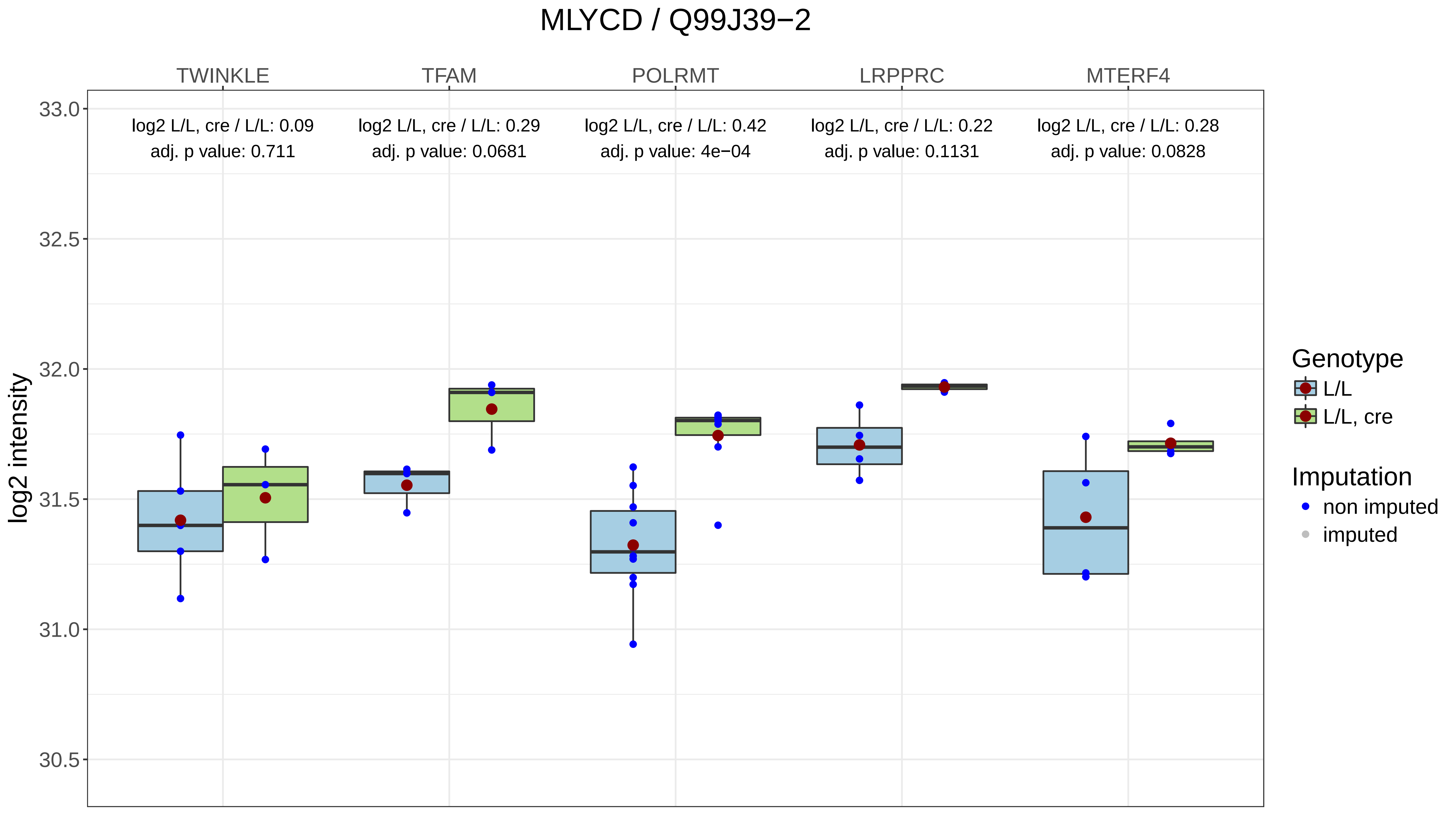
MLYCD / Q99J39−2
TWINKLE
TFAM
POLRMT
LRPPRC
MTERF4
log2 L/L, cre / L/L: 0.09
adj. p value: 0.711
log2 L/L, cre / L/L: 0.29
adj. p value: 0.0681
log2 L/L, cre / L/L: 0.42
adj. p value: 4e−04
log2 L/L, cre / L/L: 0.22
adj. p value: 0.1131
log2 L/L, cre / L/L: 0.28
adj. p value: 0.0828
log2 intensity
33.0
32.5
32.0
31.5
31.0
30.5
Genotype
L/L
L/L, cre
Imputation
non imputed
imputed

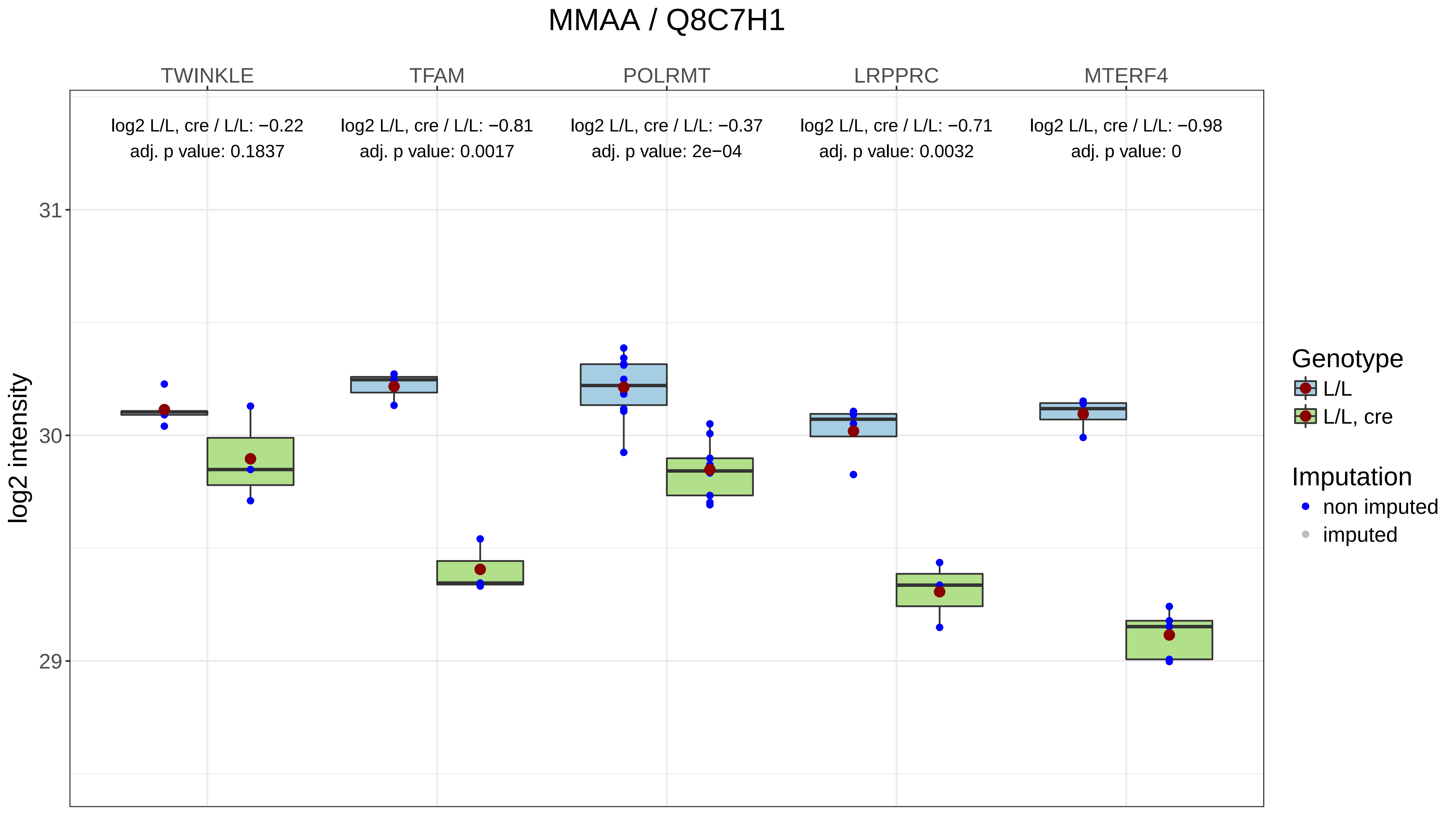
MMAA / Q8C7H1
TWINKLE
TFAM
POLRMT
LRPPRC
MTERF4
log2 L/L, cre / L/L: −0.22
adj. p value: 0.1837
log2 L/L, cre / L/L: −0.81
adj. p value: 0.0017
log2 L/L, cre / L/L: −0.37
adj. p value: 2e−04
log2 L/L, cre / L/L: −0.71
adj. p value: 0.0032
log2 L/L, cre / L/L: −0.98
adj. p value: 0
31
30
29
log2 intensity
Genotype
L/L
L/L, cre
Imputation
non imputed
imputed

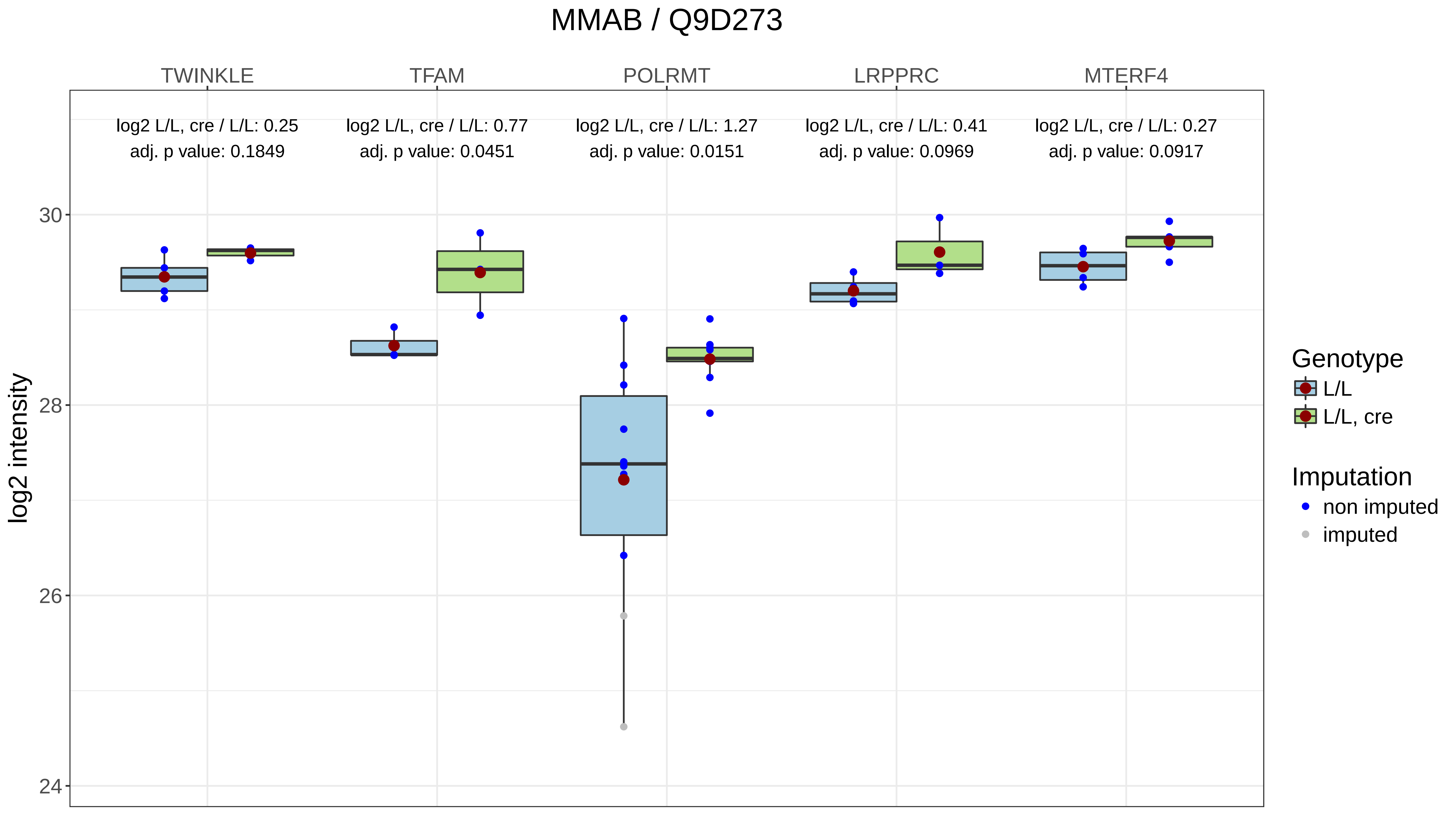
MMAB / Q9D273
TWINKLE
TFAM
POLRMT
LRPPRC
MTERF4
log2 L/L, cre / L/L: 0.25
adj. p value: 0.1849
log2 L/L, cre / L/L: 0.77
adj. p value: 0.0451
log2 L/L, cre / L/L: 1.27
adj. p value: 0.0151
log2 L/L, cre / L/L: 0.41
adj. p value: 0.0969
log2 L/L, cre / L/L: 0.27
adj. p value: 0.0917
log2 intensity
30
28
26
24
Genotype
L/L
L/L, cre
Imputation
non imputed
imputed

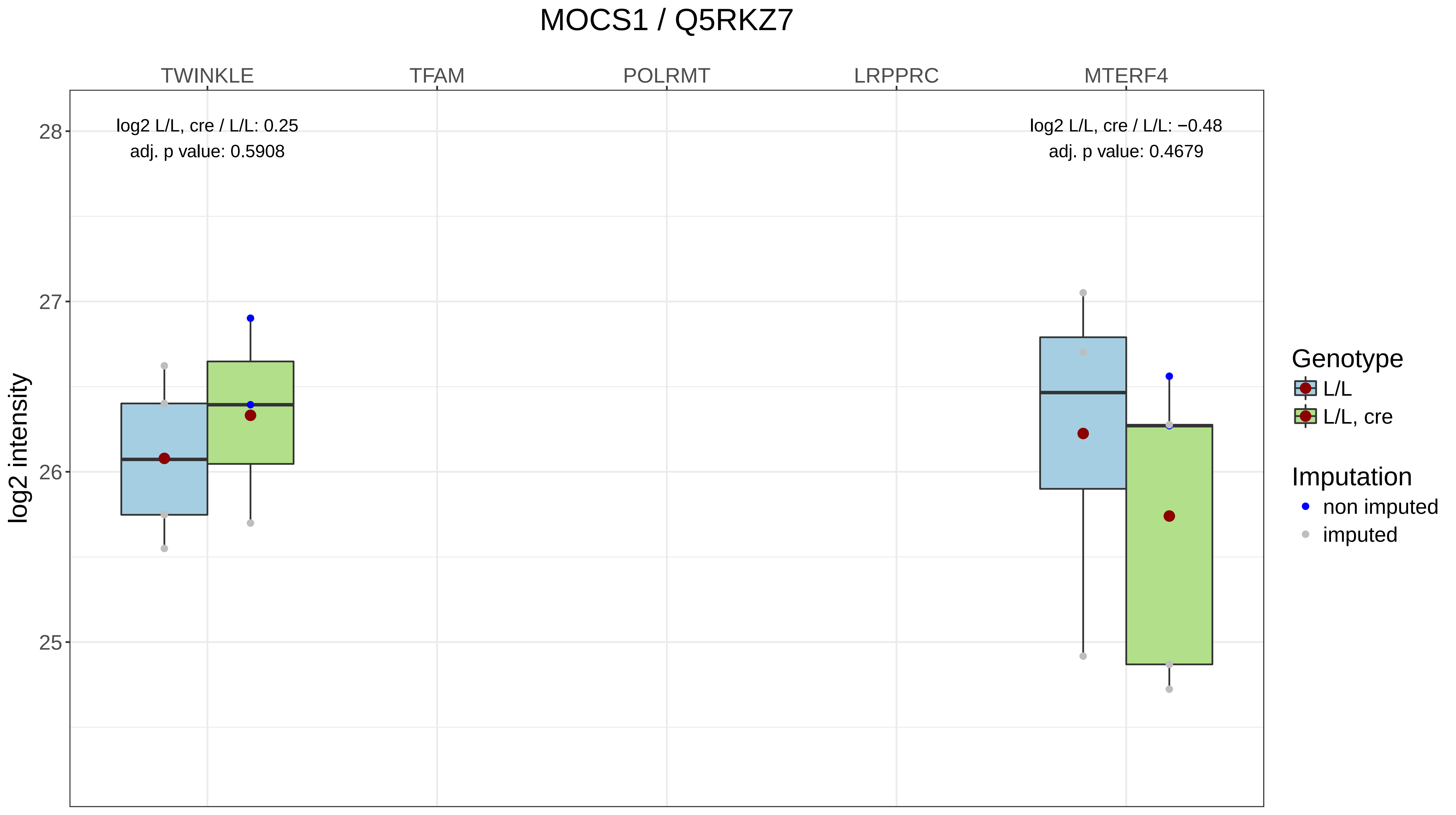
MOCS1 / Q5RKZ7
TWINKLE
TFAM
POLRMT
LRPPRC
MTERF4
log2 L/L, cre / L/L: 0.25
adj. p value: 0.5908
log2 L/L, cre / L/L: −0.48
adj. p value: 0.4679
log2 intensity
28
27
26
25
Genotype
L/L
L/L, cre
Imputation
non imputed
imputed

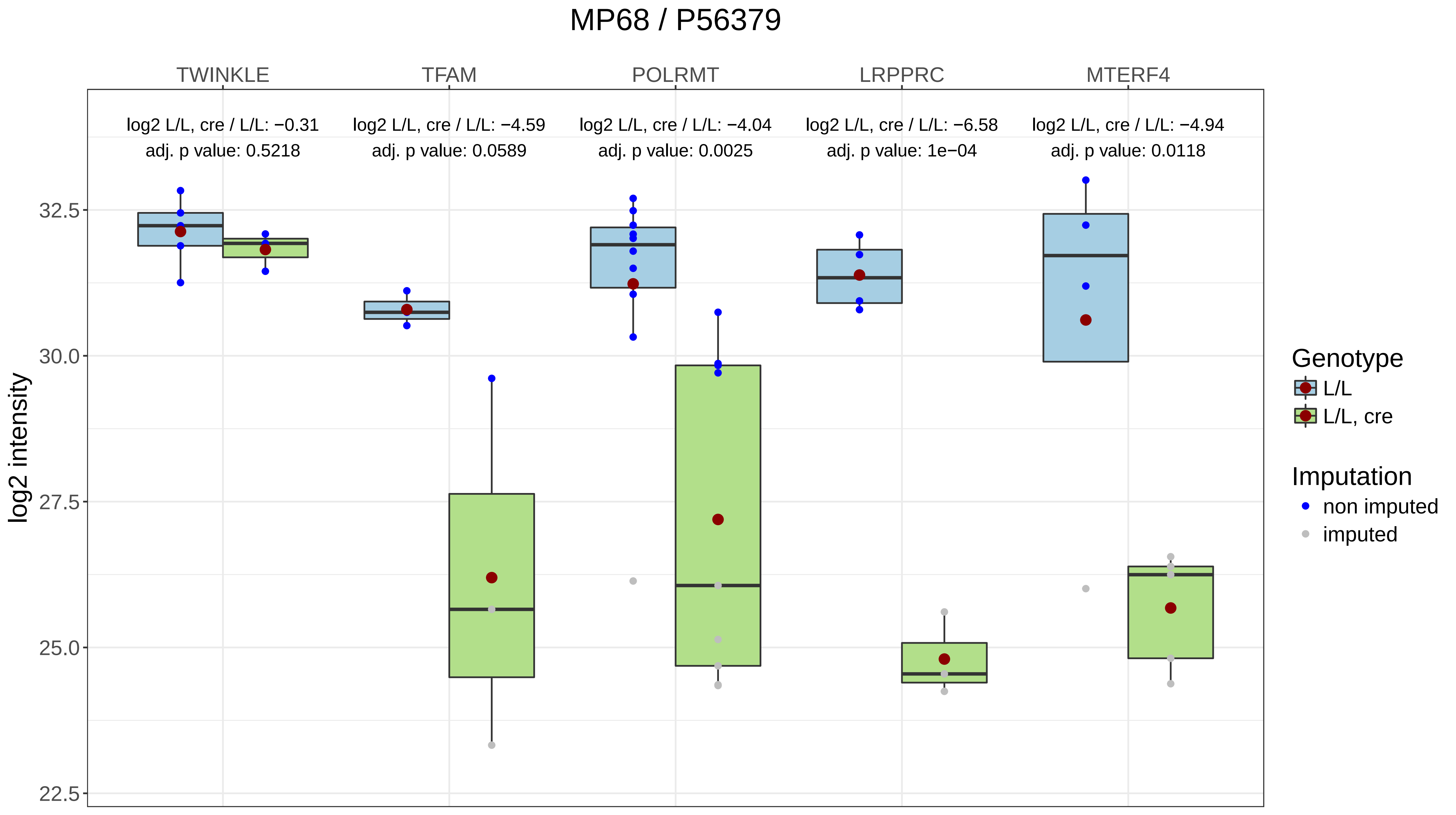
MP68 / P56379
TWINKLE
TFAM
POLRMT
LRPPRC
MTERF4
log2 L/L, cre / L/L: −0.31
adj. p value: 0.5218
log2 L/L, cre / L/L: −4.59
adj. p value: 0.0589
log2 L/L, cre / L/L: −4.04
adj. p value: 0.0025
log2 L/L, cre / L/L: −6.58
adj. p value: 1e−04
log2 L/L, cre / L/L: −4.94
adj. p value: 0.0118
log2 intensity
32.5
30.0
27.5
25.0
22.5
Genotype
L/L
L/L, cre
Imputation
non imputed
imputed

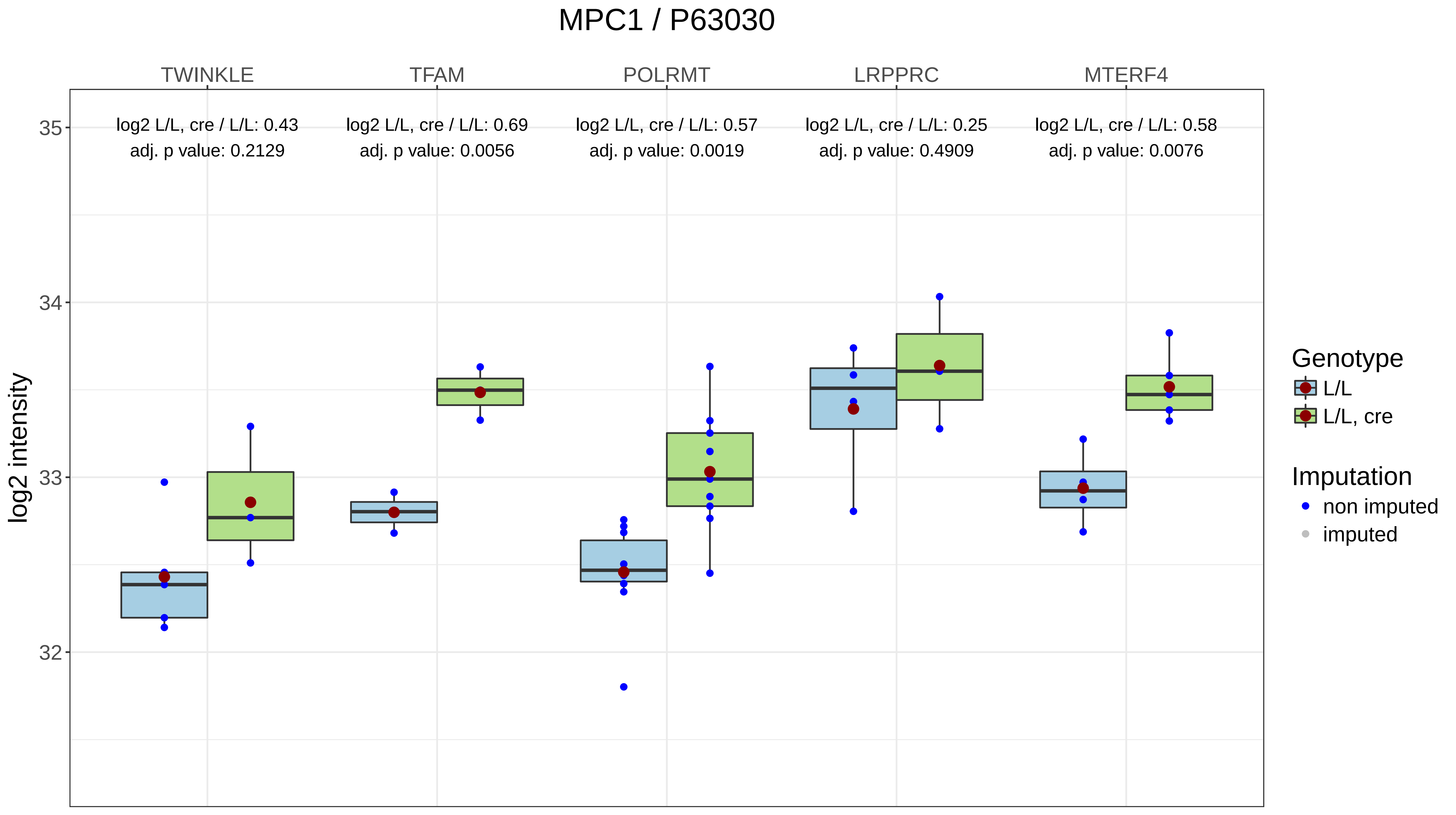
MPC1 / P63030
TWINKLE
TFAM
POLRMT
LRPPRC
MTERF4
log2 L/L, cre / L/L: 0.43
adj. p value: 0.2129
log2 L/L, cre / L/L: 0.69
adj. p value: 0.0056
log2 L/L, cre / L/L: 0.57
adj. p value: 0.0019
log2 L/L, cre / L/L: 0.25
adj. p value: 0.4909
log2 L/L, cre / L/L: 0.58
adj. p value: 0.0076
log2 intensity
35
34
33
32
Genotype
L/L
L/L, cre
Imputation
non imputed
imputed

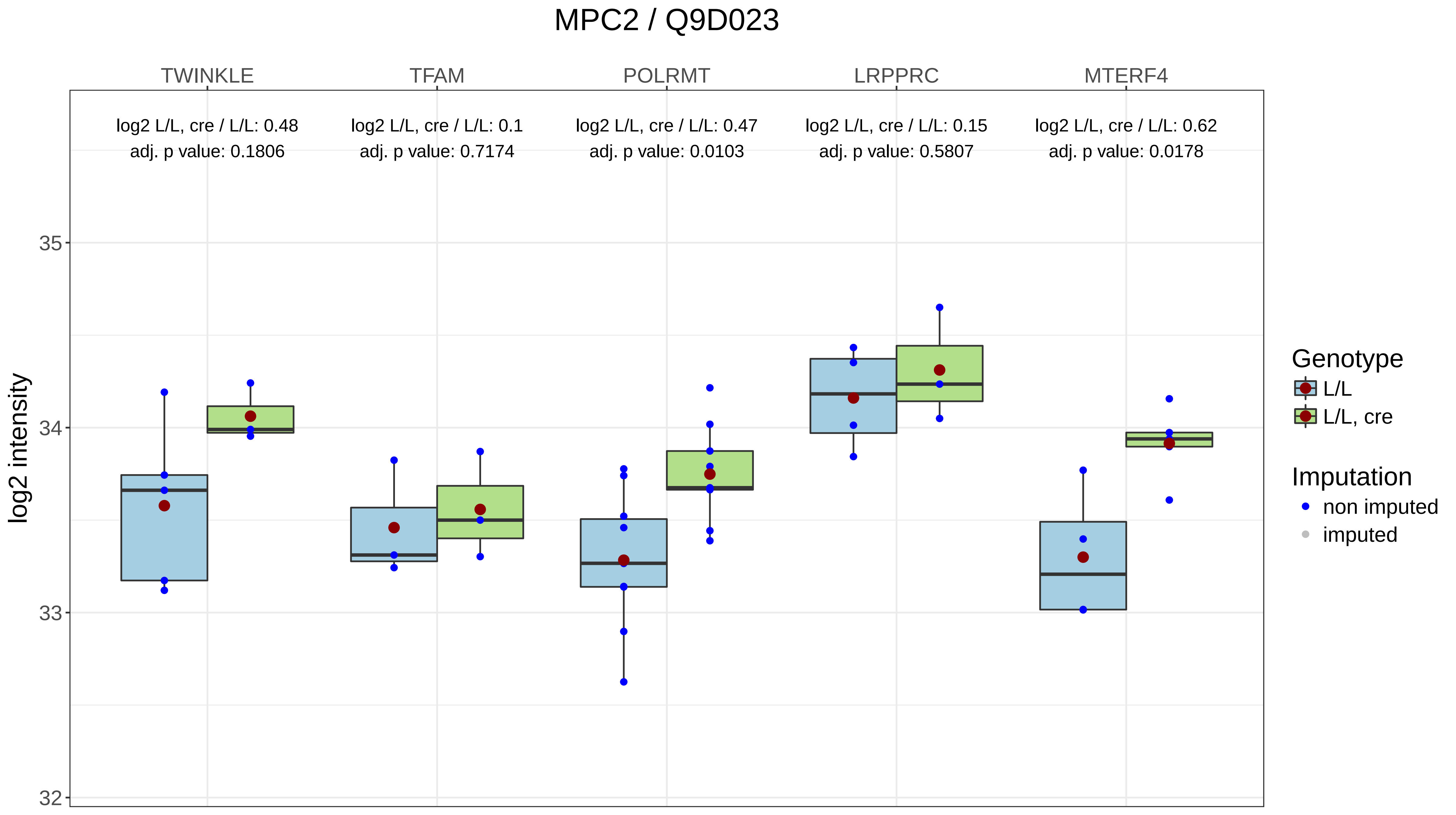
MPC2 / Q9D023
TWINKLE
TFAM
POLRMT
LRPPRC
MTERF4
log2 L/L, cre / L/L: 0.48
adj. p value: 0.1806
log2 L/L, cre / L/L: 0.1
adj. p value: 0.7174
log2 L/L, cre / L/L: 0.47
adj. p value: 0.0103
log2 L/L, cre / L/L: 0.15
adj. p value: 0.5807
log2 L/L, cre / L/L: 0.62
adj. p value: 0.0178
log2 intensity
35
34
33
32
Genotype
L/L
L/L, cre
Imputation
non imputed
imputed

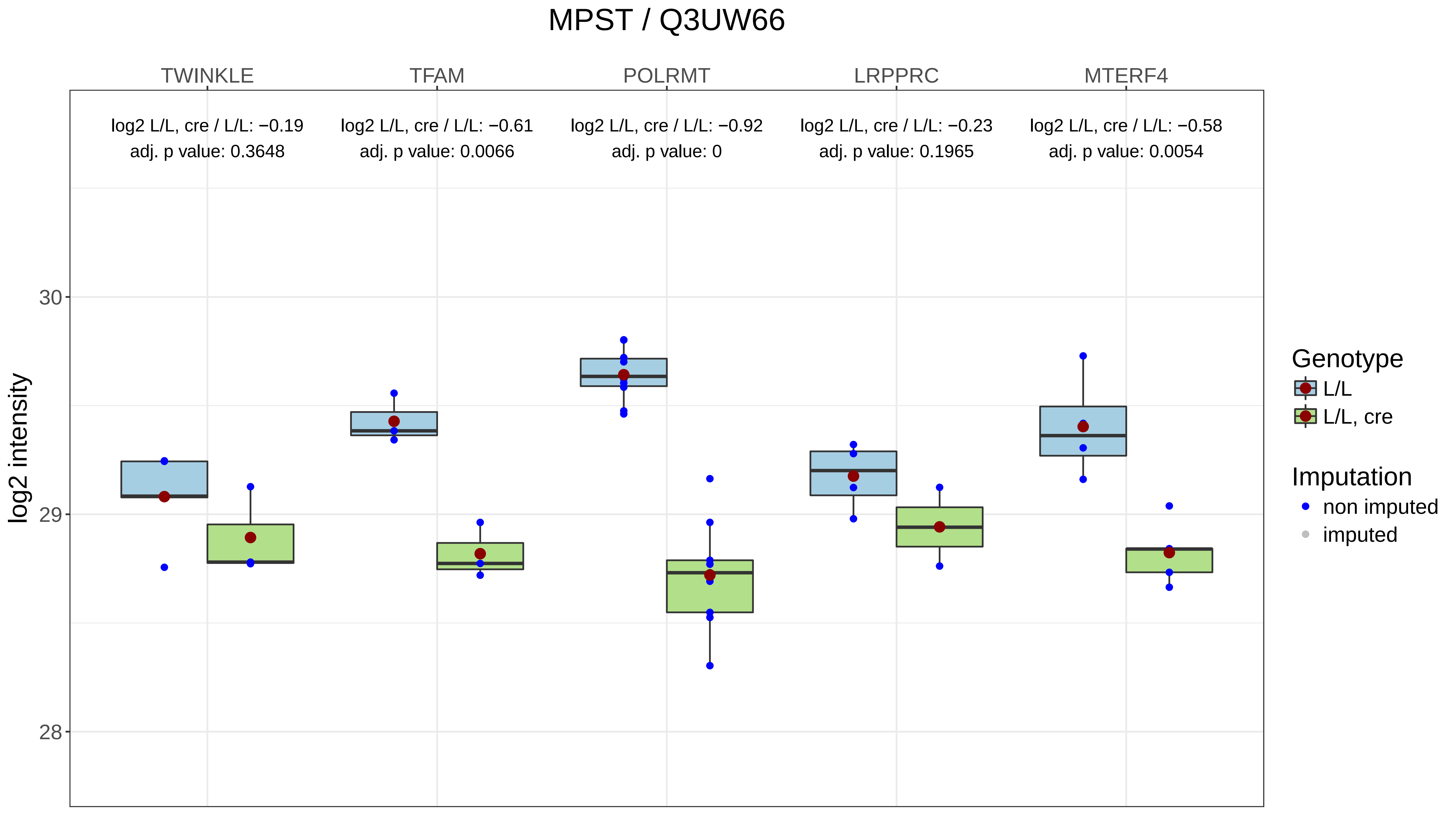

MPST / Q3UW66
TWINKLE
TFAM
POLRMT
LRPPRC
MTERF4
log2 L/L, cre / L/L: −0.19
adj. p value: 0.3648
log2 L/L, cre / L/L: −0.61
adj. p value: 0.0066
log2 L/L, cre / L/L: −0.92
adj. p value: 0
log2 L/L, cre / L/L: −0.23
adj. p value: 0.1965
log2 L/L, cre / L/L: −0.58
adj. p value: 0.0054
log2 intensity
30
29
28
Genotype
L/L
L/L, cre
Imputation
non imputed
imputed

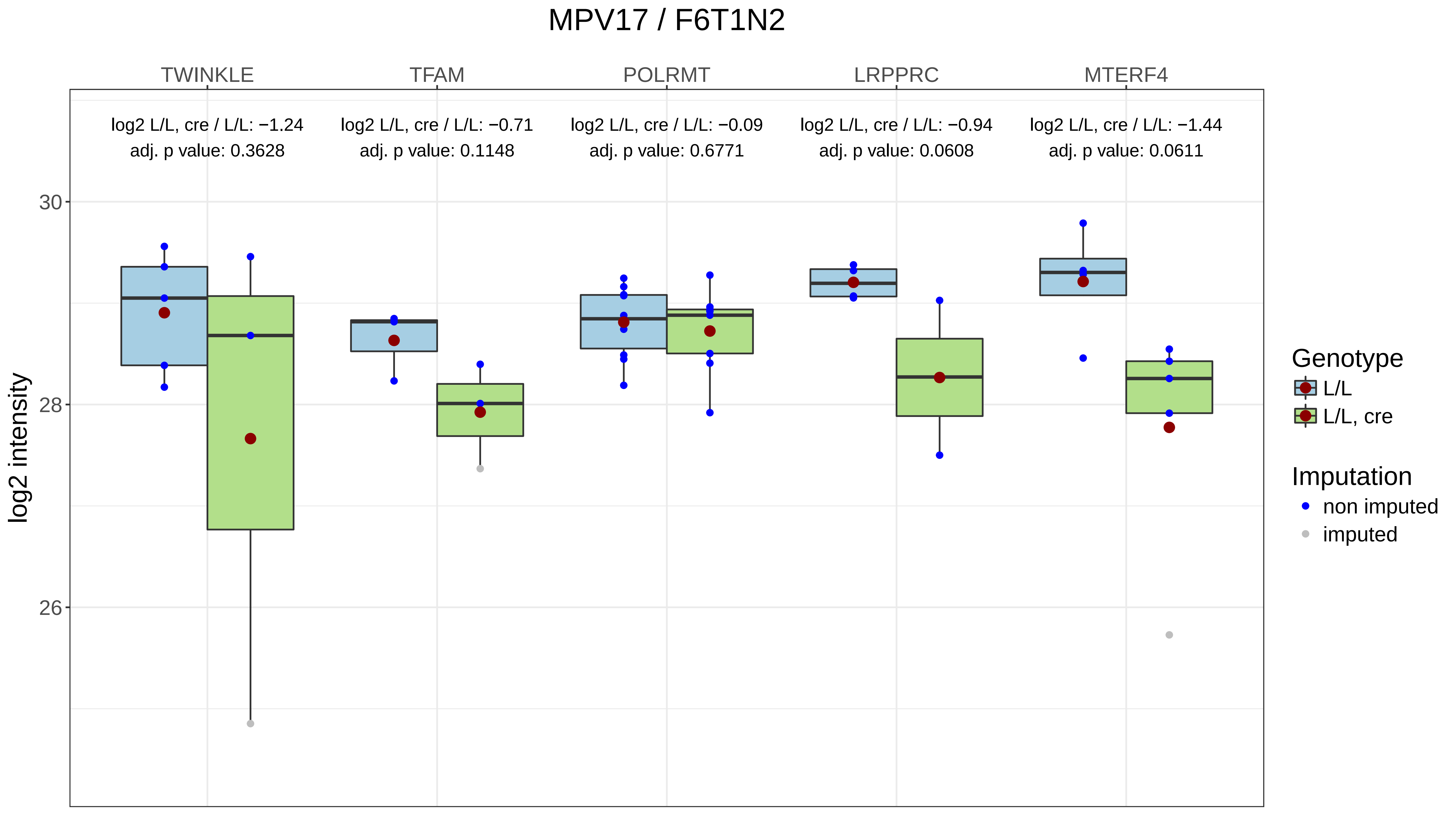
MPV17 / F6T1N2
TWINKLE
TFAM
POLRMT
LRPPRC
MTERF4
log2 L/L, cre / L/L: −1.24
adj. p value: 0.3628
log2 L/L, cre / L/L: −0.71
adj. p value: 0.1148
log2 L/L, cre / L/L: −0.09
adj. p value: 0.6771
log2 L/L, cre / L/L: −0.94
adj. p value: 0.0608
log2 L/L, cre / L/L: −1.44
adj. p value: 0.0611
log2 intensity
30
28
26
Genotype
L/L
L/L, cre
Imputation
non imputed
imputed

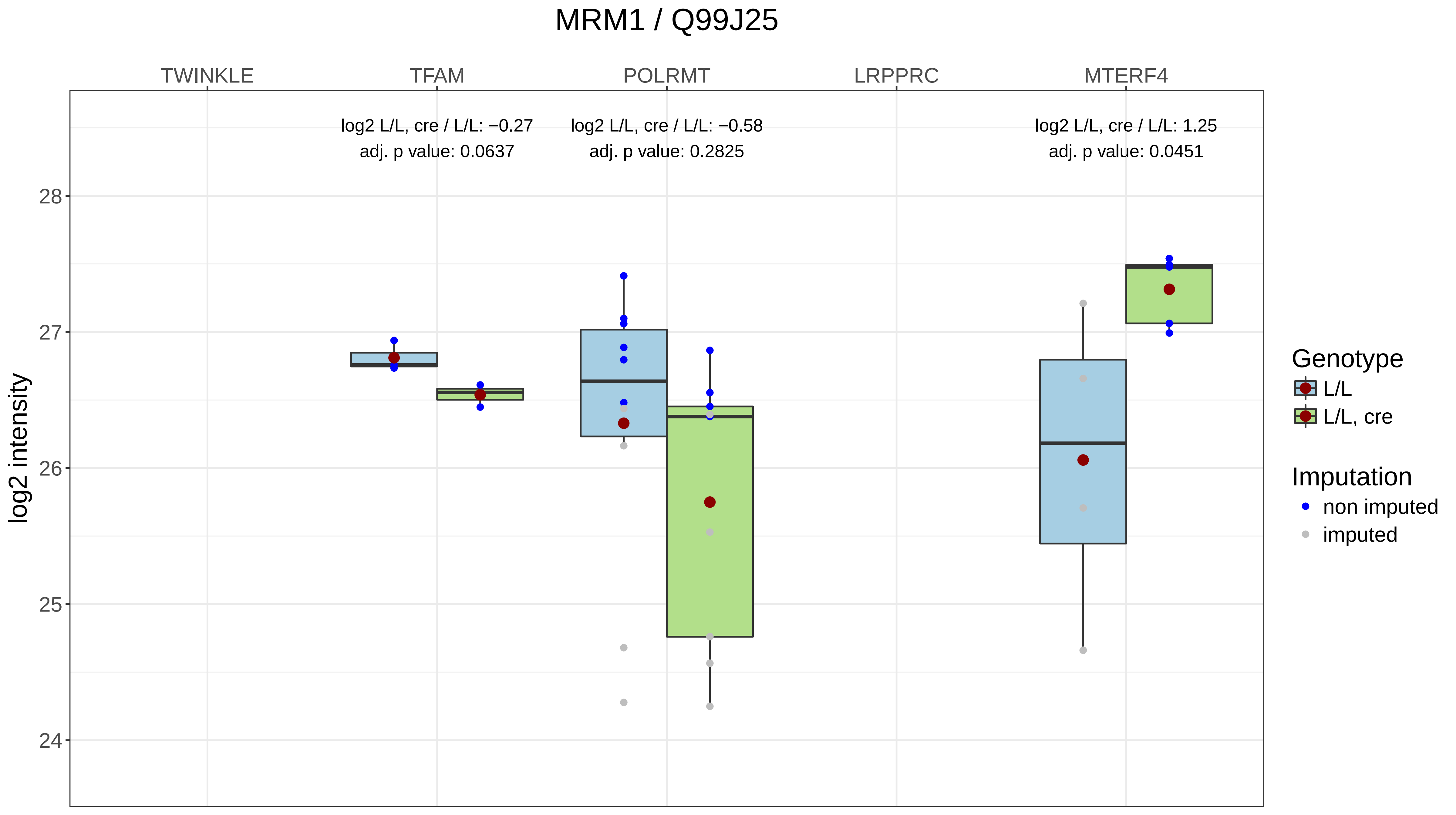
MRM1 / Q99J25
TWINKLE
TFAM
POLRMT
LRPPRC
MTERF4
log2 L/L, cre / L/L: −0.27
adj. p value: 0.0637
log2 L/L, cre / L/L: −0.58
adj. p value: 0.2825
log2 L/L, cre / L/L: 1.25
adj. p value: 0.0451
log2 intensity
28
27
26
25
24
Genotype
L/L
L/L, cre
Imputation
non imputed
imputed

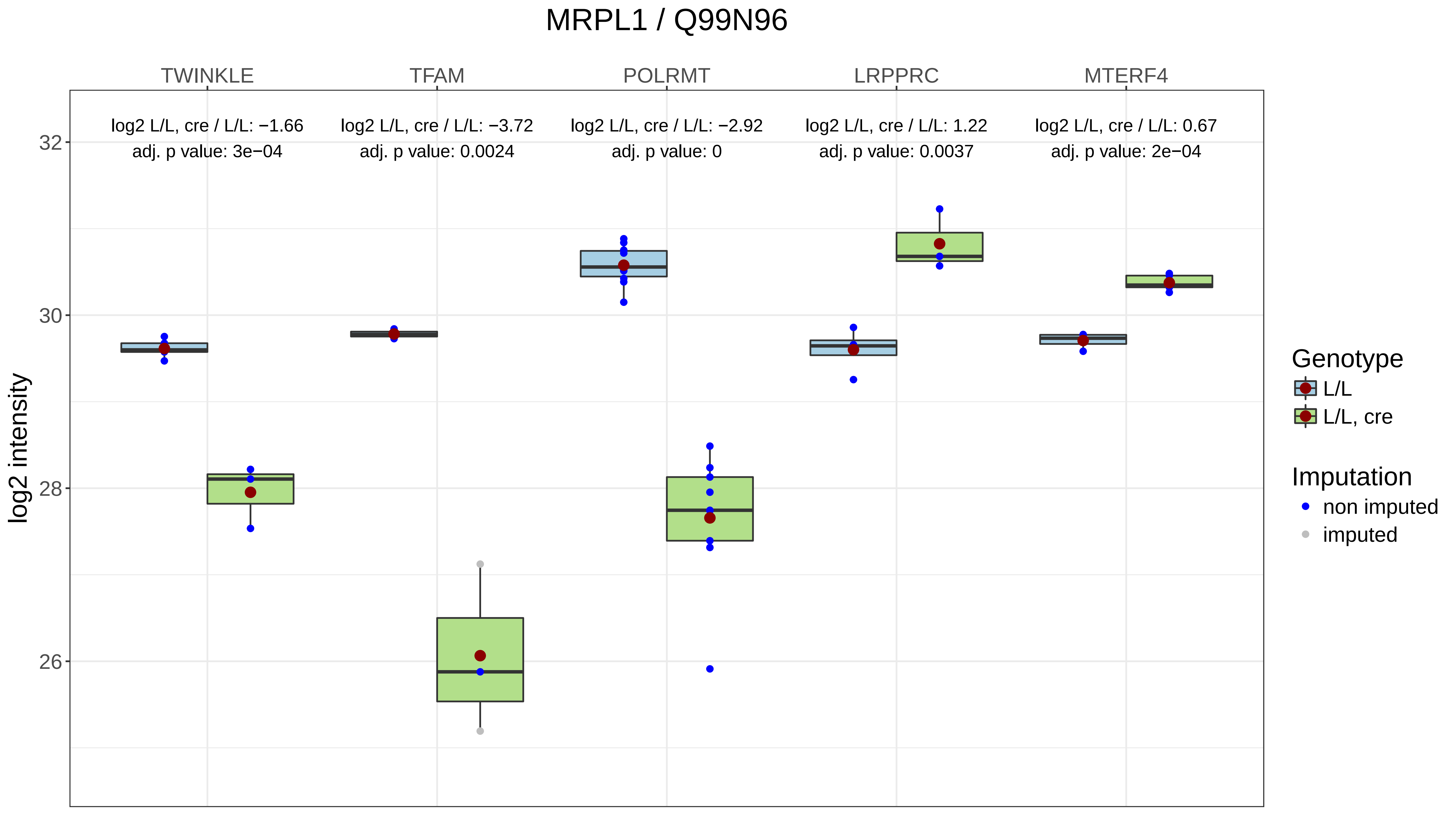
MRPL1 / Q99N96
TWINKLE
TFAM
POLRMT
LRPPRC
MTERF4
log2 L/L, cre / L/L: −1.66
adj. p value: 3e−04
log2 L/L, cre / L/L: −3.72
adj. p value: 0.0024
log2 L/L, cre / L/L: −2.92
adj. p value: 0
log2 L/L, cre / L/L: 1.22
adj. p value: 0.0037
log2 L/L, cre / L/L: 0.67
adj. p value: 2e−04
32
30
28
26
log2 intensity
Genotype
L/L
L/L, cre
Imputation
non imputed
imputed

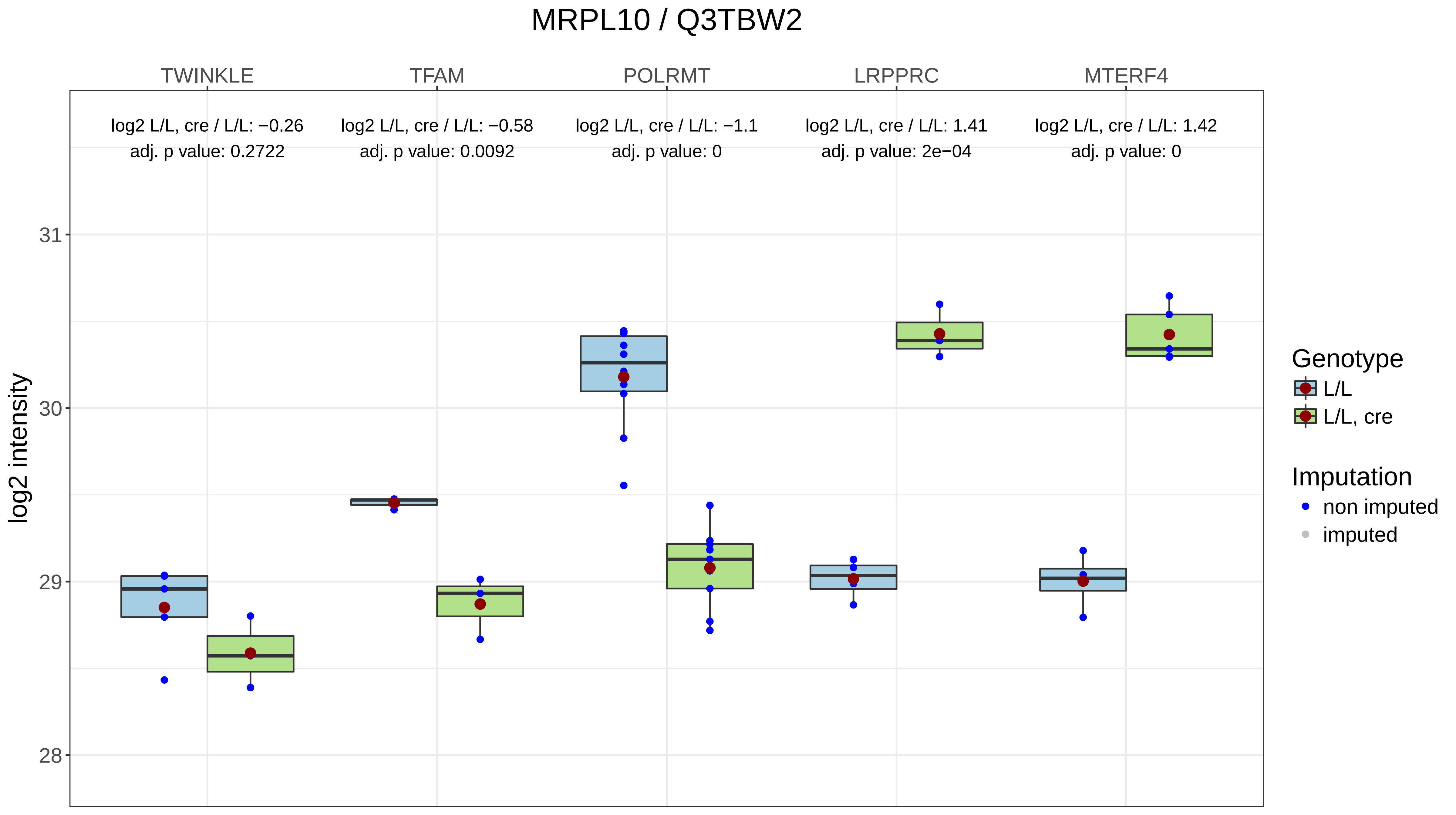

MRPL10 / Q3TBW2
TWINKLE
TFAM
POLRMT
LRPPRC
MTERF4
log2 L/L, cre / L/L: −0.26
adj. p value: 0.2722
log2 L/L, cre / L/L: −0.58
adj. p value: 0.0092
log2 L/L, cre / L/L: −1.1
adj. p value: 0
log2 L/L, cre / L/L: 1.41
adj. p value: 2e−04
log2 L/L, cre / L/L: 1.42
adj. p value: 0
log2 intensity
31
30
29
28
Genotype
L/L
L/L, cre
Imputation
non imputed
imputed

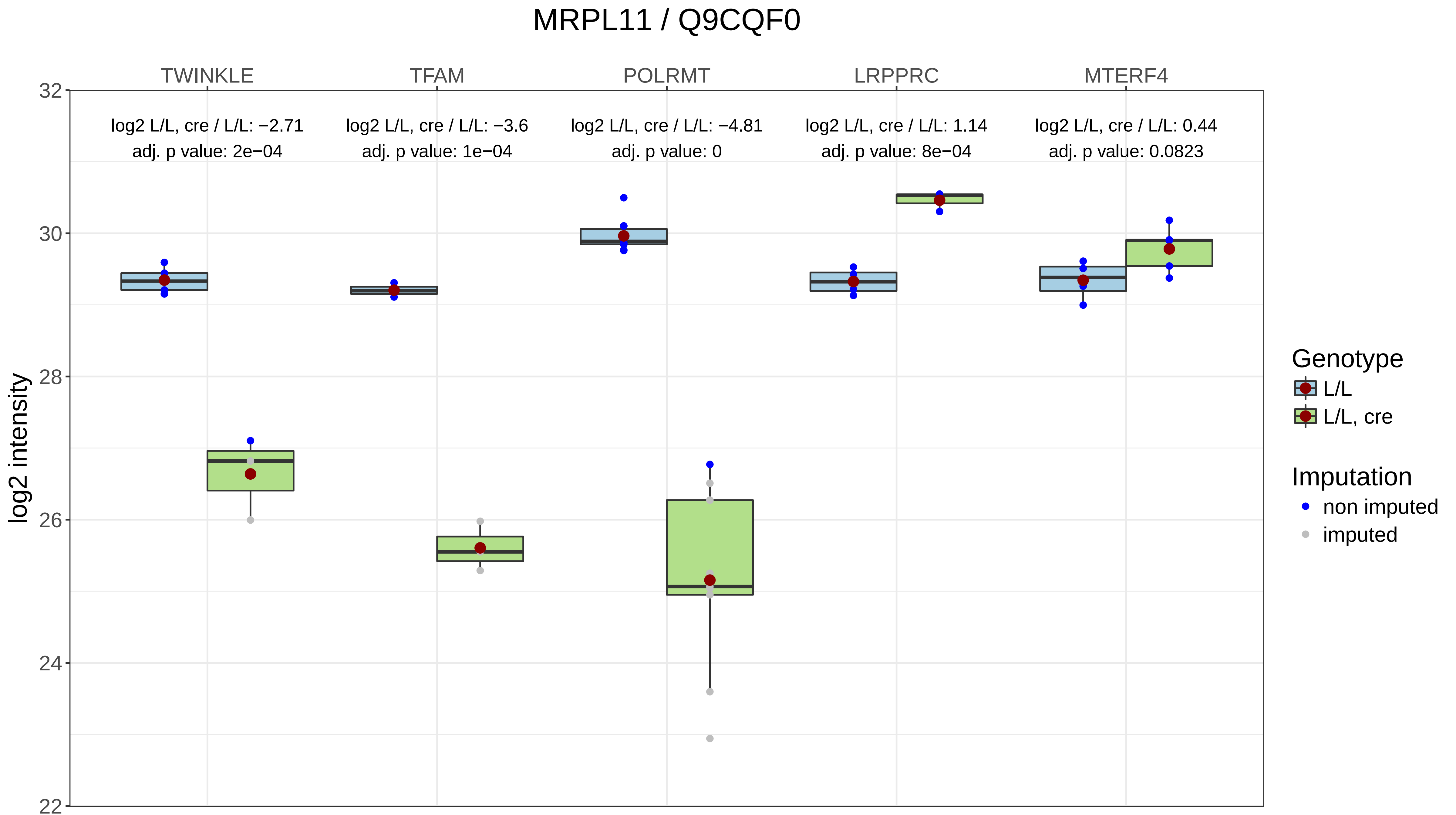
MRPL11 / Q9CQF0
TWINKLE
TFAM
POLRMT
LRPPRC
MTERF4
log2 L/L, cre / L/L: −2.71
adj. p value: 2e−04
log2 L/L, cre / L/L: −3.6
adj. p value: 1e−04
log2 L/L, cre / L/L: −4.81
adj. p value: 0
log2 L/L, cre / L/L: 1.14
adj. p value: 8e−04
log2 L/L, cre / L/L: 0.44
adj. p value: 0.0823
log2 intensity
32
30
28
26
24
22
Genotype
L/L
L/L, cre
Imputation
non imputed
imputed

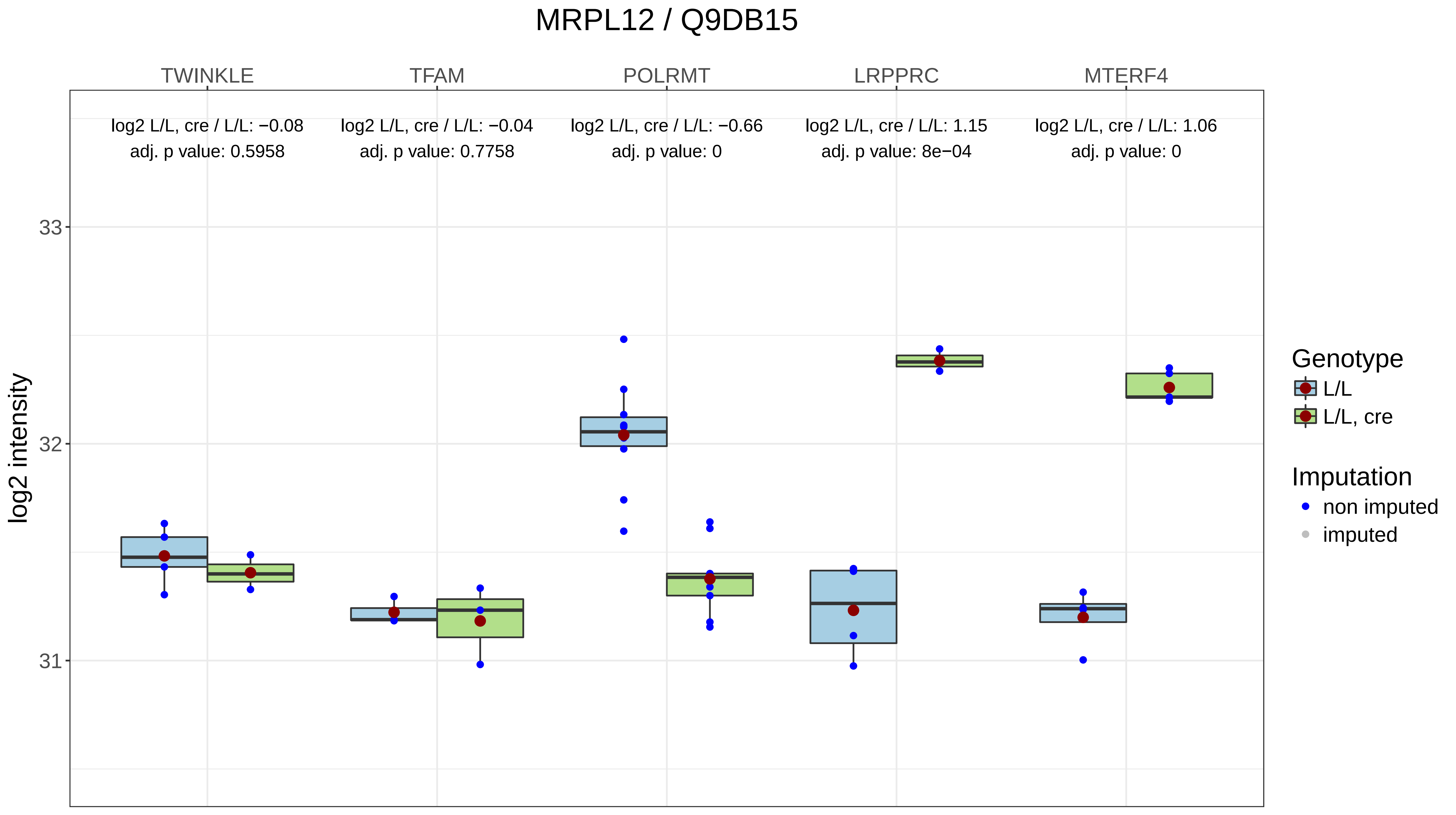
MRPL12 / Q9DB15
TWINKLE
TFAM
POLRMT
LRPPRC
MTERF4
log2 L/L, cre / L/L: −0.08
adj. p value: 0.5958
log2 L/L, cre / L/L: −0.04
adj. p value: 0.7758
log2 L/L, cre / L/L: −0.66
adj. p value: 0
log2 L/L, cre / L/L: 1.15
adj. p value: 8e−04
log2 L/L, cre / L/L: 1.06
adj. p value: 0
log2 intensity
33
32
31
Genotype
L/L
L/L, cre
Imputation
non imputed
imputed

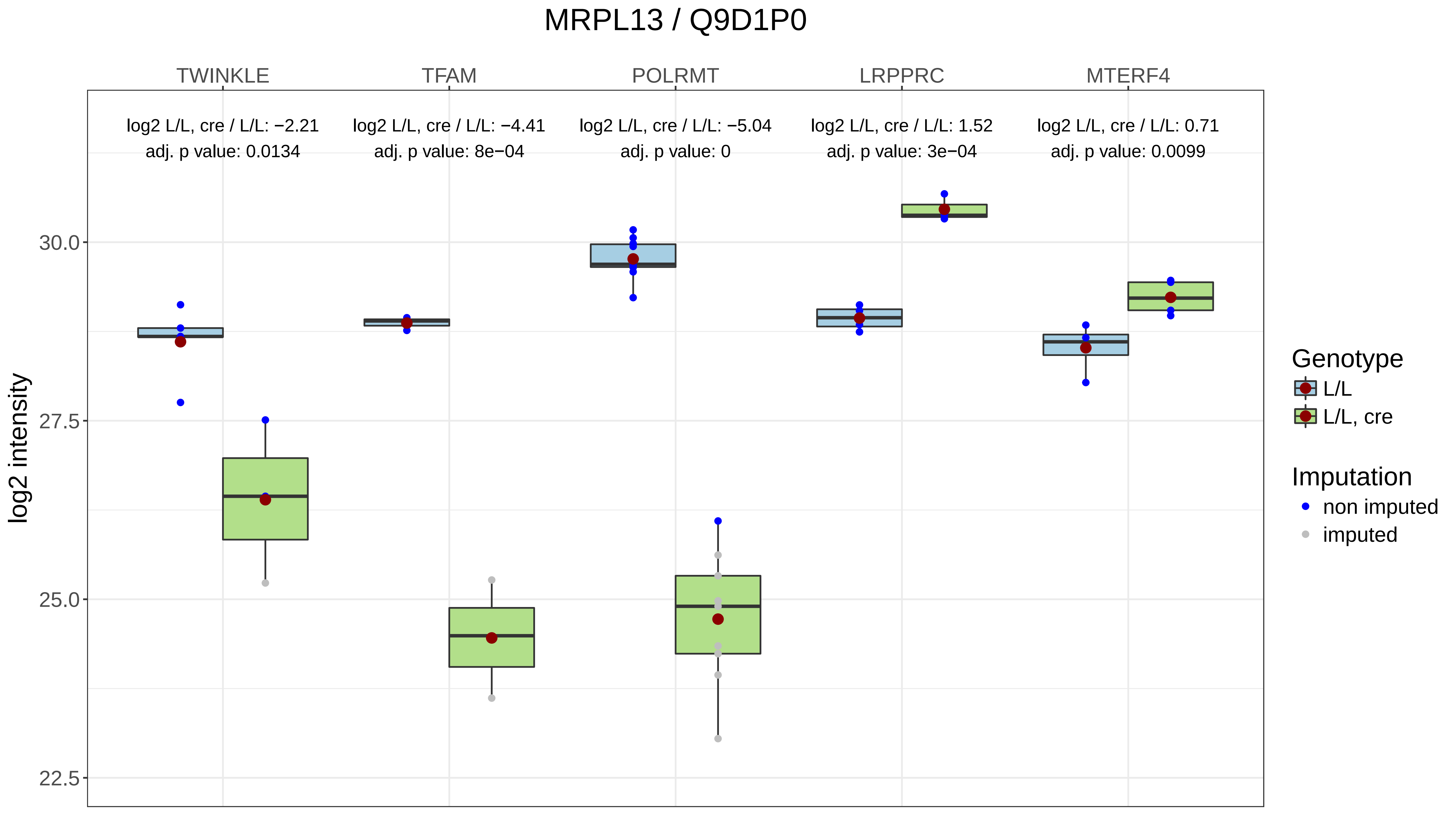
MRPL13 / Q9D1P0
TWINKLE
TFAM
POLRMT
LRPPRC
MTERF4
log2 L/L, cre / L/L: −2.21
adj. p value: 0.0134
log2 L/L, cre / L/L: −4.41
adj. p value: 8e−04
log2 L/L, cre / L/L: −5.04
adj. p value: 0
log2 L/L, cre / L/L: 1.52
adj. p value: 3e−04
log2 L/L, cre / L/L: 0.71
adj. p value: 0.0099
log2 intensity
30.0
27.5
25.0
22.5
Genotype
L/L
L/L, cre
Imputation
non imputed
imputed

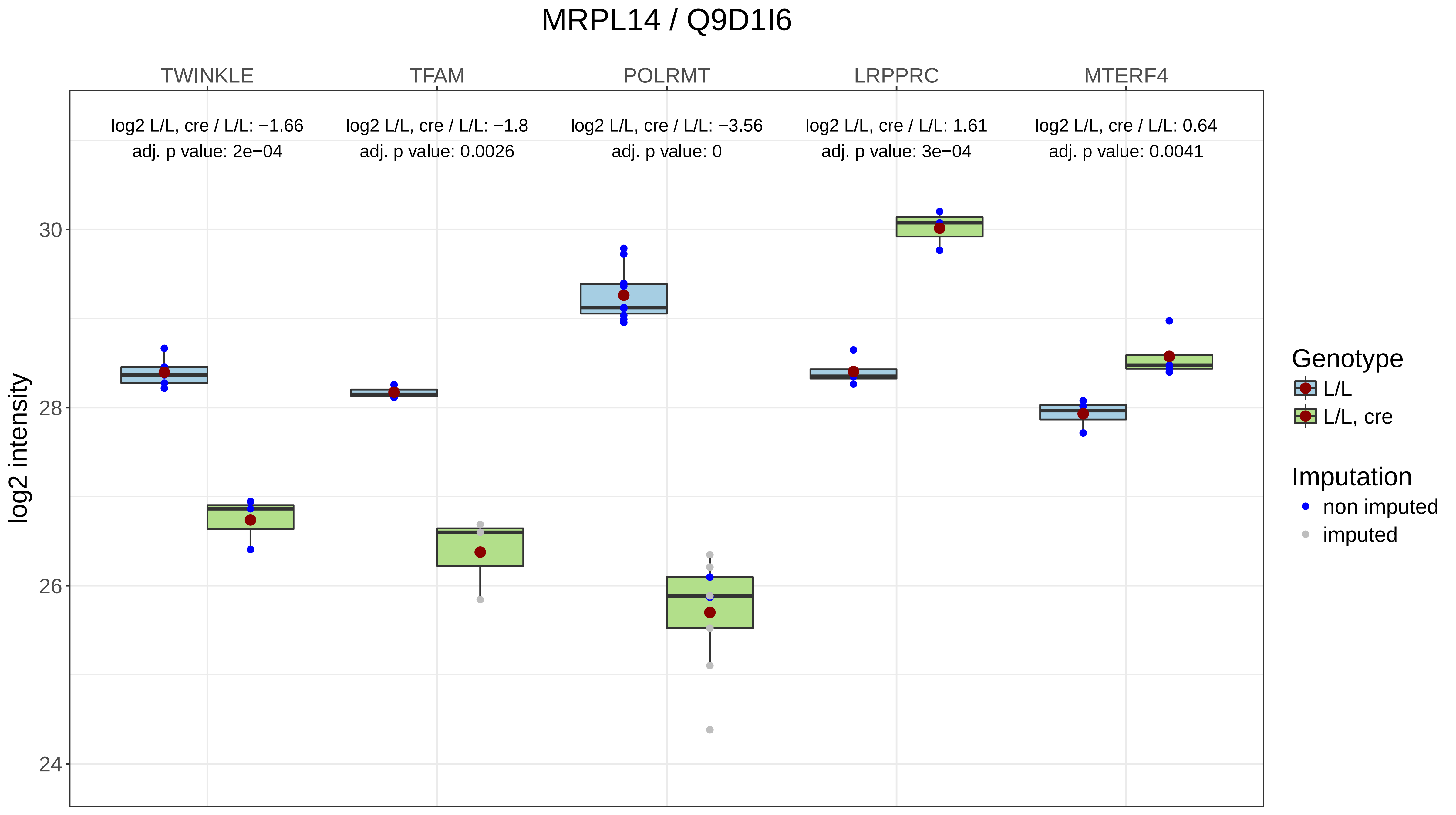

MRPL14 / Q9D1I6
TWINKLE
TFAM
POLRMT
LRPPRC
MTERF4
log2 L/L, cre / L/L: −1.66
adj. p value: 2e−04
log2 L/L, cre / L/L: −1.8
adj. p value: 0.0026
log2 L/L, cre / L/L: −3.56
adj. p value: 0
log2 L/L, cre / L/L: 1.61
adj. p value: 3e−04
log2 L/L, cre / L/L: 0.64
adj. p value: 0.0041
log2 intensity
30
28
26
24
Genotype
L/L
L/L, cre
Imputation
non imputed
imputed

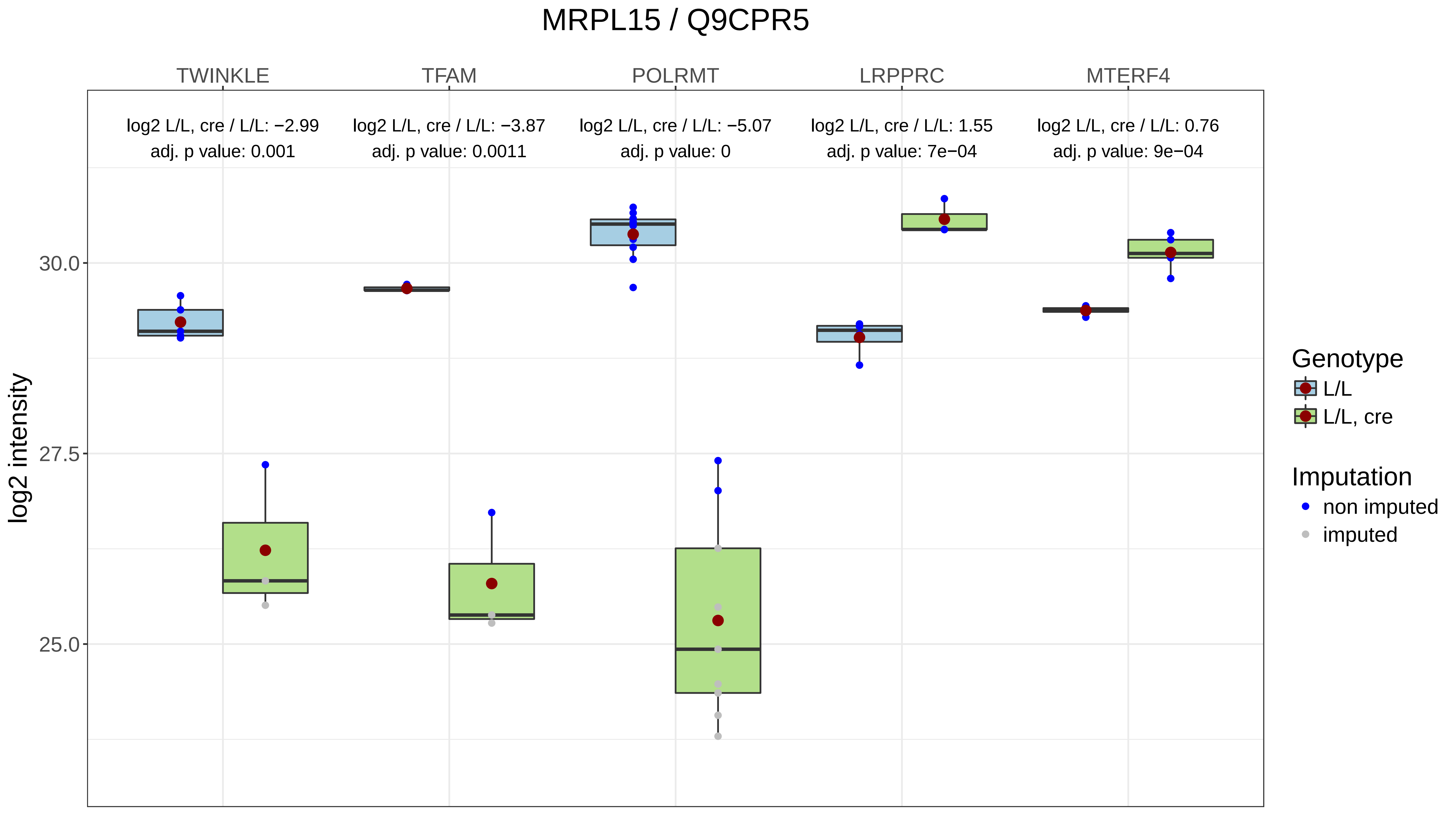

MRPL15 / Q9CPR5
TWINKLE
TFAM
POLRMT
LRPPRC
MTERF4
log2 L/L, cre / L/L: −2.99
adj. p value: 0.001
log2 L/L, cre / L/L: −3.87
adj. p value: 0.0011
log2 L/L, cre / L/L: −5.07
adj. p value: 0
log2 L/L, cre / L/L: 1.55
adj. p value: 7e−04
log2 L/L, cre / L/L: 0.76
adj. p value: 9e−04
log2 intensity
30.0
27.5
25.0
Genotype
L/L
L/L, cre
Imputation
non imputed
imputed

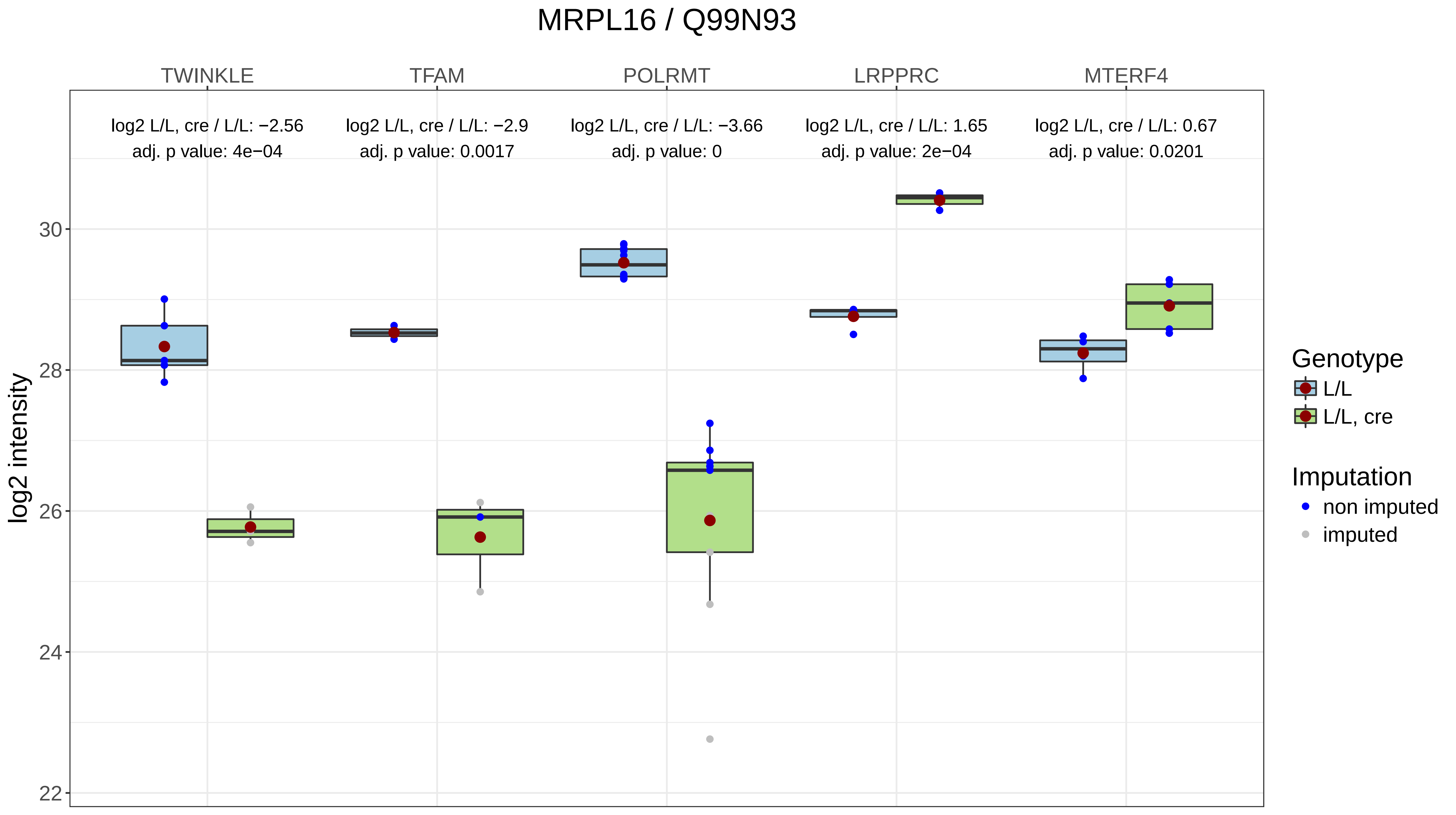
MRPL16 / Q99N93
TWINKLE
TFAM
POLRMT
LRPPRC
MTERF4
log2 L/L, cre / L/L: −2.56
adj. p value: 4e−04
log2 L/L, cre / L/L: −2.9
adj. p value: 0.0017
log2 L/L, cre / L/L: −3.66
adj. p value: 0
log2 L/L, cre / L/L: 1.65
adj. p value: 2e−04
log2 L/L, cre / L/L: 0.67
adj. p value: 0.0201
log2 intensity
30
28
26
24
22
Genotype
L/L
L/L, cre
Imputation
non imputed
imputed

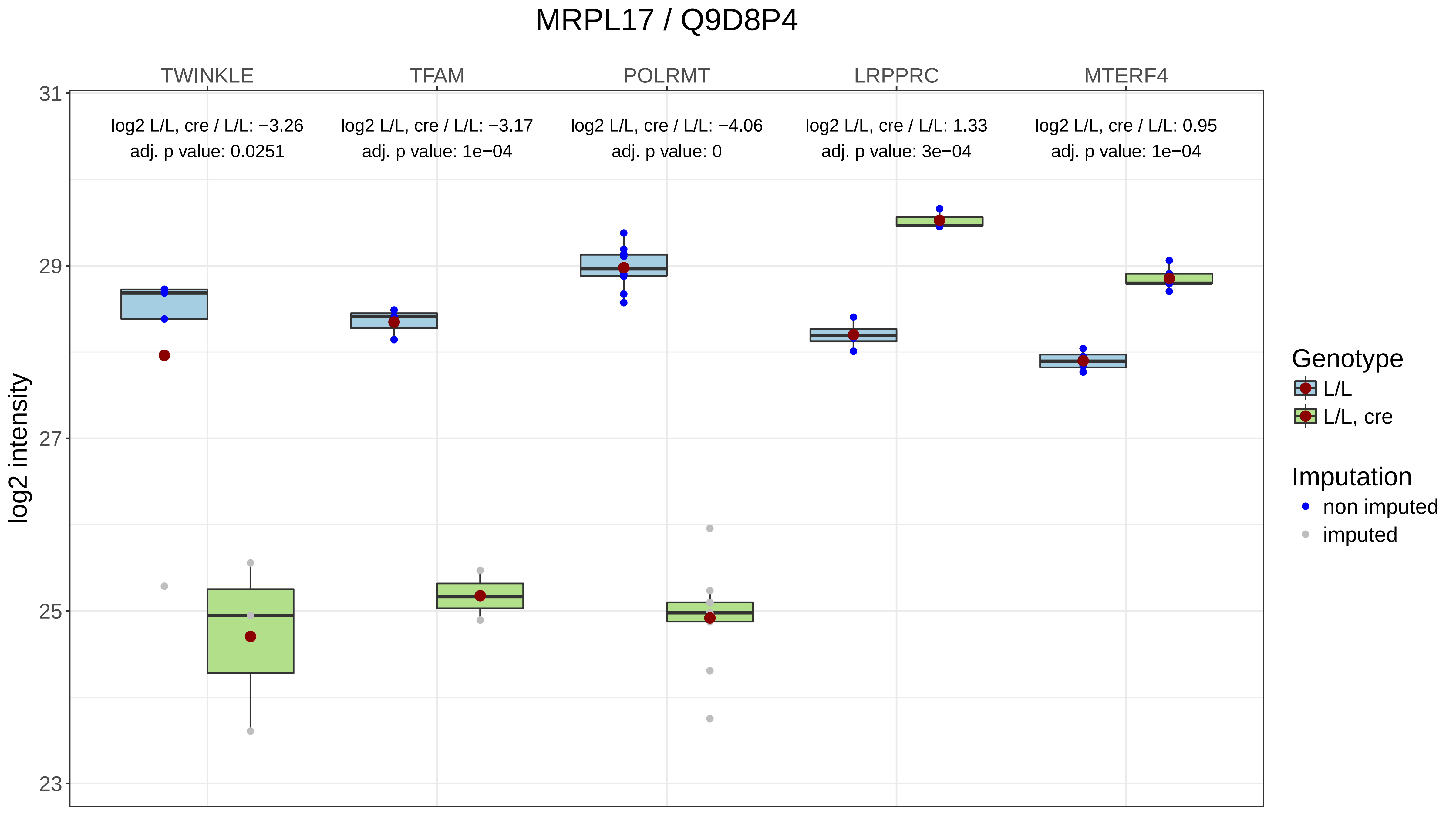

MRPL17 / Q9D8P4
TWINKLE
TFAM
POLRMT
LRPPRC
MTERF4
log2 L/L, cre / L/L: −3.26
adj. p value: 0.0251
log2 L/L, cre / L/L: −3.17
adj. p value: 1e−04
log2 L/L, cre / L/L: −4.06
adj. p value: 0
log2 L/L, cre / L/L: 1.33
adj. p value: 3e−04
log2 L/L, cre / L/L: 0.95
adj. p value: 1e−04
log2 intensity
31
29
27
25
23
Genotype
L/L
L/L, cre
Imputation
non imputed
imputed

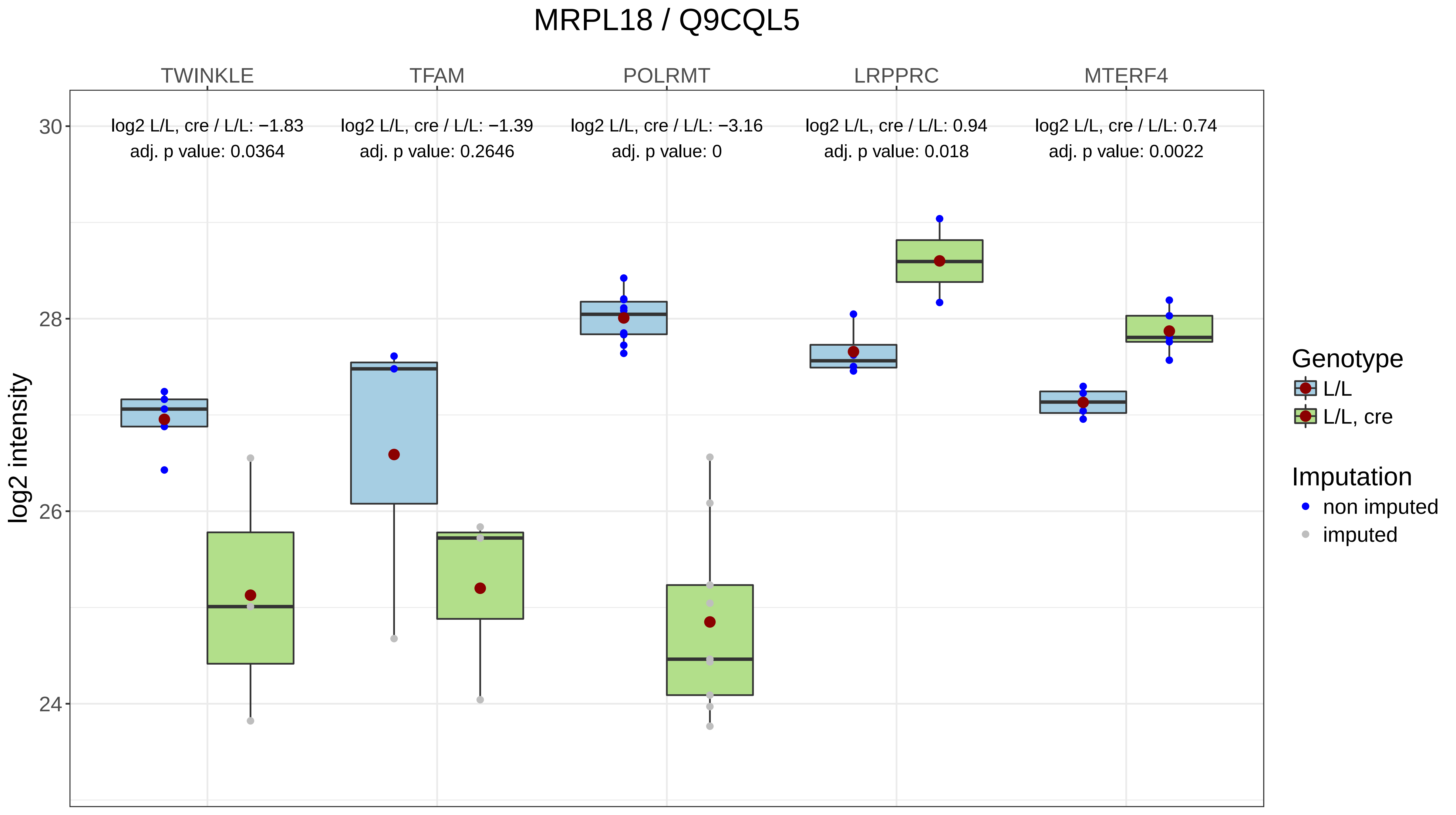

MRPL18 / Q9CQL5
TWINKLE
TFAM
POLRMT
LRPPRC
MTERF4
log2 L/L, cre / L/L: −1.83
adj. p value: 0.0364
log2 L/L, cre / L/L: −1.39
adj. p value: 0.2646
log2 L/L, cre / L/L: −3.16
adj. p value: 0
log2 L/L, cre / L/L: 0.94
adj. p value: 0.018
log2 L/L, cre / L/L: 0.74
adj. p value: 0.0022
30
28
26
24
log2 intensity
Genotype
L/L
L/L, cre
Imputation
non imputed
imputed

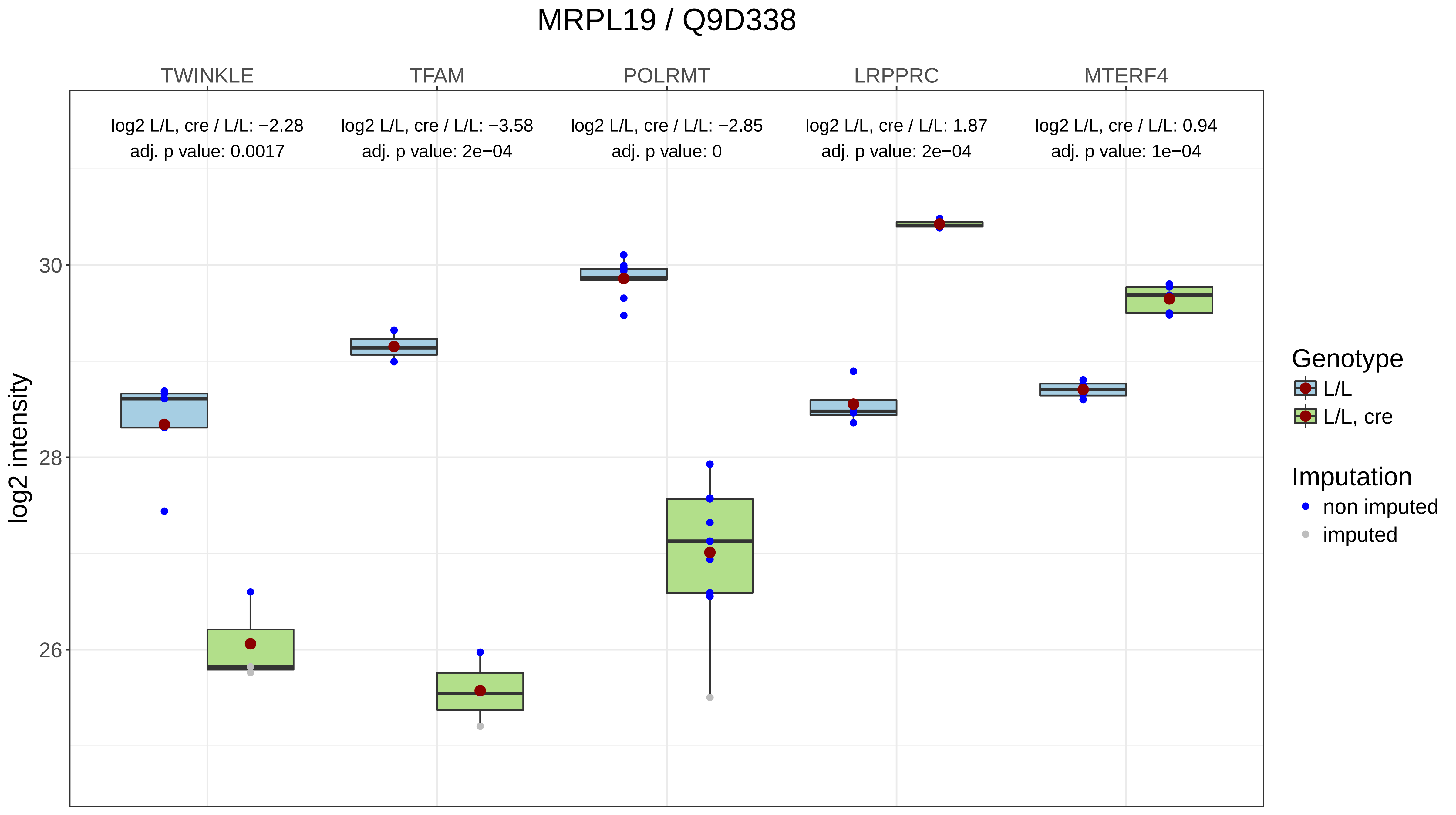
MRPL19 / Q9D338
TWINKLE
TFAM
POLRMT
LRPPRC
MTERF4
log2 L/L, cre / L/L: −2.28
adj. p value: 0.0017
log2 L/L, cre / L/L: −3.58
adj. p value: 2e−04
log2 L/L, cre / L/L: −2.85
adj. p value: 0
log2 L/L, cre / L/L: 1.87
adj. p value: 2e−04
log2 L/L, cre / L/L: 0.94
adj. p value: 1e−04
log2 intensity
30
28
26
Genotype
L/L
L/L, cre
Imputation
non imputed
imputed

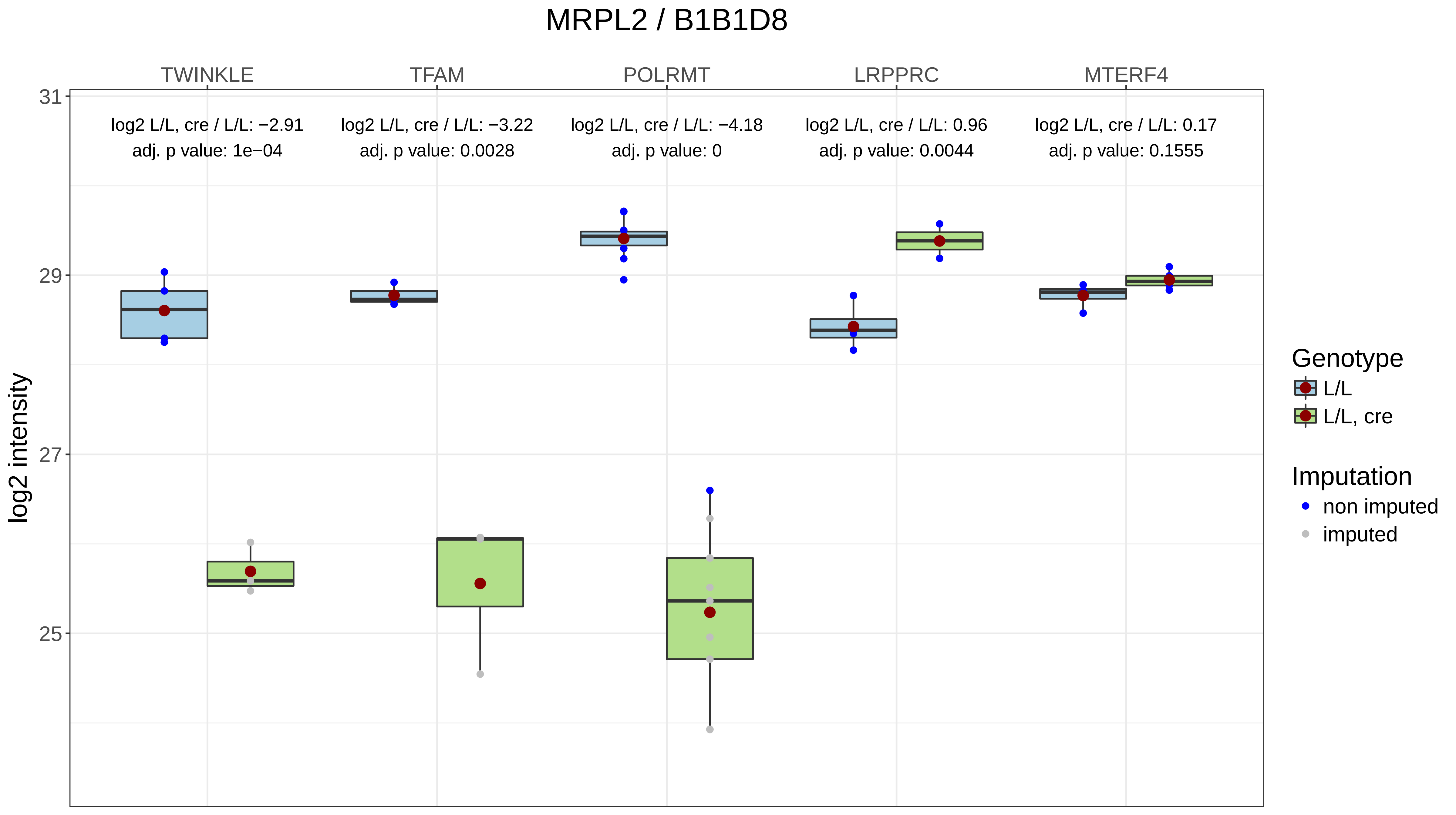
MRPL2 / B1B1D8
TWINKLE
TFAM
POLRMT
LRPPRC
MTERF4
log2 L/L, cre / L/L: −2.91
adj. p value: 1e−04
log2 L/L, cre / L/L: −3.22
adj. p value: 0.0028
log2 L/L, cre / L/L: −4.18
adj. p value: 0
log2 L/L, cre / L/L: 0.96
adj. p value: 0.0044
log2 L/L, cre / L/L: 0.17
adj. p value: 0.1555
31
29
27
25
log2 intensity
Genotype
L/L
L/L, cre
Imputation
non imputed
imputed

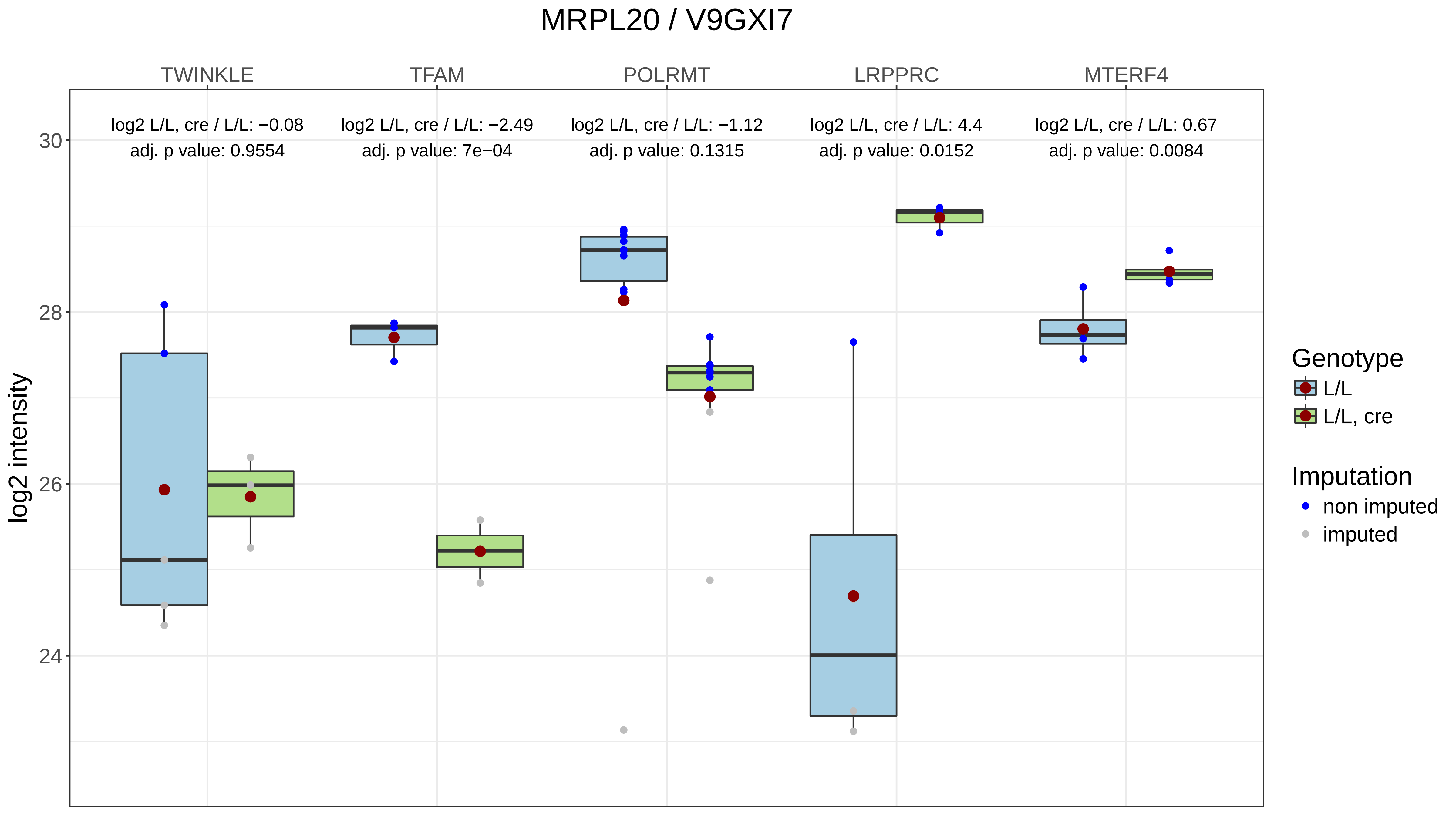
MRPL20 / V9GXI7
TWINKLE
TFAM
POLRMT
LRPPRC
MTERF4
log2 L/L, cre / L/L: −0.08
adj. p value: 0.9554
log2 L/L, cre / L/L: −2.49
adj. p value: 7e−04
log2 L/L, cre / L/L: −1.12
adj. p value: 0.1315
log2 L/L, cre / L/L: 4.4
adj. p value: 0.0152
log2 L/L, cre / L/L: 0.67
adj. p value: 0.0084
log2 intensity
30
28
26
24
Genotype
L/L
L/L, cre
Imputation
non imputed
imputed

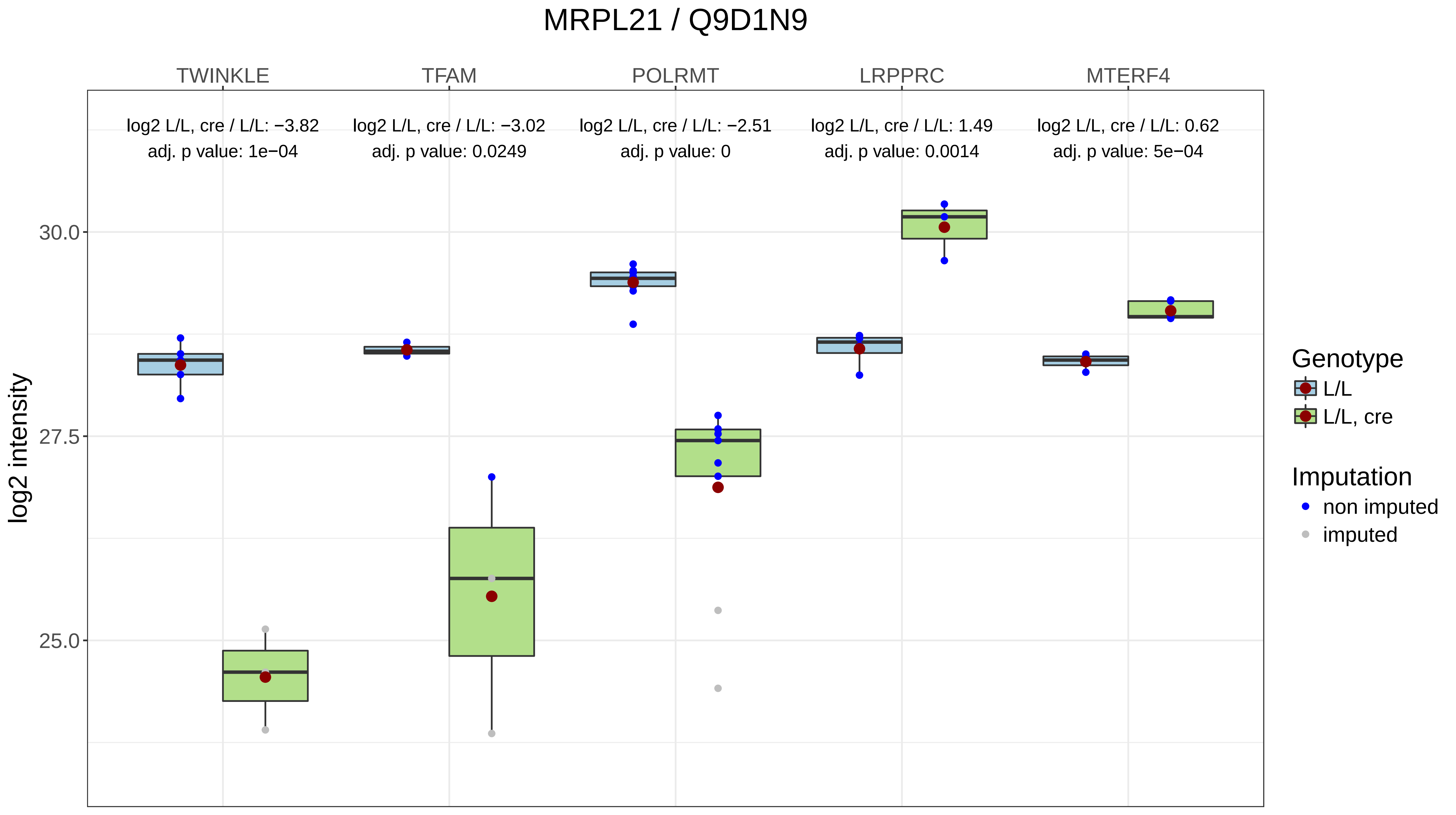
MRPL21 / Q9D1N9
TWINKLE
TFAM
POLRMT
LRPPRC
MTERF4
log2 L/L, cre / L/L: −3.82
adj. p value: 1e−04
log2 L/L, cre / L/L: −3.02
adj. p value: 0.0249
log2 L/L, cre / L/L: −2.51
adj. p value: 0
log2 L/L, cre / L/L: 1.49
adj. p value: 0.0014
log2 L/L, cre / L/L: 0.62
adj. p value: 5e−04
log2 intensity
30.0
27.5
25.0
Genotype
L/L
L/L, cre
Imputation
non imputed
imputed

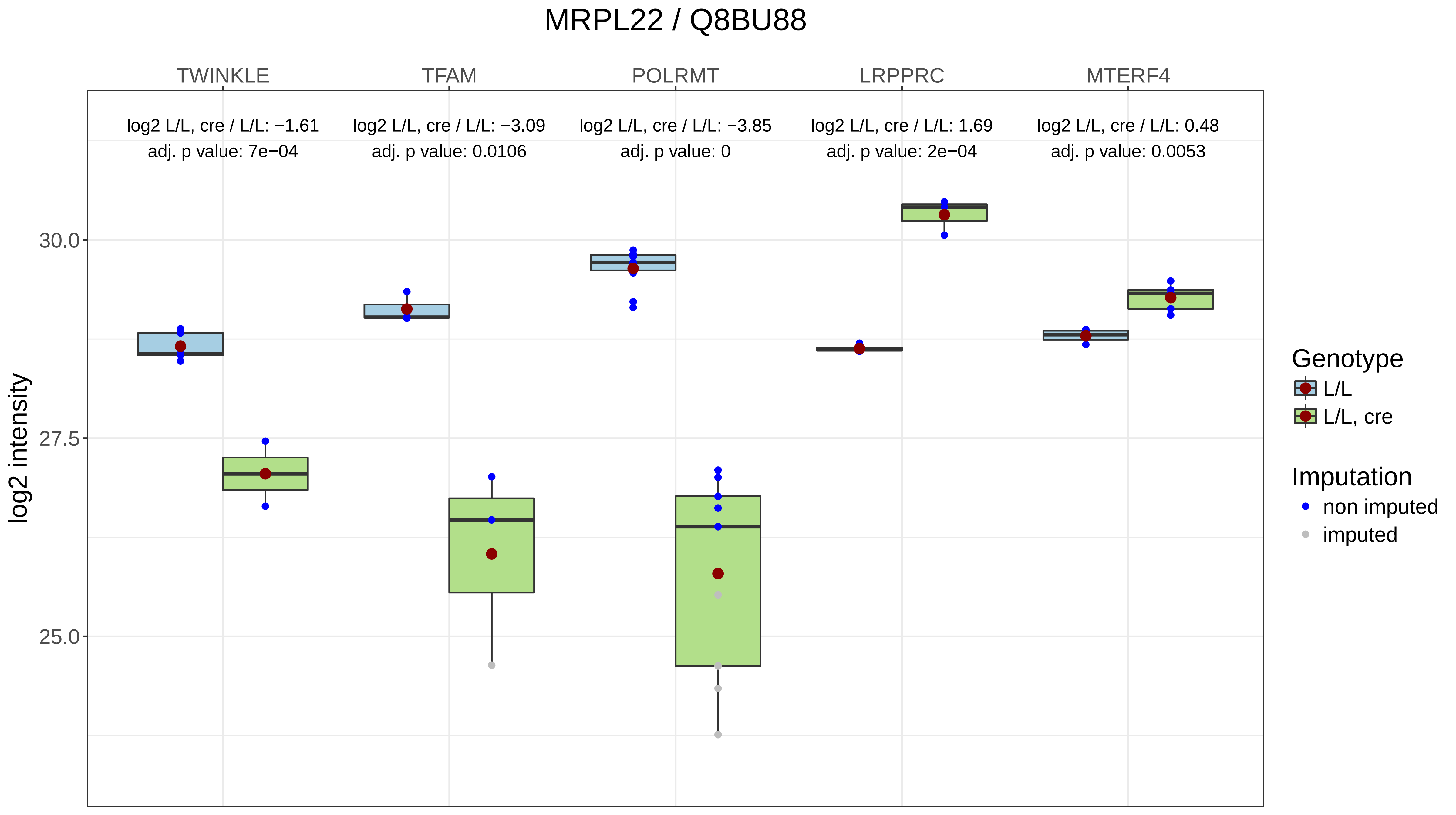
MRPL22 / Q8BU88
TWINKLE
TFAM
POLRMT
LRPPRC
MTERF4
log2 L/L, cre / L/L: −1.61
adj. p value: 7e−04
log2 L/L, cre / L/L: −3.09
adj. p value: 0.0106
log2 L/L, cre / L/L: −3.85
adj. p value: 0
log2 L/L, cre / L/L: 1.69
adj. p value: 2e−04
log2 L/L, cre / L/L: 0.48
adj. p value: 0.0053
log2 intensity
30.0
27.5
25.0
Genotype
L/L
L/L, cre
Imputation
non imputed
imputed

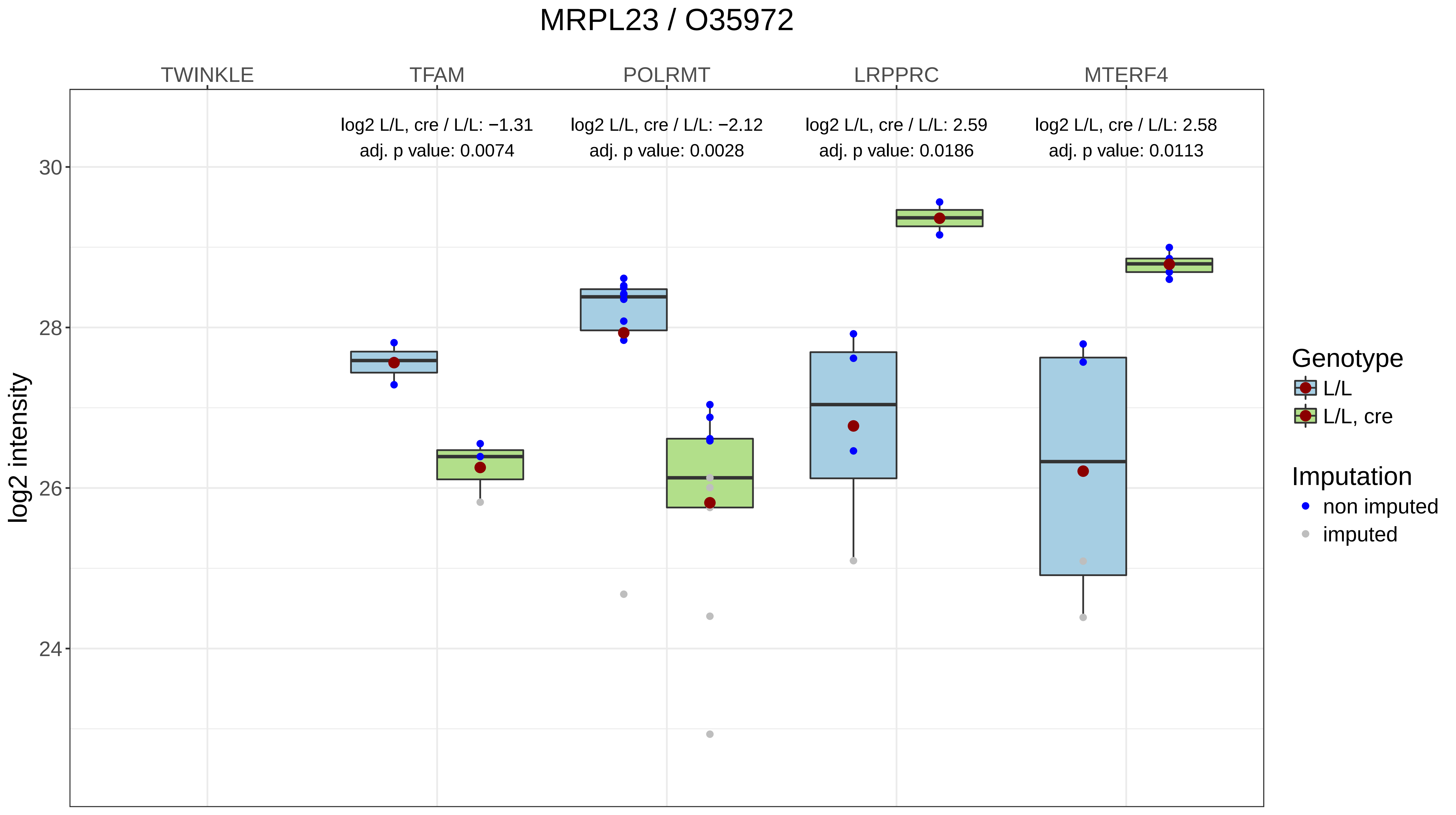

MRPL23 / O35972
TWINKLE
TFAM
POLRMT
LRPPRC
MTERF4
log2 L/L, cre / L/L: −1.31
adj. p value: 0.0074
log2 L/L, cre / L/L: −2.12
adj. p value: 0.0028
log2 L/L, cre / L/L: 2.59
adj. p value: 0.0186
log2 L/L, cre / L/L: 2.58
adj. p value: 0.0113
30
28
26
24
log2 intensity
Genotype
L/L
L/L, cre
Imputation
non imputed
imputed

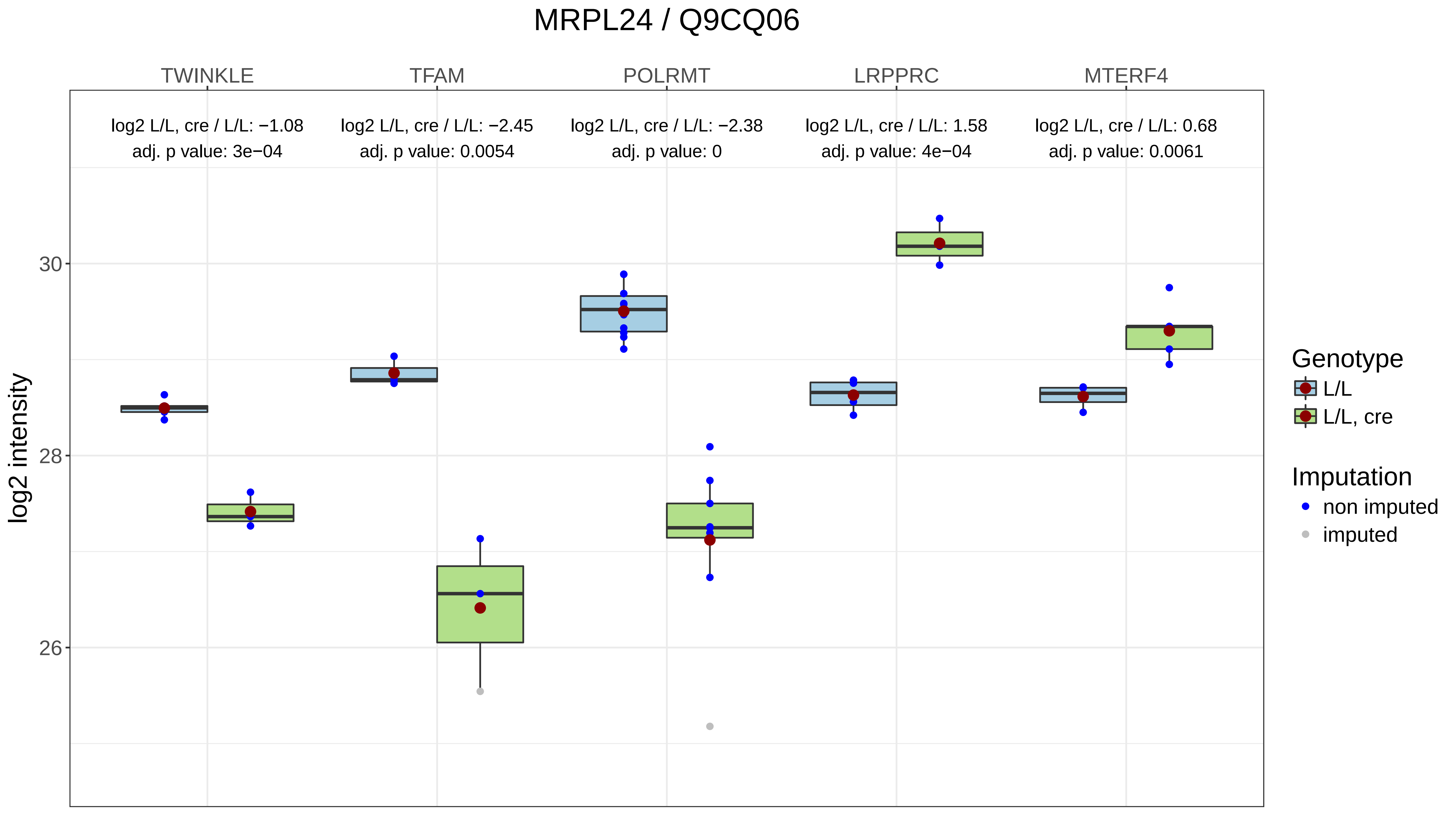
MRPL24 / Q9CQ06
TWINKLE
TFAM
POLRMT
LRPPRC
MTERF4
log2 L/L, cre / L/L: −1.08
adj. p value: 3e−04
log2 L/L, cre / L/L: −2.45
adj. p value: 0.0054
log2 L/L, cre / L/L: −2.38
adj. p value: 0
log2 L/L, cre / L/L: 1.58
adj. p value: 4e−04
log2 L/L, cre / L/L: 0.68
adj. p value: 0.0061
log2 intensity
30
28
26
Genotype
L/L
L/L, cre
Imputation
non imputed
imputed

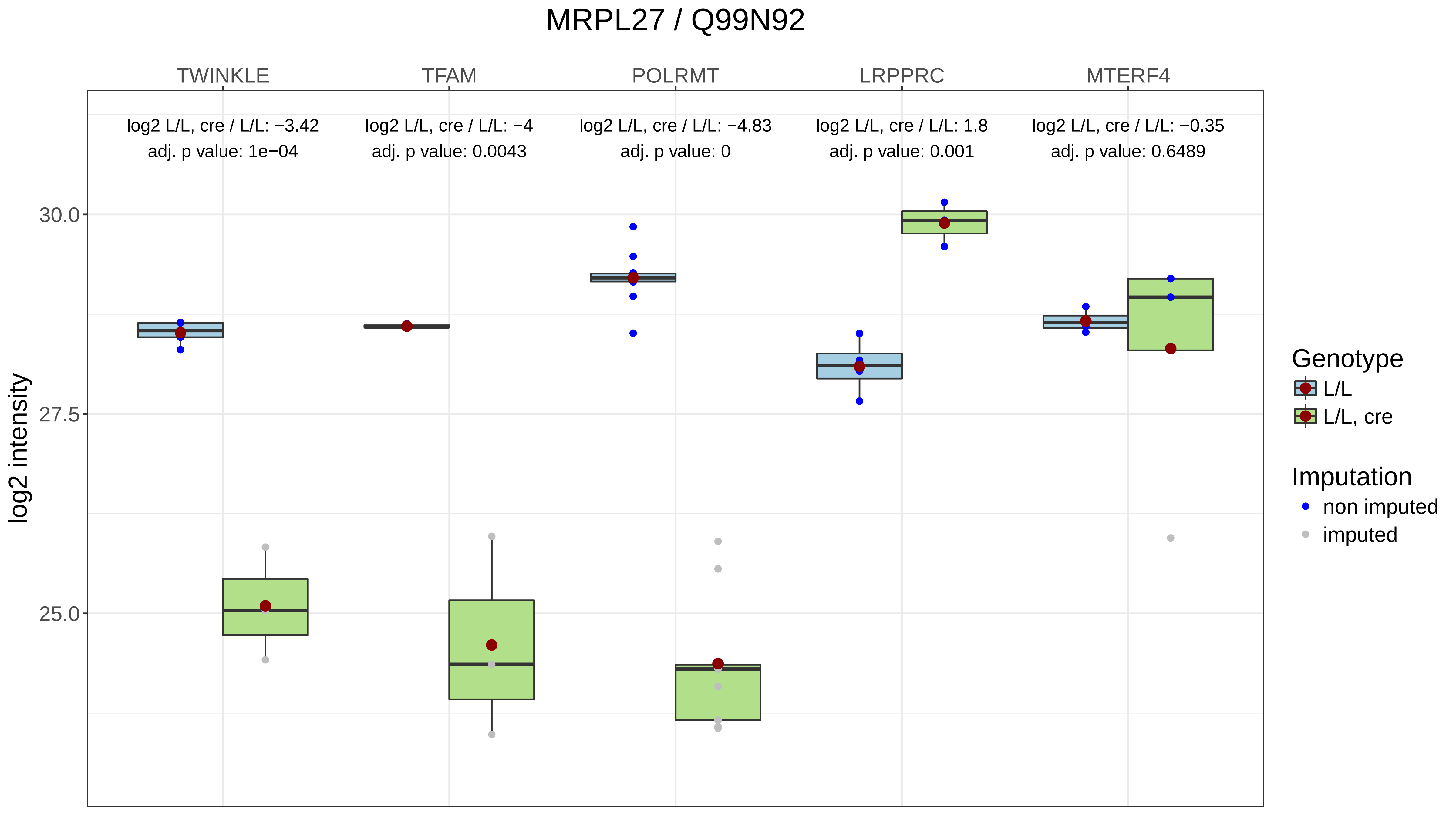
MRPL27 / Q99N92
TWINKLE
TFAM
POLRMT
LRPPRC
MTERF4
log2 L/L, cre / L/L: −3.42
adj. p value: 1e−04
log2 L/L, cre / L/L: −4
adj. p value: 0.0043
log2 L/L, cre / L/L: −4.83
adj. p value: 0
log2 L/L, cre / L/L: 1.8
adj. p value: 0.001
log2 L/L, cre / L/L: −0.35
adj. p value: 0.6489
30.0
27.5
25.0
log2 intensity
Genotype
L/L
L/L, cre
Imputation
non imputed
imputed

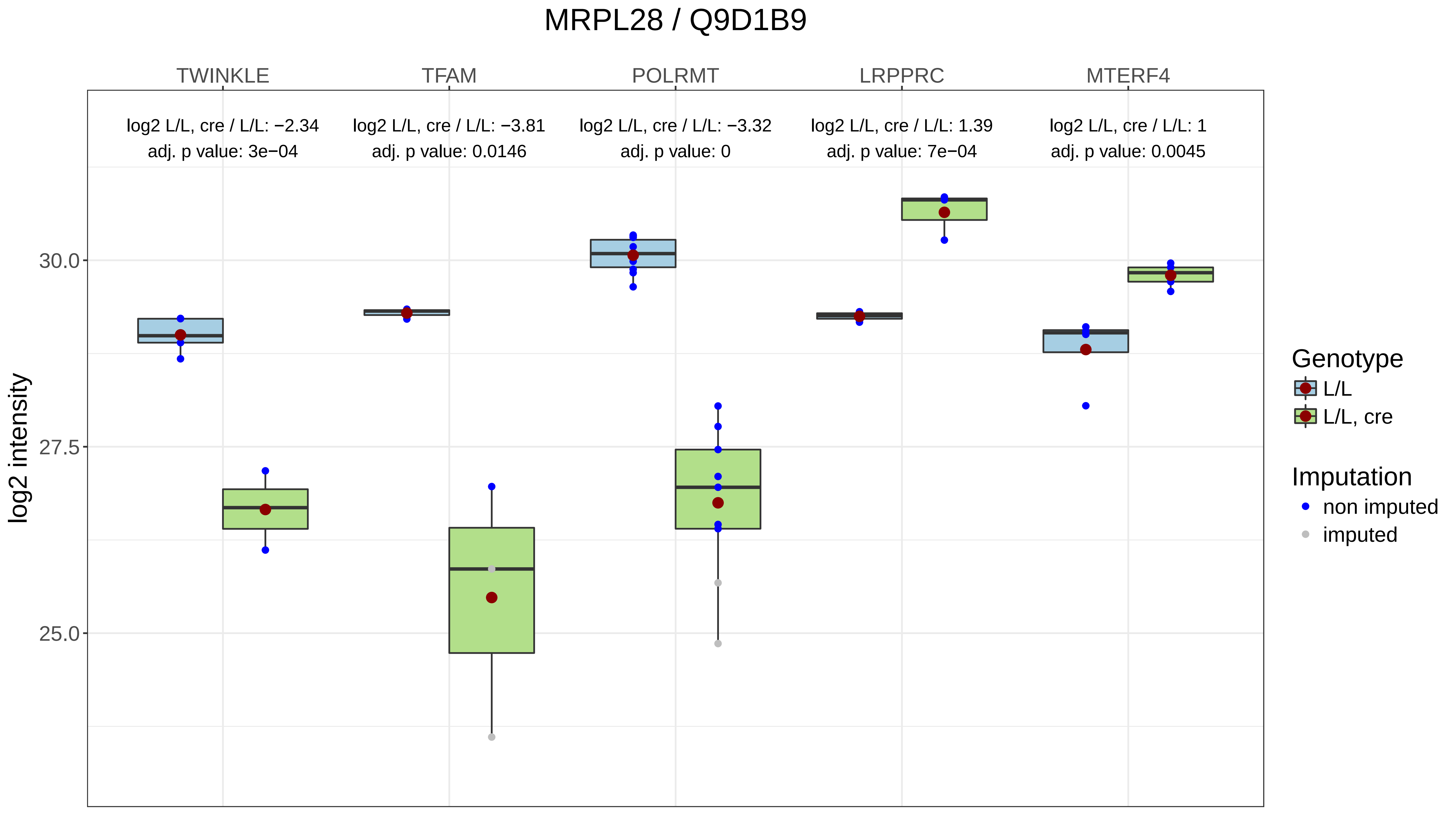
MRPL28 / Q9D1B9
TWINKLE
TFAM
POLRMT
LRPPRC
MTERF4
log2 L/L, cre / L/L: −2.34
adj. p value: 3e−04
log2 L/L, cre / L/L: −3.81
adj. p value: 0.0146
log2 L/L, cre / L/L: −3.32
adj. p value: 0
log2 L/L, cre / L/L: 1.39
adj. p value: 7e−04
log2 L/L, cre / L/L: 1
adj. p value: 0.0045
log2 intensity
30.0
27.5
25.0
Genotype
L/L
L/L, cre
Imputation
non imputed
imputed

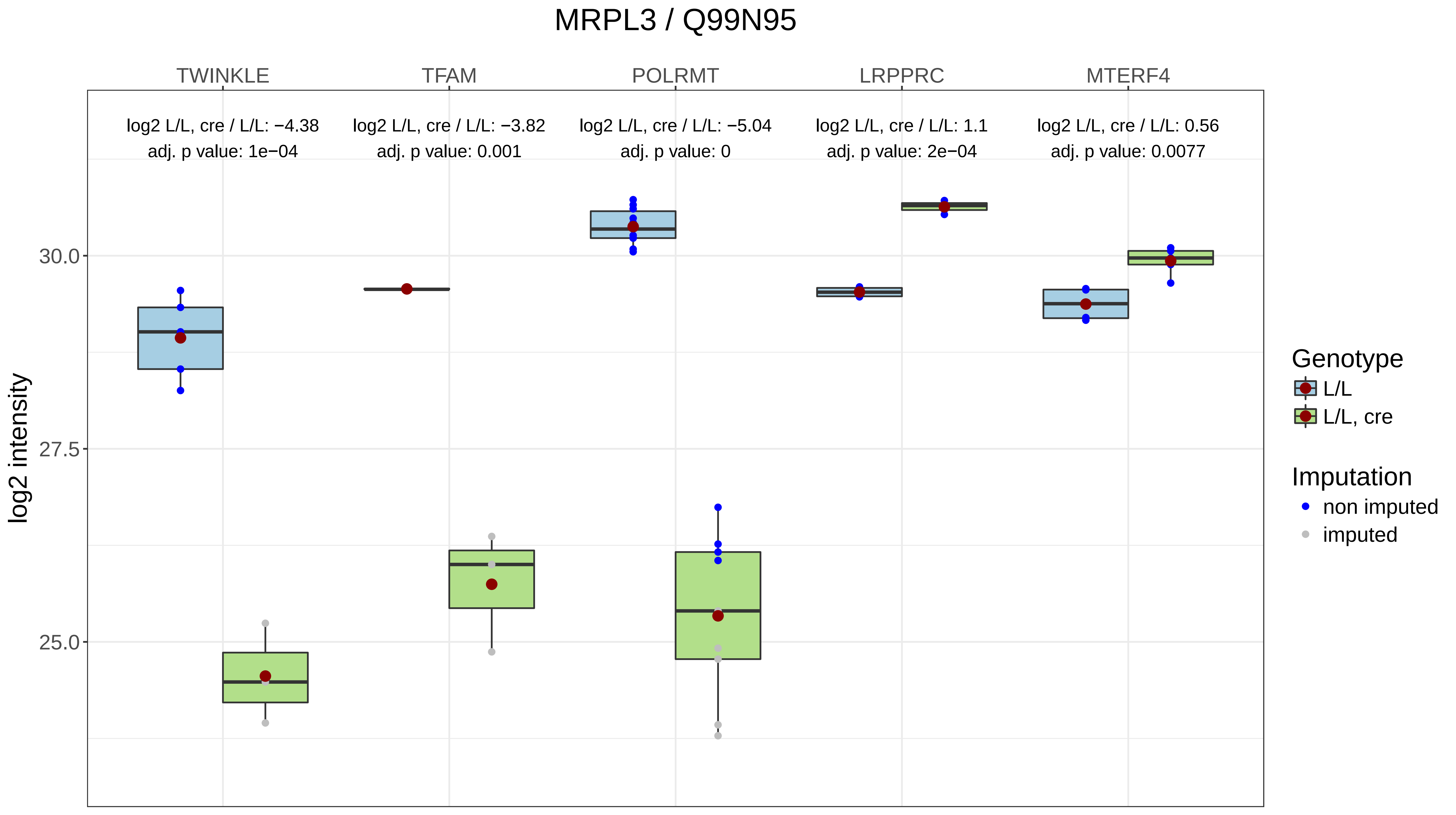
MRPL3 / Q99N95
TWINKLE
TFAM
POLRMT
LRPPRC
MTERF4
log2 L/L, cre / L/L: −4.38
adj. p value: 1e−04
log2 L/L, cre / L/L: −3.82
adj. p value: 0.001
log2 L/L, cre / L/L: −5.04
adj. p value: 0
log2 L/L, cre / L/L: 1.1
adj. p value: 2e−04
log2 L/L, cre / L/L: 0.56
adj. p value: 0.0077
log2 intensity
30.0
27.5
25.0
Genotype
L/L
L/L, cre
Imputation
non imputed
imputed

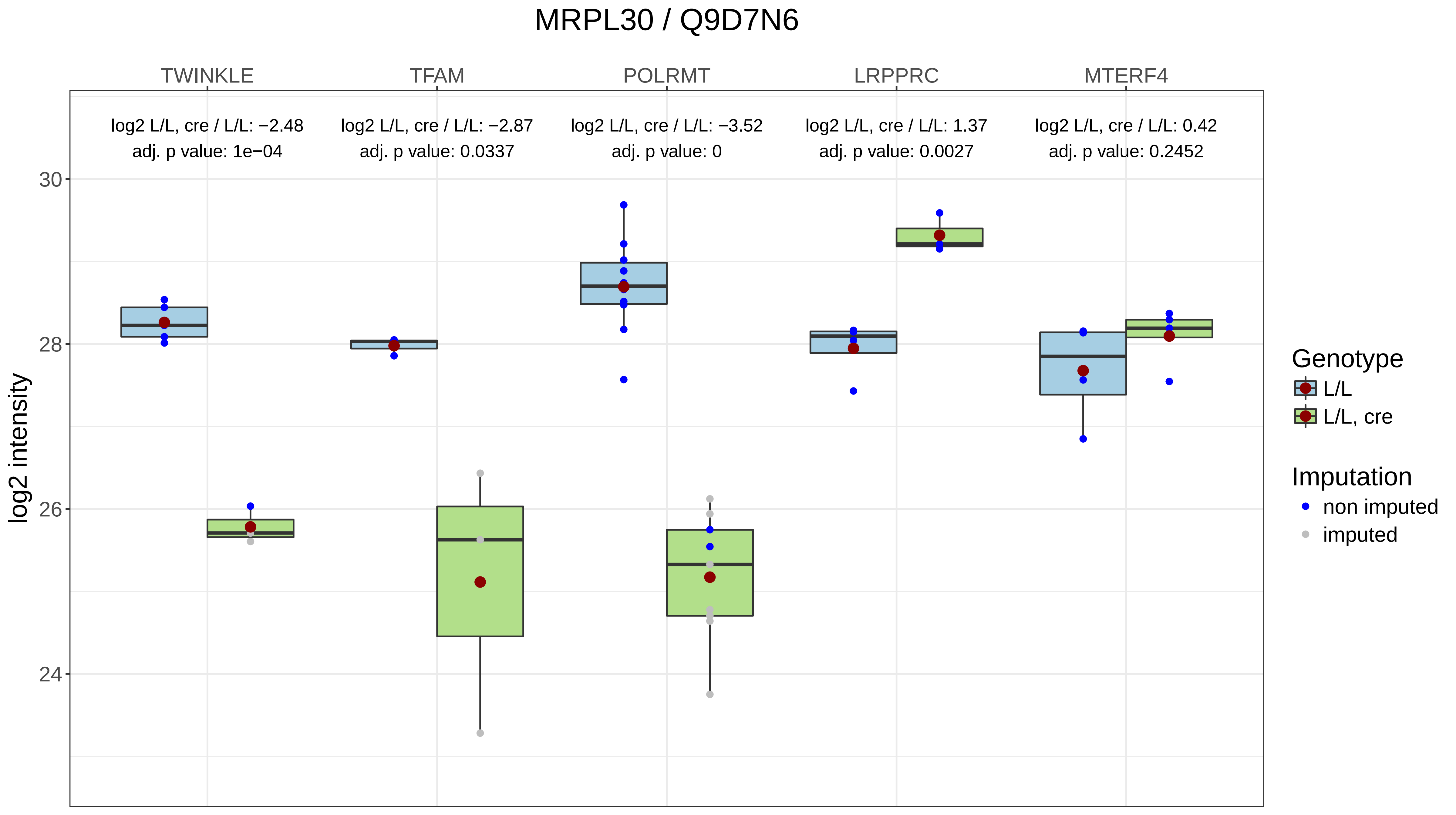

MRPL30 / Q9D7N6
TWINKLE
TFAM
POLRMT
LRPPRC
MTERF4
log2 L/L, cre / L/L: −2.48
adj. p value: 1e−04
log2 L/L, cre / L/L: −2.87
adj. p value: 0.0337
log2 L/L, cre / L/L: −3.52
adj. p value: 0
log2 L/L, cre / L/L: 1.37
adj. p value: 0.0027
log2 L/L, cre / L/L: 0.42
adj. p value: 0.2452
log2 intensity
30
28
26
24
Genotype
L/L
L/L, cre
Imputation
non imputed
imputed

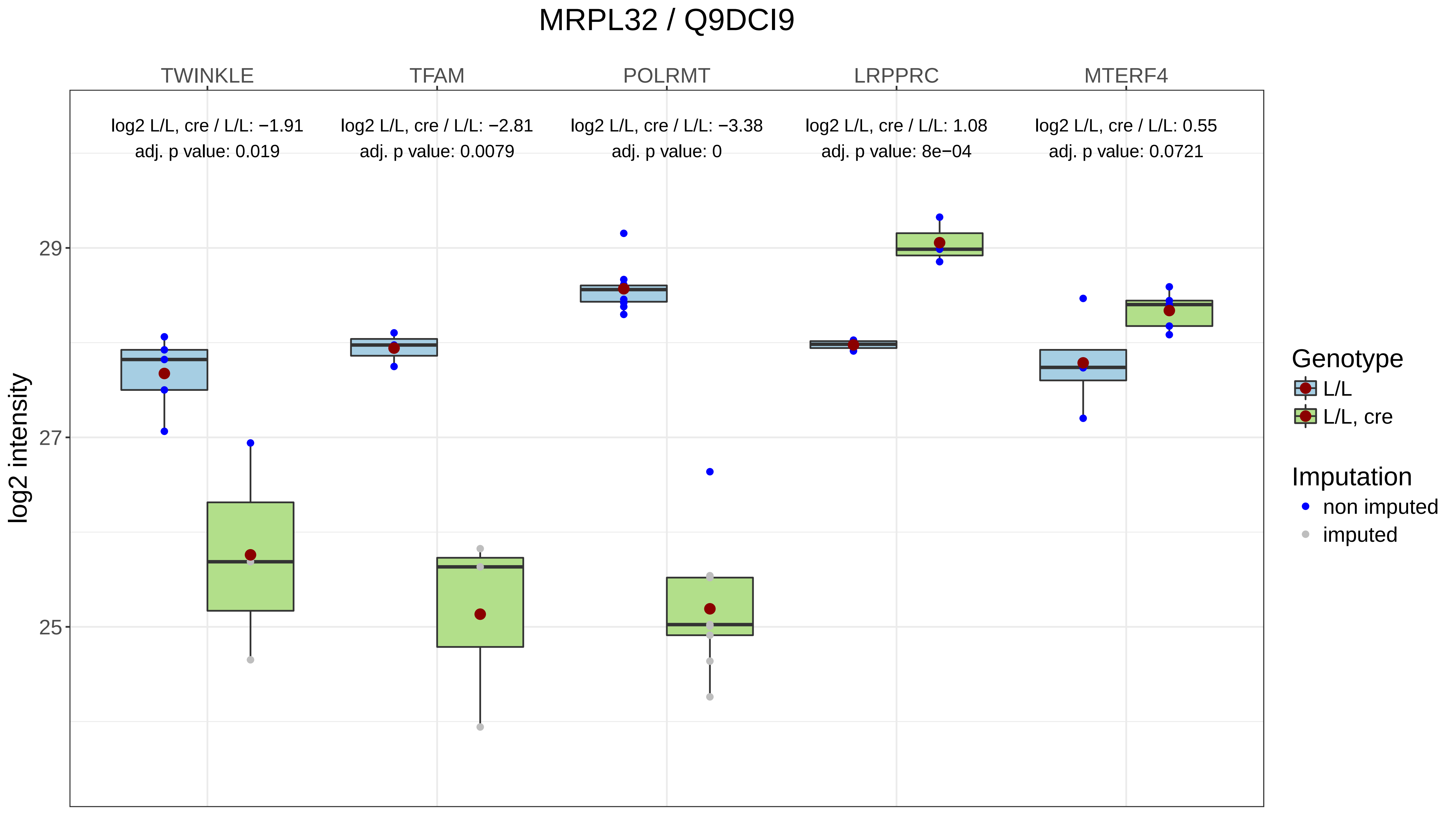

MRPL32 / Q9DCI9
TWINKLE
TFAM
POLRMT
LRPPRC
MTERF4
log2 L/L, cre / L/L: −1.91
adj. p value: 0.019
log2 L/L, cre / L/L: −2.81
adj. p value: 0.0079
log2 L/L, cre / L/L: −3.38
adj. p value: 0
log2 L/L, cre / L/L: 1.08
adj. p value: 8e−04
log2 L/L, cre / L/L: 0.55
adj. p value: 0.0721
log2 intensity
29
27
25
Genotype
L/L
L/L, cre
Imputation
non imputed
imputed

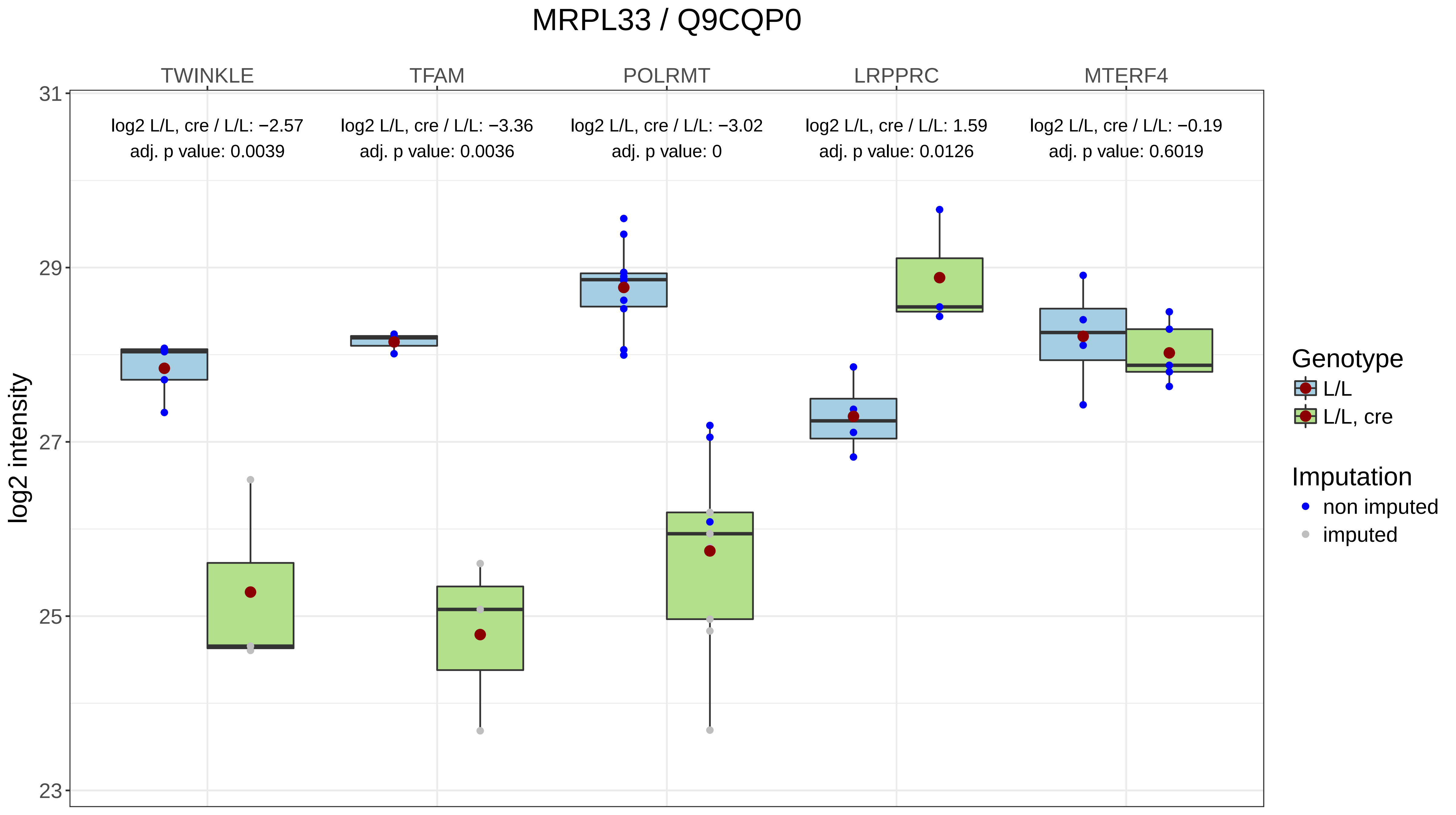
MRPL33 / Q9CQP0
TWINKLE
TFAM
POLRMT
LRPPRC
MTERF4
log2 L/L, cre / L/L: −2.57
adj. p value: 0.0039
log2 L/L, cre / L/L: −3.36
adj. p value: 0.0036
log2 L/L, cre / L/L: −3.02
adj. p value: 0
log2 L/L, cre / L/L: 1.59
adj. p value: 0.0126
log2 L/L, cre / L/L: −0.19
adj. p value: 0.6019
log2 intensity
31
29
27
25
23
Genotype
L/L
L/L, cre
Imputation
non imputed
imputed

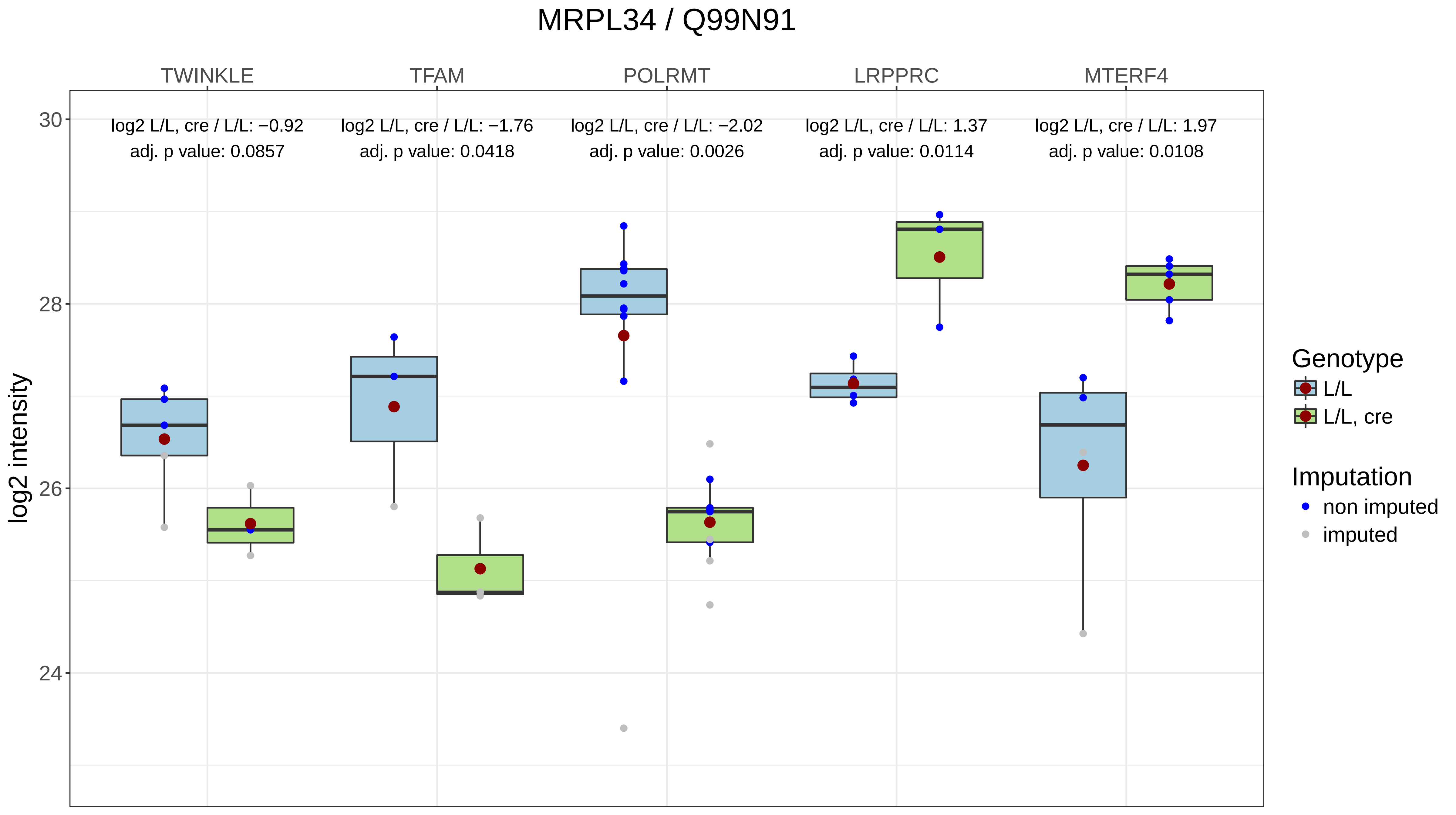
MRPL34 / Q99N91
TWINKLE
TFAM
POLRMT
LRPPRC
MTERF4
log2 L/L, cre / L/L: −0.92
adj. p value: 0.0857
log2 L/L, cre / L/L: −1.76
adj. p value: 0.0418
log2 L/L, cre / L/L: −2.02
adj. p value: 0.0026
log2 L/L, cre / L/L: 1.37
adj. p value: 0.0114
log2 L/L, cre / L/L: 1.97
adj. p value: 0.0108
log2 intensity
30
28
26
24
Genotype
L/L
L/L, cre
Imputation
non imputed
imputed

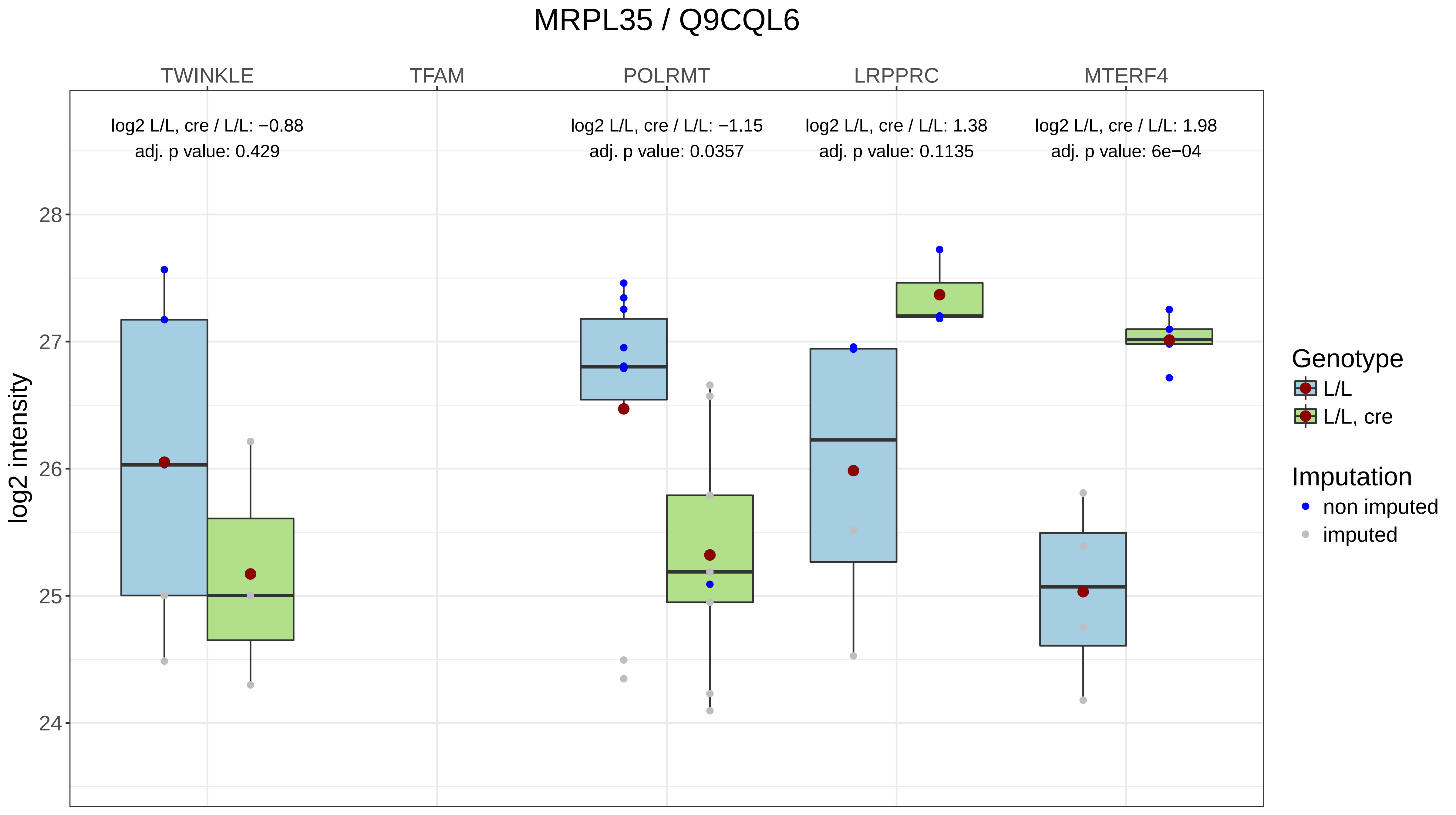
MRPL35 / Q9CQL6
TWINKLE
TFAM
POLRMT
LRPPRC
MTERF4
log2 L/L, cre / L/L: −0.88
adj. p value: 0.429
log2 L/L, cre / L/L: −1.15
adj. p value: 0.0357
log2 L/L, cre / L/L: 1.38
adj. p value: 0.1135
log2 L/L, cre / L/L: 1.98
adj. p value: 6e−04
log2 intensity
28
27
26
25
24
Genotype
L/L
L/L, cre
Imputation
non imputed
imputed

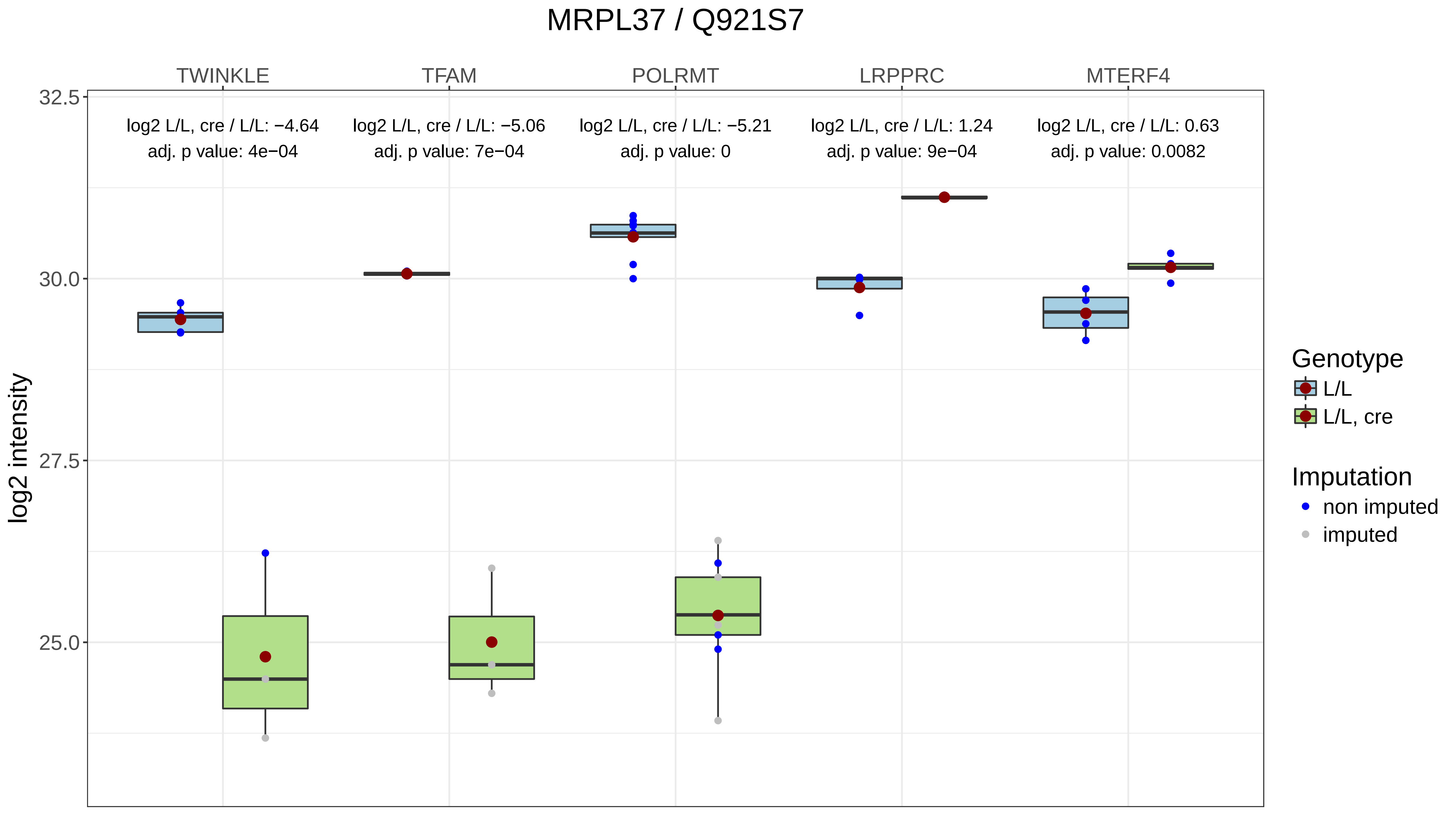

MRPL37 / Q921S7
TWINKLE
TFAM
POLRMT
LRPPRC
MTERF4
log2 L/L, cre / L/L: −4.64
adj. p value: 4e−04
log2 L/L, cre / L/L: −5.06
adj. p value: 7e−04
log2 L/L, cre / L/L: −5.21
adj. p value: 0
log2 L/L, cre / L/L: 1.24
adj. p value: 9e−04
log2 L/L, cre / L/L: 0.63
adj. p value: 0.0082
32.5
30.0
27.5
25.0
log2 intensity
Genotype
L/L
L/L, cre
Imputation
non imputed
imputed

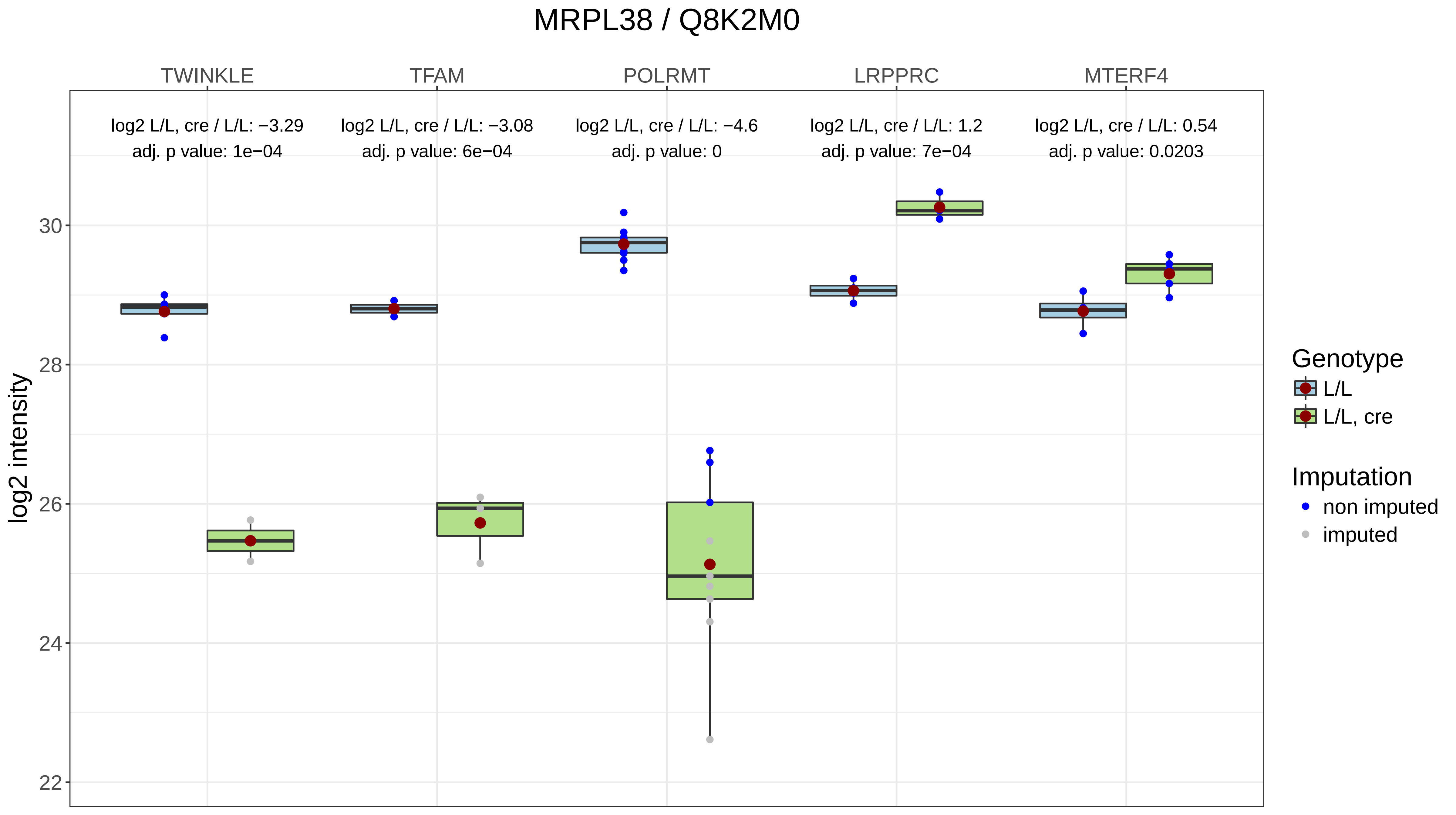

MRPL38 / Q8K2M0
TWINKLE
TFAM
POLRMT
LRPPRC
MTERF4
log2 L/L, cre / L/L: −3.29
adj. p value: 1e−04
log2 L/L, cre / L/L: −3.08
adj. p value: 6e−04
log2 L/L, cre / L/L: −4.6
adj. p value: 0
log2 L/L, cre / L/L: 1.2
adj. p value: 7e−04
log2 L/L, cre / L/L: 0.54
adj. p value: 0.0203
log2 intensity
30
28
26
24
22
Genotype
L/L
L/L, cre
Imputation
non imputed
imputed

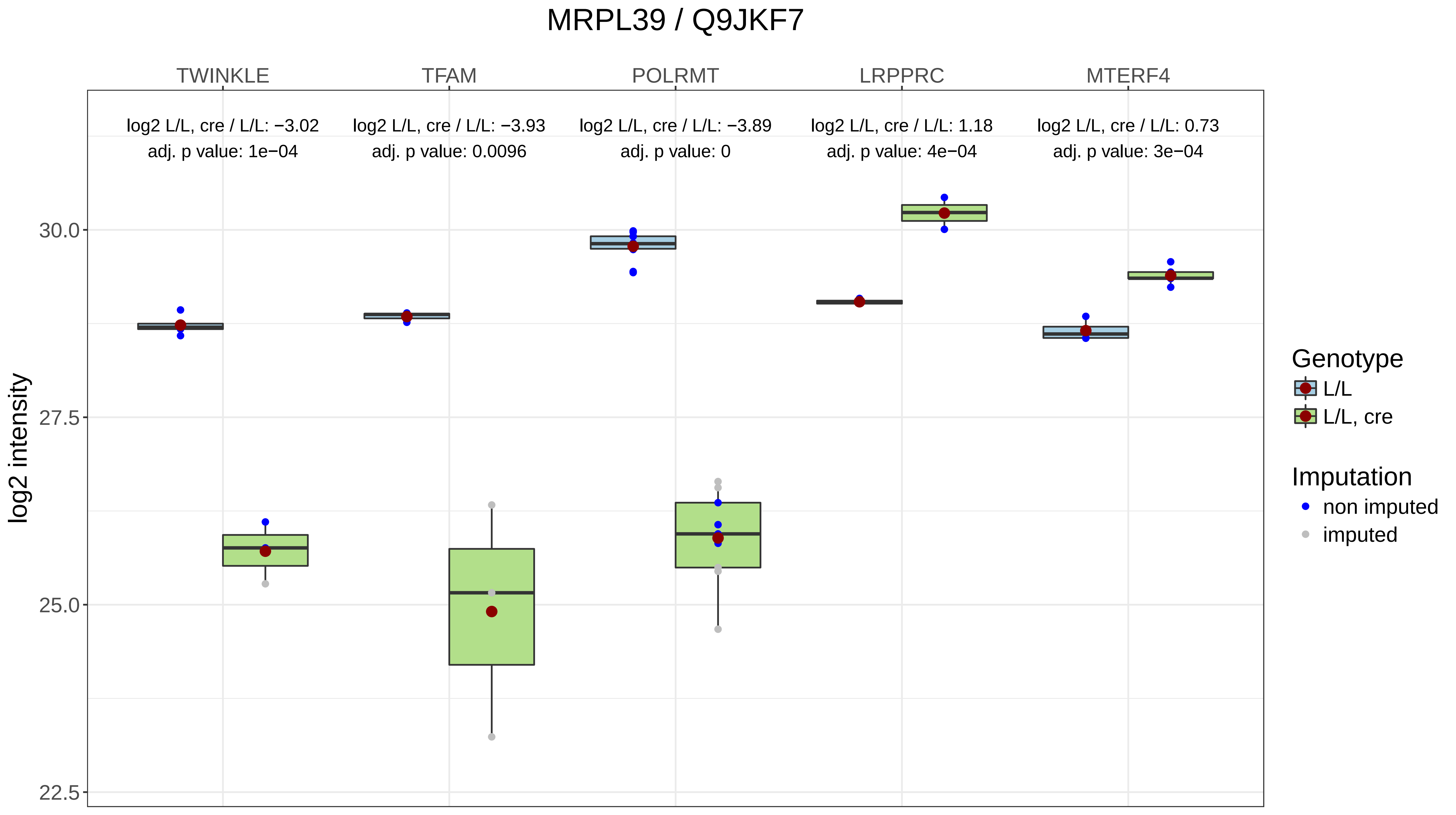
MRPL39 / Q9JKF7
TWINKLE
TFAM
POLRMT
LRPPRC
MTERF4
log2 L/L, cre / L/L: −3.02
adj. p value: 1e−04
log2 L/L, cre / L/L: −3.93
adj. p value: 0.0096
log2 L/L, cre / L/L: −3.89
adj. p value: 0
log2 L/L, cre / L/L: 1.18
adj. p value: 4e−04
log2 L/L, cre / L/L: 0.73
adj. p value: 3e−04
log2 intensity
30.0
27.5
25.0
22.5
Genotype
L/L
L/L, cre
Imputation
non imputed
imputed

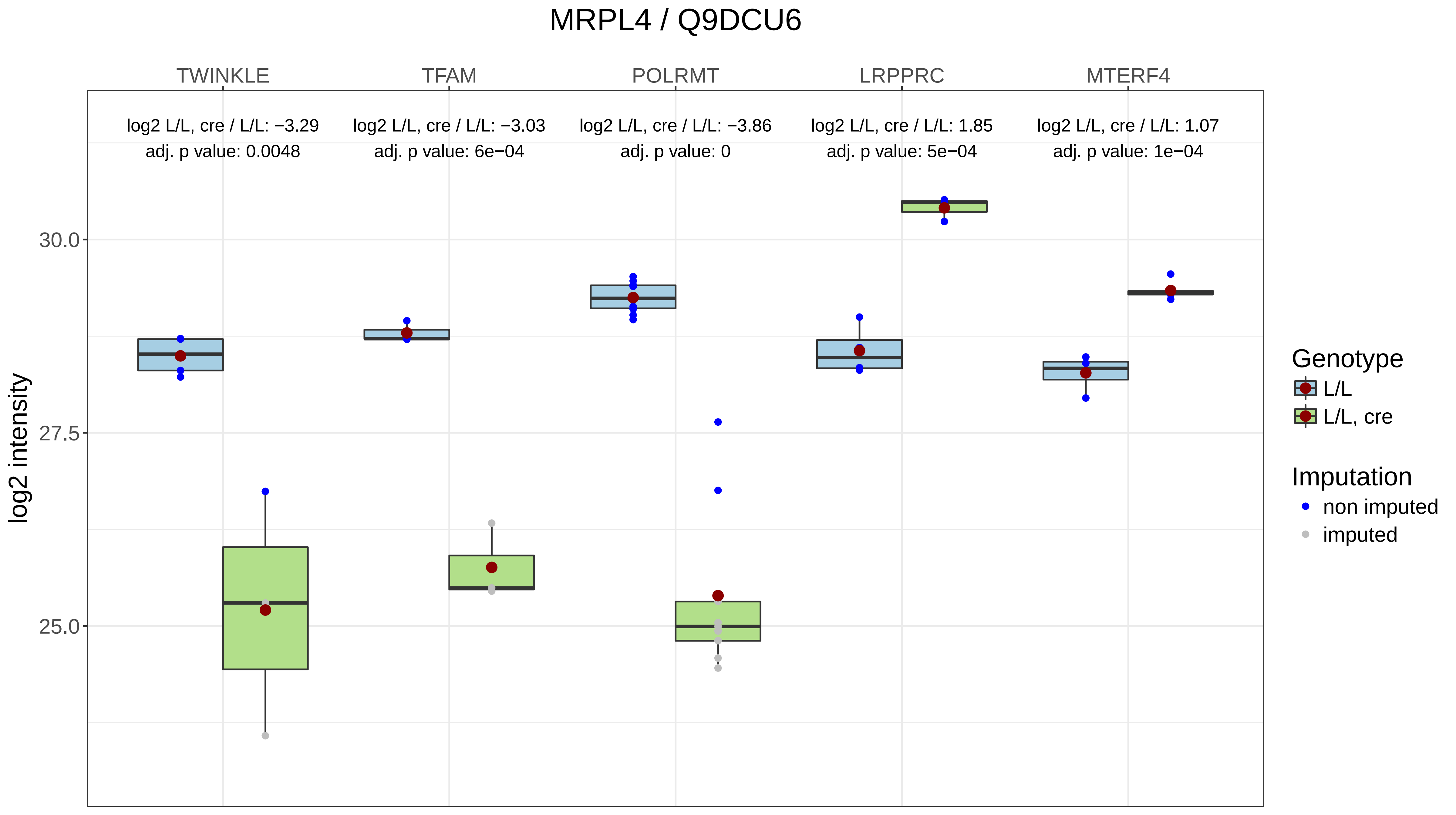

MRPL4 / Q9DCU6
TWINKLE
TFAM
POLRMT
LRPPRC
MTERF4
log2 L/L, cre / L/L: −3.29
adj. p value: 0.0048
log2 L/L, cre / L/L: −3.03
adj. p value: 6e−04
log2 L/L, cre / L/L: −3.86
adj. p value: 0
log2 L/L, cre / L/L: 1.85
adj. p value: 5e−04
log2 L/L, cre / L/L: 1.07
adj. p value: 1e−04
30.0
27.5
25.0
log2 intensity
Genotype
L/L
L/L, cre
Imputation
non imputed
imputed

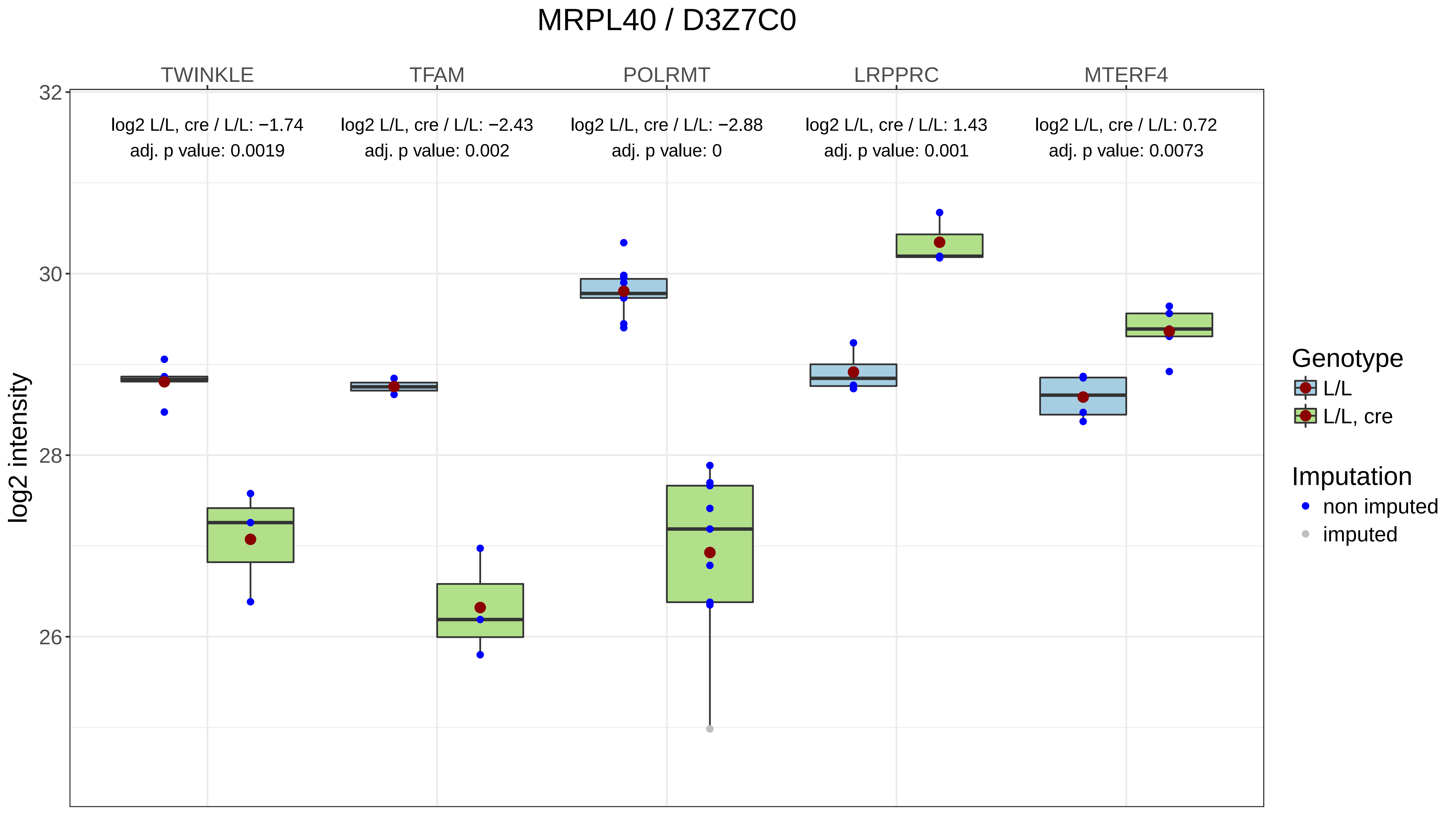
MRPL40 / D3Z7C0
TWINKLE
TFAM
POLRMT
LRPPRC
MTERF4
log2 L/L, cre / L/L: −1.74
adj. p value: 0.0019
log2 L/L, cre / L/L: −2.43
adj. p value: 0.002
log2 L/L, cre / L/L: −2.88
adj. p value: 0
log2 L/L, cre / L/L: 1.43
adj. p value: 0.001
log2 L/L, cre / L/L: 0.72
adj. p value: 0.0073
log2 intensity
32
30
28
26
Genotype
L/L
L/L, cre
Imputation
non imputed
imputed

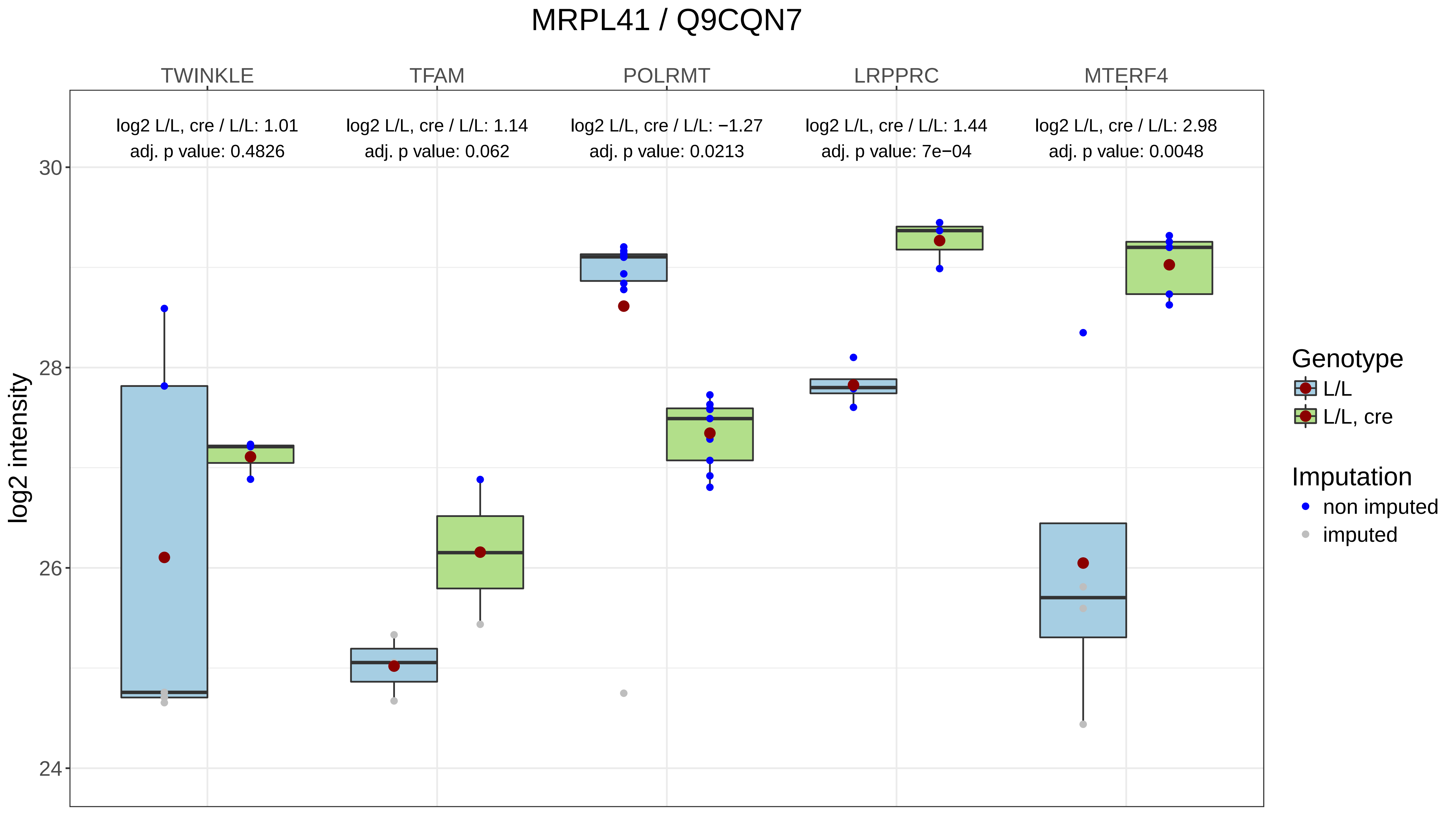

MRPL41 / Q9CQN7
TWINKLE
TFAM
POLRMT
LRPPRC
MTERF4
log2 L/L, cre / L/L: 1.01
adj. p value: 0.4826
log2 L/L, cre / L/L: 1.14
adj. p value: 0.062
log2 L/L, cre / L/L: −1.27
adj. p value: 0.0213
log2 L/L, cre / L/L: 1.44
adj. p value: 7e−04
log2 L/L, cre / L/L: 2.98
adj. p value: 0.0048
log2 intensity
30
28
26
24
Genotype
L/L
L/L, cre
Imputation
non imputed
imputed

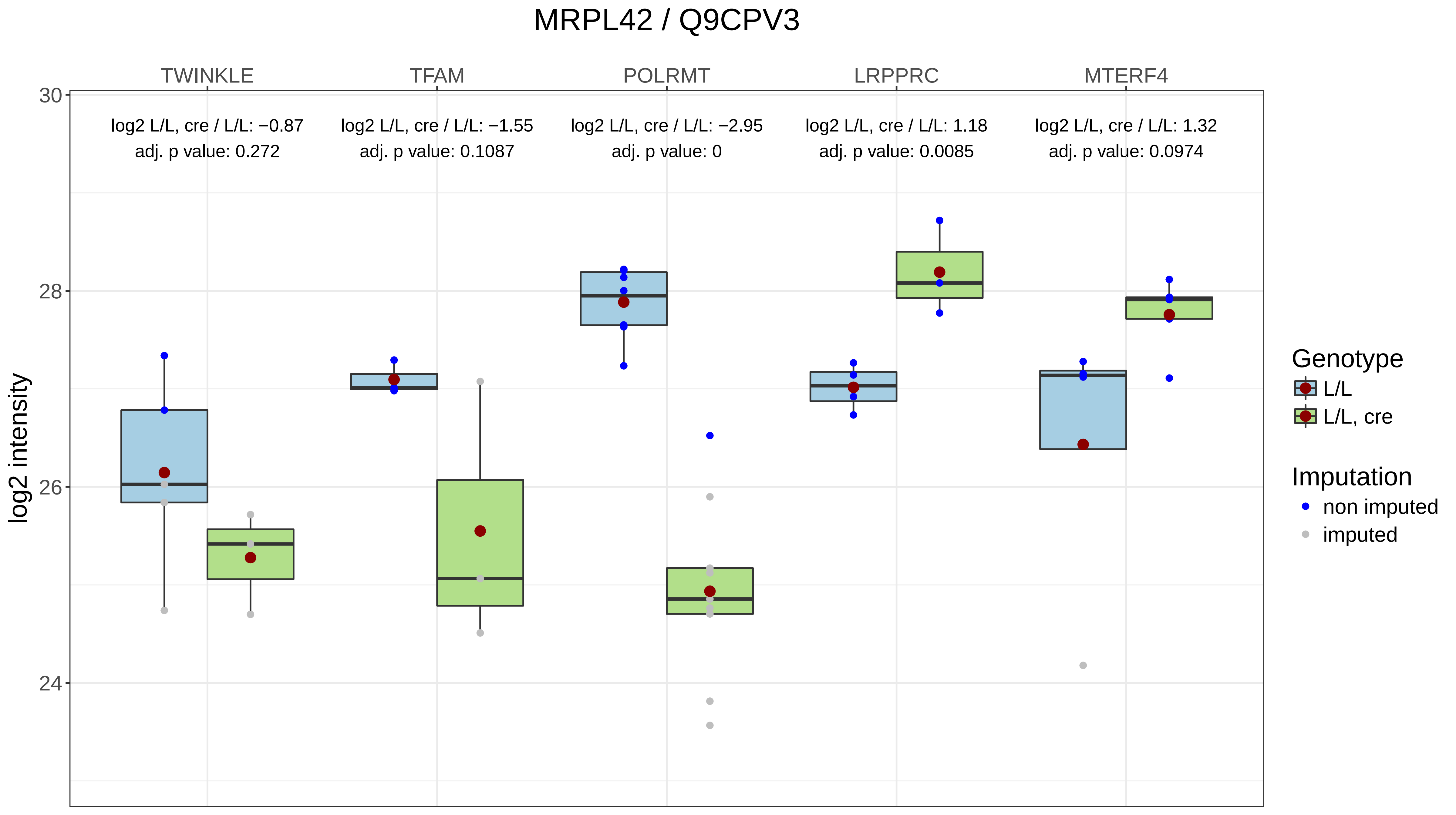
MRPL42 / Q9CPV3
TWINKLE
TFAM
POLRMT
LRPPRC
MTERF4
log2 L/L, cre / L/L: −0.87
adj. p value: 0.272
log2 L/L, cre / L/L: −1.55
adj. p value: 0.1087
log2 L/L, cre / L/L: −2.95
adj. p value: 0
log2 L/L, cre / L/L: 1.18
adj. p value: 0.0085
log2 L/L, cre / L/L: 1.32
adj. p value: 0.0974
log2 intensity
30
28
26
24
Genotype
L/L
L/L, cre
Imputation
non imputed
imputed

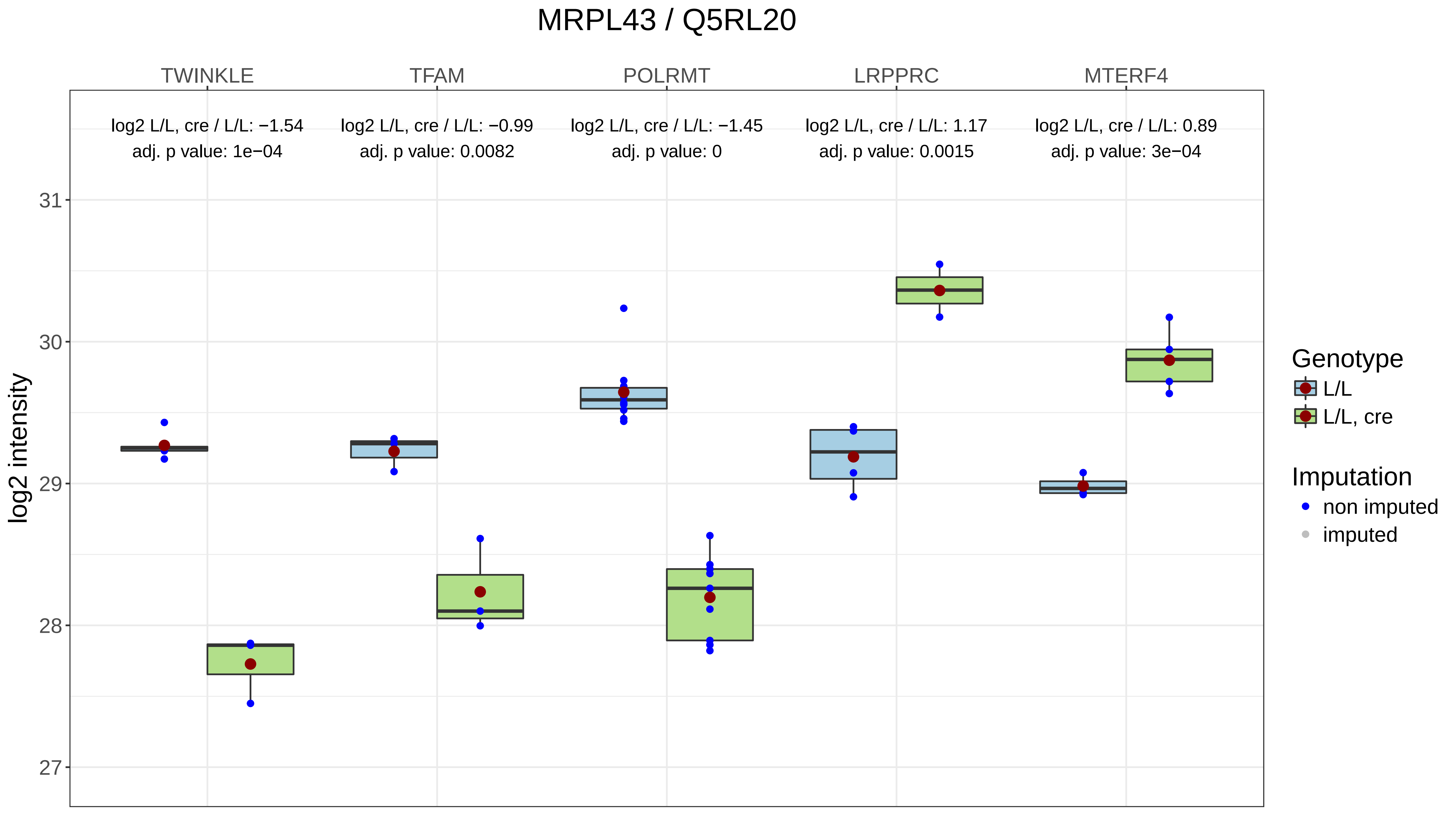
MRPL43 / Q5RL20
TWINKLE
TFAM
POLRMT
LRPPRC
MTERF4
log2 L/L, cre / L/L: −1.54
adj. p value: 1e−04
log2 L/L, cre / L/L: −0.99
adj. p value: 0.0082
log2 L/L, cre / L/L: −1.45
adj. p value: 0
log2 L/L, cre / L/L: 1.17
adj. p value: 0.0015
log2 L/L, cre / L/L: 0.89
adj. p value: 3e−04
log2 intensity
31
30
29
28
27
Genotype
L/L
L/L, cre
Imputation
non imputed
imputed

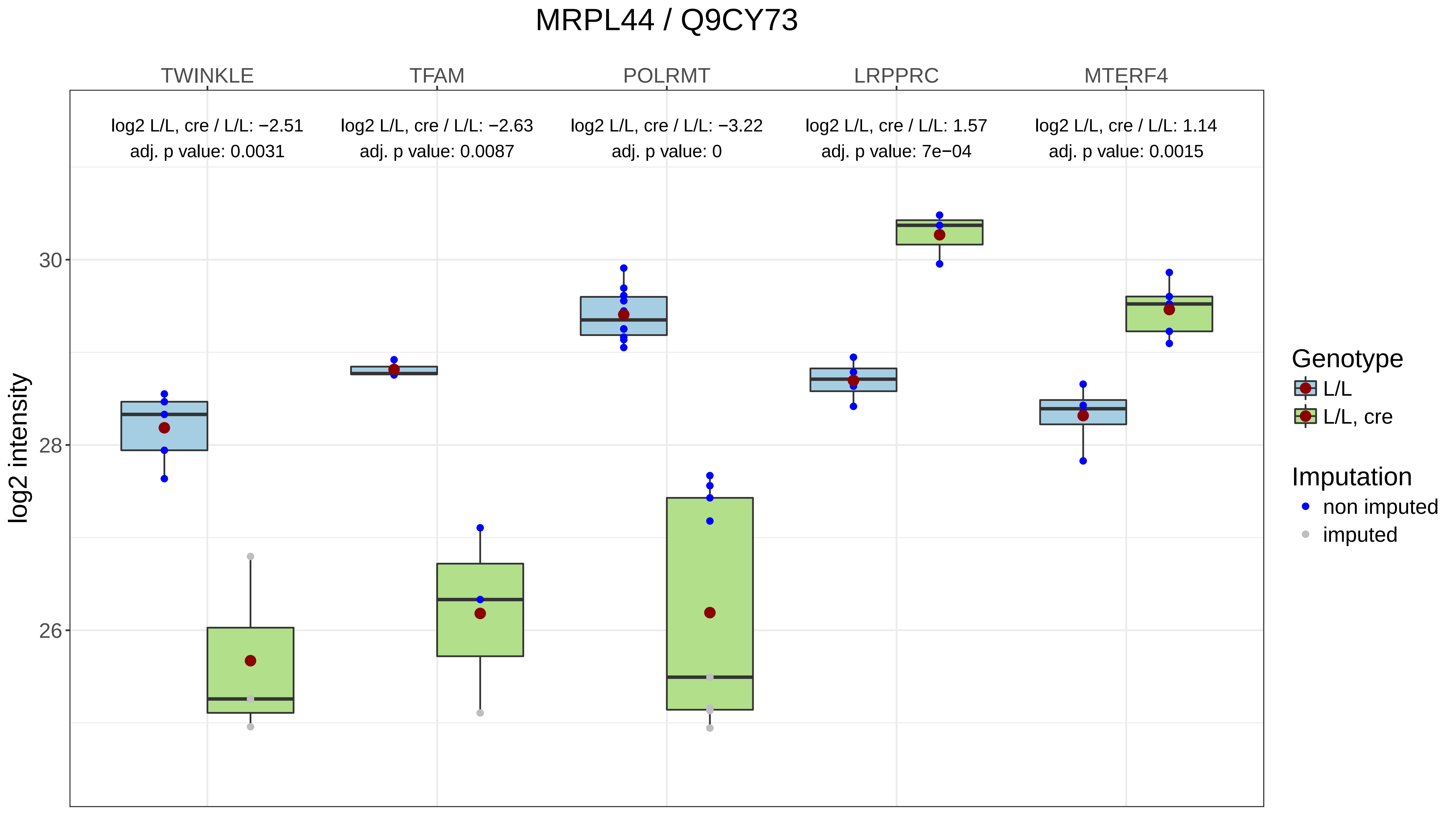
MRPL44 / Q9CY73
TWINKLE
TFAM
POLRMT
LRPPRC
MTERF4
log2 L/L, cre / L/L: −2.51
adj. p value: 0.0031
log2 L/L, cre / L/L: −2.63
adj. p value: 0.0087
log2 L/L, cre / L/L: −3.22
adj. p value: 0
log2 L/L, cre / L/L: 1.57
adj. p value: 7e−04
log2 L/L, cre / L/L: 1.14
adj. p value: 0.0015
log2 intensity
30
28
26
Genotype
L/L
L/L, cre
Imputation
non imputed
imputed

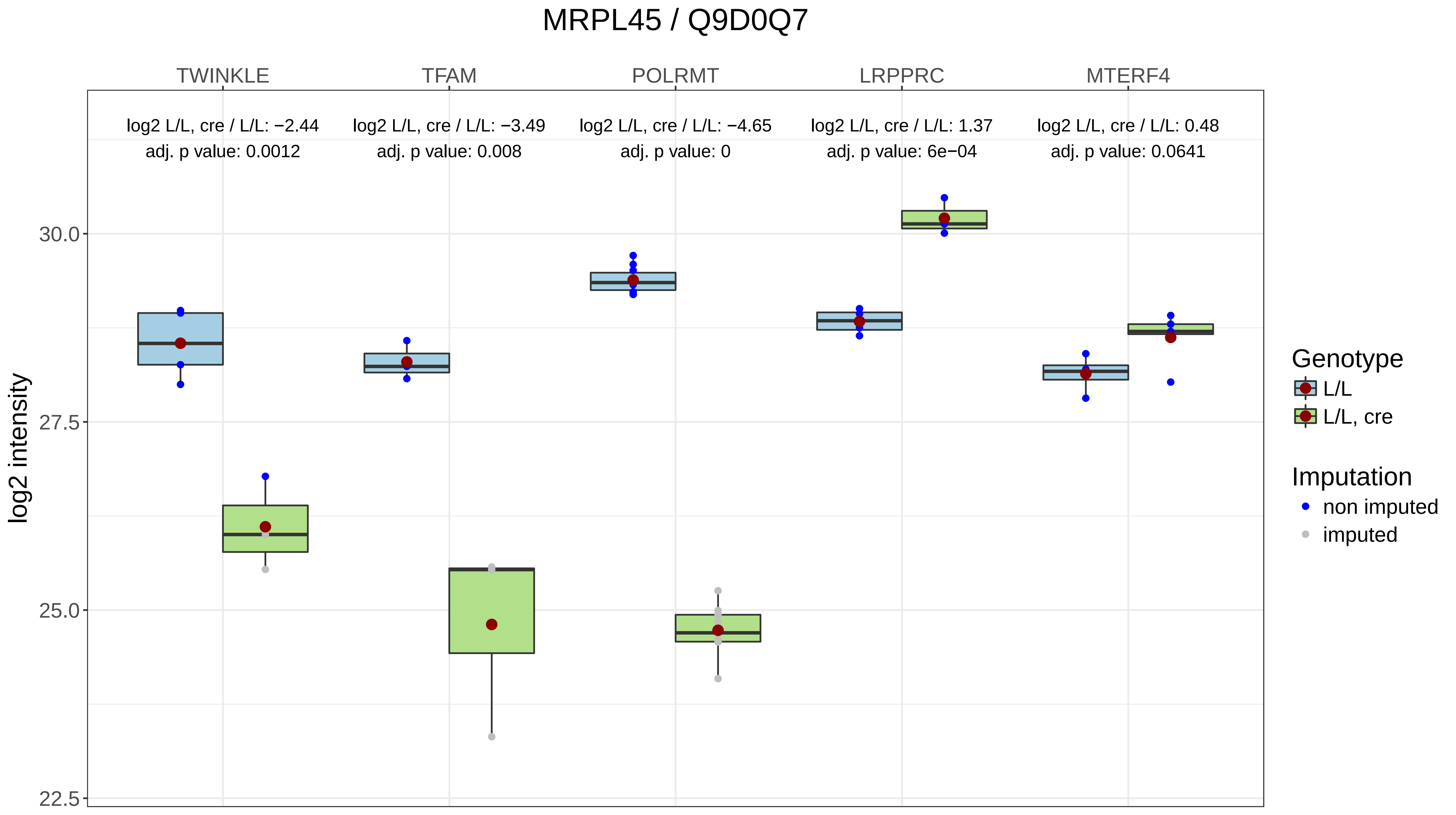

MRPL45 / Q9D0Q7
TWINKLE
TFAM
POLRMT
LRPPRC
MTERF4
log2 L/L, cre / L/L: −2.44
adj. p value: 0.0012
log2 L/L, cre / L/L: −3.49
adj. p value: 0.008
log2 L/L, cre / L/L: −4.65
adj. p value: 0
log2 L/L, cre / L/L: 1.37
adj. p value: 6e−04
log2 L/L, cre / L/L: 0.48
adj. p value: 0.0641
log2 intensity
30.0
27.5
25.0
22.5
Genotype
L/L
L/L, cre
Imputation
non imputed
imputed

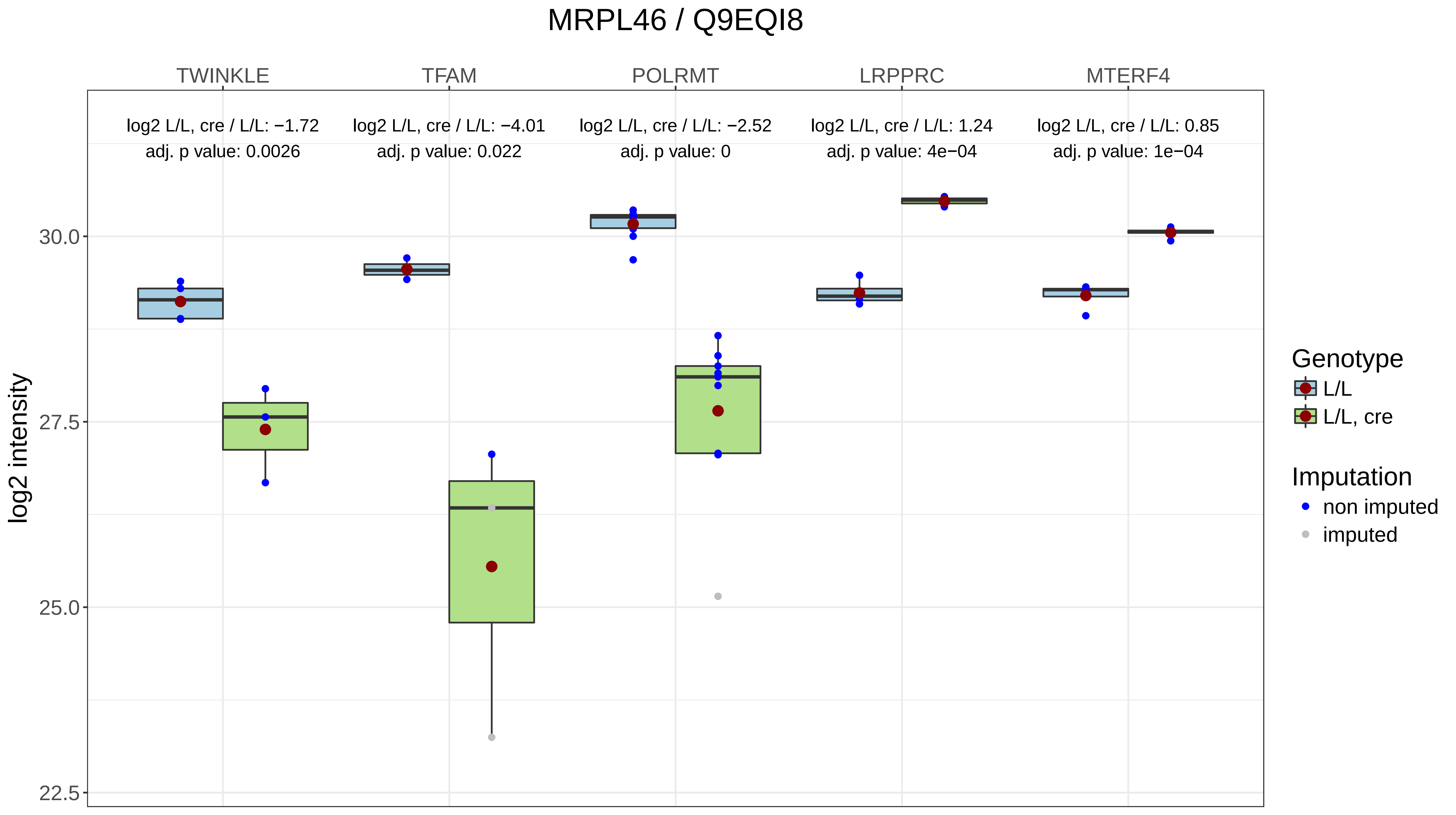
MRPL46 / Q9EQI8
TWINKLE
TFAM
POLRMT
LRPPRC
MTERF4
log2 L/L, cre / L/L: −1.72
adj. p value: 0.0026
log2 L/L, cre / L/L: −4.01
adj. p value: 0.022
log2 L/L, cre / L/L: −2.52
adj. p value: 0
log2 L/L, cre / L/L: 1.24
adj. p value: 4e−04
log2 L/L, cre / L/L: 0.85
adj. p value: 1e−04
log2 intensity
30.0
27.5
25.0
22.5
Genotype
L/L
L/L, cre
Imputation
non imputed
imputed

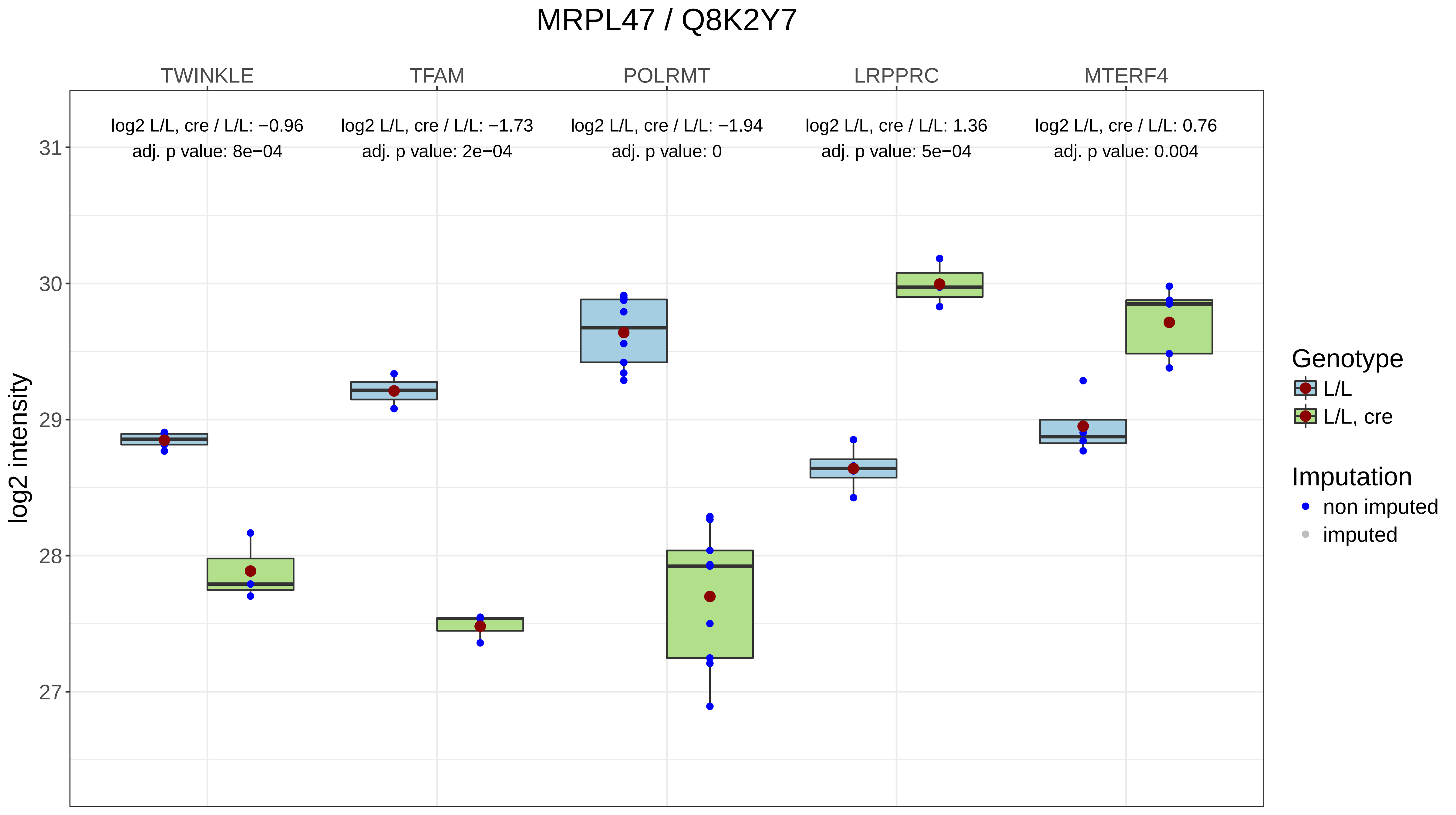
MRPL47 / Q8K2Y7
TWINKLE
TFAM
POLRMT
LRPPRC
MTERF4
log2 L/L, cre / L/L: −0.96
adj. p value: 8e−04
log2 L/L, cre / L/L: −1.73
adj. p value: 2e−04
log2 L/L, cre / L/L: −1.94
adj. p value: 0
log2 L/L, cre / L/L: 1.36
adj. p value: 5e−04
log2 L/L, cre / L/L: 0.76
adj. p value: 0.004
31
30
29
28
27
log2 intensity
Genotype
L/L
L/L, cre
Imputation
non imputed
imputed

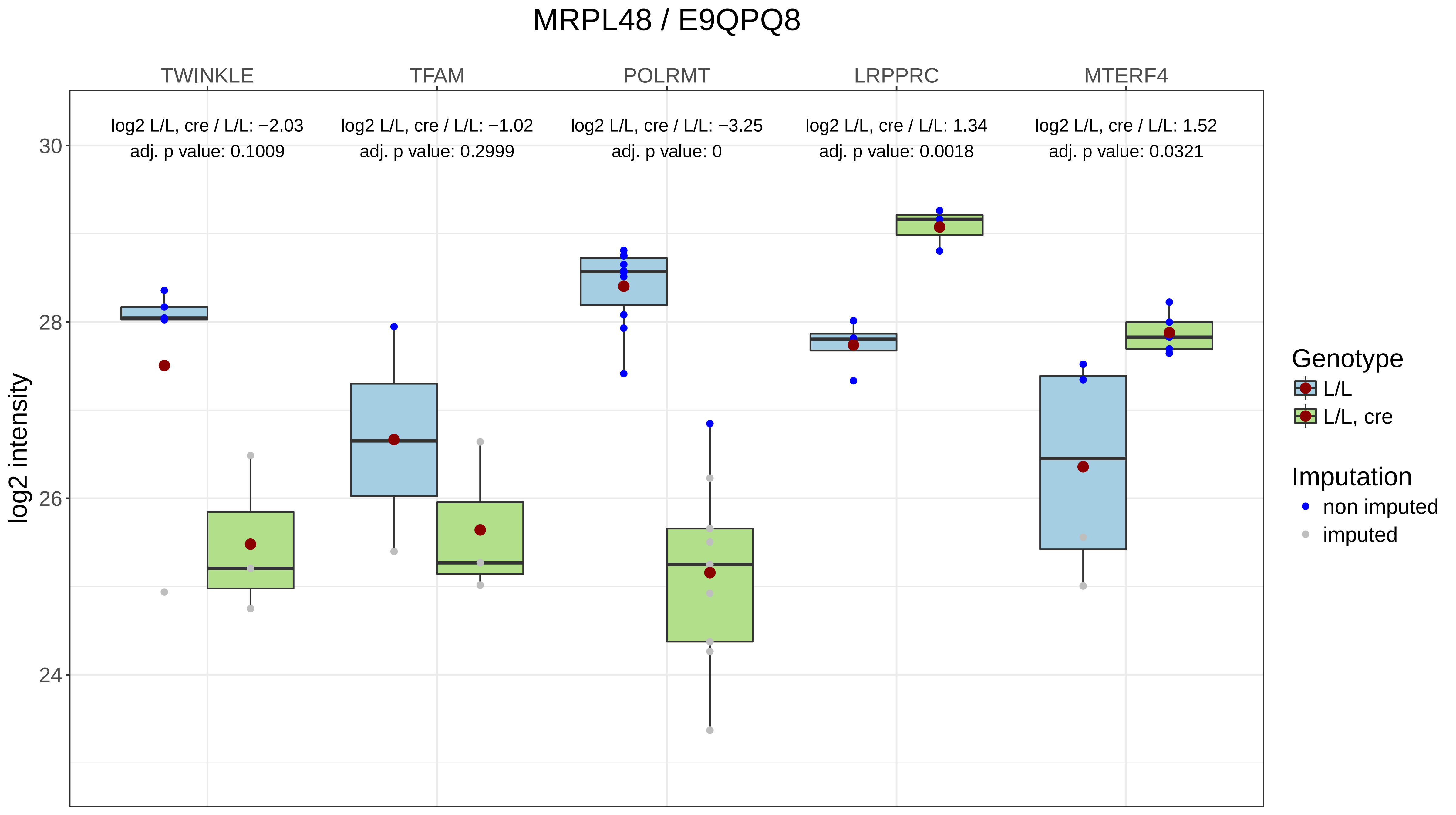

MRPL48 / E9QPQ8
TWINKLE
TFAM
POLRMT
LRPPRC
MTERF4
log2 L/L, cre / L/L: −2.03
adj. p value: 0.1009
log2 L/L, cre / L/L: −1.02
adj. p value: 0.2999
log2 L/L, cre / L/L: −3.25
adj. p value: 0
log2 L/L, cre / L/L: 1.34
adj. p value: 0.0018
log2 L/L, cre / L/L: 1.52
adj. p value: 0.0321
log2 intensity
30
28
26
24
Genotype
L/L
L/L, cre
Imputation
non imputed
imputed

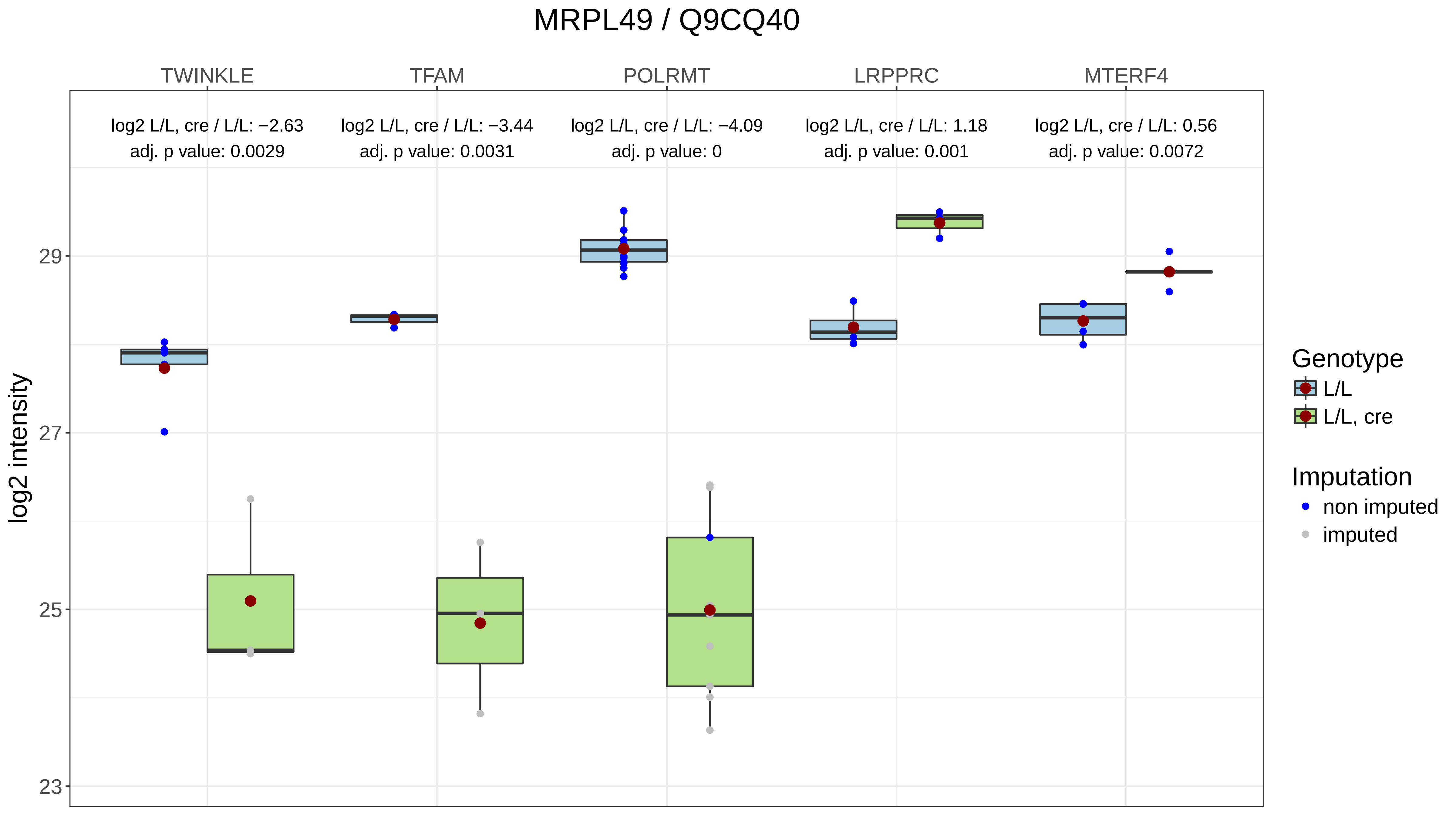
MRPL49 / Q9CQ40
TWINKLE
TFAM
POLRMT
LRPPRC
MTERF4
log2 L/L, cre / L/L: −2.63
adj. p value: 0.0029
log2 L/L, cre / L/L: −3.44
adj. p value: 0.0031
log2 L/L, cre / L/L: −4.09
adj. p value: 0
log2 L/L, cre / L/L: 1.18
adj. p value: 0.001
log2 L/L, cre / L/L: 0.56
adj. p value: 0.0072
log2 intensity
29
27
25
23
Genotype
L/L
L/L, cre
Imputation
non imputed
imputed

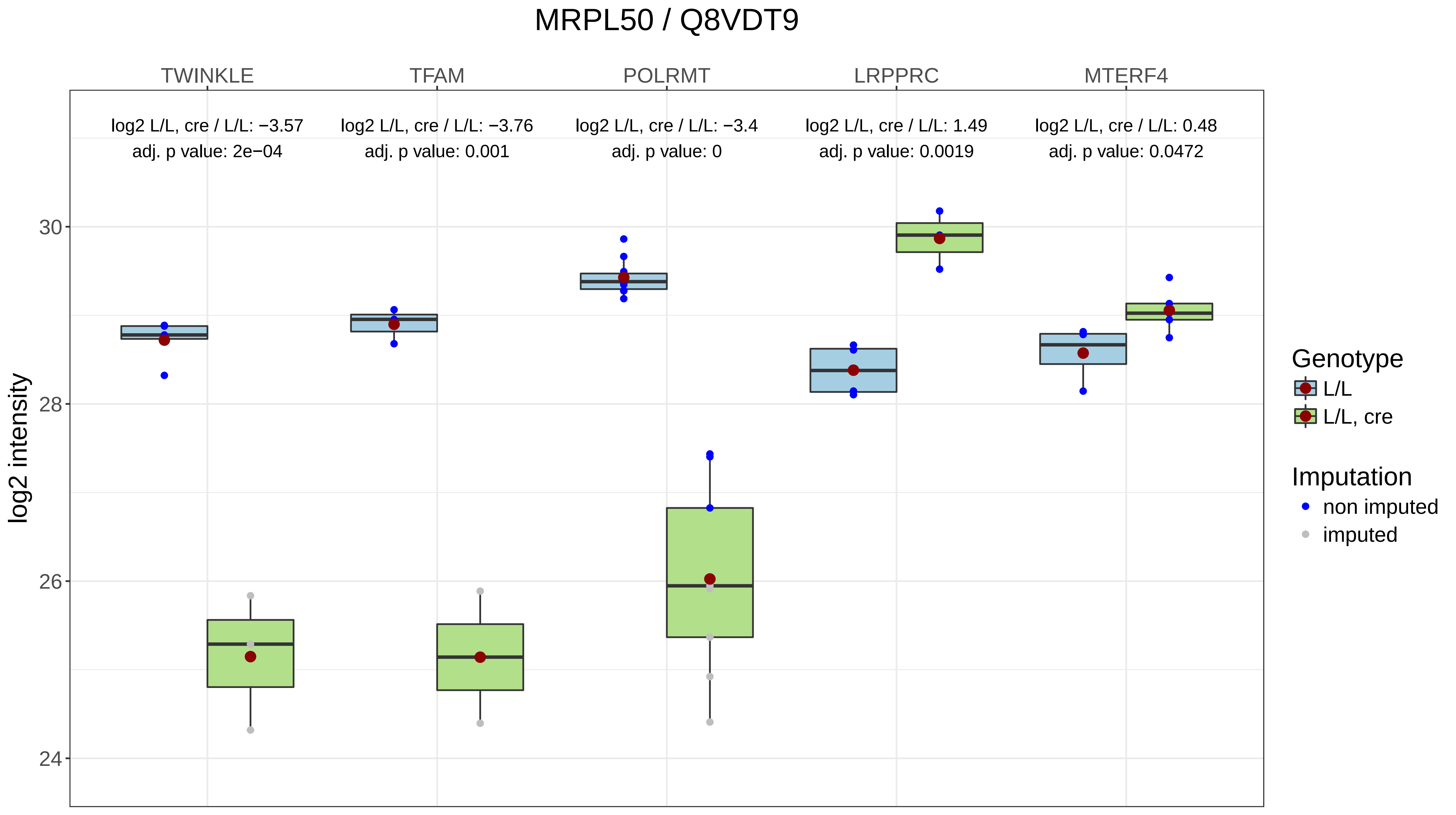
MRPL50 / Q8VDT9
TWINKLE
TFAM
POLRMT
LRPPRC
MTERF4
log2 L/L, cre / L/L: −3.57
adj. p value: 2e−04
log2 L/L, cre / L/L: −3.76
adj. p value: 0.001
log2 L/L, cre / L/L: −3.4
adj. p value: 0
log2 L/L, cre / L/L: 1.49
adj. p value: 0.0019
log2 L/L, cre / L/L: 0.48
adj. p value: 0.0472
log2 intensity
30
28
26
24
Genotype
L/L
L/L, cre
Imputation
non imputed
imputed

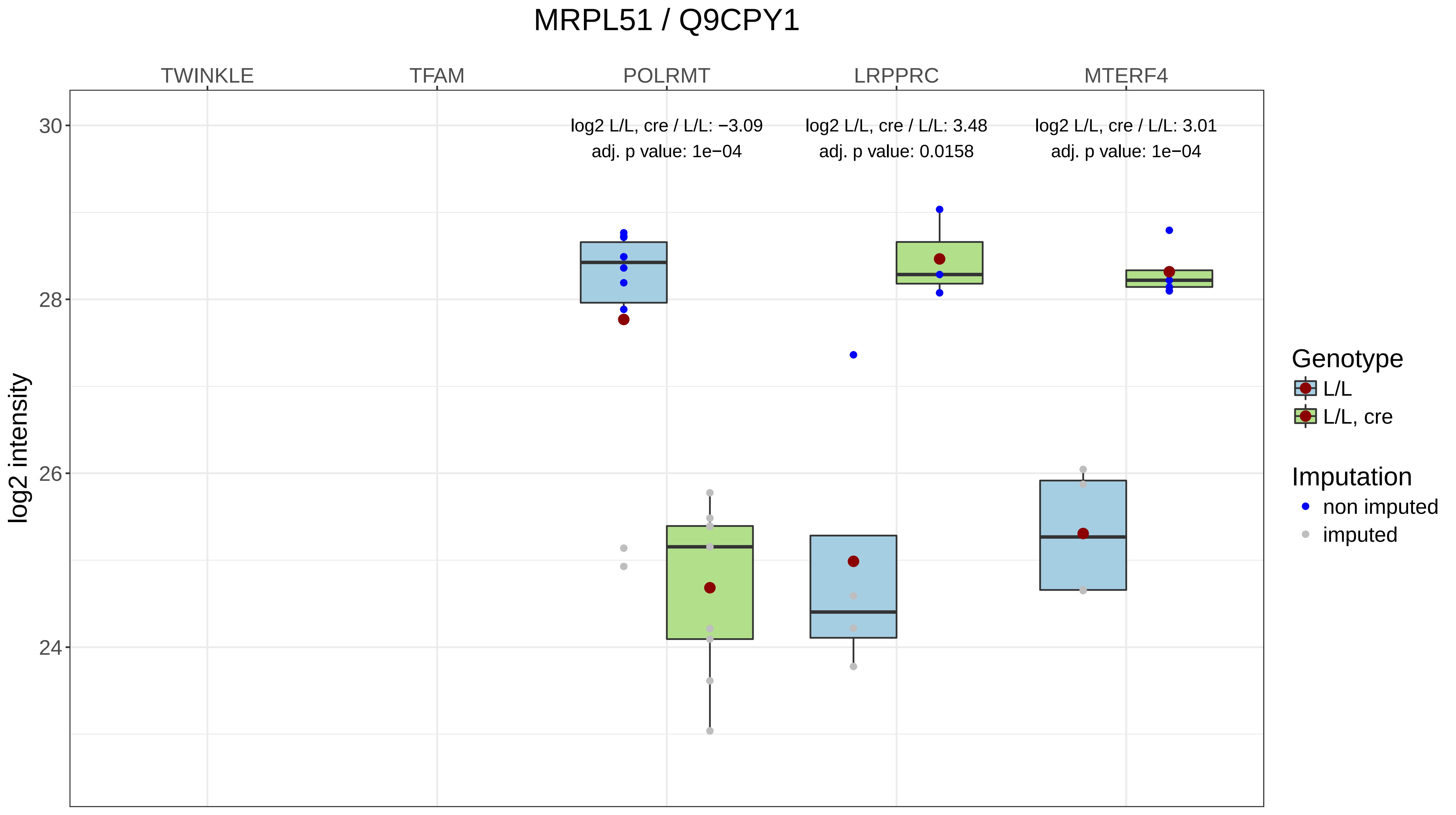

MRPL51 / Q9CPY1
TWINKLE
TFAM
POLRMT
LRPPRC
MTERF4
log2 L/L, cre / L/L: −3.09
adj. p value: 1e−04
log2 L/L, cre / L/L: 3.48
adj. p value: 0.0158
log2 L/L, cre / L/L: 3.01
adj. p value: 1e−04
30
28
26
24
log2 intensity
Genotype
L/L
L/L, cre
Imputation
non imputed
imputed

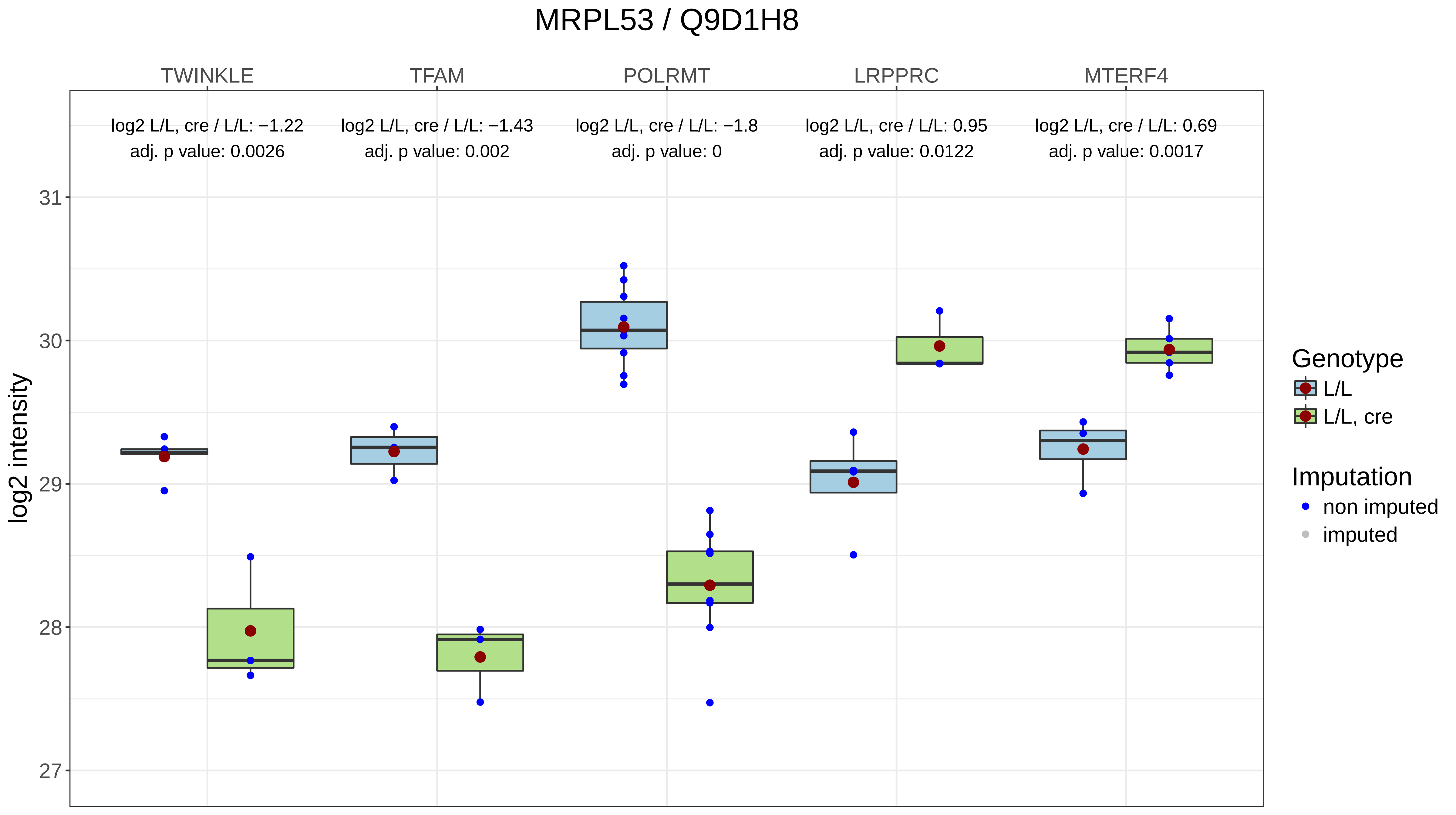

MRPL53 / Q9D1H8
TWINKLE
TFAM
POLRMT
LRPPRC
MTERF4
log2 L/L, cre / L/L: −1.22
adj. p value: 0.0026
log2 L/L, cre / L/L: −1.43
adj. p value: 0.002
log2 L/L, cre / L/L: −1.8
adj. p value: 0
log2 L/L, cre / L/L: 0.95
adj. p value: 0.0122
log2 L/L, cre / L/L: 0.69
adj. p value: 0.0017
log2 intensity
31
30
29
28
27
Genotype
L/L
L/L, cre
Imputation
non imputed
imputed

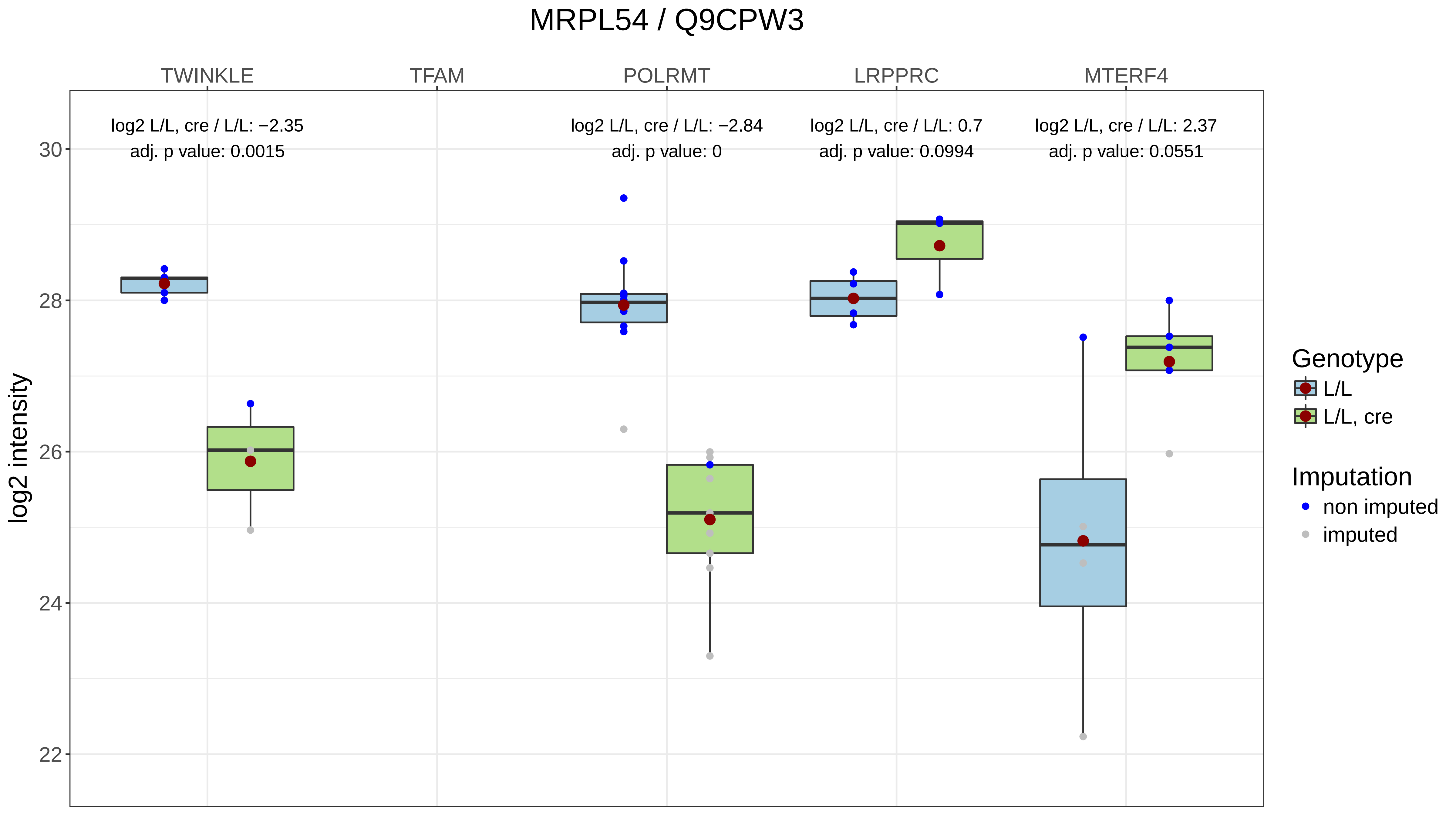
MRPL54 / Q9CPW3
TWINKLE
TFAM
POLRMT
LRPPRC
MTERF4
log2 L/L, cre / L/L: −2.35
adj. p value: 0.0015
log2 L/L, cre / L/L: −2.84
adj. p value: 0
log2 L/L, cre / L/L: 0.7
adj. p value: 0.0994
log2 L/L, cre / L/L: 2.37
adj. p value: 0.0551
log2 intensity
30
28
26
24
22
Genotype
L/L
L/L, cre
Imputation
non imputed
imputed

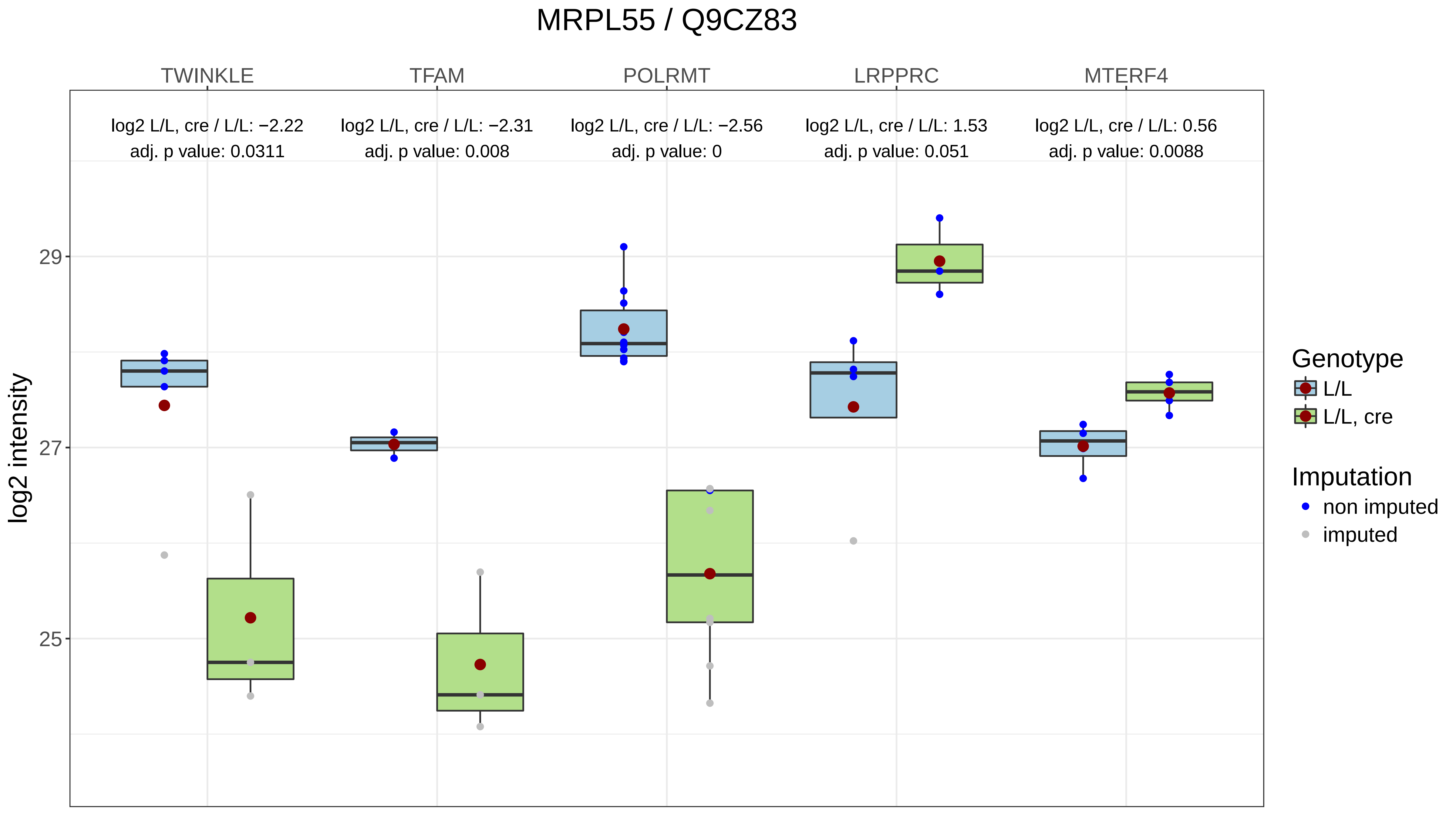

MRPL55 / Q9CZ83
TWINKLE
TFAM
POLRMT
LRPPRC
MTERF4
log2 L/L, cre / L/L: −2.22
adj. p value: 0.0311
log2 L/L, cre / L/L: −2.31
adj. p value: 0.008
log2 L/L, cre / L/L: −2.56
adj. p value: 0
log2 L/L, cre / L/L: 1.53
adj. p value: 0.051
log2 L/L, cre / L/L: 0.56
adj. p value: 0.0088
log2 intensity
29
27
25
Genotype
L/L
L/L, cre
Imputation
non imputed
imputed

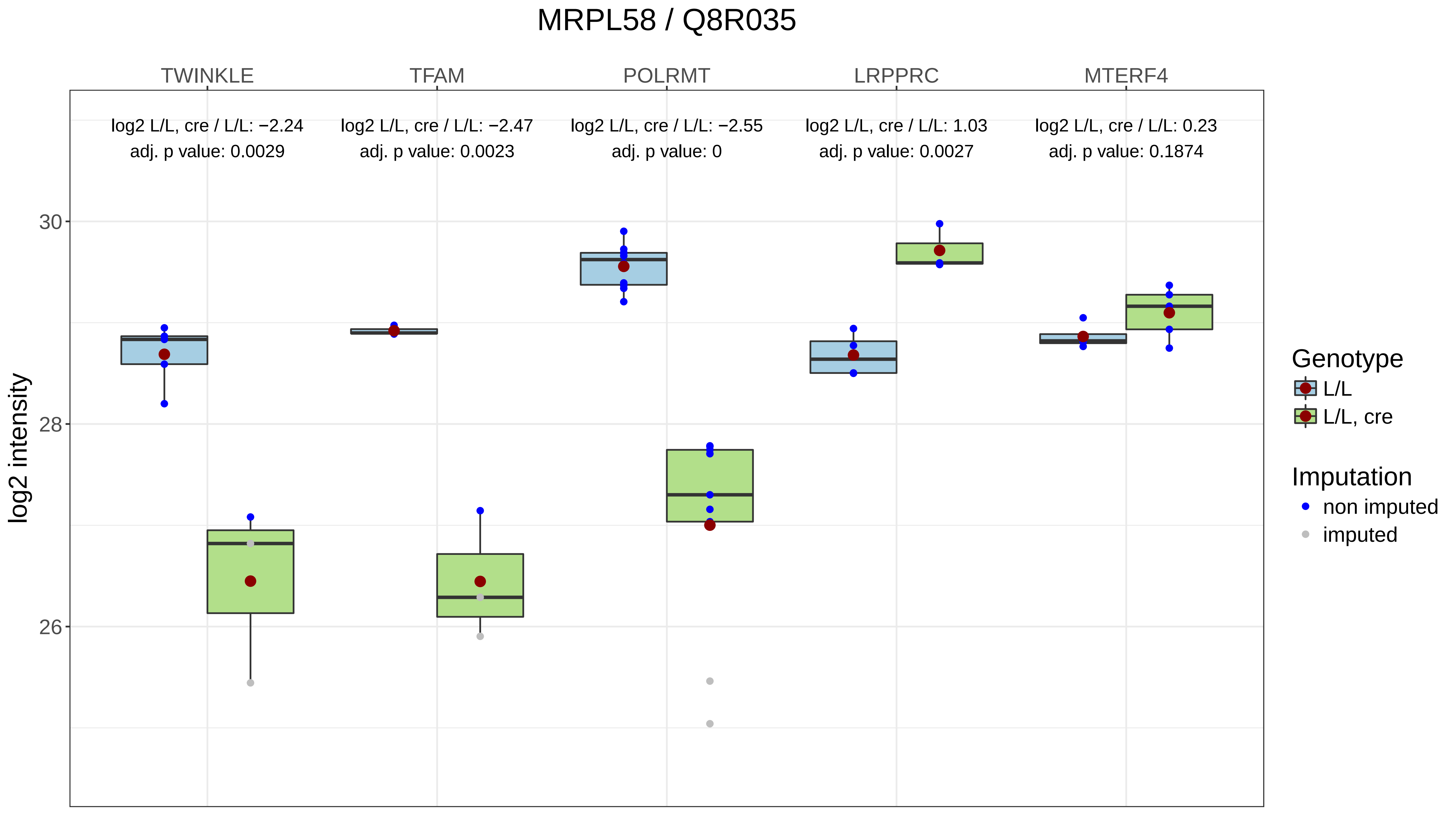
MRPL58 / Q8R035
TWINKLE
TFAM
POLRMT
LRPPRC
MTERF4
log2 L/L, cre / L/L: −2.24
adj. p value: 0.0029
log2 L/L, cre / L/L: −2.47
adj. p value: 0.0023
log2 L/L, cre / L/L: −2.55
adj. p value: 0
log2 L/L, cre / L/L: 1.03
adj. p value: 0.0027
log2 L/L, cre / L/L: 0.23
adj. p value: 0.1874
log2 intensity
30
28
26
Genotype
L/L
L/L, cre
Imputation
non imputed
imputed

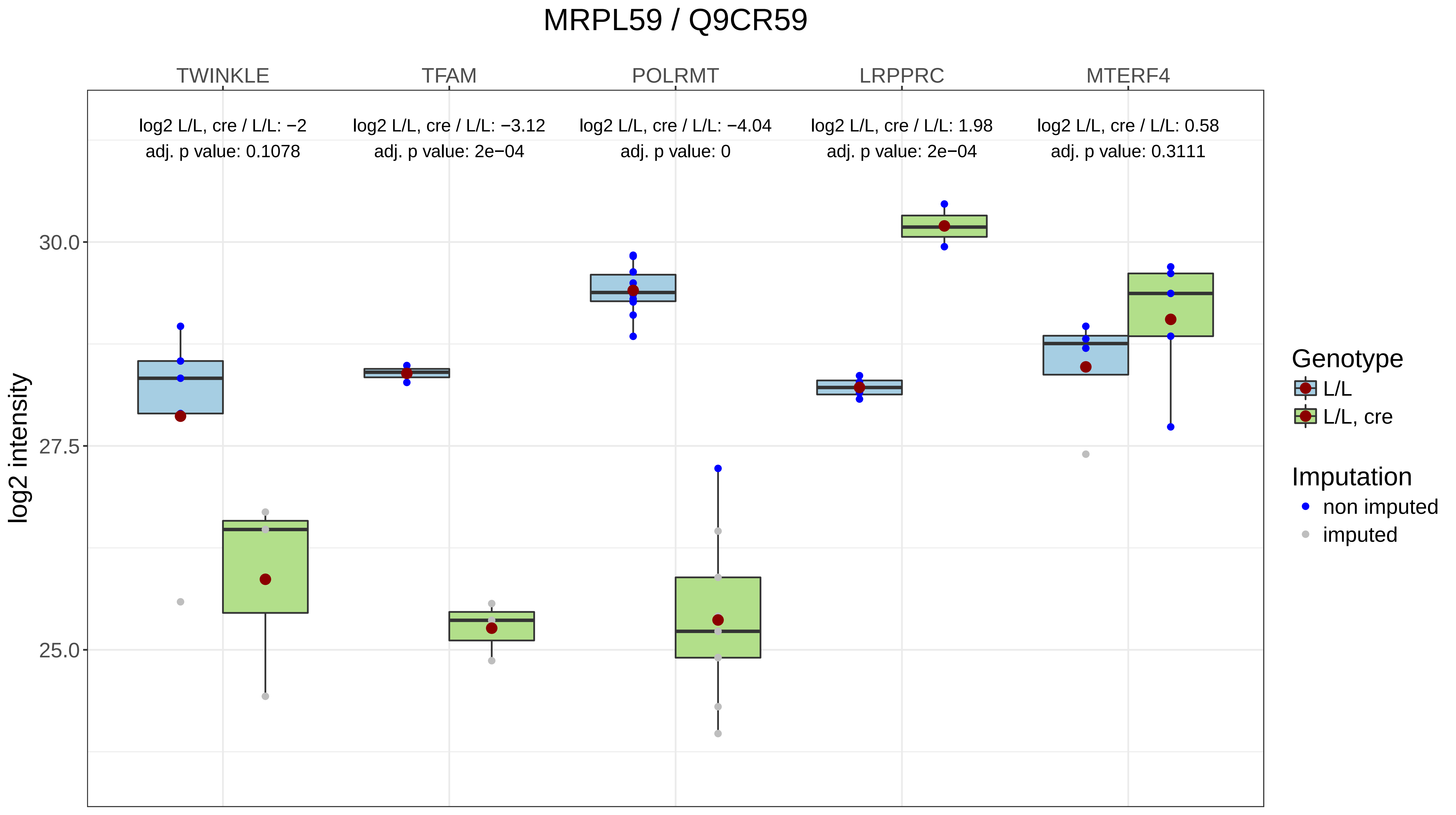
MRPL59 / Q9CR59
TWINKLE
TFAM
POLRMT
LRPPRC
MTERF4
log2 L/L, cre / L/L: −2
adj. p value: 0.1078
log2 L/L, cre / L/L: −3.12
adj. p value: 2e−04
log2 L/L, cre / L/L: −4.04
adj. p value: 0
log2 L/L, cre / L/L: 1.98
adj. p value: 2e−04
log2 L/L, cre / L/L: 0.58
adj. p value: 0.3111
log2 intensity
30.0
27.5
25.0
Genotype
L/L
L/L, cre
Imputation
non imputed
imputed

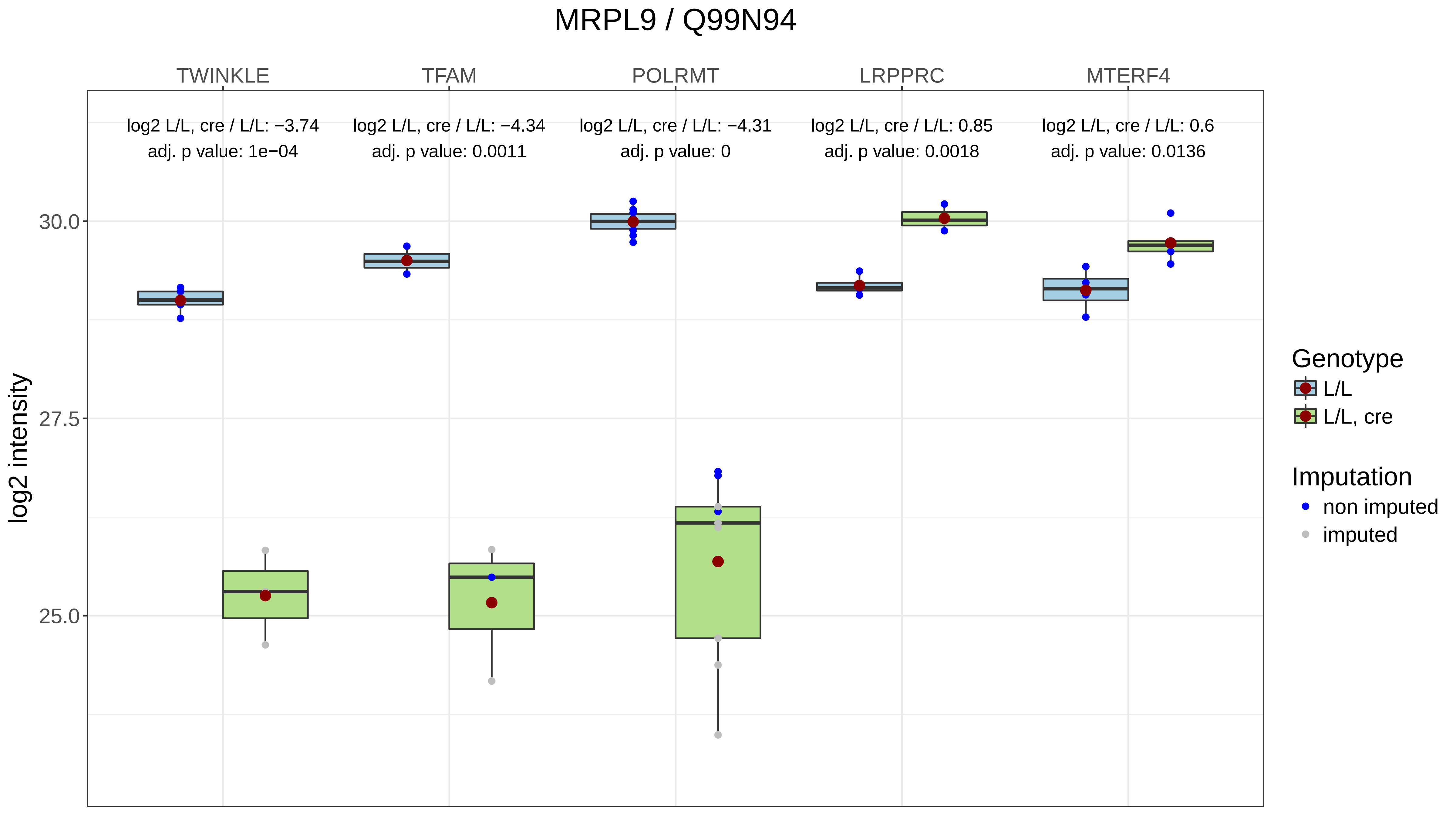
MRPL9 / Q99N94
TWINKLE
TFAM
POLRMT
LRPPRC
MTERF4
log2 L/L, cre / L/L: −3.74
adj. p value: 1e−04
log2 L/L, cre / L/L: −4.34
adj. p value: 0.0011
log2 L/L, cre / L/L: −4.31
adj. p value: 0
log2 L/L, cre / L/L: 0.85
adj. p value: 0.0018
log2 L/L, cre / L/L: 0.6
adj. p value: 0.0136
30.0
27.5
25.0
log2 intensity
Genotype
L/L
L/L, cre
Imputation
non imputed
imputed

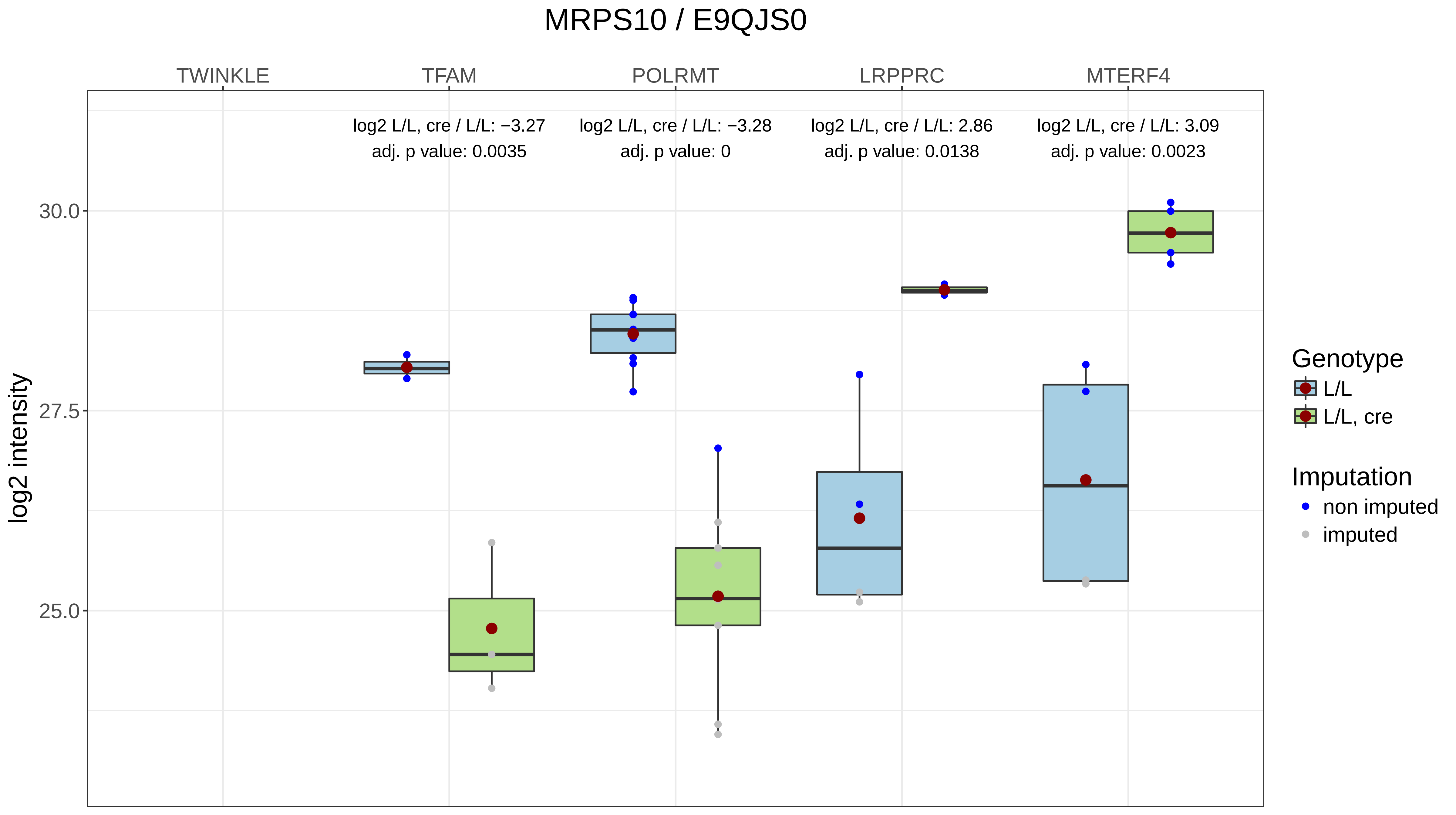

MRPS10 / E9QJS0
TWINKLE
TFAM
POLRMT
LRPPRC
MTERF4
log2 L/L, cre / L/L: −3.27
adj. p value: 0.0035
log2 L/L, cre / L/L: −3.28
adj. p value: 0
log2 L/L, cre / L/L: 2.86
adj. p value: 0.0138
log2 L/L, cre / L/L: 3.09
adj. p value: 0.0023
log2 intensity
30.0
27.5
25.0
Genotype
L/L
L/L, cre
Imputation
non imputed
imputed

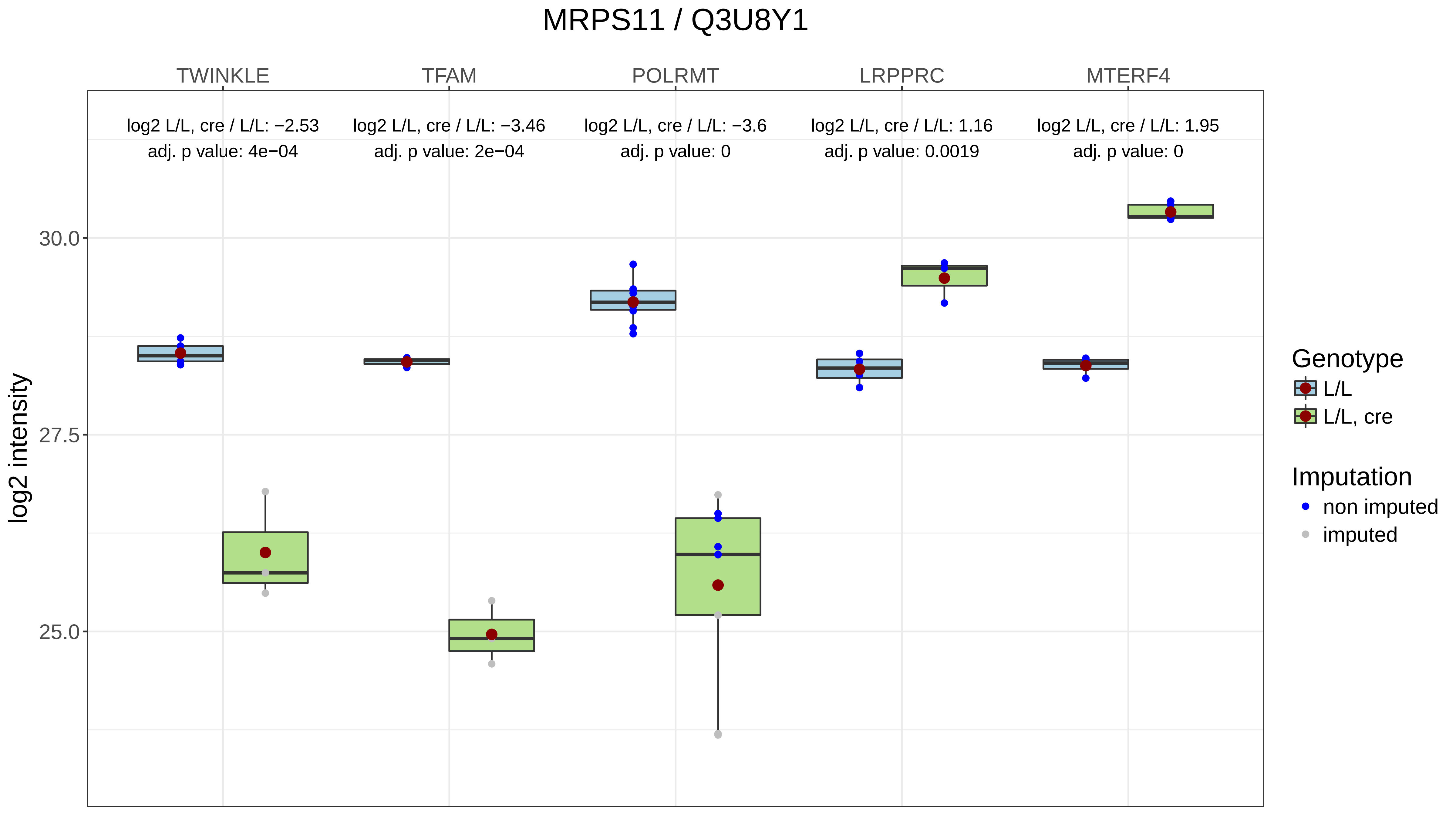

MRPS11 / Q3U8Y1
TWINKLE
TFAM
POLRMT
LRPPRC
MTERF4
log2 L/L, cre / L/L: −2.53
adj. p value: 4e−04
log2 L/L, cre / L/L: −3.46
adj. p value: 2e−04
log2 L/L, cre / L/L: −3.6
adj. p value: 0
log2 L/L, cre / L/L: 1.16
adj. p value: 0.0019
log2 L/L, cre / L/L: 1.95
adj. p value: 0
30.0
27.5
25.0
log2 intensity
Genotype
L/L
L/L, cre
Imputation
non imputed
imputed

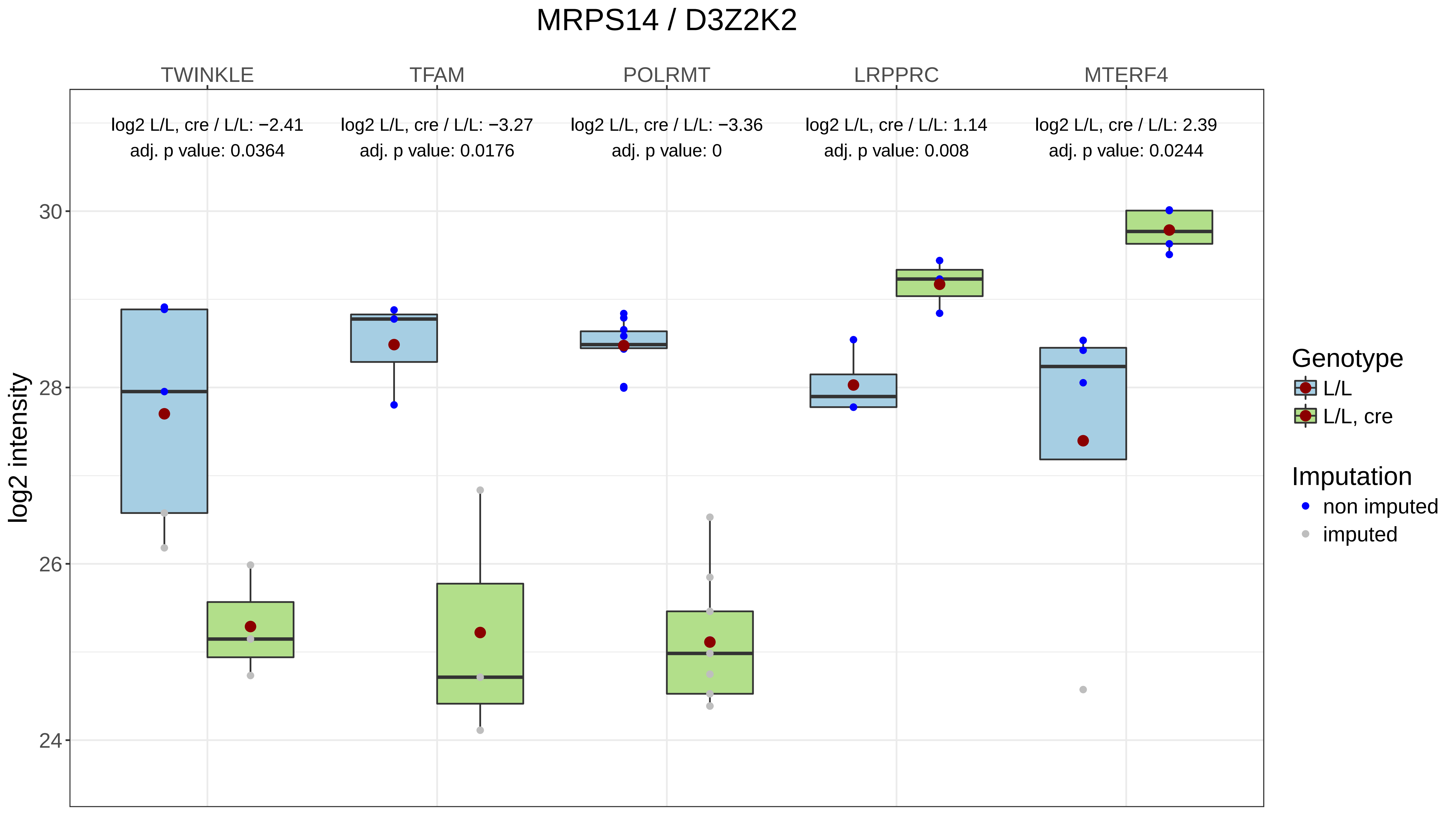

MRPS14 / D3Z2K2
TWINKLE
TFAM
POLRMT
LRPPRC
MTERF4
log2 L/L, cre / L/L: −2.41
adj. p value: 0.0364
log2 L/L, cre / L/L: −3.27
adj. p value: 0.0176
log2 L/L, cre / L/L: −3.36
adj. p value: 0
log2 L/L, cre / L/L: 1.14
adj. p value: 0.008
log2 L/L, cre / L/L: 2.39
adj. p value: 0.0244
log2 intensity
30
28
26
24
Genotype
L/L
L/L, cre
Imputation
non imputed
imputed

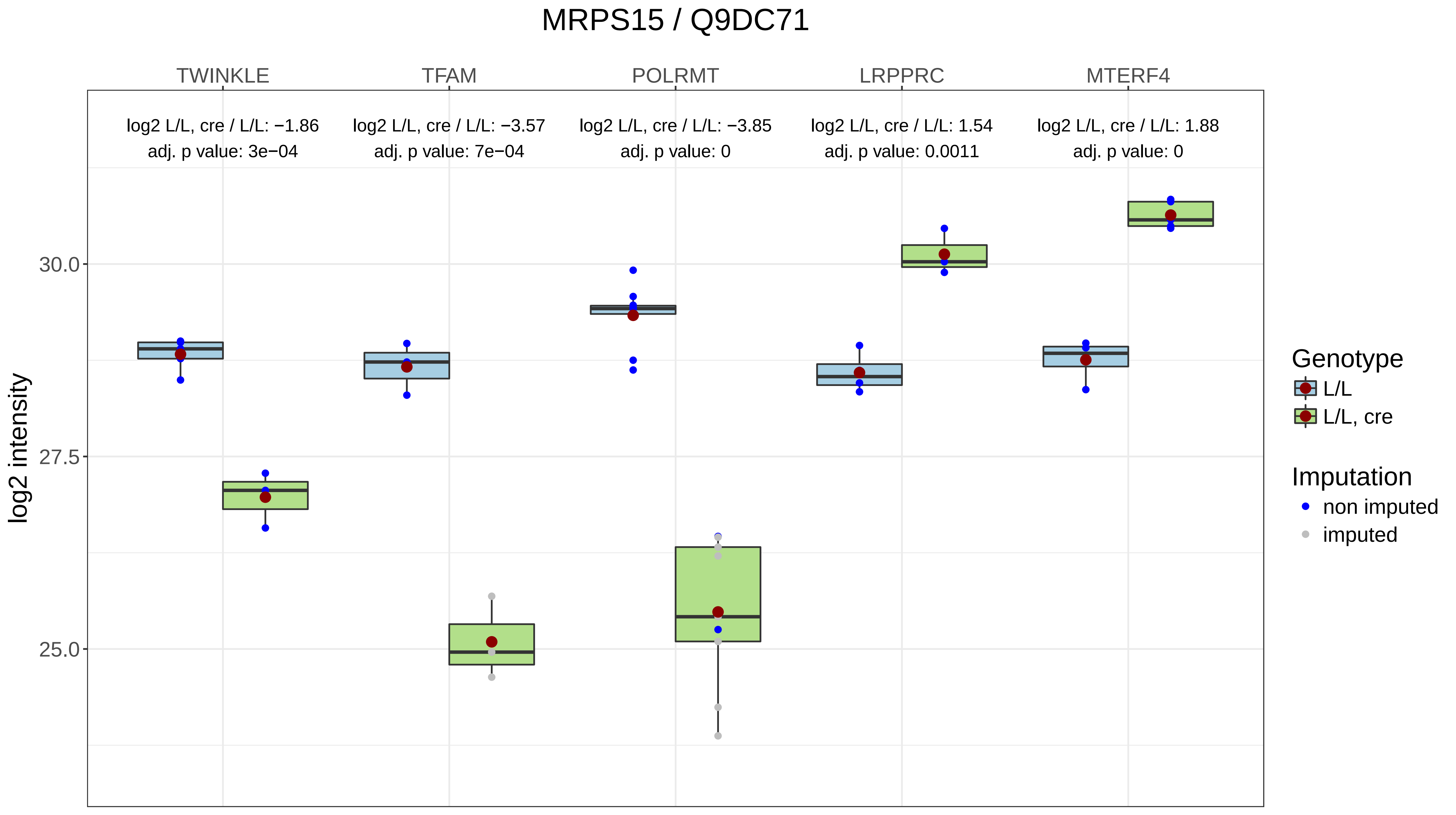
MRPS15 / Q9DC71
TWINKLE
TFAM
POLRMT
LRPPRC
MTERF4
log2 L/L, cre / L/L: −1.86
adj. p value: 3e−04
log2 L/L, cre / L/L: −3.57
adj. p value: 7e−04
log2 L/L, cre / L/L: −3.85
adj. p value: 0
log2 L/L, cre / L/L: 1.54
adj. p value: 0.0011
log2 L/L, cre / L/L: 1.88
adj. p value: 0
log2 intensity
30.0
27.5
25.0
Genotype
L/L
L/L, cre
Imputation
non imputed
imputed

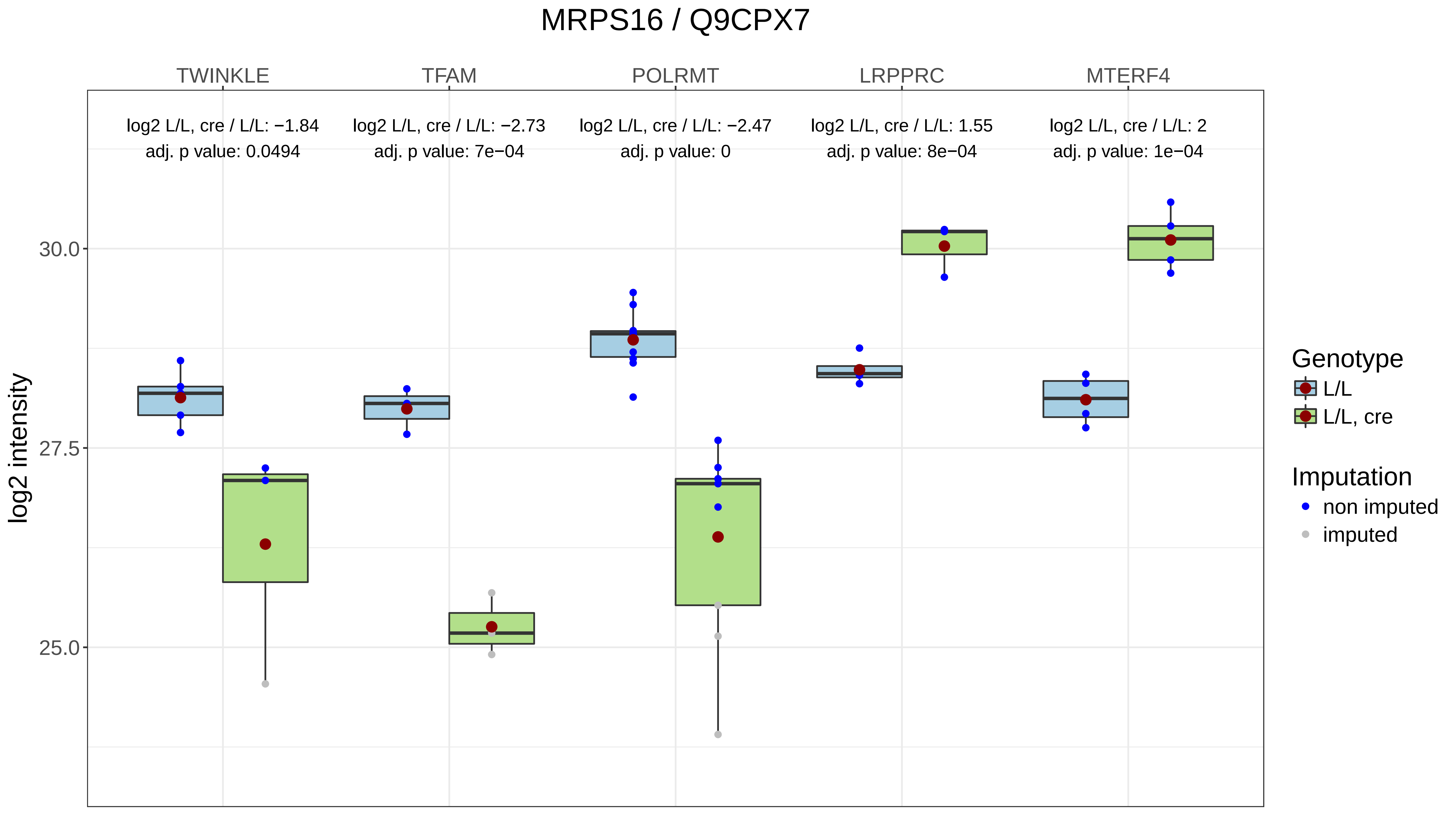

MRPS16 / Q9CPX7
TWINKLE
TFAM
POLRMT
LRPPRC
MTERF4
log2 L/L, cre / L/L: −1.84
adj. p value: 0.0494
log2 L/L, cre / L/L: −2.73
adj. p value: 7e−04
log2 L/L, cre / L/L: −2.47
adj. p value: 0
log2 L/L, cre / L/L: 1.55
adj. p value: 8e−04
log2 L/L, cre / L/L: 2
adj. p value: 1e−04
log2 intensity
30.0
27.5
25.0
Genotype
L/L
L/L, cre
Imputation
non imputed
imputed

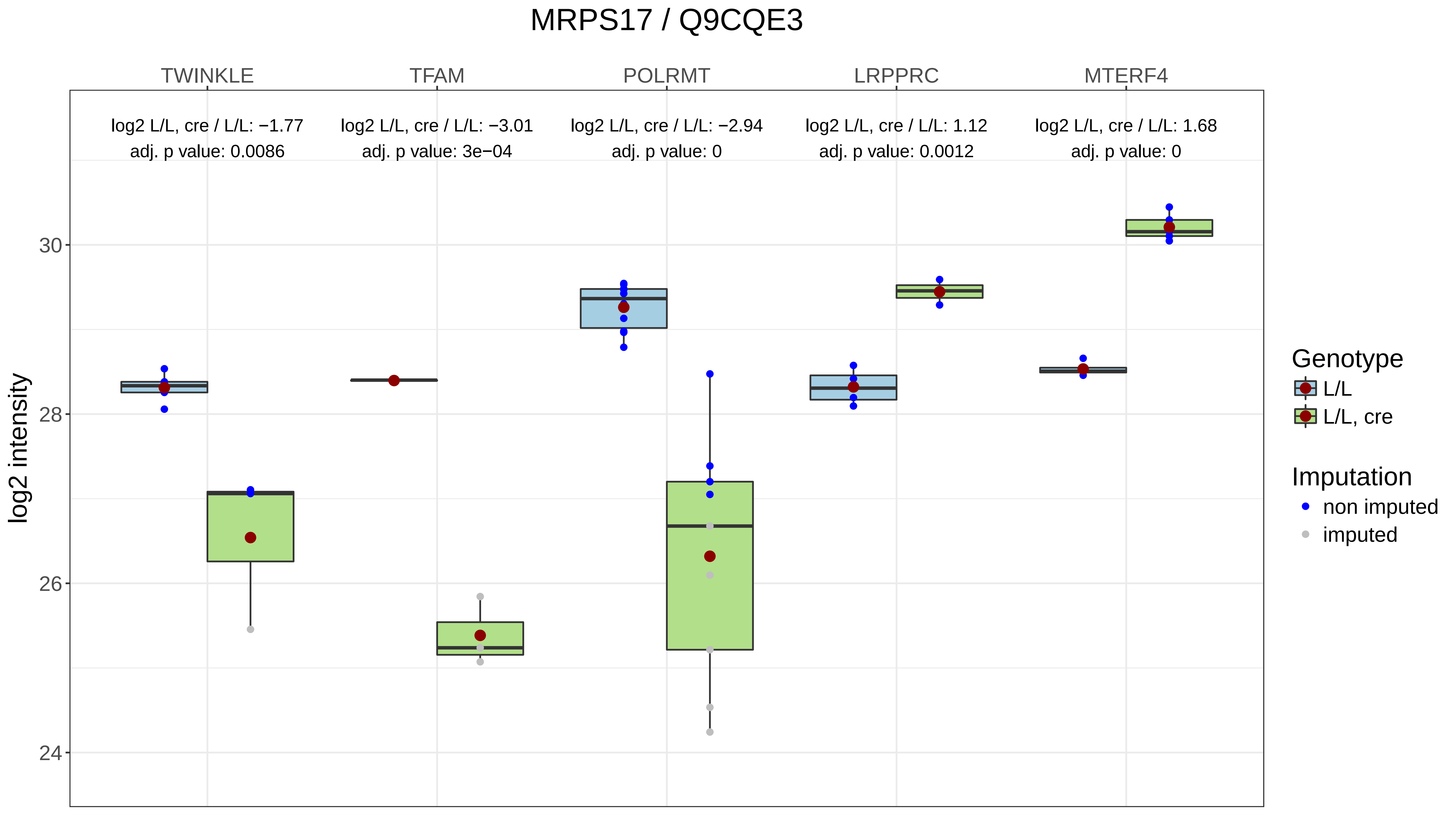
MRPS17 / Q9CQE3
TWINKLE
TFAM
POLRMT
LRPPRC
MTERF4
log2 L/L, cre / L/L: −1.77
adj. p value: 0.0086
log2 L/L, cre / L/L: −3.01
adj. p value: 3e−04
log2 L/L, cre / L/L: −2.94
adj. p value: 0
log2 L/L, cre / L/L: 1.12
adj. p value: 0.0012
log2 L/L, cre / L/L: 1.68
adj. p value: 0
log2 intensity
30
28
26
24
Genotype
L/L
L/L, cre
Imputation
non imputed
imputed

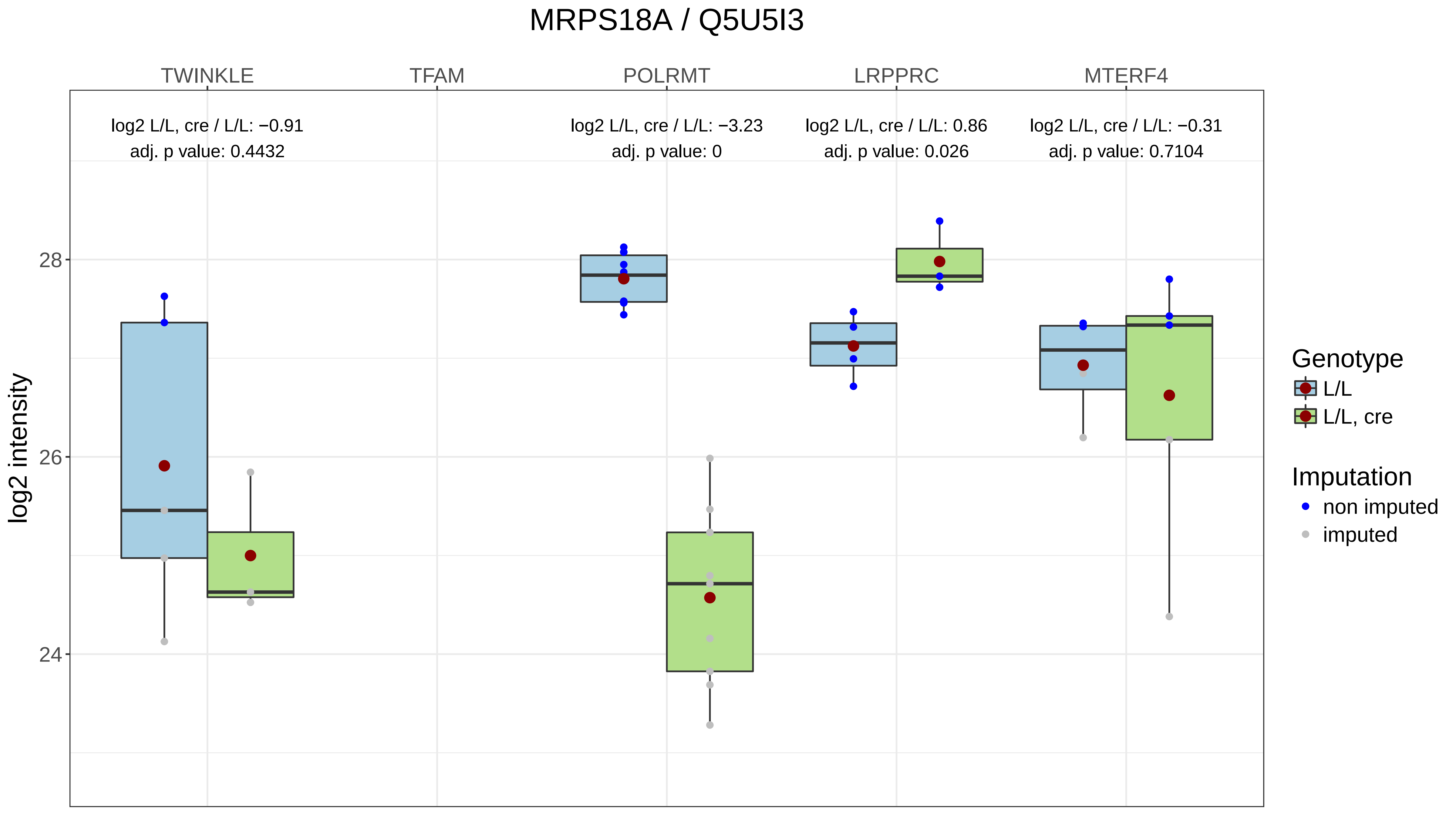
MRPS18A / Q5U5I3
TWINKLE
TFAM
POLRMT
LRPPRC
MTERF4
log2 L/L, cre / L/L: −0.91
adj. p value: 0.4432
log2 L/L, cre / L/L: −3.23
adj. p value: 0
log2 L/L, cre / L/L: 0.86
adj. p value: 0.026
log2 L/L, cre / L/L: −0.31
adj. p value: 0.7104
log2 intensity
28
26
24
Genotype
L/L
L/L, cre
Imputation
non imputed
imputed

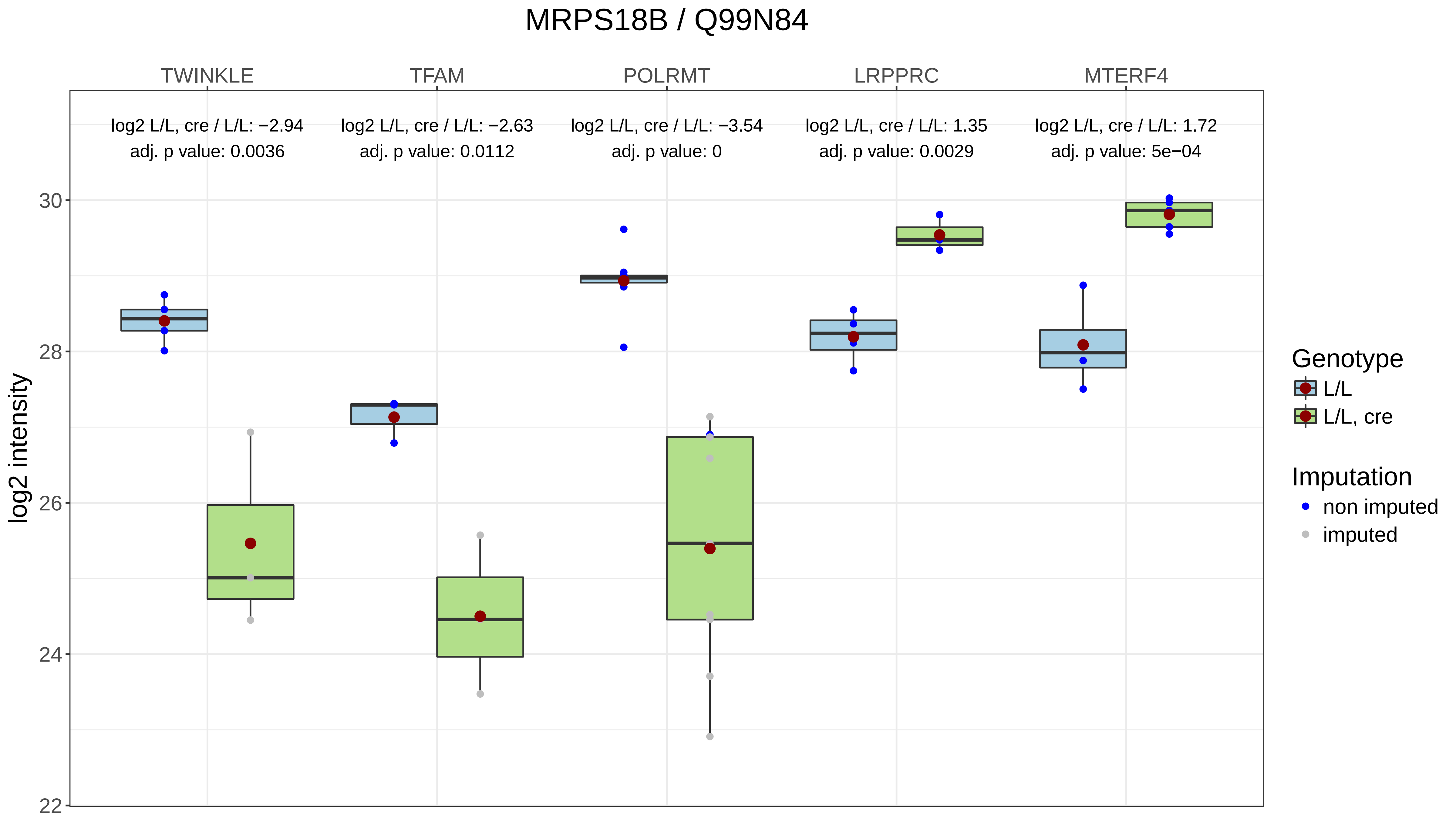

MRPS18B / Q99N84
TWINKLE
TFAM
POLRMT
LRPPRC
MTERF4
log2 L/L, cre / L/L: −2.94
adj. p value: 0.0036
log2 L/L, cre / L/L: −2.63
adj. p value: 0.0112
log2 L/L, cre / L/L: −3.54
adj. p value: 0
log2 L/L, cre / L/L: 1.35
adj. p value: 0.0029
log2 L/L, cre / L/L: 1.72
adj. p value: 5e−04
log2 intensity
30
28
26
24
22
Genotype
L/L
L/L, cre
Imputation
non imputed
imputed

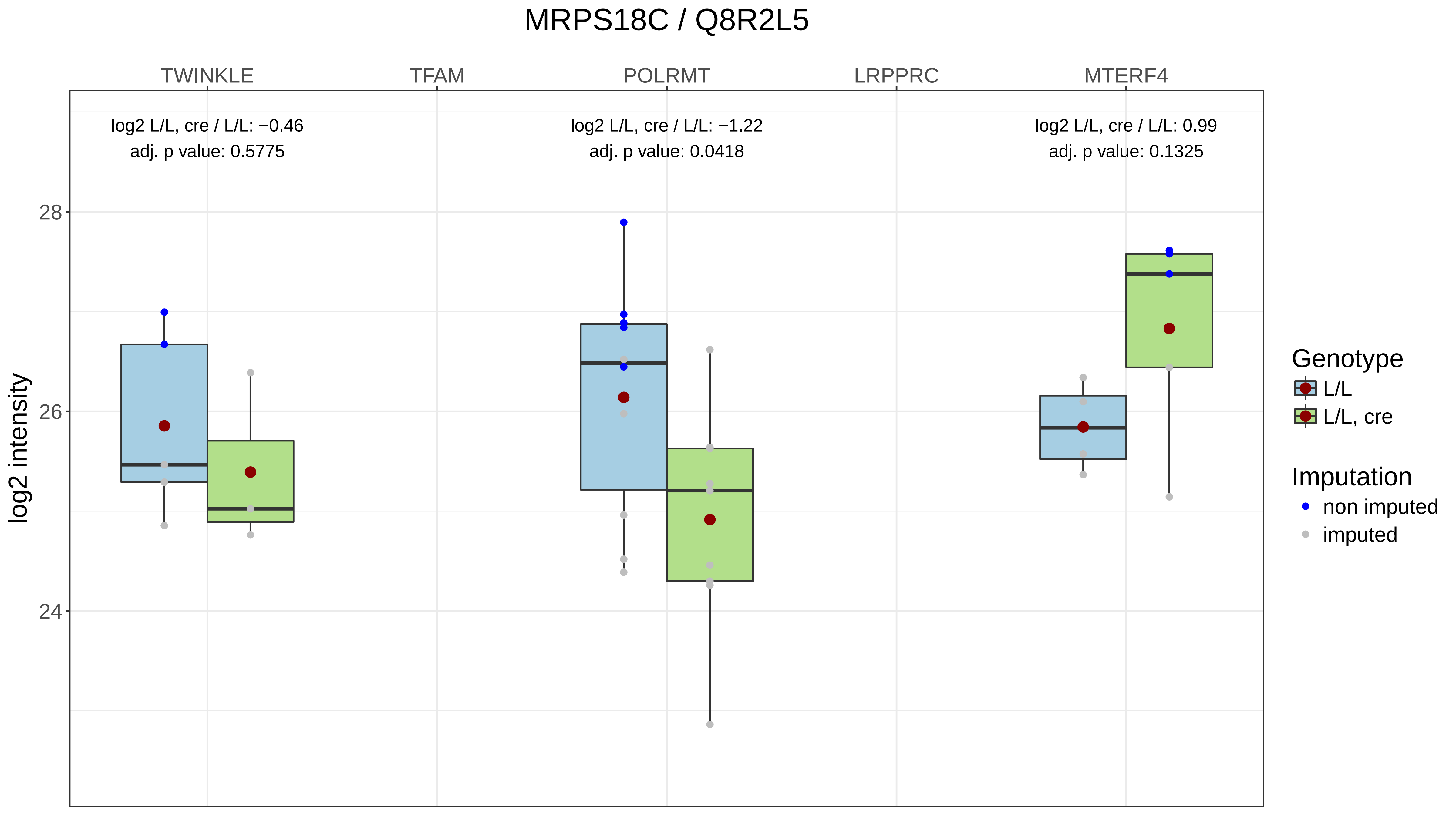
MRPS18C / Q8R2L5
TWINKLE
TFAM
POLRMT
LRPPRC
MTERF4
log2 L/L, cre / L/L: −0.46
adj. p value: 0.5775
log2 L/L, cre / L/L: −1.22
adj. p value: 0.0418
log2 L/L, cre / L/L: 0.99
adj. p value: 0.1325
28
26
24
log2 intensity
Genotype
L/L
L/L, cre
Imputation
non imputed
imputed

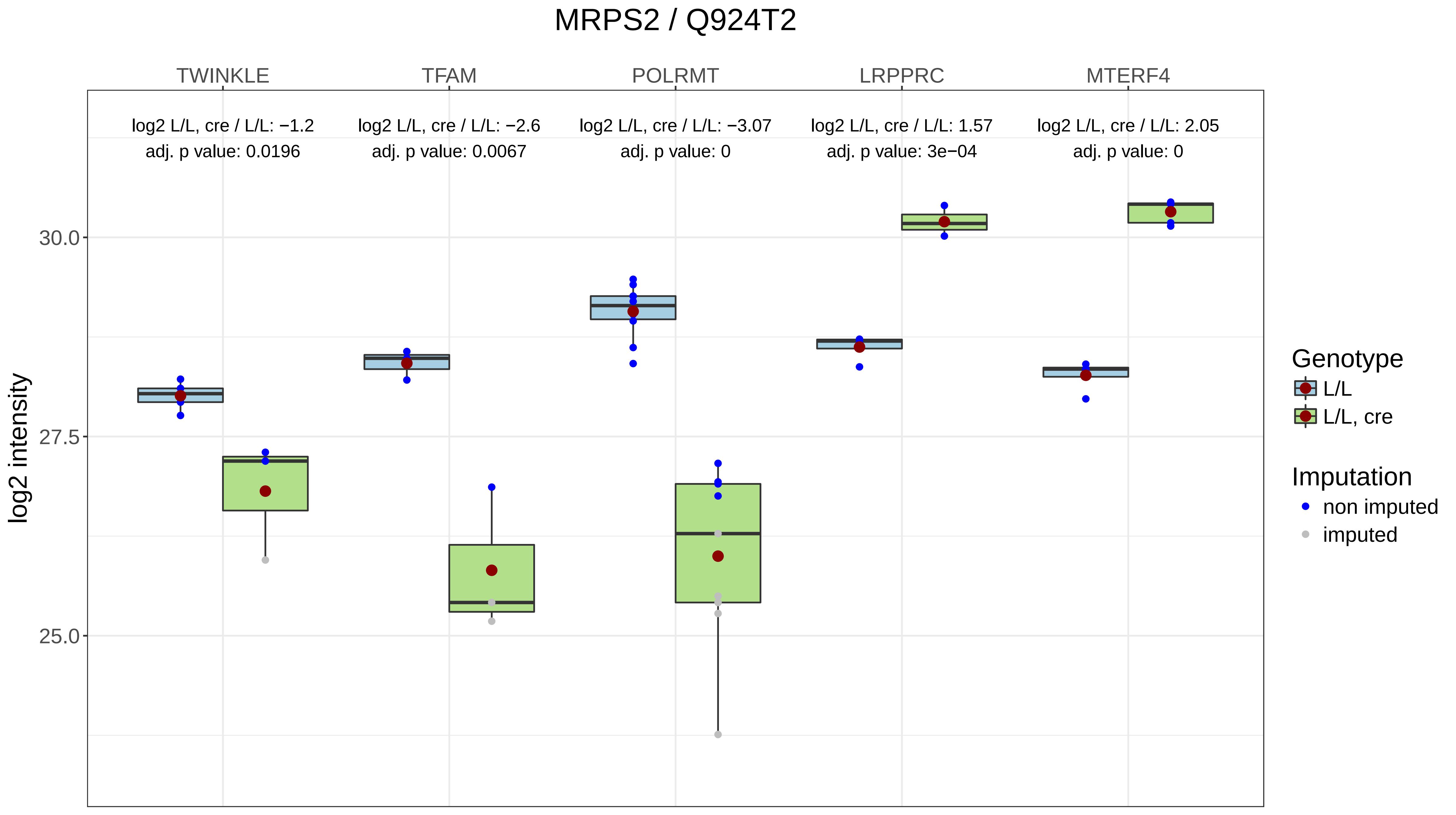

MRPS2 / Q924T2
TWINKLE
TFAM
POLRMT
LRPPRC
MTERF4
log2 L/L, cre / L/L: −1.2
adj. p value: 0.0196
log2 L/L, cre / L/L: −2.6
adj. p value: 0.0067
log2 L/L, cre / L/L: −3.07
adj. p value: 0
log2 L/L, cre / L/L: 1.57
adj. p value: 3e−04
log2 L/L, cre / L/L: 2.05
adj. p value: 0
30.0
27.5
25.0
log2 intensity
Genotype
L/L
L/L, cre
Imputation
non imputed
imputed

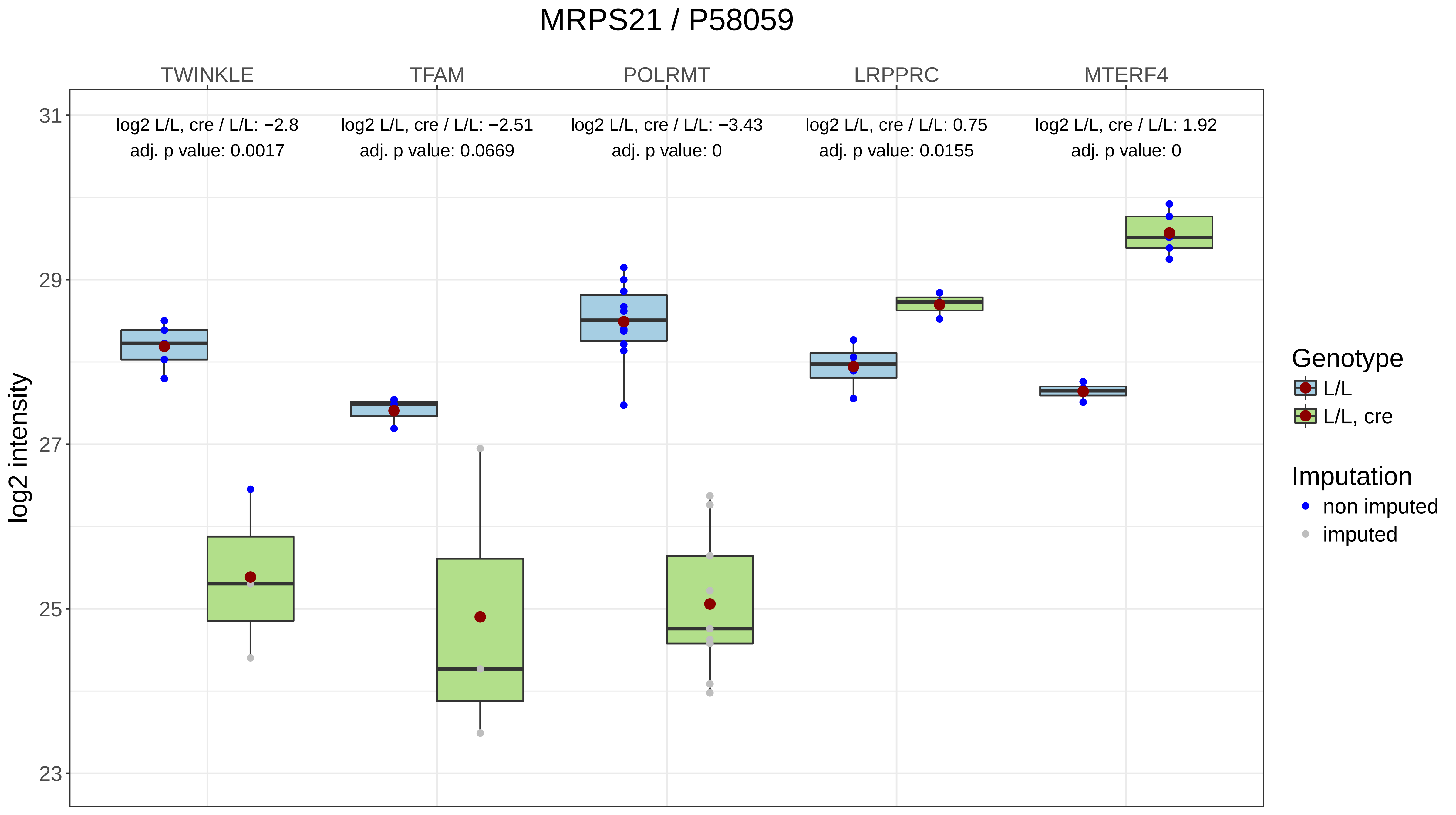
MRPS21 / P58059
TWINKLE
TFAM
POLRMT
LRPPRC
MTERF4
log2 L/L, cre / L/L: −2.8
adj. p value: 0.0017
log2 L/L, cre / L/L: −2.51
adj. p value: 0.0669
log2 L/L, cre / L/L: −3.43
adj. p value: 0
log2 L/L, cre / L/L: 0.75
adj. p value: 0.0155
log2 L/L, cre / L/L: 1.92
adj. p value: 0
log2 intensity
31
29
27
25
23
Genotype
L/L
L/L, cre
Imputation
non imputed
imputed

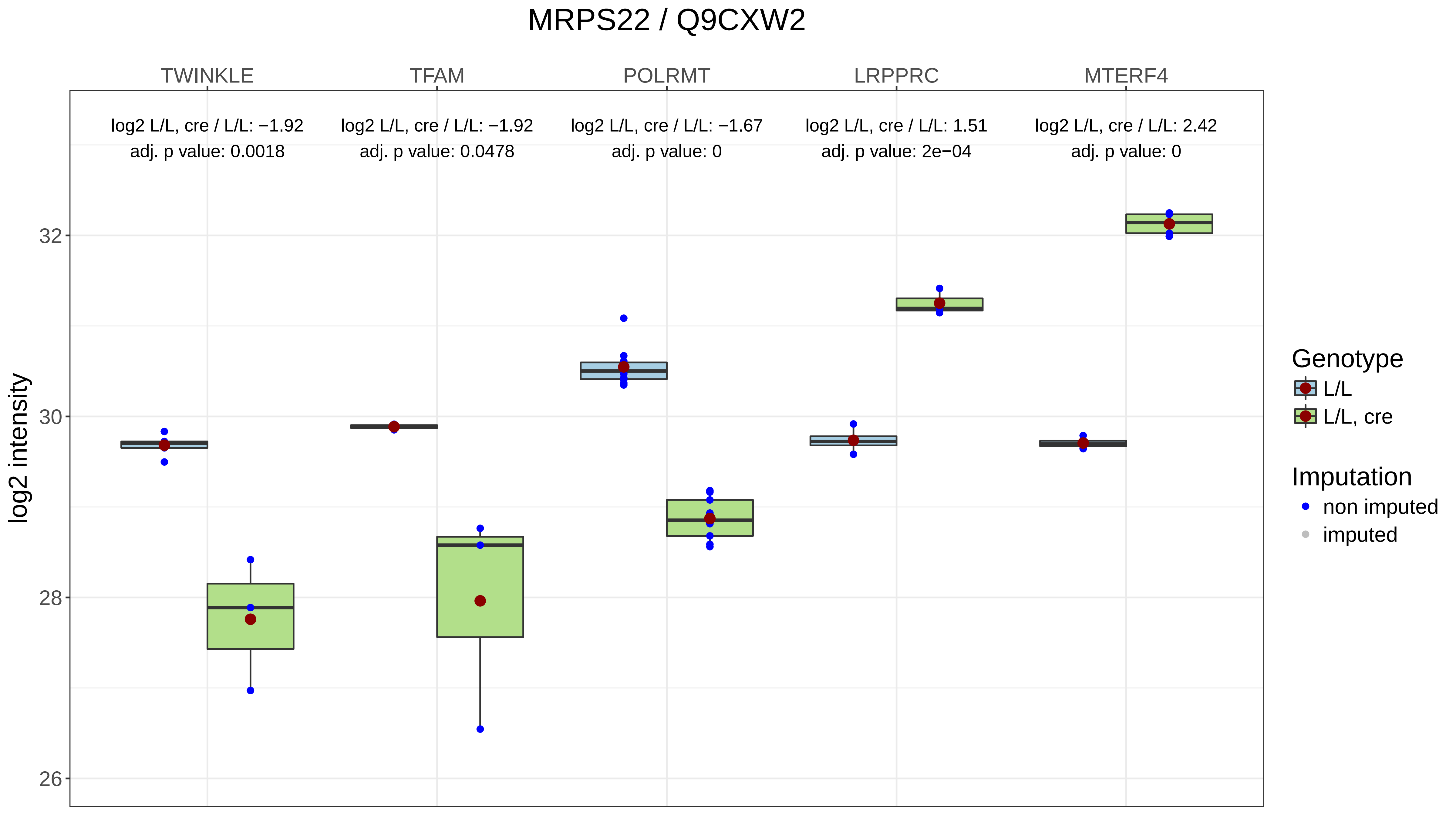
MRPS22 / Q9CXW2
TWINKLE
TFAM
POLRMT
LRPPRC
MTERF4
log2 L/L, cre / L/L: −1.92
adj. p value: 0.0018
log2 L/L, cre / L/L: −1.92
adj. p value: 0.0478
log2 L/L, cre / L/L: −1.67
adj. p value: 0
log2 L/L, cre / L/L: 1.51
adj. p value: 2e−04
log2 L/L, cre / L/L: 2.42
adj. p value: 0
log2 intensity
32
30
28
26
Genotype
L/L
L/L, cre
Imputation
non imputed
imputed

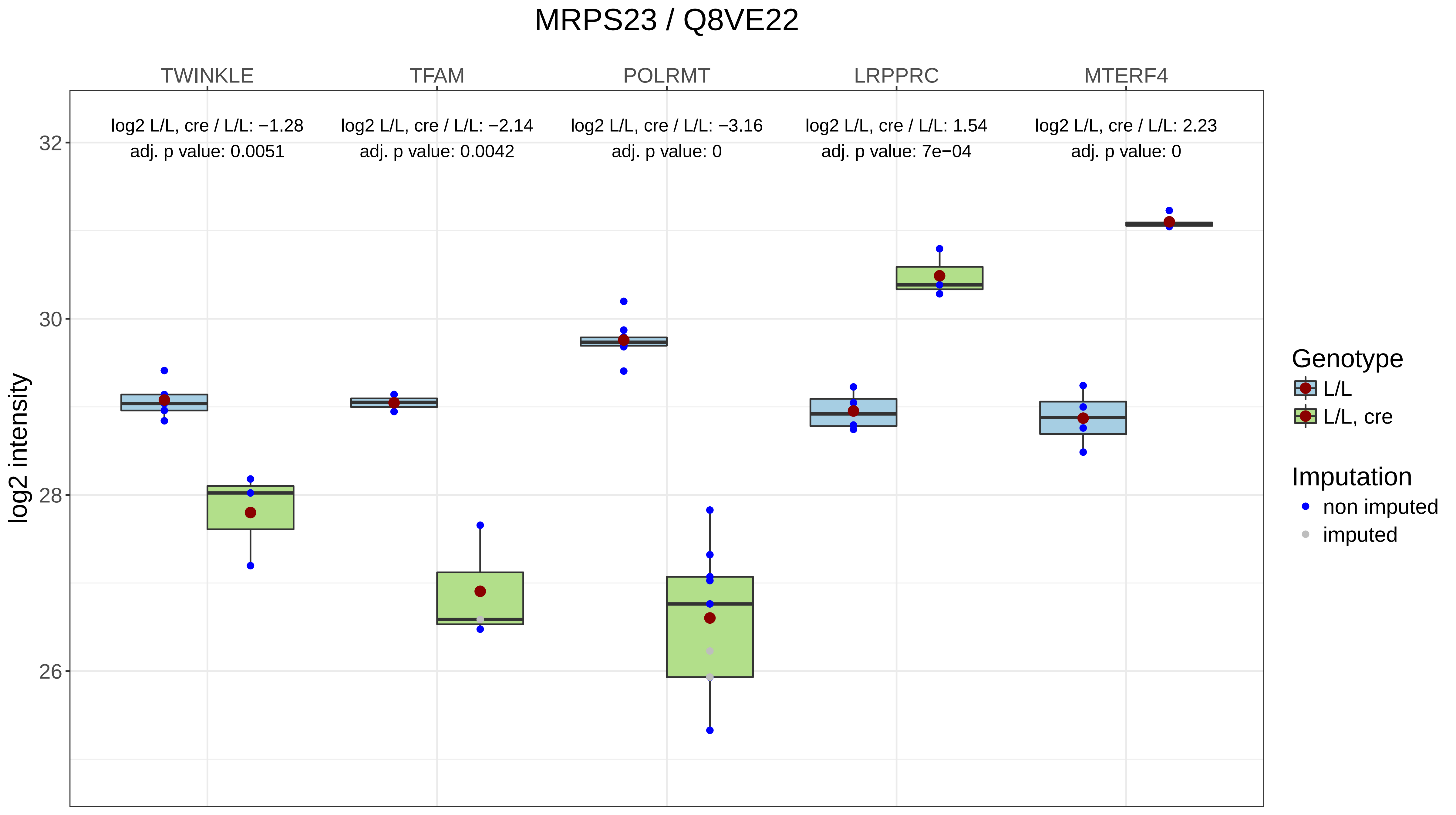
MRPS23 / Q8VE22
TWINKLE
TFAM
POLRMT
LRPPRC
MTERF4
log2 L/L, cre / L/L: −1.28
adj. p value: 0.0051
log2 L/L, cre / L/L: −2.14
adj. p value: 0.0042
log2 L/L, cre / L/L: −3.16
adj. p value: 0
log2 L/L, cre / L/L: 1.54
adj. p value: 7e−04
log2 L/L, cre / L/L: 2.23
adj. p value: 0
log2 intensity
32
30
28
26
Genotype
L/L
L/L, cre
Imputation
non imputed
imputed

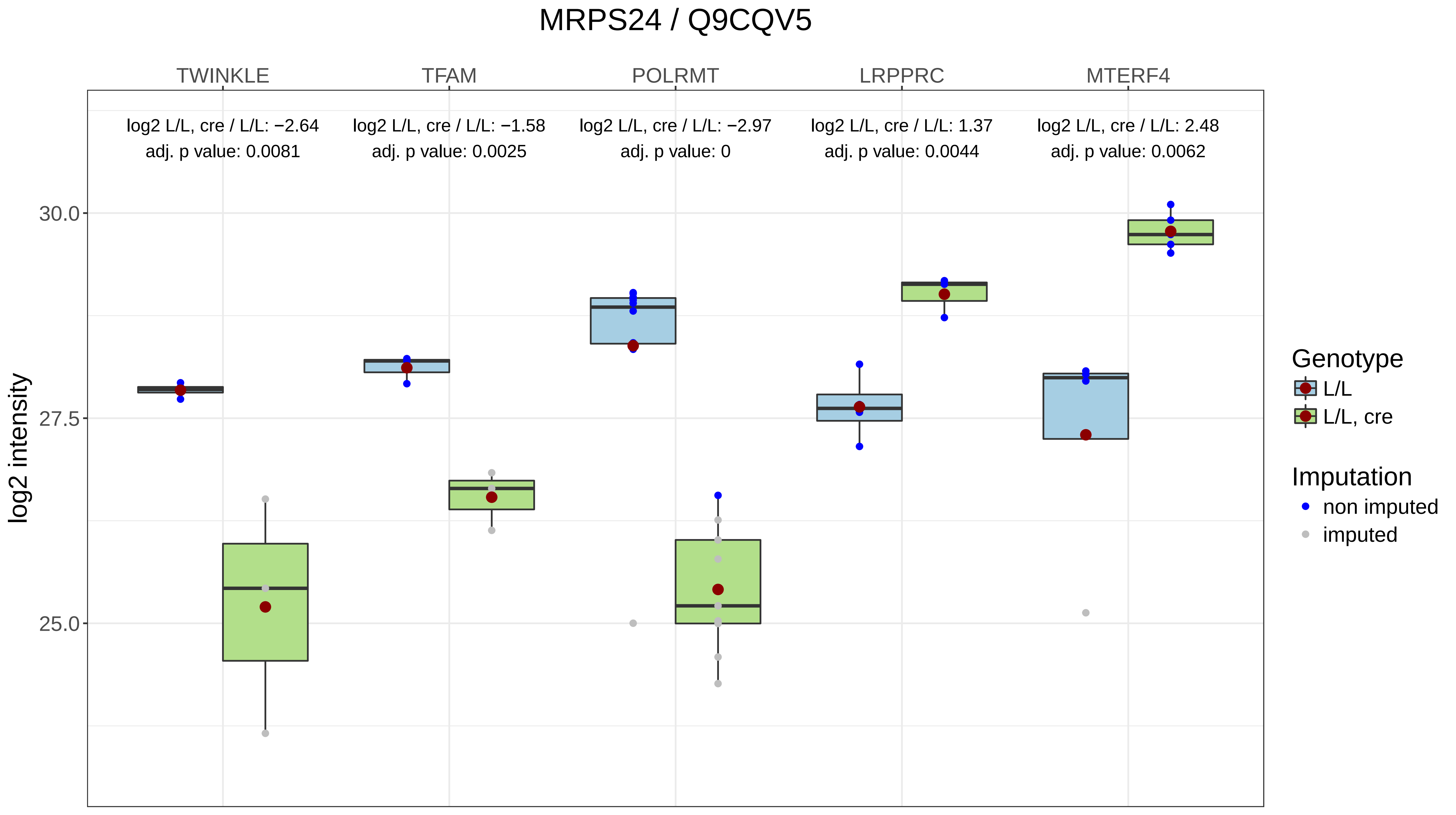

MRPS24 / Q9CQV5
TWINKLE
TFAM
POLRMT
LRPPRC
MTERF4
log2 L/L, cre / L/L: −2.64
adj. p value: 0.0081
log2 L/L, cre / L/L: −1.58
adj. p value: 0.0025
log2 L/L, cre / L/L: −2.97
adj. p value: 0
log2 L/L, cre / L/L: 1.37
adj. p value: 0.0044
log2 L/L, cre / L/L: 2.48
adj. p value: 0.0062
log2 intensity
30.0
27.5
25.0
Genotype
L/L
L/L, cre
Imputation
non imputed
imputed

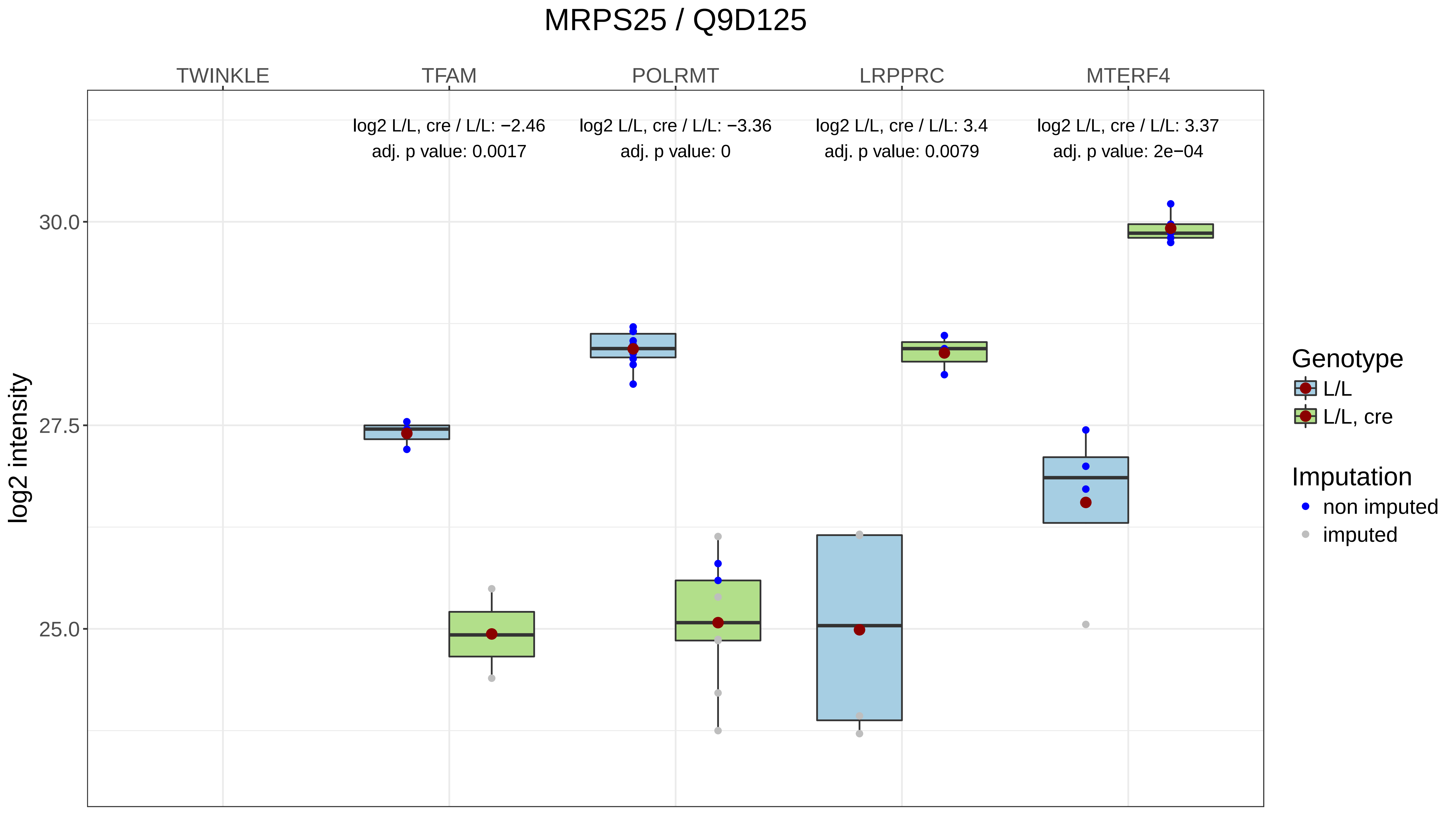
MRPS25 / Q9D125
TWINKLE
TFAM
POLRMT
LRPPRC
MTERF4
log2 L/L, cre / L/L: −2.46
adj. p value: 0.0017
log2 L/L, cre / L/L: −3.36
adj. p value: 0
log2 L/L, cre / L/L: 3.4
adj. p value: 0.0079
log2 L/L, cre / L/L: 3.37
adj. p value: 2e−04
30.0
27.5
25.0
log2 intensity
Genotype
L/L
L/L, cre
Imputation
non imputed
imputed

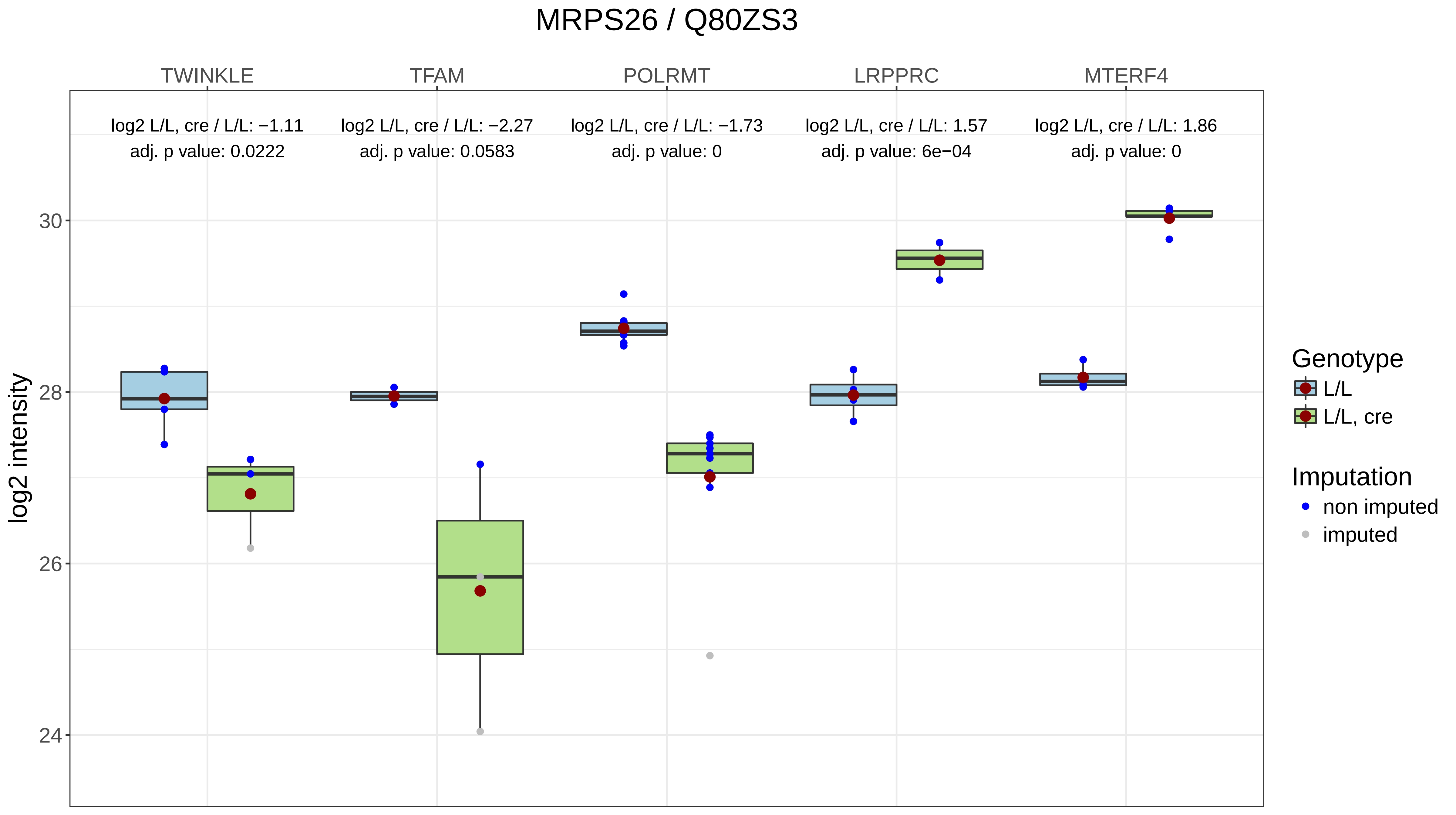
MRPS26 / Q80ZS3
TWINKLE
TFAM
POLRMT
LRPPRC
MTERF4
log2 L/L, cre / L/L: −1.11
adj. p value: 0.0222
log2 L/L, cre / L/L: −2.27
adj. p value: 0.0583
log2 L/L, cre / L/L: −1.73
adj. p value: 0
log2 L/L, cre / L/L: 1.57
adj. p value: 6e−04
log2 L/L, cre / L/L: 1.86
adj. p value: 0
log2 intensity
30
28
26
24
Genotype
L/L
L/L, cre
Imputation
non imputed
imputed

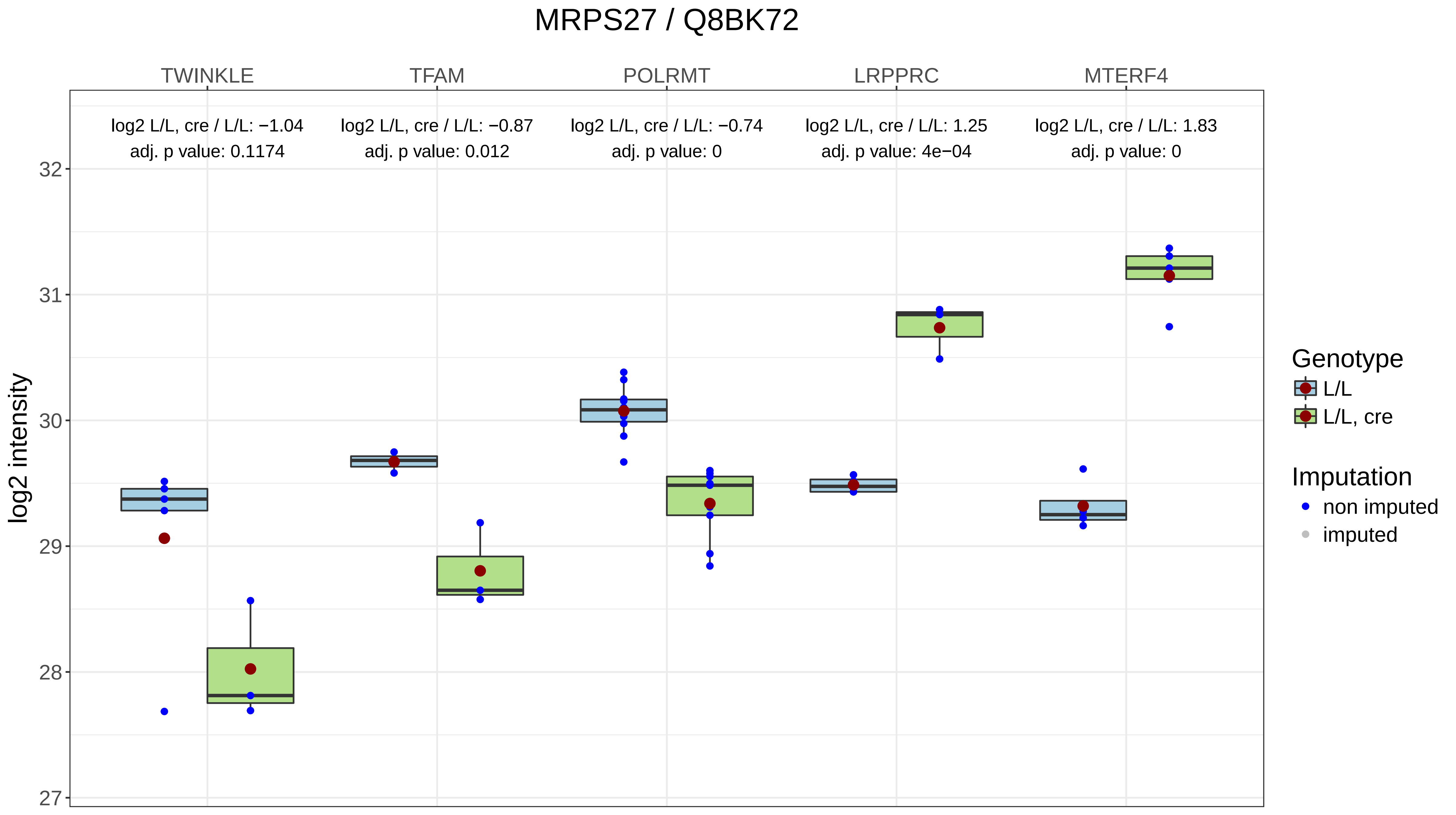
MRPS27 / Q8BK72
TWINKLE
TFAM
POLRMT
LRPPRC
MTERF4
log2 L/L, cre / L/L: −1.04
adj. p value: 0.1174
log2 L/L, cre / L/L: −0.87
adj. p value: 0.012
log2 L/L, cre / L/L: −0.74
adj. p value: 0
log2 L/L, cre / L/L: 1.25
adj. p value: 4e−04
log2 L/L, cre / L/L: 1.83
adj. p value: 0
log2 intensity
32
31
30
29
28
27
Genotype
L/L
L/L, cre
Imputation
non imputed
imputed

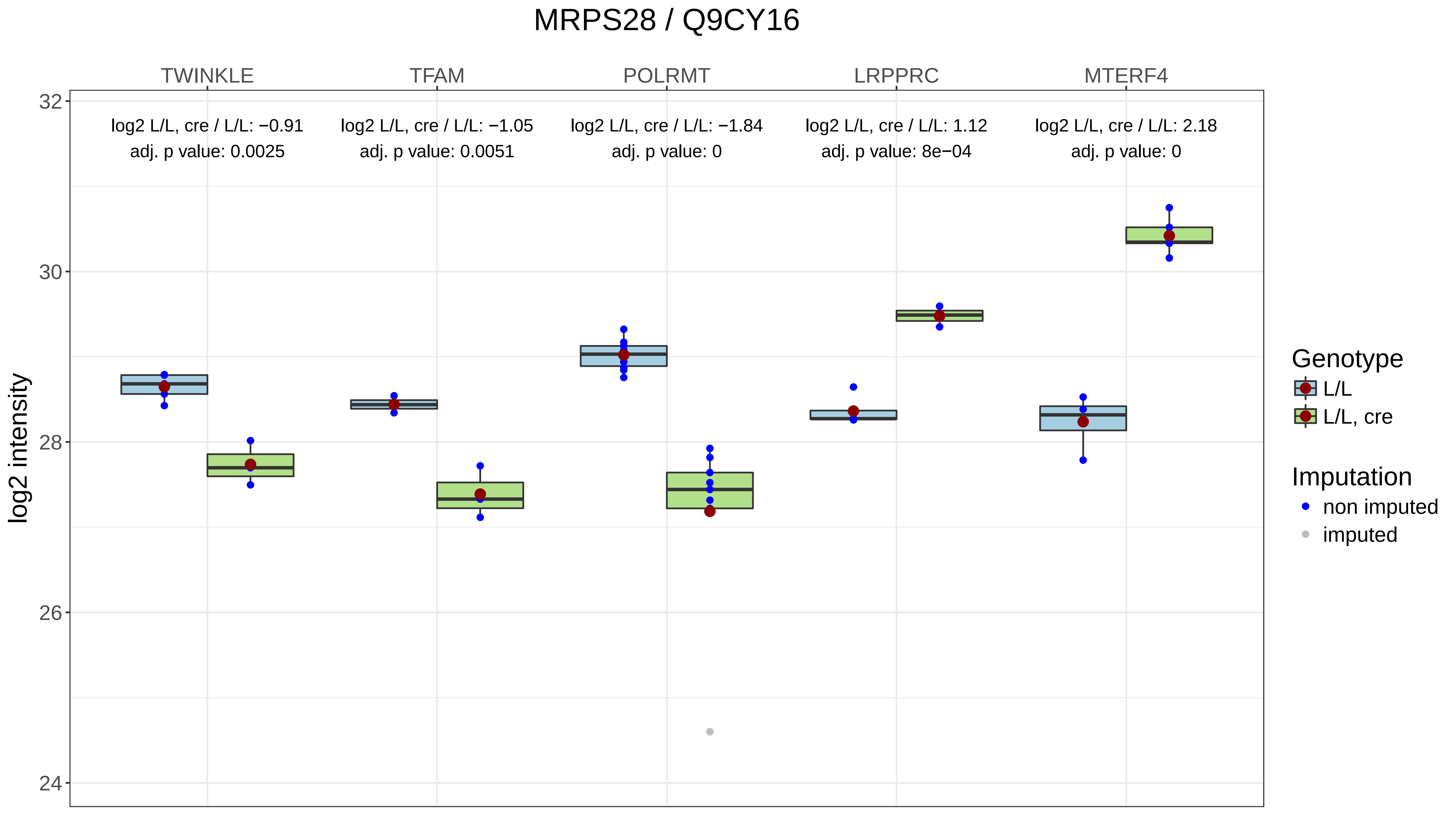
MRPS28 / Q9CY16
TWINKLE
TFAM
POLRMT
LRPPRC
MTERF4
log2 L/L, cre / L/L: −0.91
adj. p value: 0.0025
log2 L/L, cre / L/L: −1.05
adj. p value: 0.0051
log2 L/L, cre / L/L: −1.84
adj. p value: 0
log2 L/L, cre / L/L: 1.12
adj. p value: 8e−04
log2 L/L, cre / L/L: 2.18
adj. p value: 0
32
30
28
26
24
log2 intensity
Genotype
L/L
L/L, cre
Imputation
non imputed
imputed

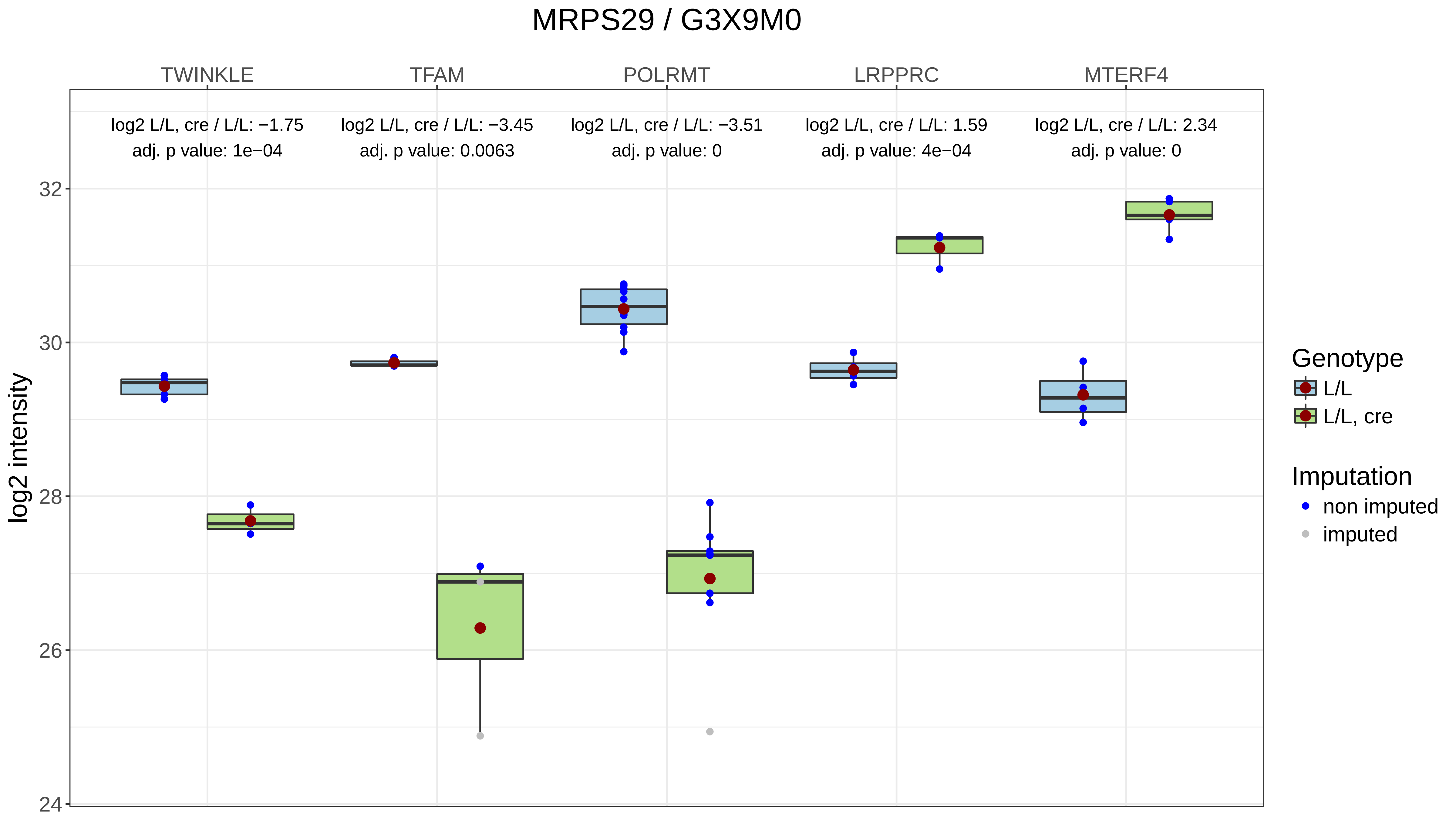
MRPS29 / G3X9M0
TWINKLE
TFAM
POLRMT
LRPPRC
MTERF4
log2 L/L, cre / L/L: −1.75
adj. p value: 1e−04
log2 L/L, cre / L/L: −3.45
adj. p value: 0.0063
log2 L/L, cre / L/L: −3.51
adj. p value: 0
log2 L/L, cre / L/L: 1.59
adj. p value: 4e−04
log2 L/L, cre / L/L: 2.34
adj. p value: 0
log2 intensity
32
30
28
26
24
Genotype
L/L
L/L, cre
Imputation
non imputed
imputed

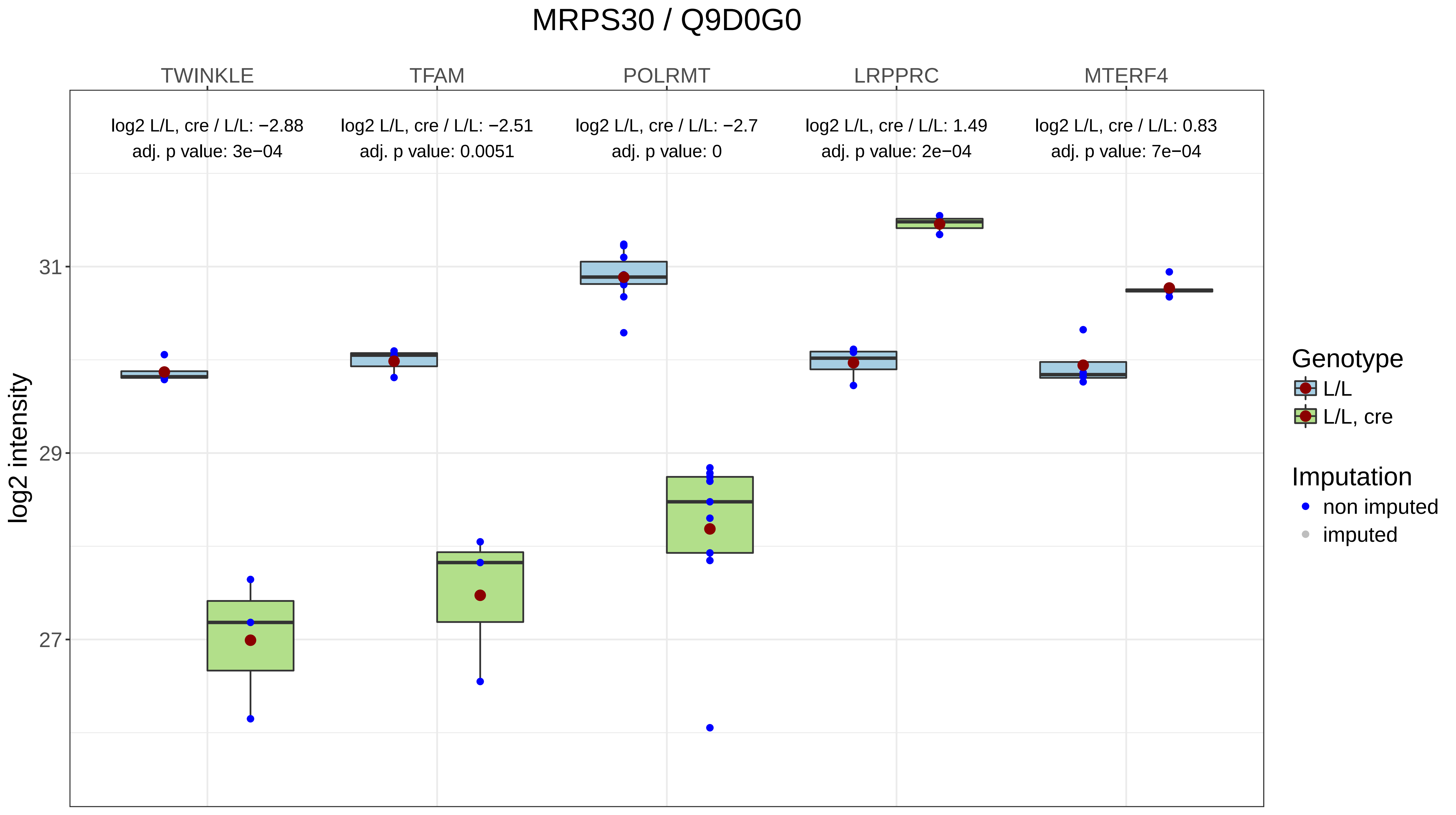
MRPS30 / Q9D0G0
TWINKLE
TFAM
POLRMT
LRPPRC
MTERF4
log2 L/L, cre / L/L: −2.88
adj. p value: 3e−04
log2 L/L, cre / L/L: −2.51
adj. p value: 0.0051
log2 L/L, cre / L/L: −2.7
adj. p value: 0
log2 L/L, cre / L/L: 1.49
adj. p value: 2e−04
log2 L/L, cre / L/L: 0.83
adj. p value: 7e−04
log2 intensity
31
29
27
Genotype
L/L
L/L, cre
Imputation
non imputed
imputed

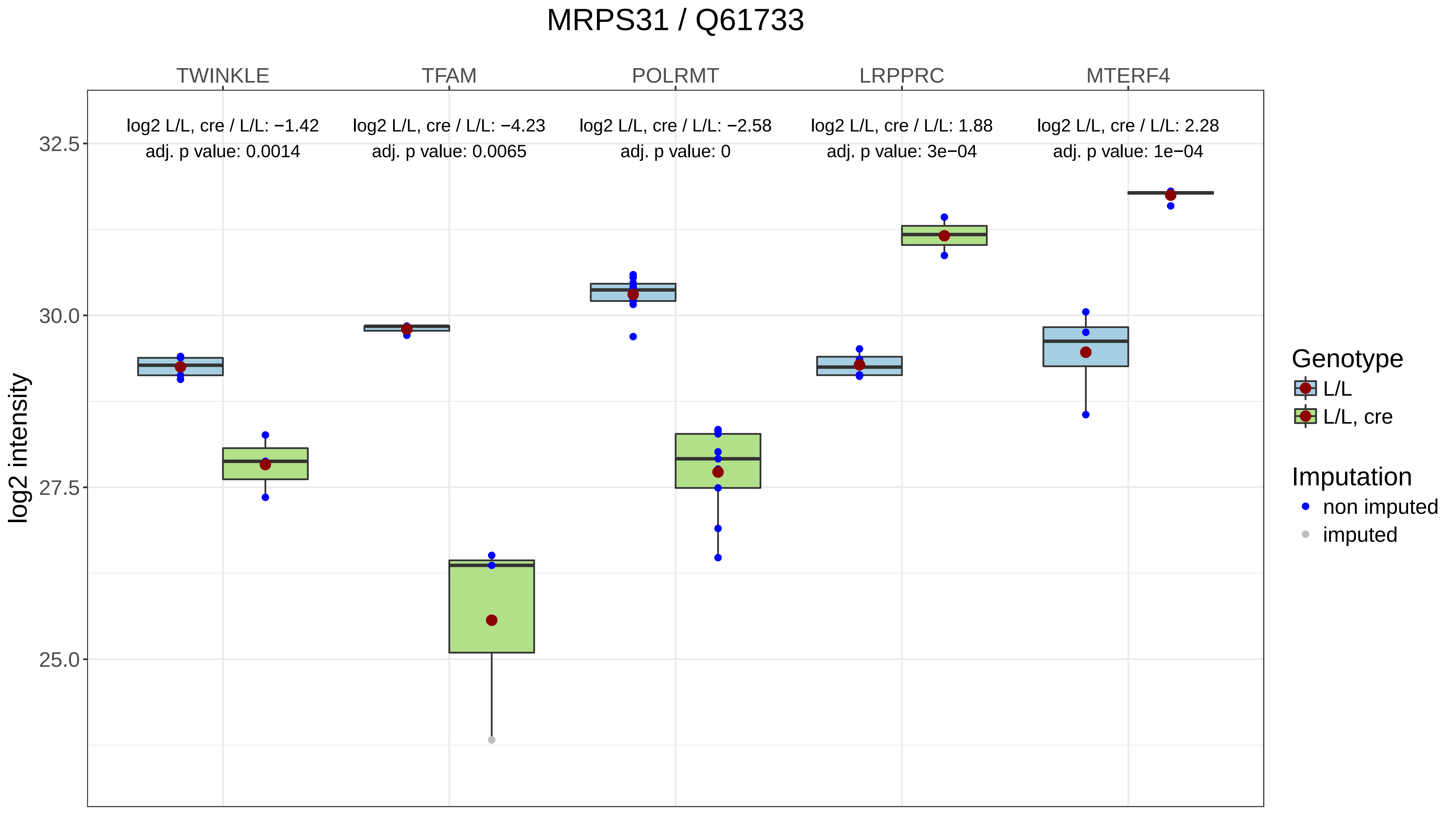
MRPS31 / Q61733
TWINKLE
TFAM
POLRMT
LRPPRC
MTERF4
log2 L/L, cre / L/L: −1.42
adj. p value: 0.0014
log2 L/L, cre / L/L: −4.23
adj. p value: 0.0065
log2 L/L, cre / L/L: −2.58
adj. p value: 0
log2 L/L, cre / L/L: 1.88
adj. p value: 3e−04
log2 L/L, cre / L/L: 2.28
adj. p value: 1e−04
32.5
30.0
27.5
25.0
log2 intensity
Genotype
L/L
L/L, cre
Imputation
non imputed
imputed

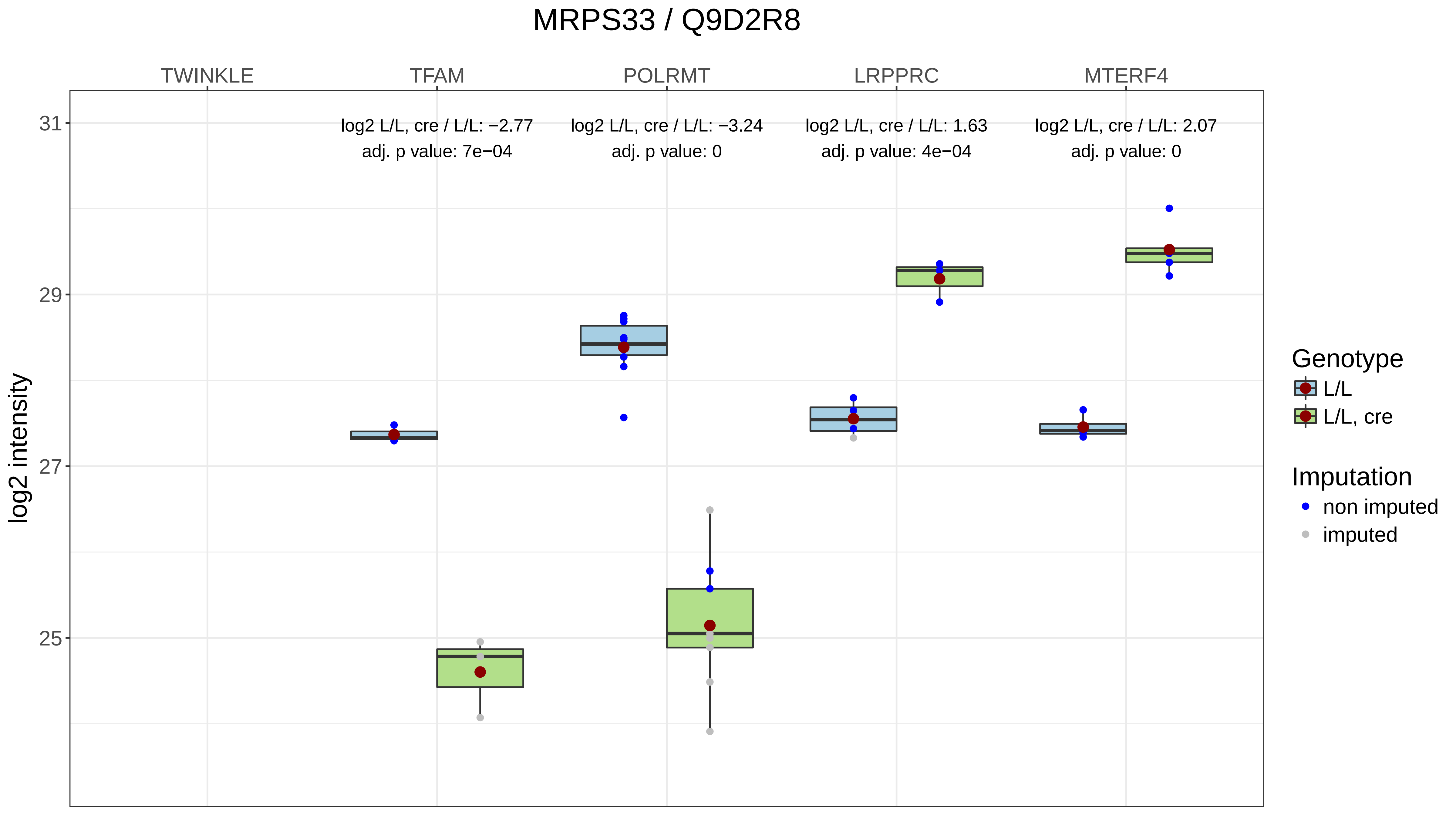
MRPS33 / Q9D2R8
TWINKLE
TFAM
POLRMT
LRPPRC
MTERF4
log2 L/L, cre / L/L: −2.77
adj. p value: 7e−04
log2 L/L, cre / L/L: −3.24
adj. p value: 0
log2 L/L, cre / L/L: 1.63
adj. p value: 4e−04
log2 L/L, cre / L/L: 2.07
adj. p value: 0
31
29
27
25
log2 intensity
Genotype
L/L
L/L, cre
Imputation
non imputed
imputed

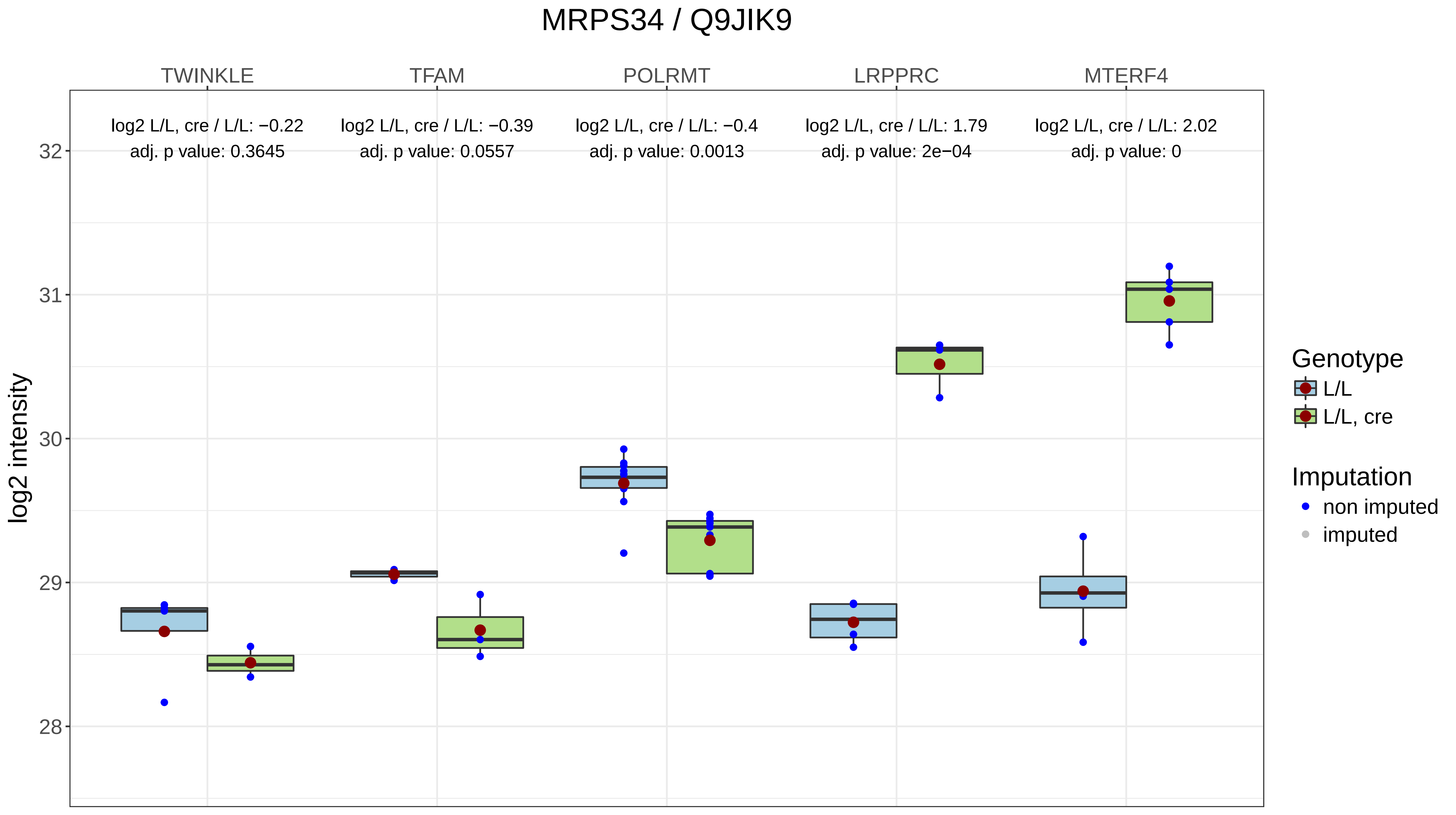

MRPS34 / Q9JIK9
TWINKLE
TFAM
POLRMT
LRPPRC
MTERF4
log2 L/L, cre / L/L: −0.22
adj. p value: 0.3645
log2 L/L, cre / L/L: −0.39
adj. p value: 0.0557
log2 L/L, cre / L/L: −0.4
adj. p value: 0.0013
log2 L/L, cre / L/L: 1.79
adj. p value: 2e−04
log2 L/L, cre / L/L: 2.02
adj. p value: 0
log2 intensity
32
31
30
29
28
Genotype
L/L
L/L, cre
Imputation
non imputed
imputed

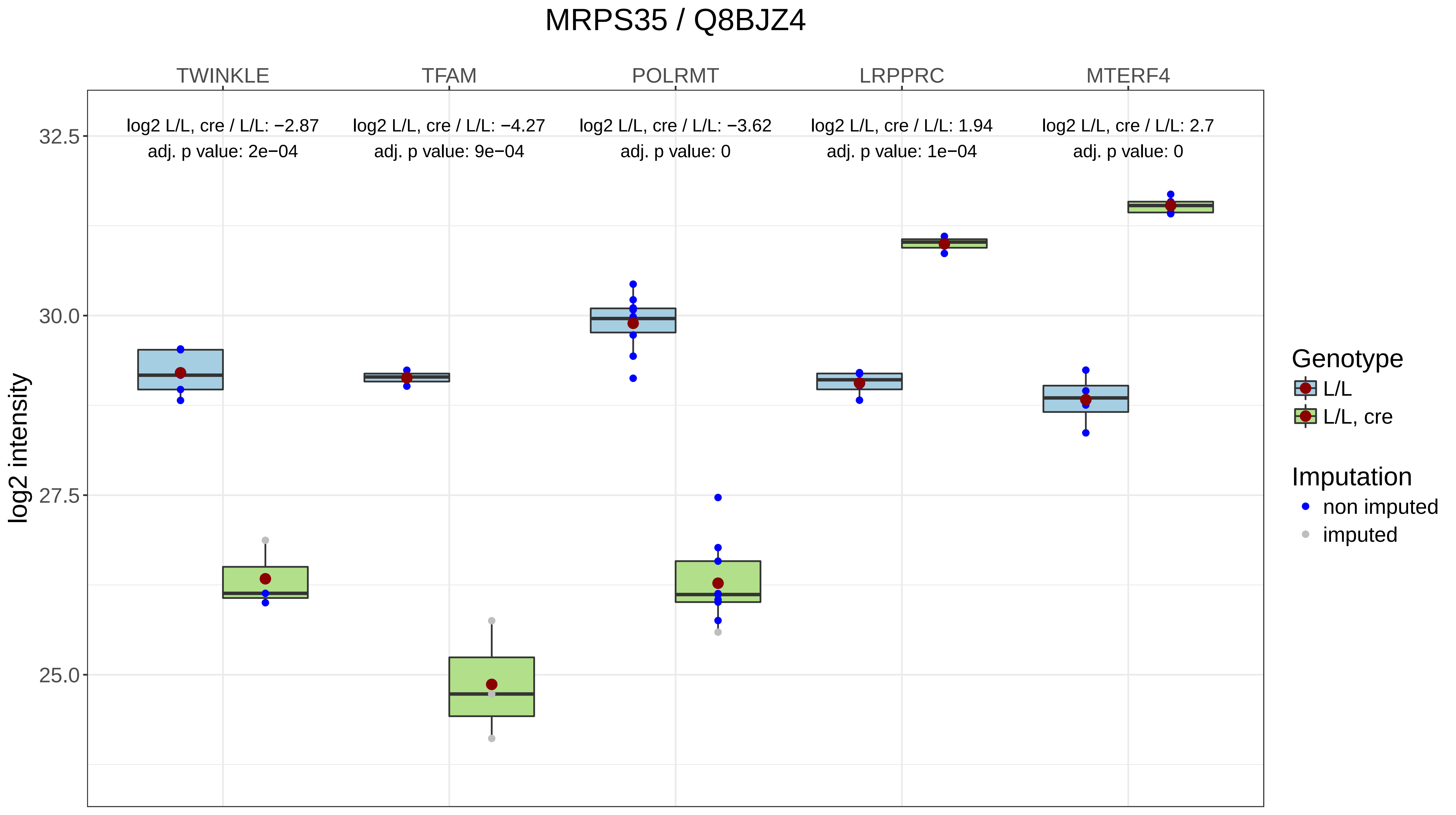
MRPS35 / Q8BJZ4
TWINKLE
TFAM
POLRMT
LRPPRC
MTERF4
log2 L/L, cre / L/L: −2.87
adj. p value: 2e−04
log2 L/L, cre / L/L: −4.27
adj. p value: 9e−04
log2 L/L, cre / L/L: −3.62
adj. p value: 0
log2 L/L, cre / L/L: 1.94
adj. p value: 1e−04
log2 L/L, cre / L/L: 2.7
adj. p value: 0
log2 intensity
32.5
30.0
27.5
25.0
Genotype
L/L
L/L, cre
Imputation
non imputed
imputed

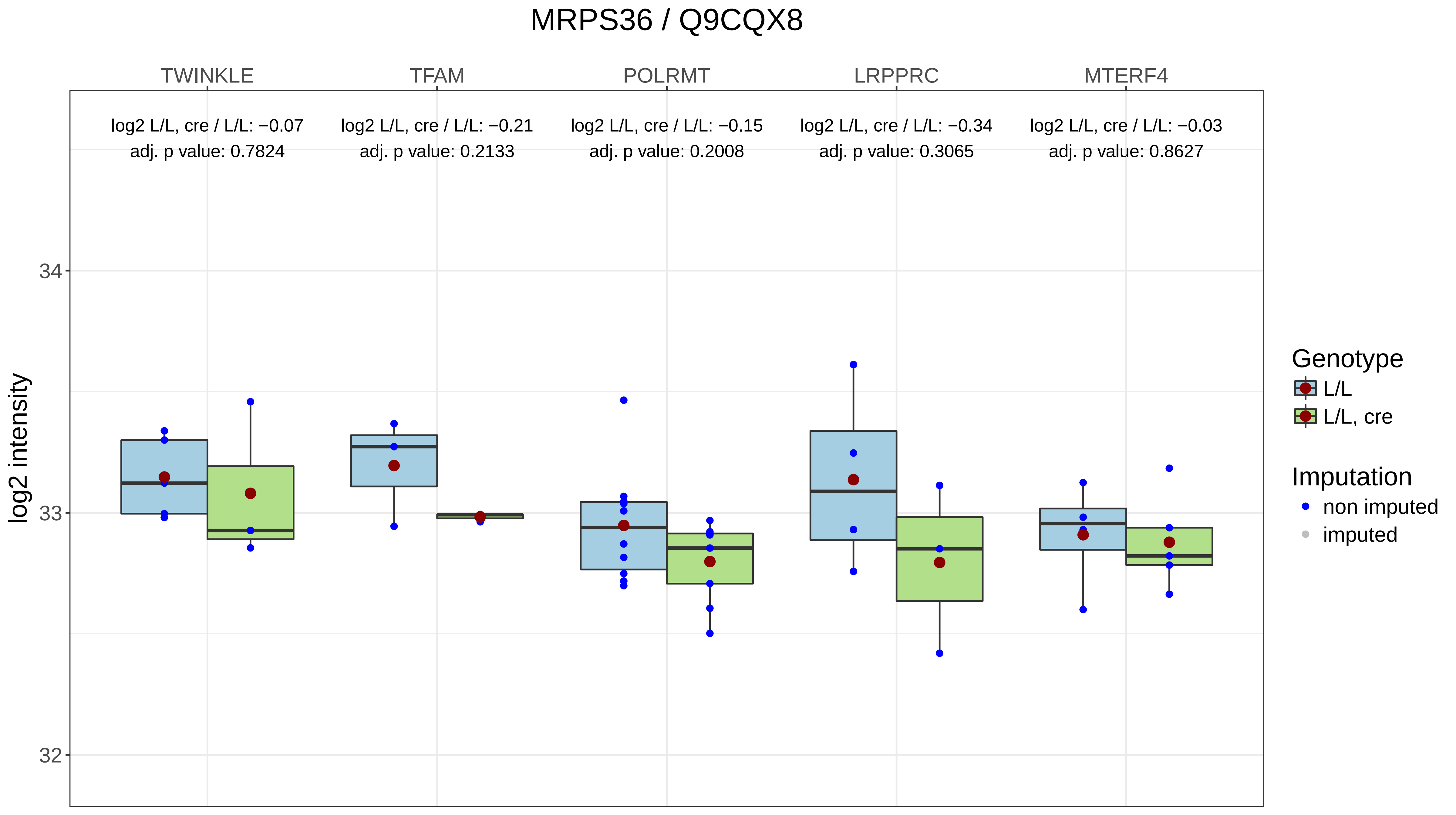
MRPS36 / Q9CQX8
TWINKLE
TFAM
POLRMT
LRPPRC
MTERF4
log2 L/L, cre / L/L: −0.07
adj. p value: 0.7824
log2 L/L, cre / L/L: −0.21
adj. p value: 0.2133
log2 L/L, cre / L/L: −0.15
adj. p value: 0.2008
log2 L/L, cre / L/L: −0.34
adj. p value: 0.3065
log2 L/L, cre / L/L: −0.03
adj. p value: 0.8627
log2 intensity
34
33
32
Genotype
L/L
L/L, cre
Imputation
non imputed
imputed

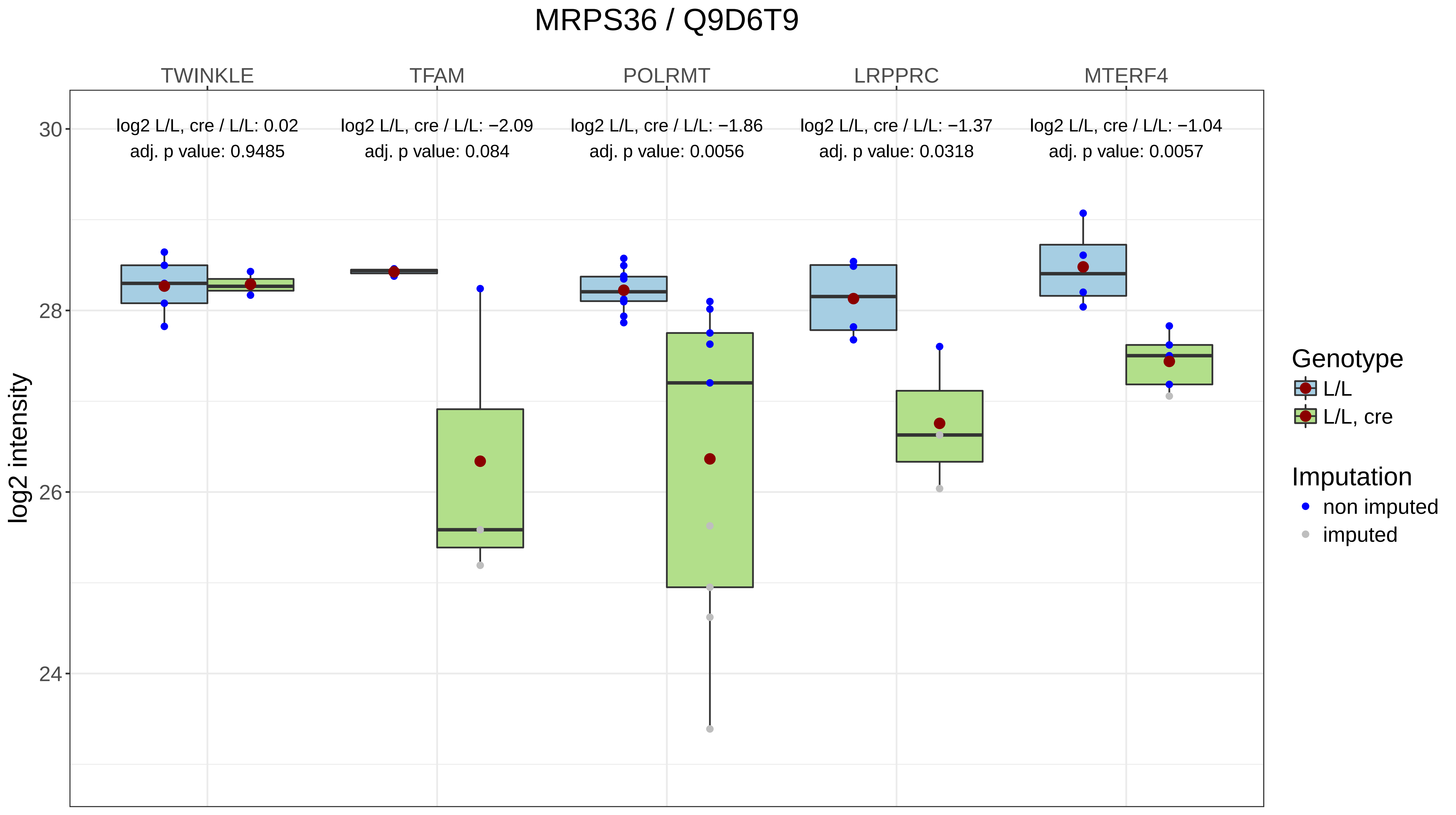
MRPS36 / Q9D6T9
TWINKLE
TFAM
POLRMT
LRPPRC
MTERF4
log2 L/L, cre / L/L: 0.02
adj. p value: 0.9485
log2 L/L, cre / L/L: −2.09
adj. p value: 0.084
log2 L/L, cre / L/L: −1.86
adj. p value: 0.0056
log2 L/L, cre / L/L: −1.37
adj. p value: 0.0318
log2 L/L, cre / L/L: −1.04
adj. p value: 0.0057
30
28
26
24
log2 intensity
Genotype
L/L
L/L, cre
Imputation
non imputed
imputed

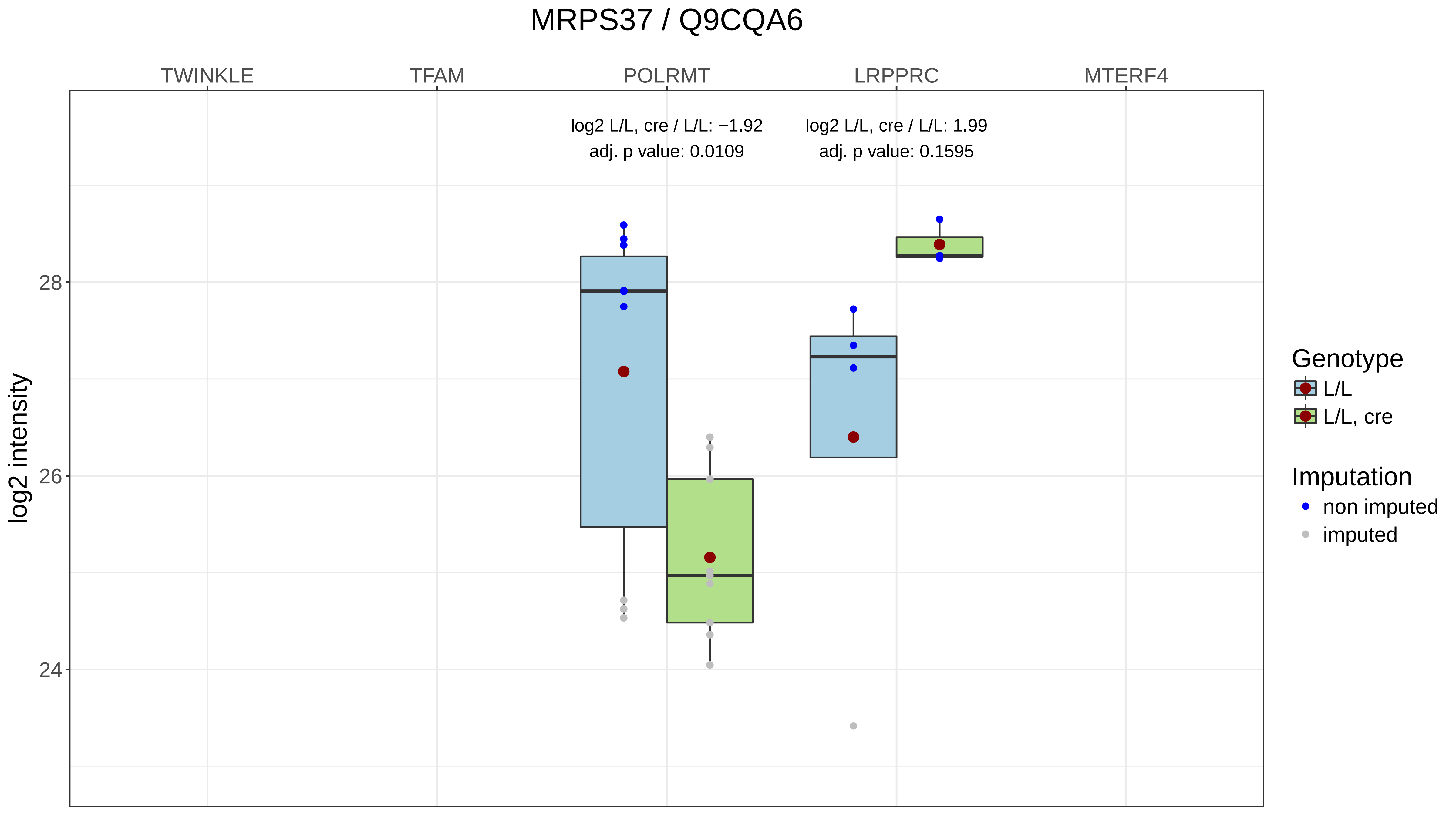
MRPS37 / Q9CQA6
TWINKLE
TFAM
POLRMT
LRPPRC
MTERF4
log2 L/L, cre / L/L: −1.92
adj. p value: 0.0109
log2 L/L, cre / L/L: 1.99
adj. p value: 0.1595
28
26
24
log2 intensity
Genotype
L/L
L/L, cre
Imputation
non imputed
imputed

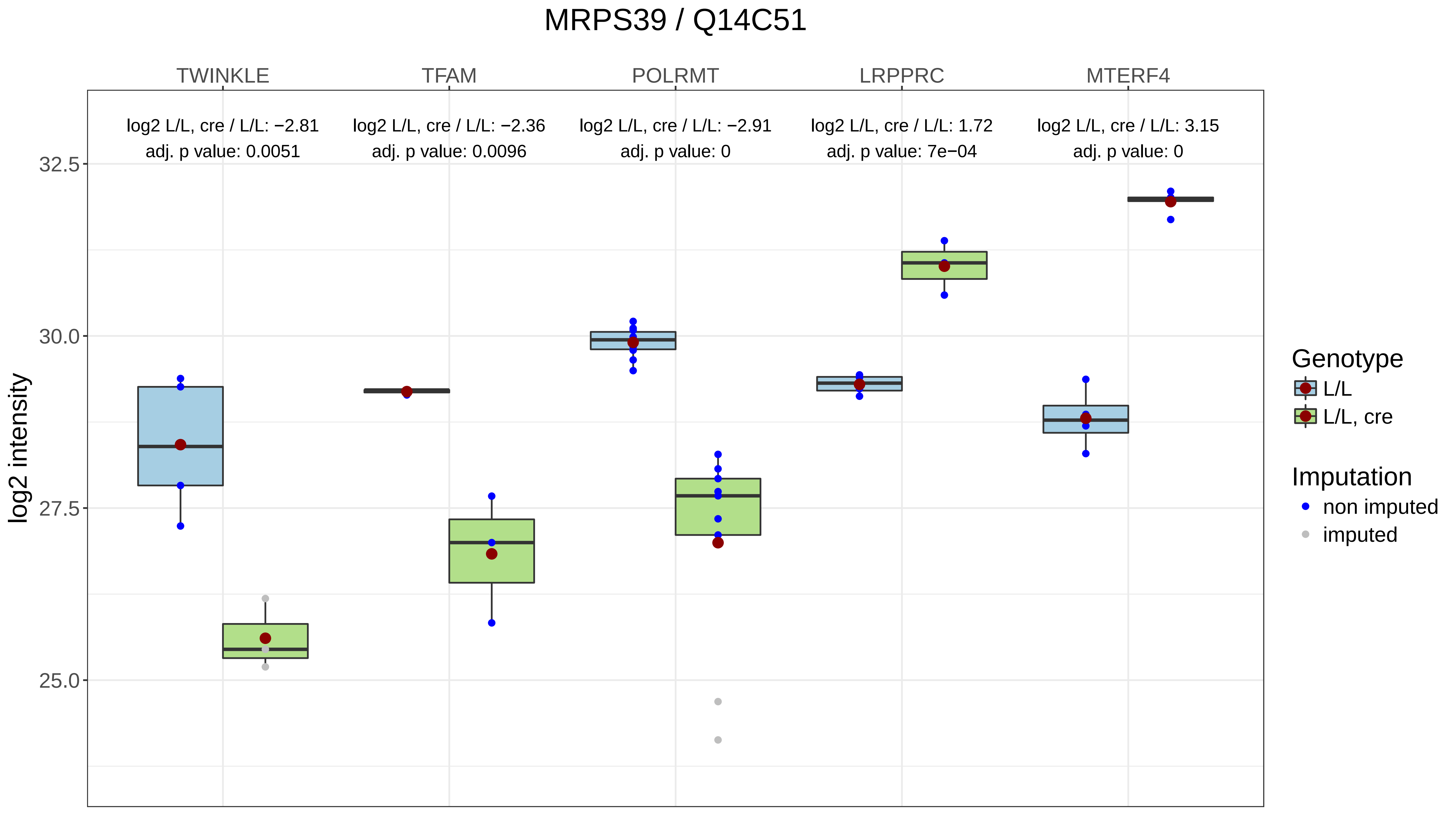
MRPS39 / Q14C51
TWINKLE
TFAM
POLRMT
LRPPRC
MTERF4
log2 L/L, cre / L/L: −2.81
adj. p value: 0.0051
log2 L/L, cre / L/L: −2.36
adj. p value: 0.0096
log2 L/L, cre / L/L: −2.91
adj. p value: 0
log2 L/L, cre / L/L: 1.72
adj. p value: 7e−04
log2 L/L, cre / L/L: 3.15
adj. p value: 0
32.5
30.0
27.5
25.0
log2 intensity
Genotype
L/L
L/L, cre
Imputation
non imputed
imputed

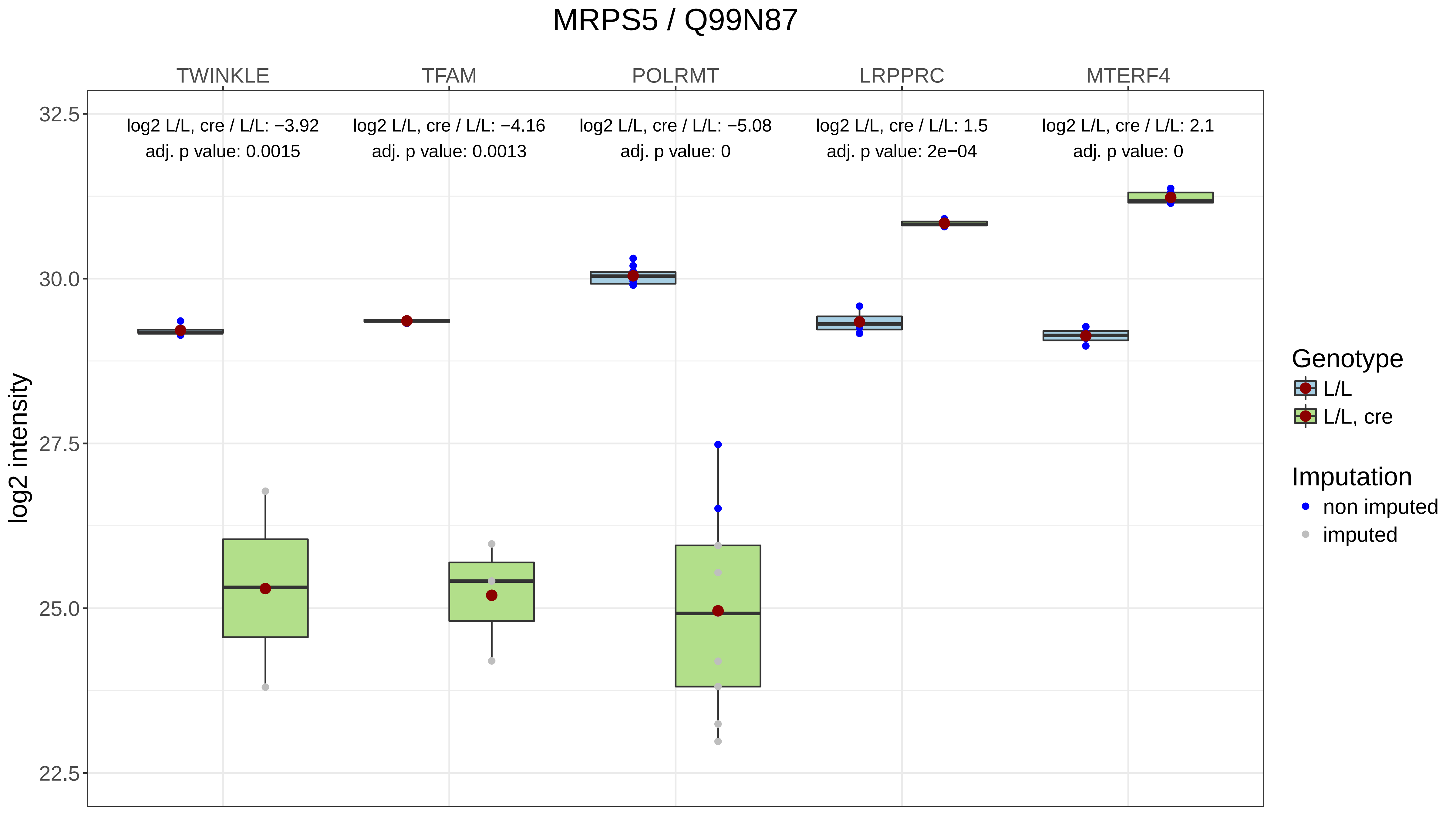

MRPS5 / Q99N87
TWINKLE
TFAM
POLRMT
LRPPRC
MTERF4
log2 L/L, cre / L/L: −3.92
adj. p value: 0.0015
log2 L/L, cre / L/L: −4.16
adj. p value: 0.0013
log2 L/L, cre / L/L: −5.08
adj. p value: 0
log2 L/L, cre / L/L: 1.5
adj. p value: 2e−04
log2 L/L, cre / L/L: 2.1
adj. p value: 0
32.5
30.0
27.5
25.0
22.5
log2 intensity
Genotype
L/L
L/L, cre
Imputation
non imputed
imputed

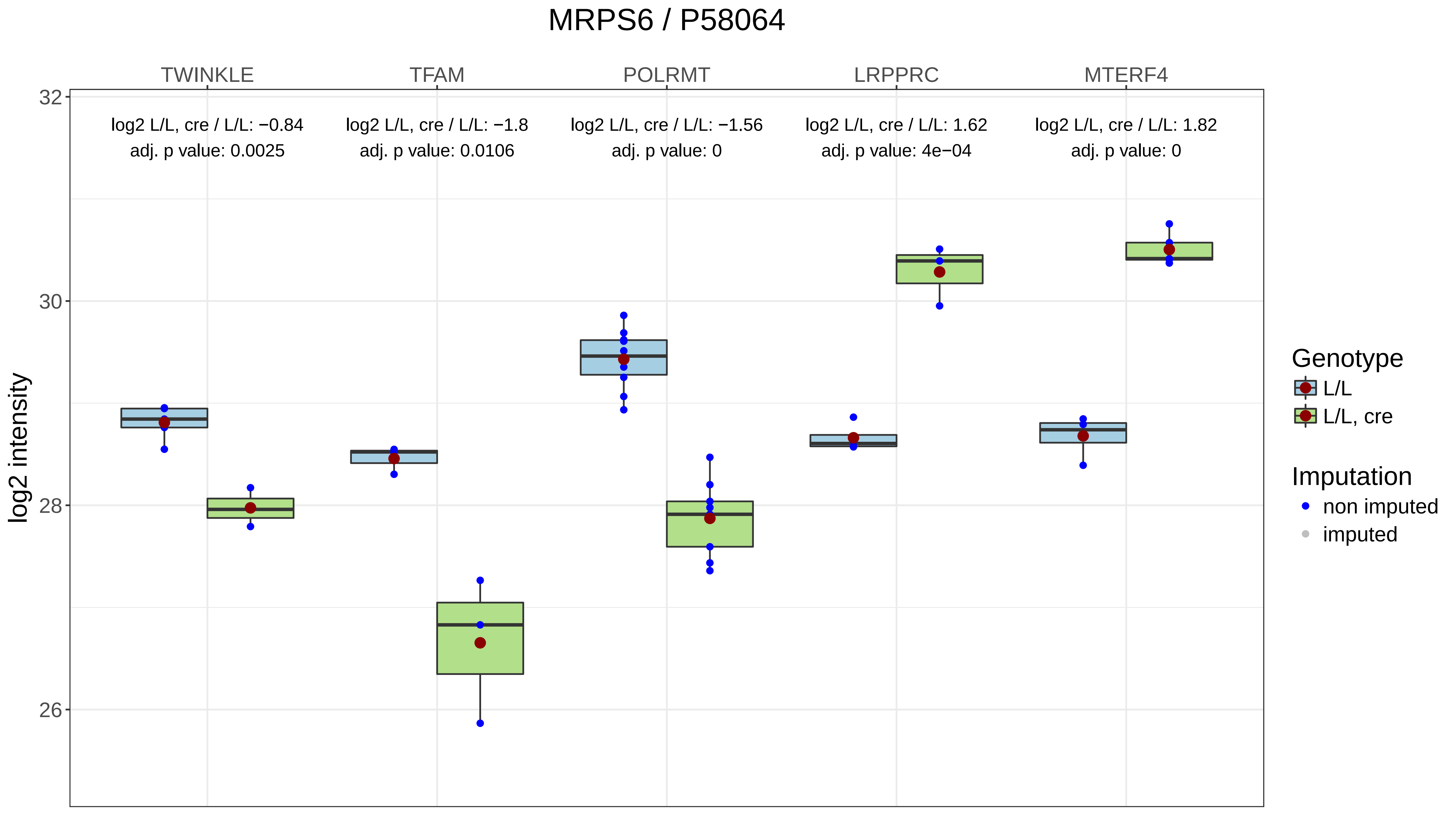
MRPS6 / P58064
TWINKLE
TFAM
POLRMT
LRPPRC
MTERF4
log2 L/L, cre / L/L: −0.84
adj. p value: 0.0025
log2 L/L, cre / L/L: −1.8
adj. p value: 0.0106
log2 L/L, cre / L/L: −1.56
adj. p value: 0
log2 L/L, cre / L/L: 1.62
adj. p value: 4e−04
log2 L/L, cre / L/L: 1.82
adj. p value: 0
32
30
28
26
log2 intensity
Genotype
L/L
L/L, cre
Imputation
non imputed
imputed

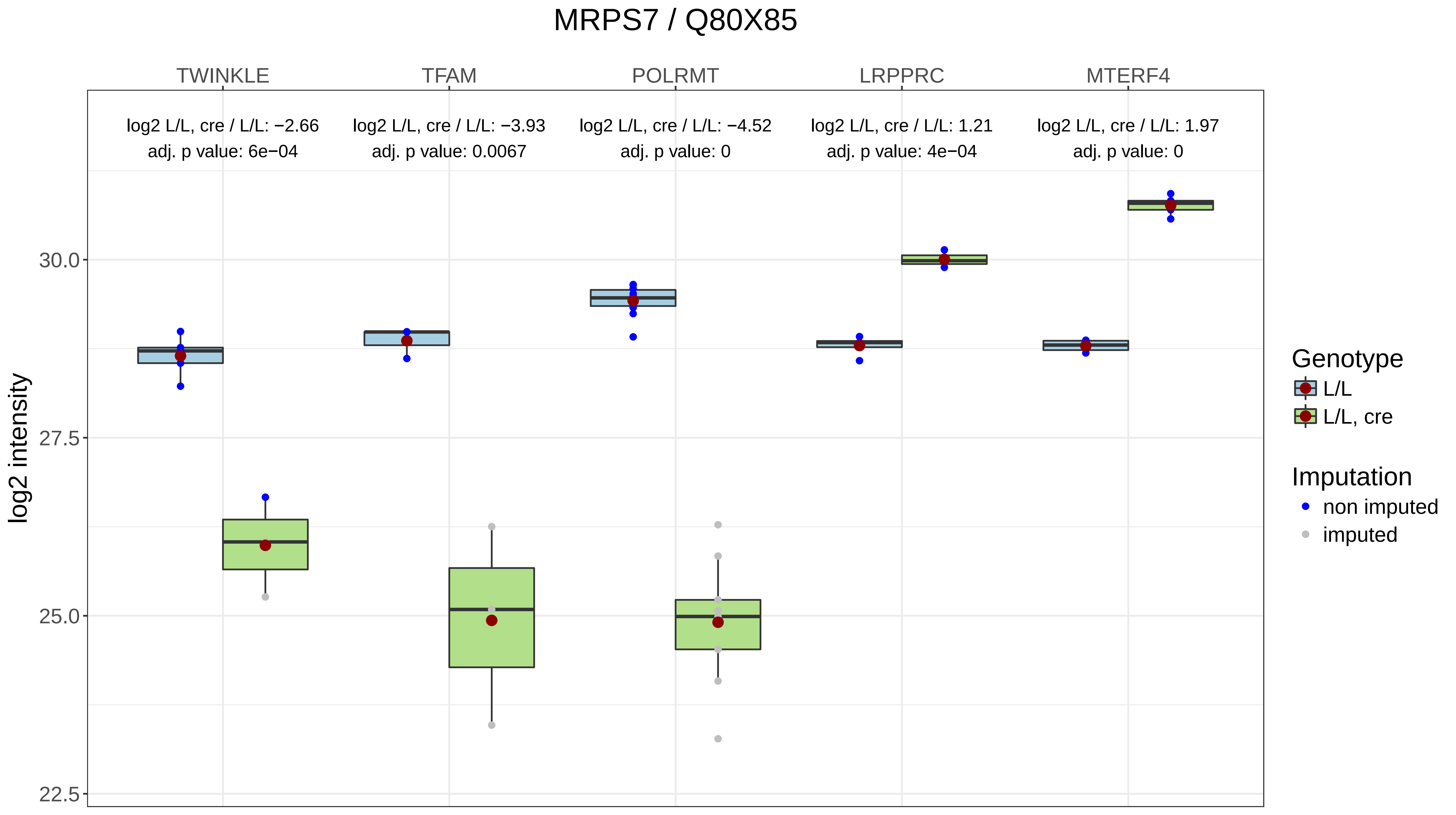

MRPS7 / Q80X85
TWINKLE
TFAM
POLRMT
LRPPRC
MTERF4
log2 L/L, cre / L/L: −2.66
adj. p value: 6e−04
log2 L/L, cre / L/L: −3.93
adj. p value: 0.0067
log2 L/L, cre / L/L: −4.52
adj. p value: 0
log2 L/L, cre / L/L: 1.21
adj. p value: 4e−04
log2 L/L, cre / L/L: 1.97
adj. p value: 0
log2 intensity
30.0
27.5
25.0
22.5
Genotype
L/L
L/L, cre
Imputation
non imputed
imputed

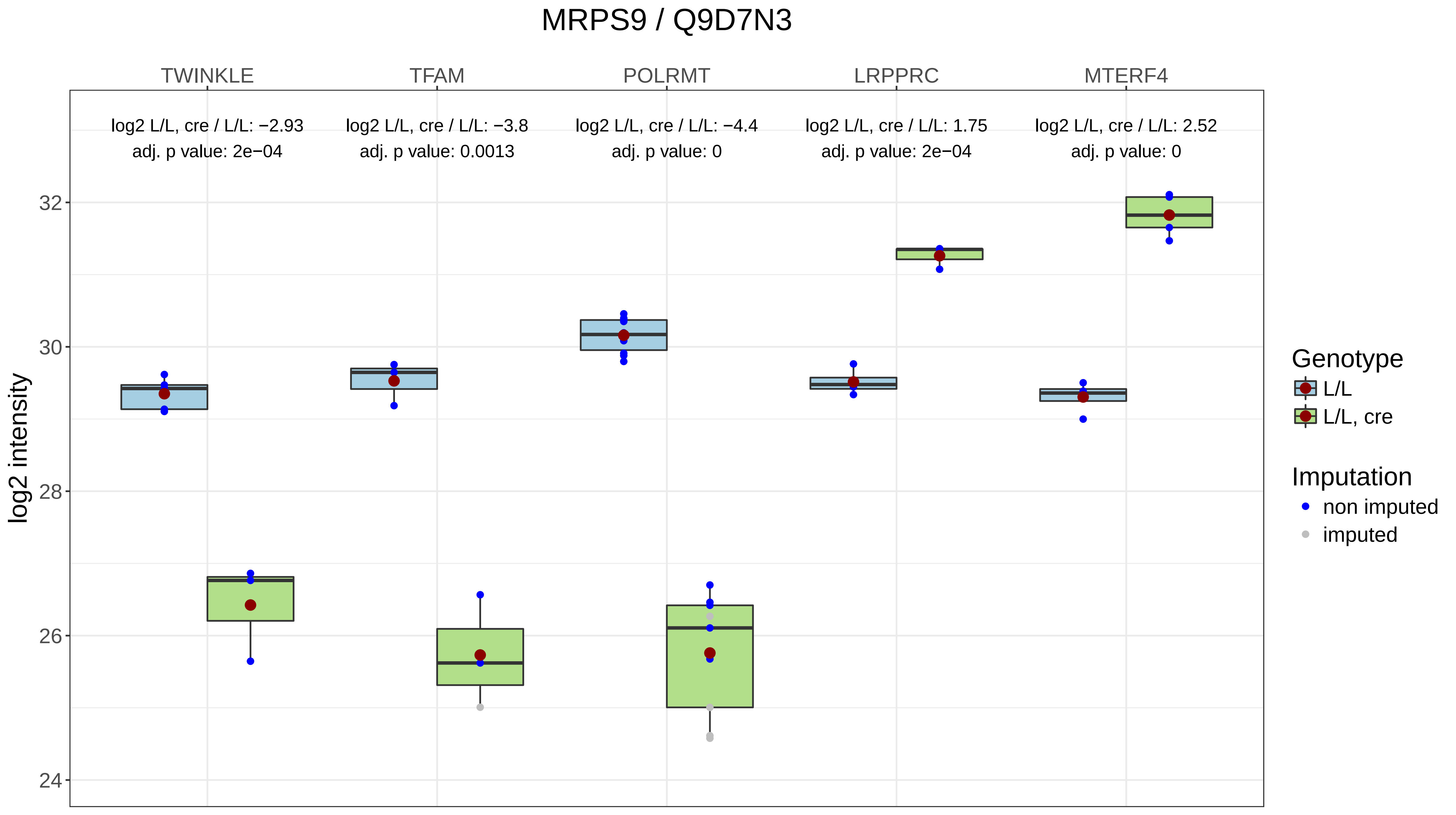
MRPS9 / Q9D7N3
TWINKLE
TFAM
POLRMT
LRPPRC
MTERF4
log2 L/L, cre / L/L: −2.93
adj. p value: 2e−04
log2 L/L, cre / L/L: −3.8
adj. p value: 0.0013
log2 L/L, cre / L/L: −4.4
adj. p value: 0
log2 L/L, cre / L/L: 1.75
adj. p value: 2e−04
log2 L/L, cre / L/L: 2.52
adj. p value: 0
log2 intensity
32
30
28
26
24
Genotype
L/L
L/L, cre
Imputation
non imputed
imputed

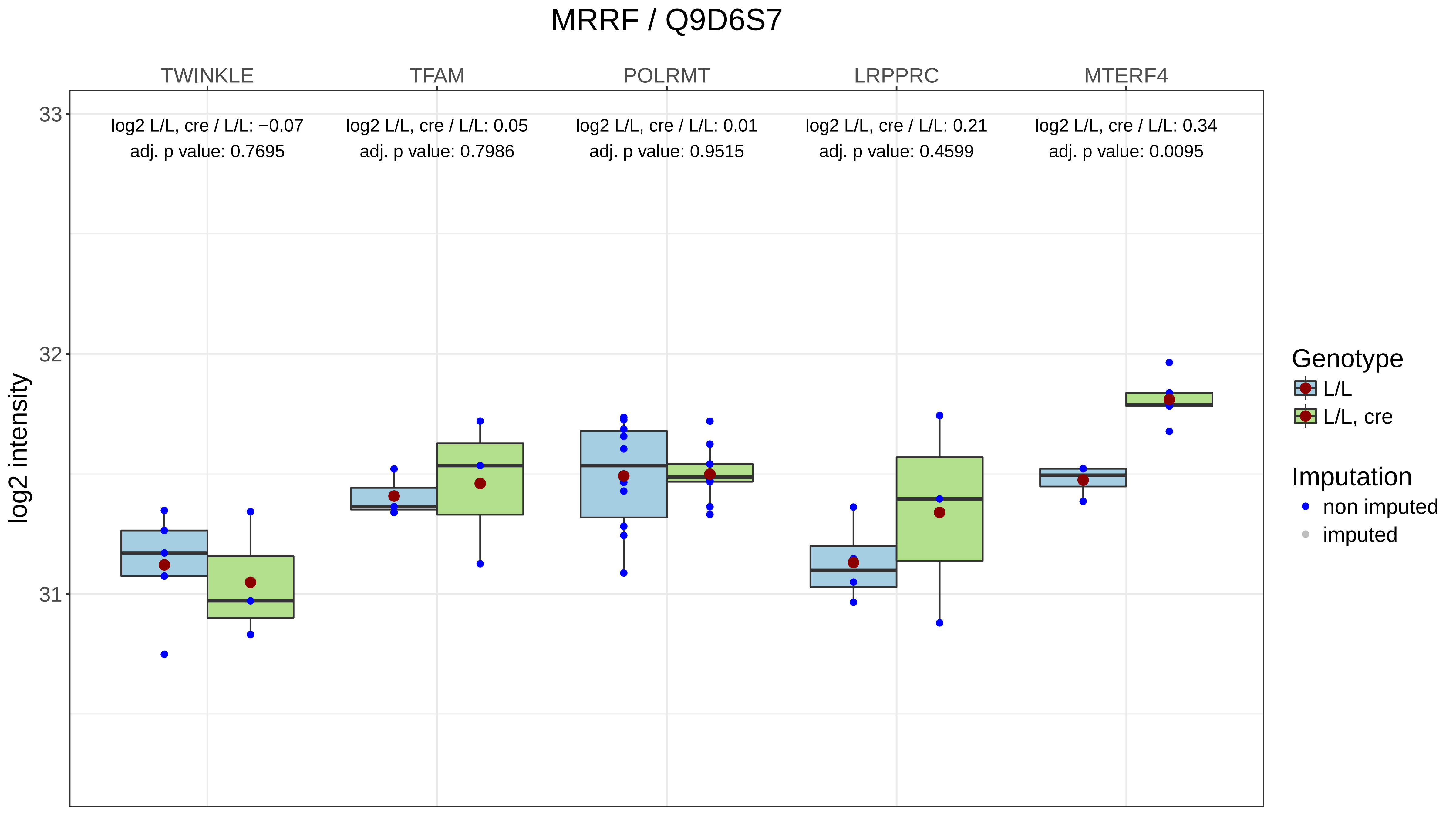

MRRF / Q9D6S7
TWINKLE
TFAM
POLRMT
LRPPRC
MTERF4
log2 L/L, cre / L/L: −0.07
adj. p value: 0.7695
log2 L/L, cre / L/L: 0.05
adj. p value: 0.7986
log2 L/L, cre / L/L: 0.01
adj. p value: 0.9515
log2 L/L, cre / L/L: 0.21
adj. p value: 0.4599
log2 L/L, cre / L/L: 0.34
adj. p value: 0.0095
33
32
31
log2 intensity
Genotype
L/L
L/L, cre
Imputation
non imputed
imputed

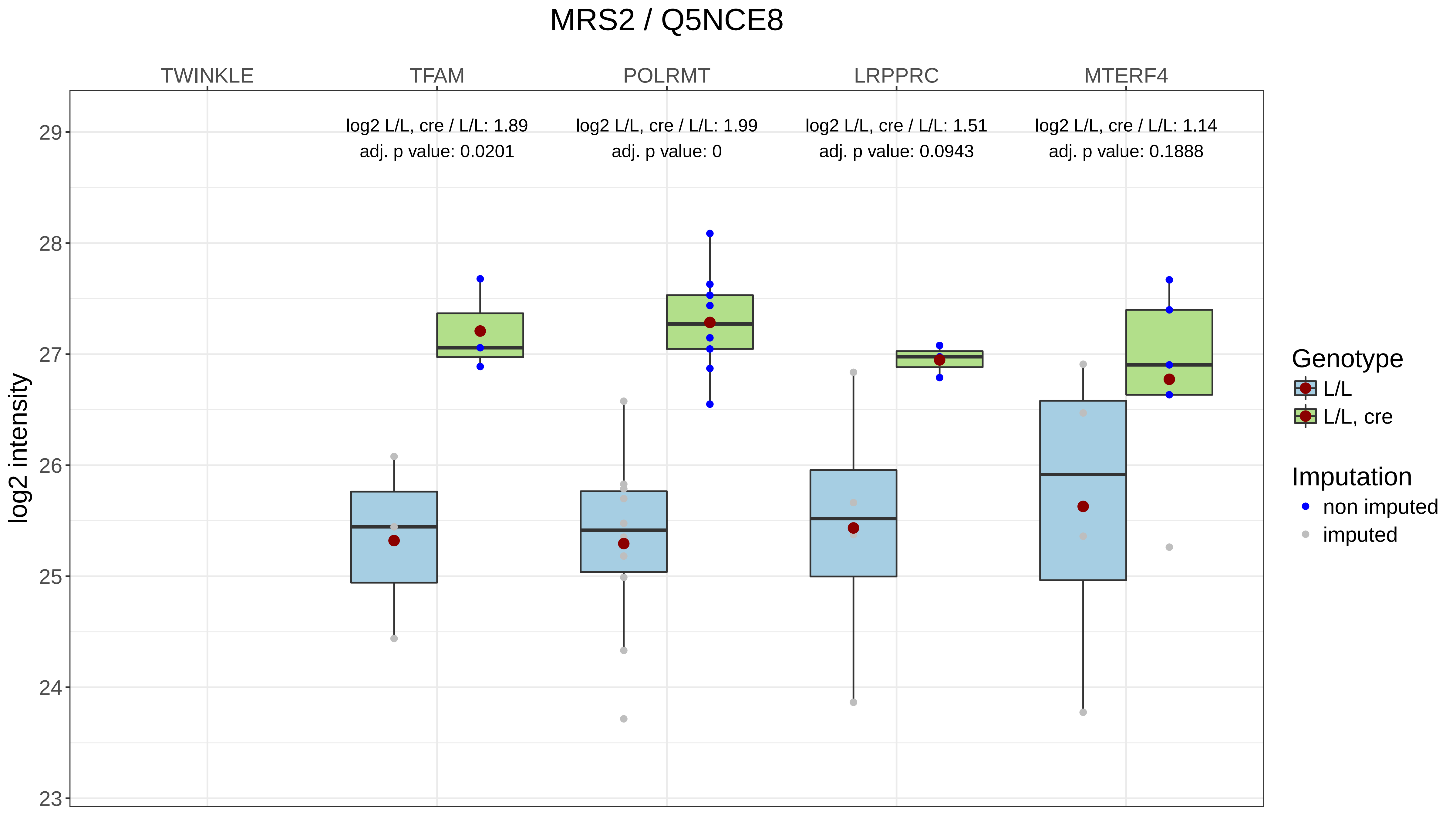

MRS2 / Q5NCE8
TWINKLE
TFAM
POLRMT
LRPPRC
MTERF4
log2 L/L, cre / L/L: 1.89
adj. p value: 0.0201
log2 L/L, cre / L/L: 1.99
adj. p value: 0
log2 L/L, cre / L/L: 1.51
adj. p value: 0.0943
log2 L/L, cre / L/L: 1.14
adj. p value: 0.1888
log2 intensity
29
28
27
26
25
24
23
Genotype
L/L
L/L, cre
Imputation
non imputed
imputed

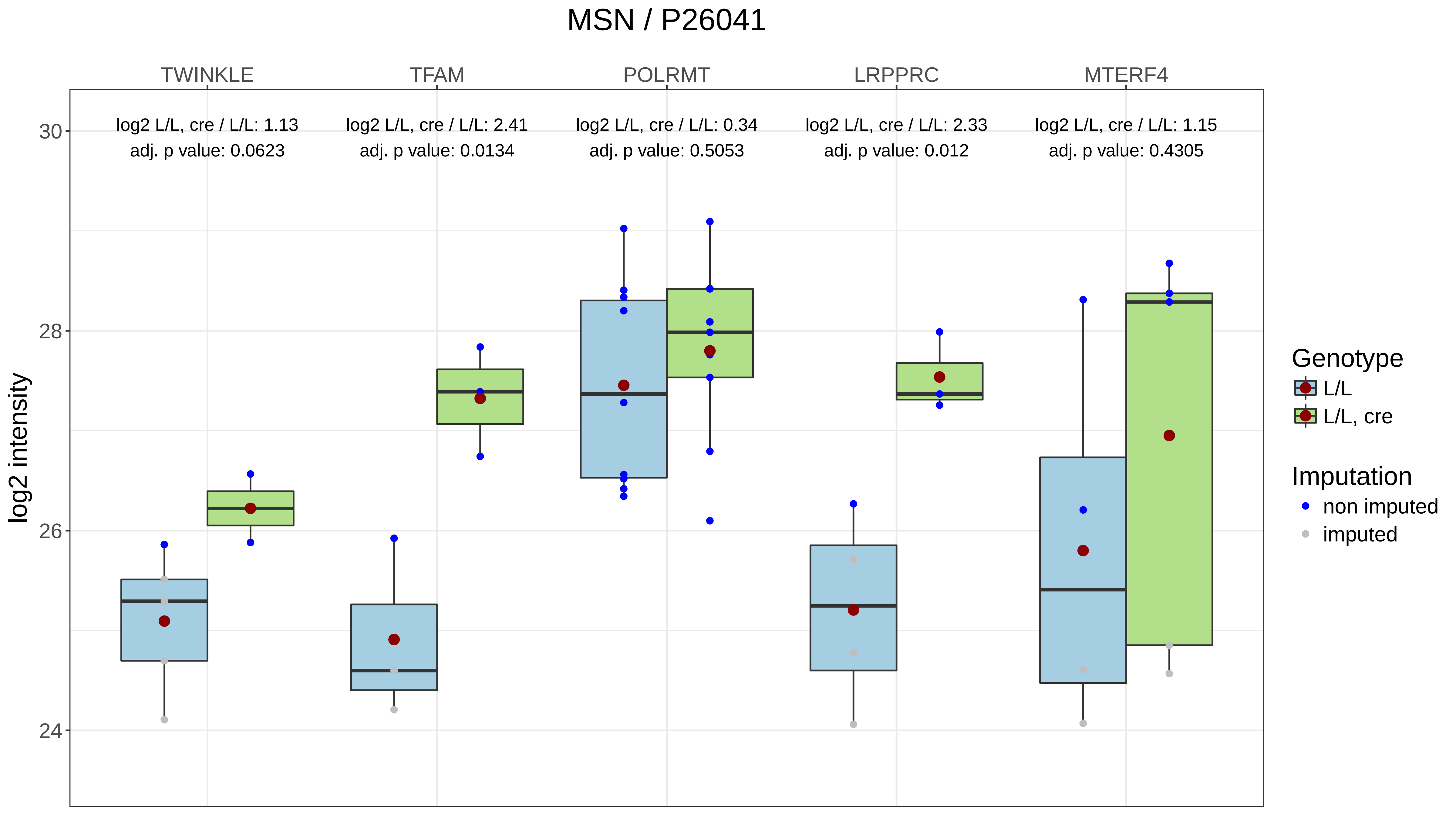
MSN / P26041
TWINKLE
TFAM
POLRMT
LRPPRC
MTERF4
log2 L/L, cre / L/L: 1.13
adj. p value: 0.0623
log2 L/L, cre / L/L: 2.41
adj. p value: 0.0134
log2 L/L, cre / L/L: 0.34
adj. p value: 0.5053
log2 L/L, cre / L/L: 2.33
adj. p value: 0.012
log2 L/L, cre / L/L: 1.15
adj. p value: 0.4305
log2 intensity
30
28
26
24
Genotype
L/L
L/L, cre
Imputation
non imputed
imputed

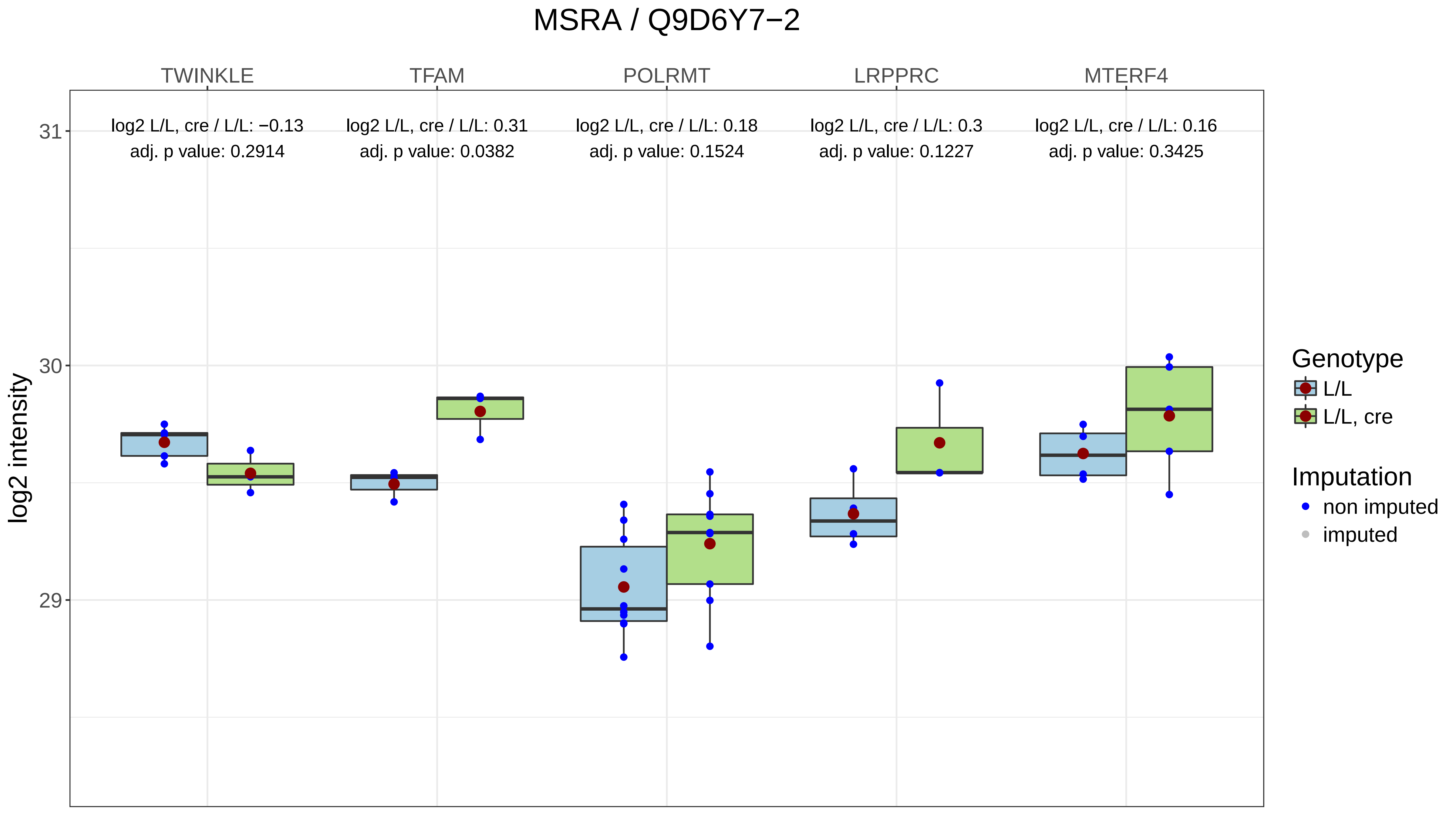

MSRA / Q9D6Y7−2
TWINKLE
TFAM
POLRMT
LRPPRC
MTERF4
log2 L/L, cre / L/L: −0.13
adj. p value: 0.2914
log2 L/L, cre / L/L: 0.31
adj. p value: 0.0382
log2 L/L, cre / L/L: 0.18
adj. p value: 0.1524
log2 L/L, cre / L/L: 0.3
adj. p value: 0.1227
log2 L/L, cre / L/L: 0.16
adj. p value: 0.3425
log2 intensity
31
30
29
Genotype
L/L
L/L, cre
Imputation
non imputed
imputed

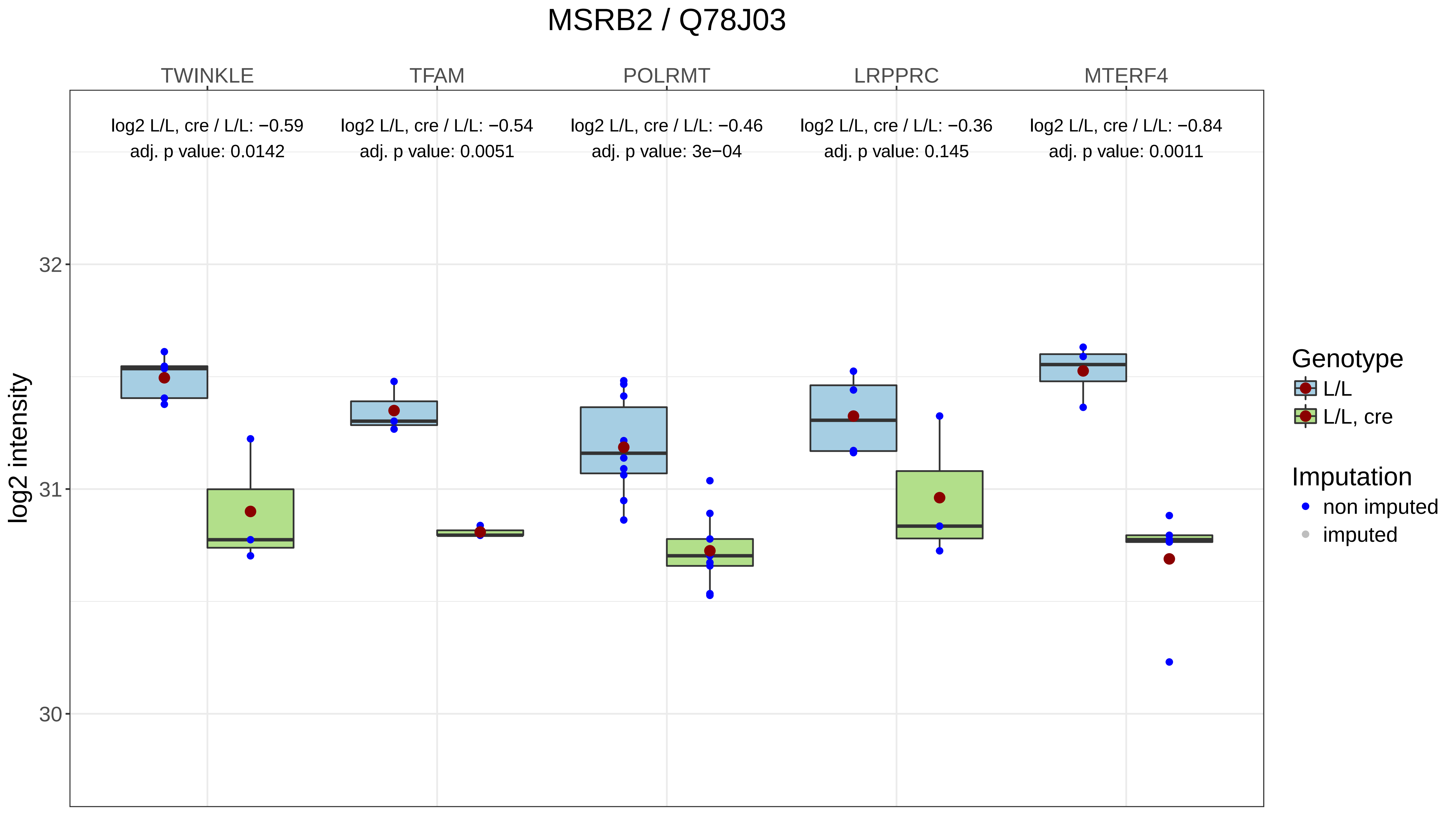
MSRB2 / Q78J03
TWINKLE
TFAM
POLRMT
LRPPRC
MTERF4
log2 L/L, cre / L/L: −0.59
adj. p value: 0.0142
log2 L/L, cre / L/L: −0.54
adj. p value: 0.0051
log2 L/L, cre / L/L: −0.46
adj. p value: 3e−04
log2 L/L, cre / L/L: −0.36
adj. p value: 0.145
log2 L/L, cre / L/L: −0.84
adj. p value: 0.0011
log2 intensity
32
31
30
Genotype
L/L
L/L, cre
Imputation
non imputed
imputed

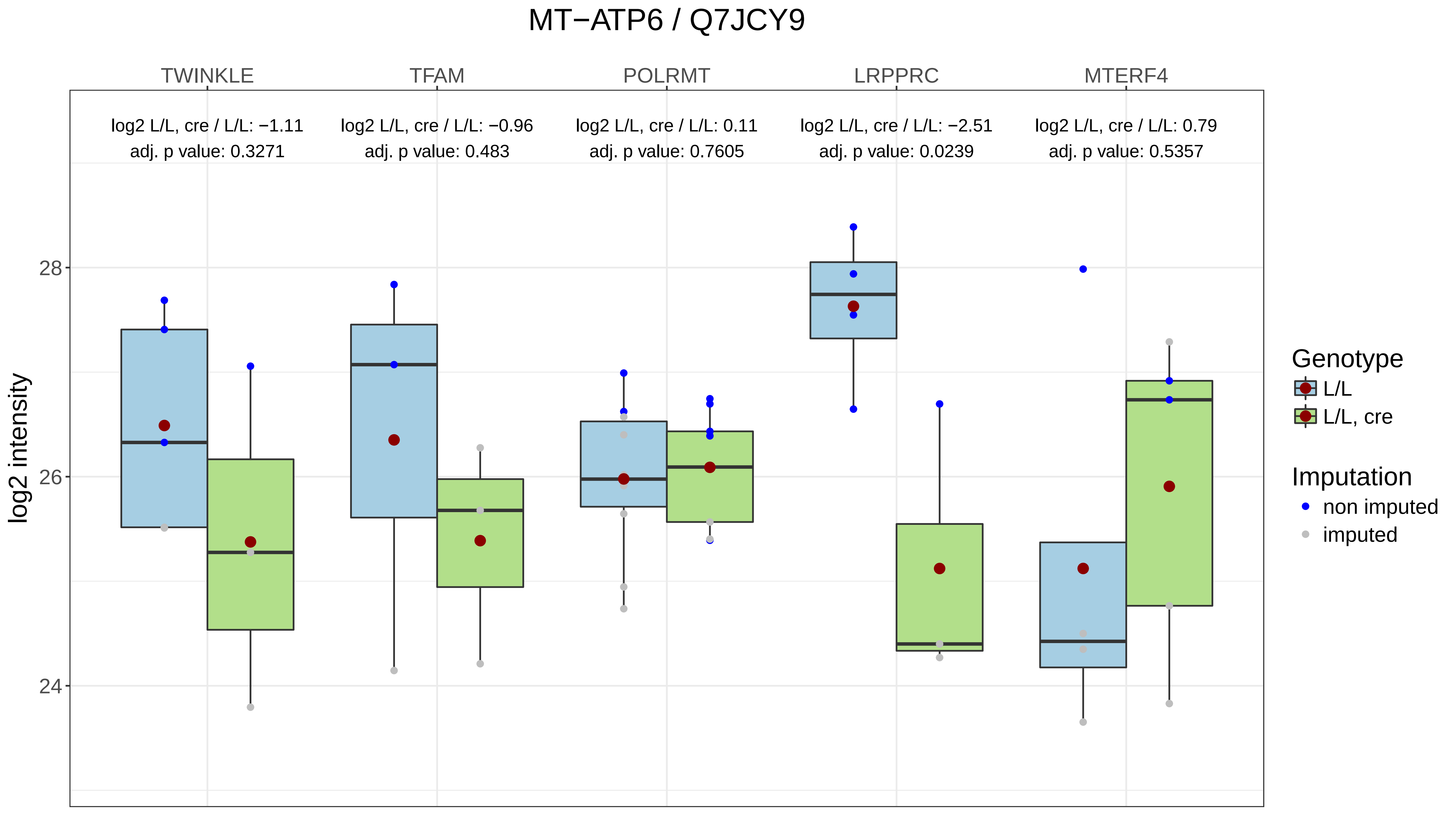
MT−ATP6 / Q7JCY9
TWINKLE
TFAM
POLRMT
LRPPRC
MTERF4
log2 L/L, cre / L/L: −1.11
adj. p value: 0.3271
log2 L/L, cre / L/L: −0.96
adj. p value: 0.483
log2 L/L, cre / L/L: 0.11
adj. p value: 0.7605
log2 L/L, cre / L/L: −2.51
adj. p value: 0.0239
log2 L/L, cre / L/L: 0.79
adj. p value: 0.5357
log2 intensity
28
26
24
Genotype
L/L
L/L, cre
Imputation
non imputed
imputed

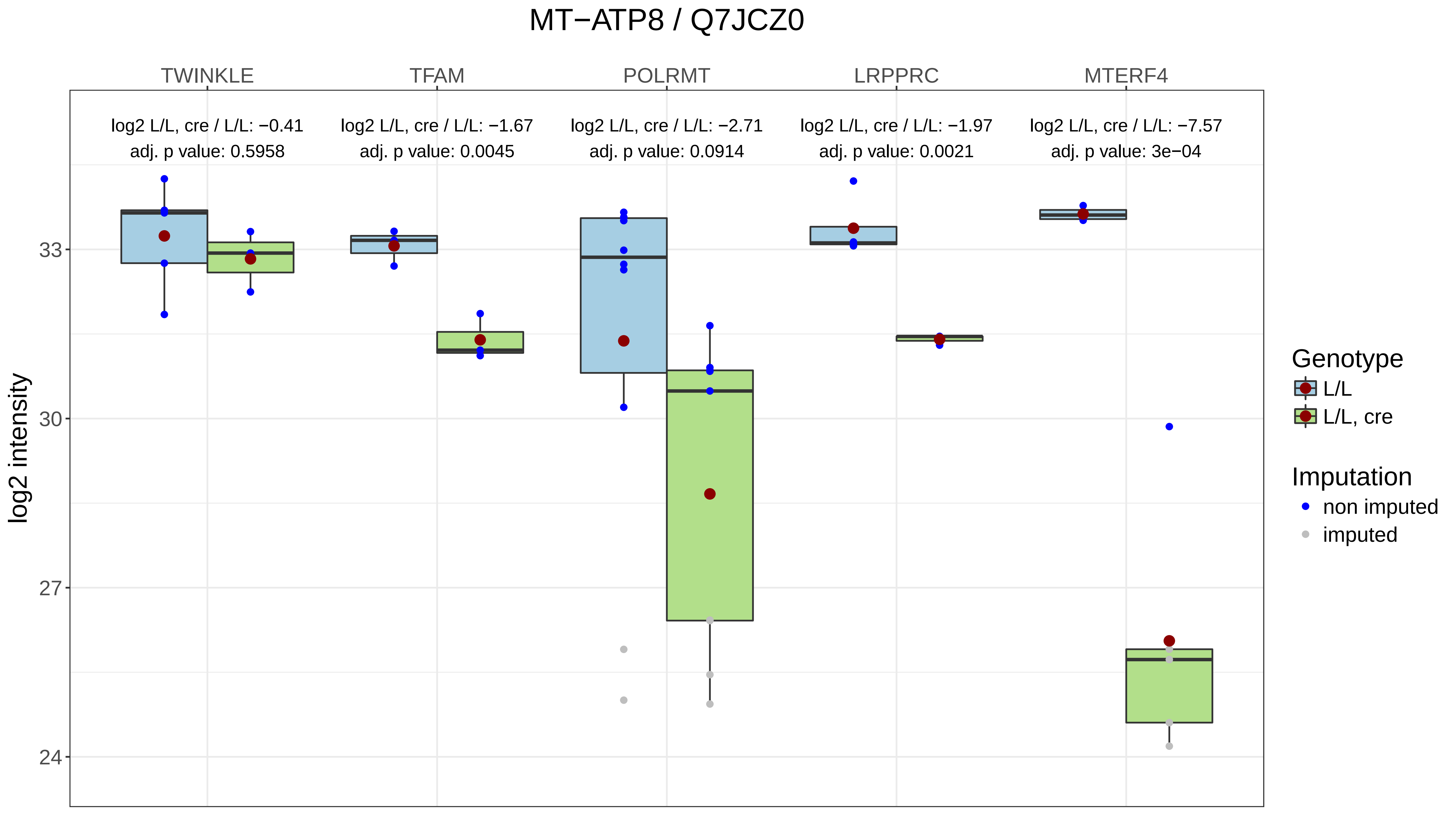
MT−ATP8 / Q7JCZ0
TWINKLE
TFAM
POLRMT
LRPPRC
MTERF4
log2 L/L, cre / L/L: −0.41
adj. p value: 0.5958
log2 L/L, cre / L/L: −1.67
adj. p value: 0.0045
log2 L/L, cre / L/L: −2.71
adj. p value: 0.0914
log2 L/L, cre / L/L: −1.97
adj. p value: 0.0021
log2 L/L, cre / L/L: −7.57
adj. p value: 3e−04
log2 intensity
33
30
27
24
Genotype
L/L
L/L, cre
Imputation
non imputed
imputed

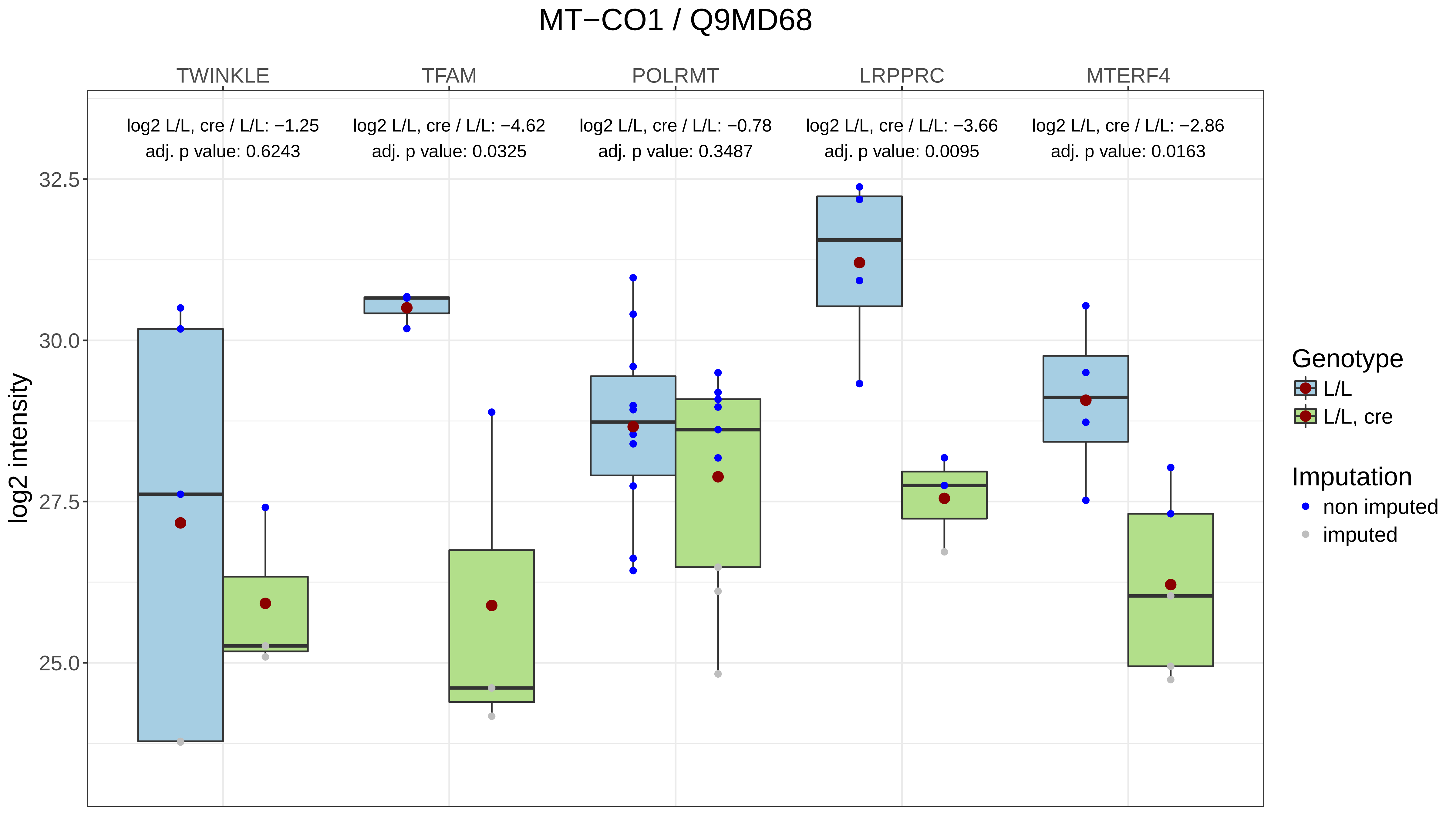

MT−CO1 / Q9MD68
TWINKLE
TFAM
POLRMT
LRPPRC
MTERF4
log2 L/L, cre / L/L: −1.25
adj. p value: 0.6243
log2 L/L, cre / L/L: −4.62
adj. p value: 0.0325
log2 L/L, cre / L/L: −0.78
adj. p value: 0.3487
log2 L/L, cre / L/L: −3.66
adj. p value: 0.0095
log2 L/L, cre / L/L: −2.86
adj. p value: 0.0163
32.5
30.0
27.5
25.0
log2 intensity
Genotype
L/L
L/L, cre
Imputation
non imputed
imputed

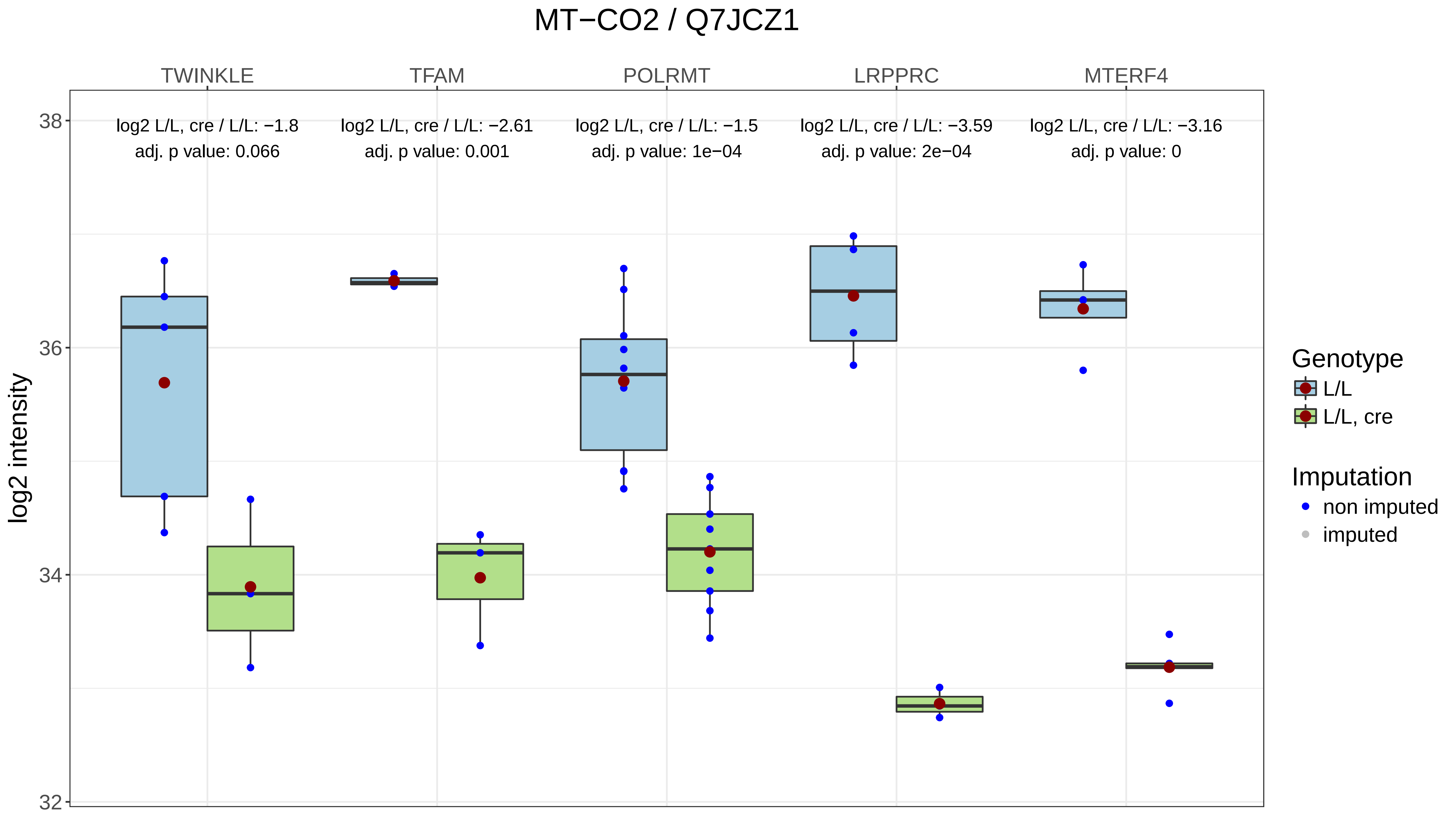
MT−CO2 / Q7JCZ1
TWINKLE
TFAM
POLRMT
LRPPRC
MTERF4
log2 L/L, cre / L/L: −1.8
adj. p value: 0.066
log2 L/L, cre / L/L: −2.61
adj. p value: 0.001
log2 L/L, cre / L/L: −1.5
adj. p value: 1e−04
log2 L/L, cre / L/L: −3.59
adj. p value: 2e−04
log2 L/L, cre / L/L: −3.16
adj. p value: 0
log2 intensity
38
36
34
32
Genotype
L/L
L/L, cre
Imputation
non imputed
imputed

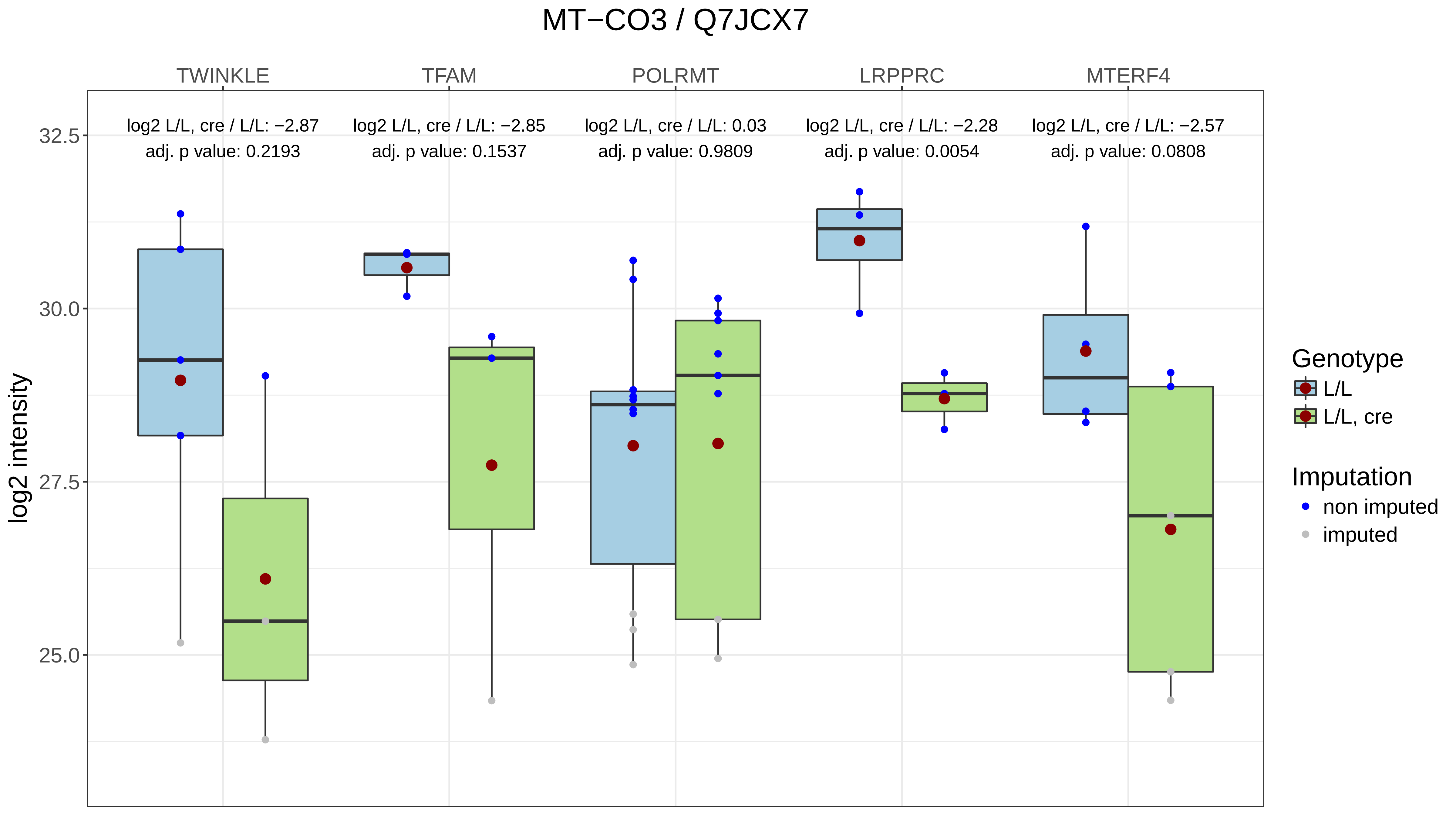

MT−CO3 / Q7JCX7
TWINKLE
TFAM
POLRMT
LRPPRC
MTERF4
log2 L/L, cre / L/L: −2.87
adj. p value: 0.2193
log2 L/L, cre / L/L: −2.85
adj. p value: 0.1537
log2 L/L, cre / L/L: 0.03
adj. p value: 0.9809
log2 L/L, cre / L/L: −2.28
adj. p value: 0.0054
log2 L/L, cre / L/L: −2.57
adj. p value: 0.0808
log2 intensity
32.5
30.0
27.5
25.0
Genotype
L/L
L/L, cre
Imputation
non imputed
imputed

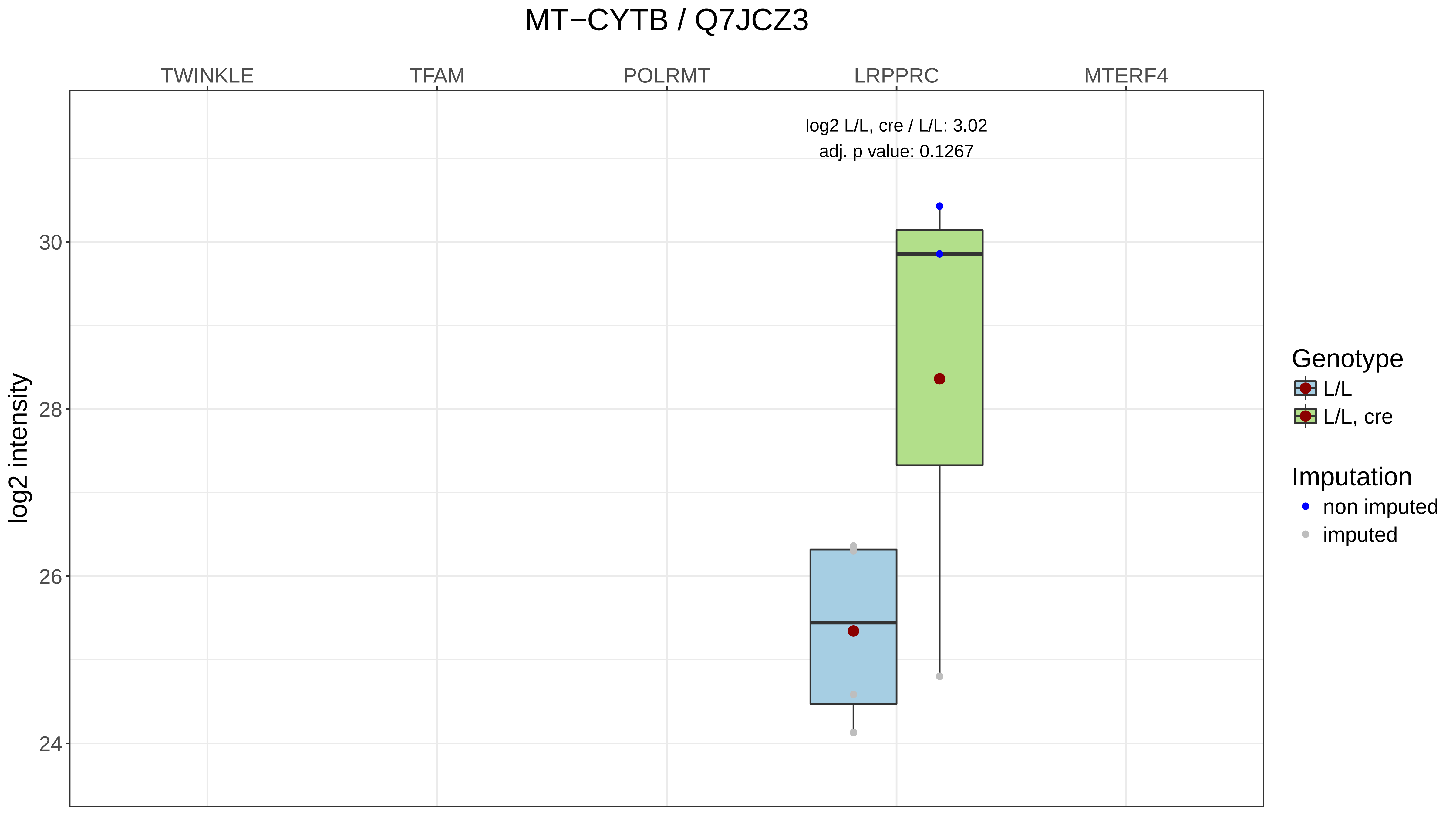
MT−CYTB / Q7JCZ3
TWINKLE
TFAM
POLRMT
LRPPRC
MTERF4
log2 L/L, cre / L/L: 3.02
adj. p value: 0.1267
log2 intensity
30
28
26
24
Genotype
L/L
L/L, cre
Imputation
non imputed
imputed

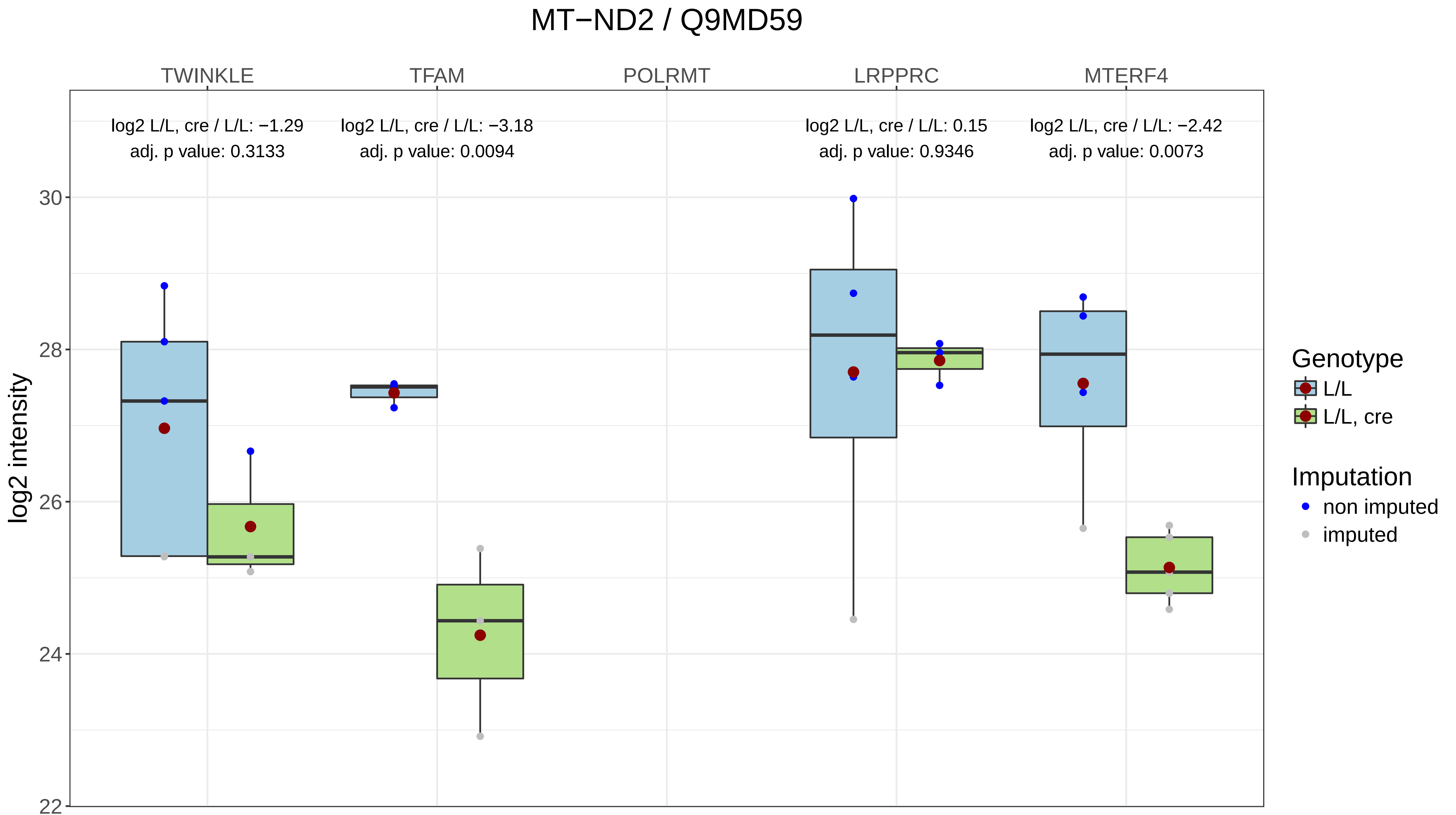
MT−ND2 / Q9MD59
TWINKLE
TFAM
POLRMT
LRPPRC
MTERF4
log2 L/L, cre / L/L: −1.29
adj. p value: 0.3133
log2 L/L, cre / L/L: −3.18
adj. p value: 0.0094
log2 L/L, cre / L/L: 0.15
adj. p value: 0.9346
log2 L/L, cre / L/L: −2.42
adj. p value: 0.0073
log2 intensity
30
28
26
24
22
Genotype
L/L
L/L, cre
Imputation
non imputed
imputed

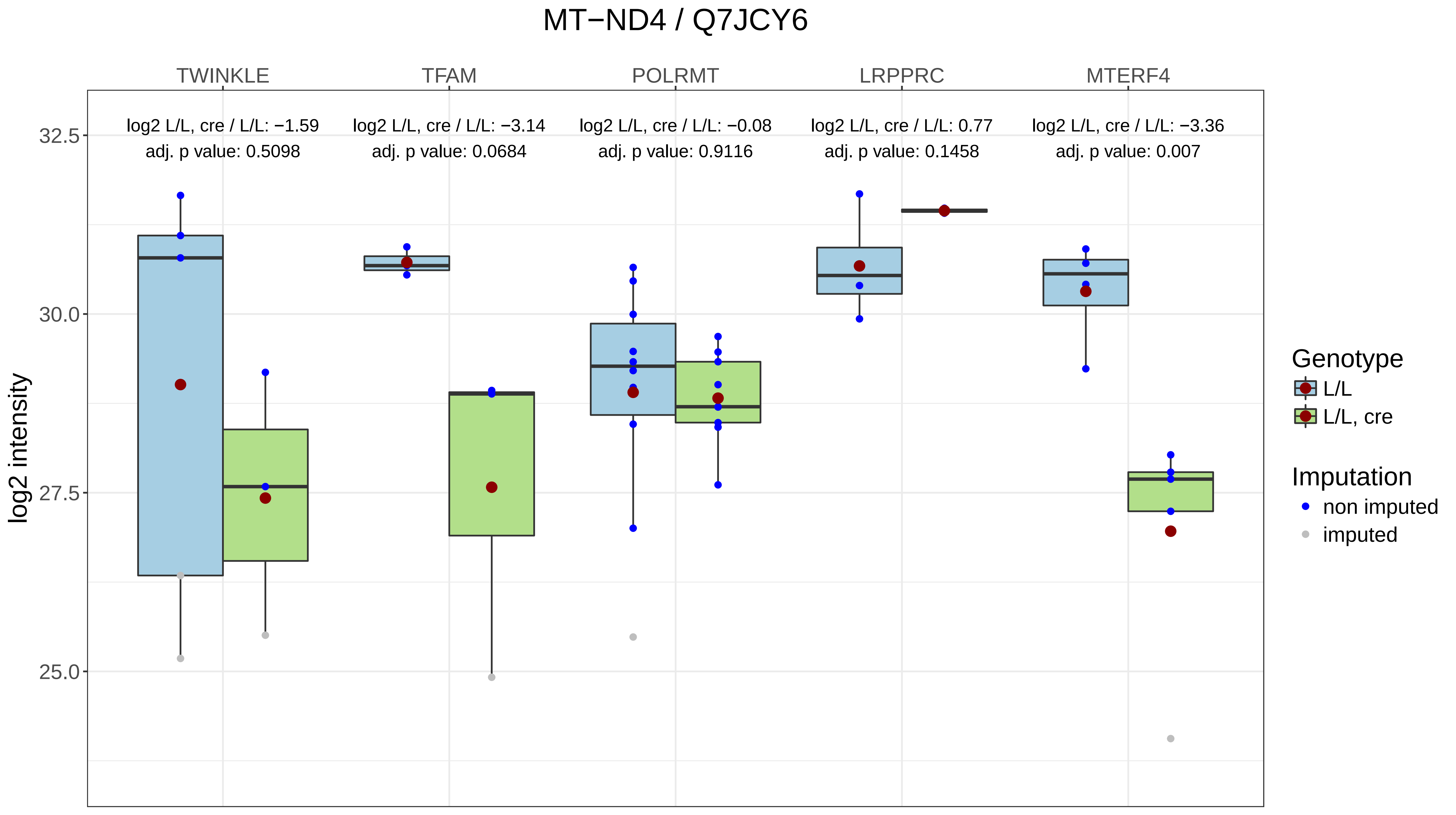
MT−ND4 / Q7JCY6
TWINKLE
TFAM
POLRMT
LRPPRC
MTERF4
log2 L/L, cre / L/L: −1.59
adj. p value: 0.5098
log2 L/L, cre / L/L: −3.14
adj. p value: 0.0684
log2 L/L, cre / L/L: −0.08
adj. p value: 0.9116
log2 L/L, cre / L/L: 0.77
adj. p value: 0.1458
log2 L/L, cre / L/L: −3.36
adj. p value: 0.007
32.5
30.0
27.5
25.0
log2 intensity
Genotype
L/L
L/L, cre
Imputation
non imputed
imputed

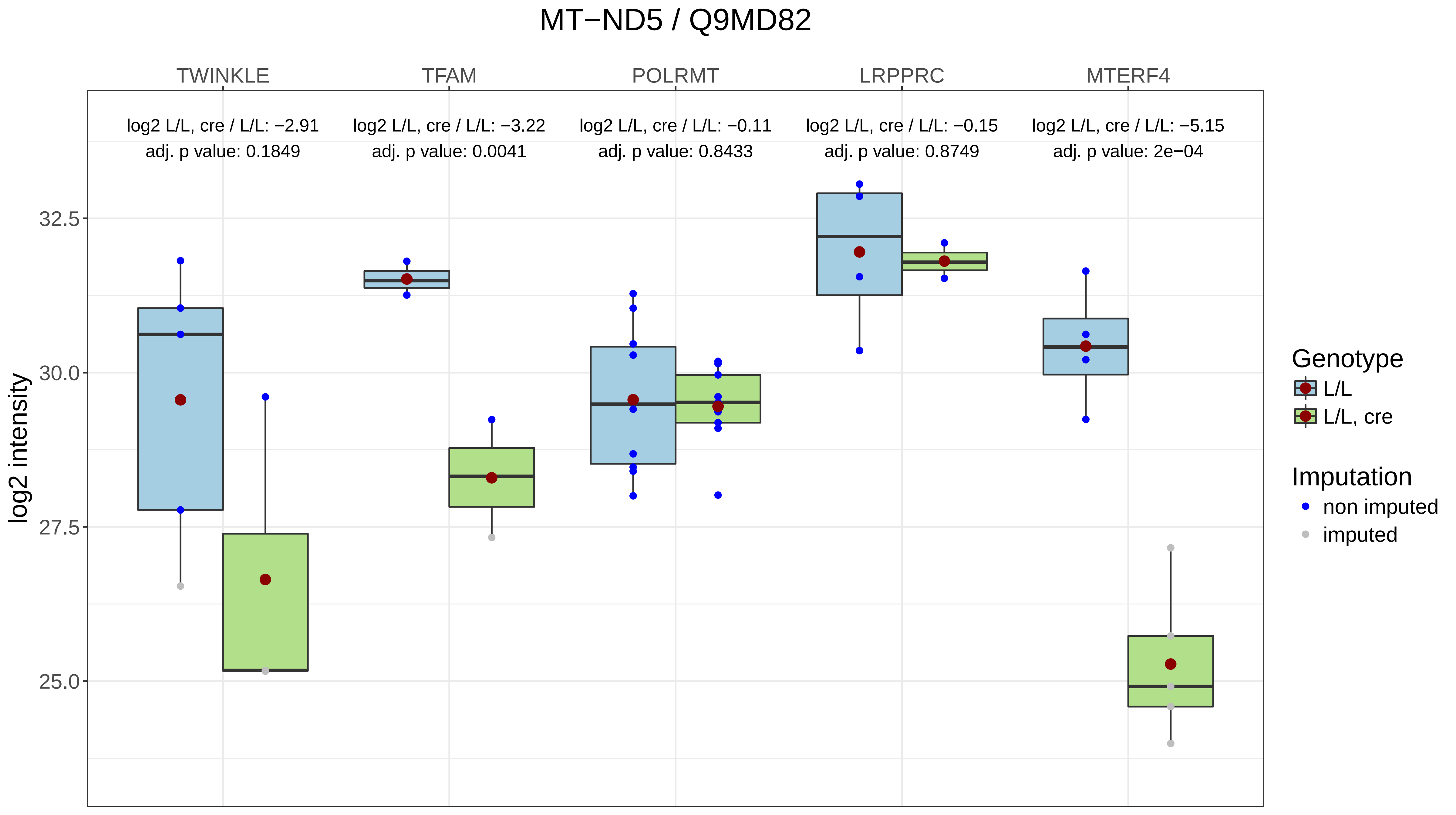
MT−ND5 / Q9MD82
TWINKLE
TFAM
POLRMT
LRPPRC
MTERF4
log2 L/L, cre / L/L: −2.91
adj. p value: 0.1849
log2 L/L, cre / L/L: −3.22
adj. p value: 0.0041
log2 L/L, cre / L/L: −0.11
adj. p value: 0.8433
log2 L/L, cre / L/L: −0.15
adj. p value: 0.8749
log2 L/L, cre / L/L: −5.15
adj. p value: 2e−04
32.5
30.0
27.5
25.0
log2 intensity
Genotype
L/L
L/L, cre
Imputation
non imputed
imputed

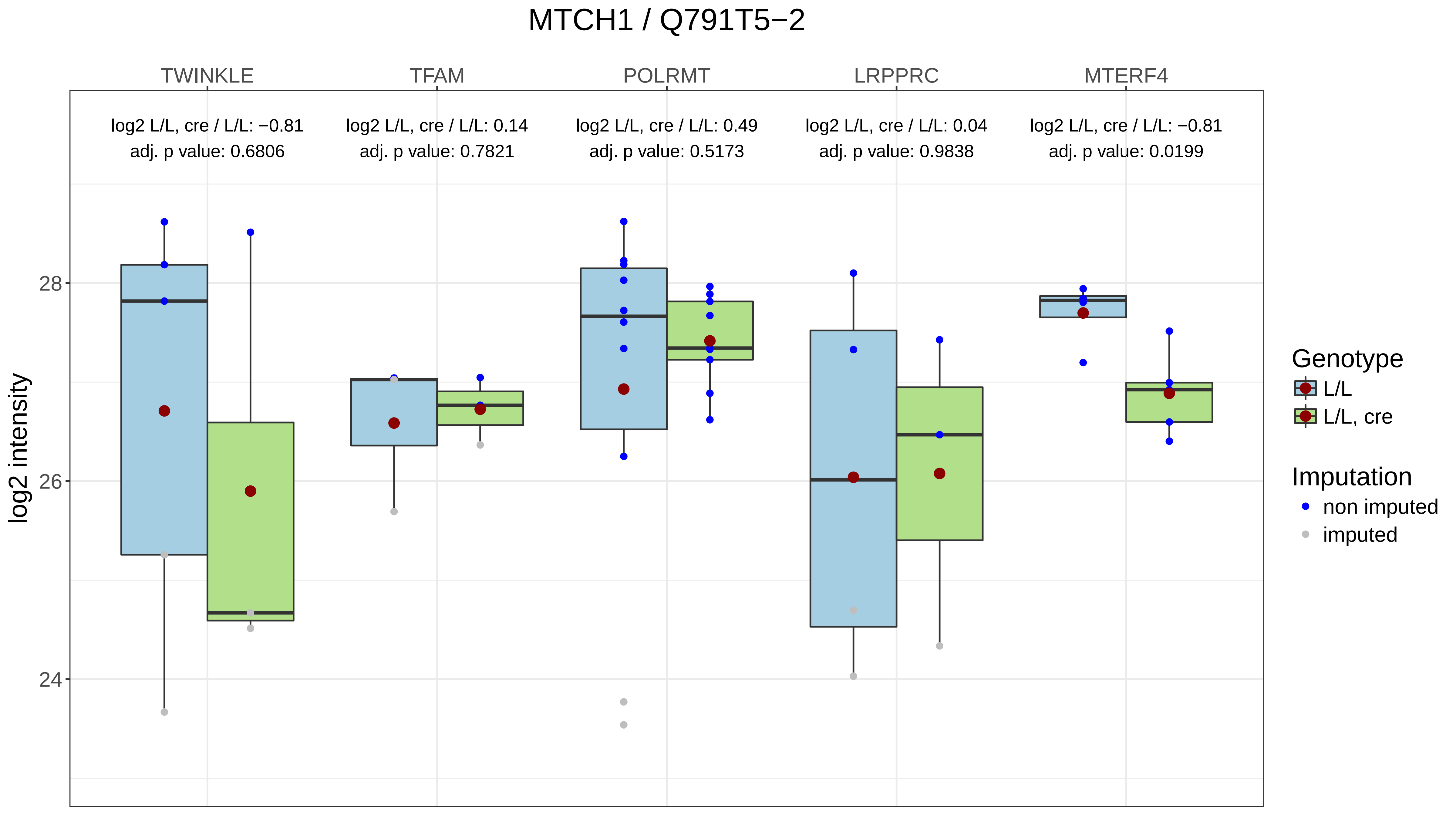
MTCH1 / Q791T5−2
TWINKLE
TFAM
POLRMT
LRPPRC
MTERF4
log2 L/L, cre / L/L: −0.81
adj. p value: 0.6806
log2 L/L, cre / L/L: 0.14
adj. p value: 0.7821
log2 L/L, cre / L/L: 0.49
adj. p value: 0.5173
log2 L/L, cre / L/L: 0.04
adj. p value: 0.9838
log2 L/L, cre / L/L: −0.81
adj. p value: 0.0199
log2 intensity
28
26
24
Genotype
L/L
L/L, cre
Imputation
non imputed
imputed

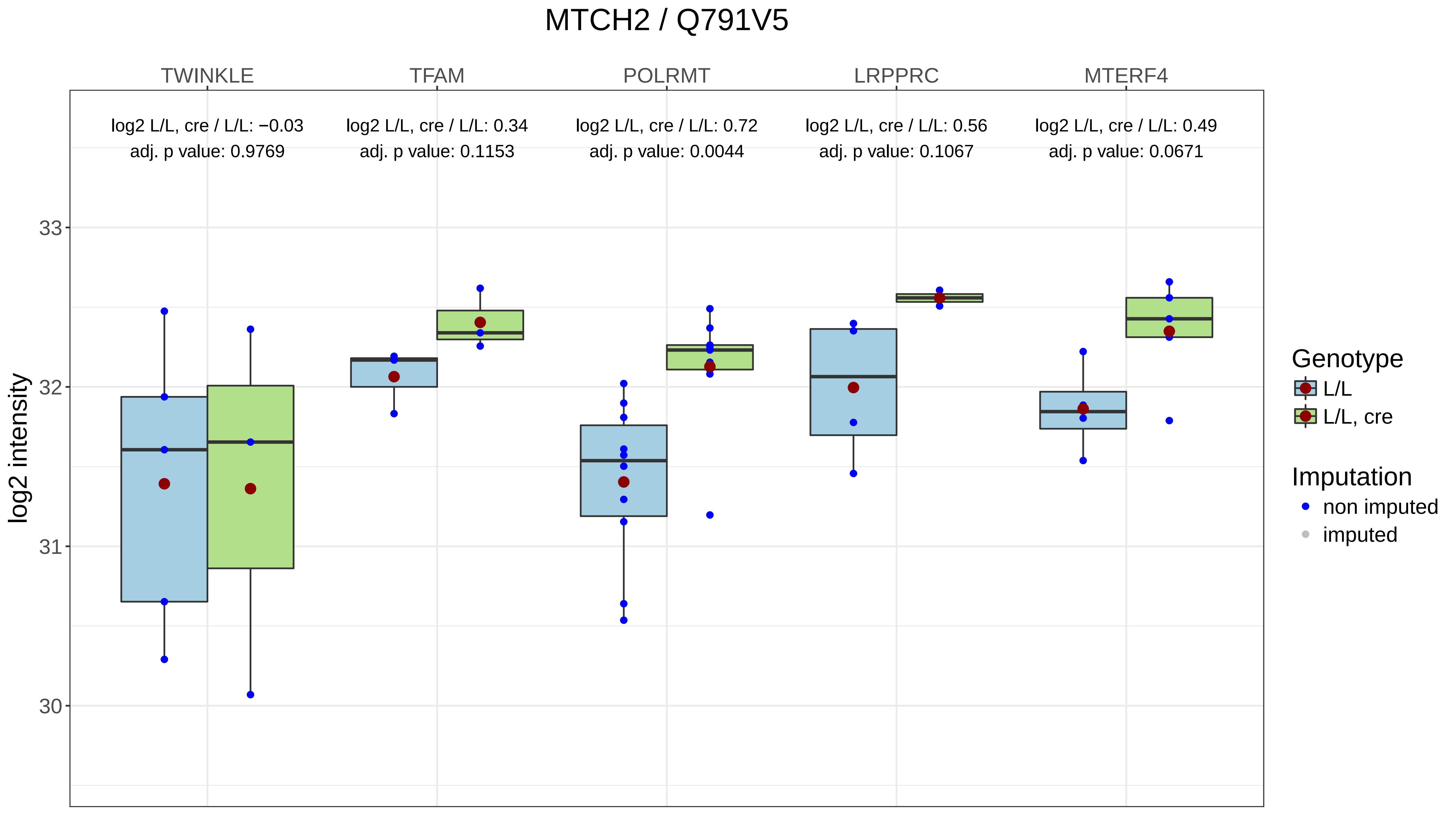
MTCH2 / Q791V5
TWINKLE
TFAM
POLRMT
LRPPRC
MTERF4
log2 L/L, cre / L/L: −0.03
adj. p value: 0.9769
log2 L/L, cre / L/L: 0.34
adj. p value: 0.1153
log2 L/L, cre / L/L: 0.72
adj. p value: 0.0044
log2 L/L, cre / L/L: 0.56
adj. p value: 0.1067
log2 L/L, cre / L/L: 0.49
adj. p value: 0.0671
log2 intensity
33
32
31
30
Genotype
L/L
L/L, cre
Imputation
non imputed
imputed

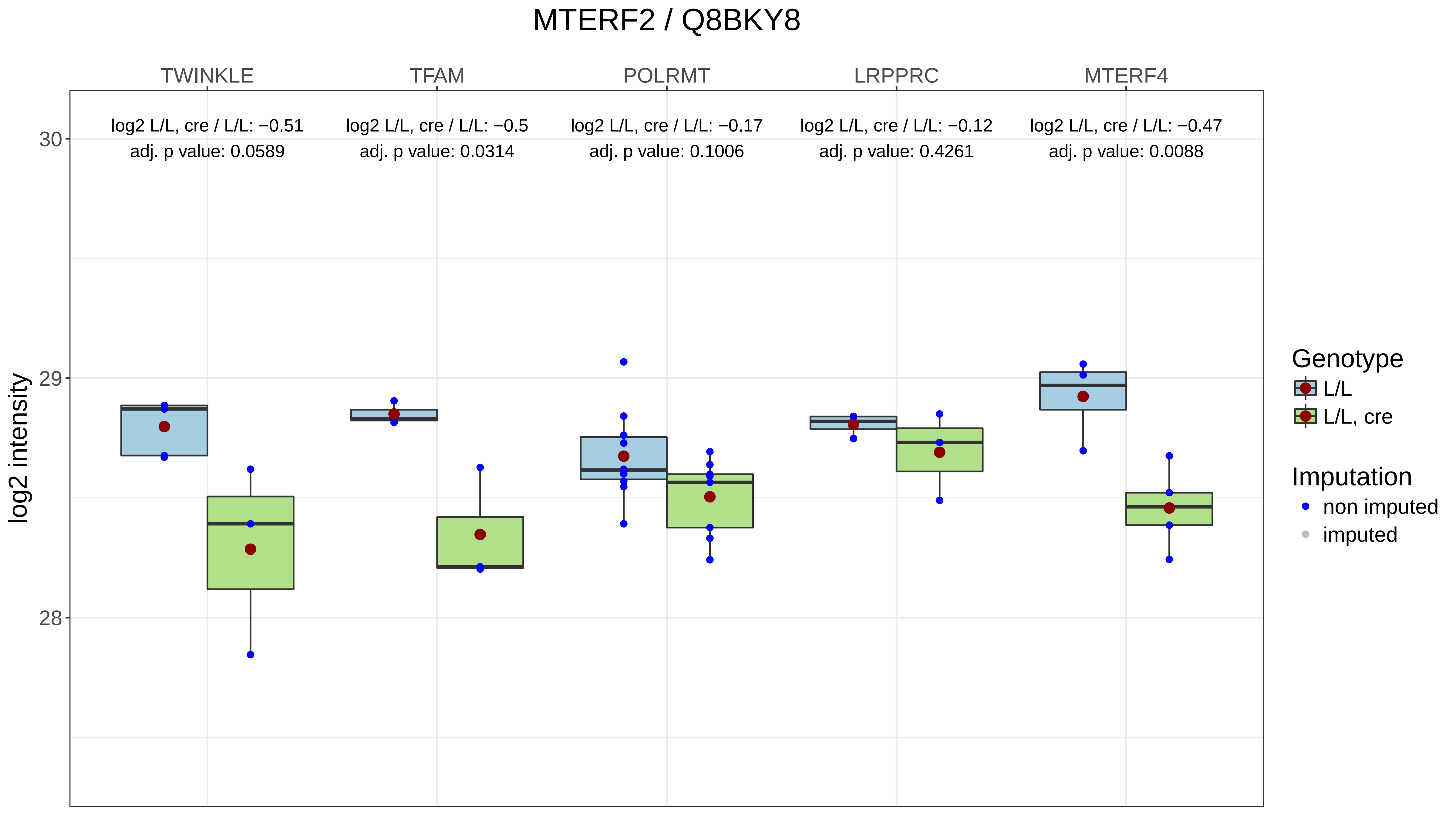
MTERF2 / Q8BKY8
TWINKLE
TFAM
POLRMT
LRPPRC
MTERF4
log2 L/L, cre / L/L: −0.51
adj. p value: 0.0589
log2 L/L, cre / L/L: −0.5
adj. p value: 0.0314
log2 L/L, cre / L/L: −0.17
adj. p value: 0.1006
log2 L/L, cre / L/L: −0.12
adj. p value: 0.4261
log2 L/L, cre / L/L: −0.47
adj. p value: 0.0088
log2 intensity
30
29
28
Genotype
L/L
L/L, cre
Imputation
non imputed
imputed

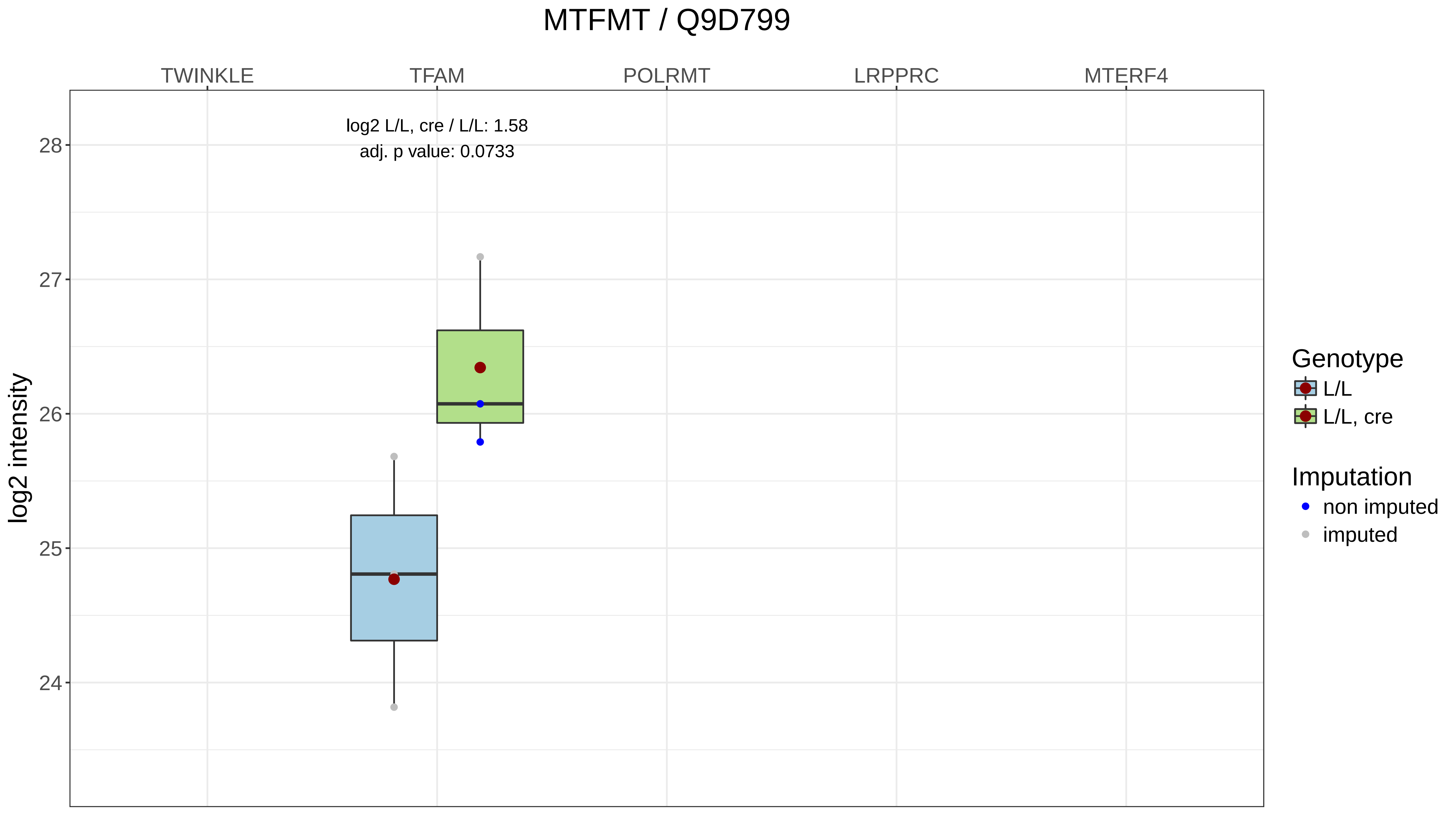
MTFMT / Q9D799
TWINKLE
TFAM
POLRMT
LRPPRC
MTERF4
log2 L/L, cre / L/L: 1.58
adj. p value: 0.0733
log2 intensity
28
27
26
25
24
Genotype
L/L
L/L, cre
Imputation
non imputed
imputed

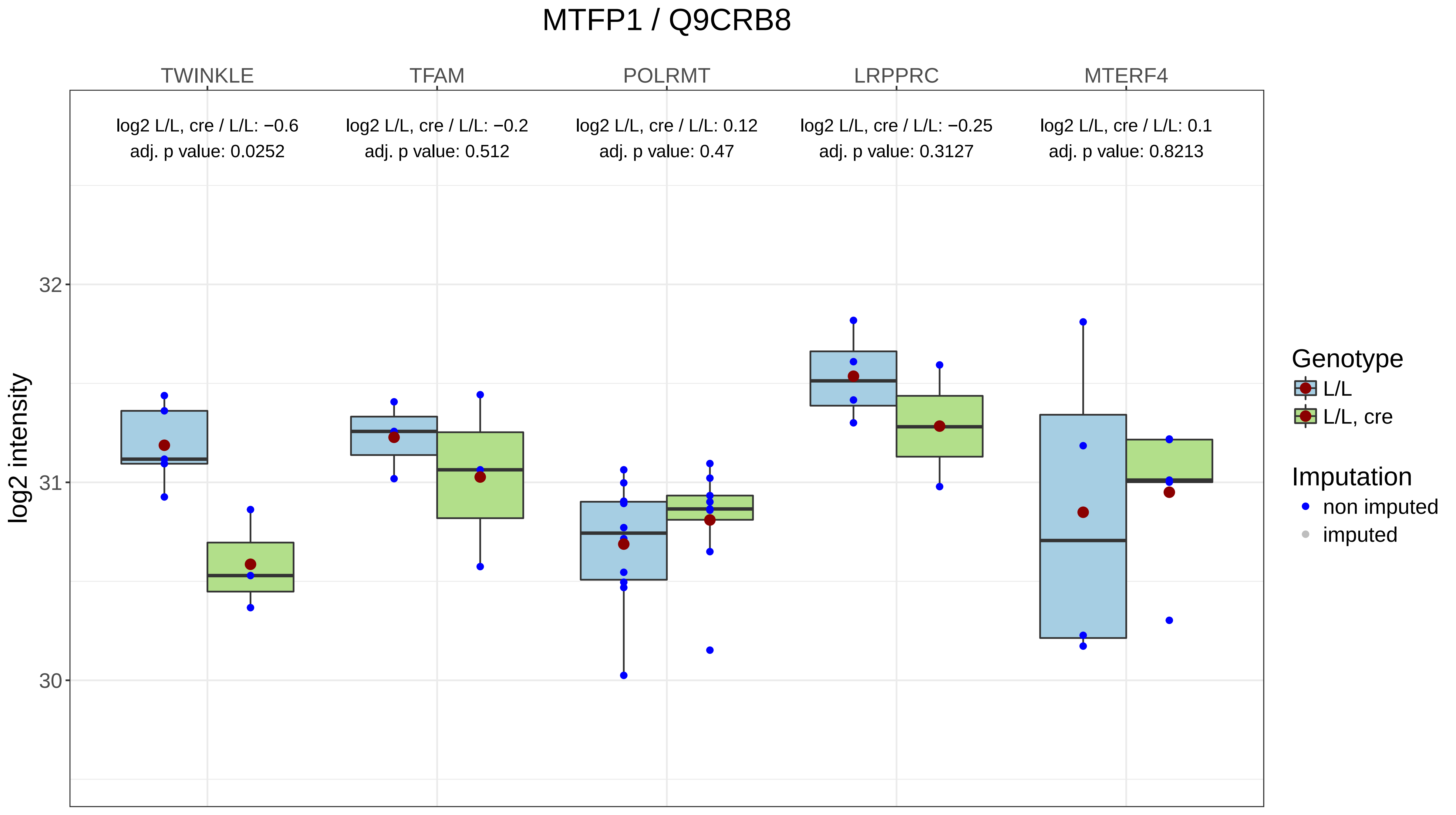
MTFP1 / Q9CRB8
TWINKLE
TFAM
POLRMT
LRPPRC
MTERF4
log2 L/L, cre / L/L: −0.6
adj. p value: 0.0252
log2 L/L, cre / L/L: −0.2
adj. p value: 0.512
log2 L/L, cre / L/L: 0.12
adj. p value: 0.47
log2 L/L, cre / L/L: −0.25
adj. p value: 0.3127
log2 L/L, cre / L/L: 0.1
adj. p value: 0.8213
log2 intensity
32
31
30
Genotype
L/L
L/L, cre
Imputation
non imputed
imputed

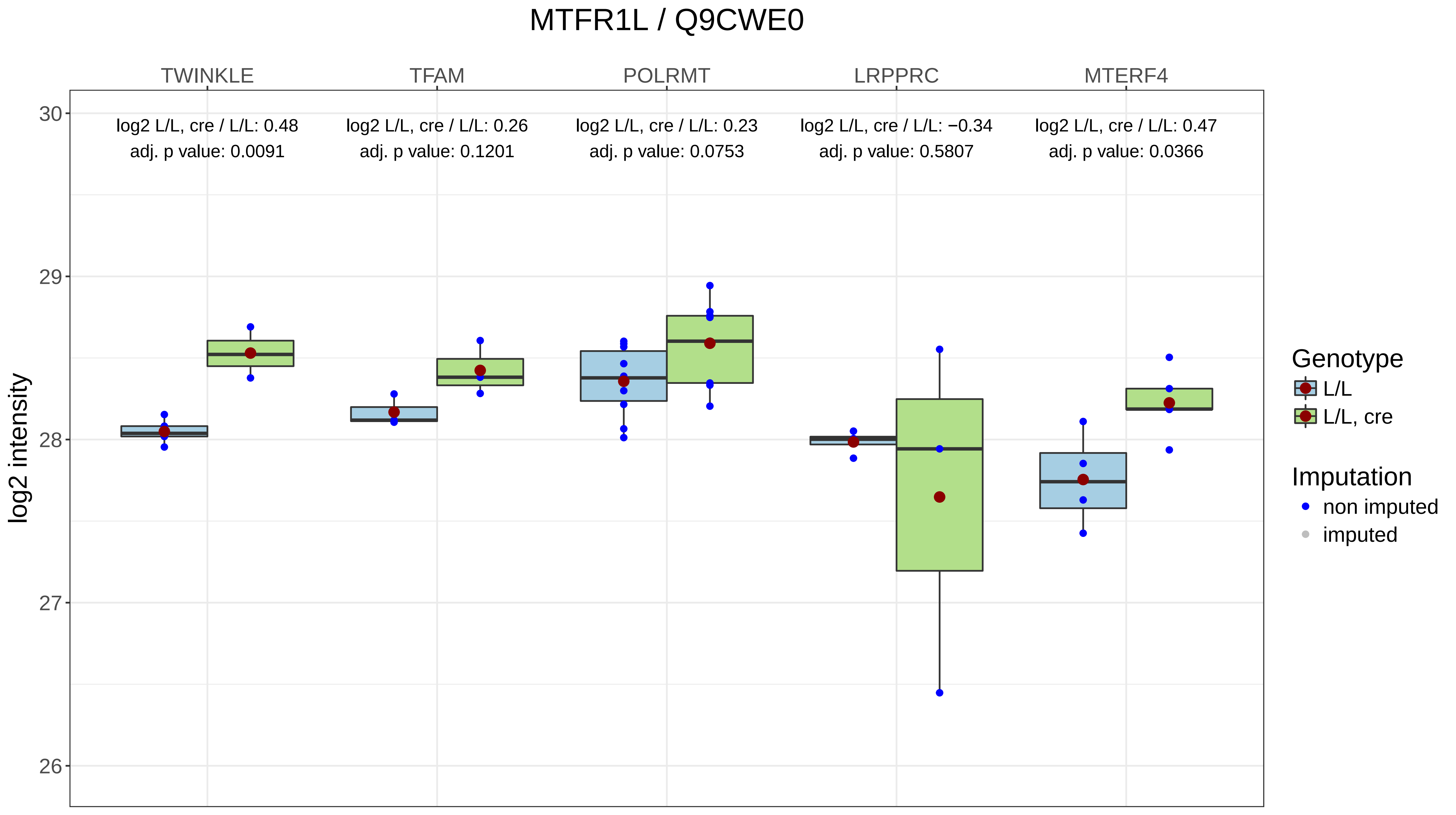

MTFR1L / Q9CWE0
TWINKLE
TFAM
POLRMT
LRPPRC
MTERF4
log2 L/L, cre / L/L: 0.48
adj. p value: 0.0091
log2 L/L, cre / L/L: 0.26
adj. p value: 0.1201
log2 L/L, cre / L/L: 0.23
adj. p value: 0.0753
log2 L/L, cre / L/L: −0.34
adj. p value: 0.5807
log2 L/L, cre / L/L: 0.47
adj. p value: 0.0366
log2 intensity
30
29
28
27
26
Genotype
L/L
L/L, cre
Imputation
non imputed
imputed

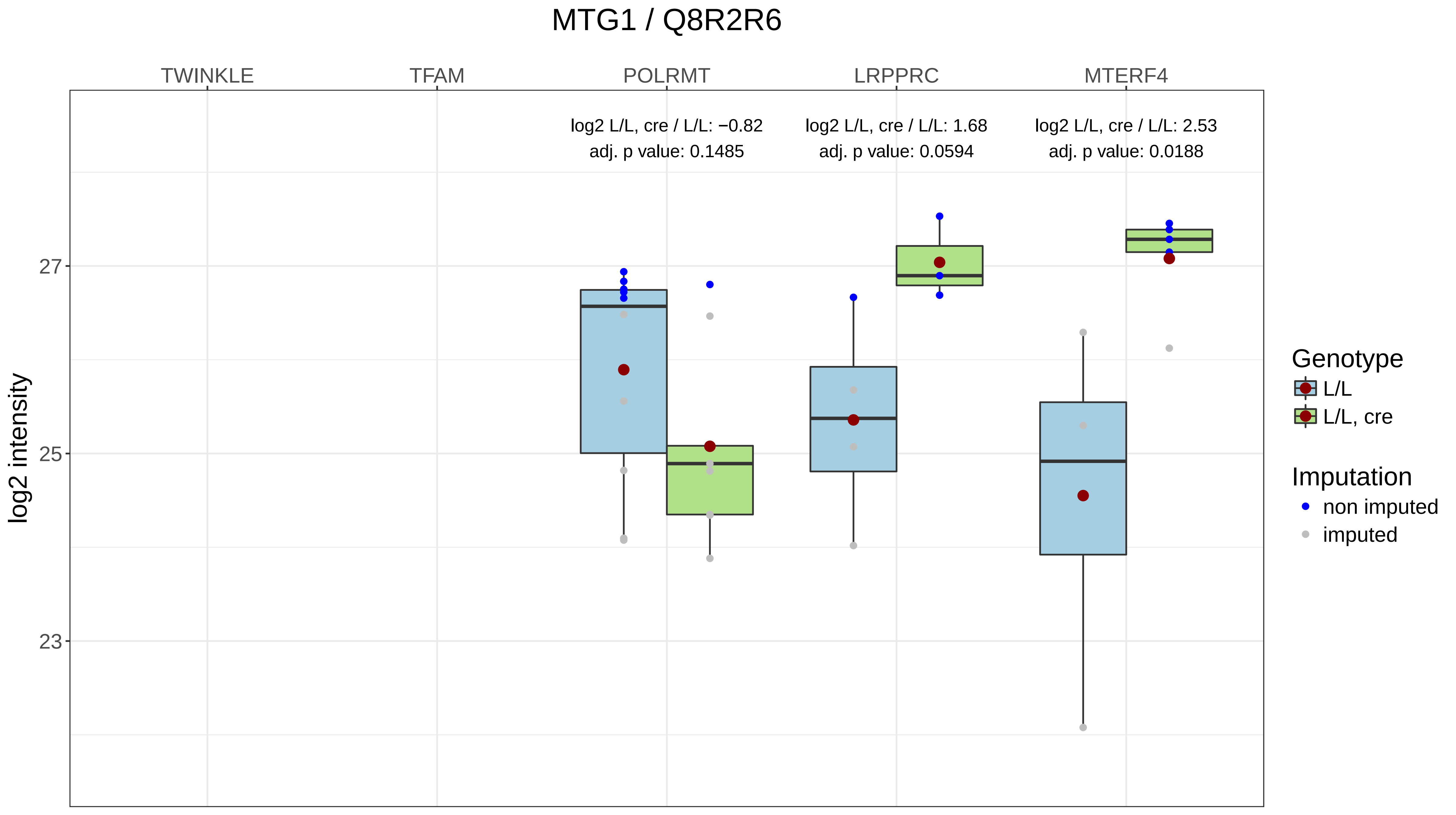
MTG1 / Q8R2R6
TWINKLE
TFAM
POLRMT
LRPPRC
MTERF4
log2 L/L, cre / L/L: −0.82
adj. p value: 0.1485
log2 L/L, cre / L/L: 1.68
adj. p value: 0.0594
log2 L/L, cre / L/L: 2.53
adj. p value: 0.0188
27
25
23
log2 intensity
Genotype
L/L
L/L, cre
Imputation
non imputed
imputed

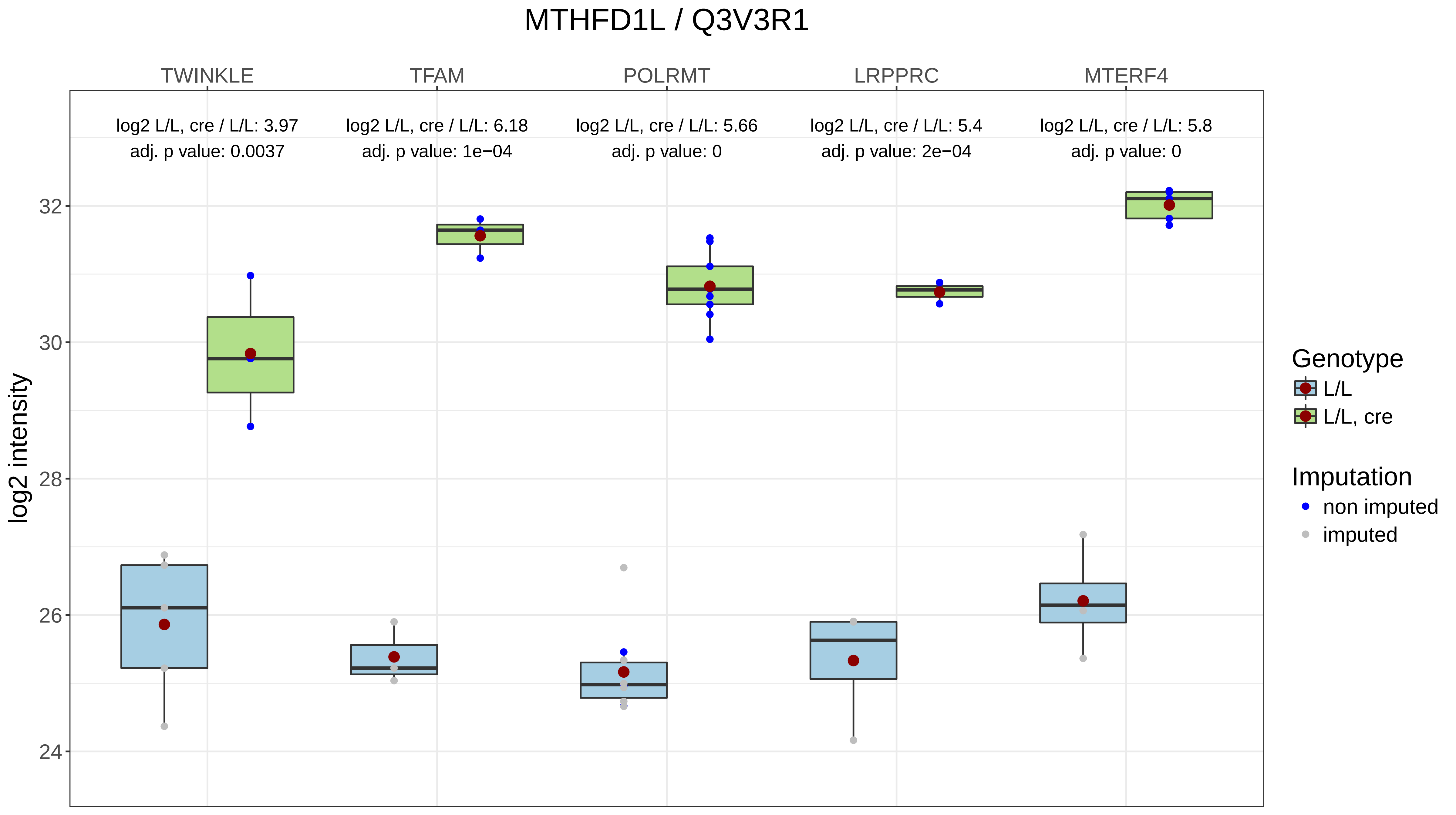

MTHFD1L / Q3V3R1
TWINKLE
TFAM
POLRMT
LRPPRC
MTERF4
log2 L/L, cre / L/L: 3.97
adj. p value: 0.0037
log2 L/L, cre / L/L: 6.18
adj. p value: 1e−04
log2 L/L, cre / L/L: 5.66
adj. p value: 0
log2 L/L, cre / L/L: 5.4
adj. p value: 2e−04
log2 L/L, cre / L/L: 5.8
adj. p value: 0
log2 intensity
32
30
28
26
24
Genotype
L/L
L/L, cre
Imputation
non imputed
imputed

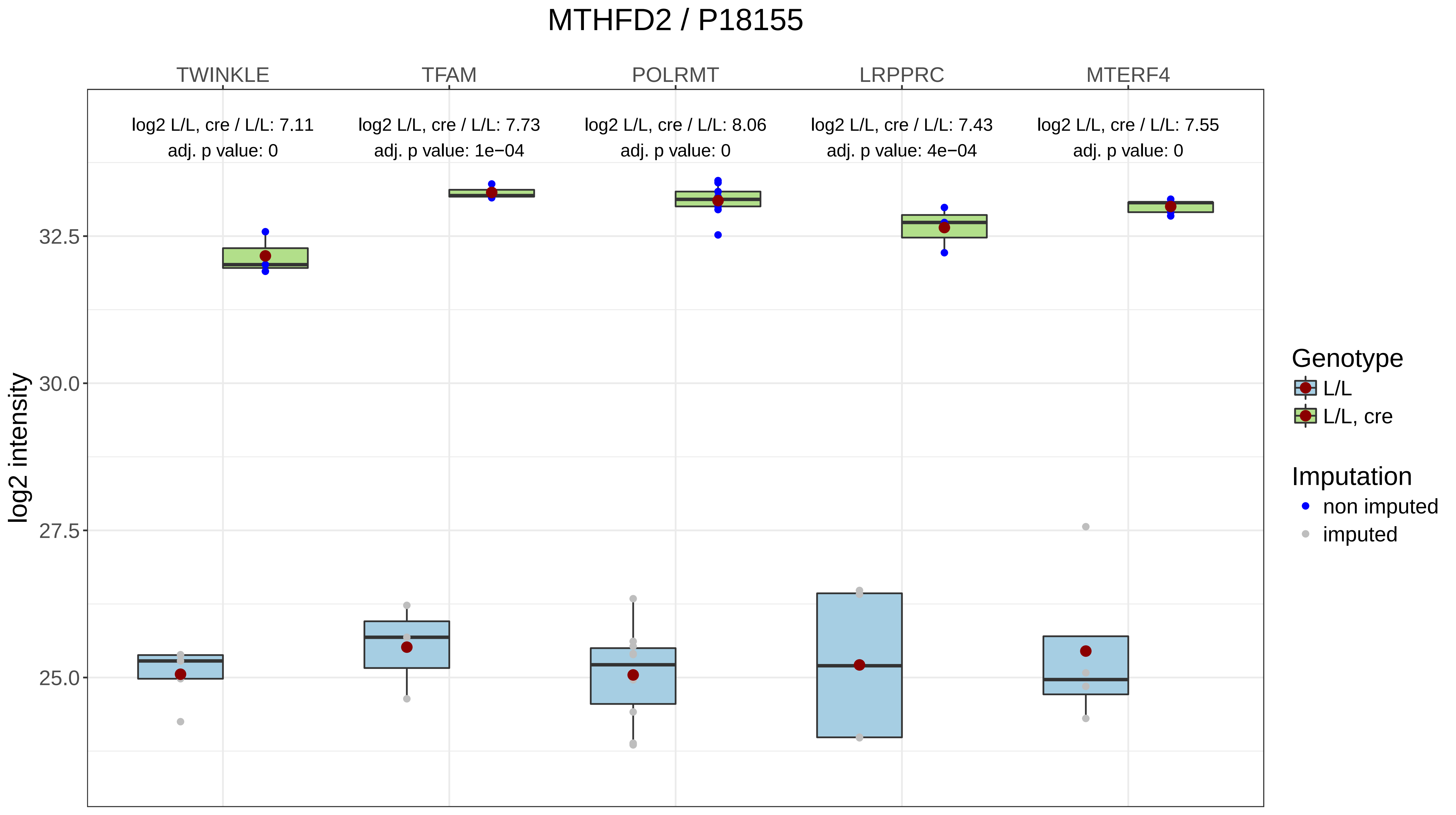
MTHFD2 / P18155
TWINKLE
TFAM
POLRMT
LRPPRC
MTERF4
log2 L/L, cre / L/L: 7.11
adj. p value: 0
log2 L/L, cre / L/L: 7.73
adj. p value: 1e−04
log2 L/L, cre / L/L: 8.06
adj. p value: 0
log2 L/L, cre / L/L: 7.43
adj. p value: 4e−04
log2 L/L, cre / L/L: 7.55
adj. p value: 0
32.5
30.0
27.5
25.0
log2 intensity
Genotype
L/L
L/L, cre
Imputation
non imputed
imputed

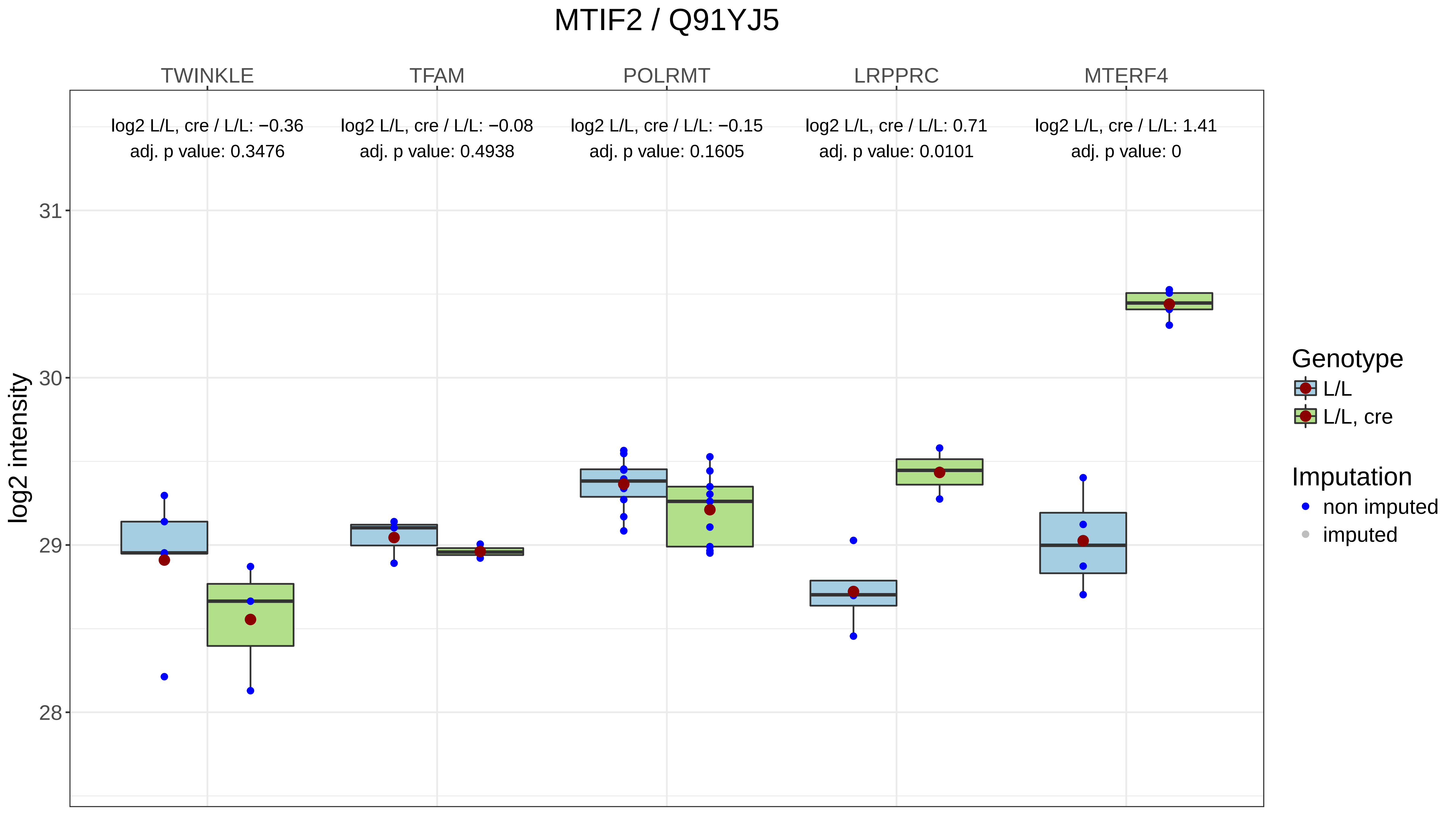

MTIF2 / Q91YJ5
TWINKLE
TFAM
POLRMT
LRPPRC
MTERF4
log2 L/L, cre / L/L: −0.36
adj. p value: 0.3476
log2 L/L, cre / L/L: −0.08
adj. p value: 0.4938
log2 L/L, cre / L/L: −0.15
adj. p value: 0.1605
log2 L/L, cre / L/L: 0.71
adj. p value: 0.0101
log2 L/L, cre / L/L: 1.41
adj. p value: 0
log2 intensity
31
30
29
28
Genotype
L/L
L/L, cre
Imputation
non imputed
imputed

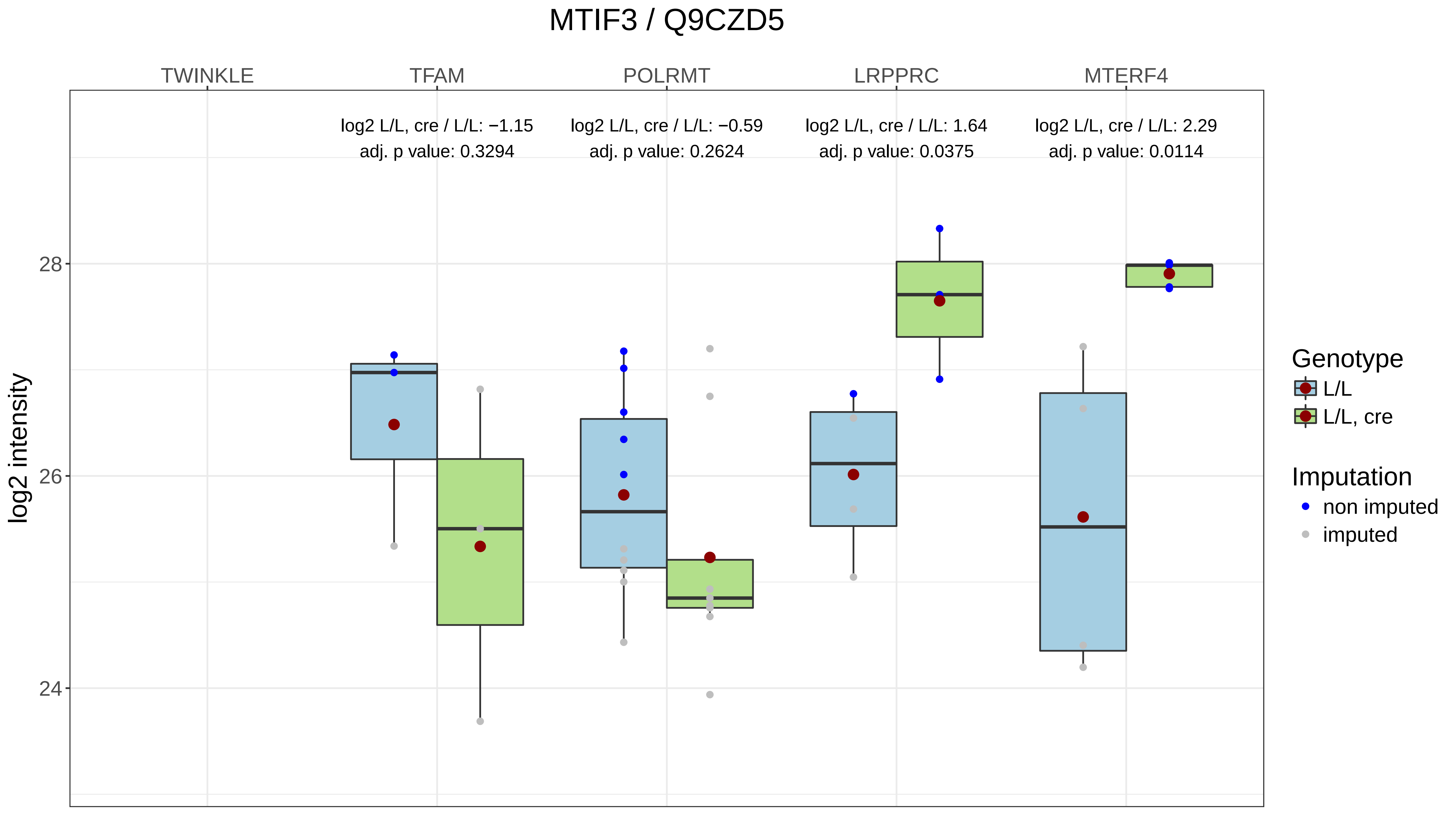
MTIF3 / Q9CZD5
TWINKLE
TFAM
POLRMT
LRPPRC
MTERF4
log2 L/L, cre / L/L: −1.15
adj. p value: 0.3294
log2 L/L, cre / L/L: −0.59
adj. p value: 0.2624
log2 L/L, cre / L/L: 1.64
adj. p value: 0.0375
log2 L/L, cre / L/L: 2.29
adj. p value: 0.0114
log2 intensity
28
26
24
Genotype
L/L
L/L, cre
Imputation
non imputed
imputed

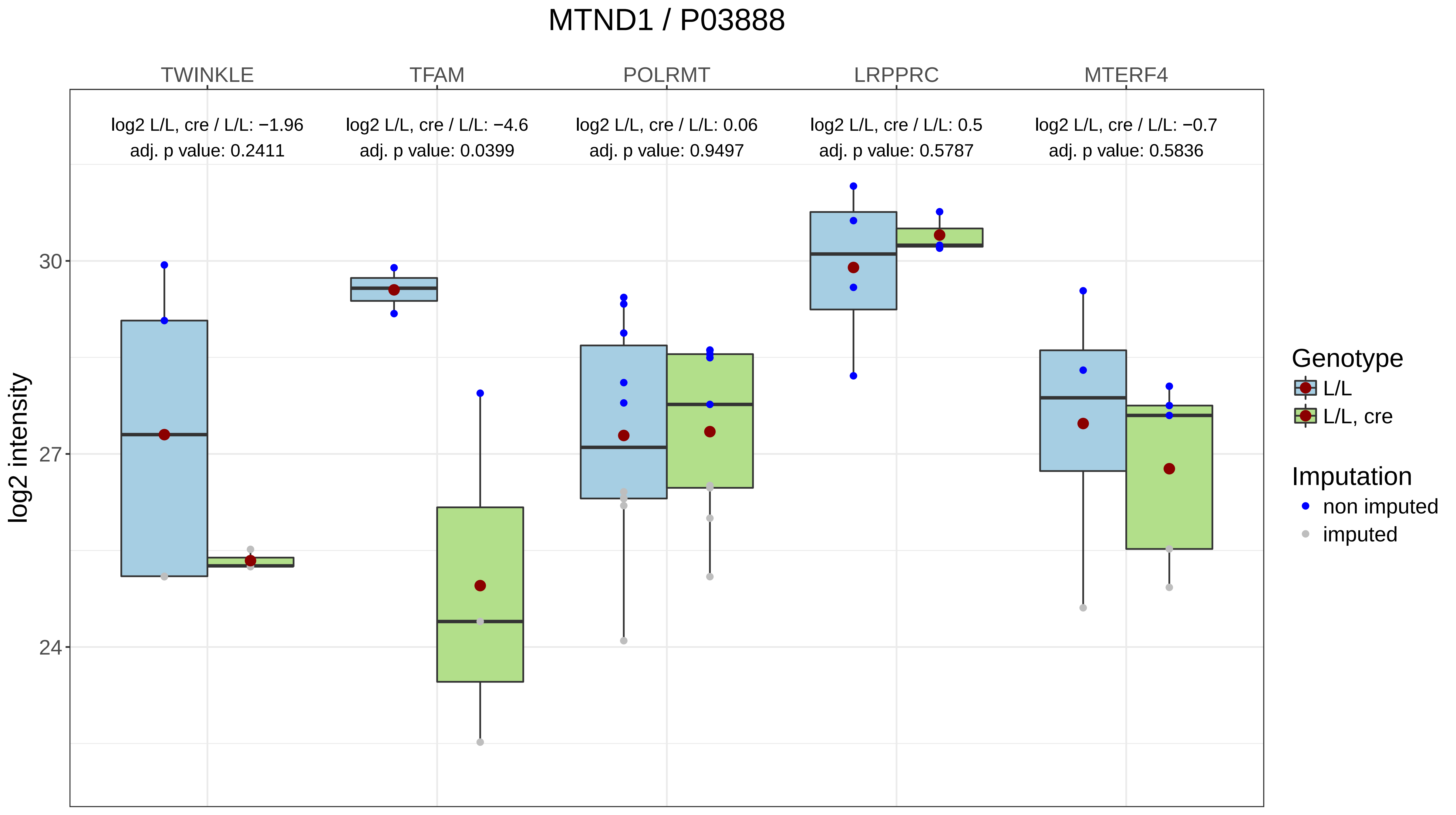
MTND1 / P03888
TWINKLE
TFAM
POLRMT
LRPPRC
MTERF4
log2 L/L, cre / L/L: −1.96
adj. p value: 0.2411
log2 L/L, cre / L/L: −4.6
adj. p value: 0.0399
log2 L/L, cre / L/L: 0.06
adj. p value: 0.9497
log2 L/L, cre / L/L: 0.5
adj. p value: 0.5787
log2 L/L, cre / L/L: −0.7
adj. p value: 0.5836
log2 intensity
30
27
24
Genotype
L/L
L/L, cre
Imputation
non imputed
imputed

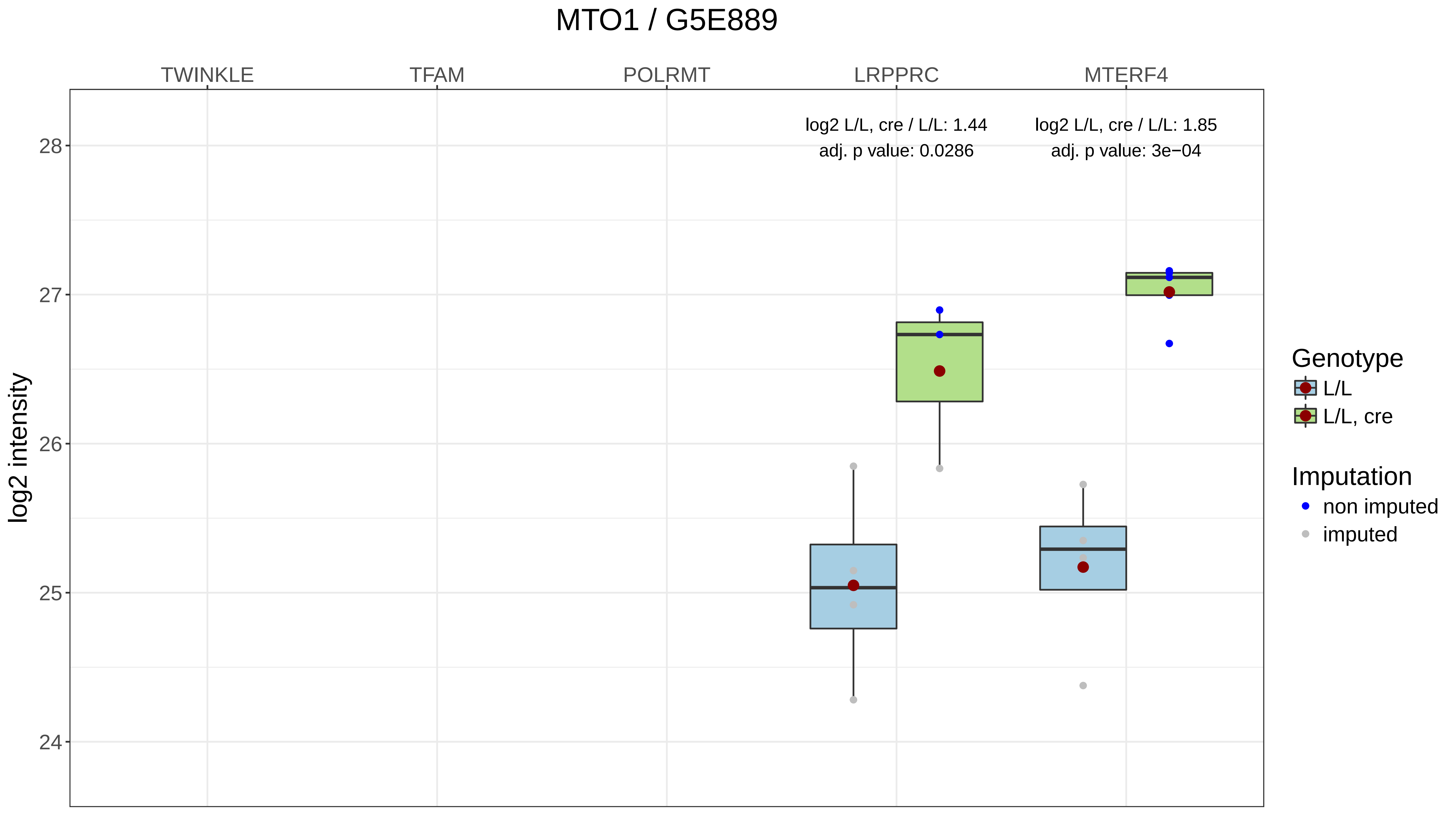

MTO1 / G5E889
TWINKLE
TFAM
POLRMT
LRPPRC
MTERF4
log2 L/L, cre / L/L: 1.44
adj. p value: 0.0286
log2 L/L, cre / L/L: 1.85
adj. p value: 3e−04
log2 intensity
28
27
26
25
24
Genotype
L/L
L/L, cre
Imputation
non imputed
imputed

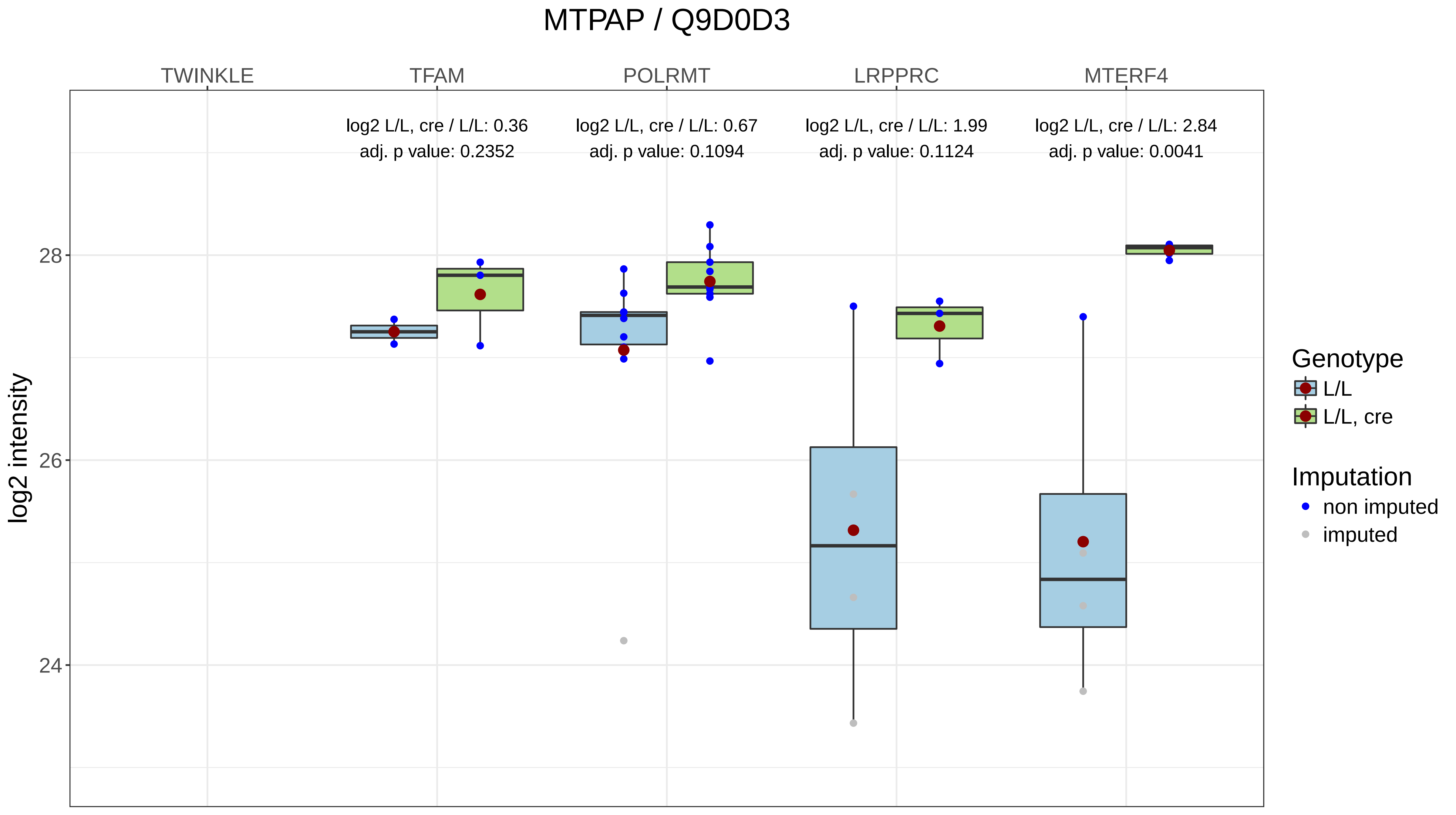

MTPAP / Q9D0D3
TWINKLE
TFAM
POLRMT
LRPPRC
MTERF4
log2 L/L, cre / L/L: 0.36
adj. p value: 0.2352
log2 L/L, cre / L/L: 0.67
adj. p value: 0.1094
log2 L/L, cre / L/L: 1.99
adj. p value: 0.1124
log2 L/L, cre / L/L: 2.84
adj. p value: 0.0041
log2 intensity
28
26
24
Genotype
L/L
L/L, cre
Imputation
non imputed
imputed

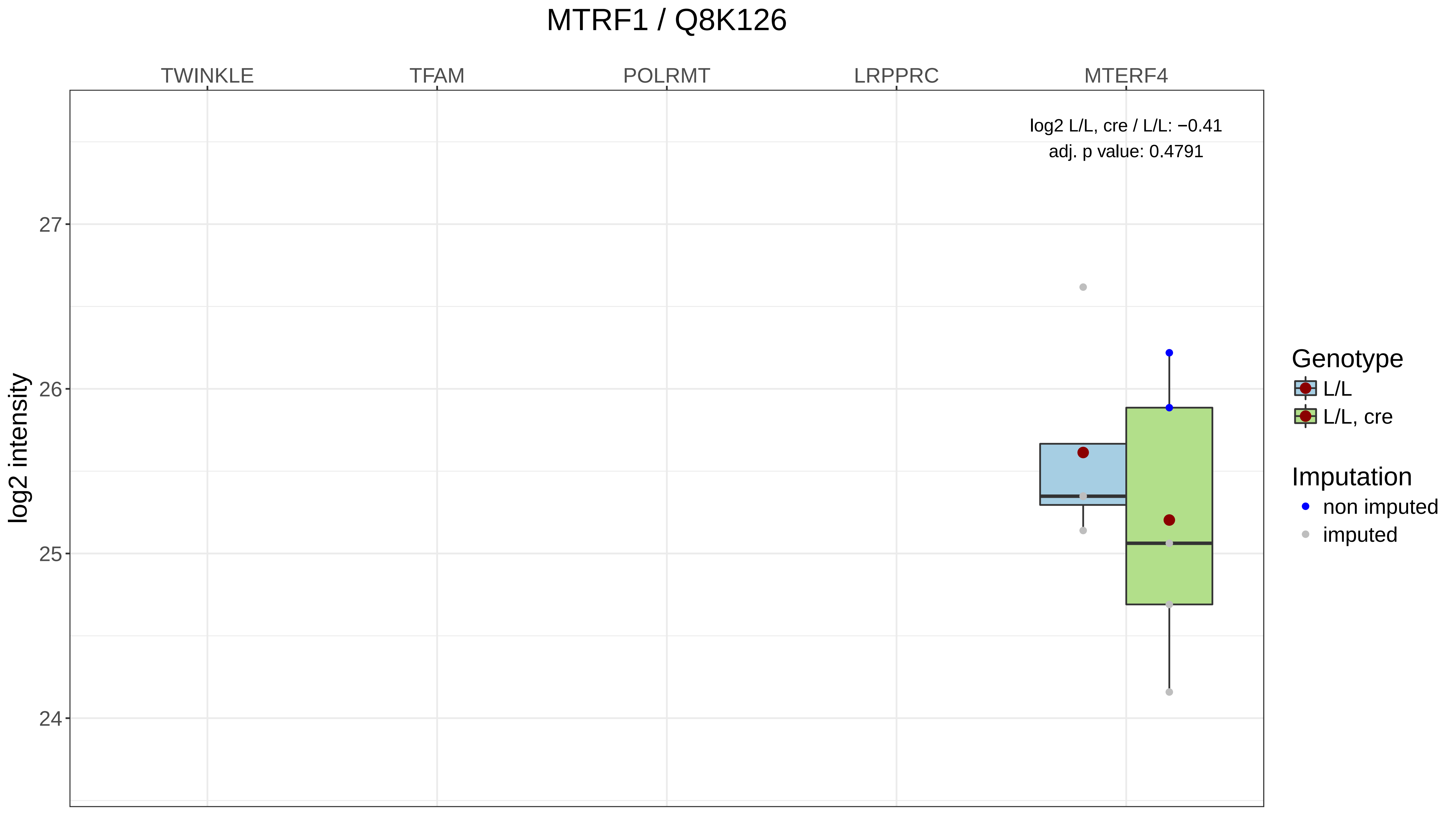

MTRF1 / Q8K126
TWINKLE
TFAM
POLRMT
LRPPRC
MTERF4
log2 L/L, cre / L/L: −0.41
adj. p value: 0.4791
log2 intensity
27
26
25
24
Genotype
L/L
L/L, cre
Imputation
non imputed
imputed

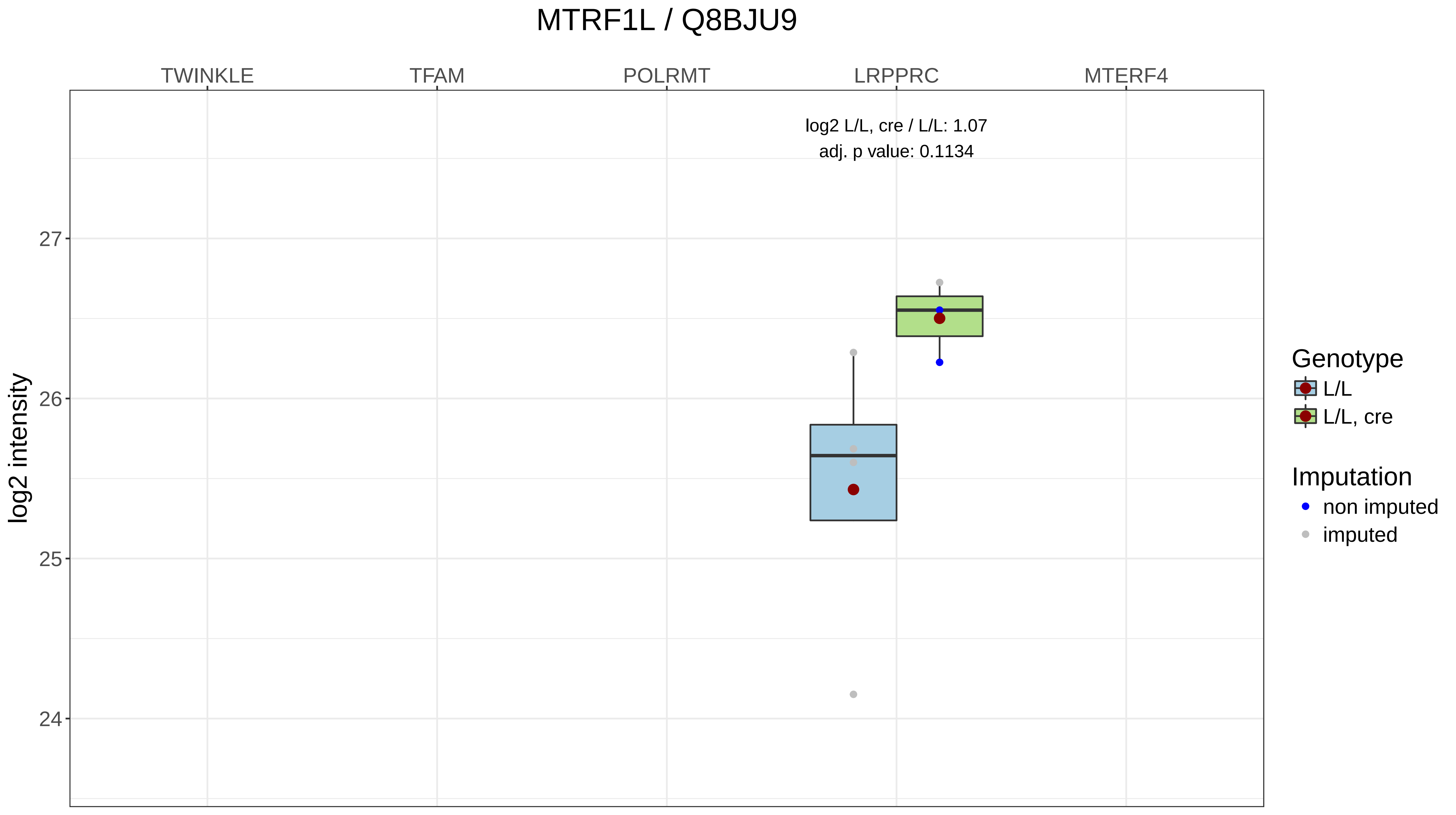
MTRF1L / Q8BJU9
TWINKLE
TFAM
POLRMT
LRPPRC
MTERF4
log2 L/L, cre / L/L: 1.07
adj. p value: 0.1134
log2 intensity
27
26
25
24
Genotype
L/L
L/L, cre
Imputation
non imputed
imputed

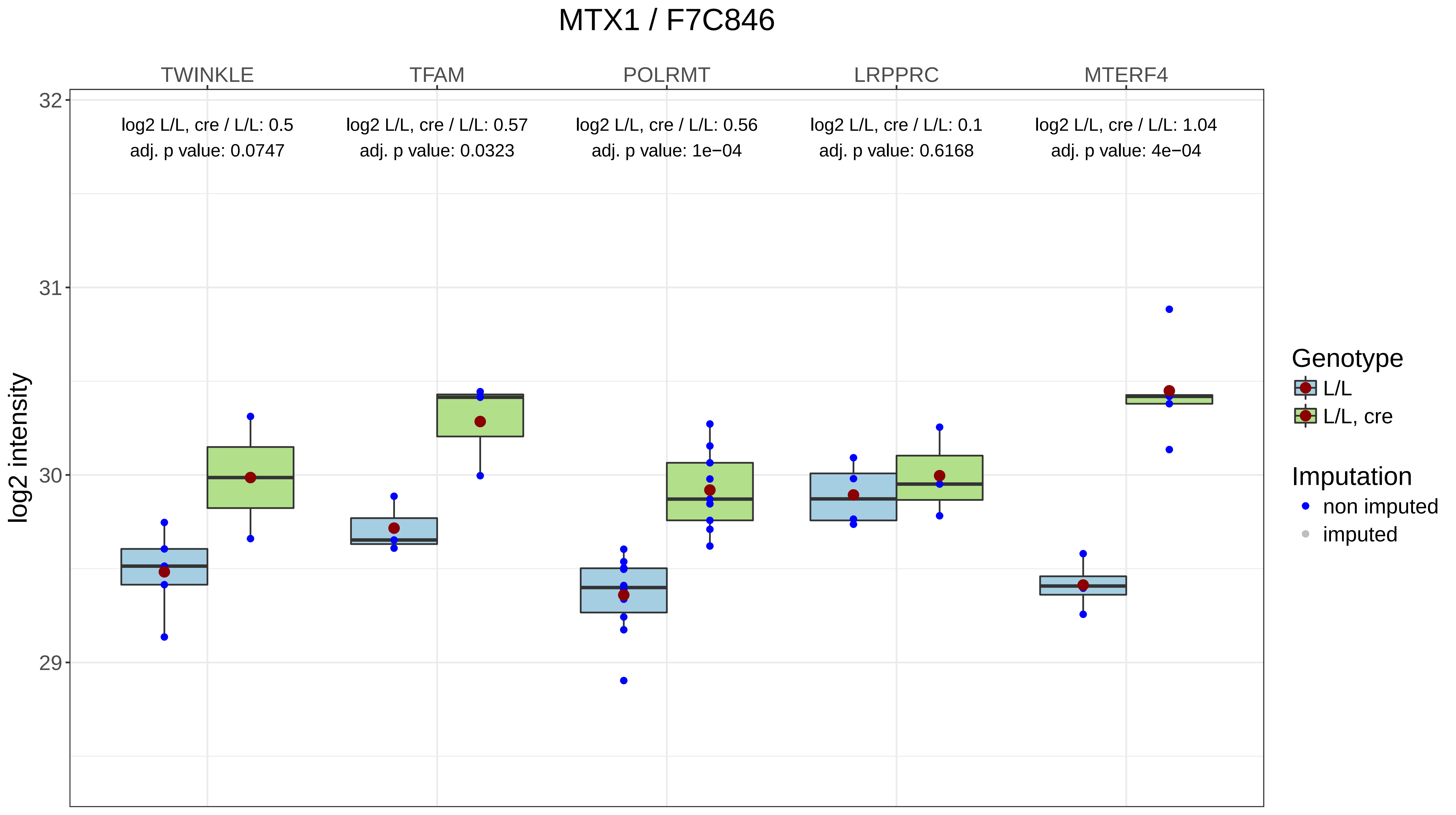
MTX1 / F7C846
TWINKLE
TFAM
POLRMT
LRPPRC
MTERF4
log2 L/L, cre / L/L: 0.5
adj. p value: 0.0747
log2 L/L, cre / L/L: 0.57
adj. p value: 0.0323
log2 L/L, cre / L/L: 0.56
adj. p value: 1e−04
log2 L/L, cre / L/L: 0.1
adj. p value: 0.6168
log2 L/L, cre / L/L: 1.04
adj. p value: 4e−04
log2 intensity
32
31
30
29
Genotype
L/L
L/L, cre
Imputation
non imputed
imputed

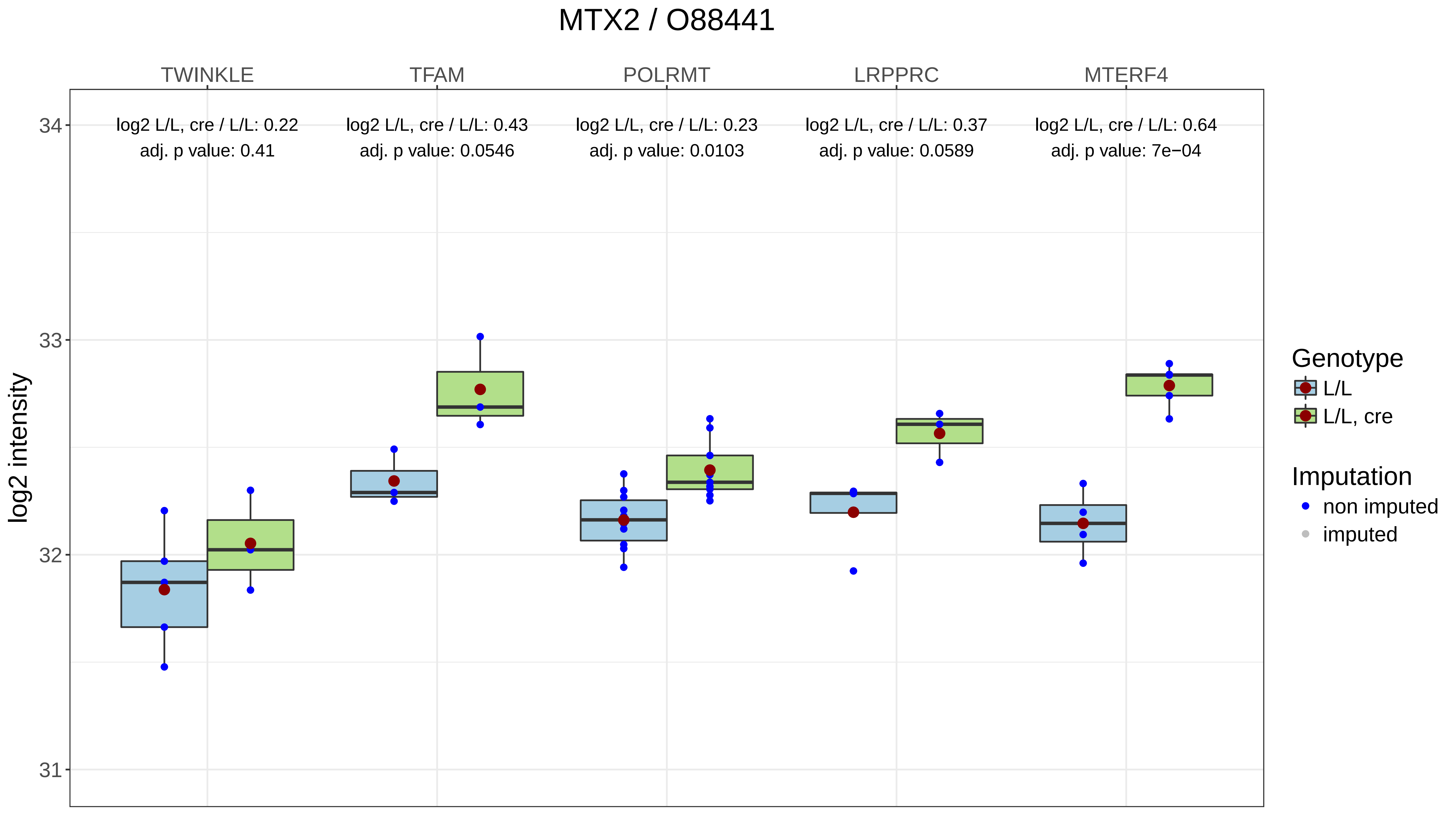
MTX2 / O88441
TWINKLE
TFAM
POLRMT
LRPPRC
MTERF4
log2 L/L, cre / L/L: 0.22
adj. p value: 0.41
log2 L/L, cre / L/L: 0.43
adj. p value: 0.0546
log2 L/L, cre / L/L: 0.23
adj. p value: 0.0103
log2 L/L, cre / L/L: 0.37
adj. p value: 0.0589
log2 L/L, cre / L/L: 0.64
adj. p value: 7e−04
log2 intensity
34
33
32
31
Genotype
L/L
L/L, cre
Imputation
non imputed
imputed

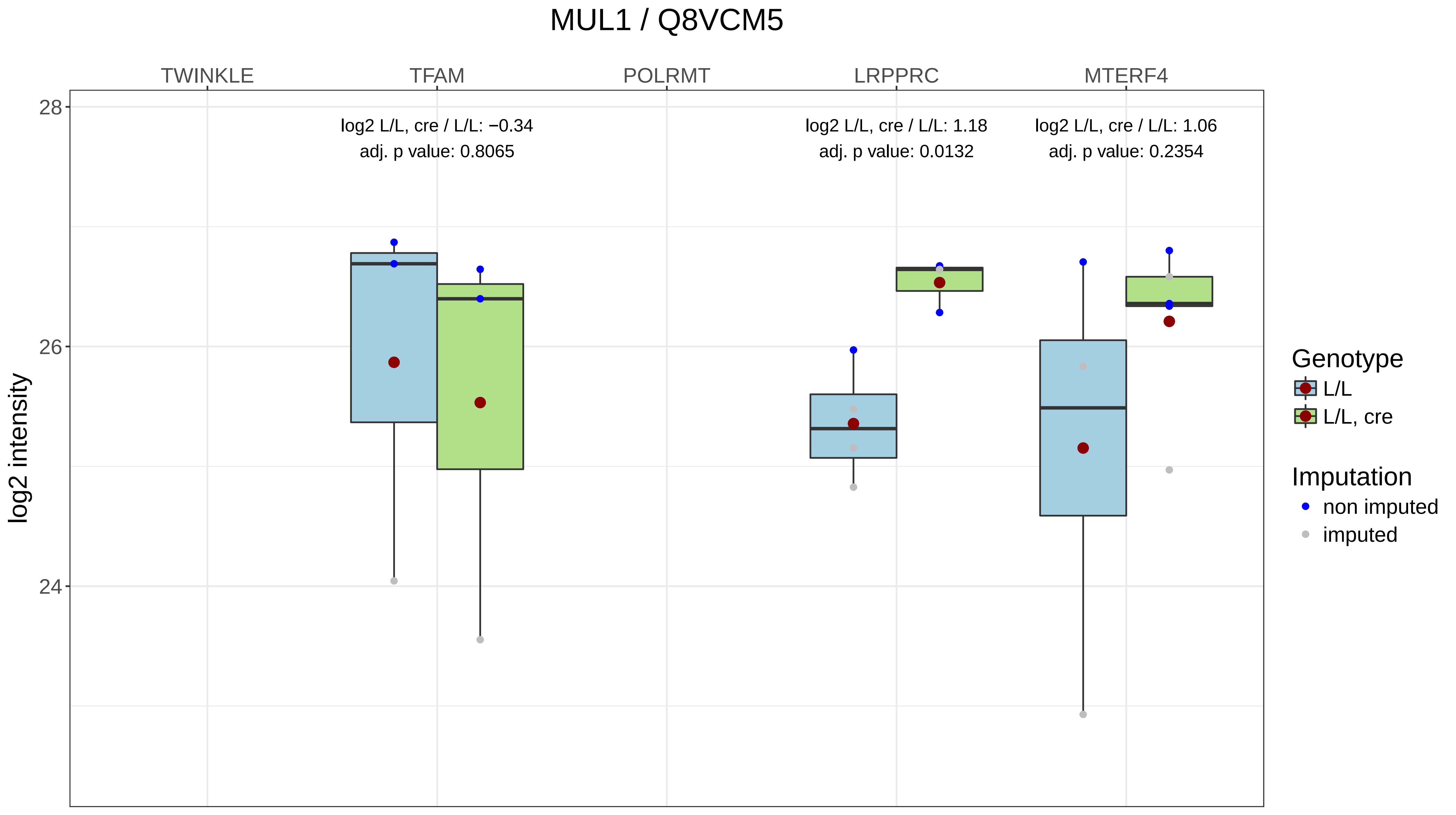
MUL1 / Q8VCM5
TWINKLE
TFAM
POLRMT
LRPPRC
MTERF4
log2 L/L, cre / L/L: −0.34
adj. p value: 0.8065
log2 L/L, cre / L/L: 1.18
adj. p value: 0.0132
log2 L/L, cre / L/L: 1.06
adj. p value: 0.2354
28
26
24
log2 intensity
Genotype
L/L
L/L, cre
Imputation
non imputed
imputed

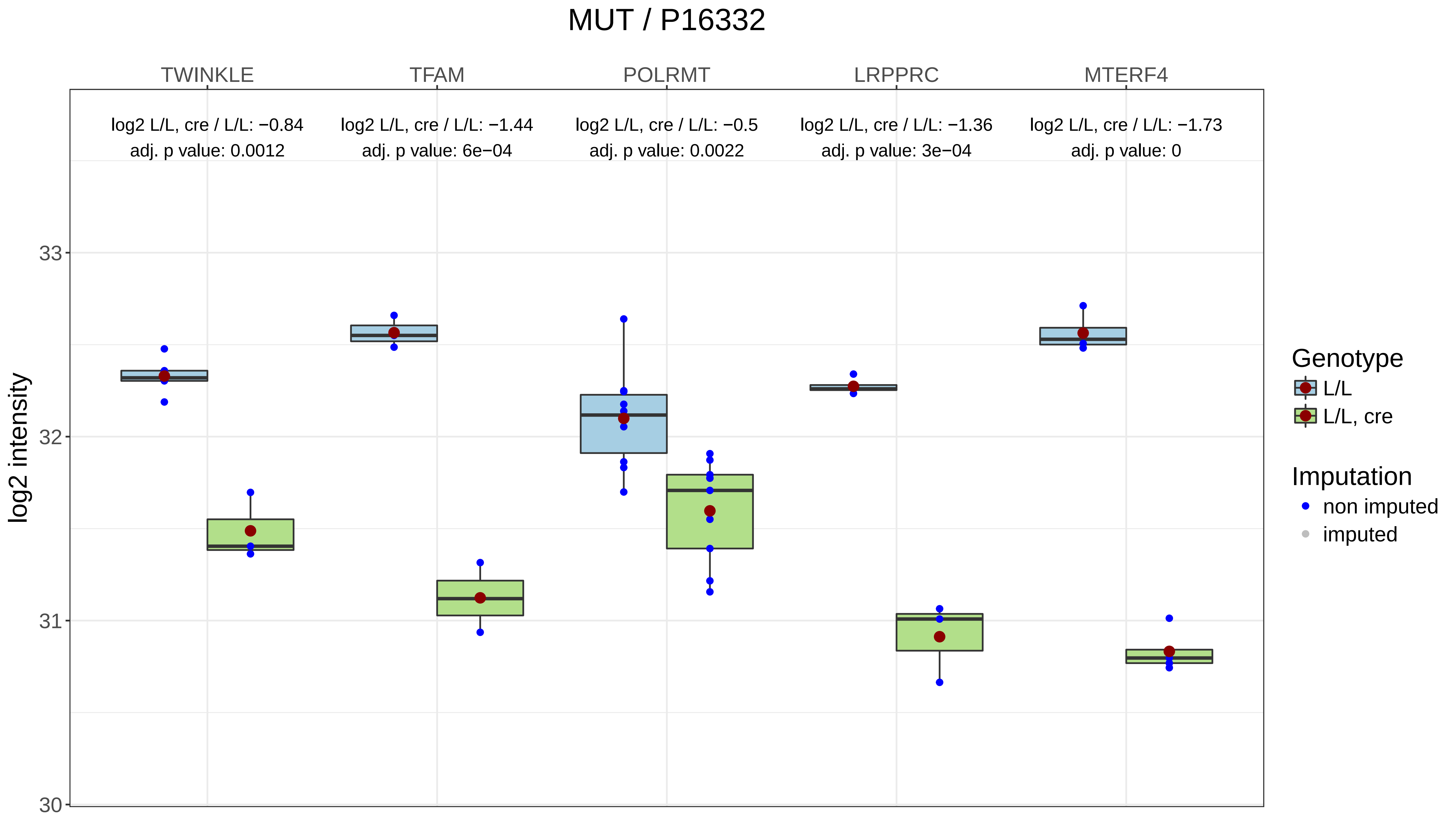
MUT / P16332
TWINKLE
TFAM
POLRMT
LRPPRC
MTERF4
log2 L/L, cre / L/L: −0.84
adj. p value: 0.0012
log2 L/L, cre / L/L: −1.44
adj. p value: 6e−04
log2 L/L, cre / L/L: −0.5
adj. p value: 0.0022
log2 L/L, cre / L/L: −1.36
adj. p value: 3e−04
log2 L/L, cre / L/L: −1.73
adj. p value: 0
log2 intensity
33
32
31
30
Genotype
L/L
L/L, cre
Imputation
non imputed
imputed

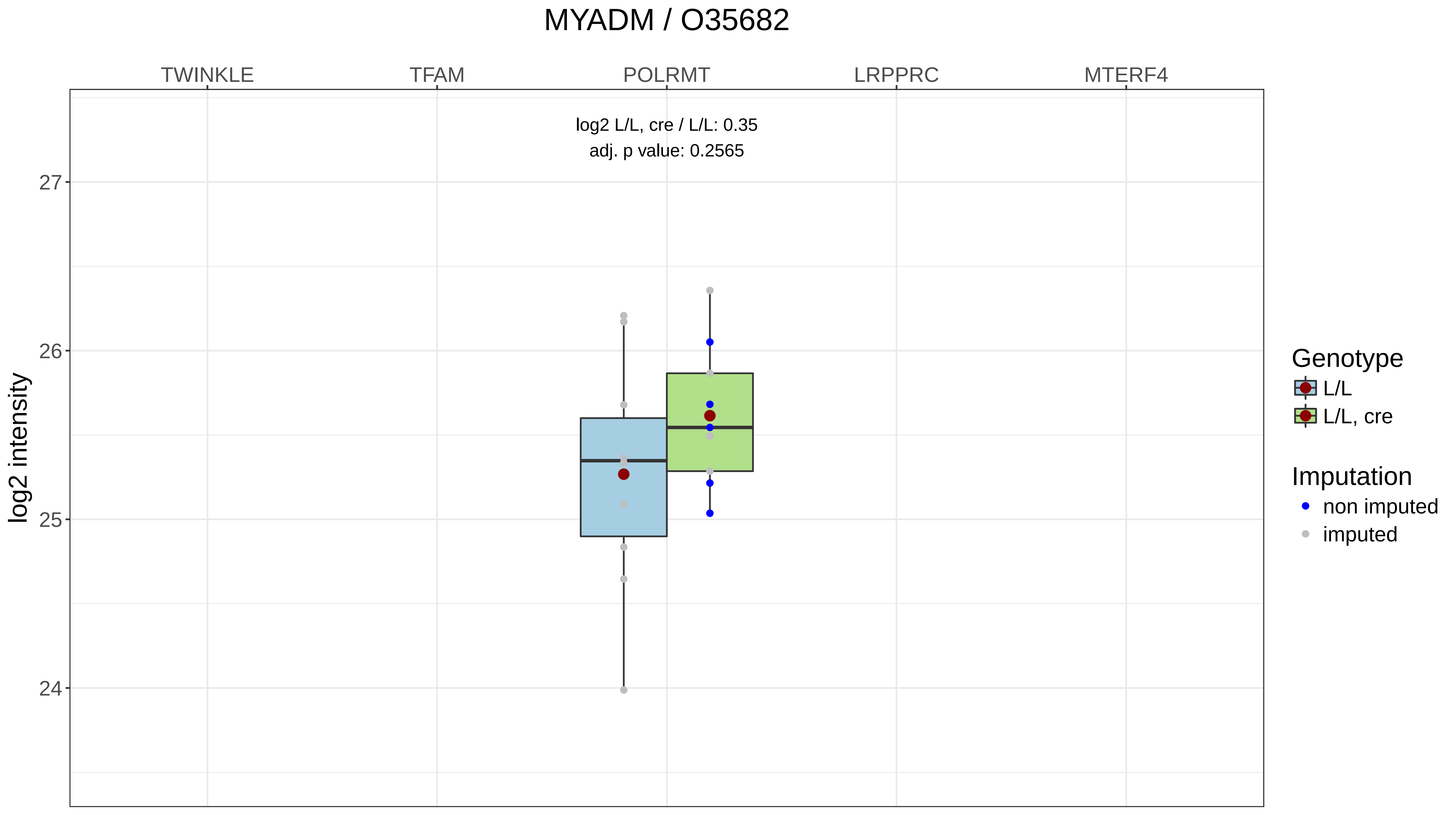
MYADM / O35682
TWINKLE
TFAM
POLRMT
LRPPRC
MTERF4
log2 L/L, cre / L/L: 0.35
adj. p value: 0.2565
log2 intensity
27
26
25
24
Genotype
L/L
L/L, cre
Imputation
non imputed
imputed

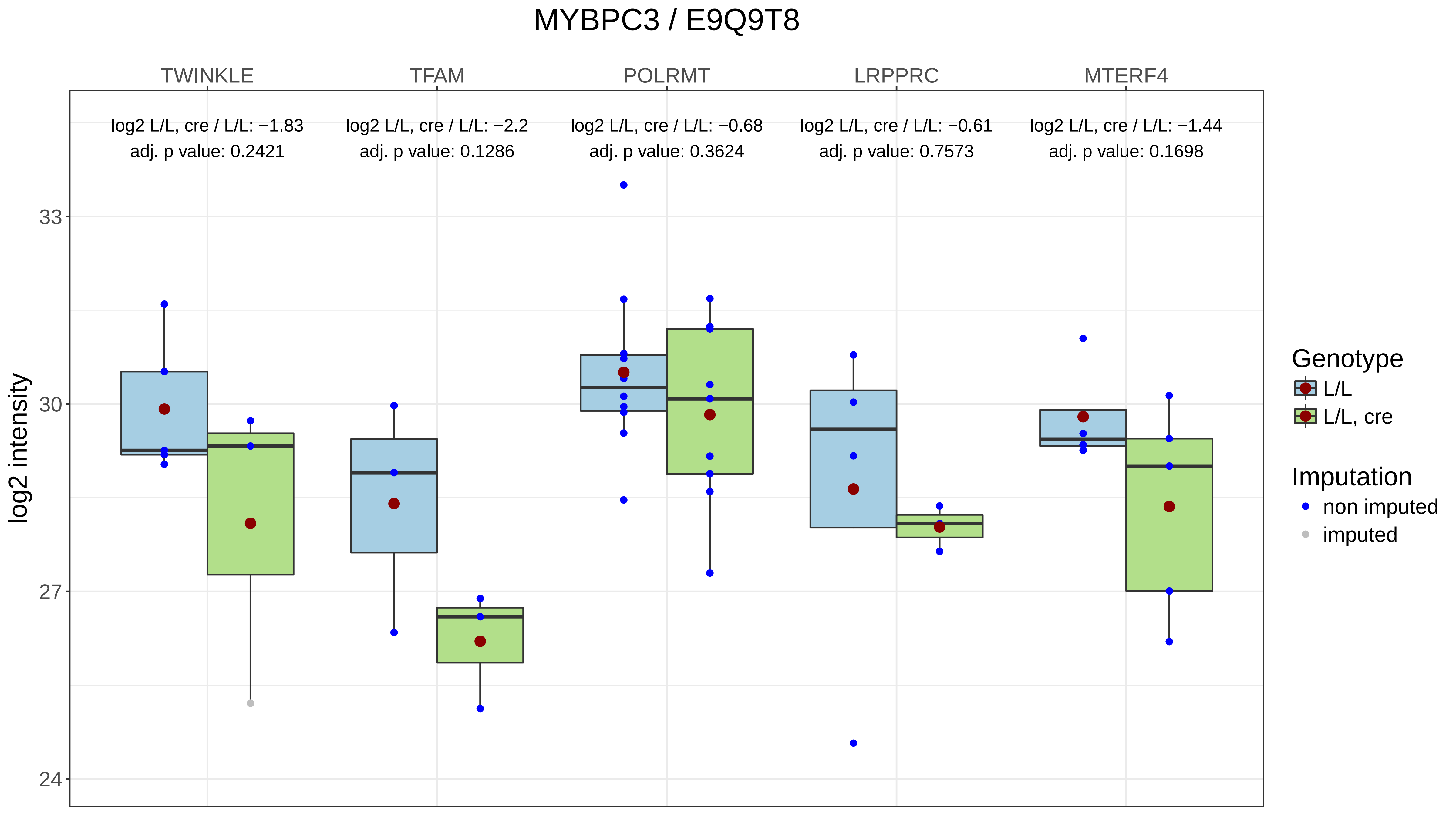

MYBPC3 / E9Q9T8
TWINKLE
TFAM
POLRMT
LRPPRC
MTERF4
log2 L/L, cre / L/L: −1.83
adj. p value: 0.2421
log2 L/L, cre / L/L: −2.2
adj. p value: 0.1286
log2 L/L, cre / L/L: −0.68
adj. p value: 0.3624
log2 L/L, cre / L/L: −0.61
adj. p value: 0.7573
log2 L/L, cre / L/L: −1.44
adj. p value: 0.1698
log2 intensity
33
30
27
24
Genotype
L/L
L/L, cre
Imputation
non imputed
imputed

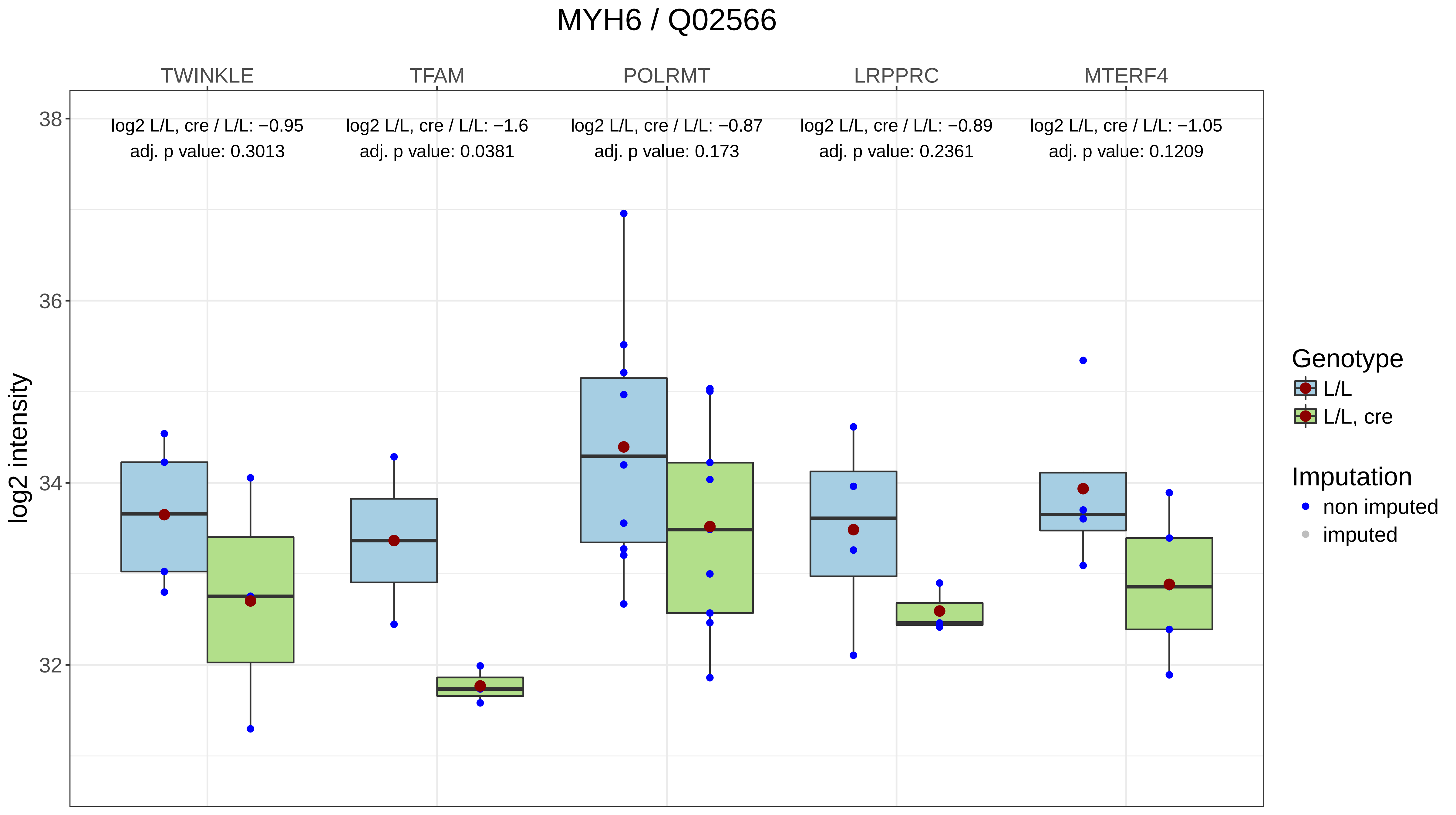
MYH6 / Q02566
TWINKLE
TFAM
POLRMT
LRPPRC
MTERF4
log2 L/L, cre / L/L: −0.95
adj. p value: 0.3013
log2 L/L, cre / L/L: −1.6
adj. p value: 0.0381
log2 L/L, cre / L/L: −0.87
adj. p value: 0.173
log2 L/L, cre / L/L: −0.89
adj. p value: 0.2361
log2 L/L, cre / L/L: −1.05
adj. p value: 0.1209
log2 intensity
38
36
34
32
Genotype
L/L
L/L, cre
Imputation
non imputed
imputed

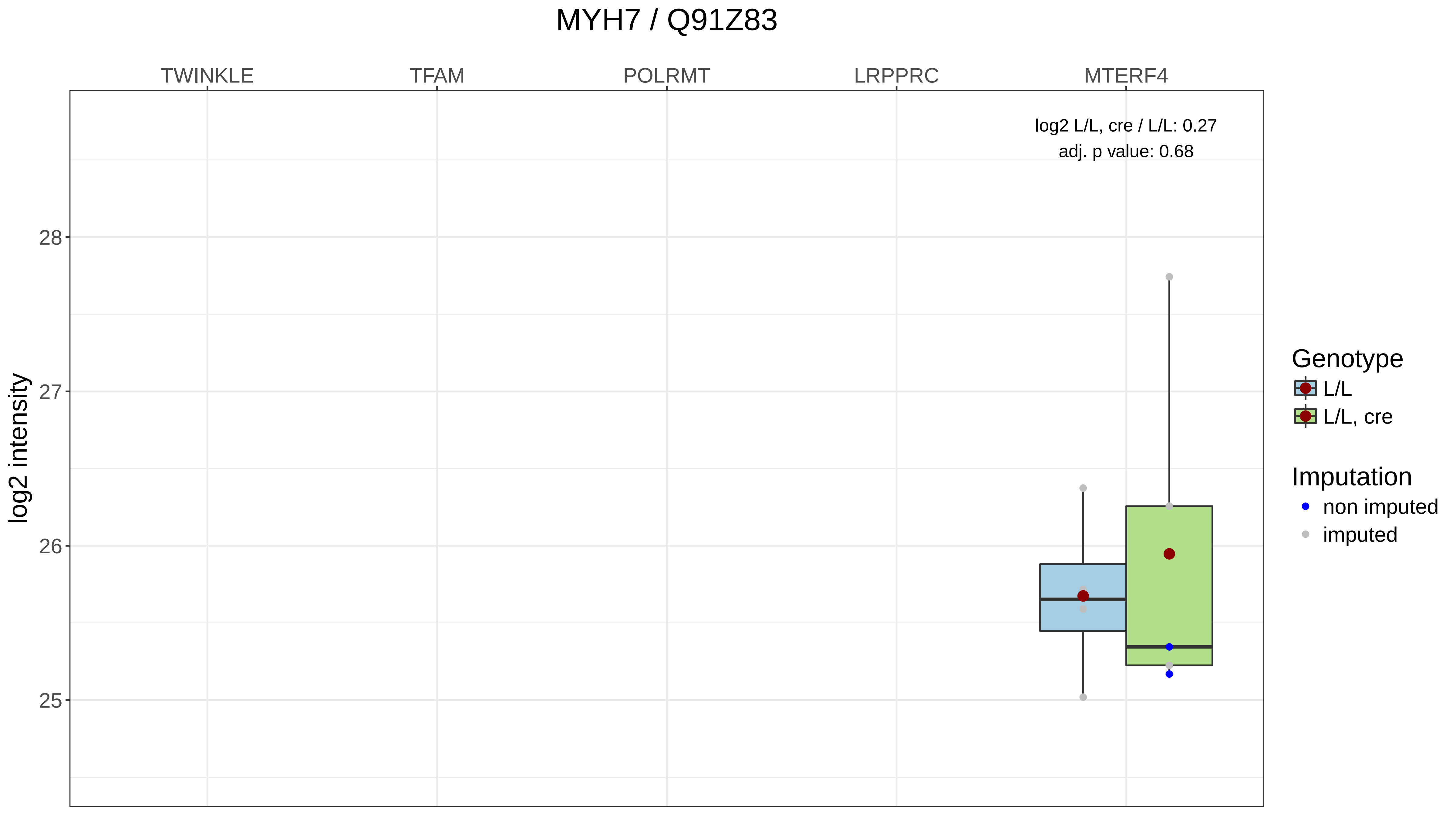
MYH7 / Q91Z83
TWINKLE
TFAM
POLRMT
LRPPRC
MTERF4
log2 L/L, cre / L/L: 0.27
adj. p value: 0.68
log2 intensity
28
27
26
25
Genotype
L/L
L/L, cre
Imputation
non imputed
imputed

MYH9 / Q8VDD5

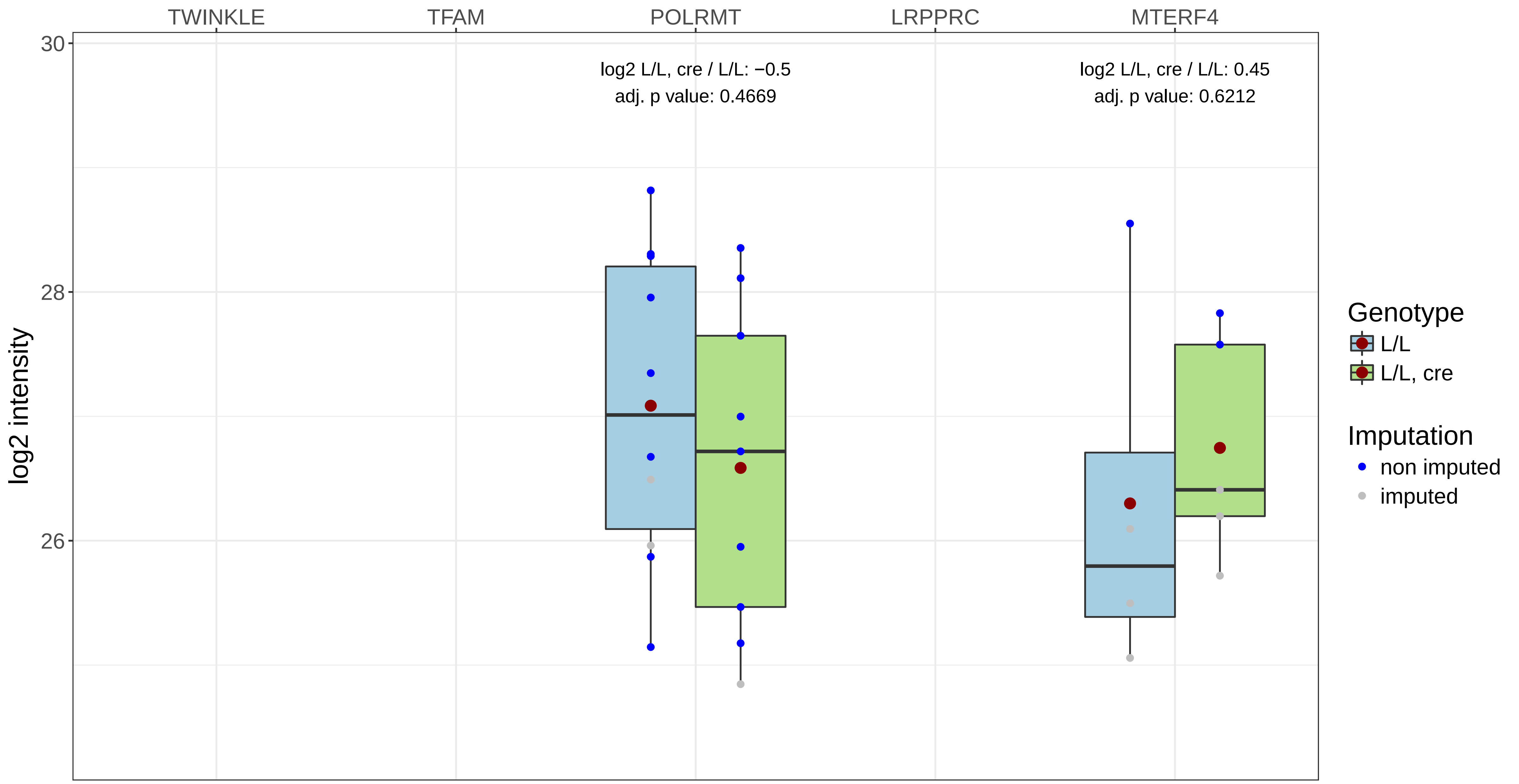

TWINKLE
TFAM
POLRMT
LRPPRC
MTERF4
30
28
26
log2 intensity
log2 L/L, cre / L/L: −0.5
adj. p value: 0.4669
log2 L/L, cre / L/L: 0.45
adj. p value: 0.6212
Genotype
L/L
L/L, cre
Imputation
non imputed
imputed

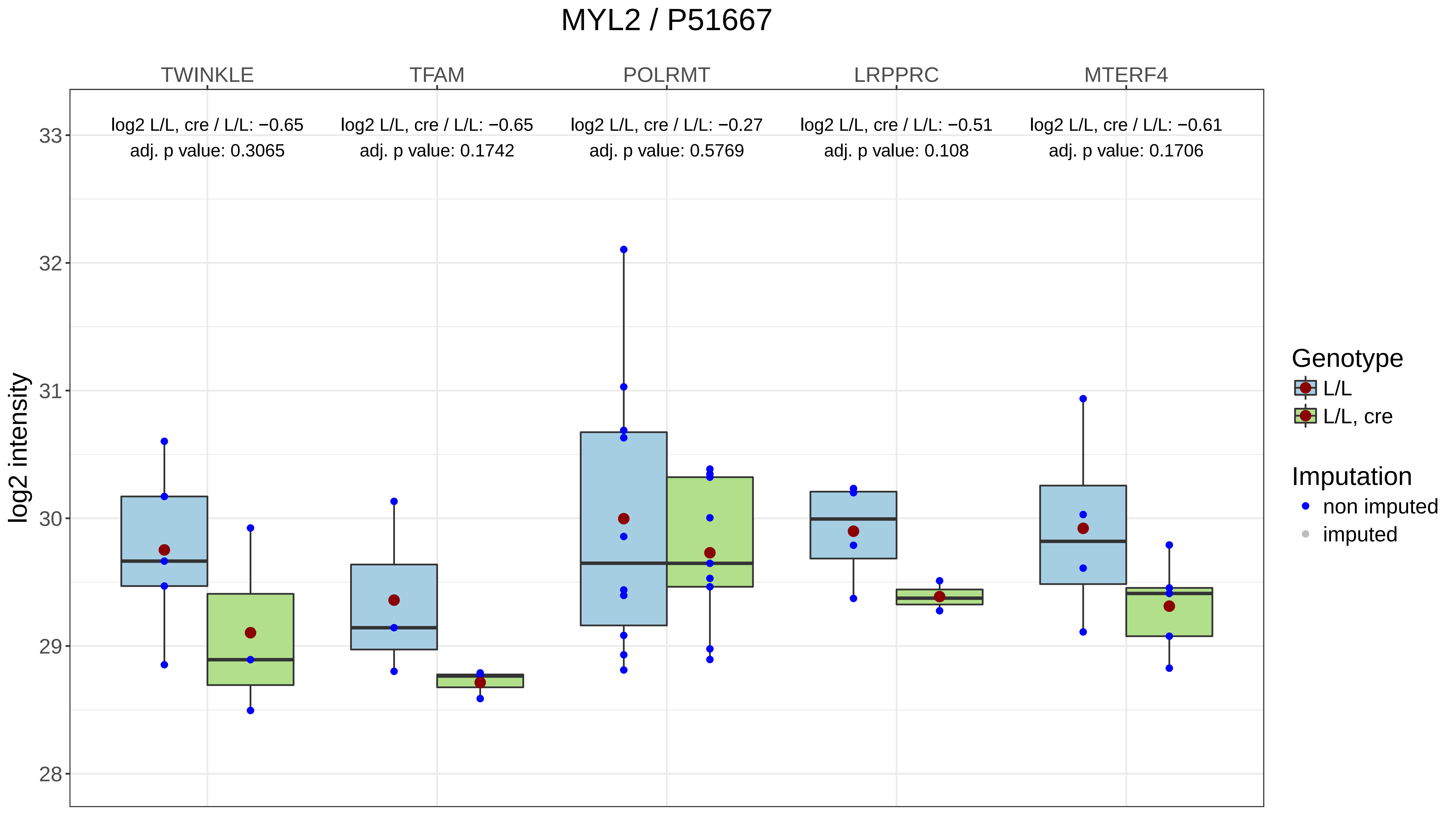

MYL2 / P51667
TWINKLE
TFAM
POLRMT
LRPPRC
MTERF4
log2 L/L, cre / L/L: −0.65
adj. p value: 0.3065
log2 L/L, cre / L/L: −0.65
adj. p value: 0.1742
log2 L/L, cre / L/L: −0.27
adj. p value: 0.5769
log2 L/L, cre / L/L: −0.51
adj. p value: 0.108
log2 L/L, cre / L/L: −0.61
adj. p value: 0.1706
log2 intensity
33
32
31
30
29
28
Genotype
L/L
L/L, cre
Imputation
non imputed
imputed

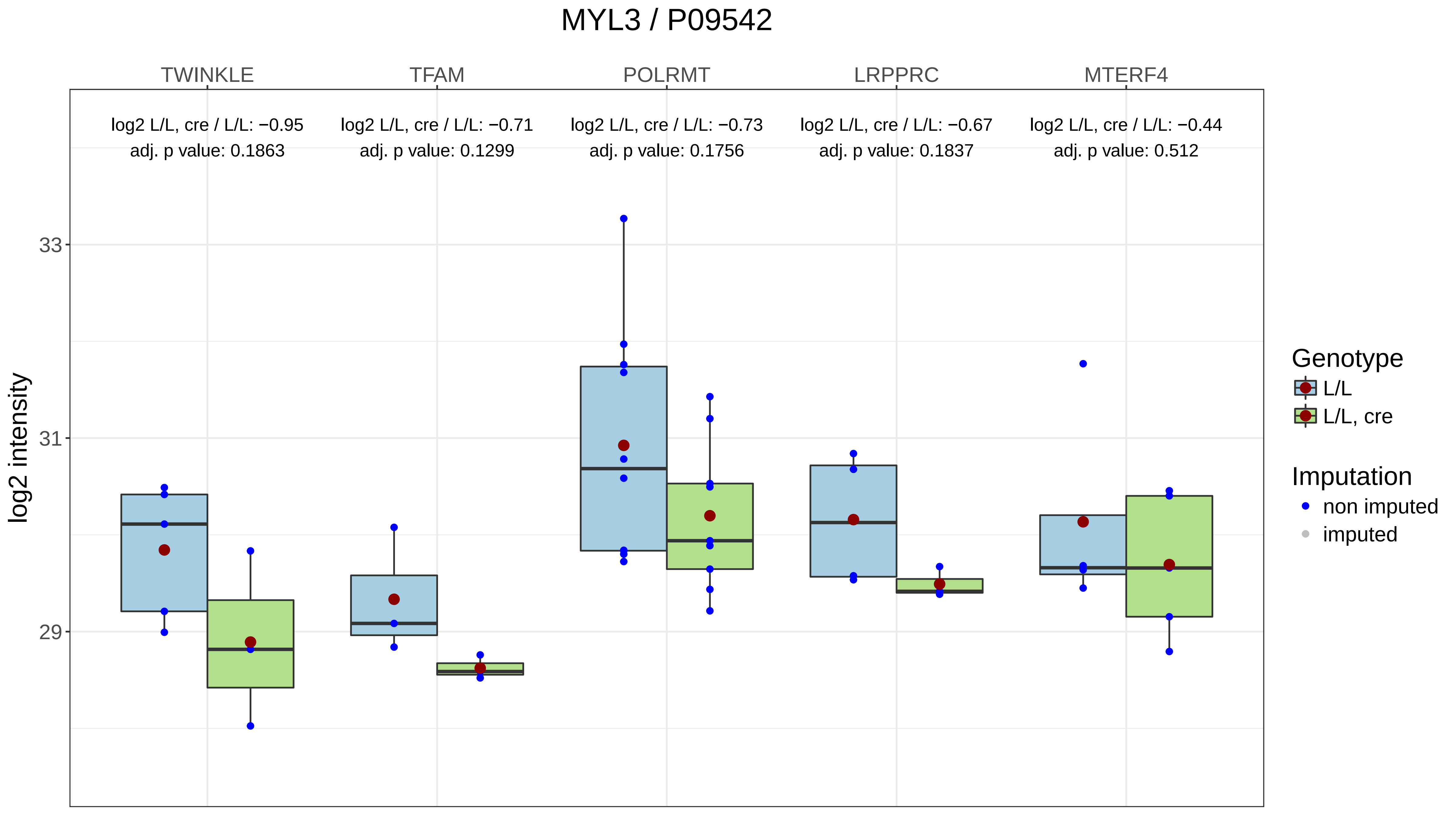
MYL3 / P09542
TWINKLE
TFAM
POLRMT
LRPPRC
MTERF4
log2 L/L, cre / L/L: −0.95
adj. p value: 0.1863
log2 L/L, cre / L/L: −0.71
adj. p value: 0.1299
log2 L/L, cre / L/L: −0.73
adj. p value: 0.1756
log2 L/L, cre / L/L: −0.67
adj. p value: 0.1837
log2 L/L, cre / L/L: −0.44
adj. p value: 0.512
log2 intensity
33
31
29
Genotype
L/L
L/L, cre
Imputation
non imputed
imputed

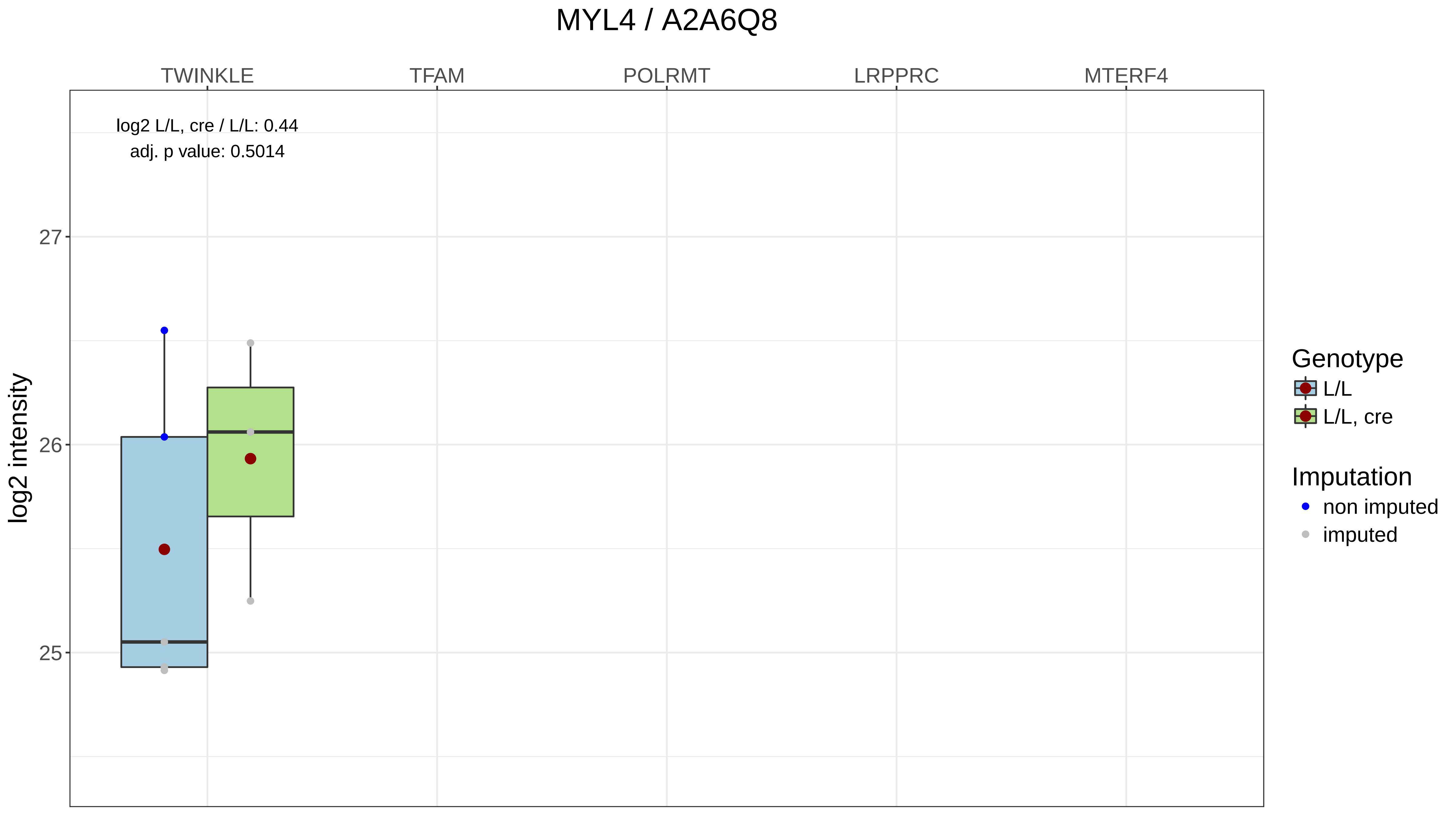

MYL4 / A2A6Q8
TWINKLE
TFAM
POLRMT
LRPPRC
MTERF4
log2 L/L, cre / L/L: 0.44
adj. p value: 0.5014
27
26
25
log2 intensity
Genotype
L/L
L/L, cre
Imputation
non imputed
imputed

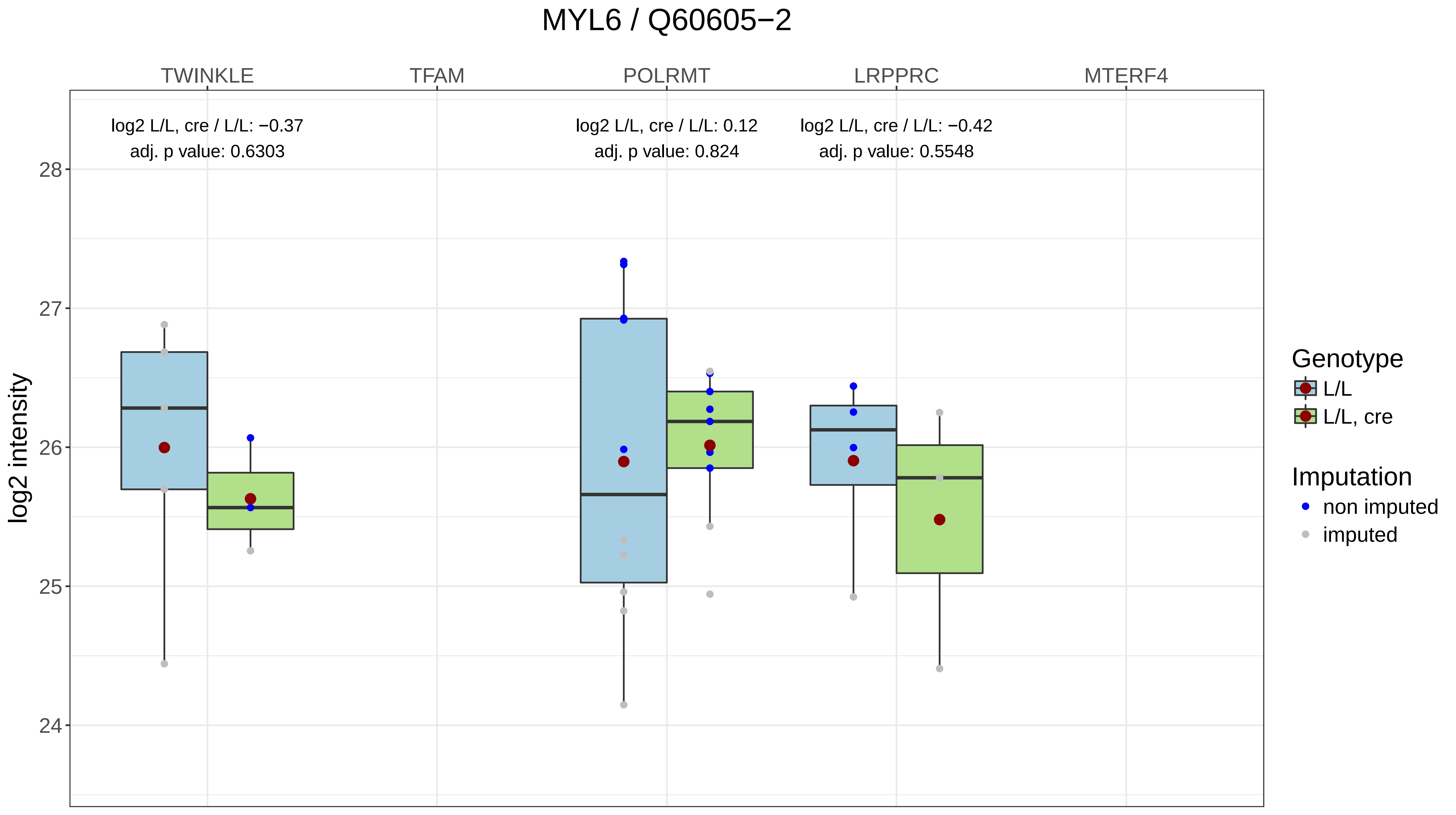
MYL6 / Q60605−2
TWINKLE
TFAM
POLRMT
LRPPRC
MTERF4
log2 L/L, cre / L/L: −0.37
adj. p value: 0.6303
log2 L/L, cre / L/L: 0.12
adj. p value: 0.824
log2 L/L, cre / L/L: −0.42
adj. p value: 0.5548
log2 intensity
28
27
26
25
24
Genotype
L/L
L/L, cre
Imputation
non imputed
imputed

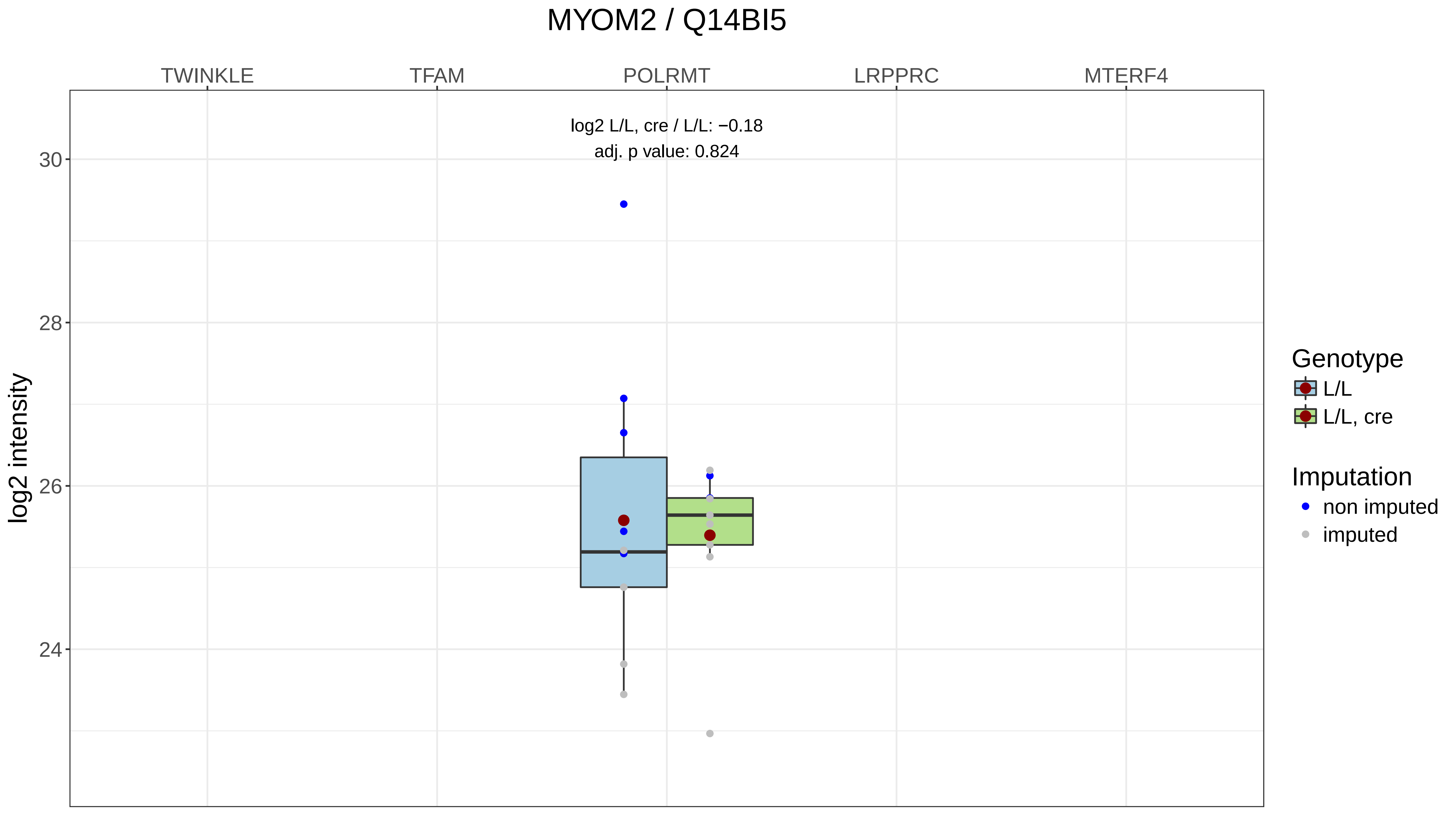

MYOM2 / Q14BI5
TWINKLE
TFAM
POLRMT
LRPPRC
MTERF4
log2 L/L, cre / L/L: −0.18
adj. p value: 0.824
30
28
26
24
log2 intensity
Genotype
L/L
L/L, cre
Imputation
non imputed
imputed

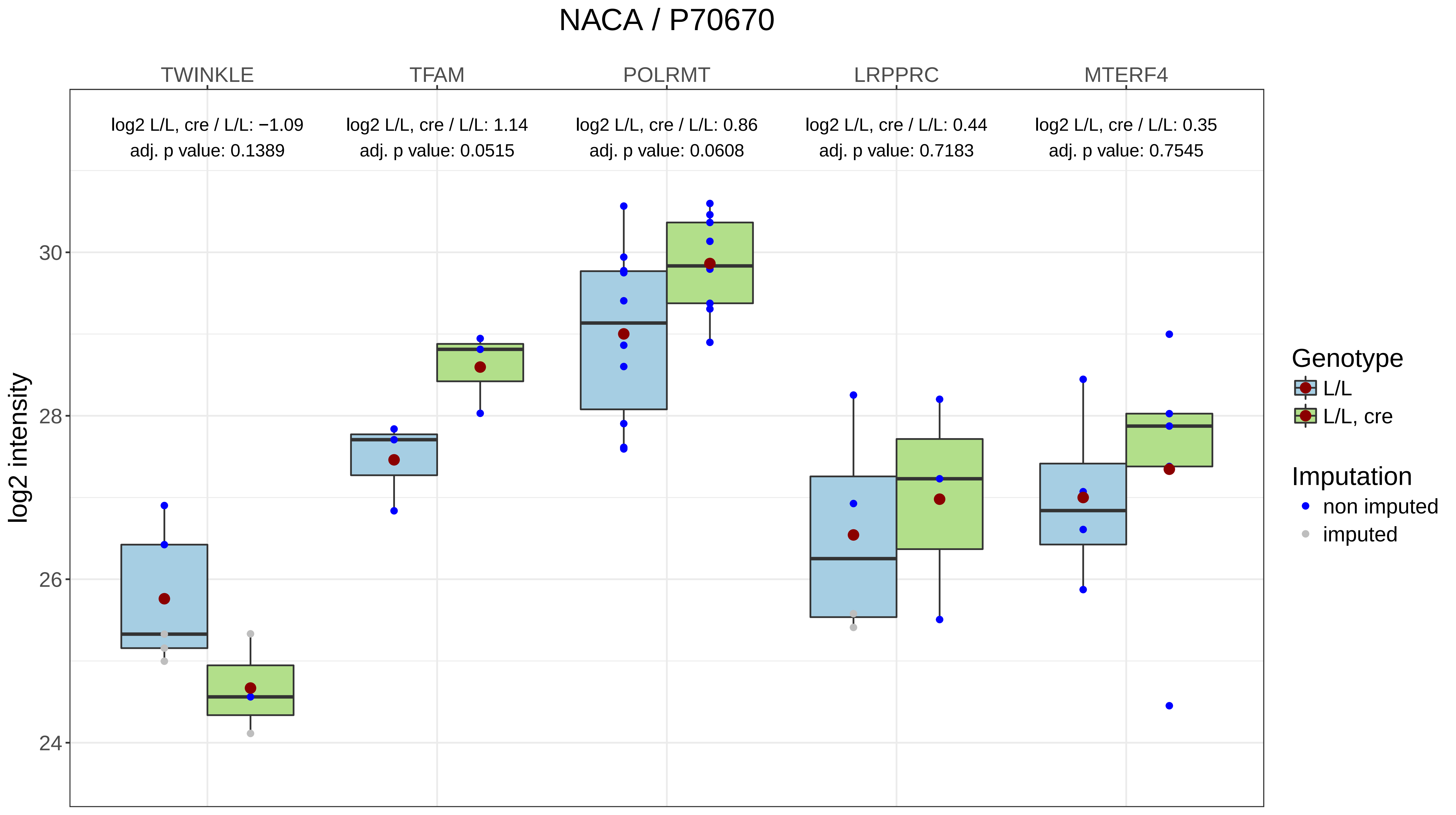
NACA / P70670
TWINKLE
TFAM
POLRMT
LRPPRC
MTERF4
log2 L/L, cre / L/L: −1.09
adj. p value: 0.1389
log2 L/L, cre / L/L: 1.14
adj. p value: 0.0515
log2 L/L, cre / L/L: 0.86
adj. p value: 0.0608
log2 L/L, cre / L/L: 0.44
adj. p value: 0.7183
log2 L/L, cre / L/L: 0.35
adj. p value: 0.7545
log2 intensity
30
28
26
24
Genotype
L/L
L/L, cre
Imputation
non imputed
imputed

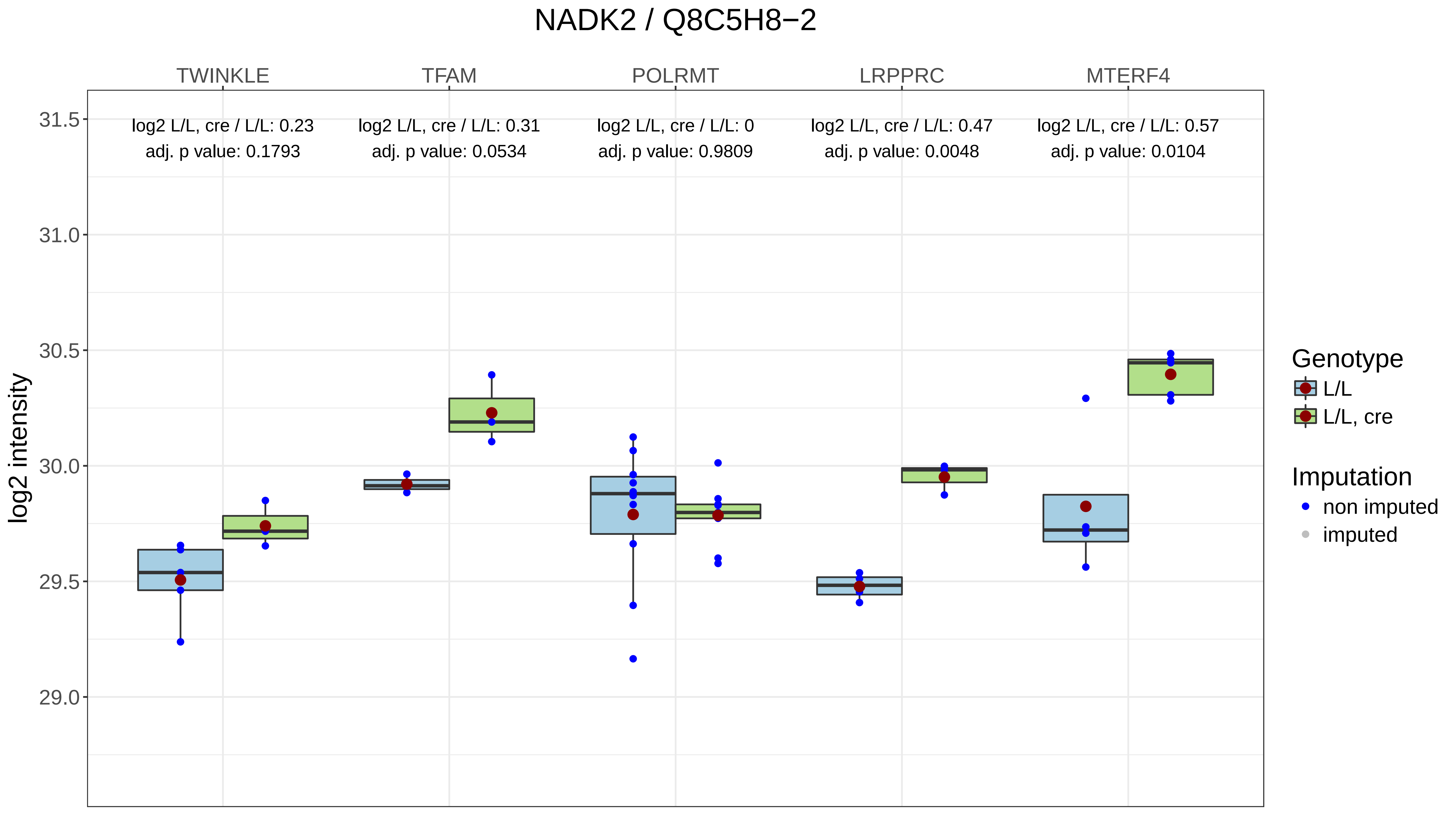

NADK2 / Q8C5H8−2
TWINKLE
TFAM
POLRMT
LRPPRC
MTERF4
log2 L/L, cre / L/L: 0.23
adj. p value: 0.1793
log2 L/L, cre / L/L: 0.31
adj. p value: 0.0534
log2 L/L, cre / L/L: 0
adj. p value: 0.9809
log2 L/L, cre / L/L: 0.47
adj. p value: 0.0048
log2 L/L, cre / L/L: 0.57
adj. p value: 0.0104
log2 intensity
31.5
31.0
30.5
30.0
29.5
29.0
Genotype
L/L
L/L, cre
Imputation
non imputed
imputed

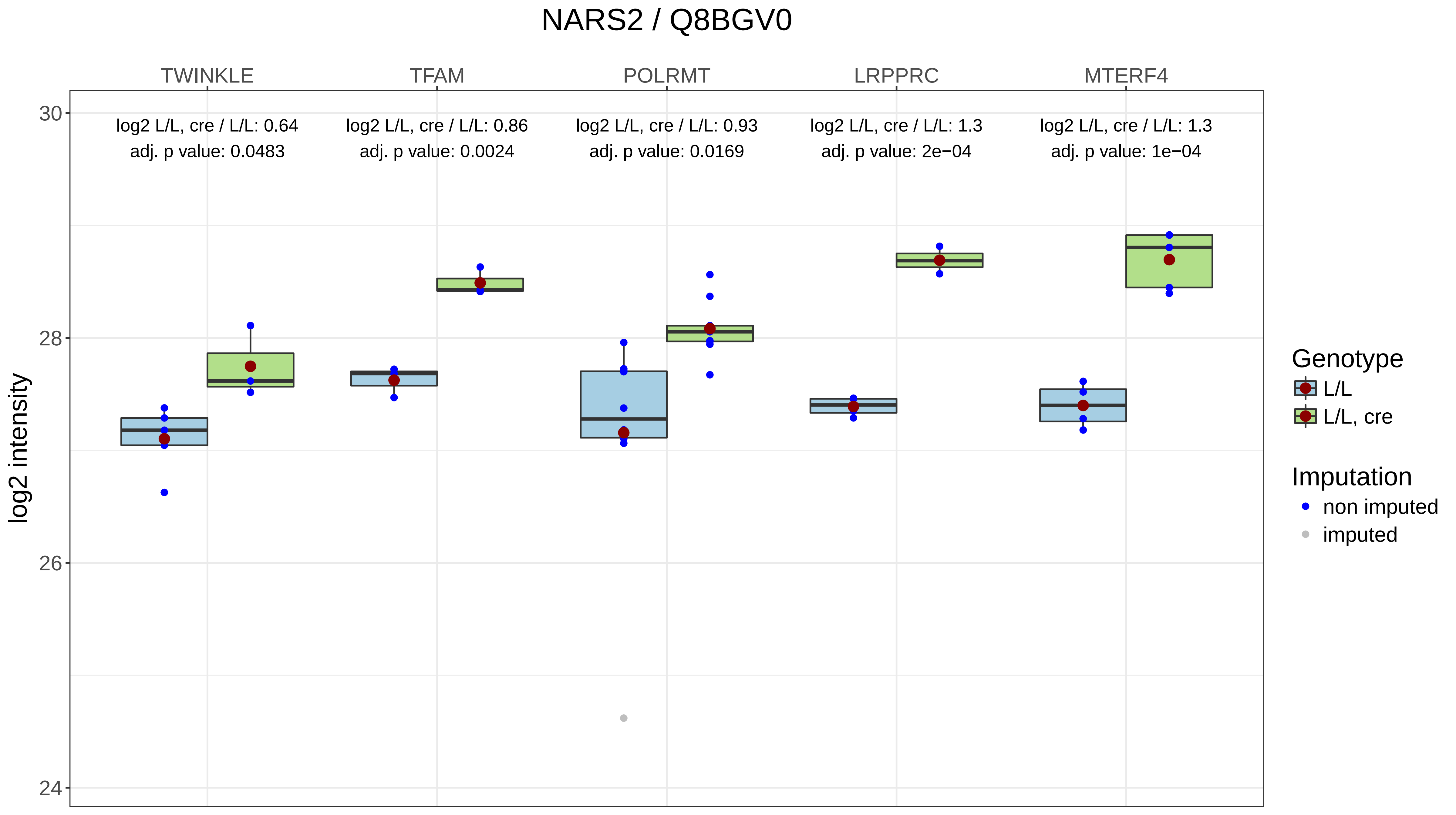
NARS2 / Q8BGV0
TWINKLE
TFAM
POLRMT
LRPPRC
MTERF4
log2 L/L, cre / L/L: 0.64
adj. p value: 0.0483
log2 L/L, cre / L/L: 0.86
adj. p value: 0.0024
log2 L/L, cre / L/L: 0.93
adj. p value: 0.0169
log2 L/L, cre / L/L: 1.3
adj. p value: 2e−04
log2 L/L, cre / L/L: 1.3
adj. p value: 1e−04
30
28
26
24
log2 intensity
Genotype
L/L
L/L, cre
Imputation
non imputed
imputed

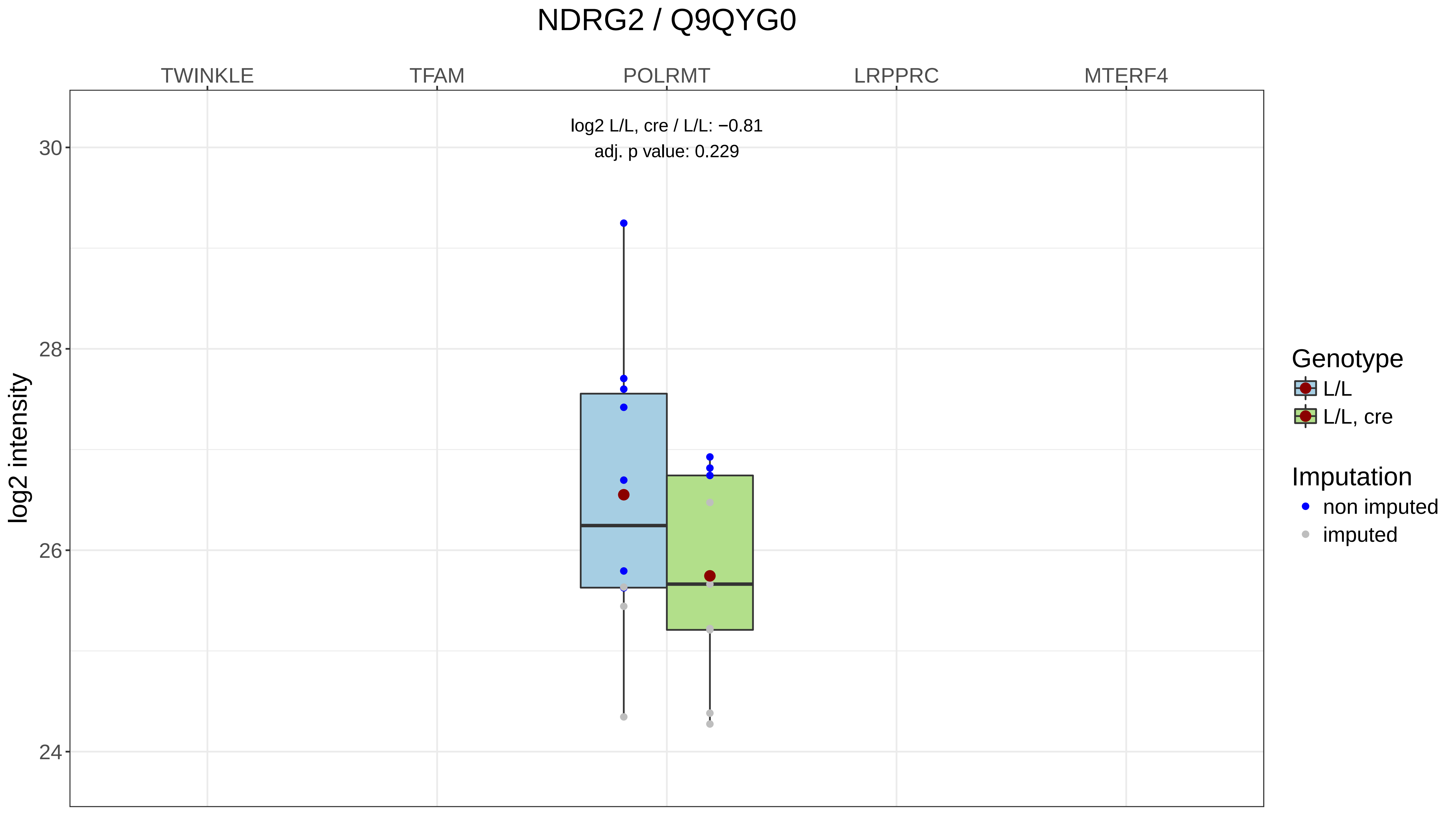

NDRG2 / Q9QYG0
TWINKLE
TFAM
POLRMT
LRPPRC
MTERF4
log2 L/L, cre / L/L: −0.81
adj. p value: 0.229
30
28
26
24
log2 intensity
Genotype
L/L
L/L, cre
Imputation
non imputed
imputed

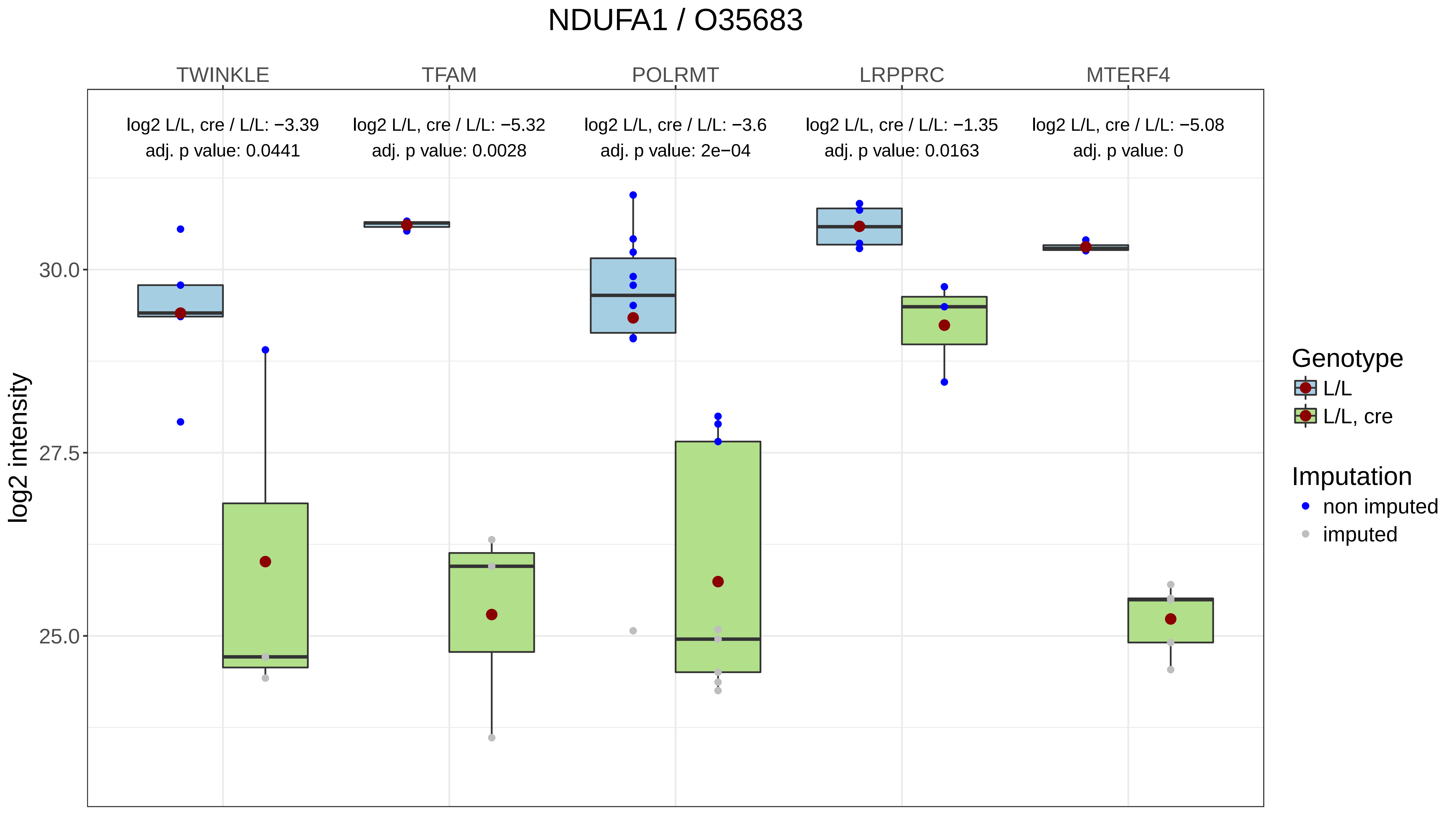
NDUFA1 / O35683
TWINKLE
TFAM
POLRMT
LRPPRC
MTERF4
log2 L/L, cre / L/L: −3.39
adj. p value: 0.0441
log2 L/L, cre / L/L: −5.32
adj. p value: 0.0028
log2 L/L, cre / L/L: −3.6
adj. p value: 2e−04
log2 L/L, cre / L/L: −1.35
adj. p value: 0.0163
log2 L/L, cre / L/L: −5.08
adj. p value: 0
log2 intensity
30.0
27.5
25.0
Genotype
L/L
L/L, cre
Imputation
non imputed
imputed

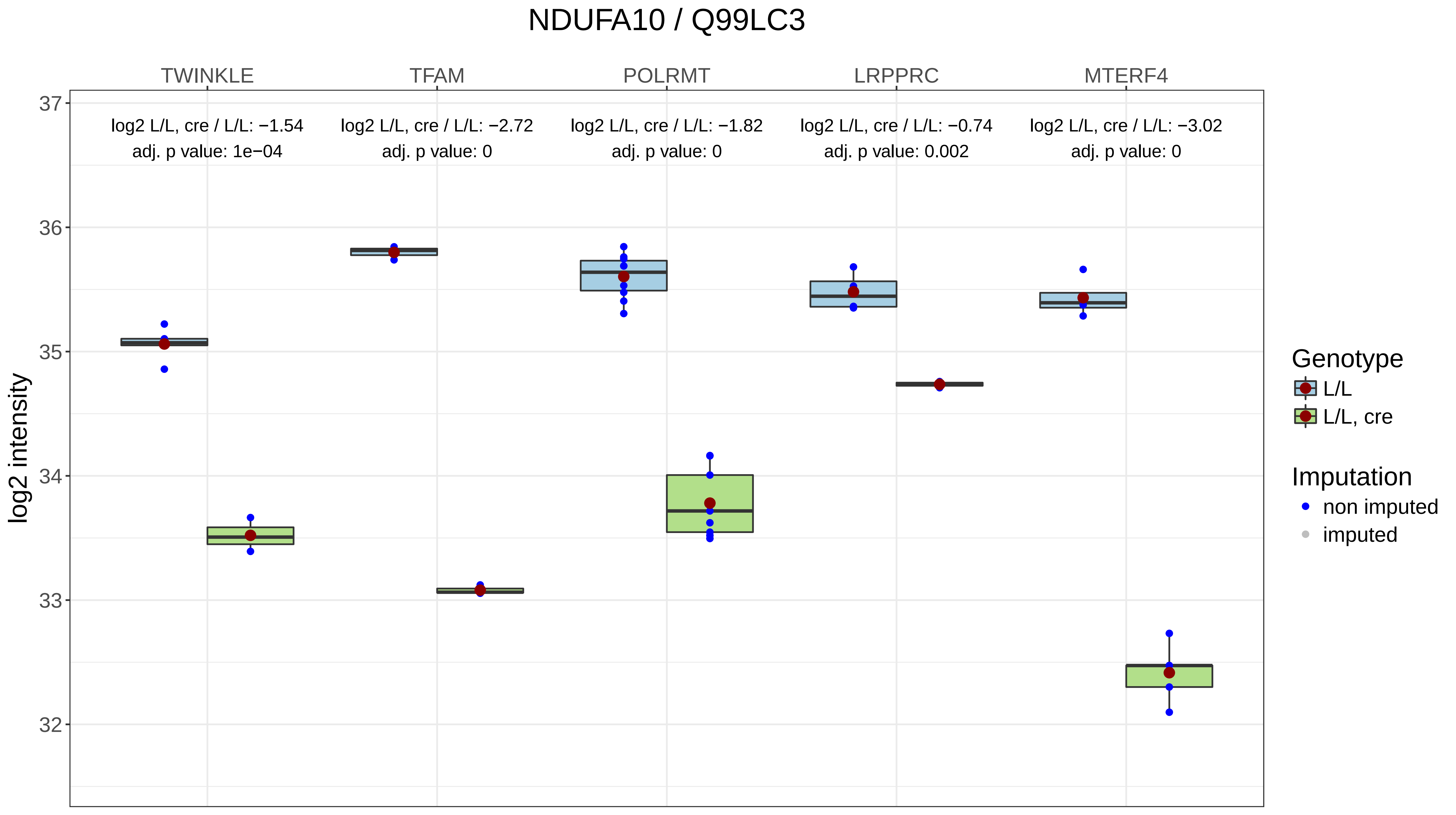

NDUFA10 / Q99LC3
TWINKLE
TFAM
POLRMT
LRPPRC
MTERF4
log2 L/L, cre / L/L: −1.54
adj. p value: 1e−04
log2 L/L, cre / L/L: −2.72
adj. p value: 0
log2 L/L, cre / L/L: −1.82
adj. p value: 0
log2 L/L, cre / L/L: −0.74
adj. p value: 0.002
log2 L/L, cre / L/L: −3.02
adj. p value: 0
log2 intensity
37
36
35
34
33
32
Genotype
L/L
L/L, cre
Imputation
non imputed
imputed

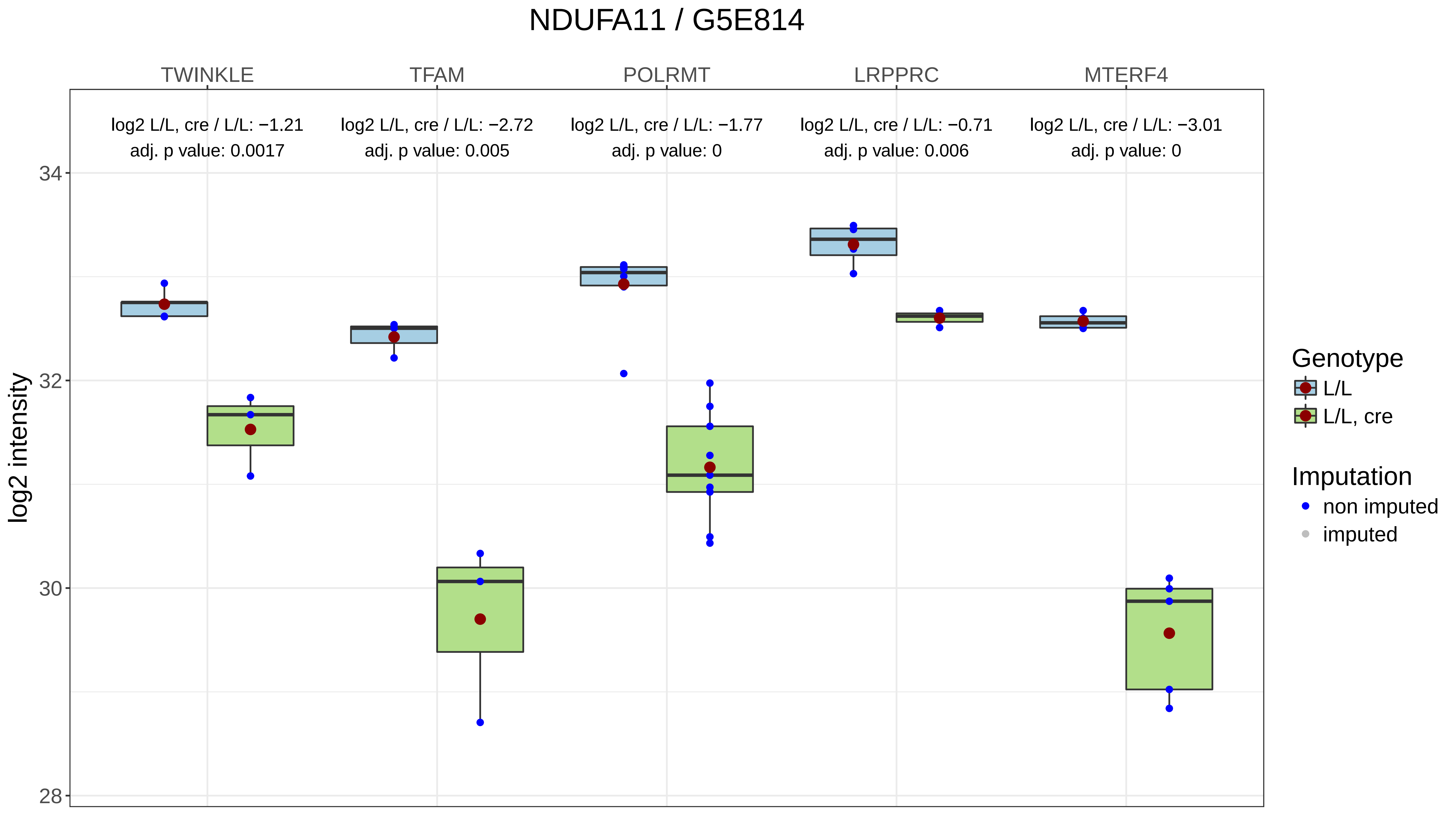
NDUFA11 / G5E814
TWINKLE
TFAM
POLRMT
LRPPRC
MTERF4
log2 L/L, cre / L/L: −1.21
adj. p value: 0.0017
log2 L/L, cre / L/L: −2.72
adj. p value: 0.005
log2 L/L, cre / L/L: −1.77
adj. p value: 0
log2 L/L, cre / L/L: −0.71
adj. p value: 0.006
log2 L/L, cre / L/L: −3.01
adj. p value: 0
log2 intensity
34
32
30
28
Genotype
L/L
L/L, cre
Imputation
non imputed
imputed

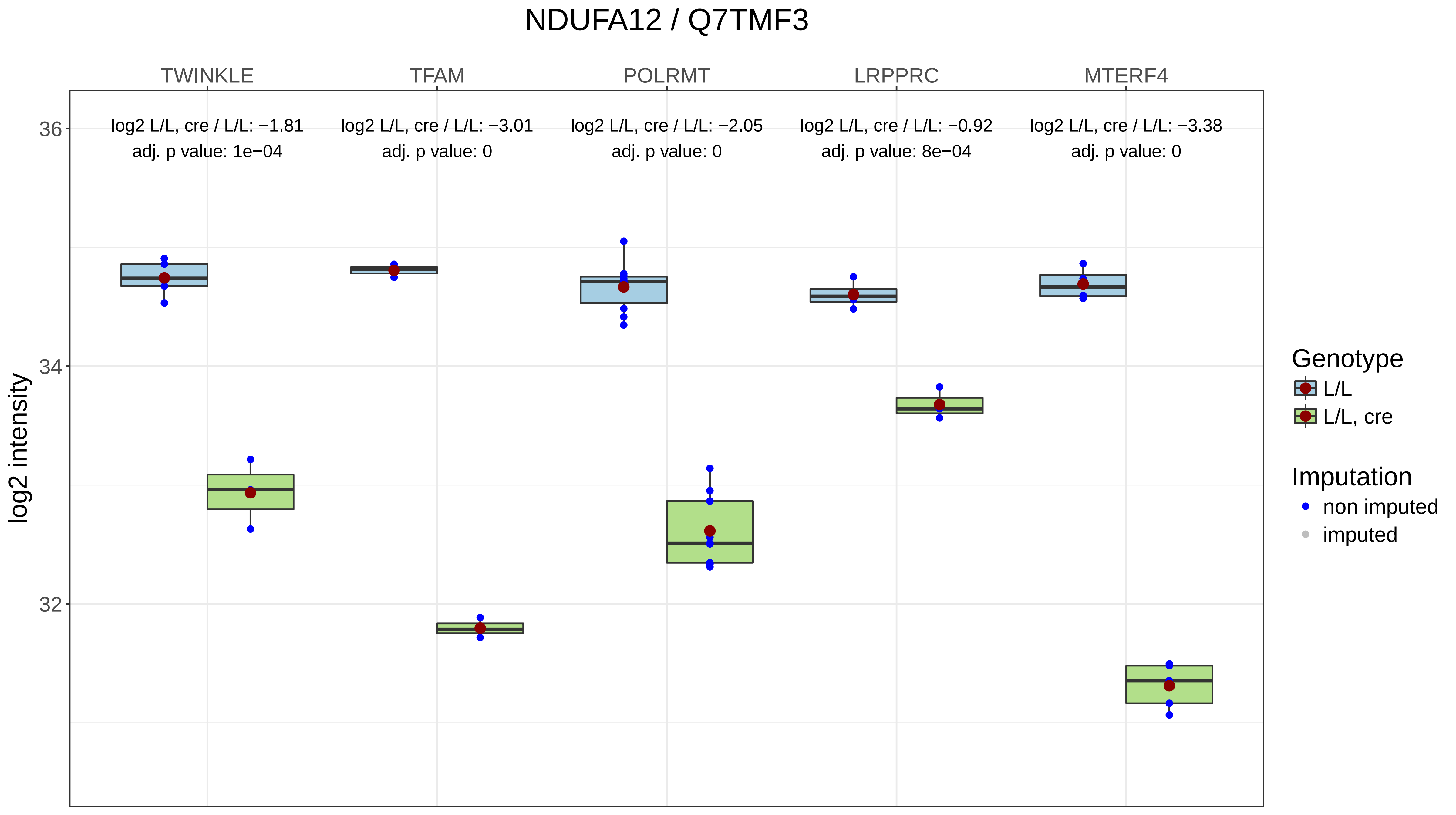
NDUFA12 / Q7TMF3
TWINKLE
TFAM
POLRMT
LRPPRC
MTERF4
log2 L/L, cre / L/L: −1.81
adj. p value: 1e−04
log2 L/L, cre / L/L: −3.01
adj. p value: 0
log2 L/L, cre / L/L: −2.05
adj. p value: 0
log2 L/L, cre / L/L: −0.92
adj. p value: 8e−04
log2 L/L, cre / L/L: −3.38
adj. p value: 0
36
34
32
log2 intensity
Genotype
L/L
L/L, cre
Imputation
non imputed
imputed

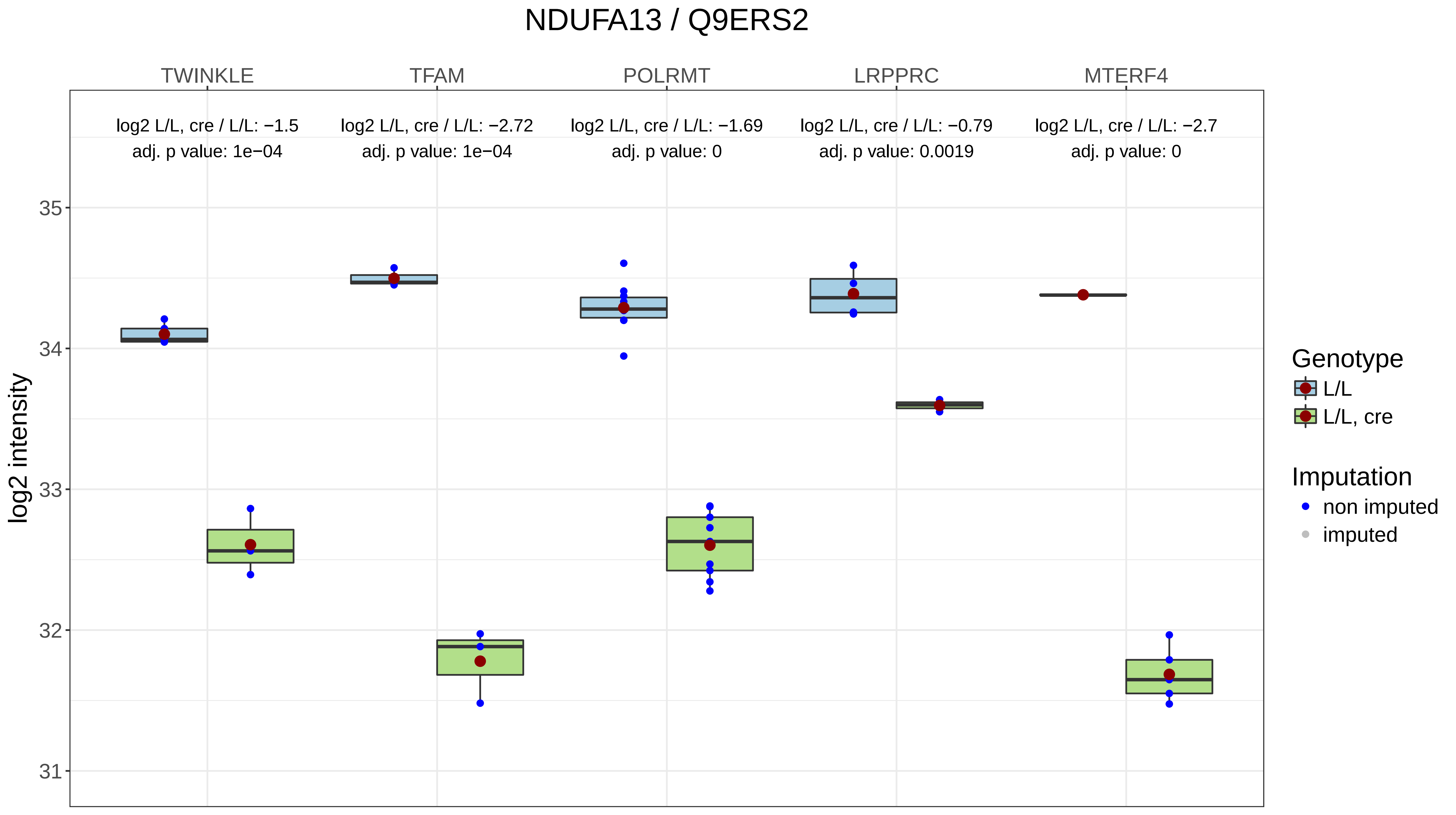
NDUFA13 / Q9ERS2
TWINKLE
TFAM
POLRMT
LRPPRC
MTERF4
log2 L/L, cre / L/L: −1.5
adj. p value: 1e−04
log2 L/L, cre / L/L: −2.72
adj. p value: 1e−04
log2 L/L, cre / L/L: −1.69
adj. p value: 0
log2 L/L, cre / L/L: −0.79
adj. p value: 0.0019
log2 L/L, cre / L/L: −2.7
adj. p value: 0
log2 intensity
35
34
33
32
31
Genotype
L/L
L/L, cre
Imputation
non imputed
imputed

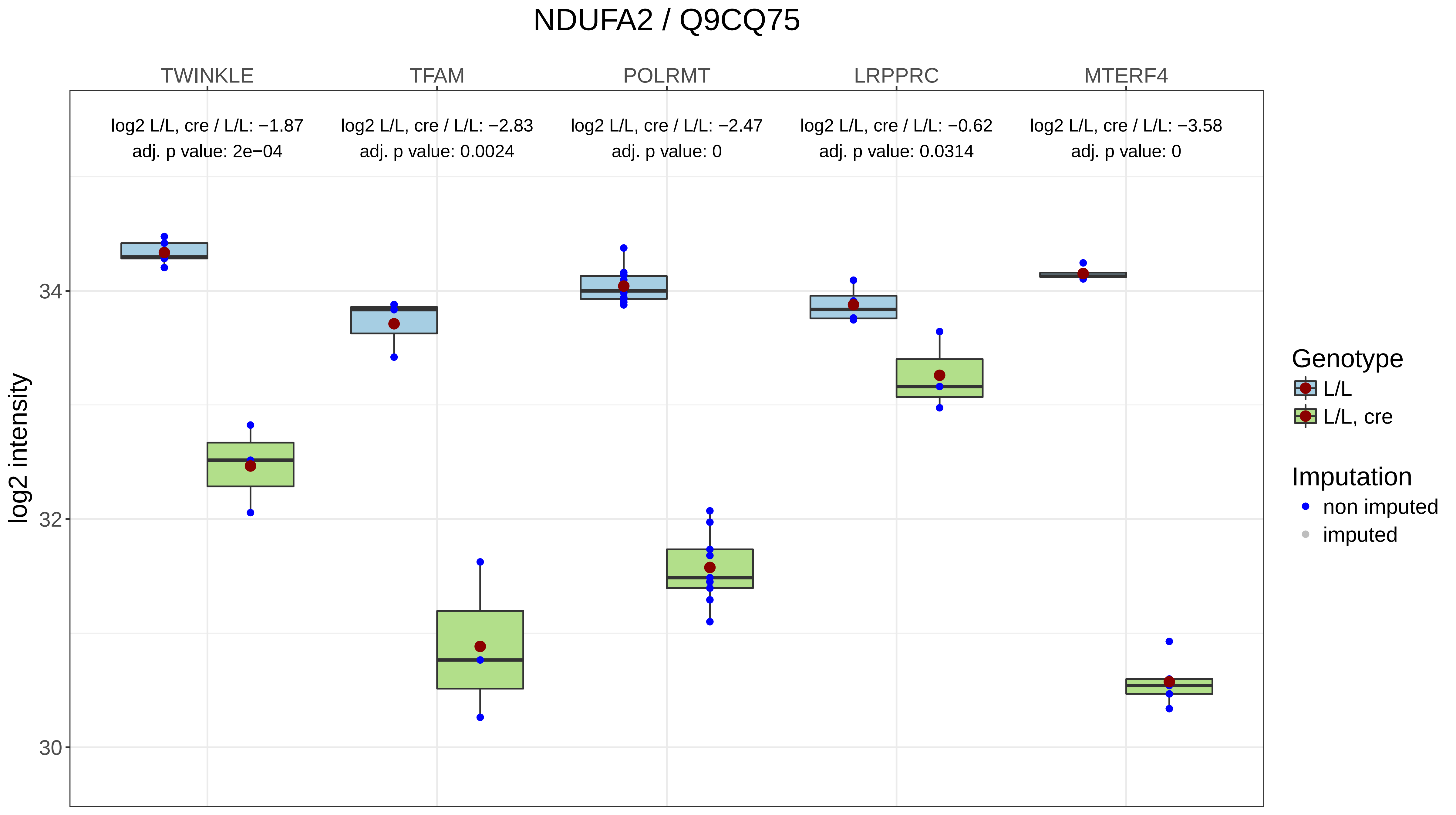

NDUFA2 / Q9CQ75
TWINKLE
TFAM
POLRMT
LRPPRC
MTERF4
log2 L/L, cre / L/L: −1.87
adj. p value: 2e−04
log2 L/L, cre / L/L: −2.83
adj. p value: 0.0024
log2 L/L, cre / L/L: −2.47
adj. p value: 0
log2 L/L, cre / L/L: −0.62
adj. p value: 0.0314
log2 L/L, cre / L/L: −3.58
adj. p value: 0
log2 intensity
34
32
30
Genotype
L/L
L/L, cre
Imputation
non imputed
imputed

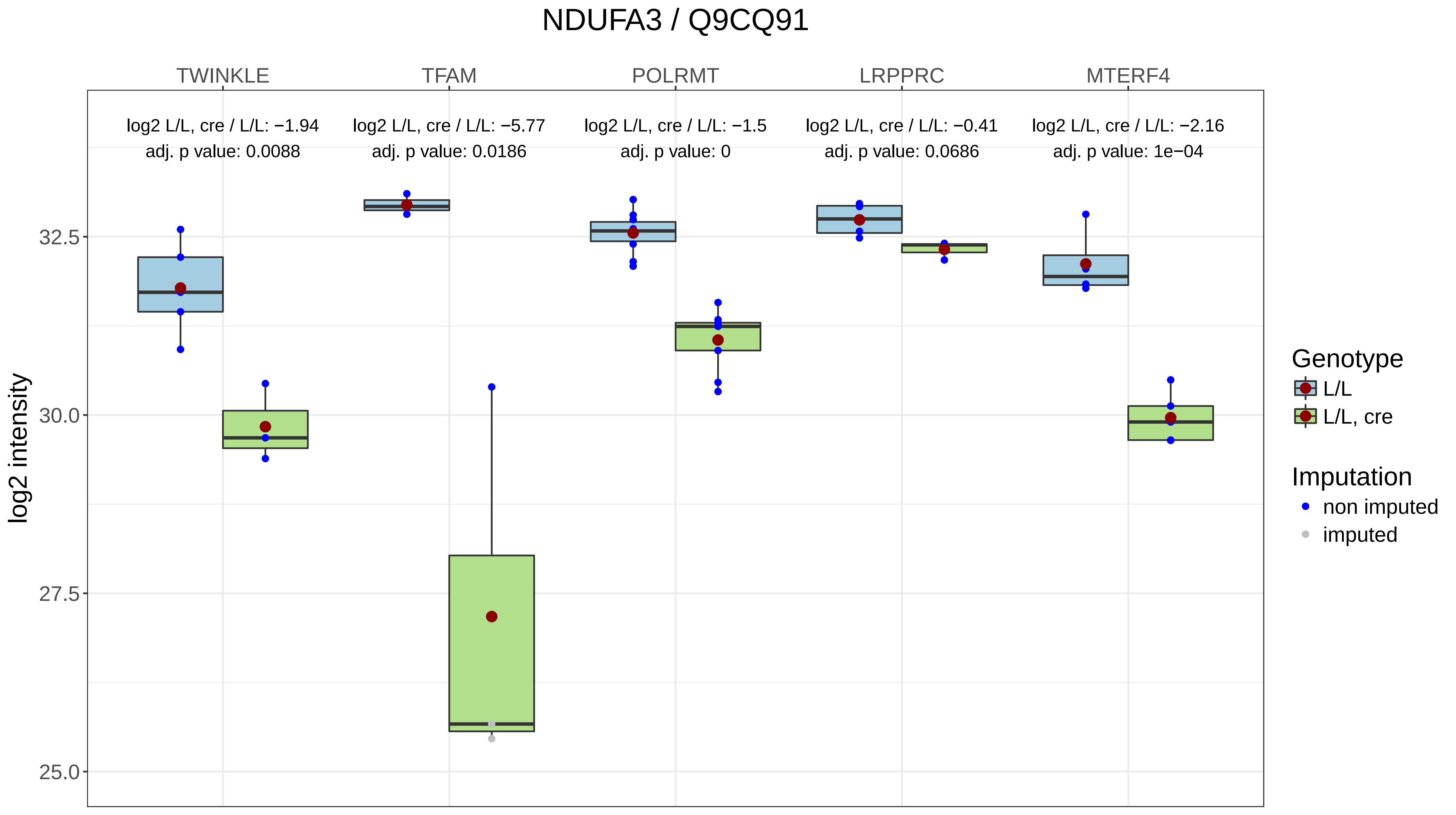
NDUFA3 / Q9CQ91
TWINKLE
TFAM
POLRMT
LRPPRC
MTERF4
log2 L/L, cre / L/L: −1.94
adj. p value: 0.0088
log2 L/L, cre / L/L: −5.77
adj. p value: 0.0186
log2 L/L, cre / L/L: −1.5
adj. p value: 0
log2 L/L, cre / L/L: −0.41
adj. p value: 0.0686
log2 L/L, cre / L/L: −2.16
adj. p value: 1e−04
log2 intensity
32.5
30.0
27.5
25.0
Genotype
L/L
L/L, cre
Imputation
non imputed
imputed

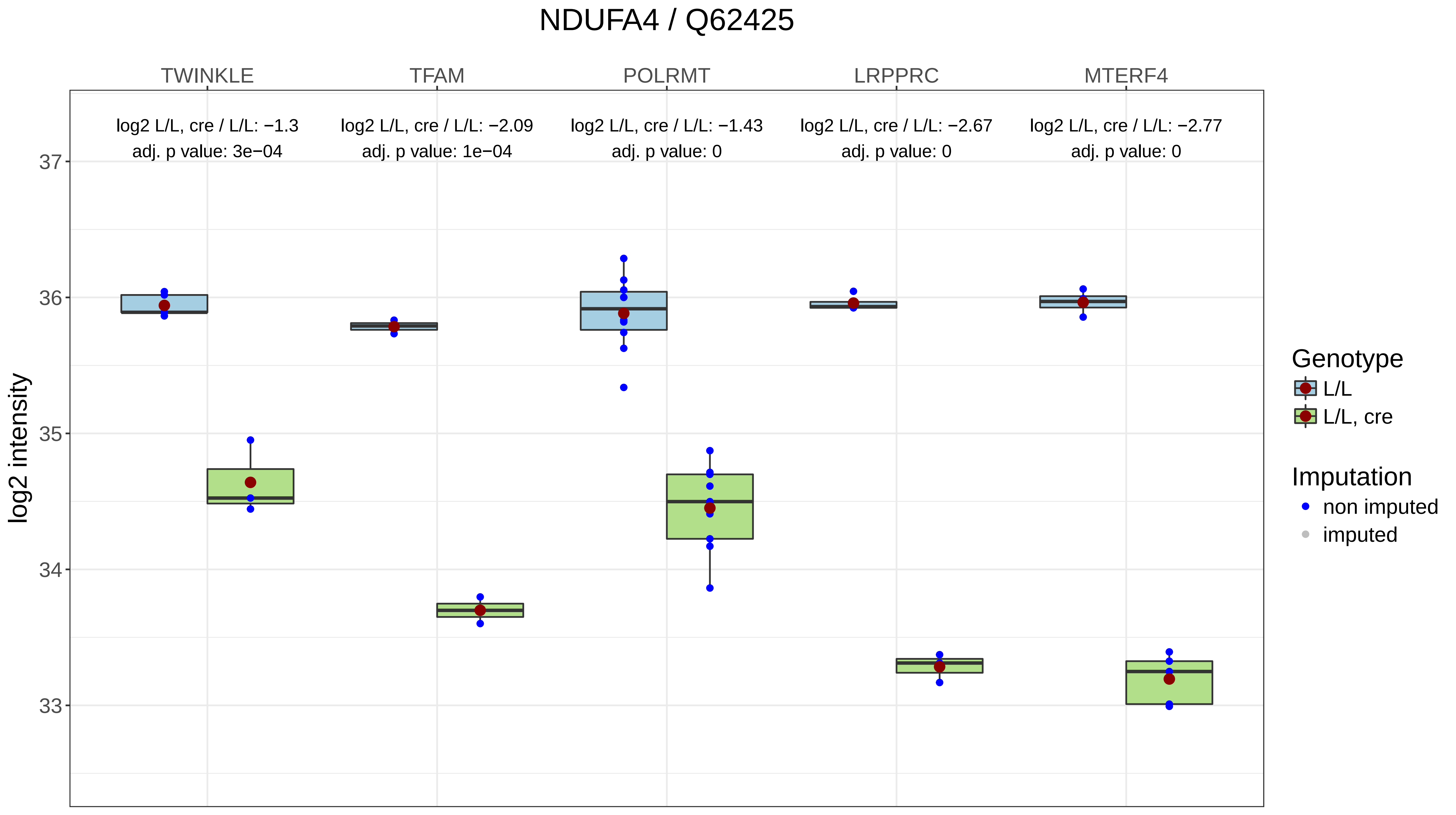

NDUFA4 / Q62425
TWINKLE
TFAM
POLRMT
LRPPRC
MTERF4
log2 L/L, cre / L/L: −1.3
adj. p value: 3e−04
log2 L/L, cre / L/L: −2.09
adj. p value: 1e−04
log2 L/L, cre / L/L: −1.43
adj. p value: 0
log2 L/L, cre / L/L: −2.67
adj. p value: 0
log2 L/L, cre / L/L: −2.77
adj. p value: 0
log2 intensity
37
36
35
34
33
Genotype
L/L
L/L, cre
Imputation
non imputed
imputed

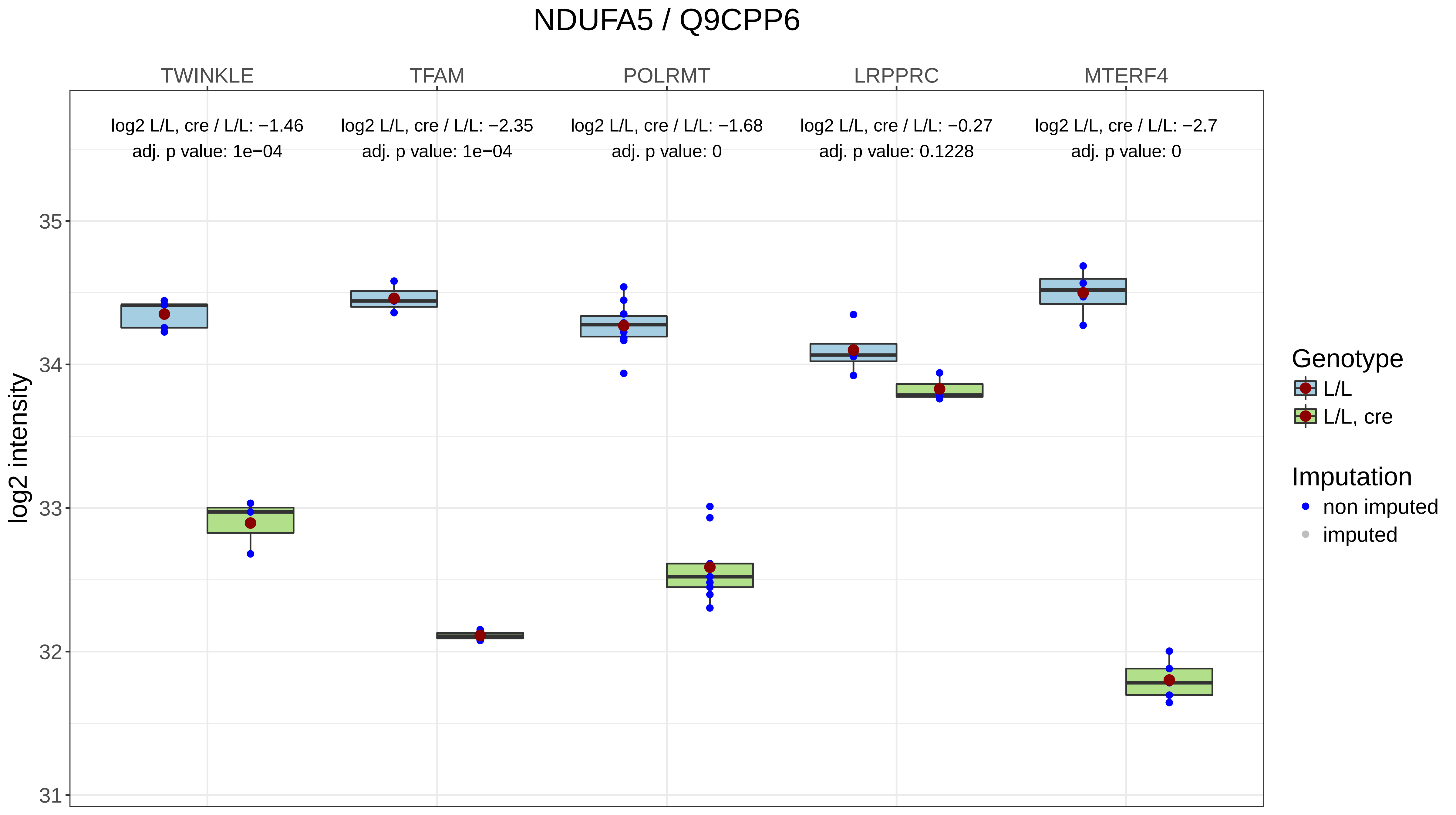
NDUFA5 / Q9CPP6
TWINKLE
TFAM
POLRMT
LRPPRC
MTERF4
log2 L/L, cre / L/L: −1.46
adj. p value: 1e−04
log2 L/L, cre / L/L: −2.35
adj. p value: 1e−04
log2 L/L, cre / L/L: −1.68
adj. p value: 0
log2 L/L, cre / L/L: −0.27
adj. p value: 0.1228
log2 L/L, cre / L/L: −2.7
adj. p value: 0
log2 intensity
35
34
33
32
31
Genotype
L/L
L/L, cre
Imputation
non imputed
imputed

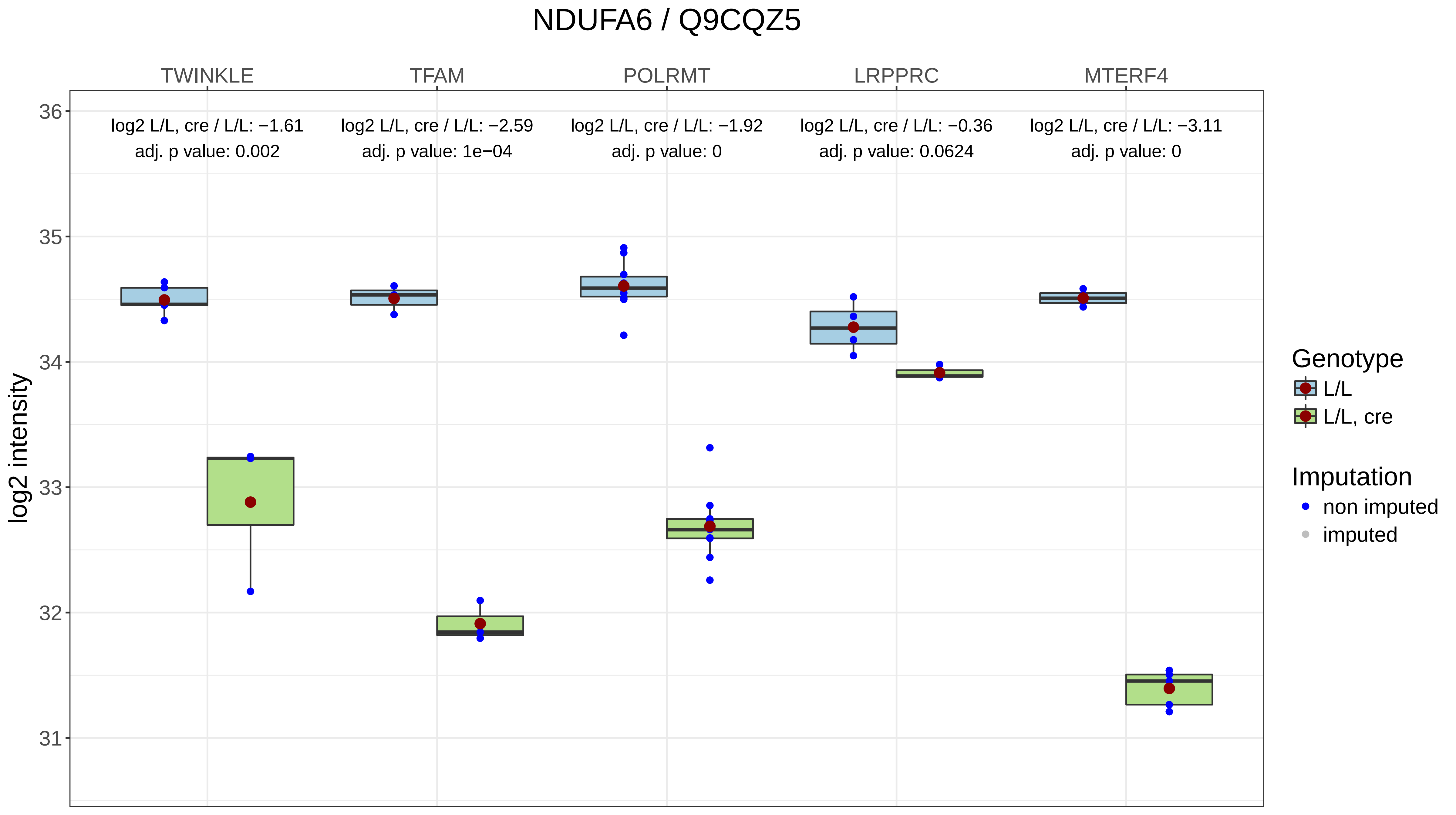
NDUFA6 / Q9CQZ5
TWINKLE
TFAM
POLRMT
LRPPRC
MTERF4
log2 L/L, cre / L/L: −1.61
adj. p value: 0.002
log2 L/L, cre / L/L: −2.59
adj. p value: 1e−04
log2 L/L, cre / L/L: −1.92
adj. p value: 0
log2 L/L, cre / L/L: −0.36
adj. p value: 0.0624
log2 L/L, cre / L/L: −3.11
adj. p value: 0
log2 intensity
36
35
34
33
32
31
Genotype
L/L
L/L, cre
Imputation
non imputed
imputed

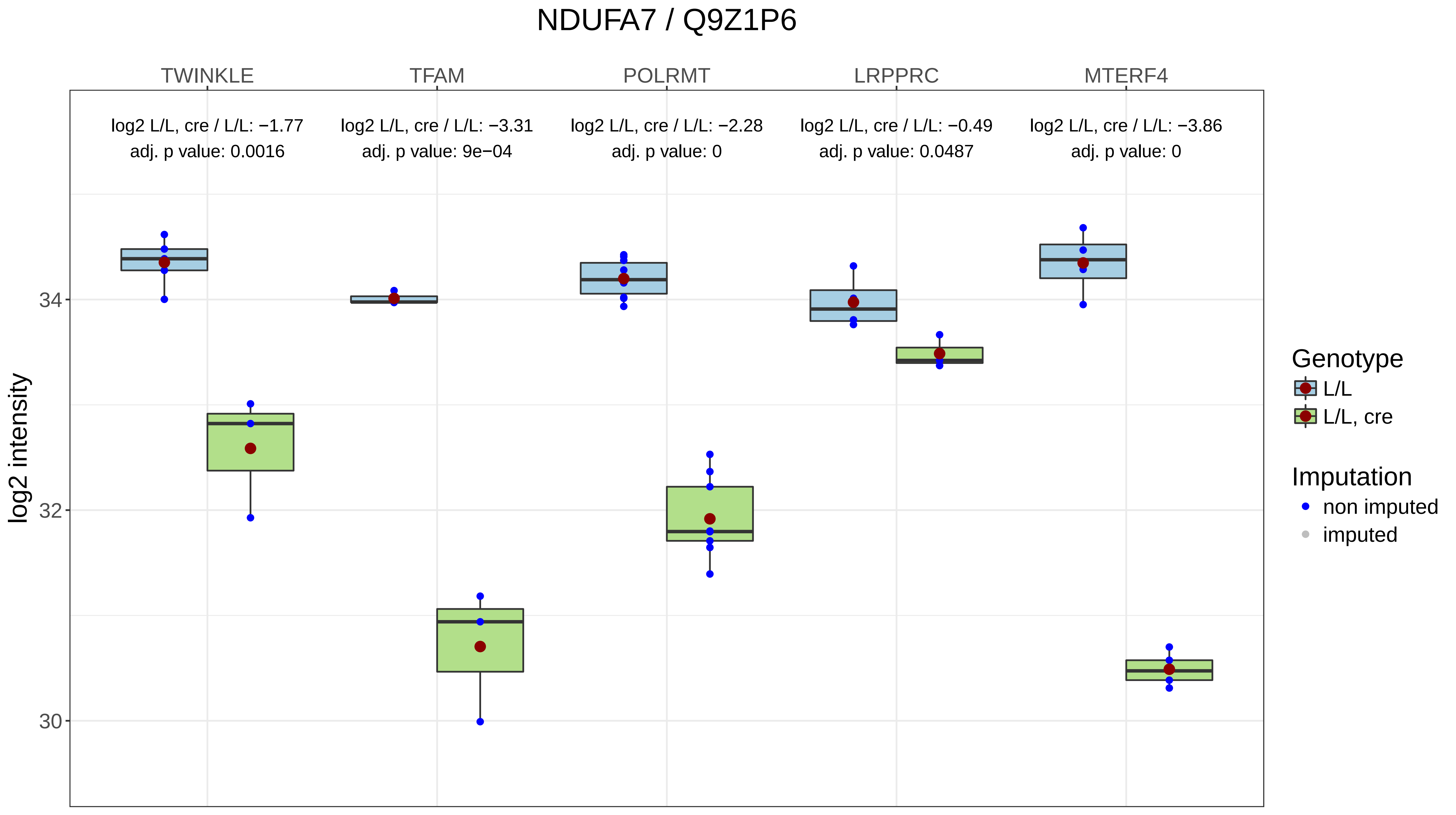
NDUFA7 / Q9Z1P6
TWINKLE
TFAM
POLRMT
LRPPRC
MTERF4
log2 L/L, cre / L/L: −1.77
adj. p value: 0.0016
log2 L/L, cre / L/L: −3.31
adj. p value: 9e−04
log2 L/L, cre / L/L: −2.28
adj. p value: 0
log2 L/L, cre / L/L: −0.49
adj. p value: 0.0487
log2 L/L, cre / L/L: −3.86
adj. p value: 0
log2 intensity
34
32
30
Genotype
L/L
L/L, cre
Imputation
non imputed
imputed

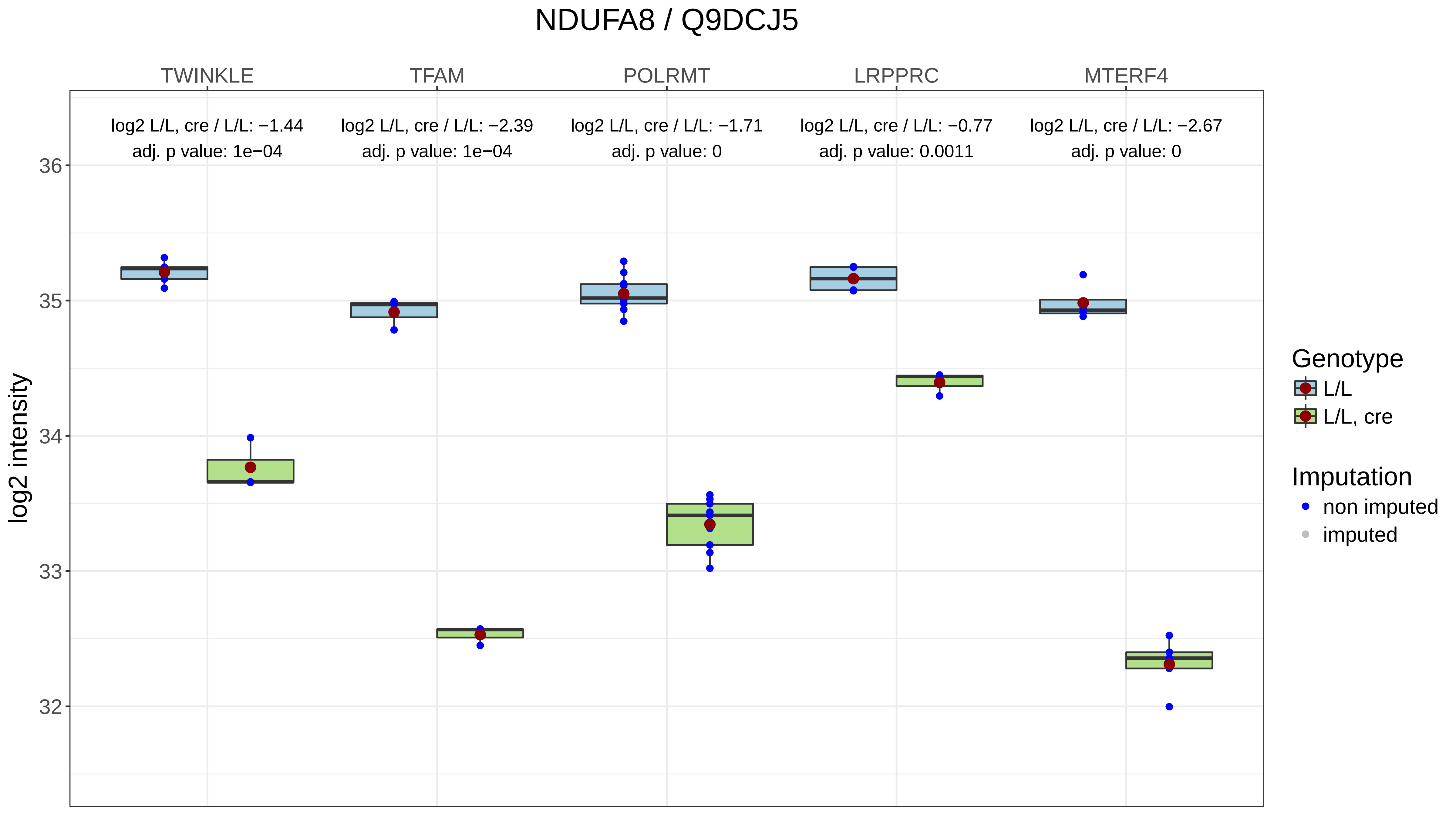

NDUFA8 / Q9DCJ5
TWINKLE
TFAM
POLRMT
LRPPRC
MTERF4
log2 L/L, cre / L/L: −1.44
adj. p value: 1e−04
log2 L/L, cre / L/L: −2.39
adj. p value: 1e−04
log2 L/L, cre / L/L: −1.71
adj. p value: 0
log2 L/L, cre / L/L: −0.77
adj. p value: 0.0011
log2 L/L, cre / L/L: −2.67
adj. p value: 0
log2 intensity
36
35
34
33
32
Genotype
L/L
L/L, cre
Imputation
non imputed
imputed

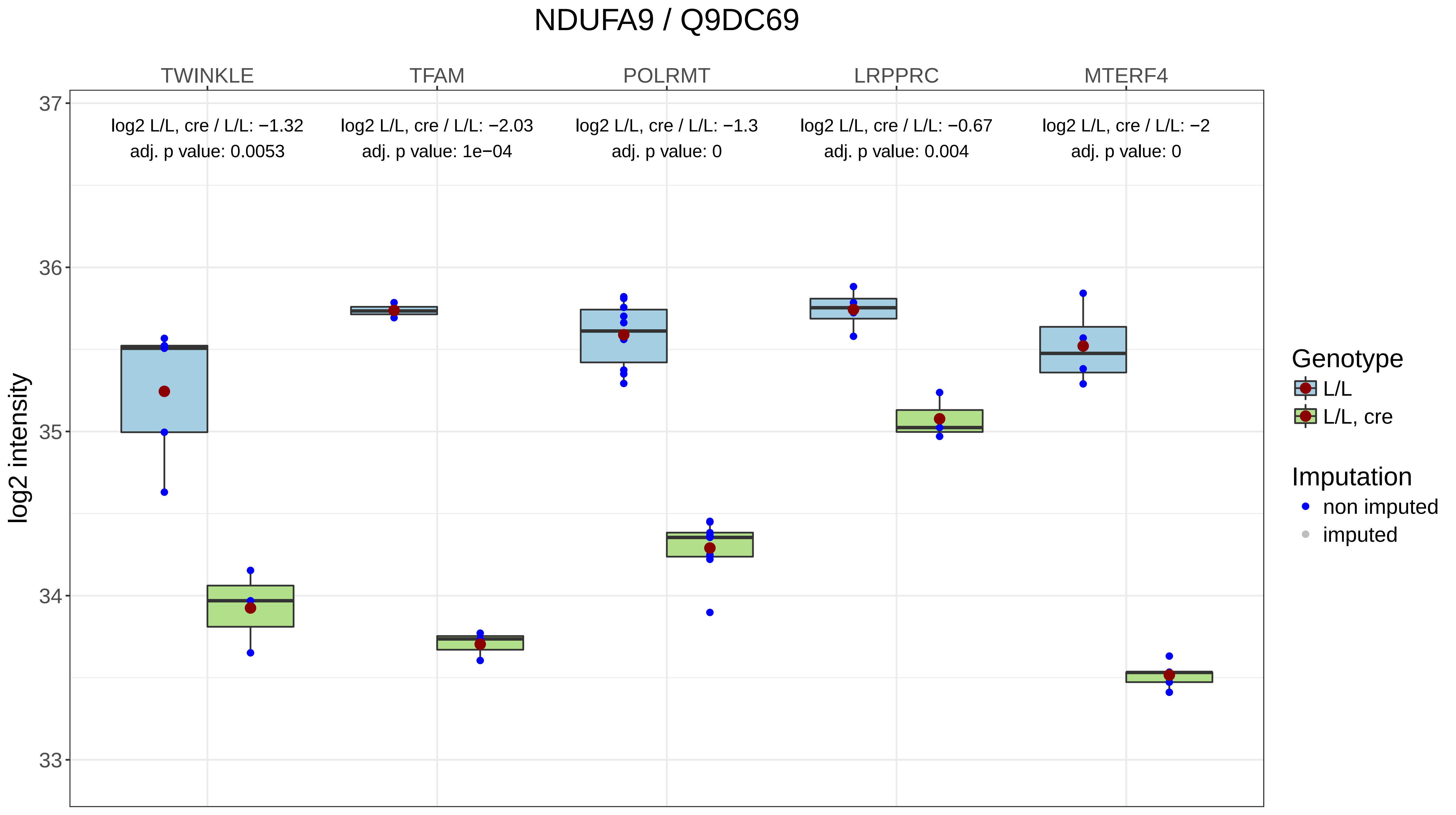

NDUFA9 / Q9DC69
TWINKLE
TFAM
POLRMT
LRPPRC
MTERF4
log2 L/L, cre / L/L: −1.32
adj. p value: 0.0053
log2 L/L, cre / L/L: −2.03
adj. p value: 1e−04
log2 L/L, cre / L/L: −1.3
adj. p value: 0
log2 L/L, cre / L/L: −0.67
adj. p value: 0.004
log2 L/L, cre / L/L: −2
adj. p value: 0
37
36
35
34
33
log2 intensity
Genotype
L/L
L/L, cre
Imputation
non imputed
imputed

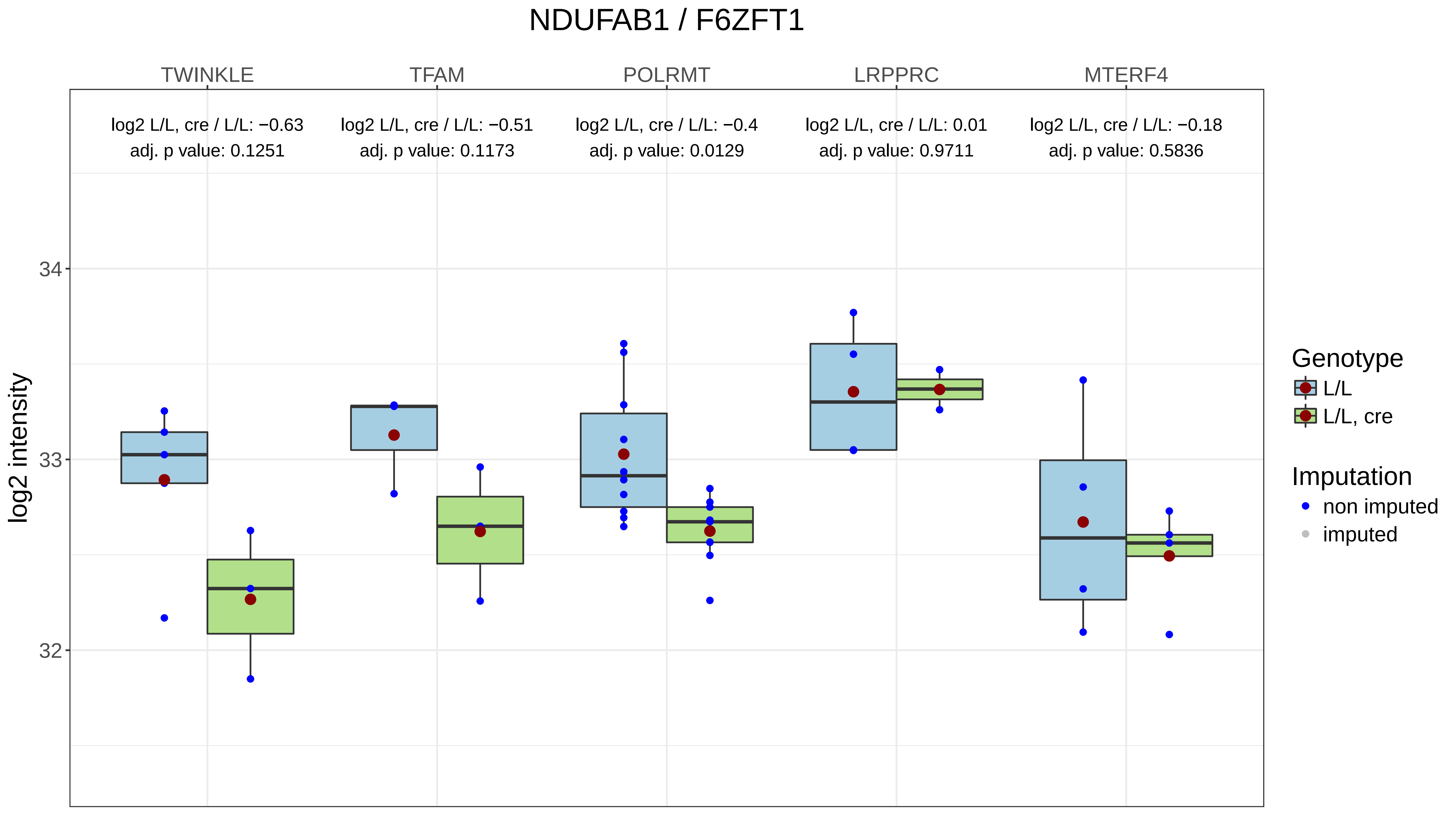
NDUFAB1 / F6ZFT1
TWINKLE
TFAM
POLRMT
LRPPRC
MTERF4
log2 L/L, cre / L/L: −0.63
adj. p value: 0.1251
log2 L/L, cre / L/L: −0.51
adj. p value: 0.1173
log2 L/L, cre / L/L: −0.4
adj. p value: 0.0129
log2 L/L, cre / L/L: 0.01
adj. p value: 0.9711
log2 L/L, cre / L/L: −0.18
adj. p value: 0.5836
log2 intensity
34
33
32
Genotype
L/L
L/L, cre
Imputation
non imputed
imputed

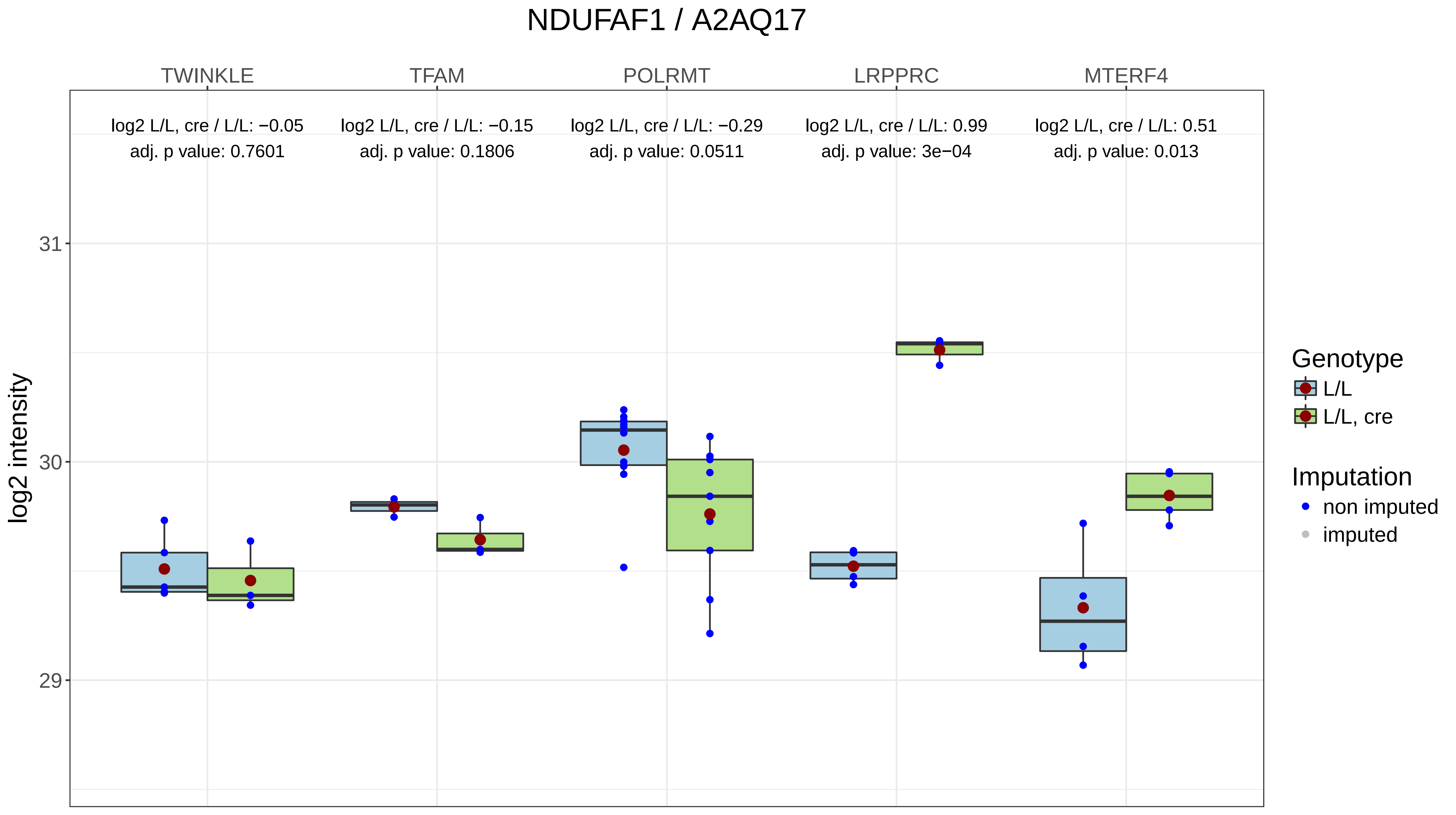
NDUFAF1 / A2AQ17
TWINKLE
TFAM
POLRMT
LRPPRC
MTERF4
log2 L/L, cre / L/L: −0.05
adj. p value: 0.7601
log2 L/L, cre / L/L: −0.15
adj. p value: 0.1806
log2 L/L, cre / L/L: −0.29
adj. p value: 0.0511
log2 L/L, cre / L/L: 0.99
adj. p value: 3e−04
log2 L/L, cre / L/L: 0.51
adj. p value: 0.013
log2 intensity
31
30
29
Genotype
L/L
L/L, cre
Imputation
non imputed
imputed

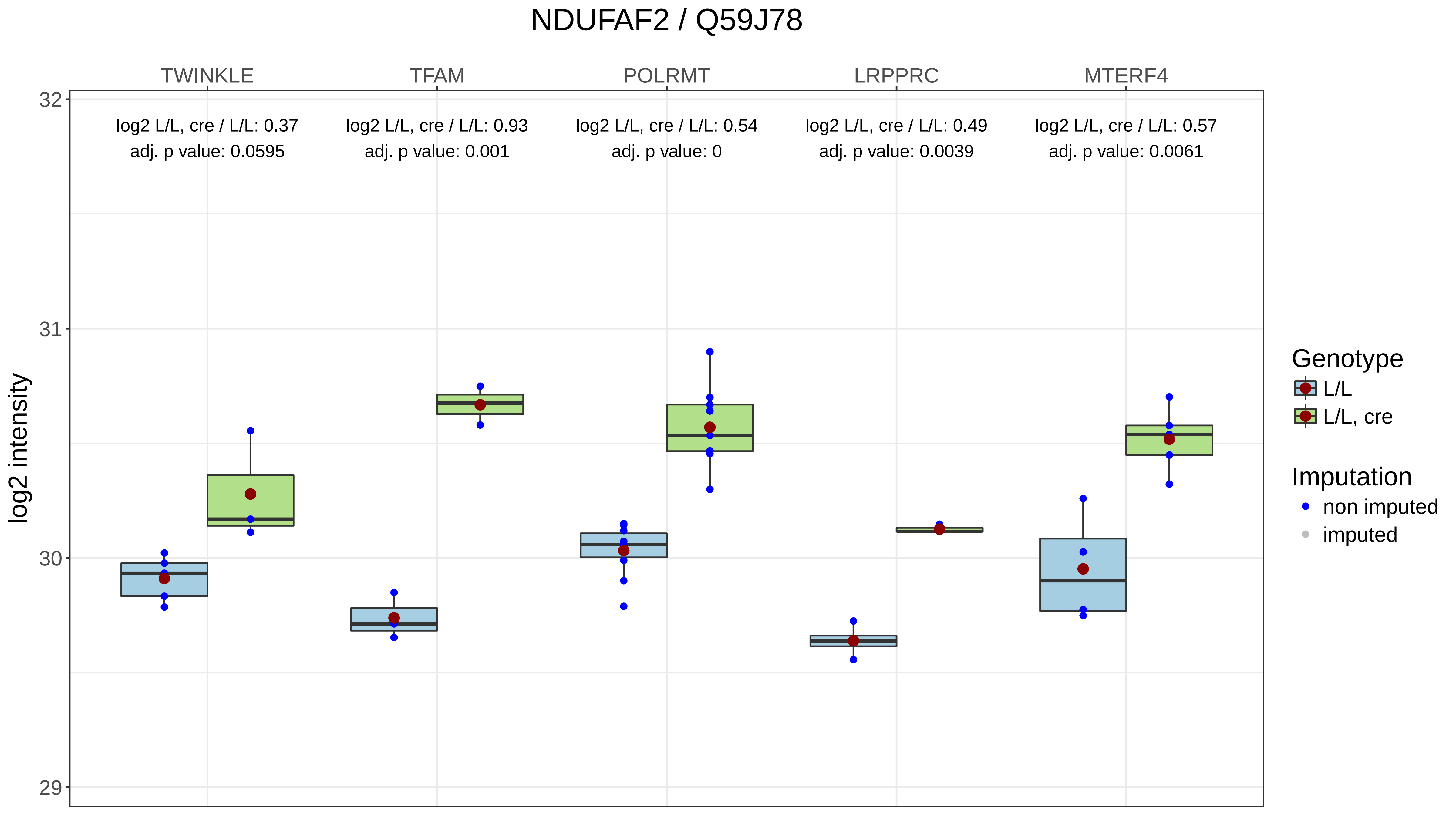
NDUFAF2 / Q59J78
TWINKLE
TFAM
POLRMT
LRPPRC
MTERF4
log2 L/L, cre / L/L: 0.37
adj. p value: 0.0595
log2 L/L, cre / L/L: 0.93
adj. p value: 0.001
log2 L/L, cre / L/L: 0.54
adj. p value: 0
log2 L/L, cre / L/L: 0.49
adj. p value: 0.0039
log2 L/L, cre / L/L: 0.57
adj. p value: 0.0061
log2 intensity
32
31
30
29
Genotype
L/L
L/L, cre
Imputation
non imputed
imputed

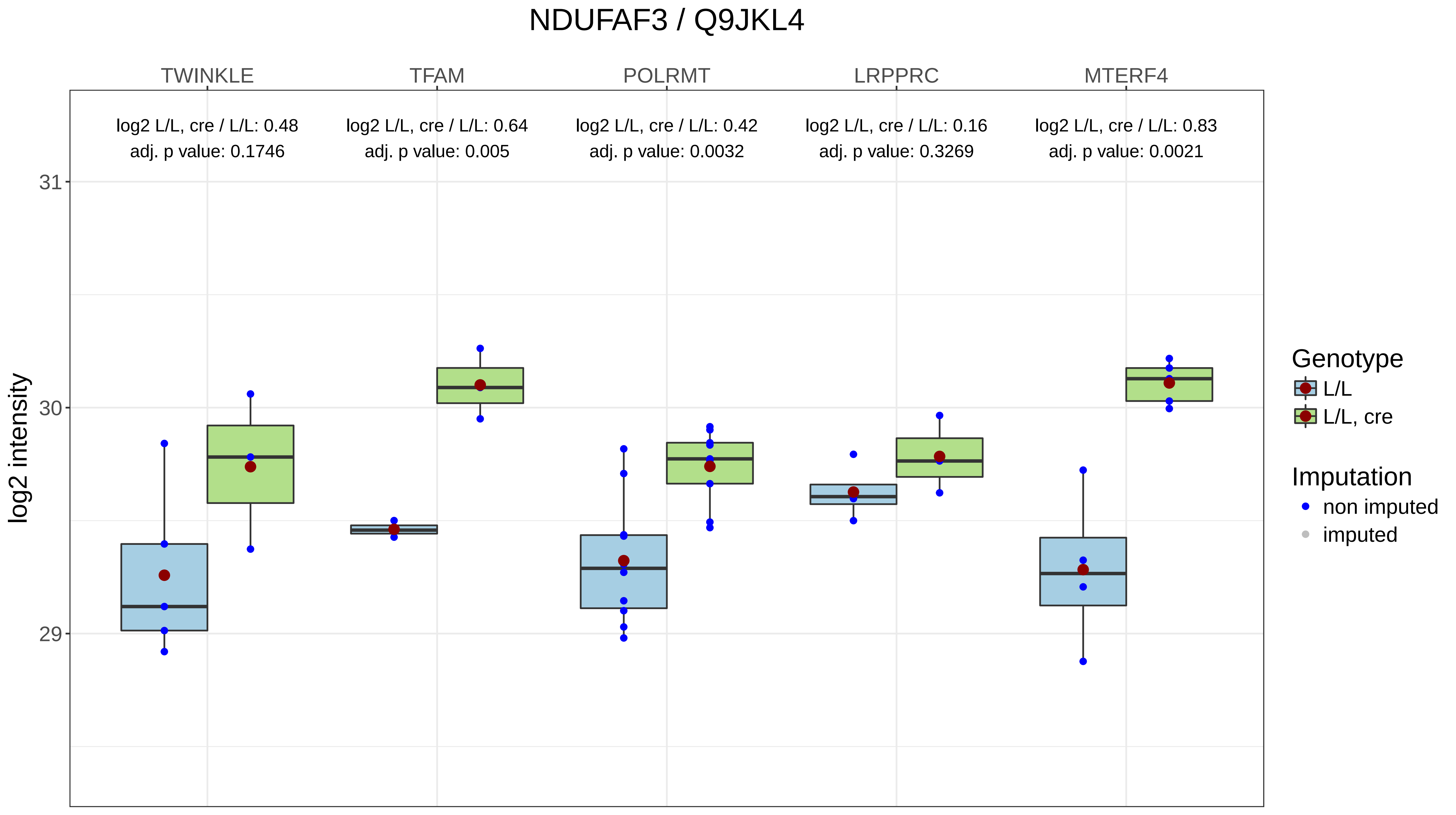

NDUFAF3 / Q9JKL4
TWINKLE
TFAM
POLRMT
LRPPRC
MTERF4
log2 L/L, cre / L/L: 0.48
adj. p value: 0.1746
log2 L/L, cre / L/L: 0.64
adj. p value: 0.005
log2 L/L, cre / L/L: 0.42
adj. p value: 0.0032
log2 L/L, cre / L/L: 0.16
adj. p value: 0.3269
log2 L/L, cre / L/L: 0.83
adj. p value: 0.0021
31
30
29
log2 intensity
Genotype
L/L
L/L, cre
Imputation
non imputed
imputed

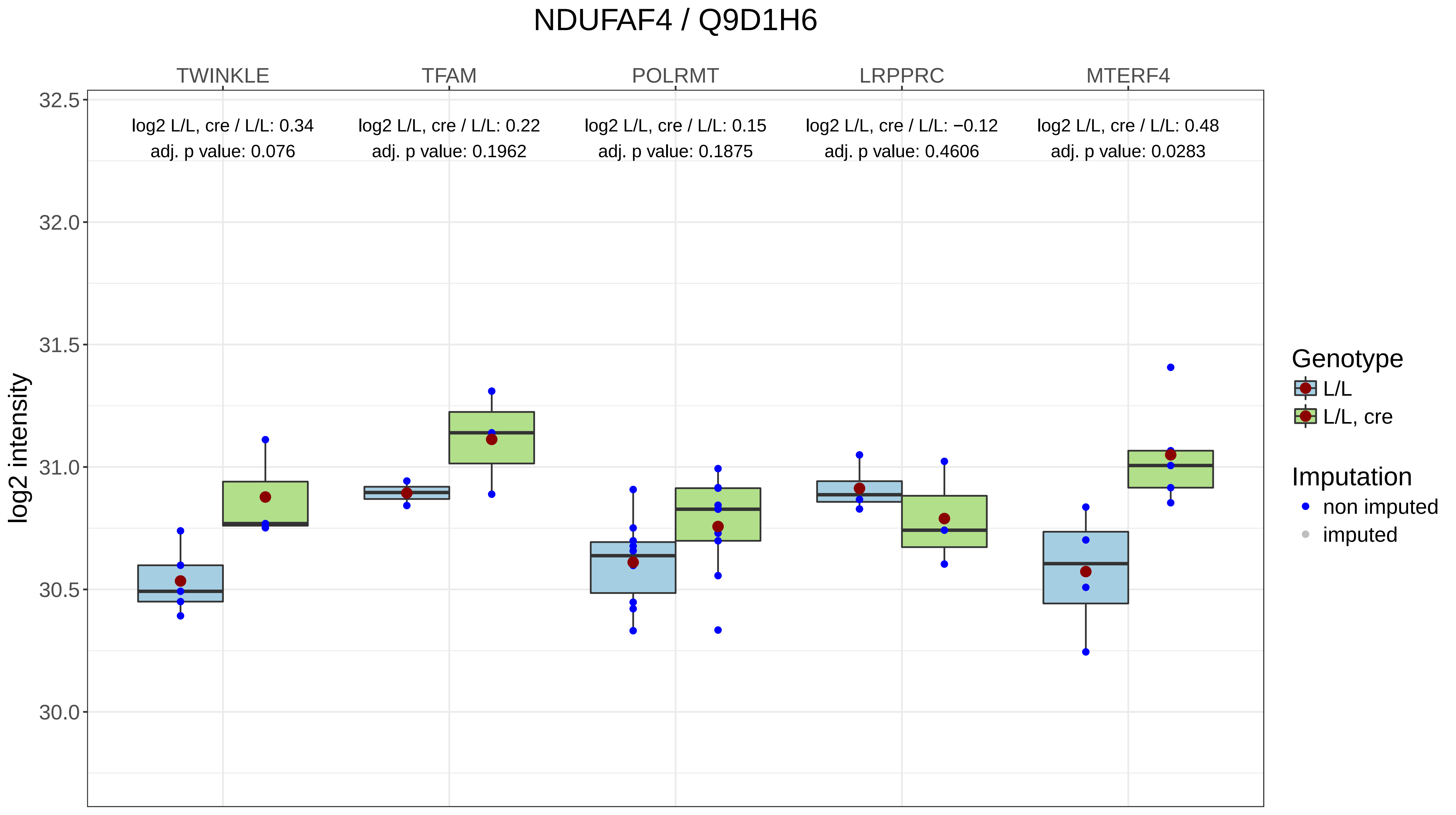
NDUFAF4 / Q9D1H6
TWINKLE
TFAM
POLRMT
LRPPRC
MTERF4
log2 L/L, cre / L/L: 0.34
adj. p value: 0.076
log2 L/L, cre / L/L: 0.22
adj. p value: 0.1962
log2 L/L, cre / L/L: 0.15
adj. p value: 0.1875
log2 L/L, cre / L/L: −0.12
adj. p value: 0.4606
log2 L/L, cre / L/L: 0.48
adj. p value: 0.0283
log2 intensity
32.5
32.0
31.5
31.0
30.5
30.0
Genotype
L/L
L/L, cre
Imputation
non imputed
imputed

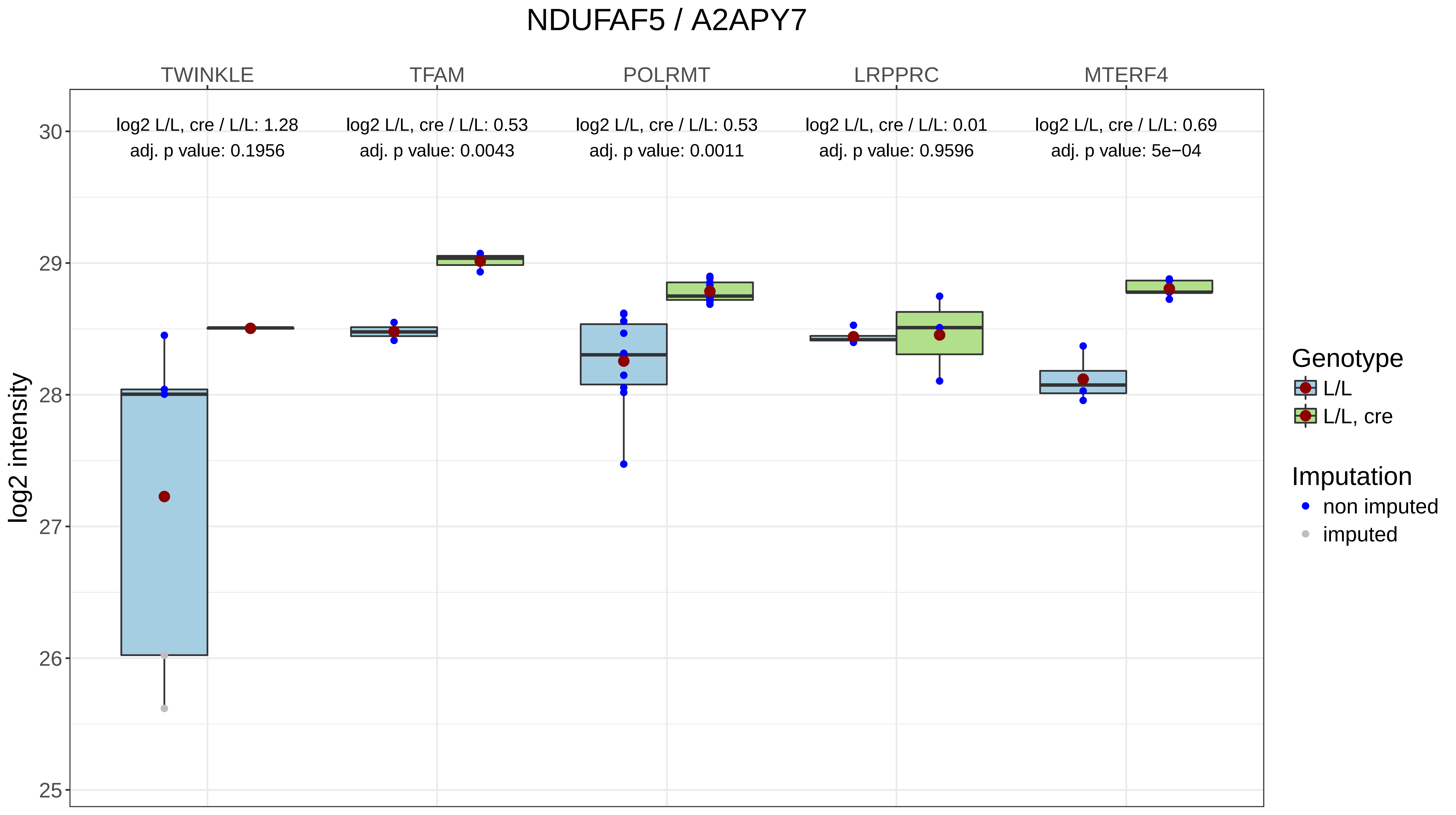
NDUFAF5 / A2APY7
TWINKLE
TFAM
POLRMT
LRPPRC
MTERF4
log2 L/L, cre / L/L: 1.28
adj. p value: 0.1956
log2 L/L, cre / L/L: 0.53
adj. p value: 0.0043
log2 L/L, cre / L/L: 0.53
adj. p value: 0.0011
log2 L/L, cre / L/L: 0.01
adj. p value: 0.9596
log2 L/L, cre / L/L: 0.69
adj. p value: 5e−04
log2 intensity
30
29
28
27
26
25
Genotype
L/L
L/L, cre
Imputation
non imputed
imputed

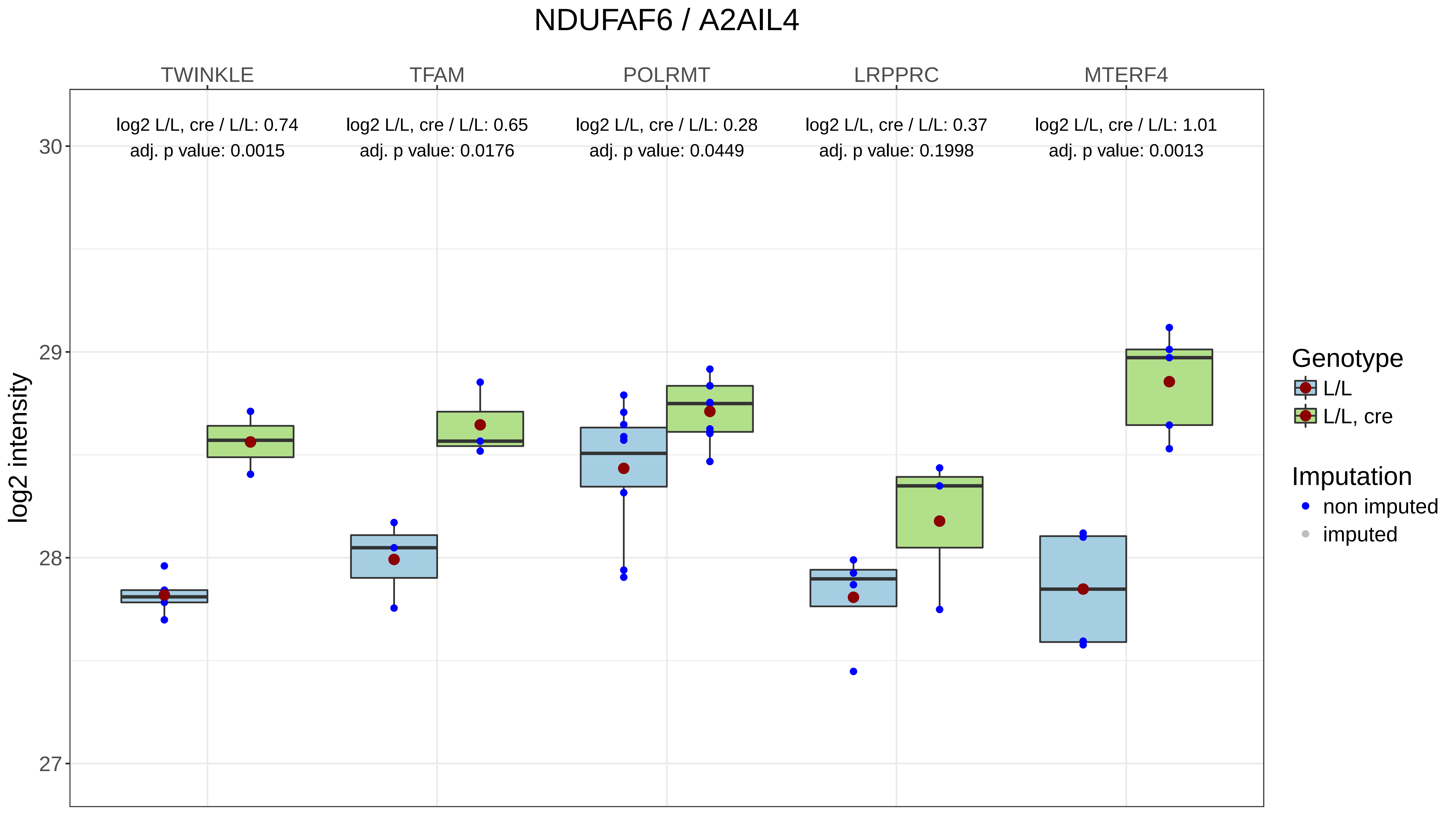

NDUFAF6 / A2AIL4
TWINKLE
TFAM
POLRMT
LRPPRC
MTERF4
log2 L/L, cre / L/L: 0.74
adj. p value: 0.0015
log2 L/L, cre / L/L: 0.65
adj. p value: 0.0176
log2 L/L, cre / L/L: 0.28
adj. p value: 0.0449
log2 L/L, cre / L/L: 0.37
adj. p value: 0.1998
log2 L/L, cre / L/L: 1.01
adj. p value: 0.0013
30
29
28
27
log2 intensity
Genotype
L/L
L/L, cre
Imputation
non imputed
imputed

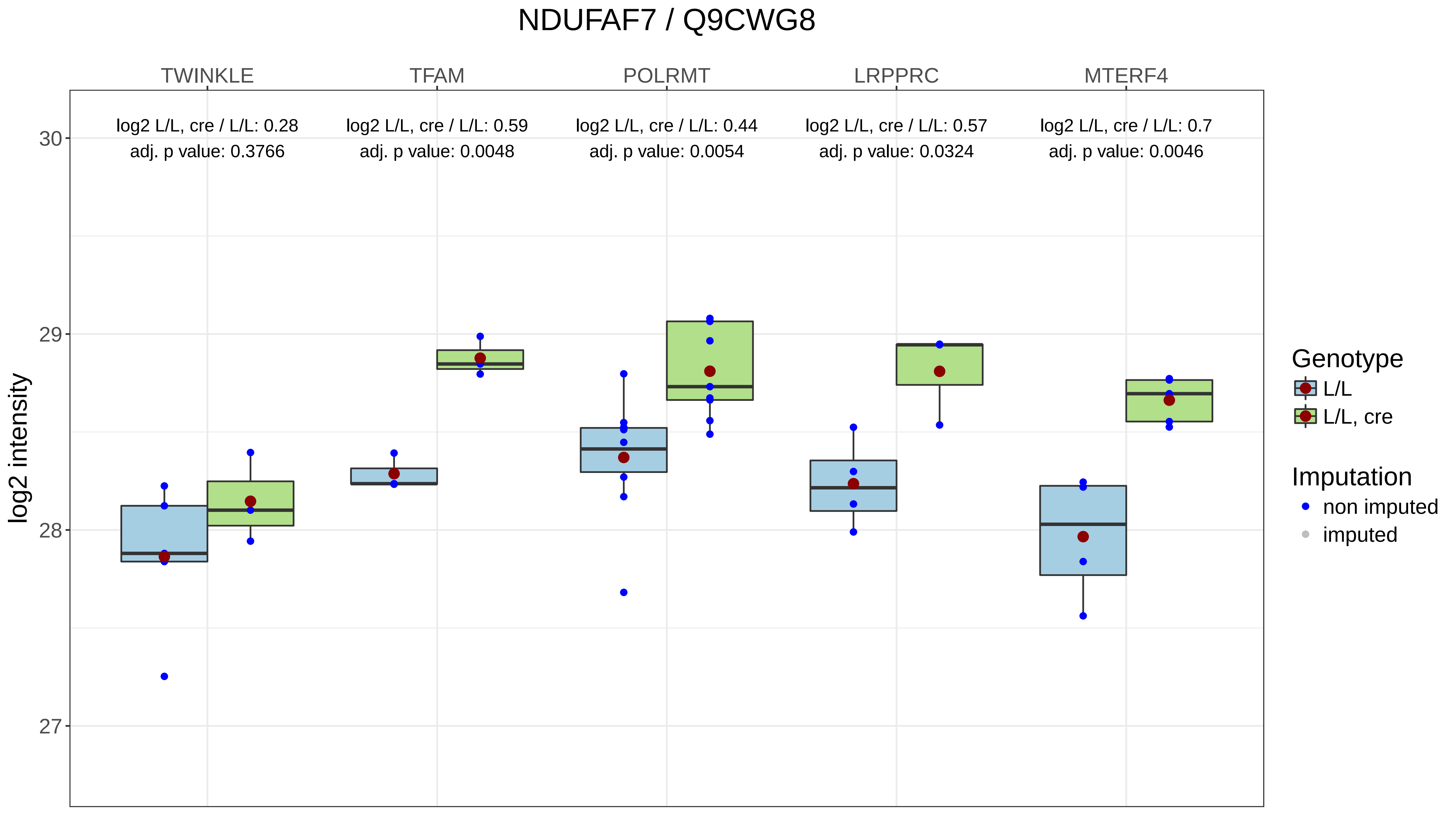
NDUFAF7 / Q9CWG8
TWINKLE
TFAM
POLRMT
LRPPRC
MTERF4
log2 L/L, cre / L/L: 0.28
adj. p value: 0.3766
log2 L/L, cre / L/L: 0.59
adj. p value: 0.0048
log2 L/L, cre / L/L: 0.44
adj. p value: 0.0054
log2 L/L, cre / L/L: 0.57
adj. p value: 0.0324
log2 L/L, cre / L/L: 0.7
adj. p value: 0.0046
log2 intensity
30
29
28
27
Genotype
L/L
L/L, cre
Imputation
non imputed
imputed

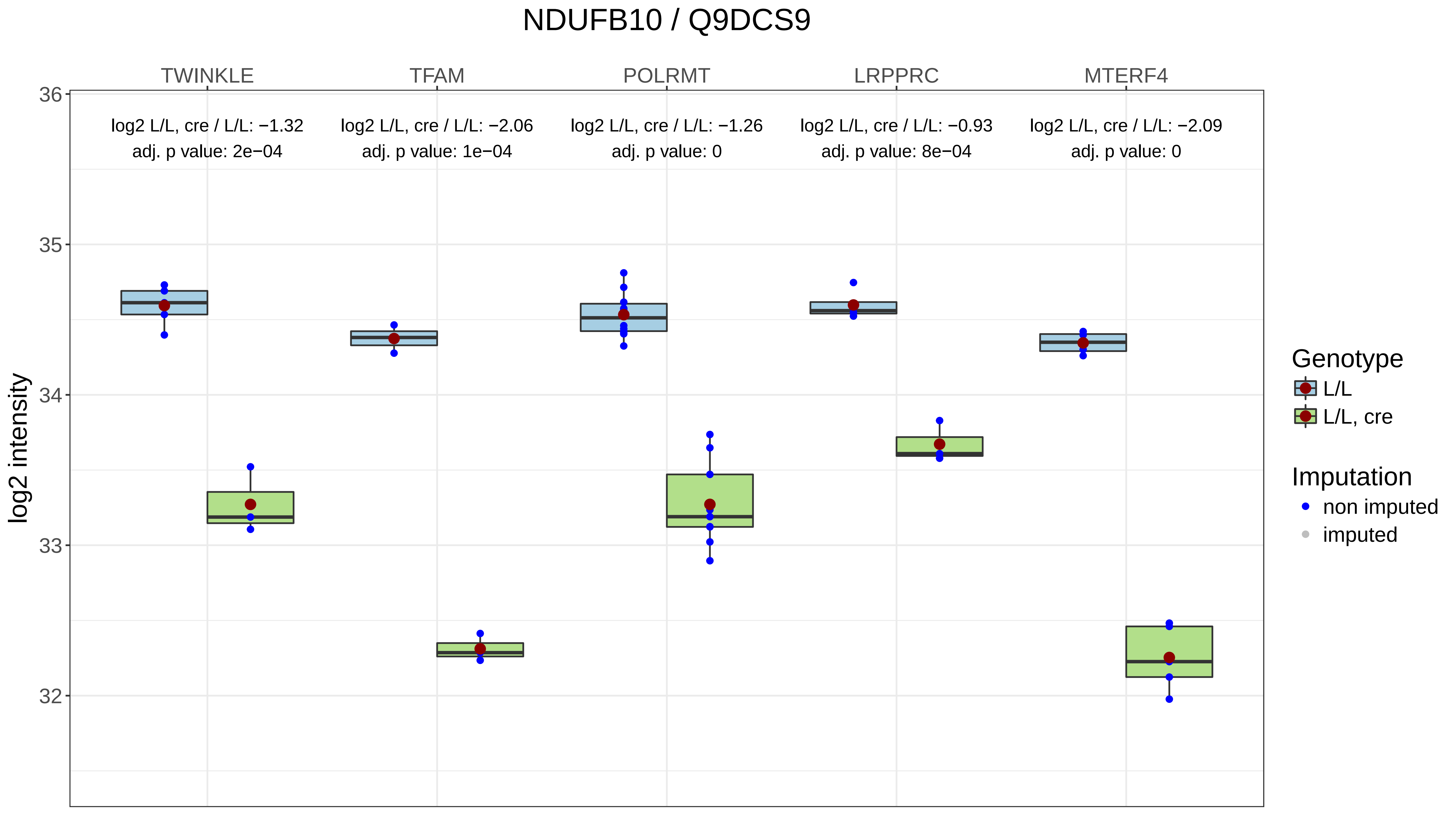
NDUFB10 / Q9DCS9
TWINKLE
TFAM
POLRMT
LRPPRC
MTERF4
log2 L/L, cre / L/L: −1.32
adj. p value: 2e−04
log2 L/L, cre / L/L: −2.06
adj. p value: 1e−04
log2 L/L, cre / L/L: −1.26
adj. p value: 0
log2 L/L, cre / L/L: −0.93
adj. p value: 8e−04
log2 L/L, cre / L/L: −2.09
adj. p value: 0
log2 intensity
36
35
34
33
32
Genotype
L/L
L/L, cre
Imputation
non imputed
imputed

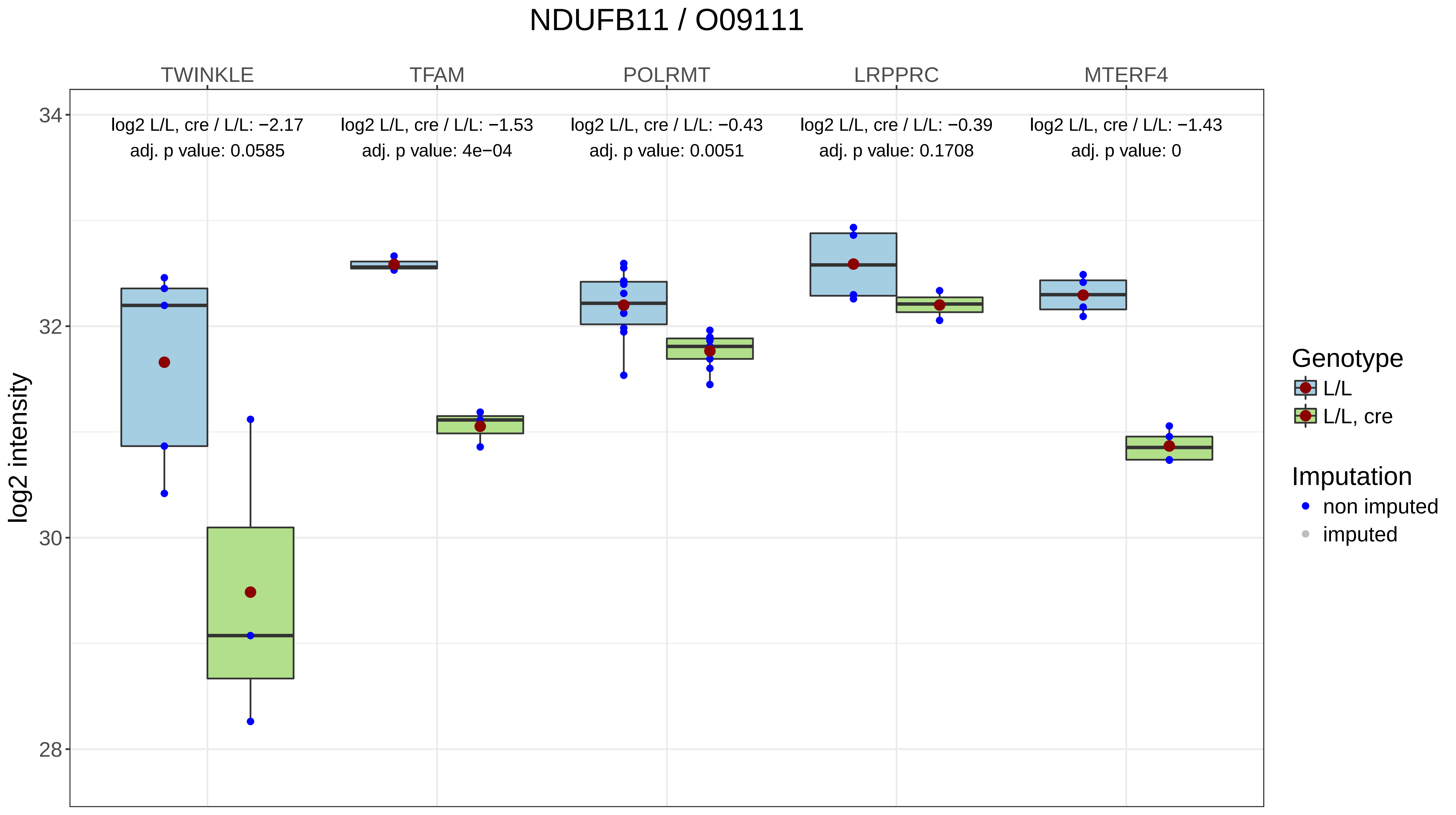

NDUFB11 / O09111
TWINKLE
TFAM
POLRMT
LRPPRC
MTERF4
log2 L/L, cre / L/L: −2.17
adj. p value: 0.0585
log2 L/L, cre / L/L: −1.53
adj. p value: 4e−04
log2 L/L, cre / L/L: −0.43
adj. p value: 0.0051
log2 L/L, cre / L/L: −0.39
adj. p value: 0.1708
log2 L/L, cre / L/L: −1.43
adj. p value: 0
34
32
30
28
log2 intensity
Genotype
L/L
L/L, cre
Imputation
non imputed
imputed

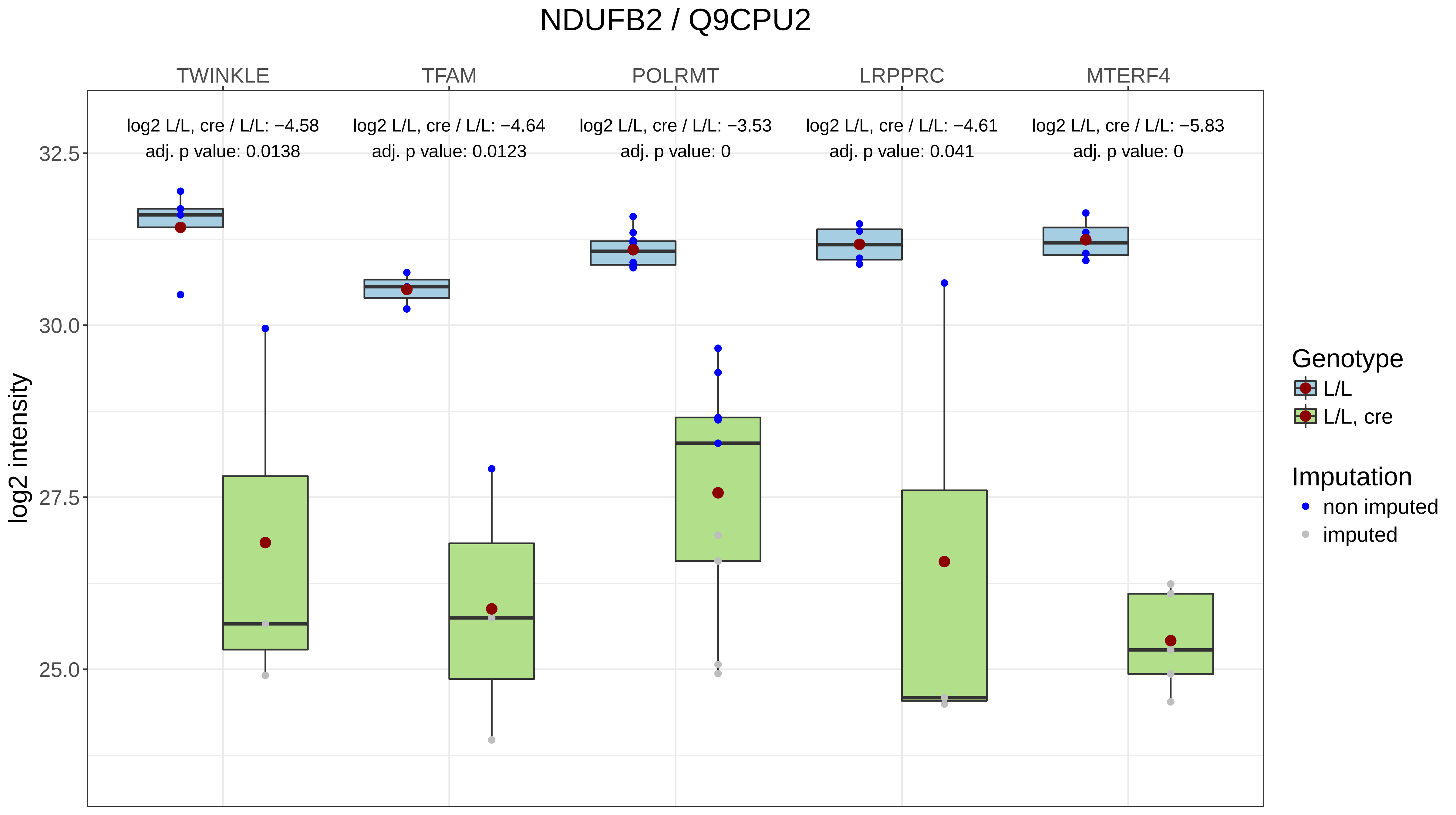
NDUFB2 / Q9CPU2
TWINKLE
TFAM
POLRMT
LRPPRC
MTERF4
log2 L/L, cre / L/L: −4.58
adj. p value: 0.0138
log2 L/L, cre / L/L: −4.64
adj. p value: 0.0123
log2 L/L, cre / L/L: −3.53
adj. p value: 0
log2 L/L, cre / L/L: −4.61
adj. p value: 0.041
log2 L/L, cre / L/L: −5.83
adj. p value: 0
32.5
30.0
27.5
25.0
log2 intensity
Genotype
L/L
L/L, cre
Imputation
non imputed
imputed

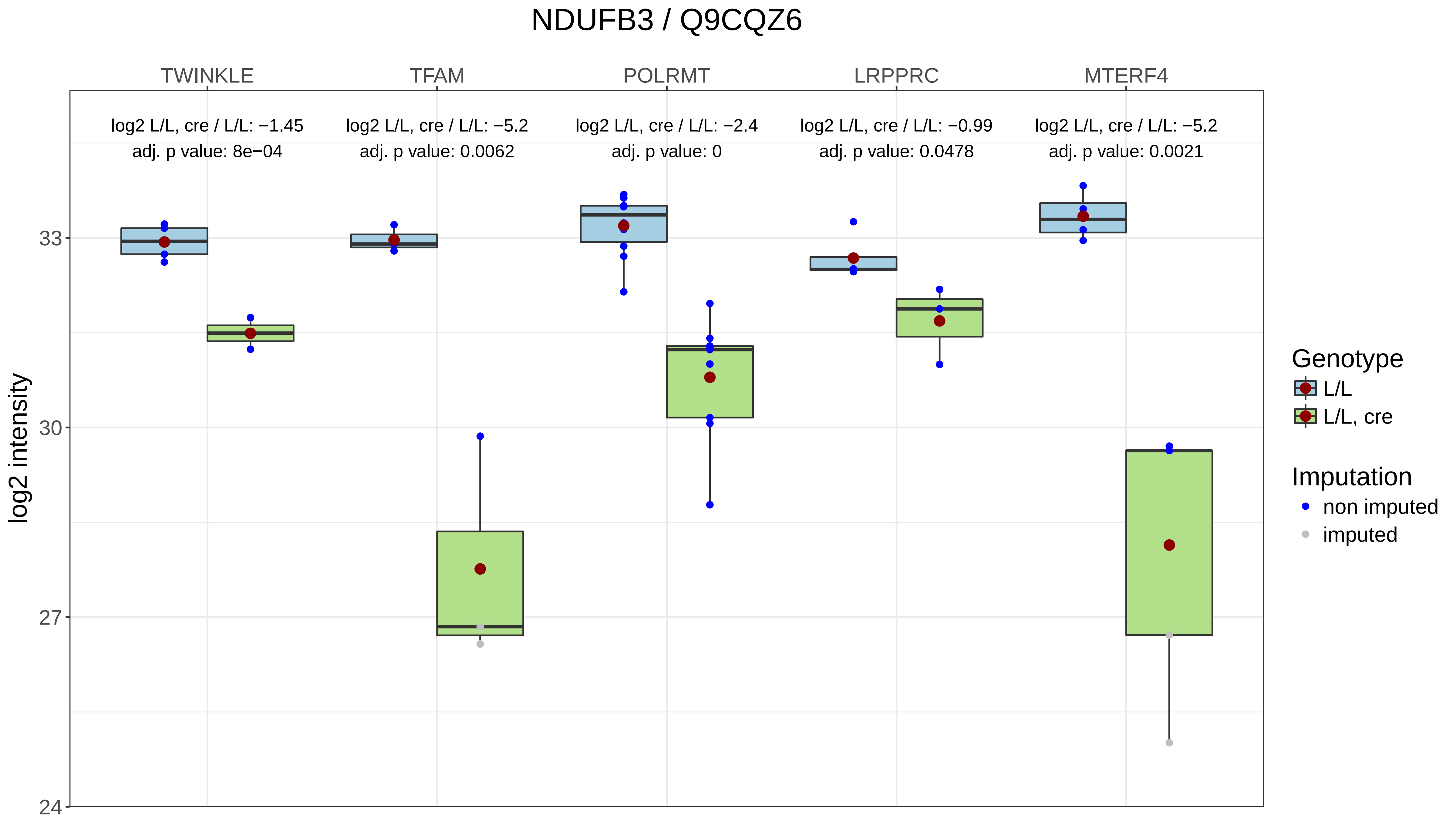
NDUFB3 / Q9CQZ6
TWINKLE
TFAM
POLRMT
LRPPRC
MTERF4
log2 L/L, cre / L/L: −1.45
adj. p value: 8e−04
log2 L/L, cre / L/L: −5.2
adj. p value: 0.0062
log2 L/L, cre / L/L: −2.4
adj. p value: 0
log2 L/L, cre / L/L: −0.99
adj. p value: 0.0478
log2 L/L, cre / L/L: −5.2
adj. p value: 0.0021
log2 intensity
33
30
27
24
Genotype
L/L
L/L, cre
Imputation
non imputed
imputed

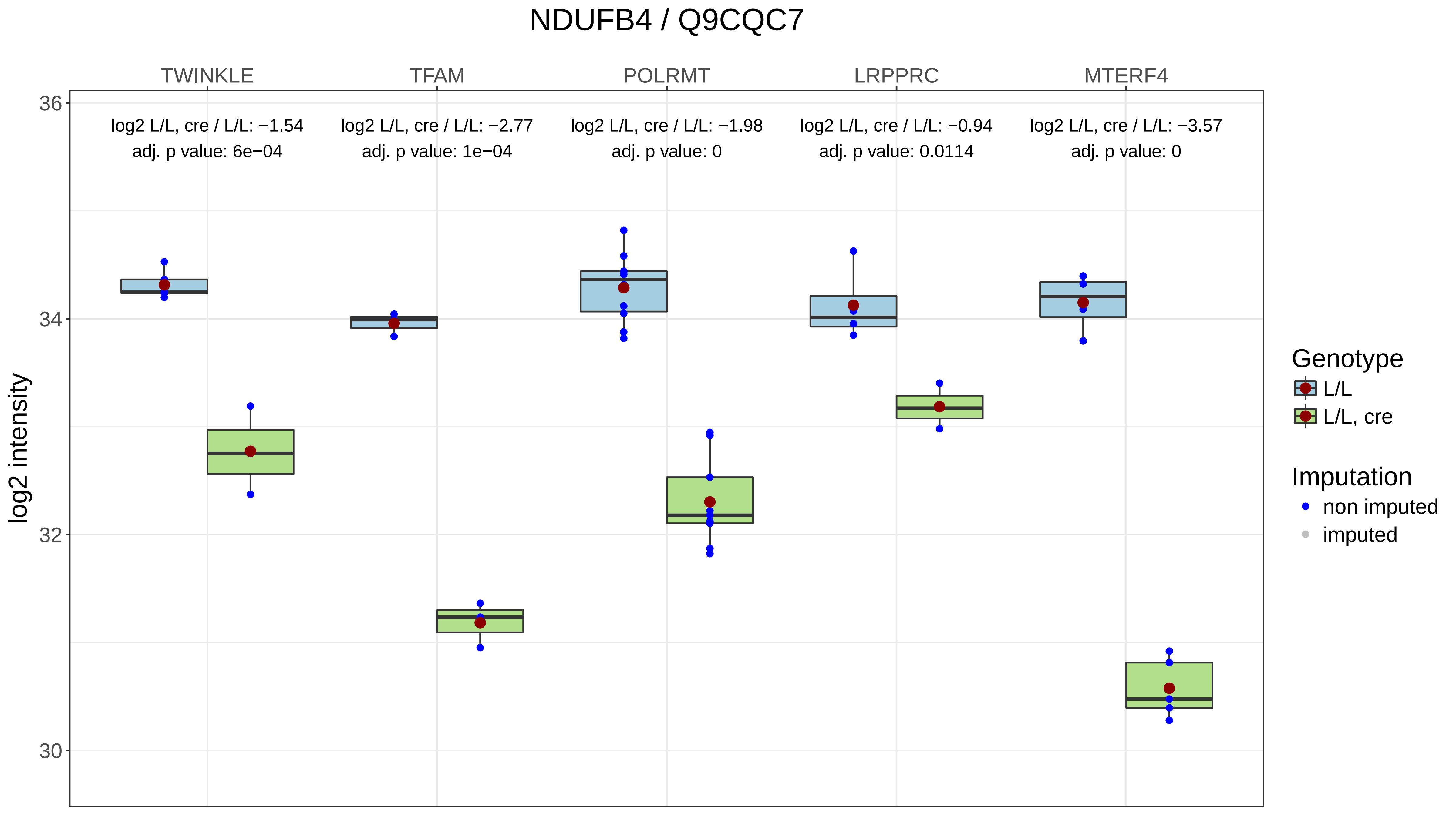
NDUFB4 / Q9CQC7
TWINKLE
TFAM
POLRMT
LRPPRC
MTERF4
log2 L/L, cre / L/L: −1.54
adj. p value: 6e−04
log2 L/L, cre / L/L: −2.77
adj. p value: 1e−04
log2 L/L, cre / L/L: −1.98
adj. p value: 0
log2 L/L, cre / L/L: −0.94
adj. p value: 0.0114
log2 L/L, cre / L/L: −3.57
adj. p value: 0
36
34
32
30
log2 intensity
Genotype
L/L
L/L, cre
Imputation
non imputed
imputed

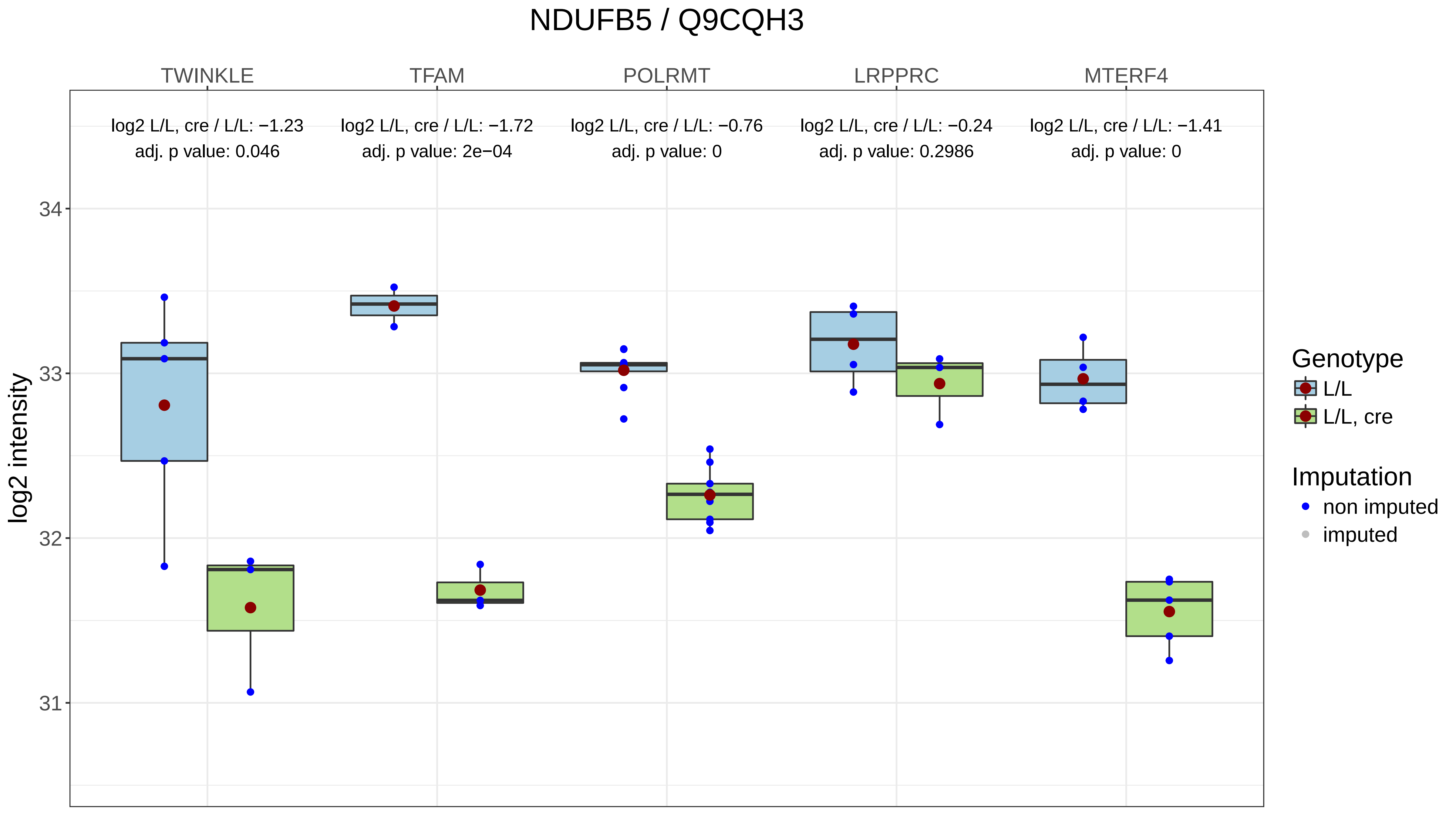
NDUFB5 / Q9CQH3
TWINKLE
TFAM
POLRMT
LRPPRC
MTERF4
log2 L/L, cre / L/L: −1.23
adj. p value: 0.046
log2 L/L, cre / L/L: −1.72
adj. p value: 2e−04
log2 L/L, cre / L/L: −0.76
adj. p value: 0
log2 L/L, cre / L/L: −0.24
adj. p value: 0.2986
log2 L/L, cre / L/L: −1.41
adj. p value: 0
log2 intensity
34
33
32
31
Genotype
L/L
L/L, cre
Imputation
non imputed
imputed

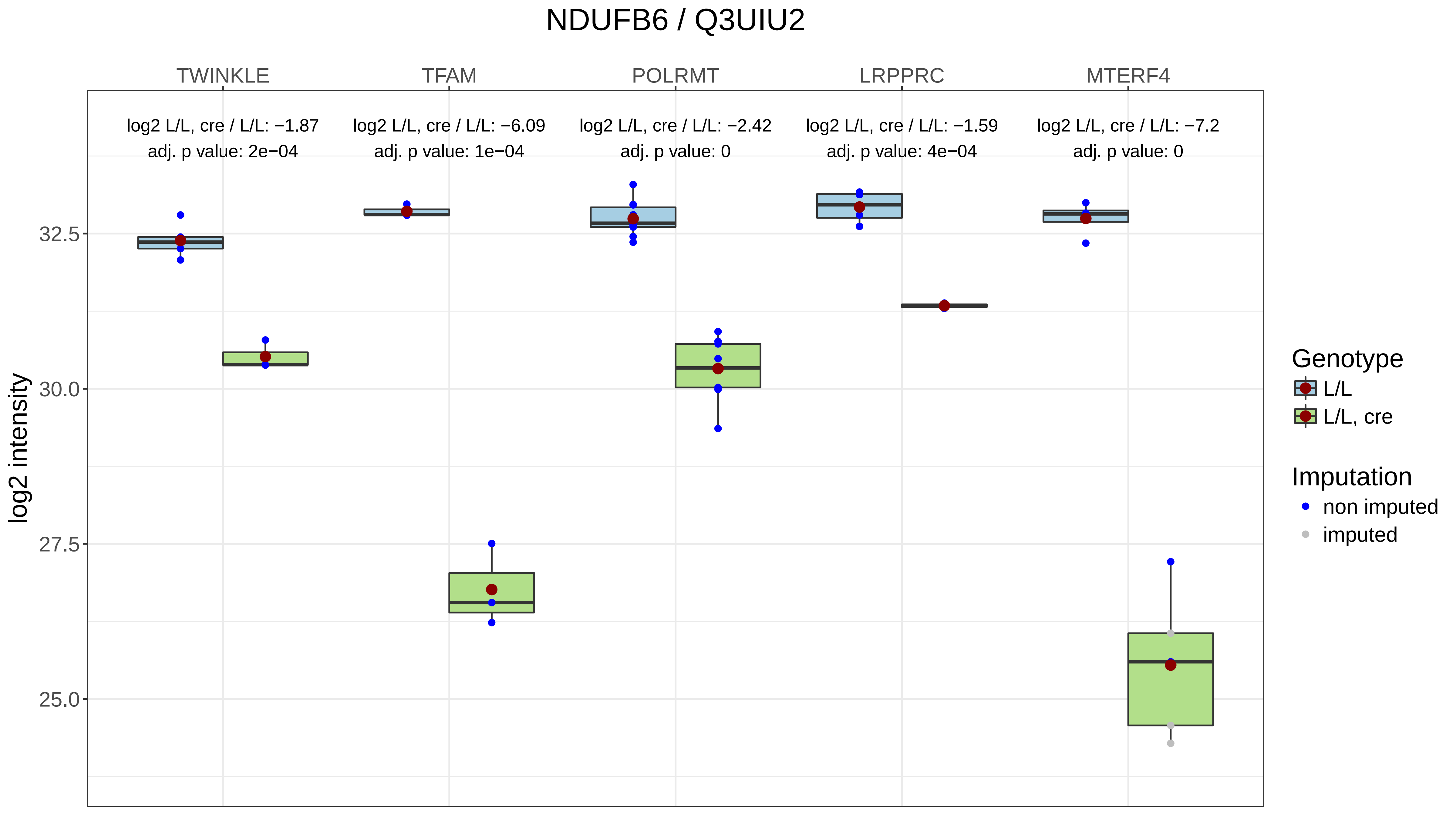
NDUFB6 / Q3UIU2
TWINKLE
TFAM
POLRMT
LRPPRC
MTERF4
log2 L/L, cre / L/L: −1.87
adj. p value: 2e−04
log2 L/L, cre / L/L: −6.09
adj. p value: 1e−04
log2 L/L, cre / L/L: −2.42
adj. p value: 0
log2 L/L, cre / L/L: −1.59
adj. p value: 4e−04
log2 L/L, cre / L/L: −7.2
adj. p value: 0
log2 intensity
32.5
30.0
27.5
25.0
Genotype
L/L
L/L, cre
Imputation
non imputed
imputed

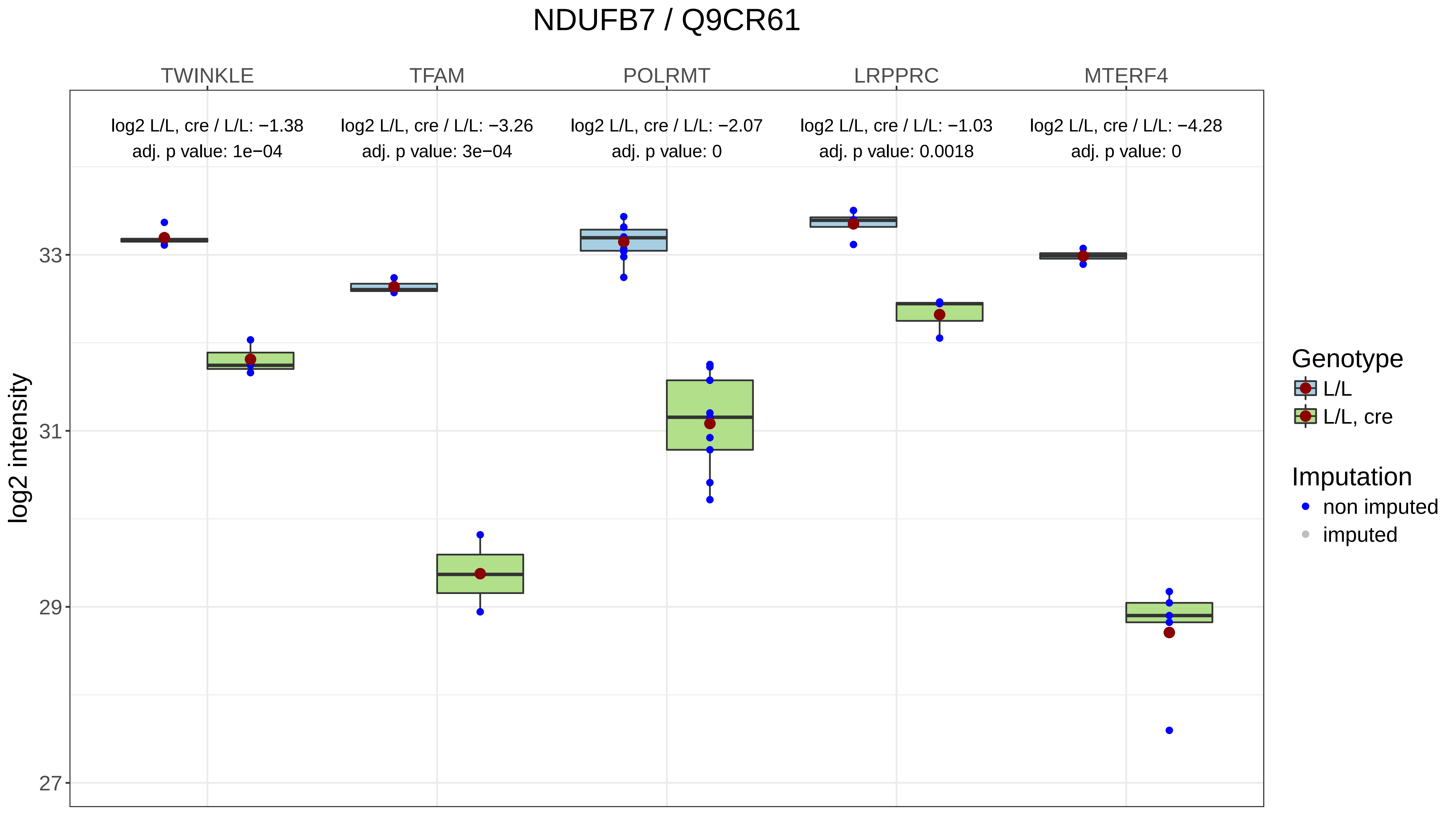
NDUFB7 / Q9CR61
TWINKLE
TFAM
POLRMT
LRPPRC
MTERF4
log2 L/L, cre / L/L: −1.38
adj. p value: 1e−04
log2 L/L, cre / L/L: −3.26
adj. p value: 3e−04
log2 L/L, cre / L/L: −2.07
adj. p value: 0
log2 L/L, cre / L/L: −1.03
adj. p value: 0.0018
log2 L/L, cre / L/L: −4.28
adj. p value: 0
log2 intensity
33
31
29
27
Genotype
L/L
L/L, cre
Imputation
non imputed
imputed

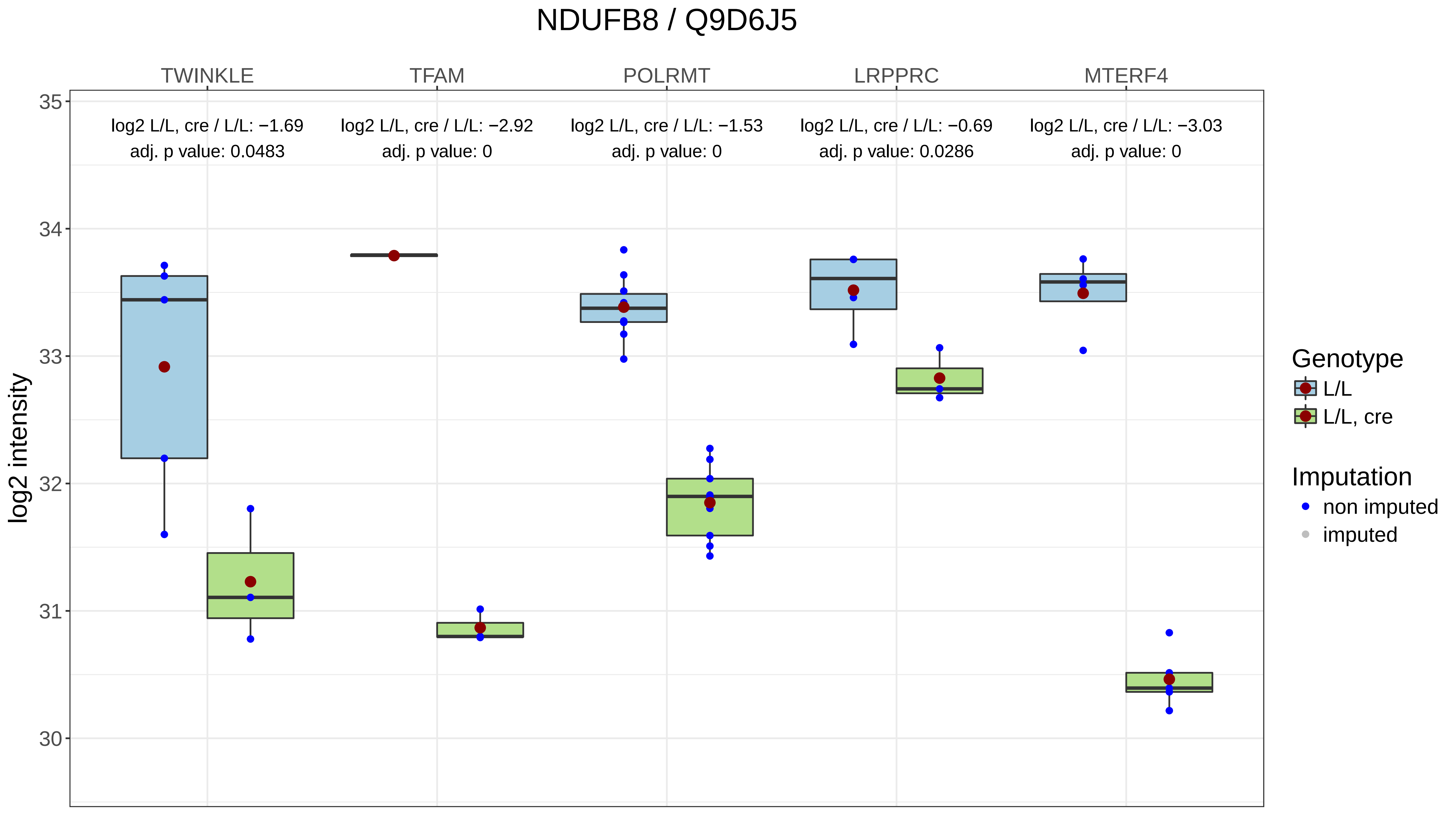
NDUFB8 / Q9D6J5
TWINKLE
TFAM
POLRMT
LRPPRC
MTERF4
log2 L/L, cre / L/L: −1.69
adj. p value: 0.0483
log2 L/L, cre / L/L: −2.92
adj. p value: 0
log2 L/L, cre / L/L: −1.53
adj. p value: 0
log2 L/L, cre / L/L: −0.69
adj. p value: 0.0286
log2 L/L, cre / L/L: −3.03
adj. p value: 0
log2 intensity
35
34
33
32
31
30
Genotype
L/L
L/L, cre
Imputation
non imputed
imputed

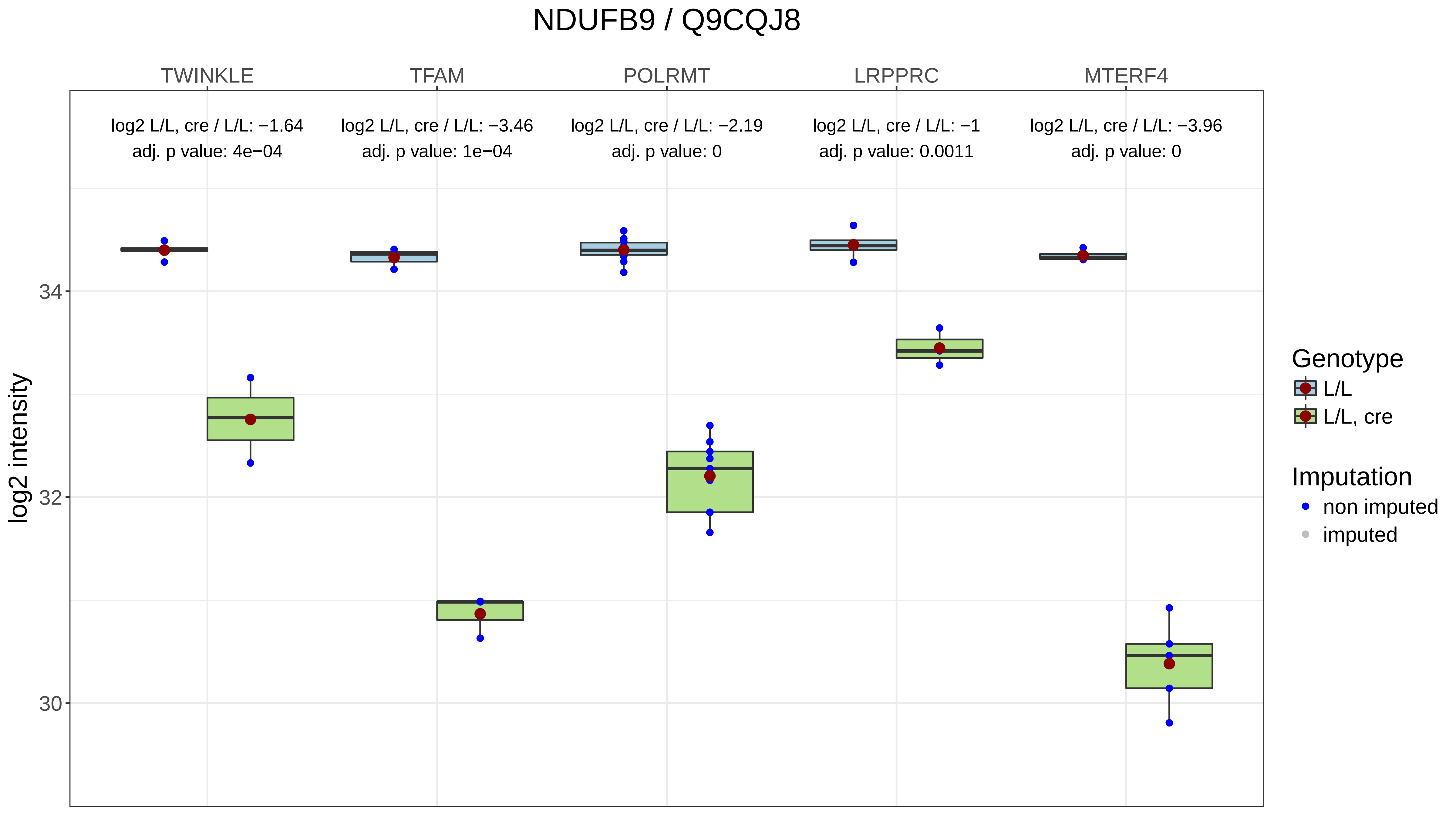
NDUFB9 / Q9CQJ8
TWINKLE
TFAM
POLRMT
LRPPRC
MTERF4
log2 L/L, cre / L/L: −1.64
adj. p value: 4e−04
log2 L/L, cre / L/L: −3.46
adj. p value: 1e−04
log2 L/L, cre / L/L: −2.19
adj. p value: 0
log2 L/L, cre / L/L: −1
adj. p value: 0.0011
log2 L/L, cre / L/L: −3.96
adj. p value: 0
log2 intensity
34
32
30
Genotype
L/L
L/L, cre
Imputation
non imputed
imputed

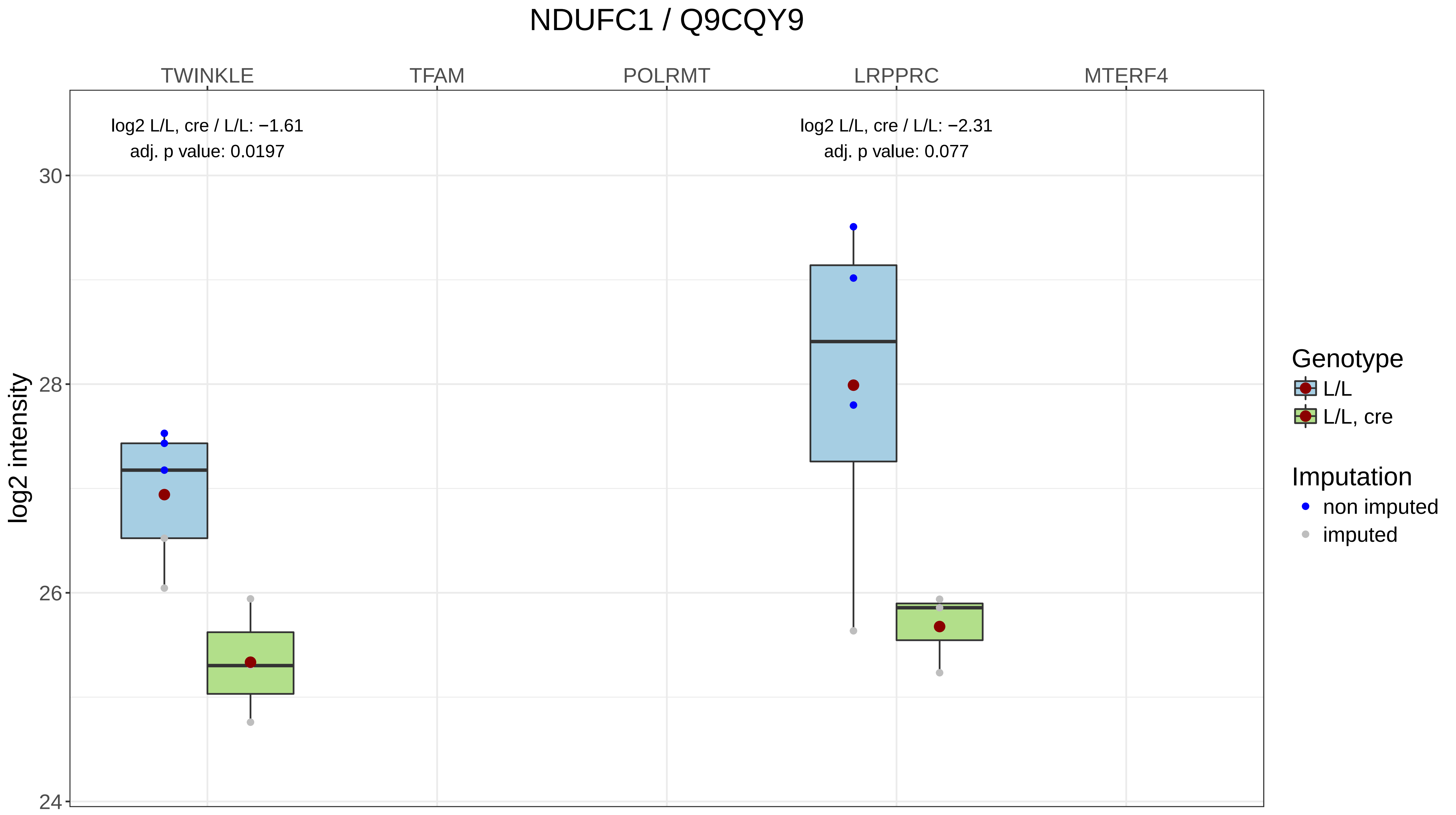
NDUFC1 / Q9CQY9
TWINKLE
TFAM
POLRMT
LRPPRC
MTERF4
log2 L/L, cre / L/L: −1.61
adj. p value: 0.0197
log2 L/L, cre / L/L: −2.31
adj. p value: 0.077
30
28
26
24
log2 intensity
Genotype
L/L
L/L, cre
Imputation
non imputed
imputed

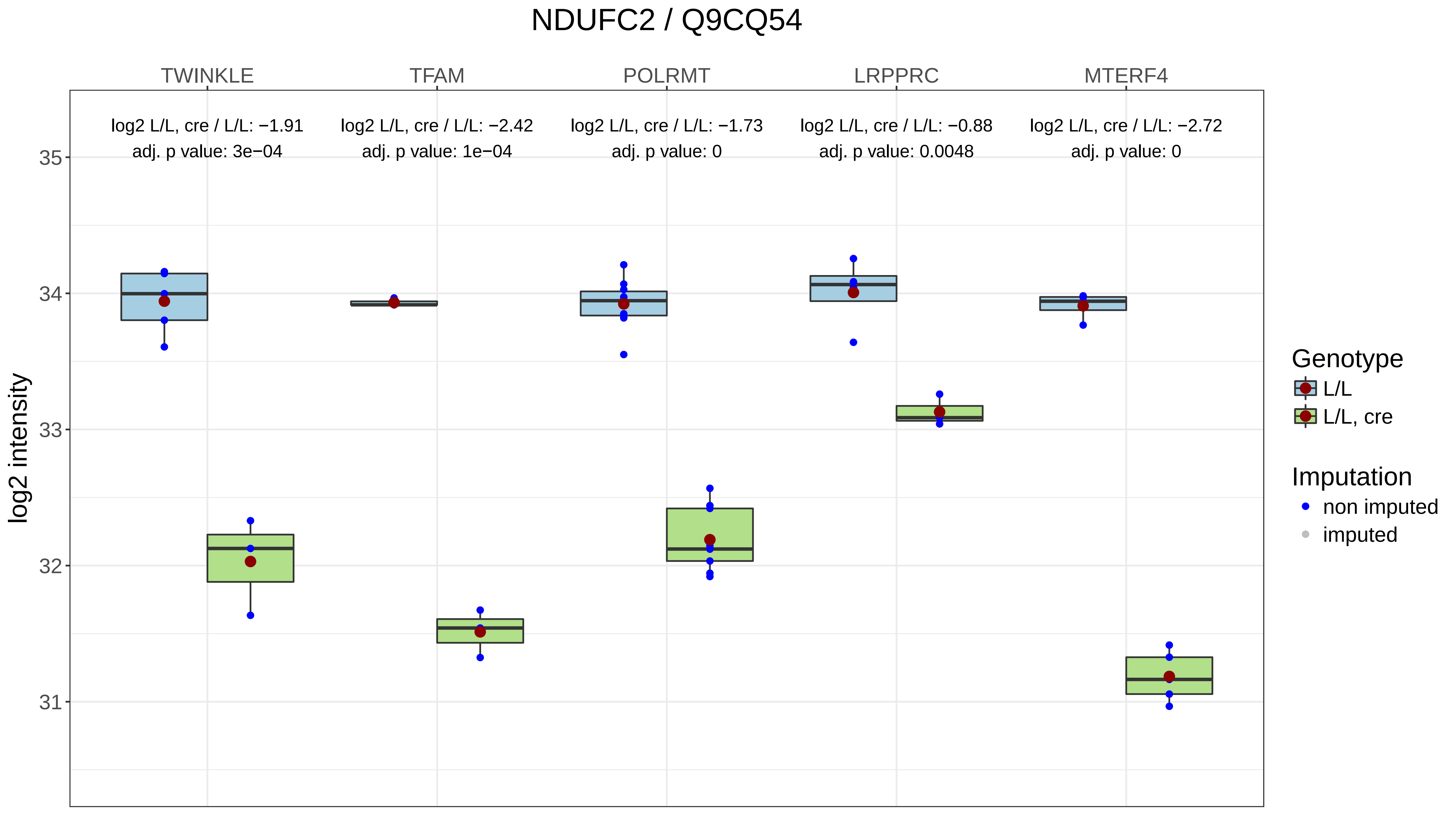
NDUFC2 / Q9CQ54
TWINKLE
TFAM
POLRMT
LRPPRC
MTERF4
log2 L/L, cre / L/L: −1.91
adj. p value: 3e−04
log2 L/L, cre / L/L: −2.42
adj. p value: 1e−04
log2 L/L, cre / L/L: −1.73
adj. p value: 0
log2 L/L, cre / L/L: −0.88
adj. p value: 0.0048
log2 L/L, cre / L/L: −2.72
adj. p value: 0
35
34
33
32
31
log2 intensity
Genotype
L/L
L/L, cre
Imputation
non imputed
imputed

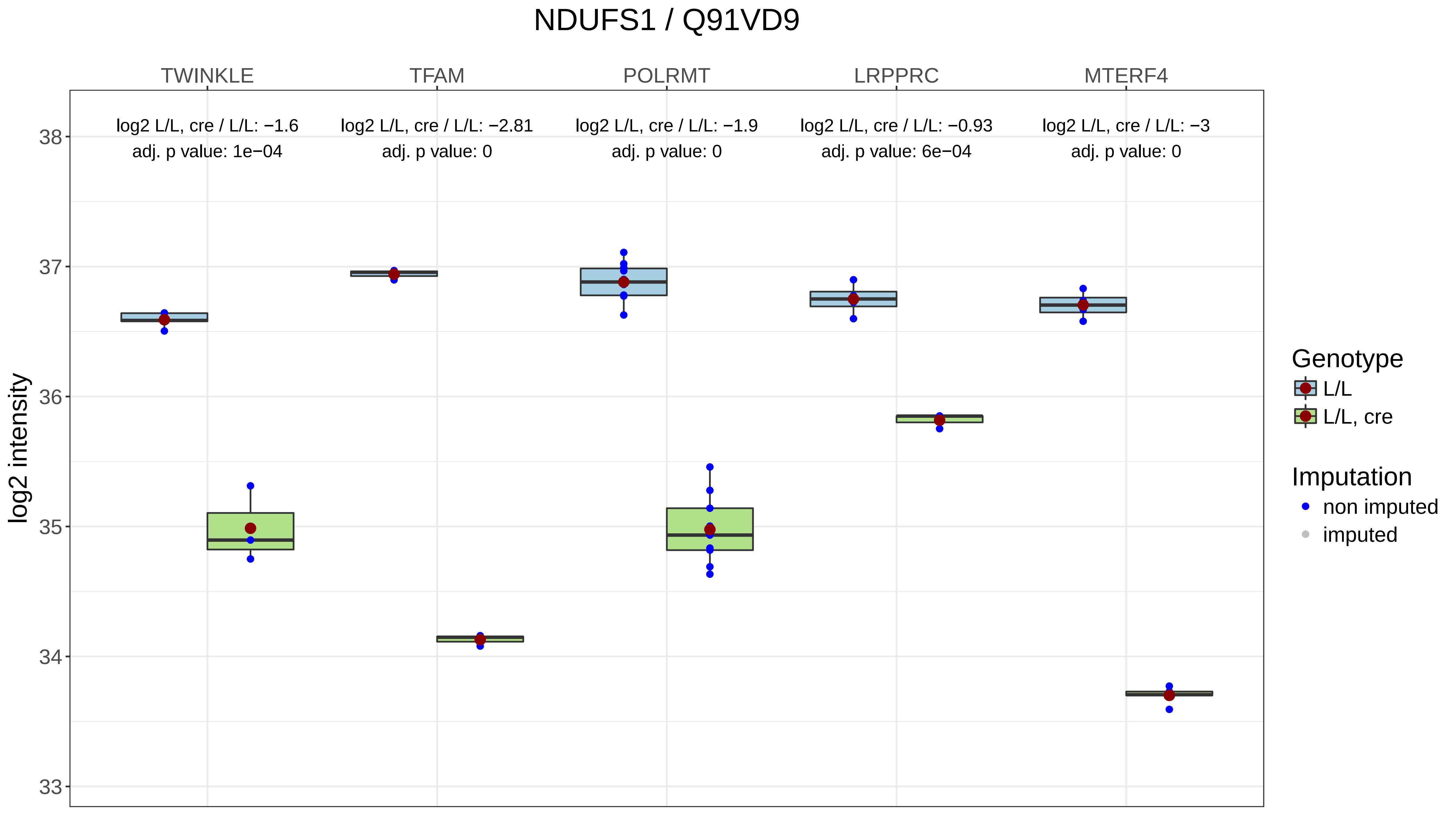
NDUFS1 / Q91VD9
TWINKLE
TFAM
POLRMT
LRPPRC
MTERF4
log2 L/L, cre / L/L: −1.6
adj. p value: 1e−04
log2 L/L, cre / L/L: −2.81
adj. p value: 0
log2 L/L, cre / L/L: −1.9
adj. p value: 0
log2 L/L, cre / L/L: −0.93
adj. p value: 6e−04
log2 L/L, cre / L/L: −3
adj. p value: 0
log2 intensity
38
37
36
35
34
33
Genotype
L/L
L/L, cre
Imputation
non imputed
imputed

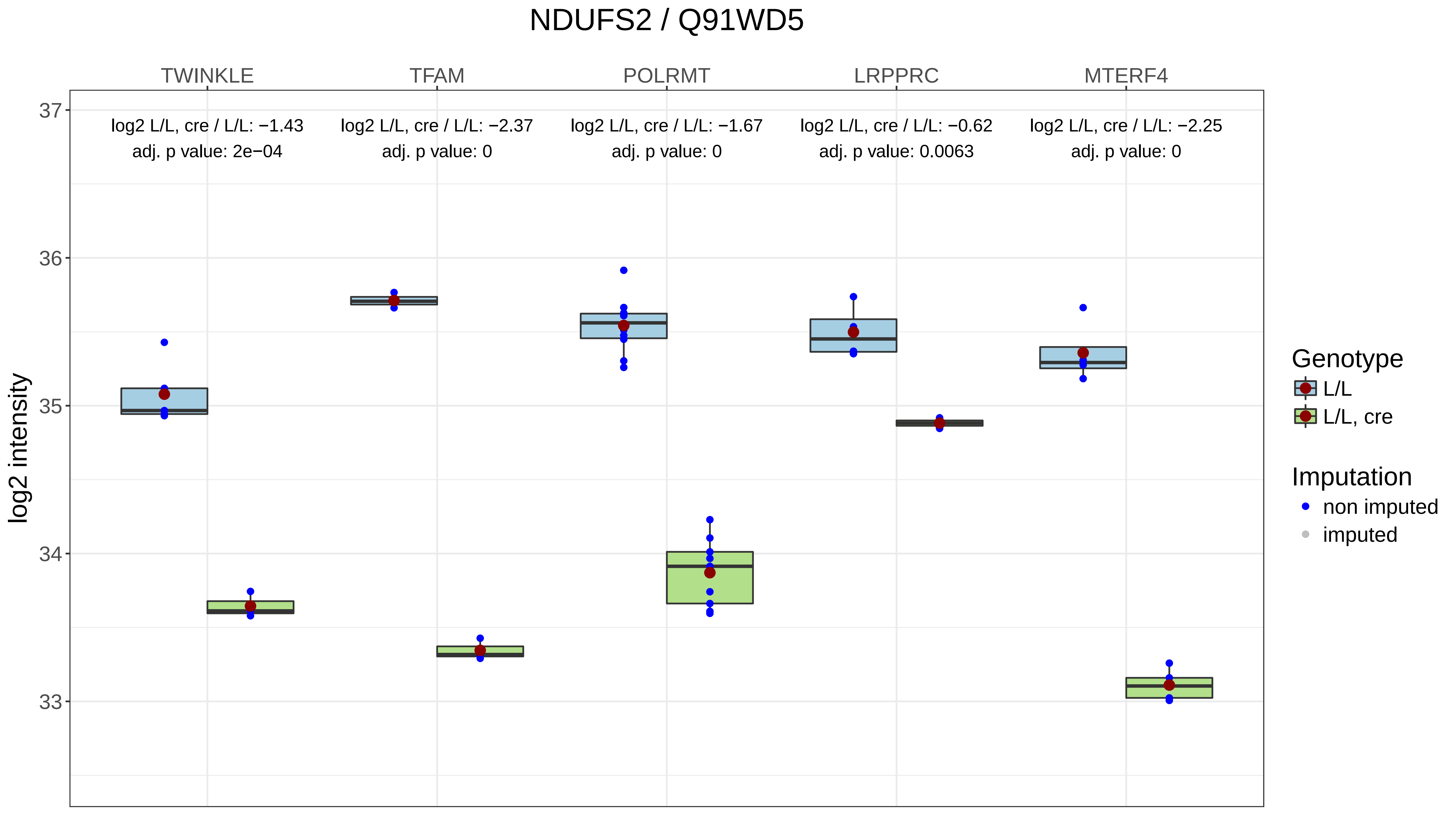
NDUFS2 / Q91WD5
TWINKLE
TFAM
POLRMT
LRPPRC
MTERF4
log2 L/L, cre / L/L: −1.43
adj. p value: 2e−04
log2 L/L, cre / L/L: −2.37
adj. p value: 0
log2 L/L, cre / L/L: −1.67
adj. p value: 0
log2 L/L, cre / L/L: −0.62
adj. p value: 0.0063
log2 L/L, cre / L/L: −2.25
adj. p value: 0
log2 intensity
37
36
35
34
33
Genotype
L/L
L/L, cre
Imputation
non imputed
imputed

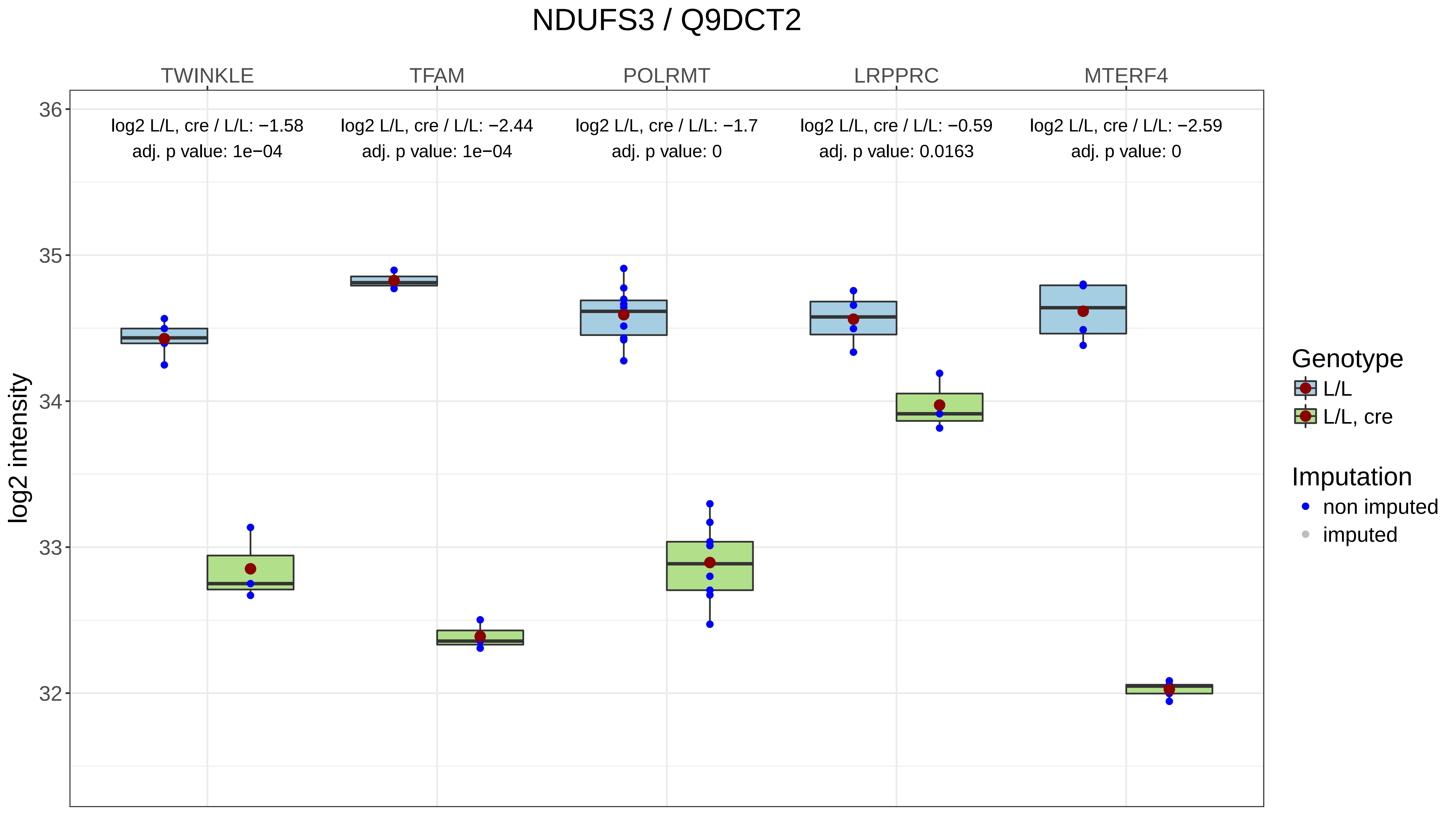
NDUFS3 / Q9DCT2
TWINKLE
TFAM
POLRMT
LRPPRC
MTERF4
log2 L/L, cre / L/L: −1.58
adj. p value: 1e−04
log2 L/L, cre / L/L: −2.44
adj. p value: 1e−04
log2 L/L, cre / L/L: −1.7
adj. p value: 0
log2 L/L, cre / L/L: −0.59
adj. p value: 0.0163
log2 L/L, cre / L/L: −2.59
adj. p value: 0
log2 intensity
36
35
34
33
32
Genotype
L/L
L/L, cre
Imputation
non imputed
imputed

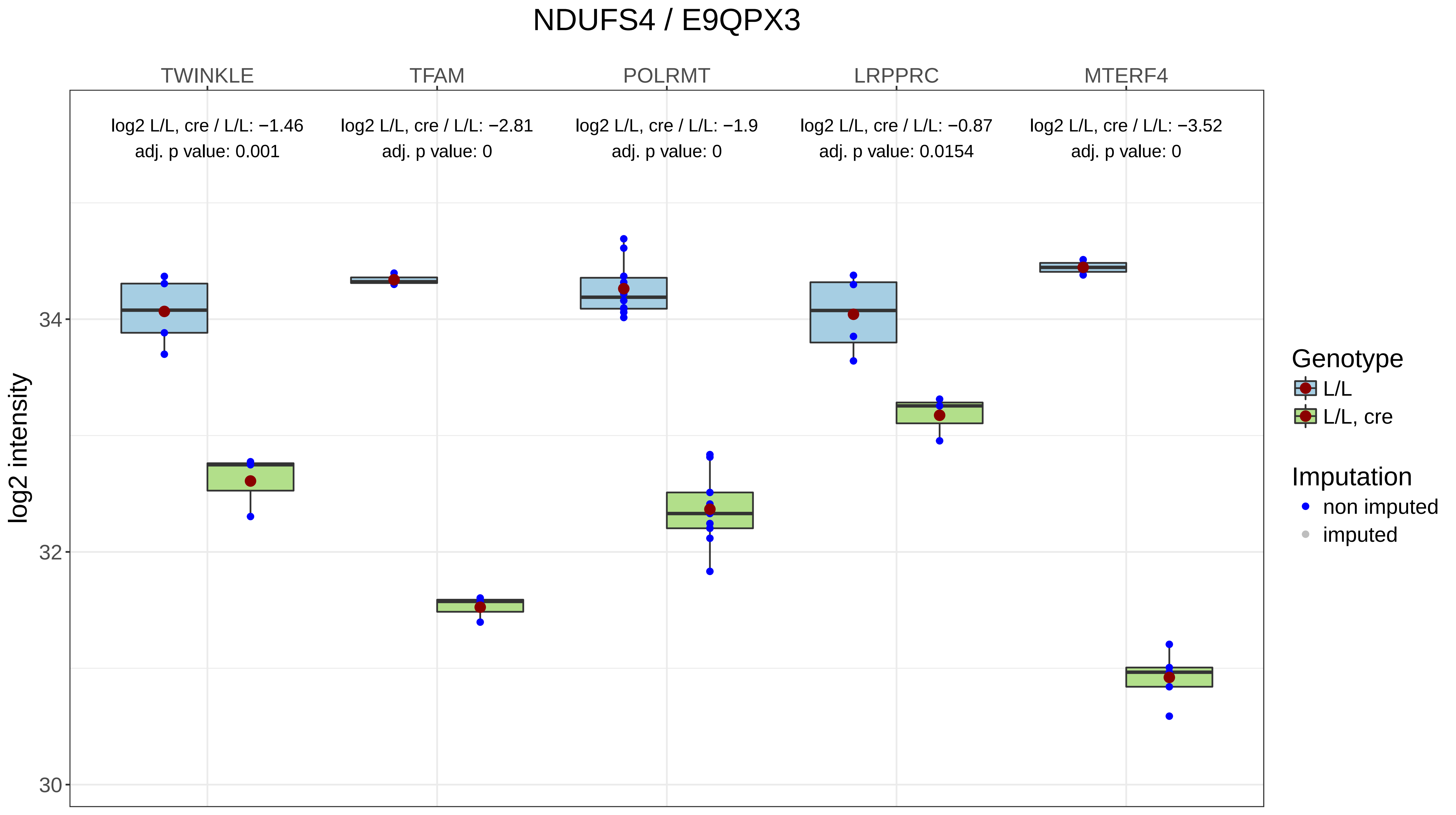
NDUFS4 / E9QPX3
TWINKLE
TFAM
POLRMT
LRPPRC
MTERF4
log2 L/L, cre / L/L: −1.46
adj. p value: 0.001
log2 L/L, cre / L/L: −2.81
adj. p value: 0
log2 L/L, cre / L/L: −1.9
adj. p value: 0
log2 L/L, cre / L/L: −0.87
adj. p value: 0.0154
log2 L/L, cre / L/L: −3.52
adj. p value: 0
log2 intensity
34
32
30
Genotype
L/L
L/L, cre
Imputation
non imputed
imputed

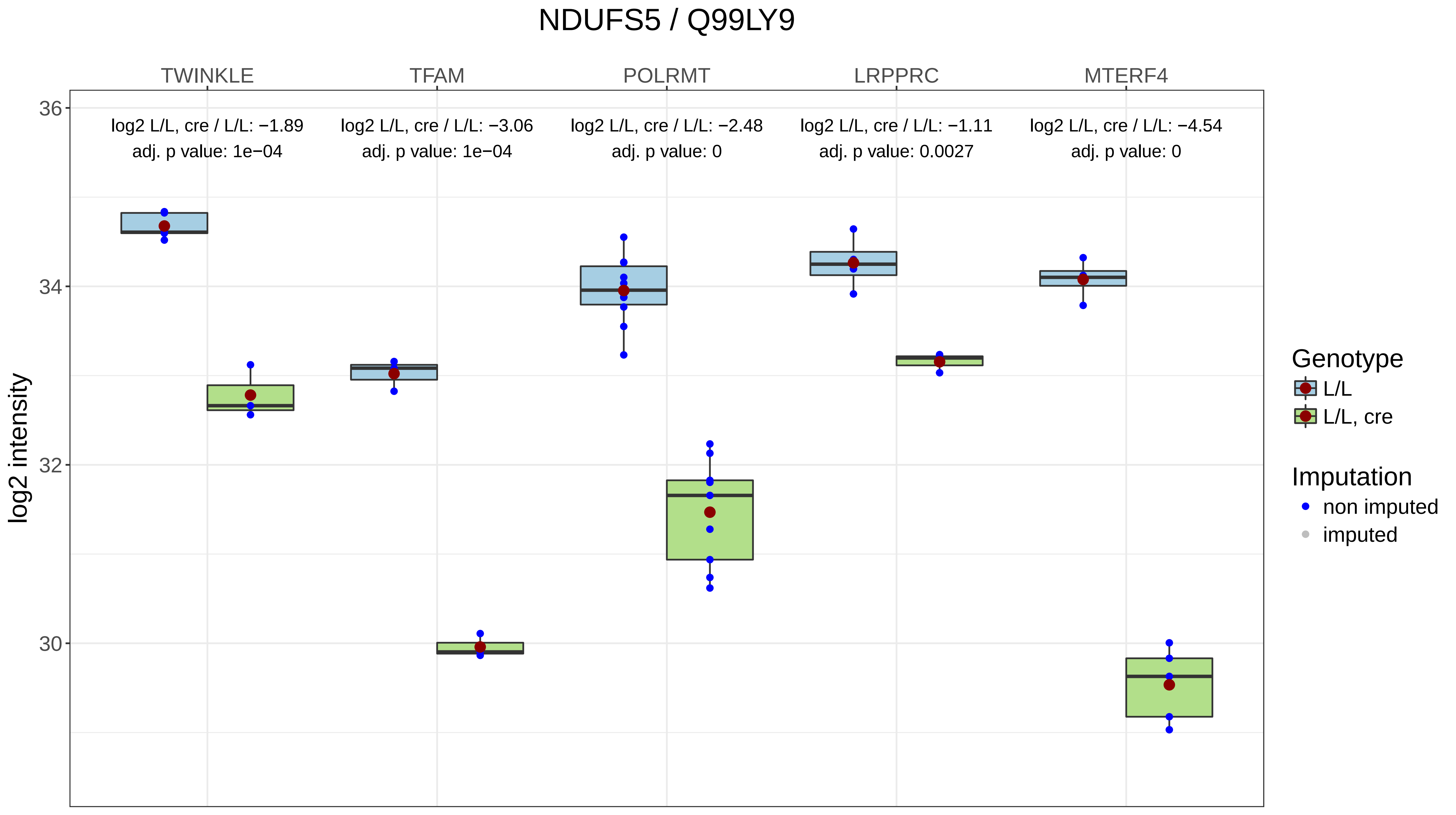

NDUFS5 / Q99LY9
TWINKLE
TFAM
POLRMT
LRPPRC
MTERF4
log2 L/L, cre / L/L: −1.89
adj. p value: 1e−04
log2 L/L, cre / L/L: −3.06
adj. p value: 1e−04
log2 L/L, cre / L/L: −2.48
adj. p value: 0
log2 L/L, cre / L/L: −1.11
adj. p value: 0.0027
log2 L/L, cre / L/L: −4.54
adj. p value: 0
log2 intensity
36
34
32
30
Genotype
L/L
L/L, cre
Imputation
non imputed
imputed

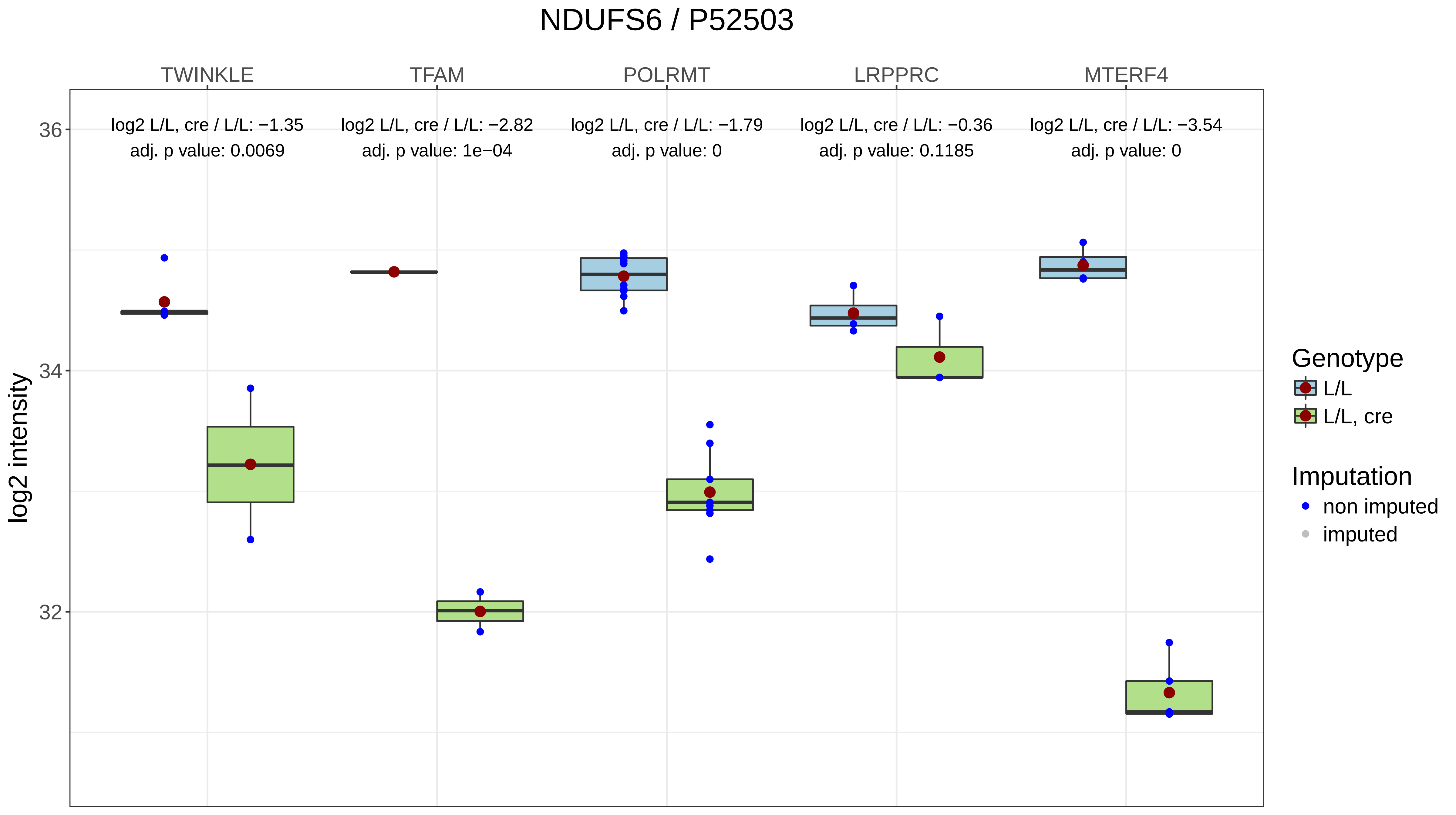

NDUFS6 / P52503
TWINKLE
TFAM
POLRMT
LRPPRC
MTERF4
log2 L/L, cre / L/L: −1.35
adj. p value: 0.0069
log2 L/L, cre / L/L: −2.82
adj. p value: 1e−04
log2 L/L, cre / L/L: −1.79
adj. p value: 0
log2 L/L, cre / L/L: −0.36
adj. p value: 0.1185
log2 L/L, cre / L/L: −3.54
adj. p value: 0
36
34
32
log2 intensity
Genotype
L/L
L/L, cre
Imputation
non imputed
imputed

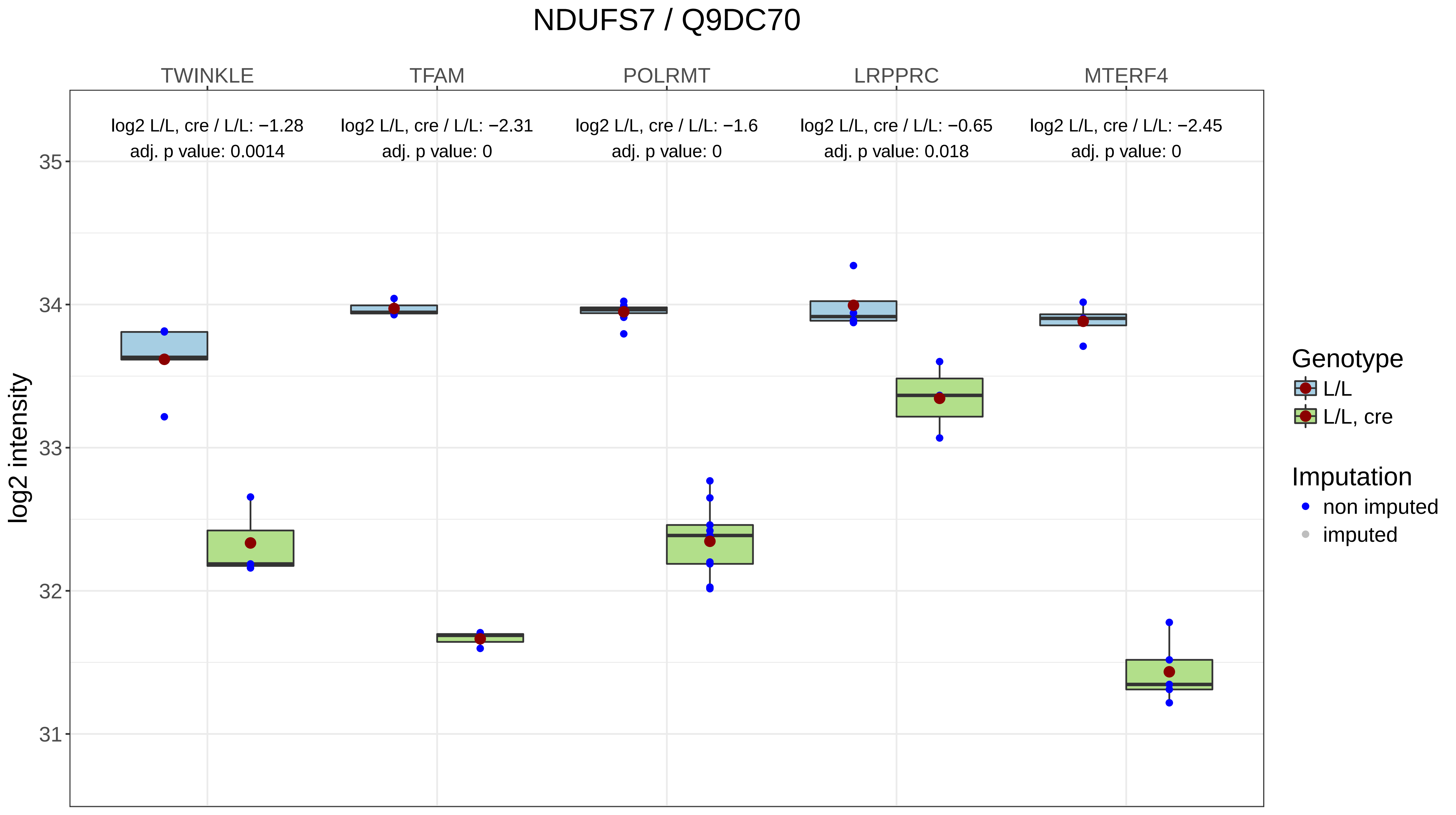
NDUFS7 / Q9DC70
TWINKLE
TFAM
POLRMT
LRPPRC
MTERF4
log2 L/L, cre / L/L: −1.28
adj. p value: 0.0014
log2 L/L, cre / L/L: −2.31
adj. p value: 0
log2 L/L, cre / L/L: −1.6
adj. p value: 0
log2 L/L, cre / L/L: −0.65
adj. p value: 0.018
log2 L/L, cre / L/L: −2.45
adj. p value: 0
log2 intensity
35
34
33
32
31
Genotype
L/L
L/L, cre
Imputation
non imputed
imputed

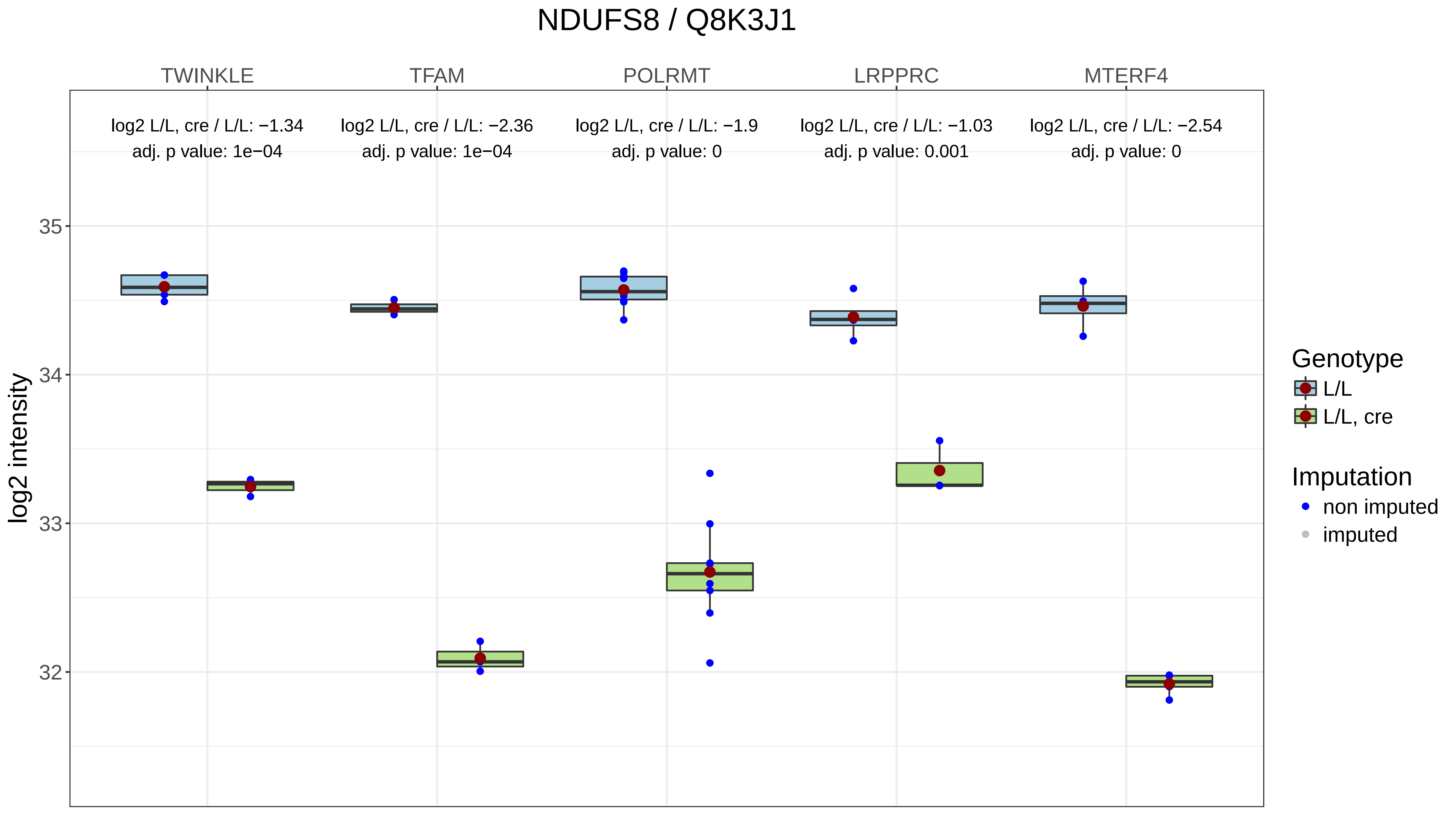
NDUFS8 / Q8K3J1
TWINKLE
TFAM
POLRMT
LRPPRC
MTERF4
log2 L/L, cre / L/L: −1.34
adj. p value: 1e−04
log2 L/L, cre / L/L: −2.36
adj. p value: 1e−04
log2 L/L, cre / L/L: −1.9
adj. p value: 0
log2 L/L, cre / L/L: −1.03
adj. p value: 0.001
log2 L/L, cre / L/L: −2.54
adj. p value: 0
log2 intensity
35
34
33
32
Genotype
L/L
L/L, cre
Imputation
non imputed
imputed

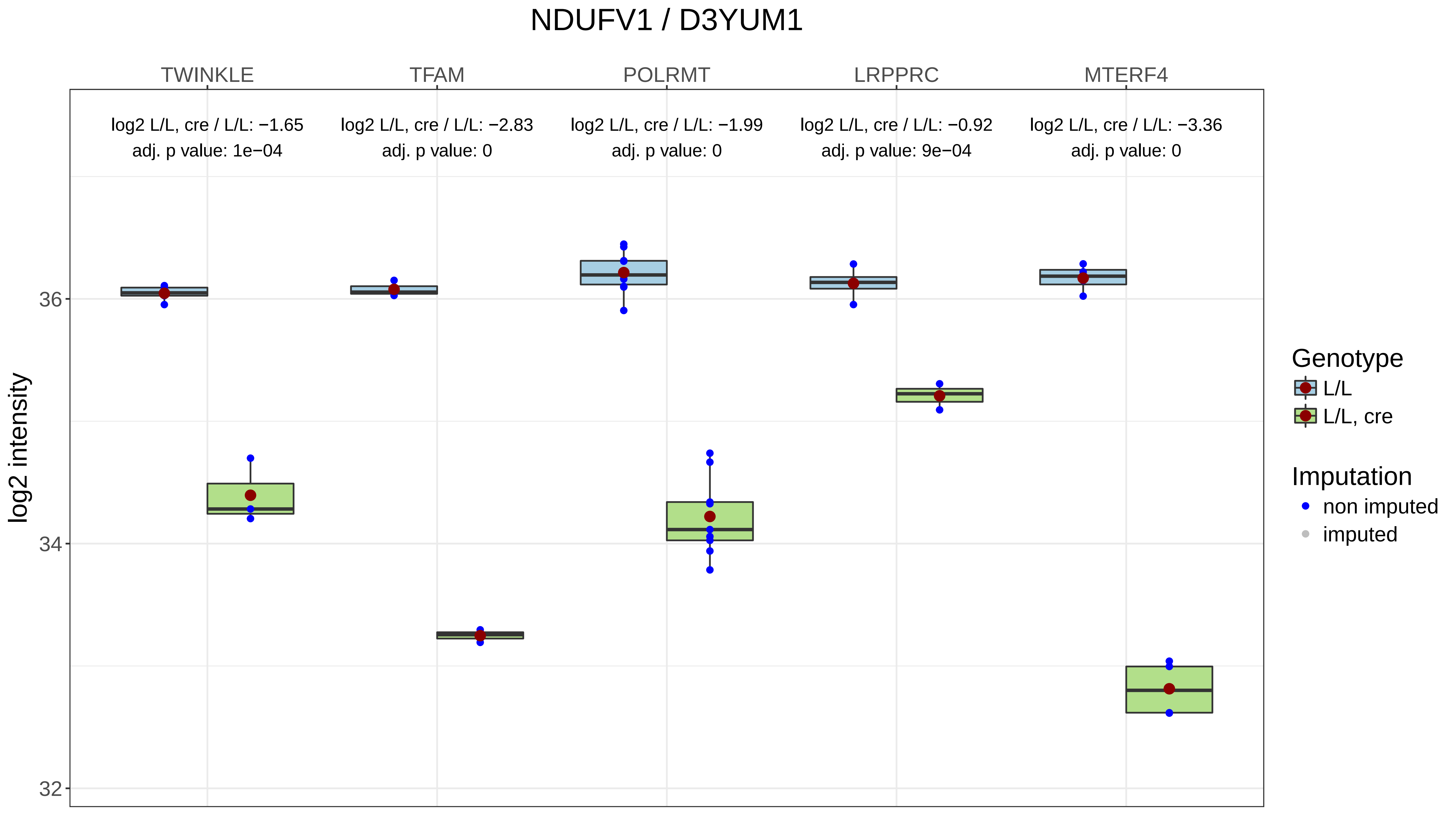
NDUFV1 / D3YUM1
TWINKLE
TFAM
POLRMT
LRPPRC
MTERF4
log2 L/L, cre / L/L: −1.65
adj. p value: 1e−04
log2 L/L, cre / L/L: −2.83
adj. p value: 0
log2 L/L, cre / L/L: −1.99
adj. p value: 0
log2 L/L, cre / L/L: −0.92
adj. p value: 9e−04
log2 L/L, cre / L/L: −3.36
adj. p value: 0
log2 intensity
36
34
32
Genotype
L/L
L/L, cre
Imputation
non imputed
imputed

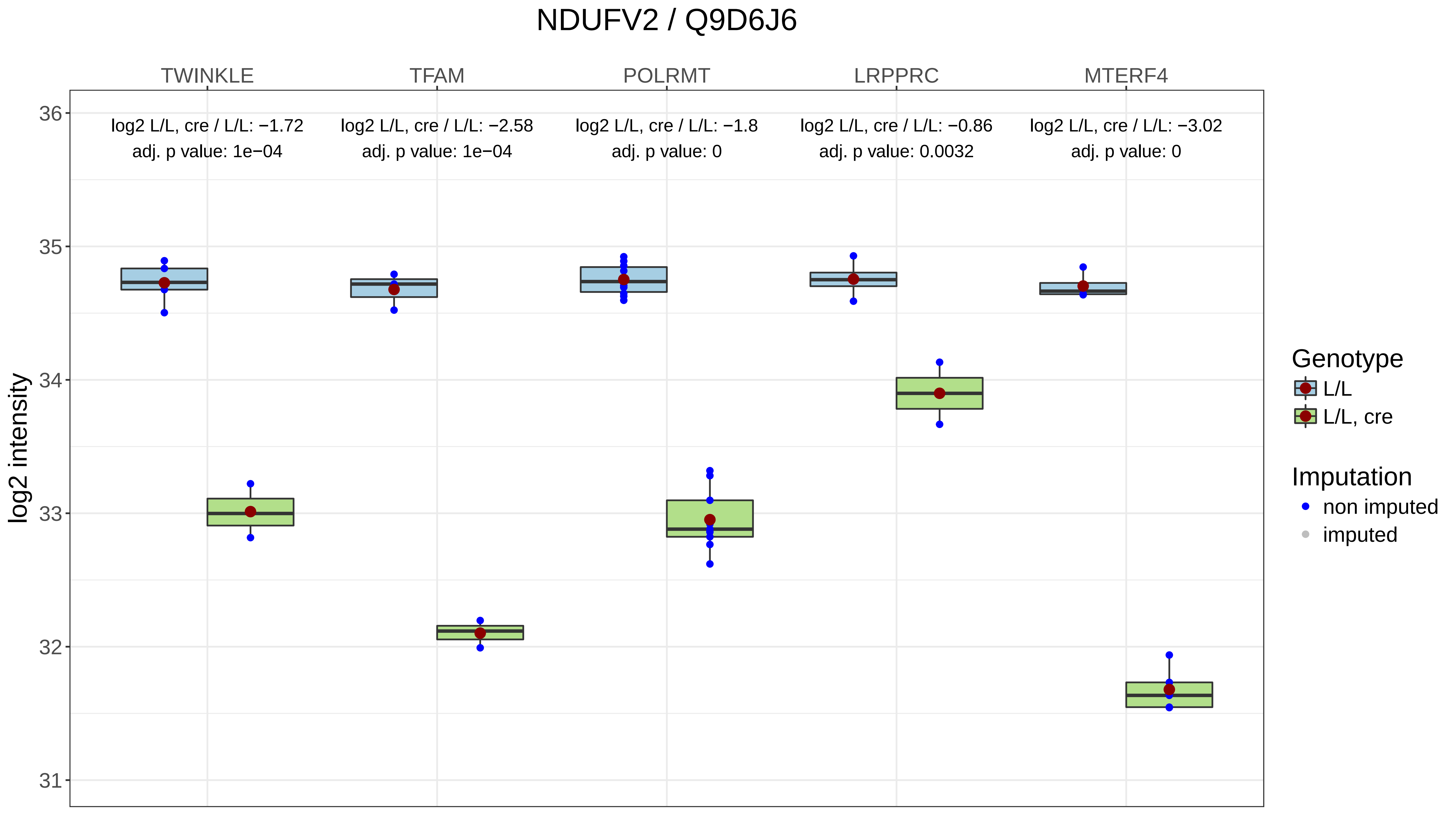
NDUFV2 / Q9D6J6
TWINKLE
TFAM
POLRMT
LRPPRC
MTERF4
log2 L/L, cre / L/L: −1.72
adj. p value: 1e−04
log2 L/L, cre / L/L: −2.58
adj. p value: 1e−04
log2 L/L, cre / L/L: −1.8
adj. p value: 0
log2 L/L, cre / L/L: −0.86
adj. p value: 0.0032
log2 L/L, cre / L/L: −3.02
adj. p value: 0
log2 intensity
36
35
34
33
32
31
Genotype
L/L
L/L, cre
Imputation
non imputed
imputed

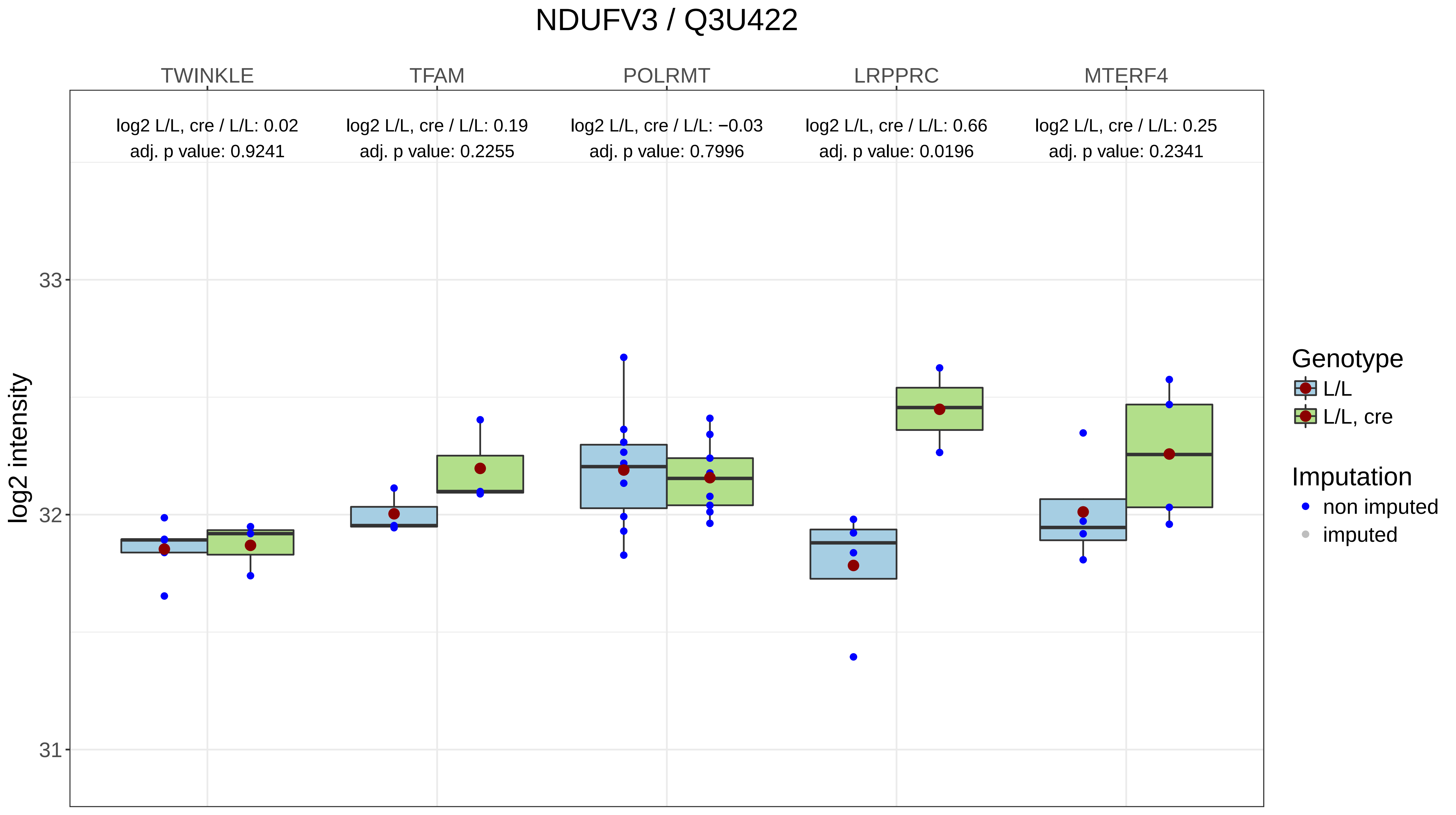
NDUFV3 / Q3U422
TWINKLE
TFAM
POLRMT
LRPPRC
MTERF4
log2 L/L, cre / L/L: 0.02
adj. p value: 0.9241
log2 L/L, cre / L/L: 0.19
adj. p value: 0.2255
log2 L/L, cre / L/L: −0.03
adj. p value: 0.7996
log2 L/L, cre / L/L: 0.66
adj. p value: 0.0196
log2 L/L, cre / L/L: 0.25
adj. p value: 0.2341
log2 intensity
33
32
31
Genotype
L/L
L/L, cre
Imputation
non imputed
imputed

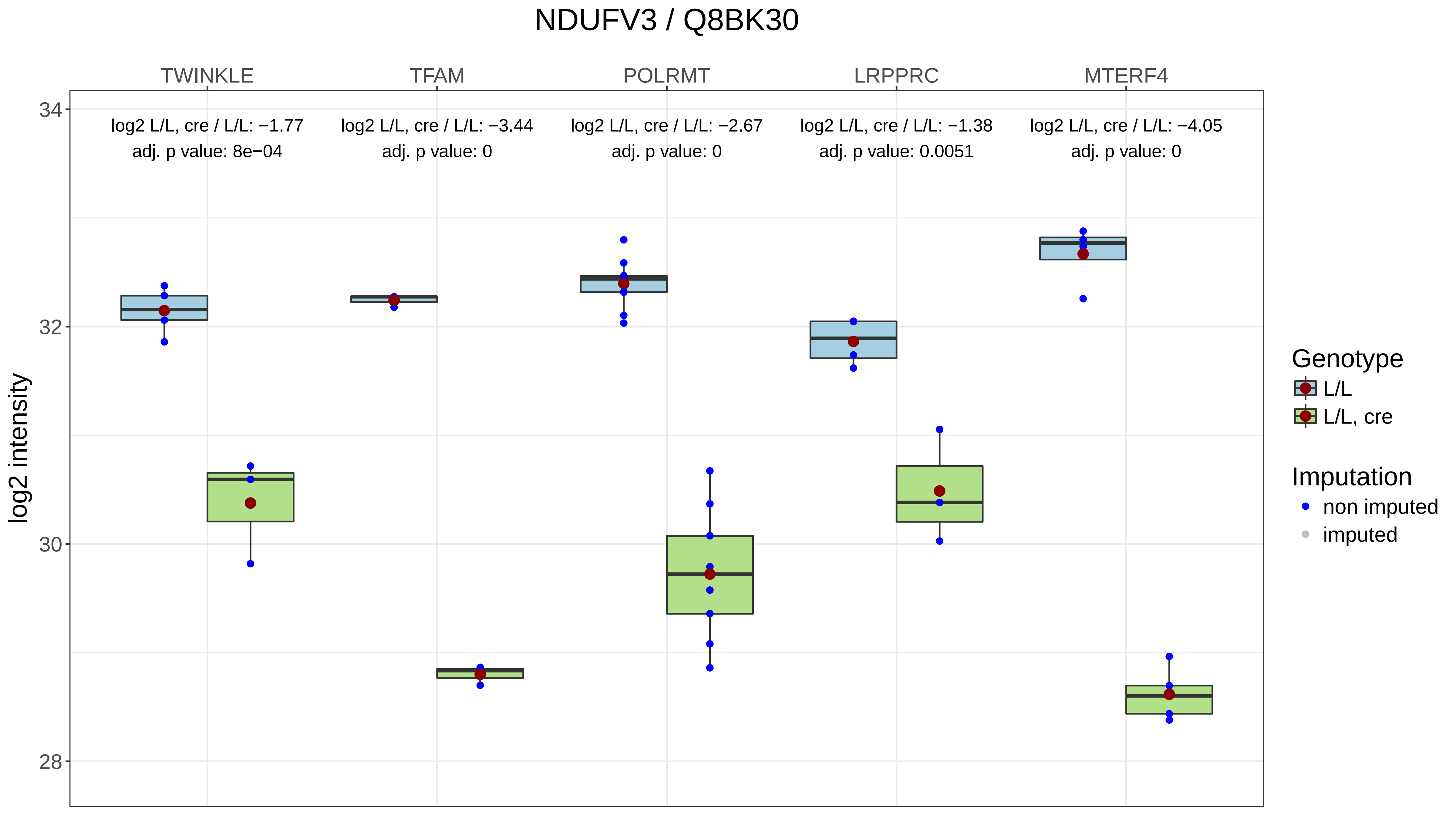

NDUFV3 / Q8BK30
TWINKLE
TFAM
POLRMT
LRPPRC
MTERF4
log2 L/L, cre / L/L: −1.77
adj. p value: 8e−04
log2 L/L, cre / L/L: −3.44
adj. p value: 0
log2 L/L, cre / L/L: −2.67
adj. p value: 0
log2 L/L, cre / L/L: −1.38
adj. p value: 0.0051
log2 L/L, cre / L/L: −4.05
adj. p value: 0
log2 intensity
34
32
30
28
Genotype
L/L
L/L, cre
Imputation
non imputed
imputed

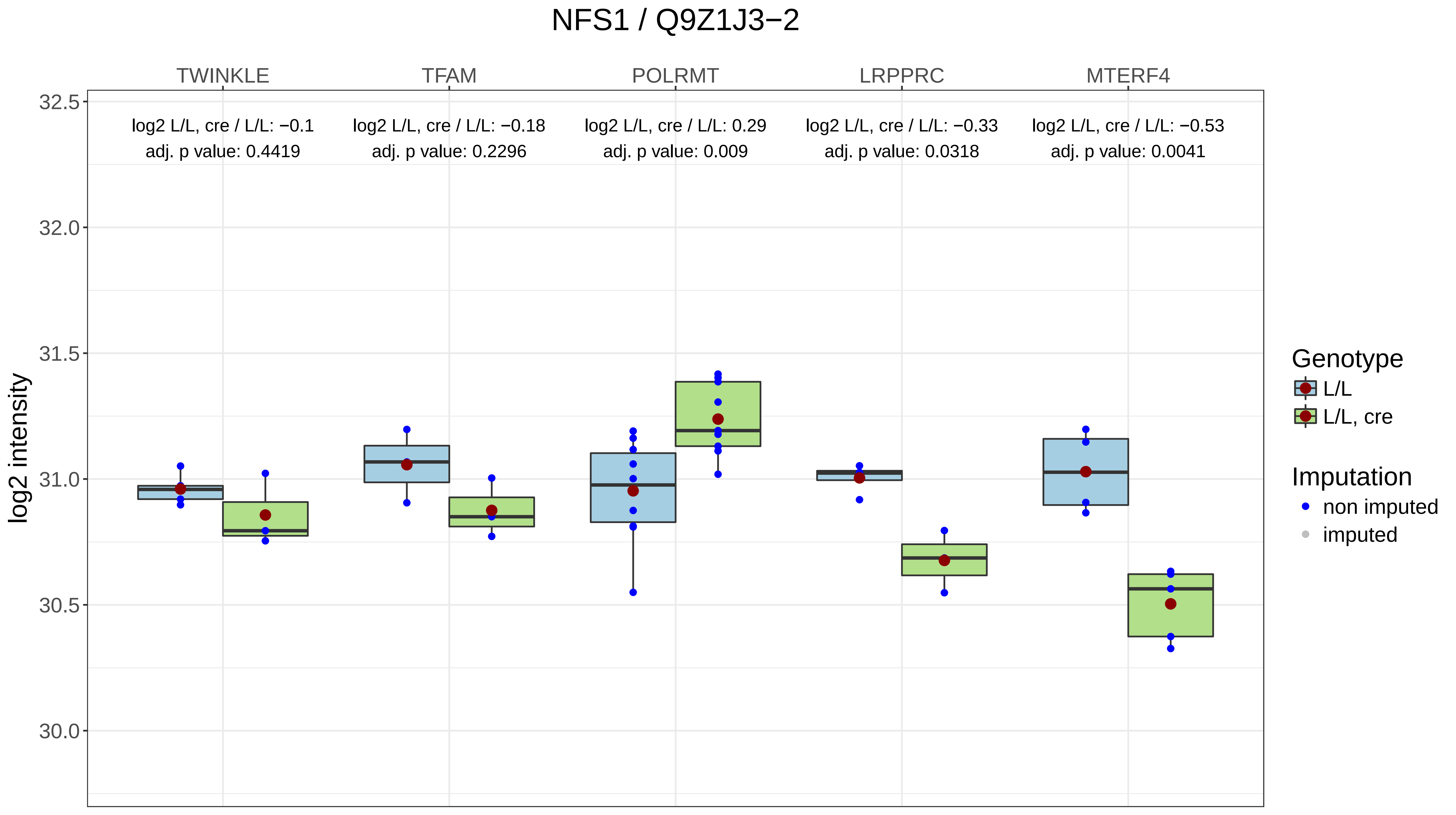
NFS1 / Q9Z1J3−2
TWINKLE
TFAM
POLRMT
LRPPRC
MTERF4
log2 L/L, cre / L/L: −0.1
adj. p value: 0.4419
log2 L/L, cre / L/L: −0.18
adj. p value: 0.2296
log2 L/L, cre / L/L: 0.29
adj. p value: 0.009
log2 L/L, cre / L/L: −0.33
adj. p value: 0.0318
log2 L/L, cre / L/L: −0.53
adj. p value: 0.0041
log2 intensity
32.5
32.0
31.5
31.0
30.5
30.0
Genotype
L/L
L/L, cre
Imputation
non imputed
imputed

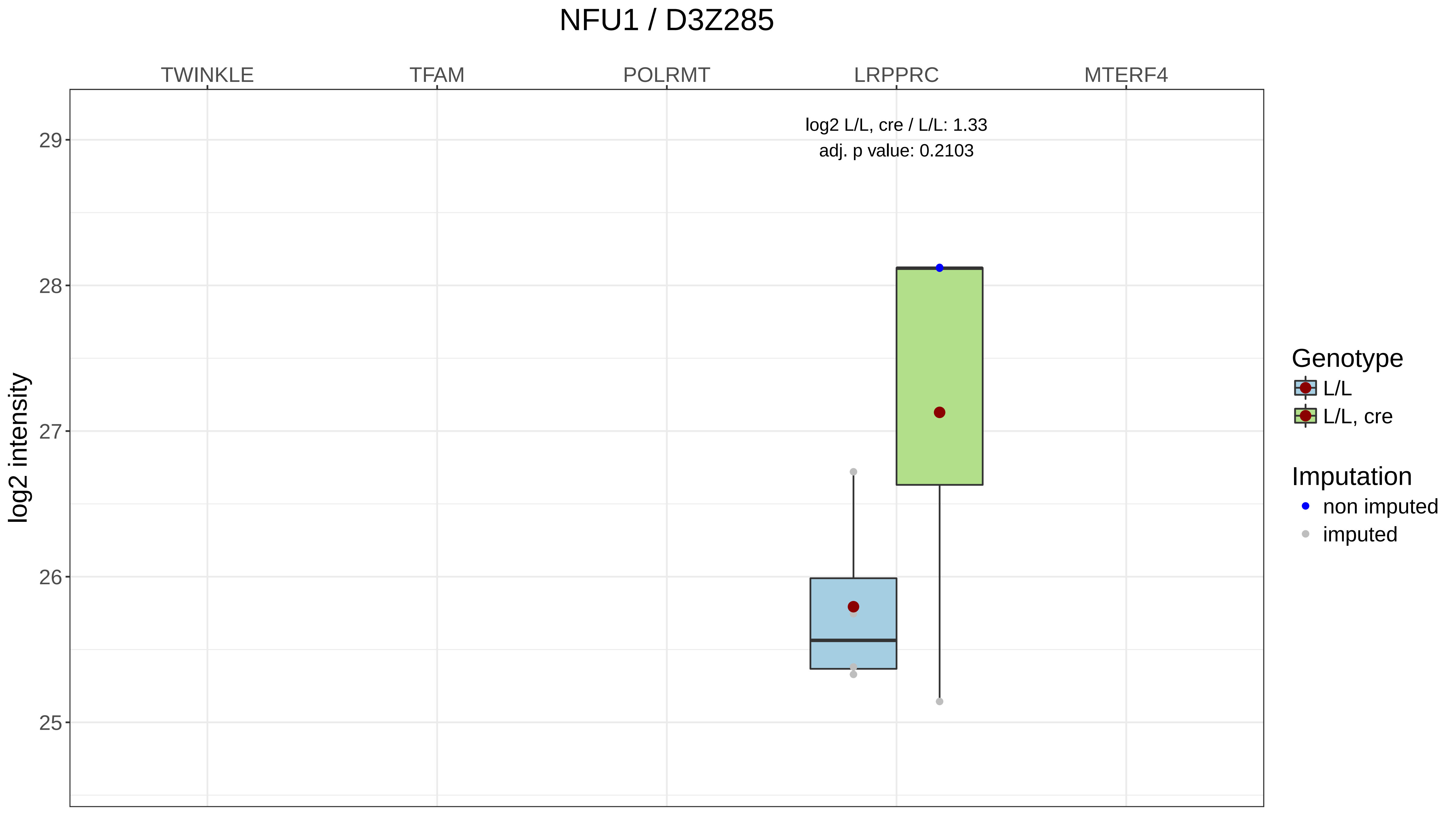
NFU1 / D3Z285
TWINKLE
TFAM
POLRMT
LRPPRC
MTERF4
log2 L/L, cre / L/L: 1.33
adj. p value: 0.2103
log2 intensity
29
28
27
26
25
Genotype
L/L
L/L, cre
Imputation
non imputed
imputed

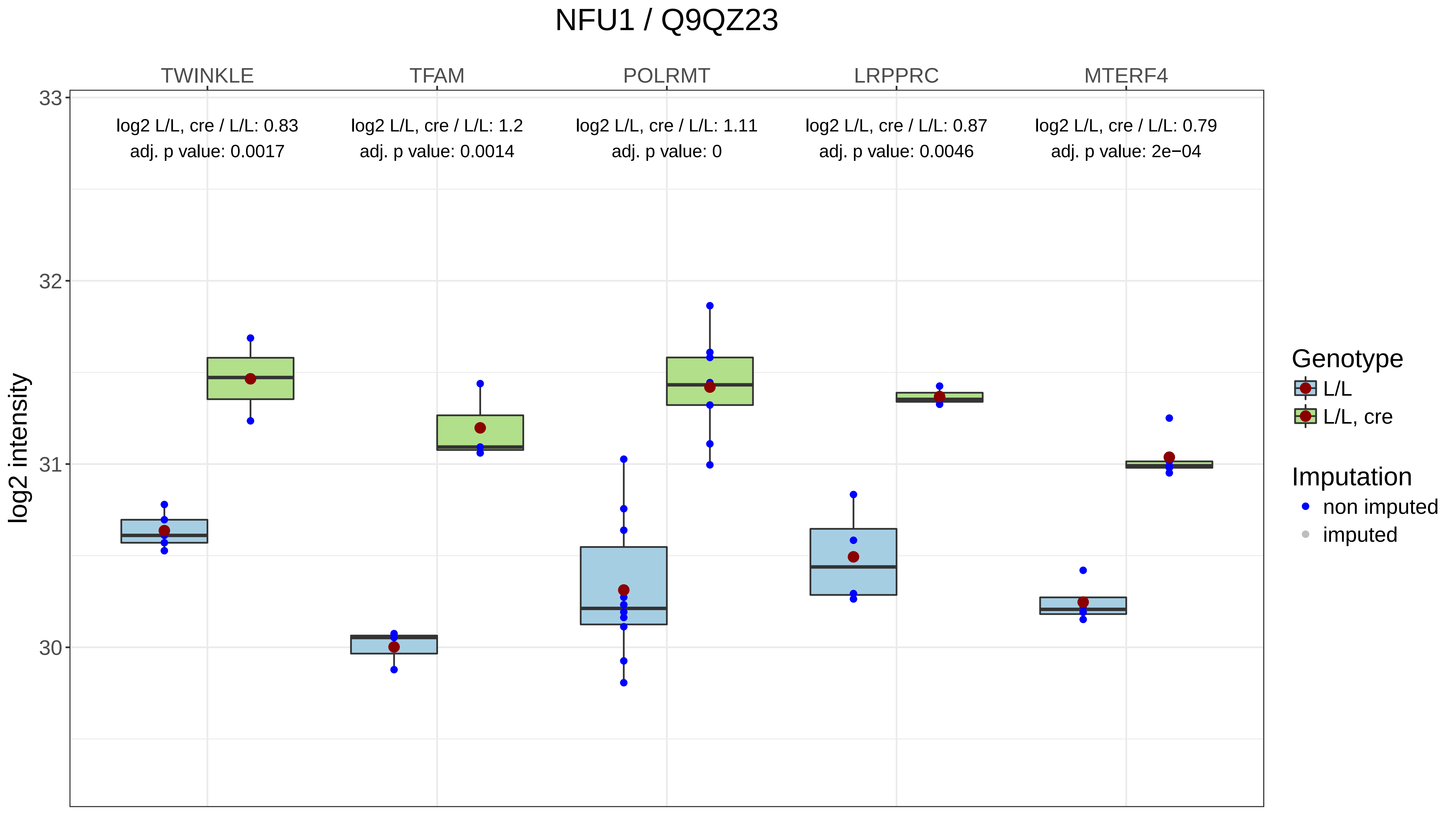

NFU1 / Q9QZ23
TWINKLE
TFAM
POLRMT
LRPPRC
MTERF4
log2 L/L, cre / L/L: 0.83
adj. p value: 0.0017
log2 L/L, cre / L/L: 1.2
adj. p value: 0.0014
log2 L/L, cre / L/L: 1.11
adj. p value: 0
log2 L/L, cre / L/L: 0.87
adj. p value: 0.0046
log2 L/L, cre / L/L: 0.79
adj. p value: 2e−04
log2 intensity
33
32
31
30
Genotype
L/L
L/L, cre
Imputation
non imputed
imputed

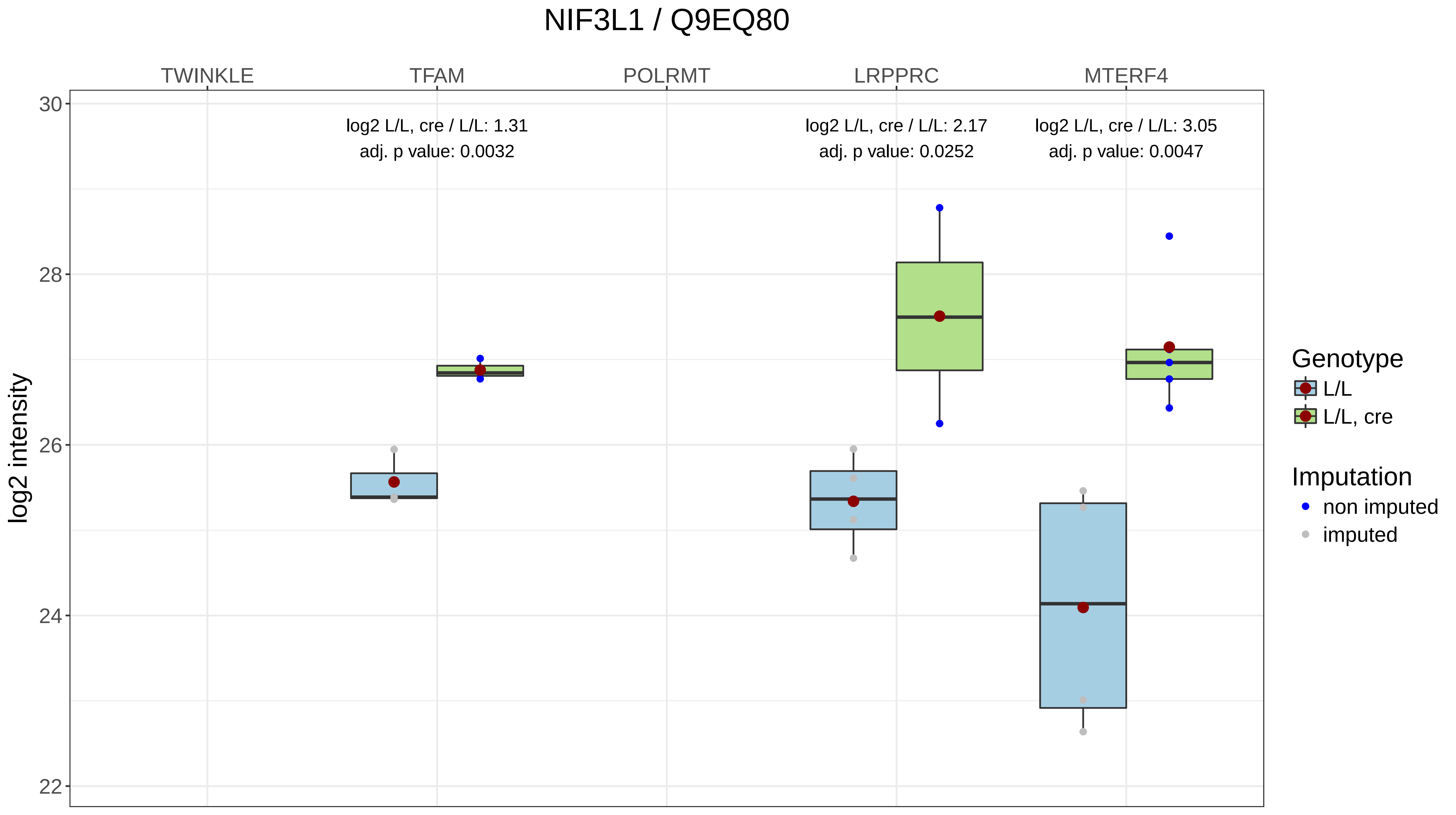
NIF3L1 / Q9EQ80
TWINKLE
TFAM
POLRMT
LRPPRC
MTERF4
log2 L/L, cre / L/L: 1.31
adj. p value: 0.0032
log2 L/L, cre / L/L: 2.17
adj. p value: 0.0252
log2 L/L, cre / L/L: 3.05
adj. p value: 0.0047
30
28
26
24
22
log2 intensity
Genotype
L/L
L/L, cre
Imputation
non imputed
imputed

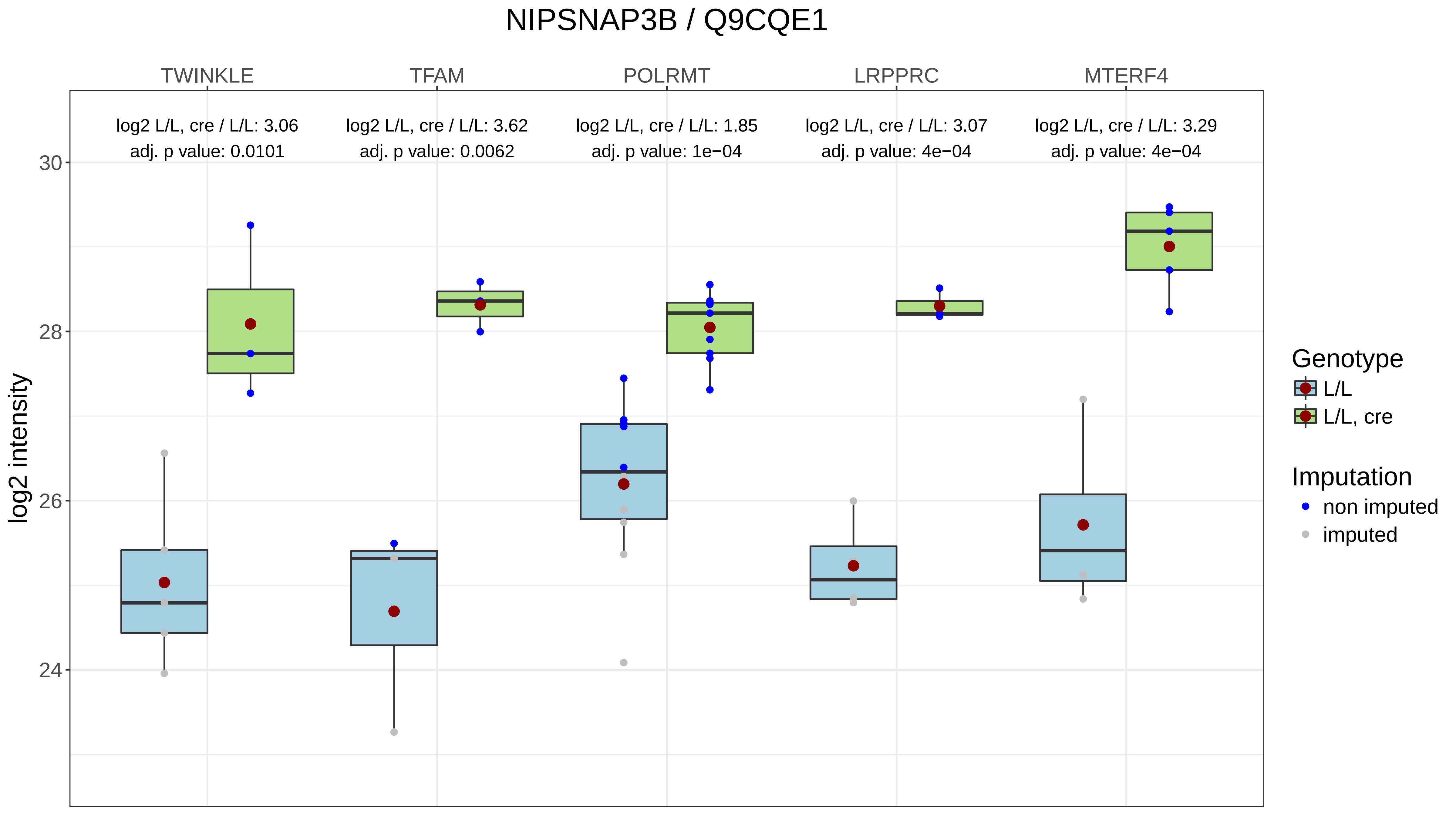
NIPSNAP3B / Q9CQE1
TWINKLE
TFAM
POLRMT
LRPPRC
MTERF4
log2 L/L, cre / L/L: 3.06
adj. p value: 0.0101
log2 L/L, cre / L/L: 3.62
adj. p value: 0.0062
log2 L/L, cre / L/L: 1.85
adj. p value: 1e−04
log2 L/L, cre / L/L: 3.07
adj. p value: 4e−04
log2 L/L, cre / L/L: 3.29
adj. p value: 4e−04
log2 intensity
30
28
26
24
Genotype
L/L
L/L, cre
Imputation
non imputed
imputed

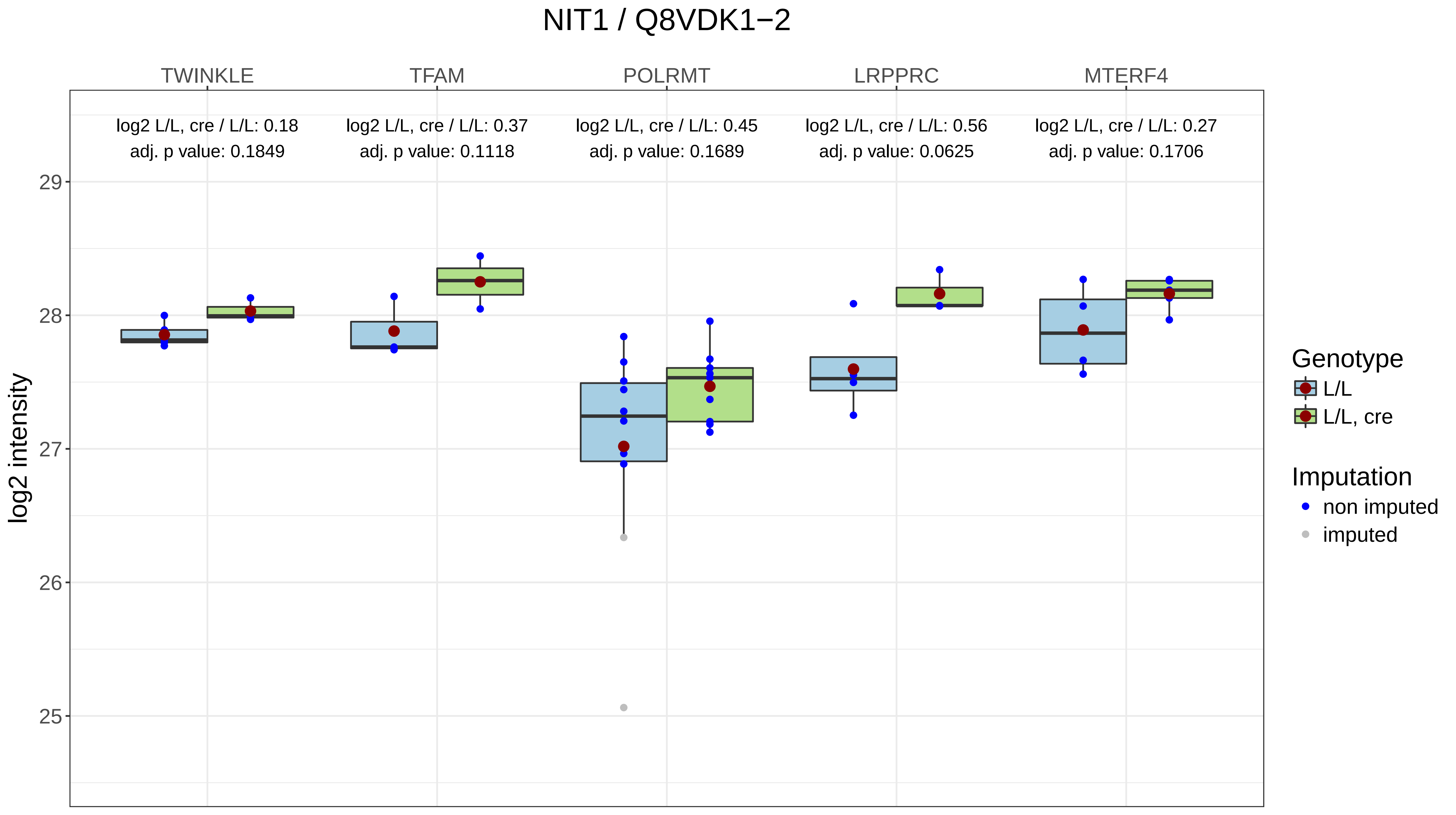
NIT1 / Q8VDK1−2
TWINKLE
TFAM
POLRMT
LRPPRC
MTERF4
log2 L/L, cre / L/L: 0.18
adj. p value: 0.1849
log2 L/L, cre / L/L: 0.37
adj. p value: 0.1118
log2 L/L, cre / L/L: 0.45
adj. p value: 0.1689
log2 L/L, cre / L/L: 0.56
adj. p value: 0.0625
log2 L/L, cre / L/L: 0.27
adj. p value: 0.1706
log2 intensity
29
28
27
26
25
Genotype
L/L
L/L, cre
Imputation
non imputed
imputed

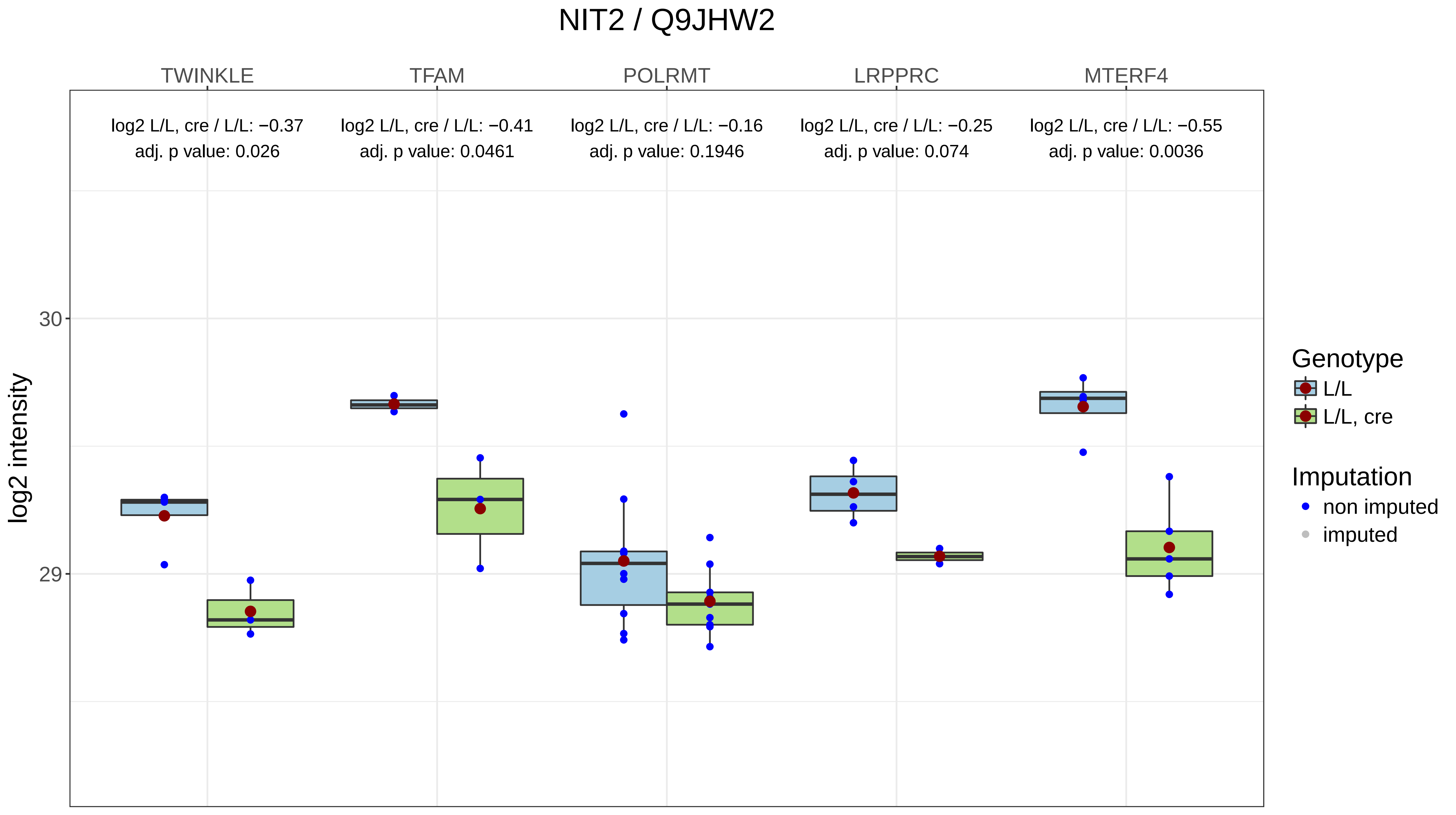
NIT2 / Q9JHW2
TWINKLE
TFAM
POLRMT
LRPPRC
MTERF4
log2 L/L, cre / L/L: −0.37
adj. p value: 0.026
log2 L/L, cre / L/L: −0.41
adj. p value: 0.0461
log2 L/L, cre / L/L: −0.16
adj. p value: 0.1946
log2 L/L, cre / L/L: −0.25
adj. p value: 0.074
log2 L/L, cre / L/L: −0.55
adj. p value: 0.0036
log2 intensity
30
29
Genotype
L/L
L/L, cre
Imputation
non imputed
imputed

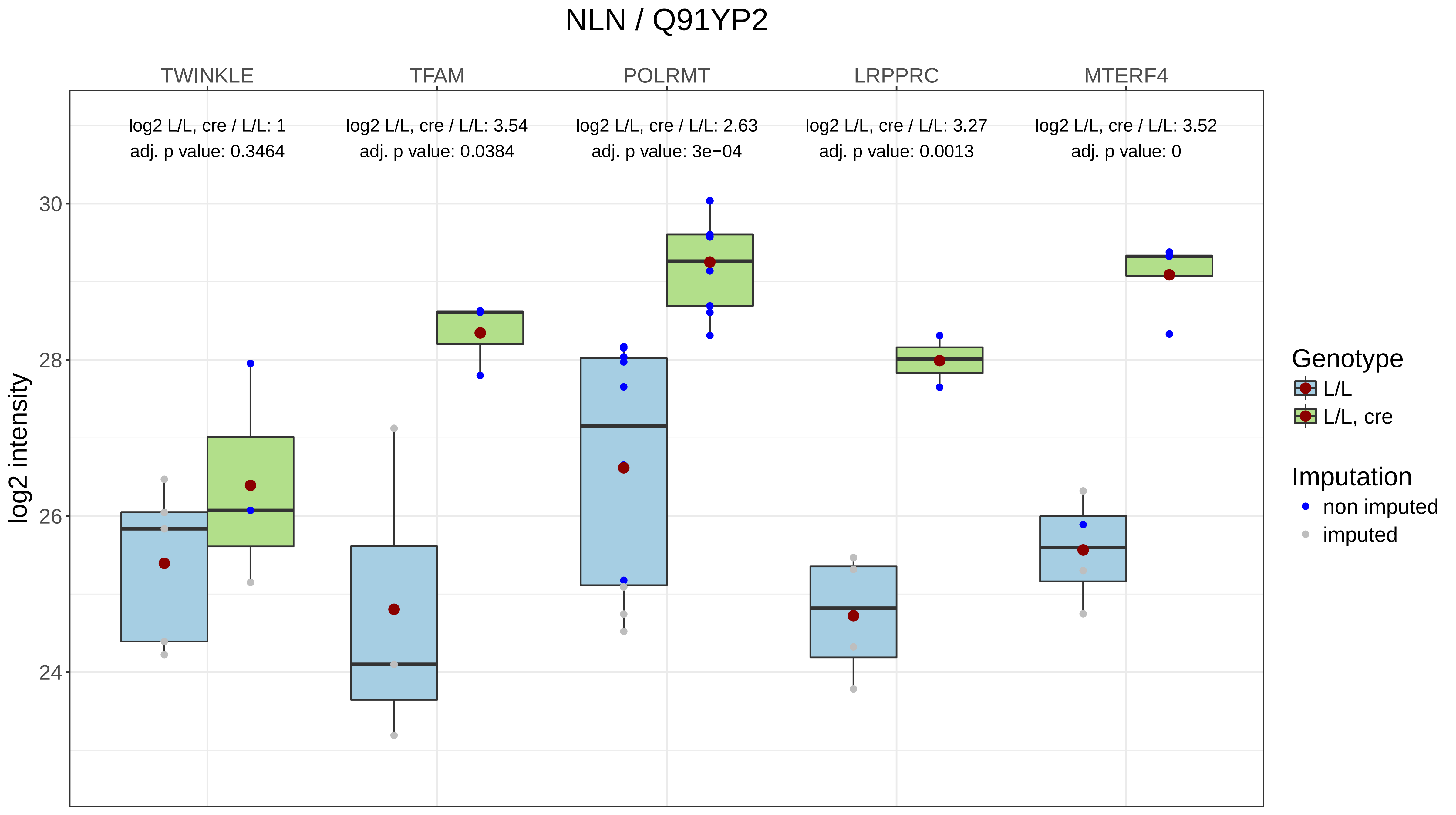
NLN / Q91YP2
TWINKLE
TFAM
POLRMT
LRPPRC
MTERF4
log2 L/L, cre / L/L: 1
adj. p value: 0.3464
log2 L/L, cre / L/L: 3.54
adj. p value: 0.0384
log2 L/L, cre / L/L: 2.63
adj. p value: 3e−04
log2 L/L, cre / L/L: 3.27
adj. p value: 0.0013
log2 L/L, cre / L/L: 3.52
adj. p value: 0
log2 intensity
30
28
26
24
Genotype
L/L
L/L, cre
Imputation
non imputed
imputed

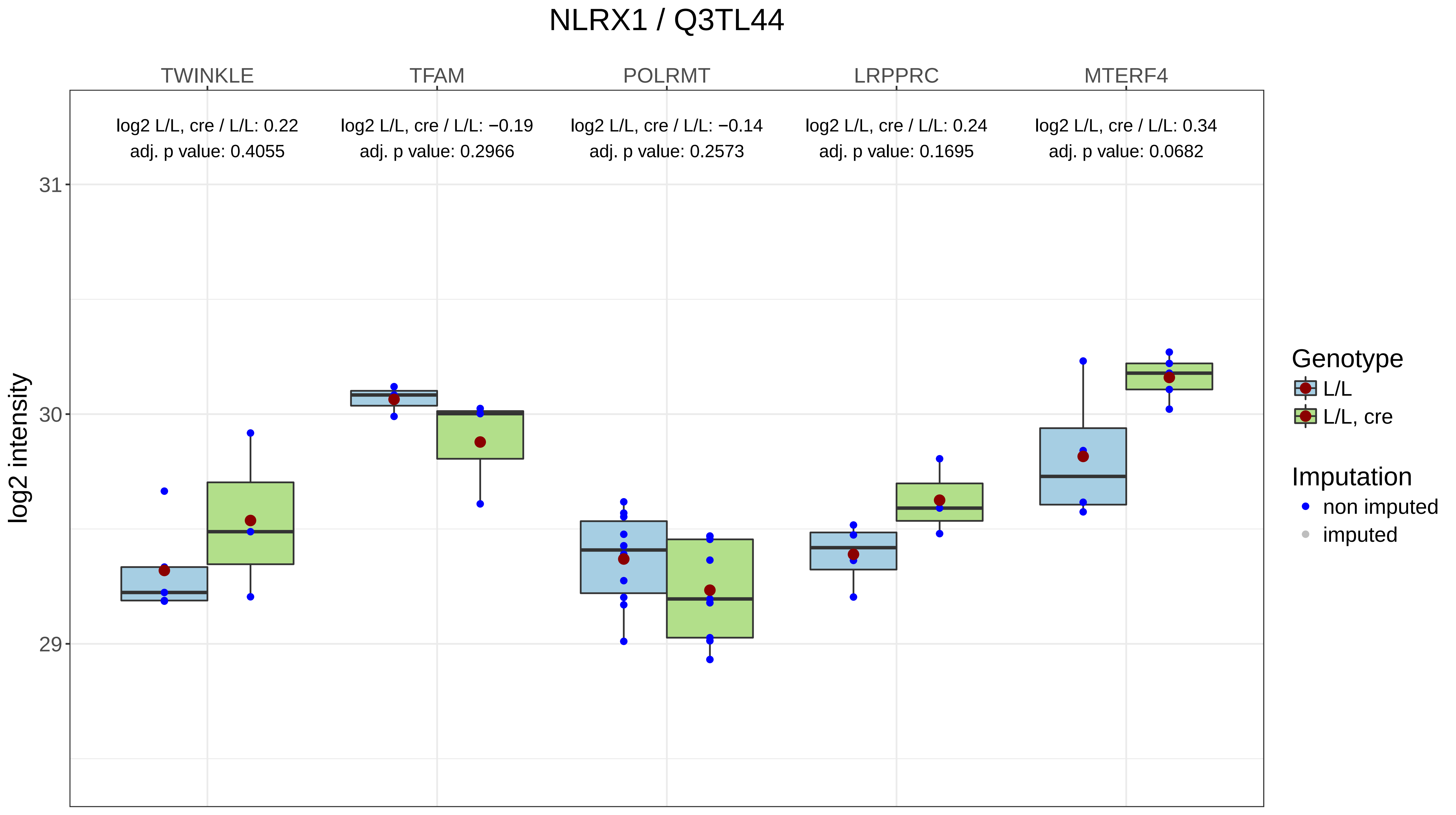
NLRX1 / Q3TL44
TWINKLE
TFAM
POLRMT
LRPPRC
MTERF4
log2 L/L, cre / L/L: 0.22
adj. p value: 0.4055
log2 L/L, cre / L/L: −0.19
adj. p value: 0.2966
log2 L/L, cre / L/L: −0.14
adj. p value: 0.2573
log2 L/L, cre / L/L: 0.24
adj. p value: 0.1695
log2 L/L, cre / L/L: 0.34
adj. p value: 0.0682
log2 intensity
31
30
29
Genotype
L/L
L/L, cre
Imputation
non imputed
imputed

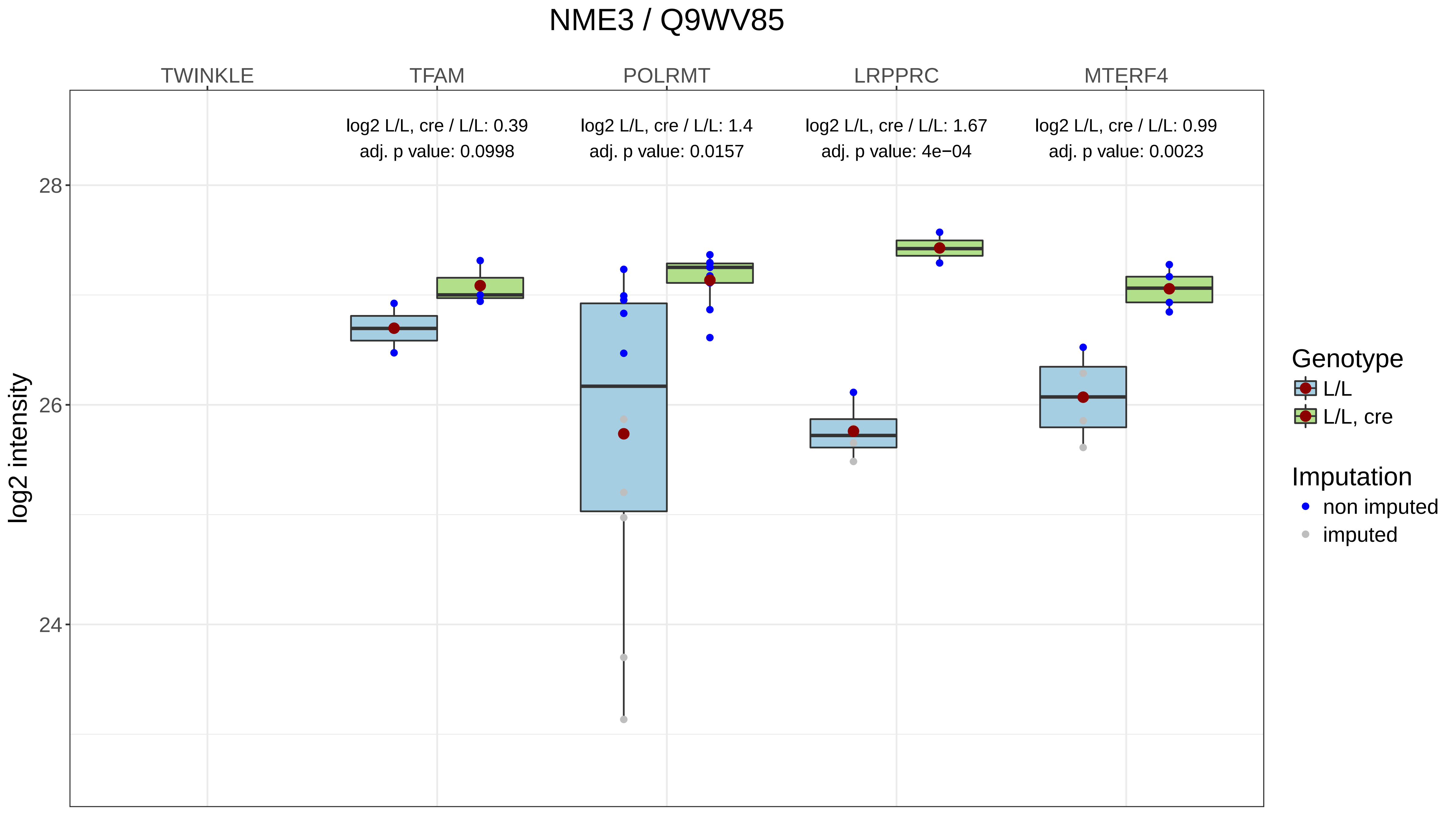
NME3 / Q9WV85
TWINKLE
TFAM
POLRMT
LRPPRC
MTERF4
log2 L/L, cre / L/L: 0.39
adj. p value: 0.0998
log2 L/L, cre / L/L: 1.4
adj. p value: 0.0157
log2 L/L, cre / L/L: 1.67
adj. p value: 4e−04
log2 L/L, cre / L/L: 0.99
adj. p value: 0.0023
28
26
24
log2 intensity
Genotype
L/L
L/L, cre
Imputation
non imputed
imputed

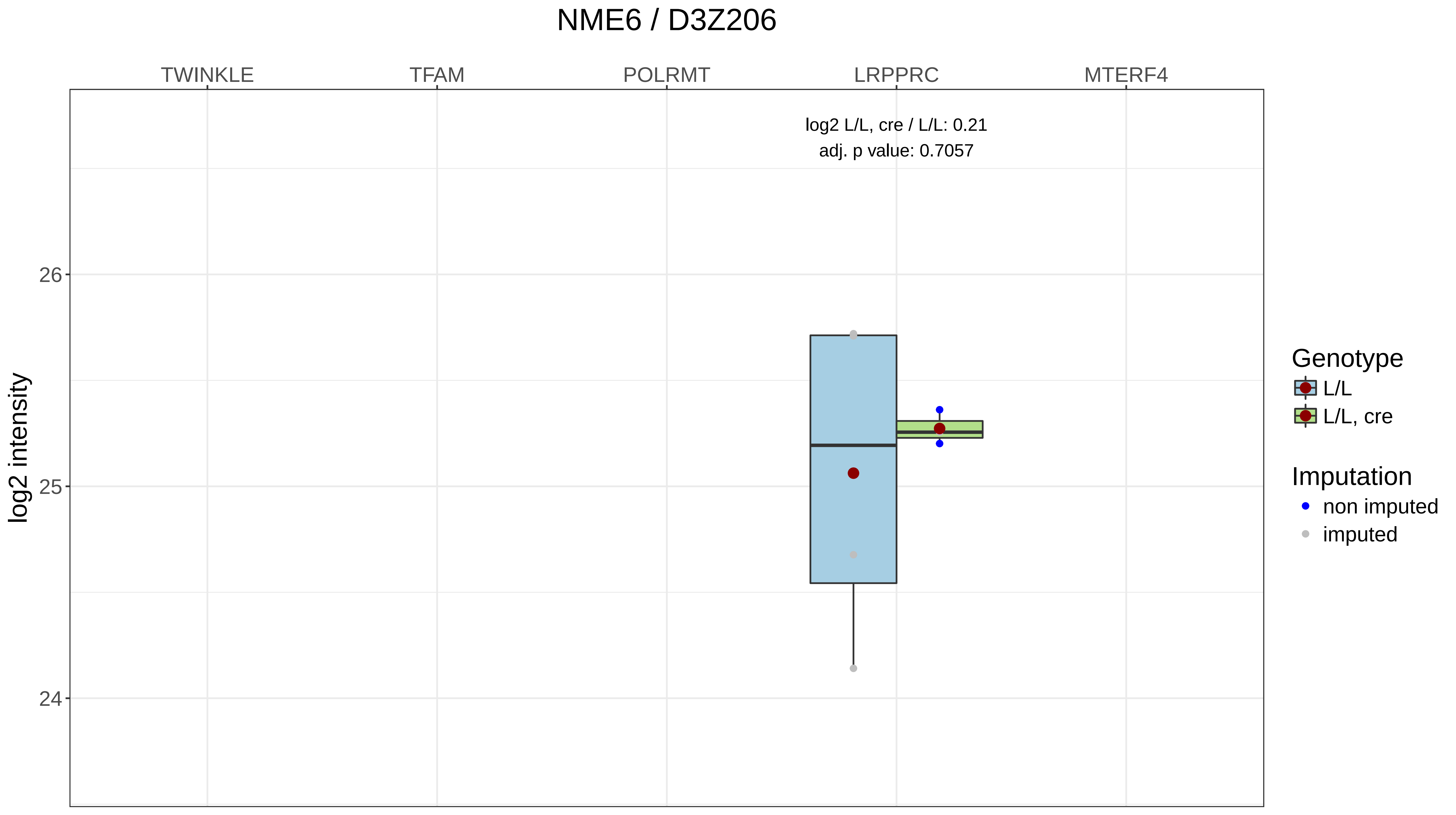
NME6 / D3Z206
TWINKLE
TFAM
POLRMT
LRPPRC
MTERF4
log2 L/L, cre / L/L: 0.21
adj. p value: 0.7057
log2 intensity
26
25
24
Genotype
L/L
L/L, cre
Imputation
non imputed
imputed

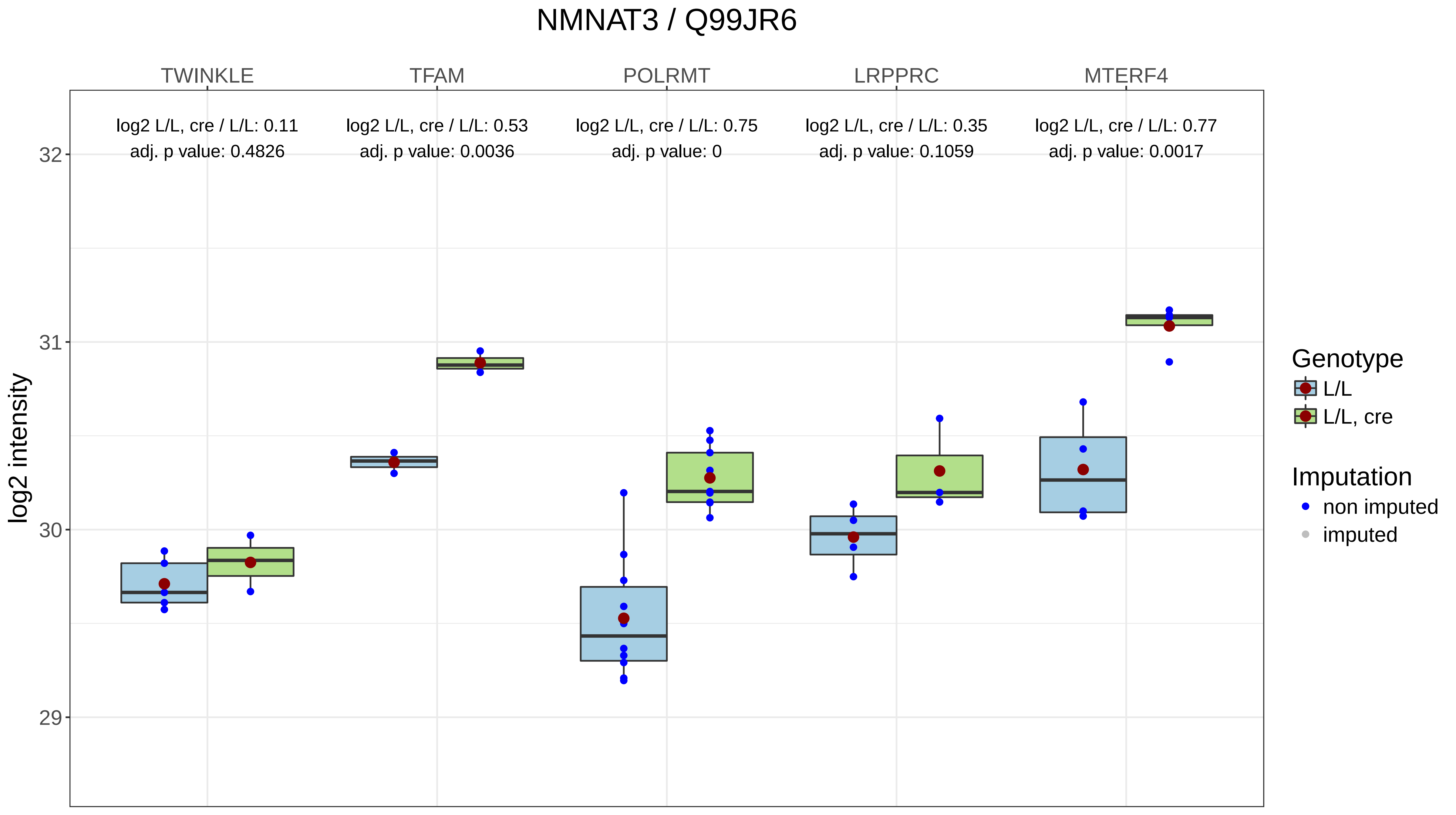

NMNAT3 / Q99JR6
TWINKLE
TFAM
POLRMT
LRPPRC
MTERF4
log2 L/L, cre / L/L: 0.11
adj. p value: 0.4826
log2 L/L, cre / L/L: 0.53
adj. p value: 0.0036
log2 L/L, cre / L/L: 0.75
adj. p value: 0
log2 L/L, cre / L/L: 0.35
adj. p value: 0.1059
log2 L/L, cre / L/L: 0.77
adj. p value: 0.0017
log2 intensity
32
31
30
29
Genotype
L/L
L/L, cre
Imputation
non imputed
imputed

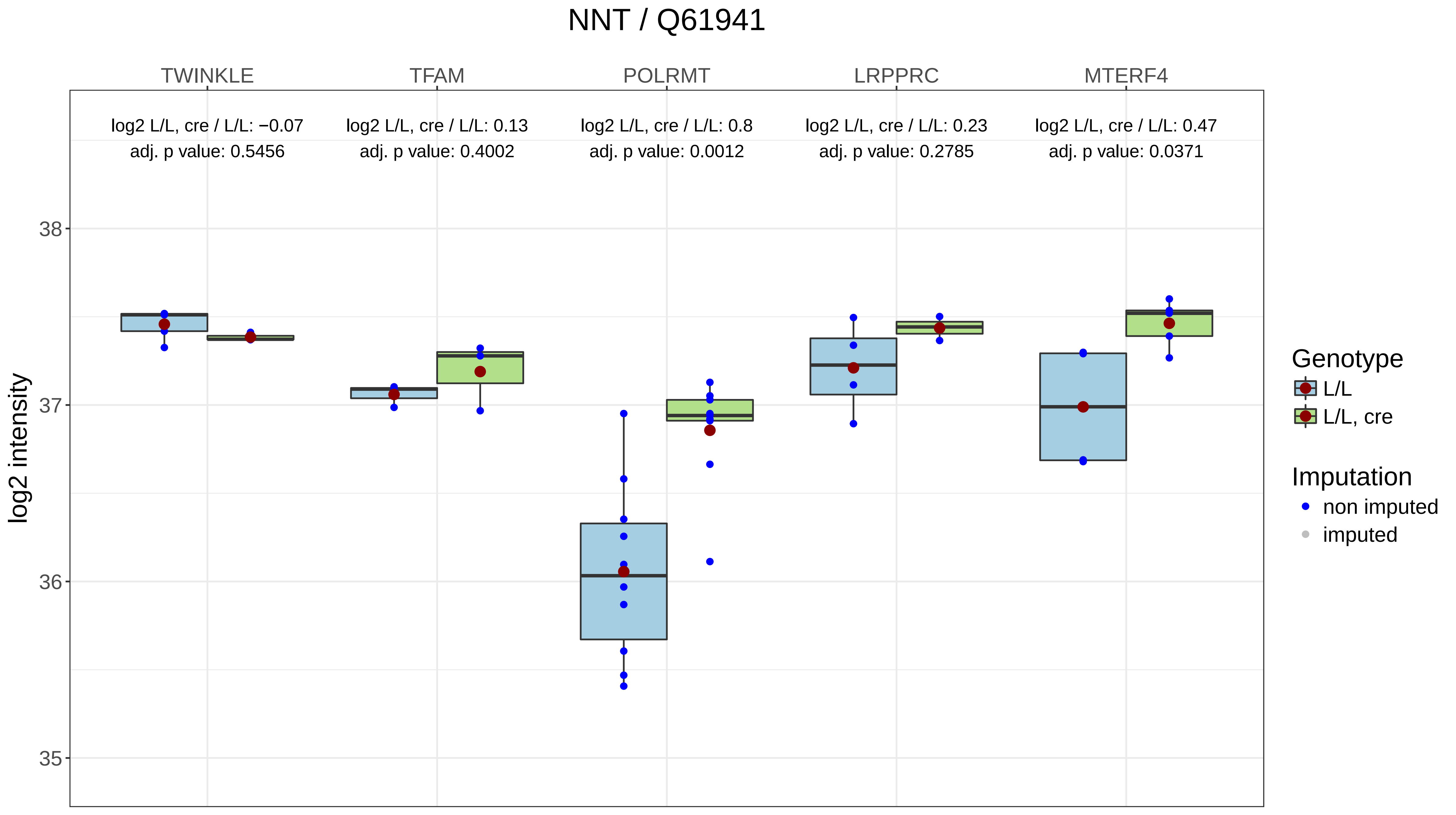

NNT / Q61941
TWINKLE
TFAM
POLRMT
LRPPRC
MTERF4
log2 L/L, cre / L/L: −0.07
adj. p value: 0.5456
log2 L/L, cre / L/L: 0.13
adj. p value: 0.4002
log2 L/L, cre / L/L: 0.8
adj. p value: 0.0012
log2 L/L, cre / L/L: 0.23
adj. p value: 0.2785
log2 L/L, cre / L/L: 0.47
adj. p value: 0.0371
log2 intensity
38
37
36
35
Genotype
L/L
L/L, cre
Imputation
non imputed
imputed

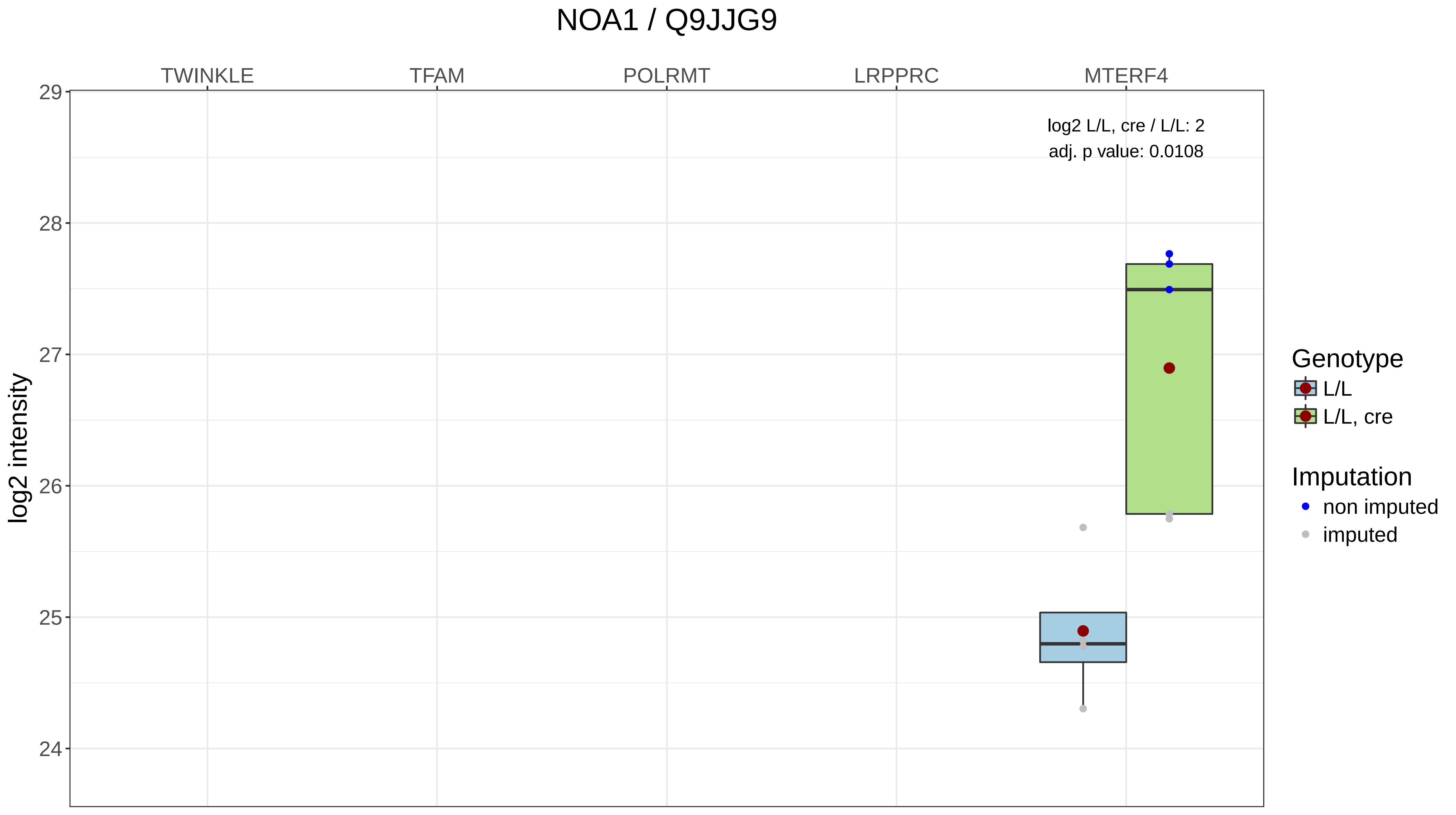
NOA1 / Q9JJG9
TWINKLE
TFAM
POLRMT
LRPPRC
MTERF4
log2 L/L, cre / L/L: 2
adj. p value: 0.0108
log2 intensity
29
28
27
26
25
24
Genotype
L/L
L/L, cre
Imputation
non imputed
imputed

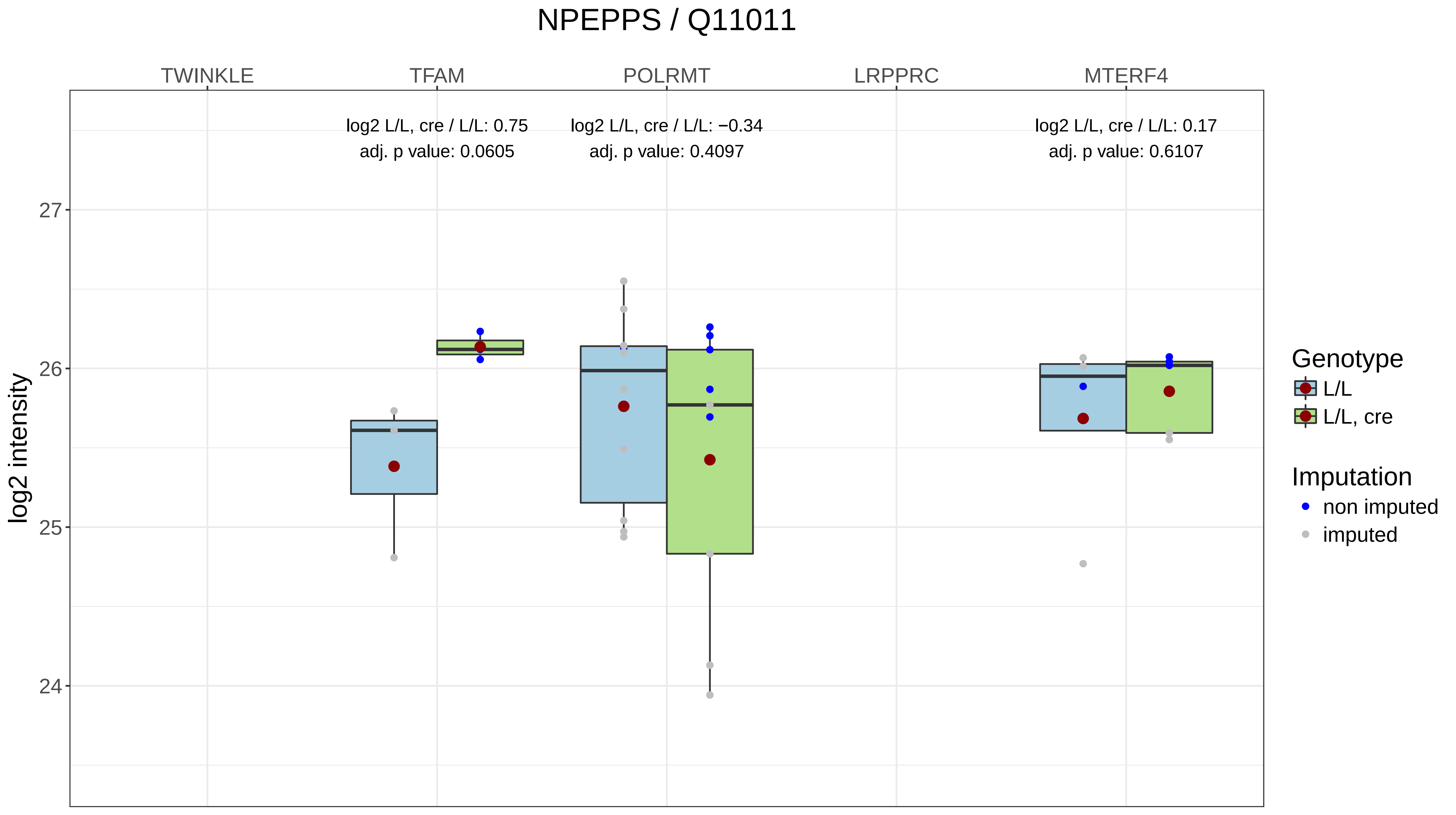

NPEPPS / Q11011
TWINKLE
TFAM
POLRMT
LRPPRC
MTERF4
log2 L/L, cre / L/L: 0.75
adj. p value: 0.0605
log2 L/L, cre / L/L: −0.34
adj. p value: 0.4097
log2 L/L, cre / L/L: 0.17
adj. p value: 0.6107
log2 intensity
27
26
25
24
Genotype
L/L
L/L, cre
Imputation
non imputed
imputed

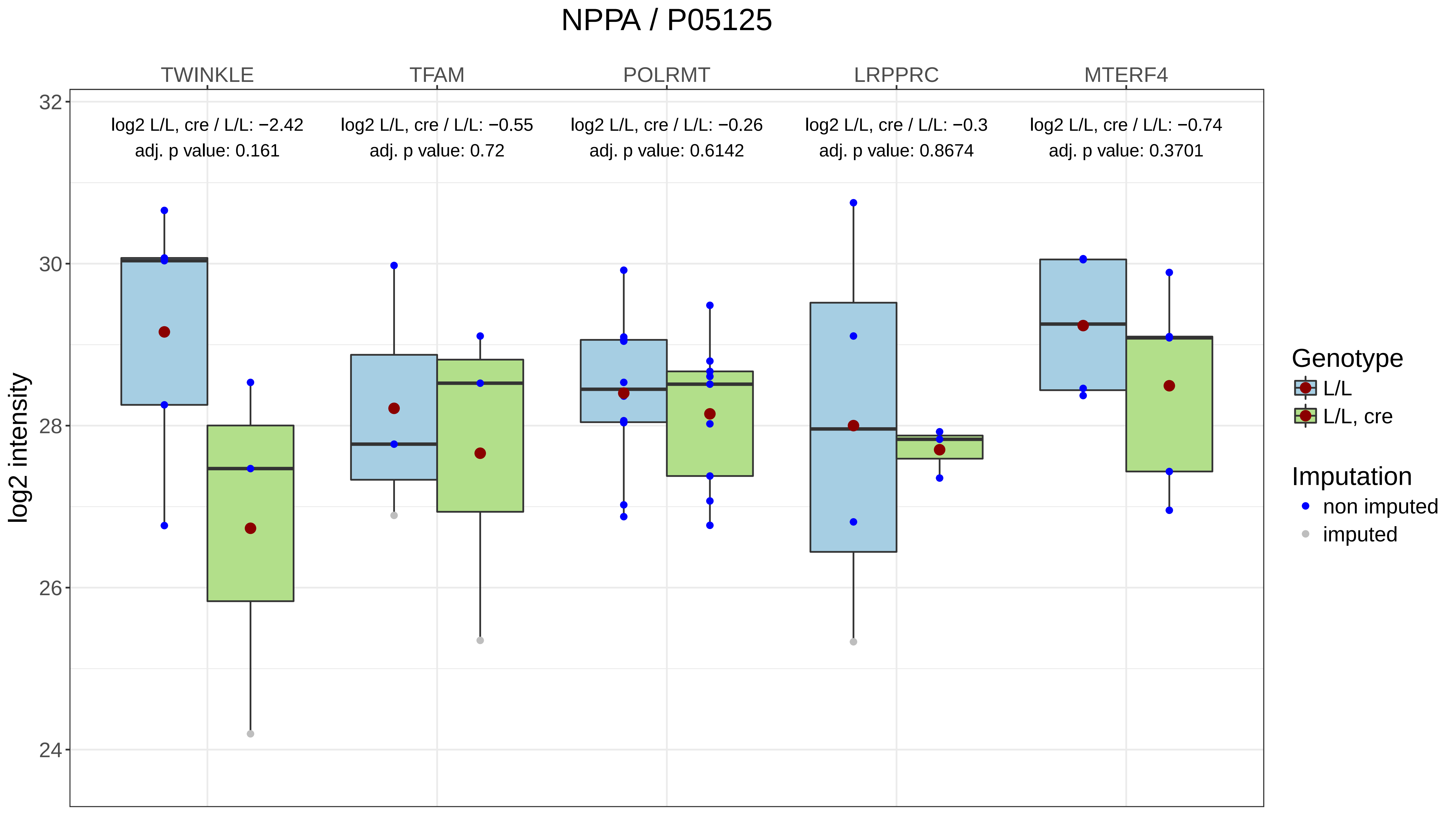

NPPA / P05125
TWINKLE
TFAM
POLRMT
LRPPRC
MTERF4
log2 L/L, cre / L/L: −2.42
adj. p value: 0.161
log2 L/L, cre / L/L: −0.55
adj. p value: 0.72
log2 L/L, cre / L/L: −0.26
adj. p value: 0.6142
log2 L/L, cre / L/L: −0.3
adj. p value: 0.8674
log2 L/L, cre / L/L: −0.74
adj. p value: 0.3701
log2 intensity
32
30
28
26
24
Genotype
L/L
L/L, cre
Imputation
non imputed
imputed

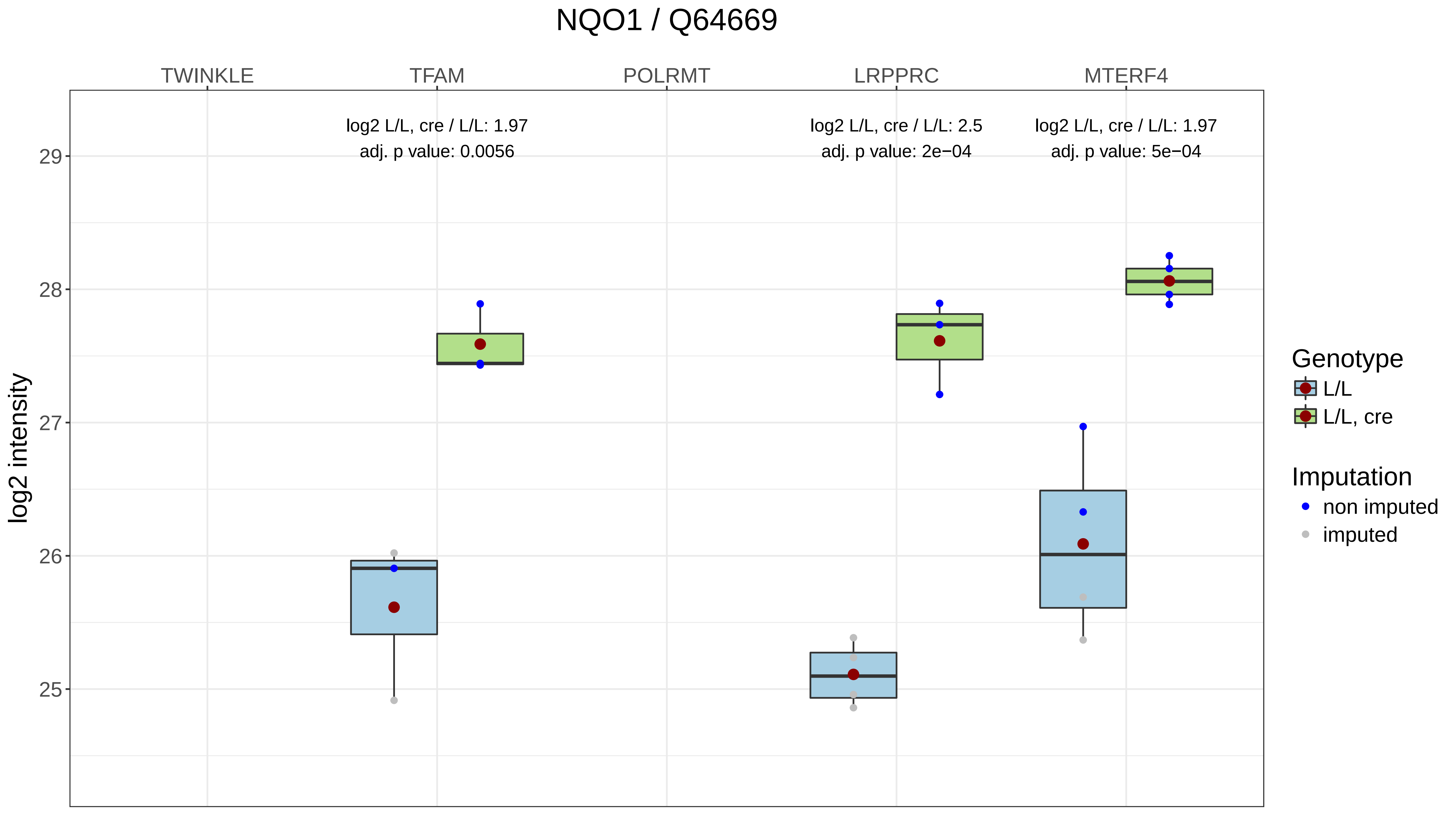
NQO1 / Q64669
TWINKLE
TFAM
POLRMT
LRPPRC
MTERF4
log2 L/L, cre / L/L: 1.97
adj. p value: 0.0056
log2 L/L, cre / L/L: 2.5
adj. p value: 2e−04
log2 L/L, cre / L/L: 1.97
adj. p value: 5e−04
29
28
27
26
25
log2 intensity
Genotype
L/L
L/L, cre
Imputation
non imputed
imputed

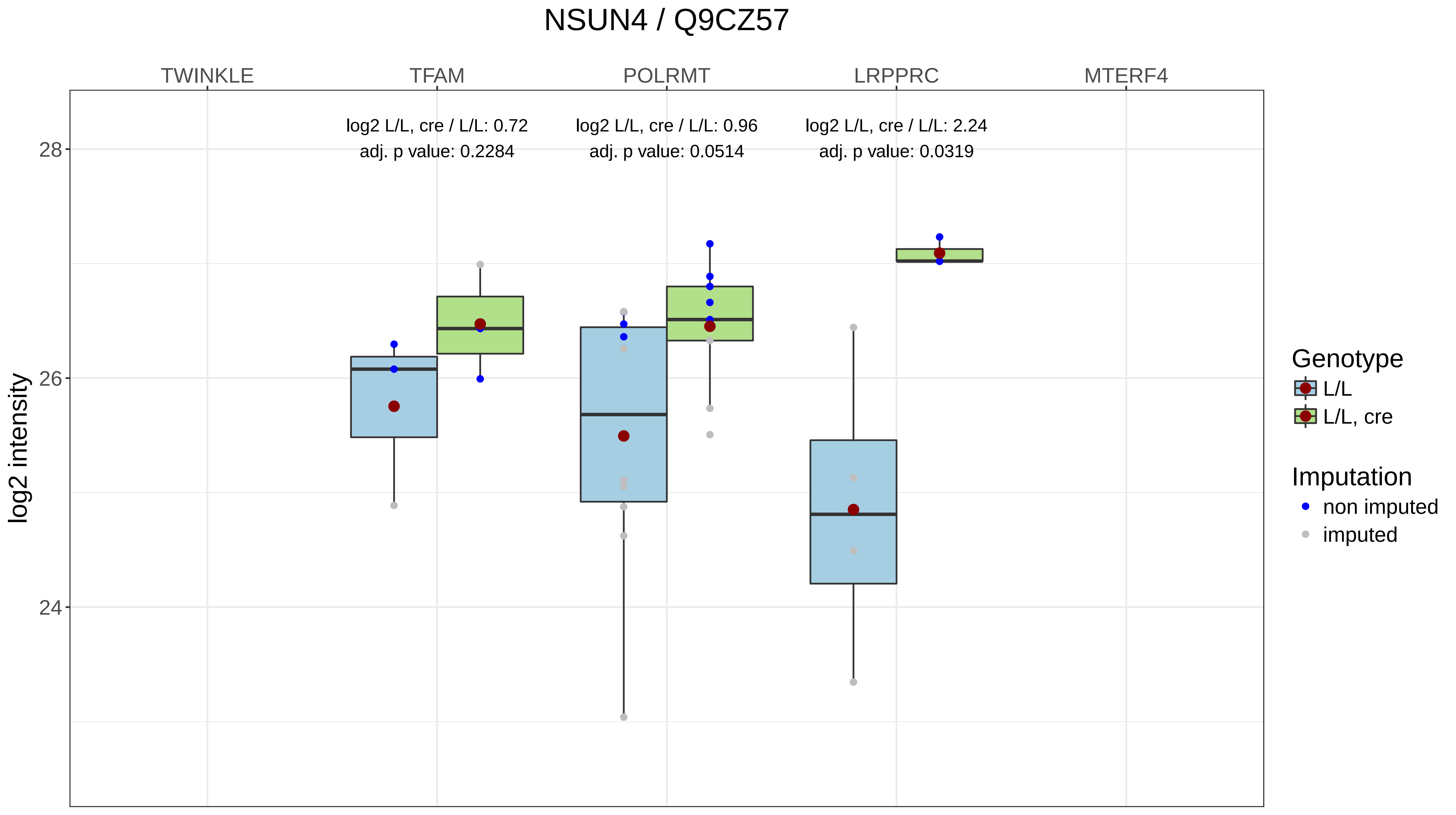
NSUN4 / Q9CZ57
TWINKLE
TFAM
POLRMT
LRPPRC
MTERF4
log2 L/L, cre / L/L: 0.72
adj. p value: 0.2284
log2 L/L, cre / L/L: 0.96
adj. p value: 0.0514
log2 L/L, cre / L/L: 2.24
adj. p value: 0.0319
28
26
24
log2 intensity
Genotype
L/L
L/L, cre
Imputation
non imputed
imputed

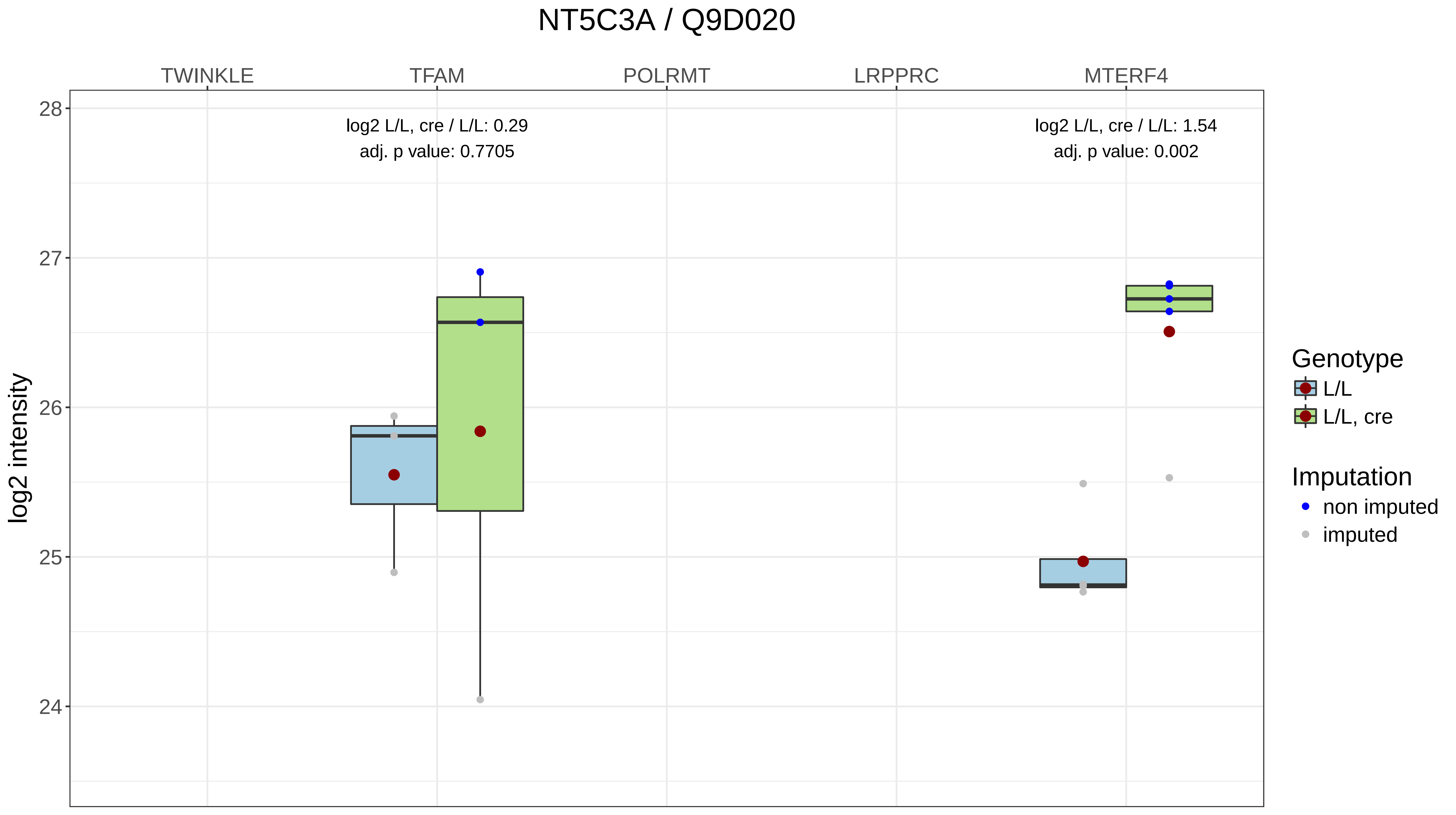
NT5C3A / Q9D020
TWINKLE
TFAM
POLRMT
LRPPRC
MTERF4
log2 L/L, cre / L/L: 0.29
adj. p value: 0.7705
log2 L/L, cre / L/L: 1.54
adj. p value: 0.002
log2 intensity
28
27
26
25
24
Genotype
L/L
L/L, cre
Imputation
non imputed
imputed

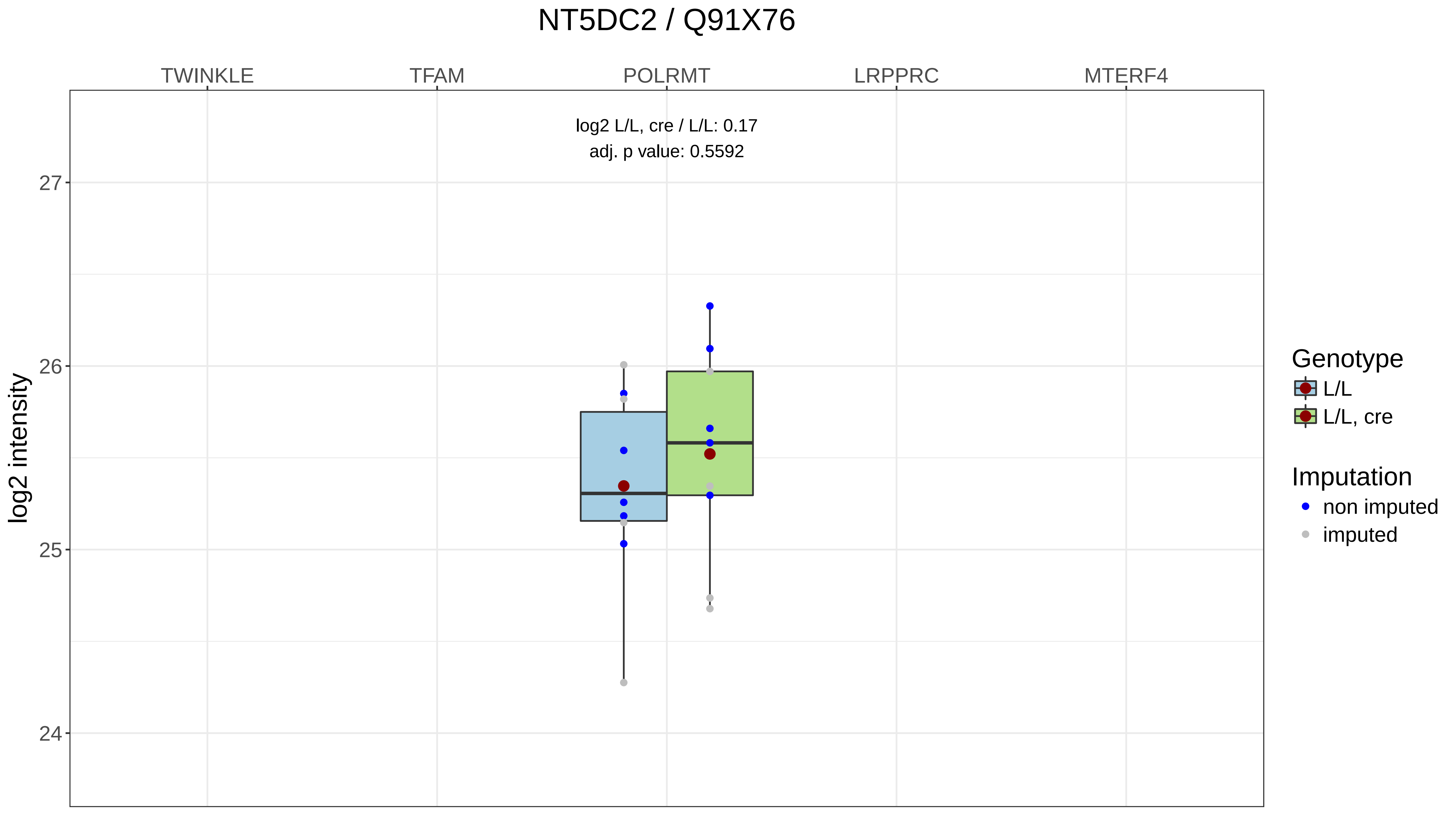
NT5DC2 / Q91X76
TWINKLE
TFAM
POLRMT
LRPPRC
MTERF4
log2 L/L, cre / L/L: 0.17
adj. p value: 0.5592
log2 intensity
27
26
25
24
Genotype
L/L
L/L, cre
Imputation
non imputed
imputed

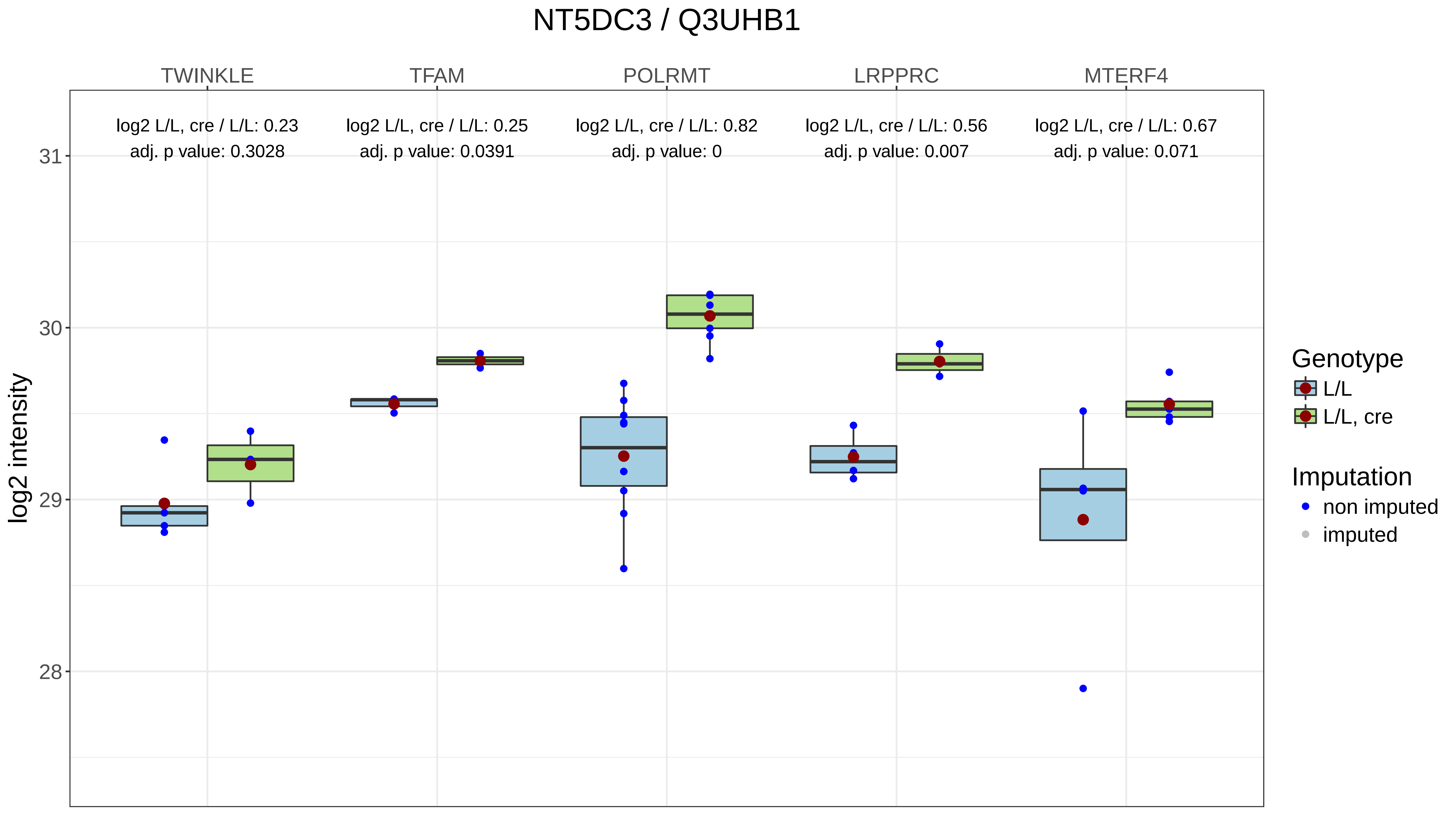
NT5DC3 / Q3UHB1
TWINKLE
TFAM
POLRMT
LRPPRC
MTERF4
log2 L/L, cre / L/L: 0.23
adj. p value: 0.3028
log2 L/L, cre / L/L: 0.25
adj. p value: 0.0391
log2 L/L, cre / L/L: 0.82
adj. p value: 0
log2 L/L, cre / L/L: 0.56
adj. p value: 0.007
log2 L/L, cre / L/L: 0.67
adj. p value: 0.071
31
30
29
28
log2 intensity
Genotype
L/L
L/L, cre
Imputation
non imputed
imputed

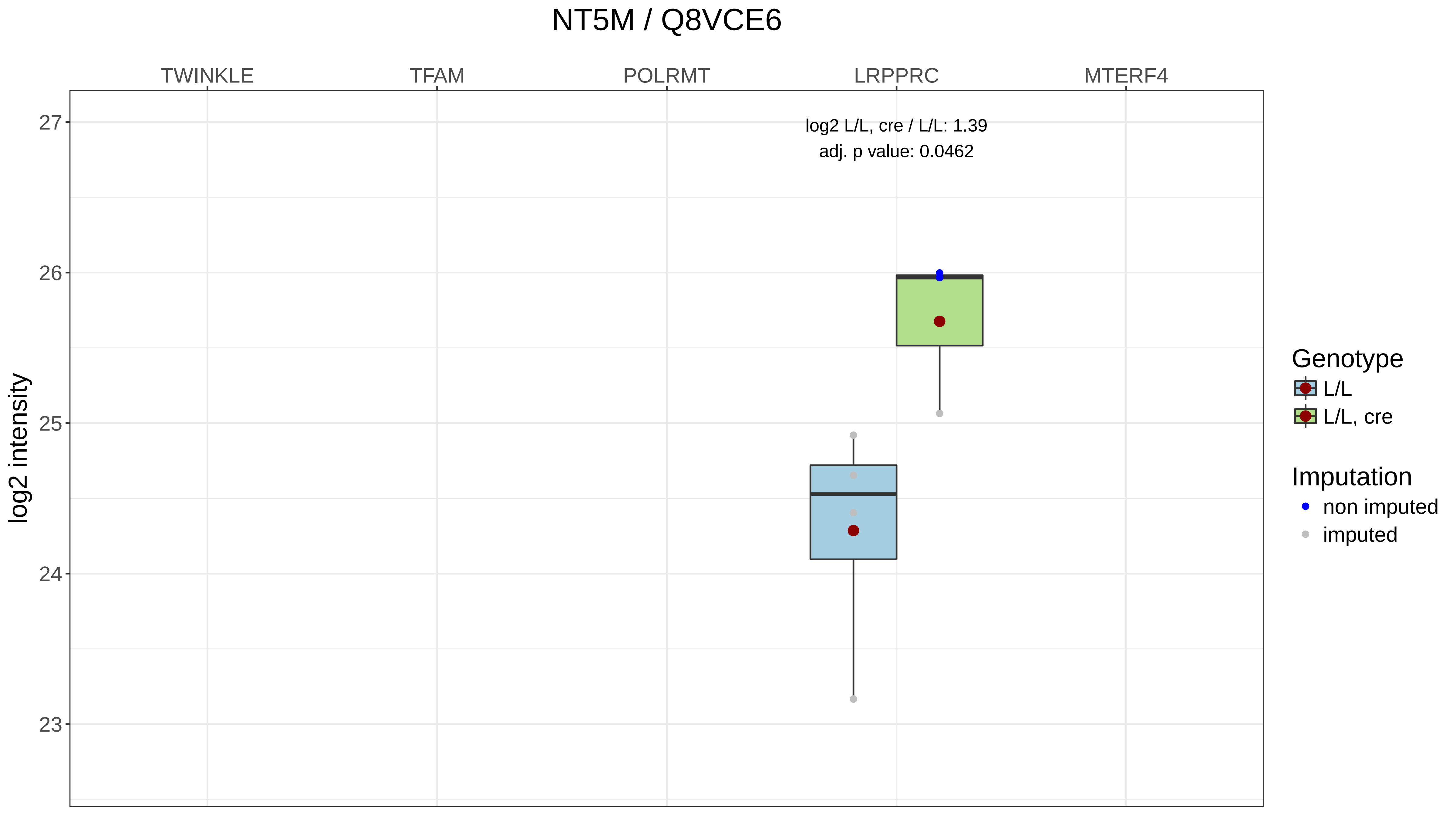
NT5M / Q8VCE6
TWINKLE
TFAM
POLRMT
LRPPRC
MTERF4
log2 L/L, cre / L/L: 1.39
adj. p value: 0.0462
log2 intensity
27
26
25
24
23
Genotype
L/L
L/L, cre
Imputation
non imputed
imputed

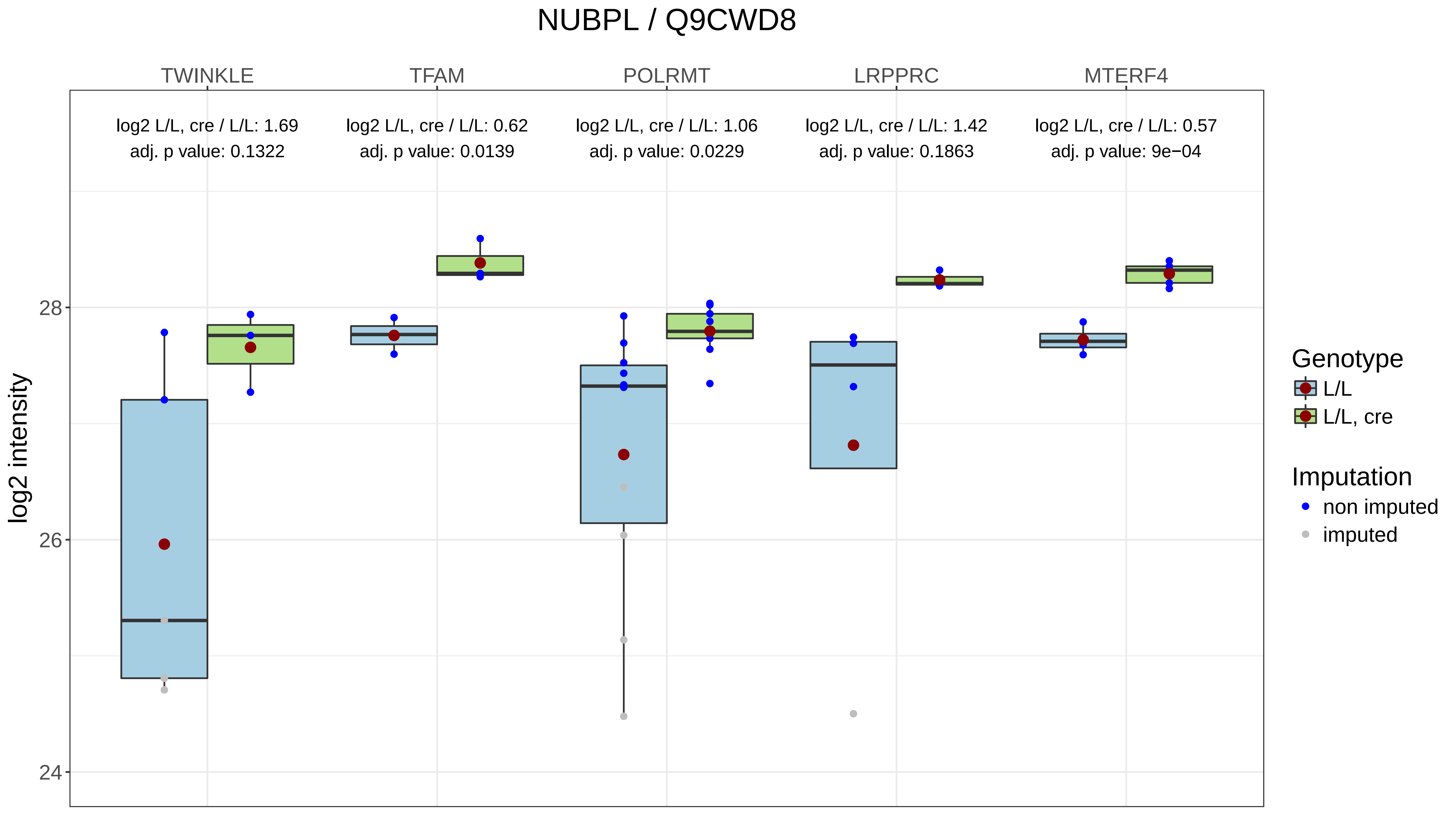

NUBPL / Q9CWD8
TWINKLE
TFAM
POLRMT
LRPPRC
MTERF4
log2 L/L, cre / L/L: 1.69
adj. p value: 0.1322
log2 L/L, cre / L/L: 0.62
adj. p value: 0.0139
log2 L/L, cre / L/L: 1.06
adj. p value: 0.0229
log2 L/L, cre / L/L: 1.42
adj. p value: 0.1863
log2 L/L, cre / L/L: 0.57
adj. p value: 9e−04
log2 intensity
28
26
24
Genotype
L/L
L/L, cre
Imputation
non imputed
imputed

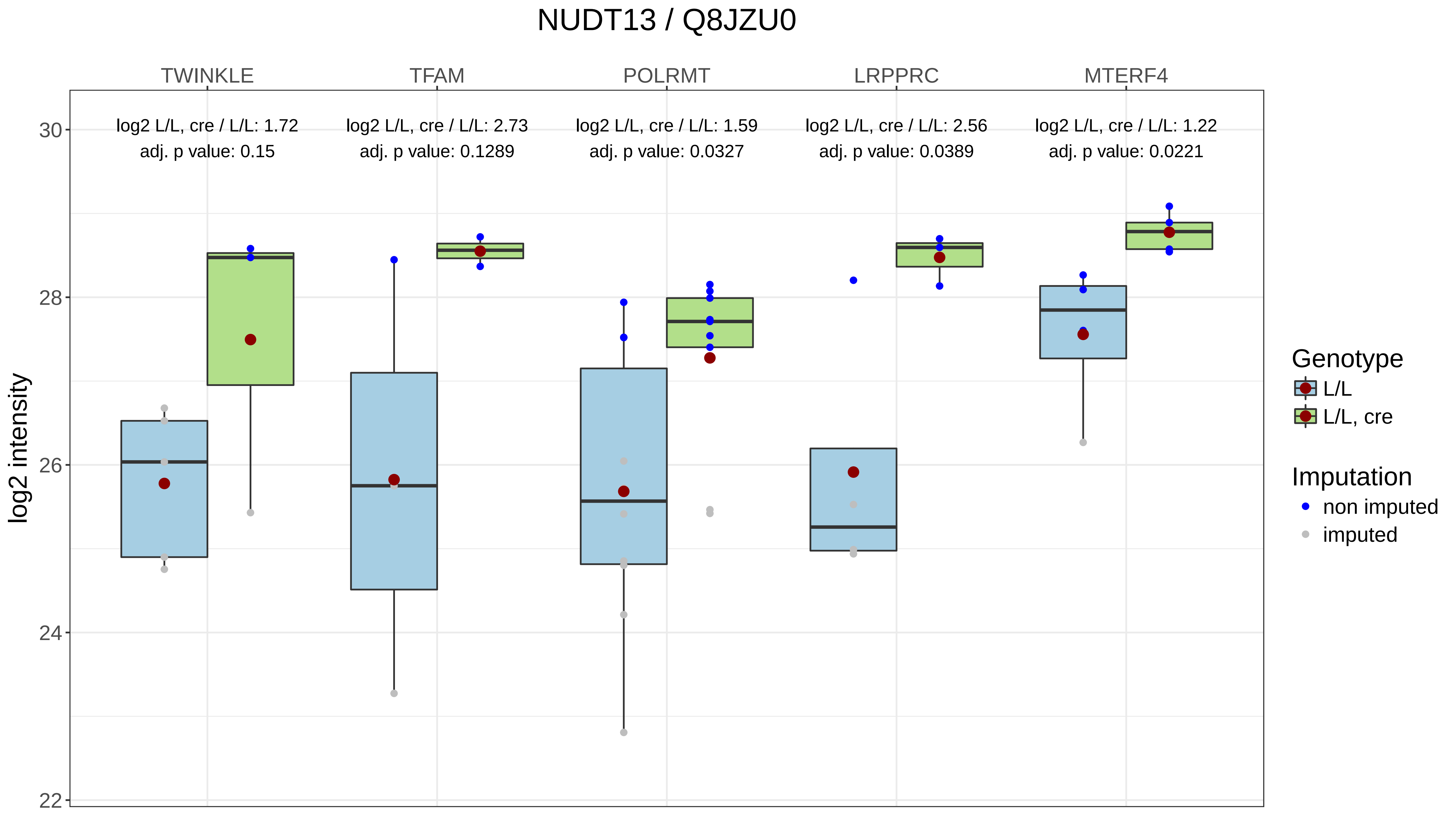
NUDT13 / Q8JZU0
TWINKLE
TFAM
POLRMT
LRPPRC
MTERF4
log2 L/L, cre / L/L: 1.72
adj. p value: 0.15
log2 L/L, cre / L/L: 2.73
adj. p value: 0.1289
log2 L/L, cre / L/L: 1.59
adj. p value: 0.0327
log2 L/L, cre / L/L: 2.56
adj. p value: 0.0389
log2 L/L, cre / L/L: 1.22
adj. p value: 0.0221
log2 intensity
30
28
26
24
22
Genotype
L/L
L/L, cre
Imputation
non imputed
imputed

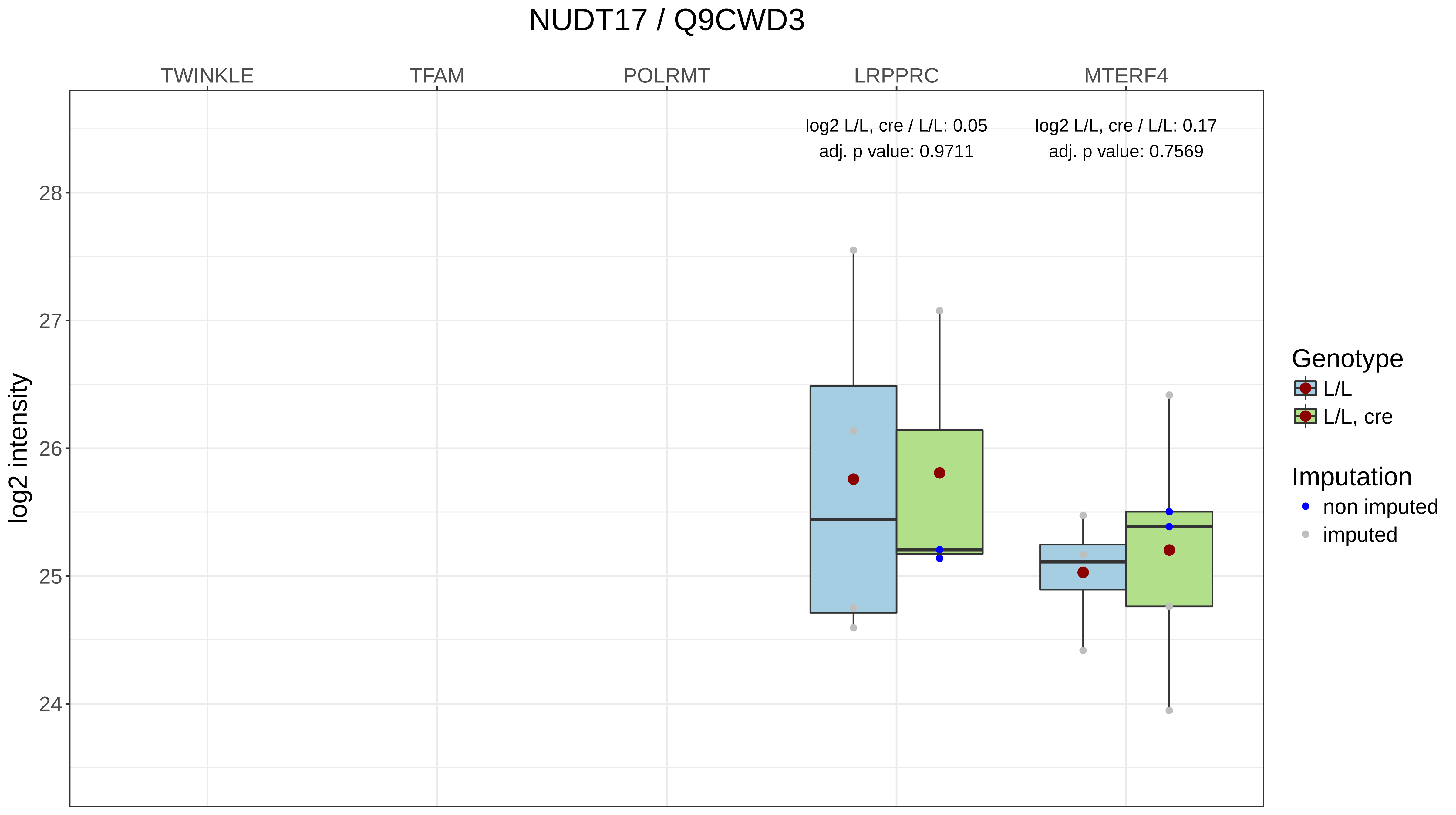
NUDT17 / Q9CWD3
TWINKLE
TFAM
POLRMT
LRPPRC
MTERF4
log2 L/L, cre / L/L: 0.05
adj. p value: 0.9711
log2 L/L, cre / L/L: 0.17
adj. p value: 0.7569
28
27
26
25
24
log2 intensity
Genotype
L/L
L/L, cre
Imputation
non imputed
imputed

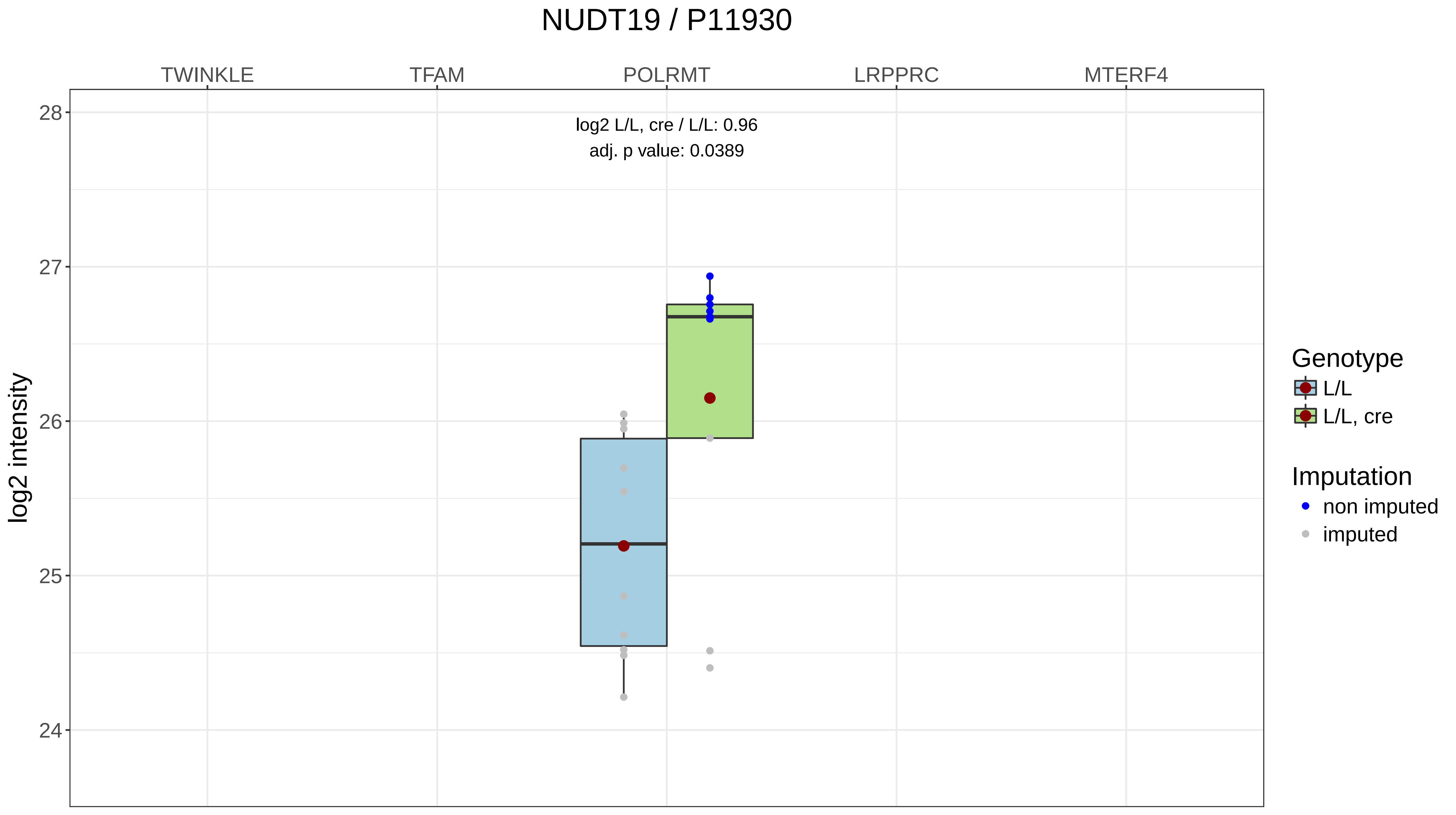
NUDT19 / P11930
TWINKLE
TFAM
POLRMT
LRPPRC
MTERF4
log2 L/L, cre / L/L: 0.96
adj. p value: 0.0389
log2 intensity
28
27
26
25
24
Genotype
L/L
L/L, cre
Imputation
non imputed
imputed

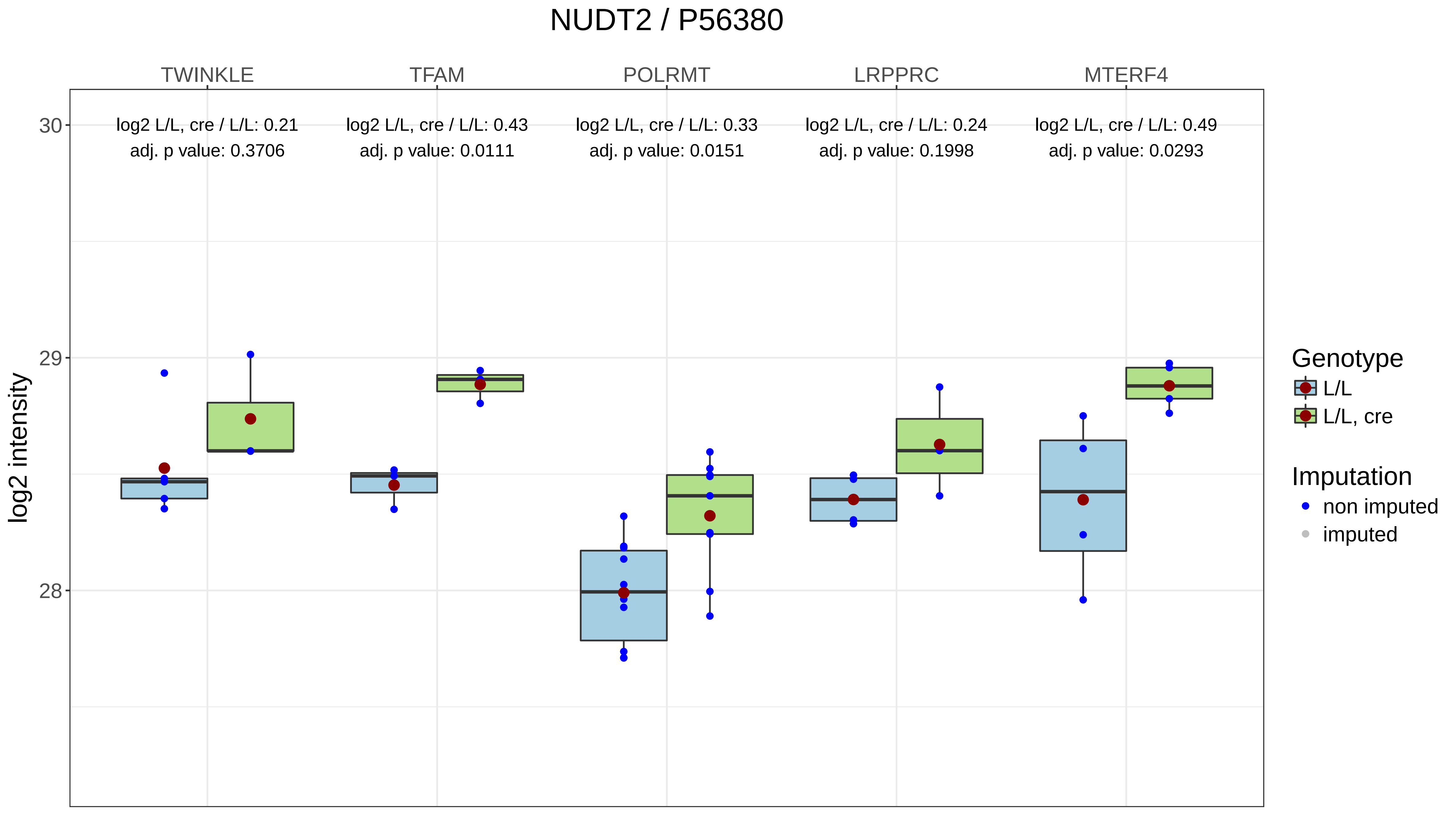
NUDT2 / P56380
TWINKLE
TFAM
POLRMT
LRPPRC
MTERF4
log2 L/L, cre / L/L: 0.21
adj. p value: 0.3706
log2 L/L, cre / L/L: 0.43
adj. p value: 0.0111
log2 L/L, cre / L/L: 0.33
adj. p value: 0.0151
log2 L/L, cre / L/L: 0.24
adj. p value: 0.1998
log2 L/L, cre / L/L: 0.49
adj. p value: 0.0293
30
29
28
log2 intensity
Genotype
L/L
L/L, cre
Imputation
non imputed
imputed

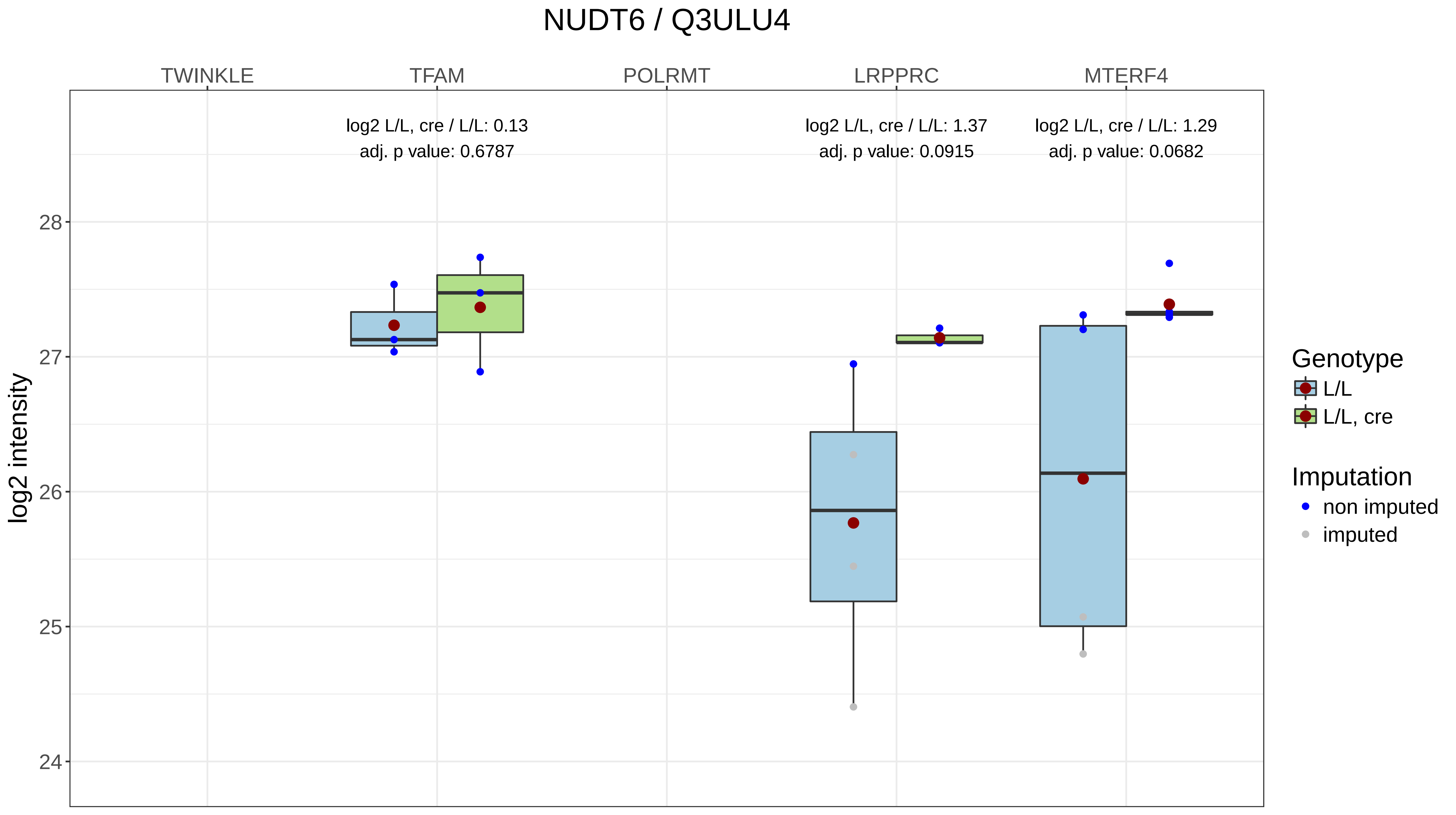
NUDT6 / Q3ULU4
TWINKLE
TFAM
POLRMT
LRPPRC
MTERF4
log2 L/L, cre / L/L: 0.13
adj. p value: 0.6787
log2 L/L, cre / L/L: 1.37
adj. p value: 0.0915
log2 L/L, cre / L/L: 1.29
adj. p value: 0.0682
28
27
26
25
24
log2 intensity
Genotype
L/L
L/L, cre
Imputation
non imputed
imputed

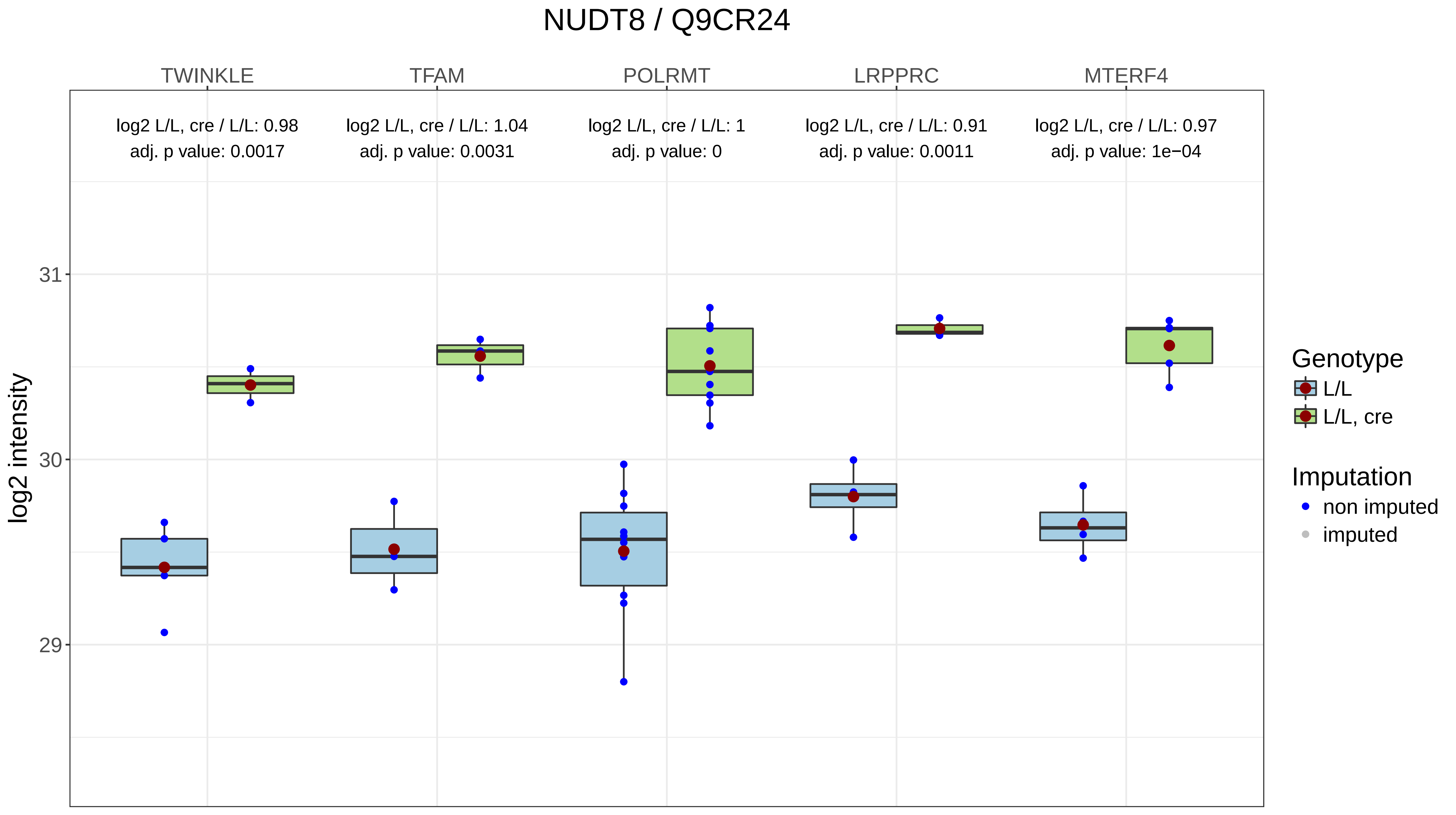

NUDT8 / Q9CR24
TWINKLE
TFAM
POLRMT
LRPPRC
MTERF4
log2 L/L, cre / L/L: 0.98
adj. p value: 0.0017
log2 L/L, cre / L/L: 1.04
adj. p value: 0.0031
log2 L/L, cre / L/L: 1
adj. p value: 0
log2 L/L, cre / L/L: 0.91
adj. p value: 0.0011
log2 L/L, cre / L/L: 0.97
adj. p value: 1e−04
log2 intensity
31
30
29
Genotype
L/L
L/L, cre
Imputation
non imputed
imputed

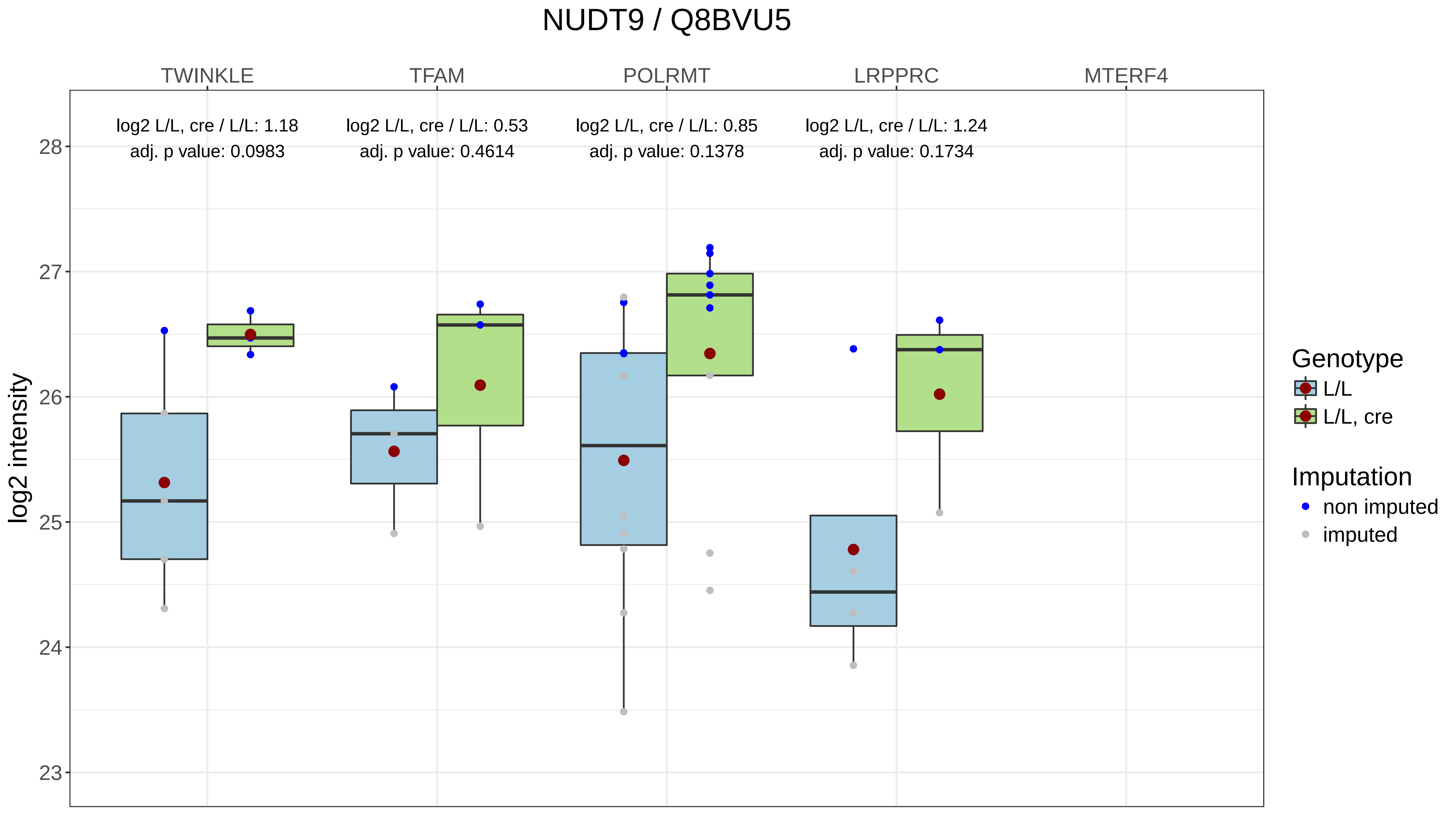
NUDT9 / Q8BVU5
TWINKLE
TFAM
POLRMT
LRPPRC
MTERF4
log2 L/L, cre / L/L: 1.18
adj. p value: 0.0983
log2 L/L, cre / L/L: 0.53
adj. p value: 0.4614
log2 L/L, cre / L/L: 0.85
adj. p value: 0.1378
log2 L/L, cre / L/L: 1.24
adj. p value: 0.1734
log2 intensity
28
27
26
25
24
23
Genotype
L/L
L/L, cre
Imputation
non imputed
imputed

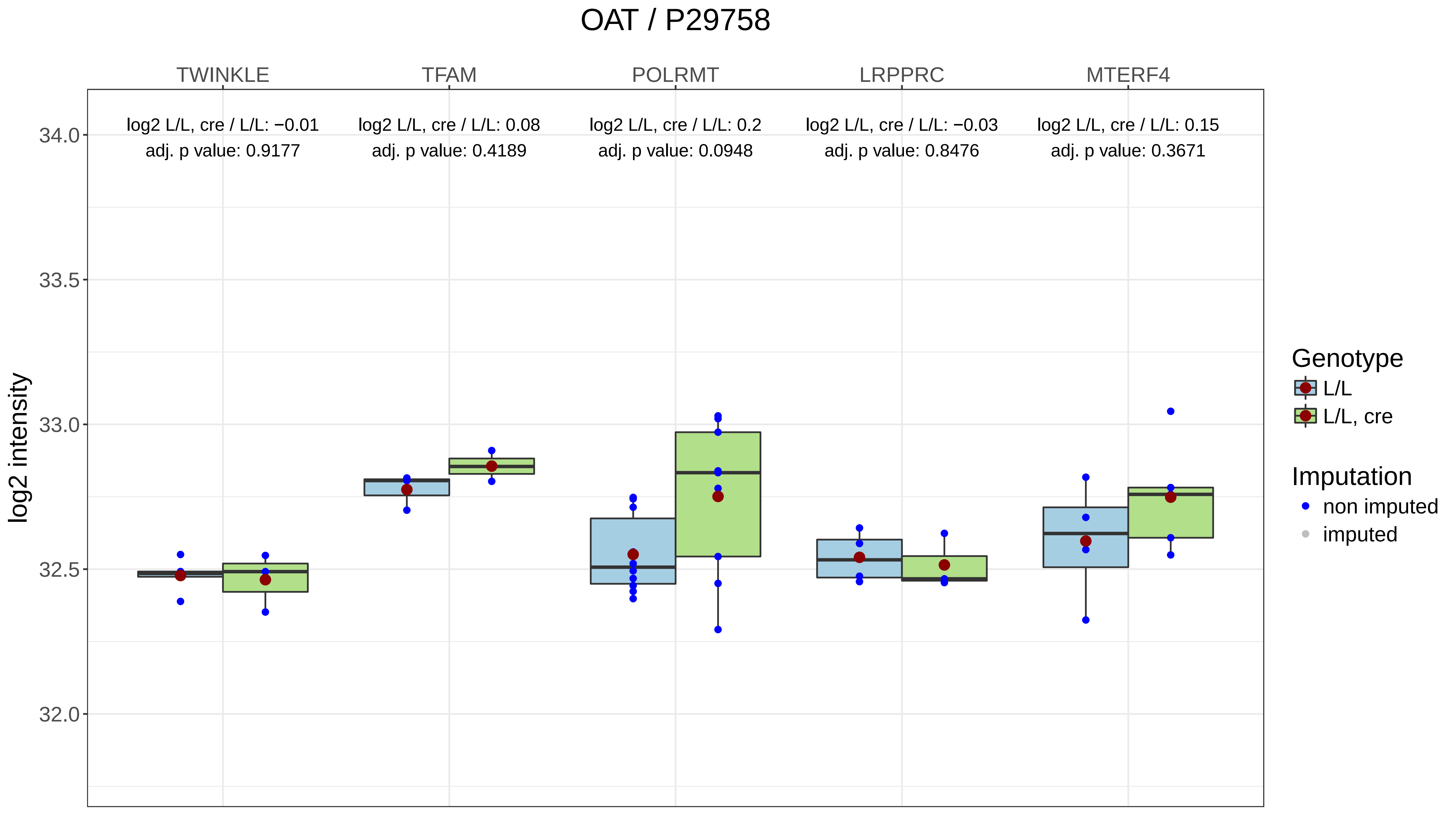

OAT / P29758
TWINKLE
TFAM
POLRMT
LRPPRC
MTERF4
log2 L/L, cre / L/L: −0.01
adj. p value: 0.9177
log2 L/L, cre / L/L: 0.08
adj. p value: 0.4189
log2 L/L, cre / L/L: 0.2
adj. p value: 0.0948
log2 L/L, cre / L/L: −0.03
adj. p value: 0.8476
log2 L/L, cre / L/L: 0.15
adj. p value: 0.3671
log2 intensity
34.0
33.5
33.0
32.5
32.0
Genotype
L/L
L/L, cre
Imputation
non imputed
imputed

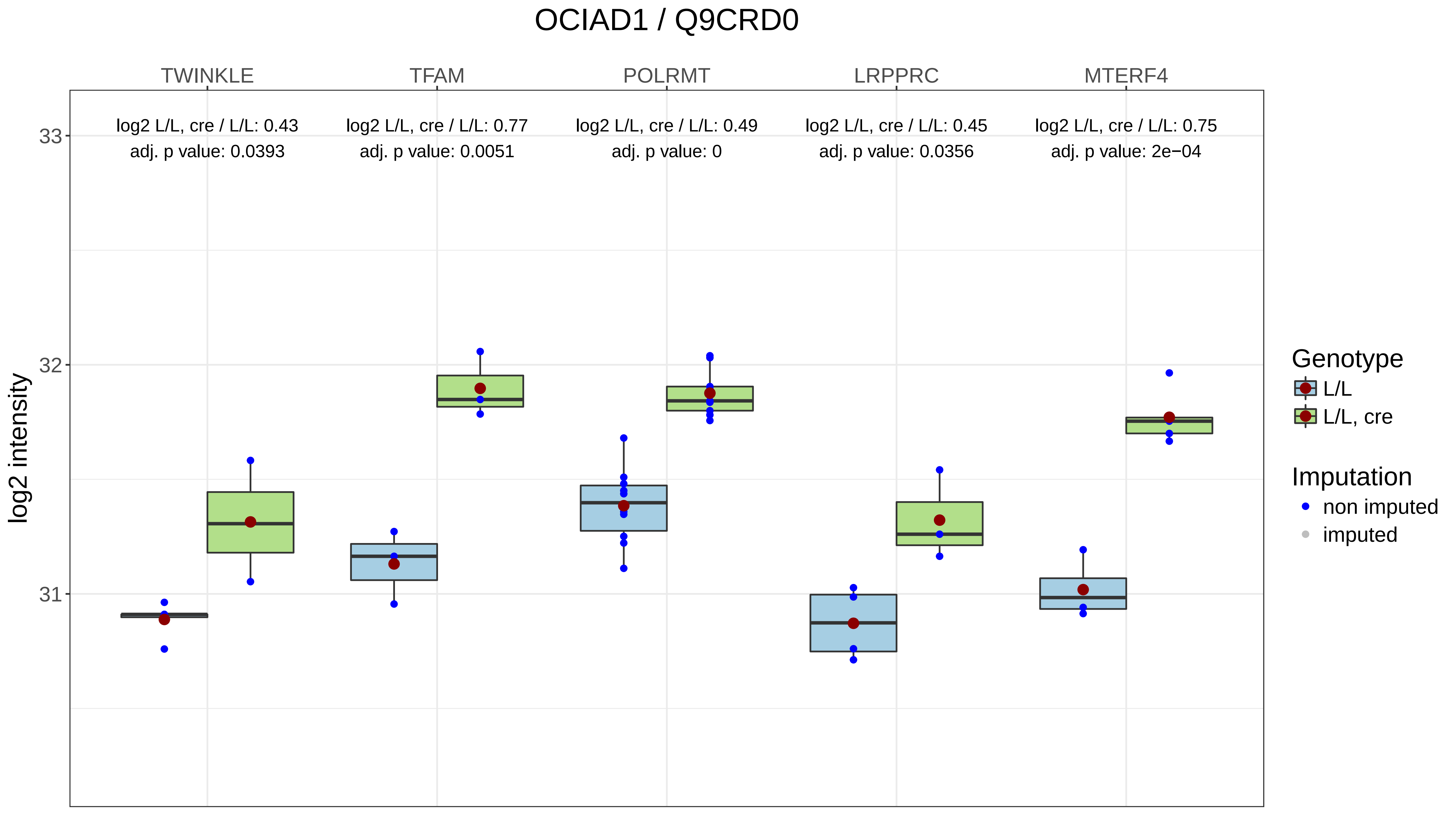
OCIAD1 / Q9CRD0
TWINKLE
TFAM
POLRMT
LRPPRC
MTERF4
log2 L/L, cre / L/L: 0.43
adj. p value: 0.0393
log2 L/L, cre / L/L: 0.77
adj. p value: 0.0051
log2 L/L, cre / L/L: 0.49
adj. p value: 0
log2 L/L, cre / L/L: 0.45
adj. p value: 0.0356
log2 L/L, cre / L/L: 0.75
adj. p value: 2e−04
33
32
31
log2 intensity
Genotype
L/L
L/L, cre
Imputation
non imputed
imputed

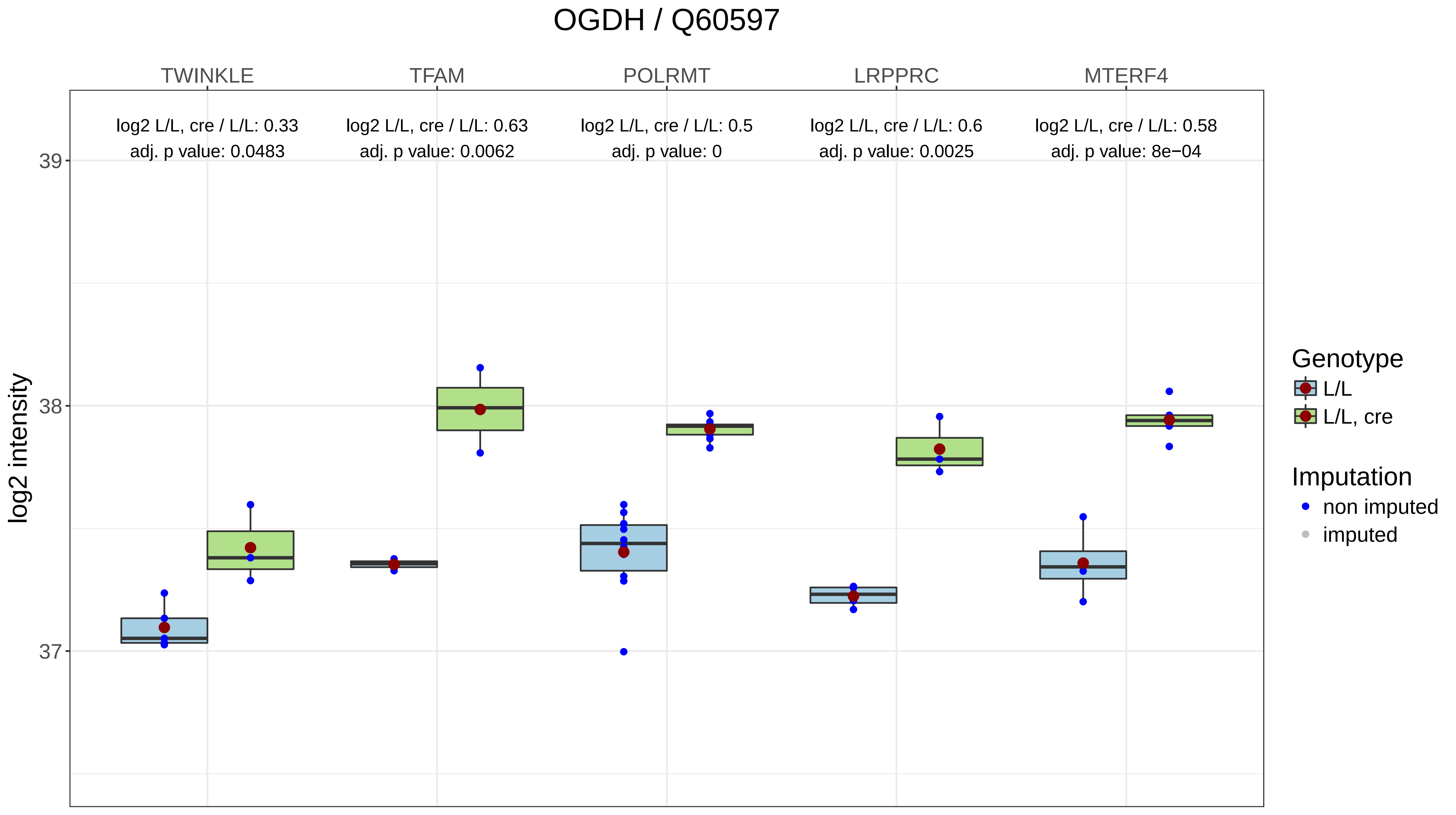
OGDH / Q60597
TWINKLE
TFAM
POLRMT
LRPPRC
MTERF4
log2 L/L, cre / L/L: 0.33
adj. p value: 0.0483
log2 L/L, cre / L/L: 0.63
adj. p value: 0.0062
log2 L/L, cre / L/L: 0.5
adj. p value: 0
log2 L/L, cre / L/L: 0.6
adj. p value: 0.0025
log2 L/L, cre / L/L: 0.58
adj. p value: 8e−04
log2 intensity
39
38
37
Genotype
L/L
L/L, cre
Imputation
non imputed
imputed

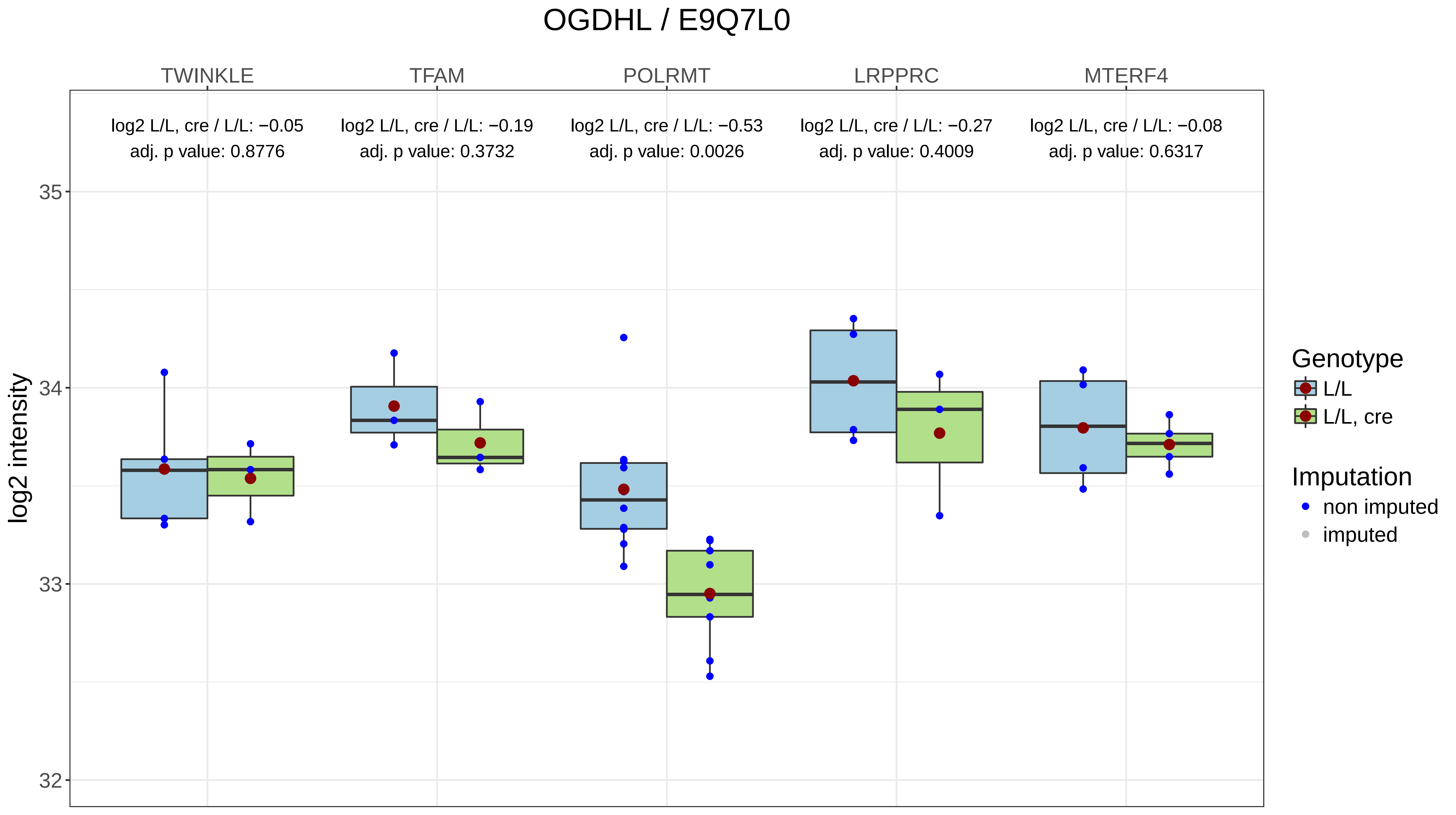
OGDHL / E9Q7L0
TWINKLE
TFAM
POLRMT
LRPPRC
MTERF4
log2 L/L, cre / L/L: −0.05
adj. p value: 0.8776
log2 L/L, cre / L/L: −0.19
adj. p value: 0.3732
log2 L/L, cre / L/L: −0.53
adj. p value: 0.0026
log2 L/L, cre / L/L: −0.27
adj. p value: 0.4009
log2 L/L, cre / L/L: −0.08
adj. p value: 0.6317
log2 intensity
35
34
33
32
Genotype
L/L
L/L, cre
Imputation
non imputed
imputed

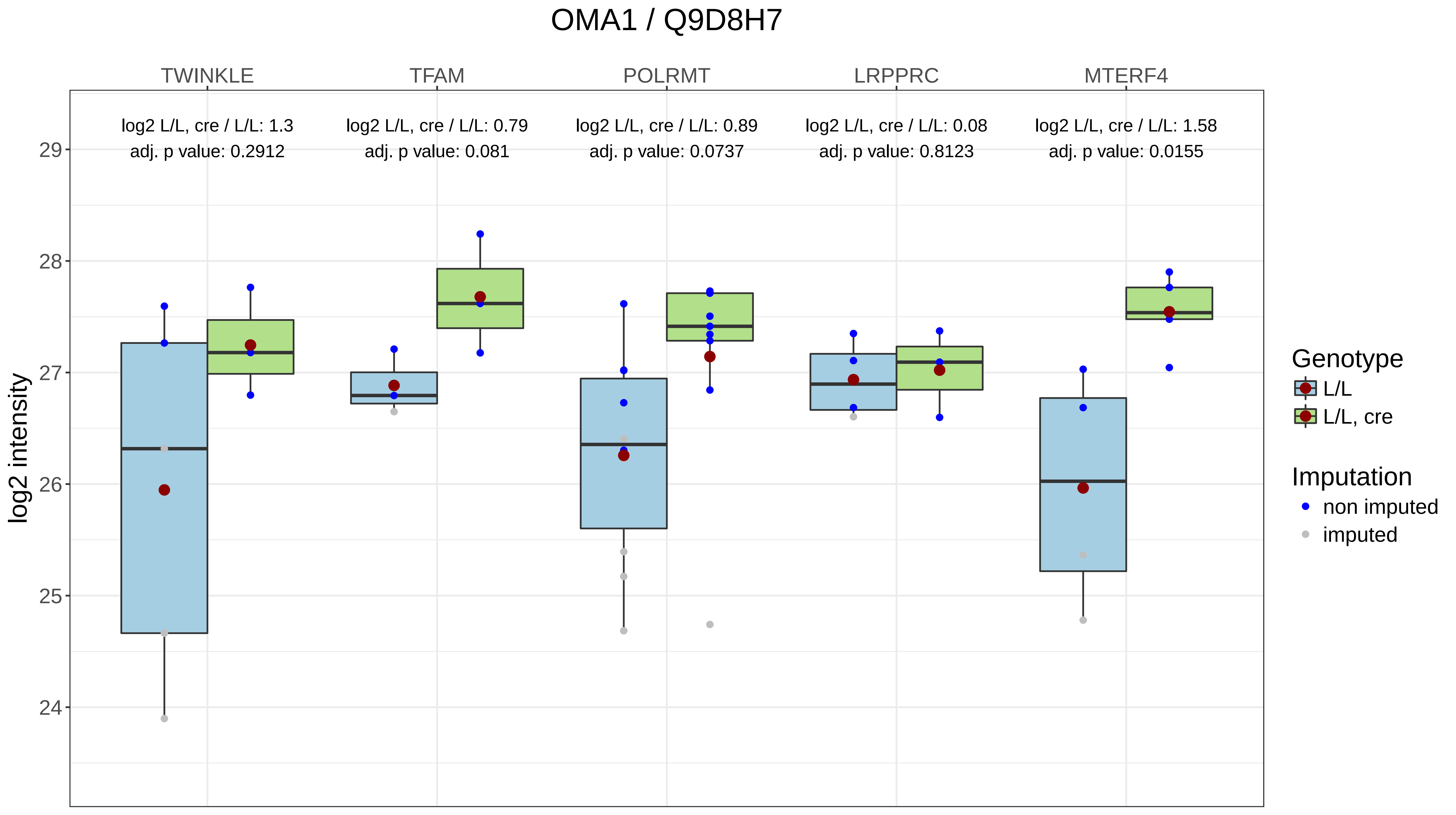

OMA1 / Q9D8H7
TWINKLE
TFAM
POLRMT
LRPPRC
MTERF4
log2 L/L, cre / L/L: 1.3
adj. p value: 0.2912
log2 L/L, cre / L/L: 0.79
adj. p value: 0.081
log2 L/L, cre / L/L: 0.89
adj. p value: 0.0737
log2 L/L, cre / L/L: 0.08
adj. p value: 0.8123
log2 L/L, cre / L/L: 1.58
adj. p value: 0.0155
log2 intensity
29
28
27
26
25
24
Genotype
L/L
L/L, cre
Imputation
non imputed
imputed

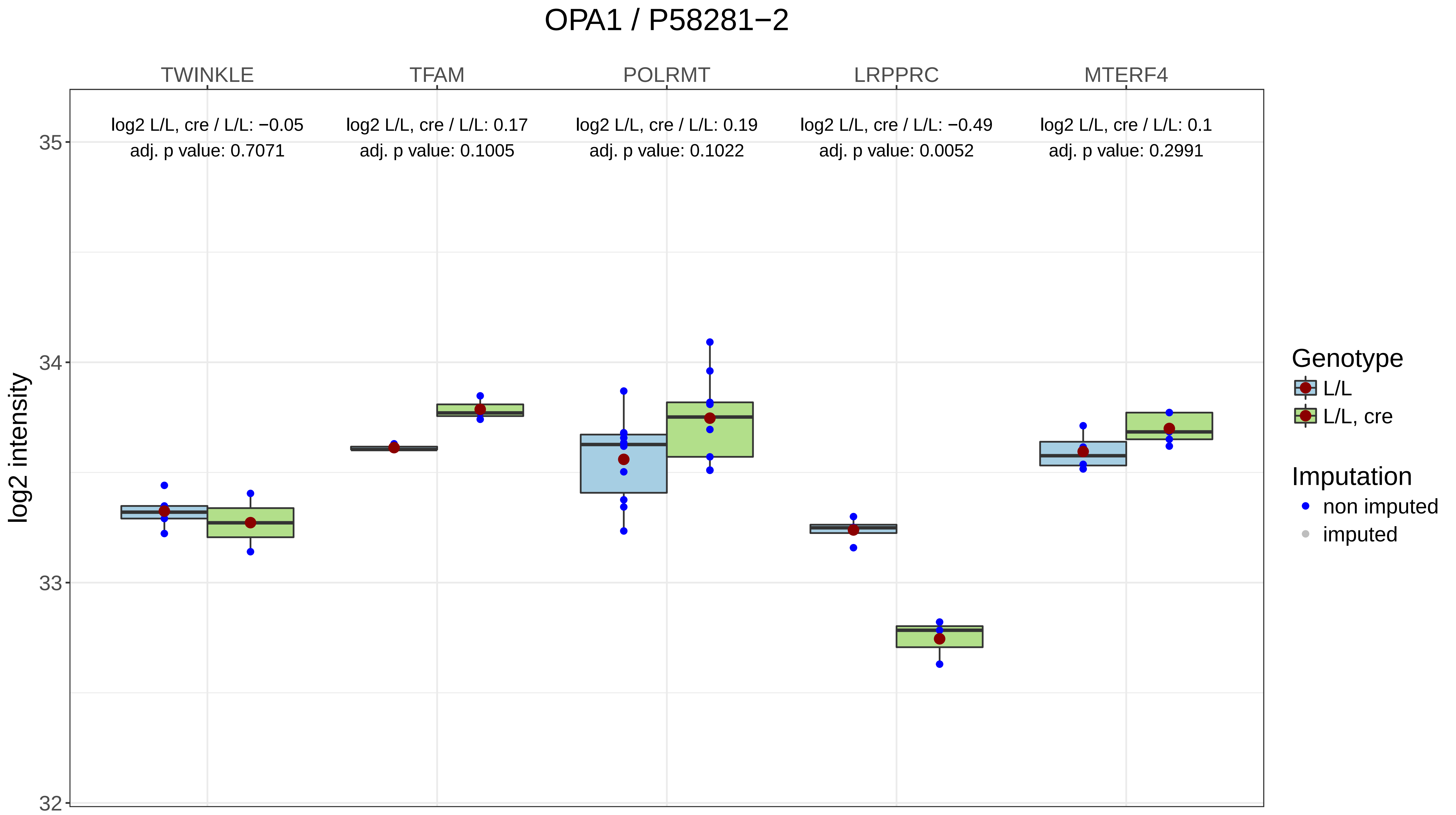
OPA1 / P58281−2
TWINKLE
TFAM
POLRMT
LRPPRC
MTERF4
log2 L/L, cre / L/L: −0.05
adj. p value: 0.7071
log2 L/L, cre / L/L: 0.17
adj. p value: 0.1005
log2 L/L, cre / L/L: 0.19
adj. p value: 0.1022
log2 L/L, cre / L/L: −0.49
adj. p value: 0.0052
log2 L/L, cre / L/L: 0.1
adj. p value: 0.2991
log2 intensity
35
34
33
32
Genotype
L/L
L/L, cre
Imputation
non imputed
imputed

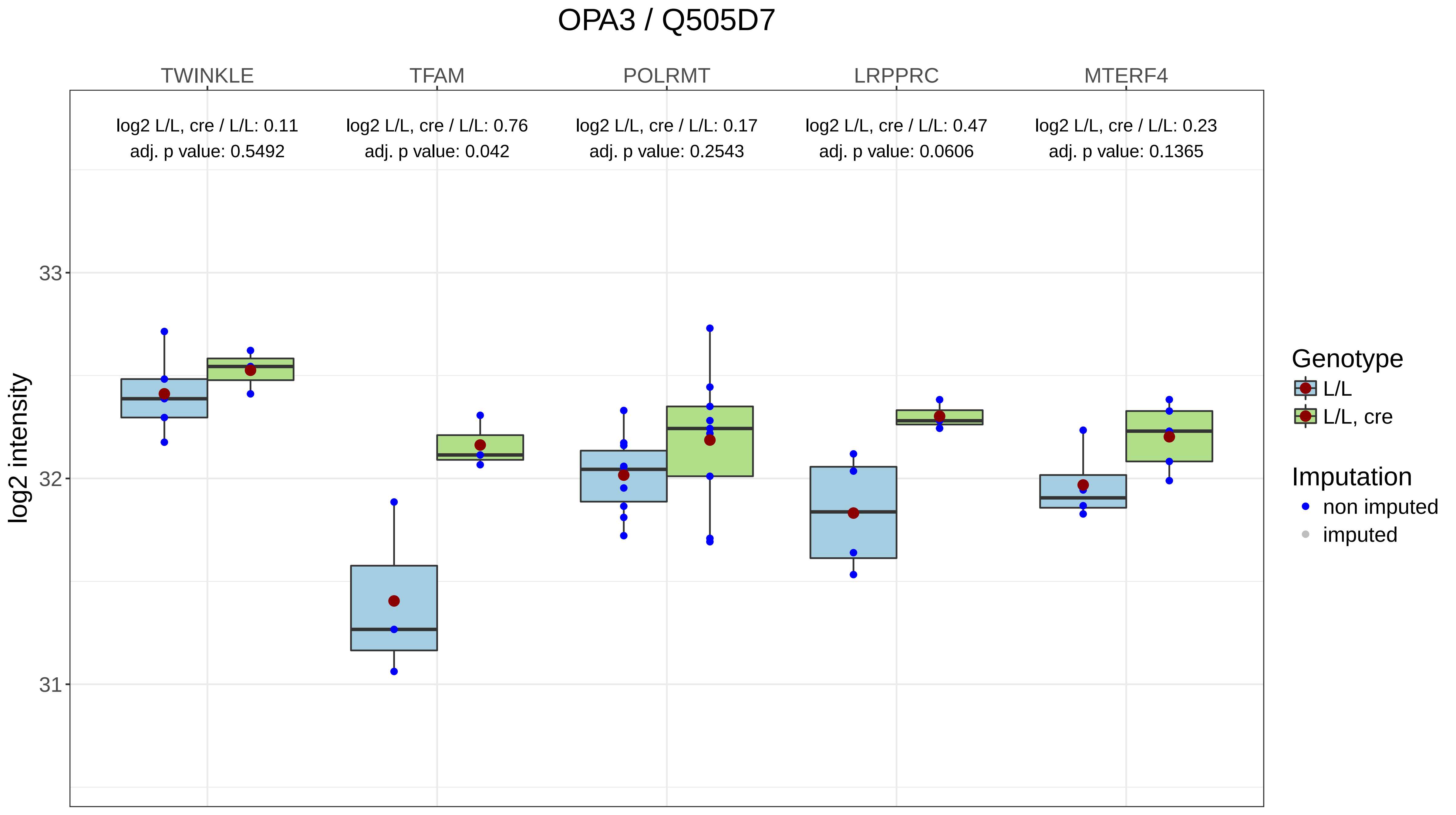
OPA3 / Q505D7
TWINKLE
TFAM
POLRMT
LRPPRC
MTERF4
log2 L/L, cre / L/L: 0.11
adj. p value: 0.5492
log2 L/L, cre / L/L: 0.76
adj. p value: 0.042
log2 L/L, cre / L/L: 0.17
adj. p value: 0.2543
log2 L/L, cre / L/L: 0.47
adj. p value: 0.0606
log2 L/L, cre / L/L: 0.23
adj. p value: 0.1365
log2 intensity
33
32
31
Genotype
L/L
L/L, cre
Imputation
non imputed
imputed

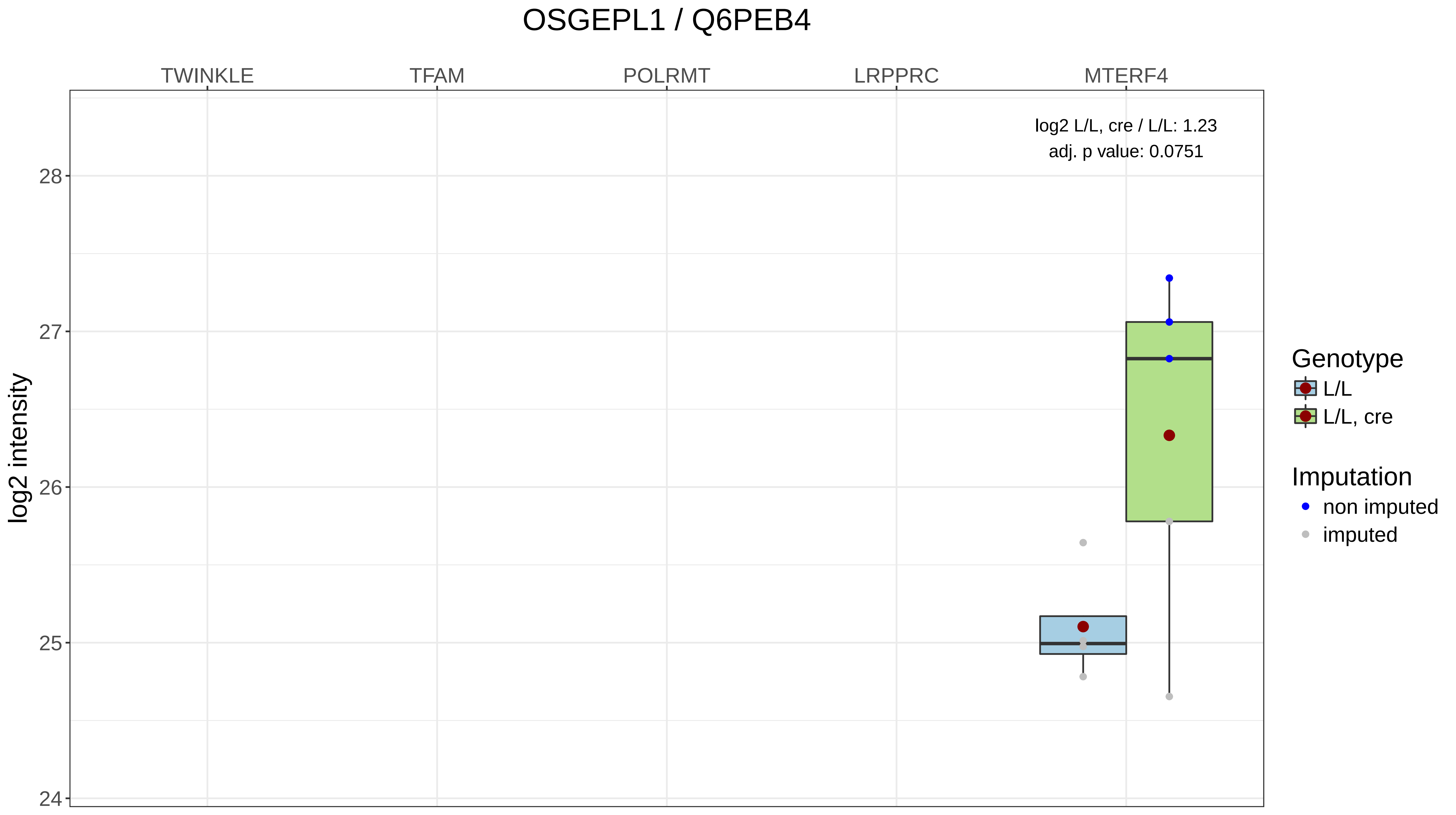
OSGEPL1 / Q6PEB4
TWINKLE
TFAM
POLRMT
LRPPRC
MTERF4
log2 L/L, cre / L/L: 1.23
adj. p value: 0.0751
log2 intensity
28
27
26
25
24
Genotype
L/L
L/L, cre
Imputation
non imputed
imputed

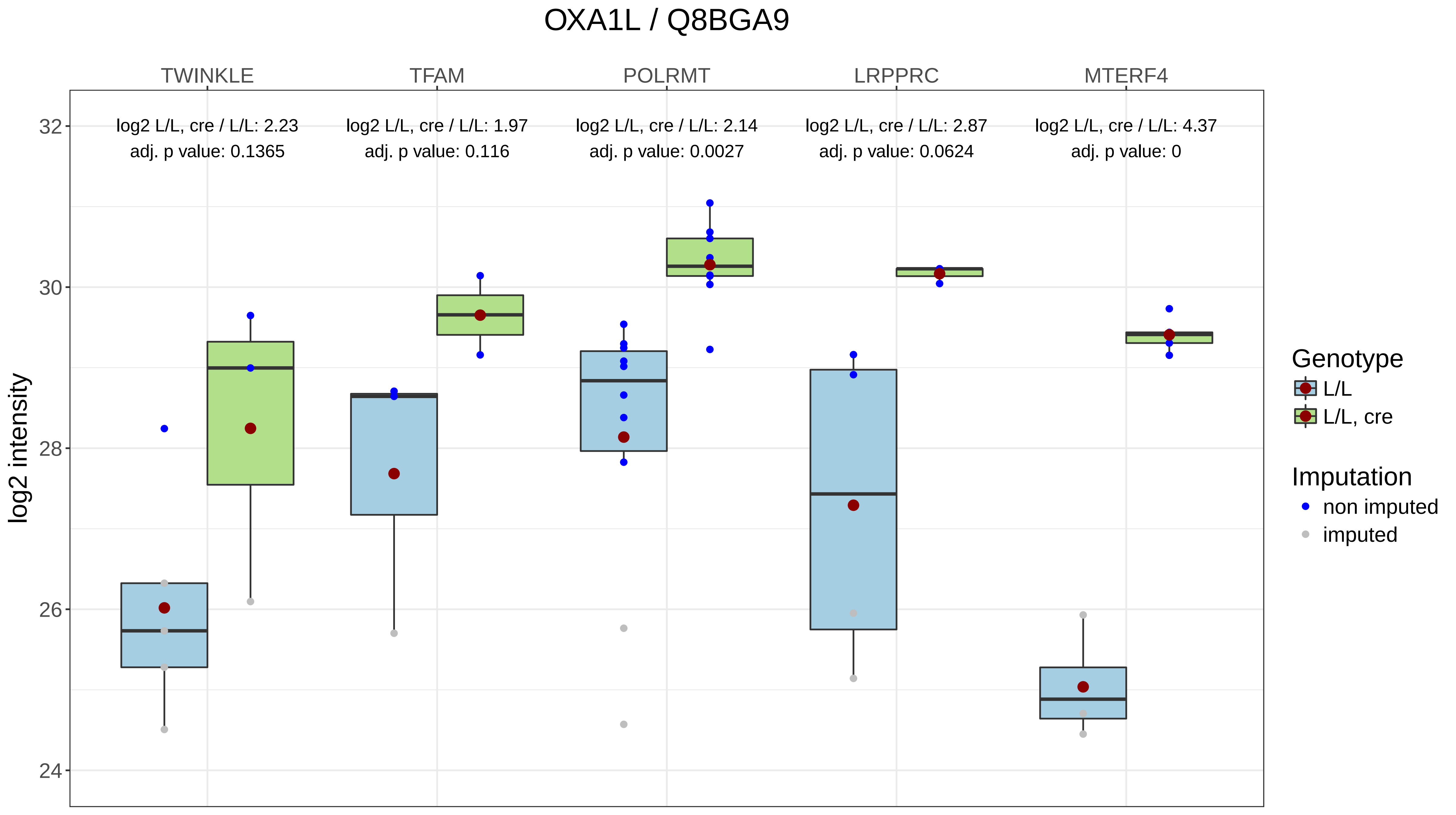
OXA1L / Q8BGA9
TWINKLE
TFAM
POLRMT
LRPPRC
MTERF4
log2 L/L, cre / L/L: 2.23
adj. p value: 0.1365
log2 L/L, cre / L/L: 1.97
adj. p value: 0.116
log2 L/L, cre / L/L: 2.14
adj. p value: 0.0027
log2 L/L, cre / L/L: 2.87
adj. p value: 0.0624
log2 L/L, cre / L/L: 4.37
adj. p value: 0
log2 intensity
32
30
28
26
24
Genotype
L/L
L/L, cre
Imputation
non imputed
imputed

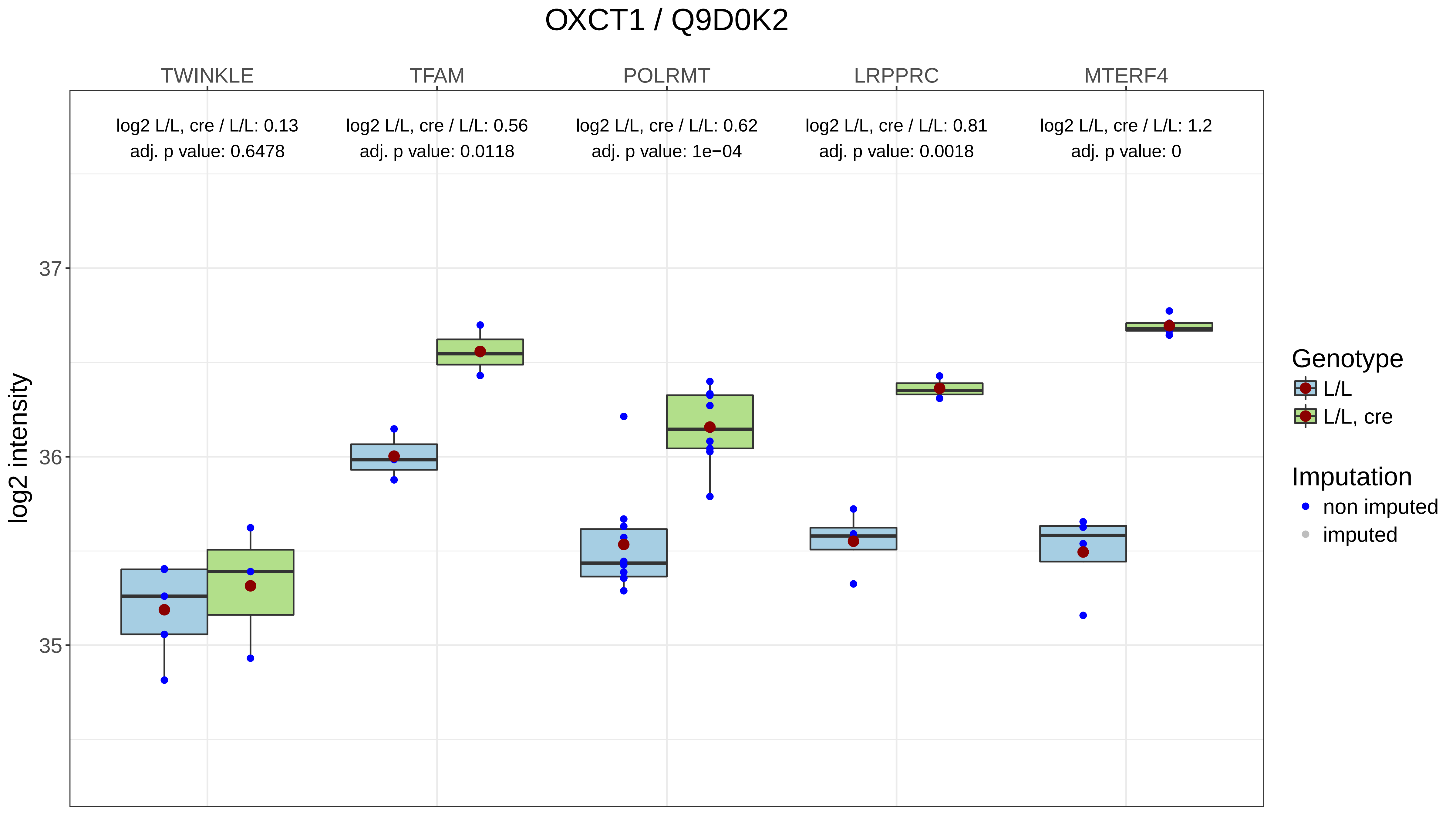
OXCT1 / Q9D0K2
TWINKLE
TFAM
POLRMT
LRPPRC
MTERF4
log2 L/L, cre / L/L: 0.13
adj. p value: 0.6478
log2 L/L, cre / L/L: 0.56
adj. p value: 0.0118
log2 L/L, cre / L/L: 0.62
adj. p value: 1e−04
log2 L/L, cre / L/L: 0.81
adj. p value: 0.0018
log2 L/L, cre / L/L: 1.2
adj. p value: 0
log2 intensity
37
36
35
Genotype
L/L
L/L, cre
Imputation
non imputed
imputed

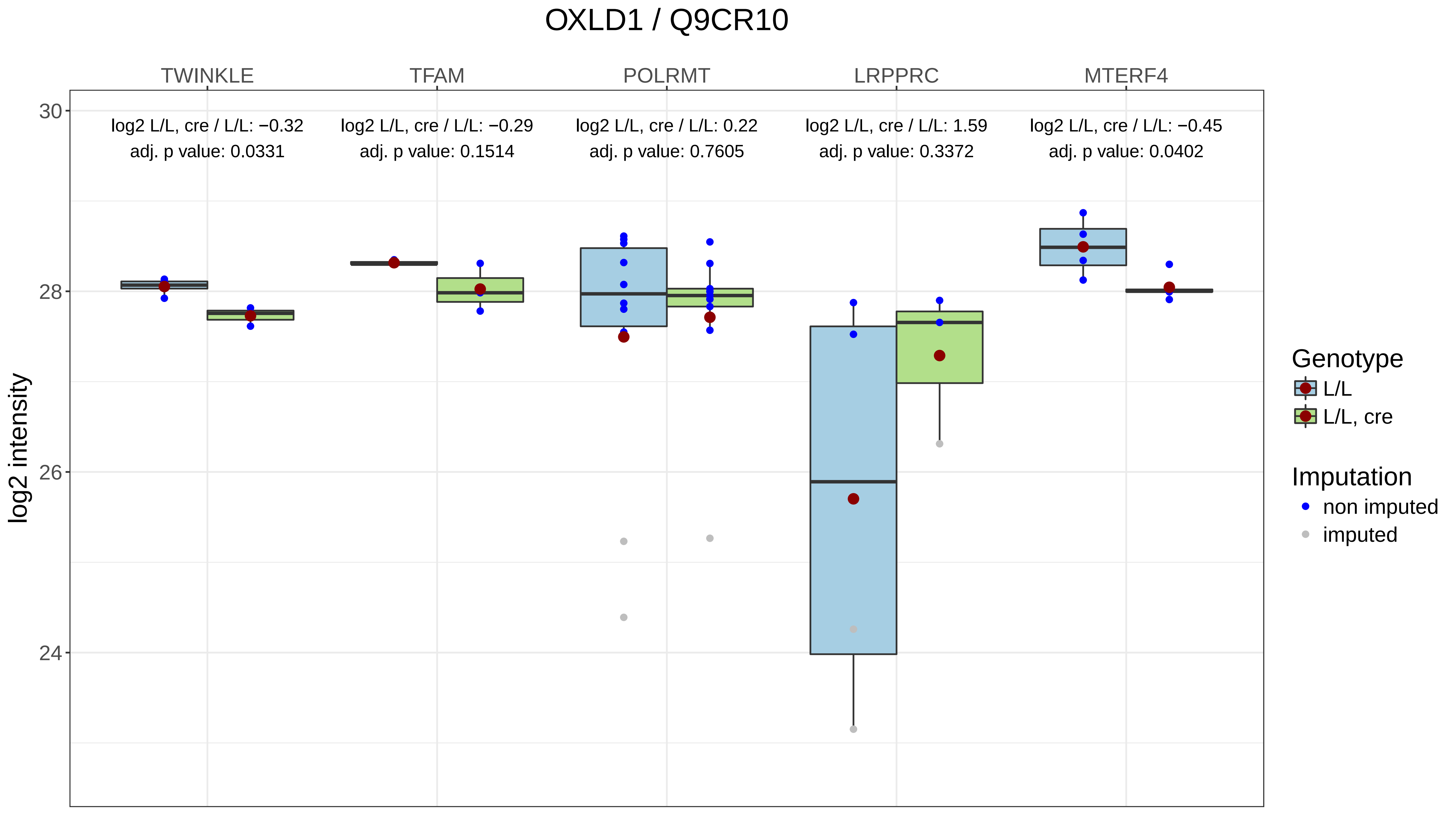
OXLD1 / Q9CR10
TWINKLE
TFAM
POLRMT
LRPPRC
MTERF4
log2 L/L, cre / L/L: −0.32
adj. p value: 0.0331
log2 L/L, cre / L/L: −0.29
adj. p value: 0.1514
log2 L/L, cre / L/L: 0.22
adj. p value: 0.7605
log2 L/L, cre / L/L: 1.59
adj. p value: 0.3372
log2 L/L, cre / L/L: −0.45
adj. p value: 0.0402
log2 intensity
30
28
26
24
Genotype
L/L
L/L, cre
Imputation
non imputed
imputed

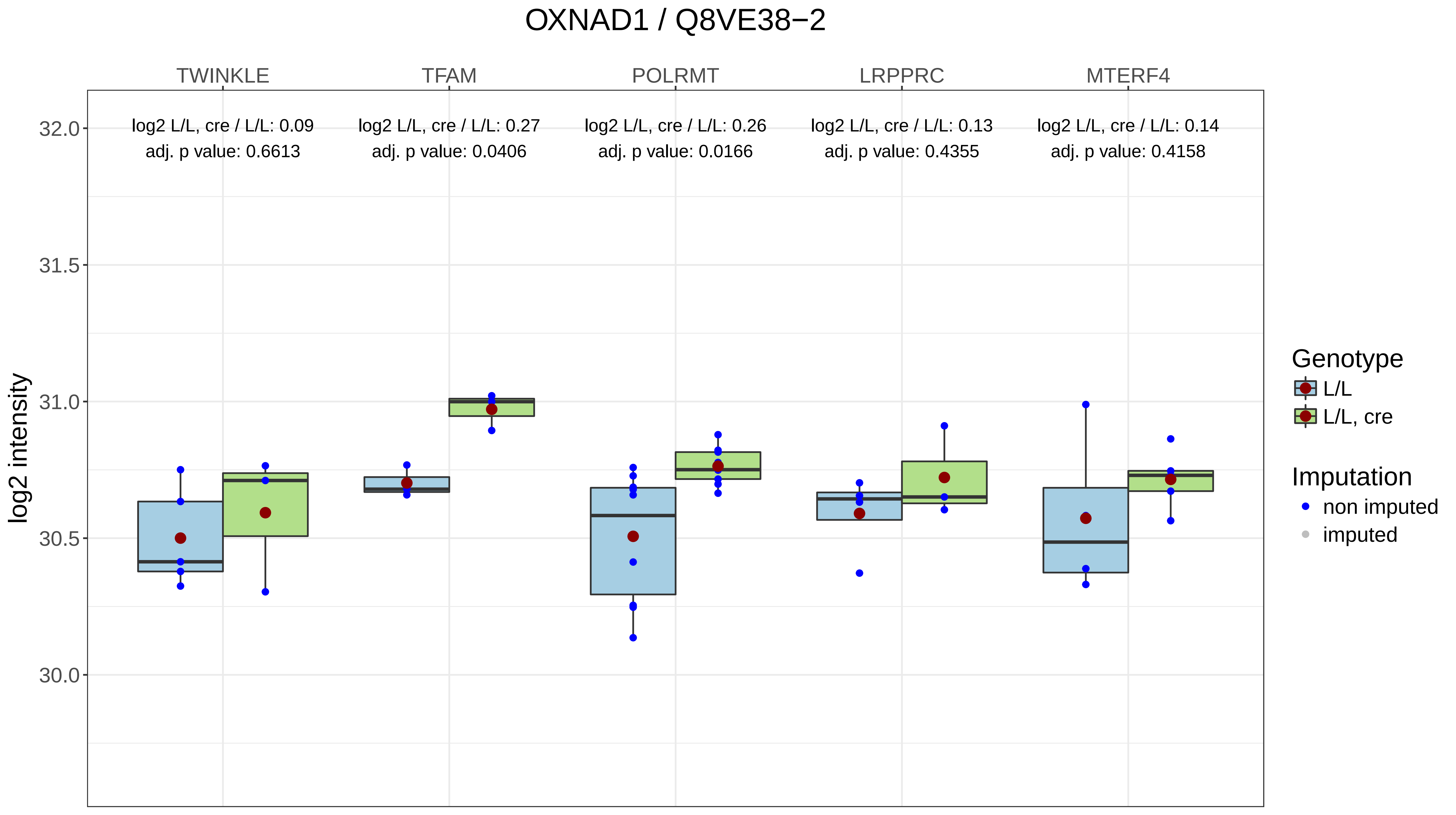
OXNAD1 / Q8VE38−2
TWINKLE
TFAM
POLRMT
LRPPRC
MTERF4
log2 L/L, cre / L/L: 0.09
adj. p value: 0.6613
log2 L/L, cre / L/L: 0.27
adj. p value: 0.0406
log2 L/L, cre / L/L: 0.26
adj. p value: 0.0166
log2 L/L, cre / L/L: 0.13
adj. p value: 0.4355
log2 L/L, cre / L/L: 0.14
adj. p value: 0.4158
log2 intensity
32.0
31.5
31.0
30.5
30.0
Genotype
L/L
L/L, cre
Imputation
non imputed
imputed

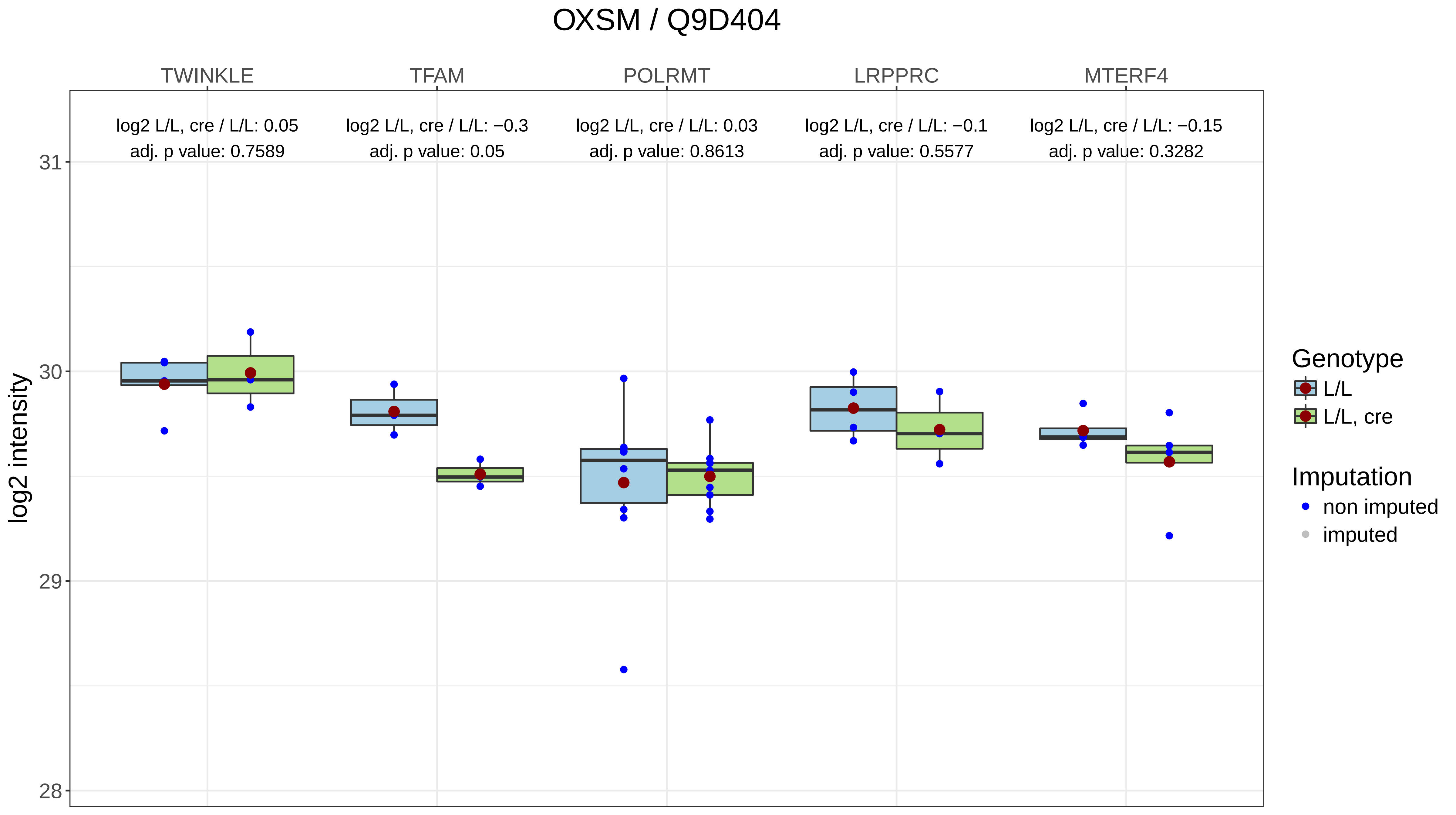
OXSM / Q9D404
TWINKLE
TFAM
POLRMT
LRPPRC
MTERF4
log2 L/L, cre / L/L: 0.05
adj. p value: 0.7589
log2 L/L, cre / L/L: −0.3
adj. p value: 0.05
log2 L/L, cre / L/L: 0.03
adj. p value: 0.8613
log2 L/L, cre / L/L: −0.1
adj. p value: 0.5577
log2 L/L, cre / L/L: −0.15
adj. p value: 0.3282
log2 intensity
31
30
29
28
Genotype
L/L
L/L, cre
Imputation
non imputed
imputed

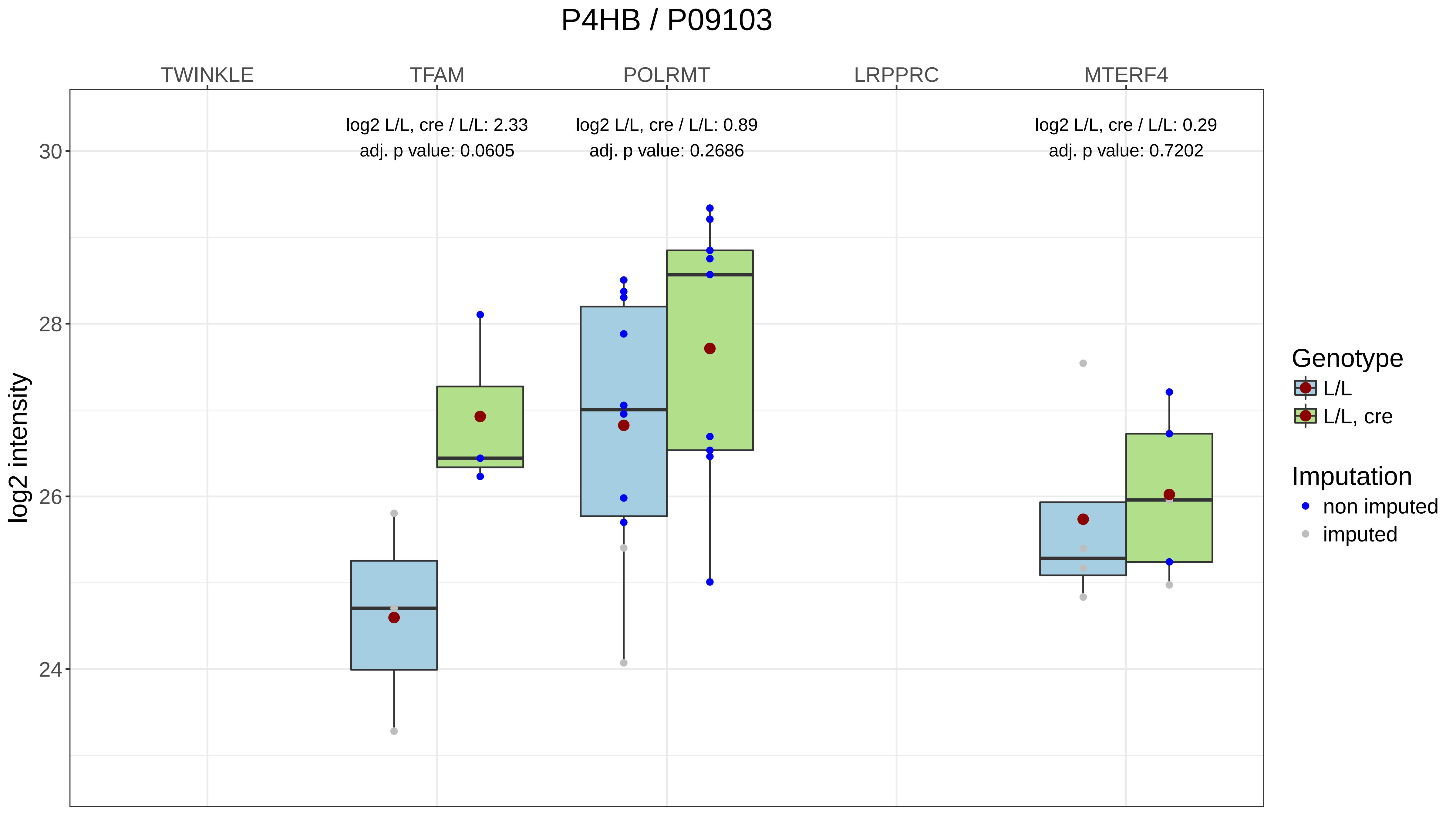
P4HB / P09103
TWINKLE
TFAM
POLRMT
LRPPRC
MTERF4
log2 L/L, cre / L/L: 2.33
adj. p value: 0.0605
log2 L/L, cre / L/L: 0.89
adj. p value: 0.2686
log2 L/L, cre / L/L: 0.29
adj. p value: 0.7202
30
28
26
24
log2 intensity
Genotype
L/L
L/L, cre
Imputation
non imputed
imputed

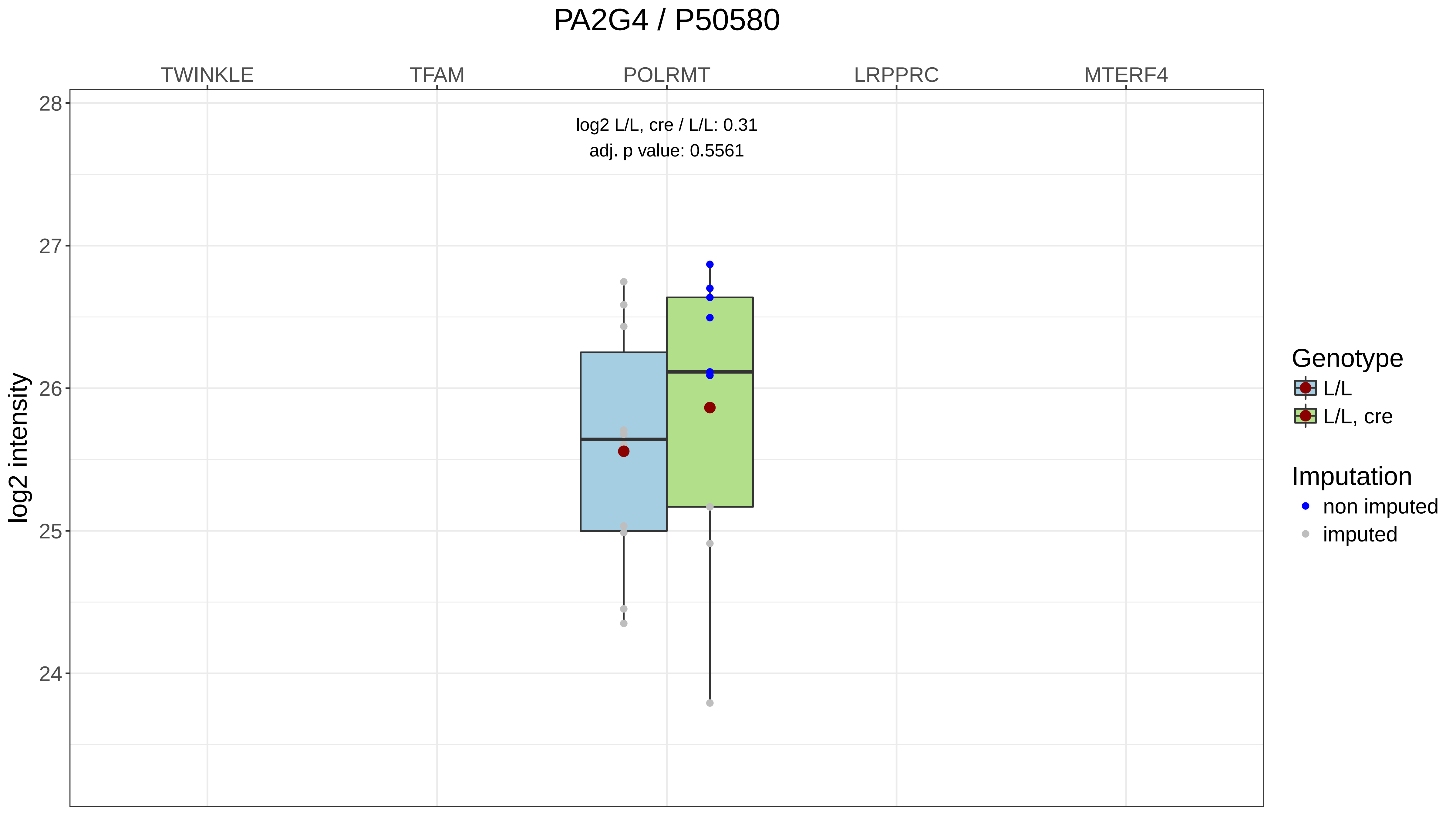
PA2G4 / P50580
TWINKLE
TFAM
POLRMT
LRPPRC
MTERF4
log2 L/L, cre / L/L: 0.31
adj. p value: 0.5561
log2 intensity
28
27
26
25
24
Genotype
L/L
L/L, cre
Imputation
non imputed
imputed

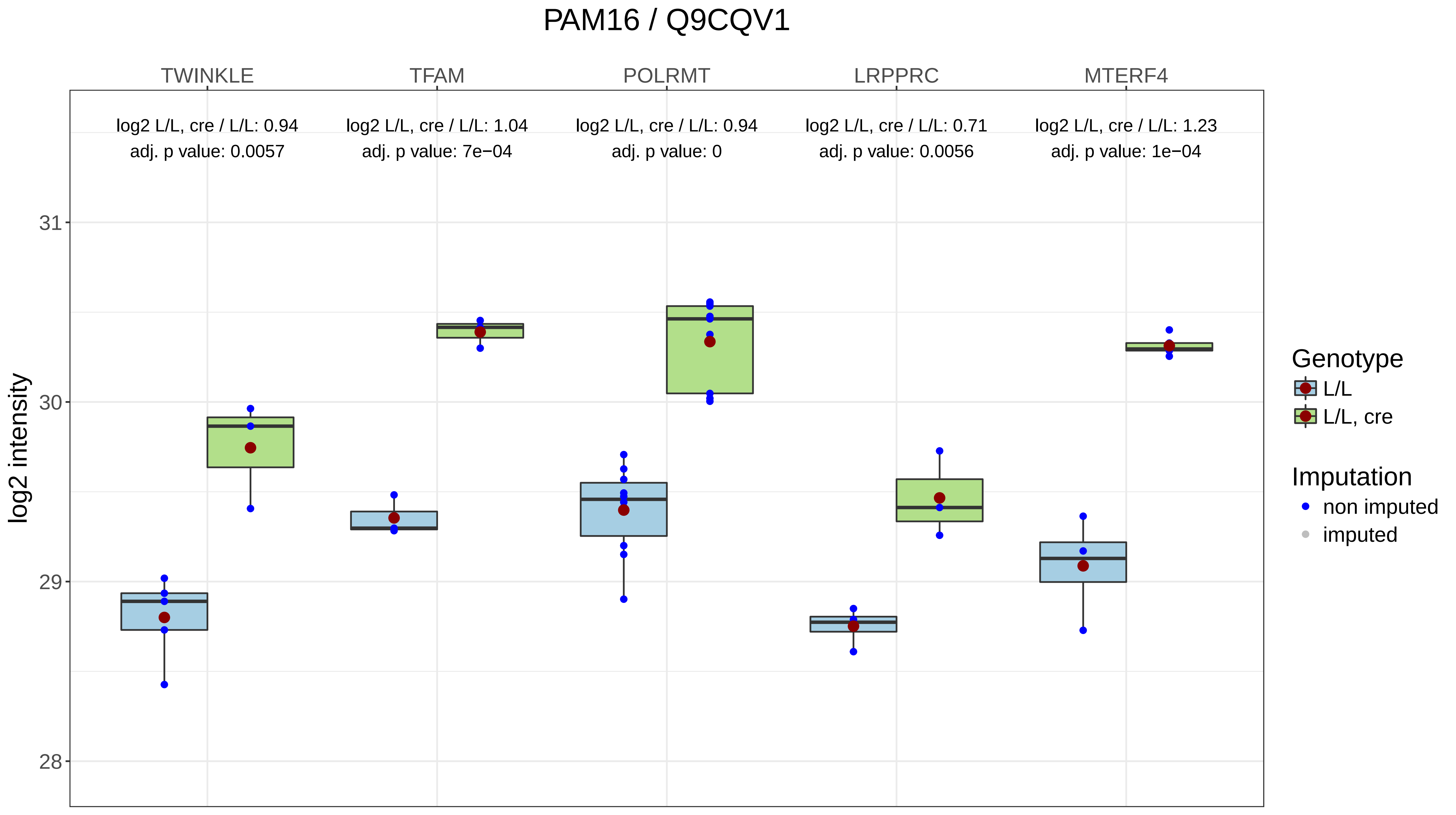
PAM16 / Q9CQV1
TWINKLE
TFAM
POLRMT
LRPPRC
MTERF4
log2 L/L, cre / L/L: 0.94
adj. p value: 0.0057
log2 L/L, cre / L/L: 1.04
adj. p value: 7e−04
log2 L/L, cre / L/L: 0.94
adj. p value: 0
log2 L/L, cre / L/L: 0.71
adj. p value: 0.0056
log2 L/L, cre / L/L: 1.23
adj. p value: 1e−04
log2 intensity
31
30
29
28
Genotype
L/L
L/L, cre
Imputation
non imputed
imputed

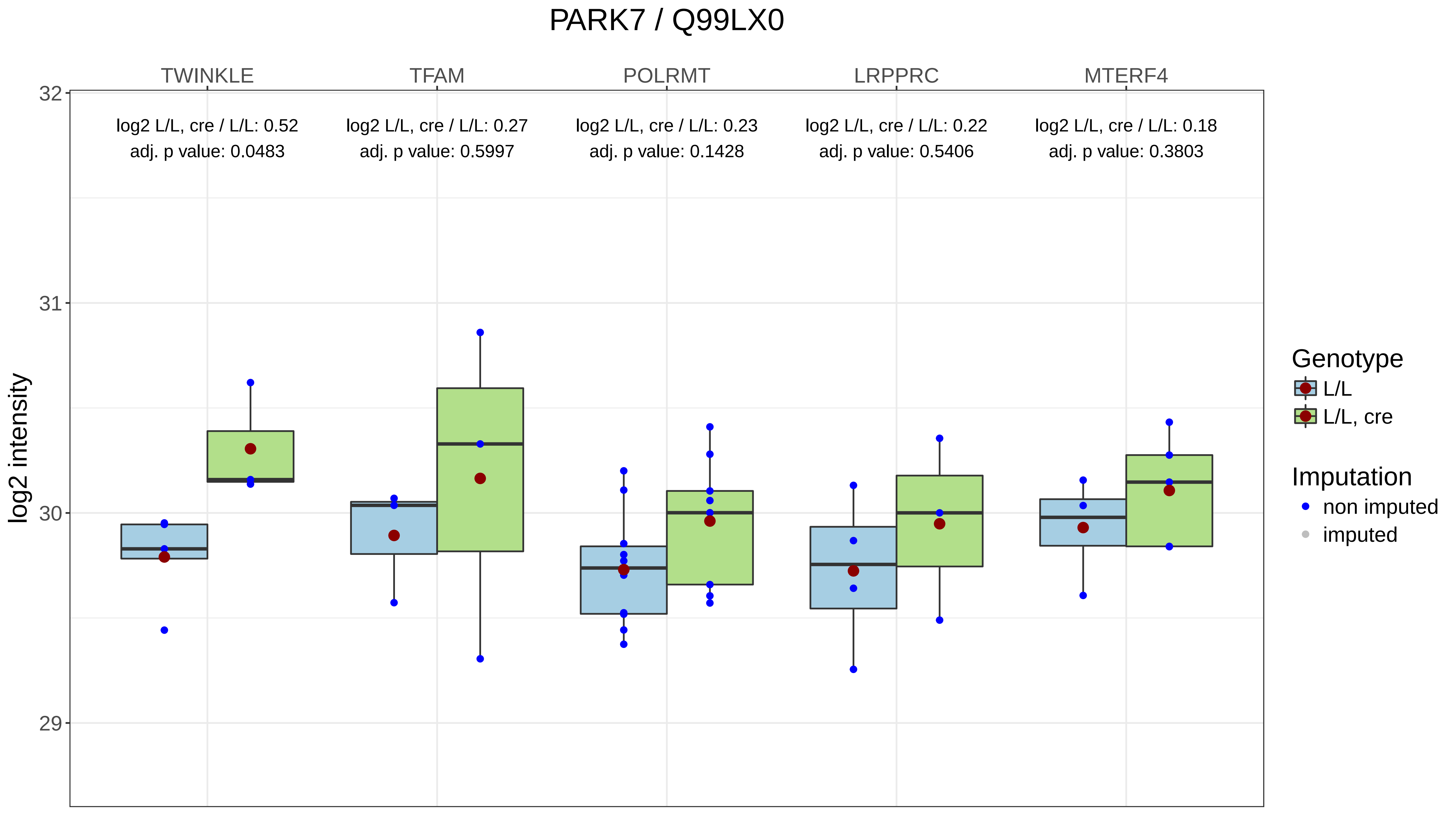

PARK7 / Q99LX0
TWINKLE
TFAM
POLRMT
LRPPRC
MTERF4
log2 L/L, cre / L/L: 0.52
adj. p value: 0.0483
log2 L/L, cre / L/L: 0.27
adj. p value: 0.5997
log2 L/L, cre / L/L: 0.23
adj. p value: 0.1428
log2 L/L, cre / L/L: 0.22
adj. p value: 0.5406
log2 L/L, cre / L/L: 0.18
adj. p value: 0.3803
log2 intensity
32
31
30
29
Genotype
L/L
L/L, cre
Imputation
non imputed
imputed

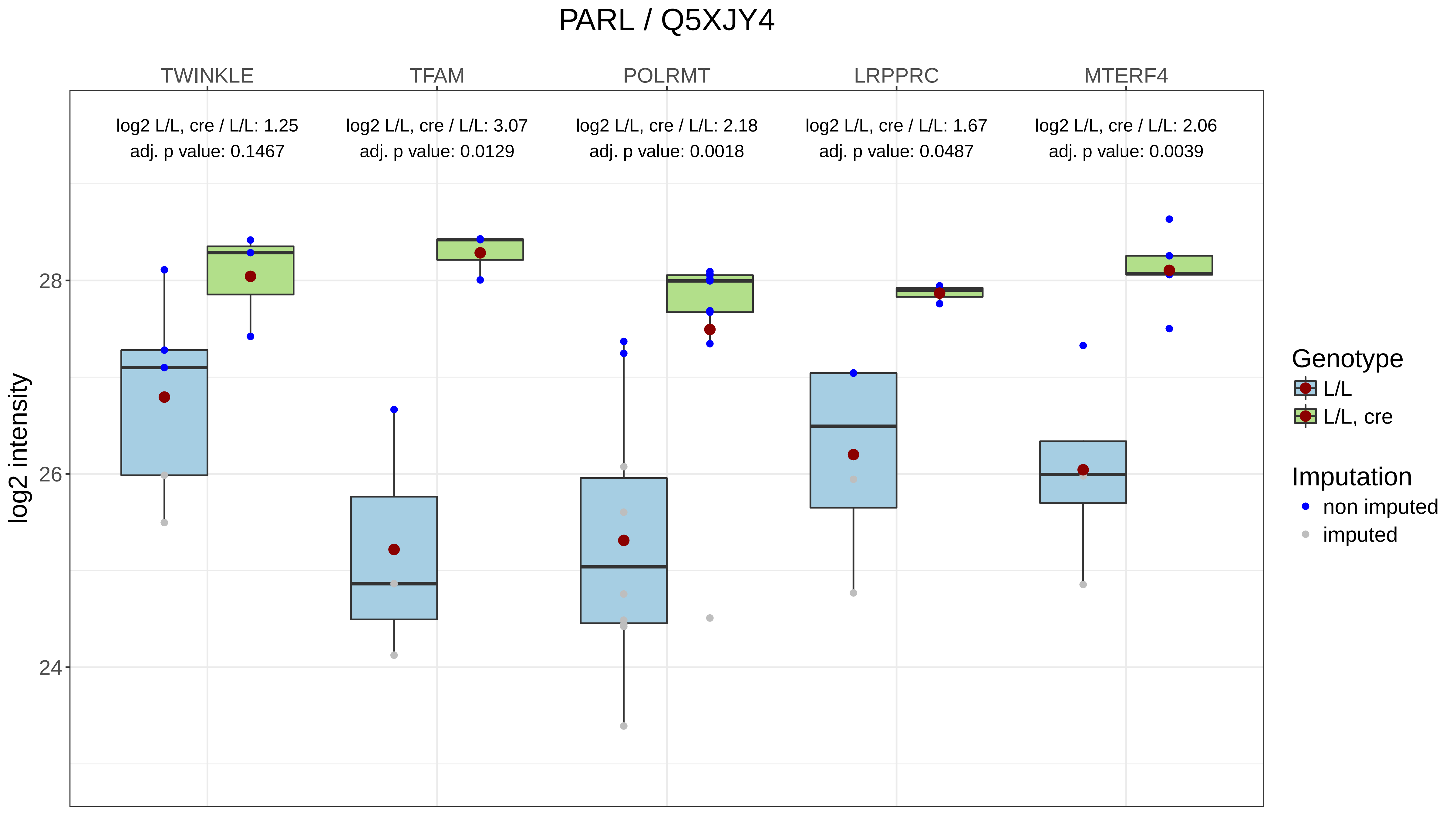
PARL / Q5XJY4
TWINKLE
TFAM
POLRMT
LRPPRC
MTERF4
log2 L/L, cre / L/L: 1.25
adj. p value: 0.1467
log2 L/L, cre / L/L: 3.07
adj. p value: 0.0129
log2 L/L, cre / L/L: 2.18
adj. p value: 0.0018
log2 L/L, cre / L/L: 1.67
adj. p value: 0.0487
log2 L/L, cre / L/L: 2.06
adj. p value: 0.0039
log2 intensity
28
26
24
Genotype
L/L
L/L, cre
Imputation
non imputed
imputed

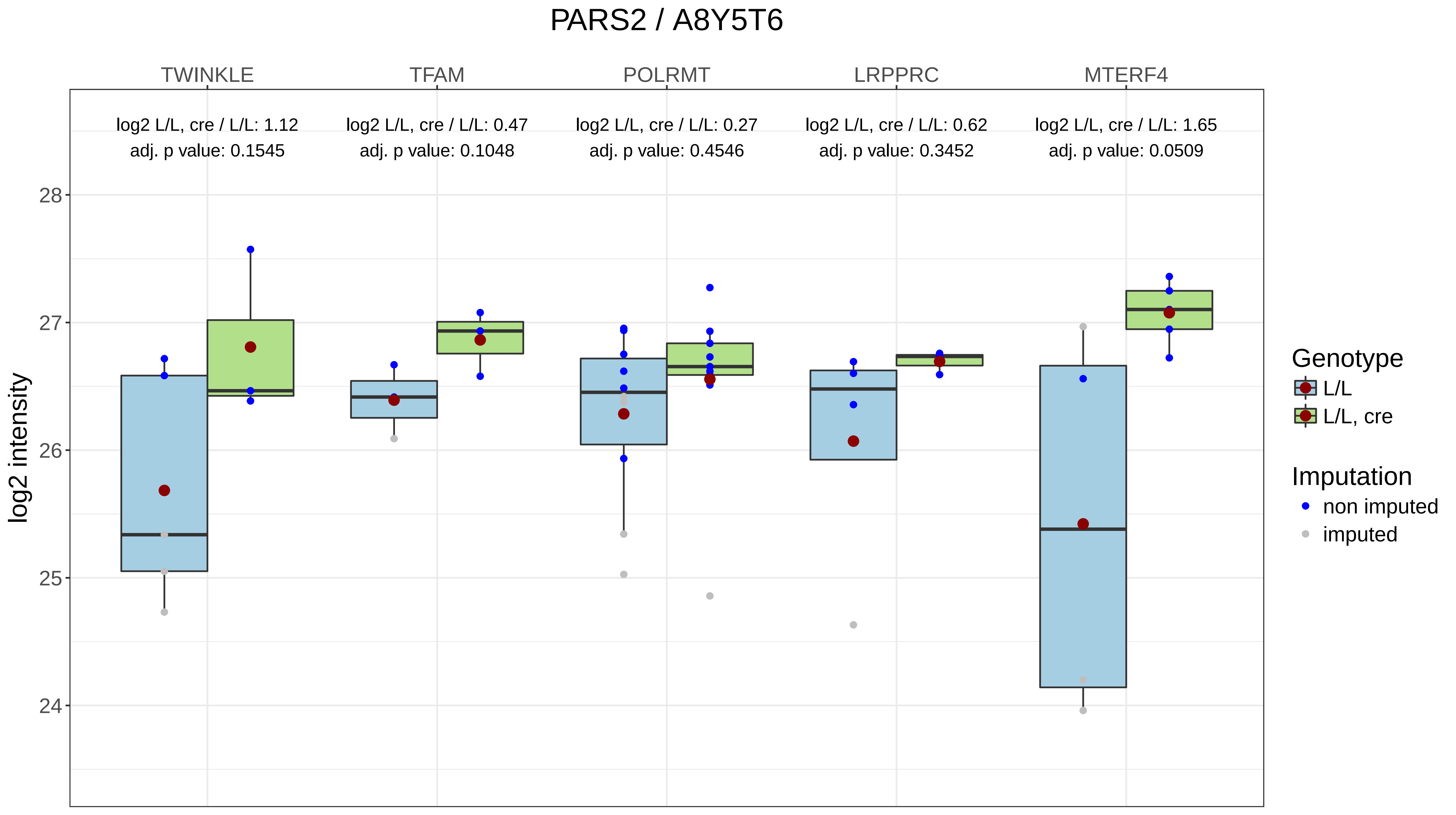
PARS2 / A8Y5T6
TWINKLE
TFAM
POLRMT
LRPPRC
MTERF4
log2 L/L, cre / L/L: 1.12
adj. p value: 0.1545
log2 L/L, cre / L/L: 0.47
adj. p value: 0.1048
log2 L/L, cre / L/L: 0.27
adj. p value: 0.4546
log2 L/L, cre / L/L: 0.62
adj. p value: 0.3452
log2 L/L, cre / L/L: 1.65
adj. p value: 0.0509
log2 intensity
28
27
26
25
24
Genotype
L/L
L/L, cre
Imputation
non imputed
imputed

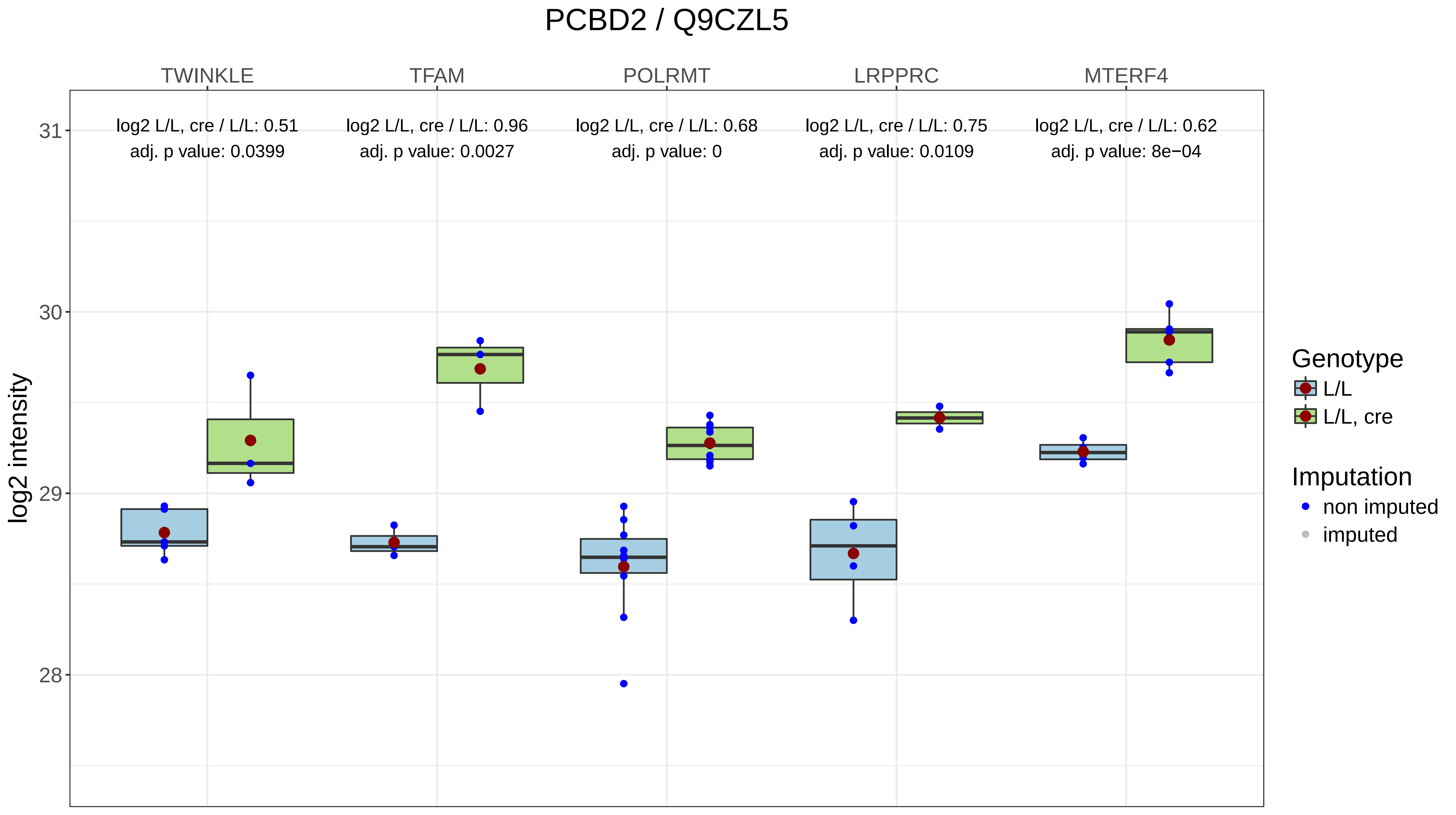
PCBD2 / Q9CZL5
TWINKLE
TFAM
POLRMT
LRPPRC
MTERF4
log2 L/L, cre / L/L: 0.51
adj. p value: 0.0399
log2 L/L, cre / L/L: 0.96
adj. p value: 0.0027
log2 L/L, cre / L/L: 0.68
adj. p value: 0
log2 L/L, cre / L/L: 0.75
adj. p value: 0.0109
log2 L/L, cre / L/L: 0.62
adj. p value: 8e−04
log2 intensity
31
30
29
28
Genotype
L/L
L/L, cre
Imputation
non imputed
imputed

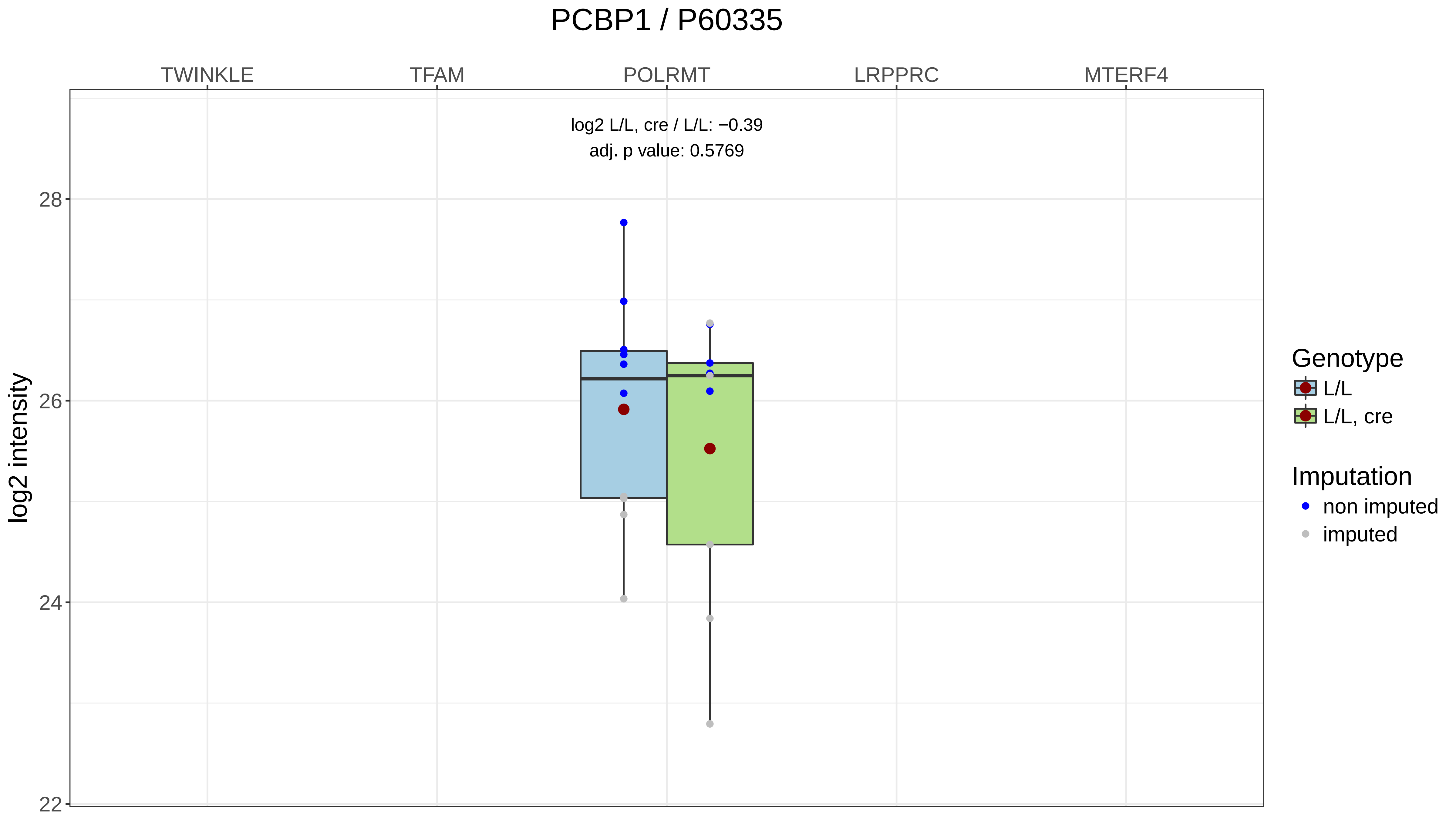

PCBP1 / P60335
TWINKLE
TFAM
POLRMT
LRPPRC
MTERF4
log2 L/L, cre / L/L: −0.39
adj. p value: 0.5769
log2 intensity
28
26
24
22
Genotype
L/L
L/L, cre
Imputation
non imputed
imputed

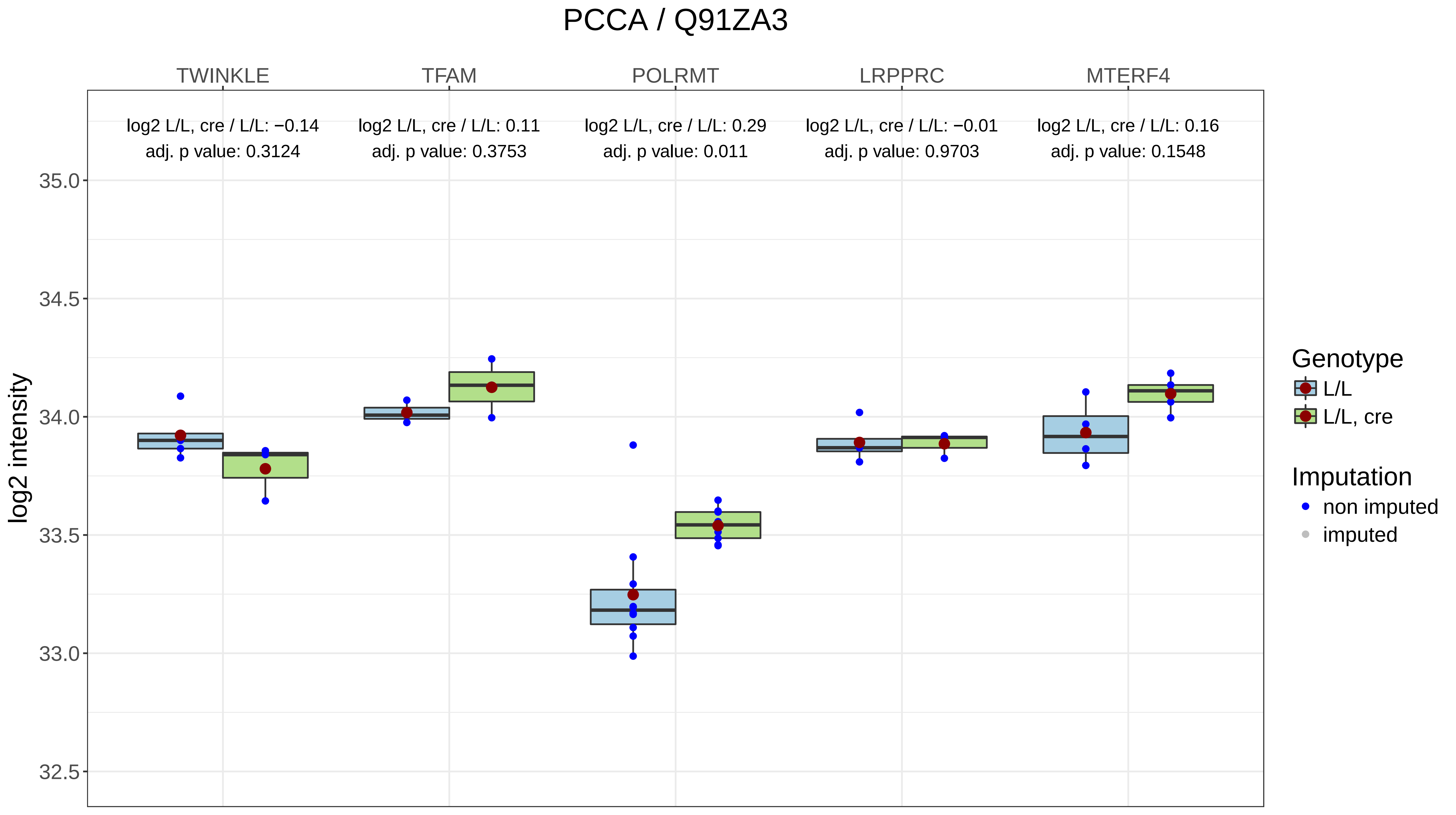
PCCA / Q91ZA3
TWINKLE
TFAM
POLRMT
LRPPRC
MTERF4
log2 L/L, cre / L/L: −0.14
adj. p value: 0.3124
log2 L/L, cre / L/L: 0.11
adj. p value: 0.3753
log2 L/L, cre / L/L: 0.29
adj. p value: 0.011
log2 L/L, cre / L/L: −0.01
adj. p value: 0.9703
log2 L/L, cre / L/L: 0.16
adj. p value: 0.1548
log2 intensity
35.0
34.5
34.0
33.5
33.0
32.5
Genotype
L/L
L/L, cre
Imputation
non imputed
imputed

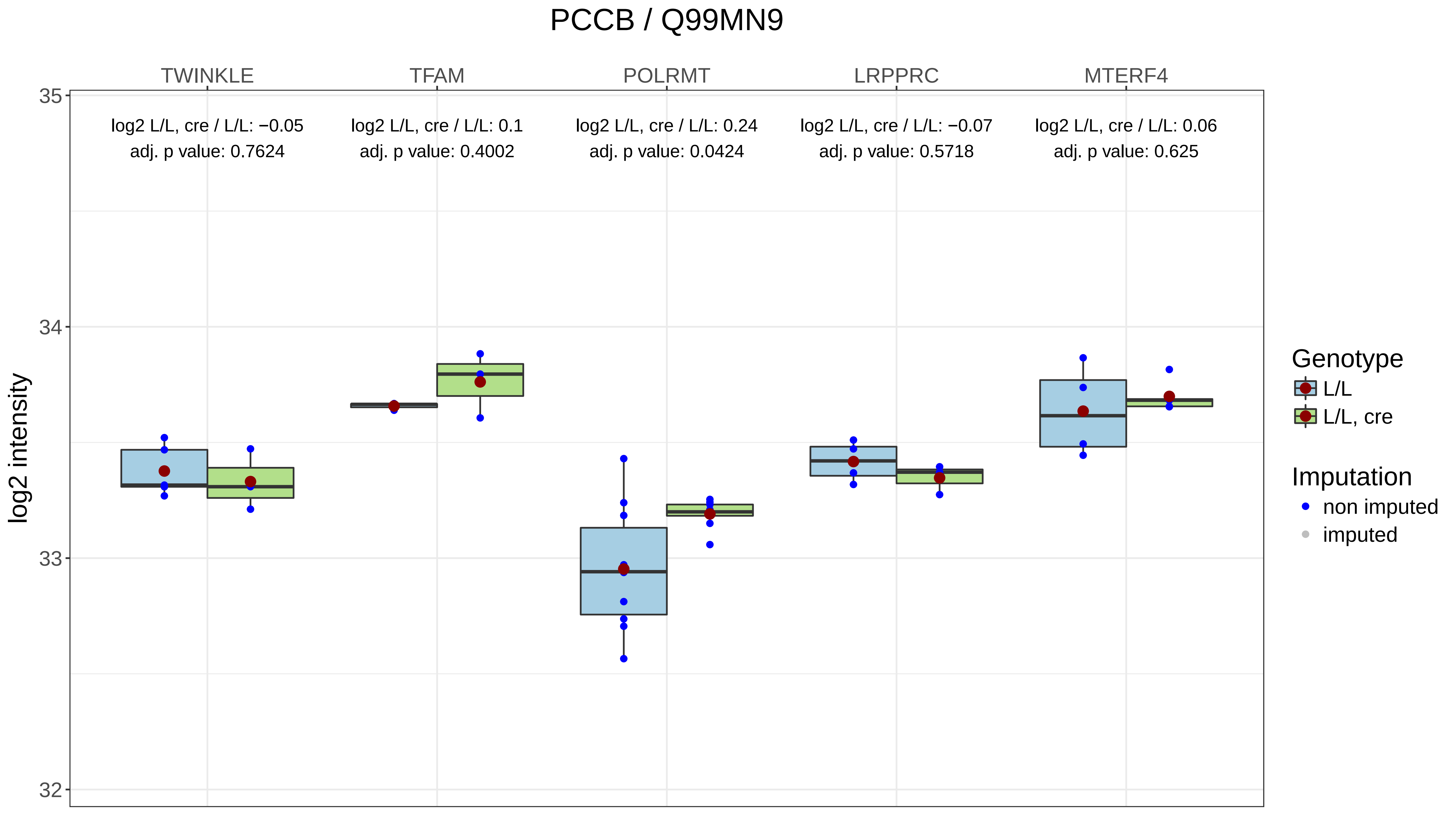
PCCB / Q99MN9
TWINKLE
TFAM
POLRMT
LRPPRC
MTERF4
log2 L/L, cre / L/L: −0.05
adj. p value: 0.7624
log2 L/L, cre / L/L: 0.1
adj. p value: 0.4002
log2 L/L, cre / L/L: 0.24
adj. p value: 0.0424
log2 L/L, cre / L/L: −0.07
adj. p value: 0.5718
log2 L/L, cre / L/L: 0.06
adj. p value: 0.625
log2 intensity
35
34
33
32
Genotype
L/L
L/L, cre
Imputation
non imputed
imputed

PCK2 / Q8BH04

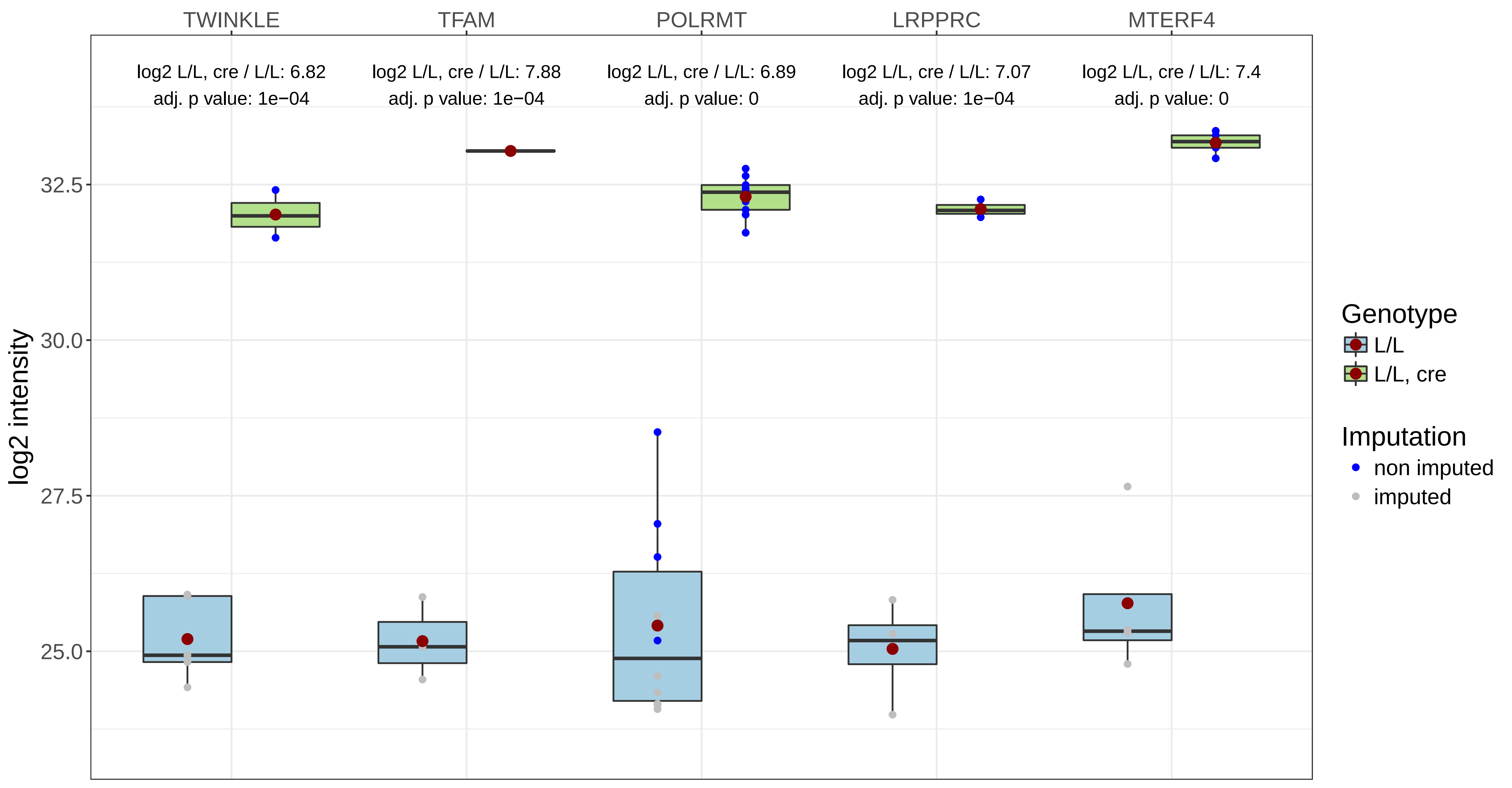

TWINKLE
TFAM
POLRMT
LRPPRC
MTERF4
log2 L/L, cre / L/L: 6.82
adj. p value: 1e−04
log2 L/L, cre / L/L: 7.88
adj. p value: 1e−04
log2 L/L, cre / L/L: 6.89
adj. p value: 0
log2 L/L, cre / L/L: 7.07
adj. p value: 1e−04
log2 L/L, cre / L/L: 7.4
adj. p value: 0
log2 intensity
32.5
30.0
27.5
25.0
Genotype
L/L
L/L, cre
Imputation
non imputed
imputed

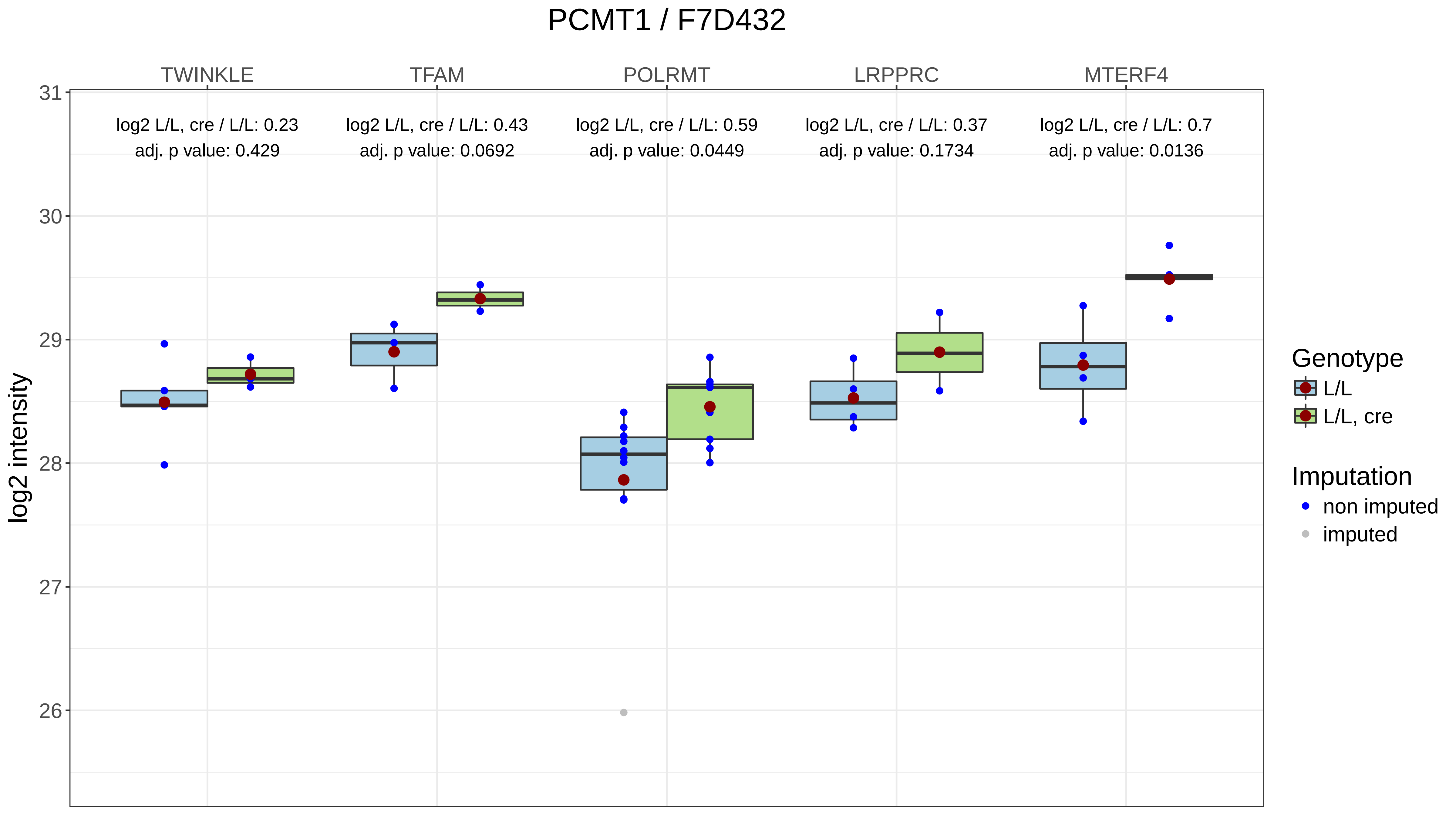
PCMT1 / F7D432
TWINKLE
TFAM
POLRMT
LRPPRC
MTERF4
log2 L/L, cre / L/L: 0.23
adj. p value: 0.429
log2 L/L, cre / L/L: 0.43
adj. p value: 0.0692
log2 L/L, cre / L/L: 0.59
adj. p value: 0.0449
log2 L/L, cre / L/L: 0.37
adj. p value: 0.1734
log2 L/L, cre / L/L: 0.7
adj. p value: 0.0136
log2 intensity
31
30
29
28
27
26
Genotype
L/L
L/L, cre
Imputation
non imputed
imputed

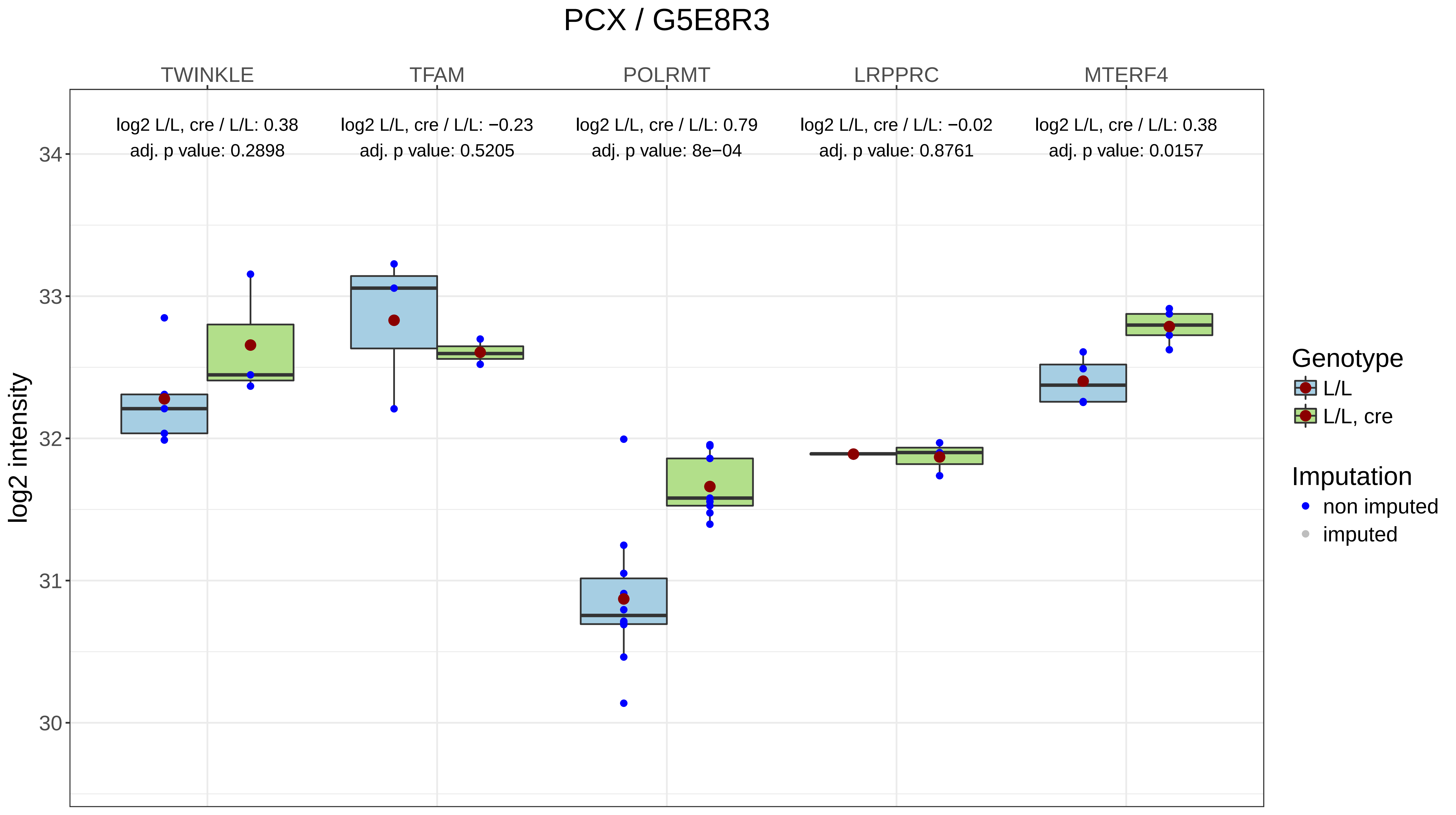
PCX / G5E8R3
TWINKLE
TFAM
POLRMT
LRPPRC
MTERF4
log2 L/L, cre / L/L: 0.38
adj. p value: 0.2898
log2 L/L, cre / L/L: −0.23
adj. p value: 0.5205
log2 L/L, cre / L/L: 0.79
adj. p value: 8e−04
log2 L/L, cre / L/L: −0.02
adj. p value: 0.8761
log2 L/L, cre / L/L: 0.38
adj. p value: 0.0157
log2 intensity
34
33
32
31
30
Genotype
L/L
L/L, cre
Imputation
non imputed
imputed

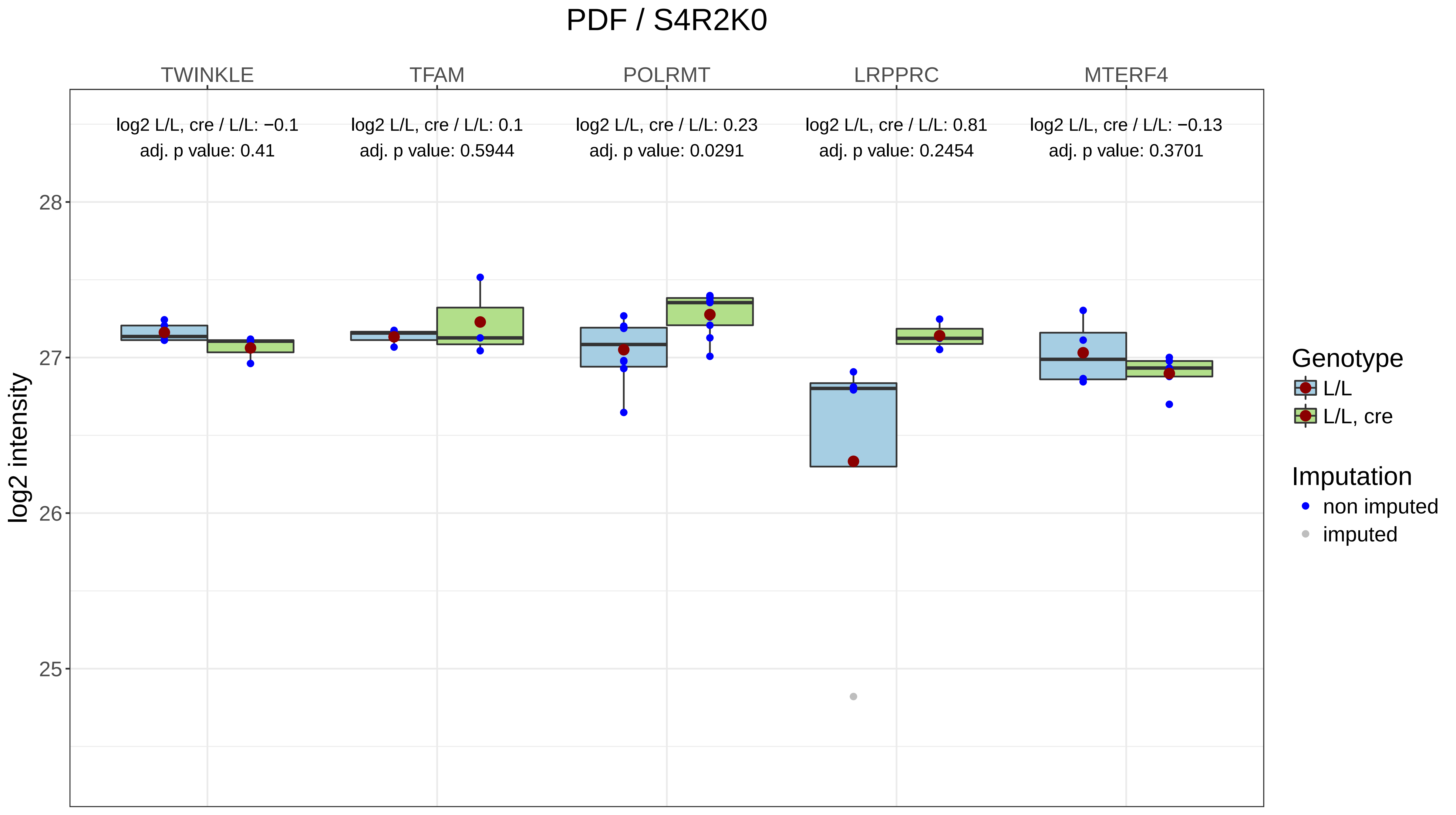
PDF / S4R2K0
TWINKLE
TFAM
POLRMT
LRPPRC
MTERF4
log2 L/L, cre / L/L: −0.1
adj. p value: 0.41
log2 L/L, cre / L/L: 0.1
adj. p value: 0.5944
log2 L/L, cre / L/L: 0.23
adj. p value: 0.0291
log2 L/L, cre / L/L: 0.81
adj. p value: 0.2454
log2 L/L, cre / L/L: −0.13
adj. p value: 0.3701
log2 intensity
28
27
26
25
Genotype
L/L
L/L, cre
Imputation
non imputed
imputed

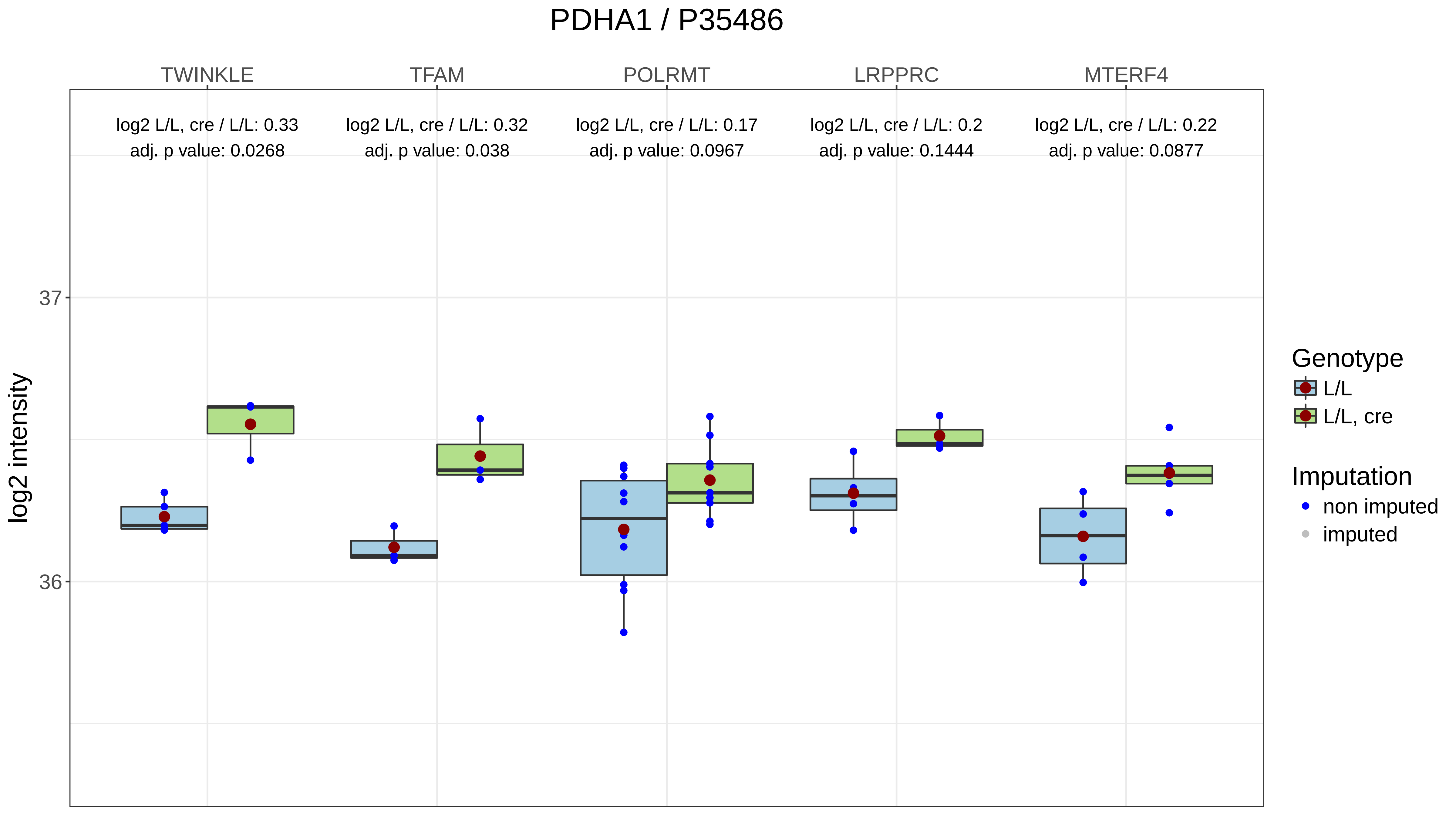

PDHA1 / P35486
TWINKLE
TFAM
POLRMT
LRPPRC
MTERF4
log2 L/L, cre / L/L: 0.33
adj. p value: 0.0268
log2 L/L, cre / L/L: 0.32
adj. p value: 0.038
log2 L/L, cre / L/L: 0.17
adj. p value: 0.0967
log2 L/L, cre / L/L: 0.2
adj. p value: 0.1444
log2 L/L, cre / L/L: 0.22
adj. p value: 0.0877
37
36
log2 intensity
Genotype
L/L
L/L, cre
Imputation
non imputed
imputed

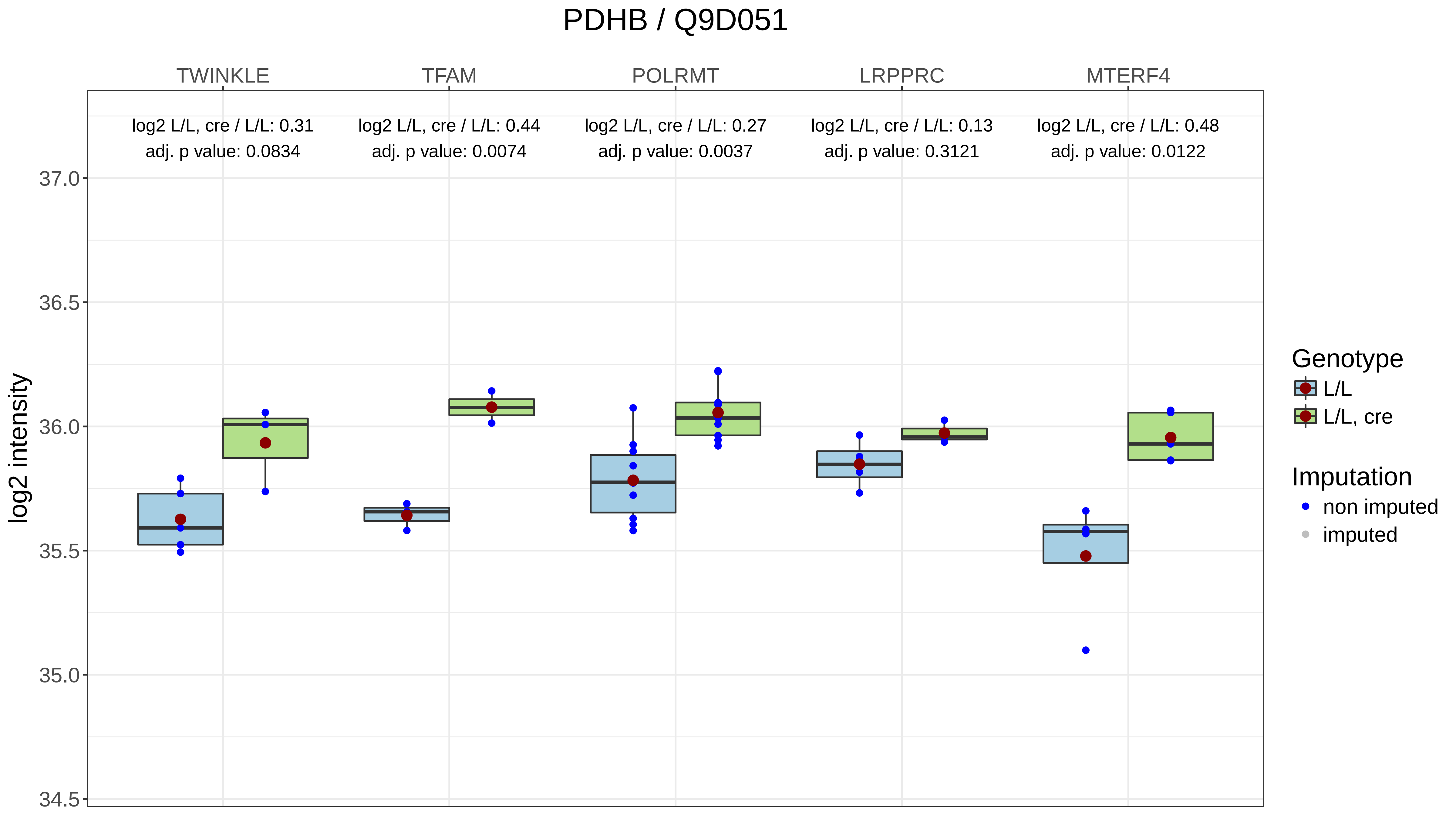
PDHB / Q9D051
TWINKLE
TFAM
POLRMT
LRPPRC
MTERF4
log2 L/L, cre / L/L: 0.31
adj. p value: 0.0834
log2 L/L, cre / L/L: 0.44
adj. p value: 0.0074
log2 L/L, cre / L/L: 0.27
adj. p value: 0.0037
log2 L/L, cre / L/L: 0.13
adj. p value: 0.3121
log2 L/L, cre / L/L: 0.48
adj. p value: 0.0122
log2 intensity
37.0
36.5
36.0
35.5
35.0
34.5
Genotype
L/L
L/L, cre
Imputation
non imputed
imputed

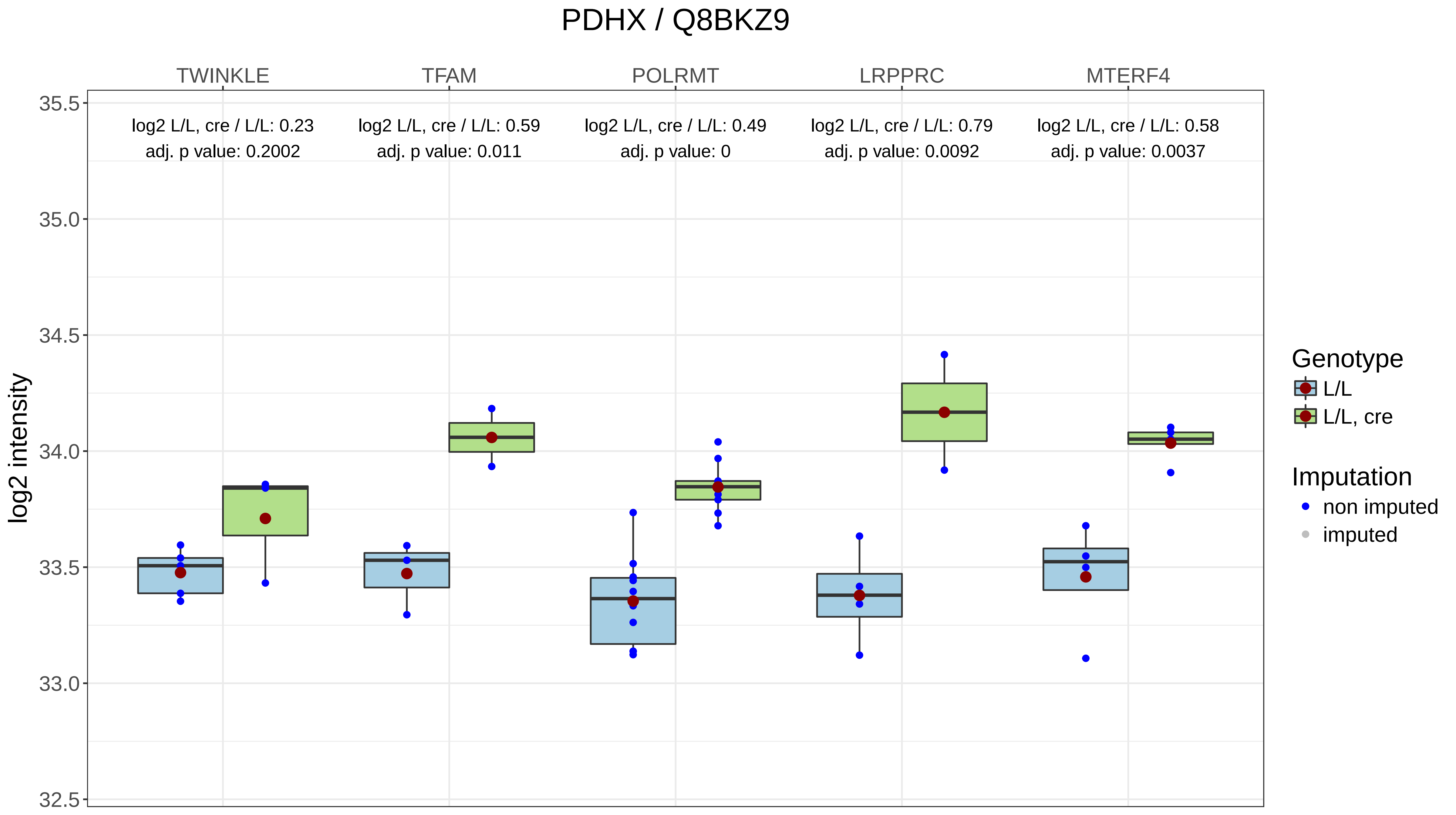
PDHX / Q8BKZ9
TWINKLE
TFAM
POLRMT
LRPPRC
MTERF4
log2 L/L, cre / L/L: 0.23
adj. p value: 0.2002
log2 L/L, cre / L/L: 0.59
adj. p value: 0.011
log2 L/L, cre / L/L: 0.49
adj. p value: 0
log2 L/L, cre / L/L: 0.79
adj. p value: 0.0092
log2 L/L, cre / L/L: 0.58
adj. p value: 0.0037
log2 intensity
35.5
35.0
34.5
34.0
33.5
33.0
32.5
Genotype
L/L
L/L, cre
Imputation
non imputed
imputed

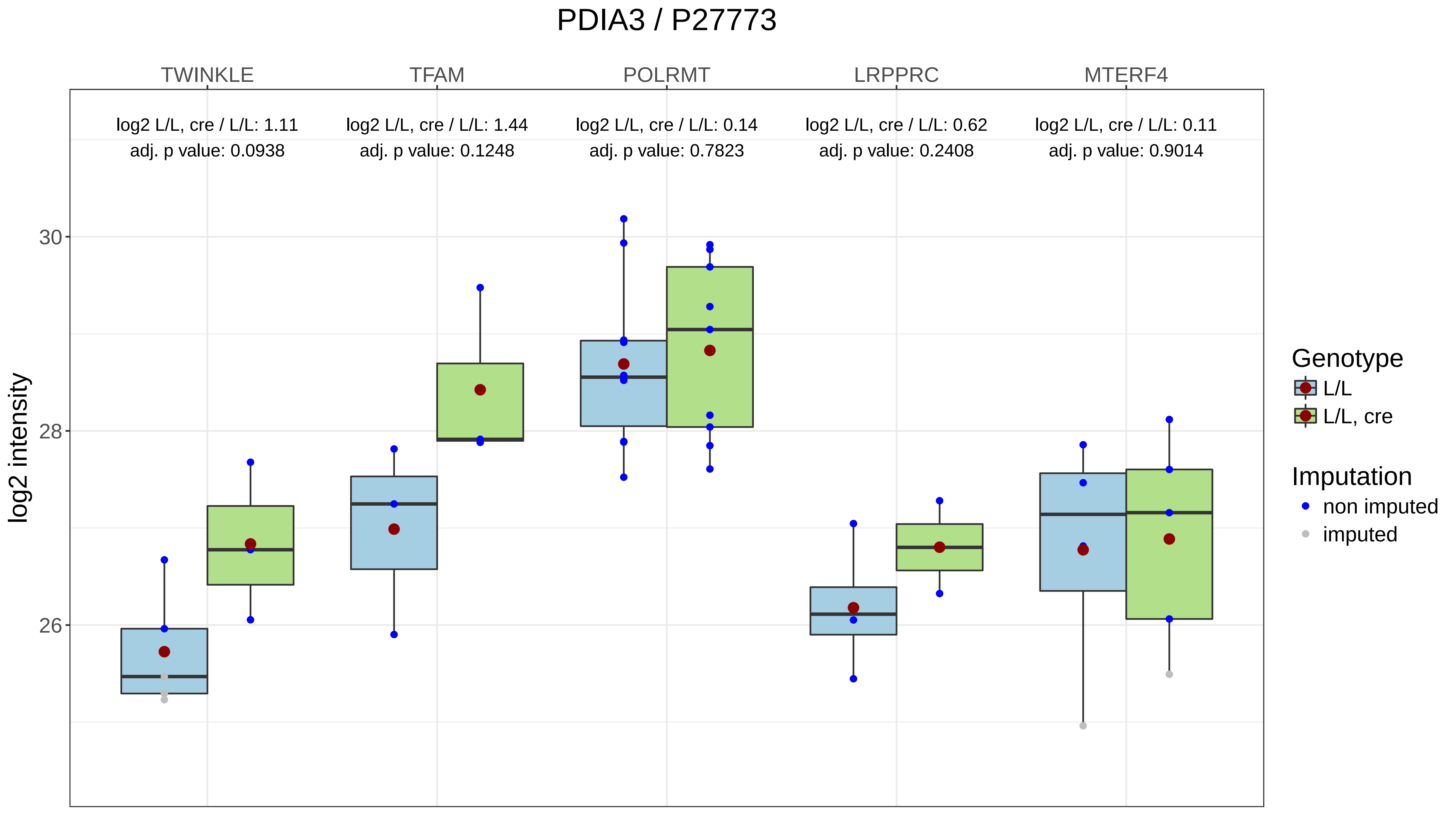
PDIA3 / P27773
TWINKLE
TFAM
POLRMT
LRPPRC
MTERF4
log2 L/L, cre / L/L: 1.11
adj. p value: 0.0938
log2 L/L, cre / L/L: 1.44
adj. p value: 0.1248
log2 L/L, cre / L/L: 0.14
adj. p value: 0.7823
log2 L/L, cre / L/L: 0.62
adj. p value: 0.2408
log2 L/L, cre / L/L: 0.11
adj. p value: 0.9014
30
28
26
log2 intensity
Genotype
L/L
L/L, cre
Imputation
non imputed
imputed

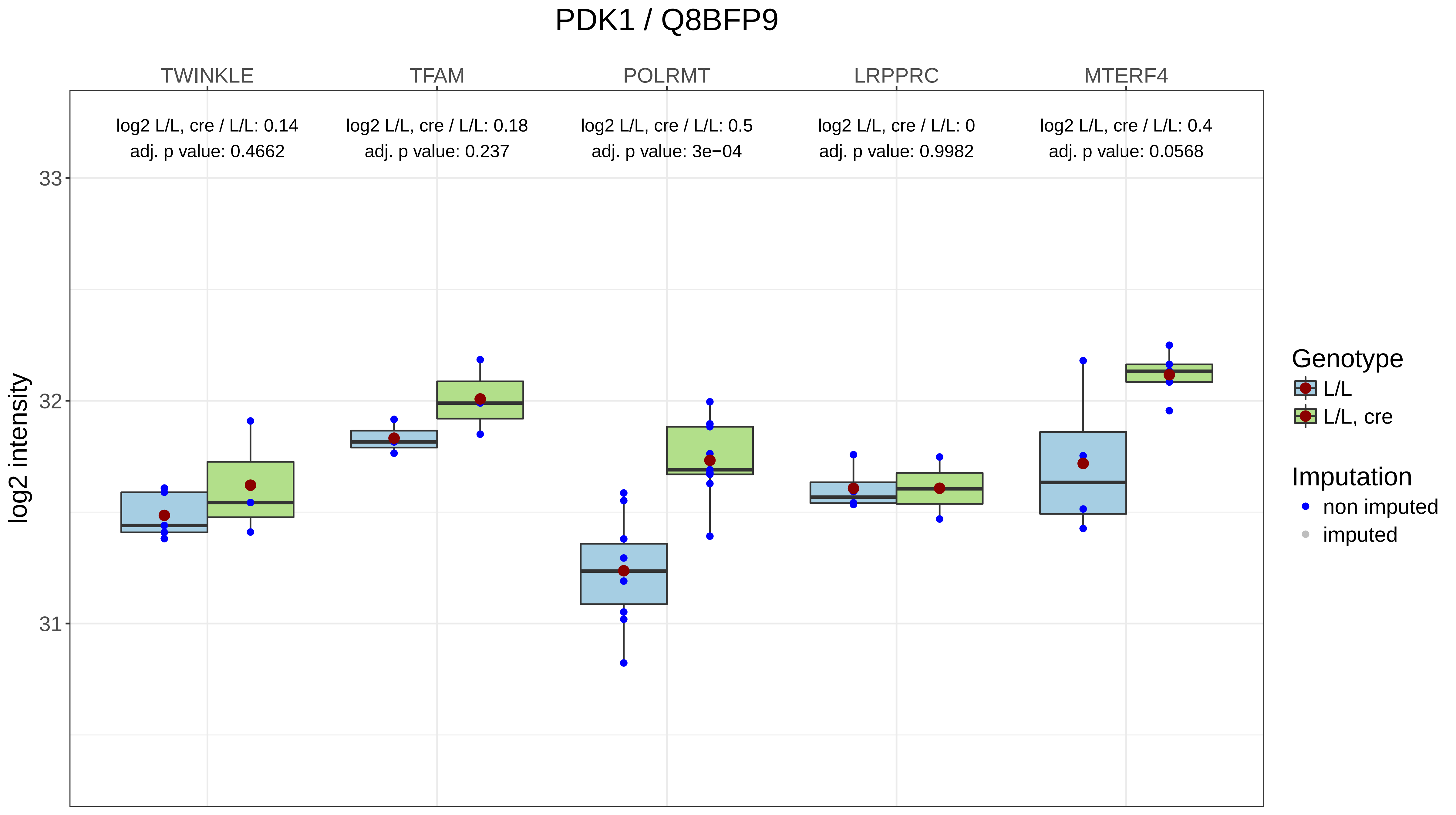
PDK1 / Q8BFP9
TWINKLE
TFAM
POLRMT
LRPPRC
MTERF4
log2 L/L, cre / L/L: 0.14
adj. p value: 0.4662
log2 L/L, cre / L/L: 0.18
adj. p value: 0.237
log2 L/L, cre / L/L: 0.5
adj. p value: 3e−04
log2 L/L, cre / L/L: 0
adj. p value: 0.9982
log2 L/L, cre / L/L: 0.4
adj. p value: 0.0568
log2 intensity
33
32
31
Genotype
L/L
L/L, cre
Imputation
non imputed
imputed

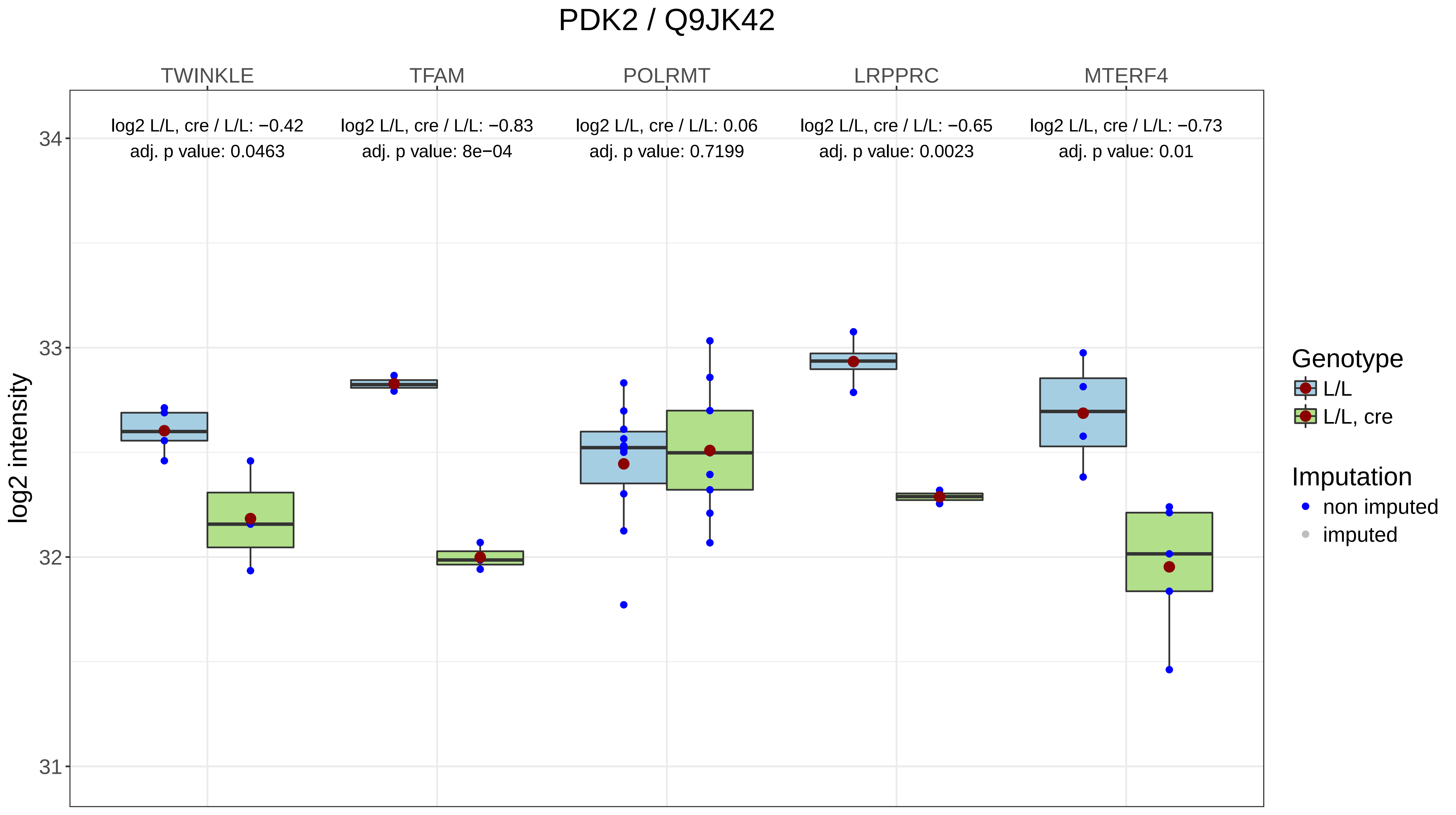
PDK2 / Q9JK42
TWINKLE
TFAM
POLRMT
LRPPRC
MTERF4
log2 L/L, cre / L/L: −0.42
adj. p value: 0.0463
log2 L/L, cre / L/L: −0.83
adj. p value: 8e−04
log2 L/L, cre / L/L: 0.06
adj. p value: 0.7199
log2 L/L, cre / L/L: −0.65
adj. p value: 0.0023
log2 L/L, cre / L/L: −0.73
adj. p value: 0.01
log2 intensity
34
33
32
31
Genotype
L/L
L/L, cre
Imputation
non imputed
imputed

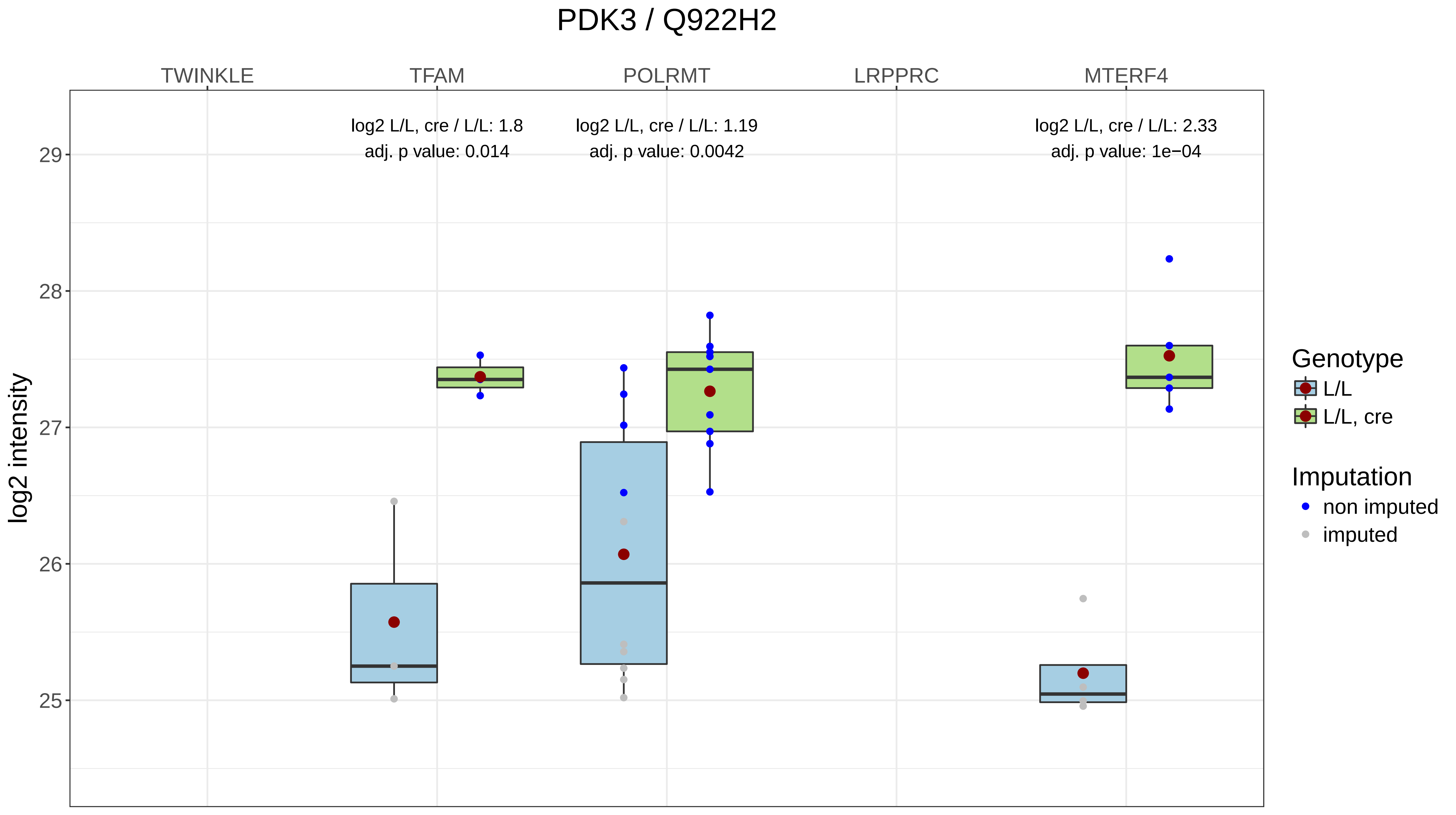
PDK3 / Q922H2
TWINKLE
TFAM
POLRMT
LRPPRC
MTERF4
log2 L/L, cre / L/L: 1.8
adj. p value: 0.014
log2 L/L, cre / L/L: 1.19
adj. p value: 0.0042
log2 L/L, cre / L/L: 2.33
adj. p value: 1e−04
log2 intensity
29
28
27
26
25
Genotype
L/L
L/L, cre
Imputation
non imputed
imputed

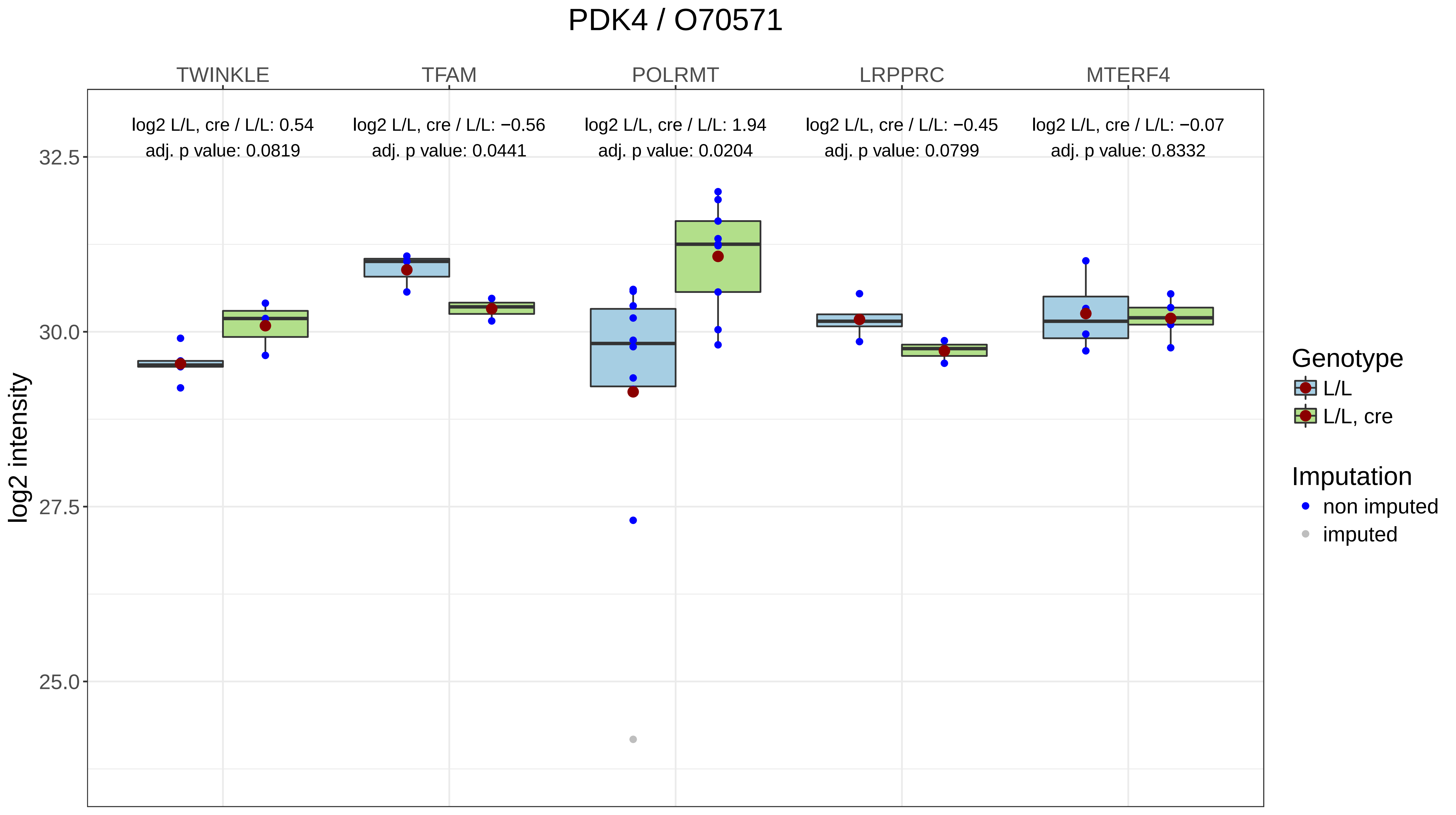
PDK4 / O70571
TWINKLE
TFAM
POLRMT
LRPPRC
MTERF4
log2 L/L, cre / L/L: 0.54
adj. p value: 0.0819
log2 L/L, cre / L/L: −0.56
adj. p value: 0.0441
log2 L/L, cre / L/L: 1.94
adj. p value: 0.0204
log2 L/L, cre / L/L: −0.45
adj. p value: 0.0799
log2 L/L, cre / L/L: −0.07
adj. p value: 0.8332
32.5
30.0
27.5
25.0
log2 intensity
Genotype
L/L
L/L, cre
Imputation
non imputed
imputed

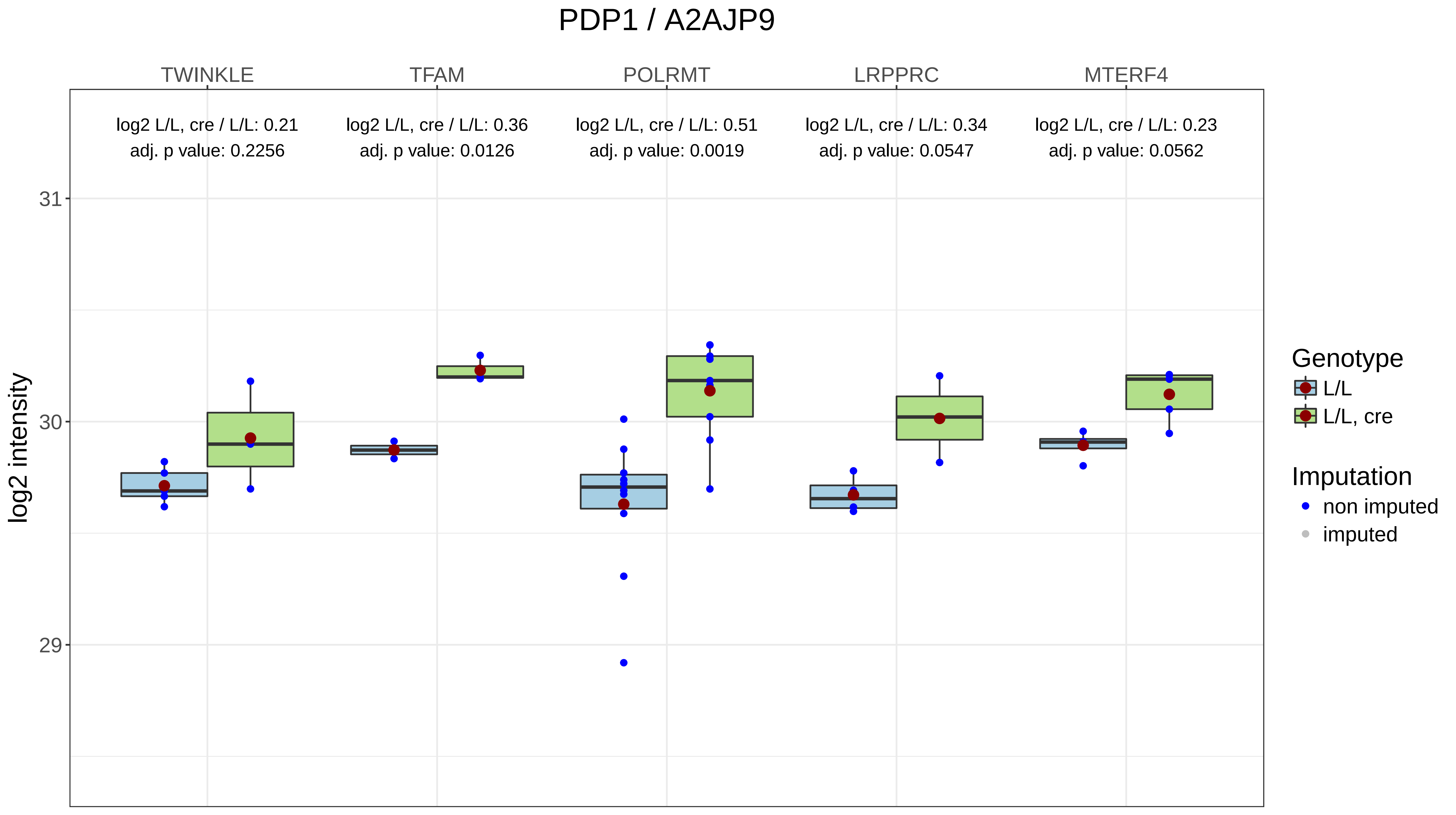
PDP1 / A2AJP9
TWINKLE
TFAM
POLRMT
LRPPRC
MTERF4
log2 L/L, cre / L/L: 0.21
adj. p value: 0.2256
log2 L/L, cre / L/L: 0.36
adj. p value: 0.0126
log2 L/L, cre / L/L: 0.51
adj. p value: 0.0019
log2 L/L, cre / L/L: 0.34
adj. p value: 0.0547
log2 L/L, cre / L/L: 0.23
adj. p value: 0.0562
log2 intensity
31
30
29
Genotype
L/L
L/L, cre
Imputation
non imputed
imputed

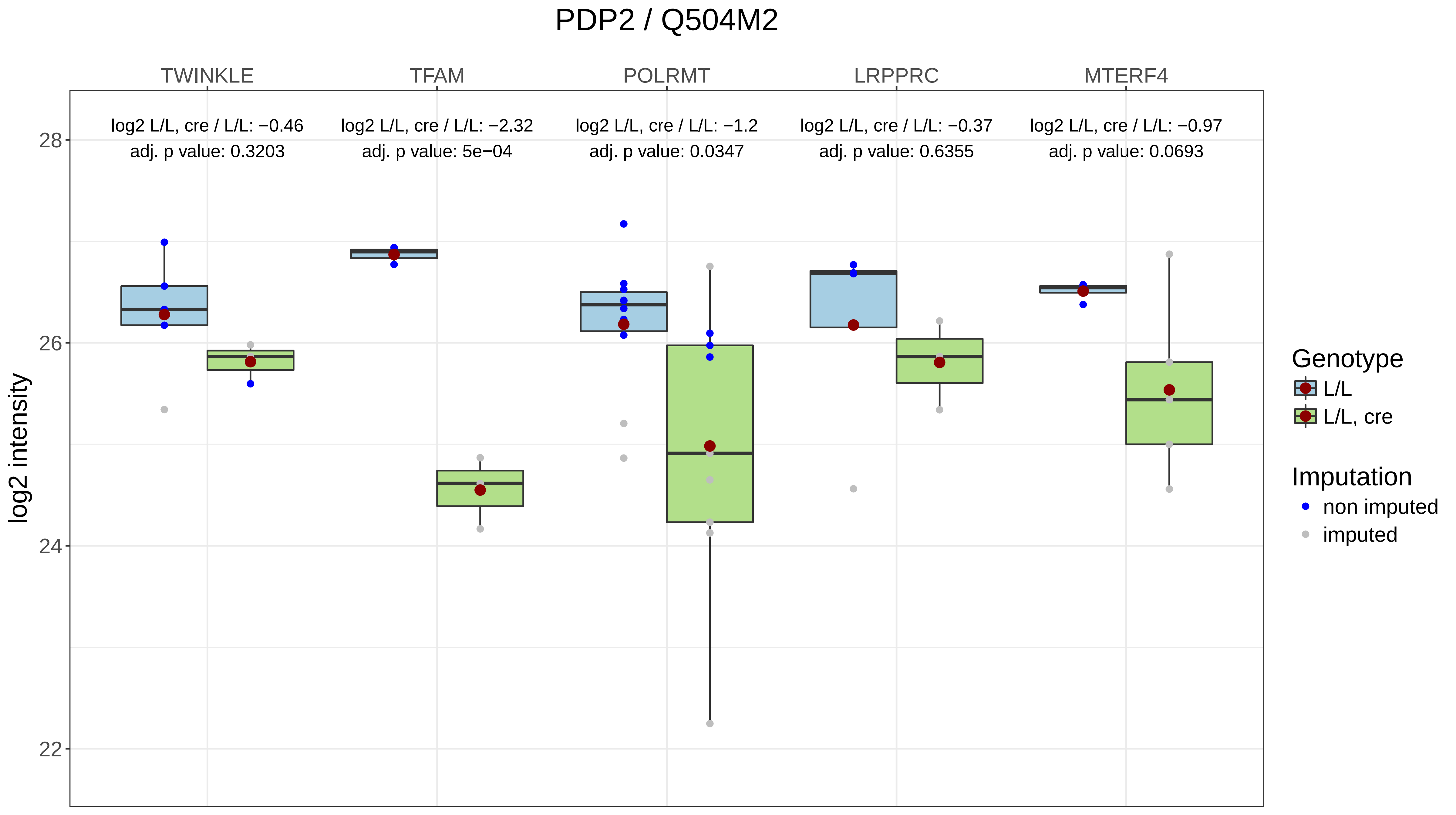

PDP2 / Q504M2
TWINKLE
TFAM
POLRMT
LRPPRC
MTERF4
log2 L/L, cre / L/L: −0.46
adj. p value: 0.3203
log2 L/L, cre / L/L: −2.32
adj. p value: 5e−04
log2 L/L, cre / L/L: −1.2
adj. p value: 0.0347
log2 L/L, cre / L/L: −0.37
adj. p value: 0.6355
log2 L/L, cre / L/L: −0.97
adj. p value: 0.0693
log2 intensity
28
26
24
22
Genotype
L/L
L/L, cre
Imputation
non imputed
imputed

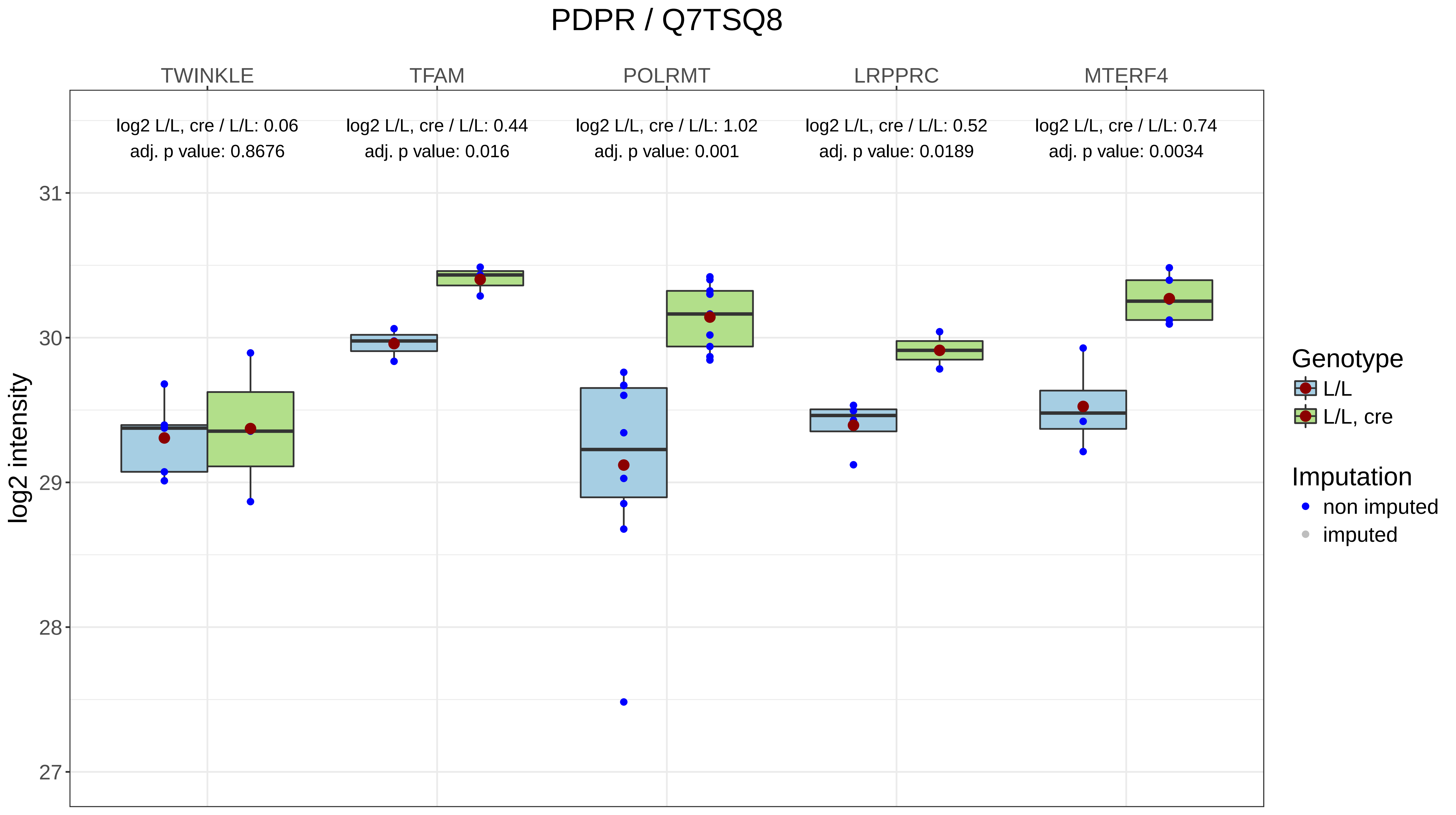
PDPR / Q7TSQ8
TWINKLE
TFAM
POLRMT
LRPPRC
MTERF4
log2 L/L, cre / L/L: 0.06
adj. p value: 0.8676
log2 L/L, cre / L/L: 0.44
adj. p value: 0.016
log2 L/L, cre / L/L: 1.02
adj. p value: 0.001
log2 L/L, cre / L/L: 0.52
adj. p value: 0.0189
log2 L/L, cre / L/L: 0.74
adj. p value: 0.0034
log2 intensity
31
30
29
28
27
Genotype
L/L
L/L, cre
Imputation
non imputed
imputed

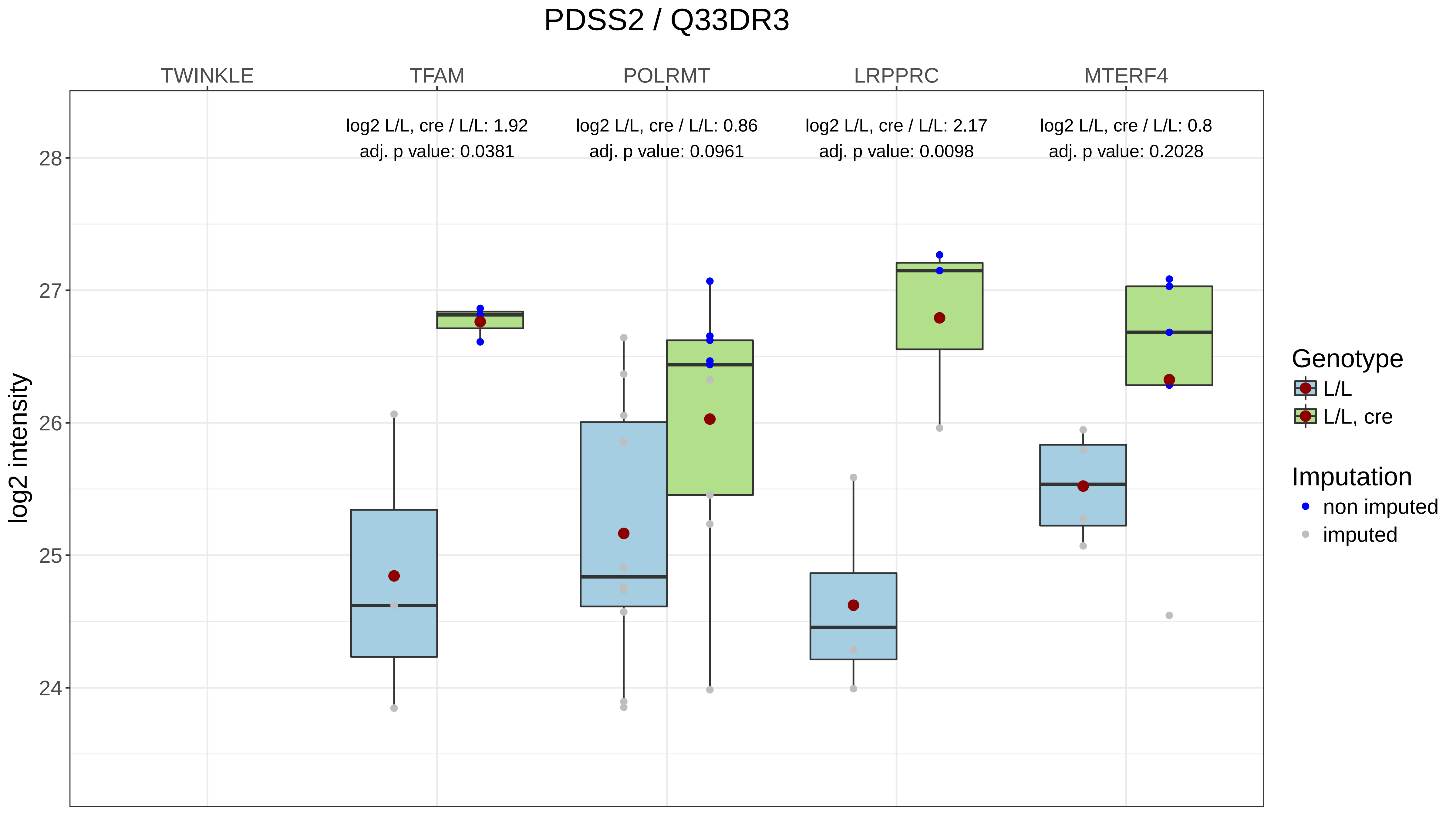
PDSS2 / Q33DR3
TWINKLE
TFAM
POLRMT
LRPPRC
MTERF4
log2 L/L, cre / L/L: 1.92
adj. p value: 0.0381
log2 L/L, cre / L/L: 0.86
adj. p value: 0.0961
log2 L/L, cre / L/L: 2.17
adj. p value: 0.0098
log2 L/L, cre / L/L: 0.8
adj. p value: 0.2028
log2 intensity
28
27
26
25
24
Genotype
L/L
L/L, cre
Imputation
non imputed
imputed

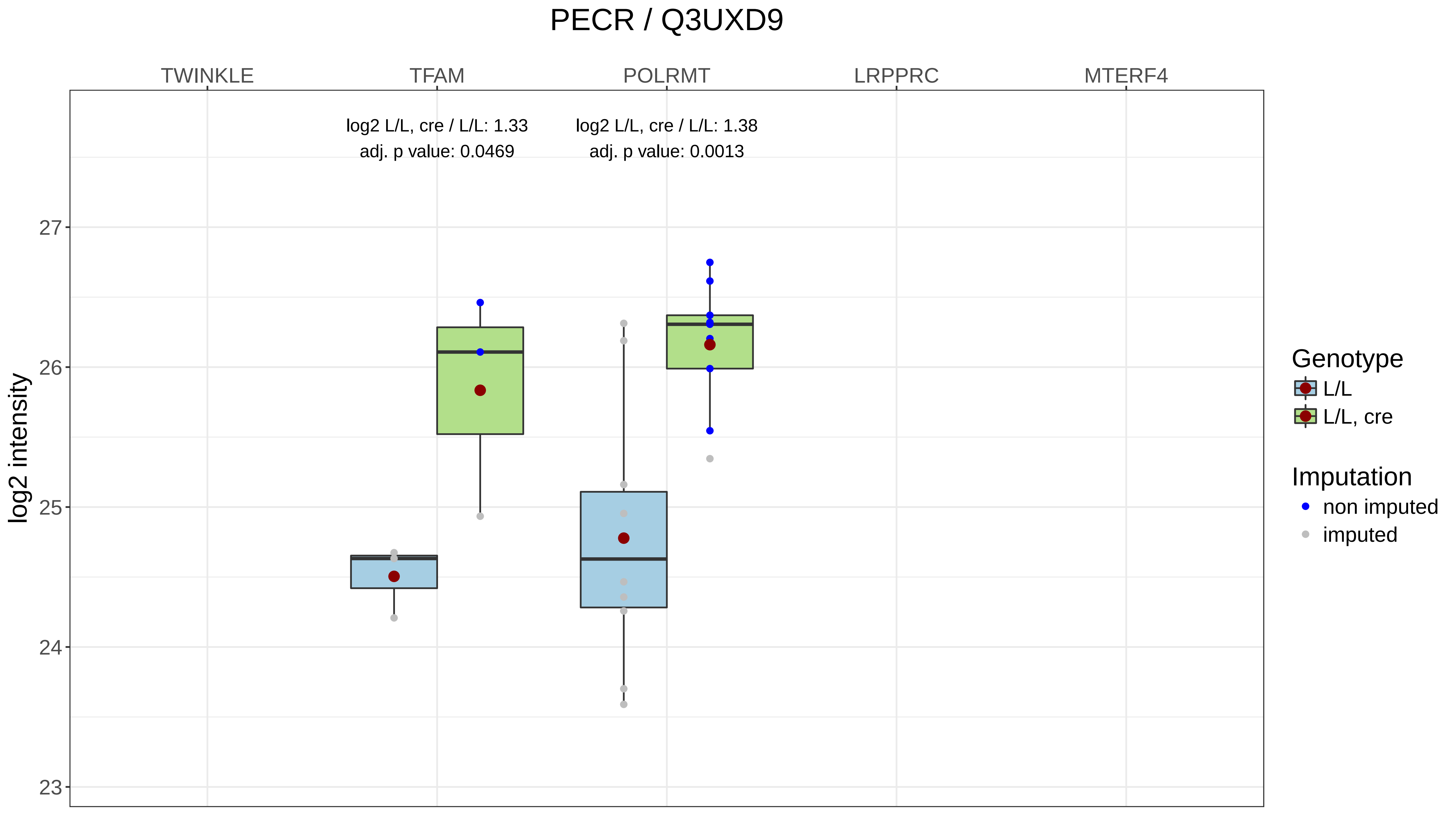
PECR / Q3UXD9
TWINKLE
TFAM
POLRMT
LRPPRC
MTERF4
log2 L/L, cre / L/L: 1.33
adj. p value: 0.0469
log2 L/L, cre / L/L: 1.38
adj. p value: 0.0013
log2 intensity
27
26
25
24
23
Genotype
L/L
L/L, cre
Imputation
non imputed
imputed

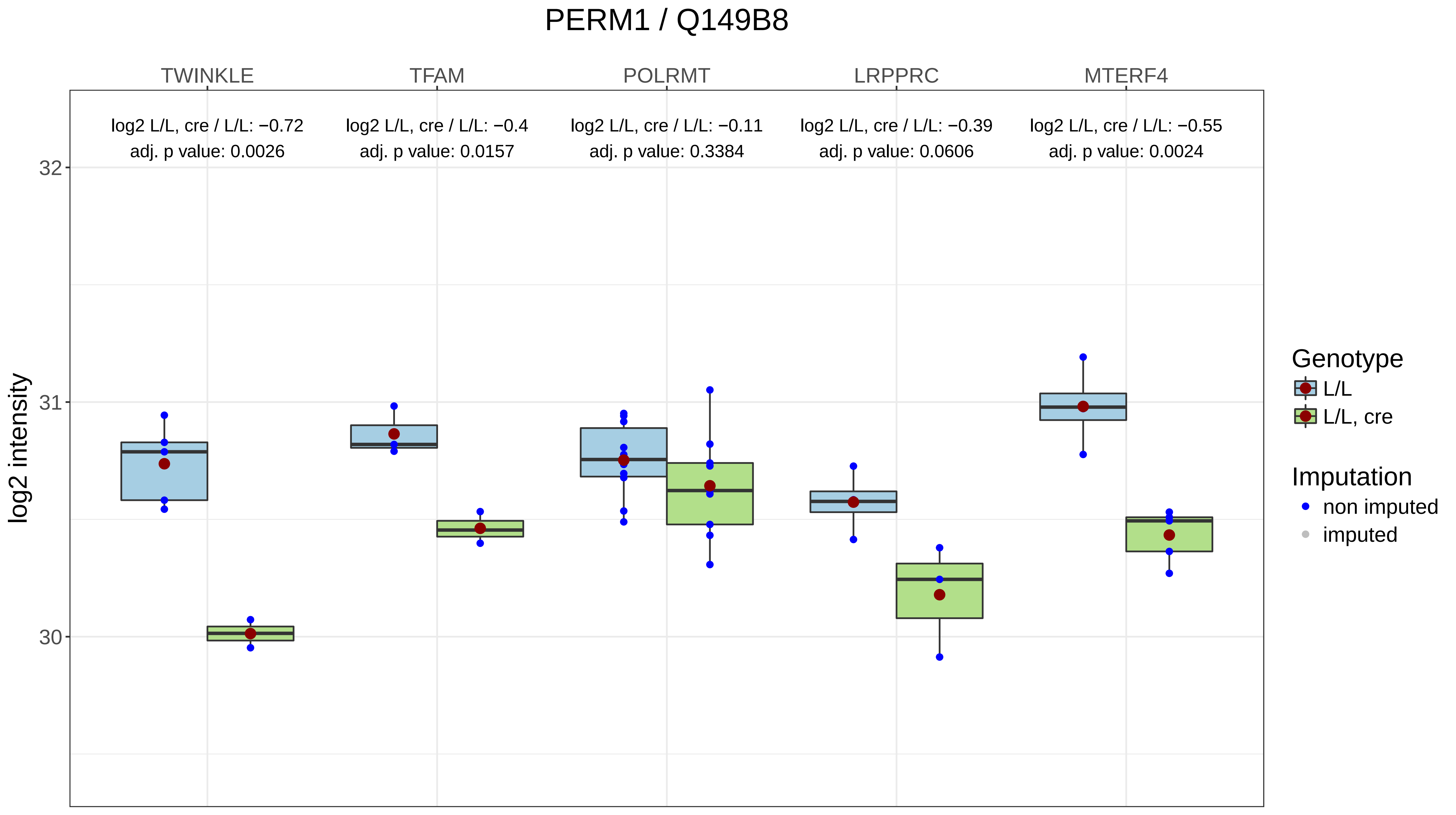
PERM1 / Q149B8
TWINKLE
TFAM
POLRMT
LRPPRC
MTERF4
log2 L/L, cre / L/L: −0.72
adj. p value: 0.0026
log2 L/L, cre / L/L: −0.4
adj. p value: 0.0157
log2 L/L, cre / L/L: −0.11
adj. p value: 0.3384
log2 L/L, cre / L/L: −0.39
adj. p value: 0.0606
log2 L/L, cre / L/L: −0.55
adj. p value: 0.0024
log2 intensity
32
31
30
Genotype
L/L
L/L, cre
Imputation
non imputed
imputed

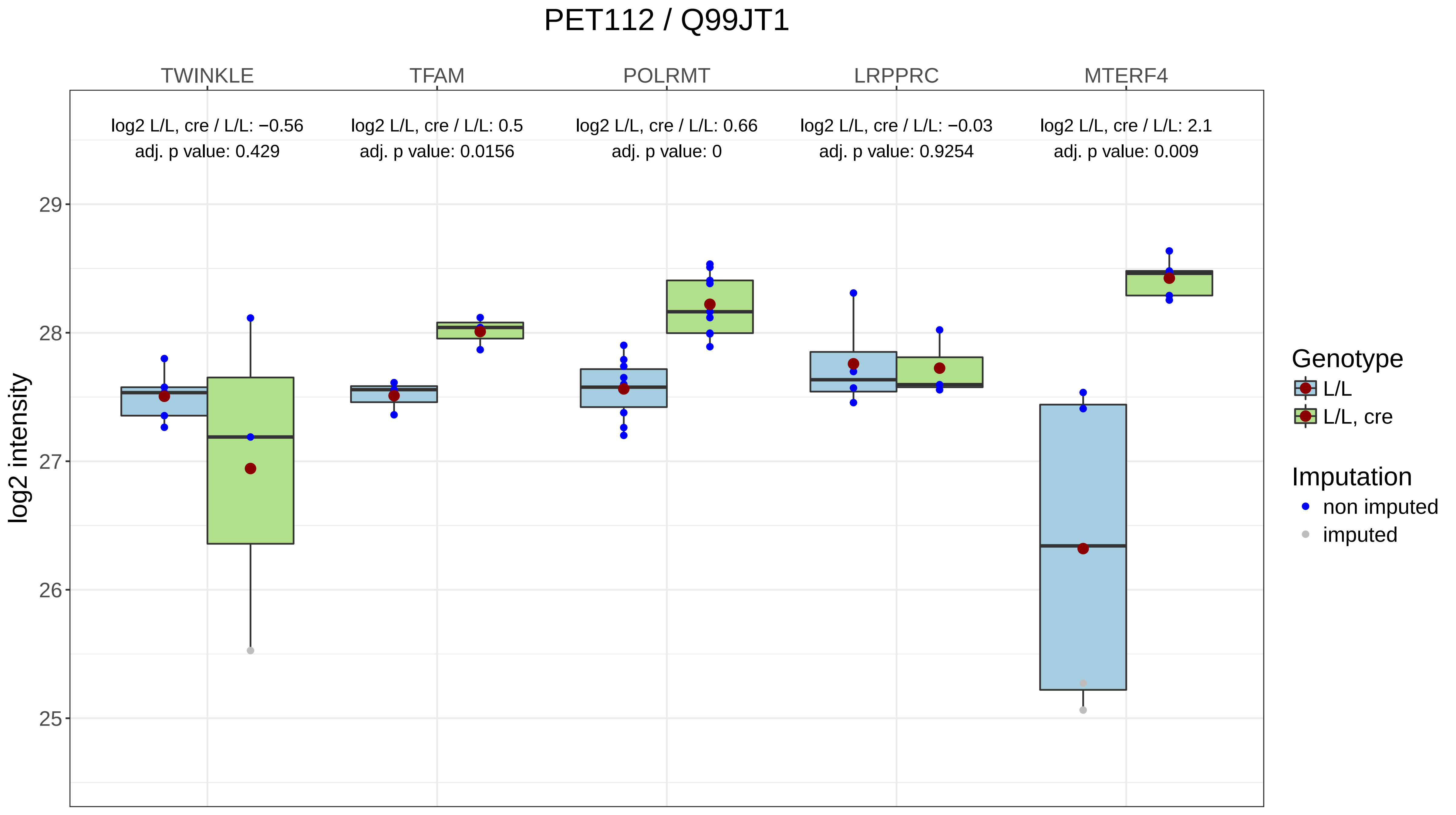
PET112 / Q99JT1
TWINKLE
TFAM
POLRMT
LRPPRC
MTERF4
log2 L/L, cre / L/L: −0.56
adj. p value: 0.429
log2 L/L, cre / L/L: 0.5
adj. p value: 0.0156
log2 L/L, cre / L/L: 0.66
adj. p value: 0
log2 L/L, cre / L/L: −0.03
adj. p value: 0.9254
log2 L/L, cre / L/L: 2.1
adj. p value: 0.009
log2 intensity
29
28
27
26
25
Genotype
L/L
L/L, cre
Imputation
non imputed
imputed

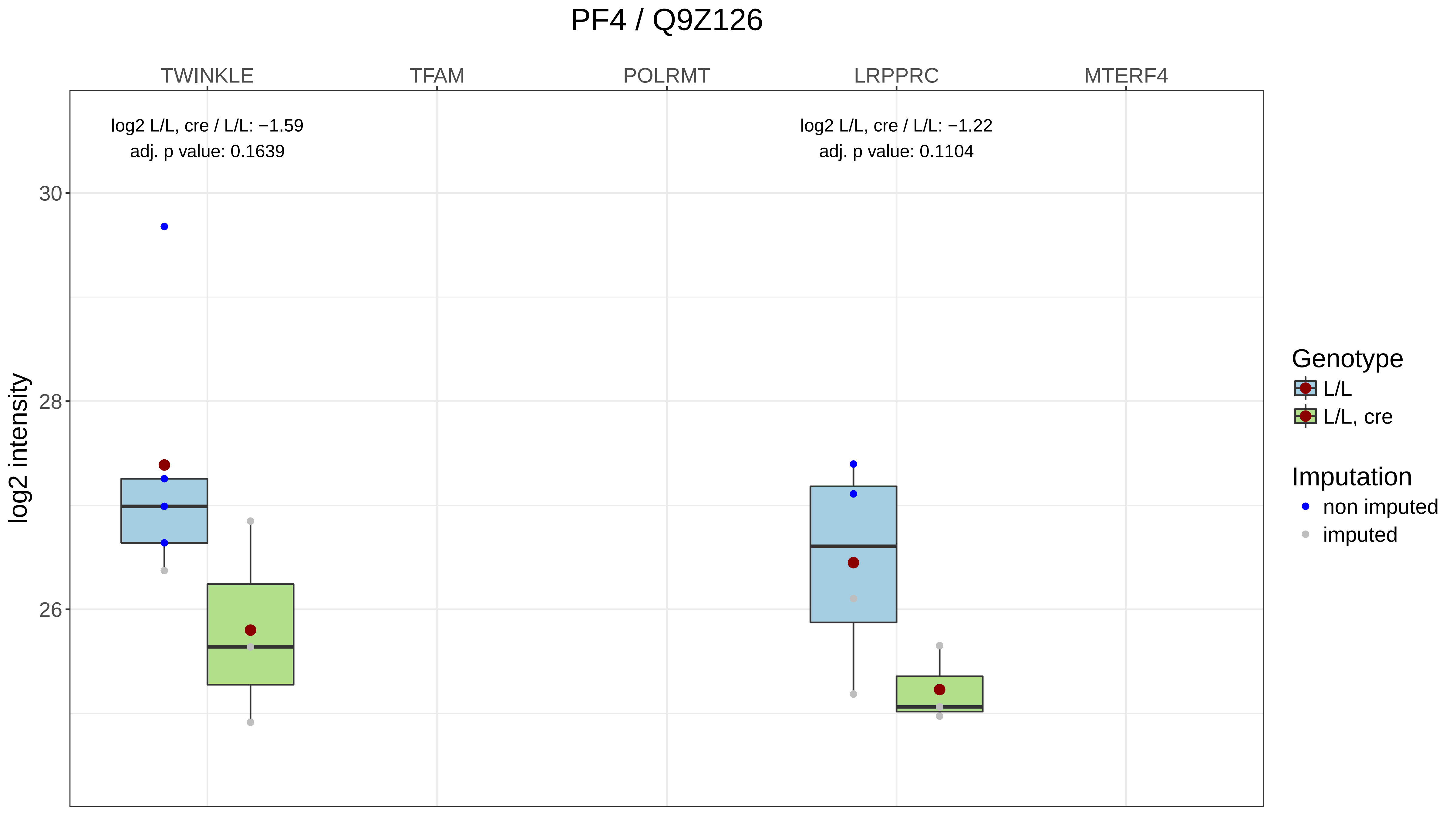
PF4 / Q9Z126
TWINKLE
TFAM
POLRMT
LRPPRC
MTERF4
log2 L/L, cre / L/L: −1.59
adj. p value: 0.1639
log2 L/L, cre / L/L: −1.22
adj. p value: 0.1104
30
28
26
log2 intensity
Genotype
L/L
L/L, cre
Imputation
non imputed
imputed

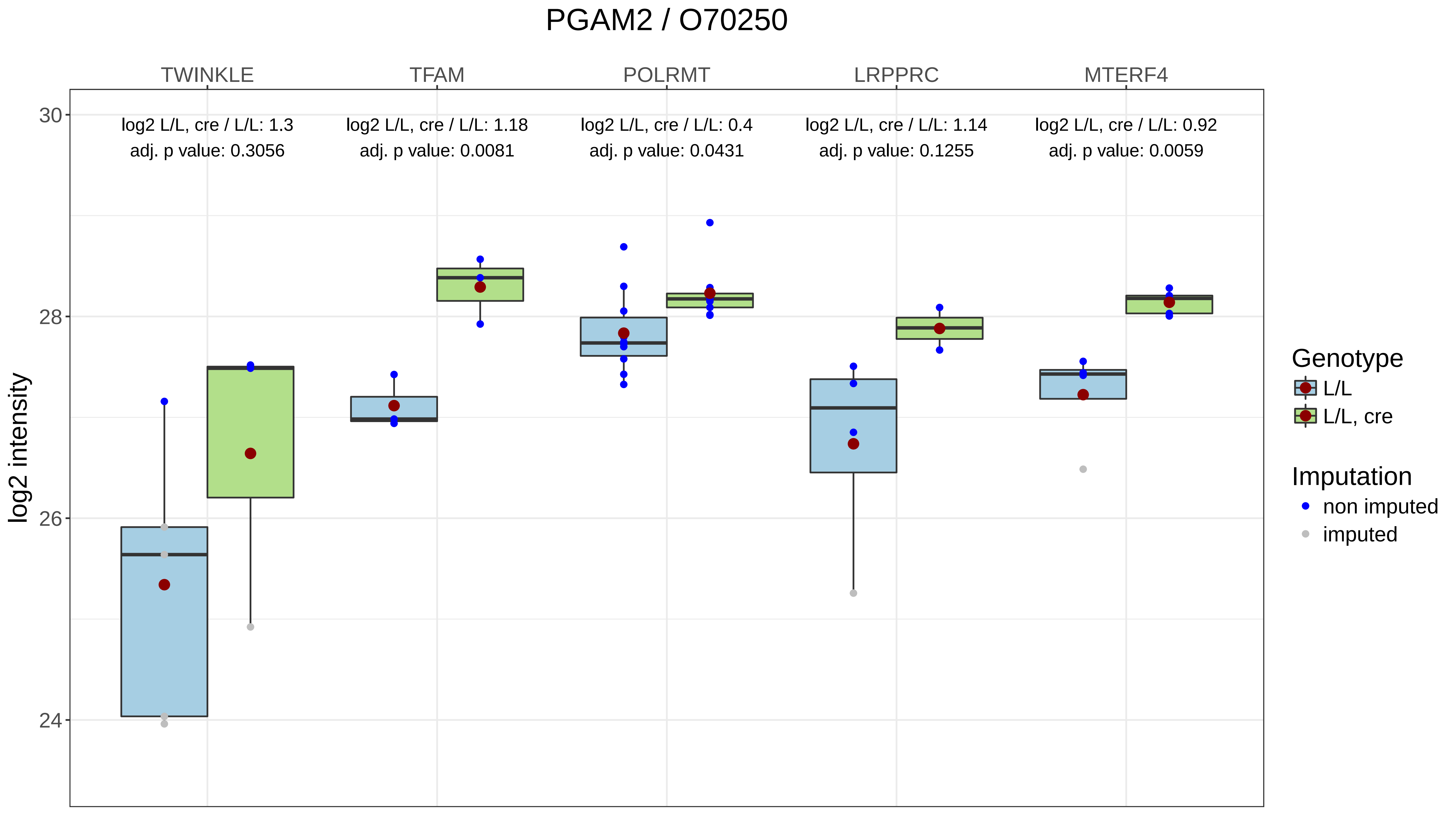
PGAM2 / O70250
TWINKLE
TFAM
POLRMT
LRPPRC
MTERF4
log2 L/L, cre / L/L: 1.3
adj. p value: 0.3056
log2 L/L, cre / L/L: 1.18
adj. p value: 0.0081
log2 L/L, cre / L/L: 0.4
adj. p value: 0.0431
log2 L/L, cre / L/L: 1.14
adj. p value: 0.1255
log2 L/L, cre / L/L: 0.92
adj. p value: 0.0059
log2 intensity
30
28
26
24
Genotype
L/L
L/L, cre
Imputation
non imputed
imputed

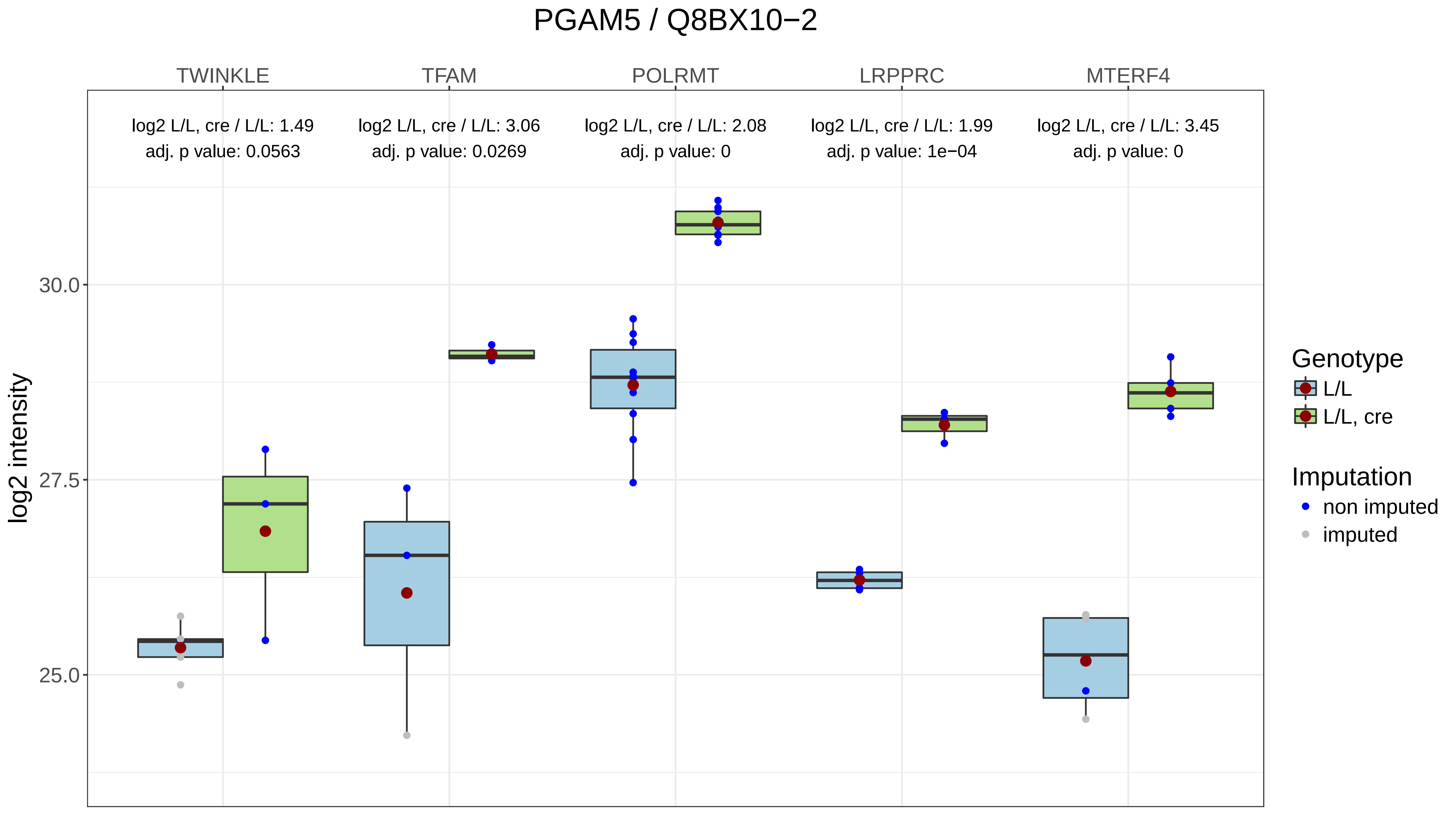
PGAM5 / Q8BX10−2
TWINKLE
TFAM
POLRMT
LRPPRC
MTERF4
log2 L/L, cre / L/L: 1.49
adj. p value: 0.0563
log2 L/L, cre / L/L: 3.06
adj. p value: 0.0269
log2 L/L, cre / L/L: 2.08
adj. p value: 0
log2 L/L, cre / L/L: 1.99
adj. p value: 1e−04
log2 L/L, cre / L/L: 3.45
adj. p value: 0
30.0
27.5
25.0
log2 intensity
Genotype
L/L
L/L, cre
Imputation
non imputed
imputed

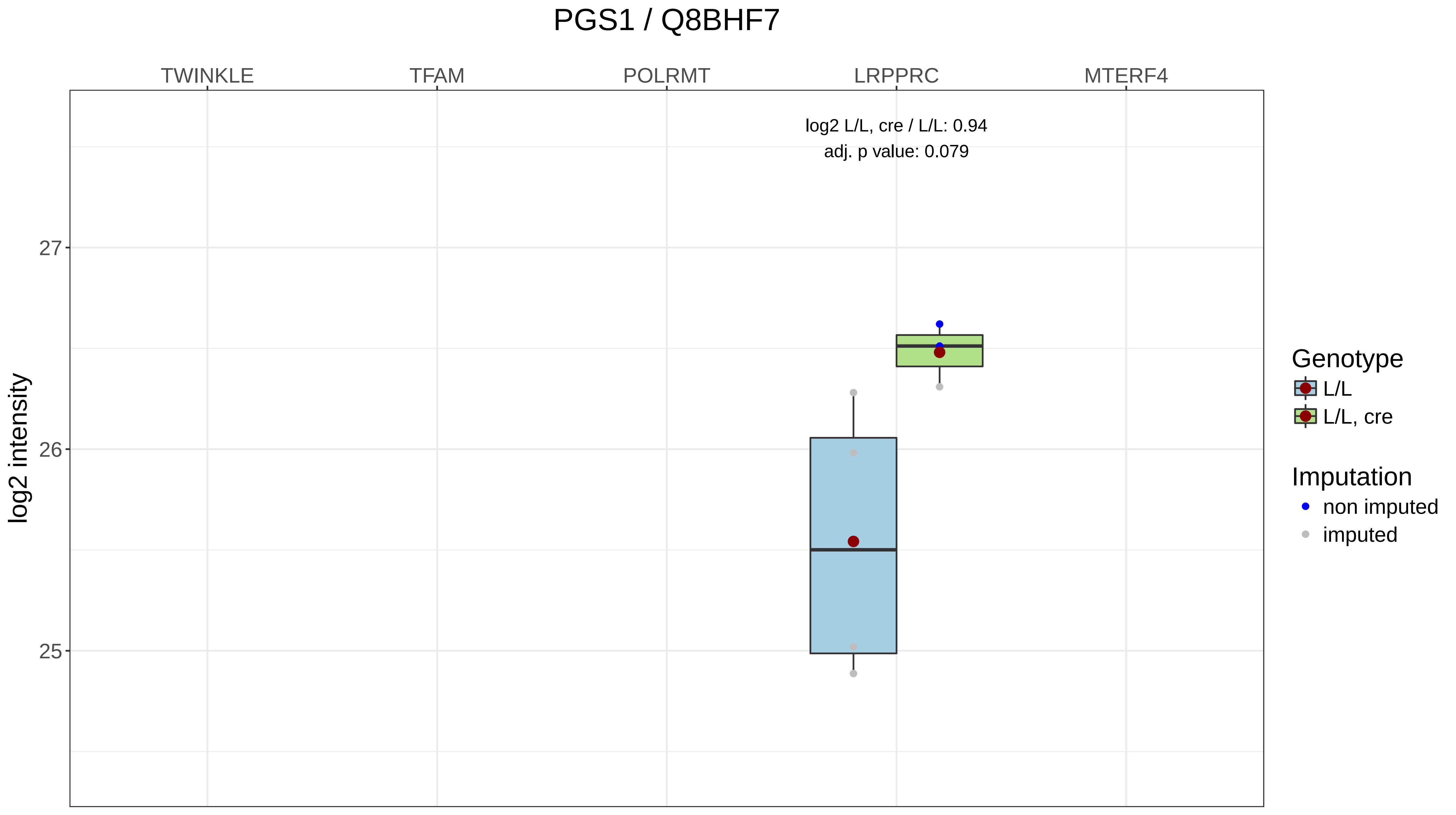
PGS1 / Q8BHF7
TWINKLE
TFAM
POLRMT
LRPPRC
MTERF4
log2 L/L, cre / L/L: 0.94
adj. p value: 0.079
27
26
25
log2 intensity
Genotype
L/L
L/L, cre
Imputation
non imputed
imputed

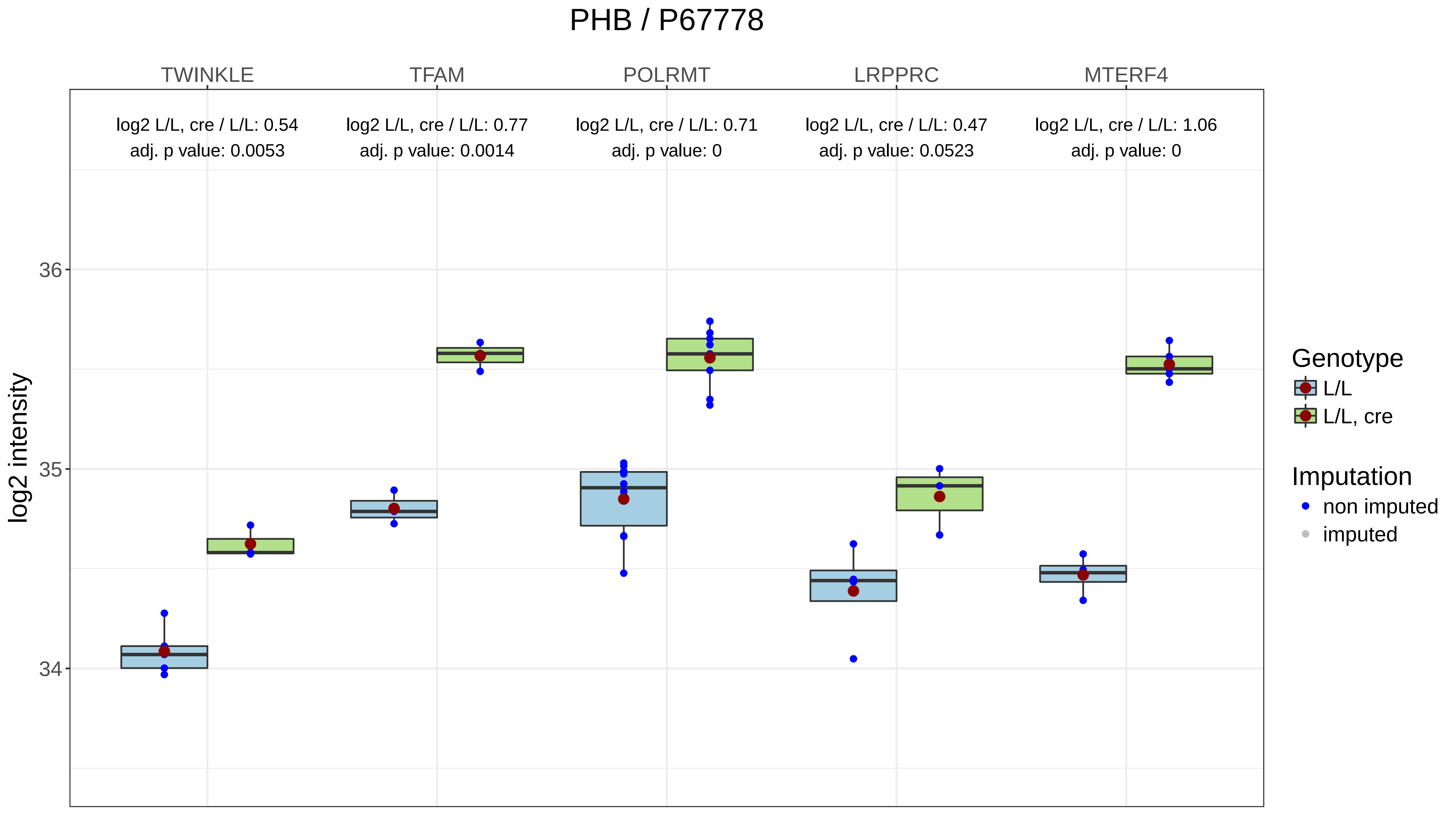

PHB / P67778
TWINKLE
TFAM
POLRMT
LRPPRC
MTERF4
log2 L/L, cre / L/L: 0.54
adj. p value: 0.0053
log2 L/L, cre / L/L: 0.77
adj. p value: 0.0014
log2 L/L, cre / L/L: 0.71
adj. p value: 0
log2 L/L, cre / L/L: 0.47
adj. p value: 0.0523
log2 L/L, cre / L/L: 1.06
adj. p value: 0
log2 intensity
36
35
34
Genotype
L/L
L/L, cre
Imputation
non imputed
imputed

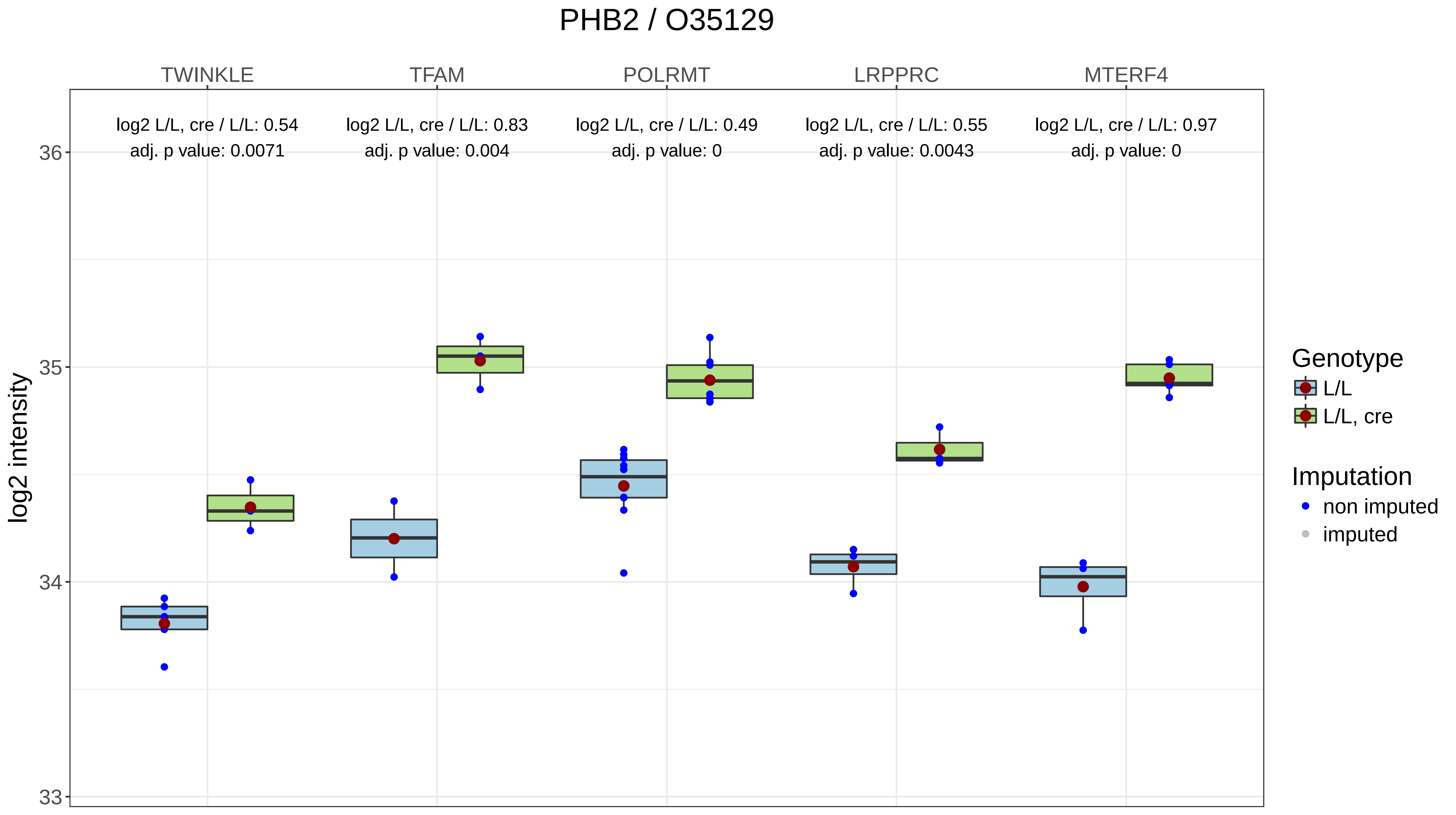
PHB2 / O35129
TWINKLE
TFAM
POLRMT
LRPPRC
MTERF4
log2 L/L, cre / L/L: 0.54
adj. p value: 0.0071
log2 L/L, cre / L/L: 0.83
adj. p value: 0.004
log2 L/L, cre / L/L: 0.49
adj. p value: 0
log2 L/L, cre / L/L: 0.55
adj. p value: 0.0043
log2 L/L, cre / L/L: 0.97
adj. p value: 0
log2 intensity
36
35
34
33
Genotype
L/L
L/L, cre
Imputation
non imputed
imputed

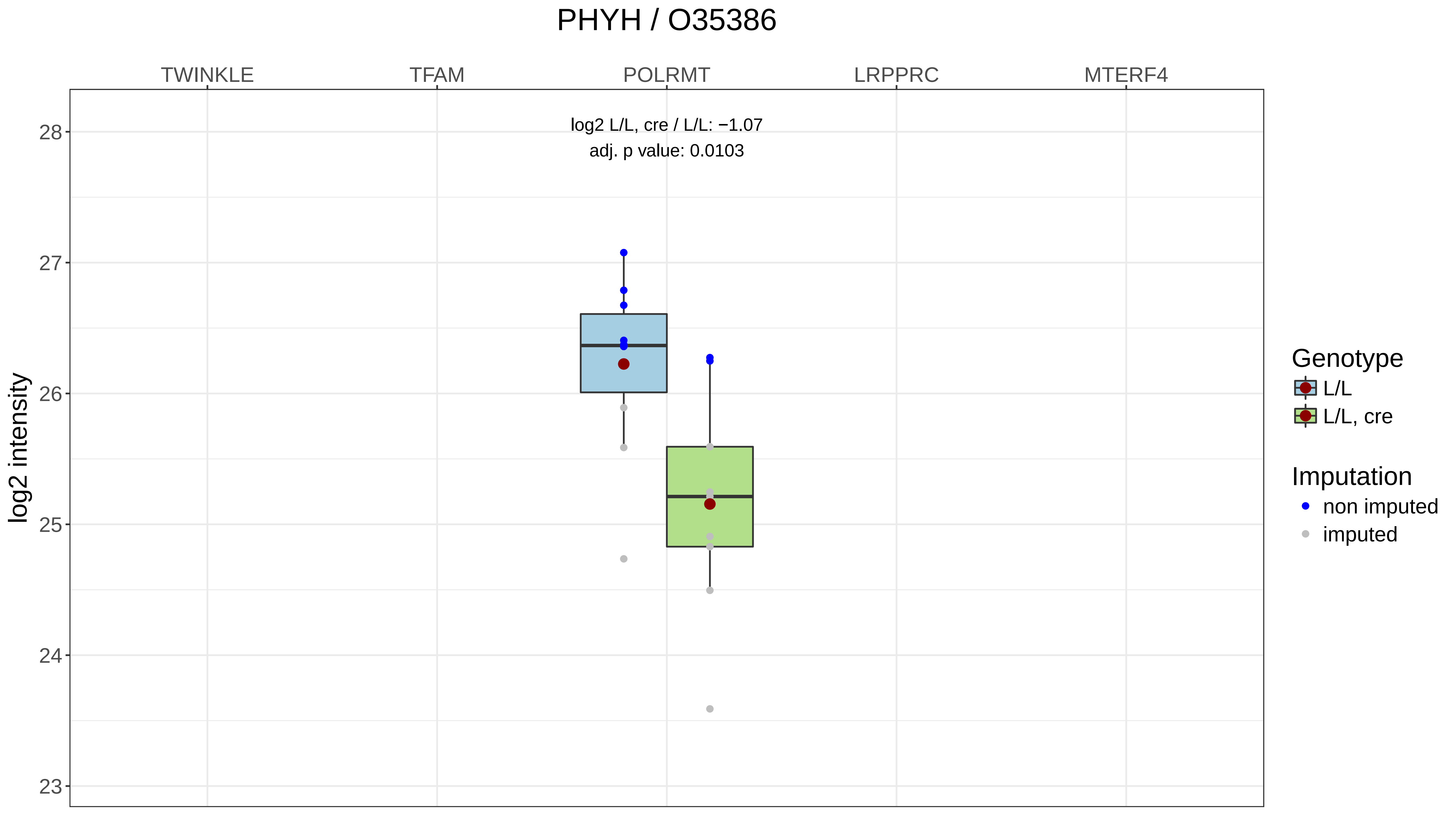

PHYH / O35386
TWINKLE
TFAM
POLRMT
LRPPRC
MTERF4
log2 L/L, cre / L/L: −1.07
adj. p value: 0.0103
log2 intensity
28
27
26
25
24
23
Genotype
L/L
L/L, cre
Imputation
non imputed
imputed

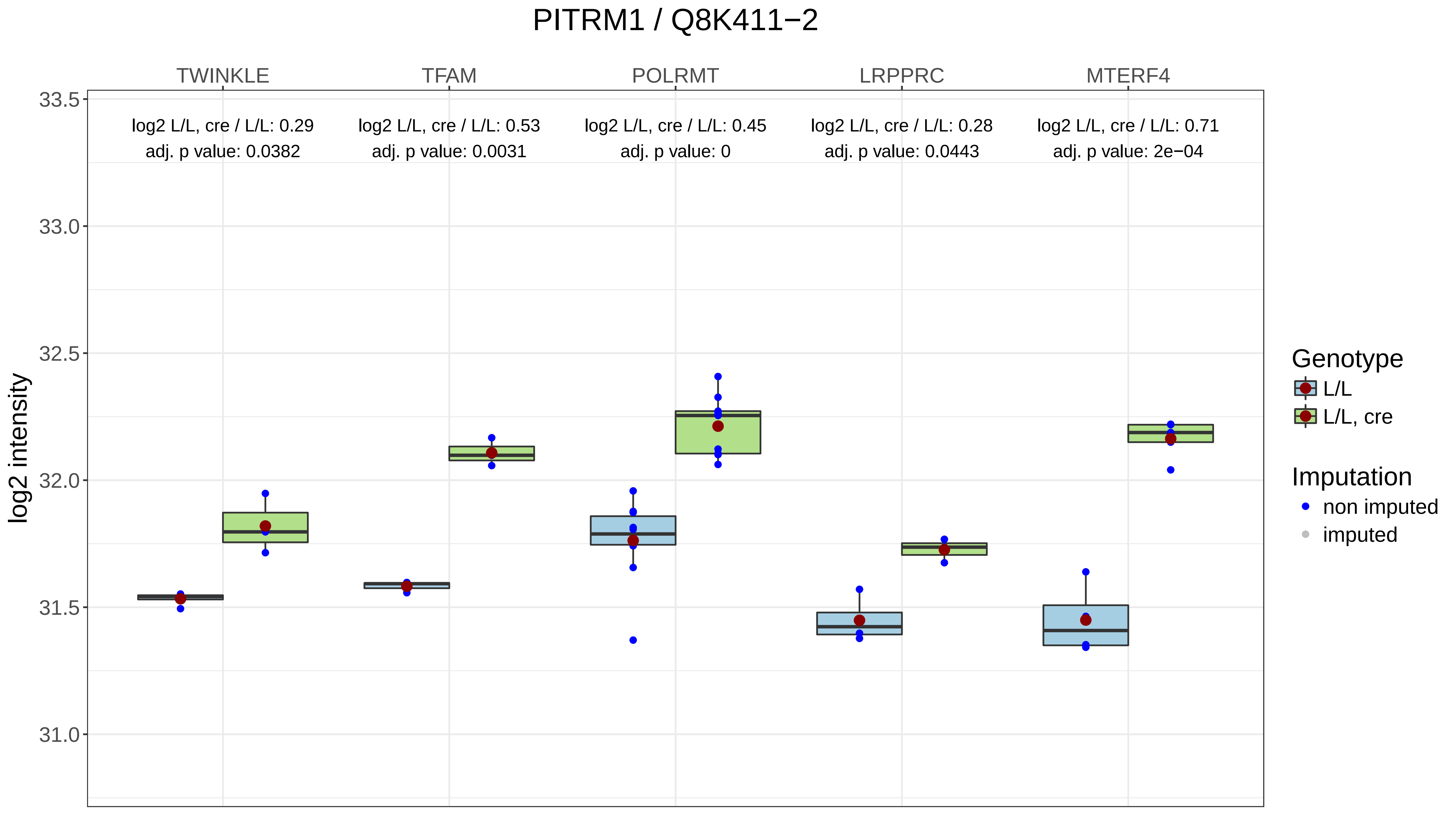

PITRM1 / Q8K411−2
TWINKLE
TFAM
POLRMT
LRPPRC
MTERF4
log2 L/L, cre / L/L: 0.29
adj. p value: 0.0382
log2 L/L, cre / L/L: 0.53
adj. p value: 0.0031
log2 L/L, cre / L/L: 0.45
adj. p value: 0
log2 L/L, cre / L/L: 0.28
adj. p value: 0.0443
log2 L/L, cre / L/L: 0.71
adj. p value: 2e−04
log2 intensity
33.5
33.0
32.5
32.0
31.5
31.0
Genotype
L/L
L/L, cre
Imputation
non imputed
imputed

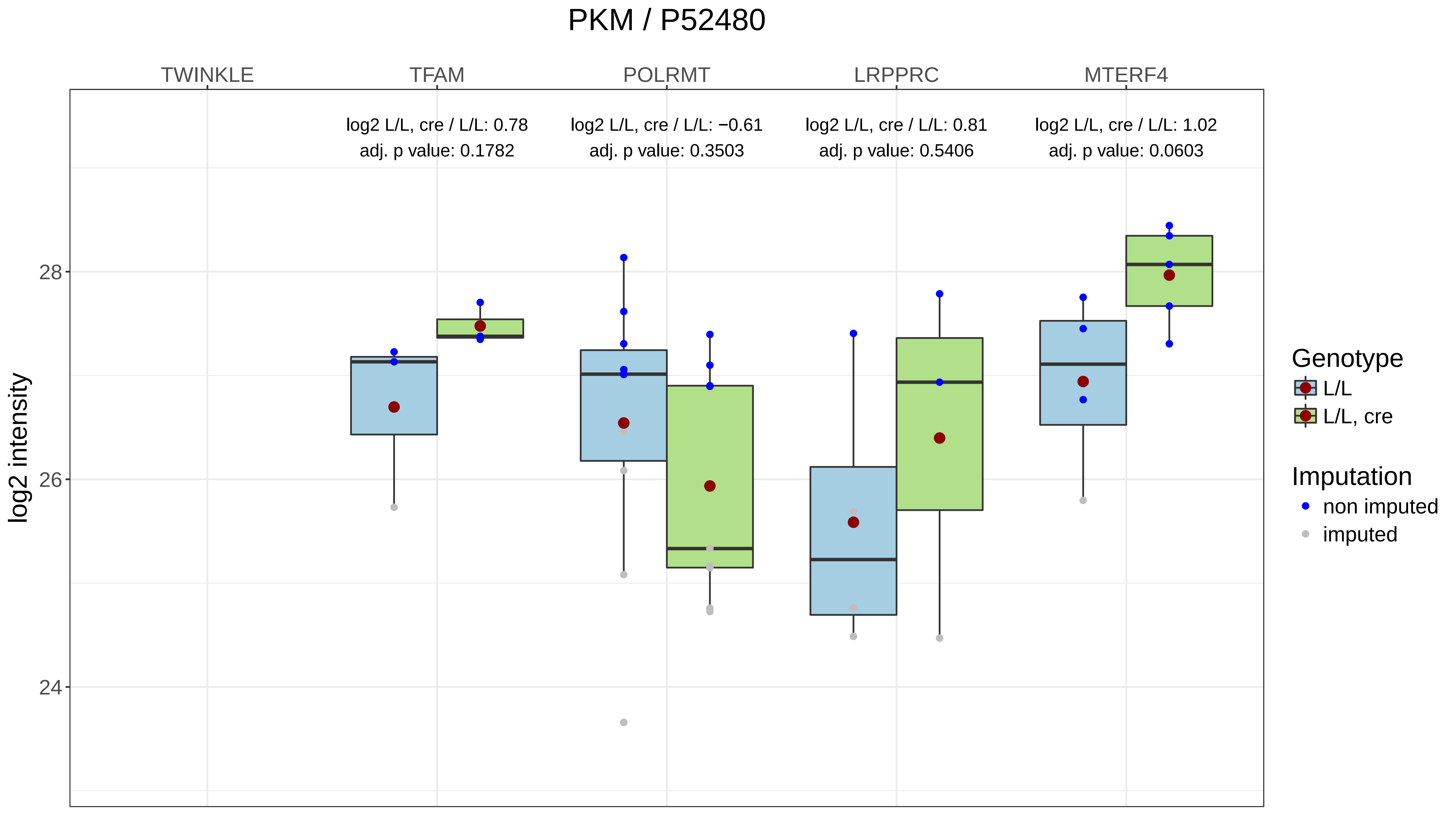
PKM / P52480
TWINKLE
TFAM
POLRMT
LRPPRC
MTERF4
log2 L/L, cre / L/L: 0.78
adj. p value: 0.1782
log2 L/L, cre / L/L: −0.61
adj. p value: 0.3503
log2 L/L, cre / L/L: 0.81
adj. p value: 0.5406
log2 L/L, cre / L/L: 1.02
adj. p value: 0.0603
log2 intensity
28
26
24
Genotype
L/L
L/L, cre
Imputation
non imputed
imputed

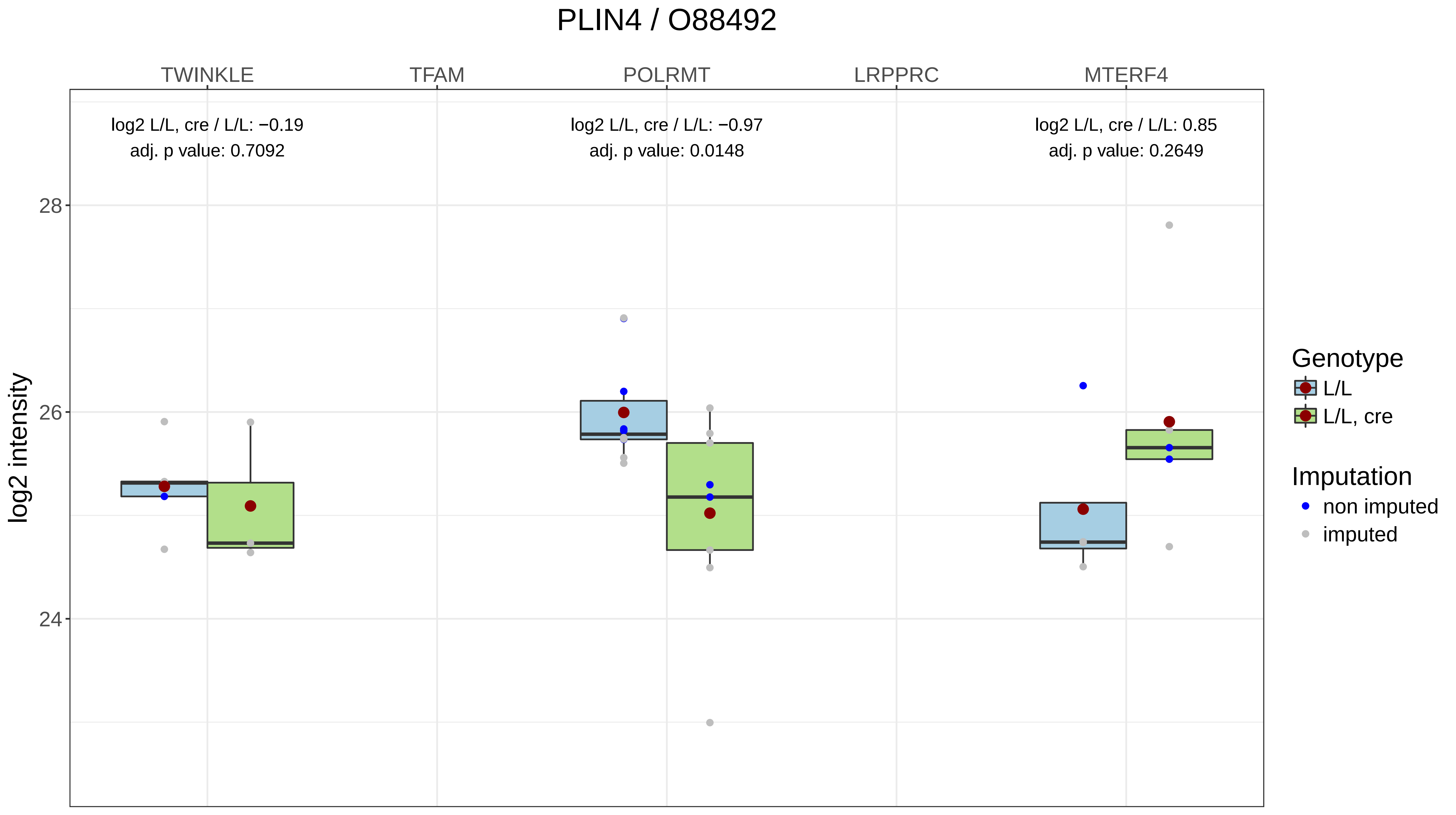
PLIN4 / O88492
TWINKLE
TFAM
POLRMT
LRPPRC
MTERF4
log2 L/L, cre / L/L: −0.19
adj. p value: 0.7092
log2 L/L, cre / L/L: −0.97
adj. p value: 0.0148
log2 L/L, cre / L/L: 0.85
adj. p value: 0.2649
log2 intensity
28
26
24
Genotype
L/L
L/L, cre
Imputation
non imputed
imputed

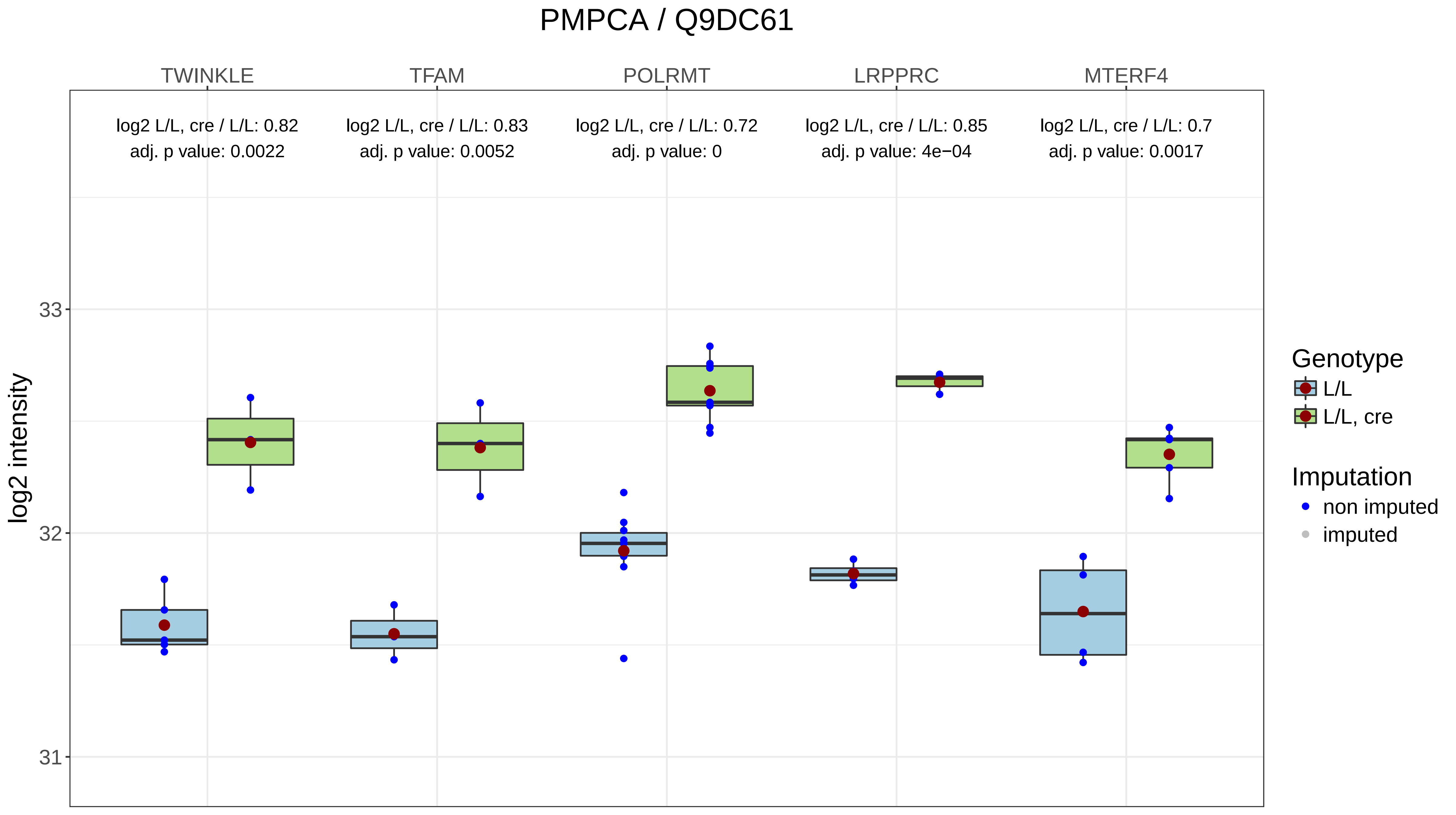
PMPCA / Q9DC61
TWINKLE
TFAM
POLRMT
LRPPRC
MTERF4
log2 L/L, cre / L/L: 0.82
adj. p value: 0.0022
log2 L/L, cre / L/L: 0.83
adj. p value: 0.0052
log2 L/L, cre / L/L: 0.72
adj. p value: 0
log2 L/L, cre / L/L: 0.85
adj. p value: 4e−04
log2 L/L, cre / L/L: 0.7
adj. p value: 0.0017
log2 intensity
33
32
31
Genotype
L/L
L/L, cre
Imputation
non imputed
imputed

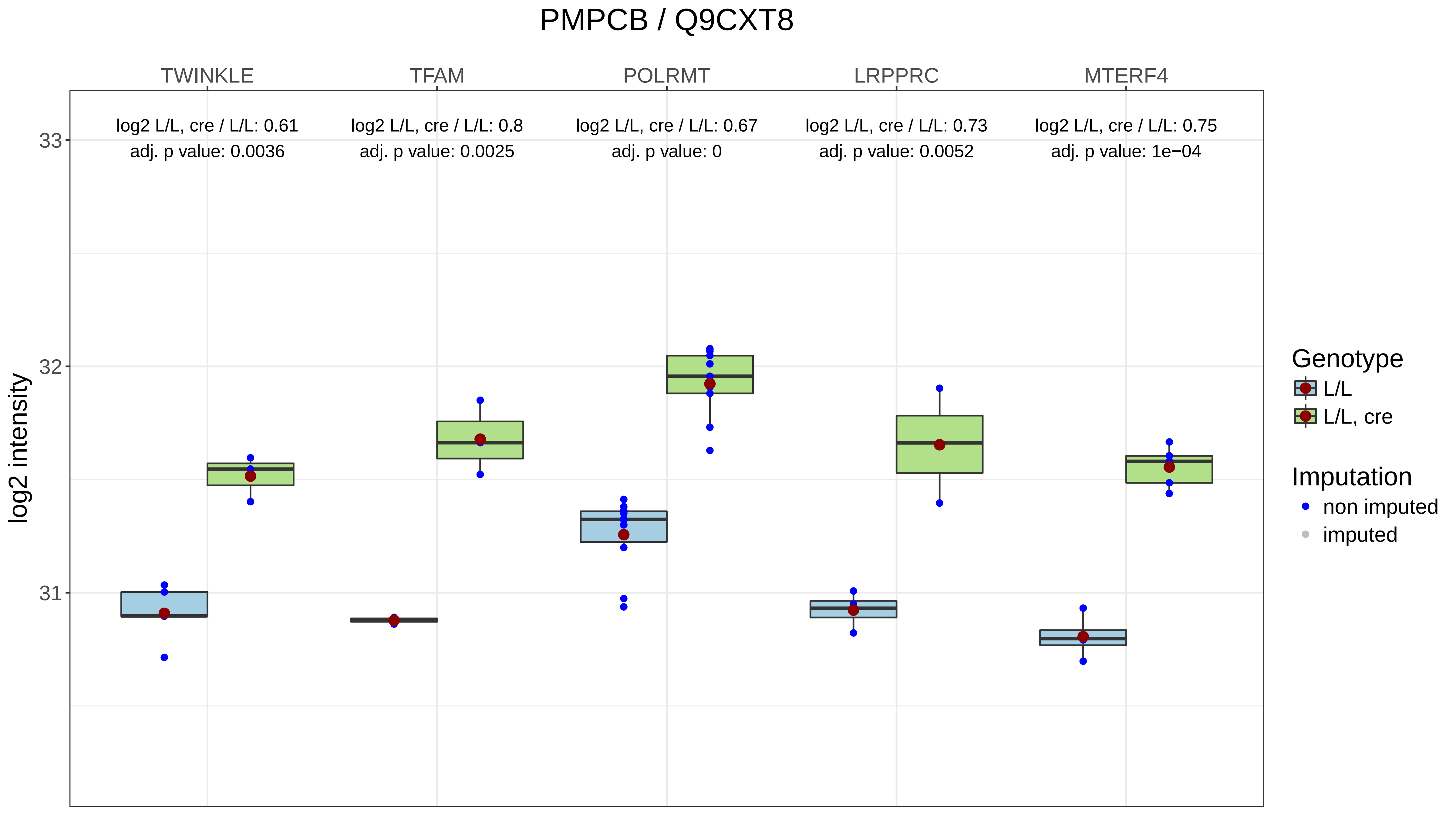
PMPCB / Q9CXT8
TWINKLE
TFAM
POLRMT
LRPPRC
MTERF4
log2 L/L, cre / L/L: 0.61
adj. p value: 0.0036
log2 L/L, cre / L/L: 0.8
adj. p value: 0.0025
log2 L/L, cre / L/L: 0.67
adj. p value: 0
log2 L/L, cre / L/L: 0.73
adj. p value: 0.0052
log2 L/L, cre / L/L: 0.75
adj. p value: 1e−04
33
32
31
log2 intensity
Genotype
L/L
L/L, cre
Imputation
non imputed
imputed

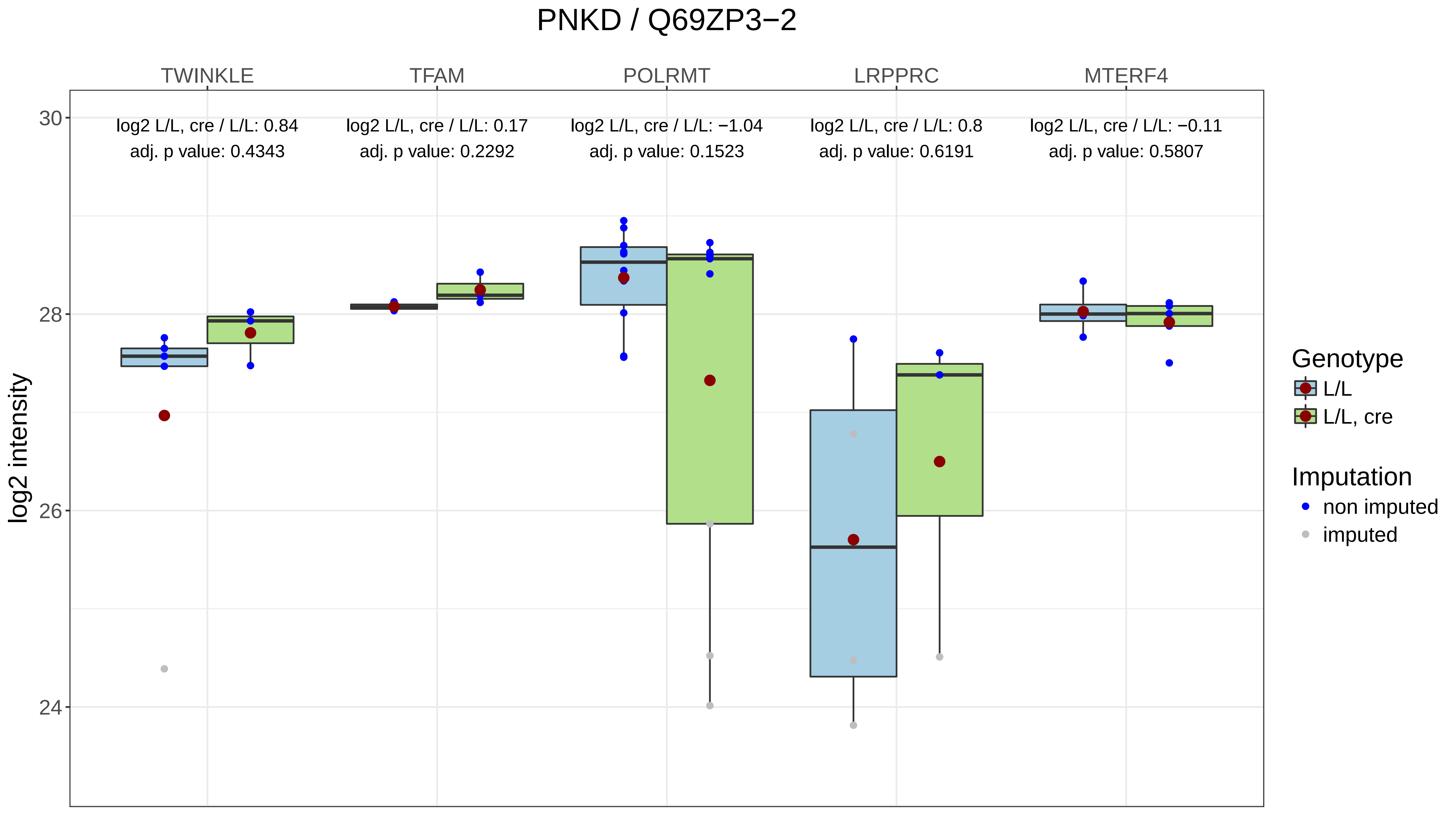
PNKD / Q69ZP3−2
TWINKLE
TFAM
POLRMT
LRPPRC
MTERF4
log2 L/L, cre / L/L: 0.84
adj. p value: 0.4343
log2 L/L, cre / L/L: 0.17
adj. p value: 0.2292
log2 L/L, cre / L/L: −1.04
adj. p value: 0.1523
log2 L/L, cre / L/L: 0.8
adj. p value: 0.6191
log2 L/L, cre / L/L: −0.11
adj. p value: 0.5807
log2 intensity
30
28
26
24
Genotype
L/L
L/L, cre
Imputation
non imputed
imputed

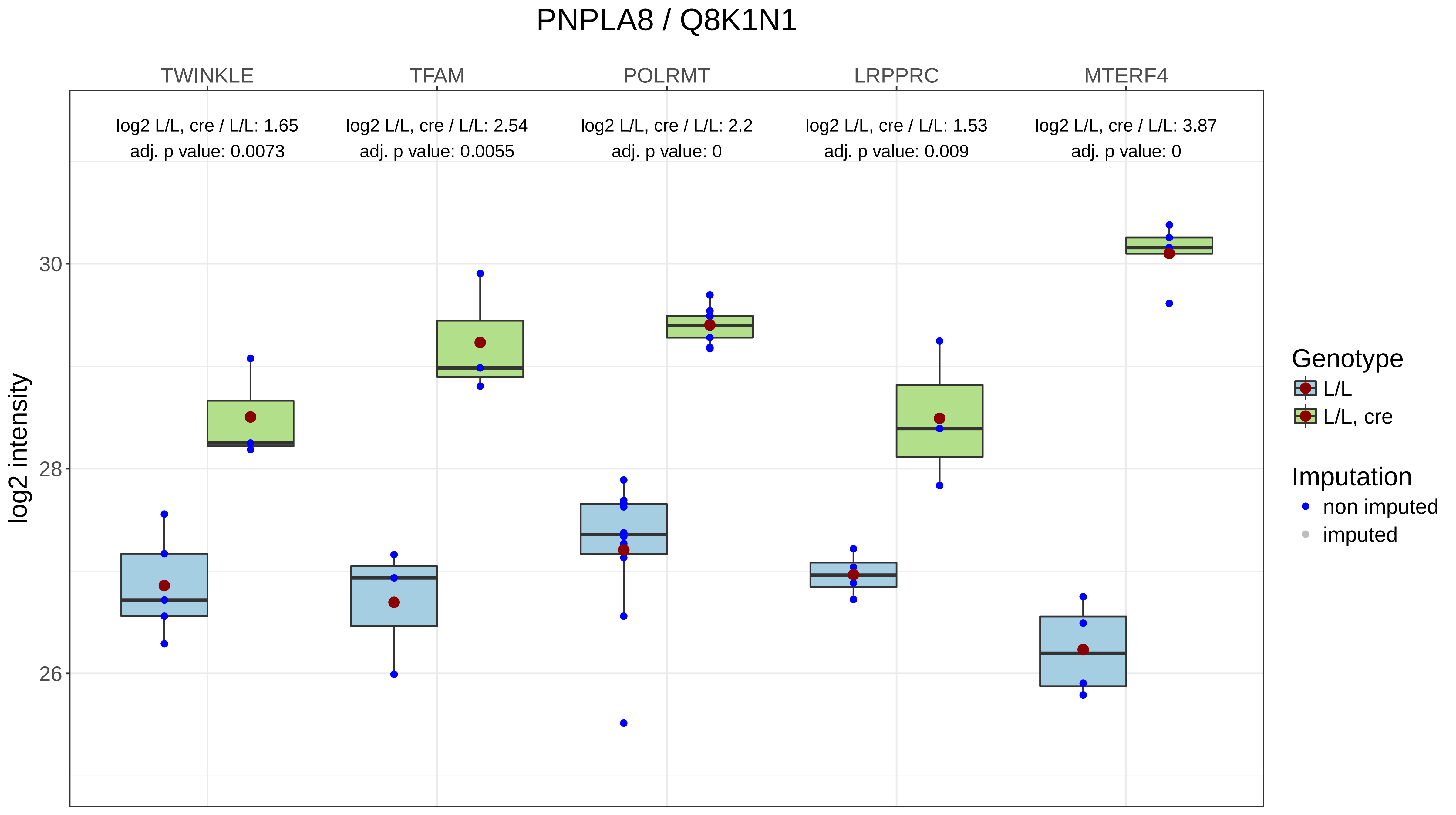
PNPLA8 / Q8K1N1
TWINKLE
TFAM
POLRMT
LRPPRC
MTERF4
log2 L/L, cre / L/L: 1.65
adj. p value: 0.0073
log2 L/L, cre / L/L: 2.54
adj. p value: 0.0055
log2 L/L, cre / L/L: 2.2
adj. p value: 0
log2 L/L, cre / L/L: 1.53
adj. p value: 0.009
log2 L/L, cre / L/L: 3.87
adj. p value: 0
log2 intensity
30
28
26
Genotype
L/L
L/L, cre
Imputation
non imputed
imputed

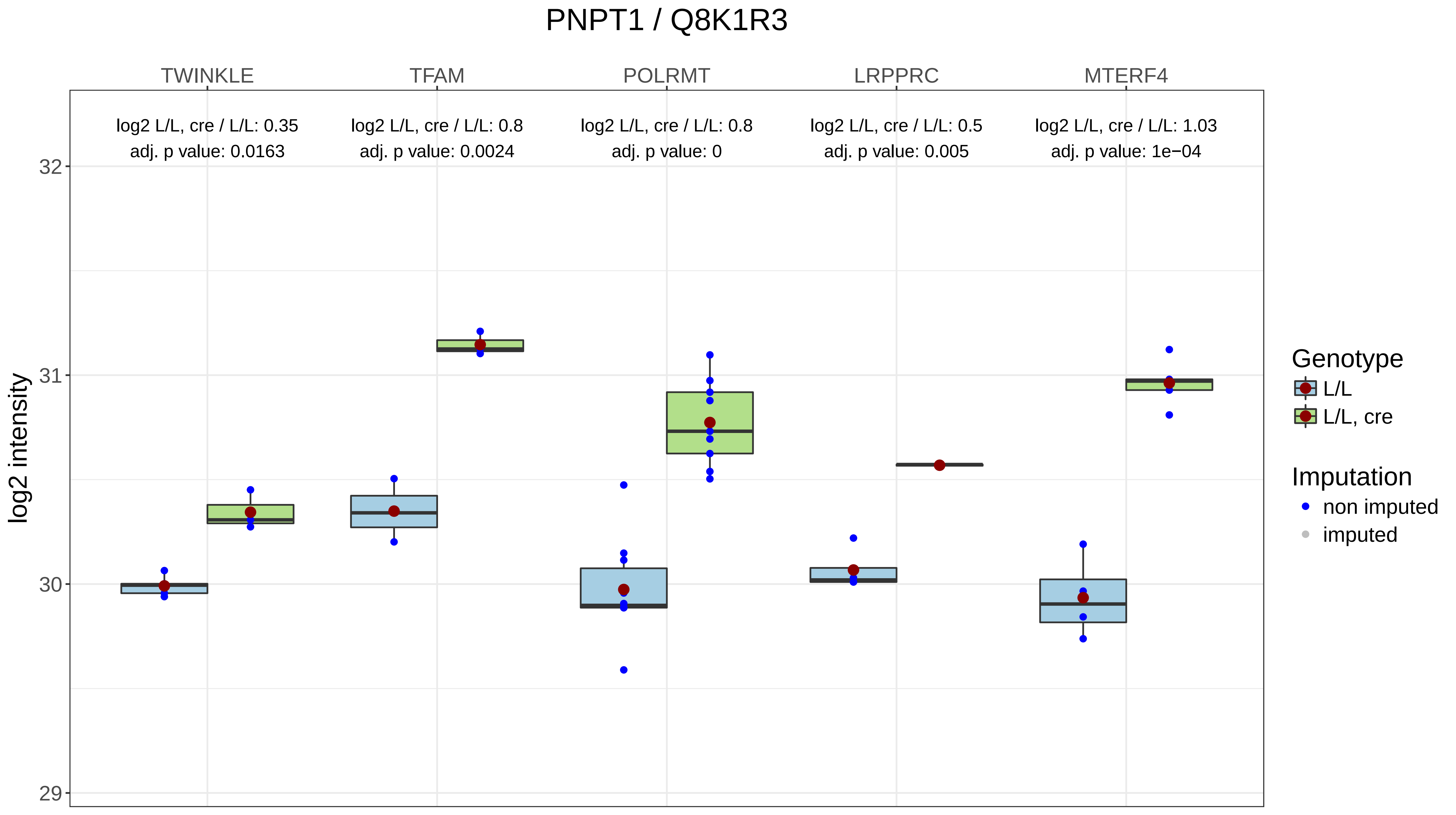
PNPT1 / Q8K1R3
TWINKLE
TFAM
POLRMT
LRPPRC
MTERF4
log2 L/L, cre / L/L: 0.35
adj. p value: 0.0163
log2 L/L, cre / L/L: 0.8
adj. p value: 0.0024
log2 L/L, cre / L/L: 0.8
adj. p value: 0
log2 L/L, cre / L/L: 0.5
adj. p value: 0.005
log2 L/L, cre / L/L: 1.03
adj. p value: 1e−04
log2 intensity
32
31
30
29
Genotype
L/L
L/L, cre
Imputation
non imputed
imputed

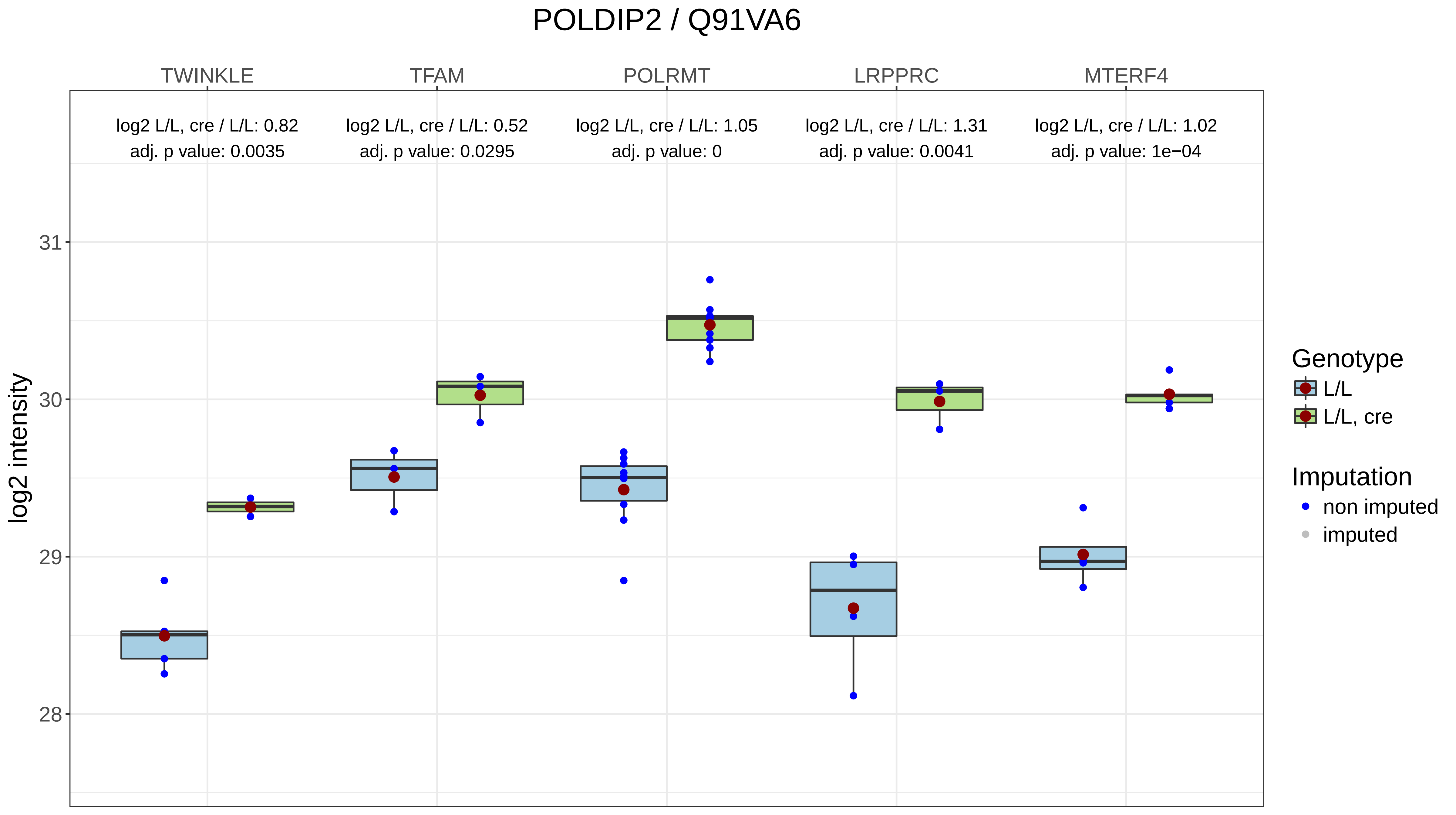
POLDIP2 / Q91VA6
TWINKLE
TFAM
POLRMT
LRPPRC
MTERF4
log2 L/L, cre / L/L: 0.82
adj. p value: 0.0035
log2 L/L, cre / L/L: 0.52
adj. p value: 0.0295
log2 L/L, cre / L/L: 1.05
adj. p value: 0
log2 L/L, cre / L/L: 1.31
adj. p value: 0.0041
log2 L/L, cre / L/L: 1.02
adj. p value: 1e−04
log2 intensity
31
30
29
28
Genotype
L/L
L/L, cre
Imputation
non imputed
imputed

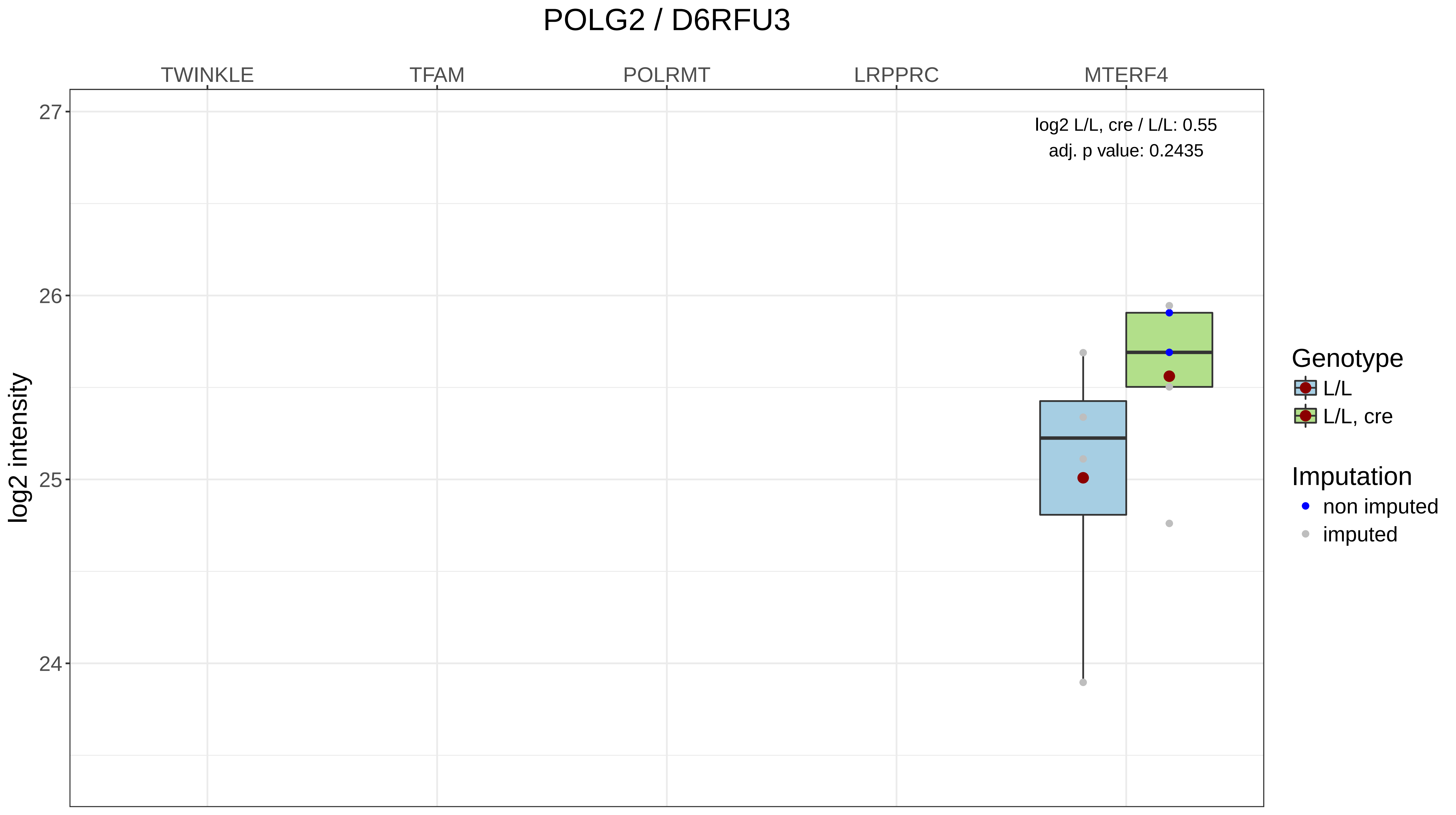
POLG2 / D6RFU3
TWINKLE
TFAM
POLRMT
LRPPRC
MTERF4
log2 L/L, cre / L/L: 0.55
adj. p value: 0.2435
log2 intensity
27
26
25
24
Genotype
L/L
L/L, cre
Imputation
non imputed
imputed

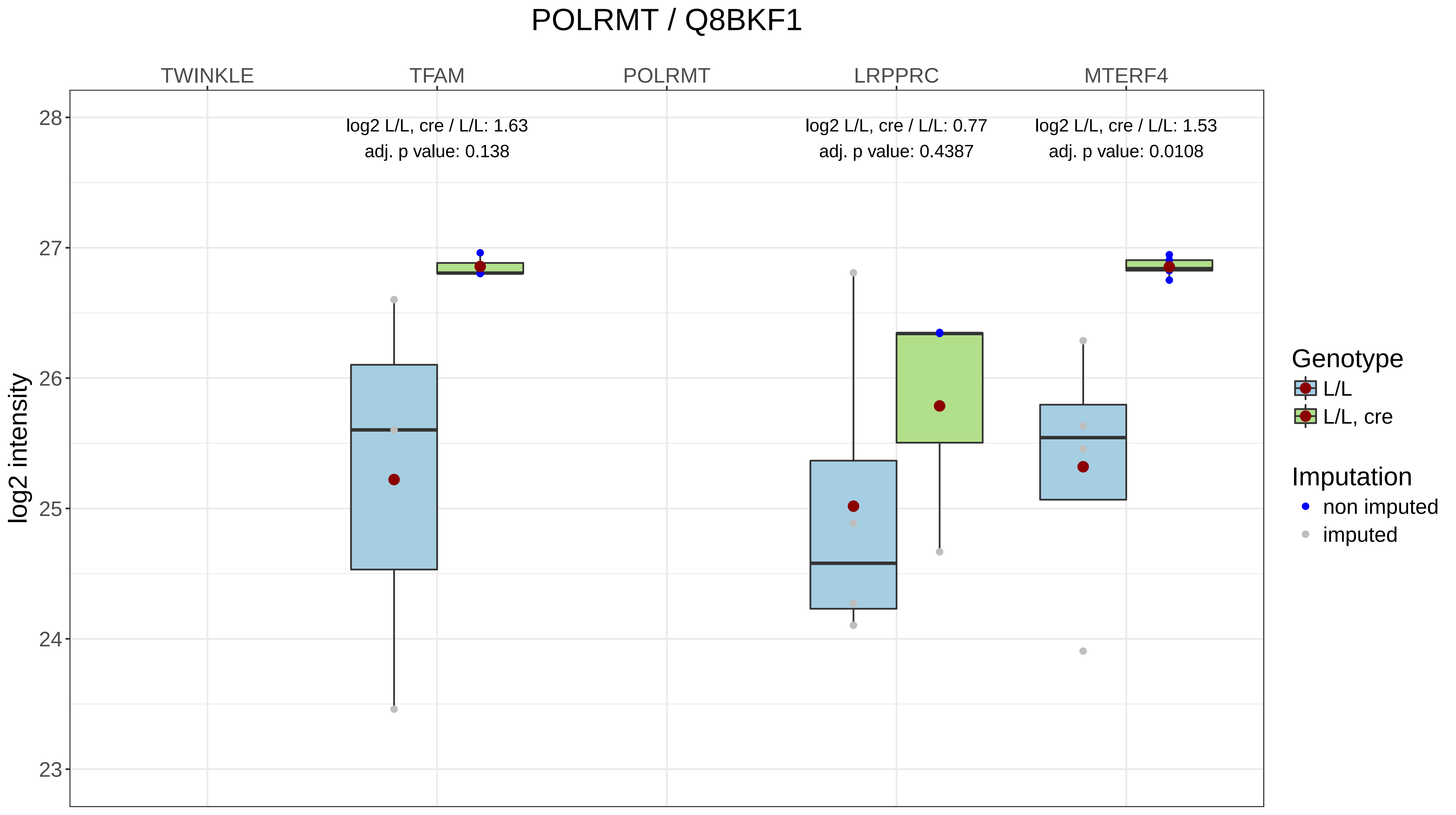

POLRMT / Q8BKF1
TWINKLE
TFAM
POLRMT
LRPPRC
MTERF4
log2 L/L, cre / L/L: 1.63
adj. p value: 0.138
log2 L/L, cre / L/L: 0.77
adj. p value: 0.4387
log2 L/L, cre / L/L: 1.53
adj. p value: 0.0108
log2 intensity
28
27
26
25
24
23
Genotype
L/L
L/L, cre
Imputation
non imputed
imputed

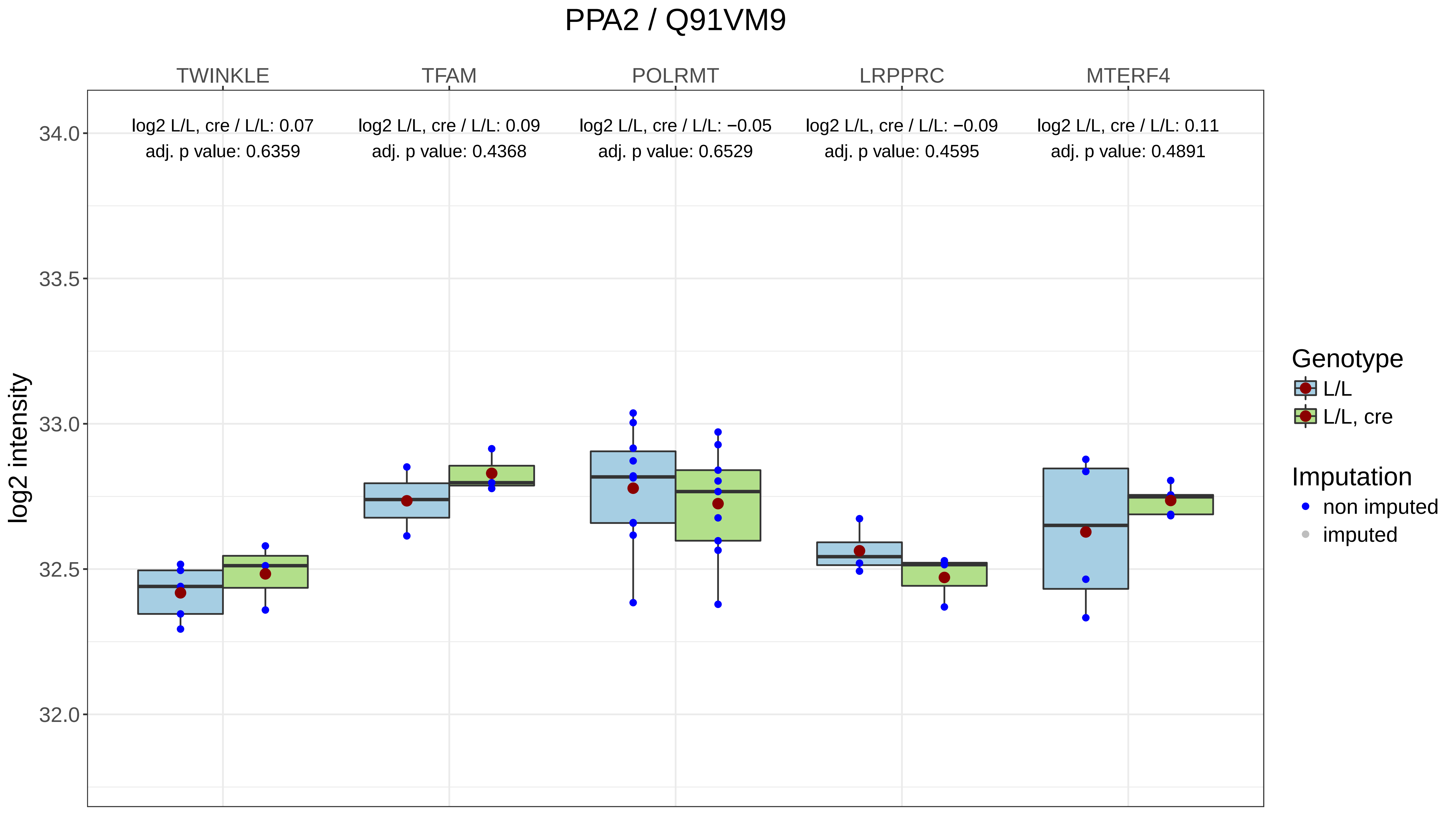
PPA2 / Q91VM9
TWINKLE
TFAM
POLRMT
LRPPRC
MTERF4
log2 L/L, cre / L/L: 0.07
adj. p value: 0.6359
log2 L/L, cre / L/L: 0.09
adj. p value: 0.4368
log2 L/L, cre / L/L: −0.05
adj. p value: 0.6529
log2 L/L, cre / L/L: −0.09
adj. p value: 0.4595
log2 L/L, cre / L/L: 0.11
adj. p value: 0.4891
log2 intensity
34.0
33.5
33.0
32.5
32.0
Genotype
L/L
L/L, cre
Imputation
non imputed
imputed

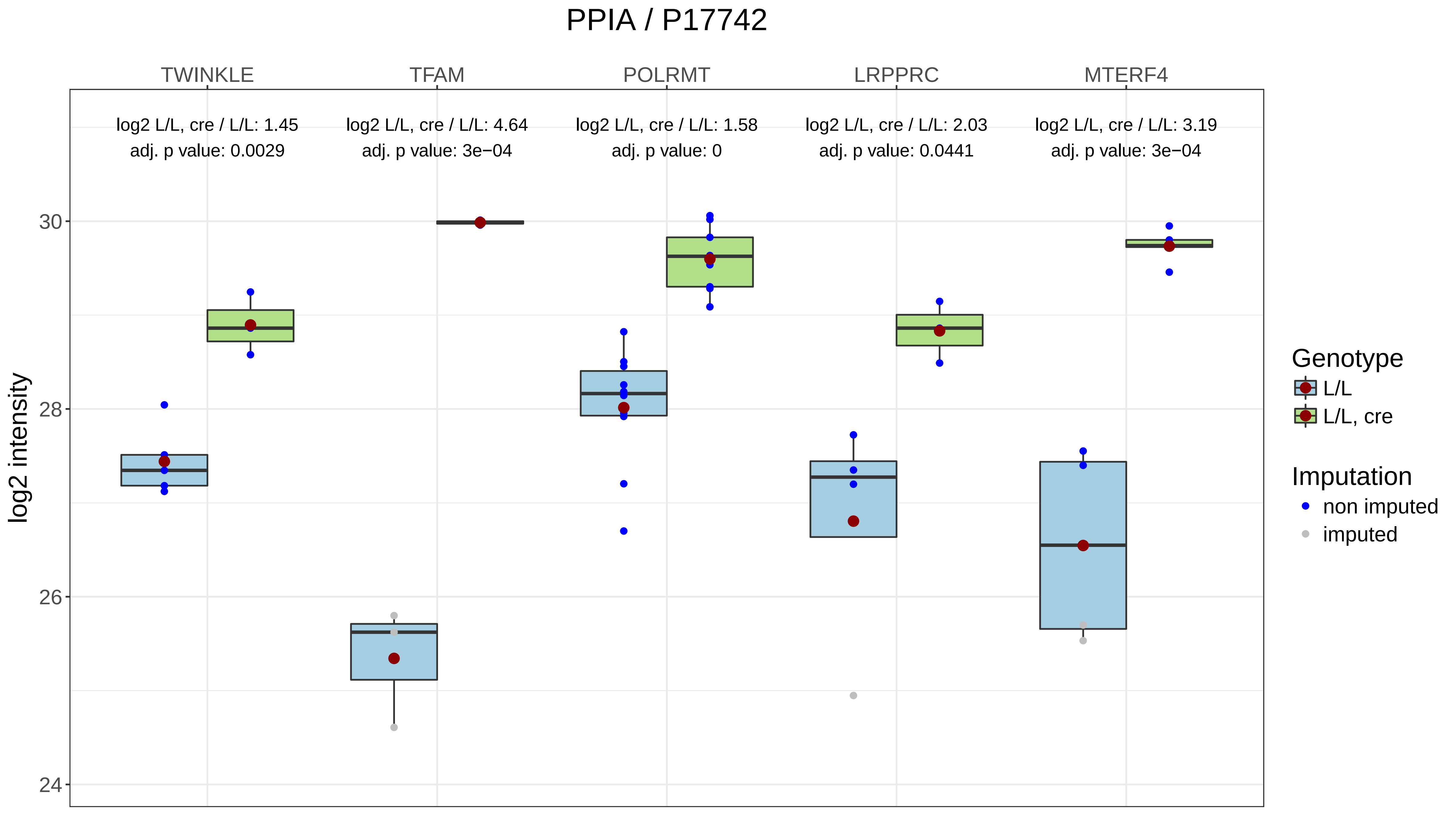
PPIA / P17742
TWINKLE
TFAM
POLRMT
LRPPRC
MTERF4
log2 L/L, cre / L/L: 1.45
adj. p value: 0.0029
log2 L/L, cre / L/L: 4.64
adj. p value: 3e−04
log2 L/L, cre / L/L: 1.58
adj. p value: 0
log2 L/L, cre / L/L: 2.03
adj. p value: 0.0441
log2 L/L, cre / L/L: 3.19
adj. p value: 3e−04
log2 intensity
30
28
26
24
Genotype
L/L
L/L, cre
Imputation
non imputed
imputed

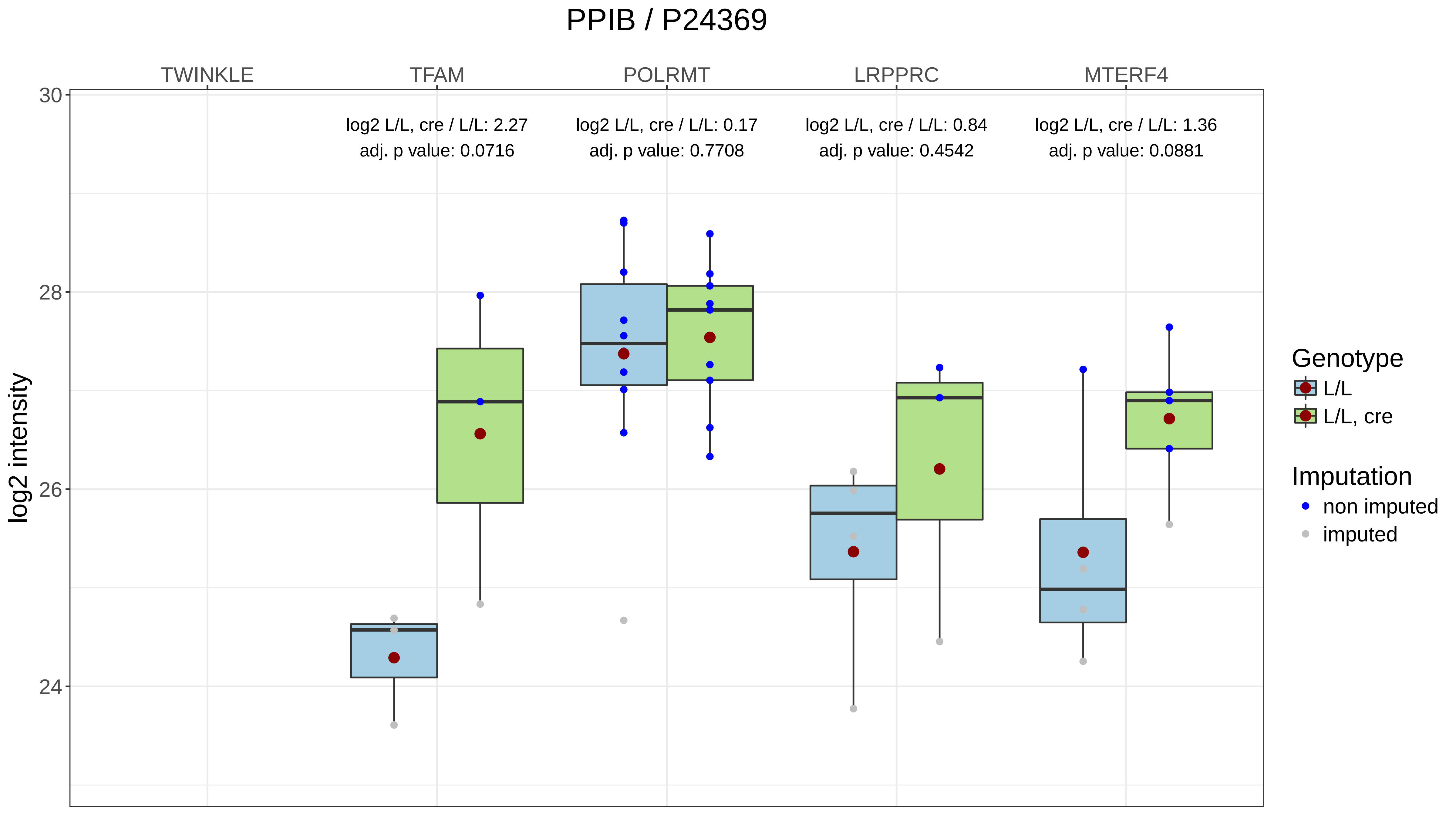

PPIB / P24369
TWINKLE
TFAM
POLRMT
LRPPRC
MTERF4
log2 L/L, cre / L/L: 2.27
adj. p value: 0.0716
log2 L/L, cre / L/L: 0.17
adj. p value: 0.7708
log2 L/L, cre / L/L: 0.84
adj. p value: 0.4542
log2 L/L, cre / L/L: 1.36
adj. p value: 0.0881
log2 intensity
30
28
26
24
Genotype
L/L
L/L, cre
Imputation
non imputed
imputed

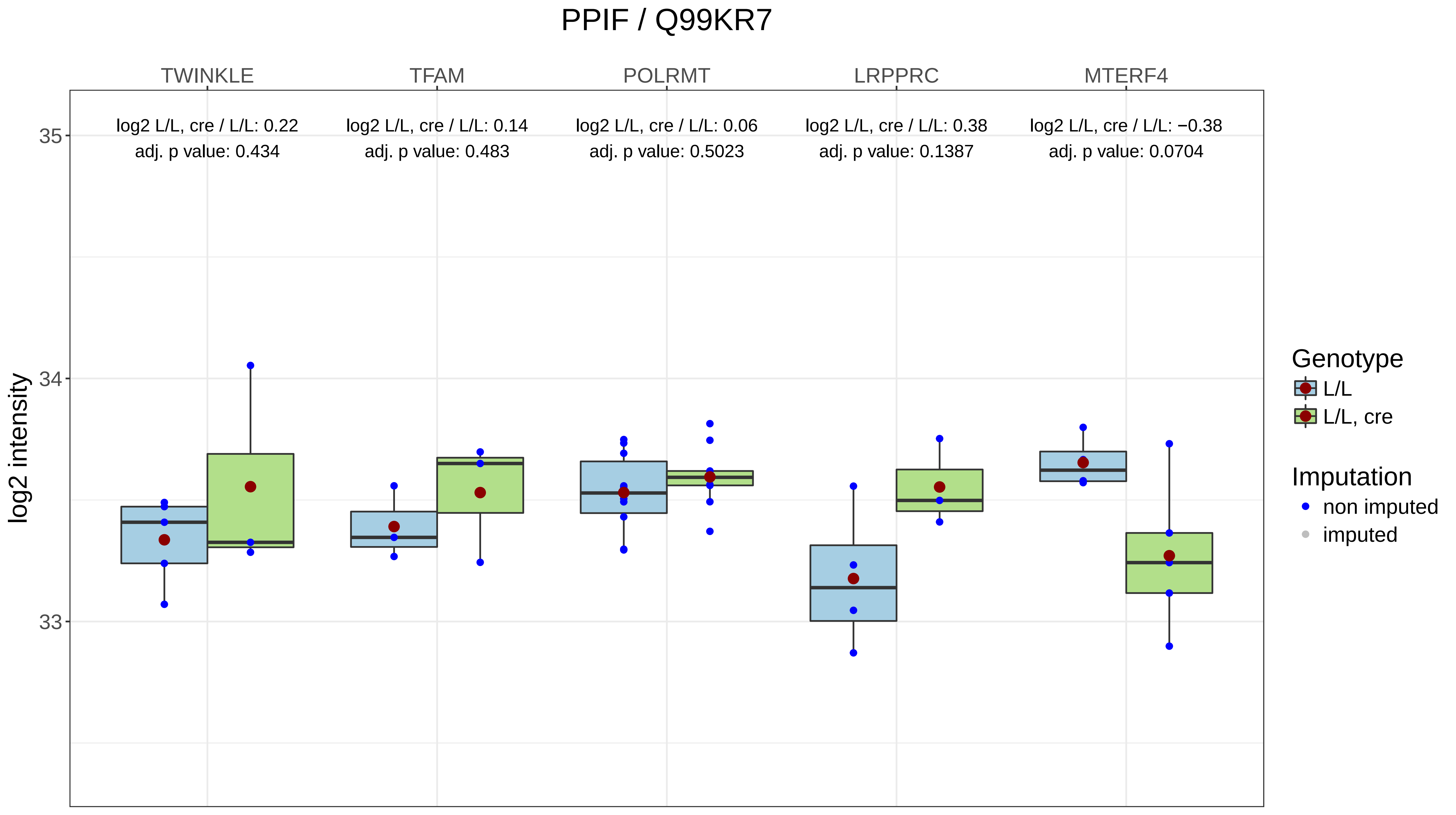
PPIF / Q99KR7
TWINKLE
TFAM
POLRMT
LRPPRC
MTERF4
log2 L/L, cre / L/L: 0.22
adj. p value: 0.434
log2 L/L, cre / L/L: 0.14
adj. p value: 0.483
log2 L/L, cre / L/L: 0.06
adj. p value: 0.5023
log2 L/L, cre / L/L: 0.38
adj. p value: 0.1387
log2 L/L, cre / L/L: −0.38
adj. p value: 0.0704
log2 intensity
35
34
33
Genotype
L/L
L/L, cre
Imputation
non imputed
imputed

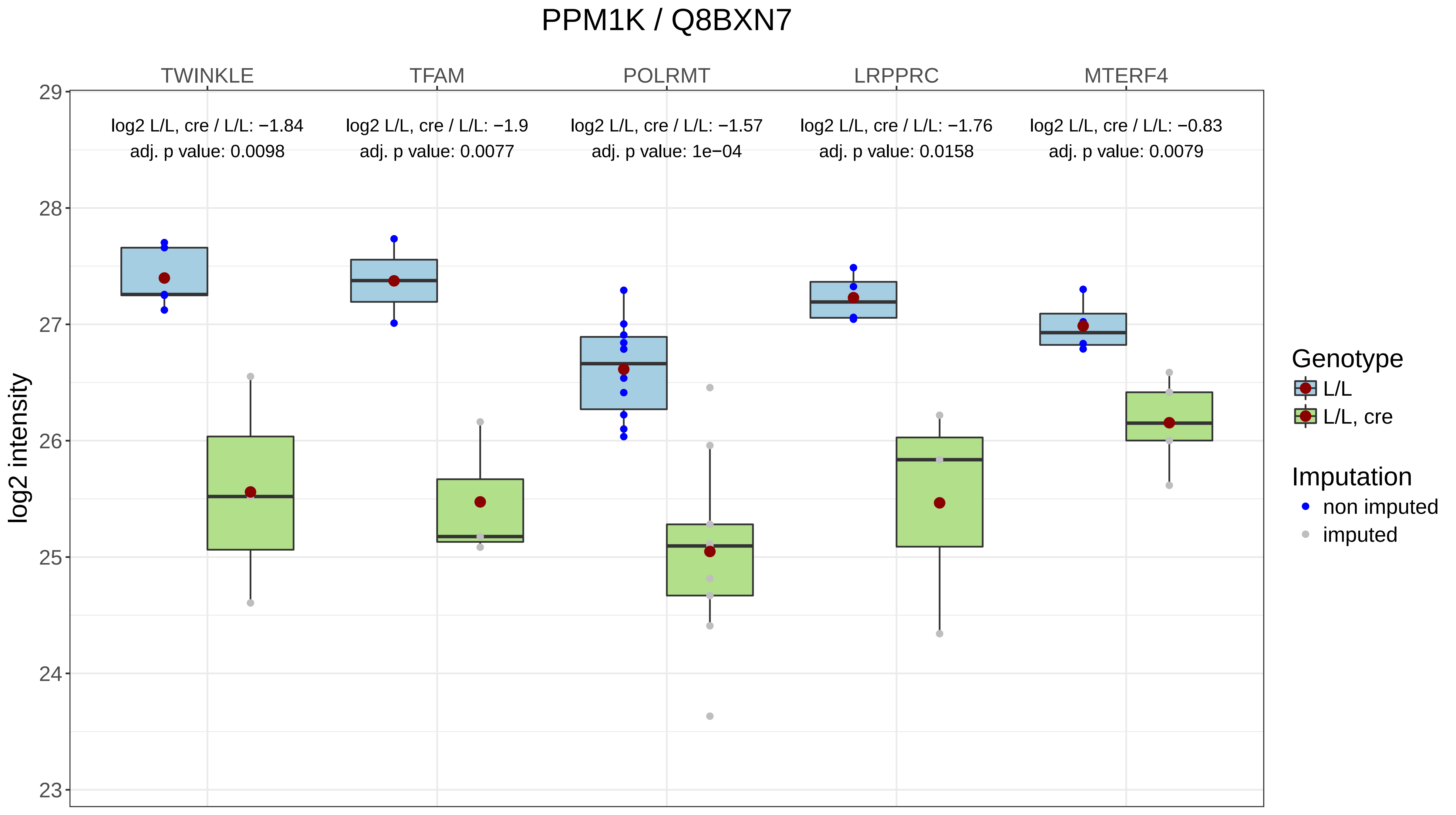
PPM1K / Q8BXN7
TWINKLE
TFAM
POLRMT
LRPPRC
MTERF4
log2 L/L, cre / L/L: −1.84
adj. p value: 0.0098
log2 L/L, cre / L/L: −1.9
adj. p value: 0.0077
log2 L/L, cre / L/L: −1.57
adj. p value: 1e−04
log2 L/L, cre / L/L: −1.76
adj. p value: 0.0158
log2 L/L, cre / L/L: −0.83
adj. p value: 0.0079
log2 intensity
29
28
27
26
25
24
23
Genotype
L/L
L/L, cre
Imputation
non imputed
imputed

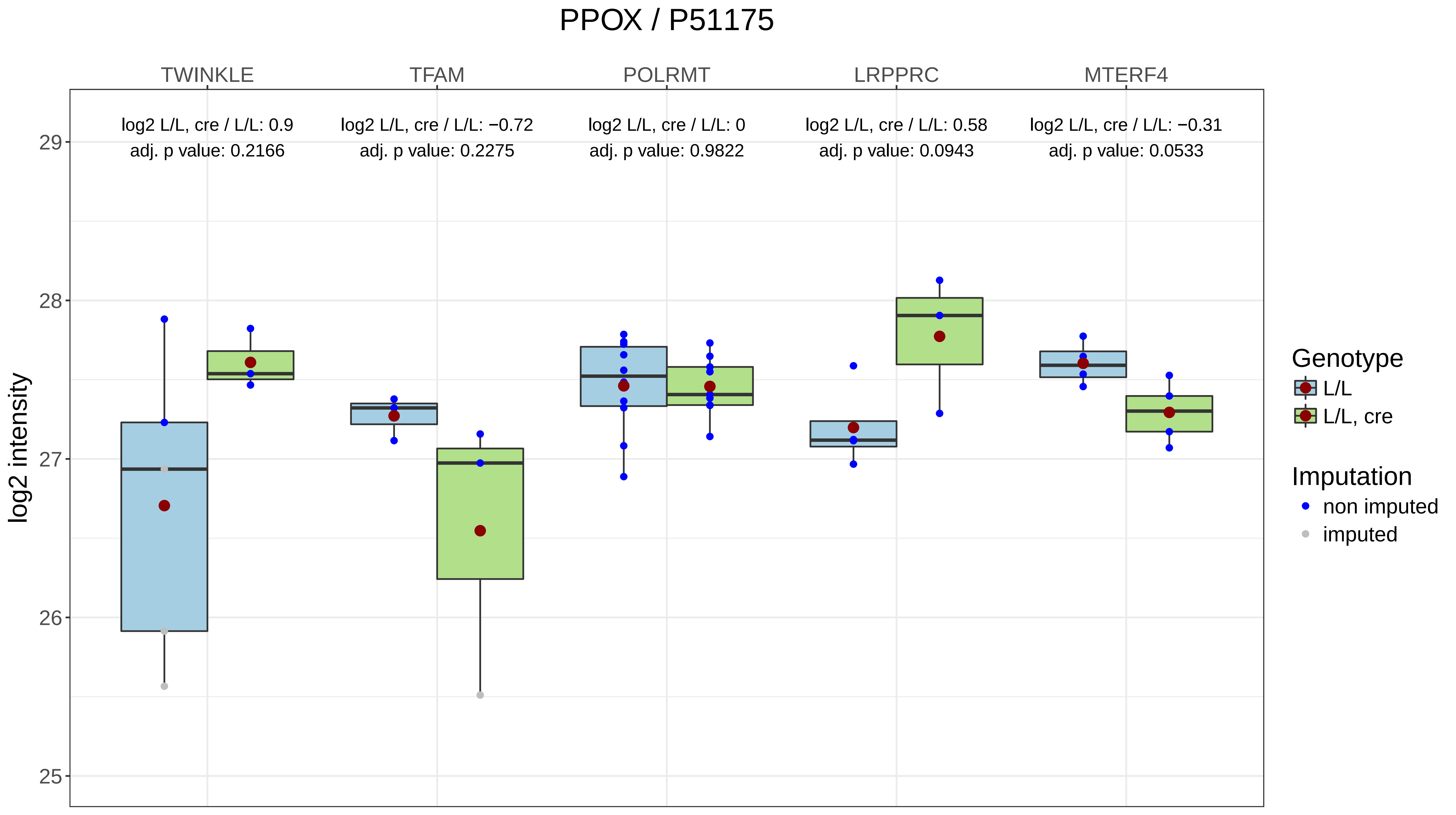
PPOX / P51175
TWINKLE
TFAM
POLRMT
LRPPRC
MTERF4
log2 L/L, cre / L/L: 0.9
adj. p value: 0.2166
log2 L/L, cre / L/L: −0.72
adj. p value: 0.2275
log2 L/L, cre / L/L: 0
adj. p value: 0.9822
log2 L/L, cre / L/L: 0.58
adj. p value: 0.0943
log2 L/L, cre / L/L: −0.31
adj. p value: 0.0533
log2 intensity
29
28
27
26
25
Genotype
L/L
L/L, cre
Imputation
non imputed
imputed

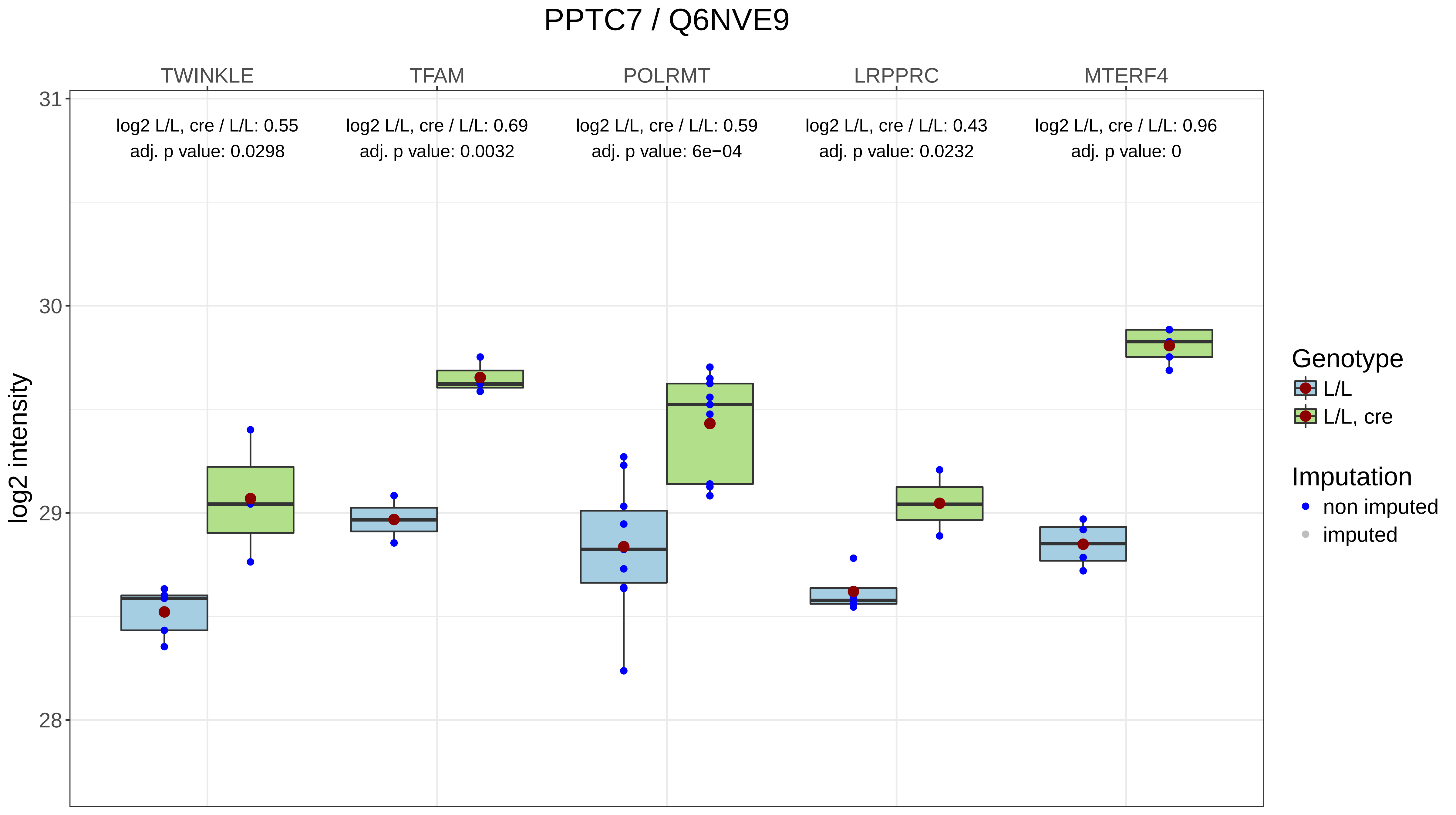
PPTC7 / Q6NVE9
TWINKLE
TFAM
POLRMT
LRPPRC
MTERF4
log2 L/L, cre / L/L: 0.55
adj. p value: 0.0298
log2 L/L, cre / L/L: 0.69
adj. p value: 0.0032
log2 L/L, cre / L/L: 0.59
adj. p value: 6e−04
log2 L/L, cre / L/L: 0.43
adj. p value: 0.0232
log2 L/L, cre / L/L: 0.96
adj. p value: 0
log2 intensity
31
30
29
28
Genotype
L/L
L/L, cre
Imputation
non imputed
imputed

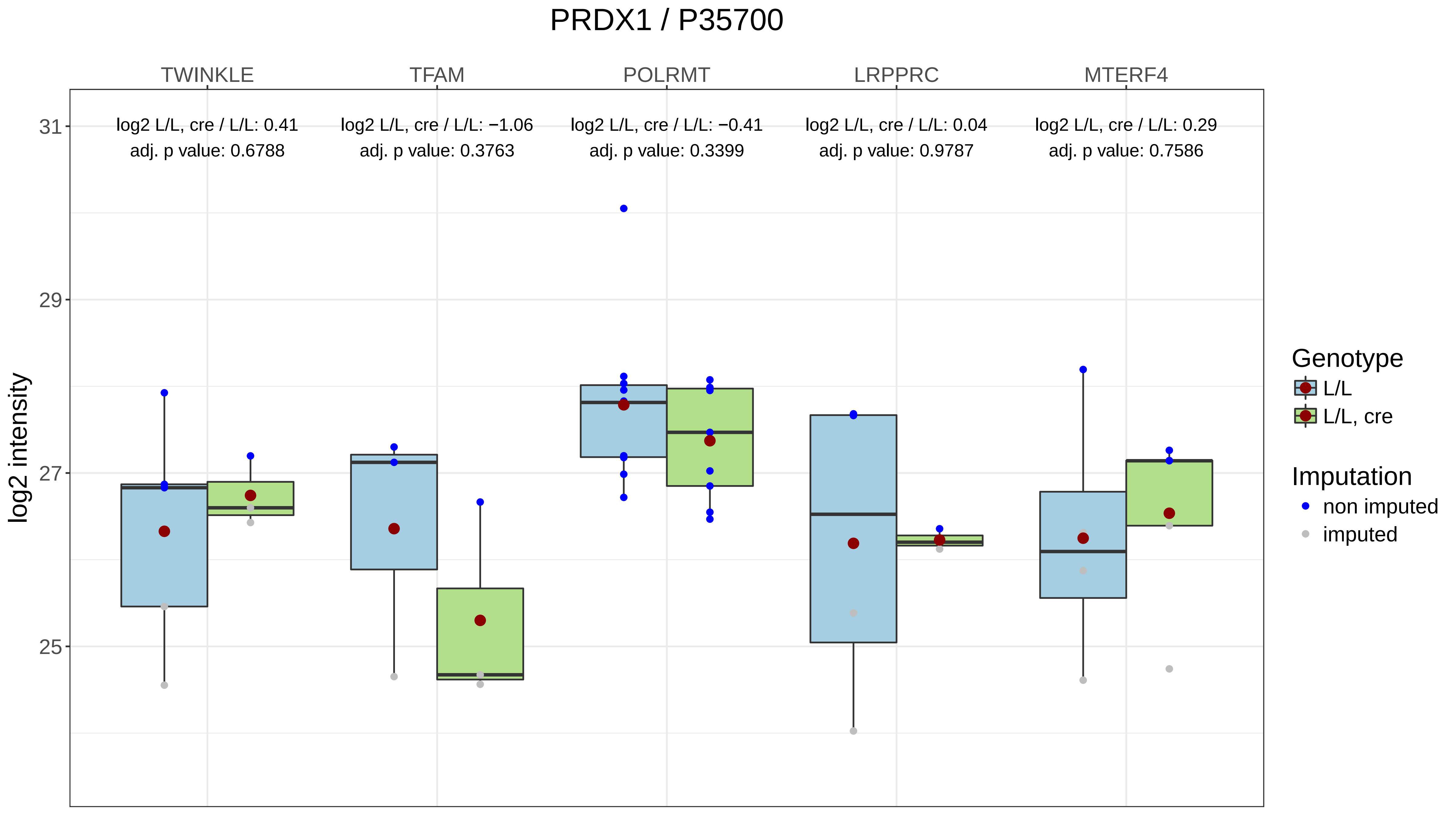
PRDX1 / P35700
TWINKLE
TFAM
POLRMT
LRPPRC
MTERF4
log2 L/L, cre / L/L: 0.41
adj. p value: 0.6788
log2 L/L, cre / L/L: −1.06
adj. p value: 0.3763
log2 L/L, cre / L/L: −0.41
adj. p value: 0.3399
log2 L/L, cre / L/L: 0.04
adj. p value: 0.9787
log2 L/L, cre / L/L: 0.29
adj. p value: 0.7586
log2 intensity
31
29
27
25
Genotype
L/L
L/L, cre
Imputation
non imputed
imputed

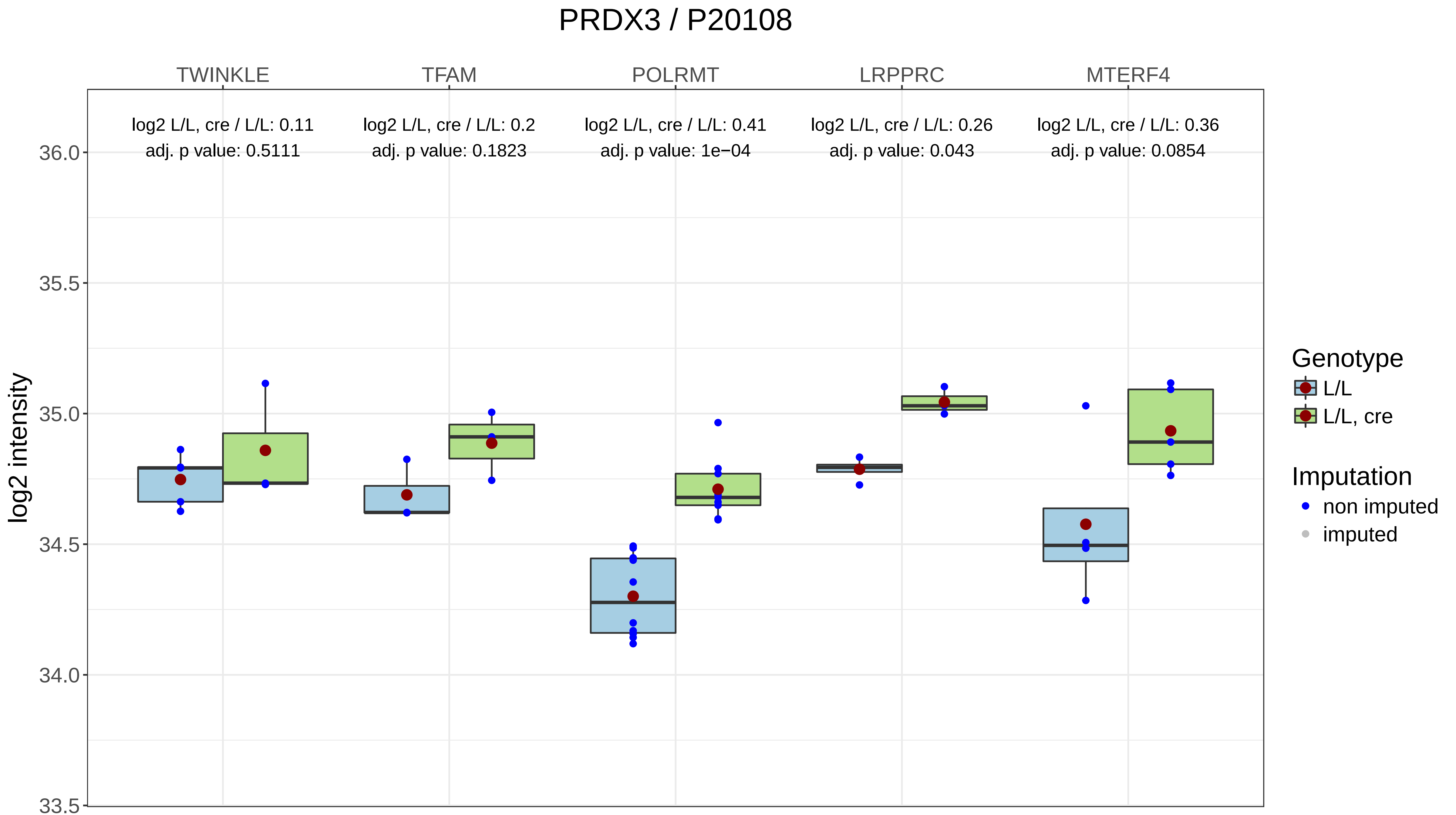
PRDX3 / P20108
TWINKLE
TFAM
POLRMT
LRPPRC
MTERF4
log2 L/L, cre / L/L: 0.11
adj. p value: 0.5111
log2 L/L, cre / L/L: 0.2
adj. p value: 0.1823
log2 L/L, cre / L/L: 0.41
adj. p value: 1e−04
log2 L/L, cre / L/L: 0.26
adj. p value: 0.043
log2 L/L, cre / L/L: 0.36
adj. p value: 0.0854
log2 intensity
36.0
35.5
35.0
34.5
34.0
33.5
Genotype
L/L
L/L, cre
Imputation
non imputed
imputed

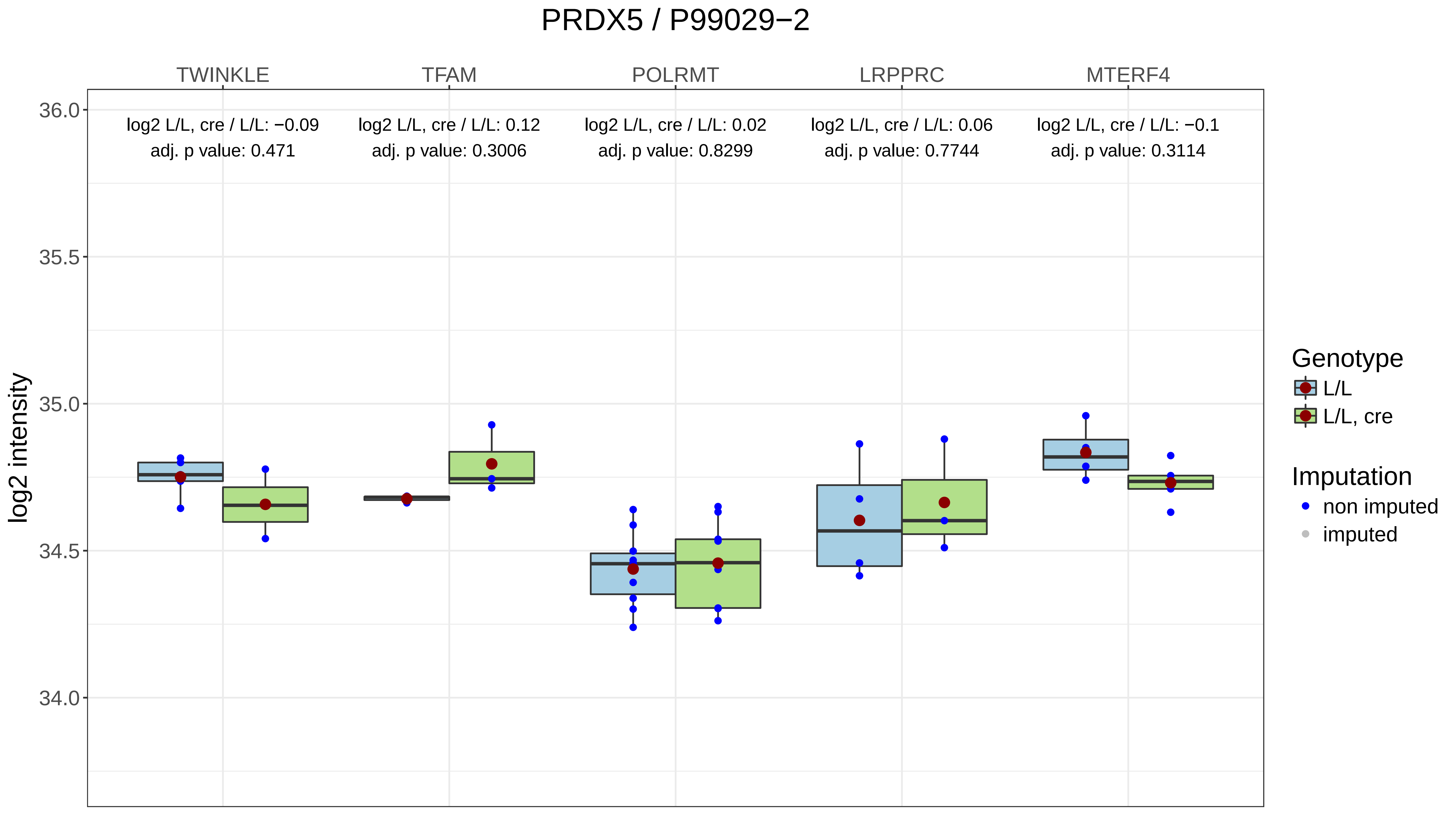
PRDX5 / P99029−2
TWINKLE
TFAM
POLRMT
LRPPRC
MTERF4
log2 L/L, cre / L/L: −0.09
adj. p value: 0.471
log2 L/L, cre / L/L: 0.12
adj. p value: 0.3006
log2 L/L, cre / L/L: 0.02
adj. p value: 0.8299
log2 L/L, cre / L/L: 0.06
adj. p value: 0.7744
log2 L/L, cre / L/L: −0.1
adj. p value: 0.3114
log2 intensity
36.0
35.5
35.0
34.5
34.0
Genotype
L/L
L/L, cre
Imputation
non imputed
imputed

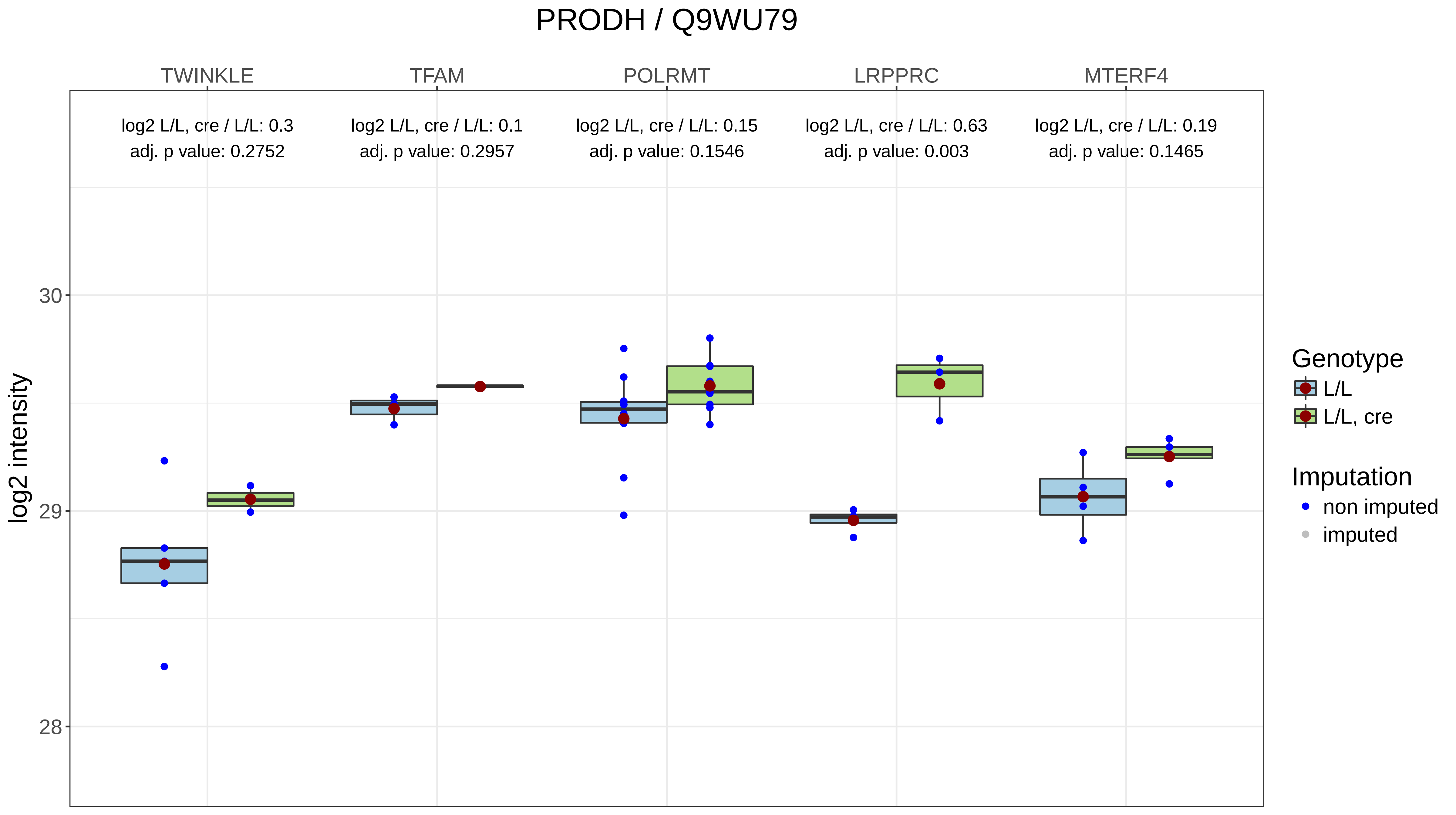

PRODH / Q9WU79
TWINKLE
TFAM
POLRMT
LRPPRC
MTERF4
log2 L/L, cre / L/L: 0.3
adj. p value: 0.2752
log2 L/L, cre / L/L: 0.1
adj. p value: 0.2957
log2 L/L, cre / L/L: 0.15
adj. p value: 0.1546
log2 L/L, cre / L/L: 0.63
adj. p value: 0.003
log2 L/L, cre / L/L: 0.19
adj. p value: 0.1465
log2 intensity
30
29
28
Genotype
L/L
L/L, cre
Imputation
non imputed
imputed

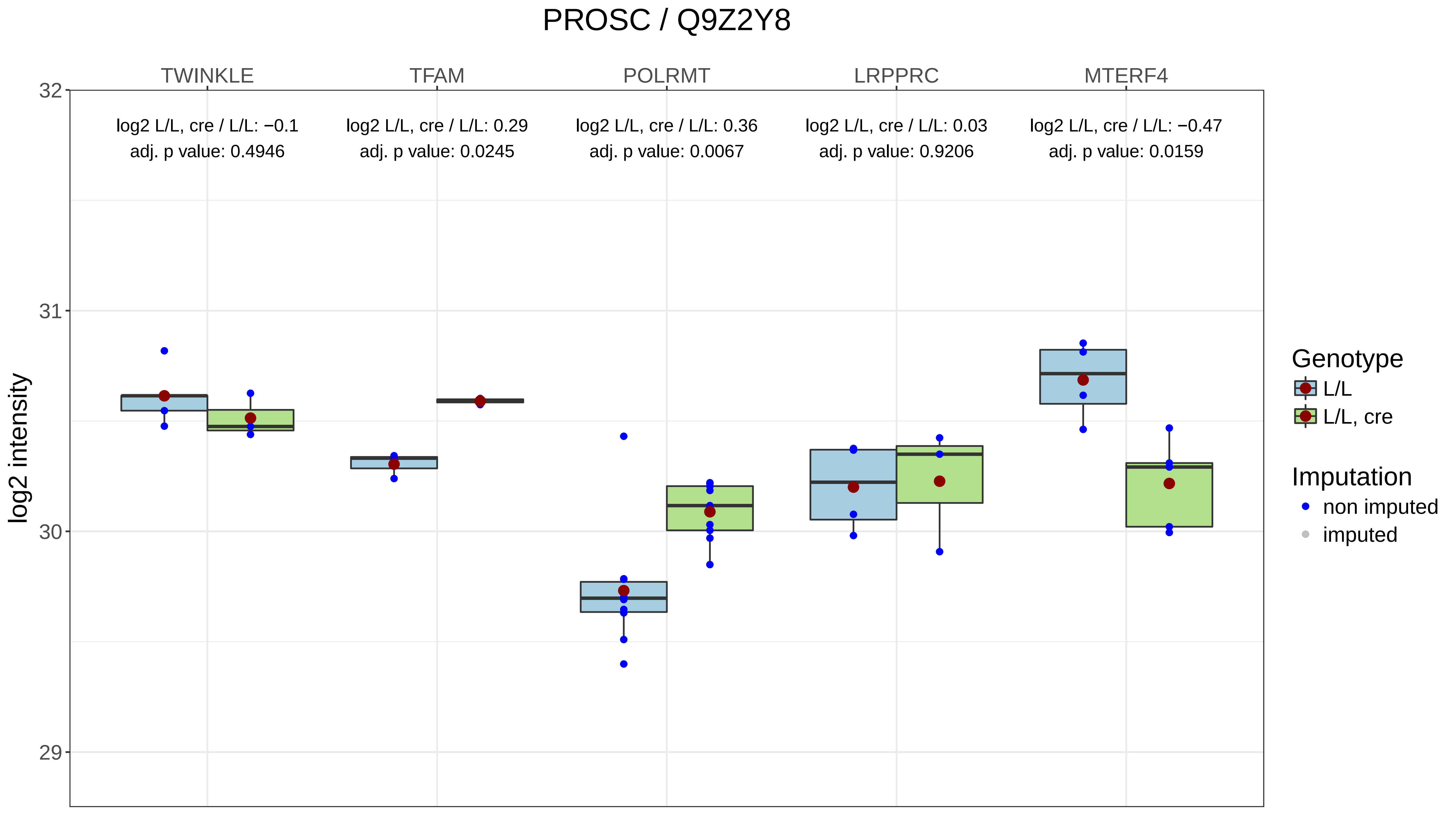
PROSC / Q9Z2Y8
TWINKLE
TFAM
POLRMT
LRPPRC
MTERF4
log2 L/L, cre / L/L: −0.1
adj. p value: 0.4946
log2 L/L, cre / L/L: 0.29
adj. p value: 0.0245
log2 L/L, cre / L/L: 0.36
adj. p value: 0.0067
log2 L/L, cre / L/L: 0.03
adj. p value: 0.9206
log2 L/L, cre / L/L: −0.47
adj. p value: 0.0159
log2 intensity
32
31
30
29
Genotype
L/L
L/L, cre
Imputation
non imputed
imputed

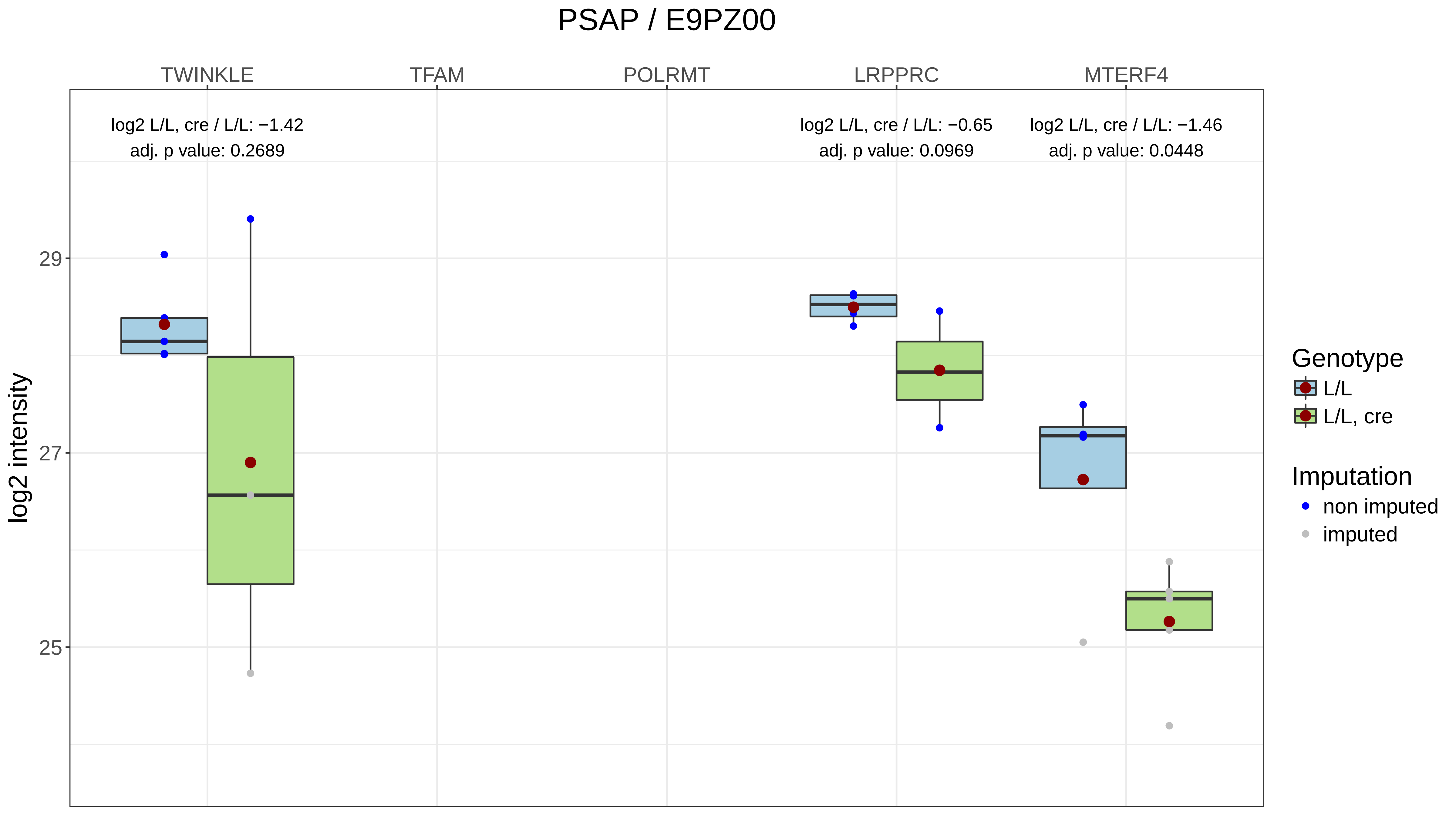
PSAP / E9PZ00
TWINKLE
TFAM
POLRMT
LRPPRC
MTERF4
log2 L/L, cre / L/L: −1.42
adj. p value: 0.2689
log2 L/L, cre / L/L: −0.65
adj. p value: 0.0969
log2 L/L, cre / L/L: −1.46
adj. p value: 0.0448
log2 intensity
29
27
25
Genotype
L/L
L/L, cre
Imputation
non imputed
imputed

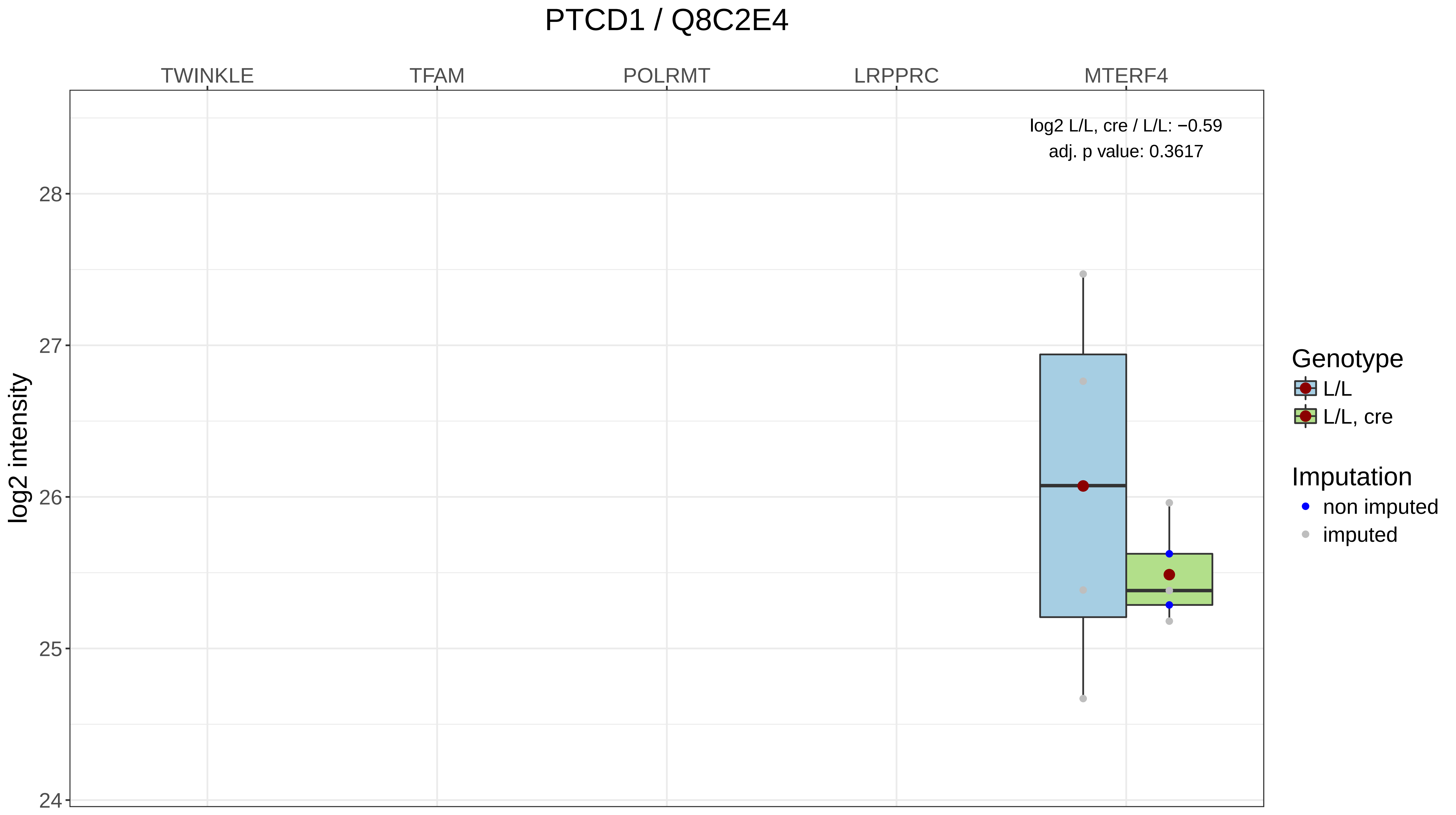
PTCD1 / Q8C2E4
TWINKLE
TFAM
POLRMT
LRPPRC
MTERF4
log2 L/L, cre / L/L: −0.59
adj. p value: 0.3617
log2 intensity
28
27
26
25
24
Genotype
L/L
L/L, cre
Imputation
non imputed
imputed

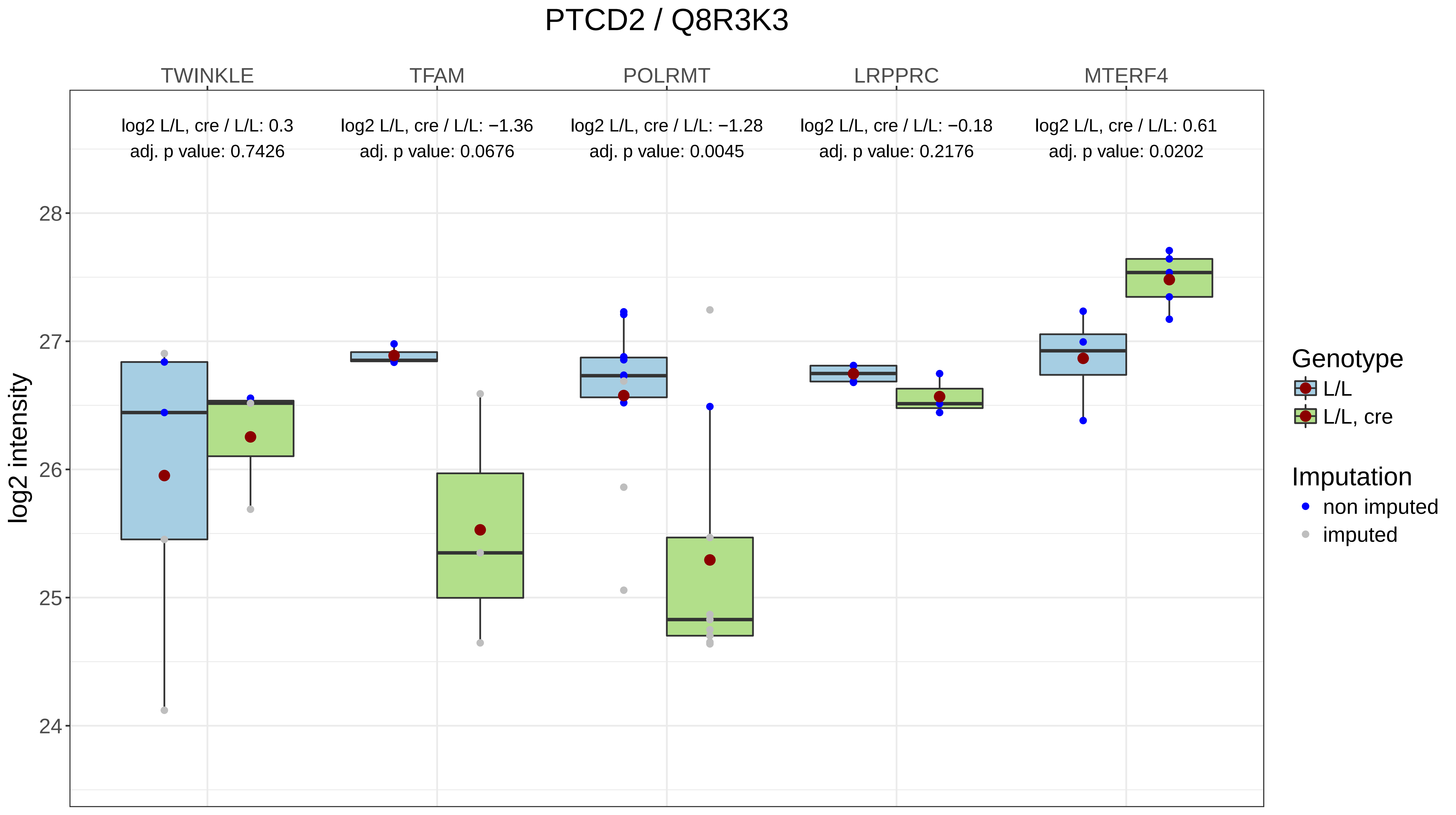

PTCD2 / Q8R3K3
TWINKLE
TFAM
POLRMT
LRPPRC
MTERF4
log2 L/L, cre / L/L: 0.3
adj. p value: 0.7426
log2 L/L, cre / L/L: −1.36
adj. p value: 0.0676
log2 L/L, cre / L/L: −1.28
adj. p value: 0.0045
log2 L/L, cre / L/L: −0.18
adj. p value: 0.2176
log2 L/L, cre / L/L: 0.61
adj. p value: 0.0202
log2 intensity
28
27
26
25
24
Genotype
L/L
L/L, cre
Imputation
non imputed
imputed

PTGES2 / Q8BWM0

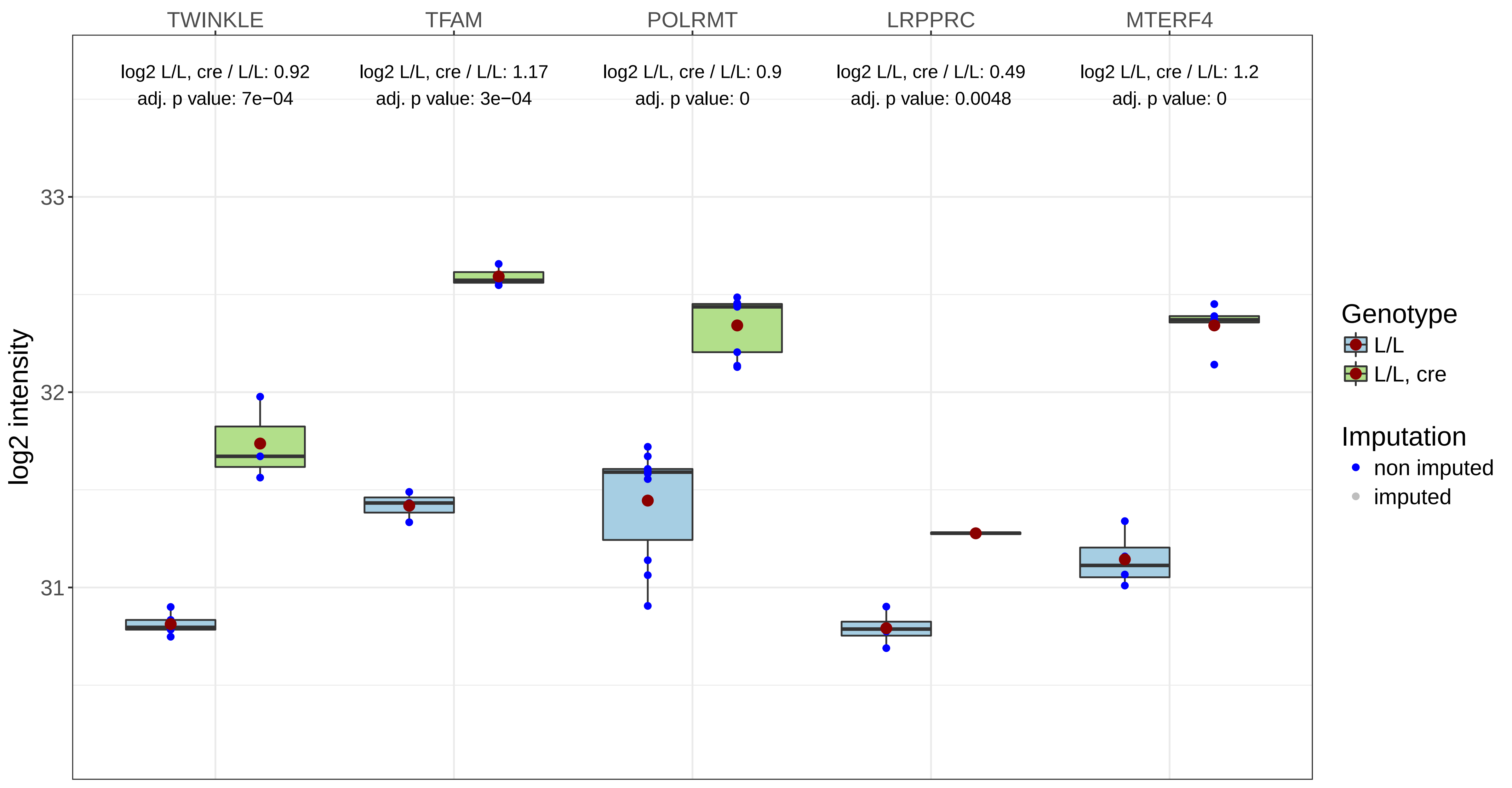

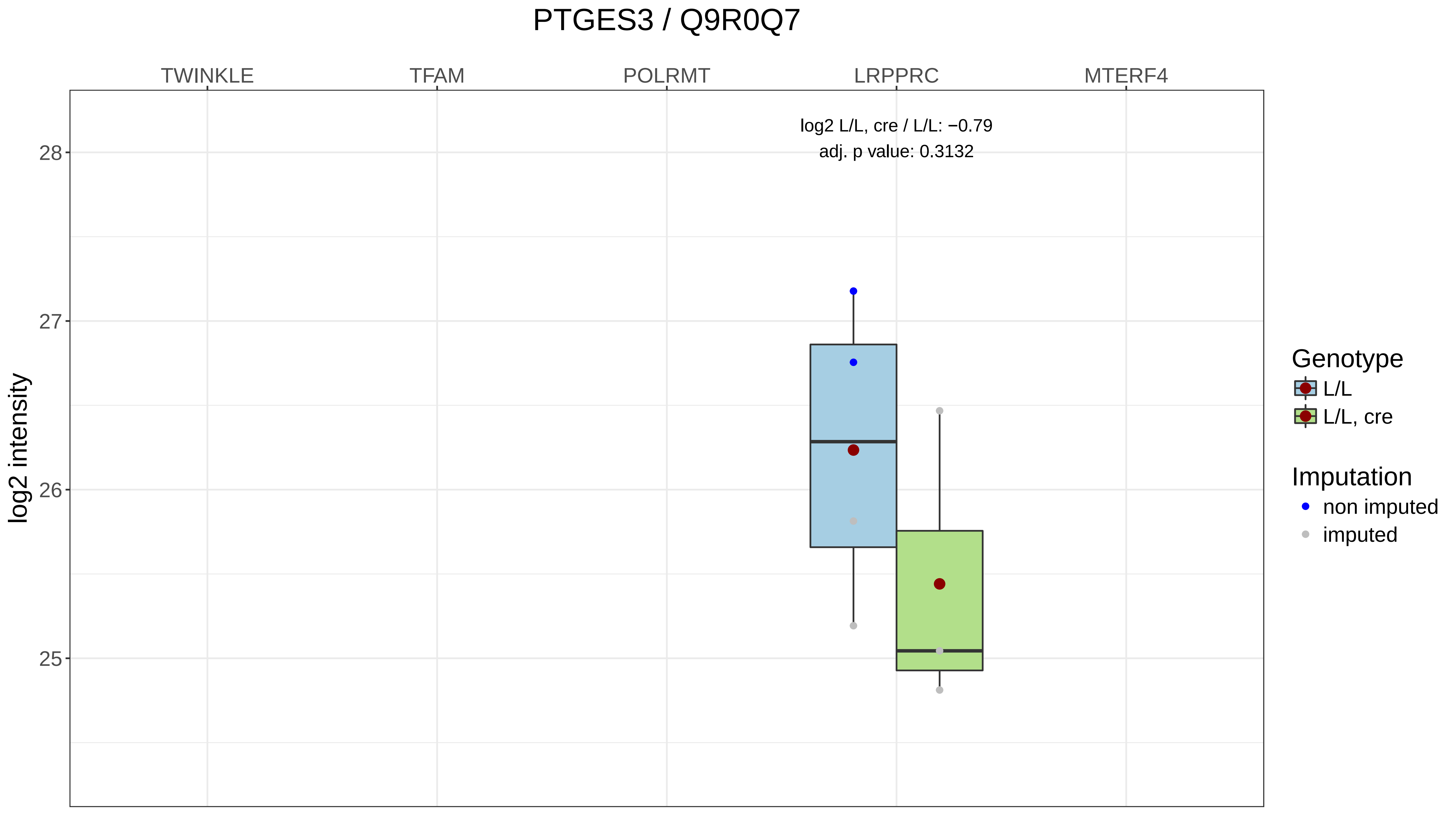

PTGES3 / Q9R0Q7
TWINKLE
TFAM
POLRMT
LRPPRC
MTERF4
log2 L/L, cre / L/L: −0.79
adj. p value: 0.3132
log2 intensity
28
27
26
25
Genotype
L/L
L/L, cre
Imputation
non imputed
imputed

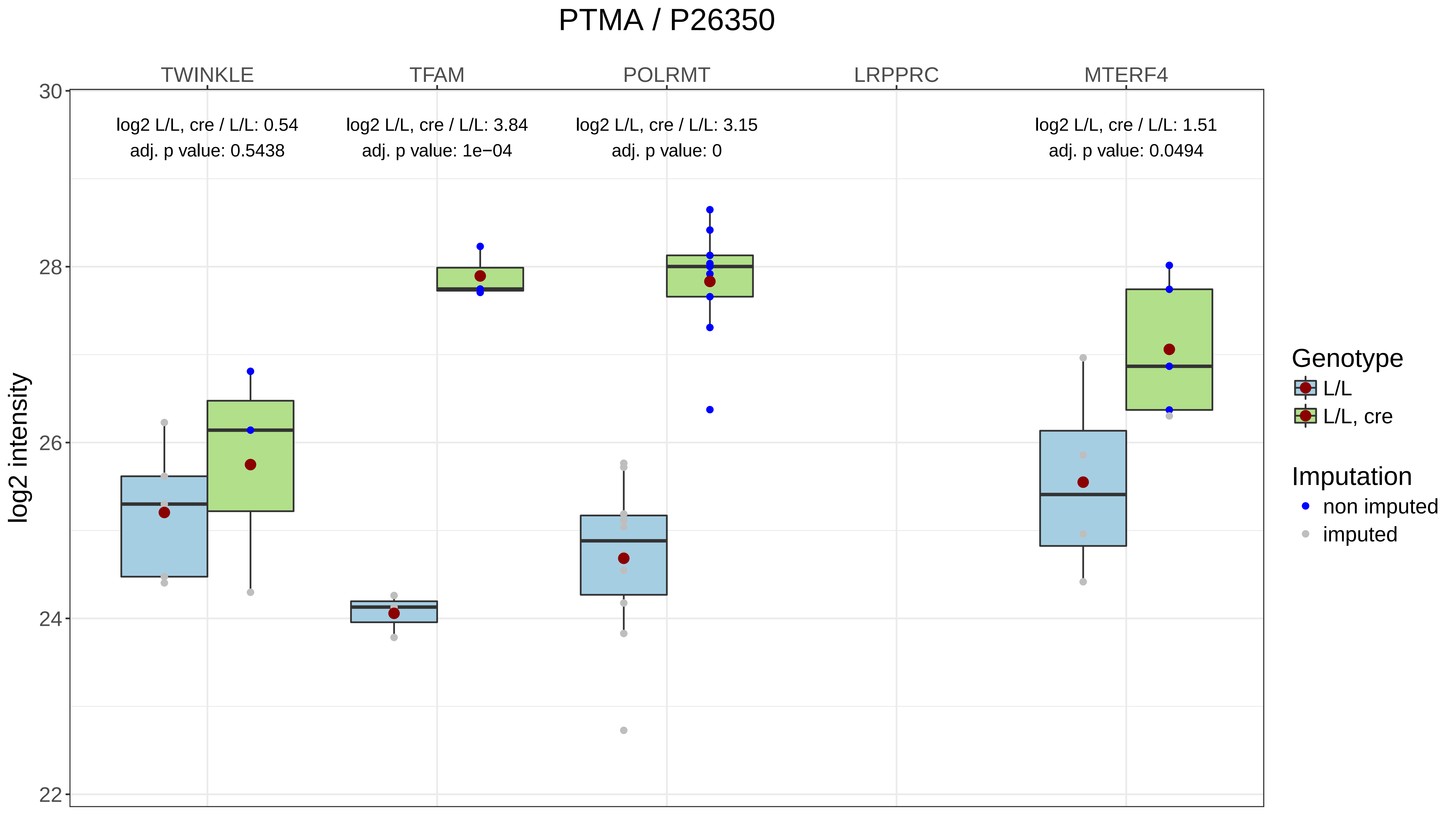
PTMA / P26350
TWINKLE
TFAM
POLRMT
LRPPRC
MTERF4
log2 L/L, cre / L/L: 0.54
adj. p value: 0.5438
log2 L/L, cre / L/L: 3.84
adj. p value: 1e−04
log2 L/L, cre / L/L: 3.15
adj. p value: 0
log2 L/L, cre / L/L: 1.51
adj. p value: 0.0494
log2 intensity
30
28
26
24
22
Genotype
L/L
L/L, cre
Imputation
non imputed
imputed

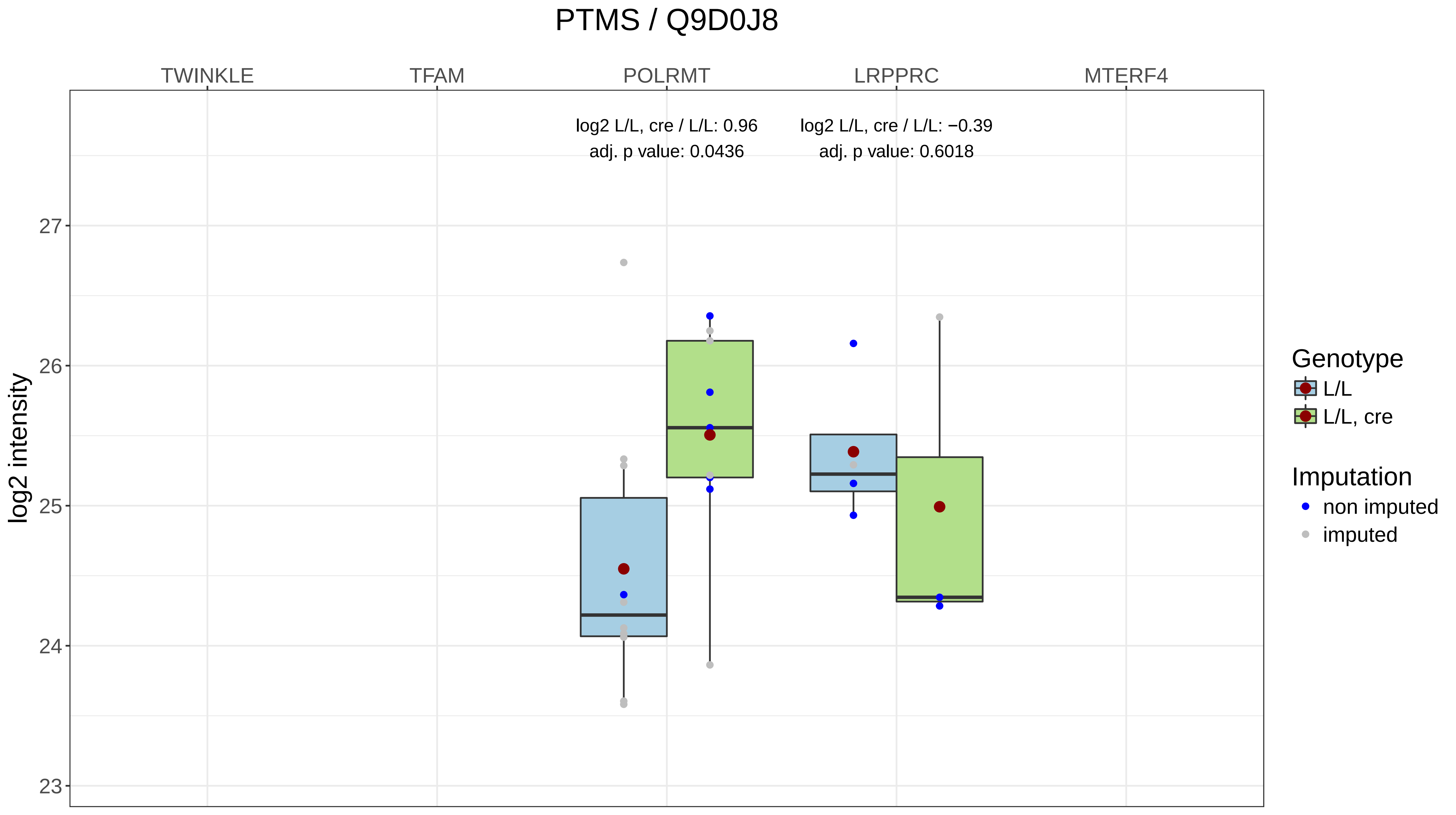
PTMS / Q9D0J8
TWINKLE
TFAM
POLRMT
LRPPRC
MTERF4
log2 L/L, cre / L/L: 0.96
adj. p value: 0.0436
log2 L/L, cre / L/L: −0.39
adj. p value: 0.6018
log2 intensity
27
26
25
24
23
Genotype
L/L
L/L, cre
Imputation
non imputed
imputed

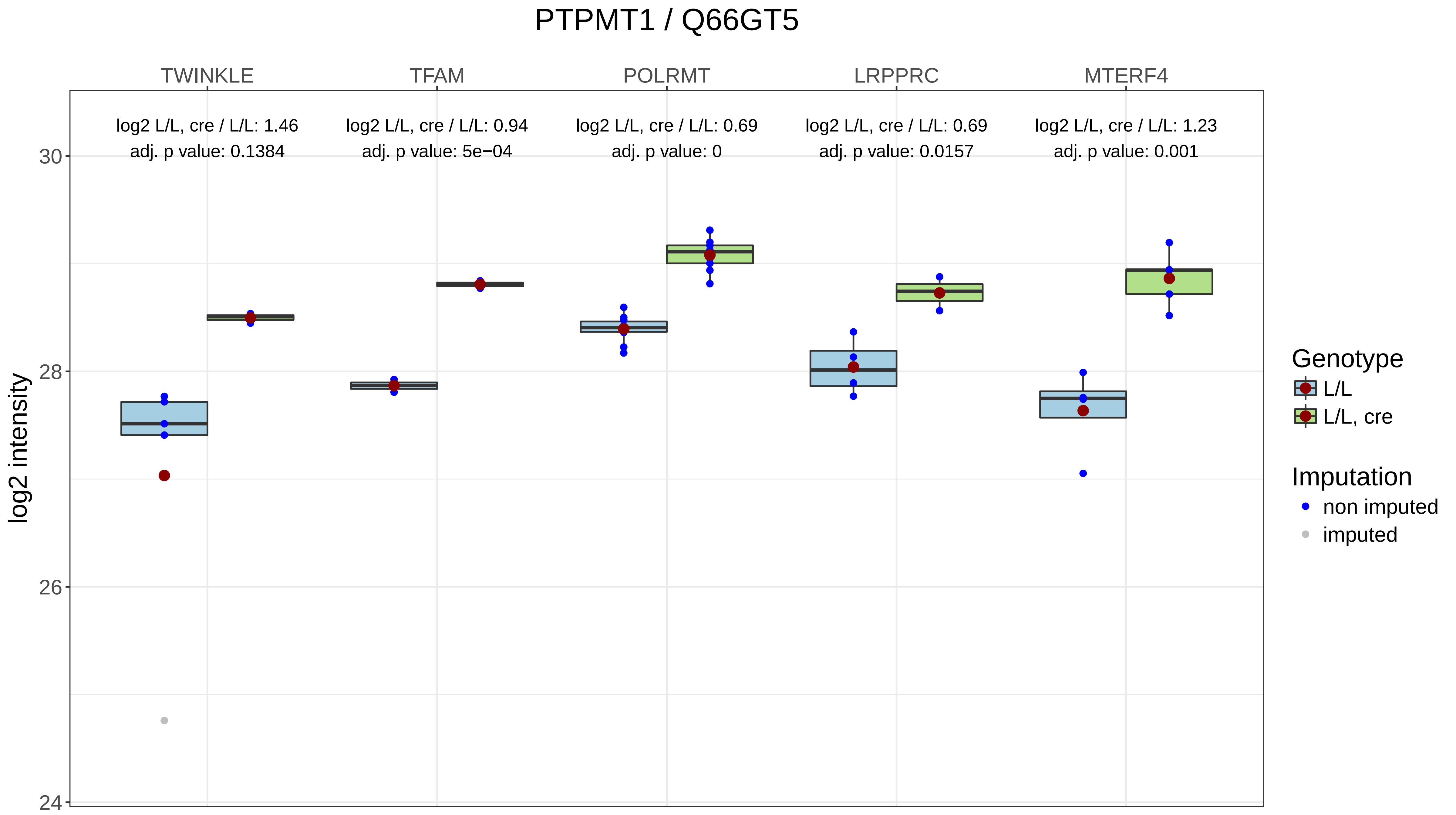
PTPMT1 / Q66GT5
TWINKLE
TFAM
POLRMT
LRPPRC
MTERF4
log2 L/L, cre / L/L: 1.46
adj. p value: 0.1384
log2 L/L, cre / L/L: 0.94
adj. p value: 5e−04
log2 L/L, cre / L/L: 0.69
adj. p value: 0
log2 L/L, cre / L/L: 0.69
adj. p value: 0.0157
log2 L/L, cre / L/L: 1.23
adj. p value: 0.001
log2 intensity
30
28
26
24
Genotype
L/L
L/L, cre
Imputation
non imputed
imputed

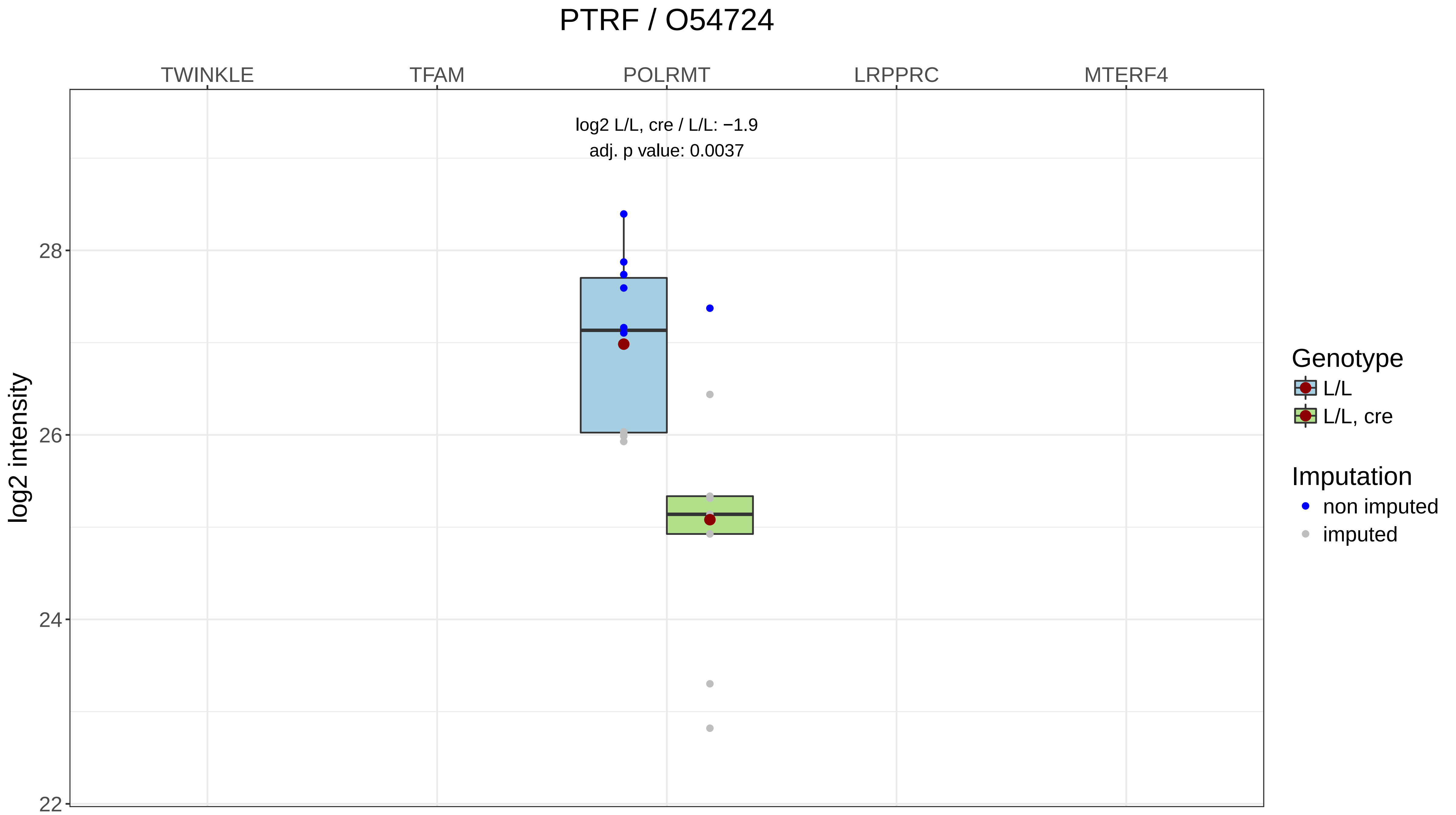
PTRF / O54724
TWINKLE
TFAM
POLRMT
LRPPRC
MTERF4
log2 L/L, cre / L/L: −1.9
adj. p value: 0.0037
log2 intensity
28
26
24
22
Genotype
L/L
L/L, cre
Imputation
non imputed
imputed

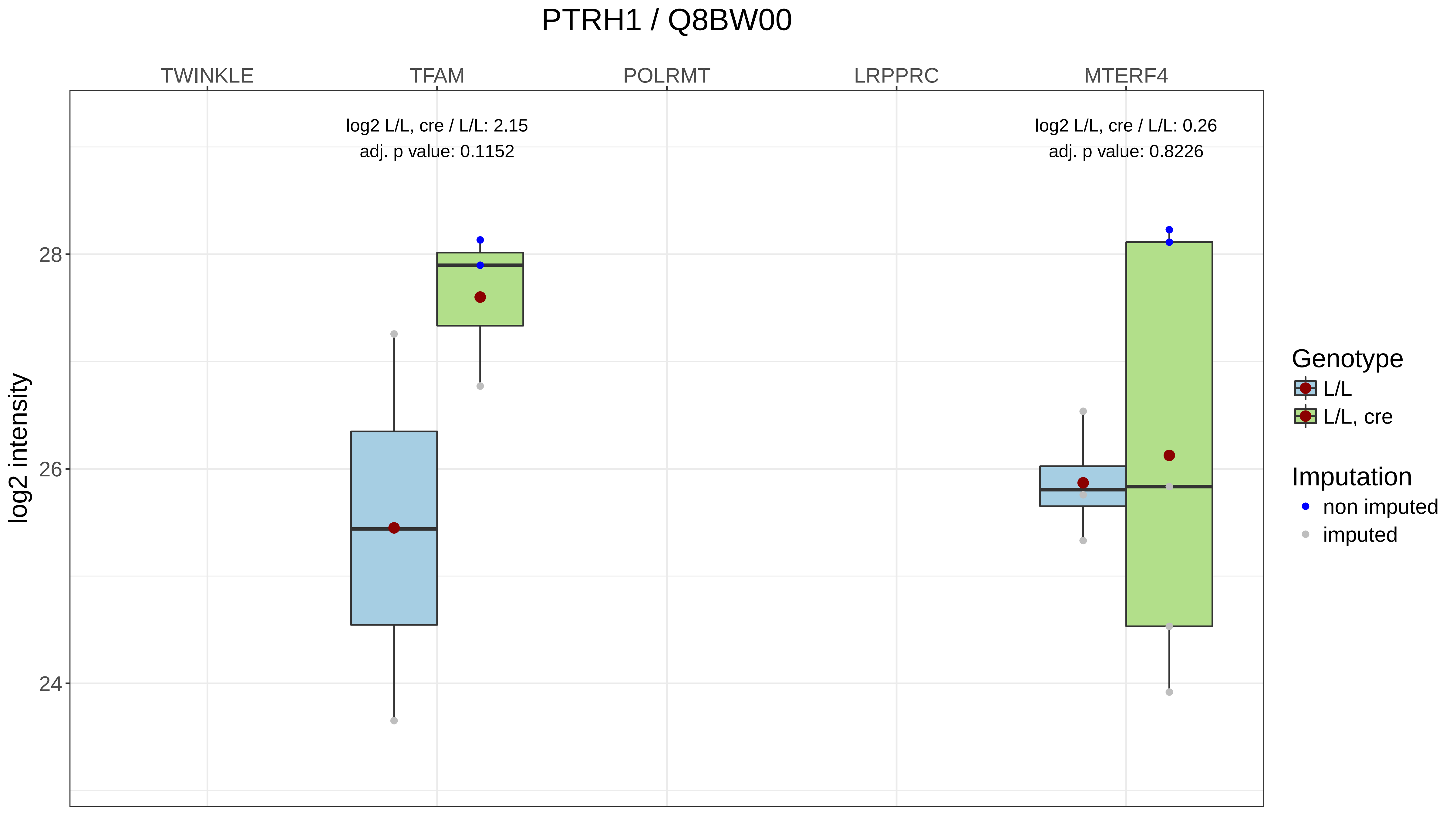

PTRH1 / Q8BW00
TWINKLE
TFAM
POLRMT
LRPPRC
MTERF4
log2 L/L, cre / L/L: 2.15
adj. p value: 0.1152
log2 L/L, cre / L/L: 0.26
adj. p value: 0.8226
log2 intensity
28
26
24
Genotype
L/L
L/L, cre
Imputation
non imputed
imputed

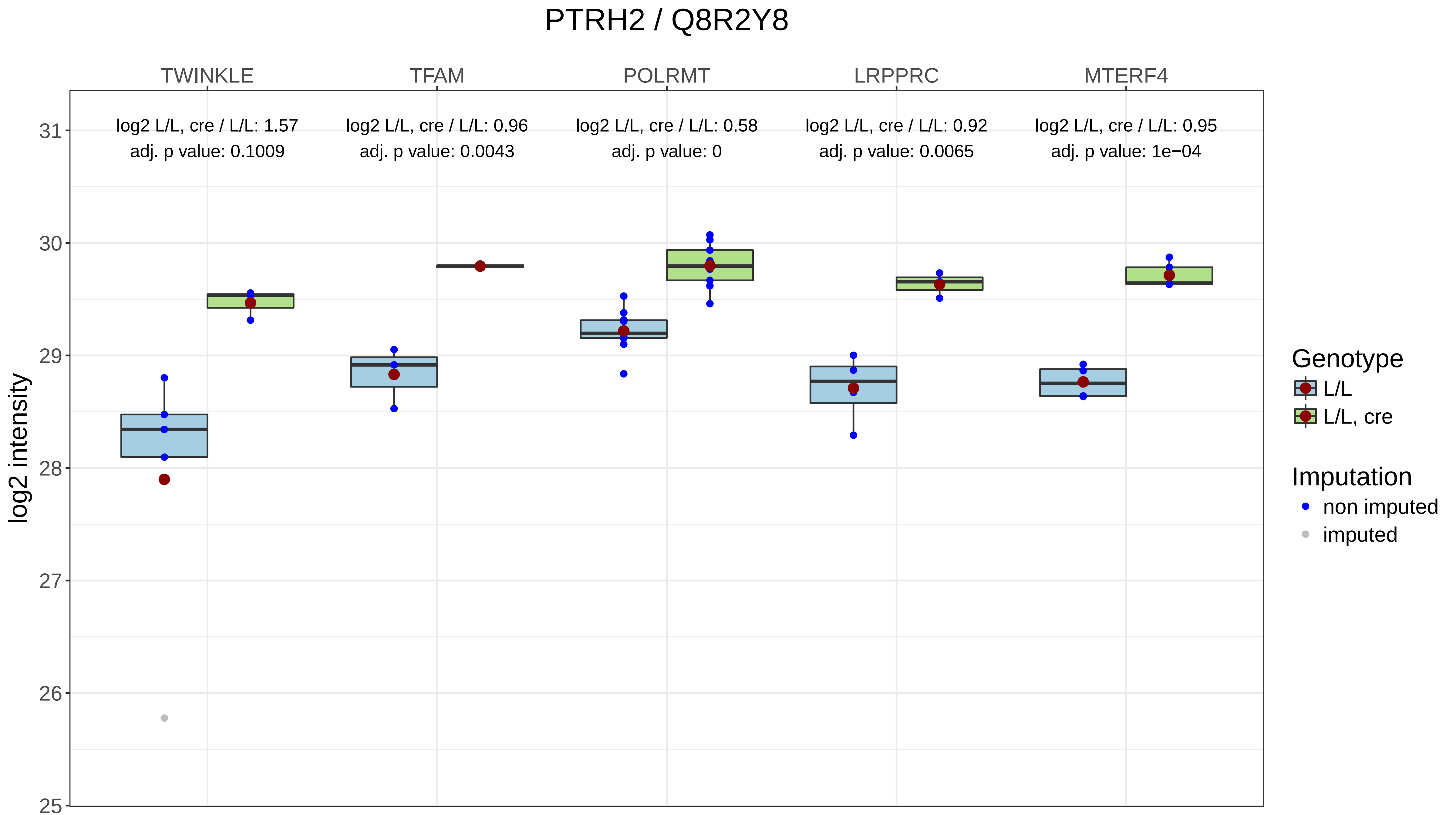

PTRH2 / Q8R2Y8
TWINKLE
TFAM
POLRMT
LRPPRC
MTERF4
log2 L/L, cre / L/L: 1.57
adj. p value: 0.1009
log2 L/L, cre / L/L: 0.96
adj. p value: 0.0043
log2 L/L, cre / L/L: 0.58
adj. p value: 0
log2 L/L, cre / L/L: 0.92
adj. p value: 0.0065
log2 L/L, cre / L/L: 0.95
adj. p value: 1e−04
log2 intensity
25
26
27
28
29
30
31
Genotype
L/L
L/L, cre
Imputation
non imputed
imputed

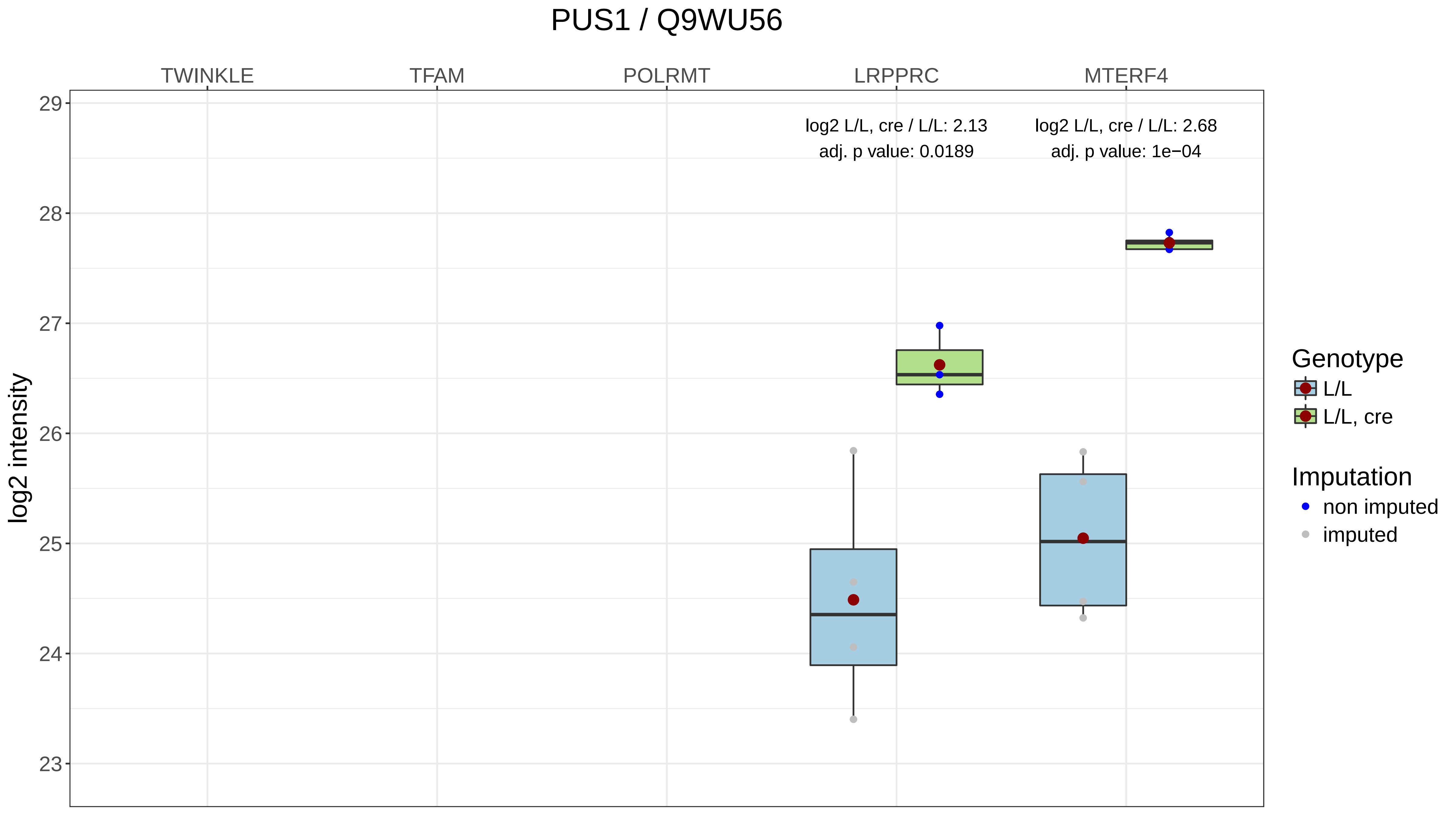
PUS1 / Q9WU56
TWINKLE
TFAM
POLRMT
LRPPRC
MTERF4
log2 L/L, cre / L/L: 2.13
adj. p value: 0.0189
log2 L/L, cre / L/L: 2.68
adj. p value: 1e−04
log2 intensity
29
28
27
26
25
24
23
Genotype
L/L
L/L, cre
Imputation
non imputed
imputed

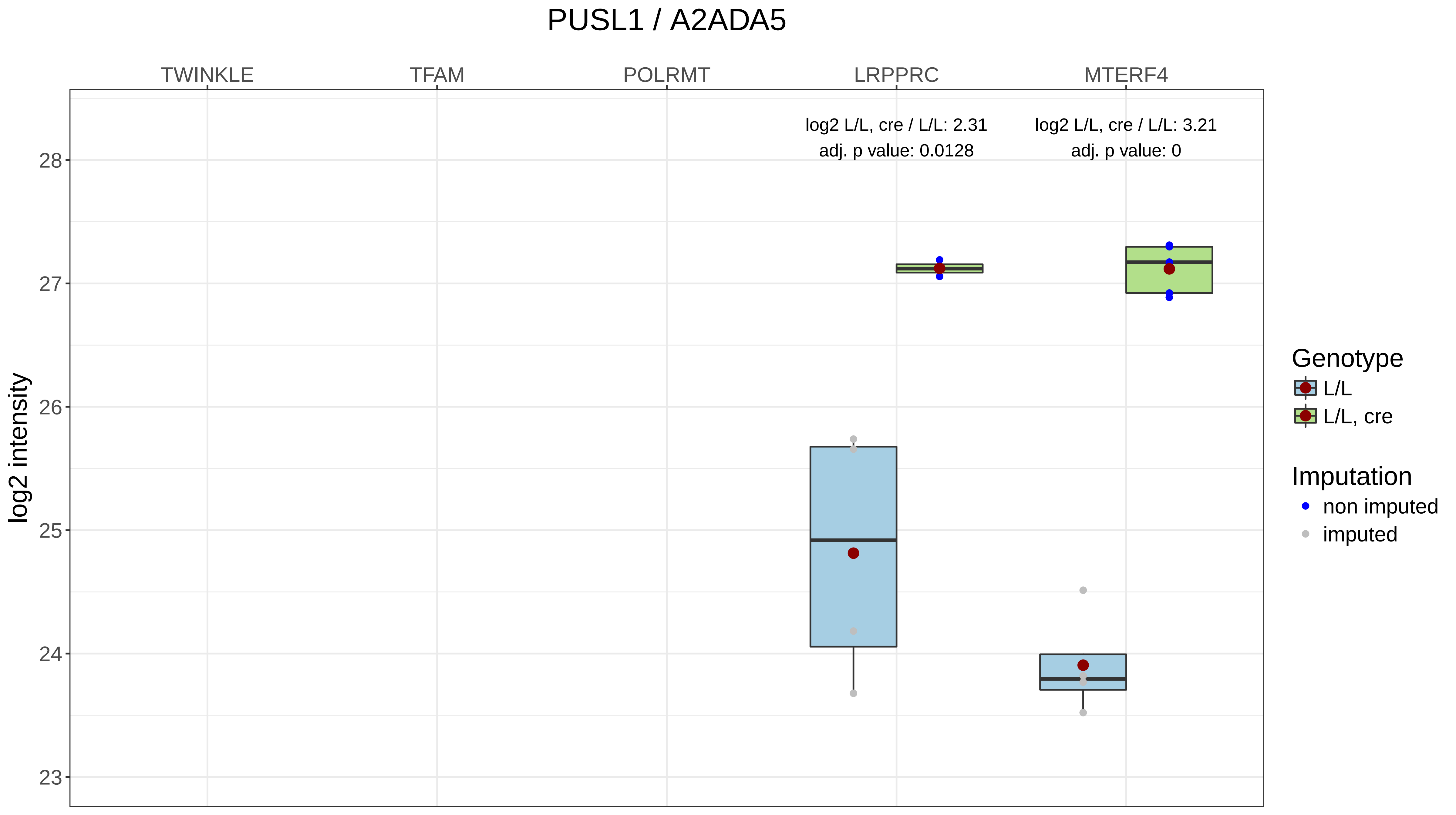
PUSL1 / A2ADA5
TWINKLE
TFAM
POLRMT
LRPPRC
MTERF4
log2 L/L, cre / L/L: 2.31
adj. p value: 0.0128
log2 L/L, cre / L/L: 3.21
adj. p value: 0
log2 intensity
28
27
26
25
24
23
Genotype
L/L
L/L, cre
Imputation
non imputed
imputed

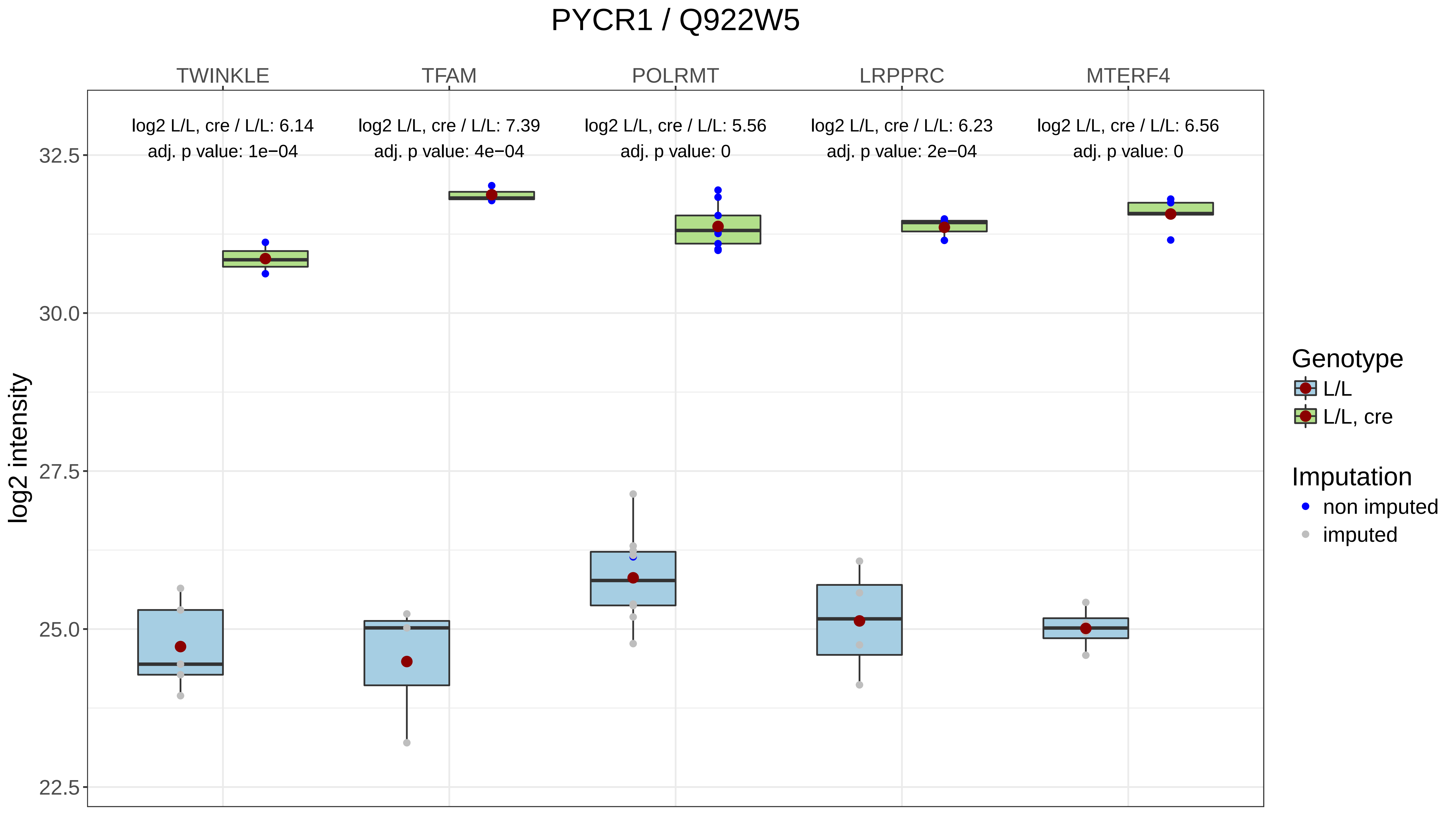
PYCR1 / Q922W5
TWINKLE
TFAM
POLRMT
LRPPRC
MTERF4
log2 L/L, cre / L/L: 6.14
adj. p value: 1e−04
log2 L/L, cre / L/L: 7.39
adj. p value: 4e−04
log2 L/L, cre / L/L: 5.56
adj. p value: 0
log2 L/L, cre / L/L: 6.23
adj. p value: 2e−04
log2 L/L, cre / L/L: 6.56
adj. p value: 0
log2 intensity
32.5
30.0
27.5
25.0
22.5
Genotype
L/L
L/L, cre
Imputation
non imputed
imputed

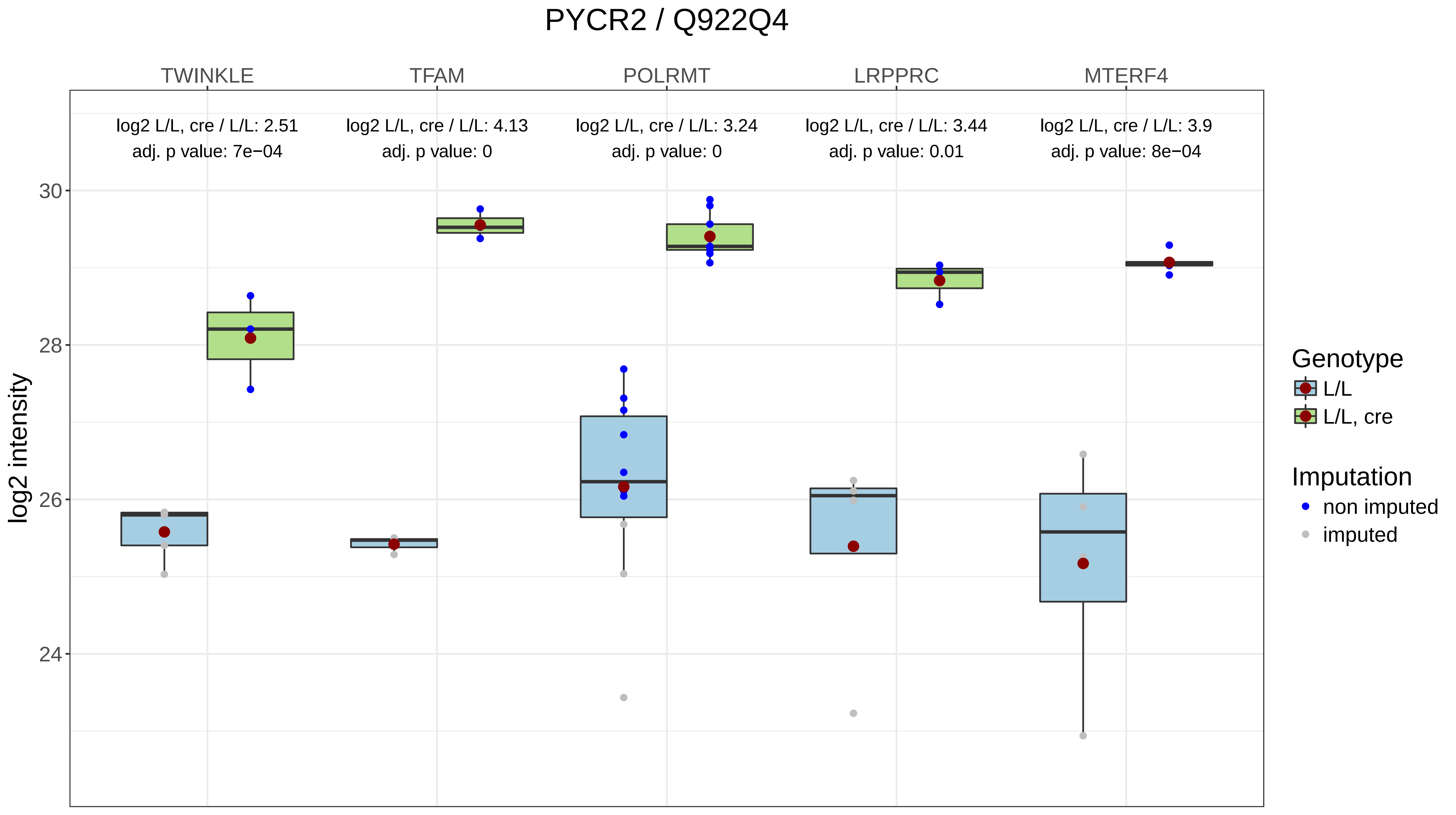
PYCR2 / Q922Q4
TWINKLE
TFAM
POLRMT
LRPPRC
MTERF4
log2 L/L, cre / L/L: 2.51
adj. p value: 7e−04
log2 L/L, cre / L/L: 4.13
adj. p value: 0
log2 L/L, cre / L/L: 3.24
adj. p value: 0
log2 L/L, cre / L/L: 3.44
adj. p value: 0.01
log2 L/L, cre / L/L: 3.9
adj. p value: 8e−04
log2 intensity
30
28
26
24
Genotype
L/L
L/L, cre
Imputation
non imputed
imputed

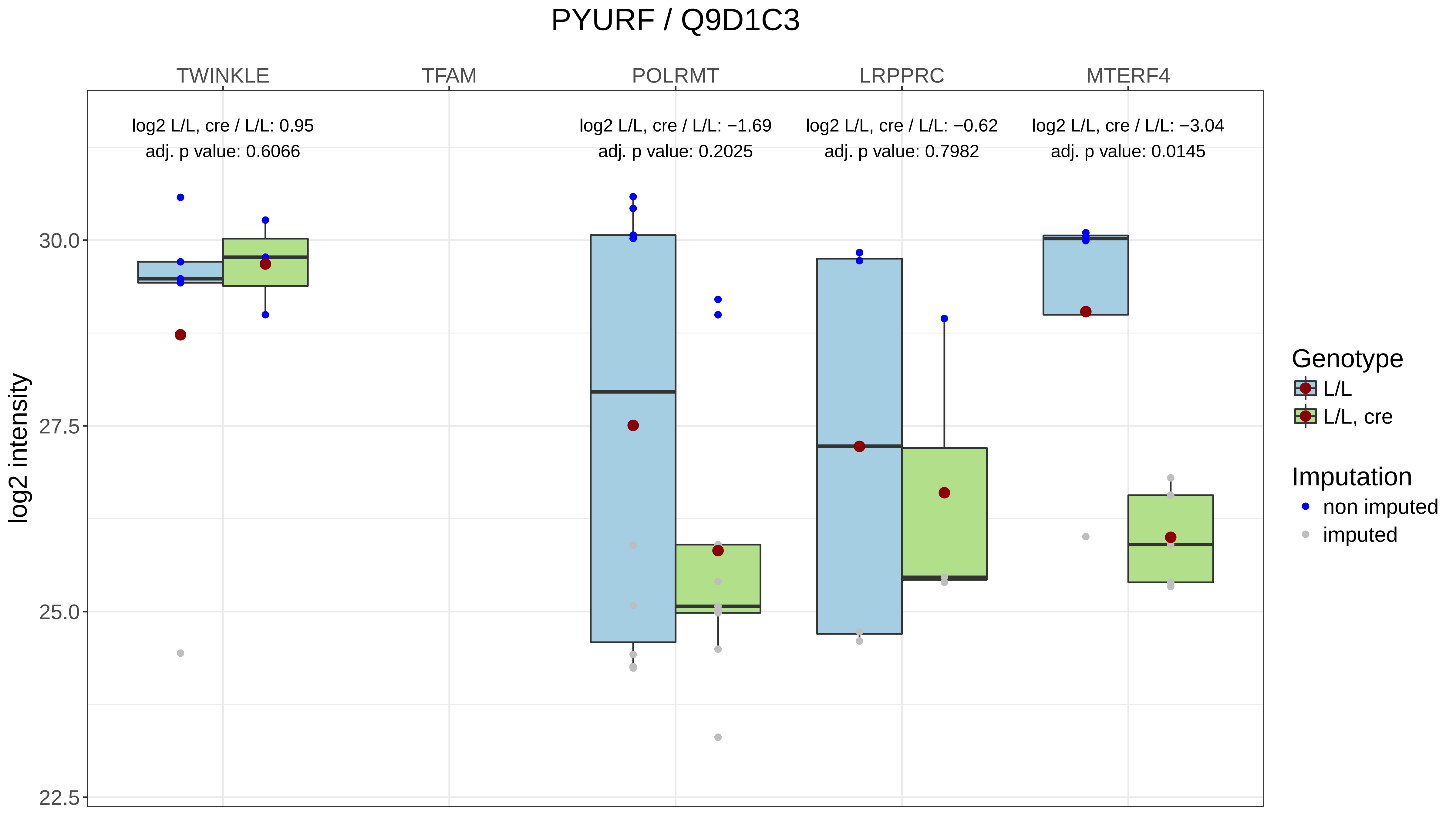
PYURF / Q9D1C3
TWINKLE
TFAM
POLRMT
LRPPRC
MTERF4
log2 L/L, cre / L/L: 0.95
adj. p value: 0.6066
log2 L/L, cre / L/L: −1.69
adj. p value: 0.2025
log2 L/L, cre / L/L: −0.62
adj. p value: 0.7982
log2 L/L, cre / L/L: −3.04
adj. p value: 0.0145
log2 intensity
30.0
27.5
25.0
22.5
Genotype
L/L
L/L, cre
Imputation
non imputed
imputed

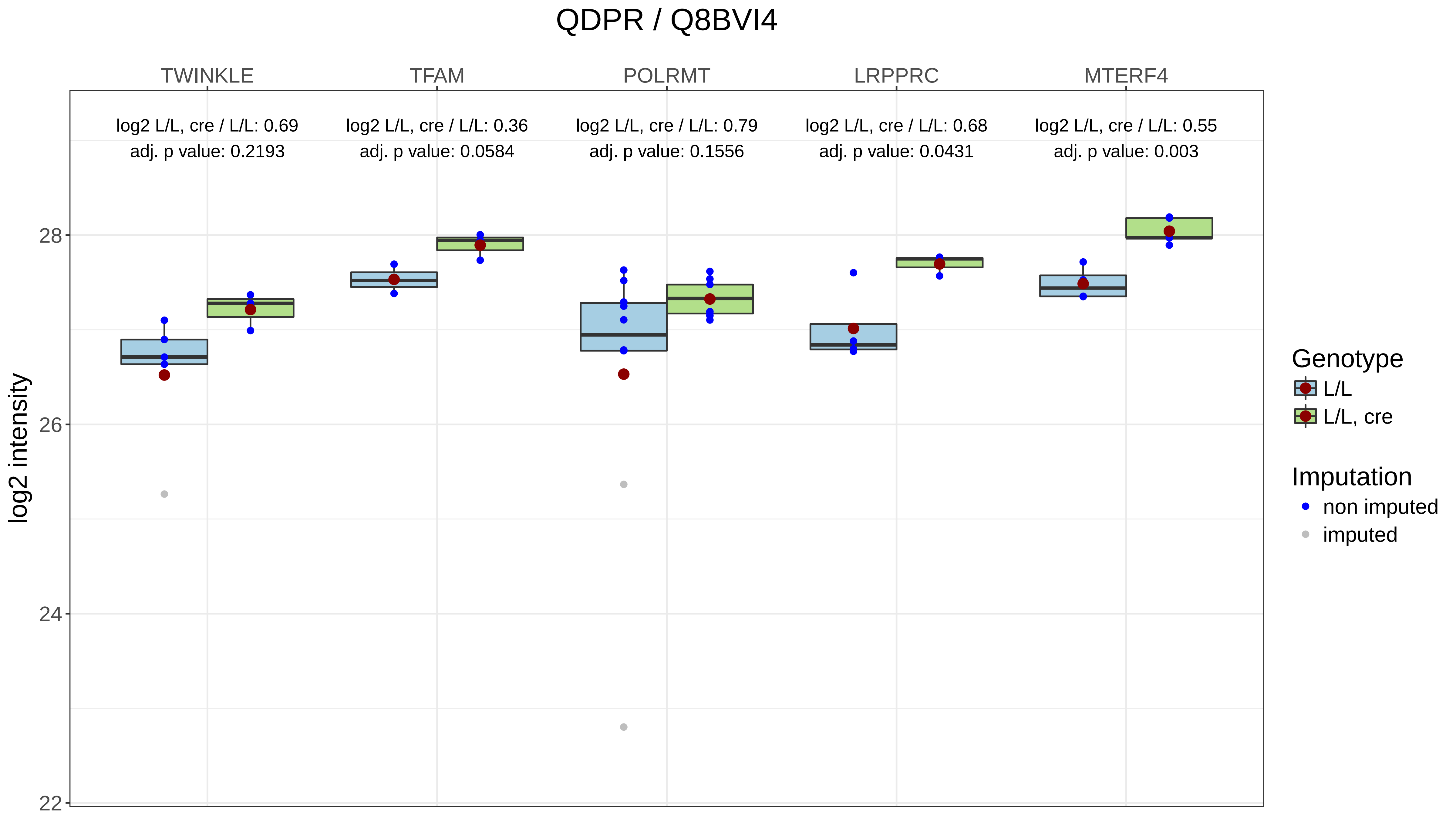
QDPR / Q8BVI4
TWINKLE
TFAM
POLRMT
LRPPRC
MTERF4
log2 L/L, cre / L/L: 0.69
adj. p value: 0.2193
log2 L/L, cre / L/L: 0.36
adj. p value: 0.0584
log2 L/L, cre / L/L: 0.79
adj. p value: 0.1556
log2 L/L, cre / L/L: 0.68
adj. p value: 0.0431
log2 L/L, cre / L/L: 0.55
adj. p value: 0.003
log2 intensity
28
26
24
22
Genotype
L/L
L/L, cre
Imputation
non imputed
imputed

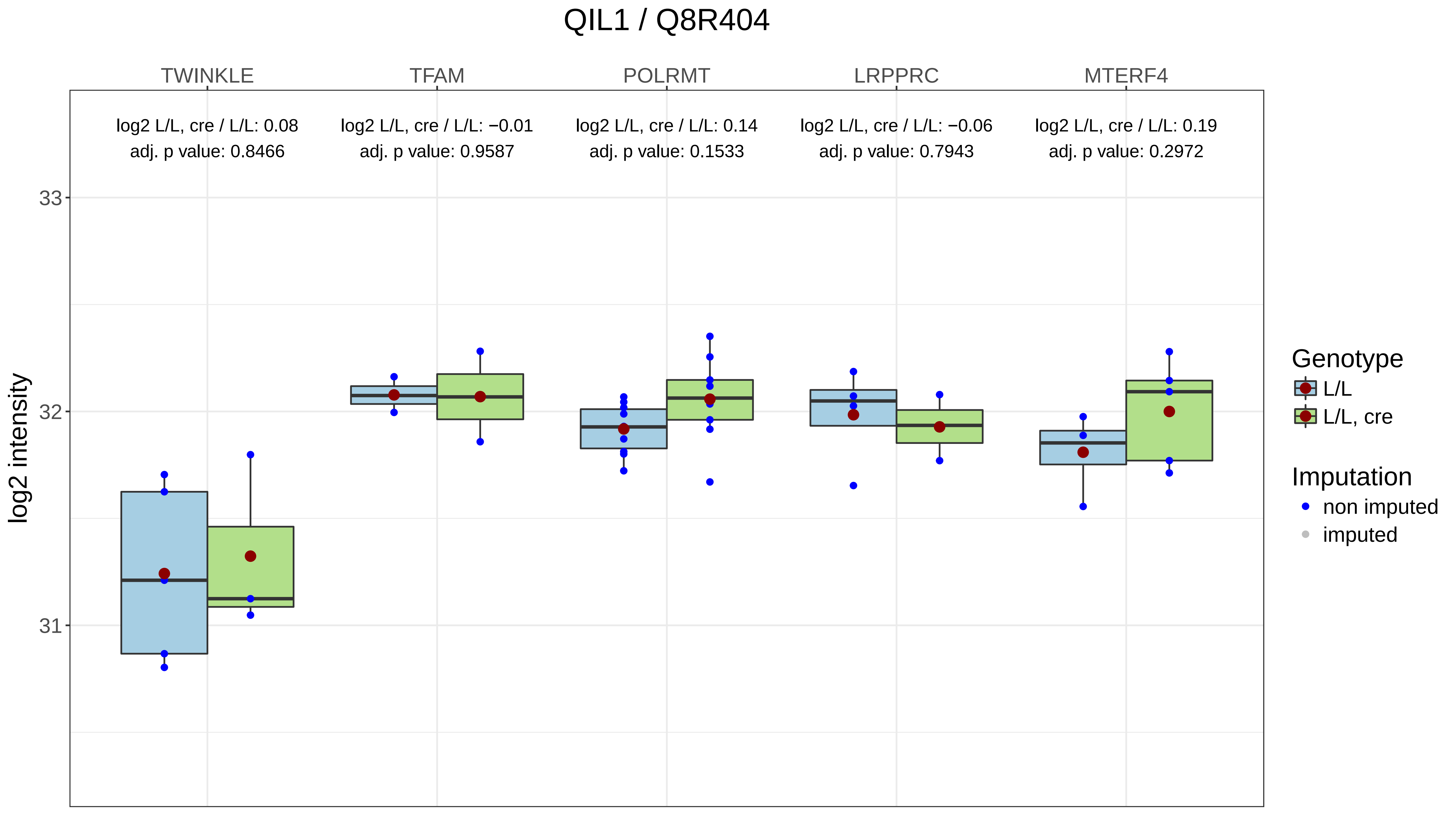
QIL1 / Q8R404
TWINKLE
TFAM
POLRMT
LRPPRC
MTERF4
log2 L/L, cre / L/L: 0.08
adj. p value: 0.8466
log2 L/L, cre / L/L: −0.01
adj. p value: 0.9587
log2 L/L, cre / L/L: 0.14
adj. p value: 0.1533
log2 L/L, cre / L/L: −0.06
adj. p value: 0.7943
log2 L/L, cre / L/L: 0.19
adj. p value: 0.2972
33
32
31
log2 intensity
Genotype
L/L
L/L, cre
Imputation
non imputed
imputed

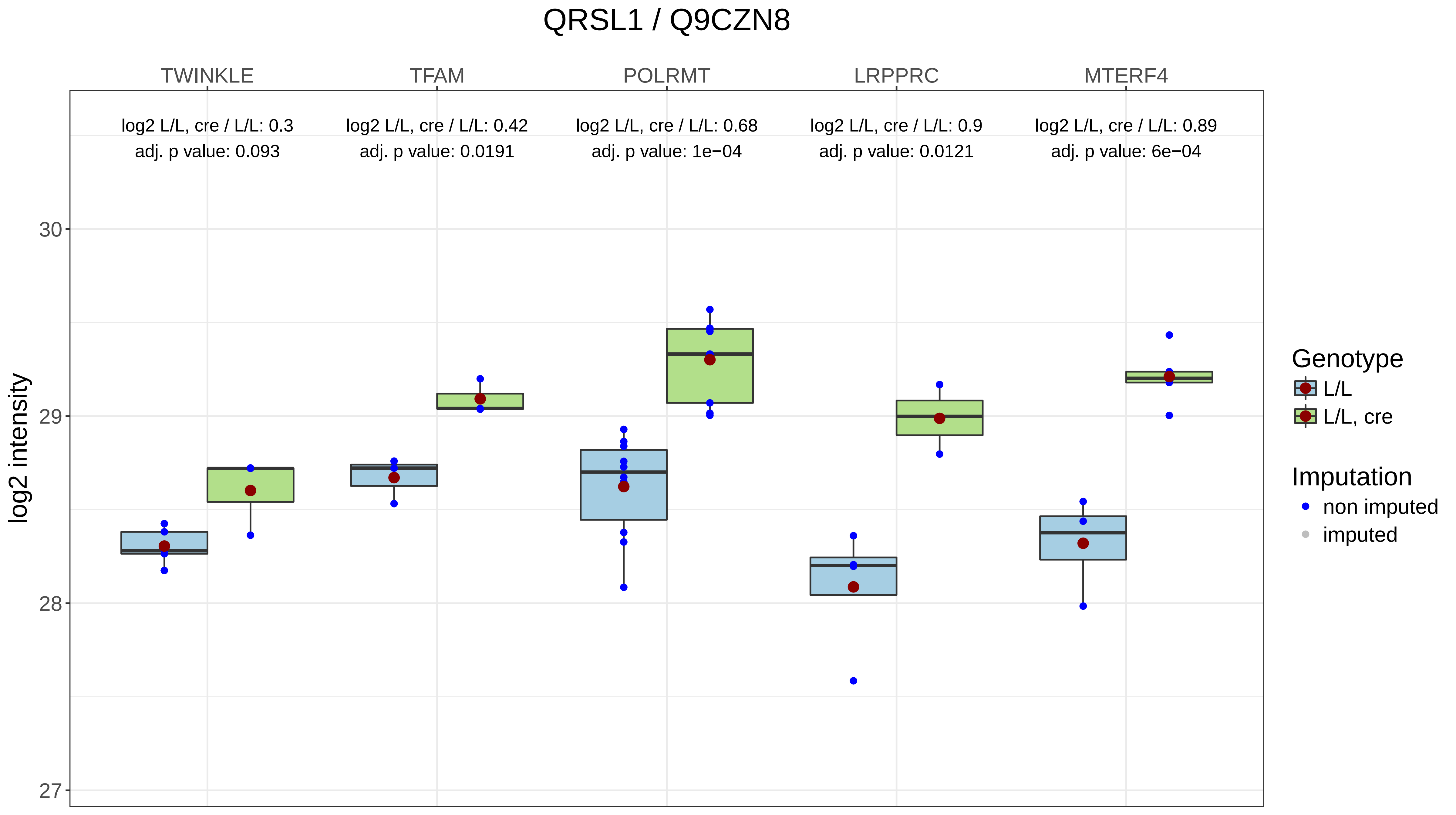
QRSL1 / Q9CZN8
TWINKLE
TFAM
POLRMT
LRPPRC
MTERF4
log2 L/L, cre / L/L: 0.3
adj. p value: 0.093
log2 L/L, cre / L/L: 0.42
adj. p value: 0.0191
log2 L/L, cre / L/L: 0.68
adj. p value: 1e−04
log2 L/L, cre / L/L: 0.9
adj. p value: 0.0121
log2 L/L, cre / L/L: 0.89
adj. p value: 6e−04
30
29
28
27
log2 intensity
Genotype
L/L
L/L, cre
Imputation
non imputed
imputed

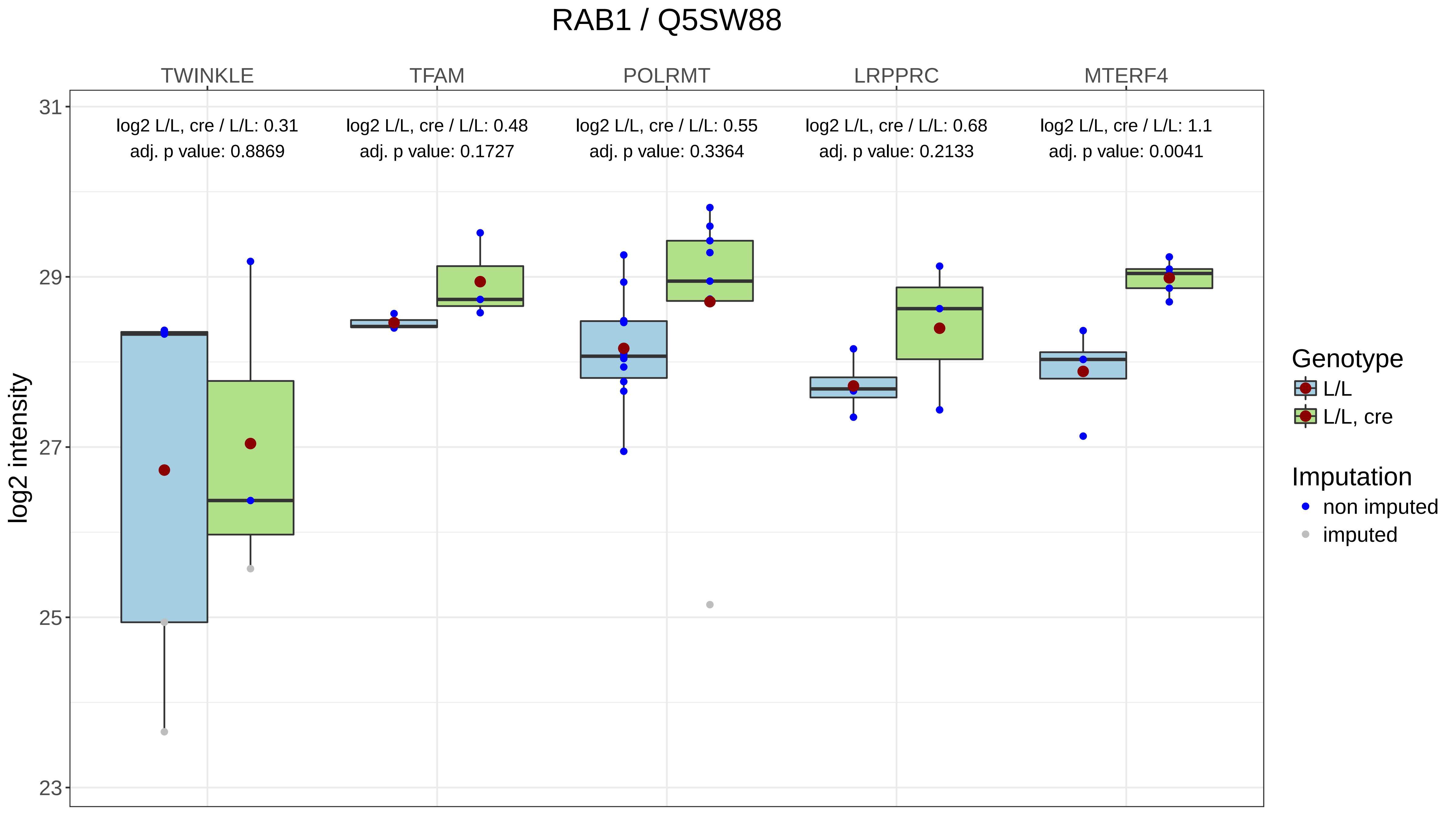

RAB1 / Q5SW88
TWINKLE
TFAM
POLRMT
LRPPRC
MTERF4
log2 L/L, cre / L/L: 0.31
adj. p value: 0.8869
log2 L/L, cre / L/L: 0.48
adj. p value: 0.1727
log2 L/L, cre / L/L: 0.55
adj. p value: 0.3364
log2 L/L, cre / L/L: 0.68
adj. p value: 0.2133
log2 L/L, cre / L/L: 1.1
adj. p value: 0.0041
log2 intensity
31
29
27
25
23
Genotype
L/L
L/L, cre
Imputation
non imputed
imputed

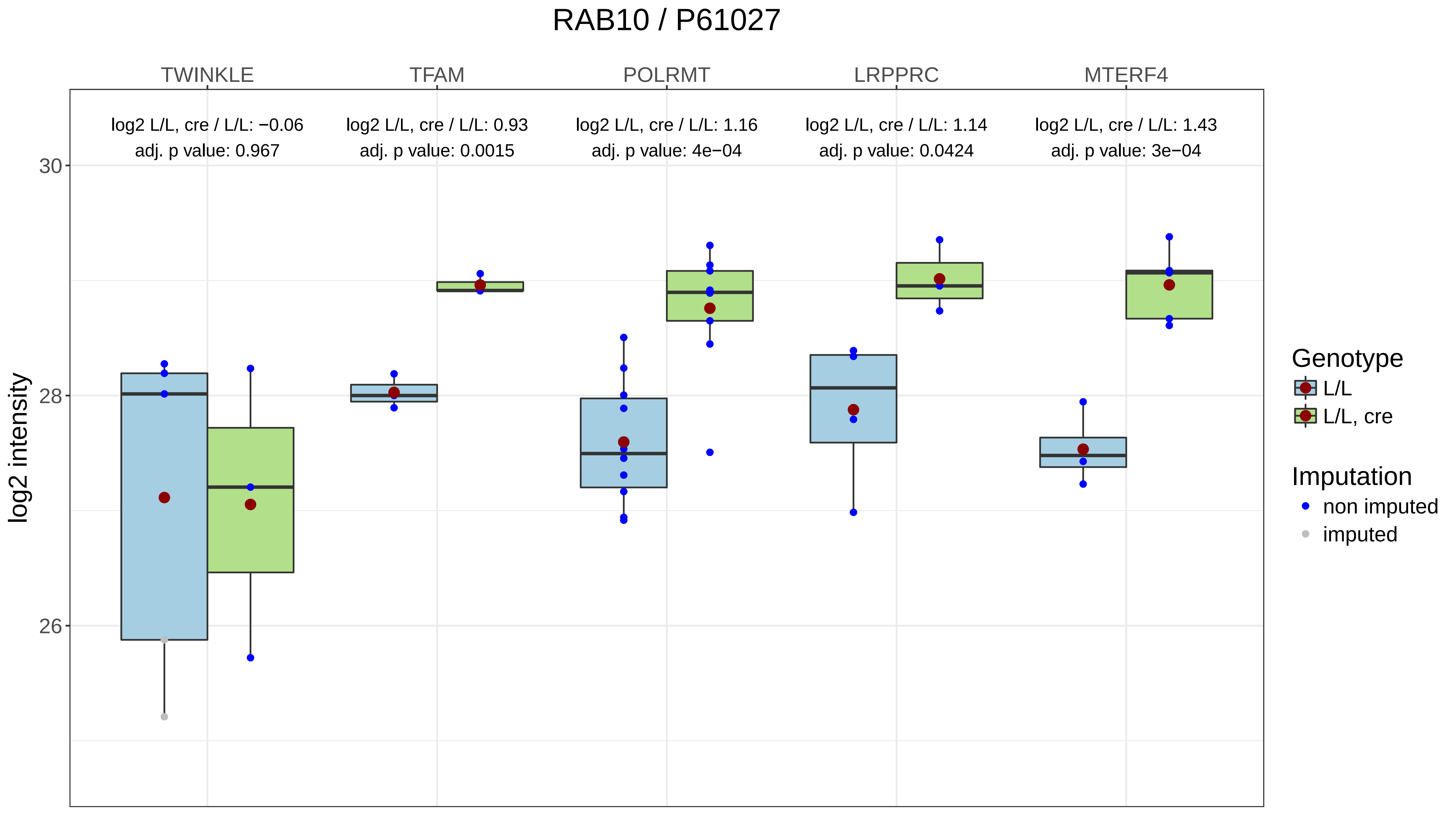

RAB10 / P61027
TWINKLE
TFAM
POLRMT
LRPPRC
MTERF4
log2 L/L, cre / L/L: −0.06
adj. p value: 0.967
log2 L/L, cre / L/L: 0.93
adj. p value: 0.0015
log2 L/L, cre / L/L: 1.16
adj. p value: 4e−04
log2 L/L, cre / L/L: 1.14
adj. p value: 0.0424
log2 L/L, cre / L/L: 1.43
adj. p value: 3e−04
30
28
26
log2 intensity
Genotype
L/L
L/L, cre
Imputation
non imputed
imputed

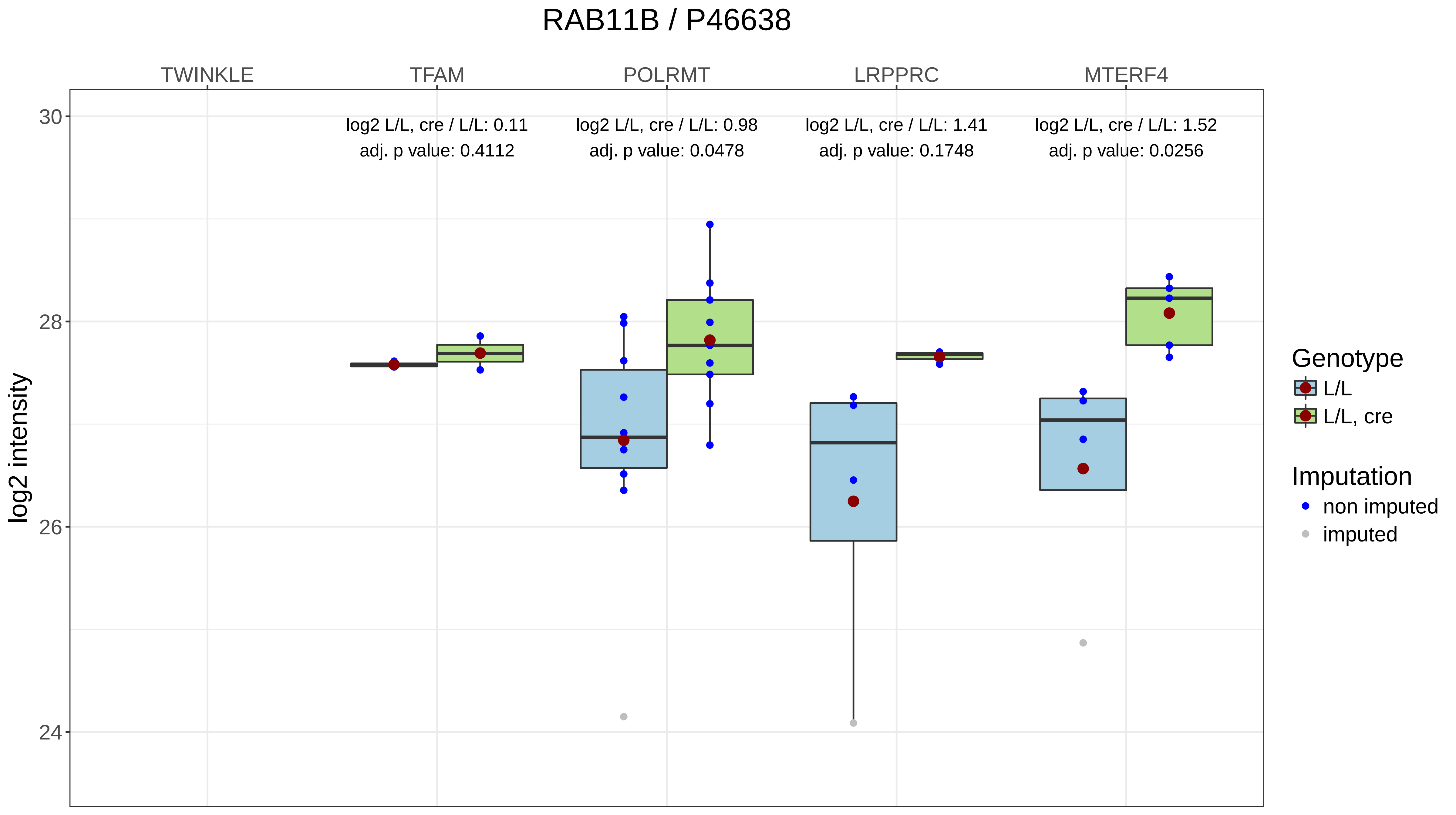
RAB11B / P46638
TWINKLE
TFAM
POLRMT
LRPPRC
MTERF4
log2 L/L, cre / L/L: 0.11
adj. p value: 0.4112
log2 L/L, cre / L/L: 0.98
adj. p value: 0.0478
log2 L/L, cre / L/L: 1.41
adj. p value: 0.1748
log2 L/L, cre / L/L: 1.52
adj. p value: 0.0256
log2 intensity
30
28
26
24
Genotype
L/L
L/L, cre
Imputation
non imputed
imputed

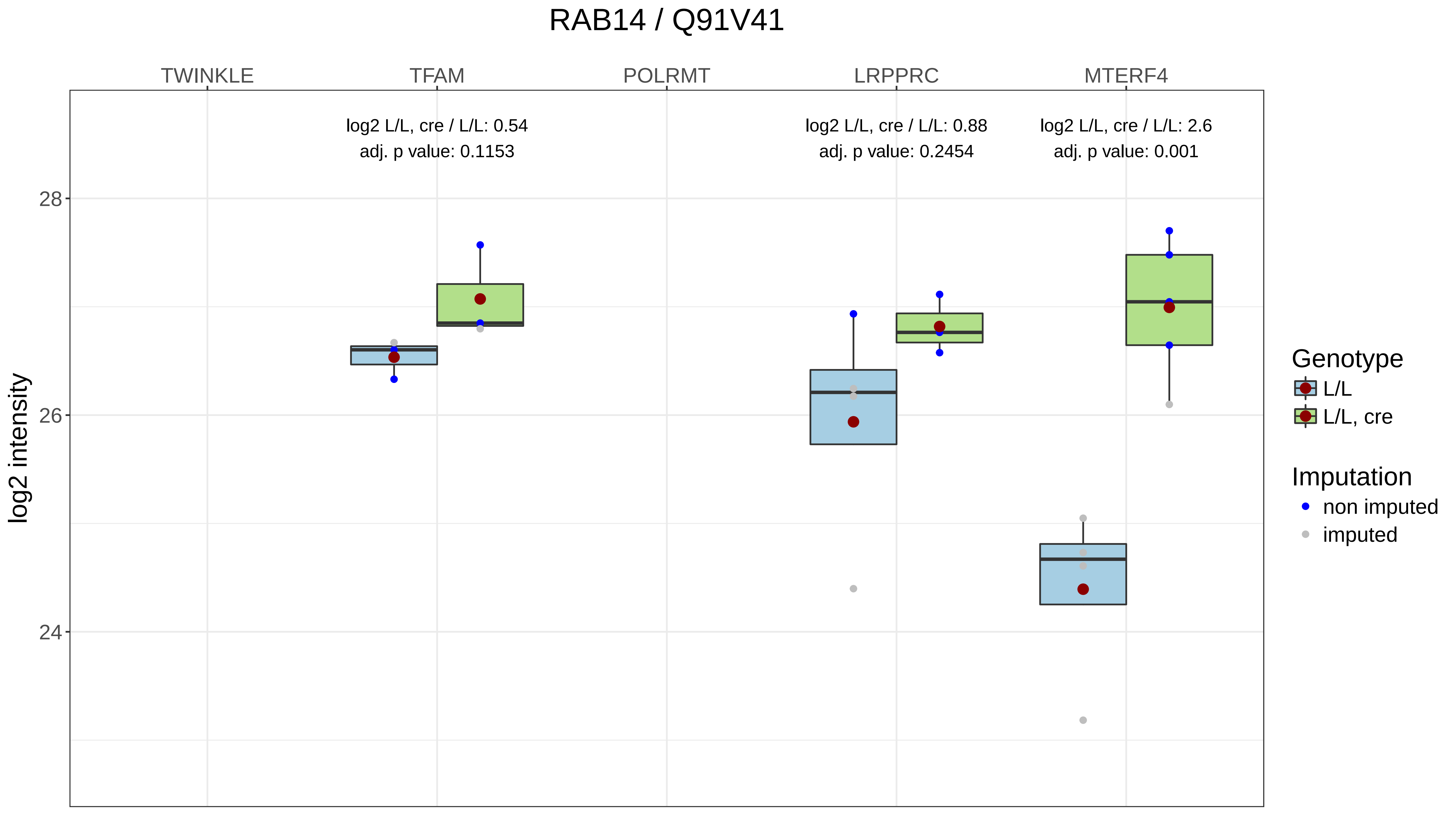
RAB14 / Q91V41
TWINKLE
TFAM
POLRMT
LRPPRC
MTERF4
log2 L/L, cre / L/L: 0.54
adj. p value: 0.1153
log2 L/L, cre / L/L: 0.88
adj. p value: 0.2454
log2 L/L, cre / L/L: 2.6
adj. p value: 0.001
28
26
24
log2 intensity
Genotype
L/L
L/L, cre
Imputation
non imputed
imputed

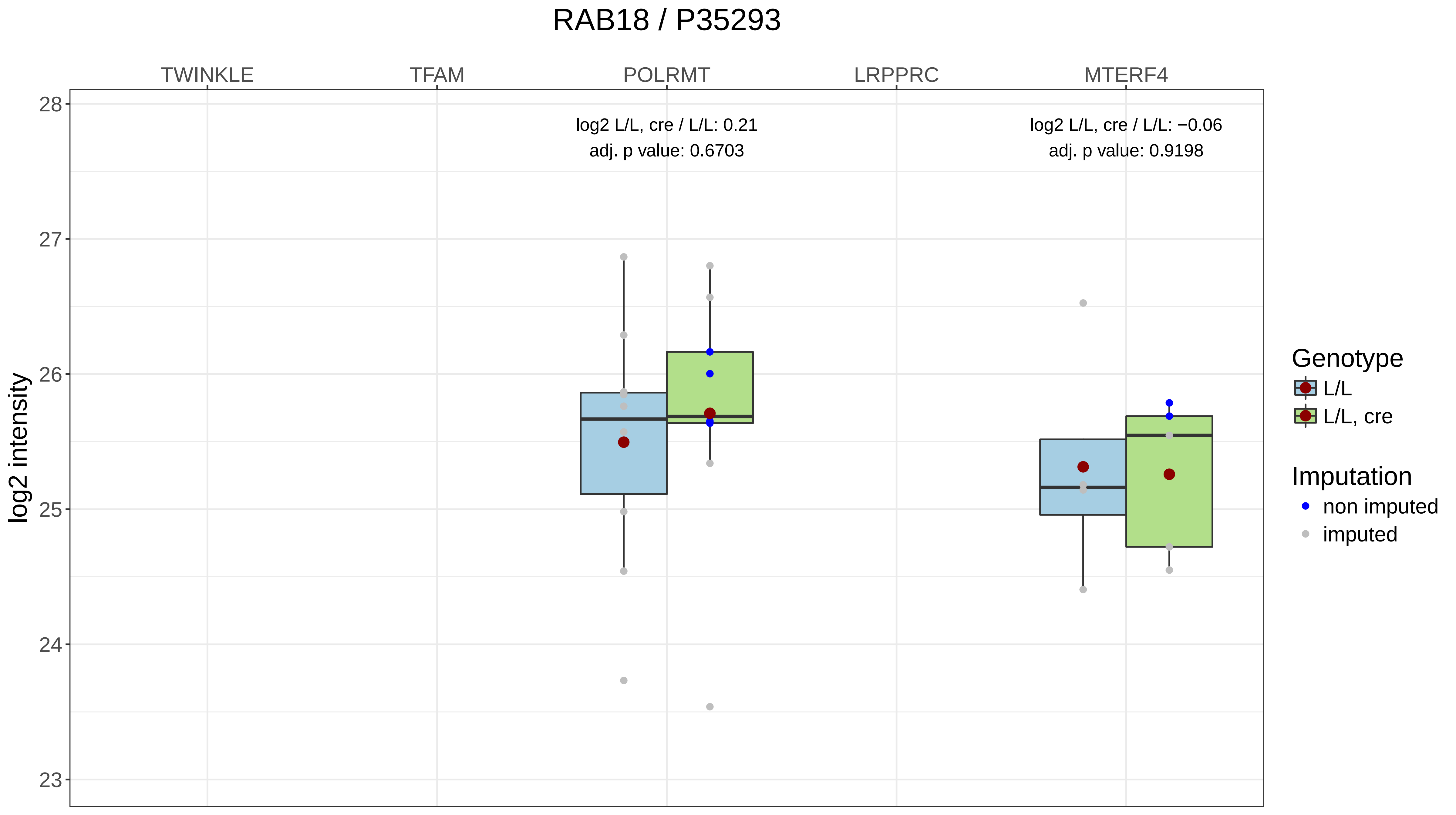
RAB18 / P35293
TWINKLE
TFAM
POLRMT
LRPPRC
MTERF4
log2 L/L, cre / L/L: 0.21
adj. p value: 0.6703
log2 L/L, cre / L/L: −0.06
adj. p value: 0.9198
log2 intensity
28
27
26
25
24
23
Genotype
L/L
L/L, cre
Imputation
non imputed
imputed

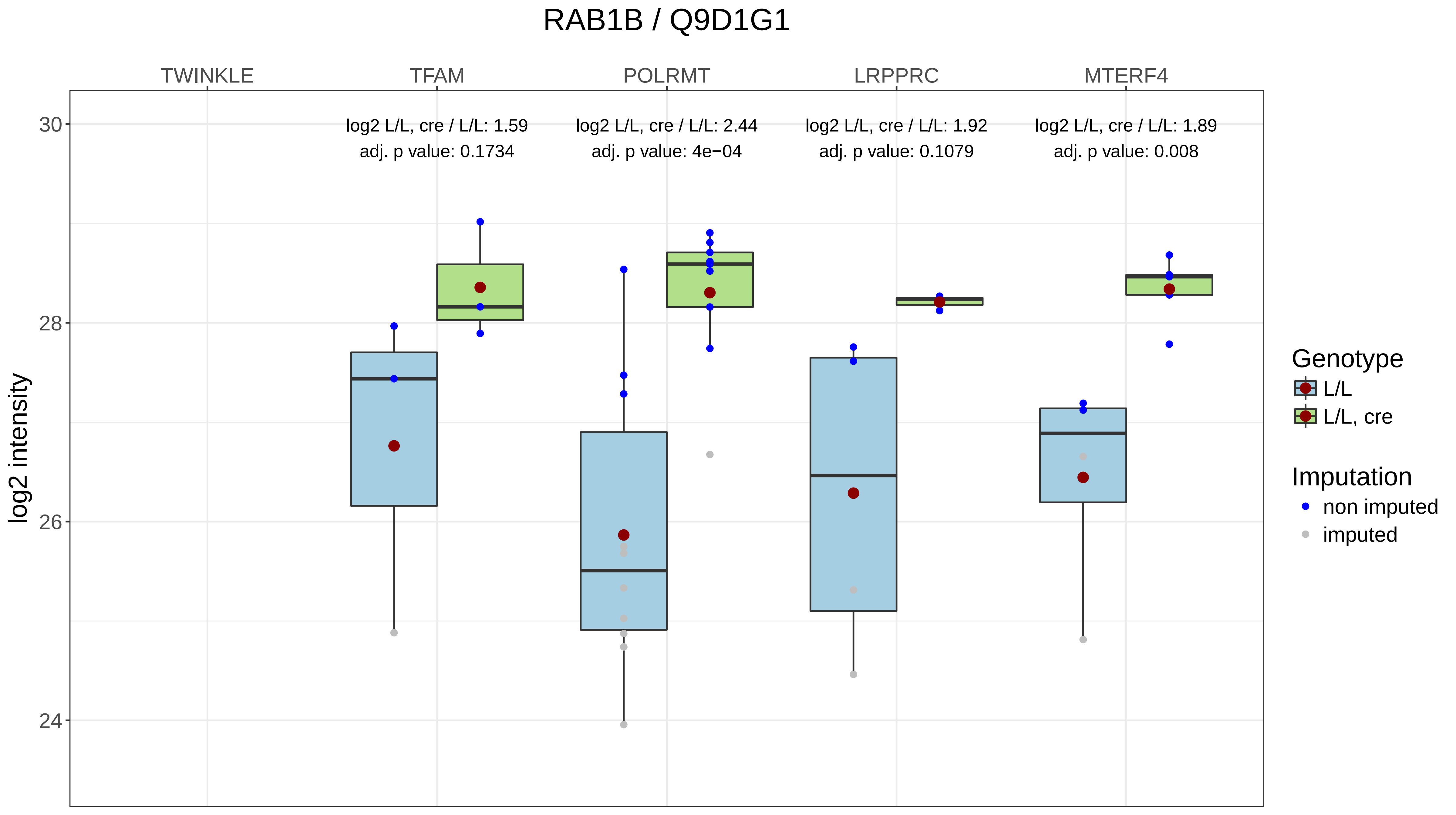
RAB1B / Q9D1G1
TWINKLE
TFAM
POLRMT
LRPPRC
MTERF4
log2 L/L, cre / L/L: 1.59
adj. p value: 0.1734
log2 L/L, cre / L/L: 2.44
adj. p value: 4e−04
log2 L/L, cre / L/L: 1.92
adj. p value: 0.1079
log2 L/L, cre / L/L: 1.89
adj. p value: 0.008
30
28
26
24
log2 intensity
Genotype
L/L
L/L, cre
Imputation
non imputed
imputed

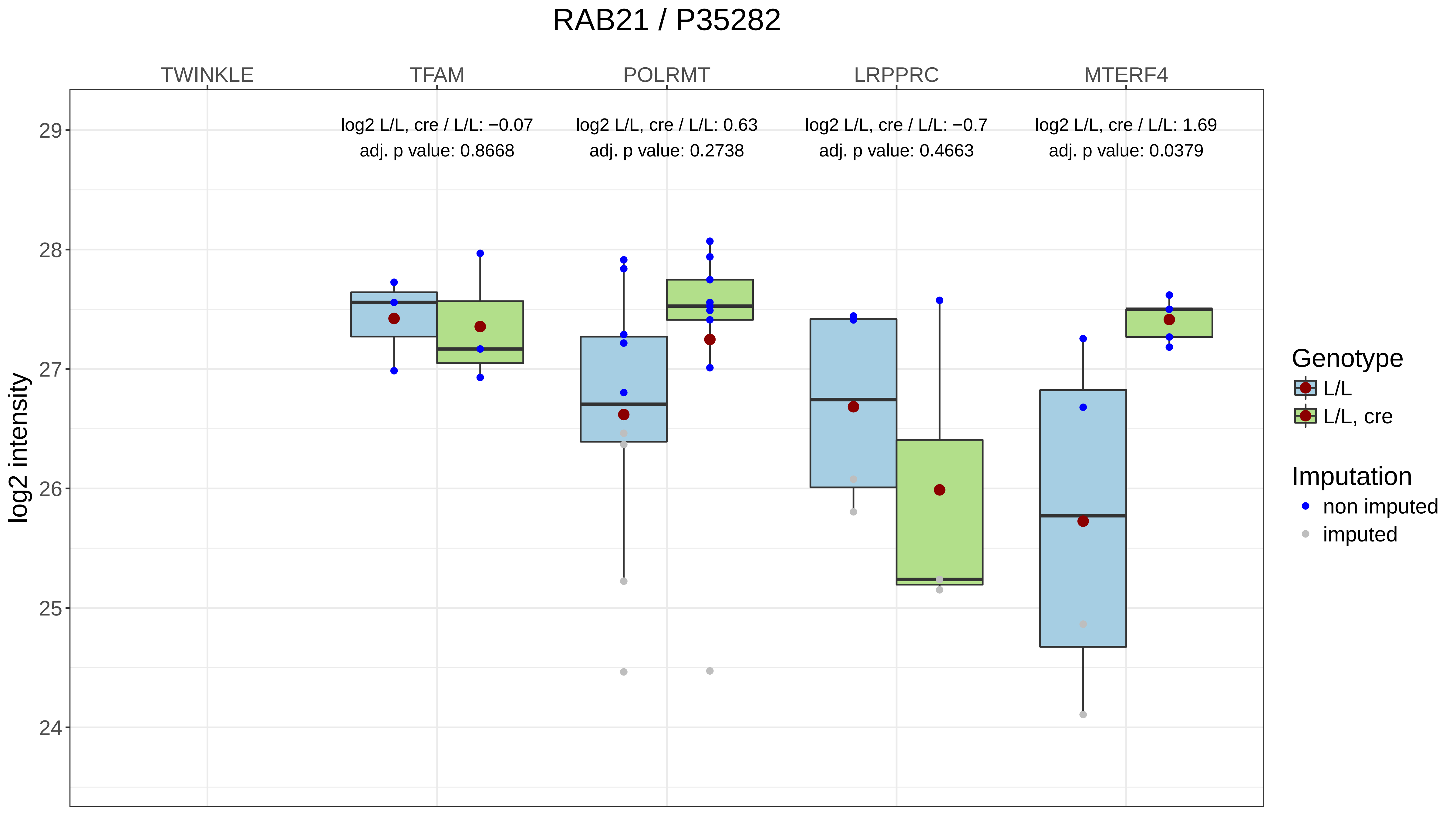
RAB21 / P35282
TWINKLE
TFAM
POLRMT
LRPPRC
MTERF4
log2 L/L, cre / L/L: −0.07
adj. p value: 0.8668
log2 L/L, cre / L/L: 0.63
adj. p value: 0.2738
log2 L/L, cre / L/L: −0.7
adj. p value: 0.4663
log2 L/L, cre / L/L: 1.69
adj. p value: 0.0379
log2 intensity
29
28
27
26
25
24
Genotype
L/L
L/L, cre
Imputation
non imputed
imputed

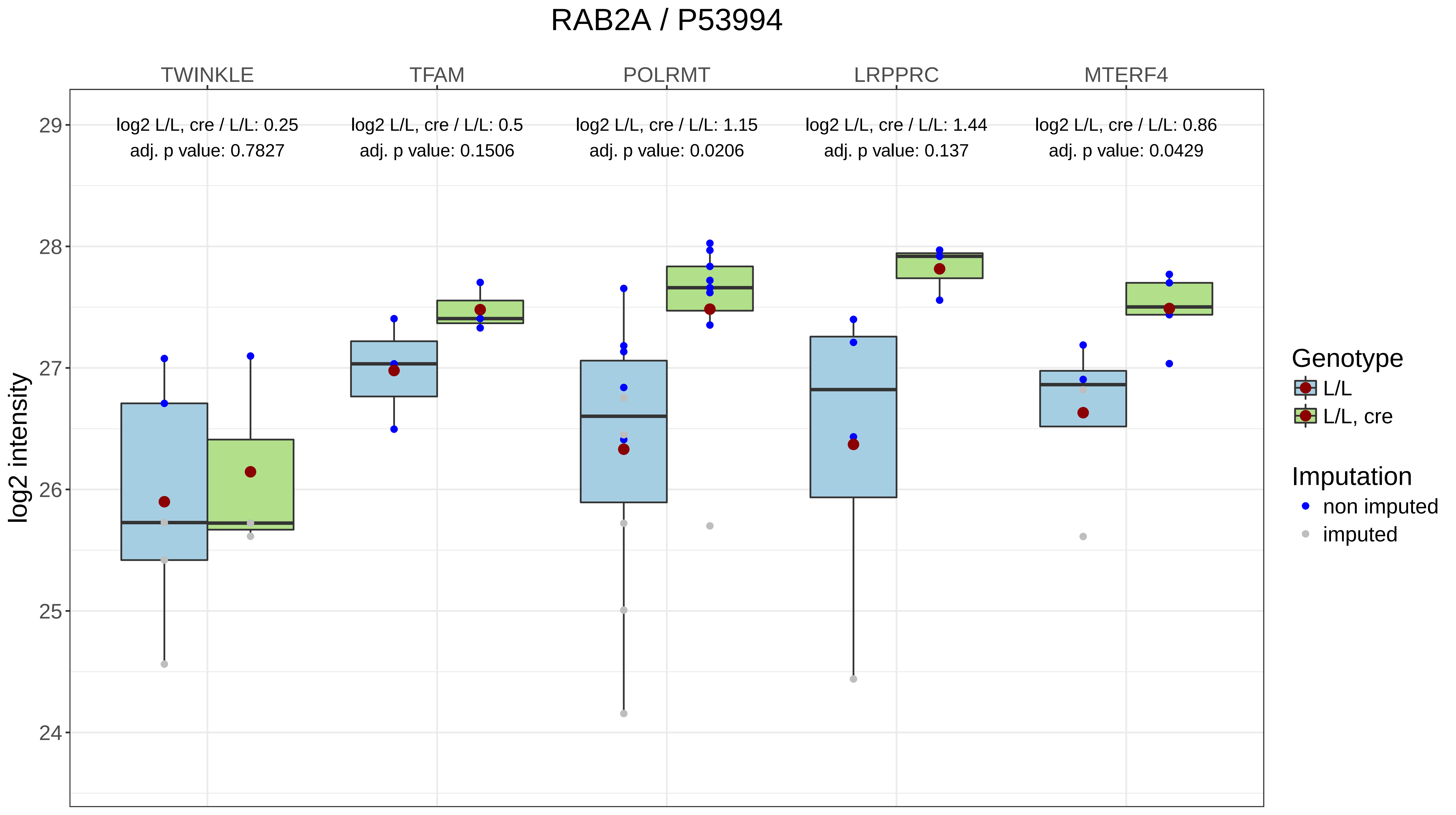
RAB2A / P53994
TWINKLE
TFAM
POLRMT
LRPPRC
MTERF4
log2 L/L, cre / L/L: 0.25
adj. p value: 0.7827
log2 L/L, cre / L/L: 0.5
adj. p value: 0.1506
log2 L/L, cre / L/L: 1.15
adj. p value: 0.0206
log2 L/L, cre / L/L: 1.44
adj. p value: 0.137
log2 L/L, cre / L/L: 0.86
adj. p value: 0.0429
log2 intensity
29
28
27
26
25
24
Genotype
L/L
L/L, cre
Imputation
non imputed
imputed

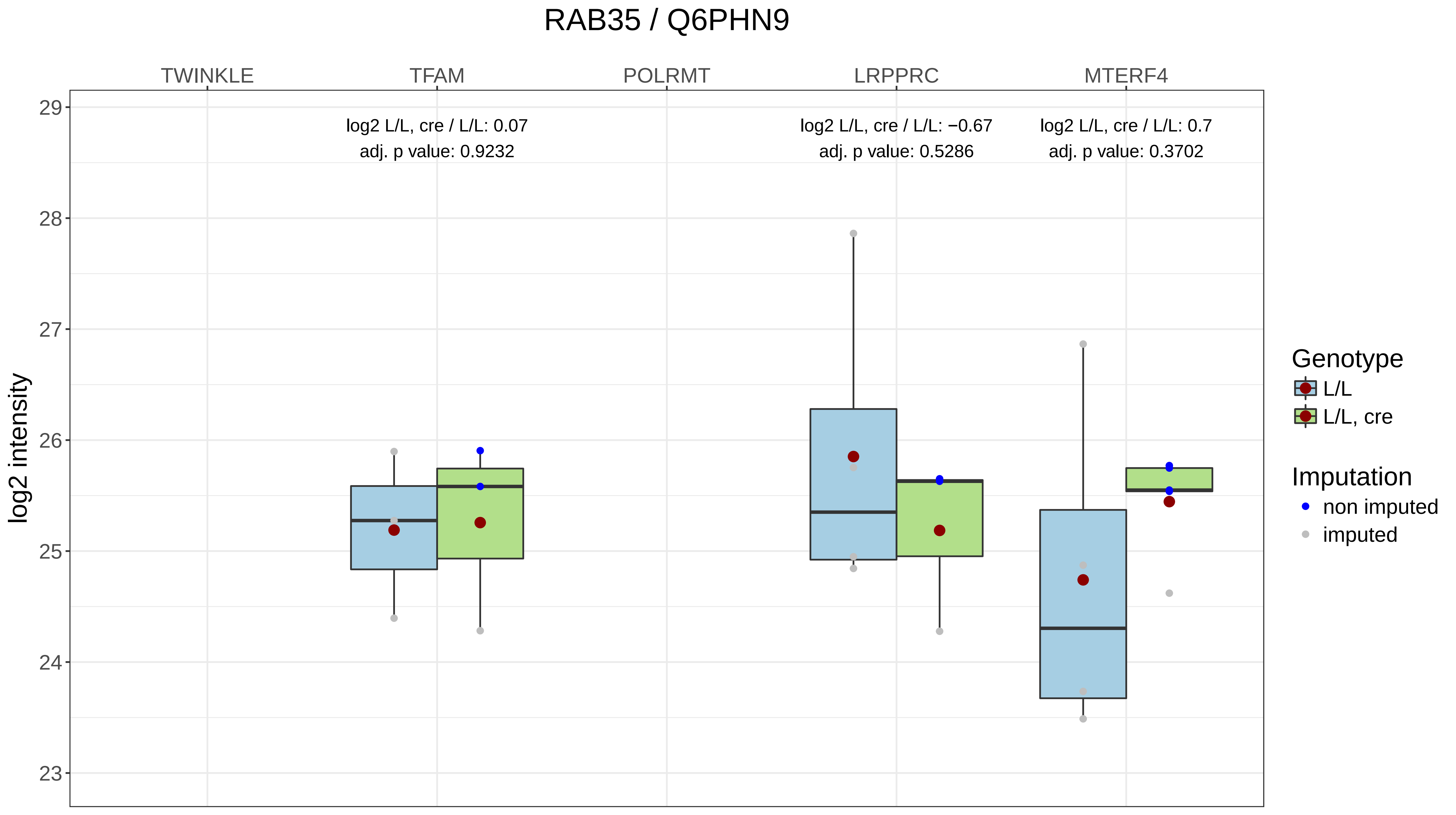
RAB35 / Q6PHN9
TWINKLE
TFAM
POLRMT
LRPPRC
MTERF4
log2 L/L, cre / L/L: 0.07
adj. p value: 0.9232
log2 L/L, cre / L/L: −0.67
adj. p value: 0.5286
log2 L/L, cre / L/L: 0.7
adj. p value: 0.3702
log2 intensity
29
28
27
26
25
24
23
Genotype
L/L
L/L, cre
Imputation
non imputed
imputed

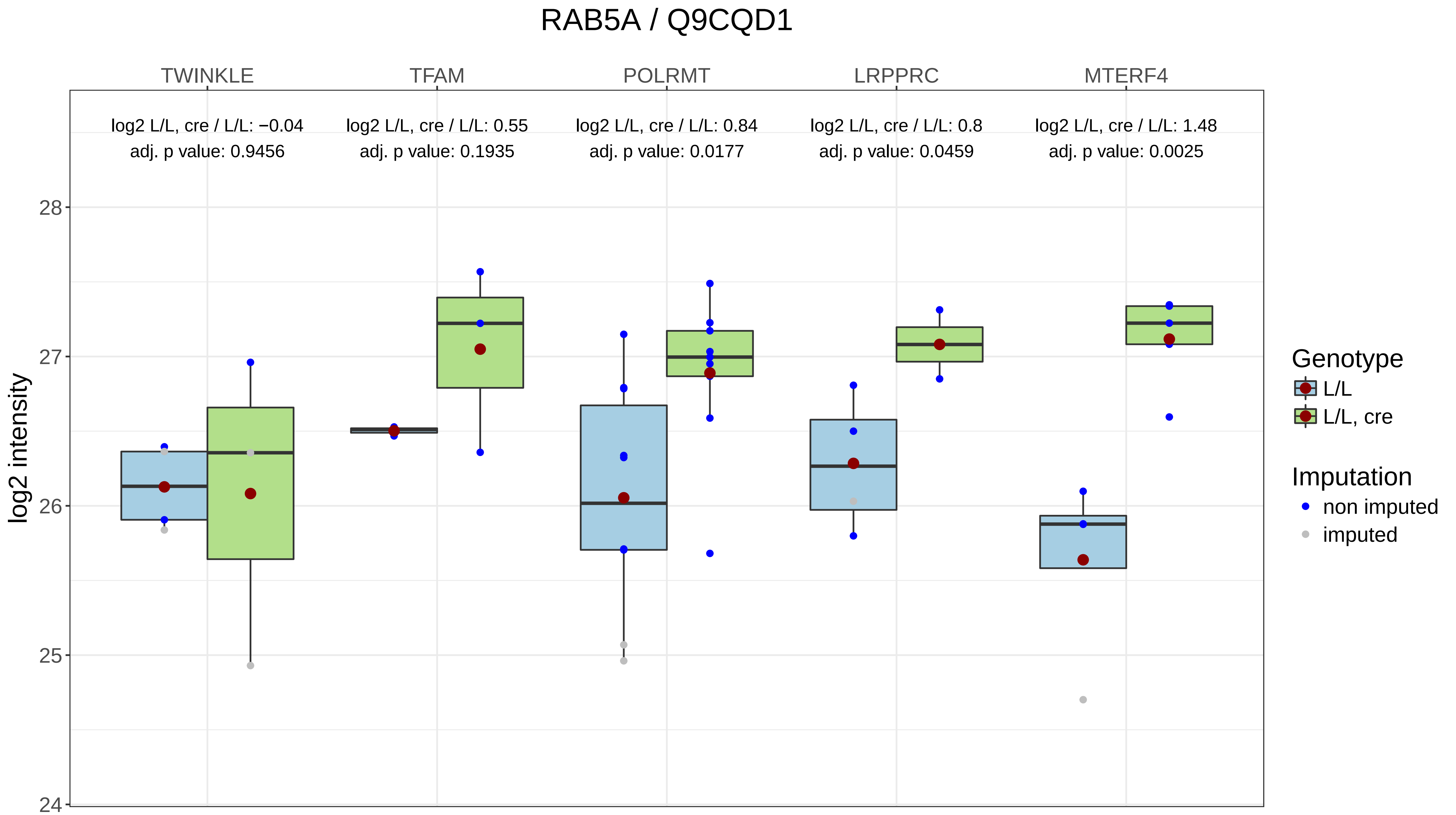

RAB5A / Q9CQD1
TWINKLE
TFAM
POLRMT
LRPPRC
MTERF4
log2 L/L, cre / L/L: −0.04
adj. p value: 0.9456
log2 L/L, cre / L/L: 0.55
adj. p value: 0.1935
log2 L/L, cre / L/L: 0.84
adj. p value: 0.0177
log2 L/L, cre / L/L: 0.8
adj. p value: 0.0459
log2 L/L, cre / L/L: 1.48
adj. p value: 0.0025
log2 intensity
28
27
26
25
24
Genotype
L/L
L/L, cre
Imputation
non imputed
imputed

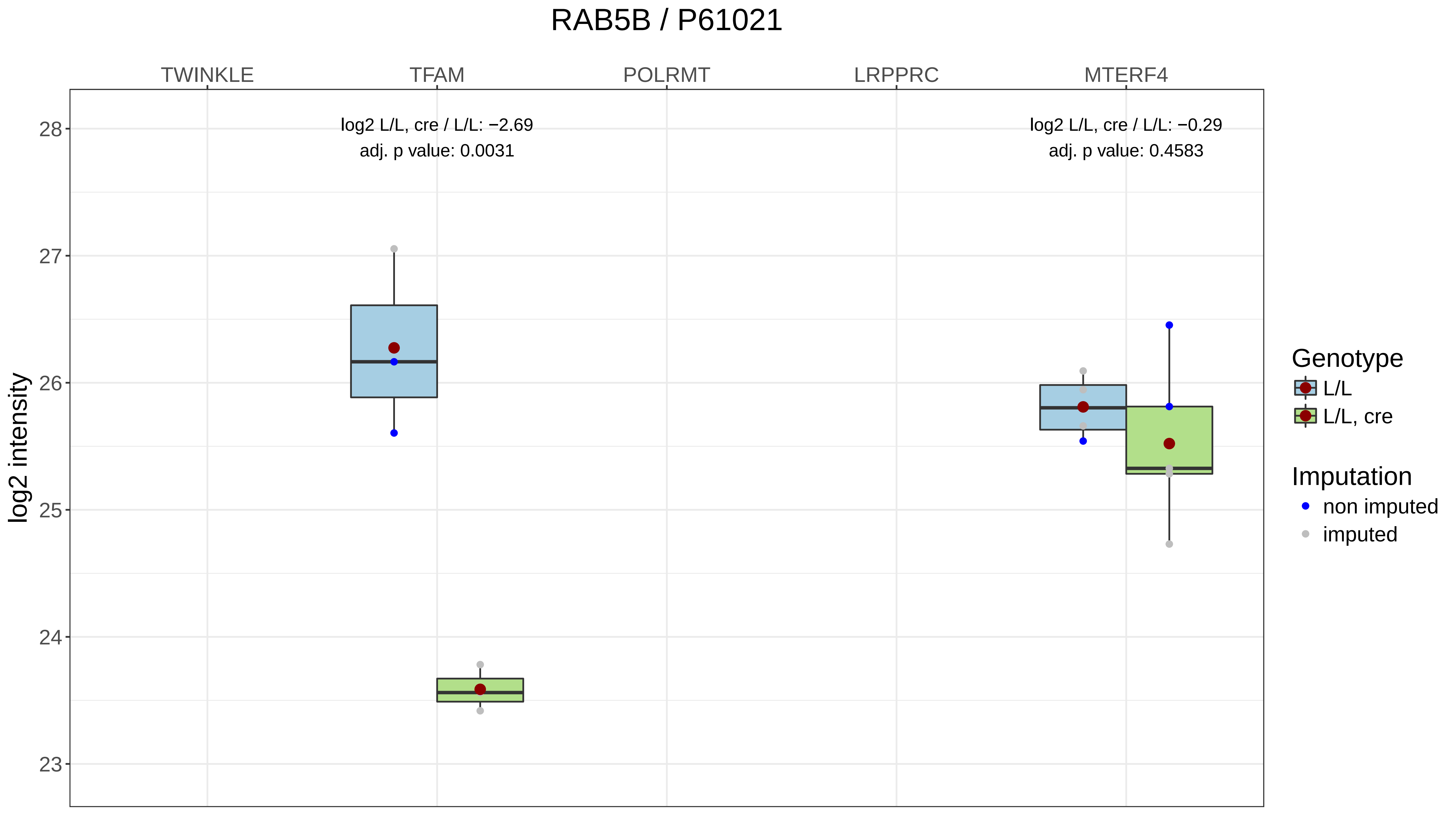

RAB5B / P61021
TWINKLE
TFAM
POLRMT
LRPPRC
MTERF4
log2 L/L, cre / L/L: −2.69
adj. p value: 0.0031
log2 L/L, cre / L/L: −0.29
adj. p value: 0.4583
log2 intensity
28
27
26
25
24
23
Genotype
L/L
L/L, cre
Imputation
non imputed
imputed

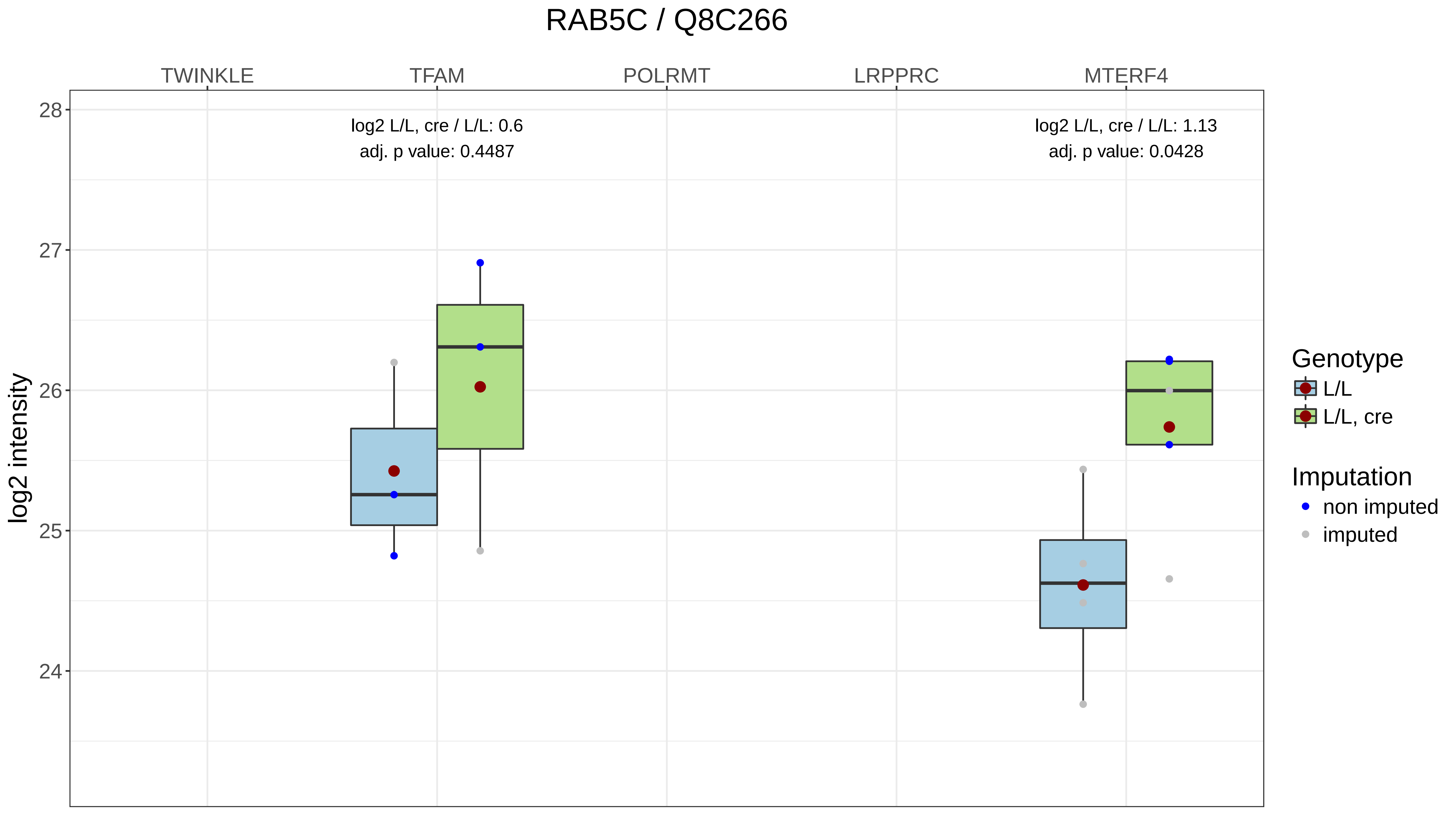

RAB5C / Q8C266
TWINKLE
TFAM
POLRMT
LRPPRC
MTERF4
log2 L/L, cre / L/L: 0.6
adj. p value: 0.4487
log2 L/L, cre / L/L: 1.13
adj. p value: 0.0428
log2 intensity
28
27
26
25
24
Genotype
L/L
L/L, cre
Imputation
non imputed
imputed

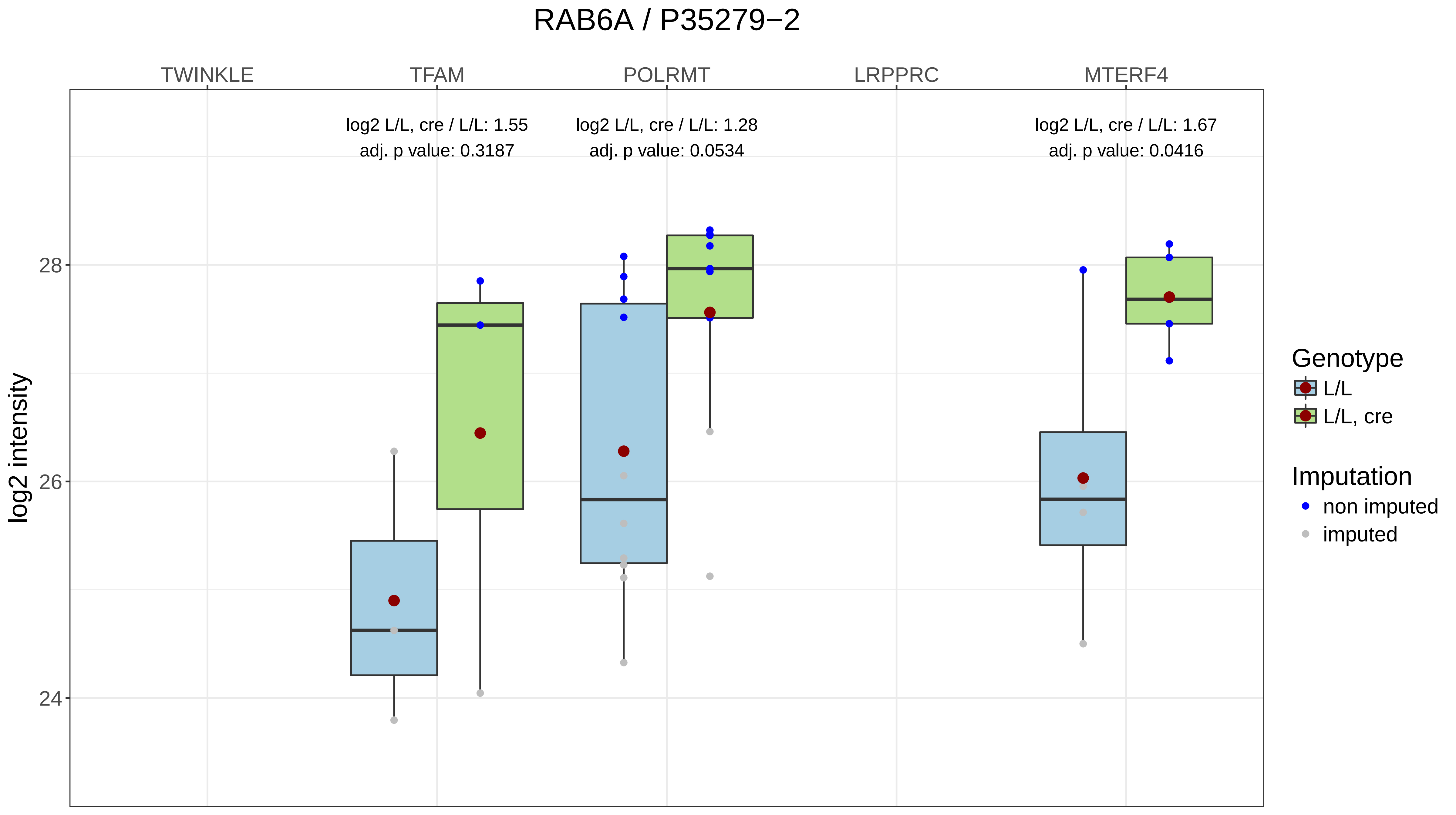

RAB6A / P35279−2
TWINKLE
TFAM
POLRMT
LRPPRC
MTERF4
log2 L/L, cre / L/L: 1.55
adj. p value: 0.3187
log2 L/L, cre / L/L: 1.28
adj. p value: 0.0534
log2 L/L, cre / L/L: 1.67
adj. p value: 0.0416
log2 intensity
28
26
24
Genotype
L/L
L/L, cre
Imputation
non imputed
imputed

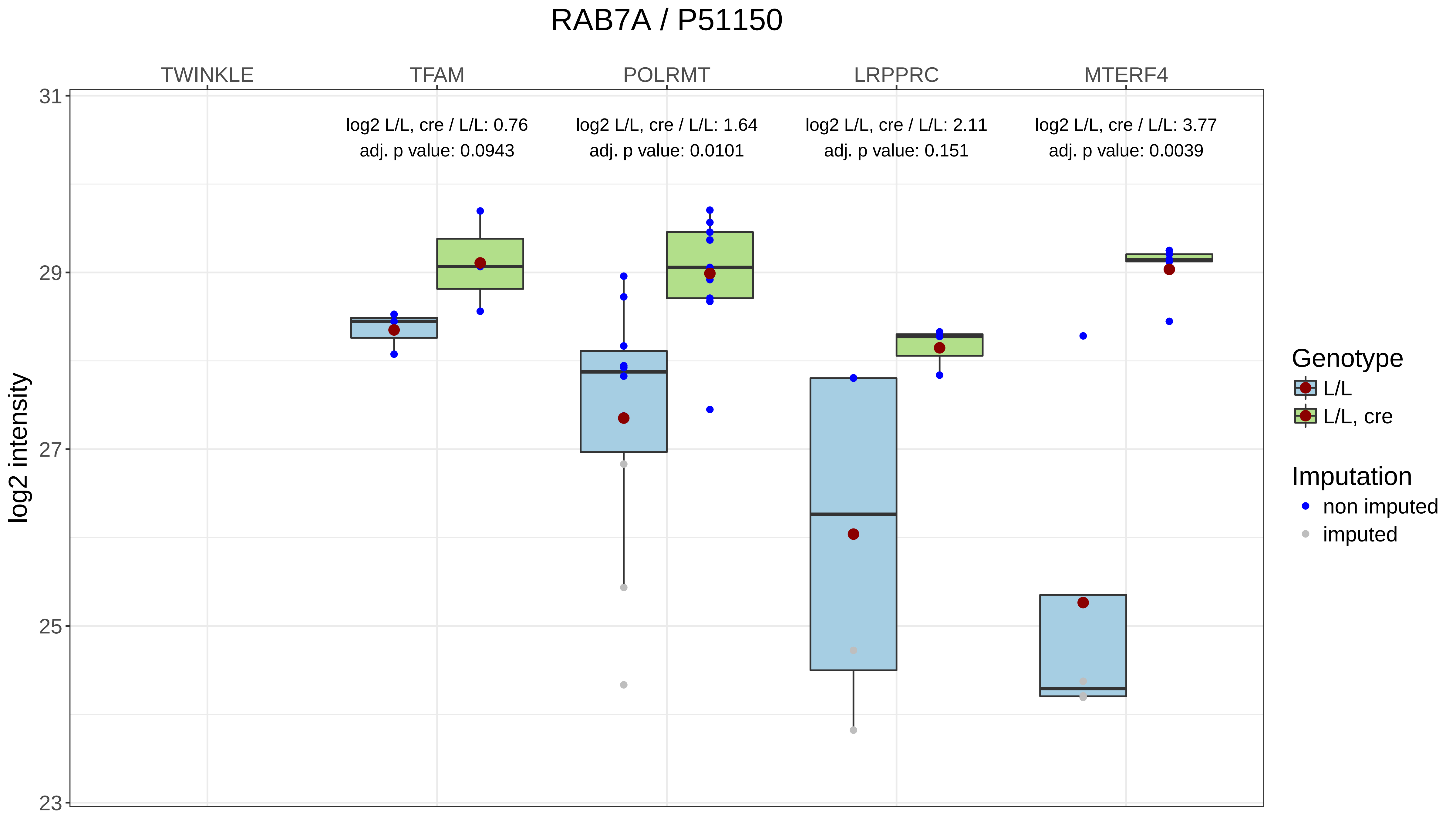
RAB7A / P51150
TWINKLE
TFAM
POLRMT
LRPPRC
MTERF4
log2 L/L, cre / L/L: 0.76
adj. p value: 0.0943
log2 L/L, cre / L/L: 1.64
adj. p value: 0.0101
log2 L/L, cre / L/L: 2.11
adj. p value: 0.151
log2 L/L, cre / L/L: 3.77
adj. p value: 0.0039
log2 intensity
31
29
27
25
23
Genotype
L/L
L/L, cre
Imputation
non imputed
imputed

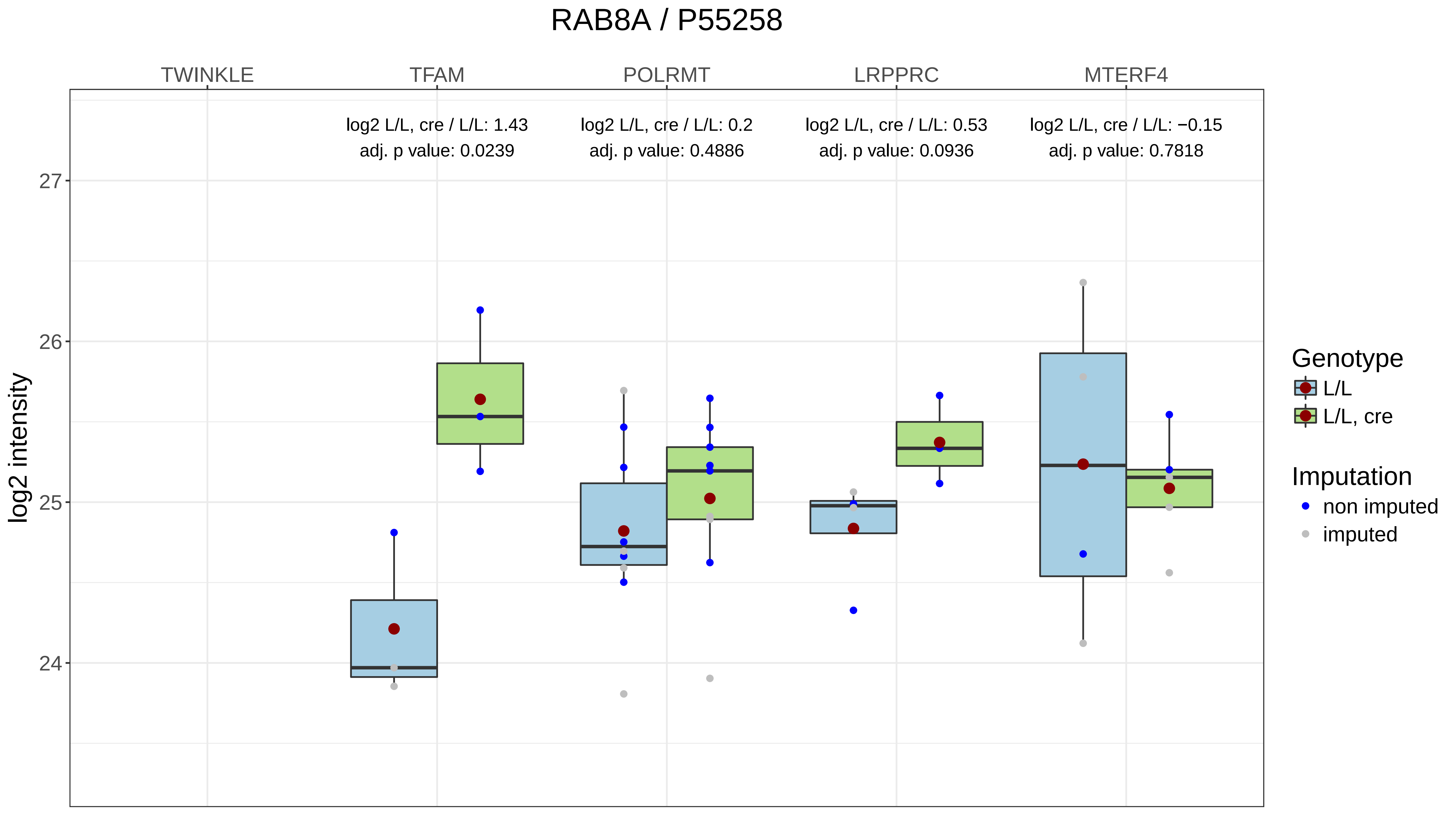
RAB8A / P55258
TWINKLE
TFAM
POLRMT
LRPPRC
MTERF4
log2 L/L, cre / L/L: 1.43
adj. p value: 0.0239
log2 L/L, cre / L/L: 0.2
adj. p value: 0.4886
log2 L/L, cre / L/L: 0.53
adj. p value: 0.0936
log2 L/L, cre / L/L: −0.15
adj. p value: 0.7818
log2 intensity
27
26
25
24
Genotype
L/L
L/L, cre
Imputation
non imputed
imputed

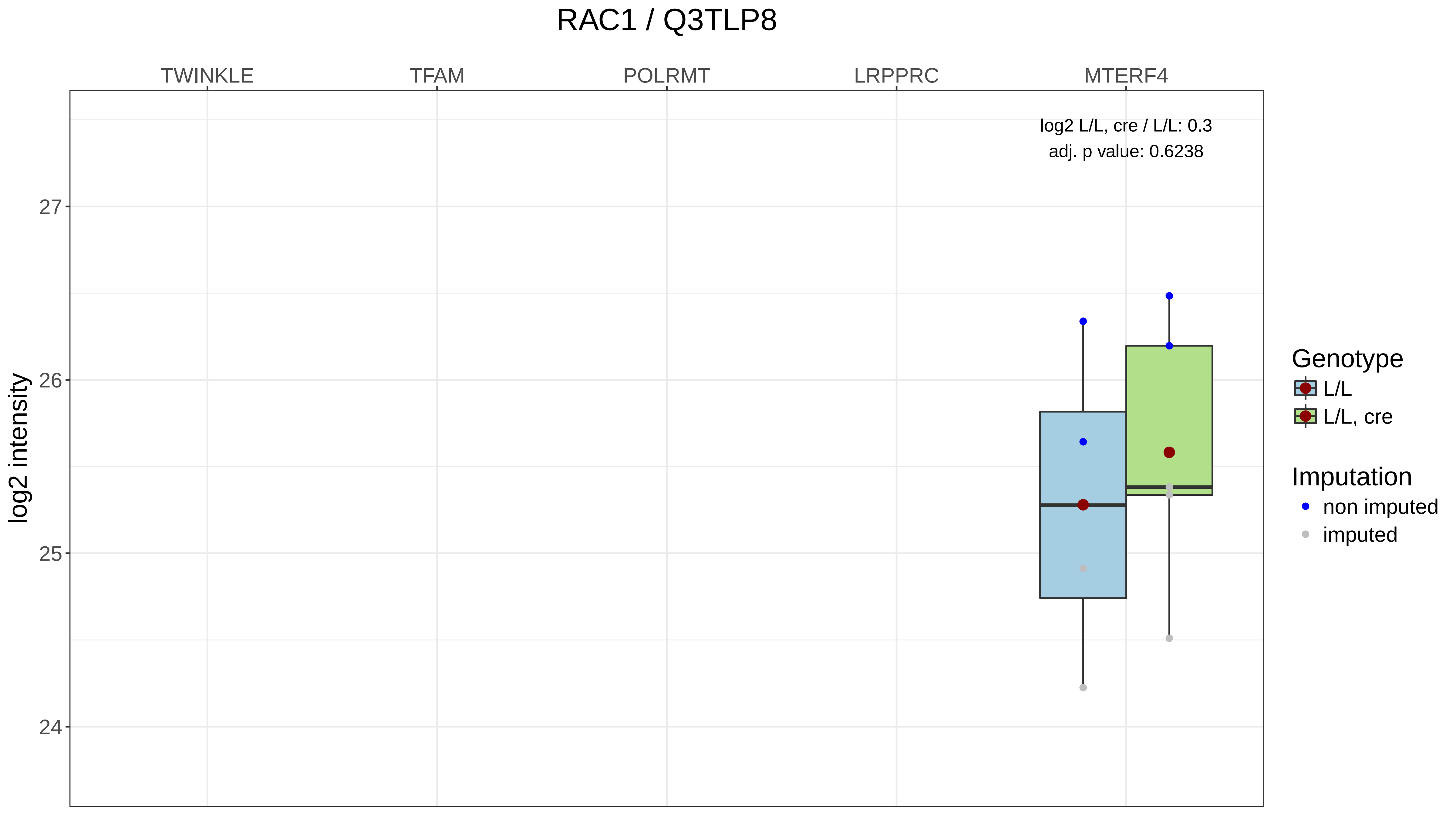
RAC1 / Q3TLP8
TWINKLE
TFAM
POLRMT
LRPPRC
MTERF4
log2 L/L, cre / L/L: 0.3
adj. p value: 0.6238
log2 intensity
27
26
25
24
Genotype
L/L
L/L, cre
Imputation
non imputed
imputed

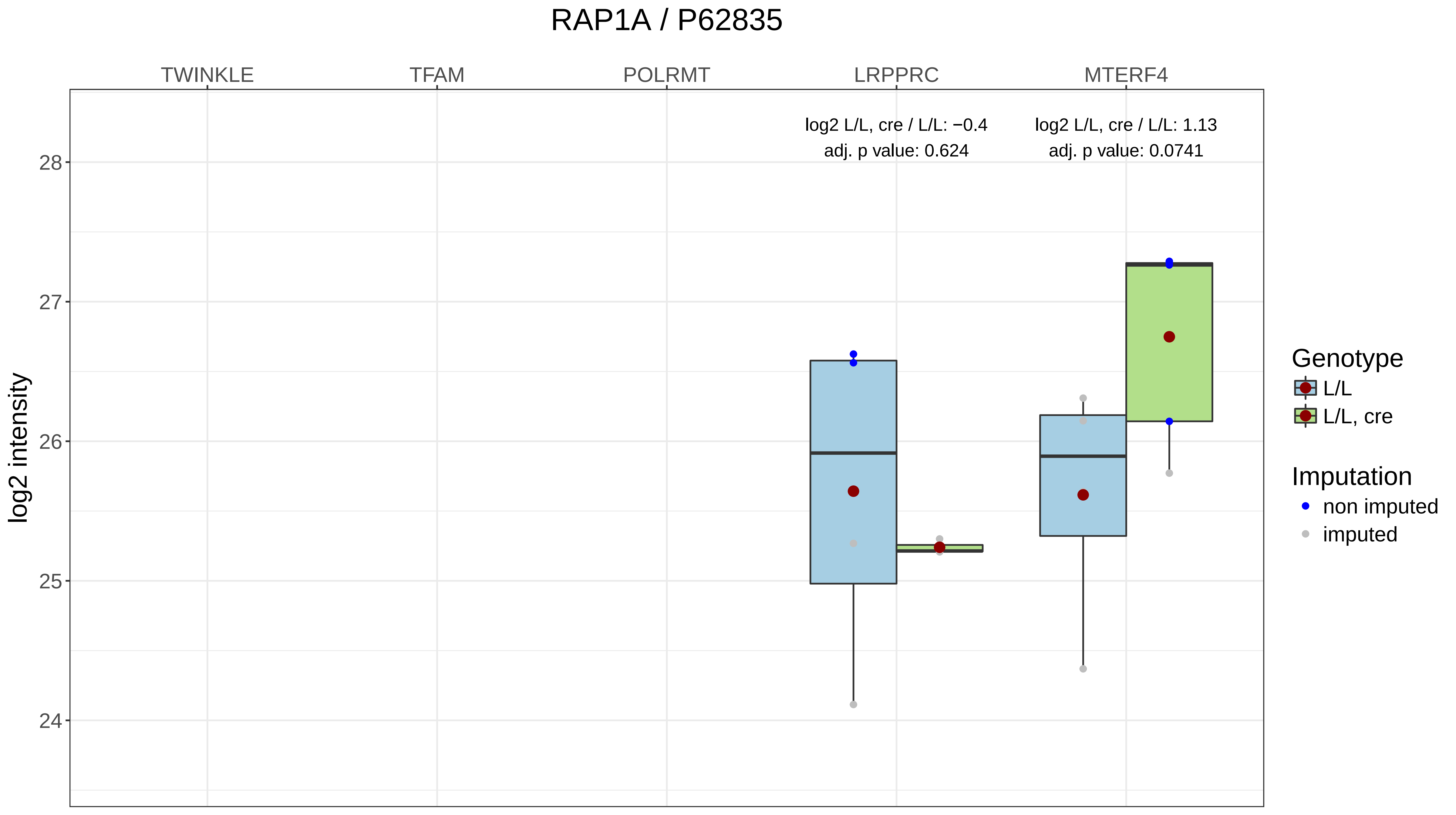
RAP1A / P62835
TWINKLE
TFAM
POLRMT
LRPPRC
MTERF4
log2 L/L, cre / L/L: −0.4
adj. p value: 0.624
log2 L/L, cre / L/L: 1.13
adj. p value: 0.0741
28
27
26
25
24
log2 intensity
Genotype
L/L
L/L, cre
Imputation
non imputed
imputed

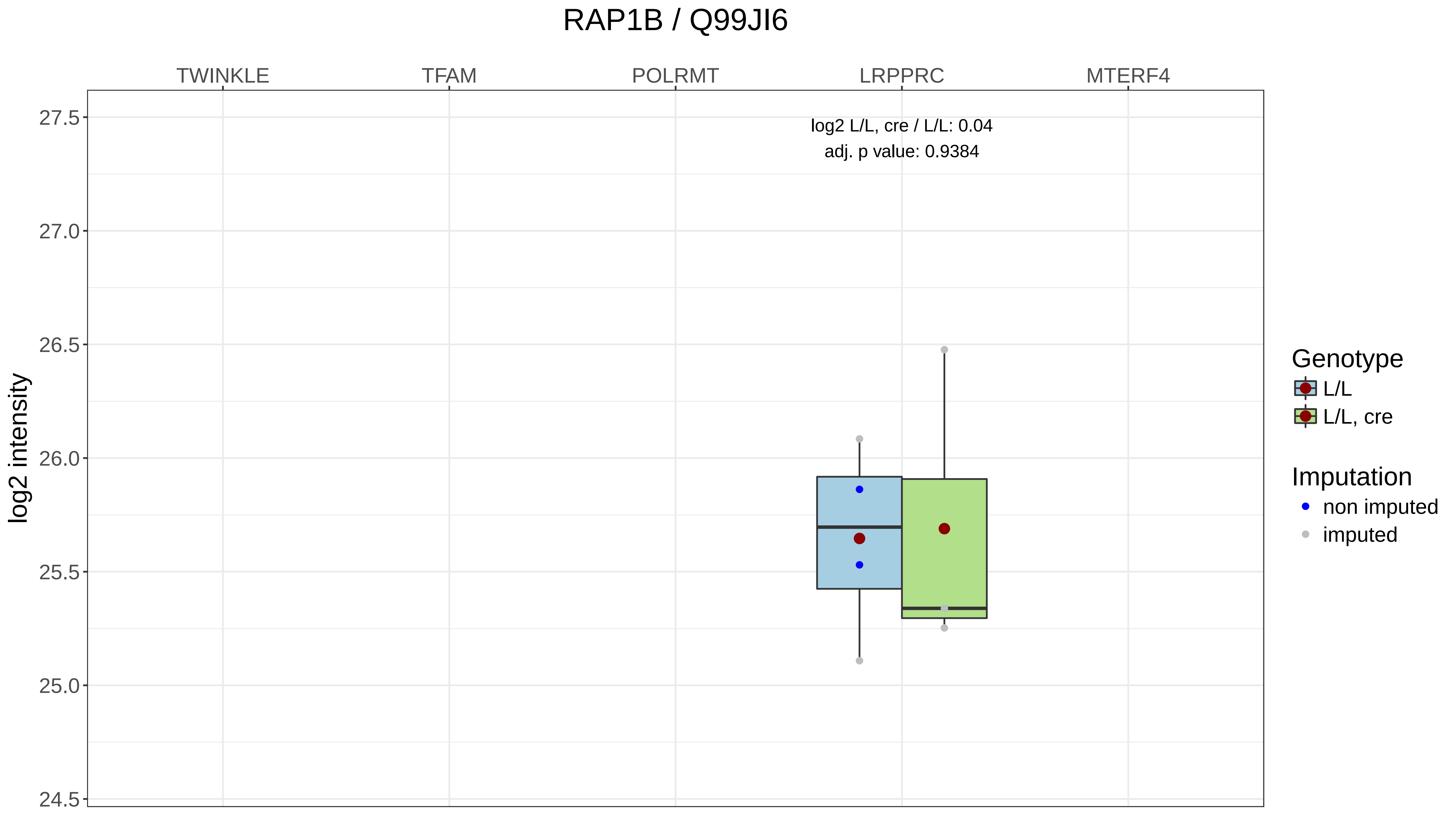

RAP1B / Q99JI6
TWINKLE
TFAM
POLRMT
LRPPRC
MTERF4
log2 L/L, cre / L/L: 0.04
adj. p value: 0.9384
log2 intensity
27.5
27.0
26.5
26.0
25.5
25.0
24.5
Genotype
L/L
L/L, cre
Imputation
non imputed
imputed

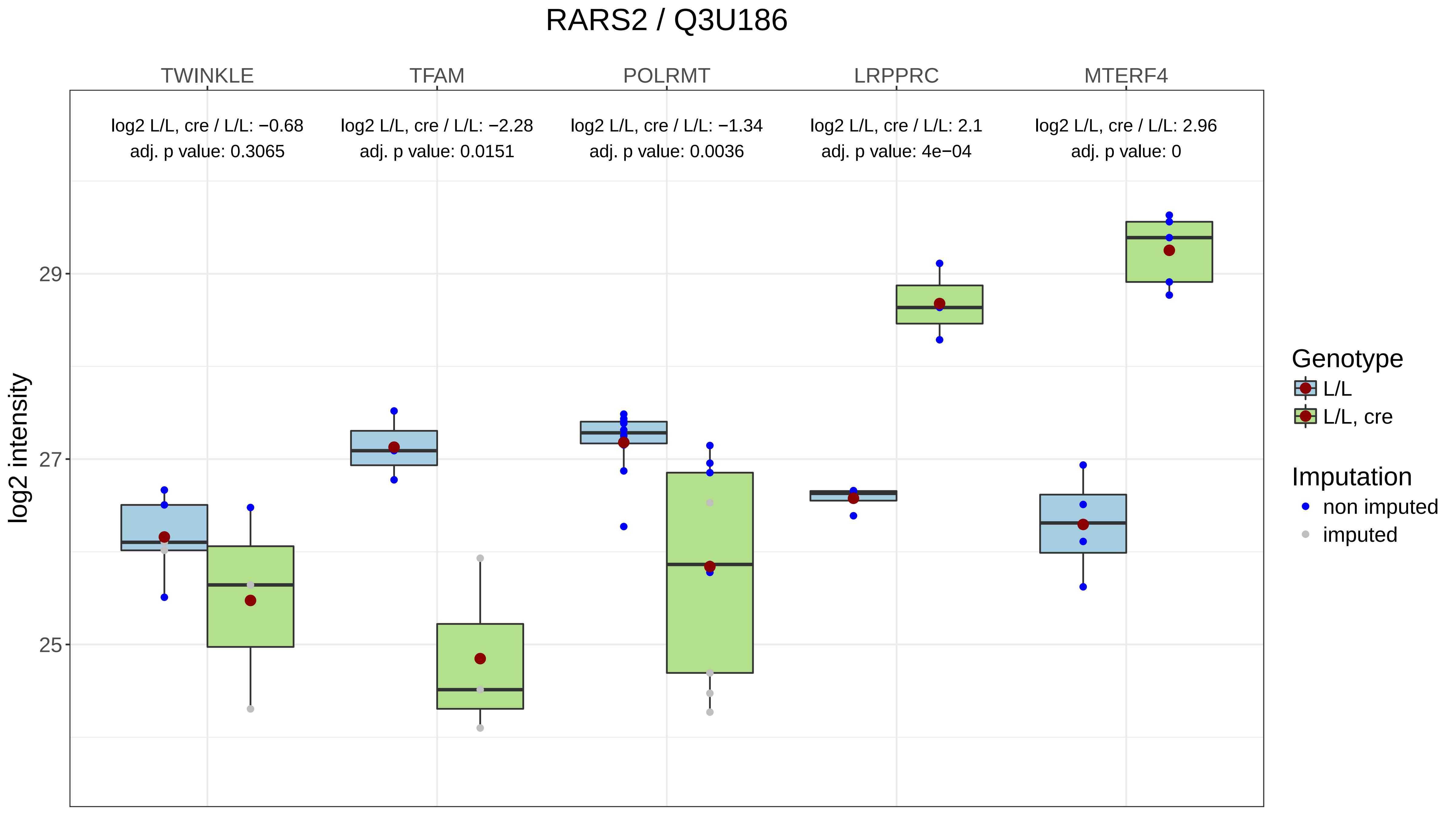
RARS2 / Q3U186
TWINKLE
TFAM
POLRMT
LRPPRC
MTERF4
log2 L/L, cre / L/L: −0.68
adj. p value: 0.3065
log2 L/L, cre / L/L: −2.28
adj. p value: 0.0151
log2 L/L, cre / L/L: −1.34
adj. p value: 0.0036
log2 L/L, cre / L/L: 2.1
adj. p value: 4e−04
log2 L/L, cre / L/L: 2.96
adj. p value: 0
log2 intensity
29
27
25
Genotype
L/L
L/L, cre
Imputation
non imputed
imputed

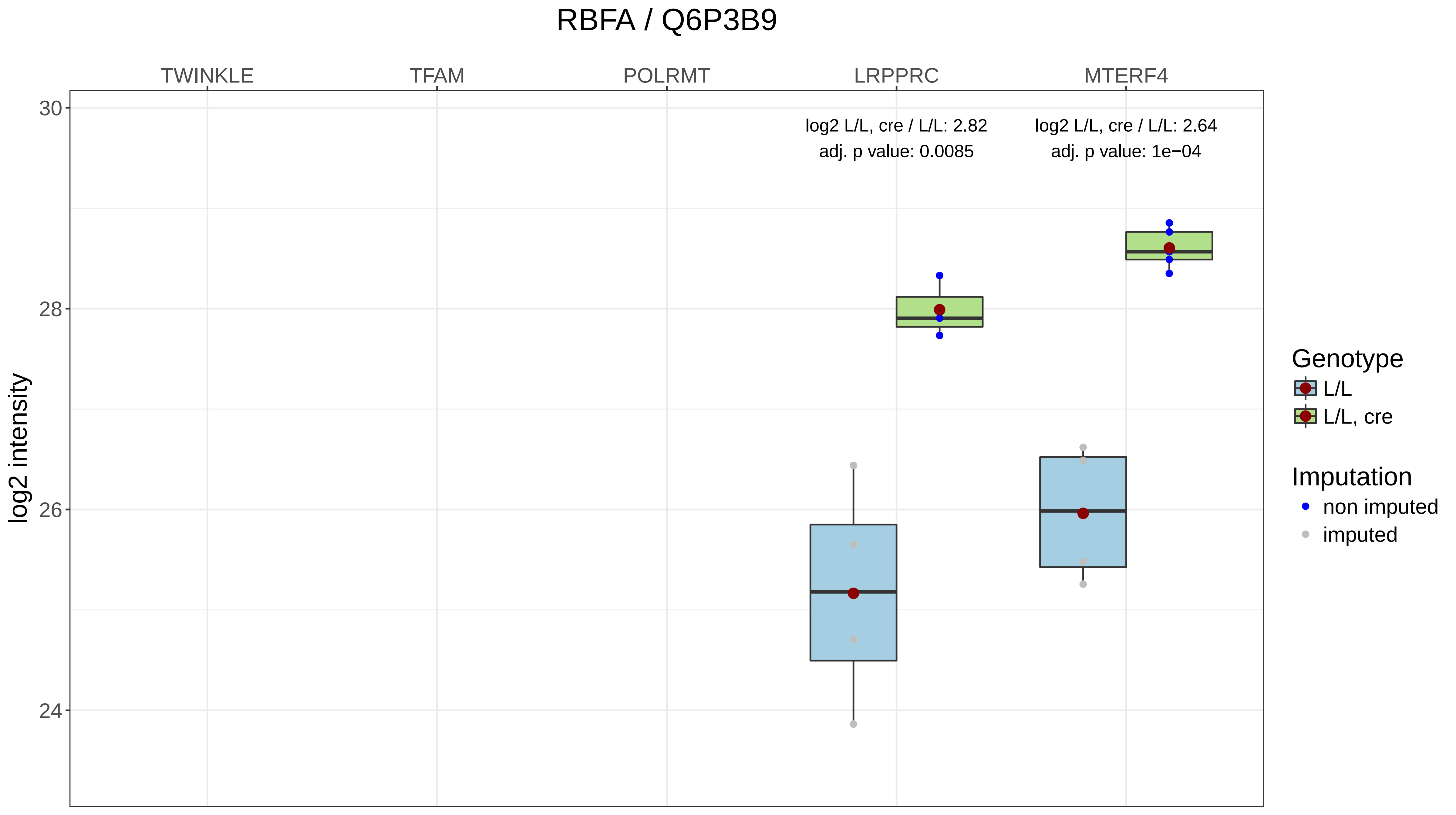

RBFA / Q6P3B9
TWINKLE
TFAM
POLRMT
LRPPRC
MTERF4
log2 L/L, cre / L/L: 2.82
adj. p value: 0.0085
log2 L/L, cre / L/L: 2.64
adj. p value: 1e−04
30
28
26
24
log2 intensity
Genotype
L/L
L/L, cre
Imputation
non imputed
imputed

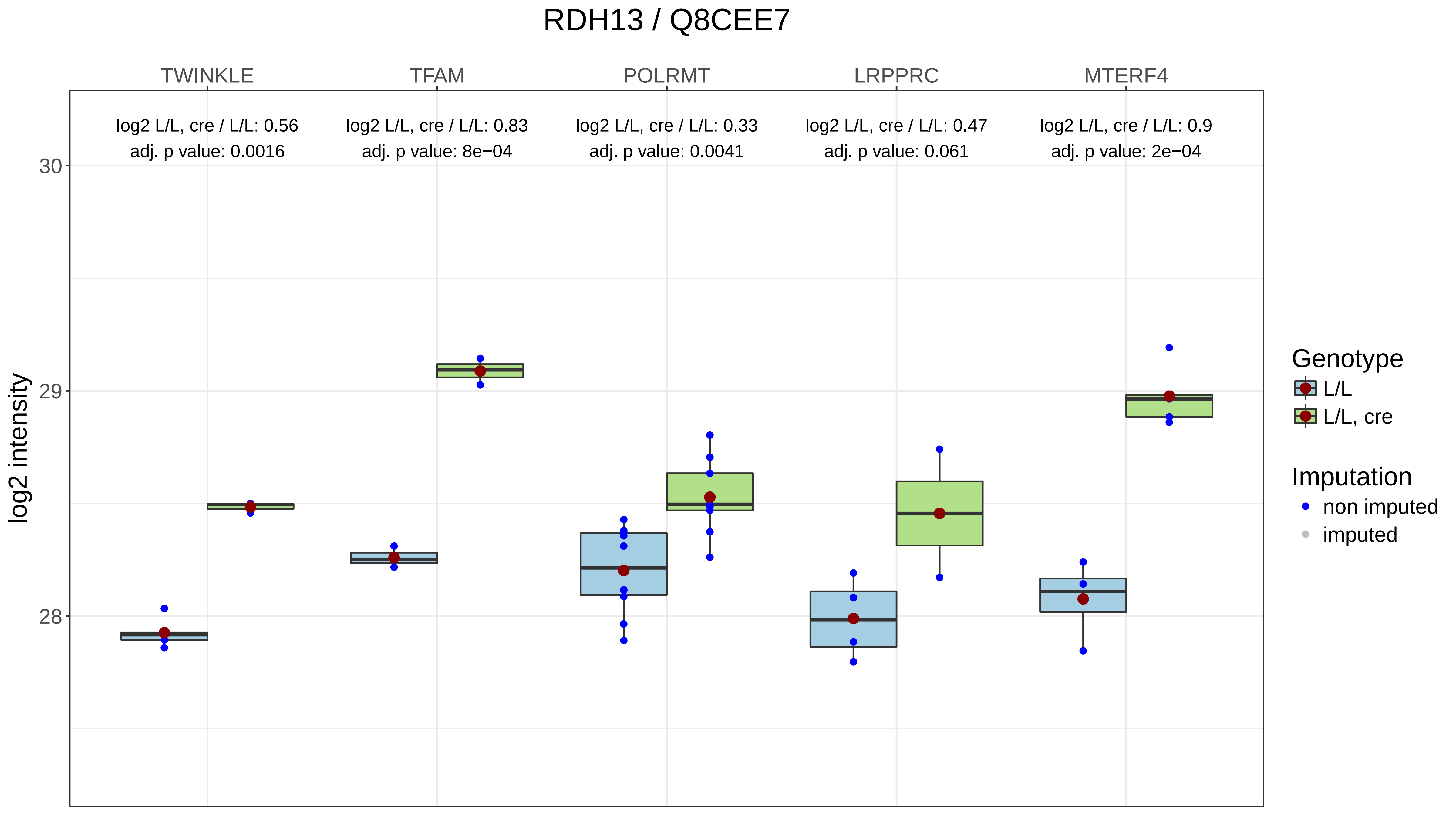
RDH13 / Q8CEE7
TWINKLE
TFAM
POLRMT
LRPPRC
MTERF4
log2 L/L, cre / L/L: 0.56
adj. p value: 0.0016
log2 L/L, cre / L/L: 0.83
adj. p value: 8e−04
log2 L/L, cre / L/L: 0.33
adj. p value: 0.0041
log2 L/L, cre / L/L: 0.47
adj. p value: 0.061
log2 L/L, cre / L/L: 0.9
adj. p value: 2e−04
log2 intensity
30
29
28
Genotype
L/L
L/L, cre
Imputation
non imputed
imputed

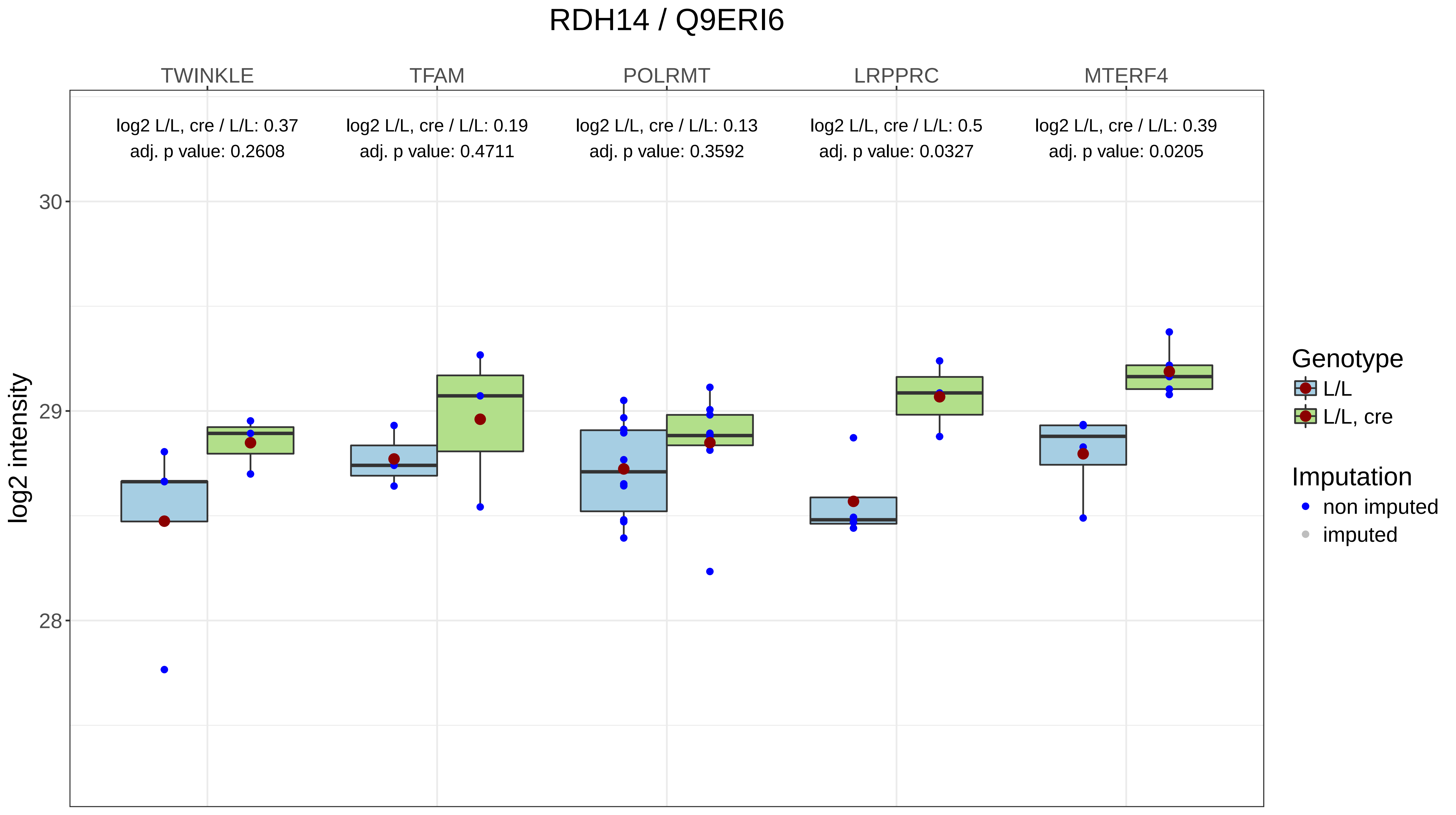
RDH14 / Q9ERI6
TWINKLE
TFAM
POLRMT
LRPPRC
MTERF4
log2 L/L, cre / L/L: 0.37
adj. p value: 0.2608
log2 L/L, cre / L/L: 0.19
adj. p value: 0.4711
log2 L/L, cre / L/L: 0.13
adj. p value: 0.3592
log2 L/L, cre / L/L: 0.5
adj. p value: 0.0327
log2 L/L, cre / L/L: 0.39
adj. p value: 0.0205
log2 intensity
30
29
28
Genotype
L/L
L/L, cre
Imputation
non imputed
imputed

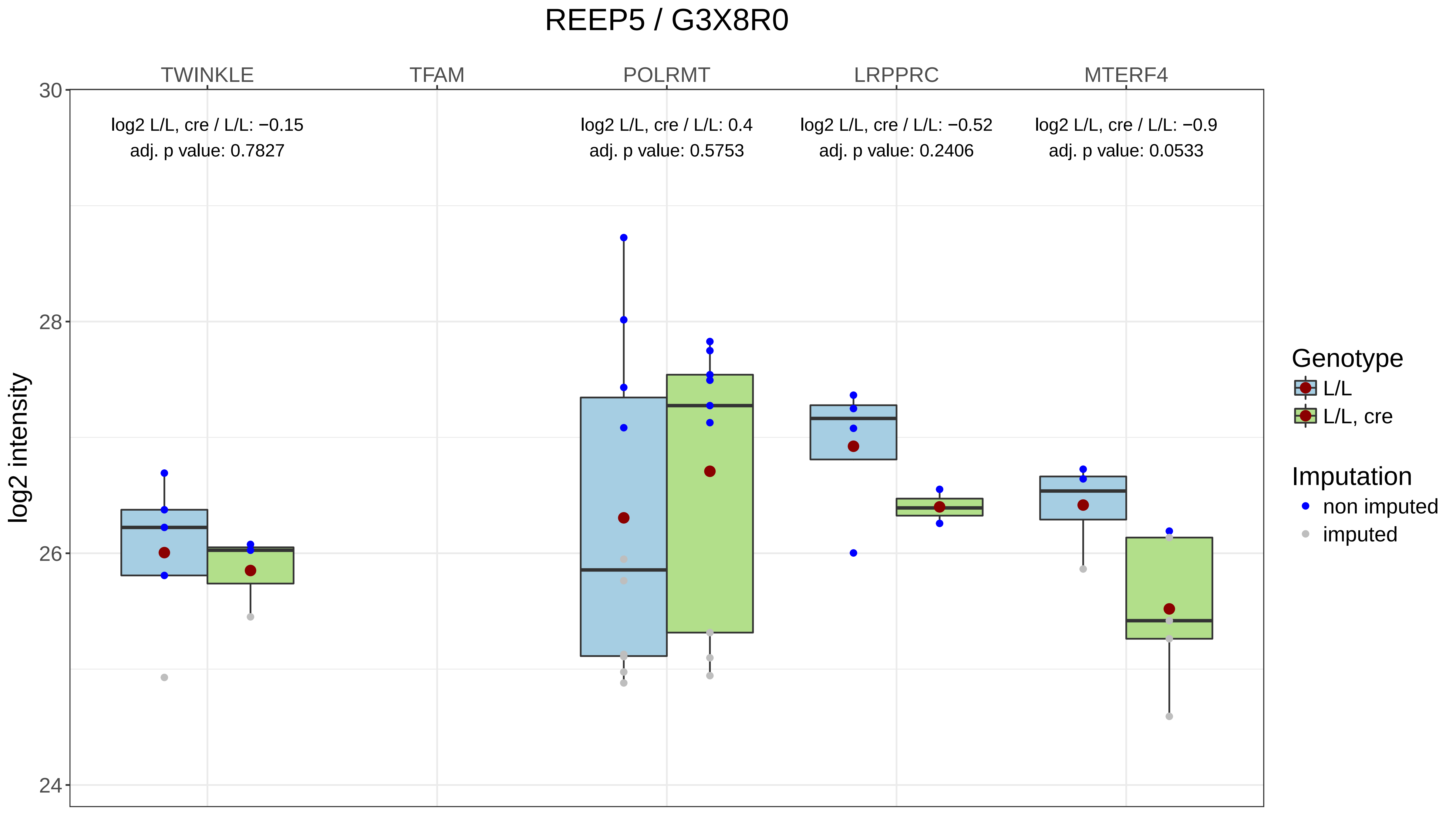

REEP5 / G3X8R0
TWINKLE
TFAM
POLRMT
LRPPRC
MTERF4
log2 L/L, cre / L/L: −0.15
adj. p value: 0.7827
log2 L/L, cre / L/L: 0.4
adj. p value: 0.5753
log2 L/L, cre / L/L: −0.52
adj. p value: 0.2406
log2 L/L, cre / L/L: −0.9
adj. p value: 0.0533
30
28
26
24
log2 intensity
Genotype
L/L
L/L, cre
Imputation
non imputed
imputed

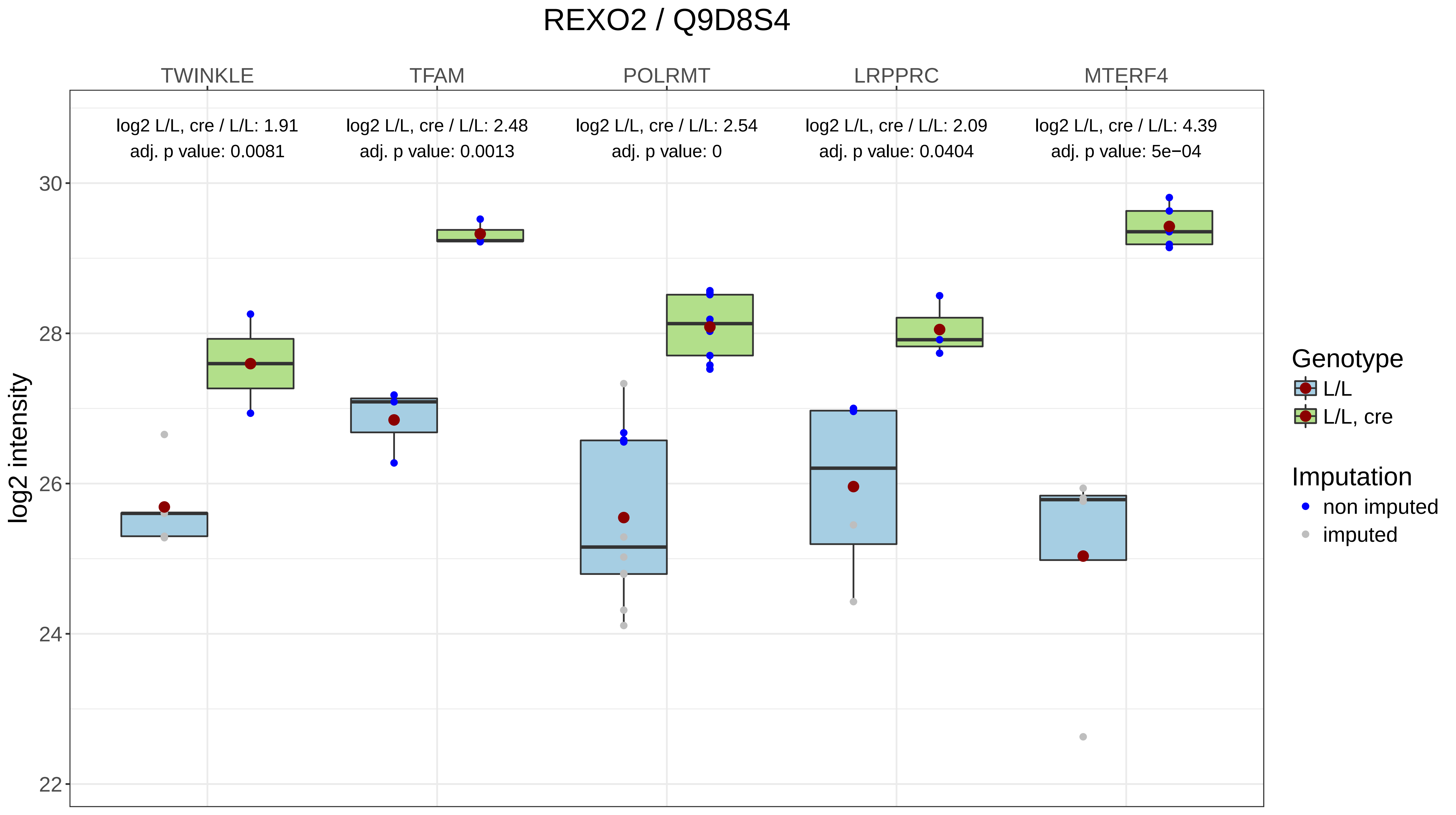
REXO2 / Q9D8S4
TWINKLE
TFAM
POLRMT
LRPPRC
MTERF4
log2 L/L, cre / L/L: 1.91
adj. p value: 0.0081
log2 L/L, cre / L/L: 2.48
adj. p value: 0.0013
log2 L/L, cre / L/L: 2.54
adj. p value: 0
log2 L/L, cre / L/L: 2.09
adj. p value: 0.0404
log2 L/L, cre / L/L: 4.39
adj. p value: 5e−04
log2 intensity
30
28
26
24
22
Genotype
L/L
L/L, cre
Imputation
non imputed
imputed

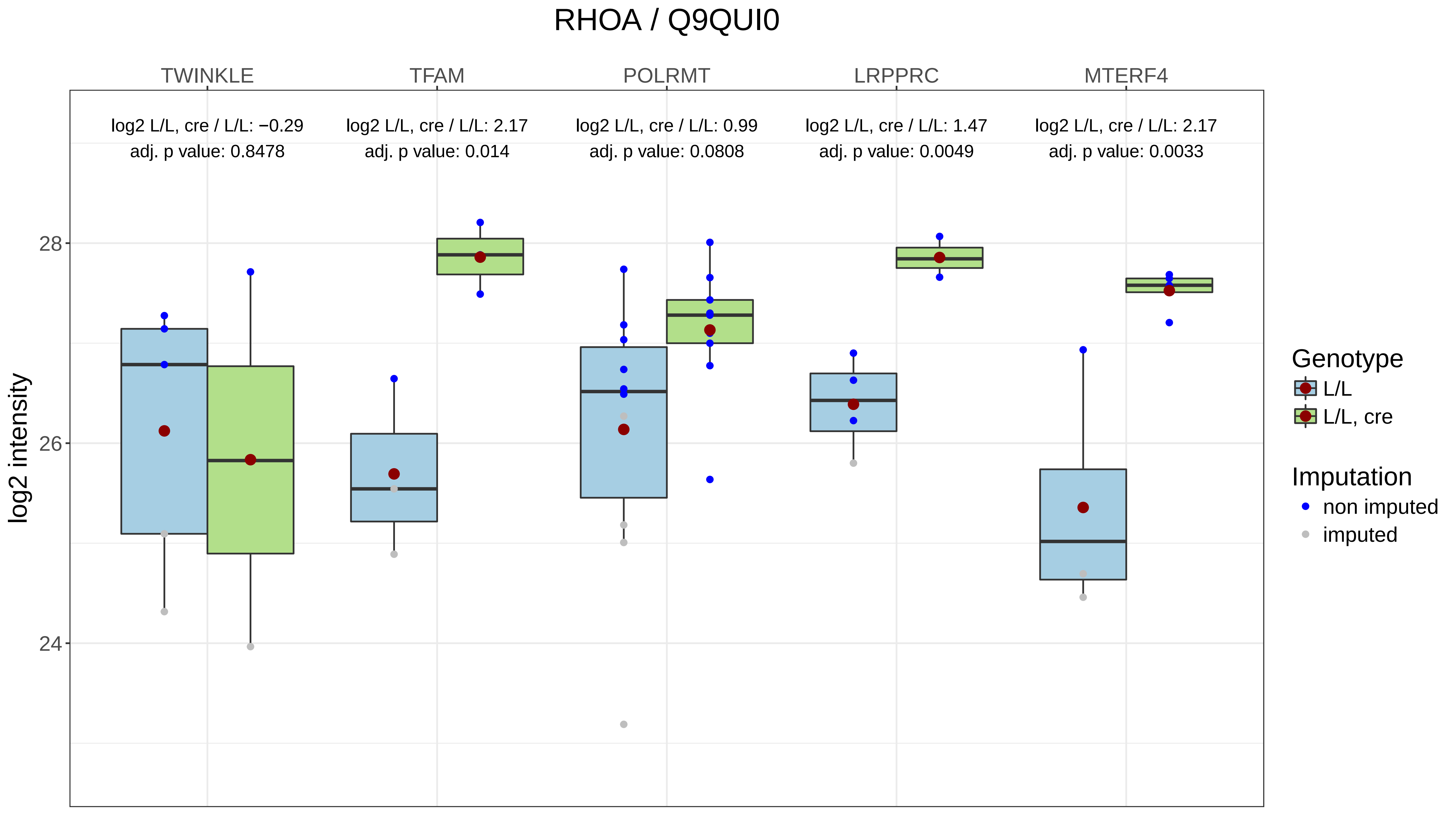
RHOA / Q9QUI0
TWINKLE
TFAM
POLRMT
LRPPRC
MTERF4
log2 L/L, cre / L/L: −0.29
adj. p value: 0.8478
log2 L/L, cre / L/L: 2.17
adj. p value: 0.014
log2 L/L, cre / L/L: 0.99
adj. p value: 0.0808
log2 L/L, cre / L/L: 1.47
adj. p value: 0.0049
log2 L/L, cre / L/L: 2.17
adj. p value: 0.0033
log2 intensity
28
26
24
Genotype
L/L
L/L, cre
Imputation
non imputed
imputed

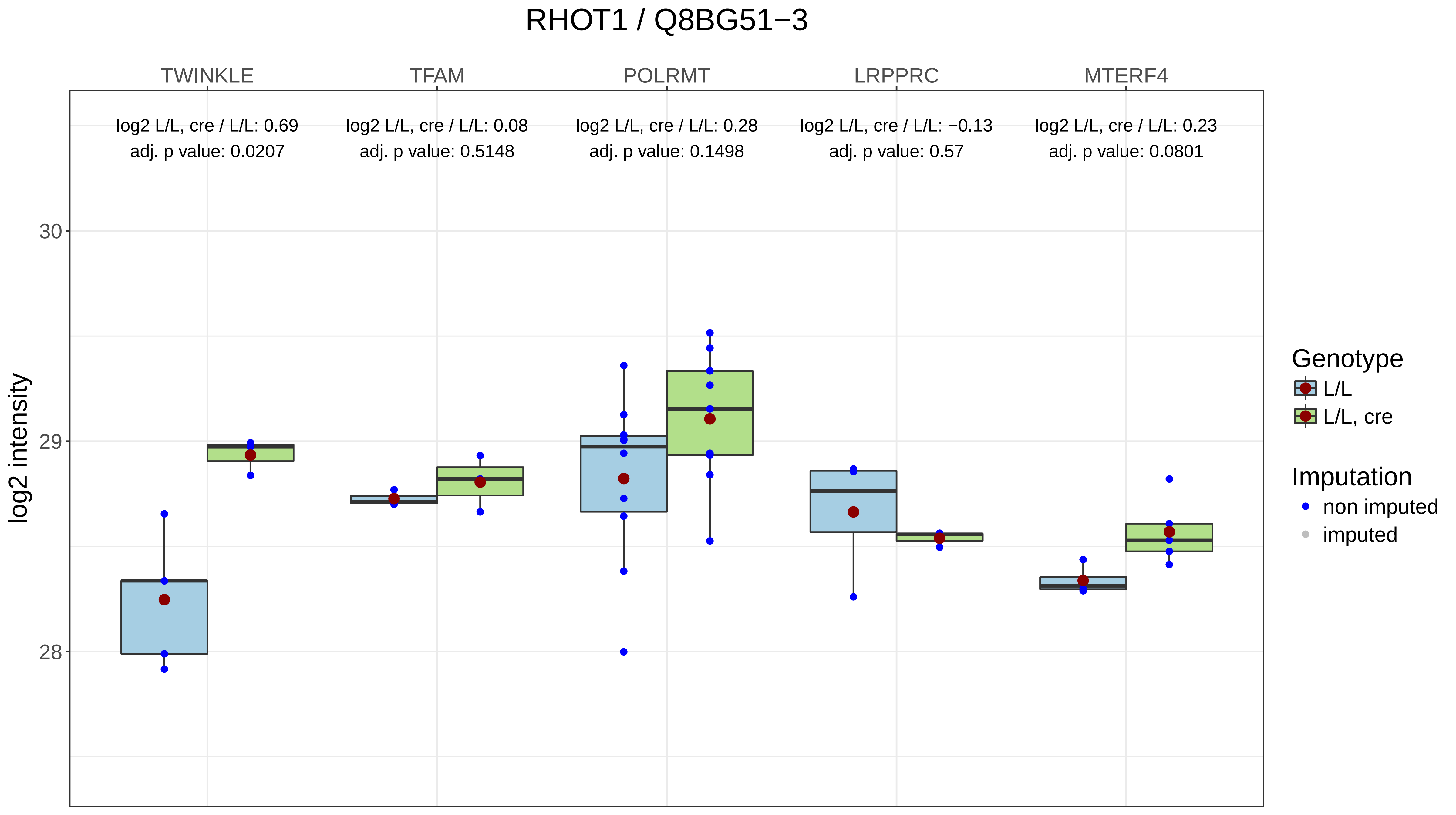
RHOT1 / Q8BG51−3
TWINKLE
TFAM
POLRMT
LRPPRC
MTERF4
log2 L/L, cre / L/L: 0.69
adj. p value: 0.0207
log2 L/L, cre / L/L: 0.08
adj. p value: 0.5148
log2 L/L, cre / L/L: 0.28
adj. p value: 0.1498
log2 L/L, cre / L/L: −0.13
adj. p value: 0.57
log2 L/L, cre / L/L: 0.23
adj. p value: 0.0801
log2 intensity
30
29
28
Genotype
L/L
L/L, cre
Imputation
non imputed
imputed

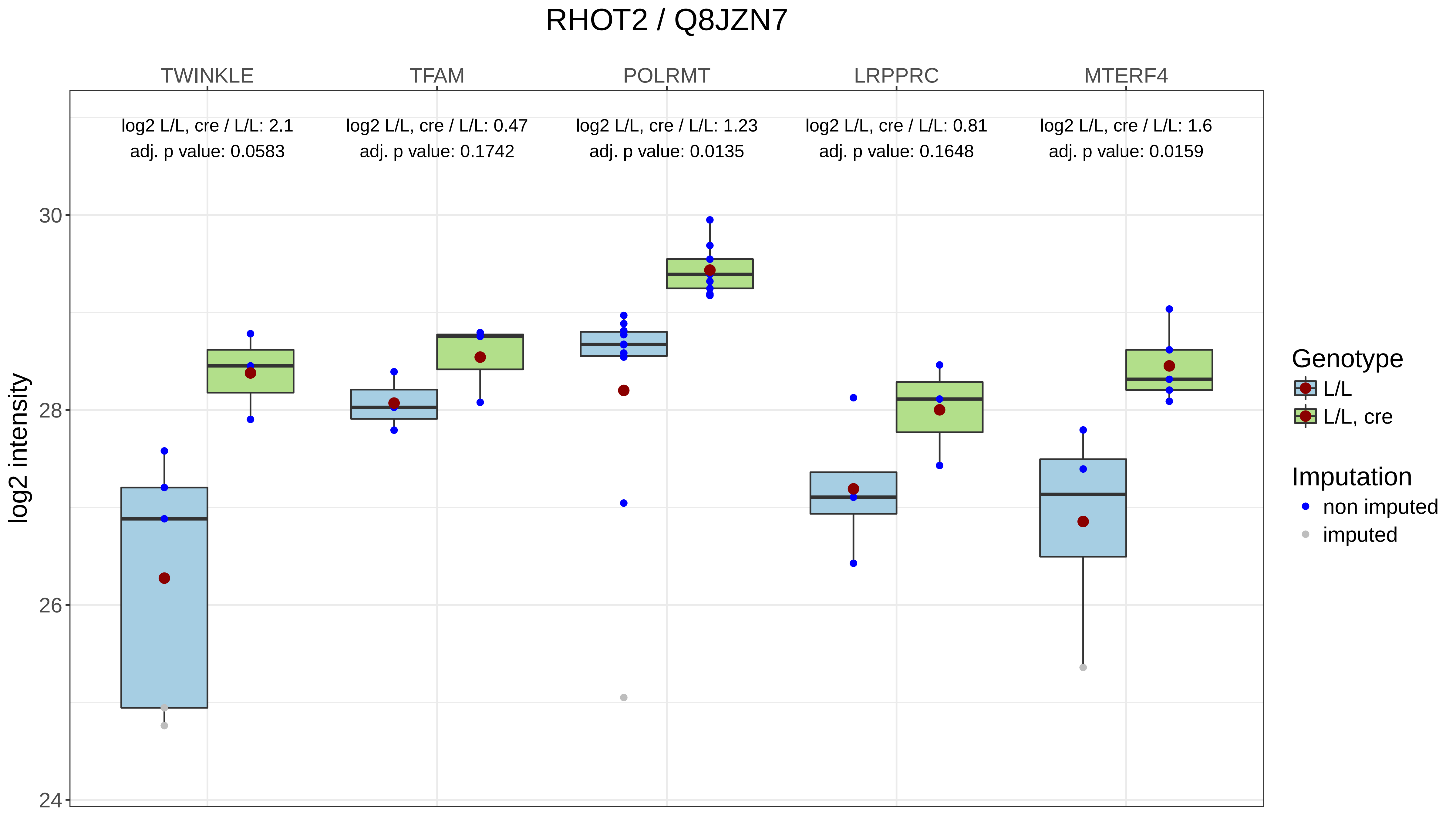
RHOT2 / Q8JZN7
TWINKLE
TFAM
POLRMT
LRPPRC
MTERF4
log2 L/L, cre / L/L: 2.1
adj. p value: 0.0583
log2 L/L, cre / L/L: 0.47
adj. p value: 0.1742
log2 L/L, cre / L/L: 1.23
adj. p value: 0.0135
log2 L/L, cre / L/L: 0.81
adj. p value: 0.1648
log2 L/L, cre / L/L: 1.6
adj. p value: 0.0159
log2 intensity
30
28
26
24
Genotype
L/L
L/L, cre
Imputation
non imputed
imputed

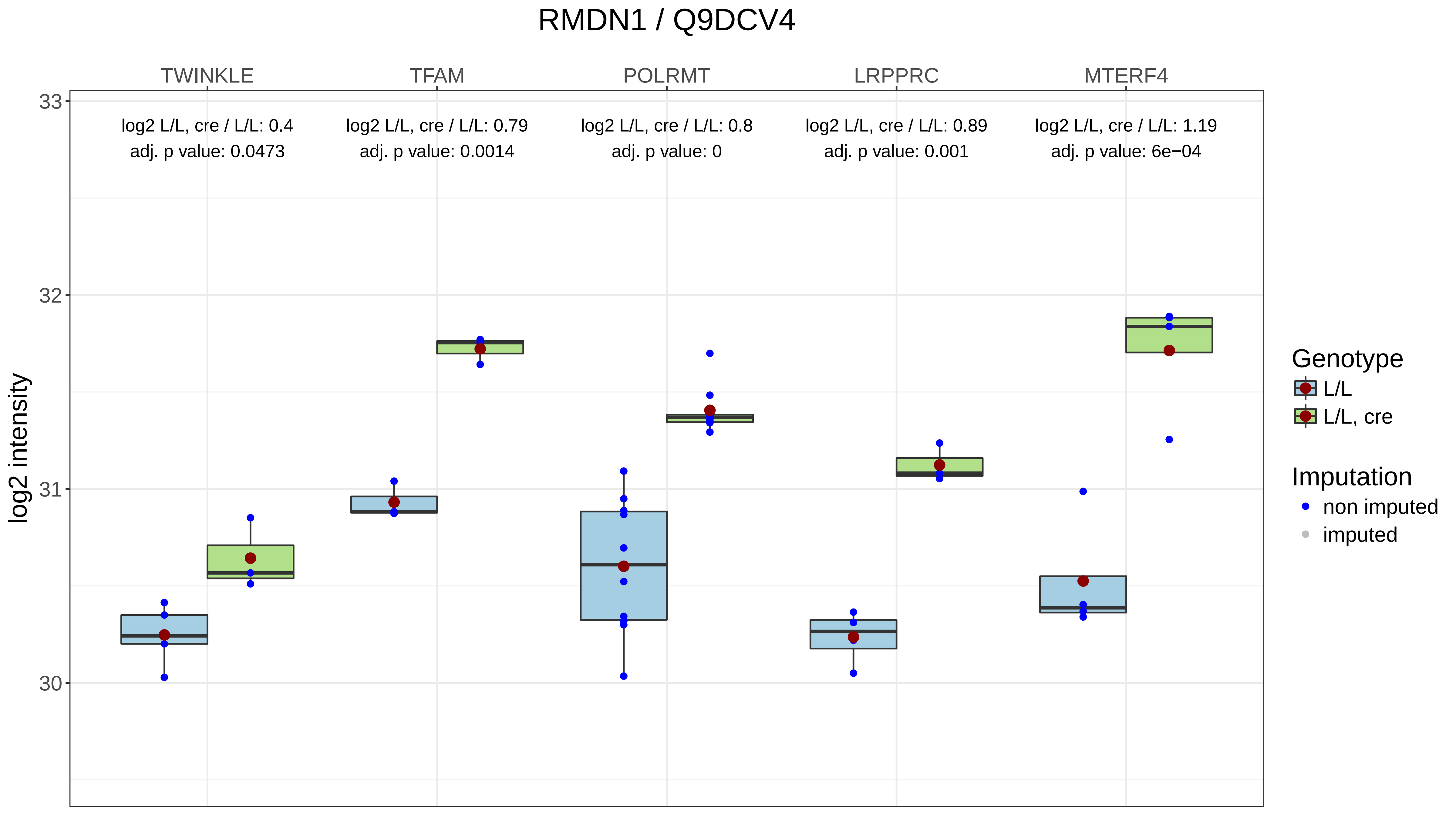
RMDN1 / Q9DCV4
TWINKLE
TFAM
POLRMT
LRPPRC
MTERF4
log2 L/L, cre / L/L: 0.4
adj. p value: 0.0473
log2 L/L, cre / L/L: 0.79
adj. p value: 0.0014
log2 L/L, cre / L/L: 0.8
adj. p value: 0
log2 L/L, cre / L/L: 0.89
adj. p value: 0.001
log2 L/L, cre / L/L: 1.19
adj. p value: 6e−04
log2 intensity
33
32
31
30
Genotype
L/L
L/L, cre
Imputation
non imputed
imputed

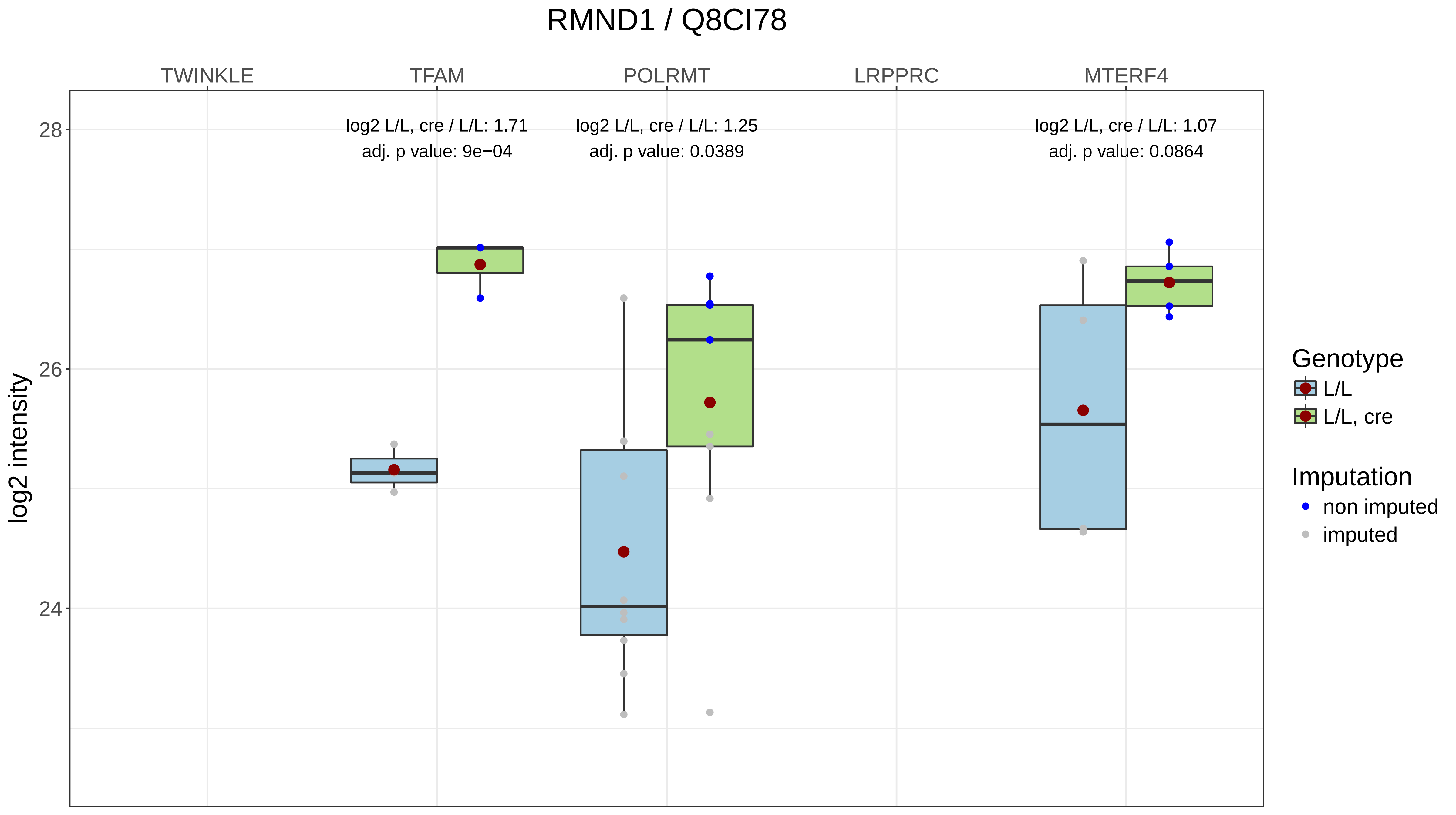
RMND1 / Q8CI78
TWINKLE
TFAM
POLRMT
LRPPRC
MTERF4
log2 L/L, cre / L/L: 1.71
adj. p value: 9e−04
log2 L/L, cre / L/L: 1.25
adj. p value: 0.0389
log2 L/L, cre / L/L: 1.07
adj. p value: 0.0864
28
26
24
log2 intensity
Genotype
L/L
L/L, cre
Imputation
non imputed
imputed

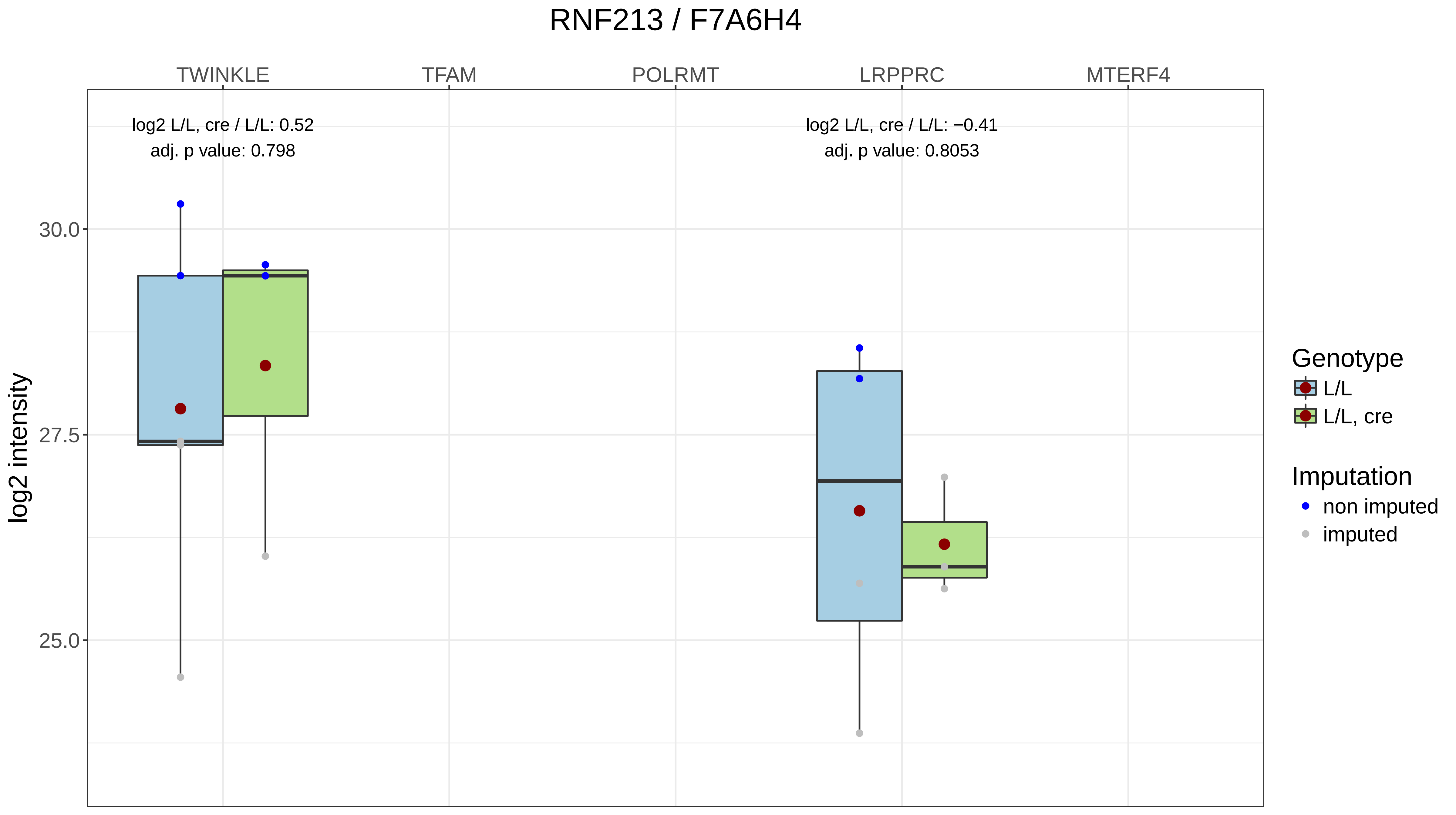
RNF213 / F7A6H4
TWINKLE
TFAM
POLRMT
LRPPRC
MTERF4
log2 L/L, cre / L/L: 0.52
adj. p value: 0.798
log2 L/L, cre / L/L: −0.41
adj. p value: 0.8053
30.0
27.5
25.0
log2 intensity
Genotype
L/L
L/L, cre
Imputation
non imputed
imputed

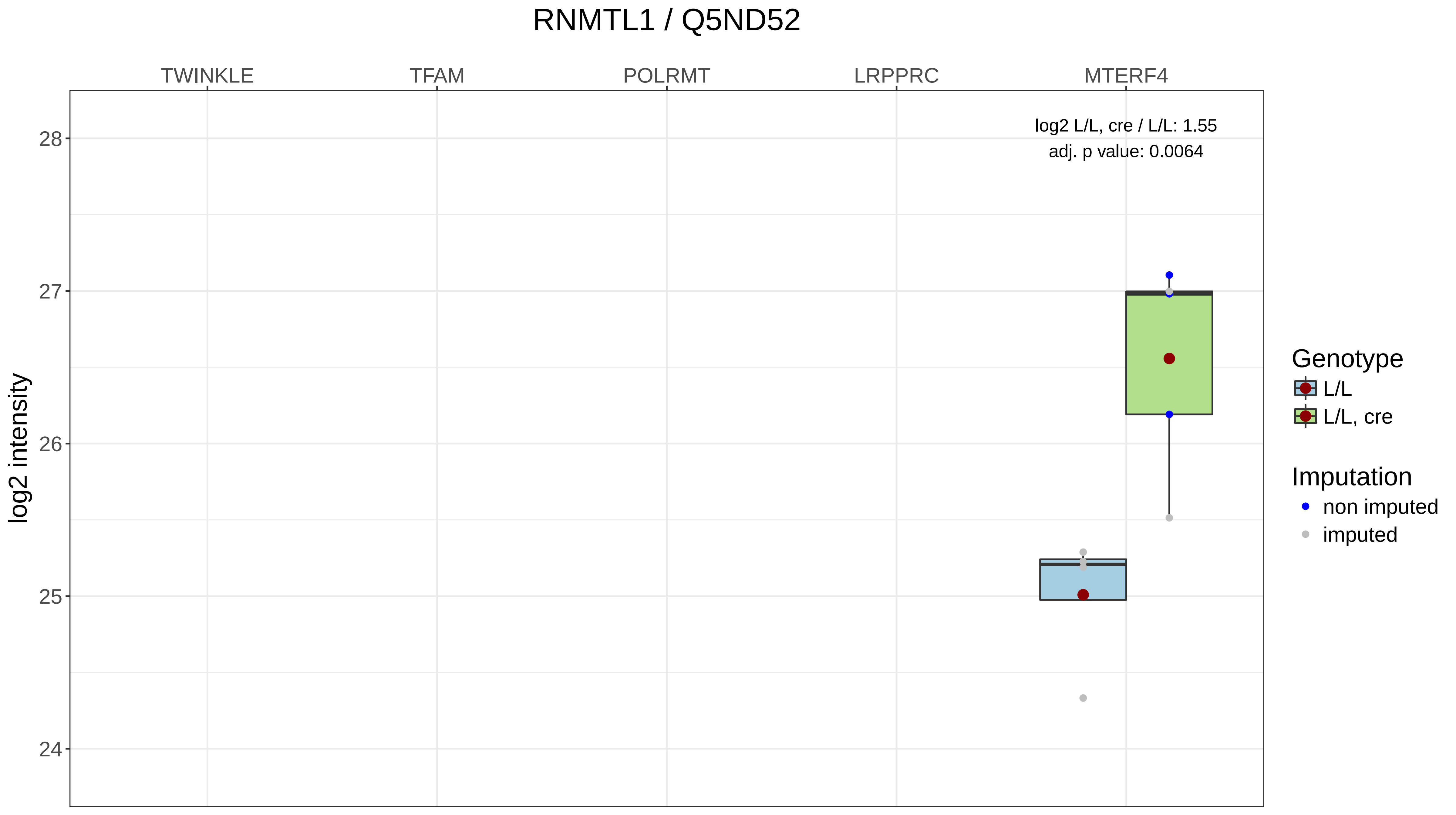
RNMTL1 / Q5ND52
TWINKLE
TFAM
POLRMT
LRPPRC
MTERF4
log2 L/L, cre / L/L: 1.55
adj. p value: 0.0064
28
27
26
25
24
log2 intensity
Genotype
L/L
L/L, cre
Imputation
non imputed
imputed

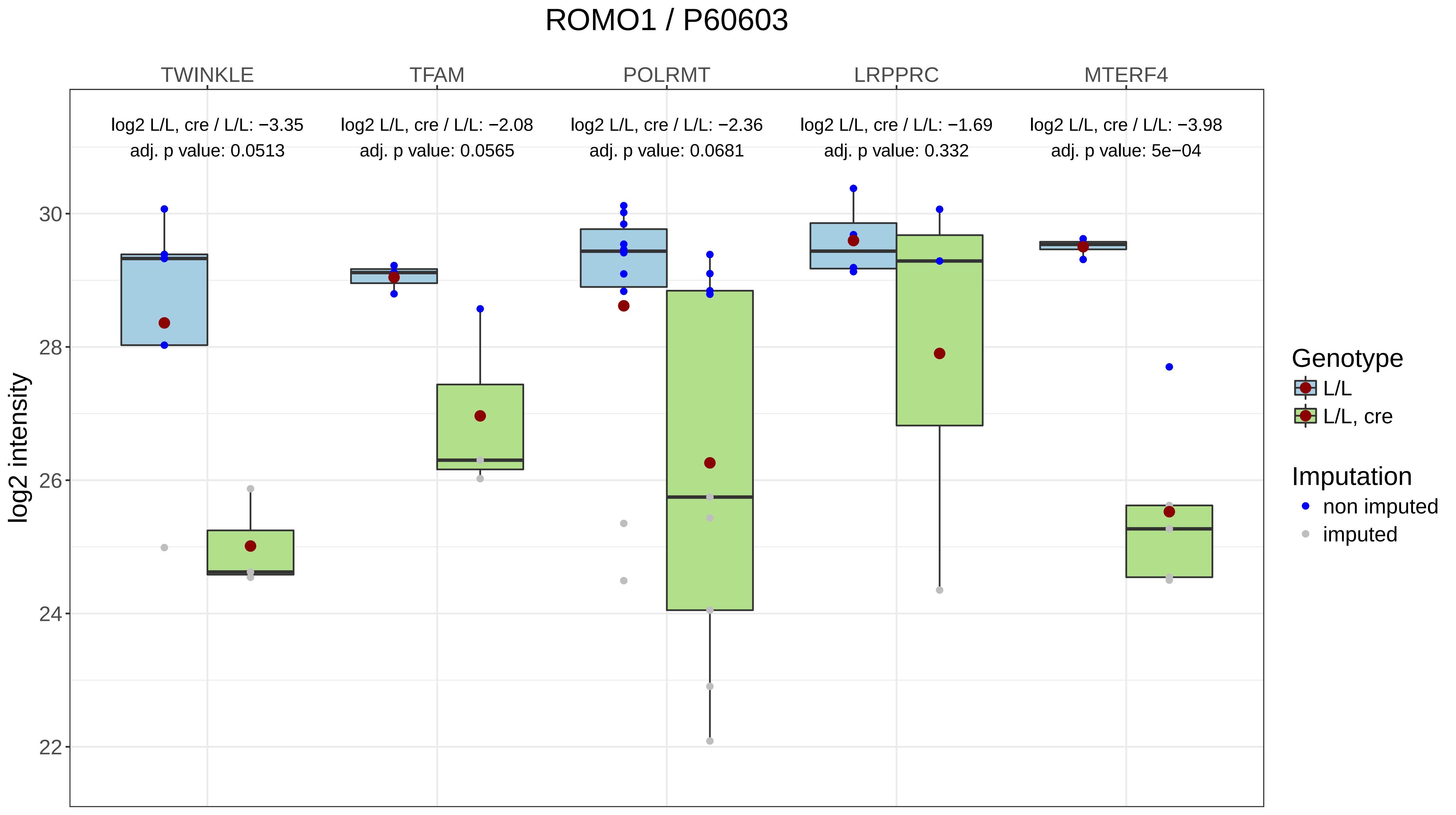
ROMO1 / P60603
TWINKLE
TFAM
POLRMT
LRPPRC
MTERF4
log2 L/L, cre / L/L: −3.35
adj. p value: 0.0513
log2 L/L, cre / L/L: −2.08
adj. p value: 0.0565
log2 L/L, cre / L/L: −2.36
adj. p value: 0.0681
log2 L/L, cre / L/L: −1.69
adj. p value: 0.332
log2 L/L, cre / L/L: −3.98
adj. p value: 5e−04
log2 intensity
30
28
26
24
22
Genotype
L/L
L/L, cre
Imputation
non imputed
imputed

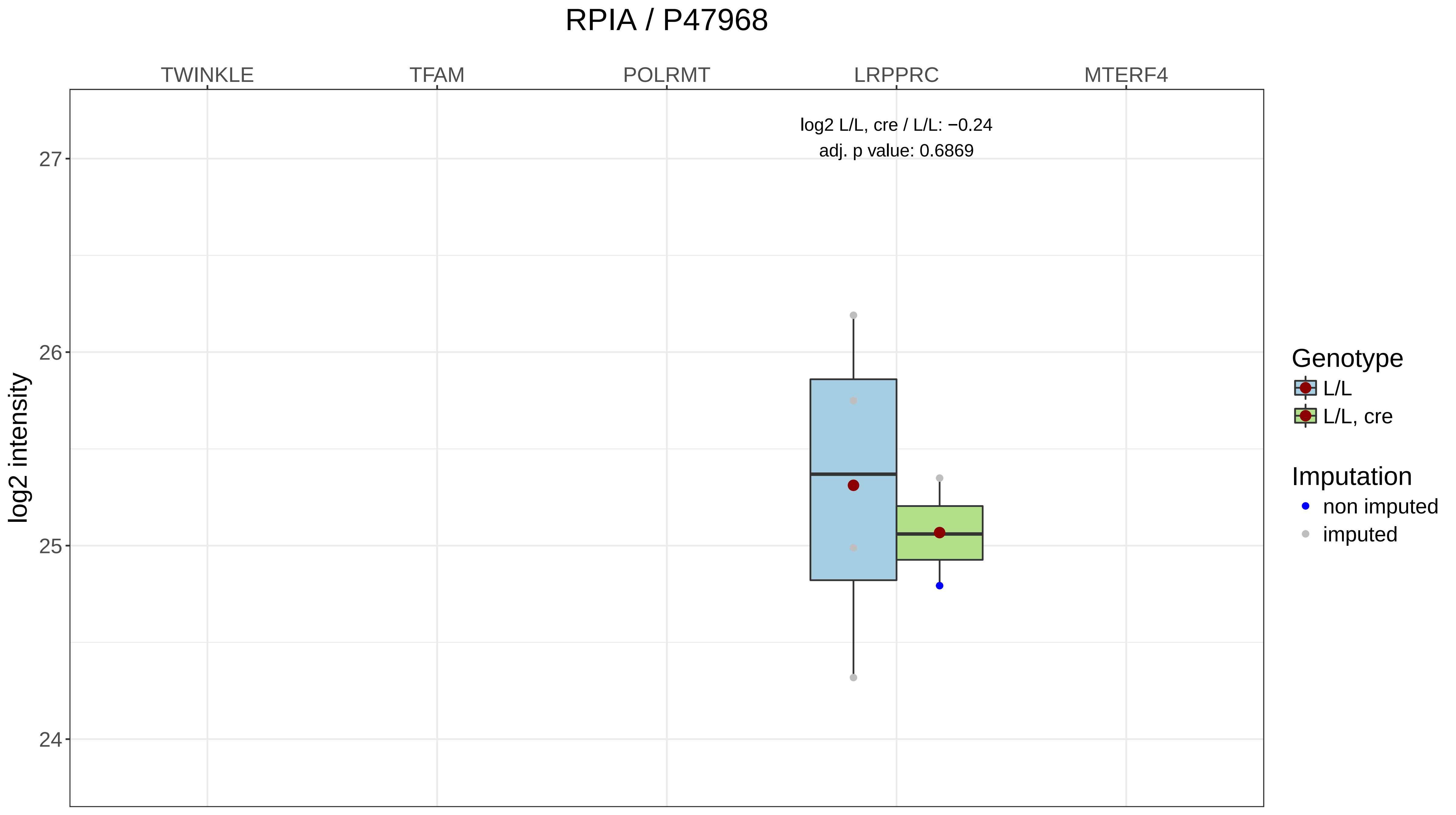

RPIA / P47968
TWINKLE
TFAM
POLRMT
LRPPRC
MTERF4
log2 L/L, cre / L/L: −0.24
adj. p value: 0.6869
27
26
25
24
log2 intensity
Genotype
L/L
L/L, cre
Imputation
non imputed
imputed

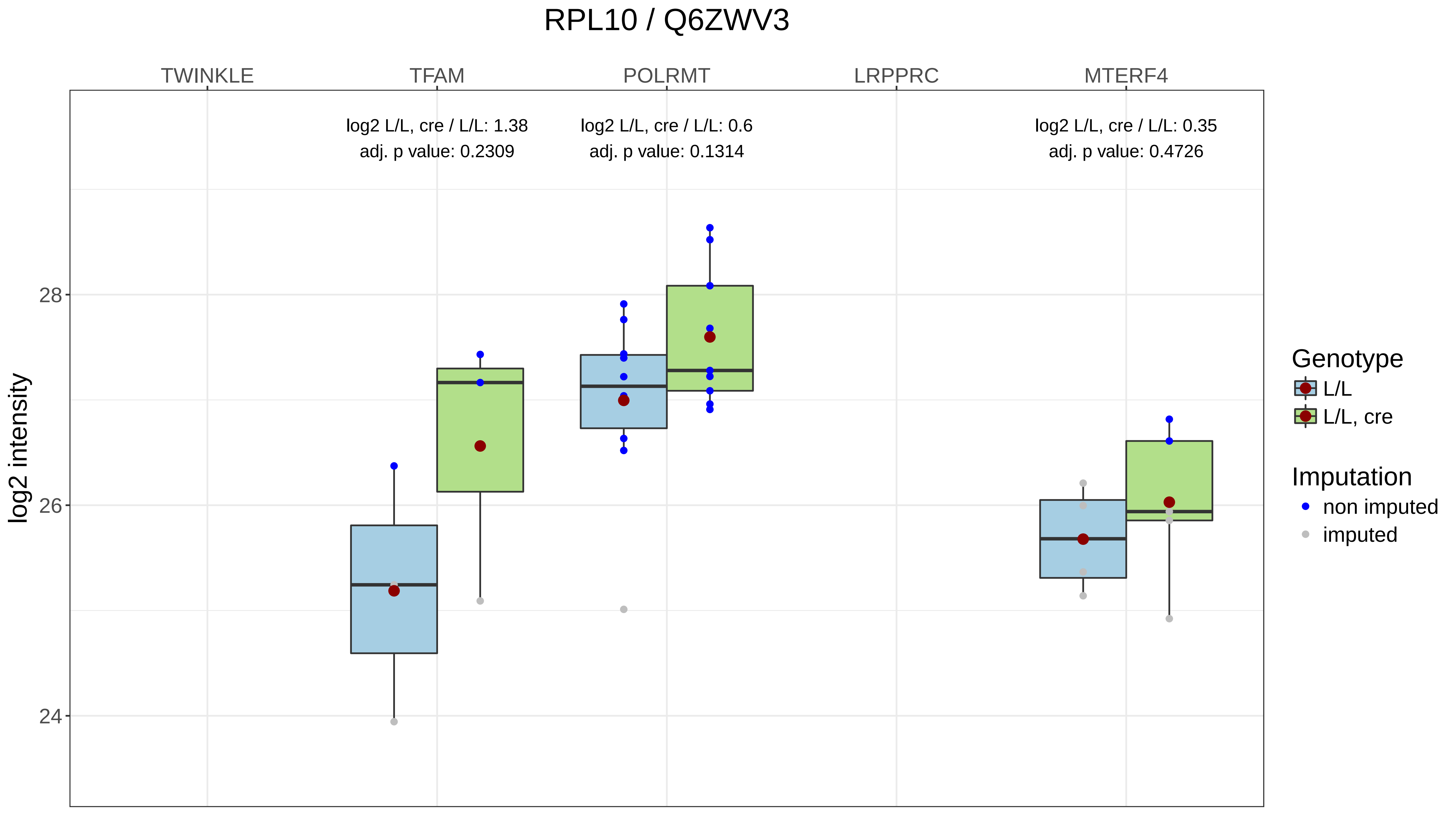

RPL10 / Q6ZWV3
TWINKLE
TFAM
POLRMT
LRPPRC
MTERF4
log2 L/L, cre / L/L: 1.38
adj. p value: 0.2309
log2 L/L, cre / L/L: 0.6
adj. p value: 0.1314
log2 L/L, cre / L/L: 0.35
adj. p value: 0.4726
log2 intensity
28
26
24
Genotype
L/L
L/L, cre
Imputation
non imputed
imputed

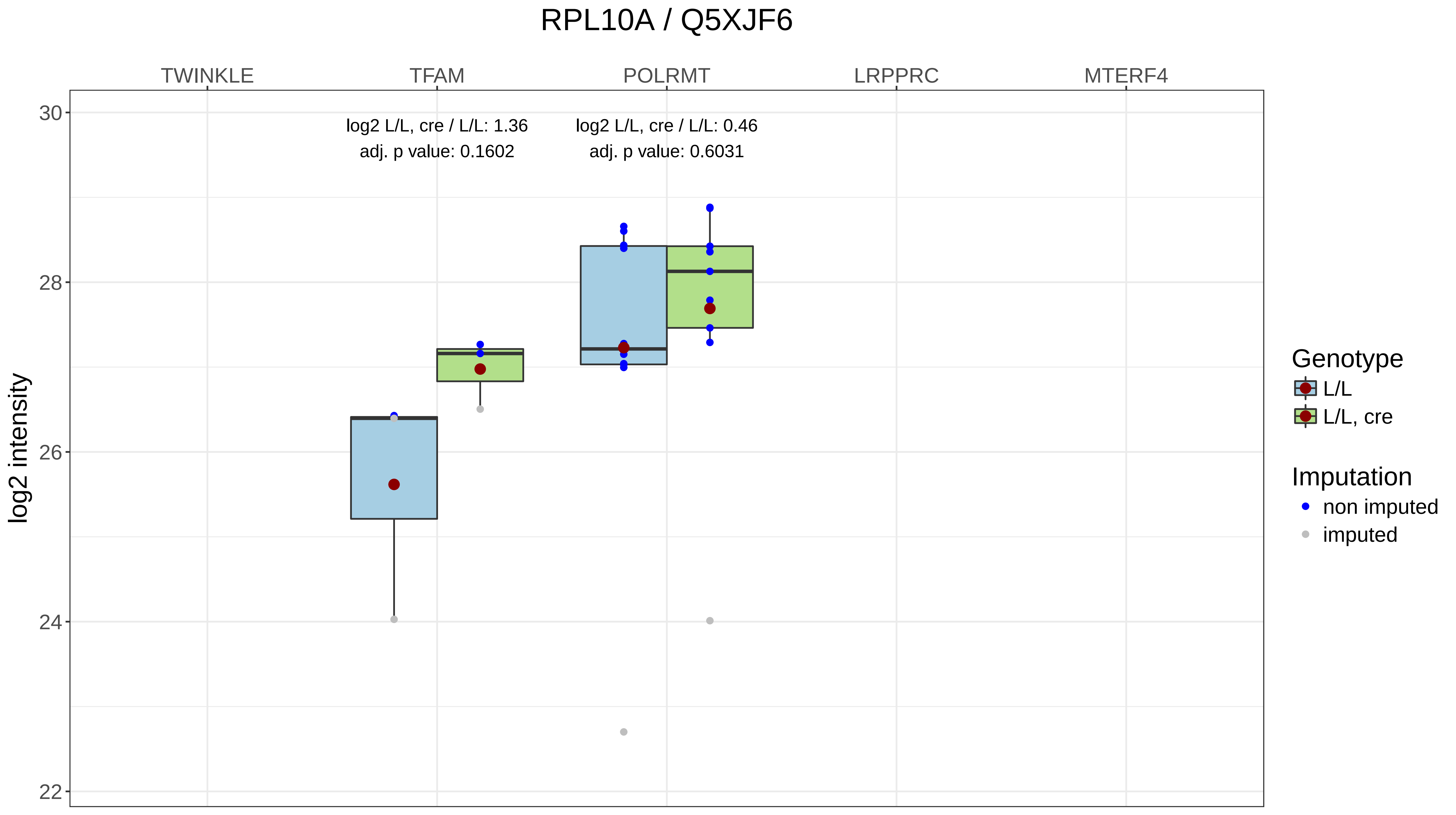
RPL10A / Q5XJF6
TWINKLE
TFAM
POLRMT
LRPPRC
MTERF4
log2 L/L, cre / L/L: 1.36
adj. p value: 0.1602
log2 L/L, cre / L/L: 0.46
adj. p value: 0.6031
30
28
26
24
22
log2 intensity
Genotype
L/L
L/L, cre
Imputation
non imputed
imputed

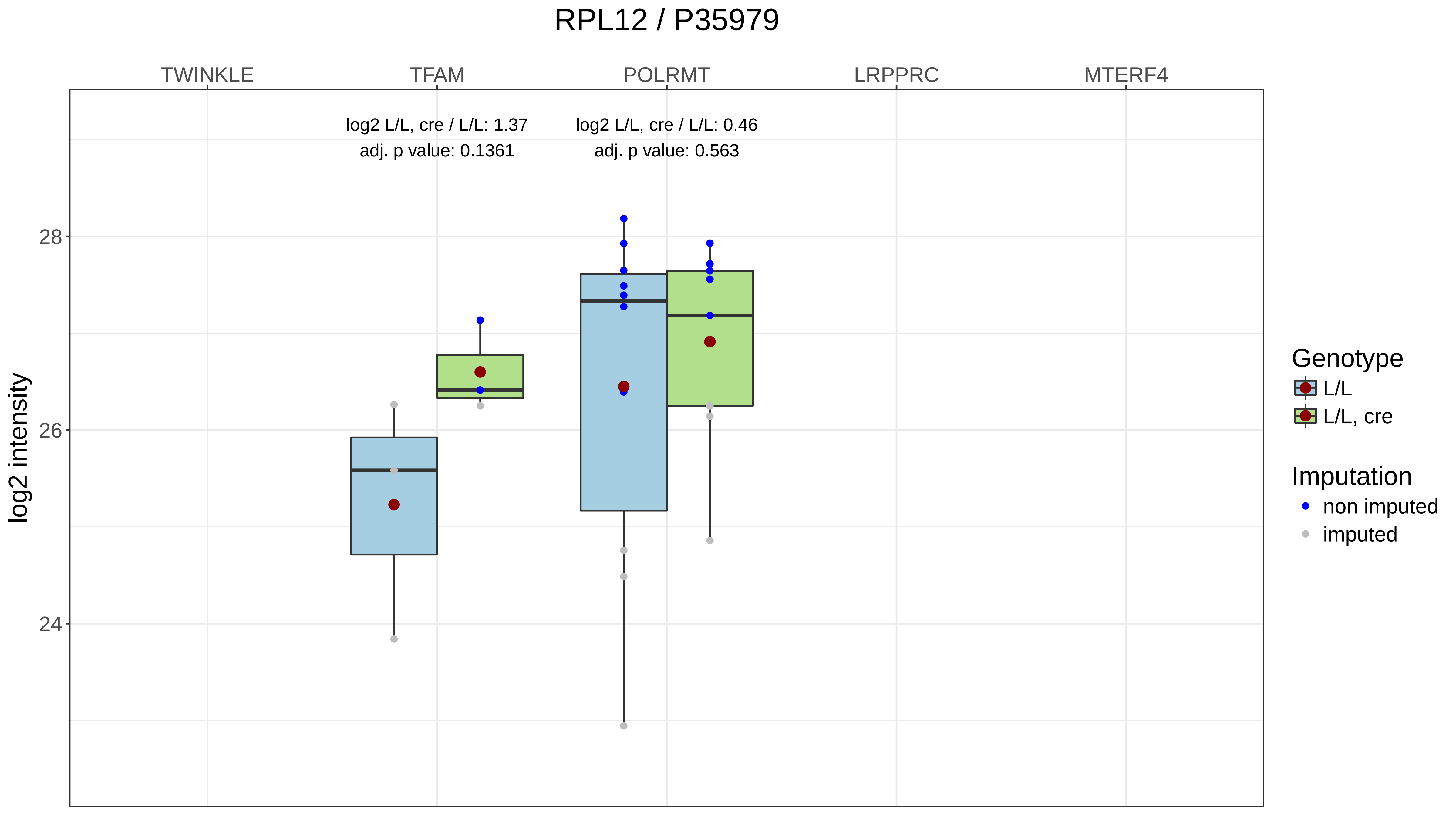
RPL12 / P35979
TWINKLE
TFAM
POLRMT
LRPPRC
MTERF4
log2 L/L, cre / L/L: 1.37
adj. p value: 0.1361
log2 L/L, cre / L/L: 0.46
adj. p value: 0.563
log2 intensity
28
26
24
Genotype
L/L
L/L, cre
Imputation
non imputed
imputed

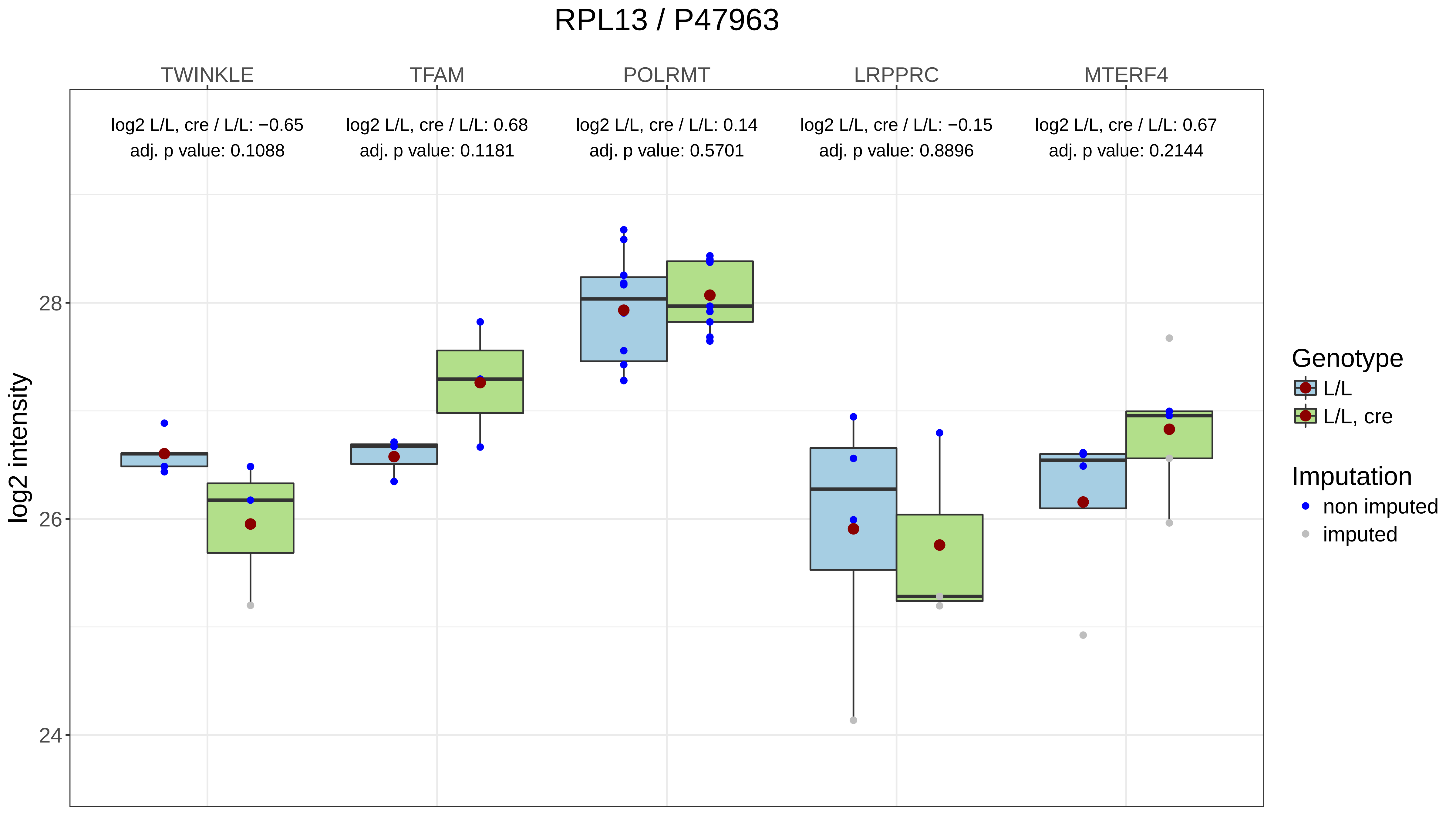
RPL13 / P47963
TWINKLE
TFAM
POLRMT
LRPPRC
MTERF4
log2 L/L, cre / L/L: −0.65
adj. p value: 0.1088
log2 L/L, cre / L/L: 0.68
adj. p value: 0.1181
log2 L/L, cre / L/L: 0.14
adj. p value: 0.5701
log2 L/L, cre / L/L: −0.15
adj. p value: 0.8896
log2 L/L, cre / L/L: 0.67
adj. p value: 0.2144
log2 intensity
28
26
24
Genotype
L/L
L/L, cre
Imputation
non imputed
imputed

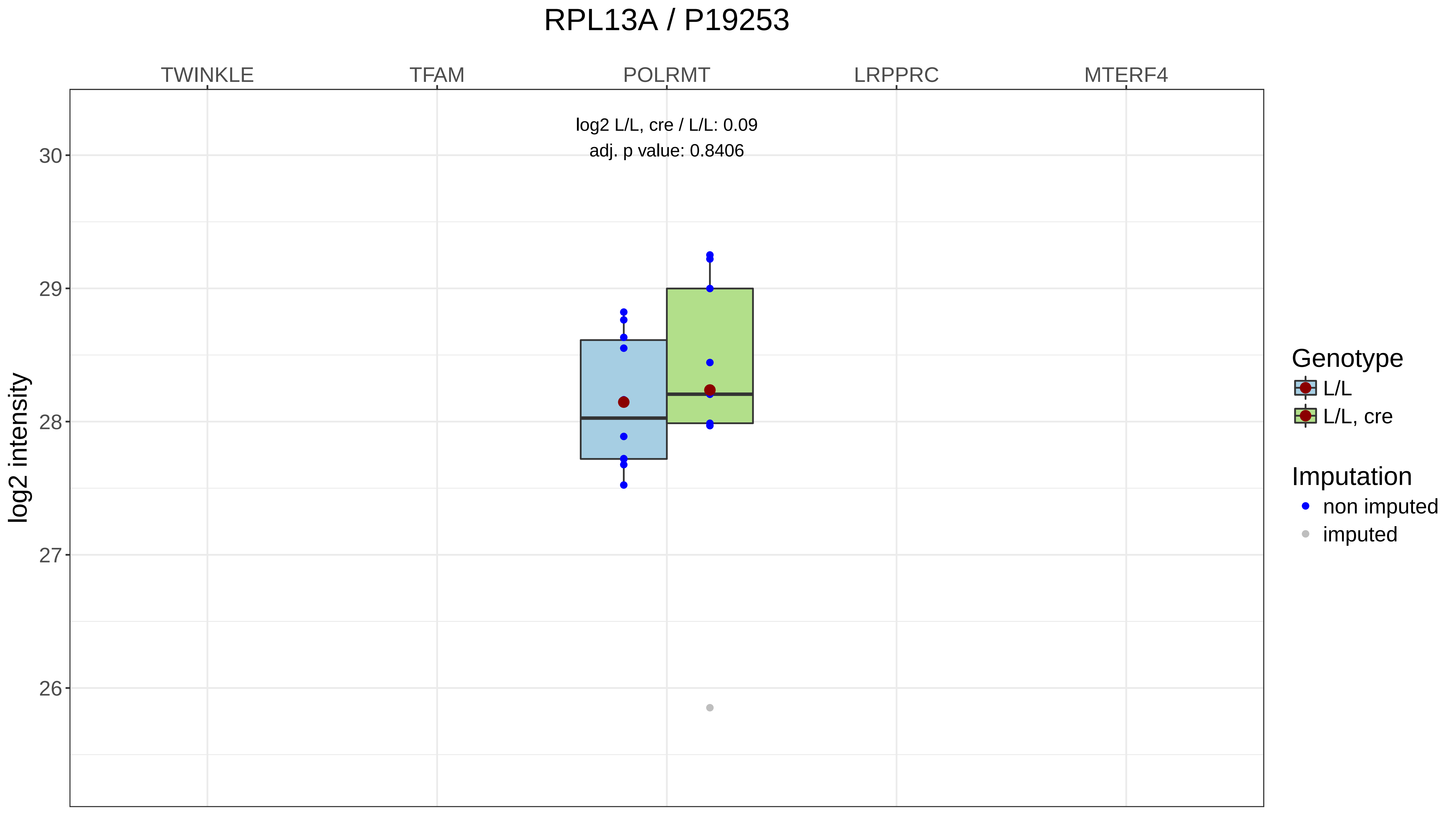
RPL13A / P19253
TWINKLE
TFAM
POLRMT
LRPPRC
MTERF4
log2 L/L, cre / L/L: 0.09
adj. p value: 0.8406
30
29
28
27
26
log2 intensity
Genotype
L/L
L/L, cre
Imputation
non imputed
imputed

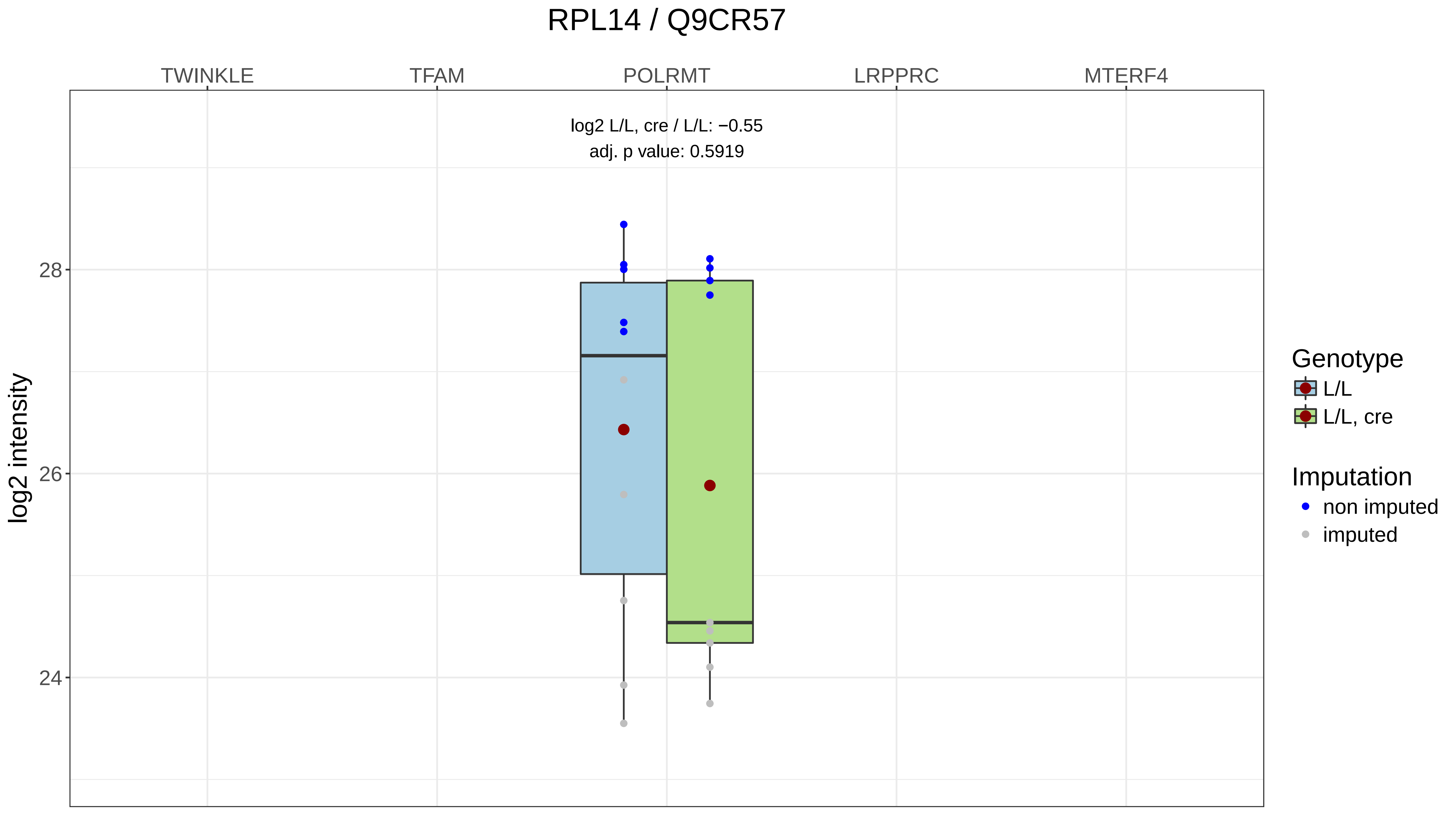
RPL14 / Q9CR57
TWINKLE
TFAM
POLRMT
LRPPRC
MTERF4
log2 L/L, cre / L/L: −0.55
adj. p value: 0.5919
28
26
24
log2 intensity
Genotype
L/L
L/L, cre
Imputation
non imputed
imputed

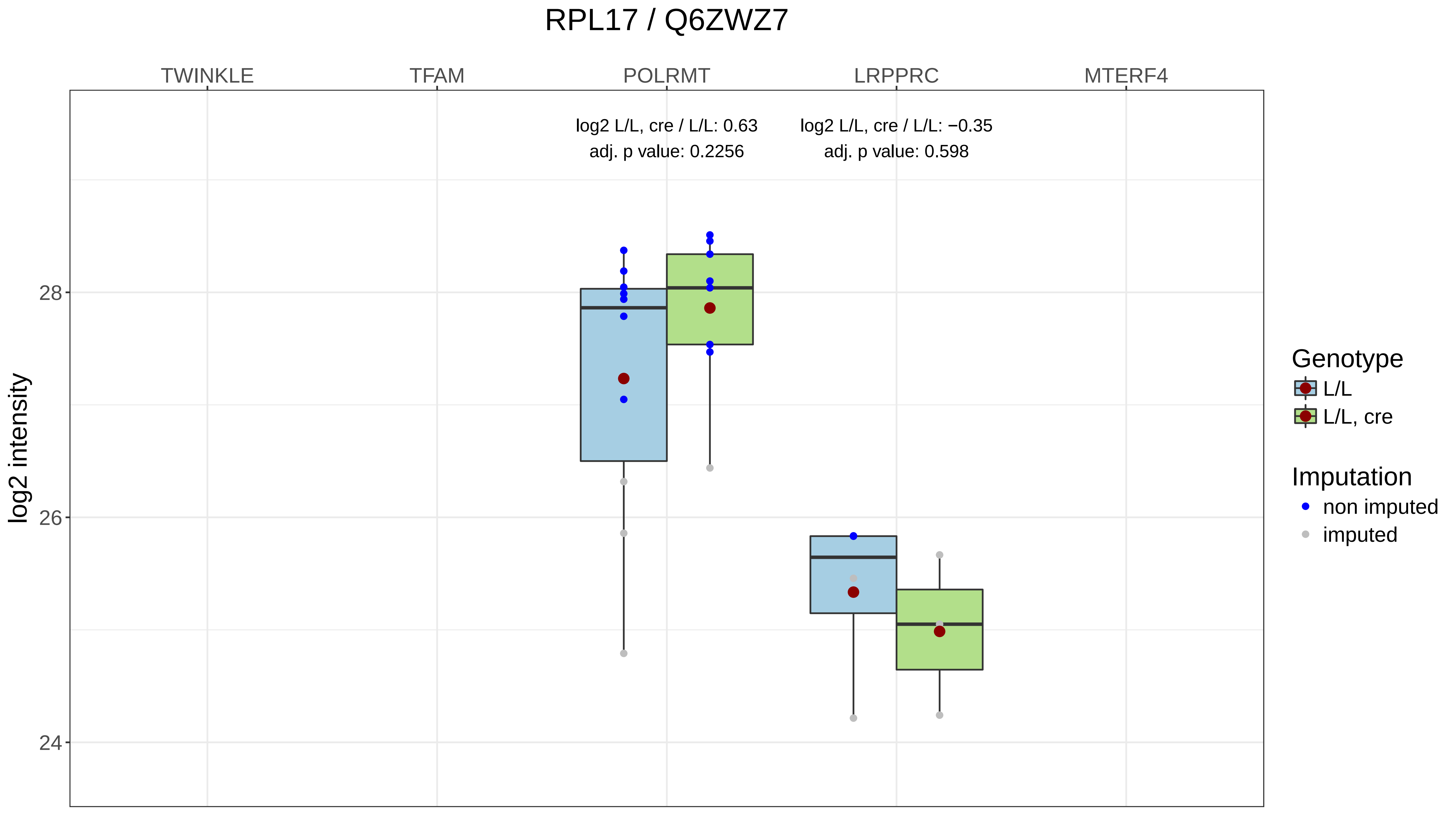
RPL17 / Q6ZWZ7
TWINKLE
TFAM
POLRMT
LRPPRC
MTERF4
log2 L/L, cre / L/L: 0.63
adj. p value: 0.2256
log2 L/L, cre / L/L: −0.35
adj. p value: 0.598
log2 intensity
28
26
24
Genotype
L/L
L/L, cre
Imputation
non imputed
imputed

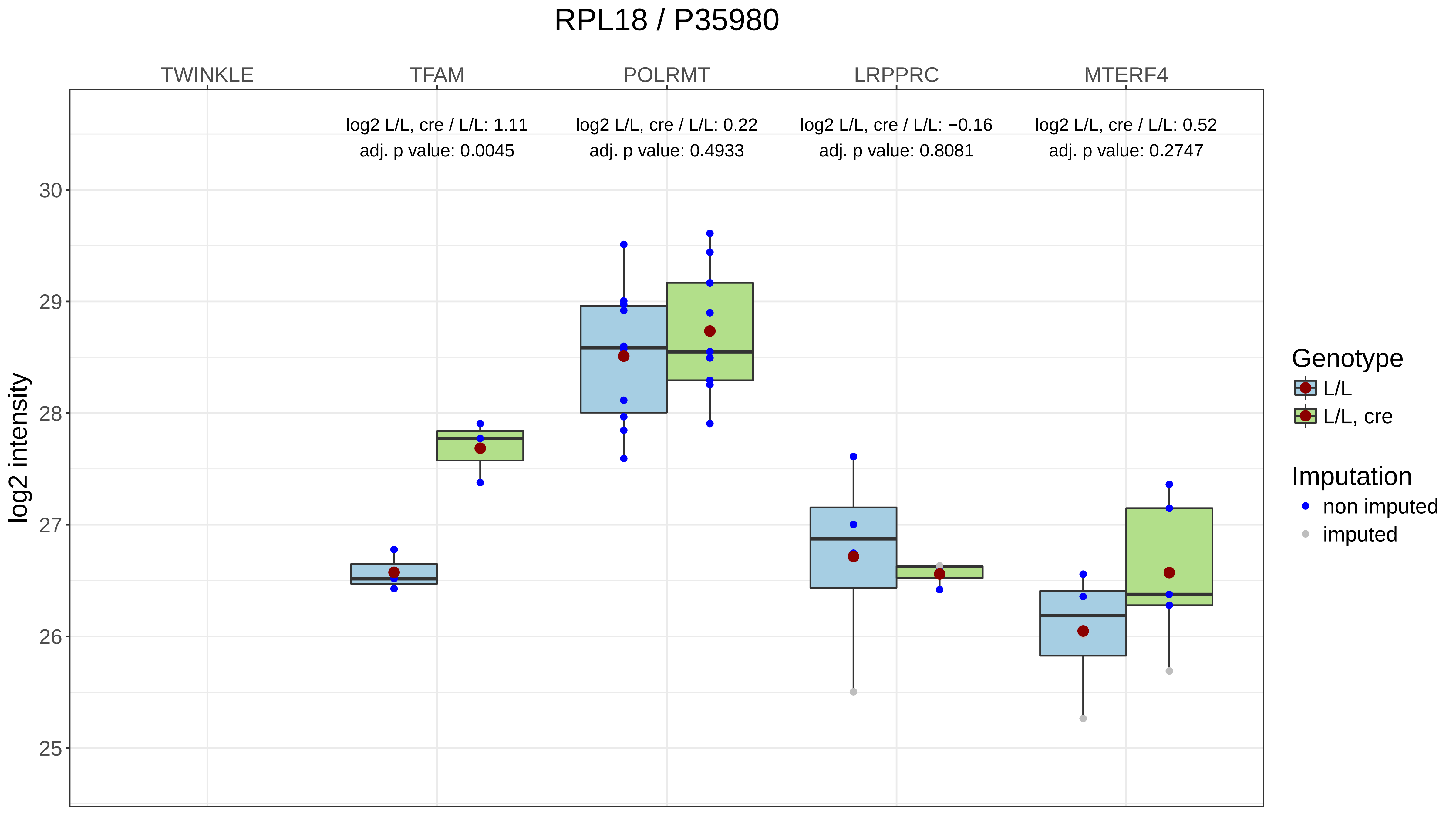
RPL18 / P35980
TWINKLE
TFAM
POLRMT
LRPPRC
MTERF4
log2 L/L, cre / L/L: 1.11
adj. p value: 0.0045
log2 L/L, cre / L/L: 0.22
adj. p value: 0.4933
log2 L/L, cre / L/L: −0.16
adj. p value: 0.8081
log2 L/L, cre / L/L: 0.52
adj. p value: 0.2747
log2 intensity
30
29
28
27
26
25
Genotype
L/L
L/L, cre
Imputation
non imputed
imputed

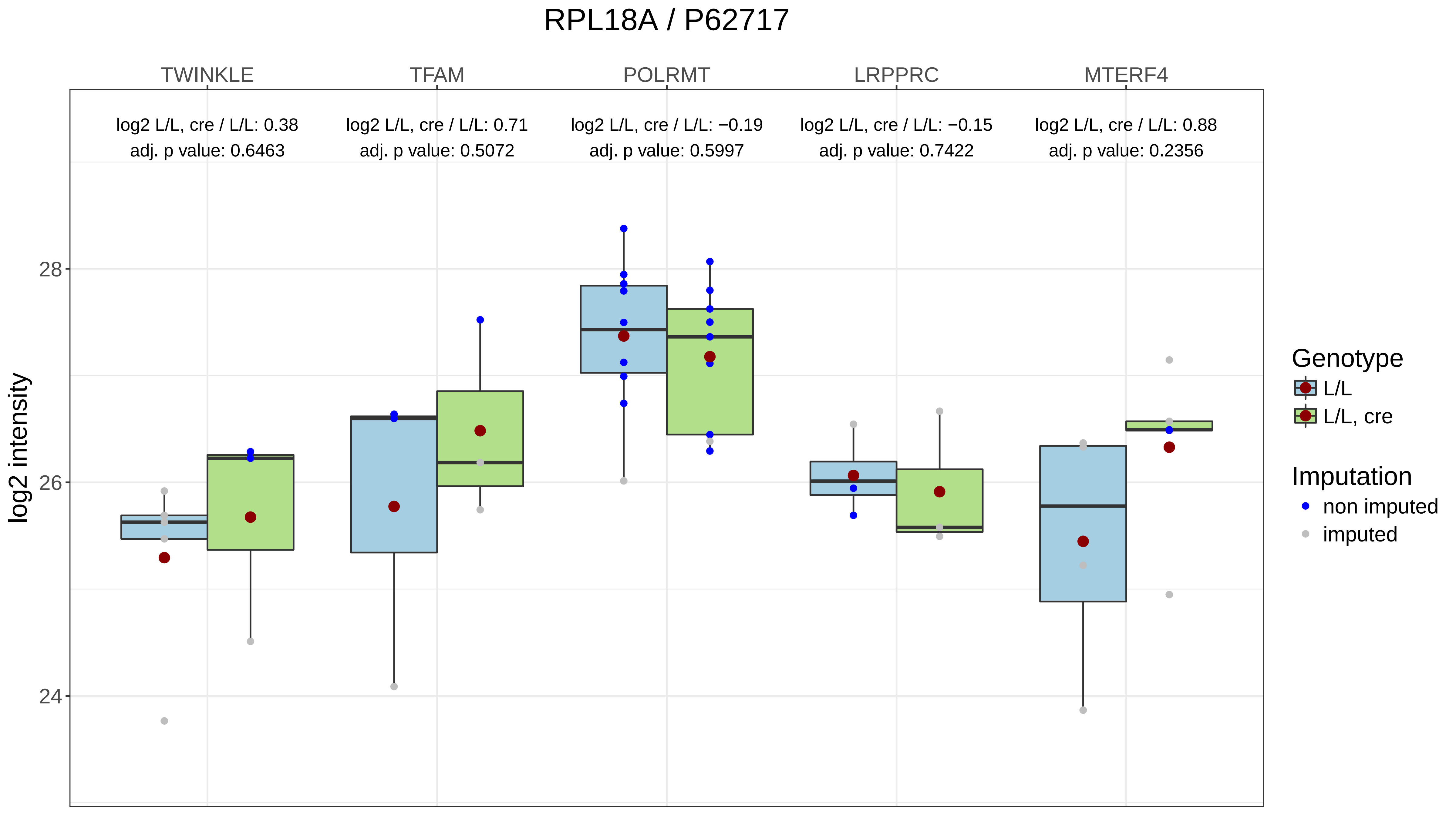

RPL18A / P62717
TWINKLE
TFAM
POLRMT
LRPPRC
MTERF4
log2 L/L, cre / L/L: 0.38
adj. p value: 0.6463
log2 L/L, cre / L/L: 0.71
adj. p value: 0.5072
log2 L/L, cre / L/L: −0.19
adj. p value: 0.5997
log2 L/L, cre / L/L: −0.15
adj. p value: 0.7422
log2 L/L, cre / L/L: 0.88
adj. p value: 0.2356
log2 intensity
28
26
24
Genotype
L/L
L/L, cre
Imputation
non imputed
imputed

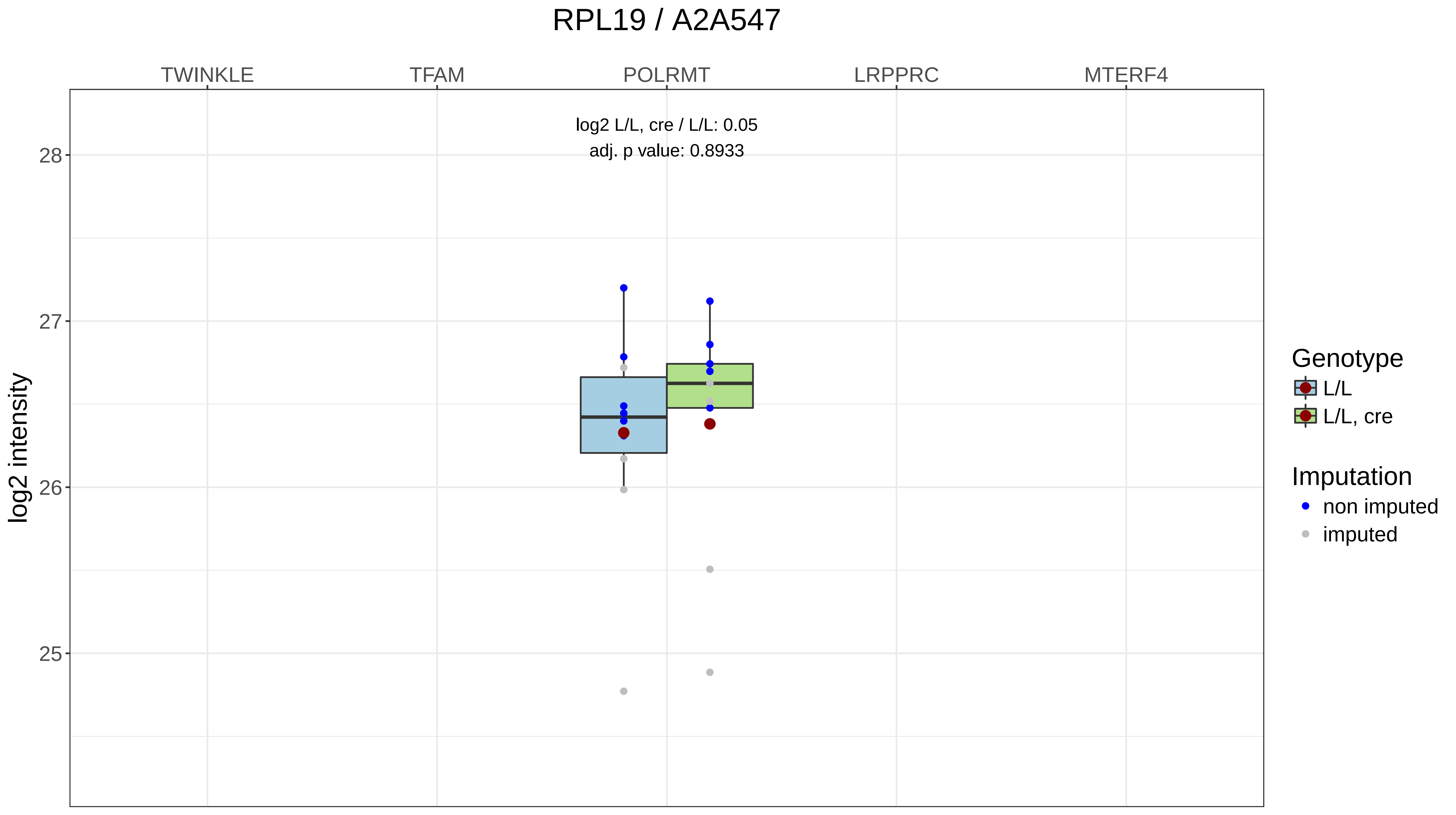
RPL19 / A2A547
TWINKLE
TFAM
POLRMT
LRPPRC
MTERF4
log2 L/L, cre / L/L: 0.05
adj. p value: 0.8933
28
27
26
25
log2 intensity
Genotype
L/L
L/L, cre
Imputation
non imputed
imputed

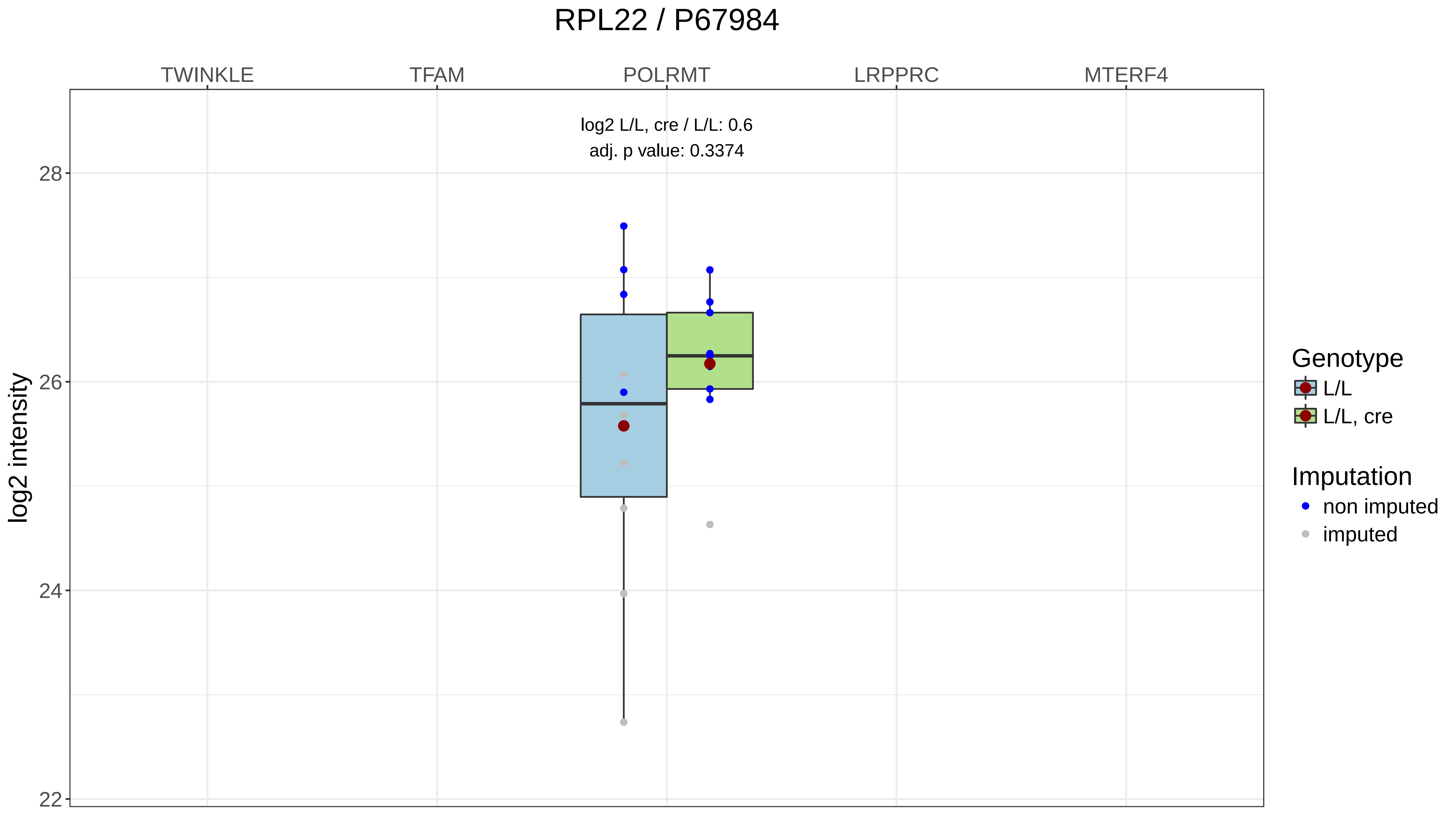
RPL22 / P67984
TWINKLE
TFAM
POLRMT
LRPPRC
MTERF4
log2 L/L, cre / L/L: 0.6
adj. p value: 0.3374
28
26
24
22
log2 intensity
Genotype
L/L
L/L, cre
Imputation
non imputed
imputed

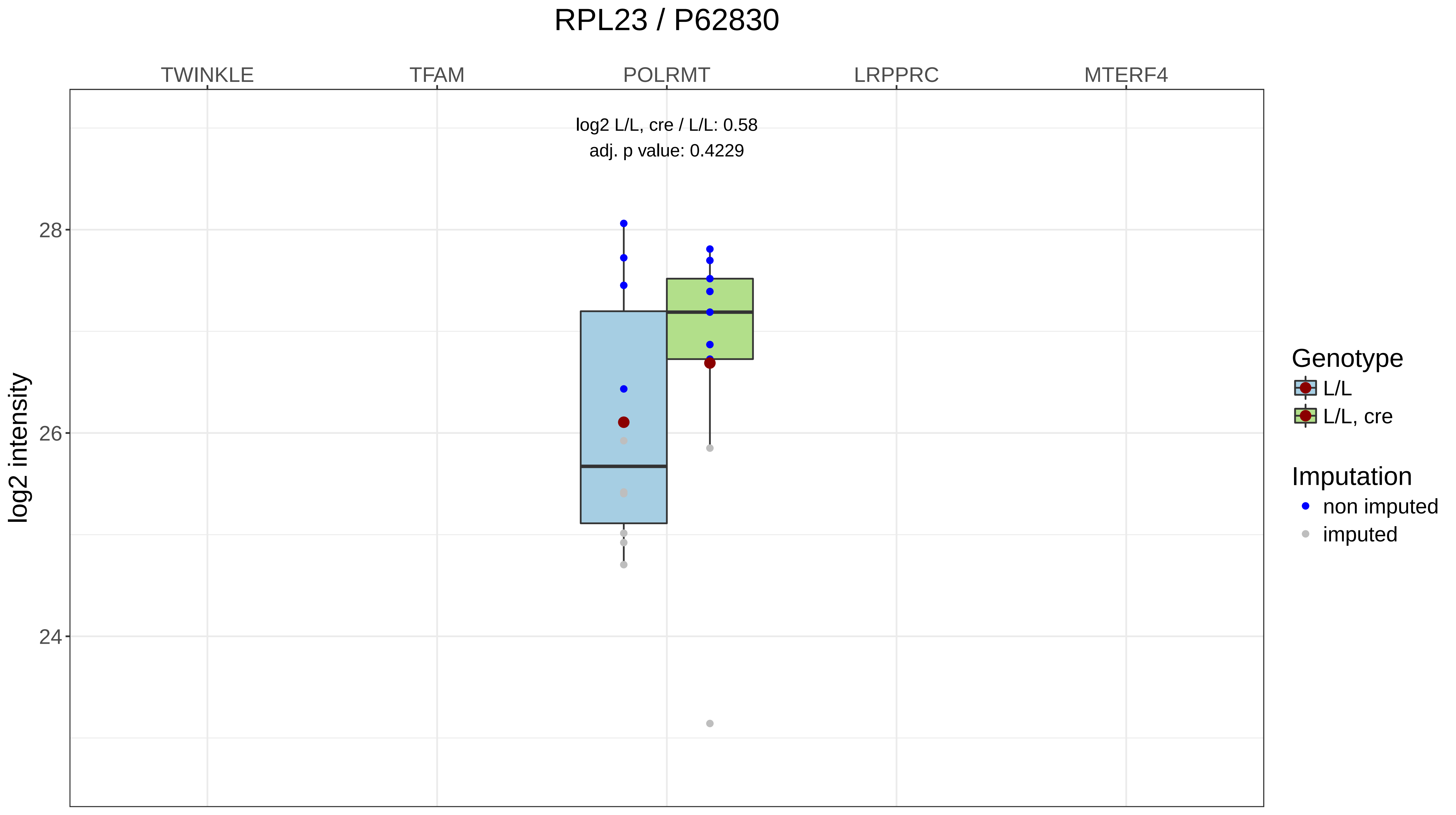
RPL23 / P62830
TWINKLE
TFAM
POLRMT
LRPPRC
MTERF4
log2 L/L, cre / L/L: 0.58
adj. p value: 0.4229
28
26
24
log2 intensity
Genotype
L/L
L/L, cre
Imputation
non imputed
imputed

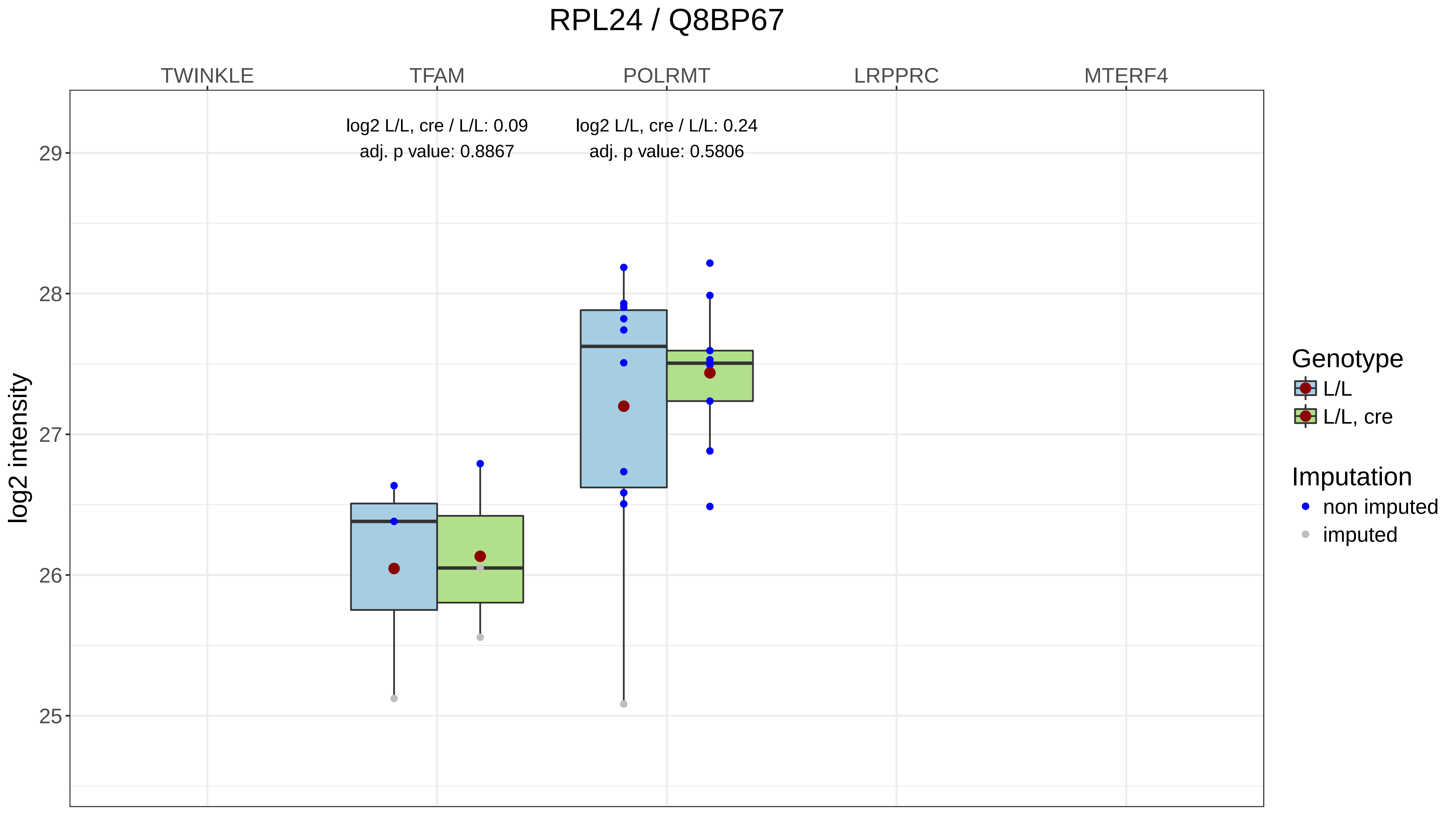
RPL24 / Q8BP67
TWINKLE
TFAM
POLRMT
LRPPRC
MTERF4
log2 L/L, cre / L/L: 0.09
adj. p value: 0.8867
log2 L/L, cre / L/L: 0.24
adj. p value: 0.5806
log2 intensity
29
28
27
26
25
Genotype
L/L
L/L, cre
Imputation
non imputed
imputed

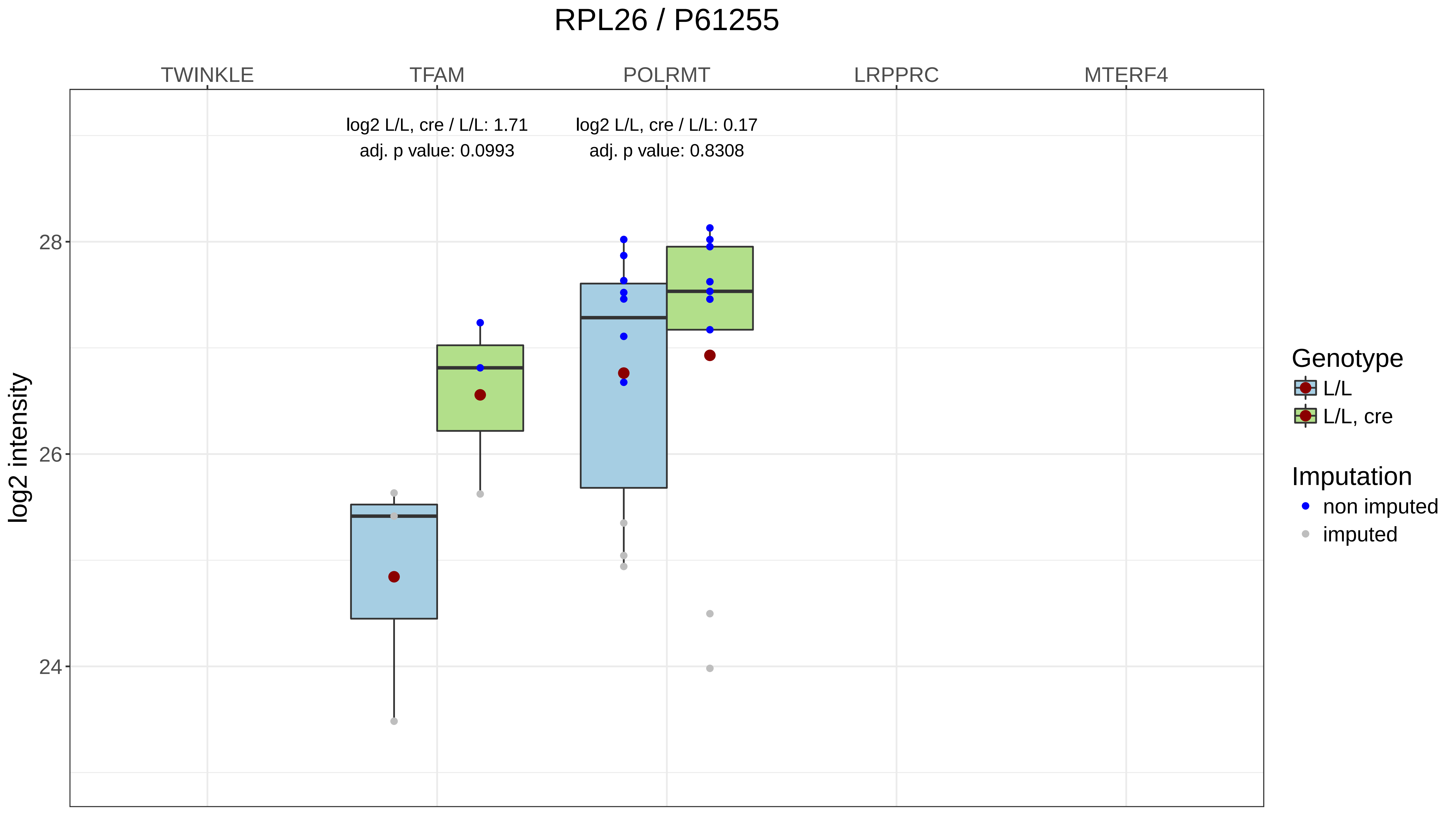

RPL26 / P61255
TWINKLE
TFAM
POLRMT
LRPPRC
MTERF4
log2 L/L, cre / L/L: 1.71
adj. p value: 0.0993
log2 L/L, cre / L/L: 0.17
adj. p value: 0.8308
log2 intensity
28
26
24
Genotype
L/L
L/L, cre
Imputation
non imputed
imputed

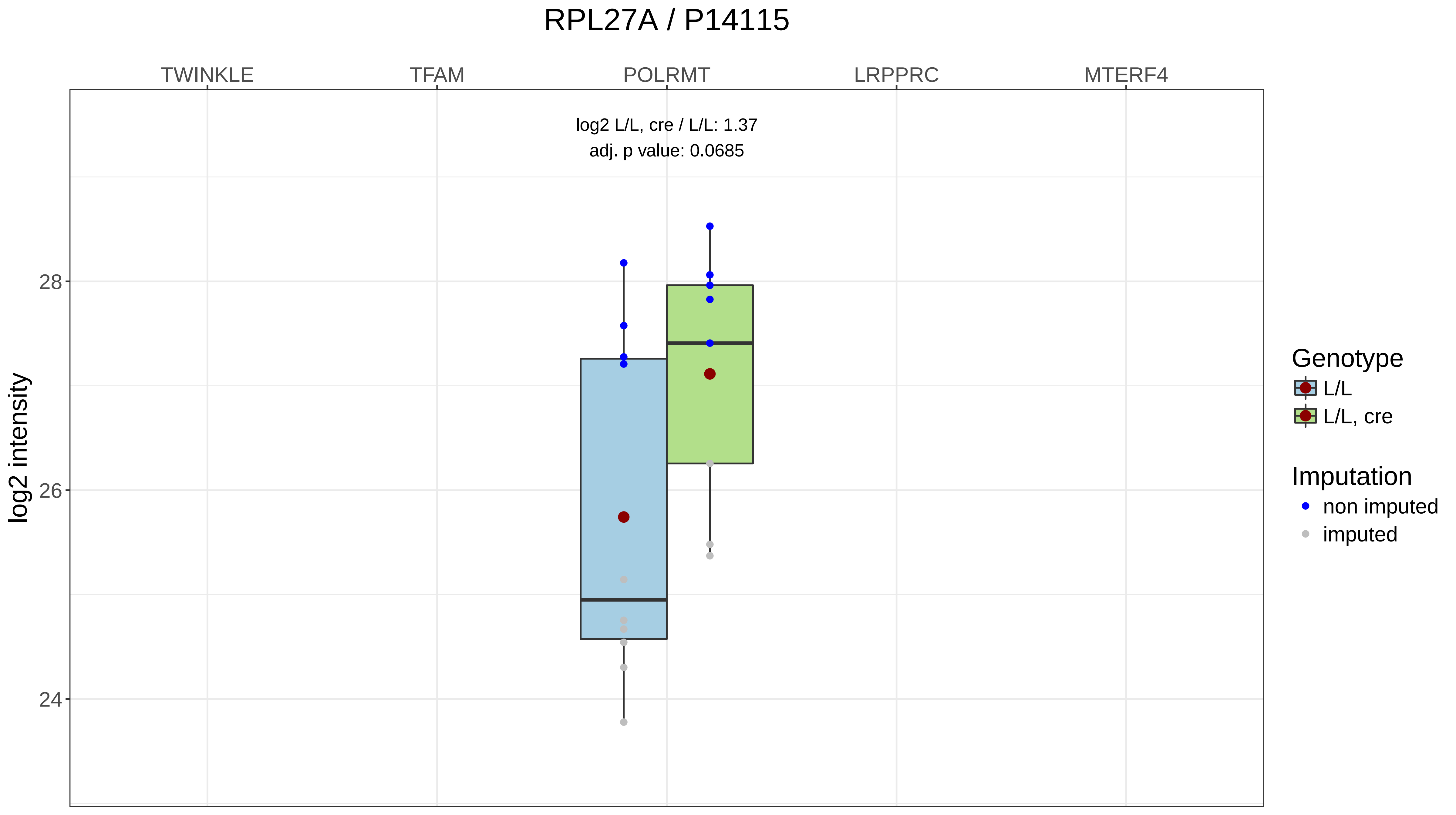
RPL27A / P14115
TWINKLE
TFAM
POLRMT
LRPPRC
MTERF4
log2 L/L, cre / L/L: 1.37
adj. p value: 0.0685
log2 intensity
28
26
24
Genotype
L/L
L/L, cre
Imputation
non imputed
imputed

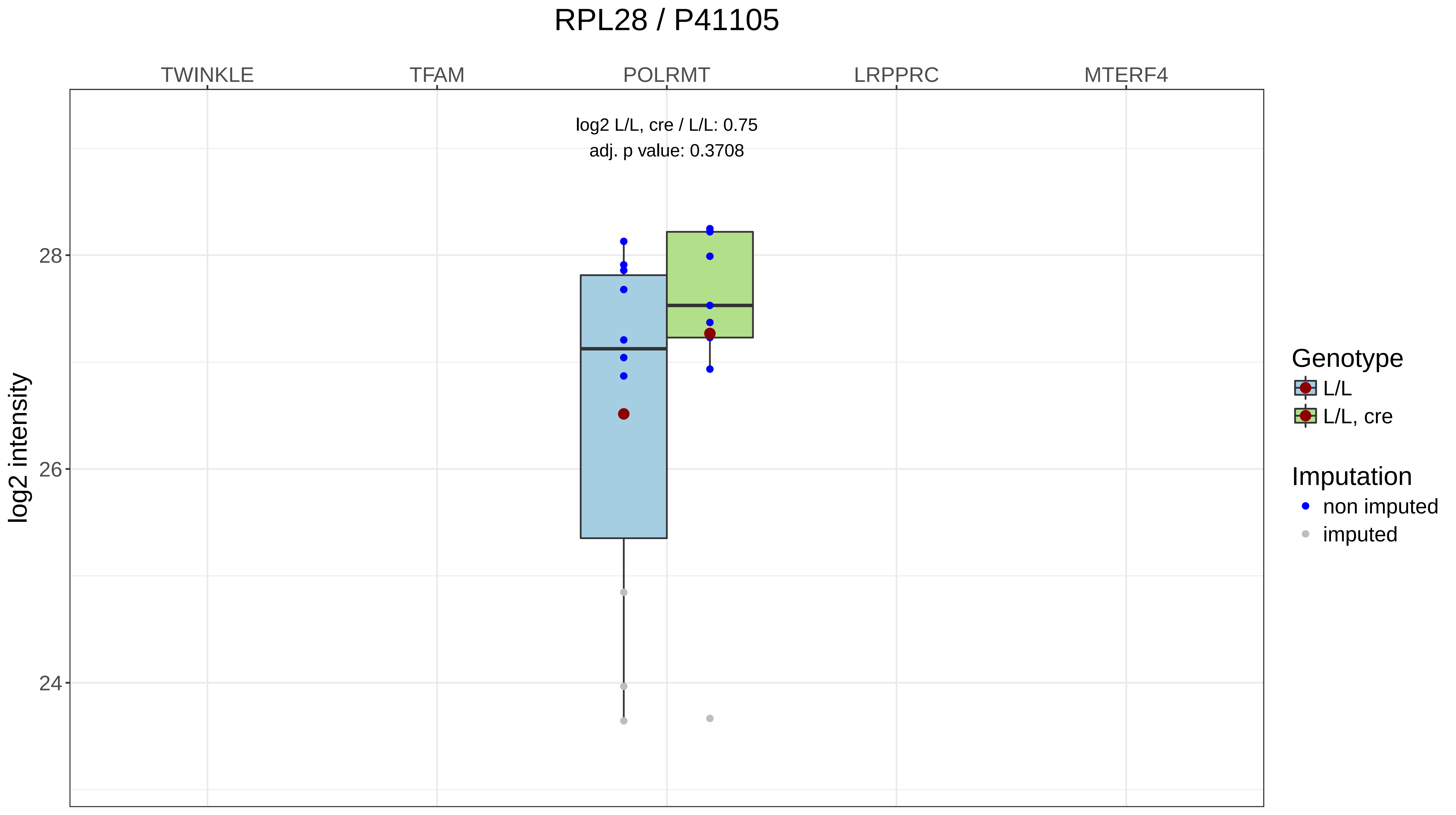
RPL28 / P41105
TWINKLE
TFAM
POLRMT
LRPPRC
MTERF4
log2 L/L, cre / L/L: 0.75
adj. p value: 0.3708
log2 intensity
28
26
24
Genotype
L/L
L/L, cre
Imputation
non imputed
imputed

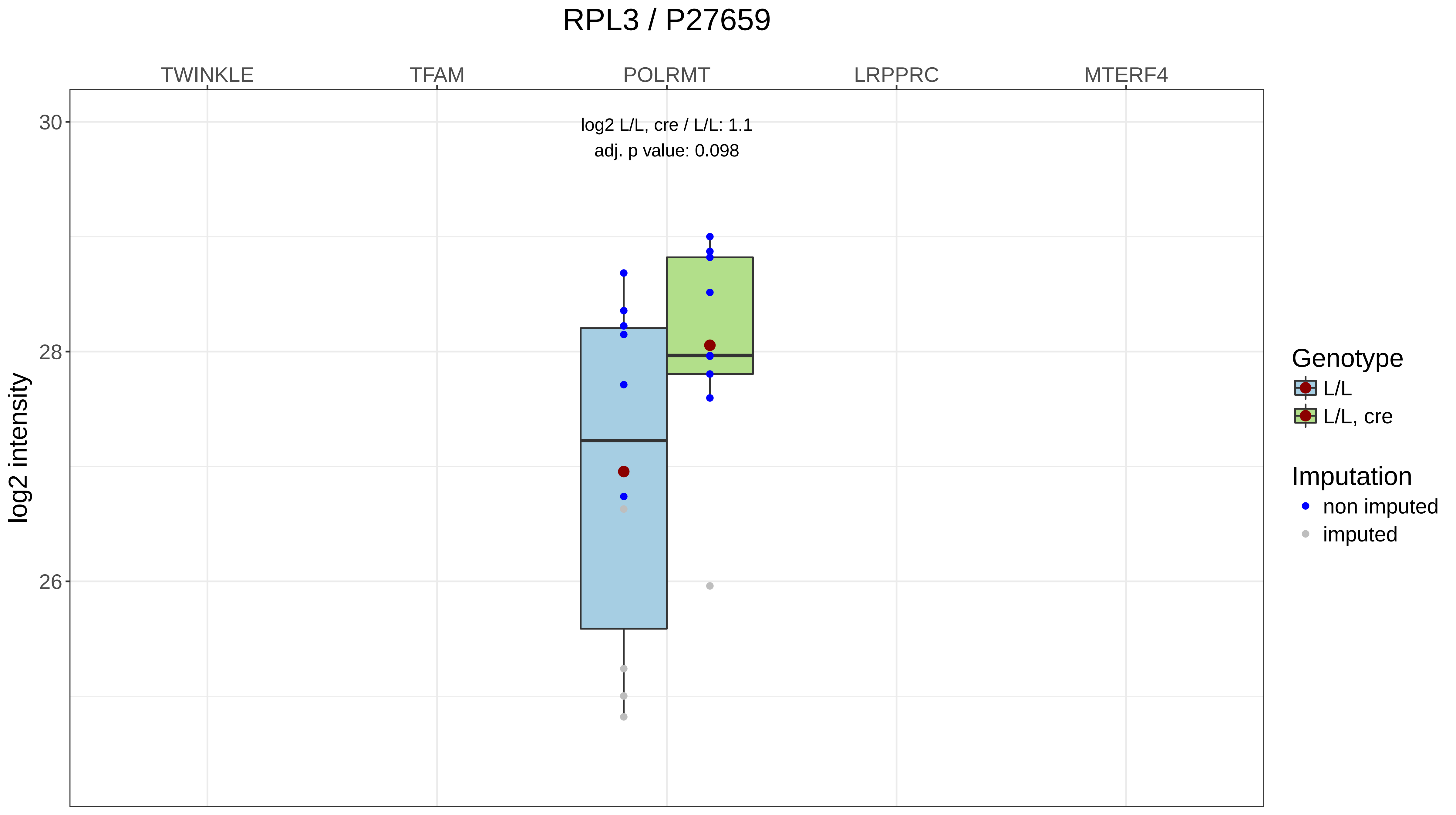

RPL3 / P27659
TWINKLE
TFAM
POLRMT
LRPPRC
MTERF4
log2 L/L, cre / L/L: 1.1
adj. p value: 0.098
30
28
26
log2 intensity
Genotype
L/L
L/L, cre
Imputation
non imputed
imputed

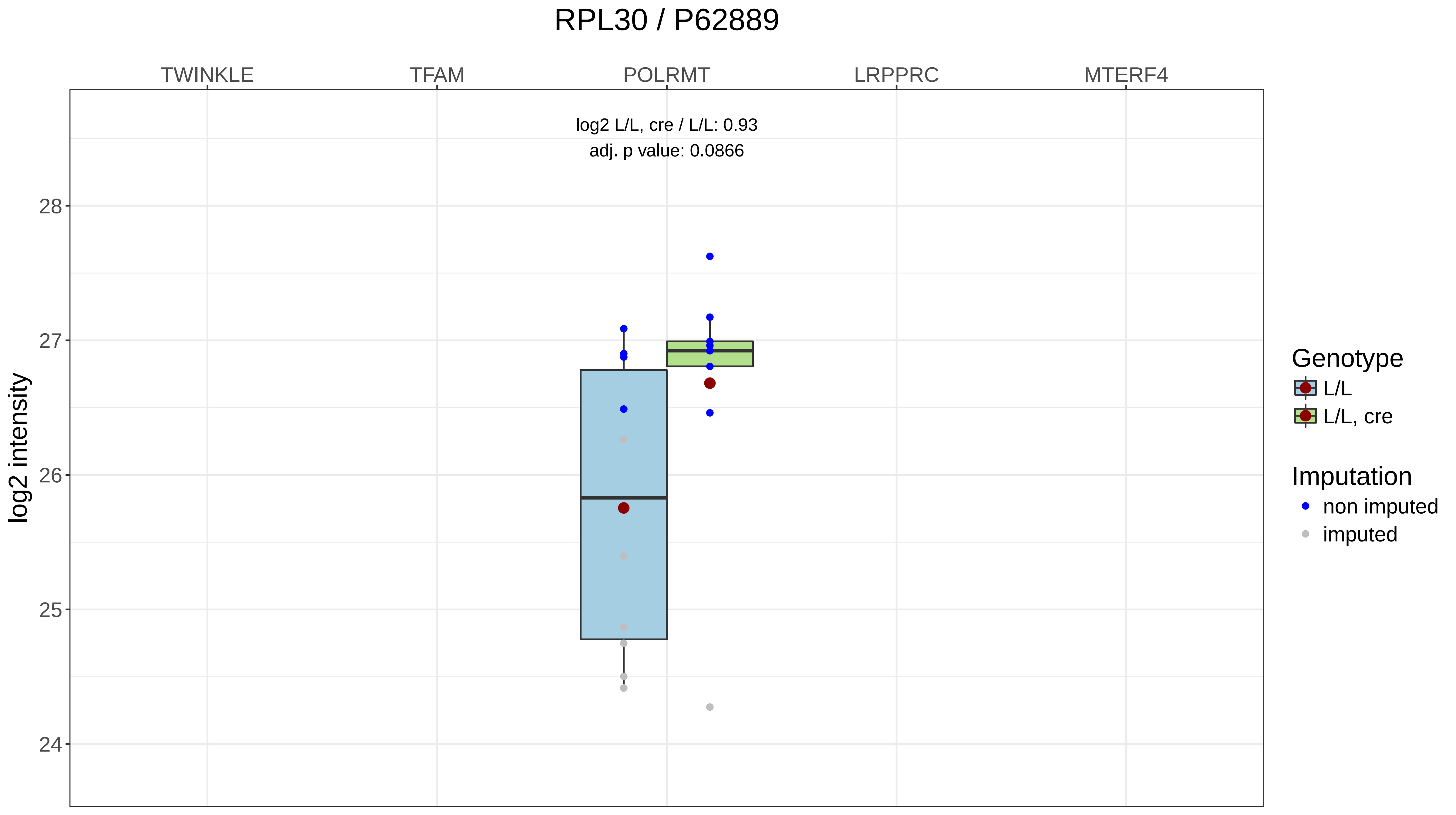
RPL30 / P62889
TWINKLE
TFAM
POLRMT
LRPPRC
MTERF4
log2 L/L, cre / L/L: 0.93
adj. p value: 0.0866
log2 intensity
28
27
26
25
24
Genotype
L/L
L/L, cre
Imputation
non imputed
imputed

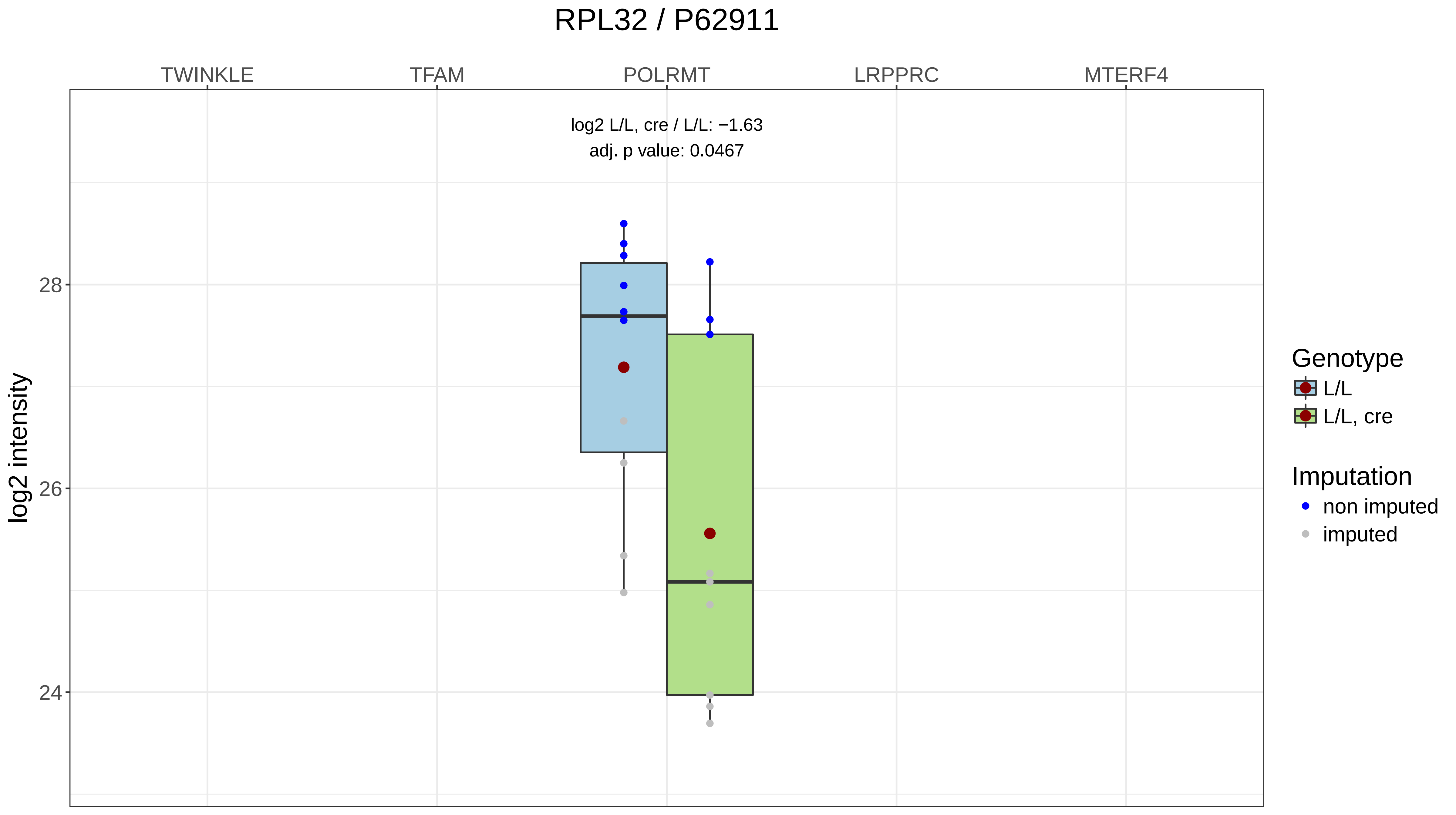
RPL32 / P62911
TWINKLE
TFAM
POLRMT
LRPPRC
MTERF4
log2 L/L, cre / L/L: −1.63
adj. p value: 0.0467
log2 intensity
28
26
24
Genotype
L/L
L/L, cre
Imputation
non imputed
imputed

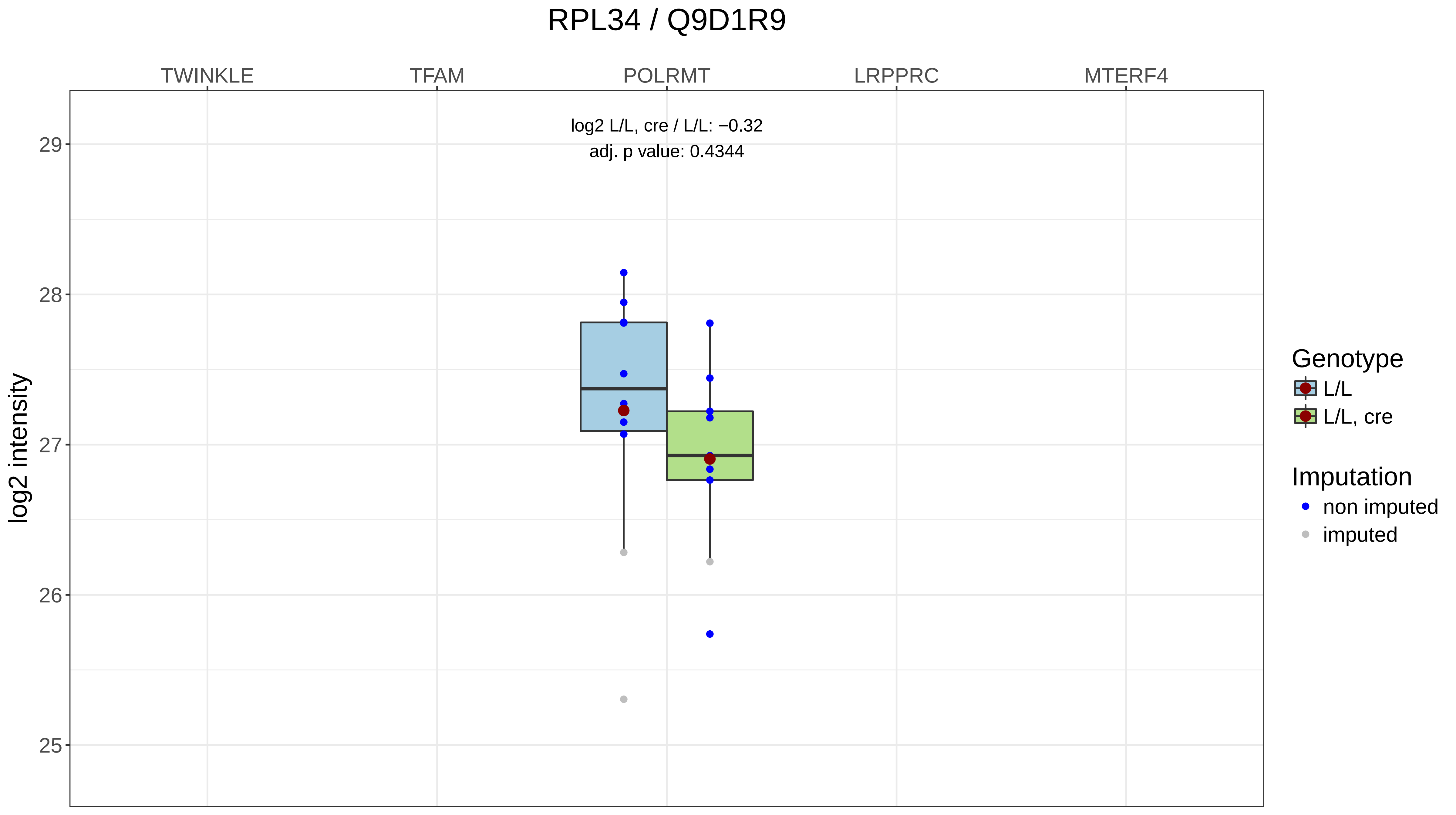
RPL34 / Q9D1R9
TWINKLE
TFAM
POLRMT
LRPPRC
MTERF4
log2 L/L, cre / L/L: −0.32
adj. p value: 0.4344
29
28
27
26
25
log2 intensity
Genotype
L/L
L/L, cre
Imputation
non imputed
imputed

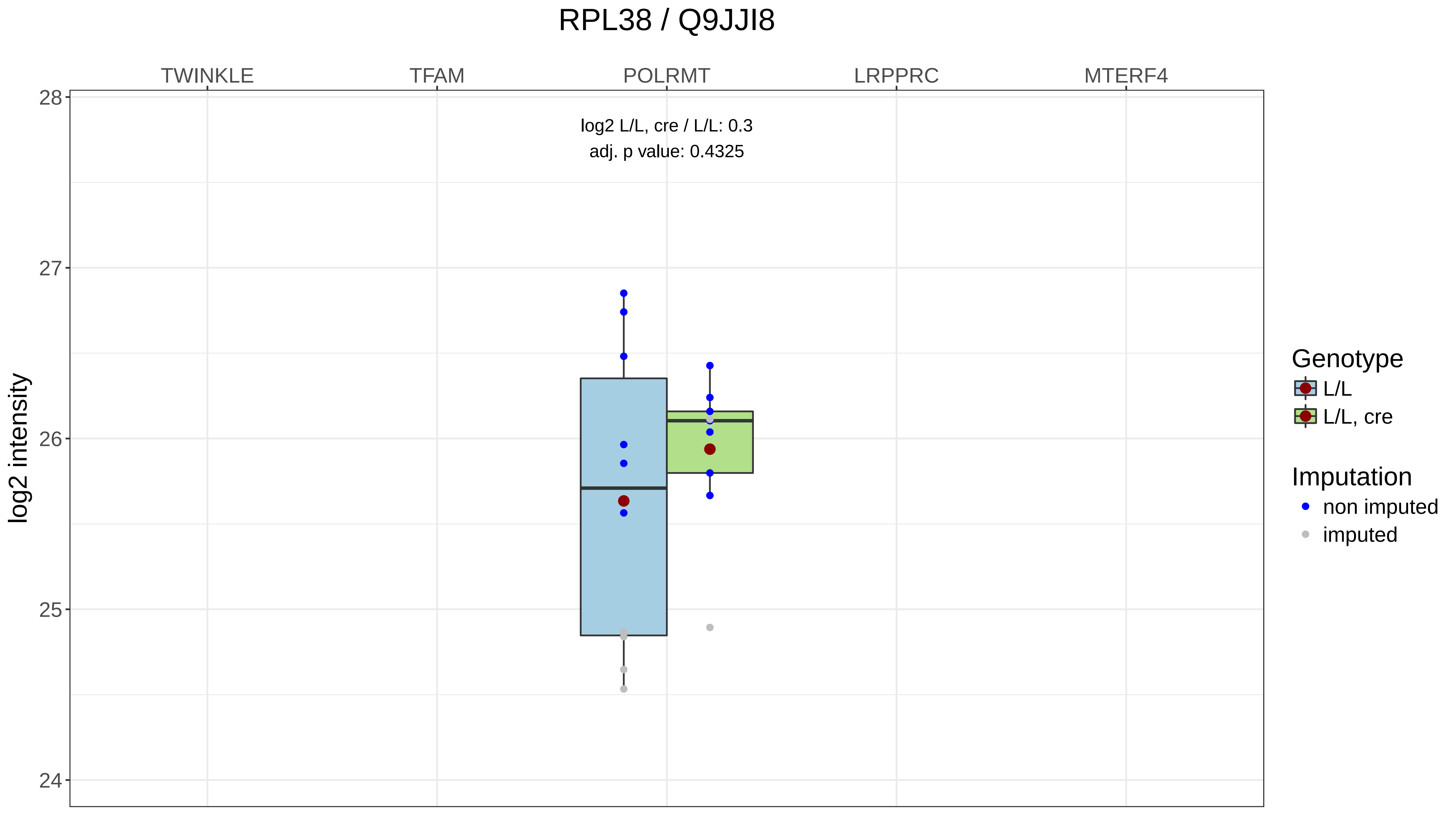
RPL38 / Q9JJI8
TWINKLE
TFAM
POLRMT
LRPPRC
MTERF4
log2 L/L, cre / L/L: 0.3
adj. p value: 0.4325
log2 intensity
28
27
26
25
24
Genotype
L/L
L/L, cre
Imputation
non imputed
imputed

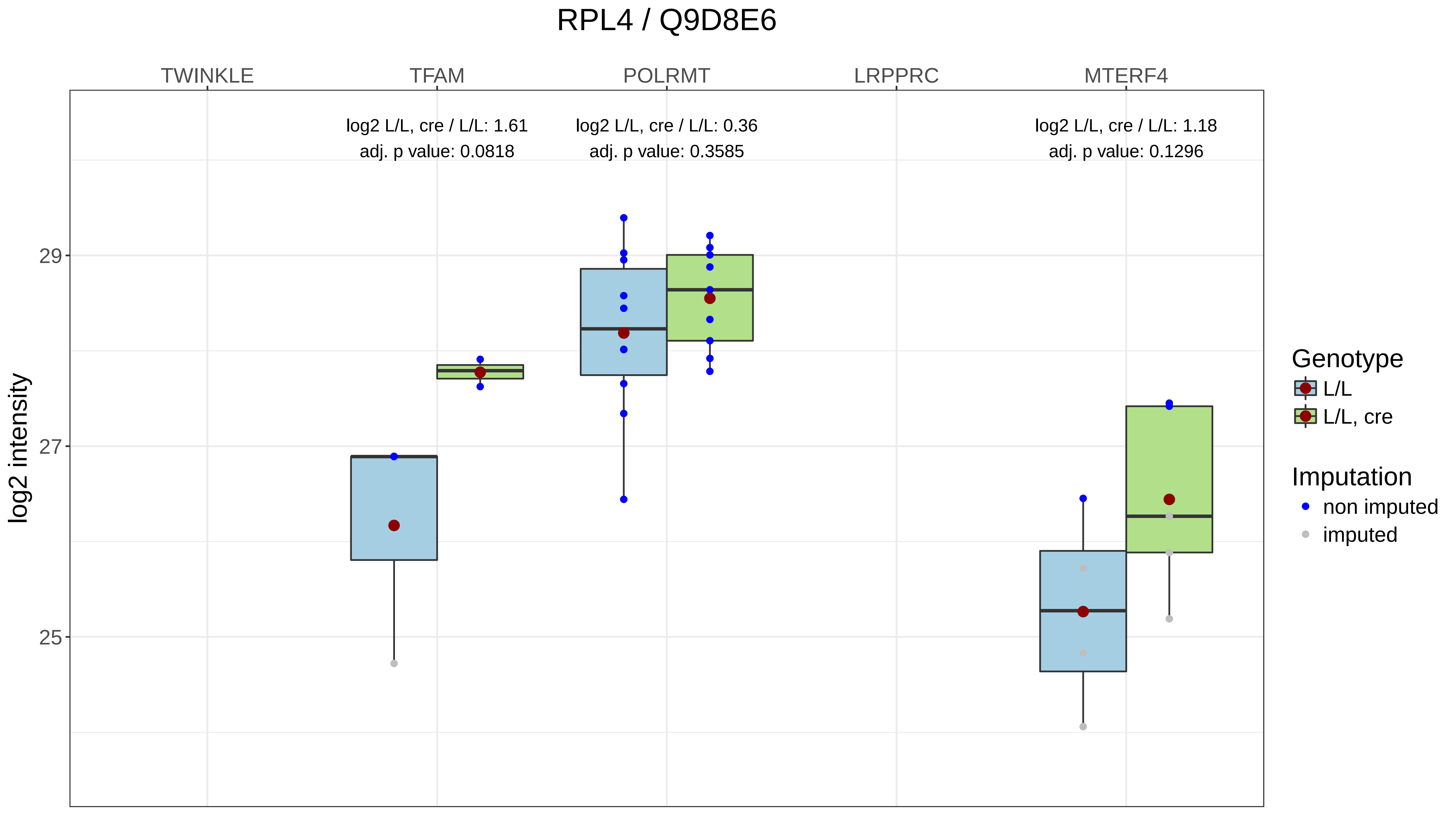
RPL4 / Q9D8E6
TWINKLE
TFAM
POLRMT
LRPPRC
MTERF4
log2 L/L, cre / L/L: 1.61
adj. p value: 0.0818
log2 L/L, cre / L/L: 0.36
adj. p value: 0.3585
log2 L/L, cre / L/L: 1.18
adj. p value: 0.1296
29
27
25
log2 intensity
Genotype
L/L
L/L, cre
Imputation
non imputed
imputed

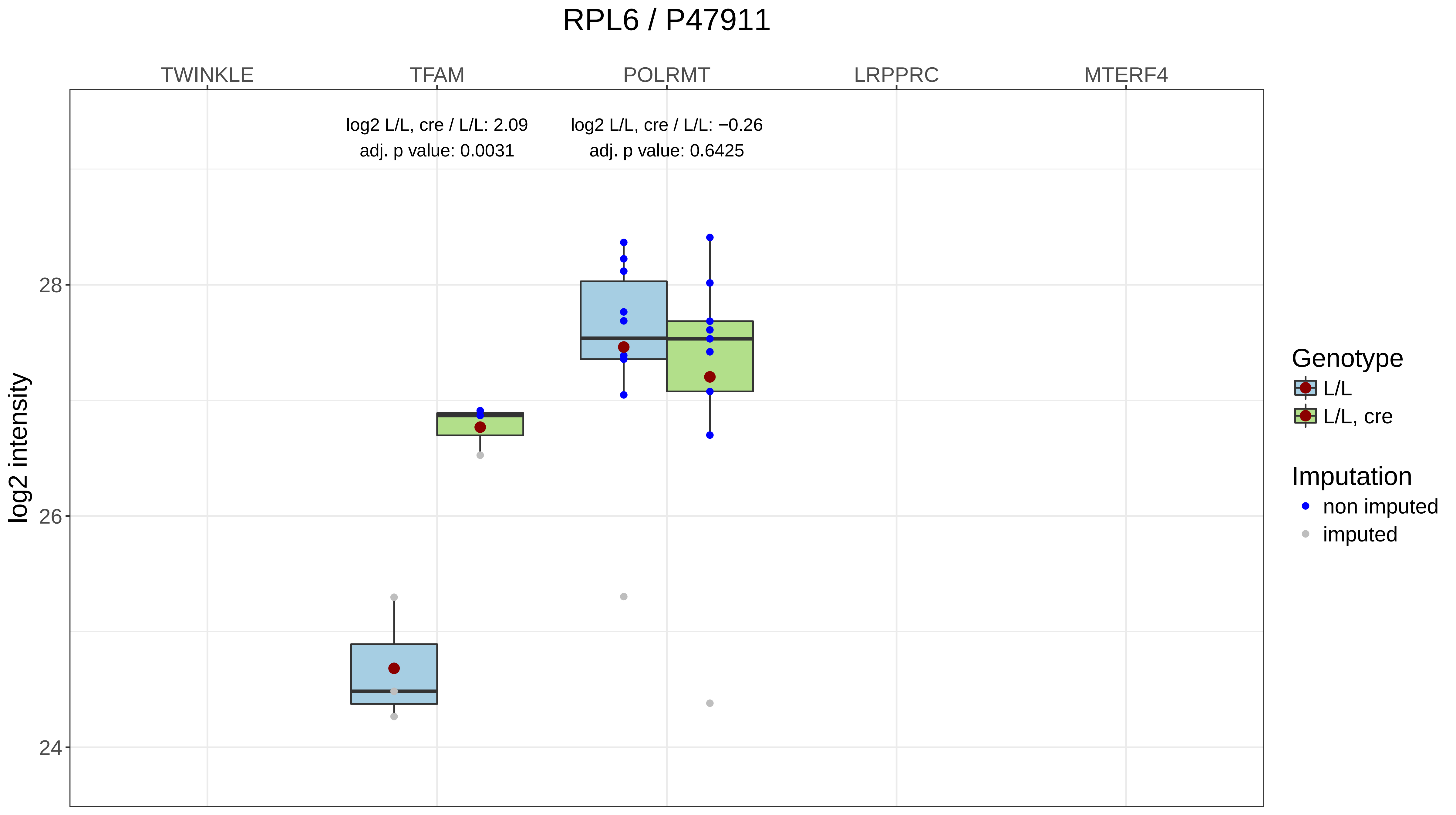
RPL6 / P47911
TWINKLE
TFAM
POLRMT
LRPPRC
MTERF4
log2 L/L, cre / L/L: 2.09
adj. p value: 0.0031
log2 L/L, cre / L/L: −0.26
adj. p value: 0.6425
log2 intensity
28
26
24
Genotype
L/L
L/L, cre
Imputation
non imputed
imputed

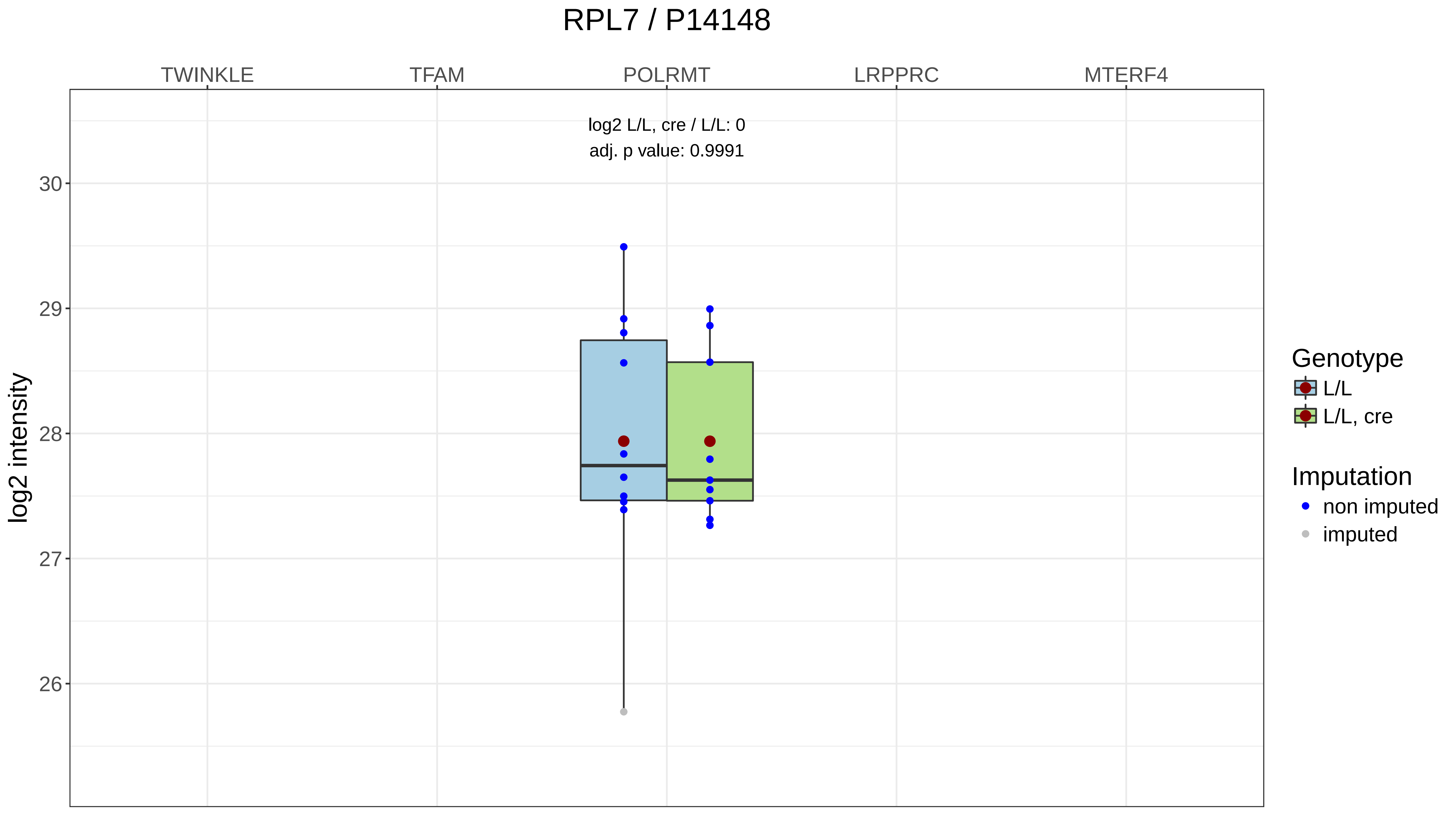

RPL7 / P14148
TWINKLE
TFAM
POLRMT
LRPPRC
MTERF4
log2 L/L, cre / L/L: 0
adj. p value: 0.9991
log2 intensity
30
29
28
27
26
Genotype
L/L
L/L, cre
Imputation
non imputed
imputed

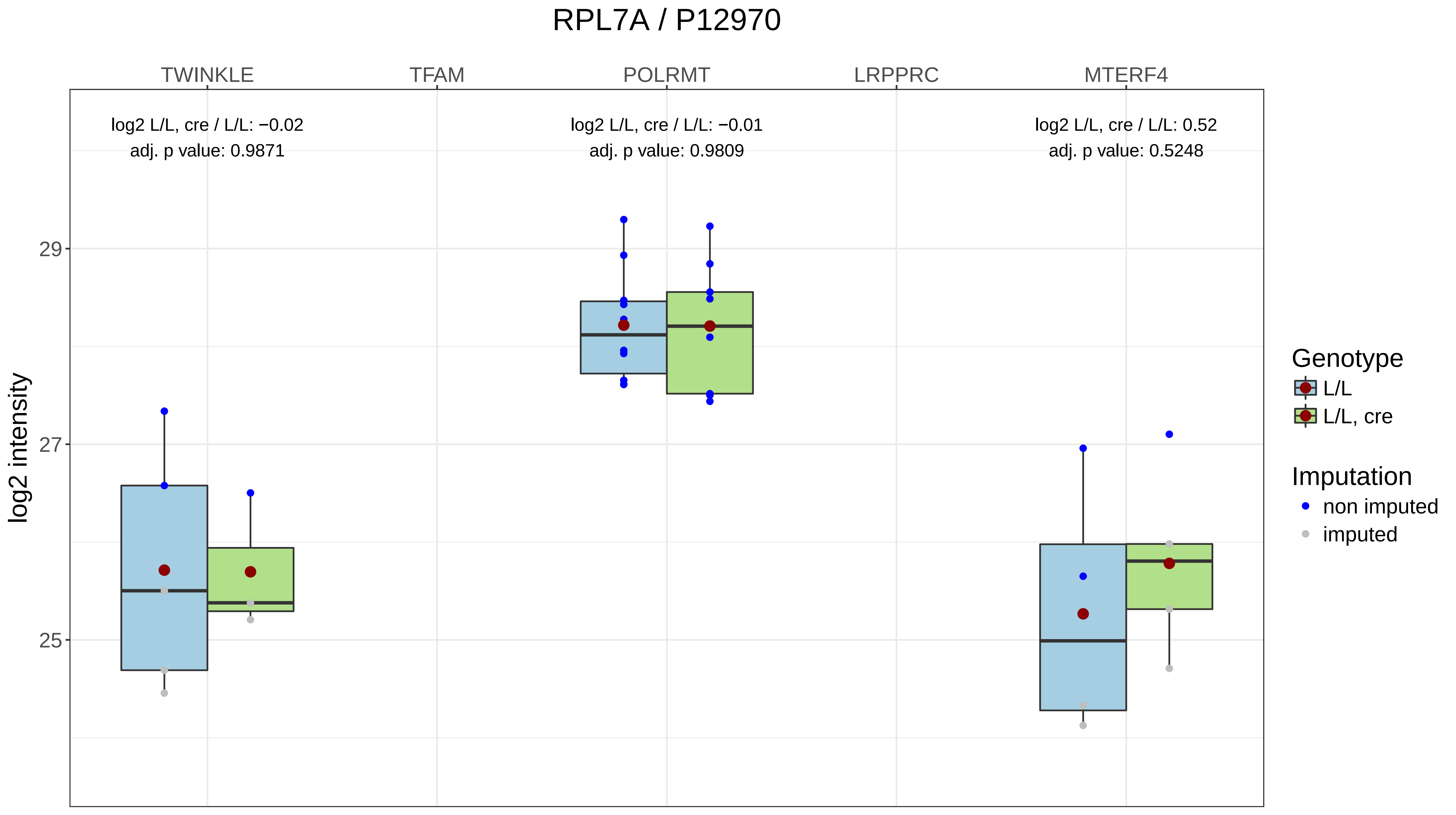
RPL7A / P12970
TWINKLE
TFAM
POLRMT
LRPPRC
MTERF4
log2 L/L, cre / L/L: −0.02
adj. p value: 0.9871
log2 L/L, cre / L/L: −0.01
adj. p value: 0.9809
log2 L/L, cre / L/L: 0.52
adj. p value: 0.5248
log2 intensity
29
27
25
Genotype
L/L
L/L, cre
Imputation
non imputed
imputed

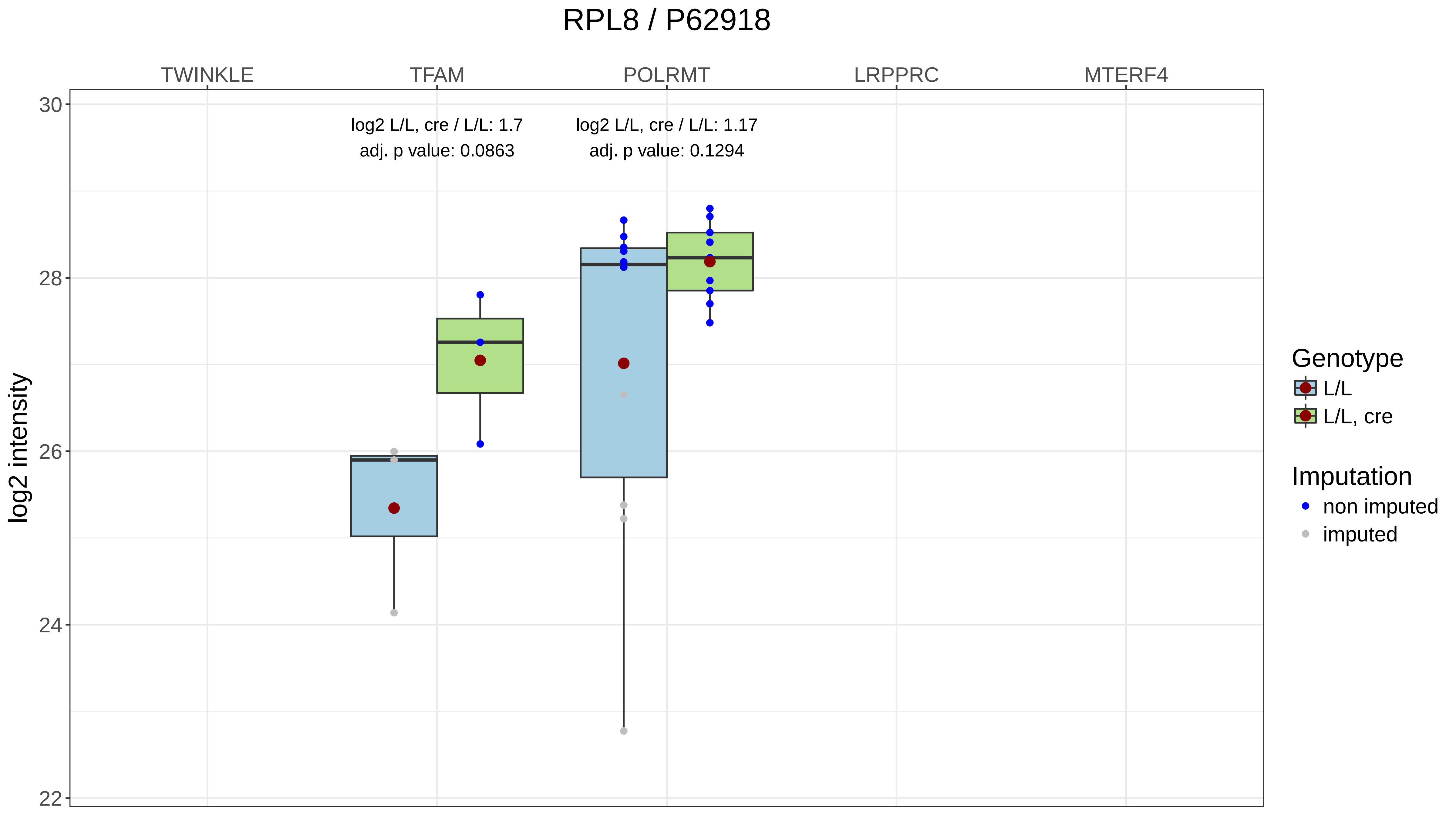

RPL8 / P62918
TWINKLE
TFAM
POLRMT
LRPPRC
MTERF4
log2 L/L, cre / L/L: 1.7
adj. p value: 0.0863
log2 L/L, cre / L/L: 1.17
adj. p value: 0.1294
log2 intensity
30
28
26
24
22
Genotype
L/L
L/L, cre
Imputation
non imputed
imputed

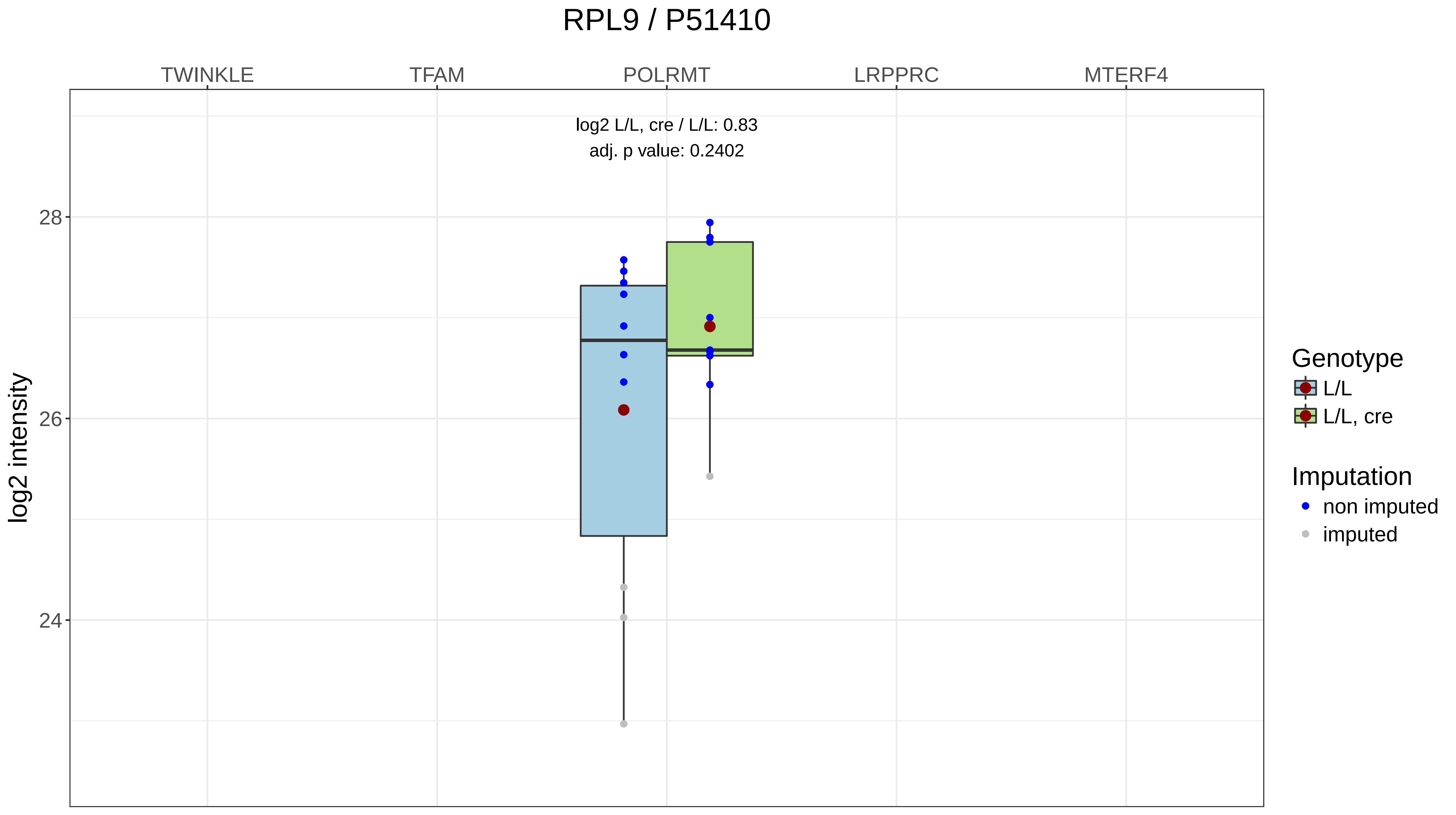
RPL9 / P51410
TWINKLE
TFAM
POLRMT
LRPPRC
MTERF4
log2 L/L, cre / L/L: 0.83
adj. p value: 0.2402
log2 intensity
28
26
24
Genotype
L/L
L/L, cre
Imputation
non imputed
imputed

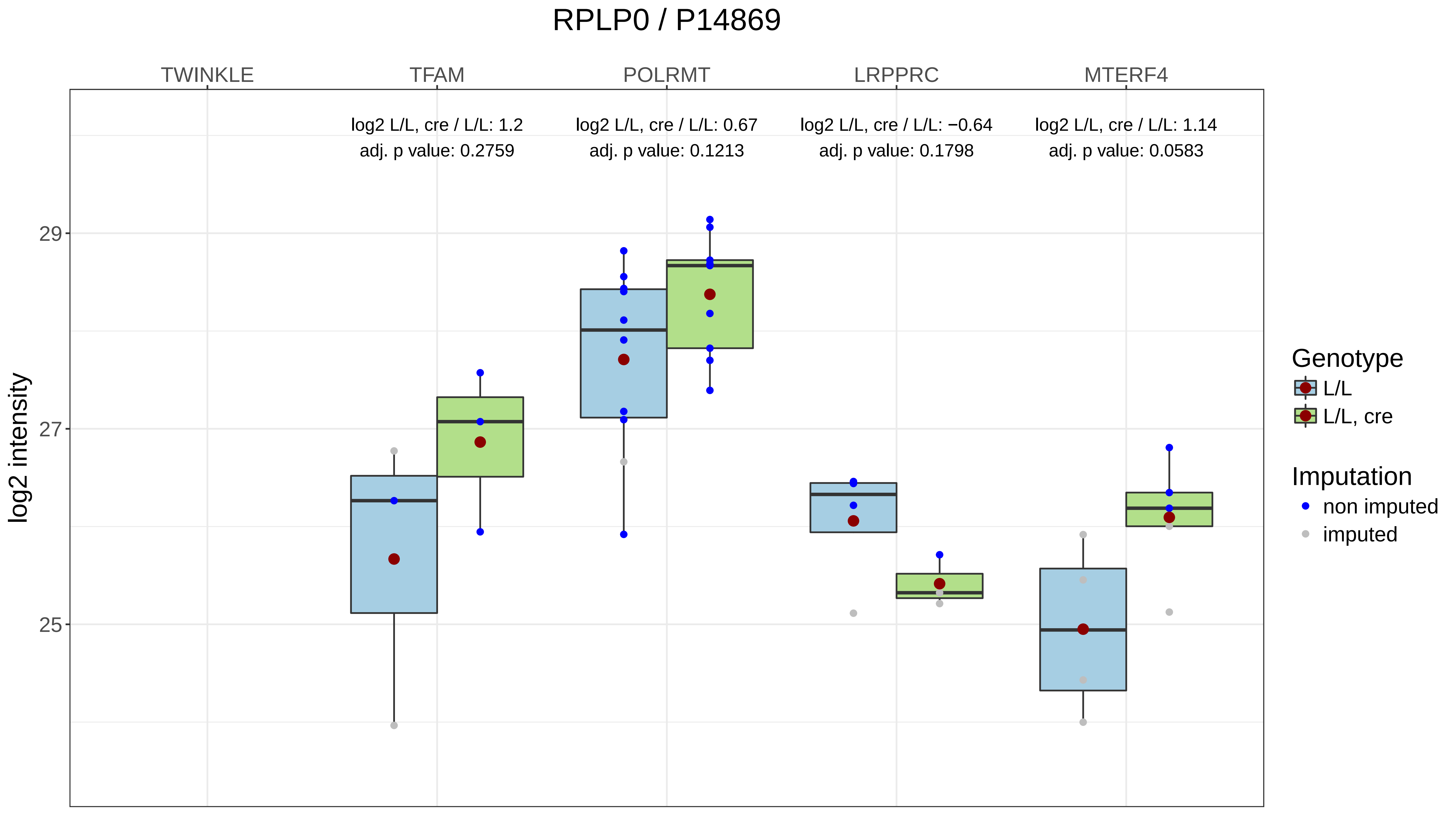

RPLP0 / P14869
TWINKLE
TFAM
POLRMT
LRPPRC
MTERF4
log2 L/L, cre / L/L: 1.2
adj. p value: 0.2759
log2 L/L, cre / L/L: 0.67
adj. p value: 0.1213
log2 L/L, cre / L/L: −0.64
adj. p value: 0.1798
log2 L/L, cre / L/L: 1.14
adj. p value: 0.0583
29
27
25
log2 intensity
Genotype
L/L
L/L, cre
Imputation
non imputed
imputed

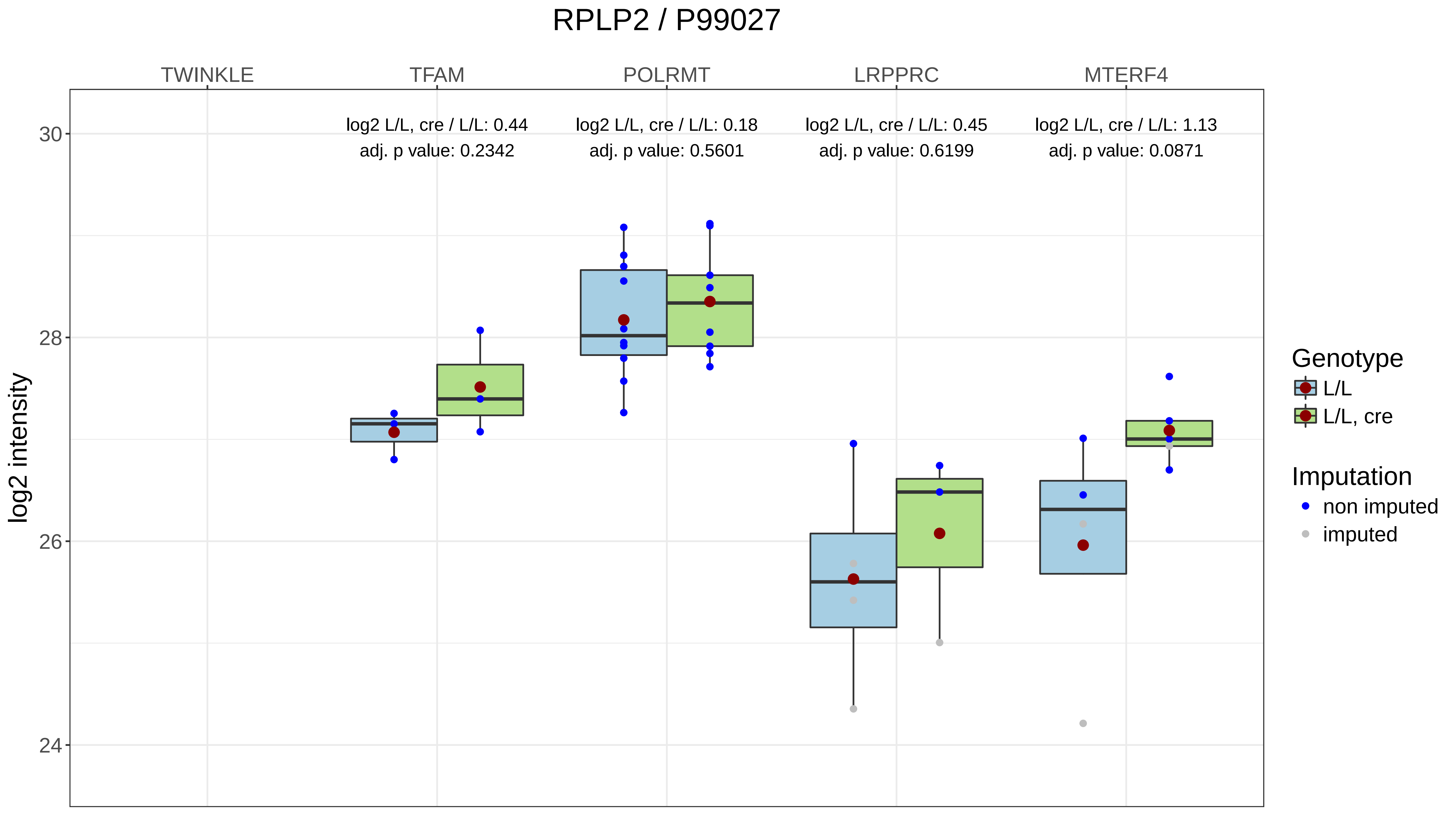
RPLP2 / P99027
TWINKLE
TFAM
POLRMT
LRPPRC
MTERF4
log2 L/L, cre / L/L: 0.44
adj. p value: 0.2342
log2 L/L, cre / L/L: 0.18
adj. p value: 0.5601
log2 L/L, cre / L/L: 0.45
adj. p value: 0.6199
log2 L/L, cre / L/L: 1.13
adj. p value: 0.0871
log2 intensity
30
28
26
24
Genotype
L/L
L/L, cre
Imputation
non imputed
imputed

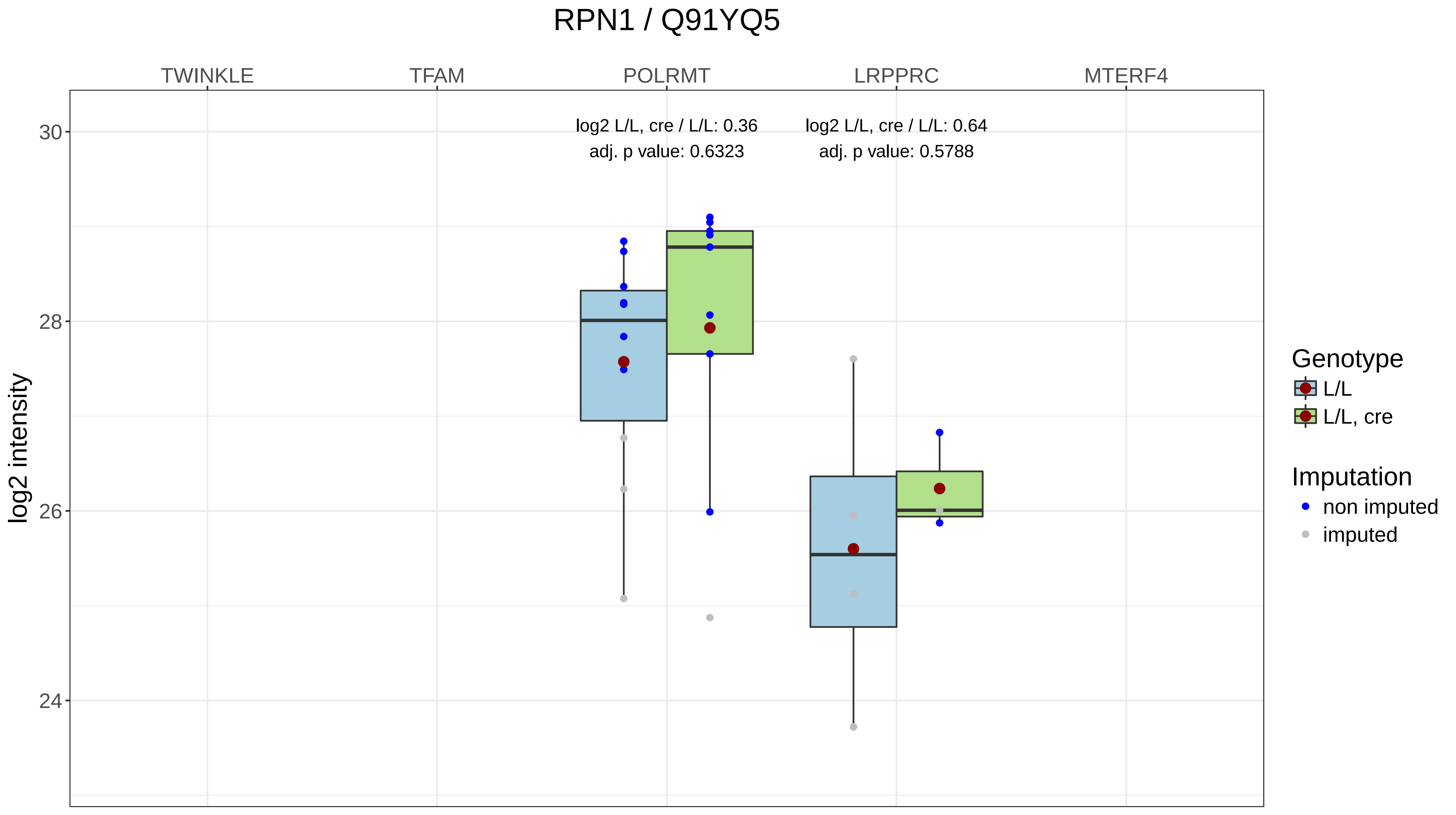

RPN1 / Q91YQ5
TWINKLE
TFAM
POLRMT
LRPPRC
MTERF4
log2 L/L, cre / L/L: 0.36
adj. p value: 0.6323
log2 L/L, cre / L/L: 0.64
adj. p value: 0.5788
30
28
26
24
log2 intensity
Genotype
L/L
L/L, cre
Imputation
non imputed
imputed

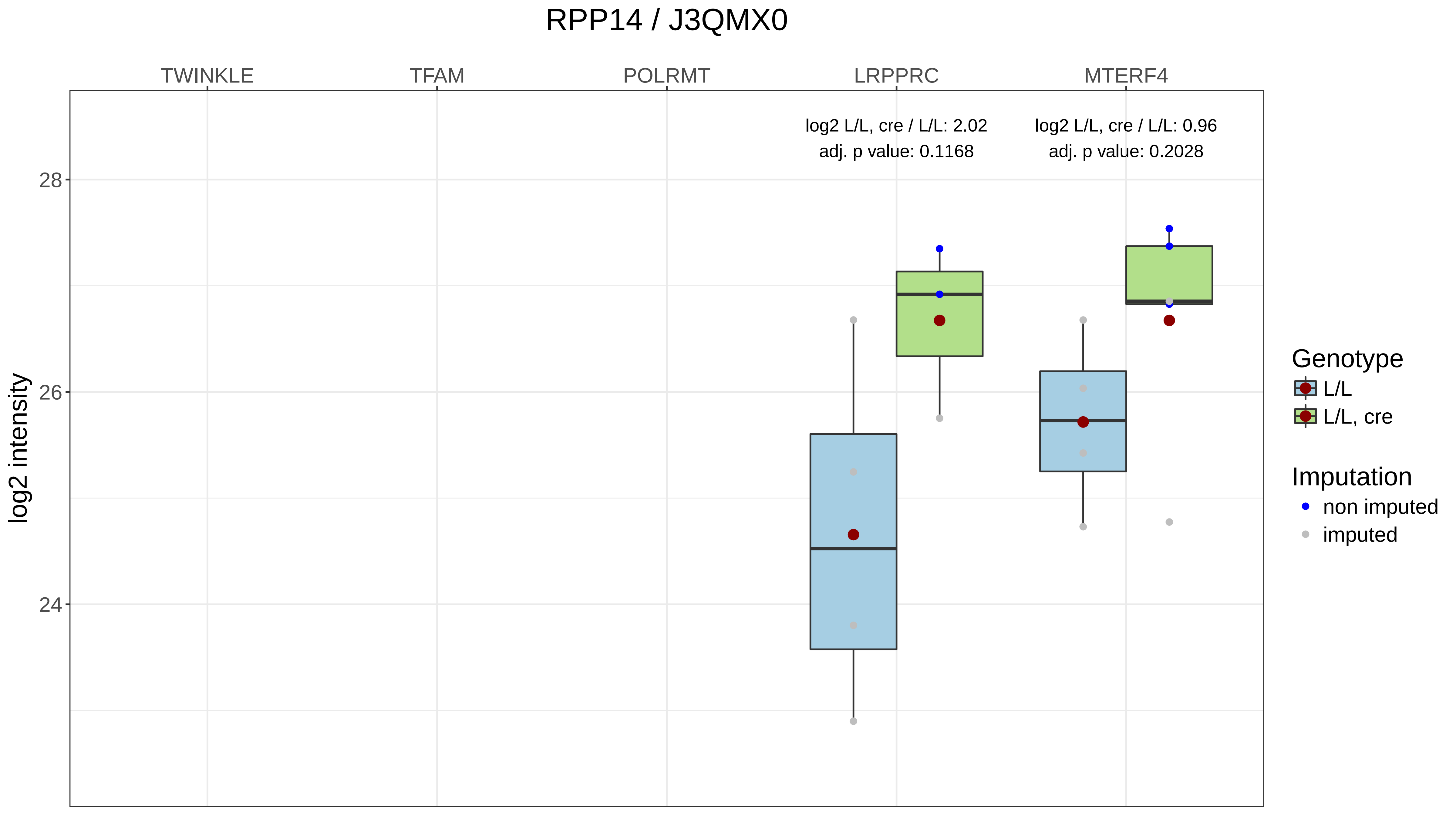
RPP14 / J3QMX0
TWINKLE
TFAM
POLRMT
LRPPRC
MTERF4
log2 L/L, cre / L/L: 2.02
adj. p value: 0.1168
log2 L/L, cre / L/L: 0.96
adj. p value: 0.2028
log2 intensity
28
26
24
Genotype
L/L
L/L, cre
Imputation
non imputed
imputed

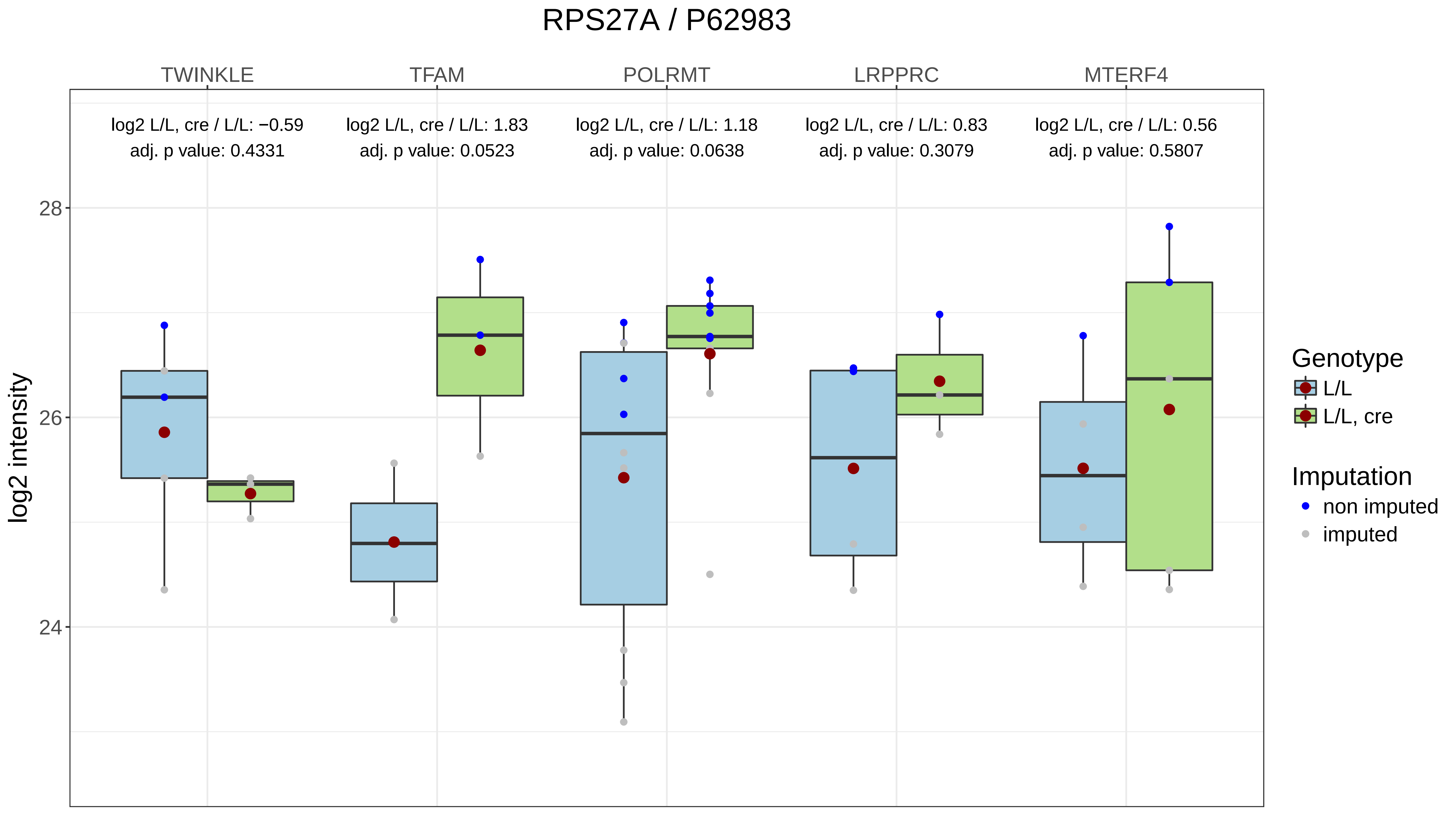
RPS27A / P62983
TWINKLE
TFAM
POLRMT
LRPPRC
MTERF4
log2 L/L, cre / L/L: −0.59
adj. p value: 0.4331
log2 L/L, cre / L/L: 1.83
adj. p value: 0.0523
log2 L/L, cre / L/L: 1.18
adj. p value: 0.0638
log2 L/L, cre / L/L: 0.83
adj. p value: 0.3079
log2 L/L, cre / L/L: 0.56
adj. p value: 0.5807
log2 intensity
28
26
24
Genotype
L/L
L/L, cre
Imputation
non imputed
imputed

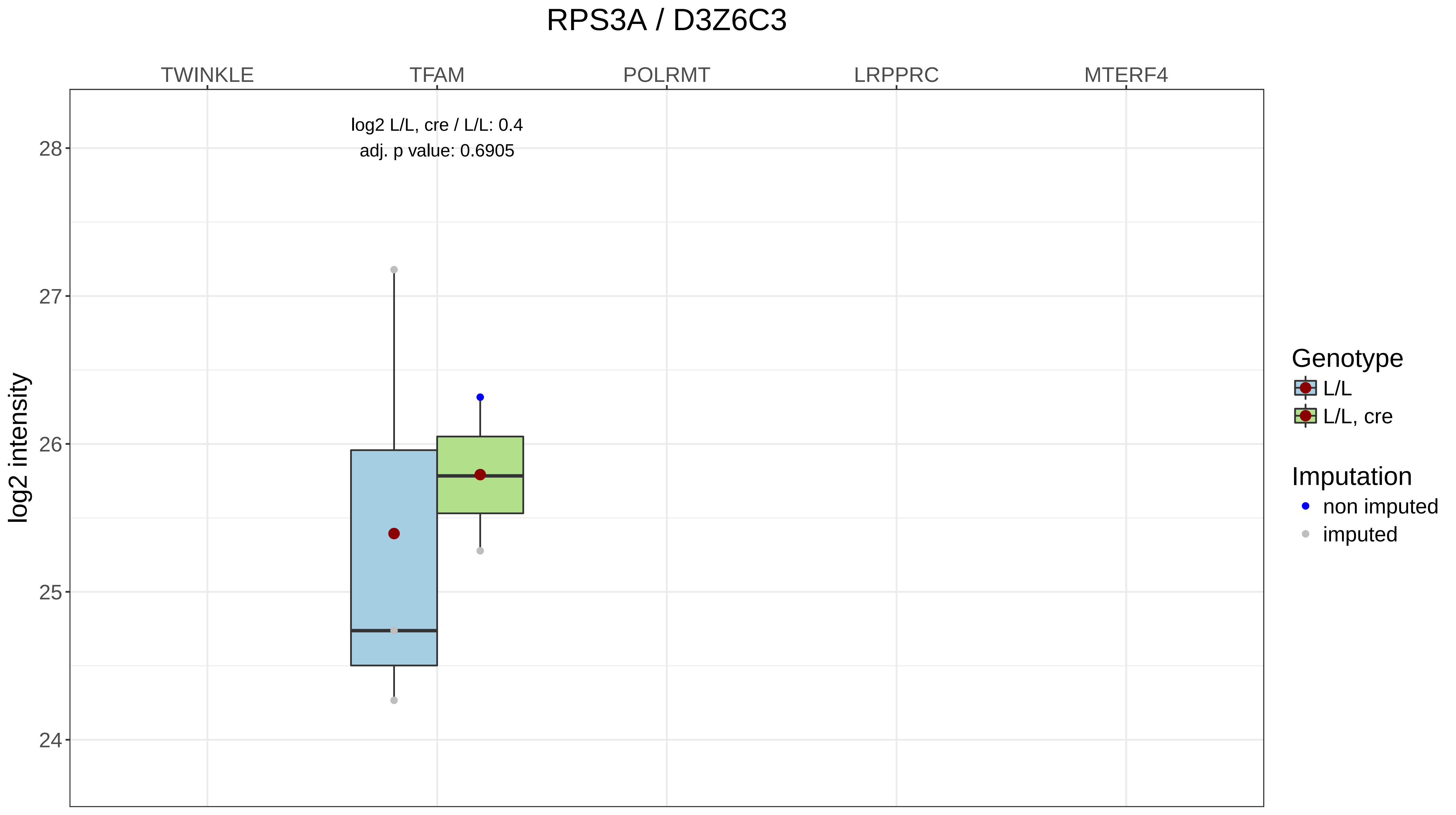

RPS3A / D3Z6C3
TWINKLE
TFAM
POLRMT
LRPPRC
MTERF4
log2 L/L, cre / L/L: 0.4
adj. p value: 0.6905
log2 intensity
24
25
26
27
28
Genotype
L/L
L/L, cre
Imputation
non imputed
imputed

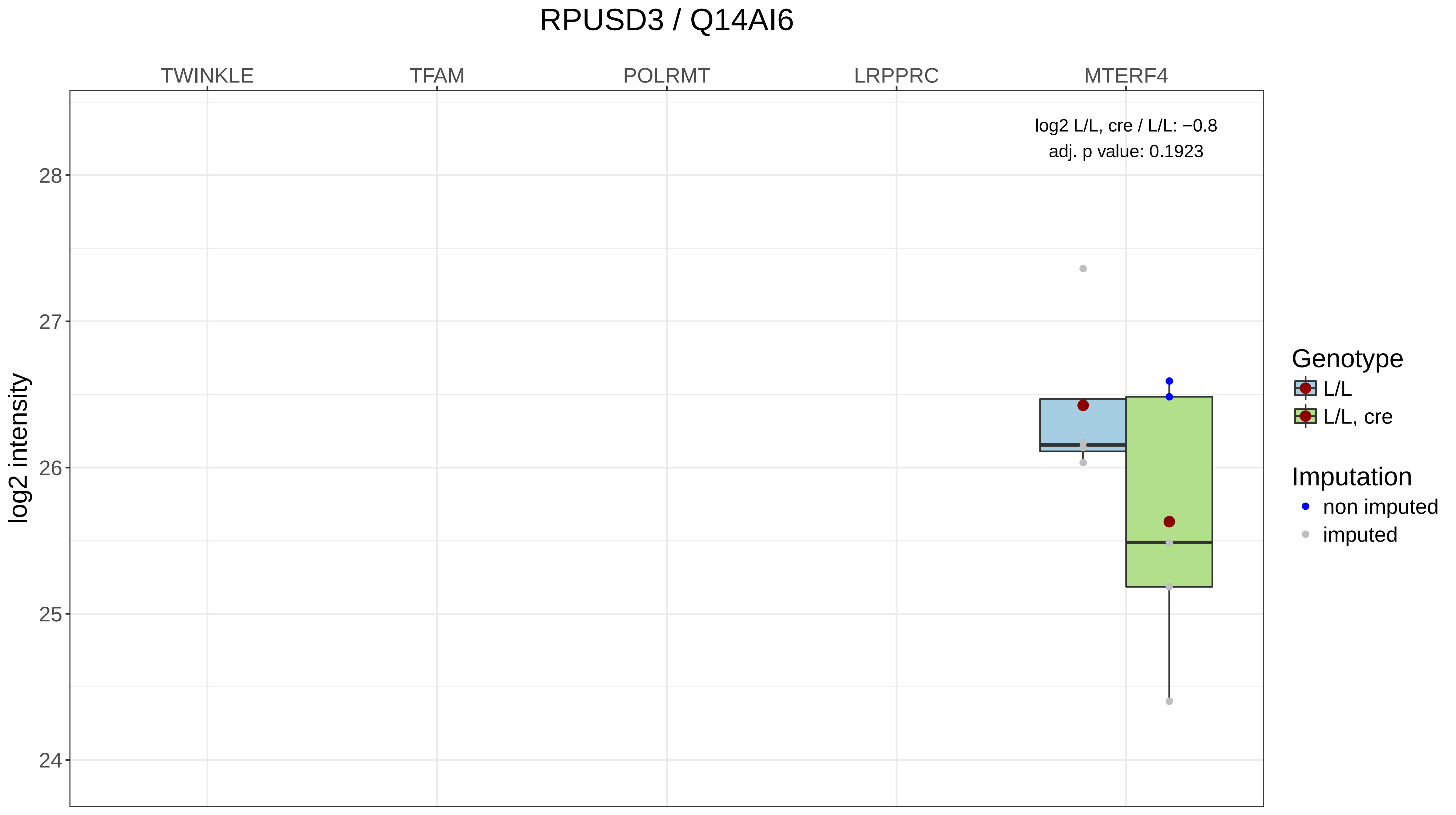
RPUSD3 / Q14AI6
TWINKLE
TFAM
POLRMT
LRPPRC
MTERF4
log2 L/L, cre / L/L: −0.8
adj. p value: 0.1923
28
27
26
25
24
log2 intensity
Genotype
L/L
L/L, cre
Imputation
non imputed
imputed

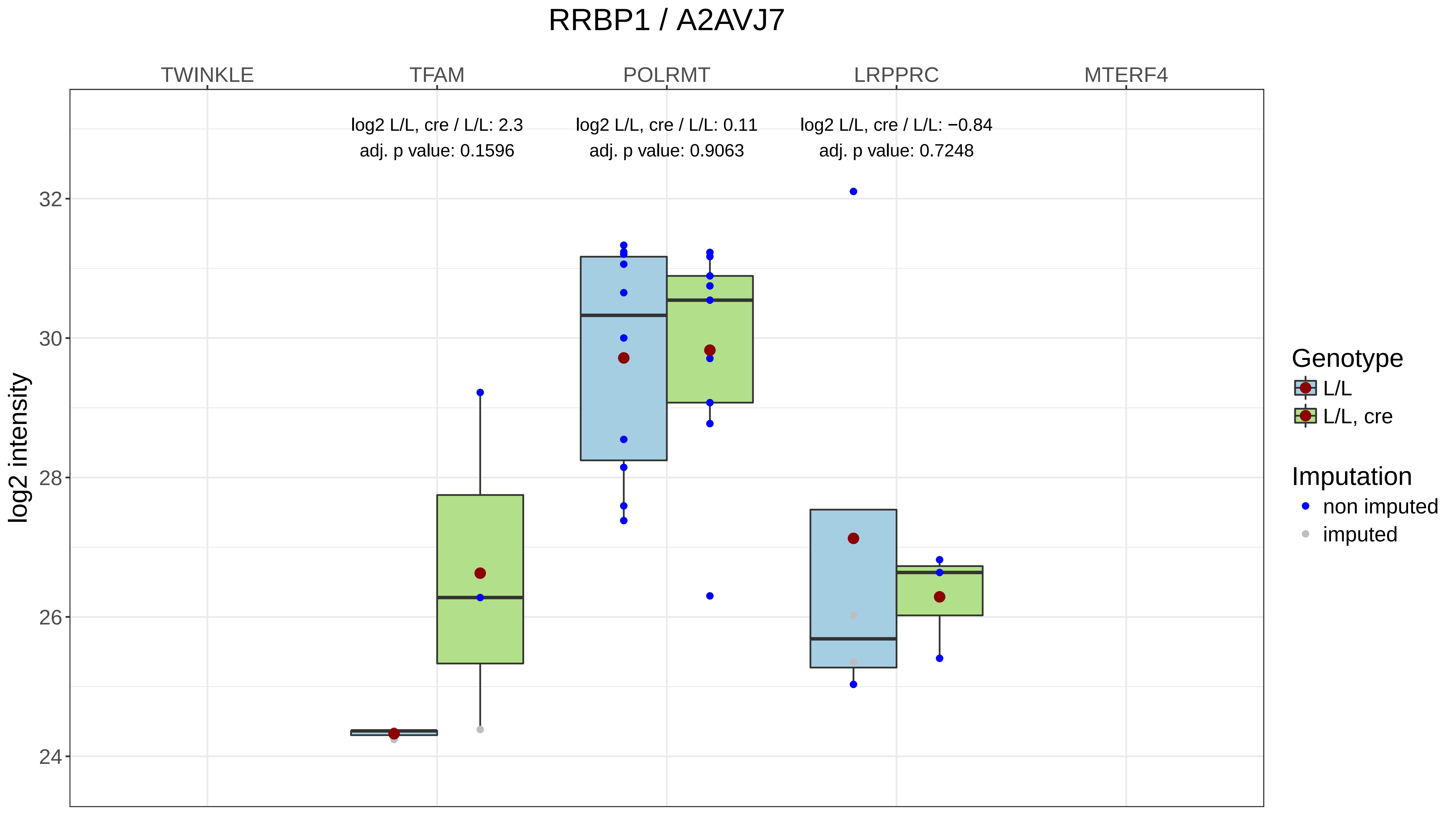
RRBP1 / A2AVJ7
TWINKLE
TFAM
POLRMT
LRPPRC
MTERF4
log2 L/L, cre / L/L: 2.3
adj. p value: 0.1596
log2 L/L, cre / L/L: 0.11
adj. p value: 0.9063
log2 L/L, cre / L/L: −0.84
adj. p value: 0.7248
log2 intensity
32
30
28
26
24
Genotype
L/L
L/L, cre
Imputation
non imputed
imputed

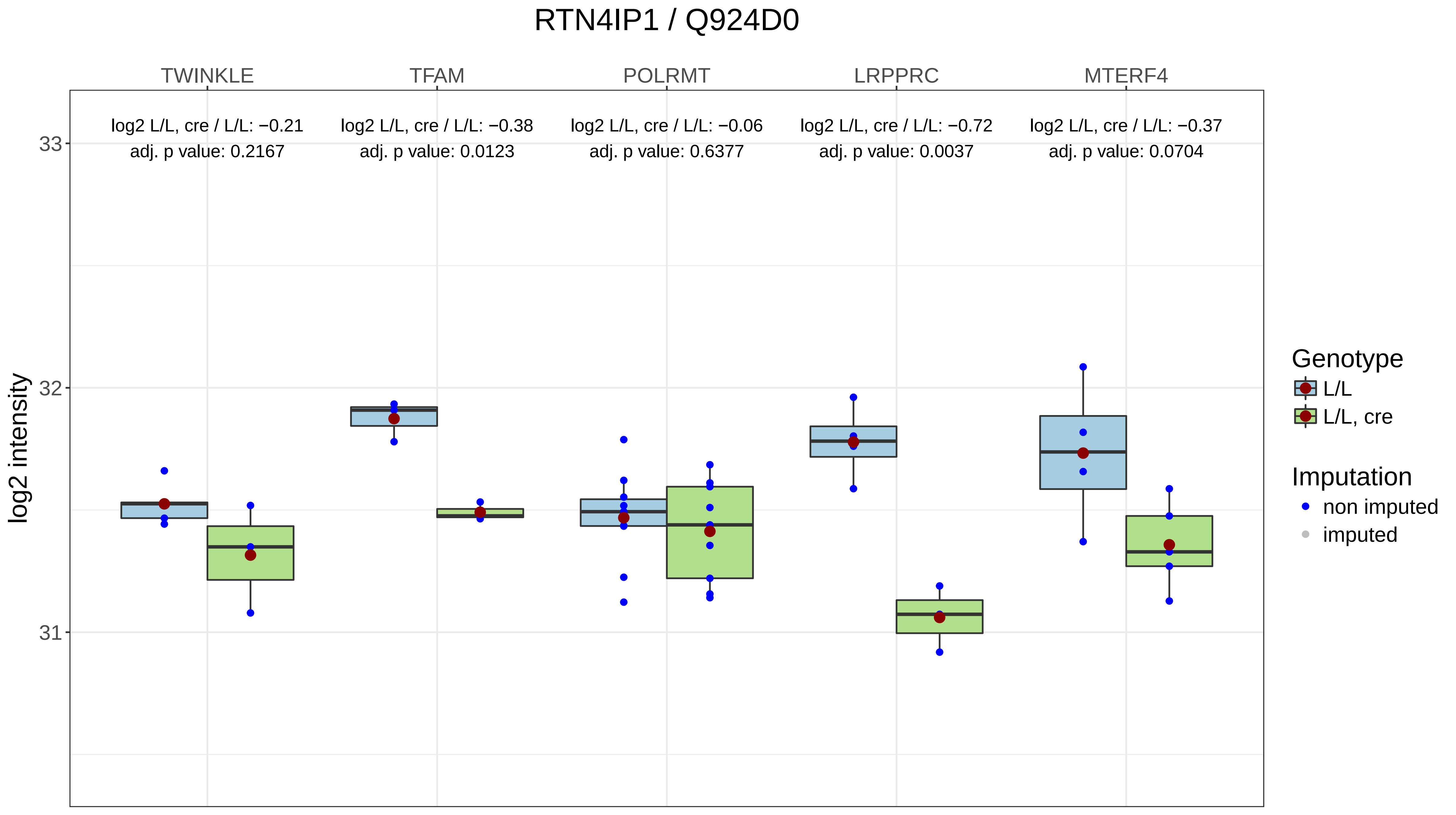

RTN4IP1 / Q924D0
TWINKLE
TFAM
POLRMT
LRPPRC
MTERF4
log2 L/L, cre / L/L: −0.21
adj. p value: 0.2167
log2 L/L, cre / L/L: −0.38
adj. p value: 0.0123
log2 L/L, cre / L/L: −0.06
adj. p value: 0.6377
log2 L/L, cre / L/L: −0.72
adj. p value: 0.0037
log2 L/L, cre / L/L: −0.37
adj. p value: 0.0704
33
32
31
log2 intensity
Genotype
L/L
L/L, cre
Imputation
non imputed
imputed

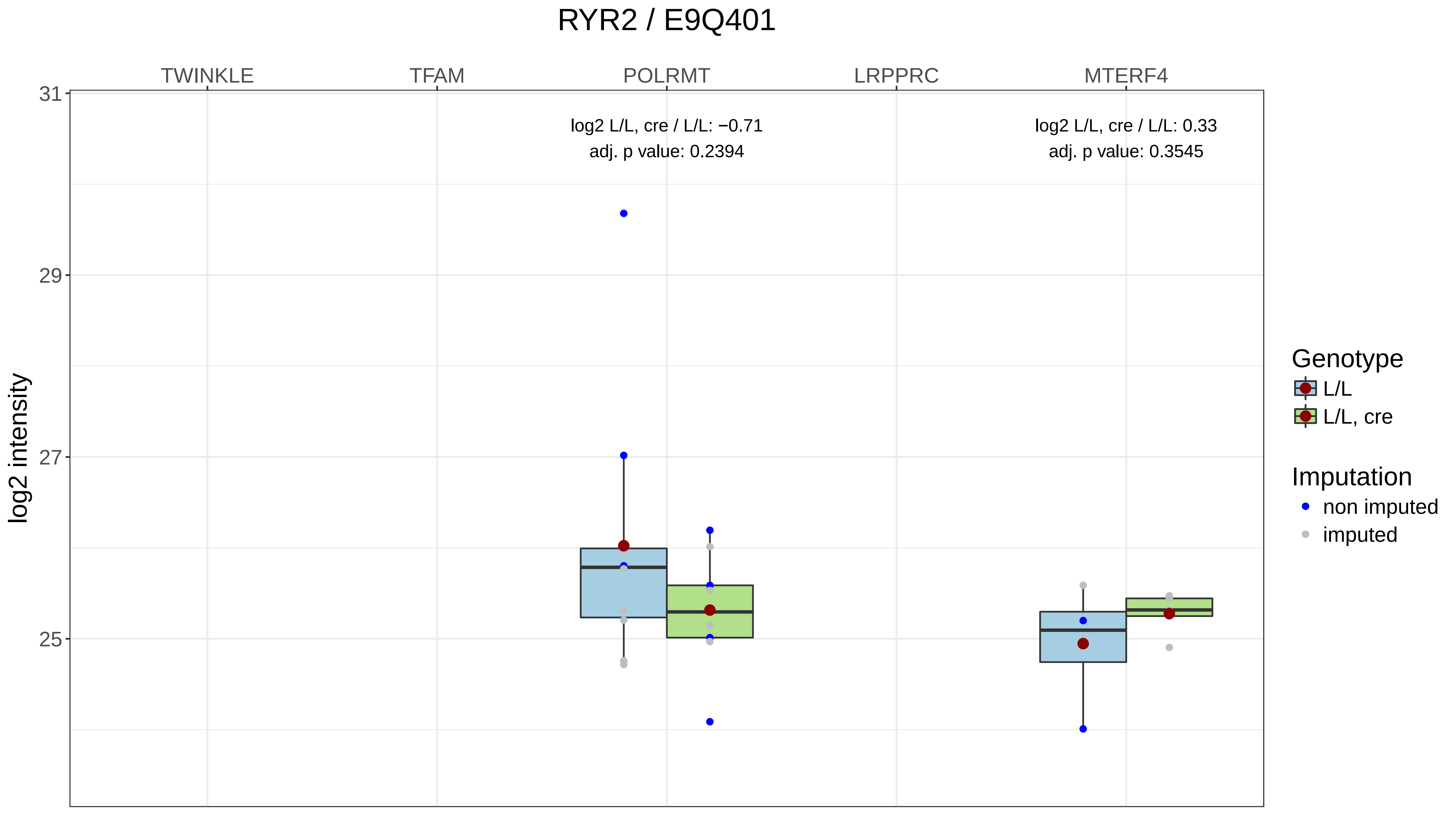
RYR2 / E9Q401
TWINKLE
TFAM
POLRMT
LRPPRC
MTERF4
log2 L/L, cre / L/L: −0.71
adj. p value: 0.2394
log2 L/L, cre / L/L: 0.33
adj. p value: 0.3545
31
29
27
25
log2 intensity
Genotype
L/L
L/L, cre
Imputation
non imputed
imputed

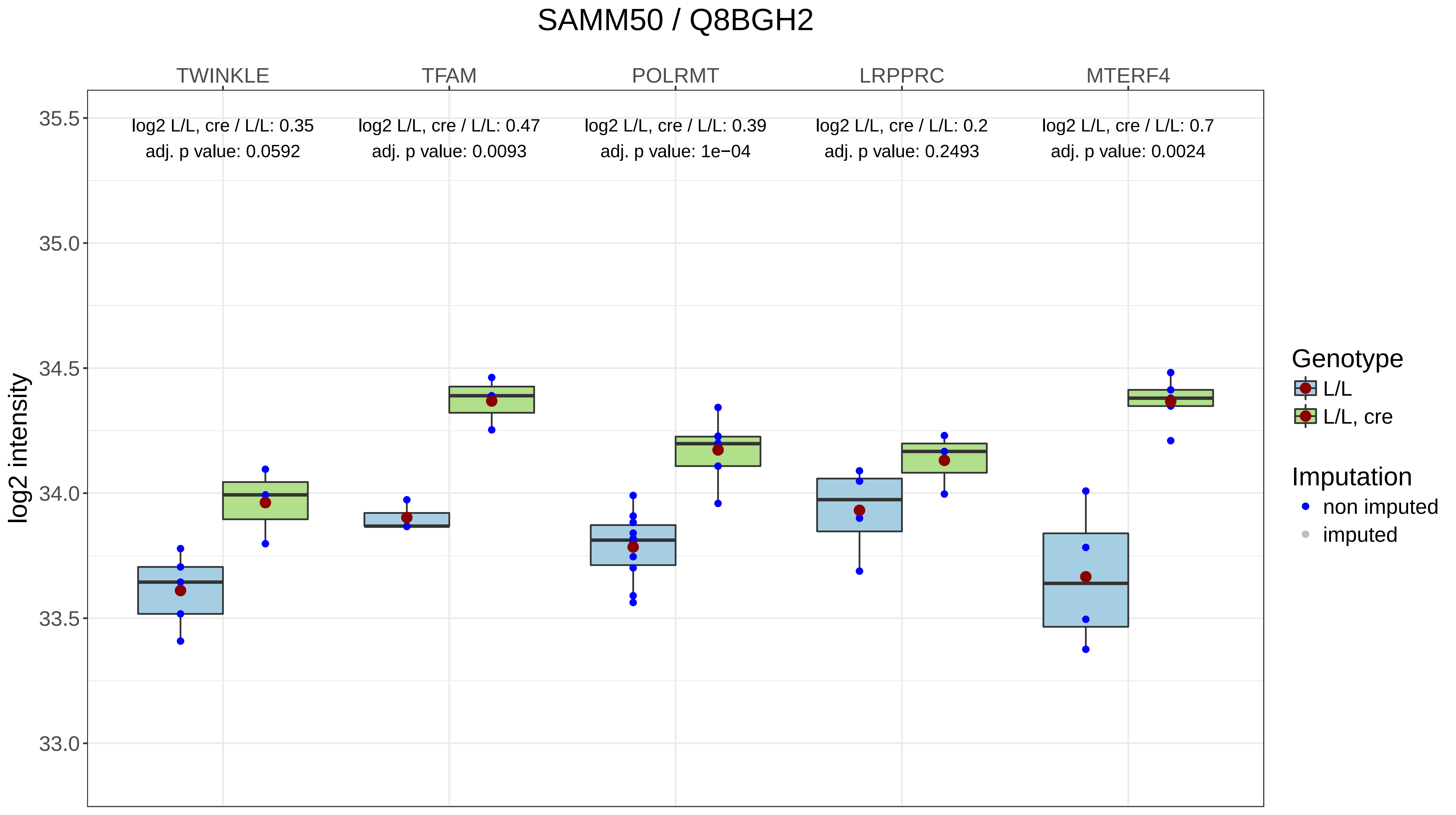
SAMM50 / Q8BGH2
TWINKLE
TFAM
POLRMT
LRPPRC
MTERF4
log2 L/L, cre / L/L: 0.35
adj. p value: 0.0592
log2 L/L, cre / L/L: 0.47
adj. p value: 0.0093
log2 L/L, cre / L/L: 0.39
adj. p value: 1e−04
log2 L/L, cre / L/L: 0.2
adj. p value: 0.2493
log2 L/L, cre / L/L: 0.7
adj. p value: 0.0024
log2 intensity
35.5
35.0
34.5
34.0
33.5
33.0
Genotype
L/L
L/L, cre
Imputation
non imputed
imputed

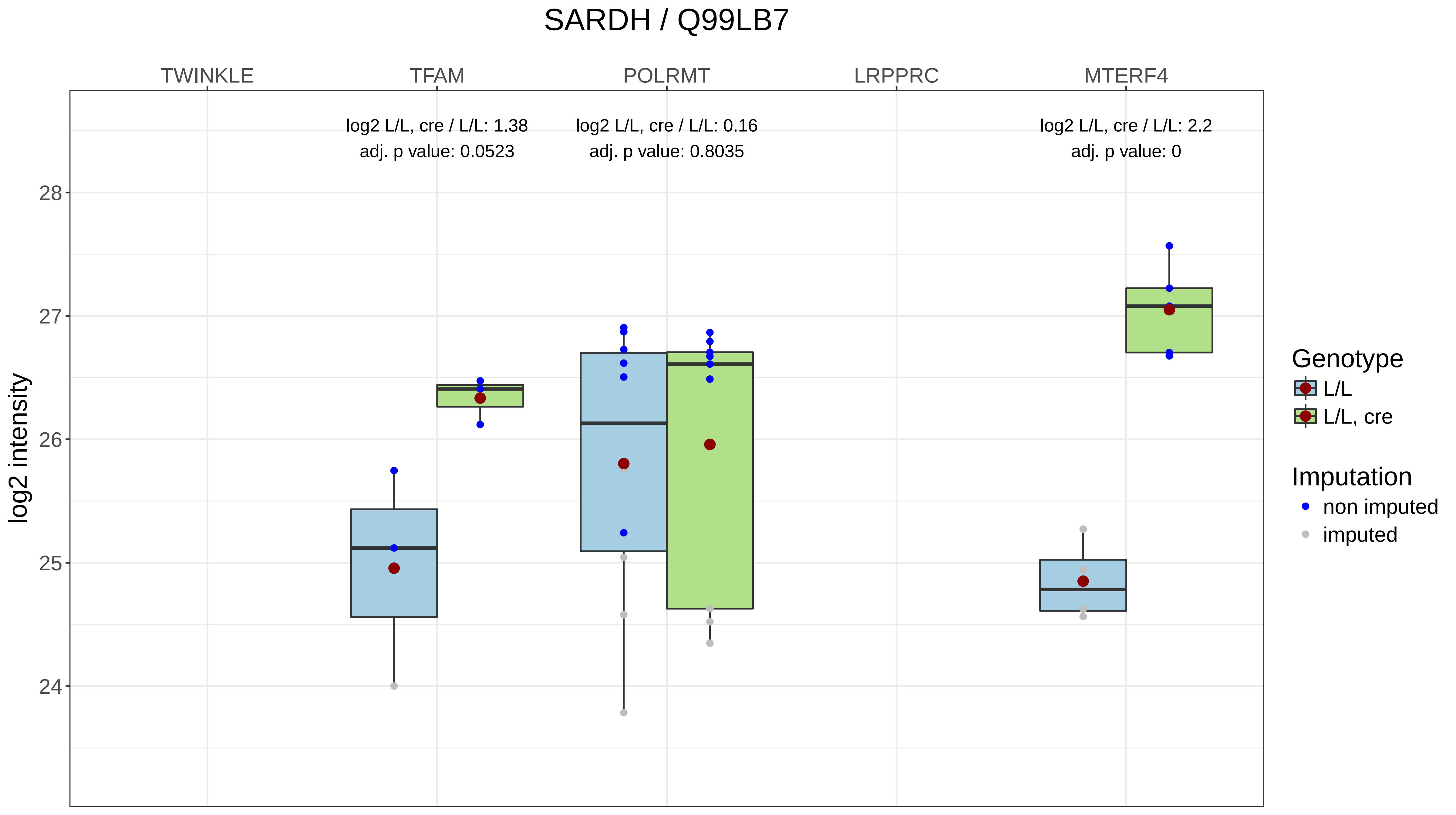
SARDH / Q99LB7
TWINKLE
TFAM
POLRMT
LRPPRC
MTERF4
log2 L/L, cre / L/L: 1.38
adj. p value: 0.0523
log2 L/L, cre / L/L: 0.16
adj. p value: 0.8035
log2 L/L, cre / L/L: 2.2
adj. p value: 0
28
27
26
25
24
log2 intensity
Genotype
L/L
L/L, cre
Imputation
non imputed
imputed

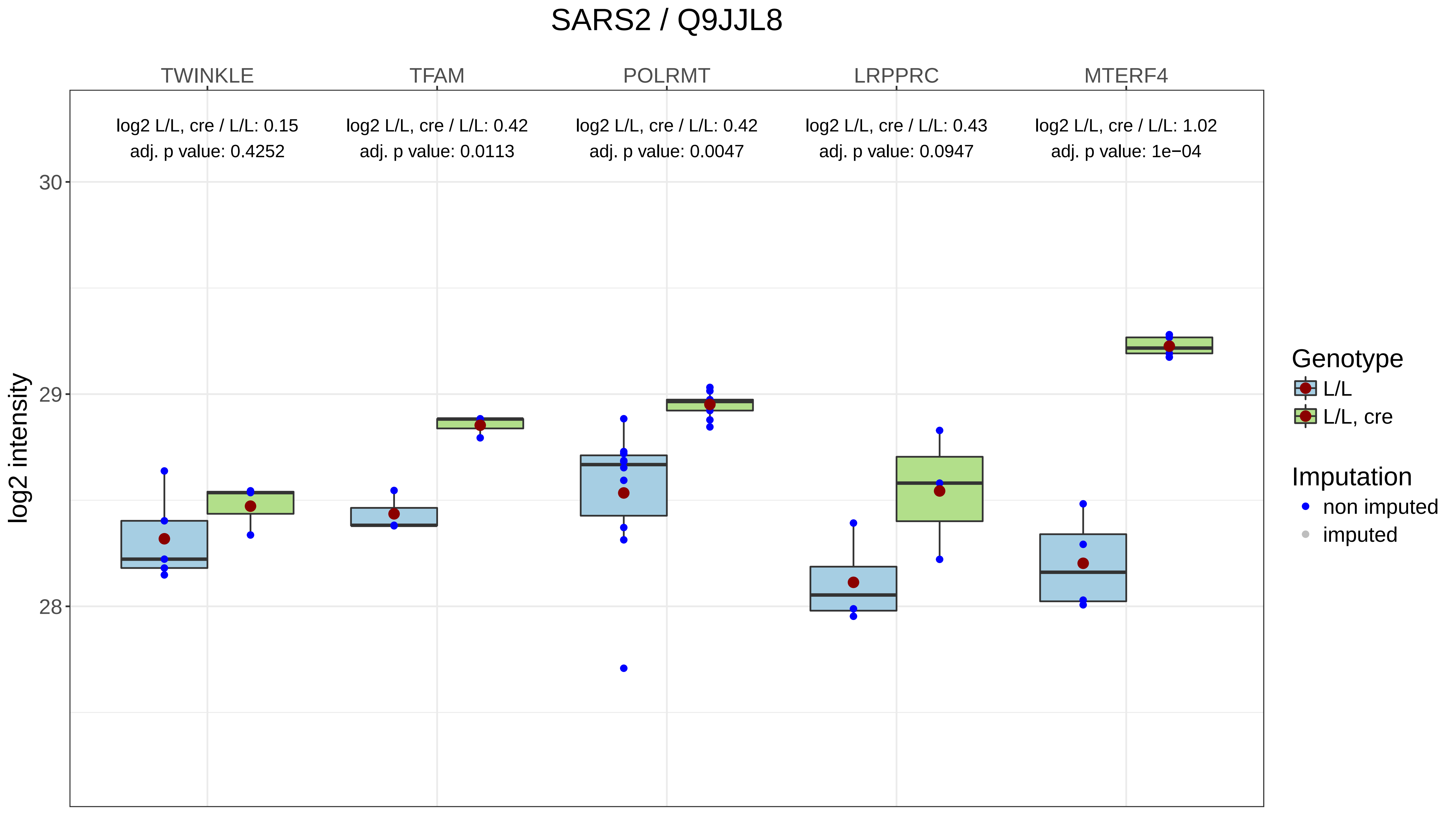
SARS2 / Q9JJL8
TWINKLE
TFAM
POLRMT
LRPPRC
MTERF4
log2 L/L, cre / L/L: 0.15
adj. p value: 0.4252
log2 L/L, cre / L/L: 0.42
adj. p value: 0.0113
log2 L/L, cre / L/L: 0.42
adj. p value: 0.0047
log2 L/L, cre / L/L: 0.43
adj. p value: 0.0947
log2 L/L, cre / L/L: 1.02
adj. p value: 1e−04
30
29
28
log2 intensity
Genotype
L/L
L/L, cre
Imputation
non imputed
imputed

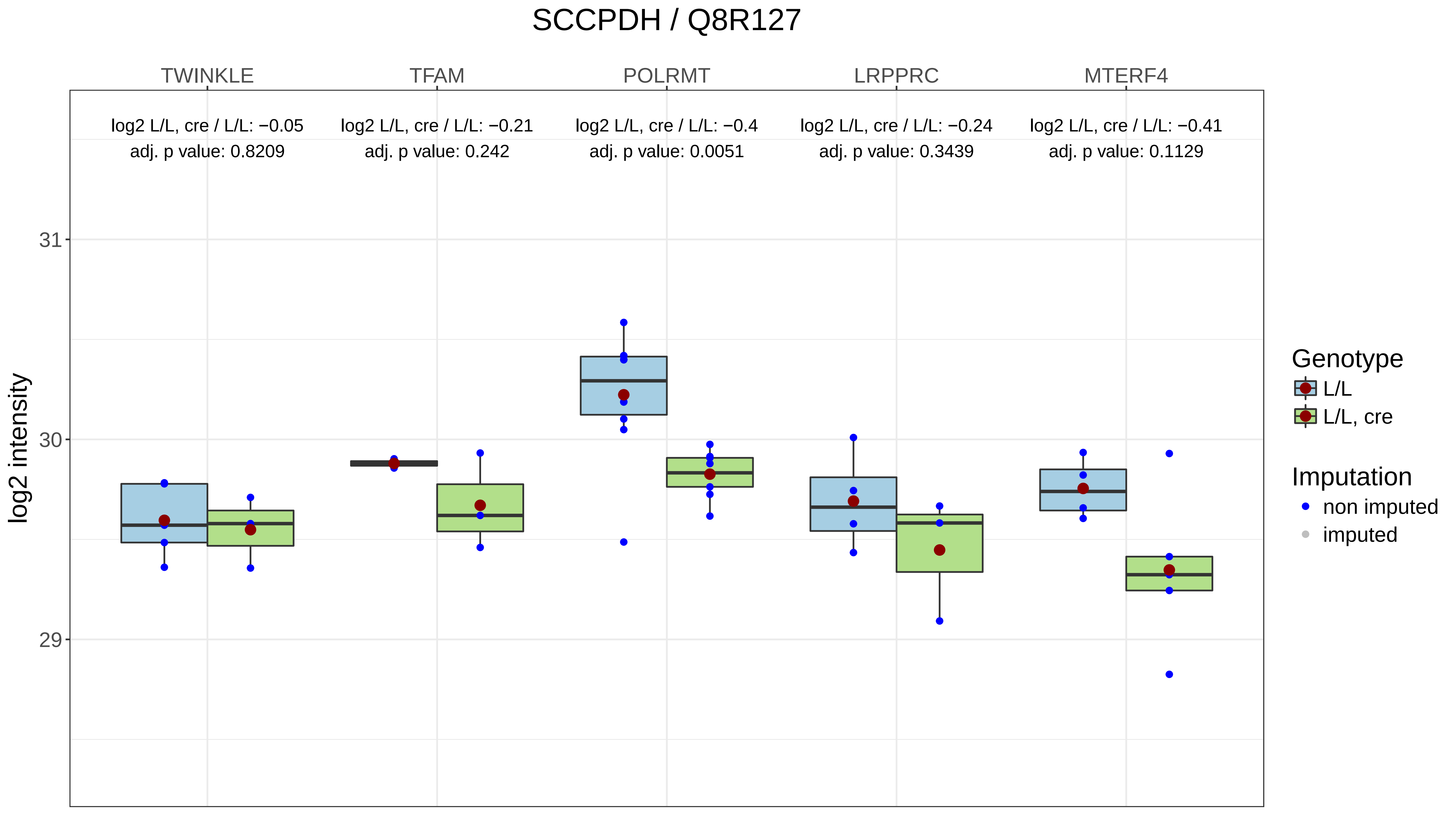
SCCPDH / Q8R127
TWINKLE
TFAM
POLRMT
LRPPRC
MTERF4
log2 L/L, cre / L/L: −0.05
adj. p value: 0.8209
log2 L/L, cre / L/L: −0.21
adj. p value: 0.242
log2 L/L, cre / L/L: −0.4
adj. p value: 0.0051
log2 L/L, cre / L/L: −0.24
adj. p value: 0.3439
log2 L/L, cre / L/L: −0.41
adj. p value: 0.1129
log2 intensity
31
30
29
Genotype
L/L
L/L, cre
Imputation
non imputed
imputed

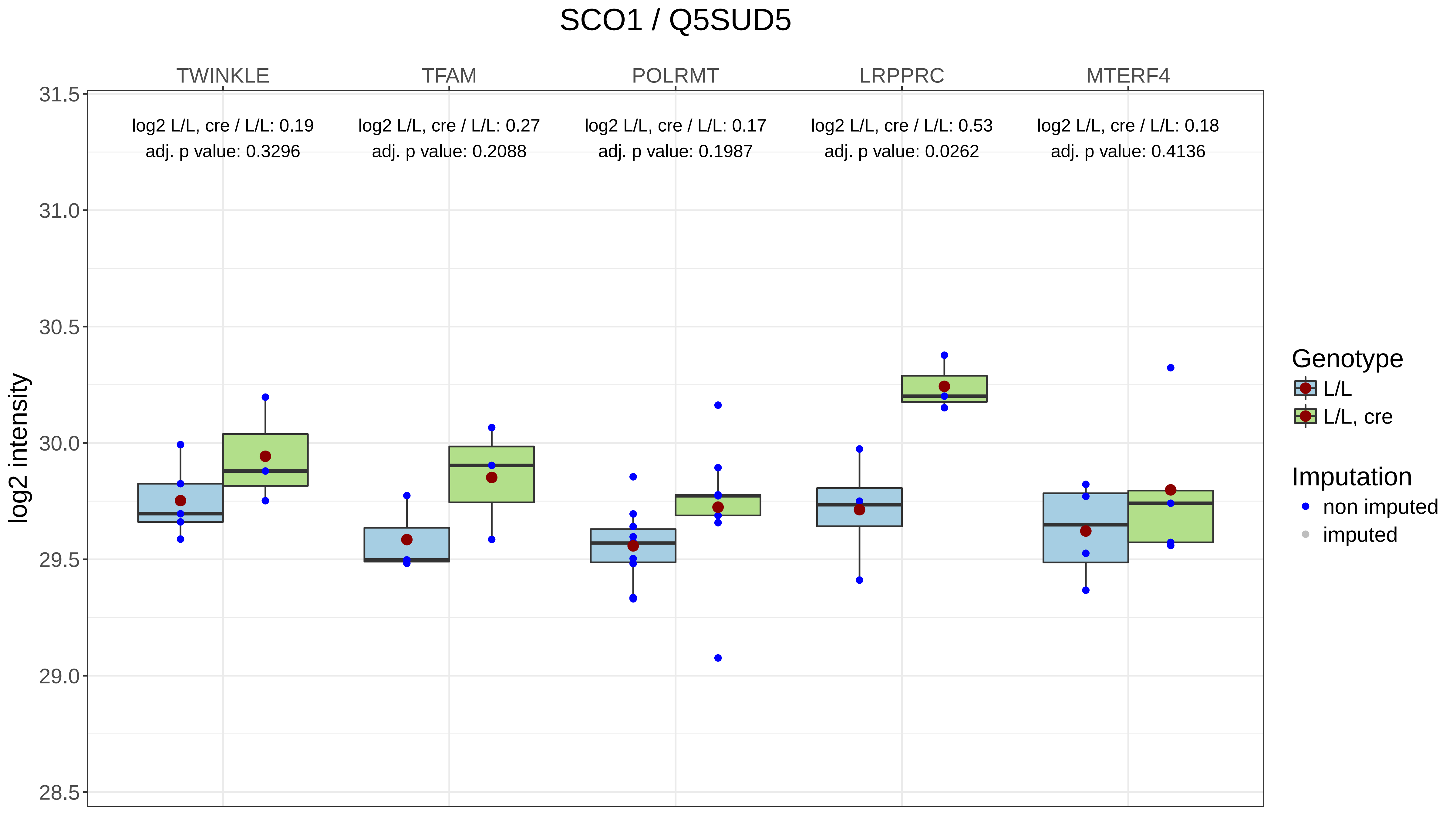
SCO1 / Q5SUD5
TWINKLE
TFAM
POLRMT
LRPPRC
MTERF4
log2 L/L, cre / L/L: 0.19
adj. p value: 0.3296
log2 L/L, cre / L/L: 0.27
adj. p value: 0.2088
log2 L/L, cre / L/L: 0.17
adj. p value: 0.1987
log2 L/L, cre / L/L: 0.53
adj. p value: 0.0262
log2 L/L, cre / L/L: 0.18
adj. p value: 0.4136
log2 intensity
31.5
31.0
30.5
30.0
29.5
29.0
28.5
Genotype
L/L
L/L, cre
Imputation
non imputed
imputed

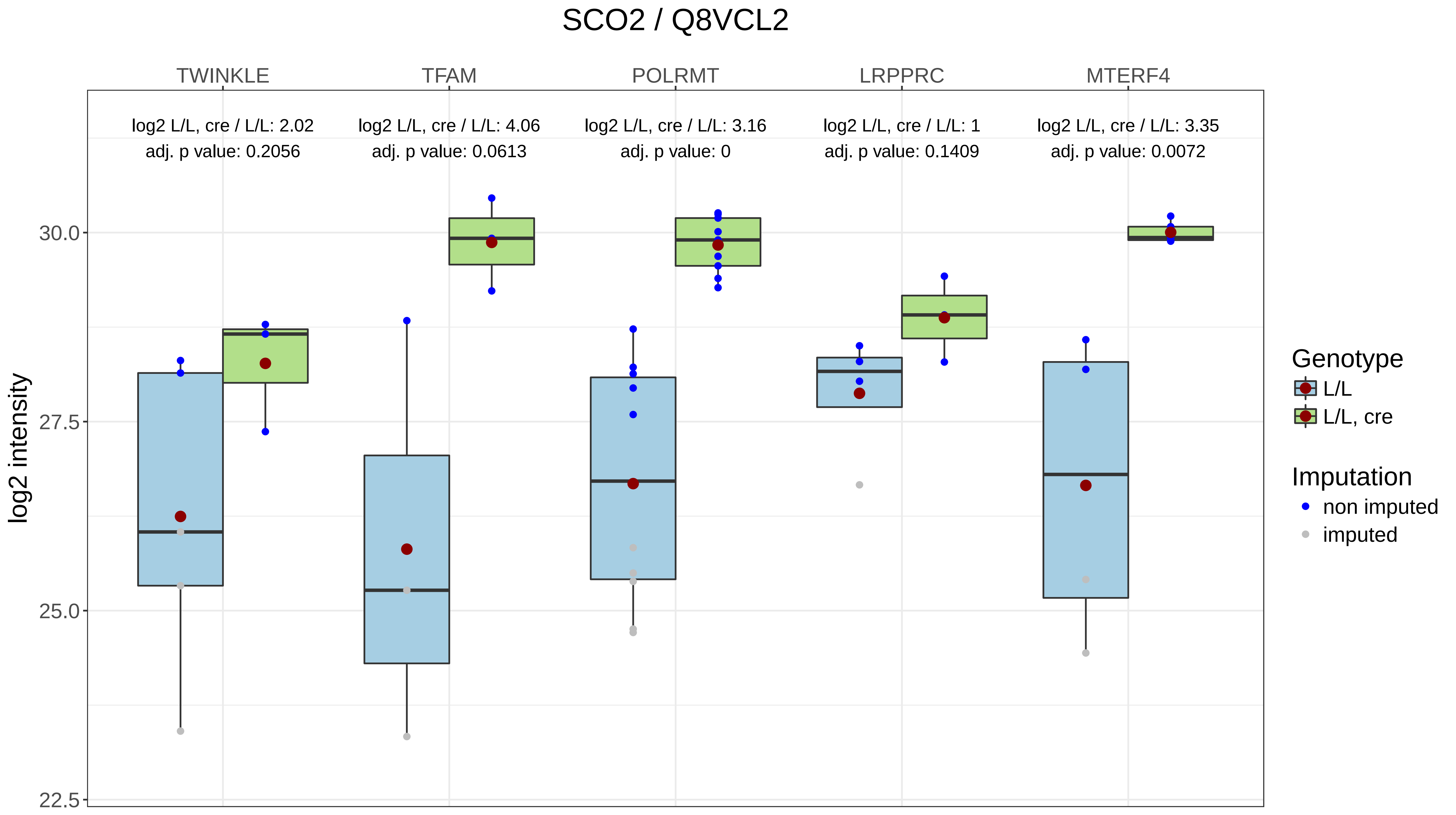
SCO2 / Q8VCL2
TWINKLE
TFAM
POLRMT
LRPPRC
MTERF4
log2 L/L, cre / L/L: 2.02
adj. p value: 0.2056
log2 L/L, cre / L/L: 4.06
adj. p value: 0.0613
log2 L/L, cre / L/L: 3.16
adj. p value: 0
log2 L/L, cre / L/L: 1
adj. p value: 0.1409
log2 L/L, cre / L/L: 3.35
adj. p value: 0.0072
log2 intensity
30.0
27.5
25.0
22.5
Genotype
L/L
L/L, cre
Imputation
non imputed
imputed

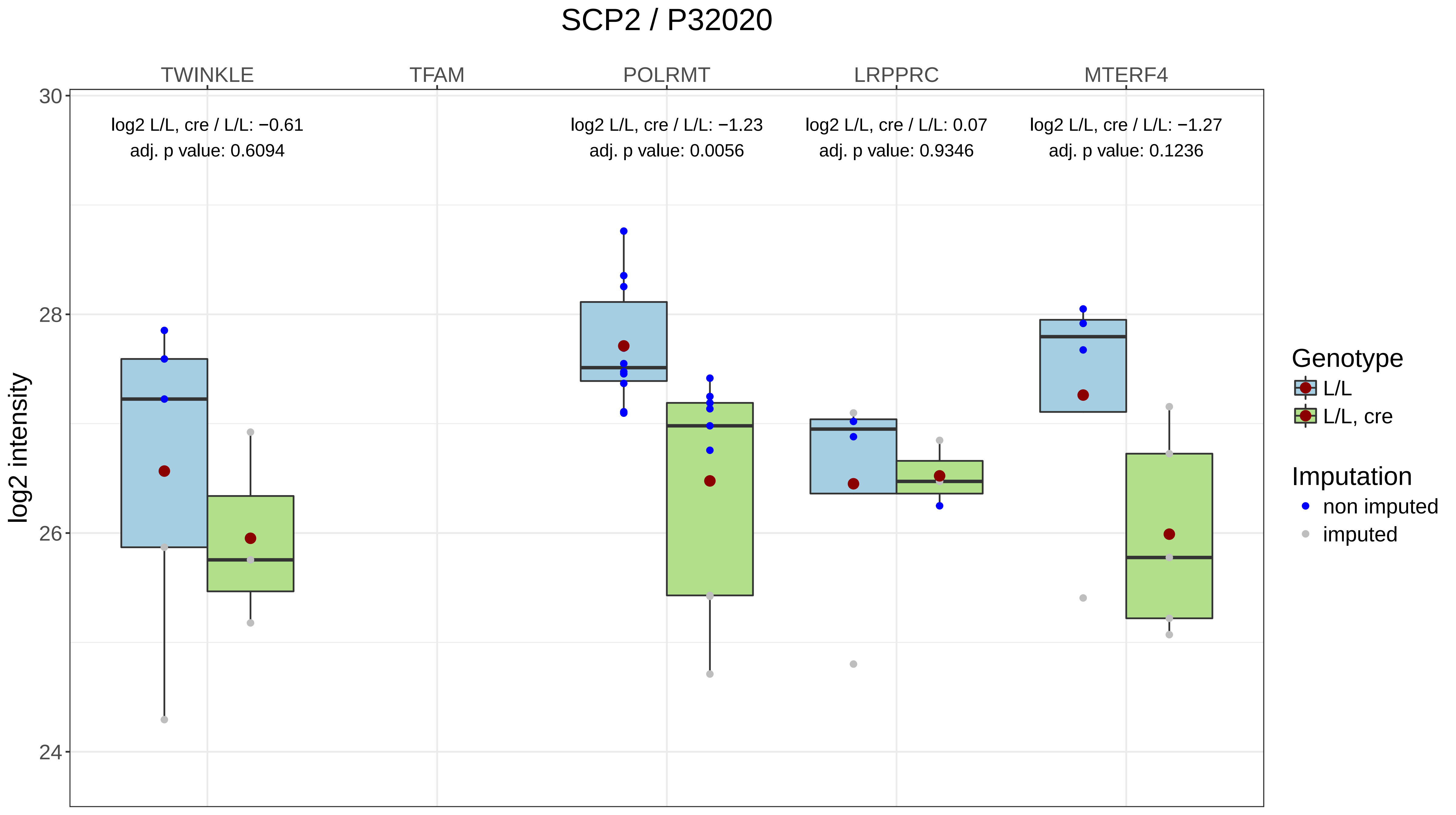
SCP2 / P32020
TWINKLE
TFAM
POLRMT
LRPPRC
MTERF4
log2 L/L, cre / L/L: −0.61
adj. p value: 0.6094
log2 L/L, cre / L/L: −1.23
adj. p value: 0.0056
log2 L/L, cre / L/L: 0.07
adj. p value: 0.9346
log2 L/L, cre / L/L: −1.27
adj. p value: 0.1236
log2 intensity
30
28
26
24
Genotype
L/L
L/L, cre
Imputation
non imputed
imputed

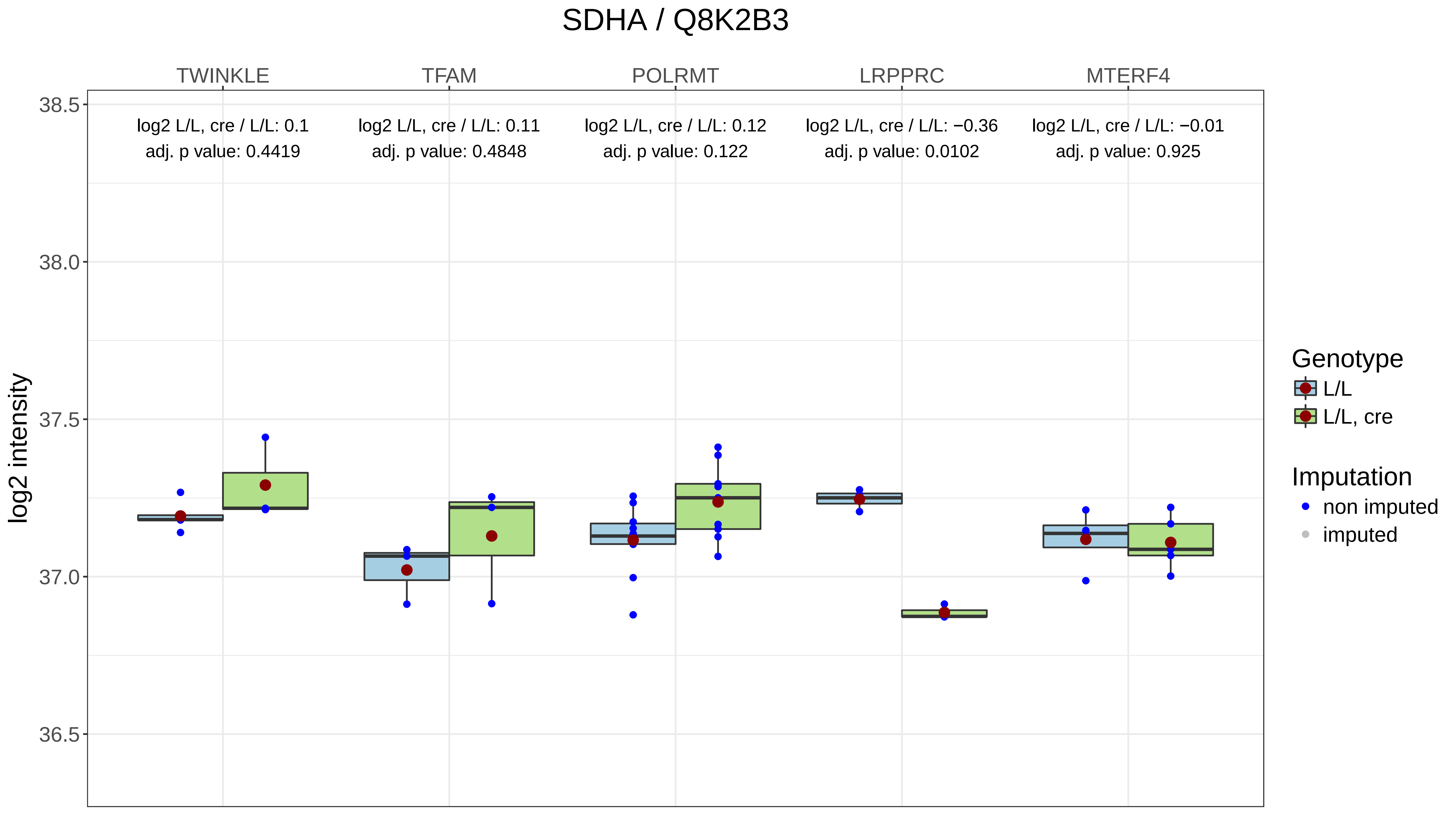
SDHA / Q8K2B3
TWINKLE
TFAM
POLRMT
LRPPRC
MTERF4
log2 L/L, cre / L/L: 0.1
adj. p value: 0.4419
log2 L/L, cre / L/L: 0.11
adj. p value: 0.4848
log2 L/L, cre / L/L: 0.12
adj. p value: 0.122
log2 L/L, cre / L/L: −0.36
adj. p value: 0.0102
log2 L/L, cre / L/L: −0.01
adj. p value: 0.925
log2 intensity
38.5
38.0
37.5
37.0
36.5
Genotype
L/L
L/L, cre
Imputation
non imputed
imputed

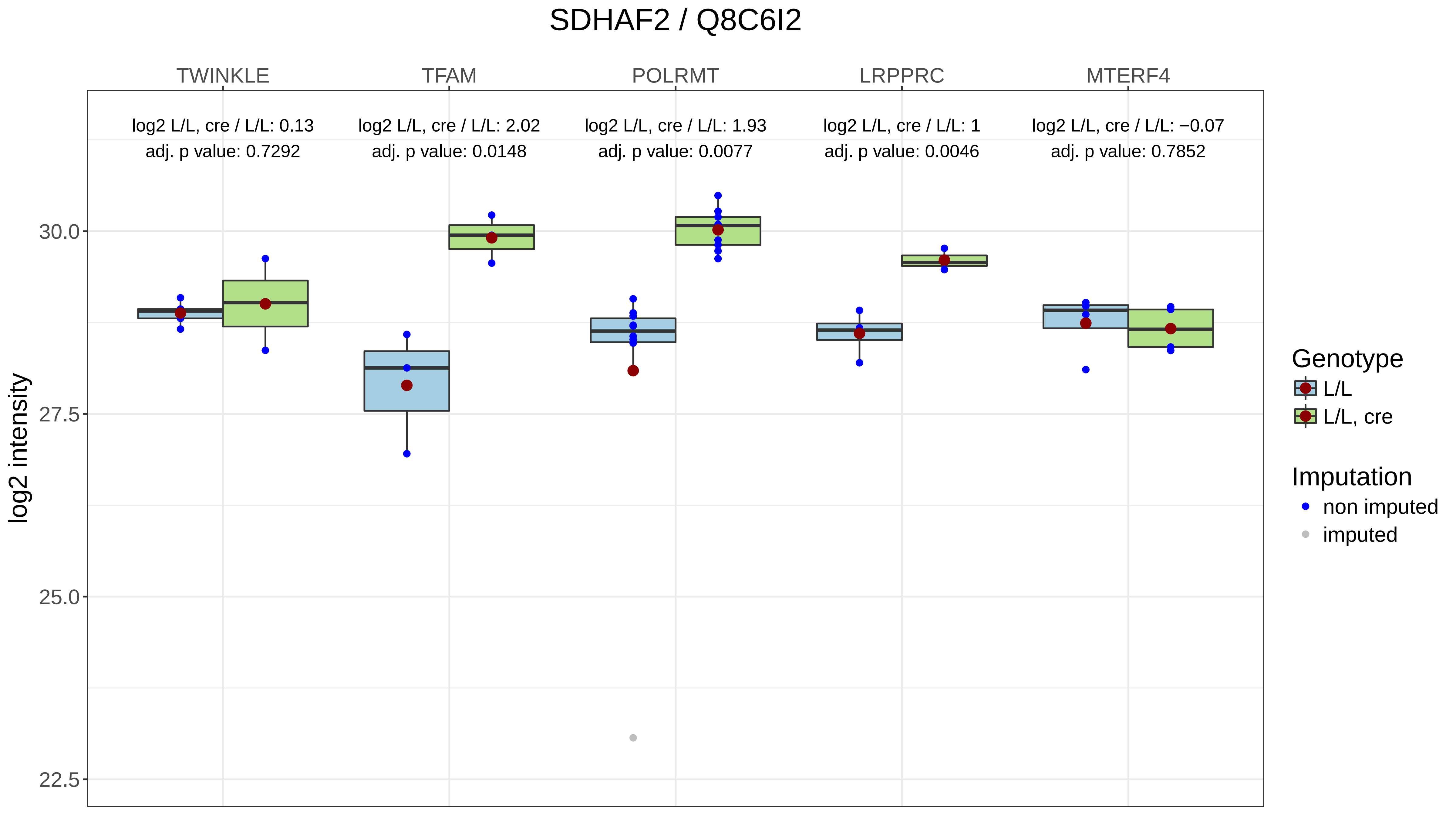

SDHAF2 / Q8C6I2
TWINKLE
TFAM
POLRMT
LRPPRC
MTERF4
log2 L/L, cre / L/L: 0.13
adj. p value: 0.7292
log2 L/L, cre / L/L: 2.02
adj. p value: 0.0148
log2 L/L, cre / L/L: 1.93
adj. p value: 0.0077
log2 L/L, cre / L/L: 1
adj. p value: 0.0046
log2 L/L, cre / L/L: −0.07
adj. p value: 0.7852
log2 intensity
30.0
27.5
25.0
22.5
Genotype
L/L
L/L, cre
Imputation
non imputed
imputed

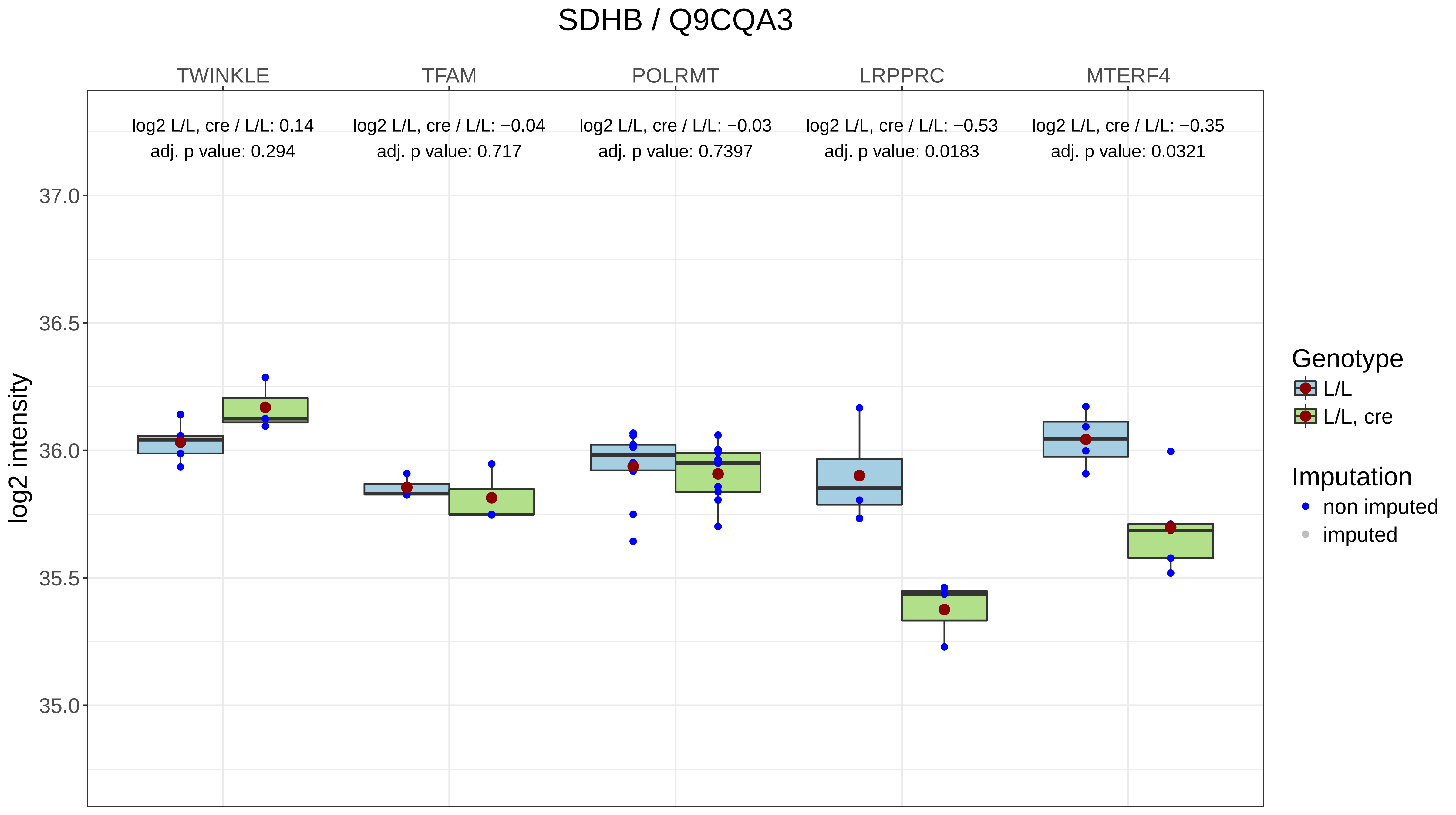
SDHB / Q9CQA3
TWINKLE
TFAM
POLRMT
LRPPRC
MTERF4
log2 L/L, cre / L/L: 0.14
adj. p value: 0.294
log2 L/L, cre / L/L: −0.04
adj. p value: 0.717
log2 L/L, cre / L/L: −0.03
adj. p value: 0.7397
log2 L/L, cre / L/L: −0.53
adj. p value: 0.0183
log2 L/L, cre / L/L: −0.35
adj. p value: 0.0321
log2 intensity
37.0
36.5
36.0
35.5
35.0
Genotype
L/L
L/L, cre
Imputation
non imputed
imputed

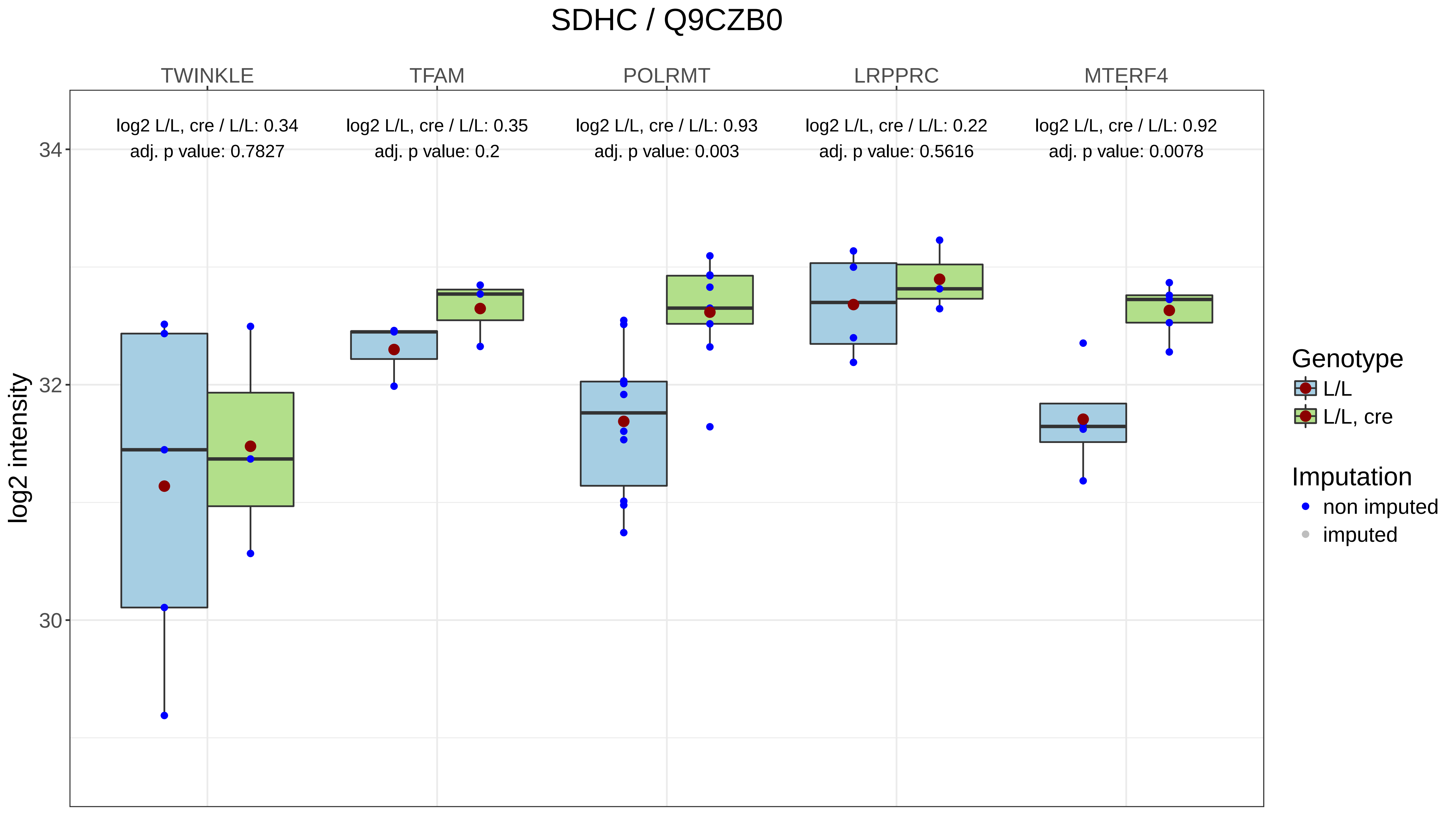
SDHC / Q9CZB0
TWINKLE
TFAM
POLRMT
LRPPRC
MTERF4
log2 L/L, cre / L/L: 0.34
adj. p value: 0.7827
log2 L/L, cre / L/L: 0.35
adj. p value: 0.2
log2 L/L, cre / L/L: 0.93
adj. p value: 0.003
log2 L/L, cre / L/L: 0.22
adj. p value: 0.5616
log2 L/L, cre / L/L: 0.92
adj. p value: 0.0078
log2 intensity
34
32
30
Genotype
L/L
L/L, cre
Imputation
non imputed
imputed

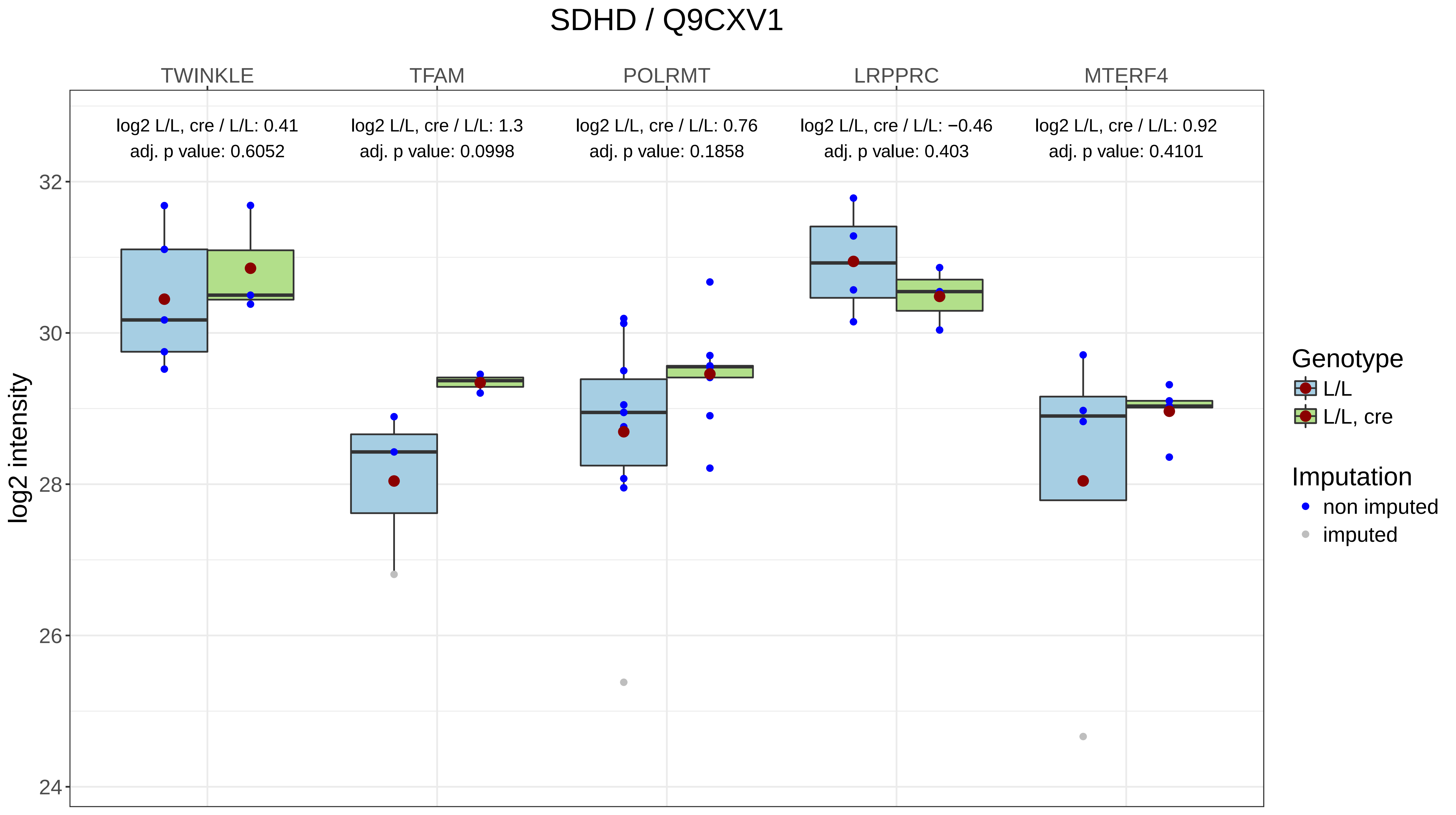

SDHD / Q9CXV1
TWINKLE
TFAM
POLRMT
LRPPRC
MTERF4
log2 L/L, cre / L/L: 0.41
adj. p value: 0.6052
log2 L/L, cre / L/L: 1.3
adj. p value: 0.0998
log2 L/L, cre / L/L: 0.76
adj. p value: 0.1858
log2 L/L, cre / L/L: −0.46
adj. p value: 0.403
log2 L/L, cre / L/L: 0.92
adj. p value: 0.4101
log2 intensity
32
30
28
26
24
Genotype
L/L
L/L, cre
Imputation
non imputed
imputed

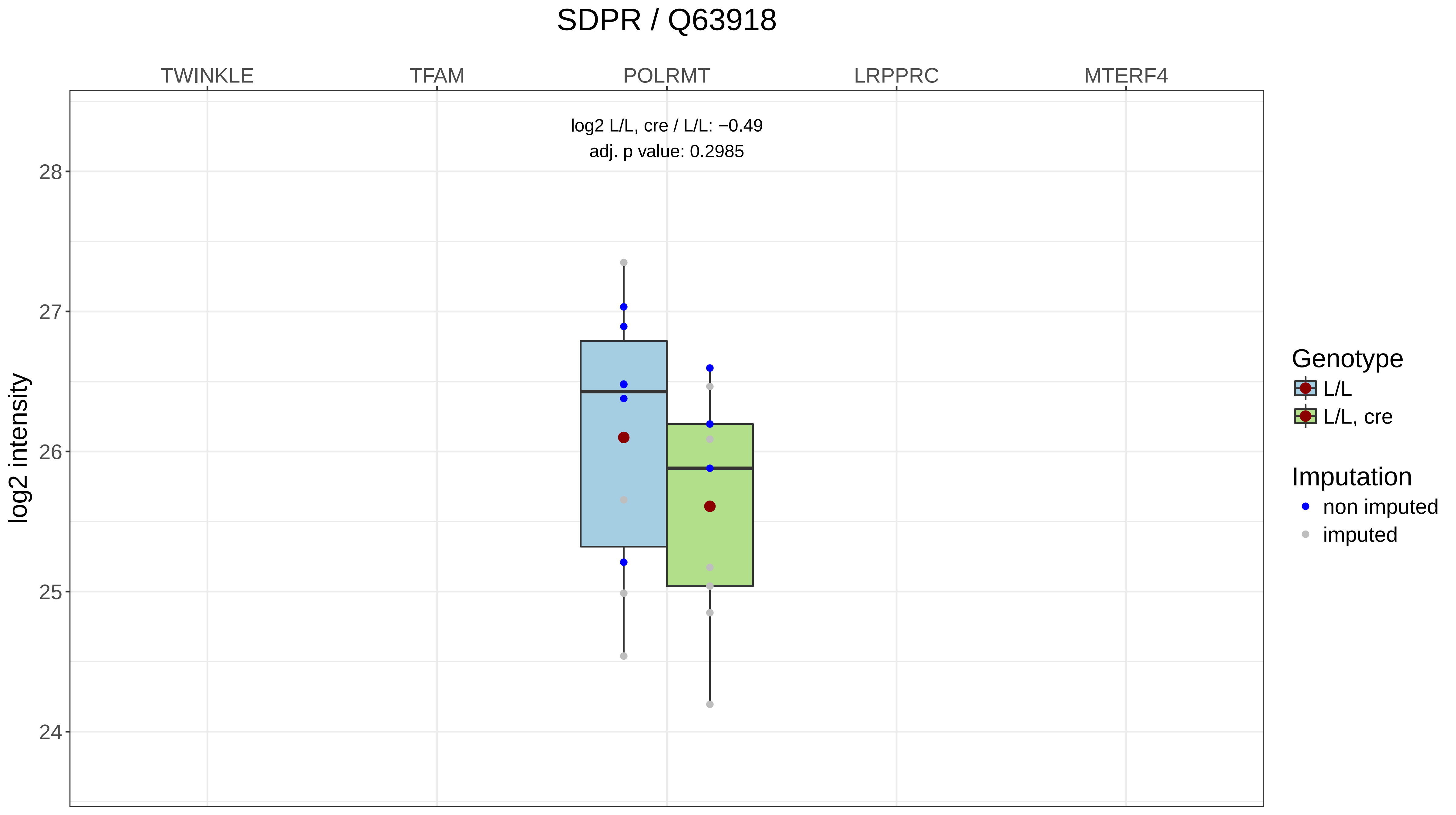
SDPR / Q63918
TWINKLE
TFAM
POLRMT
LRPPRC
MTERF4
log2 L/L, cre / L/L: −0.49
adj. p value: 0.2985
28
27
26
25
24
log2 intensity
Genotype
L/L
L/L, cre
Imputation
non imputed
imputed

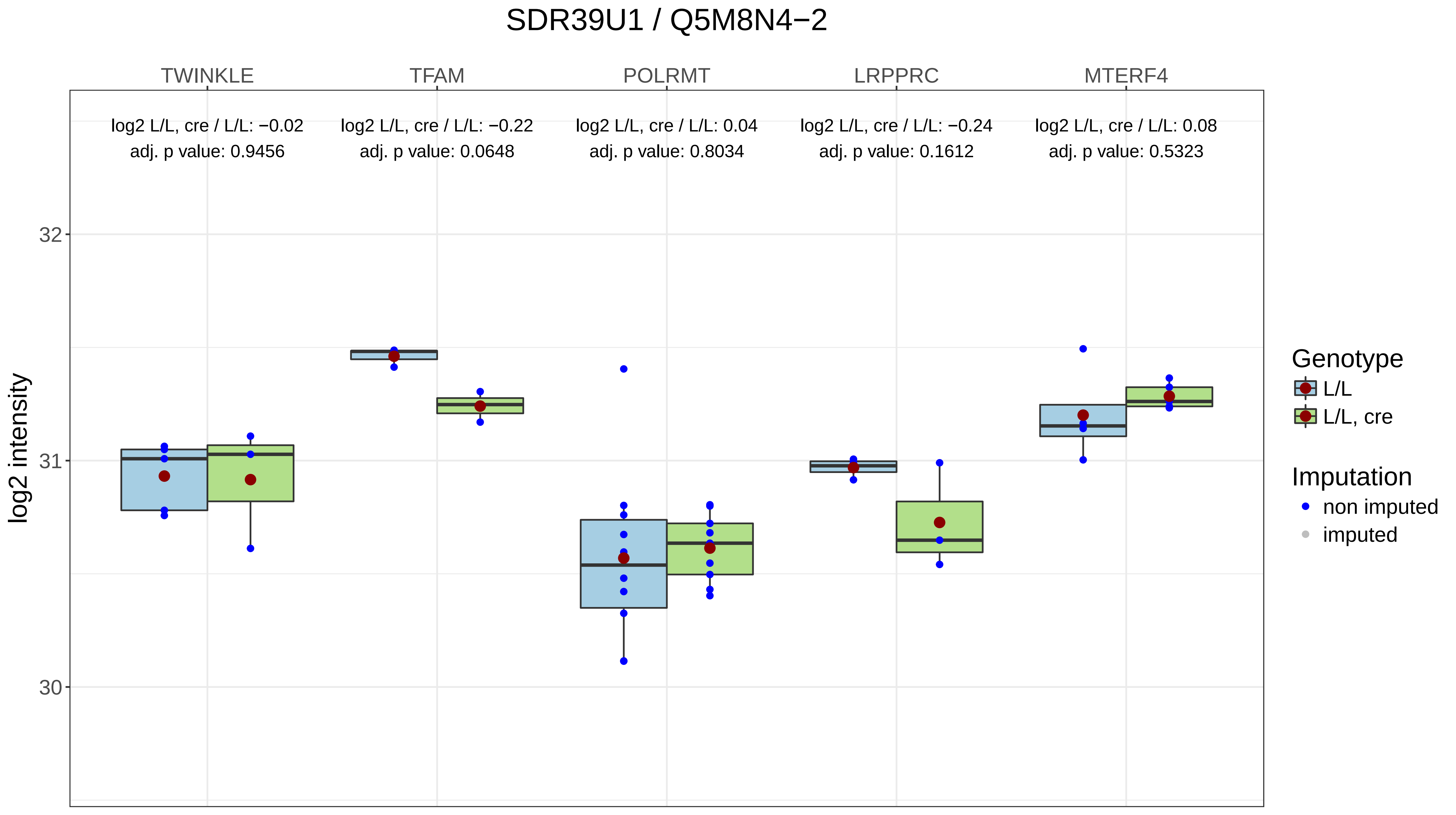
SDR39U1 / Q5M8N4−2
TWINKLE
TFAM
POLRMT
LRPPRC
MTERF4
log2 L/L, cre / L/L: −0.02
adj. p value: 0.9456
log2 L/L, cre / L/L: −0.22
adj. p value: 0.0648
log2 L/L, cre / L/L: 0.04
adj. p value: 0.8034
log2 L/L, cre / L/L: −0.24
adj. p value: 0.1612
log2 L/L, cre / L/L: 0.08
adj. p value: 0.5323
log2 intensity
32
31
30
Genotype
L/L
L/L, cre
Imputation
non imputed
imputed

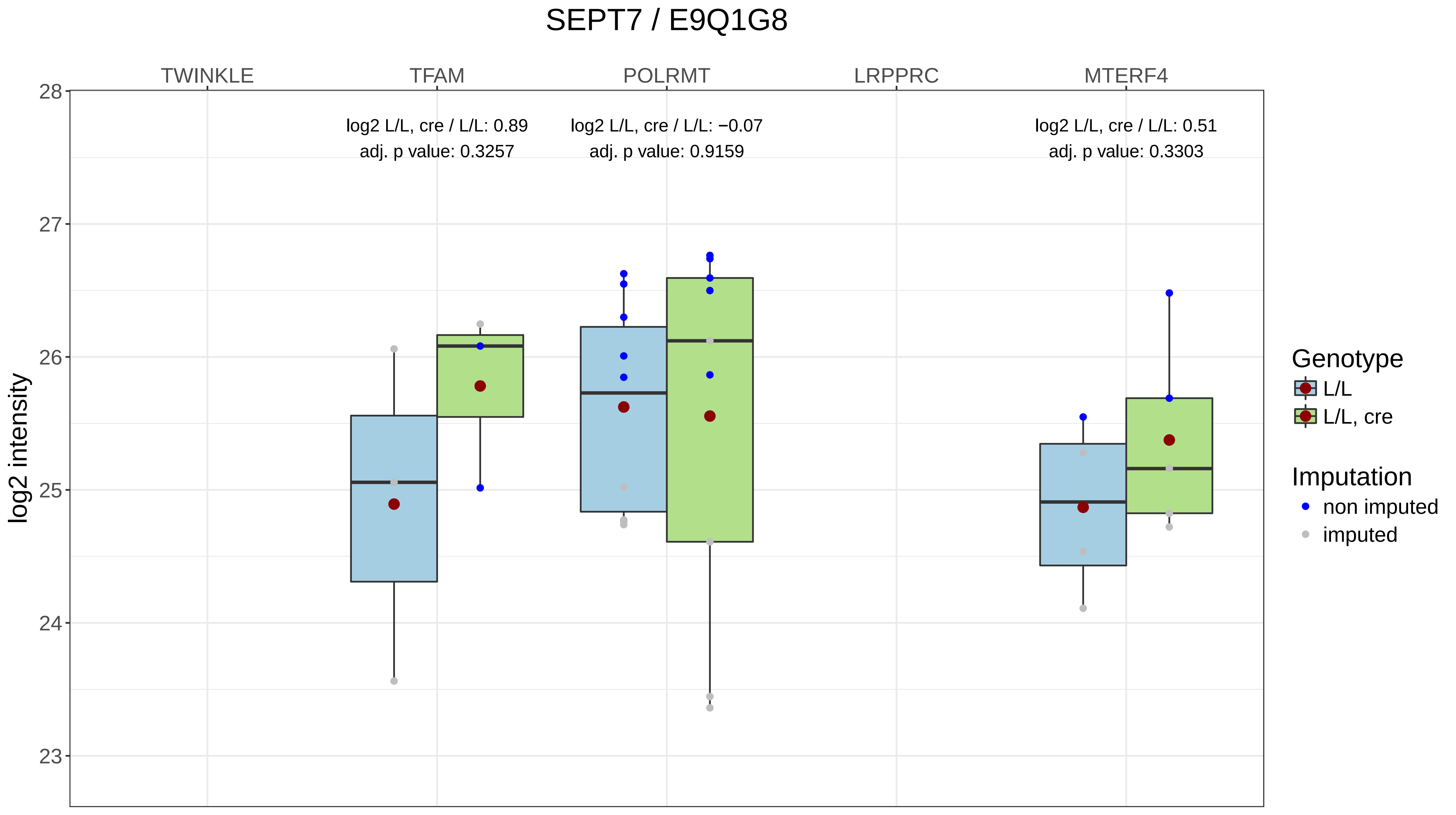
SEPT7 / E9Q1G8
TWINKLE
TFAM
POLRMT
LRPPRC
MTERF4
log2 L/L, cre / L/L: 0.89
adj. p value: 0.3257
log2 L/L, cre / L/L: −0.07
adj. p value: 0.9159
log2 L/L, cre / L/L: 0.51
adj. p value: 0.3303
log2 intensity
28
27
26
25
24
23
Genotype
L/L
L/L, cre
Imputation
non imputed
imputed

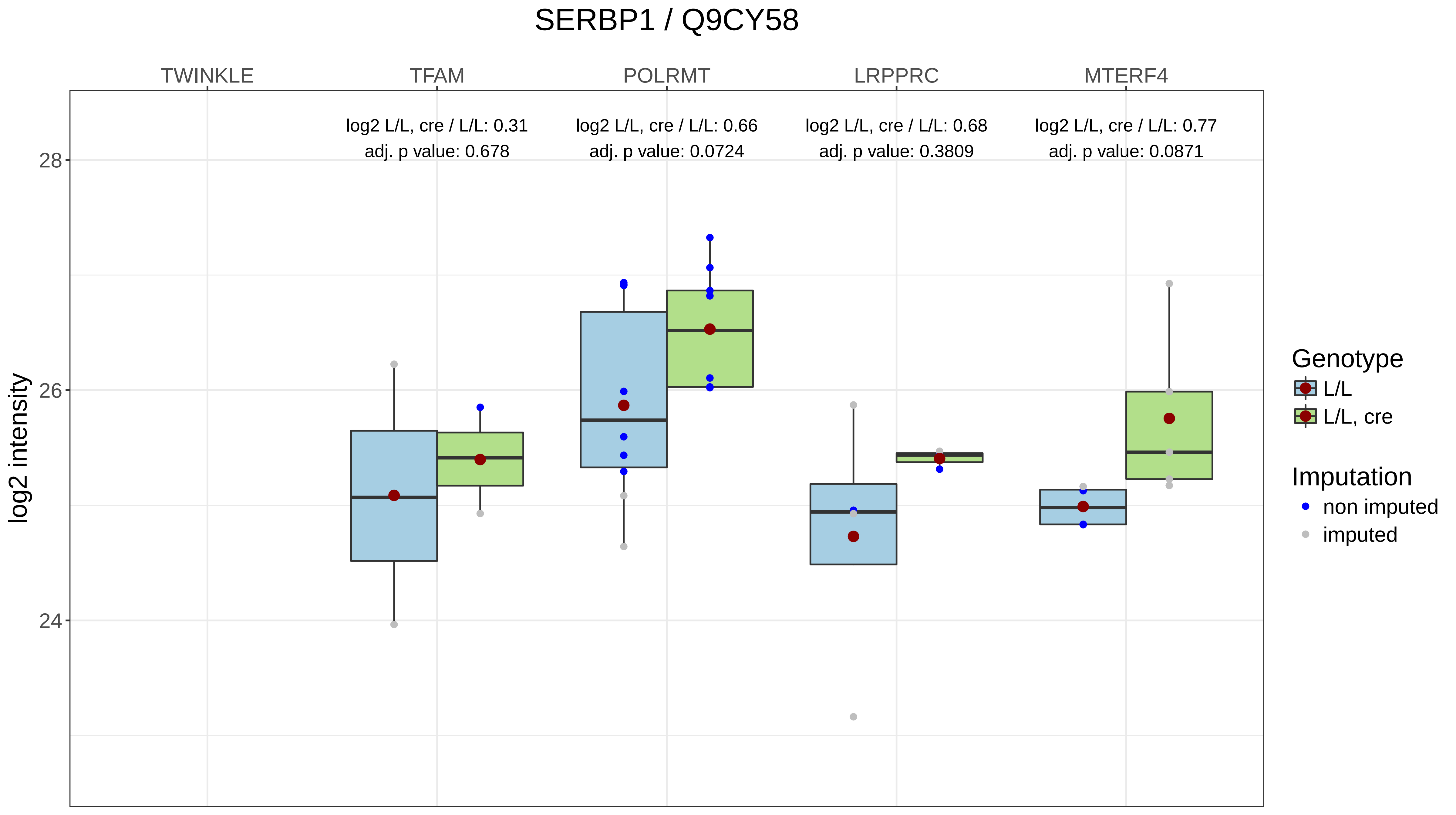
SERBP1 / Q9CY58
TWINKLE
TFAM
POLRMT
LRPPRC
MTERF4
log2 L/L, cre / L/L: 0.31
adj. p value: 0.678
log2 L/L, cre / L/L: 0.66
adj. p value: 0.0724
log2 L/L, cre / L/L: 0.68
adj. p value: 0.3809
log2 L/L, cre / L/L: 0.77
adj. p value: 0.0871
28
26
24
log2 intensity
Genotype
L/L
L/L, cre
Imputation
non imputed
imputed

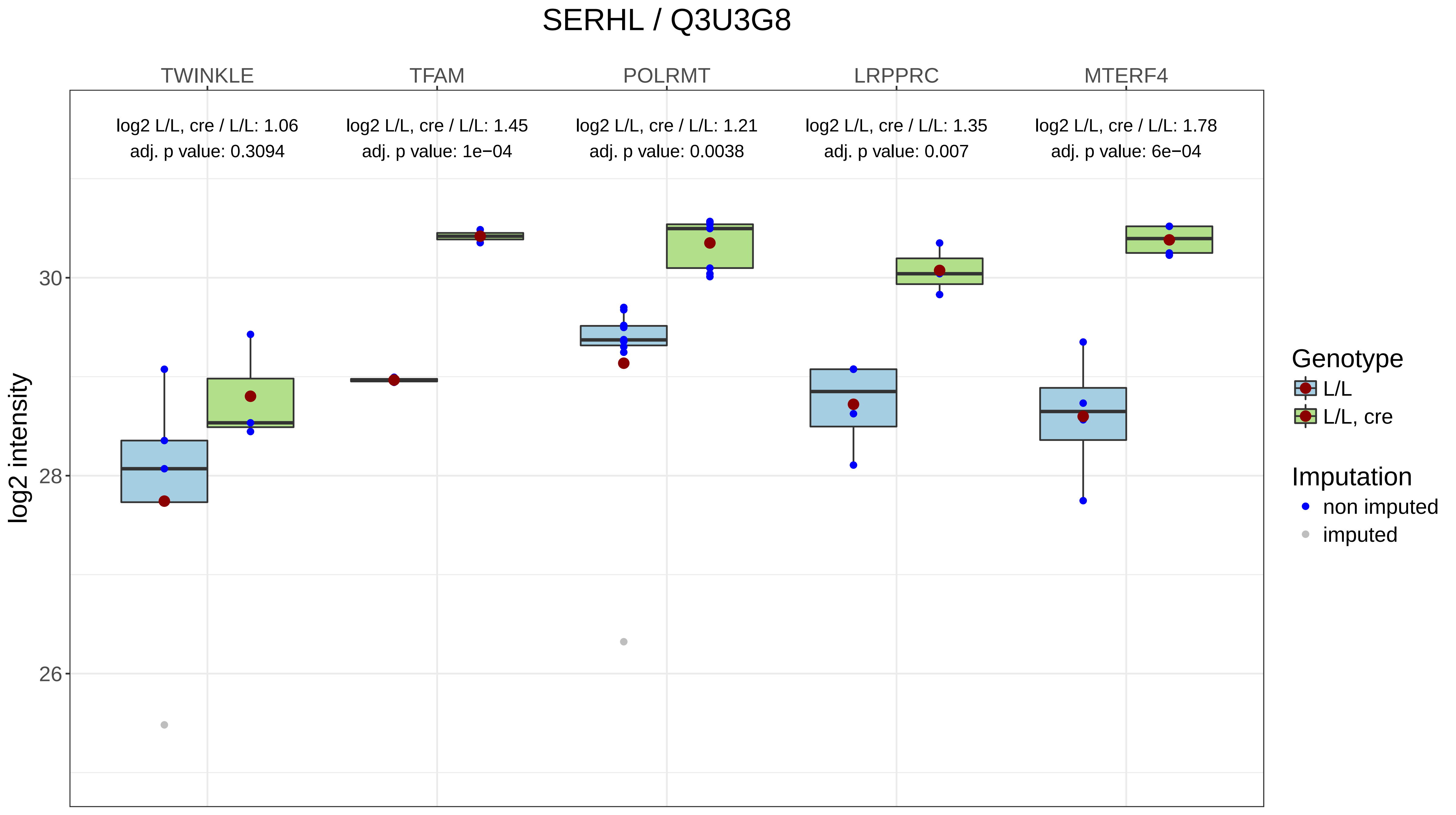
SERHL / Q3U3G8
TWINKLE
TFAM
POLRMT
LRPPRC
MTERF4
log2 L/L, cre / L/L: 1.06
adj. p value: 0.3094
log2 L/L, cre / L/L: 1.45
adj. p value: 1e−04
log2 L/L, cre / L/L: 1.21
adj. p value: 0.0038
log2 L/L, cre / L/L: 1.35
adj. p value: 0.007
log2 L/L, cre / L/L: 1.78
adj. p value: 6e−04
log2 intensity
30
28
26
Genotype
L/L
L/L, cre
Imputation
non imputed
imputed

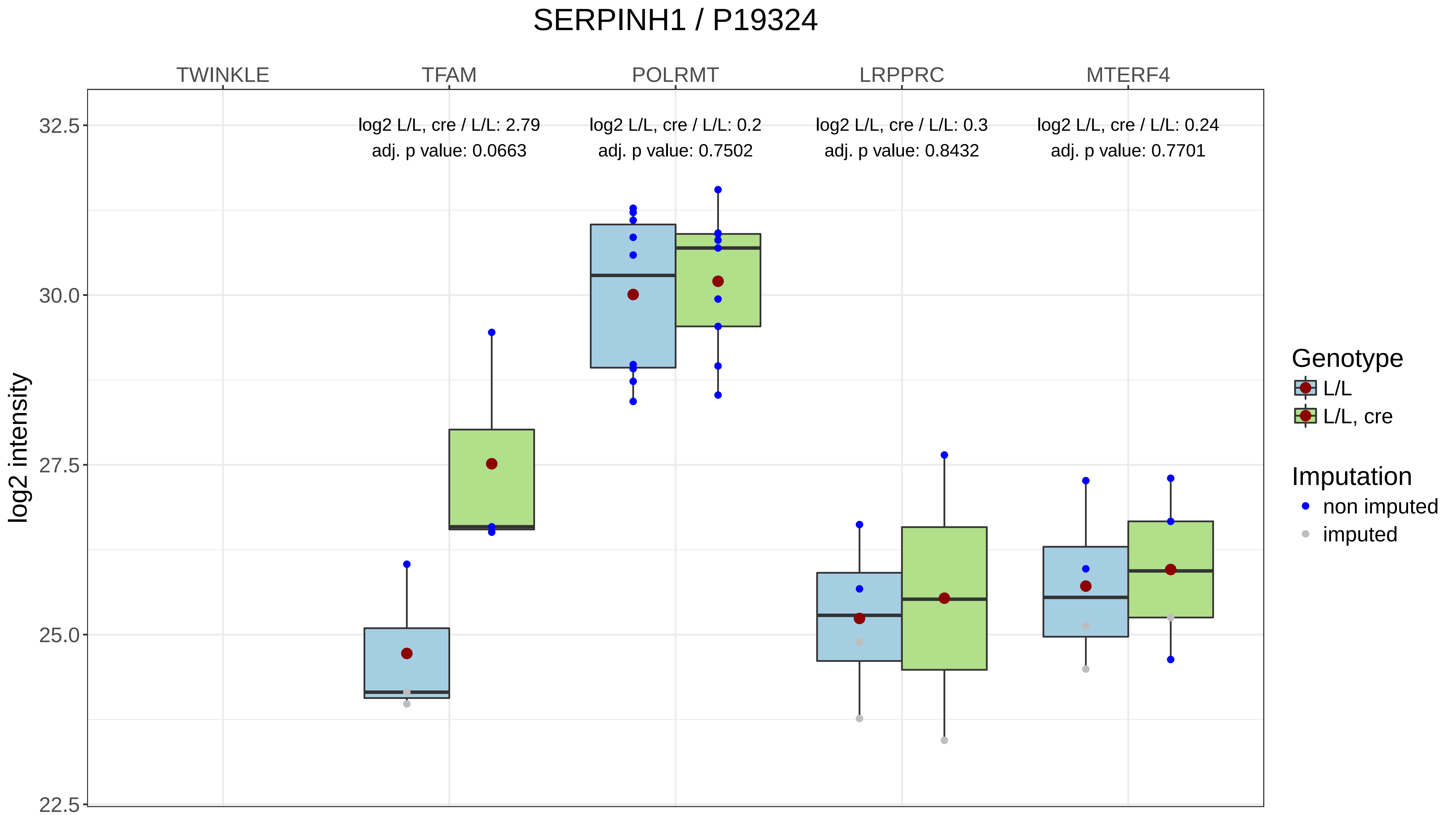

SERPINH1 / P19324
TWINKLE
TFAM
POLRMT
LRPPRC
MTERF4
log2 L/L, cre / L/L: 2.79
adj. p value: 0.0663
log2 L/L, cre / L/L: 0.2
adj. p value: 0.7502
log2 L/L, cre / L/L: 0.3
adj. p value: 0.8432
log2 L/L, cre / L/L: 0.24
adj. p value: 0.7701
32.5
30.0
27.5
25.0
22.5
log2 intensity
Genotype
L/L
L/L, cre
Imputation
non imputed
imputed

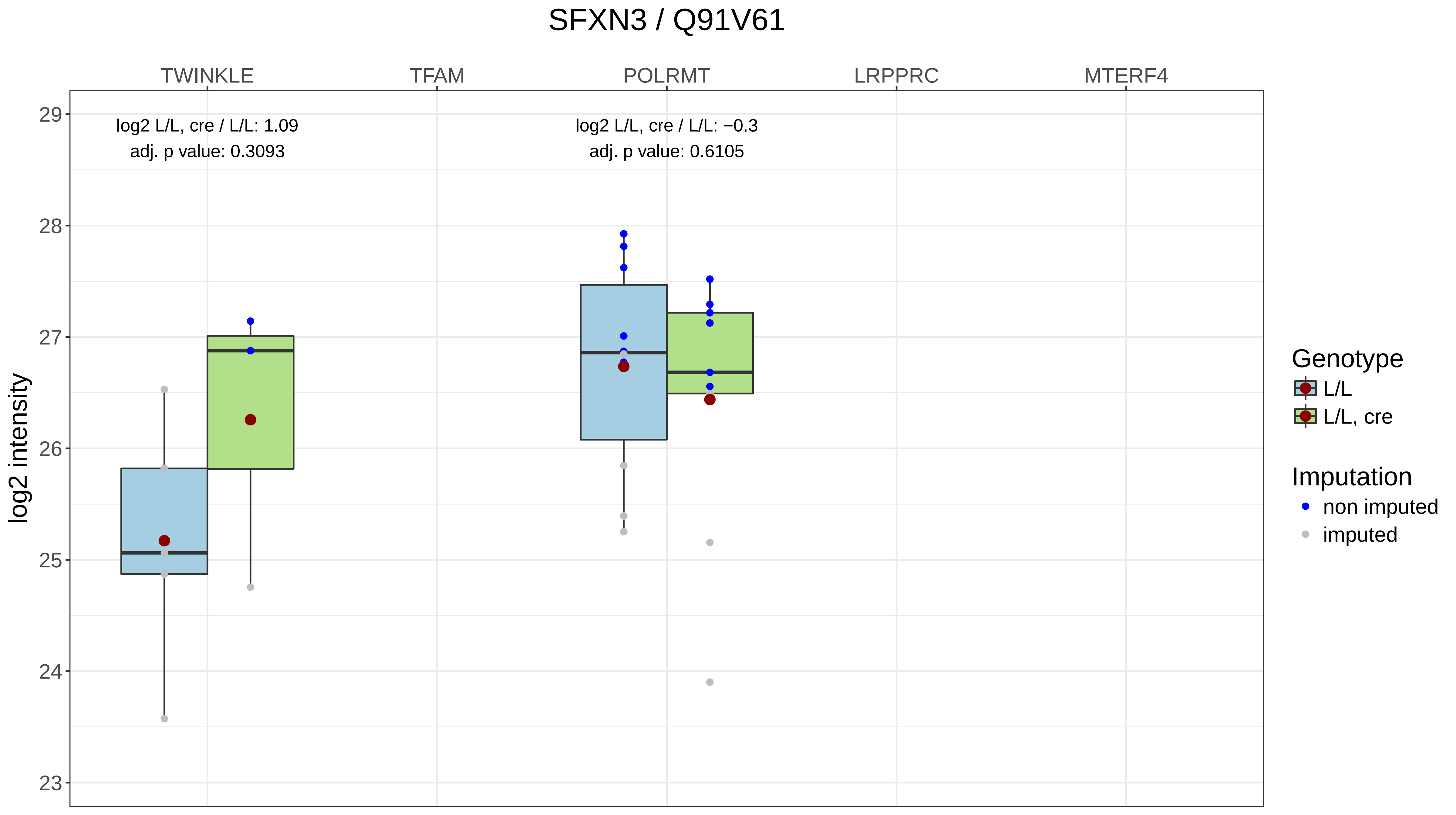
SFXN3 / Q91V61
TWINKLE
TFAM
POLRMT
LRPPRC
MTERF4
log2 L/L, cre / L/L: 1.09
adj. p value: 0.3093
log2 L/L, cre / L/L: −0.3
adj. p value: 0.6105
log2 intensity
23
24
25
26
27
28
29
Genotype
L/L
L/L, cre
Imputation
non imputed
imputed

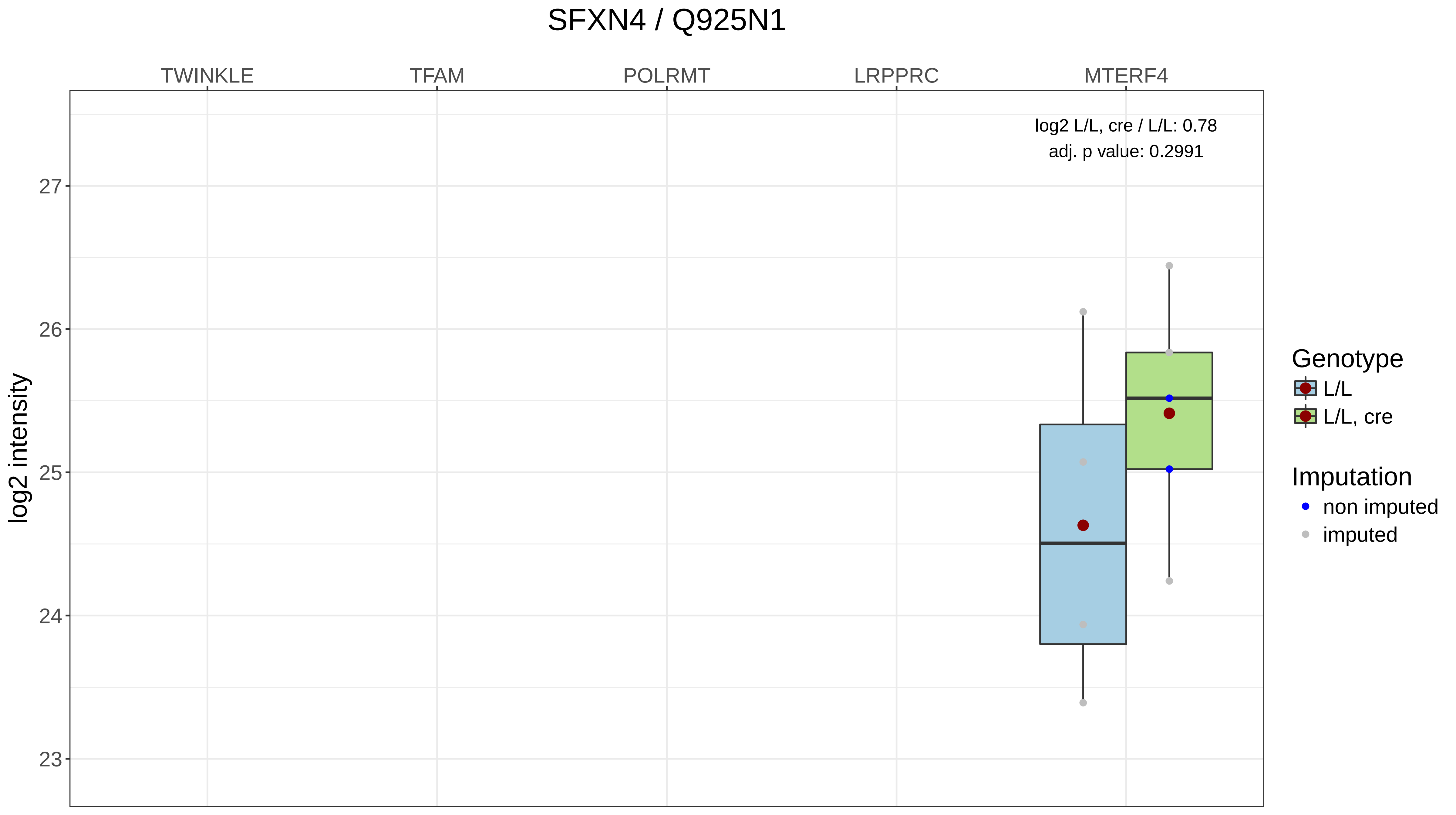

SFXN4 / Q925N1
TWINKLE
TFAM
POLRMT
LRPPRC
MTERF4
log2 L/L, cre / L/L: 0.78
adj. p value: 0.2991
log2 intensity
27
26
25
24
23
Genotype
L/L
L/L, cre
Imputation
non imputed
imputed

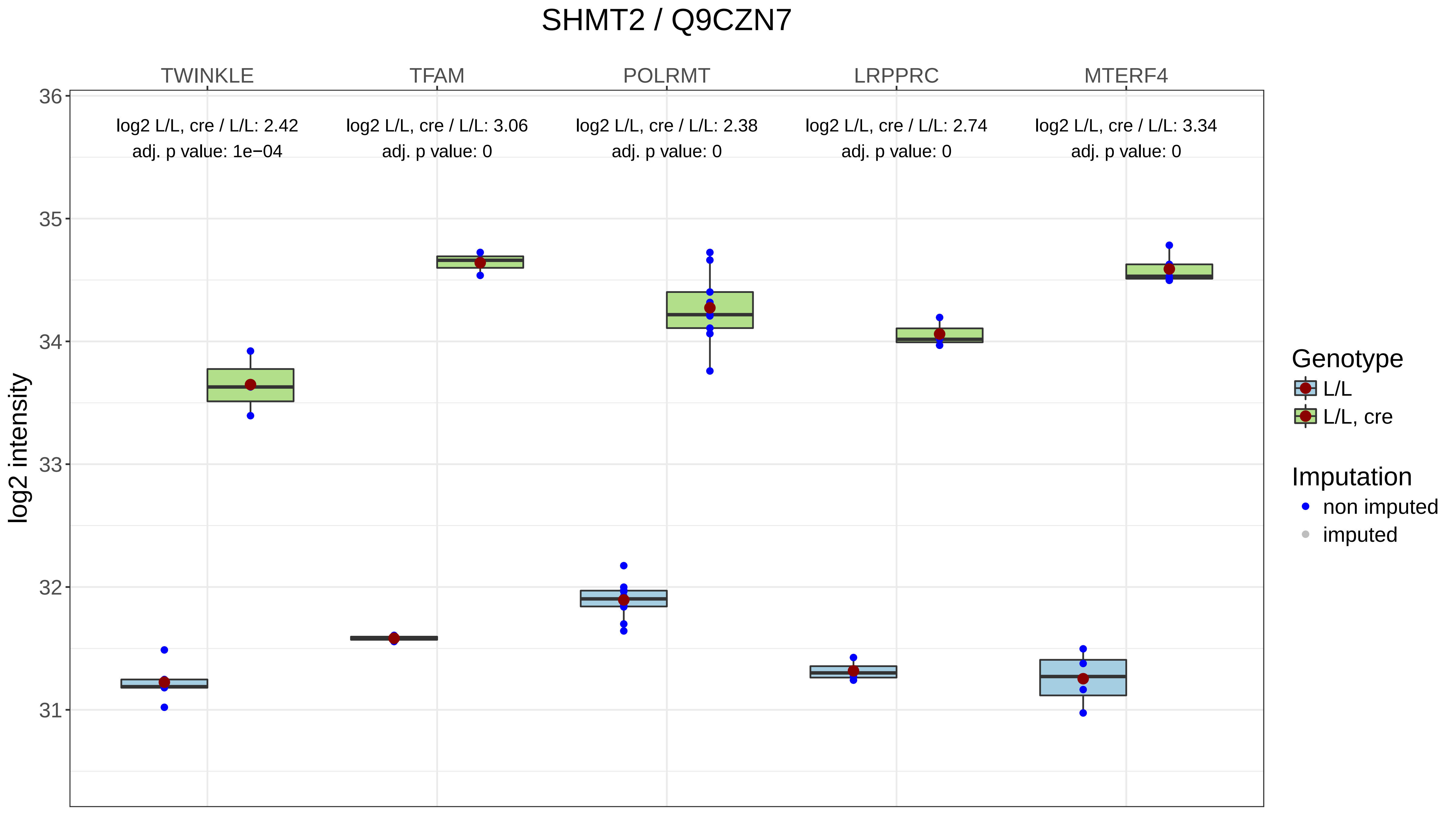
SHMT2 / Q9CZN7
TWINKLE
TFAM
POLRMT
LRPPRC
MTERF4
log2 L/L, cre / L/L: 2.42
adj. p value: 1e−04
log2 L/L, cre / L/L: 3.06
adj. p value: 0
log2 L/L, cre / L/L: 2.38
adj. p value: 0
log2 L/L, cre / L/L: 2.74
adj. p value: 0
log2 L/L, cre / L/L: 3.34
adj. p value: 0
log2 intensity
36
35
34
33
32
31
Genotype
L/L
L/L, cre
Imputation
non imputed
imputed

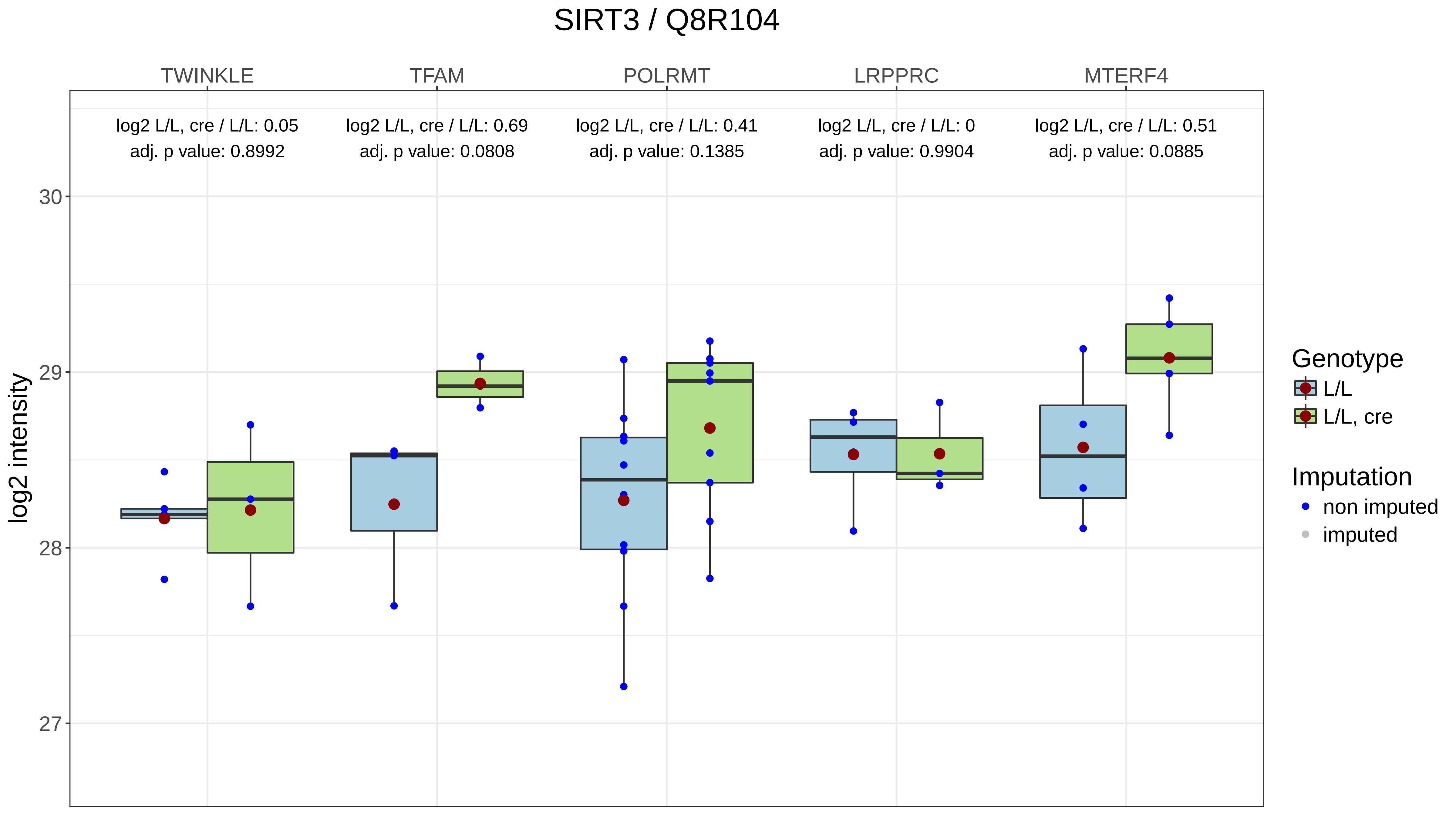

SIRT3 / Q8R104
TWINKLE
TFAM
POLRMT
LRPPRC
MTERF4
log2 L/L, cre / L/L: 0.05
adj. p value: 0.8992
log2 L/L, cre / L/L: 0.69
adj. p value: 0.0808
log2 L/L, cre / L/L: 0.41
adj. p value: 0.1385
log2 L/L, cre / L/L: 0
adj. p value: 0.9904
log2 L/L, cre / L/L: 0.51
adj. p value: 0.0885
log2 intensity
30
29
28
27
Genotype
L/L
L/L, cre
Imputation
non imputed
imputed

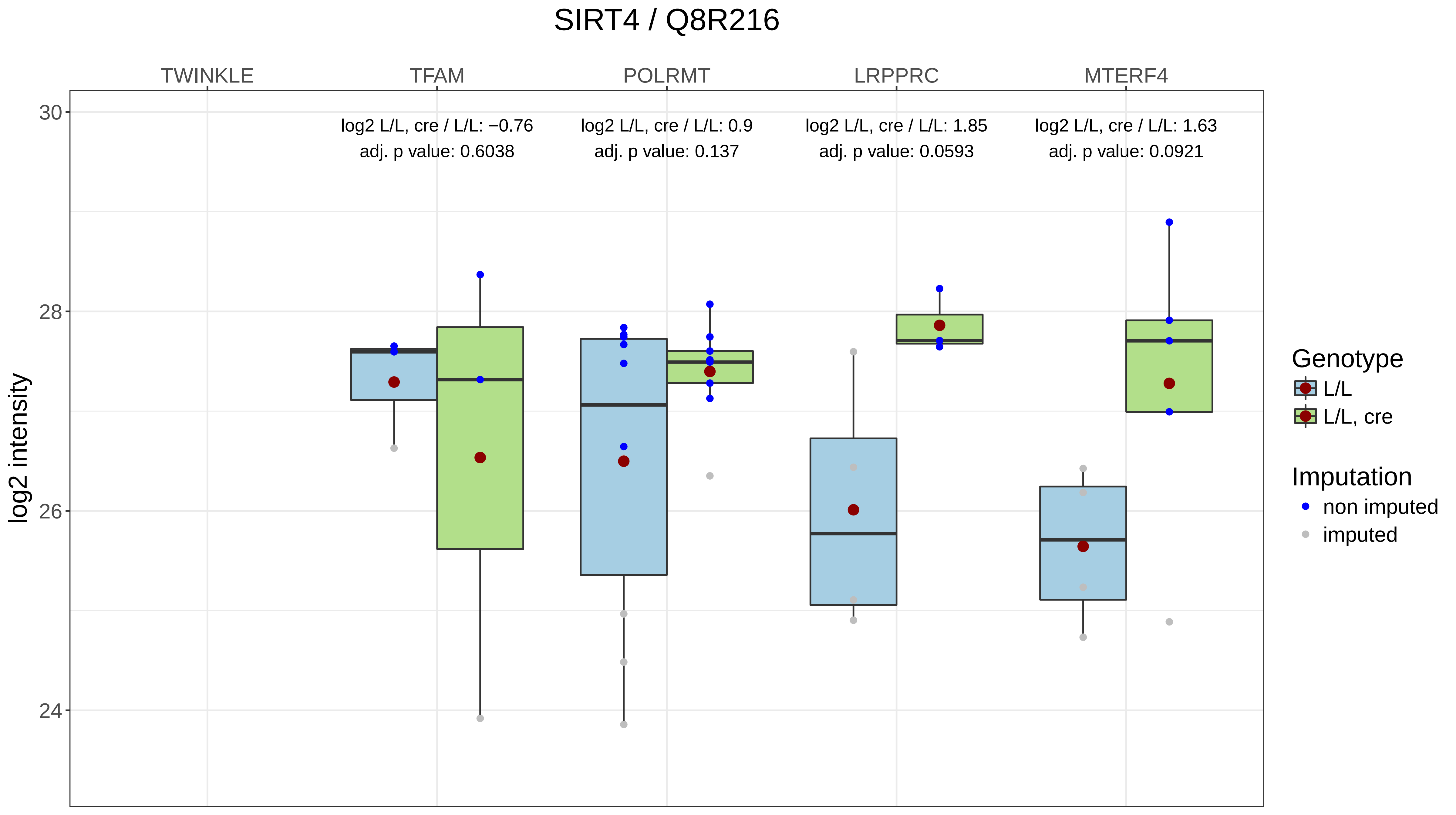
SIRT4 / Q8R216
TWINKLE
TFAM
POLRMT
LRPPRC
MTERF4
log2 L/L, cre / L/L: −0.76
adj. p value: 0.6038
log2 L/L, cre / L/L: 0.9
adj. p value: 0.137
log2 L/L, cre / L/L: 1.85
adj. p value: 0.0593
log2 L/L, cre / L/L: 1.63
adj. p value: 0.0921
log2 intensity
30
28
26
24
Genotype
L/L
L/L, cre
Imputation
non imputed
imputed

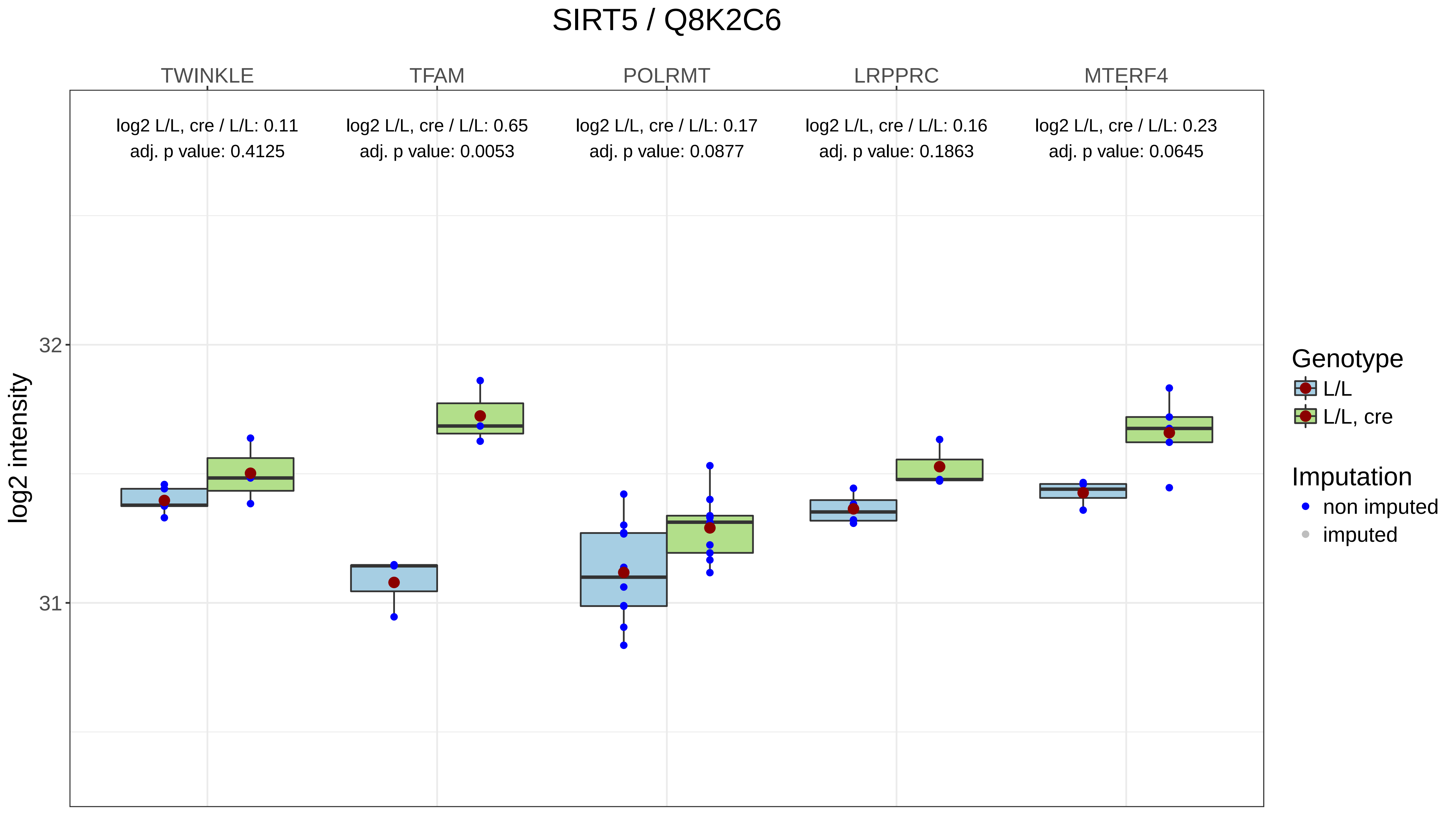
SIRT5 / Q8K2C6
TWINKLE
TFAM
POLRMT
LRPPRC
MTERF4
log2 L/L, cre / L/L: 0.11
adj. p value: 0.4125
log2 L/L, cre / L/L: 0.65
adj. p value: 0.0053
log2 L/L, cre / L/L: 0.17
adj. p value: 0.0877
log2 L/L, cre / L/L: 0.16
adj. p value: 0.1863
log2 L/L, cre / L/L: 0.23
adj. p value: 0.0645
log2 intensity
32
31
Genotype
L/L
L/L, cre
Imputation
non imputed
imputed

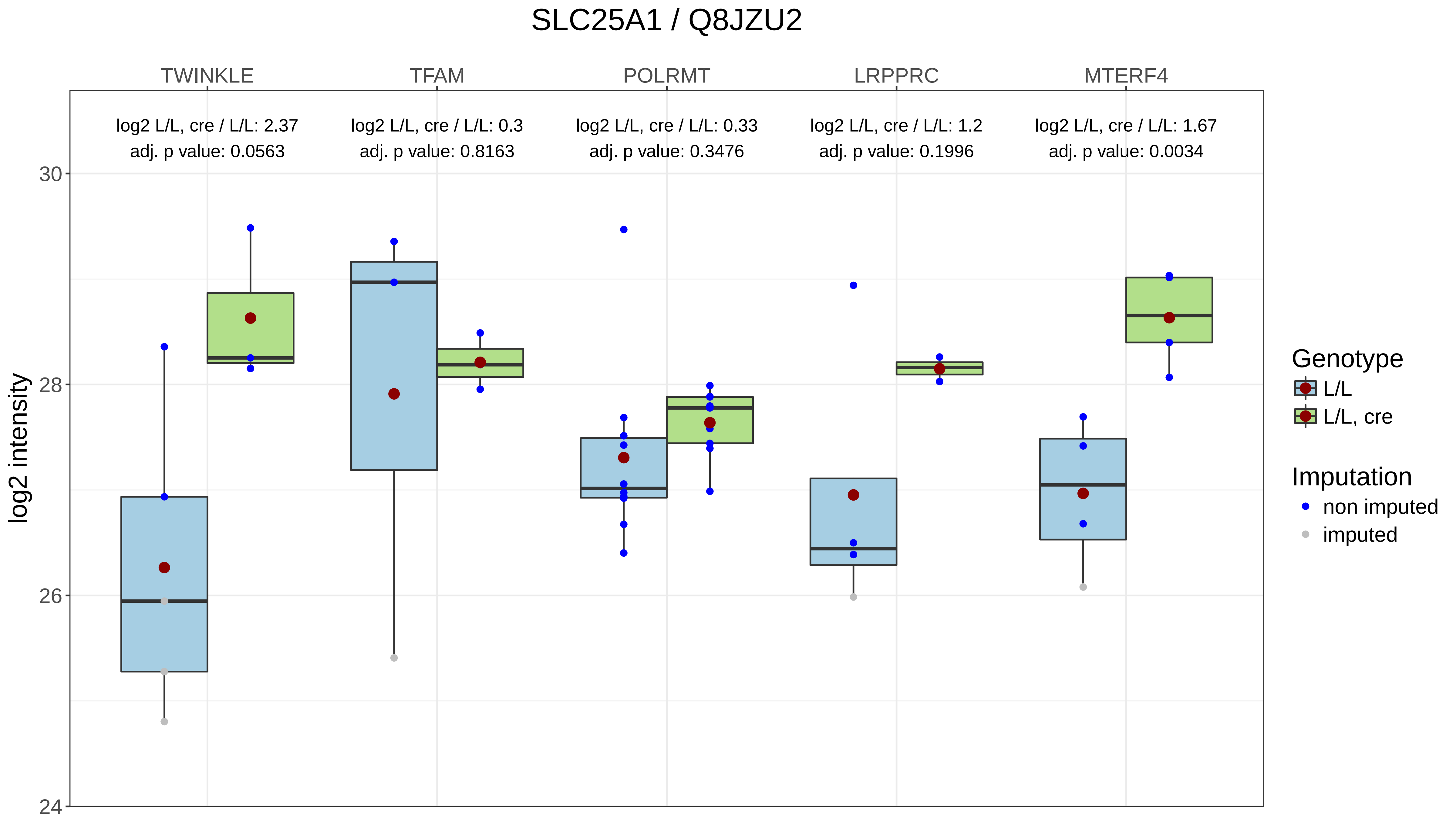
SLC25A1 / Q8JZU2
TWINKLE
TFAM
POLRMT
LRPPRC
MTERF4
log2 L/L, cre / L/L: 2.37
adj. p value: 0.0563
log2 L/L, cre / L/L: 0.3
adj. p value: 0.8163
log2 L/L, cre / L/L: 0.33
adj. p value: 0.3476
log2 L/L, cre / L/L: 1.2
adj. p value: 0.1996
log2 L/L, cre / L/L: 1.67
adj. p value: 0.0034
log2 intensity
30
28
26
24
Genotype
L/L
L/L, cre
Imputation
non imputed
imputed

SLC25A10 / Q9QZD8

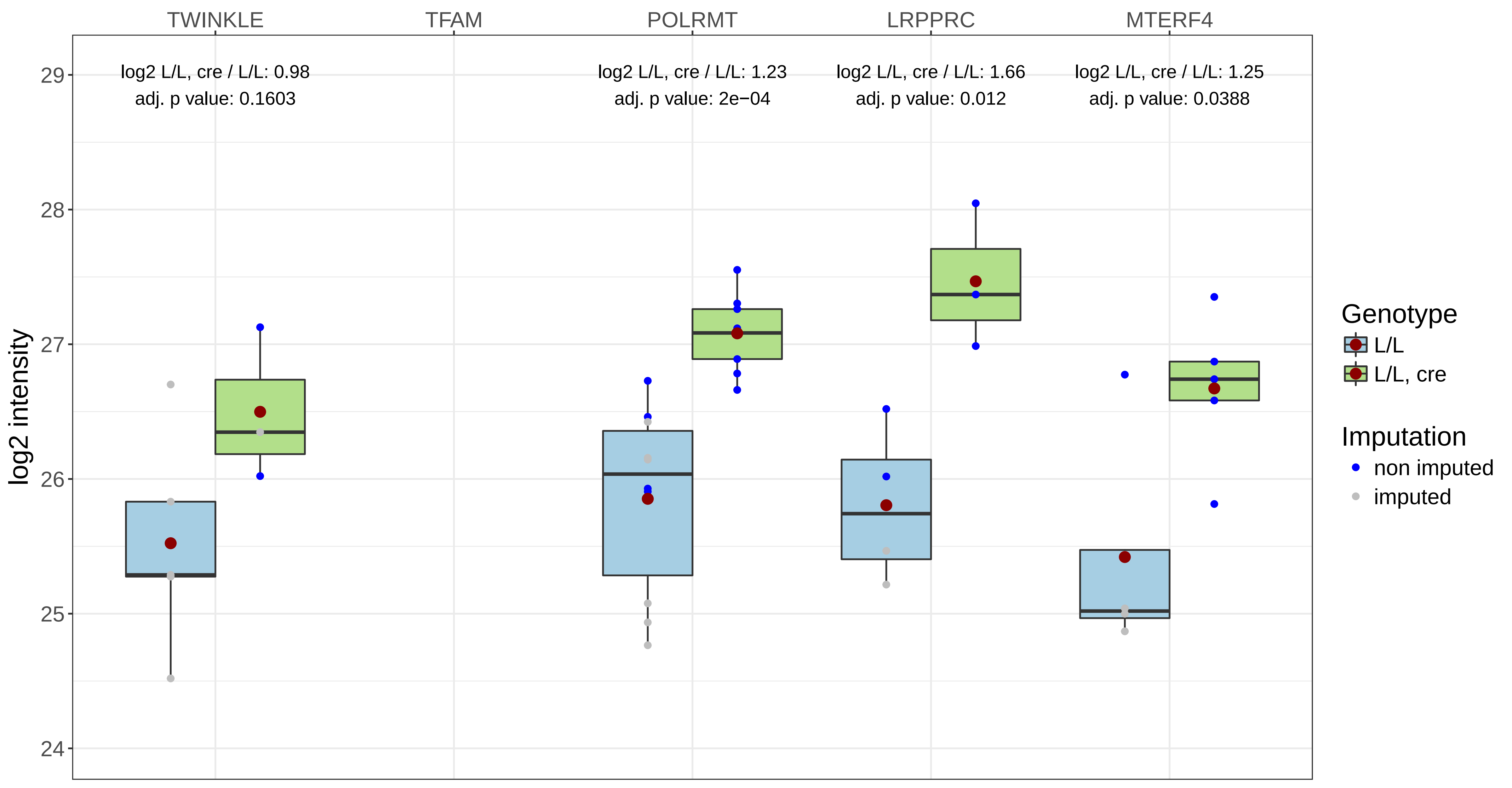

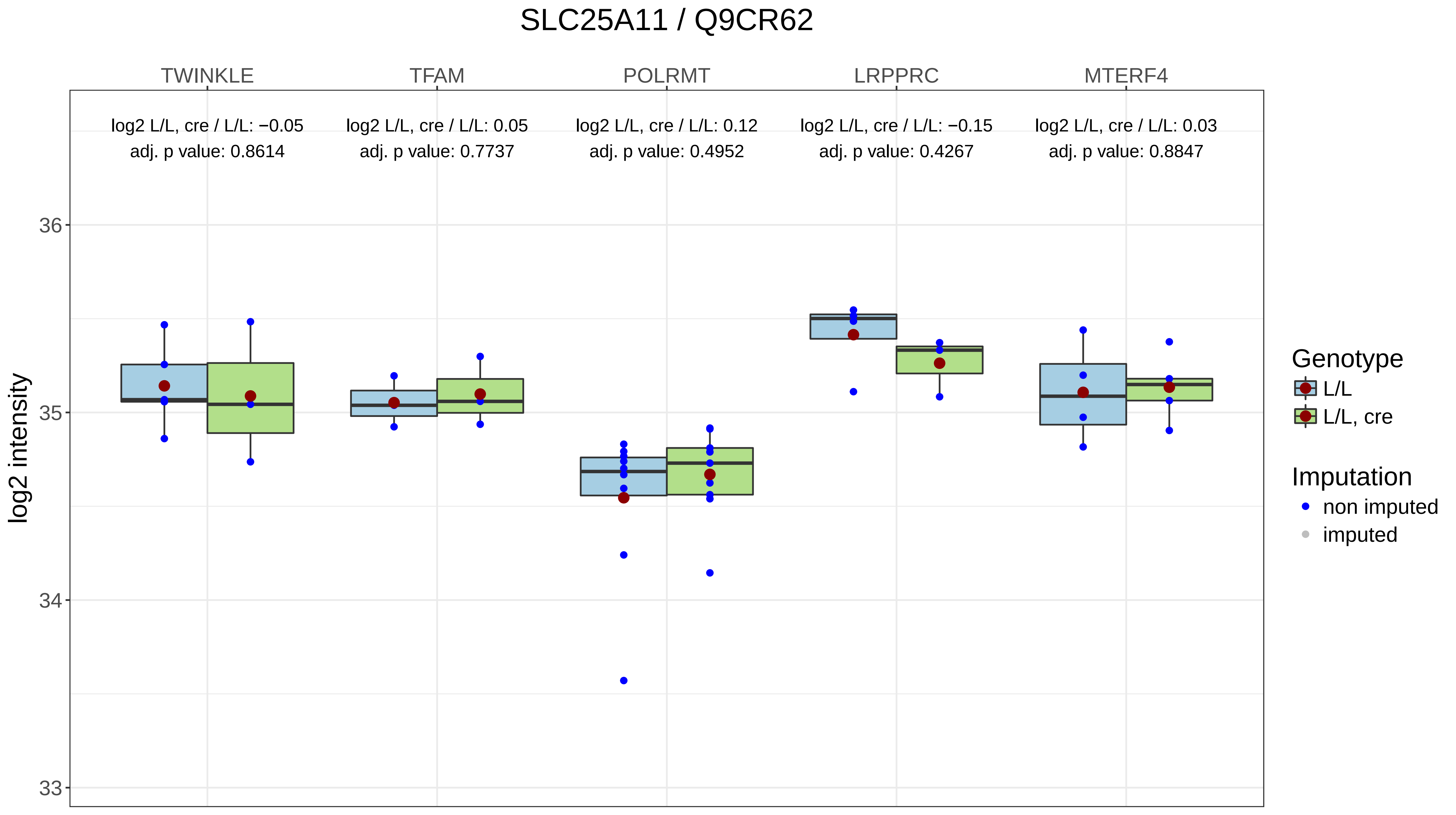

SLC25A11 / Q9CR62
TWINKLE
TFAM
POLRMT
LRPPRC
MTERF4
log2 L/L, cre / L/L: −0.05
adj. p value: 0.8614
log2 L/L, cre / L/L: 0.05
adj. p value: 0.7737
log2 L/L, cre / L/L: 0.12
adj. p value: 0.4952
log2 L/L, cre / L/L: −0.15
adj. p value: 0.4267
log2 L/L, cre / L/L: 0.03
adj. p value: 0.8847
log2 intensity
36
35
34
33
Genotype
L/L
L/L, cre
Imputation
non imputed
imputed

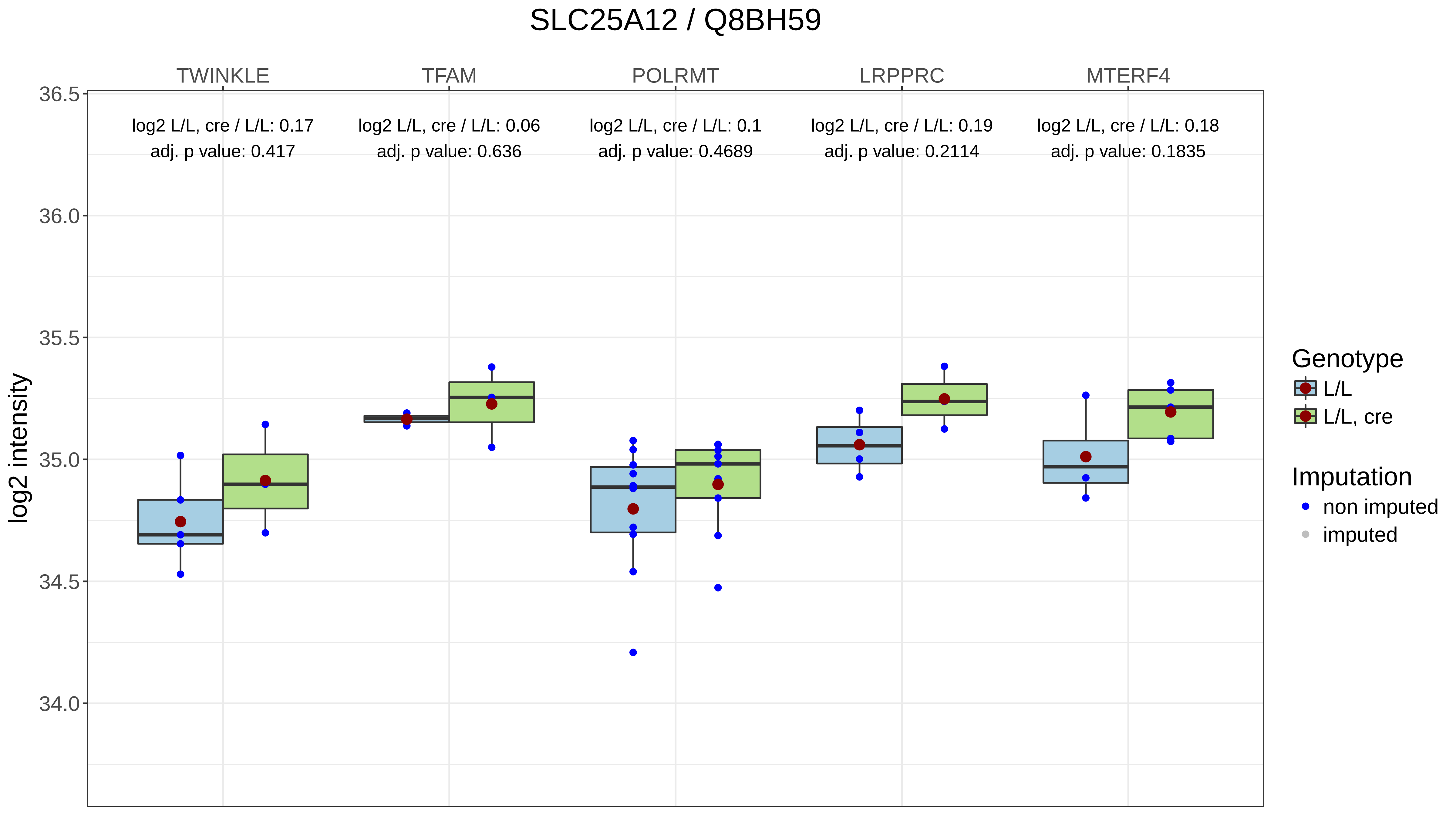
SLC25A12 / Q8BH59
TWINKLE
TFAM
POLRMT
LRPPRC
MTERF4
log2 L/L, cre / L/L: 0.17
adj. p value: 0.417
log2 L/L, cre / L/L: 0.06
adj. p value: 0.636
log2 L/L, cre / L/L: 0.1
adj. p value: 0.4689
log2 L/L, cre / L/L: 0.19
adj. p value: 0.2114
log2 L/L, cre / L/L: 0.18
adj. p value: 0.1835
log2 intensity
36.5
36.0
35.5
35.0
34.5
34.0
Genotype
L/L
L/L, cre
Imputation
non imputed
imputed

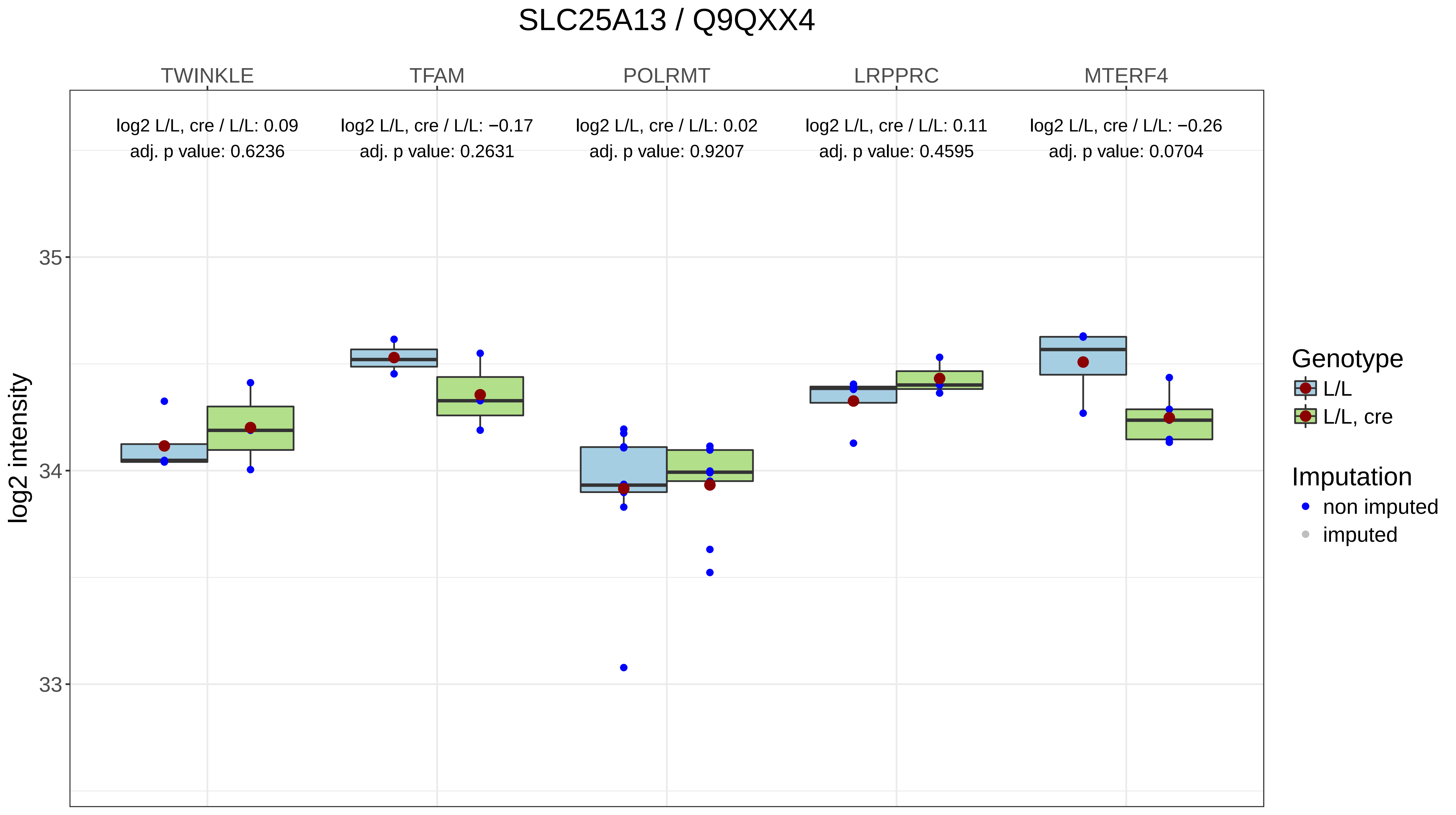
SLC25A13 / Q9QXX4
TWINKLE
TFAM
POLRMT
LRPPRC
MTERF4
log2 L/L, cre / L/L: 0.09
adj. p value: 0.6236
log2 L/L, cre / L/L: −0.17
adj. p value: 0.2631
log2 L/L, cre / L/L: 0.02
adj. p value: 0.9207
log2 L/L, cre / L/L: 0.11
adj. p value: 0.4595
log2 L/L, cre / L/L: −0.26
adj. p value: 0.0704
35
34
33
log2 intensity
Genotype
L/L
L/L, cre
Imputation
non imputed
imputed

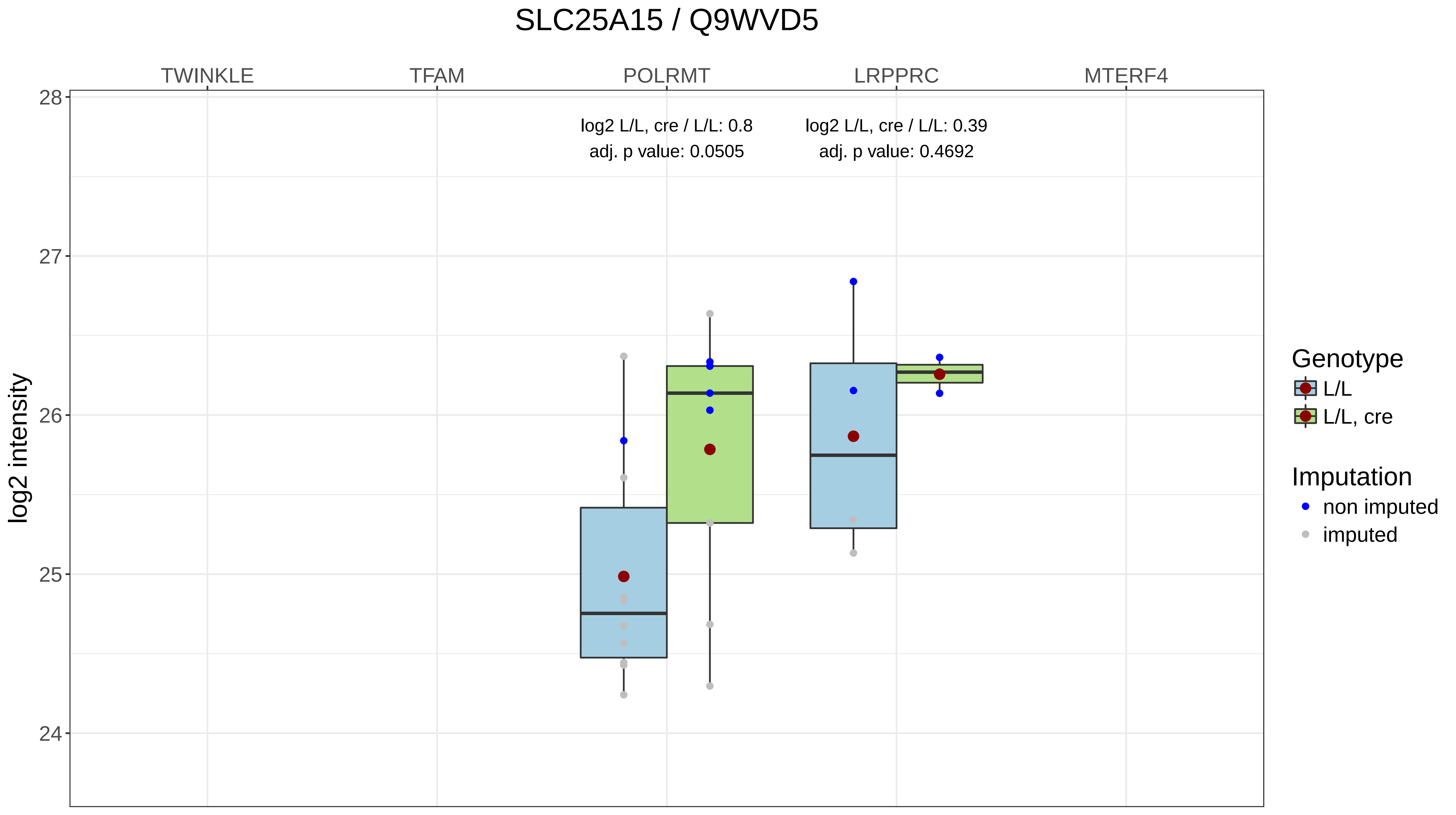

SLC25A15 / Q9WVD5
TWINKLE
TFAM
POLRMT
LRPPRC
MTERF4
log2 L/L, cre / L/L: 0.8
adj. p value: 0.0505
log2 L/L, cre / L/L: 0.39
adj. p value: 0.4692
log2 intensity
28
27
26
25
24
Genotype
L/L
L/L, cre
Imputation
non imputed
imputed

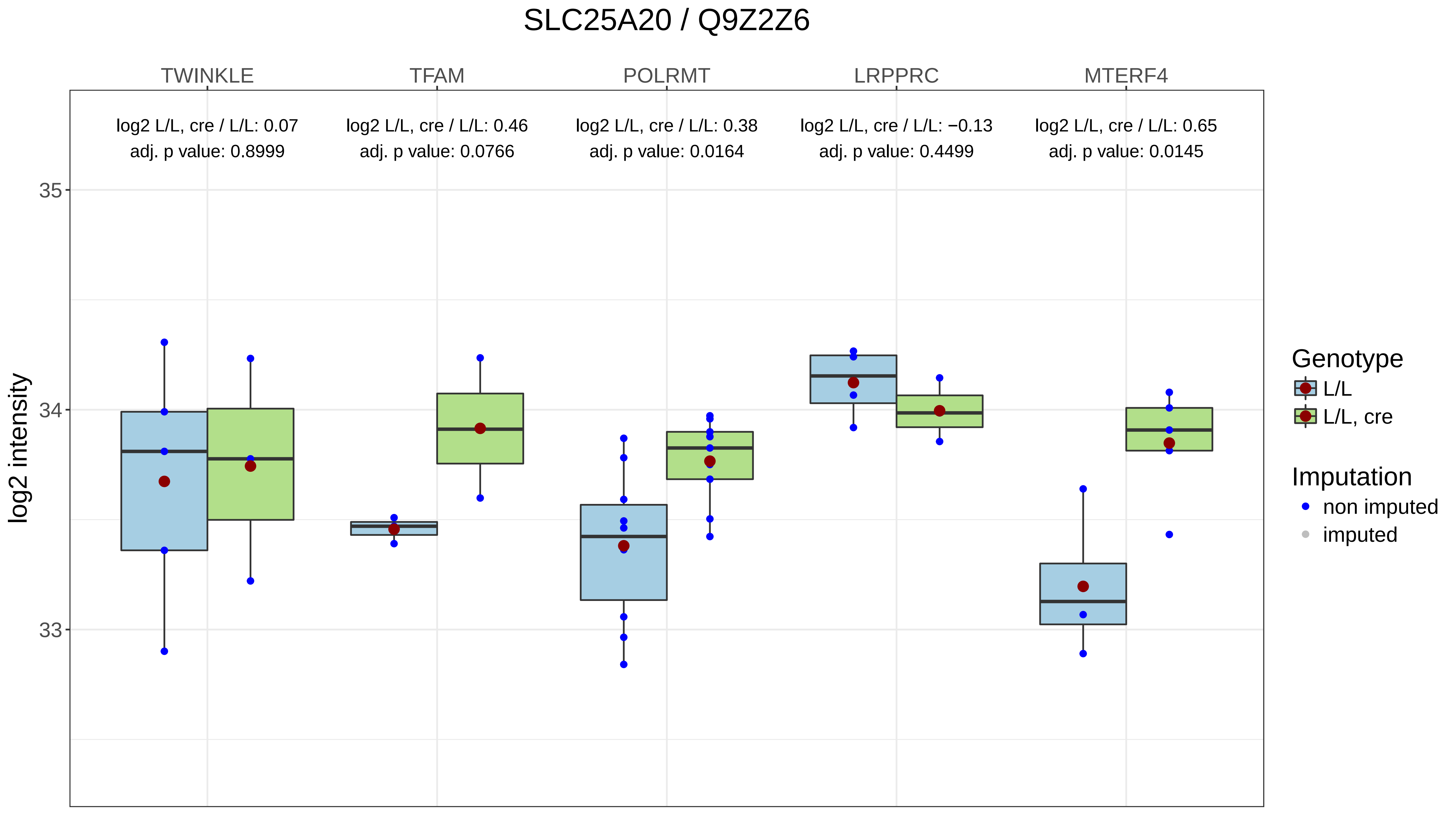
SLC25A20 / Q9Z2Z6
TWINKLE
TFAM
POLRMT
LRPPRC
MTERF4
log2 L/L, cre / L/L: 0.07
adj. p value: 0.8999
log2 L/L, cre / L/L: 0.46
adj. p value: 0.0766
log2 L/L, cre / L/L: 0.38
adj. p value: 0.0164
log2 L/L, cre / L/L: −0.13
adj. p value: 0.4499
log2 L/L, cre / L/L: 0.65
adj. p value: 0.0145
log2 intensity
35
34
33
Genotype
L/L
L/L, cre
Imputation
non imputed
imputed

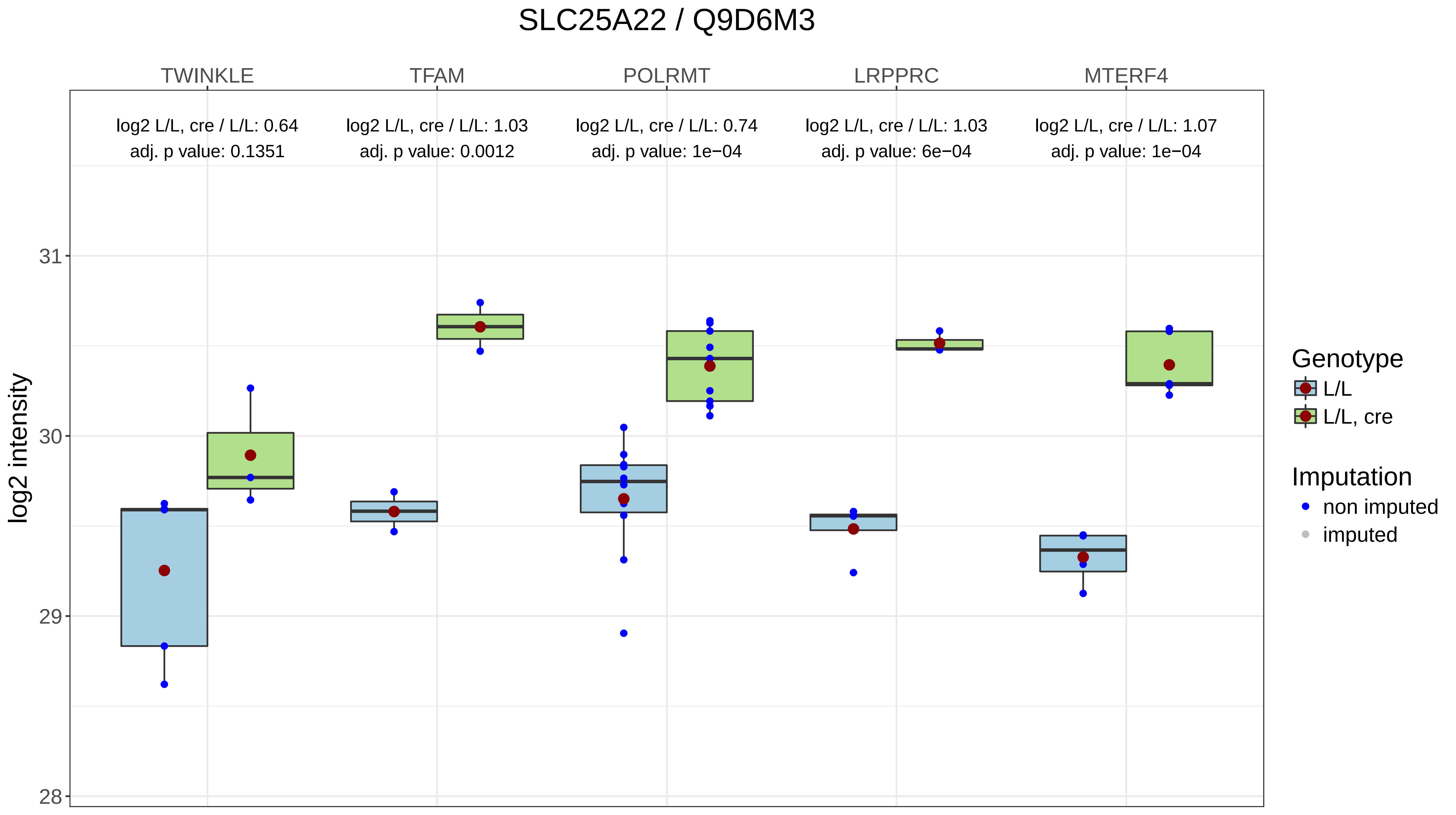
SLC25A22 / Q9D6M3
TWINKLE
TFAM
POLRMT
LRPPRC
MTERF4
log2 L/L, cre / L/L: 0.64
adj. p value: 0.1351
log2 L/L, cre / L/L: 1.03
adj. p value: 0.0012
log2 L/L, cre / L/L: 0.74
adj. p value: 1e−04
log2 L/L, cre / L/L: 1.03
adj. p value: 6e−04
log2 L/L, cre / L/L: 1.07
adj. p value: 1e−04
log2 intensity
31
30
29
28
Genotype
L/L
L/L, cre
Imputation
non imputed
imputed

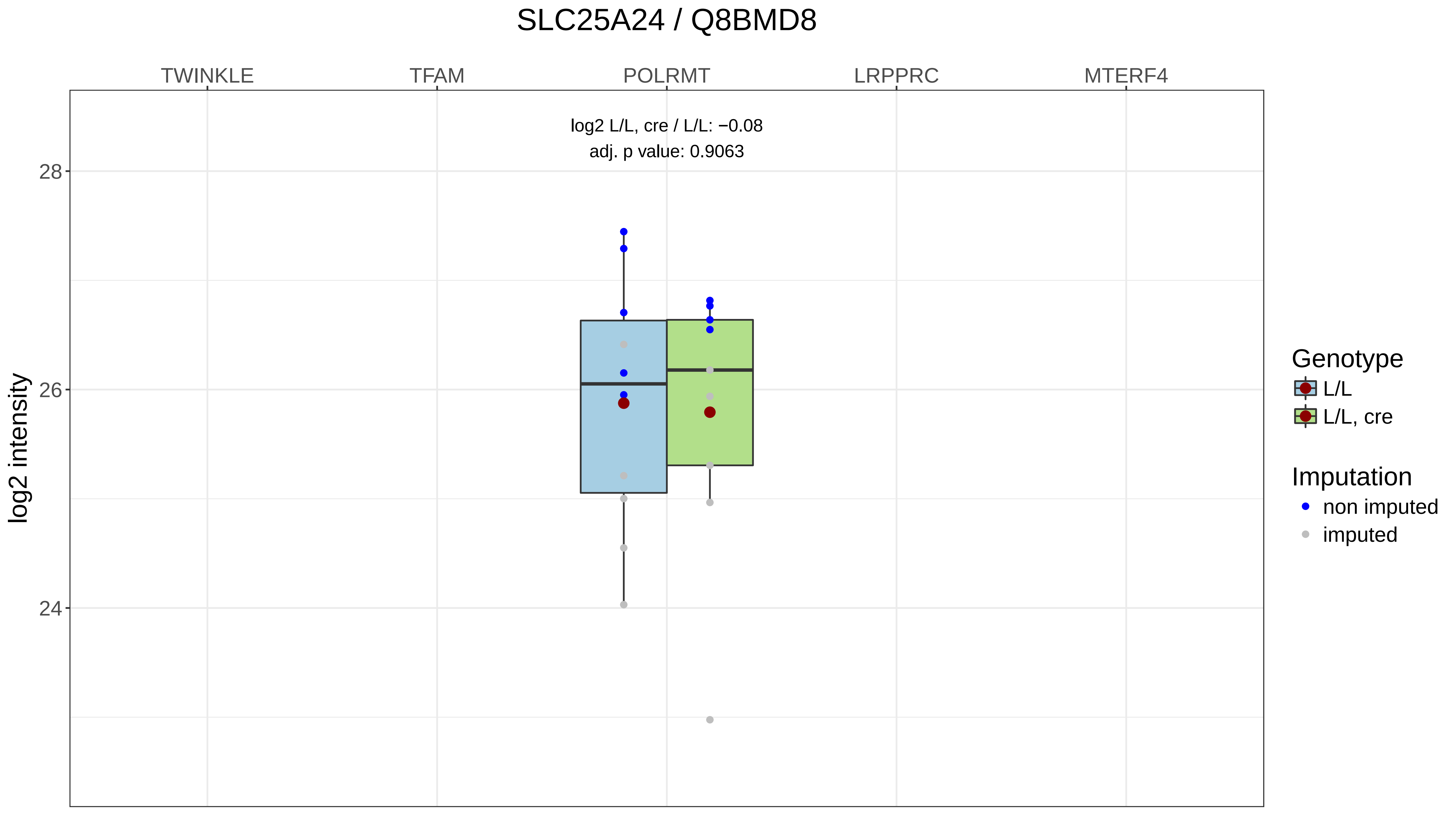
SLC25A24 / Q8BMD8
TWINKLE
TFAM
POLRMT
LRPPRC
MTERF4
log2 L/L, cre / L/L: −0.08
adj. p value: 0.9063
28
26
24
log2 intensity
Genotype
L/L
L/L, cre
Imputation
non imputed
imputed

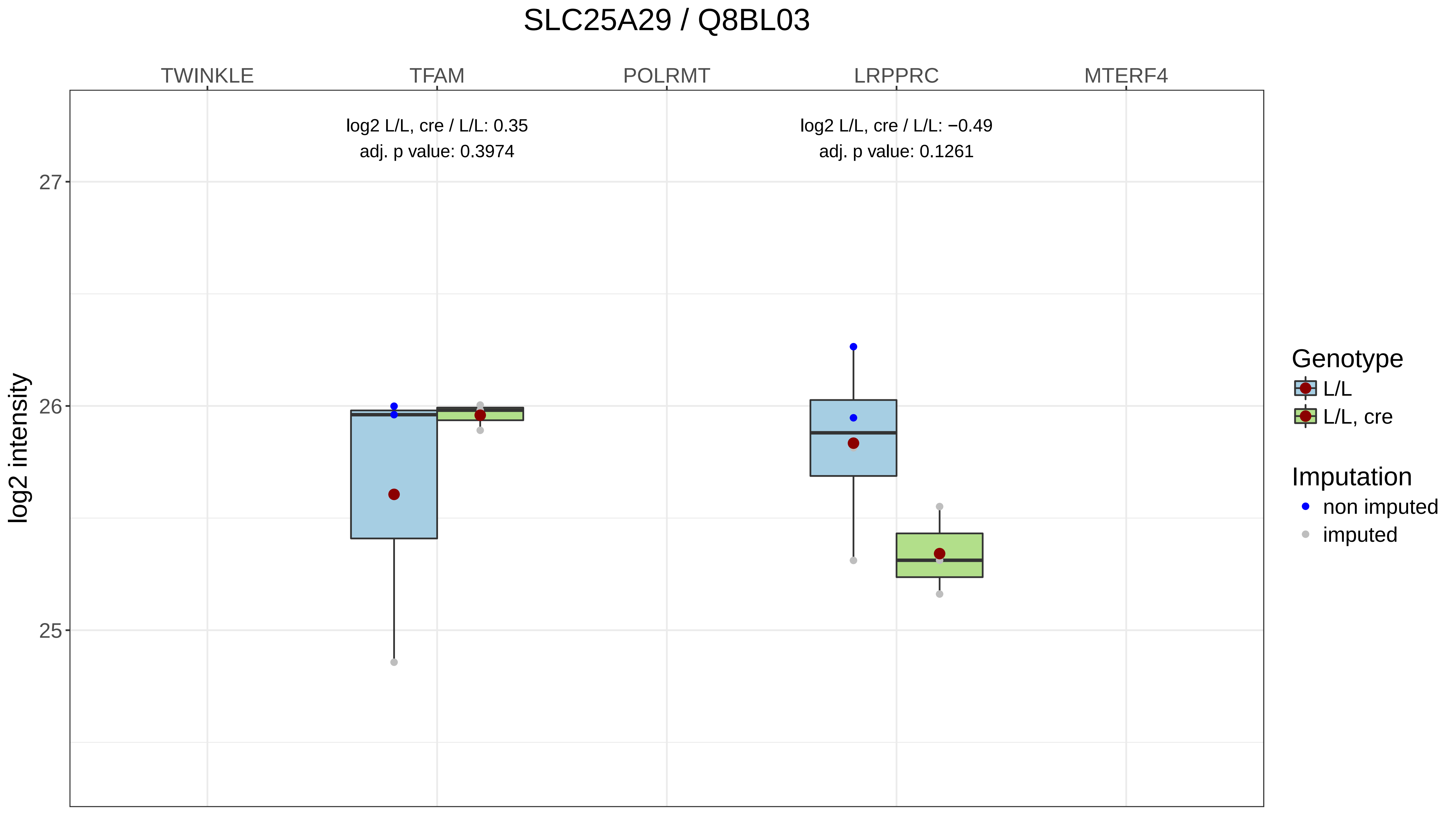
SLC25A29 / Q8BL03
TWINKLE
TFAM
POLRMT
LRPPRC
MTERF4
log2 L/L, cre / L/L: 0.35
adj. p value: 0.3974
log2 L/L, cre / L/L: −0.49
adj. p value: 0.1261
27
26
25
log2 intensity
Genotype
L/L
L/L, cre
Imputation
non imputed
imputed

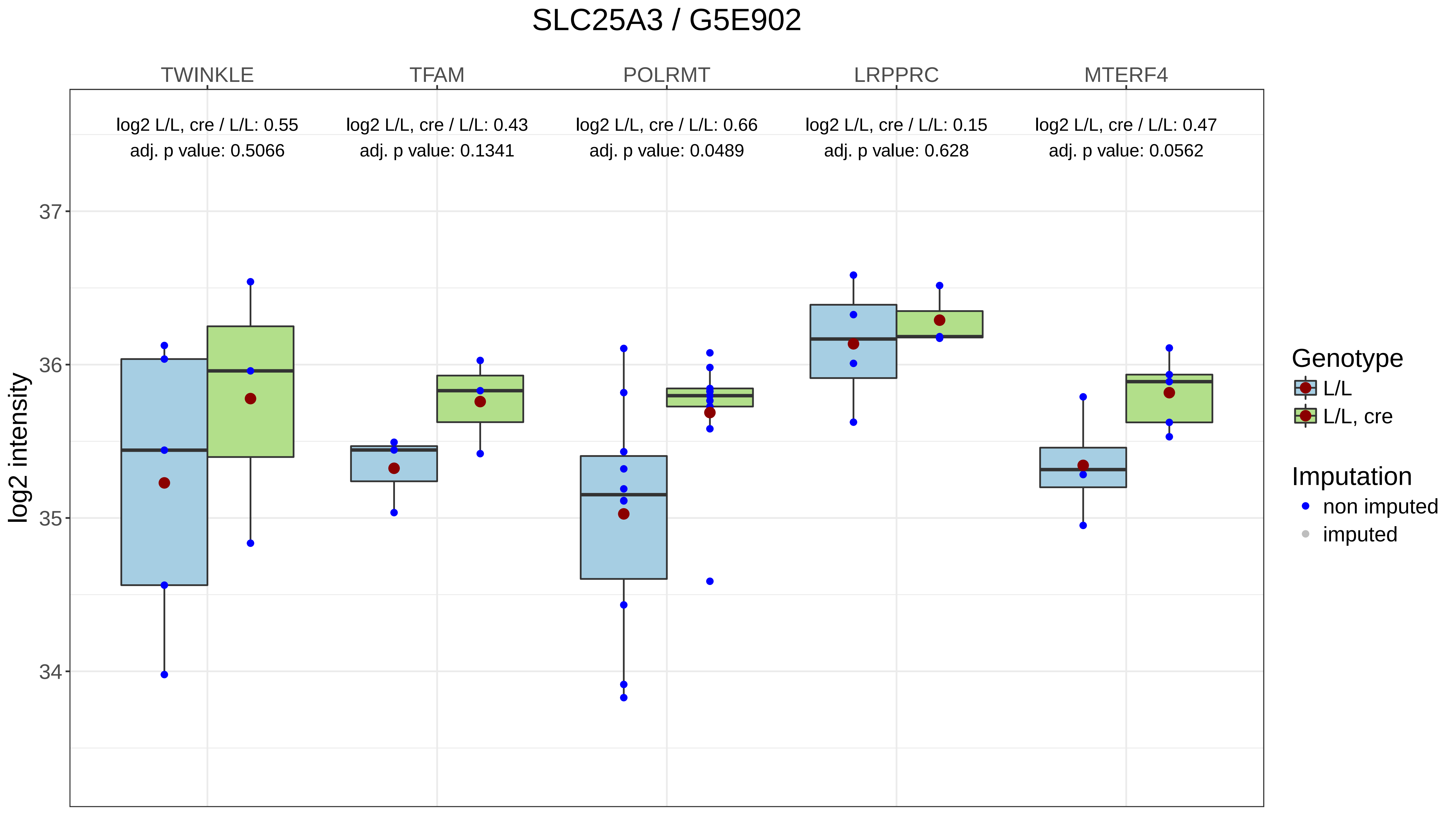
SLC25A3 / G5E902
TWINKLE
TFAM
POLRMT
LRPPRC
MTERF4
log2 L/L, cre / L/L: 0.55
adj. p value: 0.5066
log2 L/L, cre / L/L: 0.43
adj. p value: 0.1341
log2 L/L, cre / L/L: 0.66
adj. p value: 0.0489
log2 L/L, cre / L/L: 0.15
adj. p value: 0.628
log2 L/L, cre / L/L: 0.47
adj. p value: 0.0562
log2 intensity
37
36
35
34
Genotype
L/L
L/L, cre
Imputation
non imputed
imputed

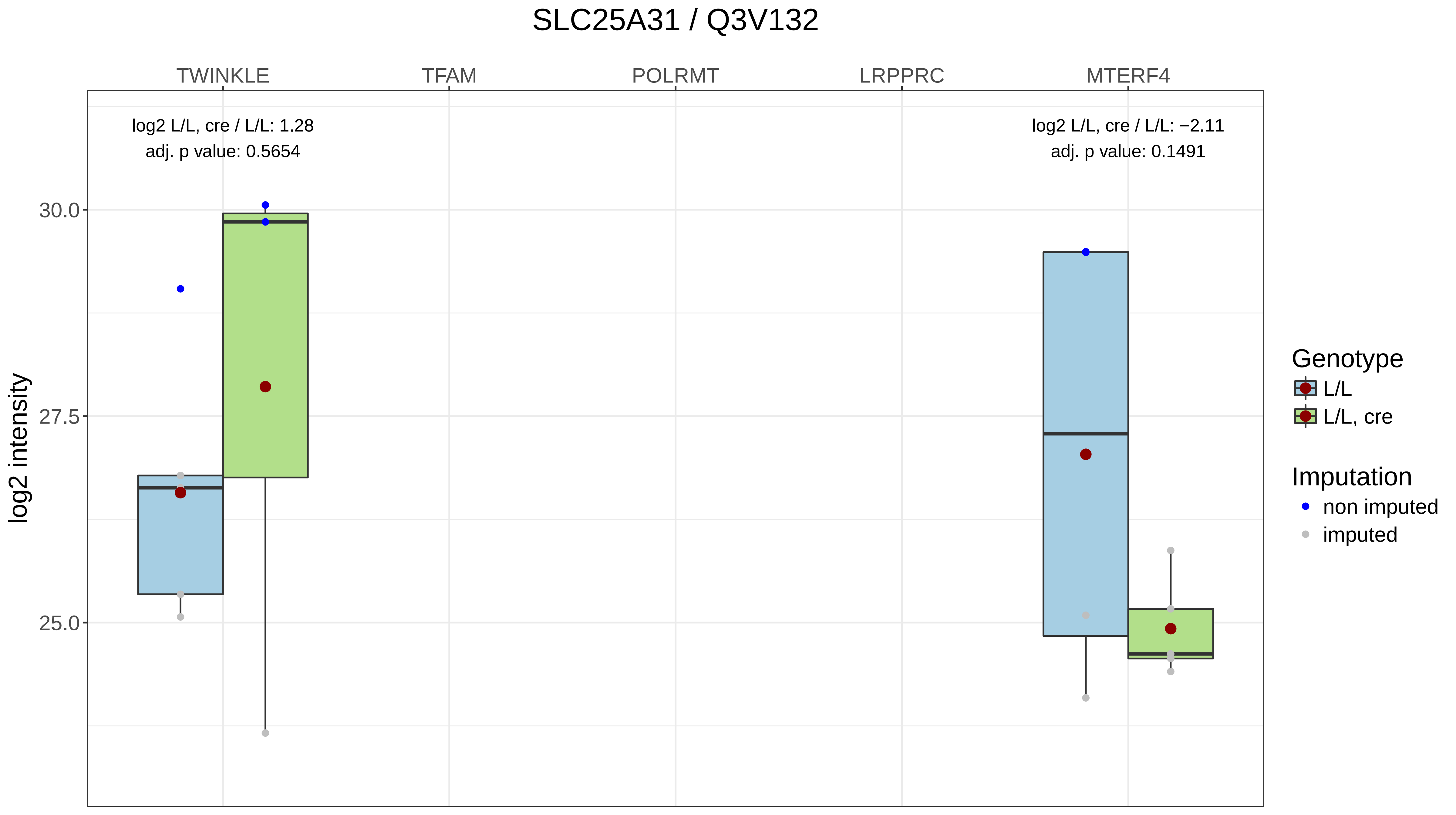
SLC25A31 / Q3V132
TWINKLE
TFAM
POLRMT
LRPPRC
MTERF4
log2 L/L, cre / L/L: 1.28
adj. p value: 0.5654
log2 L/L, cre / L/L: −2.11
adj. p value: 0.1491
log2 intensity
30.0
27.5
25.0
Genotype
L/L
L/L, cre
Imputation
non imputed
imputed

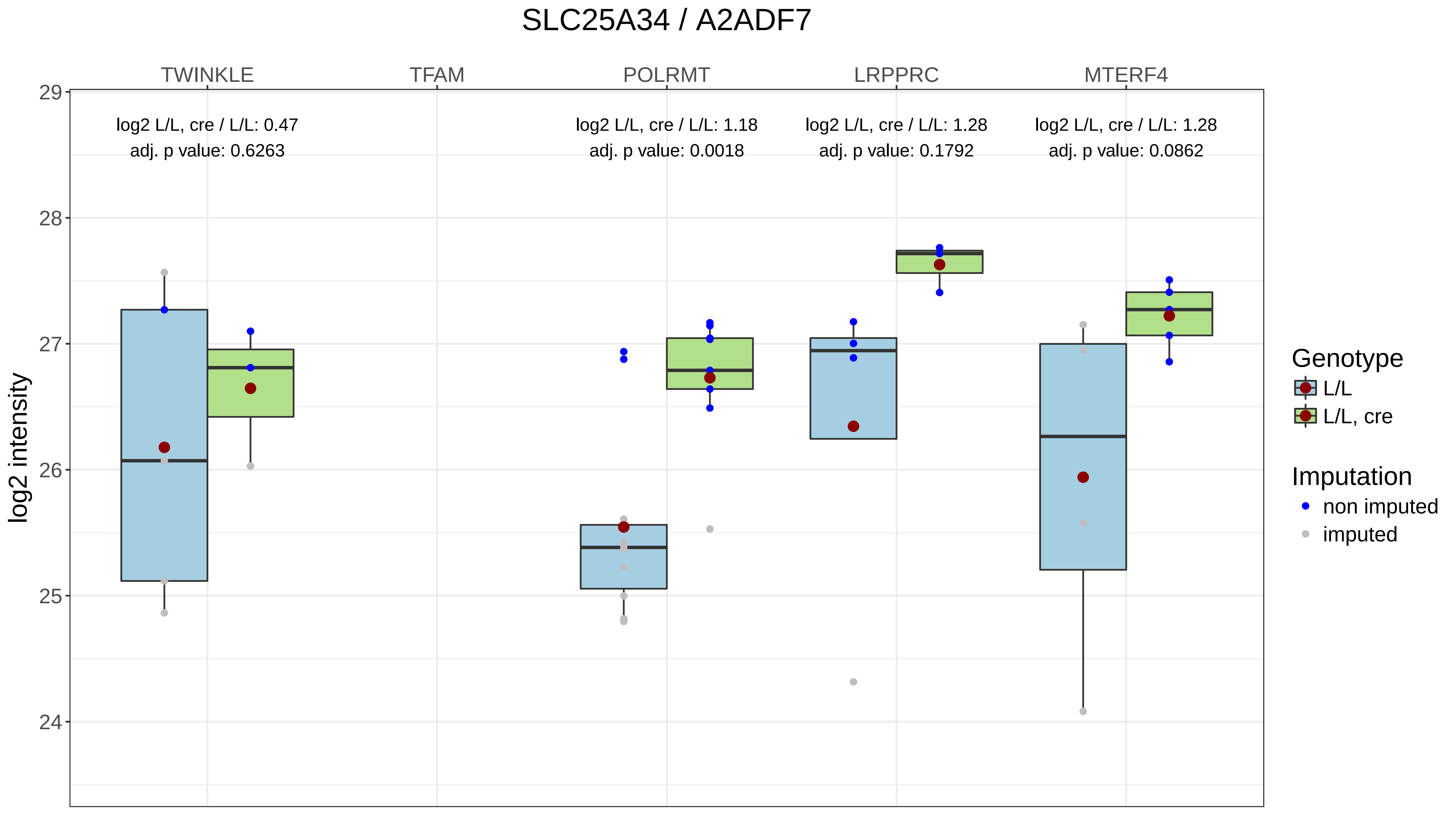
SLC25A34 / A2ADF7
TWINKLE
TFAM
POLRMT
LRPPRC
MTERF4
log2 L/L, cre / L/L: 0.47
adj. p value: 0.6263
log2 L/L, cre / L/L: 1.18
adj. p value: 0.0018
log2 L/L, cre / L/L: 1.28
adj. p value: 0.1792
log2 L/L, cre / L/L: 1.28
adj. p value: 0.0862
log2 intensity
29
28
27
26
25
24
Genotype
L/L
L/L, cre
Imputation
non imputed
imputed

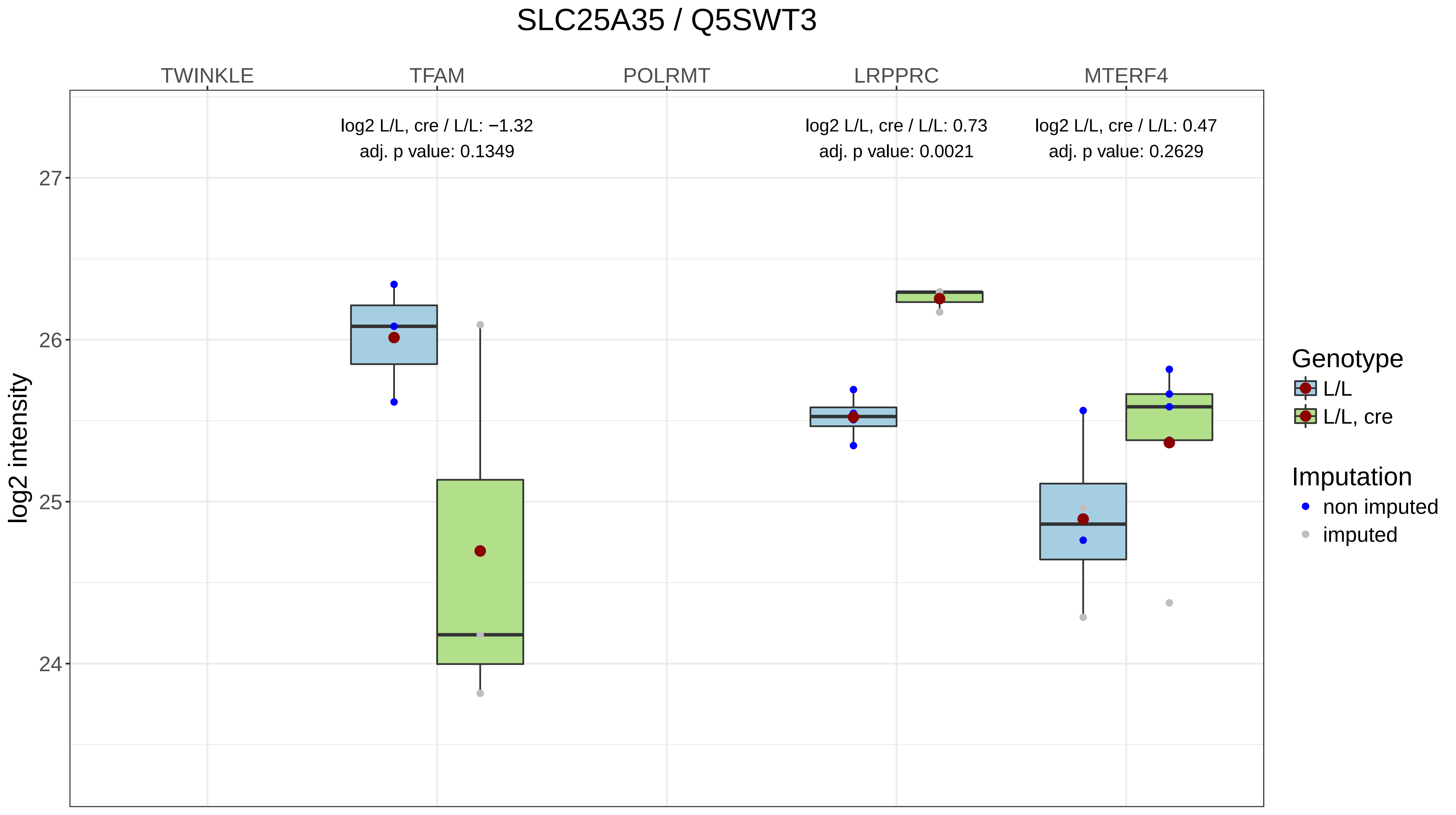
SLC25A35 / Q5SWT3
TWINKLE
TFAM
POLRMT
LRPPRC
MTERF4
log2 L/L, cre / L/L: −1.32
adj. p value: 0.1349
log2 L/L, cre / L/L: 0.73
adj. p value: 0.0021
log2 L/L, cre / L/L: 0.47
adj. p value: 0.2629
27
26
25
24
log2 intensity
Genotype
L/L
L/L, cre
Imputation
non imputed
imputed

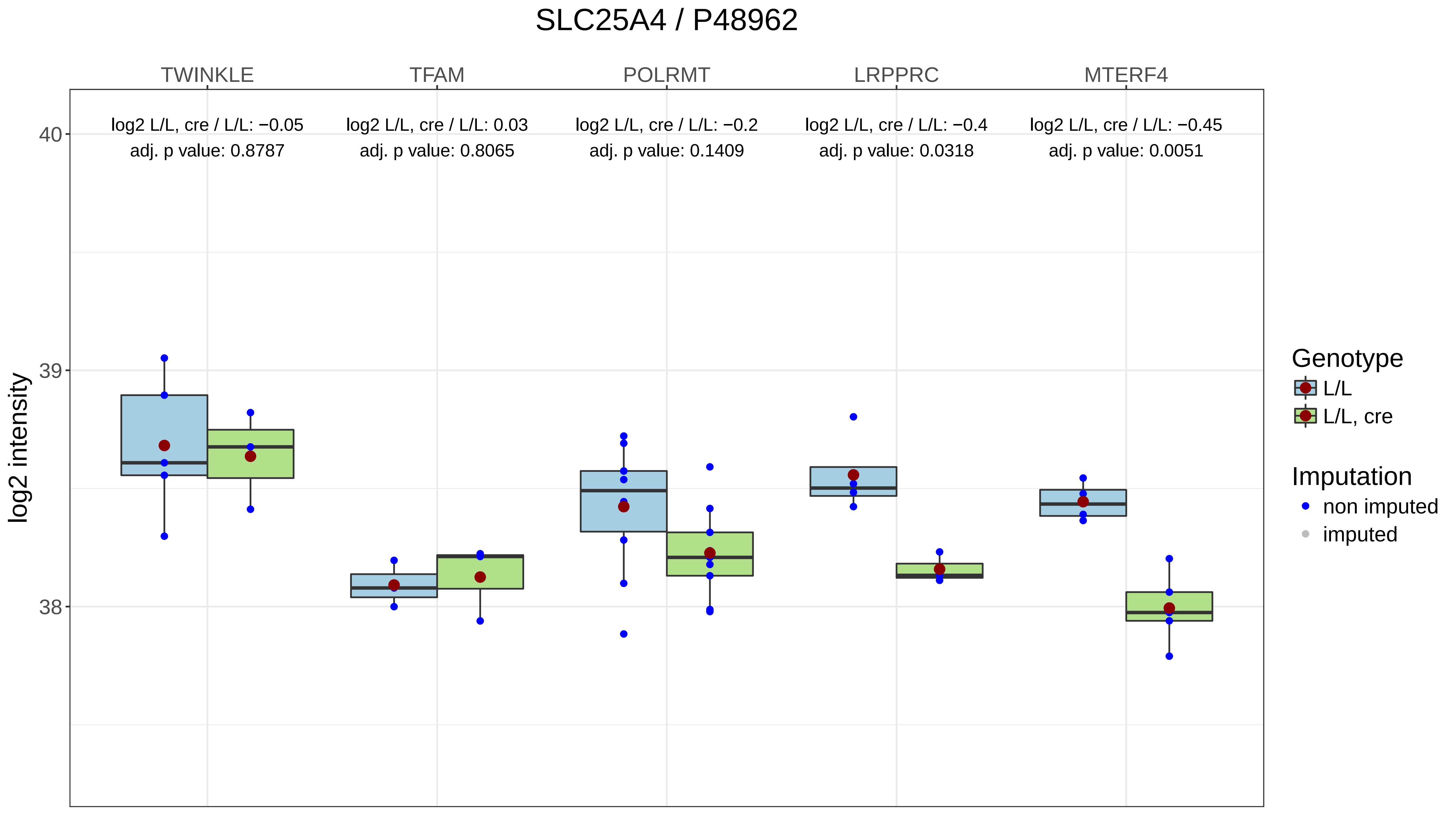

SLC25A4 / P48962
TWINKLE
TFAM
POLRMT
LRPPRC
MTERF4
log2 L/L, cre / L/L: −0.05
adj. p value: 0.8787
log2 L/L, cre / L/L: 0.03
adj. p value: 0.8065
log2 L/L, cre / L/L: −0.2
adj. p value: 0.1409
log2 L/L, cre / L/L: −0.4
adj. p value: 0.0318
log2 L/L, cre / L/L: −0.45
adj. p value: 0.0051
40
39
38
log2 intensity
Genotype
L/L
L/L, cre
Imputation
non imputed
imputed

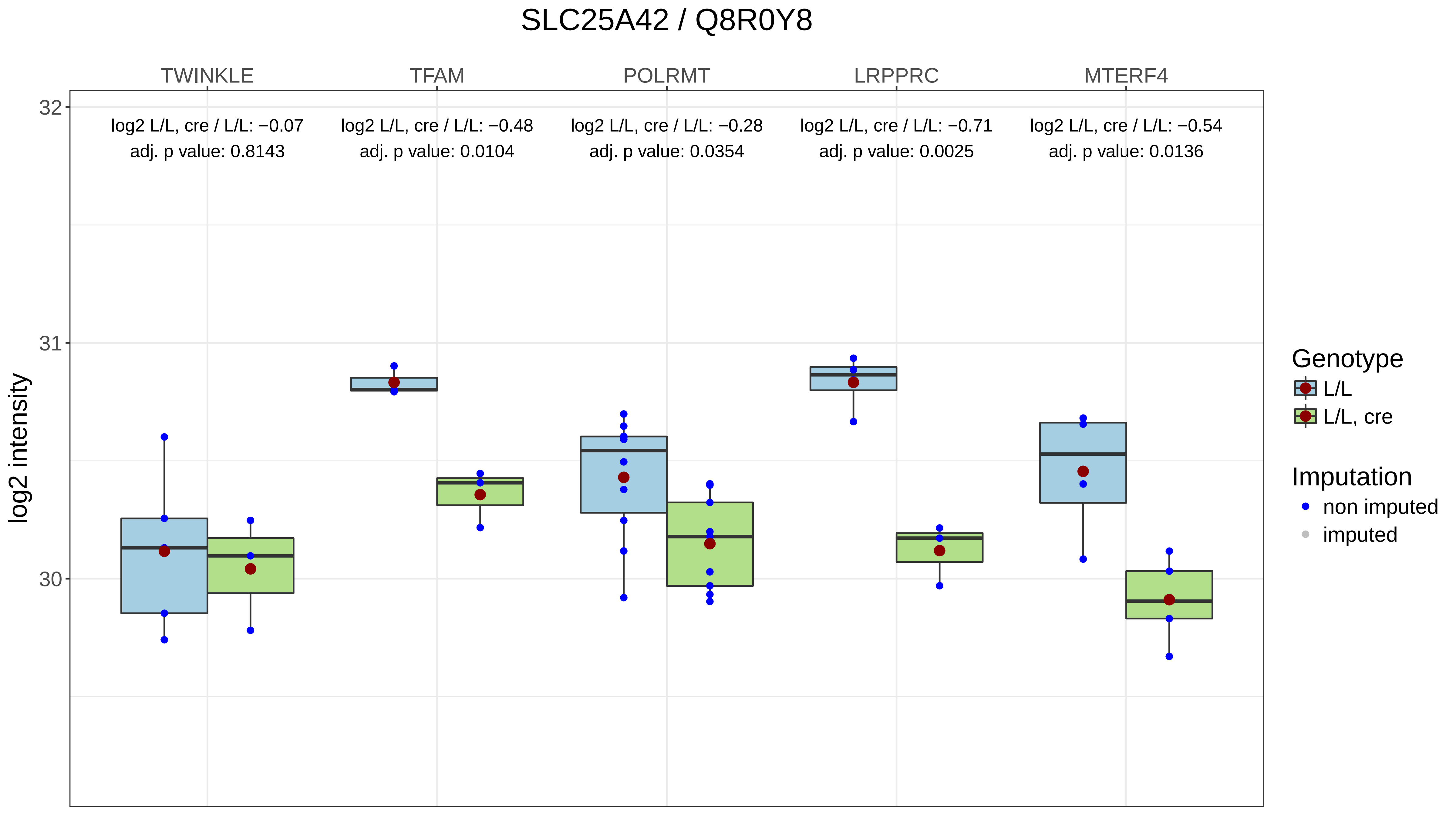
SLC25A42 / Q8R0Y8
TWINKLE
TFAM
POLRMT
LRPPRC
MTERF4
log2 L/L, cre / L/L: −0.07
adj. p value: 0.8143
log2 L/L, cre / L/L: −0.48
adj. p value: 0.0104
log2 L/L, cre / L/L: −0.28
adj. p value: 0.0354
log2 L/L, cre / L/L: −0.71
adj. p value: 0.0025
log2 L/L, cre / L/L: −0.54
adj. p value: 0.0136
32
31
30
log2 intensity
Genotype
L/L
L/L, cre
Imputation
non imputed
imputed

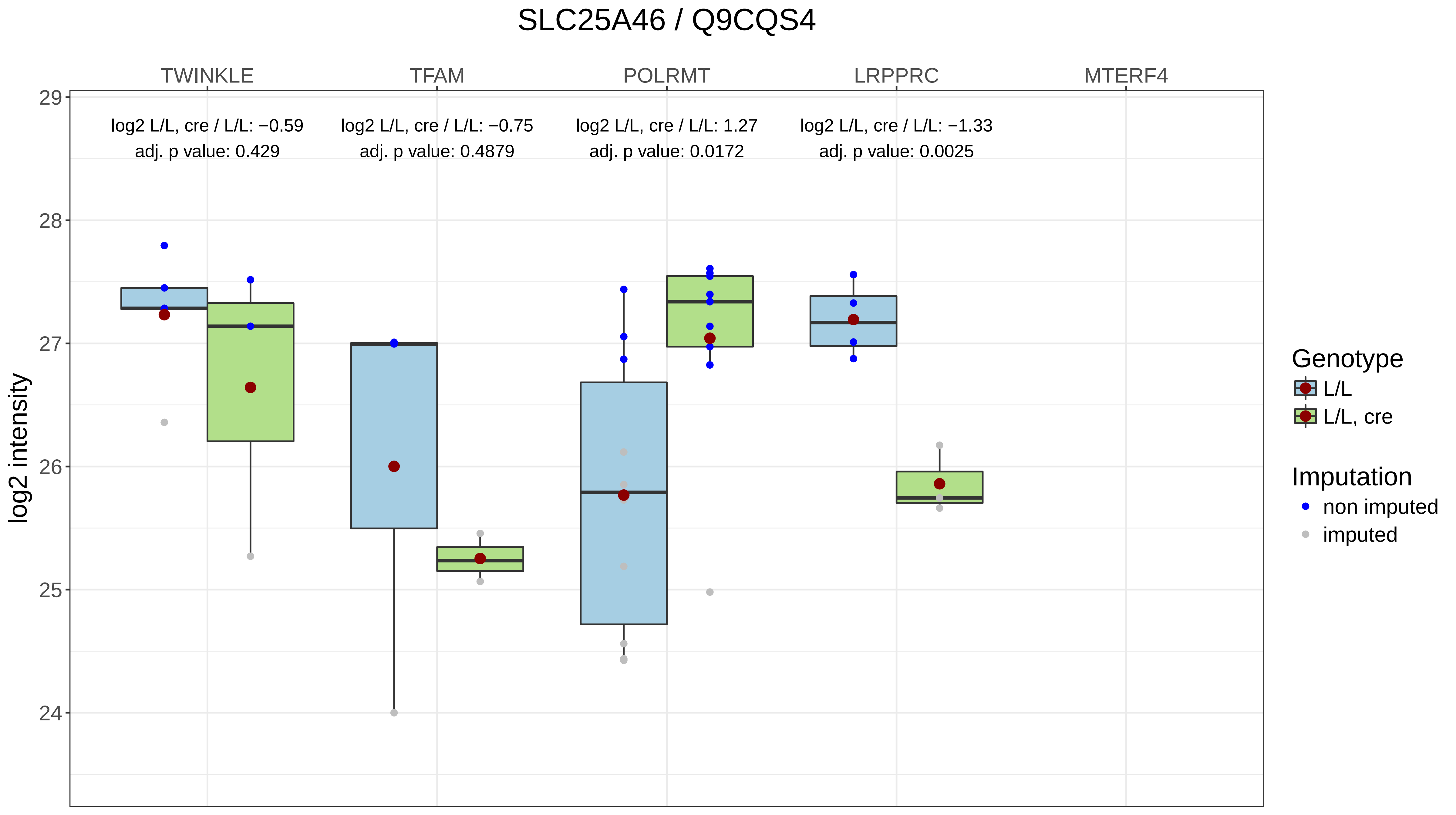
SLC25A46 / Q9CQS4
TWINKLE
TFAM
POLRMT
LRPPRC
MTERF4
log2 L/L, cre / L/L: −0.59
adj. p value: 0.429
log2 L/L, cre / L/L: −0.75
adj. p value: 0.4879
log2 L/L, cre / L/L: 1.27
adj. p value: 0.0172
log2 L/L, cre / L/L: −1.33
adj. p value: 0.0025
log2 intensity
29
28
27
26
25
24
Genotype
L/L
L/L, cre
Imputation
non imputed
imputed

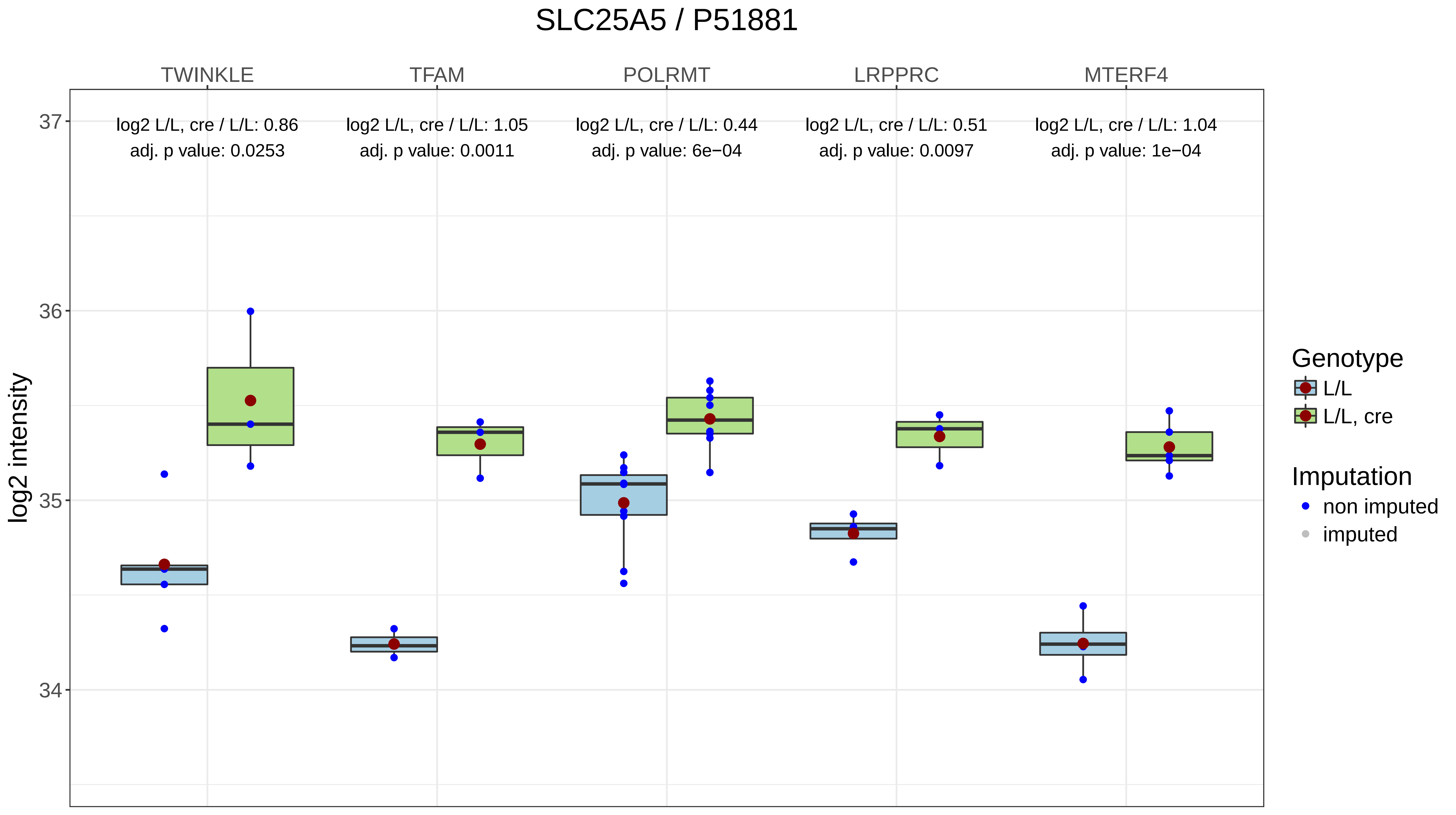
SLC25A5 / P51881
TWINKLE
TFAM
POLRMT
LRPPRC
MTERF4
log2 L/L, cre / L/L: 0.86
adj. p value: 0.0253
log2 L/L, cre / L/L: 1.05
adj. p value: 0.0011
log2 L/L, cre / L/L: 0.44
adj. p value: 6e−04
log2 L/L, cre / L/L: 0.51
adj. p value: 0.0097
log2 L/L, cre / L/L: 1.04
adj. p value: 1e−04
log2 intensity
37
36
35
34
Genotype
L/L
L/L, cre
Imputation
non imputed
imputed

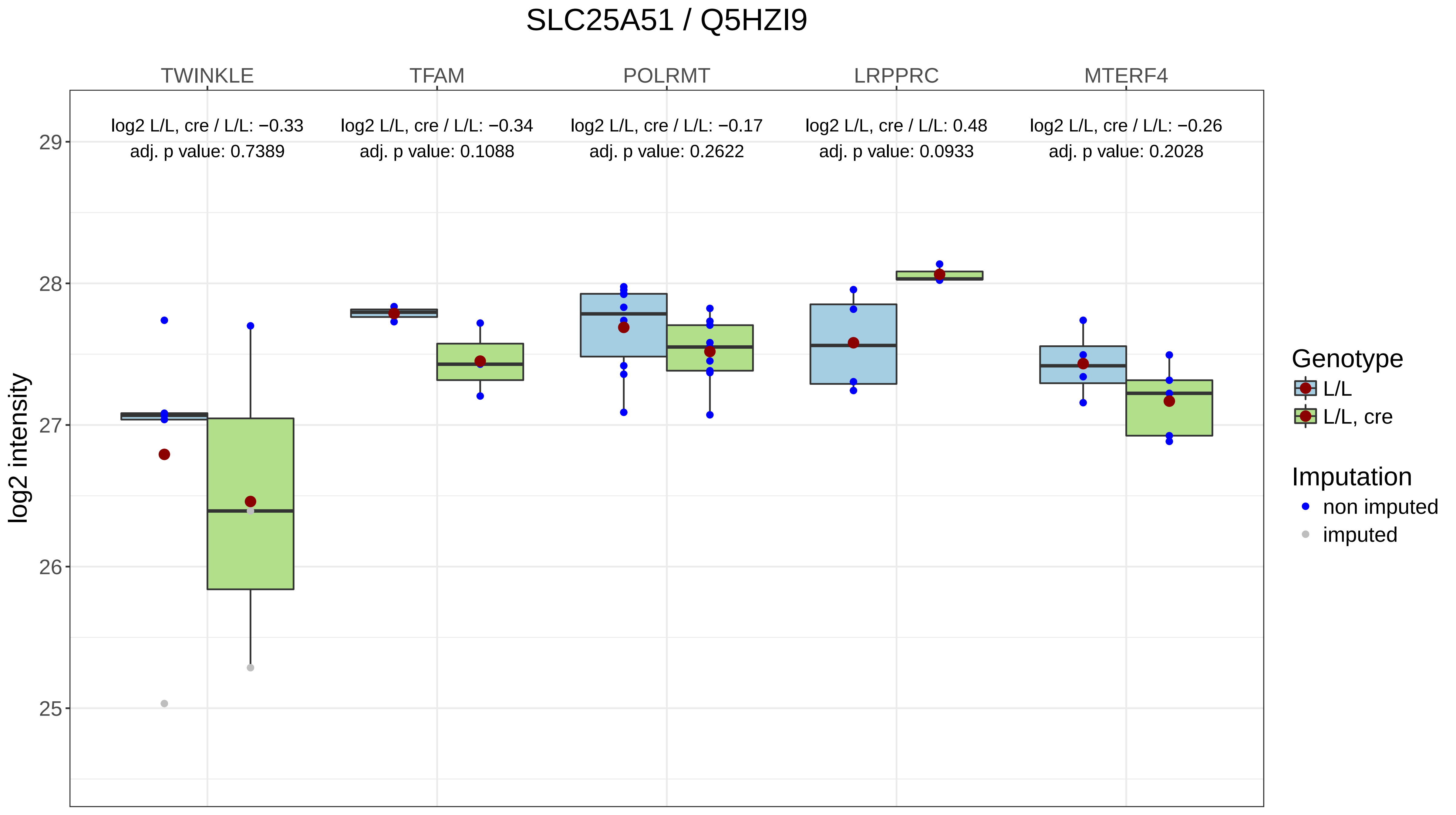

SLC25A51 / Q5HZI9
TWINKLE
TFAM
POLRMT
LRPPRC
MTERF4
log2 L/L, cre / L/L: −0.33
adj. p value: 0.7389
log2 L/L, cre / L/L: −0.34
adj. p value: 0.1088
log2 L/L, cre / L/L: −0.17
adj. p value: 0.2622
log2 L/L, cre / L/L: 0.48
adj. p value: 0.0933
log2 L/L, cre / L/L: −0.26
adj. p value: 0.2028
log2 intensity
29
28
27
26
25
Genotype
L/L
L/L, cre
Imputation
non imputed
imputed

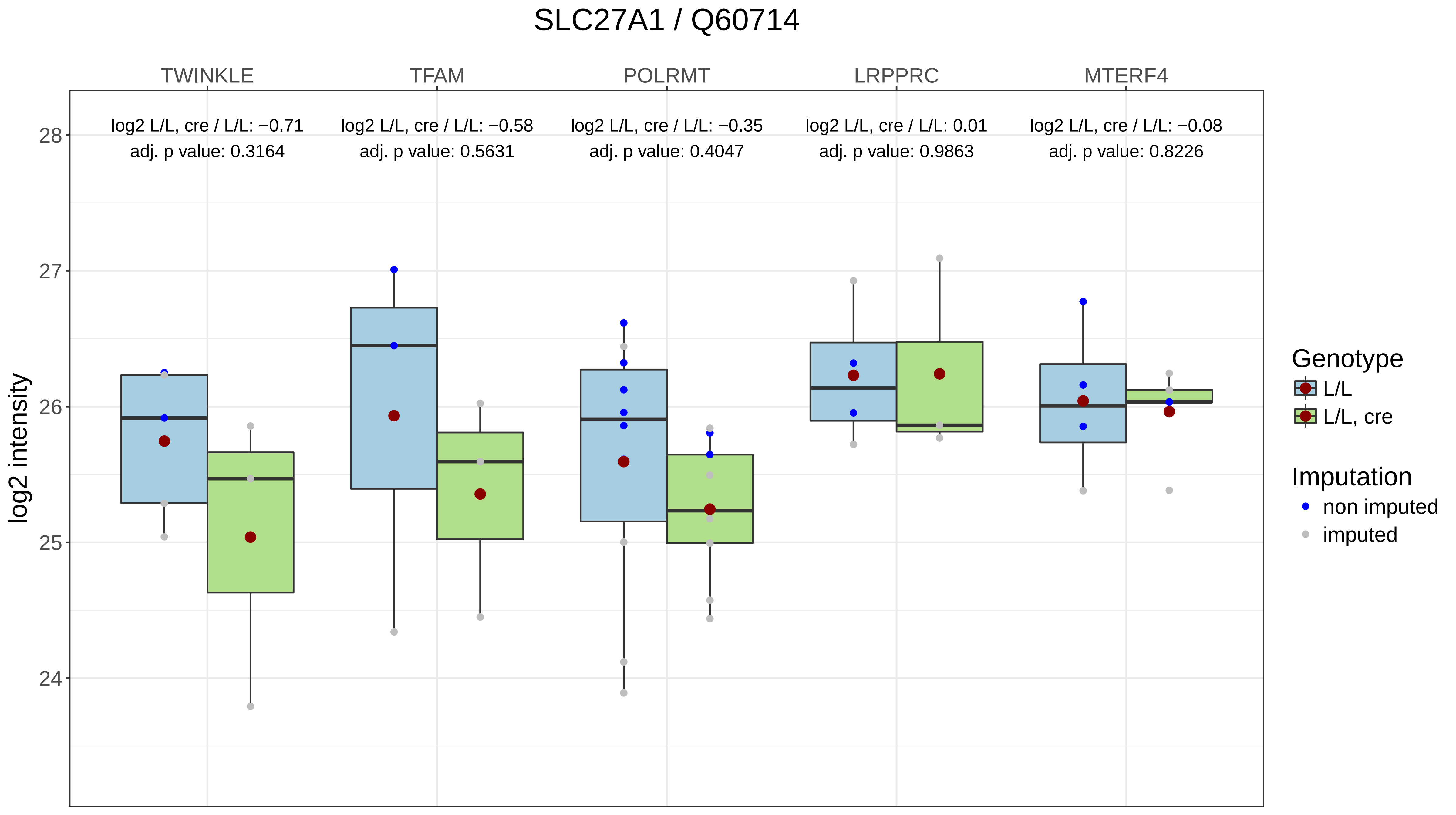
SLC27A1 / Q60714
TWINKLE
TFAM
POLRMT
LRPPRC
MTERF4
log2 L/L, cre / L/L: −0.71
adj. p value: 0.3164
log2 L/L, cre / L/L: −0.58
adj. p value: 0.5631
log2 L/L, cre / L/L: −0.35
adj. p value: 0.4047
log2 L/L, cre / L/L: 0.01
adj. p value: 0.9863
log2 L/L, cre / L/L: −0.08
adj. p value: 0.8226
log2 intensity
28
27
26
25
24
Genotype
L/L
L/L, cre
Imputation
non imputed
imputed

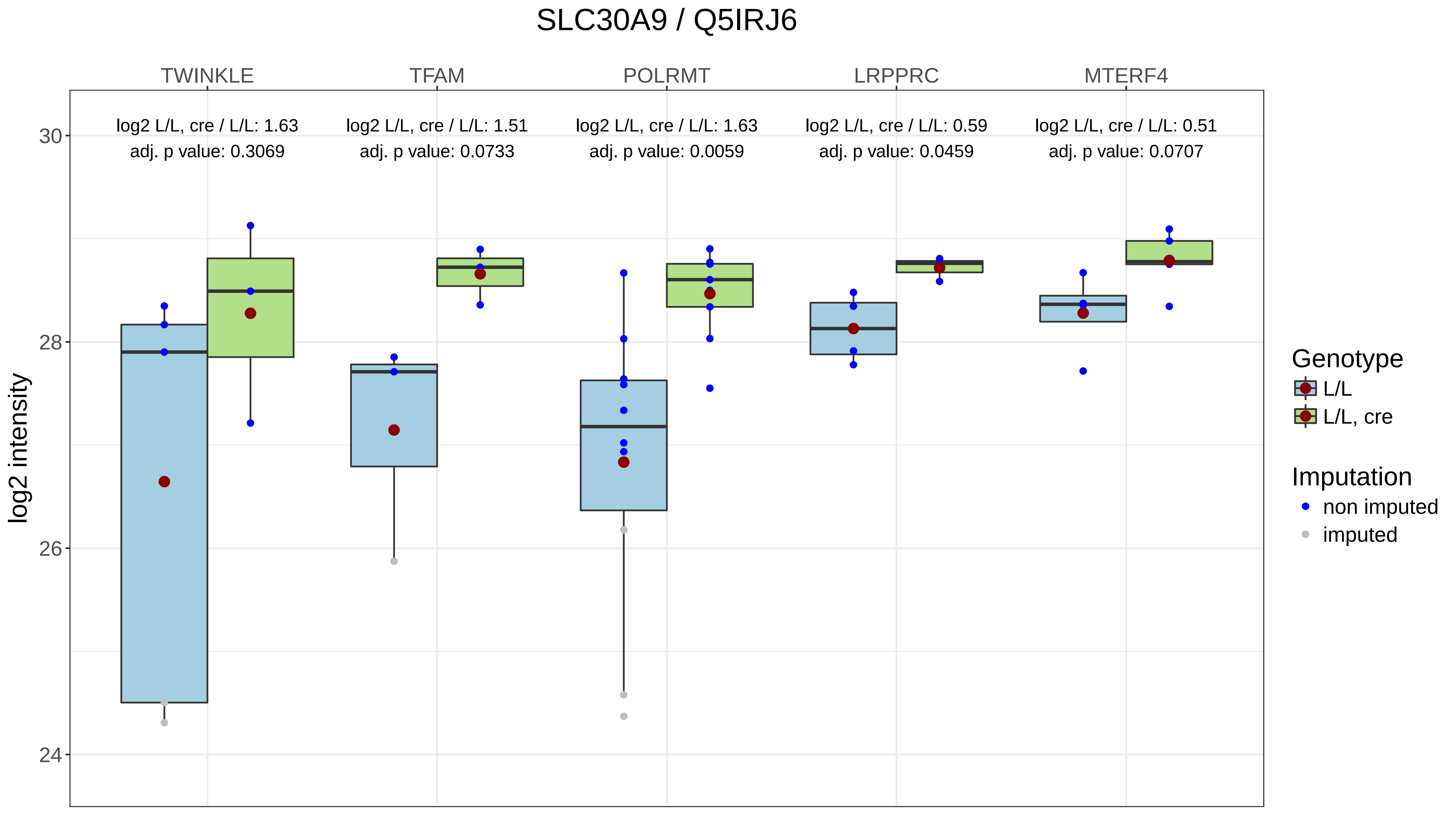
SLC30A9 / Q5IRJ6
TWINKLE
TFAM
POLRMT
LRPPRC
MTERF4
log2 L/L, cre / L/L: 1.63
adj. p value: 0.3069
log2 L/L, cre / L/L: 1.51
adj. p value: 0.0733
log2 L/L, cre / L/L: 1.63
adj. p value: 0.0059
log2 L/L, cre / L/L: 0.59
adj. p value: 0.0459
log2 L/L, cre / L/L: 0.51
adj. p value: 0.0707
log2 intensity
30
28
26
24
Genotype
L/L
L/L, cre
Imputation
non imputed
imputed

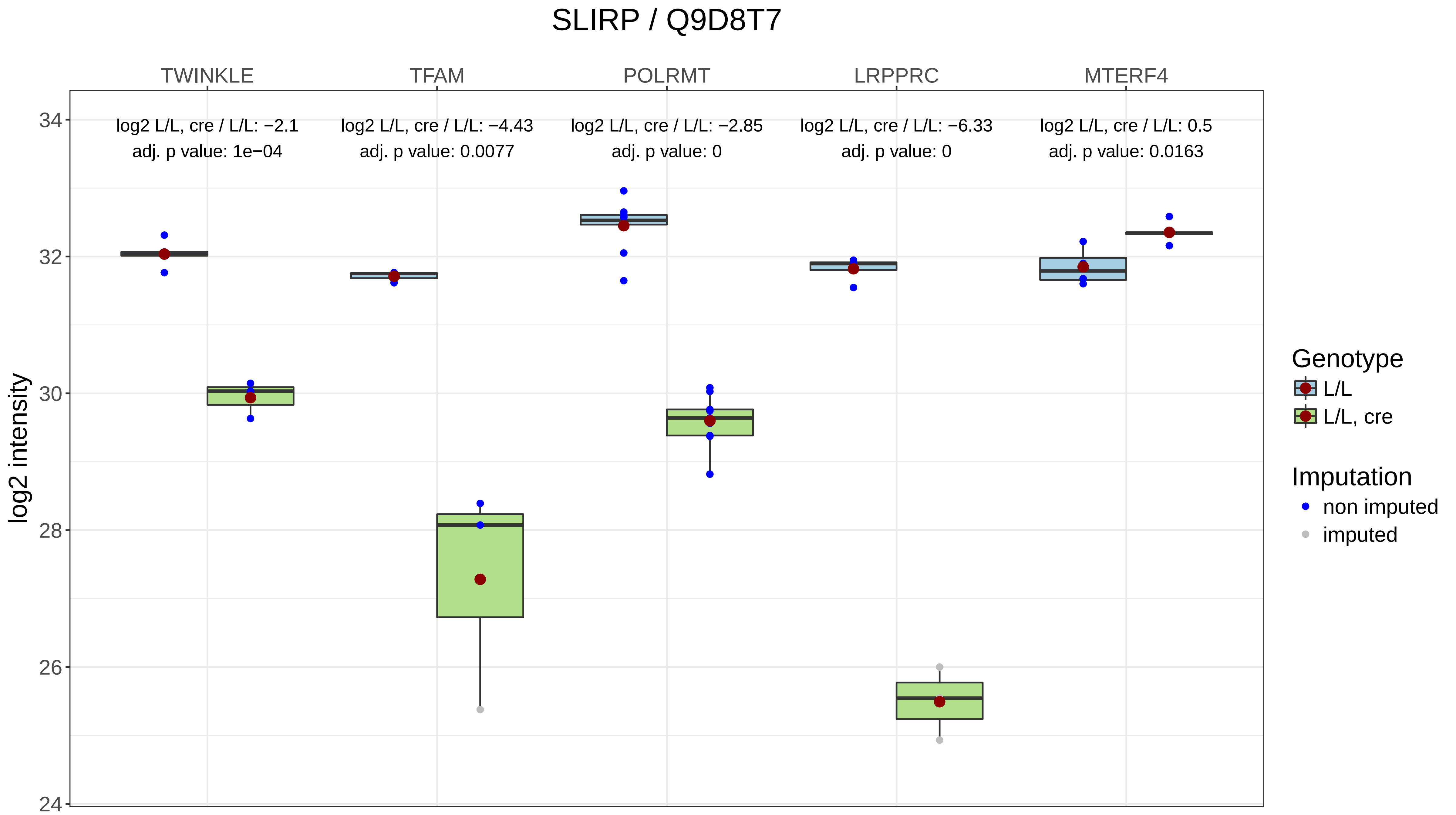
SLIRP / Q9D8T7
TWINKLE
TFAM
POLRMT
LRPPRC
MTERF4
log2 L/L, cre / L/L: −2.1
adj. p value: 1e−04
log2 L/L, cre / L/L: −4.43
adj. p value: 0.0077
log2 L/L, cre / L/L: −2.85
adj. p value: 0
log2 L/L, cre / L/L: −6.33
adj. p value: 0
log2 L/L, cre / L/L: 0.5
adj. p value: 0.0163
log2 intensity
34
32
30
28
26
24
Genotype
L/L
L/L, cre
Imputation
non imputed
imputed

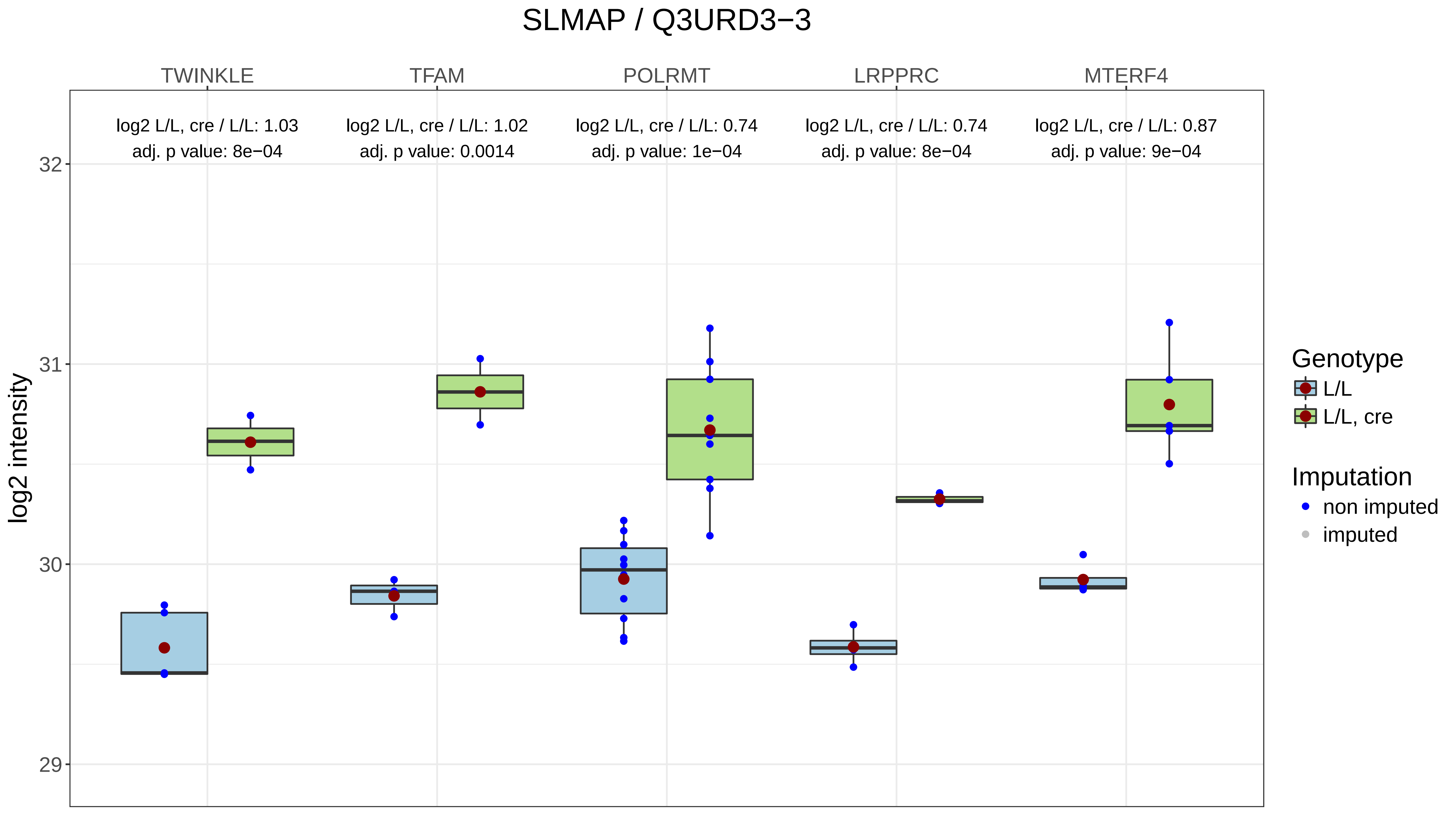
SLMAP / Q3URD3−3
TWINKLE
TFAM
POLRMT
LRPPRC
MTERF4
log2 L/L, cre / L/L: 1.03
adj. p value: 8e−04
log2 L/L, cre / L/L: 1.02
adj. p value: 0.0014
log2 L/L, cre / L/L: 0.74
adj. p value: 1e−04
log2 L/L, cre / L/L: 0.74
adj. p value: 8e−04
log2 L/L, cre / L/L: 0.87
adj. p value: 9e−04
log2 intensity
32
31
30
29
Genotype
L/L
L/L, cre
Imputation
non imputed
imputed

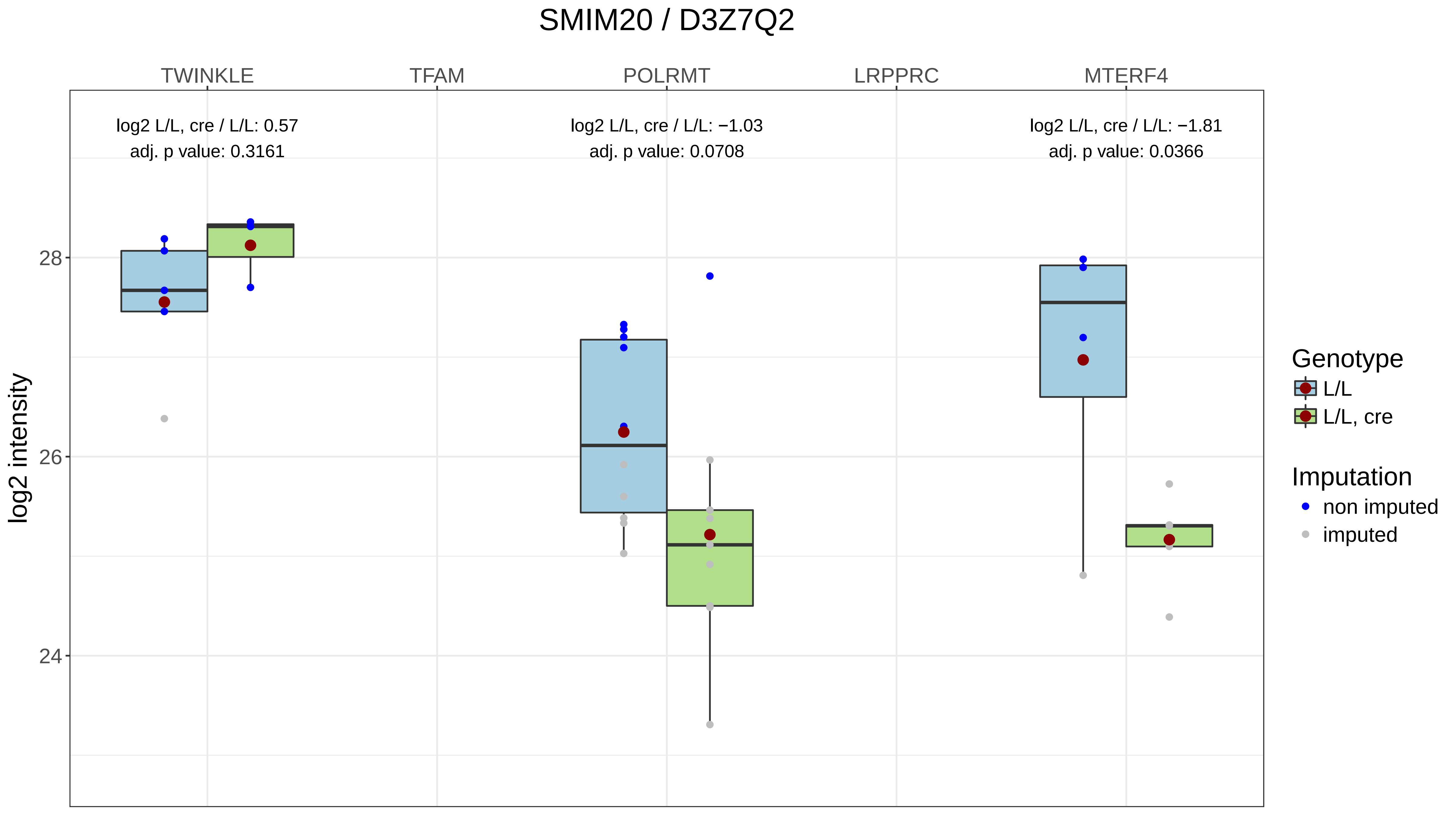

SMIM20 / D3Z7Q2
TWINKLE
TFAM
POLRMT
LRPPRC
MTERF4
log2 L/L, cre / L/L: 0.57
adj. p value: 0.3161
log2 L/L, cre / L/L: −1.03
adj. p value: 0.0708
log2 L/L, cre / L/L: −1.81
adj. p value: 0.0366
log2 intensity
28
26
24
Genotype
L/L
L/L, cre
Imputation
non imputed
imputed

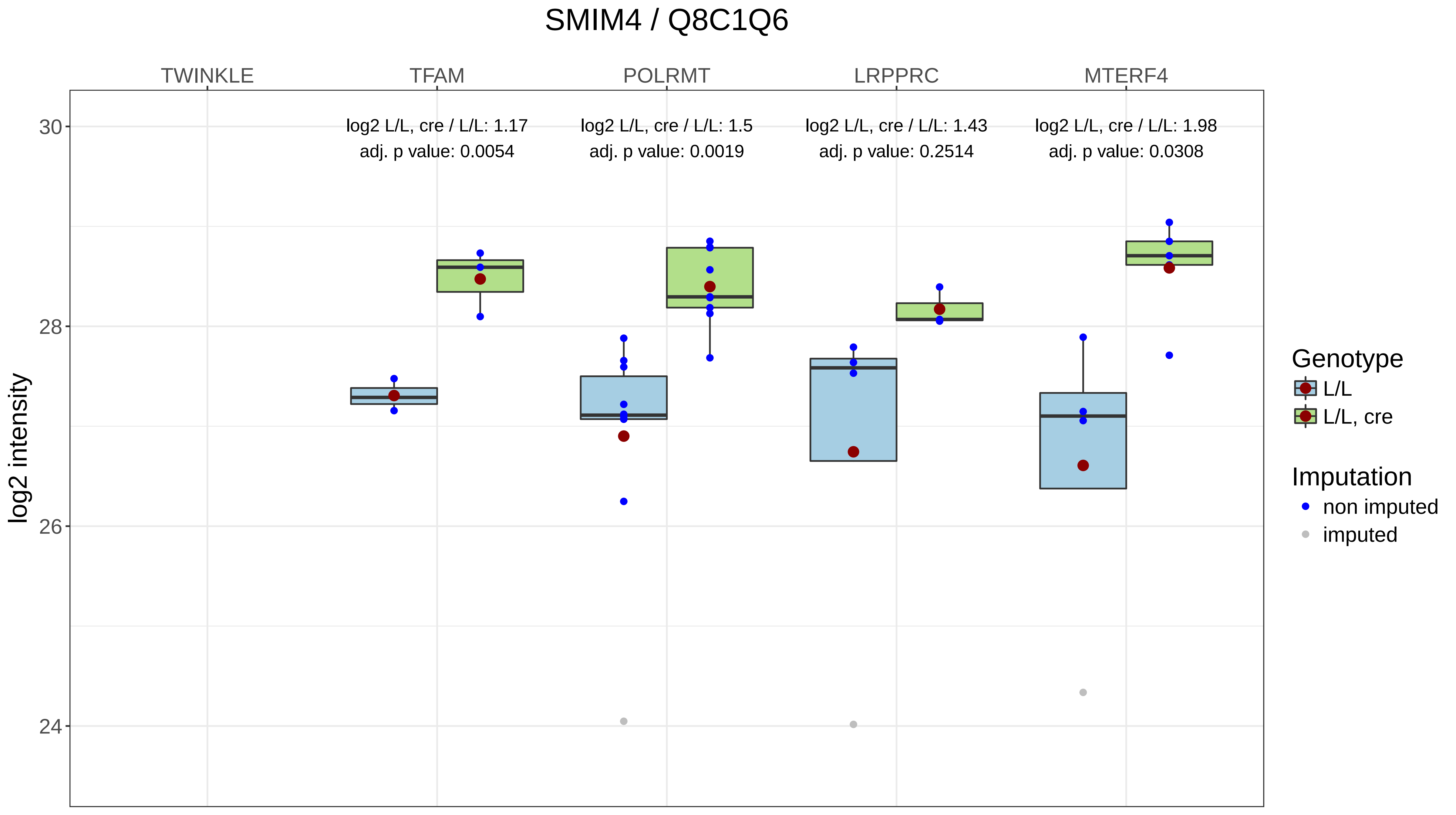

SMIM4 / Q8C1Q6
TWINKLE
TFAM
POLRMT
LRPPRC
MTERF4
log2 L/L, cre / L/L: 1.17
adj. p value: 0.0054
log2 L/L, cre / L/L: 1.5
adj. p value: 0.0019
log2 L/L, cre / L/L: 1.43
adj. p value: 0.2514
log2 L/L, cre / L/L: 1.98
adj. p value: 0.0308
30
28
26
24
log2 intensity
Genotype
L/L
L/L, cre
Imputation
non imputed
imputed

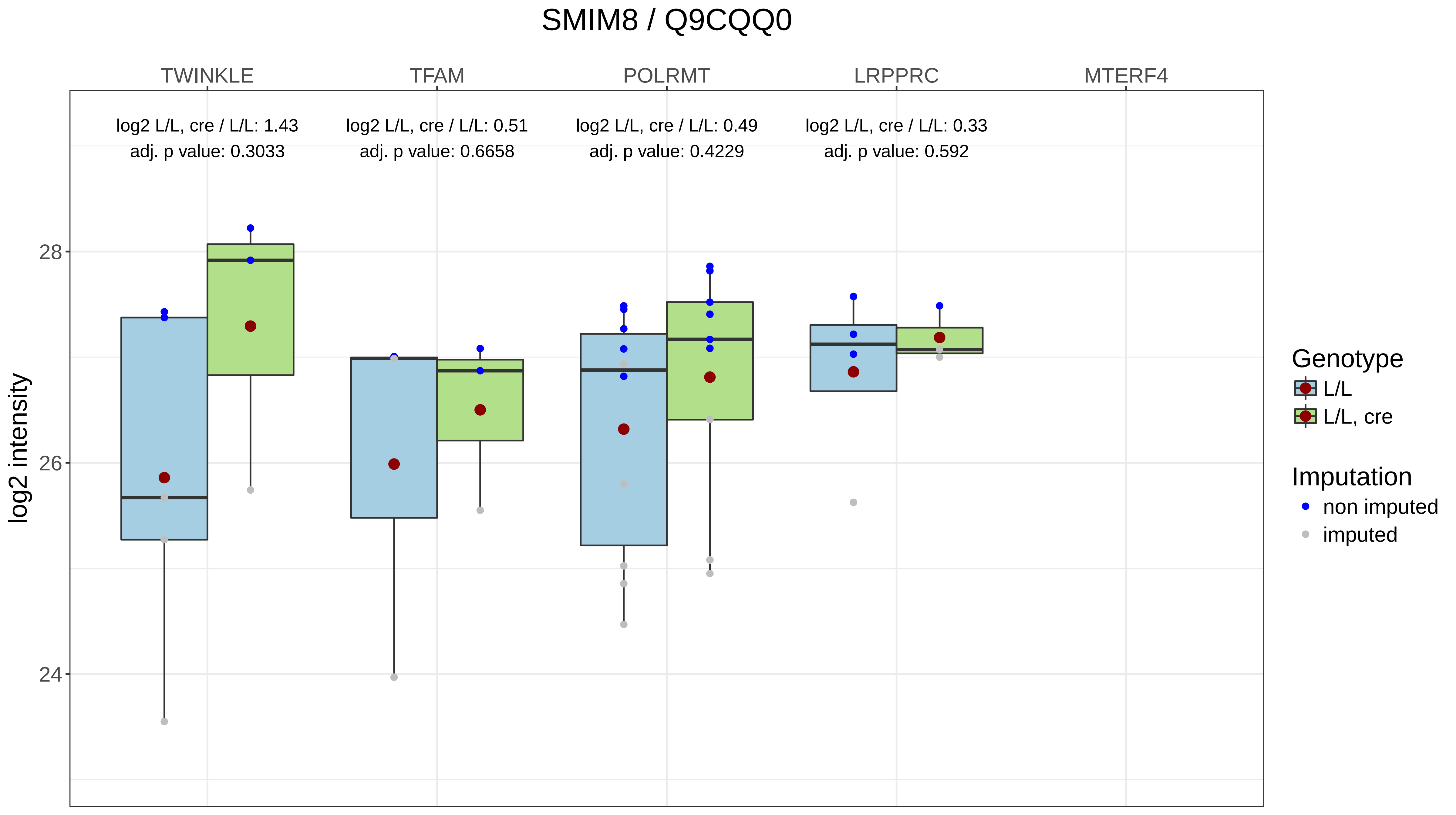
SMIM8 / Q9CQQ0
TWINKLE
TFAM
POLRMT
LRPPRC
MTERF4
log2 L/L, cre / L/L: 1.43
adj. p value: 0.3033
log2 L/L, cre / L/L: 0.51
adj. p value: 0.6658
log2 L/L, cre / L/L: 0.49
adj. p value: 0.4229
log2 L/L, cre / L/L: 0.33
adj. p value: 0.592
28
26
24
log2 intensity
Genotype
L/L
L/L, cre
Imputation
non imputed
imputed

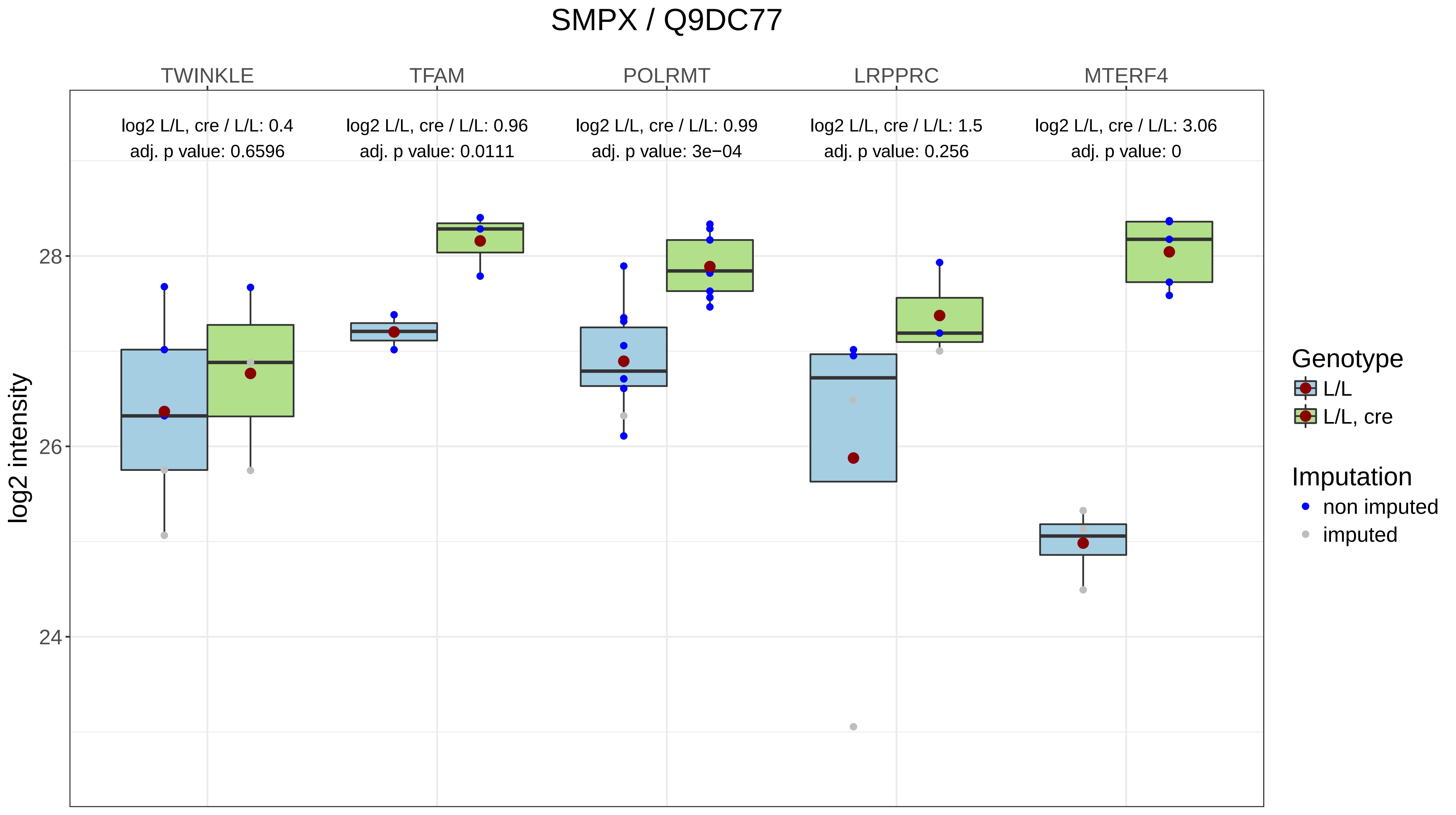
SMPX / Q9DC77
TWINKLE
TFAM
POLRMT
LRPPRC
MTERF4
log2 L/L, cre / L/L: 0.4
adj. p value: 0.6596
log2 L/L, cre / L/L: 0.96
adj. p value: 0.0111
log2 L/L, cre / L/L: 0.99
adj. p value: 3e−04
log2 L/L, cre / L/L: 1.5
adj. p value: 0.256
log2 L/L, cre / L/L: 3.06
adj. p value: 0
log2 intensity
28
26
24
Genotype
L/L
L/L, cre
Imputation
non imputed
imputed

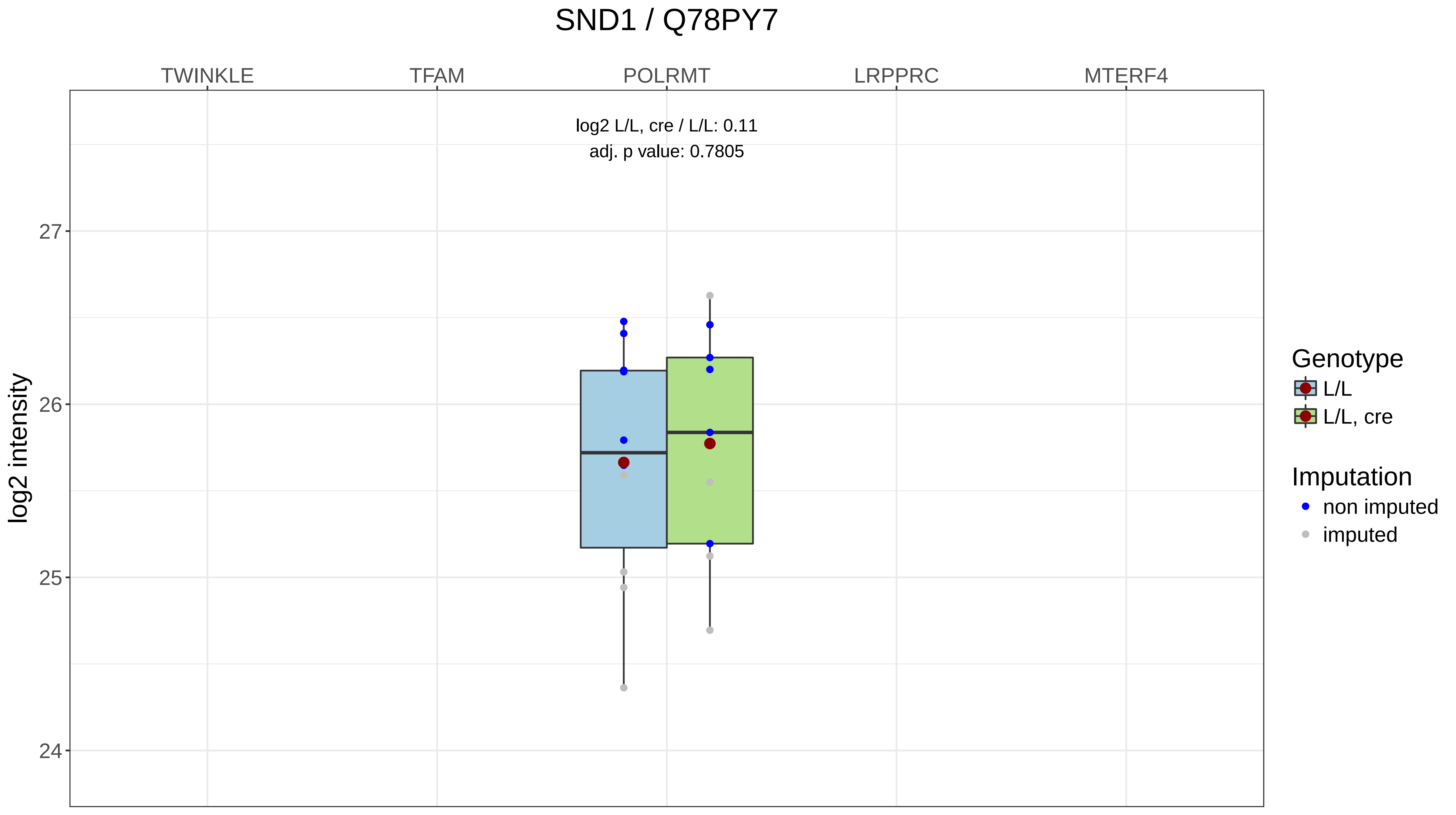
SND1 / Q78PY7
TWINKLE
TFAM
POLRMT
LRPPRC
MTERF4
log2 L/L, cre / L/L: 0.11
adj. p value: 0.7805
log2 intensity
27
26
25
24
Genotype
L/L
L/L, cre
Imputation
non imputed
imputed

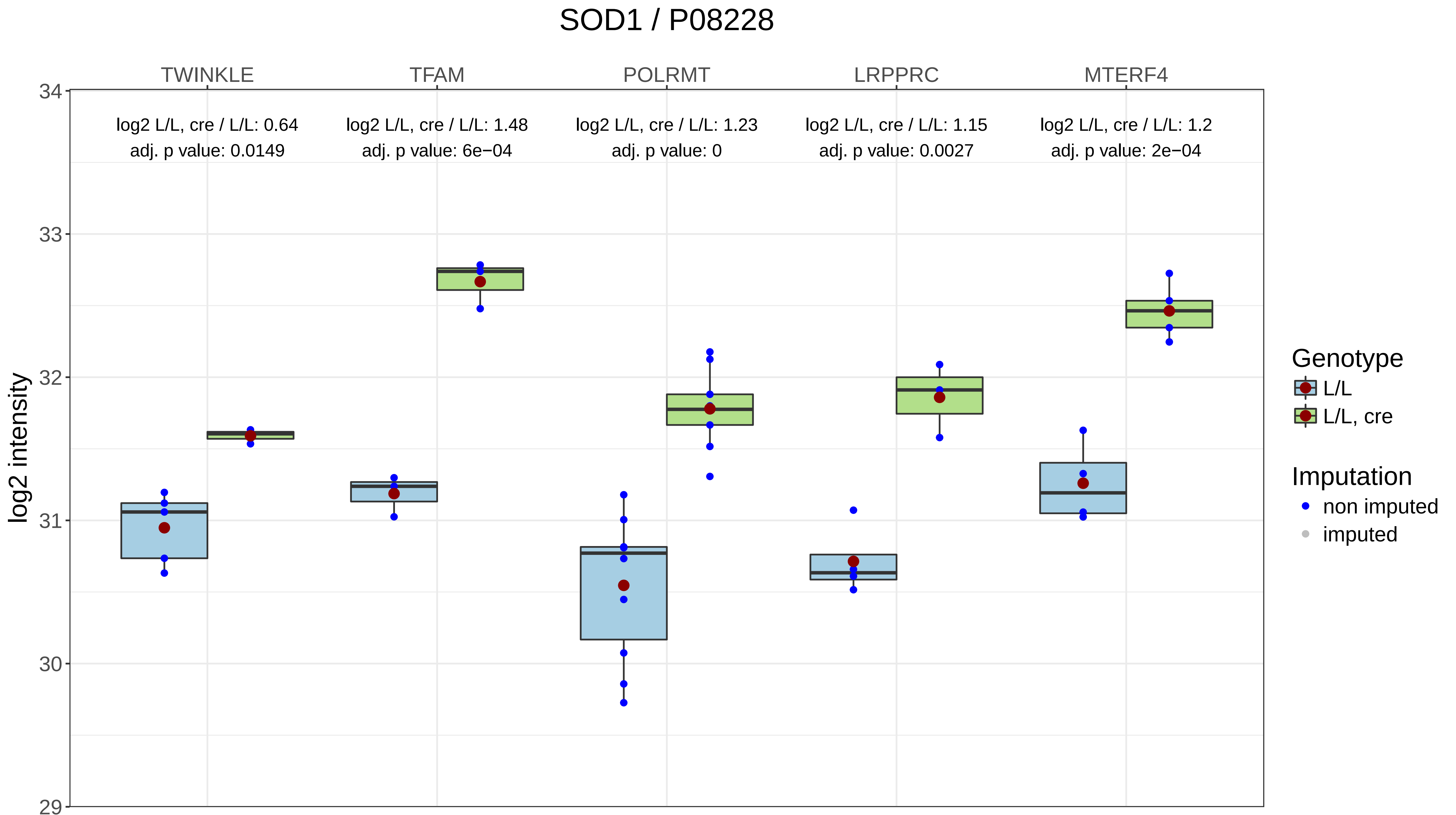
SOD1 / P08228
TWINKLE
TFAM
POLRMT
LRPPRC
MTERF4
log2 L/L, cre / L/L: 0.64
adj. p value: 0.0149
log2 L/L, cre / L/L: 1.48
adj. p value: 6e−04
log2 L/L, cre / L/L: 1.23
adj. p value: 0
log2 L/L, cre / L/L: 1.15
adj. p value: 0.0027
log2 L/L, cre / L/L: 1.2
adj. p value: 2e−04
log2 intensity
34
33
32
31
30
29
Genotype
L/L
L/L, cre
Imputation
non imputed
imputed

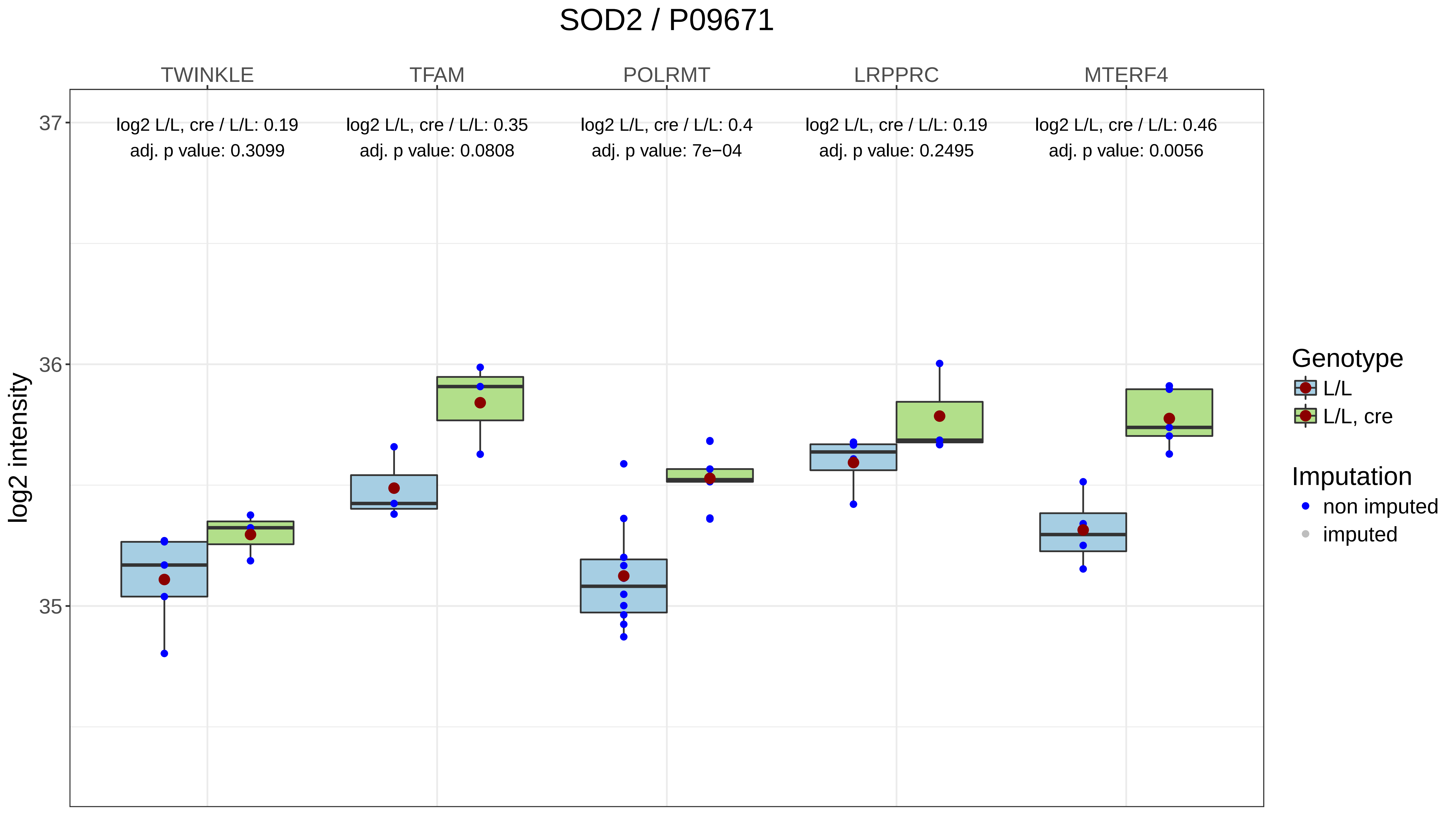
SOD2 / P09671
TWINKLE
TFAM
POLRMT
LRPPRC
MTERF4
log2 L/L, cre / L/L: 0.19
adj. p value: 0.3099
log2 L/L, cre / L/L: 0.35
adj. p value: 0.0808
log2 L/L, cre / L/L: 0.4
adj. p value: 7e−04
log2 L/L, cre / L/L: 0.19
adj. p value: 0.2495
log2 L/L, cre / L/L: 0.46
adj. p value: 0.0056
37
36
35
log2 intensity
Genotype
L/L
L/L, cre
Imputation
non imputed
imputed

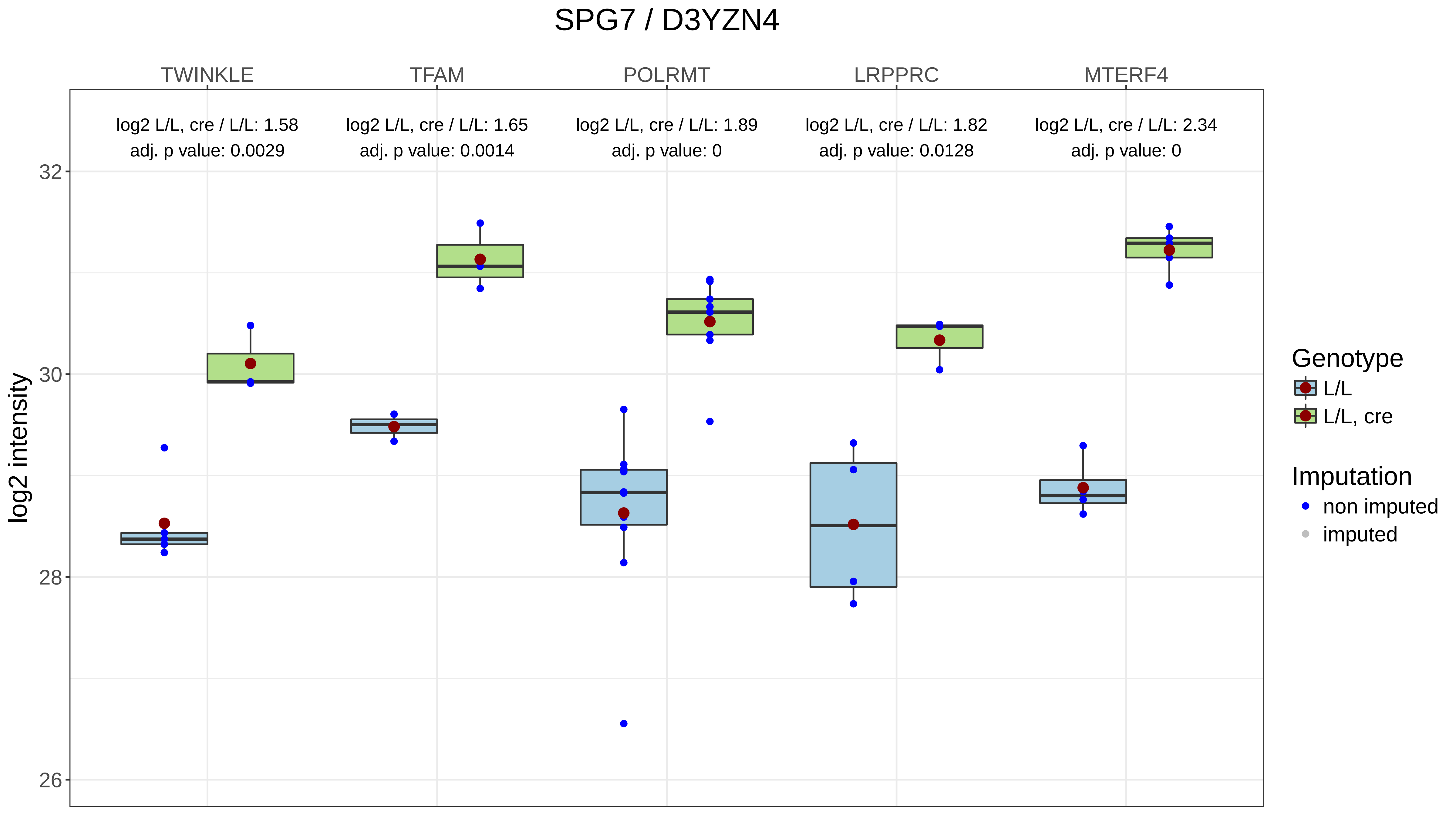
SPG7 / D3YZN4
TWINKLE
TFAM
POLRMT
LRPPRC
MTERF4
log2 L/L, cre / L/L: 1.58
adj. p value: 0.0029
log2 L/L, cre / L/L: 1.65
adj. p value: 0.0014
log2 L/L, cre / L/L: 1.89
adj. p value: 0
log2 L/L, cre / L/L: 1.82
adj. p value: 0.0128
log2 L/L, cre / L/L: 2.34
adj. p value: 0
log2 intensity
32
30
28
26
Genotype
L/L
L/L, cre
Imputation
non imputed
imputed

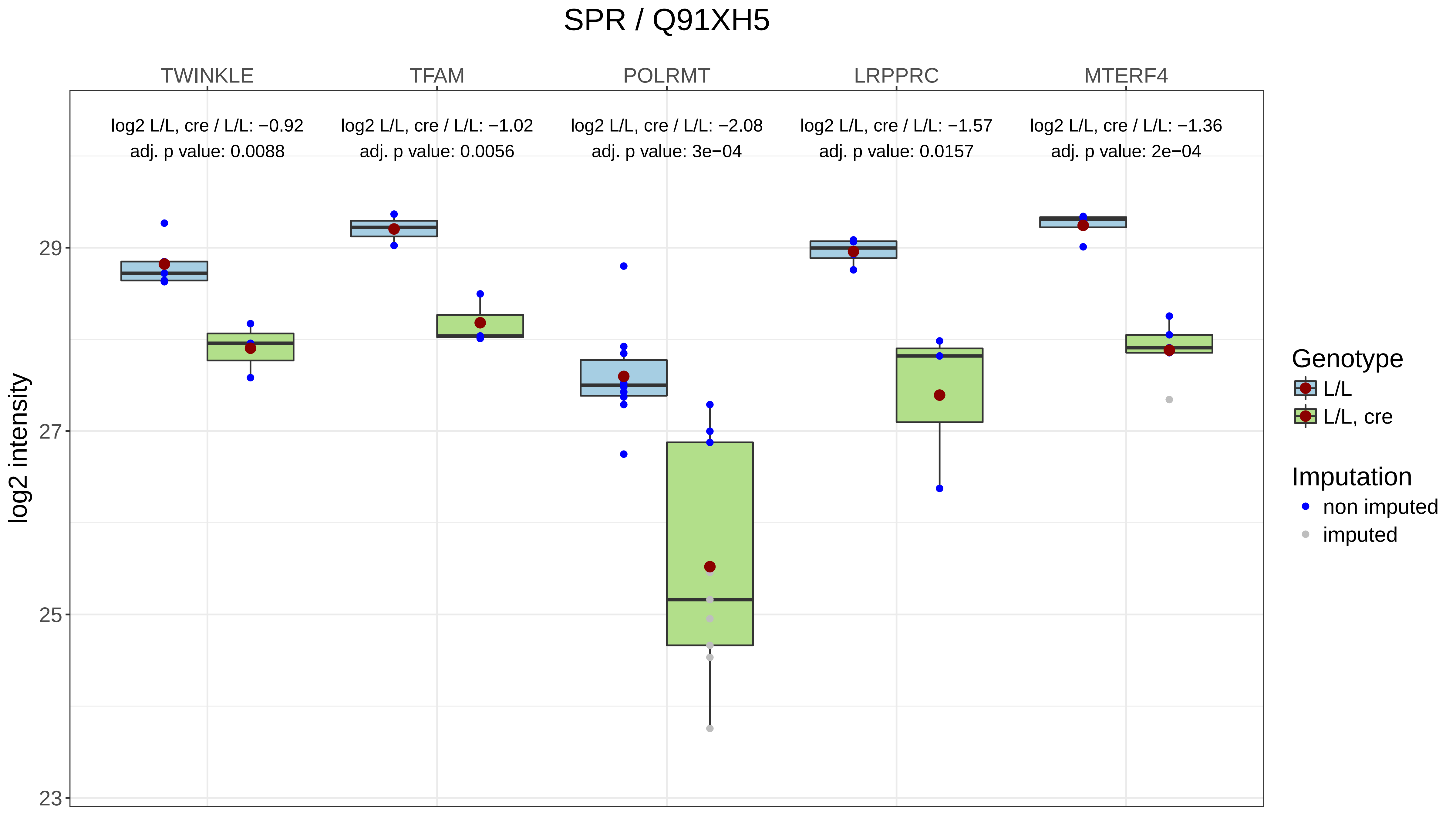

SPR / Q91XH5
TWINKLE
TFAM
POLRMT
LRPPRC
MTERF4
log2 L/L, cre / L/L: −0.92
adj. p value: 0.0088
log2 L/L, cre / L/L: −1.02
adj. p value: 0.0056
log2 L/L, cre / L/L: −2.08
adj. p value: 3e−04
log2 L/L, cre / L/L: −1.57
adj. p value: 0.0157
log2 L/L, cre / L/L: −1.36
adj. p value: 2e−04
log2 intensity
29
27
25
23
Genotype
L/L
L/L, cre
Imputation
non imputed
imputed

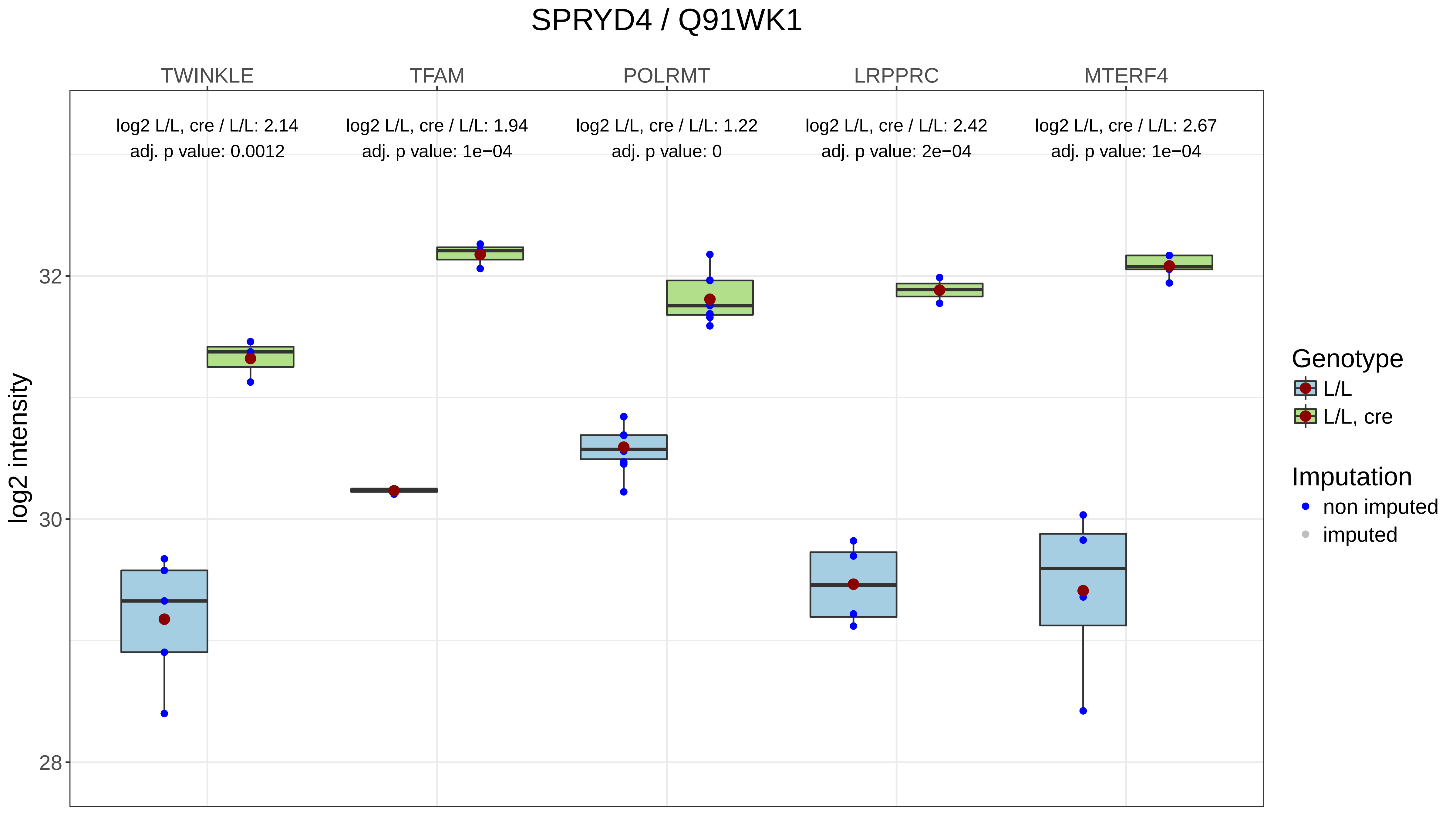
SPRYD4 / Q91WK1
TWINKLE
TFAM
POLRMT
LRPPRC
MTERF4
log2 L/L, cre / L/L: 2.14
adj. p value: 0.0012
log2 L/L, cre / L/L: 1.94
adj. p value: 1e−04
log2 L/L, cre / L/L: 1.22
adj. p value: 0
log2 L/L, cre / L/L: 2.42
adj. p value: 2e−04
log2 L/L, cre / L/L: 2.67
adj. p value: 1e−04
log2 intensity
32
30
28
Genotype
L/L
L/L, cre
Imputation
non imputed
imputed

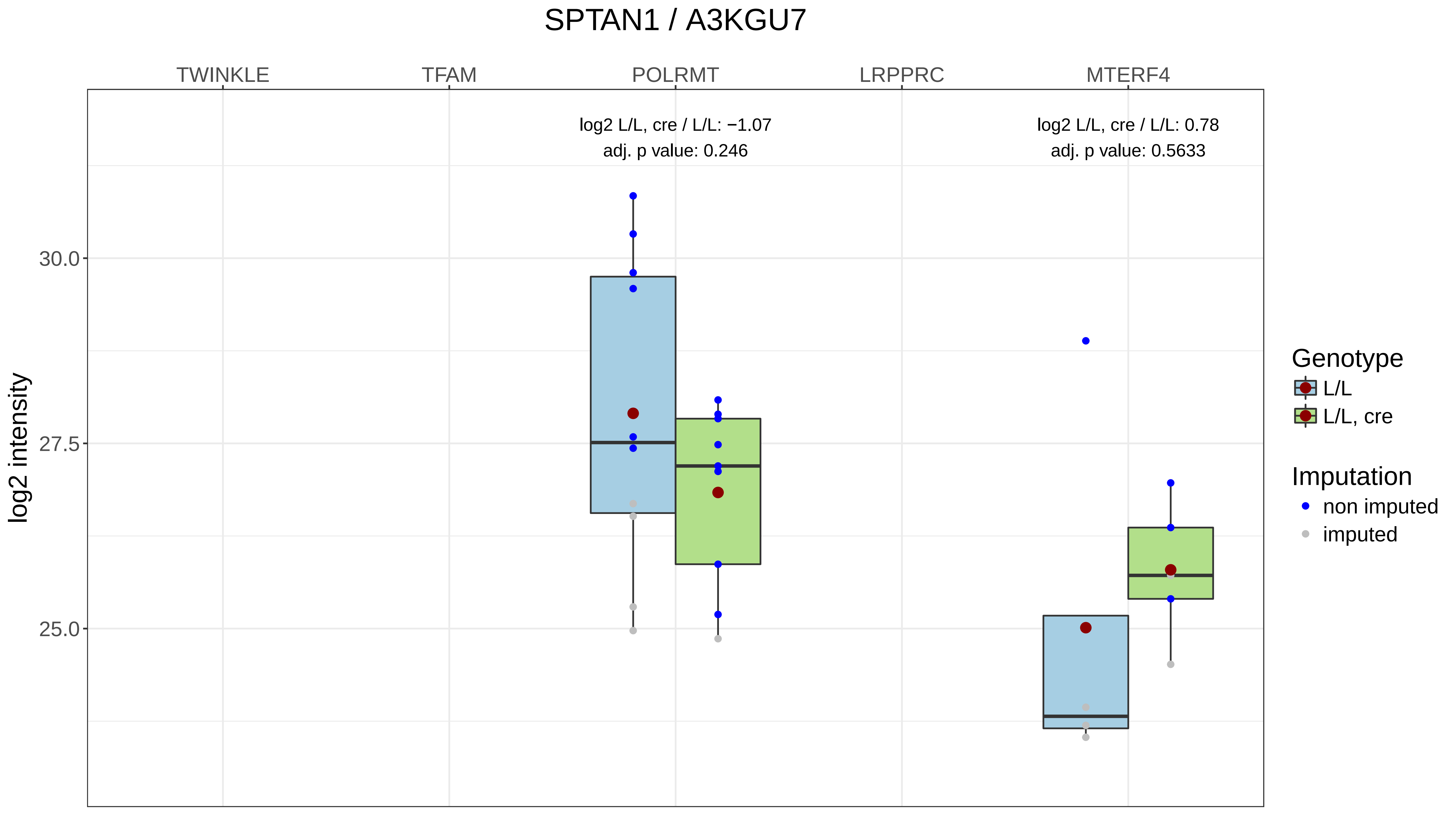

SPTAN1 / A3KGU7
TWINKLE
TFAM
POLRMT
LRPPRC
MTERF4
log2 L/L, cre / L/L: −1.07
adj. p value: 0.246
log2 L/L, cre / L/L: 0.78
adj. p value: 0.5633
30.0
27.5
25.0
log2 intensity
Genotype
L/L
L/L, cre
Imputation
non imputed
imputed

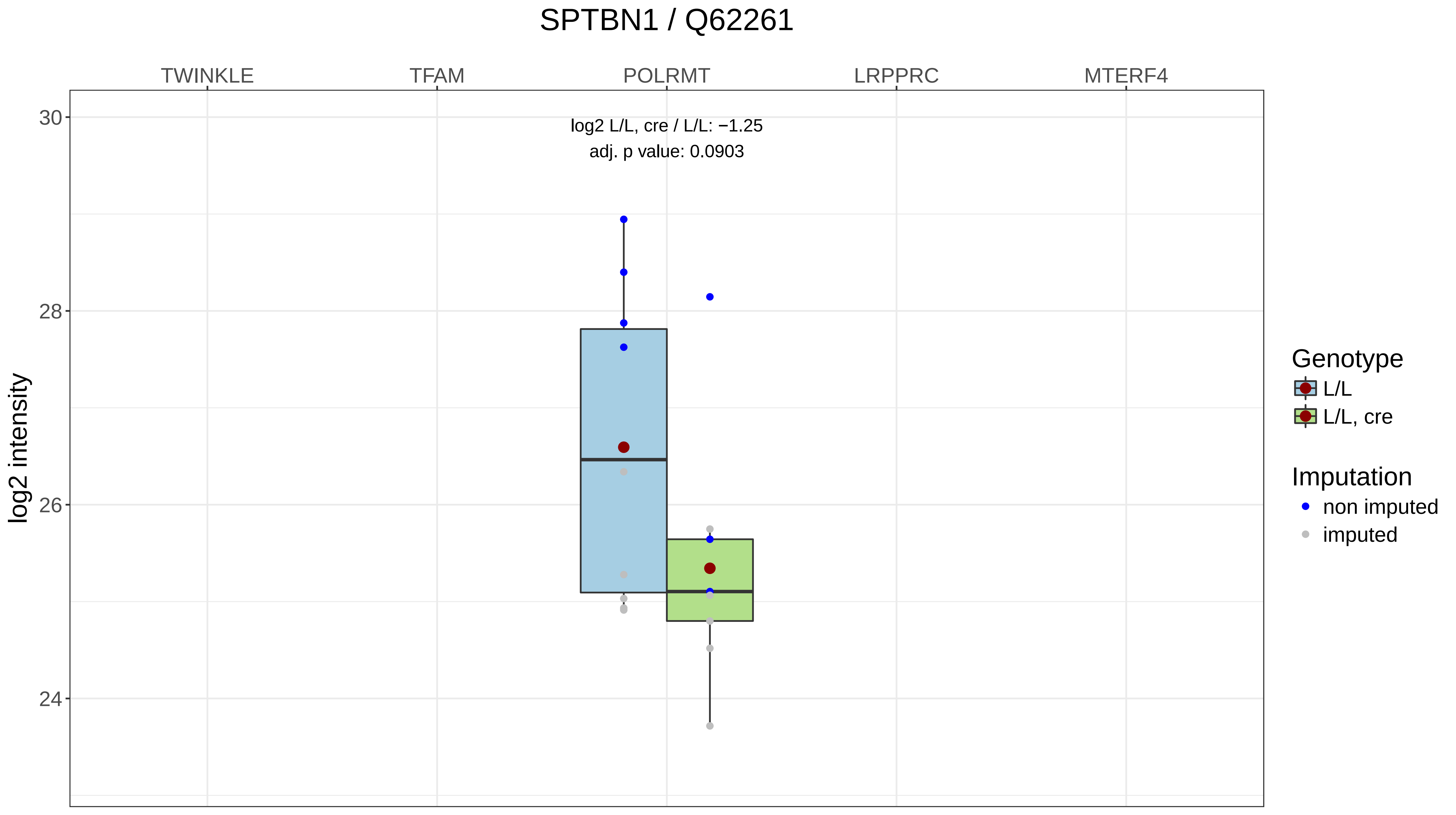
SPTBN1 / Q62261
TWINKLE
TFAM
POLRMT
LRPPRC
MTERF4
log2 L/L, cre / L/L: −1.25
adj. p value: 0.0903
30
28
26
24
log2 intensity
Genotype
L/L
L/L, cre
Imputation
non imputed
imputed

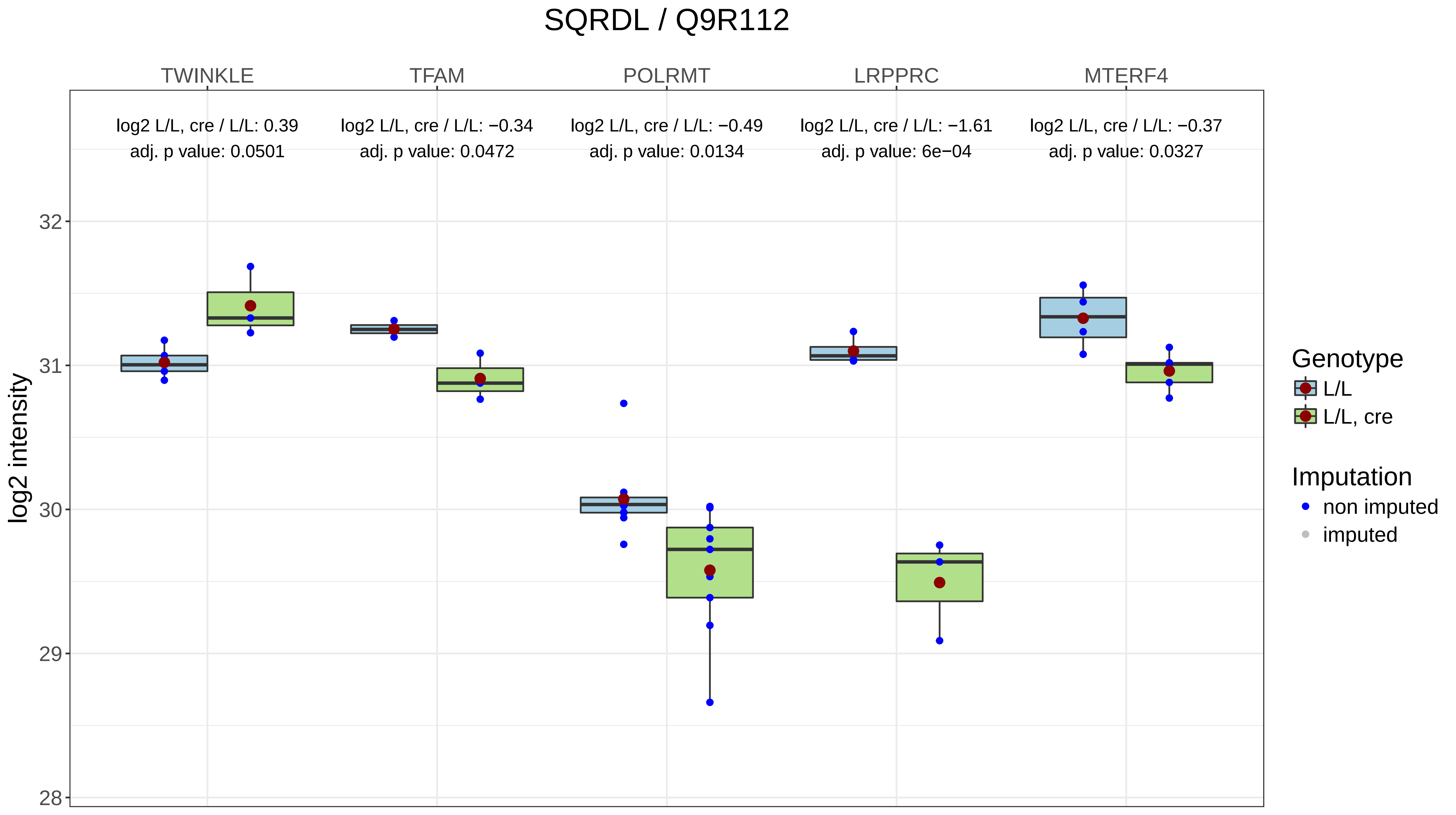
SQRDL / Q9R112
TWINKLE
TFAM
POLRMT
LRPPRC
MTERF4
log2 L/L, cre / L/L: 0.39
adj. p value: 0.0501
log2 L/L, cre / L/L: −0.34
adj. p value: 0.0472
log2 L/L, cre / L/L: −0.49
adj. p value: 0.0134
log2 L/L, cre / L/L: −1.61
adj. p value: 6e−04
log2 L/L, cre / L/L: −0.37
adj. p value: 0.0327
log2 intensity
32
31
30
29
28
Genotype
L/L
L/L, cre
Imputation
non imputed
imputed

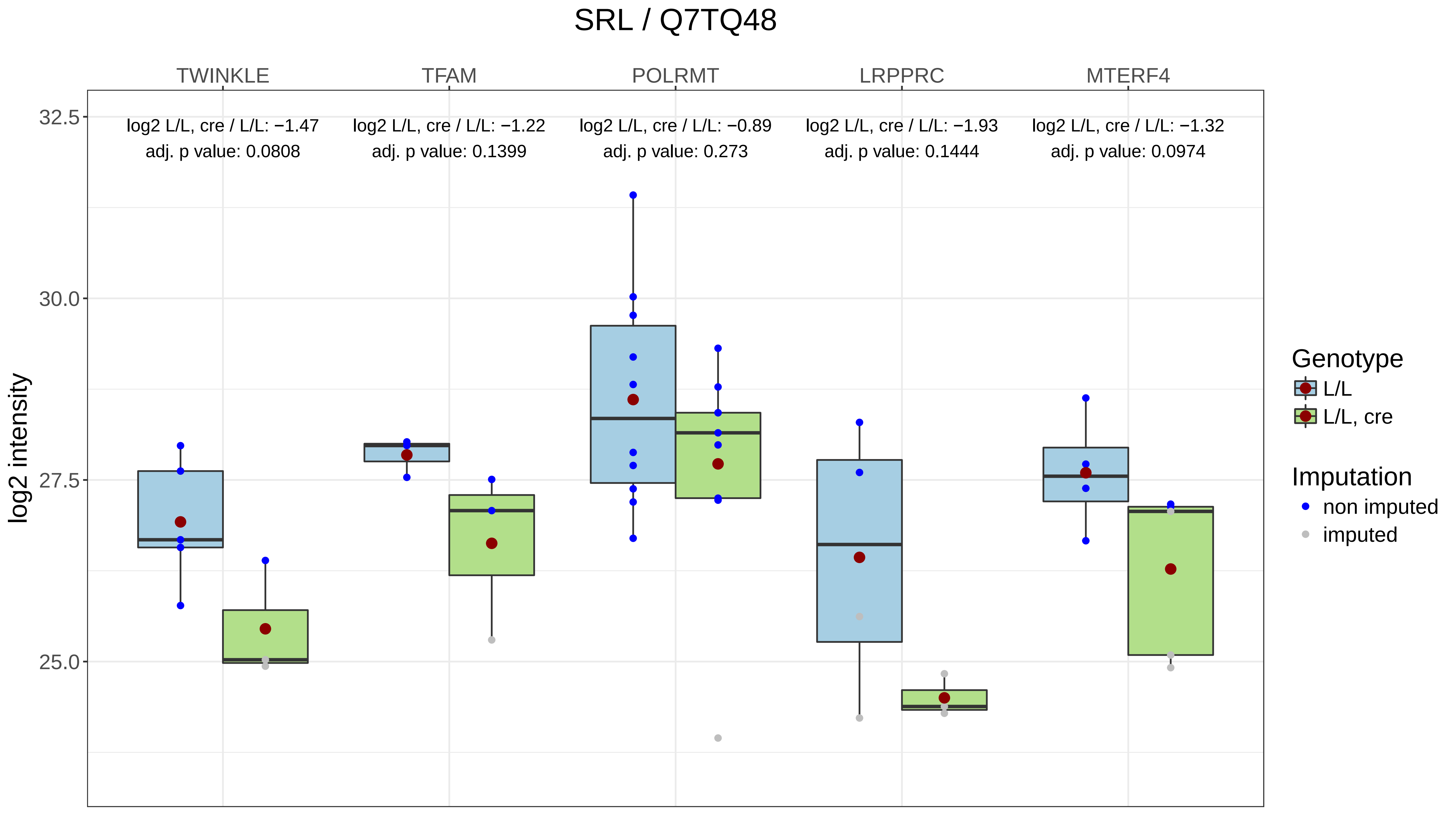
SRL / Q7TQ48
TWINKLE
TFAM
POLRMT
LRPPRC
MTERF4
log2 L/L, cre / L/L: −1.47
adj. p value: 0.0808
log2 L/L, cre / L/L: −1.22
adj. p value: 0.1399
log2 L/L, cre / L/L: −0.89
adj. p value: 0.273
log2 L/L, cre / L/L: −1.93
adj. p value: 0.1444
log2 L/L, cre / L/L: −1.32
adj. p value: 0.0974
log2 intensity
32.5
30.0
27.5
25.0
Genotype
L/L
L/L, cre
Imputation
non imputed
imputed

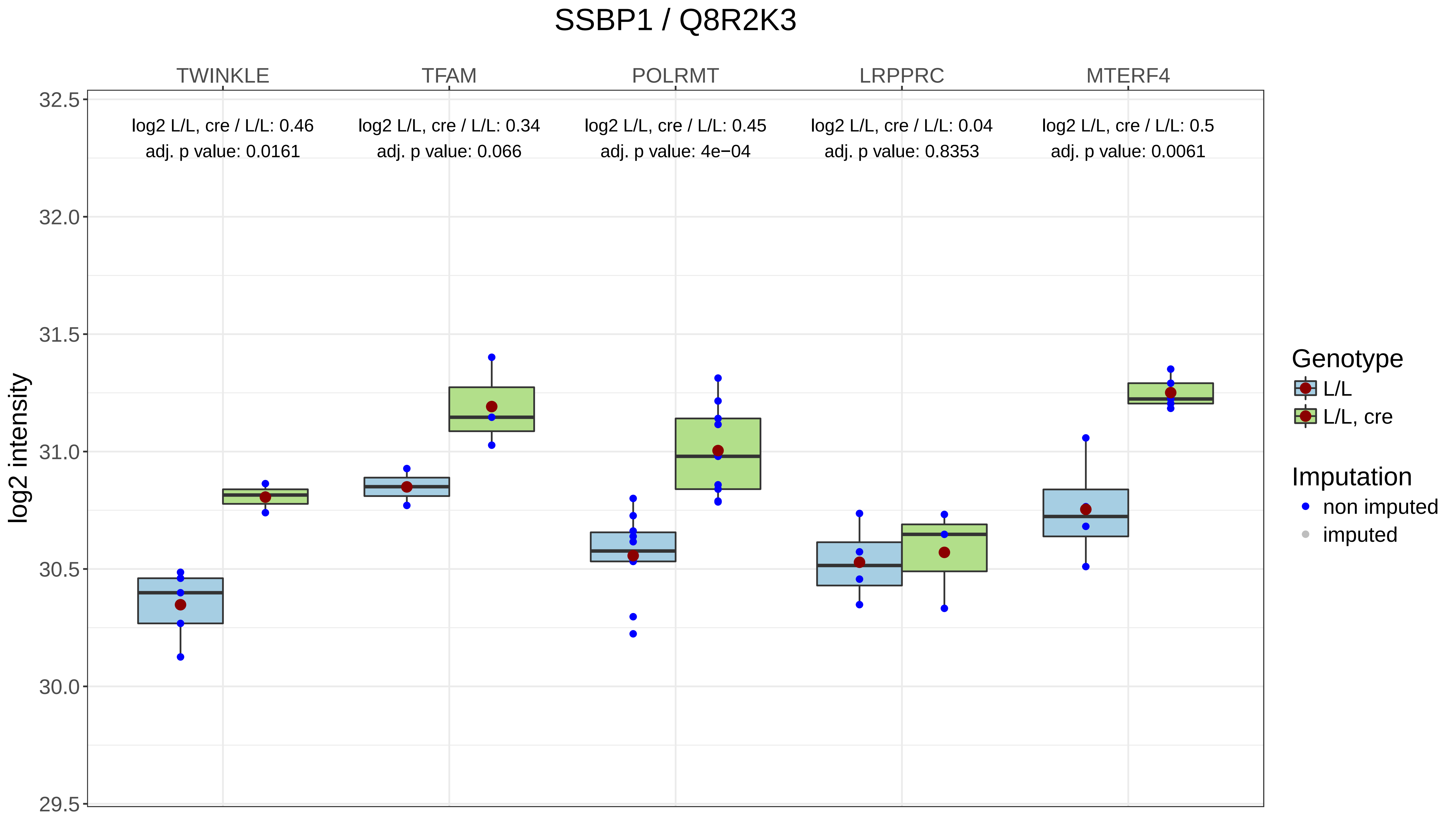
SSBP1 / Q8R2K3
TWINKLE
TFAM
POLRMT
LRPPRC
MTERF4
log2 L/L, cre / L/L: 0.46
adj. p value: 0.0161
log2 L/L, cre / L/L: 0.34
adj. p value: 0.066
log2 L/L, cre / L/L: 0.45
adj. p value: 4e−04
log2 L/L, cre / L/L: 0.04
adj. p value: 0.8353
log2 L/L, cre / L/L: 0.5
adj. p value: 0.0061
log2 intensity
32.5
32.0
31.5
31.0
30.5
30.0
29.5
Genotype
L/L
L/L, cre
Imputation
non imputed
imputed

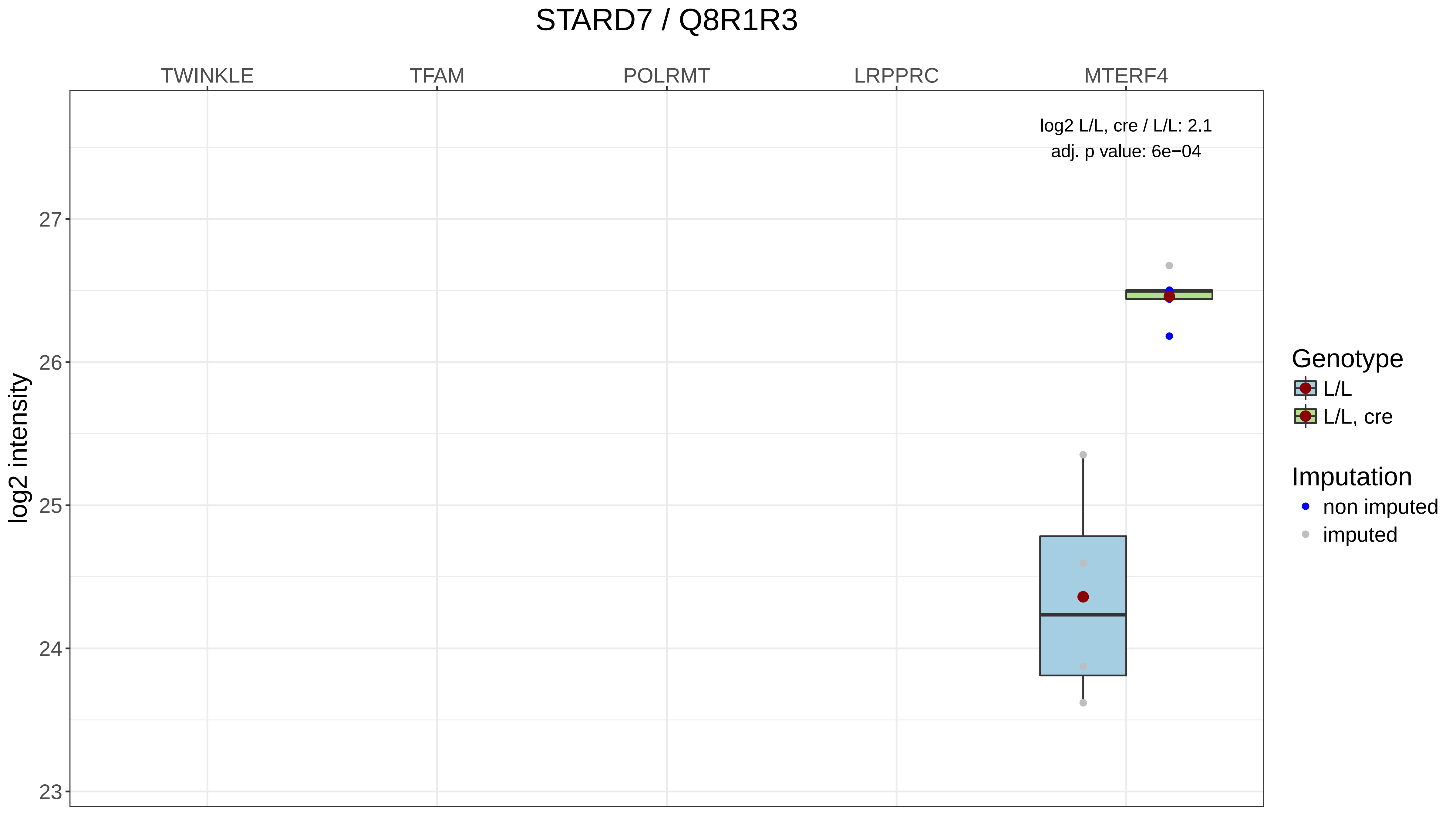
STARD7 / Q8R1R3
TWINKLE
TFAM
POLRMT
LRPPRC
MTERF4
log2 L/L, cre / L/L: 2.1
adj. p value: 6e−04
log2 intensity
27
26
25
24
23
Genotype
L/L
L/L, cre
Imputation
non imputed
imputed

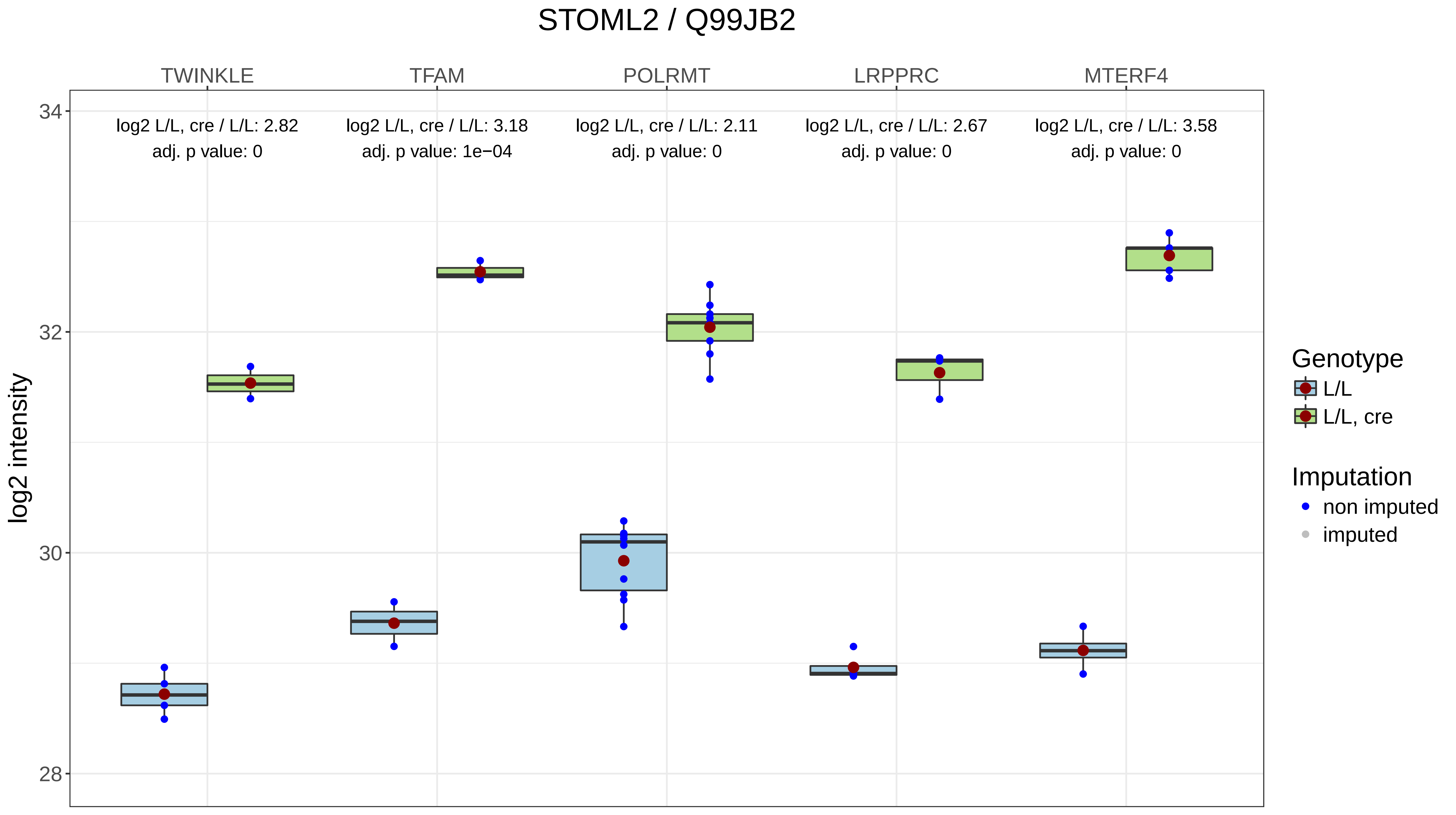
STOML2 / Q99JB2
TWINKLE
TFAM
POLRMT
LRPPRC
MTERF4
log2 L/L, cre / L/L: 2.82
adj. p value: 0
log2 L/L, cre / L/L: 3.18
adj. p value: 1e−04
log2 L/L, cre / L/L: 2.11
adj. p value: 0
log2 L/L, cre / L/L: 2.67
adj. p value: 0
log2 L/L, cre / L/L: 3.58
adj. p value: 0
log2 intensity
34
32
30
28
Genotype
L/L
L/L, cre
Imputation
non imputed
imputed

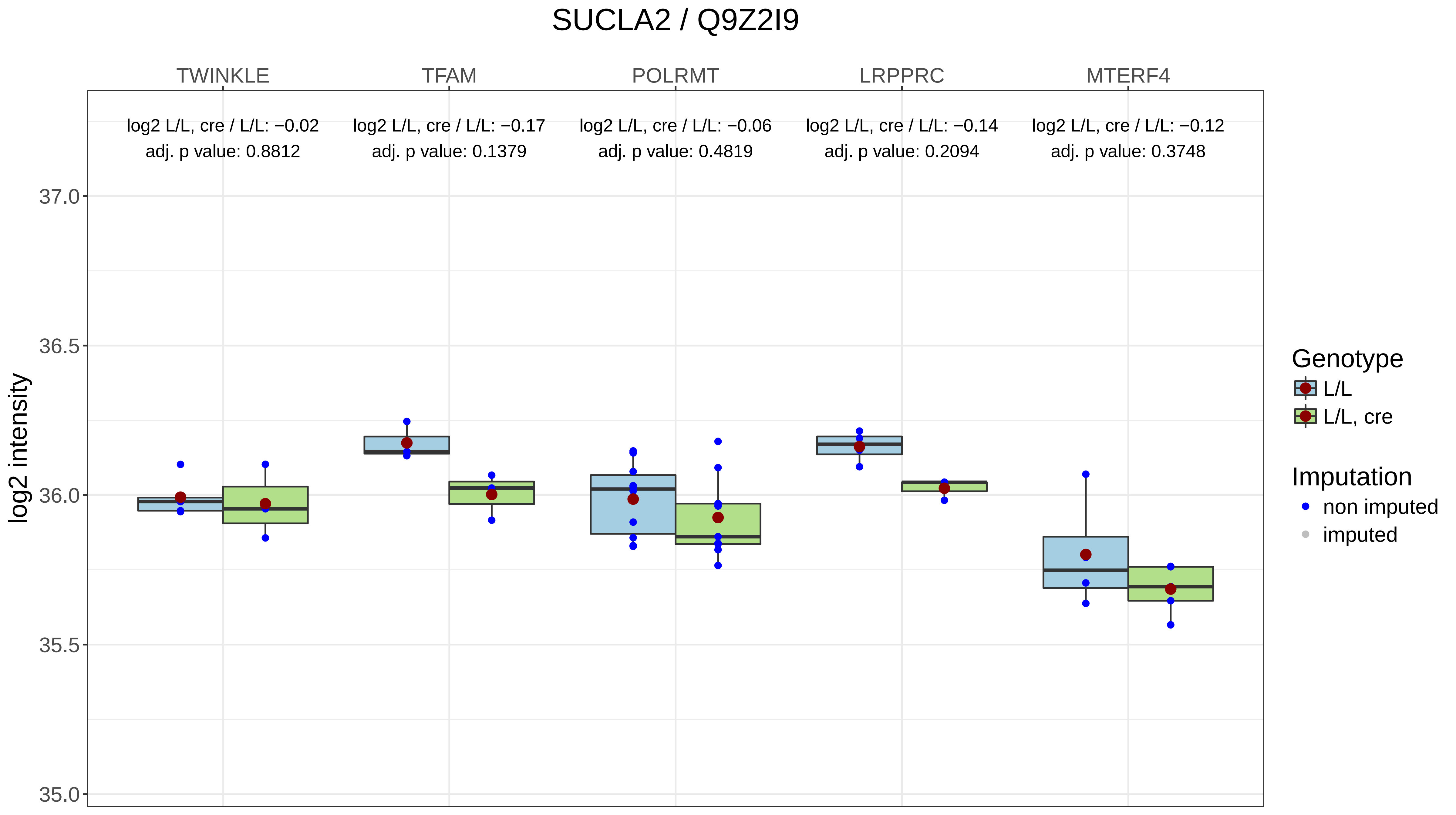
SUCLA2 / Q9Z2I9
TWINKLE
TFAM
POLRMT
LRPPRC
MTERF4
log2 L/L, cre / L/L: −0.02
adj. p value: 0.8812
log2 L/L, cre / L/L: −0.17
adj. p value: 0.1379
log2 L/L, cre / L/L: −0.06
adj. p value: 0.4819
log2 L/L, cre / L/L: −0.14
adj. p value: 0.2094
log2 L/L, cre / L/L: −0.12
adj. p value: 0.3748
log2 intensity
37.0
36.5
36.0
35.5
35.0
Genotype
L/L
L/L, cre
Imputation
non imputed
imputed

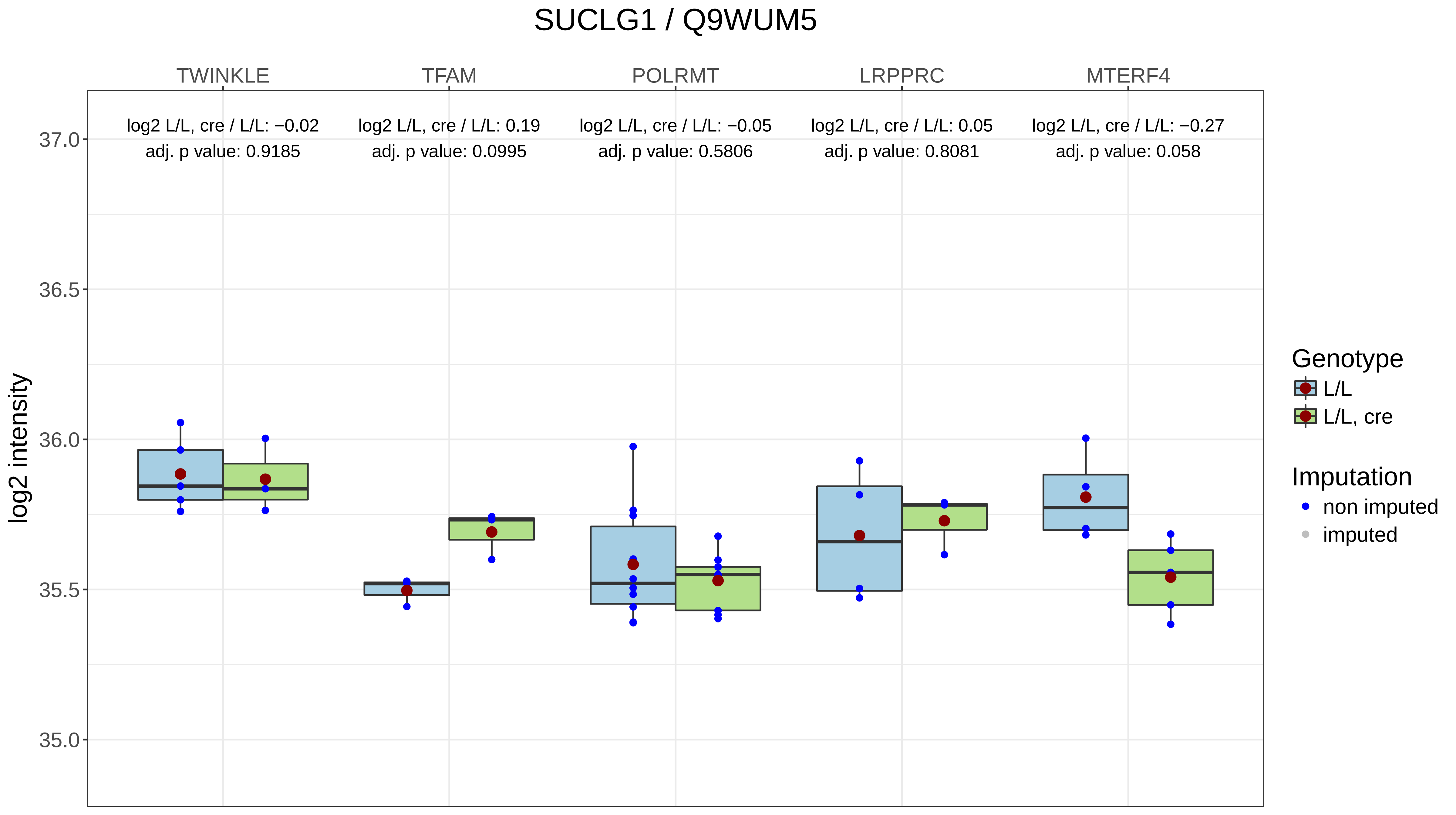

SUCLG1 / Q9WUM5
TWINKLE
TFAM
POLRMT
LRPPRC
MTERF4
log2 L/L, cre / L/L: −0.02
adj. p value: 0.9185
log2 L/L, cre / L/L: 0.19
adj. p value: 0.0995
log2 L/L, cre / L/L: −0.05
adj. p value: 0.5806
log2 L/L, cre / L/L: 0.05
adj. p value: 0.8081
log2 L/L, cre / L/L: −0.27
adj. p value: 0.058
log2 intensity
37.0
36.5
36.0
35.5
35.0
Genotype
L/L
L/L, cre
Imputation
non imputed
imputed

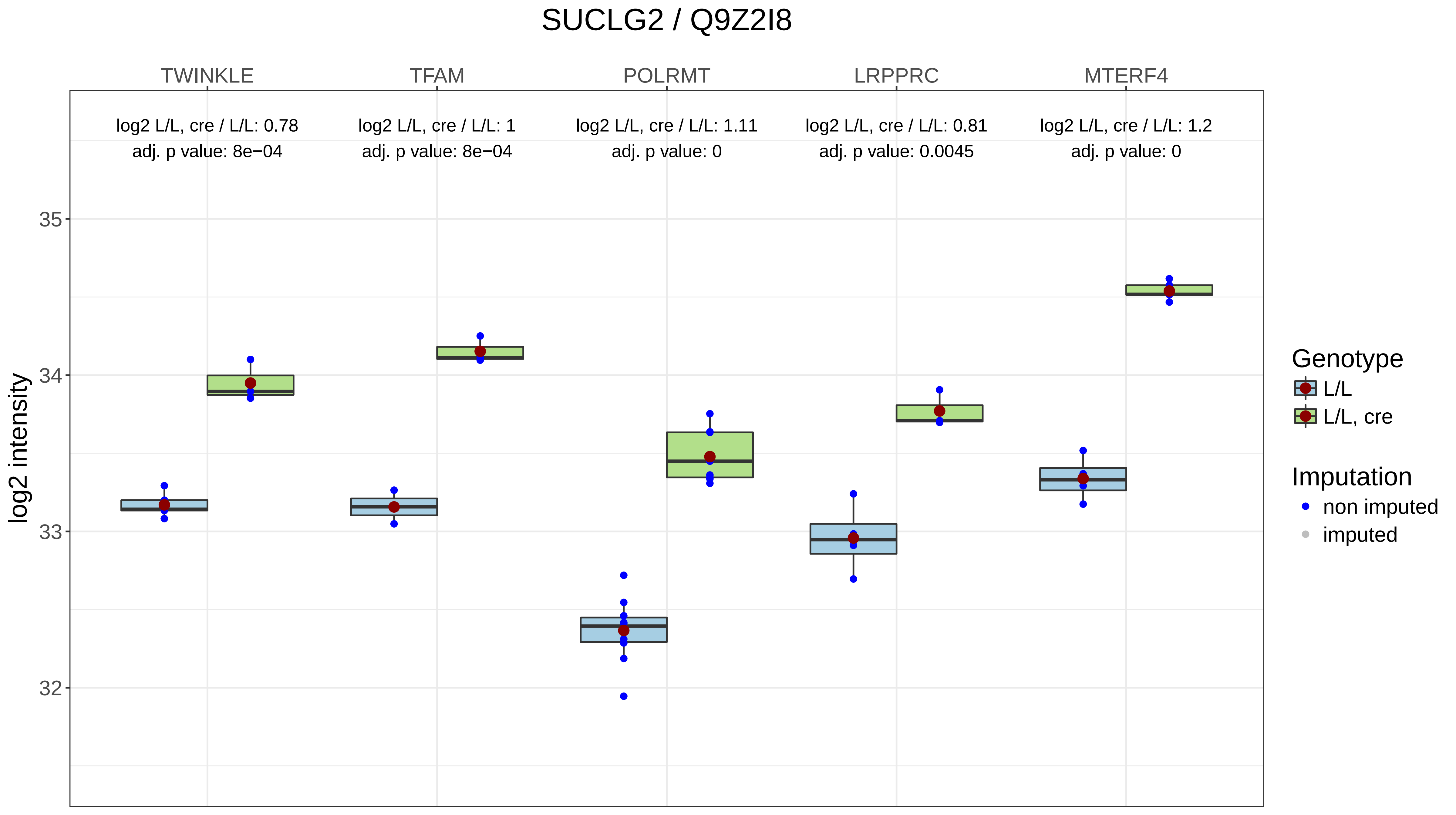
SUCLG2 / Q9Z2I8
TWINKLE
TFAM
POLRMT
LRPPRC
MTERF4
log2 L/L, cre / L/L: 0.78
adj. p value: 8e−04
log2 L/L, cre / L/L: 1
adj. p value: 8e−04
log2 L/L, cre / L/L: 1.11
adj. p value: 0
log2 L/L, cre / L/L: 0.81
adj. p value: 0.0045
log2 L/L, cre / L/L: 1.2
adj. p value: 0
log2 intensity
35
34
33
32
Genotype
L/L
L/L, cre
Imputation
non imputed
imputed

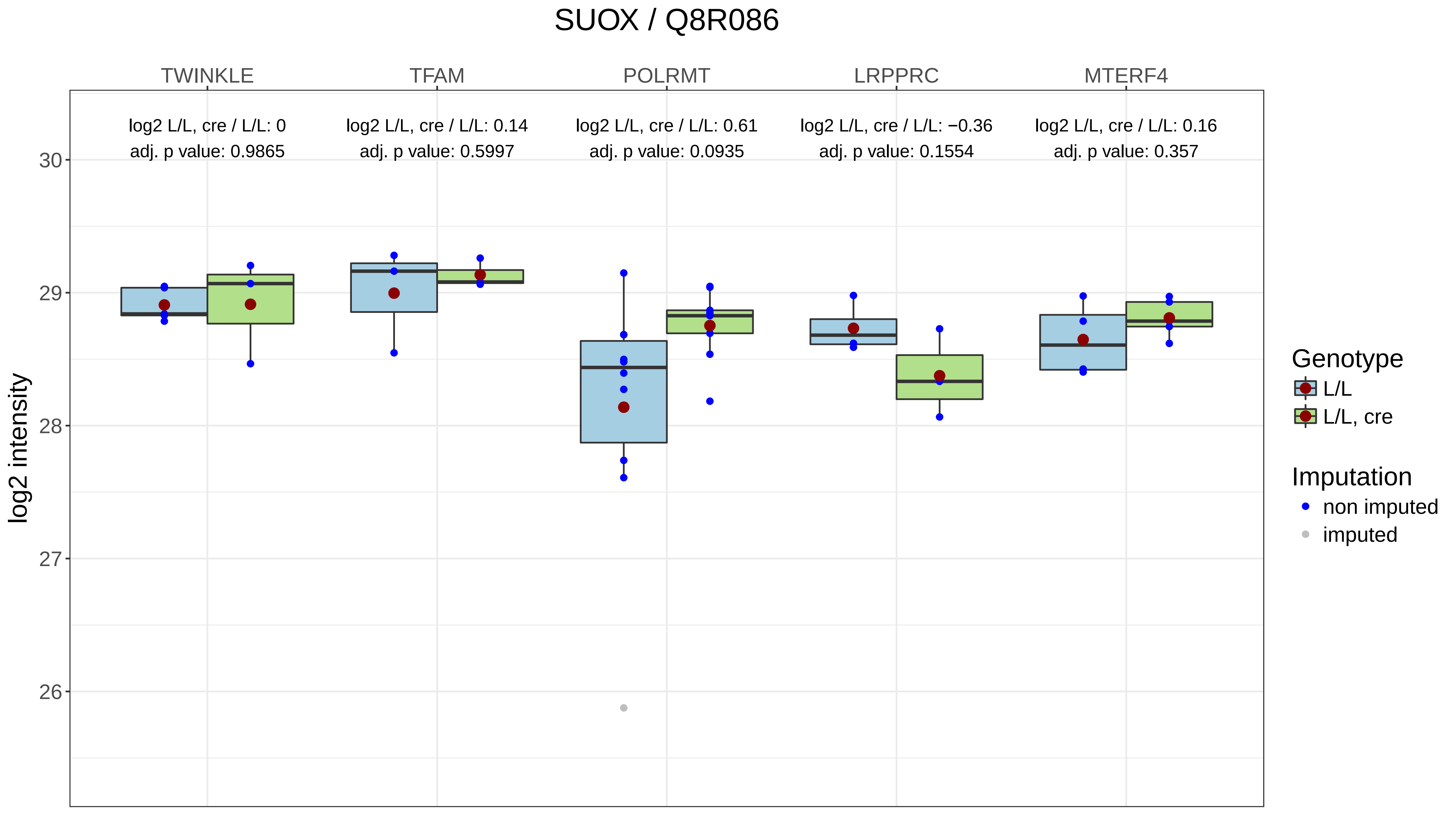
SUOX / Q8R086
TWINKLE
TFAM
POLRMT
LRPPRC
MTERF4
log2 L/L, cre / L/L: 0
adj. p value: 0.9865
log2 L/L, cre / L/L: 0.14
adj. p value: 0.5997
log2 L/L, cre / L/L: 0.61
adj. p value: 0.0935
log2 L/L, cre / L/L: −0.36
adj. p value: 0.1554
log2 L/L, cre / L/L: 0.16
adj. p value: 0.357
log2 intensity
30
29
28
27
26
Genotype
L/L
L/L, cre
Imputation
non imputed
imputed

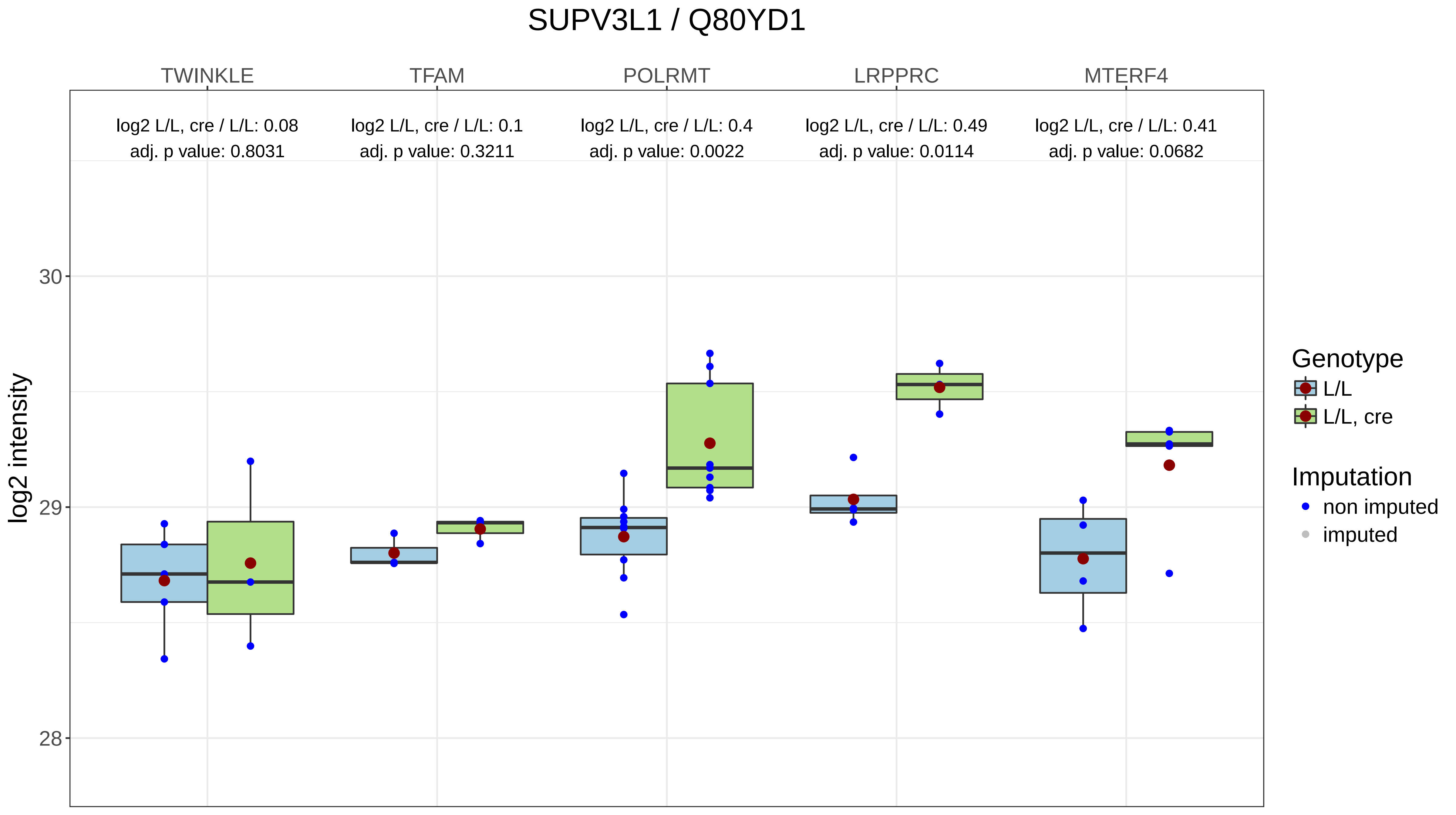
SUPV3L1 / Q80YD1
TWINKLE
TFAM
POLRMT
LRPPRC
MTERF4
log2 L/L, cre / L/L: 0.08
adj. p value: 0.8031
log2 L/L, cre / L/L: 0.1
adj. p value: 0.3211
log2 L/L, cre / L/L: 0.4
adj. p value: 0.0022
log2 L/L, cre / L/L: 0.49
adj. p value: 0.0114
log2 L/L, cre / L/L: 0.41
adj. p value: 0.0682
log2 intensity
30
29
28
Genotype
L/L
L/L, cre
Imputation
non imputed
imputed

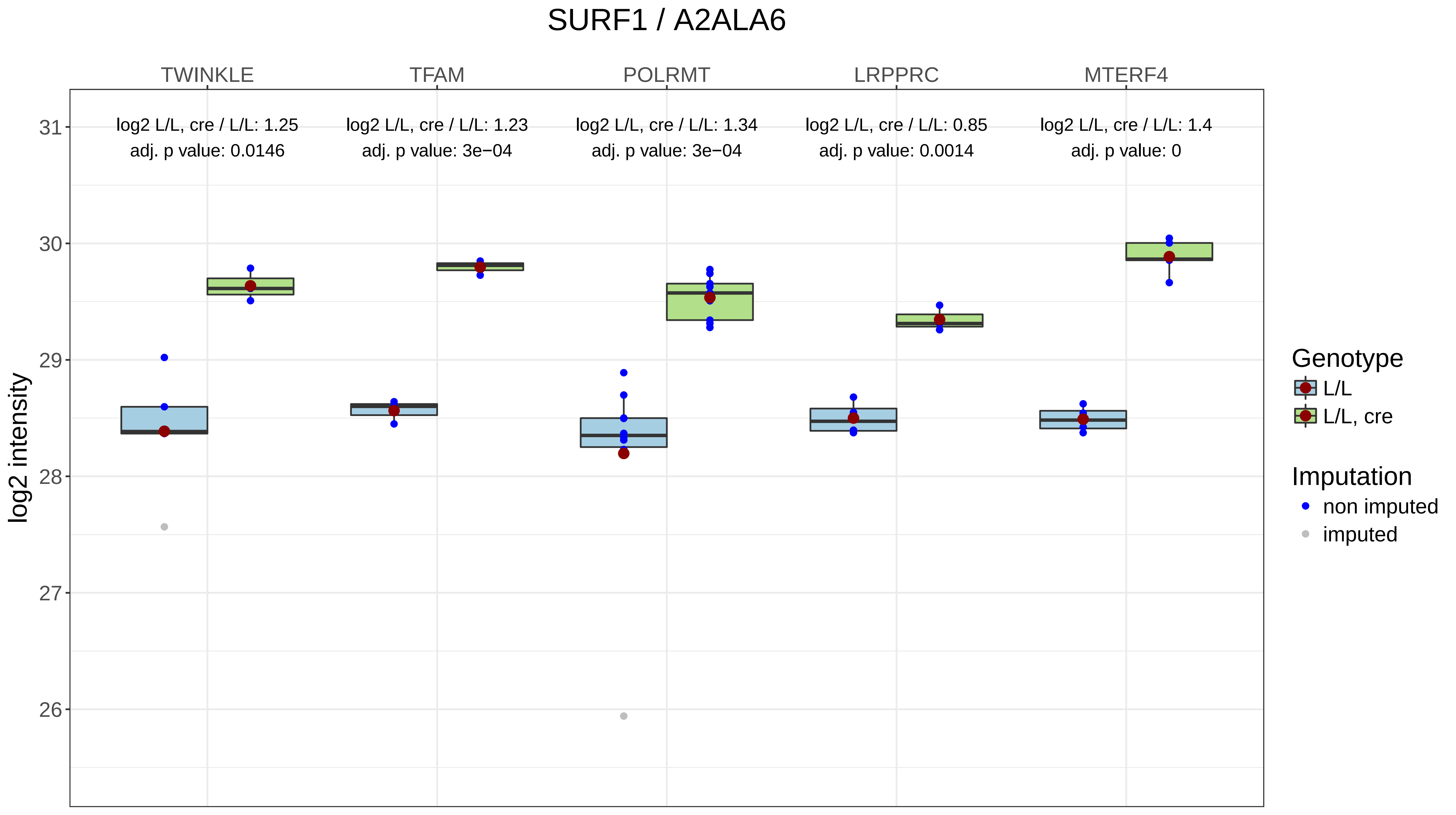

SURF1 / A2ALA6
TWINKLE
TFAM
POLRMT
LRPPRC
MTERF4
log2 L/L, cre / L/L: 1.25
adj. p value: 0.0146
log2 L/L, cre / L/L: 1.23
adj. p value: 3e−04
log2 L/L, cre / L/L: 1.34
adj. p value: 3e−04
log2 L/L, cre / L/L: 0.85
adj. p value: 0.0014
log2 L/L, cre / L/L: 1.4
adj. p value: 0
log2 intensity
31
30
29
28
27
26
Genotype
L/L
L/L, cre
Imputation
non imputed
imputed

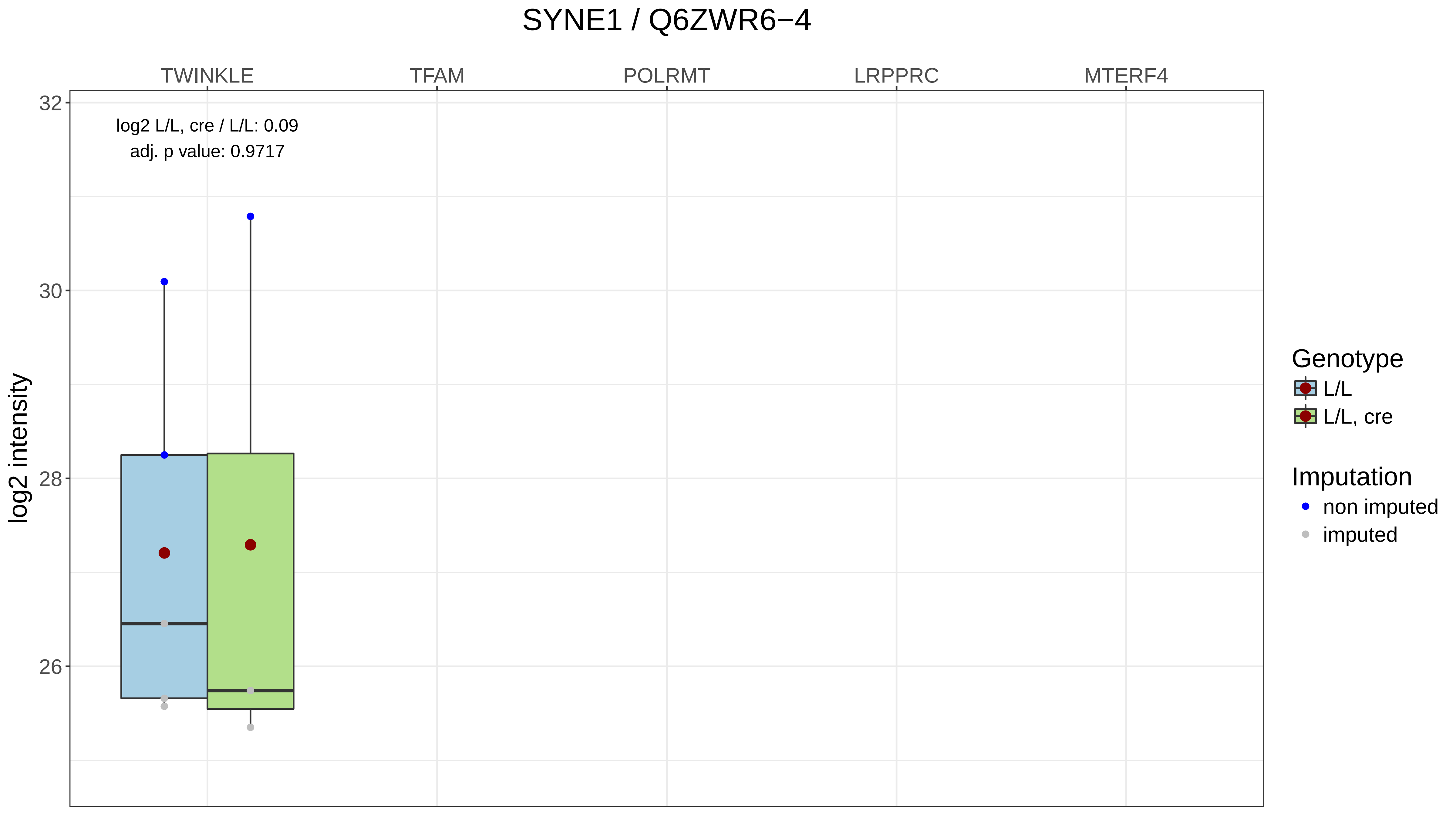

SYNE1 / Q6ZWR6−4
TWINKLE
TFAM
POLRMT
LRPPRC
MTERF4
log2 L/L, cre / L/L: 0.09
adj. p value: 0.9717
log2 intensity
32
30
28
26
Genotype
L/L
L/L, cre
Imputation
non imputed
imputed

SYNJ2BP / Q9D6K5

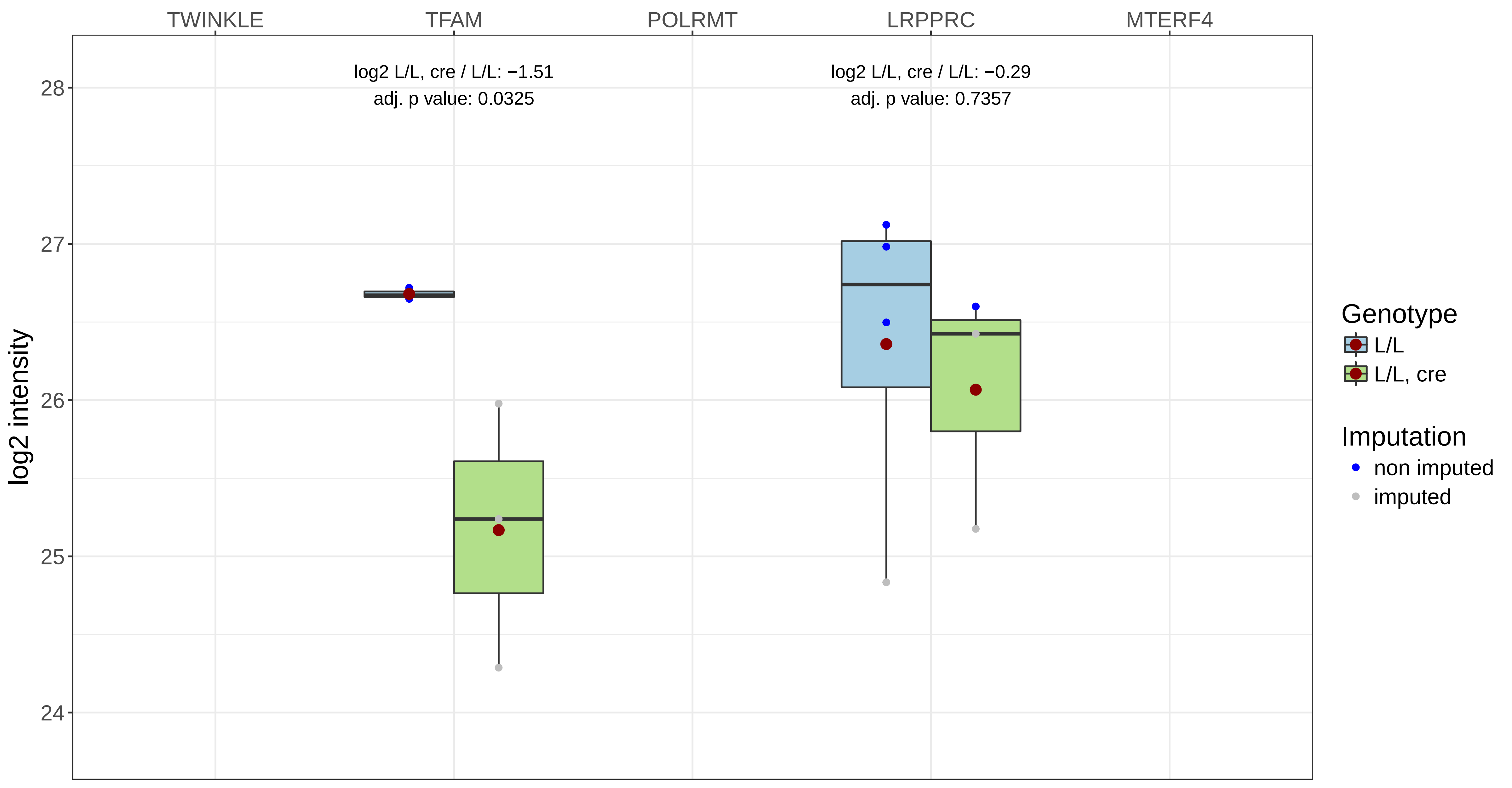

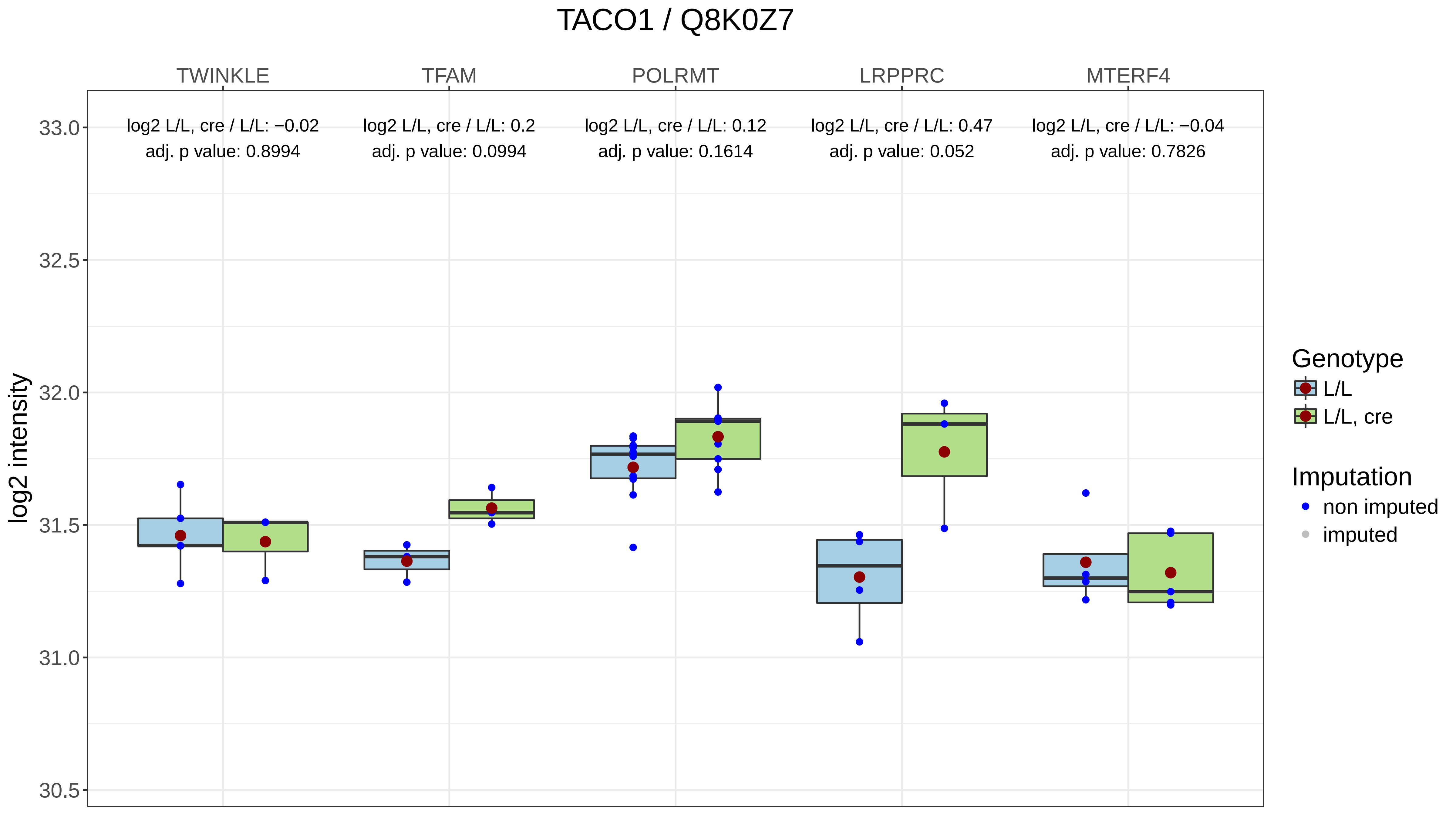
TACO1 / Q8K0Z7
TWINKLE
TFAM
POLRMT
LRPPRC
MTERF4
log2 L/L, cre / L/L: −0.02
adj. p value: 0.8994
log2 L/L, cre / L/L: 0.2
adj. p value: 0.0994
log2 L/L, cre / L/L: 0.12
adj. p value: 0.1614
log2 L/L, cre / L/L: 0.47
adj. p value: 0.052
log2 L/L, cre / L/L: −0.04
adj. p value: 0.7826
log2 intensity
33.0
32.5
32.0
31.5
31.0
30.5
Genotype
L/L
L/L, cre
Imputation
non imputed
imputed

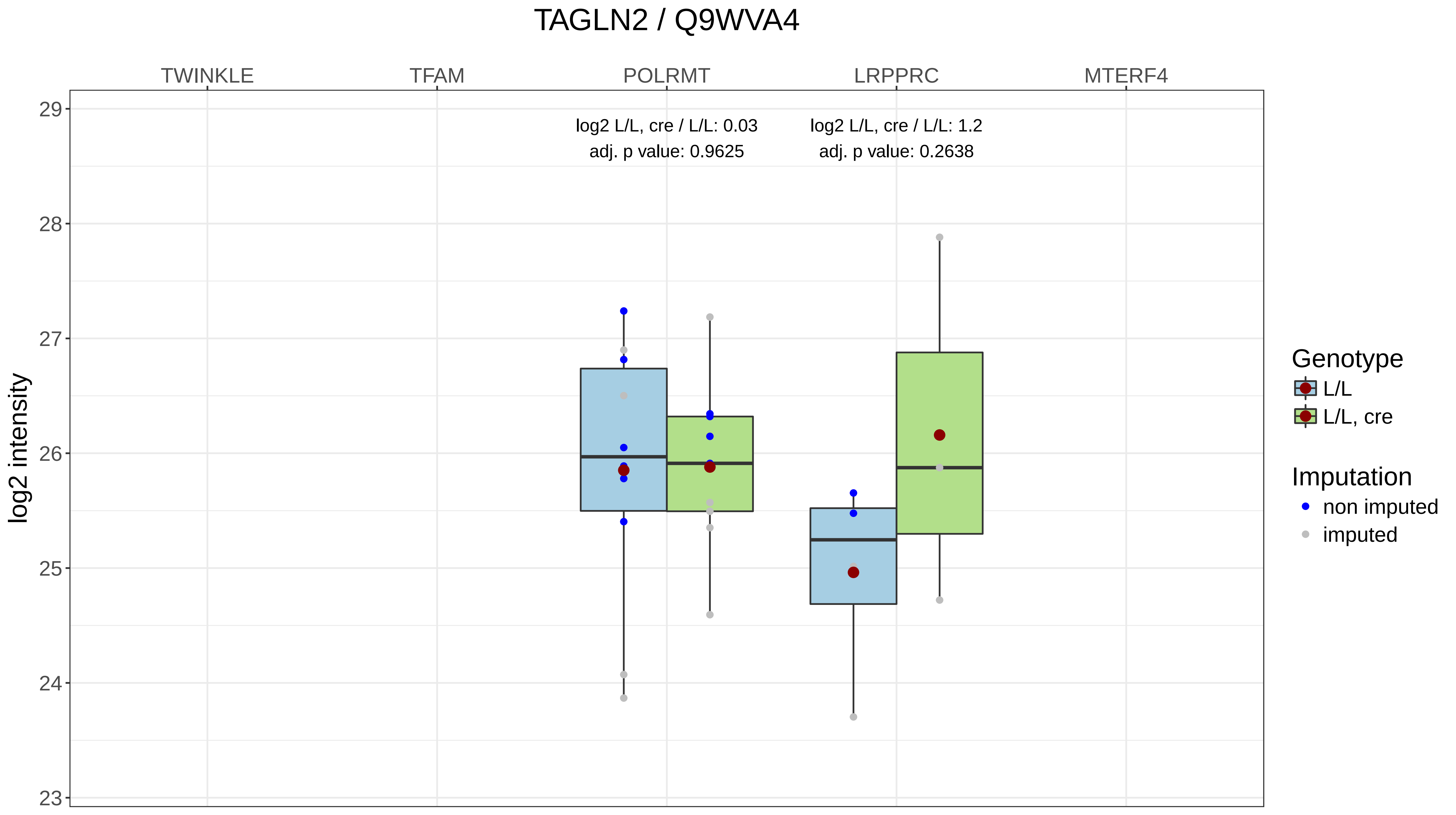
TAGLN2 / Q9WVA4
TWINKLE
TFAM
POLRMT
LRPPRC
MTERF4
log2 L/L, cre / L/L: 0.03
adj. p value: 0.9625
log2 L/L, cre / L/L: 1.2
adj. p value: 0.2638
log2 intensity
29
28
27
26
25
24
23
Genotype
L/L
L/L, cre
Imputation
non imputed
imputed

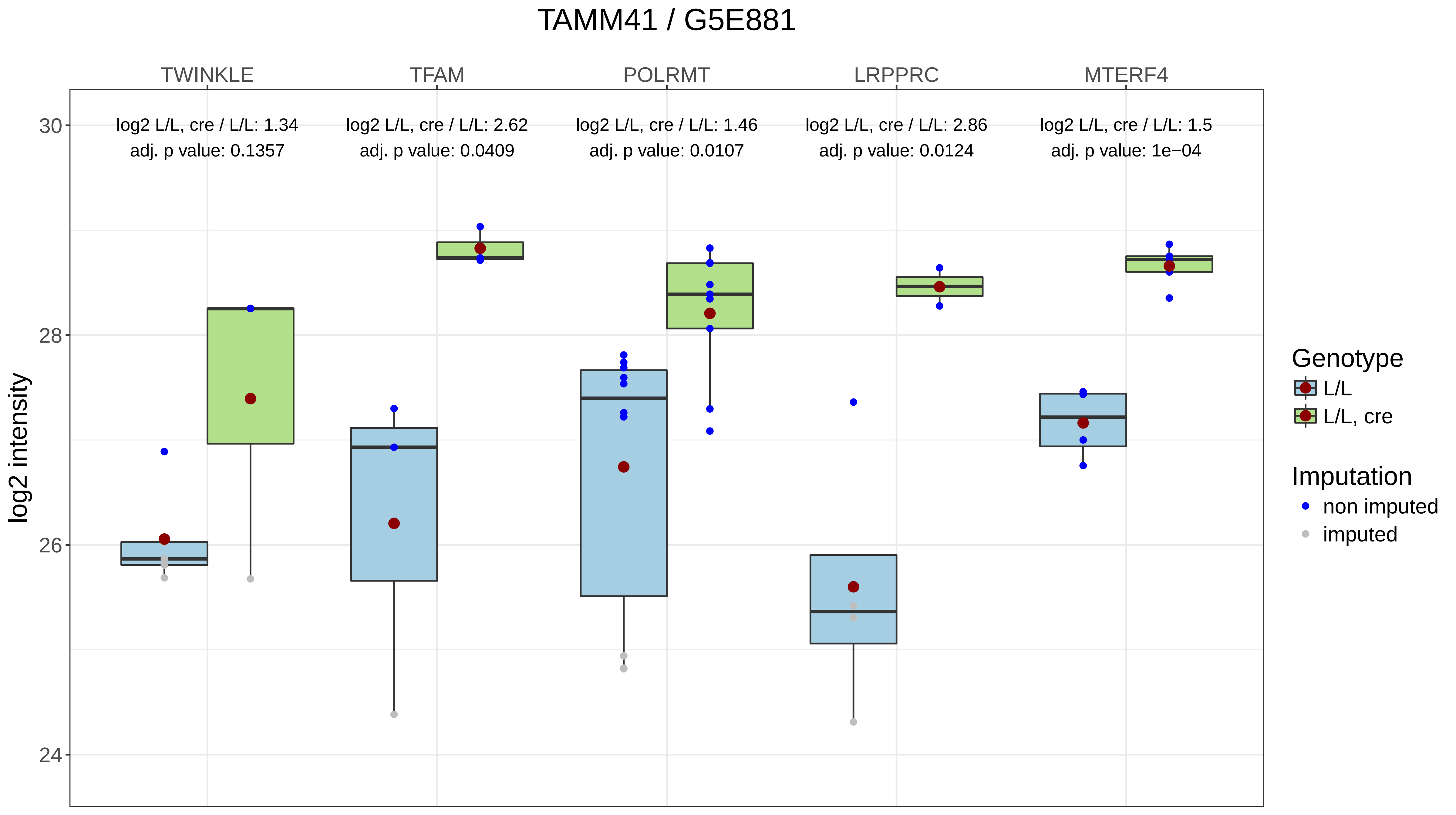

TAMM41 / G5E881
TWINKLE
TFAM
POLRMT
LRPPRC
MTERF4
log2 L/L, cre / L/L: 1.34
adj. p value: 0.1357
log2 L/L, cre / L/L: 2.62
adj. p value: 0.0409
log2 L/L, cre / L/L: 1.46
adj. p value: 0.0107
log2 L/L, cre / L/L: 2.86
adj. p value: 0.0124
log2 L/L, cre / L/L: 1.5
adj. p value: 1e−04
30
28
26
24
log2 intensity
Genotype
L/L
L/L, cre
Imputation
non imputed
imputed

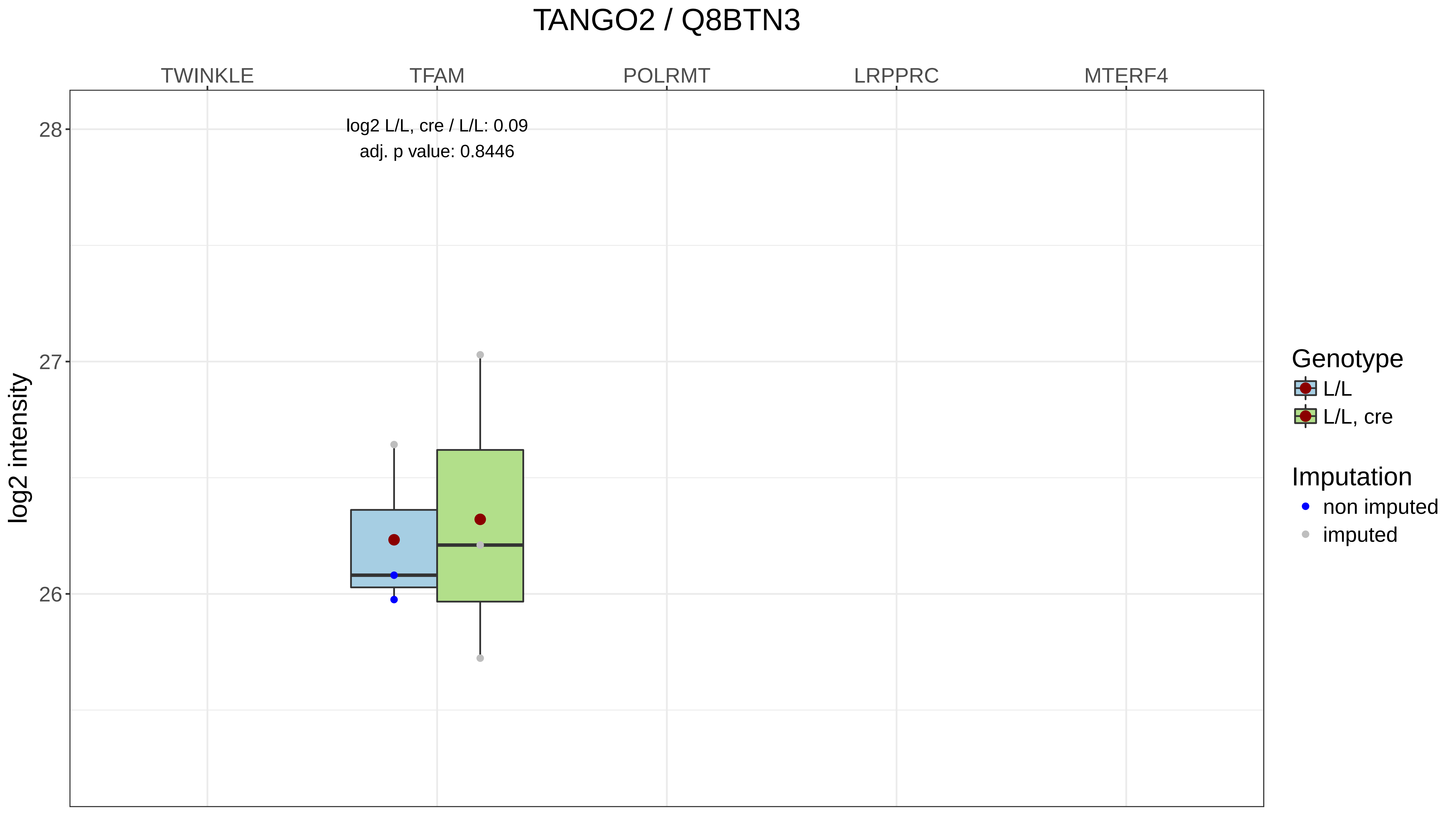

TANGO2 / Q8BTN3
TWINKLE
TFAM
POLRMT
LRPPRC
MTERF4
log2 L/L, cre / L/L: 0.09
adj. p value: 0.8446
28
27
26
log2 intensity
Genotype
L/L
L/L, cre
Imputation
non imputed
imputed

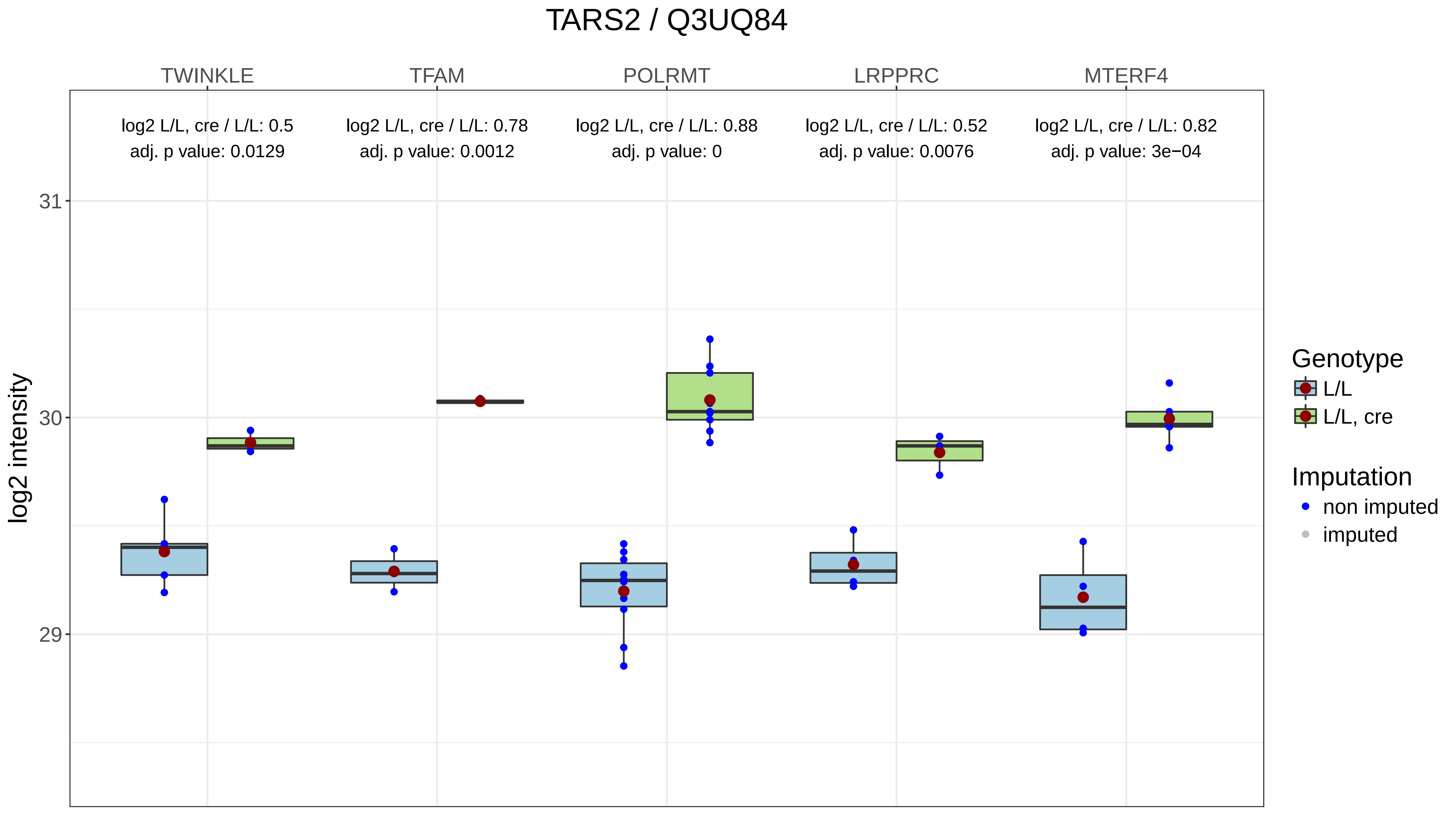

TARS2 / Q3UQ84
TWINKLE
TFAM
POLRMT
LRPPRC
MTERF4
log2 L/L, cre / L/L: 0.5
adj. p value: 0.0129
log2 L/L, cre / L/L: 0.78
adj. p value: 0.0012
log2 L/L, cre / L/L: 0.88
adj. p value: 0
log2 L/L, cre / L/L: 0.52
adj. p value: 0.0076
log2 L/L, cre / L/L: 0.82
adj. p value: 3e−04
31
30
29
log2 intensity
Genotype
L/L
L/L, cre
Imputation
non imputed
imputed

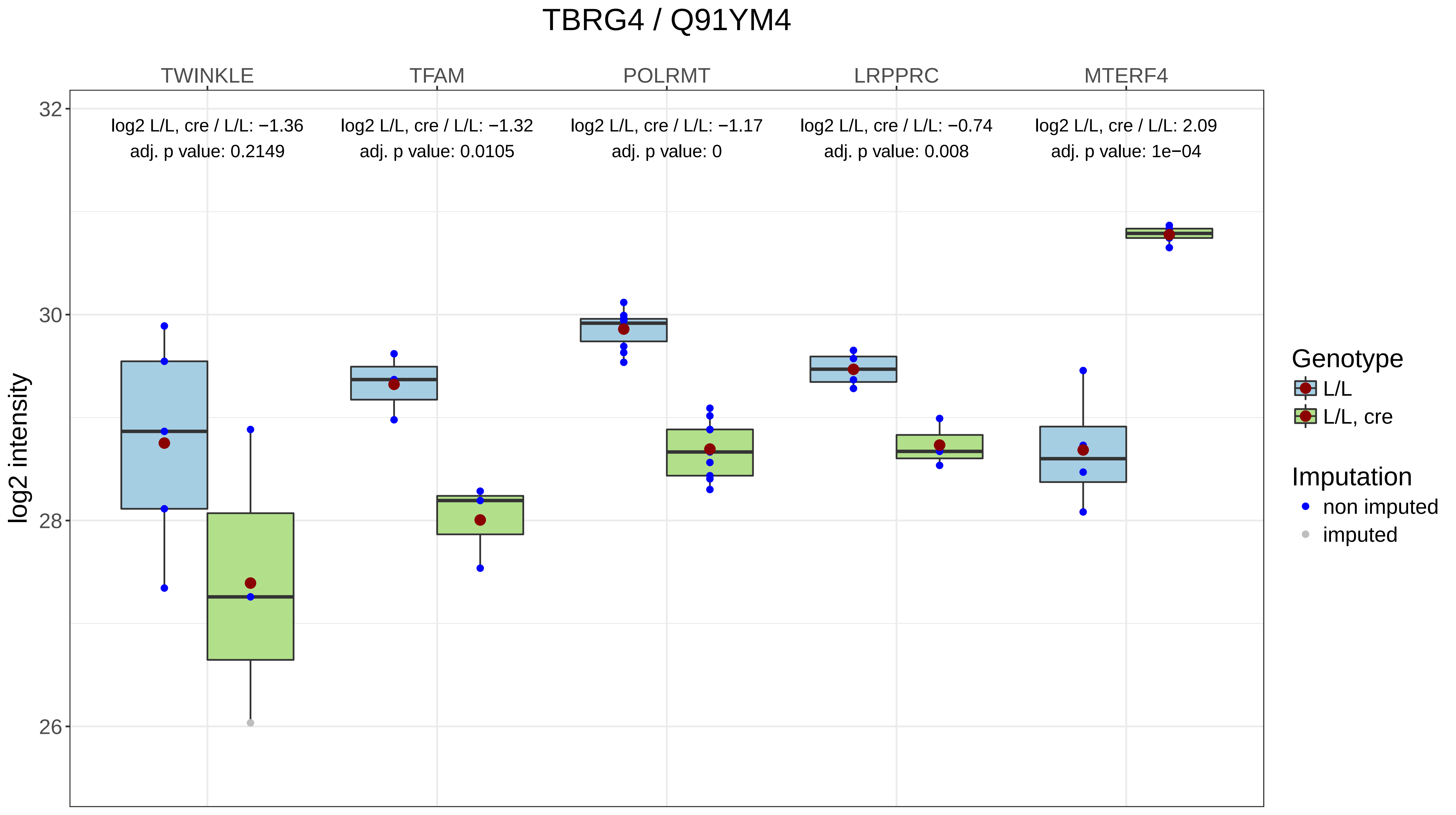
TBRG4 / Q91YM4
TWINKLE
TFAM
POLRMT
LRPPRC
MTERF4
log2 L/L, cre / L/L: −1.36
adj. p value: 0.2149
log2 L/L, cre / L/L: −1.32
adj. p value: 0.0105
log2 L/L, cre / L/L: −1.17
adj. p value: 0
log2 L/L, cre / L/L: −0.74
adj. p value: 0.008
log2 L/L, cre / L/L: 2.09
adj. p value: 1e−04
log2 intensity
32
30
28
26
Genotype
L/L
L/L, cre
Imputation
non imputed
imputed

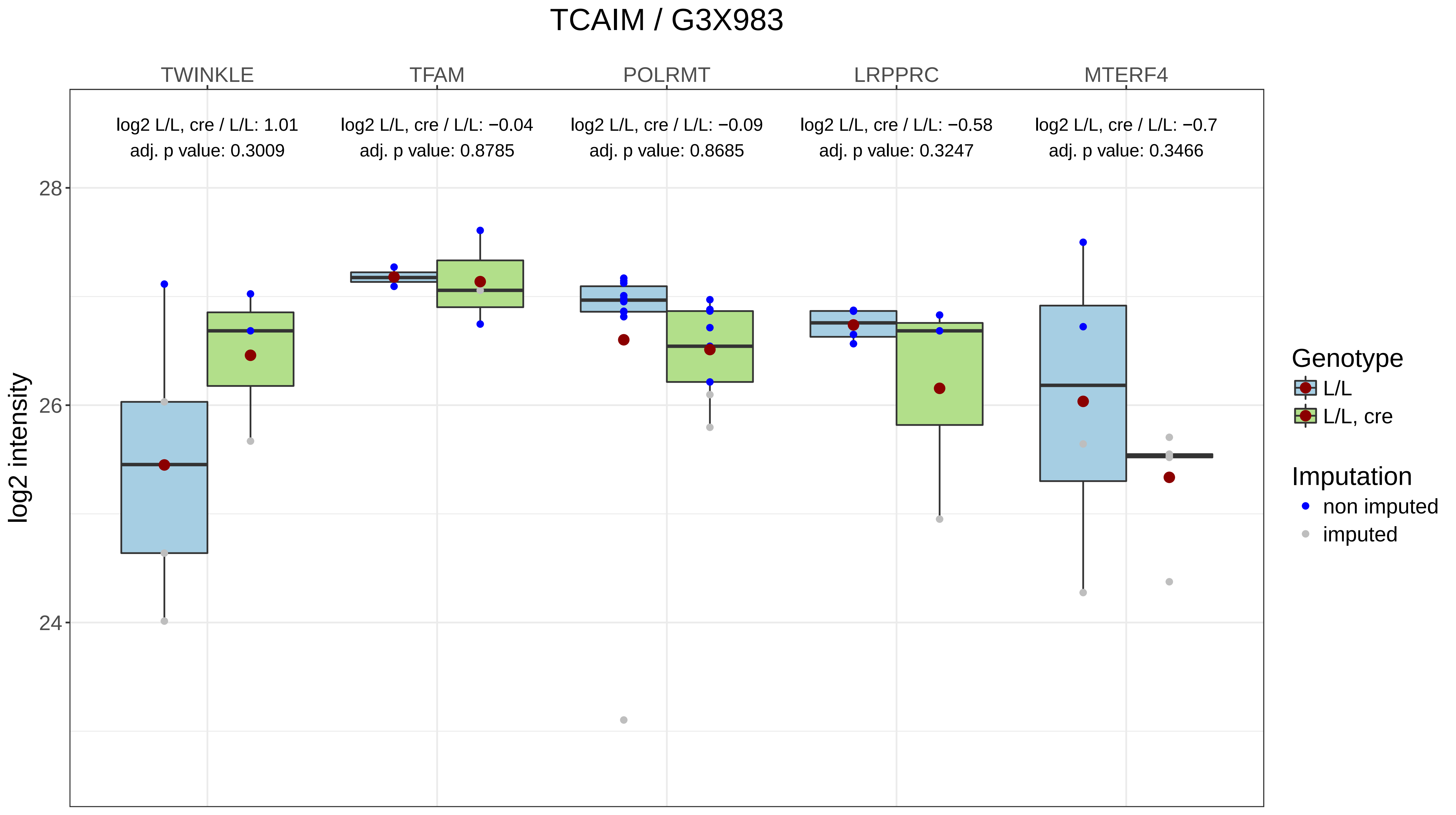
TCAIM / G3X983
TWINKLE
TFAM
POLRMT
LRPPRC
MTERF4
log2 L/L, cre / L/L: 1.01
adj. p value: 0.3009
log2 L/L, cre / L/L: −0.04
adj. p value: 0.8785
log2 L/L, cre / L/L: −0.09
adj. p value: 0.8685
log2 L/L, cre / L/L: −0.58
adj. p value: 0.3247
log2 L/L, cre / L/L: −0.7
adj. p value: 0.3466
log2 intensity
28
26
24
Genotype
L/L
L/L, cre
Imputation
non imputed
imputed

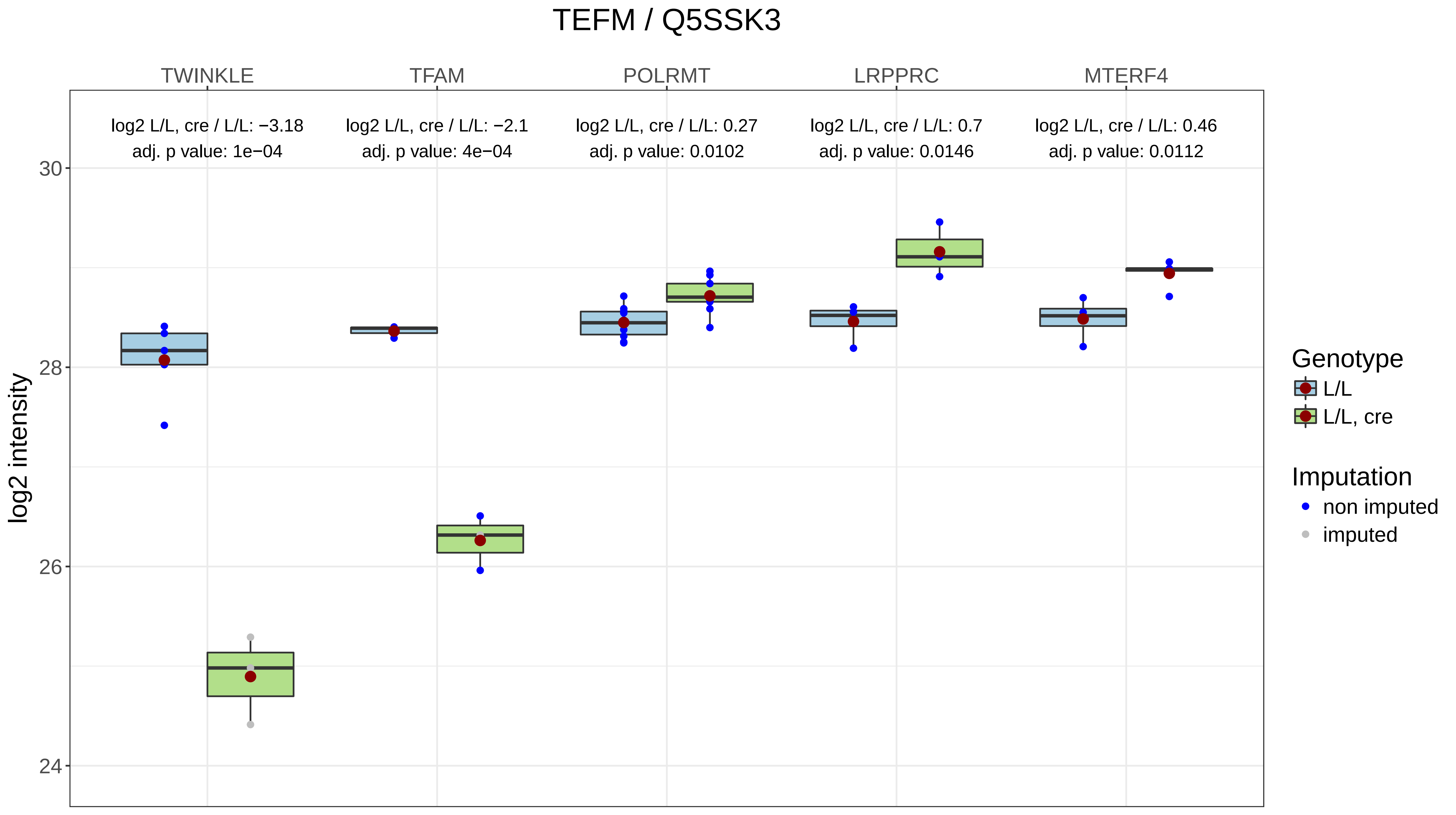
TEFM / Q5SSK3
TWINKLE
TFAM
POLRMT
LRPPRC
MTERF4
log2 L/L, cre / L/L: −3.18
adj. p value: 1e−04
log2 L/L, cre / L/L: −2.1
adj. p value: 4e−04
log2 L/L, cre / L/L: 0.27
adj. p value: 0.0102
log2 L/L, cre / L/L: 0.7
adj. p value: 0.0146
log2 L/L, cre / L/L: 0.46
adj. p value: 0.0112
log2 intensity
30
28
26
24
Genotype
L/L
L/L, cre
Imputation
non imputed
imputed

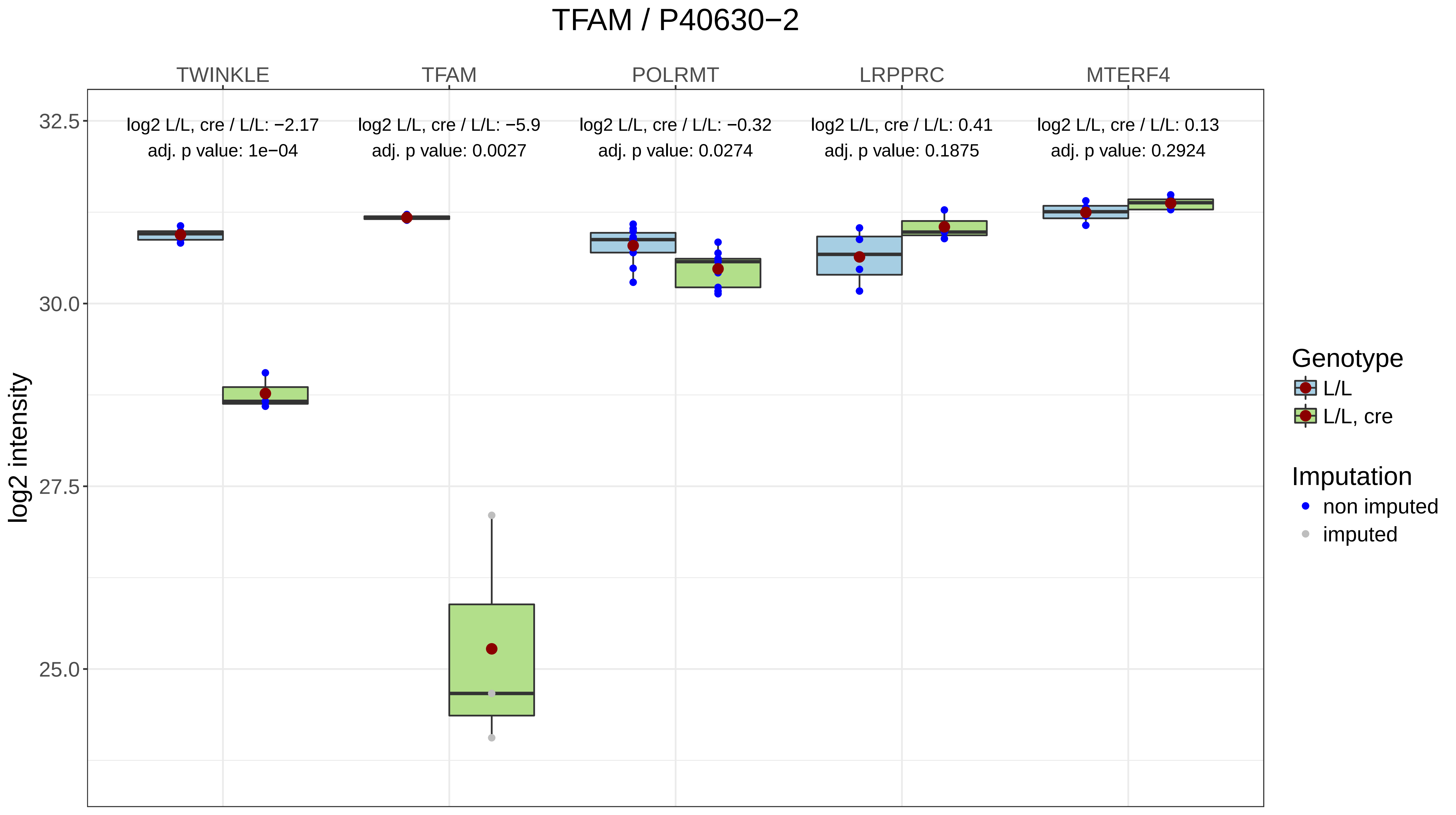
TFAM / P40630−2
TWINKLE
TFAM
POLRMT
LRPPRC
MTERF4
log2 L/L, cre / L/L: −2.17
adj. p value: 1e−04
log2 L/L, cre / L/L: −5.9
adj. p value: 0.0027
log2 L/L, cre / L/L: −0.32
adj. p value: 0.0274
log2 L/L, cre / L/L: 0.41
adj. p value: 0.1875
log2 L/L, cre / L/L: 0.13
adj. p value: 0.2924
32.5
30.0
27.5
25.0
log2 intensity
Genotype
L/L
L/L, cre
Imputation
non imputed
imputed

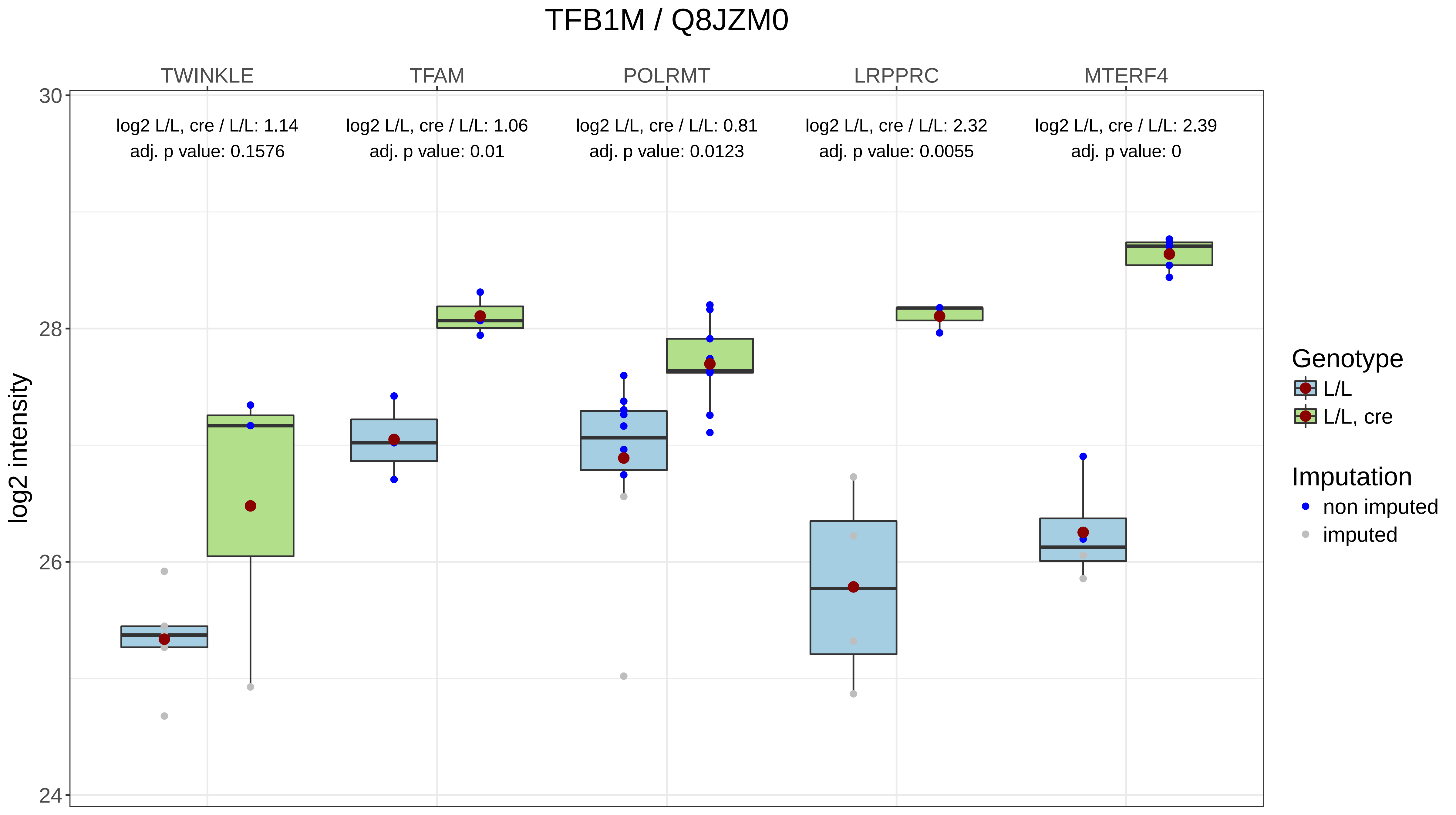
TFB1M / Q8JZM0
TWINKLE
TFAM
POLRMT
LRPPRC
MTERF4
log2 L/L, cre / L/L: 1.14
adj. p value: 0.1576
log2 L/L, cre / L/L: 1.06
adj. p value: 0.01
log2 L/L, cre / L/L: 0.81
adj. p value: 0.0123
log2 L/L, cre / L/L: 2.32
adj. p value: 0.0055
log2 L/L, cre / L/L: 2.39
adj. p value: 0
log2 intensity
30
28
26
24
Genotype
L/L
L/L, cre
Imputation
non imputed
imputed

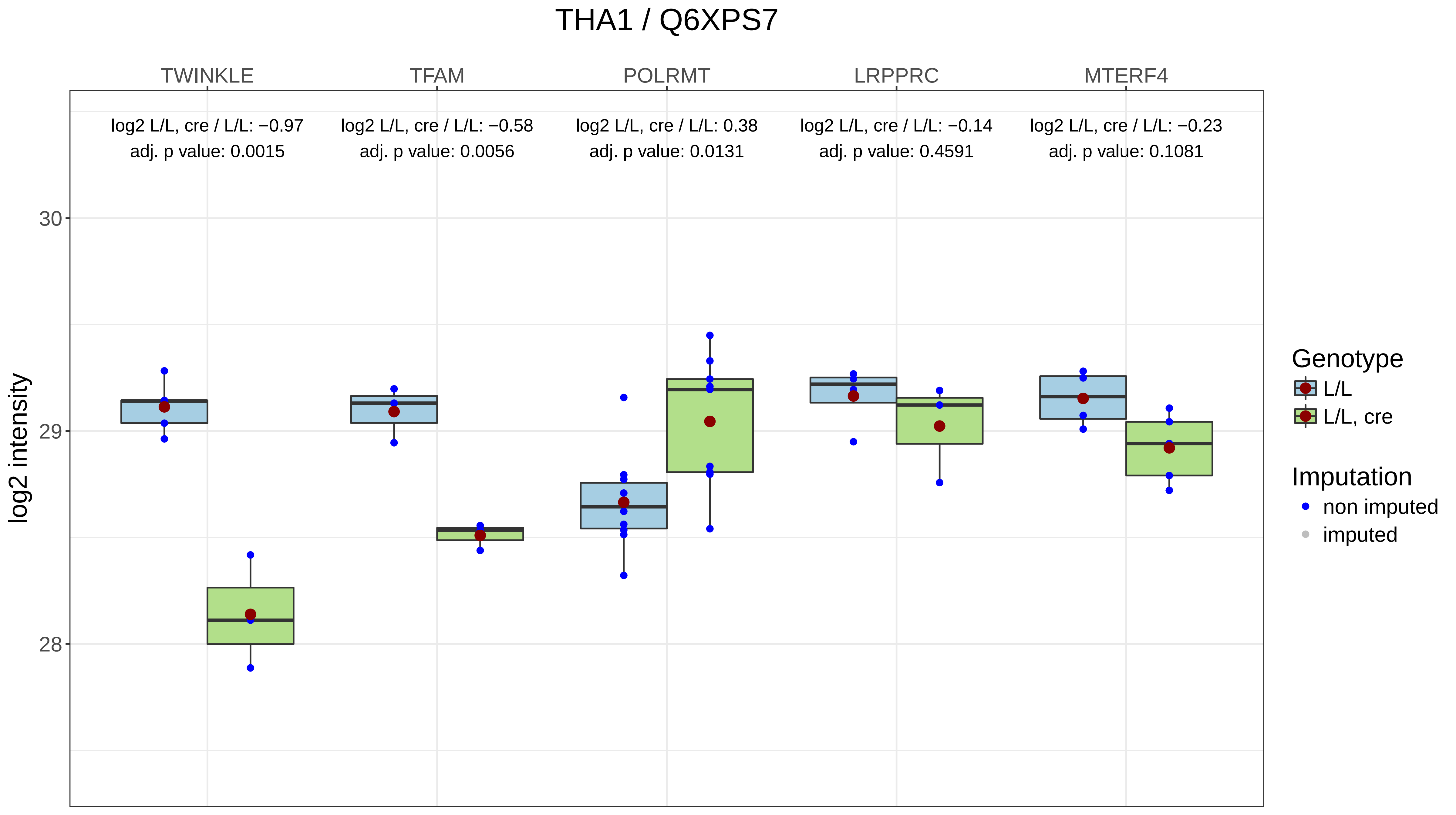
THA1 / Q6XPS7
TWINKLE
TFAM
POLRMT
LRPPRC
MTERF4
log2 L/L, cre / L/L: −0.97
adj. p value: 0.0015
log2 L/L, cre / L/L: −0.58
adj. p value: 0.0056
log2 L/L, cre / L/L: 0.38
adj. p value: 0.0131
log2 L/L, cre / L/L: −0.14
adj. p value: 0.4591
log2 L/L, cre / L/L: −0.23
adj. p value: 0.1081
30
29
28
log2 intensity
Genotype
L/L
L/L, cre
Imputation
non imputed
imputed

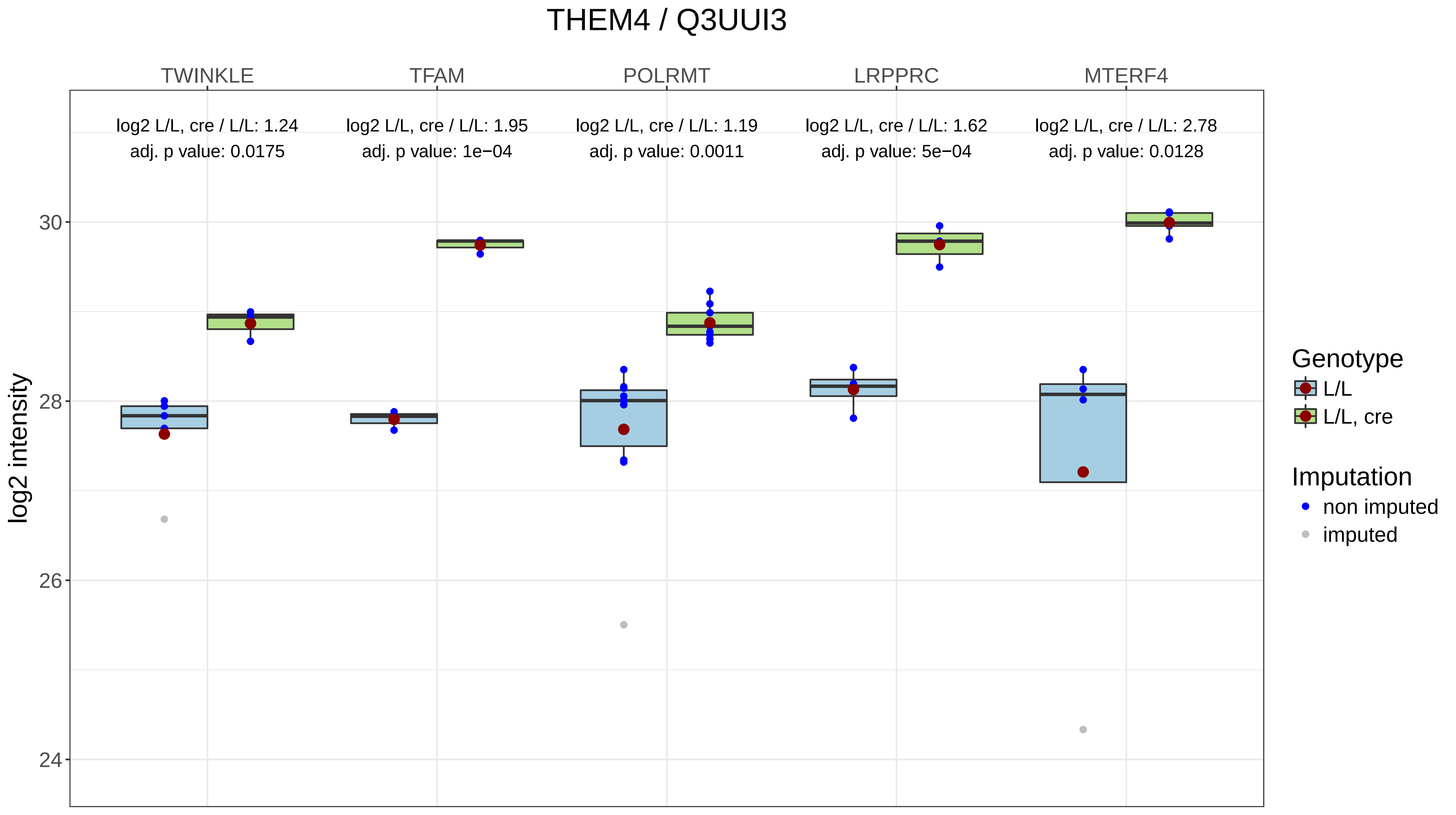
THEM4 / Q3UUI3
TWINKLE
TFAM
POLRMT
LRPPRC
MTERF4
log2 L/L, cre / L/L: 1.24
adj. p value: 0.0175
log2 L/L, cre / L/L: 1.95
adj. p value: 1e−04
log2 L/L, cre / L/L: 1.19
adj. p value: 0.0011
log2 L/L, cre / L/L: 1.62
adj. p value: 5e−04
log2 L/L, cre / L/L: 2.78
adj. p value: 0.0128
log2 intensity
30
28
26
24
Genotype
L/L
L/L, cre
Imputation
non imputed
imputed

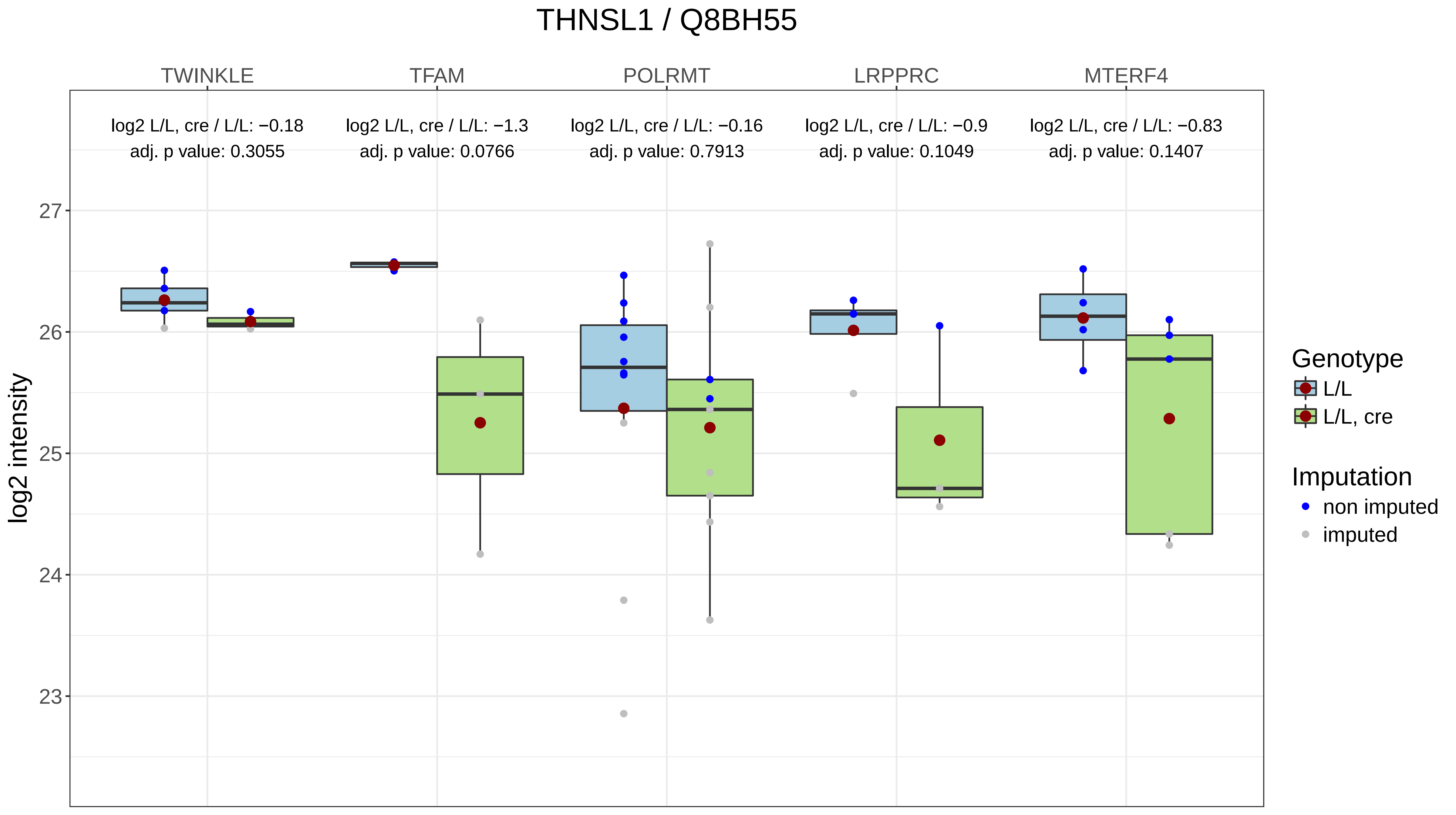

THNSL1 / Q8BH55
TWINKLE
TFAM
POLRMT
LRPPRC
MTERF4
log2 L/L, cre / L/L: −0.18
adj. p value: 0.3055
log2 L/L, cre / L/L: −1.3
adj. p value: 0.0766
log2 L/L, cre / L/L: −0.16
adj. p value: 0.7913
log2 L/L, cre / L/L: −0.9
adj. p value: 0.1049
log2 L/L, cre / L/L: −0.83
adj. p value: 0.1407
log2 intensity
27
26
25
24
23
Genotype
L/L
L/L, cre
Imputation
non imputed
imputed

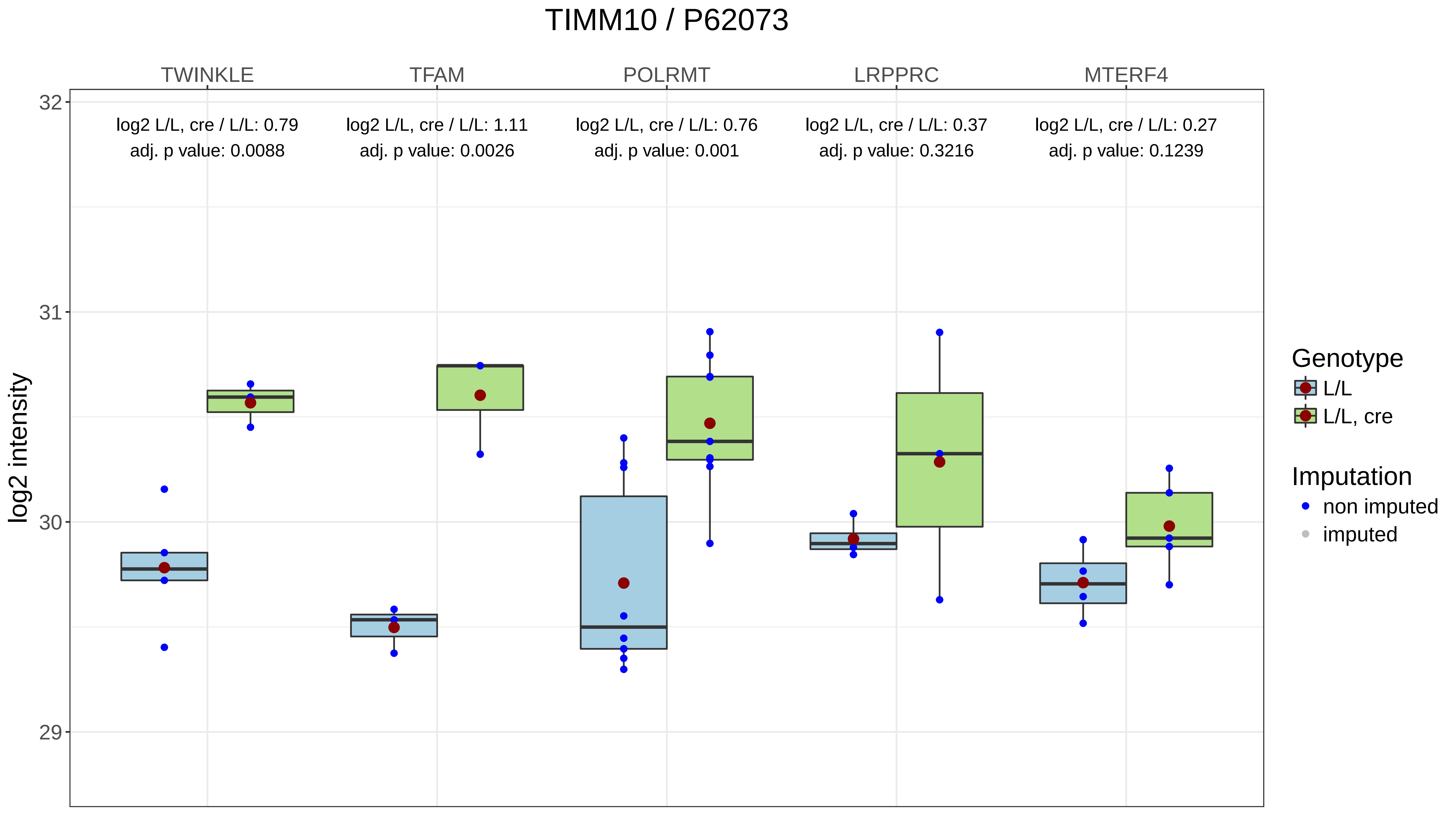

TIMM10 / P62073
TWINKLE
TFAM
POLRMT
LRPPRC
MTERF4
log2 L/L, cre / L/L: 0.79
adj. p value: 0.0088
log2 L/L, cre / L/L: 1.11
adj. p value: 0.0026
log2 L/L, cre / L/L: 0.76
adj. p value: 0.001
log2 L/L, cre / L/L: 0.37
adj. p value: 0.3216
log2 L/L, cre / L/L: 0.27
adj. p value: 0.1239
log2 intensity
32
31
30
29
Genotype
L/L
L/L, cre
Imputation
non imputed
imputed

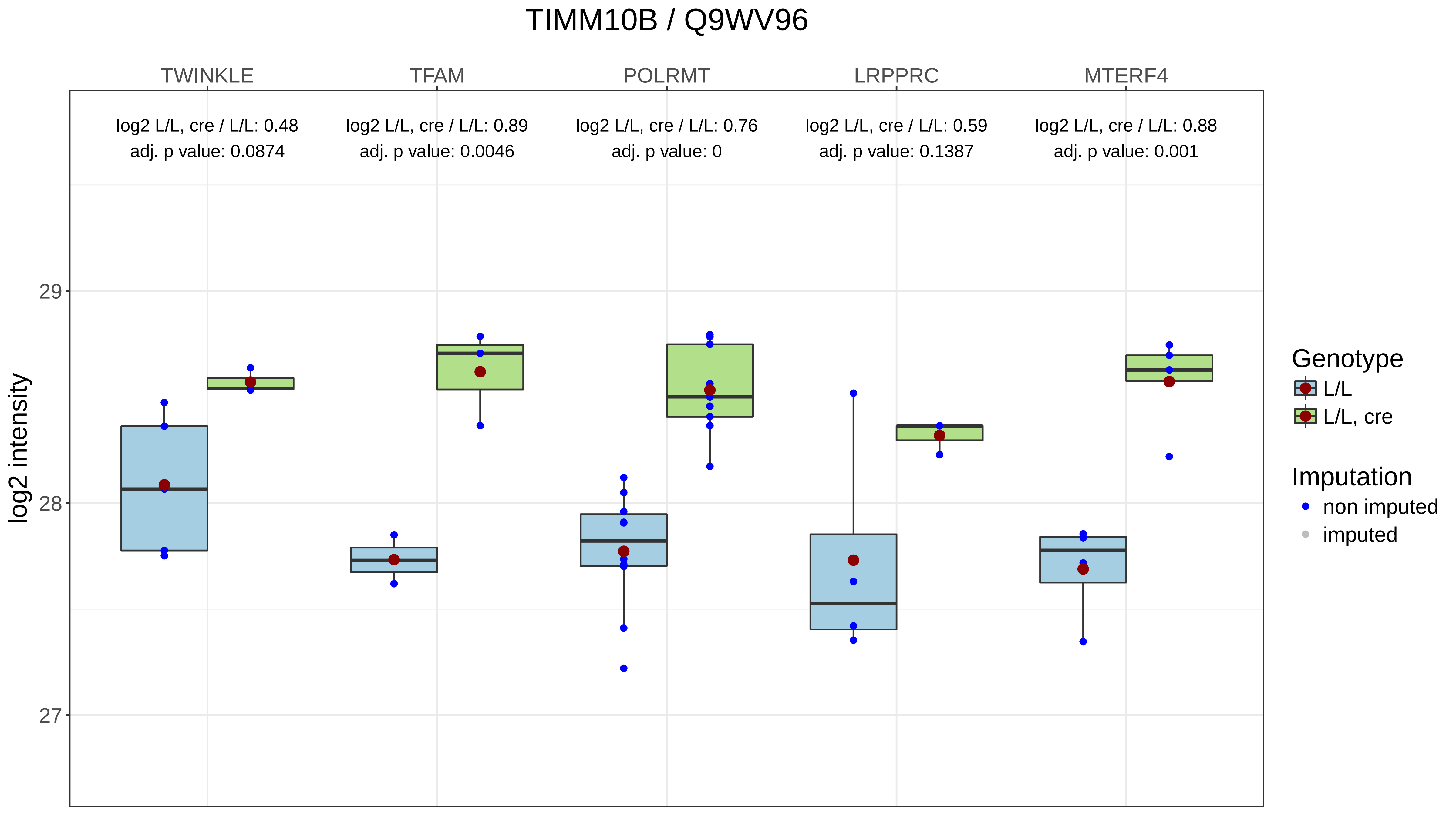

TIMM10B / Q9WV96
TWINKLE
TFAM
POLRMT
LRPPRC
MTERF4
log2 L/L, cre / L/L: 0.48
adj. p value: 0.0874
log2 L/L, cre / L/L: 0.89
adj. p value: 0.0046
log2 L/L, cre / L/L: 0.76
adj. p value: 0
log2 L/L, cre / L/L: 0.59
adj. p value: 0.1387
log2 L/L, cre / L/L: 0.88
adj. p value: 0.001
log2 intensity
29
28
27
Genotype
L/L
L/L, cre
Imputation
non imputed
imputed

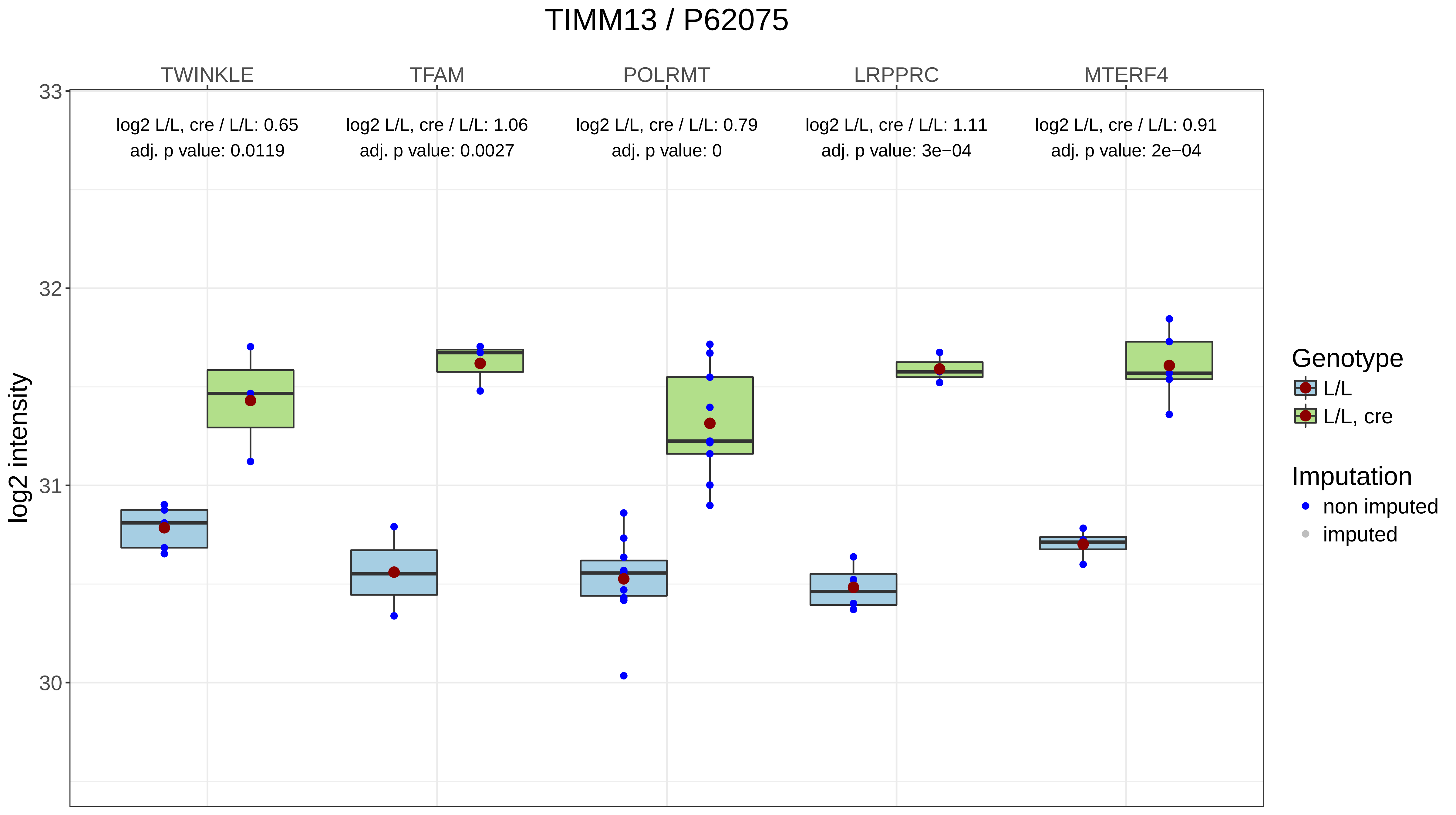
TIMM13 / P62075
TWINKLE
TFAM
POLRMT
LRPPRC
MTERF4
log2 L/L, cre / L/L: 0.65
adj. p value: 0.0119
log2 L/L, cre / L/L: 1.06
adj. p value: 0.0027
log2 L/L, cre / L/L: 0.79
adj. p value: 0
log2 L/L, cre / L/L: 1.11
adj. p value: 3e−04
log2 L/L, cre / L/L: 0.91
adj. p value: 2e−04
log2 intensity
33
32
31
30
Genotype
L/L
L/L, cre
Imputation
non imputed
imputed

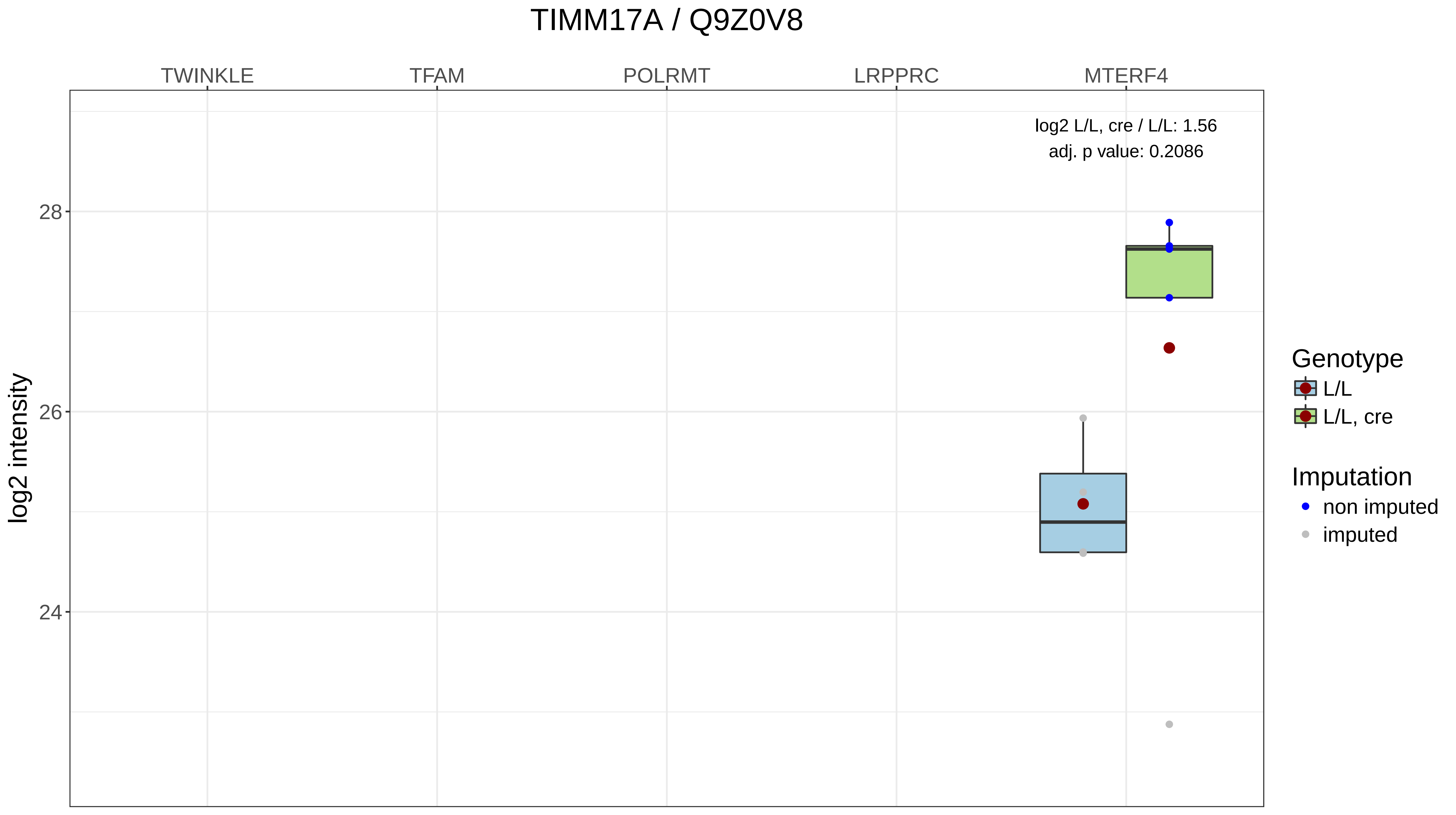
TIMM17A / Q9Z0V8
TWINKLE
TFAM
POLRMT
LRPPRC
MTERF4
log2 L/L, cre / L/L: 1.56
adj. p value: 0.2086
log2 intensity
28
26
24
Genotype
L/L
L/L, cre
Imputation
non imputed
imputed

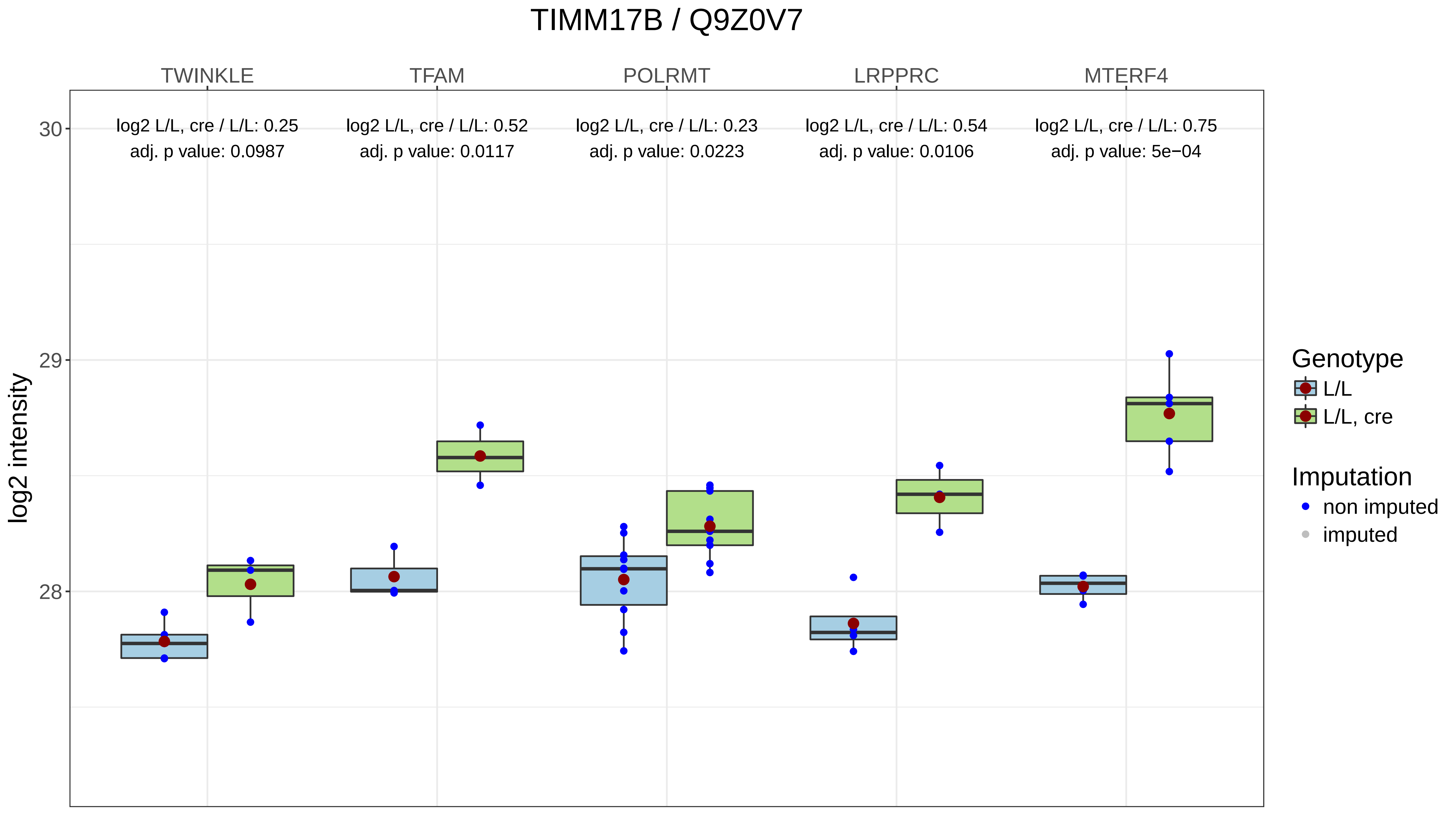
TIMM17B / Q9Z0V7
TWINKLE
TFAM
POLRMT
LRPPRC
MTERF4
log2 L/L, cre / L/L: 0.25
adj. p value: 0.0987
log2 L/L, cre / L/L: 0.52
adj. p value: 0.0117
log2 L/L, cre / L/L: 0.23
adj. p value: 0.0223
log2 L/L, cre / L/L: 0.54
adj. p value: 0.0106
log2 L/L, cre / L/L: 0.75
adj. p value: 5e−04
30
29
28
log2 intensity
Genotype
L/L
L/L, cre
Imputation
non imputed
imputed

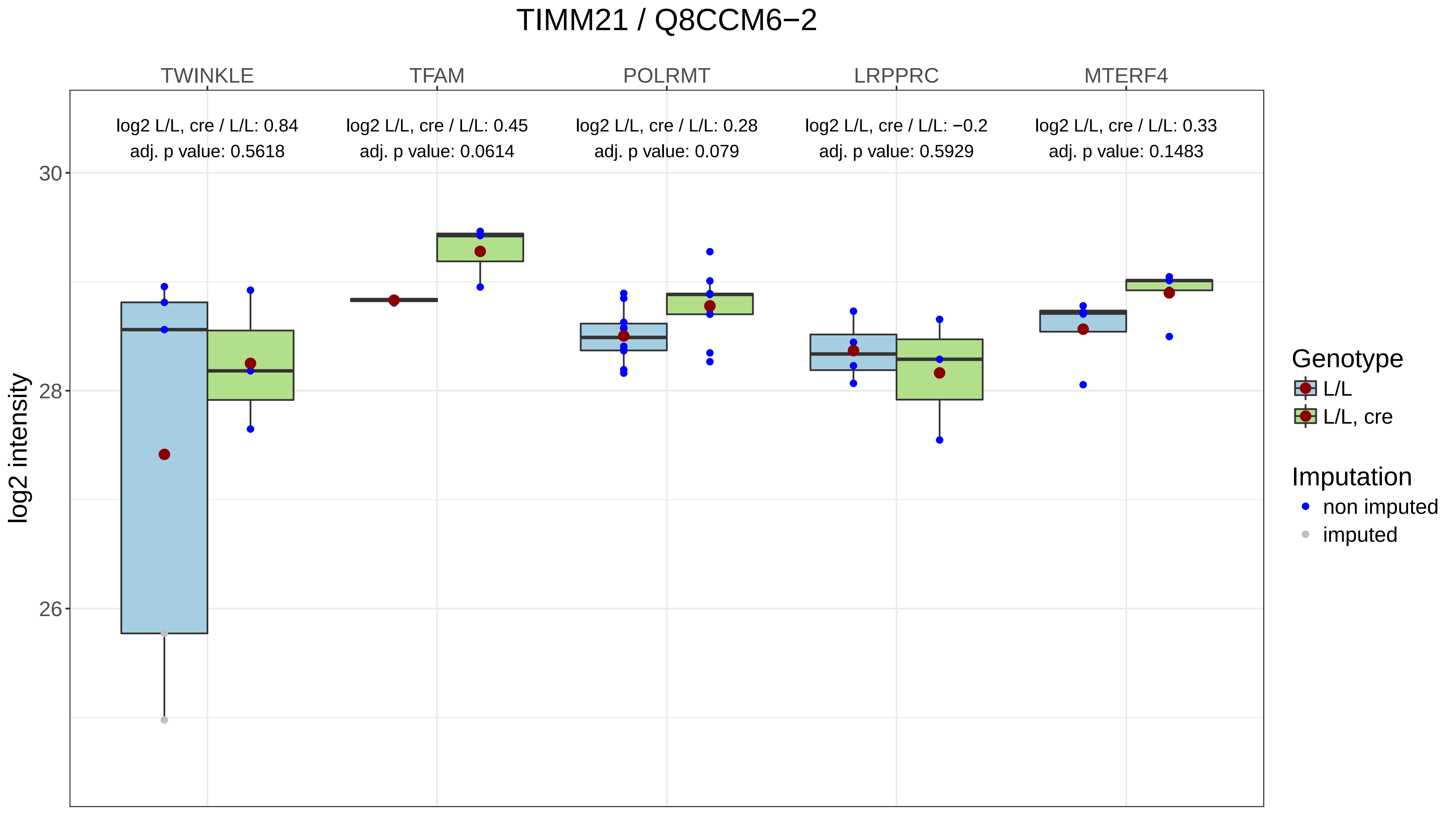

TIMM21 / Q8CCM6−2
TWINKLE
TFAM
POLRMT
LRPPRC
MTERF4
log2 L/L, cre / L/L: 0.84
adj. p value: 0.5618
log2 L/L, cre / L/L: 0.45
adj. p value: 0.0614
log2 L/L, cre / L/L: 0.28
adj. p value: 0.079
log2 L/L, cre / L/L: −0.2
adj. p value: 0.5929
log2 L/L, cre / L/L: 0.33
adj. p value: 0.1483
log2 intensity
30
28
26
Genotype
L/L
L/L, cre
Imputation
non imputed
imputed

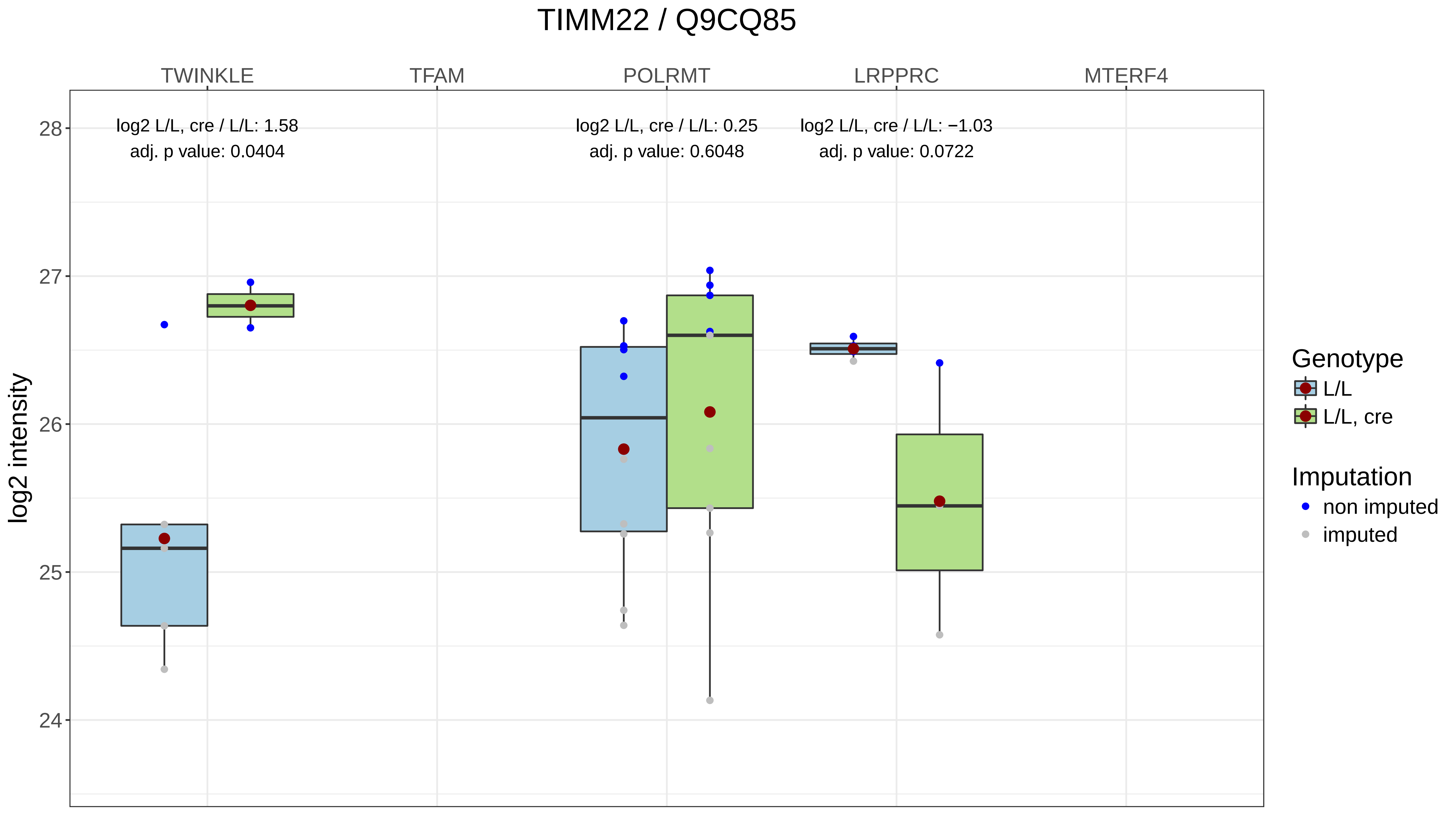
TIMM22 / Q9CQ85
TWINKLE
TFAM
POLRMT
LRPPRC
MTERF4
log2 L/L, cre / L/L: 1.58
adj. p value: 0.0404
log2 L/L, cre / L/L: 0.25
adj. p value: 0.6048
log2 L/L, cre / L/L: −1.03
adj. p value: 0.0722
log2 intensity
28
27
26
25
24
Genotype
L/L
L/L, cre
Imputation
non imputed
imputed

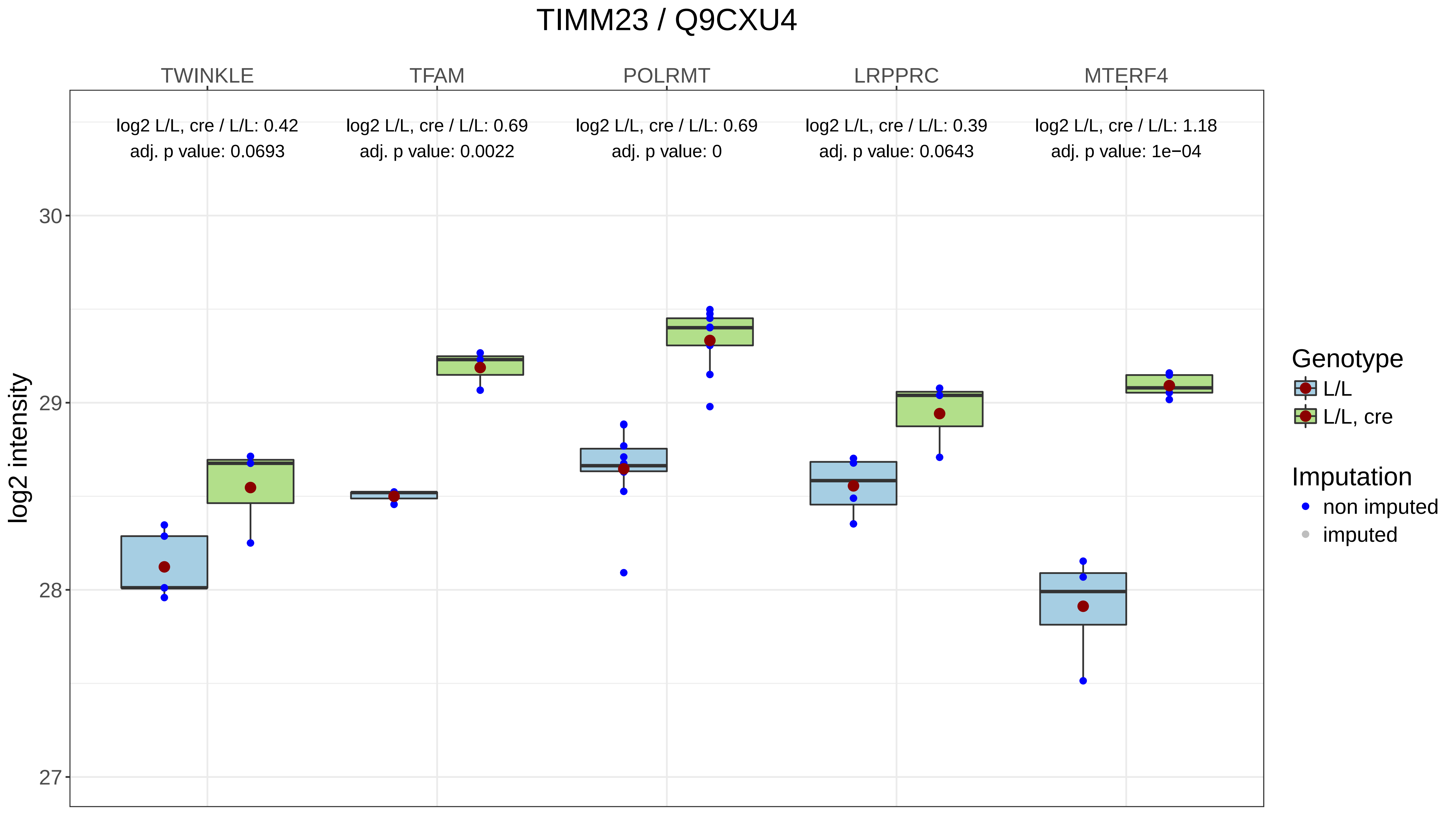
TIMM23 / Q9CXU4
TWINKLE
TFAM
POLRMT
LRPPRC
MTERF4
log2 L/L, cre / L/L: 0.42
adj. p value: 0.0693
log2 L/L, cre / L/L: 0.69
adj. p value: 0.0022
log2 L/L, cre / L/L: 0.69
adj. p value: 0
log2 L/L, cre / L/L: 0.39
adj. p value: 0.0643
log2 L/L, cre / L/L: 1.18
adj. p value: 1e−04
log2 intensity
30
29
28
27
Genotype
L/L
L/L, cre
Imputation
non imputed
imputed

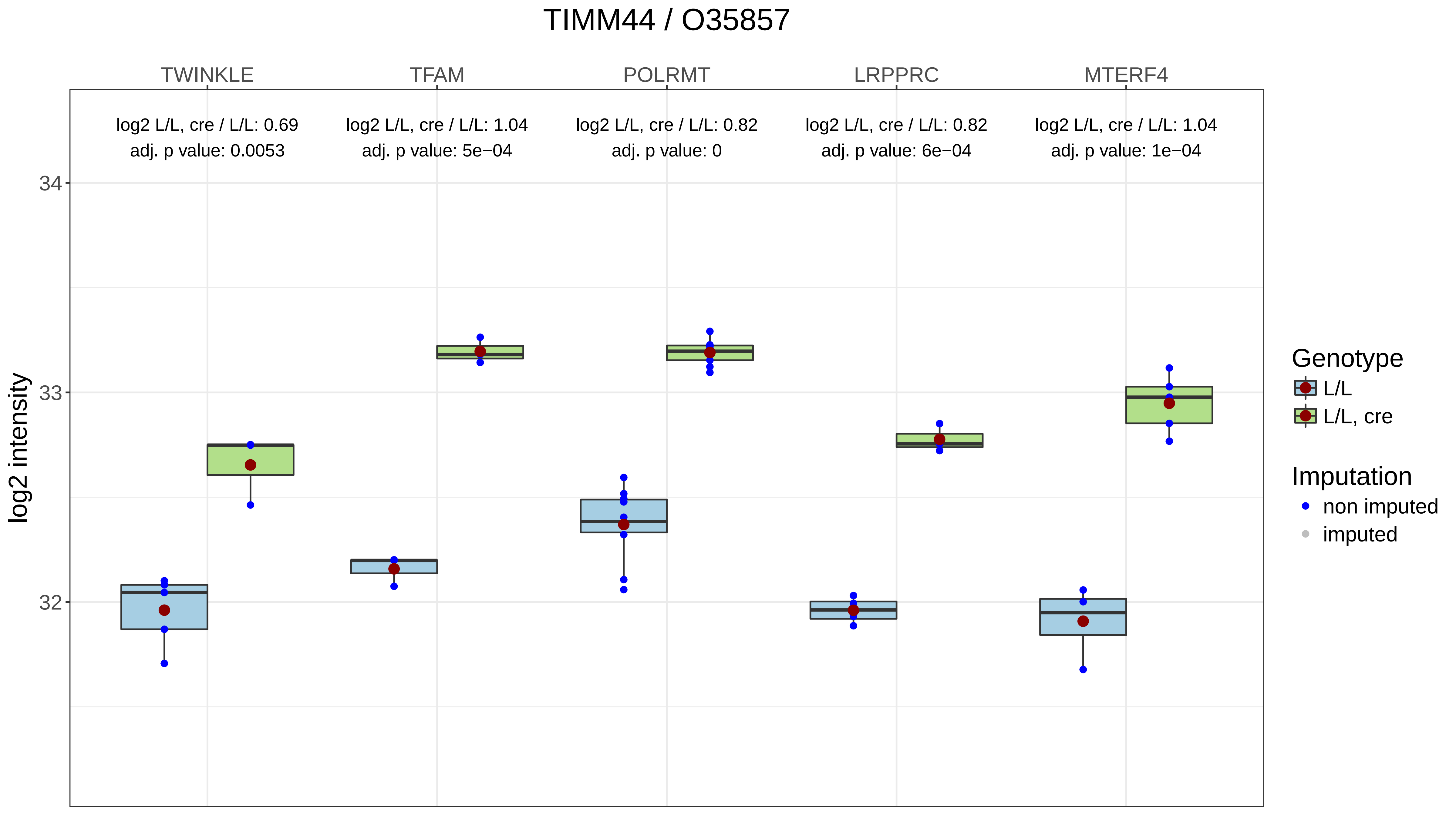

TIMM44 / O35857
TWINKLE
TFAM
POLRMT
LRPPRC
MTERF4
log2 L/L, cre / L/L: 0.69
adj. p value: 0.0053
log2 L/L, cre / L/L: 1.04
adj. p value: 5e−04
log2 L/L, cre / L/L: 0.82
adj. p value: 0
log2 L/L, cre / L/L: 0.82
adj. p value: 6e−04
log2 L/L, cre / L/L: 1.04
adj. p value: 1e−04
log2 intensity
34
33
32
Genotype
L/L
L/L, cre
Imputation
non imputed
imputed

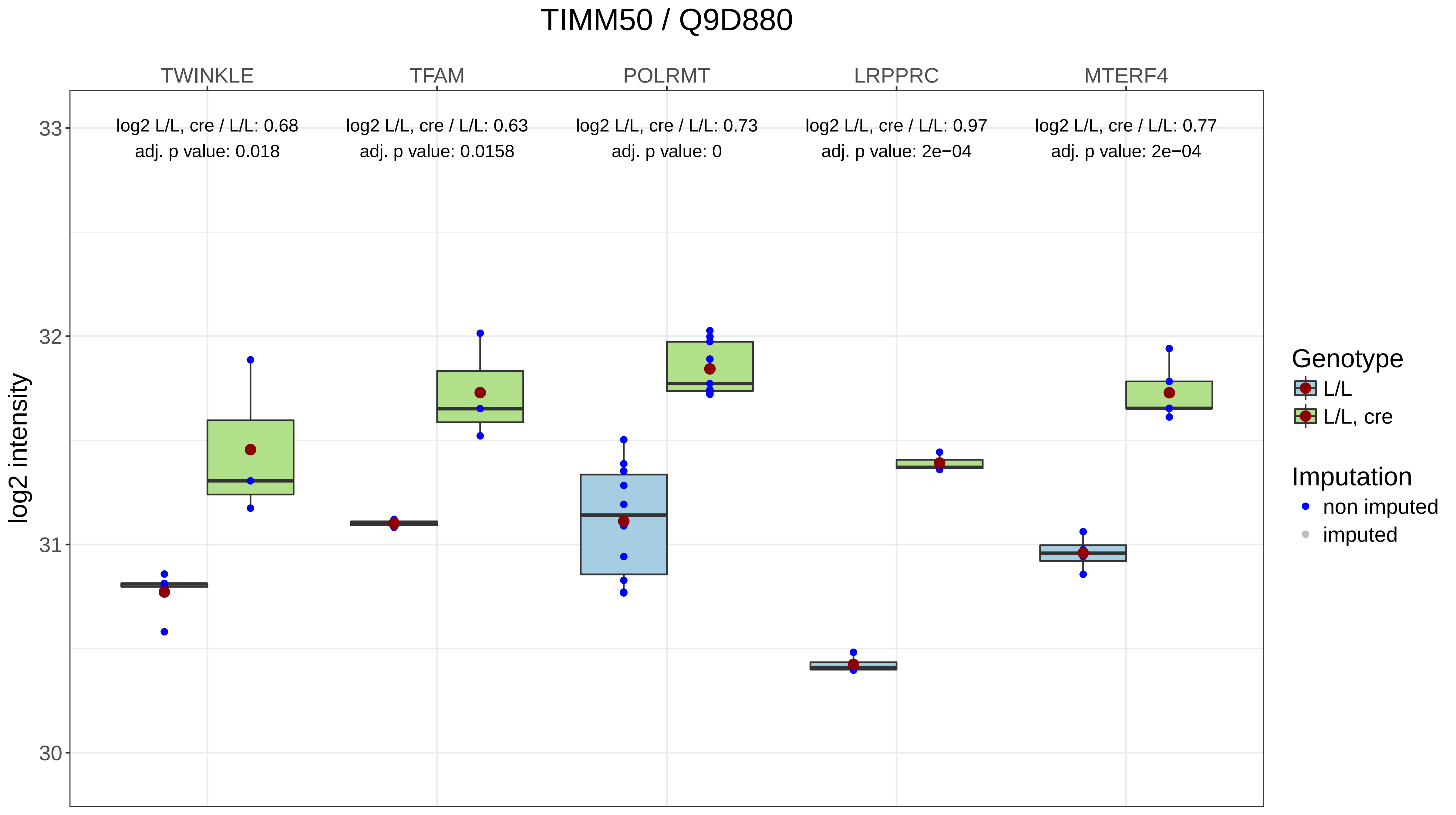
TIMM50 / Q9D880
TWINKLE
TFAM
POLRMT
LRPPRC
MTERF4
log2 L/L, cre / L/L: 0.68
adj. p value: 0.018
log2 L/L, cre / L/L: 0.63
adj. p value: 0.0158
log2 L/L, cre / L/L: 0.73
adj. p value: 0
log2 L/L, cre / L/L: 0.97
adj. p value: 2e−04
log2 L/L, cre / L/L: 0.77
adj. p value: 2e−04
log2 intensity
33
32
31
30
Genotype
L/L
L/L, cre
Imputation
non imputed
imputed

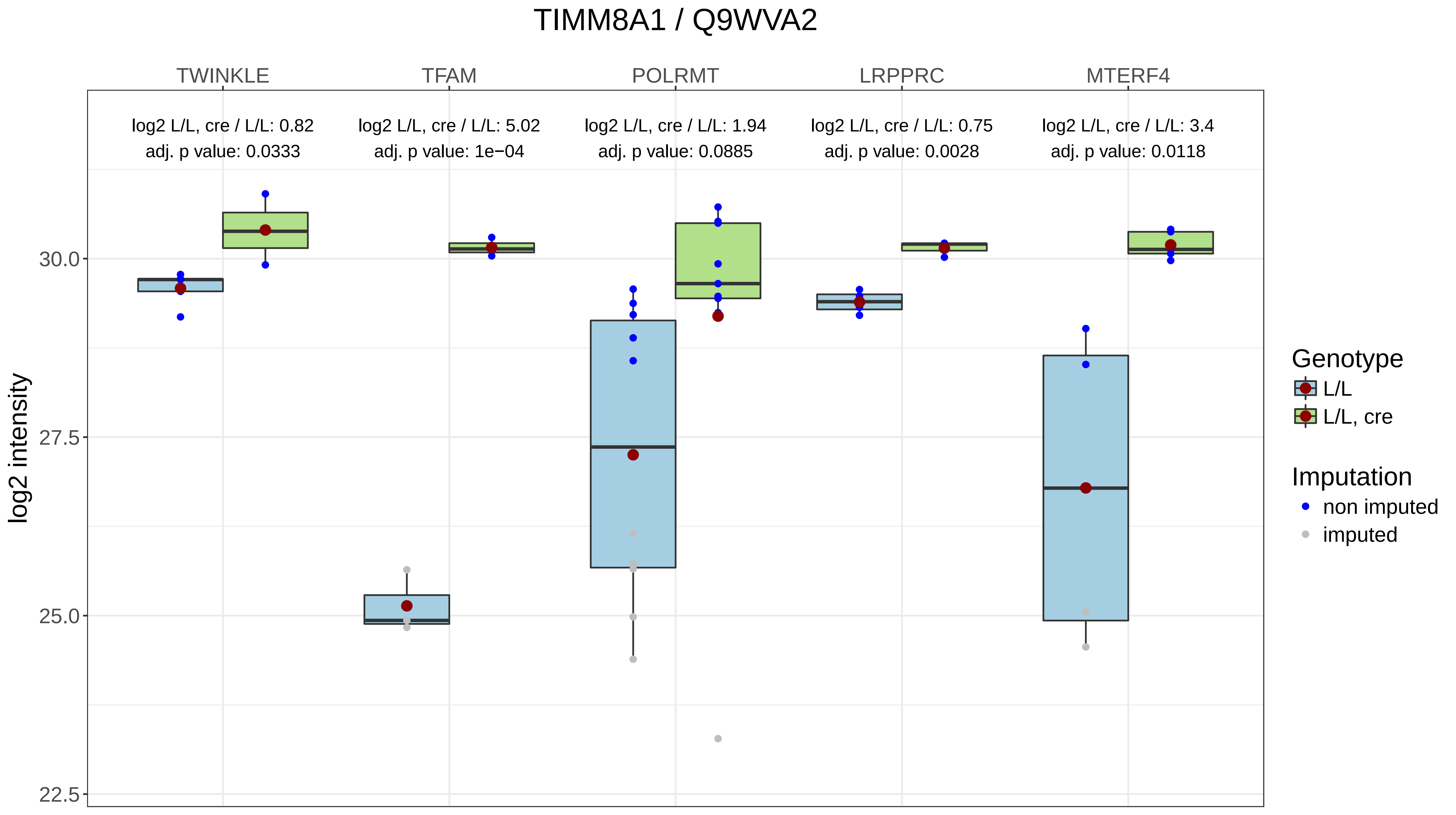
TIMM8A1 / Q9WVA2
TWINKLE
TFAM
POLRMT
LRPPRC
MTERF4
log2 L/L, cre / L/L: 0.82
adj. p value: 0.0333
log2 L/L, cre / L/L: 5.02
adj. p value: 1e−04
log2 L/L, cre / L/L: 1.94
adj. p value: 0.0885
log2 L/L, cre / L/L: 0.75
adj. p value: 0.0028
log2 L/L, cre / L/L: 3.4
adj. p value: 0.0118
log2 intensity
30.0
27.5
25.0
22.5
Genotype
L/L
L/L, cre
Imputation
non imputed
imputed

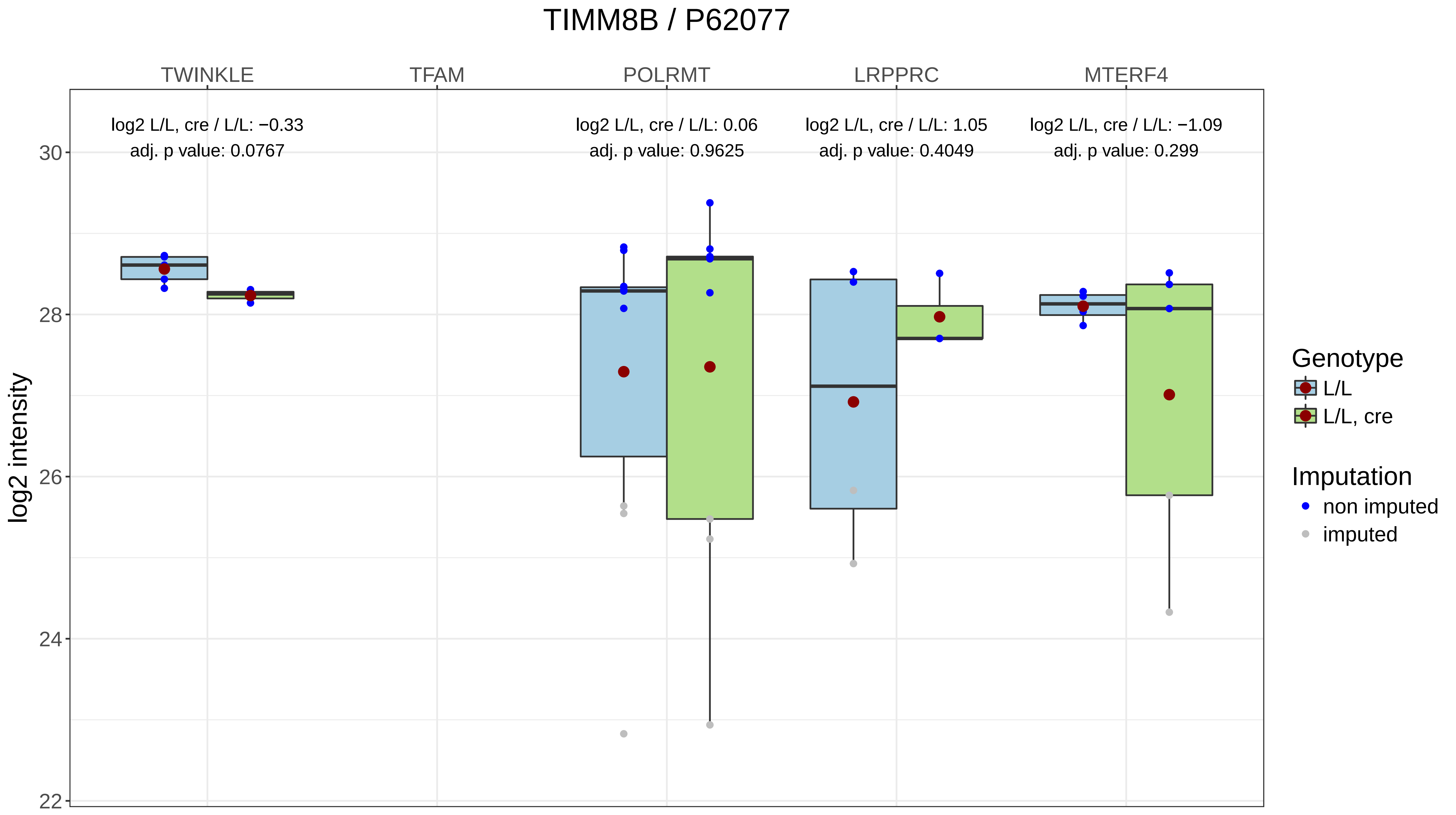
TIMM8B / P62077
TWINKLE
TFAM
POLRMT
LRPPRC
MTERF4
log2 L/L, cre / L/L: −0.33
adj. p value: 0.0767
log2 L/L, cre / L/L: 0.06
adj. p value: 0.9625
log2 L/L, cre / L/L: 1.05
adj. p value: 0.4049
log2 L/L, cre / L/L: −1.09
adj. p value: 0.299
log2 intensity
30
28
26
24
22
Genotype
L/L
L/L, cre
Imputation
non imputed
imputed

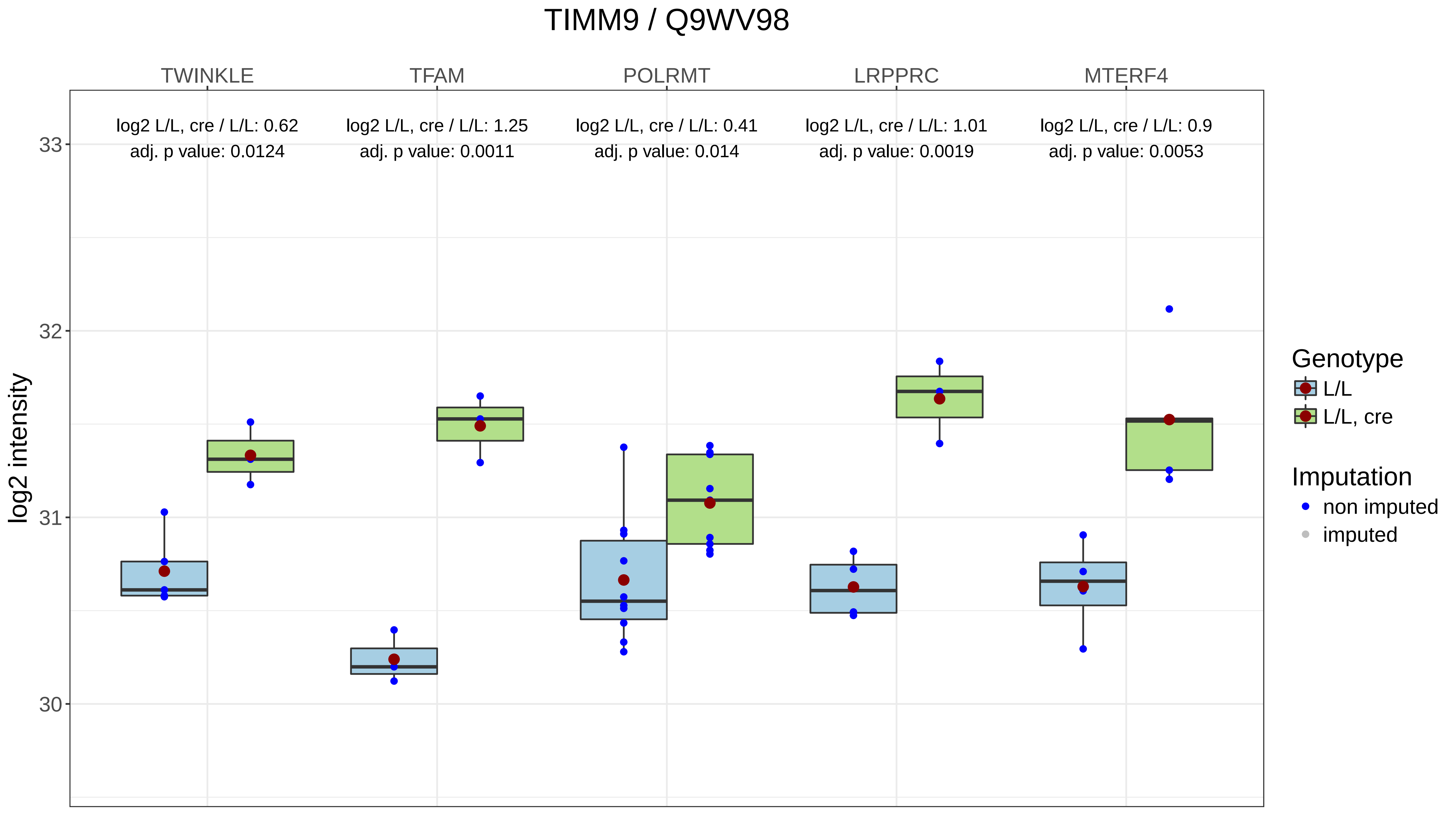
TIMM9 / Q9WV98
TWINKLE
TFAM
POLRMT
LRPPRC
MTERF4
log2 L/L, cre / L/L: 0.62
adj. p value: 0.0124
log2 L/L, cre / L/L: 1.25
adj. p value: 0.0011
log2 L/L, cre / L/L: 0.41
adj. p value: 0.014
log2 L/L, cre / L/L: 1.01
adj. p value: 0.0019
log2 L/L, cre / L/L: 0.9
adj. p value: 0.0053
log2 intensity
33
32
31
30
Genotype
L/L
L/L, cre
Imputation
non imputed
imputed

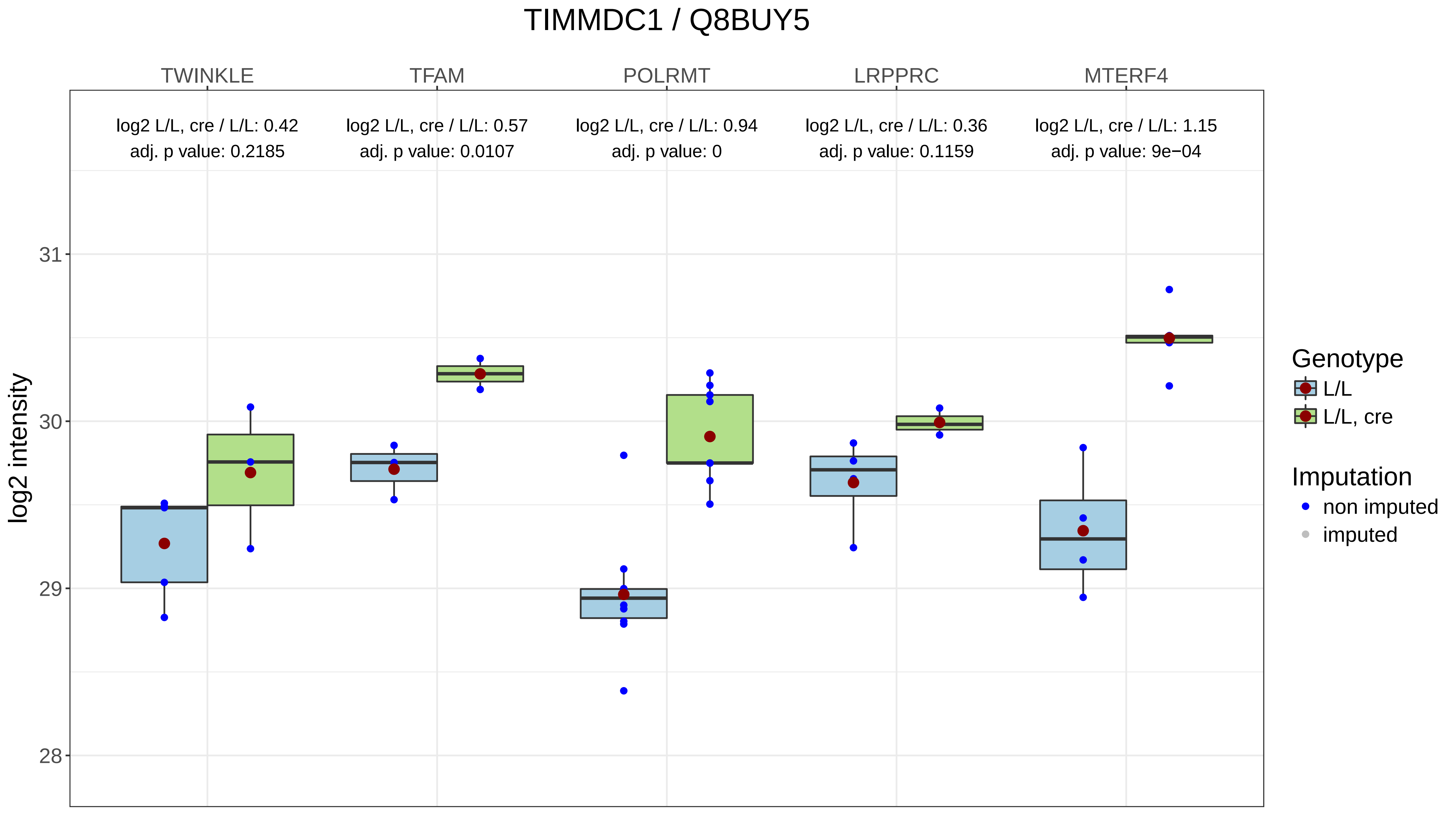
TIMMDC1 / Q8BUY5
TWINKLE
TFAM
POLRMT
LRPPRC
MTERF4
log2 L/L, cre / L/L: 0.42
adj. p value: 0.2185
log2 L/L, cre / L/L: 0.57
adj. p value: 0.0107
log2 L/L, cre / L/L: 0.94
adj. p value: 0
log2 L/L, cre / L/L: 0.36
adj. p value: 0.1159
log2 L/L, cre / L/L: 1.15
adj. p value: 9e−04
log2 intensity
31
30
29
28
Genotype
L/L
L/L, cre
Imputation
non imputed
imputed

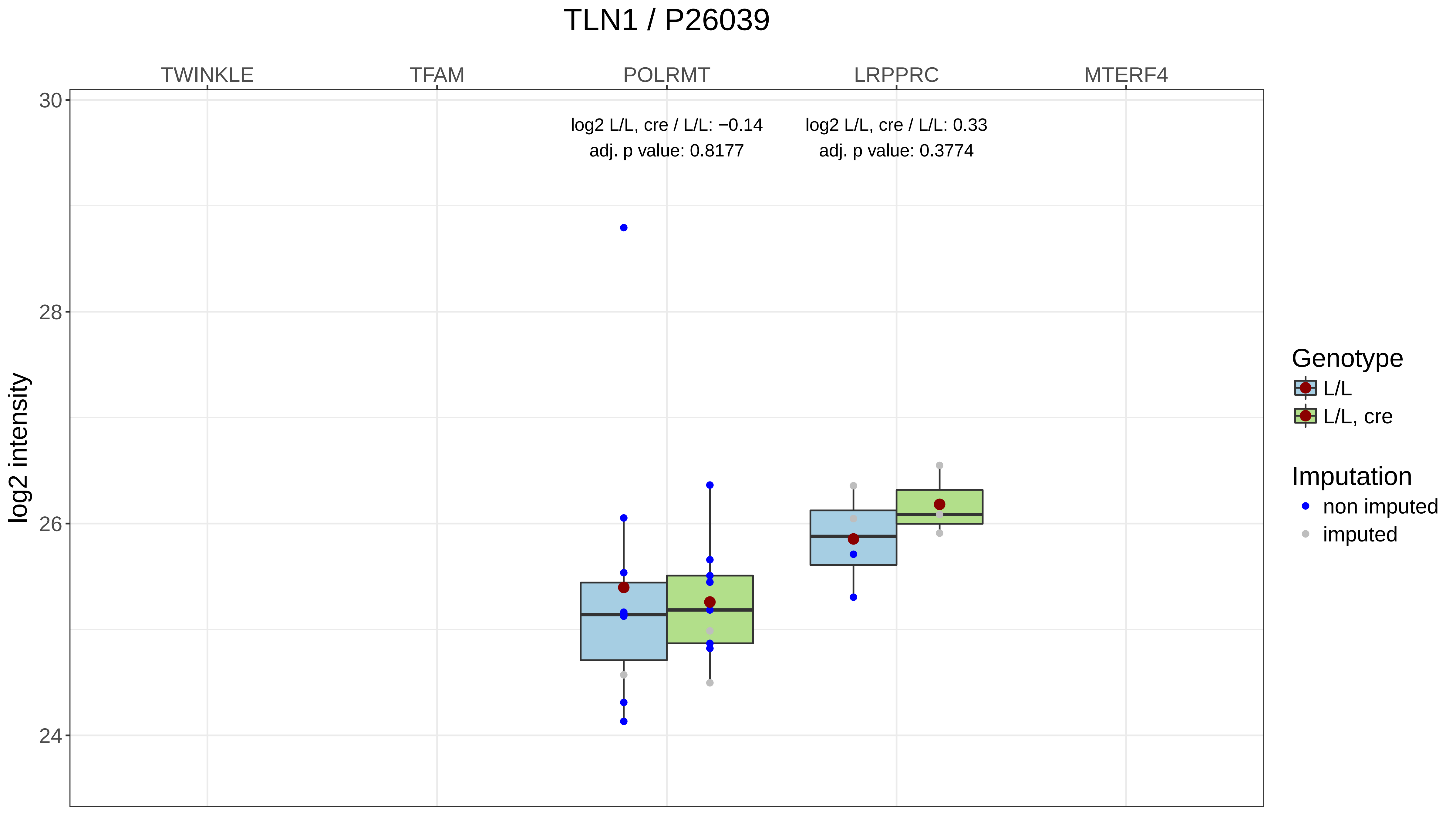
TLN1 / P26039
TWINKLE
TFAM
POLRMT
LRPPRC
MTERF4
log2 L/L, cre / L/L: −0.14
adj. p value: 0.8177
log2 L/L, cre / L/L: 0.33
adj. p value: 0.3774
30
28
26
24
log2 intensity
Genotype
L/L
L/L, cre
Imputation
non imputed
imputed

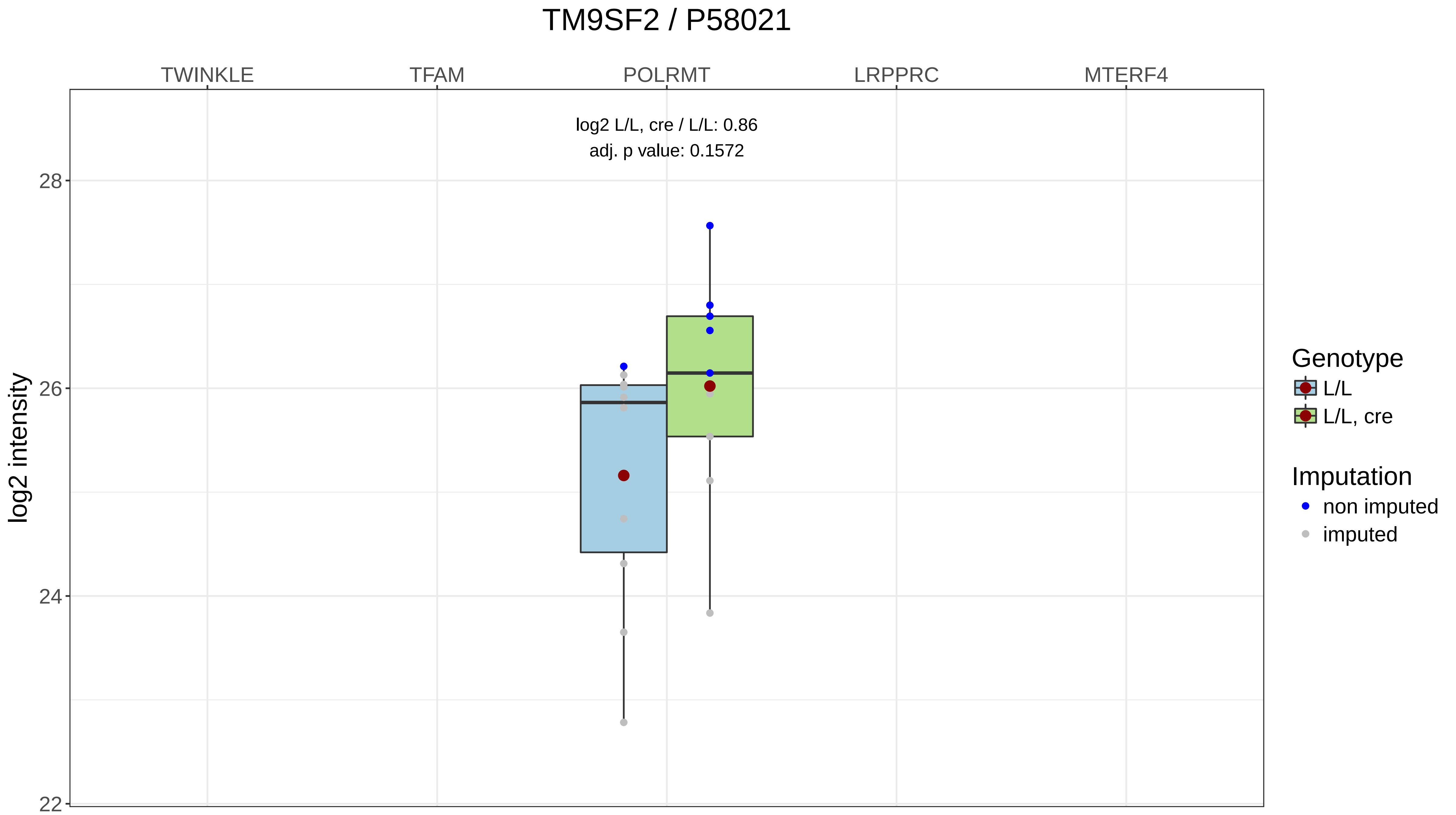

TM9SF2 / P58021
TWINKLE
TFAM
POLRMT
LRPPRC
MTERF4
log2 L/L, cre / L/L: 0.86
adj. p value: 0.1572
log2 intensity
28
26
24
22
Genotype
L/L
L/L, cre
Imputation
non imputed
imputed

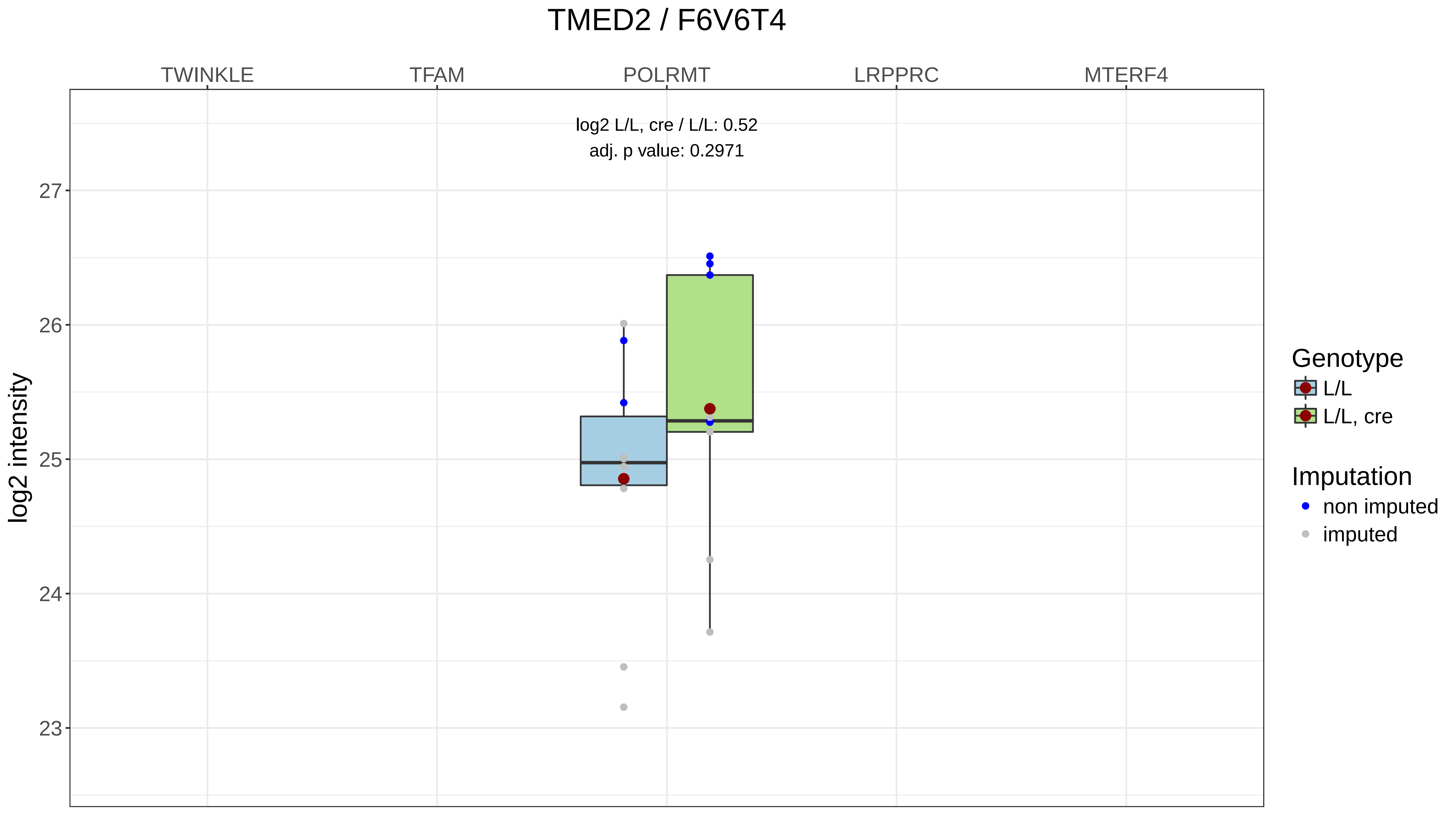
TMED2 / F6V6T4
TWINKLE
TFAM
POLRMT
LRPPRC
MTERF4
log2 L/L, cre / L/L: 0.52
adj. p value: 0.2971
log2 intensity
27
26
25
24
23
Genotype
L/L
L/L, cre
Imputation
non imputed
imputed

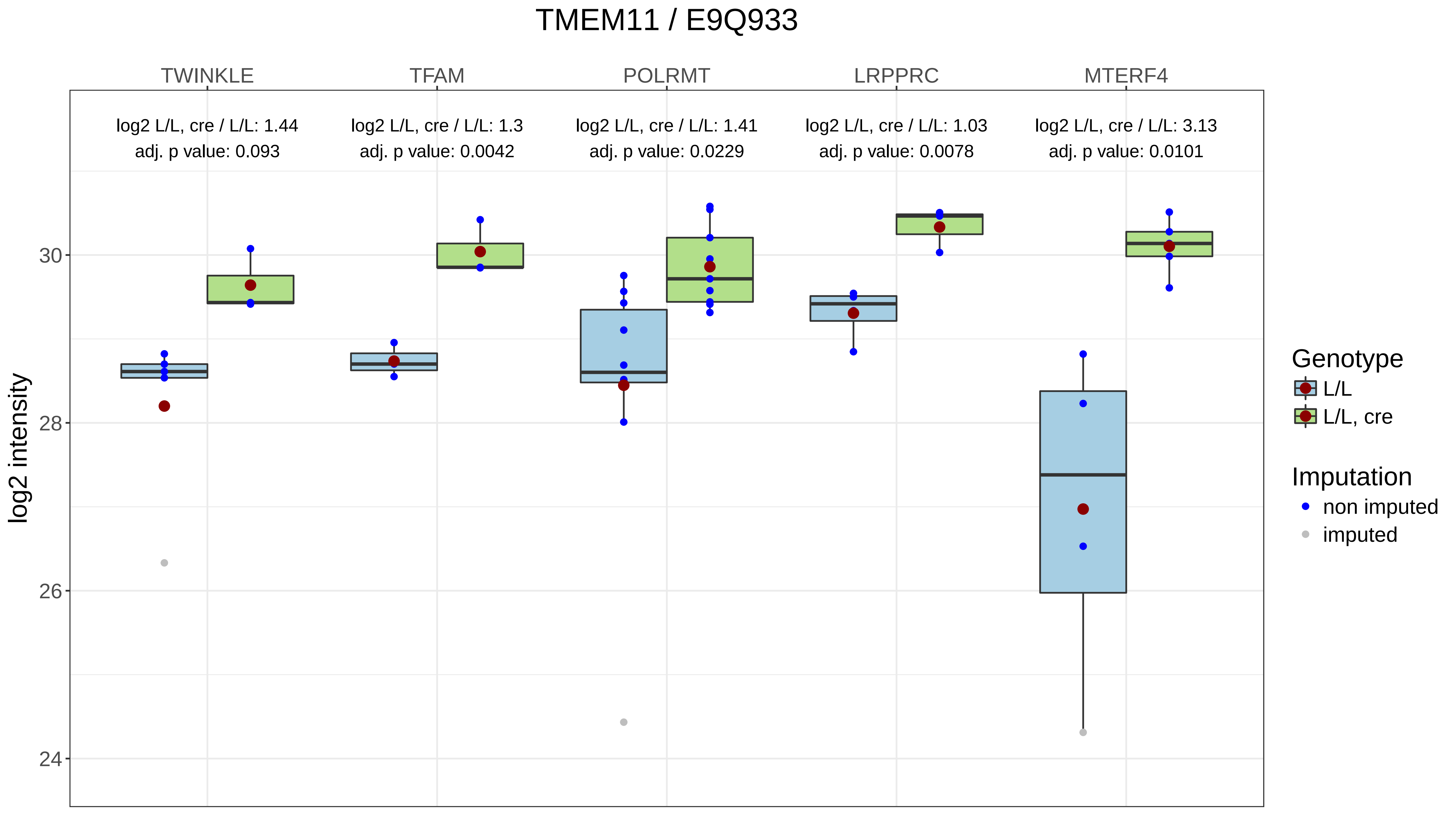
TMEM11 / E9Q933
TWINKLE
TFAM
POLRMT
LRPPRC
MTERF4
log2 L/L, cre / L/L: 1.44
adj. p value: 0.093
log2 L/L, cre / L/L: 1.3
adj. p value: 0.0042
log2 L/L, cre / L/L: 1.41
adj. p value: 0.0229
log2 L/L, cre / L/L: 1.03
adj. p value: 0.0078
log2 L/L, cre / L/L: 3.13
adj. p value: 0.0101
log2 intensity
30
28
26
24
Genotype
L/L
L/L, cre
Imputation
non imputed
imputed

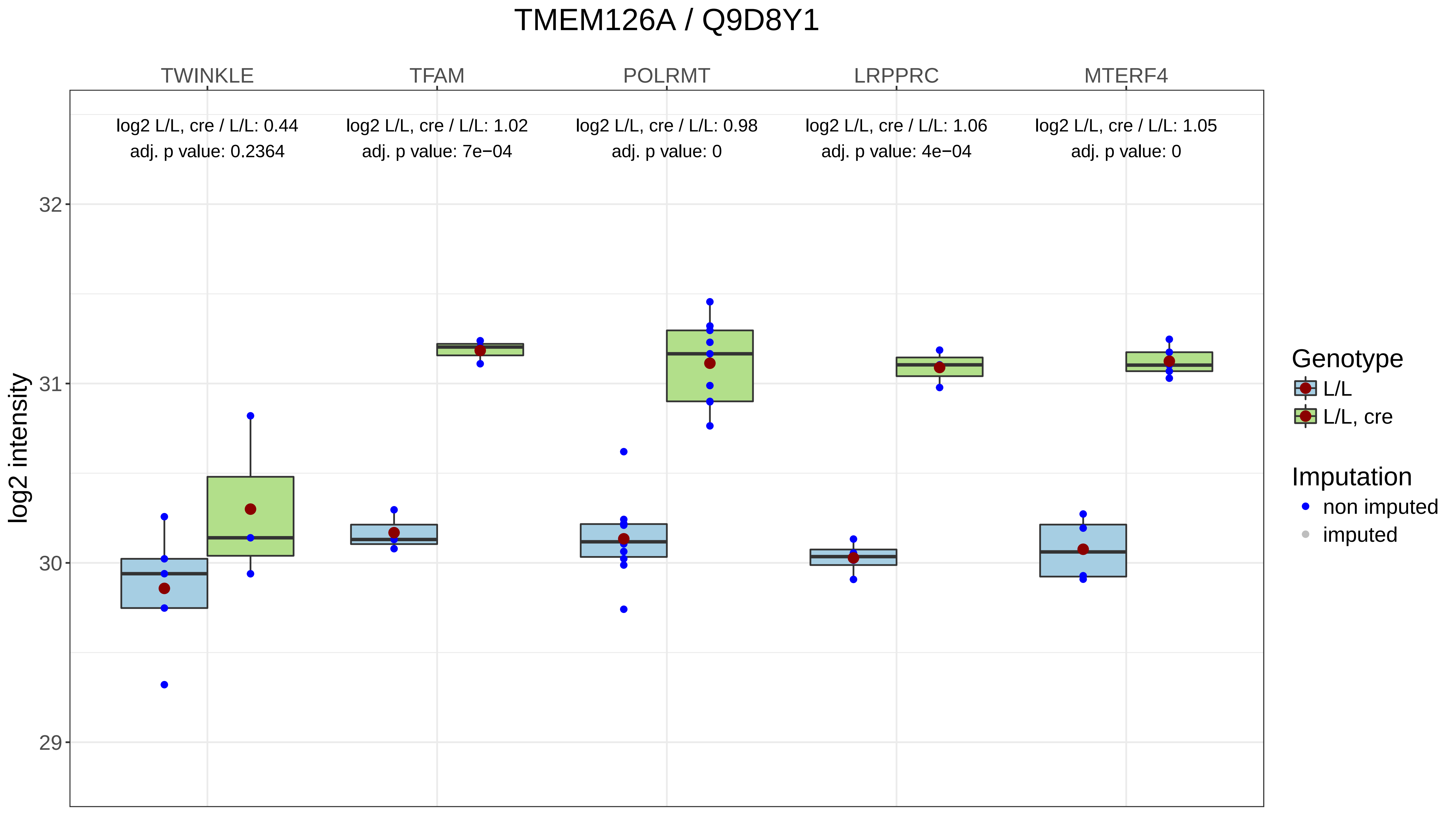
TMEM126A / Q9D8Y1
TWINKLE
TFAM
POLRMT
LRPPRC
MTERF4
log2 L/L, cre / L/L: 0.44
adj. p value: 0.2364
log2 L/L, cre / L/L: 1.02
adj. p value: 7e−04
log2 L/L, cre / L/L: 0.98
adj. p value: 0
log2 L/L, cre / L/L: 1.06
adj. p value: 4e−04
log2 L/L, cre / L/L: 1.05
adj. p value: 0
log2 intensity
32
31
30
29
Genotype
L/L
L/L, cre
Imputation
non imputed
imputed

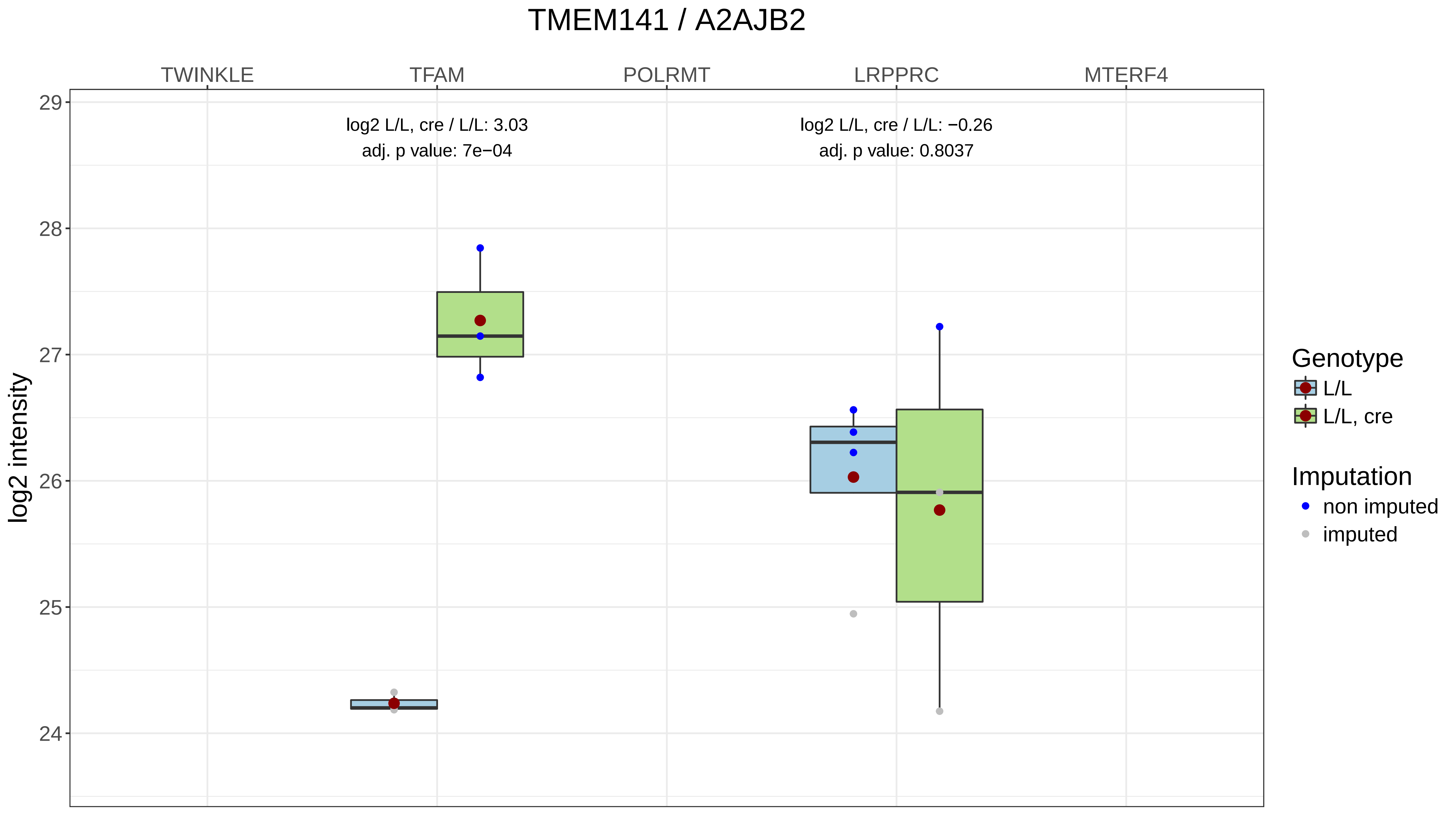
TMEM141 / A2AJB2
TWINKLE
TFAM
POLRMT
LRPPRC
MTERF4
log2 L/L, cre / L/L: 3.03
adj. p value: 7e−04
log2 L/L, cre / L/L: −0.26
adj. p value: 0.8037
log2 intensity
29
28
27
26
25
24
Genotype
L/L
L/L, cre
Imputation
non imputed
imputed

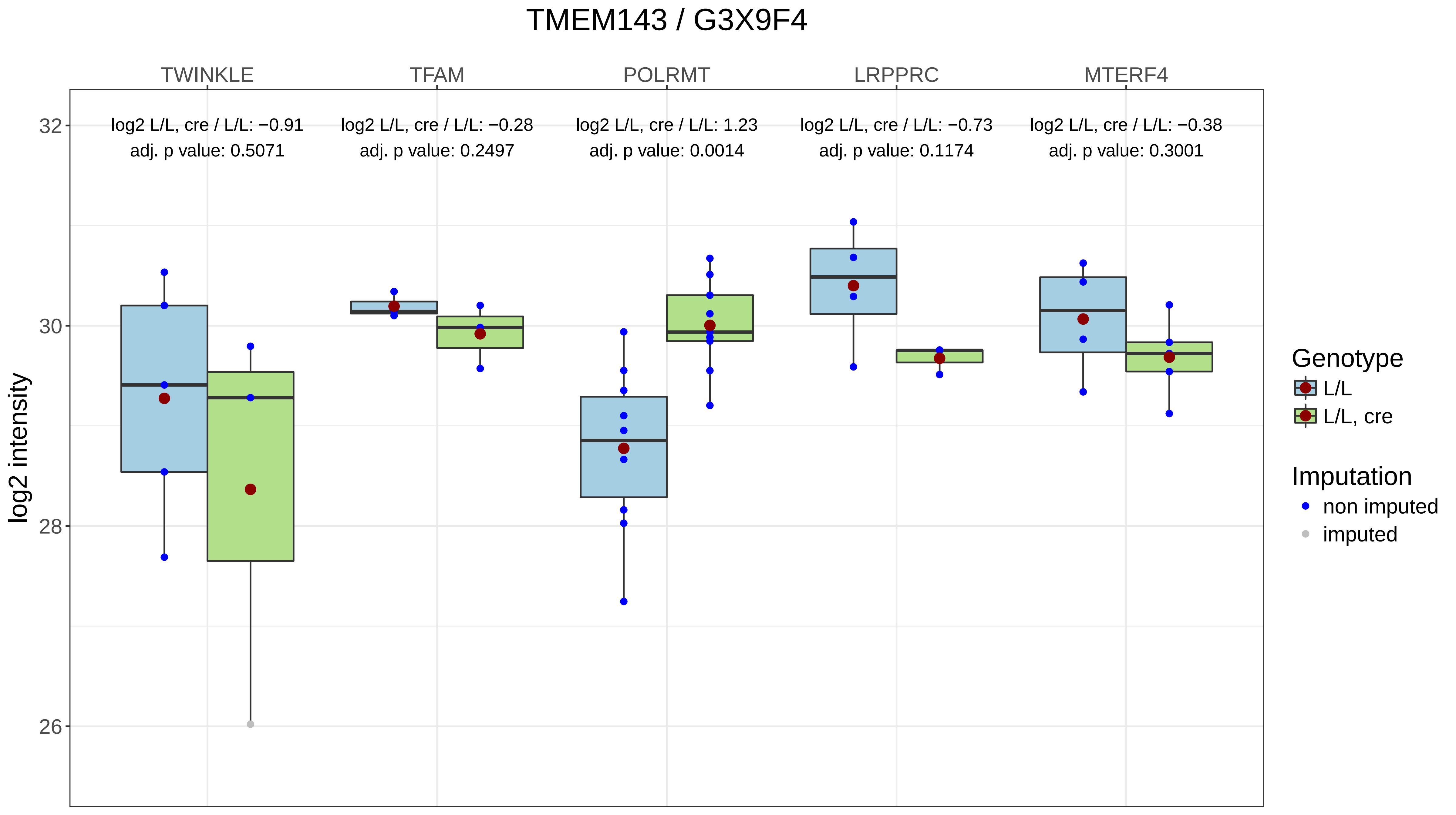

TMEM143 / G3X9F4
TWINKLE
TFAM
POLRMT
LRPPRC
MTERF4
log2 L/L, cre / L/L: −0.91
adj. p value: 0.5071
log2 L/L, cre / L/L: −0.28
adj. p value: 0.2497
log2 L/L, cre / L/L: 1.23
adj. p value: 0.0014
log2 L/L, cre / L/L: −0.73
adj. p value: 0.1174
log2 L/L, cre / L/L: −0.38
adj. p value: 0.3001
32
30
28
26
log2 intensity
Genotype
L/L
L/L, cre
Imputation
non imputed
imputed

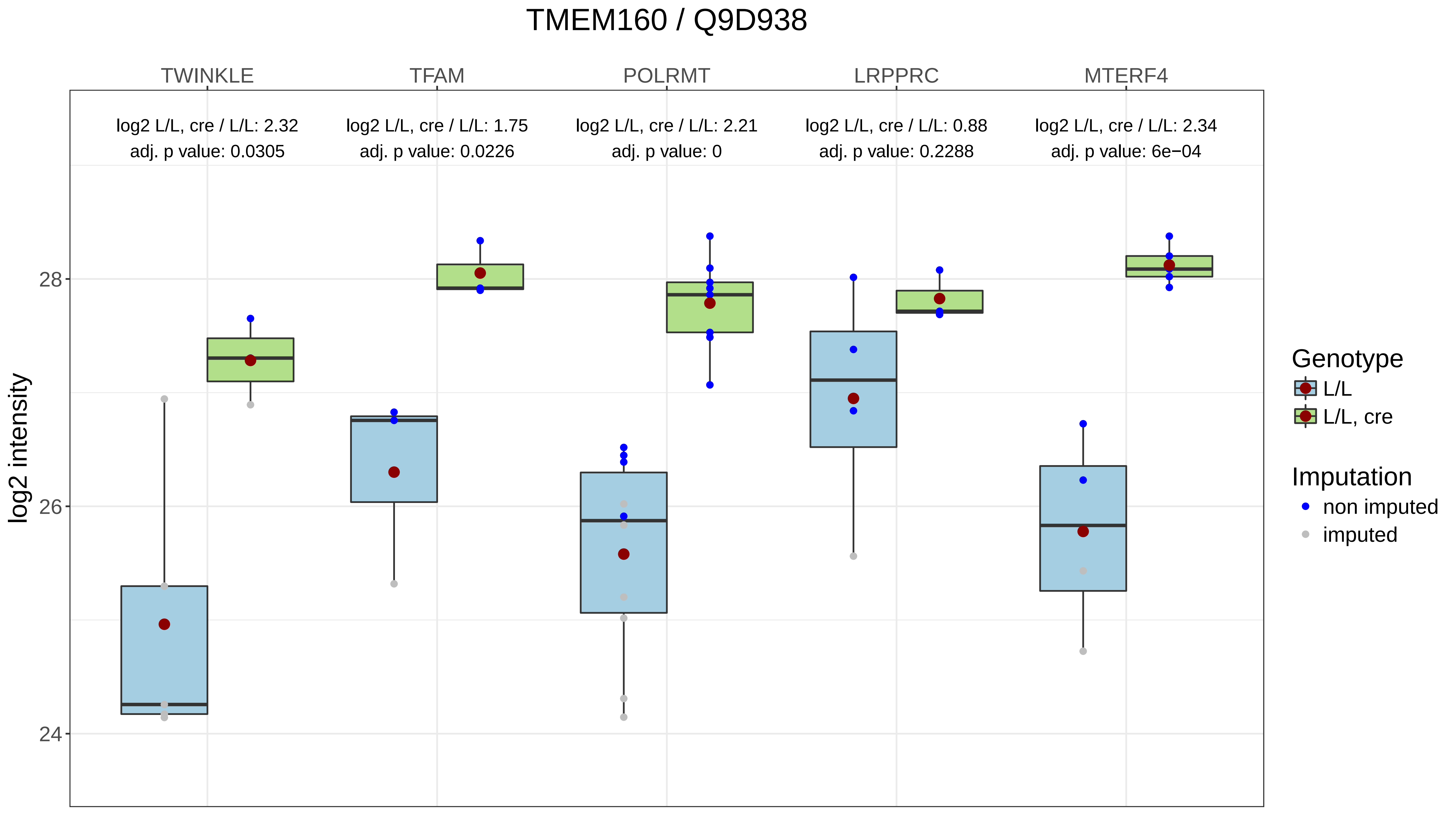
TMEM160 / Q9D938
TWINKLE
TFAM
POLRMT
LRPPRC
MTERF4
log2 L/L, cre / L/L: 2.32
adj. p value: 0.0305
log2 L/L, cre / L/L: 1.75
adj. p value: 0.0226
log2 L/L, cre / L/L: 2.21
adj. p value: 0
log2 L/L, cre / L/L: 0.88
adj. p value: 0.2288
log2 L/L, cre / L/L: 2.34
adj. p value: 6e−04
log2 intensity
28
26
24
Genotype
L/L
L/L, cre
Imputation
non imputed
imputed

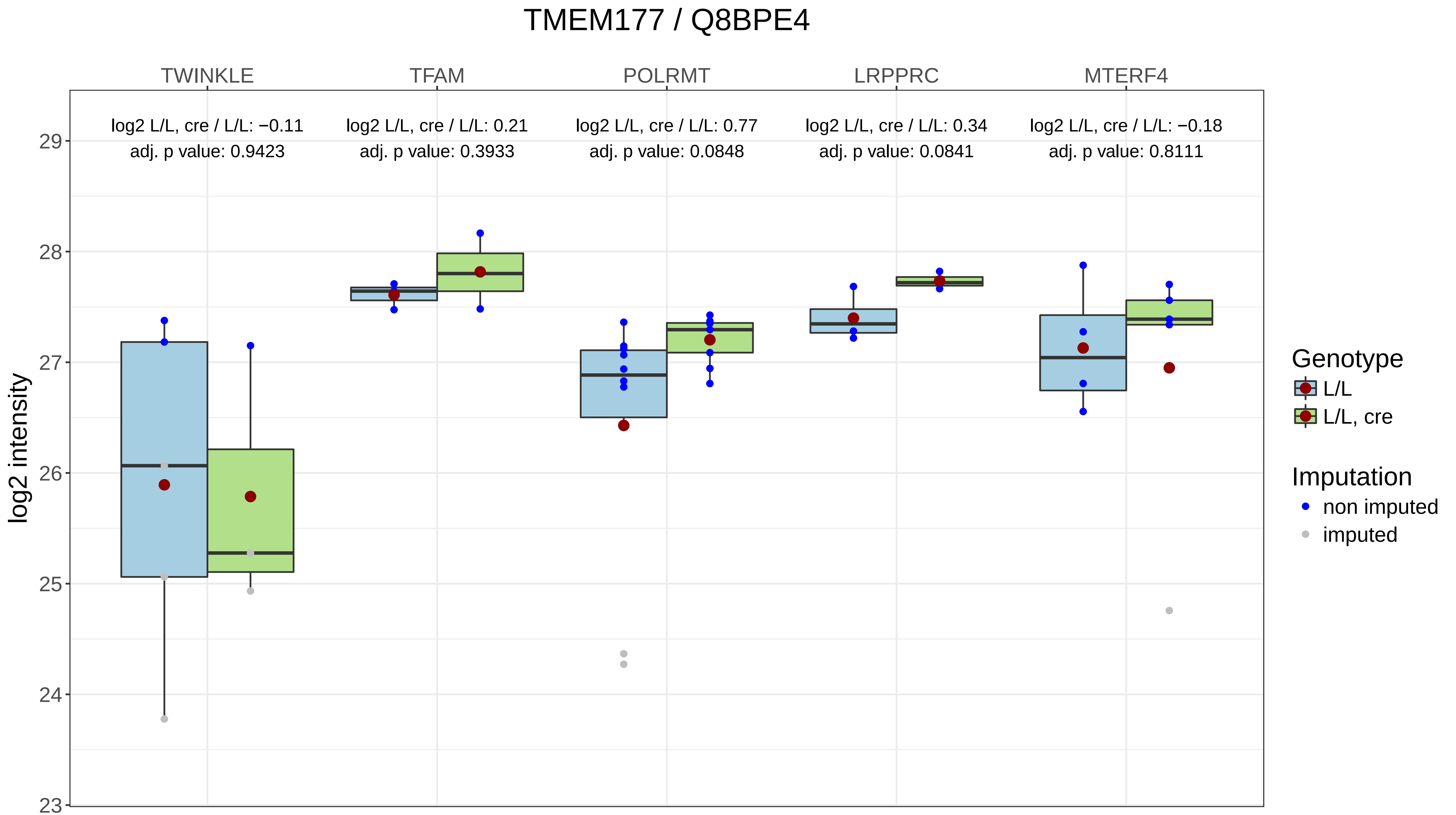

TMEM177 / Q8BPE4
TWINKLE
TFAM
POLRMT
LRPPRC
MTERF4
log2 L/L, cre / L/L: −0.11
adj. p value: 0.9423
log2 L/L, cre / L/L: 0.21
adj. p value: 0.3933
log2 L/L, cre / L/L: 0.77
adj. p value: 0.0848
log2 L/L, cre / L/L: 0.34
adj. p value: 0.0841
log2 L/L, cre / L/L: −0.18
adj. p value: 0.8111
log2 intensity
29
28
27
26
25
24
23
Genotype
L/L
L/L, cre
Imputation
non imputed
imputed

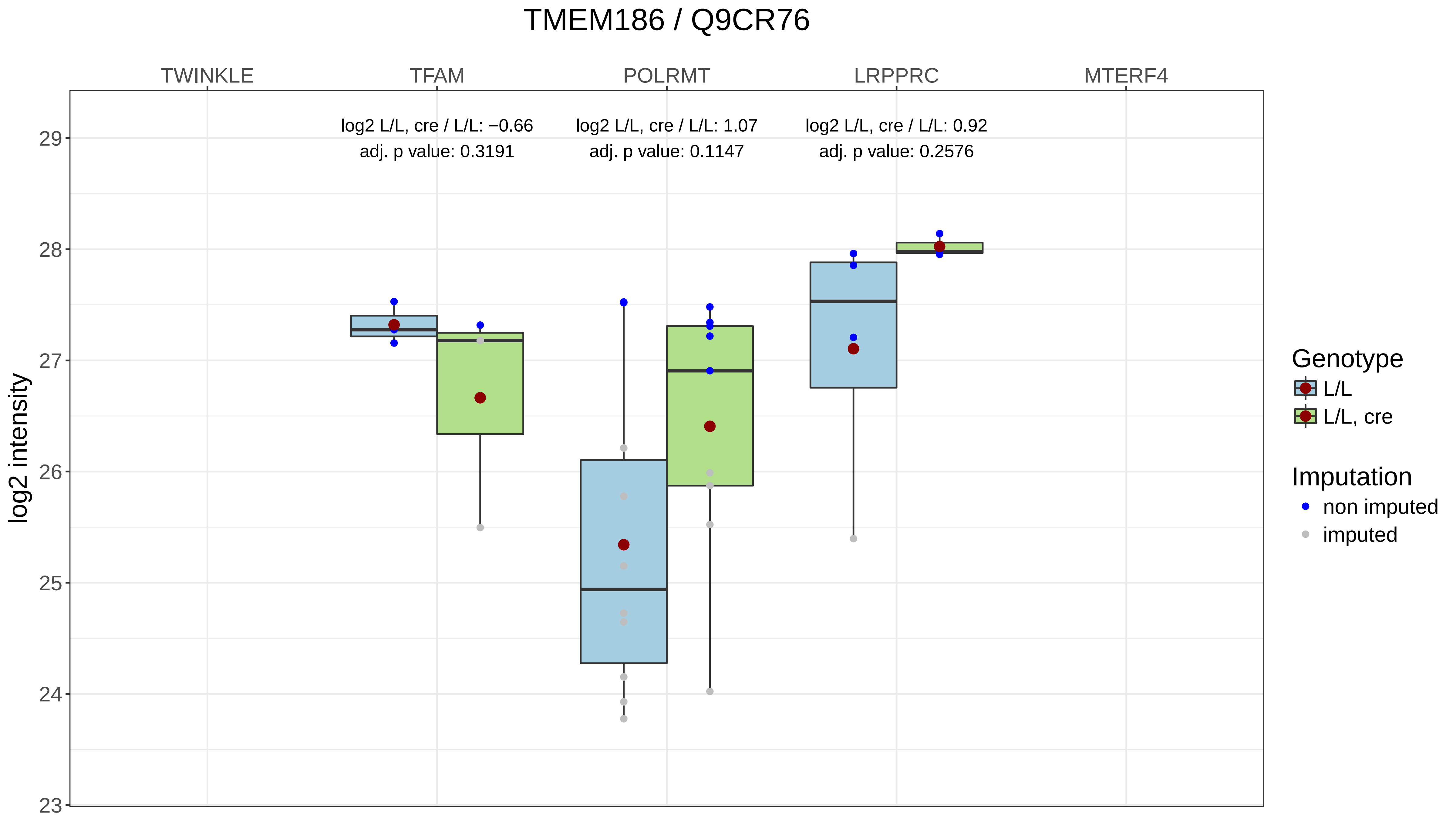
TMEM186 / Q9CR76
TWINKLE
TFAM
POLRMT
LRPPRC
MTERF4
log2 L/L, cre / L/L: −0.66
adj. p value: 0.3191
log2 L/L, cre / L/L: 1.07
adj. p value: 0.1147
log2 L/L, cre / L/L: 0.92
adj. p value: 0.2576
log2 intensity
29
28
27
26
25
24
23
Genotype
L/L
L/L, cre
Imputation
non imputed
imputed

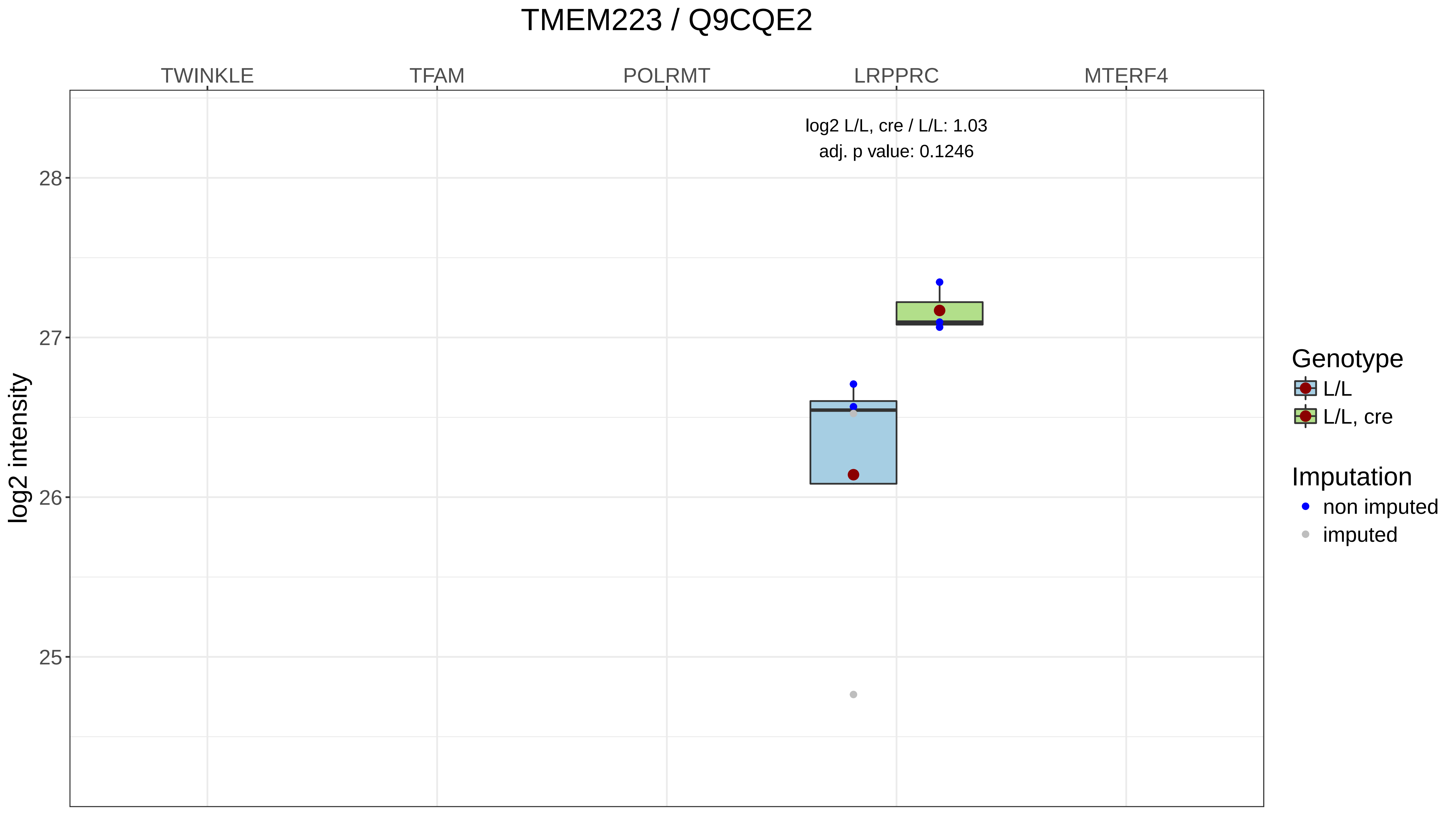

TMEM223 / Q9CQE2
TWINKLE
TFAM
POLRMT
LRPPRC
MTERF4
log2 L/L, cre / L/L: 1.03
adj. p value: 0.1246
28
27
26
25
log2 intensity
Genotype
L/L
L/L, cre
Imputation
non imputed
imputed

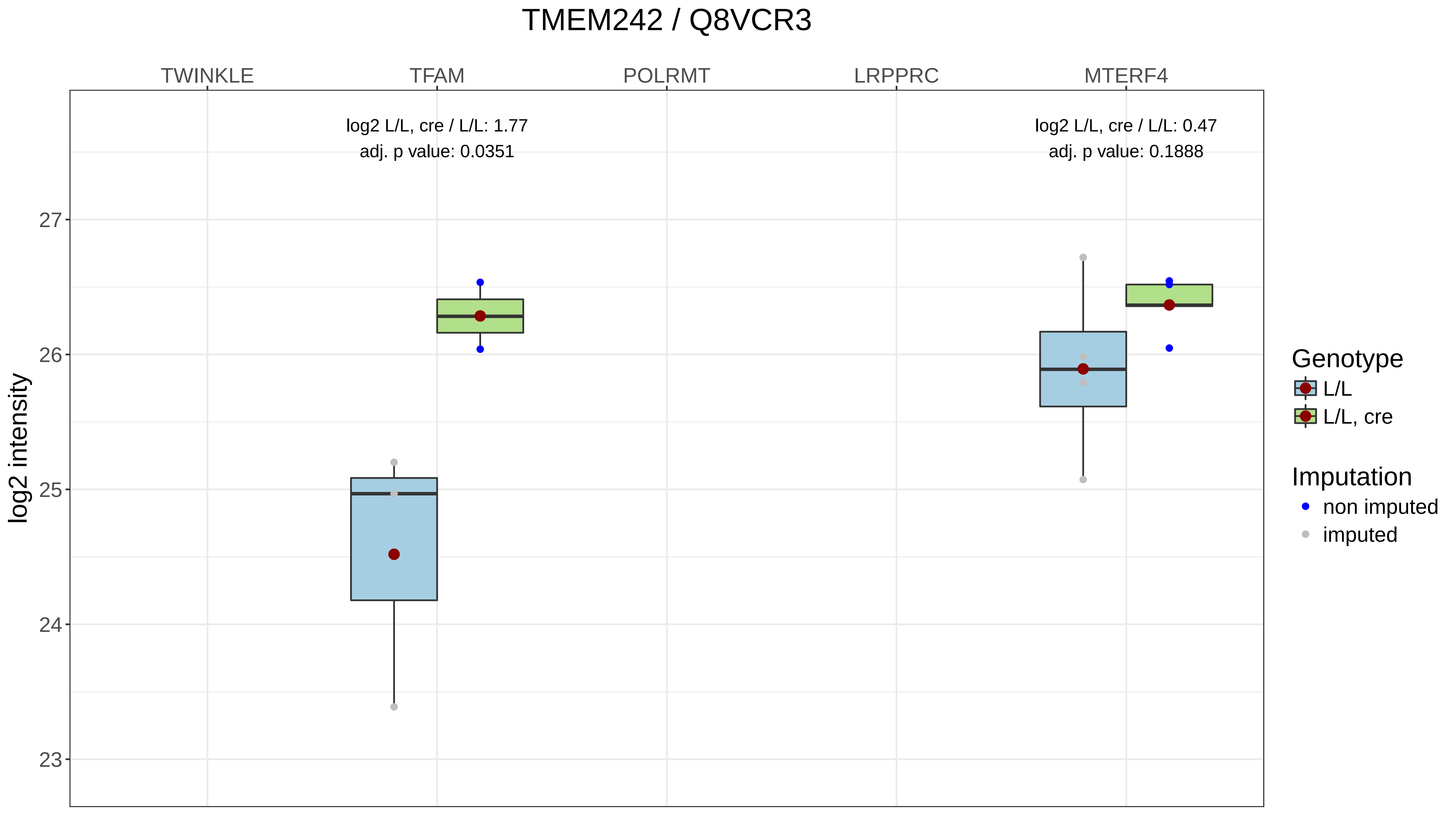
TMEM242 / Q8VCR3
TWINKLE
TFAM
POLRMT
LRPPRC
MTERF4
log2 L/L, cre / L/L: 1.77
adj. p value: 0.0351
log2 L/L, cre / L/L: 0.47
adj. p value: 0.1888
log2 intensity
27
26
25
24
23
Genotype
L/L
L/L, cre
Imputation
non imputed
imputed

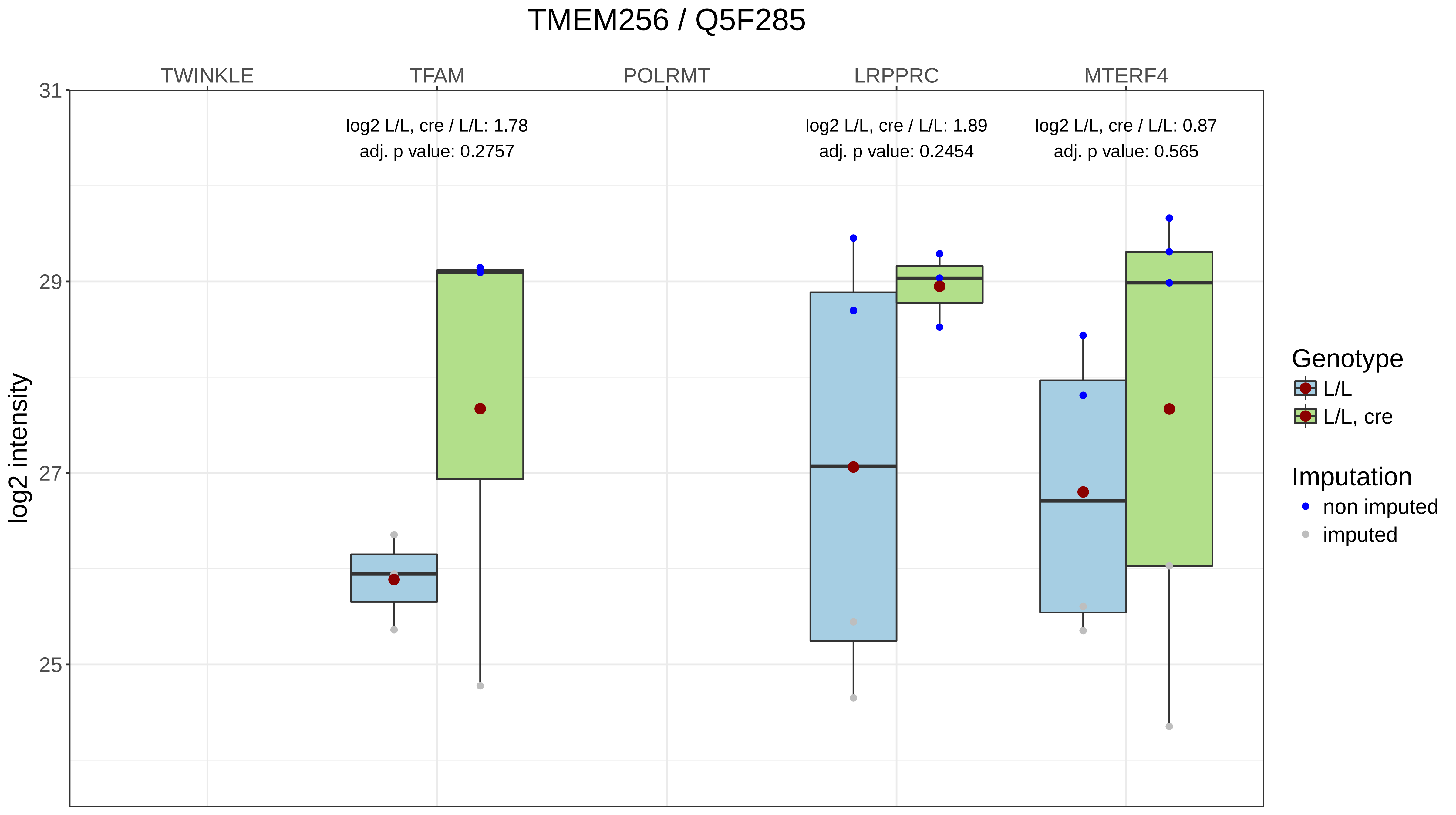
TMEM256 / Q5F285
TWINKLE
TFAM
POLRMT
LRPPRC
MTERF4
log2 L/L, cre / L/L: 1.78
adj. p value: 0.2757
log2 L/L, cre / L/L: 1.89
adj. p value: 0.2454
log2 L/L, cre / L/L: 0.87
adj. p value: 0.565
31
29
27
25
log2 intensity
Genotype
L/L
L/L, cre
Imputation
non imputed
imputed

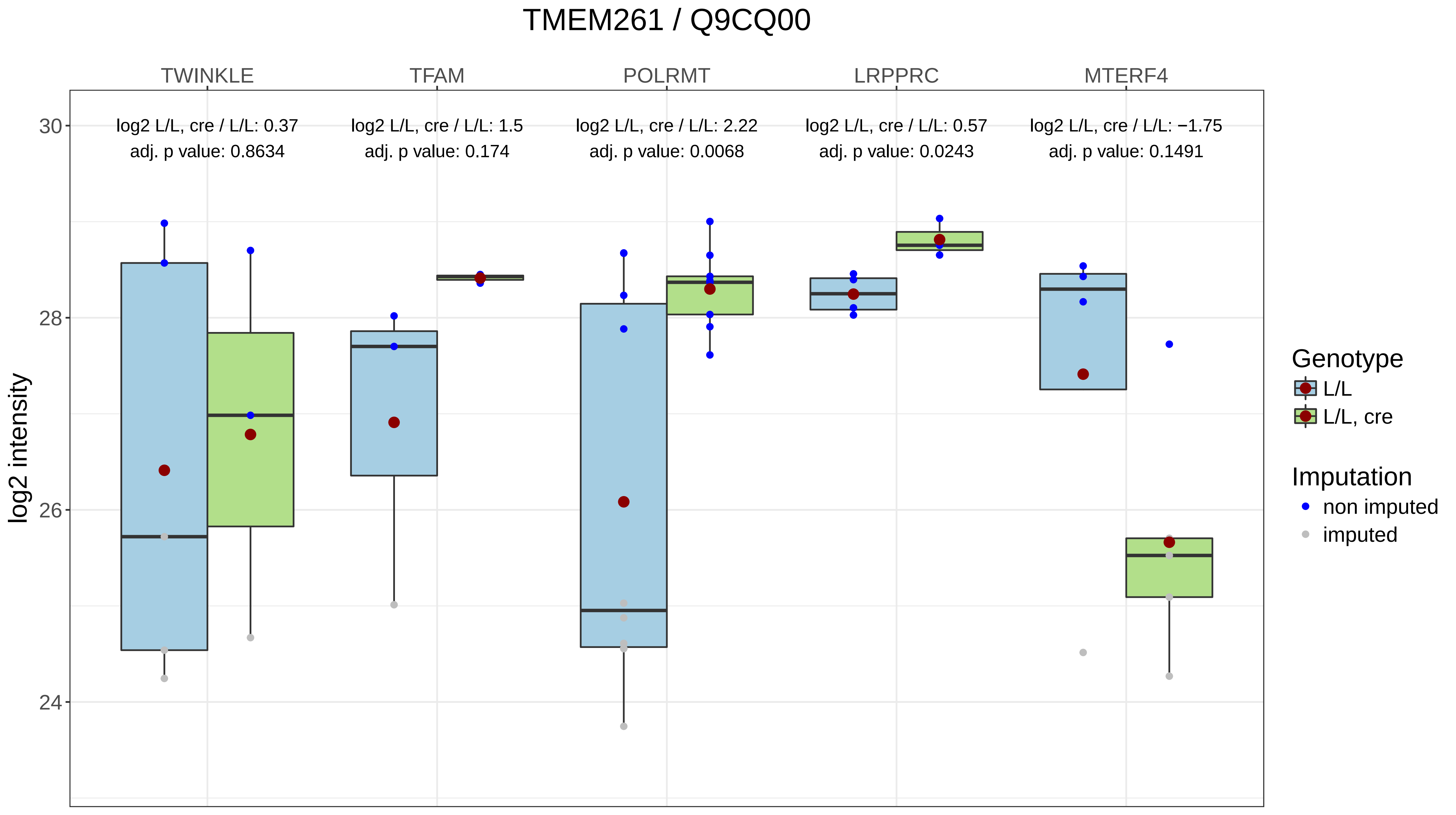
TMEM261 / Q9CQ00
TWINKLE
TFAM
POLRMT
LRPPRC
MTERF4
log2 L/L, cre / L/L: 0.37
adj. p value: 0.8634
log2 L/L, cre / L/L: 1.5
adj. p value: 0.174
log2 L/L, cre / L/L: 2.22
adj. p value: 0.0068
log2 L/L, cre / L/L: 0.57
adj. p value: 0.0243
log2 L/L, cre / L/L: −1.75
adj. p value: 0.1491
log2 intensity
30
28
26
24
Genotype
L/L
L/L, cre
Imputation
non imputed
imputed

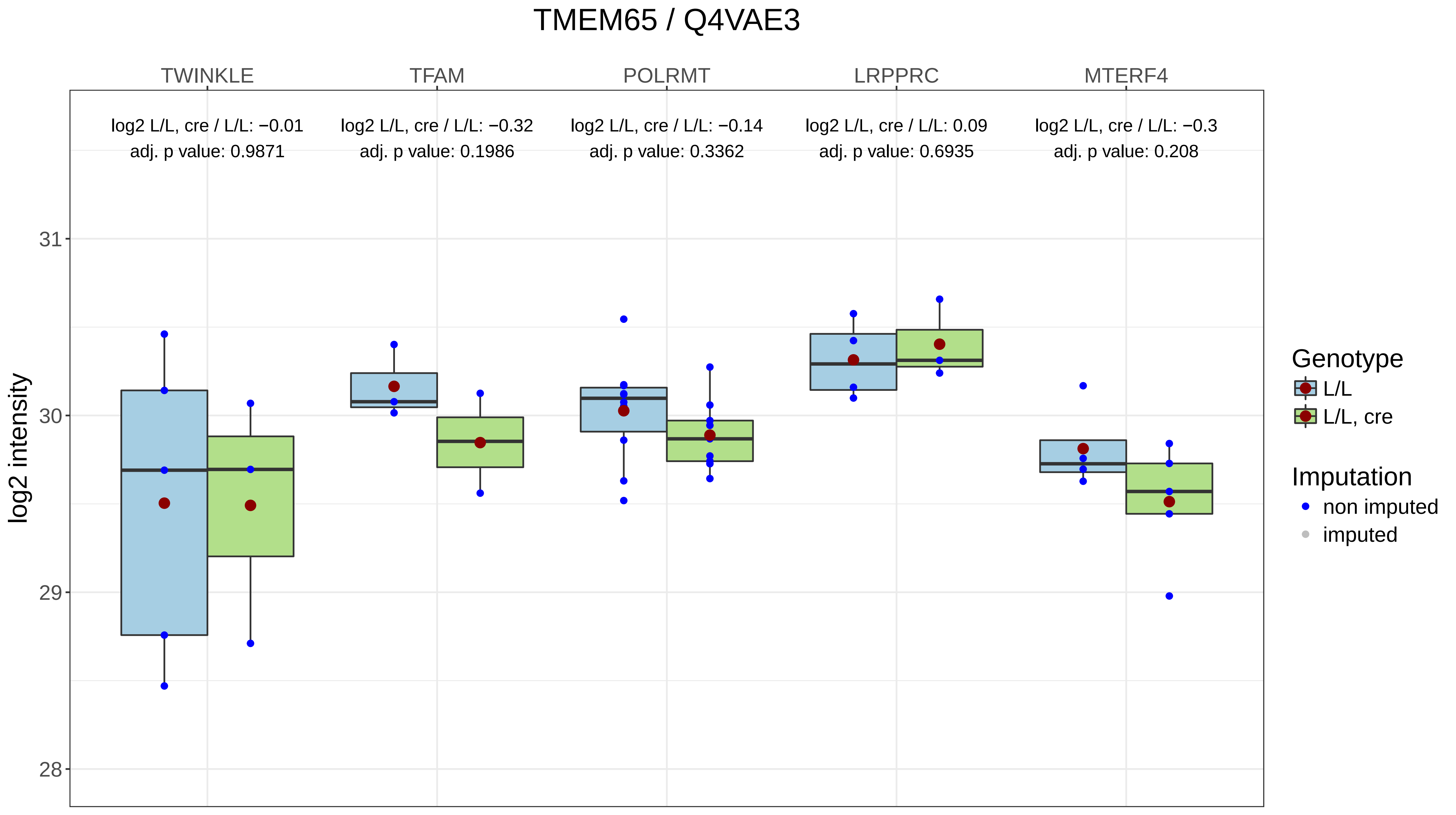

TMEM65 / Q4VAE3
TWINKLE
TFAM
POLRMT
LRPPRC
MTERF4
log2 L/L, cre / L/L: −0.01
adj. p value: 0.9871
log2 L/L, cre / L/L: −0.32
adj. p value: 0.1986
log2 L/L, cre / L/L: −0.14
adj. p value: 0.3362
log2 L/L, cre / L/L: 0.09
adj. p value: 0.6935
log2 L/L, cre / L/L: −0.3
adj. p value: 0.208
log2 intensity
31
30
29
28
Genotype
L/L
L/L, cre
Imputation
non imputed
imputed

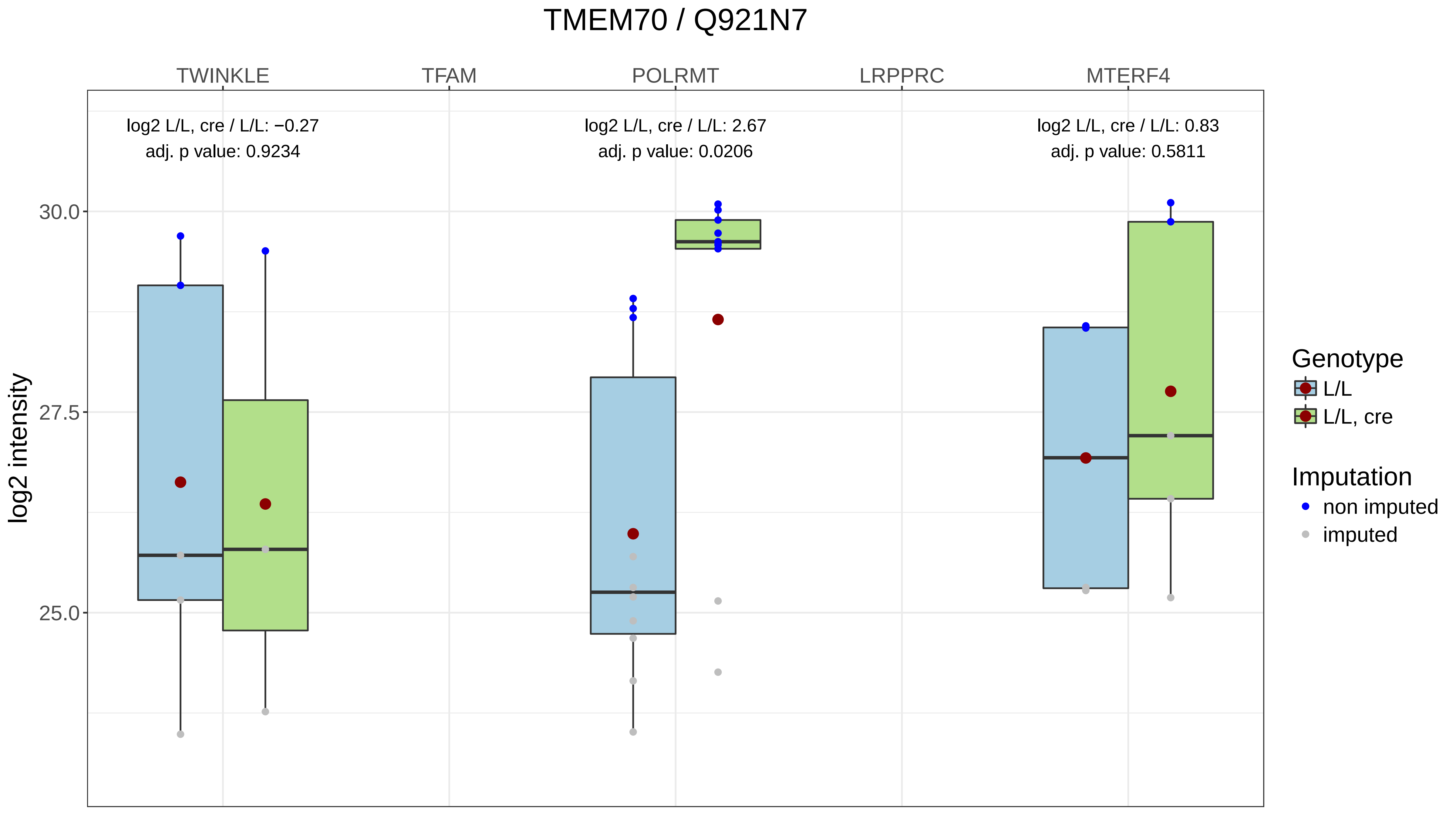
TMEM70 / Q921N7
TWINKLE
TFAM
POLRMT
LRPPRC
MTERF4
log2 L/L, cre / L/L: −0.27
adj. p value: 0.9234
log2 L/L, cre / L/L: 2.67
adj. p value: 0.0206
log2 L/L, cre / L/L: 0.83
adj. p value: 0.5811
30.0
27.5
25.0
log2 intensity
Genotype
L/L
L/L, cre
Imputation
non imputed
imputed

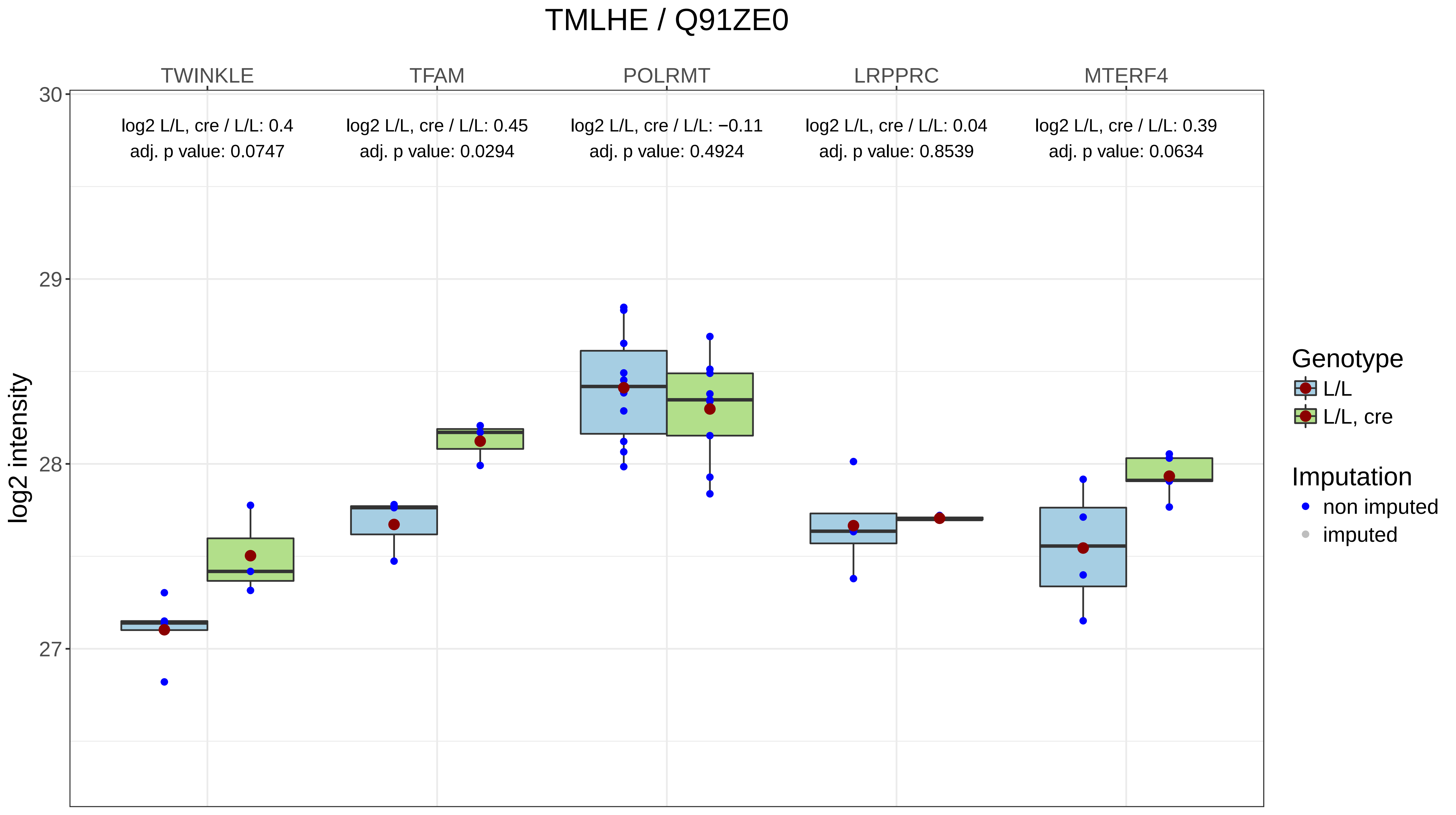
TMLHE / Q91ZE0
TWINKLE
TFAM
POLRMT
LRPPRC
MTERF4
log2 L/L, cre / L/L: 0.4
adj. p value: 0.0747
log2 L/L, cre / L/L: 0.45
adj. p value: 0.0294
log2 L/L, cre / L/L: −0.11
adj. p value: 0.4924
log2 L/L, cre / L/L: 0.04
adj. p value: 0.8539
log2 L/L, cre / L/L: 0.39
adj. p value: 0.0634
log2 intensity
30
29
28
27
Genotype
L/L
L/L, cre
Imputation
non imputed
imputed

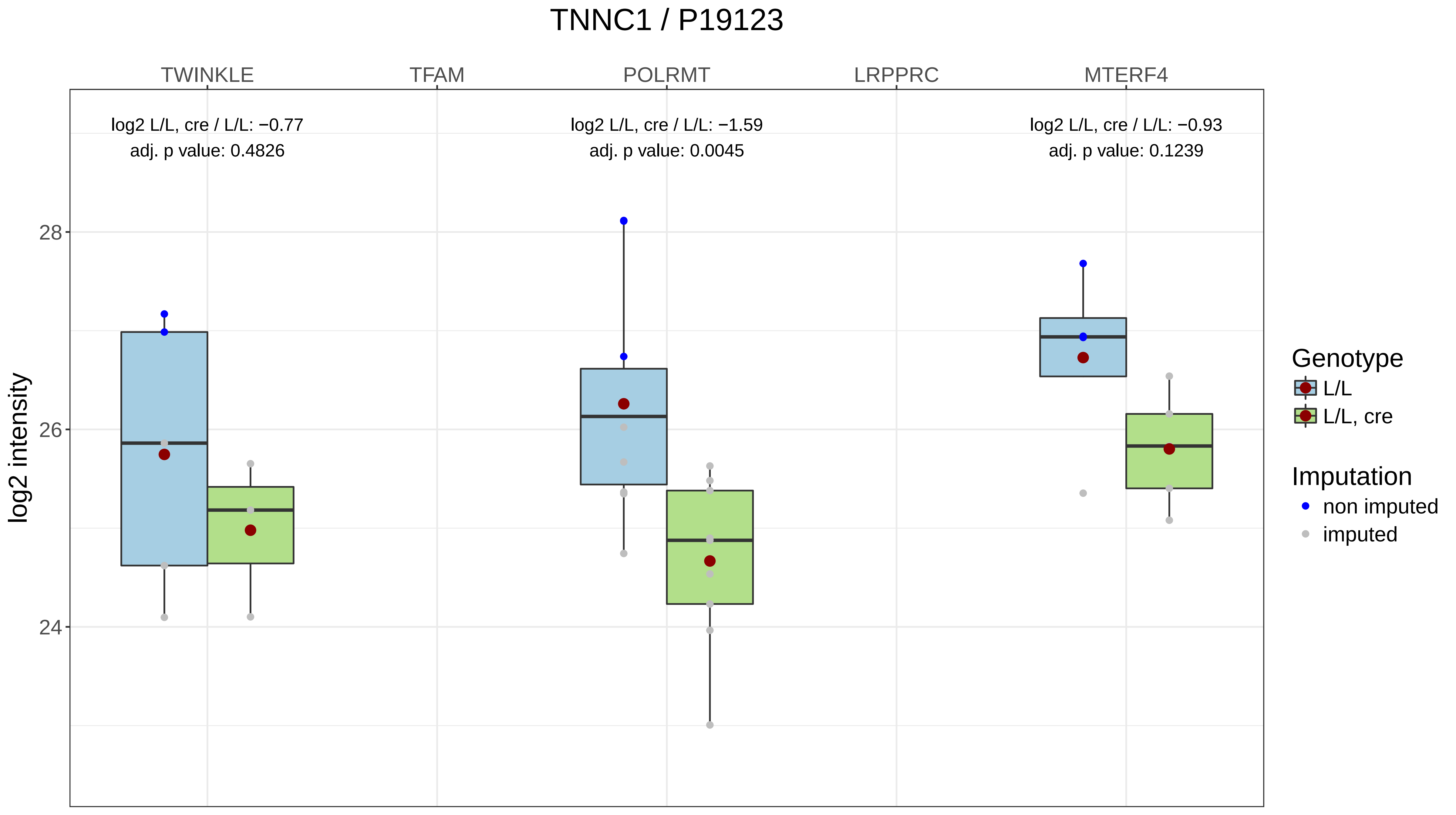

TNNC1 / P19123
TWINKLE
TFAM
POLRMT
LRPPRC
MTERF4
log2 L/L, cre / L/L: −0.77
adj. p value: 0.4826
log2 L/L, cre / L/L: −1.59
adj. p value: 0.0045
log2 L/L, cre / L/L: −0.93
adj. p value: 0.1239
log2 intensity
28
26
24
Genotype
L/L
L/L, cre
Imputation
non imputed
imputed

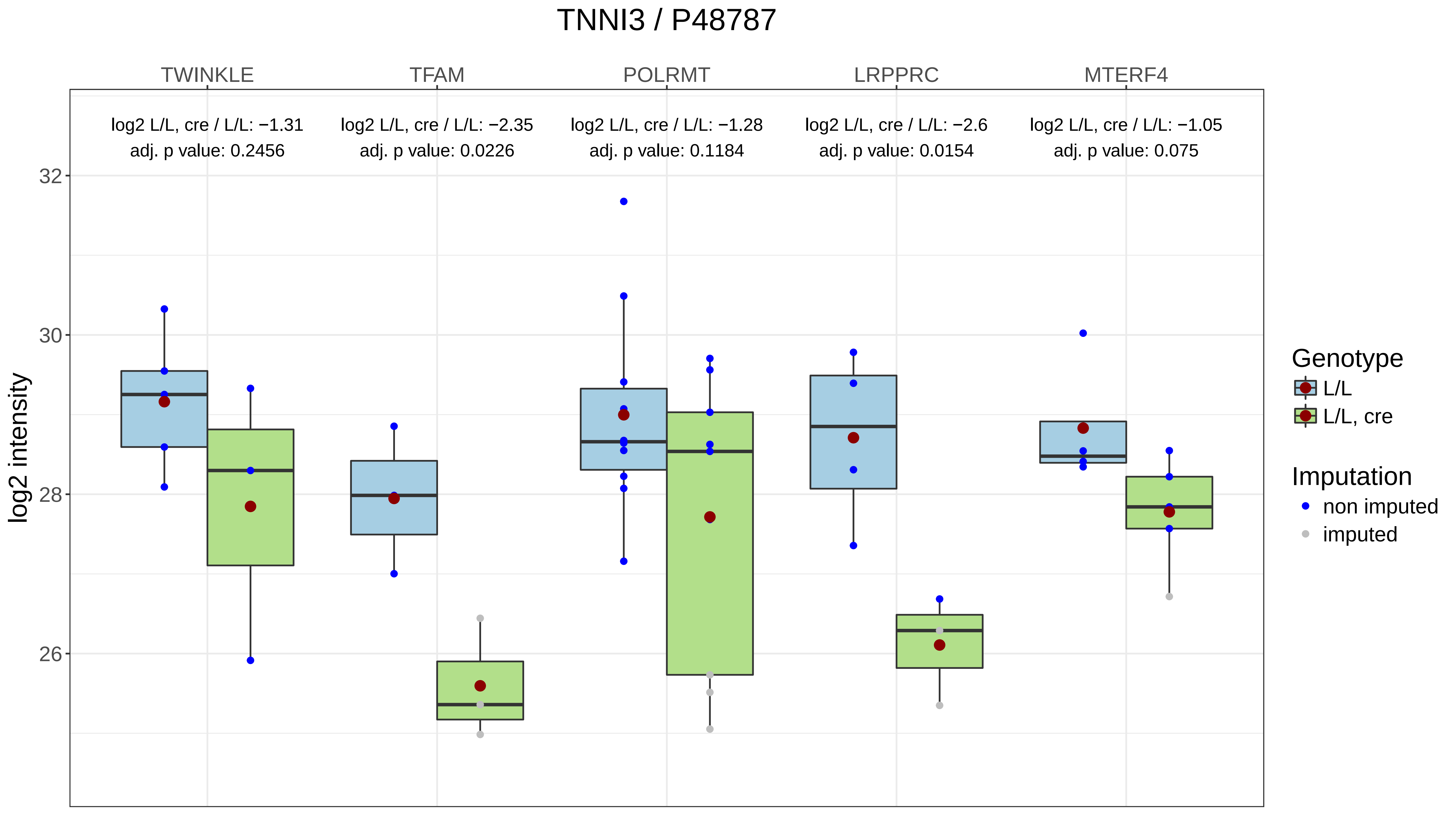

TNNI3 / P48787
TWINKLE
TFAM
POLRMT
LRPPRC
MTERF4
log2 L/L, cre / L/L: −1.31
adj. p value: 0.2456
log2 L/L, cre / L/L: −2.35
adj. p value: 0.0226
log2 L/L, cre / L/L: −1.28
adj. p value: 0.1184
log2 L/L, cre / L/L: −2.6
adj. p value: 0.0154
log2 L/L, cre / L/L: −1.05
adj. p value: 0.075
log2 intensity
32
30
28
26
Genotype
L/L
L/L, cre
Imputation
non imputed
imputed

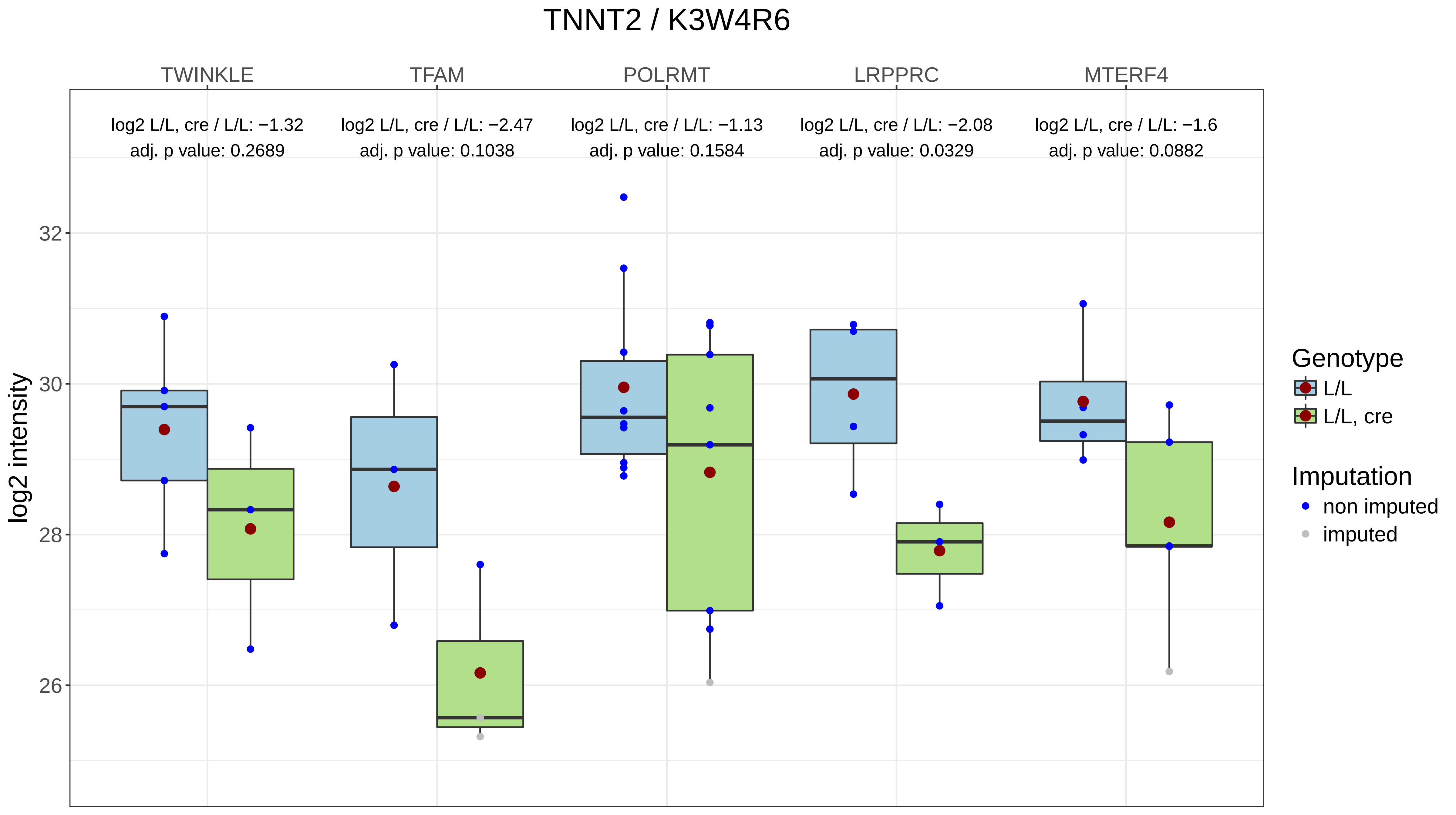

TNNT2 / K3W4R6
TWINKLE
TFAM
POLRMT
LRPPRC
MTERF4
log2 L/L, cre / L/L: −1.32
adj. p value: 0.2689
log2 L/L, cre / L/L: −2.47
adj. p value: 0.1038
log2 L/L, cre / L/L: −1.13
adj. p value: 0.1584
log2 L/L, cre / L/L: −2.08
adj. p value: 0.0329
log2 L/L, cre / L/L: −1.6
adj. p value: 0.0882
log2 intensity
32
30
28
26
Genotype
L/L
L/L, cre
Imputation
non imputed
imputed

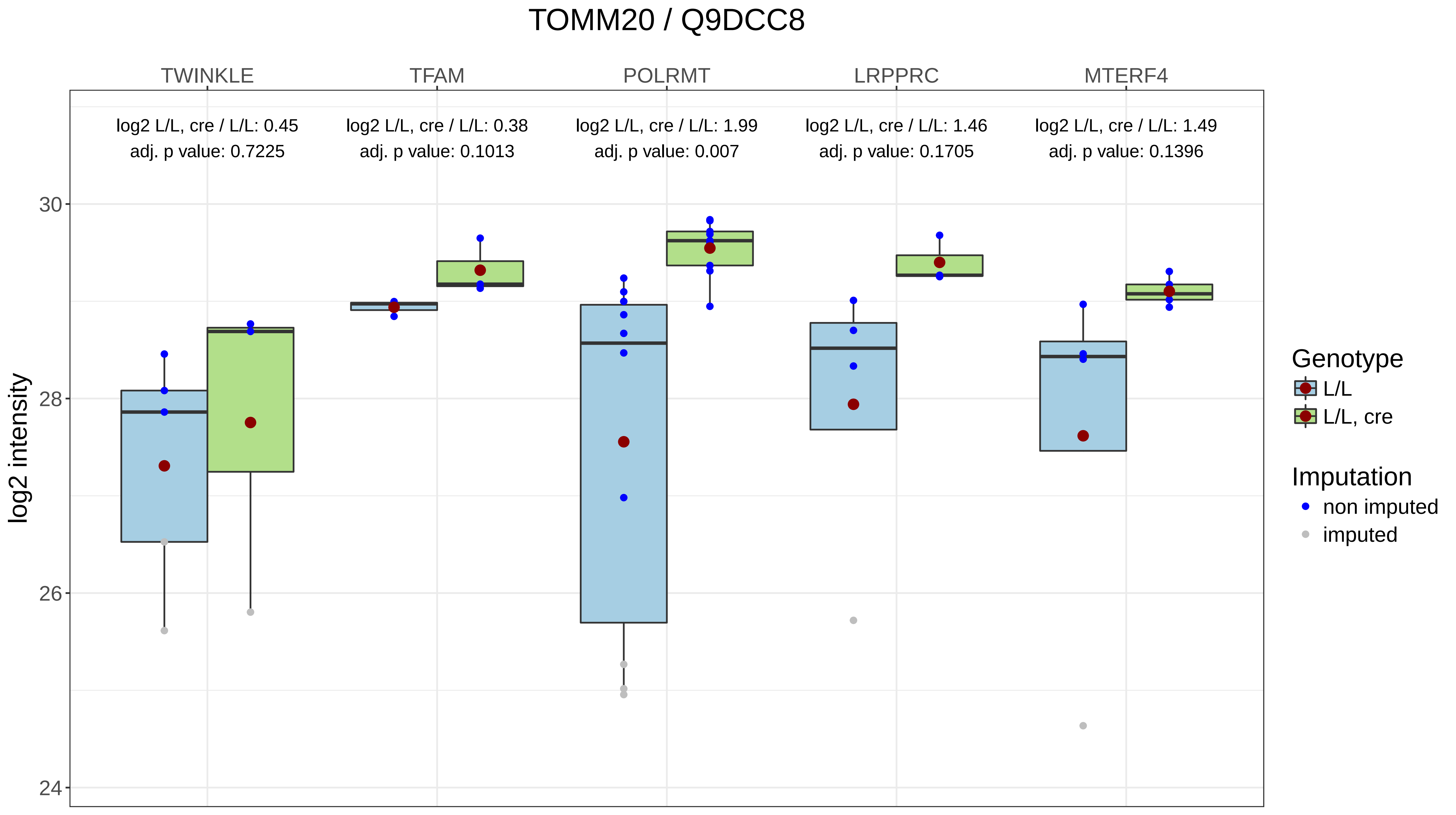

TOMM20 / Q9DCC8
TWINKLE
TFAM
POLRMT
LRPPRC
MTERF4
log2 L/L, cre / L/L: 0.45
adj. p value: 0.7225
log2 L/L, cre / L/L: 0.38
adj. p value: 0.1013
log2 L/L, cre / L/L: 1.99
adj. p value: 0.007
log2 L/L, cre / L/L: 1.46
adj. p value: 0.1705
log2 L/L, cre / L/L: 1.49
adj. p value: 0.1396
log2 intensity
30
28
26
24
Genotype
L/L
L/L, cre
Imputation
non imputed
imputed

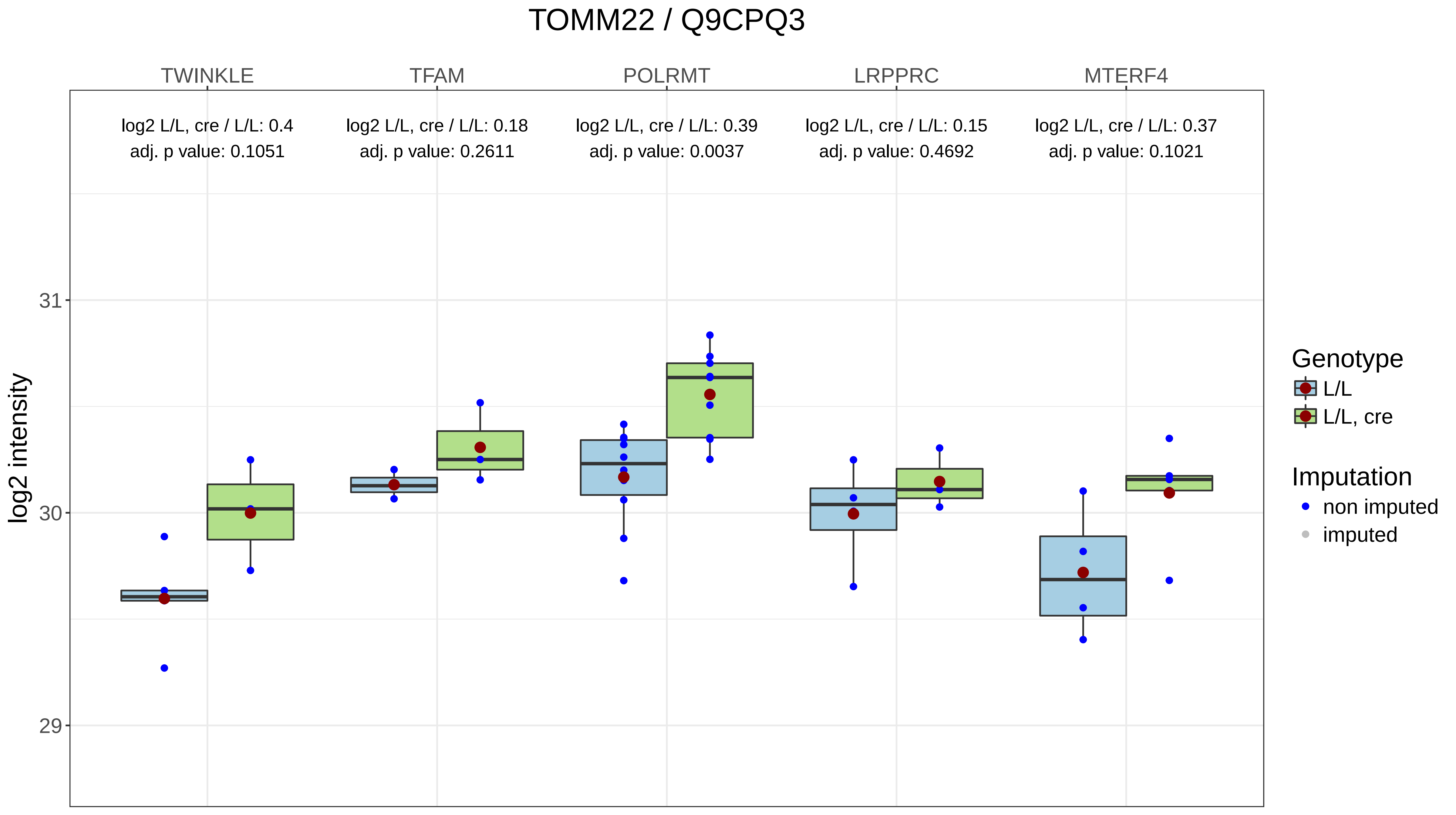
TOMM22 / Q9CPQ3
TWINKLE
TFAM
POLRMT
LRPPRC
MTERF4
log2 L/L, cre / L/L: 0.4
adj. p value: 0.1051
log2 L/L, cre / L/L: 0.18
adj. p value: 0.2611
log2 L/L, cre / L/L: 0.39
adj. p value: 0.0037
log2 L/L, cre / L/L: 0.15
adj. p value: 0.4692
log2 L/L, cre / L/L: 0.37
adj. p value: 0.1021
log2 intensity
31
30
29
Genotype
L/L
L/L, cre
Imputation
non imputed
imputed

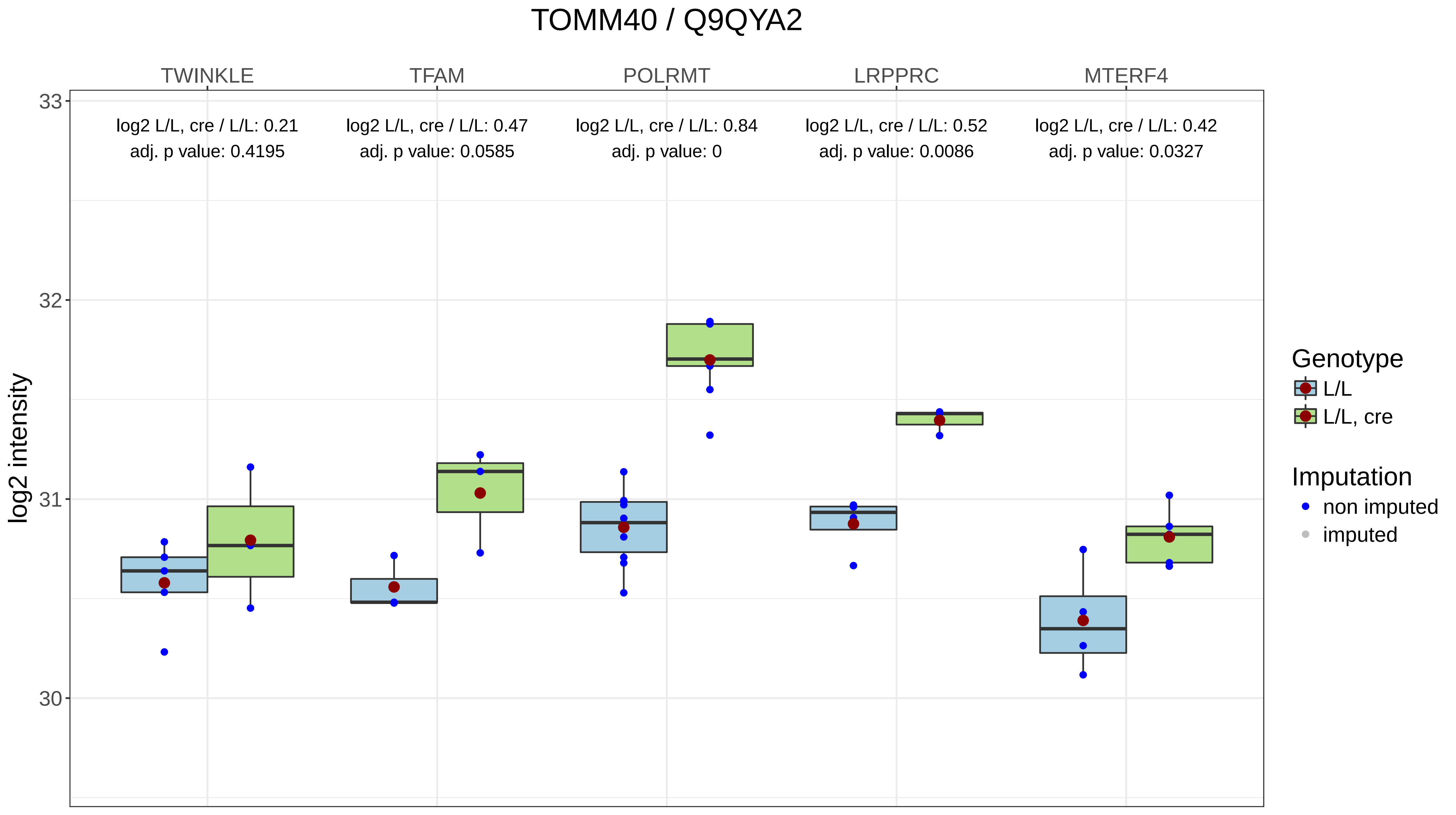
TOMM40 / Q9QYA2
TWINKLE
TFAM
POLRMT
LRPPRC
MTERF4
log2 L/L, cre / L/L: 0.21
adj. p value: 0.4195
log2 L/L, cre / L/L: 0.47
adj. p value: 0.0585
log2 L/L, cre / L/L: 0.84
adj. p value: 0
log2 L/L, cre / L/L: 0.52
adj. p value: 0.0086
log2 L/L, cre / L/L: 0.42
adj. p value: 0.0327
log2 intensity
33
32
31
30
Genotype
L/L
L/L, cre
Imputation
non imputed
imputed

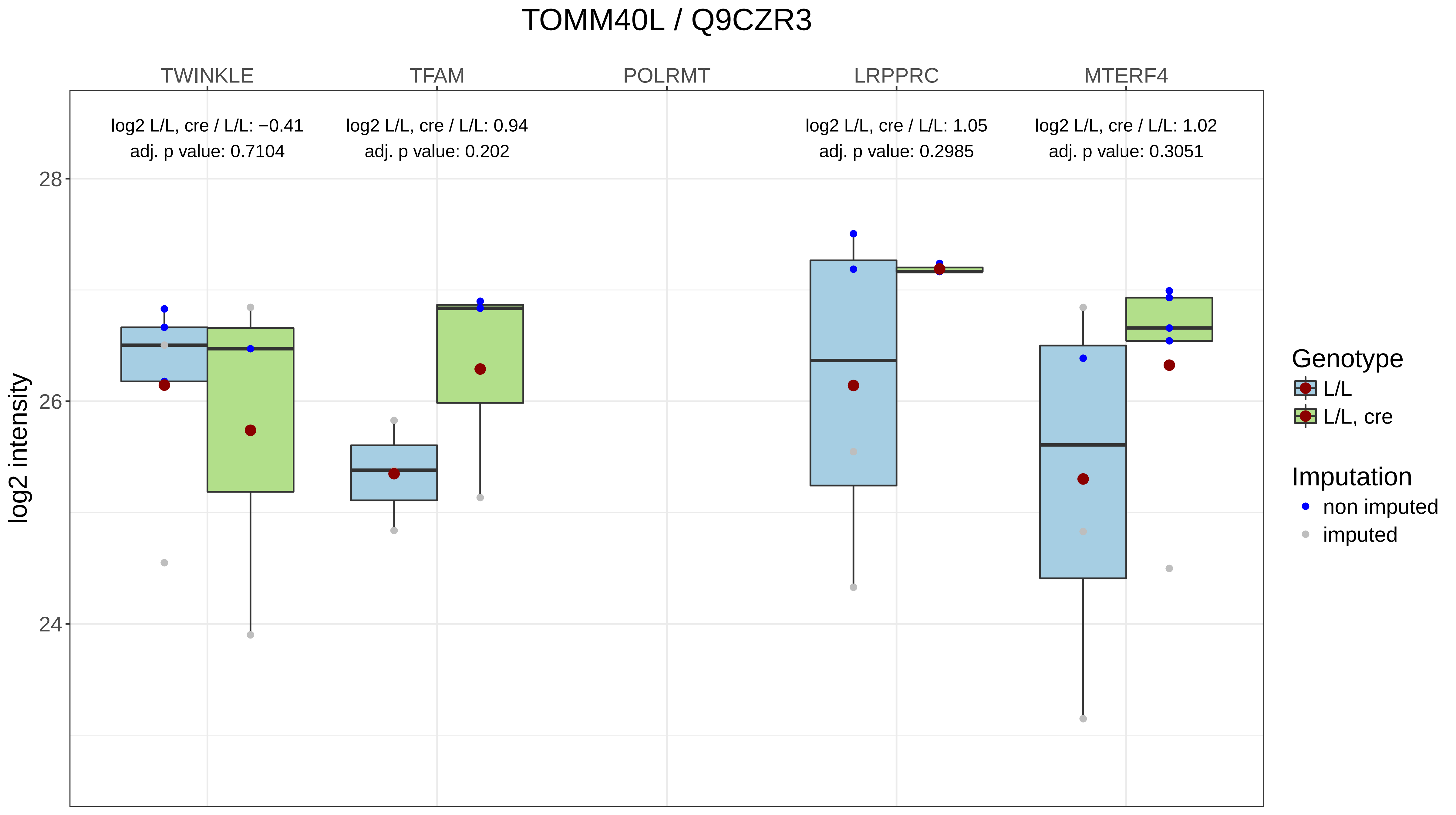
TOMM40L / Q9CZR3
TWINKLE
TFAM
POLRMT
LRPPRC
MTERF4
log2 L/L, cre / L/L: −0.41
adj. p value: 0.7104
log2 L/L, cre / L/L: 0.94
adj. p value: 0.202
log2 L/L, cre / L/L: 1.05
adj. p value: 0.2985
log2 L/L, cre / L/L: 1.02
adj. p value: 0.3051
log2 intensity
28
26
24
Genotype
L/L
L/L, cre
Imputation
non imputed
imputed

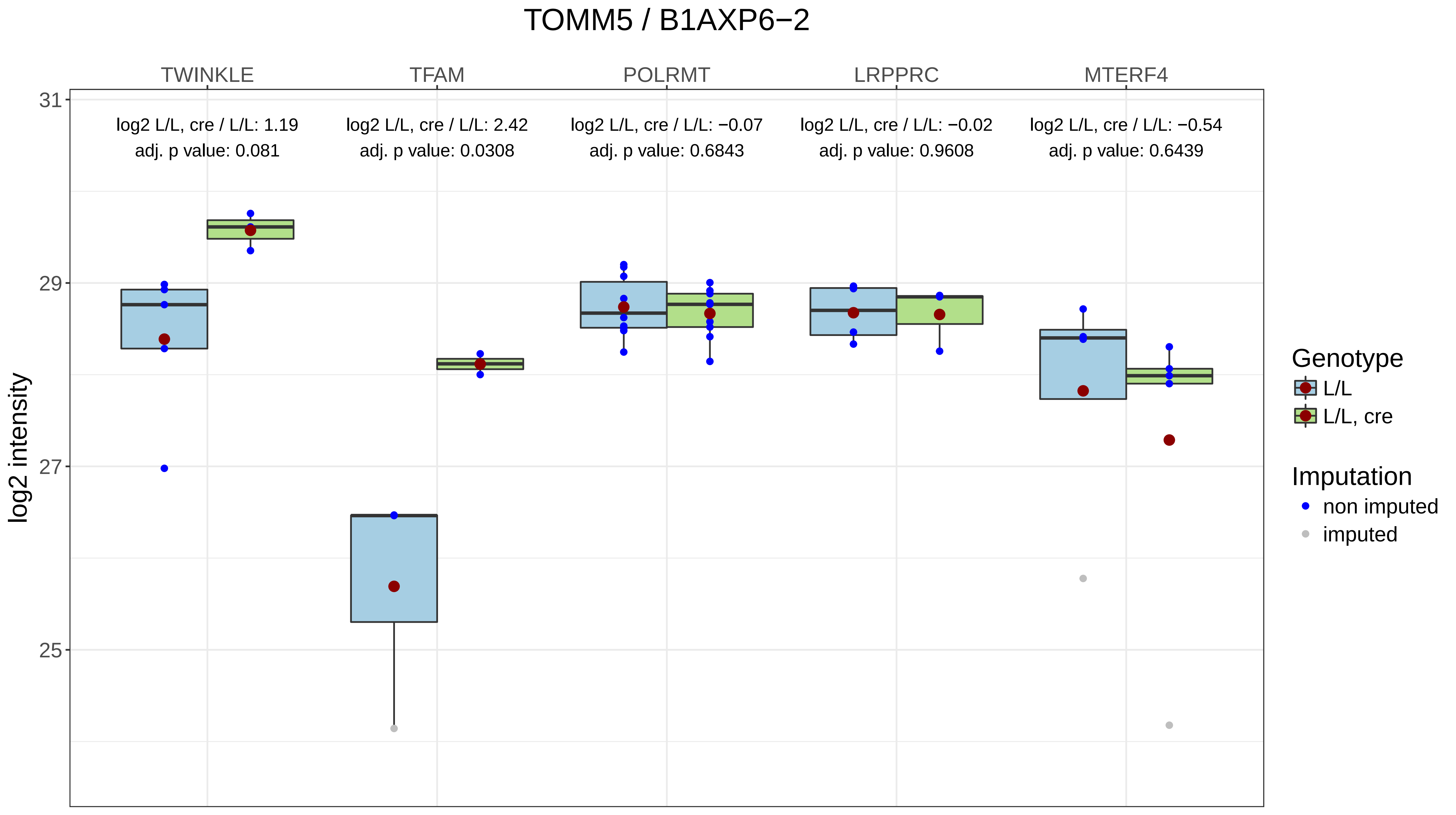
TOMM5 / B1AXP6−2
TWINKLE
TFAM
POLRMT
LRPPRC
MTERF4
log2 L/L, cre / L/L: 1.19
adj. p value: 0.081
log2 L/L, cre / L/L: 2.42
adj. p value: 0.0308
log2 L/L, cre / L/L: −0.07
adj. p value: 0.6843
log2 L/L, cre / L/L: −0.02
adj. p value: 0.9608
log2 L/L, cre / L/L: −0.54
adj. p value: 0.6439
31
29
27
25
log2 intensity
Genotype
L/L
L/L, cre
Imputation
non imputed
imputed

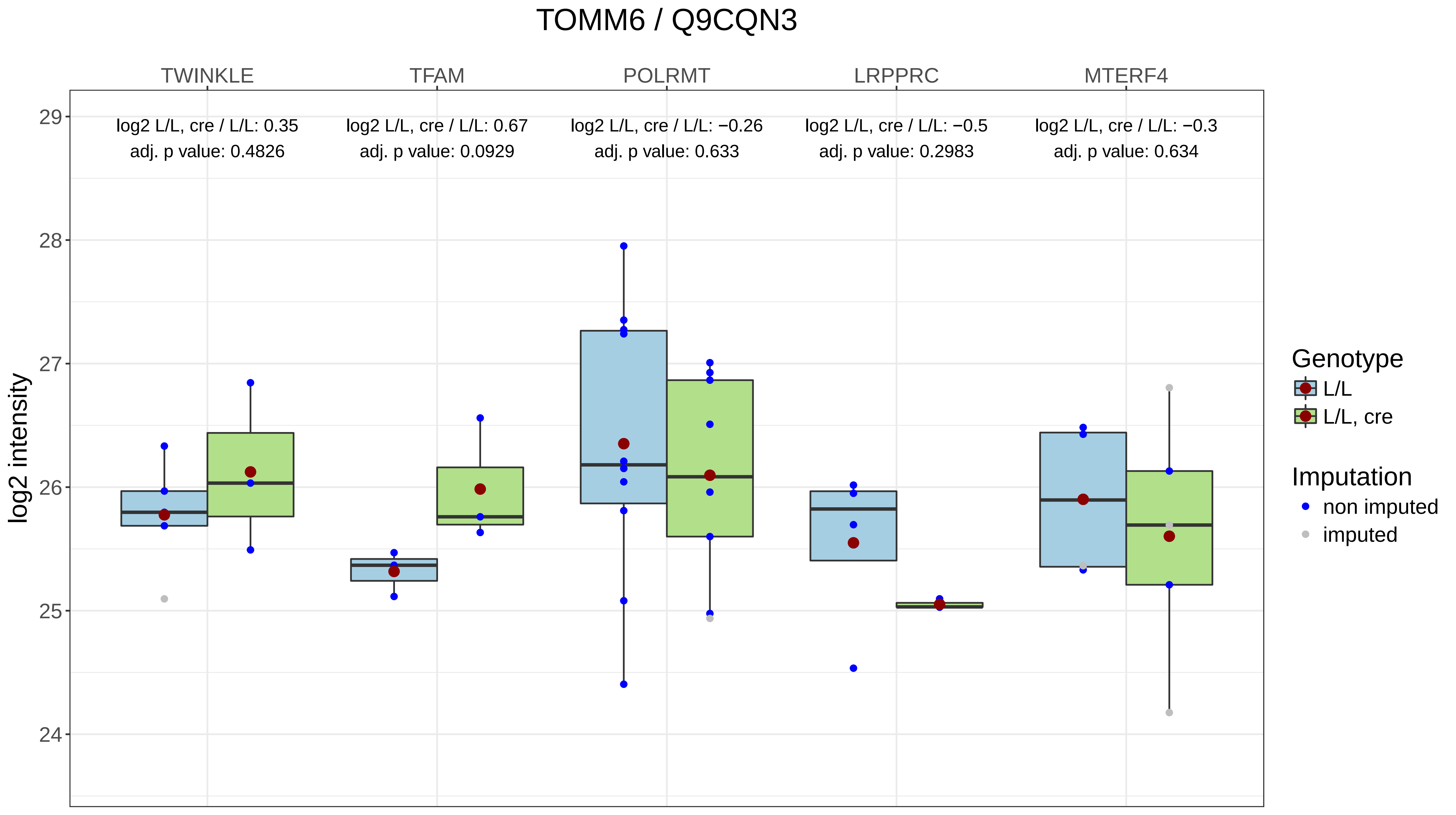
TOMM6 / Q9CQN3
TWINKLE
TFAM
POLRMT
LRPPRC
MTERF4
log2 L/L, cre / L/L: 0.35
adj. p value: 0.4826
log2 L/L, cre / L/L: 0.67
adj. p value: 0.0929
log2 L/L, cre / L/L: −0.26
adj. p value: 0.633
log2 L/L, cre / L/L: −0.5
adj. p value: 0.2983
log2 L/L, cre / L/L: −0.3
adj. p value: 0.634
log2 intensity
29
28
27
26
25
24
Genotype
L/L
L/L, cre
Imputation
non imputed
imputed

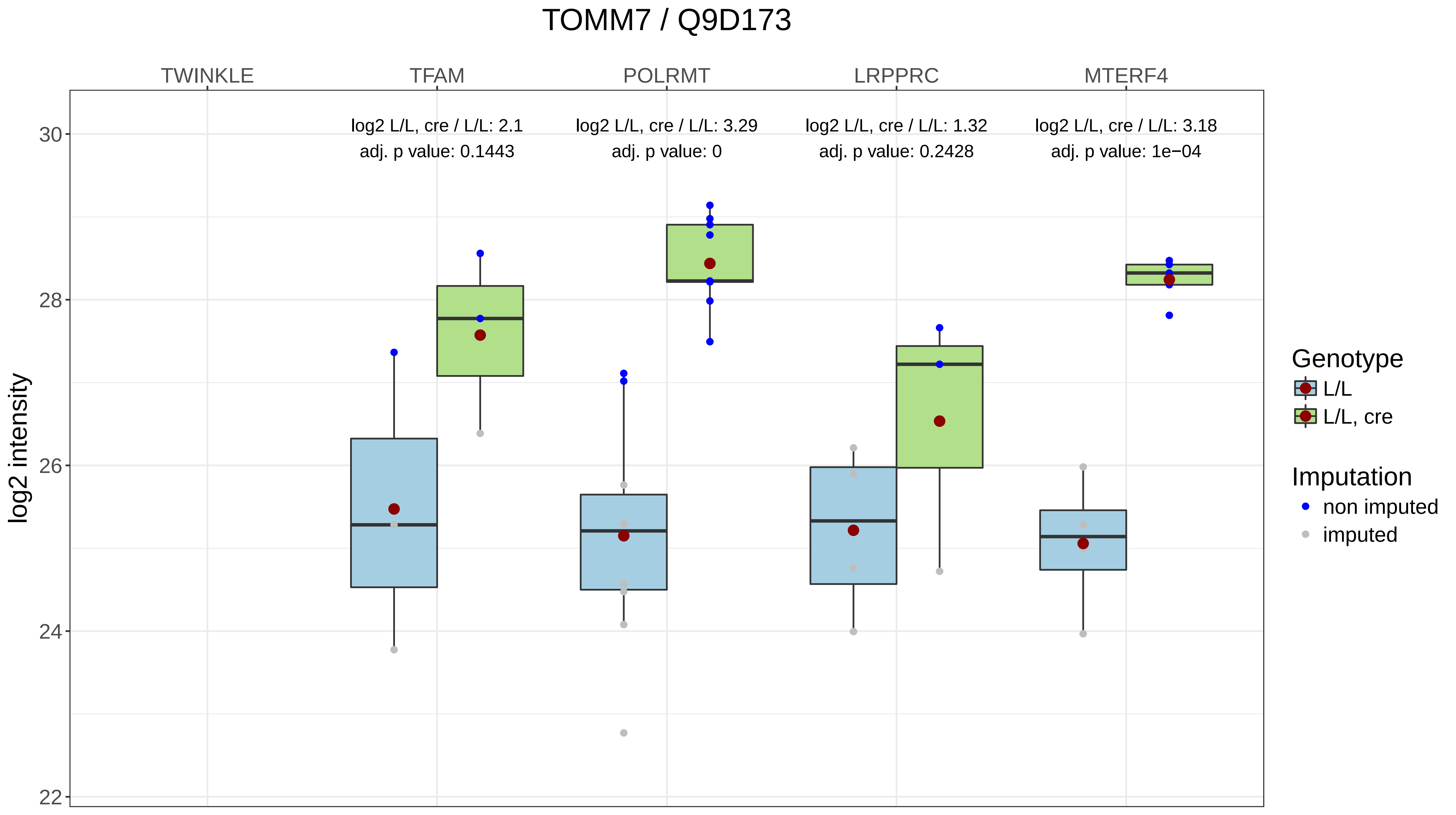

TOMM7 / Q9D173
TWINKLE
TFAM
POLRMT
LRPPRC
MTERF4
log2 L/L, cre / L/L: 2.1
adj. p value: 0.1443
log2 L/L, cre / L/L: 3.29
adj. p value: 0
log2 L/L, cre / L/L: 1.32
adj. p value: 0.2428
log2 L/L, cre / L/L: 3.18
adj. p value: 1e−04
log2 intensity
30
28
26
24
22
Genotype
L/L
L/L, cre
Imputation
non imputed
imputed

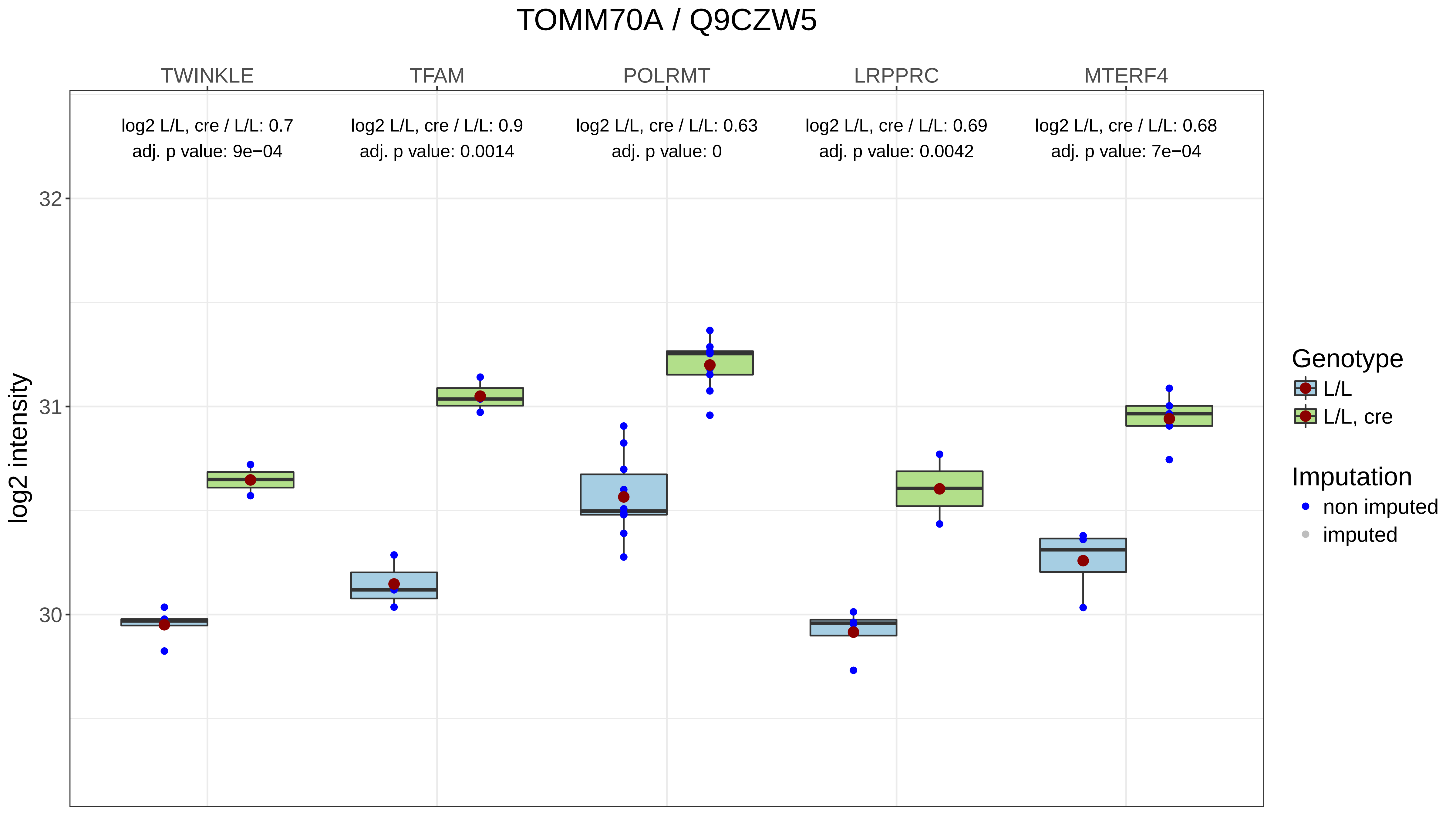
TOMM70A / Q9CZW5
TWINKLE
TFAM
POLRMT
LRPPRC
MTERF4
log2 L/L, cre / L/L: 0.7
adj. p value: 9e−04
log2 L/L, cre / L/L: 0.9
adj. p value: 0.0014
log2 L/L, cre / L/L: 0.63
adj. p value: 0
log2 L/L, cre / L/L: 0.69
adj. p value: 0.0042
log2 L/L, cre / L/L: 0.68
adj. p value: 7e−04
log2 intensity
32
31
30
Genotype
L/L
L/L, cre
Imputation
non imputed
imputed

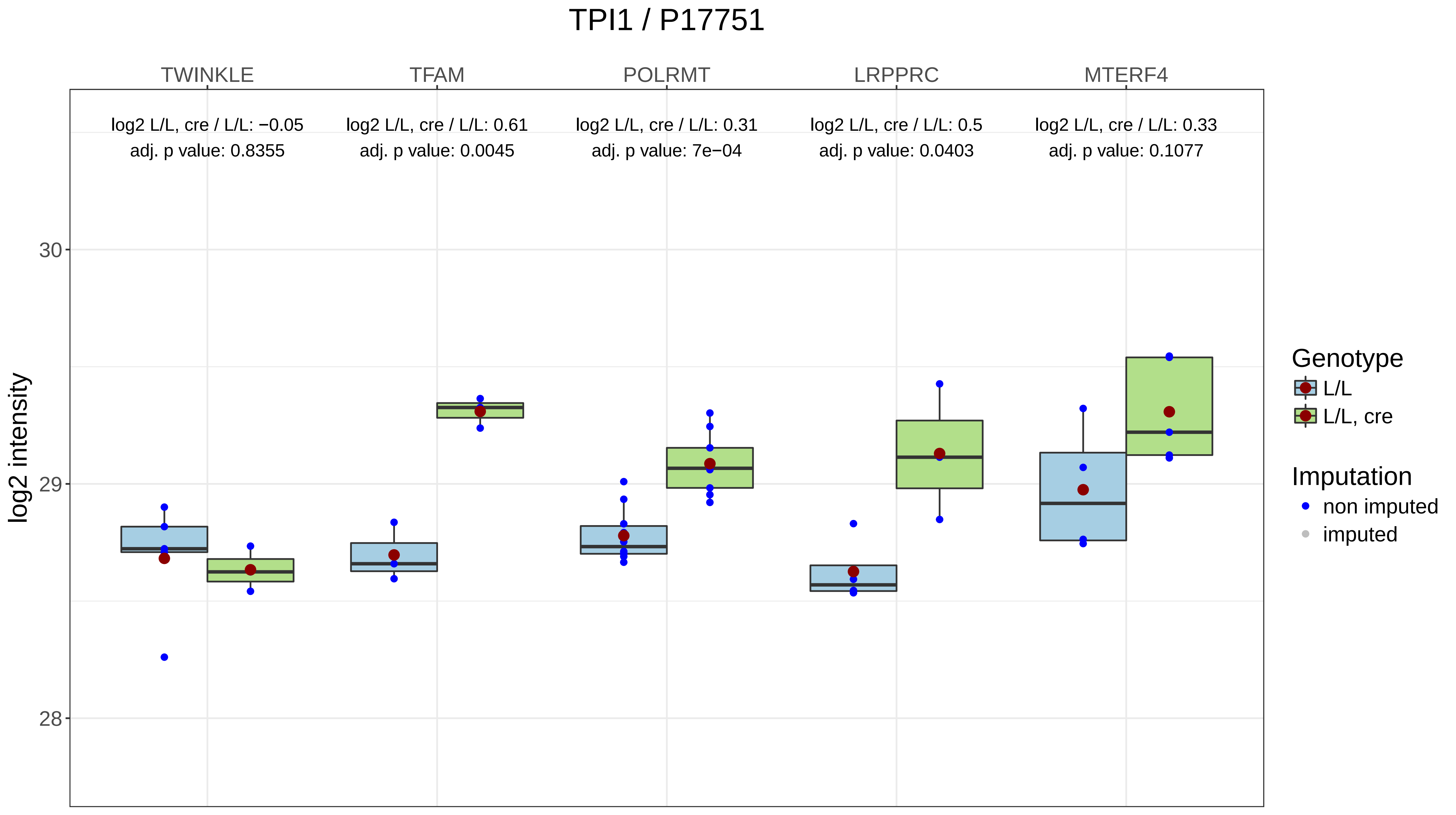
TPI1 / P17751
TWINKLE
TFAM
POLRMT
LRPPRC
MTERF4
log2 L/L, cre / L/L: −0.05
adj. p value: 0.8355
log2 L/L, cre / L/L: 0.61
adj. p value: 0.0045
log2 L/L, cre / L/L: 0.31
adj. p value: 7e−04
log2 L/L, cre / L/L: 0.5
adj. p value: 0.0403
log2 L/L, cre / L/L: 0.33
adj. p value: 0.1077
log2 intensity
30
29
28
Genotype
L/L
L/L, cre
Imputation
non imputed
imputed

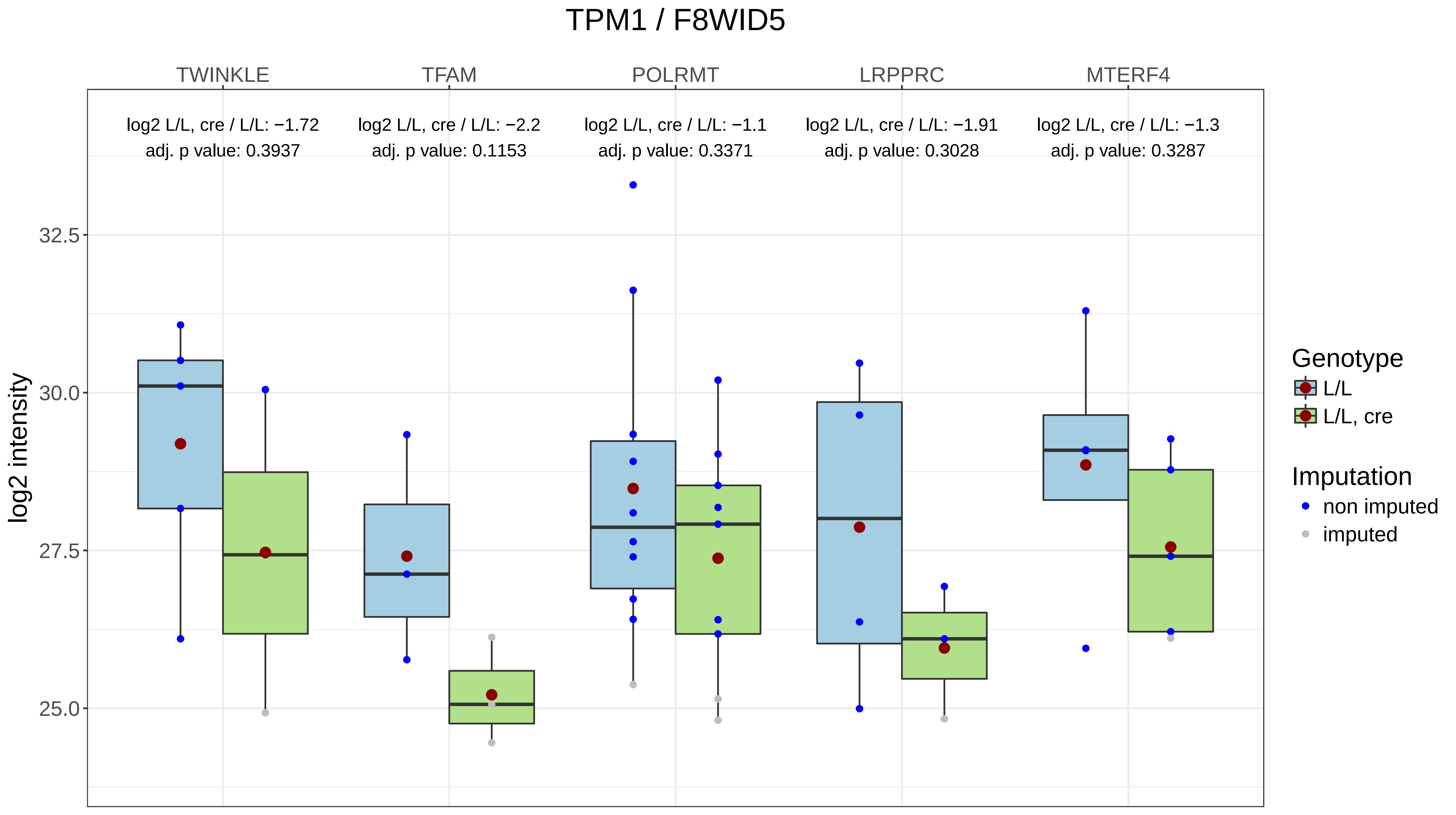
TPM1 / F8WID5
TWINKLE
TFAM
POLRMT
LRPPRC
MTERF4
log2 L/L, cre / L/L: −1.72
adj. p value: 0.3937
log2 L/L, cre / L/L: −2.2
adj. p value: 0.1153
log2 L/L, cre / L/L: −1.1
adj. p value: 0.3371
log2 L/L, cre / L/L: −1.91
adj. p value: 0.3028
log2 L/L, cre / L/L: −1.3
adj. p value: 0.3287
log2 intensity
32.5
30.0
27.5
25.0
Genotype
L/L
L/L, cre
Imputation
non imputed
imputed

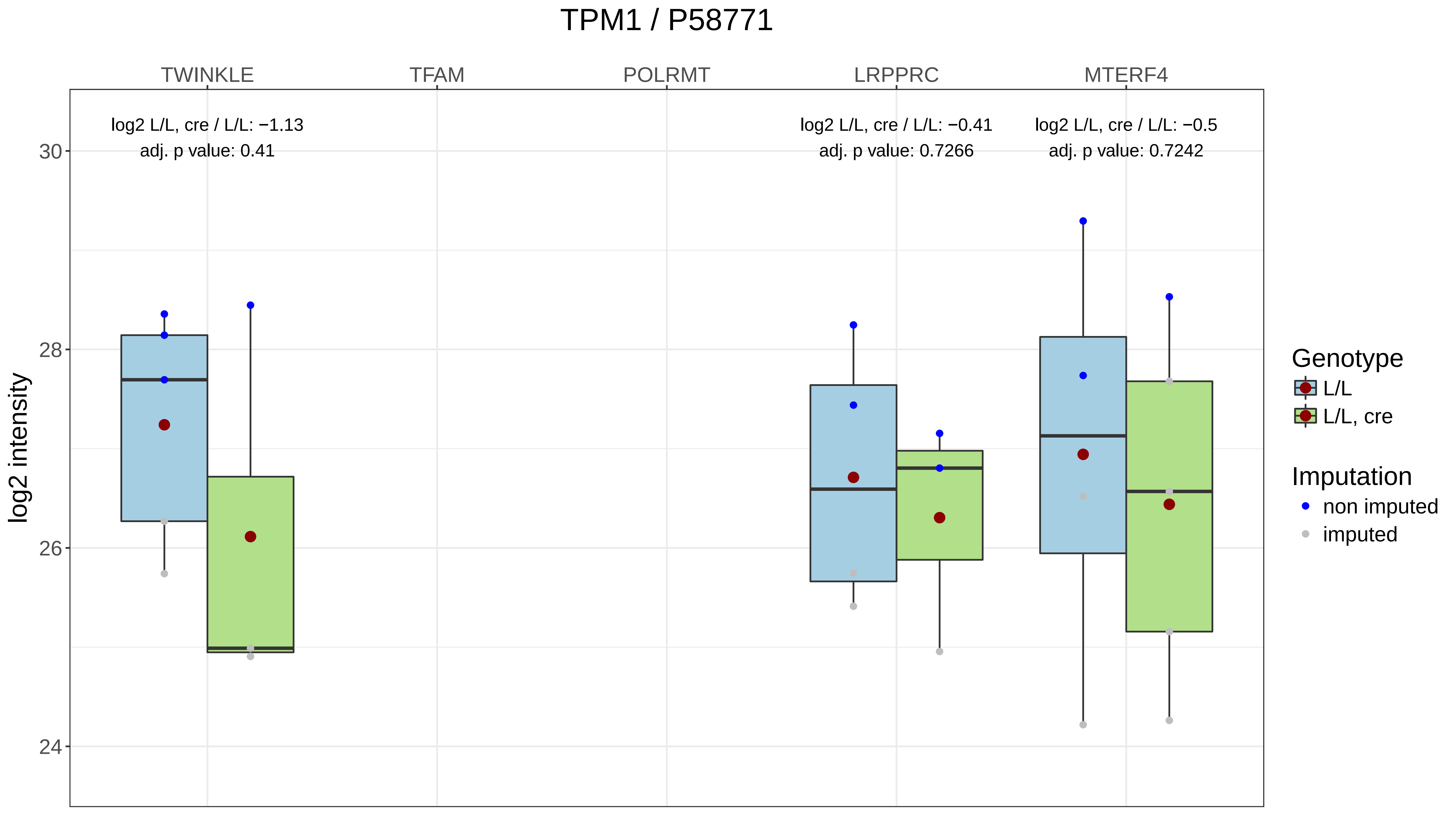

TPM1 / P58771
TWINKLE
TFAM
POLRMT
LRPPRC
MTERF4
log2 L/L, cre / L/L: −1.13
adj. p value: 0.41
log2 L/L, cre / L/L: −0.41
adj. p value: 0.7266
log2 L/L, cre / L/L: −0.5
adj. p value: 0.7242
30
28
26
24
log2 intensity
Genotype
L/L
L/L, cre
Imputation
non imputed
imputed

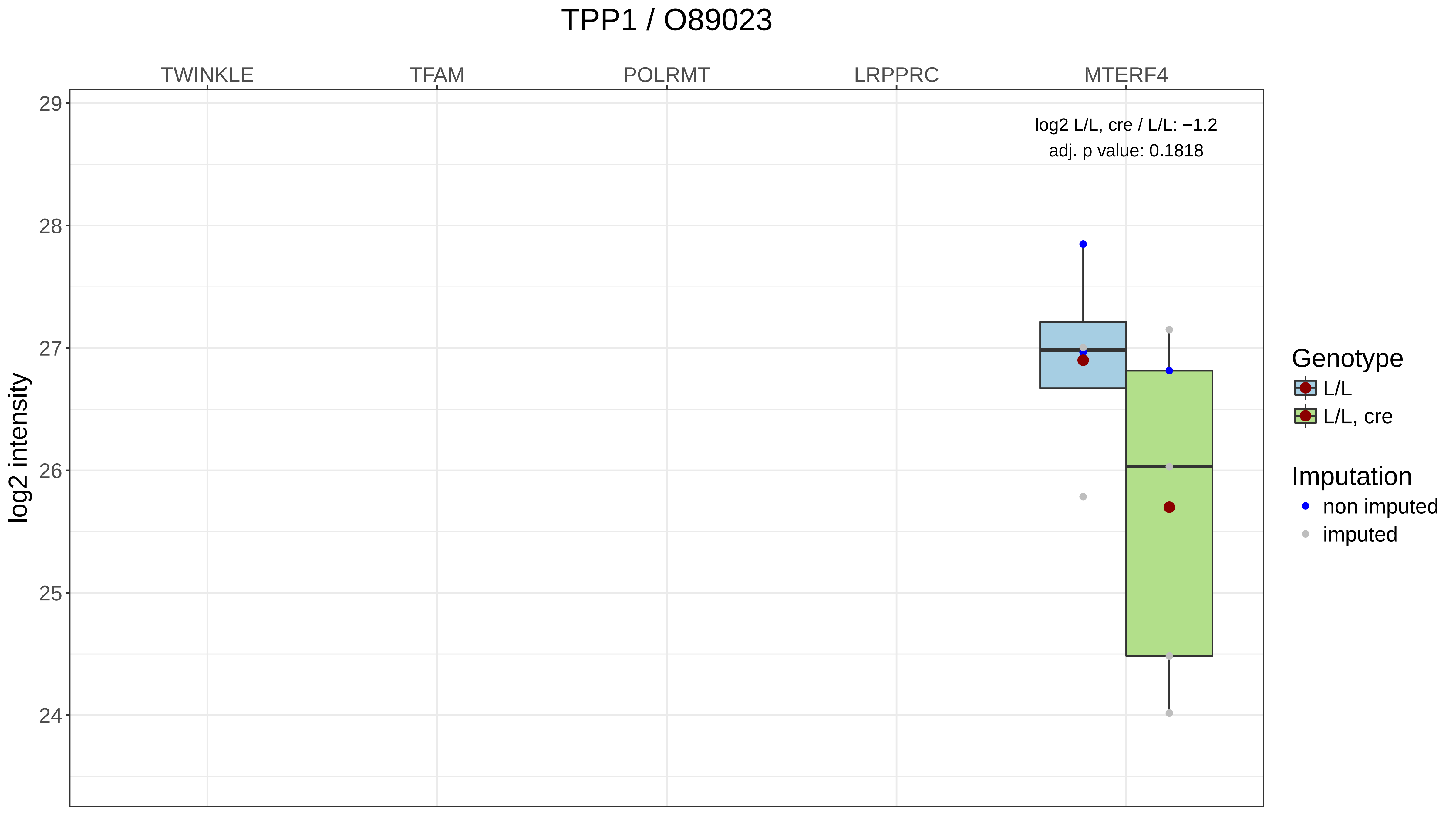

TPP1 / O89023
TWINKLE
TFAM
POLRMT
LRPPRC
MTERF4
log2 L/L, cre / L/L: −1.2
adj. p value: 0.1818
log2 intensity
29
28
27
26
25
24
Genotype
L/L
L/L, cre
Imputation
non imputed
imputed

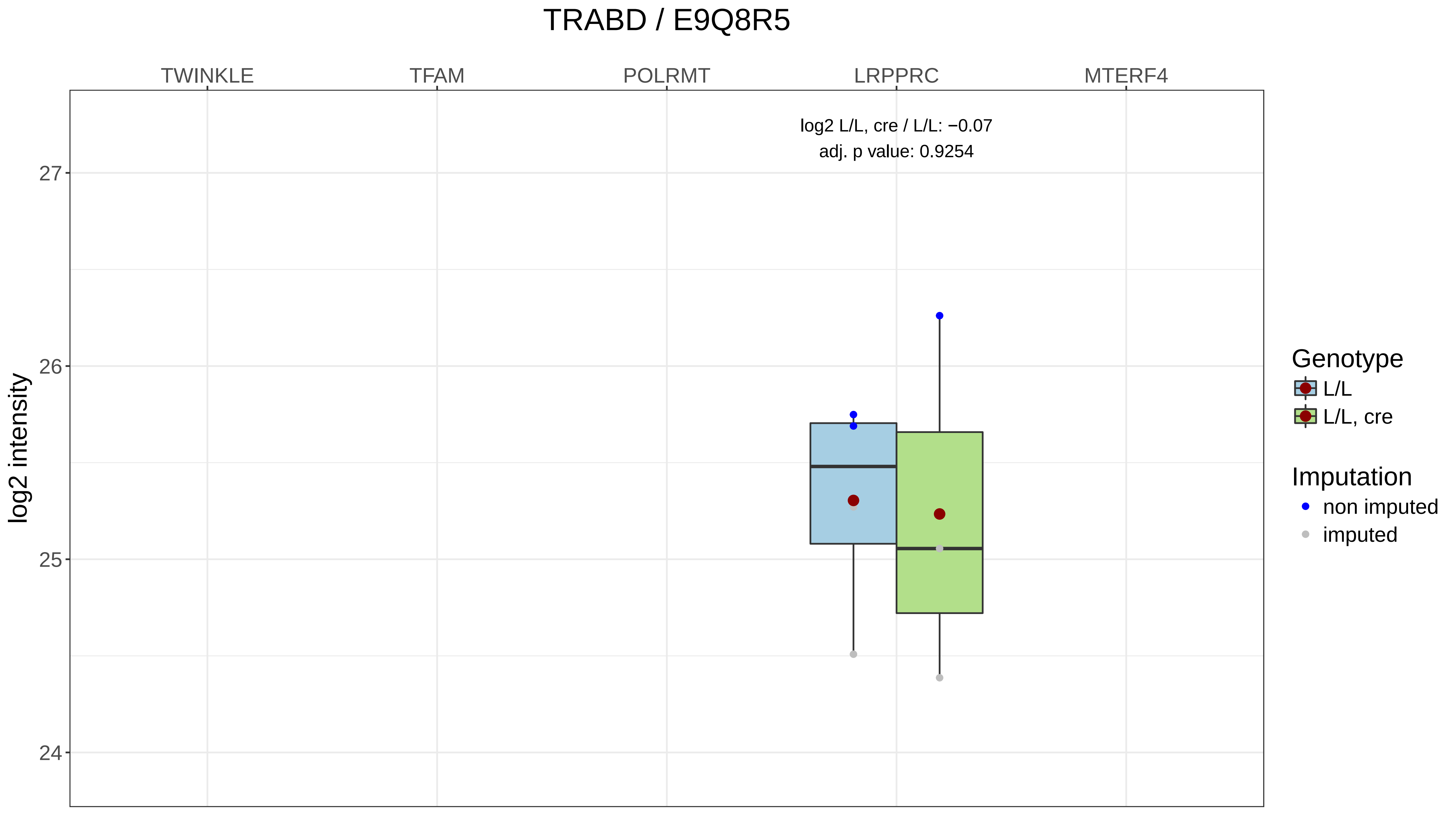
TRABD / E9Q8R5
TWINKLE
TFAM
POLRMT
LRPPRC
MTERF4
log2 L/L, cre / L/L: −0.07
adj. p value: 0.9254
27
26
25
24
log2 intensity
Genotype
L/L
L/L, cre
Imputation
non imputed
imputed

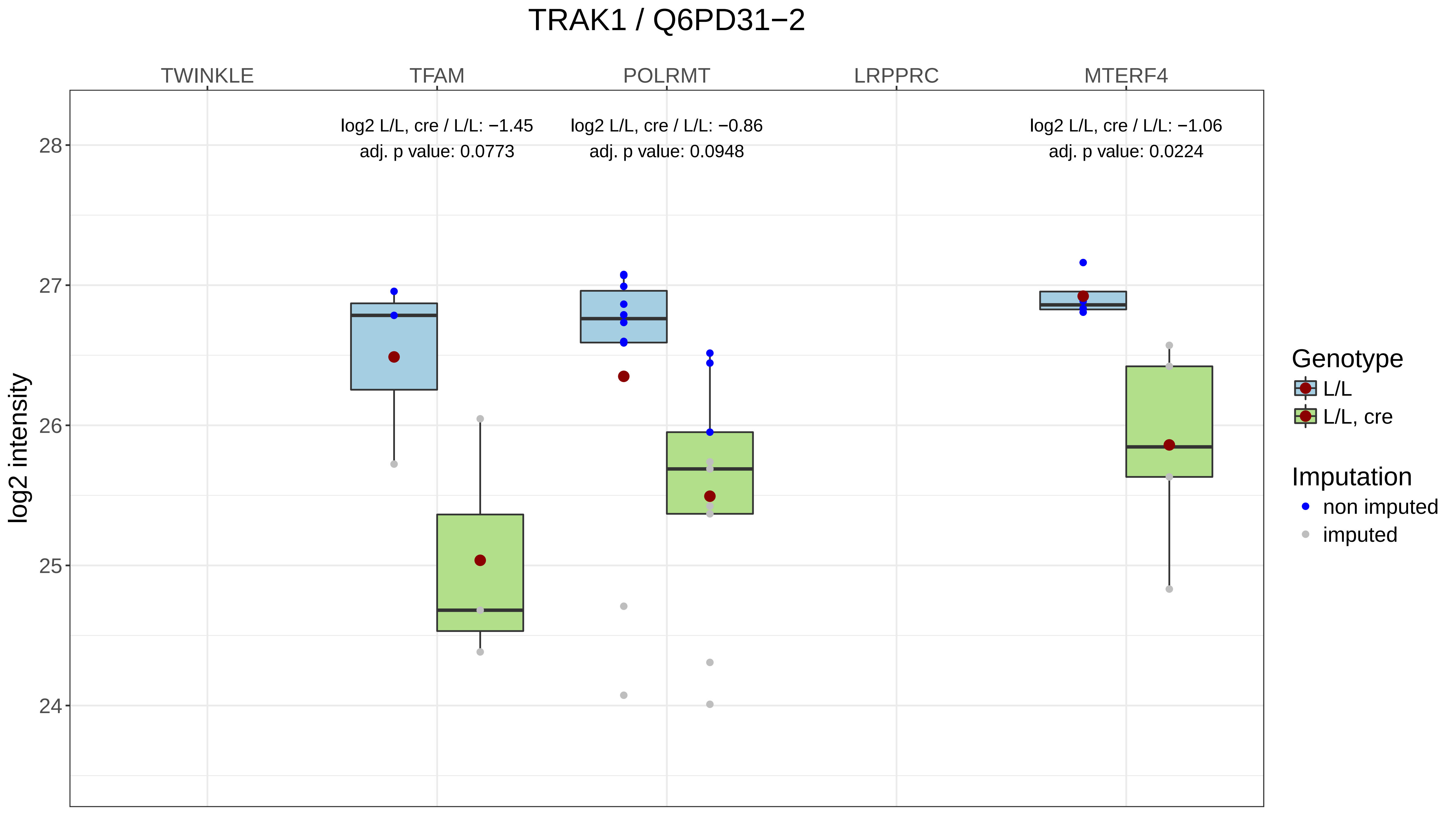

TRAK1 / Q6PD31−2
TWINKLE
TFAM
POLRMT
LRPPRC
MTERF4
log2 L/L, cre / L/L: −1.45
adj. p value: 0.0773
log2 L/L, cre / L/L: −0.86
adj. p value: 0.0948
log2 L/L, cre / L/L: −1.06
adj. p value: 0.0224
log2 intensity
28
27
26
25
24
Genotype
L/L
L/L, cre
Imputation
non imputed
imputed

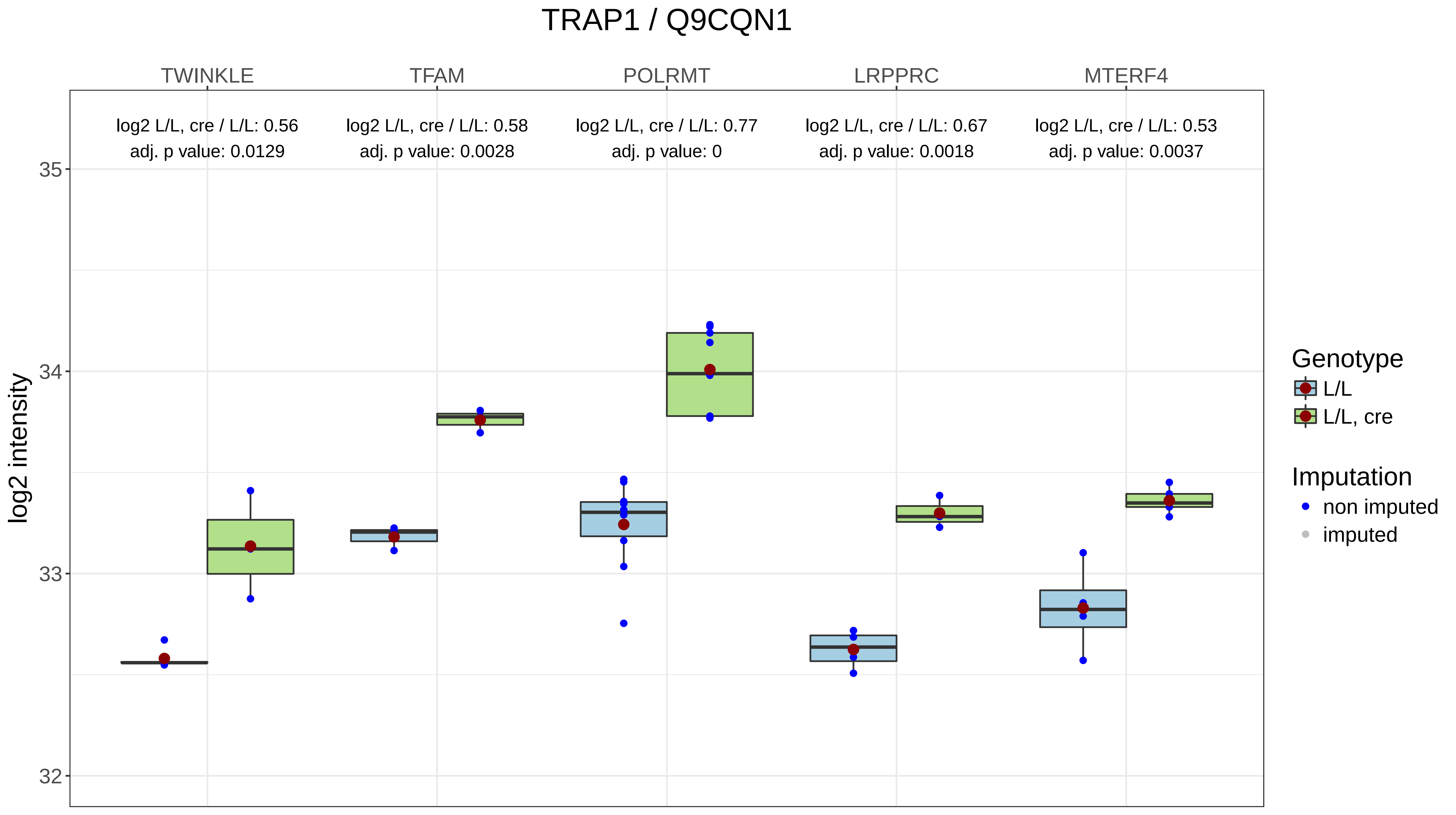
TRAP1 / Q9CQN1
TWINKLE
TFAM
POLRMT
LRPPRC
MTERF4
log2 L/L, cre / L/L: 0.56
adj. p value: 0.0129
log2 L/L, cre / L/L: 0.58
adj. p value: 0.0028
log2 L/L, cre / L/L: 0.77
adj. p value: 0
log2 L/L, cre / L/L: 0.67
adj. p value: 0.0018
log2 L/L, cre / L/L: 0.53
adj. p value: 0.0037
log2 intensity
35
34
33
32
Genotype
L/L
L/L, cre
Imputation
non imputed
imputed

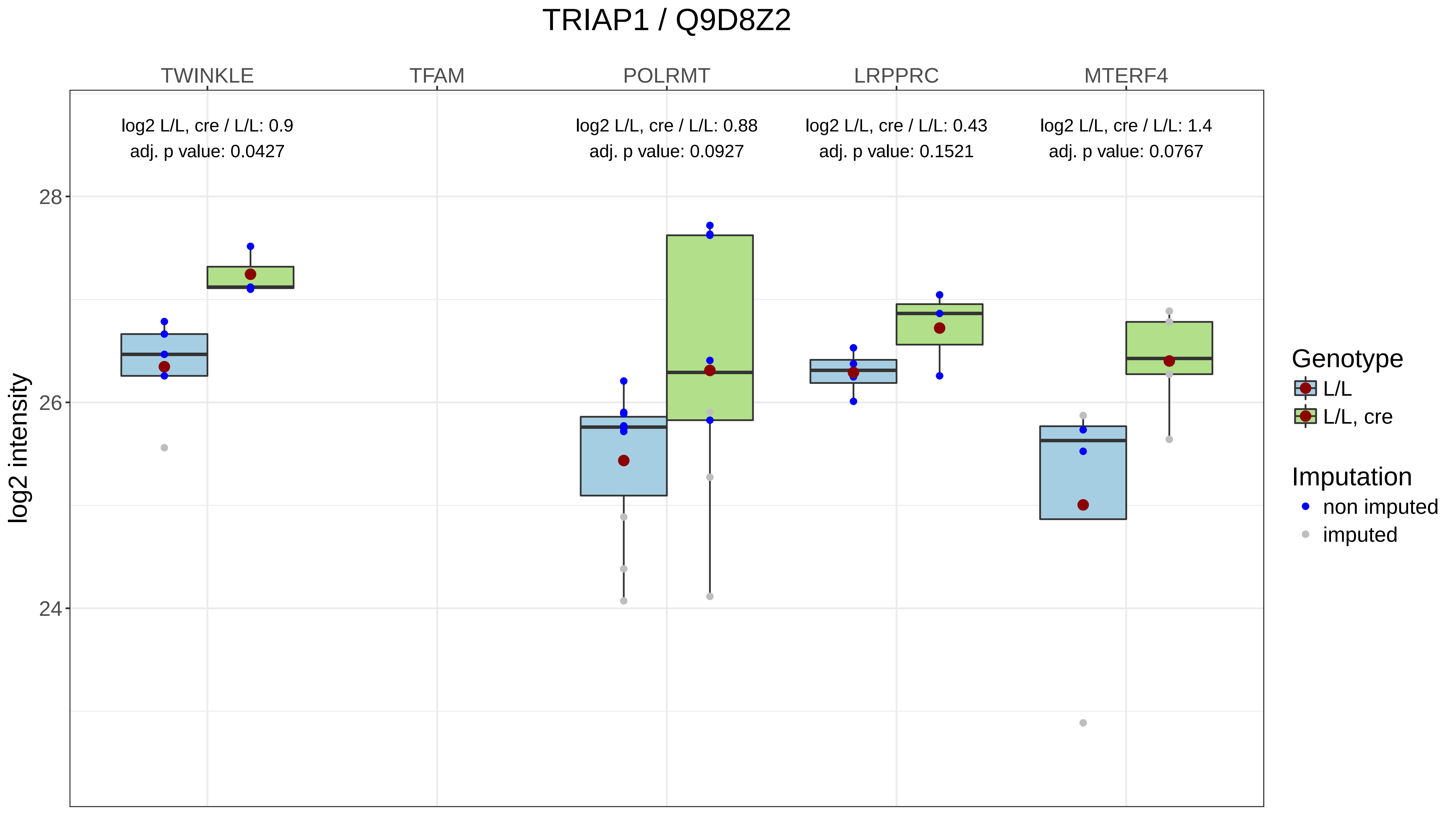
TRIAP1 / Q9D8Z2
TWINKLE
TFAM
POLRMT
LRPPRC
MTERF4
log2 L/L, cre / L/L: 0.9
adj. p value: 0.0427
log2 L/L, cre / L/L: 0.88
adj. p value: 0.0927
log2 L/L, cre / L/L: 0.43
adj. p value: 0.1521
log2 L/L, cre / L/L: 1.4
adj. p value: 0.0767
28
26
24
log2 intensity
Genotype
L/L
L/L, cre
Imputation
non imputed
imputed

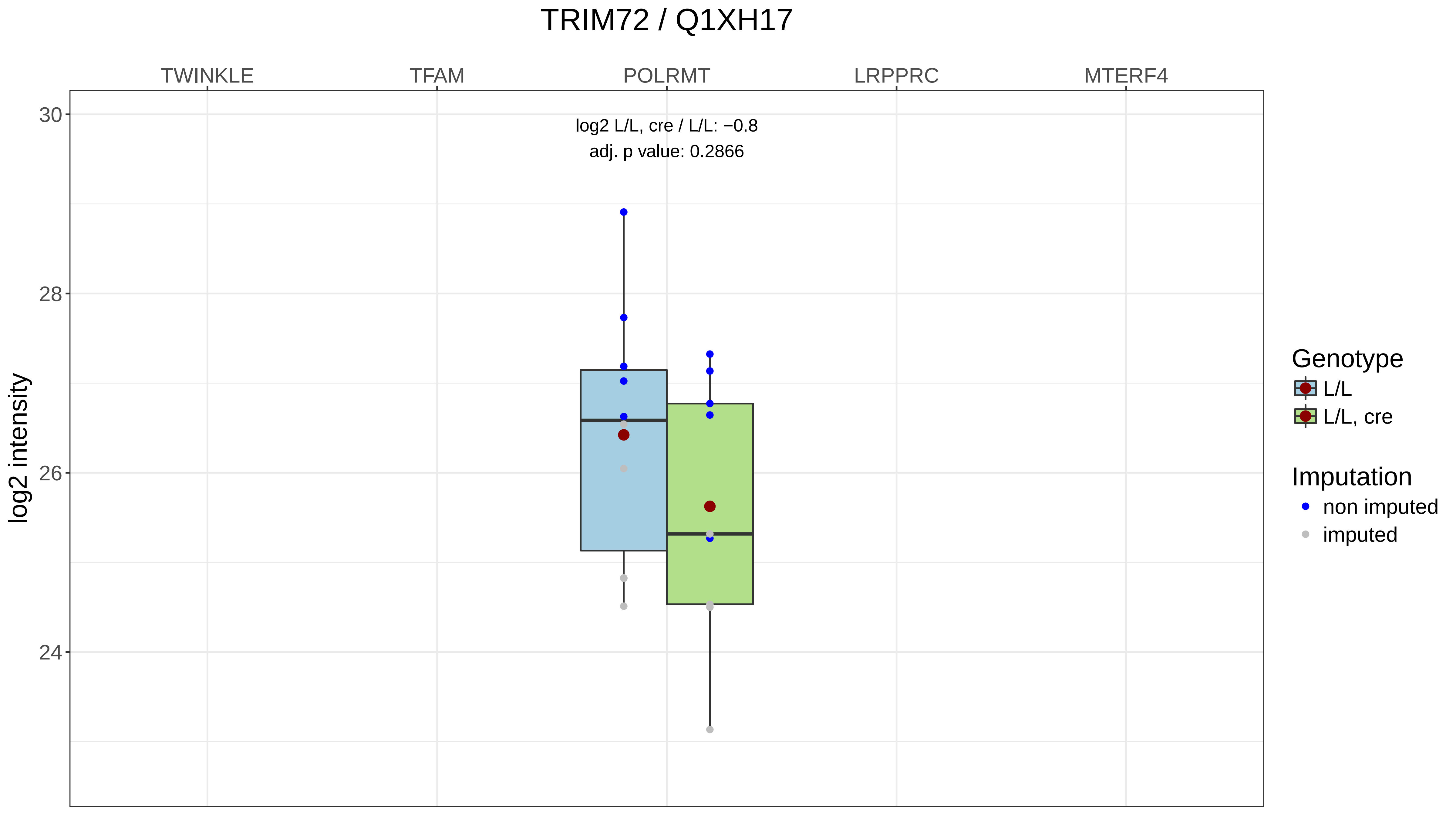

TRIM72 / Q1XH17
TWINKLE
TFAM
POLRMT
LRPPRC
MTERF4
log2 L/L, cre / L/L: −0.8
adj. p value: 0.2866
30
28
26
24
log2 intensity
Genotype
L/L
L/L, cre
Imputation
non imputed
imputed

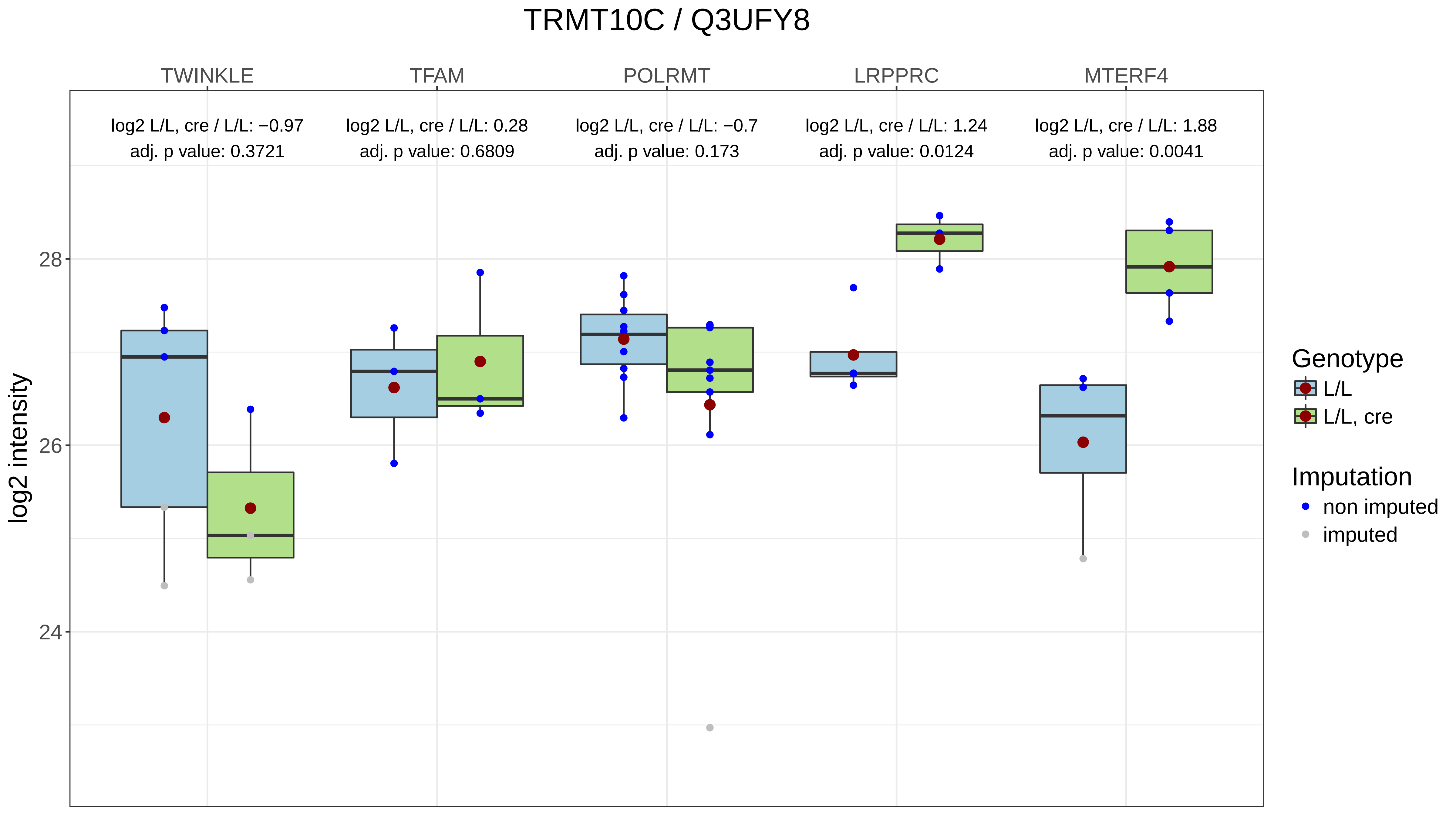
TRMT10C / Q3UFY8
TWINKLE
TFAM
POLRMT
LRPPRC
MTERF4
log2 L/L, cre / L/L: −0.97
adj. p value: 0.3721
log2 L/L, cre / L/L: 0.28
adj. p value: 0.6809
log2 L/L, cre / L/L: −0.7
adj. p value: 0.173
log2 L/L, cre / L/L: 1.24
adj. p value: 0.0124
log2 L/L, cre / L/L: 1.88
adj. p value: 0.0041
log2 intensity
28
26
24
Genotype
L/L
L/L, cre
Imputation
non imputed
imputed

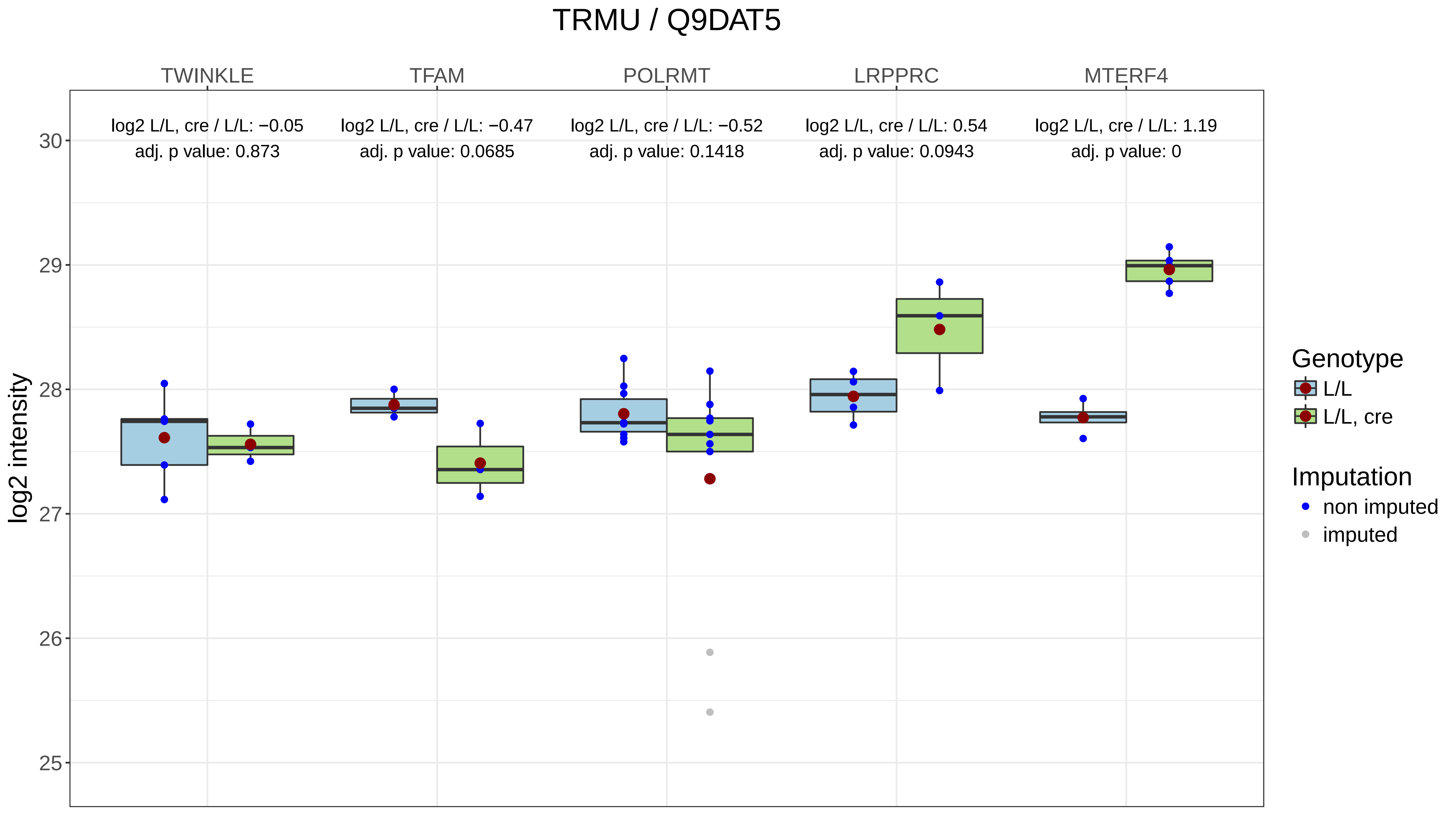

TRMU / Q9DAT5
TWINKLE
TFAM
POLRMT
LRPPRC
MTERF4
log2 L/L, cre / L/L: −0.05
adj. p value: 0.873
log2 L/L, cre / L/L: −0.47
adj. p value: 0.0685
log2 L/L, cre / L/L: −0.52
adj. p value: 0.1418
log2 L/L, cre / L/L: 0.54
adj. p value: 0.0943
log2 L/L, cre / L/L: 1.19
adj. p value: 0
log2 intensity
30
29
28
27
26
25
Genotype
L/L
L/L, cre
Imputation
non imputed
imputed

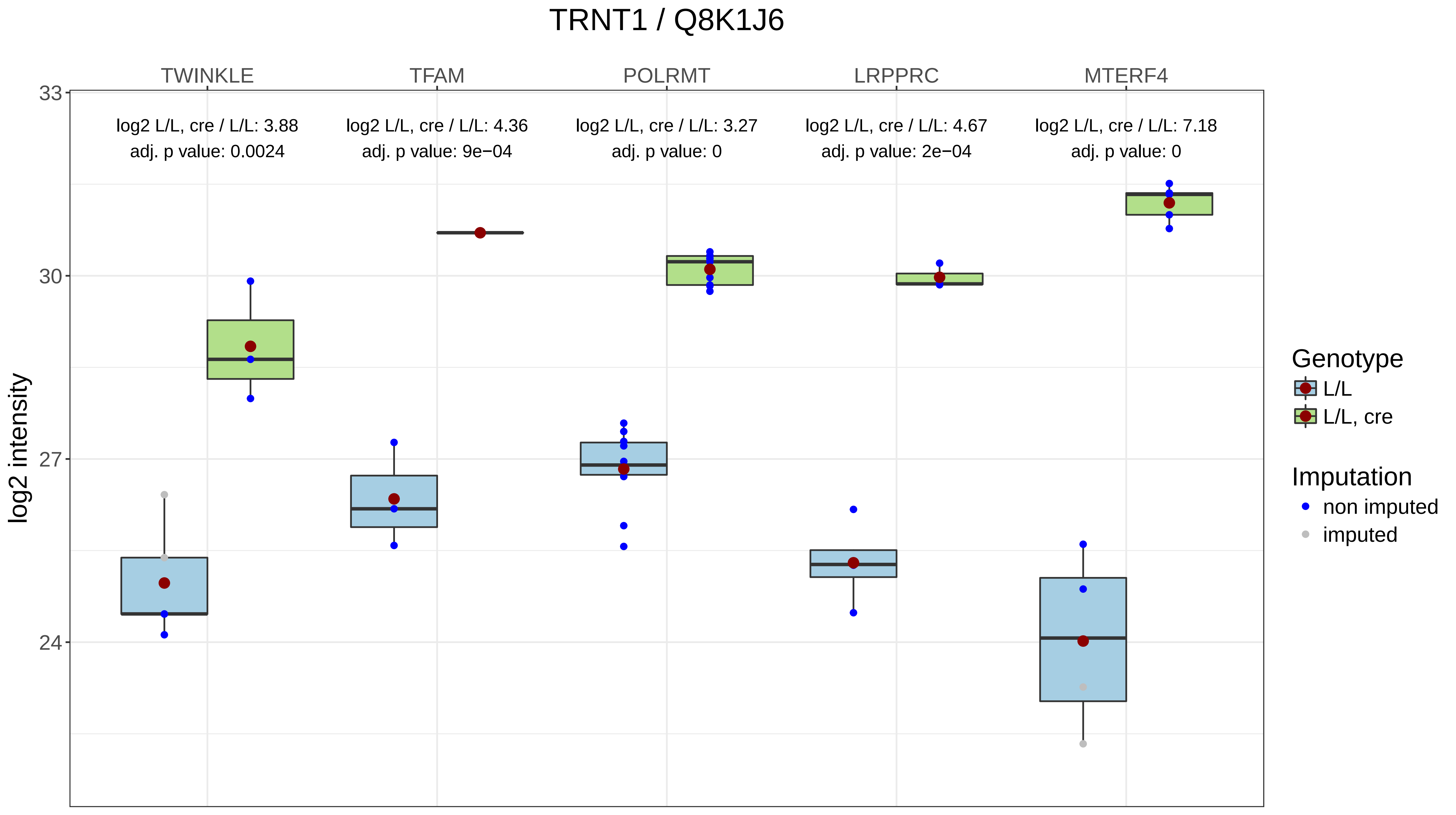
TRNT1 / Q8K1J6
TWINKLE
TFAM
POLRMT
LRPPRC
MTERF4
log2 L/L, cre / L/L: 3.88
adj. p value: 0.0024
log2 L/L, cre / L/L: 4.36
adj. p value: 9e−04
log2 L/L, cre / L/L: 3.27
adj. p value: 0
log2 L/L, cre / L/L: 4.67
adj. p value: 2e−04
log2 L/L, cre / L/L: 7.18
adj. p value: 0
log2 intensity
33
30
27
24
Genotype
L/L
L/L, cre
Imputation
non imputed
imputed

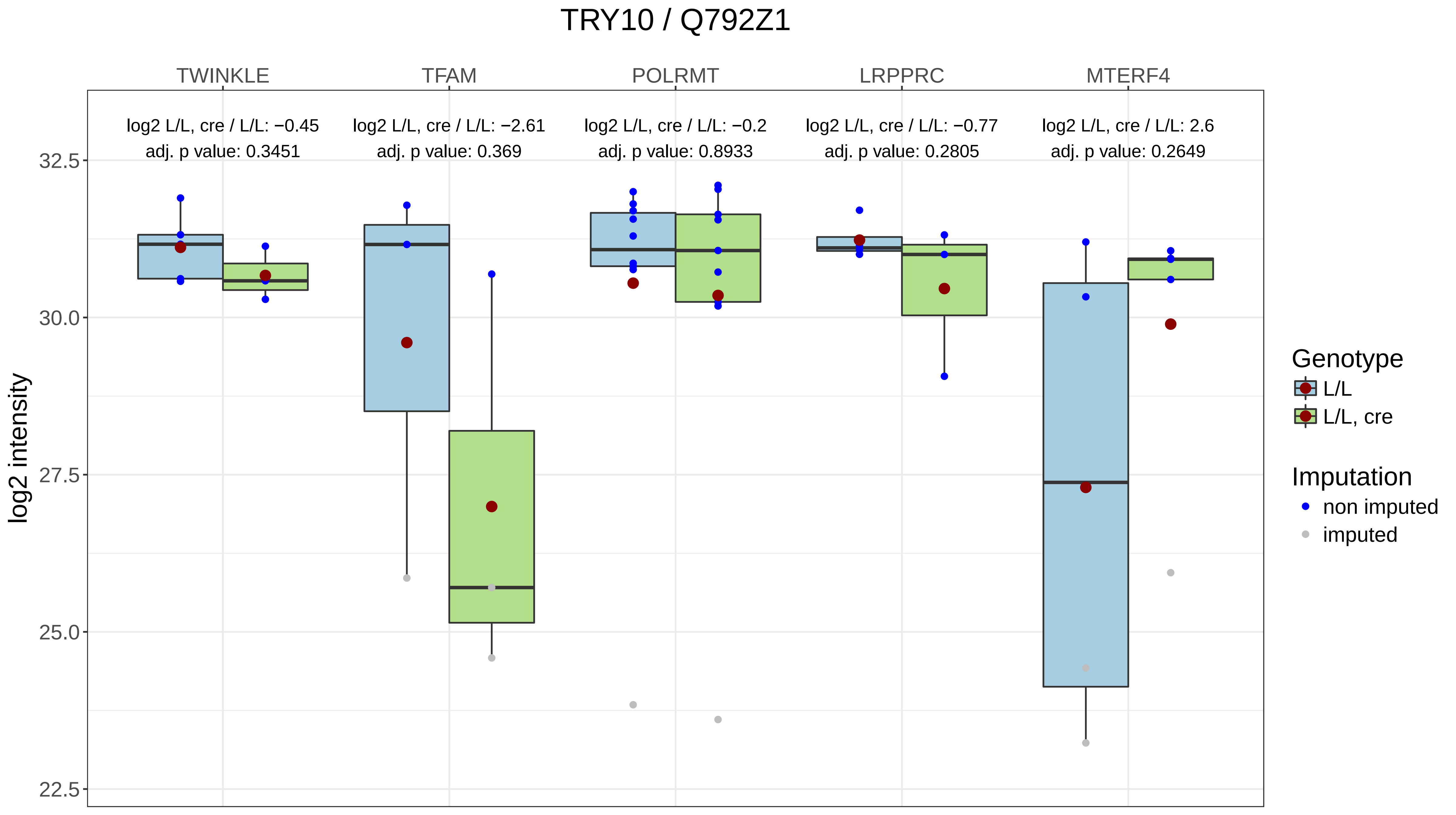
TRY10 / Q792Z1
TWINKLE
TFAM
POLRMT
LRPPRC
MTERF4
log2 L/L, cre / L/L: −0.45
adj. p value: 0.3451
log2 L/L, cre / L/L: −2.61
adj. p value: 0.369
log2 L/L, cre / L/L: −0.2
adj. p value: 0.8933
log2 L/L, cre / L/L: −0.77
adj. p value: 0.2805
log2 L/L, cre / L/L: 2.6
adj. p value: 0.2649
log2 intensity
32.5
30.0
27.5
25.0
22.5
Genotype
L/L
L/L, cre
Imputation
non imputed
imputed

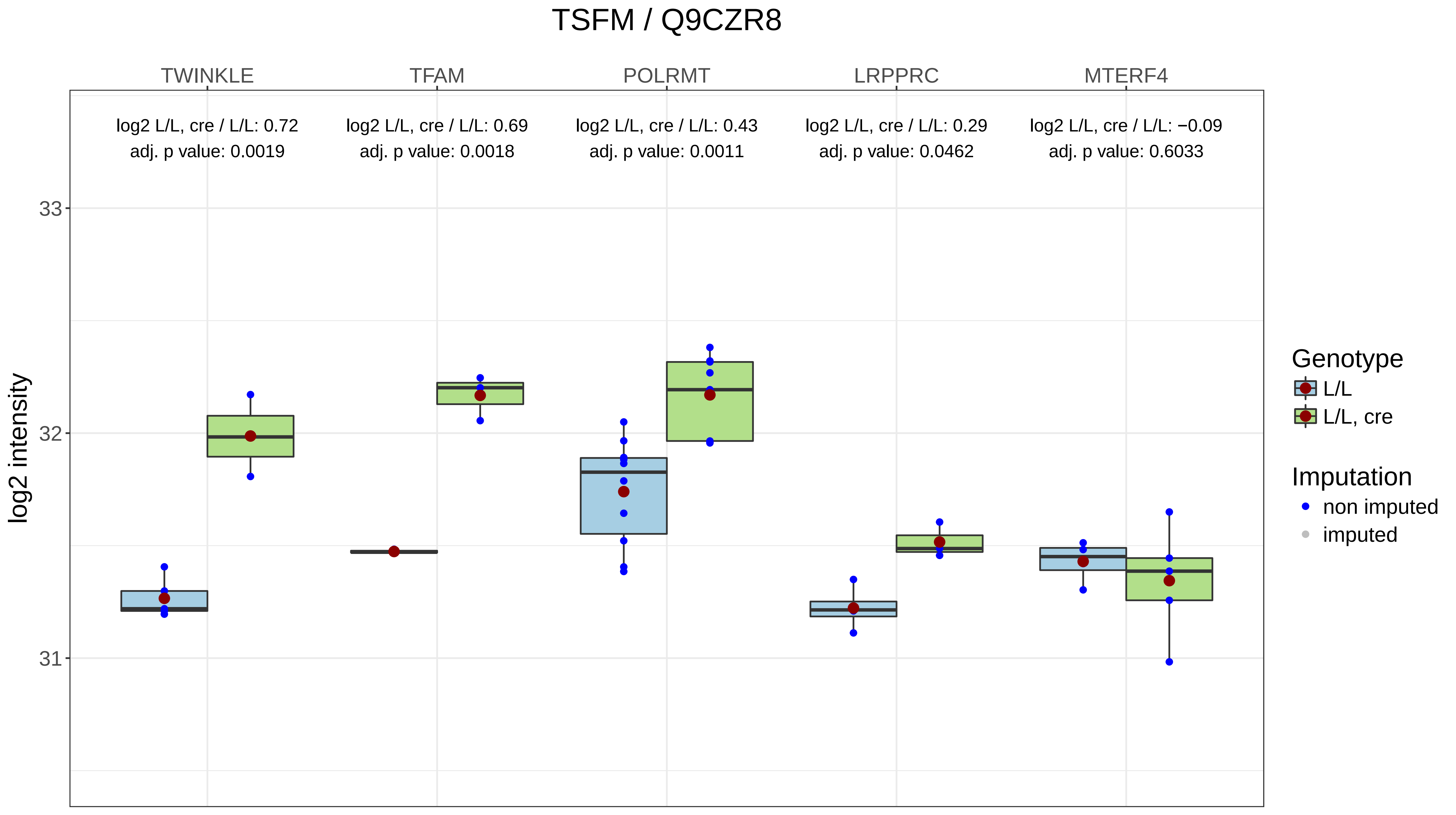
TSFM / Q9CZR8
TWINKLE
TFAM
POLRMT
LRPPRC
MTERF4
log2 L/L, cre / L/L: 0.72
adj. p value: 0.0019
log2 L/L, cre / L/L: 0.69
adj. p value: 0.0018
log2 L/L, cre / L/L: 0.43
adj. p value: 0.0011
log2 L/L, cre / L/L: 0.29
adj. p value: 0.0462
log2 L/L, cre / L/L: −0.09
adj. p value: 0.6033
33
32
31
log2 intensity
Genotype
L/L
L/L, cre
Imputation
non imputed
imputed

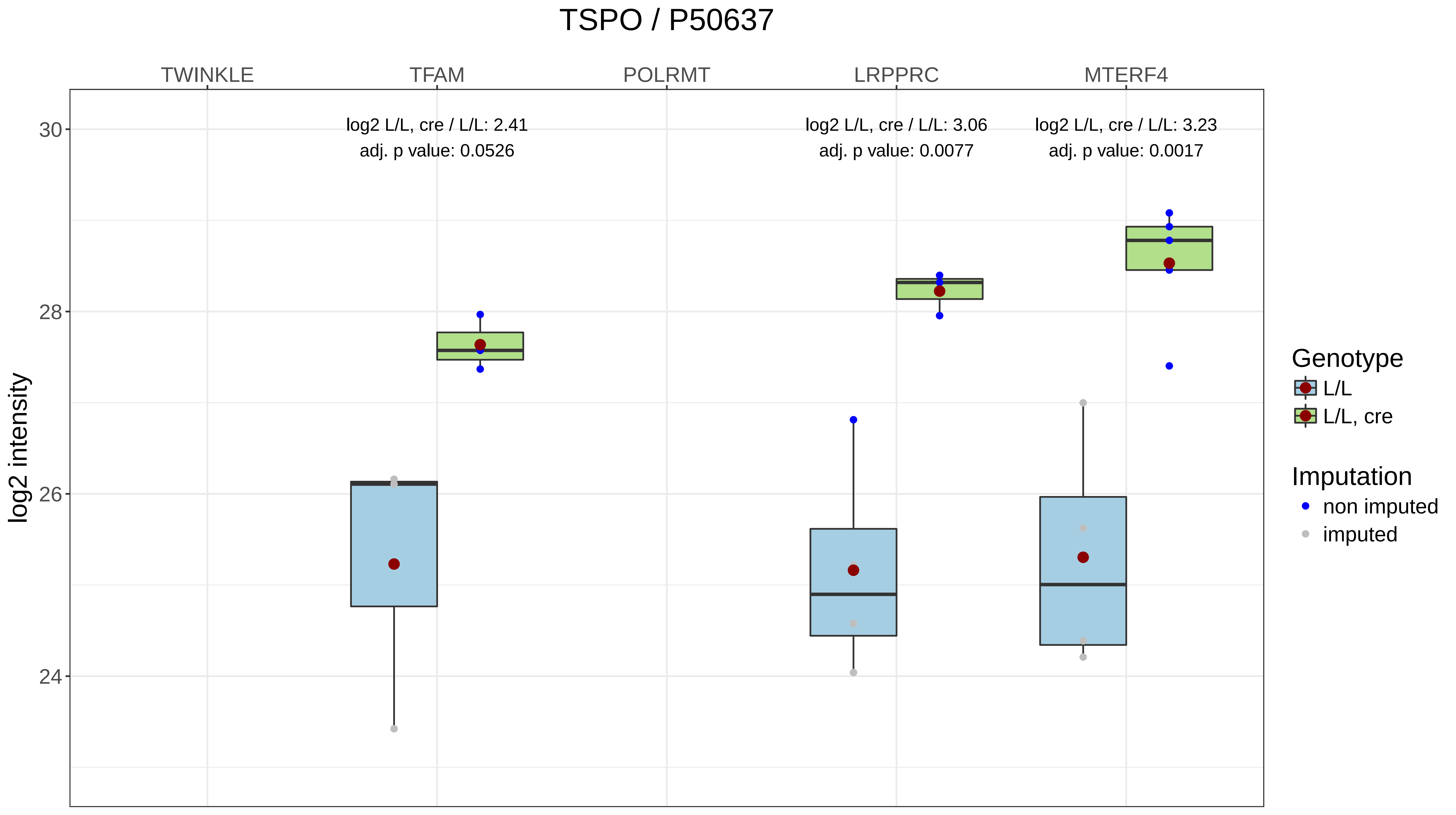
TSPO / P50637
TWINKLE
TFAM
POLRMT
LRPPRC
MTERF4
log2 L/L, cre / L/L: 2.41
adj. p value: 0.0526
log2 L/L, cre / L/L: 3.06
adj. p value: 0.0077
log2 L/L, cre / L/L: 3.23
adj. p value: 0.0017
30
28
26
24
log2 intensity
Genotype
L/L
L/L, cre
Imputation
non imputed
imputed

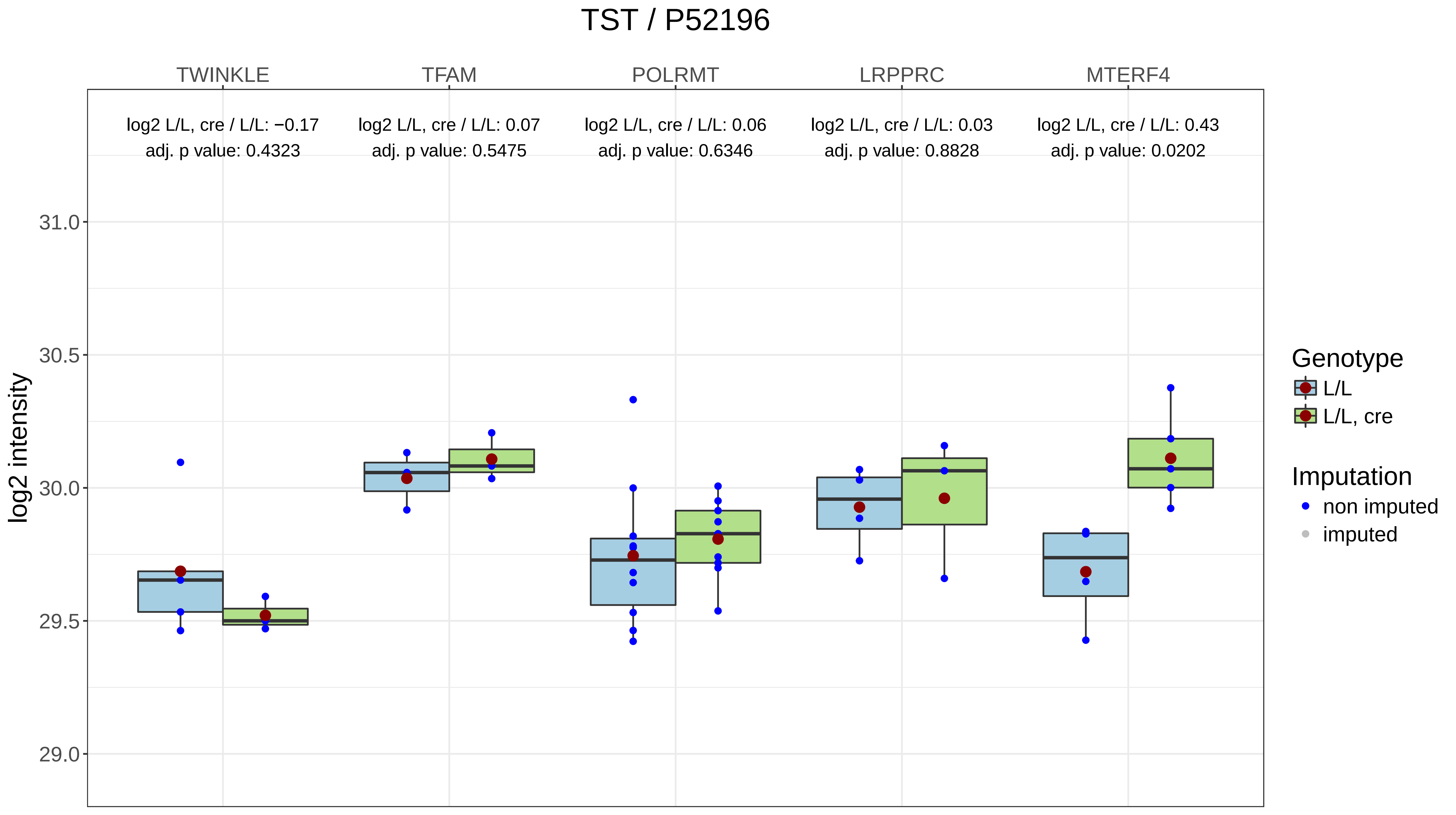
TST / P52196
TWINKLE
TFAM
POLRMT
LRPPRC
MTERF4
log2 L/L, cre / L/L: −0.17
adj. p value: 0.4323
log2 L/L, cre / L/L: 0.07
adj. p value: 0.5475
log2 L/L, cre / L/L: 0.06
adj. p value: 0.6346
log2 L/L, cre / L/L: 0.03
adj. p value: 0.8828
log2 L/L, cre / L/L: 0.43
adj. p value: 0.0202
log2 intensity
31.0
30.5
30.0
29.5
29.0
Genotype
L/L
L/L, cre
Imputation
non imputed
imputed

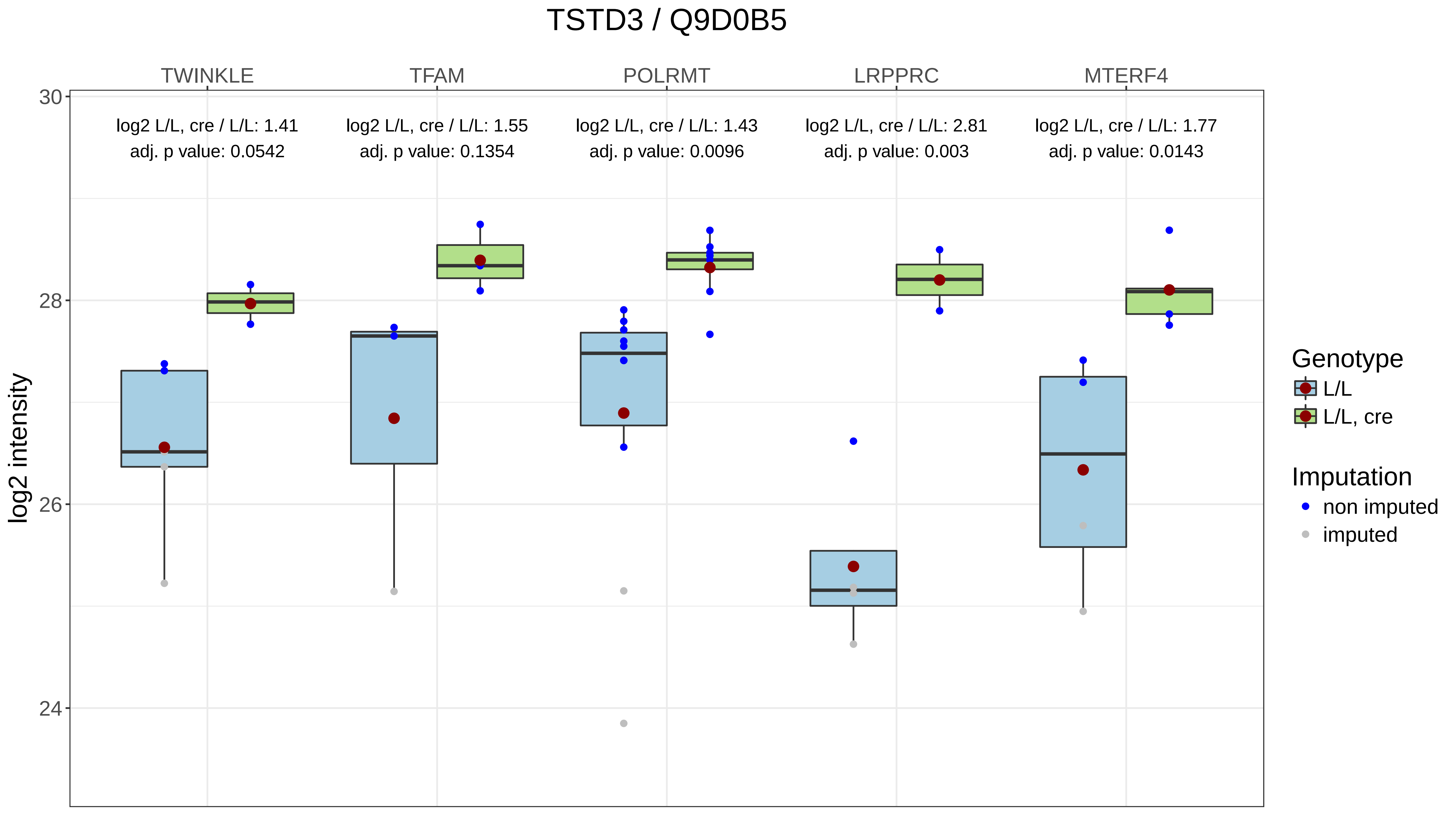

TSTD3 / Q9D0B5
TWINKLE
TFAM
POLRMT
LRPPRC
MTERF4
log2 L/L, cre / L/L: 1.41
adj. p value: 0.0542
log2 L/L, cre / L/L: 1.55
adj. p value: 0.1354
log2 L/L, cre / L/L: 1.43
adj. p value: 0.0096
log2 L/L, cre / L/L: 2.81
adj. p value: 0.003
log2 L/L, cre / L/L: 1.77
adj. p value: 0.0143
log2 intensity
30
28
26
24
Genotype
L/L
L/L, cre
Imputation
non imputed
imputed

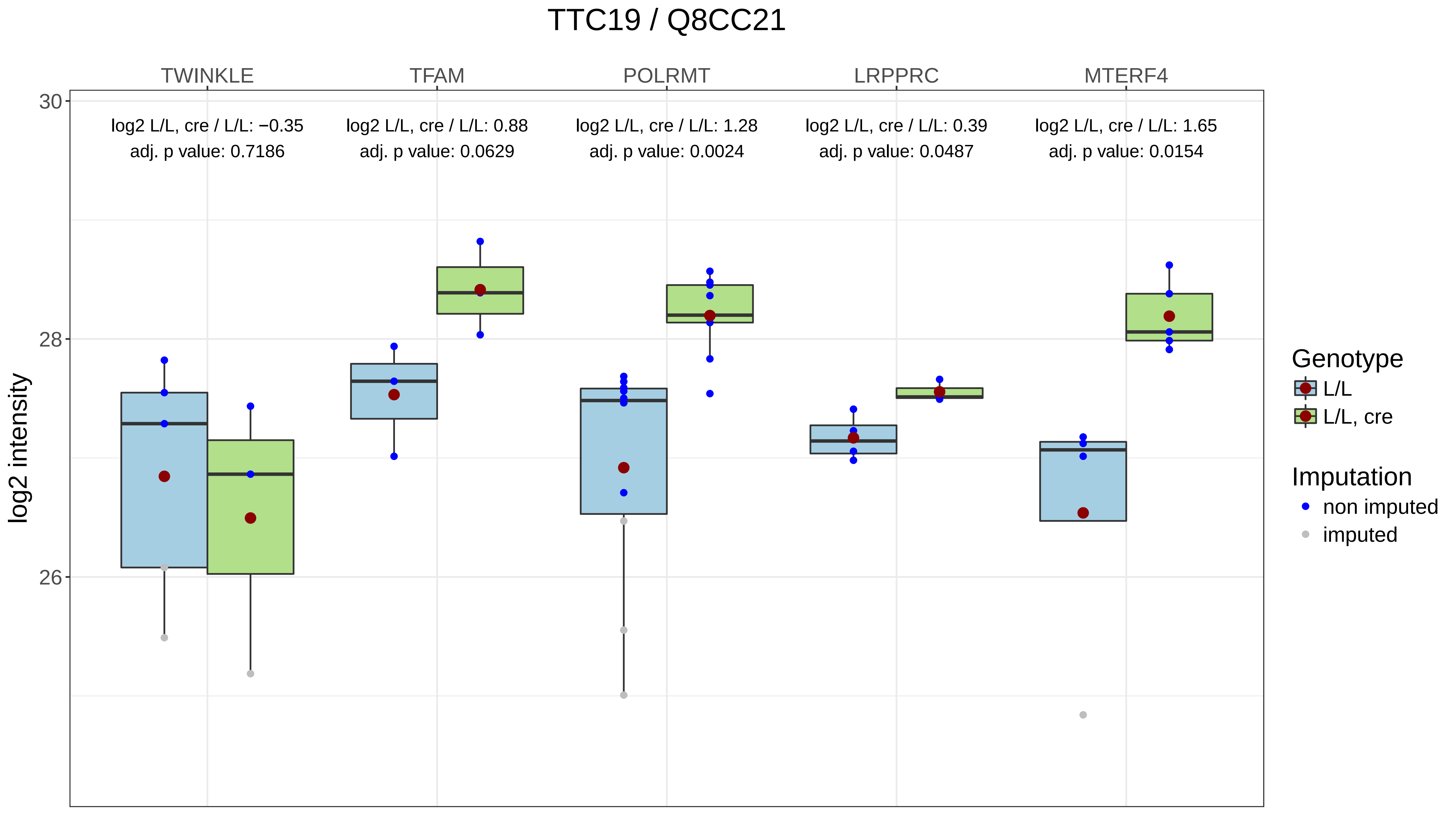
TTC19 / Q8CC21
TWINKLE
TFAM
POLRMT
LRPPRC
MTERF4
log2 L/L, cre / L/L: −0.35
adj. p value: 0.7186
log2 L/L, cre / L/L: 0.88
adj. p value: 0.0629
log2 L/L, cre / L/L: 1.28
adj. p value: 0.0024
log2 L/L, cre / L/L: 0.39
adj. p value: 0.0487
log2 L/L, cre / L/L: 1.65
adj. p value: 0.0154
30
28
26
log2 intensity
Genotype
L/L
L/L, cre
Imputation
non imputed
imputed

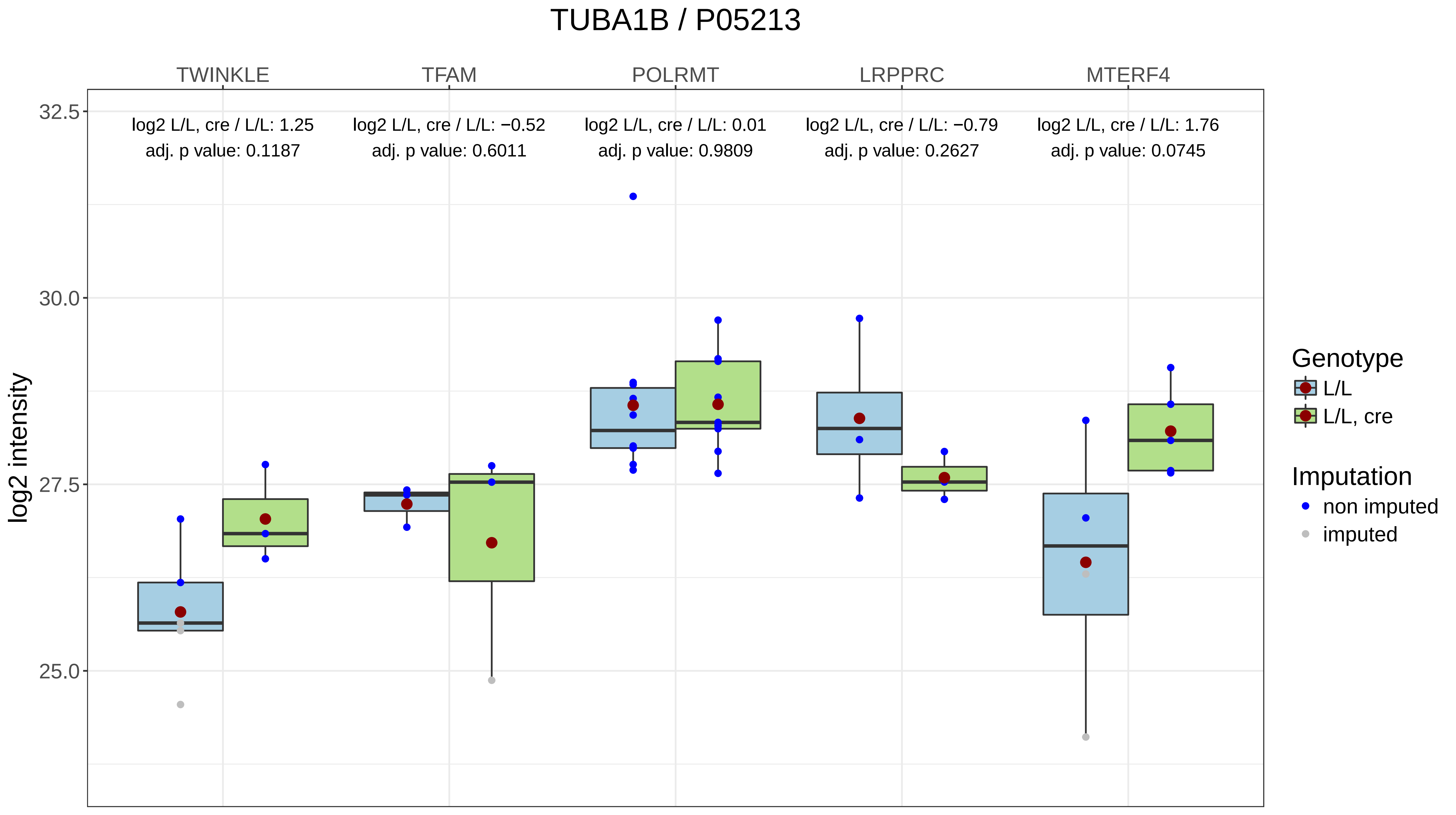
TUBA1B / P05213
TWINKLE
TFAM
POLRMT
LRPPRC
MTERF4
log2 L/L, cre / L/L: 1.25
adj. p value: 0.1187
log2 L/L, cre / L/L: −0.52
adj. p value: 0.6011
log2 L/L, cre / L/L: 0.01
adj. p value: 0.9809
log2 L/L, cre / L/L: −0.79
adj. p value: 0.2627
log2 L/L, cre / L/L: 1.76
adj. p value: 0.0745
32.5
30.0
27.5
25.0
log2 intensity
Genotype
L/L
L/L, cre
Imputation
non imputed
imputed

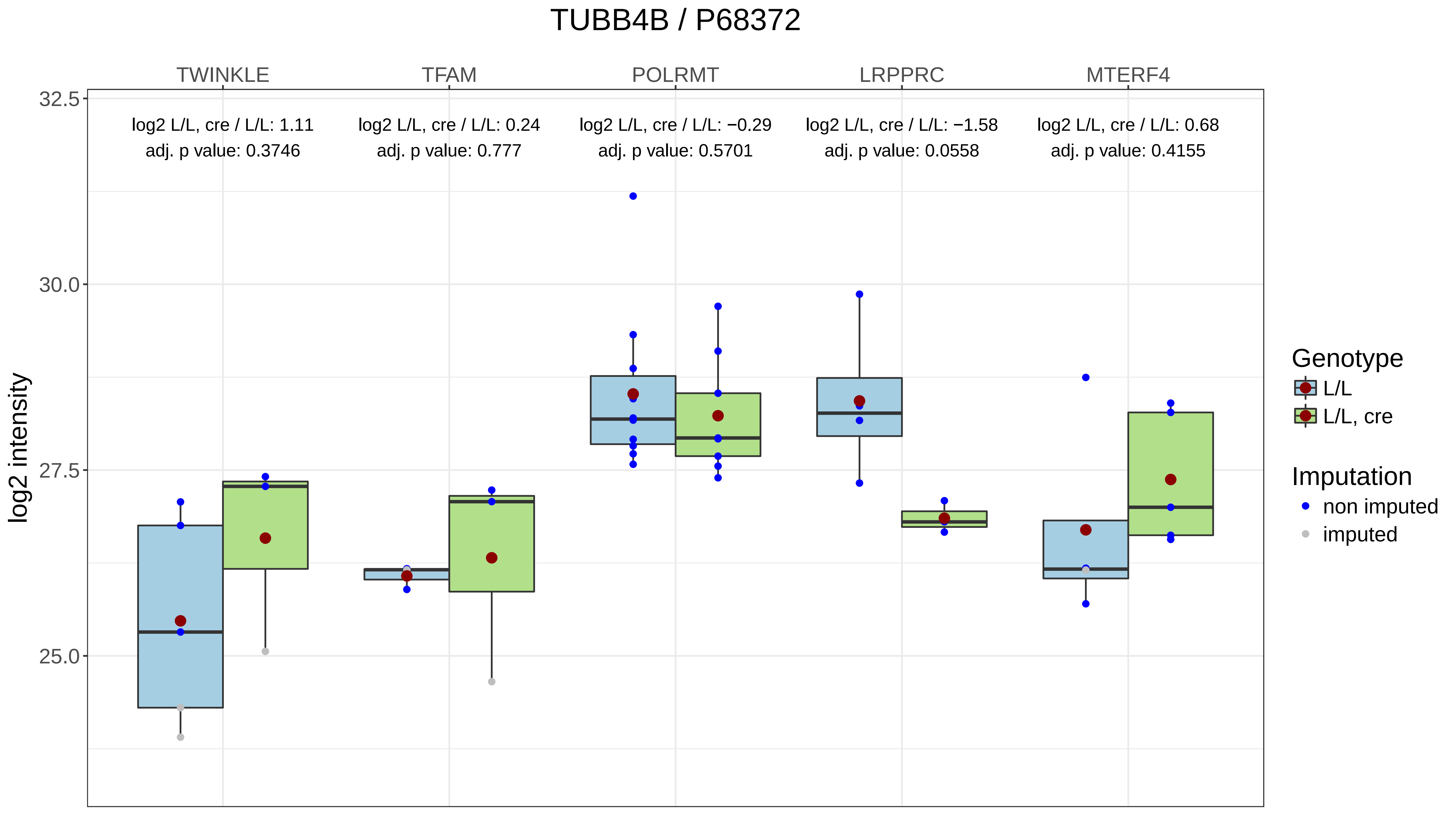
TUBB4B / P68372
TWINKLE
TFAM
POLRMT
LRPPRC
MTERF4
log2 L/L, cre / L/L: 1.11
adj. p value: 0.3746
log2 L/L, cre / L/L: 0.24
adj. p value: 0.777
log2 L/L, cre / L/L: −0.29
adj. p value: 0.5701
log2 L/L, cre / L/L: −1.58
adj. p value: 0.0558
log2 L/L, cre / L/L: 0.68
adj. p value: 0.4155
log2 intensity
32.5
30.0
27.5
25.0
Genotype
L/L
L/L, cre
Imputation
non imputed
imputed

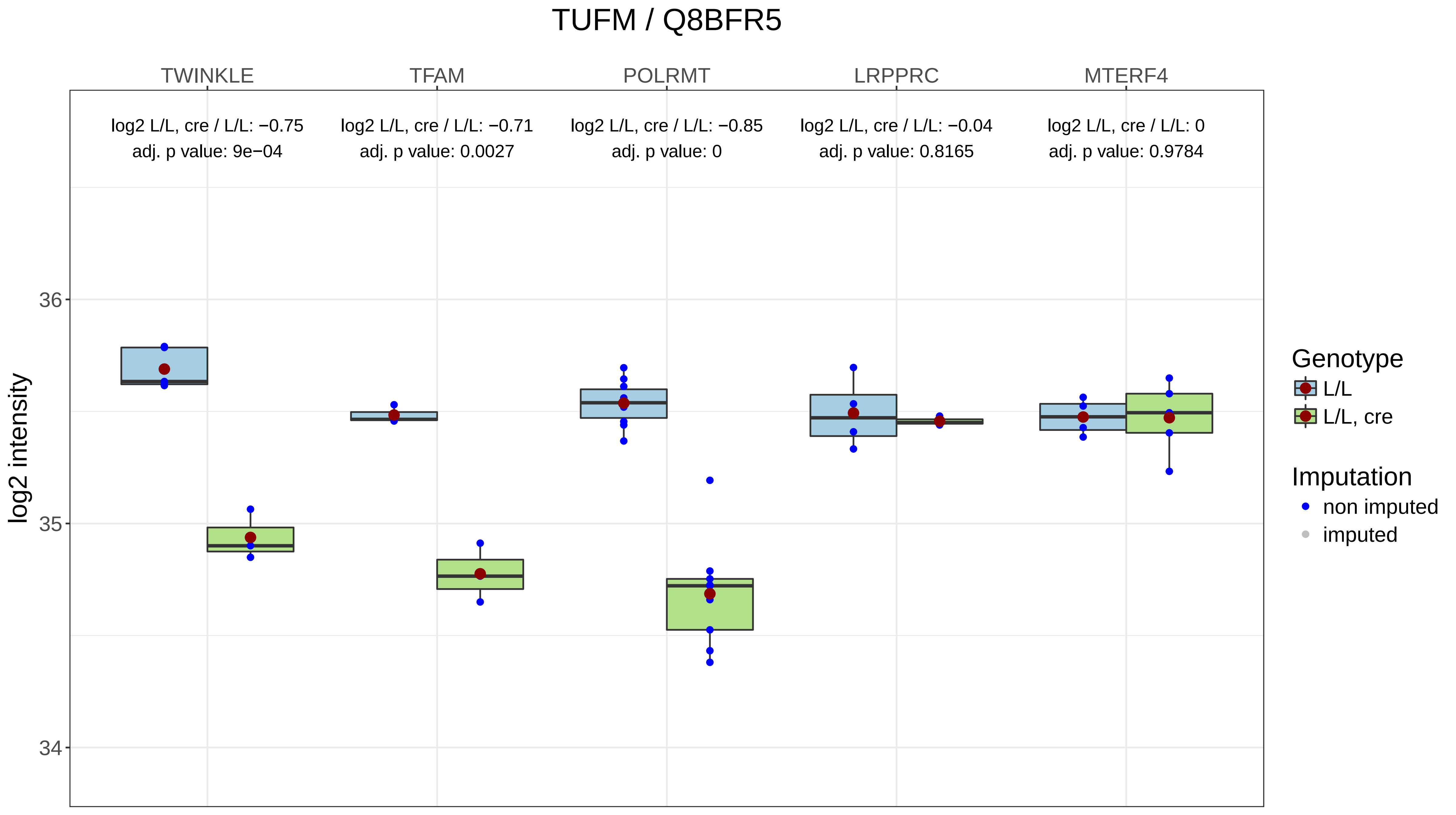
TUFM / Q8BFR5
TWINKLE
TFAM
POLRMT
LRPPRC
MTERF4
log2 L/L, cre / L/L: −0.75
adj. p value: 9e−04
log2 L/L, cre / L/L: −0.71
adj. p value: 0.0027
log2 L/L, cre / L/L: −0.85
adj. p value: 0
log2 L/L, cre / L/L: −0.04
adj. p value: 0.8165
log2 L/L, cre / L/L: 0
adj. p value: 0.9784
log2 intensity
36
35
34
Genotype
L/L
L/L, cre
Imputation
non imputed
imputed

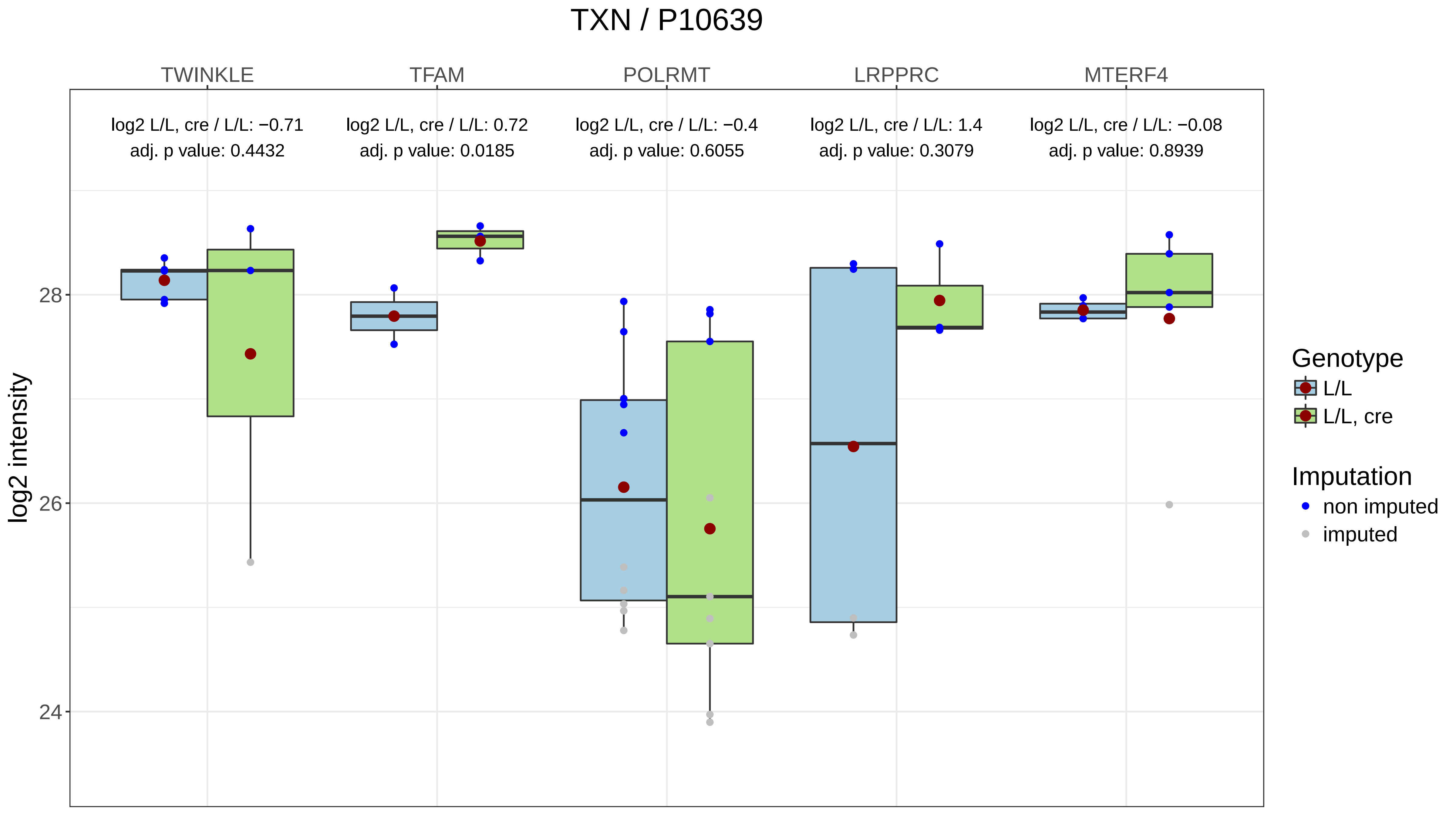
TXN / P10639
TWINKLE
TFAM
POLRMT
LRPPRC
MTERF4
log2 L/L, cre / L/L: −0.71
adj. p value: 0.4432
log2 L/L, cre / L/L: 0.72
adj. p value: 0.0185
log2 L/L, cre / L/L: −0.4
adj. p value: 0.6055
log2 L/L, cre / L/L: 1.4
adj. p value: 0.3079
log2 L/L, cre / L/L: −0.08
adj. p value: 0.8939
log2 intensity
28
26
24
Genotype
L/L
L/L, cre
Imputation
non imputed
imputed

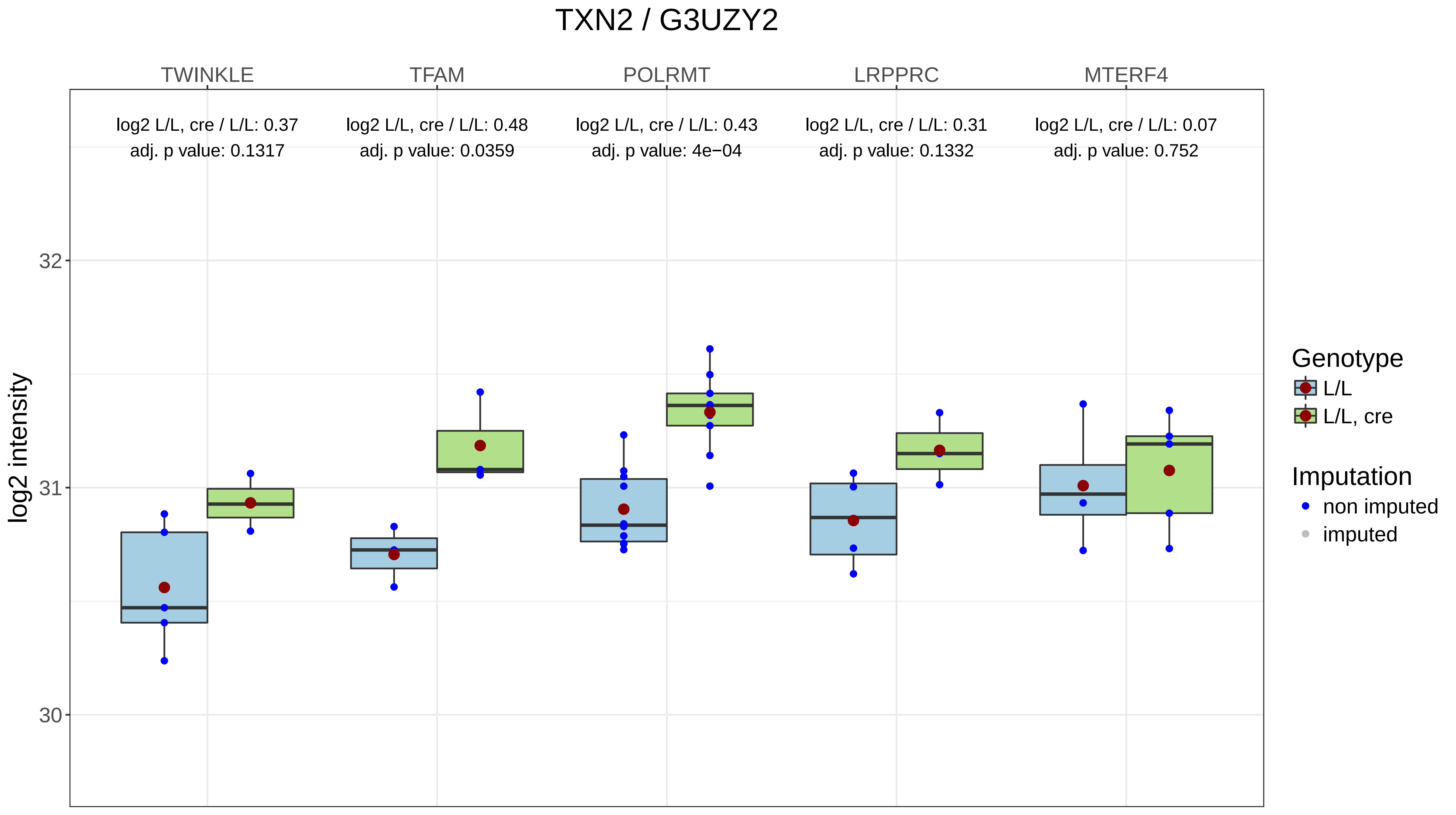
TXN2 / G3UZY2
TWINKLE
TFAM
POLRMT
LRPPRC
MTERF4
log2 L/L, cre / L/L: 0.37
adj. p value: 0.1317
log2 L/L, cre / L/L: 0.48
adj. p value: 0.0359
log2 L/L, cre / L/L: 0.43
adj. p value: 4e−04
log2 L/L, cre / L/L: 0.31
adj. p value: 0.1332
log2 L/L, cre / L/L: 0.07
adj. p value: 0.752
log2 intensity
32
31
30
Genotype
L/L
L/L, cre
Imputation
non imputed
imputed

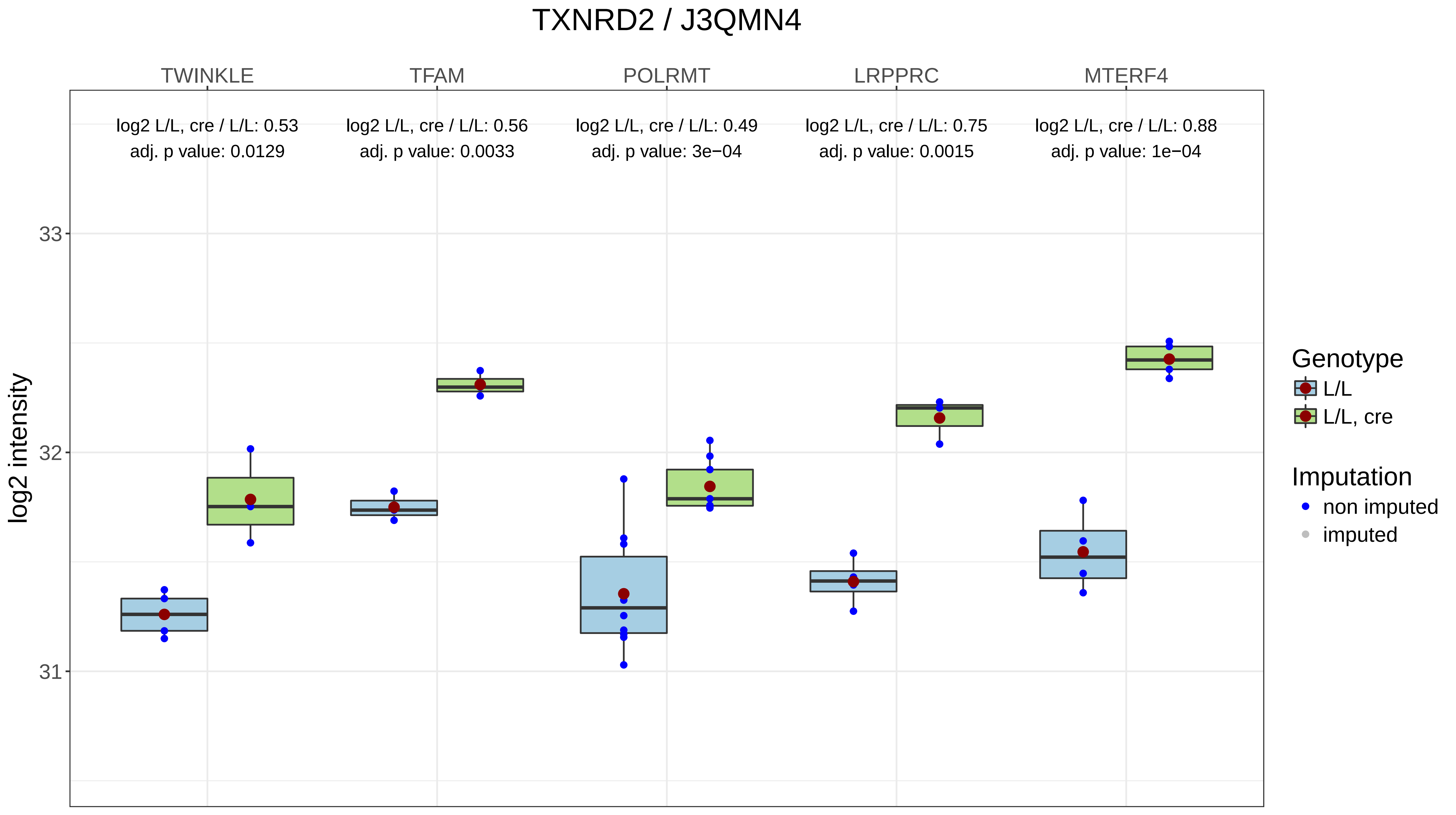
TXNRD2 / J3QMN4
TWINKLE
TFAM
POLRMT
LRPPRC
MTERF4
log2 L/L, cre / L/L: 0.53
adj. p value: 0.0129
log2 L/L, cre / L/L: 0.56
adj. p value: 0.0033
log2 L/L, cre / L/L: 0.49
adj. p value: 3e−04
log2 L/L, cre / L/L: 0.75
adj. p value: 0.0015
log2 L/L, cre / L/L: 0.88
adj. p value: 1e−04
log2 intensity
33
32
31
Genotype
L/L
L/L, cre
Imputation
non imputed
imputed

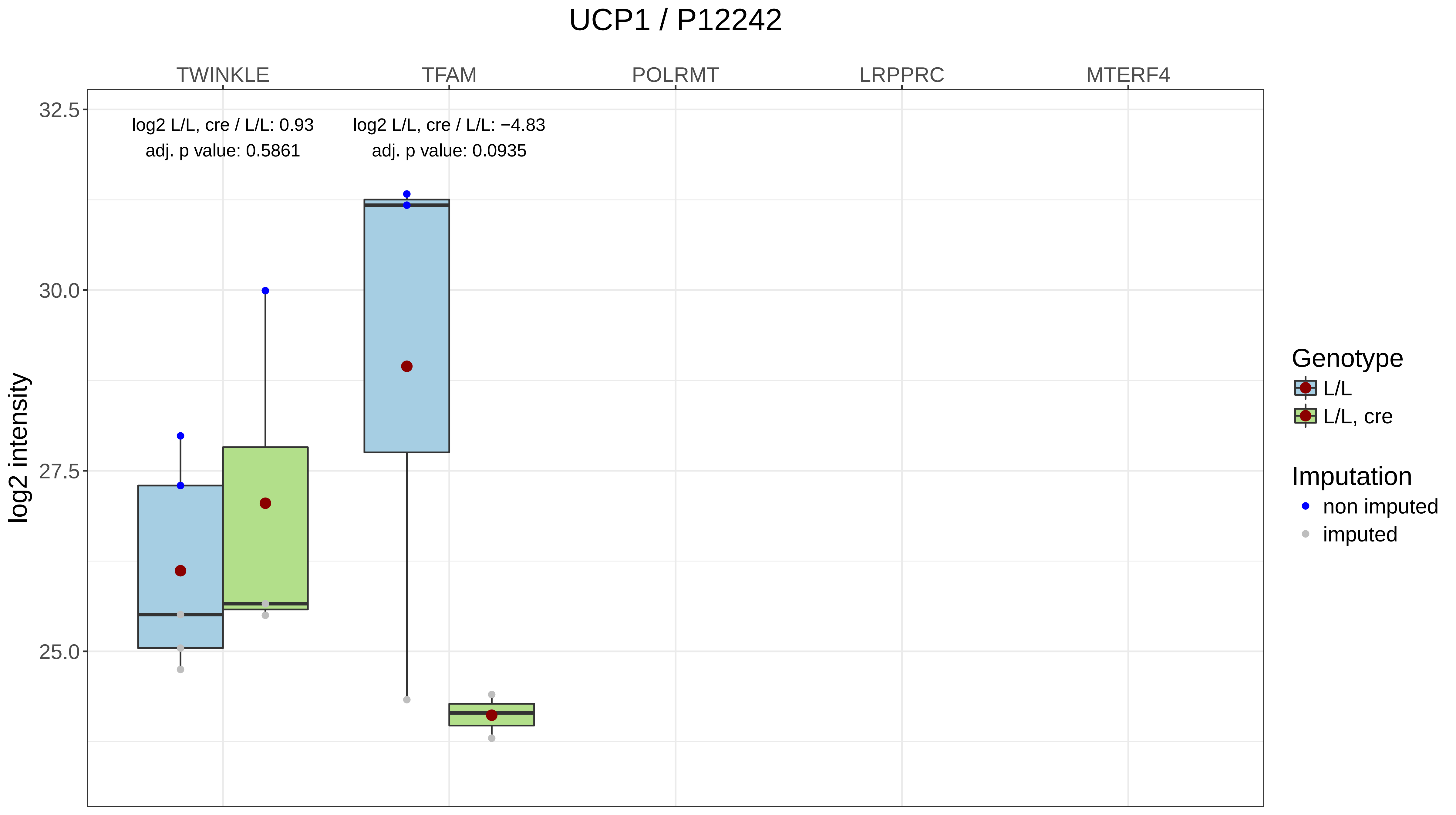
UCP1 / P12242
TWINKLE
TFAM
POLRMT
LRPPRC
MTERF4
log2 L/L, cre / L/L: 0.93
adj. p value: 0.5861
log2 L/L, cre / L/L: −4.83
adj. p value: 0.0935
32.5
30.0
27.5
25.0
log2 intensity
Genotype
L/L
L/L, cre
Imputation
non imputed
imputed

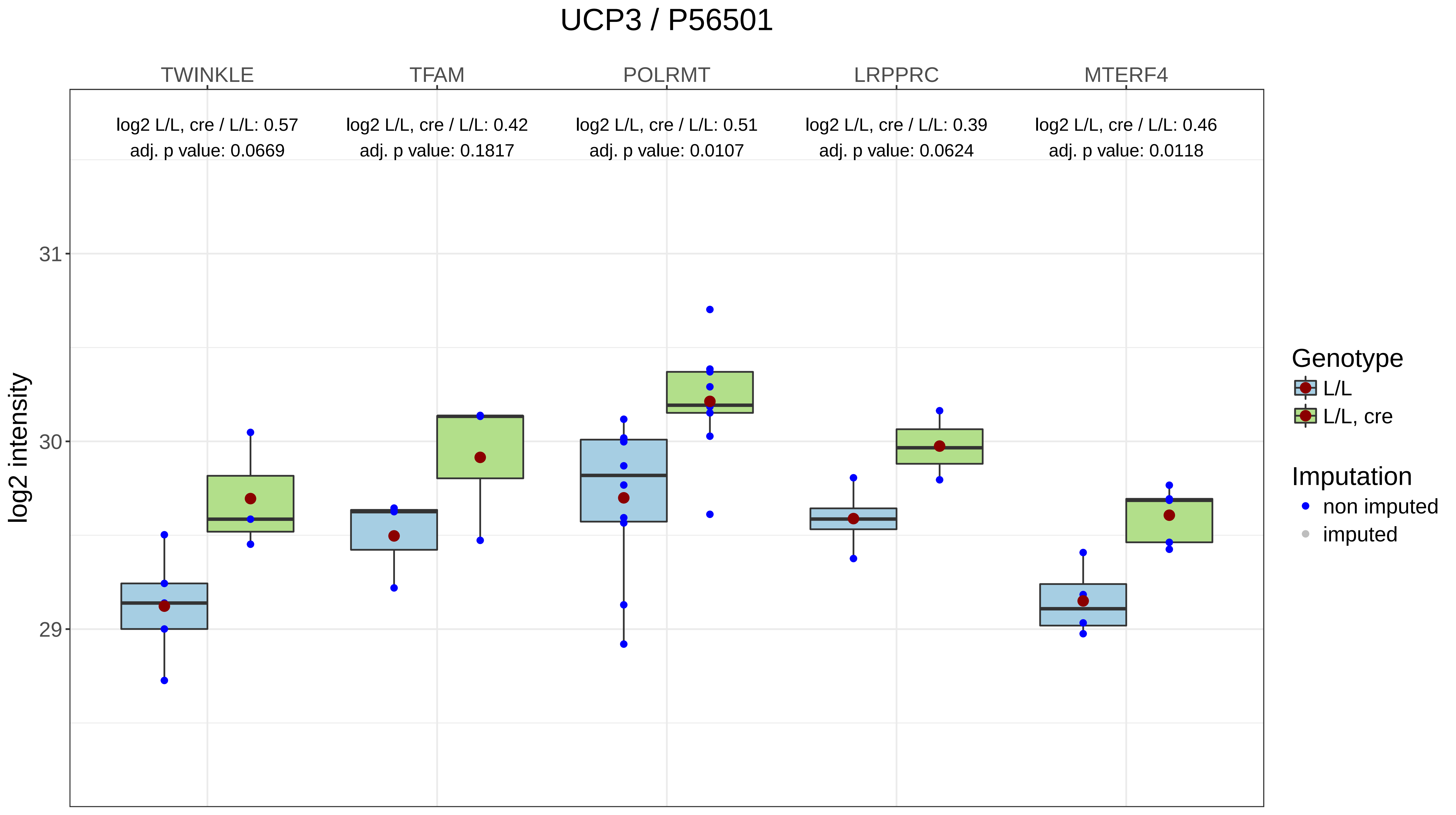

UCP3 / P56501
TWINKLE
TFAM
POLRMT
LRPPRC
MTERF4
log2 L/L, cre / L/L: 0.57
adj. p value: 0.0669
log2 L/L, cre / L/L: 0.42
adj. p value: 0.1817
log2 L/L, cre / L/L: 0.51
adj. p value: 0.0107
log2 L/L, cre / L/L: 0.39
adj. p value: 0.0624
log2 L/L, cre / L/L: 0.46
adj. p value: 0.0118
log2 intensity
31
30
29
Genotype
L/L
L/L, cre
Imputation
non imputed
imputed

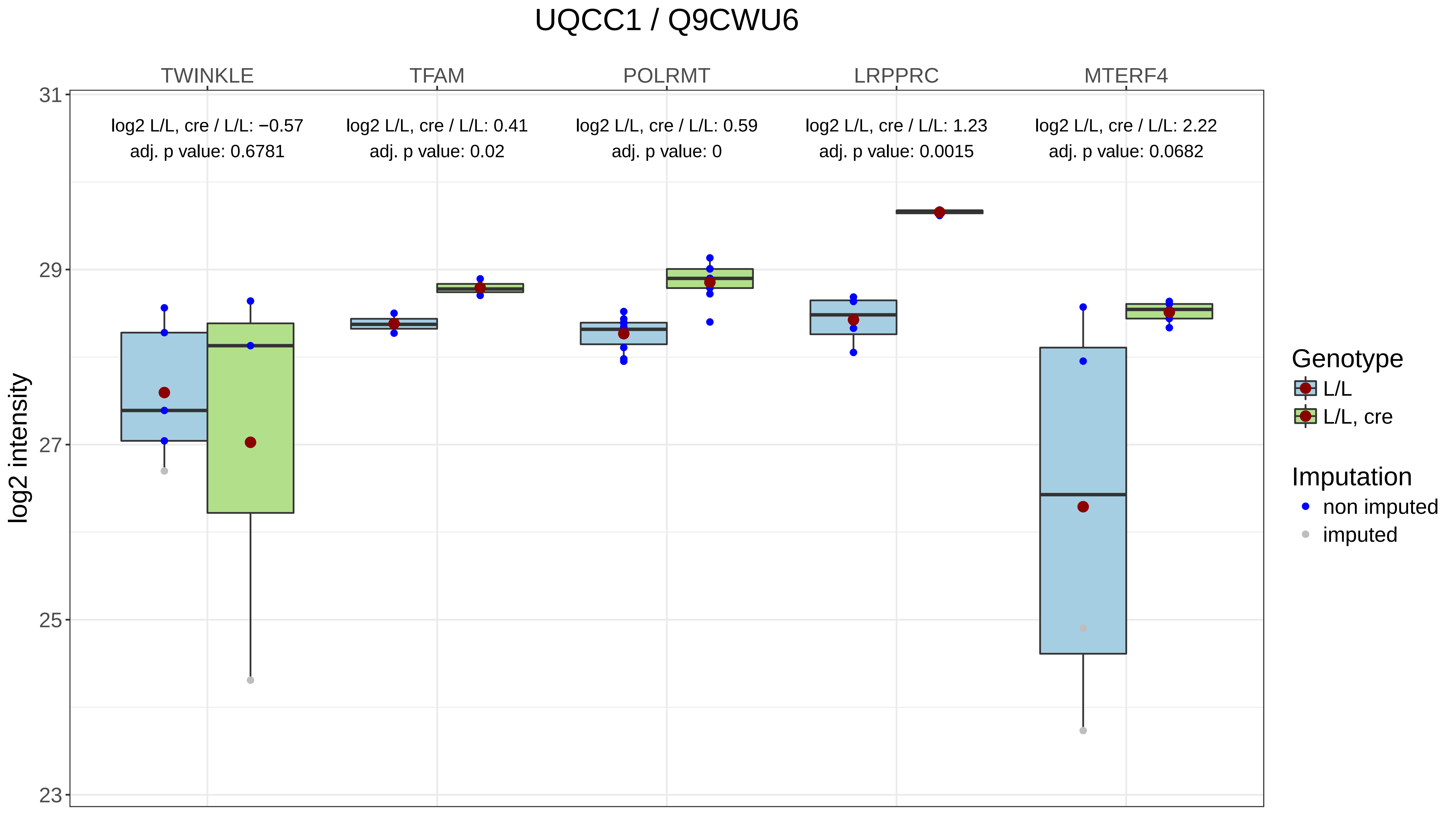
UQCC1 / Q9CWU6
TWINKLE
TFAM
POLRMT
LRPPRC
MTERF4
log2 L/L, cre / L/L: −0.57
adj. p value: 0.6781
log2 L/L, cre / L/L: 0.41
adj. p value: 0.02
log2 L/L, cre / L/L: 0.59
adj. p value: 0
log2 L/L, cre / L/L: 1.23
adj. p value: 0.0015
log2 L/L, cre / L/L: 2.22
adj. p value: 0.0682
31
29
27
25
23
log2 intensity
Genotype
L/L
L/L, cre
Imputation
non imputed
imputed

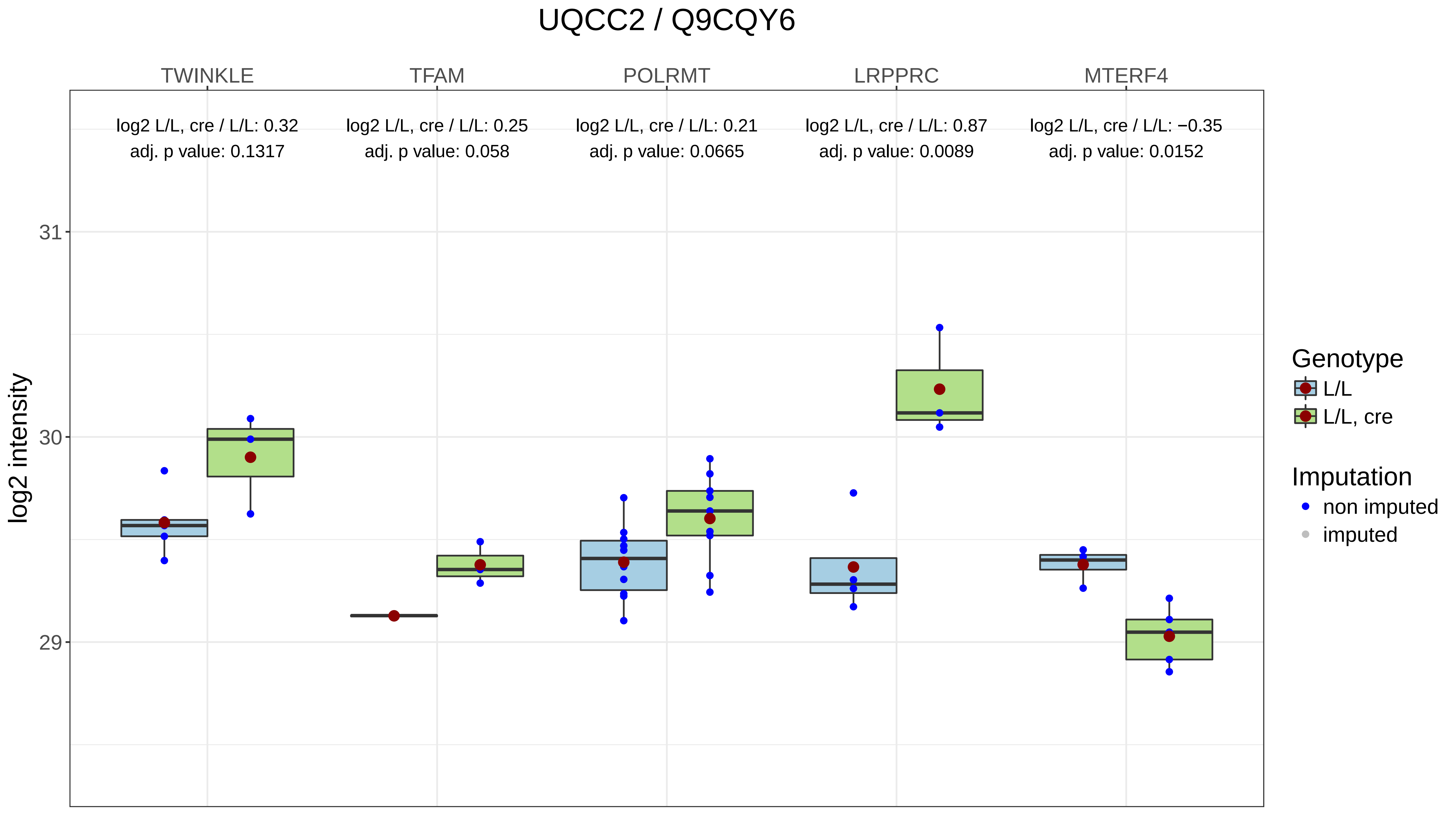

UQCC2 / Q9CQY6
TWINKLE
TFAM
POLRMT
LRPPRC
MTERF4
log2 L/L, cre / L/L: 0.32
adj. p value: 0.1317
log2 L/L, cre / L/L: 0.25
adj. p value: 0.058
log2 L/L, cre / L/L: 0.21
adj. p value: 0.0665
log2 L/L, cre / L/L: 0.87
adj. p value: 0.0089
log2 L/L, cre / L/L: −0.35
adj. p value: 0.0152
log2 intensity
31
30
29
Genotype
L/L
L/L, cre
Imputation
non imputed
imputed

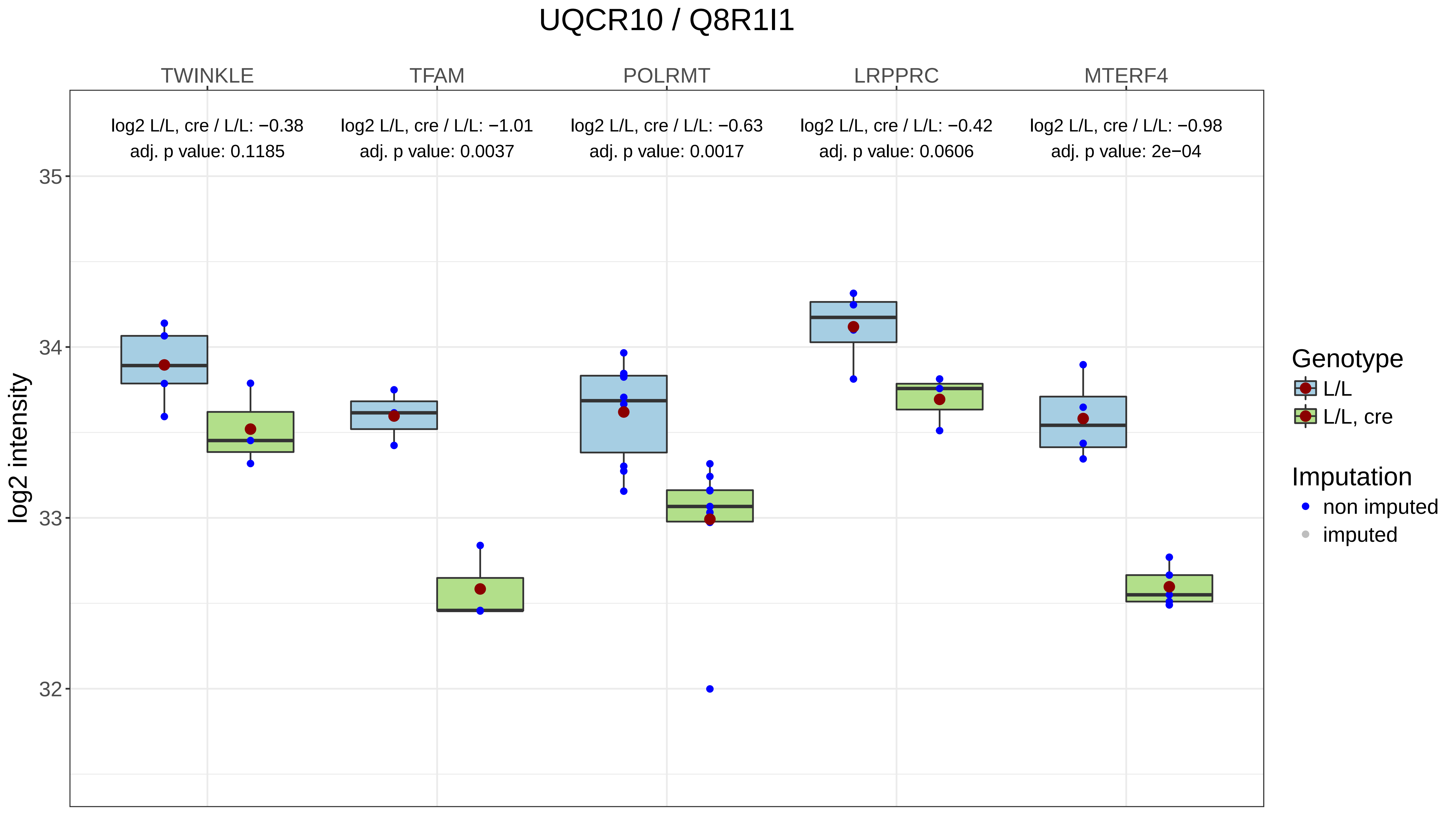
UQCR10 / Q8R1I1
TWINKLE
TFAM
POLRMT
LRPPRC
MTERF4
log2 L/L, cre / L/L: −0.38
adj. p value: 0.1185
log2 L/L, cre / L/L: −1.01
adj. p value: 0.0037
log2 L/L, cre / L/L: −0.63
adj. p value: 0.0017
log2 L/L, cre / L/L: −0.42
adj. p value: 0.0606
log2 L/L, cre / L/L: −0.98
adj. p value: 2e−04
log2 intensity
35
34
33
32
Genotype
L/L
L/L, cre
Imputation
non imputed
imputed

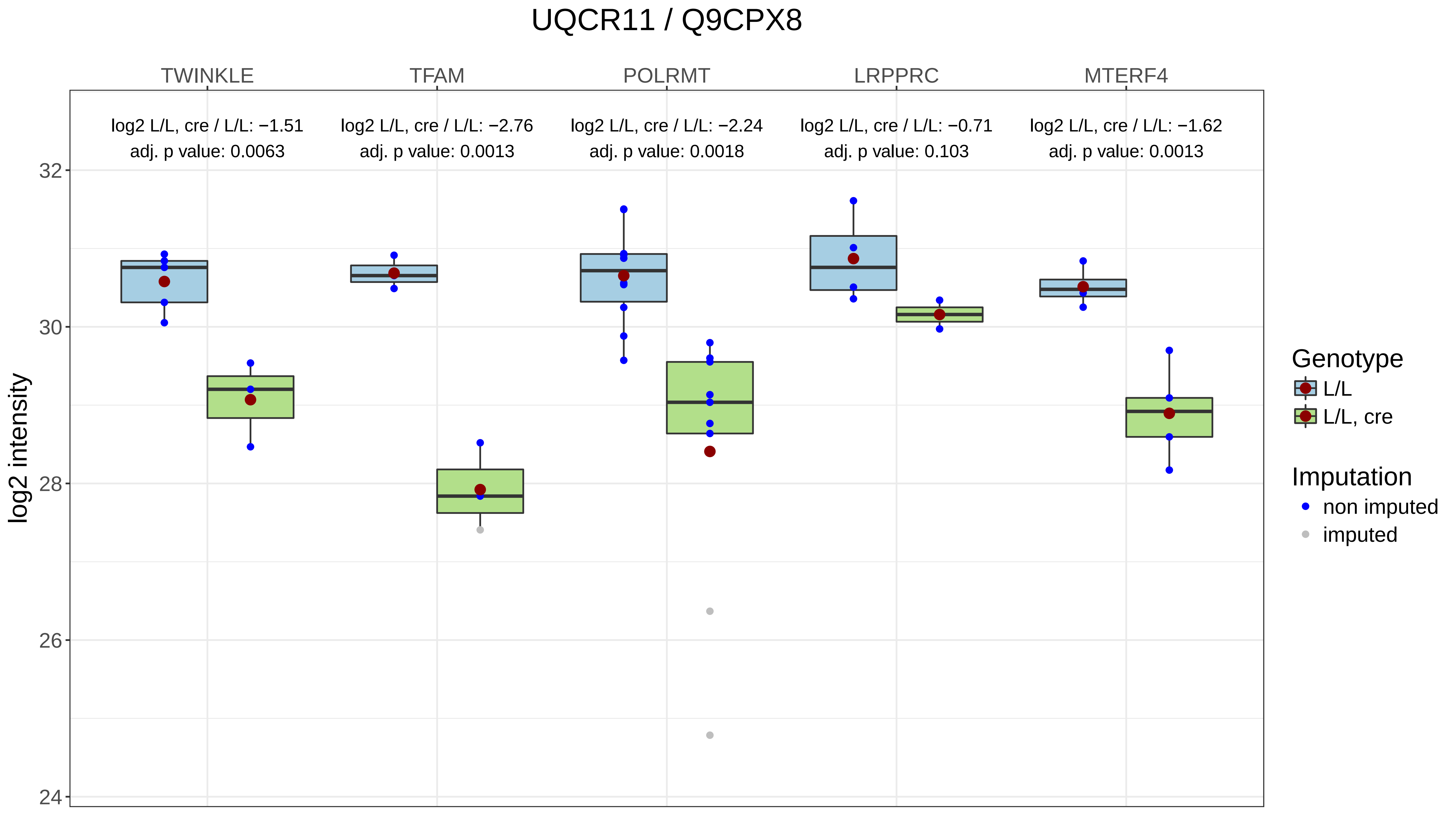
UQCR11 / Q9CPX8
TWINKLE
TFAM
POLRMT
LRPPRC
MTERF4
log2 L/L, cre / L/L: −1.51
adj. p value: 0.0063
log2 L/L, cre / L/L: −2.76
adj. p value: 0.0013
log2 L/L, cre / L/L: −2.24
adj. p value: 0.0018
log2 L/L, cre / L/L: −0.71
adj. p value: 0.103
log2 L/L, cre / L/L: −1.62
adj. p value: 0.0013
log2 intensity
32
30
28
26
24
Genotype
L/L
L/L, cre
Imputation
non imputed
imputed

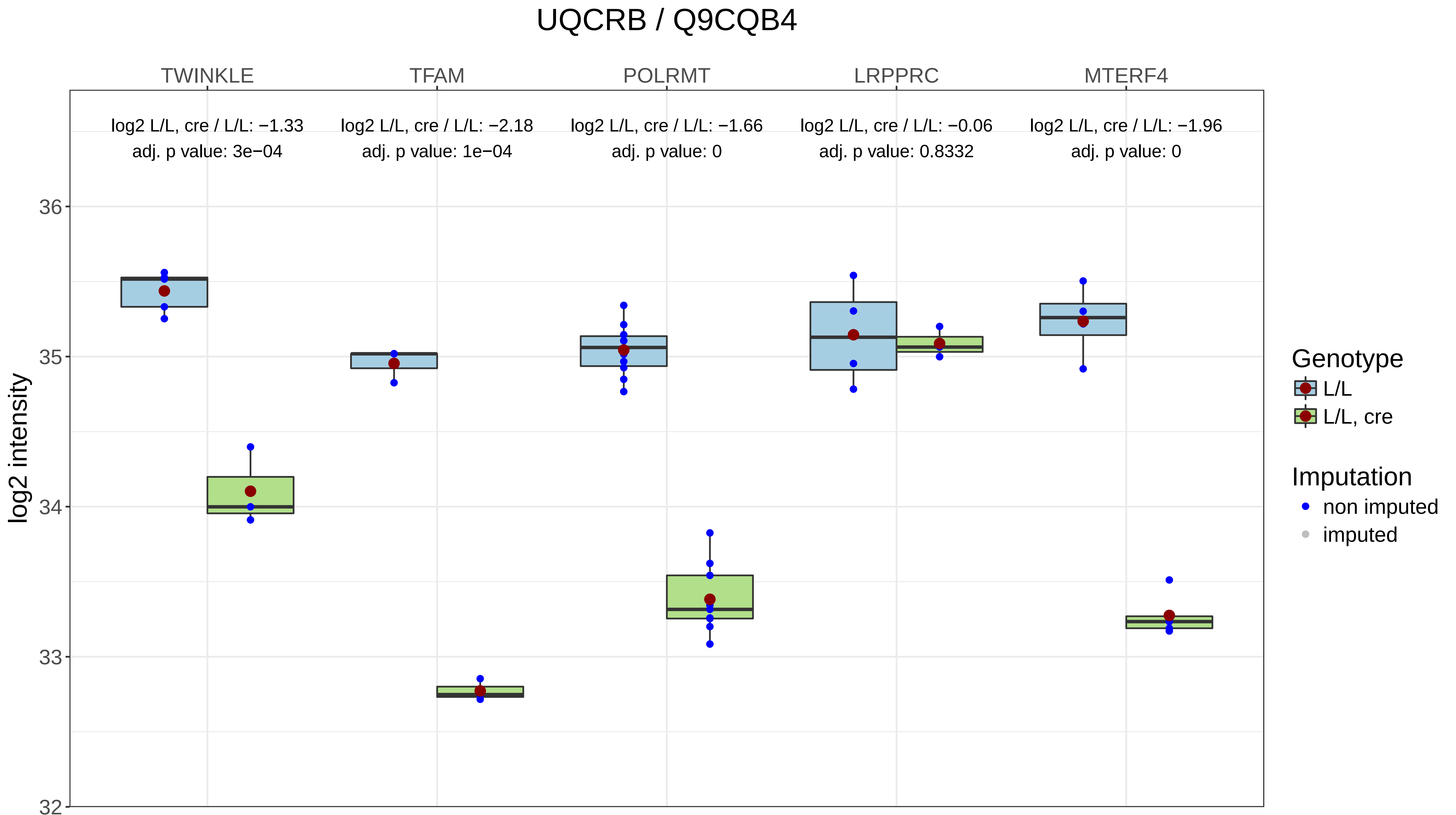
UQCRB / Q9CQB4
TWINKLE
TFAM
POLRMT
LRPPRC
MTERF4
log2 L/L, cre / L/L: −1.33
adj. p value: 3e−04
log2 L/L, cre / L/L: −2.18
adj. p value: 1e−04
log2 L/L, cre / L/L: −1.66
adj. p value: 0
log2 L/L, cre / L/L: −0.06
adj. p value: 0.8332
log2 L/L, cre / L/L: −1.96
adj. p value: 0
log2 intensity
36
35
34
33
32
Genotype
L/L
L/L, cre
Imputation
non imputed
imputed

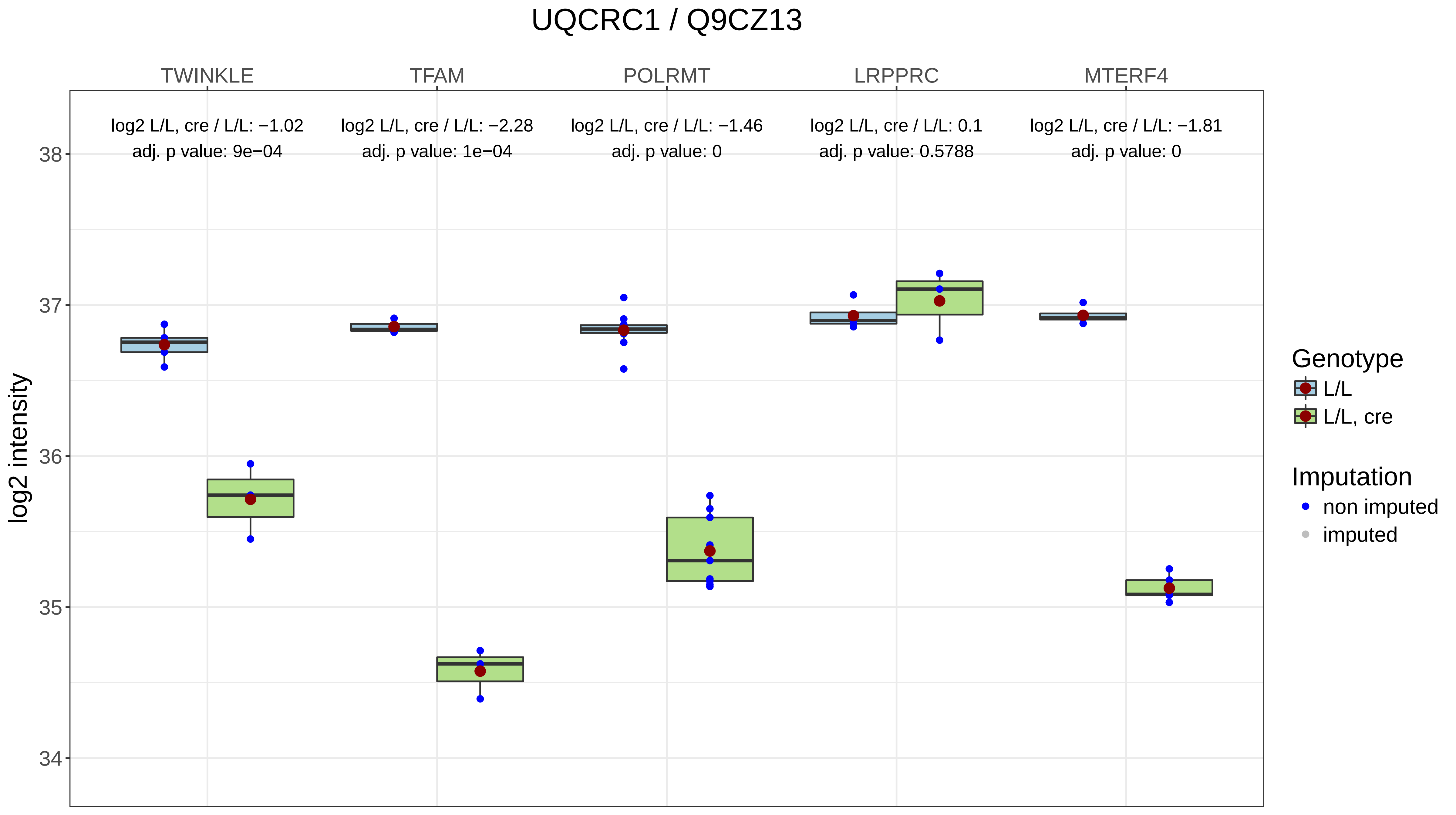
UQCRC1 / Q9CZ13
TWINKLE
TFAM
POLRMT
LRPPRC
MTERF4
log2 L/L, cre / L/L: −1.02
adj. p value: 9e−04
log2 L/L, cre / L/L: −2.28
adj. p value: 1e−04
log2 L/L, cre / L/L: −1.46
adj. p value: 0
log2 L/L, cre / L/L: 0.1
adj. p value: 0.5788
log2 L/L, cre / L/L: −1.81
adj. p value: 0
log2 intensity
38
37
36
35
34
Genotype
L/L
L/L, cre
Imputation
non imputed
imputed

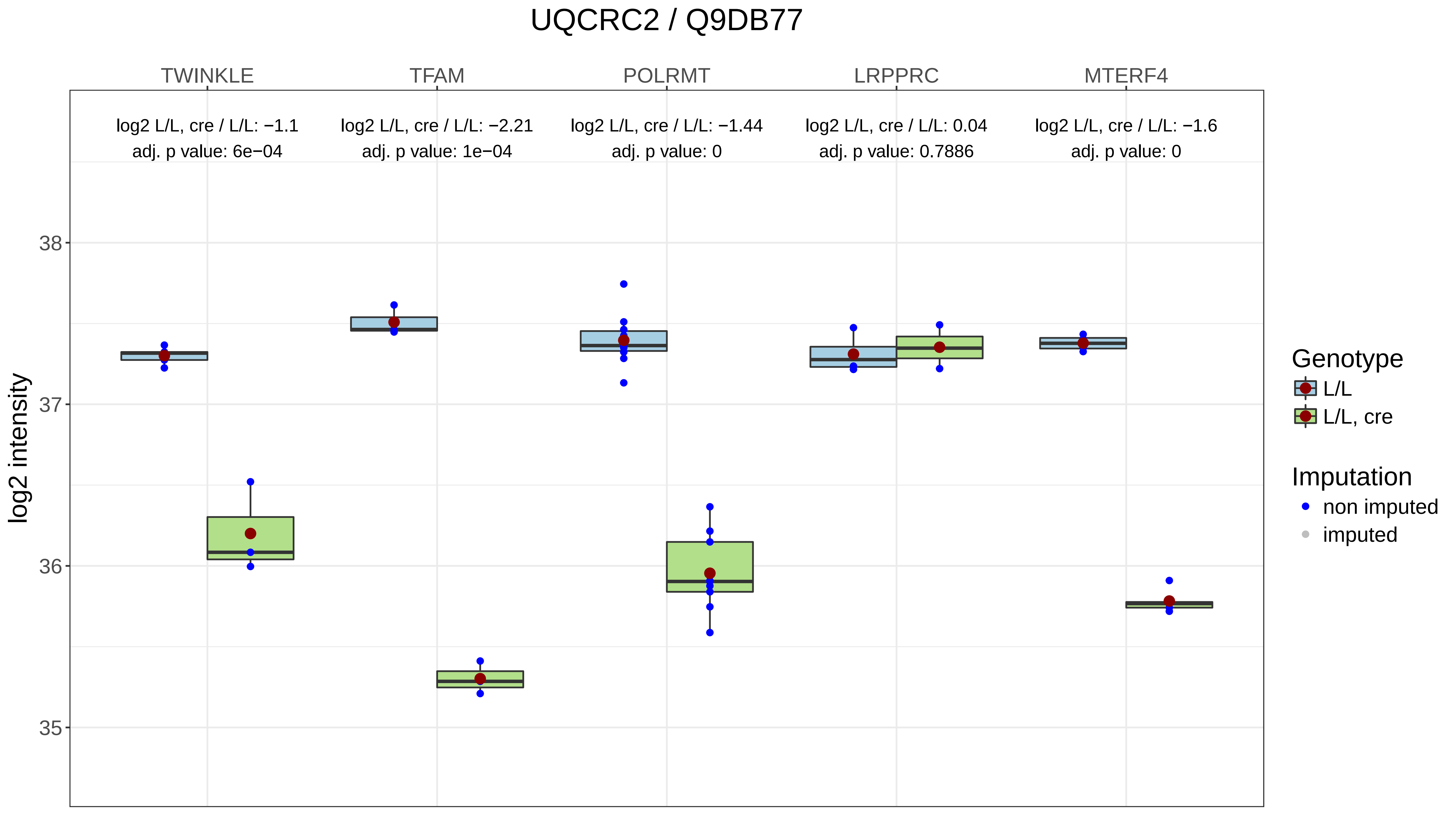

UQCRC2 / Q9DB77
TWINKLE
TFAM
POLRMT
LRPPRC
MTERF4
log2 L/L, cre / L/L: −1.1
adj. p value: 6e−04
log2 L/L, cre / L/L: −2.21
adj. p value: 1e−04
log2 L/L, cre / L/L: −1.44
adj. p value: 0
log2 L/L, cre / L/L: 0.04
adj. p value: 0.7886
log2 L/L, cre / L/L: −1.6
adj. p value: 0
log2 intensity
38
37
36
35
Genotype
L/L
L/L, cre
Imputation
non imputed
imputed

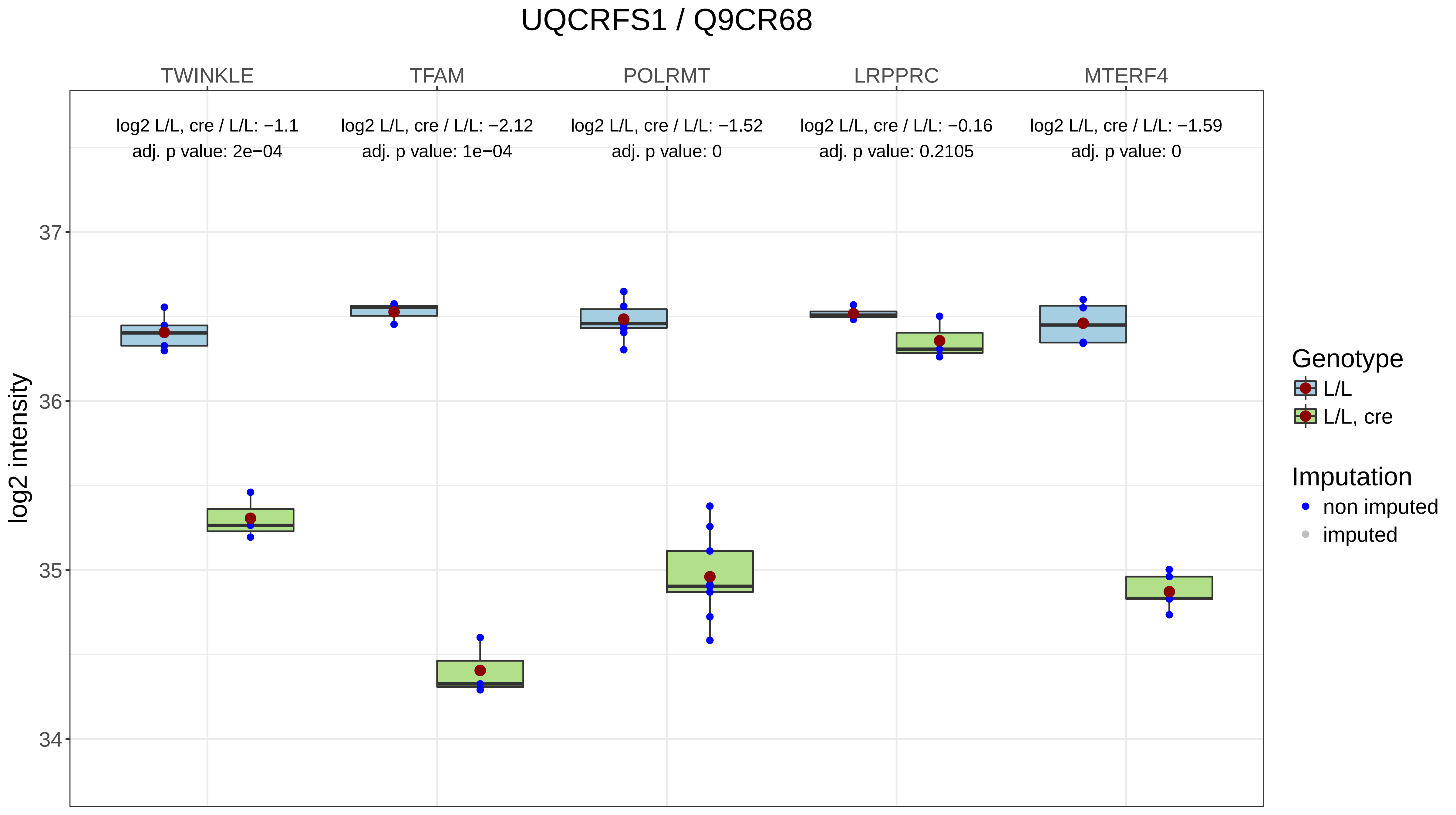
UQCRFS1 / Q9CR68
TWINKLE
TFAM
POLRMT
LRPPRC
MTERF4
log2 L/L, cre / L/L: −1.1
adj. p value: 2e−04
log2 L/L, cre / L/L: −2.12
adj. p value: 1e−04
log2 L/L, cre / L/L: −1.52
adj. p value: 0
log2 L/L, cre / L/L: −0.16
adj. p value: 0.2105
log2 L/L, cre / L/L: −1.59
adj. p value: 0
log2 intensity
37
36
35
34
Genotype
L/L
L/L, cre
Imputation
non imputed
imputed

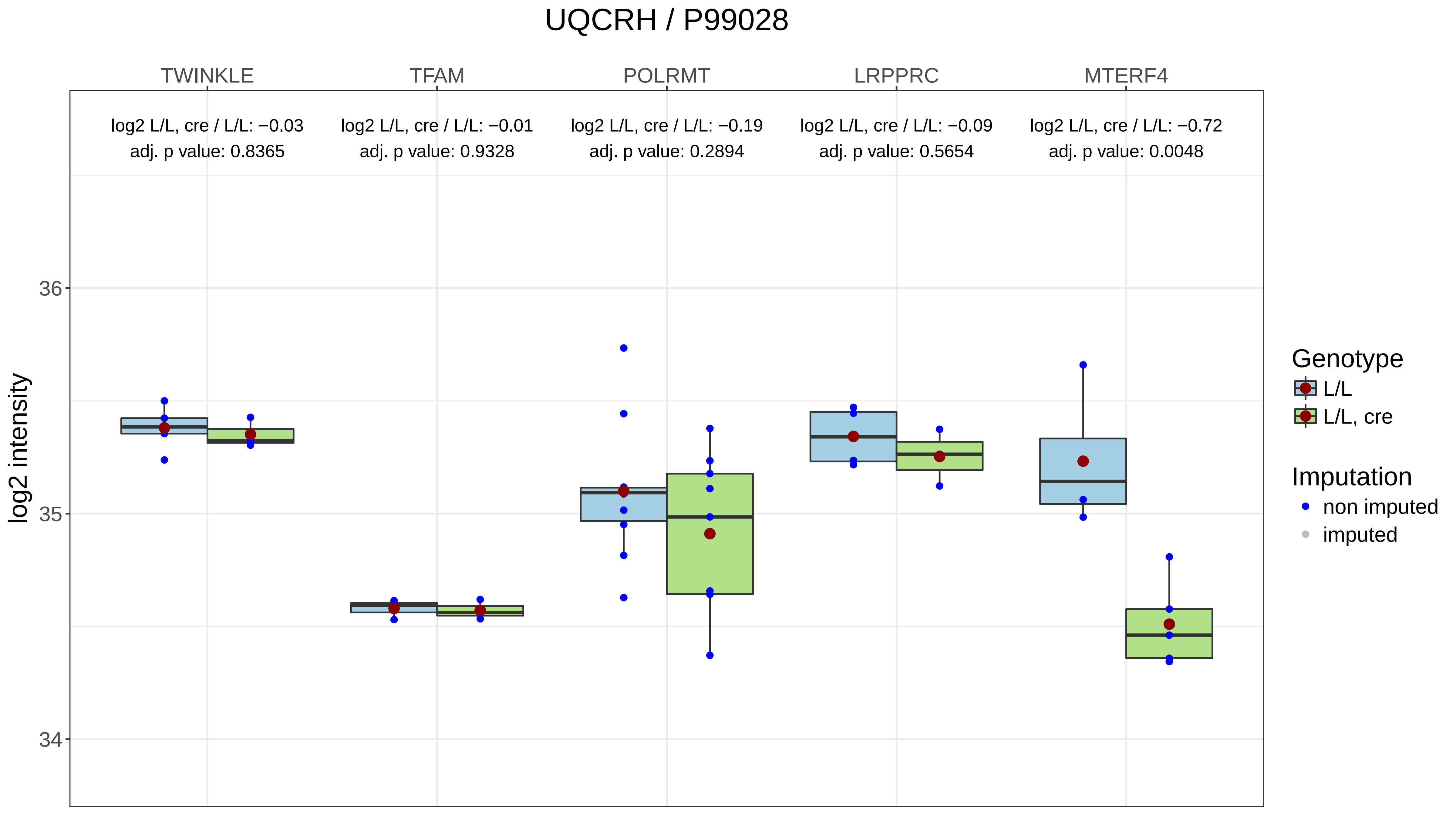

UQCRH / P99028
TWINKLE
TFAM
POLRMT
LRPPRC
MTERF4
log2 L/L, cre / L/L: −0.03
adj. p value: 0.8365
log2 L/L, cre / L/L: −0.01
adj. p value: 0.9328
log2 L/L, cre / L/L: −0.19
adj. p value: 0.2894
log2 L/L, cre / L/L: −0.09
adj. p value: 0.5654
log2 L/L, cre / L/L: −0.72
adj. p value: 0.0048
log2 intensity
36
35
34
Genotype
L/L
L/L, cre
Imputation
non imputed
imputed

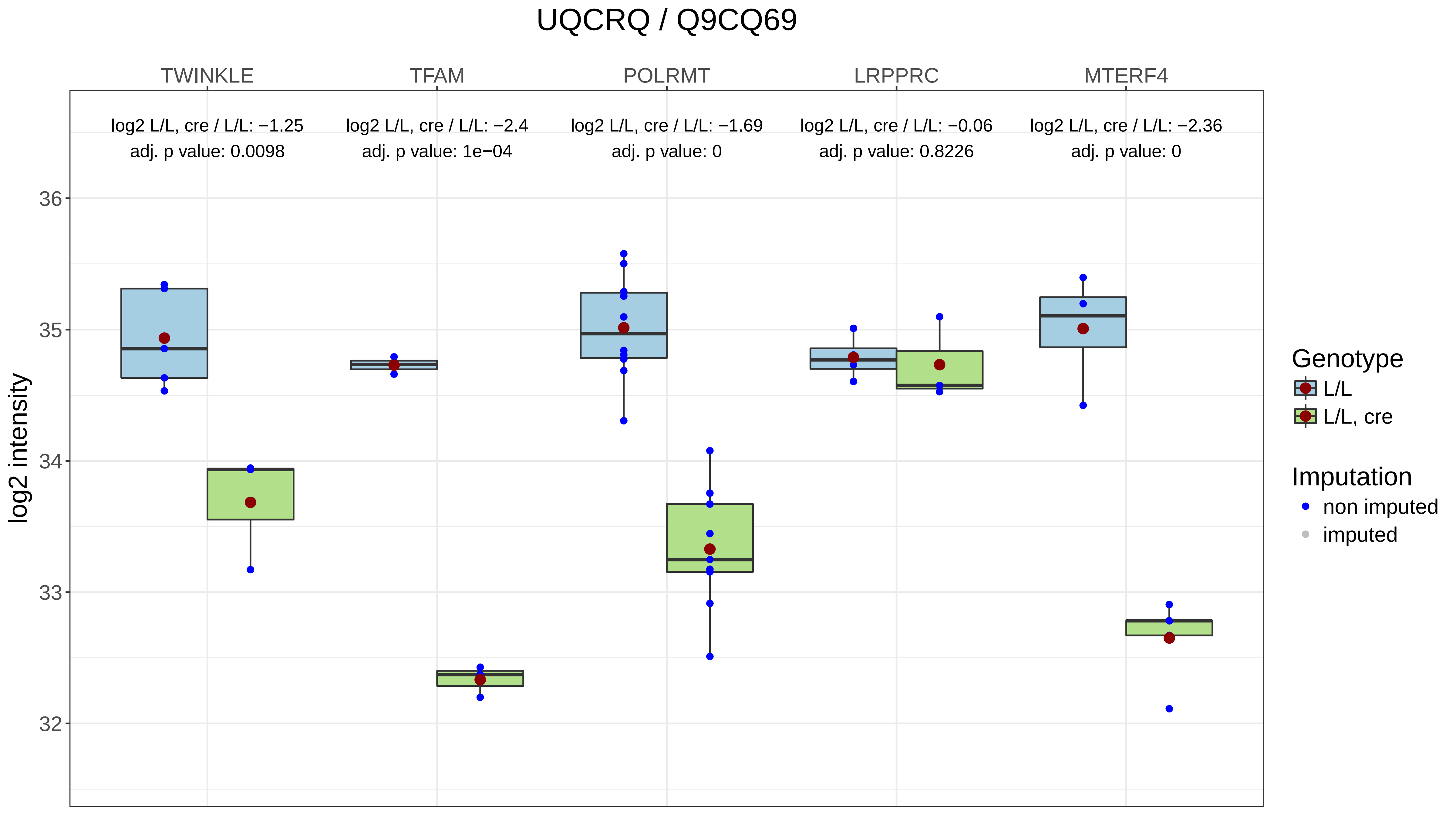
UQCRQ / Q9CQ69
TWINKLE
TFAM
POLRMT
LRPPRC
MTERF4
log2 L/L, cre / L/L: −1.25
adj. p value: 0.0098
log2 L/L, cre / L/L: −2.4
adj. p value: 1e−04
log2 L/L, cre / L/L: −1.69
adj. p value: 0
log2 L/L, cre / L/L: −0.06
adj. p value: 0.8226
log2 L/L, cre / L/L: −2.36
adj. p value: 0
log2 intensity
36
35
34
33
32
Genotype
L/L
L/L, cre
Imputation
non imputed
imputed

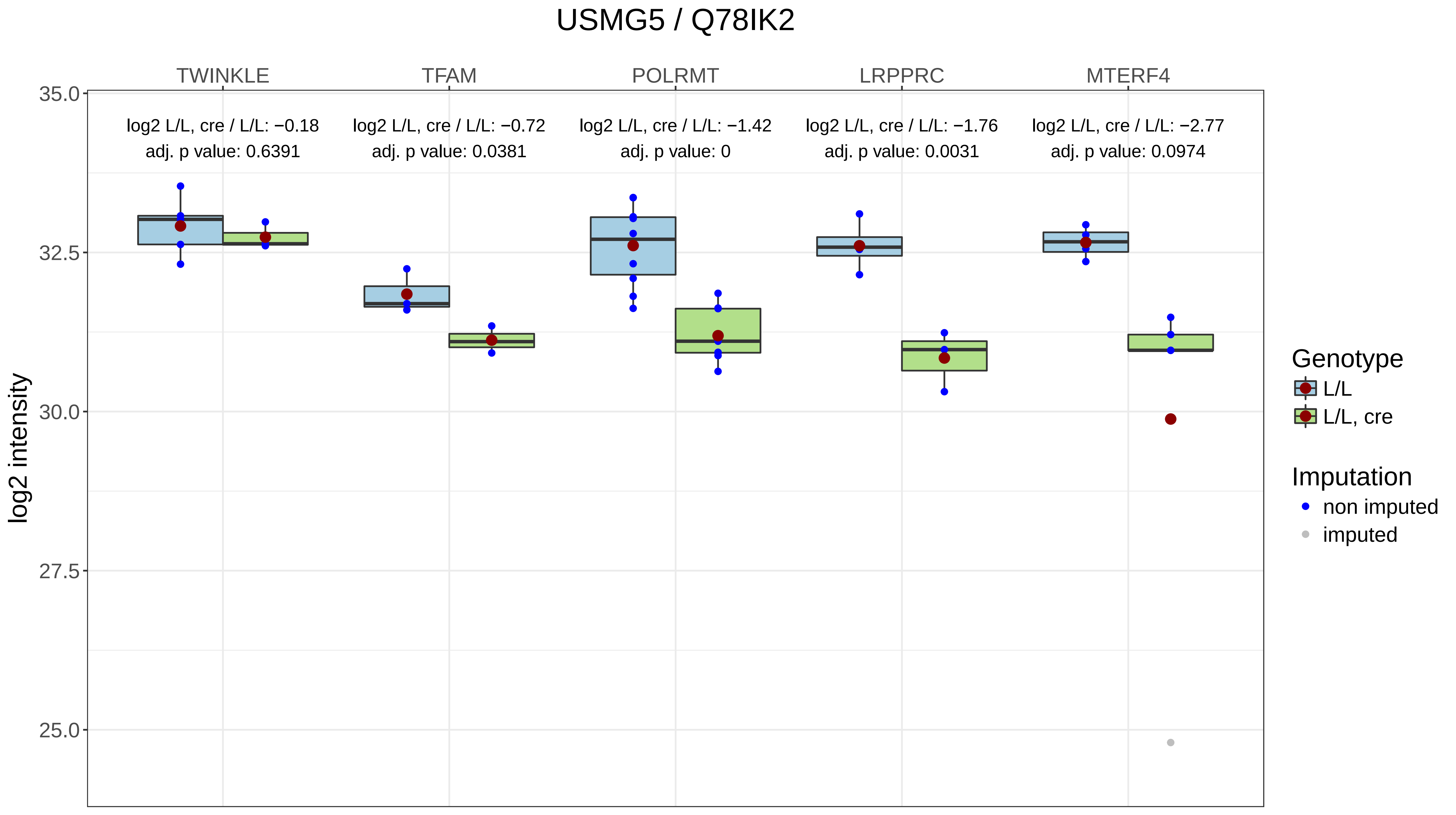

USMG5 / Q78IK2
TWINKLE
TFAM
POLRMT
LRPPRC
MTERF4
log2 L/L, cre / L/L: −0.18
adj. p value: 0.6391
log2 L/L, cre / L/L: −0.72
adj. p value: 0.0381
log2 L/L, cre / L/L: −1.42
adj. p value: 0
log2 L/L, cre / L/L: −1.76
adj. p value: 0.0031
log2 L/L, cre / L/L: −2.77
adj. p value: 0.0974
35.0
32.5
30.0
27.5
25.0
log2 intensity
Genotype
L/L
L/L, cre
Imputation
non imputed
imputed

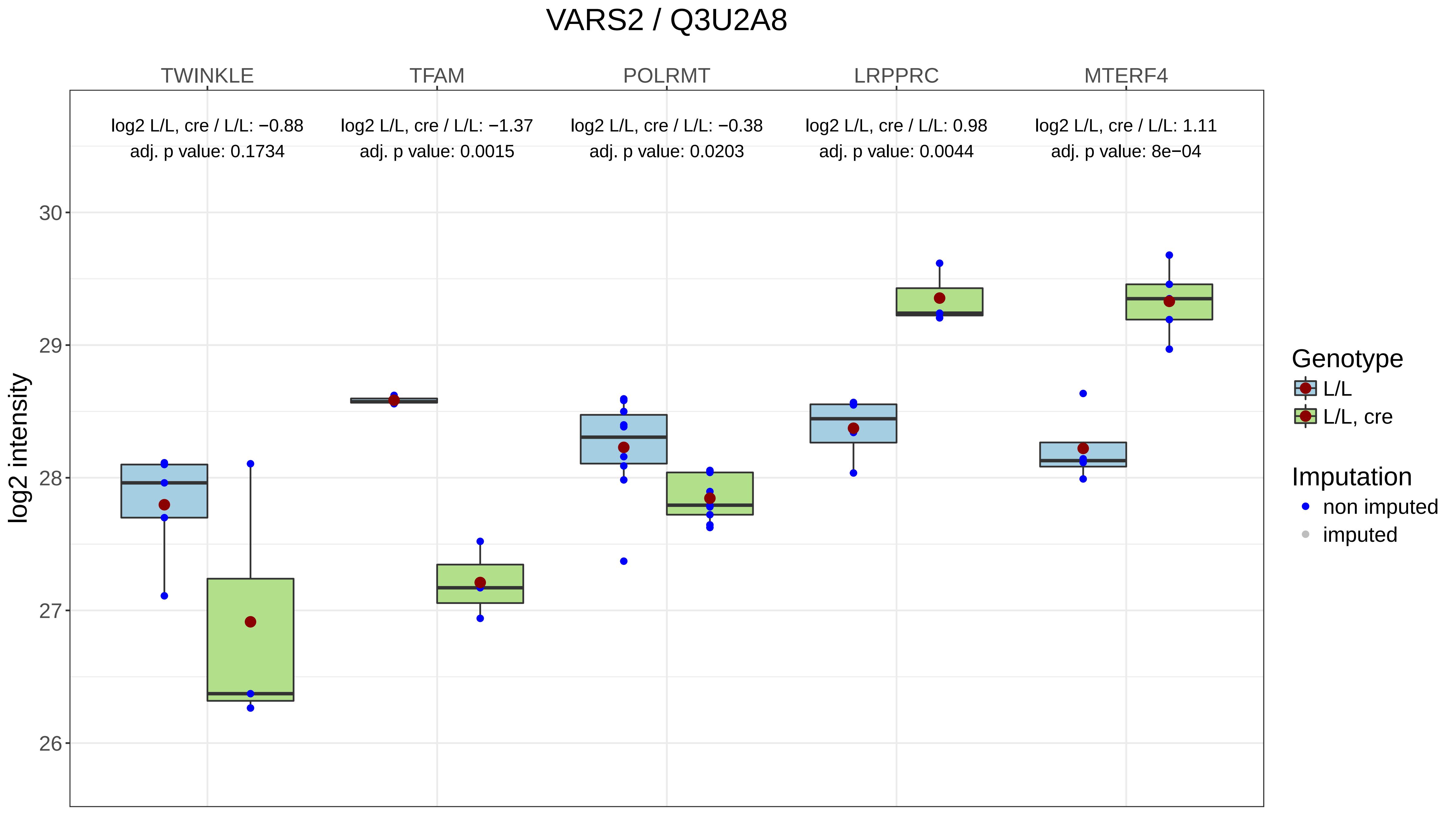

VARS2 / Q3U2A8
TWINKLE
TFAM
POLRMT
LRPPRC
MTERF4
log2 L/L, cre / L/L: −0.88
adj. p value: 0.1734
log2 L/L, cre / L/L: −1.37
adj. p value: 0.0015
log2 L/L, cre / L/L: −0.38
adj. p value: 0.0203
log2 L/L, cre / L/L: 0.98
adj. p value: 0.0044
log2 L/L, cre / L/L: 1.11
adj. p value: 8e−04
log2 intensity
30
29
28
27
26
Genotype
L/L
L/L, cre
Imputation
non imputed
imputed

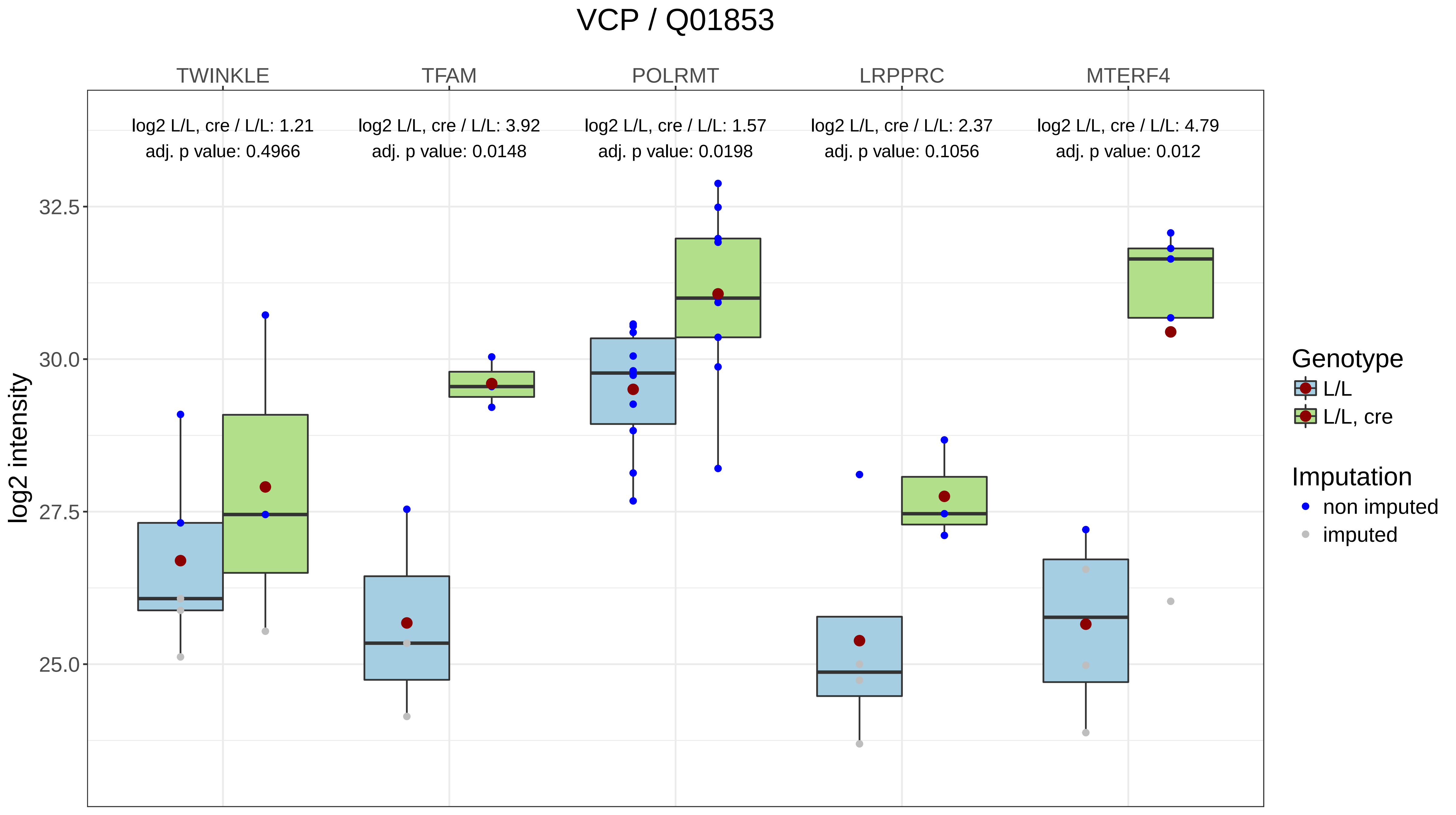

VCP / Q01853
TWINKLE
TFAM
POLRMT
LRPPRC
MTERF4
log2 L/L, cre / L/L: 1.21
adj. p value: 0.4966
log2 L/L, cre / L/L: 3.92
adj. p value: 0.0148
log2 L/L, cre / L/L: 1.57
adj. p value: 0.0198
log2 L/L, cre / L/L: 2.37
adj. p value: 0.1056
log2 L/L, cre / L/L: 4.79
adj. p value: 0.012
log2 intensity
32.5
30.0
27.5
25.0
Genotype
L/L
L/L, cre
Imputation
non imputed
imputed

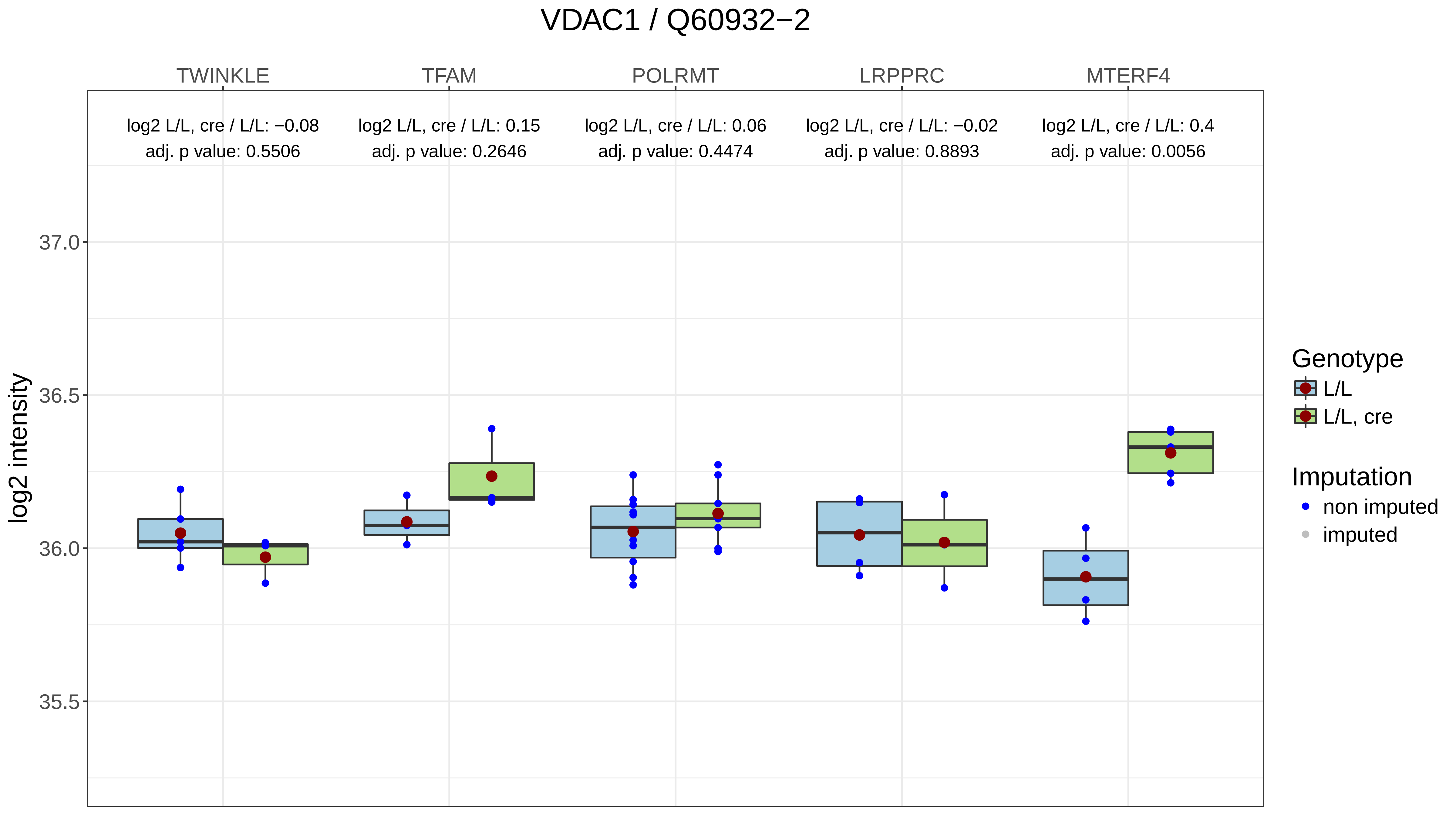
VDAC1 / Q60932−2
TWINKLE
TFAM
POLRMT
LRPPRC
MTERF4
log2 L/L, cre / L/L: −0.08
adj. p value: 0.5506
log2 L/L, cre / L/L: 0.15
adj. p value: 0.2646
log2 L/L, cre / L/L: 0.06
adj. p value: 0.4474
log2 L/L, cre / L/L: −0.02
adj. p value: 0.8893
log2 L/L, cre / L/L: 0.4
adj. p value: 0.0056
log2 intensity
37.0
36.5
36.0
35.5
Genotype
L/L
L/L, cre
Imputation
non imputed
imputed

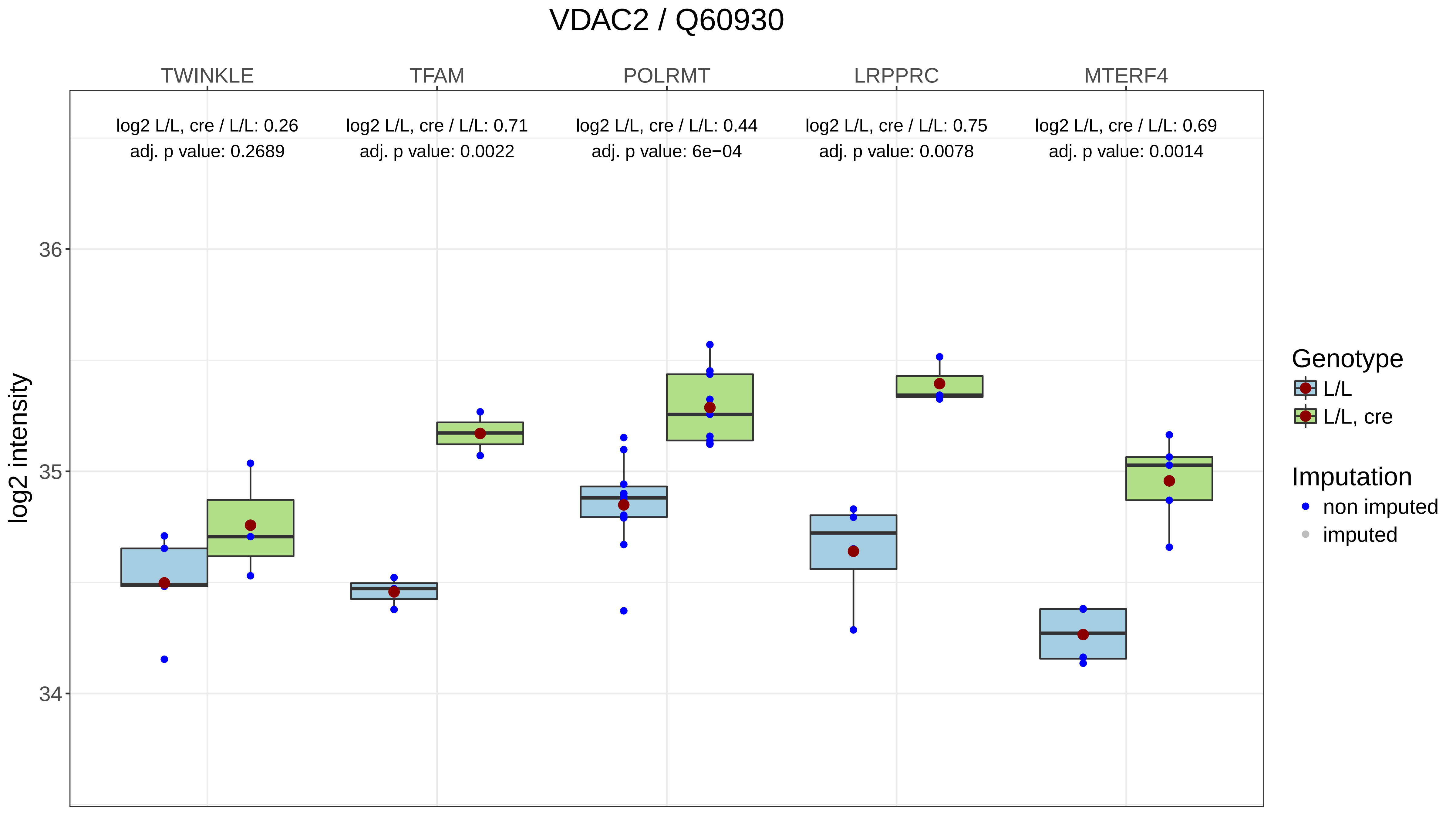
VDAC2 / Q60930
TWINKLE
TFAM
POLRMT
LRPPRC
MTERF4
log2 L/L, cre / L/L: 0.26
adj. p value: 0.2689
log2 L/L, cre / L/L: 0.71
adj. p value: 0.0022
log2 L/L, cre / L/L: 0.44
adj. p value: 6e−04
log2 L/L, cre / L/L: 0.75
adj. p value: 0.0078
log2 L/L, cre / L/L: 0.69
adj. p value: 0.0014
log2 intensity
36
35
34
Genotype
L/L
L/L, cre
Imputation
non imputed
imputed

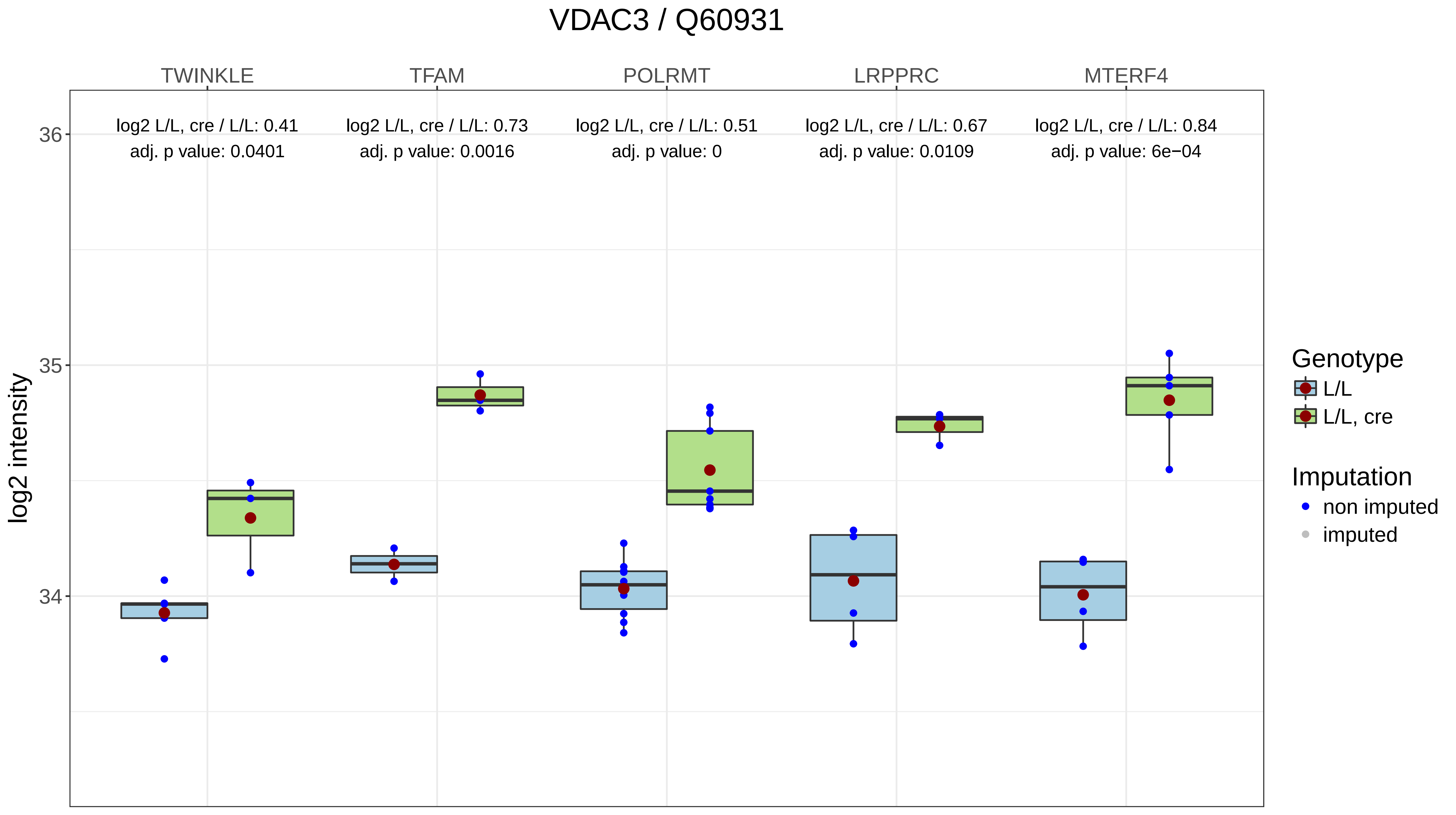

VDAC3 / Q60931
TWINKLE
TFAM
POLRMT
LRPPRC
MTERF4
log2 L/L, cre / L/L: 0.41
adj. p value: 0.0401
log2 L/L, cre / L/L: 0.73
adj. p value: 0.0016
log2 L/L, cre / L/L: 0.51
adj. p value: 0
log2 L/L, cre / L/L: 0.67
adj. p value: 0.0109
log2 L/L, cre / L/L: 0.84
adj. p value: 6e−04
log2 intensity
36
35
34
Genotype
L/L
L/L, cre
Imputation
non imputed
imputed

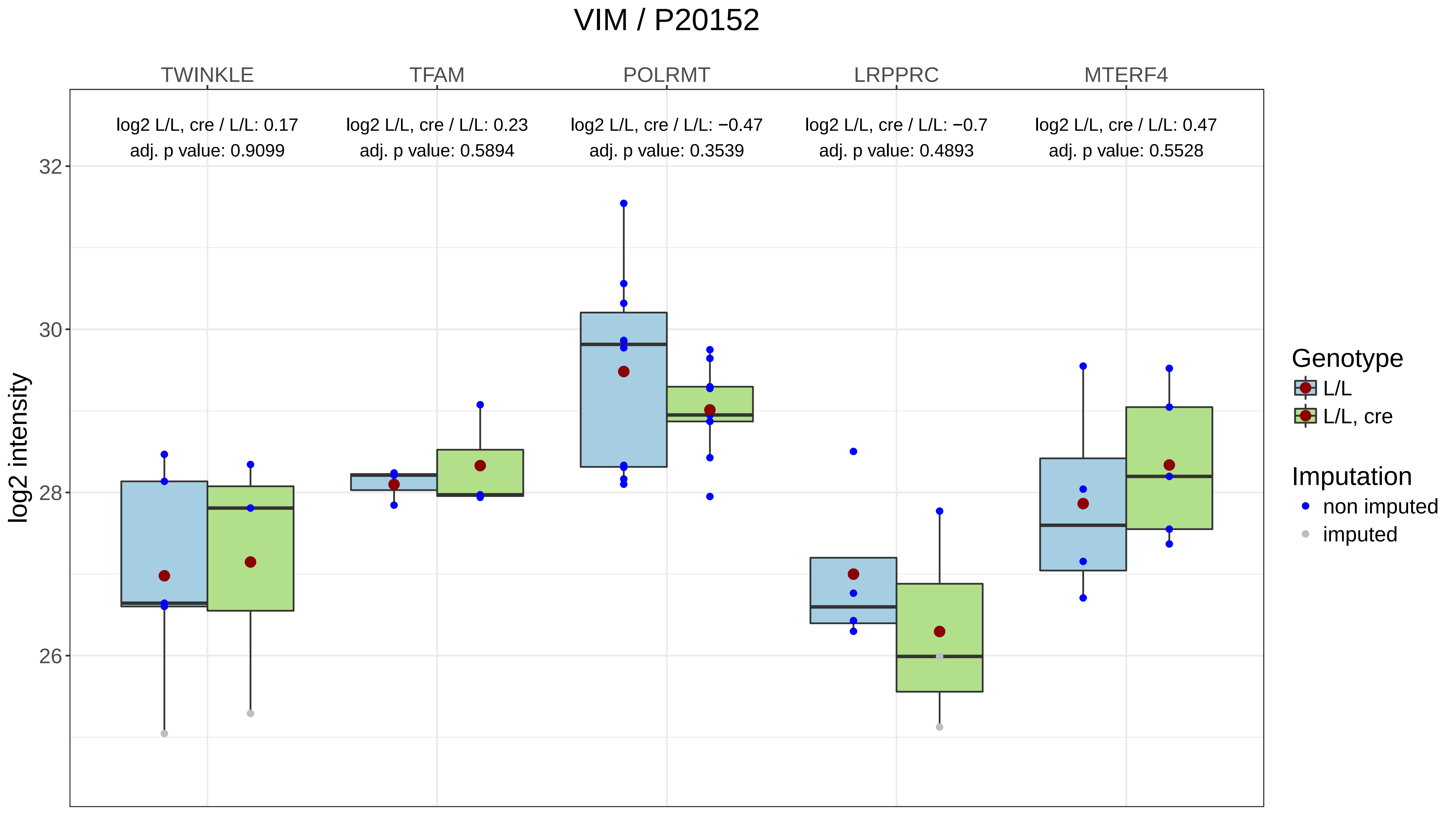
VIM / P20152
TWINKLE
TFAM
POLRMT
LRPPRC
MTERF4
log2 L/L, cre / L/L: 0.17
adj. p value: 0.9099
log2 L/L, cre / L/L: 0.23
adj. p value: 0.5894
log2 L/L, cre / L/L: −0.47
adj. p value: 0.3539
log2 L/L, cre / L/L: −0.7
adj. p value: 0.4893
log2 L/L, cre / L/L: 0.47
adj. p value: 0.5528
log2 intensity
32
30
28
26
Genotype
L/L
L/L, cre
Imputation
non imputed
imputed

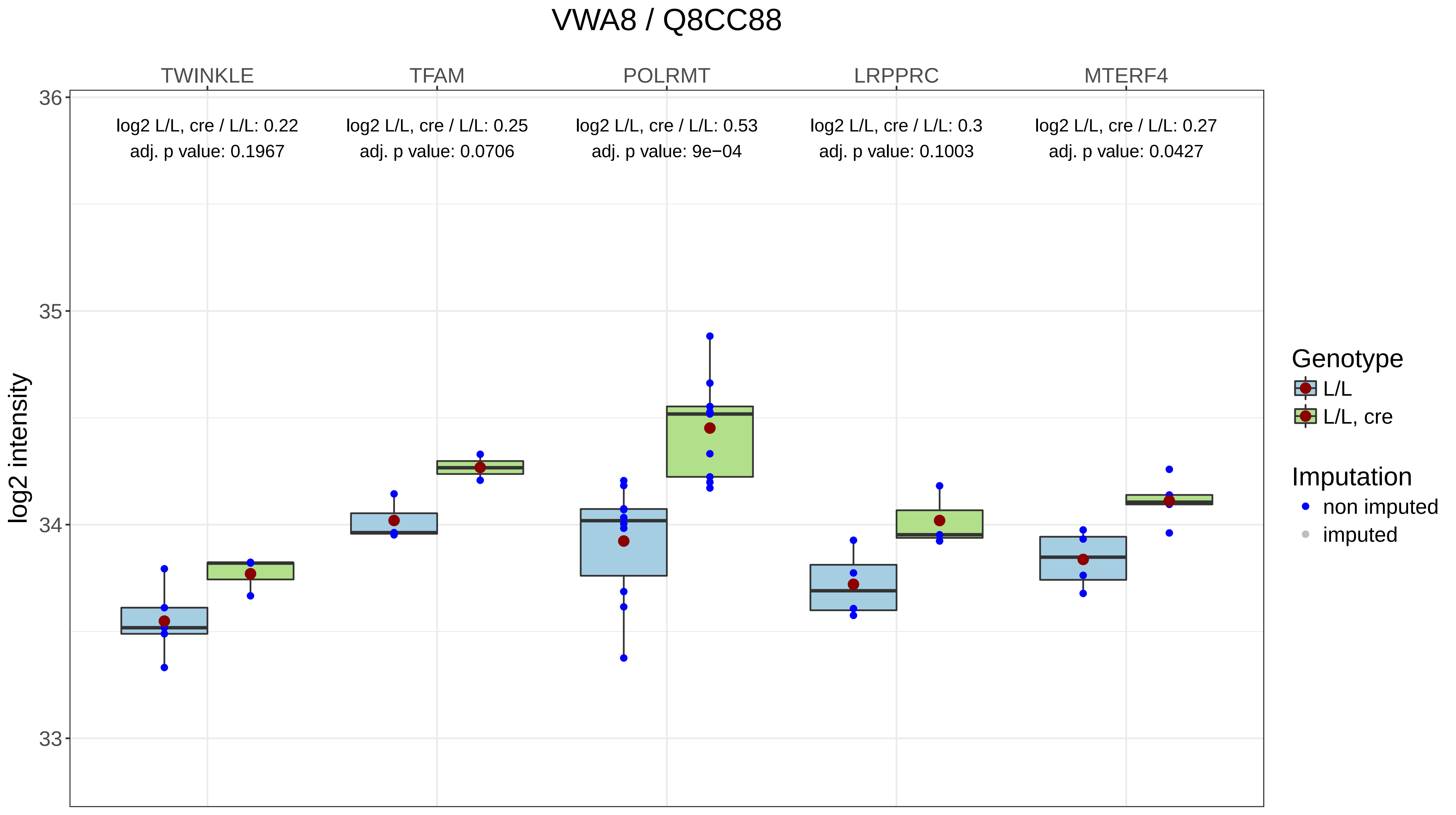
VWA8 / Q8CC88
TWINKLE
TFAM
POLRMT
LRPPRC
MTERF4
log2 L/L, cre / L/L: 0.22
adj. p value: 0.1967
log2 L/L, cre / L/L: 0.25
adj. p value: 0.0706
log2 L/L, cre / L/L: 0.53
adj. p value: 9e−04
log2 L/L, cre / L/L: 0.3
adj. p value: 0.1003
log2 L/L, cre / L/L: 0.27
adj. p value: 0.0427
log2 intensity
36
35
34
33
Genotype
L/L
L/L, cre
Imputation
non imputed
imputed

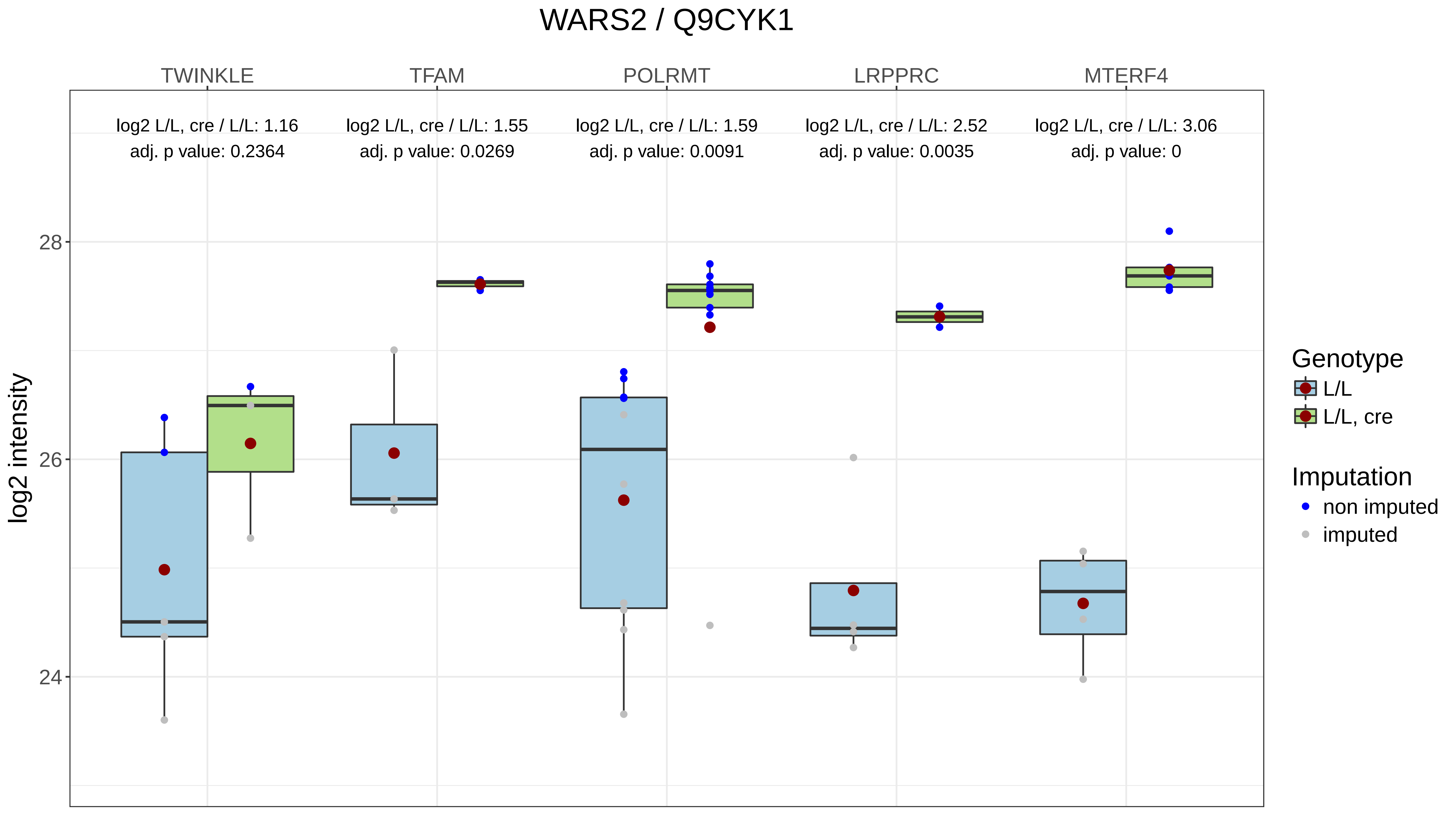
WARS2 / Q9CYK1
TWINKLE
TFAM
POLRMT
LRPPRC
MTERF4
log2 L/L, cre / L/L: 1.16
adj. p value: 0.2364
log2 L/L, cre / L/L: 1.55
adj. p value: 0.0269
log2 L/L, cre / L/L: 1.59
adj. p value: 0.0091
log2 L/L, cre / L/L: 2.52
adj. p value: 0.0035
log2 L/L, cre / L/L: 3.06
adj. p value: 0
log2 intensity
28
26
24
Genotype
L/L
L/L, cre
Imputation
non imputed
imputed

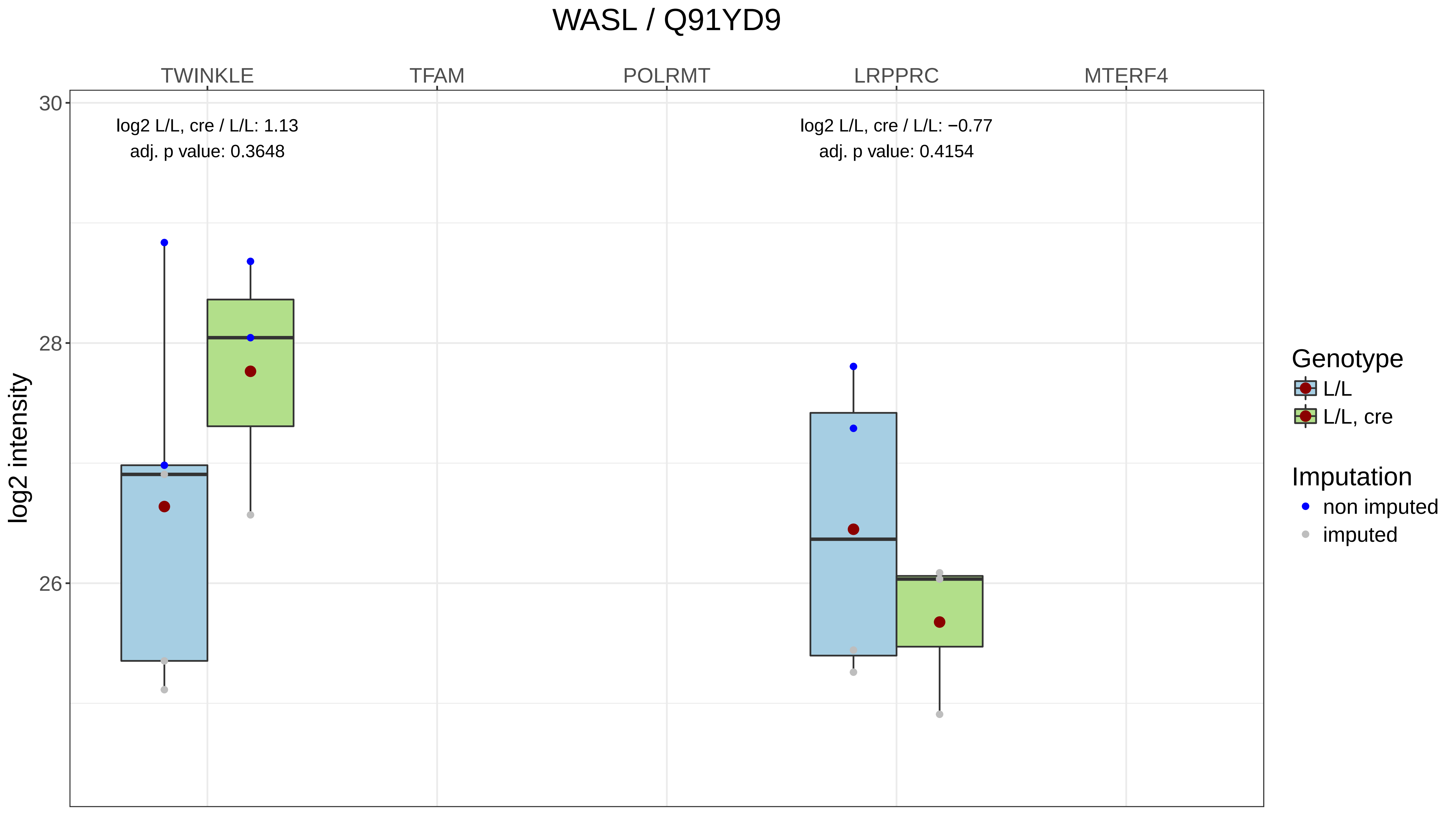

WASL / Q91YD9
TWINKLE
TFAM
POLRMT
LRPPRC
MTERF4
log2 L/L, cre / L/L: 1.13
adj. p value: 0.3648
log2 L/L, cre / L/L: −0.77
adj. p value: 0.4154
30
28
26
log2 intensity
Genotype
L/L
L/L, cre
Imputation
non imputed
imputed

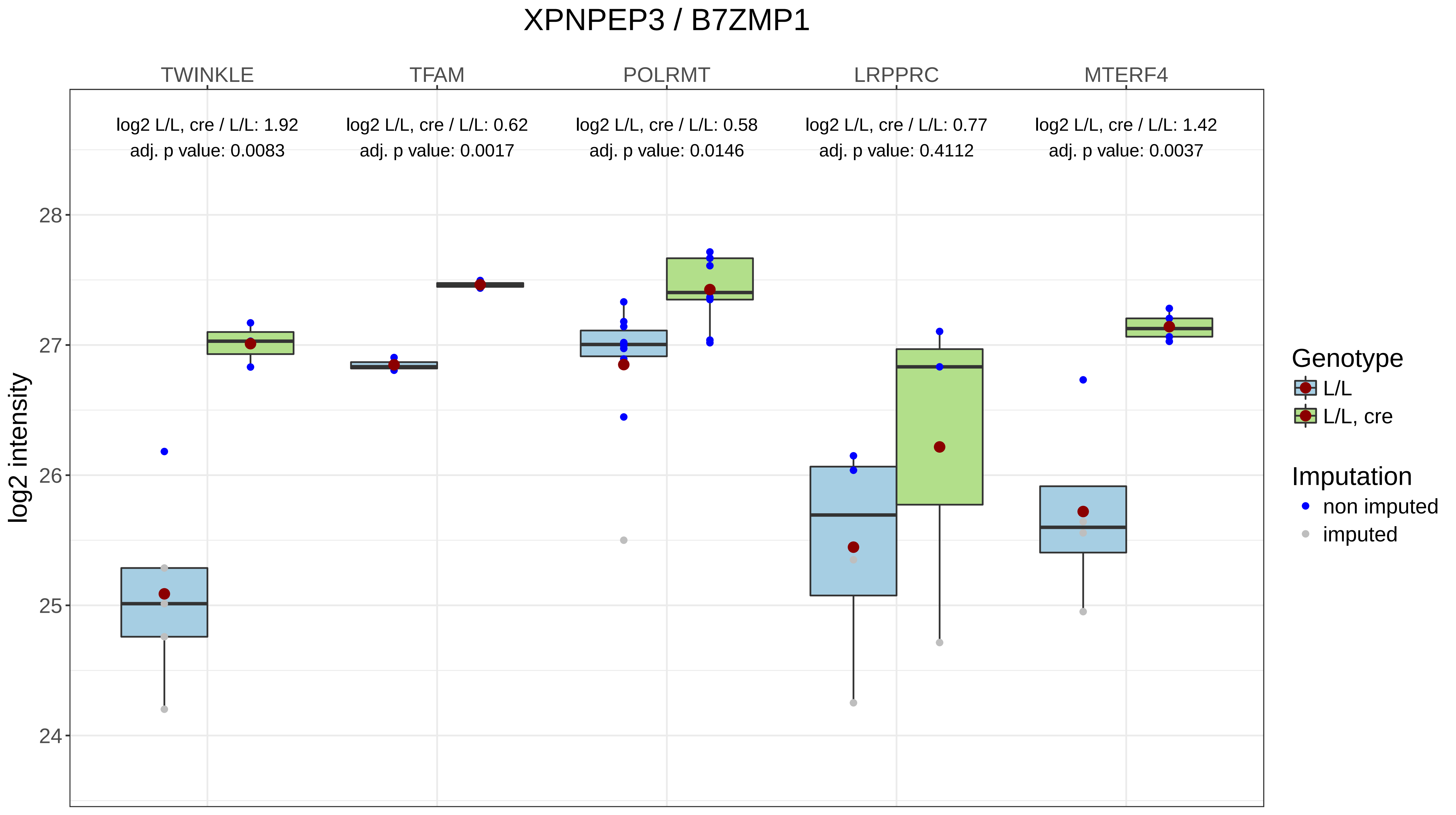

XPNPEP3 / B7ZMP1
TWINKLE
TFAM
POLRMT
LRPPRC
MTERF4
log2 L/L, cre / L/L: 1.92
adj. p value: 0.0083
log2 L/L, cre / L/L: 0.62
adj. p value: 0.0017
log2 L/L, cre / L/L: 0.58
adj. p value: 0.0146
log2 L/L, cre / L/L: 0.77
adj. p value: 0.4112
log2 L/L, cre / L/L: 1.42
adj. p value: 0.0037
log2 intensity
28
27
26
25
24
Genotype
L/L
L/L, cre
Imputation
non imputed
imputed

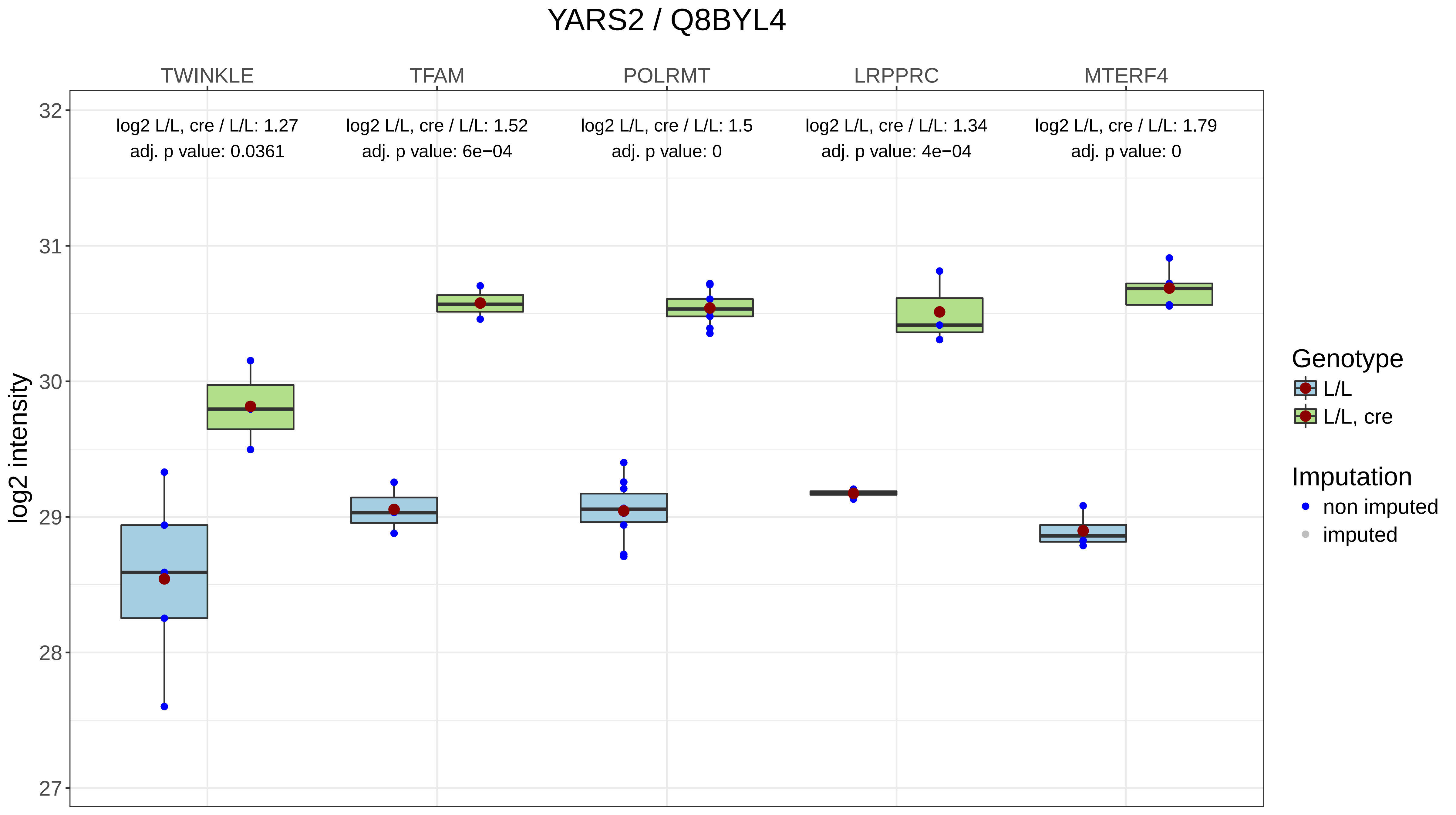

YARS2 / Q8BYL4
TWINKLE
TFAM
POLRMT
LRPPRC
MTERF4
log2 L/L, cre / L/L: 1.27
adj. p value: 0.0361
log2 L/L, cre / L/L: 1.52
adj. p value: 6e−04
log2 L/L, cre / L/L: 1.5
adj. p value: 0
log2 L/L, cre / L/L: 1.34
adj. p value: 4e−04
log2 L/L, cre / L/L: 1.79
adj. p value: 0
log2 intensity
32
31
30
29
28
27
Genotype
L/L
L/L, cre
Imputation
non imputed
imputed

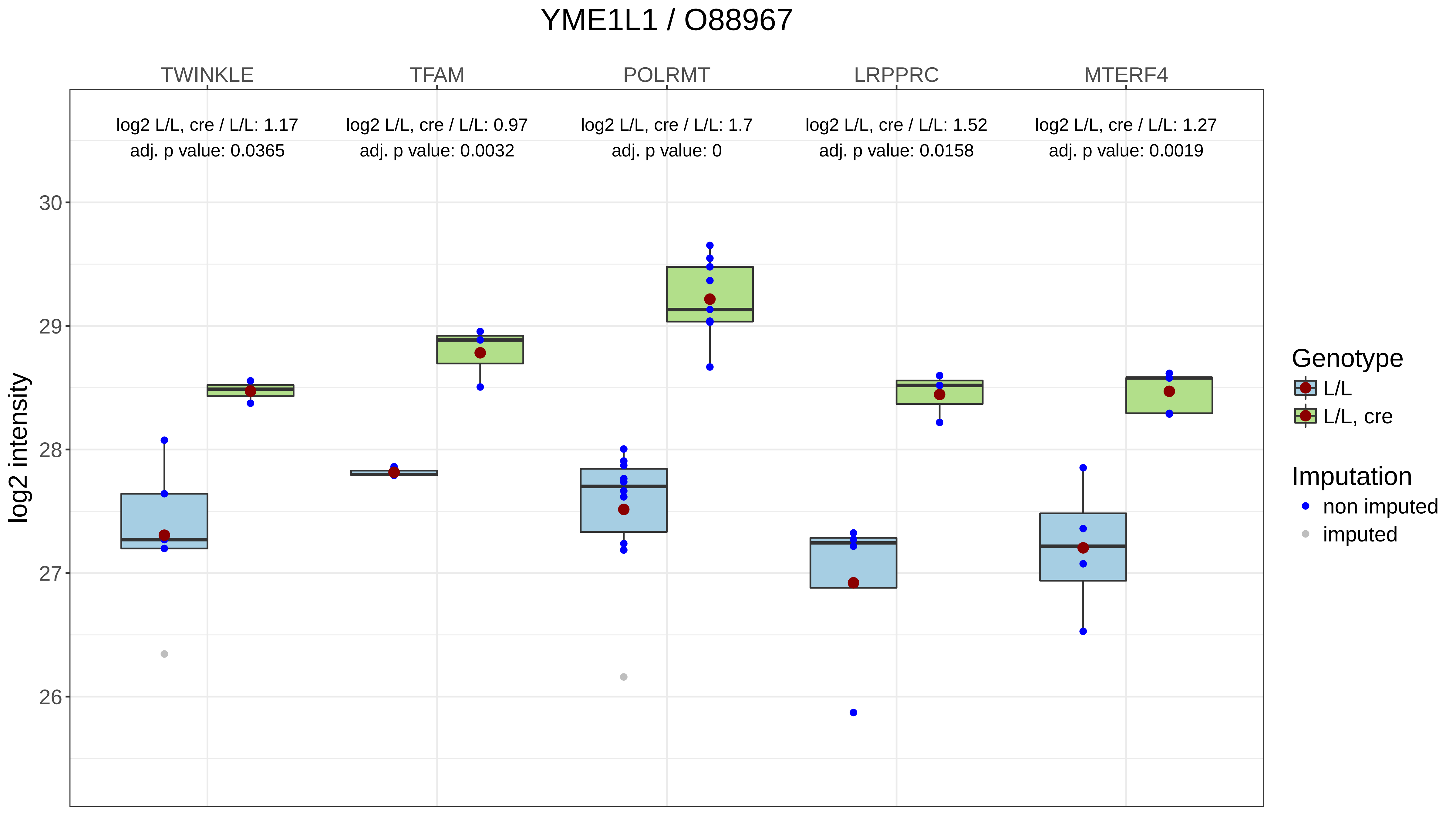

YME1L1 / O88967
TWINKLE
TFAM
POLRMT
LRPPRC
MTERF4
log2 L/L, cre / L/L: 1.17
adj. p value: 0.0365
log2 L/L, cre / L/L: 0.97
adj. p value: 0.0032
log2 L/L, cre / L/L: 1.7
adj. p value: 0
log2 L/L, cre / L/L: 1.52
adj. p value: 0.0158
log2 L/L, cre / L/L: 1.27
adj. p value: 0.0019
log2 intensity
30
29
28
27
26
Genotype
L/L
L/L, cre
Imputation
non imputed
imputed

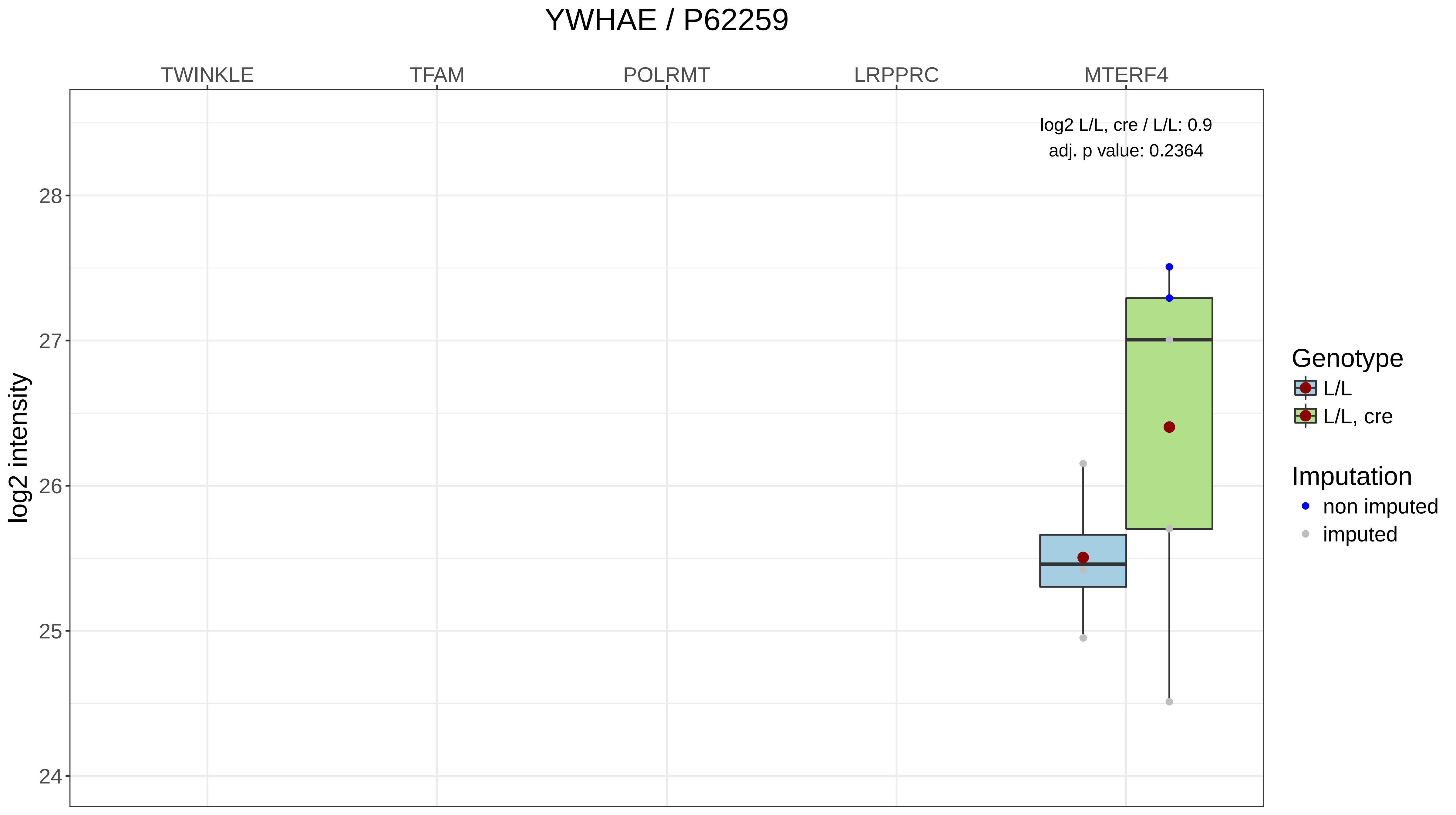
YWHAE / P62259
TWINKLE
TFAM
POLRMT
LRPPRC
MTERF4
log2 L/L, cre / L/L: 0.9
adj. p value: 0.2364
log2 intensity
28
27
26
25
24
Genotype
L/L
L/L, cre
Imputation
non imputed
imputed

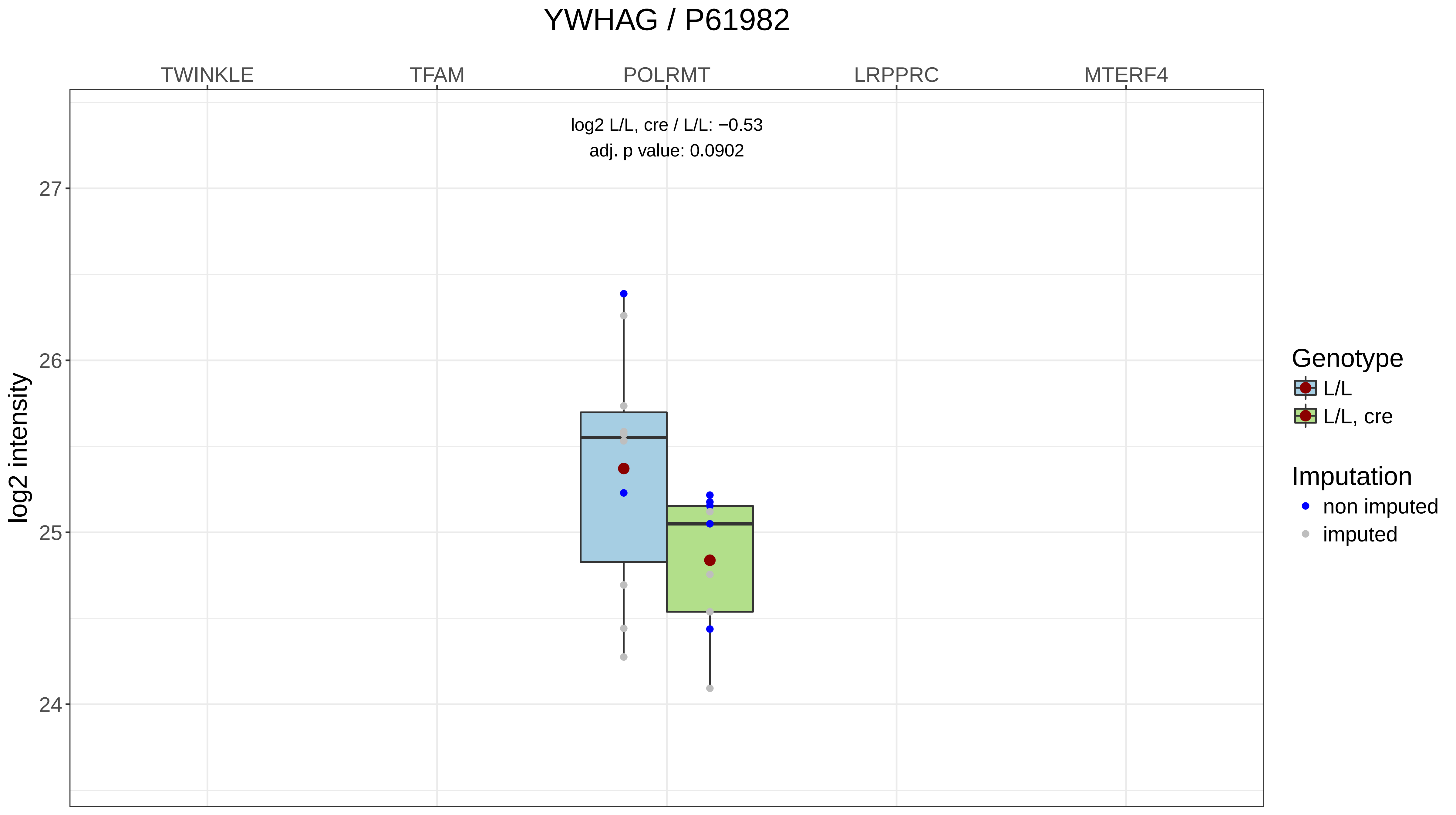
YWHAG / P61982
TWINKLE
TFAM
POLRMT
LRPPRC
MTERF4
log2 L/L, cre / L/L: −0.53
adj. p value: 0.0902
27
26
25
24
log2 intensity
Genotype
L/L
L/L, cre
Imputation
non imputed
imputed

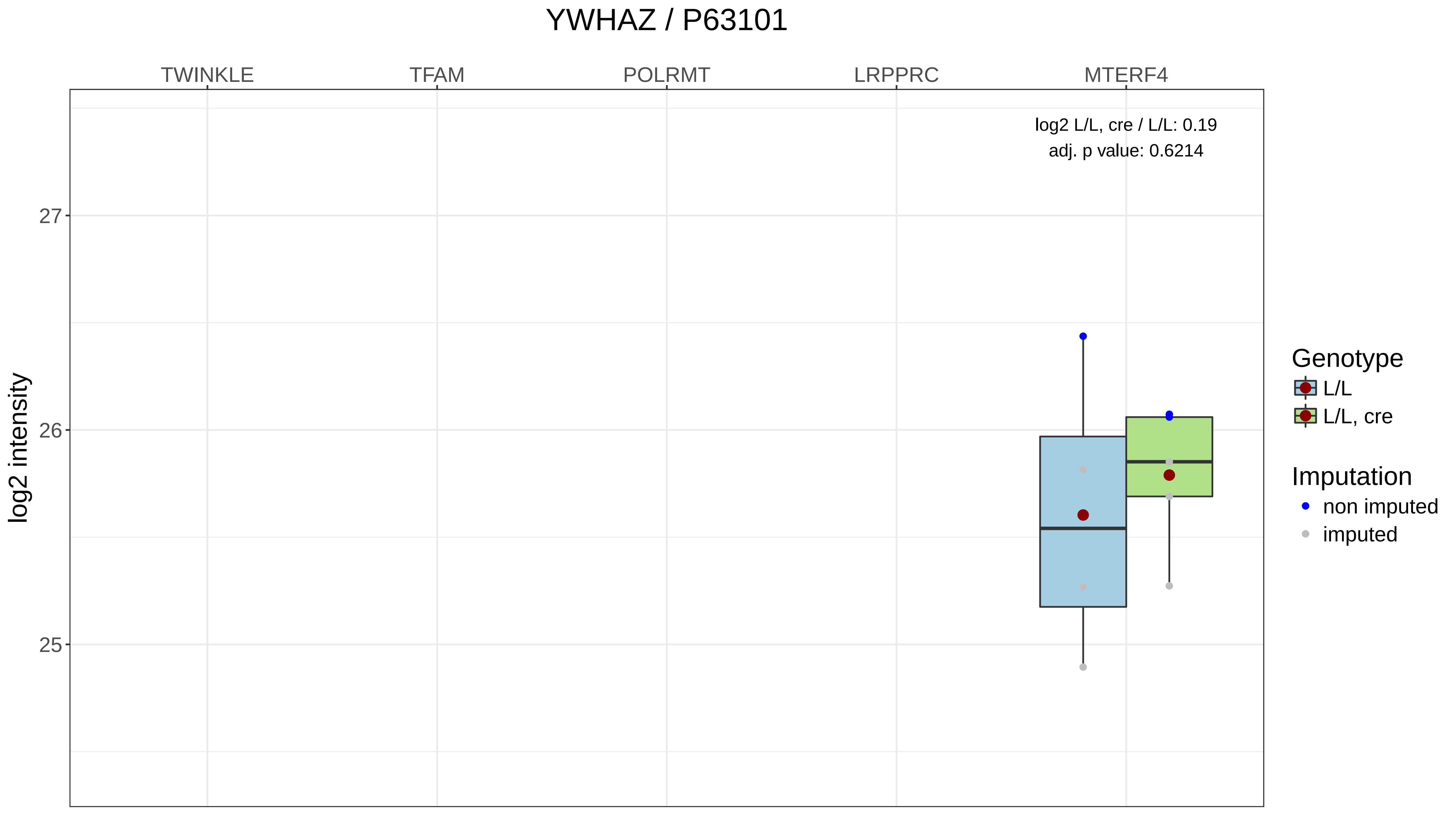
YWHAZ / P63101
TWINKLE
TFAM
POLRMT
LRPPRC
MTERF4
log2 L/L, cre / L/L: 0.19
adj. p value: 0.6214
27
26
25
log2 intensity
Genotype
L/L
L/L, cre
Imputation
non imputed
imputed

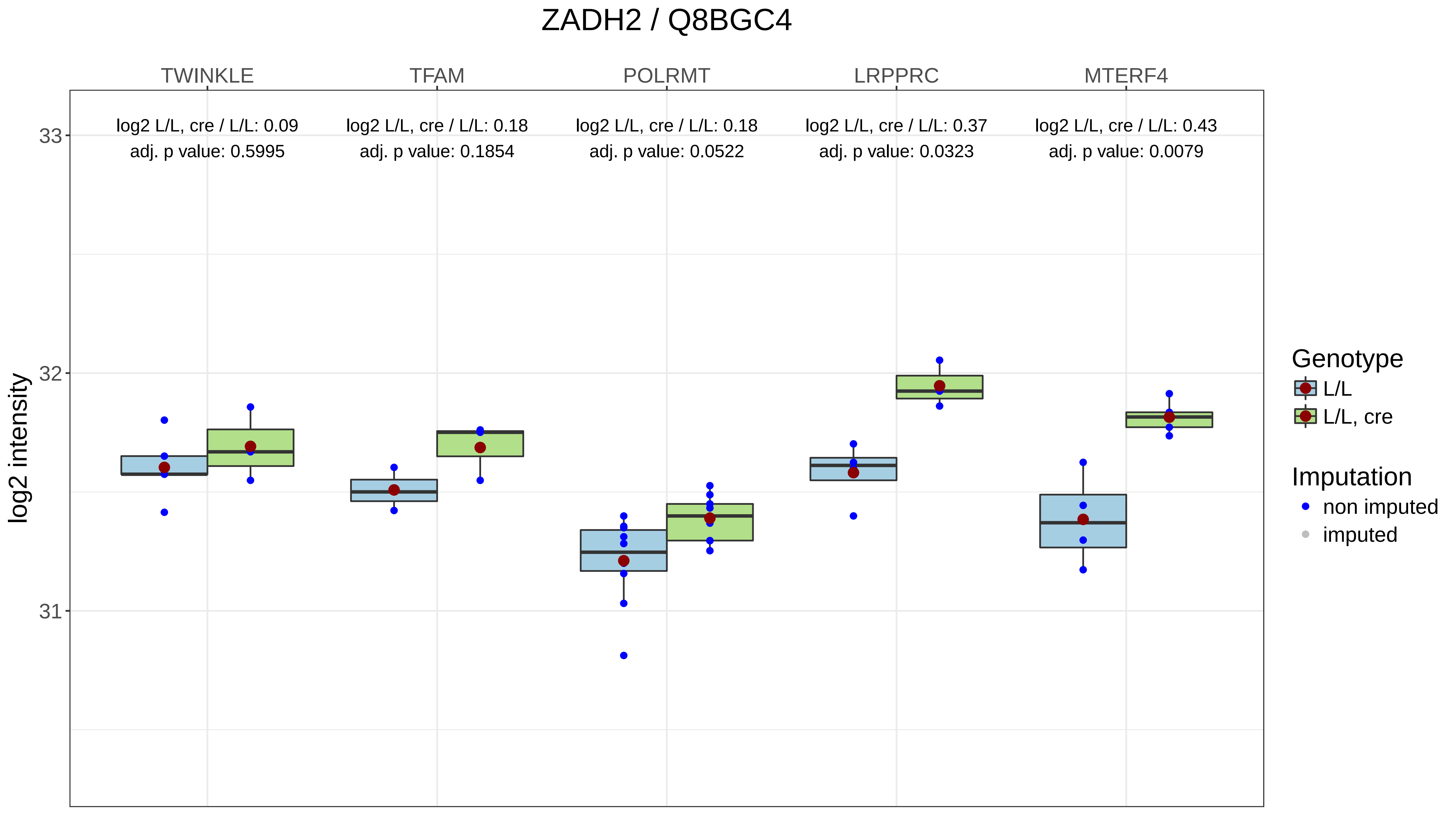
ZADH2 / Q8BGC4
TWINKLE
TFAM
POLRMT
LRPPRC
MTERF4
log2 L/L, cre / L/L: 0.09
adj. p value: 0.5995
log2 L/L, cre / L/L: 0.18
adj. p value: 0.1854
log2 L/L, cre / L/L: 0.18
adj. p value: 0.0522
log2 L/L, cre / L/L: 0.37
adj. p value: 0.0323
log2 L/L, cre / L/L: 0.43
adj. p value: 0.0079
33
32
31
log2 intensity
Genotype
L/L
L/L, cre
Imputation
non imputed
imputed

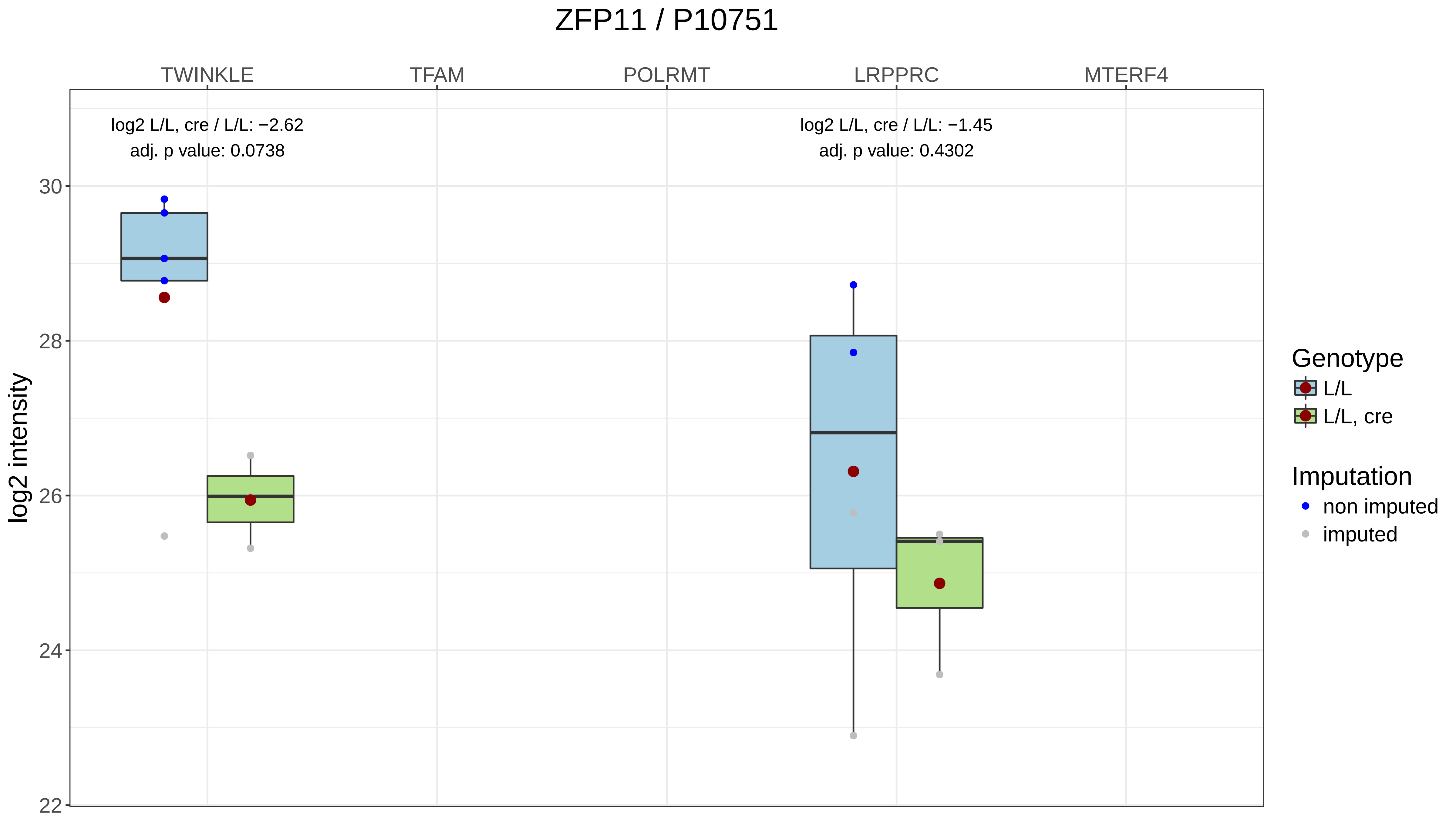

ZFP11 / P10751
TWINKLE
TFAM
POLRMT
LRPPRC
MTERF4
log2 L/L, cre / L/L: −2.62
adj. p value: 0.0738
log2 L/L, cre / L/L: −1.45
adj. p value: 0.4302
log2 intensity
30
28
26
24
22
Genotype
L/L
L/L, cre
Imputation
non imputed
imputed

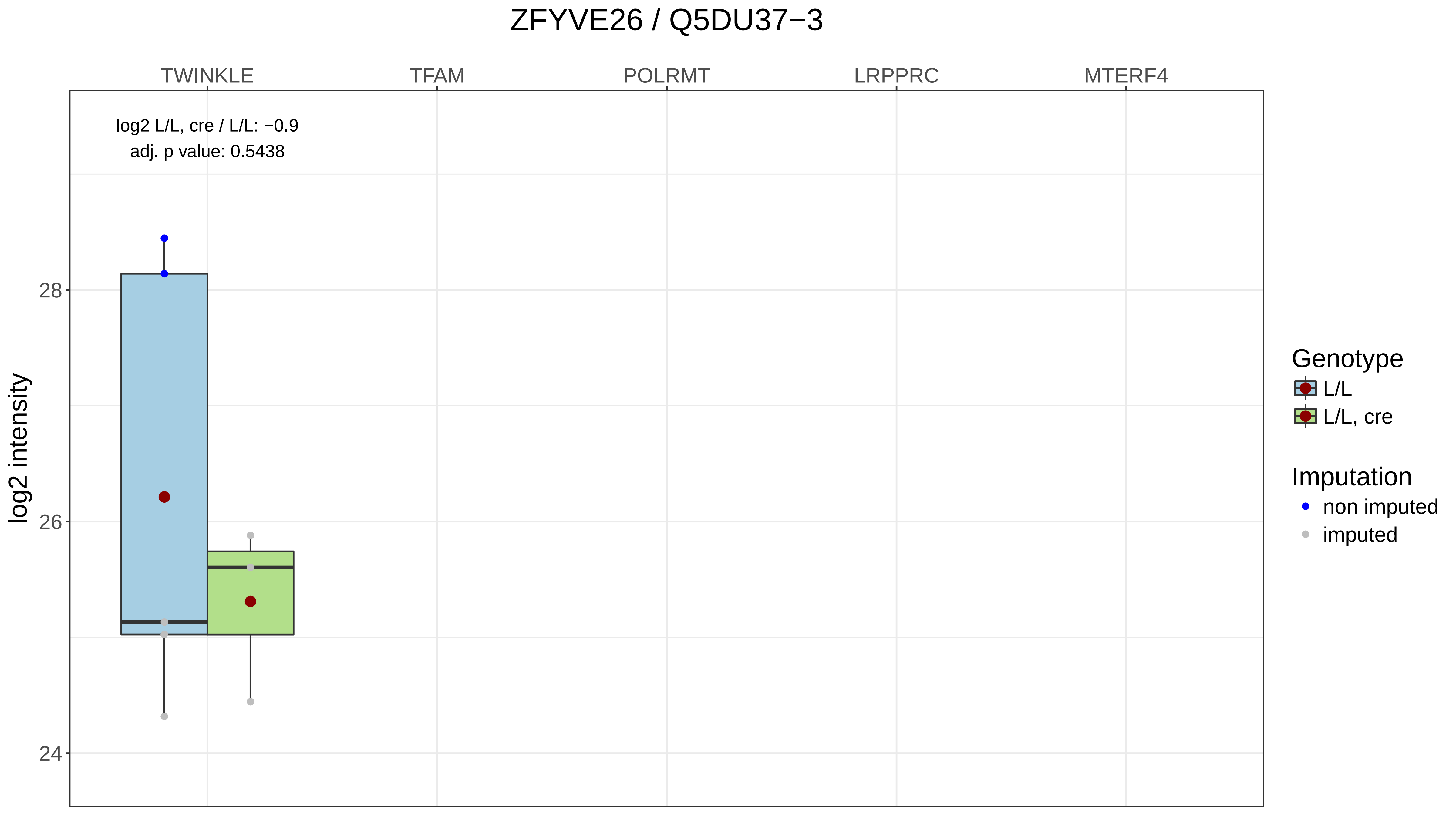
ZFYVE26 / Q5DU37−3
TWINKLE
TFAM
POLRMT
LRPPRC
MTERF4
log2 L/L, cre / L/L: −0.9
adj. p value: 0.5438
log2 intensity
28
26
24
Genotype
L/L
L/L, cre
Imputation
non imputed
imputed
